# Multiple-gene targeting and mismatch tolerance can confound analysis of genome-wide pooled CRISPR screens

**DOI:** 10.1186/s13059-019-1621-7

**Published:** 2019-01-25

**Authors:** Jean-Philippe Fortin, Jenille Tan, Karen E. Gascoigne, Peter M. Haverty, William F. Forrest, Michael R. Costa, Scott E. Martin

**Affiliations:** 1 Department of Bioinformatics and Computational Biology, Genentech, Inc., 1 DNA Way, South San Francisco, 94080 CA USA; 2 Department of Discovery Oncology, Genentech, Inc., 1 DNA Way, South San Francisco, 94080 CA USA

**Keywords:** CRISPR/Cas9, Functional genomics, Gene essentiality, Cancer vulnerability, Synthetic lethality, Achilles, Loss-of-function screen, Off-targets, Cleavage toxicity

## Abstract

**Background:**

Genome-wide loss-of-function screens using the CRISPR/Cas9 system allow the efficient discovery of cancer cell vulnerabilities. While several studies have focused on correcting for DNA cleavage toxicity biases associated with copy number alterations, the effects of sgRNAs co-targeting multiple genomic loci in CRISPR screens have not been discussed.

**Results:**

In this work, we analyze CRISPR essentiality screen data from 391 cancer cell lines to characterize biases induced by multi-target sgRNAs. We investigate two types of multi-targets: on-targets predicted through perfect sequence complementarity and off-targets predicted through sequence complementarity with up to two nucleotide mismatches. We find that the number of on-targets and off-targets both increase sgRNA activity in a cell line-specific manner and that existing additive models of gene knockout effects fail at capturing genetic interactions that may occur between co-targeted genes. We use synthetic lethality between paralog genes to show that genetic interactions can introduce biases in essentiality scores estimated from multi-target sgRNAs. We further show that single-mismatch tolerant sgRNAs can confound the analysis of gene essentiality and lead to incorrect co-essentiality functional networks. Lastly, we also find that single nucleotide polymorphisms located in protospacer regions can impair on-target activity as a result of mismatch tolerance.

**Conclusion:**

We show the impact of multi-target effects on estimating cancer cell dependencies and the impact of off-target effects caused by mismatch tolerance in sgRNA-DNA binding.

**Electronic supplementary material:**

The online version of this article (10.1186/s13059-019-1621-7) contains supplementary material, which is available to authorized users.

## Introduction

A central goal of functional genomics is to understand the complex relationship between the genotype and the phenotype of a given organism. Genome-scale forward genetic screens are powerful and unbiased experiments that help identify gene function and relationships between gene disruption and disease. In such screens, gene perturbations such as mutation or expression dysregulation are first introduced in cells or organisms, then phenotypes of interest in the cell or organism population are identified, and finally selected phenotypes are linked back to gene perturbations to establish causality. Genome-wide pooled screens exploiting the RNA interference (RNAi) pathway have significantly helped with identifying and prioritizing therapeutic targets in cancer and other diseases; several loss-of-function genome-wide RNAi screens across dozens of cancer cell lines have been conducted to study cancer genetic vulnerabilities as well as identify genes that are essential across cell lines [1–4]. However, the analysis and interpretation of results obtained from RNAi screens can be confounded by ubiquitous off-target effects and incomplete knockdown.

The discovery of the CRISPR/Cas9 genome editing system and its application to functional screens have revolutionized the field by minimizing many of the challenges and limitations observed in RNAi screens [5]. Following their previous effort in identifying cancer vulnerabilities using RNAi pooled screens [1], the Broad Institute has been performing genome-wide pooled CRISPR knockout screens across several hundreds of genomically characterized cancer cell lines [6] using the Avana guide library [7]. The screening effort using both RNAi and CRISPR technologies is referred to as Project Achilles. To date, CRISPR screening data from 391 cell lines have been released and are available to download from the Project Achilles portal (https://portals.broadinstitute.org/achilles).

Early on, it was observed that variation in genomic copy number (CN) was impacting growth measurements in loss-of-function CRISPR screens in a gene-independent manner [8–10]. Specifically, guides targeting amplified regions create a large number of DNA double-strand breaks (DSBs) resulting in a loss of cell viability, often referred to as “cleavage toxicity.” If not taken into account, gene-independent CN effects can lead to an increased number of false positives. In [6], the authors propose to delineate gene-knockout effects from CN effects in the Achilles dataset by concurrently modeling both effects in a linear regression framework; the algorithm is called CERES. In particular, the CN effects are captured in a cell-specific manner using linear splines, allowing for cell line-specific amplifications and deletions. A CN-adjusted essentiality score (CERES score) is estimated for each single-guide RNA (sgRNA) and for each cell line. Among others, CERES scores can be further analyzed to discover cancer vulnerabilities and gene essentiality.

Designing sgRNAs that uniquely map to the genome can be challenging, especially for genes sharing high homology with other genomic loci, either in coding or non-coding regions. In the Avana library, a number of sgRNAs are annotated to target multiple genes through perfect sequence complementarity between the sgRNA’s spacer sequence and genomic DNA—we refer to such guides as “multi-target” guides. The CERES model attempts to account for multi-target effects by modeling the log-fold change (LFC) of a multi-target guide as a linear combination of knockout effects from the set of genes targeted by the guide; the model assumes that gene knockout effects are additive. For instance, for a guide targeting two genes, this assumes that the phenotypic effects of a double knockout are the sum of the individual gene knockout phenotypic effects. As a limitation, genetic interactions, such as synergy, synthetic lethality, genetic buffering, and epistasis, cannot be appropriately captured by such a model. Additionally, while the CERES model attempts to account for multi-target effects, off-target effects caused by mismatch tolerance between the sgRNA’s spacer sequence and genomic DNA have not been considered in the Achilles dataset.

In this work, we investigate the impact of multi-target effects on estimating cancer cell dependencies, as well as the impact of off-target effects caused by mismatch tolerance in sgRNA-DNA binding. We take advantage of the Achilles CRISPR screening data, by far the most comprehensive CRISPR screening effort to date, to characterize these biases across cell lines and sgRNAs. First, we show that the number of on-targets of a particular sgRNA can dramatically increase the sgRNA essentiality score in a non-additive fashion. To illustrate this, we consider guides in the Avana library that co-target paralogs *MYL12A* and *MYL12B* and show that an additive model cannot capture the synthetic lethal interaction observed in a subset of cell lines in which the redundant third paralog *MYL9* is not expressed. We also show that off-target effects caused by single-mismatch sgRNA-DNA alignments can cause spurious associations between cell lineage and gene knockout. As an example, we found that several cell lines are unexpectedly reported as being dependent on *SOX9* despite the observation that *SOX9* is not expressed in these cell lines. We present evidence that off-target effects caused by single-mismatch tolerance are likely responsible for these inconsistent results. Lastly, we show that single nucleotide polymorphisms (SNPs) located in protospacer regions can impair on-target activity as a result of mismatch tolerance. We provide gene-level summaries of on-target and off-target alignments in the Avana library to help identify and interpret genes with unexpected essentiality scores.

## Results

### The impact of multiple on-target alignments on sgRNA depletion

**Fig. 1:**
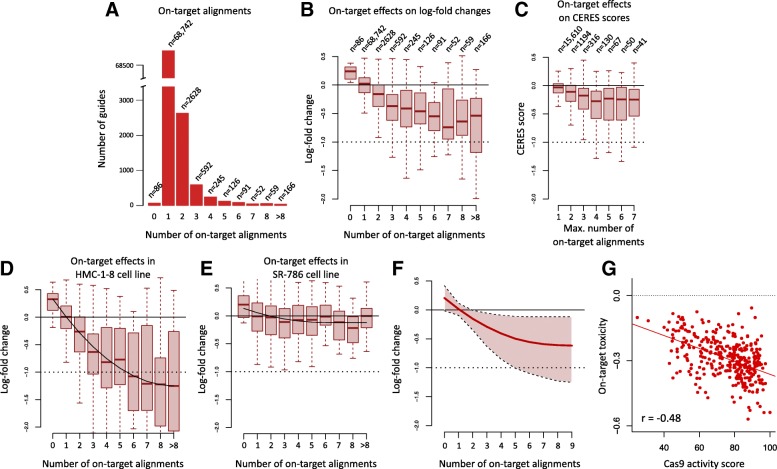
The impact of multiple on-targets on sgRNA log-fold changes. **a** Guide numbers as a function of the number of perfect alignments (on-target alignments); non-targeting controls (NTCs) were excluded from this analysis. **b** Guide-level log-fold changes (LFCs) averaged across cell lines as a function of the number of on-targets. The number of on-targets was calculated as the number of perfect alignments between the reference genome and the 20-nt spacer; we excluded guides with single-mismatch alignments to prevent the confounding effect of single-mismatch off-targets. **c** Combined effect of multiple on-targets on the CERES score. For each gene, we calculated the maximum number of targeted loci (*x*-axis) as the maximum number of perfect alignments for a guide designed to target that gene in the Avana library. **d**, **e** Effects of multiple on-target alignments on LFCs in the breast cancer cell line HMC-1-8 and in the lymphoma cell line SR-786. Solid lines represent second-degree polynomial fits (see “Material and methods”). **f** Distribution of cell-specific fitted on-target activity (average log-fold changes) as a function of the number of on-target alignments (solid line: median across cell lines; shaded area: full range of on-target activity across cell lines). **g** Average on-target toxicity as a function of Cas9 activity score

We investigated the effects of multiple on-target alignments by looking at the relationship between sgRNA alignments and LFCs. We note that negative LFCs indicate a decrease in cell proliferation, and therefore larger negative LFCs indicate greater gene essentiality. For our analyses, we also corrected LFCs for copy number variation using the methodology described in [6]. In Fig. 1a, we present the counts of guides stratified by the number of targets; the counts decay exponentially as the number of perfect alignments increases. 68,742 guides align uniquely to one target only, and 3959 guides align to more than one target (multi-target guides), resulting in 2023 genes that are targeted by at least one multi-target guide. Interestingly, 86 sgRNAs intended to target genes did not align to any genomic location in GRCh38. The median LFC for these guides is positive (Fig. 1b) and correlates with the 995 non-targeting control (NTC) guides included in the Avana library (*r*=0.69), suggesting indeed that many of the DNA sequences corresponding to those 86 sgRNA sequences are likely to be absent in the genome.

To study the impact of multiple alignments on guide essentiality scores, we averaged LFCs across cell lines to get guide-specific average essentiality scores. We restricted our analysis to guides with no single-mismatch alignments to prevent confounding with off-target effects; off-target effects caused by mismatch tolerance are further discussed below. In Fig. 1b, we report the distributions of the average LFCs as a function of the number of perfect alignments. The median LFC significantly decreases as a function of number of perfect alignments (*p* < 2.2×10^−16^, Jonckheere trend test [11, 12]). This is concordant with the hypothesis that a guide mapping to several DNA targets will introduce multiple DSBs and therefore will result in more cleavage toxicity, similar to the effect of copy number [6]. The CERES algorithm described in [6] attempts to account for these multi-target effects by explicitly modeling multi-target gene knockouts in an additive fashion. Despite this implementation, we observed that the number of perfect alignments also affects the median CERES score (*p* < 2.2×10^−16^, Jonckheere trend test, Fig. 1c).

We also found that cleavage toxicity induced by multi-target guides is cell line-specific, similar to the CN toxicity described in [6]; for instance, the on-target toxicity is quite profound for the breast cancer HMC-1-8 cell line (Fig. 1d), whereas there is minimal to no effect in the lymphoma cell line SR-786 (Fig. 1e). For both cell lines, the fitted curves were estimated using second-degree polynomials; curves for all cell lines are presented in Fig. 1f.

Using the average LFC of guides targeting 4 genomic loci as a summary metric for multiple-target cleavage toxicity, we found that the dropout associated with cleavage toxicity correlates negatively with the cell line-specific Cas9 activity score described in [9] (*r*=− 0.48, *p* < 2.2×10^−16^, Fig. 1g), and negatively correlates with the median LFC of non-targeting controls (NTCs) (*r*=− 0.67, *p* < 2.2×10^−16^). The latter association is not surprising in light of the competitive nature of CRISPR screens; cell lines with greater Cas9 activity result in more efficient DNA cleavage, potentially leading to more rapid death for cells infected with multiple-target guides or guides targeting essential genes, which in turn result in an increased proportion of cells infected with NTCs in the cell population over time. These results suggest that greater Cas9 activity leads to greater multiple-target toxicity.

#### Guides co-targeting coding regions are enriched for paralogs

Next, we sought to understand whether or not the increased lethality associated with multi-target guides is attributable to cleavage toxicity only or can also be a consequence of genetic interactions within the set of co-targeted genes. We focused on the 2628 guides in the Avana library that target exactly two genomic loci with perfect complementarity; we refer to these guides as “double-target” guides. A double-target guide can either target (a) one coding region and one non-coding region or (b) two coding regions. Because guides in the Avana library are designed to target protein-coding genes only, there are no double-target guides targeting two non-coding regions. For (a), the combined knockout effect is expected to be the sum of the gene-specific knockout effect and the cleavage toxicity effect induced by introducing DSBs at two genomic loci. For (b), the combined knockout effect is expected to be the sum of the digenic knockout effect and the cleavage toxicity effect induced by introducing DSBs at two genomic loci.

Using annotated exons from GENCODE (comprehensive gene annotation, human v28), we found that 2503 (95.2%) of the double-target guides have both targets located in coding regions. Since the annotated exons represent only 4.5% of the mappable genome, this represents a significant enrichment (exact binomial test, *p* < 2.2×10^−16^). We studied the effects of coding vs non-coding targets by averaging LFCs across cell lines for double-target guides. We excluded guides with single-mismatch off-targets, resulting in 1734 and 85 guides for coding and non-coding region secondary targets, respectively. LFCs averaged across cell lines are presented in Additional file 1: Figure S1a. For both sets of guides, the median LFC is comparable and below 0 as a result of cleavage toxicity induced by introducing DSBs at two genomic loci. However, the number of guides with high activity (guides with LFC ≤− 0.5) is significantly higher for the set of guides targeting two coding regions (OR = 4.32, *p*=0.00094, Fisher’s exact test) than for the set of guides targeting only one coding region. This suggests that a guide disrupting two genes is more likely to be more lethal than a guide targeting one coding region and one non-coding region.

For guides targeting two coding regions in the Avana library, we asked whether or not the two targets are related in terms of sequence similarity using gene paralogy as a proxy for gene similarity. Among the 2503 guides, 297 (11.9%) guides have their pair of targets annotated as being paralogs using the PANTHER database [13] (see the “Material and methods” section). In comparison, the 74,070 pairs of paralog genes annotated in the PANTHER database represent only ∼ 0.02% of all possible pairs of genes screened in the Avana library. This significant enrichment for paralog genes (exact binomial test, *p* < 2.2×10^−16^) confirms that co-targeted genes often share high homology.

To compare the effects of co-targeting a pair of paralog genes in comparison to targeting only one paralog, we further examined co-targeting guides for which there was at least one single-target guide for each of the two paralogs. We restricted our analysis to “clean” guides only, that is guides with no additional single or double-mismatch alignments, to prevent off-target effects from confounding our analysis. This left us with a set of 22 double-target guides for further quantification. For each of the 22 guides, we computed an average difference between the double-target guide log-fold change (digenic knockout effects) and each of the single-target guide log-fold change (paralog-specific knockout effects) using the delta coefficient described in the “Material and methods” section. Thus, for each double-target guide, we obtained two delta coefficients (one for each paralog). A large negative delta coefficient indicates that the digenic knockout is substantially more lethal than the single-gene knockout. On the left panel of Additional file 1: Figure S1b, we present the delta coefficient estimated using the second paralog (*y*-axis) as a function of the delta coefficient estimated using the first paralog (*x*-axis) for all 22 guides. Both delta coefficients agree overall, and several guides have greater activity in comparison to the paralog-specific single-gene knockouts.

To visualize potential genetic interactions between the pairs of paralogs, we show on the right panel of Additional file 1: Figure S1b the double-target log-fold changes as a function of the minimum expected log-fold change estimated by paralog-specific knockouts. In the absence of genetic interactions, the minimum expected log-fold change can be estimated as min(*y*_*A*_,*y*_*B*_,*y*_*A*_+*y*_*B*_) where *y*_*A*_ and *y*_*B*_ are log-fold changes associated with paralog-specific knockouts for paralogs A and B respectively. We observed that for a number of guides, the digenic knockout effects largely exceed the expected additive log-fold changes, suggesting indeed that potential synergistic or synthetic genetic interactions exist between the targeted paralogs. As an example, we present the log-fold changes for guides targeting the paralogs *RAB5B* and *RAB13* in Additional file 1: Figure S1c. Log-fold changes of paralog-specific guides are centered around 0 (gray and blue boxplots), suggesting low to no activity, while log-fold changes of the three guides co-targeting both paralogs show greater activity.

#### The interplay between multi-target guides and synthetic lethality: *MYL12A, MYL12B*, and *MYL9*

In [6], guide-level LFCs are modeled as a combination of multiple on-target gene knockout effects. The model makes the assumption that gene knockout effects are additive: the growth phenotype resulting from double mutant cells is the same as the sum of the single-mutant growth phenotypes. While this assumption is likely valid for pairs of genes/loci with no genetic interaction, such as DSB effects in non-coding regions, this can lead to erroneous estimates of gene essentiality in case of synergistic or epistatic genetic interactions. Many pairs of paralogous genes are functionally, or at least partially, redundant, and synergistic effects have been observed for such pairs [14]. We therefore expected some of the sgRNAs co-targeting paralog genes to violate the additivity assumption and result in biased estimates of gene essentiality.

**Fig. 2:**
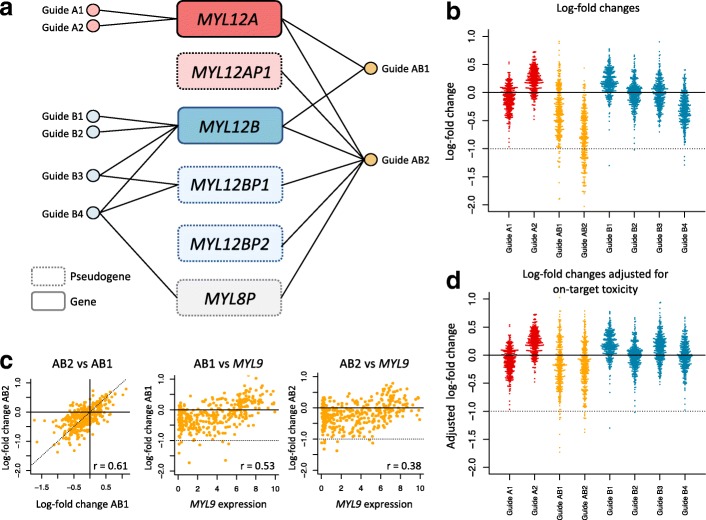
The interplay between multiple alignments and synthetic lethality in the Avana library. **a** Genomic mapping of the Avana guides targeting *MYL12A* and *MYL12B*. Guides A1 and A2 map uniquely to *MYL12A* and guides B1 and B2 map uniquely to *MYL12B*. Guides B3 and B4 map to *MYL12B*, but also to additional non-functional pseudogenes. Guides AB1 and AB2 map to both *MYL12A* and *MYL12B*. **b** Copy number-corrected LFCs for guides mapping to either *MYL12A* or *MYL12B*, or to both, in the Avana library, across 391 cell lines; each dot represents a cell line. **c** First panel: scatterplot of the log-fold changes between the two guides mapping to both *MYL12A* and *MYL12B*. Second and third panels: relationship between guide-specific log-fold changes and *MYL9* expression for guide AB1 and AB2, respectively. **d** Same as **b**, but after adjusting for cell-specific cleavage toxicity; on-target cleavage toxicity induced by multiple on-targets was first estimated for each cell line using the fitting curves from Fig. 1f and then subtracted from each guide’s log-fold change according to their respective number of multiple on-target alignments

As an example, synthetic lethality, in which deficiencies in two (or more) genes is lethal while deficiency in either one is not, is a genetic interaction that cannot be captured through additive models. The myosin light chain 12A (*MYL12A*) and myosin light chain 12B (*MYL12B*) genes, two paralogous genes that are part of the myosin II complex, are both targeted by unique and common guides in the Avana library (see Fig. 2a and Additional file 1: Table S1). Guides A1 and A2 map uniquely to *MYL12A* while guides B1 and B2 map uniquely to *MYL12B*. Guides B3 and B4 map to *MYL12B*, but also to the processed pseudogenes *MYL12BP1* and *MYL8P*. Finally, guide AB1 maps to both *MYL12A* and *MYL12B* (two genes), while AB2 maps to *MYL12A* and *MYL12B* in addition to *MYL12AP1*, *MYL12BP1*, *MYL12BP2* and *MYL8P* (six genes/pseudogenes).

LFCs for these eight guides are presented in Fig. 2b. The LFC distributions for the isoform-specific guides (A1, A2, B1, B2, and B3) are approximately centered around 0. The guide targeting two pseudogenes (B4) appears to be more active; this is consistent with the toxicity effect observed in Fig. 1b for guides targeting three loci. Interestingly, for guides targeting both *MYL12A* and *MYL12B*, LFCs are shifted downwards with more variability, indicating that these two guides targeting both isoforms are substantially more toxic. For both guides, it appears that a subset of cells lines have a depletion score comparable to that of an essential gene (around − 1). This suggests that the double knockout of *MYL12A* and *MYL12B* is potentially lethal for a subset of cell lines, suggesting context-dependent synthetic lethality between the two paralogs.

Using our estimation of cell-specific cleavage toxicity induced by multiple on-target alignments presented in Fig. 1f, we can adjust LFCs for global on-target toxicity; cleaveage toxicity-adjusted LFCs are presented in Fig. 2d. Adjusted LFCs within a guide category (A,B or AB) tend to be more similar after adjustment. It is also clear that additive on-target activity is not sufficient to fully explain greater activity of guides AB1 and AB2. This suggests that an additive model without genetic interaction does not capture the underlying biology and leads to erroneous estimates.

To formally test for a genetic interaction between *MYL12A* and *MYL12B*, we model the LFC *y*_*i*_ for guide *i* with the linear model

$$y_{i} = \left\{\begin{array}{ll} \beta_{A} & \text{if sgRNA targets } {MYL12A} \text{ only}\\ \beta_{B} & \text{if sgRNA targets } {MYL12B} \text{ only}\\ \beta_{AB} & \text{if sgRNA targets both } {MYL12A} \text{ and } {MYL12B}\\ \end{array}\right. $$

and test whether or not *β*_*A*_+*β*_*B*_=*β*_*AB*_. Using all cell lines to estimate the parameters, we obtained the isoform-specific knockout effects $\hat {\beta }_{A} = 0.06$ (*p*=3.13×10^−7^) and $\hat {\beta }_{B} = 0.07$ (*p* < 2.2×10^−16^), and the digenic knockout effect $\hat {\beta }_{AB} = -0.19$ (*p* < 2.2×10^−16^). From the theory of linear modeling, we can obtain a *t* statistic and its associated *p* value to test the additivity hypothesis *β*_*A*_+*β*_*B*_−*β*_*AB*_=0. We obtained a significant test (*p*<2.2×10^−16^) and conclude that the additivity hypothesis does not hold, therefore confirming a synergistic effect between *MYL12A* and *MYL12B*.

To investigate in which genomic context the genetic interaction between *MYL12A* and *MYL12B* appears to be maximal, we looked at the correlation between LFCs for Guides AB1 and AB2 and gene expression of 23,241 genes obtained from CCLE (see “Material and methods”). *MYL9* was the top correlate for both guides (Guide AB1: *r*=0.534, *p*<2.2×10^−16^; Guide AB2: *r*=0.377, *p*=1.6×10^−13^; see Fig. 2c). None of the guides were associated with either *MYL12A* and *MYL12B* expression. This suggests that the pair of paralogs is more essential to cell survival in the absence of *MYL9* expression. Interestingly, both *MYL12A* and *MYL12B* are non-muscle regulatory light chains (RLCs) that are highly homologous to the RLC *MYL9*. The murine orthologs (*Myl12a*, *Myl12b*, and *Myl9*) have been shown to be required to maintain the stability of myosin II and cellular integrity, and double knockdown of *Myl12a/Myl12b* using siRNA showed major alterations in cell structure that were not recapitulated by isoform-specific knockdowns [15].

#### Additive models can lead to gene essentiality scores highly dependent on guide design

Besides the problem of genetic interactions, the use of additive models to estimate gene knockout effects from multi-target guides can lead to further problems. We present in this section two guide designs that are part of the Avana library that lead to CERES scores that need to be interpreted with caution because of a violation of the additivity assumption.

**Fig. 3:**
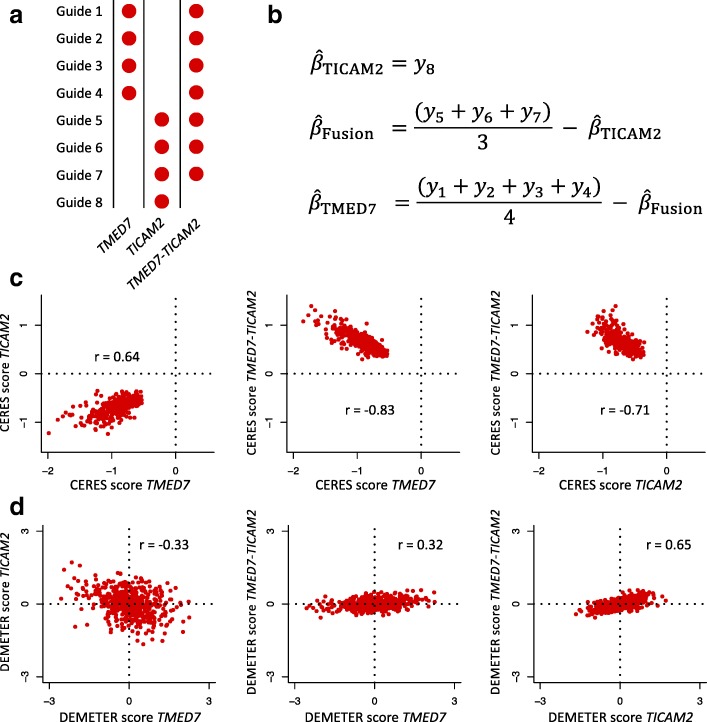
Analysis of the genetic knockout additivity constraints for *TMED7*, *TICAM2* and the *TMED7-TICAM2* readthrough. **a** Design for guides targeting *TMED7*, *TICAM2* and the readthrough *TMED7-TICAM2* in the Avana library. **b** Cell line-specific gene score solutions for an additive model using the Avana guides targeting *TMED7*, *TICAM2,* and the readthrough *TMED7-TICAM2*. *y*_*i*_ denotes the LFC for guide *i* and *β*_*Fusion*_ denotes the gene score for the readthrough *TMED7-TICAM2*. Explicit solutions are derived using ordinary least squares (OLS). **c** Pairwise scatterplots for the CERES scores for *TMED7*, *TICAM2* and the readthrough *TMED7-TICAM2*. **d** Pairwise scatterplots for the DEMETER scores (RNAi) for *TMED7*, *TICAM2*, and the readthrough *TMED7-TICAM2*

In Fig. 3a, we illustrate the guide design for the Avana guides targeting the two genes *TMED7* or *TICAM2* as well as the readthrough *TMED7-TICAM2*. Guides 1–4 target both *TMED7* and *TMED7-TICAM2*, guides 5–7 target both *TICAM2* and *TMED7-TICAM2*, while guide 8 targets only *TICAM2*. Using the additive model posited in the CERES algorithm, gene scores for *TMED7*, *TICAM2*, and *TMED7-TICAM2* can be solved using ordinary least squares (OLS). We note that in the CERES model, two additional guide-specific parameters are included in the model to capture a guide-specific activity score and offset; an iterative least squares approach is used to iteratively solve for guide-specific parameters and gene essentiality scores. These two location-scale parameters do not alter the interpretation of the gene essentiality scores derived from the additive model. Consequently, we omit them here for simplicity in order to focus on the guide-specific test of genetic interaction. For a specific cell line, let *y*_1_,*y*_2_,…,*y*_8_ denote the LFCs for guides 1–8 respectively using the guide notation presented in Fig. 3a. An additive model for the gene essentiality scores *β*_TMED7_,*β*_TICAM2_, and *β*_Fusion_ can be represented using the following system of linear equations:

$$\begin{array}{*{20}l} y_{1} &= \beta_{\text{TMED7}}\ + \; \beta_{\text{Fusion}}\\ y_{2} &= \beta_{\text{TMED7}}\ +\;\beta_{\text{Fusion}}\\ y_{3} &= \beta_{\text{TMED7}}\ +\;\beta_{\text{Fusion}}\\ y_{4} &= \beta_{\text{TMED7}}\ +\;\beta_{\text{Fusion}}\\ y_{5} &= \beta_{\text{TICAM2}}+\;\beta_{\text{Fusion}}\\ y_{6} &= \beta_{\text{TICAM2}}+\;\beta_{\text{Fusion}}\\ y_{7} &= \beta_{\text{TICAM2}}+\;\beta_{\text{Fusion}}\\ y_{8} &= \beta_{\text{TICAM2}}\\ \end{array} $$

The OLS estimates for gene essentiality scores can be written as

$$\begin{array}{*{20}l} \hat{\beta}_{\text{TICAM2}} &= y_{8}\\ \hat{\beta}_{\text{Fusion}} &= \frac{y_{5}+y_{6}+y_{7}}{3}-\hat{\beta}_{\text{TICAM2}}\\ \hat{\beta}_{\text{TMED7}} &= \frac{y_{1}+y_{2}+y_{3}+y_{4}}{4} - \hat{\beta}_{\text{Fusion}}. \end{array} $$

The gene essentiality score for *TICAM2* depends entirely on the LFC of one guide (Guide 8) and is therefore highly sensitive to outliers and off-target effects. The essentiality scores for both *TMED7* and *TMED7-TICAM2* depend on that of $\hat {\beta }_{\text {TICAM2}}$, making the scores inter-dependent and creating a correlation structure highly dependent on guide design, and not necessarily representative of true on-target effects. In the Achilles dataset, we found that the CERES scores for these three genes are highly correlated with each other (Fig. 3c) and consistent with the linear dependencies introduced by the OLS solution shown in Fig. 3b and discussed above.

With the goal of examining whether or not the CERES estimates for these three genes are biased, we compared the CERES scores to the DEMETER scores available for the Achilles RNAi dataset [1] (Fig. 3d). The DEMETER algorithm was developed to separate on- from off-target effects in pooled RNAi screens by modeling seed-based off-target effects empirically. Interestingly, the sign of the correlations between the three genes are reversed, and the three genes are broadly estimated as being non-essential genes (DEMETER score ≥− 2). This disagrees with the CERES scores of *TMED7* and *TICAM2*, which are centered at − 1 and therefore are comparable to CERES scores of essential genes. However, we note that DEMETER scores derived from RNAi screens can suffer from similar multicollinearity problems. Indeed, the dependency score solutions estimated by the DEMETER model also depend on the short hairpin RNA (shRNA) design used to target a set of genes. In particular, in the shRNA library used for the Achilles dataset, there is no unique shRNA targeting the genes *TMED7*, *TICAM2*, and the readthrough *TMED7-TICAM2*. In light of this, it is not clear which dataset represents true gene dependencies, but it is clear that both datasets and their modeling approaches suffer from the same reagent design limitations. We also note that as a general guideline, differences in RNAi and CRISPR screens results can also be of biological nature. It has been previously observed that both types of screens can reveal different aspects of biology as observed by essentiality hits falling into different orthogonal biological processes [16, 17].

The influence of guide design on correlations between essentiality scores is not limited to the triplet *TMED7*/ *TICAM2* /*TMED7-TICAM2*. The Avana library targets 36 readthrough genes, and all of them are targeted by guides that are not unique to the readthrough but also target the pairs of individual genes. The correlations of the CERES scores between two genes composing a readthrough have a distribution with two modes bounded away from 0 (Additional file 1: Figure S2, red line) which behave differently than pairs of genes chosen at random (gray line). This suggests that the guide design introduces spurious correlations between gene essentiality scores for most genes targeted in a readthrough.

Spurious correlations can also happen with pairs of genes targeted by multi-target guides, such as the pair *EIF3C*/*EIF3CL*. These two highly homologous genes are part of the eukaryotic translation initiation factor 3 complex (eIF3) and therefore are expected to be essential for cell growth. In the Avana library, five guides target both *EIF3C* and *EIF3CL*, and one additional guide targets *EIF3CL* only. All six guides have LFCs centered around − 1, indicating gene essentiality, yet the mean CERES scores for *EIF3CL* and *EIF3C* are respectively − 1.15 and 0.20, suggesting that *EIF3CL* is broadly essential and *EIF3C* broadly non-essential. This contradicts the findings of [18], which report *EIF3C* as a pan-cancer essential gene. This is a consequence of the knockout additivity assumption; the fact that the LFCs for the five guides targeting both paralogs is similar to the LFC of the guide targeting only *EIF3CL* leads to an estimate of the *EIF3C* CERES score close to 0. However, assuming both genes are broadly essential, it seems equally plausible that the double knockout does not make the cells die more in comparison to single knockout, especially since the single knockouts already induces a strong cell killing. The similar yet non-redundant function of the two genes violates the additivity assumption and leads to an incorrect estimate of the gene essentiality score for *EIF3C*. These two examples suggest that multi-targeting can lead to guide design-dependent co-dependencies and misleading biases that have to be interpreted with caution in downstream applications such as identifying gene networks and cancer cell dependencies.

### The impact of single-mismatch tolerance on sgRNA depletion

**Fig. 4:**
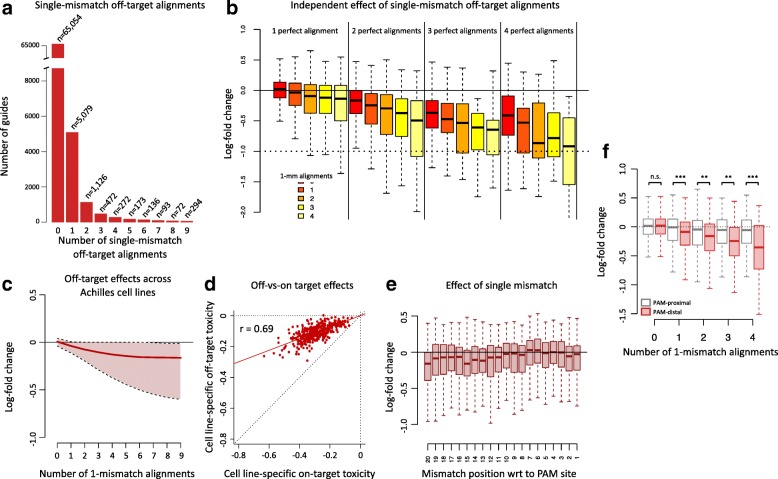
The impact of off-targets on sgRNA log-fold changes. **a** Guide counts as a function of the number of single-mismatch alignments. **b** Combined effect of multiple perfect alignments (on-targets) and single-mismatch (1-mm) alignments on LFCs. **c** Distribution of cell-specific fitted off-target activity (average log-fold changes) as a function of the number of single-mismatch off-target alignments (solid line: median across cell lines; shaded area: full range of on-target activity across cell lines). **d** Relationship between cell line-specific on-target toxicity and cell line-specific off-target toxicity. On-target and off-target toxicity were measured as the average LFC for guides with four perfect alignments and four single-mismatch alignments, respectively. **e** Effect of a single mismatch between spacer and reference genome as a function of the mismatch position. Position 1 represents the position next to the PAM site. **f** LFCs of guides with 1 perfect alignment stratified by the number of single-mismatch alignments and by spacer location: PAM-proximal (positions 1 to 10) or PAM-distal (position 11 to 20). *p* values were assessed using two-sample *t* tests. N.s.: not significant; ∗∗: *p*<0.01; ∗∗∗: *p*<0.001

We now focus on characterizing off-target effects caused by mismatch tolerance between the sgRNA’s spacer sequence and the genomic DNA. In Fig. 4a, we plot the guide counts distribution as a function of the number of single-mismatch alignments in the Avana library; a single-mismatch alignment is defined as a one-nucleotide mismatch in the sgRNA-DNA pairing. To delineate cleavage toxicity caused by multiple-target alignments (on-target effects) from off-target effects due to mismatch tolerance, we stratified guides by the number of single-mismatch alignments and the number of perfect alignments (Fig. 4b). For a fixed number of perfect alignments, additional single-mismatch alignments significantly decrease LFCs (Wald test from multiple linear regression, *p* < 2.2×10^−16^). This confirms that in addition to perfect alignments, single-mismatch off-targets contribute independently and additively to a decrease of cell viability. Similar to the cleavage toxicity associated with multiple-target guides, we found that off-target toxicity is also cell line-specific (Fig. 4c). Moreover, cell line-specific off-target toxicity correlates with cell line-specific on-target toxicity (*r*=0.69, *p* < 2.2×10^−16^); we used the fitted on-target and off-target effects for 4 perfect and single-mismatch alignments, respectively, as measures of on-target and off-target toxicity (Fig. 4d). The correlation suggests that both on-target and off-target cleavage toxicity are related effects that show specificity for cell lines but not guides, since the sets of guides used to estimate both effects are broadly different.

We also studied the relationship between mismatch position in the spacer sequence and mismatch tolerance across cell lines. To prevent the number of alignments from confounding the analysis, we only considered guides with one perfect alignment and one single-mismatch alignment. In Fig. 4e, we show the distributions of the LFCs as a function of single-mismatch position within the spacer sequence. The effect of a single mismatch is more pronounced for mismatches far away from the PAM site (PAM-distal region) as opposed to PAM-proximal nucleotides, sometimes referred to as the seed region. Mismatch tolerance appears to be maximal at the 20th position. This suggests that guides should be carefully designed to avoid mismatch at that position. Finally, to further investigate the effects of mismatch location, we considered guides with 1 perfect alignment only and stratified their LFCs by the number of single-mismatch alignments and by spacer location: PAM-proximal or PAM-distal (Fig. 4f). This confirms that single mismatches within the PAM-proximal region of the spacer are not well tolerated by the CRISPR/Cas9 system, resulting in little cleavage toxicity in comparison to single mismatches occurring far from the PAM site.

#### Mismatch-tolerant guides can confound gene essentiality: *SOX9* and *SOX10*

The CERES model does not account for off-target effects caused by single-mismatch and double-mismatch tolerance, and this may lead to erroneous conclusions when both on- and off-targets are part of the same gene family with possible genetics interactions. Importantly, we found in the Avana library that among the 4705 genes that have at least one guide with a single-mismatch alignment, 3197 (68%) such genes have at least one single-mismatch alignment located in the exon of another gene. This increases the likelihood of false positive effects caused by single-mismatch alignments. The consequences are potentially variable across cell lines and may depend on which alternative family member is affected, and whether it is expressed or not in a given subset of cell lines. We illustrate this by analyzing CERES scores and guide-level LFCs for the *SOX9* gene encoding the transcription factor SOX-9.

**Fig. 5:**
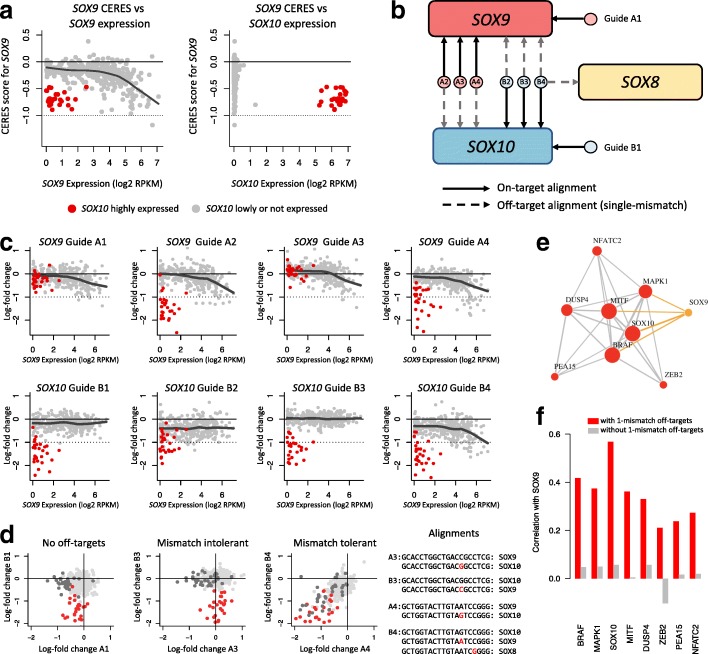
The effects of single-mismatch sgRNA-DNA tolerance on gene essentiality. **a** On the left: CERES score for *SOX9* plotted against *SOX9* expression; each dot is a cell line, and cell lines highly expressing *SOX10* (*log*_2_(rpkm+1)≥4) are colored in red. The curve represents a LOWESS estimated with cell lines lowly or not expressing *SOX10* (gray dots). On the right: CERES score for *SOX9* plotted against *SOX10* expression. **b** Guide design for guides targeting *SOX9* and *SOX10* in the Avana library. **c** Log-fold changes (LFCs) of guides targeting *SOX9* and *SOX10* as a function of *SOX9* expression. Curves represent LOWESS fit estimated with cell lines lowly or not expressing *SOX10* (gray dots). **d** Comparison of single-mismatch tolerant and intolerant guides targeting *SOX9* and *SOX10***e***BRAF*-associated coessentiality cluster using between-gene CERES Pearson correlations. A correlation cutoff of *r*=0.35 was chosen to draw edges. Edges associated with *SOX9* (orange) disappear when excluding *SOX9*-targeting guides with single-mismatch tolerant *SOX10* off-targets (dark gray indicates lines with high SOX9 expression). **f** Pearson correlations between the *SOX9*’s CERES score and CERES scores for genes that are part of *BRAF*-associated cluster, before and after removal of *SOX9*-targeting guides with single-mismatch tolerant *SOX10* off-targets

By looking at the relationship between the CERES score for *SOX9* and its expression (Fig. 5a, left panel), we noticed several cell lines with very low expression of *SOX9* that exhibit a clear and contradictory dependency on *SOX9* (CERES score close to − 1); most of these cell lines are melanoma cell lines. While all of the spacer sequences for the guides targeting *SOX9* match perfectly to only *SOX9*, an analysis of single-mismatch alignments revealed that three out of four such guides also align to *SOX10*. By plotting the *SOX9* CERES score against *SOX10* expression, it becomes clear that *SOX9*-dependent cell lines with low expression of *SOX9* are those that highly express *SOX10* (Fig. 5a, right panel).

In Fig. 5b, we show the design and alignments of guides targeting *SOX9* and *SOX10* in the Avana library. Out of four guides targeting *SOX9*, three guides also have a single-mismatch alignment to *SOX10*. Conversely, out of four guides targeting *SOX10*, three guides also have a single-mismatch alignment to *SOX10*. One of the guides (B4) has an additional single-mismatch alignment to *SOX8*. Sequence alignments for these guides are provided in Table 1. We present in Fig. 5c the LFCs of these eight guides as a function of *SOX9* expression and color cell lines highly expressing *SOX10* (*log*_2_(rpkm+1)≥4) in red. We also provide LOWESS fits for cell lines lowly or not expressing *SOX10* to visualize *SOX9* dependencies.

**Table 1:** Genomic alignments for guides targeting *SOX9* and *SOX10* in the Avana library

	Type	On-target	Off-target	Spacer sequence	BaseSub	Chr	Pos
Guide A1	PM	*SOX9*		GCTCGGACACCGAGAACACG		17	72,121,509
Guide A2	PM	*SOX9*		GCAGCACAAGAAGGACCACC		17	72,122,775
	MM		*SOX10*	GCAGCACAAGAA-GACCACC	G →A	22	37,978,058
Guide A3	PM	*SOX9*		GCACCTGGCTGACCGCCTCG		17	72,121,612
	MM		*SOX10*	GCACCTGGCTGAC-GCCTCG	C →G	22	37,983,546
Guide A4	PM	*SOX9*		GCTGGTACTTGTAATCCGGG		17	72,122,793
	MM		*SOX10*	GCTGGTACTTGTA-TCCGGG	A →G	22	37,978,040
Guide B1	PM	*SOX10*		ACAAGTACCAGCCCAGGCGG		22	37,978,032
Guide B2	PM	*SOX10*		GTAGTGGGCCTGGATGGCGG		22	37,977,942
Guide B3	PM	*SOX10*		GCACCTGGCTGACGGCCTCG		22	37,983,546
	MM		*SOX9*	GCACCTGGCTGAC-GCCTCG	G →C	17	72,121,612
Guide B4	PM	*SOX10*		GCTGGTACTTGTAGTCCGGG		22	37,978,040
	MM		*SOX9*	GCTGGTACTTGTA-TCCGGG	G →A	17	72,122,793
	MM		*SOX8*	GCTGGTACTTGTAGTC-GGG	C →G	16	983,802

*PM* perfect match alignment, *MM* single-mismatch alignment, *BaseSub* base substitution in the protospacer sequence

LFCs for guide A1, which targets *SOX9* without off-targets, show a clear dependency on *SOX9* expression, and cell lines highly expressing *SOX10* do not show activity. Conversely, LFCs for guide B1, which targets *SOX10* without off-targets, show no dependency on *SOX9* expression, and cell lines highly expressing *SOX10* are highly sensitive to knockout. LFCs for guides A2 and A4, which both have *SOX9* has an on-target and *SOX10* as an off-target, show a dependency on *SOX9* expression for cell lines lowly or not expressing *SOX10*, as seen by the LOWESS fits. In addition, cell lines highly expressing *SOX10* are also sensitive to gene knockout induced by these guides. This suggests that the CRISPR/Cas9 system tolerates single-mismatch in guides A2 and A4 and that Cas9 cutting occurs at the off-target *SOX10* and results in off-target activity.

While guide A3 also has a single-mismatch off-target alignment to *SOX10*, none of the cell lines that highly express *SOX10* is sensitive to guide A3-induced knockout. This suggests that the CRISPR/Cas9 system is intolerant to the single-mismatch occurring in guide A3. This is consistent with LFCs of guide B3. Indeed, the spacer sequence of guide B3, which targets *SOX10* and has a single-mismatch alignment to *SOX9*, is nearly identical to the spacer sequence of guide A3, except at the nucleotide position causing the single-mismatch alignments. By inspecting LFCs of guide B3, we observe that cell lines highly expressing *SOX9* are not sensitive to guide B3, therefore confirming that the CRISPR/Cas9 system does not tolerate the single-mismatch in guide B3, leading to no off-target activity. We also note that Guide B4 also shares nearly the same spacer sequence as Guide A4. As for Guide A4, the CRISPR/Cas9 system appears to tolerate the single-mismatch, as seen by the dependency of guide B4’s LFCs on SOX9 expression (Fig. 5c, bottom panel, right plot). We summarize these findings in Fig. 5d.

**Fig. 6:**
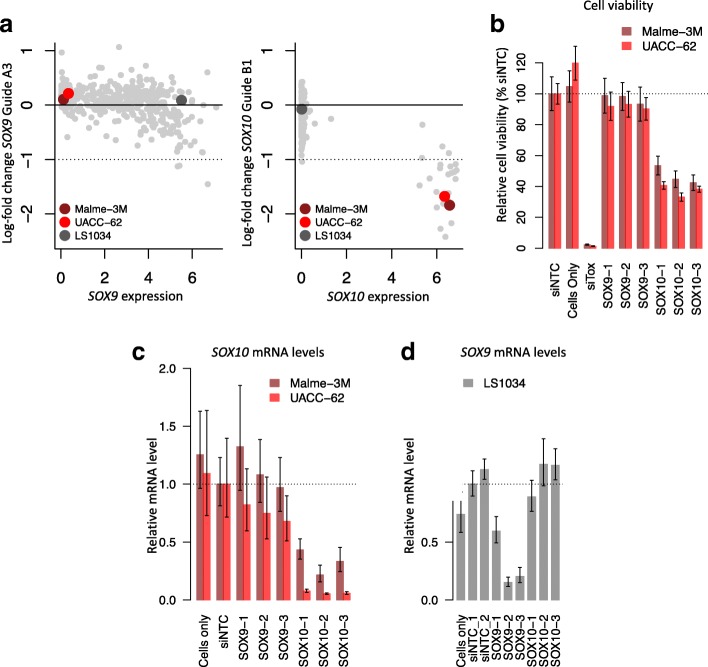
Melanoma cell lines with high expression of *SOX10* are not sensitive to *SOX9* knockdown. **a** Left panel: log-fold change for clean guide A3 targeting *SOX9* as a function of *SOX9* expression. Right panel: log-fold change for clean guide B1 targeting *SOX10* as a function of *SOX10* expression. Two melanoma cell lines were selected for *SOX9* and *SOX10* knockdowns validation: Malem-3M and UACC-62 (in red). One colorectal carcinoma (LS1034) was selected to measure *SOX9* siRNAs knockdown efficiency. **b** Cell viability of two melanoma cell lines after transfection with small interfering RNAs (siRNAs), relative to the non-targeting control (siNTC) treatment. **c** Relative *SOX10* mRNA expression levels, detected by qPCR, after transfection of the indicated siRNAs, for two melanoma cell lines. Expression levels are normalized to *GAPDH* mRNA detected in the same sample, and then expressed as a fold change with respect to the siNTC treatment. **d** Relative *SOX9* mRNA expression levels, detected by qPCR, after transfection of the indicated siRNAs, for the colorectal carcinoma cell line LS1034

Next, we sought to confirm experimentally that the observed *SOX9* dependency for cell lines highly expressing *SOX10* for two out of four guides targeting *SOX9* results from off-target activity. We selected two melanoma cell lines with low expression of *SOX9*, but high expression of *SOX10* (cell lines Malme-3M and UACC-62, see Fig. 6a). We transfected cells in a microplate format with small interfering RNAs (siRNAs) targeting *SOX9* and *SOX10*, and measured cell viability after 5 days using a CellTiter-Glo luminescence assay. In Fig. 6b, we show the relative cell viability percentages with respect to the non-targeting siRNA (siNTC) treatment. We observed a substantial decrease in viability for both melanoma cell lines after *SOX10* knockdown with three of three tested siRNAs, while *SOX9* knockdown did not lead to an apparent decrease in cell viability.

We validated knockdown efficiency for all *SOX9* and *SOX10* siRNAs by qPCR (Fig. 6c). All three *SOX10* siRNAs induced substantial knockdown of *SOX10* mRNA levels, in both cell lines, in contrast to the negative control or siRNAs targeting *SOX9*. SOX9 was not detected in these cells by qPCR (Ct > 35); this confirms low expression of SOX9 as revealed by RNA-Seq. To confirm efficacy of the *SOX9* siRNAs, we selected the colorectal cancer cell line LS1034 for knockdown validation. This cell line highly expresses *SOX9* (see Fig. 6a). *SOX9* mRNA levels are reasonably reduced after *SOX9* knockdown, but not after *SOX10* knockdown (Fig. 6d), especially for two of the three siRNAs (> 80% knockdown). Overall, there results confirm specificity of the *SOX9* and *SOX10* siRNAs, and that *SOX10* knockdown, unlike *SOX9* knockdown, reduces cell viability in melanoma cell lines.

Without analyzing single-mismatch alignments of guides targeting *SOX9*, one would have erroneously concluded that most of the melanoma cell lines in the Achilles dataset have a *SOX9*, rather than a *SOX10*, dependency. This illustrates how single-mismatch off-target effects can confound the analysis of cancer vulnerabilities, and also how it can be challenging to detect those off-target effects when they occur only in a small subset of cell lines, such as the melanoma cell lines in this case. This analysis is also complicated by the fact that some off-targets predicted by single-mismatch alignments do not necessarily lead to off-target activity because of CRISPR/Cas9 mismatch intolerance.

Recently, a coessentiality network has been derived from correlating fitness profiles across CRISPR knockout screens datasets, with the Achilles dataset being the most represented dataset [19]. The authors found a coessentiality network cluster that is highly specific to *BRAF*-mutated melanoma cell lines and contains elements of the MAP kinase pathway (*MAP2K1*, *MAPK1*, and *DUSP4*) as well as *SOX9* and *SOX10*. Using the CERES scores from the Achilles dataset, we were able to recreate the cluster entirely by taking the nine top genes correlated with the *BRAF* CERES score: *BRAF*, *MITF*, *MAPK1*, *PEA15*, *NFATC2*, *ZEB21*, *DUSP4*, *SOX9*, and *SOX10* (Fig. 5e). The correlation between the CERES score for *BRAF* and the CERES score for *SOX9* is high (*r*=0.42). We recalculated an essentiality score for *SOX9* after removing guides with mismatch-tolerant off-targets alignments to *SOX10*. As a result, *SOX9* no longer associates with the *BRAF* cluster (Fig. 5f).

To investigate how frequent Avana guides targeting transcription and lineage factors have single-mismatch alignments to alternative members of the same gene family, we studied the top genes for which the CERES score is negatively correlated with self-expression of the gene. Our rationale was to first find essential genes that are only expressed in a subset of cell lines, and then investigate off-targets when the latter are expressed in a different subset of cell lines, similar to the *SOX9*/*SOX10* case. We found that several such genes have indeed single-mismatch alignments located in the exon of another family member: *GATA2*/*GATA3*, *SOX1*/*SOX2*, *DOCK10/DOCK11*, *UBB*/*UBC*, *PAX3*/*PAX7*, *TEAD2*/*TEAD3*. For reference, we provide a table of the top 500 self-anti-correlated genes together with off-target alignments in Additional file 2. We selected three on-target/off-target pairs for which only a subset of cell lines is expressed in either gene: *GATA2*/*GATA3*, *SOX1*/*SOX* and *PAX3*/*PAX7*. We present their expression levels across Achilles cell lines in Additional file 1: Figure S3 (first column). While there exist cell lines for which these genes are expressed in a mutually exclusive fashion, only one gene for each gene pair appears to be essential (Additional file 1: Figure S3, second column) as estimated by LFCs of guides with no single-mismatch alignments (clean guides). This is in contrast with *SOX9* and *SOX9*, which are both essential genes when highly expressed. As a consequence, detecting off-target activity resulting from guides introducing DSBs at these off-target sites cannot be readily detected from knockout screens, with one exception for the *PAX7*/*PAX3* pair. Indeed, *PAX7* appears to be essential for the rhabdomyosarcoma cell line RD, but not *PAX3* (Additional file 1: Figure S3, bottom row, middle panel). One guide targeting *PAX3* has also a single-mismatch alignment to *PAX7*, and appears to be specifically lethal in the cell line RD, suggesting off-target activity (Additional file 1: Figure S3, bottom row, right panel).

#### Single-mismatch tolerance and paralogs

To investigate how often guides with single-mismatch alignments can lead to inconsistent cell line dependencies because of off-targets, we looked at guides targeting exactly one on-target and one single-mismatch off-target in the Avana library. To be able to distinguish between off-target and on-target effects, we selected guides with no double-mismatch alignments, and for which the corresponding on-target gene is also targeted by at least one “clean guide,” defined as a guide with only one on-target and no mismatch alignments, for a final set of 427 guides for off-target quantification. We note that 52% of the guides (224 guides) have the off-target locus in a coding region. In addition, among those 224 guides, 77 guides (34%) have their on-target and off-target genes annotated as paralogs in the PANTHER database.

**Fig. 7:**
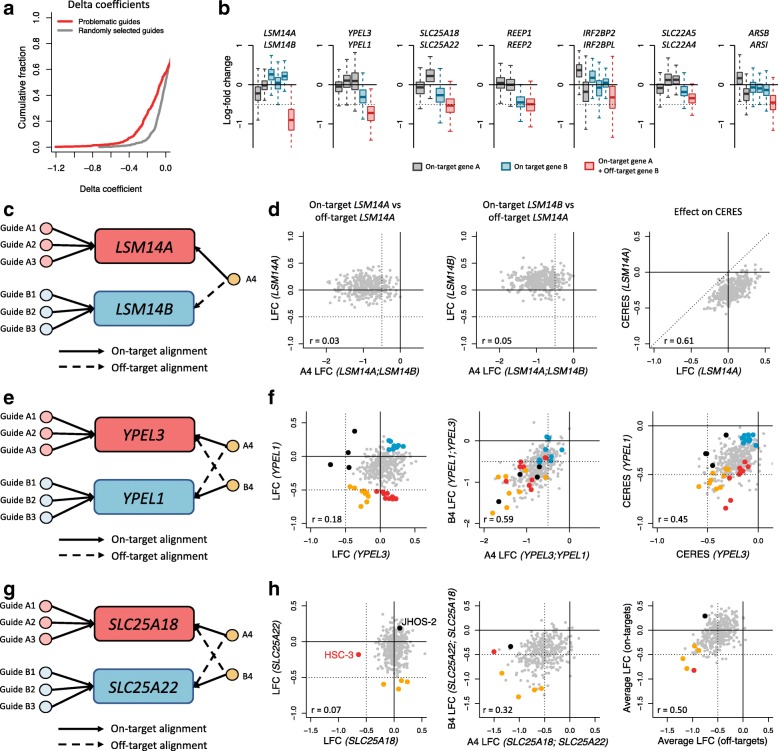
Single-mismatch tolerance and genetic interactions in the Achilles dataset. **a** Cumulative distribution of off-target delta coefficients for the set of 427 guides that have exactly one on-target and one single-mismatch off-target in the Avana library. Larger negative coefficients suggest greater off-target activity. The gray line represents the cumulative distribution of delta coefficients for 1000 guides chosen at random. **b** Log-fold changes (LFCs) for guides targeting seven pairs of paralog genes. Clean guides and double-target guides are shown. **c** Avana guide design for guides targeting *LSM14A* and *LSM14B*. **d** LFCs and CERES scores for guides targeting *LSM14A* and *LSM14B*. Notation: LFC (A;B): LFC for a guide with gene A as an on-target and gene B as a single-mismatch off-target. **e** Same as **c**, for *YPEL1* and *YPEL3*. **f** LFCs and CERES scores for guides targeting *YPEL1* and *YPEL1*. Colors indicate gene-specific cell line dependencies. Red: *YPEL1*-only dependencies; black: *YPEL3*-only dependencies; orange: both *YPEL1* and *YPEL3* dependencies; blue: no dependencies. **g** Same as **c**, for *SLC25A18* and *SLC25A22*. **h** LFCs and CERES scores for guides targeting *SLC25A18* and *SLC25A22*. Right panel: LFC averaged across all on-target guides targeting *SLC25A18* and *SLC25A22* vs LFC averaged across guides with both on-targets and off-targets. Red: *SLC25A18*-dependent cell lines. Orange: *SLC25A22*-dependent cell lines. Black: no dependencies

For each guide separately, we quantified off-target effects by measuring the average difference of the LFC between the off-target guide and the set of clean guides using the delta coefficient (see the “Material and methods” section). A delta coefficient close to 0 indicates minimal off-target effects, while a negative score indicates potential off-target effects. Delta coefficients were calculated using all cell lines screened in the Achilles dataset. A non-negligible proportion of single-mismatch guides (18%) shows substantial off-target activity, defined as an off-target delta coefficient less than − 0.25 (Fig. 7a). In comparison to 1000 clean guides chosen at random, these guides are significantly enriched for off-target effects (OR = 4.7, *p*=2.32×10^−15^, Fisher’s exact test). Looking at the 15 guides with the lowest delta coefficient, 7 of them co-target pairs of paralog genes (one on-target and one off-target): *IRF2BP2/L*, *SLC22A4/5*, *REEP1/2*, *SLC25A18/22*, *ARSB/I*, *LSM14A/B*, and *YPEL1/3*. We show in Fig. 7b the distribution of LFCs for clean guides and guides with off-targets. We discuss 3 paralog pairs below with different behaviors.

First, in Fig. 7c, we show the Avana guide design for guides targeting the paralogs *LSM14A* (mRNA processing body assembly factor) and *LSM14B* (LSM14 homolog B). Each paralog is targeted by 3 clean guides, and one additional guide (A4) targeting *LSM14A* has also an single-mismatch off-target alignment to *LSM14B*. The single-mismatch occurs at position 13 with respect to the PAM site, and therefore should be tolerated. We note that the average LFC for clean guides targeting either paralog does not correlate with the LFC for guide A4 (Fig. 7d, first and second panels). In addition, the distribution of both paralog-specific average LFCs are centered around or above 0, indicating non-essentiality, while the distribution of LFCs for guide A4 is centered around − 1. This suggests that the single-mismatch at position 13 for guide A4 is well tolerated by the CRISPR/Cas9 system, generating the hypothesis that targeting both paralogs is lethal for most cell lines. In the right panel of Fig. 7d, we show that the CERES score for *LSM14A* is shifted negatively with respect to the average LFC for clean guides targeting *LSM14A* as a result of guide A4 lethality.

Next, we discuss guides targeting the paralogs *YPEL1* (Yippee like 1) and *YPEL3* (Yippee like 3) in the Avana library. Both paralogs are targeted by three clean guides each, and each paralog is targeted by an additional guide that also targets the other paralog assuming single-mismatch tolerance. In particular, Guide A4 targets *YPEL3*, and has *YPEL1* has a single-mismatch off-target; guide B4 targets *YPEL1*, and has *YPEL3* has a single-mismatch off-target (see Fig. 7e). The single-mismatch occurs at position 13 for both guides A4 and B4. By estimating paralog-specific essentiality using LFCs of clean guides only (Fig. 7f, first panel), we found that each paralog is essential in different subsets of cell lines (black and red dots), as well as in a shared subset of cell lines (orange dots). In the middle panel of Fig. 7f, we show that LFCs of guides A4 and B4 correlate well with each other (*r*=0.59), and that paralog-specific dependent cell lines (red and black) become vulnerable when targeted by either guide A4 or B4. This suggests that knockout effects for both guides A4 and B4 are a mixture of both paralog-specific knockout effects, suggesting mismatch tolerance by the CRISPR/Cas9 system for both guides A4 and B4. As negative controls, we note that cell lines that are not dependent on either paralog (blue dots) remain unchanged when targeted by guide A4 or B4. We also observed that while paralog-specific LFCs are mostly uncorrelated for most cell lines (first panel of Fig. 7f), paralog-specific CERES scores correlate well as a consequence of including single-mismatch tolerant guides A4 and B4 (*r*=0.45, Fig. 7f, third panel).

Finally, we study the two paralog genes *SLC25A22* and *SLC25A18*, two members of the SLC25 carrier family implicated in glutamate transport across the inner mitochondrial membrane, also referred to as Mitochondrial Glutamate Carrier 1 (GC1) and Mitochondrial Glutamate Carrier 2(GC2) respectively. In the Avana library, there are four guides targeting *SLC25A22*. One of the four guides (B4) has a single-mismatch alignment to *SLC25A18* (position 16). There are also four guides targeting *SLC25A18*, and one of the four guides (A4) has a single-mismatch alignment to *SLC25A22* (position 16); see Fig. 7g. Using clean guides only, we estimated paralog-specific dependencies (Fig. 7h, left panel). Orange dots represent cell lines that are the most dependent on *SLC25A22*, but not dependent on *SLC25A18*. Conversely, the red dot represents a cell line (oral squamous cell carcinoma HSC-3) that is dependent on *SLC25A18*, but not dependent on *SLC25A22*. On the middle panel of Fig. 7h, we show LFCs for guides A4 and B4. The activity of the two guides with off-targets correlates well (*r*=0.32), and cell lines that have paralog-specific dependencies (orange and red dots) are in comparison sensitive to the knockout induced by either A4 or B4. This suggests that LFCs estimated for both A4 and B4 are a mixture of the paralog-specific LFCs occurring through mismatch tolerance. Interestingly, one cell line insensitive to both paralog-specific knockouts (ovarian serous adenocarcinoma cell line JHOS-2, colored in black) is one of the most dependent cell line for the guide targeting *SLC25A18* with an off-target to *SLC25A22*. This suggests some cell-line specific synthetic lethality, or at least some level of synergy, between the two paralogs. On the right panel of Fig. 7h, we confirm that the addition of paralog-specific knockout effects, calculated as LFCs averaged across all clean guides targeting *SLC25A18* and *SLC25A22*, can overall recapitulate the activity of guides A4 and B4 (*r*=0.50). Again, the digenic knockout for cell line JHOS-2 cannot be explained by either paralog-specific knockout.

### The impact of double-mismatch tolerance on sgRNA depletion

**Fig. 8:**
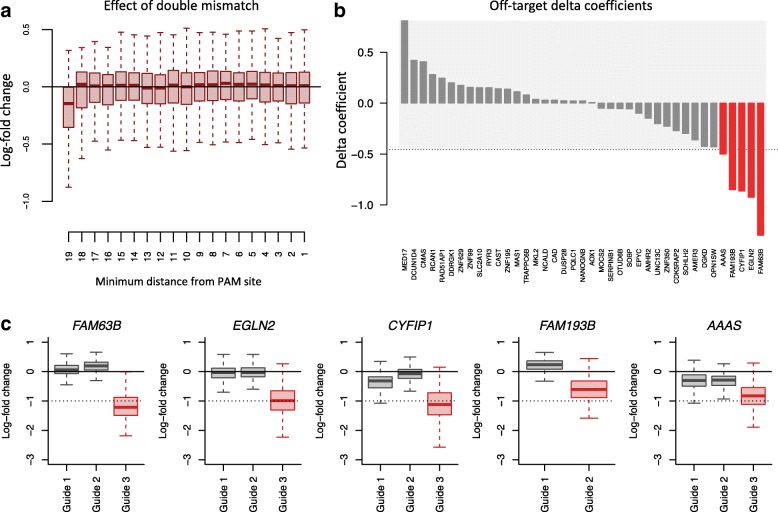
Position-specific mismatch tolerance of the spacer for double-mismatch alignments. **a** Effect of a double mismatch between spacer and reference genome as a function of the most PAM-proximal mismatch position. **b** Off-target delta coefficients for 39 genes targeted with a guide with at least 5 double-mismatch alignments with the first mismatch located at the 20th position. The gray-shaded area represents a 95% confidence interval of a null distribution of delta coefficients estimated using 1000 genes chosen at random that are targeted by clean guides only. **c** Distribution of LFCs for clean guides (gray boxplots) versus problematic guides (red boxplots) for the top 5 genes with an exceedingly large negative off-target delta coefficient from **b**

We extended our analysis to off-target effects caused by double-mismatch (sgRNA-DNA mismatch at two nucleotides) tolerance. Because the number of all possible combinations of two-nucleotide mismatches in the 20-nt spacer is large compared to the number of available guides with mismatches in the Avana library, we confined our analysis to pairs of mismatches for which the two discordant bases are located within a specified distance from the PAM site. The distributions of guide depletion are shown in Fig. 8a. Again, to prevent the number of alignments from confounding the analysis, we only considered guides with no more than one double-mismatch alignment. We can observe an apparent off-target effect for a double mismatch that occurs at the two most PAM-distal positions.

To investigate how guides with a large number of double-mismatch alignments at position affect log-fold changes, we analyzed 53 guides in the Avana library, targeting 53 different genes, with the following characteristics: one unique on-target alignment, no single-mismatch alignment, and at least 5 double-mismatch alignments with the first mismatch located at the 20th position and the second mismatch located elsewhere along the spacer sequence. To quantify off-target effects, we estimated off-target delta coefficients for each of the guide (see the “Material and methods” section). These guides should have a high probability of off-target effects, resulting in a large negative delta coefficient. To be able to distinguish between real on-target effects and off-target effects, we focused our analysis on genes with at least one additional clean guide (guide with neither single nor double-mismatch alignments), leaving us with 39 guides for further investigation. We present the off-target delta coefficients for the 39 genes targeted by the problematic guides in Fig. 8b. The gray-shaded area represents a 95% confidence interval of a null distribution of delta coefficients estimated using 1000 genes chosen at random that are targeted by clean guides only. Guides with a double-mismatch (with one at the 20th position) are more likely to produce real off-target effects (Fisher’s exact test: OR =5.71, *p* value =0.004). In Fig. 8c, we depict the distribution of LFCs for clean guides (gray boxplots) versus problematic guides (red boxplots) for the top five genes with an exceedingly large negative off-target delta coefficient from Fig. 8b. For instance, for *CYFIP1*, guides 1 and 2 do not have single or double-mismatch alignments, while guide 3 aligns to 10 different genomic loci with a double-mismatch at positions 19 and 20, resulting in a substantial decrease in LFC, most likely as a result of off-target toxicity. This biases the CERES score for *CYFIP1* towards essentiality (CERES score of − 0.41), while excluding guide 3 results in a score centered around 0 (non-essentiality).

### The effects of human genetic variation on sgRNA efficiency and specificity

In the previous sections, we analyzed the effects of on- and off-targets on sgRNA efficiency and specificity by generating a list of genomic alignments between sgRNA spacers and the reference genome, ignoring genetic variation across different cell line genomes. Genetic variation, such a single nucleotide polymophisms (SNPs) and small indels, can have a profound effect on sgRNA specificity and on-target efficiency [20–23]. For instance, the list of on- and off-target loci for a particular sgRNA depends on SNP alleles present in a particular genome as a consequence of adding or removing mismatches between sgRNA spacer sequences and the targeted genome, in comparison to the reference genome. In addition, canonical NGG PAM sites can be either destroyed or created through SNP variation.

Because SNP array data are available for 363 CCLE cell lines screened in the Achilles project, we focused on investigating the effects of SNPs on guide log-fold changes in the Achilles data. We generated genomic coordinates in GRCh38 for 904,800 SNPs measured on the Affymetrix SNP array 6.0 (see the “Material and methods” section). For each cell line and each SNP, we used the genotype call (AA, AB, or BB) estimated by Birdseed [24] to link SNP variation to sgRNA log-fold changes. We transformed the data such that the allele A represents the allele annotated in the reference genome assembly GRCh38.

#### Effect of SNP variation on sgRNA on-targets

We first studied the effects of SNP variation on on-target activity. We intersected the coordinates of all protospacer sequences targeted by the 68,742 single-target Avana guides (guides with multiple on-targets were excluded) with the array SNP locations. We found that 473 guides are targeting a protospacer sequence containing a SNP targeted by the array. No guide was targeting a sequence with more than one array SNP. One SNP was present in 3 adjacent guides targeting *C10orf82*, 31 SNPS were present in exactly 2 adjacent guides, and 408 SNPs were present in one guide only. One SNP (rs17099014) has no allele variation across the Achilles cell lines and was therefore excluded for further analyses.

**Fig. 9:**
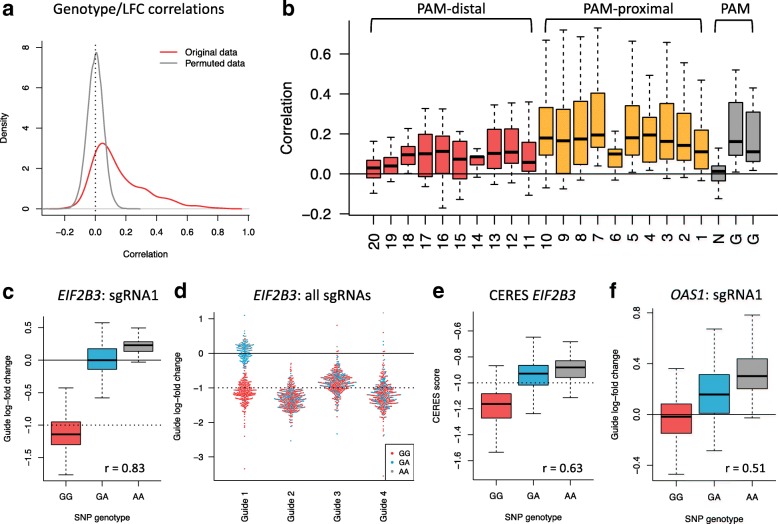
Effects of genetic variation on guide on-target activity. In the Avana library, 472 guides target protospacer sequences overlapping SNPs assayed by the Affymetrix SNP 6/0 array. **a** Distribution of correlations between guide log-fold changes and SNP genotype (genotype/LFC correlations) for the 472 affected guides (red line). A null distribution was obtained from permuting SNP genotypes *B*=100 times (gray line). **b** Genotype/LFC correlations as a function of the SNP location within the protospacer sequence. **c** Log-fold changes for a guide targeting *EIF2B3* and overlapping the SNP rs11556200. The genotype GG represents the reference allele found in the GRCh38 genome assembly. **d** Log-fold changes for all guides targeting *EIF2B3*. **e** CERES score for *EIF2B3* as a function of the SNP rs11556200 genotype. **f** Log-fold change for a guide targeting *OAS1* and overlapping the SNP rs1131454. The genotype GG represents the reference allele found in the GRCh38 genome assembly

For each SNP-guide pair, we calculated a Pearson correlation between the cell line-specific SNP genotype (0=AA, 1=AB, 2=BB) and the guide log-fold chance (Fig. 9a, red line), and also generated a null distribution of correlations by permuting cell line genotypes *B*=100 times (Fig. 9a, gray line). A large proportion of SNP-guide pairs has a genotype-LFC positive correlation greater than by chance (262 pairs, 56%), confirming the hypothesis that an alternative allele within the protospacer region results in a decrease of cleavage efficiency as observed by a less negative log-fold change. Next, we stratified the distribution of the genotype-LFC correlations by the relative position of the SNP with respect to the protospacer’s PAM site position (Fig. 9b). The genotype-LFC correlations are significantly higher for SNPs located in the PAM-proximal region of the protospacer in comparison to SNPs located in the PAM-distal region (Wilcoxon rank sum test, *p*=2.54×10^−6^). This is consistent with our analysis of single-mismatch alignments; single-mismatches located in the PAM-proximal region caused by SNP variation are less tolerated than PAM-distal single-mismatches, resulting in a more pronounced genotype-specific guide activity. Similarly, SNPs located at the second or third position of the canonical NGG PAM site are not tolerated well. As expected, a SNP located at the first position of the PAM site (nucleotide N) has virtually no effect (mean correlation =0.006).

We further illustrate the impact of SNP variation by studying the effect of the SNP rs11556200 located in one of the guides targeting *EIF2B3*. The SNP is located at position 9 with respect to the PAM site, and has a reported minor allele frequency (MAF) of 0.297 in the 1000 Genomes, which is comparable to the frequency observed in the Achilles cell lines (MAF =0.249). In Fig. 9c, we show the log-fold changes for the affected guide as a function of the SNP genotype. For cell lines with homozygous reference allele (GG), log-fold changes are centered around − 1, indicating gene essentiality. For cell lines with either heterozygous and homozygous minor allele (GA or AA), the average log-fold change is around 0 and above 0, respectively, suggesting that the guide is inefficient at inducing a homozygous gene knockout for cell lines with a minor allele at this SNP. This is further shown by comparing the guide with the SNP rs11556200 (guide 1) with the three other guides targeting *EIF2B3* (Fig. 9d); the log-fold changes of the SNP-unaffected guides do not differ by SNP genotype (*r*_2_=− 0.018, *r*_3_=0.055, *r*_4_=0.073), and are centered around − 1, confirming common essentiality of *EIF2B3* across cell lines. Not excluding guide 1 from the library results in a CERES score correlated with the SNP genotype (*r*=0.63, Fig. 9e). Overall, this shows how the presence of a common SNP within the protospacer region can alter guide activity and result in spurious log-fold changes for a subset of cell lines.

Another example is the presence of the SNP rs1131454 (MAF =0.473 in 1000 genomes, MAF=0.57 in Achilles) in one of the guides targeting *OAS1*. The SNP is located in the second position of the NGG PAM site, which destroys the PAM site for a strand containing the minor allele, and should therefore result in a complete loss of cutting efficiency for cell lines homozygous for the minor allele. The log-fold change of the corresponding guide shows an allele-dose positive association (*r*=0.51, Fig. 9f). Log-fold changes of cell lines that are homozygous for the minor allele (AA) are substantially above 0, comparable to log-fold changes of non-targeting controls (NTCs). Indeed, log-fold changes of cell lines with genotype AA correlate with log-fold changes of NTCs (*r*=0.27, *p*=0.0007), while log-fold changes of cell lines with genotype GG do not significantly correlate (*r*=− 0.07,*p*=0.48). This suggests that the minor allele results in a destruction of the PAM site that makes the guide inactive for cell lines with both copies of the minor allele.

#### SNP variation and sgRNA off-targets

In addition to the potential loss of guide efficiency due to the presence of SNPs in protospacer sequences, SNP variation can also alter the number of off-targets by increasing or decreasing the number of single-mismatch and double-mismatch alignments for a particular guide. It can also create additional on-targets, besides the designed primary on-target, in cases where a SNP is located at the single-mismatch position of a single-mismatch off-target alignments. To investigate these potential biases, we studied guides in the Avana library that have exactly one single-mismatch alignment, for a total of 5072 guides. We found 45 guides that have one SNP located within the sequence of the single-mismatch off-target.

Among these 45 guides, 9 guides satisfy the following conditions: (1) the SNP overlaps the location of the single-mismatch between the guide sequence and the reference genome and (2) the minor allele nucleotide matches the reference nucleotide single-mismatch nucleotide substitution. For such guides, the off-target becomes an additional on-target for cell lines that are homozygous for the minor allele, and log-fold changes for such guides should decrease as the number of minor alleles increases as a consequence of multiple on-target cleavage toxicity, and possibly because of genetic interactions between the two on-targets. This should be reflected in negative correlations between log-fold changes and SNP genotype. To validate our hypothesis, we generated Pearson correlations for all 45 SNP-guide pairs and used a null distribution of correlations produced from a permutation analysis (*B*=100 permutations) to assess significance. Pairs with a significant negative correlation (five pairs) are significantly enriched for pairs that generate additional minor allele-specific on-targets (three pairs out of nine, OR =7.9, Fisher’s exact test’s *p*=0.047). As an example, we consider the SNP rs2056899 located in the off-target sequence of one of the guides targeting *CYP4A11*. The guide log-fold change correlates negatively with the SNP genotype (*r*=− 0.25), with an median decrease of − 0.10 for cell lines that are homozygous for the minor allele. This is concordant with our previous observation that an additional on-target results in lower log-fold changes.

Conversely, a single-mismatch off-target with an additional single-mismatch caused by the minor allele of a SNP elsewhere in the sequence should become a double-mismatch off-target for cell lines homozygous for the minor allele. As an example, we consider the SNP rs2717932 located in the off-target sequence of one of the guides targeting *PPP1R17*. The guide log-fold change correlates positively with the SNP genotype (*r*=0.28), with an median increase of 0.16 for cell lines that are homozygous for the minor allele. This is concordant with our previous observation that double-mismatch off-targets result in less cleavage toxicity in comparison to single-mismatch off-targets.

### Comparison to other genome-wide CRISPR/Cas9 knockout libraries

To examine whether or not the problem of multiple-target and off-target effects generalizes to other CRISPR knockout libraries and datasets beside the Avana library, we analyzed three additional genome-wide libraries: GeCKO v2 [25], Brunello [7] and Toronto KnockOut v3 library (TKOv3) [26]. Each library was designed using a different set of rules and a different design. The GeCKOv2 library, one of the early genome-wide CRISPR libraries, was designed to include guides with hight specificity by calculating an off-target score based on the number of mismatches in putative off-targeted, as well as the position of the mismatches, with an average of 6 guides per gene. The Brunello library was designed based on an improvement of Rule Set 1 [27], named Rule Set 2, to maximize on-target guide efficacy. A cutting frequency determination (CFD) score was also developed to minimize potential off-target activity. Both scores (Rule Set 2 and CFD scores) were empirically derived from a tiling library targeting all possible protospacers in a set of 15 genes (over 4k sgRNAs), as well as a tiling library with mutated sgRNAs targeting the coding sequence of *CD33*. In comparison, the Avana library, which was designed using Rule Set 1 only. The TKOv3 library was developed to maximize on-target activity by leveraging sgRNA activity scores from six knockout screens performed using the previous genome-wide Toronto KnockOut v1 library (TKOv1, [28]). Discriminative power between essential and non-essential genes was used to derive activity scores based on sgRNA sequences. Guides targeting more than one gene, as well as guides with protospacer sequence overlapping a common SNP (db138), were filtered out. Guides with single and double-mismatch alignments located in intergenic regions were only included when no better guides could be found for a particular gene.

Similar to the Avana library, we generated for each additional library a list of genomic alignments with up to two mismatches between the guide sequence and the genomic DNA (GRCh38 assembly). We report several alignment summaries in Table 2, and full genomic alignments for all four libraries are provided in Additional files 3, 4, 5 and 6. For both the Brunello and GeCKOv2 libraries, CRISPR screens data were publicly available across several cell lines. For the GeCKOv2 library, LFCs across 111,227 guides were available for 33 cell lines that are also part of the Achilles project [9]. We present in Additional file 1: Figure S4a LFCs averaged across cell lines as a function of on-target alignments for the GeCKOv2 dataset. As in the Avana library, guides with no on-target alignments have LFCs greater than 0, similar to NTCS. LFCs decrease as a function of the number of perfect alignments, confirming cleavage toxicity induced by multiple on-targets observed in the Achilles-Avana dataset. For guides with one perfect alignment, we also looked at the relationship between LFCs and the number of single-mismatch alignments, stratified by single mismatch location (PAM-proximal or PAM-distal, Additional file 1: Figure S4b). The number PAM-distal single-mismatch alignments substantially increase guide activity in comparison to PAM-proximal single-mismatch alignments. We repeated the same exercise for the Brunello library with nine publicly available CRISPR knockout screens performed in primary effusion lymphoma (PEL) cell lines [29] (see the “Material and methods” section). LFCs also decrease as a function of the number of perfect alignments, and as a function of the number of single-mismatch alignments (Additional file 1: Figure S4c-d). We could not find publicly available screen data for the TKOv3 library.

**Table 2:** Summaries of sgRNA sequence alignments across four CRISPR knockout libraries

	Avana	GeCKOv2	Brunello	TKOv3
Reference	[7]	[25]	[7]	[26]
Number of unique guides	73,782	119,461	77,441	71,090
Number of unique NTC guides	995	1000	1000	0
Number of unique guides targeting miRNAs	0	6835	0	0
Number of unique guides targeting coding genes	72,787	111,626	76,441	70,948
Number of targeted coding genes	18,547	19,050	19,114	18,053
Average number of guides per gene	4	6	4	4
Number of guides with no on-targets	86	157	1	16
Number (%) of guides with 1 on-target	68,742 (94.4%)	108,368 (97.1%)	73,410 (96.0%)	68,872 (97.1%)
Number (%) of guides with 2 on-targets	2628 (3.6%)	1665 (1.5%)	1681 (2.2%)	1775 (2.5%)
Number (%) of guides with > 2 on-targets	1331(1.8%)	1436 (1.3%)	1349 (1.8%)	285 (0.4%)
Number (%) of guides with no SM off-targets	65,070 (89.4%)	104,965 (94.0%)	69,407 (90.8%)	67,584 (95.3%)
Number (%) of guides with 1 SM off-target	5079 (7.0%)	4168 (3.7%)	4352 (5.7%)	2984 (4.2%)
Number (%) of guides with 2 SM off-targets	1126 (71.5%)	1014 (70.9%)	1098 (71.4%)	281 (70.4%)
Number (%) of clean guides (up to 1 mm)	66,757 (91.7%)	106,538 (95.4%)	71,614 (93.7%)	67,886 (95.7%)

For the Avana library, we report summary statistics for the Avana library version used in the Achilles project screens (4 sgRNAs per gene and processing as described in the “Material and methods” section). For the GeCKOv2 library alignment summaries, we excluded 6835 guides targeting miRNAs. A clean guide refers to a guide with only one-target alignment and no single-mismatch alignments

*NTC* non-targeting control, *SM* single-mismatch

We also explored how other libraries compare to the Avana library in terms of multi-target guide design. To do so, we considered for each library the subset of guides targeting the 16,717 genes that are in common between the four libraries. Then, for each library, we estimated the number of genes for which all guides are multi-target guides; these genes represent genes for which the library-specific design failed at selecting uniquely targeting guides. We found 383 such genes for the Avana library, 689 for the GeCKOv2 library, 360 for the Brunello library, and 122 for the TKOv3 library. Among the 383 genes that cannot be uniquely target in the Avana library, 171 (45%), 198 (52%), and 44 (24%) are shared by the GeCKOv2, Brunello, and TKOv3 libraries, respectively. This suggests that many genes cannot be targeted uniquely by CRISPR guides, as revealed by independent guide designs.

## Discussion

In this work, we first analyzed cleavage toxicity associated with guides targeting more than one genomic locus with complete complementarity. Using LFCs from the Achilles dataset, across 342 cell lines and more than 72k sgRNAs (Avana library), we found that guide depletion increases as a function of the number of targeted loci in the genome. We observed this not only for perfect alignments between the sgRNA spacer and genomic DNA, but also for single-mismatch tolerant alignments to a lesser extent. A single-mismatch that occurs in the 10 most PAM-distal nucleotides results in more severe toxicity, and a double-mismatch that occurs only in the 2 most PAM-distal spacer positions results in greater toxicity. These biases have been reported before [7, 27, 30–41], but were only estimated using either a few cell lines or a few genes. Here, we could robustly estimate these effects across hundreds of cell lines, and found that cleavage toxicity associated with promiscuous guides substantially depends on the cell line model, similar to the copy number bias discussed in [6]. We also observed these cleavage toxicity effects in knockout screens performed with two other genome-wide libraries: Brunello and GeCKOv2.

The CERES algorithm presented in [6] implements a cell line-specific CN correction of the depletion scores, but also attempts to correct for multiple on-target effects by decomposing guide-specific LFCs as a sum of knockout effects. While this is valid for genes and genomic targets that do not interact with each other, such as DSBs introduced in non-coding DNA, the strict phenotypic additivity assumption often does not hold because of more complex genetic interactions. Two knockouts are considered to be strictly additive if the effect of the digenic knockout is the sum of the effects of the single knockout; strict additivity of cell fitness effects is rare, with most genes exhibiting some level of positive and negative genetic interaction which is substantially more frequent among essential genes [42]. Genetic interaction effects are also enhanced for guides targeting highly homologous genes that are more likely to function together in the same biological process or have some level of functional redundancy.

Synthetic lethality, for instance, is a type of genetic interaction in which double mutant cells do not survive, while single mutant cells continue to proliferate, perhaps at a slower rate. Pairs of synthetically lethal genes have been utilized to identify therapeutic targets: *BRG1-BRM* [43, 44], *ENO1-ENO2* [45], *ME2-ME3* [46], *TP53-POLR2A* [47] BRCA1/2-PARP [48]. Using guides targeting both *MYL12A* and *MYL12B*, two regulatory light chains (RLCs) essential to the Myosin II complex, we showed that there exists a subset of cell lines for which the pair of RLCs is synthetically lethal, violating the assumption of additivity and culminating in biased CERES scores for both *MYL12A* and *MYL12B*.

We also showed that the additive model can create false inter-dependencies between genes in the presence of multi-target guides. Indeed, in the presence of a non-linear effect between a multigenic knockout and individual knockouts, the estimated gene knockout effects are markedly correlated with each other in patterns determined by the library guide design used in the experiment. We used the CERES scores estimated for *TICAM2*, *TMED7*, and the readthrough *TMED7-TICAM2* to illustrate misleading CERES score correlations that are dependent on the single guide targeting *TICAM2* only.

Orthogonal to the problem of modeling multiple on-target knockout effects using an additive model, we also found that a single-mismatch in sgRNA-DNA alignments can confound phenotypic readouts because of off-target effects. We found that according to the CERES score, the transcription factor *SOX9* is essential in melanoma cell lines, despite the fact these cell lines lack expression of *SOX9* but highly express *SOX10*. We showed this was apparently caused by guides with a single-mismatch alignment to *SOX10*, causing off-target effects that confound the growth phenotype for *SOX9* non-expressing cell lines. Downstream consequences of such confounding factors were exemplified in a recent publication [19], in which the authors inferred a coessentiality network using the Achilles dataset and reported that *SOX9* is part of a gene cluster highly specific to *BRAF*-mutated melanoma cell lines. We showed that removing single-mismatch tolerant guides from the analysis removes *SOX9* membership in the cluster.

These observations suggest that multi-target guides as well as mismatch-tolerant guides can lead to false positives and biased essentiality scores. This is compounded by the bias for targeting paralogs with high degrees of sequence homology. When interpreting essentiality score for a given gene, one should also investigate guide-level LFCs to detect abnormalities, such as guides with multiple on-target and off-target single-mismatch alignments. We provide in Additional files 7, 8, 9 and 10 gene-level tables summarizing the number of on-target and off-target alignments for all four CRISPR libraries analyzed in this paper to help readers with flagging potentially problematic genes.

We also investigated the effects of SNPs located in protospacer sequences on guide activity by correlating LFCs with SNP genotypes using available SNP array data from CCLE. We have shown that SNPs located in the PAM-proximal region of the spacer, as well as in one of the two guanines of the PAM site, are not well tolerated by the CRISPR/Cas9 system. This leads to a significant decrease of on-target activity for cell lines carrying two minor alleles. While our analysis is limited to SNPs assayed on the Affymetrix SNP array 6.0, it confirms and shows the importance of considering cell line-specific or patient-specific genomes in the analysis of CRISPR screens and in the design of CRISPR guides. Small indels and large structural variants are also likely to affect CRISPR binding; this is part of our future work.

Similar to the CN correction algorithm implemented in [6], one could attempt to systematically correct for multi-target and off-target toxicity by removing the observed effects for each cell line separately. This apppears to be a sensible approach when additional targets are located in intergenic regions. Indeed, for such regions, introducing additional DSBs are not likely to cause spurious phenotypic effects besides the toxicity induced by DSBs, and predicted cleavage toxicity can be subtracted from the observed LFCs. This rationale was used recently in the design of a genome-wide CRISPR library [26], in which inclusion of guides with additional on-targets located in intergenic regions were allowed, in contrast to guides with additional on-targets located in other genes.

Because of genetic interactions, correcting depletion scores for multi-target guides targeting several proteincoding regions is not straightforward. As opposed to cleavage toxicity induced by differential CN, the increased activity observed in multi-target guides depends on the set of targeted genes; these genes can interact with each other in a cell line-specific manner, excluding the possibility of fitting a global genetic interaction correction model across cell lines. Therefore, we do not recommend to correct LFCs for these multi-target guides. One solution is to remove guides that do not map uniquely to the genome when calculating a gene-level essentiality score; 383 genes would have to be excluded in the Avana library because of the absence of uniquely targeting guides for these genes. A large number of these genes also cannot be targeted uniquely by the three other libraries that we analyzed. On the other hand, we note that multi-target guides could be still further analyzed separately, since such guides can be informative about multigenic knockout, such as co-targeting known paralogs. These guides should be annotated separately, and may potentially be further utilized to help design a library of guides co-targeting paralogs.

In terms of library design, including guides that neither have multiple protein-coding on-targets nor predicted single-mismatch off-targets for all targeted genes is not an easy task. This is even more challenging when additional constraints, such as on-target efficiency threshold or exclusion of guides targeting protospacer sequence overlapping common variants, are considered. For instance, while the two paralogs *MYL12A* and *MYL12B* are targeted by a total of 8 guides in the Avana library, *MYL12A* and *MYL12B* are respectively targeted by 0 and 1 guides in the TKOv3 library; no other guides satisfied the library design criteria in terms of specificity and on-target efficacy. When possible, we recommend to lower criteria related to on-target efficiency and allow less active but specific guides to be included for genes that are more challenging to target, such as highly homologous genes.

## Material and methods

### Datasets

**Achilles CRISPR dataset (Avana)** From the Achilles data portal (https://portals.broadinstitute.org/achilles), we downloaded CERES scores for 391 cell lines across 17,655 genes (file: gene_effect.csv); CERES scores are not provided for genes on sex chromosomes. We also obtained guide-level raw log-fold changes (LFCs) from the Achilles portal (logfold_change.csv along with a guide-to-gene mapping file, for a total of 73,782 unique and annotated guides; 995 guides are non-targeting controls (NTCs). As detailed in [6], fold changes were first calculated by dividing sample read counts by their representation in the starting plasmid DNA library (pDNA). As in [6], we normalized LFCs for each cell line replicate by centering the distribution using the median LFC value, and then dividing by the median absolute deviation (MAD). Since we are interested in visualizing and analyzing LFCs, we further scaled LFCs by the absolute average LFC value across cell lines for guides targeting essential genes (212 genes total, [18]), such that a value of -1 roughly indicates essentiality. We then averaged normalized LFCs across replicates by taking the mean. For each cell line separately, we corrected LFCs for gene copy number alteration using relative copy numbers provided by CCLE using the methodology described in [6]. Throughout the manuscript, all log-fold changes for the Achilles dataset are corrected for copy number, and we therefore refer to the CN-corrected log-fold changes simply as “log-fold changes” or “LFCs.’.

**Achilles CRISPR dataset (GeCKOv2)** From the Achilles data portal (https://portals.broadinstitute.org/achilles), we downloaded guide-level log-fold changes across 33 cell lines (file: Achilles_v3.3.8.gct for a total of 111,227 guides). As described in [9], log-fold changes were normalized around the median of negative controls and z-score normalized across cell lines. We further processed the data by correcting the log-fold changes for gene copy number alteration, for each cell line separately, using relative copy numbers provided by CCLE, using the methodology described in [6].

**Brunello CRISPR library and Brunello-PEL dataset** We downloaded the publicly-available Brunello library guide annotation, described in [7], from the Addgene website (catalog number: 73179; file: broadgpp-brunello-library-contents.txt). We downloaded raw read counts for 9 CRISPR knockout screens performed in primary effusion lymphoma (PEL) cell lines, publicly available through the Supplementary material of [29]; we refer to the dataset as the Brunello-PEL dataset. We filtered out sgRNAs for which there was less than 30 reads in the plasmid library, and then log-transformed the raw read counts (log_2_(counts+1)). We normalized the data across cell lines by centering around the median of guides targeting non-essential genes, and computed log-fold changes (LFCs) by subtracting the log-transformed normalized counts from the plasmid library. Finally, we scaled LFCs using the median LFC of guides targeting essential genes, and averaged LFCs across replicates. The final dataset has LFCs for 75,006 guides across 9 samples.

**Toronto KnockOut CRISPR library v3** We downloaded the publicly-available Toronto KnockOut Library v3 (TKOv3) guide annotation, described in [26], from the Addgene website (catalog number: 90294; file: tkov3_guide_sequence.xlsx).

**Achilles RNAi** From the Achilles data portal (https://portals.broadinstitute.org/achilles), we downloaded gene-level DEMETER scores for 501 cell lines across 17,098 genes (file: ExpandedGeneZSolsCleaned.csv).

**CCLE RNA-Seq and CN datasets** From the Cancer Cell Line Encyclopedia (CCLE) portal (https://portals.broadinstitute.org/ccle), we downloaded gene-level RNA-Seq data (file: CCLE_RNAseq_081117.rpkm.gct) and gene-level relative copy number data (file CCLE_copynumber_byGene_2013-12-03.txt). We applied the transformation log2(rpkm+1) to the RNA-Seq data.

**CCLP SNP array data** We downloaded preprocessed SNP genotype calls from the CCLE portal (file: CCLE_SNP.Birdseed.Calls_2013-07-29.tar.gz). SNPs were assayed using the Affymetrix Genome-Wide Human SNP Array 6.0, and SNP genotype calls were previously generated using the Birdseed algorithm implemented in Birdsuite [24]. We generated an updated SNP annotation for the Array 6.0 in GRCh38 coordinates using the R packages pd.genomewidesnp.6 and rtracklayer, for a total of 904,800 SNPs available for further analysis across 363 cell lines screened in the Achilles project.

### sgRNA sequence alignments

For each genome-wide human CRISPR library, we used bowtie (v.1.2.2, [49]) to align guide sequences to the human genome assembly GRCh38, allowing up to two mismatches between the reference sequence (DNA) and the sgRNA’s spacer sequence (bowtie with options -v 2 -k 10000). Using the R package BSgenome [50] we filtered out alignments that did not have the canonical NGG PAM site. Using the comprehensive gene annotation from GENCODE v28 [51], we added gene and exon annotation for all alignments. Sequence alignments are provided in the Additional file 3.

### List of paralog gene pairs

We downloaded human gene paralog pairs from the Protein ANalysis THrough Evolutionary Relationships (PANTHER) database [13] using the file ftp://ftp.pantherdb.org/ortholog/13.1/RefGenomeOrthologs.tar.gz. Paralogs, defined as genes that diverged via a duplication event, were predicted using phylogenetic trees of protein-coding genes across 104 organisms. We only considered genes screened in the Achilles dataset, resulting in 74,070 paralog pairs.

### Delta coefficient for measuring sgRNA discrepancy

To quantify how the activity of a given sgRNA compare to the activity of another set of sgRNAs, for instance other guides targeting the same gene, we propose a simple metric based on guide-level log-fold changes. The metric is particularly useful to quantify off-target activity for guides with single and double-mismatch alignments in comparison to “clean guides” (guides that have neither predicted multiple on-targets nor off-targets).

For a fixed CRISPR library, and for a given gene *g*, let *j*=1 index the guide of interest to be examined, and *j*=2,…,*n*_*g*_ index all (*n*_*g*_−1) clean guides targeting gene *g*. We assume here that there exists at least one clean guide targeting gene *g*. Let *i*=1,2,…,*n* index cell lines for which log-fold changes are available for all *n*_*g*_ guides and let y _*ij*_ be the log-fold change for guide *j* for cell line *i*. Using data for *j*∈{1,2,…,*n*_*g*_}, we fit the following fixed-effects linear model using ordinary least squares (OLS):

$$\begin{array}{*{20}l} y_{ij} = \alpha + \beta_{i} + \delta\mathbbm{1}(j = 1) +r_{ij} \end{array} $$

where *α* is the average log-fold change across the clean guides targeting gene *g*, *β*_*i*_ is an offset accounting for cell line-specific knockout effect of gene *g*, *δ* is the average change in the log-fold change associated with the guide of interest in comparison to clean guides, and *r*_*ij*_ are residuals. We simply refer to the estimated coefficient $\hat {\delta }$ as “delta coefficient”. A negative coefficient indicates that the guide of interest has greater activity in comparison to the remaining clean guides targeting gene *g*, possibly because of better cleavage efficiency or because of off-target effects.

### Experimental validation

#### Tissue culture

Cells were cultured in RPMI 1640 media supplemented with 10% FBS. Cell identity and quality is ensured by an internal cell line repository, which performs short tandem repeat profiling, mycoplasma testing, and rigorous tracking of all cell lines.

#### siRNA transfections

siRNAs were reverse transfected in 384-well plate format. Briefly, 1.2 pmol of siRNA was spotted into individual plate wells (*n*=5) followed by the addition of 0.15 µL of RNAiMax in 20 µL of serum free RPMI. After a 30-min incubation at ambient temperature, cells were added in 20 µL of RPMI supplemented with 20% serum to yield a final concentrations of 30 nM siRNA and 10% FBS. Malme-3M, UACC-62, and LS1034 cells were seeded at 3000, 1000, and 3000 cells per well respectively. Transfections were incubated for 48 h prior to harvesting for qPCR or 120 h prior to assaying for viability. Viability was assessed by adding 30 µL of CellTiter Glo (Promega) and reading on an Envision 2104 plate reader (PerkinElmer) after a 10-min incubation at ambient temperature (Table 3).

**Table 3:** List of siRNAs used for *SOX9* and *SOX10* knockdown

siRNA	Vendor	Cat#	Sense	Antisense
SOX9-1	Ambion	s13306	AGACCUUCGAUGUCAACGATT	UCGUUGACAUCGAAGGUCUCG
SOX9-2	Ambion	s532658	CCUUCGAUGUCAACGAGUUTT	AACUCGUUGACAUCGAAGGTC
SOX9-3	Ambion	s532659	CCCGCUCACAGUACGACUATT	UAGUCGUACUGUGAGCGGGTG
SOX10-1	Ambion	s13309	CCACCUCACAGAUCGCCUATT	UAGGCGAUCUGUGAGGUGGAT
SOX10-2	Ambion	s13310	CCGUAUGCAGCACAAGAAATT	UUUCUUGUGCUGCAUACGGAG
SOX10-3	Ambion	s13311	GCAACGUGGACAUUGGUGATT	UCACCAAUGUCCACGUUGCCG
Silencer Select Negative Control #2	Ambion	4390846		
AllStars Hs Cell Death Control	Qiagen	SI04381048		

#### TaqMan gene expression assays

After 48 h of siRNA knockdown, cells were lysed and RNA was extracted using RNeasy Mini QIAcube Kit (Qiagen 74116). After quantification, equal amounts of RNA were reverse transcribed using High Capacity cDNA Reverse Transcription Kit (Applied Biosystems 4368814) on a Applied Biosystems ProFlex PCR System. TaqMan assays were conducted using TaqMan Universal PCR Master Mix (Applied Biosystems 4304437) using TaqMan primers from Life Technologies (GAPDH Hs99999905_m1, SOX9 Hs00165814_m1, SOX10 Hs00366918_m1). Amplification and analysis were performed on a Bio Rad CFX384 Real-Time System.

## Additional files

Additional file 1 **Table S1** Genomic alignments for MYL12A and MYL12B guides in the Avana library. **Figure S1** Log-fold changes for double-target guides. **Figure S2** The effect of multi-target guides in readthrough genes. **Figure S3** Off-target effects for guides targeting 3 self-correlated genes. **Figure S4** On-target and off-target cleavage toxicity in the GeCKOv2-Achilles and Brunello datasets. (PDF 350 kb)

Additional file 2 Top 500 self-correlated genes and single-mismatch alignments. (CSV 30 kb)

Additional file 3 Genomic alignments to GRCh38 for the Avana guide sequences. (CSV 33.4 mb)

Additional file 4 Genomic alignments to GRCh38 for the GeckoV2 guide sequences. (CSV 186.6 mb)

Additional file 5 Genomic alignments to GRCh38 for the Brunello guide sequences. (CSV 33.1 mb)

Additional file 6 Genomic alignments to GRCh38 for the TKOv3 guide sequences. (CSV 15.6 mb)

Additional file 7 Gene-level summary table for multiple on-target and off-target alignments (Avana library). (CSV 665 kb)

Additional file 8 Gene-level summary table for multiple on-target and off-target alignments (GeckoV2 library). (CSV 829 kb)

Additional file 9 Gene-level summary table for multiple on-target and off-target alignments (Brunello library). (CSV 715 kb)

Additional file 10 Gene-level summary table for multiple on-target and off-target alignments (TKOv3 library). (CSV 671 kb)

Additional file 11 Review history. (DOCX 2.8 mb)

## Abbreviations

CCLE: Cancer cell line encyclopedia
CFD: Cutting frequency determination
CRISPR: Clustered regularly interspaced short palindromic repeats
DSB: DNA double-strand break
LOWESS: Locally weighted scatterplot smoothing
MAF: Minor allele freqency
NSCLC: Non-small cell lung cancer
OLS: Ordinary least squares
PAM: Protospacer adjacent motif
PEL: Primary effusion lymphoma
qPCR: Quantitative polymerase chain reaction
RLC: Regulatory light chain
RNAi: RNA interference
sgRNA: Single guide RNA
shRNA: Short hairpin RNA siRNA: Small interfering RNA
SNP: Single nucleotide polymorphism
