# Supplementary material for: Multiple-gene targeting and mismatch tolerance can confound analysis of genome-wide pooled CRISPR screens
Source: Genome Biol. 2019 Jan 25;20:21. doi: 10.1186/s13059-019-1621-7 (PMC6346559; doi:10.1186/s13059-019-1621-7)
Supplement: Supplementary file 10 — Gene-level summary table for multiple on-target and off-target alignments (TKOv3 library). (CSV 671 kb) [file 13059_2019_1621_MOESM10_ESM.csv]

**First round of review**

**Reviewer 1**

**Are the methods appropriate to the aims of the study, are they well described, and are necessary controls included?**
Yes, the methods are appropriate and well described.

**Are the conclusions adequately supported by the data shown?**

Some conclusions appear to be drawn based on selected examples. It would be nice to demonstrate how generalizable the results are.

**Are sufficient details provided to allow replication and comparison with related analyses that may have been performed?**

Yes, sufficient details are provided.

**Does the work represent a significant advance over previously published studies? Please explain.**

Yes, the work represents a significant advance by highlighting the need to design sgRNAs properly in a CRISPR screen and the need to be careful of false positives in the downstream analysis.

**Is the paper of broad interest to others in the field, or of outstanding interest to a broad audience of biologists?**

Yes: CRISPR screens are gaining popularity and many biologists are switching over from RNAi screens.

**Comments to authors**

In the submitted manuscript, the authors analyzed CRISPR screen data from hundreds of cancer cell lines and demonstarted how particular sgRNA designs might create problems in the downstream analysis. Overall, I think that the topic is interesting and may potentially be a nice fit for Genome Biology. Below are some suggestions that I have for improving the work. 

Major comments:

1) The dependency of cleavage toxicity on cellular context is intriguing. 

(a) Page 4: The authors found that cleavage toxicity induced by multi-target guides is cell line-specific, similar to the copy number toxicity described in Meyers et al. [2017]. I understand the cell line-specificity for copy number, since there can be different gene duplications in different cell lines. But why is there cell line-specificity for multi-target guides? After all, every cell line should in general have the same set of genes. Is the cell line-specificity caused by variable expression of the different targeted genes? Can the authors check the expression levels of all the genes hit by each multi-target guide?

(b) Page 12: Similar to the cleavage toxicity associated with multiple-target guides, the authors also found that off-target toxicity is cell line-specific (Figure 4c). The same off-targets exist in all cell lines, so does this result indicate that the off-targets are mainly located in genes (or some functional elements), that these genes are expressed differently in different cell lines, and that the off-targets are performing some novel context-dependent biology?

2) Despite the authors writing in the discussion that "the large number of cell lines and targeted genes made it possible to robustly generalize" their results, there are several instances in the manuscript where this supposed generalization is unclear. In other words, the authors have highlighted in several parts of the manuscript that there are potential problems with specific guides, but how widespread are these problems? 

(a) 297 double-target guides have their pair of targets annotated as being paralogs in PANTHER. But in the results, the authors have focused on only MYL12A-MYL12B. How about all the other potential synthetic lethal interactions?

(b) The authors discussed EIF3C and EIF3CL - two paralogs with essential but non-redundant functions - but how about other pairs of paralogs? How generalizable is the finding?

(c) The CYFIP1 example is a nice illustration. But how generalizable is the finding? How many guides are there with one double-mismatch alignment and what fraction of them are problematic?

(d) 3197 genes have at least one single-mismatch alignment located in the exon of another gene. But the authors' discussion has focused largely on SOX9-SOX10. How about all the other genes? For example, out of the 3197 genes, how many of them exhibit strong essentiality scores but low expression levels in cell lines where their off-targets are highly expressed (in a similar manner to SOX9-SOX10)? 

3) The submitted work is essentially a body of computational analysis, but I think there is a need for some independent experimental validation of results. The CRISPR screen and RNAi screen data are from Broad, but the RNA-seq and copy number data are from CCLE. A couple of examples where it would be nice to have some validations:

(a) The authors found that that the pair of paralogs (MYL12A and MYL12B) is more essential to cell survival in the absence of MYL9 expression. Can the authors confirm this prediction? For example, take a cell line where MYL9 is expressed and (i) knockout MYL12A and MYL12B only and (ii) knockout MYL9 as well as MYL12A and MYL12B (is the triple knockout viable?). 

(b) The authors found that in melanoma cell lines, SOX10 is the important gene, not SOX9, since SOX9 is barely expressed in these cell lines. However, the CRISPR screen data and the RNA-seq data are from different sources. Can the authors knockout SOX9/ SOX10 in a melanoma cell line and show that SOX10 has a stronger effect than SOX9? 

4) Can the authors consider the effect of SNPs across the different cell lines? Cancer genomes are typically riddled with mutations, other labs have shown that SNPs can have an effect on CRISPR targeting (e.g. PMID 28759051), and the authors have demonstrated that single mismatch off-targets can confound analysis. Hence, I would imagine that the issue of genetic variation would be important to consider for the analysis of CRISPR screen results. 

5) Overall, how does this work help the typical CRISPR screen user? Supplementary Table 3 is useful, but it is restricted to the Avana library. Can the authors also examine the sgRNA sequences in other CRISPR libraries (e.g. Gecko v2, Toronto v3 etc)? Additionally, I suggest creating a web interface as well where users can query whether the hits that they obtain for their screens may be false positives due to poor guide design. To be even more useful, the web interface can incorporate a feature where users can upload any customized library of sgRNA sequences and the website will flag any potential problematic designs (e.g. multi-target, potential off-targets etc). 

Minor comments:

1) Can the authors discuss more about the DEMETER scores from the Achilles RNAi dataset? On page 9, the authors noted that TMED7, TICAM2, and TMED7-TICAM2 are essential in the CRISPR screen, but non-essential in the RNAi screen and posited that this is because of the additivity assumption in the CERES score. How does the DEMETER score handle the problem? Also, RNAi screens and CRISPR screens are well-known to frequently produce different outcomes, partly because one is a partial knockdown and the other is a complete knockout - it is not clear whether the difference in result between CRISPR screen and RNAi screen at the TMED7-TICAM2 locus may also be due to this. 

2) I'm puzzled by Supplementary Table 2, which contains the top 200 self-anti-correlated genes (presumably strong CERES scores indicating that they are essential but low expression levels). Most of these genes do not have anything listed under off-target (column E). Does it mean that these genes do not have any single-mismatch alignments to an alternative member within their gene family or do not have any single-mismatch alignments at all? 

3) Page 4: A double-target guide doesn't hit two non-coding regions - is this because the Avana library is designed against protein-coding genes only? It will be good to clarify in the main text. 

4) Page 5: The authors concluded that a guide disrupting two protein-coding genes is likely to be more lethal than a guide targeting one coding region and one non-coding region. However, there 1734 and 85 guides for coding and non-coding region secondary targets, respectively. Could this simply be due to a >10-fold difference in the number of guides between the two categories? 

5) Page 11: The authors wrote "mismatch tolerance between the sgRNA's protospacer sequence and the genomic DNA". It should be "mismatch tolerance between the sgRNA's spacer sequence and the genomic DNA".

6) Page 15: The authors wrote "perfect alignments between the sgRNA protospacer and genomic DNA". It should be "perfect alignments between the sgRNA spacer and genomic DNA".

7) Page 12: The authors wrote "One can observe an apparent off-target effect". I suggest "We can observe an apparent off-target effect".

8) Page 16: The authors wrote "Downstream consequences of such confounding was exemplified". It should be "Downstream consequences of such confounding factors were exemplified".

9) Page 16: The authors wrote "We provide in the Supplementary material a gene-level table summarizing the number of on-target and off-target alignments for the Avana library to help readers with flagging potentially problematic genes." Presumably, the authors are referring to Supplementary Table 3?

**Reviewer 2**

**Are the methods appropriate to the aims of the study, are they well described, and are necessary controls included?**
Yes.

**Are the conclusions adequately supported by the data shown?**

Yes.

**Are sufficient details provided to allow replication and comparison with related analyses that may have been performed?**

Yes.

**Does the work represent a significant advance over previously published studies? Please explain.**

Yes.

**Is the paper of broad interest to others in the field, or of outstanding interest to a broad audience of biologists?**

Yes.

**Comments to authors**

The submission by Martin and colleagues describes the importance of correcting for the effects of sgRNAs that align with multiple genomic loci in characterizing gene function in large-scale, pooled viability screens. They focus their analysis on characterizing and improving the CERES model, which corrects for copy number variation and has been used in the compilation of the Project Achilles dataset of cancer dependencies in 391 cell lines. They present ways in which the model does not fully capture the nuances of multi-target guides, both those with perfect on-target alignment, and those with near-perfect alignment (up to two nucleotide mismatches). They show that without additional correction for multiple alignments, the current model can lead to the mischaracterization of the essentiality of some genes and the interaction between genes. They propose a correction, and show that it helps to avoid false positive conclusions.

Overall this paper is well-written and the analyses convincing; the many users of these data will find the results helpful. I have several suggestions for the authors to consider to further improve the manuscript.

Major Points
1) The authors note the potential pitfalls of guides with multiple targets, but how much of this dual-gene targeting is actually avoidable in library design? In other words, for some gene pairs, it may impossible to target one without also targeting another. Could the authors characterize the extent of this phenomenon? Related, to what extent do other, more-recent libraries show this level of multi-gene targeting in their designs? Although this analysis is not relevant to the Achilles data per se, it would be very helpful for people using those other libraries, as well as future library development. Indeed, they conclude section 3.4 by noting "These two examples suggest that multi-targeting can lead to guide design-dependent co-dependencies and misleading biases that have to be interpreted with caution in downstream applications such as identifying gene networks and cancer cell dependencies." For such gene pairs, the authors should provide designs that avoid this problem. 

Minor Points
1) It is often confusing as to when the authors are using log-fold change (LFC) vs copy-number corrected LFC vs CERES score and other similar-sounding terms. I would recommend defining those terms more explicitly and sticking with a consistent nomenclature. Related, in figure 1b,c the y-axis is explicit as to whether the LFC or CERES score is being plotted. However, in figure 2b,d, they annotate the y-axis as LFC, but is this copy-number corrected or not? If not, the cutoff of -1 (dotted horizontal line) is only meaningful for CERES corrected values. 
2) In figure 1a it is hard to tell the actual number of guides is, as the count of single-target aligned guides surpasses the y-axis. 
3) Figure 1f may be easier to interpret as one median line with a shaded interval representing the range of possible cell-line-specific curves. 
4) The second paragraph of section 3.4, the authors use 'iterative' redundantly. 
5) For the data in figure 2b and 2d, could the authors also compare Guide AB1 and Guide AB2 as a scatter plot, to see how consistent this effect is within each cell line?
6) In figure 2d how was the adjustment for LFC made? Is it just a subtraction of the curves in fig. 1f?
7) In section 3.4 the authors compare essentiality predictions between CERES and DEMETER scores. In this analysis, the authors should mention that these differences could be due to biological differences between CRISPR and RNAi screens as opposed to erroneous model assumptions. 
8) In Figure 6, please show all 4 guides for SOX9, not just two of them. Also, what does the guide/target alignment look like for these four guides and SOX10 (i.e. where in the sequence are the mismatches)? 
9) Everywhere in the paper except the discussion the authors cite data from 391 cell lines and 72,787 guides, but in the discussion they cite 342 cell lines and 75,000+ guides. Could the authors clarify?

**Authors’ response to reviewers**

***Reviewer #1****:*

1. The dependency of cleavage toxicity on cellular context is intriguing.
2. Page 4: The authors found that cleavage toxicity induced by multi-target guides is cell line-specific, similar to the copy number toxicity described in Meyers et al. [2017]. I understand the cell line-specificity for copy number, since there can be different gene duplications in different cell lines. But why is there cell line-specificity for multi-target guides? After all, every cell line should in general have the same set of genes. Is the cell line-specificity caused by variable expression of the different targeted genes? Can the authors check the expression levels of all the genes hit by each multi-target guide?
3. Page 12: Similar to the cleavage toxicity associated with multiple-target guides, the authors also found that off-target toxicity is cell line-specific (Figure 4c). The same off-targets exist in all cell lines, so does this result indicate that the off-targets are mainly located in genes (or some functional elements), that these genes are expressed differently in different cell lines, and that the off-targets are performing some novel context-dependent biology?

**Response:** We also found this quite intriguing. As suggested by the reviewer, we looked at the expression levels of all genes hit by multi-target guides, as well as the expression levels of genes hit by off-target guides. We have now found any significant associations (see Figure [1](#_bookmark0) included below). However, we found that cell line-specific Cas9 activity, as measured previously by [Aguirre et al.](#_bookmark12) [[2016](#_bookmark12)], and also provided in the Achilles data release, correlates negatively and significantly with cleavage toxicity (*r* = 0*.*48), suggesting that cell lines with more expressed Cas9 are more sensitive to cleavage and lead to greater off-target activity. We have now added the following discussion in the Results section:

−

“Using the average LFC of guides targeting 4 genomic loci as a summary metric for multiple-target cleavage toxicity, we found that the dropout associated with cleavage toxicity correlates negatively with the cell line-specific Cas9 activity score described in [Aguirre et al.](#_bookmark12) [[2016](#_bookmark12)] (r = 0.48, p < 2.2 x 10^−16^, Figure [6](#_bookmark6)g), and negatively correlates with the median LFC of non-targeting controls (NTCs) (r = 0.67, p

−

−

< 2.2 x 10^−16^). The latter association is not surprising in light of the competitive nature of CRISPR screens; cell lines with greater Cas9 activity result in more effi- cient DNA cleavage, potentially leading to more rapid death for cells infected with multiple-target guides or guides targeting essential genes, which in turn result in an increased proportion of cells infected with NTCs in the cell population over time. These results suggest that greater Cas9 activity leads to greater multiple-target tox- icity. ”


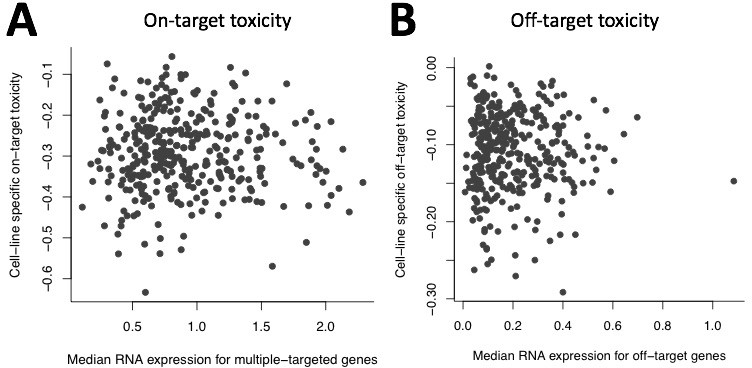


Figure 1: **Relationship between cleavage toxicity and expression of multi- and off-target genes. (a)** y- axis: cell-line specific cleavage toxicity associated with multiple on-targets. x-axis: median RNA expression for genes targeted my multiple-target guides. **(a)** y-axis: cell-line specific cleavage toxicity associated with off-target activity. x-axis: median RNA expression for genes targeted my off-target guides.

### Despite the authors writing in the discussion that ”the large number of cell lines and targeted genes made it possible to robustly generalize” their results, there are several instances in the manuscript where this supposed generalization is unclear. In other words, the authors have highlighted in several parts of the manuscript that there are potential problems with specific guides, but how widespread are these problems?

**Response:** We agree with the reviewer that several analyses in the manuscript are illustrations of potential artifacts in CRISPR screens using specific genes, with guides specific to the Avana library; we have now generalized these analyses and this will be discussed below. We must say that our statement *“the large number of cell lines and targeted genes made it possible to robustly generalize”* is indeed vague and does not provide much information. Our statement was with respect to global non-specific cleavage toxicity associated with an increase number of DSBs induced by both multi-target guides and mismatch-tolerant guides. Besides perhaps in publications studying the effects of copy number on guide toxicity, several previous studies have mainly analyzed these effects in a very targeted manner by looking at a small set of genes in one or two cell lines. Therefore it was unclear how these effects would translate to other cell lines and if it is widespread across the genome. To make sure that these effects are also observed in screens performed in other labs with other CRISPR libraries, we have now extended our analyses of cleavage toxicity to the Brunello and GeCKOv2 libraries (we could not find publicly available screen dafa for the TKOv3 library); this will be discussed further below in response to other comments. We have now changed the sentence to the following:

“We also observed these cleavage toxicity effects in knockout screens performed with two other genome-wide libraries (Brunello and GeCKOv2). ”

### We now answer the two following specific questions from the reviewer as one response.:

1. 297 double-target guides have their pair of targets annotated as being paralogs in PANTHER. But in the results, the authors have focused on only MYL12A-MYL12B. How about all the other potential synthetic

lethal interactions? and (b) The authors discussed EIF3C and EIF3CL - two paralogs with essential but non-redundant functions - but how about other pairs of paralogs? How generalizable is the finding?

**Response:** First, to be able to quantify off-target effects exceeding cleavage toxicity induced by DSBs, we made use of the following delta coefficient described in the Methods section:

***Delta coefficient for measuring sgRNA discrepancy***

“To quantify how the activity of a given sgRNA compare to the activity of another set of sgRNAs, for instance other guides targeting the same gene, we propose a simple metric based on guide-level log-fold changes. The metric is particularly useful to quantify off-target activity for guides with single and double-mismatch alignments in comparison to “clean guides” (guides that have neither predicted multiple on- targets nor off-targets).”

“For a fixed CRISPR library, and for a given gene g, let j = 1 index the guide of interest to be examined, and j = 2, . . . , n_g_ index all (n_g_ 1) clean guides targeting gene g. We assume here that there exists at least one clean guide targeting gene g. Let i = 1, 2, . . . , n index cell lines for which log-fold changes are available for all n_g_ guides and let y_ij_ be the CN-corrected log-fold change for guide j for cell line

−

- 1. *Using data for j* 1*,* 2*, . . . , n_g_ , we fit the following fixed-effects linear model using ordinary least squares (OLS):*

∈ { }

*y_ij_* = *α* + *β_i_* + *δ*1(*j* = 1) + *r_ij_*

where α is the average log-fold change across the clean guides targeting gene g, β_i_ is an offset accounting for cell line-specific knockout effect of gene g, δ is the average change in the log-fold change associated with the guide of interest in comparison to clean guides, and r_ij_ are residuals. We simply refer to the estimated coefficient

δˆ as “delta coefficient”. A negative coefficient indicates that the guide of interest has greater activity in comparison to the remaining clean guides targeting gene g, possibly because of better cleavage efficiency or because of off-target effects.”

### To generalize the effects of co-targeting paralogs to other genes, we have performed the follow- ing analysis (with an additional Supplementary Figure, included below):

“For guides targeting two coding regions in the Avana library, we asked whether or not the two targets are related in terms of sequence similarity using gene paralogy as a proxy for gene similarity. Among the 2503 guides, 297 (11.9%) guides have their pair of targets annotated as being paralogs using the PANTHER database [[Mi et al.](#_bookmark23), [2016](#_bookmark23)] (see Methods). In comparison, the 74,070 pairs of paralog genes annotated in the PANTHER database represent only 0.02% of all possible pairs of genes screened in the Avana library. This significant enrichment for paralog genes (exact binomial test, p < 2.2 x 10^−16^) confirms that co-targeted genes often share high homology.”

∼

“To compare the effects of co-targeting a pair of paralog genes in comparison to targeting only one paralog, we further examined co-targeting guides for which there

was at least one single-target guide for each of the two paralogs. We restricted our analysis to “clean” guides only, that is guides with no additional single or double- mismatch alignments, to prevent off-target effects from confounding our analysis. This left us with a set of 22 double-target guides for further quantification. For each of the 22 guides, we computed an average difference between the double-target guide log-fold change (digenic knockout effects) and each of the single-target guide log- fold change (paralog-specific knockout effects) using the delta coefficient described in the Methods section. Thus, for each double-target guide, we obtained two delta coefficients (one for each paralog). A large negative delta coefficient indicates that the digenic knockout is substantially more lethal than the single-gene knockout. On the left panel of Supplementary Figure [S1](#_bookmark9)b, we present the delta coefficient estimated using the second paralog (y-axis) as a function of the delta coefficient estimated using the first paralog (x-axis) for all 22 guides. Both delta coefficients agree overall, and several guides have greater activity in comparison to the paralog-specific single- gene knockouts.”

“To visualize potential genetic interactions between the pairs of paralogs, we show on the right panel of Supplementary Figure [S1](#_bookmark9)b the double-target log-fold changes as a function of the minimum expected log-fold change estimated by paralog-specific knockouts. In the absence of genetic interactions, the minimum expected log-fold change can be estimated as min(y_A_, y_B_, y_A_ + y_B_) where y_A_ and y_B_ are log-fold changes associated with paralog-specific knockouts for paralogs A and B respec- tively. We observed that for a number of guides, the digenic knockout effects largely exceed the expected additive log-fold changes, suggesting indeed that potential syn- ergistic or synthetic genetic interactions exist between the targeted paralogs. As an example, we present the log-fold changes for guides targeting the paralogs RAB5B and RAB13 in Supplementary Figure [S1](#_bookmark9)c. Log-fold changes of paralog-specific guides are centered around 0 (grey and blue boxplots), suggesting low to no activ- ity, while log-fold changes of the 3 guides co-targeting both paralogs show greater activity.”

### We have also generated a whole new section in the Results describing our analysis of paralogs for guides with a single-mismatch alignments to a gene paralog to the on-target gene, together with a new figure, also included below. We have incorporated more examples that hopefully show that the delta coefficient methods is useful at detecting guides with problematic behav- iors. While there are a lot of guides with single-mismatch alignments in the Avana library, distinguishing off-target activity from on-target activity in an automatic manner genome-wide is difficult because of the small number of guides targeting each gene and because of the lack of ground truth (is an off-target guide seemingly active because of off-target activity, or are the other guides inactive because of a lack of on-target activity?). However, we have tried to circumvent that by choosing genes that are at least targeted by one “clean guide”, that is a guide with no multiple-target alignments and no single-mismatch alignments to establish a baseline level of activity.

***Single-mismatch tolerance and paralogs***

“To investigate how often guides with single-mismatch alignments can lead to incon-

sistent cell line dependencies because of off-targets, we looked at guides targeting exactly one on-target and one single-mismatch off-target in the Avana library. To be able to distinguish between off-target and on-target effects, we selected guides with no double-mismatch alignments, and for which the corresponding on-target gene is also targeted by at least one “clean guide”, defined as a guide with only one on-target and no mismatch alignments, for a final set of 427 guides for off-target quantification. We note that 52% of the guides (224 guides) have the off-target locus in a coding region. In addition, among those 224 guides, 77 guides (34%) have their on-target and off-target genes annotated as paralogs in the PANTHER database.”

“For each guide separately, we quantified off-target effects by measuring the aver- age difference of the LFC between the off-target guide and the set of clean guides using the delta coefficient (see Methods). A delta coefficient close to 0 indicates minimal off-target effects, while a negative score indicates potential off-target ef- fects. Delta coefficients were calculated using all cell lines screened in the Achilles dataset. A non-negligible proportion of single-mismatch guides (18%) shows sub- stantial off-target activity, defined as an off-target delta coefficient less than -0.25 (Figure [2](#_bookmark1)a). In comparison to 1000 clean guides chosen at random, these guides are significantly enriched for off-target effects (OR = 4.7, p = 2.32 x 10^−15^, Fisher’s exact test). Looking at the 15 guides with the lowest delta coefficient, 7 of them co-target pairs of paralog genes (one on-target and one off-target): IRF2BP2/L, SLC22A4/5, REEP1/2, SLC25A18/22, ARSB/I, LSM14A/B, YPEL1/3. We show in

Figure [2](#_bookmark1)b the distribution of LFCs for clean guides and guides with off-targets. We discuss 3 paralog pairs below with different behaviors.”

“First, in Figure [2](#_bookmark1)c, we show the Avana guide design for guides targeting the par- alogs LSM14A (mRNA processing body assembly factor) and LSM14B (LSM14 ho- molog B). Each paralog is targeted by 3 clean guides, and one additional guide (A4) targeting LSM14A has also an single-mismatch off-target alignment to LSM14B. The single-mismatch occurs at position 13 with respect to the PAM site, and there- fore should be tolerated. We note that the average LFC for clean guides targeting either paralog does not correlate with the LFC for guide A4 (Figure [2](#_bookmark1)d, first and second panels). In addition, the distribution of both paralog-specific average LFCs are centered around or above 0, indicating non-essentiality, while the distribution of LFCs for guide A4 is centered around -1. This suggests that the single-mismatch at position 13 for guide A4 is well tolerated by the CRISPR/Cas9 system, generating the hypothesis that targeting both paralogs is lethal for most cell lines. In the right panel of Figure [2](#_bookmark1)d, we show that the CERES score for LSM14A is shifted negatively with respect to the average LFC for clean guides targeting LSM14A as a result of Guide A4 lethality.”

“Next, we discuss guides targeting the paralogs YPEL1 (Yippee like 1) and YPEL3 (Yippee like 3) in the Avana library. Both paralogs are targeted by 3 clean guides each, and each paralog is targeted by an additional guide that also targets the other paralog assuming single-mismatch tolerance. In particular, Guide A4 tar- gets YPEL3, and has YPEL1 has a single-mismatch off-target; guide B4 targets YPEL1, and has YPEL3 has a single-mismatch off-target (see Figure [2](#_bookmark1)e). The single-

mismatch occurs at position 13 for both guides A4 and B4. By estimating paralog- specific essentiality using LFCs of clean guides only (Figure [2](#_bookmark1)f, first panel), we found that each paralog is essential in different subsets of cell lines (black and red dots), as well as in a shared subset of cell lines (orange dots). In the middle panel of Figure [2](#_bookmark1)f, we show that LFCs of guides A4 and B4 correlate well with each other (r = 0.59), and that paralog-specific dependent cell lines (red and black) become vulnerable when targeted by either guide A4 or B4. This suggests that knockout effects for both guides A4 and B4 are a mixture of both paralog-specific knockout effects, suggest- ing mismatch tolerance by the CRISPR/Cas9 system for both guides A4 and B4. As negative controls, we note that cell lines that are not dependent on either paralog (blue dots) remain unchanged when targeted by guide A4 or B4. We also observed that while paralog-specific LFCs are mostly uncorrelated for most cell lines (first panel of Figure [2](#_bookmark1)f), paralog-specific CERES scores correlate well as a consequence of including single-mismatch tolerant guides A4 and B4 (r = 0.45, Figure [2](#_bookmark1)f, third panel).”

“Finally, we study the two paralog genes SLC25A22 and SLC25A18, two members of the SLC25 carrier family implicated in glutamate transport across the inner mito- chondrial membrane, also referred to as Mitochondrial Glutamate Carrier 1 (GC1) and Mitochondrial Glutamate Carrier 2(GC2) respectively. In the Avana library, there are 4 guides targeting SLC25A22. One of the 4 guides (B4) has a single- mismatch alignment to SLC25A18 (position 16). There are also 4 guides target- ing SLC25A18, and one of the 4 guides (A4) has a single-mismatch alignment to SLC25A22 (position 16); see Figure [2](#_bookmark1)g. Using clean guides only, we estimated paralog-specific dependencies (Figure [2](#_bookmark1)h, left panel). Orange dots represent cell lines that are the most dependent on SLC25A22, but not dependent on SLC25A18. Conversely, the red dot represents a cell line (oral squamous cell carcinoma HSC-3) that is dependent on SLC25A18, but not dependent on SLC25A22. On the mid- dle panel of Figure [2](#_bookmark1)h, we show LFCs for guides A4 and B4. The activity of the two guides with off-targets correlates well (r = 0.32), and cell lines that have paralog-specific dependencies (orange and red dots) are in comparison sensitive to the knockout induced by either A4 or B4. This suggests that LFCs estimated for both A4 and B4 are a mixture of the paralog-specific LFCs occurring through mismatch tolerance. Interestingly, one cell line insensitive to both paralog-specific knockouts (ovarian serous adenocarcinoma cell line JHOS-2, colored in black) is one of the most dependent cell line for the guide targeting SLC25A18 with an off- target to SLC25A22. This suggests some cell-line specific synthetic lethality, or at least some level of synergy, between the two paralogs. On the right panel of Fig- ure [2](#_bookmark1)h, we confirm that the addition of paralog-specific knockout effects, calculated as LFCs averaged across all clean guides targeting SLC25A18 and SLC25A22, can overall recapitulate the activity of guides A4 and B4 (r = 0.50). Again, the di- genic knockout for cell line JHOS-2 cannot be explained by either paralog-specific knockout. ”

### (c) The CYFIP1 example is a nice illustration. But how generalizable is the finding? How many guides are there with one double-mismatch alignment and what fraction of them are problematic?


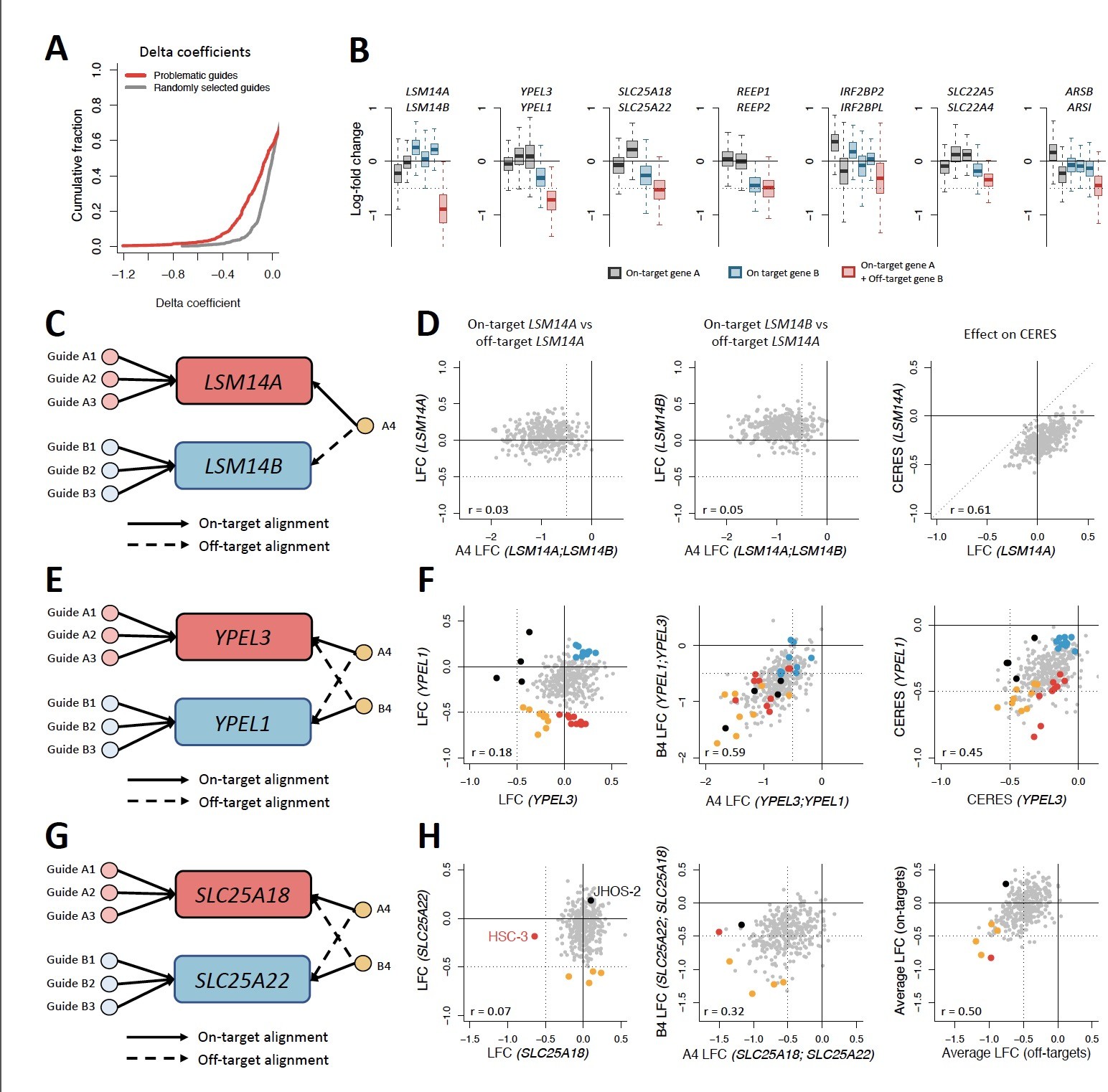


Figure 2: **Single-mismatch tolerance and genetic interactions in the Achilles dataset (a)** Cumulative distribution of off-target delta coefficients for the set of 427 guides that have exactly one on-target and one single-mismatch off-target in the Avana library. Larger negative coefficients suggest greater off-target activity. The grey line represents the cumulative distribution of delta coefficients for 1000 guides chosen at random. **(b)** Log-fold changes (LFCs) for guides targeting 7 pairs of paralog genes. Clean guides and double-target guides are shown. **(c)** Avana guide design for guides targeting *LSM14A* and *LSM14B*. **d** LFCs and CERES scores for guides targeting *LSM14A* and *LSM14B*. Notation: LFC(A;B): LFC for a guide with gene A as an on-target and gene B as a single-mismatch off-target. **(e)** Same as (c), for *YPEL1* and *YPEL3*.

**(f)** LFCs and CERES scores for guides targeting *YPEL1* and *YPEL1*. Colors indicate gene-specific cell line dependencies. Red: *YPEL1*-only dependencies; black: *YPEL3*-only dependencies; orange: both *YPEL1* and *YPEL3* dependencies; blue: no dependencies. **(g)** Same as (c), for *SLC25A18* and *SLC25A22*. **(h)** LFCs and CERES scores for guides targeting *SLC25A18* and *SLC25A22*. Right panel: LFC averaged across all on-target guides targeting *SLC25A18* and *SLC25A22* vs LFC averaged across guides with both on-targets

and off-targets. Red: *SLC25A18*-dependent cell lines8. Orange: *SLC25A22*-dependent cell lines. Black: no dependencies.

**Response:**

### Our position-specific analysis of double-mismatch alignments (see Figure 8a in the current manuscript version) suggests that overall, double-mismatches are substantially less tolerated by the CRISPR/Cas9 system in comparison to single-mismatches, which makes the effect size of cleavage toxicity associated with double-mismatch tolerance in comparison to on-target activ- ity and off-target activity caused by single-mismatch alignments. This hinders our an analy- sis of double-mismatch alignments in existing libraries because of the confounding effects of single-mismatch alignments. In this case, an experiment designed to analyze double-mismatch alignments would be helpful. However, we now include in our manuscript an analysis of guides with double-mismatch alignments that are more likely to cause a dropout in guide activity as a result of cleavage toxicity. We have now added the following paragraph, along with a new figure (also included below), describing our computational analysis:

”To investigate how guides with a large number of double-mismatch alignments at position 20th position affect log-fold changes, we analyzed 53 guides in the Avana library, targeting 53 different genes, with the following characteristics: one unique on-target alignment, no single-mismatch alignment, and at least 5 double-mismatch alignments with the first mismatch located at the 20th position and the second mis- match located elsewhere along the spacer sequence. To quantify off-target effects, we estimated off-target delta coefficients for each of the guide (see Methods). These guides should have a high probability of off-target effects, resulting in a large neg- ative delta coefficient. To be able to distinguish between real on-target effects and off-target effects, we focused our analysis on genes with at least one additional clean guide (guide with neither single nor double-mismatch alignments), leaving us with 39 guides for further investigation. We present the off-target delta coefficients for the 39 genes targeted by the problematic guides in Figure [3](#_bookmark2)b. The grey shaded area rep- resents a 95% confidence interval of a null distribution of delta coefficients estimated using 1000 genes chosen at random that are targeted by clean guides only. Guides with a double-mismatch with 1 mismatch at the 20th position are more likely to pro- duce real off-target effects (Fisher’s exact test: OR = 5.71, p-value = 0.004). In Figure [3](#_bookmark2)c, we depict the distribution of LFCs for clean guides (grey boxplots) versus problematic guides (red boxplots) for the top 5 genes with an exceedingly large neg- ative off-target delta coefficient from Figure [3](#_bookmark2)b. For instance, for CYFIP1, Guides 1 and 2 do not have single or double-mismatch alignments, while Guide 3 aligns to 10 different genomic loci with a double-mismatch at positions 19 and 20, resulting in a substantial decrease in LFC, most likely as a result of off-target toxicity. This biases the CERES score for CYFIP1 towards essentiality (CERES score of 0.41), while excluding guide 3 results in a score centered around 0 (non-essentiality). ”

−

### (d) 3197 genes have at least one single-mismatch alignment located in the exon of another gene. But the authors’ discussion has focused largely on SOX9-SOX10. How about all the other genes?

**Response:** We believe our new section entitled ***Single-mismatch tolerance and paralogs*** de- scribed in response to the reviewer’s question 2 above answers this comment.


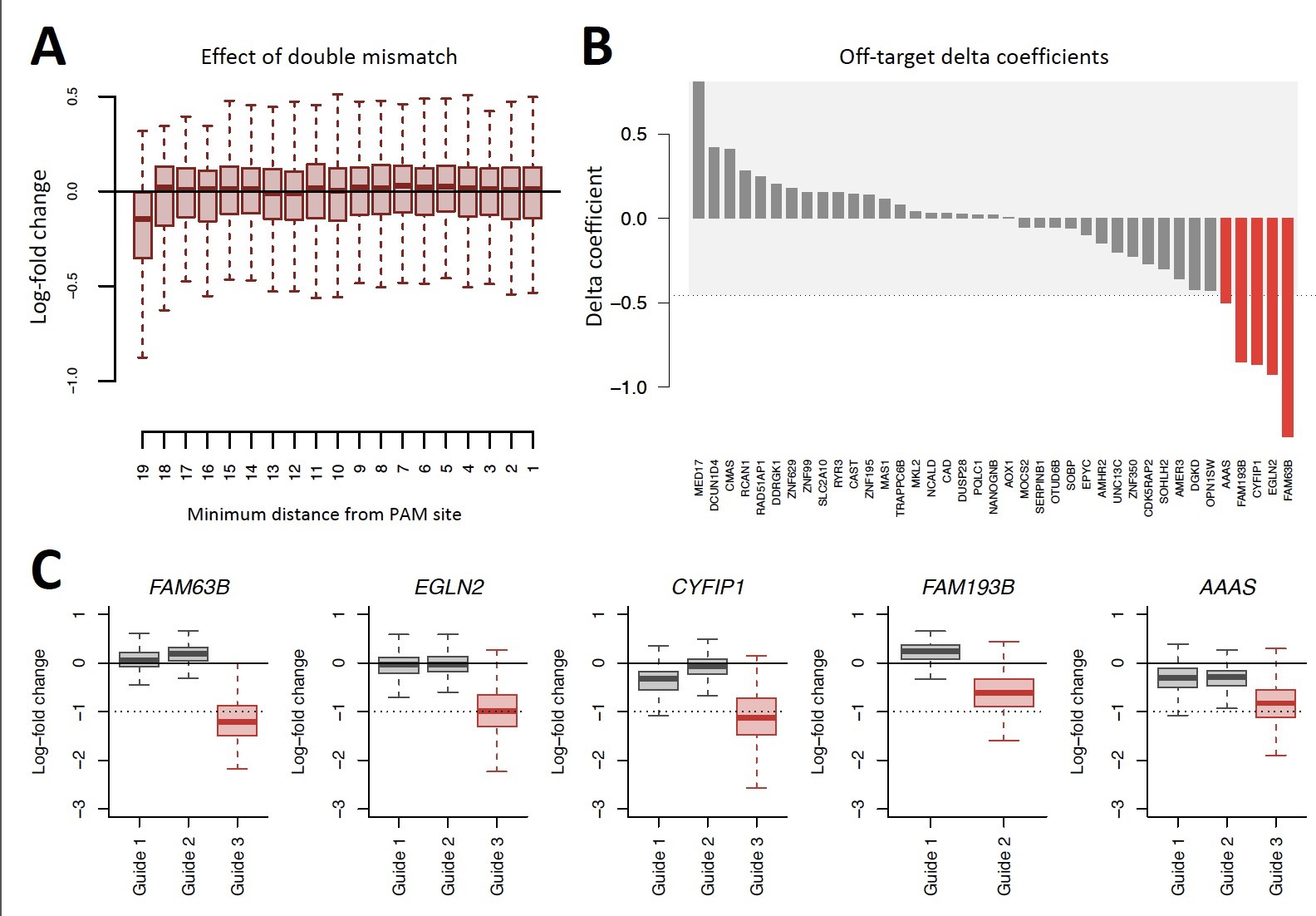


### Figure 3: **Position-specific mismatch tolerance of the spacer for double-mismatch alignments (a)** Ef- fect of a double mismatch between spacer and reference genome as a function of the most PAM-proximal mismatch position. **(b)** Off-target delta coefficients for 39 genes targeted with a guide with at least 5 double- mismatch alignments with the first mismatch located at the 20th position. The grey shaded area represents a 95% confidence interval of a null distribution of delta coefficients estimated using 1000 genes chosen at random that are targeted by clean guides only. **(c)** Distribution of LFCs for clean guides (grey boxplots) versus problematic guides (red boxplots) for the top 5 genes with an exceedingly large negative off-target delta coefficient from (b).

1. The submitted work is essentially a body of computational analysis, but I think there is a need for some independent experimental validation of results. The CRISPR screen and RNAi screen data are from Broad, but the RNA-seq and copy number data are from CCLE. A couple of examples where it would be nice to have some validations:
2. The authors found that that the pair of paralogs (MYL12A and MYL12B) is more essential to cell survival in the absence of MYL9 expression. Can the authors confirm this prediction? For example, take a cell line where MYL9 is expressed and (i) knockout MYL12A and MYL12B only and (ii) knockout MYL9 as well as MYL12A and MYL12B (is the triple knockout viable?).
3. The authors found that in melanoma cell lines, SOX10 is the important gene, not SOX9, since SOX9 is barely expressed in these cell lines. However, the CRISPR screen data and the RNA-seq data are from different sources. Can the authors knockout SOX9/ SOX10 in a melanoma cell line and show that SOX10 has a stronger effect than SOX9?

**Response:** We thank the reviewer for suggesting these experiments – we also believe that experimental validations of computational results add great and significant value to results. Because of time constraints, we decided to focus on experimentally validating the *SOX9*/*SOX10* story. Investigating the double vs triple knockout of *MYL12A*/*MYL12B*/*MYL9* could be done using a sequential approach of gene knockdowns, but this would require much more time. We have added the following sections in the current manuscript to describe our experimental validations:

“Next, we sought to confirm experimentally that the observed SOX9 dependency for cell lines highly expressing SOX10 for 2 out of 4 guides targeting SOX9 results from off-target activity. We selected two melanoma cell lines with low expression of SOX9, but high expression of SOX10 (cell lines Malme-3M and UACC-62, see Figure [4](#_bookmark3)a). We transfected cells in a microplate format with small interfering RNAs (siRNAs) targeting SOX9 and SOX10, and measured cell viability after 5 days using a CellTiter-Glo luminescence assay. In Figure [4](#_bookmark3)b, we show the relative cell viability percentages with respect to the non-targeting siRNA (siNTC) treatment. We observed a substantial decrease in viability for both melanoma cell lines after SOX10 knock- down with 3 of 3 tested siRNAs, while SOX9 knockdown did not lead to an apparent decrease in cell viability.

We validated knockdown efficiency for all SOX9 and SOX10 siRNAs by qPCR (Fig- ure [4](#_bookmark3)c). All three SOX10 siRNAs induced substantial knockdown of SOX10 mRNA levels, in both cell lines, in contrast to the negative control or siRNAs targeting SOX9. SOX9 was not detected in these cells by qPCR (Ct>35); this confirms low expression of SOX9 as revealed by RNA-Seq. To confirm efficacy of the SOX9 siR- NAs, we selected the colorectal cancer cell line LS1034 for knockdown validation. This cell line highly expresses SOX9 (see Figure [4](#_bookmark3)a). SOX9 mRNA levels are reason- ably reduced after SOX9 knockdown, but not after SOX10 knockdown (Figure [4](#_bookmark3)d), especially for two of the three siRNAs (> 80% knockdown). Overall, there results confirm specificity of the SOX9 and SOX10 siRNAs, and that SOX10 knockdown, unlike SOX9 knockdown, reduces cell viability in melanoma cell lines.”

### We have also added the description of the experimental setup and reagents used in the Methods section:

***Tissue culture:***

Cells were cultured in RPMI 1640 media supplemented with 10% FBS. Cell iden- tity and quality is ensured by an internal cell line repository, which performs short tandem repeat profiling, mycoplasma testing, and rigorous tracking of all cell lines.

## siRNA Transfections

siRNAs were reverse transfected in 384-well plate format. Briefly, 1.2 pmol of siRNA was spotted into individual plate wells (n = 5) followed by the addition of 0.15 µL of RNAiMax in 20 µL of serum free RPMI. After a 30-minute incubation at ambient temperature, cells were added in 20 µL of RPMI supplemented with 20% serum to yield a final concentrations of 30 nM siRNA and 10% FBS. Malme-3M, UACC-62, and LS1034 cells were seeded at 3,000, 1,000, and 3,000 cells per well respectively. Transfections were incubated for 48 h prior to harvesting for qPCR or 120 h prior to assaying for viability. Viability was assessed by adding 30 µL of CellTiter Glo (Promega) and reading on an Envision 2104 plate reader (PerkinElmer) after a 10- minute incubation at ambient temperature.

|  |  | | | |  |  |
| --- | --- | --- | --- | --- | --- | --- |
|  |  | | | |  |  |
| siRNA | | Vendor | Cat# | Sense | | Antisense |
| SOX9-1 | | Ambion | s13306 | AGACCUUCGAUGUCAACGATT | | UCGUUGACAUCGAAGGUCUCG |
| SOX9-2 | | Ambion | s532658 | CCUUCGAUGUCAACGAGUUTT | | AACUCGUUGACAUCGAAGGTC |
| SOX9-3 | | Ambion | s532659 | CCCGCUCACAGUACGACUATT | | UAGUCGUACUGUGAGCGGGTG |
| SOX10-1 | | Ambion | s13309 | CCACCUCACAGAUCGCCUATT | | UAGGCGAUCUGUGAGGUGGAT |
| SOX10-2 | | Ambion | s13310 | CCGUAUGCAGCACAAGAAATT | | UUUCUUGUGCUGCAUACGGAG |
| SOX10-3 | | Ambion | s13311 | GCAACGUGGACAUUGGUGATT | | UCACCAAUGUCCACGUUGCCG |
| Silencer Select Negative Control #2 | | Ambion | 4390846 |  | |  |
| AllStars Hs Cell Death Control | | Qiagen | SI04381048 |  | |  |

### Table 1: List of siRNAs used for *SOX9* and *SOX10* knockdown

***TaqMan Gene Expression Assays***

After 48 h of siRNA knockdown, cells were lysed and RNA was extracted using RNeasy Mini QIAcube Kit (Qiagen 74116). After quantification, equal amounts of RNA were reverse transcribed using High Capacity cDNA Reverse Transcription Kit (Applied Biosystems 4368814) on a Applied Biosystems ProFlex PCR System. TaqMan assays were conducted using TaqMan Universal PCR Master Mix (Ap- plied Biosystems 4304437) using TaqMan primers from Life Technologies (GAPDH Hs99999905 m1, SOX9 Hs00165814 m1, SOX10 Hs00366918 m1). Amplification

and analysis were performed on a Bio Rad CFX384 Real-Time System.

### Can the authors consider the effect of SNPs across the different cell lines? Cancer genomes are typically riddled with mutations, other labs have shown that SNPs can have an effect on CRISPR targeting (e.g. see [[Scott and Zhang](#_bookmark26), [2017](#_bookmark26)]), and the authors have demonstrated that single mismatch off-targets can confound analysis. Hence, I would imagine that the issue of genetic variation would be important to consider for the analysis of CRISPR screen results.


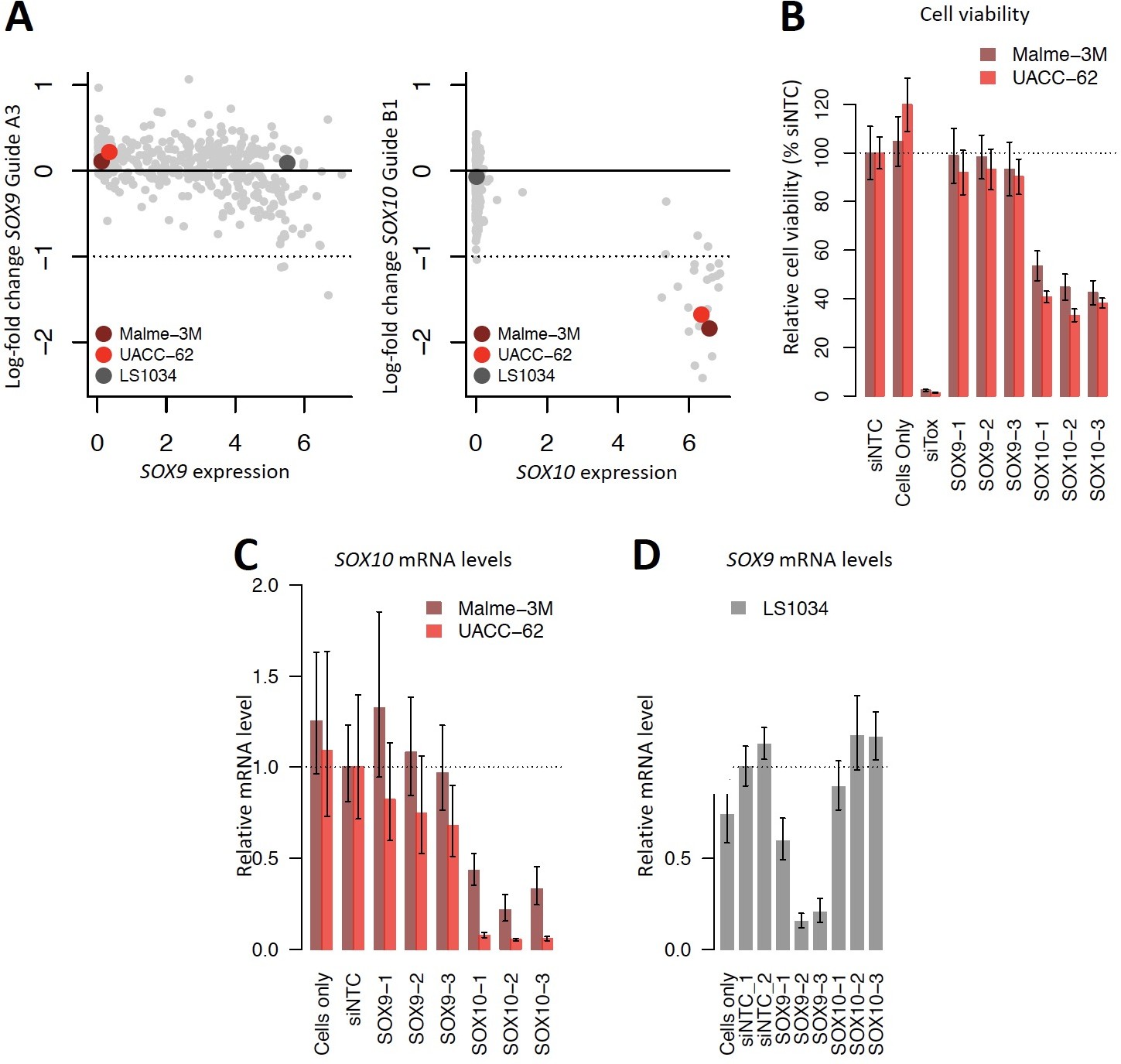


Figure 4: **Melanoma cell lines with high expression of *SOX10* are not sensitive to *SOX9* knockdown**

### Left panel: log-fold change for clean guide A3 targeting *SOX9* as a function of *SOX9* expression. Right panel: log-fold change for clean guide B1 targeting *SOX10* as a function of *SOX10* expression. Two melanoma cell lines were selected for *SOX9* and SOX10 knockdowns validation: Malem-3M and UACC-62 (in red). One colorectal carcinoma (LS1034) was selected to measure *SOX9* siRNAs knockdown efficiency.

1. Cell viability of two melanoma cell lines after transfection with small interfering RNAs (siRNAs), rela- tive to the non-targeting control (siNTC) treatment. **(c)** Relative *SOX10* mRNA expression levels, detected by qPCR, after transfection of the indicated siRNAs, for two melanoma cell lines. Expression levels are nor- malized to *GAPDH* mRNA detected in the same sample, and then expressed as a fold change with respect to the siNTC treatment. **(d)** Relative *SOX9* mRNA expression levels, detected by qPCR, after transfection of the indicated siRNAs, for the colorectal carcinoma cell line LS1034.

**Response:** This is an excellent point, and we thank the reviewer for the suggestion. We have downloaded SNP arrays available for CCLE cell lines (more than 900k measured SNPs), and we have analyzed how SNPs affect on-target activity for guides targeting a protospacer region that contains one of the measured array SNP. We have now added an entire section to the manuscript, together with a figure (included below) describing our analysis:

***The effects of human genetic variation on sgRNA efficiency and specificity***

“In the previous sections, we analyzed the effects of on- and off-targets on sgRNA efficiency and specificity by generating a list of genomic alignments between sgRNA spacers and the reference genome, ignoring genetic variation across different cell line genomes. Genetic variation, such a single-nucleotide polymophisms (SNPs) and small indels, can have a profound effect on sgRNA specificity and on-target efficiency [[Scott and Zhang](#_bookmark26), [2017](#_bookmark26), [Lessard et al.](#_bookmark20), [2017](#_bookmark20), [Canver et al.](#_bookmark13), [2017](#_bookmark13), [Wang](#_bookmark29) [et al.](#_bookmark29), [2018](#_bookmark29)]. For instance, the list of on- and off-target loci for a particular sgRNA depends on SNP alleles present in a particular genome as a consequence of adding or removing mismatches between sgRNA spacer sequences and the targeted genome, in comparison to the reference genome. In addition, canonical NGG PAM sites can be either destroyed or created through SNP variation.” “Because SNP array data

are available for 363 CCLE cell lines screened in the Achilles project, we focused on investigating the effects of SNPs on guide log-fold changes in the Achilles data. We generated genomic coordinates in GRCh38 for 904,800 SNPs measured on the Affymetrix SNP array 6.0 (see Methods). For each cell line and each SNP, we used the genotype call (AA, AB or BB) estimated by Birdseed [[Korn et al.](#_bookmark19), [2008](#_bookmark19)] to link SNP variation to sgRNA log-fold changes. We transformed the data such that the allele A represents the allele annotated in the reference genome assembly GRCh38.”

## Effect of SNP variation on sgRNA on-targets

“We first studied the effects of SNP variation on on-target activity. We intersected the coordinates of all protospacer sequences targeted by the 68,742 single-target Avana guides (guides with multiple on-targets were excluded) with the array SNP locations. We found that 473 guides are targeting a protospacer sequence contain- ing a SNP targeted by the array. No guide was targeting a sequence with more than one array SNP. One SNP was present in 3 adjacent guides targeting C10orf82, 31 SNPS were present in exactly 2 adjacent guides, and 408 SNPs were present in one guide only. One SNP (rs17099014) has no allele variation across the Achilles cell lines and was therefore excluded for further analyses.” “For each SNP-guide pair,

we calculated a Pearson correlation between the cell line-specific SNP genotype (0 =AA,1 =AB, 2 =BB) and the guide log-fold chance (Figure Figure [5](#_bookmark4)a, red line), and also generated a null distribution of correlations by permuting cell line geno- types B = 100 times (Figure [5](#_bookmark4)a, grey line). A large proportion of SNP-guide pairs has a genotype-LFC positive correlation greater than by chance (262 pairs, 56%), confirming the hypothesis that an alternative allele within the protospacer region results in a decrease of cleavage efficiency as observed by a less negative log-fold change. Next, we stratified the distribution of the genotype-LFC correlations by the

relative position of the SNP with respect to the protospacer’s PAM site position (Fig- ure [5](#_bookmark4)b). The genotype-LFC correlations are significantly higher for SNPs located in the PAM-proximal region of the protospacer in comparison to SNPs located in the PAM-distal region (Wilcoxon rank sum test, p = 2.54 10^−6^). This is consistent with our analysis of single-mismatch alignments; single-mismatches located in the PAM-proximal region caused by SNP variation are less tolerated than PAM-distal single-mismatches, resulting in a more pronounced genotype-specific guide activity. Similarly, SNPs located at the second or third position of the canonical NGG PAM site are not tolerated well. As expected, a SNP located at the first position of the PAM site (nucleotide N) has virtually no effect (mean correlation = 0.006).”

×

“We further illustrate the impact of SNP variation by studying the effect of the SNP rs11556200 located in one of the guides targeting EIF2B3. The SNP is located at position 9 with respect to the PAM site, and has a reported minor allele frequency (MAF) of 0.297 in the 1000 Genomes, which is comparable to the frequency ob- served in the Achilles cell lines (MAF = 0.249). In Figure [5](#_bookmark4)c, we show the log-fold changes for the affected guide as a function of the SNP genotype. For cell lines with homozygous reference allele (GG), log-fold changes are centered around -1, indicat- ing gene essentiality. For cell lines with either heterozygous and homozygous minor allele (GA or AA), the average log-fold change is around 0 and above 0, respectively, suggesting that the guide is inefficient at inducing a homozygous gene knockout for cell lines with a minor allele at this SNP. This is further shown by comparing the guide with the SNP rs11556200 (Guide 1) with the 3 other guides targeting EIF2B3 (Figure [5](#_bookmark4)d); the log-fold changes of the SNP-unaffected guides do not differ by SNP genotype (r_2_ = 0.018, r_3_ = 0.055, r_4_ = 0.073), and are centered around -1, con- firming common essentiality of EIF2B3 across cell lines. Not excluding Guide 1 from the library results in a CERES score correlated with the SNP genotype (r = 0.63, Figure [5](#_bookmark4)e). Overall, this shows how the presence of a common SNP within the pro- tospacer region can alter guide activity and result in spurious log-fold changes for a subset of cell lines.” “Another example is the presence of the SNP rs1131454 (MAF

−

= 0.473 in 1000 Genomes, MAF= 0.57 in Achilles) in one of the guides targeting OAS1. The SNP is located in the second position of the NGG PAM site, which de- stroys the PAM site for a strand containing the minor allele, and should therefore result in a complete loss of cutting efficiency for cell lines homozygous for the mi- nor allele. The log-fold change of the corresponding guide shows an allele-dose positive association ( r = 0.51, Figure [5](#_bookmark4)f). Log-fold changes of cell lines that are homozygous for the minor allele (AA) are substantially above 0, comparable to log- fold changes of non-targeting controls (NTCs). Indeed, log-fold changes of cell lines with genotype AA correlate with log-fold changes of NTCs (r = 0.27, p=0.0007), while log-fold changes of cell lines with genotype GG do not significantly correlate (r = 0.07, p = 0.48). This suggests that the minor allele results in a destruction of the PAM site that makes the guide inactive for cell lines with both copies of the minor allele.”

−

## SNP variation and sgRNA off-targets

“In addition to the potential loss of guide efficiency due to the presence of SNPs in

protospacer sequences, SNP variation can also alter the number of off-targets by in- creasing or decreasing the number of single-mismatch and double-mismatch align- ments for a particular guide. It can also create additional on-targets, besides the designed primary on-target, in cases where a SNP is located at the single-mismatch position of a single-mismatch off-target alignments. To investigate these potential bi- ases, we studied guides in the Avana library that have exactly one single-mismatch alignment, for a total of 5072 guides. We found 45 guides that have one SNP located within the sequence of the single-mismatch off-target.” “Among these 45 guides, 9

guides satisfy the following conditions: (1) the SNP overlaps the location of the single-mismatch between the guide sequence and the reference genome and (2) the minor allele nucleotide matches the reference nucleotide single-mismatch nucleotide substitution. For such guides, the off-target becomes an additional on-target for cell lines that are homozygous for the minor allele, and log-fold changes for such guides should decrease as the number of minor alleles increases as a consequence of multiple on-target cleavage toxicity, and possibly because of genetic interactions between the two on-targets. This should be reflected in negative correlations be- tween log-fold changes and SNP genotype. To validate our hypothesis, we generated Pearson correlations for all 45 SNP-guide pairs and used a null distribution of cor- relations produced from a permutation analysis (B = 100 permutations) to assess significance. Pairs with a significant negative correlation (5 pairs) are significantly enriched for pairs that generate additional minor allele-specific on-targets (3 pairs out of 9, OR = 7.9, Fisher’s exact test’s p = 0.047). As an example, we consider the SNP rs2056899 located in the off-target sequence of one of the guides targeting CYP4A11. The guide log-fold change correlates negatively with the SNP genotype (r = 0.25), with an median decrease of -0.10 for cell lines that are homozygous for the minor allele. This is concordant with our previous observation that an addi- tional on-target results in lower log-fold changes.” “Conversely, a single-mismatch

−

off-target with an additional single-mismatch caused by the minor allele of a SNP elsewhere in the sequence should become a double-mismatch off-target for cell lines homozygous for the minor allele. As an example, we consider the SNP rs2717932 located in the off-target sequence of one of the guides targeting PPP1R17. The guide log-fold change correlates positively with the SNP genotype (r = 0.28), with an me- dian increase of 0.16 for cell lines that are homozygous for the minor allele. This is concordant with our previous observation that double-mismatch off-targets result in less cleavage toxicity in comparison to single-mismatch off-targets.”

### Overall, how does this work help the typical CRISPR screen user? Supplementary Table 3 is useful, but it is restricted to the Avana library. Can the authors also examine the sgRNA sequences in other CRISPR libraries (e.g. Gecko v2, Toronto v3 etc)? Additionally, I suggest creating a web interface as well where users can query whether the hits that they obtain for their screens may be false positives due to poor guide design. To be even more useful, the web interface can incorporate a feature where users can upload any customized library of sgRNA sequences and the website will flag any potential problematic designs (e.g. multi-target, potential off-targets etc).


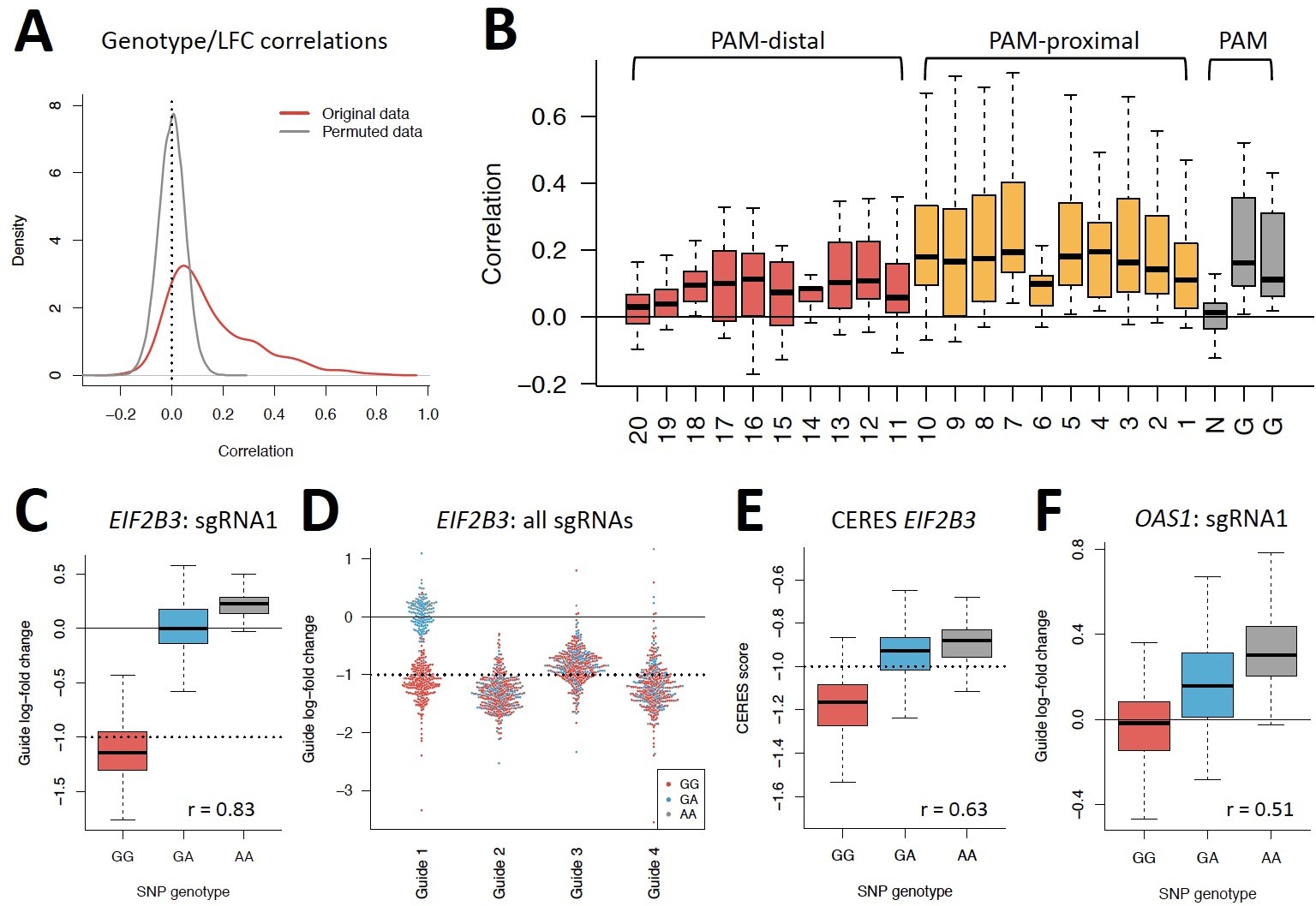


Figure 5: **Effects of genetic variation on guide on-target activity** In the Avana library, 472 guides target protospacer sequences overlapping SNPs assayed by the Affymetrix SNP 6/0 array. **(a)** Distribution of correlations between guide log-fold changes and SNP genotype (genotype/LFC correlations) for the 472 affected guides (red line). A null distribution was obtained from permuting SNP genotypes *B* = 100 times (grey line). **(b)** Genotype/LFC correlations as a function of the SNP location within the protospacer sequence. **(c)** Log-fold changes for a guide targeting *EIF2B3* and overlapping the SNP rs11556200. The genotype GG represents the reference allele found in the GRCh38 genome assembly. **(d)** Log-fold changes for all guides targeting *EIF2B3*. **(e)** CERES score for *EIF2B3* as a function of the SNP rs11556200 genotype.

**(f)** Log-fold change for a guide targeting *OAS1* and overlapping the SNP rs1131454. The genotype GG represents the reference allele found in the GRCh38 genome assembly.

**Response:** We thank the reviewer for suggesting to extend our work to other libraries. We believe generalizing our findings to other libraries adds significant value to the manuscript. We have generated genomic alignments for 3 additional libraries: GeCKOv2, Brunello and Toronto KnockOut v3 (TKOv3), that we provide in the Supplementary Material. We have also downloaded and analyzed publicly available data for both the Brunello and GeCKOv2 libraries to investigate the cleavage toxicity biases observed in the Avana-Achilles dataset. The results hold in these two additional datasets. We could not find a publicly available dataset for the TKOv3 library. We have added the following paragraphs in the Results section, together with a Table and a Supplementary Figure, both provided below for reference. We have also added a description of the datasets in the Methods section.

**Achilles CRISPR dataset (GeCKOv2)** From the Achilles data portal ([https://](https://portals.broadinstitute.org/achilles) [portals.broadinstitute.org/achilles](https://portals.broadinstitute.org/achilles)), we downloaded guide-level log- fold changes across 33 cell lines (file: Achilles v3.3.8.gct for a total of 111,227 guides). As described in [Aguirre et al.](#_bookmark12) [[2016](#_bookmark12)], log-fold changes were nor- malized around the median of negative controls and z-score normalized across cell lines. We further processed the data by correcting the log-fold changes for gene copy number alteration, for each cell line separately, using relative copy numbers provided by CCLE, using the methodology described in [Meyers et al.](#_bookmark22) [[2017](#_bookmark22)].

***Brunello CRISPR library and Brunello-PEL dataset:*** *We downloaded the publicly- available Brunello library guide annotation, described in* [*Doench et al.*](#_bookmark15) *[*[*2016*](#_bookmark15)*], from*

the Addgene website (catalog number: 73179; file: broadgpp-brunello-library-contents.txt). We downloaded raw read counts for 9 CRISPR knockout screens performed in pri-

mary effusion lymphoma (PEL) cell lines, publicly available through the supplemen- tary material of [Manzano et al.](#_bookmark21) [[2018](#_bookmark21)]; we refer to the dataset as the Brunello-PEL

dataset. We filtered out sgRNAs for which there was less than 30 reads in the plas- mid library, and then log-transformed the raw read counts (log2(counts + 1)). We normalized the data across cell lines by centering around the median of guides tar-

geting non-essential genes, and computed log-fold changes (LFCs) by subtracting the log-transformed normalized counts from the plasmid library. Finally, we scaled LFCs using the median LFC of guides targeting essential genes, and averaged LFCs across replicates. The final dataset has LFCs for 75,006 guides across 9 samples.

***Toronto KnockOut CRISPR library v3:*** *We downloaded the publicly-available Toronto KnockOut Library v3 (TKOv3) guide annotation, described in* [*Hart et al.*](#_bookmark18)

[[2017](#_bookmark18)], from the Addgene website (catalog number: 90294; file: tkov3 guide sequence.xlsx).

## Comparison to other genome-wide CRISPR/Cas9 knockout libraries

“To examine whether or not the problem of multiple-target and off-target effects gen- eralizes to other CRISPR knockout libraries and datasets beside the Avana library, we analyzed three additional genome-wide libraries: GeCKO v2 [[Sanjana et al.](#_bookmark25), [2014](#_bookmark25)], Brunello [[Doench et al.](#_bookmark15), [2016](#_bookmark15)] and Toronto KnockOut v3 library (TKOv3) [[Hart et al.](#_bookmark18), [2017](#_bookmark18)]. Each library was designed using a different set of rules and a different design. The GeCKOv2 library, one of the early genome-wide CRISPR

libraries, was designed to include guides with hight specificity by calculating an off- target score based on the number of mismatches in putative off-targeted, as well as the position of the mismatches, with an average of 6 guides per gene. The Brunello library was designed based on an improvement of Rule Set 1 [[Doench et al.](#_bookmark14), [2014](#_bookmark14)], named Rule Set 2, to maximize on-target guide efficacy. A cutting frequency deter- mination (CFD) score was also developed to minimize potential off-target activity. Both scores (Rule Set 2 and CFD scores) were empirically derived from a tiling li- brary targeting all possible protospacers in a set of 15 genes (over 4k sgRNAs), as well as a tiling library with mutated sgRNAs targeting the coding sequence of CD33. In comparison, the Avana library, which was designed using Rule Set 1 only. The TKOv3 library was developed to maximize on-target activity by leveraging sgRNA activity scores from six knockout screens performed using the previous genome-wide Toronto KnockOut v1 library (TKOv1, [[Hart et al.](#_bookmark17), [2015](#_bookmark17)]). Discriminative power between essential and non-essential genes was used to derive activity scores based on sgRNA sequences. Guides targeting more than one gene, as well as guides with protospacer sequence overlapping a common SNP (db138), were filtered out. Guides with single and double-mismatch alignments located in intergenic regions were only included when no better guides could be found for a particular gene.”

“Similar to the Avana library, we generated for each additional library a list of genomic alignments with up to 2 mismatches between the guide sequence and the genomic DNA (GRCh38 assembly). We report several alignment summaries in Ta- ble [2](#_bookmark5). We provide in the Supplementary Material genomic alignments for all three additional libraries. For both the Brunello and GeCKOv2 libraries, CRISPR screens data were publicly available across several cell lines. For the GeCKOv2 library, LFCs across 111,227 guides were available for 33 cell lines that are also part of the Achilles project [[Aguirre et al.](#_bookmark12), [2016](#_bookmark12)]. We present in Supplementary Fig- ure [S3](#_bookmark11)a LFCs averaged across cell lines as a function of on-target alignments for the GeCKOv2 dataset. As in the Avana library, guides with no on-target alignments have LFCs greater than 0, similar to NTCS. LFCs decrease as a function of the number of perfect alignments, confirming cleavage toxicity induced by multiple on-targets ob- served in the Achilles-Avana dataset. For guides with 1 perfect alignment, we also looked at the relationship between LFCs and the number of single-mismatch align- ments, stratified by single mismatch location (PAM-proximal or PAM-distal, Sup- plementary Figure [S3](#_bookmark11)b). The number PAM-distal single-mismatch alignments sub- stantially increase guide activity in comparison to PAM-proximal single-mismatch alignments. We repeated the same exercise for the Brunello library with 9 pub- licly available CRISPR knockout screens performed in primary effusion lymphoma (PEL) cell lines [Manzano et al.](#_bookmark21) [[2018](#_bookmark21)] (see Methods). LFCs also decrease as a function of the number of perfect alignments, and as a function of the number of single-mismatch alignments (Supplementary Figure [S3](#_bookmark11)c-d). We could not find pub- licly available screen data for the TKOv3 library.”

“ We also explored how other libraries compare to the Avana library in terms of multi-target guide design. To do so, we considered for each library the subset of guides targeting the 16,717 genes that are in common between the four libraries. Then, for each library, we estimated the number of genes for which all guides are

multi-target guides; these genes represent genes for which the library-specific design failed at selecting uniquely targeting guides. We found 383 such genes for the Avana library, 689 for the GeCKOv2 library, 360 for the Brunello library, and 122 for the TKOv3 library. Among the 383 genes that cannot be uniquely target in the Avana library, 171 (45%), 198 (52%) and 44 (24%) are shared by the GeCKOv2, Brunello and TKOv3 libraries, respectively. This suggests that many genes cannot be targeted uniquely by CRISPR guides, as revealed by independent guide designs.”

|  | Avana | GeCKOv2 | Brunello | TKOv3 |
| --- | --- | --- | --- | --- |
| Reference | [[Doench et al., 2016](#_bookmark15)] | [[Sanjana et al., 2014](#_bookmark25)] | [[Doench et al., 2016](#_bookmark15)] | [[Hart et al., 2017](#_bookmark18)] |
| Number of unique guides | 73,782 | 119,461 | 77,441 | 71,090 |
| Number of unique NTC guides | 995 | 1,000 | 1,000 | 0 |
| Number of unique guides targeting miRNAs | 0 | 6,835 | 0 | 0 |
| Number of unique guides targeting coding genes | 72,787 | 111,626 | 76,441 | 70,948 |
| Number of targeted coding genes | 18,547 | 19,050 | 19,114 | 18,053 |
| Average number of guides per gene | 4 | 6 | 4 | 4 |
| Number of guides with no on-targets | 86 | 157 | 1 | 16 |
| Number (%) of guides with 1 on-target | 68,742 (94.4%) | 108,368 (97.1%) | 73,410 (96.0%) | 68,872 (97.1%) |
| Number (%) of guides with 2 on-targets | 2,628 (3.6%) | 1,665 (1.5%) | 1,681 (2.2%) | 1,775 (2.5%) |
| Number (%) of guides with *>*2 on-targets | 1,331( 1.8%) | 1,436 (1.3%) | 1,349 (1.8%) | 285 (0.4%) |
| Number (%) of guides with no SM off-targets | 65,070 (89.4%) | 104,965 (94.0%) | 69,407 (90.8%) | 67,584 (95.3%) |
| Number (%) of guides with 1 SM off-target | 5,079 (7.0%) | 4,168 (3.7%) | 4,352 (5.7%) | 2,984 (4.2%) |
| Number (%) of guides with 2 SM off-targets | 1,126 (71.5%) | 1,014 (70.9%) | 1,098 (71.4%) | 281 (70.4%) |
| Number (%) of clean guides (up to 1mm) | 66,757 (91.7%) | 106,538 (95.4%) | 71,614 (93.7%) | 67,886 (95.7%) |

### Table 2: **Summaries of sgRNA sequence alignments across four CRISPR knockout libraries**. For the Avana library, we report summary statistics for the Avana library version used in the Achilles project screens (4 sgRNAs per gene, and processing as described in the Methods section). For the GeCKOv2 library alignment summaries, we excluded 6835 guides targeting miRNAs. A clean guide refers to a guide with only one-target alignment and no single-mismatch alignments. NTC: non-targeting control; SM: single- mismatch.

**Minor comments:**

1. Can the authors discuss more about the DEMETER scores from the Achilles RNAi dataset? On page 9, the authors noted that TMED7, TICAM2, and TMED7-TICAM2 are essential in the CRISPR screen, but non-essential in the RNAi screen and posited that this is because of the additivity assumption in the CERES score. How does the DEMETER score handle the problem? Also, RNAi screens and CRISPR screens are well-known to frequently produce different outcomes, partly because one is a partial knockdown and the other is a complete knockout - it is not clear whether the difference in result between CRISPR screen and RNAi screen at the TMED7-TICAM2 locus may also be due to this.

**Response:** We definitely agree with the reviewer, and we are thankful for the suggestions. We have now rewritten the text to include a much more nuanced discussion of the results:

“With the goal of examining whether or not the CERES estimates for these three genes are biased, we compared the CERES scores to the DEMETER scores avail- able for the Achilles RNAi dataset [[Tsherniak et al.](#_bookmark28), [2017](#_bookmark28)] (Figure 3d). The DEME- TER algorithm was developed to separate on- from off-target effects in pooled RNAi

screens by modeling seed-based off-target effects empirically. Interestingly, the sign of the correlations between the three genes are reversed, and the three genes are broadly estimated as being non-essential genes (DEMETER score 2). This dis- agrees with the CERES scores of TMED7 and TICAM2, which are centered at -1 and therefore are comparable to CERES scores of essential genes. However, we note that DEMETER scores derived from RNAi screens can suffer from similar multicollinear- ity problems. Indeed, the dependency score solutions estimated by the DEMETER model also depend on the short hairpin RNA (shRNA) shRNA design used to tar- get a set of genes. In particular, in the shRNA library used for the Achilles dataset, there is no unique shRNA targeting the genes TMED7, TICAM2 and the readthrough TMED7-TICAM2. In light of this, it is not clear which dataset represents true gene dependencies, but it is clear that both datasets and their modeling approaches suffer from the same reagent design limitations. We also note that as a general guideline, differences in RNAi and CRISPR screens results can also be of biological nature. It has been previously observed that both types of screens can reveal different aspects of biology as observed by essentiality hits falling into different orthogonal biological processes [[Morgens et al.](#_bookmark24), [2016](#_bookmark24), [Smith et al.](#_bookmark27), [2017](#_bookmark27)]. ”

≥ −

### I’m puzzled by Supplementary Table 2, which contains the top 200 self-anti-correlated genes (presumably strong CERES scores indicating that they are essential but low expression levels). Most of these genes do not have anything listed under off-target (column E). Does it mean that these genes do not have any single- mismatch alignments to an alternative member within their gene family or do not have any single-mismatch alignments at all?

**Response:** The Supplementary Table 2 lists the top negatively and self-correlated genes, whether or not they are targeted by guides with off-targets; for this reason, some genes do not have any listed off-targets. For transparency, we have improved the supplementary table in the following way: for each gene, we listed all guides with a single-mismatch alignment with an off-target located in an exon of another gene. We have also added the following paragraph in the Results section, along with a Supplementary Figure (included at the end of the present document) to explain the rationale behind our approach and analyses:

“To investigate how frequent Avana guides targeting transcription and lineage factors have single-mismatch alignments to alternative members of the same gene family, we studied the top genes for which the CERES score is negatively correlated with self-expression of the gene. Our rationale was to first find essential genes that are only expressed in a subset of cell lines, and then investigate off-targets when the latter are expressed in a different subset of cell lines, similar to the SOX9/SOX10 case. We found that several such genes have indeed single-mismatch

alignments located in the exon of another family member: GATA2/GATA3, SOX1/SOX2, DOCK10/DOCK11, UBB/UBC, PAX3/PAX7, TEAD2/TEAD3. For reference, we provide a table of the top 500 self-

anti-correlated genes together with off-target alignments in the Supplementary File 9 . We selected 3 on-target/off-target pairs for which only a subset of cell lines is expressed in either gene: GATA2/GATA3, SOX1/SOX and PAX3/PAX7. We present their expression levels across Achilles cell lines in Supplementary Figure [S2](#_bookmark10) (first column). While there exist cell lines for which these genes are expressed in a mutually exclusive fashion, only one gene for each gene

pair appears to be essential (Supplementary Figure [S2](#_bookmark10), second column) as estimated by LFCs of guides with no single-mismatch alignments (clean guides). This is in contrast with SOX9 and SOX9, which are both essential genes when highly expressed. As a consequence, detecting off- target activity resulting from guides introducing DSBs at these off-target sites cannot be readily detected from knockout screens, with one exception for the PAX7PAX3 pair. Indeed, PAX7 ap- pears to be essential for the rhabdomyosarcoma cell line RD, but not PAX3 (Supplementary Figure [S2](#_bookmark10), bottom row, middle panel). One guide targeting PAX3 has also a single-mismatch alignment to PAX7, and appears to be specifically lethal in the cell line RD, suggesting off-target activity (Supplementary Figure [S2](#_bookmark10), bottom row, right panel).”

### Page 4: A double-target guide doesn’t hit two non-coding regions - is this because the Avana library is designed against protein-coding genes only? It will be good to clarify in the main text.

**Response:** Yes, the Avana library was designed to target protein-coding genes only. We thank the reviewer for suggesting this clarification. We have now added the following statement to the text:

“A double-target guide can either target (a) one coding region and one non-coding region, or

(b) two coding regions. Because guides in the Avana library are designed to target protein- coding genes only, there are no double-target guides targeting two non-coding regions. ”

### Page 5: The authors concluded that a guide disrupting two protein-coding genes is likely to be more lethal than a guide targeting one coding region and one non-coding region. However, there 1734 and 85 guides for coding and non-coding region secondary targets, respectively. Could this simply be due to a *>*10-fold difference in the number of guides between the two categories?

**Response:** This is a good point. The odd-ratios (OR) controls for this *>*10-fold difference by calculating odds of having a lethal guide with a category (coding vs non-coding secondary targets), therefore adjusting for the number of guides in each category, and then comparing the odds between the two categories. This is why we used the Fisher’s exact test to assess whether or not the OR is different from 1: “the number of guides with high activity (guides with LFC

? ?0.5) is significantly higher for the set of guides targeting two coding regions (OR = 4.32, p = 0.00094, Fisher?s exact test) than for the set of guides targeting only one coding region.” instead of comparing directly the number of guides with high activity between categories.

1. Page 11: The authors wrote ”mismatch tolerance between the sgRNA’s protospacer sequence and the genomic DNA”. It should be ”mismatch tolerance between the sgRNA’s spacer sequence and the genomic DNA”.

**Response:** We thank the reviewer for pointing this out. We have now corrected the sentence accordingly. We also went through the manuscript to ensure the proper use of spacer and pro- tospacer. We made sure to use protospacer when we refer to the (spacer+PAM) sequence in the genomic DNA.

1. Page 15: The authors wrote ”perfect alignments between the sgRNA protospacer and genomic DNA”. It should be ”perfect alignments between the sgRNA spacer and genomic DNA”.

**Response:** We thank the reviewer for pointing this out. We have now corrected the sentence accordingly.

1. Page 12: The authors wrote ”One can observe an apparent off-target effect”. I suggest ”We can observe an apparent off-target effect”.

**Response:** We agree with the suggestion, and have changed the text accordingly.

1. Page 16: The authors wrote ”Downstream consequences of such confounding was exemplified”. It should be ”Downstream consequences of such confounding factors were exemplified”.

**Response:** We agree and have changed the text accordingly.

1. Page 16: The authors wrote ”We provide in the Supplementary material a gene-level table summariz- ing the number of on-target and off-target alignments for the Avana library to help readers with flagging potentially problematic genes.” Presumably, the authors are referring to Supplementary Table 3?

**Response:** We thank the reviewer for pointing this out. We have now changed the sentence accordingly: *“We provide in Supplementary Table 3 a gene-level table summarizing the num- ber of on-target and off-target alignments for the Avana library to help readers with flagging potentially problematic genes. ”*

***Reviewer #2****:*

1) The authors note the potential pitfalls of guides with multiple targets, but how much of this dual-gene targeting is actually avoidable in library design? In other words, for some gene pairs, it may impossible to target one without also targeting another. Could the authors characterize the extent of this phenomenon? Re- lated, to what extent do other, more-recent libraries show this level of multi-gene targeting in their designs? Although this analysis is not relevant to the Achilles data per se, it would be very helpful for people using those other libraries, as well as future library development. Indeed, they conclude section 3.4 by noting ”These two examples suggest that multi-targeting can lead to guide design-dependent co-dependencies and misleading biases that have to be interpreted with caution in downstream applications such as identifying gene networks and cancer cell dependencies.” For such gene pairs, the authors should provide designs that avoid this problem.

**Response:** We agree with the reviewer that for many pairs of genes, it is hard to design guides that uniquely map to either gene because of high homology. We have attempted in our new re- vised manuscript to nuance our discussion of the matter by acknowledging the problem and the fact that there might not be a solution. However, we believe it is still important to be aware of limitations of current guide designs, especially when large efforts such as the Achilles project is limited to one guide design. To investigate the extent of the problem in other libraries, we have extended our analyses to three other libraries, Brunello, GeCKOv2 and Toronto KnockOut v3 (TKOv3), for which we have generated genomic alignments for all guides; these genomic align- ments are included in the Supplementary files. We have added a new section in the manuscript describing our analyses of these other libraries, which was also a suggestion proposed by Re- viewer 1. Please see our answer to Reviewer 1’s Question 5. It appears that all libraries suffer from multiple-target alignments, and that perhaps not surprisingly, a number of genes shared across all 4 libraries cannot be uniquely targeted at all. We have added the following paragraph in the Results section:

We also explored how other libraries compare to the Avana library in terms of multi- target guide design. To do so, we considered for each library the subset of guides

targeting the 16,717 genes that are in common between the four libraries. Then, for each library, we estimated the number of genes for which all guides are multi-target guides; these genes represent genes for which the library-specific design failed at selecting uniquely targeting guides. We found 383 such genes for the Avana library, 689 for the GeCKOv2 library, 360 for the Brunello library, and 122 for the TKOv3 library. Among the 383 genes that cannot be uniquely target in the Avana library, 171 (45%), 198 (52%) and 44 (24%) are shared by the GeCKOv2, Brunello and TKOv3 libraries, respectively. This suggests that many genes cannot be targeted uniquely by CRISPR guides, as revealed by independent guide designs.

### We have also added the following discussions and recommendations in the Discussion section:

Similar to the CN correction algorithm implemented in [Meyers et al.](#_bookmark22) [[2017](#_bookmark22)], one could attempt to systematically correct for multi-target and off-target toxicity by re- moving the observed effects for each cell line separately. This apppears to be a sensible approach when additional targets are located in intergenic regions. Indeed, for such regions, introducing additional DSBs are not likely to cause spurious phe- notypic effects besides the toxicity induced by DSBs, and predicted cleavage toxicity can be subtracted from the observed LFCs. This rationale was used recently in the design of a genome-wide CRISPR library [[Hart et al.](#_bookmark18), [2017](#_bookmark18)], in which inclusion of guides with additional on-targets located in intergenic regions were allowed, in contrast to guides with additional on-targets located in other genes.

Because of genetic interactions, correcting depletion scores for multi-target guides targeting several protein-coding regions is not straightforward. As opposed to cleav- age toxicity induced by differential CN, the increased activity observed in multi- target guides depends on the set of targeted genes; these genes can interact with each other in a cell line-specific manner, excluding the possibility of fitting a global genetic interaction correction model across cell lines. Therefore, we do not rec- ommend to correct LFCs for these multi-target guides. One solution is to remove guides that do not map uniquely to the genome when calculating a gene-level essen- tiality score; 383 genes would have to be excluded in the Avana library because of the absence of uniquely-targeting guides for these genes. A large number of these genes also cannot be targeted uniquely by the three other libraries that we analyzed. On the other hand, we note that multi-target guides could be still further analyzed separately, since such guides can be informative about multigenic knockout, such as co-targeting known paralogs. These guides should be annotated separately, and may potentially be further utilized to help design a library of guides co-targeting paralogs.

In terms of library design, including guides that neither have multiple protein- coding on-targets nor predicted single-mismatch off-targets for all targeted genes is not an easy task. This is even more challenging when additional constraints, such as on-target efficiency threshold or exclusion of guides targeting protospacer sequence overlapping common variants, are considered. For instance, while the two par- alogs MYL12A and MYL12B are targeted by a total of 8 guides in the Avana library, MYL12A and MYL12B are respectively targeted by 0 and 1 guides in the TKOv3

library; no other guides satisfied the library design criteria in terms of specificity and on-target efficacy. When possible, we recommend to lower criteria related to on-target efficiency and allow less active but specific guides to be included for genes that are more challenging to target, such as highly homologous genes.

**Minor comments:**

### It is often confusing as to when the authors are using log-fold change (LFC) vs copy-number corrected LFC vs CERES score and other similar-sounding terms. I would recommend defining those terms more explicitly and sticking with a consistent nomenclature. Related, in Figure 1b,c the y-axis is explicit as to whether the LFC or CERES score is being plotted. However, in Figure 2b,d, they annotate the y-axis as LFC, but is this copy-number corrected or not? If not, the cutoff of -1 (dotted horizontal line) is only meaningful for CERES corrected values.

**Response:** We agree with the reviewer that our nomenclature was ambiguous and would benefit from clarifications. In our analyses and figures, we only use copy-number corrected log-fold changes (LFCs) throughout the manuscript, and these LFCs were also scaled so that the median CN-corrected LFC for essential genes is -1, similar to the scaling transformation used for the CERES score. To make this clear to readers, we have added the following statements in the Methods section:

“ Since we are interested in visualizing and analyzing LFCs, we further scaled LFCs by the absolute average LFC value across cell lines for guides targeting essential genes (212 genes total, [[Hart et al.](#_bookmark16), [2014](#_bookmark16)]), such that a value of -1 roughly indicates essentiality. For each cell line separately, we corrected LFCs for gene copy number alteration using relative copy numbers provided by CCLE using the methodology described in [Meyers et al.](#_bookmark22) [[2017](#_bookmark22)]. Throughout the manuscript, all log-fold changes for the Achilles dataset are corrected for copy number, and we therefore refer to the CN-corrected log-fold changes simply as “log-fold changes” or “LFCs”.”

### Accordingly, we have changed all axis labels to reflect these changes, and have removed the terminology “CN-corrected” throughout the manuscript. The only time in the manuscript where we apply an additional transformation to the log-fold changes is in Figure 2. We further adjusted LFCs for cleavage toxicity induced by multiple on-targets DSBs. We have changed the label and title to make this additional transformation explicit using “log-fold changes adjusted for on-target toxicity”. We hope this clarifies our terminology.

1. In figure 1a it is hard to tell the actual number of guides is, as the count of single-target aligned guides surpasses the y-axis.

**Response:** We agree with the reviewer. We have fixed the figure by adding the number of guides in top of each histogram bin (figure included below).

1. Figure 1f may be easier to interpret as one median line with a shaded interval representing the range of possible cell-line-specific curves.

**Response:** This is a great suggestion. We have now changed the figure accordingly (included below).


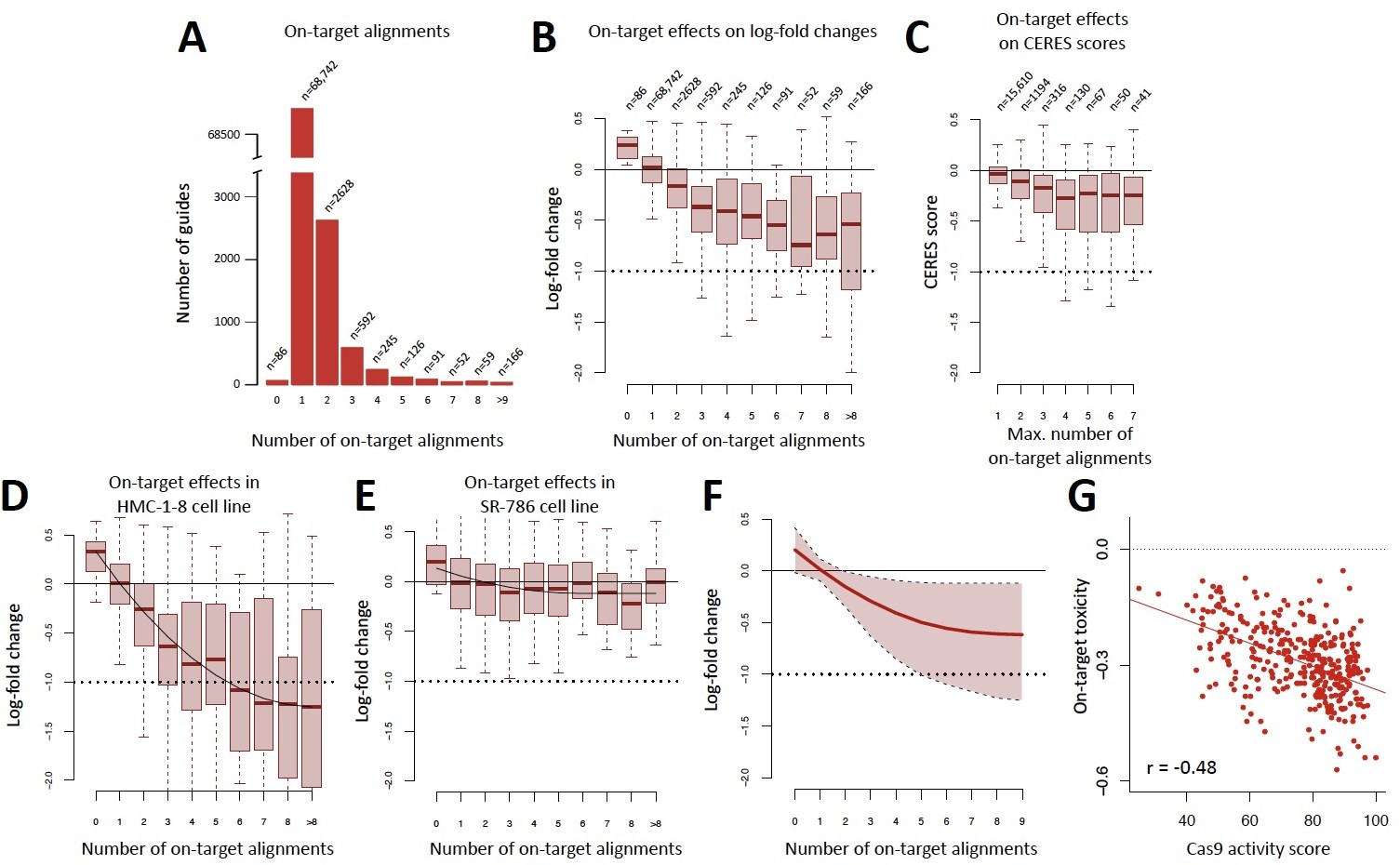


Figure 6: **The impact of multiple on-targets on sgRNA log-fold changes. (a)** Guide numbers as a function of the number of perfect alignments (on-target alignments); non-targeting controls (NTCs) were excluded from this analysis. **(b)** Guide-level log-fold changes (LFCs) averaged across cell lines as a function of the number of on-targets. The number of on-targets was calculated as the number of perfect alignments between the reference genome and the 20-nt spacer; we excluded guides with single-mismatch alignments to prevent the confounding effect of single-mismatch off-targets. **(c)** Combined effect of multiple on-targets on the CERES score. For each gene, we calculated the maximum number of targeted loci (x-axis) as the maximum number of perfect alignments for a guide designed to target that gene in the Avana library. **(d-e)** Effects of multiple on-target alignments on LFCs in the breast cancer cell line HMC-1-8 and in the lymphoma cell line SR-786. Solid lines represent second-degree polynomial fits (see Methods). **(f)** Distribution of cell-specific fitted on-target activity (average log-fold changes) as a function of the number of on-target alignments (solid line: median across cell lines; shaded area: full range of on-target activity across cell lines). **(g)** Average on-target toxicity as a function of Cas9 activity score.

1. The second paragraph of section 3.4, the authors use “iterative” redundantly.

**Response:** We thank the reviewer for pointing this out; we have now changed the following sentence *“an iterative least squares approach is used to iteratively solve for guide-specific parameters and gene essentiality scores.”*

### for

“an iterative least squares approach is used to solve for guide-specific parameters and gene essentiality scores. ”

### For the data in figure 2b and 2d, could the authors also compare Guide AB1 and Guide AB2 as a scatter plot, to see how consistent this effect is within each cell line?

**Response:** We have now added a scatterplot comparing LFCs for Guides AB1 and AB2. The two guides correlate well (*r* = 0*.*61). We have included the updated figure below for reference.

1. In figure 2d how was the adjustment for LFC made? Is it just a subtraction of the curves in fig. 1f?

**Response:** Yes, and we apologize for the lack of details in the previous version of the manuscript. On-target cleavage toxicity induced by multiple on-targets was estimated for each cell line us- ing the fitted curves from Fig 1f, and then subtracted. We have now included the following sentence in the manuscript for clarification:

“” On-target cleavage toxicity induced by multiple on-targets was first estimated for each cell line using the fitting curves from Fig 1f, and then subtracted from each guide’s log-fold change according to their respective number of multiple on-target alignments.

### In section 3.4 the authors compare essentiality predictions between CERES and DEMETER scores. In this analysis, the authors should mention that these differences could be due to biological differences between CRISPR and RNAi screens as opposed to erroneous model assumptions.

**Response:** We agree with the reviewer. This question was also raised by Reviewer 1. We thank both reviewers for suggesting a nuance interpretation of the results. We have now added the following discussion in the Results section:

“With the goal of examining whether or not the CERES estimates for these three genes are biased, we compared the CERES scores to the DEMETER scores avail- able for the Achilles RNAi dataset [[Tsherniak et al.](#_bookmark28), [2017](#_bookmark28)] (Figure 3d). The DEME- TER algorithm was developed to separate on- from off-target effects in pooled RNAi screens by modeling seed-based off-target effects empirically. Interestingly, the sign of the correlations between the three genes are reversed, and the three genes are broadly estimated as being non-essential genes (DEMETER score 2). This dis- agrees with the CERES scores of TMED7 and TICAM2, which are centered at -1 and therefore are comparable to CERES scores of essential genes. However, we note that DEMETER scores derived from RNAi screens can suffer from similar multicollinear- ity problems. Indeed, the dependency score solutions estimated by the DEMETER model also depend on the short hairpin RNA (shRNA) shRNA design used to tar- get a set of genes. In particular, in the shRNA library used for the Achilles dataset, there is no unique shRNA targeting the genes TMED7, TICAM2 and the readthrough TMED7-TICAM2. In light of this, it is not clear which dataset represents true gene

≥ −

dependencies, but it is clear that both datasets and their modeling approaches suffer from the same reagent design limitations. We also note that as a general guideline, differences in RNAi and CRISPR screens results can also be of biological nature. It has been previously observed that both types of screens can reveal different aspects of biology as observed by essentiality hits falling into different orthogonal biological processes [[Morgens et al.](#_bookmark24), [2016](#_bookmark24), [Smith et al.](#_bookmark27), [2017](#_bookmark27)]. ”

### In Figure 6, please show all 4 guides for SOX9, not just two of them. Also, what does the guide/target alignment look like for these four guides and SOX10 (i.e. where in the sequence are the mismatches)?

**Response:** This is a great suggestion, and we have now performed a more comprehensive analysis of the *SOX9* and *SOX10* guides. We have now added the following section in the Results section with an improved figure and table describing the different alignments for these guides:

“In Figure [8](#_bookmark8)b, we show the design and alignments of guides targeting SOX9 and SOX10 in the Avana library. Out of 4 guides targeting SOX9, 3 guides also have a single-mismatch alignment to SOX10. Conversely, out of 4 guides targeting SOX10, 3 guides also have a single-mismatch alignment to SOX10. One of the guides (B4) has an additional single-mismatch alignment to SOX8. Sequence alignments for these guides are provided in Table [3](#_bookmark7). We present in Figure [8](#_bookmark8)c LFCs of these 8 guides as a function of SOX9 expression, and color cell lines highly expressing SOX10 (log_2_(rpkm + 1) 4) in red. We also provide LOWESS fits for cell lines lowly or not expressing SOX10 to visualize SOX9 dependencies.

≥

“LFCs for guide A1, which targets SOX9 without off-targets, show a clear dependency on SOX9 expression, and cell lines highly expressing SOX10 do not show activity. Conversely, LFCs for guide B1, which targets SOX10 without off-targets, show no dependency on SOX9 expression, and cell lines highly expressing SOX10 are highly sensitive to knockout. LFCs for guides A2 and A4, which both have SOX9 has an on-target and SOX10 as an off-target, show a dependency on SOX9 expression for cell lines lowly or not expressing SOX10, as seen by the LOWESS fits. In addition, cell lines highly expressing SOX10 are also sensitive to gene knockout induced by these guides. This suggests that the CRISPR/Cas9 system tolerates single- mismatch in guides A2 and A4, and that Cas9 cutting occurs at the off-target SOX10 and results in off-target activity.”

“While guide A3 also has a single-mismatch off-target alignment to SOX10, none of the cell lines that highly express SOX10 is sensitive to guide A3-induced knockout. This suggests that the CRISPR/Cas9 system is intolerant to the single-mismatch occurring in Guide A3. This is consistent with LFCs of guide B3. Indeed, the spacer sequence of guide B3, which targets SOX10 and has a single-mismatch alignment to SOX9, is nearly identical to the spacer sequence of guide A3, except at the nucleotide position causing the single-mismatch alignments. By inspecting LFCs of guide B3, we observe that cell lines highly expressing SOX9 are not sensitive to guide B3, therefore confirming that the CRISPR/Cas9 system does not tolerate the single- mismatch in guide B3 because, leading to no off-target activity. We also note that Guide B4 also shares nearly the same spacer sequence as Guide A4. As for Guide A4, the CRISPR/Cas9 system appears to tolerate the single-mismatch, as seen by the dependency of guide B4’s LFCs

on SOX9 expression (Figure [8](#_bookmark8)c, bottom panel, right plot). We summarize these findings in Figure [8](#_bookmark8)d.

| Type | | On-target | Off-target | Spacer sequence | BaseSub | Chr | Pos |
| --- | --- | --- | --- | --- | --- | --- | --- |
| Guide A1 | PM | *SOX9* |  | GCTCGGACACCGAGAACACG | G → A C → G A → G | 17  17  22  17  22  17  22 | 72,121,509  72,122,775  37,978,058  72,121,612  37,983,546  72,122,793  37,978,040 |
| Guide A2 | PM | *SOX9* |  | GCAGCACAAGAAGGACCACC |  |  |  |
|  | MM |  | *SOX10* | GCAGCACAAGAA-GACCACC |  |  |  |
| Guide A3 | PM | *SOX9* |  | GCACCTGGCTGACCGCCTCG |  |  |  |
|  | MM |  | *SOX10* | GCACCTGGCTGAC-GCCTCG |  |  |  |
| Guide A4 | PM | *SOX9* |  | GCTGGTACTTGTAATCCGGG |  |  |  |
|  | MM |  | *SOX10* | GCTGGTACTTGTA-TCCGGG |  |  |  |
| Guide B1 | PM | *SOX10* |  | ACAAGTACCAGCCCAGGCGG | G → C  G → A C → G | 22  22  22  17  22  17  16 | 37,978,032  37,977,942  37,983,546  72,121,612  37,978,040  72,122,793  983,802 |
| Guide B2 | PM | *SOX10* |  | GTAGTGGGCCTGGATGGCGG |  |  |  |
| Guide B3 | PM | *SOX10* |  | GCACCTGGCTGACGGCCTCG |  |  |  |
|  | MM |  | *SOX9* | GCACCTGGCTGAC-GCCTCG |  |  |  |
| Guide B4 | PM | *SOX10* |  | GCTGGTACTTGTAGTCCGGG |  |  |  |
|  | MM |  | *SOX9* | GCTGGTACTTGTA-TCCGGG |  |  |  |
|  | MM |  | *SOX8* | GCTGGTACTTGTAGTC-GGG |  |  |  |

### Table 3: Genomic alignments for guides targeting *SOX9* and *SOX10* in the Avana library. PM: perfect match alignment; MM: single-mismatch alignment; BaseSub: base substitution in the protospacer sequence.

1. Everywhere in the paper except the discussion the authors cite data from 391 cell lines and 72,787 guides, but in the discussion they cite 342 cell lines and 75,000+ guides. Could the authors clarify?

**Response:** We have now corrected the typo in the present manuscript: *“Using LFCs from the Achilles dataset, across 342 cell lines and more than 72k sgRNAs (Avana library), we found that guide depletion increases as a function of the number of targeted loci in the genome. ”*


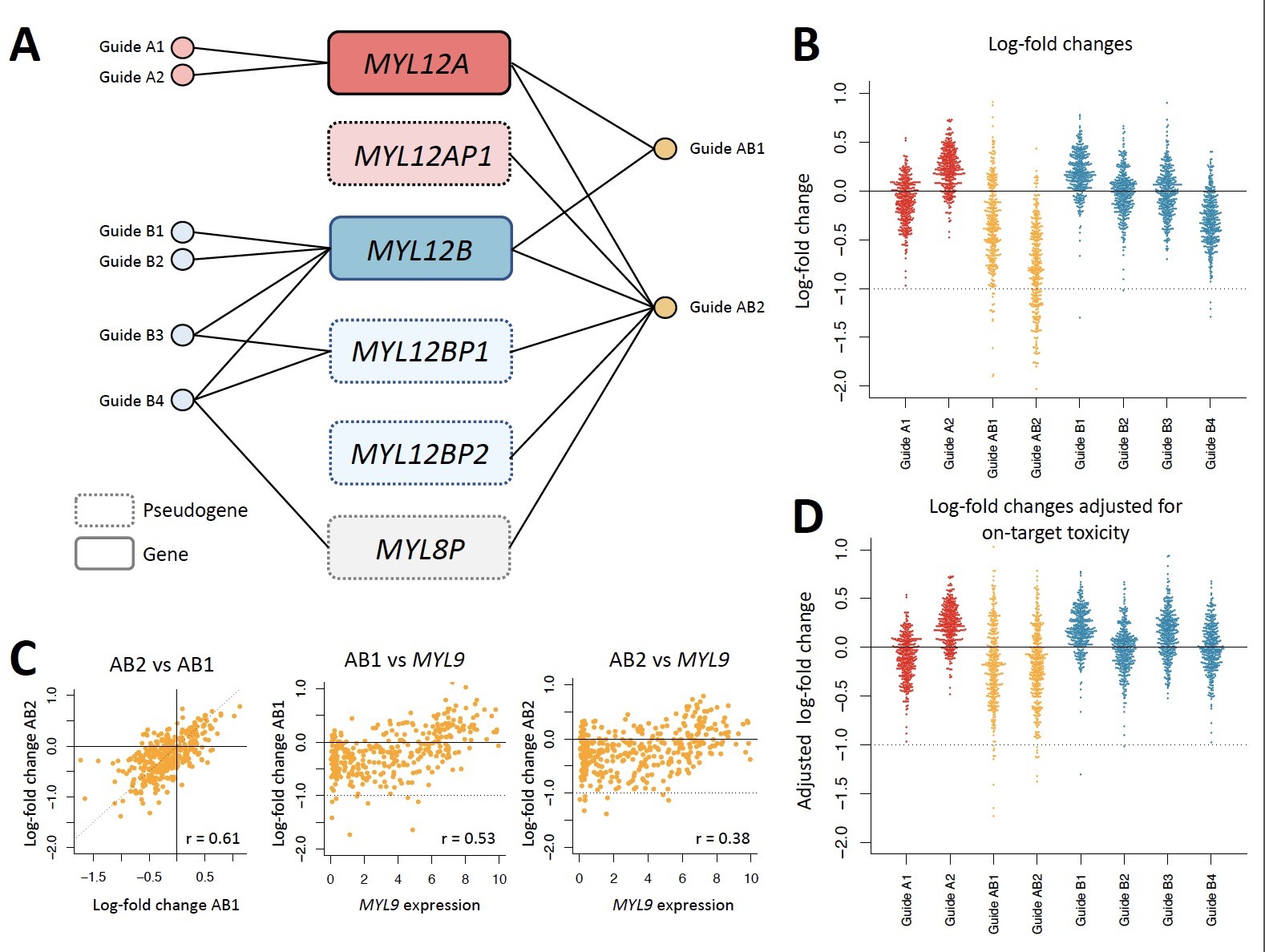


### Figure 7: **The interplay between multiple alignments and synthetic lethality in the Avana library (a)** Genomic mapping of the Avana guides targeting *MYL12A* and *MYL12B*. Guides A1 and A2 map uniquely to *MYL12A* and guides B1 and B2 map uniquely to *MYL12B*. Guides B3 and B4 map to *MYL12B*, but also to additional non-functional pseudogenes. Guides AB1 and AB2 map to both *MYL12A* and *MYL12B*. **(b)** Copy number-corrected LFCs for guides mapping to either *MYL12A* or *MYL12B*, or to both, in the Avana library, across 391 cell lines; each dot represents a cell line. **(c)** First panel: scatterplot of the log-fold changes between the two guides mapping to both *MYL12A* and *MYL12B*. Second and third panels: relationship between guide-specific log-fold changes and *MYL9* expression for guide AB1 and AB2, respectively. **(d)** Same as (b), but after adjusting for cell-specific cleavage toxicity induced by multiple on-target alignments.


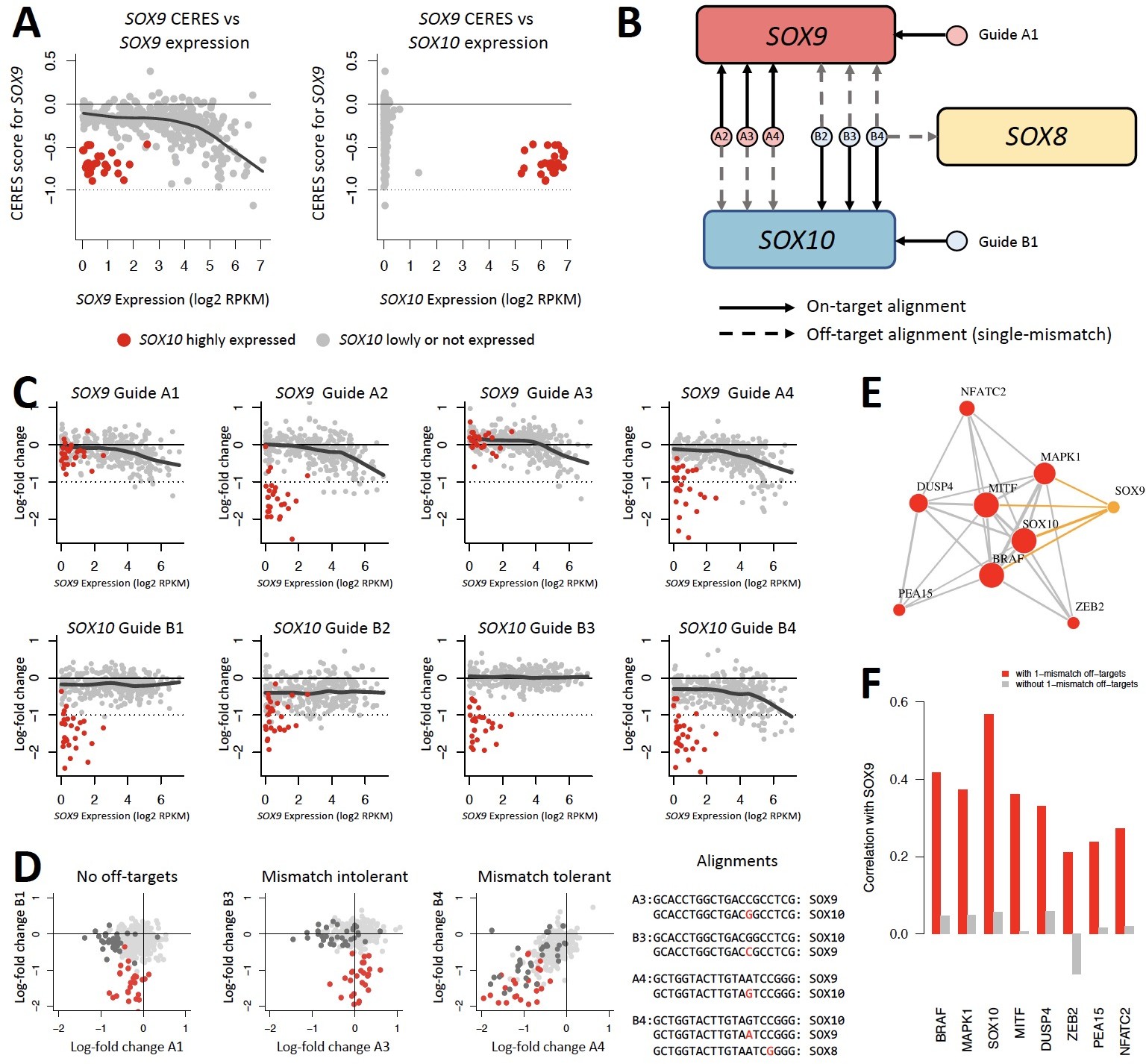


Figure 8: **The effects of single-mismatch sgRNA-DNA tolerance on gene essentiality (a)** On the left: CERES score for *SOX9* plotted against *SOX9* expression; each dot is a cell line, and cell lines highly ex- pressing *SOX10* (*log*_2_(rpkm + 1) 4) are colored in red. The curve represents a LOWESS estimated with cell lines lowly or not expressing *SOX10* (grey dots). On the right: CERES score for *SOX9* plotted against *SOX10* expression. **(b)** Guide design for guides targeting *SOX9* and *SOX10* in the Avana library. **(c)** Log-fold changes (LFCs) of guides targeting *SOX9* and *SOX10* as a function of *SOX9* expression. Curves represent LOWESS fit estimated with cell lines lowly or not expressing *SOX10* (grey dots). **(d)** *BRAF*-associated coessentiality cluster using between-gene CERES Pearson correlations. A correlation cutoff of *r* = 0*.*35 was chosen to draw edges. Edges associated with *SOX9* (orange) disappear when excluding *SOX9*-targeting guides with single-mismatch tolerant *SOX10* off-targets. **(e)** Comparison of single-mismatch tolerant and intolerant guides targeting *SOX9* and *SOX10*/ **(f)** Pearson correlations between the *SOX9*’s CERES score and CERES scores for genes that are part of *BRAF*-associated cluster, before and after removal of *SOX9*- targeting guides with single-mismatch tolerant *SOX10* off-targets.

≥

**Supplementary Tables and Figures**


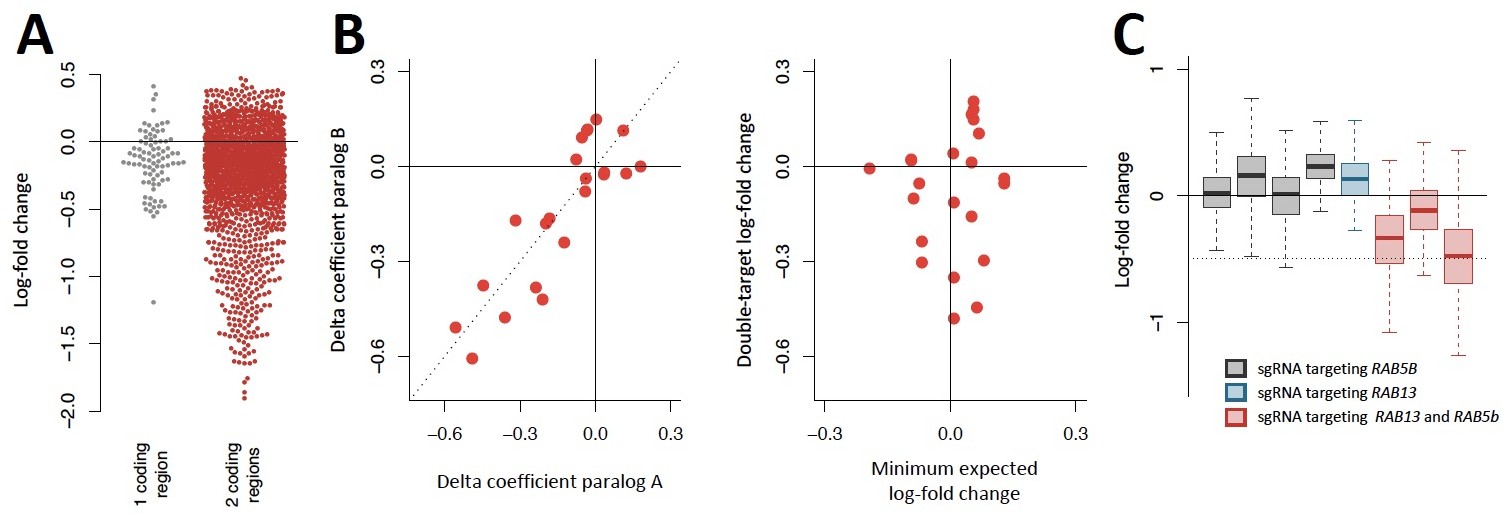


Supplementary Figure S1: **Log-fold changes for double-target guides** (a) Log-fold changes averaged across cell lines for guides mapping to two genomic loci (double-target guides). The grey dots represent double-guides for which only one of the two targets is located in a coding region, and they red dots repre- sent double-target guides for which both targets are located in coding regions. (b) Among the 297 guides co-targeting a pair of paralog genes, we further examined 22 such guides for which there was at least one additional “clean” guide (guide with no single or double-mismatch alignments) for each of the gene in the paralog pair to be able to compare the effects of a digenic knockout in comparison to single-gene knockouts. For each guide, we computed an average log-fold change difference between the double-target guide and each of the single-knockout guide using the delta coefficient described in the Methods section. A larger negative delta coefficient indicates greater activity for the double-target knockout. On the left panel, we plot the delta coefficient estimated with respect to the set of clean guides targeting the second paralog (y- axis) versus the delta coefficient estimated with respect to the set of clean guides targeting the first paralog (x-axis). On the right panel, we plot log-fold changes of double-target guides (y-axis) as a function of the minimum additive log-fold change predicted from the set of clean guides targeting each paralog separately (x-axis). (c) Log-fold changes for single-target guides targeting the paralogs *RAB5B* and *RAB13*, as well as double-target guides targeting both *RAB5B* and *RAB13*. Each boxplot shows log-fold changes across the Achilles cell lines.


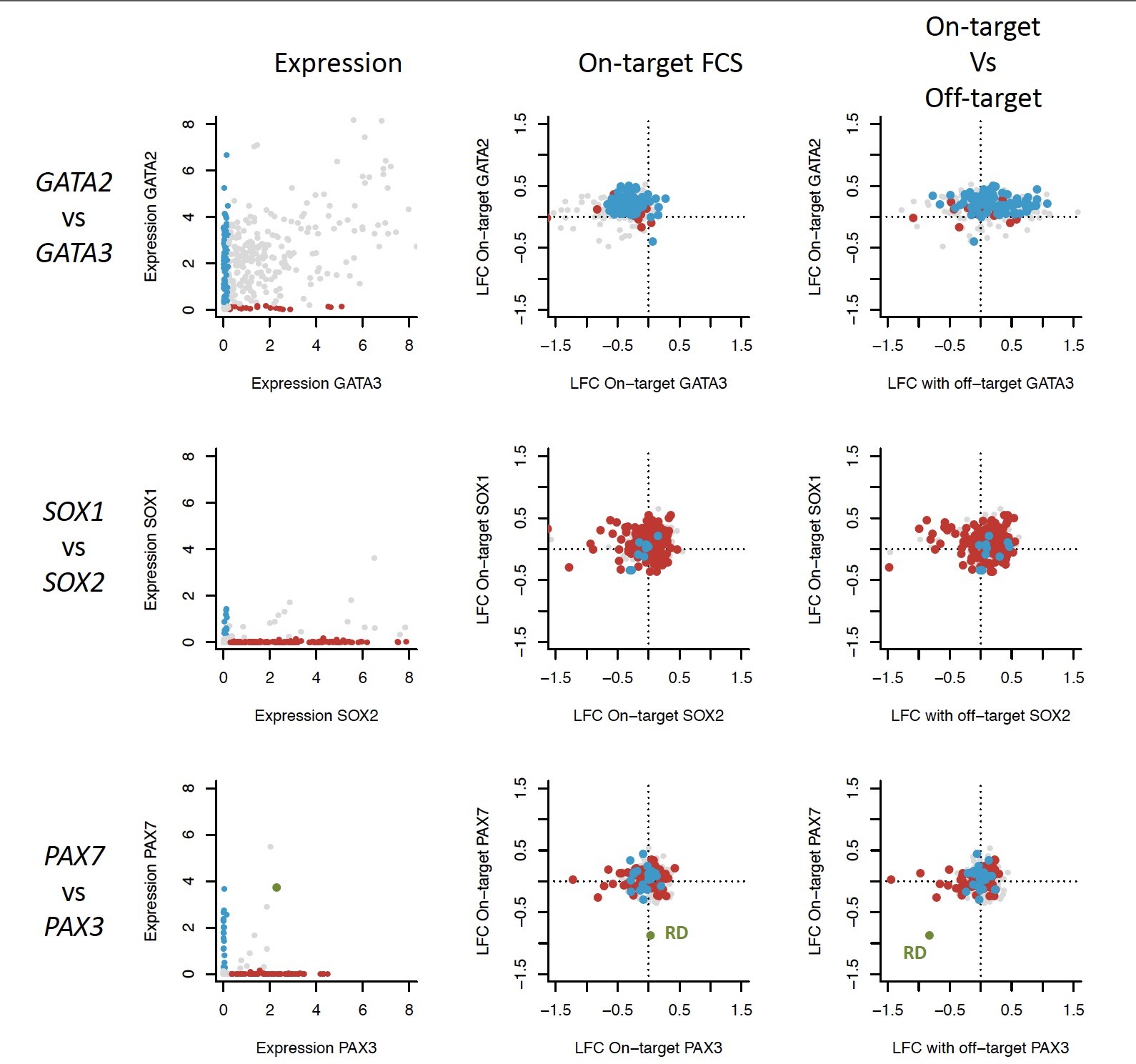


Supplementary Figure S2: **Off-target effects for guides targeting 3 self-correlated genes** The CERES score for *GATA3*, *SOX2* and *PAX3* are negatively and highly correlated with their own gene expression. For each gene, one guide in the Avana library has a single-mismatch alignment to another member of their gene family (*GATA2*, *SOX1* and *PAX7*, respectively). For each row, we show in the left panel the expression levels of each pair of genes. We draw in blue cell lines for which the off-target gene is expressed, but not the on-target gene, and vice-versa for cell lines drawn in red. On the middle panel, we plot the average log-fold change (LFC) of clean guides targeting the off-target gene with perfect alignments against the average LFC for clean guide targeting the on-target gene with perfect alignments. In the right panel, we plot on the y-axis the average LFC for clean guides targeting the off-target gene, and we plot on the x-axis the LFC for the problematic guide targeting the on-target gene with an off-target single-mismatch alignment to the off-target gene; in case of off-target activity, these LFCs should correlate better than the LFCs presented in the center plots.


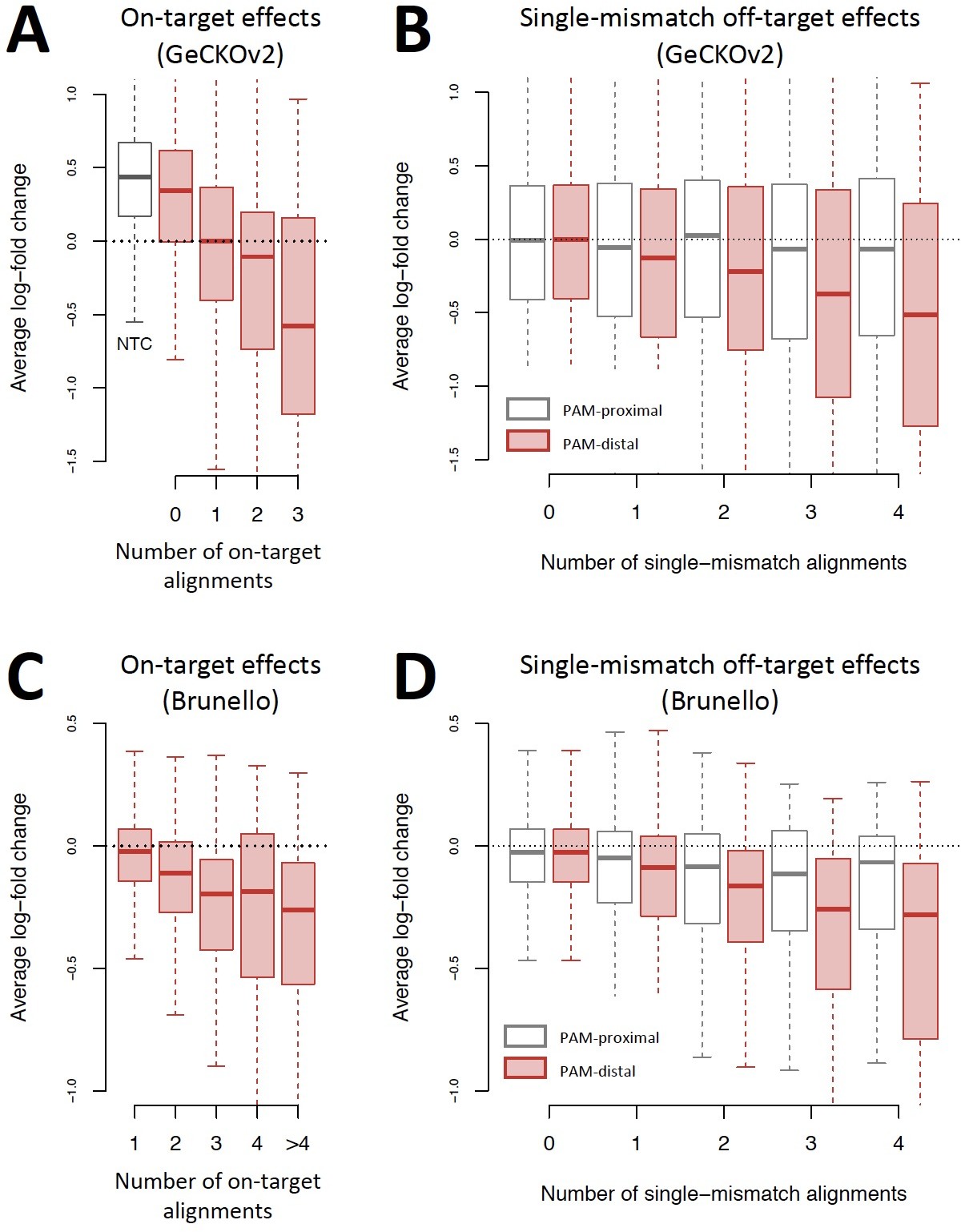


Supplementary Figure S3: **On-target and off-target cleavage toxicity in the GeCKOv2-Achilles and Brunello datasets. (a)** Guide-level log-fold changes (LFCs) as a function of the number of on-target align- ments in the GeCKOv2-Achilles dataset. LFCs were averaged across all 33 cell lines. NTC: non-targeting controls (*n* = 1000). **(b)** LFCs of guides with 1 perfect alignment stratified by the number of single- mismatch alignments and by spacer location: PAM-proximal (positions 1 to 10) or PAM-distal (position 11 to 20), in the GeCKOv2-Achille dataset. **(c)** Same as (a), but for the Brunello dataset (9 primary effusion lymphoma (PEL) cell lines). **(d)** Same as (b), but for the Brunello dataset.

**References**

Andrew J Aguirre, Robin M Meyers, Barbara A Weir, Francisca Vazquez, Cheng-Zhong Zhang, Uri Ben- David, April Cook, Gavin Ha, William F Harrington, Mihir B Doshi, et al. Genomic copy number dictates a gene-independent cell response to crispr/cas9 targeting. *Cancer discovery*, 6(8):914–929, 2016.

Matthew C Canver, Samuel Lessard, Luca Pinello, Yuxuan Wu, Yann Ilboudo, Emily N Stern, Austen J Needleman, Fre´de´ric Galacte´ros, Carlo Brugnara, Abdullah Kutlar, et al. Variant-aware saturating mu- tagenesis using multiple cas9 nucleases identifies regulatory elements at trait-associated loci. *Nature genetics*, 49(4):625, 2017.

John G Doench, Ella Hartenian, Daniel B Graham, Zuzana Tothova, Mudra Hegde, Ian Smith, Meagan Sullender, Benjamin L Ebert, Ramnik J Xavier, and David E Root. Rational design of highly active sgrnas for crispr-cas9–mediated gene inactivation. *Nature biotechnology*, 32(12):1262, 2014.

John G Doench, Nicolo Fusi, Meagan Sullender, Mudra Hegde, Emma W Vaimberg, Katherine F Donovan, Ian Smith, Zuzana Tothova, Craig Wilen, Robert Orchard, et al. Optimized sgrna design to maximize activity and minimize off-target effects of crispr-cas9. *Nature biotechnology*, 34(2):184, 2016.

Traver Hart, Kevin R Brown, Fabrice Sircoulomb, Robert Rottapel, and Jason Moffat. Measuring error rates in genomic perturbation screens: gold standards for human functional genomics. *Molecular systems biology*, 10(7):733, 2014.

Traver Hart, Megha Chandrashekhar, Michael Aregger, Zachary Steinhart, Kevin R Brown, Graham MacLeod, Monika Mis, Michal Zimmermann, Amelie Fradet-Turcotte, Song Sun, et al. High-resolution crispr screens reveal fitness genes and genotype-specific cancer liabilities. *Cell*, 163(6):1515–1526, 2015.

Traver Hart, Amy Hin Yan Tong, Katie Chan, Jolanda Van Leeuwen, Ashwin Seetharaman, Michael Areg- ger, Megha Chandrashekhar, Nicole Hustedt, Sahil Seth, Avery Noonan, et al. Evaluation and design of genome-wide crispr/spcas9 knockout screens. *G3: Genes, Genomes, Genetics*, pages g3–117, 2017.

Joshua M Korn, Finny G Kuruvilla, Steven A McCarroll, Alec Wysoker, James Nemesh, Simon Cawley, Earl Hubbell, Jim Veitch, Patrick J Collins, Katayoon Darvishi, et al. Integrated genotype calling and association analysis of snps, common copy number polymorphisms and rare cnvs. *Nature genetics*, 40 (10):1253, 2008.

Samuel Lessard, Laurent Francioli, Jessica Alfoldi, Jean-Claude Tardif, Patrick T Ellinor, Daniel G MacArthur, Guillaume Lettre, Stuart H Orkin, and Matthew C Canver. Human genetic variation alters crispr-cas9 on-and off-targeting specificity at therapeutically implicated loci. *Proceedings of the National Academy of Sciences*, page 201714640, 2017.

Mark Manzano, Ajinkya Patil, Alexander Waldrop, Sandeep S Dave, Amir Behdad, and Eva Gottwein. Gene essentiality landscape and druggable oncogenic dependencies in herpesviral primary effusion lymphoma. *Nature communications*, 9(1):3263, 2018.

Robin M Meyers, Jordan G Bryan, James M McFarland, Barbara A Weir, Ann E Sizemore, Han Xu, Neekesh V Dharia, Phillip G Montgomery, Glenn S Cowley, Sasha Pantel, et al. Computational correc- tion of copy number effect improves specificity of crispr-cas9 essentiality screens in cancer cells. *Nature Genetics*, 2017.

Huaiyu Mi, Xiaosong Huang, Anushya Muruganujan, Haiming Tang, Caitlin Mills, Diane Kang, and Paul D Thomas. Panther version 11: expanded annotation data from gene ontology and reactome pathways, and data analysis tool enhancements. *Nucleic acids research*, 45(D1):D183–D189, 2016.

David W Morgens, Richard M Deans, Amy Li, and Michael C Bassik. Systematic comparison of crispr/cas9 and rnai screens for essential genes. *Nature biotechnology*, 34(6):634, 2016.

Neville E Sanjana, Ophir Shalem, and Feng Zhang. Improved vectors and genome-wide libraries for crispr screening. *Nature methods*, 11(8):783, 2014.

David A Scott and Feng Zhang. Implications of human genetic variation in crispr-based therapeutic genome editing. *Nature medicine*, 23(9):1095, 2017.

Ian Smith, Peyton G Greenside, Ted Natoli, David L Lahr, David Wadden, Itay Tirosh, Rajiv Narayan, David E Root, Todd R Golub, Aravind Subramanian, et al. Evaluation of rnai and crispr technologies by large-scale gene expression profiling in the connectivity map. *PLoS biology*, 15(11):e2003213, 2017.

Aviad Tsherniak, Francisca Vazquez, Phil G Montgomery, Barbara A Weir, Gregory Kryukov, Glenn S Cowley, Stanley Gill, William F Harrington, Sasha Pantel, John M Krill-Burger, et al. Defining a cancer dependency map. *Cell*, 170(3):564–576, 2017.

Guanqun Wang, Meijie Du, Jianbin Wang, and Ting F Zhu. Genetic variation may confound analysis of crispr-cas9 off-target mutations. *Cell discovery*, 4(1):18, 2018.
