# Supplementary material for: Multiple-gene targeting and mismatch tolerance can confound analysis of genome-wide pooled CRISPR screens
Source: Genome Biol. 2019 Jan 25;20:21. doi: 10.1186/s13059-019-1621-7 (PMC6346559; doi:10.1186/s13059-019-1621-7)
Supplement: Supplementary file 11 — Review history. (DOCX 2.8 mb) [file 13059_2019_1621_MOESM11_ESM.docx]

"","sgrna","chr","start","strand","mismatches","pam","ref","n.mismatches","pos1","pos2","pos3","gene_id","gene_symbol","isExonic","targetedGene"

"1","ACCAATTCCCTCTGCAGCCT","chr9",115401292,"-","none","TGG","ACCAATTCCCTCTGCAGCCTTGG",0,NA,NA,NA,"GeneID:50514;GeneID:101928775","DEC1;LOC101928775",TRUE,"1-Dec"

"2","ACCAATTCCCTCTGCAGCCT","chr10",98355664,"-","14:A>G,15:C>T","GGG","ACCAATTCCCTCTGTGGCCTGGG",2,15,16,NA,"","",FALSE,"1-Dec"

"3","ACCAATTCCCTCTGCAGCCT","chr1",116532695,"-","0:A>T,6:T>A","TGG","TCCAATACCCTCTGCAGCCTTGG",2,1,7,NA,"GeneID:965","CD58",FALSE,"1-Dec"

"4","ACCAATTCCCTCTGCAGCCT","chr3",14254375,"-","4:C>T,9:A>G","GGG","ACCAGTTCCTTCTGCAGCCTGGG",2,5,10,NA,"","",FALSE,"1-Dec"

"5","ACCAATTCCCTCTGCAGCCT","chr6",107744735,"+","4:C>A","GGG","ACCACTTCCCTCTGCAGCCTGGG",1,5,NA,NA,"GeneID:256380","SCML4",FALSE,"1-Dec"

"6","ACCAATTCCCTCTGCAGCCT","chr16",53154793,"+","3:C>A,9:A>C","GGG","ACCCATTCCATCTGCAGCCTGGG",2,4,10,NA,"GeneID:80205","CHD9",FALSE,"1-Dec"

"7","ACCTACCATCATGTGTAACA","chr9",115400418,"-","none","CGG","ACCTACCATCATGTGTAACACGG",0,NA,NA,NA,"GeneID:50514;GeneID:101928775","DEC1;LOC101928775",TRUE,"1-Dec"

"8","ACCTACCATCATGTGTAACA","chr4",116190219,"+","1:G>C,2:T>C","TGG","AGTTACCATCATGTGTAACATGG",2,2,3,NA,"","",FALSE,"1-Dec"

"9","AGCCCAGTTATGACAATGAA","chr9",115376693,"+","none","TGG","AGCCCAGTTATGACAATGAATGG",0,NA,NA,NA,"GeneID:50514;GeneID:101928775","DEC1;LOC101928775",TRUE,"1-Dec"

"10","AGCCCAGTTATGACAATGAA","chrX",30759255,"+","7:C>T,15:C>A","TGG","AGCCCAGCTATGACACTGAATGG",2,8,16,NA,"","",FALSE,"1-Dec"

"11","AGCCCAGTTATGACAATGAA","chr10",10640854,"+","0:G>A,18:G>A","TGG","GGCCCAGTTATGACAATGGATGG",2,1,19,NA,"","",FALSE,"1-Dec"

"12","AGCCCAGTTATGACAATGAA","chr16",68805497,"-","1:T>C,11:G>C","AGG","AACCCAGTTATCACAATGAAAGG",2,2,12,NA,"GeneID:999","CDH1",FALSE,"1-Dec"

"13","CCCAAGGCTGCAGAGGGAAT","chr9",115401288,"+","none","TGG","CCCAAGGCTGCAGAGGGAATTGG",0,NA,NA,NA,"GeneID:50514;GeneID:101928775","DEC1;LOC101928775",TRUE,"1-Dec"

"14","CCCAAGGCTGCAGAGGGAAT","chr4",44415565,"-","12:G>C,19:C>A","TGG","CCCAAGGCTGCACAGGGAAGTGG",2,13,20,NA,"GeneID:386617","KCTD8",FALSE,"1-Dec"

"15","CCCAAGGCTGCAGAGGGAAT","chr5",84782357,"+","15:A>G,18:G>A","GGG","CCCAAGGCTGCAGAGAGAGTGGG",2,16,19,NA,"","",FALSE,"1-Dec"

"16","CCCAAGGCTGCAGAGGGAAT","chr17",17654027,"+","12:C>G,19:A>T","CGG","CCCAAGGCTGCACAGGGAAACGG",2,13,20,NA,"","",FALSE,"1-Dec"

"17","CCCAAGGCTGCAGAGGGAAT","chr10",91093560,"+","12:C>G,17:T>A","TGG","CCCAAGGCTGCACAGGGTATTGG",2,13,18,NA,"","",FALSE,"1-Dec"

"18","CCCAAGGCTGCAGAGGGAAT","chr20",60189648,"+","7:T>C,16:C>G","AGG","CCCAAGGTTGCAGAGGCAATAGG",2,8,17,NA,"GeneID:284757","MIR646HG",FALSE,"1-Dec"

"19","CCCAAGGCTGCAGAGGGAAT","chr1",116532691,"+","0:A>C,17:T>A","TGG","ACCAAGGCTGCAGAGGGTATTGG",2,1,18,NA,"GeneID:965","CD58",FALSE,"1-Dec"

"20","CCCAAGGCTGCAGAGGGAAT","chr6",107744739,"-","3:G>T,19:C>A","TGG","CCCCAGGCTGCAGAGGGAAGTGG",2,4,20,NA,"GeneID:256380","SCML4",FALSE,"1-Dec"

"21","CCCAAGGCTGCAGAGGGAAT","chr16",53154797,"-","3:G>T,14:A>C","GGG","CCCCAGGCTGCAGATGGAATGGG",2,4,15,NA,"GeneID:80205","CHD9",FALSE,"1-Dec"

"22","TCTTGCCGTGTTACACATGA","chr9",115400410,"+","none","TGG","TCTTGCCGTGTTACACATGATGG",0,NA,NA,NA,"GeneID:50514;GeneID:101928775","DEC1;LOC101928775",TRUE,"1-Dec"

"23","TCTTGCCGTGTTACACATGA","chr15",72595366,"+","12:G>A,14:C>A","TGG","TCTTGCCGTGTTGCCCATGATGG",2,13,15,NA,"GeneID:25820","ARIH1",TRUE,"1-Dec"

"24","TCTTGCCGTGTTACACATGA","chr17",61074079,"-","6:A>G,19:G>T","TGG","TCTTGCTGTGTTACACATGCTGG",2,7,20,NA,"GeneID:54828","BCAS3",FALSE,"1-Dec"

"25","TTACAGACCTCTCTCTGTGC","chr9",115401209,"-","none","AGG","TTACAGACCTCTCTCTGTGCAGG",0,NA,NA,NA,"GeneID:50514;GeneID:101928775","DEC1;LOC101928775",TRUE,"1-Dec"

"26","TTACAGACCTCTCTCTGTGC","chr5",62671509,"+","4:T>A,9:A>T","TGG","TTACTGACCACTCTCTGTGCTGG",2,5,10,NA,"","",FALSE,"1-Dec"

"27","TTACAGACCTCTCTCTGTGC","chr16",27822057,"-","3:C>G,11:G>A","AGG","TTAGAGACCTCCCTCTGTGCAGG",2,4,12,NA,"GeneID:146395","GSG1L",FALSE,"1-Dec"

"28","AGAAGAAACGCGATCAGTTT","chr17",62788539,"-","none","TGG","AGAAGAAACGCGATCAGTTTTGG",0,NA,NA,NA,"GeneID:162333","MARCH10",TRUE,"10-Mar"

"29","CGAAAAAGAGCCCTCTTTCG","chr17",62736661,"-","none","TGG","CGAAAAAGAGCCCTCTTTCGTGG",0,NA,NA,NA,"GeneID:162333","MARCH10",TRUE,"10-Mar"

"30","CTCACCTGGAGAGGGTTCGC","chr17",62759829,"+","none","TGG","CTCACCTGGAGAGGGTTCGCTGG",0,NA,NA,NA,"GeneID:162333","MARCH10",TRUE,"10-Mar"

"31","CTCACCTGGAGAGGGTTCGC","chr10",23696291,"+","9:G>A,18:T>G","AGG","CTCACCTGGGGAGGGTTCTCAGG",2,10,19,NA,"GeneID:56243","KIAA1217",FALSE,"10-Mar"

"32","CTGCAAAGGTGCCGGTTCCC","chr17",62744385,"-","none","AGG","CTGCAAAGGTGCCGGTTCCCAGG",0,NA,NA,NA,"GeneID:162333","MARCH10",TRUE,"10-Mar"

"33","GATTTGGTGTCCCACGAAAG","chr17",62736647,"+","none","AGG","GATTTGGTGTCCCACGAAAGAGG",0,NA,NA,NA,"GeneID:162333","MARCH10",TRUE,"10-Mar"

"34","TCGCACAGCCCGAGAGCATC","chr17",62744437,"-","none","TGG","TCGCACAGCCCGAGAGCATCTGG",0,NA,NA,NA,"GeneID:162333","MARCH10",TRUE,"10-Mar"

"35","AAATCCCACTGTATTCACAA","chr2",109585151,"+","none","TGG","AAATCCCACTGTATTCACAATGG",0,NA,NA,NA,"GeneID:151011","SEPT10",TRUE,"10-Sep"

"36","AAATCCCACTGTATTCACAA","chr9",116593877,"+","3:G>T,10:T>G","AGG","AAAGCCCACTTTATTCACAAAGG",2,4,11,NA,"GeneID:23245","ASTN2",FALSE,"10-Sep"

"37","ATTCGTTCGTTGACTATGTC","chr2",109585806,"-","none","TGG","ATTCGTTCGTTGACTATGTCTGG",0,NA,NA,NA,"GeneID:151011","SEPT10",TRUE,"10-Sep"

"38","ATTCGTTCGTTGACTATGTC","chr13",38492867,"+","4:T>G,8:C>G","AGG","ATTCTTTCCTTGACTATGTCAGG",2,5,9,NA,"","",FALSE,"10-Sep"

"39","ATTCGTTCGTTGACTATGTC","chr8",56478058,"-","8:G>C,15:C>T","TGG","ATTCGTTCCTTGACTGTGTCTGG",2,9,16,NA,"GeneID:101929415","LOC101929415",FALSE,"10-Sep"

"40","CTCAACCCACCTTGCTGTCA","chr2",109574570,"+","none","AGG","CTCAACCCACCTTGCTGTCAAGG",0,NA,NA,NA,"GeneID:151011","SEPT10",TRUE,"10-Sep"

"41","CTCTACTTCATTTCACCGAC","chr2",109574635,"-","none","AGG","CTCTACTTCATTTCACCGACAGG",0,NA,NA,NA,"GeneID:151011","SEPT10",TRUE,"10-Sep"

"42","CTCTACTTCATTTCACCGAC","chr8",56477646,"-","16:A>G","AGG","CTCTACTTCATTTCACTGACAGG",1,17,NA,NA,"GeneID:101929415","LOC101929415",TRUE,"10-Sep"

"43","CTCTACTTCATTTCACCGAC","chr7",55842996,"-","2:A>G,17:A>C","AGG","CTTTACTTCATTTCACCTACAGG",2,3,18,NA,"GeneID:346288","SEPT14",TRUE,"10-Sep"

"44","TGAGAGTTTGCCTGATCAGC","chr2",109585771,"-","none","TGG","TGAGAGTTTGCCTGATCAGCTGG",0,NA,NA,NA,"GeneID:151011","SEPT10",TRUE,"10-Sep"

"45","TGAGAGTTTGCCTGATCAGC","chr8",56478023,"-","5:T>C","TGG","TGAGAATTTGCCTGATCAGCTGG",1,6,NA,NA,"GeneID:101929415","LOC101929415",FALSE,"10-Sep"

"46","TTAGCTCTTTCAGTCTCACA","chr2",109593103,"-","none","TGG","TTAGCTCTTTCAGTCTCACATGG",0,NA,NA,NA,"GeneID:151011","SEPT10",TRUE,"10-Sep"

"47","TTAGCTCTTTCAGTCTCACA","chr5",32585364,"+","12:T>G,17:T>A","AGG","TTAGCTCTTTCATTCTCTCAAGG",2,13,18,NA,"GeneID:10923","SUB1",FALSE,"10-Sep"

"48","TTAGCTCTTTCAGTCTCACA","chrX",125808089,"-","1:G>A,12:A>C","GGG","TCAGCTCTTTCATTCTCACAGGG",2,2,13,NA,"","",FALSE,"10-Sep"

"49","ACCGAACTGACCCATCACAT","chr5",16177838,"+","none","CGG","ACCGAACTGACCCATCACATCGG",0,NA,NA,NA,"GeneID:441061","MARCH11",TRUE,"11-Mar"

"50","AGACCAGCCCGAGACGCGCT","chr5",16179148,"-","none","CGG","AGACCAGCCCGAGACGCGCTCGG",0,NA,NA,NA,"GeneID:441061","MARCH11",TRUE,"11-Mar"

"51","AGCTCTGCGGAACAGAAACT","chr5",16067613,"-","none","TGG","AGCTCTGCGGAACAGAAACTTGG",0,NA,NA,NA,"GeneID:441061","MARCH11",TRUE,"11-Mar"

"52","AGCTCTGCGGAACAGAAACT","chr14",91242073,"+","13:T>A,18:G>C","AGG","AGCTCTGCGGAACTGAAAGTAGG",2,14,19,NA,"GeneID:8111","GPR68",FALSE,"11-Mar"

"53","AGCTCTGCGGAACAGAAACT","chrX",17918043,"-","5:T>A,13:A>T","AGG","AGCTCAGCGGAACTGAAACTAGG",2,6,14,NA,"","",FALSE,"11-Mar"

"54","GCTGCACACCGAGCGCGTCT","chr5",16179137,"+","none","CGG","GCTGCACACCGAGCGCGTCTCGG",0,NA,NA,NA,"GeneID:441061","MARCH11",TRUE,"11-Mar"

"55","TTGAACCCCTGCCGATGTGA","chr5",16177852,"-","none","TGG","TTGAACCCCTGCCGATGTGATGG",0,NA,NA,NA,"GeneID:441061","MARCH11",TRUE,"11-Mar"

"56","TTTACAGAGTGTTTAAGCGC","chr5",16067746,"-","none","TGG","TTTACAGAGTGTTTAAGCGCTGG",0,NA,NA,NA,"GeneID:441061","MARCH11",TRUE,"11-Mar"

"57","AAATCCCACGGTGTCAACAA","chr4",77005750,"-","none","TGG","AAATCCCACGGTGTCAACAATGG",0,NA,NA,NA,"GeneID:55752","SEPT11",TRUE,"11-Sep"

"58","AAGTAGACTTGTTGACCAGC","chr4",76996486,"-","none","TGG","AAGTAGACTTGTTGACCAGCTGG",0,NA,NA,NA,"GeneID:55752","SEPT11",TRUE,"11-Sep"

"59","AAGTAGACTTGTTGACCAGC","chr3",122618851,"-","3:T>A,6:C>T","GGG","AAGAAGGCTTGTTGACCAGCGGG",2,4,7,NA,"GeneID:165631","PARP15",FALSE,"11-Sep"

"60","ATATATTGATGCCCAGTTCG","chr4",77011752,"+","none","AGG","ATATATTGATGCCCAGTTCGAGG",0,NA,NA,NA,"GeneID:55752","SEPT11",TRUE,"11-Sep"

"61","CTCTACTTTATTGCCCCTAC","chr4",77011846,"+","none","TGG","CTCTACTTTATTGCCCCTACTGG",0,NA,NA,NA,"GeneID:55752","SEPT11",TRUE,"11-Sep"

"62","GAATTTGTGCAGTTCATTCT","chr4",77014898,"-","none","TGG","GAATTTGTGCAGTTCATTCTTGG",0,NA,NA,NA,"GeneID:55752","SEPT11",TRUE,"11-Sep"

"63","GTTAACCATTGTTGACACCG","chr4",77005742,"+","none","TGG","GTTAACCATTGTTGACACCGTGG",0,NA,NA,NA,"GeneID:55752","SEPT11",TRUE,"11-Sep"

"64","AAGTCAAACCCACCGGGCTT","chr16",4786026,"-","none","GGG","AAGTCAAACCCACCGGGCTTGGG",0,NA,NA,NA,"GeneID:124404","SEPT12",TRUE,"12-Sep"

"65","ATGTGGTGCCCGTGATTGCC","chr16",4783696,"-","none","AGG","ATGTGGTGCCCGTGATTGCCAGG",0,NA,NA,NA,"GeneID:124404","SEPT12",TRUE,"12-Sep"

"66","ATTGCTCGTTGATGTAGCCC","chr16",4784033,"+","none","AGG","ATTGCTCGTTGATGTAGCCCAGG",0,NA,NA,NA,"GeneID:124404","SEPT12",TRUE,"12-Sep"

"67","CCAGATCACCCTGCGCCTGA","chr16",4783639,"+","none","AGG","CCAGATCACCCTGCGCCTGAAGG",0,NA,NA,NA,"GeneID:124404","SEPT12",TRUE,"12-Sep"

"68","CGCTGTTCAAGTCCAAAGTG","chr16",4786049,"-","none","TGG","CGCTGTTCAAGTCCAAAGTGTGG",0,NA,NA,NA,"GeneID:124404","SEPT12",TRUE,"12-Sep"

"69","GCAATACGAGCAGTACCTGC","chr16",4784018,"-","none","AGG","GCAATACGAGCAGTACCTGCAGG",0,NA,NA,NA,"GeneID:124404","SEPT12",TRUE,"12-Sep"

"70","AGAATCATGGTACTCAAACA","chr7",55843031,"+","none","AGG","AGAATCATGGTACTCAAACAAGG",0,NA,NA,NA,"GeneID:346288","SEPT14",TRUE,"14-Sep"

"71","AGATCTGCTCACCAACTGAT","chr7",55846553,"+","none","TGG","AGATCTGCTCACCAACTGATTGG",0,NA,NA,NA,"GeneID:346288","SEPT14",TRUE,"14-Sep"

"72","ATTACAGGGGAGACTGGAAT","chr7",55844705,"-","none","TGG","ATTACAGGGGAGACTGGAATTGG",0,NA,NA,NA,"GeneID:346288","SEPT14",TRUE,"14-Sep"

"73","ATTACAGGGGAGACTGGAAT","chr7",90654750,"-","2:T>A,11:T>C","AGG","ATAACAGGGGAAACTGGAATAGG",2,3,12,NA,"GeneID:5218","CDK14",FALSE,"14-Sep"

"74","ATTCGTTGTTTAACTACGAT","chr7",55846602,"-","none","TGG","ATTCGTTGTTTAACTACGATTGG",0,NA,NA,NA,"GeneID:346288","SEPT14",TRUE,"14-Sep"

"75","CTACATAGATGCCCAATTTG","chr7",55843090,"-","none","AGG","CTACATAGATGCCCAATTTGAGG",0,NA,NA,NA,"GeneID:346288","SEPT14",TRUE,"14-Sep"

"76","CTACATAGATGCCCAATTTG","chr8",56477740,"-","12:A>G,15:C>T","AGG","CTACATAGATGCTCAGTTTGAGG",2,13,16,NA,"GeneID:101929415","LOC101929415",TRUE,"14-Sep"

"77","CTACATAGATGCCCAATTTG","chr2",109574729,"-","12:A>G,15:C>T","AGG","CTACATAGATGCTCAGTTTGAGG",2,13,16,NA,"GeneID:151011","SEPT10",TRUE,"14-Sep"

"78","TCAGTTGAAATTGACTGTTG","chr7",55844565,"-","none","TGG","TCAGTTGAAATTGACTGTTGTGG",0,NA,NA,NA,"GeneID:346288","SEPT14",TRUE,"14-Sep"

"79","AAATCTGTTGTTCGTAAAAC","chr1",86863156,"+","none","TGG","AAATCTGTTGTTCGTAAAACTGG",0,NA,NA,NA,"GeneID:9403","SEP15",TRUE,"15-Sep"

"80","AAATCTGTTGTTCGTAAAAC","chr14",40394138,"-","13:A>C,19:A>G","GGG","AAATCTGTTGTTCTTAAAATGGG",2,14,20,NA,"","",FALSE,"15-Sep"

"81","ACAGTTCAACCTGCTTCAGC","chr1",86903336,"-","none","TGG","ACAGTTCAACCTGCTTCAGCTGG",0,NA,NA,NA,"GeneID:9403","SEP15",TRUE,"15-Sep"

"82","GATAAACCCAAACTGTTCAG","chr1",86868068,"-","none","AGG","GATAAACCCAAACTGTTCAGAGG",0,NA,NA,NA,"GeneID:9403","SEP15",TRUE,"15-Sep"

"83","GATAAACCCAAACTGTTCAG","chr6",3492156,"-","11:G>T,12:A>G","TGG","GATAAACCCAACTTGTTCAGTGG",2,12,13,NA,"","",FALSE,"15-Sep"

"84","TCGTCCAAAAGCTTTAATAC","chr1",86863564,"+","none","AGG","TCGTCCAAAAGCTTTAATACAGG",0,NA,NA,NA,"GeneID:9403","SEP15",TRUE,"15-Sep"

"85","TGATTGCAGAGGATGCTGTC","chr1",86903309,"-","none","AGG","TGATTGCAGAGGATGCTGTCAGG",0,NA,NA,NA,"GeneID:9403","SEP15",TRUE,"15-Sep"

"86","TTGCAGTCCTCTGAACAGTT","chr1",86868058,"+","none","TGG","TTGCAGTCCTCTGAACAGTTTGG",0,NA,NA,NA,"GeneID:9403","SEP15",TRUE,"15-Sep"

"87","CCATCCTTCCCCTCAGCCCA","chr1",8431559,"+","none","TGG","CCATCCTTCCCCTCAGCCCATGG",0,NA,NA,NA,"GeneID:473","RERE",FALSE,"2-Mar"

"88","CCATCCTTCCCCTCAGCCCA","chr2",218136114,"+","7:G>T,13:G>C","TGG","CCATCCTGCCCCTGAGCCCATGG",2,8,14,NA,"GeneID:3579","CXCR2",TRUE,"2-Mar"

"89","CTGAGGGGAAGGATGGTTTC","chr1",8431555,"-","none","AGG","CTGAGGGGAAGGATGGTTTCAGG",0,NA,NA,NA,"GeneID:473","RERE",FALSE,"2-Mar"

"90","CTGAGGGGAAGGATGGTTTC","chr15",57496497,"-","0:A>G,9:C>T","AGG","TTGAGGGGAGGGATGGTTTCAGG",2,1,10,NA,"GeneID:84952","CGNL1",FALSE,"2-Mar"

"91","CTGAGGGGAAGGATGGTTTC","chr19",38475957,"+","0:A>C,3:C>A","AGG","ATGCGGGGAAGGATGGTTTCAGG",2,1,4,NA,"GeneID:6261","RYR1",FALSE,"2-Mar"

"92","CTGAGGGGAAGGATGGTTTC","chr11",130130862,"-","3:A>T,13:C>A","TGG","CTGTGGGGAAGGAGGGTTTCTGG",2,4,14,NA,"GeneID:334","APLP2",FALSE,"2-Mar"

"93","CTGTGCTGTACCTGTCCCGC","chr1",220748794,"-","none","AGG","CTGTGCTGTACCTGTCCCGCAGG",0,NA,NA,NA,"GeneID:54996","MARC2",TRUE,"2-Mar"

"94","GGATGGTGTAGTTCCTCTAG","chr1",8435549,"-","none","TGG","GGATGGTGTAGTTCCTCTAGTGG",0,NA,NA,NA,"GeneID:473","RERE",FALSE,"2-Mar"

"95","GTAGATCCAGAGCTTCGCCA","chr1",220748700,"-","none","CGG","GTAGATCCAGAGCTTCGCCACGG",0,NA,NA,NA,"GeneID:54996","MARC2",TRUE,"2-Mar"

"96","TCTTCGTTCAAACCCACTAG","chr1",8435533,"+","none","AGG","TCTTCGTTCAAACCCACTAGAGG",0,NA,NA,NA,"GeneID:473","RERE",FALSE,"2-Mar"

"97","TCTTCGTTCAAACCCACTAG","chr18",8986433,"+","7:C>T,11:G>A","TGG","TCTTCGTCCAAGCCCACTAGTGG",2,8,12,NA,"","",FALSE,"2-Mar"

"98","ATGACGAGAGCGGCTTGAAC","chr2",241337431,"+","none","AGG","ATGACGAGAGCGGCTTGAACAGG",0,NA,NA,NA,"GeneID:4735","SEPT2",TRUE,"2-Sep"

"99","GGTCAAGCTACGCCTGACAG","chr2",241336032,"+","none","TGG","GGTCAAGCTACGCCTGACAGTGG",0,NA,NA,NA,"GeneID:4735","SEPT2",TRUE,"2-Sep"

"100","TACCCAGAAAGAGTCATACC","chr2",241335181,"+","none","TGG","TACCCAGAAAGAGTCATACCTGG",0,NA,NA,NA,"GeneID:4735","SEPT2",TRUE,"2-Sep"

"101","TCAAGCCGCTCTCGTCATGC","chr2",241337429,"-","none","AGG","TCAAGCCGCTCTCGTCATGCAGG",0,NA,NA,NA,"GeneID:4735","SEPT2",TRUE,"2-Sep"

"102","TTGATAGCGTCACCATAGCC","chr2",241336066,"-","none","AGG","TTGATAGCGTCACCATAGCCAGG",0,NA,NA,NA,"GeneID:4735","SEPT2",TRUE,"2-Sep"

"103","TTTAAAGGTGAATCAGGTCT","chr2",241335118,"+","none","AGG","TTTAAAGGTGAATCAGGTCTAGG",0,NA,NA,NA,"GeneID:4735","SEPT2",TRUE,"2-Sep"

"104","TTTAAAGGTGAATCAGGTCT","chr1",160259576,"-","6:T>C,17:C>A","GGG","TTTAAAAGTGAATCAGGGCTGGG",2,7,18,NA,"GeneID:50717","DCAF8",FALSE,"2-Sep"

"105","ACTCGTTGTACAATCGACAG","chr5",126870754,"+","none","TGG","ACTCGTTGTACAATCGACAGTGG",0,NA,NA,NA,"GeneID:115123","MARCH3",TRUE,"3-Mar"

"106","ACTGTCGATTGTACAACGAG","chr5",126870755,"-","none","TGG","ACTGTCGATTGTACAACGAGTGG",0,NA,NA,NA,"GeneID:115123","MARCH3",TRUE,"3-Mar"

"107","AGTGAGTGCAATCAGTCCGA","chr5",126878220,"+","none","CGG","AGTGAGTGCAATCAGTCCGACGG",0,NA,NA,NA,"GeneID:115123","MARCH3",TRUE,"3-Mar"

"108","CCAGCCGACTACTAAAGTGC","chr5",126878246,"+","none","AGG","CCAGCCGACTACTAAAGTGCAGG",0,NA,NA,NA,"GeneID:115123","MARCH3",TRUE,"3-Mar"

"109","GCTCGACTGCAAACCTGAAG","chr5",126914952,"+","none","TGG","GCTCGACTGCAAACCTGAAGTGG",0,NA,NA,NA,"GeneID:115123","MARCH3",TRUE,"3-Mar"

"110","TTGCAGTCGAGCGCAAACCC","chr5",126914944,"-","none","AGG","TTGCAGTCGAGCGCAAACCCAGG",0,NA,NA,NA,"GeneID:115123","MARCH3",TRUE,"3-Mar"

"111","AAAGCCTGGGGTGTCGATGA","chr22",41987241,"-","none","CGG","AAAGCCTGGGGTGTCGATGACGG",0,NA,NA,NA,"GeneID:55964","SEPT3",TRUE,"3-Sep"

"112","CAAATCCCAAGTGAGCCGCA","chr22",41986026,"+","none","AGG","CAAATCCCAAGTGAGCCGCAAGG",0,NA,NA,NA,"GeneID:55964","SEPT3",TRUE,"3-Sep"

"113","CTGACCGTCATCGACACCCC","chr22",41987234,"+","none","AGG","CTGACCGTCATCGACACCCCAGG",0,NA,NA,NA,"GeneID:55964","SEPT3",TRUE,"3-Sep"

"114","CTTGCGCATCTGCTCGATGA","chr22",41981773,"-","none","TGG","CTTGCGCATCTGCTCGATGATGG",0,NA,NA,NA,"GeneID:55964","SEPT3",TRUE,"3-Sep"

"115","TGACATGGCTGCGTCCGTCC","chr22",41981659,"-","none","TGG","TGACATGGCTGCGTCCGTCCTGG",0,NA,NA,NA,"GeneID:55964","SEPT3",TRUE,"3-Sep"

"116","TTGATTTGCCCAGTCCACTC","chr22",41985987,"-","none","TGG","TTGATTTGCCCAGTCCACTCTGG",0,NA,NA,NA,"GeneID:55964","SEPT3",TRUE,"3-Sep"

"117","CAACAACACCCTTCCGGCTC","chr2",216370016,"-","none","TGG","CAACAACACCCTTCCGGCTCTGG",0,NA,NA,NA,"GeneID:57574","MARCH4",TRUE,"4-Mar"

"118","CATACATCCCGTAGCAGATC","chr2",216277695,"+","none","TGG","CATACATCCCGTAGCAGATCTGG",0,NA,NA,NA,"GeneID:57574","MARCH4",TRUE,"4-Mar"

"119","CGGTGTACCGCATCTTTAAA","chr2",216259635,"-","none","CGG","CGGTGTACCGCATCTTTAAACGG",0,NA,NA,NA,"GeneID:57574","MARCH4",TRUE,"4-Mar"

"120","CTGAGCCCATGCCGCTGTGA","chr2",216283700,"-","none","TGG","CTGAGCCCATGCCGCTGTGATGG",0,NA,NA,NA,"GeneID:57574","MARCH4",TRUE,"4-Mar"

"121","GACCCTGGTGGCGCAACATC","chr2",216370151,"+","none","TGG","GACCCTGGTGGCGCAACATCTGG",0,NA,NA,NA,"GeneID:57574","MARCH4",TRUE,"4-Mar"

"122","GATGTATGGCTTCATGGACG","chr2",216277683,"-","none","TGG","GATGTATGGCTTCATGGACGTGG",0,NA,NA,NA,"GeneID:57574","MARCH4",TRUE,"4-Mar"

"123","AGACCAGCAGCCCAGCTGCT","chr17",58539128,"+","none","TGG","AGACCAGCAGCCCAGCTGCTTGG",0,NA,NA,NA,"GeneID:101927688;GeneID:5414","LOC101927688;SEPT4",TRUE,"4-Sep"

"124","AGACCAGCAGCCCAGCTGCT","chr18",78123462,"-","2:C>T,9:A>C","GGG","AGGCCAGCATCCCAGCTGCTGGG",2,3,10,NA,"","",FALSE,"4-Sep"

"125","AGACCAGCAGCCCAGCTGCT","chr1",226688531,"+","1:C>G,3:G>C","CGG","ACAGCAGCAGCCCAGCTGCTCGG",2,2,4,NA,"GeneID:3707","ITPKB",FALSE,"4-Sep"

"126","CCCCCAGATCAAGCGTTTCC","chr17",58526965,"-","none","TGG","CCCCCAGATCAAGCGTTTCCTGG",0,NA,NA,NA,"GeneID:101927688;GeneID:5414","LOC101927688;SEPT4",TRUE,"4-Sep"

"127","CTCTACCTCAGAGGAATCAT","chr17",58526675,"+","none","AGG","CTCTACCTCAGAGGAATCATAGG",0,NA,NA,NA,"GeneID:101927688;GeneID:5414","LOC101927688;SEPT4",TRUE,"4-Sep"

"128","GAGCAGCTCTGCTGCTGAAC","chr17",58539176,"+","none","AGG","GAGCAGCTCTGCTGCTGAACAGG",0,NA,NA,NA,"GeneID:101927688;GeneID:5414","LOC101927688;SEPT4",TRUE,"4-Sep"

"129","GAGCAGCTCTGCTGCTGAAC","chr1",225433194,"-","3:T>G,18:C>T","AGG","GAGAAGCTCTGCTGCTGAGCAGG",2,4,19,NA,"","",FALSE,"4-Sep"

"130","GAGCAGCTCTGCTGCTGAAC","chr13",61233562,"-","0:G>C,18:C>T","AGG","CAGCAGCTCTGCTGCTGAGCAGG",2,1,19,NA,"","",FALSE,"4-Sep"

"131","GCAAGGGAATTCTGTCCCTG","chr17",58529134,"-","none","AGG","GCAAGGGAATTCTGTCCCTGAGG",0,NA,NA,NA,"GeneID:101927688;GeneID:5414","LOC101927688;SEPT4",TRUE,"4-Sep"

"132","GTGGGACATGGACCGTTCAC","chr17",58529161,"-","none","TGG","GTGGGACATGGACCGTTCACTGG",0,NA,NA,NA,"GeneID:101927688;GeneID:5414","LOC101927688;SEPT4",TRUE,"4-Sep"

"133","AGATCCAAGACGTAAACCAC","chr10",92340677,"-","none","TGG","AGATCCAAGACGTAAACCACTGG",0,NA,NA,NA,"GeneID:54708","MARCH5",TRUE,"5-Mar"

"134","AGGTCCAGTGGTTTACGTCT","chr10",92340670,"+","none","TGG","AGGTCCAGTGGTTTACGTCTTGG",0,NA,NA,NA,"GeneID:54708","MARCH5",TRUE,"5-Mar"

"135","ATCCACCCAGCGTTGTAGAC","chr10",92311235,"-","none","AGG","ATCCACCCAGCGTTGTAGACAGG",0,NA,NA,NA,"GeneID:54708","MARCH5",TRUE,"5-Mar"

"136","CAGGTTGTAGGTCATAAAGA","chr10",92349345,"+","none","AGG","CAGGTTGTAGGTCATAAAGAAGG",0,NA,NA,NA,"GeneID:54708","MARCH5",TRUE,"5-Mar"

"137","CCTACTATTGCTACAATAGT","chr10",92349765,"+","none","TGG","CCTACTATTGCTACAATAGTTGG",0,NA,NA,NA,"GeneID:54708","MARCH5",TRUE,"5-Mar"

"138","TCTGCTACTCGAATCTTGTG","chr10",92349729,"+","none","TGG","TCTGCTACTCGAATCTTGTGTGG",0,NA,NA,NA,"GeneID:54708","MARCH5",TRUE,"5-Mar"

"139","AGCCTTTCTTCACCGACTTG","chr22",19719654,"-","none","CGG","AGCCTTTCTTCACCGACTTGCGG",0,NA,NA,NA,"GeneID:5413","SEPT5",TRUE,"5-Sep"

"140","AGCCTTTCTTCACCGACTTG","chr17",58526243,"+","13:G>C,19:T>G","CGG","AGCCTTTCTTCACGGACTTTCGG",2,14,20,NA,"GeneID:101927688;GeneID:5414","LOC101927688;SEPT4",TRUE,"5-Sep"

"141","AGCCTTTCTTCACCGACTTG","chr1",152816946,"+","9:A>T,14:C>G","AGG","AGCCTTTCTACACCCACTTGAGG",2,10,15,NA,"","",FALSE,"5-Sep"

"142","CACCGCAAGTCGGTGAAGAA","chr22",19719649,"+","none","AGG","CACCGCAAGTCGGTGAAGAAAGG",0,NA,NA,NA,"GeneID:5413","SEPT5",TRUE,"5-Sep"

"143","CACCGCAAGTCGGTGAAGAA","chr16",30382303,"-","11:A>C,14:G>C","GGG","CACCGCAAGTCTGTCAAGAAGGG",2,12,15,NA,"GeneID:197407;GeneID:1731","ZNF48;SEPT1",TRUE,"5-Sep"

"144","CACCGCAAGTCGGTGAAGAA","chr17",58526248,"-","5:T>G,11:G>C","AGG","CACCGAAAGTCCGTGAAGAAAGG",2,6,12,NA,"GeneID:101927688;GeneID:5414","LOC101927688;SEPT4",TRUE,"5-Sep"

"145","CACGCACCTTGAGCCGCCTC","chr22",19718805,"-","none","TGG","CACGCACCTTGAGCCGCCTCTGG",0,NA,NA,NA,"GeneID:5413","SEPT5",TRUE,"5-Sep"

"146","CGACCCCGCAGAGGACAAGC","chr22",19714769,"+","none","AGG","CGACCCCGCAGAGGACAAGCAGG",0,NA,NA,NA,"GeneID:5413","SEPT5",TRUE,"5-Sep"

"147","CTTCCTGACAGACTTGTACA","chr22",19719845,"+","none","AGG","CTTCCTGACAGACTTGTACAAGG",0,NA,NA,NA,"GeneID:5413","SEPT5",TRUE,"5-Sep"

"148","GGTCGCCGCGATGGACTCGC","chr22",19718727,"+","none","TGG","GGTCGCCGCGATGGACTCGCTGG",0,NA,NA,NA,"GeneID:5413","SEPT5",TRUE,"5-Sep"

"149","ATACACTTGTGGCCTTTGCA","chr5",10381895,"+","none","TGG","ATACACTTGTGGCCTTTGCATGG",0,NA,NA,NA,"GeneID:10299","MARCH6",TRUE,"6-Mar"

"150","CACGTTGACAGCATATCTAA","chr5",10387049,"-","none","TGG","CACGTTGACAGCATATCTAATGG",0,NA,NA,NA,"GeneID:10299","MARCH6",TRUE,"6-Mar"

"151","CAGCGTCAGTAGTGAGCTCA","chr5",10387026,"-","none","CGG","CAGCGTCAGTAGTGAGCTCACGG",0,NA,NA,NA,"GeneID:10299","MARCH6",TRUE,"6-Mar"

"152","CAGCGTCAGTAGTGAGCTCA","chr10",132633171,"+","4:T>G,19:C>A","CGG","CAGCTTCAGTAGTGAGCTCCCGG",2,5,20,NA,"GeneID:3632","INPP5A",FALSE,"6-Mar"

"153","GTATGACTTACCATTCTTGA","chr5",10377885,"-","none","TGG","GTATGACTTACCATTCTTGATGG",0,NA,NA,NA,"GeneID:10299","MARCH6",TRUE,"6-Mar"

"154","GTATGACTTACCATTCTTGA","chr17",37007979,"+","15:T>C,19:C>A","GGG","GTATGACTTACCATTTTTGCGGG",2,16,20,NA,"GeneID:26574","AATF",FALSE,"6-Mar"

"155","TATCATCCTTGTGTATGTAC","chr5",10377844,"+","none","TGG","TATCATCCTTGTGTATGTACTGG",0,NA,NA,NA,"GeneID:10299","MARCH6",TRUE,"6-Mar"

"156","TTGGCACTGCAATACGATAT","chr5",10381865,"+","none","TGG","TTGGCACTGCAATACGATATTGG",0,NA,NA,NA,"GeneID:10299","MARCH6",TRUE,"6-Mar"

"157","ACCTGTCCTCTTTGTTGATC","chrX",119663479,"+","none","TGG","ACCTGTCCTCTTTGTTGATCTGG",0,NA,NA,NA,"GeneID:23157","SEPT6",TRUE,"6-Sep"

"158","ATTCATCGATGCACAATTCG","chrX",119653002,"-","none","AGG","ATTCATCGATGCACAATTCGAGG",0,NA,NA,NA,"GeneID:23157","SEPT6",TRUE,"6-Sep"

"159","CTCACGATCGTTAGCACAGT","chrX",119663514,"-","none","TGG","CTCACGATCGTTAGCACAGTTGG",0,NA,NA,NA,"GeneID:23157","SEPT6",TRUE,"6-Sep"

"160","CTGCTTCAACATCCTGTGCG","chrX",119675556,"-","none","TGG","CTGCTTCAACATCCTGTGCGTGG",0,NA,NA,NA,"GeneID:23157","SEPT6",TRUE,"6-Sep"

"161","GCATCGATGAATTCCACGAT","chrX",119653010,"+","none","AGG","GCATCGATGAATTCCACGATAGG",0,NA,NA,NA,"GeneID:23157","SEPT6",TRUE,"6-Sep"

"162","TGACGGACTTATTCACCAGC","chrX",119675586,"+","none","TGG","TGACGGACTTATTCACCAGCTGG",0,NA,NA,NA,"GeneID:23157","SEPT6",TRUE,"6-Sep"

"163","ACAAACTGTACTACCTCAGC","chr2",159743195,"+","none","TGG","ACAAACTGTACTACCTCAGCTGG",0,NA,NA,NA,"GeneID:64844","MARCH7",TRUE,"7-Mar"

"164","AGTACCATTGATGAAGATCT","chr2",159745798,"-","none","AGG","AGTACCATTGATGAAGATCTAGG",0,NA,NA,NA,"GeneID:64844","MARCH7",TRUE,"7-Mar"

"165","ATCCATAAGATTACTAATAG","chr2",159745880,"-","none","AGG","ATCCATAAGATTACTAATAGAGG",0,NA,NA,NA,"GeneID:64844","MARCH7",TRUE,"7-Mar"

"166","CGTCACCATTTCAATCTGCA","chr2",159743082,"+","none","TGG","CGTCACCATTTCAATCTGCATGG",0,NA,NA,NA,"GeneID:64844","MARCH7",TRUE,"7-Mar"

"167","CTTCTGAAGTTCCCGATAAT","chr2",159748287,"+","none","AGG","CTTCTGAAGTTCCCGATAATAGG",0,NA,NA,NA,"GeneID:64844","MARCH7",TRUE,"7-Mar"

"168","TCCCGAAGCAATACGCAGCC","chr2",159747989,"+","none","TGG","TCCCGAAGCAATACGCAGCCTGG",0,NA,NA,NA,"GeneID:64844","MARCH7",TRUE,"7-Mar"

"169","AAATCAAGTATACAGAAAAT","chr7",35832841,"+","none","CGG","AAATCAAGTATACAGAAAATCGG",0,NA,NA,NA,"GeneID:989","SEPT7",TRUE,"7-Sep"

"170","AAATCAAGTATACAGAAAAT","chr3",127228152,"-","10:G>A,14:T>C","AGG","AAATCAAGTACACAAAAAATAGG",2,11,15,NA,"","",FALSE,"7-Sep"

"171","AAATCAAGTATACAGAAAAT","chr17",60256763,"+","14:A>G,19:C>T","TGG","AAATCAAGTATACAAAAAACTGG",2,15,20,NA,"GeneID:84669","USP32",TRUE,"7-Sep"

"172","AAATCAAGTATACAGAAAAT","chr4",27396138,"-","4:T>G,7:T>C","TGG","AAATAAAATATACAGAAAATTGG",2,5,8,NA,"","",FALSE,"7-Sep"

"173","AAATCAAGTATACAGAAAAT","chr1",188040685,"+","5:T>A,7:C>G","AGG","AAATCTACTATACAGAAAATAGG",2,6,8,NA,"","",FALSE,"7-Sep"

"174","AAATCAAGTATACAGAAAAT","chr6",149560982,"+","1:C>A,8:A>T","AGG","ACATCAAGAATACAGAAAATAGG",2,2,9,NA,"","",FALSE,"7-Sep"

"175","AAATCAAGTATACAGAAAAT","chr12",73036717,"-","5:A>T,10:C>A","TGG","AAATCTAGTAGACAGAAAATTGG",2,6,11,NA,"","",FALSE,"7-Sep"

"176","AAATCAAGTATACAGAAAAT","chr2",113807293,"-","1:A>T,10:T>A","TGG","ATATCAAGTAAACAGAAAATTGG",2,2,11,NA,"","",FALSE,"7-Sep"

"177","CAGCAACAGAAGAACCTTGA","chr7",35832794,"+","none","AGG","CAGCAACAGAAGAACCTTGAAGG",0,NA,NA,NA,"GeneID:989","SEPT7",TRUE,"7-Sep"

"178","CAGCAACAGAAGAACCTTGA","chr4",38239669,"+","0:G>C,3:G>C","GGG","GAGGAACAGAAGAACCTTGAGGG",2,1,4,NA,"","",FALSE,"7-Sep"

"179","CAGCAACAGAAGAACCTTGA","chr2",16042190,"+","2:A>G,14:G>C","GGG","CAACAACAGAAGAAGCTTGAGGG",2,3,15,NA,"","",FALSE,"7-Sep"

"180","CAGCAACAGAAGAACCTTGA","chr2",37572220,"-","4:G>T,18:G>C","TGG","CAGCCACAGAAGAACCTTCATGG",2,5,19,NA,"","",FALSE,"7-Sep"

"181","CTACATTGATAGTAAATTTG","chr7",35873658,"+","none","AGG","CTACATTGATAGTAAATTTGAGG",0,NA,NA,NA,"GeneID:989","SEPT7",TRUE,"7-Sep"

"182","CTACATTGATAGTAAATTTG","chr14",35158666,"-","17:G>A","TGG","CTACATTGATAGTAAATCTGTGG",1,18,NA,NA,"GeneID:9692","KIAA0391",TRUE,"7-Sep"

"183","CTACATTGATAGTAAATTTG","chr9",98607225,"+","11:A>G,16:C>T","AGG","CTACATTGATAATAAACTTGAGG",2,12,17,NA,"GeneID:9568","GABBR2",TRUE,"7-Sep"

"184","CTACATTGATAGTAAATTTG","chr11",50293596,"-","0:A>G","AGG","TTACATTGATAGTAAATTTGAGG",1,1,NA,NA,"GeneID:441601","LOC441601",TRUE,"7-Sep"

"185","CTACATTGATAGTAAATTTG","chr19",53773141,"-","0:A>G","AGG","TTACATTGATAGTAAATTTGAGG",1,1,NA,NA,"","",TRUE,"7-Sep"

"186","CTACATTGATAGTAAATTTG","chr7",45758139,"-","0:A>G","AGG","TTACATTGATAGTAAATTTGAGG",1,1,NA,NA,"GeneID:641977","SEPT7P2",TRUE,"7-Sep"

"187","CTACATTGATAGTAAATTTG","chr10",38392079,"-","0:A>G,3:A>G","AGG","TTATATTGATAGTAAATTTGAGG",2,1,4,NA,"","",TRUE,"7-Sep"

"188","CTACATTGATAGTAAATTTG","chr1",224003537,"+","0:T>C","AGG","TTACATTGATAGTAAATTTGAGG",1,1,NA,NA,"","",TRUE,"7-Sep"

"189","CTACATTGATAGTAAATTTG","chr4",119461734,"+","0:T>C,11:T>G","AGG","TTACATTGATATTAAATTTGAGG",2,1,12,NA,"GeneID:645513","LOC645513",TRUE,"7-Sep"

"190","CTACATTGATAGTAAATTTG","chr7",152367744,"+","0:T>C,17:C>T","AGG","TTACATTGATAGTAAATCTGAGG",2,1,18,NA,"GeneID:58508","KMT2C",TRUE,"7-Sep"

"191","CTATCAATGTAGTCGATAAC","chr7",35873650,"-","none","AGG","CTATCAATGTAGTCGATAACAGG",0,NA,NA,NA,"GeneID:989","SEPT7",TRUE,"7-Sep"

"192","CTATCAATGTAGTCGATAAC","chr7",152367736,"-","11:T>C,19:C>G","AGG","CTATCAATGTAATCGATAAGAGG",2,12,20,NA,"GeneID:58508","KMT2C",TRUE,"7-Sep"

"193","CTATCAATGTAGTCGATAAC","chr19",53773149,"+","11:A>G","AGG","CTATCAATGTAATCGATAACAGG",1,12,NA,NA,"","",TRUE,"7-Sep"

"194","CTATCAATGTAGTCGATAAC","chr11",50293604,"+","11:A>G","AGG","CTATCAATGTAATCGATAACAGG",1,12,NA,NA,"GeneID:441601","LOC441601",TRUE,"7-Sep"

"195","CTATCAATGTAGTCGATAAC","chr7",45758147,"+","11:A>G,13:T>C","AGG","CTATCAATGTAATTGATAACAGG",2,12,14,NA,"GeneID:641977","SEPT7P2",TRUE,"7-Sep"

"196","CTGGAGAATACAAATCTGTG","chr7",57074598,"+","none","AGG","CTGGAGAATACAAATCTGTGAGG",0,NA,NA,NA,"","",TRUE,"7-Sep"

"197","CTGGAGAATACAAATCTGTG","chr7",45765746,"+","none","AGG","CTGGAGAATACAAATCTGTGAGG",0,NA,NA,NA,"GeneID:641977","SEPT7P2",TRUE,"7-Sep"

"198","CTGGAGAATACAAATCTGTG","chr10",38399664,"+","none","AGG","CTGGAGAATACAAATCTGTGAGG",0,NA,NA,NA,"","",TRUE,"7-Sep"

"199","CTGGAGAATACAAATCTGTG","chr7",63248236,"+","none","AGG","CTGGAGAATACAAATCTGTGAGG",0,NA,NA,NA,"","",TRUE,"7-Sep"

"200","CTGGAGAATACAAATCTGTG","chr7",35863597,"-","none","AGG","CTGGAGAATACAAATCTGTGAGG",0,NA,NA,NA,"GeneID:989","SEPT7",TRUE,"7-Sep"

"201","CTGGAGAATACAAATCTGTG","chr7",63495863,"-","none","AGG","CTGGAGAATACAAATCTGTGAGG",0,NA,NA,NA,"","",TRUE,"7-Sep"

"202","CTGGAGAATACAAATCTGTG","chr1",223995940,"-","none","AGG","CTGGAGAATACAAATCTGTGAGG",0,NA,NA,NA,"","",TRUE,"7-Sep"

"203","CTGGAGAATACAAATCTGTG","chr1",781982,"-","none","AGG","CTGGAGAATACAAATCTGTGAGG",0,NA,NA,NA,"GeneID:101928626","LOC101928626",TRUE,"7-Sep"

"204","CTGGAGAATACAAATCTGTG","chr4",119458016,"-","18:C>A","AGG","CTGGAGAATACAAATCTGGGAGG",1,19,NA,NA,"GeneID:645513","LOC645513",TRUE,"7-Sep"

"205","CTGGAGAATACAAATCTGTG","chr17",60256863,"-","10:C>G","AGG","CTGGAGAATAGAAATCTGTGAGG",1,11,NA,NA,"GeneID:84669","USP32",TRUE,"7-Sep"

"206","CTGGAGAATACAAATCTGTG","chr11",50295268,"+","17:A>G","AGG","CTGGAGAATACAAATCTATGAGG",1,18,NA,NA,"GeneID:441601","LOC441601",TRUE,"7-Sep"

"207","CTGGAGAATACAAATCTGTG","chr14",35158854,"+","9:G>A,17:A>G","AGG","CTGGAGAATGCAAATCTATGAGG",2,10,18,NA,"GeneID:9692","KIAA0391",TRUE,"7-Sep"

"208","CTGGAGAATACAAATCTGTG","chr7",152367565,"-","6:G>T,19:T>C","AGG","CTGGAGCATACAAATCTGTAAGG",2,7,20,NA,"GeneID:58508","KMT2C",TRUE,"7-Sep"

"209","TTGTATTCTCCAGAGTATCC","chr4",119458023,"+","none","GGG","TTGTATTCTCCAGAGTATCCGGG",0,NA,NA,NA,"GeneID:645513","LOC645513",TRUE,"7-Sep"

"210","TTGTATTCTCCAGAGTATCC","chr1",223995947,"+","none","AGG","TTGTATTCTCCAGAGTATCCAGG",0,NA,NA,NA,"","",TRUE,"7-Sep"

"211","TTGTATTCTCCAGAGTATCC","chr1",781989,"+","none","AGG","TTGTATTCTCCAGAGTATCCAGG",0,NA,NA,NA,"GeneID:101928626","LOC101928626",TRUE,"7-Sep"

"212","TTGTATTCTCCAGAGTATCC","chr7",35863604,"+","none","AGG","TTGTATTCTCCAGAGTATCCAGG",0,NA,NA,NA,"GeneID:989","SEPT7",TRUE,"7-Sep"

"213","TTGTATTCTCCAGAGTATCC","chr7",45765739,"-","none","AGG","TTGTATTCTCCAGAGTATCCAGG",0,NA,NA,NA,"GeneID:641977","SEPT7P2",TRUE,"7-Sep"

"214","TTGTATTCTCCAGAGTATCC","chr10",38399657,"-","none","AGG","TTGTATTCTCCAGAGTATCCAGG",0,NA,NA,NA,"","",TRUE,"7-Sep"

"215","TTGTATTCTCCAGAGTATCC","chr11",50295261,"-","none","AGG","TTGTATTCTCCAGAGTATCCAGG",0,NA,NA,NA,"GeneID:441601","LOC441601",TRUE,"7-Sep"

"216","TTGTATTCTCCAGAGTATCC","chr7",57074591,"-","17:G>A","AGG","TTGTATTCTCCAGAGTACCCAGG",1,18,NA,NA,"","",TRUE,"7-Sep"

"217","TTGTATTCTCCAGAGTATCC","chr7",63248229,"-","17:G>A","AGG","TTGTATTCTCCAGAGTACCCAGG",1,18,NA,NA,"","",TRUE,"7-Sep"

"218","TTGTATTCTCCAGAGTATCC","chr7",63495870,"+","17:C>T","AGG","TTGTATTCTCCAGAGTACCCAGG",1,18,NA,NA,"","",TRUE,"7-Sep"

"219","TTGTATTCTCCAGAGTATCC","chr17",60256870,"+","2:C>G","AGG","TTCTATTCTCCAGAGTATCCAGG",1,3,NA,NA,"GeneID:84669","USP32",TRUE,"7-Sep"

"220","TTGTATTCTCCAGAGTATCC","chr9",77546944,"+","3:C>T","TGG","TTGCATTCTCCAGAGTATCCTGG",1,4,NA,NA,"GeneID:9630","GNA14",FALSE,"7-Sep"

"221","TTGTATTCTCCAGAGTATCC","chr7",152367572,"+","6:G>T","AGG","TTGTATGCTCCAGAGTATCCAGG",1,7,NA,NA,"GeneID:58508","KMT2C",TRUE,"7-Sep"

"222","AAGCCTCCACTTCGTGCACC","chr10",45461331,"-","none","AGG","AAGCCTCCACTTCGTGCACCAGG",0,NA,NA,NA,"GeneID:220972","MARCH8",TRUE,"8-Mar"

"223","CATGATCTTCCTGCGCTCGC","chr10",45459222,"+","none","TGG","CATGATCTTCCTGCGCTCGCTGG",0,NA,NA,NA,"GeneID:220972","MARCH8",TRUE,"8-Mar"

"224","CGTGATAGAAGTGCGAGAGA","chr10",45464261,"+","none","AGG","CGTGATAGAAGTGCGAGAGAAGG",0,NA,NA,NA,"GeneID:220972","MARCH8",TRUE,"8-Mar"

"225","GAGCTCGCAGCAGCGCGTGT","chr10",45461278,"+","none","CGG","GAGCTCGCAGCAGCGCGTGTCGG",0,NA,NA,NA,"GeneID:220972","MARCH8",TRUE,"8-Mar"

"226","GCTCATTGACCGTACTGCTG","chr10",45459146,"-","none","AGG","GCTCATTGACCGTACTGCTGAGG",0,NA,NA,NA,"GeneID:220972","MARCH8",TRUE,"8-Mar"

"227","TTCTATCACGCCATCCAGCC","chr10",45464251,"-","none","AGG","TTCTATCACGCCATCCAGCCAGG",0,NA,NA,NA,"GeneID:220972","MARCH8",TRUE,"8-Mar"

"228","ATGAGGCATGCGTGCGCCTG","chr5",132764321,"-","none","CGG","ATGAGGCATGCGTGCGCCTGCGG",0,NA,NA,NA,"GeneID:23176","SEPT8",TRUE,"8-Sep"

"229","CAACACGACCTTCGAGACTG","chr5",132764356,"-","none","AGG","CAACACGACCTTCGAGACTGAGG",0,NA,NA,NA,"GeneID:23176","SEPT8",TRUE,"8-Sep"

"230","CATTATTCCCATCATCGCCA","chr5",132762620,"-","none","AGG","CATTATTCCCATCATCGCCAAGG",0,NA,NA,NA,"GeneID:23176","SEPT8",TRUE,"8-Sep"

"231","CGCATCGATGTAGTCAACTA","chr5",132763861,"+","none","TGG","CGCATCGATGTAGTCAACTATGG",0,NA,NA,NA,"GeneID:23176","SEPT8",TRUE,"8-Sep"

"232","CTCTACTTCATCACGCCCAC","chr5",132763760,"-","none","AGG","CTCTACTTCATCACGCCCACAGG",0,NA,NA,NA,"GeneID:23176","SEPT8",TRUE,"8-Sep"

"233","CTCTACTTCATCACGCCCAC","chr1",22286753,"-","10:G>A,18:G>T","TGG","CTCTACTTCACCACGCCCCCTGG",2,11,19,NA,"","",FALSE,"8-Sep"

"234","TGCAACAGCCTCATCATCCG","chr5",132762504,"+","none","TGG","TGCAACAGCCTCATCATCCGTGG",0,NA,NA,NA,"GeneID:23176","SEPT8",TRUE,"8-Sep"

"235","TGCAACAGCCTCATCATCCG","chr3",88190544,"-","14:A>G,18:A>G","GGG","TGCAACAGCCTCATTATCTGGGG",2,15,19,NA,"","",FALSE,"8-Sep"

"236","AGCGCACTGAGCCGTCGCAG","chr12",57756951,"-","none","CGG","AGCGCACTGAGCCGTCGCAGCGG",0,NA,NA,NA,"GeneID:92979","MARCH9",TRUE,"9-Mar"

"237","CAAGAGCGTCCGCATGCGCT","chr12",57758763,"-","none","GGG","CAAGAGCGTCCGCATGCGCTGGG",0,NA,NA,NA,"GeneID:92979","MARCH9",TRUE,"9-Mar"

"238","GCTCTGCTACTTCAAGTACC","chr12",57757029,"+","none","AGG","GCTCTGCTACTTCAAGTACCAGG",0,NA,NA,NA,"GeneID:92979","MARCH9",TRUE,"9-Mar"

"239","GCTCTGCTACTTCAAGTACC","chr3",62372345,"-","5:T>C,15:G>C","TGG","GCTCTACTACTTCAACTACCTGG",2,6,16,NA,"GeneID:55079","FEZF2",TRUE,"9-Mar"

"240","GGACCTTCTCGATGACCGTC","chr12",57758124,"-","none","AGG","GGACCTTCTCGATGACCGTCAGG",0,NA,NA,NA,"GeneID:92979","MARCH9",TRUE,"9-Mar"

"241","TCCGGACGATGCTCAAGTCT","chr12",57755520,"+","none","CGG","TCCGGACGATGCTCAAGTCTCGG",0,NA,NA,NA,"GeneID:1019;GeneID:92979","CDK4;MARCH9",TRUE,"9-Mar"

"242","TCTACCGCATCTTCAAGCGC","chr12",57758589,"+","none","TGG","TCTACCGCATCTTCAAGCGCTGG",0,NA,NA,NA,"GeneID:92979","MARCH9",TRUE,"9-Mar"

"243","AGCCATCATGTCGGACCCCG","chr17",77373490,"+","none","CGG","AGCCATCATGTCGGACCCCGCGG",0,NA,NA,NA,"GeneID:10801","SEPT9",TRUE,"9-Sep"

"244","AGTCACCAAGCCTCCGGAGC","chr17",77307165,"-","none","CGG","AGTCACCAAGCCTCCGGAGCCGG",0,NA,NA,NA,"GeneID:10801","SEPT9",TRUE,"9-Sep"

"245","AGTCACCAAGCCTCCGGAGC","chr17",36936797,"+","3:G>C,12:G>T","TGG","AGTGACCAAGCCGCCGGAGCTGG",2,4,13,NA,"GeneID:3975","LHX1",TRUE,"9-Sep"

"246","ATCCAGCTGCGCGTTGACCG","chr17",77373510,"-","none","CGG","ATCCAGCTGCGCGTTGACCGCGG",0,NA,NA,NA,"GeneID:10801","SEPT9",TRUE,"9-Sep"

"247","CACCCTCTTGGGGGCTGCGT","chr17",77402526,"-","none","CGG","CACCCTCTTGGGGGCTGCGTCGG",0,NA,NA,NA,"GeneID:10801","SEPT9",TRUE,"9-Sep"

"248","GGAGGCACGCGGACCTCCAG","chr17",77307139,"+","none","TGG","GGAGGCACGCGGACCTCCAGTGG",0,NA,NA,NA,"GeneID:10801","SEPT9",TRUE,"9-Sep"

"249","GGAGGCACGCGGACCTCCAG","chr3",194691586,"+","2:C>A,12:T>A","GGG","GGCGGCACGCGGTCCTCCAGGGG",2,3,13,NA,"","",FALSE,"9-Sep"

"250","TGAGGCTACACCCCGGAGCC","chr17",77402677,"+","none","AGG","TGAGGCTACACCCCGGAGCCAGG",0,NA,NA,NA,"GeneID:10801","SEPT9",TRUE,"9-Sep"

"251","ACCTGTAGTTGCCGGCGTGC","chr19",58347424,"+","none","TGG","ACCTGTAGTTGCCGGCGTGCTGG",0,NA,NA,NA,"GeneID:1","A1BG",TRUE,"A1BG"

"252","CAATGTGACGCTGACGTGCC","chr19",58353119,"-","none","AGG","CAATGTGACGCTGACGTGCCAGG",0,NA,NA,NA,"GeneID:1;GeneID:503538","A1BG;A1BG-AS1",TRUE,"A1BG"

"253","CAATGTGACGCTGACGTGCC","chr12",118073923,"-","12:A>C,15:T>C","AGG","CAATGTGACGCTTACATGCCAGG",2,13,16,NA,"GeneID:54621","VSIG10",TRUE,"A1BG"

"254","CAATGTGACGCTGACGTGCC","chr1",168409966,"+","8:G>C,14:G>C","AGG","CAATGTGAGGCTGAGGTGCCAGG",2,9,15,NA,"GeneID:101928565","LOC101928565",FALSE,"A1BG"

"255","CATATGGCTGCTTCTGTCAC","chr19",58353290,"+","none","TGG","CATATGGCTGCTTCTGTCACTGG",0,NA,NA,NA,"GeneID:1;GeneID:503538","A1BG;A1BG-AS1",TRUE,"A1BG"

"256","CGTCAGCGTCACATTGGCCA","chr19",58353123,"+","none","AGG","CGTCAGCGTCACATTGGCCAAGG",0,NA,NA,NA,"GeneID:1;GeneID:503538","A1BG;A1BG-AS1",TRUE,"A1BG"

"257","GTCGCTGAGCTCCGATTCGA","chr19",58347374,"+","none","AGG","GTCGCTGAGCTCCGATTCGAAGG",0,NA,NA,NA,"GeneID:1","A1BG",TRUE,"A1BG"

"258","TGTCTCCGCAGGTGTCACCT","chr19",58353318,"-","none","GGG","TGTCTCCGCAGGTGTCACCTGGG",0,NA,NA,NA,"GeneID:1;GeneID:503538","A1BG;A1BG-AS1",TRUE,"A1BG"

"259","AACTTAATAATTATGAAATT","chr10",50841863,"-","none","AGG","AACTTAATAATTATGAAATTAGG",0,NA,NA,NA,"GeneID:29974","A1CF",TRUE,"A1CF"

"260","AACTTAATAATTATGAAATT","chr20",41879879,"+","2:T>C,14:C>G","TGG","AATTTAATAATTATCAAATTTGG",2,3,15,NA,"","",FALSE,"A1CF"

"261","AACTTAATAATTATGAAATT","chr8",71915936,"-","2:T>G,11:T>A","GGG","AAATTAATAATAATGAAATTGGG",2,3,12,NA,"GeneID:100132891","LOC100132891",FALSE,"A1CF"

"262","AAGTTGCTTGATTGCATTCT","chr10",50841878,"+","none","TGG","AAGTTGCTTGATTGCATTCTTGG",0,NA,NA,NA,"GeneID:29974","A1CF",TRUE,"A1CF"

"263","CCAAGCTATATCCTGTGCGC","chr10",50859846,"+","none","TGG","CCAAGCTATATCCTGTGCGCTGG",0,NA,NA,NA,"GeneID:29974","A1CF",TRUE,"A1CF"

"264","CGCGCACTGGTCCAGCGCAC","chr10",50859860,"-","none","AGG","CGCGCACTGGTCCAGCGCACAGG",0,NA,NA,NA,"GeneID:29974","A1CF",TRUE,"A1CF"

"265","GGGATGCTGCACCCCCTGAA","chr10",50844062,"-","none","AGG","GGGATGCTGCACCCCCTGAAAGG",0,NA,NA,NA,"GeneID:29974","A1CF",TRUE,"A1CF"

"266","GGTGCAGCATCCCAACCAGG","chr10",50844069,"+","none","TGG","GGTGCAGCATCCCAACCAGGTGG",0,NA,NA,NA,"GeneID:29974","A1CF",TRUE,"A1CF"

"267","CAAACTCCTTCATCCAAGTC","chr12",9115818,"-","none","TGG","CAAACTCCTTCATCCAAGTCTGG",0,NA,NA,NA,"GeneID:2","A2M",TRUE,"A2M"

"268","CAAACTCCTTCATCCAAGTC","chr6",129310408,"-","9:T>A,10:C>G","TGG","CAAACTCCTAGATCCAAGTCTGG",2,10,11,NA,"GeneID:3908","LAMA2",FALSE,"A2M"

"269","CCCAAAGTCTTCATCCAATG","chr12",9112514,"-","none","AGG","CCCAAAGTCTTCATCCAATGAGG",0,NA,NA,NA,"GeneID:2","A2M",TRUE,"A2M"

"270","CCCAAAGTCTTCATCCAATG","chrY",15480827,"-","17:A>T,19:A>C","AGG","CCCAAAGTCTTCATCCATTTAGG",2,18,20,NA,"","",FALSE,"A2M"

"271","CCCACAGACGCCTCAGTCTC","chr12",9115772,"-","none","TGG","CCCACAGACGCCTCAGTCTCTGG",0,NA,NA,NA,"GeneID:2","A2M",TRUE,"A2M"

"272","CGCTTCTTAAATTCTTGGGT","chr12",9112456,"+","none","TGG","CGCTTCTTAAATTCTTGGGTTGG",0,NA,NA,NA,"GeneID:2","A2M",TRUE,"A2M"

"273","TCACAGCGAAGGCGACACAG","chr12",9113355,"+","none","TGG","TCACAGCGAAGGCGACACAGTGG",0,NA,NA,NA,"GeneID:2","A2M",TRUE,"A2M"

"274","TCACAGCGAAGGCGACACAG","chr12",9203763,"+","6:T>C,12:A>C","TGG","TCACAGTGAAGGAGACACAGTGG",2,7,13,NA,"GeneID:5858","PZP",TRUE,"A2M"

"275","TCCCAGGCAGTATATGGTTC","chr12",9113529,"-","none","TGG","TCCCAGGCAGTATATGGTTCTGG",0,NA,NA,NA,"GeneID:2","A2M",TRUE,"A2M"

"276","TCCCAGGCAGTATATGGTTC","chr12",9203937,"-","18:C>A","TGG","TCCCAGGCAGTATATGGTGCTGG",1,19,NA,NA,"GeneID:5858","PZP",TRUE,"A2M"

"277","AAATTTCCCCTCCGTTCAGA","chr12",8823211,"+","none","AGG","AAATTTCCCCTCCGTTCAGAAGG",0,NA,NA,NA,"GeneID:144568","A2ML1",TRUE,"A2ML1"

"278","GACAAACCTCTCTACACCCC","chr12",8823851,"+","none","AGG","GACAAACCTCTCTACACCCCAGG",0,NA,NA,NA,"GeneID:144568","A2ML1",TRUE,"A2ML1"

"279","GCCGGGCTGGTAATGTCACC","chr12",8823190,"-","none","AGG","GCCGGGCTGGTAATGTCACCAGG",0,NA,NA,NA,"GeneID:144568","A2ML1",TRUE,"A2ML1"

"280","GCCGGGCTGGTAATGTCACC","chr18",13532150,"+","9:T>G,13:A>T","CGG","GCCGGGCTGTTAAAGTCACCCGG",2,10,14,NA,"GeneID:753","LDLRAD4",FALSE,"A2ML1"

"281","GCTTACCTGTAGTTCCACCA","chr12",8834668,"-","none","TGG","GCTTACCTGTAGTTCCACCATGG",0,NA,NA,NA,"GeneID:144568","A2ML1",TRUE,"A2ML1"

"282","GTATTTCCGCATTGTCACCA","chr12",8829727,"+","none","TGG","GTATTTCCGCATTGTCACCATGG",0,NA,NA,NA,"GeneID:144568","A2ML1",TRUE,"A2ML1"

"283","TATTTCCAACTCCCGACACC","chr12",8823763,"-","none","CGG","TATTTCCAACTCCCGACACCCGG",0,NA,NA,NA,"GeneID:144568","A2ML1",TRUE,"A2ML1"

"284","TATTTCCAACTCCCGACACC","chr12",26750544,"+","12:A>C,13:G>C","TGG","TATTTCCAACTCAGGACACCTGG",2,13,14,NA,"GeneID:3709","ITPR2",FALSE,"A2ML1"

"285","AAGTTCTGACCTGTACCCCC","chr1",33312158,"-","none","TGG","AAGTTCTGACCTGTACCCCCTGG",0,NA,NA,NA,"GeneID:127550","A3GALT2",TRUE,"A3GALT2"

"286","AGCAAAGATAGTCAGCCCAA","chr1",33312059,"+","none","TGG","AGCAAAGATAGTCAGCCCAATGG",0,NA,NA,NA,"GeneID:127550","A3GALT2",TRUE,"A3GALT2"

"287","AGCAAAGATAGTCAGCCCAA","chr4",53976166,"-","2:C>G,3:A>T","GGG","AGGTAAGATAGTCAGCCCAAGGG",2,3,4,NA,"","",FALSE,"A3GALT2"

"288","CGAATGCGCGTCGCGTTCGA","chr1",33307068,"+","none","AGG","CGAATGCGCGTCGCGTTCGAAGG",0,NA,NA,NA,"GeneID:127550","A3GALT2",TRUE,"A3GALT2"

"289","CGGCAGATCCTACTTACACT","chr1",33312848,"-","none","TGG","CGGCAGATCCTACTTACACTTGG",0,NA,NA,NA,"GeneID:127550","A3GALT2",TRUE,"A3GALT2"

"290","CGGCAGATCCTACTTACACT","chr3",66804096,"+","0:G>C,6:C>A","GGG","GGGCAGCTCCTACTTACACTGGG",2,1,7,NA,"","",FALSE,"A3GALT2"

"291","CTTCGAACGCGACGCGCATT","chr1",33307070,"-","none","CGG","CTTCGAACGCGACGCGCATTCGG",0,NA,NA,NA,"GeneID:127550","A3GALT2",TRUE,"A3GALT2"

"292","GGCCGAAGGGCAGACGCCCA","chr1",33312547,"+","none","TGG","GGCCGAAGGGCAGACGCCCATGG",0,NA,NA,NA,"GeneID:127550","A3GALT2",TRUE,"A3GALT2"

"293","GGCCGAAGGGCAGACGCCCA","chr7",10779975,"+","6:C>A,10:A>C","GGG","GGCCGACGGGAAGACGCCCAGGG",2,7,11,NA,"","",FALSE,"A3GALT2"

"294","CAGTCCCGCTACGTCCTCAA","chr22",42693301,"-","none","CGG","CAGTCCCGCTACGTCCTCAACGG",0,NA,NA,NA,"GeneID:53947","A4GALT",TRUE,"A4GALT"

"295","CGTGCGTCGTGGGGCAGTAG","chr22",42692912,"+","none","CGG","CGTGCGTCGTGGGGCAGTAGCGG",0,NA,NA,NA,"GeneID:53947","A4GALT",TRUE,"A4GALT"

"296","CTACTGCCCCACGACGCACG","chr22",42692912,"-","none","AGG","CTACTGCCCCACGACGCACGAGG",0,NA,NA,NA,"GeneID:53947","A4GALT",TRUE,"A4GALT"

"297","CTTCCACATGAGTGCGATCC","chr22",42693396,"+","none","TGG","CTTCCACATGAGTGCGATCCTGG",0,NA,NA,NA,"GeneID:53947","A4GALT",TRUE,"A4GALT"

"298","CTTTGAGGACATCAACCCCG","chr22",42693047,"-","none","AGG","CTTTGAGGACATCAACCCCGAGG",0,NA,NA,NA,"GeneID:53947","A4GALT",TRUE,"A4GALT"

"299","GAACAATGAAGTCCGTGTCC","chr22",42693359,"+","none","AGG","GAACAATGAAGTCCGTGTCCAGG",0,NA,NA,NA,"GeneID:53947","A4GALT",TRUE,"A4GALT"

"300","AAACGTTGTCTATTGCTGAC","chr3",138130913,"+","none","AGG","AAACGTTGTCTATTGCTGACAGG",0,NA,NA,NA,"GeneID:51146","A4GNT",TRUE,"A4GNT"

"301","ACCCCATCTCCTATCGAGAG","chr3",138124485,"-","none","TGG","ACCCCATCTCCTATCGAGAGTGG",0,NA,NA,NA,"GeneID:51146","A4GNT",TRUE,"A4GNT"

"302","CAGCTCGGATGCATCCCGCC","chr3",138124823,"-","none","TGG","CAGCTCGGATGCATCCCGCCTGG",0,NA,NA,NA,"GeneID:51146","A4GNT",TRUE,"A4GNT"

"303","GAAACACAATGCCACGTCTG","chr3",138131093,"+","none","TGG","GAAACACAATGCCACGTCTGTGG",0,NA,NA,NA,"GeneID:51146","A4GNT",TRUE,"A4GNT"

"304","GCTTCTCGGTACTCTAGTAA","chr3",138124711,"-","none","TGG","GCTTCTCGGTACTCTAGTAATGG",0,NA,NA,NA,"GeneID:51146","A4GNT",TRUE,"A4GNT"

"305","GTAGCGCCTCCACTCTCGAT","chr3",138124473,"+","none","AGG","GTAGCGCCTCCACTCTCGATAGG",0,NA,NA,NA,"GeneID:51146","A4GNT",TRUE,"A4GNT"

"306","AATTTGCCCAAGTCACAAAT","chr12",53315095,"-","none","TGG","AATTTGCCCAAGTCACAAATTGG",0,NA,NA,NA,"GeneID:8086","AAAS",TRUE,"AAAS"

"307","AATTTGCCCAAGTCACAAAT","chrX",128218157,"+","3:A>T,13:A>C","TGG","AATATGCCCAAGTAACAAATTGG",2,4,14,NA,"","",FALSE,"AAAS"

"308","CCCAGACGGCAGCAAAATCC","chr12",53309184,"-","none","TGG","CCCAGACGGCAGCAAAATCCTGG",0,NA,NA,NA,"GeneID:8086","AAAS",TRUE,"AAAS"

"309","CCCAGACGGCAGCAAAATCC","chrX",45345825,"-","6:T>G,15:C>T","TGG","CCCAGAAGGCAGCAAGATCCTGG",2,7,16,NA,"","",FALSE,"AAAS"

"310","GCACTCACCAATTTGTGACT","chr12",53315085,"+","none","TGG","GCACTCACCAATTTGTGACTTGG",0,NA,NA,NA,"GeneID:8086","AAAS",TRUE,"AAAS"

"311","GTGGGGTGACCAACCTGCTC","chr12",53309209,"-","none","TGG","GTGGGGTGACCAACCTGCTCTGG",0,NA,NA,NA,"GeneID:8086","AAAS",TRUE,"AAAS"

"312","TCCCTTATCAGGCGTGATGT","chr12",53315773,"-","none","GGG","TCCCTTATCAGGCGTGATGTGGG",0,NA,NA,NA,"GeneID:8086","AAAS",TRUE,"AAAS"

"313","TTTCATTTAGCACCCCAAAA","chr12",53315747,"+","none","AGG","TTTCATTTAGCACCCCAAAAAGG",0,NA,NA,NA,"GeneID:8086","AAAS",TRUE,"AAAS"

"314","TTTCATTTAGCACCCCAAAA","chr18",32618765,"-","12:A>G,14:A>G","TGG","TTTCATTTAGCATCTCAAAATGG",2,13,15,NA,"","",FALSE,"AAAS"

"315","TTTCATTTAGCACCCCAAAA","chr15",46760680,"+","6:C>T,15:A>C","AGG","TTTCATCTAGCACCCAAAAAAGG",2,7,16,NA,"","",FALSE,"AAAS"

"316","CCCCGGTCGGGAGGAGATCC","chr12",125065601,"+","none","TGG","CCCCGGTCGGGAGGAGATCCTGG",0,NA,NA,NA,"GeneID:65985","AACS",TRUE,"AACS"

"317","CGCCGCCGCCATGTCCAAGG","chr12",125065574,"+","none","AGG","CGCCGCCGCCATGTCCAAGGAGG",0,NA,NA,NA,"GeneID:65985","AACS",TRUE,"AACS"

"318","CGCCGCCGCCATGTCCAAGG","chrX",1391939,"-","12:G>C,19:T>C","CGG","CGCCGCCGCCATCTCCAAGACGG",2,13,20,NA,"GeneID:293","SLC25A6",TRUE,"AACS"

"319","CGCCGCCGCCATGTCCAAGG","chr8",102863661,"+","14:T>C,16:G>A","CGG","CGCCGCCGCCATGTTCGAGGCGG",2,15,17,NA,"GeneID:51582","AZIN1",FALSE,"AACS"

"320","CTTCCCAGGGGAAGGCAAAG","chr12",125086321,"+","none","AGG","CTTCCCAGGGGAAGGCAAAGAGG",0,NA,NA,NA,"GeneID:65985","AACS",TRUE,"AACS"

"321","CTTCCCAGGGGAAGGCAAAG","chr6",20476519,"-","13:T>C,17:C>T","TGG","CTTCCCAGGGGAAAGCAGAGTGG",2,14,18,NA,"GeneID:1871","E2F3",FALSE,"AACS"

"322","CTTCTCACGTGTGTATGATG","chr12",125073957,"+","none","AGG","CTTCTCACGTGTGTATGATGAGG",0,NA,NA,NA,"GeneID:65985","AACS",TRUE,"AACS"

"323","CTTTGTTTGCAGCAGCAATG","chr12",125086386,"+","none","AGG","CTTTGTTTGCAGCAGCAATGAGG",0,NA,NA,NA,"GeneID:65985","AACS",TRUE,"AACS"

"324","GTTATGATGACTTGTACCAT","chr12",125073878,"+","none","TGG","GTTATGATGACTTGTACCATTGG",0,NA,NA,NA,"GeneID:65985","AACS",TRUE,"AACS"

"325","AAAGCTCCCGACAACCTTAA","chr3",151817411,"-","none","AGG","AAAGCTCCCGACAACCTTAAAGG",0,NA,NA,NA,"GeneID:13","AADAC",TRUE,"AADAC"

"326","CCAGAAATACCGATTCTCTC","chr3",151824771,"-","none","AGG","CCAGAAATACCGATTCTCTCAGG",0,NA,NA,NA,"GeneID:13","AADAC",TRUE,"AADAC"

"327","CCTGAGAGAATCGGTATTTC","chr3",151824768,"+","none","TGG","CCTGAGAGAATCGGTATTTCTGG",0,NA,NA,NA,"GeneID:13","AADAC",TRUE,"AADAC"

"328","GAACCCCAGTGTTGCGAAGT","chr3",151828036,"-","none","CGG","GAACCCCAGTGTTGCGAAGTCGG",0,NA,NA,NA,"GeneID:13","AADAC",TRUE,"AADAC"

"329","GTCACCCGACTTCGCAACAC","chr3",151828028,"+","none","TGG","GTCACCCGACTTCGCAACACTGG",0,NA,NA,NA,"GeneID:13","AADAC",TRUE,"AADAC"

"330","GTTATGACTTGCTGTCAAGA","chr3",151820391,"+","none","TGG","GTTATGACTTGCTGTCAAGATGG",0,NA,NA,NA,"GeneID:13","AADAC",TRUE,"AADAC"

"331","GTTATGACTTGCTGTCAAGA","chrX",140945293,"+","5:C>G,15:G>C","AGG","GTTATCACTTGCTGTGAAGAAGG",2,6,16,NA,"","",FALSE,"AADAC"

"332","AATATATCACAGCTCGCCTT","chr3",151740815,"-","none","CGG","AATATATCACAGCTCGCCTTCGG",0,NA,NA,NA,"GeneID:344752","AADACL2",TRUE,"AADACL2"

"333","ACGCTTGATGCTGTTGTTGT","chr3",151744133,"+","none","AGG","ACGCTTGATGCTGTTGTTGTAGG",0,NA,NA,NA,"GeneID:344752","AADACL2",TRUE,"AADACL2"

"334","ATGCAAGTCTTACTTTACCC","chr3",151757027,"+","none","TGG","ATGCAAGTCTTACTTTACCCTGG",0,NA,NA,NA,"GeneID:344752","AADACL2",TRUE,"AADACL2"

"335","TCCCACCCGAATCTGCATTG","chr3",151745613,"+","none","CGG","TCCCACCCGAATCTGCATTGCGG",0,NA,NA,NA,"GeneID:344752","AADACL2",TRUE,"AADACL2"

"336","TGTTTGCCGTCCATCTATTC","chr3",151744114,"-","none","AGG","TGTTTGCCGTCCATCTATTCAGG",0,NA,NA,NA,"GeneID:344752","AADACL2",TRUE,"AADACL2"

"337","TTTGGCAAGTACAATCGTAC","chr3",151740777,"-","none","TGG","TTTGGCAAGTACAATCGTACTGG",0,NA,NA,NA,"GeneID:344752","AADACL2",TRUE,"AADACL2"

"338","AAATCCGTGACCACAACATC","chr1",12719561,"-","none","GGG","AAATCCGTGACCACAACATCGGG",0,NA,NA,NA,"GeneID:126767","AADACL3",TRUE,"AADACL3"

"339","ACCTGGGAGTGCCCGTGACC","chr1",12725856,"+","none","TGG","ACCTGGGAGTGCCCGTGACCTGG",0,NA,NA,NA,"GeneID:126767","AADACL3",TRUE,"AADACL3"

"340","ATCGTGTACTACCACGGTGG","chr1",12719648,"+","none","CGG","ATCGTGTACTACCACGGTGGCGG",0,NA,NA,NA,"GeneID:126767","AADACL3",TRUE,"AADACL3"

"341","CATATGCTCTCGTTTGTGCA","chr1",12720895,"+","none","AGG","CATATGCTCTCGTTTGTGCAAGG",0,NA,NA,NA,"GeneID:126767","AADACL3",TRUE,"AADACL3"

"342","GACTCCGTGGTTCTGGCAGT","chr1",12720923,"+","none","TGG","GACTCCGTGGTTCTGGCAGTTGG",0,NA,NA,NA,"GeneID:126767","AADACL3",TRUE,"AADACL3"

"343","GATCCACTCCATATGCATCC","chr1",12725302,"-","none","AGG","GATCCACTCCATATGCATCCAGG",0,NA,NA,NA,"GeneID:126767","AADACL3",TRUE,"AADACL3"

"344","ACCATGTCACGCGGACCCCC","chr1",12666599,"-","none","TGG","ACCATGTCACGCGGACCCCCTGG",0,NA,NA,NA,"GeneID:343066","AADACL4",TRUE,"AADACL4"

"345","CGGGTATCGTCCCAAAACGC","chr1",12651229,"-","none","AGG","CGGGTATCGTCCCAAAACGCAGG",0,NA,NA,NA,"GeneID:343066","AADACL4",TRUE,"AADACL4"

"346","GTGGTGACCGACCTGCGTTT","chr1",12651215,"+","none","TGG","GTGGTGACCGACCTGCGTTTTGG",0,NA,NA,NA,"GeneID:343066","AADACL4",TRUE,"AADACL4"

"347","TCGTACTTCCTCCAGACGTC","chr1",12666336,"-","none","TGG","TCGTACTTCCTCCAGACGTCTGG",0,NA,NA,NA,"GeneID:343066","AADACL4",TRUE,"AADACL4"

"348","TGCCAAGAGTAGCACTAGCC","chr1",12644559,"-","none","AGG","TGCCAAGAGTAGCACTAGCCAGG",0,NA,NA,NA,"GeneID:343066","AADACL4",TRUE,"AADACL4"

"349","TGTCTTGCAGATTGTTACCA","chr1",12661780,"+","none","TGG","TGTCTTGCAGATTGTTACCATGG",0,NA,NA,NA,"GeneID:343066","AADACL4",TRUE,"AADACL4"

"350","ACCAAAATCGATGATCTCCT","chr4",170088525,"-","none","TGG","ACCAAAATCGATGATCTCCTTGG",0,NA,NA,NA,"GeneID:51166","AADAT",TRUE,"AADAT"

"351","ACCAAAATCGATGATCTCCT","chr2",118963505,"-","8:T>G,15:T>G","AGG","ACCAAAATAGATGATATCCTAGG",2,9,16,NA,"GeneID:8685","MARCO",FALSE,"AADAT"

"352","ATTAACCAGTGAACGCAAAA","chr4",170073149,"-","none","AGG","ATTAACCAGTGAACGCAAAAAGG",0,NA,NA,NA,"GeneID:51166","AADAT",TRUE,"AADAT"

"353","ATTAACCAGTGAACGCAAAA","chr3",144972799,"+","13:A>C,15:T>C","AGG","ATTAACCAGTGAAAGTAAAAAGG",2,14,16,NA,"","",FALSE,"AADAT"

"354","CAGCCAACAAGGTCTTTGTA","chr4",170087117,"-","none","AGG","CAGCCAACAAGGTCTTTGTAAGG",0,NA,NA,NA,"GeneID:51166","AADAT",TRUE,"AADAT"

"355","CCAGTAGGGTTGTTGCCATT","chr4",170073175,"+","none","TGG","CCAGTAGGGTTGTTGCCATTTGG",0,NA,NA,NA,"GeneID:51166","AADAT",TRUE,"AADAT"

"356","CTTCAGTATTCTCCGAGTGC","chr4",170088398,"-","none","TGG","CTTCAGTATTCTCCGAGTGCTGG",0,NA,NA,NA,"GeneID:51166","AADAT",TRUE,"AADAT"

"357","TAGATCCATTTGTCCTTGAC","chr4",170087151,"+","none","TGG","TAGATCCATTTGTCCTTGACTGG",0,NA,NA,NA,"GeneID:51166","AADAT",TRUE,"AADAT"

"358","AATGTCACCCTTATAGTGAT","chr9",96651686,"-","none","TGG","AATGTCACCCTTATAGTGATTGG",0,NA,NA,NA,"GeneID:195827","AAED1",TRUE,"AAED1"

"359","ACTCACTTGTAAGAAACTCC","chr9",96654698,"+","none","TGG","ACTCACTTGTAAGAAACTCCTGG",0,NA,NA,NA,"GeneID:195827","AAED1",TRUE,"AAED1"

"360","ACTCACTTGTAAGAAACTCC","chr1",73521291,"-","5:T>G,16:A>G","AGG","ACTCAATTGTAAGAAATTCCAGG",2,6,17,NA,"","",FALSE,"AAED1"

"361","AGGATCTGGCCAAAATCCCC","chr9",96654719,"-","none","AGG","AGGATCTGGCCAAAATCCCCAGG",0,NA,NA,NA,"GeneID:195827","AAED1",TRUE,"AAED1"

"362","ATGACTGTCCAATCACTATA","chr9",96651675,"+","none","AGG","ATGACTGTCCAATCACTATAAGG",0,NA,NA,NA,"GeneID:195827","AAED1",TRUE,"AAED1"

"363","GCTTACCTGGACCTAAAATG","chr9",96645886,"+","none","AGG","GCTTACCTGGACCTAAAATGAGG",0,NA,NA,NA,"GeneID:195827","AAED1",TRUE,"AAED1"

"364","TATCCAGTCAGCTTGCAAAA","chr9",96651472,"+","none","AGG","TATCCAGTCAGCTTGCAAAAAGG",0,NA,NA,NA,"GeneID:195827","AAED1",TRUE,"AAED1"

"365","ACCATTCTTCATCACTACAT","chr15",67231811,"+","none","TGG","ACCATTCTTCATCACTACATTGG",0,NA,NA,NA,"GeneID:79719","AAGAB",TRUE,"AAGAB"

"366","AGCAGACATCAATCTATGTG","chr15",67236709,"-","none","TGG","AGCAGACATCAATCTATGTGTGG",0,NA,NA,NA,"GeneID:79719","AAGAB",TRUE,"AAGAB"

"367","AGCAGACATCAATCTATGTG","chr11",28471863,"+","11:T>A,15:C>A","GGG","AGCAGACATCATTCTCTGTGGGG",2,12,16,NA,"GeneID:196074","METTL15",FALSE,"AAGAB"

"368","CAGACCAAGATCATCACCTC","chr15",67236427,"+","none","AGG","CAGACCAAGATCATCACCTCAGG",0,NA,NA,NA,"GeneID:79719","AAGAB",TRUE,"AAGAB"

"369","CGCTTTACTCCTGTAGATTC","chr15",67231866,"+","none","TGG","CGCTTTACTCCTGTAGATTCTGG",0,NA,NA,NA,"GeneID:79719","AAGAB",TRUE,"AAGAB"

"370","GCCTTGATAGTGTCTCCTCA","chr15",67236477,"-","none","TGG","GCCTTGATAGTGTCTCCTCATGG",0,NA,NA,NA,"GeneID:79719","AAGAB",TRUE,"AAGAB"

"371","TGGAACAGAAGATCTTATTG","chr15",67236793,"-","none","TGG","TGGAACAGAAGATCTTATTGTGG",0,NA,NA,NA,"GeneID:79719","AAGAB",TRUE,"AAGAB"

"372","TGGAACAGAAGATCTTATTG","chr2",237347904,"+","15:C>T,17:C>T","AGG","TGGAACAGAAGATCTCACTGAGG",2,16,18,NA,"GeneID:1293","COL6A3",FALSE,"AAGAB"

"373","AGTCAGGTGGCAATTTGTGA","chr2",69530066,"-","none","TGG","AGTCAGGTGGCAATTTGTGATGG",0,NA,NA,NA,"GeneID:22848","AAK1",TRUE,"AAK1"

"374","CAACAATGAGCATGATCTCC","chr2",69556888,"-","none","AGG","CAACAATGAGCATGATCTCCAGG",0,NA,NA,NA,"GeneID:22848","AAK1",TRUE,"AAK1"

"375","GACCATTTCTGGTGCTCGAT","chr2",69530669,"+","none","AGG","GACCATTTCTGGTGCTCGATAGG",0,NA,NA,NA,"GeneID:22848","AAK1",TRUE,"AAK1"

"376","GTCCTATCGAGCACCAGAAA","chr2",69530674,"-","none","TGG","GTCCTATCGAGCACCAGAAATGG",0,NA,NA,NA,"GeneID:22848","AAK1",TRUE,"AAK1"

"377","GTTGACAAACATGCGTTTCA","chr2",69556904,"+","none","AGG","GTTGACAAACATGCGTTTCAAGG",0,NA,NA,NA,"GeneID:22848","AAK1",TRUE,"AAK1"

"378","GTTGACAAACATGCGTTTCA","chr19",31278055,"+","4:C>A,13:G>C","GGG","GTTGCCAAACATGGGTTTCAGGG",2,5,14,NA,"GeneID:57616","TSHZ3",TRUE,"AAK1"

"379","TGAGAATATCGAGAATTATC","chr2",69530027,"+","none","AGG","TGAGAATATCGAGAATTATCAGG",0,NA,NA,NA,"GeneID:22848","AAK1",TRUE,"AAK1"

"380","AGGCTCTAATACAACCTATA","chr11",77842546,"+","none","AGG","AGGCTCTAATACAACCTATAAGG",0,NA,NA,NA,"GeneID:28971","AAMDC",TRUE,"AAMDC"

"381","CCAGGTGCCTTCATCAACTG","chr11",77872170,"+","none","TGG","CCAGGTGCCTTCATCAACTGTGG",0,NA,NA,NA,"GeneID:28971","AAMDC",TRUE,"AAMDC"

"382","CCAGGTGCCTTCATCAACTG","chr15",44812449,"-","9:G>A,19:G>C","GGG","CCAGGTGCCCTCATCAACTCGGG",2,10,20,NA,"","",FALSE,"AAMDC"

"383","GGTGTACAGACTCTTGTGAT","chr11",77869769,"+","none","TGG","GGTGTACAGACTCTTGTGATTGG",0,NA,NA,NA,"GeneID:28971","AAMDC",TRUE,"AAMDC"

"384","TATAATGCCTTGGTTGCCCA","chr11",77872255,"+","none","AGG","TATAATGCCTTGGTTGCCCAAGG",0,NA,NA,NA,"GeneID:28971","AAMDC",TRUE,"AAMDC"

"385","TCAAGGCCTCACTCATCCCT","chr11",77869795,"-","none","CGG","TCAAGGCCTCACTCATCCCTCGG",0,NA,NA,NA,"GeneID:28971","AAMDC",TRUE,"AAMDC"

"386","TCAAGGCCTCACTCATCCCT","chr6",20790569,"+","2:C>A,15:G>T","GGG","TCCAGGCCTCACTCAGCCCTGGG",2,3,16,NA,"GeneID:54901","CDKAL1",FALSE,"AAMDC"

"387","TCCCAAGTCCGACTACCCCC","chr11",77842586,"-","none","TGG","TCCCAAGTCCGACTACCCCCTGG",0,NA,NA,NA,"GeneID:28971","AAMDC",TRUE,"AAMDC"

"388","AAGATGACAAAGCCTTCGTA","chr2",218267533,"-","none","TGG","AAGATGACAAAGCCTTCGTATGG",0,NA,NA,NA,"GeneID:14","AAMP",TRUE,"AAMP"

"389","AAGATGACAAAGCCTTCGTA","chr12",2142581,"-","10:A>T,17:A>C","TGG","AAGATGACAATGCCTTCTTATGG",2,11,18,NA,"GeneID:775","CACNA1C",FALSE,"AAMP"

"390","AAGATGACAAAGCCTTCGTA","chr6",94084327,"+","17:A>G,18:C>T","AGG","AAGATGACAAAGCCTTCACAAGG",2,18,19,NA,"","",FALSE,"AAMP"

"391","ATCGCTGAGCCGCCATACGA","chr2",218267518,"+","none","AGG","ATCGCTGAGCCGCCATACGAAGG",0,NA,NA,NA,"GeneID:14","AAMP",TRUE,"AAMP"

"392","CGTCAGCTGTGCCCGCCAAC","chr2",218266535,"+","none","AGG","CGTCAGCTGTGCCCGCCAACAGG",0,NA,NA,NA,"GeneID:14","AAMP",TRUE,"AAMP"

"393","CTCCAGGTCTCCCGCTTCAA","chr2",218266846,"+","none","AGG","CTCCAGGTCTCCCGCTTCAAAGG",0,NA,NA,NA,"GeneID:14","AAMP",TRUE,"AAMP"

"394","GGAGGGCCCCGACGATAGCG","chr2",218269405,"-","none","AGG","GGAGGGCCCCGACGATAGCGAGG",0,NA,NA,NA,"GeneID:14","AAMP",TRUE,"AAMP"

"395","TCCTCGGGCACCTGTCCTGT","chr2",218266553,"-","none","TGG","TCCTCGGGCACCTGTCCTGTTGG",0,NA,NA,NA,"GeneID:14","AAMP",TRUE,"AAMP"

"396","ACCTTCACGCTCGATCTCAA","chr17",76468891,"-","none","AGG","ACCTTCACGCTCGATCTCAAAGG",0,NA,NA,NA,"GeneID:15","AANAT",TRUE,"AANAT"

"397","CCGGGCCGCGCTCATGTGCG","chr17",76469807,"+","none","AGG","CCGGGCCGCGCTCATGTGCGAGG",0,NA,NA,NA,"GeneID:15","AANAT",TRUE,"AANAT"

"398","CCTTCATCATCGGCTCGCTC","chr17",76469283,"+","none","TGG","CCTTCATCATCGGCTCGCTCTGG",0,NA,NA,NA,"GeneID:15","AANAT",TRUE,"AANAT"

"399","CGCGTCCTCGCACATGAGCG","chr17",76469815,"-","none","CGG","CGCGTCCTCGCACATGAGCGCGG",0,NA,NA,NA,"GeneID:15","AANAT",TRUE,"AANAT"

"400","GACGCCCAAGACGGAGATGA","chr17",76469175,"-","none","AGG","GACGCCCAAGACGGAGATGAAGG",0,NA,NA,NA,"GeneID:15","AANAT",TRUE,"AANAT"

"401","GCCTTTGAGATCGAGCGTGA","chr17",76468887,"+","none","AGG","GCCTTTGAGATCGAGCGTGAAGG",0,NA,NA,NA,"GeneID:15","AANAT",TRUE,"AANAT"

"402","AAAGTCCCACCGGAACTTCT","chr20",36255659,"-","none","TGG","AAAGTCCCACCGGAACTTCTTGG",0,NA,NA,NA,"GeneID:25980","AAR2",TRUE,"AAR2"

"403","GAGGTCGGGCCCAAGTTCCG","chr20",36239985,"+","none","GGG","GAGGTCGGGCCCAAGTTCCGGGG",0,NA,NA,NA,"GeneID:25980","AAR2",TRUE,"AAR2"

"404","GGACTTTGCTGCGGAACCTG","chr20",36255672,"+","none","AGG","GGACTTTGCTGCGGAACCTGAGG",0,NA,NA,NA,"GeneID:25980","AAR2",TRUE,"AAR2"

"405","GGACTTTGCTGCGGAACCTG","chr5",2010333,"-","10:G>C,16:A>G","TGG","GGACTTTGCTCCGGAATCTGTGG",2,11,17,NA,"","",FALSE,"AAR2"

"406","GGCACAGATCTGTCGATTCT","chr20",36240319,"-","none","CGG","GGCACAGATCTGTCGATTCTCGG",0,NA,NA,NA,"GeneID:25980","AAR2",TRUE,"AAR2"

"407","TCTCACCAAGCTGGTGGTAC","chr20",36244844,"-","none","AGG","TCTCACCAAGCTGGTGGTACAGG",0,NA,NA,NA,"GeneID:25980","AAR2",TRUE,"AAR2"

"408","TGTACGAGGCATTTGAGCAT","chr20",36244738,"+","none","TGG","TGTACGAGGCATTTGAGCATTGG",0,NA,NA,NA,"GeneID:25980","AAR2",TRUE,"AAR2"

"409","AAAAGATGATGCTGCGAATC","chr8",116942673,"+","none","CGG","AAAAGATGATGCTGCGAATCCGG",0,NA,NA,NA,"GeneID:441376","AARD",TRUE,"AARD"

"410","AACCAAAGCTCCGCTCTACC","chr8",116938306,"-","none","TGG","AACCAAAGCTCCGCTCTACCTGG",0,NA,NA,NA,"GeneID:441376","AARD",TRUE,"AARD"

"411","CCAGTAAAGTTCTAGCCAGC","chr8",116942583,"-","none","TGG","CCAGTAAAGTTCTAGCCAGCTGG",0,NA,NA,NA,"GeneID:441376","AARD",TRUE,"AARD"

"412","CCAGTAAAGTTCTAGCCAGC","chr12",113805628,"-","4:C>A,13:C>T","AGG","CCAGGAAAGTTCTGGCCAGCAGG",2,5,14,NA,"","",FALSE,"AARD"

"413","CGGTGCAGCGCGCGATCTCG","chr8",116938451,"+","none","AGG","CGGTGCAGCGCGCGATCTCGAGG",0,NA,NA,NA,"GeneID:441376","AARD",TRUE,"AARD"

"414","CGTCCCGCGTGCGACTTCTT","chr8",116938333,"+","none","CGG","CGTCCCGCGTGCGACTTCTTCGG",0,NA,NA,NA,"GeneID:441376","AARD",TRUE,"AARD"

"415","GTCCCCGAAGAAGTCGCACG","chr8",116938340,"-","none","CGG","GTCCCCGAAGAAGTCGCACGCGG",0,NA,NA,NA,"GeneID:441376","AARD",TRUE,"AARD"

"416","AGCTGGCTTAGAAGCAGATC","chr16",70276516,"-","none","TGG","AGCTGGCTTAGAAGCAGATCTGG",0,NA,NA,NA,"GeneID:16","AARS",TRUE,"AARS"

"417","CCACGCTCGGACCATCACTG","chr16",70269660,"-","none","TGG","CCACGCTCGGACCATCACTGTGG",0,NA,NA,NA,"GeneID:16","AARS",TRUE,"AARS"

"418","GAACTCCTCACCCAAGAGTT","chr16",70276587,"-","none","TGG","GAACTCCTCACCCAAGAGTTTGG",0,NA,NA,NA,"GeneID:16","AARS",TRUE,"AARS"

"419","GGTGGCCGGCCTGACAACAC","chr16",70269626,"-","none","AGG","GGTGGCCGGCCTGACAACACAGG",0,NA,NA,NA,"GeneID:16","AARS",TRUE,"AARS"

"420","TCACACCTTCTTCGAGATGC","chr16",70276997,"-","none","TGG","TCACACCTTCTTCGAGATGCTGG",0,NA,NA,NA,"GeneID:16","AARS",TRUE,"AARS"

"421","TGGGGGCAAACATAATGACC","chr16",70277045,"-","none","TGG","TGGGGGCAAACATAATGACCTGG",0,NA,NA,NA,"GeneID:16","AARS",TRUE,"AARS"

"422","AAGCCTGCCATCTCGCTTCG","chr6",44312210,"+","none","TGG","AAGCCTGCCATCTCGCTTCGTGG",0,NA,NA,NA,"GeneID:57505","AARS2",TRUE,"AARS2"

"423","CCATTCGAAGGTCGCCCGCA","chr6",44313260,"-","none","TGG","CCATTCGAAGGTCGCCCGCATGG",0,NA,NA,NA,"GeneID:57505","AARS2",TRUE,"AARS2"

"424","CGTGGATCCACGAAGCGAGA","chr6",44312220,"-","none","TGG","CGTGGATCCACGAAGCGAGATGG",0,NA,NA,NA,"GeneID:57505","AARS2",TRUE,"AARS2"

"425","CTCTGGATCTCCTACTTTGA","chr6",44311452,"-","none","TGG","CTCTGGATCTCCTACTTTGATGG",0,NA,NA,NA,"GeneID:57505","AARS2",TRUE,"AARS2"

"426","CTCTGGATCTCCTACTTTGA","chr6",572538,"-","15:C>A,18:T>C","TGG","CTCTGGATCTCCTACGTTAATGG",2,16,19,NA,"GeneID:55770","EXOC2",TRUE,"AARS2"

"427","GAACTGCTGACTCAGGTCTA","chr6",44311491,"-","none","TGG","GAACTGCTGACTCAGGTCTATGG",0,NA,NA,NA,"GeneID:57505","AARS2",TRUE,"AARS2"

"428","GAACTGCTGACTCAGGTCTA","chr10",92577055,"-","5:G>C,15:G>C","TGG","GAACTCCTGACTCAGCTCTATGG",2,6,16,NA,"","",FALSE,"AARS2"

"429","GCCGCGGGGCCGCACGGAAG","chr6",44313119,"+","none","CGG","GCCGCGGGGCCGCACGGAAGCGG",0,NA,NA,NA,"GeneID:57505","AARS2",TRUE,"AARS2"

"430","AGATGTCATTGATTGTACCA","chr17",42961320,"+","none","CGG","AGATGTCATTGATTGTACCACGG",0,NA,NA,NA,"GeneID:100885850;GeneID:80755","PTGES3L-AARSD1;AARSD1",TRUE,"AARSD1"

"431","CTTAGGGCAGCATCTCATCA","chr17",42957180,"-","none","CGG","CTTAGGGCAGCATCTCATCACGG",0,NA,NA,NA,"GeneID:100885850;GeneID:80755","PTGES3L-AARSD1;AARSD1",TRUE,"AARSD1"

"432","GTGCTGAGAGTGACTCGCCG","chr17",42961298,"-","none","TGG","GTGCTGAGAGTGACTCGCCGTGG",0,NA,NA,NA,"GeneID:100885850;GeneID:80755","PTGES3L-AARSD1;AARSD1",TRUE,"AARSD1"

"433","TACTTGCTCTGCAGTCATAG","chr17",42956496,"+","none","AGG","TACTTGCTCTGCAGTCATAGAGG",0,NA,NA,NA,"GeneID:100885850;GeneID:80755","PTGES3L-AARSD1;AARSD1",TRUE,"AARSD1"

"434","TGTTCAGTTCACCACCACCG","chr17",42964224,"-","none","TGG","TGTTCAGTTCACCACCACCGTGG",0,NA,NA,NA,"GeneID:100885850;GeneID:80755","PTGES3L-AARSD1;AARSD1",TRUE,"AARSD1"

"435","TTCATTGACGCTCTGCTCAA","chr17",42956469,"+","none","TGG","TTCATTGACGCTCTGCTCAATGG",0,NA,NA,NA,"GeneID:100885850;GeneID:80755","PTGES3L-AARSD1;AARSD1",TRUE,"AARSD1"

"436","ATATTTCTTGCTCTATCAAG","chr4",56371547,"-","none","TGG","ATATTTCTTGCTCTATCAAGTGG",0,NA,NA,NA,"GeneID:132949","AASDH",TRUE,"AASDH"

"437","ATATTTCTTGCTCTATCAAG","chr3",109575985,"+","14:T>A,16:T>C","TGG","ATATTTCTTGCTCTTTTAAGTGG",2,15,17,NA,"","",FALSE,"AASDH"

"438","CTTTAGAATTCTCCAAGTCC","chr4",56382583,"-","none","CGG","CTTTAGAATTCTCCAAGTCCCGG",0,NA,NA,NA,"GeneID:132949","AASDH",TRUE,"AASDH"

"439","CTTTAGAATTCTCCAAGTCC","chr2",144546943,"-","12:C>G,18:C>G","TGG","CTTTAGAATTCTGCAAGTGCTGG",2,13,19,NA,"","",FALSE,"AASDH"

"440","CTTTAGAATTCTCCAAGTCC","chr7",110727084,"+","5:A>G,9:C>T","AGG","CTTTAAAATCCTCCAAGTCCAGG",2,6,10,NA,"GeneID:83943","IMMP2L",FALSE,"AASDH"

"441","CTTTAGAATTCTCCAAGTCC","chr6",90207834,"-","4:C>T,15:A>T","TGG","CTTTGGAATTCTCCATGTCCTGG",2,5,16,NA,"GeneID:60468","BACH2",FALSE,"AASDH"

"442","CTTTAGAATTCTCCAAGTCC","chr7",16319957,"-","2:C>A,13:T>G","AGG","CTGTAGAATTCTCAAAGTCCAGG",2,3,14,NA,"GeneID:729920","ISPD",FALSE,"AASDH"

"443","GACCTTCGATCCTTCTGTTG","chr4",56371572,"-","none","TGG","GACCTTCGATCCTTCTGTTGTGG",0,NA,NA,NA,"GeneID:132949","AASDH",TRUE,"AASDH"

"444","TAAGGCTGCCTCCTGTTATA","chr4",56384256,"-","none","TGG","TAAGGCTGCCTCCTGTTATATGG",0,NA,NA,NA,"GeneID:132949","AASDH",TRUE,"AASDH"

"445","TCGATAGGTACATAAGCAGC","chr4",56382561,"+","none","CGG","TCGATAGGTACATAAGCAGCCGG",0,NA,NA,NA,"GeneID:132949","AASDH",TRUE,"AASDH"

"446","TTACTACACCTACAAGACTG","chr4",56384187,"-","none","TGG","TTACTACACCTACAAGACTGTGG",0,NA,NA,NA,"GeneID:132949","AASDH",TRUE,"AASDH"

"447","ACCCGATGGATCTAGACATC","chr11",106094629,"+","none","AGG","ACCCGATGGATCTAGACATCAGG",0,NA,NA,NA,"GeneID:60496","AASDHPPT",TRUE,"AASDHPPT"

"448","AGGCTGGTCGTCTGATGATA","chr11",106079464,"+","none","AGG","AGGCTGGTCGTCTGATGATAAGG",0,NA,NA,NA,"GeneID:60496","AASDHPPT",TRUE,"AASDHPPT"

"449","ATCCTGATGTCTAGATCCAT","chr11",106094634,"-","none","CGG","ATCCTGATGTCTAGATCCATCGG",0,NA,NA,NA,"GeneID:60496","AASDHPPT",TRUE,"AASDHPPT"

"450","ATCCTGATGTCTAGATCCAT","chr5",39694369,"+","10:T>C,16:A>C","GGG","ATCCTGATGTTTAGATACATGGG",2,11,17,NA,"","",FALSE,"AASDHPPT"

"451","GACTAGGATTTGAATTGCAG","chr11",106091352,"+","none","CGG","GACTAGGATTTGAATTGCAGCGG",0,NA,NA,NA,"GeneID:60496","AASDHPPT",TRUE,"AASDHPPT"

"452","GACTAGGATTTGAATTGCAG","chr11",33856261,"-","4:A>T,16:G>C","TGG","GACTTGGATTTGAATTCCAGTGG",2,5,17,NA,"","",FALSE,"AASDHPPT"

"453","GATGAGTCCTTTGCAAGAAC","chr11",106079559,"-","none","TGG","GATGAGTCCTTTGCAAGAACTGG",0,NA,NA,NA,"GeneID:60496","AASDHPPT",TRUE,"AASDHPPT"

"454","GATGAGTCCTTTGCAAGAAC","chr9",21611410,"-","1:G>T,13:T>G","AGG","GCTGAGTCCTTTGAAAGAACAGG",2,2,14,NA,"","",FALSE,"AASDHPPT"

"455","TAAAGAAACACGTTTATTCC","chr11",106091422,"+","none","TGG","TAAAGAAACACGTTTATTCCTGG",0,NA,NA,NA,"GeneID:60496","AASDHPPT",TRUE,"AASDHPPT"

"456","TAAAGAAACACGTTTATTCC","chr8",132674589,"+","16:C>T,18:A>C","CGG","TAAAGAAACACGTTTACTACCGG",2,17,19,NA,"GeneID:23639","LRRC6",FALSE,"AASDHPPT"

"457","ACAAGAGGGTGCAATACATA","chr7",122093128,"+","none","AGG","ACAAGAGGGTGCAATACATAAGG",0,NA,NA,NA,"GeneID:10157","AASS",TRUE,"AASS"

"458","ACAATACTTACCTGCTCTCC","chr7",122101359,"+","none","CGG","ACAATACTTACCTGCTCTCCCGG",0,NA,NA,NA,"GeneID:10157","AASS",TRUE,"AASS"

"459","GCTAACATGTGATCCAGACC","chr7",122092882,"+","none","AGG","GCTAACATGTGATCCAGACCAGG",0,NA,NA,NA,"GeneID:10157","AASS",TRUE,"AASS"

"460","GTTAACTTTGTTTGTGATGC","chr7",122093097,"+","none","AGG","GTTAACTTTGTTTGTGATGCAGG",0,NA,NA,NA,"GeneID:10157","AASS",TRUE,"AASS"

"461","TAGGCAGTGATTACATCCAA","chr7",122101421,"-","none","CGG","TAGGCAGTGATTACATCCAACGG",0,NA,NA,NA,"GeneID:10157","AASS",TRUE,"AASS"

"462","TCTGGATCACATGTTAGCAA","chr7",122092880,"-","none","TGG","TCTGGATCACATGTTAGCAATGG",0,NA,NA,NA,"GeneID:10157","AASS",TRUE,"AASS"

"463","AGTCAAGATTGAGCGTTCAA","chr17",36989259,"-","none","AGG","AGTCAAGATTGAGCGTTCAAAGG",0,NA,NA,NA,"GeneID:26574","AATF",TRUE,"AATF"

"464","CGCTCTGTCTATCGAGTTCT","chr17",36989336,"+","none","TGG","CGCTCTGTCTATCGAGTTCTTGG",0,NA,NA,NA,"GeneID:26574","AATF",TRUE,"AATF"

"465","CTCCTTGGATCCCAACGATC","chr17",37019036,"+","none","AGG","CTCCTTGGATCCCAACGATCAGG",0,NA,NA,NA,"GeneID:26574","AATF",TRUE,"AATF"

"466","GGACCCTGAAGCGGACCCCG","chr17",36949190,"+","none","AGG","GGACCCTGAAGCGGACCCCGAGG",0,NA,NA,NA,"GeneID:26574","AATF",TRUE,"AATF"

"467","GGACCCTGAAGCGGACCCCG","chr8",23144671,"-","12:A>C,15:C>G","GGG","GGACCCTGAAGCTGAGCCCGGGG",2,13,16,NA,"GeneID:8793","TNFRSF10D",FALSE,"AATF"

"468","GTCTACTCACCAGACGATCC","chr17",36950395,"-","none","TGG","GTCTACTCACCAGACGATCCTGG",0,NA,NA,NA,"GeneID:26574","AATF",TRUE,"AATF"

"469","GTTGAACCCGCGACCAAGCG","chr17",36949166,"+","none","AGG","GTTGAACCCGCGACCAAGCGAGG",0,NA,NA,NA,"GeneID:26574","AATF",TRUE,"AATF"

"470","AGTCACCAATCTTCACCGTC","chr17",81124955,"+","none","AGG","AGTCACCAATCTTCACCGTCAGG",0,NA,NA,NA,"GeneID:9625","AATK",TRUE,"AATK"

"471","CCGTGAGCAGGCAGTTCCGC","chr17",81124982,"+","none","AGG","CCGTGAGCAGGCAGTTCCGCAGG",0,NA,NA,NA,"GeneID:9625","AATK",TRUE,"AATK"

"472","GCAGGCTAGTGCCAGCGTGC","chr17",81127831,"-","none","AGG","GCAGGCTAGTGCCAGCGTGCAGG",0,NA,NA,NA,"GeneID:9625","AATK",TRUE,"AATK"

"473","GCAGGCTAGTGCCAGCGTGC","chr11",534762,"+","16:C>G","AGG","GCAGGCTAGTGCCAGCCTGCAGG",1,17,NA,NA,"GeneID:3265","HRAS",FALSE,"AATK"

"474","GCGCTGGATCGCGCCAGAGC","chr17",81124787,"-","none","TGG","GCGCTGGATCGCGCCAGAGCTGG",0,NA,NA,NA,"GeneID:9625","AATK",TRUE,"AATK"

"475","TCGTGACTGCCGACCAGCTG","chr17",81124818,"-","none","TGG","TCGTGACTGCCGACCAGCTGTGG",0,NA,NA,NA,"GeneID:9625","AATK",TRUE,"AATK"

"476","TGTAGGGCTGCACCTCCTCC","chr17",81127792,"+","none","AGG","TGTAGGGCTGCACCTCCTCCAGG",0,NA,NA,NA,"GeneID:9625","AATK",TRUE,"AATK"

"477","CATCAACCAGGTAATTGCCT","chr16",8750465,"-","none","CGG","CATCAACCAGGTAATTGCCTCGG",0,NA,NA,NA,"GeneID:18","ABAT",TRUE,"ABAT"

"478","CATCAACCAGGTAATTGCCT","chr3",55620143,"-","0:C>G,12:G>T","GGG","GATCAACCAGGTCATTGCCTGGG",2,1,13,NA,"GeneID:26059","ERC2",FALSE,"ABAT"

"479","CATGTTGCTCGCCCAGCGCC","chr16",8735747,"+","none","TGG","CATGTTGCTCGCCCAGCGCCTGG",0,NA,NA,NA,"GeneID:18","ABAT",TRUE,"ABAT"

"480","CCAGCACAGCTACCGCCTGC","chr16",8735780,"+","none","TGG","CCAGCACAGCTACCGCCTGCTGG",0,NA,NA,NA,"GeneID:18","ABAT",TRUE,"ABAT"

"481","GAAACAGCTGAATATAATTC","chr16",8748115,"+","none","AGG","GAAACAGCTGAATATAATTCAGG",0,NA,NA,NA,"GeneID:18","ABAT",TRUE,"ABAT"

"482","GAAACAGCTGAATATAATTC","chr7",93988739,"+","4:A>C,19:T>C","AGG","GAAAAAGCTGAATATAATTTAGG",2,5,20,NA,"GeneID:10282","BET1",FALSE,"ABAT"

"483","GCTCTTACCTATGGGGACAG","chr16",8750527,"-","none","AGG","GCTCTTACCTATGGGGACAGAGG",0,NA,NA,NA,"GeneID:18","ABAT",TRUE,"ABAT"

"484","GCTCTTACCTATGGGGACAG","chr16",27573109,"+","11:A>T,19:C>G","TGG","GCTCTTACCTAAGGGGACACTGG",2,12,20,NA,"GeneID:23247","KIAA0556",FALSE,"ABAT"

"485","TGCAATTACGAAGAGAGCCG","chr16",8750445,"+","none","AGG","TGCAATTACGAAGAGAGCCGAGG",0,NA,NA,NA,"GeneID:18","ABAT",TRUE,"ABAT"

"486","AGCTCAGCCGAACAGAGATC","chr9",104889125,"+","none","AGG","AGCTCAGCCGAACAGAGATCAGG",0,NA,NA,NA,"GeneID:19","ABCA1",TRUE,"ABCA1"

"487","CCTCCCCAGGAGTCGGGTAA","chr9",104884460,"+","none","CGG","CCTCCCCAGGAGTCGGGTAACGG",0,NA,NA,NA,"GeneID:19","ABCA1",TRUE,"ABCA1"

"488","CCTCCCCAGGAGTCGGGTAA","chr6",42932588,"-","13:C>G,17:T>A","GGG","CCTCCCCAGGAGTGGGGAAAGGG",2,14,18,NA,"GeneID:10695","CNPY3",FALSE,"ABCA1"

"489","CCTCCCCAGGAGTCGGGTAA","chr1",248279737,"+","9:A>G,13:T>C","TGG","CCTCCCCAGAAGTTGGGTAATGG",2,10,14,NA,"","",FALSE,"ABCA1"

"490","GCCTCCGAGCATCTGAGAAC","chr9",104883125,"+","none","AGG","GCCTCCGAGCATCTGAGAACAGG",0,NA,NA,NA,"GeneID:19","ABCA1",TRUE,"ABCA1"

"491","TCCTGATCCTGATCTCTGTT","chr9",104889135,"-","none","CGG","TCCTGATCCTGATCTCTGTTCGG",0,NA,NA,NA,"GeneID:19","ABCA1",TRUE,"ABCA1"

"492","TCCTGATCCTGATCTCTGTT","chr7",113958895,"-","16:T>A,17:A>C","TGG","TCCTGATCCTGATCTCATTTTGG",2,17,18,NA,"GeneID:5506","PPP1R3A",FALSE,"ABCA1"

"493","TCCTGATCCTGATCTCTGTT","chr4",81047130,"-","12:G>A,19:C>A","GGG","TCCTGATCCTGACCTCTGTGGGG",2,13,20,NA,"GeneID:651","BMP3",FALSE,"ABCA1"

"494","TCCTGATCCTGATCTCTGTT","chr14",80020353,"+","6:G>T,13:A>C","AGG","TCCTGAGCCTGATATCTGTTAGG",2,7,14,NA,"","",FALSE,"ABCA1"

"495","TGTTTCCGTTACCCGACTCC","chr9",104884468,"-","none","TGG","TGTTTCCGTTACCCGACTCCTGG",0,NA,NA,NA,"GeneID:19","ABCA1",TRUE,"ABCA1"

"496","TTTGCGCATGTCCTTCATGC","chr9",104883078,"+","none","TGG","TTTGCGCATGTCCTTCATGCTGG",0,NA,NA,NA,"GeneID:19","ABCA1",TRUE,"ABCA1"

"497","TTTGCGCATGTCCTTCATGC","chr1",246929505,"-","5:A>C,18:G>C","TGG","TTTGCTCATGTCCTTCATCCTGG",2,6,19,NA,"GeneID:25909","AHCTF1",FALSE,"ABCA1"

"498","ACAAAAAATTTCACCATGCA","chr17",69221860,"+","none","TGG","ACAAAAAATTTCACCATGCATGG",0,NA,NA,NA,"GeneID:10349","ABCA10",TRUE,"ABCA10"

"499","ACAAAAAATTTCACCATGCA","chr15",29422111,"+","11:A>C,12:G>A","TGG","ACAAAAAATTTAGCCATGCATGG",2,12,13,NA,"GeneID:23359","FAM189A1",FALSE,"ABCA10"

"500","ACAAAAAATTTCACCATGCA","chr2",121048563,"+","1:A>C,8:A>T","TGG","AAAAAAAAATTCACCATGCATGG",2,2,9,NA,"","",FALSE,"ABCA10"

"501","ACAAAAAATTTCACCATGCA","chr5",137851678,"+","8:A>T,16:A>T","TGG","ACAAAAAAATTCACCAAGCATGG",2,9,17,NA,"","",FALSE,"ABCA10"

"502","ACAAAAAATTTCACCATGCA","chr19",19031544,"+","1:A>C,16:G>T","TGG","AAAAAAAATTTCACCAGGCATGG",2,2,17,NA,"GeneID:10147","SUGP2",FALSE,"ABCA10"

"503","ACAAAAAATTTCACCATGCA","chr3",186327005,"+","8:A>T,16:G>T","TGG","ACAAAAAAATTCACCAGGCATGG",2,9,17,NA,"GeneID:1608","DGKG",FALSE,"ABCA10"

"504","ACAAAAAATTTCACCATGCA","chr5",138320757,"+","8:A>T,16:G>T","TGG","ACAAAAAAATTCACCAGGCATGG",2,9,17,NA,"GeneID:995","CDC25C",FALSE,"ABCA10"

"505","ACAAAAAATTTCACCATGCA","chr3",142110794,"+","8:A>T,16:G>T","TGG","ACAAAAAAATTCACCAGGCATGG",2,9,17,NA,"GeneID:7029","TFDP2",FALSE,"ABCA10"

"506","ACAAAAAATTTCACCATGCA","chr9",109059523,"+","8:A>T,16:G>T","TGG","ACAAAAAAATTCACCAGGCATGG",2,9,17,NA,"GeneID:23731","TMEM245",FALSE,"ABCA10"

"507","ACAAAAAATTTCACCATGCA","chr7",72905541,"+","8:A>T,16:G>T","TGG","ACAAAAAAATTCACCAGGCATGG",2,9,17,NA,"GeneID:9883","POM121",FALSE,"ABCA10"

"508","ACAAAAAATTTCACCATGCA","chr2",75696941,"-","8:T>A,16:C>A","TGG","ACAAAAAAATTCACCAGGCATGG",2,9,17,NA,"GeneID:6936","GCFC2",FALSE,"ABCA10"

"509","ACAAAAAATTTCACCATGCA","chr7",56715715,"-","8:T>A,11:T>G","TGG","ACAAAAAAATTAACCATGCATGG",2,9,12,NA,"","",FALSE,"ABCA10"

"510","CATATCGCCTGAAGTTTAAT","chr17",69222578,"-","none","TGG","CATATCGCCTGAAGTTTAATTGG",0,NA,NA,NA,"GeneID:10349","ABCA10",TRUE,"ABCA10"

"511","CTTCCTTAAAGAACACTGTT","chr17",69221886,"-","none","GGG","CTTCCTTAAAGAACACTGTTGGG",0,NA,NA,NA,"GeneID:10349","ABCA10",TRUE,"ABCA10"

"512","GATACCACCTTTCATTTCTA","chr17",69219692,"-","none","AGG","GATACCACCTTTCATTTCTAAGG",0,NA,NA,NA,"GeneID:10349","ABCA10",TRUE,"ABCA10"

"513","TCAGAGTGCTCCTTTATAAC","chr17",69222540,"+","none","TGG","TCAGAGTGCTCCTTTATAACTGG",0,NA,NA,NA,"GeneID:10349","ABCA10",TRUE,"ABCA10"

"514","TTGCATCATTAAATGTTGCA","chr17",69219606,"-","none","AGG","TTGCATCATTAAATGTTGCAAGG",0,NA,NA,NA,"GeneID:10349","ABCA10",TRUE,"ABCA10"

"515","ATAAACTGCTGTCCTTATCC","chr2",215054617,"+","none","AGG","ATAAACTGCTGTCCTTATCCAGG",0,NA,NA,NA,"GeneID:26154","ABCA12",TRUE,"ABCA12"

"516","CAAGTGCTCGCACGAATTCT","chr2",215052499,"-","none","TGG","CAAGTGCTCGCACGAATTCTTGG",0,NA,NA,NA,"GeneID:26154","ABCA12",TRUE,"ABCA12"

"517","CCCGGAACATATACTTTCAA","chr2",215052526,"-","none","TGG","CCCGGAACATATACTTTCAATGG",0,NA,NA,NA,"GeneID:26154","ABCA12",TRUE,"ABCA12"

"518","CCTCGAAACCTTCCTAGTAC","chr2",215064188,"-","none","TGG","CCTCGAAACCTTCCTAGTACTGG",0,NA,NA,NA,"GeneID:26154","ABCA12",TRUE,"ABCA12"

"519","GGACACTTGTCTTGATCTTA","chr2",215111666,"-","none","TGG","GGACACTTGTCTTGATCTTATGG",0,NA,NA,NA,"GeneID:26154","ABCA12",TRUE,"ABCA12"

"520","TCTGAGAAAGTCATCCAACC","chr2",215054638,"-","none","TGG","TCTGAGAAAGTCATCCAACCTGG",0,NA,NA,NA,"GeneID:26154","ABCA12",TRUE,"ABCA12"

"521","TCTGAGAAAGTCATCCAACC","chr6",99894352,"-","12:A>T,19:T>G","TGG","TCTGAGAAAGTCTTCCAACATGG",2,13,20,NA,"","",FALSE,"ABCA12"

"522","ACCCACACTCTTCCAATATG","chr7",48387866,"-","none","AGG","ACCCACACTCTTCCAATATGAGG",0,NA,NA,NA,"GeneID:154664","ABCA13",TRUE,"ABCA13"

"523","AGACCAAATCACCGCCCTGC","chr7",48389166,"+","none","TGG","AGACCAAATCACCGCCCTGCTGG",0,NA,NA,NA,"GeneID:154664","ABCA13",TRUE,"ABCA13"

"524","GAACAAGGGGGCATGACATT","chr7",48376472,"+","none","TGG","GAACAAGGGGGCATGACATTTGG",0,NA,NA,NA,"GeneID:154664","ABCA13",TRUE,"ABCA13"

"525","GAACAAGGGGGCATGACATT","chr8",10205251,"+","3:A>C,10:T>G","TGG","GAAAAAGGGGTCATGACATTTGG",2,4,11,NA,"GeneID:4482","MSRA",FALSE,"ABCA13"

"526","GAACAAGGGGGCATGACATT","chr8",119535948,"+","9:A>G,13:A>T","AGG","GAACAAGGGAGCAAGACATTAGG",2,10,14,NA,"","",FALSE,"ABCA13"

"527","TACTTGAGCAACTTGATTCC","chr7",48376550,"+","none","TGG","TACTTGAGCAACTTGATTCCTGG",0,NA,NA,NA,"GeneID:154664","ABCA13",TRUE,"ABCA13"

"528","TCCCCTTTACTGCCTCATAT","chr7",48387851,"+","none","TGG","TCCCCTTTACTGCCTCATATTGG",0,NA,NA,NA,"GeneID:154664","ABCA13",TRUE,"ABCA13"

"529","TCCCCTTTACTGCCTCATAT","chr4",41142283,"+","1:T>C,3:A>C","GGG","TTCACTTTACTGCCTCATATGGG",2,2,4,NA,"GeneID:323","APBB2",FALSE,"ABCA13"

"530","TCCCCTTTACTGCCTCATAT","chr10",14943984,"+","7:G>T,11:A>G","TGG","TCCCCTTGACTACCTCATATTGG",2,8,12,NA,"GeneID:64421","DCLRE1C",FALSE,"ABCA13"

"531","TTTGGTCTCTGTAGAAGGTC","chr7",48389154,"-","none","AGG","TTTGGTCTCTGTAGAAGGTCAGG",0,NA,NA,NA,"GeneID:154664","ABCA13",TRUE,"ABCA13"

"532","TTTGGTCTCTGTAGAAGGTC","chr11",68216532,"+","10:C>G,14:C>A","TGG","TTTGGTCTCTCTAGCAGGTCTGG",2,11,15,NA,"","",FALSE,"ABCA13"

"533","CAGGGGGATGAAGATCTCGA","chr9",137024203,"+","none","AGG","CAGGGGGATGAAGATCTCGAAGG",0,NA,NA,NA,"GeneID:20","ABCA2",TRUE,"ABCA2"

"534","CTCGGTGGCCAGAAACCCGC","chr9",137022439,"-","none","AGG","CTCGGTGGCCAGAAACCCGCAGG",0,NA,NA,NA,"GeneID:20","ABCA2",TRUE,"ABCA2"

"535","CTTCCTCCACCACGCGGTCC","chr9",137022821,"+","none","AGG","CTTCCTCCACCACGCGGTCCAGG",0,NA,NA,NA,"GeneID:20","ABCA2",TRUE,"ABCA2"

"536","CTTCCTCCACCACGCGGTCC","chr9",88936859,"+","7:A>C,14:T>C","AGG","CTTCCTCAACCACGTGGTCCAGG",2,8,15,NA,"","",FALSE,"ABCA2"

"537","GAAGCCCACCATCTCCGTGA","chr9",137024148,"-","none","AGG","GAAGCCCACCATCTCCGTGAAGG",0,NA,NA,NA,"GeneID:20","ABCA2",TRUE,"ABCA2"

"538","GAGTGCTTGGGCCGTGCTAT","chr9",137022377,"+","none","TGG","GAGTGCTTGGGCCGTGCTATTGG",0,NA,NA,NA,"GeneID:20","ABCA2",TRUE,"ABCA2"

"539","TGGGCCGCGCTGGGTCAAAC","chr9",137022794,"+","none","AGG","TGGGCCGCGCTGGGTCAAACAGG",0,NA,NA,NA,"GeneID:20","ABCA2",TRUE,"ABCA2"

"540","CAACTGCTCGTCCAGCGTGC","chr16",2324462,"-","none","TGG","CAACTGCTCGTCCAGCGTGCTGG",0,NA,NA,NA,"GeneID:21","ABCA3",TRUE,"ABCA3"

"541","CCAGGAGTACATGCGCATGA","chr16",2317748,"-","none","TGG","CCAGGAGTACATGCGCATGATGG",0,NA,NA,NA,"GeneID:21","ABCA3",TRUE,"ABCA3"

"542","CCAGGAGTACATGCGCATGA","chrX",80572833,"-","2:A>T,13:C>G","AGG","CCTGGAGTACATGGGCATGAAGG",2,3,14,NA,"","",TRUE,"ABCA3"

"543","GCGCGGCTTTCCCTCCGAGA","chr16",2324510,"-","none","AGG","GCGCGGCTTTCCCTCCGAGAAGG",0,NA,NA,NA,"GeneID:21","ABCA3",TRUE,"ABCA3"

"544","TCACCAGGTTCTCCGCCATC","chr16",2323518,"+","none","AGG","TCACCAGGTTCTCCGCCATCAGG",0,NA,NA,NA,"GeneID:21","ABCA3",TRUE,"ABCA3"

"545","TCATGAAGGAGGCGGCGATG","chr16",2317667,"+","none","AGG","TCATGAAGGAGGCGGCGATGAGG",0,NA,NA,NA,"GeneID:21","ABCA3",TRUE,"ABCA3"

"546","TCCGTGTGTAACTGAACCGT","chr16",2323654,"+","none","AGG","TCCGTGTGTAACTGAACCGTAGG",0,NA,NA,NA,"GeneID:21","ABCA3",TRUE,"ABCA3"

"547","ATGCCACCGTAGCAGGCAAG","chr1",94007635,"-","none","AGG","ATGCCACCGTAGCAGGCAAGAGG",0,NA,NA,NA,"GeneID:24","ABCA4",TRUE,"ABCA4"

"548","CACCTCGAAGCCGGGCATAA","chr1",94005463,"+","none","AGG","CACCTCGAAGCCGGGCATAAAGG",0,NA,NA,NA,"GeneID:24","ABCA4",TRUE,"ABCA4"

"549","CATTCATGATGGCTGTAGAG","chr1",94112971,"+","none","TGG","CATTCATGATGGCTGTAGAGTGG",0,NA,NA,NA,"GeneID:24","ABCA4",TRUE,"ABCA4"

"550","CATTCATGATGGCTGTAGAG","chr7",84200332,"+","4:A>C,13:G>T","TGG","CATTAATGATGGCGGTAGAGTGG",2,5,14,NA,"GeneID:10371","SEMA3A",FALSE,"ABCA4"

"551","GCAATTGATGAGCTGCTCAC","chr1",94005504,"-","none","AGG","GCAATTGATGAGCTGCTCACAGG",0,NA,NA,NA,"GeneID:24","ABCA4",TRUE,"ABCA4"

"552","GCAATTGATGAGCTGCTCAC","chr22",19161778,"-","6:G>C,14:T>C","AGG","GCAATTCATGAGCTACTCACAGG",2,7,15,NA,"","",FALSE,"ABCA4"

"553","GCATCGCCTTGTTGGGGAAA","chr1",94111555,"+","none","TGG","GCATCGCCTTGTTGGGGAAATGG",0,NA,NA,NA,"GeneID:24","ABCA4",TRUE,"ABCA4"

"554","GCATCGCCTTGTTGGGGAAA","chr1",59077660,"-","6:A>G,13:T>C","TGG","GCATCGTCTTGTTAGGGAAATGG",2,7,14,NA,"","",FALSE,"ABCA4"

"555","TGCCGGCACCATTCACTCCC","chr1",94007706,"+","none","AGG","TGCCGGCACCATTCACTCCCAGG",0,NA,NA,NA,"GeneID:24","ABCA4",TRUE,"ABCA4"

"556","ACCACACCTACAAAGTTGCT","chr17",69309341,"+","none","CGG","ACCACACCTACAAAGTTGCTCGG",0,NA,NA,NA,"GeneID:23461","ABCA5",TRUE,"ABCA5"

"557","ACCACACCTACAAAGTTGCT","chr11",8553431,"+","2:A>C,13:T>A","AGG","ACAACACCTACAATGTTGCTAGG",2,3,14,NA,"GeneID:65975","STK33",FALSE,"ABCA5"

"558","CTGCTTGTAATATTAGTCAC","chr17",69313129,"+","none","TGG","CTGCTTGTAATATTAGTCACTGG",0,NA,NA,NA,"GeneID:23461","ABCA5",TRUE,"ABCA5"

"559","GAAATAGATACCTTTCCCCG","chr17",69306859,"-","none","AGG","GAAATAGATACCTTTCCCCGAGG",0,NA,NA,NA,"GeneID:23461","ABCA5",TRUE,"ABCA5"

"560","GTCAACTAAAGCTGTTATTA","chr17",69306899,"-","none","TGG","GTCAACTAAAGCTGTTATTATGG",0,NA,NA,NA,"GeneID:23461","ABCA5",TRUE,"ABCA5"

"561","TCTAAGCCGAGCAACTTTGT","chr17",69309350,"-","none","AGG","TCTAAGCCGAGCAACTTTGTAGG",0,NA,NA,NA,"GeneID:23461","ABCA5",TRUE,"ABCA5"

"562","TCTACTGATCATCTACCTGA","chr17",69313093,"-","none","TGG","TCTACTGATCATCTACCTGATGG",0,NA,NA,NA,"GeneID:23461","ABCA5",TRUE,"ABCA5"

"563","AAGATGATTCCCATAGCATA","chr17",69136165,"+","none","TGG","AAGATGATTCCCATAGCATATGG",0,NA,NA,NA,"GeneID:23460","ABCA6",TRUE,"ABCA6"

"564","ATTCTTGCACAGAAGTGCTT","chr17",69140646,"+","none","TGG","ATTCTTGCACAGAAGTGCTTTGG",0,NA,NA,NA,"GeneID:23460","ABCA6",TRUE,"ABCA6"

"565","GATGAAAAGAGAGAGCTTAT","chr17",69140609,"-","none","TGG","GATGAAAAGAGAGAGCTTATTGG",0,NA,NA,NA,"GeneID:23460","ABCA6",TRUE,"ABCA6"

"566","GATGAAAAGAGAGAGCTTAT","chr10",86788319,"-","12:T>C,16:G>A","AGG","GATGAAAAGAGAAAGCCTATAGG",2,13,17,NA,"GeneID:657","BMPR1A",FALSE,"ABCA6"

"567","GTACACCTACCTTTCAAAAG","chr17",69137285,"+","none","AGG","GTACACCTACCTTTCAAAAGAGG",0,NA,NA,NA,"GeneID:23460","ABCA6",TRUE,"ABCA6"

"568","TGGGGCCTCTCAATACTTCT","chr17",69137477,"-","none","AGG","TGGGGCCTCTCAATACTTCTAGG",0,NA,NA,NA,"GeneID:23460","ABCA6",TRUE,"ABCA6"

"569","TTCACAGGAACAAGTGTCAT","chr17",69136237,"-","none","TGG","TTCACAGGAACAAGTGTCATTGG",0,NA,NA,NA,"GeneID:23460","ABCA6",TRUE,"ABCA6"

"570","TTCACAGGAACAAGTGTCAT","chr1",205421819,"-","3:A>T,13:T>C","AGG","TTCTCAGGAACAAATGTCATAGG",2,4,14,NA,"GeneID:93273","LEMD1",FALSE,"ABCA6"

"571","ACTCACTGCAGATTCGCGGC","chr19",1064919,"+","none","GGG","ACTCACTGCAGATTCGCGGCGGG",0,NA,NA,NA,"GeneID:10347","ABCA7",TRUE,"ABCA7"

"572","AGCCGATGCCCGCACTGTGC","chr19",1042078,"+","none","TGG","AGCCGATGCCCGCACTGTGCTGG",0,NA,NA,NA,"GeneID:10347","ABCA7",TRUE,"ABCA7"

"573","AGCCGATGCCCGCACTGTGC","chr22",40811582,"-","3:T>G,10:T>G","TGG","AGCAGATGCCAGCACTGTGCTGG",2,4,11,NA,"GeneID:10478","SLC25A17",FALSE,"ABCA7"

"574","GAGGCCACAGCAATTCGACC","chr19",1041520,"-","none","AGG","GAGGCCACAGCAATTCGACCAGG",0,NA,NA,NA,"GeneID:10347","ABCA7",TRUE,"ABCA7"

"575","GCTCTCCAAAGACGCGCGCC","chr19",1065086,"-","none","AGG","GCTCTCCAAAGACGCGCGCCAGG",0,NA,NA,NA,"GeneID:10347","ABCA7",TRUE,"ABCA7"

"576","GGAAGCTGATCGCCACGCTG","chr19",1042137,"+","none","AGG","GGAAGCTGATCGCCACGCTGAGG",0,NA,NA,NA,"GeneID:10347","ABCA7",TRUE,"ABCA7"

"577","GGAAGCTGATCGCCACGCTG","chr10",101771459,"-","15:T>G,17:C>G","AGG","GGAAGCTGATCGCCAAGGTGAGG",2,16,18,NA,"GeneID:2253","FGF8",TRUE,"ABCA7"

"578","GGAAGCTGATCGCCACGCTG","chr6",152122784,"-","2:G>T,15:C>G","GGG","GGCAGCTGATCGCCAGGCTGGGG",2,3,16,NA,"GeneID:2099;GeneID:23345","ESR1;SYNE1",FALSE,"ABCA7"

"579","GGGAGTCGTTGAAGTTGCTC","chr19",1041951,"-","none","AGG","GGGAGTCGTTGAAGTTGCTCAGG",0,NA,NA,NA,"GeneID:10347","ABCA7",TRUE,"ABCA7"

"580","ACTGTGAAGTTTCAGTATTT","chr17",68933221,"-","none","TGG","ACTGTGAAGTTTCAGTATTTTGG",0,NA,NA,NA,"GeneID:10351","ABCA8",TRUE,"ABCA8"

"581","ACTGTGAAGTTTCAGTATTT","chr6",32851678,"+","6:C>A,15:C>T","AGG","ACTGTGCAGTTTCAGCATTTAGG",2,7,16,NA,"GeneID:5698;GeneID:6890","PSMB9;TAP1",FALSE,"ABCA8"

"582","ATATGAGTATGTATTAGTAA","chr17",68937011,"+","none","AGG","ATATGAGTATGTATTAGTAAAGG",0,NA,NA,NA,"GeneID:10351","ABCA8",TRUE,"ABCA8"

"583","ATATGAGTATGTATTAGTAA","chr3",147725832,"-","6:G>C,18:G>T","GGG","ATATGACTATGTATTAGTCAGGG",2,7,19,NA,"","",FALSE,"ABCA8"

"584","TAAATGTATCTACCCGTCCC","chr17",68940847,"+","none","AGG","TAAATGTATCTACCCGTCCCAGG",0,NA,NA,NA,"GeneID:10351","ABCA8",TRUE,"ABCA8"

"585","TCATTTACTTGATGACTATG","chr17",68940894,"+","none","AGG","TCATTTACTTGATGACTATGAGG",0,NA,NA,NA,"GeneID:10351","ABCA8",TRUE,"ABCA8"

"586","TCATTTACTTGATGACTATG","chr11",116530809,"-","0:T>A,13:A>C","TGG","ACATTTACTTGATTACTATGTGG",2,1,14,NA,"","",FALSE,"ABCA8"

"587","TCTTTAATACTTTCCTCATC","chr17",68937072,"+","none","TGG","TCTTTAATACTTTCCTCATCTGG",0,NA,NA,NA,"GeneID:10351","ABCA8",TRUE,"ABCA8"

"588","TTCTATAATAGCAGCATTAA","chr17",68933167,"+","none","TGG","TTCTATAATAGCAGCATTAATGG",0,NA,NA,NA,"GeneID:10351","ABCA8",TRUE,"ABCA8"

"589","TTCTATAATAGCAGCATTAA","chr8",53690974,"+","4:G>A","TGG","TTCTGTAATAGCAGCATTAATGG",1,5,NA,NA,"","",FALSE,"ABCA8"

"590","TTCTATAATAGCAGCATTAA","chr17",69044496,"+","6:G>A","TGG","TTCTATGATAGCAGCATTAATGG",1,7,NA,NA,"GeneID:10350","ABCA9",TRUE,"ABCA8"

"591","TTCTATAATAGCAGCATTAA","chr4",18591867,"+","6:T>A,13:C>G","AGG","TTCTATTATAGCACCATTAAAGG",2,7,14,NA,"","",FALSE,"ABCA8"

"592","TTCTATAATAGCAGCATTAA","chr18",70127389,"+","6:G>A,16:G>T","GGG","TTCTATGATAGCAGCAGTAAGGG",2,7,17,NA,"GeneID:25914","RTTN",FALSE,"ABCA8"

"593","TTCTATAATAGCAGCATTAA","chr6",119811153,"-","4:C>T,14:T>G","AGG","TTCTGTAATAGCAGAATTAAAGG",2,5,15,NA,"","",FALSE,"ABCA8"

"594","AAAGAGCACAGAGACCATTC","chr17",69045173,"-","none","AGG","AAAGAGCACAGAGACCATTCAGG",0,NA,NA,NA,"GeneID:10350","ABCA9",TRUE,"ABCA9"

"595","AAAGAGCACAGAGACCATTC","chr13",33265015,"+","2:T>A,14:T>C","AGG","AATGAGCACAGAGATCATTCAGG",2,3,15,NA,"GeneID:90627;GeneID:100874241","STARD13;STARD13-AS",FALSE,"ABCA9"

"596","AAAGAGCACAGAGACCATTC","chr7",32293926,"+","1:G>A,17:G>T","TGG","AGAGAGCACAGAGACCAGTCTGG",2,2,18,NA,"GeneID:5137","PDE1C",FALSE,"ABCA9"

"597","AATCAGTTGCAACTCCTCCT","chr17",69043615,"+","none","TGG","AATCAGTTGCAACTCCTCCTTGG",0,NA,NA,NA,"GeneID:10350","ABCA9",TRUE,"ABCA9"

"598","AATCAGTTGCAACTCCTCCT","chrX",74461827,"+","5:C>G,8:A>G","AGG","AATCACTTACAACTCCTCCTAGG",2,6,9,NA,"GeneID:6567","SLC16A2",FALSE,"ABCA9"

"599","AATGAAAAAATGAAGTGTGA","chr17",69044563,"-","none","AGG","AATGAAAAAATGAAGTGTGAAGG",0,NA,NA,NA,"GeneID:10350","ABCA9",TRUE,"ABCA9"

"600","AATGAAAAAATGAAGTGTGA","chr3",3621512,"+","14:T>G,15:A>T","AGG","AATGAAAAAATGAATAGTGAAGG",2,15,16,NA,"","",FALSE,"ABCA9"

"601","AATGAAAAAATGAAGTGTGA","chr5",54058329,"-","0:A>T,2:C>A","GGG","TAGGAAAAAATGAAGTGTGAGGG",2,1,3,NA,"GeneID:54622","ARL15",FALSE,"ABCA9"

"602","AATGAAAAAATGAAGTGTGA","chr2",44666172,"+","2:G>T,14:A>G","GGG","AAGGAAAAAATGAAATGTGAGGG",2,3,15,NA,"GeneID:79823","CAMKMT",FALSE,"ABCA9"

"603","AATGAAAAAATGAAGTGTGA","chr2",23198745,"+","9:C>A,14:A>G","TGG","AATGAAAAACTGAAATGTGATGG",2,10,15,NA,"","",FALSE,"ABCA9"

"604","AATGAAAAAATGAAGTGTGA","chr7",28725689,"+","2:A>T,14:C>G","AGG","AAAGAAAAAATGAACTGTGAAGG",2,3,15,NA,"GeneID:9586","CREB5",FALSE,"ABCA9"

"605","AATGAAAAAATGAAGTGTGA","chr3",165009291,"-","8:C>T,16:A>C","TGG","AATGAAAAGATGAAGTTTGATGG",2,9,17,NA,"GeneID:6476","SI",TRUE,"ABCA9"

"606","AATGAAAAAATGAAGTGTGA","chr5",120425939,"-","7:A>T,17:T>A","AGG","AATGAAATAATGAAGTGAGAAGG",2,8,18,NA,"","",FALSE,"ABCA9"

"607","AATGAAAAAATGAAGTGTGA","chr11",129241065,"-","1:C>T,18:A>C","AGG","AGTGAAAAAATGAAGTGTTAAGG",2,2,19,NA,"GeneID:9743","ARHGAP32",FALSE,"ABCA9"

"608","GTGGCCTGATGAAAAAAGCA","chr17",69045300,"-","none","TGG","GTGGCCTGATGAAAAAAGCATGG",0,NA,NA,NA,"GeneID:10350","ABCA9",TRUE,"ABCA9"

"609","GTGGCCTGATGAAAAAAGCA","chrX",102883717,"+","0:A>G,8:G>A","GGG","ATGGCCTGGTGAAAAAAGCAGGG",2,1,9,NA,"GeneID:100287765","LINC00630",FALSE,"ABCA9"

"610","GTGGCCTGATGAAAAAAGCA","chr11",7701806,"+","9:A>T,15:G>A","AGG","GTGGCCTGAAGAAAAGAGCAAGG",2,10,16,NA,"GeneID:341277","OVCH2",TRUE,"ABCA9"

"611","TACGTCATTGATGACAATGA","chr17",69043513,"-","none","TGG","TACGTCATTGATGACAATGATGG",0,NA,NA,NA,"GeneID:10350","ABCA9",TRUE,"ABCA9"

"612","TACGTCATTGATGACAATGA","chr4",176241914,"-","11:C>A,17:T>A","TGG","TACGTCATTGAGGACAAAGATGG",2,12,18,NA,"GeneID:140458","ASB5",FALSE,"ABCA9"

"613","TTCTATGATAGCAGCATTAA","chr17",69044496,"+","none","TGG","TTCTATGATAGCAGCATTAATGG",0,NA,NA,NA,"GeneID:10350","ABCA9",TRUE,"ABCA9"

"614","TTCTATGATAGCAGCATTAA","chr18",70127389,"+","16:G>T","GGG","TTCTATGATAGCAGCAGTAAGGG",1,17,NA,NA,"GeneID:25914","RTTN",FALSE,"ABCA9"

"615","TTCTATGATAGCAGCATTAA","chr7",4546707,"+","16:A>T,17:A>T","TGG","TTCTATGATAGCAGCAAAAATGG",2,17,18,NA,"","",FALSE,"ABCA9"

"616","TTCTATGATAGCAGCATTAA","chr17",68933167,"+","6:A>G","TGG","TTCTATAATAGCAGCATTAATGG",1,7,NA,NA,"GeneID:10351","ABCA8",TRUE,"ABCA9"

"617","TTCTATGATAGCAGCATTAA","chr8",53690974,"+","4:G>A,6:A>G","TGG","TTCTGTAATAGCAGCATTAATGG",2,5,7,NA,"","",FALSE,"ABCA9"

"618","TTCTATGATAGCAGCATTAA","chr4",18591867,"+","6:T>G,13:C>G","AGG","TTCTATTATAGCACCATTAAAGG",2,7,14,NA,"","",FALSE,"ABCA9"

"619","TTCTATGATAGCAGCATTAA","chr3",178130831,"-","2:A>G,17:T>A","TGG","TTTTATGATAGCAGCATAAATGG",2,3,18,NA,"","",FALSE,"ABCA9"

"620","ATCTGGAGGAAGACATGACC","chr7",87570173,"-","none","AGG","ATCTGGAGGAAGACATGACCAGG",0,NA,NA,NA,"GeneID:5243","ABCB1",TRUE,"ABCB1"

"621","ATCTGGAGGAAGACATGACC","chr2",219801791,"+","1:C>T,16:C>G","TGG","ACCTGGAGGAAGACATCACCTGG",2,2,17,NA,"","",FALSE,"ABCB1"

"622","CCAAACACCAGCATCATGAG","chr7",87585579,"+","none","AGG","CCAAACACCAGCATCATGAGAGG",0,NA,NA,NA,"GeneID:5243","ABCB1",TRUE,"ABCB1"

"623","CCAAACACCAGCATCATGAG","chr9",89854707,"+","3:C>A,15:C>A","AGG","CCACACACCAGCATCCTGAGAGG",2,4,16,NA,"","",FALSE,"ABCB1"

"624","CCAAACACCAGCATCATGAG","chr5",31799217,"-","3:C>T,17:G>C","AGG","CCAGACACCAGCATCATCAGAGG",2,4,18,NA,"GeneID:23037","PDZD2",TRUE,"ABCB1"

"625","CCAAACACCAGCATCATGAG","chr9",128137578,"-","3:G>T,15:G>T","AGG","CCACACACCAGCATCCTGAGAGG",2,4,16,NA,"","",FALSE,"ABCB1"

"626","CTAGGTGATATCAATGATAC","chr7",87570207,"-","none","AGG","CTAGGTGATATCAATGATACAGG",0,NA,NA,NA,"GeneID:5243","ABCB1",TRUE,"ABCB1"

"627","CTAGGTGATATCAATGATAC","chr6",109386689,"-","11:T>G,19:T>G","TGG","CTAGGTGATATAAATGATAATGG",2,12,20,NA,"","",FALSE,"ABCB1"

"628","GAAGCTAACCCTTGTGATTT","chr7",87566116,"-","none","TGG","GAAGCTAACCCTTGTGATTTTGG",0,NA,NA,NA,"GeneID:5243","ABCB1",TRUE,"ABCB1"

"629","TGGACTTCCTCTCATGATGC","chr7",87585589,"-","none","TGG","TGGACTTCCTCTCATGATGCTGG",0,NA,NA,NA,"GeneID:5243","ABCB1",TRUE,"ABCB1"

"630","TGGACTTCCTCTCATGATGC","chr3",104643554,"-","8:T>G,15:T>C","CGG","TGGACTTCATCTCATAATGCCGG",2,9,16,NA,"","",FALSE,"ABCB1"

"631","TTGGACTGTCAGCTGCTGTC","chr7",87566078,"-","none","TGG","TTGGACTGTCAGCTGCTGTCTGG",0,NA,NA,NA,"GeneID:5243","ABCB1",TRUE,"ABCB1"

"632","CAAGGAACTATTAGTCTTGA","chr1",229527292,"-","none","TGG","CAAGGAACTATTAGTCTTGATGG",0,NA,NA,NA,"GeneID:23456","ABCB10",TRUE,"ABCB10"

"633","CAGCTAGCTGAGGAACGTAT","chr1",229540735,"-","none","TGG","CAGCTAGCTGAGGAACGTATTGG",0,NA,NA,NA,"GeneID:23456","ABCB10",TRUE,"ABCB10"

"634","GTCTACCTCATGCAAACTTC","chr1",229549235,"-","none","AGG","GTCTACCTCATGCAAACTTCAGG",0,NA,NA,NA,"GeneID:23456","ABCB10",TRUE,"ABCB10"

"635","GTCTACCTCATGCAAACTTC","chr2",82169388,"-","12:T>G,18:C>A","TGG","GTCTACCTCATGAAAACTGCTGG",2,13,19,NA,"","",FALSE,"ABCB10"

"636","TACCATCATACTGATGCCTA","chr1",229547495,"+","none","CGG","TACCATCATACTGATGCCTACGG",0,NA,NA,NA,"GeneID:23456","ABCB10",TRUE,"ABCB10"

"637","TCCTTGCTAACTGCATTACA","chr1",229540640,"+","none","TGG","TCCTTGCTAACTGCATTACATGG",0,NA,NA,NA,"GeneID:23456","ABCB10",TRUE,"ABCB10"

"638","TTCTTTGACAAGACTCGCAC","chr1",229547616,"-","none","AGG","TTCTTTGACAAGACTCGCACAGG",0,NA,NA,NA,"GeneID:23456","ABCB10",TRUE,"ABCB10"

"639","TTCTTTGACAAGACTCGCAC","chr15",28508034,"-","12:C>T,15:C>G","AGG","TTCTTTGACAAGGCTGGCACAGG",2,13,16,NA,"","",TRUE,"ABCB10"

"640","TTCTTTGACAAGACTCGCAC","chr16",48104327,"-","10:G>T,16:G>C","TGG","TTCTTTGACACGACTCCCACTGG",2,11,17,NA,"GeneID:94160","ABCC12",TRUE,"ABCB10"

"641","TTCTTTGACAAGACTCGCAC","chr15",28403603,"+","12:G>A,15:G>C","AGG","TTCTTTGACAAGGCTGGCACAGG",2,13,16,NA,"","",TRUE,"ABCB10"

"642","AAGAATTACTGAGTCAGACA","chr2",169018104,"+","none","TGG","AAGAATTACTGAGTCAGACATGG",0,NA,NA,NA,"GeneID:8647","ABCB11",TRUE,"ABCB11"

"643","AAGAATTACTGAGTCAGACA","chr1",178002056,"-","9:T>A,13:G>A","AGG","AAGAATTACAGAGCCAGACAAGG",2,10,14,NA,"","",FALSE,"ABCB11"

"644","AAGAATTACTGAGTCAGACA","chr1",238026606,"-","0:A>T,18:C>G","TGG","TAGAATTACTGAGTCAGAGATGG",2,1,19,NA,"","",FALSE,"ABCB11"

"645","ATAATAATGATAAGAAATCA","chr2",169016779,"-","none","AGG","ATAATAATGATAAGAAATCAAGG",0,NA,NA,NA,"GeneID:8647","ABCB11",TRUE,"ABCB11"

"646","ATAATAATGATAAGAAATCA","chr1",209058598,"+","13:T>G,18:T>C","AGG","ATAATAATGATAATAAATTAAGG",2,14,19,NA,"","",FALSE,"ABCB11"

"647","ATAATAATGATAAGAAATCA","chr5",150830513,"+","10:A>T,15:G>A","AGG","ATAATAATGAAAAGAGATCAAGG",2,11,16,NA,"","",FALSE,"ABCB11"

"648","ATAATAATGATAAGAAATCA","chr10",19772283,"+","7:A>T,10:G>T","AGG","ATAATAAAGAGAAGAAATCAAGG",2,8,11,NA,"GeneID:340895","MALRD1",FALSE,"ABCB11"

"649","ATAATAATGATAAGAAATCA","chr1",212776967,"+","7:A>T,11:T>A","AGG","ATAATAAAGATTAGAAATCAAGG",2,8,12,NA,"GeneID:25936","NSL1",FALSE,"ABCB11"

"650","ATAATAATGATAAGAAATCA","chr17",51022959,"+","8:A>G,13:T>G","GGG","ATAATAATAATAATAAATCAGGG",2,9,14,NA,"GeneID:9043","SPAG9",FALSE,"ABCB11"

"651","ATAATAATGATAAGAAATCA","chr12",9922916,"-","8:T>C,13:A>C","TGG","ATAATAATAATAATAAATCATGG",2,9,14,NA,"GeneID:387836","CLEC2A",FALSE,"ABCB11"

"652","ATAATAATGATAAGAAATCA","chr6",32358409,"-","8:T>C,10:T>A","AGG","ATAATAATAAAAAGAAATCAAGG",2,9,11,NA,"GeneID:10665","C6orf10",FALSE,"ABCB11"

"653","CCAAAAATGAGTAGCACGCC","chr2",169013409,"+","none","TGG","CCAAAAATGAGTAGCACGCCTGG",0,NA,NA,NA,"GeneID:8647","ABCB11",TRUE,"ABCB11"

"654","CTTCGAAGTATAAAGAAATT","chr2",169018087,"-","none","TGG","CTTCGAAGTATAAAGAAATTTGG",0,NA,NA,NA,"GeneID:8647","ABCB11",TRUE,"ABCB11"

"655","GGAACTGTTAGTCCATACAA","chr2",169013306,"+","none","TGG","GGAACTGTTAGTCCATACAATGG",0,NA,NA,NA,"GeneID:8647","ABCB11",TRUE,"ABCB11"

"656","GTGTGAATAACACCATTGTA","chr2",169013321,"-","none","TGG","GTGTGAATAACACCATTGTATGG",0,NA,NA,NA,"GeneID:8647","ABCB11",TRUE,"ABCB11"

"657","AAAGTCGCCCTCCGCGCTCG","chr7",87475402,"+","none","TGG","AAAGTCGCCCTCCGCGCTCGTGG",0,NA,NA,NA,"GeneID:5244","ABCB4",TRUE,"ABCB4"

"658","ACGAAGACAGTGAAAATGAT","chr7",87472636,"-","none","TGG","ACGAAGACAGTGAAAATGATTGG",0,NA,NA,NA,"GeneID:5244","ABCB4",TRUE,"ABCB4"

"659","ACGAAGACAGTGAAAATGAT","chr3",14170453,"-","1:C>G,7:T>G","TGG","AGGAAGAAAGTGAAAATGATTGG",2,2,8,NA,"GeneID:7508","XPC",TRUE,"ABCB4"

"660","ACGAAGACAGTGAAAATGAT","chr10",69088931,"+","2:T>G,9:A>G","AGG","ACTAAGACAATGAAAATGATAGG",2,3,10,NA,"GeneID:5552","SRGN",FALSE,"ABCB4"

"661","CGCGGAGGGCGACTTTGAAC","chr7",87475398,"-","none","TGG","CGCGGAGGGCGACTTTGAACTGG",0,NA,NA,NA,"GeneID:5244","ABCB4",TRUE,"ABCB4"

"662","CGGCAAAGAACGGAACAGCC","chr7",87475432,"-","none","TGG","CGGCAAAGAACGGAACAGCCTGG",0,NA,NA,NA,"GeneID:5244","ABCB4",TRUE,"ABCB4"

"663","GAGACCTGATCCGTGAGCTA","chr7",87462830,"+","none","TGG","GAGACCTGATCCGTGAGCTATGG",0,NA,NA,NA,"GeneID:5244","ABCB4",TRUE,"ABCB4"

"664","TTTAGTTTCGATACTCCGAT","chr7",87462893,"-","none","TGG","TTTAGTTTCGATACTCCGATTGG",0,NA,NA,NA,"GeneID:5244","ABCB4",TRUE,"ABCB4"

"665","ACTGCTTCCTTTCTCAGTTT","chr7",20626575,"-","none","TGG","ACTGCTTCCTTTCTCAGTTTTGG",0,NA,NA,NA,"GeneID:340273","ABCB5",TRUE,"ABCB5"

"666","AGAACAGCCAAAACTGAGAA","chr7",20626565,"+","none","AGG","AGAACAGCCAAAACTGAGAAAGG",0,NA,NA,NA,"GeneID:340273","ABCB5",TRUE,"ABCB5"

"667","AGAACAGCCAAAACTGAGAA","chrX",18758788,"+","4:T>C,9:C>A","TGG","AGAATAGCCCAAACTGAGAATGG",2,5,10,NA,"GeneID:5475","PPEF1",FALSE,"ABCB5"

"668","AGAACAGCCAAAACTGAGAA","chr5",155560521,"+","8:A>C,11:G>A","AGG","AGAACAGCAAAGACTGAGAAAGG",2,9,12,NA,"","",FALSE,"ABCB5"

"669","AGGTTGACCCTGTATTATGT","chr7",20643181,"+","none","TGG","AGGTTGACCCTGTATTATGTTGG",0,NA,NA,NA,"GeneID:340273","ABCB5",TRUE,"ABCB5"

"670","AGGTTGACCCTGTATTATGT","chr2",144178466,"+","8:A>C,15:C>T","TGG","AGGTTGACACTGTATCATGTTGG",2,9,16,NA,"GeneID:79712","GTDC1",TRUE,"ABCB5"

"671","ATACTGGCATCACTGGTCAA","chr7",20628732,"+","none","TGG","ATACTGGCATCACTGGTCAATGG",0,NA,NA,NA,"GeneID:340273","ABCB5",TRUE,"ABCB5"

"672","CTGCAGCACGACAGACCAAG","chr7",20643260,"+","none","AGG","CTGCAGCACGACAGACCAAGAGG",0,NA,NA,NA,"GeneID:340273","ABCB5",TRUE,"ABCB5"

"673","GGACATCACACTCATGATCC","chr7",20628707,"+","none","TGG","GGACATCACACTCATGATCCTGG",0,NA,NA,NA,"GeneID:340273","ABCB5",TRUE,"ABCB5"

"674","CTTCCTGCCAAAATCTCGCC","chr2",219216784,"+","none","AGG","CTTCCTGCCAAAATCTCGCCAGG",0,NA,NA,NA,"GeneID:10058","ABCB6",TRUE,"ABCB6"

"675","GGAACGGGCACTCAATGTGT","chr2",219216678,"-","none","TGG","GGAACGGGCACTCAATGTGTTGG",0,NA,NA,NA,"GeneID:10058","ABCB6",TRUE,"ABCB6"

"676","GGGTGCAGCAGTTCACGTCT","chr2",219216124,"-","none","CGG","GGGTGCAGCAGTTCACGTCTCGG",0,NA,NA,NA,"GeneID:10058","ABCB6",TRUE,"ABCB6"

"677","GTGCTGCGGTATGTGGTCTC","chr2",219217769,"-","none","TGG","GTGCTGCGGTATGTGGTCTCTGG",0,NA,NA,NA,"GeneID:10058","ABCB6",TRUE,"ABCB6"

"678","TCCCCAGTCCTATACATTGC","chr2",219217707,"-","none","AGG","TCCCCAGTCCTATACATTGCAGG",0,NA,NA,NA,"GeneID:10058","ABCB6",TRUE,"ABCB6"

"679","TGCACGAGCTCTCACTGCGC","chr2",219216073,"-","none","TGG","TGCACGAGCTCTCACTGCGCTGG",0,NA,NA,NA,"GeneID:10058","ABCB6",TRUE,"ABCB6"

"680","AACAAAGCAGATAATGATGC","chrX",75073734,"-","none","AGG","AACAAAGCAGATAATGATGCAGG",0,NA,NA,NA,"GeneID:22","ABCB7",TRUE,"ABCB7"

"681","AACAAAGCAGATAATGATGC","chr11",105799475,"+","7:A>C,12:G>A","TGG","AACAAAGAAGATGATGATGCTGG",2,8,13,NA,"GeneID:2893","GRIA4",FALSE,"ABCB7"

"682","CACCTTGTTTACTCTACTCA","chrX",75070384,"-","none","AGG","CACCTTGTTTACTCTACTCAAGG",0,NA,NA,NA,"GeneID:22","ABCB7",TRUE,"ABCB7"

"683","CACCTTGTTTACTCTACTCA","chr10",35211412,"+","0:G>C,16:A>C","AGG","GACCTTGTTTACTCTAATCAAGG",2,1,17,NA,"GeneID:1390","CREM",TRUE,"ABCB7"

"684","CTGTATTTGGTGCATCACTC","chrX",75076554,"+","none","AGG","CTGTATTTGGTGCATCACTCAGG",0,NA,NA,NA,"GeneID:22","ABCB7",TRUE,"ABCB7"

"685","GCTGAATTATGAAACTGTGA","chrX",75073690,"-","none","AGG","GCTGAATTATGAAACTGTGAAGG",0,NA,NA,NA,"GeneID:22","ABCB7",TRUE,"ABCB7"

"686","GCTGAATTATGAAACTGTGA","chr4",32121694,"+","12:G>A,14:A>C","TGG","GCTGAATTATGAGAATGTGATGG",2,13,15,NA,"GeneID:102723828","LOC102723828",FALSE,"ABCB7"

"687","GCTGAATTATGAAACTGTGA","chr13",70290872,"+","3:T>G,17:A>T","GGG","GCTTAATTATGAAACTGAGAGGG",2,4,18,NA,"","",FALSE,"ABCB7"

"688","TCAGCATGTTTCCCGACATC","chrX",75076578,"+","none","TGG","TCAGCATGTTTCCCGACATCTGG",0,NA,NA,NA,"GeneID:22","ABCB7",TRUE,"ABCB7"

"689","TTACTAGATCTCCAACAGTA","chrX",75070495,"+","none","AGG","TTACTAGATCTCCAACAGTAAGG",0,NA,NA,NA,"GeneID:22","ABCB7",TRUE,"ABCB7"

"690","CAGTCATGAAACTCCCTACG","chr7",151034361,"-","none","TGG","CAGTCATGAAACTCCCTACGTGG",0,NA,NA,NA,"GeneID:11194","ABCB8",TRUE,"ABCB8"

"691","CCGCTTGACAACTGACGTGC","chr7",151034771,"+","none","AGG","CCGCTTGACAACTGACGTGCAGG",0,NA,NA,NA,"GeneID:11194","ABCB8",TRUE,"ABCB8"

"692","CCTGCACGTCAGTTGTCAAG","chr7",151034774,"-","none","CGG","CCTGCACGTCAGTTGTCAAGCGG",0,NA,NA,NA,"GeneID:11194","ABCB8",TRUE,"ABCB8"

"693","GGGATGCTTACTCAGTACCA","chr7",151033753,"-","none","TGG","GGGATGCTTACTCAGTACCATGG",0,NA,NA,NA,"GeneID:11194","ABCB8",TRUE,"ABCB8"

"694","TAAAGCGAGACCCCACGACA","chr7",151033835,"-","none","TGG","TAAAGCGAGACCCCACGACATGG",0,NA,NA,NA,"GeneID:11194","ABCB8",TRUE,"ABCB8"

"695","TGGCCACGACCTCTACCAGC","chr7",151034325,"-","none","TGG","TGGCCACGACCTCTACCAGCTGG",0,NA,NA,NA,"GeneID:11194","ABCB8",TRUE,"ABCB8"

"696","AGCGGAAGAGACAGTTTCGA","chr12",122949832,"+","none","AGG","AGCGGAAGAGACAGTTTCGAAGG",0,NA,NA,NA,"GeneID:23457","ABCB9",TRUE,"ABCB9"

"697","GCAATTAGCTCATTTGCCGC","chr12",122949906,"-","none","AGG","GCAATTAGCTCATTTGCCGCAGG",0,NA,NA,NA,"GeneID:23457","ABCB9",TRUE,"ABCB9"

"698","GTAGTACTTGCCGTAGATGT","chr12",122948626,"+","none","TGG","GTAGTACTTGCCGTAGATGTTGG",0,NA,NA,NA,"GeneID:23457","ABCB9",TRUE,"ABCB9"

"699","TGACCTTGACTGTGTTCCGC","chr12",122948733,"+","none","AGG","TGACCTTGACTGTGTTCCGCAGG",0,NA,NA,NA,"GeneID:23457","ABCB9",TRUE,"ABCB9"

"700","TGACGATGCCATCAATGGCG","chr12",122950514,"+","none","CGG","TGACGATGCCATCAATGGCGCGG",0,NA,NA,NA,"GeneID:23457","ABCB9",TRUE,"ABCB9"

"701","TGTCGTCATCGTGTGCCTGC","chr12",122950463,"-","none","TGG","TGTCGTCATCGTGTGCCTGCTGG",0,NA,NA,NA,"GeneID:23457","ABCB9",TRUE,"ABCB9"

"702","CAAGTTCGTGAATGACACGA","chr16",16045844,"+","none","AGG","CAAGTTCGTGAATGACACGAAGG",0,NA,NA,NA,"GeneID:4363","ABCC1",TRUE,"ABCC1"

"703","CCAGCCGAAAGAGAGTTCCA","chr16",16044488,"+","none","AGG","CCAGCCGAAAGAGAGTTCCAAGG",0,NA,NA,NA,"GeneID:4363","ABCC1",TRUE,"ABCC1"

"704","CCGATGGCTCCGACCCGCTC","chr16",15949776,"+","none","TGG","CCGATGGCTCCGACCCGCTCTGG",0,NA,NA,NA,"GeneID:4363","ABCC1",TRUE,"ABCC1"

"705","CGGAAAACATCATCAGGTCG","chr16",16044644,"-","none","TGG","CGGAAAACATCATCAGGTCGTGG",0,NA,NA,NA,"GeneID:4363","ABCC1",TRUE,"ABCC1"

"706","GCGCCACCGGCATGGCGCTC","chr16",15949740,"+","none","CGG","GCGCCACCGGCATGGCGCTCCGG",0,NA,NA,NA,"GeneID:4363","ABCC1",TRUE,"ABCC1"

"707","TAGAAGTAGCCCTGCCAGTC","chr16",16045872,"-","none","TGG","TAGAAGTAGCCCTGCCAGTCTGG",0,NA,NA,NA,"GeneID:4363","ABCC1",TRUE,"ABCC1"

"708","AATGGACAAGAGTCTGACTC","chr6",43443988,"+","none","AGG","AATGGACAAGAGTCTGACTCAGG",0,NA,NA,NA,"GeneID:89845","ABCC10",TRUE,"ABCC10"

"709","ATCCGAGACAACATCCTCTT","chr6",43438705,"+","none","TGG","ATCCGAGACAACATCCTCTTTGG",0,NA,NA,NA,"GeneID:89845","ABCC10",TRUE,"ABCC10"

"710","ATTTGATGCACAGCTGTACA","chr6",43438734,"+","none","AGG","ATTTGATGCACAGCTGTACAAGG",0,NA,NA,NA,"GeneID:89845","ABCC10",TRUE,"ABCC10"

"711","GTAAGCTTGGTACACGTGCA","chr6",43444272,"-","none","AGG","GTAAGCTTGGTACACGTGCAAGG",0,NA,NA,NA,"GeneID:89845","ABCC10",TRUE,"ABCC10"

"712","TGCACGTGTACCAAGCTTAC","chr6",43444272,"+","none","TGG","TGCACGTGTACCAAGCTTACTGG",0,NA,NA,NA,"GeneID:89845","ABCC10",TRUE,"ABCC10"

"713","TTGGGGACAGCTTGTACCAG","chr6",43443955,"-","none","TGG","TTGGGGACAGCTTGTACCAGTGG",0,NA,NA,NA,"GeneID:89845","ABCC10",TRUE,"ABCC10"

"714","CGCTCATGATCCAAAGCTTA","chr16",48227876,"-","none","CGG","CGCTCATGATCCAAAGCTTACGG",0,NA,NA,NA,"GeneID:85320","ABCC11",TRUE,"ABCC11"

"715","CTAAGCGACTCCGTAAGCTT","chr16",48227863,"+","none","TGG","CTAAGCGACTCCGTAAGCTTTGG",0,NA,NA,NA,"GeneID:85320","ABCC11",TRUE,"ABCC11"

"716","GGACTCACCTCGGCTTGGGA","chr16",48230428,"+","none","CGG","GGACTCACCTCGGCTTGGGACGG",0,NA,NA,NA,"GeneID:85320","ABCC11",TRUE,"ABCC11"

"717","GTGAATCGTGGCATCGACAT","chr16",48231853,"-","none","AGG","GTGAATCGTGGCATCGACATAGG",0,NA,NA,NA,"GeneID:85320","ABCC11",TRUE,"ABCC11"

"718","TGTCGATGCCACGATTCACG","chr16",48231854,"+","none","AGG","TGTCGATGCCACGATTCACGAGG",0,NA,NA,NA,"GeneID:85320","ABCC11",TRUE,"ABCC11"

"719","TTCCCTTCCGTCCCAAGCCG","chr16",48230438,"-","none","AGG","TTCCCTTCCGTCCCAAGCCGAGG",0,NA,NA,NA,"GeneID:85320","ABCC11",TRUE,"ABCC11"

"720","TTCCCTTCCGTCCCAAGCCG","chr12",121531171,"+","9:A>G,18:A>C","GGG","TTCCCTTCCATCCCAAGCAGGGG",2,10,19,NA,"GeneID:84678","KDM2B",FALSE,"ABCC11"

"721","CACCATCACCGGCGTGAGCC","chr16",48143989,"+","none","AGG","CACCATCACCGGCGTGAGCCAGG",0,NA,NA,NA,"GeneID:94160","ABCC12",TRUE,"ABCC12"

"722","CCAGAGGACACGCGTGTTGA","chr16",48141255,"-","none","TGG","CCAGAGGACACGCGTGTTGATGG",0,NA,NA,NA,"GeneID:94160","ABCC12",TRUE,"ABCC12"

"723","CGTGCGGTAGTTGATGGCCC","chr16",48140773,"+","none","AGG","CGTGCGGTAGTTGATGGCCCAGG",0,NA,NA,NA,"GeneID:94160","ABCC12",TRUE,"ABCC12"

"724","CTGGGCCATCAACTACCGCA","chr16",48140775,"-","none","CGG","CTGGGCCATCAACTACCGCACGG",0,NA,NA,NA,"GeneID:94160","ABCC12",TRUE,"ABCC12"

"725","GATGAGTCATATGTCGACAA","chr16",48143927,"+","none","TGG","GATGAGTCATATGTCGACAATGG",0,NA,NA,NA,"GeneID:94160","ABCC12",TRUE,"ABCC12"

"726","TGCCGCACTCACCGGCCCTA","chr16",48141193,"+","none","TGG","TGCCGCACTCACCGGCCCTATGG",0,NA,NA,NA,"GeneID:94160","ABCC12",TRUE,"ABCC12"

"727","GGATTCCCTTGGGCTACCTA","chr10",99784674,"+","none","TGG","GGATTCCCTTGGGCTACCTATGG",0,NA,NA,NA,"GeneID:1244","ABCC2",TRUE,"ABCC2"

"728","GGATTGGTATATCGAACAGC","chr10",99792317,"-","none","AGG","GGATTGGTATATCGAACAGCAGG",0,NA,NA,NA,"GeneID:1244","ABCC2",TRUE,"ABCC2"

"729","GTATCGAGAGAATCCAGAAT","chr10",99793618,"-","none","AGG","GTATCGAGAGAATCCAGAATAGG",0,NA,NA,NA,"GeneID:1244","ABCC2",TRUE,"ABCC2"

"730","TAGGTCTTACCCATGTGCCT","chr10",99792349,"-","none","AGG","TAGGTCTTACCCATGTGCCTAGG",0,NA,NA,NA,"GeneID:1244","ABCC2",TRUE,"ABCC2"

"731","TCCTTACCTGTAAGAGTGTC","chr10",99793672,"-","none","CGG","TCCTTACCTGTAAGAGTGTCCGG",0,NA,NA,NA,"GeneID:1244","ABCC2",TRUE,"ABCC2"

"732","TTCTCCACGTGTATAAATCC","chr10",99784716,"+","none","AGG","TTCTCCACGTGTATAAATCCAGG",0,NA,NA,NA,"GeneID:1244","ABCC2",TRUE,"ABCC2"

"733","TTCTCCACGTGTATAAATCC","chr14",78524268,"-","10:A>C,15:A>T","CGG","TTCTCCACGTTTATATATCCCGG",2,11,16,NA,"GeneID:9369","NRXN3",FALSE,"ABCC2"

"734","CCCGGCCATGGACCAGGCCA","chr17",50656757,"-","none","TGG","CCCGGCCATGGACCAGGCCATGG",0,NA,NA,NA,"GeneID:8714","ABCC3",TRUE,"ABCC3"

"735","CCCGGCCATGGACCAGGCCA","chr1",21585133,"+","13:A>C,16:T>G","AGG","CCCGGCCATGGACAAGTCCAAGG",2,14,17,NA,"","",FALSE,"ABCC3"

"736","CCCGGCCATGGACCAGGCCA","chr1",16135001,"-","2:A>G,5:C>G","GGG","CCTGGGCATGGACCAGGCCAGGG",2,3,6,NA,"GeneID:1969","EPHA2",FALSE,"ABCC3"

"737","CCCGGCCATGGACCAGGCCA","chr2",33495496,"+","0:A>C,2:A>C","TGG","ACAGGCCATGGACCAGGCCATGG",2,1,3,NA,"GeneID:25780","RASGRP3",FALSE,"ABCC3"

"738","CCCTGCTGATACAGTATGAG","chr17",50657055,"+","none","CGG","CCCTGCTGATACAGTATGAGCGG",0,NA,NA,NA,"GeneID:8714","ABCC3",TRUE,"ABCC3"

"739","CCCTGCTGATACAGTATGAG","chr9",77795105,"-","11:T>G,14:C>A","TGG","CCCTGCTGATAAAGGATGAGTGG",2,12,15,NA,"GeneID:2776","GNAQ",FALSE,"ABCC3"

"740","GCCGCTCATACTGTATCAGC","chr17",50657059,"-","none","AGG","GCCGCTCATACTGTATCAGCAGG",0,NA,NA,NA,"GeneID:8714","ABCC3",TRUE,"ABCC3"

"741","GGAGTAAAAAAGGTCCGCCC","chr17",50656732,"-","none","AGG","GGAGTAAAAAAGGTCCGCCCAGG",0,NA,NA,NA,"GeneID:8714","ABCC3",TRUE,"ABCC3"

"742","GGATGATGTAGCCACGACAA","chr17",50655962,"-","none","TGG","GGATGATGTAGCCACGACAATGG",0,NA,NA,NA,"GeneID:8714","ABCC3",TRUE,"ABCC3"

"743","GGCGACCCACAGGTAGATGC","chr17",50655910,"-","none","AGG","GGCGACCCACAGGTAGATGCAGG",0,NA,NA,NA,"GeneID:8714","ABCC3",TRUE,"ABCC3"

"744","ATGATTGCTCTTGTTAAAGA","chr13",95247026,"+","none","AGG","ATGATTGCTCTTGTTAAAGAAGG",0,NA,NA,NA,"GeneID:10257","ABCC4",TRUE,"ABCC4"

"745","ATGATTGCTCTTGTTAAAGA","chr11",29920425,"+","10:G>T,19:G>A","GGG","ATGATTGCTCGTGTTAAAGGGGG",2,11,20,NA,"","",FALSE,"ABCC4"

"746","ATGATTGCTCTTGTTAAAGA","chr15",40607638,"+","3:T>A","AGG","ATGTTTGCTCTTGTTAAAGAAGG",1,4,NA,NA,"GeneID:57082","CASC5",FALSE,"ABCC4"

"747","CTTCCTCTAATCTCCGTTTA","chr13",95247703,"+","none","TGG","CTTCCTCTAATCTCCGTTTATGG",0,NA,NA,NA,"GeneID:10257","ABCC4",TRUE,"ABCC4"

"748","GAAGACCGCTCACAGCACCT","chr13",95247660,"-","none","TGG","GAAGACCGCTCACAGCACCTTGG",0,NA,NA,NA,"GeneID:10257","ABCC4",TRUE,"ABCC4"

"749","GAGCAATCATAAAGTGTTAC","chr13",95247016,"-","none","TGG","GAGCAATCATAAAGTGTTACTGG",0,NA,NA,NA,"GeneID:10257","ABCC4",TRUE,"ABCC4"

"750","GGCACTTCGTCTTAGTAACA","chr13",95210762,"-","none","TGG","GGCACTTCGTCTTAGTAACATGG",0,NA,NA,NA,"GeneID:10257","ABCC4",TRUE,"ABCC4"

"751","TGATGTGAACAAGTTTGATC","chr13",95210693,"-","none","AGG","TGATGTGAACAAGTTTGATCAGG",0,NA,NA,NA,"GeneID:10257","ABCC4",TRUE,"ABCC4"

"752","AAACATTCTCTGCCCATCGT","chr3",183982585,"+","none","TGG","AAACATTCTCTGCCCATCGTTGG",0,NA,NA,NA,"GeneID:10057","ABCC5",TRUE,"ABCC5"

"753","AAACATTCTCTGCCCATCGT","chr10",53596466,"+","16:C>T,17:A>C","GGG","AAACATTCTCTGCCCACAGTGGG",2,17,18,NA,"","",FALSE,"ABCC5"

"754","AGATCCACACAACCCTTCGC","chr3",183987840,"+","none","AGG","AGATCCACACAACCCTTCGCAGG",0,NA,NA,NA,"GeneID:10057","ABCC5",TRUE,"ABCC5"

"755","ATCAACATTTGCTCCAACGA","chr3",183982601,"-","none","TGG","ATCAACATTTGCTCCAACGATGG",0,NA,NA,NA,"GeneID:10057","ABCC5",TRUE,"ABCC5"

"756","CATCTTACTTGGAAGTAGTC","chr3",183989217,"+","none","CGG","CATCTTACTTGGAAGTAGTCCGG",0,NA,NA,NA,"GeneID:10057","ABCC5",TRUE,"ABCC5"

"757","CCCAAGGGAAAGTACCATCA","chr3",183989264,"-","none","TGG","CCCAAGGGAAAGTACCATCATGG",0,NA,NA,NA,"GeneID:10057","ABCC5",TRUE,"ABCC5"

"758","GTGCCTGATGATCACGCAGC","chr3",183987789,"-","none","TGG","GTGCCTGATGATCACGCAGCTGG",0,NA,NA,NA,"GeneID:10057","ABCC5",TRUE,"ABCC5"

"759","AGTCCTGTGTACCTCCAGCG","chr16",18574780,"+","none","TGG","AGTCCTGTGTACCTCCAGCGTGG",0,NA,NA,NA,"GeneID:653190","ABCC6P1",TRUE,"ABCC6"

"760","AGTCCTGTGTACCTCCAGCG","chr16",14821145,"-","none","TGG","AGTCCTGTGTACCTCCAGCGTGG",0,NA,NA,NA,"GeneID:730013","ABCC6P2",TRUE,"ABCC6"

"761","AGTCCTGTGTACCTCCAGCG","chr16",16219907,"-","none","TGG","AGTCCTGTGTACCTCCAGCGTGG",0,NA,NA,NA,"GeneID:368","ABCC6",TRUE,"ABCC6"

"762","AGTCCTGTGTACCTCCAGCG","chr12",100381399,"-","1:T>C,17:T>C","TGG","AATCCTGTGTACCTCCAACGTGG",2,2,18,NA,"GeneID:246213","SLC17A8",FALSE,"ABCC6"

"763","ATCGTGGTGAGCCACACAGT","chr16",16219822,"+","none","AGG","ATCGTGGTGAGCCACACAGTAGG",0,NA,NA,NA,"GeneID:368","ABCC6",TRUE,"ABCC6"

"764","ATCGTGGTGAGCCACACAGT","chr16",14821060,"+","none","AGG","ATCGTGGTGAGCCACACAGTAGG",0,NA,NA,NA,"GeneID:730013","ABCC6P2",TRUE,"ABCC6"

"765","ATCGTGGTGAGCCACACAGT","chr16",18574865,"-","none","AGG","ATCGTGGTGAGCCACACAGTAGG",0,NA,NA,NA,"GeneID:653190","ABCC6P1",TRUE,"ABCC6"

"766","ATCGTGGTGAGCCACACAGT","chr4",75281517,"-","3:T>C,11:T>G","GGG","ATCATGGTGAGACACACAGTGGG",2,4,12,NA,"","",FALSE,"ABCC6"

"767","CACCTACCTATGCCTGTCTC","chr16",18580241,"+","none","TGG","CACCTACCTATGCCTGTCTCTGG",0,NA,NA,NA,"GeneID:653190","ABCC6P1",TRUE,"ABCC6"

"768","CACCTACCTATGCCTGTCTC","chr16",16214397,"-","none","TGG","CACCTACCTATGCCTGTCTCTGG",0,NA,NA,NA,"GeneID:368","ABCC6",TRUE,"ABCC6"

"769","TGGTGACTTACAGACTGCTG","chr16",16214312,"+","none","GGG","TGGTGACTTACAGACTGCTGGGG",0,NA,NA,NA,"GeneID:368","ABCC6",TRUE,"ABCC6"

"770","TGGTGACTTACAGACTGCTG","chr16",18580326,"-","none","GGG","TGGTGACTTACAGACTGCTGGGG",0,NA,NA,NA,"GeneID:653190","ABCC6P1",TRUE,"ABCC6"

"771","TGGTGACTTACAGACTGCTG","chrX",90492337,"+","16:C>G,17:T>C","AGG","TGGTGACTTACAGACTCTTGAGG",2,17,18,NA,"","",FALSE,"ABCC6"

"772","TGGTGACTTACAGACTGCTG","chrY",4070141,"+","16:C>G,17:T>C","AGG","TGGTGACTTACAGACTCTTGAGG",2,17,18,NA,"","",FALSE,"ABCC6"

"773","TGGTGACTTACAGACTGCTG","chr12",109008330,"-","9:A>T,16:A>C","TGG","TGGTGACTTTCAGACTTCTGTGG",2,10,17,NA,"GeneID:55530","SVOP",FALSE,"ABCC6"

"774","TGTTCCTGATTCACACCGAG","chr16",16219652,"-","none","AGG","TGTTCCTGATTCACACCGAGAGG",0,NA,NA,NA,"GeneID:368","ABCC6",TRUE,"ABCC6"

"775","TGTTCCTGATTCACACCGAG","chr16",14820890,"-","none","AGG","TGTTCCTGATTCACACCGAGAGG",0,NA,NA,NA,"GeneID:730013","ABCC6P2",TRUE,"ABCC6"

"776","TGTTCCTGATTCACACCGAG","chr16",18575035,"+","17:A>G","AGG","TGTTCCTGATTCACACCAAGAGG",1,18,NA,NA,"GeneID:653190","ABCC6P1",TRUE,"ABCC6"

"777","TTACCGCTCCGGAGGCCTGC","chr16",16219549,"+","none","TGG","TTACCGCTCCGGAGGCCTGCTGG",0,NA,NA,NA,"GeneID:368","ABCC6",TRUE,"ABCC6"

"778","TTACCGCTCCGGAGGCCTGC","chr16",18575138,"-","none","TGG","TTACCGCTCCGGAGGCCTGCTGG",0,NA,NA,NA,"GeneID:653190","ABCC6P1",TRUE,"ABCC6"

"779","TTACCGCTCCGGAGGCCTGC","chr16",14820787,"+","5:A>G","TGG","TTACCACTCCGGAGGCCTGCTGG",1,6,NA,NA,"GeneID:730013","ABCC6P2",TRUE,"ABCC6"

"780","ATCCACCGCAGGTTGTGCCC","chr11",17474948,"+","none","AGG","ATCCACCGCAGGTTGTGCCCAGG",0,NA,NA,NA,"GeneID:6833","ABCC8",TRUE,"ABCC8"

"781","ATCCACCGCAGGTTGTGCCC","chr12",117347723,"+","7:C>G,11:C>G","TGG","ATCCACCCCAGCTTGTGCCCTGG",2,8,12,NA,"GeneID:4842","NOS1",FALSE,"ABCC8"

"782","CTTCATGCTGCTCTTCGTCC","chr11",17474922,"-","none","TGG","CTTCATGCTGCTCTTCGTCCTGG",0,NA,NA,NA,"GeneID:6833","ABCC8",TRUE,"ABCC8"

"783","GATGTTGTGATAGTAGACCA","chr11",17470131,"+","none","CGG","GATGTTGTGATAGTAGACCACGG",0,NA,NA,NA,"GeneID:6833","ABCC8",TRUE,"ABCC8"

"784","GCGTAGCTGCGAGAAGCCGA","chr11",17463512,"+","none","TGG","GCGTAGCTGCGAGAAGCCGATGG",0,NA,NA,NA,"GeneID:6833","ABCC8",TRUE,"ABCC8"

"785","TCACCTACCAATTAGCAGCT","chr11",17470092,"+","none","TGG","TCACCTACCAATTAGCAGCTTGG",0,NA,NA,NA,"GeneID:6833","ABCC8",TRUE,"ABCC8"

"786","TGATGACATTGACCTCCACG","chr11",17463445,"+","none","AGG","TGATGACATTGACCTCCACGAGG",0,NA,NA,NA,"GeneID:6833","ABCC8",TRUE,"ABCC8"

"787","CCTCTTTATGCCAGCCGTGA","chr12",21926019,"-","none","TGG","CCTCTTTATGCCAGCCGTGATGG",0,NA,NA,NA,"GeneID:10060","ABCC9",TRUE,"ABCC9"

"788","CGAATGTAAGAATCCATCTC","chr12",21933833,"+","none","AGG","CGAATGTAAGAATCCATCTCAGG",0,NA,NA,NA,"GeneID:10060","ABCC9",TRUE,"ABCC9"

"789","TACTTACCAATAAACAATAT","chr12",21936525,"+","none","TGG","TACTTACCAATAAACAATATTGG",0,NA,NA,NA,"GeneID:10060","ABCC9",TRUE,"ABCC9"

"790","TACTTACCAATAAACAATAT","chr18",30931769,"+","6:A>C,14:A>C","GGG","TACTTAACAATAAAAAATATGGG",2,7,15,NA,"","",FALSE,"ABCC9"

"791","TACTTACCAATAAACAATAT","chr12",121656934,"+","5:G>A,18:T>A","GGG","TACTTGCCAATAAACAATTTGGG",2,6,19,NA,"GeneID:283385","MORN3",FALSE,"ABCC9"

"792","TCTTCATATAATATCAACGA","chr12",21936624,"-","none","TGG","TCTTCATATAATATCAACGATGG",0,NA,NA,NA,"GeneID:10060","ABCC9",TRUE,"ABCC9"

"793","TTCCGGGACATAACCTGAGA","chr12",21933849,"-","none","TGG","TTCCGGGACATAACCTGAGATGG",0,NA,NA,NA,"GeneID:10060","ABCC9",TRUE,"ABCC9"

"794","TTTCTAGGCGGCGGGAATCA","chr12",21926050,"-","none","AGG","TTTCTAGGCGGCGGGAATCAAGG",0,NA,NA,NA,"GeneID:10060","ABCC9",TRUE,"ABCC9"

"795","AAGGATGAGGTTGATCTGCG","chrX",153729274,"-","none","AGG","AAGGATGAGGTTGATCTGCGAGG",0,NA,NA,NA,"GeneID:215","ABCD1",TRUE,"ABCD1"

"796","CCTGGTAGGAGCGCTGTAGC","chrX",153729245,"-","none","AGG","CCTGGTAGGAGCGCTGTAGCAGG",0,NA,NA,NA,"GeneID:215","ABCD1",TRUE,"ABCD1"

"797","CCTGTCGTTCCGCAGCCGTC","chrX",153725742,"+","none","TGG","CCTGTCGTTCCGCAGCCGTCTGG",0,NA,NA,NA,"GeneID:215","ABCD1",TRUE,"ABCD1"

"798","CGACGACATGATCCGCTCAA","chrX",153736225,"-","none","TGG","CGACGACATGATCCGCTCAATGG",0,NA,NA,NA,"GeneID:215","ABCD1",TRUE,"ABCD1"

"799","GCGCTACATGCACTCGCGTG","chrX",153726102,"+","none","TGG","GCGCTACATGCACTCGCGTGTGG",0,NA,NA,NA,"GeneID:215","ABCD1",TRUE,"ABCD1"

"800","GCGGATCATGTCGTCGTACA","chrX",153736229,"+","none","AGG","GCGGATCATGTCGTCGTACAAGG",0,NA,NA,NA,"GeneID:215","ABCD1",TRUE,"ABCD1"

"801","CAGCAGTGGACTAATTATGG","chr12",39617029,"-","none","TGG","CAGCAGTGGACTAATTATGGTGG",0,NA,NA,NA,"GeneID:225","ABCD2",TRUE,"ABCD2"

"802","CATGATGTACCACAAACGTT","chr12",39617081,"+","none","TGG","CATGATGTACCACAAACGTTTGG",0,NA,NA,NA,"GeneID:225","ABCD2",TRUE,"ABCD2"

"803","CCTTATTGCACTGTTGACGA","chr12",39619120,"+","none","AGG","CCTTATTGCACTGTTGACGAAGG",0,NA,NA,NA,"GeneID:225","ABCD2",TRUE,"ABCD2"

"804","TAAATTTCGAGCAGTGGTAA","chr12",39607649,"+","none","AGG","TAAATTTCGAGCAGTGGTAAAGG",0,NA,NA,NA,"GeneID:225","ABCD2",TRUE,"ABCD2"

"805","TACCACTGCTCGAAATTTAC","chr12",39607648,"-","none","TGG","TACCACTGCTCGAAATTTACTGG",0,NA,NA,NA,"GeneID:225","ABCD2",TRUE,"ABCD2"

"806","TTTCATAGGCGTGGTCTACT","chr12",39619059,"+","none","AGG","TTTCATAGGCGTGGTCTACTAGG",0,NA,NA,NA,"GeneID:225","ABCD2",TRUE,"ABCD2"

"807","AAAATGGGACACTAATTGAA","chr1",94467985,"+","none","AGG","AAAATGGGACACTAATTGAAAGG",0,NA,NA,NA,"GeneID:5825","ABCD3",TRUE,"ABCD3"

"808","AAAATGGGACACTAATTGAA","chr13",103659647,"-","15:C>A,19:A>T","AGG","AAAATGGGACACTAAGTGATAGG",2,16,20,NA,"","",FALSE,"ABCD3"

"809","ACCACCATTACAGAACAATG","chr1",94458621,"+","none","AGG","ACCACCATTACAGAACAATGAGG",0,NA,NA,NA,"GeneID:5825","ABCD3",TRUE,"ABCD3"

"810","ACCACCATTACAGAACAATG","chr1",234927008,"+","4:A>C,8:C>T","AGG","ACCAACATCACAGAACAATGAGG",2,5,9,NA,"","",FALSE,"ABCD3"

"811","ACCTCTTTACAAAATGTTCT","chr1",94464855,"-","none","AGG","ACCTCTTTACAAAATGTTCTAGG",0,NA,NA,NA,"GeneID:5825","ABCD3",TRUE,"ABCD3"

"812","CTCGAACATATTGTGATGTT","chr1",94467955,"+","none","TGG","CTCGAACATATTGTGATGTTTGG",0,NA,NA,NA,"GeneID:5825","ABCD3",TRUE,"ABCD3"

"813","CTCGAACATATTGTGATGTT","chrY",13762879,"-","2:T>G,8:G>A","TGG","CTAGAACACATTGTGATGTTTGG",2,3,9,NA,"","",FALSE,"ABCD3"

"814","GTGGTTTGCAGTGGTATCAT","chr1",94473754,"+","none","TGG","GTGGTTTGCAGTGGTATCATTGG",0,NA,NA,NA,"GeneID:5825","ABCD3",TRUE,"ABCD3"

"815","GTGGTTTGCAGTGGTATCAT","chr8",126068413,"+","1:G>T,4:A>T","GGG","GGGGATTGCAGTGGTATCATGGG",2,2,5,NA,"","",FALSE,"ABCD3"

"816","TTTACCTCATTGTTCTGTAA","chr1",94458628,"-","none","TGG","TTTACCTCATTGTTCTGTAATGG",0,NA,NA,NA,"GeneID:5825","ABCD3",TRUE,"ABCD3"

"817","TTTACCTCATTGTTCTGTAA","chr5",11269543,"+","4:A>C,13:A>T","TGG","TTTAACTCATTGTACTGTAATGG",2,5,14,NA,"GeneID:1501","CTNND2",FALSE,"ABCD3"

"818","AGAGCAATTTGTGATCTACC","chr14",74299657,"-","none","AGG","AGAGCAATTTGTGATCTACCAGG",0,NA,NA,NA,"GeneID:5826","ABCD4",TRUE,"ABCD4"

"819","CTCACTCACCCAGTAGGGTC","chr14",74300140,"+","none","AGG","CTCACTCACCCAGTAGGGTCAGG",0,NA,NA,NA,"GeneID:5826","ABCD4",TRUE,"ABCD4"

"820","CTCACTCACCCAGTAGGGTC","chr9",98520588,"+","2:G>C,13:G>T","TGG","CTGACTCACCCAGGAGGGTCTGG",2,3,14,NA,"GeneID:9568","GABBR2",FALSE,"ABCD4"

"821","GAGACCTCACCTGCGTACTT","chr14",74287799,"+","none","CGG","GAGACCTCACCTGCGTACTTCGG",0,NA,NA,NA,"GeneID:5826","ABCD4",TRUE,"ABCD4"

"822","GTCTCAGGTCATGGCGGTCG","chr14",74302902,"-","none","CGG","GTCTCAGGTCATGGCGGTCGCGG",0,NA,NA,NA,"GeneID:5826","ABCD4",TRUE,"ABCD4"

"823","TACCTGCAGCCGAAGTACGC","chr14",74287811,"-","none","AGG","TACCTGCAGCCGAAGTACGCAGG",0,NA,NA,NA,"GeneID:5826","ABCD4",TRUE,"ABCD4"

"824","TTGATCCCCAGTCAGTACTA","chr14",74299629,"-","none","TGG","TTGATCCCCAGTCAGTACTATGG",0,NA,NA,NA,"GeneID:5826","ABCD4",TRUE,"ABCD4"

"825","ACAAATGTACCTCCTTCATC","chr4",145121223,"-","none","AGG","ACAAATGTACCTCCTTCATCAGG",0,NA,NA,NA,"GeneID:6059;GeneID:54726","ABCE1;OTUD4",TRUE,"ABCE1"

"826","ACAAATGTACCTCCTTCATC","chr5",133342521,"-","0:G>T,8:A>T","AGG","CCAAATGTTCCTCCTTCATCAGG",2,1,9,NA,"GeneID:23105","FSTL4",FALSE,"ABCE1"

"827","AGACTTAAACCTGATGAAGG","chr4",145121211,"+","none","AGG","AGACTTAAACCTGATGAAGGAGG",0,NA,NA,NA,"GeneID:6059;GeneID:54726","ABCE1;OTUD4",TRUE,"ABCE1"

"828","AGACTTAAACCTGATGAAGG","chr1",192247302,"-","14:C>A","AGG","AGACTTAAACCTGAGGAAGGAGG",1,15,NA,NA,"","",TRUE,"ABCE1"

"829","AGACTTAAACCTGATGAAGG","chr7",23563055,"-","6:C>T,19:T>C","AGG","AGACTTGAACCTGATGAAGAAGG",2,7,20,NA,"","",TRUE,"ABCE1"

"830","ATGTGTATGTATAAATATCC","chr4",145120041,"+","none","AGG","ATGTGTATGTATAAATATCCAGG",0,NA,NA,NA,"GeneID:6059;GeneID:54726","ABCE1;OTUD4",TRUE,"ABCE1"

"831","ATGTGTATGTATAAATATCC","chr1",192247452,"-","none","AGG","ATGTGTATGTATAAATATCCAGG",0,NA,NA,NA,"","",TRUE,"ABCE1"

"832","ATGTGTATGTATAAATATCC","chr7",23563207,"-","18:A>G","AGG","ATGTGTATGTATAAATATTCAGG",1,19,NA,NA,"","",TRUE,"ABCE1"

"833","ATGTGTATGTATAAATATCC","chr1",42472935,"-","13:A>T,19:A>G","TGG","ATGTGTATGTATATATATCTTGG",2,14,20,NA,"GeneID:79717;GeneID:728621","PPCS;CCDC30",FALSE,"ABCE1"

"834","ATGTGTATGTATAAATATCC","chr10",100402197,"-","2:T>C,5:G>A","AGG","ATATGCATGTATAAATATCCAGG",2,3,6,NA,"GeneID:90271","LINC00263",FALSE,"ABCE1"

"835","ATGTGTATGTATAAATATCC","chr3",140927069,"+","4:A>G,13:T>A","TGG","ATGTATATGTATATATATCCTGG",2,5,14,NA,"","",FALSE,"ABCE1"

"836","ATGTGTATGTATAAATATCC","chr8",89087077,"+","5:C>T,13:T>A","AGG","ATGTGCATGTATATATATCCAGG",2,6,14,NA,"","",FALSE,"ABCE1"

"837","ATGTGTATGTATAAATATCC","chrY",26390236,"+","4:A>G,15:A>T","TGG","ATGTATATGTATAAAAATCCTGG",2,5,16,NA,"","",FALSE,"ABCE1"

"838","ATGTGTATGTATAAATATCC","chr7",29605756,"+","6:G>A,18:G>C","AGG","ATGTGTGTGTATAAATATGCAGG",2,7,19,NA,"","",FALSE,"ABCE1"

"839","ATGTGTATGTATAAATATCC","chr1",202381053,"-","4:T>C,15:T>A","CGG","ATGTATATGTATAAAAATCCCGG",2,5,16,NA,"GeneID:4660","PPP1R12B",FALSE,"ABCE1"

"840","TAATAACTCACAGTTGATTT","chr4",145121382,"-","none","GGG","TAATAACTCACAGTTGATTTGGG",0,NA,NA,NA,"GeneID:6059;GeneID:54726","ABCE1;OTUD4",TRUE,"ABCE1"

"841","TTATAACTGACATTTAGAAC","chr7",23563022,"+","none","TGG","TTATAACTGACATTTAGAACTGG",0,NA,NA,NA,"","",TRUE,"ABCE1"

"842","TTATAACTGACATTTAGAAC","chr1",192247269,"+","none","TGG","TTATAACTGACATTTAGAACTGG",0,NA,NA,NA,"","",TRUE,"ABCE1"

"843","TTATAACTGACATTTAGAAC","chr4",145121343,"-","none","TGG","TTATAACTGACATTTAGAACTGG",0,NA,NA,NA,"GeneID:6059;GeneID:54726","ABCE1;OTUD4",TRUE,"ABCE1"

"844","TTTGAGCTAGCAATTGTAGC","chr4",145120086,"+","none","TGG","TTTGAGCTAGCAATTGTAGCTGG",0,NA,NA,NA,"GeneID:6059;GeneID:54726","ABCE1;OTUD4",TRUE,"ABCE1"

"845","TTTGAGCTAGCAATTGTAGC","chr7",23563163,"-","none","TGG","TTTGAGCTAGCAATTGTAGCTGG",0,NA,NA,NA,"","",TRUE,"ABCE1"

"846","AAAAAGCGAGATACCCGAAA","chr6",30578081,"+","none","AGG","AAAAAGCGAGATACCCGAAAAGG",0,NA,NA,NA,"GeneID:23","ABCF1",TRUE,"ABCF1"

"847","CAGTAGAAGATAAACAGGCT","chr6",30577833,"+","none","GGG","CAGTAGAAGATAAACAGGCTGGG",0,NA,NA,NA,"GeneID:23","ABCF1",TRUE,"ABCF1"

"848","CAGTAGAAGATAAACAGGCT","chr1",9289067,"-","2:A>C,7:A>T","TGG","CATTAGATGATAAACAGGCTTGG",2,3,8,NA,"","",FALSE,"ABCF1"

"849","CTAGACAAAGTGGTGAAGAA","chr6",30577404,"+","none","AGG","CTAGACAAAGTGGTGAAGAAAGG",0,NA,NA,NA,"GeneID:23","ABCF1",TRUE,"ABCF1"

"850","CTAGACAAAGTGGTGAAGAA","chr6",144969469,"-","4:C>T,14:A>C","TGG","CTAGGCAAAGTGGTTAAGAATGG",2,5,15,NA,"","",FALSE,"ABCF1"

"851","CTCAGTGCCAACCAGTGATG","chr6",30578170,"+","none","AGG","CTCAGTGCCAACCAGTGATGAGG",0,NA,NA,NA,"GeneID:23","ABCF1",TRUE,"ABCF1"

"852","GTACCCGCCCCAAAACCCCG","chr6",30578346,"+","none","CGG","GTACCCGCCCCAAAACCCCGCGG",0,NA,NA,NA,"GeneID:23","ABCF1",TRUE,"ABCF1"

"853","TTTATAGTTCTTTGAAGAGC","chr6",30577810,"+","none","TGG","TTTATAGTTCTTTGAAGAGCTGG",0,NA,NA,NA,"GeneID:23","ABCF1",TRUE,"ABCF1"

"854","TTTATAGTTCTTTGAAGAGC","chr2",101080074,"-","9:T>G,16:T>C","AGG","TTTATAGTTATTTGAAAAGCAGG",2,10,17,NA,"GeneID:11138","TBC1D8",FALSE,"ABCF1"

"855","AGATCAGATTGCACACATGA","chr7",151219065,"-","none","AGG","AGATCAGATTGCACACATGAAGG",0,NA,NA,NA,"GeneID:10061","ABCF2",TRUE,"ABCF2"

"856","ATGATCAGTACGTGAAGACG","chr7",151219132,"-","none","CGG","ATGATCAGTACGTGAAGACGCGG",0,NA,NA,NA,"GeneID:10061","ABCF2",TRUE,"ABCF2"

"857","ATGATCAGTACGTGAAGACG","chr3",88318079,"+","10:T>C,18:T>C","TGG","ATGATCAGTATGTGAAGATGTGG",2,11,19,NA,"","",TRUE,"ABCF2"

"858","CAAGAAACTGAAGTATTATA","chr7",151221579,"-","none","CGG","CAAGAAACTGAAGTATTATACGG",0,NA,NA,NA,"GeneID:10061","ABCF2",TRUE,"ABCF2"

"859","CAAGAAACTGAAGTATTATA","chr3",88318050,"+","17:T>A","TGG","CAAGAAACTGAAGTATTTTATGG",1,18,NA,NA,"","",TRUE,"ABCF2"

"860","CAAGAAACTGAAGTATTATA","chr8",4608478,"+","11:T>A,19:T>A","TGG","CAAGAAACTGATGTATTATTTGG",2,12,20,NA,"GeneID:64478","CSMD1",FALSE,"ABCF2"

"861","CCTAGATGCTTGCGTGTGGT","chr7",151222539,"-","none","TGG","CCTAGATGCTTGCGTGTGGTTGG",0,NA,NA,NA,"GeneID:10061","ABCF2",TRUE,"ABCF2"

"862","CCTAGATGCTTGCGTGTGGT","chr3",88317930,"+","13:A>G","TGG","CCTAGATGCTTGCATGTGGTTGG",1,14,NA,NA,"","",TRUE,"ABCF2"

"863","GGTGGTTGGTAGGCTCATCC","chr7",151222563,"+","none","AGG","GGTGGTTGGTAGGCTCATCCAGG",0,NA,NA,NA,"GeneID:10061","ABCF2",TRUE,"ABCF2"

"864","GGTGGTTGGTAGGCTCATCC","chr3",88317906,"-","10:C>T","AGG","GGTGGTTGGTGGGCTCATCCAGG",1,11,NA,NA,"","",TRUE,"ABCF2"

"865","GGTGGTTGGTAGGCTCATCC","chr5",172522139,"-","0:A>C,8:A>C","TGG","TGTGGTTGTTAGGCTCATCCTGG",2,1,9,NA,"","",FALSE,"ABCF2"

"866","GGTGGTTGGTAGGCTCATCC","chr3",88318769,"-","4:T>C,10:C>T","AGG","GGTGATTGGTGGGCTCATCCAGG",2,5,11,NA,"","",TRUE,"ABCF2"

"867","GTTGTGCATGTGAATGATAT","chr7",151221598,"+","none","TGG","GTTGTGCATGTGAATGATATTGG",0,NA,NA,NA,"GeneID:10061","ABCF2",TRUE,"ABCF2"

"868","GTTGTGCATGTGAATGATAT","chr3",88318031,"-","18:C>T","TGG","GTTGTGCATGTGAATGATGTTGG",1,19,NA,NA,"","",TRUE,"ABCF2"

"869","CCTTTAAAGAGTCTTAGAAG","chr3",184187851,"+","none","AGG","CCTTTAAAGAGTCTTAGAAGAGG",0,NA,NA,NA,"GeneID:55324","ABCF3",TRUE,"ABCF3"

"870","CCTTTAAAGAGTCTTAGAAG","chr8",80318029,"-","12:C>G,14:T>A","AGG","CCTTTAAAGAGTGTAAGAAGAGG",2,13,15,NA,"","",FALSE,"ABCF3"

"871","GCCGCCGTTACGGGCTGGTG","chr3",184188178,"+","none","GGG","GCCGCCGTTACGGGCTGGTGGGG",0,NA,NA,NA,"GeneID:55324","ABCF3",TRUE,"ABCF3"

"872","GTTCTCAATTCGCACATCAT","chr3",184187940,"-","none","AGG","GTTCTCAATTCGCACATCATAGG",0,NA,NA,NA,"GeneID:55324","ABCF3",TRUE,"ABCF3"

"873","TAGGGGCGTCCAGTAGCACC","chr3",184186825,"-","none","TGG","TAGGGGCGTCCAGTAGCACCTGG",0,NA,NA,NA,"GeneID:55324","ABCF3",TRUE,"ABCF3"

"874","TCCGCCCCACCAGCCCGTAA","chr3",184188185,"-","none","CGG","TCCGCCCCACCAGCCCGTAACGG",0,NA,NA,NA,"GeneID:55324","ABCF3",TRUE,"ABCF3"

"875","TCTCCGTTATCTTTGACAAC","chr3",184186849,"-","none","TGG","TCTCCGTTATCTTTGACAACTGG",0,NA,NA,NA,"GeneID:55324","ABCF3",TRUE,"ABCF3"

"876","ACTGAGACGGACCTGCTGAA","chr21",42225757,"+","none","TGG","ACTGAGACGGACCTGCTGAATGG",0,NA,NA,NA,"GeneID:9619","ABCG1",TRUE,"ABCG1"

"877","AGAAGCGCTGGGCTTCCGTG","chr21",42225807,"-","none","AGG","AGAAGCGCTGGGCTTCCGTGAGG",0,NA,NA,NA,"GeneID:9619","ABCG1",TRUE,"ABCG1"

"878","ATTTCCGGGAAGTTCAATAG","chr21",42271092,"+","none","TGG","ATTTCCGGGAAGTTCAATAGTGG",0,NA,NA,NA,"GeneID:9619","ABCG1",TRUE,"ABCG1"

"879","GTCCACGCTGATGAACATCC","chr21",42271154,"+","none","TGG","GTCCACGCTGATGAACATCCTGG",0,NA,NA,NA,"GeneID:9619","ABCG1",TRUE,"ABCG1"

"880","GTTCTTGATGCTGGGAACGC","chr21",42201668,"+","none","AGG","GTTCTTGATGCTGGGAACGCAGG",0,NA,NA,NA,"GeneID:9619","ABCG1",TRUE,"ABCG1"

"881","GTTCTTGATGCTGGGAACGC","chr22",45982630,"-","5:T>A,19:A>G","GGG","GTTCTAGATGCTGGGAACGTGGG",2,6,20,NA,"","",FALSE,"ABCG1"

"882","TCTGACCACACCTGAGGGCA","chr21",42201714,"-","none","GGG","TCTGACCACACCTGAGGGCAGGG",0,NA,NA,NA,"GeneID:9619","ABCG1",TRUE,"ABCG1"

"883","TCTGACCACACCTGAGGGCA","chr14",68898763,"-","1:A>G,4:A>T","AGG","TTTGTCCACACCTGAGGGCAAGG",2,2,5,NA,"GeneID:87","ACTN1",FALSE,"ABCG1"

"884","TCTGACCACACCTGAGGGCA","chr3",114810401,"+","1:T>C,13:A>G","TGG","TTTGACCACACCTAAGGGCATGG",2,2,14,NA,"GeneID:26137","ZBTB20",FALSE,"ABCG1"

"885","ACATCTCCAGATAATCCACT","chr4",88131863,"+","none","TGG","ACATCTCCAGATAATCCACTTGG",0,NA,NA,NA,"GeneID:9429","ABCG2",TRUE,"ABCG2"

"886","AGCTGTCGCGGGGAAGCCAT","chr4",88139923,"+","none","TGG","AGCTGTCGCGGGGAAGCCATTGG",0,NA,NA,NA,"GeneID:9429","ABCG2",TRUE,"ABCG2"

"887","CGAAGATTTGCCTCCACCTG","chr4",88132574,"+","none","TGG","CGAAGATTTGCCTCCACCTGTGG",0,NA,NA,NA,"GeneID:9429","ABCG2",TRUE,"ABCG2"

"888","GACAGCTTCCAATGACCTGA","chr4",88139910,"-","none","AGG","GACAGCTTCCAATGACCTGAAGG",0,NA,NA,NA,"GeneID:9429","ABCG2",TRUE,"ABCG2"

"889","TACAGTGGGATCATGAAACC","chr4",88132620,"-","none","TGG","TACAGTGGGATCATGAAACCTGG",0,NA,NA,NA,"GeneID:9429","ABCG2",TRUE,"ABCG2"

"890","TATTAGATGTCTTAGCTGCA","chr4",88131895,"-","none","AGG","TATTAGATGTCTTAGCTGCAAGG",0,NA,NA,NA,"GeneID:9429","ABCG2",TRUE,"ABCG2"

"891","TATTAGATGTCTTAGCTGCA","chr2",31304250,"-","12:G>A,17:T>C","AGG","TATTAGATGTCTCAGCTACAAGG",2,13,18,NA,"","",FALSE,"ABCG2"

"892","TATTAGATGTCTTAGCTGCA","chr15",69022480,"+","15:T>C,17:T>G","AGG","TATTAGATGTCTTAGTTTCAAGG",2,16,18,NA,"GeneID:79400","NOX5",TRUE,"ABCG2"

"893","CCTGAAGCTGAGTGAGAAGC","chr11",119154535,"+","none","AGG","CCTGAAGCTGAGTGAGAAGCAGG",0,NA,NA,NA,"GeneID:64137","ABCG4",TRUE,"ABCG4"

"894","CCTGAAGCTGAGTGAGAAGC","chr15",79990213,"+","0:T>C,1:G>C","AGG","TGTGAAGCTGAGTGAGAAGCAGG",2,1,2,NA,"","",TRUE,"ABCG4"

"895","CCTGCTTCTCACTCAGCTTC","chr11",119154538,"-","none","AGG","CCTGCTTCTCACTCAGCTTCAGG",0,NA,NA,NA,"GeneID:64137","ABCG4",TRUE,"ABCG4"

"896","CCTGCTTCTCACTCAGCTTC","chr2",75605465,"-","10:G>T,13:A>G","TGG","CCTGCTTCTCCCTTAGCTTCTGG",2,11,14,NA,"","",FALSE,"ABCG4"

"897","CCTGCTTCTCACTCAGCTTC","chr19",3160760,"+","1:A>C,10:T>A","TGG","CATGCTTCTCTCTCAGCTTCTGG",2,2,11,NA,"GeneID:2769","GNA15",FALSE,"ABCG4"

"898","CCTGCTTCTCACTCAGCTTC","chrX",39447036,"+","5:C>T,12:C>T","TGG","CCTGCCTCTCACCCAGCTTCTGG",2,6,13,NA,"","",FALSE,"ABCG4"

"899","CCTGCTTCTCACTCAGCTTC","chr2",223690718,"+","5:C>T,12:C>T","TGG","CCTGCCTCTCACCCAGCTTCTGG",2,6,13,NA,"","",FALSE,"ABCG4"

"900","CCTGCTTCTCACTCAGCTTC","chr12",109983964,"-","5:G>A,16:C>G","TGG","CCTGCCTCTCACTCAGGTTCTGG",2,6,17,NA,"GeneID:9815","GIT2",FALSE,"ABCG4"

"901","CCTGCTTCTCACTCAGCTTC","chr11",76912261,"-","1:T>G,10:A>T","AGG","CATGCTTCTCTCTCAGCTTCAGG",2,2,11,NA,"GeneID:55331","ACER3",FALSE,"ABCG4"

"902","CGGAAGGTCCTCAGCTCCCG","chr11",119154302,"-","none","TGG","CGGAAGGTCCTCAGCTCCCGTGG",0,NA,NA,NA,"GeneID:64137","ABCG4",TRUE,"ABCG4"

"903","TCATGGCTTCCAACACCGTG","chr11",119154370,"-","none","AGG","TCATGGCTTCCAACACCGTGAGG",0,NA,NA,NA,"GeneID:64137","ABCG4",TRUE,"ABCG4"

"904","TCATGGCTTCCAACACCGTG","chr22",25205314,"+","12:G>A,13:G>C","TGG","TCATGGCTTCCAGGACCGTGTGG",2,13,14,NA,"GeneID:1417","CRYBB3",TRUE,"ABCG4"

"905","TCGTGTGGGAGCACGACATC","chr11",119154798,"-","none","AGG","TCGTGTGGGAGCACGACATCAGG",0,NA,NA,NA,"GeneID:64137","ABCG4",TRUE,"ABCG4"

"906","TGATGTCGTGCTCCCACACG","chr11",119154797,"+","none","AGG","TGATGTCGTGCTCCCACACGAGG",0,NA,NA,NA,"GeneID:64137","ABCG4",TRUE,"ABCG4"

"907","CTCCGGGAAAACCACGCTGC","chr2",43832062,"-","none","TGG","CTCCGGGAAAACCACGCTGCTGG",0,NA,NA,NA,"GeneID:64240;GeneID:64241","ABCG5;ABCG8",TRUE,"ABCG5"

"908","CTGCCCGCTCTCCACGTACA","chr2",43837858,"+","none","AGG","CTGCCCGCTCTCCACGTACAAGG",0,NA,NA,NA,"GeneID:64240;GeneID:64241","ABCG5;ABCG8",TRUE,"ABCG5"

"909","GCCGGGATTGCCGCGGCGGA","chr2",43831783,"+","none","TGG","GCCGGGATTGCCGCGGCGGATGG",0,NA,NA,NA,"GeneID:64240","ABCG5",TRUE,"ABCG5"

"910","GTAGTGCAGCGTCTCGCGCA","chr2",43831819,"+","none","CGG","GTAGTGCAGCGTCTCGCGCACGG",0,NA,NA,NA,"GeneID:64240","ABCG5",TRUE,"ABCG5"

"911","TCCTTTAAAGCCACCGCGTG","chr2",43837945,"-","none","AGG","TCCTTTAAAGCCACCGCGTGAGG",0,NA,NA,NA,"GeneID:64240;GeneID:64241","ABCG5;ABCG8",TRUE,"ABCG5"

"912","TGAACGGCCGGGCGCTGCGC","chr2",43831985,"-","none","CGG","TGAACGGCCGGGCGCTGCGCCGG",0,NA,NA,NA,"GeneID:64240;GeneID:64241","ABCG5;ABCG8",TRUE,"ABCG5"

"913","CACGGCGGCAAGATCAAGTC","chr2",43851627,"+","none","AGG","CACGGCGGCAAGATCAAGTCAGG",0,NA,NA,NA,"GeneID:64241","ABCG8",TRUE,"ABCG8"

"914","CTGCCAGAATTCTTGTGAGC","chr2",43846225,"+","none","TGG","CTGCCAGAATTCTTGTGAGCTGG",0,NA,NA,NA,"GeneID:64241","ABCG8",TRUE,"ABCG8"

"915","GCTGCCCATTGATCCAGATC","chr2",43851653,"-","none","TGG","GCTGCCCATTGATCCAGATCTGG",0,NA,NA,NA,"GeneID:64241","ABCG8",TRUE,"ABCG8"

"916","GGCCACTCCCCAGGATACCT","chr2",43839094,"+","none","CGG","GGCCACTCCCCAGGATACCTCGG",0,NA,NA,NA,"GeneID:64241","ABCG8",TRUE,"ABCG8"

"917","GGCCACTCCCCAGGATACCT","chr2",72133822,"+","11:T>A,15:G>T","GGG","GGCCACTCCCCTGGAGACCTGGG",2,12,16,NA,"GeneID:56603","CYP26B1",FALSE,"ABCG8"

"918","GGTACCTGAGCTCCCTATGA","chr2",43846296,"-","none","TGG","GGTACCTGAGCTCCCTATGATGG",0,NA,NA,NA,"GeneID:64241","ABCG8",TRUE,"ABCG8"

"919","GGTACCTGAGCTCCCTATGA","chr13",97621679,"+","10:G>C,11:G>T","AGG","GGTACCTGAGGGCCCTATGAAGG",2,11,12,NA,"","",FALSE,"ABCG8"

"920","TGTGGGCCCCATGGCCGGGA","chr2",43839043,"+","none","AGG","TGTGGGCCCCATGGCCGGGAAGG",0,NA,NA,NA,"GeneID:64241","ABCG8",TRUE,"ABCG8"

"921","AGACTTTCTACCCAACGCTG","chr2",27128507,"+","none","TGG","AGACTTTCTACCCAACGCTGTGG",0,NA,NA,NA,"GeneID:84696","ABHD1",TRUE,"ABHD1"

"922","CCCAGTAGTAGCCCAAGTAG","chr2",27124028,"-","none","AGG","CCCAGTAGTAGCCCAAGTAGAGG",0,NA,NA,NA,"GeneID:84696","ABHD1",TRUE,"ABHD1"

"923","CTTTGAGACCACTCGCTCCC","chr2",27129837,"+","none","TGG","CTTTGAGACCACTCGCTCCCTGG",0,NA,NA,NA,"GeneID:84696","ABHD1",TRUE,"ABHD1"

"924","GACCCTACCGTTCCACAAGT","chr2",27129915,"-","none","TGG","GACCCTACCGTTCCACAAGTTGG",0,NA,NA,NA,"GeneID:84696","ABHD1",TRUE,"ABHD1"

"925","GGTGCAAGACGTATGTATCC","chr2",27129076,"-","16:G>T","TGG","GGTGCAAGACGTATGTCTCCTGG",1,17,NA,NA,"GeneID:84696","ABHD1",TRUE,"ABHD1"

"926","GTAGAAAGTCTCGGTGGTGA","chr2",27128498,"-","none","TGG","GTAGAAAGTCTCGGTGGTGATGG",0,NA,NA,NA,"GeneID:84696","ABHD1",TRUE,"ABHD1"

"927","AACACAGTGATCACCGAATG","chr3",111991623,"+","none","AGG","AACACAGTGATCACCGAATGAGG",0,NA,NA,NA,"GeneID:55347","ABHD10",TRUE,"ABHD10"

"928","ACCTGCATTGATGTATGCCA","chr3",111991559,"-","none","AGG","ACCTGCATTGATGTATGCCAAGG",0,NA,NA,NA,"GeneID:55347","ABHD10",TRUE,"ABHD10"

"929","ATTCTTGTTGGATCTAGCCT","chr3",111986913,"+","none","TGG","ATTCTTGTTGGATCTAGCCTTGG",0,NA,NA,NA,"GeneID:55347","ABHD10",TRUE,"ABHD10"

"930","TACAAAAGCGTTGGCGATTG","chr3",111981910,"+","none","AGG","TACAAAAGCGTTGGCGATTGAGG",0,NA,NA,NA,"GeneID:55347","ABHD10",TRUE,"ABHD10"

"931","TGCAATTGCACGACCAGAGA","chr3",111986957,"+","none","AGG","TGCAATTGCACGACCAGAGAAGG",0,NA,NA,NA,"GeneID:55347","ABHD10",TRUE,"ABHD10"

"932","TGCAATTGCACGACCAGAGA","chr9",80951737,"+","16:A>G,17:T>A","TGG","TGCAATTGCACGACCAATGATGG",2,17,18,NA,"","",FALSE,"ABHD10"

"933","TTGGAAGGTCTGGTCGATTA","chr3",111981814,"-","none","AGG","TTGGAAGGTCTGGTCGATTAAGG",0,NA,NA,NA,"GeneID:55347","ABHD10",TRUE,"ABHD10"

"934","AGCGCCCGTCTACCTCTACC","chr7",73737055,"+","none","AGG","AGCGCCCGTCTACCTCTACCAGG",0,NA,NA,NA,"GeneID:83451","ABHD11",TRUE,"ABHD11"

"935","ATGATCTCGTAGCTCATGTC","chr7",73737667,"+","none","TGG","ATGATCTCGTAGCTCATGTCTGG",0,NA,NA,NA,"GeneID:83451","ABHD11",TRUE,"ABHD11"

"936","CCGCTCCCGTGCCCGAAAAC","chr7",73737252,"-","none","TGG","CCGCTCCCGTGCCCGAAAACTGG",0,NA,NA,NA,"GeneID:83451","ABHD11",TRUE,"ABHD11"

"937","CTGGTGCCCTGCGTCGTCGT","chr7",73737610,"-","none","TGG","CTGGTGCCCTGCGTCGTCGTTGG",0,NA,NA,NA,"GeneID:83451","ABHD11",TRUE,"ABHD11"

"938","GAGCCCGTGCAAAAAGACGA","chr7",73738390,"+","none","CGG","GAGCCCGTGCAAAAAGACGACGG",0,NA,NA,NA,"GeneID:83451","ABHD11",TRUE,"ABHD11"

"939","GGCTAGCTTACTGCACGAAC","chr7",73736917,"+","none","TGG","GGCTAGCTTACTGCACGAACTGG",0,NA,NA,NA,"GeneID:83451","ABHD11",TRUE,"ABHD11"

"940","AAGACGTGACCATTGGAGTC","chr20",25323326,"-","none","TGG","AAGACGTGACCATTGGAGTCTGG",0,NA,NA,NA,"GeneID:26090","ABHD12",TRUE,"ABHD12"

"941","AAGACGTGACCATTGGAGTC","chr17",40676292,"-","11:A>T,16:C>T","CGG","AAGACGTGACCTTTGGGGTCCGG",2,12,17,NA,"","",FALSE,"ABHD12"

"942","CTTGCAGGCGAAAGGGCGTG","chr20",25339338,"-","none","TGG","CTTGCAGGCGAAAGGGCGTGTGG",0,NA,NA,NA,"GeneID:26090","ABHD12",TRUE,"ABHD12"

"943","GCGTTCTTCCACCAGACTGC","chr20",25320285,"+","none","AGG","GCGTTCTTCCACCAGACTGCAGG",0,NA,NA,NA,"GeneID:26090","ABHD12",TRUE,"ABHD12"

"944","GCGTTCTTCCACCAGACTGC","chr10",84586746,"+","5:G>C,6:C>T","TGG","GCGTTGCTCCACCAGACTGCTGG",2,6,7,NA,"","",FALSE,"ABHD12"

"945","TGATTCAAACCTTGATCCTG","chr20",25323375,"+","none","TGG","TGATTCAAACCTTGATCCTGTGG",0,NA,NA,NA,"GeneID:26090","ABHD12",TRUE,"ABHD12"

"946","TGGTACCTGCGTTCCCATGC","chr20",25320199,"+","none","AGG","TGGTACCTGCGTTCCCATGCAGG",0,NA,NA,NA,"GeneID:26090","ABHD12",TRUE,"ABHD12"

"947","TTTCTCATCAAACTATGTCC","chr20",25339261,"-","none","TGG","TTTCTCATCAAACTATGTCCTGG",0,NA,NA,NA,"GeneID:26090","ABHD12",TRUE,"ABHD12"

"948","ACATGGTCGACCGCAACCTG","chr14",50872256,"+","none","CGG","ACATGGTCGACCGCAACCTGCGG",0,NA,NA,NA,"GeneID:5836;GeneID:145447","PYGL;ABHD12B",TRUE,"ABHD12B"

"949","ACCAGGCGGCCACGCAGCTA","chr14",50872233,"-","none","CGG","ACCAGGCGGCCACGCAGCTACGG",0,NA,NA,NA,"GeneID:5836;GeneID:145447","PYGL;ABHD12B",TRUE,"ABHD12B"

"950","AGCTTCGCACAGACTGAAGC","chr14",50881598,"+","none","TGG","AGCTTCGCACAGACTGAAGCTGG",0,NA,NA,NA,"GeneID:5836;GeneID:145447","PYGL;ABHD12B",TRUE,"ABHD12B"

"951","AGCTTCGCACAGACTGAAGC","chr3",197023235,"-","5:A>G,8:A>T","TGG","AGCTTTGCTCAGACTGAAGCTGG",2,6,9,NA,"GeneID:4241","MFI2",FALSE,"ABHD12B"

"952","CCAAGGGGAAGGACTGTTGC","chr14",50880483,"+","none","TGG","CCAAGGGGAAGGACTGTTGCTGG",0,NA,NA,NA,"GeneID:5836;GeneID:145447","PYGL;ABHD12B",TRUE,"ABHD12B"

"953","CCAAGGGGAAGGACTGTTGC","chr10",49094180,"-","0:A>G,2:G>T","TGG","TCCAGGGGAAGGACTGTTGCTGG",2,1,3,NA,"GeneID:196740","VSTM4",FALSE,"ABHD12B"

"954","GCCGCATCGCCCGAGCCGCC","chr14",50872198,"+","none","CGG","GCCGCATCGCCCGAGCCGCCCGG",0,NA,NA,NA,"GeneID:5836;GeneID:145447","PYGL;ABHD12B",TRUE,"ABHD12B"

"955","TATGAAGCAGCCCTTCGTGA","chr14",50880506,"+","none","TGG","TATGAAGCAGCCCTTCGTGATGG",0,NA,NA,NA,"GeneID:5836;GeneID:145447","PYGL;ABHD12B",TRUE,"ABHD12B"

"956","CAAGTGCAGTGAAATAGCCT","chr13",108230132,"-","none","TGG","CAAGTGCAGTGAAATAGCCTTGG",0,NA,NA,NA,"GeneID:84945","ABHD13",TRUE,"ABHD13"

"957","CGATGCGTTACCTTCCTTTA","chr13",108229918,"+","none","TGG","CGATGCGTTACCTTCCTTTATGG",0,NA,NA,NA,"GeneID:84945","ABHD13",TRUE,"ABHD13"

"958","CTCGATAATCAACCAGCAAA","chr13",108229652,"-","none","AGG","CTCGATAATCAACCAGCAAAAGG",0,NA,NA,NA,"GeneID:84945","ABHD13",TRUE,"ABHD13"

"959","CTTTATGTTCCCATGCCCAC","chr13",108229443,"+","none","TGG","CTTTATGTTCCCATGCCCACTGG",0,NA,NA,NA,"GeneID:84945","ABHD13",TRUE,"ABHD13"

"960","GCTAATCTCTTAGTCCGAGA","chr13",108230070,"-","none","TGG","GCTAATCTCTTAGTCCGAGATGG",0,NA,NA,NA,"GeneID:84945","ABHD13",TRUE,"ABHD13"

"961","GTGTCATTGTGAGTCCCATC","chr13",108230100,"-","none","TGG","GTGTCATTGTGAGTCCCATCTGG",0,NA,NA,NA,"GeneID:84945","ABHD13",TRUE,"ABHD13"

"962","ACTCCAACCCTTATCCTGTA","chr3",51980835,"+","none","TGG","ACTCCAACCCTTATCCTGTATGG",0,NA,NA,NA,"GeneID:84836;GeneID:25864;GeneID:100526760","ABHD14B;ABHD14A;ABHD14A-ACY1",TRUE,"ABHD14A"

"963","AGGCCTTTAACTCTCACACG","chr3",51978284,"+","none","TGG","AGGCCTTTAACTCTCACACGTGG",0,NA,NA,NA,"GeneID:84836;GeneID:25864;GeneID:100526760","ABHD14B;ABHD14A;ABHD14A-ACY1",TRUE,"ABHD14A"

"964","CTCCCACGTGTGAGAGTTAA","chr3",51978290,"-","none","AGG","CTCCCACGTGTGAGAGTTAAAGG",0,NA,NA,NA,"GeneID:84836;GeneID:25864;GeneID:100526760","ABHD14B;ABHD14A;ABHD14A-ACY1",TRUE,"ABHD14A"

"965","GAGCACCTCGCGGTAAAAGA","chr3",51978042,"-","none","TGG","GAGCACCTCGCGGTAAAAGATGG",0,NA,NA,NA,"GeneID:84836;GeneID:25864;GeneID:100526760","ABHD14B;ABHD14A;ABHD14A-ACY1",TRUE,"ABHD14A"

"966","GCCGCAGTGACTCTCGAGCC","chr3",51980876,"-","none","AGG","GCCGCAGTGACTCTCGAGCCAGG",0,NA,NA,NA,"GeneID:84836;GeneID:25864;GeneID:100526760","ABHD14B;ABHD14A;ABHD14A-ACY1",TRUE,"ABHD14A"

"967","GGCATAGTGGCCACTCAGCG","chr3",51980506,"-","none","AGG","GGCATAGTGGCCACTCAGCGAGG",0,NA,NA,NA,"GeneID:84836;GeneID:25864;GeneID:100526760","ABHD14B;ABHD14A;ABHD14A-ACY1",TRUE,"ABHD14A"

"968","CTGTTACCTGGACAAACCAG","chr3",51969472,"-","none","AGG","CTGTTACCTGGACAAACCAGAGG",0,NA,NA,NA,"GeneID:57060;GeneID:84836","PCBP4;ABHD14B",TRUE,"ABHD14B"

"969","GTGATCAGTCCATCACTGAG","chr3",51970057,"-","none","TGG","GTGATCAGTCCATCACTGAGTGG",0,NA,NA,NA,"GeneID:57060;GeneID:84836","PCBP4;ABHD14B",TRUE,"ABHD14B"

"970","TACCTTCACACTGGCATAGT","chr3",51969939,"+","none","TGG","TACCTTCACACTGGCATAGTTGG",0,NA,NA,NA,"GeneID:57060;GeneID:84836","PCBP4;ABHD14B",TRUE,"ABHD14B"

"971","TGAAGCAGCTGCCCAACCAC","chr3",51969524,"-","none","CGG","TGAAGCAGCTGCCCAACCACCGG",0,NA,NA,NA,"GeneID:57060;GeneID:84836","PCBP4;ABHD14B",TRUE,"ABHD14B"

"972","TTAAGTACCTGGCAGGTCAA","chr3",51971451,"+","none","TGG","TTAAGTACCTGGCAGGTCAATGG",0,NA,NA,NA,"GeneID:57060;GeneID:84836;GeneID:25864","PCBP4;ABHD14B;ABHD14A",TRUE,"ABHD14B"

"973","TTCGCTTCTCCTCCGAGACC","chr3",51971529,"-","none","TGG","TTCGCTTCTCCTCCGAGACCTGG",0,NA,NA,NA,"GeneID:57060;GeneID:84836;GeneID:25864","PCBP4;ABHD14B;ABHD14A",TRUE,"ABHD14B"

"974","TTCGCTTCTCCTCCGAGACC","chr12",68863341,"+","2:T>C,13:A>C","GGG","TTTGCTTCTCCTCAGAGACCGGG",2,3,14,NA,"GeneID:1368","CPM",FALSE,"ABHD14B"

"975","CCCCACGACGACGGCCCCCA","chr17",29562640,"+","none","AGG","CCCCACGACGACGGCCCCCAAGG",0,NA,NA,NA,"GeneID:116236","ABHD15",TRUE,"ABHD15"

"976","CCGCGGCGATGGAAGATGAC","chr17",29566382,"+","none","CGG","CCGCGGCGATGGAAGATGACCGG",0,NA,NA,NA,"GeneID:116236;GeneID:90313","ABHD15;TP53I13",TRUE,"ABHD15"

"977","CCGCTGTTCGCGGTGAGCGA","chr17",29566259,"-","none","AGG","CCGCTGTTCGCGGTGAGCGAAGG",0,NA,NA,NA,"GeneID:116236;GeneID:90313","ABHD15;TP53I13",TRUE,"ABHD15"

"978","CCTTCGCTCACCGCGAACAG","chr17",29566256,"+","none","CGG","CCTTCGCTCACCGCGAACAGCGG",0,NA,NA,NA,"GeneID:116236;GeneID:90313","ABHD15;TP53I13",TRUE,"ABHD15"

"979","CGGGTCGTTGCGGTCCCAGT","chr17",29562944,"+","none","AGG","CGGGTCGTTGCGGTCCCAGTAGG",0,NA,NA,NA,"GeneID:116236","ABHD15",TRUE,"ABHD15"

"980","TTCGTCCTGCCCGTAGCGCC","chr17",29566613,"-","none","TGG","TTCGTCCTGCCCGTAGCGCCTGG",0,NA,NA,NA,"GeneID:116236;GeneID:90313","ABHD15;TP53I13",TRUE,"ABHD15"

"981","ACATTGCTGCTACTTCTGGC","chr6",31700961,"-","none","AGG","ACATTGCTGCTACTTCTGGCAGG",0,NA,NA,NA,"GeneID:7920","ABHD16A",TRUE,"ABHD16A"

"982","ACATTGCTGCTACTTCTGGC","chr15",98965838,"-","1:A>G,11:G>T","TGG","ATATTGCTGCTCCTTCTGGCTGG",2,2,12,NA,"","",FALSE,"ABHD16A"

"983","AGGGGAGGACCGACGGATAC","chr6",31702675,"+","none","AGG","AGGGGAGGACCGACGGATACAGG",0,NA,NA,NA,"GeneID:7920","ABHD16A",TRUE,"ABHD16A"

"984","GTACTATCAGCCCCGTGCCC","chr6",31702105,"-","none","TGG","GTACTATCAGCCCCGTGCCCTGG",0,NA,NA,NA,"GeneID:7920","ABHD16A",TRUE,"ABHD16A"

"985","TCGCCTTCTTCTACTTGTAC","chr6",31701280,"-","none","AGG","TCGCCTTCTTCTACTTGTACAGG",0,NA,NA,NA,"GeneID:7920","ABHD16A",TRUE,"ABHD16A"

"986","TCTCCACCTACCTCGGAGGC","chr6",31700930,"+","none","AGG","TCTCCACCTACCTCGGAGGCAGG",0,NA,NA,NA,"GeneID:7920","ABHD16A",TRUE,"ABHD16A"

"987","TTTACAGATCCTGTATCCGT","chr6",31702687,"-","none","CGG","TTTACAGATCCTGTATCCGTCGG",0,NA,NA,NA,"GeneID:7920","ABHD16A",TRUE,"ABHD16A"

"988","CGCCTCGTGGAGCGCTACCA","chr20",63861936,"+","none","CGG","CGCCTCGTGGAGCGCTACCACGG",0,NA,NA,NA,"GeneID:140701","ABHD16B",TRUE,"ABHD16B"

"989","CTACCGTGCACGCTGCGAAG","chr20",63862739,"+","none","AGG","CTACCGTGCACGCTGCGAAGAGG",0,NA,NA,NA,"GeneID:140701","ABHD16B",TRUE,"ABHD16B"

"990","CTTCGACGACCTTGTGCCGC","chr20",63862352,"+","none","TGG","CTTCGACGACCTTGTGCCGCTGG",0,NA,NA,NA,"GeneID:140701","ABHD16B",TRUE,"ABHD16B"

"991","CTTCTATGCACGCTACCGCG","chr20",63862685,"+","none","TGG","CTTCTATGCACGCTACCGCGTGG",0,NA,NA,NA,"GeneID:140701","ABHD16B",TRUE,"ABHD16B"

"992","GCACCGCTACCCCGTCGTGA","chr20",63862598,"+","none","TGG","GCACCGCTACCCCGTCGTGATGG",0,NA,NA,NA,"GeneID:140701","ABHD16B",TRUE,"ABHD16B"

"993","TCAGCACGACGCCAACGCCA","chr20",63862187,"+","none","TGG","TCAGCACGACGCCAACGCCATGG",0,NA,NA,NA,"GeneID:140701","ABHD16B",TRUE,"ABHD16B"

"994","AGGGAAGGCGTCGAAGCAGT","chr22",20668045,"+","none","AGG","AGGGAAGGCGTCGAAGCAGTAGG",0,NA,NA,NA,"","",TRUE,"ABHD17A"

"995","AGGGAAGGCGTCGAAGCAGT","chr22",22115071,"+","none","AGG","AGGGAAGGCGTCGAAGCAGTAGG",0,NA,NA,NA,"","",TRUE,"ABHD17A"

"996","AGGGAAGGCGTCGAAGCAGT","chr1",214605901,"+","none","AGG","AGGGAAGGCGTCGAAGCAGTAGG",0,NA,NA,NA,"GeneID:1063","CENPF",TRUE,"ABHD17A"

"997","AGGGAAGGCGTCGAAGCAGT","chr19",1877509,"+","none","AGG","AGGGAAGGCGTCGAAGCAGTAGG",0,NA,NA,NA,"GeneID:81926","ABHD17A",TRUE,"ABHD17A"

"998","AGGGAAGGCGTCGAAGCAGT","chr1",148149318,"-","none","AGG","AGGGAAGGCGTCGAAGCAGTAGG",0,NA,NA,NA,"GeneID:200030","NBPF11",TRUE,"ABHD17A"

"999","AGGGAAGGCGTCGAAGCAGT","chr17",20843914,"-","none","AGG","AGGGAAGGCGTCGAAGCAGTAGG",0,NA,NA,NA,"GeneID:339184","CCDC144NL",TRUE,"ABHD17A"

"1000","AGGGAAGGCGTCGAAGCAGT","chr16",32202482,"-","0:C>T","AGG","GGGGAAGGCGTCGAAGCAGTAGG",1,1,NA,NA,"","",TRUE,"ABHD17A"

"1001","AGGGAAGGCGTCGAAGCAGT","chr16",33143458,"-","0:C>T,9:T>C","AGG","GGGGAAGGCATCGAAGCAGTAGG",2,1,10,NA,"","",FALSE,"ABHD17A"

"1002","AGGGAAGGCGTCGAAGCAGT","chr15",28455181,"+","0:G>A","AGG","GGGGAAGGCGTCGAAGCAGTAGG",1,1,NA,NA,"","",TRUE,"ABHD17A"

"1003","AGGGAAGGCGTCGAAGCAGT","chr15",23226213,"+","0:G>A","AGG","GGGGAAGGCGTCGAAGCAGTAGG",1,1,NA,NA,"","",TRUE,"ABHD17A"

"1004","AGGGAAGGCGTCGAAGCAGT","chr16_KI270728v1_random",987251,"+","0:G>A","AGG","GGGGAAGGCGTCGAAGCAGTAGG",1,1,NA,NA,"","",FALSE,"ABHD17A"

"1005","AGGGAAGGCGTCGAAGCAGT","chr16",32726929,"+","0:G>A,9:A>G","AGG","GGGGAAGGCATCGAAGCAGTAGG",2,1,10,NA,"","",TRUE,"ABHD17A"

"1006","CAGAACCCCACCTGGCGAGA","chr19",1880884,"+","none","AGG","CAGAACCCCACCTGGCGAGAAGG",0,NA,NA,NA,"GeneID:81926","ABHD17A",TRUE,"ABHD17A"

"1007","CAGAACCCCACCTGGCGAGA","chr15",45184946,"+","11:A>C,15:G>C","AGG","CAGAACCCCACATGGGGAGAAGG",2,12,16,NA,"GeneID:90525","SHF",FALSE,"ABHD17A"

"1008","CGCCAGGTACGGCATCAGCC","chr1",148149154,"+","none","CGG","CGCCAGGTACGGCATCAGCCCGG",0,NA,NA,NA,"GeneID:200030","NBPF11",TRUE,"ABHD17A"

"1009","CGCCAGGTACGGCATCAGCC","chr19",1877673,"-","none","CGG","CGCCAGGTACGGCATCAGCCCGG",0,NA,NA,NA,"GeneID:81926","ABHD17A",TRUE,"ABHD17A"

"1010","CGCCAGGTACGGCATCAGCC","chr22",20668206,"-","none","CGG","CGCCAGGTACGGCATCAGCCCGG",0,NA,NA,NA,"","",TRUE,"ABHD17A"

"1011","CGCCAGGTACGGCATCAGCC","chr22",22115231,"-","none","CGG","CGCCAGGTACGGCATCAGCCCGG",0,NA,NA,NA,"","",TRUE,"ABHD17A"

"1012","CGCCAGGTACGGCATCAGCC","chr15",28455344,"-","10:G>C","CGG","CGCCAGGTACCGCATCAGCCCGG",1,11,NA,NA,"","",TRUE,"ABHD17A"

"1013","CGCCAGGTACGGCATCAGCC","chr15",23226376,"-","10:G>C","CGG","CGCCAGGTACCGCATCAGCCCGG",1,11,NA,NA,"","",TRUE,"ABHD17A"

"1014","CGCCAGGTACGGCATCAGCC","chr16",32727089,"-","10:G>C","CGG","CGCCAGGTACCGCATCAGCCCGG",1,11,NA,NA,"","",TRUE,"ABHD17A"

"1015","CGCCAGGTACGGCATCAGCC","chr16_KI270728v1_random",987411,"-","10:G>C","CGG","CGCCAGGTACCGCATCAGCCCGG",1,11,NA,NA,"","",FALSE,"ABHD17A"

"1016","CGCCAGGTACGGCATCAGCC","chr16",33143298,"+","10:C>G","CGG","CGCCAGGTACCGCATCAGCCCGG",1,11,NA,NA,"","",TRUE,"ABHD17A"

"1017","CGCCAGGTACGGCATCAGCC","chr16",32202322,"+","10:C>G","CGG","CGCCAGGTACCGCATCAGCCCGG",1,11,NA,NA,"","",TRUE,"ABHD17A"

"1018","CGCCAGGTACGGCATCAGCC","chr1",214606065,"-","1:A>C","CGG","CTCCAGGTACGGCATCAGCCCGG",1,2,NA,NA,"GeneID:1063","CENPF",TRUE,"ABHD17A"

"1019","CGCCAGGTACGGCATCAGCC","chr17",20843750,"+","0:T>C","CGG","TGCCAGGTACGGCATCAGCCCGG",1,1,NA,NA,"GeneID:339184","CCDC144NL",TRUE,"ABHD17A"

"1020","CGCCAGGTACGGCATCAGCC","chr6",42722025,"+","0:A>C,13:T>A","AGG","AGCCAGGTACGGCTTCAGCCAGG",2,1,14,NA,"GeneID:5961","PRPH2",TRUE,"ABHD17A"

"1021","GACTCCAACAACCCAGCGCC","chr19",1880970,"-","none","TGG","GACTCCAACAACCCAGCGCCTGG",0,NA,NA,NA,"GeneID:81926","ABHD17A",TRUE,"ABHD17A"

"1022","GTCGCCCTCACCTGGTGCGC","chr19",1879909,"+","none","AGG","GTCGCCCTCACCTGGTGCGCAGG",0,NA,NA,NA,"GeneID:81926","ABHD17A",TRUE,"ABHD17A"

"1023","GTCGCCCTCACCTGGTGCGC","chr22",22117473,"+","none","AGG","GTCGCCCTCACCTGGTGCGCAGG",0,NA,NA,NA,"","",TRUE,"ABHD17A"

"1024","GTCGCCCTCACCTGGTGCGC","chr16_KI270728v1_random",989668,"+","none","AGG","GTCGCCCTCACCTGGTGCGCAGG",0,NA,NA,NA,"","",FALSE,"ABHD17A"

"1025","GTCGCCCTCACCTGGTGCGC","chr16",32729354,"+","none","AGG","GTCGCCCTCACCTGGTGCGCAGG",0,NA,NA,NA,"","",TRUE,"ABHD17A"

"1026","GTCGCCCTCACCTGGTGCGC","chr15",28457600,"+","none","AGG","GTCGCCCTCACCTGGTGCGCAGG",0,NA,NA,NA,"","",TRUE,"ABHD17A"

"1027","GTCGCCCTCACCTGGTGCGC","chr22",20670449,"+","none","AGG","GTCGCCCTCACCTGGTGCGCAGG",0,NA,NA,NA,"","",TRUE,"ABHD17A"

"1028","GTCGCCCTCACCTGGTGCGC","chr16",32200064,"-","none","AGG","GTCGCCCTCACCTGGTGCGCAGG",0,NA,NA,NA,"","",TRUE,"ABHD17A"

"1029","GTCGCCCTCACCTGGTGCGC","chr17",20841515,"-","none","AGG","GTCGCCCTCACCTGGTGCGCAGG",0,NA,NA,NA,"GeneID:339184","CCDC144NL",TRUE,"ABHD17A"

"1030","GTCGCCCTCACCTGGTGCGC","chr1",148146912,"-","none","AGG","GTCGCCCTCACCTGGTGCGCAGG",0,NA,NA,NA,"GeneID:200030","NBPF11",TRUE,"ABHD17A"

"1031","GTCGCCCTCACCTGGTGCGC","chr16",33141033,"-","none","AGG","GTCGCCCTCACCTGGTGCGCAGG",0,NA,NA,NA,"","",TRUE,"ABHD17A"

"1032","GTCGCCCTCACCTGGTGCGC","chr1",214608302,"+","2:T>C","AGG","GTTGCCCTCACCTGGTGCGCAGG",1,3,NA,NA,"GeneID:1063","CENPF",TRUE,"ABHD17A"

"1033","TACACGGTCCTCTTCTCGCA","chr1",148146727,"+","none","CGG","TACACGGTCCTCTTCTCGCACGG",0,NA,NA,NA,"GeneID:200030","NBPF11",TRUE,"ABHD17A"

"1034","TACACGGTCCTCTTCTCGCA","chr19",1880094,"-","none","CGG","TACACGGTCCTCTTCTCGCACGG",0,NA,NA,NA,"GeneID:81926","ABHD17A",TRUE,"ABHD17A"

"1035","TACACGGTCCTCTTCTCGCA","chr22",22117655,"-","none","CGG","TACACGGTCCTCTTCTCGCACGG",0,NA,NA,NA,"","",TRUE,"ABHD17A"

"1036","TACACGGTCCTCTTCTCGCA","chr22",20670631,"-","9:A>G","CGG","TACACGGTCTTCTTCTCGCACGG",1,10,NA,NA,"","",TRUE,"ABHD17A"

"1037","TACACGGTCCTCTTCTCGCA","chr15",80695825,"+","6:C>G,8:G>C","CGG","TACACGCTGCTCTTCTCGCACGG",2,7,9,NA,"GeneID:58489","ABHD17C",TRUE,"ABHD17A"

"1038","ACTCGCATTCCCGAAGTCAG","chr9",71870130,"+","none","AGG","ACTCGCATTCCCGAAGTCAGAGG",0,NA,NA,NA,"GeneID:51104","ABHD17B",TRUE,"ABHD17B"

"1039","ATCTGTCTGAACGAGCAGAC","chr9",71874915,"-","none","TGG","ATCTGTCTGAACGAGCAGACTGG",0,NA,NA,NA,"GeneID:51104","ABHD17B",TRUE,"ABHD17B"

"1040","GAGTAAAGTGTATTTCGCAT","chr9",71874795,"+","none","TGG","GAGTAAAGTGTATTTCGCATTGG",0,NA,NA,NA,"GeneID:51104","ABHD17B",TRUE,"ABHD17B"

"1041","GGCAACGTTCAAACAATGCG","chr9",71866902,"+","none","AGG","GGCAACGTTCAAACAATGCGAGG",0,NA,NA,NA,"GeneID:51104","ABHD17B",TRUE,"ABHD17B"

"1042","GTCCCATGAATTATTAATAC","chr9",71866955,"+","none","TGG","GTCCCATGAATTATTAATACTGG",0,NA,NA,NA,"GeneID:51104","ABHD17B",TRUE,"ABHD17B"

"1043","TATAGGGACAGTACCGTCTG","chr9",71870200,"-","none","TGG","TATAGGGACAGTACCGTCTGTGG",0,NA,NA,NA,"GeneID:51104","ABHD17B",TRUE,"ABHD17B"

"1044","ACCTCAGCGAGCGCGCCGAC","chr15",80695697,"+","none","TGG","ACCTCAGCGAGCGCGCCGACTGG",0,NA,NA,NA,"GeneID:58489","ABHD17C",TRUE,"ABHD17C"

"1045","CGCTGCGCATTCATACCTCG","chr15",80749586,"-","none","AGG","CGCTGCGCATTCATACCTCGAGG",0,NA,NA,NA,"GeneID:58489","ABHD17C",TRUE,"ABHD17C"

"1046","CTCGAGGTATGAATGCGCAG","chr15",80749584,"+","none","CGG","CTCGAGGTATGAATGCGCAGCGG",0,NA,NA,NA,"GeneID:58489","ABHD17C",TRUE,"ABHD17C"

"1047","GACAGCGCTCGTACATCGCT","chr15",80754234,"-","none","AGG","GACAGCGCTCGTACATCGCTAGG",0,NA,NA,NA,"GeneID:58489","ABHD17C",TRUE,"ABHD17C"

"1048","GCCCAGCCCCTTCAACCCAA","chr15",80754273,"-","none","AGG","GCCCAGCCCCTTCAACCCAAAGG",0,NA,NA,NA,"GeneID:58489","ABHD17C",TRUE,"ABHD17C"

"1049","GCCCAGCCCCTTCAACCCAA","chr11",2072558,"-","8:T>G,13:G>T","GGG","GCCCAGCCACTTCCACCCAAGGG",2,9,14,NA,"","",FALSE,"ABHD17C"

"1050","GCGCGAGAAGAAGACCTCGA","chr15",80695748,"-","none","CGG","GCGCGAGAAGAAGACCTCGACGG",0,NA,NA,NA,"GeneID:58489","ABHD17C",TRUE,"ABHD17C"

"1051","AGAAGATCATCCTCTCGCAC","chr15",89185494,"+","none","AGG","AGAAGATCATCCTCTCGCACAGG",0,NA,NA,NA,"GeneID:11057","ABHD2",TRUE,"ABHD2"

"1052","ATTCGACCTCTTCGAGCCCT","chr15",89151811,"+","none","TGG","ATTCGACCTCTTCGAGCCCTTGG",0,NA,NA,NA,"GeneID:11057","ABHD2",TRUE,"ABHD2"

"1053","GATACATTCCACCGTTGATC","chr15",89151675,"+","none","TGG","GATACATTCCACCGTTGATCTGG",0,NA,NA,NA,"GeneID:11057","ABHD2",TRUE,"ABHD2"

"1054","GTGCTGCGTCAGCGTGTGCC","chr15",89175956,"+","none","AGG","GTGCTGCGTCAGCGTGTGCCAGG",0,NA,NA,NA,"GeneID:11057","ABHD2",TRUE,"ABHD2"

"1055","TCGCCACGCATGTTCACCTA","chr15",89155512,"+","none","TGG","TCGCCACGCATGTTCACCTATGG",0,NA,NA,NA,"GeneID:11057","ABHD2",TRUE,"ABHD2"

"1056","TTACCATAGGTGAACATGCG","chr15",89155518,"-","none","TGG","TTACCATAGGTGAACATGCGTGG",0,NA,NA,NA,"GeneID:11057","ABHD2",TRUE,"ABHD2"

"1057","ACTCCTTGCTTGTTCCCGTG","chr18",21702361,"+","none","AGG","ACTCCTTGCTTGTTCCCGTGAGG",0,NA,NA,NA,"GeneID:171586","ABHD3",TRUE,"ABHD3"

"1058","ACTCCTTGCTTGTTCCCGTG","chr6",18295114,"-","11:G>C,17:G>C","GGG","ACTCCTTGCTTCTTCCCCTGGGG",2,12,18,NA,"","",FALSE,"ABHD3"

"1059","CCGAGCCGAAGAACCCCACC","chr18",21704574,"+","none","CGG","CCGAGCCGAAGAACCCCACCCGG",0,NA,NA,NA,"GeneID:171586","ABHD3",TRUE,"ABHD3"

"1060","TACAGAAACGTACTACCCGA","chr18",21703662,"-","none","CGG","TACAGAAACGTACTACCCGACGG",0,NA,NA,NA,"GeneID:171586","ABHD3",TRUE,"ABHD3"

"1061","TCTCACCCCCGGTCACTAAC","chr18",21703719,"+","none","TGG","TCTCACCCCCGGTCACTAACTGG",0,NA,NA,NA,"GeneID:171586","ABHD3",TRUE,"ABHD3"

"1062","TGATAACAGTACGTGTTATA","chr18",21702424,"-","none","TGG","TGATAACAGTACGTGTTATATGG",0,NA,NA,NA,"GeneID:171586","ABHD3",TRUE,"ABHD3"

"1063","TGGACCTGCGGATGTTGTCC","chr18",21704629,"-","none","CGG","TGGACCTGCGGATGTTGTCCCGG",0,NA,NA,NA,"GeneID:171586","ABHD3",TRUE,"ABHD3"

"1064","ACGTCCTAGGACAGATGCCA","chr14",22604019,"-","none","CGG","ACGTCCTAGGACAGATGCCACGG",0,NA,NA,NA,"GeneID:63874","ABHD4",TRUE,"ABHD4"

"1065","CGGAATCGCTGCACCAGACC","chr14",22606426,"-","none","AGG","CGGAATCGCTGCACCAGACCAGG",0,NA,NA,NA,"GeneID:63874","ABHD4",TRUE,"ABHD4"

"1066","CTGCACACCTTCGATCTGCT","chr14",22603565,"+","none","TGG","CTGCACACCTTCGATCTGCTTGG",0,NA,NA,NA,"GeneID:63874","ABHD4",TRUE,"ABHD4"

"1067","GCAAAACGACCGCACCCCCT","chr14",22603468,"+","none","TGG","GCAAAACGACCGCACCCCCTTGG",0,NA,NA,NA,"GeneID:63874","ABHD4",TRUE,"ABHD4"

"1068","TGGGATTCTGTGCGTTGCAG","chr14",22606512,"-","none","TGG","TGGGATTCTGTGCGTTGCAGTGG",0,NA,NA,NA,"GeneID:63874","ABHD4",TRUE,"ABHD4"

"1069","TTGGCTGTTCTTCGAGTAGC","chr14",22604048,"+","none","TGG","TTGGCTGTTCTTCGAGTAGCTGG",0,NA,NA,NA,"GeneID:63874","ABHD4",TRUE,"ABHD4"

"1070","TTGGCTGTTCTTCGAGTAGC","chr2",6664471,"-","13:G>C,16:T>A","TGG","TTGGCTGTTCTTCCAGAAGCTGG",2,14,17,NA,"","",FALSE,"ABHD4"

"1071","ACTCCCTTTAACCCTTTAGC","chr3",43711817,"+","none","TGG","ACTCCCTTTAACCCTTTAGCTGG",0,NA,NA,NA,"GeneID:51099","ABHD5",TRUE,"ABHD5"

"1072","ACTCGCTGAAGTACCCATCA","chr3",43702565,"+","none","AGG","ACTCGCTGAAGTACCCATCAAGG",0,NA,NA,NA,"GeneID:51099","ABHD5",TRUE,"ABHD5"

"1073","GCACAACCTAGGTGGATTCT","chr3",43702533,"+","none","TGG","GCACAACCTAGGTGGATTCTTGG",0,NA,NA,NA,"GeneID:51099","ABHD5",TRUE,"ABHD5"

"1074","TAAGTCTAGTGCAGCGTTTA","chr3",43714949,"+","none","AGG","TAAGTCTAGTGCAGCGTTTAAGG",0,NA,NA,NA,"GeneID:51099","ABHD5",TRUE,"ABHD5"

"1075","TAAGTCTAGTGCAGCGTTTA","chr18",43524677,"-","5:C>G,8:T>C","AGG","TAAGTGTAATGCAGCGTTTAAGG",2,6,9,NA,"","",FALSE,"ABHD5"

"1076","TATAGACGTAGGGCACCATG","chr3",43699304,"-","none","TGG","TATAGACGTAGGGCACCATGTGG",0,NA,NA,NA,"GeneID:51099","ABHD5",TRUE,"ABHD5"

"1077","TCTAGGTCAGGATGGCTAAC","chr3",43699270,"+","none","TGG","TCTAGGTCAGGATGGCTAACTGG",0,NA,NA,NA,"GeneID:51099","ABHD5",TRUE,"ABHD5"

"1078","AACATGTTTGTGATTGCGGG","chr3",58256604,"+","none","CGG","AACATGTTTGTGATTGCGGGCGG",0,NA,NA,NA,"GeneID:57406","ABHD6",TRUE,"ABHD6"

"1079","CAGAGAATCCGTGGAGCATG","chr3",58267293,"-","none","AGG","CAGAGAATCCGTGGAGCATGAGG",0,NA,NA,NA,"GeneID:57406","ABHD6",TRUE,"ABHD6"

"1080","CAGAGAATCCGTGGAGCATG","chr9",129858663,"+","1:T>A,5:C>A","GGG","CTGAGCATCCGTGGAGCATGGGG",2,2,6,NA,"GeneID:10868","USP20",FALSE,"ABHD6"

"1081","CCCCTTCCAGGTACTGGCGG","chr3",58267178,"+","none","AGG","CCCCTTCCAGGTACTGGCGGAGG",0,NA,NA,NA,"GeneID:57406","ABHD6",TRUE,"ABHD6"

"1082","CCTCTTAACTTGCCCATCTA","chr3",58269405,"-","none","TGG","CCTCTTAACTTGCCCATCTATGG",0,NA,NA,NA,"GeneID:57406","ABHD6",TRUE,"ABHD6"

"1083","GAGCAGGATGGATCTTGATG","chr3",58256579,"+","none","TGG","GAGCAGGATGGATCTTGATGTGG",0,NA,NA,NA,"GeneID:57406","ABHD6",TRUE,"ABHD6"

"1084","GCAAGTTAAGAGGATACACC","chr3",58269412,"+","none","AGG","GCAAGTTAAGAGGATACACCAGG",0,NA,NA,NA,"GeneID:57406","ABHD6",TRUE,"ABHD6"

"1085","ACCTTTGTAGAGGTCAAGCC","chr19",17301497,"-","none","CGG","ACCTTTGTAGAGGTCAAGCCCGG",0,NA,NA,NA,"GeneID:79575;GeneID:64981","ABHD8;MRPL34",TRUE,"ABHD8"

"1086","ATTCCTGAAGCTCATCGACG","chr19",17292797,"-","none","AGG","ATTCCTGAAGCTCATCGACGAGG",0,NA,NA,NA,"GeneID:79575;GeneID:64981","ABHD8;MRPL34",TRUE,"ABHD8"

"1087","CAAACTTATCGTGCATGCCG","chr19",17294319,"+","none","TGG","CAAACTTATCGTGCATGCCGTGG",0,NA,NA,NA,"GeneID:79575;GeneID:64981","ABHD8;MRPL34",TRUE,"ABHD8"

"1088","CCATGATGAGCGGCCAGTAC","chr19",17294398,"-","none","TGG","CCATGATGAGCGGCCAGTACTGG",0,NA,NA,NA,"GeneID:79575;GeneID:64981","ABHD8;MRPL34",TRUE,"ABHD8"

"1089","TCGGCGGTTCCCTGGCCATC","chr19",17301046,"-","none","TGG","TCGGCGGTTCCCTGGCCATCTGG",0,NA,NA,NA,"GeneID:79575;GeneID:64981","ABHD8;MRPL34",TRUE,"ABHD8"

"1090","TGCCCTCGTCGATGAGCTTC","chr19",17292791,"+","none","AGG","TGCCCTCGTCGATGAGCTTCAGG",0,NA,NA,NA,"GeneID:79575;GeneID:64981","ABHD8;MRPL34",TRUE,"ABHD8"

"1091","ACAGGGCGCTCCATATTCGC","chr10",26777125,"+","none","AGG","ACAGGGCGCTCCATATTCGCAGG",0,NA,NA,NA,"GeneID:10006","ABI1",TRUE,"ABI1"

"1092","AGCGCCCTGTAAGGTATATT","chr10",26777115,"-","none","CGG","AGCGCCCTGTAAGGTATATTCGG",0,NA,NA,NA,"GeneID:10006","ABI1",TRUE,"ABI1"

"1093","AGGAGATCCCGTCTGGCAAG","chr10",26860815,"-","none","AGG","AGGAGATCCCGTCTGGCAAGAGG",0,NA,NA,NA,"GeneID:10006","ABI1",TRUE,"ABI1"

"1094","AGTAGTCTGCCACCCGAGTC","chr10",26860766,"+","none","AGG","AGTAGTCTGCCACCCGAGTCAGG",0,NA,NA,NA,"GeneID:10006","ABI1",TRUE,"ABI1"

"1095","CAACAATGTACTCCAGTTGC","chr10",26823196,"-","none","TGG","CAACAATGTACTCCAGTTGCTGG",0,NA,NA,NA,"GeneID:10006","ABI1",TRUE,"ABI1"

"1096","CTCCATTCTCCGAAGCTGAG","chr10",26823161,"+","none","AGG","CTCCATTCTCCGAAGCTGAGAGG",0,NA,NA,NA,"GeneID:10006","ABI1",TRUE,"ABI1"

"1097","CTCCATTCTCCGAAGCTGAG","chr1",239198570,"+","11:A>G,17:C>G","CGG","CTCCATTCTCCAAAGCTCAGCGG",2,12,18,NA,"","",FALSE,"ABI1"

"1098","AACACTTGCTAAGGATTGGG","chr2",203366922,"-","none","TGG","AACACTTGCTAAGGATTGGGTGG",0,NA,NA,NA,"GeneID:10152","ABI2",TRUE,"ABI2"

"1099","AAGATTCCATCCTTCGTAGC","chr2",203367004,"-","none","TGG","AAGATTCCATCCTTCGTAGCTGG",0,NA,NA,NA,"GeneID:10152","ABI2",TRUE,"ABI2"

"1100","CCCCCTAGTCCCCCTATGTC","chr2",203391105,"+","none","AGG","CCCCCTAGTCCCCCTATGTCAGG",0,NA,NA,NA,"GeneID:10152","ABI2",TRUE,"ABI2"

"1101","CCCCCTAGTCCCCCTATGTC","chr15",76973450,"-","8:T>A,15:C>T","AGG","CCCCCTAGACCCCCTGTGTCAGG",2,9,16,NA,"","",FALSE,"ABI2"

"1102","GCACTCCCCCTATCGCACAC","chr2",203394702,"+","none","TGG","GCACTCCCCCTATCGCACACTGG",0,NA,NA,NA,"GeneID:10152;GeneID:65059","ABI2;RAPH1",TRUE,"ABI2"

"1103","GGGCTAGGTACGTAATCATT","chr2",203394751,"-","none","TGG","GGGCTAGGTACGTAATCATTTGG",0,NA,NA,NA,"GeneID:10152;GeneID:65059","ABI2;RAPH1",TRUE,"ABI2"

"1104","TATAACGAACTGGTCGTTCA","chr2",203380311,"-","none","AGG","TATAACGAACTGGTCGTTCAAGG",0,NA,NA,NA,"GeneID:10152","ABI2",TRUE,"ABI2"

"1105","AGCACGCAGCTGTCAAGAAC","chr17",49219545,"+","none","AGG","AGCACGCAGCTGTCAAGAACAGG",0,NA,NA,NA,"GeneID:51225","ABI3",TRUE,"ABI3"

"1106","AGTAGTCAGCGACCCGCAGC","chr17",49210801,"-","none","AGG","AGTAGTCAGCGACCCGCAGCAGG",0,NA,NA,NA,"GeneID:51225","ABI3",TRUE,"ABI3"

"1107","CCTTGATGCTCTTTCGAGAC","chr17",49219574,"-","none","AGG","CCTTGATGCTCTTTCGAGACAGG",0,NA,NA,NA,"GeneID:51225","ABI3",TRUE,"ABI3"

"1108","CGACATTGGCCATGGGATCA","chr17",49217893,"+","none","AGG","CGACATTGGCCATGGGATCAAGG",0,NA,NA,NA,"GeneID:51225","ABI3",TRUE,"ABI3"

"1109","CTCCTGCAGTAGGGCGTGAG","chr17",49217849,"-","none","AGG","CTCCTGCAGTAGGGCGTGAGAGG",0,NA,NA,NA,"GeneID:51225","ABI3",TRUE,"ABI3"

"1110","CTGCGAGGACAACTATGTGC","chr17",49210819,"+","none","AGG","CTGCGAGGACAACTATGTGCAGG",0,NA,NA,NA,"GeneID:51225","ABI3",TRUE,"ABI3"

"1111","AGTCAAAAGAAGTCATGTTC","chr3",100902619,"-","none","AGG","AGTCAAAAGAAGTCATGTTCAGG",0,NA,NA,NA,"GeneID:25890","ABI3BP",TRUE,"ABI3BP"

"1112","AGTCAAAAGAAGTCATGTTC","chr3",99757516,"-","16:G>C,19:A>G","TGG","AGTCAAAAGAAGTCATCTTTTGG",2,17,20,NA,"GeneID:1295","COL8A1",FALSE,"ABI3BP"

"1113","CAAGTCACTGTCCCAATGAC","chr3",100898763,"-","none","AGG","CAAGTCACTGTCCCAATGACAGG",0,NA,NA,NA,"GeneID:25890","ABI3BP",TRUE,"ABI3BP"

"1114","CGCACAACTATCAGATATTT","chr3",100902655,"+","none","CGG","CGCACAACTATCAGATATTTCGG",0,NA,NA,NA,"GeneID:25890","ABI3BP",TRUE,"ABI3BP"

"1115","CGCACAACTATCAGATATTT","chr2",202831901,"+","0:G>C,4:G>C","AGG","GGCAGAACTATCAGATATTTAGG",2,1,5,NA,"GeneID:130026","ICA1L",FALSE,"ABI3BP"

"1116","GTTGCATTTGCCACCTCGCA","chr3",100993384,"+","none","TGG","GTTGCATTTGCCACCTCGCATGG",0,NA,NA,NA,"GeneID:25890","ABI3BP",TRUE,"ABI3BP"

"1117","TGGAAGTATTACACTAGCCC","chr3",100993332,"-","none","TGG","TGGAAGTATTACACTAGCCCTGG",0,NA,NA,NA,"GeneID:25890","ABI3BP",TRUE,"ABI3BP"

"1118","TGGCACTCTGACACCGAGCT","chr3",100898837,"-","none","CGG","TGGCACTCTGACACCGAGCTCGG",0,NA,NA,NA,"GeneID:25890","ABI3BP",TRUE,"ABI3BP"

"1119","ACTCACCAAGGAGCTGCACC","chr9",130872199,"-","none","AGG","ACTCACCAAGGAGCTGCACCAGG",0,NA,NA,NA,"GeneID:25","ABL1",TRUE,"ABL1"

"1120","AGAGATCAAACACCCTAACC","chr9",130872178,"+","none","TGG","AGAGATCAAACACCCTAACCTGG",0,NA,NA,NA,"GeneID:25","ABL1",TRUE,"ABL1"

"1121","CCGGCAGGAGGTGAACGCCG","chr9",130872944,"+","none","TGG","CCGGCAGGAGGTGAACGCCGTGG",0,NA,NA,NA,"GeneID:25","ABL1",TRUE,"ABL1"

"1122","CCGGCAGGAGGTGAACGCCG","chr2",176131530,"+","15:G>C,17:A>C","GGG","CCGGCAGGAGGTGAAGGACGGGG",2,16,18,NA,"GeneID:3234","HOXD8",TRUE,"ABL1"

"1123","CCTCGTACACCTCCCCGTAC","chr9",130862968,"-","none","TGG","CCTCGTACACCTCCCCGTACTGG",0,NA,NA,NA,"GeneID:25","ABL1",TRUE,"ABL1"

"1124","TGCACTCCCTCAGGTAGTCC","chr9",130872923,"-","none","AGG","TGCACTCCCTCAGGTAGTCCAGG",0,NA,NA,NA,"GeneID:25","ABL1",TRUE,"ABL1"

"1125","TGCACTCCCTCAGGTAGTCC","chr11",13239743,"+","10:A>C,18:A>C","AGG","TGCACTCCCTAAGGTAGTACAGG",2,11,19,NA,"","",FALSE,"ABL1"

"1126","TGTCCCCCAACTACGACAAG","chr9",130862895,"+","none","TGG","TGTCCCCCAACTACGACAAGTGG",0,NA,NA,NA,"GeneID:25","ABL1",TRUE,"ABL1"

"1127","CACTGCTGCCCCGGATCCCG","chr1",179229318,"+","none","CGG","CACTGCTGCCCCGGATCCCGCGG",0,NA,NA,NA,"GeneID:27","ABL2",TRUE,"ABL2"

"1128","CACTGCTGCCCCGGATCCCG","chr16",77191502,"+","14:C>A,15:C>T","GGG","CACTGCTGCCCCGGCCCCCGGGG",2,15,16,NA,"GeneID:22879","MON1B",TRUE,"ABL2"

"1129","GGCTCCGAGCAAGTTCTCCT","chr1",179131389,"+","none","TGG","GGCTCCGAGCAAGTTCTCCTTGG",0,NA,NA,NA,"GeneID:27","ABL2",TRUE,"ABL2"

"1130","GGCTCCGAGCAAGTTCTCCT","chr10",95365462,"-","0:T>C,13:G>A","TGG","AGCTCCGAGCAAGCTCTCCTTGG",2,1,14,NA,"GeneID:10580","SORBS1",FALSE,"ABL2"

"1131","GTCCATCTCGCTCAGGTACG","chr1",179126423,"-","none","AGG","GTCCATCTCGCTCAGGTACGAGG",0,NA,NA,NA,"GeneID:27","ABL2",TRUE,"ABL2"

"1132","TCCCTCGTACCTGAGCGAGA","chr1",179126418,"+","none","TGG","TCCCTCGTACCTGAGCGAGATGG",0,NA,NA,NA,"GeneID:27","ABL2",TRUE,"ABL2"

"1133","TCTCTGTGGTGCGCCCCGCC","chr1",179229267,"+","none","GGG","TCTCTGTGGTGCGCCCCGCCGGG",0,NA,NA,NA,"GeneID:27","ABL2",TRUE,"ABL2"

"1134","TGATAGCCTCATTTAGTGCC","chr1",179131421,"+","none","TGG","TGATAGCCTCATTTAGTGCCTGG",0,NA,NA,NA,"GeneID:27","ABL2",TRUE,"ABL2"

"1135","CATTTATGGCAGTGAATGAC","chr10",114601900,"+","none","AGG","CATTTATGGCAGTGAATGACAGG",0,NA,NA,NA,"GeneID:3983","ABLIM1",TRUE,"ABLIM1"

"1136","CGAAGCACTTCACCCTTGCA","chr10",114601870,"+","none","AGG","CGAAGCACTTCACCCTTGCAAGG",0,NA,NA,NA,"GeneID:3983","ABLIM1",TRUE,"ABLIM1"

"1137","CGTGAGCTCAGTCATTTCCA","chr10",114684320,"+","none","AGG","CGTGAGCTCAGTCATTTCCAAGG",0,NA,NA,NA,"GeneID:3983","ABLIM1",TRUE,"ABLIM1"

"1138","CTAACCTTTGTAGTCTCCCA","chr10",114684284,"+","none","TGG","CTAACCTTTGTAGTCTCCCATGG",0,NA,NA,NA,"GeneID:3983","ABLIM1",TRUE,"ABLIM1"

"1139","CTTGATATCTCTTCCGCAGC","chr10",114547747,"+","none","CGG","CTTGATATCTCTTCCGCAGCCGG",0,NA,NA,NA,"GeneID:3983","ABLIM1",TRUE,"ABLIM1"

"1140","TTACTTGCTGATGTACTCCC","chr10",114547645,"+","none","CGG","TTACTTGCTGATGTACTCCCCGG",0,NA,NA,NA,"GeneID:3983","ABLIM1",TRUE,"ABLIM1"

"1141","CATGCATTCCTTCCCGTTGA","chr4",8088232,"+","none","AGG","CATGCATTCCTTCCCGTTGAAGG",0,NA,NA,NA,"GeneID:84448","ABLIM2",TRUE,"ABLIM2"

"1142","CATGCATTCCTTCCCGTTGA","chr4",26709599,"+","11:G>T,14:T>C","GGG","CATGCATTCCTGCCTGTTGAGGG",2,12,15,NA,"GeneID:55296","TBC1D19",FALSE,"ABLIM2"

"1143","CATGCATTCCTTCCCGTTGA","chr3",64681211,"+","7:G>T,14:T>C","GGG","CATGCATGCCTTCCTGTTGAGGG",2,8,15,NA,"GeneID:56999","ADAMTS9",TRUE,"ABLIM2"

"1144","CCGAGTGACCTTCAACGGGA","chr4",8088243,"-","none","AGG","CCGAGTGACCTTCAACGGGAAGG",0,NA,NA,NA,"GeneID:84448","ABLIM2",TRUE,"ABLIM2"

"1145","CGCGCTGCTGCCCACCGATA","chr4",8088190,"+","none","CGG","CGCGCTGCTGCCCACCGATACGG",0,NA,NA,NA,"GeneID:84448","ABLIM2",TRUE,"ABLIM2"

"1146","GAAGTGTTCCCTGCCCGTAT","chr4",8088207,"-","none","CGG","GAAGTGTTCCCTGCCCGTATCGG",0,NA,NA,NA,"GeneID:84448","ABLIM2",TRUE,"ABLIM2"

"1147","GCTGACTATCACGCCAAGTT","chr4",8077679,"-","none","CGG","GCTGACTATCACGCCAAGTTCGG",0,NA,NA,NA,"GeneID:84448","ABLIM2",TRUE,"ABLIM2"

"1148","GCTGACTATCACGCCAAGTT","chr3",12954564,"+","4:T>A,12:A>G","AGG","GCTGTCTATCACACCAAGTTAGG",2,5,13,NA,"GeneID:9922","IQSEC1",FALSE,"ABLIM2"

"1149","GTGATAGTCAGCTTCGCAGT","chr4",8077687,"+","none","AGG","GTGATAGTCAGCTTCGCAGTAGG",0,NA,NA,NA,"GeneID:84448","ABLIM2",TRUE,"ABLIM2"

"1150","AGCGGGTGCCATAGAGTTGC","chr5",149198291,"-","none","TGG","AGCGGGTGCCATAGAGTTGCTGG",0,NA,NA,NA,"GeneID:22885","ABLIM3",TRUE,"ABLIM3"

"1151","CATCACAGGCGAAGTCATCT","chr5",149198330,"+","none","CGG","CATCACAGGCGAAGTCATCTCGG",0,NA,NA,NA,"GeneID:22885","ABLIM3",TRUE,"ABLIM3"

"1152","CCACTTACTGCTAGTGTTCA","chr5",149142096,"-","none","TGG","CCACTTACTGCTAGTGTTCATGG",0,NA,NA,NA,"GeneID:22885","ABLIM3",TRUE,"ABLIM3"

"1153","CTTCGCCTGTGATGAAGTCC","chr5",149198324,"-","none","CGG","CTTCGCCTGTGATGAAGTCCCGG",0,NA,NA,NA,"GeneID:22885","ABLIM3",TRUE,"ABLIM3"

"1154","GCGGACCACTTCCCCTTTGC","chr5",149183526,"-","none","AGG","GCGGACCACTTCCCCTTTGCAGG",0,NA,NA,NA,"GeneID:22885","ABLIM3",TRUE,"ABLIM3"

"1155","GTCATCCAGTGCTACCGCTG","chr5",149183495,"+","none","TGG","GTCATCCAGTGCTACCGCTGTGG",0,NA,NA,NA,"GeneID:22885","ABLIM3",TRUE,"ABLIM3"

"1156","GTCATCCAGTGCTACCGCTG","chr11",133913973,"+","9:G>T,12:G>T","GGG","GTCATCCAGGGCGACCGCTGGGG",2,10,13,NA,"GeneID:22997","IGSF9B",FALSE,"ABLIM3"

"1157","ACGGTGTCAGCACCTTTGAC","chr9",133258097,"+","18:G>A","TGG","ACGGTGTCAGCACCTTTGGCTGG",1,19,NA,NA,"GeneID:28","ABO",TRUE,"ABO"

"1158","AGCCCCAGAAGTCTAATGCC","chr9",133261341,"-","none","AGG","AGCCCCAGAAGTCTAATGCCAGG",0,NA,NA,NA,"GeneID:28","ABO",TRUE,"ABO"

"1159","GATGGTCTACCCCCAGTCAA","chr9",133258112,"-","16:G>A","AGG","GATGGTCTACCCCCAGCCAAAGG",1,17,NA,NA,"GeneID:28","ABO",TRUE,"ABO"

"1160","GCAACGAGACATGCTGCAGA","chr9",133259822,"+","10:G>A,11:C>T","TGG","GCAACGAGACGCGCTGCAGATGG",2,11,12,NA,"GeneID:28","ABO",TRUE,"ABO"

"1161","GCAACGAGACATGCTGCAGA","chr5",159561091,"+","9:G>C,11:G>T","GGG","GCAACGAGAGAGGCTGCAGAGGG",2,10,12,NA,"GeneID:285626","LOC285626",FALSE,"ABO"

"1162","GGATCATAGGTCGAAGTGCG","chr9",133262132,"+","none","TGG","GGATCATAGGTCGAAGTGCGTGG",0,NA,NA,NA,"GeneID:28","ABO",TRUE,"ABO"

"1163","TGCAGCATGTCTCGTTGCCA","chr9",133259820,"-","6:C>T,7:G>A","AGG","TGCAGCGCGTCTCGTTGCCAAGG",2,7,8,NA,"GeneID:28","ABO",TRUE,"ABO"

"1164","TGCAGCATGTCTCGTTGCCA","chr2",201701328,"-","4:A>C,13:A>C","AGG","TGCATCATGTCTCTTTGCCAAGG",2,5,14,NA,"GeneID:57679","ALS2",TRUE,"ABO"

"1165","CGGAGAGCGCATTGCCTATG","chr7",150857495,"+","none","AGG","CGGAGAGCGCATTGCCTATGAGG",0,NA,NA,NA,"GeneID:26","AOC1",TRUE,"ABP1"

"1166","GATCCAGCGCTGGACGTAGT","chr7",150861123,"-","none","TGG","GATCCAGCGCTGGACGTAGTTGG",0,NA,NA,NA,"GeneID:26","AOC1",TRUE,"ABP1"

"1167","GCAGGCGGTACGTGCGCTTG","chr7",150858958,"-","none","TGG","GCAGGCGGTACGTGCGCTTGTGG",0,NA,NA,NA,"GeneID:26","AOC1",TRUE,"ABP1"

"1168","GCCCCAACTACGTCCAGCGC","chr7",150861117,"+","none","TGG","GCCCCAACTACGTCCAGCGCTGG",0,NA,NA,NA,"GeneID:26","AOC1",TRUE,"ABP1"

"1169","GGTGCACTACCGCGTAGACC","chr7",150858008,"+","none","TGG","GGTGCACTACCGCGTAGACCTGG",0,NA,NA,NA,"GeneID:26","AOC1",TRUE,"ABP1"

"1170","TCTACCACCAGAACGACCCC","chr7",150860553,"+","none","TGG","TCTACCACCAGAACGACCCCTGG",0,NA,NA,NA,"GeneID:26","AOC1",TRUE,"ABP1"

"1171","ACGCTTTGTACACACCGAGC","chr17",1083599,"+","none","TGG","ACGCTTTGTACACACCGAGCTGG",0,NA,NA,NA,"GeneID:29","ABR",TRUE,"ABR"

"1172","AGAGATCTACATTAACCAGC","chr17",1100653,"-","none","TGG","AGAGATCTACATTAACCAGCTGG",0,NA,NA,NA,"GeneID:29","ABR",TRUE,"ABR"

"1173","CGACTCATCGATGTACGGCA","chr17",1125269,"+","none","TGG","CGACTCATCGATGTACGGCATGG",0,NA,NA,NA,"GeneID:29","ABR",TRUE,"ABR"

"1174","CGATAACTATAAAGTCGCTC","chr17",1083575,"-","none","TGG","CGATAACTATAAAGTCGCTCTGG",0,NA,NA,NA,"GeneID:29","ABR",TRUE,"ABR"

"1175","CGTCTCCCCGACTCCACCTG","chr17",1125199,"-","none","AGG","CGTCTCCCCGACTCCACCTGAGG",0,NA,NA,NA,"GeneID:29","ABR",TRUE,"ABR"

"1176","CGTTTGTGCAGGTGGAAGCA","chr17",1100726,"-","none","GGG","CGTTTGTGCAGGTGGAAGCAGGG",0,NA,NA,NA,"GeneID:29","ABR",TRUE,"ABR"

"1177","AGTCTTGCTGACCACCGTTT","chr8",106769839,"+","none","TGG","AGTCTTGCTGACCACCGTTTTGG",0,NA,NA,NA,"GeneID:137735","ABRA",TRUE,"ABRA"

"1178","AGTCTTGCTGACCACCGTTT","chr11",48000526,"-","8:T>A,15:A>G","GGG","AGTCTTGCAGACCACTGTTTGGG",2,9,16,NA,"GeneID:5795","PTPRJ",FALSE,"ABRA"

"1179","ATGTCCACCCGCCTACACAA","chr8",106761340,"-","none","AGG","ATGTCCACCCGCCTACACAAAGG",0,NA,NA,NA,"GeneID:137735","ABRA",TRUE,"ABRA"

"1180","CAGCATGGCTCCGGGCGAAA","chr8",106770174,"-","none","AGG","CAGCATGGCTCCGGGCGAAAAGG",0,NA,NA,NA,"GeneID:137735","ABRA",TRUE,"ABRA"

"1181","GATGTGCTCCTCAGCACGCT","chr8",106761264,"+","none","TGG","GATGTGCTCCTCAGCACGCTTGG",0,NA,NA,NA,"GeneID:137735","ABRA",TRUE,"ABRA"

"1182","GGCTATGGCCGCCCCAAAGA","chr8",106761310,"-","none","AGG","GGCTATGGCCGCCCCAAAGAAGG",0,NA,NA,NA,"GeneID:137735","ABRA",TRUE,"ABRA"

"1183","GGGCGAATGAGAACAGCATC","chr8",106770064,"-","none","AGG","GGGCGAATGAGAACAGCATCAGG",0,NA,NA,NA,"GeneID:137735","ABRA",TRUE,"ABRA"

"1184","AGTGGAGGAAATTCATCGTT","chr6",139034189,"+","none","TGG","AGTGGAGGAAATTCATCGTTTGG",0,NA,NA,NA,"GeneID:58527","ABRACL",TRUE,"ABRACL"

"1185","CACATTTATCATCACGGAAG","chr6",139042755,"-","none","AGG","CACATTTATCATCACGGAAGAGG",0,NA,NA,NA,"GeneID:58527","ABRACL",TRUE,"ABRACL"

"1186","CACATTTATCATCACGGAAG","chr11",60998022,"-","0:A>G,1:G>T","TGG","TCCATTTATCATCACGGAAGTGG",2,1,2,NA,"GeneID:923","CD6",FALSE,"ABRACL"

"1187","CTCTTAAAGCTGCAAAACGA","chr6",139042802,"+","none","AGG","CTCTTAAAGCTGCAAAACGAAGG",0,NA,NA,NA,"GeneID:58527","ABRACL",TRUE,"ABRACL"

"1188","GATGAATTTCCTCCACTAAG","chr6",139034186,"-","none","AGG","GATGAATTTCCTCCACTAAGAGG",0,NA,NA,NA,"GeneID:58527","ABRACL",TRUE,"ABRACL"

"1189","GGAAAGTTAAGCGTGAAATT","chr6",139042726,"+","none","TGG","GGAAAGTTAAGCGTGAAATTTGG",0,NA,NA,NA,"GeneID:58527","ABRACL",TRUE,"ABRACL"

"1190","TGCCAACCTCTTTGAAGCAT","chr6",139042773,"+","none","TGG","TGCCAACCTCTTTGAAGCATTGG",0,NA,NA,NA,"GeneID:58527","ABRACL",TRUE,"ABRACL"

"1191","CAAGCGCATAGCCAAGCGCG","chr6",26598013,"+","none","TGG","CAAGCGCATAGCCAAGCGCGTGG",0,NA,NA,NA,"GeneID:29777","ABT1",TRUE,"ABT1"

"1192","CAAGCGCATAGCCAAGCGCG","chr4",99022772,"-","16:T>G,18:A>G","TGG","CAAGCGCATAGCCAAGAGTGTGG",2,17,19,NA,"GeneID:23173","METAP1",TRUE,"ABT1"

"1193","CGAGCCCGTTCACGTCCCCC","chr6",26598510,"-","none","TGG","CGAGCCCGTTCACGTCCCCCTGG",0,NA,NA,NA,"GeneID:29777","ABT1",TRUE,"ABT1"

"1194","GACGCGTCCGACCTCGCCAT","chr6",26597187,"-","none","AGG","GACGCGTCCGACCTCGCCATAGG",0,NA,NA,NA,"GeneID:29777","ABT1",TRUE,"ABT1"

"1195","GGGACGTGAACGGGCTCGCC","chr6",26598512,"+","none","TGG","GGGACGTGAACGGGCTCGCCTGG",0,NA,NA,NA,"GeneID:29777","ABT1",TRUE,"ABT1"

"1196","GGTCGGACGCGTCTTCTTTC","chr6",26597194,"+","none","AGG","GGTCGGACGCGTCTTCTTTCAGG",0,NA,NA,NA,"GeneID:29777","ABT1",TRUE,"ABT1"

"1197","TAGACTGGCCGCCACGCGCT","chr6",26598027,"-","none","TGG","TAGACTGGCCGCCACGCGCTTGG",0,NA,NA,NA,"GeneID:29777","ABT1",TRUE,"ABT1"

"1198","TAGACTGGCCGCCACGCGCT","chr4",99022758,"+","15:A>G,17:T>G","TGG","TAGACTGGCCGCCACACTCTTGG",2,16,18,NA,"GeneID:23173","METAP1",TRUE,"ABT1"

"1199","AGGTGAATGTGCGGGACAAG","chr3",127674416,"+","none","TGG","AGGTGAATGTGCGGGACAAGTGG",0,NA,NA,NA,"GeneID:80325","ABTB1",TRUE,"ABTB1"

"1200","ATAGTAATCCCGCCTCCTGC","chr3",127676077,"-","none","AGG","ATAGTAATCCCGCCTCCTGCAGG",0,NA,NA,NA,"GeneID:80325","ABTB1",TRUE,"ABTB1"

"1201","CCTGCTGGAGCAGCGAGACG","chr3",127674393,"+","none","TGG","CCTGCTGGAGCAGCGAGACGTGG",0,NA,NA,NA,"GeneID:80325","ABTB1",TRUE,"ABTB1"

"1202","CTGCCCACAGATCAACCCCG","chr3",127676525,"+","none","TGG","CTGCCCACAGATCAACCCCGTGG",0,NA,NA,NA,"GeneID:80325","ABTB1",TRUE,"ABTB1"

"1203","CTGCTGCAGTACCTGTACAC","chr3",127676559,"+","none","AGG","CTGCTGCAGTACCTGTACACAGG",0,NA,NA,NA,"GeneID:80325","ABTB1",TRUE,"ABTB1"

"1204","GCGCTCACCATCGAAGGTGT","chr3",127675987,"-","none","TGG","GCGCTCACCATCGAAGGTGTTGG",0,NA,NA,NA,"GeneID:80325","ABTB1",TRUE,"ABTB1"

"1205","ACACGCTCTACTACTTTCTG","chr11",34197395,"-","none","CGG","ACACGCTCTACTACTTTCTGCGG",0,NA,NA,NA,"GeneID:25841","ABTB2",TRUE,"ABTB2"

"1206","CGCCGGCGCTCATGCTGTAC","chr11",34357012,"+","none","AGG","CGCCGGCGCTCATGCTGTACAGG",0,NA,NA,NA,"GeneID:25841","ABTB2",TRUE,"ABTB2"

"1207","CGGCTCGCTCGTCATGGCCC","chr11",34204621,"+","none","AGG","CGGCTCGCTCGTCATGGCCCAGG",0,NA,NA,NA,"GeneID:25841","ABTB2",TRUE,"ABTB2"

"1208","CTCCGAGATGCTGCCCACGC","chr11",34204547,"+","none","AGG","CTCCGAGATGCTGCCCACGCAGG",0,NA,NA,NA,"GeneID:25841","ABTB2",TRUE,"ABTB2"

"1209","TCCCTACCGTTCATTGTTCA","chr11",34197317,"+","none","AGG","TCCCTACCGTTCATTGTTCAAGG",0,NA,NA,NA,"GeneID:25841","ABTB2",TRUE,"ABTB2"

"1210","TGCGTACTCGTGGATGCGCA","chr11",34356902,"+","none","CGG","TGCGTACTCGTGGATGCGCACGG",0,NA,NA,NA,"GeneID:25841","ABTB2",TRUE,"ABTB2"

"1211","AGGGCTGACAGTGAGTGACG","chr3",38125863,"-","none","TGG","AGGGCTGACAGTGAGTGACGTGG",0,NA,NA,NA,"GeneID:30","ACAA1",TRUE,"ACAA1"

"1212","AGGGCTGACAGTGAGTGACG","chr2",219489461,"-","0:C>T,14:G>C","GGG","GGGGCTGACAGTGACTGACGGGG",2,1,15,NA,"GeneID:10290","SPEG",TRUE,"ACAA1"

"1213","ATTCACGTCCTTGAGAACCG","chr3",38136625,"+","none","CGG","ATTCACGTCCTTGAGAACCGCGG",0,NA,NA,NA,"GeneID:30","ACAA1",TRUE,"ACAA1"

"1214","CACCCCCGACGAGCTTCTCT","chr3",38136660,"-","none","CGG","CACCCCCGACGAGCTTCTCTCGG",0,NA,NA,NA,"GeneID:30","ACAA1",TRUE,"ACAA1"

"1215","GATTCGGGCCATGATTGCCC","chr3",38133965,"+","none","CGG","GATTCGGGCCATGATTGCCCCGG",0,NA,NA,NA,"GeneID:30","ACAA1",TRUE,"ACAA1"

"1216","TATCCGCCCCAGCACCACCA","chr3",38126566,"-","none","TGG","TATCCGCCCCAGCACCACCATGG",0,NA,NA,NA,"GeneID:30","ACAA1",TRUE,"ACAA1"

"1217","TGGGGCGGATACCCTCATCC","chr3",38126575,"+","none","TGG","TGGGGCGGATACCCTCATCCTGG",0,NA,NA,NA,"GeneID:30","ACAA1",TRUE,"ACAA1"

"1218","ATTGTGGGCTACTTTGTATC","chr18",49791498,"-","none","TGG","ATTGTGGGCTACTTTGTATCTGG",0,NA,NA,NA,"GeneID:10449","ACAA2",TRUE,"ACAA2"

"1219","CAAGCTTGGATCAGATATCA","chr18",49795766,"-","none","AGG","CAAGCTTGGATCAGATATCAAGG",0,NA,NA,NA,"GeneID:10449","ACAA2",TRUE,"ACAA2"

"1220","CTTACCAATACCCATGATAG","chr18",49791464,"+","none","AGG","CTTACCAATACCCATGATAGAGG",0,NA,NA,NA,"GeneID:10449","ACAA2",TRUE,"ACAA2"

"1221","TACCTGACATCCATTCACAA","chr18",49797462,"+","none","TGG","TACCTGACATCCATTCACAATGG",0,NA,NA,NA,"GeneID:10449","ACAA2",TRUE,"ACAA2"

"1222","TACCTGACATCCATTCACAA","chr10",69563158,"-","19:G>T","AGG","TACCTGACATCCATTCACACAGG",1,20,NA,NA,"","",FALSE,"ACAA2"

"1223","TGTGTCAGAAATGTGCGTTT","chr18",49795792,"-","none","TGG","TGTGTCAGAAATGTGCGTTTTGG",0,NA,NA,NA,"GeneID:10449","ACAA2",TRUE,"ACAA2"

"1224","TGTGTCAGAAATGTGCGTTT","chrX",48776767,"-","15:A>G","TGG","TGTGTCAGAAATGTGTGTTTTGG",1,16,NA,NA,"","",TRUE,"ACAA2"

"1225","TTCTTCAGATGCTATATATT","chr18",49797572,"-","none","TGG","TTCTTCAGATGCTATATATTTGG",0,NA,NA,NA,"GeneID:10449","ACAA2",TRUE,"ACAA2"

"1226","TTCTTCAGATGCTATATATT","chr2",230450292,"+","2:A>C,16:C>T","TGG","TTATTCAGATGCTATACATTTGG",2,3,17,NA,"GeneID:6672","SP100",FALSE,"ACAA2"

"1227","TTCTTCAGATGCTATATATT","chr4",11982083,"-","0:C>A,15:A>T","AGG","GTCTTCAGATGCTATTTATTAGG",2,1,16,NA,"","",FALSE,"ACAA2"

"1228","CAACAACAACTATGCAAATG","chr17",37277936,"-","none","TGG","CAACAACAACTATGCAAATGTGG",0,NA,NA,NA,"GeneID:31","ACACA",TRUE,"ACACA"

"1229","CAACAACAACTATGCAAATG","chr10",49104309,"+","14:A>C,16:C>A","AGG","CAACAACAACTATGAACATGAGG",2,15,17,NA,"GeneID:196740","VSTM4",FALSE,"ACACA"

"1230","CAACAACAACTATGCAAATG","chr7",157267433,"+","10:A>T,12:A>T","TGG","CAACAACAACAAAGCAAATGTGG",2,11,13,NA,"GeneID:9690","UBE3C",FALSE,"ACACA"

"1231","CAACAACAACTATGCAAATG","chrX",102128425,"+","1:C>A,14:A>C","TGG","CCACAACAACTATGAAAATGTGG",2,2,15,NA,"","",FALSE,"ACACA"

"1232","GAAGACCTTAAAGCCAATGC","chr17",37283268,"-","none","AGG","GAAGACCTTAAAGCCAATGCAGG",0,NA,NA,NA,"GeneID:31","ACACA",TRUE,"ACACA"

"1233","GATCACTATGTGCCAGTGCC","chr17",37277968,"-","none","TGG","GATCACTATGTGCCAGTGCCTGG",0,NA,NA,NA,"GeneID:31","ACACA",TRUE,"ACACA"

"1234","GCAGAATTTGTTACTCGCTT","chr17",37284862,"-","none","TGG","GCAGAATTTGTTACTCGCTTTGG",0,NA,NA,NA,"GeneID:31","ACACA",TRUE,"ACACA"

"1235","TGCAATTAGATTCGTTGTCA","chr17",37283299,"-","none","TGG","TGCAATTAGATTCGTTGTCATGG",0,NA,NA,NA,"GeneID:31","ACACA",TRUE,"ACACA"

"1236","TGGCTTGCACCTAGTAAAGC","chr17",37284938,"-","none","AGG","TGGCTTGCACCTAGTAAAGCAGG",0,NA,NA,NA,"GeneID:31","ACACA",TRUE,"ACACA"

"1237","CCCTTGTCATAAACATCTTC","chr12",109175989,"-","none","TGG","CCCTTGTCATAAACATCTTCTGG",0,NA,NA,NA,"GeneID:32","ACACB",TRUE,"ACACB"

"1238","CGTTGTCGCCCAGACGCTAC","chr12",109174180,"+","none","AGG","CGTTGTCGCCCAGACGCTACAGG",0,NA,NA,NA,"GeneID:32","ACACB",TRUE,"ACACB"

"1239","GATCATTACGTCCCCGTCCC","chr12",109171821,"+","none","AGG","GATCATTACGTCCCCGTCCCAGG",0,NA,NA,NA,"GeneID:32","ACACB",TRUE,"ACACB"

"1240","GGGTTGGGACCTGTAGCGTC","chr12",109174192,"-","none","TGG","GGGTTGGGACCTGTAGCGTCTGG",0,NA,NA,NA,"GeneID:32","ACACB",TRUE,"ACACB"

"1241","TACCTGCACGGGGATTCTCT","chr12",109171897,"-","none","TGG","TACCTGCACGGGGATTCTCTTGG",0,NA,NA,NA,"GeneID:32","ACACB",TRUE,"ACACB"

"1242","TTGCGTGAAAGACGTAGATG","chr12",109176009,"+","none","AGG","TTGCGTGAAAGACGTAGATGAGG",0,NA,NA,NA,"GeneID:32","ACACB",TRUE,"ACACB"

"1243","AAGCACAGCTTGTAGATCCT","chr12",111709567,"-","none","AGG","AAGCACAGCTTGTAGATCCTAGG",0,NA,NA,NA,"GeneID:80724","ACAD10",TRUE,"ACAD10"

"1244","ATAACTCAAATTCGGGCAAA","chr12",111705824,"+","none","AGG","ATAACTCAAATTCGGGCAAAAGG",0,NA,NA,NA,"GeneID:80724","ACAD10",TRUE,"ACAD10"

"1245","ATAACTCAAATTCGGGCAAA","chr18",38551373,"+","5:C>T,12:A>C","GGG","ATAACCCAAATTAGGGCAAAGGG",2,6,13,NA,"","",FALSE,"ACAD10"

"1246","GTACAGAATCGTATCCCTTC","chr12",111702169,"+","none","TGG","GTACAGAATCGTATCCCTTCTGG",0,NA,NA,NA,"GeneID:80724","ACAD10",TRUE,"ACAD10"

"1247","GTTAAAGACCTCCGTGCCTG","chr12",111705736,"+","none","TGG","GTTAAAGACCTCCGTGCCTGTGG",0,NA,NA,NA,"GeneID:80724","ACAD10",TRUE,"ACAD10"

"1248","TCAGATTGTGGAGTCCTGCA","chr12",111709521,"+","none","TGG","TCAGATTGTGGAGTCCTGCATGG",0,NA,NA,NA,"GeneID:80724","ACAD10",TRUE,"ACAD10"

"1249","TCAGATTGTGGAGTCCTGCA","chr17",65391655,"-","10:T>C,18:C>G","GGG","TCAGATTGTGAAGTCCTGGAGGG",2,11,19,NA,"","",FALSE,"ACAD10"

"1250","TGCTCTCATAAATCTCATCC","chr12",111702236,"-","none","AGG","TGCTCTCATAAATCTCATCCAGG",0,NA,NA,NA,"GeneID:80724","ACAD10",TRUE,"ACAD10"

"1251","AGAACGTTCAGCCATATATG","chr3",132642069,"-","none","TGG","AGAACGTTCAGCCATATATGTGG",0,NA,NA,NA,"GeneID:84129;GeneID:100532724","ACAD11;NPHP3-ACAD11",TRUE,"ACAD11"

"1252","CCACTCCGATAGCTGTTGCA","chr3",132639581,"+","none","TGG","CCACTCCGATAGCTGTTGCATGG",0,NA,NA,NA,"GeneID:84129;GeneID:100532724","ACAD11;NPHP3-ACAD11",TRUE,"ACAD11"

"1253","CTAACTGTACCTCTTTAGGG","chr3",132639481,"+","none","TGG","CTAACTGTACCTCTTTAGGGTGG",0,NA,NA,NA,"GeneID:84129;GeneID:100532724","ACAD11;NPHP3-ACAD11",TRUE,"ACAD11"

"1254","CTGAGCCAATGTTTCTACCG","chr3",132642043,"+","none","TGG","CTGAGCCAATGTTTCTACCGTGG",0,NA,NA,NA,"GeneID:84129;GeneID:100532724","ACAD11;NPHP3-ACAD11",TRUE,"ACAD11"

"1255","TGCAGTGATACTTCTGTCAT","chr3",132642710,"-","none","TGG","TGCAGTGATACTTCTGTCATTGG",0,NA,NA,NA,"GeneID:84129;GeneID:100532724","ACAD11;NPHP3-ACAD11",TRUE,"ACAD11"

"1256","TTACGTAATGGAACATGTGC","chr3",132642678,"-","none","AGG","TTACGTAATGGAACATGTGCAGG",0,NA,NA,NA,"GeneID:84129;GeneID:100532724","ACAD11;NPHP3-ACAD11",TRUE,"ACAD11"

"1257","AGACCCCTCCGAAGCCTAGC","chr11",134257125,"-","none","TGG","AGACCCCTCCGAAGCCTAGCTGG",0,NA,NA,NA,"GeneID:27034","ACAD8",TRUE,"ACAD8"

"1258","GCCTTCCGCATCACATCCAC","chr11",134257099,"-","none","TGG","GCCTTCCGCATCACATCCACTGG",0,NA,NA,NA,"GeneID:27034","ACAD8",TRUE,"ACAD8"

"1259","TCCGAAGCTATCAATCATCC","chr11",134258525,"-","none","AGG","TCCGAAGCTATCAATCATCCAGG",0,NA,NA,NA,"GeneID:27034","ACAD8",TRUE,"ACAD8"

"1260","TCTGACCTCCGCTAAGAAAC","chr11",134259032,"+","none","AGG","TCTGACCTCCGCTAAGAAACAGG",0,NA,NA,NA,"GeneID:27034","ACAD8",TRUE,"ACAD8"

"1261","TGACTTTGCTGCCCGAGAGA","chr11",134256596,"+","none","TGG","TGACTTTGCTGCCCGAGAGATGG",0,NA,NA,NA,"GeneID:27034","ACAD8",TRUE,"ACAD8"

"1262","TGACTTTGCTGCCCGAGAGA","chr2",218642341,"-","15:C>T,19:G>T","GGG","TGACTTTGCTGCCCGGGAGCGGG",2,16,20,NA,"GeneID:7701","ZNF142",TRUE,"ACAD8"

"1263","TTGCCCACCGCTCTGTACCA","chr11",134258568,"+","none","TGG","TTGCCCACCGCTCTGTACCATGG",0,NA,NA,NA,"GeneID:27034","ACAD8",TRUE,"ACAD8"

"1264","AGCCAAATACTTGCCTAAAC","chr3",128896467,"+","none","TGG","AGCCAAATACTTGCCTAAACTGG",0,NA,NA,NA,"GeneID:28976","ACAD9",TRUE,"ACAD9"

"1265","AGCCAAATACTTGCCTAAAC","chr7",24933694,"-","2:C>G,16:G>T","TGG","AGGCAAATACTTGCCTCAACTGG",2,3,17,NA,"GeneID:26031","OSBPL3",FALSE,"ACAD9"

"1266","ATCCTTGGAGAGGTCGGAGA","chr3",128901319,"+","none","TGG","ATCCTTGGAGAGGTCGGAGATGG",0,NA,NA,NA,"GeneID:28976","ACAD9",TRUE,"ACAD9"

"1267","ATCCTTGGAGAGGTCGGAGA","chr4",186590181,"+","13:C>T,14:G>C","AGG","ATCCTTGGAGAGGCGGGAGAAGG",2,14,15,NA,"GeneID:2195","FAT1",FALSE,"ACAD9"

"1268","ATCCTTGGAGAGGTCGGAGA","chr10",47325695,"-","7:A>C,13:T>A","TGG","ATCCTTGTAGAGGACGGAGATGG",2,8,14,NA,"GeneID:2658","GDF2",TRUE,"ACAD9"

"1269","CGCCAGTTTAGGCAAGTATT","chr3",128896472,"-","none","TGG","CGCCAGTTTAGGCAAGTATTTGG",0,NA,NA,NA,"GeneID:28976","ACAD9",TRUE,"ACAD9"

"1270","CTCCCCTAGTCTTGAGTACA","chr3",128895333,"-","none","TGG","CTCCCCTAGTCTTGAGTACATGG",0,NA,NA,NA,"GeneID:28976","ACAD9",TRUE,"ACAD9"

"1271","TGAAAACACCAAGATACCTG","chr3",128901291,"+","none","TGG","TGAAAACACCAAGATACCTGTGG",0,NA,NA,NA,"GeneID:28976","ACAD9",TRUE,"ACAD9"

"1272","TGACCTTGAGGCCAATAGCC","chr3",128895400,"-","none","TGG","TGACCTTGAGGCCAATAGCCTGG",0,NA,NA,NA,"GeneID:28976","ACAD9",TRUE,"ACAD9"

"1273","CGCGTAGGGACCCTCGGAGA","chr2",210225229,"+","none","AGG","CGCGTAGGGACCCTCGGAGAAGG",0,NA,NA,NA,"GeneID:33","ACADL",TRUE,"ACADL"

"1274","CTCACCGCGCGGCGGGCAGC","chr2",210225181,"+","none","TGG","CTCACCGCGCGGCGGGCAGCTGG",0,NA,NA,NA,"GeneID:33","ACADL",TRUE,"ACADL"

"1275","CTCACCGCGCGGCGGGCAGC","chr9",76910992,"-","6:T>C,13:A>C","TGG","CTCACCACGCGGCTGGCAGCTGG",2,7,14,NA,"","",FALSE,"ACADL"

"1276","GCCACTTACTCTGAGTGATG","chr2",210220637,"+","none","AGG","GCCACTTACTCTGAGTGATGAGG",0,NA,NA,NA,"GeneID:33","ACADL",TRUE,"ACADL"

"1277","GCCACTTACTCTGAGTGATG","chr17",37836274,"-","2:T>G,3:A>T","TGG","GCATCTTACTCTGAGTGATGTGG",2,3,4,NA,"GeneID:284100","YWHAEP7",FALSE,"ACADL"

"1278","GCTGGAAAACAAGGACTGCT","chr2",210218039,"-","none","TGG","GCTGGAAAACAAGGACTGCTTGG",0,NA,NA,NA,"GeneID:33","ACADL",TRUE,"ACADL"

"1279","TGTACTCCGCAGCTATTGTC","chr2",210217975,"-","none","TGG","TGTACTCCGCAGCTATTGTCTGG",0,NA,NA,NA,"GeneID:33","ACADL",TRUE,"ACADL"

"1280","TTCTAGACGTTCTTCCCCTC","chr2",210220768,"+","none","CGG","TTCTAGACGTTCTTCCCCTCCGG",0,NA,NA,NA,"GeneID:33","ACADL",TRUE,"ACADL"

"1281","ACTGTAGGAGGTCTTGGACT","chr1",75733520,"+","none","TGG","ACTGTAGGAGGTCTTGGACTTGG",0,NA,NA,NA,"GeneID:34","ACADM",TRUE,"ACADM"

"1282","GAAAGCTTACCACAGTTCTC","chr1",75732912,"-","none","TGG","GAAAGCTTACCACAGTTCTCTGG",0,NA,NA,NA,"GeneID:34","ACADM",TRUE,"ACADM"

"1283","GCCAATCGACAACGTGAACC","chr1",75728448,"+","none","AGG","GCCAATCGACAACGTGAACCAGG",0,NA,NA,NA,"GeneID:34","ACADM",TRUE,"ACADM"

"1284","GGGGTTCAGACTGCTATTGA","chr1",75733592,"+","none","AGG","GGGGTTCAGACTGCTATTGAAGG",0,NA,NA,NA,"GeneID:34","ACADM",TRUE,"ACADM"

"1285","GGGGTTCAGACTGCTATTGA","chr13",28638164,"+","3:A>G,10:T>C","AGG","GGGATTCAGATTGCTATTGAAGG",2,4,11,NA,"","",FALSE,"ACADM"

"1286","GGGGTTCAGACTGCTATTGA","chr12",108765253,"-","6:T>G,13:C>G","AGG","GGGGTTAAGACTGGTATTGAAGG",2,7,14,NA,"","",FALSE,"ACADM"

"1287","TCCCCCTAATTAGAAGAGCC","chr1",75732859,"+","none","TGG","TCCCCCTAATTAGAAGAGCCTGG",0,NA,NA,NA,"GeneID:34","ACADM",TRUE,"ACADM"

"1288","TGCTCGTAAATTTGCCAGAG","chr1",75732677,"+","none","AGG","TGCTCGTAAATTTGCCAGAGAGG",0,NA,NA,NA,"GeneID:34","ACADM",TRUE,"ACADM"

"1289","CAGAACCCATGAGTCGCCCT","chr12",120737881,"-","none","CGG","CAGAACCCATGAGTCGCCCTCGG",0,NA,NA,NA,"GeneID:35","ACADS",TRUE,"ACADS"

"1290","CCTGGCCTACGCCATCGCCA","chr12",120737059,"+","none","TGG","CCTGGCCTACGCCATCGCCATGG",0,NA,NA,NA,"GeneID:35","ACADS",TRUE,"ACADS"

"1291","GGCAGGTACCTGGTTCGCTG","chr12",120737456,"-","none","AGG","GGCAGGTACCTGGTTCGCTGAGG",0,NA,NA,NA,"GeneID:35","ACADS",TRUE,"ACADS"

"1292","GGCGACTCATGGGTTCTGAA","chr12",120737883,"+","none","TGG","GGCGACTCATGGGTTCTGAATGG",0,NA,NA,NA,"GeneID:35","ACADS",TRUE,"ACADS"

"1293","TGCTTTGCCCTCAGCGAACC","chr12",120737445,"+","none","AGG","TGCTTTGCCCTCAGCGAACCAGG",0,NA,NA,NA,"GeneID:35","ACADS",TRUE,"ACADS"

"1294","TGGCGTAGGCCAGGTAATCG","chr12",120737053,"-","none","AGG","TGGCGTAGGCCAGGTAATCGAGG",0,NA,NA,NA,"GeneID:35","ACADS",TRUE,"ACADS"

"1295","ATCCACTGTGCTCGTGATAG","chr10",123040518,"+","none","AGG","ATCCACTGTGCTCGTGATAGAGG",0,NA,NA,NA,"GeneID:36","ACADSB",TRUE,"ACADSB"

"1296","ATCTCCCTCTTTATCAGCTC","chr10",123041269,"-","none","TGG","ATCTCCCTCTTTATCAGCTCTGG",0,NA,NA,NA,"GeneID:36","ACADSB",TRUE,"ACADSB"

"1297","ATCTCCCTCTTTATCAGCTC","chr14",84949575,"-","7:T>A,8:A>G","TGG","ATCTCCCATTTTATCAGCTCTGG",2,8,9,NA,"","",FALSE,"ACADSB"

"1298","TATTGAGTAGAGCTTCTGAC","chr10",123034423,"-","none","TGG","TATTGAGTAGAGCTTCTGACTGG",0,NA,NA,NA,"GeneID:36","ACADSB",TRUE,"ACADSB"

"1299","TCCACATCTTTGATCCATTG","chr10",123041300,"-","none","AGG","TCCACATCTTTGATCCATTGAGG",0,NA,NA,NA,"GeneID:36","ACADSB",TRUE,"ACADSB"

"1300","TGCACCTTTGGTTTCAACCA","chr10",123037771,"+","none","TGG","TGCACCTTTGGTTTCAACCATGG",0,NA,NA,NA,"GeneID:36","ACADSB",TRUE,"ACADSB"

"1301","TGCACCTTTGGTTTCAACCA","chr12",34305825,"-","12:C>A,14:C>G","GGG","TGCACCTTTGGTGTGAACCAGGG",2,13,15,NA,"","",FALSE,"ACADSB"

"1302","TTCCTCTATCACGAGCACAG","chr10",123040523,"-","none","TGG","TTCCTCTATCACGAGCACAGTGG",0,NA,NA,NA,"GeneID:36","ACADSB",TRUE,"ACADSB"

"1303","ACAGTCCATGTTGGGGGGCC","chr17",7217138,"+","none","TGG","ACAGTCCATGTTGGGGGGCCTGG",0,NA,NA,NA,"GeneID:1742;GeneID:37","DLG4;ACADVL",TRUE,"ACADVL"

"1304","CACAGCAGAGGTTCGGATGG","chr17",7222008,"-","none","AGG","CACAGCAGAGGTTCGGATGGAGG",0,NA,NA,NA,"GeneID:37","ACADVL",TRUE,"ACADVL"

"1305","CACAGCAGAGGTTCGGATGG","chr10",16631406,"-","6:G>T,14:A>C","TGG","CACAGCCGAGGTTCTGATGGTGG",2,7,15,NA,"GeneID:6251","RSU1",FALSE,"ACADVL"

"1306","CGATGACAGCAAAGACGATG","chr17",7217796,"+","none","AGG","CGATGACAGCAAAGACGATGAGG",0,NA,NA,NA,"GeneID:1742;GeneID:37","DLG4;ACADVL",TRUE,"ACADVL"

"1307","GAGCCCCTTACCCGCCGCCG","chr17",7217164,"-","none","CGG","GAGCCCCTTACCCGCCGCCGCGG",0,NA,NA,NA,"GeneID:1742;GeneID:37","DLG4;ACADVL",TRUE,"ACADVL"

"1308","GAGCCCCTTACCCGCCGCCG","chr9",99906697,"-","12:A>G,13:G>C","GGG","GAGCCCCTTACCTCCCGCCGGGG",2,13,14,NA,"GeneID:55014","STX17",TRUE,"ACADVL"

"1309","GAGCCCCTTACCCGCCGCCG","chr10",35337223,"+","7:C>T,16:C>G","CGG","GAGCCCCCTACCCGCCCCCGCGG",2,8,17,NA,"GeneID:219771","CCNY",TRUE,"ACADVL"

"1310","GCTGGCAGGAACCCGGATAA","chr17",7217707,"+","none","TGG","GCTGGCAGGAACCCGGATAATGG",0,NA,NA,NA,"GeneID:1742;GeneID:37","DLG4;ACADVL",TRUE,"ACADVL"

"1311","TGTCTAACCGAGCCCTCAAG","chr17",7221971,"+","none","CGG","TGTCTAACCGAGCCCTCAAGCGG",0,NA,NA,NA,"GeneID:37","ACADVL",TRUE,"ACADVL"

"1312","CACGAAAACCCAGAGTAAAG","chr15",88836213,"-","none","TGG","CACGAAAACCCAGAGTAAAGTGG",0,NA,NA,NA,"GeneID:176","ACAN",TRUE,"ACAN"

"1313","CCCCAGAGTCATTGGAGCGC","chr15",88838960,"-","none","AGG","CCCCAGAGTCATTGGAGCGCAGG",0,NA,NA,NA,"GeneID:176","ACAN",TRUE,"ACAN"

"1314","GAACTATGACCACTTTACTC","chr15",88836201,"+","none","TGG","GAACTATGACCACTTTACTCTGG",0,NA,NA,NA,"GeneID:176","ACAN",TRUE,"ACAN"

"1315","GCGGGTCAACAGTGCCTATC","chr15",88838873,"+","none","AGG","GCGGGTCAACAGTGCCTATCAGG",0,NA,NA,NA,"GeneID:176","ACAN",TRUE,"ACAN"

"1316","GCTACACCCTCGACTTTGAC","chr15",88840044,"+","none","AGG","GCTACACCCTCGACTTTGACAGG",0,NA,NA,NA,"GeneID:176","ACAN",TRUE,"ACAN"

"1317","GCTGCGCCCTGTCAAAGTCG","chr15",88840054,"-","none","AGG","GCTGCGCCCTGTCAAAGTCGAGG",0,NA,NA,NA,"GeneID:176","ACAN",TRUE,"ACAN"

"1318","AAATGCCGACAACGAAGGCG","chr17",7342006,"-","none","CGG","AAATGCCGACAACGAAGGCGCGG",0,NA,NA,NA,"GeneID:9744","ACAP1",TRUE,"ACAP1"

"1319","CCCCATCTCACTCCTTGACC","chr17",7342465,"-","none","AGG","CCCCATCTCACTCCTTGACCAGG",0,NA,NA,NA,"GeneID:9744","ACAP1",TRUE,"ACAP1"

"1320","CCCCATCTCACTCCTTGACC","chr16",24844924,"-","16:G>C,19:A>G","GGG","CCCCATCTCACTCCTTCACTGGG",2,17,20,NA,"","",FALSE,"ACAP1"

"1321","CCCCATCTCACTCCTTGACC","chrX",93439917,"-","0:A>G,4:G>T","TGG","TCCCCTCTCACTCCTTGACCTGG",2,1,5,NA,"","",FALSE,"ACAP1"

"1322","CCCCATCTCACTCCTTGACC","chr7",102253275,"+","9:C>A,17:G>A","AGG","CCCCATCTCCCTCCTTGGCCAGG",2,10,18,NA,"GeneID:1523","CUX1",TRUE,"ACAP1"

"1323","GCCAGCCGCGCCTTCGTTGT","chr17",7341998,"+","none","CGG","GCCAGCCGCGCCTTCGTTGTCGG",0,NA,NA,NA,"GeneID:9744","ACAP1",TRUE,"ACAP1"

"1324","GGCTTCCACCAGCTCAATAG","chr17",7337316,"-","none","AGG","GGCTTCCACCAGCTCAATAGAGG",0,NA,NA,NA,"GeneID:9744","ACAP1",TRUE,"ACAP1"

"1325","TCCCAGAGCCTCTATTGAGC","chr17",7337305,"+","none","TGG","TCCCAGAGCCTCTATTGAGCTGG",0,NA,NA,NA,"GeneID:9744","ACAP1",TRUE,"ACAP1"

"1326","TCCCAGAGCCTCTATTGAGC","chr3",15493727,"-","5:G>C,13:C>T","TGG","TCCCACAGCCTCTGTTGAGCTGG",2,6,14,NA,"GeneID:8292","COLQ",FALSE,"ACAP1"

"1327","TCTGCTGCTGCAGTGTGTGT","chr17",7342435,"-","none","TGG","TCTGCTGCTGCAGTGTGTGTTGG",0,NA,NA,NA,"GeneID:9744","ACAP1",TRUE,"ACAP1"

"1328","TCTGCTGCTGCAGTGTGTGT","chr2",72908327,"-","4:T>G,5:T>A","TGG","TCTGAAGCTGCAGTGTGTGTTGG",2,5,6,NA,"","",FALSE,"ACAP1"

"1329","AGCCCTCGATTATGTGCTTC","chr3",195342472,"-","none","AGG","AGCCCTCGATTATGTGCTTCAGG",0,NA,NA,NA,"GeneID:23527","ACAP2",TRUE,"ACAP2"

"1330","AGCCCTCGATTATGTGCTTC","chr1",26303945,"-","4:A>G,15:G>C","AGG","AGCCTTCGATTATGTCCTTCAGG",2,5,16,NA,"GeneID:91544","UBXN11",FALSE,"ACAP2"

"1331","CATTACTAGAATACTGAGCC","chr3",195381916,"+","none","AGG","CATTACTAGAATACTGAGCCAGG",0,NA,NA,NA,"GeneID:23527","ACAP2",TRUE,"ACAP2"

"1332","CCAAACTCAGAGATCAATTA","chr3",195345286,"-","none","AGG","CCAAACTCAGAGATCAATTAAGG",0,NA,NA,NA,"GeneID:23527","ACAP2",TRUE,"ACAP2"

"1333","CCAAACTCAGAGATCAATTA","chr3",40889749,"+","16:G>A,18:C>T","GGG","CCAAACTCAGAGATCAGTCAGGG",2,17,19,NA,"","",FALSE,"ACAP2"

"1334","CCTTAATTGATCTCTGAGTT","chr3",195345283,"+","none","TGG","CCTTAATTGATCTCTGAGTTTGG",0,NA,NA,NA,"GeneID:23527","ACAP2",TRUE,"ACAP2"

"1335","TAGTAATGATGCTGTCGTTG","chr3",195381904,"-","none","AGG","TAGTAATGATGCTGTCGTTGAGG",0,NA,NA,NA,"GeneID:23527","ACAP2",TRUE,"ACAP2"

"1336","TCTTGTTGCTGTCAGAATGT","chr3",195342509,"+","none","TGG","TCTTGTTGCTGTCAGAATGTTGG",0,NA,NA,NA,"GeneID:23527","ACAP2",TRUE,"ACAP2"

"1337","CAAGAAGAAGTTTGAGATCC","chr1",1300165,"-","none","TGG","CAAGAAGAAGTTTGAGATCCTGG",0,NA,NA,NA,"GeneID:116983","ACAP3",TRUE,"ACAP3"

"1338","CGCTGACAGCCTACAGGAGG","chr1",1302938,"-","none","TGG","CGCTGACAGCCTACAGGAGGTGG",0,NA,NA,NA,"GeneID:116983","ACAP3",TRUE,"ACAP3"

"1339","CGCTGACAGCCTACAGGAGG","chr16",35454968,"-","5:C>T,12:A>T","TGG","CGCTGGCAGCCTTCAGGAGGTGG",2,6,13,NA,"","",FALSE,"ACAP3"

"1340","CTGGCAGATCAATGTTCTGC","chr1",1300189,"-","none","AGG","CTGGCAGATCAATGTTCTGCAGG",0,NA,NA,NA,"GeneID:116983","ACAP3",TRUE,"ACAP3"

"1341","CTGGCAGATCAATGTTCTGC","chrY",13980132,"-","8:T>A,9:T>G","TGG","CTGGCAGAAAAATGTTCTGCTGG",2,9,10,NA,"","",FALSE,"ACAP3"

"1342","CTGGCAGATCAATGTTCTGC","chrX",9011957,"+","8:A>T,9:A>C","TGG","CTGGCAGAAAAATGTTCTGCTGG",2,9,10,NA,"","",FALSE,"ACAP3"

"1343","GGTAGTTCACCACCTCCTGT","chr1",1302926,"+","none","AGG","GGTAGTTCACCACCTCCTGTAGG",0,NA,NA,NA,"GeneID:116983","ACAP3",TRUE,"ACAP3"

"1344","GGTAGTTCACCACCTCCTGT","chr8",139252092,"+","2:G>T,5:C>T","GGG","GGGAGCTCACCACCTCCTGTGGG",2,3,6,NA,"","",FALSE,"ACAP3"

"1345","GTCCACGTCAAACTCCACTT","chr1",1298637,"+","none","TGG","GTCCACGTCAAACTCCACTTTGG",0,NA,NA,NA,"GeneID:116983","ACAP3",TRUE,"ACAP3"

"1346","TTTGACGTGGACGCGCCCAG","chr1",1298629,"-","none","TGG","TTTGACGTGGACGCGCCCAGTGG",0,NA,NA,NA,"GeneID:116983","ACAP3",TRUE,"ACAP3"

"1347","AAGTGCCGCCAGCACAGCCA","chr11",108121607,"-","none","TGG","AAGTGCCGCCAGCACAGCCATGG",0,NA,NA,NA,"GeneID:38","ACAT1",TRUE,"ACAT1"

"1348","AAGTGCCGCCAGCACAGCCA","chr10",130871515,"+","1:T>A,7:T>G","GGG","ATGTGCCTCCAGCACAGCCAGGG",2,2,8,NA,"","",FALSE,"ACAT1"

"1349","AATGCCAGTACACTGAATGA","chr11",108142453,"+","none","TGG","AATGCCAGTACACTGAATGATGG",0,NA,NA,NA,"GeneID:38","ACAT1",TRUE,"ACAT1"

"1350","CCCAATACTGCCTGCCTTGT","chr11",108134291,"-","none","AGG","CCCAATACTGCCTGCCTTGTAGG",0,NA,NA,NA,"GeneID:38","ACAT1",TRUE,"ACAT1"

"1351","TCCTACAAGGCAGGCAGTAT","chr11",108134287,"+","none","TGG","TCCTACAAGGCAGGCAGTATTGG",0,NA,NA,NA,"GeneID:38","ACAT1",TRUE,"ACAT1"

"1352","TCCTACAAGGCAGGCAGTAT","chrX",84323079,"-","1:C>G,14:T>G","AGG","TGCTACAAGGCAGGAAGTATAGG",2,2,15,NA,"GeneID:139324","HDX",FALSE,"ACAT1"

"1353","TGCTCCATCATTCAGTGTAC","chr11",108142460,"-","none","TGG","TGCTCCATCATTCAGTGTACTGG",0,NA,NA,NA,"GeneID:38","ACAT1",TRUE,"ACAT1"

"1354","TGTATCAAAACCCACTTTGA","chr11",108131932,"+","none","AGG","TGTATCAAAACCCACTTTGAAGG",0,NA,NA,NA,"GeneID:38","ACAT1",TRUE,"ACAT1"

"1355","TGTATCAAAACCCACTTTGA","chr5",128595774,"+","18:T>G","TGG","TGTATCAAAACCCACTTTTATGG",1,19,NA,NA,"GeneID:2201;GeneID:28965","FBN2;SLC27A6",FALSE,"ACAT1"

"1356","TGTATCAAAACCCACTTTGA","chr5",69268549,"+","0:G>T,10:A>C","AGG","GGTATCAAAAACCACTTTGAAGG",2,1,11,NA,"GeneID:1022","CDK7",FALSE,"ACAT1"

"1357","TGTATCAAAACCCACTTTGA","chr22",46977115,"+","5:T>C,15:G>T","CGG","TGTATTAAAACCCACGTTGACGG",2,6,16,NA,"GeneID:25771","TBC1D22A",FALSE,"ACAT1"

"1358","AGTCTCCTATCCCTATTGAC","chr6",159767125,"-","none","TGG","AGTCTCCTATCCCTATTGACTGG",0,NA,NA,NA,"GeneID:39","ACAT2",TRUE,"ACAT2"

"1359","AGTCTCCTATCCCTATTGAC","chr1",19020053,"-","8:A>T,19:T>G","TGG","AGTCTCCTTTCCCTATTGAATGG",2,9,20,NA,"","",FALSE,"ACAT2"

"1360","CAGAGTATACTGTCAGTCAG","chr6",159768561,"-","none","TGG","CAGAGTATACTGTCAGTCAGTGG",0,NA,NA,NA,"GeneID:39","ACAT2",TRUE,"ACAT2"

"1361","CAGAGTATACTGTCAGTCAG","chr1",23352138,"-","6:A>T,17:C>G","TGG","CAGAGTTTACTGTCAGTGAGTGG",2,7,18,NA,"","",FALSE,"ACAT2"

"1362","GCCGCCGAGACGATGACCAC","chr6",159762108,"-","none","AGG","GCCGCCGAGACGATGACCACAGG",0,NA,NA,NA,"GeneID:39","ACAT2",TRUE,"ACAT2"

"1363","TCGGCGGCGCGGACCATCAT","chr6",159762120,"+","none","AGG","TCGGCGGCGCGGACCATCATAGG",0,NA,NA,NA,"GeneID:39","ACAT2",TRUE,"ACAT2"

"1364","TGCATTTCACAACTGTCATA","chr6",159768596,"+","none","TGG","TGCATTTCACAACTGTCATATGG",0,NA,NA,NA,"GeneID:39","ACAT2",TRUE,"ACAT2"

"1365","TGCCTTGCAGTCCAGTCAAT","chr6",159767111,"+","none","AGG","TGCCTTGCAGTCCAGTCAATAGG",0,NA,NA,NA,"GeneID:39","ACAT2",TRUE,"ACAT2"

"1366","AAGGCTTCGACGGGAGGAAG","chr1",226161579,"-","none","AGG","AAGGCTTCGACGGGAGGAAGAGG",0,NA,NA,NA,"GeneID:64746","ACBD3",TRUE,"ACBD3"

"1367","CATGTCTAAAGAGGATGCCA","chr1",226164885,"-","none","TGG","CATGTCTAAAGAGGATGCCATGG",0,NA,NA,NA,"GeneID:64746","ACBD3",TRUE,"ACBD3"

"1368","CATGTCTAAAGAGGATGCCA","chr15",86273766,"+","5:T>C,7:T>A","GGG","CATGTTTTAAGAGGATGCCAGGG",2,6,8,NA,"GeneID:123624","AGBL1",FALSE,"ACBD3"

"1369","GATAGTGTTGCTCCTGCAAC","chr1",226159230,"+","none","TGG","GATAGTGTTGCTCCTGCAACTGG",0,NA,NA,NA,"GeneID:64746","ACBD3",TRUE,"ACBD3"

"1370","TAATCCAGACACTTGTCCTG","chr1",226165895,"-","none","AGG","TAATCCAGACACTTGTCCTGAGG",0,NA,NA,NA,"GeneID:64746","ACBD3",TRUE,"ACBD3"

"1371","TAATCCAGACACTTGTCCTG","chr1",82299233,"+","4:G>C,7:T>G","AGG","TAATGCATACACTTGTCCTGAGG",2,5,8,NA,"","",FALSE,"ACBD3"

"1372","TAATCCAGACACTTGTCCTG","chr12",5792868,"-","1:A>T,13:C>A","GGG","TTATCCAGACACTGGTCCTGGGG",2,2,14,NA,"GeneID:57101","ANO2",FALSE,"ACBD3"

"1373","TATGCAGCCCAACAGTATCC","chr1",226159286,"-","none","AGG","TATGCAGCCCAACAGTATCCAGG",0,NA,NA,NA,"GeneID:64746","ACBD3",TRUE,"ACBD3"

"1374","TGCGTCCCACAAAATAGAGA","chr1",226164810,"-","none","AGG","TGCGTCCCACAAAATAGAGAAGG",0,NA,NA,NA,"GeneID:64746","ACBD3",TRUE,"ACBD3"

"1375","TGCGTCCCACAAAATAGAGA","chr8",33600429,"+","3:C>G,15:G>A","AGG","TGCCTCCCACAAAATGGAGAAGG",2,4,16,NA,"","",FALSE,"ACBD3"

"1376","ATTCTACAGTTACTACAAGC","chr17",45136533,"+","none","AGG","ATTCTACAGTTACTACAAGCAGG",0,NA,NA,NA,"GeneID:79777","ACBD4",TRUE,"ACBD4"

"1377","CAGCCTCACCGTTCTTGGGC","chr17",45136221,"-","none","AGG","CAGCCTCACCGTTCTTGGGCAGG",0,NA,NA,NA,"GeneID:79777","ACBD4",TRUE,"ACBD4"

"1378","CAGGGACGCCTGGAACAGTC","chr17",45136688,"+","none","TGG","CAGGGACGCCTGGAACAGTCTGG",0,NA,NA,NA,"GeneID:79777","ACBD4",TRUE,"ACBD4"

"1379","CAGTTTCATTTCAGTGATGT","chr17",45136744,"-","none","AGG","CAGTTTCATTTCAGTGATGTAGG",0,NA,NA,NA,"GeneID:79777","ACBD4",TRUE,"ACBD4"

"1380","CAGTTTCATTTCAGTGATGT","chr16",53938059,"+","1:T>A,3:C>T","AGG","CTGCTTCATTTCAGTGATGTAGG",2,2,4,NA,"GeneID:79068","FTO",FALSE,"ACBD4"

"1381","TCACCACTTATATCGTCCAA","chr17",45136604,"-","none","TGG","TCACCACTTATATCGTCCAATGG",0,NA,NA,NA,"GeneID:79777","ACBD4",TRUE,"ACBD4"

"1382","TGTTTCTGGCAGTCGGGCTC","chr17",45136168,"-","none","TGG","TGTTTCTGGCAGTCGGGCTCTGG",0,NA,NA,NA,"GeneID:79777","ACBD4",TRUE,"ACBD4"

"1383","TGTTTCTGGCAGTCGGGCTC","chr1",71307667,"+","13:G>C,14:T>G","TGG","TGTTTCTGGCAGTGTGGCTCTGG",2,14,15,NA,"GeneID:100852410","ZRANB2-AS2",FALSE,"ACBD4"

"1384","AGGAGTTCTGATATAACCTC","chr10",27223339,"-","none","AGG","AGGAGTTCTGATATAACCTCAGG",0,NA,NA,NA,"GeneID:91452","ACBD5",TRUE,"ACBD5"

"1385","AGGAGTTCTGATATAACCTC","chrUn_KI270743v1",127649,"+","4:T>G,15:T>A","TGG","AGGATTTCTGATATATCCTCTGG",2,5,16,NA,"","",FALSE,"ACBD5"

"1386","AGGAGTTCTGATATAACCTC","chr4_GL000008v2_random",142760,"+","4:T>G,15:T>A","TGG","AGGATTTCTGATATATCCTCTGG",2,5,16,NA,"","",FALSE,"ACBD5"

"1387","AGGAGTTCTGATATAACCTC","chr14_GL000009v2_random",70790,"-","4:A>C,15:A>T","TGG","AGGATTTCTGATATATCCTCTGG",2,5,16,NA,"","",FALSE,"ACBD5"

"1388","GAAGAATTGCTGCGTGTCAT","chr10",27223399,"-","none","AGG","GAAGAATTGCTGCGTGTCATAGG",0,NA,NA,NA,"GeneID:91452","ACBD5",TRUE,"ACBD5"

"1389","GATCATGTTGAAGATGTTAC","chr10",27215610,"-","none","AGG","GATCATGTTGAAGATGTTACAGG",0,NA,NA,NA,"GeneID:91452","ACBD5",TRUE,"ACBD5"

"1390","GATCATGTTGAAGATGTTAC","chr2",239152089,"+","5:C>T,7:A>T","TGG","GATCACGATGAAGATGTTACTGG",2,6,8,NA,"GeneID:9759","HDAC4",FALSE,"ACBD5"

"1391","TAGGGATGCTTGGAGTTCAC","chr10",27231803,"-","none","TGG","TAGGGATGCTTGGAGTTCACTGG",0,NA,NA,NA,"GeneID:91452","ACBD5",TRUE,"ACBD5"

"1392","TGAAGTTTACTGTGATTCTA","chr10",27215557,"-","none","TGG","TGAAGTTTACTGTGATTCTATGG",0,NA,NA,NA,"GeneID:91452","ACBD5",TRUE,"ACBD5"

"1393","TGAAGTTTACTGTGATTCTA","chr12",40238963,"+","1:T>G,14:T>A","AGG","TTAAGTTTACTGTGTTTCTAAGG",2,2,15,NA,"GeneID:120892","LRRK2",FALSE,"ACBD5"

"1394","TTCTTCAACATATGCAATCA","chr10",27231756,"+","none","TGG","TTCTTCAACATATGCAATCATGG",0,NA,NA,NA,"GeneID:91452","ACBD5",TRUE,"ACBD5"

"1395","CAGCTCTCCACCGCTGTCGC","chr1",180502209,"+","none","CGG","CAGCTCTCCACCGCTGTCGCCGG",0,NA,NA,NA,"GeneID:84320","ACBD6",TRUE,"ACBD6"

"1396","CCCCCATAGCCCTGAGATCG","chr1",180502157,"-","none","AGG","CCCCCATAGCCCTGAGATCGAGG",0,NA,NA,NA,"GeneID:84320","ACBD6",TRUE,"ACBD6"

"1397","CCTTCAAAATCAAAGAAGCT","chr1",180495472,"+","none","TGG","CCTTCAAAATCAAAGAAGCTTGG",0,NA,NA,NA,"GeneID:84320","ACBD6",TRUE,"ACBD6"

"1398","CCTTCAAAATCAAAGAAGCT","chr3",79742811,"+","9:G>T,16:G>A","TGG","CCTTCAAAAGCAAAGAGGCTTGG",2,10,17,NA,"GeneID:6091","ROBO1",FALSE,"ACBD6"

"1399","CGATATATTCCTGCATTGCT","chr1",180492306,"+","none","TGG","CGATATATTCCTGCATTGCTTGG",0,NA,NA,NA,"GeneID:84320","ACBD6",TRUE,"ACBD6"

"1400","CTTACCTGAGGATTCCAACC","chr1",180492263,"+","none","TGG","CTTACCTGAGGATTCCAACCTGG",0,NA,NA,NA,"GeneID:84320","ACBD6",TRUE,"ACBD6"

"1401","GCCATCACCGGCGACAGCGG","chr1",180502219,"-","none","TGG","GCCATCACCGGCGACAGCGGTGG",0,NA,NA,NA,"GeneID:84320","ACBD6",TRUE,"ACBD6"

"1402","CCCGGGTACCTGCAGGGCCA","chr10",15088707,"+","none","TGG","CCCGGGTACCTGCAGGGCCATGG",0,NA,NA,NA,"GeneID:414149","ACBD7",TRUE,"ACBD7"

"1403","CCCGGGTACCTGCAGGGCCA","chr19",33820419,"+","7:C>A,10:C>T","AGG","CCCGGGTCCCCGCAGGGCCAAGG",2,8,11,NA,"","",FALSE,"ACBD7"

"1404","CTGAAAGCAAGACCAGATGA","chr10",15078984,"-","none","TGG","CTGAAAGCAAGACCAGATGATGG",0,NA,NA,NA,"GeneID:414149","ACBD7",TRUE,"ACBD7"

"1405","CTTTACAAACAAGCAATAGT","chr10",15078939,"-","none","TGG","CTTTACAAACAAGCAATAGTTGG",0,NA,NA,NA,"GeneID:414149","ACBD7",TRUE,"ACBD7"

"1406","CTTTACAAACAAGCAATAGT","chr16",24606991,"-","14:A>T,18:T>C","TGG","CTTTACAAACAAGCTATAATTGG",2,15,19,NA,"","",FALSE,"ACBD7"

"1407","CTTTACAAACAAGCAATAGT","chr15",50556224,"+","5:A>C,17:T>A","TGG","CTTTAAAAACAAGCAATTGTTGG",2,6,18,NA,"GeneID:54822","TRPM7",FALSE,"ACBD7"

"1408","GAAGCATGGAACCTCAAAAA","chr10",15078692,"-","none","AGG","GAAGCATGGAACCTCAAAAAAGG",0,NA,NA,NA,"GeneID:414149","ACBD7",TRUE,"ACBD7"

"1409","GAGGTTCCATGCTTCCCATT","chr10",15078697,"+","none","TGG","GAGGTTCCATGCTTCCCATTTGG",0,NA,NA,NA,"GeneID:414149","ACBD7",TRUE,"ACBD7"

"1410","TATCTTTCTAGGGTTGTCGA","chr10",15078594,"-","none","CGG","TATCTTTCTAGGGTTGTCGACGG",0,NA,NA,NA,"GeneID:414149","ACBD7",TRUE,"ACBD7"

"1411","TATCTTTCTAGGGTTGTCGA","chr6",77168114,"+","11:T>G,18:A>G","TGG","TATCTTTCTAGTGTTGTCAATGG",2,12,19,NA,"","",FALSE,"ACBD7"

"1412","ACTTGGCCACTTCCTCCCGG","chr11",44074614,"-","none","AGG","ACTTGGCCACTTCCTCCCGGAGG",0,NA,NA,NA,"GeneID:84680","ACCS",TRUE,"ACCS"

"1413","CTGCAGGACTTCGGGATGTC","chr11",44081172,"+","none","TGG","CTGCAGGACTTCGGGATGTCTGG",0,NA,NA,NA,"GeneID:84680","ACCS",TRUE,"ACCS"

"1414","CTGCAGGACTTCGGGATGTC","chr11",117594628,"-","7:A>T,10:C>A","AGG","CTGCAGGTCTGCGGGATGTCAGG",2,8,11,NA,"GeneID:57453","DSCAML1",FALSE,"ACCS"

"1415","CTGTGTACAGCGTGCCAAAG","chr11",44081201,"-","none","CGG","CTGTGTACAGCGTGCCAAAGCGG",0,NA,NA,NA,"GeneID:84680","ACCS",TRUE,"ACCS"

"1416","GACTTACCCGCCAGGACAGC","chr11",44071302,"-","none","AGG","GACTTACCCGCCAGGACAGCAGG",0,NA,NA,NA,"GeneID:84680","ACCS",TRUE,"ACCS"

"1417","GGCTCTTGCAGTAGAAAGAC","chr11",44074638,"-","none","AGG","GGCTCTTGCAGTAGAAAGACAGG",0,NA,NA,NA,"GeneID:84680","ACCS",TRUE,"ACCS"

"1418","TCTCCAGGGCATCATTAACT","chr11",44071248,"+","none","TGG","TCTCCAGGGCATCATTAACTTGG",0,NA,NA,NA,"GeneID:84680","ACCS",TRUE,"ACCS"

"1419","TCTCCAGGGCATCATTAACT","chr1",112462472,"+","12:T>C,16:T>A","TGG","TCTCCAGGGCATTATTTACTTGG",2,13,17,NA,"GeneID:55917","CTTNBP2NL",FALSE,"ACCS"

"1420","TCTCCAGGGCATCATTAACT","chr12",52812113,"+","11:C>T,16:C>A","CGG","TCTCCAGGGCACCATTCACTCGG",2,12,17,NA,"GeneID:3851","KRT4",FALSE,"ACCS"

"1421","AATGGCTGCCCTCTCCAATC","chr11",44050602,"-","none","AGG","AATGGCTGCCCTCTCCAATCAGG",0,NA,NA,NA,"GeneID:390110","ACCSL",TRUE,"ACCSL"

"1422","AATGGCTGCCCTCTCCAATC","chr6",7341874,"+","2:C>T,5:G>C","AGG","AACGGGTGCCCTCTCCAATCAGG",2,3,6,NA,"GeneID:6745;GeneID:285782","SSR1;CAGE1",TRUE,"ACCSL"

"1423","ACCGGGCCACTTCTTCCCGC","chr11",44051337,"-","none","AGG","ACCGGGCCACTTCTTCCCGCAGG",0,NA,NA,NA,"GeneID:390110","ACCSL",TRUE,"ACCSL"

"1424","ATGGTTCTGTGTGATCCAGG","chr11",44051697,"+","none","CGG","ATGGTTCTGTGTGATCCAGGCGG",0,NA,NA,NA,"GeneID:390110","ACCSL",TRUE,"ACCSL"

"1425","CTCCAGGGTTTCATTAACCT","chr11",44050055,"+","none","TGG","CTCCAGGGTTTCATTAACCTTGG",0,NA,NA,NA,"GeneID:390110","ACCSL",TRUE,"ACCSL"

"1426","CTTCAGGTGGTGGTTCTAAA","chr11",44051646,"+","none","TGG","CTTCAGGTGGTGGTTCTAAATGG",0,NA,NA,NA,"GeneID:390110","ACCSL",TRUE,"ACCSL"

"1427","GGTTCCTGACCTACTACTGC","chr11",44051354,"+","none","AGG","GGTTCCTGACCTACTACTGCAGG",0,NA,NA,NA,"GeneID:390110","ACCSL",TRUE,"ACCSL"

"1428","ACCAACCAGGCACCCGTAGC","chr16",67659534,"+","none","CGG","ACCAACCAGGCACCCGTAGCCGG",0,NA,NA,NA,"GeneID:65057","ACD",TRUE,"ACD"

"1429","CAGCCCCGGCTACGGGTGCC","chr16",67659542,"-","none","TGG","CAGCCCCGGCTACGGGTGCCTGG",0,NA,NA,NA,"GeneID:65057","ACD",TRUE,"ACD"

"1430","CCCCGACGTCGGACGTATCA","chr16",67659978,"+","none","GGG","CCCCGACGTCGGACGTATCAGGG",0,NA,NA,NA,"GeneID:65057","ACD",TRUE,"ACD"

"1431","CCGCGCCGCCCTCAGCGACC","chr16",67659700,"+","none","TGG","CCGCGCCGCCCTCAGCGACCTGG",0,NA,NA,NA,"GeneID:65057","ACD",TRUE,"ACD"

"1432","CCGCGCCGCCCTCAGCGACC","chr6",52577504,"-","16:G>C,17:G>T","GGG","CCGCGCCGCCCTCAGCCCCCGGG",2,17,18,NA,"GeneID:401264","TRAM2-AS1",TRUE,"ACD"

"1433","CCGCGCCGCCCTCAGCGACC","chr7",137846600,"+","13:G>A,17:C>A","AGG","CCGCGCCGCCCTCGGCGCCCAGG",2,14,18,NA,"GeneID:9162","DGKI",TRUE,"ACD"

"1434","GACCCACAGTGTCCGATGCC","chr16",67659936,"-","none","TGG","GACCCACAGTGTCCGATGCCTGG",0,NA,NA,NA,"GeneID:65057","ACD",TRUE,"ACD"

"1435","GGTTCATGTCCAGGTCGCTG","chr16",67659712,"-","none","AGG","GGTTCATGTCCAGGTCGCTGAGG",0,NA,NA,NA,"GeneID:65057","ACD",TRUE,"ACD"

"1436","GGTTCATGTCCAGGTCGCTG","chr16",58507810,"-","4:T>G,16:A>C","CGG","GGTTAATGTCCAGGTCTCTGCGG",2,5,17,NA,"GeneID:65009","NDRG4",TRUE,"ACD"

"1437","AAGGGCCGTACCTGGGTCCA","chr17",63479091,"-","none","GGG","AAGGGCCGTACCTGGGTCCAGGG",0,NA,NA,NA,"GeneID:1636","ACE",TRUE,"ACE"

"1438","AGGAGCTGTATGAACCGATC","chr17",63477985,"+","none","TGG","AGGAGCTGTATGAACCGATCTGG",0,NA,NA,NA,"GeneID:1636","ACE",TRUE,"ACE"

"1439","CCCGCTGAAACCGCTGTACG","chr17",63479850,"+","none","AGG","CCCGCTGAAACCGCTGTACGAGG",0,NA,NA,NA,"GeneID:1636","ACE",TRUE,"ACE"

"1440","CCCTGCTAAGCAACATGAGC","chr17",63479013,"+","none","AGG","CCCTGCTAAGCAACATGAGCAGG",0,NA,NA,NA,"GeneID:1636","ACE",TRUE,"ACE"

"1441","CCGATGATCCTGCGCAGCTG","chr17",63478026,"-","none","CGG","CCGATGATCCTGCGCAGCTGCGG",0,NA,NA,NA,"GeneID:1636","ACE",TRUE,"ACE"

"1442","GAGCATGGCGTAGCTTCGCG","chr17",63479792,"-","none","AGG","GAGCATGGCGTAGCTTCGCGAGG",0,NA,NA,NA,"GeneID:1636","ACE",TRUE,"ACE"

"1443","ATGAGCACCATCTACAGTAC","chrX",15592281,"-","none","TGG","ATGAGCACCATCTACAGTACTGG",0,NA,NA,NA,"GeneID:59272","ACE2",TRUE,"ACE2"

"1444","CAAGTGAACTTTGATAGAAC","chrX",15600775,"+","none","AGG","CAAGTGAACTTTGATAGAACAGG",0,NA,NA,NA,"GeneID:59272","ACE2",TRUE,"ACE2"

"1445","CACAGTCAAGCTTCAGCTGC","chrX",15594897,"-","none","AGG","CACAGTCAAGCTTCAGCTGCAGG",0,NA,NA,NA,"GeneID:59272","ACE2",TRUE,"ACE2"

"1446","CACAGTCAAGCTTCAGCTGC","chr11",71333123,"+","0:A>C,1:T>A","AGG","ATCAGTCAAGCTTCAGCTGCAGG",2,1,2,NA,"","",FALSE,"ACE2"

"1447","TCAAGTAATAAGCATTCTTG","chrX",15592233,"+","none","TGG","TCAAGTAATAAGCATTCTTGTGG",0,NA,NA,NA,"GeneID:59272","ACE2",TRUE,"ACE2"

"1448","TGCTGCTCAGTCCACCATTG","chrX",15600847,"-","none","AGG","TGCTGCTCAGTCCACCATTGAGG",0,NA,NA,NA,"GeneID:59272","ACE2",TRUE,"ACE2"

"1449","TGCTGCTCAGTCCACCATTG","chr8",140347024,"-","6:G>A,17:C>A","TGG","TGCTGCCCAGTCCACCAGTGTGG",2,7,18,NA,"GeneID:83696","TRAPPC9",FALSE,"ACE2"

"1450","TGTGGACTGTTCCTTTAAAA","chrX",15594955,"+","none","AGG","TGTGGACTGTTCCTTTAAAAAGG",0,NA,NA,NA,"GeneID:59272","ACE2",TRUE,"ACE2"

"1451","TGTGGACTGTTCCTTTAAAA","chr1",70117069,"+","4:T>G,9:G>T","AGG","TGTGTACTGGTCCTTTAAAAAGG",2,5,10,NA,"GeneID:57554","LRRC7",FALSE,"ACE2"

"1452","CATTCTCTACATCGTGTGCC","chr19",6309709,"-","none","AGG","CATTCTCTACATCGTGTGCCAGG",0,NA,NA,NA,"GeneID:125981","ACER1",TRUE,"ACER1"

"1453","CCCGCTACATTTACGTTGTC","chr19",6312406,"-","none","TGG","CCCGCTACATTTACGTTGTCTGG",0,NA,NA,NA,"GeneID:125981","ACER1",TRUE,"ACER1"

"1454","GAGGGCGTAGGCGTTGACCG","chr19",6309746,"+","none","TGG","GAGGGCGTAGGCGTTGACCGTGG",0,NA,NA,NA,"GeneID:125981","ACER1",TRUE,"ACER1"

"1455","GCACCTGATTGAGGTCTCCG","chr19",6307249,"-","none","TGG","GCACCTGATTGAGGTCTCCGTGG",0,NA,NA,NA,"GeneID:125981","ACER1",TRUE,"ACER1"

"1456","GTGCCGAAGCTCCTTATTGC","chr19",6307265,"+","none","TGG","GTGCCGAAGCTCCTTATTGCTGG",0,NA,NA,NA,"GeneID:125981","ACER1",TRUE,"ACER1"

"1457","TGGACGAGATCGCCATCCTG","chr19",6312219,"-","none","TGG","TGGACGAGATCGCCATCCTGTGG",0,NA,NA,NA,"GeneID:125981","ACER1",TRUE,"ACER1"

"1458","ACGAAACAAGCACATGCAGA","chr2",206410747,"+","none","TGG","ACGAAACAAGCACATGCAGATGG",0,NA,NA,NA,"","",TRUE,"ACER2"

"1459","ACGAAACAAGCACATGCAGA","chr9",19423895,"-","none","TGG","ACGAAACAAGCACATGCAGATGG",0,NA,NA,NA,"GeneID:340485","ACER2",TRUE,"ACER2"

"1460","CCATGCAACCCTTAGTTTCT","chr9",19424718,"+","none","TGG","CCATGCAACCCTTAGTTTCTTGG",0,NA,NA,NA,"GeneID:340485","ACER2",TRUE,"ACER2"

"1461","CCATGCAACCCTTAGTTTCT","chr2",206410649,"-","19:G>A","TGG","CCATGCAACCCTTAGTTTCCTGG",1,20,NA,NA,"","",TRUE,"ACER2"

"1462","CCGCACCGTGTTGTAGAACT","chr9",19409178,"-","none","CGG","CCGCACCGTGTTGTAGAACTCGG",0,NA,NA,NA,"GeneID:340485","ACER2",TRUE,"ACER2"

"1463","CCGCACCGTGTTGTAGAACT","chr19",6333452,"+","0:A>C,2:T>G","CGG","ACTCACCGTGTTGTAGAACTCGG",2,1,3,NA,"GeneID:125981","ACER1",TRUE,"ACER2"

"1464","CTACTTAATCTGGACTCTTT","chr9",19423944,"+","none","TGG","CTACTTAATCTGGACTCTTTTGG",0,NA,NA,NA,"GeneID:340485","ACER2",TRUE,"ACER2"

"1465","CTACTTAATCTGGACTCTTT","chr6",122144793,"+","6:C>A,8:A>T","GGG","CTACTTCAACTGGACTCTTTGGG",2,7,9,NA,"","",FALSE,"ACER2"

"1466","CTACTTAATCTGGACTCTTT","chr11",85875632,"-","8:C>A,19:G>A","TGG","CTACTTAAGCTGGACTCTTCTGG",2,9,20,NA,"GeneID:220047","CCDC83",FALSE,"ACER2"

"1467","GCATCTGACCCAAGAAACTA","chr9",19424730,"-","none","AGG","GCATCTGACCCAAGAAACTAAGG",0,NA,NA,NA,"GeneID:340485","ACER2",TRUE,"ACER2"

"1468","GCATCTGACCCAAGAAACTA","chr2",206410637,"+","12:G>A","AGG","GCATCTGACCCAGGAAACTAAGG",1,13,NA,NA,"","",TRUE,"ACER2"

"1469","TATCGCCGAGTTCTACAACA","chr9",19409170,"+","none","CGG","TATCGCCGAGTTCTACAACACGG",0,NA,NA,NA,"GeneID:340485","ACER2",TRUE,"ACER2"

"1470","ACACAGGAACTTTCGAAAGA","chr11",77016668,"+","none","AGG","ACACAGGAACTTTCGAAAGAAGG",0,NA,NA,NA,"GeneID:55331","ACER3",TRUE,"ACER3"

"1471","ACACAGGAACTTTCGAAAGA","chr4",5620413,"+","1:G>C,12:C>T","AGG","AGACAGGAACTTCCGAAAGAAGG",2,2,13,NA,"GeneID:132884","EVC2",FALSE,"ACER3"

"1472","ATGATTATACCTCCAATGTT","chr11",76926579,"+","none","CGG","ATGATTATACCTCCAATGTTCGG",0,NA,NA,NA,"GeneID:55331","ACER3",TRUE,"ACER3"

"1473","ATGCAACAGCTGTATATCAT","chr11",76976306,"-","none","TGG","ATGCAACAGCTGTATATCATTGG",0,NA,NA,NA,"GeneID:55331","ACER3",TRUE,"ACER3"

"1474","CCACCATGCATGAAATTGTG","chr11",77016715,"-","none","TGG","CCACCATGCATGAAATTGTGTGG",0,NA,NA,NA,"GeneID:55331","ACER3",TRUE,"ACER3"

"1475","CTCTGAACTGCACCGAACAT","chr11",76926594,"-","none","TGG","CTCTGAACTGCACCGAACATTGG",0,NA,NA,NA,"GeneID:55331","ACER3",TRUE,"ACER3"

"1476","TTTCATATTTCAGAGTCATG","chr11",76959006,"-","none","TGG","TTTCATATTTCAGAGTCATGTGG",0,NA,NA,NA,"GeneID:55331","ACER3",TRUE,"ACER3"

"1477","CCACAATGTCGTGTGCCCCG","chr7",100892562,"-","none","TGG","CCACAATGTCGTGTGCCCCGTGG",0,NA,NA,NA,"GeneID:43","ACHE",TRUE,"ACHE"

"1478","CCCACAATGGCCCCCGTACA","chr7",100891288,"-","none","CGG","CCCACAATGGCCCCCGTACACGG",0,NA,NA,NA,"GeneID:43","ACHE",TRUE,"ACHE"

"1479","CCCAGCGACTGATGCGATAC","chr7",100892356,"-","none","TGG","CCCAGCGACTGATGCGATACTGG",0,NA,NA,NA,"GeneID:43","ACHE",TRUE,"ACHE"

"1480","GGCCGCAGACACGCTCGACG","chr7",100890323,"-","none","AGG","GGCCGCAGACACGCTCGACGAGG",0,NA,NA,NA,"GeneID:43","ACHE",TRUE,"ACHE"

"1481","GGCTCAGCAGTACGTTAGTC","chr7",100891261,"-","none","TGG","GGCTCAGCAGTACGTTAGTCTGG",0,NA,NA,NA,"GeneID:43","ACHE",TRUE,"ACHE"

"1482","GTTCGACCACTACAGCAAGC","chr7",100890236,"-","none","AGG","GTTCGACCACTACAGCAAGCAGG",0,NA,NA,NA,"GeneID:43","ACHE",TRUE,"ACHE"

"1483","AAAAGCAGCAGGAGCTACTT","chr14",23090567,"-","none","AGG","AAAAGCAGCAGGAGCTACTTAGG",0,NA,NA,NA,"GeneID:22985","ACIN1",TRUE,"ACIN1"

"1484","AAAAGCAGCAGGAGCTACTT","chr8",101581353,"-","11:T>C,13:A>C","GGG","AAAAGCAGCAGAATCTACTTGGG",2,12,14,NA,"GeneID:79977","GRHL2",FALSE,"ACIN1"

"1485","AAAAGCAGCAGGAGCTACTT","chr5",150795692,"+","14:G>C,15:G>T","TGG","AAAAGCAGCAGGAGGGACTTTGG",2,15,16,NA,"GeneID:85027","SMIM3",TRUE,"ACIN1"

"1486","AAAAGCAGCAGGAGCTACTT","chr4",127174270,"-","7:T>C,9:A>T","AGG","AAAAGCAACTGGAGCTACTTAGG",2,8,10,NA,"","",FALSE,"ACIN1"

"1487","AAAAGCAGCAGGAGCTACTT","chr11",80184684,"-","1:G>T,14:T>G","AGG","ACAAGCAGCAGGAGATACTTAGG",2,2,15,NA,"","",FALSE,"ACIN1"

"1488","AAGGAGAAAGACGATCATCT","chr14",23081759,"-","none","AGG","AAGGAGAAAGACGATCATCTAGG",0,NA,NA,NA,"GeneID:22985","ACIN1",TRUE,"ACIN1"

"1489","ACCTTTCTCTTCAGAAATTG","chr14",23081804,"+","none","AGG","ACCTTTCTCTTCAGAAATTGAGG",0,NA,NA,NA,"GeneID:22985","ACIN1",TRUE,"ACIN1"

"1490","ACCTTTCTCTTCAGAAATTG","chr12",61585105,"+","13:C>G,14:C>A","TGG","ACCTTTCTCTTCACCAATTGTGG",2,14,15,NA,"","",FALSE,"ACIN1"

"1491","CAGTTTCATAAAACAGTATC","chr14",23090590,"-","none","TGG","CAGTTTCATAAAACAGTATCTGG",0,NA,NA,NA,"GeneID:22985","ACIN1",TRUE,"ACIN1"

"1492","CAGTTTCATAAAACAGTATC","chr4",104027898,"-","0:T>G,2:G>C","TGG","AACTTTCATAAAACAGTATCTGG",2,1,3,NA,"","",FALSE,"ACIN1"

"1493","CAGTTTCATAAAACAGTATC","chr14",82670486,"-","0:C>G,15:T>C","TGG","GAGTTTCATAAAACAATATCTGG",2,1,16,NA,"GeneID:101928599","LOC101928599",FALSE,"ACIN1"

"1494","GGTTGGCATGAGTACGTGCC","chr14",23079641,"+","none","TGG","GGTTGGCATGAGTACGTGCCTGG",0,NA,NA,NA,"GeneID:22985","ACIN1",TRUE,"ACIN1"

"1495","TTTCGATCTTCAGTCAGTCG","chr14",23080318,"+","none","AGG","TTTCGATCTTCAGTCAGTCGAGG",0,NA,NA,NA,"GeneID:22985","ACIN1",TRUE,"ACIN1"

"1496","ATCTCAACCATCCGCCTGCG","chr3",42864730,"-","none","AGG","ATCTCAACCATCCGCCTGCGAGG",0,NA,NA,NA,"GeneID:1238;GeneID:100506243;GeneID:1582","ACKR2;KRBOX1;CYP8B1",TRUE,"ACKR2"

"1497","CATGCCCGCCGAAATCTGCG","chr3",42865089,"-","none","TGG","CATGCCCGCCGAAATCTGCGTGG",0,NA,NA,NA,"GeneID:1238;GeneID:100506243;GeneID:1582","ACKR2;KRBOX1;CYP8B1",TRUE,"ACKR2"

"1498","GCATCTAGACTACGCACTCC","chr3",42865365,"+","none","AGG","GCATCTAGACTACGCACTCCAGG",0,NA,NA,NA,"GeneID:1238;GeneID:100506243;GeneID:1582","ACKR2;KRBOX1;CYP8B1",TRUE,"ACKR2"

"1499","GCTGAGCATGAACGATCTCC","chr3",42864933,"-","none","AGG","GCTGAGCATGAACGATCTCCAGG",0,NA,NA,NA,"GeneID:1238;GeneID:100506243;GeneID:1582","ACKR2;KRBOX1;CYP8B1",TRUE,"ACKR2"

"1500","TGCTCCGTTACGTGCCTCGC","chr3",42864713,"+","none","AGG","TGCTCCGTTACGTGCCTCGCAGG",0,NA,NA,NA,"GeneID:1238;GeneID:100506243;GeneID:1582","ACKR2;KRBOX1;CYP8B1",TRUE,"ACKR2"

"1501","TGCTCCGTTACGTGCCTCGC","chr3",118880418,"+","6:A>G,13:T>G","AGG","TGCTCCATTACGTTCCTCGCAGG",2,7,14,NA,"","",FALSE,"ACKR2"

"1502","TGGTGGCCTTCTTCGTGCTA","chr3",42865268,"+","none","TGG","TGGTGGCCTTCTTCGTGCTATGG",0,NA,NA,NA,"GeneID:1238;GeneID:100506243;GeneID:1582","ACKR2;KRBOX1;CYP8B1",TRUE,"ACKR2"

"1503","ACGCAGACGTGACGGTCTTC","chr2",236581013,"-","none","AGG","ACGCAGACGTGACGGTCTTCAGG",0,NA,NA,NA,"GeneID:57007","ACKR3",TRUE,"ACKR3"

"1504","ACTGGACGCCGAGATGGCTC","chr2",236581174,"-","none","TGG","ACTGGACGCCGAGATGGCTCTGG",0,NA,NA,NA,"GeneID:57007","ACKR3",TRUE,"ACKR3"

"1505","CAACAGCAGCGACTGCATCG","chr2",236580527,"+","none","TGG","CAACAGCAGCGACTGCATCGTGG",0,NA,NA,NA,"GeneID:57007","ACKR3",TRUE,"ACKR3"

"1506","CATCTCGGCGTCCAGTGACC","chr2",236581178,"+","none","AGG","CATCTCGGCGTCCAGTGACCAGG",0,NA,NA,NA,"GeneID:57007","ACKR3",TRUE,"ACKR3"

"1507","CATGATTGCCAACTCCGTGG","chr2",236580638,"+","none","TGG","CATGATTGCCAACTCCGTGGTGG",0,NA,NA,NA,"GeneID:57007","ACKR3",TRUE,"ACKR3"

"1508","TTTGCACGTGAGCTCGCCCA","chr2",236580799,"-","none","TGG","TTTGCACGTGAGCTCGCCCATGG",0,NA,NA,NA,"GeneID:57007","ACKR3",TRUE,"ACKR3"

"1509","ACTAAAGTCCCCAGCCAATC","chr3",132600820,"+","none","AGG","ACTAAAGTCCCCAGCCAATCAGG",0,NA,NA,NA,"GeneID:84129;GeneID:100532724;GeneID:51554","ACAD11;NPHP3-ACAD11;ACKR4",TRUE,"ACKR4"

"1510","ACTAAAGTCCCCAGCCAATC","chr6",138823169,"+","5:T>A","AGG","ACTAATGTCCCCAGCCAATCAGG",1,6,NA,NA,"GeneID:345930","ECT2L",TRUE,"ACKR4"

"1511","ACTAAAGTCCCCAGCCAATC","chr5",170801618,"+","9:T>C,17:G>A","TGG","ACTAAAGTCTCCAGCCAGTCTGG",2,10,18,NA,"GeneID:2568","GABRP",FALSE,"ACKR4"

"1512","ACTAAAGTCCCCAGCCAATC","chr15",93659470,"-","6:T>C,13:A>C","TGG","ACTAAAATCCCCATCCAATCTGG",2,7,14,NA,"","",FALSE,"ACKR4"

"1513","CAATGACGAAAACTATTGTG","chr3",132600540,"-","none","AGG","CAATGACGAAAACTATTGTGAGG",0,NA,NA,NA,"GeneID:84129;GeneID:100532724;GeneID:51554","ACAD11;NPHP3-ACAD11;ACKR4",TRUE,"ACKR4"

"1514","CAATGACGAAAACTATTGTG","chr6",138822889,"-","11:C>T","AGG","CAATGACGAAAGCTATTGTGAGG",1,12,NA,NA,"GeneID:345930","ECT2L",TRUE,"ACKR4"

"1515","CCTTTAAATGCTAAAAGTAC","chr6",138823782,"-","none","TGG","CCTTTAAATGCTAAAAGTACTGG",0,NA,NA,NA,"GeneID:345930","ECT2L",TRUE,"ACKR4"

"1516","CCTTTAAATGCTAAAAGTAC","chr3",132601433,"-","none","TGG","CCTTTAAATGCTAAAAGTACTGG",0,NA,NA,NA,"GeneID:84129;GeneID:100532724;GeneID:51554","ACAD11;NPHP3-ACAD11;ACKR4",TRUE,"ACKR4"

"1517","GTGCGATGCTTTCTGTGACT","chr3",132601254,"-","none","TGG","GTGCGATGCTTTCTGTGACTTGG",0,NA,NA,NA,"GeneID:84129;GeneID:100532724;GeneID:51554","ACAD11;NPHP3-ACAD11;ACKR4",TRUE,"ACKR4"

"1518","GTGCGATGCTTTCTGTGACT","chr6",138823603,"-","4:T>C","TGG","GTGCAATGCTTTCTGTGACTTGG",1,5,NA,NA,"GeneID:345930","ECT2L",TRUE,"ACKR4"

"1519","TATCAGCATAGACAGATATG","chr3",132600792,"+","none","TGG","TATCAGCATAGACAGATATGTGG",0,NA,NA,NA,"GeneID:84129;GeneID:100532724;GeneID:51554","ACAD11;NPHP3-ACAD11;ACKR4",TRUE,"ACKR4"

"1520","TATCAGCATAGACAGATATG","chr6",138823141,"+","0:C>T","TGG","CATCAGCATAGACAGATATGTGG",1,1,NA,NA,"GeneID:345930","ECT2L",TRUE,"ACKR4"

"1521","TGTGCTACTTTATCACAGCA","chr3",132601049,"+","none","AGG","TGTGCTACTTTATCACAGCAAGG",0,NA,NA,NA,"GeneID:84129;GeneID:100532724;GeneID:51554","ACAD11;NPHP3-ACAD11;ACKR4",TRUE,"ACKR4"

"1522","TGTGCTACTTTATCACAGCA","chr6",138823398,"+","16:G>A","AGG","TGTGCTACTTTATCACGGCAAGG",1,17,NA,NA,"GeneID:345930","ECT2L",TRUE,"ACKR4"

"1523","AACTTCCTCCTCAACGCCAG","chr17",41898643,"-","none","CGG","AACTTCCTCCTCAACGCCAGCGG",0,NA,NA,NA,"GeneID:47","ACLY",TRUE,"ACLY"

"1524","ACCTACCGATGTGCTCCCGC","chr17",41898624,"+","none","TGG","ACCTACCGATGTGCTCCCGCTGG",0,NA,NA,NA,"GeneID:47","ACLY",TRUE,"ACLY"

"1525","GACCAGCTGATCAAACGTCG","chr17",41912504,"-","none","TGG","GACCAGCTGATCAAACGTCGTGG",0,NA,NA,NA,"GeneID:47","ACLY",TRUE,"ACLY"

"1526","GAGAGCAATTCGAGATTACC","chr17",41901785,"-","none","AGG","GAGAGCAATTCGAGATTACCAGG",0,NA,NA,NA,"GeneID:47","ACLY",TRUE,"ACLY"

"1527","TTCCACGACGTTTGATCAGC","chr17",41912499,"+","none","TGG","TTCCACGACGTTTGATCAGCTGG",0,NA,NA,NA,"GeneID:47","ACLY",TRUE,"ACLY"

"1528","TTGTGACTTCGTGCTCCTTC","chr17",41901755,"+","none","AGG","TTGTGACTTCGTGCTCCTTCAGG",0,NA,NA,NA,"GeneID:47","ACLY",TRUE,"ACLY"

"1529","ATTTCTCTAATACGAACTTC","chr2",134859326,"-","none","TGG","ATTTCTCTAATACGAACTTCTGG",0,NA,NA,NA,"GeneID:130013","ACMSD",TRUE,"ACMSD"

"1530","CCACTGCAGAGGTTTGGCTA","chr2",134845223,"+","none","CGG","CCACTGCAGAGGTTTGGCTACGG",0,NA,NA,NA,"GeneID:130013","ACMSD",TRUE,"ACMSD"

"1531","CCACTGCAGAGGTTTGGCTA","chr5",34791858,"+","7:C>A,13:A>T","GGG","CCACTGCCGAGGTATGGCTAGGG",2,8,14,NA,"GeneID:26064","RAI14",FALSE,"ACMSD"

"1532","CCGCTACTCACCGCATAGAC","chr2",134863622,"-","none","AGG","CCGCTACTCACCGCATAGACAGG",0,NA,NA,NA,"GeneID:130013","ACMSD",TRUE,"ACMSD"

"1533","GAGTGGTGCGAGAGAATTGC","chr2",134859297,"+","none","TGG","GAGTGGTGCGAGAGAATTGCTGG",0,NA,NA,NA,"GeneID:130013","ACMSD",TRUE,"ACMSD"

"1534","TCAGGTCCCACTCGTTGACG","chr2",134863582,"-","none","TGG","TCAGGTCCCACTCGTTGACGTGG",0,NA,NA,NA,"GeneID:130013","ACMSD",TRUE,"ACMSD"

"1535","TTCCTGTCATGTTTAGCTAC","chr2",134861995,"+","none","TGG","TTCCTGTCATGTTTAGCTACTGG",0,NA,NA,NA,"GeneID:130013","ACMSD",TRUE,"ACMSD"

"1536","ACTCGAGAAACGTGCCGCCC","chr7",97117729,"-","none","CGG","ACTCGAGAAACGTGCCGCCCCGG",0,NA,NA,NA,"GeneID:57001","ACN9",TRUE,"ACN9"

"1537","AGTACGTGAAAGACGAATTT","chr7",97117820,"+","none","AGG","AGTACGTGAAAGACGAATTTAGG",0,NA,NA,NA,"GeneID:57001","ACN9",TRUE,"ACN9"

"1538","CGTGGGGCGCTATGCCGGGG","chr7",97117712,"+","none","CGG","CGTGGGGCGCTATGCCGGGGCGG",0,NA,NA,NA,"GeneID:57001","ACN9",TRUE,"ACN9"

"1539","GACTTTCGTGATGAACAAAT","chr7",97181110,"+","none","TGG","GACTTTCGTGATGAACAAATTGG",0,NA,NA,NA,"GeneID:57001","ACN9",TRUE,"ACN9"

"1540","GACTTTCGTGATGAACAAAT","chr1",150521859,"-","7:G>C,11:T>A","TGG","GACTTTCCTGAAGAACAAATTGG",2,8,12,NA,"","",FALSE,"ACN9"

"1541","GACTTTCGTGATGAACAAAT","chr1",92580560,"-","6:C>G,16:C>T","TGG","GACTTTGGTGATGAACGAATTGG",2,7,17,NA,"GeneID:7813","EVI5",TRUE,"ACN9"

"1542","TAAATTCGTCTTTCACGTAC","chr7",97117821,"-","none","TGG","TAAATTCGTCTTTCACGTACTGG",0,NA,NA,NA,"GeneID:57001","ACN9",TRUE,"ACN9"

"1543","TGCAACAGCGTTATTGCAAC","chr7",97181016,"+","none","AGG","TGCAACAGCGTTATTGCAACAGG",0,NA,NA,NA,"GeneID:57001","ACN9",TRUE,"ACN9"

"1544","TGCAACAGCGTTATTGCAAC","chr5",142404621,"+","8:G>C,19:T>C","AGG","TGCAACAGGGTTATTGCAATAGG",2,9,20,NA,"GeneID:101926941","LOC101926941",FALSE,"ACN9"

"1545","AATGGTGAGCACGATGTCAG","chr9",32419151,"-","none","TGG","AATGGTGAGCACGATGTCAGTGG",0,NA,NA,NA,"GeneID:48","ACO1",TRUE,"ACO1"

"1546","ACCTTCTGTTGAAATCAACC","chr9",32408633,"-","none","TGG","ACCTTCTGTTGAAATCAACCTGG",0,NA,NA,NA,"GeneID:48","ACO1",TRUE,"ACO1"

"1547","AGAAGAACTCTGATCGAAAA","chr9",32407271,"-","none","TGG","AGAAGAACTCTGATCGAAAATGG",0,NA,NA,NA,"GeneID:48","ACO1",TRUE,"ACO1"

"1548","ATGACACGAGCAGGCTTAAA","chr9",32407394,"-","none","TGG","ATGACACGAGCAGGCTTAAATGG",0,NA,NA,NA,"GeneID:48","ACO1",TRUE,"ACO1"

"1549","CGCACACCTTGCTGAGCCAT","chr9",32405520,"+","none","TGG","CGCACACCTTGCTGAGCCATTGG",0,NA,NA,NA,"GeneID:48","ACO1",TRUE,"ACO1"

"1550","TGATCTATTACAAGATCAGC","chr9",32408604,"-","none","AGG","TGATCTATTACAAGATCAGCAGG",0,NA,NA,NA,"GeneID:48","ACO1",TRUE,"ACO1"

"1551","TGATCTATTACAAGATCAGC","chr19",14807257,"-","5:T>A,16:A>G","AGG","TGATCAATTACAAGATTAGCAGG",2,6,17,NA,"GeneID:26664","OR7C1",FALSE,"ACO1"

"1552","ATGCTGCCCGCTGCTGTCCT","chr22",41525232,"-","none","TGG","ATGCTGCCCGCTGCTGTCCTTGG",0,NA,NA,NA,"GeneID:50","ACO2",TRUE,"ACO2"

"1553","CACATAGCCGTCCCGCTCAA","chr22",41522970,"-","none","TGG","CACATAGCCGTCCCGCTCAATGG",0,NA,NA,NA,"GeneID:50","ACO2",TRUE,"ACO2"

"1554","CTGCTGCAGGAGTTTGACCC","chr22",41525183,"+","none","AGG","CTGCTGCAGGAGTTTGACCCAGG",0,NA,NA,NA,"GeneID:50","ACO2",TRUE,"ACO2"

"1555","CTGCTGCAGGAGTTTGACCC","chr6",3625836,"-","7:A>T,16:A>T","AGG","CTGCTGCTGGAGTTTGTCCCAGG",2,8,17,NA,"","",FALSE,"ACO2"

"1556","GCGCGGATCTGCTCGGAACC","chr22",41522945,"-","none","TGG","GCGCGGATCTGCTCGGAACCTGG",0,NA,NA,NA,"GeneID:50","ACO2",TRUE,"ACO2"

"1557","GCGCGGATCTGCTCGGAACC","chr11",849877,"-","9:G>A,17:G>T","CGG","GCGCGGATCCGCTCGGACCCCGG",2,10,18,NA,"GeneID:7106","TSPAN4",TRUE,"ACO2"

"1558","GCGCGGATCTGCTCGGAACC","chr1",236111286,"-","3:A>G,17:C>T","TGG","GCGTGGATCTGCTCGGAGCCTGG",2,4,18,NA,"","",TRUE,"ACO2"

"1559","TCTCGGGGTTTGCGTCGTTG","chr22",41523895,"-","none","CGG","TCTCGGGGTTTGCGTCGTTGCGG",0,NA,NA,NA,"GeneID:50","ACO2",TRUE,"ACO2"

"1560","TGCCTTTGTCACGTCCCCAG","chr22",41523919,"+","none","AGG","TGCCTTTGTCACGTCCCCAGAGG",0,NA,NA,NA,"GeneID:50","ACO2",TRUE,"ACO2"

"1561","TGCCTTTGTCACGTCCCCAG","chr16",80290768,"+","7:C>G,12:C>G","TGG","TGCCTTTCTCACCTCCCCAGTGG",2,8,13,NA,"","",FALSE,"ACO2"

"1562","AGTAGCGCTCGTGCCGCACC","chr14",73537786,"-","none","CGG","AGTAGCGCTCGTGCCGCACCCGG",0,NA,NA,NA,"GeneID:399671;GeneID:641371","HEATR4;ACOT1",TRUE,"ACOT1"

"1563","AGTAGCGCTCGTGCCGCACC","chr14",73569791,"-","17:C>T,18:A>G","TGG","AGTAGCGCTCGTGCCGCGTCTGG",2,18,19,NA,"GeneID:10965","ACOT2",TRUE,"ACOT1"

"1564","CCGAACATGTCCACAATGCC","chr14",73541509,"-","none","AGG","CCGAACATGTCCACAATGCCAGG",0,NA,NA,NA,"GeneID:399671;GeneID:641371","HEATR4;ACOT1",TRUE,"ACOT1"

"1565","CCGAACATGTCCACAATGCC","chr14",73573404,"-","17:G>C","AGG","CCGAACATGTCCACAATCCCAGG",1,18,NA,NA,"GeneID:10965","ACOT2",TRUE,"ACOT1"

"1566","CGCTACCGCGCCGACACCCT","chr14",73537571,"+","none","TGG","CGCTACCGCGCCGACACCCTTGG",0,NA,NA,NA,"GeneID:399671;GeneID:641371","HEATR4;ACOT1",TRUE,"ACOT1"

"1567","CGCTACCGCGCCGACACCCT","chr14",73569576,"+","17:T>C","TGG","CGCTACCGCGCCGACACTCTTGG",1,18,NA,NA,"GeneID:10965","ACOT2",TRUE,"ACOT1"

"1568","CTACTTGCTCAGTCATCCTG","chr14",73541673,"+","none","AGG","CTACTTGCTCAGTCATCCTGAGG",0,NA,NA,NA,"GeneID:399671;GeneID:641371","HEATR4;ACOT1",TRUE,"ACOT1"

"1569","CTACTTGCTCAGTCATCCTG","chr14",73573568,"+","18:C>T","AGG","CTACTTGCTCAGTCATCCCGAGG",1,19,NA,NA,"GeneID:10965","ACOT2",TRUE,"ACOT1"

"1570","CTACTTGCTCAGTCATCCTG","chr22",46344226,"-","5:C>A,15:C>A","GGG","CTACTGGCTCAGTCAGCCTGGGG",2,6,16,NA,"GeneID:55687","TRMU",FALSE,"ACOT1"

"1571","GGTCTCGCCCTTGTAGCGTA","chr14",73543182,"-","none","AGG","GGTCTCGCCCTTGTAGCGTAAGG",0,NA,NA,NA,"GeneID:399671;GeneID:641371","HEATR4;ACOT1",TRUE,"ACOT1"

"1572","GGTCTCGCCCTTGTAGCGTA","chr14",73575040,"-","16:A>G","AGG","GGTCTCGCCCTTGTAGTGTAAGG",1,17,NA,NA,"GeneID:10965","ACOT2",TRUE,"ACOT1"

"1573","TGGGGGAACCTTACGCTACA","chr14",73543171,"+","none","AGG","TGGGGGAACCTTACGCTACAAGG",0,NA,NA,NA,"GeneID:399671;GeneID:641371","HEATR4;ACOT1",TRUE,"ACOT1"

"1574","TGGGGGAACCTTACGCTACA","chr14",73575029,"+","14:A>G","AGG","TGGGGGAACCTTACACTACAAGG",1,15,NA,NA,"GeneID:10965","ACOT2",TRUE,"ACOT1"

"1575","ACTGTCGTTCCCCGCACGTA","chr1",54584700,"-","none","AGG","ACTGTCGTTCCCCGCACGTAAGG",0,NA,NA,NA,"GeneID:26027","ACOT11",TRUE,"ACOT11"

"1576","AGCTGAAGCAGATCACGCCG","chr1",54594559,"+","none","CGG","AGCTGAAGCAGATCACGCCGCGG",0,NA,NA,NA,"GeneID:26027","ACOT11",TRUE,"ACOT11"

"1577","CAAGGCCTTGGCCACCTTCG","chr1",54593996,"+","none","TGG","CAAGGCCTTGGCCACCTTCGTGG",0,NA,NA,NA,"GeneID:26027","ACOT11",TRUE,"ACOT11"

"1578","CATAGACAAGGCGCATGCGC","chr1",54594626,"-","none","CGG","CATAGACAAGGCGCATGCGCCGG",0,NA,NA,NA,"GeneID:26027","ACOT11",TRUE,"ACOT11"

"1579","CTTACCGGACAGGCAAGCCG","chr1",54584847,"-","none","TGG","CTTACCGGACAGGCAAGCCGTGG",0,NA,NA,NA,"GeneID:26027","ACOT11",TRUE,"ACOT11"

"1580","TCCTCTCCAGGTGGGCATCC","chr1",54593930,"+","none","AGG","TCCTCTCCAGGTGGGCATCCAGG",0,NA,NA,NA,"GeneID:26027","ACOT11",TRUE,"ACOT11"

"1581","TCCTCTCCAGGTGGGCATCC","chr19",1037614,"+","18:G>C,19:A>C","CGG","TCCTCTCCAGGTGGGCATGACGG",2,19,20,NA,"GeneID:1265","CNN2",TRUE,"ACOT11"

"1582","TCCTCTCCAGGTGGGCATCC","chr12",57041487,"-","6:A>G,19:C>G","GGG","TCCTCTTCAGGTGGGCATCGGGG",2,7,20,NA,"GeneID:4640","MYO1A",TRUE,"ACOT11"

"1583","AAACTGTATGTCATCCACTG","chr5",81385770,"+","none","AGG","AAACTGTATGTCATCCACTGAGG",0,NA,NA,NA,"GeneID:134526","ACOT12",TRUE,"ACOT12"

"1584","ATGACCACCTCGCCGGGCGC","chr5",81394082,"+","none","CGG","ATGACCACCTCGCCGGGCGCCGG",0,NA,NA,NA,"GeneID:134526","ACOT12",TRUE,"ACOT12"

"1585","CGTGCGCCGGCTGGATGGCT","chr5",81394056,"+","none","TGG","CGTGCGCCGGCTGGATGGCTTGG",0,NA,NA,NA,"GeneID:134526","ACOT12",TRUE,"ACOT12"

"1586","GCTCAGCTCGCCGCGCGCAG","chr5",81394033,"+","none","TGG","GCTCAGCTCGCCGCGCGCAGTGG",0,NA,NA,NA,"GeneID:134526","ACOT12",TRUE,"ACOT12"

"1587","TAGAGCATTCAGCACAAGCA","chr5",81371754,"-","none","TGG","TAGAGCATTCAGCACAAGCATGG",0,NA,NA,NA,"GeneID:134526","ACOT12",TRUE,"ACOT12"

"1588","TAGAGCATTCAGCACAAGCA","chr6",53458844,"+","14:G>C,15:G>A","TGG","TAGAGCATTCAGCAGGAGCATGG",2,15,16,NA,"","",FALSE,"ACOT12"

"1589","TTTGAAGCTGAGAAACATGC","chr5",81385813,"-","none","TGG","TTTGAAGCTGAGAAACATGCTGG",0,NA,NA,NA,"GeneID:134526","ACOT12",TRUE,"ACOT12"

"1590","TTTGAAGCTGAGAAACATGC","chr3",183700199,"-","6:T>C,19:A>G","GGG","TTTGAAACTGAGAAACATGTGGG",2,7,20,NA,"GeneID:55689","YEATS2",FALSE,"ACOT12"

"1591","AACACTTGCATTTACCTCTG","chr6",24701524,"+","none","TGG","AACACTTGCATTTACCTCTGTGG",0,NA,NA,NA,"GeneID:55856","ACOT13",TRUE,"ACOT13"

"1592","AACACTTGCATTTACCTCTG","chr20",53310265,"+","4:A>C,5:A>T","TGG","AACAAATGCATTTACCTCTGTGG",2,5,6,NA,"GeneID:128553","TSHZ2",FALSE,"ACOT13"

"1593","AACACTTGCATTTACCTCTG","chr3",78065457,"-","6:C>A,13:C>T","GGG","AACACTGGCATTTGCCTCTGGGG",2,7,14,NA,"","",FALSE,"ACOT13"

"1594","CTTGTTGGTCAGATCCACAG","chr6",24701541,"-","none","AGG","CTTGTTGGTCAGATCCACAGAGG",0,NA,NA,NA,"GeneID:55856","ACOT13",TRUE,"ACOT13"

"1595","GGTGATAAAGGCCATGACCA","chr6",24667292,"+","none","AGG","GGTGATAAAGGCCATGACCAAGG",0,NA,NA,NA,"GeneID:55856","ACOT13",TRUE,"ACOT13"

"1596","GTGGAGAGTGCCTATTGCAT","chr6",24697949,"-","none","TGG","GTGGAGAGTGCCTATTGCATTGG",0,NA,NA,NA,"GeneID:55856","ACOT13",TRUE,"ACOT13"

"1597","TCTCTCAAAATTGCGAGCCT","chr6",24667312,"-","none","TGG","TCTCTCAAAATTGCGAGCCTTGG",0,NA,NA,NA,"GeneID:55856","ACOT13",TRUE,"ACOT13"

"1598","TGATATGTTATCTACTAACG","chr6",24697985,"-","none","TGG","TGATATGTTATCTACTAACGTGG",0,NA,NA,NA,"GeneID:55856","ACOT13",TRUE,"ACOT13"

"1599","CCGAACATGTCCACAATCCC","chr14",73573404,"-","none","AGG","CCGAACATGTCCACAATCCCAGG",0,NA,NA,NA,"GeneID:10965","ACOT2",TRUE,"ACOT2"

"1600","CCGAACATGTCCACAATCCC","chr14",73541509,"-","17:C>G","AGG","CCGAACATGTCCACAATGCCAGG",1,18,NA,NA,"GeneID:399671;GeneID:641371","HEATR4;ACOT1",TRUE,"ACOT2"

"1601","CCGAACATGTCCACAATCCC","chrX",22281719,"+","1:A>C,8:A>G","CGG","CAGAACATATCCACAATCCCCGG",2,2,9,NA,"","",FALSE,"ACOT2"

"1602","CTACTTGCTCAGTCATCCCG","chr14",73573568,"+","none","AGG","CTACTTGCTCAGTCATCCCGAGG",0,NA,NA,NA,"GeneID:10965","ACOT2",TRUE,"ACOT2"

"1603","CTACTTGCTCAGTCATCCCG","chr14",73541673,"+","18:T>C","AGG","CTACTTGCTCAGTCATCCTGAGG",1,19,NA,NA,"GeneID:399671;GeneID:641371","HEATR4;ACOT1",TRUE,"ACOT2"

"1604","GGAACTGCGCCGAACTCTTC","chr14",73569353,"-","none","AGG","GGAACTGCGCCGAACTCTTCAGG",0,NA,NA,NA,"GeneID:10965","ACOT2",TRUE,"ACOT2"

"1605","GGTACAGCCGGGACGCTCGA","chr14",73569323,"-","none","AGG","GGTACAGCCGGGACGCTCGAAGG",0,NA,NA,NA,"GeneID:10965","ACOT2",TRUE,"ACOT2"

"1606","GGTCTCGCCCTTGTAGTGTA","chr14",73575040,"-","none","AGG","GGTCTCGCCCTTGTAGTGTAAGG",0,NA,NA,NA,"GeneID:10965","ACOT2",TRUE,"ACOT2"

"1607","GGTCTCGCCCTTGTAGTGTA","chr14",73543182,"-","16:G>A","AGG","GGTCTCGCCCTTGTAGCGTAAGG",1,17,NA,NA,"GeneID:399671;GeneID:641371","HEATR4;ACOT1",TRUE,"ACOT2"

"1608","TGGGGGAACCTTACACTACA","chr14",73575029,"+","none","AGG","TGGGGGAACCTTACACTACAAGG",0,NA,NA,NA,"GeneID:10965","ACOT2",TRUE,"ACOT2"

"1609","TGGGGGAACCTTACACTACA","chr14",73543171,"+","14:G>A","AGG","TGGGGGAACCTTACGCTACAAGG",1,15,NA,NA,"GeneID:399671;GeneID:641371","HEATR4;ACOT1",TRUE,"ACOT2"

"1610","ATAAGGAATGCTCTCGTAGG","chr14",73595270,"+","none","AGG","ATAAGGAATGCTCTCGTAGGAGG",0,NA,NA,NA,"GeneID:122970","ACOT4",TRUE,"ACOT4"

"1611","CCGCGCACGGCAATGCGCAC","chr14",73592013,"-","none","CGG","CCGCGCACGGCAATGCGCACCGG",0,NA,NA,NA,"GeneID:122970","ACOT4",TRUE,"ACOT4"

"1612","CCGCGCACGGCAATGCGCAC","chr14",73537475,"-","11:C>T,14:A>C","CGG","CCGCGCACGGCGATTCGCACCGG",2,12,15,NA,"GeneID:399671;GeneID:641371","HEATR4;ACOT1",TRUE,"ACOT4"

"1613","CCGCGCACGGCAATGCGCAC","chr14",73569480,"-","11:C>T,14:A>C","CGG","CCGCGCACGGCGATTCGCACCGG",2,12,15,NA,"GeneID:10965","ACOT2",TRUE,"ACOT4"

"1614","CGCAGCGTAACCCGCTGCTC","chr14",73592043,"-","none","CGG","CGCAGCGTAACCCGCTGCTCCGG",0,NA,NA,NA,"GeneID:122970","ACOT4",TRUE,"ACOT4"

"1615","CGCAGCGTAACCCGCTGCTC","chr14",73569510,"-","8:C>T,12:C>G","CGG","CGCAGCGTGACCGGCTGCTCCGG",2,9,13,NA,"GeneID:10965","ACOT2",TRUE,"ACOT4"

"1616","CGCAGCGTAACCCGCTGCTC","chr14",73537505,"-","8:C>T,12:C>G","CGG","CGCAGCGTGACCGGCTGCTCCGG",2,9,13,NA,"GeneID:399671;GeneID:641371","HEATR4;ACOT1",TRUE,"ACOT4"

"1617","GGATATGTTGTCCATGTTAT","chr14",73593836,"-","none","TGG","GGATATGTTGTCCATGTTATTGG",0,NA,NA,NA,"GeneID:122970","ACOT4",TRUE,"ACOT4"

"1618","TGAAGATCTCCCCAATAACA","chr14",73593822,"+","none","TGG","TGAAGATCTCCCCAATAACATGG",0,NA,NA,NA,"GeneID:122970","ACOT4",TRUE,"ACOT4"

"1619","TGAAGATCTCCCCAATAACA","chr19",36422104,"+","15:G>T,19:G>A","TGG","TGAAGATCTCCCCAAGAACGTGG",2,16,20,NA,"","",TRUE,"ACOT4"

"1620","TTATATCCACAATGTCCACG","chr14",73595254,"-","none","AGG","TTATATCCACAATGTCCACGAGG",0,NA,NA,NA,"GeneID:122970","ACOT4",TRUE,"ACOT4"

"1621","TTATATCCACAATGTCCACG","chr19",36422392,"-","10:C>T,12:G>A","AGG","TTATATCCACGACGTCCACGAGG",2,11,13,NA,"","",TRUE,"ACOT4"

"1622","AATCCAAGAAGCGCAATACT","chr14",73619245,"-","none","AGG","AATCCAAGAAGCGCAATACTAGG",0,NA,NA,NA,"GeneID:641372","ACOT6",TRUE,"ACOT6"

"1623","CCTAAACTTGTCGATGATCT","chr14",73619389,"+","none","AGG","CCTAAACTTGTCGATGATCTAGG",0,NA,NA,NA,"GeneID:641372","ACOT6",TRUE,"ACOT6"

"1624","CCTAGATCATCGACAAGTTT","chr14",73619392,"-","none","AGG","CCTAGATCATCGACAAGTTTAGG",0,NA,NA,NA,"GeneID:641372","ACOT6",TRUE,"ACOT6"

"1625","CTTTATGCTGCAGCATCCAA","chr14",73617170,"+","none","AGG","CTTTATGCTGCAGCATCCAAAGG",0,NA,NA,NA,"GeneID:641372","ACOT6",TRUE,"ACOT6"

"1626","CTTTATGCTGCAGCATCCAA","chr20",2480549,"-","12:A>C,18:G>T","TGG","CTTTATGCTGCATCATCCCATGG",2,13,19,NA,"","",FALSE,"ACOT6"

"1627","CTTTATGCTGCAGCATCCAA","chr8",65420909,"+","12:T>G","TGG","CTTTATGCTGCATCATCCAATGG",1,13,NA,NA,"","",FALSE,"ACOT6"

"1628","CTTTATGCTGCAGCATCCAA","chr7",129490987,"+","12:T>G,18:C>A","TGG","CTTTATGCTGCATCATCCCATGG",2,13,19,NA,"","",FALSE,"ACOT6"

"1629","CTTTATGCTGCAGCATCCAA","chr3",75031043,"-","4:C>T,12:A>C","TGG","CTTTGTGCTGCATCATCCAATGG",2,5,13,NA,"","",FALSE,"ACOT6"

"1630","CTTTATGCTGCAGCATCCAA","chr17",41645406,"-","3:G>A,18:G>T","TGG","CTTCATGCTGCAGCATCCCATGG",2,4,19,NA,"","",FALSE,"ACOT6"

"1631","GCGTGCACAGAAGCTCTAGA","chr14",73619680,"-","none","AGG","GCGTGCACAGAAGCTCTAGAAGG",0,NA,NA,NA,"GeneID:641372","ACOT6",TRUE,"ACOT6"

"1632","GGTCCTAGTATTGCGCTTCT","chr14",73619239,"+","none","TGG","GGTCCTAGTATTGCGCTTCTTGG",0,NA,NA,NA,"GeneID:641372","ACOT6",TRUE,"ACOT6"

"1633","CCTCGAGGTGCCTCCTGTTG","chr1",6333478,"-","none","TGG","CCTCGAGGTGCCTCCTGTTGTGG",0,NA,NA,NA,"GeneID:11332","ACOT7",TRUE,"ACOT7"

"1634","CGGTGCGCTCGACACGAGCC","chr1",6339549,"+","none","AGG","CGGTGCGCTCGACACGAGCCAGG",0,NA,NA,NA,"GeneID:11332","ACOT7",TRUE,"ACOT7"

"1635","CTCTTACCTGGGTTGAGGAC","chr1",6327291,"+","none","TGG","CTCTTACCTGGGTTGAGGACTGG",0,NA,NA,NA,"GeneID:11332","ACOT7",TRUE,"ACOT7"

"1636","GTCTCCACAGGAGCGCTGTG","chr1",6339579,"-","none","TGG","GTCTCCACAGGAGCGCTGTGTGG",0,NA,NA,NA,"GeneID:11332","ACOT7",TRUE,"ACOT7"

"1637","GTCTCCACAGGAGCGCTGTG","chr14",46802533,"+","9:A>G,13:G>C","TGG","GTCTCCACAAGAGGGCTGTGTGG",2,10,14,NA,"","",FALSE,"ACOT7"

"1638","TGTCCACATTCTTCAGCGAC","chr1",6333502,"+","none","AGG","TGTCCACATTCTTCAGCGACAGG",0,NA,NA,NA,"GeneID:11332","ACOT7",TRUE,"ACOT7"

"1639","TGTCCACATTCTTCAGCGAC","chr4",40043755,"+","7:G>A,11:G>T","AGG","TGTCCACGTTCGTCAGCGACAGG",2,8,12,NA,"GeneID:344967","LOC344967",TRUE,"ACOT7"

"1640","TTTCTCGCAGTATTCCCGGC","chr1",6327403,"-","none","AGG","TTTCTCGCAGTATTCCCGGCAGG",0,NA,NA,NA,"GeneID:11332","ACOT7",TRUE,"ACOT7"

"1641","ACGAACAGGGTCGAGCTTCT","chr20",45848615,"-","none","CGG","ACGAACAGGGTCGAGCTTCTCGG",0,NA,NA,NA,"GeneID:10005","ACOT8",TRUE,"ACOT8"

"1642","CGGTCGTGACCAAGACGCTA","chr20",45857224,"+","none","CGG","CGGTCGTGACCAAGACGCTACGG",0,NA,NA,NA,"GeneID:10005","ACOT8",TRUE,"ACOT8"

"1643","CTGTTCGTGTCCGCTCCACT","chr20",45848627,"+","none","TGG","CTGTTCGTGTCCGCTCCACTTGG",0,NA,NA,NA,"GeneID:10005","ACOT8",TRUE,"ACOT8"

"1644","GAACTAGATGTCGTCCCCGC","chr20",45857302,"-","none","AGG","GAACTAGATGTCGTCCCCGCAGG",0,NA,NA,NA,"GeneID:10005","ACOT8",TRUE,"ACOT8"

"1645","GCGCCAATGGGTACCTCTTT","chr20",45844385,"+","none","TGG","GCGCCAATGGGTACCTCTTTTGG",0,NA,NA,NA,"GeneID:10005","ACOT8",TRUE,"ACOT8"

"1646","GCTCAACCGAATTGCTGCTC","chr20",45844367,"-","none","AGG","GCTCAACCGAATTGCTGCTCAGG",0,NA,NA,NA,"GeneID:10005","ACOT8",TRUE,"ACOT8"

"1647","AAATATTTCTCTCGTAATTC","chrX",23730836,"+","none","AGG","AAATATTTCTCTCGTAATTCAGG",0,NA,NA,NA,"GeneID:23597","ACOT9",TRUE,"ACOT9"

"1648","AAATATTTCTCTCGTAATTC","chr6",140305987,"-","10:A>G,19:A>G","GGG","AAATATTTCTTTCGTAATTTGGG",2,11,20,NA,"","",FALSE,"ACOT9"

"1649","AAATATTTCTCTCGTAATTC","chr3",166674722,"+","13:A>G,18:A>T","TGG","AAATATTTCTCTCATAATACTGG",2,14,19,NA,"","",FALSE,"ACOT9"

"1650","AAATATTTCTCTCGTAATTC","chr2",191533705,"+","10:G>C,19:T>C","GGG","AAATATTTCTGTCGTAATTTGGG",2,11,20,NA,"","",FALSE,"ACOT9"

"1651","CACAGGATGGACTGCCTCCT","chrX",23730910,"-","none","AGG","CACAGGATGGACTGCCTCCTAGG",0,NA,NA,NA,"GeneID:23597","ACOT9",TRUE,"ACOT9"

"1652","CAGATTTGGCAGGATTCTTG","chrX",23730547,"-","none","AGG","CAGATTTGGCAGGATTCTTGAGG",0,NA,NA,NA,"GeneID:23597","ACOT9",TRUE,"ACOT9"

"1653","CAGATTTGGCAGGATTCTTG","chr10",35448836,"+","3:T>A,17:C>T","TGG","CAGTTTTGGCAGGATTCCTGTGG",2,4,18,NA,"GeneID:219771","CCNY",FALSE,"ACOT9"

"1654","CAGATTTGGCAGGATTCTTG","chr9",128380173,"+","0:G>C,19:C>G","TGG","GAGATTTGGCAGGATTCTTCTGG",2,1,20,NA,"GeneID:81605","URM1",FALSE,"ACOT9"

"1655","CTCCATCCACACTGCATTAG","chrX",23705806,"+","none","AGG","CTCCATCCACACTGCATTAGAGG",0,NA,NA,NA,"GeneID:23597","ACOT9",TRUE,"ACOT9"

"1656","CTCCATCCACACTGCATTAG","chr1",152687540,"-","10:G>T,18:A>T","AGG","CTCCATCCACCCTGCATTTGAGG",2,11,19,NA,"","",FALSE,"ACOT9"

"1657","GAATTCAAAACTGAAGAGTT","chrX",23705787,"-","none","TGG","GAATTCAAAACTGAAGAGTTTGG",0,NA,NA,NA,"GeneID:23597","ACOT9",TRUE,"ACOT9"

"1658","GAATTCAAAACTGAAGAGTT","chr7",151571883,"-","10:A>G","TGG","GAATTCAAAATTGAAGAGTTTGG",1,11,NA,NA,"GeneID:51422","PRKAG2",FALSE,"ACOT9"

"1659","GAATTCAAAACTGAAGAGTT","chr2",55521985,"+","9:G>A,18:C>T","AGG","GAATTCAAAGCTGAAGAGCTAGG",2,10,19,NA,"GeneID:112942","CFAP36",FALSE,"ACOT9"

"1660","GAATTCAAAACTGAAGAGTT","chr2",49940919,"+","5:G>C,19:G>T","AGG","GAATTGAAAACTGAAGAGTGAGG",2,6,20,NA,"GeneID:9378","NRXN1",FALSE,"ACOT9"

"1661","TGACTGTTCAAAACACCGTA","chrX",23730817,"-","none","AGG","TGACTGTTCAAAACACCGTAAGG",0,NA,NA,NA,"GeneID:23597","ACOT9",TRUE,"ACOT9"

"1662","ATCGTACCTATTCGTGAAAT","chr17",75955850,"-","none","CGG","ATCGTACCTATTCGTGAAATCGG",0,NA,NA,NA,"GeneID:51","ACOX1",TRUE,"ACOX1"

"1663","ATCGTACCTATTCGTGAAAT","chrX",70832570,"-","12:A>G,17:C>T","TGG","ATCGTACCTATTTGTGAGATTGG",2,13,18,NA,"GeneID:56159","TEX11",FALSE,"ACOX1"

"1664","CACGTATGACCCTGAAACCC","chr17",75957515,"-","none","AGG","CACGTATGACCCTGAAACCCAGG",0,NA,NA,NA,"GeneID:51","ACOX1",TRUE,"ACOX1"

"1665","CACTCGCAGCCAGCGTTATG","chr17",75978621,"-","none","AGG","CACTCGCAGCCAGCGTTATGAGG",0,NA,NA,NA,"GeneID:51","ACOX1",TRUE,"ACOX1"

"1666","TCCTTAGGAACTCACCTTCG","chr17",75957553,"-","none","AGG","TCCTTAGGAACTCACCTTCGAGG",0,NA,NA,NA,"GeneID:51","ACOX1",TRUE,"ACOX1"

"1667","TGACAGCCACCTCATAACGC","chr17",75978609,"+","none","TGG","TGACAGCCACCTCATAACGCTGG",0,NA,NA,NA,"GeneID:51","ACOX1",TRUE,"ACOX1"

"1668","TTCACGAATAGGTACGATAA","chr17",75955852,"+","none","AGG","TTCACGAATAGGTACGATAAAGG",0,NA,NA,NA,"GeneID:51","ACOX1",TRUE,"ACOX1"

"1669","AGGCGCTCACCTTAGCAGCC","chr3",58522485,"+","none","TGG","AGGCGCTCACCTTAGCAGCCTGG",0,NA,NA,NA,"GeneID:8309","ACOX2",TRUE,"ACOX2"

"1670","CTCCATGACGCCTTCCTGTC","chr3",58517262,"-","none","TGG","CTCCATGACGCCTTCCTGTCTGG",0,NA,NA,NA,"GeneID:8309","ACOX2",TRUE,"ACOX2"

"1671","CTGCCACTCACCGGATCAGG","chr3",58517194,"+","none","CGG","CTGCCACTCACCGGATCAGGCGG",0,NA,NA,NA,"GeneID:8309","ACOX2",TRUE,"ACOX2"

"1672","GCCGAGCTATCAACCGGATG","chr3",58534405,"+","none","TGG","GCCGAGCTATCAACCGGATGTGG",0,NA,NA,NA,"GeneID:8309","ACOX2",TRUE,"ACOX2"

"1673","TCTGGTTCCAAGCCTCGTGC","chr3",58522524,"+","none","TGG","TCTGGTTCCAAGCCTCGTGCTGG",0,NA,NA,NA,"GeneID:8309","ACOX2",TRUE,"ACOX2"

"1674","TGATAGCTCGGCGCCTGGGT","chr3",58534397,"-","none","TGG","TGATAGCTCGGCGCCTGGGTTGG",0,NA,NA,NA,"GeneID:8309","ACOX2",TRUE,"ACOX2"

"1675","AGCAAACACCACCGCGTGAG","chr4",8410265,"+","none","TGG","AGCAAACACCACCGCGTGAGTGG",0,NA,NA,NA,"GeneID:8310","ACOX3",TRUE,"ACOX3"

"1676","ATGTCGCCAACCATCACTCC","chr4",8405990,"+","none","AGG","ATGTCGCCAACCATCACTCCAGG",0,NA,NA,NA,"GeneID:8310","ACOX3",TRUE,"ACOX3"

"1677","CAGCTCGCGATACTTCTCCA","chr4",8415912,"+","none","AGG","CAGCTCGCGATACTTCTCCAAGG",0,NA,NA,NA,"GeneID:8310","ACOX3",TRUE,"ACOX3"

"1678","CGAAGATCCGCTTGCATCGA","chr4",8415884,"+","none","AGG","CGAAGATCCGCTTGCATCGAAGG",0,NA,NA,NA,"GeneID:8310","ACOX3",TRUE,"ACOX3"

"1679","GAAGAAGGGTCTTCGGGTCC","chr4",8406018,"+","none","CGG","GAAGAAGGGTCTTCGGGTCCCGG",0,NA,NA,NA,"GeneID:8310","ACOX3",TRUE,"ACOX3"

"1680","GCTGCATCCCTTTATCGTGC","chr4",8410213,"-","none","AGG","GCTGCATCCCTTTATCGTGCAGG",0,NA,NA,NA,"GeneID:8310","ACOX3",TRUE,"ACOX3"

"1681","GCTGCATCCCTTTATCGTGC","chr1",3235320,"+","5:T>A,13:C>A","TGG","GCTGCTTCCCTTTCTCGTGCTGG",2,6,14,NA,"GeneID:63976","PRDM16",FALSE,"ACOX3"

"1682","AGAGGACTTCTCCTATGACA","chr2",110784766,"-","none","AGG","AGAGGACTTCTCCTATGACAAGG",0,NA,NA,NA,"GeneID:55289","ACOXL",TRUE,"ACOXL"

"1683","AGAGGACTTCTCCTATGACA","chrX",152720550,"-","11:T>G,18:C>G","AGG","AGAGGACTTCTACTATGAGAAGG",2,12,19,NA,"","",FALSE,"ACOXL"

"1684","AGAGGACTTCTCCTATGACA","chrX",152747897,"+","11:A>C,18:G>C","AGG","AGAGGACTTCTACTATGAGAAGG",2,12,19,NA,"","",FALSE,"ACOXL"

"1685","CTGCCTCTGTTAAAACGTGC","chr2",110768431,"+","none","AGG","CTGCCTCTGTTAAAACGTGCAGG",0,NA,NA,NA,"GeneID:55289","ACOXL",TRUE,"ACOXL"

"1686","GCCCTGAACATGTTACTAAG","chr2",110793698,"+","none","TGG","GCCCTGAACATGTTACTAAGTGG",0,NA,NA,NA,"GeneID:55289","ACOXL",TRUE,"ACOXL"

"1687","GCCCTGAACATGTTACTAAG","chr15",38764007,"+","8:T>C,16:A>T","AGG","GCCCTGAATATGTTACAAAGAGG",2,9,17,NA,"GeneID:400359","C15orf53",FALSE,"ACOXL"

"1688","GTCAGCCGAAGCCTTGTCAT","chr2",110784752,"+","none","AGG","GTCAGCCGAAGCCTTGTCATAGG",0,NA,NA,NA,"GeneID:55289","ACOXL",TRUE,"ACOXL"

"1689","TCAGAGAGTGAAGTTTGCCA","chr2",110768406,"+","none","TGG","TCAGAGAGTGAAGTTTGCCATGG",0,NA,NA,NA,"GeneID:55289","ACOXL",TRUE,"ACOXL"

"1690","TCCAGTGCGGAATAATTTAT","chr2",110793644,"+","none","TGG","TCCAGTGCGGAATAATTTATTGG",0,NA,NA,NA,"GeneID:55289","ACOXL",TRUE,"ACOXL"

"1691","TCCAGTGCGGAATAATTTAT","chr14",54042336,"+","5:G>T,7:A>C","TGG","TCCAGGGAGGAATAATTTATTGG",2,6,8,NA,"","",FALSE,"ACOXL"

"1692","AAAGCTAAAATTGAACTACT","chr10",85709023,"+","none","TGG","AAAGCTAAAATTGAACTACTTGG",0,NA,NA,NA,"GeneID:2894","GRID1",TRUE,"ACP1"

"1693","AAAGCTAAAATTGAACTACT","chr2",277016,"+","none","TGG","AAAGCTAAAATTGAACTACTTGG",0,NA,NA,NA,"GeneID:52","ACP1",TRUE,"ACP1"

"1694","AAAGCTAAAATTGAACTACT","chr5",160248077,"+","none","TGG","AAAGCTAAAATTGAACTACTTGG",0,NA,NA,NA,"","",TRUE,"ACP1"

"1695","AAAGCTAAAATTGAACTACT","chr16",14862443,"-","17:G>T","TGG","AAAGCTAAAATTGAACTCCTTGG",1,18,NA,NA,"GeneID:23420","NOMO1",TRUE,"ACP1"

"1696","AAAGCTAAAATTGAACTACT","chr16",16260932,"-","17:G>T","TGG","AAAGCTAAAATTGAACTCCTTGG",1,18,NA,NA,"GeneID:408050","NOMO3",TRUE,"ACP1"

"1697","AAAGCTAAAATTGAACTACT","chr1",233836203,"-","14:C>T","TGG","AAAGCTAAAATTGAGCTACTTGG",1,15,NA,NA,"","",TRUE,"ACP1"

"1698","AAAGCTAAAATTGAACTACT","chrX",18216198,"-","10:G>A,17:A>T","AGG","AAAGCTAAAACTGAACTTCTAGG",2,11,18,NA,"GeneID:139105","BEND2",FALSE,"ACP1"

"1699","AAAGCTAAAATTGAACTACT","chr16",18533728,"+","17:C>A","TGG","AAAGCTAAAATTGAACTCCTTGG",1,18,NA,NA,"GeneID:283820","NOMO2",TRUE,"ACP1"

"1700","ACGGTACCTGCCGGGCAACG","chr2",272137,"-","none","TGG","ACGGTACCTGCCGGGCAACGTGG",0,NA,NA,NA,"GeneID:52","ACP1",TRUE,"ACP1"

"1701","CAACTTCCGGGTATGAGATA","chr2",272055,"+","none","GGG","CAACTTCCGGGTATGAGATAGGG",0,NA,NA,NA,"GeneID:52","ACP1",TRUE,"ACP1"

"1702","CAACTTCCGGGTATGAGATA","chr1",233836398,"-","1:C>T,13:C>A","GGG","CGACTTCCGGGTAGGAGATAGGG",2,2,14,NA,"","",TRUE,"ACP1"

"1703","CCTGAAAACTGCTTCTGCAA","chr2",271886,"-","none","TGG","CCTGAAAACTGCTTCTGCAATGG",0,NA,NA,NA,"GeneID:52","ACP1",TRUE,"ACP1"

"1704","CCTGAAAACTGCTTCTGCAA","chr6",147434548,"+","1:T>C","TGG","CTTGAAAACTGCTTCTGCAATGG",1,2,NA,NA,"","",FALSE,"ACP1"

"1705","CCTGAAAACTGCTTCTGCAA","chr5",160247580,"-","0:A>G,19:C>T","TGG","TCTGAAAACTGCTTCTGCAGTGG",2,1,20,NA,"","",TRUE,"ACP1"

"1706","GTGAGCTGCCTAAGAAATCA","chr2",272254,"+","none","TGG","GTGAGCTGCCTAAGAAATCATGG",0,NA,NA,NA,"GeneID:52","ACP1",TRUE,"ACP1"

"1707","GTGAGCTGCCTAAGAAATCA","chr17",42159465,"+","3:G>A,6:G>T","TGG","GTGGGCGGCCTAAGAAATCATGG",2,4,7,NA,"GeneID:23415","KCNH4",FALSE,"ACP1"

"1708","TGCTGTTTCTGACTGGAACG","chr2",272208,"+","none","TGG","TGCTGTTTCTGACTGGAACGTGG",0,NA,NA,NA,"GeneID:52","ACP1",TRUE,"ACP1"

"1709","AGCCGTGATAGCGCTGCCGC","chr11",47247669,"+","none","AGG","AGCCGTGATAGCGCTGCCGCAGG",0,NA,NA,NA,"GeneID:53","ACP2",TRUE,"ACP2"

"1710","CGTTCTGCAGCTGCTCATAA","chr11",47245520,"+","none","CGG","CGTTCTGCAGCTGCTCATAACGG",0,NA,NA,NA,"GeneID:53","ACP2",TRUE,"ACP2"

"1711","GCCCTGCGGCAGCGCTATCA","chr11",47247674,"-","none","CGG","GCCCTGCGGCAGCGCTATCACGG",0,NA,NA,NA,"GeneID:53","ACP2",TRUE,"ACP2"

"1712","GCTTCAACCCGAACATCTCG","chr11",47245726,"-","none","TGG","GCTTCAACCCGAACATCTCGTGG",0,NA,NA,NA,"GeneID:53","ACP2",TRUE,"ACP2"

"1713","GCTTCAACCCGAACATCTCG","chr2",22469348,"-","9:A>G,19:G>C","TGG","GCTTCAACCTGAACATCTCCTGG",2,10,20,NA,"","",FALSE,"ACP2"

"1714","GTTGAAGCGCTGCATCCCGT","chr11",47245738,"+","none","TGG","GTTGAAGCGCTGCATCCCGTTGG",0,NA,NA,NA,"GeneID:53","ACP2",TRUE,"ACP2"

"1715","TTCTCTCAGCTGTACCGCCA","chr11",47248122,"-","none","TGG","TTCTCTCAGCTGTACCGCCATGG",0,NA,NA,NA,"GeneID:53","ACP2",TRUE,"ACP2"

"1716","AGCCGTTGGGGACCTTGCGC","chr19",11575155,"+","none","TGG","AGCCGTTGGGGACCTTGCGCTGG",0,NA,NA,NA,"GeneID:199692;GeneID:54","ZNF627;ACP5",TRUE,"ACP5"

"1717","CACAGTCCGAGCGATCTCCT","chr19",11577143,"+","none","TGG","CACAGTCCGAGCGATCTCCTTGG",0,NA,NA,NA,"GeneID:199692;GeneID:54","ZNF627;ACP5",TRUE,"ACP5"

"1718","GGTAGGCCCGTGCTCGGCTA","chr19",11576326,"+","none","TGG","GGTAGGCCCGTGCTCGGCTATGG",0,NA,NA,NA,"GeneID:199692;GeneID:54","ZNF627;ACP5",TRUE,"ACP5"

"1719","GTGTGGTCCATAGCCGAGCA","chr19",11576336,"-","none","CGG","GTGTGGTCCATAGCCGAGCACGG",0,NA,NA,NA,"GeneID:199692;GeneID:54","ZNF627;ACP5",TRUE,"ACP5"

"1720","TGCCCTGCGCTTTGTAGCCG","chr19",11577226,"-","none","TGG","TGCCCTGCGCTTTGTAGCCGTGG",0,NA,NA,NA,"GeneID:199692;GeneID:54","ZNF627;ACP5",TRUE,"ACP5"

"1721","TTTCCGGCTAGCACGTACCA","chr19",11576775,"+","none","GGG","TTTCCGGCTAGCACGTACCAGGG",0,NA,NA,NA,"GeneID:199692;GeneID:54","ZNF627;ACP5",TRUE,"ACP5"

"1722","ACGAACCTTCTTTCTGACAC","chr1",147658953,"+","none","TGG","ACGAACCTTCTTTCTGACACTGG",0,NA,NA,NA,"GeneID:51205","ACP6",TRUE,"ACP6"

"1723","CAGACTGCCTCTTTACAGCC","chr1",147654296,"-","none","AGG","CAGACTGCCTCTTTACAGCCAGG",0,NA,NA,NA,"GeneID:51205","ACP6",TRUE,"ACP6"

"1724","CAGACTGCCTCTTTACAGCC","chr7",74679733,"-","2:T>C,4:A>G","AGG","CAAATTGCCTCTTTACAGCCAGG",2,3,5,NA,"GeneID:2969","GTF2I",FALSE,"ACP6"

"1725","CATTTGCTGCATGCCCACCT","chr1",147659478,"+","none","TGG","CATTTGCTGCATGCCCACCTTGG",0,NA,NA,NA,"GeneID:51205","ACP6",TRUE,"ACP6"

"1726","CTTCATCCTCCTGGACAACG","chr1",147654207,"-","none","TGG","CTTCATCCTCCTGGACAACGTGG",0,NA,NA,NA,"GeneID:51205","ACP6",TRUE,"ACP6"

"1727","CTTTAGGGGGGCATGTTTGC","chr1",147659512,"-","none","TGG","CTTTAGGGGGGCATGTTTGCTGG",0,NA,NA,NA,"GeneID:51205","ACP6",TRUE,"ACP6"

"1728","GGAGTCCACCCGTTGTTTGC","chr1",147658989,"-","none","TGG","GGAGTCCACCCGTTGTTTGCTGG",0,NA,NA,NA,"GeneID:51205","ACP6",TRUE,"ACP6"

"1729","ACCAGAAATTGACTGCACTC","chr3",141279471,"+","none","TGG","ACCAGAAATTGACTGCACTCTGG",0,NA,NA,NA,"GeneID:92370","PXYLP1",TRUE,"ACPL2"

"1730","CGTTTCAGTCCACCTGATCC","chr3",141278333,"+","none","CGG","CGTTTCAGTCCACCTGATCCCGG",0,NA,NA,NA,"GeneID:92370","PXYLP1",TRUE,"ACPL2"

"1731","GAAACCGTATCACCCAAAAC","chr3",141287313,"+","none","TGG","GAAACCGTATCACCCAAAACTGG",0,NA,NA,NA,"GeneID:92370","PXYLP1",TRUE,"ACPL2"

"1732","GCAGCAGCAAGAAGCGGTTG","chr3",141260186,"-","none","CGG","GCAGCAGCAAGAAGCGGTTGCGG",0,NA,NA,NA,"GeneID:92370","PXYLP1",TRUE,"ACPL2"

"1733","GCAGCAGCAAGAAGCGGTTG","chr7",20461823,"+","17:C>T,19:T>G","AGG","GCAGCAGCAAGAAGCGGCTTAGG",2,18,20,NA,"","",FALSE,"ACPL2"

"1734","GTCACAGGGGGCTCCGTCAC","chr3",141278415,"-","none","AGG","GTCACAGGGGGCTCCGTCACAGG",0,NA,NA,NA,"GeneID:92370","PXYLP1",TRUE,"ACPL2"

"1735","GTTCAAGGGGCTTTCGAAAG","chr3",141287375,"-","none","AGG","GTTCAAGGGGCTTTCGAAAGAGG",0,NA,NA,NA,"GeneID:92370","PXYLP1",TRUE,"ACPL2"

"1736","AGACCGAAGTGTACTAGCCA","chr3",132317533,"+","none","AGG","AGACCGAAGTGTACTAGCCAAGG",0,NA,NA,NA,"GeneID:55","ACPP",TRUE,"ACPP"

"1737","AGGGCAGCAAGCCTTAGCCT","chr3",132317483,"+","none","TGG","AGGGCAGCAAGCCTTAGCCTTGG",0,NA,NA,NA,"GeneID:55","ACPP",TRUE,"ACPP"

"1738","ATGGAGCAGCATTATGAACT","chr3",132331652,"+","none","TGG","ATGGAGCAGCATTATGAACTTGG",0,NA,NA,NA,"GeneID:55","ACPP",TRUE,"ACPP"

"1739","TACTTTCAGGTGTTTCGGCA","chr3",132328257,"+","none","TGG","TACTTTCAGGTGTTTCGGCATGG",0,NA,NA,NA,"GeneID:55","ACPP",TRUE,"ACPP"

"1740","TCCTCATGGCCACAAGGATT","chr3",132328326,"+","none","TGG","TCCTCATGGCCACAAGGATTTGG",0,NA,NA,NA,"GeneID:55","ACPP",TRUE,"ACPP"

"1741","TGAGTCCTATAAACATGAAC","chr3",132331711,"+","none","AGG","TGAGTCCTATAAACATGAACAGG",0,NA,NA,NA,"GeneID:55","ACPP",TRUE,"ACPP"

"1742","ACAGTCCCGTGAAGTTCTCC","chr19",50792258,"-","none","AGG","ACAGTCCCGTGAAGTTCTCCAGG",0,NA,NA,NA,"GeneID:93650","ACPT",TRUE,"ACPT"

"1743","ACCCCTGGTGTTCGTGGCTC","chr19",50790503,"+","none","TGG","ACCCCTGGTGTTCGTGGCTCTGG",0,NA,NA,NA,"GeneID:93650","ACPT",TRUE,"ACPT"

"1744","CACCTCCTTGTGTGGGTCCA","chr19",50790636,"-","none","TGG","CACCTCCTTGTGTGGGTCCATGG",0,NA,NA,NA,"GeneID:93650","ACPT",TRUE,"ACPT"

"1745","CACCTCCTTGTGTGGGTCCA","chr9",14168593,"-","11:T>C,15:A>C","AGG","CACCTCCTTGTATGGTTCCAAGG",2,12,16,NA,"GeneID:4781","NFIB",FALSE,"ACPT"

"1746","CGCTTCTCACCGTGGTCAGC","chr19",50790688,"-","none","TGG","CGCTTCTCACCGTGGTCAGCTGG",0,NA,NA,NA,"GeneID:93650","ACPT",TRUE,"ACPT"

"1747","CTGGGTGGAAATGGCCGGCC","chr19",50790404,"+","none","TGG","CTGGGTGGAAATGGCCGGCCTGG",0,NA,NA,NA,"GeneID:93650","ACPT",TRUE,"ACPT"

"1748","CTGGGTGGAAATGGCCGGCC","chrX",85004189,"+","7:A>G,15:T>C","AGG","CTGGGTGAAAATGGCTGGCCAGG",2,8,16,NA,"GeneID:139322","APOOL",FALSE,"ACPT"

"1749","GAACTTCACGGGACTGTCGC","chr19",50792261,"+","none","TGG","GAACTTCACGGGACTGTCGCTGG",0,NA,NA,NA,"GeneID:93650","ACPT",TRUE,"ACPT"

"1750","AAAATACAACTCTGCGACAG","chr22",50739807,"+","none","AGG","AAAATACAACTCTGCGACAGAGG",0,NA,NA,NA,"GeneID:49","ACR",TRUE,"ACR"

"1751","AATGAAGCGCCCACACGAAA","chr22",50739866,"-","none","TGG","AATGAAGCGCCCACACGAAATGG",0,NA,NA,NA,"GeneID:49","ACR",TRUE,"ACR"

"1752","GAACGCGCCCATTGTACCAC","chr22",50744133,"-","none","TGG","GAACGCGCCCATTGTACCACTGG",0,NA,NA,NA,"GeneID:49","ACR",TRUE,"ACR"

"1753","GAACGCGCCCATTGTACCAC","chr2",113668532,"+","5:T>C,8:T>C","TGG","GAACGTGCTCATTGTACCACTGG",2,6,9,NA,"","",TRUE,"ACR"

"1754","GGCGTTATCTTTAGCAACCA","chr22",50738284,"-","none","CGG","GGCGTTATCTTTAGCAACCACGG",0,NA,NA,NA,"GeneID:49","ACR",TRUE,"ACR"

"1755","GTGGTGTCCGCATCGTCGGC","chr22",50739308,"+","none","GGG","GTGGTGTCCGCATCGTCGGCGGG",0,NA,NA,NA,"GeneID:49","ACR",TRUE,"ACR"

"1756","TTGCCTGAACCGTAACCCAC","chr22",50739278,"-","none","AGG","TTGCCTGAACCGTAACCCACAGG",0,NA,NA,NA,"GeneID:49","ACR",TRUE,"ACR"

"1757","CACAGCCTTTCGTGGCAAGC","chr12",6640130,"+","none","CGG","CACAGCCTTTCGTGGCAAGCCGG",0,NA,NA,NA,"GeneID:84519","ACRBP",TRUE,"ACRBP"

"1758","CACCATCGGGCACTAAGCCG","chr12",6646790,"+","none","TGG","CACCATCGGGCACTAAGCCGTGG",0,NA,NA,NA,"GeneID:84519","ACRBP",TRUE,"ACRBP"

"1759","GGGTGCTCACCTTCCGATTG","chr12",6638943,"+","none","CGG","GGGTGCTCACCTTCCGATTGCGG",0,NA,NA,NA,"GeneID:84519","ACRBP",TRUE,"ACRBP"

"1760","GTCTGATGAGAAACCGCAAT","chr12",6638959,"-","none","CGG","GTCTGATGAGAAACCGCAATCGG",0,NA,NA,NA,"GeneID:84519","ACRBP",TRUE,"ACRBP"

"1761","TGCGAAGAAGCGTTCGTATT","chr12",6646911,"+","none","CGG","TGCGAAGAAGCGTTCGTATTCGG",0,NA,NA,NA,"GeneID:84519","ACRBP",TRUE,"ACRBP"

"1762","TGTGAAGATGTCCGAGTCTC","chr12",6640114,"-","none","TGG","TGTGAAGATGTCCGAGTCTCTGG",0,NA,NA,NA,"GeneID:84519","ACRBP",TRUE,"ACRBP"

"1763","TGTGAAGATGTCCGAGTCTC","chr4",184869832,"+","12:A>C,16:A>T","GGG","TGTGAAGATGTCAGAGACTCGGG",2,13,17,NA,"","",FALSE,"ACRBP"

"1764","ACTCACCTCGCCTGTCTCTT","chrX",71594765,"-","none","CGG","ACTCACCTCGCCTGTCTCTTCGG",0,NA,NA,NA,"GeneID:93953","ACRC",TRUE,"ACRC"

"1765","ACTCACCTCGCCTGTCTCTT","chrX",71592556,"-","none","CGG","ACTCACCTCGCCTGTCTCTTCGG",0,NA,NA,NA,"GeneID:93953","ACRC",TRUE,"ACRC"

"1766","ACTCACCTCGCCTGTCTCTT","chr2",2150032,"+","0:T>A,13:C>G","TGG","TCTCACCTCGCCTCTCTCTTTGG",2,1,14,NA,"GeneID:23040","MYT1L",FALSE,"ACRC"

"1767","ACTCTACTTACAAGACCCAC","chrX",71594368,"-","none","TGG","ACTCTACTTACAAGACCCACTGG",0,NA,NA,NA,"GeneID:93953","ACRC",TRUE,"ACRC"

"1768","ACTCTACTTACAAGACCCAC","chrX",71592159,"-","none","TGG","ACTCTACTTACAAGACCCACTGG",0,NA,NA,NA,"GeneID:93953","ACRC",TRUE,"ACRC"

"1769","CACTGCTTGACTGAACATTA","chrX",71592130,"-","none","AGG","CACTGCTTGACTGAACATTAAGG",0,NA,NA,NA,"GeneID:93953","ACRC",TRUE,"ACRC"

"1770","CACTGCTTGACTGAACATTA","chr3",152225641,"-","8:T>C,17:T>A","AGG","CACTGCTTAACTGAACAATAAGG",2,9,18,NA,"","",FALSE,"ACRC"

"1771","CACTGCTTGACTGAACATTA","chrX",71594339,"-","4:G>C,13:C>T","AGG","CACTCCTTGACTGGACATTAAGG",2,5,14,NA,"GeneID:93953","ACRC",TRUE,"ACRC"

"1772","CAGAAGAGCTCCGAAGAGAC","chrX",71594752,"+","none","AGG","CAGAAGAGCTCCGAAGAGACAGG",0,NA,NA,NA,"GeneID:93953","ACRC",TRUE,"ACRC"

"1773","CAGAAGAGCTCCGAAGAGAC","chrX",71592543,"+","none","AGG","CAGAAGAGCTCCGAAGAGACAGG",0,NA,NA,NA,"GeneID:93953","ACRC",TRUE,"ACRC"

"1774","CAGAAGAGCTCCGAAGAGAC","chrX",23201430,"-","10:C>G,12:T>C","CGG","CAGAAGAGCTGCAAAGAGACCGG",2,11,13,NA,"","",FALSE,"ACRC"

"1775","CAGAAGAGCTCCGAAGAGAC","chr11",97078229,"+","11:A>C,15:A>G","TGG","CAGAAGAGCTCAGAAAAGACTGG",2,12,16,NA,"","",FALSE,"ACRC"

"1776","TCAAGCAGTGATGACACCAG","chrX",71592140,"+","none","TGG","TCAAGCAGTGATGACACCAGTGG",0,NA,NA,NA,"GeneID:93953","ACRC",TRUE,"ACRC"

"1777","TCAAGCAGTGATGACACCAG","chrX",71594349,"+","5:G>C,10:G>A","TGG","TCAAGGAGTGGTGACACCAGTGG",2,6,11,NA,"GeneID:93953","ACRC",TRUE,"ACRC"

"1778","ACTTCAGTTCAGCAACTTCC","chr11",125678230,"-","none","AGG","ACTTCAGTTCAGCAACTTCCAGG",0,NA,NA,NA,"GeneID:56","ACRV1",TRUE,"ACRV1"

"1779","ACTTCAGTTCAGCAACTTCC","chr10",16934003,"-","1:A>G,3:G>A","TGG","ATTCCAGTTCAGCAACTTCCTGG",2,2,4,NA,"GeneID:8029","CUBN",FALSE,"ACRV1"

"1780","ACTTCAGTTCAGCAACTTCC","chr11",65910721,"+","1:T>C,13:C>A","TGG","ATTTCAGTTCAGCCACTTCCTGG",2,2,14,NA,"","",FALSE,"ACRV1"

"1781","AGTCAGCCTAATGAGCTTTC","chr11",125678269,"-","none","TGG","AGTCAGCCTAATGAGCTTTCTGG",0,NA,NA,NA,"GeneID:56","ACRV1",TRUE,"ACRV1"

"1782","AGTCAGCCTAATGAGCTTTC","chr2",21642892,"-","6:C>G,19:C>G","TGG","AGTCAGGCTAATGAGCTTTGTGG",2,7,20,NA,"","",FALSE,"ACRV1"

"1783","CACACCCTTGAACCATGAAT","chr11",125672684,"+","none","TGG","CACACCCTTGAACCATGAATTGG",0,NA,NA,NA,"GeneID:1111;GeneID:56","CHEK1;ACRV1",TRUE,"ACRV1"

"1784","CCTTTGAGTGAGCTTGAGTC","chr11",125677945,"-","none","AGG","CCTTTGAGTGAGCTTGAGTCAGG",0,NA,NA,NA,"GeneID:56","ACRV1",TRUE,"ACRV1"

"1785","CCTTTGAGTGAGCTTGAGTC","chr5",86709692,"+","11:C>G,19:G>C","AGG","CCTTTGAGTGACCTTGAGTGAGG",2,12,20,NA,"","",FALSE,"ACRV1"

"1786","CCTTTGAGTGAGCTTGAGTC","chr12",109727199,"+","6:T>A,18:C>T","GGG","CCTTTGTGTGAGCTTGAGCCGGG",2,7,19,NA,"GeneID:84915","FAM222A",FALSE,"ACRV1"

"1787","CTCACCAGAAGGCTGTTCAC","chr11",125677878,"+","none","CGG","CTCACCAGAAGGCTGTTCACCGG",0,NA,NA,NA,"GeneID:56","ACRV1",TRUE,"ACRV1"

"1788","TAATTTGCATCCTCGTTCCA","chr11",125672630,"+","none","TGG","TAATTTGCATCCTCGTTCCATGG",0,NA,NA,NA,"GeneID:1111;GeneID:56","CHEK1;ACRV1",TRUE,"ACRV1"

"1789","TAATTTGCATCCTCGTTCCA","chr3",68136729,"+","13:A>C,14:C>G","TGG","TAATTTGCATCCTACTTCCATGG",2,14,15,NA,"GeneID:407738","FAM19A1",FALSE,"ACRV1"

"1790","AGAACCTCCTCCAAACAAGA","chr15",78182721,"-","none","TGG","AGAACCTCCTCCAAACAAGATGG",0,NA,NA,NA,"GeneID:23205","ACSBG1",TRUE,"ACSBG1"

"1791","ATCACGACTGCCTTTAGATG","chr15",78182747,"+","none","TGG","ATCACGACTGCCTTTAGATGTGG",0,NA,NA,NA,"GeneID:23205","ACSBG1",TRUE,"ACSBG1"

"1792","CCGCCCAGATCTACGACCTG","chr15",78182025,"-","none","TGG","CCGCCCAGATCTACGACCTGTGG",0,NA,NA,NA,"GeneID:23205","ACSBG1",TRUE,"ACSBG1"

"1793","CTGTCCACAGGTCGTAGATC","chr15",78182018,"+","none","TGG","CTGTCCACAGGTCGTAGATCTGG",0,NA,NA,NA,"GeneID:23205","ACSBG1",TRUE,"ACSBG1"

"1794","TGTGTGCTAGTCTACACTTC","chr15",78182511,"-","none","CGG","TGTGTGCTAGTCTACACTTCCGG",0,NA,NA,NA,"GeneID:23205","ACSBG1",TRUE,"ACSBG1"

"1795","TTGACTCAGCATCACGCCCT","chr15",78182471,"+","none","TGG","TTGACTCAGCATCACGCCCTTGG",0,NA,NA,NA,"GeneID:23205","ACSBG1",TRUE,"ACSBG1"

"1796","ATGACCTTACCCGGCTAGGA","chr19",6151785,"-","none","TGG","ATGACCTTACCCGGCTAGGATGG",0,NA,NA,NA,"GeneID:5990;GeneID:81616","RFX2;ACSBG2",TRUE,"ACSBG2"

"1797","CGAGAGTCAGTCAACCGATT","chr19",6147549,"+","none","TGG","CGAGAGTCAGTCAACCGATTTGG",0,NA,NA,NA,"GeneID:5990;GeneID:81616","RFX2;ACSBG2",TRUE,"ACSBG2"

"1798","CTGGGTTTGGAGCGTTTCCA","chr19",6151706,"+","none","CGG","CTGGGTTTGGAGCGTTTCCACGG",0,NA,NA,NA,"GeneID:5990;GeneID:81616","RFX2;ACSBG2",TRUE,"ACSBG2"

"1799","GGAACCCCAAAGACTCAAGA","chr19",6141549,"+","none","AGG","GGAACCCCAAAGACTCAAGAAGG",0,NA,NA,NA,"GeneID:5990;GeneID:81616","RFX2;ACSBG2",TRUE,"ACSBG2"

"1800","GGAACCCCAAAGACTCAAGA","chr7",30823700,"-","3:G>T,4:A>G","TGG","GGACTCCCAAAGACTCAAGATGG",2,4,5,NA,"GeneID:100526825;GeneID:84182","INMT-FAM188B;FAM188B",FALSE,"ACSBG2"

"1801","TCCGACAAGCCTCATAGTAC","chr19",6147632,"-","none","TGG","TCCGACAAGCCTCATAGTACTGG",0,NA,NA,NA,"GeneID:5990;GeneID:81616","RFX2;ACSBG2",TRUE,"ACSBG2"

"1802","TTAGCTCCTTCTTGAGTCTT","chr19",6141558,"-","none","TGG","TTAGCTCCTTCTTGAGTCTTTGG",0,NA,NA,NA,"GeneID:5990;GeneID:81616","RFX2;ACSBG2",TRUE,"ACSBG2"

"1803","TTAGCTCCTTCTTGAGTCTT","chr7",30823691,"+","2:T>A,8:A>T","TGG","TTTGCTCCATCTTGAGTCTTTGG",2,3,9,NA,"GeneID:100526825;GeneID:84182","INMT-FAM188B;FAM188B",FALSE,"ACSBG2"

"1804","ACGACCAAGGCCTCTCGTTC","chr17",50460806,"-","none","TGG","ACGACCAAGGCCTCTCGTTCTGG",0,NA,NA,NA,"GeneID:80221","ACSF2",TRUE,"ACSF2"

"1805","CAAGATCGTGGGCCGCTCTA","chr17",50473697,"+","none","AGG","CAAGATCGTGGGCCGCTCTAAGG",0,NA,NA,NA,"GeneID:80221","ACSF2",TRUE,"ACSF2"

"1806","CATCCCGACGTAGACAGCCA","chr17",50426262,"-","none","TGG","CATCCCGACGTAGACAGCCATGG",0,NA,NA,NA,"GeneID:80221","ACSF2",TRUE,"ACSF2"

"1807","CATCCCGACGTAGACAGCCA","chr15",81865447,"+","6:T>G,9:C>G","TGG","CATCCCTACCTAGACAGCCATGG",2,7,10,NA,"","",FALSE,"ACSF2"

"1808","CATCTACCCCGCAGAGCTCG","chr17",50473745,"+","none","AGG","CATCTACCCCGCAGAGCTCGAGG",0,NA,NA,NA,"GeneID:80221","ACSF2",TRUE,"ACSF2"

"1809","CATCTACCCCGCAGAGCTCG","chr1",223247149,"+","10:A>G,13:C>G","TGG","CATCTACCCCACACAGCTCGTGG",2,11,14,NA,"GeneID:55061","SUSD4",FALSE,"ACSF2"

"1810","CGAGCCATGGCTGTCTACGT","chr17",50426255,"+","none","CGG","CGAGCCATGGCTGTCTACGTCGG",0,NA,NA,NA,"GeneID:80221","ACSF2",TRUE,"ACSF2"

"1811","TGCACCCCTGAACGTAGCTG","chr17",50460721,"-","none","AGG","TGCACCCCTGAACGTAGCTGAGG",0,NA,NA,NA,"GeneID:80221","ACSF2",TRUE,"ACSF2"

"1812","AAACTGCACGCAAGAAATCC","chr16",89112210,"-","none","TGG","AAACTGCACGCAAGAAATCCTGG",0,NA,NA,NA,"GeneID:197322","ACSF3",TRUE,"ACSF3"

"1813","CGGTATGGCATGACCGAGAT","chr16",89114420,"+","none","CGG","CGGTATGGCATGACCGAGATCGG",0,NA,NA,NA,"GeneID:197322","ACSF3",TRUE,"ACSF3"

"1814","CTCCCGCTGCACCACGTCCA","chr16",89102660,"+","none","TGG","CTCCCGCTGCACCACGTCCATGG",0,NA,NA,NA,"GeneID:197322","ACSF3",TRUE,"ACSF3"

"1815","CTCCCGCTGCACCACGTCCA","chr8",55452572,"+","13:G>A,15:C>G","GGG","CTCCCGCTGCACCGCCTCCAGGG",2,14,16,NA,"GeneID:114786;GeneID:100133234","XKR4;SBF1P1",TRUE,"ACSF3"

"1816","GCAGCGCGTTGACCACACCA","chr16",89102680,"-","none","TGG","GCAGCGCGTTGACCACACCATGG",0,NA,NA,NA,"GeneID:197322","ACSF3",TRUE,"ACSF3"

"1817","GGACAGAGCCATGCCGATCT","chr16",89114436,"-","none","CGG","GGACAGAGCCATGCCGATCTCGG",0,NA,NA,NA,"GeneID:197322","ACSF3",TRUE,"ACSF3"

"1818","TCTTAAGTTCTGAAACGCCG","chr16",89112104,"+","none","CGG","TCTTAAGTTCTGAAACGCCGCGG",0,NA,NA,NA,"GeneID:197322","ACSF3",TRUE,"ACSF3"

"1819","AACCAGACCAACCCTATGAA","chr4",184783944,"-","none","TGG","AACCAGACCAACCCTATGAATGG",0,NA,NA,NA,"GeneID:2180","ACSL1",TRUE,"ACSL1"

"1820","ACTTGACAGCGACGAGCCCT","chr4",184788682,"-","none","TGG","ACTTGACAGCGACGAGCCCTTGG",0,NA,NA,NA,"GeneID:2180","ACSL1",TRUE,"ACSL1"

"1821","CTCGTCGCTGTCAAGTAGTG","chr4",184788686,"+","none","CGG","CTCGTCGCTGTCAAGTAGTGCGG",0,NA,NA,NA,"GeneID:2180","ACSL1",TRUE,"ACSL1"

"1822","GGGTCTCGTGGCGTACCAGA","chr4",184803370,"+","none","AGG","GGGTCTCGTGGCGTACCAGAAGG",0,NA,NA,NA,"GeneID:2180","ACSL1",TRUE,"ACSL1"

"1823","GGTTTCCGAGAGCCTAAACA","chr4",184783960,"+","none","AGG","GGTTTCCGAGAGCCTAAACAAGG",0,NA,NA,NA,"GeneID:2180","ACSL1",TRUE,"ACSL1"

"1824","TCTTCCGACCAACACGCTTA","chr4",184803423,"-","none","TGG","TCTTCCGACCAACACGCTTATGG",0,NA,NA,NA,"GeneID:2180","ACSL1",TRUE,"ACSL1"

"1825","CAACAGTGATGATGTGCCGC","chr2",222919089,"-","none","AGG","CAACAGTGATGATGTGCCGCAGG",0,NA,NA,NA,"GeneID:2181","ACSL3",TRUE,"ACSL3"

"1826","CGAGTGGATGATAGCTGCAC","chr2",222916449,"+","none","AGG","CGAGTGGATGATAGCTGCACAGG",0,NA,NA,NA,"GeneID:2181","ACSL3",TRUE,"ACSL3"

"1827","CGAGTGGATGATAGCTGCAC","chr8",128482180,"+","1:A>G,8:A>T","AGG","CAAGTGGAAGATAGCTGCACAGG",2,2,9,NA,"","",FALSE,"ACSL3"

"1828","CTCACAGAAGATGGCGATGT","chr2",222916421,"-","none","TGG","CTCACAGAAGATGGCGATGTTGG",0,NA,NA,NA,"GeneID:2181","ACSL3",TRUE,"ACSL3"

"1829","CTCACAGAAGATGGCGATGT","chr3",133760774,"-","9:G>C,15:T>C","TGG","CTCACAGAACATGGCAATGTTGG",2,10,16,NA,"GeneID:7018","TF",TRUE,"ACSL3"

"1830","GCAATGGTTTGCTATGAGGT","chr2",222921286,"-","none","TGG","GCAATGGTTTGCTATGAGGTTGG",0,NA,NA,NA,"GeneID:2181","ACSL3",TRUE,"ACSL3"

"1831","TAACATACCCATGCTGGCCT","chr2",222919190,"-","none","TGG","TAACATACCCATGCTGGCCTTGG",0,NA,NA,NA,"GeneID:2181","ACSL3",TRUE,"ACSL3"

"1832","TCACATAGTAACATTATTGC","chr2",222921371,"+","none","TGG","TCACATAGTAACATTATTGCTGG",0,NA,NA,NA,"GeneID:2181","ACSL3",TRUE,"ACSL3"

"1833","AATCGCAGAGTGAATAACTT","chrX",109682834,"-","none","TGG","AATCGCAGAGTGAATAACTTTGG",0,NA,NA,NA,"GeneID:2182","ACSL4",TRUE,"ACSL4"

"1834","AGGAAAGTTGTACTTAAAGC","chrX",109682719,"+","none","AGG","AGGAAAGTTGTACTTAAAGCAGG",0,NA,NA,NA,"GeneID:2182","ACSL4",TRUE,"ACSL4"

"1835","CATGCAATCAGTAGAAGAGT","chrX",109681018,"-","none","TGG","CATGCAATCAGTAGAAGAGTTGG",0,NA,NA,NA,"GeneID:2182","ACSL4",TRUE,"ACSL4"

"1836","CATGCAATCAGTAGAAGAGT","chr11",79976403,"+","5:T>A,19:C>T","TGG","CATGCTATCAGTAGAAGAGCTGG",2,6,20,NA,"","",FALSE,"ACSL4"

"1837","CATGCAATCAGTAGAAGAGT","chr7",12616882,"-","0:A>G,11:G>A","AGG","TATGCAATCAGCAGAAGAGTAGG",2,1,12,NA,"GeneID:85477","SCIN",FALSE,"ACSL4"

"1838","CTGTGAATCTCAAATCCTTC","chrX",109681038,"+","none","AGG","CTGTGAATCTCAAATCCTTCAGG",0,NA,NA,NA,"GeneID:2182","ACSL4",TRUE,"ACSL4"

"1839","GATTACCAGTGTTGAACTTC","chrX",109681282,"-","none","TGG","GATTACCAGTGTTGAACTTCTGG",0,NA,NA,NA,"GeneID:2182","ACSL4",TRUE,"ACSL4"

"1840","GATTACCAGTGTTGAACTTC","chr12",125073342,"+","2:C>T,10:T>G","TGG","GACTACCAGTTTTGAACTTCTGG",2,3,11,NA,"GeneID:65985","AACS",FALSE,"ACSL4"

"1841","TCATGGGCTAAATGAATCTG","chrX",109681315,"-","none","AGG","TCATGGGCTAAATGAATCTGAGG",0,NA,NA,NA,"GeneID:2182","ACSL4",TRUE,"ACSL4"

"1842","TCATGGGCTAAATGAATCTG","chr6",49886327,"-","12:C>A,15:G>T","GGG","TCATGGGCTAAAGGACTCTGGGG",2,13,16,NA,"","",FALSE,"ACSL4"

"1843","TCATGGGCTAAATGAATCTG","chr18",4850274,"+","11:C>A,17:A>C","GGG","TCATGGGCTAACTGAATATGGGG",2,12,18,NA,"","",FALSE,"ACSL4"

"1844","AGAAGAGGTAAGACGGGTTG","chr10",112395051,"-","none","AGG","AGAAGAGGTAAGACGGGTTGAGG",0,NA,NA,NA,"GeneID:51703","ACSL5",TRUE,"ACSL5"

"1845","CAAAGAGGACTCGCTGTGTC","chr10",112398987,"+","none","TGG","CAAAGAGGACTCGCTGTGTCTGG",0,NA,NA,NA,"GeneID:51703","ACSL5",TRUE,"ACSL5"

"1846","CAGACACAGCGAGTCCTCTT","chr10",112398989,"-","none","TGG","CAGACACAGCGAGTCCTCTTTGG",0,NA,NA,NA,"GeneID:51703","ACSL5",TRUE,"ACSL5"

"1847","GTCAGGATGCAGATCAACGC","chr10",112394991,"-","none","CGG","GTCAGGATGCAGATCAACGCCGG",0,NA,NA,NA,"GeneID:51703","ACSL5",TRUE,"ACSL5"

"1848","TTTAGACAATGGGCCCTGCT","chr10",112404505,"+","none","TGG","TTTAGACAATGGGCCCTGCTTGG",0,NA,NA,NA,"GeneID:51703","ACSL5",TRUE,"ACSL5"

"1849","TTTGTAAGATAGCCATCTGT","chr10",112404552,"-","none","AGG","TTTGTAAGATAGCCATCTGTAGG",0,NA,NA,NA,"GeneID:51703","ACSL5",TRUE,"ACSL5"

"1850","TTTGTAAGATAGCCATCTGT","chr7",6376103,"+","10:G>A,11:C>G","CGG","TTTGTAAGATGCCCATCTGTCGG",2,11,12,NA,"GeneID:5879","RAC1",FALSE,"ACSL5"

"1851","AGCCTAAGCAGCCTTACCAG","chr5",131990117,"-","none","TGG","AGCCTAAGCAGCCTTACCAGTGG",0,NA,NA,NA,"GeneID:23305","ACSL6",TRUE,"ACSL6"

"1852","ATCCGCTACATCATCAATAC","chr5",131988806,"-","none","AGG","ATCCGCTACATCATCAATACAGG",0,NA,NA,NA,"GeneID:23305","ACSL6",TRUE,"ACSL6"

"1853","ATGTACCAGGTGTTCCGCCG","chr5",131990869,"-","none","TGG","ATGTACCAGGTGTTCCGCCGTGG",0,NA,NA,NA,"GeneID:23305","ACSL6",TRUE,"ACSL6"

"1854","CCTCCTGGTAGGACAGCCAC","chr5",131990098,"+","none","TGG","CCTCCTGGTAGGACAGCCACTGG",0,NA,NA,NA,"GeneID:23305","ACSL6",TRUE,"ACSL6"

"1855","CGGCGATCTGTGATTGGGTC","chr5",131990932,"-","none","TGG","CGGCGATCTGTGATTGGGTCTGG",0,NA,NA,NA,"GeneID:23305","ACSL6",TRUE,"ACSL6"

"1856","GGCCTGCTACACATATTCCA","chr5",131988867,"-","none","TGG","GGCCTGCTACACATATTCCATGG",0,NA,NA,NA,"GeneID:23305","ACSL6",TRUE,"ACSL6"

"1857","AACGTCTTCACACAGACCTG","chr16",20685278,"-","none","TGG","AACGTCTTCACACAGACCTGTGG",0,NA,NA,NA,"GeneID:6296;GeneID:116285","ACSM3;ACSM1",TRUE,"ACSM1"

"1858","ACAGGTCTGTGTGAAGACGT","chr16",20685277,"+","none","TGG","ACAGGTCTGTGTGAAGACGTTGG",0,NA,NA,NA,"GeneID:6296;GeneID:116285","ACSM3;ACSM1",TRUE,"ACSM1"

"1859","ACAGGTCTGTGTGAAGACGT","chr10",120408128,"-","6:C>G,17:T>G","GGG","ACAGGTGTGTGTGAAGAAGTGGG",2,7,18,NA,"","",FALSE,"ACSM1"

"1860","AGGGTTGTAAGGCCAACCCA","chr16",20671543,"+","none","TGG","AGGGTTGTAAGGCCAACCCATGG",0,NA,NA,NA,"GeneID:6296;GeneID:116285","ACSM3;ACSM1",TRUE,"ACSM1"

"1861","CAAGGTCTTTGACTTAACAC","chr16",20671628,"+","none","AGG","CAAGGTCTTTGACTTAACACAGG",0,NA,NA,NA,"GeneID:6296;GeneID:116285","ACSM3;ACSM1",TRUE,"ACSM1"

"1862","CAATCCATCCTGAGTCCGAC","chr16",20669921,"+","none","AGG","CAATCCATCCTGAGTCCGACAGG",0,NA,NA,NA,"GeneID:6296;GeneID:116285","ACSM3;ACSM1",TRUE,"ACSM1"

"1863","GTCGGACTCAGGATGGATTG","chr16",20669921,"-","none","TGG","GTCGGACTCAGGATGGATTGTGG",0,NA,NA,NA,"GeneID:6296;GeneID:116285","ACSM3;ACSM1",TRUE,"ACSM1"

"1864","AACGTCCTCTCGGGAGCCTG","chr16",20465621,"+","none","TGG","AACGTCCTCTCGGGAGCCTGTGG",0,NA,NA,NA,"GeneID:123876","ACSM2A",TRUE,"ACSM2A"

"1865","AACGTCCTCTCGGGAGCCTG","chr16",20559322,"-","3:T>C","TGG","AACATCCTCTCGGGAGCCTGTGG",1,4,NA,NA,"GeneID:348158","ACSM2B",TRUE,"ACSM2A"

"1866","AACGTCCTCTCGGGAGCCTG","chr10",25879384,"-","1:C>T,10:A>G","AGG","AGCGTCCTCTTGGGAGCCTGAGG",2,2,11,NA,"","",FALSE,"ACSM2A"

"1867","ACAGGCTCCCGAGAGGACGT","chr16",20465622,"-","none","TGG","ACAGGCTCCCGAGAGGACGTTGG",0,NA,NA,NA,"GeneID:123876","ACSM2A",TRUE,"ACSM2A"

"1868","ACAGGCTCCCGAGAGGACGT","chr16",20559321,"+","17:T>C","TGG","ACAGGCTCCCGAGAGGATGTTGG",1,18,NA,NA,"GeneID:348158","ACSM2B",TRUE,"ACSM2A"

"1869","AGTAGCTTAATTCTCAGAGA","chr16",20555319,"+","none","AGG","AGTAGCTTAATTCTCAGAGAAGG",0,NA,NA,NA,"GeneID:348158","ACSM2B",TRUE,"ACSM2A"

"1870","AGTAGCTTAATTCTCAGAGA","chr16",20469648,"-","none","AGG","AGTAGCTTAATTCTCAGAGAAGG",0,NA,NA,NA,"GeneID:123876","ACSM2A",TRUE,"ACSM2A"

"1871","AGTAGCTTAATTCTCAGAGA","chr20",1576510,"-","2:C>A,5:T>G","AGG","AGGAGATTAATTCTCAGAGAAGG",2,3,6,NA,"GeneID:10326","SIRPB1",FALSE,"ACSM2A"

"1872","AGTAGCTTAATTCTCAGAGA","chr9",74291116,"-","4:A>C,9:G>T","TGG","AGTATCTTACTTCTCAGAGATGG",2,5,10,NA,"","",FALSE,"ACSM2A"

"1873","GAACATCTTGTGCTCACTTA","chr16",20471595,"+","none","TGG","GAACATCTTGTGCTCACTTATGG",0,NA,NA,NA,"GeneID:123876","ACSM2A",TRUE,"ACSM2A"

"1874","GAACATCTTGTGCTCACTTA","chr16",20552217,"-","10:C>A,19:A>T","TGG","GAACATCTTGGGCTCACTTTTGG",2,11,20,NA,"GeneID:348158","ACSM2B",TRUE,"ACSM2A"

"1875","GTGCATGCTCCTAATGCCCA","chr16",20471623,"-","none","AGG","GTGCATGCTCCTAATGCCCAAGG",0,NA,NA,NA,"GeneID:123876","ACSM2A",TRUE,"ACSM2A"

"1876","GTGCATGCTCCTAATGCCCA","chr6",32973390,"+","5:G>T,7:T>C","GGG","GTGCAGGTTCCTAATGCCCAGGG",2,6,8,NA,"GeneID:6046","BRD2",FALSE,"ACSM2A"

"1877","TGTCTGAGAAAAGCTGTGAT","chr16",20469670,"+","none","GGG","TGTCTGAGAAAAGCTGTGATGGG",0,NA,NA,NA,"GeneID:123876","ACSM2A",TRUE,"ACSM2A"

"1878","TGTCTGAGAAAAGCTGTGAT","chr16",20555297,"-","16:G>A","GGG","TGTCTGAGAAAAGCTGCGATGGG",1,17,NA,NA,"GeneID:348158","ACSM2B",TRUE,"ACSM2A"

"1879","TGTCTGAGAAAAGCTGTGAT","chr13",30681894,"-","9:A>T","AGG","TGTCTGAGATAAGCTGTGATAGG",1,10,NA,NA,"","",FALSE,"ACSM2A"

"1880","AACATCCTCTCGGGAGCCTG","chr16",20559322,"-","none","TGG","AACATCCTCTCGGGAGCCTGTGG",0,NA,NA,NA,"GeneID:348158","ACSM2B",TRUE,"ACSM2B"

"1881","AACATCCTCTCGGGAGCCTG","chr16",20465621,"+","3:G>A","TGG","AACGTCCTCTCGGGAGCCTGTGG",1,4,NA,NA,"GeneID:123876","ACSM2A",TRUE,"ACSM2B"

"1882","AACATCCTCTCGGGAGCCTG","chr11",33715059,"-","4:C>A,16:C>G","AGG","AACAGCCTCTCGGGAGGCTGAGG",2,5,17,NA,"GeneID:966","CD59",FALSE,"ACSM2B"

"1883","ACAGGCTCCCGAGAGGATGT","chr16",20559321,"+","none","TGG","ACAGGCTCCCGAGAGGATGTTGG",0,NA,NA,NA,"GeneID:348158","ACSM2B",TRUE,"ACSM2B"

"1884","ACAGGCTCCCGAGAGGATGT","chr16",20465622,"-","17:G>A","TGG","ACAGGCTCCCGAGAGGACGTTGG",1,18,NA,NA,"GeneID:123876","ACSM2A",TRUE,"ACSM2B"

"1885","AGCTTACCCAGCATCCATCT","chr16",20553769,"+","none","TGG","AGCTTACCCAGCATCCATCTTGG",0,NA,NA,NA,"GeneID:348158","ACSM2B",TRUE,"ACSM2B"

"1886","AGCTTACCCAGCATCCATCT","chr16",20471203,"-","none","TGG","AGCTTACCCAGCATCCATCTTGG",0,NA,NA,NA,"GeneID:123876","ACSM2A",TRUE,"ACSM2B"

"1887","AGCTTACCCAGCATCCATCT","chr3",81033831,"+","3:A>T,10:T>G","GGG","AGCATACCCATCATCCATCTGGG",2,4,11,NA,"GeneID:728290","LOC728290",FALSE,"ACSM2B"

"1888","AGCTTACCCAGCATCCATCT","chr14",80526321,"-","3:G>A,11:A>G","GGG","AGCCTACCCAGTATCCATCTGGG",2,4,12,NA,"GeneID:145508","CEP128",FALSE,"ACSM2B"

"1889","ATCCACCACTCATCACTGTG","chr16",20471078,"+","none","TGG","ATCCACCACTCATCACTGTGTGG",0,NA,NA,NA,"GeneID:123876","ACSM2A",TRUE,"ACSM2B"

"1890","ATCCACCACTCATCACTGTG","chr16",20553894,"-","none","TGG","ATCCACCACTCATCACTGTGTGG",0,NA,NA,NA,"GeneID:348158","ACSM2B",TRUE,"ACSM2B"

"1891","TGTCTGAGAAAAGCTGCGAT","chr16",20555297,"-","none","GGG","TGTCTGAGAAAAGCTGCGATGGG",0,NA,NA,NA,"GeneID:348158","ACSM2B",TRUE,"ACSM2B"

"1892","TGTCTGAGAAAAGCTGCGAT","chr16",20469670,"+","16:T>C","GGG","TGTCTGAGAAAAGCTGTGATGGG",1,17,NA,NA,"GeneID:123876","ACSM2A",TRUE,"ACSM2B"

"1893","TGTCTGAGAAAAGCTGCGAT","chr13",30681894,"-","9:A>T,16:A>G","AGG","TGTCTGAGATAAGCTGTGATAGG",2,10,17,NA,"","",FALSE,"ACSM2B"

"1894","TTAGGTCTCATCTTTATGCC","chr16",20469507,"+","none","TGG","TTAGGTCTCATCTTTATGCCTGG",0,NA,NA,NA,"GeneID:123876","ACSM2A",TRUE,"ACSM2B"

"1895","TTAGGTCTCATCTTTATGCC","chr16",20555460,"-","none","TGG","TTAGGTCTCATCTTTATGCCTGG",0,NA,NA,NA,"GeneID:348158","ACSM2B",TRUE,"ACSM2B"

"1896","TTAGGTCTCATCTTTATGCC","chr1",246874263,"-","6:C>G,19:T>G","AGG","TTAGGTGTCATCTTTATGCAAGG",2,7,20,NA,"GeneID:25909","AHCTF1",FALSE,"ACSM2B"

"1897","GATGCAACAGCGTCTACTGC","chr16",20777479,"-","none","TGG","GATGCAACAGCGTCTACTGCTGG",0,NA,NA,NA,"GeneID:6296","ACSM3",TRUE,"ACSM3"

"1898","GGCATGACGTAGCATCTTCC","chr16",20770053,"-","none","TGG","GGCATGACGTAGCATCTTCCTGG",0,NA,NA,NA,"GeneID:6296","ACSM3",TRUE,"ACSM3"

"1899","GTTCTCTGATACAATCAGCT","chr16",20777522,"-","none","TGG","GTTCTCTGATACAATCAGCTTGG",0,NA,NA,NA,"GeneID:6296","ACSM3",TRUE,"ACSM3"

"1900","TGATGCTAGCTCGTGTCACC","chr16",20770032,"+","none","AGG","TGATGCTAGCTCGTGTCACCAGG",0,NA,NA,NA,"GeneID:6296","ACSM3",TRUE,"ACSM3"

"1901","TTACCCGATCTCCTCTTTGT","chr16",20775969,"-","none","AGG","TTACCCGATCTCCTCTTTGTAGG",0,NA,NA,NA,"GeneID:6296","ACSM3",TRUE,"ACSM3"

"1902","TTACTAACCTGTTCGCAGAC","chr16",20776037,"-","none","AGG","TTACTAACCTGTTCGCAGACAGG",0,NA,NA,NA,"GeneID:6296","ACSM3",TRUE,"ACSM3"

"1903","AATACAATGGACTCCACCGC","chr12",7310645,"-","none","TGG","AATACAATGGACTCCACCGCTGG",0,NA,NA,NA,"GeneID:341392","ACSM4",TRUE,"ACSM4"

"1904","AATGTAGCTTGCATACGAAC","chr12",7306721,"+","none","AGG","AATGTAGCTTGCATACGAACAGG",0,NA,NA,NA,"GeneID:341392","ACSM4",TRUE,"ACSM4"

"1905","CCCAGGGATCATCTTCATGC","chr12",7310533,"+","none","CGG","CCCAGGGATCATCTTCATGCCGG",0,NA,NA,NA,"GeneID:341392","ACSM4",TRUE,"ACSM4"

"1906","TCAACCTACCTGGACATCAT","chr12",7323303,"-","none","AGG","TCAACCTACCTGGACATCATAGG",0,NA,NA,NA,"GeneID:341392","ACSM4",TRUE,"ACSM4"

"1907","TGAAGTCCTCAGATATCATA","chr12",7318039,"+","none","TGG","TGAAGTCCTCAGATATCATATGG",0,NA,NA,NA,"GeneID:341392","ACSM4",TRUE,"ACSM4"

"1908","TGGGCAGAATCACGGCCAAA","chr12",7306678,"-","none","CGG","TGGGCAGAATCACGGCCAAACGG",0,NA,NA,NA,"GeneID:341392","ACSM4",TRUE,"ACSM4"

"1909","CGTACAGCTCCACACCAGTC","chr16",20429758,"-","none","TGG","CGTACAGCTCCACACCAGTCTGG",0,NA,NA,NA,"GeneID:54988","ACSM5",TRUE,"ACSM5"

"1910","CGTACAGCTCCACACCAGTC","chr16",20593852,"+","16:G>A","TGG","CGTACAGCTCCACACCGGTCTGG",1,17,NA,NA,"","",TRUE,"ACSM5"

"1911","CTGCACAAGCAGCCGAAAGA","chr16",20595778,"+","none","TGG","CTGCACAAGCAGCCGAAAGATGG",0,NA,NA,NA,"","",TRUE,"ACSM5"

"1912","CTGCACAAGCAGCCGAAAGA","chr16",20427833,"-","none","TGG","CTGCACAAGCAGCCGAAAGATGG",0,NA,NA,NA,"GeneID:54988","ACSM5",TRUE,"ACSM5"

"1913","CTGCACAAGCAGCCGAAAGA","chr15",79463102,"+","4:C>A,14:C>G","TGG","CTGCCCAAGCAGCCCAAAGATGG",2,5,15,NA,"GeneID:23251","KIAA1024",TRUE,"ACSM5"

"1914","GAGAAGTGGAAACACCAGAC","chr16",20429741,"+","none","TGG","GAGAAGTGGAAACACCAGACTGG",0,NA,NA,NA,"GeneID:54988","ACSM5",TRUE,"ACSM5"

"1915","GAGAAGTGGAAACACCAGAC","chr16",20593869,"-","13:C>T","CGG","GAGAAGTGGAAACGCCAGACCGG",1,14,NA,NA,"","",TRUE,"ACSM5"

"1916","GAGAAGTGGAAACACCAGAC","chr11",111526335,"+","0:C>G,12:A>C","TGG","CAGAAGTGGAAAAACCAGACTGG",2,1,13,NA,"GeneID:399949","C11orf88",FALSE,"ACSM5"

"1917","GAGAAGTGGAAACACCAGAC","chr8",134230749,"-","6:C>A,18:A>T","AGG","GAGAAGGGGAAACACCAGTCAGG",2,7,19,NA,"","",FALSE,"ACSM5"

"1918","GCTGTGTCCCAACCATCTTT","chr16",20427818,"+","none","CGG","GCTGTGTCCCAACCATCTTTCGG",0,NA,NA,NA,"GeneID:54988","ACSM5",TRUE,"ACSM5"

"1919","GCTGTGTCCCAACCATCTTT","chr16",20595793,"-","7:A>G","CGG","GCTGTGTTCCAACCATCTTTCGG",1,8,NA,NA,"","",TRUE,"ACSM5"

"1920","TCTTACATTCAGGATAACTT","chr16",20424055,"-","none","TGG","TCTTACATTCAGGATAACTTTGG",0,NA,NA,NA,"GeneID:54988","ACSM5",TRUE,"ACSM5"

"1921","TCTTACATTCAGGATAACTT","chr16",20599556,"+","17:A>C","TGG","TCTTACATTCAGGATAAATTTGG",1,18,NA,NA,"","",TRUE,"ACSM5"

"1922","TGACCGAATCTGACATCTTC","chr16",20423929,"+","none","TGG","TGACCGAATCTGACATCTTCTGG",0,NA,NA,NA,"GeneID:54988","ACSM5",TRUE,"ACSM5"

"1923","TGACCGAATCTGACATCTTC","chr16",20599682,"-","10:C>A,14:C>T","TGG","TGACCGAATCGGACGTCTTCTGG",2,11,15,NA,"","",TRUE,"ACSM5"

"1924","ACGAGAGTGTCCCGCGCCAG","chr20",25057857,"+","none","AGG","ACGAGAGTGTCCCGCGCCAGAGG",0,NA,NA,NA,"GeneID:84532","ACSS1",TRUE,"ACSS1"

"1925","CCAGATCAAAGCAACGCTCT","chr20",25048125,"+","none","CGG","CCAGATCAAAGCAACGCTCTCGG",0,NA,NA,NA,"GeneID:84532","ACSS1",TRUE,"ACSS1"

"1926","CCGAGAGCGTTGCTTTGATC","chr20",25048128,"-","none","TGG","CCGAGAGCGTTGCTTTGATCTGG",0,NA,NA,NA,"GeneID:84532","ACSS1",TRUE,"ACSS1"

"1927","GCCGGCCATGCGGTGCGAAG","chr20",25032366,"+","none","TGG","GCCGGCCATGCGGTGCGAAGTGG",0,NA,NA,NA,"GeneID:84532","ACSS1",TRUE,"ACSS1"

"1928","TGGCGCGGGACACTCTCGTG","chr20",25057856,"-","none","TGG","TGGCGCGGGACACTCTCGTGTGG",0,NA,NA,NA,"GeneID:84532","ACSS1",TRUE,"ACSS1"

"1929","TTCCTCCACTTCGCACCGCA","chr20",25032374,"-","none","TGG","TTCCTCCACTTCGCACCGCATGG",0,NA,NA,NA,"GeneID:84532","ACSS1",TRUE,"ACSS1"

"1930","CCACAGTAGACATGTCCCCG","chr20",34927138,"-","none","AGG","CCACAGTAGACATGTCCCCGAGG",0,NA,NA,NA,"GeneID:55902","ACSS2",TRUE,"ACSS2"

"1931","GACTACATCCAGAATGCACC","chr20",34926908,"+","none","TGG","GACTACATCCAGAATGCACCTGG",0,NA,NA,NA,"GeneID:55902","ACSS2",TRUE,"ACSS2"

"1932","GCTCAGAATGACCATGACCT","chr20",34927118,"+","none","CGG","GCTCAGAATGACCATGACCTCGG",0,NA,NA,NA,"GeneID:55902","ACSS2",TRUE,"ACSS2"

"1933","GGTGCATTCTGGATGTAGTC","chr20",34926908,"-","none","TGG","GGTGCATTCTGGATGTAGTCTGG",0,NA,NA,NA,"GeneID:55902","ACSS2",TRUE,"ACSS2"

"1934","GGTGCATTCTGGATGTAGTC","chr19",17436564,"+","15:C>T,18:G>T","AGG","GGTGCATTCTGGATGCAGGCAGG",2,16,19,NA,"GeneID:100130519","TMEM221",TRUE,"ACSS2"

"1935","TACAACTTTGATGTGACTAA","chr20",34882858,"+","none","AGG","TACAACTTTGATGTGACTAAAGG",0,NA,NA,NA,"GeneID:55902","ACSS2",TRUE,"ACSS2"

"1936","TCACATCAAAGTTGTACCGA","chr20",34882854,"-","none","AGG","TCACATCAAAGTTGTACCGAAGG",0,NA,NA,NA,"GeneID:55902","ACSS2",TRUE,"ACSS2"

"1937","CTCCCGGTCGTGACCTTGAT","chr12",81143122,"+","none","TGG","CTCCCGGTCGTGACCTTGATTGG",0,NA,NA,NA,"GeneID:79611","ACSS3",TRUE,"ACSS3"

"1938","GTTGATCGTCATATTGAAAA","chr12",81109596,"+","none","TGG","GTTGATCGTCATATTGAAAATGG",0,NA,NA,NA,"GeneID:79611","ACSS3",TRUE,"ACSS3"

"1939","GTTGCTTTAGTGTTTGTAAC","chr12",81109656,"-","none","AGG","GTTGCTTTAGTGTTTGTAACAGG",0,NA,NA,NA,"GeneID:79611","ACSS3",TRUE,"ACSS3"

"1940","TACATCGCCTGTGGGATCAT","chr12",81134887,"-","none","AGG","TACATCGCCTGTGGGATCATAGG",0,NA,NA,NA,"GeneID:79611","ACSS3",TRUE,"ACSS3"

"1941","TAGTCGCATTGATCATGTAA","chr12",81134982,"+","none","AGG","TAGTCGCATTGATCATGTAAAGG",0,NA,NA,NA,"GeneID:79611","ACSS3",TRUE,"ACSS3"

"1942","TGTGTAGGAGGCGGTTCCTT","chr12",81143099,"+","none","TGG","TGTGTAGGAGGCGGTTCCTTTGG",0,NA,NA,NA,"GeneID:79611","ACSS3",TRUE,"ACSS3"

"1943","TGTGTAGGAGGCGGTTCCTT","chr1",2150218,"-","0:C>A,4:C>A","GGG","GGTGGAGGAGGCGGTTCCTTGGG",2,1,5,NA,"GeneID:5590","PRKCZ",FALSE,"ACSS3"

"1944","CAACGAGCGCTTCCGCTGCC","chr1",229432023,"-","none","CGG","CAACGAGCGCTTCCGCTGCCCGG",0,NA,NA,NA,"GeneID:58","ACTA1",TRUE,"ACTA1"

"1945","CAACGTGCCCGCCATGTACG","chr1",229432603,"-","none","TGG","CAACGTGCCCGCCATGTACGTGG",0,NA,NA,NA,"GeneID:58","ACTA1",TRUE,"ACTA1"

"1946","CGGTGAGATCGCGGCCCGCC","chr1",229432320,"+","none","AGG","CGGTGAGATCGCGGCCCGCCAGG",0,NA,NA,NA,"GeneID:58","ACTA1",TRUE,"ACTA1"

"1947","GCCCCCCGACATGACGTTGT","chr1",229431798,"+","none","TGG","GCCCCCCGACATGACGTTGTTGG",0,NA,NA,NA,"GeneID:58","ACTA1",TRUE,"ACTA1"

"1948","GGGGCACCACGATGTACCCT","chr1",229431783,"-","none","GGG","GGGGCACCACGATGTACCCTGGG",0,NA,NA,NA,"GeneID:58","ACTA1",TRUE,"ACTA1"

"1949","GTTGTGGGTGACGCCGTCGC","chr1",229432393,"+","none","CGG","GTTGTGGGTGACGCCGTCGCCGG",0,NA,NA,NA,"GeneID:58","ACTA1",TRUE,"ACTA1"

"1950","CCTACCAGTTGTGCGTCCAG","chr10",88941779,"+","none","AGG","CCTACCAGTTGTGCGTCCAGAGG",0,NA,NA,NA,"GeneID:57559;GeneID:59","STAMBPL1;ACTA2",TRUE,"ACTA2"

"1951","CTGAAGTACCCGATAGAACA","chr10",88947291,"-","none","TGG","CTGAAGTACCCGATAGAACATGG",0,NA,NA,NA,"GeneID:57559;GeneID:59","STAMBPL1;ACTA2",TRUE,"ACTA2"

"1952","CTGAAGTACCCGATAGAACA","chr2",130075153,"-","11:G>C,14:C>T","CGG","CTGAAGTACCCCATGGAACACGG",2,12,15,NA,"GeneID:728378","POTEF",TRUE,"ACTA2"

"1953","CTGAAGTACCCGATAGAACA","chr2",130463725,"-","11:G>C,14:C>T","CGG","CTGAAGTACCCCATGGAACACGG",2,12,15,NA,"GeneID:653269","POTEI",TRUE,"ACTA2"

"1954","CTGAAGTACCCGATAGAACA","chr2",130656947,"+","11:C>G,14:G>A","CGG","CTGAAGTACCCCATGGAACACGG",2,12,15,NA,"GeneID:653781","POTEJ",TRUE,"ACTA2"

"1955","CTGAAGTACCCGATAGAACA","chr2",131263753,"+","11:C>G,14:G>A","CGG","CTGAAGTACCCCATGGAACACGG",2,12,15,NA,"GeneID:55041;GeneID:445582","PLEKHB2;POTEE",TRUE,"ACTA2"

"1956","GGCCTCACCAGTAGTAACGA","chr10",88941220,"+","none","AGG","GGCCTCACCAGTAGTAACGAAGG",0,NA,NA,NA,"GeneID:57559;GeneID:59","STAMBPL1;ACTA2",TRUE,"ACTA2"

"1957","GGGACAAAAAGACAGCTACG","chr10",88947349,"-","none","TGG","GGGACAAAAAGACAGCTACGTGG",0,NA,NA,NA,"GeneID:57559;GeneID:59","STAMBPL1;ACTA2",TRUE,"ACTA2"

"1958","TCAGGGGTGCCTCCGTGAGC","chr10",88943828,"+","none","AGG","TCAGGGGTGCCTCCGTGAGCAGG",0,NA,NA,NA,"GeneID:57559;GeneID:59","STAMBPL1;ACTA2",TRUE,"ACTA2"

"1959","TCAGGGGTGCCTCCGTGAGC","chr22",15721797,"-","13:C>G,16:G>C","AGG","TCAGGGGTGCCTCGGTCAGCAGG",2,14,17,NA,"","",TRUE,"ACTA2"

"1960","TCAGGGGTGCCTCCGTGAGC","chr14",18999178,"-","13:C>G,16:G>C","AGG","TCAGGGGTGCCTCGGTCAGCAGG",2,14,17,NA,"","",FALSE,"ACTA2"

"1961","TCAGGGGTGCCTCCGTGAGC","chr5",57482373,"+","13:G>C,19:G>C","AGG","TCAGGGGTGCCTCGGTGAGGAGG",2,14,20,NA,"GeneID:345651","ACTBL2",TRUE,"ACTA2"

"1962","TCAGGGGTGCCTCCGTGAGC","chr14",19402577,"+","13:G>C,16:C>G","AGG","TCAGGGGTGCCTCGGTCAGCAGG",2,14,17,NA,"GeneID:404785","POTEG",TRUE,"ACTA2"

"1963","TCAGGGGTGCCTCCGTGAGC","chr14",101841984,"-","2:A>T,7:C>A","TGG","TCTGGGGGGCCTCCGTGAGCTGG",2,3,8,NA,"GeneID:5527","PPP2R5C",FALSE,"ACTA2"

"1964","TCAGGGGTGCCTCCGTGAGC","chr17",81655143,"-","6:T>C,19:A>G","GGG","TCAGGGATGCCTCCGTGAGTGGG",2,7,20,NA,"GeneID:5148","PDE6G",FALSE,"ACTA2"

"1965","TTGTCTCCCAGGCATCGTGC","chr10",88941381,"-","none","TGG","TTGTCTCCCAGGCATCGTGCTGG",0,NA,NA,NA,"GeneID:57559;GeneID:59","STAMBPL1;ACTA2",TRUE,"ACTA2"

"1966","AGCCATGTACGTTGCTATCC","chr7",5528673,"-","none","AGG","AGCCATGTACGTTGCTATCCAGG",0,NA,NA,NA,"GeneID:60","ACTB",TRUE,"ACTB"

"1967","AGCCATGTACGTTGCTATCC","chr2",130074962,"-","12:C>A,15:G>A","AGG","AGCCATGTACGTGGCCATCCAGG",2,13,16,NA,"GeneID:728378","POTEF",TRUE,"ACTB"

"1968","AGCCATGTACGTTGCTATCC","chr2",130463534,"-","12:C>A,15:G>A","AGG","AGCCATGTACGTGGCCATCCAGG",2,13,16,NA,"GeneID:653269","POTEI",TRUE,"ACTB"

"1969","AGCCATGTACGTTGCTATCC","chr1",223864443,"-","12:C>A,15:G>A","AGG","AGCCATGTACGTGGCCATCCAGG",2,13,16,NA,"","",TRUE,"ACTB"

"1970","AGCCATGTACGTTGCTATCC","chr15",43989773,"-","12:C>A,15:G>A","AGG","AGCCATGTACGTGGCCATCCAGG",2,13,16,NA,"GeneID:84978","FRMD5",TRUE,"ACTB"

"1971","AGCCATGTACGTTGCTATCC","chr2",131626527,"+","12:G>T,15:C>T","AGG","AGCCATGTACGTGGCCATCCAGG",2,13,16,NA,"","",TRUE,"ACTB"

"1972","AGCCATGTACGTTGCTATCC","chr2",130657138,"+","12:G>T,15:C>T","AGG","AGCCATGTACGTGGCCATCCAGG",2,13,16,NA,"GeneID:653781","POTEJ",TRUE,"ACTB"

"1973","AGCCATGTACGTTGCTATCC","chr2",131263944,"+","12:G>T,15:C>T","AGG","AGCCATGTACGTGGCCATCCAGG",2,13,16,NA,"GeneID:55041;GeneID:445582","PLEKHB2;POTEE",TRUE,"ACTB"

"1974","AGCCATGTACGTTGCTATCC","chr10",88941823,"-","9:A>G,12:C>A","AGG","AGCCATGTATGTGGCTATCCAGG",2,10,13,NA,"GeneID:57559;GeneID:59","STAMBPL1;ACTA2",TRUE,"ACTB"

"1975","CAACCGCGAGAAGATGACCC","chr7",5529162,"-","none","AGG","CAACCGCGAGAAGATGACCCAGG",0,NA,NA,NA,"GeneID:60","ACTB",TRUE,"ACTB"

"1976","CACCTTGATCTTCATTGTGC","chr7",5528000,"+","none","TGG","CACCTTGATCTTCATTGTGCTGG",0,NA,NA,NA,"GeneID:60","ACTB",TRUE,"ACTB"

"1977","CACCTTGATCTTCATTGTGC","chr2",73916748,"-","15:C>A","TGG","CACCTTGATCTTCATGGTGCTGG",1,16,NA,NA,"GeneID:72","ACTG2",TRUE,"ACTB"

"1978","CACCTTGATCTTCATTGTGC","chr17",81510923,"+","15:G>T","TGG","CACCTTGATCTTCATGGTGCTGG",1,16,NA,NA,"GeneID:71","ACTG1",TRUE,"ACTB"

"1979","CACCTTGATCTTCATTGTGC","chr1",229431717,"+","15:G>T","TGG","CACCTTGATCTTCATGGTGCTGG",1,16,NA,NA,"GeneID:58","ACTA1",TRUE,"ACTB"

"1980","CACCTTGATCTTCATTGTGC","chr15",43989201,"+","0:G>C,2:T>C","TGG","GATCTTGATCTTCATTGTGCTGG",2,1,3,NA,"GeneID:84978","FRMD5",TRUE,"ACTB"

"1981","CACCTTGATCTTCATTGTGC","chr10",88938057,"+","0:T>C,15:G>T","TGG","TACCTTGATCTTCATGGTGCTGG",2,1,16,NA,"GeneID:57559;GeneID:100132116;GeneID:59","STAMBPL1;ACTA2-AS1;ACTA2",TRUE,"ACTB"

"1982","CGTACAGGGATAGCACAGCC","chr7",5528652,"+","none","TGG","CGTACAGGGATAGCACAGCCTGG",0,NA,NA,NA,"GeneID:60","ACTB",TRUE,"ACTB"

"1983","CGTACAGGGATAGCACAGCC","chr5",80300374,"+","10:C>T,16:G>A","TGG","CGTACAGGGACAGCACGGCCTGG",2,11,17,NA,"","",TRUE,"ACTB"

"1984","CGTACAGGGATAGCACAGCC","chr1",223864422,"+","10:C>T,15:G>C","TGG","CGTACAGGGACAGCAGAGCCTGG",2,11,16,NA,"","",TRUE,"ACTB"

"1985","CGTACAGGGATAGCACAGCC","chr2",130074941,"+","0:T>C,10:C>T","TGG","TGTACAGGGACAGCACAGCCTGG",2,1,11,NA,"GeneID:728378","POTEF",TRUE,"ACTB"

"1986","CGTACAGGGATAGCACAGCC","chr15",34793262,"+","1:A>G,16:T>A","TGG","CATACAGGGATAGCACTGCCTGG",2,2,17,NA,"GeneID:101928174;GeneID:70","LOC101928174;ACTC1",TRUE,"ACTB"

"1987","CGTACAGGGATAGCACAGCC","chr4",93328879,"+","3:C>A,19:A>C","AGG","CGTCCAGGGATAGCACAGCAAGG",2,4,20,NA,"GeneID:2895","GRID2",FALSE,"ACTB"

"1988","CGTACAGGGATAGCACAGCC","chr1",202873696,"-","0:A>G,10:G>A","TGG","TGTACAGGGACAGCACAGCCTGG",2,1,11,NA,"GeneID:148709","LOC148709",TRUE,"ACTB"

"1989","CTCTCAGGCATGGAGTCCTG","chr7",5528172,"-","none","TGG","CTCTCAGGCATGGAGTCCTGTGG",0,NA,NA,NA,"GeneID:60","ACTB",TRUE,"ACTB"

"1990","CTCTCAGGCATGGAGTCCTG","chr9",126977981,"-","9:C>T","TGG","CTCTCAGGCGTGGAGTCCTGTGG",1,10,NA,NA,"GeneID:9649","RALGPS1",TRUE,"ACTB"

"1991","CTCTCAGGCATGGAGTCCTG","chr1",76239087,"+","0:T>C,9:C>A","GGG","TTCTCAGGCCTGGAGTCCTGGGG",2,1,10,NA,"GeneID:256435","ST6GALNAC3",FALSE,"ACTB"

"1992","TCCTTCCCAGGGCGTGATGG","chr7",5529390,"-","none","TGG","TCCTTCCCAGGGCGTGATGGTGG",0,NA,NA,NA,"GeneID:60","ACTB",TRUE,"ACTB"

"1993","GCCTCATGCCATTCTACGCC","chr5",57482172,"-","none","TGG","GCCTCATGCCATTCTACGCCTGG",0,NA,NA,NA,"GeneID:345651","ACTBL2",TRUE,"ACTBL2"

"1994","GGAAGTTCATAGCTCCGTTC","chr5",57481976,"+","none","CGG","GGAAGTTCATAGCTCCGTTCCGG",0,NA,NA,NA,"GeneID:345651","ACTBL2",TRUE,"ACTBL2"

"1995","TCCTCGACACCAGGGCGTTA","chr5",57482574,"-","none","TGG","TCCTCGACACCAGGGCGTTATGG",0,NA,NA,NA,"GeneID:345651","ACTBL2",TRUE,"ACTBL2"

"1996","TCGAGGACGCCCTATCATGG","chr5",57482587,"+","none","AGG","TCGAGGACGCCCTATCATGGAGG",0,NA,NA,NA,"GeneID:345651","ACTBL2",TRUE,"ACTBL2"

"1997","TCGGAAGCGTTCATTCCCAA","chr5",57481936,"+","none","TGG","TCGGAAGCGTTCATTCCCAATGG",0,NA,NA,NA,"GeneID:345651","ACTBL2",TRUE,"ACTBL2"

"1998","TGCCAGATCCAGGCGTAGAA","chr5",57482161,"+","none","TGG","TGCCAGATCCAGGCGTAGAATGG",0,NA,NA,NA,"GeneID:345651","ACTBL2",TRUE,"ACTBL2"

"1999","CTACAACTCACCAATGAAGG","chr15",34792078,"+","none","AGG","CTACAACTCACCAATGAAGGAGG",0,NA,NA,NA,"GeneID:101928174;GeneID:70","LOC101928174;ACTC1",TRUE,"ACTC1"

"2000","GTGCTATCCCTGTATGCTTC","chr15",34793258,"-","none","TGG","GTGCTATCCCTGTATGCTTCTGG",0,NA,NA,NA,"GeneID:101928174;GeneID:70","LOC101928174;ACTC1",TRUE,"ACTC1"

"2001","GTGCTATCCCTGTATGCTTC","chr7",5528648,"-","14:G>A,17:G>A","TGG","GTGCTATCCCTGTACGCCTCTGG",2,15,18,NA,"GeneID:60","ACTB",TRUE,"ACTC1"

"2002","GTGCTATCCCTGTATGCTTC","chr6",88276573,"-","5:C>T,17:G>A","TGG","GTGCTGTCCCTGTATGCCTCTGG",2,6,18,NA,"","",TRUE,"ACTC1"

"2003","GTGTGACATTGATATCCGCA","chr15",34791226,"-","none","AGG","GTGTGACATTGATATCCGCAAGG",0,NA,NA,NA,"GeneID:101928174;GeneID:70","LOC101928174;ACTC1",TRUE,"ACTC1"

"2004","GTGTGACATTGATATCCGCA","chr7",100646752,"-","15:A>G,19:G>T","CGG","GTGTGACATTGATATTCGCCCGG",2,16,20,NA,"GeneID:51412","ACTL6B",TRUE,"ACTC1"

"2005","GTGTGACATTGATATCCGCA","chr2",73916632,"+","12:C>T,18:T>C","AGG","GTGTGACATTGACATCCGTAAGG",2,13,19,NA,"GeneID:72","ACTG2",TRUE,"ACTC1"

"2006","TCCCCGGGCAGGCATTGTTC","chr15",34792560,"-","none","TGG","TCCCCGGGCAGGCATTGTTCTGG",0,NA,NA,NA,"GeneID:101928174;GeneID:70","LOC101928174;ACTC1",TRUE,"ACTC1"

"2007","TGCATACGATCAGCAATACC","chr15",34791156,"+","none","AGG","TGCATACGATCAGCAATACCAGG",0,NA,NA,NA,"GeneID:101928174;GeneID:70","LOC101928174;ACTC1",TRUE,"ACTC1"

"2008","TGGTACGGCCAGAAGCATAC","chr15",34793247,"+","none","AGG","TGGTACGGCCAGAAGCATACAGG",0,NA,NA,NA,"GeneID:101928174;GeneID:70","LOC101928174;ACTC1",TRUE,"ACTC1"

"2009","TGGTACGGCCAGAAGCATAC","chr5",77785311,"-","13:C>T,16:C>T","AGG","TGGTACGGCCAGAGGCGTACAGG",2,14,17,NA,"GeneID:6902","TBCA",TRUE,"ACTC1"

"2010","TGGTACGGCCAGAAGCATAC","chr9",100731656,"-","13:C>T,16:C>T","AGG","TGGTACGGCCAGAGGCGTACAGG",2,14,17,NA,"","",TRUE,"ACTC1"

"2011","TGGTACGGCCAGAAGCATAC","chr1",223864407,"+","13:G>A,16:G>A","AGG","TGGTACGGCCAGAGGCGTACAGG",2,14,17,NA,"","",TRUE,"ACTC1"

"2012","TGGTACGGCCAGAAGCATAC","chr7",5528637,"+","13:G>A,16:G>A","AGG","TGGTACGGCCAGAGGCGTACAGG",2,14,17,NA,"GeneID:60","ACTB",TRUE,"ACTC1"

"2013","AGCTGTAGCCTCGCTCAGTG","chr17",81511391,"+","none","AGG","AGCTGTAGCCTCGCTCAGTGAGG",0,NA,NA,NA,"GeneID:71","ACTG1",TRUE,"ACTG1"

"2014","AGCTGTAGCCTCGCTCAGTG","chr1",237927413,"-","10:T>A,16:C>T","AGG","AGCTGTAGCCACGCTCGGTGAGG",2,11,17,NA,"GeneID:100130331","LOC100130331",TRUE,"ACTG1"

"2015","AGCTGTAGCCTCGCTCAGTG","chr18",62442588,"-","10:C>A,16:C>T","AGG","AGCTGTAGCCGCGCTCGGTGAGG",2,11,17,NA,"","",TRUE,"ACTG1"

"2016","AGCTGTAGCCTCGCTCAGTG","chr5",61832456,"+","11:T>C,16:T>A","AGG","AGCTGTAGCCTTGCTCTGTGAGG",2,12,17,NA,"","",TRUE,"ACTG1"

"2017","AGCTGTAGCCTCGCTCAGTG","chr15",34374073,"+","10:A>T,16:G>A","AGG","AGCTGTAGCCACGCTCGGTGAGG",2,11,17,NA,"","",TRUE,"ACTG1"

"2018","AGCTGTAGCCTCGCTCAGTG","chr7",5528484,"+","10:G>T,16:G>A","AGG","AGCTGTAGCCGCGCTCGGTGAGG",2,11,17,NA,"GeneID:60","ACTB",TRUE,"ACTG1"

"2019","AGCTGTAGCCTCGCTCAGTG","chr1",223864254,"+","10:G>T,16:C>A","AGG","AGCTGTAGCCGCGCTCCGTGAGG",2,11,17,NA,"","",TRUE,"ACTG1"

"2020","AGCTGTAGCCTCGCTCAGTG","chr22",41074686,"+","10:G>T,11:T>C","AGG","AGCTGTAGCCGTGCTCAGTGAGG",2,11,12,NA,"","",TRUE,"ACTG1"

"2021","CAACACCTACCCAGGAAGGA","chr17",81511177,"+","none","AGG","CAACACCTACCCAGGAAGGAAGG",0,NA,NA,NA,"GeneID:71","ACTG1",TRUE,"ACTG1"

"2022","CAAGCAGGAGTACGACGAGT","chr17",81510724,"-","none","CGG","CAAGCAGGAGTACGACGAGTCGG",0,NA,NA,NA,"GeneID:71","ACTG1",TRUE,"ACTG1"

"2023","CAAGCAGGAGTACGACGAGT","chr1",92229047,"-","12:A>G,16:T>C","TGG","CAAGCAGGAGTATGACAAGTTGG",2,13,17,NA,"GeneID:388649","C1orf146",TRUE,"ACTG1"

"2024","CAAGCAGGAGTACGACGAGT","chr6",46206046,"+","13:A>G","CGG","CAAGCAGGAGTACAACGAGTCGG",1,14,NA,NA,"","",TRUE,"ACTG1"

"2025","CAAGCAGGAGTACGACGAGT","chr3",12071309,"+","13:A>G,16:A>G","TGG","CAAGCAGGAGTACAACAAGTTGG",2,14,17,NA,"GeneID:6854","SYN2",TRUE,"ACTG1"

"2026","CAAGCAGGAGTACGACGAGT","chr20",1161643,"+","12:T>C,16:A>G","CGG","CAAGCAGGAGTATGACAAGTCGG",2,13,17,NA,"GeneID:9491","PSMF1",TRUE,"ACTG1"

"2027","CAAGCAGGAGTACGACGAGT","chr3",139494889,"+","12:T>C,15:T>C","TGG","CAAGCAGGAGTATGATGAGTTGG",2,13,16,NA,"GeneID:100507291","LOC100507291",TRUE,"ACTG1"

"2028","CCCCTAGGTATGGAATCTTG","chr17",81511095,"-","none","CGG","CCCCTAGGTATGGAATCTTGCGG",0,NA,NA,NA,"GeneID:71","ACTG1",TRUE,"ACTG1"

"2029","CCCCTAGGTATGGAATCTTG","chr5",24988647,"-","1:T>G,3:A>G","AGG","CACTTAGGTATGGAATCTTGAGG",2,2,4,NA,"","",FALSE,"ACTG1"

"2030","TCCCCTGCAGGGCGTCATGG","chr17",81512132,"-","none","TGG","TCCCCTGCAGGGCGTCATGGTGG",0,NA,NA,NA,"GeneID:71","ACTG1",TRUE,"ACTG1"

"2031","TCCCCTGCAGGGCGTCATGG","chr5",83817044,"-","12:T>G,17:C>A","AGG","TCCCCTGCAGGGAGTCAGGGAGG",2,13,18,NA,"","",FALSE,"ACTG1"

"2032","TCGACTCACCTTGATCTTCA","chr17",81510917,"+","none","TGG","TCGACTCACCTTGATCTTCATGG",0,NA,NA,NA,"GeneID:71","ACTG1",TRUE,"ACTG1"

"2033","ACCGCGCTCGTGTGTGACAA","chr2",73901329,"+","none","TGG","ACCGCGCTCGTGTGTGACAATGG",0,NA,NA,NA,"GeneID:72","ACTG2",TRUE,"ACTG2"

"2034","ACCGCGCTCGTGTGTGACAA","chr1",229433074,"-","5:G>C,14:G>A","TGG","ACCGCCCTCGTGTGCGACAATGG",2,6,15,NA,"GeneID:58","ACTA1",TRUE,"ACTG2"

"2035","CCACTAGGCATGGAGTCCGC","chr2",73916576,"+","none","TGG","CCACTAGGCATGGAGTCCGCTGG",0,NA,NA,NA,"GeneID:72","ACTG2",TRUE,"ACTG2"

"2036","GGTCACGGCCAGCCAAGTCC","chr2",73913569,"-","none","AGG","GGTCACGGCCAGCCAAGTCCAGG",0,NA,NA,NA,"GeneID:72","ACTG2",TRUE,"ACTG2"

"2037","GGTCACGGCCAGCCAAGTCC","chr1",11444959,"+","6:T>G,16:A>G","TGG","GGTCACTGCCAGCCAAATCCTGG",2,7,17,NA,"","",FALSE,"ACTG2"

"2038","GGTCACGGCCAGCCAAGTCC","chr14",94930541,"-","5:T>G,18:C>G","AGG","GGTCAAGGCCAGCCAAGTGCAGG",2,6,19,NA,"","",FALSE,"ACTG2"

"2039","GTGTGACATTGACATCCGTA","chr2",73916632,"+","none","AGG","GTGTGACATTGACATCCGTAAGG",0,NA,NA,NA,"GeneID:72","ACTG2",TRUE,"ACTG2"

"2040","GTGTGACATTGACATCCGTA","chr15",34791226,"-","12:A>G,18:G>A","AGG","GTGTGACATTGATATCCGCAAGG",2,13,19,NA,"GeneID:101928174;GeneID:70","LOC101928174;ACTC1",TRUE,"ACTG2"

"2041","TCTGTCCACAGGCATCGTCC","chr2",73913473,"+","none","TGG","TCTGTCCACAGGCATCGTCCTGG",0,NA,NA,NA,"GeneID:72","ACTG2",TRUE,"ACTG2"

"2042","TGAGCACGCACCTGGTGGCG","chr2",73901428,"-","none","AGG","TGAGCACGCACCTGGTGGCGAGG",0,NA,NA,NA,"GeneID:72","ACTG2",TRUE,"ACTG2"

"2043","ACATGGCCCGAGTTATCCAG","chr20",33668171,"-","none","CGG","ACATGGCCCGAGTTATCCAGCGG",0,NA,NA,NA,"GeneID:63941;GeneID:170487","NECAB3;ACTL10",TRUE,"ACTL10"

"2044","CATGGCTAGCACCGCGTTGC","chr20",33667496,"+","none","TGG","CATGGCTAGCACCGCGTTGCTGG",0,NA,NA,NA,"GeneID:63941;GeneID:170487","NECAB3;ACTL10",TRUE,"ACTL10"

"2045","CCACGAGTGACCCGCGTAGA","chr20",33667591,"-","none","TGG","CCACGAGTGACCCGCGTAGATGG",0,NA,NA,NA,"GeneID:63941;GeneID:170487","NECAB3;ACTL10",TRUE,"ACTL10"

"2046","CCCGTTACCCACGCTGAAAC","chr20",33667810,"-","none","TGG","CCCGTTACCCACGCTGAAACTGG",0,NA,NA,NA,"GeneID:63941;GeneID:170487","NECAB3;ACTL10",TRUE,"ACTL10"

"2047","GGGCCGCGTAGCCGTGCCGC","chr20",33668060,"-","none","CGG","GGGCCGCGTAGCCGTGCCGCCGG",0,NA,NA,NA,"GeneID:63941;GeneID:170487","NECAB3;ACTL10",TRUE,"ACTL10"

"2048","TGCTCCACCGGCGCGTTCAG","chr20",33667524,"+","none","CGG","TGCTCCACCGGCGCGTTCAGCGG",0,NA,NA,NA,"GeneID:63941;GeneID:170487","NECAB3;ACTL10",TRUE,"ACTL10"

"2049","GTACCGAAGCTTGAAAATCC","chr3",179580650,"-","none","TGG","GTACCGAAGCTTGAAAATCCTGG",0,NA,NA,NA,"GeneID:86","ACTL6A",TRUE,"ACTL6A"

"2050","GTTGAAGGACATAGCCATCG","chr3",179576288,"-","none","TGG","GTTGAAGGACATAGCCATCGTGG",0,NA,NA,NA,"GeneID:86","ACTL6A",TRUE,"ACTL6A"

"2051","TCGTTCTACTGGGCTGATTT","chr3",179576228,"+","none","TGG","TCGTTCTACTGGGCTGATTTTGG",0,NA,NA,NA,"GeneID:86","ACTL6A",TRUE,"ACTL6A"

"2052","TCTCAGGAAGCTGTTCGTGA","chr3",179576817,"+","none","AGG","TCTCAGGAAGCTGTTCGTGAAGG",0,NA,NA,NA,"GeneID:86","ACTL6A",TRUE,"ACTL6A"

"2053","TCTCAGGAAGCTGTTCGTGA","chr8",19853968,"-","0:C>A,3:C>G","TGG","GCTGAGGAAGCTGTTCGTGATGG",2,1,4,NA,"","",FALSE,"ACTL6A"

"2054","TGCCAAGACCTCGTAACCTG","chr3",179576877,"-","none","AGG","TGCCAAGACCTCGTAACCTGAGG",0,NA,NA,NA,"GeneID:86","ACTL6A",TRUE,"ACTL6A"

"2055","TGCCAAGACCTCGTAACCTG","chr2",130028466,"+","11:G>C,13:A>T","GGG","TGCCAAGACCTGGAAACCTGGGG",2,12,14,NA,"GeneID:440905","FAR2P1",TRUE,"ACTL6A"

"2056","TTACTCACAGTGTGTTATCC","chr3",179580629,"+","none","AGG","TTACTCACAGTGTGTTATCCAGG",0,NA,NA,NA,"GeneID:86","ACTL6A",TRUE,"ACTL6A"

"2057","ACTATGAGCGGGGGCGTCTA","chr7",100656337,"-","none","CGG","ACTATGAGCGGGGGCGTCTACGG",0,NA,NA,NA,"GeneID:51412","ACTL6B",TRUE,"ACTL6B"

"2058","ATGACCTCCGCTCCATCCCG","chr7",100655443,"+","none","AGG","ATGACCTCCGCTCCATCCCGAGG",0,NA,NA,NA,"GeneID:51412","ACTL6B",TRUE,"ACTL6B"

"2059","GCAGGGCATTGGTGTCGATG","chr7",100655471,"+","none","TGG","GCAGGGCATTGGTGTCGATGTGG",0,NA,NA,NA,"GeneID:51412","ACTL6B",TRUE,"ACTL6B"

"2060","GCAGGGCATTGGTGTCGATG","chr21",9866098,"+","8:C>T,15:A>C","GGG","GCAGGGCACTGGTGTAGATGGGG",2,9,16,NA,"","",FALSE,"ACTL6B"

"2061","GCAGGGCATTGGTGTCGATG","chr9",67754860,"+","8:C>T,15:A>C","GGG","GCAGGGCACTGGTGTAGATGGGG",2,9,16,NA,"","",FALSE,"ACTL6B"

"2062","GCAGGGCATTGGTGTCGATG","chr9",61825956,"-","8:G>A,15:T>G","GGG","GCAGGGCACTGGTGTAGATGGGG",2,9,16,NA,"","",FALSE,"ACTL6B"

"2063","GCAGGGCATTGGTGTCGATG","chr9",41615802,"-","8:G>A,15:T>G","GGG","GCAGGGCACTGGTGTAGATGGGG",2,9,16,NA,"","",FALSE,"ACTL6B"

"2064","GGCTCCTTCTCAGTCCGCGC","chr7",100655830,"-","none","TGG","GGCTCCTTCTCAGTCCGCGCTGG",0,NA,NA,NA,"GeneID:51412","ACTL6B",TRUE,"ACTL6B"

"2065","GTCATGTCGCCCCTCAAGAA","chr7",100655428,"-","none","TGG","GTCATGTCGCCCCTCAAGAATGG",0,NA,NA,NA,"GeneID:51412","ACTL6B",TRUE,"ACTL6B"

"2066","TCCCCGCACAGATGAGGTGG","chr7",100655870,"-","none","GGG","TCCCCGCACAGATGAGGTGGGGG",0,NA,NA,NA,"GeneID:51412","ACTL6B",TRUE,"ACTL6B"

"2067","ACCCACACGGCCCGCTTCGC","chr9",108862433,"-","none","CGG","ACCCACACGGCCCGCTTCGCCGG",0,NA,NA,NA,"GeneID:10881","ACTL7A",TRUE,"ACTL7A"

"2068","CAAAGGATAACCCTCGTAGA","chr9",108863007,"-","none","TGG","CAAAGGATAACCCTCGTAGATGG",0,NA,NA,NA,"GeneID:10881","ACTL7A",TRUE,"ACTL7A"

"2069","CGTGTTCCTCGTACTCAAAG","chr9",108863578,"-","none","CGG","CGTGTTCCTCGTACTCAAAGCGG",0,NA,NA,NA,"GeneID:10881","ACTL7A",TRUE,"ACTL7A"

"2070","GGTCCACCGCTTTGAGTACG","chr9",108863569,"+","none","AGG","GGTCCACCGCTTTGAGTACGAGG",0,NA,NA,NA,"GeneID:10881","ACTL7A",TRUE,"ACTL7A"

"2071","GTGTCCCAATGACACCCCGC","chr9",108863464,"+","none","AGG","GTGTCCCAATGACACCCCGCAGG",0,NA,NA,NA,"GeneID:10881","ACTL7A",TRUE,"ACTL7A"

"2072","TACGATCCGCTACGTGCTGC","chr9",108863212,"+","none","CGG","TACGATCCGCTACGTGCTGCCGG",0,NA,NA,NA,"GeneID:10881","ACTL7A",TRUE,"ACTL7A"

"2073","ACCAGCCGCGCCGACTACGC","chr9",108855256,"-","none","TGG","ACCAGCCGCGCCGACTACGCTGG",0,NA,NA,NA,"GeneID:10880","ACTL7B",TRUE,"ACTL7B"

"2074","ATAGTGATGAGTTTGCCGTC","chr9",108855058,"+","none","CGG","ATAGTGATGAGTTTGCCGTCCGG",0,NA,NA,NA,"GeneID:10880","ACTL7B",TRUE,"ACTL7B"

"2075","ATCCGAGGGCGACGTGCTGC","chr9",108855284,"-","none","CGG","ATCCGAGGGCGACGTGCTGCCGG",0,NA,NA,NA,"GeneID:10880","ACTL7B",TRUE,"ACTL7B"

"2076","CGTAGCCGCACTTGCAGTAC","chr9",108855734,"+","none","TGG","CGTAGCCGCACTTGCAGTACTGG",0,NA,NA,NA,"GeneID:10880","ACTL7B",TRUE,"ACTL7B"

"2077","CTTGCGGGTGTCGCCAGCGT","chr9",108855648,"+","none","CGG","CTTGCGGGTGTCGCCAGCGTCGG",0,NA,NA,NA,"GeneID:10880","ACTL7B",TRUE,"ACTL7B"

"2078","GCCCGGCAGCACGTCGCCCT","chr9",108855279,"+","none","CGG","GCCCGGCAGCACGTCGCCCTCGG",0,NA,NA,NA,"GeneID:10880","ACTL7B",TRUE,"ACTL7B"

"2079","ACGGATTCCACAATGGCCCG","chr1",17826237,"-","none","TGG","ACGGATTCCACAATGGCCCGTGG",0,NA,NA,NA,"GeneID:81569","ACTL8",TRUE,"ACTL8"

"2080","CACACGCCTACGGGCATAGC","chr1",17823141,"-","none","TGG","CACACGCCTACGGGCATAGCTGG",0,NA,NA,NA,"GeneID:81569","ACTL8",TRUE,"ACTL8"

"2081","CCGCTCGATGGGGTAGCTAA","chr1",17823195,"-","none","AGG","CCGCTCGATGGGGTAGCTAAAGG",0,NA,NA,NA,"GeneID:81569","ACTL8",TRUE,"ACTL8"

"2082","GAACTCCAGCGTCTTGCCGC","chr1",17825923,"-","none","TGG","GAACTCCAGCGTCTTGCCGCTGG",0,NA,NA,NA,"GeneID:81569","ACTL8",TRUE,"ACTL8"

"2083","GATAACGGTTCTTGCAGCCA","chr1",17823009,"-","none","TGG","GATAACGGTTCTTGCAGCCATGG",0,NA,NA,NA,"GeneID:81569","ACTL8",TRUE,"ACTL8"

"2084","GTGCTACGTGCCGCAGAATC","chr1",17826050,"+","none","TGG","GTGCTACGTGCCGCAGAATCTGG",0,NA,NA,NA,"GeneID:81569","ACTL8",TRUE,"ACTL8"

"2085","CCACGACGATGCCGCTGCGC","chr19",8698340,"+","none","AGG","CCACGACGATGCCGCTGCGCAGG",0,NA,NA,NA,"GeneID:284382","ACTL9",TRUE,"ACTL9"

"2086","CCTGCGCAGCGGCATCGTCG","chr19",8698343,"-","none","TGG","CCTGCGCAGCGGCATCGTCGTGG",0,NA,NA,NA,"GeneID:284382","ACTL9",TRUE,"ACTL9"

"2087","CGACTGCGATGCCACGTACA","chr19",8698158,"+","none","TGG","CGACTGCGATGCCACGTACATGG",0,NA,NA,NA,"GeneID:284382","ACTL9",TRUE,"ACTL9"

"2088","GTCCGCTTGTACTCCTGCTC","chr19",8697865,"+","none","CGG","GTCCGCTTGTACTCCTGCTCCGG",0,NA,NA,NA,"GeneID:284382","ACTL9",TRUE,"ACTL9"

"2089","TAAGCACCACTATTGCTACG","chr19",8697920,"-","none","TGG","TAAGCACCACTATTGCTACGTGG",0,NA,NA,NA,"GeneID:284382","ACTL9",TRUE,"ACTL9"

"2090","TGGGGAGCGCAGCGACTCGA","chr19",8698182,"+","none","AGG","TGGGGAGCGCAGCGACTCGAAGG",0,NA,NA,NA,"GeneID:284382","ACTL9",TRUE,"ACTL9"

"2091","CCAGCGGACCGAGAAACTGC","chr14",68884292,"-","none","TGG","CCAGCGGACCGAGAAACTGCTGG",0,NA,NA,NA,"GeneID:87","ACTN1",TRUE,"ACTN1"

"2092","GATCCAAACAGGTGAACGCT","chr14",68921116,"-","none","TGG","GATCCAAACAGGTGAACGCTTGG",0,NA,NA,NA,"GeneID:87","ACTN1",TRUE,"ACTN1"

"2093","GCTGGCCTCACCGTCAGTCC","chr14",68890127,"+","none","AGG","GCTGGCCTCACCGTCAGTCCAGG",0,NA,NA,NA,"GeneID:87","ACTN1",TRUE,"ACTN1"

"2094","GGCCTTACCTTCGGCTCCGA","chr14",68920997,"+","none","TGG","GGCCTTACCTTCGGCTCCGATGG",0,NA,NA,NA,"GeneID:87","ACTN1",TRUE,"ACTN1"

"2095","TAACTCCCACCTCCGGAAGG","chr14",68925635,"-","none","CGG","TAACTCCCACCTCCGGAAGGCGG",0,NA,NA,NA,"GeneID:87","ACTN1",TRUE,"ACTN1"

"2096","TGGCATACTCCAAGTACAGC","chr14",68884256,"+","none","TGG","TGGCATACTCCAAGTACAGCTGG",0,NA,NA,NA,"GeneID:87","ACTN1",TRUE,"ACTN1"

"2097","AAAGGCTGCCCAAACCTGAC","chr1",236718896,"+","none","CGG","AAAGGCTGCCCAAACCTGACCGG",0,NA,NA,NA,"GeneID:88","ACTN2",TRUE,"ACTN2"

"2098","AAAGGCTGCCCAAACCTGAC","chr13",36345786,"+","13:C>A,15:T>C","GGG","AAAGGCTGCCCAACCTTGACGGG",2,14,16,NA,"GeneID:23111","SPG20",FALSE,"ACTN2"

"2099","CACGTAGTTGTACTGCACGC","chr1",236686692,"-","none","CGG","CACGTAGTTGTACTGCACGCCGG",0,NA,NA,NA,"GeneID:88","ACTN2",TRUE,"ACTN2"

"2100","CATCCTTCGCTTTGCTATTC","chr1",236720153,"+","none","AGG","CATCCTTCGCTTTGCTATTCAGG",0,NA,NA,NA,"GeneID:88","ACTN2",TRUE,"ACTN2"

"2101","CCTCTCACCTTCAGCGCCAA","chr1",236719001,"-","none","TGG","CCTCTCACCTTCAGCGCCAATGG",0,NA,NA,NA,"GeneID:88","ACTN2",TRUE,"ACTN2"

"2102","CCTCTCACCTTCAGCGCCAA","chr16",66461856,"-","14:T>G,18:G>T","GGG","CCTCTCACCTTCAGAGCCCAGGG",2,15,19,NA,"GeneID:146227","BEAN1",FALSE,"ACTN2"

"2103","CCTCTCACCTTCAGCGCCAA","chr1",164912296,"+","15:A>G,16:G>C","TGG","CCTCTCACCTTCAGCAGCAATGG",2,16,17,NA,"","",FALSE,"ACTN2"

"2104","GTACAACTACGTGTACGACG","chr1",236686699,"+","none","AGG","GTACAACTACGTGTACGACGAGG",0,NA,NA,NA,"GeneID:88","ACTN2",TRUE,"ACTN2"

"2105","TATCCTGAATAGCAAAGCGA","chr1",236720159,"-","none","AGG","TATCCTGAATAGCAAAGCGAAGG",0,NA,NA,NA,"GeneID:88","ACTN2",TRUE,"ACTN2"

"2106","ACTCTCATCTTCCCCCGCTC","chr19",38701021,"-","none","CGG","ACTCTCATCTTCCCCCGCTCCGG",0,NA,NA,NA,"GeneID:81","ACTN4",TRUE,"ACTN4"

"2107","ACTCTCATCTTCCCCCGCTC","chr14",68921085,"+","11:G>C,14:T>C","TGG","ACTCTCATCTTGCCTCGCTCTGG",2,12,15,NA,"GeneID:87","ACTN1",TRUE,"ACTN4"

"2108","ACTCTCATCTTCCCCCGCTC","chr11",34719257,"-","4:T>A,15:T>G","AGG","ACTCACATCTTCCCCAGCTCAGG",2,5,16,NA,"","",FALSE,"ACTN4"

"2109","CAACAATGTGAACAAAGCGC","chr19",38701050,"+","none","TGG","CAACAATGTGAACAAAGCGCTGG",0,NA,NA,NA,"GeneID:81","ACTN4",TRUE,"ACTN4"

"2110","CATCCTTAGGTTCGCCATCC","chr19",38704982,"+","none","AGG","CATCCTTAGGTTCGCCATCCAGG",0,NA,NA,NA,"GeneID:81","ACTN4",TRUE,"ACTN4"

"2111","CATCCTTAGGTTCGCCATCC","chr11",66554093,"+","7:C>A,9:C>G","AGG","CATCCTTCGCTTCGCCATCCAGG",2,8,10,NA,"GeneID:89","ACTN3",TRUE,"ACTN4"

"2112","GCATGAGCTTGAGCCCGTCT","chr19",38700673,"-","none","CGG","GCATGAGCTTGAGCCCGTCTCGG",0,NA,NA,NA,"GeneID:81","ACTN4",TRUE,"ACTN4"

"2113","GGAGTTGCACCATGCCGTGA","chr19",38700603,"-","none","AGG","GGAGTTGCACCATGCCGTGAAGG",0,NA,NA,NA,"GeneID:81","ACTN4",TRUE,"ACTN4"

"2114","TGCACATTGACGTTCTTATA","chr19",38706096,"-","none","CGG","TGCACATTGACGTTCTTATACGG",0,NA,NA,NA,"GeneID:81","ACTN4",TRUE,"ACTN4"

"2115","TGCACATTGACGTTCTTATA","chr11",22350289,"-","10:A>G,18:T>A","TGG","TGCACATTGATGTTCTTAAATGG",2,11,19,NA,"GeneID:57084","SLC17A6",FALSE,"ACTN4"

"2116","AATGTTGACTATCCATTAGA","chr14",58223800,"+","none","TGG","AATGTTGACTATCCATTAGATGG",0,NA,NA,NA,"GeneID:145407;GeneID:55860","C14orf37;ACTR10",TRUE,"ACTR10"

"2117","AATGTTGACTATCCATTAGA","chr10",72939914,"+","4:G>T,13:A>C","TGG","AATGGTGACTATCAATTAGATGG",2,5,14,NA,"GeneID:84647","PLA2G12B",FALSE,"ACTR10"

"2118","CCTAGTGTGATAAAAAGAGC","chr14",58202894,"+","none","TGG","CCTAGTGTGATAAAAAGAGCTGG",0,NA,NA,NA,"GeneID:145407;GeneID:55860","C14orf37;ACTR10",TRUE,"ACTR10"

"2119","CTTACCTGAAATATAGTATG","chr14",58208003,"-","none","TGG","CTTACCTGAAATATAGTATGTGG",0,NA,NA,NA,"GeneID:145407;GeneID:55860","C14orf37;ACTR10",TRUE,"ACTR10"

"2120","CTTACCTGAAATATAGTATG","chr5",126997094,"+","2:G>T,12:T>A","AGG","CTGACCTGAAATTTAGTATGAGG",2,3,13,NA,"GeneID:115123","MARCH3",FALSE,"ACTR10"

"2121","GATCTAACCTGATTGATCCA","chr14",58223844,"-","none","AGG","GATCTAACCTGATTGATCCAAGG",0,NA,NA,NA,"GeneID:145407;GeneID:55860","C14orf37;ACTR10",TRUE,"ACTR10"

"2122","GCTCCCCAACAATTTAGAAC","chr14",58213648,"-","none","TGG","GCTCCCCAACAATTTAGAACTGG",0,NA,NA,NA,"GeneID:145407;GeneID:55860","C14orf37;ACTR10",TRUE,"ACTR10"

"2123","TTATACTGAACAACTCTGAC","chr14",58207938,"-","none","AGG","TTATACTGAACAACTCTGACAGG",0,NA,NA,NA,"GeneID:145407;GeneID:55860","C14orf37;ACTR10",TRUE,"ACTR10"

"2124","AAACTTCGGCAGCTCGTTCC","chr10",102485666,"+","none","CGG","AAACTTCGGCAGCTCGTTCCCGG",0,NA,NA,NA,"GeneID:10121","ACTR1A",TRUE,"ACTR1A"

"2125","ACCCAAGCACGTTCGTGTCA","chr10",102489109,"-","none","TGG","ACCCAAGCACGTTCGTGTCATGG",0,NA,NA,NA,"GeneID:10121","ACTR1A",TRUE,"ACTR1A"

"2126","AGCATGGCATCGTCAAGGAT","chr10",102488218,"-","none","TGG","AGCATGGCATCGTCAAGGATTGG",0,NA,NA,NA,"GeneID:10121","ACTR1A",TRUE,"ACTR1A"

"2127","AGCATGGCATCGTCAAGGAT","chr2",110184281,"+","10:T>C,19:C>T","TGG","AGCATGGCATTGTCAAGGACTGG",2,11,20,NA,"GeneID:4867","NPHP1",TRUE,"ACTR1A"

"2128","GCTTTCAATCCGCTATCCCA","chr10",102488241,"-","none","TGG","GCTTTCAATCCGCTATCCCATGG",0,NA,NA,NA,"GeneID:10121","ACTR1A",TRUE,"ACTR1A"

"2129","GGTTTAAAGGCGCCTCAGTC","chr10",102485699,"+","none","AGG","GGTTTAAAGGCGCCTCAGTCAGG",0,NA,NA,NA,"GeneID:10121","ACTR1A",TRUE,"ACTR1A"

"2130","GGTTTAAAGGCGCCTCAGTC","chr2",110184384,"-","10:A>G,19:C>G","AGG","GGTTTAAAGGTGCCTCAGTGAGG",2,11,20,NA,"GeneID:4867","NPHP1",TRUE,"ACTR1A"

"2131","TGCCATGACACGAACGTGCT","chr10",102489104,"+","none","TGG","TGCCATGACACGAACGTGCTTGG",0,NA,NA,NA,"GeneID:10121","ACTR1A",TRUE,"ACTR1A"

"2132","AGCACGGCGTGGTGCGAGAC","chr2",97659420,"-","none","TGG","AGCACGGCGTGGTGCGAGACTGG",0,NA,NA,NA,"GeneID:10120","ACTR1B",TRUE,"ACTR1B"

"2133","CGCTCAACCCGAGTAAGAAC","chr2",97658958,"-","none","CGG","CGCTCAACCCGAGTAAGAACCGG",0,NA,NA,NA,"GeneID:10120","ACTR1B",TRUE,"ACTR1B"

"2134","CTGGCAGTACGTCTACTCCA","chr2",97659380,"-","none","AGG","CTGGCAGTACGTCTACTCCAAGG",0,NA,NA,NA,"GeneID:10120","ACTR1B",TRUE,"ACTR1B"

"2135","GAACAGGGCCGGCACGTTGA","chr2",97658904,"+","none","AGG","GAACAGGGCCGGCACGTTGAAGG",0,NA,NA,NA,"GeneID:10120","ACTR1B",TRUE,"ACTR1B"

"2136","GGTTCGGGGGTGATTAAAGC","chr2",97661926,"-","none","TGG","GGTTCGGGGGTGATTAAAGCTGG",0,NA,NA,NA,"GeneID:10120","ACTR1B",TRUE,"ACTR1B"

"2137","TCGGGCGGCCGAAGCACATG","chr2",97660624,"-","none","CGG","TCGGGCGGCCGAAGCACATGCGG",0,NA,NA,NA,"GeneID:10120","ACTR1B",TRUE,"ACTR1B"

"2138","CACATTTGCCCAGTATATGA","chr2",65253771,"+","none","AGG","CACATTTGCCCAGTATATGAAGG",0,NA,NA,NA,"GeneID:10097","ACTR2",TRUE,"ACTR2"

"2139","CACATTTGCCCAGTATATGA","chr22",24687414,"+","2:T>C","AGG","CATATTTGCCCAGTATATGAAGG",1,3,NA,NA,"","",TRUE,"ACTR2"

"2140","CGTACAAAGTCAGAACTGCC","chr2",65251073,"-","none","TGG","CGTACAAAGTCAGAACTGCCTGG",0,NA,NA,NA,"GeneID:10097","ACTR2",TRUE,"ACTR2"

"2141","CGTACAAAGTCAGAACTGCC","chr19",20258655,"-","none","TGG","CGTACAAAGTCAGAACTGCCTGG",0,NA,NA,NA,"","",TRUE,"ACTR2"

"2142","CGTACAAAGTCAGAACTGCC","chr22",24687344,"-","16:G>A,18:A>G","TGG","CGTACAAAGTCAGAACCGTCTGG",2,17,19,NA,"","",TRUE,"ACTR2"

"2143","CGTACAAAGTCAGAACTGCC","chr1",200706998,"+","0:G>C,8:A>G","TGG","GGTACAAAATCAGAACTGCCTGG",2,1,9,NA,"","",FALSE,"ACTR2"

"2144","CTACATATACACCGGAAAAC","chr19",20258628,"-","none","TGG","CTACATATACACCGGAAAACTGG",0,NA,NA,NA,"","",TRUE,"ACTR2"

"2145","CTACATATACACCGGAAAAC","chr2",65251046,"-","none","TGG","CTACATATACACCGGAAAACTGG",0,NA,NA,NA,"GeneID:10097","ACTR2",TRUE,"ACTR2"

"2146","CTACATATACACCGGAAAAC","chr14",98187974,"+","7:A>T,19:T>C","AGG","CTACATAAACACCGGAAAATAGG",2,8,20,NA,"","",FALSE,"ACTR2"

"2147","GATTTCAATGTTTCCCACTT","chr2",65239939,"-","none","TGG","GATTTCAATGTTTCCCACTTTGG",0,NA,NA,NA,"GeneID:10097","ACTR2",TRUE,"ACTR2"

"2148","GATTTCAATGTTTCCCACTT","chr19",20258369,"-","none","TGG","GATTTCAATGTTTCCCACTTTGG",0,NA,NA,NA,"","",TRUE,"ACTR2"

"2149","TATAACTAGATATCTTATCA","chr2",65253842,"+","none","AGG","TATAACTAGATATCTTATCAAGG",0,NA,NA,NA,"GeneID:10097","ACTR2",TRUE,"ACTR2"

"2150","TCATTCCAGTTTGTGAAGTG","chr2",65239842,"+","none","TGG","TCATTCCAGTTTGTGAAGTGTGG",0,NA,NA,NA,"GeneID:10097","ACTR2",TRUE,"ACTR2"

"2151","AAGTGGGTGATCAAGCTCAA","chr2",113916907,"+","none","AGG","AAGTGGGTGATCAAGCTCAAAGG",0,NA,NA,NA,"GeneID:10096","ACTR3",TRUE,"ACTR3"

"2152","AAGTGGGTGATCAAGCTCAA","chrX",68771445,"+","14:T>G","AGG","AAGTGGGTGATCAATCTCAAAGG",1,15,NA,NA,"","",TRUE,"ACTR3"

"2153","ACGTCCAAAGTCCCTGAACA","chr2",113951747,"-","none","TGG","ACGTCCAAAGTCCCTGAACATGG",0,NA,NA,NA,"GeneID:10096","ACTR3",TRUE,"ACTR3"

"2154","CACTAGAATATTGTCCTCTC","chr2",113951713,"+","none","TGG","CACTAGAATATTGTCCTCTCTGG",0,NA,NA,NA,"GeneID:10096","ACTR3",TRUE,"ACTR3"

"2155","CATACCTTTGTTGCATATGT","chr2",113916993,"-","none","AGG","CATACCTTTGTTGCATATGTAGG",0,NA,NA,NA,"GeneID:10096","ACTR3",TRUE,"ACTR3"

"2156","GCTATAAATACCTTGTAGAG","chr2",113951562,"-","none","AGG","GCTATAAATACCTTGTAGAGAGG",0,NA,NA,NA,"GeneID:10096","ACTR3",TRUE,"ACTR3"

"2157","TGTCAGACGTCCTCTCTACA","chr2",113951549,"+","none","AGG","TGTCAGACGTCCTCTCTACAAGG",0,NA,NA,NA,"GeneID:10096","ACTR3",TRUE,"ACTR3"

"2158","TGTCAGACGTCCTCTCTACA","chrX",49217808,"-","2:T>A,8:T>C","GGG","TGACAGACATCCTCTCTACAGGG",2,3,9,NA,"GeneID:778","CACNA1F",TRUE,"ACTR3"

"2159","AGGCTCCCTGCCTCCCTGCG","chr7",152759887,"+","none","TGG","AGGCTCCCTGCCTCCCTGCGTGG",0,NA,NA,NA,"GeneID:57180","ACTR3B",TRUE,"ACTR3B"

"2160","AGGCTCCCTGCCTCCCTGCG","chr10",26360905,"-","14:A>G,19:T>C","TGG","AGGCTCCCTGCCTCTCTGCATGG",2,15,20,NA,"","",FALSE,"ACTR3B"

"2161","AGGCTCCCTGCCTCCCTGCG","chr21",18245122,"-","2:G>C,19:G>C","GGG","AGCCTCCCTGCCTCCCTGCCGGG",2,3,20,NA,"GeneID:140578","CHODL",TRUE,"ACTR3B"

"2162","CTCCAGTGGCCGATACGACA","chr7",152801614,"+","none","TGG","CTCCAGTGGCCGATACGACATGG",0,NA,NA,NA,"GeneID:57180","ACTR3B",TRUE,"ACTR3B"

"2163","CTCCAGTGGCCGATACGACA","chr7",149891287,"-","none","TGG","CTCCAGTGGCCGATACGACATGG",0,NA,NA,NA,"","",TRUE,"ACTR3B"

"2164","GGTGCCACAGTCCACCACGC","chr7",152759904,"-","none","AGG","GGTGCCACAGTCCACCACGCAGG",0,NA,NA,NA,"GeneID:57180","ACTR3B",TRUE,"ACTR3B"

"2165","GGTGCCACAGTCCACCACGC","chrX",55658031,"-","10:G>A,17:C>G","AGG","GGTGCCACAGCCCACCAGGCAGG",2,11,18,NA,"","",FALSE,"ACTR3B"

"2166","GGTGCCACAGTCCACCACGC","chr2",113890301,"-","0:G>C,18:T>C","AGG","CGTGCCACAGTCCACCACACAGG",2,1,19,NA,"GeneID:10096","ACTR3",TRUE,"ACTR3B"

"2167","TACTCACCATTAAAAAATAA","chr7",149891183,"+","none","TGG","TACTCACCATTAAAAAATAATGG",0,NA,NA,NA,"","",TRUE,"ACTR3B"

"2168","TACTCACCATTAAAAAATAA","chr7",152801718,"-","none","TGG","TACTCACCATTAAAAAATAATGG",0,NA,NA,NA,"GeneID:57180","ACTR3B",TRUE,"ACTR3B"

"2169","TACTCACCATTAAAAAATAA","chr3",162032319,"+","8:T>A","TGG","TACTCACCTTTAAAAAATAATGG",1,9,NA,NA,"","",FALSE,"ACTR3B"

"2170","TACTCACCATTAAAAAATAA","chr6",13140442,"+","6:T>C,17:G>T","AGG","TACTCATCATTAAAAAAGAAAGG",2,7,18,NA,"GeneID:221692","PHACTR1",FALSE,"ACTR3B"

"2171","TGAACTGCAATGTAGAGTCC","chr7",150295252,"+","none","TGG","TGAACTGCAATGTAGAGTCCTGG",0,NA,NA,NA,"GeneID:653857","ACTR3C",TRUE,"ACTR3B"

"2172","TGAACTGCAATGTAGAGTCC","chr7",152814624,"-","none","TGG","TGAACTGCAATGTAGAGTCCTGG",0,NA,NA,NA,"GeneID:57180","ACTR3B",TRUE,"ACTR3B"

"2173","TGAACTGCAATGTAGAGTCC","chrY",11396239,"-","16:T>C","TGG","TGAACTGCAATGTAGAATCCTGG",1,17,NA,NA,"","",TRUE,"ACTR3B"

"2174","TGAACTGCAATGTAGAGTCC","chr10",38696965,"-","16:T>C","TGG","TGAACTGCAATGTAGAATCCTGG",1,17,NA,NA,"","",TRUE,"ACTR3B"

"2175","TGAACTGCAATGTAGAGTCC","chr2",91941207,"-","16:T>C","TGG","TGAACTGCAATGTAGAATCCTGG",1,17,NA,NA,"","",TRUE,"ACTR3B"

"2176","TGAACTGCAATGTAGAGTCC","chr4",102962494,"-","16:T>C","TGG","TGAACTGCAATGTAGAATCCTGG",1,17,NA,NA,"GeneID:150159","SLC9B1",TRUE,"ACTR3B"

"2177","TGAACTGCAATGTAGAGTCC","chr16_KI270728v1_random",137872,"-","16:T>C","TGG","TGAACTGCAATGTAGAATCCTGG",1,17,NA,NA,"","",FALSE,"ACTR3B"

"2178","TGAACTGCAATGTAGAGTCC","chr22",16487059,"-","16:T>C","TGG","TGAACTGCAATGTAGAATCCTGG",1,17,NA,NA,"GeneID:440888","ACTR3BP2",TRUE,"ACTR3B"

"2179","TGAACTGCAATGTAGAGTCC","chr22",11124876,"-","16:T>C","TGG","TGAACTGCAATGTAGAATCCTGG",1,17,NA,NA,"","",TRUE,"ACTR3B"

"2180","TGAACTGCAATGTAGAGTCC","chr16",32379883,"+","16:A>G","TGG","TGAACTGCAATGTAGAATCCTGG",1,17,NA,NA,"","",TRUE,"ACTR3B"

"2181","TTTGAATCATTTAACGTACC","chr7",152814603,"+","none","AGG","TTTGAATCATTTAACGTACCAGG",0,NA,NA,NA,"GeneID:57180","ACTR3B",TRUE,"ACTR3B"

"2182","TTTGAATCATTTAACGTACC","chrY",11396218,"+","7:T>C,14:T>C","AGG","TTTGAATTATTTAATGTACCAGG",2,8,15,NA,"","",TRUE,"ACTR3B"

"2183","TTTGAATCATTTAACGTACC","chr10",38696944,"+","7:T>C,14:T>C","AGG","TTTGAATTATTTAATGTACCAGG",2,8,15,NA,"","",TRUE,"ACTR3B"

"2184","TTTGAATCATTTAACGTACC","chr2",91941186,"+","7:T>C,14:T>C","AGG","TTTGAATTATTTAATGTACCAGG",2,8,15,NA,"","",TRUE,"ACTR3B"

"2185","TTTGAATCATTTAACGTACC","chr4",102962473,"+","7:T>C,14:T>C","AGG","TTTGAATTATTTAATGTACCAGG",2,8,15,NA,"GeneID:150159","SLC9B1",TRUE,"ACTR3B"

"2186","TTTGAATCATTTAACGTACC","chr7",150295273,"-","2:G>A,17:A>T","AGG","TTCGAATCATTTAACGTTCCAGG",2,3,18,NA,"GeneID:653857","ACTR3C",TRUE,"ACTR3B"

"2187","ATCAAACACATCCCGATTGC","chr7",152820325,"+","none","AGG","ATCAAACACATCCCGATTGCAGG",0,NA,NA,NA,"GeneID:57180","ACTR3B",TRUE,"ACTR3C"

"2188","ATCAAACACATCCCGATTGC","chr7",150289546,"-","none","AGG","ATCAAACACATCCCGATTGCAGG",0,NA,NA,NA,"GeneID:653857","ACTR3C",TRUE,"ACTR3C"

"2189","ATCAAACACATCCCGATTGC","chr8",91308179,"-","8:A>G,19:T>G","GGG","ATCAAACATATCCCGATTGAGGG",2,9,20,NA,"GeneID:115111","SLC26A7",FALSE,"ACTR3C"

"2190","TAGGCAGAAGGTTATGTAAT","chr7",152820295,"+","none","TGG","TAGGCAGAAGGTTATGTAATTGG",0,NA,NA,NA,"GeneID:57180","ACTR3B",TRUE,"ACTR3C"

"2191","TAGGCAGAAGGTTATGTAAT","chr7",150289576,"-","none","TGG","TAGGCAGAAGGTTATGTAATTGG",0,NA,NA,NA,"GeneID:653857","ACTR3C",TRUE,"ACTR3C"

"2192","TCTCCAGGCAGTGCTGGCCT","chr7",152816473,"+","none","TGG","TCTCCAGGCAGTGCTGGCCTTGG",0,NA,NA,NA,"GeneID:57180","ACTR3B",TRUE,"ACTR3C"

"2193","TCTCCAGGCAGTGCTGGCCT","chr7",150293406,"-","none","TGG","TCTCCAGGCAGTGCTGGCCTTGG",0,NA,NA,NA,"GeneID:653857","ACTR3C",TRUE,"ACTR3C"

"2194","TGAACTGCAATGTAGAGTCC","chr7",150295252,"+","none","TGG","TGAACTGCAATGTAGAGTCCTGG",0,NA,NA,NA,"GeneID:653857","ACTR3C",TRUE,"ACTR3C"

"2195","TGAACTGCAATGTAGAGTCC","chr7",152814624,"-","none","TGG","TGAACTGCAATGTAGAGTCCTGG",0,NA,NA,NA,"GeneID:57180","ACTR3B",TRUE,"ACTR3C"

"2196","TGAACTGCAATGTAGAGTCC","chrY",11396239,"-","16:T>C","TGG","TGAACTGCAATGTAGAATCCTGG",1,17,NA,NA,"","",TRUE,"ACTR3C"

"2197","TGAACTGCAATGTAGAGTCC","chr10",38696965,"-","16:T>C","TGG","TGAACTGCAATGTAGAATCCTGG",1,17,NA,NA,"","",TRUE,"ACTR3C"

"2198","TGAACTGCAATGTAGAGTCC","chr2",91941207,"-","16:T>C","TGG","TGAACTGCAATGTAGAATCCTGG",1,17,NA,NA,"","",TRUE,"ACTR3C"

"2199","TGAACTGCAATGTAGAGTCC","chr4",102962494,"-","16:T>C","TGG","TGAACTGCAATGTAGAATCCTGG",1,17,NA,NA,"GeneID:150159","SLC9B1",TRUE,"ACTR3C"

"2200","TGAACTGCAATGTAGAGTCC","chr16_KI270728v1_random",137872,"-","16:T>C","TGG","TGAACTGCAATGTAGAATCCTGG",1,17,NA,NA,"","",FALSE,"ACTR3C"

"2201","TGAACTGCAATGTAGAGTCC","chr22",16487059,"-","16:T>C","TGG","TGAACTGCAATGTAGAATCCTGG",1,17,NA,NA,"GeneID:440888","ACTR3BP2",TRUE,"ACTR3C"

"2202","TGAACTGCAATGTAGAGTCC","chr22",11124876,"-","16:T>C","TGG","TGAACTGCAATGTAGAATCCTGG",1,17,NA,NA,"","",TRUE,"ACTR3C"

"2203","TGAACTGCAATGTAGAGTCC","chr16",32379883,"+","16:A>G","TGG","TGAACTGCAATGTAGAATCCTGG",1,17,NA,NA,"","",TRUE,"ACTR3C"

"2204","TGCTTACCACTGGGATAACA","chr7",150293304,"+","none","TGG","TGCTTACCACTGGGATAACATGG",0,NA,NA,NA,"GeneID:653857","ACTR3C",TRUE,"ACTR3C"

"2205","TGCTTACCACTGGGATAACA","chr7",152816575,"-","none","TGG","TGCTTACCACTGGGATAACATGG",0,NA,NA,NA,"GeneID:57180","ACTR3B",TRUE,"ACTR3C"

"2206","TTCGAATCATTTAACGTTCC","chr7",150295273,"-","none","AGG","TTCGAATCATTTAACGTTCCAGG",0,NA,NA,NA,"GeneID:653857","ACTR3C",TRUE,"ACTR3C"

"2207","TTCGAATCATTTAACGTTCC","chr7",152814603,"+","2:T>C,17:A>T","AGG","TTTGAATCATTTAACGTACCAGG",2,3,18,NA,"GeneID:57180","ACTR3B",TRUE,"ACTR3C"

"2208","AACTGCAAGCGCATCAATCT","chr20",38752143,"+","none","TGG","AACTGCAAGCGCATCAATCTTGG",0,NA,NA,NA,"GeneID:79913","ACTR5",TRUE,"ACTR5"

"2209","CCAAAGAACTCGATGTGCAG","chr20",38750159,"+","none","TGG","CCAAAGAACTCGATGTGCAGTGG",0,NA,NA,NA,"GeneID:79913","ACTR5",TRUE,"ACTR5"

"2210","CCACTGCACATCGAGTTCTT","chr20",38750162,"-","none","TGG","CCACTGCACATCGAGTTCTTTGG",0,NA,NA,NA,"GeneID:79913","ACTR5",TRUE,"ACTR5"

"2211","CCACTGCACATCGAGTTCTT","chr4",45322956,"-","12:G>C,17:T>G","GGG","CCACTGCACATCCAGTTATTGGG",2,13,18,NA,"","",FALSE,"ACTR5"

"2212","TGAGCACAGCTACATCGCTG","chr20",38752265,"+","none","AGG","TGAGCACAGCTACATCGCTGAGG",0,NA,NA,NA,"GeneID:79913","ACTR5",TRUE,"ACTR5"

"2213","TGTCTACCACATCCACCTCG","chr20",38756008,"-","none","AGG","TGTCTACCACATCCACCTCGAGG",0,NA,NA,NA,"GeneID:79913","ACTR5",TRUE,"ACTR5"

"2214","TTACCTGCACATATAGCAGT","chr20",38755158,"-","none","CGG","TTACCTGCACATATAGCAGTCGG",0,NA,NA,NA,"GeneID:79913","ACTR5",TRUE,"ACTR5"

"2215","ACTCTTAACCAATCATCTAA","chr12",100210312,"+","none","AGG","ACTCTTAACCAATCATCTAAAGG",0,NA,NA,NA,"GeneID:64431;GeneID:120863","ACTR6;DEPDC4",TRUE,"ACTR6"

"2216","AGATGTATGCTATGTGTCTC","chr12",100212303,"+","none","AGG","AGATGTATGCTATGTGTCTCAGG",0,NA,NA,NA,"GeneID:64431;GeneID:120863","ACTR6;DEPDC4",TRUE,"ACTR6"

"2217","GGAGCTTACAACGCCAAAAT","chr12",100200875,"+","none","CGG","GGAGCTTACAACGCCAAAATCGG",0,NA,NA,NA,"GeneID:64431","ACTR6",TRUE,"ACTR6"

"2218","TCCCCTTGCAGGATAAATGT","chr12",100210283,"+","none","GGG","TCCCCTTGCAGGATAAATGTGGG",0,NA,NA,NA,"GeneID:64431;GeneID:120863","ACTR6;DEPDC4",TRUE,"ACTR6"

"2219","TGAGATGACGACCTTAGTGC","chr12",100200847,"+","none","TGG","TGAGATGACGACCTTAGTGCTGG",0,NA,NA,NA,"GeneID:64431","ACTR6",TRUE,"ACTR6"

"2220","TGTTAGGCAGCTACATGTTA","chr12",100212249,"+","none","TGG","TGTTAGGCAGCTACATGTTATGG",0,NA,NA,NA,"GeneID:64431;GeneID:120863","ACTR6;DEPDC4",TRUE,"ACTR6"

"2221","TGTTAGGCAGCTACATGTTA","chr9",30839315,"+","9:C>G,10:A>C","AGG","TGTTAGGCACATACATGTTAAGG",2,10,11,NA,"","",FALSE,"ACTR6"

"2222","TGTTAGGCAGCTACATGTTA","chr7",37802616,"+","9:C>G,10:A>C","AGG","TGTTAGGCACATACATGTTAAGG",2,10,11,NA,"GeneID:353345;GeneID:54749","GPR141;EPDR1",FALSE,"ACTR6"

"2223","AAATGGTGGATCAAGCAATA","chr3",53878410,"-","none","TGG","AAATGGTGGATCAAGCAATATGG",0,NA,NA,NA,"GeneID:93973","ACTR8",TRUE,"ACTR8"

"2224","AAATGGTGGATCAAGCAATA","chr1",51316095,"-","8:T>C,10:T>A","AGG","AAATGGTGAAACAAGCAATAAGG",2,9,11,NA,"GeneID:22996","TTC39A",FALSE,"ACTR8"

"2225","CATCACCCTGAGTATTTAGT","chr3",53877656,"-","none","AGG","CATCACCCTGAGTATTTAGTAGG",0,NA,NA,NA,"GeneID:93973","ACTR8",TRUE,"ACTR8"

"2226","CGAACCTGTTCAGGGGACAC","chr3",53878351,"+","none","AGG","CGAACCTGTTCAGGGGACACAGG",0,NA,NA,NA,"GeneID:93973","ACTR8",TRUE,"ACTR8"

"2227","CGAACCTGTTCAGGGGACAC","chr11",32549030,"-","0:A>G,7:A>C","AGG","TGAACCTTTTCAGGGGACACAGG",2,1,8,NA,"","",FALSE,"ACTR8"

"2228","CGAACCTGTTCAGGGGACAC","chr7",98526041,"-","9:G>A,11:A>T","TGG","CGAACCTGTCCTGGGGACACTGG",2,10,12,NA,"","",FALSE,"ACTR8"

"2229","CGCACCTCTTGCAGCGACTC","chr3",53881973,"+","none","CGG","CGCACCTCTTGCAGCGACTCCGG",0,NA,NA,NA,"GeneID:93973","ACTR8",TRUE,"ACTR8"

"2230","GCGGCCCATCGTGCCCGCGC","chr3",53882001,"-","none","TGG","GCGGCCCATCGTGCCCGCGCTGG",0,NA,NA,NA,"GeneID:93973","ACTR8",TRUE,"ACTR8"

"2231","TCTTCTCCTACTAAATACTC","chr3",53877647,"+","none","AGG","TCTTCTCCTACTAAATACTCAGG",0,NA,NA,NA,"GeneID:93973","ACTR8",TRUE,"ACTR8"

"2232","TCTTCTCCTACTAAATACTC","chr12",55750342,"-","12:C>T,17:C>G","AGG","TCTTCTCCTACTGAATAGTCAGG",2,13,18,NA,"GeneID:10220","GDF11",TRUE,"ACTR8"

"2233","AGTTAAACCCGCTAGCAAAG","chrX",128051599,"+","none","AGG","AGTTAAACCCGCTAGCAAAGAGG",0,NA,NA,NA,"GeneID:139741","ACTRT1",TRUE,"ACTRT1"

"2234","CCATGTCATCAGCTCCGTCT","chrX",128052091,"-","none","TGG","CCATGTCATCAGCTCCGTCTTGG",0,NA,NA,NA,"GeneID:139741","ACTRT1",TRUE,"ACTRT1"

"2235","CGCAGTCACCAAACTCTGTA","chrX",128051659,"-","none","TGG","CGCAGTCACCAAACTCTGTATGG",0,NA,NA,NA,"GeneID:139741","ACTRT1",TRUE,"ACTRT1"

"2236","GAGGGCTCGGTCATAAGTAC","chrX",128051871,"+","none","AGG","GAGGGCTCGGTCATAAGTACAGG",0,NA,NA,NA,"GeneID:139741","ACTRT1",TRUE,"ACTRT1"

"2237","TGTTACCAGTCCACGCTCAA","chrX",128051966,"+","none","TGG","TGTTACCAGTCCACGCTCAATGG",0,NA,NA,NA,"GeneID:139741","ACTRT1",TRUE,"ACTRT1"

"2238","TTGCACTACCCCATTGAGCG","chrX",128051979,"-","none","TGG","TTGCACTACCCCATTGAGCGTGG",0,NA,NA,NA,"GeneID:139741","ACTRT1",TRUE,"ACTRT1"

"2239","AGATCACGGCTCCCCCCGAC","chr1",3022674,"+","none","CGG","AGATCACGGCTCCCCCCGACCGG",0,NA,NA,NA,"GeneID:140625","ACTRT2",TRUE,"ACTRT2"

"2240","CTGCACTCCCCTTTCGAGCG","chr1",3021896,"+","none","TGG","CTGCACTCCCCTTTCGAGCGTGG",0,NA,NA,NA,"GeneID:140625","ACTRT2",TRUE,"ACTRT2"

"2241","CTGCACTCCCCTTTCGAGCG","chr8",142632432,"-","16:G>T,17:A>C","TGG","CTGCACTCCCCTTTCGCTCGTGG",2,17,18,NA,"","",FALSE,"ACTRT2"

"2242","GAGCCCCGGGCTCTCGAATA","chr1",3022501,"+","none","TGG","GAGCCCCGGGCTCTCGAATATGG",0,NA,NA,NA,"GeneID:140625","ACTRT2",TRUE,"ACTRT2"

"2243","GGAGACCATATTCGAGAGCC","chr1",3022509,"-","none","CGG","GGAGACCATATTCGAGAGCCCGG",0,NA,NA,NA,"GeneID:140625","ACTRT2",TRUE,"ACTRT2"

"2244","GGAGTTTGGACCCCGGCACA","chr1",3021766,"+","none","TGG","GGAGTTTGGACCCCGGCACATGG",0,NA,NA,NA,"GeneID:140625","ACTRT2",TRUE,"ACTRT2"

"2245","TGATGTCCCTGCCCGCCACG","chr1",3022234,"-","none","TGG","TGATGTCCCTGCCCGCCACGTGG",0,NA,NA,NA,"GeneID:140625","ACTRT2",TRUE,"ACTRT2"

"2246","ACTAACCGCTTGTCTAAACC","chr3",169767618,"+","none","AGG","ACTAACCGCTTGTCTAAACCAGG",0,NA,NA,NA,"GeneID:84517","ACTRT3",TRUE,"ACTRT3"

"2247","CAACGGCTCGGGAATGATCA","chr3",169769468,"-","none","AGG","CAACGGCTCGGGAATGATCAAGG",0,NA,NA,NA,"GeneID:84517","ACTRT3",TRUE,"ACTRT3"

"2248","CGCAGAGTTCTAGCCCGCCC","chr3",169769363,"+","none","TGG","CGCAGAGTTCTAGCCCGCCCTGG",0,NA,NA,NA,"GeneID:84517","ACTRT3",TRUE,"ACTRT3"

"2249","GAGCCGTTGTCGATCACCAC","chr3",169769479,"+","none","CGG","GAGCCGTTGTCGATCACCACCGG",0,NA,NA,NA,"GeneID:84517","ACTRT3",TRUE,"ACTRT3"

"2250","GGGCGGGCTAGAACTCTGCG","chr3",169769363,"-","none","TGG","GGGCGGGCTAGAACTCTGCGTGG",0,NA,NA,NA,"GeneID:84517","ACTRT3",TRUE,"ACTRT3"

"2251","TCCGTGTCATATGAACCTTG","chr3",169767751,"-","none","AGG","TCCGTGTCATATGAACCTTGAGG",0,NA,NA,NA,"GeneID:84517","ACTRT3",TRUE,"ACTRT3"

"2252","AGCAGCCTTTCTGGTAGACG","chr2",157780456,"+","none","TGG","AGCAGCCTTTCTGGTAGACGTGG",0,NA,NA,NA,"GeneID:90","ACVR1",TRUE,"ACVR1"

"2253","AGTCACTTACCTTCCATACT","chr2",157799416,"+","none","AGG","AGTCACTTACCTTCCATACTAGG",0,NA,NA,NA,"GeneID:90","ACVR1",TRUE,"ACVR1"

"2254","CAAGCCGTGGAGTGCTGCCA","chr2",157780383,"-","none","AGG","CAAGCCGTGGAGTGCTGCCAAGG",0,NA,NA,NA,"GeneID:90","ACVR1",TRUE,"ACVR1"

"2255","GGCTCGCCAGATTACACTGT","chr2",157774099,"-","none","TGG","GGCTCGCCAGATTACACTGTTGG",0,NA,NA,NA,"GeneID:90","ACVR1",TRUE,"ACVR1"

"2256","TGCTCTCCCCTCCCCTAGTA","chr2",157799432,"-","none","TGG","TGCTCTCCCCTCCCCTAGTATGG",0,NA,NA,NA,"GeneID:90","ACVR1",TRUE,"ACVR1"

"2257","TTGGATCATTCGTGTACATC","chr2",157774161,"-","none","AGG","TTGGATCATTCGTGTACATCAGG",0,NA,NA,NA,"GeneID:90","ACVR1",TRUE,"ACVR1"

"2258","ACACCTGCACATGGAGATCG","chr12",51984134,"+","none","TGG","ACACCTGCACATGGAGATCGTGG",0,NA,NA,NA,"GeneID:91","ACVR1B",TRUE,"ACVR1B"

"2259","AGCCTGAGCACCCGTCCATG","chr12",51976338,"+","none","TGG","AGCCTGAGCACCCGTCCATGTGG",0,NA,NA,NA,"GeneID:91","ACVR1B",TRUE,"ACVR1B"

"2260","CCTCTTTGTCCAGCGCACAG","chr12",51980975,"+","none","TGG","CCTCTTTGTCCAGCGCACAGTGG",0,NA,NA,NA,"GeneID:91","ACVR1B",TRUE,"ACVR1B"

"2261","GAGCTGGTAGGCATCATCGC","chr12",51976370,"+","none","CGG","GAGCTGGTAGGCATCATCGCCGG",0,NA,NA,NA,"GeneID:91","ACVR1B",TRUE,"ACVR1B"

"2262","GCTACACTGACTACTGCAAC","chr12",51975459,"+","none","AGG","GCTACACTGACTACTGCAACAGG",0,NA,NA,NA,"GeneID:91","ACVR1B",TRUE,"ACVR1B"

"2263","TGCCCACGATCTCCATGTGC","chr12",51984140,"-","none","AGG","TGCCCACGATCTCCATGTGCAGG",0,NA,NA,NA,"GeneID:91","ACVR1B",TRUE,"ACVR1B"

"2264","AAGACTTGAGCATTCAGTTC","chr2",157587269,"+","none","TGG","AAGACTTGAGCATTCAGTTCTGG",0,NA,NA,NA,"GeneID:130399","ACVR1C",TRUE,"ACVR1C"

"2265","AGGGTCGACAGTGCTCCTAC","chr2",157556207,"-","none","AGG","AGGGTCGACAGTGCTCCTACAGG",0,NA,NA,NA,"GeneID:130399","ACVR1C",TRUE,"ACVR1C"

"2266","ATTTATGATGTGACCGCCTC","chr2",157556106,"-","none","TGG","ATTTATGATGTGACCGCCTCTGG",0,NA,NA,NA,"GeneID:130399","ACVR1C",TRUE,"ACVR1C"

"2267","GCATCAGTCATGCTAACCAA","chr2",157587329,"-","none","TGG","GCATCAGTCATGCTAACCAATGG",0,NA,NA,NA,"GeneID:130399","ACVR1C",TRUE,"ACVR1C"

"2268","TGCAAGGACGATTGTGCTTC","chr2",157550344,"-","none","AGG","TGCAAGGACGATTGTGCTTCAGG",0,NA,NA,NA,"GeneID:130399","ACVR1C",TRUE,"ACVR1C"

"2269","TTGGTTAGGTCTACCTCTGT","chr2",157550380,"-","none","TGG","TTGGTTAGGTCTACCTCTGTTGG",0,NA,NA,NA,"GeneID:130399","ACVR1C",TRUE,"ACVR1C"

"2270","ACTTGAGTTGGAACAAGTAC","chr2",147899880,"-","none","AGG","ACTTGAGTTGGAACAAGTACAGG",0,NA,NA,NA,"GeneID:92","ACVR2A",TRUE,"ACVR2A"

"2271","CAGCAACAAAAATATACTTC","chr2",147899488,"-","none","AGG","CAGCAACAAAAATATACTTCAGG",0,NA,NA,NA,"GeneID:92","ACVR2A",TRUE,"ACVR2A"

"2272","CAGCAACAAAAATATACTTC","chr13",98623691,"-","15:G>T,16:A>G","AGG","CAGCAACAAAAATATCTTTCAGG",2,16,17,NA,"","",FALSE,"ACVR2A"

"2273","CAGCAACAAAAATATACTTC","chr8",35965137,"-","0:C>G,3:A>G","AGG","GAGTAACAAAAATATACTTCAGG",2,1,4,NA,"","",FALSE,"ACVR2A"

"2274","CAGCAACAAAAATATACTTC","chr14",39804353,"+","3:T>C,4:C>A","AGG","CAGTCACAAAAATATACTTCAGG",2,4,5,NA,"","",FALSE,"ACVR2A"

"2275","CAGCAACAAAAATATACTTC","chrX",137345912,"-","2:T>C,15:G>T","GGG","CAACAACAAAAATATCCTTCGGG",2,3,16,NA,"","",FALSE,"ACVR2A"

"2276","CAGCAACAAAAATATACTTC","chr3",68721847,"-","2:T>C,12:T>A","AGG","CAACAACAAAAAAATACTTCAGG",2,3,13,NA,"","",FALSE,"ACVR2A"

"2277","CAGCAACAAAAATATACTTC","chr11",96860137,"-","2:T>C,12:T>A","AGG","CAACAACAAAAAAATACTTCAGG",2,3,13,NA,"","",FALSE,"ACVR2A"

"2278","CAGCAACAAAAATATACTTC","chr2",163314061,"-","0:T>G,10:G>T","TGG","AAGCAACAAACATATACTTCTGG",2,1,11,NA,"","",FALSE,"ACVR2A"

"2279","CCACATATTCGTTAAGCAAC","chr2",147915291,"-","none","TGG","CCACATATTCGTTAAGCAACTGG",0,NA,NA,NA,"GeneID:92","ACVR2A",TRUE,"ACVR2A"

"2280","CCAGTTGCTTAACGAATATG","chr2",147915288,"+","none","TGG","CCAGTTGCTTAACGAATATGTGG",0,NA,NA,NA,"GeneID:92","ACVR2A",TRUE,"ACVR2A"

"2281","CGCTGTGTGACTTCCATCTC","chr2",147899548,"-","none","CGG","CGCTGTGTGACTTCCATCTCCGG",0,NA,NA,NA,"GeneID:92","ACVR2A",TRUE,"ACVR2A"

"2282","TGCCACTTATGTTAATTGCG","chr2",147899803,"+","none","GGG","TGCCACTTATGTTAATTGCGGGG",0,NA,NA,NA,"GeneID:92","ACVR2A",TRUE,"ACVR2A"

"2283","ACGCTTCACTCATTTGCCAG","chr3",38477932,"+","none","AGG","ACGCTTCACTCATTTGCCAGAGG",0,NA,NA,NA,"GeneID:93","ACVR2B",TRUE,"ACVR2B"

"2284","ACGCTTCACTCATTTGCCAG","chr7",38935756,"+","2:A>G,8:T>C","TGG","ACACTTCATTCATTTGCCAGTGG",2,3,9,NA,"","",FALSE,"ACVR2B"

"2285","CACAGCAGCAGAAGTACACC","chr3",38477893,"-","none","TGG","CACAGCAGCAGAAGTACACCTGG",0,NA,NA,NA,"GeneID:93","ACVR2B",TRUE,"ACVR2B"

"2286","CGCGGAACATGACGGCGCCC","chr3",38454314,"+","none","TGG","CGCGGAACATGACGGCGCCCTGG",0,NA,NA,NA,"GeneID:100128640;GeneID:93","ACVR2B-AS1;ACVR2B",TRUE,"ACVR2B"

"2287","CGGCATCGCAAGCCCCCCTA","chr3",38478250,"+","none","CGG","CGGCATCGCAAGCCCCCCTACGG",0,NA,NA,NA,"GeneID:93","ACVR2B",TRUE,"ACVR2B"

"2288","CGGCGCACAGCGATCCCCAG","chr3",38454354,"-","none","AGG","CGGCGCACAGCGATCCCCAGAGG",0,NA,NA,NA,"GeneID:100128640;GeneID:93","ACVR2B-AS1;ACVR2B",TRUE,"ACVR2B"

"2289","GCGATGCCGGTACATCCAAA","chr3",38478239,"-","none","AGG","GCGATGCCGGTACATCCAAAAGG",0,NA,NA,NA,"GeneID:93","ACVR2B",TRUE,"ACVR2B"

"2290","CATCGGCAACAACCCGAGAG","chr12",51916075,"+","none","TGG","CATCGGCAACAACCCGAGAGTGG",0,NA,NA,NA,"GeneID:94","ACVRL1",TRUE,"ACVRL1"

"2291","CCCGCCACACTTCGCCATAG","chr12",51914448,"-","none","CGG","CCCGCCACACTTCGCCATAGCGG",0,NA,NA,NA,"GeneID:94","ACVRL1",TRUE,"ACVRL1"

"2292","CTCAGACACGACAACATCCT","chr12",51914563,"+","none","AGG","CTCAGACACGACAACATCCTAGG",0,NA,NA,NA,"GeneID:94","ACVRL1",TRUE,"ACVRL1"

"2293","CTTGAAGTCGCGGTGGGCAA","chr12",51915428,"-","none","TGG","CTTGAAGTCGCGGTGGGCAATGG",0,NA,NA,NA,"GeneID:94","ACVRL1",TRUE,"ACVRL1"

"2294","GCAGTGTTGCATCGCCGACC","chr12",51915477,"+","none","TGG","GCAGTGTTGCATCGCCGACCTGG",0,NA,NA,NA,"GeneID:94","ACVRL1",TRUE,"ACVRL1"

"2295","TGGGCCCTCACCATTCACGA","chr12",51916224,"-","none","TGG","TGGGCCCTCACCATTCACGATGG",0,NA,NA,NA,"GeneID:94","ACVRL1",TRUE,"ACVRL1"

"2296","CCTGGGCTGTCAGAAAGTAG","chr3",51985249,"+","none","AGG","CCTGGGCTGTCAGAAAGTAGAGG",0,NA,NA,NA,"GeneID:100526760;GeneID:95","ABHD14A-ACY1;ACY1",TRUE,"ACY1"

"2297","GCTGGACAGTGCGGATACGC","chr3",51984123,"-","none","AGG","GCTGGACAGTGCGGATACGCAGG",0,NA,NA,NA,"GeneID:100526760;GeneID:95","ABHD14A-ACY1;ACY1",TRUE,"ACY1"

"2298","GGTCACCCTAGAGTTTGCTC","chr3",51987602,"+","none","AGG","GGTCACCCTAGAGTTTGCTCAGG",0,NA,NA,NA,"GeneID:100526760;GeneID:95","ABHD14A-ACY1;ACY1",TRUE,"ACY1"

"2299","TGACCCCCTCGCCAGCTGCC","chr3",51987587,"-","none","TGG","TGACCCCCTCGCCAGCTGCCTGG",0,NA,NA,NA,"GeneID:100526760;GeneID:95","ABHD14A-ACY1;ACY1",TRUE,"ACY1"

"2300","TGACCCCCTCGCCAGCTGCC","chr4",103719585,"-","5:C>G,12:A>G","GGG","TGACCGCCTCGCTAGCTGCCGGG",2,6,13,NA,"GeneID:6870","TACR3",TRUE,"ACY1"

"2301","TGAGGAGACAGCCCGCCAGC","chr3",51985225,"+","none","TGG","TGAGGAGACAGCCCGCCAGCTGG",0,NA,NA,NA,"GeneID:100526760;GeneID:95","ABHD14A-ACY1;ACY1",TRUE,"ACY1"

"2302","TGGCGGAAGAGCGTCACCGA","chr3",51984097,"-","none","TGG","TGGCGGAAGAGCGTCACCGATGG",0,NA,NA,NA,"GeneID:100526760;GeneID:95","ABHD14A-ACY1;ACY1",TRUE,"ACY1"

"2303","CTATAGACCCGTGGGCGTCG","chr11",67644818,"-","none","TGG","CTATAGACCCGTGGGCGTCGTGG",0,NA,NA,NA,"GeneID:91703","ACY3",TRUE,"ACY3"

"2304","GGCAACGCCCTTCTCATAGT","chr11",67642801,"+","none","AGG","GGCAACGCCCTTCTCATAGTAGG",0,NA,NA,NA,"GeneID:91703","ACY3",TRUE,"ACY3"

"2305","GGGGAAGTCCACGACGCCCA","chr11",67644807,"+","none","CGG","GGGGAAGTCCACGACGCCCACGG",0,NA,NA,NA,"GeneID:91703","ACY3",TRUE,"ACY3"

"2306","GGTGCATGGCAAAGACTTCG","chr11",67645708,"+","none","TGG","GGTGCATGGCAAAGACTTCGTGG",0,NA,NA,NA,"GeneID:91703","ACY3",TRUE,"ACY3"

"2307","GTCACCTCATATGGGTCGTC","chr11",67645851,"+","none","CGG","GTCACCTCATATGGGTCGTCCGG",0,NA,NA,NA,"GeneID:91703","ACY3",TRUE,"ACY3"

"2308","TCTTCCTGTACCAGCGGTCT","chr11",67645332,"-","none","GGG","TCTTCCTGTACCAGCGGTCTGGG",0,NA,NA,NA,"GeneID:91703","ACY3",TRUE,"ACY3"

"2309","AGCTTTGCTACCAGTTATGC","chr14",75061685,"-","none","AGG","AGCTTTGCTACCAGTTATGCAGG",0,NA,NA,NA,"GeneID:97","ACYP1",TRUE,"ACYP1"

"2310","AGCTTTGCTACCAGTTATGC","chr7",150247424,"+","8:C>T,16:T>A","AGG","AGCTTTGCCACCAGTTTTGCAGG",2,9,17,NA,"GeneID:653857","ACTR3C",TRUE,"ACYP1"

"2311","AGCTTTGCTACCAGTTATGC","chr7",153722034,"-","8:G>A,16:A>T","AGG","AGCTTTGCCACCAGTTTTGCAGG",2,9,17,NA,"","",FALSE,"ACYP1"

"2312","AGGAAACACCCTGATATCAG","chr14",75063525,"-","none","TGG","AGGAAACACCCTGATATCAGTGG",0,NA,NA,NA,"GeneID:97","ACYP1",TRUE,"ACYP1"

"2313","AGGAAACACCCTGATATCAG","chr14",67120563,"+","2:A>G,16:A>T","AGG","AGAAAACACCCTGATAACAGAGG",2,3,17,NA,"GeneID:10243","GPHN",FALSE,"ACYP1"

"2314","AGGAAACACCCTGATATCAG","chr10",124207396,"-","6:C>G,12:T>C","TGG","AGGAAAGACCCTAATATCAGTGG",2,7,13,NA,"","",FALSE,"ACYP1"

"2315","CACATACCTGAGTATGCTTA","chr14",75063462,"+","none","CGG","CACATACCTGAGTATGCTTACGG",0,NA,NA,NA,"GeneID:97","ACYP1",TRUE,"ACYP1"

"2316","CACATACCTGAGTATGCTTA","chr9",118396549,"+","3:T>A,5:T>A","GGG","CACTTTCCTGAGTATGCTTAGGG",2,4,6,NA,"","",FALSE,"ACYP1"

"2317","CACATACCTGAGTATGCTTA","chr2",83853317,"+","4:A>T,17:C>T","AGG","CACAAACCTGAGTATGCCTAAGG",2,5,18,NA,"","",FALSE,"ACYP1"

"2318","CAGGAACTTACCTGCATAAC","chr14",75061672,"+","none","TGG","CAGGAACTTACCTGCATAACTGG",0,NA,NA,NA,"GeneID:97","ACYP1",TRUE,"ACYP1"

"2319","TTTCATAATCCACTGATATC","chr14",75063513,"+","none","AGG","TTTCATAATCCACTGATATCAGG",0,NA,NA,NA,"GeneID:97","ACYP1",TRUE,"ACYP1"

"2320","AGAGTATTCAAGCTTAGAGA","chr2",54304761,"-","none","TGG","AGAGTATTCAAGCTTAGAGATGG",0,NA,NA,NA,"GeneID:98","ACYP2",TRUE,"ACYP2"

"2321","AGAGTATTCAAGCTTAGAGA","chr10",92303339,"-","6:T>A,7:C>A","TGG","AGAGTAAGCAAGCTTAGAGATGG",2,7,8,NA,"GeneID:54708","MARCH5",FALSE,"ACYP2"

"2322","ATGGAATTGACTTTGTCTTC","chr2",54138728,"-","none","TGG","ATGGAATTGACTTTGTCTTCTGG",0,NA,NA,NA,"GeneID:98","ACYP2",TRUE,"ACYP2"

"2323","ATGGAATTGACTTTGTCTTC","chr7",154824041,"-","19:C>G","AGG","ATGGAATTGACTTTGTCTTGAGG",1,20,NA,NA,"GeneID:1804","DPP6",FALSE,"ACYP2"

"2324","ATGGAATTGACTTTGTCTTC","chr9",119635278,"-","4:G>T,5:G>T","AGG","ATGGCCTTGACTTTGTCTTCAGG",2,5,6,NA,"","",FALSE,"ACYP2"

"2325","ATGGAATTGACTTTGTCTTC","chr21",6257593,"+","9:G>A,18:C>T","AGG","ATGGAATTGGCTTTGTCTCCAGG",2,10,19,NA,"","",FALSE,"ACYP2"

"2326","ATGGAATTGACTTTGTCTTC","chr21",5563359,"-","9:C>T,18:G>A","AGG","ATGGAATTGGCTTTGTCTCCAGG",2,10,19,NA,"","",FALSE,"ACYP2"

"2327","ATGGAATTGACTTTGTCTTC","chr21",7058581,"-","9:C>T,18:G>A","AGG","ATGGAATTGGCTTTGTCTCCAGG",2,10,19,NA,"","",FALSE,"ACYP2"

"2328","ATGGAATTGACTTTGTCTTC","chr21",7440359,"-","9:C>T,18:G>A","AGG","ATGGAATTGGCTTTGTCTCCAGG",2,10,19,NA,"","",FALSE,"ACYP2"

"2329","ATGGAATTGACTTTGTCTTC","chr21",6731512,"-","9:C>T,18:G>A","AGG","ATGGAATTGGCTTTGTCTCCAGG",2,10,19,NA,"","",FALSE,"ACYP2"

"2330","ATGGAATTGACTTTGTCTTC","chr11",811292,"-","3:T>C,15:T>A","AGG","ATGAAATTGACTTTGACTTCAGG",2,4,16,NA,"GeneID:6181","RPLP2",FALSE,"ACYP2"

"2331","CGAGGTGTTCGGAAGAGTGC","chr2",54115733,"+","none","AGG","CGAGGTGTTCGGAAGAGTGCAGG",0,NA,NA,NA,"GeneID:98","ACYP2",TRUE,"ACYP2"

"2332","GCTTCCAACCTTGCTCAGCC","chr2",54304695,"-","none","AGG","GCTTCCAACCTTGCTCAGCCAGG",0,NA,NA,NA,"GeneID:98","ACYP2",TRUE,"ACYP2"

"2333","TCCGAACACCTCGTAGTCCA","chr2",54115726,"-","none","CGG","TCCGAACACCTCGTAGTCCACGG",0,NA,NA,NA,"GeneID:98","ACYP2",TRUE,"ACYP2"

"2334","TGGGTGAAGAATACCAGCAA","chr2",54138677,"+","none","AGG","TGGGTGAAGAATACCAGCAAAGG",0,NA,NA,NA,"GeneID:98","ACYP2",TRUE,"ACYP2"

"2335","TGGGTGAAGAATACCAGCAA","chr8",73001630,"+","0:G>T,2:T>G","AGG","GGTGTGAAGAATACCAGCAAAGG",2,1,3,NA,"","",FALSE,"ACYP2"

"2336","AGCCCCTCTGCTGTGTTAGC","chr20",44629127,"+","none","TGG","AGCCCCTCTGCTGTGTTAGCTGG",0,NA,NA,NA,"GeneID:100","ADA",TRUE,"ADA"

"2337","AGGCATGTAGTAGTCAAACT","chr20",44629054,"+","none","TGG","AGGCATGTAGTAGTCAAACTTGG",0,NA,NA,NA,"GeneID:100","ADA",TRUE,"ADA"

"2338","AGGCATGTAGTAGTCAAACT","chr14",53983634,"-","11:G>T,12:A>C","GGG","AGGCATGTAGTCTTCAAACTGGG",2,12,13,NA,"","",FALSE,"ADA"

"2339","CAGGCTTGATGGATCCGTCT","chr20",44636248,"+","none","AGG","CAGGCTTGATGGATCCGTCTAGG",0,NA,NA,NA,"GeneID:100","ADA",TRUE,"ADA"

"2340","GAACTGCATGTCCACCTAGA","chr20",44636265,"-","none","CGG","GAACTGCATGTCCACCTAGACGG",0,NA,NA,NA,"GeneID:100","ADA",TRUE,"ADA"

"2341","GCTCACTTTGGGCTTGTCGA","chr20",44651568,"+","none","AGG","GCTCACTTTGGGCTTGTCGAAGG",0,NA,NA,NA,"GeneID:100","ADA",TRUE,"ADA"

"2342","TTACCTGCCATAGTATAAGA","chr20",44636222,"+","none","TGG","TTACCTGCCATAGTATAAGATGG",0,NA,NA,NA,"GeneID:100","ADA",TRUE,"ADA"

"2343","TTACCTGCCATAGTATAAGA","chr6",3006543,"-","13:G>A,14:C>T","AGG","TTACCTGCCATAGCGTAAGAAGG",2,14,15,NA,"GeneID:4835","NQO2",TRUE,"ADA"

"2344","CCAATGAACTGCTATATTTC","chr4",122396336,"-","none","AGG","CCAATGAACTGCTATATTTCAGG",0,NA,NA,NA,"GeneID:132612","ADAD1",TRUE,"ADAD1"

"2345","CCAATGAACTGCTATATTTC","chr1",175195994,"-","4:T>A,6:G>T","AGG","CCAAAGCACTGCTATATTTCAGG",2,5,7,NA,"","",FALSE,"ADAD1"

"2346","CCAATGAACTGCTATATTTC","chr6",16942638,"-","2:A>T,8:T>G","TGG","CCTATGAAATGCTATATTTCTGG",2,3,9,NA,"","",FALSE,"ADAD1"

"2347","CCTGAAATATAGCAGTTCAT","chr4",122396333,"+","none","TGG","CCTGAAATATAGCAGTTCATTGG",0,NA,NA,NA,"GeneID:132612","ADAD1",TRUE,"ADAD1"

"2348","CCTGAAATATAGCAGTTCAT","chr1",175195991,"+","16:G>T,18:T>A","TGG","CCTGAAATATAGCAGTGCTTTGG",2,17,19,NA,"","",FALSE,"ADAD1"

"2349","CCTGAAATATAGCAGTTCAT","chr6",16942635,"+","2:A>T,14:T>G","AGG","CCAGAAATATAGCATTTCATAGG",2,3,15,NA,"","",FALSE,"ADAD1"

"2350","CTCGCTGCATTTGTGCAAAC","chr4",122381127,"-","none","TGG","CTCGCTGCATTTGTGCAAACTGG",0,NA,NA,NA,"GeneID:132612","ADAD1",TRUE,"ADAD1"

"2351","CTCGCTGCATTTGTGCAAAC","chr12",9090398,"-","3:T>C,10:G>A","GGG","CTCACTGCATCTGTGCAAACGGG",2,4,11,NA,"GeneID:2","A2M",TRUE,"ADAD1"

"2352","GCGAGTTCAGCTTGACCTTA","chr4",122381142,"+","none","AGG","GCGAGTTCAGCTTGACCTTAAGG",0,NA,NA,NA,"GeneID:132612","ADAD1",TRUE,"ADAD1"

"2353","GCTAGTTCAGATAAAACAAC","chr4",122393614,"-","none","AGG","GCTAGTTCAGATAAAACAACAGG",0,NA,NA,NA,"GeneID:132612","ADAD1",TRUE,"ADAD1"

"2354","TATGTTTCAAAAGTACATTA","chr4",122393635,"+","none","TGG","TATGTTTCAAAAGTACATTATGG",0,NA,NA,NA,"GeneID:132612","ADAD1",TRUE,"ADAD1"

"2355","TATGTTTCAAAAGTACATTA","chr6",103957388,"+","14:G>A,18:G>T","AGG","TATGTTTCAAAAGTGCATGAAGG",2,15,19,NA,"","",FALSE,"ADAD1"

"2356","ACGCTGCGCAGCGCTGCTCA","chr16",84195084,"-","none","TGG","ACGCTGCGCAGCGCTGCTCATGG",0,NA,NA,NA,"GeneID:161931","ADAD2",TRUE,"ADAD2"

"2357","ATGGCGAGCGCTCGTCCAAC","chr16",84195129,"-","none","AGG","ATGGCGAGCGCTCGTCCAACAGG",0,NA,NA,NA,"GeneID:161931","ADAD2",TRUE,"ADAD2"

"2358","CAGTTCCGCGCTCACCGAGA","chr16",84194456,"-","none","AGG","CAGTTCCGCGCTCACCGAGAAGG",0,NA,NA,NA,"GeneID:161931","ADAD2",TRUE,"ADAD2"

"2359","CGGTTTGCCCAACTTGAGTC","chr16",84194142,"-","none","AGG","CGGTTTGCCCAACTTGAGTCAGG",0,NA,NA,NA,"GeneID:161931","ADAD2",TRUE,"ADAD2"

"2360","TGCTCACCTACCTACGCTCA","chr16",84194971,"-","none","GGG","TGCTCACCTACCTACGCTCAGGG",0,NA,NA,NA,"GeneID:161931","ADAD2",TRUE,"ADAD2"

"2361","TTGCTATTCGCAGTGCCCGC","chr16",84194494,"-","none","AGG","TTGCTATTCGCAGTGCCCGCAGG",0,NA,NA,NA,"GeneID:161931","ADAD2",TRUE,"ADAD2"

"2362","ATAAAAGAATTTGCAGATGA","chr15",43340279,"+","none","CGG","ATAAAAGAATTTGCAGATGACGG",0,NA,NA,NA,"GeneID:161823","ADAL",TRUE,"ADAL"

"2363","ATAAAAGAATTTGCAGATGA","chr8",24334527,"+","12:C>G,15:A>G","GGG","ATAAAAGAATTTCCAAATGAGGG",2,13,16,NA,"GeneID:10863","ADAM28",FALSE,"ADAL"

"2364","ATAAAAGAATTTGCAGATGA","chr4",47283273,"+","7:C>A,13:T>C","GGG","ATAAAAGCATTTGTAGATGAGGG",2,8,14,NA,"GeneID:2560","GABRB1",FALSE,"ADAL"

"2365","ATAAAAGAATTTGCAGATGA","chr3",154005423,"+","7:T>A,18:A>G","AGG","ATAAAAGTATTTGCAGATAAAGG",2,8,19,NA,"","",FALSE,"ADAL"

"2366","ATAAAAGAATTTGCAGATGA","chr1",108076025,"-","0:C>T,19:C>T","TGG","GTAAAAGAATTTGCAGATGGTGG",2,1,20,NA,"","",TRUE,"ADAL"

"2367","CAATCACAGTCATCTGATCG","chr15",43335778,"-","none","TGG","CAATCACAGTCATCTGATCGTGG",0,NA,NA,NA,"GeneID:161823","ADAL",TRUE,"ADAL"

"2368","CCCAGAAGAGAAAATGCTAC","chr15",43340330,"+","none","TGG","CCCAGAAGAGAAAATGCTACTGG",0,NA,NA,NA,"GeneID:161823","ADAL",TRUE,"ADAL"

"2369","CCCAGAAGAGAAAATGCTAC","chr16",2261355,"+","7:C>G,13:G>A","CGG","CCCAGAACAGAAAGTGCTACCGG",2,8,14,NA,"GeneID:10921","RNPS1",FALSE,"ADAL"

"2370","CTATTCTGAATTGCCAAAAG","chr15",43335148,"+","none","TGG","CTATTCTGAATTGCCAAAAGTGG",0,NA,NA,NA,"GeneID:161823","ADAL",TRUE,"ADAL"

"2371","CTATTCTGAATTGCCAAAAG","chr7",91100814,"-","7:T>C,10:G>A","TGG","CTATTCTAAACTGCCAAAAGTGG",2,8,11,NA,"GeneID:5218","CDK14",FALSE,"ADAL"

"2372","GAATAGAAGTCTGTCTTGCA","chr15",43335134,"-","none","AGG","GAATAGAAGTCTGTCTTGCAAGG",0,NA,NA,NA,"GeneID:161823","ADAL",TRUE,"ADAL"

"2373","GAATAGAAGTCTGTCTTGCA","chr2",96269246,"+","0:C>G,3:G>T","GGG","CAAGAGAAGTCTGTCTTGCAGGG",2,1,4,NA,"GeneID:9391","CIAO1",TRUE,"ADAL"

"2374","TCAGATGACTGTGATTGACA","chr15",43335781,"+","none","AGG","TCAGATGACTGTGATTGACAAGG",0,NA,NA,NA,"GeneID:161823","ADAL",TRUE,"ADAL"

"2375","TCAGATGACTGTGATTGACA","chr10",3531473,"-","13:A>T,19:A>T","CGG","TCAGATGACTGTGTTTGACTCGG",2,14,20,NA,"","",FALSE,"ADAL"

"2376","AAAATGTGCCACCACGAGTC","chr15",58679201,"+","none","TGG","AAAATGTGCCACCACGAGTCTGG",0,NA,NA,NA,"GeneID:102","ADAM10",TRUE,"ADAM10"

"2377","AAGTGTCCCTCTTCATTCGT","chr15",58682282,"+","none","AGG","AAGTGTCCCTCTTCATTCGTAGG",0,NA,NA,NA,"GeneID:102","ADAM10",TRUE,"ADAM10"

"2378","AAGTGTCCCTCTTCATTCGT","chr1",73329159,"+","13:T>C,17:T>C","AGG","AAGTGTCCCTCTTTATTTGTAGG",2,14,18,NA,"GeneID:101927295","LINC01360",FALSE,"ADAM10"

"2379","CGTCTAGATTTCCATGCCCA","chr15",58717582,"-","none","TGG","CGTCTAGATTTCCATGCCCATGG",0,NA,NA,NA,"GeneID:102","ADAM10",TRUE,"ADAM10"

"2380","GATACCTCTCATATTTACAC","chr15",58682209,"-","none","TGG","GATACCTCTCATATTTACACTGG",0,NA,NA,NA,"GeneID:102","ADAM10",TRUE,"ADAM10"

"2381","TGATCTGCACAGCCCCCCTG","chr15",58665154,"+","none","AGG","TGATCTGCACAGCCCCCCTGAGG",0,NA,NA,NA,"GeneID:102","ADAM10",TRUE,"ADAM10"

"2382","TGATCTGCACAGCCCCCCTG","chr2",10137124,"+","14:A>C,17:G>C","GGG","TGATCTGCACAGCCACCGTGGGG",2,15,18,NA,"","",FALSE,"ADAM10"

"2383","TTAGACTATCCCCATAAATA","chr15",58665181,"-","none","CGG","TTAGACTATCCCCATAAATACGG",0,NA,NA,NA,"GeneID:102","ADAM10",TRUE,"ADAM10"

"2384","CCTCCTCTCCTCGCAATACG","chr17",44769984,"+","none","TGG","CCTCCTCTCCTCGCAATACGTGG",0,NA,NA,NA,"GeneID:4185","ADAM11",TRUE,"ADAM11"

"2385","CGGAGCTCTCCCTAACCAGA","chr17",44759815,"-","none","CGG","CGGAGCTCTCCCTAACCAGACGG",0,NA,NA,NA,"GeneID:4185","ADAM11",TRUE,"ADAM11"

"2386","CTGGGATGACGAAACTCACC","chr17",44769734,"-","none","TGG","CTGGGATGACGAAACTCACCTGG",0,NA,NA,NA,"GeneID:4185","ADAM11",TRUE,"ADAM11"

"2387","CTTCAACTCAAACTTCACCC","chr17",44769755,"+","none","TGG","CTTCAACTCAAACTTCACCCTGG",0,NA,NA,NA,"GeneID:4185","ADAM11",TRUE,"ADAM11"

"2388","CTTCAACTCAAACTTCACCC","chr13",94191400,"-","15:C>G,18:T>G","TGG","CTTCAACTCAAACTTGACACTGG",2,16,19,NA,"GeneID:10082","GPC6",FALSE,"ADAM11"

"2389","CTTCAACTCAAACTTCACCC","chr1",39731649,"+","5:T>A,8:A>C","TGG","CTTCATCTAAAACTTCACCCTGG",2,6,9,NA,"GeneID:10450","PPIE",FALSE,"ADAM11"

"2390","CTTCAACTCAAACTTCACCC","chr11",10209341,"+","2:G>T,12:G>C","TGG","CTGCAACTCAAAGTTCACCCTGG",2,3,13,NA,"GeneID:81846","SBF2",FALSE,"ADAM11"

"2391","GCTCCACGTATTGCGAGGAG","chr17",44769990,"-","none","AGG","GCTCCACGTATTGCGAGGAGAGG",0,NA,NA,NA,"GeneID:4185","ADAM11",TRUE,"ADAM11"

"2392","GGTCACGGAACCCAGCCGTC","chr17",44759797,"+","none","TGG","GGTCACGGAACCCAGCCGTCTGG",0,NA,NA,NA,"GeneID:4185","ADAM11",TRUE,"ADAM11"

"2393","AATATCCCCGTACATGTCCA","chr10",126135623,"+","none","TGG","AATATCCCCGTACATGTCCATGG",0,NA,NA,NA,"GeneID:8038","ADAM12",TRUE,"ADAM12"

"2394","AGTCGAATATTCAGCACTTC","chr10",126278959,"+","none","TGG","AGTCGAATATTCAGCACTTCTGG",0,NA,NA,NA,"GeneID:8038","ADAM12",TRUE,"ADAM12"

"2395","AGTCGAATATTCAGCACTTC","chr1",184949091,"+","3:G>C,19:T>C","GGG","AGTGGAATATTCAGCACTTTGGG",2,4,20,NA,"GeneID:116496","FAM129A",FALSE,"ADAM12"

"2396","AGTGAAGAGCTTCGACTCCA","chr10",126330413,"-","none","AGG","AGTGAAGAGCTTCGACTCCAAGG",0,NA,NA,NA,"GeneID:8038","ADAM12",TRUE,"ADAM12"

"2397","GTCAGTCTCAGCACGTGTTC","chr10",126135592,"-","none","TGG","GTCAGTCTCAGCACGTGTTCTGG",0,NA,NA,NA,"GeneID:8038","ADAM12",TRUE,"ADAM12"

"2398","TGCTGAATATTCGACTACAA","chr10",126278955,"-","none","CGG","TGCTGAATATTCGACTACAACGG",0,NA,NA,NA,"GeneID:8038","ADAM12",TRUE,"ADAM12"

"2399","TTGGAGTCGAAGCTCTTCAC","chr10",126330412,"+","none","TGG","TTGGAGTCGAAGCTCTTCACTGG",0,NA,NA,NA,"GeneID:8038","ADAM12",TRUE,"ADAM12"

"2400","CACAGCTTGCCCGCCAGCGC","chr1",155057968,"-","none","AGG","CACAGCTTGCCCGCCAGCGCAGG",0,NA,NA,NA,"GeneID:8751","ADAM15",TRUE,"ADAM15"

"2401","GCAGGTCACCCGATGGGCAC","chr1",155062306,"-","none","CGG","GCAGGTCACCCGATGGGCACCGG",0,NA,NA,NA,"GeneID:8751","ADAM15",TRUE,"ADAM15"

"2402","GCAGGTCACCCGATGGGCAC","chr6",32214158,"+","7:C>A,11:C>G","AGG","GCAGGTCCCCCCATGGGCACAGG",2,8,12,NA,"GeneID:4855","NOTCH4",TRUE,"ADAM15"

"2403","GGACGGTGACAGTCATATCC","chr1",155053454,"+","none","TGG","GGACGGTGACAGTCATATCCTGG",0,NA,NA,NA,"GeneID:8751","ADAM15",TRUE,"ADAM15"

"2404","TAATTGGGAGATCGTCCTGA","chr1",155052737,"-","none","AGG","TAATTGGGAGATCGTCCTGAAGG",0,NA,NA,NA,"GeneID:8751","ADAM15",TRUE,"ADAM15"

"2405","TGCCCATCGGGTGACCTGCC","chr1",155062307,"+","none","CGG","TGCCCATCGGGTGACCTGCCCGG",0,NA,NA,NA,"GeneID:8751","ADAM15",TRUE,"ADAM15"

"2406","TTCCCCGAGTATTAGCTGTC","chr1",155058077,"-","none","TGG","TTCCCCGAGTATTAGCTGTCTGG",0,NA,NA,NA,"GeneID:8751","ADAM15",TRUE,"ADAM15"

"2407","AAAGCGAGTACACTGTAAAA","chr2",9536728,"-","none","TGG","AAAGCGAGTACACTGTAAAATGG",0,NA,NA,NA,"GeneID:6868","ADAM17",TRUE,"ADAM17"

"2408","AACGTTCAGTACTTGATGTC","chr2",9536790,"+","none","AGG","AACGTTCAGTACTTGATGTCAGG",0,NA,NA,NA,"GeneID:6868","ADAM17",TRUE,"ADAM17"

"2409","ATCTAATATCCAGCAGCATT","chr2",9543222,"-","none","CGG","ATCTAATATCCAGCAGCATTCGG",0,NA,NA,NA,"GeneID:6868","ADAM17",TRUE,"ADAM17"

"2410","CTTAAGGTGAGCCTGACTCT","chr2",9535908,"-","none","AGG","CTTAAGGTGAGCCTGACTCTAGG",0,NA,NA,NA,"GeneID:6868","ADAM17",TRUE,"ADAM17"

"2411","GGACTTCTTCACTGGACACG","chr2",9536703,"-","none","TGG","GGACTTCTTCACTGGACACGTGG",0,NA,NA,NA,"GeneID:6868","ADAM17",TRUE,"ADAM17"

"2412","TAACATCATCATCTCTTATG","chr2",9535874,"+","none","TGG","TAACATCATCATCTCTTATGTGG",0,NA,NA,NA,"GeneID:6868","ADAM17",TRUE,"ADAM17"

"2413","TAACATCATCATCTCTTATG","chr7",43953349,"-","0:T>A,6:T>G","GGG","AAACATAATCATCTCTTATGGGG",2,1,7,NA,"GeneID:51619;GeneID:84820","UBE2D4;POLR2J4",TRUE,"ADAM17"

"2414","CAACCTTACACTCTACATCT","chr8",39606333,"+","none","CGG","CAACCTTACACTCTACATCTCGG",0,NA,NA,NA,"GeneID:8749","ADAM18",TRUE,"ADAM18"

"2415","CTCGCCCTCCTCACTGAGCT","chr8",39584634,"+","none","TGG","CTCGCCCTCCTCACTGAGCTTGG",0,NA,NA,NA,"GeneID:8749","ADAM18",TRUE,"ADAM18"

"2416","CTCGCCCTCCTCACTGAGCT","chr14",36574066,"-","3:G>C,5:A>G","TGG","CTCCCTCTCCTCACTGAGCTTGG",2,4,6,NA,"","",FALSE,"ADAM18"

"2417","CTCGCCCTCCTCACTGAGCT","chr17",41579101,"-","2:C>G,7:T>A","TGG","CTGGCCCACCTCACTGAGCTTGG",2,3,8,NA,"","",FALSE,"ADAM18"

"2418","CTCGCCCTCCTCACTGAGCT","chr17",31677237,"+","1:A>T,7:C>T","GGG","CACGCCCCCCTCACTGAGCTGGG",2,2,8,NA,"","",FALSE,"ADAM18"

"2419","GCAGTCTTCCAAGCTCAGTG","chr8",39584645,"-","none","AGG","GCAGTCTTCCAAGCTCAGTGAGG",0,NA,NA,NA,"GeneID:8749","ADAM18",TRUE,"ADAM18"

"2420","TCATTTGACTTAATCTTCCG","chr8",39585310,"-","none","TGG","TCATTTGACTTAATCTTCCGTGG",0,NA,NA,NA,"GeneID:8749","ADAM18",TRUE,"ADAM18"

"2421","TCATTTGACTTAATCTTCCG","chr5",90246109,"+","6:C>G,19:A>G","AGG","TCATTTCACTTAATCTTCCAAGG",2,7,20,NA,"","",FALSE,"ADAM18"

"2422","TTCTGCATGTCACAGTTCCA","chr8",39585290,"+","none","CGG","TTCTGCATGTCACAGTTCCACGG",0,NA,NA,NA,"GeneID:8749","ADAM18",TRUE,"ADAM18"

"2423","TTTCCGAGATGTAGAGTGTA","chr8",39606339,"-","none","AGG","TTTCCGAGATGTAGAGTGTAAGG",0,NA,NA,NA,"GeneID:8749","ADAM18",TRUE,"ADAM18"

"2424","ACTCACCTCCAATTTCCGTG","chr5",157537906,"+","none","TGG","ACTCACCTCCAATTTCCGTGTGG",0,NA,NA,NA,"GeneID:8728","ADAM19",TRUE,"ADAM19"

"2425","ATCCACTCAAAGCTGAGCTC","chr5",157564422,"-","none","AGG","ATCCACTCAAAGCTGAGCTCAGG",0,NA,NA,NA,"GeneID:8728","ADAM19",TRUE,"ADAM19"

"2426","ATCCACTCAAAGCTGAGCTC","chr2",205928663,"-","2:C>G,5:A>G","GGG","ATGCATTCAAAGCTGAGCTCGGG",2,3,6,NA,"","",FALSE,"ADAM19"

"2427","ATGAACTTATCATACCTCAG","chr5",157570927,"-","none","TGG","ATGAACTTATCATACCTCAGTGG",0,NA,NA,NA,"GeneID:8728","ADAM19",TRUE,"ADAM19"

"2428","ATGAACTTATCATACCTCAG","chr17",67473809,"+","9:A>T,13:T>A","TGG","ATGAACTTAACATTCCTCAGTGG",2,10,14,NA,"GeneID:26207","PITPNC1",FALSE,"ADAM19"

"2429","ATGAACTTATCATACCTCAG","chrX",135076628,"-","4:C>T,10:A>G","GGG","ATGAGCTTATTATACCTCAGGGG",2,5,11,NA,"","",FALSE,"ADAM19"

"2430","GATAAGTTCATGCTGCAGCT","chr5",157570936,"+","none","TGG","GATAAGTTCATGCTGCAGCTTGG",0,NA,NA,NA,"GeneID:8728","ADAM19",TRUE,"ADAM19"

"2431","TCAAACCACCACACGGAAAT","chr5",157537917,"-","none","TGG","TCAAACCACCACACGGAAATTGG",0,NA,NA,NA,"GeneID:8728","ADAM19",TRUE,"ADAM19"

"2432","TCAAACCACCACACGGAAAT","chr6",29292529,"-","7:G>T,14:A>C","AGG","TCAAACCCCCACACTGAAATAGG",2,8,15,NA,"GeneID:101929006","LOC101929006",TRUE,"ADAM19"

"2433","TGAGGGGCGAGAACTGATCC","chr5",157564391,"-","none","TGG","TGAGGGGCGAGAACTGATCCTGG",0,NA,NA,NA,"GeneID:8728","ADAM19",TRUE,"ADAM19"

"2434","ACCTGTGCAAATTACAGTTC","chr8",39837179,"-","none","CGG","ACCTGTGCAAATTACAGTTCCGG",0,NA,NA,NA,"GeneID:2515","ADAM2",TRUE,"ADAM2"

"2435","CAGAAACAAGACGCGCCACA","chr8",39838164,"+","none","TGG","CAGAAACAAGACGCGCCACATGG",0,NA,NA,NA,"GeneID:2515","ADAM2",TRUE,"ADAM2"

"2436","CGCGTCTTGTTTCTGCTCAG","chr8",39838159,"-","none","CGG","CGCGTCTTGTTTCTGCTCAGCGG",0,NA,NA,NA,"GeneID:2515","ADAM2",TRUE,"ADAM2"

"2437","GAAAATACGGTCAATAATAA","chr8",39837155,"-","none","AGG","GAAAATACGGTCAATAATAAAGG",0,NA,NA,NA,"GeneID:2515","ADAM2",TRUE,"ADAM2"

"2438","GAAAATACGGTCAATAATAA","chr13",32089001,"+","0:A>G,9:T>G","TGG","AAAAATACGTTCAATAATAATGG",2,1,10,NA,"GeneID:10129","FRY",FALSE,"ADAM2"

"2439","TCCTACAAAATTGTAATTGA","chr8",39833976,"-","none","AGG","TCCTACAAAATTGTAATTGAAGG",0,NA,NA,NA,"GeneID:2515","ADAM2",TRUE,"ADAM2"

"2440","TGCATTAAATTCACAGTATA","chr8",39833946,"+","none","TGG","TGCATTAAATTCACAGTATATGG",0,NA,NA,NA,"GeneID:2515","ADAM2",TRUE,"ADAM2"

"2441","ACCCTCCCACACATGATATC","chr14",70523066,"+","none","AGG","ACCCTCCCACACATGATATCAGG",0,NA,NA,NA,"GeneID:8748","ADAM20",TRUE,"ADAM20"

"2442","ACTGCAGTTATGCCCAATAT","chr14",70523605,"-","none","TGG","ACTGCAGTTATGCCCAATATTGG",0,NA,NA,NA,"GeneID:8748","ADAM20",TRUE,"ADAM20"

"2443","CCAAATTATGACCAAGCTCG","chr14",70523712,"+","none","TGG","CCAAATTATGACCAAGCTCGTGG",0,NA,NA,NA,"GeneID:8748","ADAM20",TRUE,"ADAM20"

"2444","CCATCAGCGGTTTGTTGAGC","chr14",70524126,"-","none","TGG","CCATCAGCGGTTTGTTGAGCTGG",0,NA,NA,NA,"GeneID:8748","ADAM20",TRUE,"ADAM20"

"2445","CCGTAAGAAGTGTGCCAGTA","chr14",70522875,"-","none","TGG","CCGTAAGAAGTGTGCCAGTATGG",0,NA,NA,NA,"GeneID:8748","ADAM20",TRUE,"ADAM20"

"2446","GTAGCTCGCACACACACCAC","chr14",70523673,"+","none","TGG","GTAGCTCGCACACACACCACTGG",0,NA,NA,NA,"GeneID:8748","ADAM20",TRUE,"ADAM20"

"2447","ACGACATATTCCTGCAACAT","chr14",70458430,"-","none","AGG","ACGACATATTCCTGCAACATAGG",0,NA,NA,NA,"GeneID:8747","ADAM21",TRUE,"ADAM21"

"2448","ACGACATATTCCTGCAACAT","chr14",70246709,"+","17:T>C,18:G>A","AGG","ACGACATATTCCTGCAATGTAGG",2,18,19,NA,"GeneID:145241","ADAM21P1",TRUE,"ADAM21"

"2449","CCCAATGACAAGCATAGAAC","chr14",70458299,"+","none","AGG","CCCAATGACAAGCATAGAACAGG",0,NA,NA,NA,"GeneID:8747","ADAM21",TRUE,"ADAM21"

"2450","GAGTGACTCTGATGTACACG","chr14",70457519,"-","none","AGG","GAGTGACTCTGATGTACACGAGG",0,NA,NA,NA,"GeneID:8747","ADAM21",TRUE,"ADAM21"

"2451","GAGTGACTCTGATGTACACG","chr14",70247620,"+","7:C>T,19:T>G","AGG","GAGTGACCCTGATGTACACTAGG",2,8,20,NA,"GeneID:145241","ADAM21P1",TRUE,"ADAM21"

"2452","TAAGAAGCCCGACAGTAAAC","chr14",70459592,"-","none","AGG","TAAGAAGCCCGACAGTAAACAGG",0,NA,NA,NA,"GeneID:8747","ADAM21",TRUE,"ADAM21"

"2453","TAAGAAGCCCGACAGTAAAC","chr14",70245546,"+","10:A>G,19:G>C","AGG","TAAGAAGCCCAACAGTAAAGAGG",2,11,20,NA,"GeneID:145241","ADAM21P1",TRUE,"ADAM21"

"2454","TCCATTGAGAGAAATCGTTC","chr14",70458326,"-","none","AGG","TCCATTGAGAGAAATCGTTCAGG",0,NA,NA,NA,"GeneID:8747","ADAM21",TRUE,"ADAM21"

"2455","TCCTGAACGATTTCTCTCAA","chr14",70458322,"+","none","TGG","TCCTGAACGATTTCTCTCAATGG",0,NA,NA,NA,"GeneID:8747","ADAM21",TRUE,"ADAM21"

"2456","TCCTGAACGATTTCTCTCAA","chr14",70246817,"-","4:T>C,7:A>G","CGG","TCCTAAATGATTTCTCTCAACGG",2,5,8,NA,"GeneID:145241","ADAM21P1",TRUE,"ADAM21"

"2457","AGGAGAGCACTGTTACTACC","chr7",88108177,"+","none","AGG","AGGAGAGCACTGTTACTACCAGG",0,NA,NA,NA,"GeneID:53616","ADAM22",TRUE,"ADAM22"

"2458","ATAGAGAGACACATTGAACA","chr7",88075647,"+","none","TGG","ATAGAGAGACACATTGAACATGG",0,NA,NA,NA,"GeneID:53616","ADAM22",TRUE,"ADAM22"

"2459","ATAGAGAGACACATTGAACA","chr18",6053051,"-","5:T>C,13:T>A","AGG","ATAGAAAGACACAATGAACAAGG",2,6,14,NA,"GeneID:91133","L3MBTL4",FALSE,"ADAM22"

"2460","GAGAATGAATGACGTTCCAA","chr7",87978375,"-","none","AGG","GAGAATGAATGACGTTCCAAAGG",0,NA,NA,NA,"GeneID:53616","ADAM22",TRUE,"ADAM22"

"2461","GAGAATGAATGACGTTCCAA","chr5",174648274,"+","8:G>A,12:T>C","GGG","GAGAATGAGTGATGTTCCAAGGG",2,9,13,NA,"","",FALSE,"ADAM22"

"2462","GTGTCTCTCTATGTATTCAG","chr7",88075639,"-","none","AGG","GTGTCTCTCTATGTATTCAGAGG",0,NA,NA,NA,"GeneID:53616","ADAM22",TRUE,"ADAM22"

"2463","GTGTCTCTCTATGTATTCAG","chr7",106732366,"+","4:G>C,10:T>A","TGG","GTGTGTCTCTTTGTATTCAGTGG",2,5,11,NA,"","",TRUE,"ADAM22"

"2464","GTGTCTCTCTATGTATTCAG","chr14",100846229,"+","4:G>C,18:T>A","GGG","GTGTGTCTCTATGTATTCTGGGG",2,5,19,NA,"GeneID:55384","MEG3",TRUE,"ADAM22"

"2465","TGAACACTTACTGAAGTCCG","chr7",88108249,"-","none","TGG","TGAACACTTACTGAAGTCCGTGG",0,NA,NA,NA,"GeneID:53616","ADAM22",TRUE,"ADAM22"

"2466","TGAACACTTACTGAAGTCCG","chr15",52753973,"+","6:T>C,18:T>C","GGG","TGAACATTTACTGAAGTCTGGGG",2,7,19,NA,"","",FALSE,"ADAM22"

"2467","TGAACACTTACTGAAGTCCG","chr6",17923436,"-","4:C>G,15:A>C","GGG","TGAAGACTTACTGAATTCCGGGG",2,5,16,NA,"GeneID:63971","KIF13A",FALSE,"ADAM22"

"2468","TGTTGACCAAGCAAGCTTCC","chr7",87978343,"+","none","AGG","TGTTGACCAAGCAAGCTTCCAGG",0,NA,NA,NA,"GeneID:53616","ADAM22",TRUE,"ADAM22"

"2469","TGTTGACCAAGCAAGCTTCC","chr10",28394427,"-","6:T>G,15:T>G","TGG","TGTTGAACAAGCAAGATTCCTGG",2,7,16,NA,"","",FALSE,"ADAM22"

"2470","AACCGTACCTTAGAGTACTG","chr2",206530936,"-","none","TGG","AACCGTACCTTAGAGTACTGTGG",0,NA,NA,NA,"GeneID:8745","ADAM23",TRUE,"ADAM23"

"2471","CACTTACTGAAGTCCATTGC","chr2",206542121,"-","none","AGG","CACTTACTGAAGTCCATTGCAGG",0,NA,NA,NA,"GeneID:8745","ADAM23",TRUE,"ADAM23"

"2472","CACTTACTGAAGTCCATTGC","chr2",99810030,"-","9:A>T,16:C>A","AGG","CACTTACTGTAGTCCAGTGCAGG",2,10,17,NA,"GeneID:3899","AFF3",FALSE,"ADAM23"

"2473","CAGAGGCGTCAAAGACTCCA","chr2",206542083,"+","none","AGG","CAGAGGCGTCAAAGACTCCAAGG",0,NA,NA,NA,"GeneID:8745","ADAM23",TRUE,"ADAM23"

"2474","TATGATAGAGCCACTAGAGC","chr2",206543282,"+","none","TGG","TATGATAGAGCCACTAGAGCTGG",0,NA,NA,NA,"GeneID:8745","ADAM23",TRUE,"ADAM23"

"2475","TCATCATGAACCAGCTCTAG","chr2",206543295,"-","none","TGG","TCATCATGAACCAGCTCTAGTGG",0,NA,NA,NA,"GeneID:8745","ADAM23",TRUE,"ADAM23"

"2476","TGGGAAACCACAGTACTCTA","chr2",206530926,"+","none","AGG","TGGGAAACCACAGTACTCTAAGG",0,NA,NA,NA,"GeneID:8745","ADAM23",TRUE,"ADAM23"

"2477","AAAAGCTGTACCTGTTGCTC","chr8",24300068,"-","none","TGG","AAAAGCTGTACCTGTTGCTCTGG",0,NA,NA,NA,"GeneID:10863","ADAM28",TRUE,"ADAM28"

"2478","AAAAGCTGTACCTGTTGCTC","chr2",180513076,"+","17:T>C,19:A>C","TGG","AAAAGCTGTACCTGTTGTTATGG",2,18,20,NA,"","",FALSE,"ADAM28"

"2479","AAAAGCTGTACCTGTTGCTC","chr10",8131798,"+","1:T>A,5:G>C","AGG","ATAAGGTGTACCTGTTGCTCAGG",2,2,6,NA,"","",FALSE,"ADAM28"

"2480","AAGTATAAAATGACAATTAA","chr8",24309914,"+","none","TGG","AAGTATAAAATGACAATTAATGG",0,NA,NA,NA,"GeneID:10863","ADAM28",TRUE,"ADAM28"

"2481","AAGTATAAAATGACAATTAA","chr5",161788900,"-","15:A>T,16:C>A","TGG","AAGTATAAAATGACATGTAATGG",2,16,17,NA,"","",FALSE,"ADAM28"

"2482","AAGTATAAAATGACAATTAA","chr6",91843227,"-","13:A>G,18:C>T","AGG","AAGTATAAAATGATAATTGAAGG",2,14,19,NA,"","",FALSE,"ADAM28"

"2483","AAGTATAAAATGACAATTAA","chr13",104112306,"-","10:C>A,17:T>A","TGG","AAGTATAAAAGGACAATAAATGG",2,11,18,NA,"","",FALSE,"ADAM28"

"2484","AAGTATAAAATGACAATTAA","chr12",68120513,"+","11:C>G,12:T>A","TGG","AAGTATAAAATCTCAATTAATGG",2,12,13,NA,"GeneID:100885789","IFNG-AS1",FALSE,"ADAM28"

"2485","AAGTATAAAATGACAATTAA","chr13",43266122,"-","0:A>T,3:C>A","AGG","TAGGATAAAATGACAATTAAAGG",2,1,4,NA,"GeneID:55068","ENOX1",FALSE,"ADAM28"

"2486","AAGTATAAAATGACAATTAA","chr11",44131270,"-","2:G>C,4:G>T","GGG","AACTCTAAAATGACAATTAAGGG",2,3,5,NA,"GeneID:2132","EXT2",FALSE,"ADAM28"

"2487","AAGTATAAAATGACAATTAA","chr9",102684536,"+","4:T>A,13:A>C","AGG","AAGTTTAAAATGAAAATTAAAGG",2,5,14,NA,"","",FALSE,"ADAM28"

"2488","AAGTATAAAATGACAATTAA","chr15",45928714,"+","7:C>A,14:T>A","AGG","AAGTATACAATGACTATTAAAGG",2,8,15,NA,"","",FALSE,"ADAM28"

"2489","AAGTATAAAATGACAATTAA","chr5",81068536,"+","4:C>A,16:C>T","GGG","AAGTCTAAAATGACAACTAAGGG",2,5,17,NA,"GeneID:5924","RASGRF2",FALSE,"ADAM28"

"2490","AAGTATAAAATGACAATTAA","chr8",34094743,"+","3:A>T,18:T>A","AGG","AAGAATAAAATGACAATTTAAGG",2,4,19,NA,"","",FALSE,"ADAM28"

"2491","AAGTATAAAATGACAATTAA","chr19",21073292,"-","3:G>A,15:C>T","TGG","AAGCATAAAATGACAGTTAATGG",2,4,16,NA,"","",FALSE,"ADAM28"

"2492","AATATGTACCTGCAACAGAG","chr8",24294184,"-","none","AGG","AATATGTACCTGCAACAGAGAGG",0,NA,NA,NA,"GeneID:10863","ADAM28",TRUE,"ADAM28"

"2493","AATATGTACCTGCAACAGAG","chr10",99499006,"+","15:G>C,19:A>G","TGG","AATATGTACCTGCAAGAGAATGG",2,16,20,NA,"","",FALSE,"ADAM28"

"2494","AATATGTACCTGCAACAGAG","chr16",80020843,"+","1:T>A,3:C>A","TGG","ATTCTGTACCTGCAACAGAGTGG",2,2,4,NA,"","",FALSE,"ADAM28"

"2495","AATATGTACCTGCAACAGAG","chr2",197398088,"-","8:A>G,15:C>G","TGG","AATATGTATCTGCAAGAGAGTGG",2,9,16,NA,"GeneID:23451","SF3B1",TRUE,"ADAM28"

"2496","ACTTCATACTTCTTCACCCC","chr8",24299996,"-","none","AGG","ACTTCATACTTCTTCACCCCAGG",0,NA,NA,NA,"GeneID:10863","ADAM28",TRUE,"ADAM28"

"2497","ACTTCATACTTCTTCACCCC","chr12",16325296,"+","1:T>C,8:T>C","AGG","ATTTCATATTTCTTCACCCCAGG",2,2,9,NA,"","",FALSE,"ADAM28"

"2498","ACTTCATACTTCTTCACCCC","chr7",18123679,"-","0:G>T,10:C>A","AGG","CCTTCATACTGCTTCACCCCAGG",2,1,11,NA,"GeneID:9734","HDAC9",FALSE,"ADAM28"

"2499","AGTCTCCTCCTCTCTGTTGC","chr8",24294173,"+","none","AGG","AGTCTCCTCCTCTCTGTTGCAGG",0,NA,NA,NA,"GeneID:10863","ADAM28",TRUE,"ADAM28"

"2500","AGTCTCCTCCTCTCTGTTGC","chr10",106213948,"+","6:T>C,18:T>G","TGG","AGTCTCTTCCTCTCTGTTTCTGG",2,7,19,NA,"","",FALSE,"ADAM28"

"2501","TCATCCACTGCATAAAAGAG","chr8",24300034,"+","none","AGG","TCATCCACTGCATAAAAGAGAGG",0,NA,NA,NA,"GeneID:10863","ADAM28",TRUE,"ADAM28"

"2502","TCATCCACTGCATAAAAGAG","chr2",102532538,"-","13:C>T,17:G>C","AGG","TCATCCACTGCATGAAACAGAGG",2,14,18,NA,"GeneID:389015","SLC9A4",TRUE,"ADAM28"

"2503","AGTAGTATGATCACTCATAT","chr4",174977215,"-","none","TGG","AGTAGTATGATCACTCATATTGG",0,NA,NA,NA,"GeneID:11086","ADAM29",TRUE,"ADAM29"

"2504","AGTCGGAGAACATTACGCCC","chr4",174976342,"+","none","CGG","AGTCGGAGAACATTACGCCCCGG",0,NA,NA,NA,"GeneID:11086","ADAM29",TRUE,"ADAM29"

"2505","ATCTTTAATGTGAAGCGCTG","chr4",174976704,"+","none","TGG","ATCTTTAATGTGAAGCGCTGTGG",0,NA,NA,NA,"GeneID:11086","ADAM29",TRUE,"ADAM29"

"2506","ATCTTTAATGTGAAGCGCTG","chr19",12132190,"+","12:G>A,16:A>G","GGG","ATCTTTAATGTGGAGCACTGGGG",2,13,17,NA,"GeneID:388507;GeneID:7568","ZNF788;ZNF20",TRUE,"ADAM29"

"2507","ATGACCGCAATGAACAGTGT","chr4",174977020,"+","none","AGG","ATGACCGCAATGAACAGTGTAGG",0,NA,NA,NA,"GeneID:11086","ADAM29",TRUE,"ADAM29"

"2508","CCGACTTCCACTTGCAATAC","chr4",174976328,"-","none","AGG","CCGACTTCCACTTGCAATACAGG",0,NA,NA,NA,"GeneID:11086","ADAM29",TRUE,"ADAM29"

"2509","GTGGTGCCAGTTATCCTCAC","chr4",174975633,"-","none","AGG","GTGGTGCCAGTTATCCTCACAGG",0,NA,NA,NA,"GeneID:11086","ADAM29",TRUE,"ADAM29"

"2510","ACTTTCAAGATGTTCGTATG","chr1",119895598,"-","none","AGG","ACTTTCAAGATGTTCGTATGAGG",0,NA,NA,NA,"GeneID:11085","ADAM30",TRUE,"ADAM30"

"2511","ATCGCCCCTCTGAGCAGTCC","chr1",119894291,"+","none","TGG","ATCGCCCCTCTGAGCAGTCCTGG",0,NA,NA,NA,"GeneID:11085","ADAM30",TRUE,"ADAM30"

"2512","ATGCTCGCCTGTCATCAGAT","chr1",119895463,"-","none","TGG","ATGCTCGCCTGTCATCAGATTGG",0,NA,NA,NA,"GeneID:11085","ADAM30",TRUE,"ADAM30"

"2513","GACCTTGCAGAGTACTGCGA","chr1",119894924,"-","none","CGG","GACCTTGCAGAGTACTGCGACGG",0,NA,NA,NA,"GeneID:11085","ADAM30",TRUE,"ADAM30"

"2514","GCATTGTCGGAACTAGTAGA","chr1",119896272,"+","none","AGG","GCATTGTCGGAACTAGTAGAAGG",0,NA,NA,NA,"GeneID:11085","ADAM30",TRUE,"ADAM30"

"2515","GGAACCCCTTGCAAGTATGA","chr1",119894861,"-","none","AGG","GGAACCCCTTGCAAGTATGAAGG",0,NA,NA,NA,"GeneID:11085","ADAM30",TRUE,"ADAM30"

"2516","AACTGTCATTTGTATTTGTT","chr8",39118131,"-","none","TGG","AACTGTCATTTGTATTTGTTTGG",0,NA,NA,NA,"GeneID:203102","ADAM32",TRUE,"ADAM32"

"2517","AACTGTCATTTGTATTTGTT","chr8",26423175,"+","13:T>A,15:G>T","GGG","AACTGTCATTTGTTTGTGTTGGG",2,14,16,NA,"GeneID:665","BNIP3L",FALSE,"ADAM32"

"2518","AACTGTCATTTGTATTTGTT","chr21",15667031,"+","2:G>C,4:T>G","TGG","AAGTTTCATTTGTATTTGTTTGG",2,3,5,NA,"","",FALSE,"ADAM32"

"2519","AACTGTCATTTGTATTTGTT","chr7",53839554,"-","2:T>G,12:C>A","AGG","AAATGTCATTTGGATTTGTTAGG",2,3,13,NA,"","",FALSE,"ADAM32"

"2520","ACACTGTGCACCTTAAACAA","chr8",39136696,"+","none","AGG","ACACTGTGCACCTTAAACAAAGG",0,NA,NA,NA,"GeneID:203102","ADAM32",TRUE,"ADAM32"

"2521","ACACTGTGCACCTTAAACAA","chr19",56611742,"+","1:G>C,10:G>C","AGG","AGACTGTGCAGCTTAAACAAAGG",2,2,11,NA,"GeneID:58491","ZNF71",FALSE,"ADAM32"

"2522","GCCCGGCCAGCAGCAACCAG","chr8",39107786,"-","none","AGG","GCCCGGCCAGCAGCAACCAGAGG",0,NA,NA,NA,"GeneID:203102","ADAM32",TRUE,"ADAM32"

"2523","GCCCGGCCAGCAGCAACCAG","chr14",104093489,"+","11:C>A","AGG","GCCCGGCCAGCCGCAACCAGAGG",1,12,NA,NA,"GeneID:374569","ASPG",TRUE,"ADAM32"

"2524","GCCCGGCCAGCAGCAACCAG","chr16",253218,"+","1:T>C,13:G>C","GGG","GTCCGGCCAGCAGGAACCAGGGG",2,2,14,NA,"GeneID:83986","ITFG3",FALSE,"ADAM32"

"2525","GCCGCACCATGTTCCGCCTC","chr8",39107767,"+","none","TGG","GCCGCACCATGTTCCGCCTCTGG",0,NA,NA,NA,"GeneID:203102","ADAM32",TRUE,"ADAM32"

"2526","GCTCACCGGGTCTTGACGCC","chr8",39107819,"-","none","AGG","GCTCACCGGGTCTTGACGCCAGG",0,NA,NA,NA,"GeneID:203102","ADAM32",TRUE,"ADAM32"

"2527","GTGTACAGTTTCTCATCTAT","chr8",39136680,"-","none","TGG","GTGTACAGTTTCTCATCTATTGG",0,NA,NA,NA,"GeneID:203102","ADAM32",TRUE,"ADAM32"

"2528","AAGCATCTCACCGTGTGGTT","chr20",3675015,"+","none","GGG","AAGCATCTCACCGTGTGGTTGGG",0,NA,NA,NA,"GeneID:80332","ADAM33",TRUE,"ADAM33"

"2529","AAGCATCTCACCGTGTGGTT","chr20",49102602,"+","5:G>T,11:T>C","GGG","AAGCAGCTCACTGTGTGGTTGGG",2,6,12,NA,"","",FALSE,"ADAM33"

"2530","ACCCTGGCGCACCGTCAGCC","chr20",3679502,"-","none","TGG","ACCCTGGCGCACCGTCAGCCTGG",0,NA,NA,NA,"GeneID:80332","ADAM33",TRUE,"ADAM33"

"2531","CCAGGTCTCGAAGCCAGACA","chr20",3677127,"-","none","TGG","CCAGGTCTCGAAGCCAGACATGG",0,NA,NA,NA,"GeneID:80332","ADAM33",TRUE,"ADAM33"

"2532","GAAACCCACTACGGCCCAGA","chr20",3675060,"-","none","TGG","GAAACCCACTACGGCCCAGATGG",0,NA,NA,NA,"GeneID:80332","ADAM33",TRUE,"ADAM33"

"2533","GCAGCCAGTCACCCCGCACT","chr20",3679538,"-","none","GGG","GCAGCCAGTCACCCCGCACTGGG",0,NA,NA,NA,"GeneID:80332","ADAM33",TRUE,"ADAM33"

"2534","GCAGTCTCGCCTGACCCCCA","chr20",3669300,"-","none","AGG","GCAGTCTCGCCTGACCCCCAAGG",0,NA,NA,NA,"GeneID:80332","ADAM33",TRUE,"ADAM33"

"2535","GCAGTCTCGCCTGACCCCCA","chr22",49700199,"+","8:T>G,14:G>C","TGG","GCAGTCTCTCCTGAGCCCCATGG",2,9,15,NA,"","",FALSE,"ADAM33"

"2536","AATCACTCAGAATGCTTCCC","chr8",24441097,"+","none","GGG","AATCACTCAGAATGCTTCCCGGG",0,NA,NA,NA,"GeneID:8756","ADAM7",TRUE,"ADAM7"

"2537","AATCACTCAGAATGCTTCCC","chr11",134148900,"-","12:T>A,13:A>C","AGG","AATCACTCAGAAATCTTCCCAGG",2,13,14,NA,"GeneID:83700","JAM3",FALSE,"ADAM7"

"2538","AATCACTCAGAATGCTTCCC","chr8",126087898,"-","11:C>T,12:C>A","AGG","AATCACTCAGAGGGCTTCCCAGG",2,12,13,NA,"","",FALSE,"ADAM7"

"2539","AATCACTCAGAATGCTTCCC","chr8",114265090,"+","5:A>C,13:T>G","TGG","AATCAATCAGAATTCTTCCCTGG",2,6,14,NA,"","",FALSE,"ADAM7"

"2540","AATCACTCAGAATGCTTCCC","chr4",42304858,"-","0:G>T,13:G>C","TGG","CATCACTCAGAATCCTTCCCTGG",2,1,14,NA,"","",FALSE,"ADAM7"

"2541","ACCTGGATCTTAGAAGATGA","chr8",24447244,"-","none","AGG","ACCTGGATCTTAGAAGATGAAGG",0,NA,NA,NA,"GeneID:8756","ADAM7",TRUE,"ADAM7"

"2542","ACCTGGATCTTAGAAGATGA","chr2",197208404,"-","8:T>G,16:A>T","AGG","ACCTGGATATTAGAAGTTGAAGG",2,9,17,NA,"GeneID:91526","ANKRD44",FALSE,"ADAM7"

"2543","ACCTGGATCTTAGAAGATGA","chr6",140263164,"-","6:G>T,13:A>T","TGG","ACCTGGCTCTTAGTAGATGATGG",2,7,14,NA,"","",FALSE,"ADAM7"

"2544","CAAGACATACCTTTAACCTG","chr8",24441150,"-","none","AGG","CAAGACATACCTTTAACCTGAGG",0,NA,NA,NA,"GeneID:8756","ADAM7",TRUE,"ADAM7"

"2545","CTGATACAGAAGCGAGATAC","chr8",24442528,"+","none","TGG","CTGATACAGAAGCGAGATACTGG",0,NA,NA,NA,"GeneID:8756","ADAM7",TRUE,"ADAM7"

"2546","GTATCTCGCTTCTGTATCAG","chr8",24442528,"-","none","AGG","GTATCTCGCTTCTGTATCAGAGG",0,NA,NA,NA,"GeneID:8756","ADAM7",TRUE,"ADAM7"

"2547","TCCTTCATCTTCTAAGATCC","chr8",24447240,"+","none","AGG","TCCTTCATCTTCTAAGATCCAGG",0,NA,NA,NA,"GeneID:8756","ADAM7",TRUE,"ADAM7"

"2548","TCCTTCATCTTCTAAGATCC","chr7",39215933,"+","7:C>T,14:G>A","AGG","TCCTTCACCTTCTAGGATCCAGG",2,8,15,NA,"GeneID:11281","POU6F2",FALSE,"ADAM7"

"2549","TCCTTCATCTTCTAAGATCC","chr13",107708730,"+","7:A>T,16:T>A","AGG","TCCTTCAACTTCTAAGTTCCAGG",2,8,17,NA,"GeneID:728215","FAM155A",FALSE,"ADAM7"

"2550","AGGACGTAGCTCACCCTCTC","chr10",133274203,"+","none","TGG","AGGACGTAGCTCACCCTCTCTGG",0,NA,NA,NA,"GeneID:101","ADAM8",TRUE,"ADAM8"

"2551","CCACTCACCTGTTCTTCCGC","chr10",133274150,"+","none","AGG","CCACTCACCTGTTCTTCCGCAGG",0,NA,NA,NA,"GeneID:101","ADAM8",TRUE,"ADAM8"

"2552","CCGACCCATACCTGCCCGCG","chr10",133273939,"+","none","AGG","CCGACCCATACCTGCCCGCGAGG",0,NA,NA,NA,"GeneID:101","ADAM8",TRUE,"ADAM8"

"2553","CGGCTACACAGAGACCTATA","chr10",133273994,"-","none","CGG","CGGCTACACAGAGACCTATACGG",0,NA,NA,NA,"GeneID:101","ADAM8",TRUE,"ADAM8"

"2554","CTGGGCGCGATGATGCTGCC","chr10",133276773,"-","none","TGG","CTGGGCGCGATGATGCTGCCTGG",0,NA,NA,NA,"GeneID:101","ADAM8",TRUE,"ADAM8"

"2555","GACCCGGCCATGCGCGGCCT","chr10",133276806,"-","none","CGG","GACCCGGCCATGCGCGGCCTCGG",0,NA,NA,NA,"GeneID:101","ADAM8",TRUE,"ADAM8"

"2556","AAAAGAGCATATTATTCACT","chr8",39011683,"+","none","TGG","AAAAGAGCATATTATTCACTTGG",0,NA,NA,NA,"GeneID:8754","ADAM9",TRUE,"ADAM9"

"2557","AAAAGAGCATATTATTCACT","chr7",133603007,"-","12:G>A,16:T>G","GGG","AAAAGAGCATATCATTAACTGGG",2,13,17,NA,"GeneID:60412","EXOC4",FALSE,"ADAM9"

"2558","AAAAGAGCATATTATTCACT","chr8",66580754,"-","7:A>G,12:T>A","GGG","AAAAGAGTATATAATTCACTGGG",2,8,13,NA,"GeneID:4603","MYBL1",FALSE,"ADAM9"

"2559","AAATACATTACCTGTATATT","chr8",39014034,"-","none","GGG","AAATACATTACCTGTATATTGGG",0,NA,NA,NA,"GeneID:8754","ADAM9",TRUE,"ADAM9"

"2560","CACTGACCATCCCAATATAC","chr8",39014021,"+","none","AGG","CACTGACCATCCCAATATACAGG",0,NA,NA,NA,"GeneID:8754","ADAM9",TRUE,"ADAM9"

"2561","CTTATGAAATTATAACTCCT","chr8",39007912,"+","none","TGG","CTTATGAAATTATAACTCCTTGG",0,NA,NA,NA,"GeneID:8754","ADAM9",TRUE,"ADAM9"

"2562","CTTATGAAATTATAACTCCT","chr8",138528294,"+","3:G>A,12:A>T","TGG","CTTGTGAAATTAAAACTCCTTGG",2,4,13,NA,"","",FALSE,"ADAM9"

"2563","CTTTCTCTAGTTAATCTCCA","chr8",39007932,"-","none","AGG","CTTTCTCTAGTTAATCTCCAAGG",0,NA,NA,NA,"GeneID:8754","ADAM9",TRUE,"ADAM9"

"2564","CTTTCTCTAGTTAATCTCCA","chrX",16838024,"-","5:T>A,6:T>G","AGG","CTTTCAATAGTTAATCTCCAAGG",2,6,7,NA,"GeneID:55787","TXLNG",FALSE,"ADAM9"

"2565","CTTTCTCTAGTTAATCTCCA","chr3",180570316,"-","0:C>G,19:A>T","GGG","GTTTCTCTAGTTAATCTCCTGGG",2,1,20,NA,"","",FALSE,"ADAM9"

"2566","TTAGGTATCTTATGTTATTC","chr8",39011653,"+","none","AGG","TTAGGTATCTTATGTTATTCAGG",0,NA,NA,NA,"GeneID:8754","ADAM9",TRUE,"ADAM9"

"2567","TTAGGTATCTTATGTTATTC","chr7",128453609,"+","4:T>G,13:C>G","AGG","TTAGTTATCTTATCTTATTCAGG",2,5,14,NA,"","",FALSE,"ADAM9"

"2568","ACCACAACTTCATGCTGCGT","chr8",24384493,"+","none","GGG","ACCACAACTTCATGCTGCGTGGG",0,NA,NA,NA,"GeneID:27299","ADAMDEC1",TRUE,"ADAMDEC1"

"2569","CAATATCAGATGATCTTAAA","chr8",24393282,"+","none","TGG","CAATATCAGATGATCTTAAATGG",0,NA,NA,NA,"GeneID:27299","ADAMDEC1",TRUE,"ADAMDEC1"

"2570","CAATATCAGATGATCTTAAA","chr3",112098893,"-","12:C>T,18:C>T","GGG","CAATATCAGATGGTCTTAGAGGG",2,13,19,NA,"GeneID:79669","C3orf52",FALSE,"ADAMDEC1"

"2571","CAATATCAGATGATCTTAAA","chrX",24480179,"-","2:G>T,4:A>T","TGG","CACTTTCAGATGATCTTAAATGG",2,3,5,NA,"GeneID:5165","PDK3",FALSE,"ADAMDEC1"

"2572","TACAATGTTTCAGTGTAGTC","chr8",24394084,"-","none","TGG","TACAATGTTTCAGTGTAGTCTGG",0,NA,NA,NA,"GeneID:27299","ADAMDEC1",TRUE,"ADAMDEC1"

"2573","TACAATGTTTCAGTGTAGTC","chr13",59745650,"-","4:A>T,15:T>A","TGG","TACATTGTTTCAGTGAAGTCTGG",2,5,16,NA,"GeneID:81624","DIAPH3",FALSE,"ADAMDEC1"

"2574","TACCACGAAACCTGAGAACA","chr8",24394125,"+","none","TGG","TACCACGAAACCTGAGAACATGG",0,NA,NA,NA,"GeneID:27299","ADAMDEC1",TRUE,"ADAMDEC1"

"2575","TCGGACCCTACCATGTTCTC","chr8",24394138,"-","none","AGG","TCGGACCCTACCATGTTCTCAGG",0,NA,NA,NA,"GeneID:27299","ADAMDEC1",TRUE,"ADAMDEC1"

"2576","TGAACAATGAGCCAAAGTAC","chr8",24384564,"-","none","AGG","TGAACAATGAGCCAAAGTACAGG",0,NA,NA,NA,"GeneID:27299","ADAMDEC1",TRUE,"ADAMDEC1"

"2577","AAAGCATTGACGCCATCATG","chr21",26841072,"+","none","TGG","AAAGCATTGACGCCATCATGTGG",0,NA,NA,NA,"GeneID:9510","ADAMTS1",TRUE,"ADAMTS1"

"2578","AATCCTGGTTCACACCATTA","chr21",26841096,"+","none","AGG","AATCCTGGTTCACACCATTAAGG",0,NA,NA,NA,"GeneID:9510","ADAMTS1",TRUE,"ADAMTS1"

"2579","CTGCCGGTTGGCATCGTACG","chr21",26840479,"+","none","AGG","CTGCCGGTTGGCATCGTACGAGG",0,NA,NA,NA,"GeneID:9510","ADAMTS1",TRUE,"ADAMTS1"

"2580","GCACCTCGTACGATGCCAAC","chr21",26840485,"-","none","CGG","GCACCTCGTACGATGCCAACCGG",0,NA,NA,NA,"GeneID:9510","ADAMTS1",TRUE,"ADAMTS1"

"2581","GCTTGTGGCAGACCAGTCGA","chr21",26842607,"-","none","TGG","GCTTGTGGCAGACCAGTCGATGG",0,NA,NA,NA,"GeneID:9510","ADAMTS1",TRUE,"ADAMTS1"

"2582","TGTGTCCAGTCACCGCTATG","chr21",26842637,"-","none","TGG","TGTGTCCAGTCACCGCTATGTGG",0,NA,NA,NA,"GeneID:9510","ADAMTS1",TRUE,"ADAMTS1"

"2583","CAGACAGTACTTGCCCCCGA","chr19",8591835,"+","none","TGG","CAGACAGTACTTGCCCCCGATGG",0,NA,NA,NA,"GeneID:81794","ADAMTS10",TRUE,"ADAMTS10"

"2584","CCAGACGCACACCATCGACA","chr19",8592767,"-","none","AGG","CCAGACGCACACCATCGACAAGG",0,NA,NA,NA,"GeneID:81794","ADAMTS10",TRUE,"ADAMTS10"

"2585","CGCTCACACATTCCGCCCAC","chr19",8596381,"+","none","CGG","CGCTCACACATTCCGCCCACCGG",0,NA,NA,NA,"GeneID:81794","ADAMTS10",TRUE,"ADAMTS10"

"2586","CTCACGTGTGCCCGATCTCG","chr19",8596301,"+","none","TGG","CTCACGTGTGCCCGATCTCGTGG",0,NA,NA,NA,"GeneID:81794","ADAMTS10",TRUE,"ADAMTS10"

"2587","GGAACTCACATCCGTGTTGC","chr19",8591790,"+","none","AGG","GGAACTCACATCCGTGTTGCAGG",0,NA,NA,NA,"GeneID:81794","ADAMTS10",TRUE,"ADAMTS10"

"2588","GGCGCTCACCCCCTTGTCGA","chr19",8592753,"+","none","TGG","GGCGCTCACCCCCTTGTCGATGG",0,NA,NA,NA,"GeneID:81794","ADAMTS10",TRUE,"ADAMTS10"

"2589","ACCACCCGCACATCGTTTAC","chr5",33751452,"-","none","AGG","ACCACCCGCACATCGTTTACAGG",0,NA,NA,NA,"GeneID:81792","ADAMTS12",TRUE,"ADAMTS12"

"2590","CATTCTACTCGAAGAAGAAG","chr5",33683019,"-","none","AGG","CATTCTACTCGAAGAAGAAGAGG",0,NA,NA,NA,"GeneID:81792","ADAMTS12",TRUE,"ADAMTS12"

"2591","CCCGCCATAGCTCTTGCTTC","chr5",33684016,"+","none","TGG","CCCGCCATAGCTCTTGCTTCTGG",0,NA,NA,NA,"GeneID:81792","ADAMTS12",TRUE,"ADAMTS12"

"2592","CTCGGACTGGACCCACCACG","chr5",33881429,"+","none","TGG","CTCGGACTGGACCCACCACGTGG",0,NA,NA,NA,"GeneID:81792","ADAMTS12",TRUE,"ADAMTS12"

"2593","GTACTACAGAATTTCTCACG","chr5",33881315,"-","none","AGG","GTACTACAGAATTTCTCACGAGG",0,NA,NA,NA,"GeneID:81792","ADAMTS12",TRUE,"ADAMTS12"

"2594","TGCCTCCTGTAAACGATGTG","chr5",33751444,"+","none","CGG","TGCCTCCTGTAAACGATGTGCGG",0,NA,NA,NA,"GeneID:81792","ADAMTS12",TRUE,"ADAMTS12"

"2595","AAGTAAGAAGACACGGCCTG","chr9",133423124,"-","none","TGG","AAGTAAGAAGACACGGCCTGTGG",0,NA,NA,NA,"GeneID:11093","ADAMTS13",TRUE,"ADAMTS13"

"2596","CATAGCGCTCTGTGTCCTCC","chr9",133424438,"-","none","TGG","CATAGCGCTCTGTGTCCTCCTGG",0,NA,NA,NA,"GeneID:11093","ADAMTS13",TRUE,"ADAMTS13"

"2597","GGTCATTCTGACAGAGCCTG","chr9",133425590,"+","none","AGG","GGTCATTCTGACAGAGCCTGAGG",0,NA,NA,NA,"GeneID:11093","ADAMTS13",TRUE,"ADAMTS13"

"2598","GGTCATTCTGACAGAGCCTG","chr14",59619330,"+","2:G>T,13:C>G","GGG","GGGCATTCTGACACAGCCTGGGG",2,3,14,NA,"GeneID:6252","RTN1",FALSE,"ADAMTS13"

"2599","GTGTCTTCTTACTTGAGCCC","chr9",133423130,"+","none","TGG","GTGTCTTCTTACTTGAGCCCTGG",0,NA,NA,NA,"GeneID:11093","ADAMTS13",TRUE,"ADAMTS13"

"2600","TCAGAATGACCATCTTCACC","chr9",133425581,"-","none","AGG","TCAGAATGACCATCTTCACCAGG",0,NA,NA,NA,"GeneID:11093","ADAMTS13",TRUE,"ADAMTS13"

"2601","TGCAGGCGGCATCCTACACC","chr9",133424372,"+","none","TGG","TGCAGGCGGCATCCTACACCTGG",0,NA,NA,NA,"GeneID:11093","ADAMTS13",TRUE,"ADAMTS13"

"2602","TGCAGGCGGCATCCTACACC","chr17",77877603,"+","7:A>G,15:G>A","AGG","TGCAGGCAGCATCCTGCACCAGG",2,8,16,NA,"","",FALSE,"ADAMTS13"

"2603","AAGCCGGACCTTCACCGCCA","chr10",70749879,"+","none","TGG","AAGCCGGACCTTCACCGCCATGG",0,NA,NA,NA,"GeneID:140766","ADAMTS14",TRUE,"ADAMTS14"

"2604","AAGCCGGACCTTCACCGCCA","chr7",45497164,"-","15:T>G,16:T>C","AGG","AAGCCGGACCTTCACAACCAAGG",2,16,17,NA,"","",FALSE,"ADAMTS14"

"2605","ACACTTGTCATCCGCCTTCA","chr10",70744096,"-","none","TGG","ACACTTGTCATCCGCCTTCATGG",0,NA,NA,NA,"GeneID:140766","ADAMTS14",TRUE,"ADAMTS14"

"2606","ACCTCCTTGTCACAGCCGAC","chr10",70744068,"-","none","AGG","ACCTCCTTGTCACAGCCGACAGG",0,NA,NA,NA,"GeneID:140766","ADAMTS14",TRUE,"ADAMTS14"

"2607","ACTTCTCCAGTGCCTCAATC","chr10",70745271,"-","none","TGG","ACTTCTCCAGTGCCTCAATCTGG",0,NA,NA,NA,"GeneID:140766","ADAMTS14",TRUE,"ADAMTS14"

"2608","CCAGCACTCACCAATGCGGT","chr10",70745297,"-","none","GGG","CCAGCACTCACCAATGCGGTGGG",0,NA,NA,NA,"GeneID:140766","ADAMTS14",TRUE,"ADAMTS14"

"2609","GGCGGTGAAGGTCCGGCTTG","chr10",70749878,"-","none","TGG","GGCGGTGAAGGTCCGGCTTGTGG",0,NA,NA,NA,"GeneID:140766","ADAMTS14",TRUE,"ADAMTS14"

"2610","AAGAAGAGATTCGACAAGTG","chr11",130471300,"+","none","TGG","AAGAAGAGATTCGACAAGTGTGG",0,NA,NA,NA,"GeneID:170689","ADAMTS15",TRUE,"ADAMTS15"

"2611","ACACTTGTCGAATCTCTTCT","chr11",130471301,"-","none","TGG","ACACTTGTCGAATCTCTTCTTGG",0,NA,NA,NA,"GeneID:170689","ADAMTS15",TRUE,"ADAMTS15"

"2612","AGGGGTTGGCACGGTCGATC","chr11",130462183,"-","none","TGG","AGGGGTTGGCACGGTCGATCTGG",0,NA,NA,NA,"GeneID:170689","ADAMTS15",TRUE,"ADAMTS15"

"2613","CGCGGAGCCTTTGGCTACCG","chr11",130449342,"+","none","AGG","CGCGGAGCCTTTGGCTACCGAGG",0,NA,NA,NA,"GeneID:170689","ADAMTS15",TRUE,"ADAMTS15"

"2614","GCTTACCGTGCCCGCTGTCC","chr11",130462240,"-","none","AGG","GCTTACCGTGCCCGCTGTCCAGG",0,NA,NA,NA,"GeneID:170689","ADAMTS15",TRUE,"ADAMTS15"

"2615","TTAACGGCCGCCGCTACTAC","chr11",130449076,"+","none","TGG","TTAACGGCCGCCGCTACTACTGG",0,NA,NA,NA,"GeneID:170689","ADAMTS15",TRUE,"ADAMTS15"

"2616","AGCCCCGCCATCCGCGCGCG","chr5",5140478,"-","none","CGG","AGCCCCGCCATCCGCGCGCGCGG",0,NA,NA,NA,"GeneID:170690","ADAMTS16",TRUE,"ADAMTS16"

"2617","GCAGGCACCTGCGTGCGCCA","chr5",5140659,"+","none","TGG","GCAGGCACCTGCGTGCGCCATGG",0,NA,NA,NA,"GeneID:170690","ADAMTS16",TRUE,"ADAMTS16"

"2618","GCGGCCGGGCTGGATGGAAA","chr5",5140740,"+","none","AGG","GCGGCCGGGCTGGATGGAAAAGG",0,NA,NA,NA,"GeneID:170690","ADAMTS16",TRUE,"ADAMTS16"

"2619","GCGGCCGGGCTGGATGGAAA","chr1",41395551,"+","5:A>C,19:G>A","TGG","GCGGCAGGGCTGGATGGAAGTGG",2,6,20,NA,"","",FALSE,"ADAMTS16"

"2620","GGGACACGTAATCGCCCCTG","chr5",5146163,"-","none","TGG","GGGACACGTAATCGCCCCTGTGG",0,NA,NA,NA,"GeneID:170690","ADAMTS16",TRUE,"ADAMTS16"

"2621","TGGATGAAGCCCCGCGCGCG","chr5",5140464,"+","none","CGG","TGGATGAAGCCCCGCGCGCGCGG",0,NA,NA,NA,"GeneID:170690","ADAMTS16",TRUE,"ADAMTS16"

"2622","TGTGCAGACTTTACCGCCAG","chr5",5146358,"+","none","AGG","TGTGCAGACTTTACCGCCAGAGG",0,NA,NA,NA,"GeneID:170690","ADAMTS16",TRUE,"ADAMTS16"

"2623","AGATGTTCTCGTCCACTGAA","chr15",100330968,"+","none","TGG","AGATGTTCTCGTCCACTGAATGG",0,NA,NA,NA,"GeneID:170691","ADAMTS17",TRUE,"ADAMTS17"

"2624","AGATGTTCTCGTCCACTGAA","chr11",65531633,"+","10:A>G,19:C>A","CGG","AGATGTTCTCATCCACTGACCGG",2,11,20,NA,"GeneID:57410","SCYL1",TRUE,"ADAMTS17"

"2625","CCCGTGGTACTGCACCATGT","chr15",100281270,"+","none","CGG","CCCGTGGTACTGCACCATGTCGG",0,NA,NA,NA,"GeneID:170691","ADAMTS17",TRUE,"ADAMTS17"

"2626","CCGGCTCACCAGCGAGCACA","chr15",100281320,"-","none","CGG","CCGGCTCACCAGCGAGCACACGG",0,NA,NA,NA,"GeneID:170691","ADAMTS17",TRUE,"ADAMTS17"

"2627","GTGGACGAGAACATCTGATC","chr15",100330964,"-","none","AGG","GTGGACGAGAACATCTGATCAGG",0,NA,NA,NA,"GeneID:170691","ADAMTS17",TRUE,"ADAMTS17"

"2628","TATGTTTCAGCACCAGAGCC","chr15",100262407,"-","none","TGG","TATGTTTCAGCACCAGAGCCTGG",0,NA,NA,NA,"GeneID:170691","ADAMTS17",TRUE,"ADAMTS17"

"2629","TATGTTTCAGCACCAGAGCC","chr3",5316429,"+","1:C>A,10:A>C","AGG","TCTGTTTCAGAACCAGAGCCAGG",2,2,11,NA,"","",FALSE,"ADAMTS17"

"2630","TATGTTTCAGCACCAGAGCC","chr11",23744122,"-","2:T>A,13:A>G","AGG","TAAGTTTCAGCACTAGAGCCAGG",2,3,14,NA,"","",FALSE,"ADAMTS17"

"2631","TTACGGGACGTTGTCGTAGC","chr15",100262347,"+","none","AGG","TTACGGGACGTTGTCGTAGCAGG",0,NA,NA,NA,"GeneID:170691","ADAMTS17",TRUE,"ADAMTS17"

"2632","ATATTCATCAAACCTTAGAT","chr16",77364331,"+","none","AGG","ATATTCATCAAACCTTAGATAGG",0,NA,NA,NA,"GeneID:170692","ADAMTS18",TRUE,"ADAMTS18"

"2633","ATATTCATCAAACCTTAGAT","chr19",33601440,"+","1:C>T,15:A>T","TGG","ACATTCATCAAACCTAAGATTGG",2,2,16,NA,"","",FALSE,"ADAMTS18"

"2634","GAAGGCACACGCGAGCAGGA","chr16",77434662,"+","none","GGG","GAAGGCACACGCGAGCAGGAGGG",0,NA,NA,NA,"GeneID:170692","ADAMTS18",TRUE,"ADAMTS18"

"2635","GAAGGCACACGCGAGCAGGA","chr11",2579304,"+","9:G>C,12:C>G","GGG","GAAGGCACAGGCCAGCAGGAGGG",2,10,13,NA,"GeneID:3784","KCNQ1",FALSE,"ADAMTS18"

"2636","GGCGGGCCCGAACCCGCAGC","chr16",77434639,"+","none","CGG","GGCGGGCCCGAACCCGCAGCCGG",0,NA,NA,NA,"GeneID:170692","ADAMTS18",TRUE,"ADAMTS18"

"2637","GGGCGACCCAGAAGATCAGC","chr16",77364302,"-","none","TGG","GGGCGACCCAGAAGATCAGCTGG",0,NA,NA,NA,"GeneID:170692","ADAMTS18",TRUE,"ADAMTS18"

"2638","GTCGTGTGAAATATATGACC","chr16",77431552,"+","none","CGG","GTCGTGTGAAATATATGACCCGG",0,NA,NA,NA,"GeneID:170692","ADAMTS18",TRUE,"ADAMTS18"

"2639","GTGACTGCTCAAAATCGCCG","chr16",77431426,"+","none","AGG","GTGACTGCTCAAAATCGCCGAGG",0,NA,NA,NA,"GeneID:170692","ADAMTS18",TRUE,"ADAMTS18"

"2640","CACTGATTAGAGCCCGAGAG","chr5",129701482,"+","none","AGG","CACTGATTAGAGCCCGAGAGAGG",0,NA,NA,NA,"GeneID:171019","ADAMTS19",TRUE,"ADAMTS19"

"2641","GAGAGGACACACTTCTTTCT","chr5",129704295,"-","none","TGG","GAGAGGACACACTTCTTTCTTGG",0,NA,NA,NA,"GeneID:171019","ADAMTS19",TRUE,"ADAMTS19"

"2642","GAGAGGACACACTTCTTTCT","chr2",12884799,"-","0:G>C,6:A>T","TGG","CAGAGGTCACACTTCTTTCTTGG",2,1,7,NA,"","",FALSE,"ADAMTS19"

"2643","GAGAGGACACACTTCTTTCT","chr2",17581670,"-","5:T>C,10:C>T","AGG","GAGAGAACACGCTTCTTTCTAGG",2,6,11,NA,"GeneID:7447","VSNL1",FALSE,"ADAMTS19"

"2644","GCCAGGACTGCATGACCGTG","chr5",129701548,"+","none","TGG","GCCAGGACTGCATGACCGTGTGG",0,NA,NA,NA,"GeneID:171019","ADAMTS19",TRUE,"ADAMTS19"

"2645","GCCAGGACTGCATGACCGTG","chr4",5931511,"-","6:C>T,17:A>C","GGG","GCCAGGGCTGCATGACCTTGGGG",2,7,18,NA,"GeneID:389197","C4orf50",FALSE,"ADAMTS19"

"2646","TAGAGTTACCTTGTTTGACA","chr5",129694844,"-","none","TGG","TAGAGTTACCTTGTTTGACATGG",0,NA,NA,NA,"GeneID:171019","ADAMTS19",TRUE,"ADAMTS19"

"2647","TCATTGCACTTTCGAATCTG","chr5",129694817,"-","none","TGG","TCATTGCACTTTCGAATCTGTGG",0,NA,NA,NA,"GeneID:171019","ADAMTS19",TRUE,"ADAMTS19"

"2648","TCATTGCACTTTCGAATCTG","chr6",87428632,"+","13:T>G,15:C>A","TGG","TCATTGCACTTTCTACTCTGTGG",2,14,16,NA,"GeneID:154313","C6orf165",TRUE,"ADAMTS19"

"2649","TTCCAGTGTTCAGTCAAGTG","chr5",129704232,"+","none","TGG","TTCCAGTGTTCAGTCAAGTGTGG",0,NA,NA,NA,"GeneID:171019","ADAMTS19",TRUE,"ADAMTS19"

"2650","TTCCAGTGTTCAGTCAAGTG","chr12",52775082,"+","4:T>A,9:C>T","TGG","TTCCTGTGTCCAGTCAAGTGTGG",2,5,10,NA,"GeneID:51350","KRT76",FALSE,"ADAMTS19"

"2651","TTCCAGTGTTCAGTCAAGTG","chr9",94851911,"+","9:G>T,19:T>G","GGG","TTCCAGTGTGCAGTCAAGTTGGG",2,10,20,NA,"GeneID:84909","C9orf3",FALSE,"ADAMTS19"

"2652","ACGTAGCTGCCGACACCACG","chr5",179344062,"+","none","TGG","ACGTAGCTGCCGACACCACGTGG",0,NA,NA,NA,"GeneID:9509","ADAMTS2",TRUE,"ADAMTS2"

"2653","AGGAGCACGCCAACAGCTCG","chr5",179207644,"-","none","AGG","AGGAGCACGCCAACAGCTCGAGG",0,NA,NA,NA,"GeneID:9509","ADAMTS2",TRUE,"ADAMTS2"

"2654","CGAGAAGGGCACCACCCGCG","chr5",179343858,"-","none","TGG","CGAGAAGGGCACCACCCGCGTGG",0,NA,NA,NA,"GeneID:9509","ADAMTS2",TRUE,"ADAMTS2"

"2655","CTACAACATCGAGGTCCTGC","chr5",179207589,"-","none","TGG","CTACAACATCGAGGTCCTGCTGG",0,NA,NA,NA,"GeneID:9509","ADAMTS2",TRUE,"ADAMTS2"

"2656","CTACAACATCGAGGTCCTGC","chr10",70708681,"+","5:G>A,15:G>C","TGG","CTACAGCATCGAGGTGCTGCTGG",2,6,16,NA,"GeneID:140766","ADAMTS14",TRUE,"ADAMTS2"

"2657","GGAGTTCTTCATCGAACCCT","chr5",179273018,"-","none","TGG","GGAGTTCTTCATCGAACCCTTGG",0,NA,NA,NA,"GeneID:9509","ADAMTS2",TRUE,"ADAMTS2"

"2658","GGCGATACACCACATGCACA","chr5",179272958,"+","none","CGG","GGCGATACACCACATGCACACGG",0,NA,NA,NA,"GeneID:9509","ADAMTS2",TRUE,"ADAMTS2"

"2659","ACGAAGTAGTGATCCCCGAG","chr12",43551223,"-","none","CGG","ACGAAGTAGTGATCCCCGAGCGG",0,NA,NA,NA,"GeneID:80070","ADAMTS20",TRUE,"ADAMTS20"

"2660","ATTAGTTATGATTCACCGTG","chr12",43493174,"-","none","AGG","ATTAGTTATGATTCACCGTGAGG",0,NA,NA,NA,"GeneID:80070","ADAMTS20",TRUE,"ADAMTS20"

"2661","CTGAAGTATTGCAGTGTGTC","chr12",43532037,"-","none","AGG","CTGAAGTATTGCAGTGTGTCAGG",0,NA,NA,NA,"GeneID:80070","ADAMTS20",TRUE,"ADAMTS20"

"2662","GACCGCCGATGCATCCTTTC","chr12",43551063,"-","none","TGG","GACCGCCGATGCATCCTTTCTGG",0,NA,NA,NA,"GeneID:80070","ADAMTS20",TRUE,"ADAMTS20"

"2663","GCTAAAGTGGTTTCTGCTCA","chr12",43502194,"-","none","TGG","GCTAAAGTGGTTTCTGCTCATGG",0,NA,NA,NA,"GeneID:80070","ADAMTS20",TRUE,"ADAMTS20"

"2664","TTTCTTAGAACCTATAATGA","chr12",43532143,"-","none","AGG","TTTCTTAGAACCTATAATGAAGG",0,NA,NA,NA,"GeneID:80070","ADAMTS20",TRUE,"ADAMTS20"

"2665","TTTCTTAGAACCTATAATGA","chr18",77434416,"-","11:T>G,18:A>C","AGG","TTTCTTAGAACATATAATTAAGG",2,12,19,NA,"","",FALSE,"ADAMTS20"

"2666","AATGAGACGCCGCAGACACG","chr4",72339603,"-","none","CGG","AATGAGACGCCGCAGACACGCGG",0,NA,NA,NA,"GeneID:9508","ADAMTS3",TRUE,"ADAMTS3"

"2667","ACCTCTGTAGTGGAAGTCTT","chr4",72414812,"+","none","TGG","ACCTCTGTAGTGGAAGTCTTTGG",0,NA,NA,NA,"GeneID:9508","ADAMTS3",TRUE,"ADAMTS3"

"2668","AGAAAACGATTACAATATCG","chr4",72339579,"-","none","AGG","AGAAAACGATTACAATATCGAGG",0,NA,NA,NA,"GeneID:9508","ADAMTS3",TRUE,"ADAMTS3"

"2669","CCAGGCTGGTAATGAAGAAA","chr4",72567385,"-","none","TGG","CCAGGCTGGTAATGAAGAAATGG",0,NA,NA,NA,"GeneID:9508","ADAMTS3",TRUE,"ADAMTS3"

"2670","CCAGGCTGGTAATGAAGAAA","chr7",3922068,"-","12:T>A,18:C>T","AGG","CCAGGCTGGTAAAGAAGAGAAGG",2,13,19,NA,"GeneID:221935","SDK1",FALSE,"ADAMTS3"

"2671","CCAGGCTGGTAATGAAGAAA","chr8",18052113,"+","10:G>A,17:C>A","TGG","CCAGGCTGGTGATGAAGCAATGG",2,11,18,NA,"","",FALSE,"ADAMTS3"

"2672","TCCAAAGACTTCCACTACAG","chr4",72414816,"-","none","AGG","TCCAAAGACTTCCACTACAGAGG",0,NA,NA,NA,"GeneID:9508","ADAMTS3",TRUE,"ADAMTS3"

"2673","TCTCTTATTAGAGTCGGACC","chr4",72339684,"-","none","TGG","TCTCTTATTAGAGTCGGACCTGG",0,NA,NA,NA,"GeneID:9508","ADAMTS3",TRUE,"ADAMTS3"

"2674","AGCCATTCTGTTTACCCGTC","chr1",161196558,"-","none","AGG","AGCCATTCTGTTTACCCGTCAGG",0,NA,NA,NA,"GeneID:9507","ADAMTS4",TRUE,"ADAMTS4"

"2675","AGTCTCCACTTGCGACACGC","chr1",161196272,"-","none","TGG","AGTCTCCACTTGCGACACGCTGG",0,NA,NA,NA,"GeneID:9507","ADAMTS4",TRUE,"ADAMTS4"

"2676","CATACCCAGCGTGTCGCAAG","chr1",161196264,"+","none","TGG","CATACCCAGCGTGTCGCAAGTGG",0,NA,NA,NA,"GeneID:9507","ADAMTS4",TRUE,"ADAMTS4"

"2677","GACTGGGTTCCTCGCTACAC","chr1",161193291,"-","none","AGG","GACTGGGTTCCTCGCTACACAGG",0,NA,NA,NA,"GeneID:9507","ADAMTS4",TRUE,"ADAMTS4"

"2678","GCTTACCATAGCCATTGTCC","chr1",161195458,"+","none","AGG","GCTTACCATAGCCATTGTCCAGG",0,NA,NA,NA,"GeneID:9507","ADAMTS4",TRUE,"ADAMTS4"

"2679","GGTGACTCGGCTAGTGATCC","chr1",161196699,"-","none","TGG","GGTGACTCGGCTAGTGATCCTGG",0,NA,NA,NA,"GeneID:9507","ADAMTS4",TRUE,"ADAMTS4"

"2680","CATTAAGCGCTTATCTTCTG","chr21",26943467,"+","none","TGG","CATTAAGCGCTTATCTTCTGTGG",0,NA,NA,NA,"GeneID:11096","ADAMTS5",TRUE,"ADAMTS5"

"2681","CCGGCCATACAACCGCGCCA","chr21",26965536,"+","none","TGG","CCGGCCATACAACCGCGCCATGG",0,NA,NA,NA,"GeneID:11096","ADAMTS5",TRUE,"ADAMTS5"

"2682","CTCCTGGGCAGGATTTATGT","chr21",26954862,"-","none","GGG","CTCCTGGGCAGGATTTATGTGGG",0,NA,NA,NA,"GeneID:11096","ADAMTS5",TRUE,"ADAMTS5"

"2683","CTCCTGGGCAGGATTTATGT","chr5",168137152,"-","11:G>C,13:T>A","GGG","CTCCTGGGCAGCAATTATGTGGG",2,12,14,NA,"GeneID:57451","TENM2",FALSE,"ADAMTS5"

"2684","GGACGCGGGGGTTTCGCAGC","chr21",26965749,"+","none","TGG","GGACGCGGGGGTTTCGCAGCTGG",0,NA,NA,NA,"GeneID:11096","ADAMTS5",TRUE,"ADAMTS5"

"2685","GGGCTTAGATGCATCAATGC","chr21",26943431,"+","none","TGG","GGGCTTAGATGCATCAATGCTGG",0,NA,NA,NA,"GeneID:11096","ADAMTS5",TRUE,"ADAMTS5"

"2686","GGGCTTAGATGCATCAATGC","chr4",62286867,"+","14:G>C,18:T>G","TGG","GGGCTTAGATGCATGAATTCTGG",2,15,19,NA,"","",FALSE,"ADAMTS5"

"2687","TCCCAACGTCTGCCATTCCC","chr21",26954819,"+","none","AGG","TCCCAACGTCTGCCATTCCCAGG",0,NA,NA,NA,"GeneID:11096","ADAMTS5",TRUE,"ADAMTS5"

"2688","ACCAGCTAACTATTCCAATA","chr5",65471095,"-","none","AGG","ACCAGCTAACTATTCCAATAAGG",0,NA,NA,NA,"GeneID:11174","ADAMTS6",TRUE,"ADAMTS6"

"2689","CATACAGATGTCGTTGTTGA","chr5",65460193,"+","none","AGG","CATACAGATGTCGTTGTTGAAGG",0,NA,NA,NA,"GeneID:11174","ADAMTS6",TRUE,"ADAMTS6"

"2690","CTGTATGATCACTCTCATTG","chr5",65460180,"-","none","TGG","CTGTATGATCACTCTCATTGTGG",0,NA,NA,NA,"GeneID:11174","ADAMTS6",TRUE,"ADAMTS6"

"2691","CTTTACCGTGATTCCAGCCT","chr5",65452187,"-","none","AGG","CTTTACCGTGATTCCAGCCTAGG",0,NA,NA,NA,"GeneID:11174","ADAMTS6",TRUE,"ADAMTS6"

"2692","CTTTACCGTGATTCCAGCCT","chr15",89395451,"+","3:C>T,10:T>A","GGG","CTTCACCGTGTTTCCAGCCTGGG",2,4,11,NA,"GeneID:254559","LINC00925",TRUE,"ADAMTS6"

"2693","TAATATTCACAACGTTTCCT","chr5",65452167,"+","none","AGG","TAATATTCACAACGTTTCCTAGG",0,NA,NA,NA,"GeneID:11174","ADAMTS6",TRUE,"ADAMTS6"

"2694","TAATATTCACAACGTTTCCT","chrX",108596687,"-","2:G>T,6:C>A","GGG","TACTATGCACAACGTTTCCTGGG",2,3,7,NA,"GeneID:1287","COL4A5",FALSE,"ADAMTS6"

"2695","TCAACGTAGTACAACTAAAG","chr5",65470803,"-","none","TGG","TCAACGTAGTACAACTAAAGTGG",0,NA,NA,NA,"GeneID:11174","ADAMTS6",TRUE,"ADAMTS6"

"2696","ACACCCACCTGGTGCGGGTC","chr15",78811112,"+","none","CGG","ACACCCACCTGGTGCGGGTCCGG",0,NA,NA,NA,"GeneID:11173;GeneID:10933","ADAMTS7;MORF4L1",TRUE,"ADAMTS7"

"2697","CCCCCCGCGTCGACTCGAAC","chr15",78800483,"+","none","CGG","CCCCCCGCGTCGACTCGAACCGG",0,NA,NA,NA,"GeneID:11173","ADAMTS7",TRUE,"ADAMTS7"

"2698","CCGGCTTTGTGAGCGAGACG","chr15",78800317,"-","none","CGG","CCGGCTTTGTGAGCGAGACGCGG",0,NA,NA,NA,"GeneID:11173","ADAMTS7",TRUE,"ADAMTS7"

"2699","CGCCGGGAGCCAGAGCGCAG","chr15",78811138,"+","none","AGG","CGCCGGGAGCCAGAGCGCAGAGG",0,NA,NA,NA,"GeneID:11173;GeneID:10933","ADAMTS7;MORF4L1",TRUE,"ADAMTS7"

"2700","CGCCGGGAGCCAGAGCGCAG","chr13",113143742,"-","0:T>G,9:C>G","GGG","AGCCGGGAGGCAGAGCGCAGGGG",2,1,10,NA,"GeneID:2159","F10",FALSE,"ADAMTS7"

"2701","CGCCGGGAGCCAGAGCGCAG","chr12",120368910,"+","7:C>A,13:G>A","GGG","CGCCGGGCGCCAGGGCGCAGGGG",2,8,14,NA,"GeneID:4440","MSI1",FALSE,"ADAMTS7"

"2702","CTCAATGAAGTAGTCCTCGT","chr15",78798071,"+","none","TGG","CTCAATGAAGTAGTCCTCGTTGG",0,NA,NA,NA,"GeneID:11173","ADAMTS7",TRUE,"ADAMTS7"

"2703","CTCAATGAAGTAGTCCTCGT","chr15",82301021,"-","2:C>G,17:A>G","TGG","CTGAATGAAGTAGTCCTTGTTGG",2,3,18,NA,"GeneID:390660","ADAMTS7P1",TRUE,"ADAMTS7"

"2704","GAGCATACCTTGCACTCCAC","chr15",78797939,"+","none","AGG","GAGCATACCTTGCACTCCACAGG",0,NA,NA,NA,"GeneID:11173","ADAMTS7",TRUE,"ADAMTS7"

"2705","GAGCATACCTTGCACTCCAC","chr15",82301153,"-","15:G>A","AGG","GAGCATACCTTGCACCCCACAGG",1,16,NA,NA,"GeneID:390660","ADAMTS7P1",TRUE,"ADAMTS7"

"2706","ATGGCCGTGTCGTAGTGCTC","chr11",130419068,"+","none","TGG","ATGGCCGTGTCGTAGTGCTCTGG",0,NA,NA,NA,"GeneID:11095","ADAMTS8",TRUE,"ADAMTS8"

"2707","CAGTGTAAGCCCCCCATTGT","chr11",130419142,"+","none","CGG","CAGTGTAAGCCCCCCATTGTCGG",0,NA,NA,NA,"GeneID:11095","ADAMTS8",TRUE,"ADAMTS8"

"2708","CGAACAGCGGTGCCATCACG","chr11",130416237,"+","none","TGG","CGAACAGCGGTGCCATCACGTGG",0,NA,NA,NA,"GeneID:11095","ADAMTS8",TRUE,"ADAMTS8"

"2709","GAGCCCCTCATCCTCGATCA","chr11",130416970,"+","none","CGG","GAGCCCCTCATCCTCGATCACGG",0,NA,NA,NA,"GeneID:11095","ADAMTS8",TRUE,"ADAMTS8"

"2710","GCAAGTTGTTCTGCCGAGCC","chr11",130408803,"-","none","CGG","GCAAGTTGTTCTGCCGAGCCCGG",0,NA,NA,NA,"GeneID:11095","ADAMTS8",TRUE,"ADAMTS8"

"2711","GGGGGACACCCCAGCATACT","chr11",130408833,"+","none","TGG","GGGGGACACCCCAGCATACTTGG",0,NA,NA,NA,"GeneID:11095","ADAMTS8",TRUE,"ADAMTS8"

"2712","ATGCTGCTACGTCACCAGCC","chr3",64658701,"+","none","AGG","ATGCTGCTACGTCACCAGCCAGG",0,NA,NA,NA,"GeneID:56999","ADAMTS9",TRUE,"ADAMTS9"

"2713","CGCTGCGCCTATAAATGATG","chr3",64681248,"+","none","TGG","CGCTGCGCCTATAAATGATGTGG",0,NA,NA,NA,"GeneID:56999","ADAMTS9",TRUE,"ADAMTS9"

"2714","GACGTAGCAGCATTAAACAG","chr3",64658694,"-","none","CGG","GACGTAGCAGCATTAAACAGCGG",0,NA,NA,NA,"GeneID:56999","ADAMTS9",TRUE,"ADAMTS9"

"2715","TCTAATAGGCCTGGCTGAAC","chr3",64654446,"-","none","TGG","TCTAATAGGCCTGGCTGAACTGG",0,NA,NA,NA,"GeneID:56999","ADAMTS9",TRUE,"ADAMTS9"

"2716","TGTTCATCCATAGACTGTAG","chr3",64681298,"+","none","TGG","TGTTCATCCATAGACTGTAGTGG",0,NA,NA,NA,"GeneID:56999","ADAMTS9",TRUE,"ADAMTS9"

"2717","TGTTCTATTAGTGAAGATAG","chr3",64654397,"-","none","TGG","TGTTCTATTAGTGAAGATAGTGG",0,NA,NA,NA,"GeneID:56999","ADAMTS9",TRUE,"ADAMTS9"

"2718","AGGCATCCCATAGGCCGTCC","chr9",18504860,"-","none","CGG","AGGCATCCCATAGGCCGTCCCGG",0,NA,NA,NA,"GeneID:92949","ADAMTSL1",TRUE,"ADAMTSL1"

"2719","CAAGCATGGAATGCTGCCGT","chr9",18474227,"+","none","CGG","CAAGCATGGAATGCTGCCGTCGG",0,NA,NA,NA,"GeneID:92949","ADAMTSL1",TRUE,"ADAMTSL1"

"2720","CAAGCATGGAATGCTGCCGT","chr16",7067117,"-","11:C>A,19:G>A","TGG","CAAGCATGGAAGGCTGCCGCTGG",2,12,20,NA,"GeneID:54715","RBFOX1",FALSE,"ADAMTSL1"

"2721","CATTACTGCATGTTCTGTAT","chr9",18533270,"-","none","CGG","CATTACTGCATGTTCTGTATCGG",0,NA,NA,NA,"GeneID:92949","ADAMTSL1",TRUE,"ADAMTSL1"

"2722","CTCCTCGGAGCGTGCGGTCC","chr9",18504835,"-","none","TGG","CTCCTCGGAGCGTGCGGTCCTGG",0,NA,NA,NA,"GeneID:92949","ADAMTSL1",TRUE,"ADAMTSL1"

"2723","GTGTGCCAGGAGTTGCCCGA","chr9",18474246,"-","none","CGG","GTGTGCCAGGAGTTGCCCGACGG",0,NA,NA,NA,"GeneID:92949","ADAMTSL1",TRUE,"ADAMTSL1"

"2724","GTGTGCCAGGAGTTGCCCGA","chr9",125131491,"-","1:T>A,17:T>G","GGG","GAGTGCCAGGAGTTGCCAGAGGG",2,2,18,NA,"GeneID:286205","SCAI",FALSE,"ADAMTSL1"

"2725","TTGCAACTTAGGAGCTGTGA","chr9",18533235,"+","none","AGG","TTGCAACTTAGGAGCTGTGAAGG",0,NA,NA,NA,"GeneID:92949","ADAMTSL1",TRUE,"ADAMTSL1"

"2726","AAGCACCCCGTCACAGCCGA","chr9",133540881,"-","none","TGG","AAGCACCCCGTCACAGCCGATGG",0,NA,NA,NA,"GeneID:9719","ADAMTSL2",TRUE,"ADAMTSL2"

"2727","GACCAACGAGGTGTGCGAGC","chr9",133554622,"+","none","AGG","GACCAACGAGGTGTGCGAGCAGG",0,NA,NA,NA,"GeneID:9719","ADAMTSL2",TRUE,"ADAMTSL2"

"2728","GACCGGAATCGAGTACATCG","chr9",133547155,"+","none","TGG","GACCGGAATCGAGTACATCGTGG",0,NA,NA,NA,"GeneID:9719","ADAMTSL2",TRUE,"ADAMTSL2"

"2729","GGCCGAACAGCCGGTTGTCC","chr9",133554559,"-","none","AGG","GGCCGAACAGCCGGTTGTCCAGG",0,NA,NA,NA,"GeneID:9719","ADAMTSL2",TRUE,"ADAMTSL2"

"2730","TGCCACGATGTACTCGATTC","chr9",133547160,"-","none","CGG","TGCCACGATGTACTCGATTCCGG",0,NA,NA,NA,"GeneID:9719","ADAMTSL2",TRUE,"ADAMTSL2"

"2731","TGCGATAGTTGCCCGTCACG","chr9",133540963,"-","none","TGG","TGCGATAGTTGCCCGTCACGTGG",0,NA,NA,NA,"GeneID:9719","ADAMTSL2",TRUE,"ADAMTSL2"

"2732","ACTGGGATGCTTGGGGCGAC","chr15",83773562,"+","none","TGG","ACTGGGATGCTTGGGGCGACTGG",0,NA,NA,NA,"GeneID:57188","ADAMTSL3",TRUE,"ADAMTSL3"

"2733","ACTGGGATGCTTGGGGCGAC","chr3",38542477,"-","8:G>C,14:A>C","TGG","ACTGGGATCCTTGGTGCGACTGG",2,9,15,NA,"","",FALSE,"ADAMTSL3"

"2734","CACTTATCGCTATGATGACC","chr15",83704486,"+","none","AGG","CACTTATCGCTATGATGACCAGG",0,NA,NA,NA,"GeneID:57188","ADAMTSL3",TRUE,"ADAMTSL3"

"2735","CATCCCTATCAGCACCCACC","chr15",83655783,"-","none","AGG","CATCCCTATCAGCACCCACCAGG",0,NA,NA,NA,"GeneID:57188","ADAMTSL3",TRUE,"ADAMTSL3"

"2736","GAGGAGCATCATATTCTCTG","chr15",83773610,"+","none","CGG","GAGGAGCATCATATTCTCTGCGG",0,NA,NA,NA,"GeneID:57188","ADAMTSL3",TRUE,"ADAMTSL3"

"2737","GGTCATCATAGCGATAAGTG","chr15",83704486,"-","none","AGG","GGTCATCATAGCGATAAGTGAGG",0,NA,NA,NA,"GeneID:57188","ADAMTSL3",TRUE,"ADAMTSL3"

"2738","TATGACCTTACCTGCGGGAG","chr15",83655821,"-","none","GGG","TATGACCTTACCTGCGGGAGGGG",0,NA,NA,NA,"GeneID:57188","ADAMTSL3",TRUE,"ADAMTSL3"

"2739","TATGACCTTACCTGCGGGAG","chr14",106270654,"+","10:T>C,14:G>C","AGG","TATGACCTTATCTGGGGGAGAGG",2,11,15,NA,"","",FALSE,"ADAMTSL3"

"2740","AAGCAGCAGCAGATACAGCC","chr1",150552547,"-","none","AGG","AAGCAGCAGCAGATACAGCCAGG",0,NA,NA,NA,"GeneID:54507","ADAMTSL4",TRUE,"ADAMTSL4"

"2741","AAGCAGCAGCAGATACAGCC","chr17",44088393,"-","12:G>T,19:A>G","GGG","AAGCAGCAGCAGCTACAGCTGGG",2,13,20,NA,"GeneID:10014","HDAC5",TRUE,"ADAMTSL4"

"2742","AAGCAGCAGCAGATACAGCC","chr12",47983529,"+","12:T>A,13:G>T","AGG","AAGCAGCAGCAGTGACAGCCAGG",2,13,14,NA,"GeneID:1280","COL2A1",FALSE,"ADAMTSL4"

"2743","AAGCAGCAGCAGATACAGCC","chr7",158795877,"+","11:C>G,18:G>C","AGG","AAGCAGCAGCACATACAGGCAGG",2,12,19,NA,"GeneID:57488","ESYT2",FALSE,"ADAMTSL4"

"2744","AAGCAGCAGCAGATACAGCC","chr1",231026165,"+","3:G>C,12:G>A","AGG","AAGGAGCAGCAGGTACAGCCAGG",2,4,13,NA,"GeneID:375061","FAM89A",FALSE,"ADAMTSL4"

"2745","AAGCAGCAGCAGATACAGCC","chr4",10724749,"+","6:G>C,12:G>A","TGG","AAGCAGGAGCAGGTACAGCCTGG",2,7,13,NA,"","",FALSE,"ADAMTSL4"

"2746","AAGCAGCAGCAGATACAGCC","chr5",136216823,"+","6:A>C,13:C>T","GGG","AAGCAGAAGCAGACACAGCCGGG",2,7,14,NA,"GeneID:57113","TRPC7",FALSE,"ADAMTSL4"

"2747","AAGCAGCAGCAGATACAGCC","chr6",123636900,"-","3:T>G,13:T>A","AGG","AAGAAGCAGCAGAAACAGCCAGG",2,4,14,NA,"GeneID:10345","TRDN",TRUE,"ADAMTSL4"

"2748","AGGACGGTTATATCGAAAGA","chr1",150556704,"-","none","CGG","AGGACGGTTATATCGAAAGACGG",0,NA,NA,NA,"GeneID:54507","ADAMTSL4",TRUE,"ADAMTSL4"

"2749","CAGAAACCCTCCCCTTGTAC","chr1",150553141,"+","none","AGG","CAGAAACCCTCCCCTTGTACAGG",0,NA,NA,NA,"GeneID:54507","ADAMTSL4",TRUE,"ADAMTSL4"

"2750","TCCTGATCCAAGCAGAGCTG","chr1",150552579,"-","none","AGG","TCCTGATCCAAGCAGAGCTGAGG",0,NA,NA,NA,"GeneID:54507","ADAMTSL4",TRUE,"ADAMTSL4"

"2751","TCCTGATCCAAGCAGAGCTG","chr1",246274789,"-","1:A>G,17:T>G","AGG","TTCTGATCCAAGCAGAGATGAGG",2,2,18,NA,"GeneID:64754","SMYD3",FALSE,"ADAMTSL4"

"2752","TCCTGGCCGCTCGAATCTCC","chr1",150553232,"-","none","TGG","TCCTGGCCGCTCGAATCTCCTGG",0,NA,NA,NA,"GeneID:54507","ADAMTSL4",TRUE,"ADAMTSL4"

"2753","TTCGATATAACCGTCCTCCC","chr1",150556707,"+","none","AGG","TTCGATATAACCGTCCTCCCAGG",0,NA,NA,NA,"GeneID:54507","ADAMTSL4",TRUE,"ADAMTSL4"

"2754","CACGCCTTCTACCACAGCTT","chr19",1508512,"-","none","CGG","CACGCCTTCTACCACAGCTTCGG",0,NA,NA,NA,"GeneID:339366","ADAMTSL5",TRUE,"ADAMTSL5"

"2755","GCACACTGTAGGTCTCGGAA","chr19",1510214,"+","none","GGG","GCACACTGTAGGTCTCGGAAGGG",0,NA,NA,NA,"GeneID:339366","ADAMTSL5",TRUE,"ADAMTSL5"

"2756","GCGGCCGAAGCTGTGGTAGA","chr19",1508505,"+","none","AGG","GCGGCCGAAGCTGTGGTAGAAGG",0,NA,NA,NA,"GeneID:339366","ADAMTSL5",TRUE,"ADAMTSL5"

"2757","GGACAGGGCGGCCATTGTAC","chr19",1510191,"+","none","AGG","GGACAGGGCGGCCATTGTACAGG",0,NA,NA,NA,"GeneID:339366","ADAMTSL5",TRUE,"ADAMTSL5"

"2758","GGCTTACTGGCAACTGGCAG","chr19",1510360,"+","none","AGG","GGCTTACTGGCAACTGGCAGAGG",0,NA,NA,NA,"GeneID:339366","ADAMTSL5",TRUE,"ADAMTSL5"

"2759","TTCCTGGGGAAGAACCGTGC","chr19",1510406,"-","none","TGG","TTCCTGGGGAAGAACCGTGCTGG",0,NA,NA,NA,"GeneID:339366","ADAMTSL5",TRUE,"ADAMTSL5"

"2760","AATCCACCGGAATATCCCCC","chr7",935430,"-","none","AGG","AATCCACCGGAATATCCCCCAGG",0,NA,NA,NA,"GeneID:11033;GeneID:90639","ADAP1;COX19",TRUE,"ADAP1"

"2761","CAAGGTGAAGTCCGTCCGCC","chr7",935403,"-","none","TGG","CAAGGTGAAGTCCGTCCGCCTGG",0,NA,NA,NA,"GeneID:11033;GeneID:90639","ADAP1;COX19",TRUE,"ADAP1"

"2762","CAAGGTGAAGTCCGTCCGCC","chr13",98448134,"-","12:T>G,14:C>A","TGG","CAAGGTGAAGTCAGGCCGCCTGG",2,13,15,NA,"GeneID:10160;GeneID:8428","FARP1;STK24",TRUE,"ADAP1"

"2763","CCGGGCCAAGTACGAGCGAC","chr7",920009,"-","none","AGG","CCGGGCCAAGTACGAGCGACAGG",0,NA,NA,NA,"GeneID:11033;GeneID:90639","ADAP1;COX19",TRUE,"ADAP1"

"2764","GAACTCCTGTCGCTCGTACT","chr7",920001,"+","none","TGG","GAACTCCTGTCGCTCGTACTTGG",0,NA,NA,NA,"GeneID:11033;GeneID:90639","ADAP1;COX19",TRUE,"ADAP1"

"2765","TCAAAAACTGCCCGTTGTCC","chr7",905118,"+","none","CGG","TCAAAAACTGCCCGTTGTCCCGG",0,NA,NA,NA,"GeneID:11033;GeneID:90639","ADAP1;COX19",TRUE,"ADAP1"

"2766","TTCCCCCACAGGGTACCGTG","chr7",905163,"-","none","AGG","TTCCCCCACAGGGTACCGTGAGG",0,NA,NA,NA,"GeneID:11033;GeneID:90639","ADAP1;COX19",TRUE,"ADAP1"

"2767","AACTTTACCTGGGAGCGAGA","chr17",30931956,"-","none","TGG","AACTTTACCTGGGAGCGAGATGG",0,NA,NA,NA,"GeneID:55803","ADAP2",TRUE,"ADAP2"

"2768","AACTTTACCTGGGAGCGAGA","chr2",41035581,"-","14:A>C,15:T>G","GGG","AACTTTACCTGGGATAGAGAGGG",2,15,16,NA,"","",FALSE,"ADAP2"

"2769","AATCTGTGCGACTTGACTTC","chr17",30923029,"+","none","TGG","AATCTGTGCGACTTGACTTCTGG",0,NA,NA,NA,"GeneID:55803","ADAP2",TRUE,"ADAP2"

"2770","ATCCCCTGCAGATCCCGACT","chr17",30922928,"+","none","GGG","ATCCCCTGCAGATCCCGACTGGG",0,NA,NA,NA,"GeneID:55803","ADAP2",TRUE,"ADAP2"

"2771","ATCCCCTGCAGATCCCGACT","chr18",47602740,"+","11:C>A,15:T>C","TGG","ATCCCCTGCAGCTCCTGACTTGG",2,12,16,NA,"","",FALSE,"ADAP2"

"2772","ATCCCCTGCAGATCCCGACT","chr7",935496,"-","0:A>T,5:A>G","GGG","TTCCCTTGCAGATCCCGACTGGG",2,1,6,NA,"GeneID:11033;GeneID:90639","ADAP1;COX19",TRUE,"ADAP2"

"2773","ATTCCTGTGGAAGCGAGGAA","chr17",30934197,"+","none","GGG","ATTCCTGTGGAAGCGAGGAAGGG",0,NA,NA,NA,"GeneID:55803","ADAP2",TRUE,"ADAP2"

"2774","GCTGCTTAAGGTAACCGAGA","chr17",30934174,"+","none","AGG","GCTGCTTAAGGTAACCGAGAAGG",0,NA,NA,NA,"GeneID:55803","ADAP2",TRUE,"ADAP2"

"2775","GGGGAAACCATCTCGCTCCC","chr17",30931946,"+","none","AGG","GGGGAAACCATCTCGCTCCCAGG",0,NA,NA,NA,"GeneID:55803","ADAP2",TRUE,"ADAP2"

"2776","AATAGTATCCGCGCAGCACC","chr1",154601232,"-","none","AGG","AATAGTATCCGCGCAGCACCAGG",0,NA,NA,NA,"GeneID:103","ADAR",TRUE,"ADAR"

"2777","AGCCATGACAATTCTGCTAG","chr1",154598475,"-","none","AGG","AGCCATGACAATTCTGCTAGAGG",0,NA,NA,NA,"GeneID:103","ADAR",TRUE,"ADAR"

"2778","CATGGGTGTCGTCGTCAGCT","chr1",154589765,"-","none","TGG","CATGGGTGTCGTCGTCAGCTTGG",0,NA,NA,NA,"GeneID:103","ADAR",TRUE,"ADAR"

"2779","CTCGGCCATTGATGACAACC","chr1",154598553,"+","none","TGG","CTCGGCCATTGATGACAACCTGG",0,NA,NA,NA,"GeneID:103","ADAR",TRUE,"ADAR"

"2780","GTGCATACACTCAAGCAGTG","chr1",154597889,"+","none","TGG","GTGCATACACTCAAGCAGTGTGG",0,NA,NA,NA,"GeneID:103","ADAR",TRUE,"ADAR"

"2781","TGGTCTCTTACCGAGGTTCA","chr1",154601029,"+","none","TGG","TGGTCTCTTACCGAGGTTCATGG",0,NA,NA,NA,"GeneID:103","ADAR",TRUE,"ADAR"

"2782","AGCTGACGCTGTCTCACGCC","chr21",45180334,"+","none","TGG","AGCTGACGCTGTCTCACGCCTGG",0,NA,NA,NA,"GeneID:104","ADARB1",TRUE,"ADARB1"

"2783","CGTCTCATACCTTCCAGGAT","chr21",45183500,"-","none","TGG","CGTCTCATACCTTCCAGGATTGG",0,NA,NA,NA,"GeneID:104","ADARB1",TRUE,"ADARB1"

"2784","CTCACCACATGAGCCAATCC","chr21",45183484,"+","none","TGG","CTCACCACATGAGCCAATCCTGG",0,NA,NA,NA,"GeneID:104","ADARB1",TRUE,"ADARB1"

"2785","TCACGCTCGCAGAAAAGTGC","chr21",45180400,"+","none","TGG","TCACGCTCGCAGAAAAGTGCTGG",0,NA,NA,NA,"GeneID:104","ADARB1",TRUE,"ADARB1"

"2786","TCAGGTCACCAAACTTACCC","chr21",45180361,"-","none","AGG","TCAGGTCACCAAACTTACCCAGG",0,NA,NA,NA,"GeneID:104","ADARB1",TRUE,"ADARB1"

"2787","TTCTTCATCTTCTATATCCA","chr21",45171657,"-","none","TGG","TTCTTCATCTTCTATATCCATGG",0,NA,NA,NA,"GeneID:104","ADARB1",TRUE,"ADARB1"

"2788","TTCTTCATCTTCTATATCCA","chr4",171423169,"+","15:C>A,17:A>C","AGG","TTCTTCATCTTCTATCTACAAGG",2,16,18,NA,"","",FALSE,"ADARB1"

"2789","TTCTTCATCTTCTATATCCA","chr2",81606088,"+","14:A>T,17:T>C","AGG","TTCTTCATCTTCTAAATTCAAGG",2,15,18,NA,"","",FALSE,"ADARB1"

"2790","TTCTTCATCTTCTATATCCA","chrX",37063697,"-","8:A>G,10:C>A","TGG","TTCTTCATTTGCTATATCCATGG",2,9,11,NA,"","",FALSE,"ADARB1"

"2791","TTCTTCATCTTCTATATCCA","chr12",40270196,"-","3:G>A,16:C>A","GGG","TTCCTCATCTTCTATAGCCAGGG",2,4,17,NA,"GeneID:120892","LRRK2",FALSE,"ADARB1"

"2792","CGATTCCTGCCAGCGCTTTA","chr10",1270966,"+","none","TGG","CGATTCCTGCCAGCGCTTTATGG",0,NA,NA,NA,"GeneID:105","ADARB2",TRUE,"ADARB2"

"2793","CGTGGACGGCAGGACGTTCG","chr10",1363152,"-","none","AGG","CGTGGACGGCAGGACGTTCGAGG",0,NA,NA,NA,"GeneID:105","ADARB2",TRUE,"ADARB2"

"2794","CTCGGCCATGGCCTCGGTCC","chr10",1737137,"-","none","TGG","CTCGGCCATGGCCTCGGTCCTGG",0,NA,NA,NA,"GeneID:105","ADARB2",TRUE,"ADARB2"

"2795","CTCGGCCATGGCCTCGGTCC","chr12",5079005,"+","11:G>C,18:G>C","TGG","CTCGGCCATGGGCTCGGTGCTGG",2,12,19,NA,"","",FALSE,"ADARB2"

"2796","GCACGCCCGCCATAAAGCGC","chr10",1270978,"-","none","TGG","GCACGCCCGCCATAAAGCGCTGG",0,NA,NA,NA,"GeneID:105","ADARB2",TRUE,"ADARB2"

"2797","GCGGTCCTTACCTTTCCGCT","chr10",1737039,"+","none","TGG","GCGGTCCTTACCTTTCCGCTTGG",0,NA,NA,NA,"GeneID:105","ADARB2",TRUE,"ADARB2"

"2798","GGTGAACGGGCTCACGTTCG","chr10",1363602,"-","none","AGG","GGTGAACGGGCTCACGTTCGAGG",0,NA,NA,NA,"GeneID:105","ADARB2",TRUE,"ADARB2"

"2799","ATTTGAACAGAGCCGCAGTG","chr16",75608896,"-","none","CGG","ATTTGAACAGAGCCGCAGTGCGG",0,NA,NA,NA,"GeneID:23536","ADAT1",TRUE,"ADAT1"

"2800","CCTCAATGATAGCCATGCTG","chr16",75618613,"-","none","AGG","CCTCAATGATAGCCATGCTGAGG",0,NA,NA,NA,"GeneID:23536","ADAT1",TRUE,"ADAT1"

"2801","CCTCAATGATAGCCATGCTG","chr2",21465210,"+","3:G>C,5:G>A","AGG","CCTGAGTGATAGCCATGCTGAGG",2,4,6,NA,"","",FALSE,"ADAT1"

"2802","CCTCAGCATGGCTATCATTG","chr16",75618610,"+","none","AGG","CCTCAGCATGGCTATCATTGAGG",0,NA,NA,NA,"GeneID:23536","ADAT1",TRUE,"ADAT1"

"2803","CCTCAGCATGGCTATCATTG","chr2",21465213,"-","17:G>A,19:G>C","AGG","CCTCAGCATGGCTATCACTCAGG",2,18,20,NA,"","",FALSE,"ADAT1"

"2804","CTTACCGTTCTTCCTCATTT","chr16",75620260,"+","none","TGG","CTTACCGTTCTTCCTCATTTTGG",0,NA,NA,NA,"GeneID:23536","ADAT1",TRUE,"ADAT1"

"2805","GCATAGGACAGTCCAAAATG","chr16",75620275,"-","none","AGG","GCATAGGACAGTCCAAAATGAGG",0,NA,NA,NA,"GeneID:23536","ADAT1",TRUE,"ADAT1"

"2806","GCATAGGACAGTCCAAAATG","chr3",147342326,"-","1:T>G,7:C>T","TGG","GAATAGGGCAGTCCAAAATGTGG",2,2,8,NA,"","",FALSE,"ADAT1"

"2807","TCTTGAACTCCGAAGCCTTT","chr16",75608943,"+","none","TGG","TCTTGAACTCCGAAGCCTTTTGG",0,NA,NA,NA,"GeneID:23536","ADAT1",TRUE,"ADAT1"

"2808","CACTTTGACGACACCAATCG","chr6",143433920,"+","none","AGG","CACTTTGACGACACCAATCGAGG",0,NA,NA,NA,"GeneID:134637","ADAT2",TRUE,"ADAT2"

"2809","CCATCGATCAGGTCCTCGAT","chr6",143433936,"-","none","TGG","CCATCGATCAGGTCCTCGATTGG",0,NA,NA,NA,"GeneID:134637","ADAT2",TRUE,"ADAT2"

"2810","CCATCGATCAGGTCCTCGAT","chr7",111394908,"+","4:T>C,16:T>C","TGG","CCATTGATCAGGTCCTTGATTGG",2,5,17,NA,"GeneID:83943","IMMP2L",TRUE,"ADAT2"

"2811","CGCGTGCTCGGTGTCGGCAG","chr6",143450600,"-","none","AGG","CGCGTGCTCGGTGTCGGCAGAGG",0,NA,NA,NA,"GeneID:134637","ADAT2",TRUE,"ADAT2"

"2812","CGCGTGCTCGGTGTCGGCAG","chr1",41422828,"-","3:G>C,13:C>A","AGG","CGCCTGCTCGGTGGCGGCAGAGG",2,4,14,NA,"","",FALSE,"ADAT2"

"2813","GAGCACGCGCCGCTTGCAGC","chr6",143450611,"+","none","TGG","GAGCACGCGCCGCTTGCAGCTGG",0,NA,NA,NA,"GeneID:134637","ADAT2",TRUE,"ADAT2"

"2814","GGCTGTCAGAATGAACGATT","chr6",143432571,"-","none","TGG","GGCTGTCAGAATGAACGATTTGG",0,NA,NA,NA,"GeneID:134637","ADAT2",TRUE,"ADAT2"

"2815","GTCTACAACAATGAAGTTGT","chr6",143438626,"-","none","AGG","GTCTACAACAATGAAGTTGTAGG",0,NA,NA,NA,"GeneID:134637","ADAT2",TRUE,"ADAT2"

"2816","CGCACACCATGACGGCGTGC","chr19",1912760,"-","none","AGG","CGCACACCATGACGGCGTGCAGG",0,NA,NA,NA,"GeneID:113178;GeneID:113179","SCAMP4;ADAT3",TRUE,"ADAT3"

"2817","CGCATCCTGCGCGTCTTCTA","chr19",1912998,"+","none","CGG","CGCATCCTGCGCGTCTTCTACGG",0,NA,NA,NA,"GeneID:113178;GeneID:113179","SCAMP4;ADAT3",TRUE,"ADAT3"

"2818","GCGCACCGTAGAAGACGCGC","chr19",1913006,"-","none","AGG","GCGCACCGTAGAAGACGCGCAGG",0,NA,NA,NA,"GeneID:113178;GeneID:113179","SCAMP4;ADAT3",TRUE,"ADAT3"

"2819","GCGTAAACTGGACGCAGACG","chr19",1912889,"+","none","AGG","GCGTAAACTGGACGCAGACGAGG",0,NA,NA,NA,"GeneID:113178;GeneID:113179","SCAMP4;ADAT3",TRUE,"ADAT3"

"2820","GTCTGCGTCCAGTTTACGCA","chr19",1912888,"-","none","CGG","GTCTGCGTCCAGTTTACGCACGG",0,NA,NA,NA,"GeneID:113178;GeneID:113179","SCAMP4;ADAT3",TRUE,"ADAT3"

"2821","TGCGTGGACCTCGTGGCGCG","chr19",1912776,"+","none","CGG","TGCGTGGACCTCGTGGCGCGCGG",0,NA,NA,NA,"GeneID:113178;GeneID:113179","SCAMP4;ADAT3",TRUE,"ADAT3"

"2822","CATCGTGTACCACCTTGATG","chr1",33098091,"+","none","AGG","CATCGTGTACCACCTTGATGAGG",0,NA,NA,NA,"GeneID:113451","AZIN2",TRUE,"ADC"

"2823","CGAGGTAGCTGCCTTCTTCG","chr1",33083955,"+","none","TGG","CGAGGTAGCTGCCTTCTTCGTGG",0,NA,NA,NA,"GeneID:113451","AZIN2",TRUE,"ADC"

"2824","GATCTGTGCAATTTGCTTAC","chr1",33092113,"-","none","AGG","GATCTGTGCAATTTGCTTACAGG",0,NA,NA,NA,"GeneID:113451","AZIN2",TRUE,"ADC"

"2825","GTACACGATGGTCTTGGAGG","chr1",33098081,"-","none","TGG","GTACACGATGGTCTTGGAGGTGG",0,NA,NA,NA,"GeneID:113451","AZIN2",TRUE,"ADC"

"2826","GTTGGCGCAGATGATCTTAC","chr1",33092089,"-","none","TGG","GTTGGCGCAGATGATCTTACTGG",0,NA,NA,NA,"GeneID:113451","AZIN2",TRUE,"ADC"

"2827","TGAAGTGCCTGCCACGAGTC","chr1",33084017,"+","none","CGG","TGAAGTGCCTGCCACGAGTCCGG",0,NA,NA,NA,"GeneID:113451","AZIN2",TRUE,"ADC"

"2828","AAGACCAACGACCTGCTGCG","chr14",77931607,"+","none","TGG","AAGACCAACGACCTGCTGCGTGG",0,NA,NA,NA,"GeneID:57143","ADCK1",TRUE,"ADCK1"

"2829","AAGTACTCCTCTGAGCCATA","chr14",77822482,"-","none","AGG","AAGTACTCCTCTGAGCCATAAGG",0,NA,NA,NA,"GeneID:57143","ADCK1",TRUE,"ADCK1"

"2830","AAGTACTCCTCTGAGCCATA","chr9",109694211,"-","8:T>G,17:G>T","TGG","AAGTACTCATCTGAGCCCTATGG",2,9,18,NA,"GeneID:114299","PALM2",FALSE,"ADCK1"

"2831","CTAATGACTTTGGCGCTGTC","chr14",77819069,"+","none","AGG","CTAATGACTTTGGCGCTGTCAGG",0,NA,NA,NA,"GeneID:57143","ADCK1",TRUE,"ADCK1"

"2832","CTTAGAGATTCGCAACAACG","chr14",77931519,"+","none","CGG","CTTAGAGATTCGCAACAACGCGG",0,NA,NA,NA,"GeneID:57143","ADCK1",TRUE,"ADCK1"

"2833","GCTTGCTTCGTGGACCAGCA","chr14",77818998,"+","none","TGG","GCTTGCTTCGTGGACCAGCATGG",0,NA,NA,NA,"GeneID:57143","ADCK1",TRUE,"ADCK1"

"2834","GTACTTGCAGCTGAGATCTA","chr14",77822496,"+","none","AGG","GTACTTGCAGCTGAGATCTAAGG",0,NA,NA,NA,"GeneID:57143","ADCK1",TRUE,"ADCK1"

"2835","AATTCCTGCCTGCTGGTAAC","chr7",140681060,"-","none","TGG","AATTCCTGCCTGCTGGTAACTGG",0,NA,NA,NA,"GeneID:90956","ADCK2",TRUE,"ADCK2"

"2836","ACTTGACGGCTTTCACATTC","chr7",140679207,"-","none","CGG","ACTTGACGGCTTTCACATTCCGG",0,NA,NA,NA,"GeneID:90956","ADCK2",TRUE,"ADCK2"

"2837","ATCCTGGTTCAGGGTGCCAA","chr7",140687034,"+","none","CGG","ATCCTGGTTCAGGGTGCCAACGG",0,NA,NA,NA,"GeneID:90956","ADCK2",TRUE,"ADCK2"

"2838","GAATTCCCGTGGACTTGAAA","chr7",140681075,"+","none","AGG","GAATTCCCGTGGACTTGAAAAGG",0,NA,NA,NA,"GeneID:90956","ADCK2",TRUE,"ADCK2"

"2839","GATTCTGAGCTTCGTAACGC","chr7",140679162,"-","none","AGG","GATTCTGAGCTTCGTAACGCAGG",0,NA,NA,NA,"GeneID:90956","ADCK2",TRUE,"ADCK2"

"2840","TTCCCTCTGCCCGCTGCGAC","chr7",140687129,"+","none","TGG","TTCCCTCTGCCCGCTGCGACTGG",0,NA,NA,NA,"GeneID:90956","ADCK2",TRUE,"ADCK2"

"2841","AGCCGGCCAATCCTCGTCAC","chr1",226965703,"-","none","AGG","AGCCGGCCAATCCTCGTCACAGG",0,NA,NA,NA,"GeneID:56997","ADCK3",TRUE,"ADCK3"

"2842","ATTGGCCGGCTGGCCAACTT","chr1",226965712,"+","none","CGG","ATTGGCCGGCTGGCCAACTTCGG",0,NA,NA,NA,"GeneID:56997","ADCK3",TRUE,"ADCK3"

"2843","CAAGAAGAGCCTGCGCTCCG","chr1",226977491,"+","none","AGG","CAAGAAGAGCCTGCGCTCCGAGG",0,NA,NA,NA,"GeneID:56997","ADCK3",TRUE,"ADCK3"

"2844","CAAGAAGAGCCTGCGCTCCG","chr8",104361577,"-","13:T>G,16:G>A","AGG","CAAGAAGAGCCTGAGCCCCGAGG",2,14,17,NA,"GeneID:1807","DPYS",FALSE,"ADCK3"

"2845","GATCGTGCGCACGCTCTGCA","chr1",226982081,"+","none","AGG","GATCGTGCGCACGCTCTGCAAGG",0,NA,NA,NA,"GeneID:56997","ADCK3",TRUE,"ADCK3"

"2846","GCGTGGTGCGGCACTCAAGC","chr1",226982105,"+","none","TGG","GCGTGGTGCGGCACTCAAGCTGG",0,NA,NA,NA,"GeneID:56997","ADCK3",TRUE,"ADCK3"

"2847","GGGGAACCCGGCTTCTCTAA","chr1",226965195,"-","none","AGG","GGGGAACCCGGCTTCTCTAAAGG",0,NA,NA,NA,"GeneID:56997","ADCK3",TRUE,"ADCK3"

"2848","CATGGACTTCTTAGCCATCT","chr19",40710080,"+","none","CGG","CATGGACTTCTTAGCCATCTCGG",0,NA,NA,NA,"GeneID:79934","ADCK4",TRUE,"ADCK4"

"2849","CATGGACTTCTTAGCCATCT","chr2",70781003,"-","13:G>C,17:C>A","TGG","CATGGACTTCTTACCCAGCTTGG",2,14,18,NA,"GeneID:344018","FIGLA",FALSE,"ADCK4"

"2850","GCCCATCCTGGTAAAACTTT","chr19",40714351,"+","none","TGG","GCCCATCCTGGTAAAACTTTTGG",0,NA,NA,NA,"GeneID:79934","ADCK4",TRUE,"ADCK4"

"2851","GCCCATCCTGGTAAAACTTT","chr5",90097177,"-","5:T>A,10:T>C","AGG","GCCCAACCTGATAAAACTTTAGG",2,6,11,NA,"","",FALSE,"ADCK4"

"2852","GCTAGGAGTACTGGCCGAGA","chr19",40710097,"-","none","TGG","GCTAGGAGTACTGGCCGAGATGG",0,NA,NA,NA,"GeneID:79934","ADCK4",TRUE,"ADCK4"

"2853","GGACATTCGCAGGGCACGGG","chr19",40714309,"-","none","AGG","GGACATTCGCAGGGCACGGGAGG",0,NA,NA,NA,"GeneID:79934","ADCK4",TRUE,"ADCK4"

"2854","TGACCGCTCTCGAGAACGCA","chr19",40714108,"-","none","AGG","TGACCGCTCTCGAGAACGCAAGG",0,NA,NA,NA,"GeneID:79934","ADCK4",TRUE,"ADCK4"

"2855","TGCCTCCCGCATCAGCCGCT","chr19",40714081,"-","none","TGG","TGCCTCCCGCATCAGCCGCTTGG",0,NA,NA,NA,"GeneID:79934","ADCK4",TRUE,"ADCK4"

"2856","AAACCTCTTCCACCCCTCGA","chr8",144390730,"-","none","AGG","AAACCTCTTCCACCCCTCGAAGG",0,NA,NA,NA,"GeneID:203054","ADCK5",TRUE,"ADCK5"

"2857","CCTCGGAGCCCGCTATGTCA","chr8",144383146,"+","none","TGG","CCTCGGAGCCCGCTATGTCATGG",0,NA,NA,NA,"GeneID:203054","ADCK5",TRUE,"ADCK5"

"2858","CGTCCCCTGCTGTGTTCTTC","chr8",144379441,"+","none","AGG","CGTCCCCTGCTGTGTTCTTCAGG",0,NA,NA,NA,"GeneID:203054","ADCK5",TRUE,"ADCK5"

"2859","GGACTCGTTACCTTGGAGGA","chr8",144379481,"-","none","AGG","GGACTCGTTACCTTGGAGGAAGG",0,NA,NA,NA,"GeneID:203054","ADCK5",TRUE,"ADCK5"

"2860","GGTGCTCTCCACCGCGGTAG","chr8",144383110,"+","none","TGG","GGTGCTCTCCACCGCGGTAGTGG",0,NA,NA,NA,"GeneID:203054","ADCK5",TRUE,"ADCK5"

"2861","TGCACCAATGTTGTCCTTCG","chr8",144390713,"+","none","AGG","TGCACCAATGTTGTCCTTCGAGG",0,NA,NA,NA,"GeneID:203054","ADCK5",TRUE,"ADCK5"

"2862","ACTTGCCGAAGAGCTCATTG","chr7",45622706,"-","none","AGG","ACTTGCCGAAGAGCTCATTGAGG",0,NA,NA,NA,"GeneID:107","ADCY1",TRUE,"ADCY1"

"2863","CAAGTTCGATGAATTAGCCA","chr7",45622721,"+","none","CGG","CAAGTTCGATGAATTAGCCACGG",0,NA,NA,NA,"GeneID:107","ADCY1",TRUE,"ADCY1"

"2864","GGACTGCTACTACTGCGTGT","chr7",45648701,"+","none","CGG","GGACTGCTACTACTGCGTGTCGG",0,NA,NA,NA,"GeneID:107","ADCY1",TRUE,"ADCY1"

"2865","GTTCACACCGACGAAGAGCA","chr7",45592774,"-","none","AGG","GTTCACACCGACGAAGAGCAAGG",0,NA,NA,NA,"GeneID:107","ADCY1",TRUE,"ADCY1"

"2866","TGTCGCCGCATCAAGATTCT","chr7",45648678,"+","none","CGG","TGTCGCCGCATCAAGATTCTCGG",0,NA,NA,NA,"GeneID:107","ADCY1",TRUE,"ADCY1"

"2867","TTCTGACTGAGCGTTCACAG","chr7",45592816,"+","none","AGG","TTCTGACTGAGCGTTCACAGAGG",0,NA,NA,NA,"GeneID:107","ADCY1",TRUE,"ADCY1"

"2868","AAATTCATCAAAATTAAAAT","chr1",167896646,"+","none","TGG","AAATTCATCAAAATTAAAATTGG",0,NA,NA,NA,"GeneID:55811","ADCY10",TRUE,"ADCY10"

"2869","AAATTCATCAAAATTAAAAT","chr10",84971260,"-","1:A>T,2:A>T","GGG","ATTTTCATCAAAATTAAAATGGG",2,2,3,NA,"","",FALSE,"ADCY10"

"2870","AAATTCATCAAAATTAAAAT","chr17",37279226,"+","2:T>A,6:T>A","AGG","AATTTCTTCAAAATTAAAATAGG",2,3,7,NA,"GeneID:31","ACACA",FALSE,"ADCY10"

"2871","AAATTCATCAAAATTAAAAT","chr11",37156893,"-","5:C>G,19:C>A","AGG","AAATTGATCAAAATTAAAAGAGG",2,6,20,NA,"","",FALSE,"ADCY10"

"2872","AAATTCATCAAAATTAAAAT","chr11",108688410,"-","4:G>A,13:T>A","GGG","AAATCCATCAAAAATAAAATGGG",2,5,14,NA,"GeneID:1662","DDX10",FALSE,"ADCY10"

"2873","AAATTCATCAAAATTAAAAT","chrX",55978532,"-","3:G>A,12:G>T","GGG","AAACTCATCAAACTTAAAATGGG",2,4,13,NA,"","",FALSE,"ADCY10"

"2874","AAATTCATCAAAATTAAAAT","chr5",129069843,"-","2:A>T,11:A>T","GGG","AATTTCATCAATATTAAAATGGG",2,3,12,NA,"","",FALSE,"ADCY10"

"2875","AAATTCATCAAAATTAAAAT","chr9",96454113,"-","0:C>T,12:G>T","AGG","GAATTCATCAAACTTAAAATAGG",2,1,13,NA,"GeneID:22927","HABP4",FALSE,"ADCY10"

"2876","ACCTTAACTGCTCTCTGATC","chr1",167899420,"+","none","TGG","ACCTTAACTGCTCTCTGATCTGG",0,NA,NA,NA,"GeneID:55811","ADCY10",TRUE,"ADCY10"

"2877","CCCCCATACCTGCAAATTTC","chr1",167902006,"+","none","AGG","CCCCCATACCTGCAAATTTCAGG",0,NA,NA,NA,"GeneID:55811","ADCY10",TRUE,"ADCY10"

"2878","CCTGACCAATCACCAGAAAG","chr1",167899553,"+","none","TGG","CCTGACCAATCACCAGAAAGTGG",0,NA,NA,NA,"GeneID:55811","ADCY10",TRUE,"ADCY10"

"2879","GGAGACATCCTGAAATTTGC","chr1",167902017,"-","none","AGG","GGAGACATCCTGAAATTTGCAGG",0,NA,NA,NA,"GeneID:55811","ADCY10",TRUE,"ADCY10"

"2880","TTCATGCATTATTATCCTTC","chr1",167896608,"-","none","TGG","TTCATGCATTATTATCCTTCTGG",0,NA,NA,NA,"GeneID:55811","ADCY10",TRUE,"ADCY10"

"2881","ACACAGACTCGATGCAGACC","chr5",7414646,"-","none","AGG","ACACAGACTCGATGCAGACCAGG",0,NA,NA,NA,"GeneID:108","ADCY2",TRUE,"ADCY2"

"2882","ACACAGACTCGATGCAGACC","chr9",75519169,"+","10:C>G,19:A>C","TGG","ACACAGACTCCATGCAGACATGG",2,11,20,NA,"","",FALSE,"ADCY2"

"2883","CCGCGACTTGATGCAATTAC","chr5",7626266,"-","none","AGG","CCGCGACTTGATGCAATTACAGG",0,NA,NA,NA,"GeneID:108","ADCY2",TRUE,"ADCY2"

"2884","GCTTATGGTAGGCTCCCGCC","chr5",7626204,"-","none","AGG","GCTTATGGTAGGCTCCCGCCAGG",0,NA,NA,NA,"GeneID:108","ADCY2",TRUE,"ADCY2"

"2885","TCCACAACCTGTATGTGAAG","chr5",7690802,"+","none","CGG","TCCACAACCTGTATGTGAAGCGG",0,NA,NA,NA,"GeneID:108","ADCY2",TRUE,"ADCY2"

"2886","TCCACAACCTGTATGTGAAG","chr1",58356239,"+","16:A>G,19:A>G","TGG","TCCACAACCTGTATGTAAAATGG",2,17,20,NA,"","",FALSE,"ADCY2"

"2887","TCCATATCACCAACGAGAAG","chr5",7414685,"-","none","AGG","TCCATATCACCAACGAGAAGAGG",0,NA,NA,NA,"GeneID:108","ADCY2",TRUE,"ADCY2"

"2888","TTGTATGCCGCTTCACATAC","chr5",7690812,"-","none","AGG","TTGTATGCCGCTTCACATACAGG",0,NA,NA,NA,"GeneID:108","ADCY2",TRUE,"ADCY2"

"2889","AGCCCACGATGTCGGCAAAG","chr2",24841641,"+","none","AGG","AGCCCACGATGTCGGCAAAGAGG",0,NA,NA,NA,"GeneID:109","ADCY3",TRUE,"ADCY3"

"2890","AGCCCACGATGTCGGCAAAG","chr16",50294645,"-","13:G>C,17:A>T","AGG","AGCCCACGATGTCCGCATAGAGG",2,14,18,NA,"GeneID:113","ADCY7",TRUE,"ADCY3"

"2891","CACGCGCGAAGTTCAGGCCC","chr2",24918503,"+","none","AGG","CACGCGCGAAGTTCAGGCCCAGG",0,NA,NA,NA,"GeneID:109","ADCY3",TRUE,"ADCY3"

"2892","CATCCTCTTTGCCGACATCG","chr2",24841647,"-","none","TGG","CATCCTCTTTGCCGACATCGTGG",0,NA,NA,NA,"GeneID:109","ADCY3",TRUE,"ADCY3"

"2893","CATCCTCTTTGCCGACATCG","chr16",50294639,"+","8:A>T,12:G>C","TGG","CATCCTCTATGCGGACATCGTGG",2,9,13,NA,"GeneID:113","ADCY7",TRUE,"ADCY3"

"2894","CATCCTCTTTGCCGACATCG","chr7",45622631,"+","6:G>C,12:T>C","TGG","CATCCTGTTTGCTGACATCGTGG",2,7,13,NA,"GeneID:107","ADCY1",TRUE,"ADCY3"

"2895","CATCCTCTTTGCCGACATCG","chr3",123332655,"-","6:C>G,12:A>G","AGG","CATCCTGTTTGCTGACATCGAGG",2,7,13,NA,"GeneID:111","ADCY5",TRUE,"ADCY3"

"2896","CGCCGTCTGCTCCATCCTCA","chr2",24841286,"-","none","TGG","CGCCGTCTGCTCCATCCTCATGG",0,NA,NA,NA,"GeneID:109","ADCY3",TRUE,"ADCY3"

"2897","CTTCTCCGAGCCCGAATACT","chr2",24918950,"-","none","CGG","CTTCTCCGAGCCCGAATACTCGG",0,NA,NA,NA,"GeneID:109","ADCY3",TRUE,"ADCY3"

"2898","TTACGAGATGGCCTCCACCA","chr2",24841254,"+","none","TGG","TTACGAGATGGCCTCCACCATGG",0,NA,NA,NA,"GeneID:109","ADCY3",TRUE,"ADCY3"

"2899","CAGCGCGACCCATACCAAGC","chr14",24332847,"+","none","CGG","CAGCGCGACCCATACCAAGCCGG",0,NA,NA,NA,"GeneID:196883","ADCY4",TRUE,"ADCY4"

"2900","CTCGTAGAAGAGGTCTTCGC","chr14",24334598,"+","none","TGG","CTCGTAGAAGAGGTCTTCGCTGG",0,NA,NA,NA,"GeneID:196883","ADCY4",TRUE,"ADCY4"

"2901","GATCGTGCTCTGTGCGCTCG","chr14",24334528,"-","none","CGG","GATCGTGCTCTGTGCGCTCGCGG",0,NA,NA,NA,"GeneID:196883","ADCY4",TRUE,"ADCY4"

"2902","GATCGTGCTCTGTGCGCTCG","chr18",14260854,"-","1:G>T,3:T>G","CGG","GCTAGTGCTCTGTGCGCTCGCGG",2,2,4,NA,"","",FALSE,"ADCY4"

"2903","GCTGTATCTTGGGCCACAGC","chr14",24332550,"-","none","CGG","GCTGTATCTTGGGCCACAGCCGG",0,NA,NA,NA,"GeneID:196883","ADCY4",TRUE,"ADCY4"

"2904","GCTGTATCTTGGGCCACAGC","chr14",31913453,"+","15:C>A,19:A>C","GGG","GCTGTATCTTGGGCCCCAGAGGG",2,16,20,NA,"","",FALSE,"ADCY4"

"2905","GGCGGCGTCCCGCATGCCCA","chr14",24332614,"+","none","AGG","GGCGGCGTCCCGCATGCCCAAGG",0,NA,NA,NA,"GeneID:196883","ADCY4",TRUE,"ADCY4"

"2906","TGGGTCGCGCTGCTAGCGCT","chr14",24332839,"-","none","AGG","TGGGTCGCGCTGCTAGCGCTAGG",0,NA,NA,NA,"GeneID:196883","ADCY4",TRUE,"ADCY4"

"2907","ACCTCACGTTGTCATGTTTC","chr3",123347779,"+","none","TGG","ACCTCACGTTGTCATGTTTCTGG",0,NA,NA,NA,"GeneID:111","ADCY5",TRUE,"ADCY5"

"2908","AGCAGACATCAACGCCAAGC","chr3",123347833,"-","none","AGG","AGCAGACATCAACGCCAAGCAGG",0,NA,NA,NA,"GeneID:111","ADCY5",TRUE,"ADCY5"

"2909","CTGCACCCACTATCCGGCTG","chr3",123352514,"-","none","AGG","CTGCACCCACTATCCGGCTGAGG",0,NA,NA,NA,"GeneID:111","ADCY5",TRUE,"ADCY5"

"2910","GCGCCCCTTCTACAACCACC","chr3",123325359,"-","none","TGG","GCGCCCCTTCTACAACCACCTGG",0,NA,NA,NA,"GeneID:111","ADCY5",TRUE,"ADCY5"

"2911","GCGGTTCATCTTGGCGATCA","chr3",123325426,"+","none","TGG","GCGGTTCATCTTGGCGATCATGG",0,NA,NA,NA,"GeneID:111","ADCY5",TRUE,"ADCY5"

"2912","GGTGCAGACACCCACGATGT","chr3",123352527,"+","none","TGG","GGTGCAGACACCCACGATGTTGG",0,NA,NA,NA,"GeneID:111","ADCY5",TRUE,"ADCY5"

"2913","ACGCGTTGCGCTCGCCACCA","chr12",48776264,"+","none","CGG","ACGCGTTGCGCTCGCCACCACGG",0,NA,NA,NA,"GeneID:112","ADCY6",TRUE,"ADCY6"

"2914","CCCTGAATGAGCTCTTTGCC","chr12",48777430,"-","none","CGG","CCCTGAATGAGCTCTTTGCCCGG",0,NA,NA,NA,"GeneID:112","ADCY6",TRUE,"ADCY6"

"2915","CCGCAGCCAGCTTGTCAAAC","chr12",48777408,"+","none","CGG","CCGCAGCCAGCTTGTCAAACCGG",0,NA,NA,NA,"GeneID:112","ADCY6",TRUE,"ADCY6"

"2916","CCTGACTTACTTGTCTTTGC","chr12",48775662,"+","none","TGG","CCTGACTTACTTGTCTTTGCTGG",0,NA,NA,NA,"GeneID:112","ADCY6",TRUE,"ADCY6"

"2917","CCTGACTTACTTGTCTTTGC","chr2",13083934,"+","8:C>A,13:G>T","GGG","CCTGACTTCCTTGGCTTTGCGGG",2,9,14,NA,"","",FALSE,"ADCY6"

"2918","GCAACACTGCAGTACCTGAA","chr12",48776311,"-","none","CGG","GCAACACTGCAGTACCTGAACGG",0,NA,NA,NA,"GeneID:112","ADCY6",TRUE,"ADCY6"

"2919","GCAGTGTTGCCCGAGTGATG","chr12",48776321,"+","none","TGG","GCAGTGTTGCCCGAGTGATGTGG",0,NA,NA,NA,"GeneID:112","ADCY6",TRUE,"ADCY6"

"2920","ACTTCTCGTAGAGCGCATCT","chr16",50288232,"-","none","TGG","ACTTCTCGTAGAGCGCATCTTGG",0,NA,NA,NA,"GeneID:113","ADCY7",TRUE,"ADCY7"

"2921","CTACTTCCTCAACGAGGGCG","chr16",50288196,"+","none","AGG","CTACTTCCTCAACGAGGGCGAGG",0,NA,NA,NA,"GeneID:113","ADCY7",TRUE,"ADCY7"

"2922","GACAGCGCCCCGCATGCTGA","chr16",50291784,"-","none","AGG","GACAGCGCCCCGCATGCTGAAGG",0,NA,NA,NA,"GeneID:113","ADCY7",TRUE,"ADCY7"

"2923","GCTTCACGACACCCAGTGTC","chr16",50291862,"+","none","CGG","GCTTCACGACACCCAGTGTCCGG",0,NA,NA,NA,"GeneID:113","ADCY7",TRUE,"ADCY7"

"2924","GCTTCACGACACCCAGTGTC","chr19",5222276,"-","8:G>T,18:G>A","AGG","GCTTCACGCCACCCAGTGCCAGG",2,9,19,NA,"GeneID:5802","PTPRS",FALSE,"ADCY7"

"2925","GTTCGACGCATGGACAAAGG","chr16",50290620,"+","none","CGG","GTTCGACGCATGGACAAAGGCGG",0,NA,NA,NA,"GeneID:113","ADCY7",TRUE,"ADCY7"

"2926","TGTTACCTGCTCCCACGCAC","chr16",50290646,"-","none","AGG","TGTTACCTGCTCCCACGCACAGG",0,NA,NA,NA,"GeneID:113","ADCY7",TRUE,"ADCY7"

"2927","TGTTACCTGCTCCCACGCAC","chr4",185574162,"+","5:A>C,16:T>G","CGG","TGTTAACTGCTCCCACTCACCGG",2,6,17,NA,"","",FALSE,"ADCY7"

"2928","ATGGGCCAGTCGATCAAATC","chr8",130943350,"+","none","TGG","ATGGGCCAGTCGATCAAATCTGG",0,NA,NA,NA,"GeneID:114","ADCY8",TRUE,"ADCY8"

"2929","CCACTTCCGATTTGCGCCTT","chr8",131039783,"+","none","TGG","CCACTTCCGATTTGCGCCTTTGG",0,NA,NA,NA,"GeneID:114","ADCY8",TRUE,"ADCY8"

"2930","CGTAGCCTATGCCGTCGCCC","chr8",131039513,"+","none","AGG","CGTAGCCTATGCCGTCGCCCAGG",0,NA,NA,NA,"GeneID:114","ADCY8",TRUE,"ADCY8"

"2931","CTTTGCCAGATTTGATCGAC","chr8",130943358,"-","none","TGG","CTTTGCCAGATTTGATCGACTGG",0,NA,NA,NA,"GeneID:114","ADCY8",TRUE,"ADCY8"

"2932","GATCAACGACATGACCAATG","chr8",130951931,"-","none","TGG","GATCAACGACATGACCAATGTGG",0,NA,NA,NA,"GeneID:114","ADCY8",TRUE,"ADCY8"

"2933","TCCATCGCTATGAGAACGTC","chr8",130951869,"-","none","AGG","TCCATCGCTATGAGAACGTCAGG",0,NA,NA,NA,"GeneID:114","ADCY8",TRUE,"ADCY8"

"2934","ACAGCTGCAGCGAGACTCCT","chr16",4007502,"+","none","TGG","ACAGCTGCAGCGAGACTCCTTGG",0,NA,NA,NA,"GeneID:115","ADCY9",TRUE,"ADCY9"

"2935","ACAGCTGCAGCGAGACTCCT","chrX",20374257,"-","0:A>T,10:T>G","GGG","TCAGCTGCAGAGAGACTCCTGGG",2,1,11,NA,"","",FALSE,"ADCY9"

"2936","CGGCCCCCACGACGGTGGCG","chr16",3977521,"+","none","AGG","CGGCCCCCACGACGGTGGCGAGG",0,NA,NA,NA,"GeneID:115","ADCY9",TRUE,"ADCY9"

"2937","CTTACCTGATGGACACCGCG","chr16",3983226,"+","none","AGG","CTTACCTGATGGACACCGCGAGG",0,NA,NA,NA,"GeneID:115","ADCY9",TRUE,"ADCY9"

"2938","GAAGTTACAGTAGTGCACGA","chr16",3977573,"+","none","CGG","GAAGTTACAGTAGTGCACGACGG",0,NA,NA,NA,"GeneID:115","ADCY9",TRUE,"ADCY9"

"2939","GGACAGGGGACAGCGTCATC","chr16",4007419,"-","none","AGG","GGACAGGGGACAGCGTCATCAGG",0,NA,NA,NA,"GeneID:115","ADCY9",TRUE,"ADCY9"

"2940","GGACAGGGGACAGCGTCATC","chr10",126187880,"-","1:G>C,2:A>T","TGG","GCTCAGGGGACAGCGTCATCTGG",2,2,3,NA,"GeneID:8038","ADAM12",FALSE,"ADCY9"

"2941","TGTCCCTCGCGGTGTCCATC","chr16",3983233,"-","none","AGG","TGTCCCTCGCGGTGTCCATCAGG",0,NA,NA,NA,"GeneID:115","ADCY9",TRUE,"ADCY9"

"2942","AACCCGCTGCCAGACTTCGA","chr18",907689,"+","none","TGG","AACCCGCTGCCAGACTTCGATGG",0,NA,NA,NA,"GeneID:116","ADCYAP1",TRUE,"ADCYAP1"

"2943","CAGCCTCGCTCCGCTACACA","chr18",905393,"-","none","TGG","CAGCCTCGCTCCGCTACACATGG",0,NA,NA,NA,"GeneID:116","ADCYAP1",TRUE,"ADCYAP1"

"2944","CCCGTTAGAGATGTCGCCCA","chr18",908256,"+","none","CGG","CCCGTTAGAGATGTCGCCCACGG",0,NA,NA,NA,"GeneID:116","ADCYAP1",TRUE,"ADCYAP1"

"2945","CCGTGAAGATCCCGTCCGAG","chr18",909499,"-","none","TGG","CCGTGAAGATCCCGTCCGAGTGG",0,NA,NA,NA,"GeneID:116","ADCYAP1",TRUE,"ADCYAP1"

"2946","GAAGCACCTGCAGTCGCTCG","chr18",908327,"+","none","TGG","GAAGCACCTGCAGTCGCTCGTGG",0,NA,NA,NA,"GeneID:116","ADCYAP1",TRUE,"ADCYAP1"

"2947","GGCTCCGAGCCATCGAAGTC","chr18",907701,"-","none","TGG","GGCTCCGAGCCATCGAAGTCTGG",0,NA,NA,NA,"GeneID:116","ADCYAP1",TRUE,"ADCYAP1"

"2948","ATTGAAGCCCATCAGCTCAT","chr7",31064903,"-","none","TGG","ATTGAAGCCCATCAGCTCATTGG",0,NA,NA,NA,"GeneID:117","ADCYAP1R1",TRUE,"ADCYAP1R1"

"2949","CCCCTTACCTGAGAGATCTA","chr7",31081742,"-","none","AGG","CCCCTTACCTGAGAGATCTAAGG",0,NA,NA,NA,"GeneID:117","ADCYAP1R1",TRUE,"ADCYAP1R1"

"2950","CGAATCTTCAACCCAGACCA","chr7",31078076,"+","none","AGG","CGAATCTTCAACCCAGACCAAGG",0,NA,NA,NA,"GeneID:117","ADCYAP1R1",TRUE,"ADCYAP1R1"

"2951","GTCTGGGAGACCGAAACCAT","chr7",31080611,"+","none","TGG","GTCTGGGAGACCGAAACCATTGG",0,NA,NA,NA,"GeneID:117","ADCYAP1R1",TRUE,"ADCYAP1R1"

"2952","GTGTTGGAAGCCCGCCCATG","chr7",31078018,"+","none","TGG","GTGTTGGAAGCCCGCCCATGTGG",0,NA,NA,NA,"GeneID:117","ADCYAP1R1",TRUE,"ADCYAP1R1"

"2953","GTTCCTCTTACCAATGGTTT","chr7",31080624,"-","none","CGG","GTTCCTCTTACCAATGGTTTCGG",0,NA,NA,NA,"GeneID:117","ADCYAP1R1",TRUE,"ADCYAP1R1"

"2954","GTTCCTCTTACCAATGGTTT","chr12",68285291,"-","7:T>A,13:G>T","GGG","GTTCCTCATACCACTGGTTTGGG",2,8,14,NA,"GeneID:56890","MDM1",FALSE,"ADCYAP1R1"

"2955","CATGGCACCAGACCTTCGCC","chr4",2876034,"+","none","AGG","CATGGCACCAGACCTTCGCCAGG",0,NA,NA,NA,"GeneID:118","ADD1",TRUE,"ADD1"

"2956","CCTCTTGCCAATCTCCCCGG","chr4",2898203,"+","none","AGG","CCTCTTGCCAATCTCCCCGGAGG",0,NA,NA,NA,"GeneID:118","ADD1",TRUE,"ADD1"

"2957","GAAAGCGCCTCCGGGGAGAT","chr4",2898213,"-","none","TGG","GAAAGCGCCTCCGGGGAGATTGG",0,NA,NA,NA,"GeneID:118","ADD1",TRUE,"ADD1"

"2958","GAATGGTGATTCTCGTGCTG","chr4",2875917,"+","none","CGG","GAATGGTGATTCTCGTGCTGCGG",0,NA,NA,NA,"GeneID:118","ADD1",TRUE,"ADD1"

"2959","GACCAGAGTGAACTCCGAGC","chr4",2894011,"+","none","AGG","GACCAGAGTGAACTCCGAGCAGG",0,NA,NA,NA,"GeneID:118","ADD1",TRUE,"ADD1"

"2960","TGAAGTGACTGCATCCAGTT","chr4",2894071,"+","none","TGG","TGAAGTGACTGCATCCAGTTTGG",0,NA,NA,NA,"GeneID:118","ADD1",TRUE,"ADD1"

"2961","TGAAGTGACTGCATCCAGTT","chr16",72247498,"+","13:G>T,17:A>G","AGG","TGAAGTGACTGCAGCCAATTAGG",2,14,18,NA,"","",FALSE,"ADD1"

"2962","TGAAGTGACTGCATCCAGTT","chr10",116463302,"+","1:C>G,3:G>A","GGG","TCAGGTGACTGCATCCAGTTGGG",2,2,4,NA,"GeneID:119548","PNLIPRP3",FALSE,"ADD1"

"2963","AGTACATGCGCCTTCGCAAC","chr2",70706300,"-","none","CGG","AGTACATGCGCCTTCGCAACCGG",0,NA,NA,NA,"GeneID:119","ADD2",TRUE,"ADD2"

"2964","CAGCAGTGTCTACCGACTCC","chr2",70696288,"-","none","TGG","CAGCAGTGTCTACCGACTCCTGG",0,NA,NA,NA,"GeneID:119","ADD2",TRUE,"ADD2"

"2965","CCGCATGAGCCGCTCCCCTT","chr2",70696316,"+","none","TGG","CCGCATGAGCCGCTCCCCTTTGG",0,NA,NA,NA,"GeneID:119","ADD2",TRUE,"ADD2"

"2966","GCGACAGATCGCGGACTTCA","chr2",70704362,"-","none","TGG","GCGACAGATCGCGGACTTCATGG",0,NA,NA,NA,"GeneID:119","ADD2",TRUE,"ADD2"

"2967","TGAAGTCCGCGATCTGTCGC","chr2",70704362,"+","none","AGG","TGAAGTCCGCGATCTGTCGCAGG",0,NA,NA,NA,"GeneID:119","ADD2",TRUE,"ADD2"

"2968","TTGCGAAGGCGCATGTACTC","chr2",70706301,"+","none","GGG","TTGCGAAGGCGCATGTACTCGGG",0,NA,NA,NA,"GeneID:119","ADD2",TRUE,"ADD2"

"2969","ACAAGTCTACAAGTCTGTAC","chr10",110116355,"-","none","AGG","ACAAGTCTACAAGTCTGTACAGG",0,NA,NA,NA,"GeneID:120","ADD3",TRUE,"ADD3"

"2970","ACACCTATCAATGACCTTCC","chr10",110116272,"+","none","TGG","ACACCTATCAATGACCTTCCTGG",0,NA,NA,NA,"GeneID:120","ADD3",TRUE,"ADD3"

"2971","ACACCTATCAATGACCTTCC","chr8",23857230,"-","7:C>A,14:T>G","AGG","ACACCTAGCAATGAACTTCCAGG",2,8,15,NA,"","",FALSE,"ADD3"

"2972","ACAGGCCTTTCGGGAAGACT","chr10",110112772,"+","none","TGG","ACAGGCCTTTCGGGAAGACTTGG",0,NA,NA,NA,"GeneID:120","ADD3",TRUE,"ADD3"

"2973","ACAGGCCTTTCGGGAAGACT","chr17",82887266,"-","8:G>A,19:T>A","GGG","ACAGGCCTCTCGGGAAGACAGGG",2,9,20,NA,"GeneID:6904","TBCD",FALSE,"ADD3"

"2974","GTGTTGAATAGATTGCAGCA","chr10",110118666,"-","none","TGG","GTGTTGAATAGATTGCAGCATGG",0,NA,NA,NA,"GeneID:120","ADD3",TRUE,"ADD3"

"2975","TGAAGCTACAGCCTCCAATT","chr10",110117400,"+","none","TGG","TGAAGCTACAGCCTCCAATTTGG",0,NA,NA,NA,"GeneID:120","ADD3",TRUE,"ADD3"

"2976","TGAAGCTACAGCCTCCAATT","chr10",68496304,"+","11:T>C,16:C>A","GGG","TGAAGCTACAGTCTCCCATTGGG",2,12,17,NA,"GeneID:8034","SLC25A16",FALSE,"ADD3"

"2977","TGTAATGCTAGTAATCCAGT","chr10",110112836,"-","none","TGG","TGTAATGCTAGTAATCCAGTTGG",0,NA,NA,NA,"GeneID:120","ADD3",TRUE,"ADD3"

"2978","TGTAATGCTAGTAATCCAGT","chr1",216170741,"-","18:T>C,19:C>A","TGG","TGTAATGCTAGTAATCCAAGTGG",2,19,20,NA,"GeneID:7399","USH2A",FALSE,"ADD3"

"2979","ACTCTGTAGATGGTGGCTGT","chr4",99286977,"-","none","AGG","ACTCTGTAGATGGTGGCTGTAGG",0,NA,NA,NA,"GeneID:100507053;GeneID:124","LOC100507053;ADH1A",TRUE,"ADH1A"

"2980","ACTCTGTAGATGGTGGCTGT","chr4",99318173,"-","1:A>G","AGG","ATTCTGTAGATGGTGGCTGTAGG",1,2,NA,NA,"GeneID:125","ADH1B",TRUE,"ADH1A"

"2981","ACTCTGTAGATGGTGGCTGT","chr12",21981850,"-","4:T>A,12:G>C","TGG","ACTCAGTAGATGCTGGCTGTTGG",2,5,13,NA,"","",FALSE,"ADH1A"

"2982","ACTCTGTAGATGGTGGCTGT","chr4",99347133,"-","1:A>G,19:G>A","AGG","ATTCTGTAGATGGTGGCTGCAGG",2,2,20,NA,"GeneID:125;GeneID:126","ADH1B;ADH1C",TRUE,"ADH1A"

"2983","AGAATCAACATGAGCACAGC","chr4",99290903,"-","none","AGG","AGAATCAACATGAGCACAGCAGG",0,NA,NA,NA,"GeneID:100507053;GeneID:124","LOC100507053;ADH1A",TRUE,"ADH1A"

"2984","AGAATCAACATGAGCACAGC","chr4",15706260,"+","14:G>C,18:T>G","AGG","AGAATCAACATGAGGACATCAGG",2,15,19,NA,"GeneID:683","BST1",FALSE,"ADH1A"

"2985","AGAATCAACATGAGCACAGC","chr4",99321320,"-","4:T>A,6:C>T","AGG","AGAAACGACATGAGCACAGCAGG",2,5,7,NA,"GeneID:125","ADH1B",TRUE,"ADH1A"

"2986","AGAATCAACATGAGCACAGC","chr4",99352664,"-","8:A>G","AGG","AGAATCAATATGAGCACAGCAGG",1,9,NA,NA,"GeneID:125;GeneID:126","ADH1B;ADH1C",TRUE,"ADH1A"

"2987","AGAATCAACATGAGCACAGC","chr4",3422866,"-","9:C>T,12:C>T","AGG","AGAATCAACGTGGGCACAGCAGG",2,10,13,NA,"GeneID:6002","RGS12",FALSE,"ADH1A"

"2988","CCATGGTACCACTAACCACG","chr4",99286933,"+","none","TGG","CCATGGTACCACTAACCACGTGG",0,NA,NA,NA,"GeneID:100507053;GeneID:124","LOC100507053;ADH1A",TRUE,"ADH1A"

"2989","CTGTGGCACAGATGACCACG","chr4",99286951,"-","none","TGG","CTGTGGCACAGATGACCACGTGG",0,NA,NA,NA,"GeneID:100507053;GeneID:124","LOC100507053;ADH1A",TRUE,"ADH1A"

"2990","CTGTGGCACAGATGACCACG","chr4",99318147,"-","4:G>C,5:T>C","TGG","CTGTCACACAGATGACCACGTGG",2,5,6,NA,"GeneID:125","ADH1B",TRUE,"ADH1A"

"2991","CTGTGGCACAGATGACCACG","chr17",31550954,"+","4:T>G,18:T>C","AGG","CTGTTGCACAGATGACCATGAGG",2,5,19,NA,"","",FALSE,"ADH1A"

"2992","CTGTGGCACAGATGACCACG","chr2",131034734,"-","1:T>A,14:C>T","GGG","CAGTGGCACAGATGGCCACGGGG",2,2,15,NA,"GeneID:50649","ARHGEF4",FALSE,"ADH1A"

"2993","GGTGGAGGTTGCACCTCCTA","chr4",99287586,"-","none","AGG","GGTGGAGGTTGCACCTCCTAAGG",0,NA,NA,NA,"GeneID:100507053;GeneID:124","LOC100507053;ADH1A",TRUE,"ADH1A"

"2994","GGTGGAGGTTGCACCTCCTA","chr1",246379928,"+","18:C>T","GGG","GGTGGAGGTTGCACCTCCCAGGG",1,19,NA,NA,"GeneID:64754","SMYD3",FALSE,"ADH1A"

"2995","GGTGGAGGTTGCACCTCCTA","chr4",99318807,"-","0:A>C","AGG","TGTGGAGGTTGCACCTCCTAAGG",1,1,NA,NA,"GeneID:125","ADH1B",TRUE,"ADH1A"

"2996","GGTGGAGGTTGCACCTCCTA","chr4",99347767,"-","3:T>C","AGG","GGTAGAGGTTGCACCTCCTAAGG",1,4,NA,NA,"GeneID:125;GeneID:126","ADH1B;ADH1C",TRUE,"ADH1A"

"2997","GGTGGAGGTTGCACCTCCTA","chr14",29942776,"-","2:C>A,9:C>A","TGG","GGGGGAGGTGGCACCTCCTATGG",2,3,10,NA,"GeneID:5587","PRKD1",FALSE,"ADH1A"

"2998","TCACCTTAATACGAACTTCA","chr4",99287559,"+","none","TGG","TCACCTTAATACGAACTTCATGG",0,NA,NA,NA,"GeneID:100507053;GeneID:124","LOC100507053;ADH1A",TRUE,"ADH1A"

"2999","ATGCGAACTTCATAAGCCTT","chr4",99318788,"+","none","AGG","ATGCGAACTTCATAAGCCTTAGG",0,NA,NA,NA,"GeneID:125","ADH1B",TRUE,"ADH1B"

"3000","ATGCGAACTTCATAAGCCTT","chr4",99347748,"+","13:G>A","AGG","ATGCGAACTTCATGAGCCTTAGG",1,14,NA,NA,"GeneID:125;GeneID:126","ADH1B;ADH1C",TRUE,"ADH1B"

"3001","ATGCGAACTTCATAAGCCTT","chr4",99142682,"+","13:G>A","GGG","ATGCGAACTTCATGAGCCTTGGG",1,14,NA,NA,"GeneID:100507053;GeneID:127","LOC100507053;ADH4",TRUE,"ADH1B"

"3002","ATGCGAACTTCATAAGCCTT","chr4",87307982,"-","2:T>C,4:A>C","GGG","ATACTAACTTCATAAGCCTTGGG",2,3,5,NA,"GeneID:345275","HSD17B13",FALSE,"ADH1B"

"3003","ATGCGAACTTCATAAGCCTT","chr4",99085118,"+","2:T>G,13:G>A","TGG","ATTCGAACTTCATGAGCCTTTGG",2,3,14,NA,"GeneID:128","ADH5",TRUE,"ADH1B"

"3004","ATGCGAACTTCATAAGCCTT","chr6",65837944,"+","2:T>G,13:G>A","TGG","ATTCGAACTTCATGAGCCTTTGG",2,3,14,NA,"","",TRUE,"ADH1B"

"3005","ATGCGAACTTCATAAGCCTT","chr5",23980678,"-","2:A>C,13:A>T","TGG","ATTCGAACTTCATTAGCCTTTGG",2,3,14,NA,"","",TRUE,"ADH1B"

"3006","CCAGGTTGCCACTAACCACG","chr4",99318129,"+","none","TGG","CCAGGTTGCCACTAACCACGTGG",0,NA,NA,NA,"GeneID:125","ADH1B",TRUE,"ADH1B"

"3007","CTGTCACACAGATGACCACG","chr4",99318147,"-","none","TGG","CTGTCACACAGATGACCACGTGG",0,NA,NA,NA,"GeneID:125","ADH1B",TRUE,"ADH1B"

"3008","CTGTCACACAGATGACCACG","chr4",99286951,"-","4:C>G,5:C>T","TGG","CTGTGGCACAGATGACCACGTGG",2,5,6,NA,"GeneID:100507053;GeneID:124","LOC100507053;ADH1A",TRUE,"ADH1B"

"3009","GTCTGCAGTTAACGTTGCCA","chr4",99315899,"-","none","AGG","GTCTGCAGTTAACGTTGCCAAGG",0,NA,NA,NA,"GeneID:125","ADH1B",TRUE,"ADH1B"

"3010","GTCTGCAGTTAACGTTGCCA","chr4",99344863,"-","9:G>A,12:T>G","AGG","GTCTGCAGTCAAAGTTGCCAAGG",2,10,13,NA,"GeneID:125;GeneID:126","ADH1B;ADH1C",TRUE,"ADH1B"

"3011","GTCTGCAGTTAACGTTGCCA","chr4",99284400,"-","9:G>A,12:A>G","AGG","GTCTGCAGTCAATGTTGCCAAGG",2,10,13,NA,"GeneID:100507053;GeneID:124","LOC100507053;ADH1A",TRUE,"ADH1B"

"3012","GTCTGCAGTTAACGTTGCCA","chr10",45100990,"-","0:T>C,14:G>A","GGG","ATCTGCAGTTAACGCTGCCAGGG",2,1,15,NA,"","",FALSE,"ADH1B"

"3013","TCTCATCCACCACCGTGTAC","chr4",99315998,"+","none","TGG","TCTCATCCACCACCGTGTACTGG",0,NA,NA,NA,"GeneID:125","ADH1B",TRUE,"ADH1B"

"3014","TCTCATCCACCACCGTGTAC","chr4",99344962,"+","13:T>C","TGG","TCTCATCCACCACTGTGTACTGG",1,14,NA,NA,"GeneID:125;GeneID:126","ADH1B;ADH1C",TRUE,"ADH1B"

"3015","TGTGGAGGTTGCACCTCCTA","chr4",99318807,"-","none","AGG","TGTGGAGGTTGCACCTCCTAAGG",0,NA,NA,NA,"GeneID:125","ADH1B",TRUE,"ADH1B"

"3016","TGTGGAGGTTGCACCTCCTA","chr4",99287586,"-","0:C>A","AGG","GGTGGAGGTTGCACCTCCTAAGG",1,1,NA,NA,"GeneID:100507053;GeneID:124","LOC100507053;ADH1A",TRUE,"ADH1B"

"3017","TGTGGAGGTTGCACCTCCTA","chr4",99347767,"-","0:C>A,3:T>C","AGG","GGTAGAGGTTGCACCTCCTAAGG",2,1,4,NA,"GeneID:125;GeneID:126","ADH1B;ADH1C",TRUE,"ADH1B"

"3018","TGTGGAGGTTGCACCTCCTA","chr1",246379928,"+","0:G>T,18:C>T","GGG","GGTGGAGGTTGCACCTCCCAGGG",2,1,19,NA,"GeneID:64754","SMYD3",FALSE,"ADH1B"

"3019","AAGACAGCACAAGTCGAACC","chr4",99131735,"+","none","AGG","AAGACAGCACAAGTCGAACCAGG",0,NA,NA,NA,"GeneID:100507053;GeneID:127","LOC100507053;ADH4",TRUE,"ADH4"

"3020","AAGACAGCACAAGTCGAACC","chr10",22004088,"+","7:G>C,14:T>C","AGG","AAGACAGGACAAGTTGAACCAGG",2,8,15,NA,"","",FALSE,"ADH4"

"3021","AGACTTACATAAACCGATCC","chr4",99131577,"-","none","AGG","AGACTTACATAAACCGATCCAGG",0,NA,NA,NA,"GeneID:100507053;GeneID:127","LOC100507053;ADH4",TRUE,"ADH4"

"3022","AGACTTACATAAACCGATCC","chrX",31337410,"-","14:A>G,16:A>T","TGG","AGACTTACATAAACTGTTCCTGG",2,15,17,NA,"GeneID:1756","DMD",FALSE,"ADH4"

"3023","CTAGCTTTCCCAGTGATCGT","chr4",99141602,"-","none","TGG","CTAGCTTTCCCAGTGATCGTTGG",0,NA,NA,NA,"GeneID:100507053;GeneID:127","LOC100507053;ADH4",TRUE,"ADH4"

"3024","CTTCAACCTCTTCAATGCAA","chr4",99142713,"+","none","AGG","CTTCAACCTCTTCAATGCAAAGG",0,NA,NA,NA,"GeneID:100507053;GeneID:127","LOC100507053;ADH4",TRUE,"ADH4"

"3025","GGAGTGACCAACGTCAAACC","chr4",99141542,"-","none","AGG","GGAGTGACCAACGTCAAACCAGG",0,NA,NA,NA,"GeneID:100507053;GeneID:127","LOC100507053;ADH4",TRUE,"ADH4"

"3026","TTCCCACACAAATTTGTGAG","chr4",99139066,"+","none","TGG","TTCCCACACAAATTTGTGAGTGG",0,NA,NA,NA,"GeneID:100507053;GeneID:127","LOC100507053;ADH4",TRUE,"ADH4"

"3027","TTCCCACACAAATTTGTGAG","chr19",47275735,"-","12:G>A,16:C>A","GGG","TTCCCACACAAACTTGGGAGGGG",2,13,17,NA,"","",FALSE,"ADH4"

"3028","AAGATACTAACCTTATCTTC","chr4",99081353,"+","none","TGG","AAGATACTAACCTTATCTTCTGG",0,NA,NA,NA,"GeneID:128","ADH5",TRUE,"ADH5"

"3029","ACTTCAGGTTATCAAGTGCA","chr4",99085203,"-","none","AGG","ACTTCAGGTTATCAAGTGCAAGG",0,NA,NA,NA,"GeneID:128","ADH5",TRUE,"ADH5"

"3030","ACTTCAGGTTATCAAGTGCA","chr8",96671164,"-","4:T>G,17:T>C","AGG","ACTTAAGGTTATCAAGTACAAGG",2,5,18,NA,"GeneID:10404","CPQ",FALSE,"ADH5"

"3031","AGTCGGATTGGCAGTTATCA","chr4",99076491,"-","none","TGG","AGTCGGATTGGCAGTTATCATGG",0,NA,NA,NA,"GeneID:128","ADH5",TRUE,"ADH5"

"3032","AGTCGGATTGGCAGTTATCA","chr6",65837429,"-","3:A>G","AGG","AGTTGGATTGGCAGTTATCAAGG",1,4,NA,NA,"","",TRUE,"ADH5"

"3033","ATCGGTGTGGCAAACCGCAG","chr4",99082086,"+","none","TGG","ATCGGTGTGGCAAACCGCAGTGG",0,NA,NA,NA,"GeneID:128","ADH5",TRUE,"ADH5"

"3034","ATCGGTGTGGCAAACCGCAG","chr6",65837910,"+","0:G>A","TGG","GTCGGTGTGGCAAACCGCAGTGG",1,1,NA,NA,"","",TRUE,"ADH5"

"3035","ATCGGTGTGGCAAACCGCAG","chr7",140788062,"+","3:T>G,18:T>A","TGG","ATCTGTGTGGCAAACCGCTGTGG",2,4,19,NA,"GeneID:673","BRAF",FALSE,"ADH5"

"3036","ATCGGTGTGGCAAACCGCAG","chr5",23980712,"-","0:C>T,16:T>C","TGG","GTCGGTGTGGCAAACCACAGTGG",2,1,17,NA,"","",TRUE,"ADH5"

"3037","CTGATAGATCATTGCCACTG","chr4",99082103,"-","none","CGG","CTGATAGATCATTGCCACTGCGG",0,NA,NA,NA,"GeneID:128","ADH5",TRUE,"ADH5"

"3038","GTGCTCATTGAGATGACCGA","chr4",99076340,"-","none","TGG","GTGCTCATTGAGATGACCGATGG",0,NA,NA,NA,"GeneID:128","ADH5",TRUE,"ADH5"

"3039","ACACTCTTTCTGCCACAGTG","chr4",99210468,"-","none","TGG","ACACTCTTTCTGCCACAGTGTGG",0,NA,NA,NA,"GeneID:100507053;GeneID:130","LOC100507053;ADH6",TRUE,"ADH6"

"3040","GAGGTACATTCTCCACACTG","chr4",99210453,"+","none","TGG","GAGGTACATTCTCCACACTGTGG",0,NA,NA,NA,"GeneID:100507053;GeneID:130","LOC100507053;ADH6",TRUE,"ADH6"

"3041","GAGGTACATTCTCCACACTG","chr15",85724591,"+","5:G>A,15:G>C","AGG","GAGGTGCATTCTCCAGACTGAGG",2,6,16,NA,"GeneID:11214","AKAP13",FALSE,"ADH6"

"3042","GATGCAAAGCAGCCATACTC","chr4",99216235,"-","none","TGG","GATGCAAAGCAGCCATACTCTGG",0,NA,NA,NA,"GeneID:100507053;GeneID:130","LOC100507053;ADH6",TRUE,"ADH6"

"3043","GCTGGAATCGTTGAGAGTAT","chr4",99213634,"-","none","TGG","GCTGGAATCGTTGAGAGTATTGG",0,NA,NA,NA,"GeneID:100507053;GeneID:130","LOC100507053;ADH6",TRUE,"ADH6"

"3044","GCTGGAATCGTTGAGAGTAT","chr4",99476775,"-","8:A>G,11:T>A","TGG","GCTGGAATTGTAGAGAGTATTGG",2,9,12,NA,"","",TRUE,"ADH6"

"3045","TCCATACAGGTTGTGGCCAC","chr4",99213736,"-","none","CGG","TCCATACAGGTTGTGGCCACCGG",0,NA,NA,NA,"GeneID:100507053;GeneID:130","LOC100507053;ADH6",TRUE,"ADH6"

"3046","TGCACCAGGCTTCCAGAGTA","chr4",99216220,"+","none","TGG","TGCACCAGGCTTCCAGAGTATGG",0,NA,NA,NA,"GeneID:100507053;GeneID:130","LOC100507053;ADH6",TRUE,"ADH6"

"3047","AATGCTTGTCGCAACCCAGA","chr4",99428110,"-","none","TGG","AATGCTTGTCGCAACCCAGATGG",0,NA,NA,NA,"GeneID:131","ADH7",TRUE,"ADH7"

"3048","ACTGGGATTGTAGAGAGCAT","chr4",99428520,"-","none","TGG","ACTGGGATTGTAGAGAGCATTGG",0,NA,NA,NA,"GeneID:131","ADH7",TRUE,"ADH7"

"3049","ATCTGTGCGACAGATTCCTG","chr4",99428600,"+","none","TGG","ATCTGTGCGACAGATTCCTGTGG",0,NA,NA,NA,"GeneID:131","ADH7",TRUE,"ADH7"

"3050","ATGCGAACTTCTTTAGTCTT","chr4",99429535,"+","none","TGG","ATGCGAACTTCTTTAGTCTTTGG",0,NA,NA,NA,"GeneID:131","ADH7",TRUE,"ADH7"

"3051","ATGCGAACTTCTTTAGTCTT","chr10",56014095,"+","3:A>C,4:A>G","GGG","ATGAAAACTTCTTTAGTCTTGGG",2,4,5,NA,"","",FALSE,"ADH7"

"3052","CCTACTCGCTCCTAATGCAA","chr4",99428081,"+","none","AGG","CCTACTCGCTCCTAATGCAAAGG",0,NA,NA,NA,"GeneID:131","ADH7",TRUE,"ADH7"

"3053","GACTAAAGAAGTTCGCATTA","chr4",99429533,"-","none","AGG","GACTAAAGAAGTTCGCATTAAGG",0,NA,NA,NA,"GeneID:131","ADH7",TRUE,"ADH7"

"3054","AGGATCAATCAGTCCCAGTG","chr8",66448891,"-","none","TGG","AGGATCAATCAGTCCCAGTGTGG",0,NA,NA,NA,"GeneID:137872","ADHFE1",TRUE,"ADHFE1"

"3055","CGACCACTCGGGCAGGCATG","chr8",66448925,"-","none","TGG","CGACCACTCGGGCAGGCATGTGG",0,NA,NA,NA,"GeneID:137872","ADHFE1",TRUE,"ADHFE1"

"3056","GCAGCAGTTACAAAGGAAGT","chr8",66444393,"+","none","AGG","GCAGCAGTTACAAAGGAAGTAGG",0,NA,NA,NA,"GeneID:137872","ADHFE1",TRUE,"ADHFE1"

"3057","GCAGCAGTTACAAAGGAAGT","chrX",10971288,"-","6:A>C,16:G>T","GGG","GCAGCATTTACAAAGGCAGTGGG",2,7,17,NA,"","",FALSE,"ADHFE1"

"3058","GCAGCAGTTACAAAGGAAGT","chr22",33542195,"-","1:A>G,13:A>T","GGG","GTAGCAGTTACAATGGAAGTGGG",2,2,14,NA,"GeneID:9215","LARGE",FALSE,"ADHFE1"

"3059","GCAGCAGTTACAAAGGAAGT","chr3",61907773,"-","0:A>C,18:T>C","CGG","TCAGCAGTTACAAAGGAAATCGG",2,1,19,NA,"GeneID:5793","PTPRG",FALSE,"ADHFE1"

"3060","GCTGTTTCAAATATTAGATA","chr8",66444369,"+","none","TGG","GCTGTTTCAAATATTAGATATGG",0,NA,NA,NA,"GeneID:137872","ADHFE1",TRUE,"ADHFE1"

"3061","GCTGTTTCAAATATTAGATA","chr1",69521899,"+","17:C>A,19:G>A","CGG","GCTGTTTCAAATATTAGCTGCGG",2,18,20,NA,"","",FALSE,"ADHFE1"

"3062","GCTGTTTCAAATATTAGATA","chr20",11788514,"-","7:T>G,16:T>C","TGG","GCTGTTTAAAATATTAAATATGG",2,8,17,NA,"","",FALSE,"ADHFE1"

"3063","GGAATGTCTTACCCAATTTC","chr8",66456820,"+","none","AGG","GGAATGTCTTACCCAATTTCAGG",0,NA,NA,NA,"GeneID:137872","ADHFE1",TRUE,"ADHFE1"

"3064","GGAATGTCTTACCCAATTTC","chr1",234912877,"+","7:A>C,16:A>T","AGG","GGAATGTATTACCCAAATTCAGG",2,8,17,NA,"","",FALSE,"ADHFE1"

"3065","TTACAATGTGGATCACCCAC","chr8",66456870,"+","none","TGG","TTACAATGTGGATCACCCACTGG",0,NA,NA,NA,"GeneID:137872","ADHFE1",TRUE,"ADHFE1"

"3066","ACGACGCCCCGGGCGACCCG","chr2",3519445,"-","none","CGG","ACGACGCCCCGGGCGACCCGCGG",0,NA,NA,NA,"GeneID:55256","ADI1",TRUE,"ADI1"

"3067","CTTCAAAATGGTCAGCGGGC","chr2",3499003,"+","none","CGG","CTTCAAAATGGTCAGCGGGCCGG",0,NA,NA,NA,"GeneID:55256","ADI1",TRUE,"ADI1"

"3068","CTTCAAAATGGTCAGCGGGC","chr17",9003741,"+","0:T>C,5:G>A","TGG","TTTCAGAATGGTCAGCGGGCTGG",2,1,6,NA,"","",FALSE,"ADI1"

"3069","GAAATTTCACGTACTGCCCG","chr2",3498979,"+","none","CGG","GAAATTTCACGTACTGCCCGCGG",0,NA,NA,NA,"GeneID:55256","ADI1",TRUE,"ADI1"

"3070","GCAGTGGGTACTTCGATGTG","chr2",3500912,"-","none","AGG","GCAGTGGGTACTTCGATGTGAGG",0,NA,NA,NA,"GeneID:55256","ADI1",TRUE,"ADI1"

"3071","GGGGATCTATCACCGCTTCA","chr2",3500827,"-","none","CGG","GGGGATCTATCACCGCTTCACGG",0,NA,NA,NA,"GeneID:55256","ADI1",TRUE,"ADI1"

"3072","TGGGGTTGCCGCGGGTCGCC","chr2",3519434,"+","none","CGG","TGGGGTTGCCGCGGGTCGCCCGG",0,NA,NA,NA,"GeneID:55256","ADI1",TRUE,"ADI1"

"3073","AAATGACTCCAGTGTGTGCT","chr20",38586038,"+","none","TGG","AAATGACTCCAGTGTGTGCTTGG",0,NA,NA,NA,"GeneID:149685","ADIG",TRUE,"ADIG"

"3074","CCCTTCCAGCAAAACTCAGC","chr20",38586087,"-","none","TGG","CCCTTCCAGCAAAACTCAGCTGG",0,NA,NA,NA,"GeneID:149685","ADIG",TRUE,"ADIG"

"3075","CCCTTCCAGCAAAACTCAGC","chr5",41141534,"-","18:G>C,19:A>G","GGG","CCCTTCCAGCAAAACTCACTGGG",2,19,20,NA,"","",FALSE,"ADIG"

"3076","CCCTTCCAGCAAAACTCAGC","chr11",70231871,"-","4:G>A,18:A>C","AGG","CCCTCCCAGCAAAACTCATCAGG",2,5,19,NA,"","",FALSE,"ADIG"

"3077","CTACGCTTCTTACTTAGCCA","chr20",38581352,"+","none","AGG","CTACGCTTCTTACTTAGCCAAGG",0,NA,NA,NA,"GeneID:149685","ADIG",TRUE,"ADIG"

"3078","GAAGTACCCTTTGATGCCGC","chr20",38581252,"+","none","TGG","GAAGTACCCTTTGATGCCGCTGG",0,NA,NA,NA,"GeneID:149685","ADIG",TRUE,"ADIG"

"3079","GACACTCCACGGCCAAGAGA","chr20",38586107,"+","none","AGG","GACACTCCACGGCCAAGAGAAGG",0,NA,NA,NA,"GeneID:149685","ADIG",TRUE,"ADIG"

"3080","TCGTTCACCAGCGGCATCAA","chr20",38581262,"-","none","AGG","TCGTTCACCAGCGGCATCAAAGG",0,NA,NA,NA,"GeneID:149685","ADIG",TRUE,"ADIG"

"3081","ATACCGCTCAGCATTCAGTG","chr3",186854298,"+","none","TGG","ATACCGCTCAGCATTCAGTGTGG",0,NA,NA,NA,"GeneID:9370;GeneID:100874095","ADIPOQ;ADIPOQ-AS1",TRUE,"ADIPOQ"

"3082","ATCGGTGAAACCGGAGTACC","chr3",186854206,"+","none","CGG","ATCGGTGAAACCGGAGTACCCGG",0,NA,NA,NA,"GeneID:9370;GeneID:100874095","ADIPOQ;ADIPOQ-AS1",TRUE,"ADIPOQ"

"3083","CCAGGGCATCCGGGCCATAA","chr3",186853187,"+","none","TGG","CCAGGGCATCCGGGCCATAATGG",0,NA,NA,NA,"GeneID:9370;GeneID:100874095","ADIPOQ;ADIPOQ-AS1",TRUE,"ADIPOQ"

"3084","GCCCGCCATCCAACCTGTGC","chr3",186853164,"-","none","AGG","GCCCGCCATCCAACCTGTGCAGG",0,NA,NA,NA,"GeneID:9370;GeneID:100874095","ADIPOQ;ADIPOQ-AS1",TRUE,"ADIPOQ"

"3085","GGGCGACCAAGTCTGGCTCC","chr3",186854589,"+","none","AGG","GGGCGACCAAGTCTGGCTCCAGG",0,NA,NA,NA,"GeneID:9370;GeneID:100874095","ADIPOQ;ADIPOQ-AS1",TRUE,"ADIPOQ"

"3086","TAGGCAAAGTAGTACAGCCC","chr3",186854434,"-","none","AGG","TAGGCAAAGTAGTACAGCCCAGG",0,NA,NA,NA,"GeneID:9370;GeneID:100874095","ADIPOQ;ADIPOQ-AS1",TRUE,"ADIPOQ"

"3087","AAAACCACCTTCTCCTGTAG","chr1",202945081,"+","none","AGG","AAAACCACCTTCTCCTGTAGAGG",0,NA,NA,NA,"GeneID:51094","ADIPOR1",TRUE,"ADIPOR1"

"3088","CCTGCAAGCCCACCACGCCA","chr1",202948332,"-","none","TGG","CCTGCAAGCCCACCACGCCATGG",0,NA,NA,NA,"GeneID:51094","ADIPOR1",TRUE,"ADIPOR1"

"3089","CCTGCAAGCCCACCACGCCA","chr14",94632076,"+","16:A>G","TGG","CCTGCAAGCCCACCACACCATGG",1,17,NA,NA,"","",FALSE,"ADIPOR1"

"3090","CTGCCATCATTGTGGCGCAG","chr1",202943800,"-","none","TGG","CTGCCATCATTGTGGCGCAGTGG",0,NA,NA,NA,"GeneID:51094","ADIPOR1",TRUE,"ADIPOR1"

"3091","GACAACGACTATCTGCTACA","chr1",202946533,"-","none","TGG","GACAACGACTATCTGCTACATGG",0,NA,NA,NA,"GeneID:51094","ADIPOR1",TRUE,"ADIPOR1"

"3092","GCTCTTGAAGCAAGCCCGAA","chr1",202946487,"+","none","AGG","GCTCTTGAAGCAAGCCCGAAAGG",0,NA,NA,NA,"GeneID:51094","ADIPOR1",TRUE,"ADIPOR1"

"3093","GCTCTTGAAGCAAGCCCGAA","chr14",94633762,"-","17:T>C,18:C>T","AGG","GCTCTTGAAGCAAGCCCAGAAGG",2,18,19,NA,"","",TRUE,"ADIPOR1"

"3094","GTCCCACTGCGCCACAATGA","chr1",202943794,"+","none","TGG","GTCCCACTGCGCCACAATGATGG",0,NA,NA,NA,"GeneID:51094","ADIPOR1",TRUE,"ADIPOR1"

"3095","ATACAGTGATGAAGCTCCTC","chr12",1772858,"+","none","AGG","ATACAGTGATGAAGCTCCTCAGG",0,NA,NA,NA,"GeneID:79602","ADIPOR2",TRUE,"ADIPOR2"

"3096","CTCATGATGTACTACCAGAC","chr12",1777881,"+","none","TGG","CTCATGATGTACTACCAGACTGG",0,NA,NA,NA,"GeneID:79602","ADIPOR2",TRUE,"ADIPOR2"

"3097","CTCTTCGTGTACCATCCAGT","chr12",1754420,"-","none","TGG","CTCTTCGTGTACCATCCAGTTGG",0,NA,NA,NA,"GeneID:79602","ADIPOR2",TRUE,"ADIPOR2"

"3098","CTTACAAGCCCATCATGCTA","chr12",1772912,"+","none","TGG","CTTACAAGCCCATCATGCTATGG",0,NA,NA,NA,"GeneID:79602","ADIPOR2",TRUE,"ADIPOR2"

"3099","GAGTCCTGCTGCACCCCAAT","chr12",1754366,"-","none","CGG","GAGTCCTGCTGCACCCCAATCGG",0,NA,NA,NA,"GeneID:79602","ADIPOR2",TRUE,"ADIPOR2"

"3100","GAGTCCTGCTGCACCCCAAT","chrX",146341858,"-","10:G>C,18:C>T","GGG","GAGTCCTGCTCCACCCCAGTGGG",2,11,19,NA,"","",FALSE,"ADIPOR2"

"3101","TCGCCCAAATATCTCCTTTG","chr12",1780490,"+","none","TGG","TCGCCCAAATATCTCCTTTGTGG",0,NA,NA,NA,"GeneID:79602","ADIPOR2",TRUE,"ADIPOR2"

"3102","CACTTGCTGAGCTGCCGCTC","chr10",86970211,"-","none","CGG","CACTTGCTGAGCTGCCGCTCCGG",0,NA,NA,NA,"GeneID:10974","ADIRF",TRUE,"ADIRF"

"3103","CATCGACAAGACTGCTAACC","chr10",86970509,"+","none","AGG","CATCGACAAGACTGCTAACCAGG",0,NA,NA,NA,"GeneID:10974","ADIRF",TRUE,"ADIRF"

"3104","CTGCAAGCCCTTGCTTGCCA","chr10",86968545,"-","none","TGG","CTGCAAGCCCTTGCTTGCCATGG",0,NA,NA,NA,"GeneID:79812;GeneID:10974","MMRN2;ADIRF",TRUE,"ADIRF"

"3105","CTGCAAGCCCTTGCTTGCCA","chr9",84383500,"+","5:G>A,17:A>C","CGG","CTGCAGGCCCTTGCTTGACACGG",2,6,18,NA,"","",FALSE,"ADIRF"

"3106","CTGGTTAGCAGTCTTGTCGA","chr10",86970511,"-","none","TGG","CTGGTTAGCAGTCTTGTCGATGG",0,NA,NA,NA,"GeneID:10974","ADIRF",TRUE,"ADIRF"

"3107","GCTATCCTCACCGGCTTCCT","chr10",86968596,"-","none","GGG","GCTATCCTCACCGGCTTCCTGGG",0,NA,NA,NA,"GeneID:79812;GeneID:10974","MMRN2;ADIRF",TRUE,"ADIRF"

"3108","TCTGTTCCAGTGTCAGCGGC","chr10",86970189,"+","none","CGG","TCTGTTCCAGTGTCAGCGGCCGG",0,NA,NA,NA,"GeneID:10974","ADIRF",TRUE,"ADIRF"

"3109","TCTGTTCCAGTGTCAGCGGC","chr8",41253304,"+","4:A>T,17:T>G","TGG","TCTGATCCAGTGTCAGCTGCTGG",2,5,18,NA,"","",FALSE,"ADIRF"

"3110","TCTGTTCCAGTGTCAGCGGC","chr9",8822759,"-","9:A>C,17:A>C","GGG","TCTGTTCCATTGTCAGCTGCGGG",2,10,18,NA,"GeneID:5789","PTPRD",FALSE,"ADIRF"

"3111","ACAGCAGAGATGTCAAGCAG","chr10",74200794,"-","none","AGG","ACAGCAGAGATGTCAAGCAGAGG",0,NA,NA,NA,"GeneID:132","ADK",TRUE,"ADK"

"3112","ACAGCAGAGATGTCAAGCAG","chr2",143204879,"-","1:C>G,13:T>G","AGG","AGAGCAGAGATGTAAAGCAGAGG",2,2,14,NA,"GeneID:55843","ARHGAP15",FALSE,"ADK"

"3113","AGCGCTCACCTCAGCGCTTG","chr10",74151332,"-","none","CGG","AGCGCTCACCTCAGCGCTTGCGG",0,NA,NA,NA,"GeneID:132","ADK",TRUE,"ADK"

"3114","GAGCCACTTTAATTGAATTC","chr10",74314723,"-","none","TGG","GAGCCACTTTAATTGAATTCTGG",0,NA,NA,NA,"GeneID:132","ADK",TRUE,"ADK"

"3115","GAGCCACTTTAATTGAATTC","chr1",95642094,"+","7:C>T,18:C>T","TGG","GAGCCACCTTAATTGAATCCTGG",2,8,19,NA,"GeneID:100996635","LOC100996635",FALSE,"ADK"

"3116","GCTTGACATCTCTGCTGTAG","chr10",74200796,"+","none","TGG","GCTTGACATCTCTGCTGTAGTGG",0,NA,NA,NA,"GeneID:132","ADK",TRUE,"ADK"

"3117","TGGAGGCGCCGCAAGCGCTG","chr10",74151321,"+","none","AGG","TGGAGGCGCCGCAAGCGCTGAGG",0,NA,NA,NA,"GeneID:132","ADK",TRUE,"ADK"

"3118","TTCAAAGTCGAATATCATGC","chr10",74314688,"+","none","TGG","TTCAAAGTCGAATATCATGCTGG",0,NA,NA,NA,"GeneID:132","ADK",TRUE,"ADK"

"3119","ACAAGGACAACGTCGCCCCC","chr11",10306474,"+","none","AGG","ACAAGGACAACGTCGCCCCCAGG",0,NA,NA,NA,"GeneID:133","ADM",TRUE,"ADM"

"3120","CGACGCGACATCCAACCGAG","chr11",10305764,"-","none","CGG","CGACGCGACATCCAACCGAGCGG",0,NA,NA,NA,"GeneID:133","ADM",TRUE,"ADM"

"3121","GGCCGAATAAGGGTCTGGGC","chr11",10306039,"-","none","AGG","GGCCGAATAAGGGTCTGGGCAGG",0,NA,NA,NA,"GeneID:133","ADM",TRUE,"ADM"

"3122","GGCGTAGTTACCTGTCTTCG","chr11",10306089,"-","none","GGG","GGCGTAGTTACCTGTCTTCGGGG",0,NA,NA,NA,"GeneID:133","ADM",TRUE,"ADM"

"3123","GGCGTAGTTACCTGTCTTCG","chr4",4309496,"+","6:A>G,18:T>C","GGG","GGCGTAATTACCTGTCTTTGGGG",2,7,19,NA,"GeneID:166793","ZBTB49",FALSE,"ADM"

"3124","TCCTAGGCGCTGACACCGCT","chr11",10305746,"+","none","CGG","TCCTAGGCGCTGACACCGCTCGG",0,NA,NA,NA,"GeneID:133","ADM",TRUE,"ADM"

"3125","TCTGGCGGTAGCGCTTGACT","chr11",10306355,"-","none","CGG","TCTGGCGGTAGCGCTTGACTCGG",0,NA,NA,NA,"GeneID:133","ADM",TRUE,"ADM"

"3126","AACTCATGGGACCGGCCGGC","chr22",50482832,"+","none","CGG","AACTCATGGGACCGGCCGGCCGG",0,NA,NA,NA,"GeneID:79924","ADM2",TRUE,"ADM2"

"3127","ACCCGCGACCCGTCAAACCC","chr22",50481935,"+","none","AGG","ACCCGCGACCCGTCAAACCCAGG",0,NA,NA,NA,"GeneID:79924","ADM2",TRUE,"ADM2"

"3128","CCGGTCCCATGAGTTGCCAC","chr22",50482827,"-","none","AGG","CCGGTCCCATGAGTTGCCACAGG",0,NA,NA,NA,"GeneID:79924","ADM2",TRUE,"ADM2"

"3129","CGCCATGGCCCGGATCCCGA","chr22",50481843,"+","none","CGG","CGCCATGGCCCGGATCCCGACGG",0,NA,NA,NA,"GeneID:79924","ADM2",TRUE,"ADM2"

"3130","GGCCGTCGGGATCCGGGCCA","chr22",50481848,"-","none","TGG","GGCCGTCGGGATCCGGGCCATGG",0,NA,NA,NA,"GeneID:79924","ADM2",TRUE,"ADM2"

"3131","GTCTTCGGGGGCCCGAGTGT","chr22",50482719,"-","none","TGG","GTCTTCGGGGGCCCGAGTGTTGG",0,NA,NA,NA,"GeneID:79924","ADM2",TRUE,"ADM2"

"3132","CCCCTTGGGCGGAGAAGGCG","chr19",49689872,"-","none","AGG","CCCCTTGGGCGGAGAAGGCGAGG",0,NA,NA,NA,"GeneID:199800","ADM5",TRUE,"ADM5"

"3133","CCTACAGCTATGGGCGCCGC","chr19",49690252,"+","none","CGG","CCTACAGCTATGGGCGCCGCCGG",0,NA,NA,NA,"GeneID:199800","ADM5",TRUE,"ADM5"

"3134","CTCCTCGCCTTCTCCGCCCA","chr19",49689867,"+","none","AGG","CTCCTCGCCTTCTCCGCCCAAGG",0,NA,NA,NA,"GeneID:199800","ADM5",TRUE,"ADM5"

"3135","CTCCTCGCCTTCTCCGCCCA","chr6",113859969,"+","7:A>C,13:T>C","AGG","CTCCTCGACTTCTTCGCCCAAGG",2,8,14,NA,"GeneID:4082","MARCKS",TRUE,"ADM5"

"3136","CTTCCGCCTCGCGCCACCGG","chr19",49690271,"-","none","CGG","CTTCCGCCTCGCGCCACCGGCGG",0,NA,NA,NA,"GeneID:199800","ADM5",TRUE,"ADM5"

"3137","GGTGGGTCCGGCATACGCCC","chr19",49690153,"-","none","AGG","GGTGGGTCCGGCATACGCCCAGG",0,NA,NA,NA,"GeneID:199800","ADM5",TRUE,"ADM5"

"3138","TCCAGTATATGATCTCCGCC","chr19",49690177,"-","none","AGG","TCCAGTATATGATCTCCGCCAGG",0,NA,NA,NA,"GeneID:199800","ADM5",TRUE,"ADM5"

"3139","AAACTGCCAAGATTGTTGAC","chr20",50903961,"+","none","AGG","AAACTGCCAAGATTGTTGACAGG",0,NA,NA,NA,"GeneID:23394","ADNP",TRUE,"ADNP"

"3140","ATCGAAGACCATGAACGTAT","chr20",50893973,"-","none","AGG","ATCGAAGACCATGAACGTATAGG",0,NA,NA,NA,"GeneID:23394","ADNP",TRUE,"ADNP"

"3141","CAACTTCCTGTCAACAATCT","chr20",50903970,"-","none","TGG","CAACTTCCTGTCAACAATCTTGG",0,NA,NA,NA,"GeneID:23394","ADNP",TRUE,"ADNP"

"3142","CCCATCACTTACGAAAAACC","chr20",50902018,"-","none","AGG","CCCATCACTTACGAAAAACCAGG",0,NA,NA,NA,"GeneID:23394","ADNP",TRUE,"ADNP"

"3143","TGCTCGTAAGTGCGCTTCAC","chr20",50892551,"+","none","AGG","TGCTCGTAAGTGCGCTTCACAGG",0,NA,NA,NA,"GeneID:23394","ADNP",TRUE,"ADNP"

"3144","TTTATTTGAAAAACACTACA","chr20",50902061,"-","none","TGG","TTTATTTGAAAAACACTACATGG",0,NA,NA,NA,"GeneID:23394","ADNP",TRUE,"ADNP"

"3145","TTTATTTGAAAAACACTACA","chr1",72287086,"-","16:T>A,18:C>G","TGG","TTTATTTGAAAAACACAAGATGG",2,17,19,NA,"","",FALSE,"ADNP"

"3146","TTTATTTGAAAAACACTACA","chr6",132421615,"-","12:G>T,15:C>G","GGG","TTTATTTGAAAACCAGTACAGGG",2,13,16,NA,"","",FALSE,"ADNP"

"3147","TTTATTTGAAAAACACTACA","chr2",47425254,"+","12:G>A","GGG","TTTATTTGAAAAGCACTACAGGG",1,13,NA,NA,"GeneID:4436","MSH2",FALSE,"ADNP"

"3148","TTTATTTGAAAAACACTACA","chr2",78810361,"+","3:C>A,18:T>C","TGG","TTTCTTTGAAAAACACTATATGG",2,4,19,NA,"","",FALSE,"ADNP"

"3149","TTTATTTGAAAAACACTACA","chr4",131255619,"-","8:C>T,18:T>G","AGG","TTTATTTGGAAAACACTAAAAGG",2,9,19,NA,"","",FALSE,"ADNP"

"3150","TTTATTTGAAAAACACTACA","chr4",73163158,"-","1:T>A,12:G>T","GGG","TATATTTGAAAACCACTACAGGG",2,2,13,NA,"GeneID:26057","ANKRD17",FALSE,"ADNP"

"3151","AAAGGTGAAAGGTATTCTTG","chr18",80117592,"+","none","TGG","AAAGGTGAAAGGTATTCTTGTGG",0,NA,NA,NA,"GeneID:22850","ADNP2",TRUE,"ADNP2"

"3152","AAAGGTGAAAGGTATTCTTG","chr4",157700887,"+","5:A>T,19:A>G","GGG","AAAGGAGAAAGGTATTCTTAGGG",2,6,20,NA,"","",FALSE,"ADNP2"

"3153","AAAGGTGAAAGGTATTCTTG","chr3",43236605,"-","8:C>T,16:T>G","TGG","AAAGGTGAGAGGTATTATTGTGG",2,9,17,NA,"","",FALSE,"ADNP2"

"3154","CCGTCACGGGTTCTTCCCCC","chr18",80136892,"+","none","AGG","CCGTCACGGGTTCTTCCCCCAGG",0,NA,NA,NA,"GeneID:22850","ADNP2",TRUE,"ADNP2"

"3155","CTTTCCGTCCAACGTCTACC","chr18",80137518,"+","none","AGG","CTTTCCGTCCAACGTCTACCAGG",0,NA,NA,NA,"GeneID:22850","ADNP2",TRUE,"ADNP2"

"3156","GACCTTAAAGGCTTTGATCC","chr18",80133102,"+","none","AGG","GACCTTAAAGGCTTTGATCCAGG",0,NA,NA,NA,"GeneID:22850","ADNP2",TRUE,"ADNP2"

"3157","GACCTTAAAGGCTTTGATCC","chr6",42263575,"+","3:A>C,4:A>T","TGG","GACAATAAAGGCTTTGATCCTGG",2,4,5,NA,"GeneID:55809","TRERF1",TRUE,"ADNP2"

"3158","GTTTCTCTCTGGGAACCTTC","chr18",80133159,"+","none","TGG","GTTTCTCTCTGGGAACCTTCTGG",0,NA,NA,NA,"GeneID:22850","ADNP2",TRUE,"ADNP2"

"3159","GTTTCTCTCTGGGAACCTTC","chr19",16037179,"-","3:C>A,18:G>A","GGG","GTTGCTCTCTGGGAACCTCCGGG",2,4,19,NA,"GeneID:148231","LINC00905",TRUE,"ADNP2"

"3160","TATTGGGCTTGACAGCTGCA","chr18",80117616,"+","none","AGG","TATTGGGCTTGACAGCTGCAAGG",0,NA,NA,NA,"GeneID:22850","ADNP2",TRUE,"ADNP2"

"3161","ACAAGCTAGACGCGGGCGGC","chr10",62805459,"+","none","GGG","ACAAGCTAGACGCGGGCGGCGGG",0,NA,NA,NA,"GeneID:84890","ADO",TRUE,"ADO"

"3162","CCTGCACCAGATCGACGCCG","chr10",62805631,"+","none","TGG","CCTGCACCAGATCGACGCCGTGG",0,NA,NA,NA,"GeneID:84890","ADO",TRUE,"ADO"

"3163","CGGCGCTTCCGATCGCGACG","chr10",62805142,"+","none","CGG","CGGCGCTTCCGATCGCGACGCGG",0,NA,NA,NA,"GeneID:84890","ADO",TRUE,"ADO"

"3164","GATCCAACGGATCGCCCGCC","chr10",62805085,"+","none","AGG","GATCCAACGGATCGCCCGCCAGG",0,NA,NA,NA,"GeneID:84890","ADO",TRUE,"ADO"

"3165","GCCCTTCCACGGCGTCGATC","chr10",62805640,"-","none","TGG","GCCCTTCCACGGCGTCGATCTGG",0,NA,NA,NA,"GeneID:84890","ADO",TRUE,"ADO"

"3166","TTCGCGGGCCGAGTACACCG","chr10",62805571,"+","none","AGG","TTCGCGGGCCGAGTACACCGAGG",0,NA,NA,NA,"GeneID:84890","ADO",TRUE,"ADO"

"3167","CTCGAACTCGCACTTGATCA","chr1",203165415,"-","none","CGG","CTCGAACTCGCACTTGATCACGG",0,NA,NA,NA,"GeneID:134","ADORA1",TRUE,"ADORA1"

"3168","CTTCTGCTTCATCGTGTCGC","chr1",203128972,"+","none","TGG","CTTCTGCTTCATCGTGTCGCTGG",0,NA,NA,NA,"GeneID:134","ADORA1",TRUE,"ADORA1"

"3169","GAAGGCGGGGTCTAGTCATC","chr1",203165891,"-","none","AGG","GAAGGCGGGGTCTAGTCATCAGG",0,NA,NA,NA,"GeneID:134","ADORA1",TRUE,"ADORA1"

"3170","GGACCGGACAGGCAACCATG","chr1",203129083,"-","none","AGG","GGACCGGACAGGCAACCATGAGG",0,NA,NA,NA,"GeneID:134","ADORA1",TRUE,"ADORA1"

"3171","GGCGACCCGCAGAAGTACTA","chr1",203165576,"+","none","TGG","GGCGACCCGCAGAAGTACTATGG",0,NA,NA,NA,"GeneID:134","ADORA1",TRUE,"ADORA1"

"3172","TGGCCGAGTTGCCGTGCGTG","chr1",203165746,"-","none","AGG","TGGCCGAGTTGCCGTGCGTGAGG",0,NA,NA,NA,"GeneID:134","ADORA1",TRUE,"ADORA1"

"3173","CGTGGCTGCGAATGATCTTG","chr22",24441149,"-","none","CGG","CGTGGCTGCGAATGATCTTGCGG",0,NA,NA,NA,"GeneID:101730217;GeneID:135;GeneID:646023","SPECC1L-ADORA2A;ADORA2A;ADORA2A-AS1",TRUE,"ADORA2A"

"3174","CTCCACCGTGATGTACACCG","chr22",24433423,"-","none","AGG","CTCCACCGTGATGTACACCGAGG",0,NA,NA,NA,"GeneID:101730217;GeneID:135;GeneID:646023","SPECC1L-ADORA2A;ADORA2A;ADORA2A-AS1",TRUE,"ADORA2A"

"3175","CTCCTCGGTGTACATCACGG","chr22",24433418,"+","none","TGG","CTCCTCGGTGTACATCACGGTGG",0,NA,NA,NA,"GeneID:101730217;GeneID:135;GeneID:646023","SPECC1L-ADORA2A;ADORA2A;ADORA2A-AS1",TRUE,"ADORA2A"

"3176","GAAGGGATTCACAACCGAAT","chr22",24441088,"-","none","TGG","GAAGGGATTCACAACCGAATTGG",0,NA,NA,NA,"GeneID:101730217;GeneID:135;GeneID:646023","SPECC1L-ADORA2A;ADORA2A;ADORA2A-AS1",TRUE,"ADORA2A"

"3177","TAGCCATTGGGCCTCCGCTC","chr22",24441312,"-","none","AGG","TAGCCATTGGGCCTCCGCTCAGG",0,NA,NA,NA,"GeneID:101730217;GeneID:135;GeneID:646023","SPECC1L-ADORA2A;ADORA2A;ADORA2A-AS1",TRUE,"ADORA2A"

"3178","TCTGGCGGAACTCGCGGATA","chr22",24441122,"-","none","CGG","TCTGGCGGAACTCGCGGATACGG",0,NA,NA,NA,"GeneID:101730217;GeneID:135;GeneID:646023","SPECC1L-ADORA2A;ADORA2A;ADORA2A-AS1",TRUE,"ADORA2A"

"3179","CAGGTATCTGTCGACTGCCA","chr17",15945543,"-","none","CGG","CAGGTATCTGTCGACTGCCACGG",0,NA,NA,NA,"GeneID:136","ADORA2B",TRUE,"ADORA2B"

"3180","CATAGGCCCACACCGAGAGC","chr17",15975321,"-","none","AGG","CATAGGCCCACACCGAGAGCAGG",0,NA,NA,NA,"GeneID:136","ADORA2B",TRUE,"ADORA2B"

"3181","CATCGCCGCGCTTTCGGTGG","chr17",15945295,"+","none","CGG","CATCGCCGCGCTTTCGGTGGCGG",0,NA,NA,NA,"GeneID:136","ADORA2B",TRUE,"ADORA2B"

"3182","GACGCCCACCAACTACTTCC","chr17",15945364,"+","none","TGG","GACGCCCACCAACTACTTCCTGG",0,NA,NA,NA,"GeneID:136","ADORA2B",TRUE,"ADORA2B"

"3183","GTTCCGGTAAGCATAGACAA","chr17",15975205,"-","none","TGG","GTTCCGGTAAGCATAGACAATGG",0,NA,NA,NA,"GeneID:136","ADORA2B",TRUE,"ADORA2B"

"3184","GTTGCCCGCCACCGAAAGCG","chr17",15945303,"-","none","CGG","GTTGCCCGCCACCGAAAGCGCGG",0,NA,NA,NA,"GeneID:136","ADORA2B",TRUE,"ADORA2B"

"3185","ACCTGGAGCGGTCAGCCTTG","chr1",111486583,"+","none","CGG","ACCTGGAGCGGTCAGCCTTGCGG",0,NA,NA,NA,"","",TRUE,"ADORA3"

"3186","ATAGACGATAGGGTTCATCA","chr1",111500060,"+","none","TGG","ATAGACGATAGGGTTCATCATGG",0,NA,NA,NA,"GeneID:140","ADORA3",TRUE,"ADORA3"

"3187","CATTGGACTCTGCGCCATAG","chr1",111503272,"-","none","TGG","CATTGGACTCTGCGCCATAGTGG",0,NA,NA,NA,"GeneID:140","ADORA3",TRUE,"ADORA3"

"3188","GTGGGATTGACCCCCATGTT","chr1",111500466,"-","none","TGG","GTGGGATTGACCCCCATGTTTGG",0,NA,NA,NA,"GeneID:140","ADORA3",TRUE,"ADORA3"

"3189","GTGGGATTGACCCCCATGTT","chr1",203165330,"+","6:C>T,14:T>C","TGG","GTGGGACTGACCCCTATGTTTGG",2,7,15,NA,"GeneID:134","ADORA1",TRUE,"ADORA3"

"3190","GTGGGATTGACCCCCATGTT","chr18",46089309,"-","5:A>T,10:C>G","GGG","GTGGGTTTGAGCCCCATGTTGGG",2,6,11,NA,"GeneID:498","ATP5A1",FALSE,"ADORA3"

"3191","TCCGCAAGGCTGACCGCTCC","chr1",111486587,"-","none","AGG","TCCGCAAGGCTGACCGCTCCAGG",0,NA,NA,NA,"","",TRUE,"ADORA3"

"3192","TCGCTGTGGACCGATACTTG","chr1",111503024,"-","none","CGG","TCGCTGTGGACCGATACTTGCGG",0,NA,NA,NA,"GeneID:140","ADORA3",TRUE,"ADORA3"

"3193","AGTCAATGCATGTGTTGATG","chr15",72775077,"-","none","TGG","AGTCAATGCATGTGTTGATGTGG",0,NA,NA,NA,"GeneID:83440","ADPGK",TRUE,"ADPGK"

"3194","CTCTTACAGCACTATGTAGG","chr15",72771834,"-","none","AGG","CTCTTACAGCACTATGTAGGAGG",0,NA,NA,NA,"GeneID:83440","ADPGK",TRUE,"ADPGK"

"3195","CTCTTACAGCACTATGTAGG","chr12",122669972,"-","4:T>A,16:T>A","AGG","CTCTAACAGCACTATGAAGGAGG",2,5,17,NA,"","",FALSE,"ADPGK"

"3196","CTGCTTGATACTCTAAAATG","chr15",72760406,"+","none","AGG","CTGCTTGATACTCTAAAATGAGG",0,NA,NA,NA,"GeneID:83440","ADPGK",TRUE,"ADPGK"

"3197","CTGCTTGATACTCTAAAATG","chr2",217553367,"+","7:T>A,14:C>A","CGG","CTGCTTGTTACTCTCAAATGCGG",2,8,15,NA,"","",FALSE,"ADPGK"

"3198","GGTCGTGAGAGAAGATGAAT","chr15",72756387,"+","none","CGG","GGTCGTGAGAGAAGATGAATCGG",0,NA,NA,NA,"GeneID:83440","ADPGK",TRUE,"ADPGK"

"3199","GGTCGTGAGAGAAGATGAAT","chr1",172883327,"+","4:A>G,13:A>G","GGG","GGTCATGAGAGAAAATGAATGGG",2,5,14,NA,"","",FALSE,"ADPGK"

"3200","TGTTCCACCAGAGTCATTGC","chr15",72760448,"-","none","AGG","TGTTCCACCAGAGTCATTGCAGG",0,NA,NA,NA,"GeneID:83440","ADPGK",TRUE,"ADPGK"

"3201","TTCTCTCACGACCTCTCCAA","chr15",72756380,"-","none","CGG","TTCTCTCACGACCTCTCCAACGG",0,NA,NA,NA,"GeneID:83440","ADPGK",TRUE,"ADPGK"

"3202","ATCCAAGTGAGCATCGAGAG","chr3",119586439,"+","none","TGG","ATCCAAGTGAGCATCGAGAGTGG",0,NA,NA,NA,"GeneID:141","ADPRH",TRUE,"ADPRH"

"3203","CGGCTTGGATGCCCTAGACG","chr3",119582291,"+","none","TGG","CGGCTTGGATGCCCTAGACGTGG",0,NA,NA,NA,"GeneID:141","ADPRH",TRUE,"ADPRH"

"3204","GACCACTCTCGATGCTCACT","chr3",119586444,"-","none","TGG","GACCACTCTCGATGCTCACTTGG",0,NA,NA,NA,"GeneID:141","ADPRH",TRUE,"ADPRH"

"3205","GGAGAAGATACACCGGCAGT","chr3",119582258,"+","none","TGG","GGAGAAGATACACCGGCAGTTGG",0,NA,NA,NA,"GeneID:141","ADPRH",TRUE,"ADPRH"

"3206","GGTCCTACTTCCAAACCAAA","chr3",119587462,"+","none","TGG","GGTCCTACTTCCAAACCAAATGG",0,NA,NA,NA,"GeneID:141","ADPRH",TRUE,"ADPRH"

"3207","TGCTGGTGGGGAGTTATGTA","chr3",119587737,"+","none","TGG","TGCTGGTGGGGAGTTATGTATGG",0,NA,NA,NA,"GeneID:141","ADPRH",TRUE,"ADPRH"

"3208","TGCTGGTGGGGAGTTATGTA","chr12",92552450,"-","8:A>C,17:A>C","TGG","TGCTGGTGTGGAGTTATTTATGG",2,9,18,NA,"","",FALSE,"ADPRH"

"3209","ACTCCACGCTGACCTCGATG","chr13",113433771,"+","none","AGG","ACTCCACGCTGACCTCGATGAGG",0,NA,NA,NA,"GeneID:113622","ADPRHL1",TRUE,"ADPRHL1"

"3210","ACTCCACGCTGACCTCGATG","chr1",161072840,"-","14:T>A,17:C>T","AGG","ACTCCACGCTGACCACGGTGAGG",2,15,18,NA,"GeneID:81607","PVRL4",TRUE,"ADPRHL1"

"3211","CCAGGCGAGAAGGTAGTTAT","chr13",113444446,"+","none","TGG","CCAGGCGAGAAGGTAGTTATTGG",0,NA,NA,NA,"GeneID:113622","ADPRHL1",TRUE,"ADPRHL1"

"3212","CTCTTCTGCATCATAATTGT","chr13",113425060,"+","none","CGG","CTCTTCTGCATCATAATTGTCGG",0,NA,NA,NA,"GeneID:113622","ADPRHL1",TRUE,"ADPRHL1"

"3213","CTCTTCTGCATCATAATTGT","chr10",58344458,"+","8:A>C,18:T>G","AGG","CTCTTCTGAATCATAATTTTAGG",2,9,19,NA,"GeneID:7321","UBE2D1",FALSE,"ADPRHL1"

"3214","GACCCTCATCGAGGTCAGCG","chr13",113433777,"-","none","TGG","GACCCTCATCGAGGTCAGCGTGG",0,NA,NA,NA,"GeneID:113622","ADPRHL1",TRUE,"ADPRHL1"

"3215","GCTGGGTCTGGCCGGCGTTC","chr13",113444498,"+","none","TGG","GCTGGGTCTGGCCGGCGTTCTGG",0,NA,NA,NA,"GeneID:113622","ADPRHL1",TRUE,"ADPRHL1"

"3216","GTTTAGAATACCAGGAGCAC","chr13",113425165,"-","none","TGG","GTTTAGAATACCAGGAGCACTGG",0,NA,NA,NA,"GeneID:113622","ADPRHL1",TRUE,"ADPRHL1"

"3217","AGGAGCCTTTCCCGTTAAAC","chr1",36091734,"-","none","TGG","AGGAGCCTTTCCCGTTAAACTGG",0,NA,NA,NA,"GeneID:54936","ADPRHL2",TRUE,"ADPRHL2"

"3218","CTCAAAGACATCGCGACATT","chr1",36091700,"-","none","TGG","CTCAAAGACATCGCGACATTTGG",0,NA,NA,NA,"GeneID:54936","ADPRHL2",TRUE,"ADPRHL2"

"3219","GGGTGATGCCCAGTCCGTCT","chr1",36092061,"+","none","TGG","GGGTGATGCCCAGTCCGTCTTGG",0,NA,NA,NA,"GeneID:54936","ADPRHL2",TRUE,"ADPRHL2"

"3220","GGTGCAGTCCCTGCTAGCCA","chr1",36091286,"+","none","AGG","GGTGCAGTCCCTGCTAGCCAAGG",0,NA,NA,NA,"GeneID:54936","ADPRHL2",TRUE,"ADPRHL2"

"3221","GGTGCAGTCCCTGCTAGCCA","chr5",159347913,"+","1:T>G,17:T>C","GGG","GTTGCAGTCCCTGCTAGTCAGGG",2,2,18,NA,"GeneID:285626","LOC285626",FALSE,"ADPRHL2"

"3222","TCCAGCCGCCTGAAGAAGAT","chr1",36092446,"+","none","TGG","TCCAGCCGCCTGAAGAAGATTGG",0,NA,NA,NA,"GeneID:54936","ADPRHL2",TRUE,"ADPRHL2"

"3223","TCCAGCCGCCTGAAGAAGAT","chr8",38580806,"-","6:A>G,12:G>T","GGG","TCCAGCTGCCTGCAGAAGATGGG",2,7,13,NA,"","",FALSE,"ADPRHL2"

"3224","TTACAATGGCGCCATCCTGC","chr1",36091953,"+","none","AGG","TTACAATGGCGCCATCCTGCAGG",0,NA,NA,NA,"GeneID:54936","ADPRHL2",TRUE,"ADPRHL2"

"3225","AACATCTGCGATGACGCCAA","chr17",10704984,"-","none","AGG","AACATCTGCGATGACGCCAAAGG",0,NA,NA,NA,"GeneID:56985;GeneID:388335","ADPRM;TMEM220",TRUE,"ADPRM"

"3226","ATGTCTGTAGTATCGCCGCC","chr17",10705047,"-","none","TGG","ATGTCTGTAGTATCGCCGCCTGG",0,NA,NA,NA,"GeneID:56985;GeneID:388335","ADPRM;TMEM220",TRUE,"ADPRM"

"3227","CACACATTGTCAGAGGCGTC","chr17",10710853,"-","none","CGG","CACACATTGTCAGAGGCGTCCGG",0,NA,NA,NA,"GeneID:56985;GeneID:388335","ADPRM;TMEM220",TRUE,"ADPRM"

"3228","CCCCAGTTTGTCCAGTTTAA","chr17",10706448,"+","none","TGG","CCCCAGTTTGTCCAGTTTAATGG",0,NA,NA,NA,"GeneID:56985;GeneID:388335","ADPRM;TMEM220",TRUE,"ADPRM"

"3229","TCAGCCAAGAACAGCTAAAC","chr17",10706476,"+","none","TGG","TCAGCCAAGAACAGCTAAACTGG",0,NA,NA,NA,"GeneID:56985;GeneID:388335","ADPRM;TMEM220",TRUE,"ADPRM"

"3230","TTGACGTGGTATACACCAAA","chr17",10710979,"-","none","AGG","TTGACGTGGTATACACCAAAAGG",0,NA,NA,NA,"GeneID:56985;GeneID:388335","ADPRM;TMEM220",TRUE,"ADPRM"

"3231","CATTCTGAACCCTTTCTCCA","chr8",26766054,"+","none","CGG","CATTCTGAACCCTTTCTCCACGG",0,NA,NA,NA,"GeneID:148","ADRA1A",TRUE,"ADRA1A"

"3232","CATTCTGAACCCTTTCTCCA","chr11",88444266,"+","8:G>A,10:T>C","GGG","CATTCTGAGCTCTTTCTCCAGGG",2,9,11,NA,"","",FALSE,"ADRA1A"

"3233","CGAAGTCTCGCTCTGTCACC","chr11",43597667,"+","none","CGG","CGAAGTCTCGCTCTGTCACCCGG",0,NA,NA,NA,"","",FALSE,"ADRA1A"

"3234","CGAAGTCTCGCTCTGTCACC","chr5",480912,"+","none","AGG","CGAAGTCTCGCTCTGTCACCAGG",0,NA,NA,NA,"GeneID:6550","SLC9A3",FALSE,"ADRA1A"

"3235","CGAAGTCTCGCTCTGTCACC","chr18",15076781,"+","none","AGG","CGAAGTCTCGCTCTGTCACCAGG",0,NA,NA,NA,"","",FALSE,"ADRA1A"

"3236","CGAAGTCTCGCTCTGTCACC","chr3",9967944,"+","none","AGG","CGAAGTCTCGCTCTGTCACCAGG",0,NA,NA,NA,"GeneID:55831","EMC3",FALSE,"ADRA1A"

"3237","CGAAGTCTCGCTCTGTCACC","chr16",85804379,"+","none","AGG","CGAAGTCTCGCTCTGTCACCAGG",0,NA,NA,NA,"GeneID:1327","COX4I1",TRUE,"ADRA1A"

"3238","CGAAGTCTCGCTCTGTCACC","chr17",42265393,"+","none","AGG","CGAAGTCTCGCTCTGTCACCAGG",0,NA,NA,NA,"GeneID:6777","STAT5B",FALSE,"ADRA1A"

"3239","CGAAGTCTCGCTCTGTCACC","chr6",125718541,"+","none","AGG","CGAAGTCTCGCTCTGTCACCAGG",0,NA,NA,NA,"GeneID:643623","LOC643623",TRUE,"ADRA1A"

"3240","CGAAGTCTCGCTCTGTCACC","chr2",208161959,"+","none","AGG","CGAAGTCTCGCTCTGTCACCAGG",0,NA,NA,NA,"GeneID:1418","CRYGA",FALSE,"ADRA1A"

"3241","CGAAGTCTCGCTCTGTCACC","chr3",44485925,"+","none","AGG","CGAAGTCTCGCTCTGTCACCAGG",0,NA,NA,NA,"","",FALSE,"ADRA1A"

"3242","CGAAGTCTCGCTCTGTCACC","chr13",57442518,"+","none","AGG","CGAAGTCTCGCTCTGTCACCAGG",0,NA,NA,NA,"","",FALSE,"ADRA1A"

"3243","CGAAGTCTCGCTCTGTCACC","chr17",43132601,"+","none","AGG","CGAAGTCTCGCTCTGTCACCAGG",0,NA,NA,NA,"GeneID:672;GeneID:10230","BRCA1;NBR2",TRUE,"ADRA1A"

"3244","CGAAGTCTCGCTCTGTCACC","chr20",43229208,"+","none","AGG","CGAAGTCTCGCTCTGTCACCAGG",0,NA,NA,NA,"","",FALSE,"ADRA1A"

"3245","CGAAGTCTCGCTCTGTCACC","chr20",53176709,"+","none","AGG","CGAAGTCTCGCTCTGTCACCAGG",0,NA,NA,NA,"GeneID:128553","TSHZ2",FALSE,"ADRA1A"

"3246","CGAAGTCTCGCTCTGTCACC","chr3",174403510,"+","none","AGG","CGAAGTCTCGCTCTGTCACCAGG",0,NA,NA,NA,"","",FALSE,"ADRA1A"

"3247","CGAAGTCTCGCTCTGTCACC","chr11",73835164,"+","none","AGG","CGAAGTCTCGCTCTGTCACCAGG",0,NA,NA,NA,"GeneID:51642","MRPL48",FALSE,"ADRA1A"

"3248","CGAAGTCTCGCTCTGTCACC","chr19",55776398,"+","none","AGG","CGAAGTCTCGCTCTGTCACCAGG",0,NA,NA,NA,"","",FALSE,"ADRA1A"

"3249","CGAAGTCTCGCTCTGTCACC","chr17",43222792,"+","none","AGG","CGAAGTCTCGCTCTGTCACCAGG",0,NA,NA,NA,"GeneID:100874261","LINC00854",FALSE,"ADRA1A"

"3250","CGAAGTCTCGCTCTGTCACC","chr17",64143377,"-","none","AGG","CGAAGTCTCGCTCTGTCACCAGG",0,NA,NA,NA,"","",FALSE,"ADRA1A"

"3251","CGAAGTCTCGCTCTGTCACC","chr10",63392799,"-","none","AGG","CGAAGTCTCGCTCTGTCACCAGG",0,NA,NA,NA,"GeneID:221037","JMJD1C",FALSE,"ADRA1A"

"3252","CGAAGTCTCGCTCTGTCACC","chr21",28003174,"-","none","AGG","CGAAGTCTCGCTCTGTCACCAGG",0,NA,NA,NA,"","",FALSE,"ADRA1A"

"3253","CGAAGTCTCGCTCTGTCACC","chr21",14077589,"-","none","CGG","CGAAGTCTCGCTCTGTCACCCGG",0,NA,NA,NA,"","",FALSE,"ADRA1A"

"3254","CGAAGTCTCGCTCTGTCACC","chr12",55907608,"-","none","AGG","CGAAGTCTCGCTCTGTCACCAGG",0,NA,NA,NA,"GeneID:84305","WIBG",FALSE,"ADRA1A"

"3255","CGAAGTCTCGCTCTGTCACC","chr8",26748727,"-","none","AGG","CGAAGTCTCGCTCTGTCACCAGG",0,NA,NA,NA,"GeneID:148","ADRA1A",TRUE,"ADRA1A"

"3256","CGAAGTCTCGCTCTGTCACC","chr15",75448152,"-","none","AGG","CGAAGTCTCGCTCTGTCACCAGG",0,NA,NA,NA,"GeneID:25942","SIN3A",TRUE,"ADRA1A"

"3257","CGAAGTCTCGCTCTGTCACC","chr12",31965865,"-","none","AGG","CGAAGTCTCGCTCTGTCACCAGG",0,NA,NA,NA,"GeneID:55196","KIAA1551",FALSE,"ADRA1A"

"3258","CGAAGTCTCGCTCTGTCACC","chr1",240646155,"-","none","AGG","CGAAGTCTCGCTCTGTCACCAGG",0,NA,NA,NA,"","",FALSE,"ADRA1A"

"3259","CGAAGTCTCGCTCTGTCACC","chr19",19680894,"-","none","AGG","CGAAGTCTCGCTCTGTCACCAGG",0,NA,NA,NA,"GeneID:94039","ZNF101",TRUE,"ADRA1A"

"3260","CGAAGTCTCGCTCTGTCACC","chr5",141890142,"-","19:T>G","AGG","CGAAGTCTCGCTCTGTCACAAGG",1,20,NA,NA,"","",FALSE,"ADRA1A"

"3261","CGAAGTCTCGCTCTGTCACC","chr19",57246750,"-","18:A>G","AGG","CGAAGTCTCGCTCTGTCATCAGG",1,19,NA,NA,"GeneID:390980","ZNF805",FALSE,"ADRA1A"

"3262","CGAAGTCTCGCTCTGTCACC","chr5",61377691,"-","17:C>T","AGG","CGAAGTCTCGCTCTGTCGCCAGG",1,18,NA,NA,"GeneID:57688","ZSWIM6",FALSE,"ADRA1A"

"3263","CGAAGTCTCGCTCTGTCACC","chr4",154257222,"-","17:C>T","AGG","CGAAGTCTCGCTCTGTCGCCAGG",1,18,NA,NA,"GeneID:54798","DCHS2",FALSE,"ADRA1A"

"3264","CGAAGTCTCGCTCTGTCACC","chrX",32156354,"-","17:C>T","AGG","CGAAGTCTCGCTCTGTCGCCAGG",1,18,NA,NA,"GeneID:1756","DMD",FALSE,"ADRA1A"

"3265","CGAAGTCTCGCTCTGTCACC","chr10",73627036,"-","17:C>T","AGG","CGAAGTCTCGCTCTGTCGCCAGG",1,18,NA,NA,"","",FALSE,"ADRA1A"

"3266","CGAAGTCTCGCTCTGTCACC","chr2",121707272,"-","17:C>T","AGG","CGAAGTCTCGCTCTGTCGCCAGG",1,18,NA,NA,"GeneID:254128","NIFK-AS1",FALSE,"ADRA1A"

"3267","CGAAGTCTCGCTCTGTCACC","chr3",150695642,"-","17:C>T","AGG","CGAAGTCTCGCTCTGTCGCCAGG",1,18,NA,NA,"GeneID:131831","ERICH6",FALSE,"ADRA1A"

"3268","CGAAGTCTCGCTCTGTCACC","chr6",39256177,"-","17:C>T","AGG","CGAAGTCTCGCTCTGTCGCCAGG",1,18,NA,NA,"","",FALSE,"ADRA1A"

"3269","CGAAGTCTCGCTCTGTCACC","chr10",74959764,"-","17:C>T","AGG","CGAAGTCTCGCTCTGTCGCCAGG",1,18,NA,NA,"GeneID:23522","KAT6B",FALSE,"ADRA1A"

"3270","CGAAGTCTCGCTCTGTCACC","chr7",134150436,"-","17:C>T","AGG","CGAAGTCTCGCTCTGTCGCCAGG",1,18,NA,NA,"GeneID:136332","LRGUK",FALSE,"ADRA1A"

"3271","CGAAGTCTCGCTCTGTCACC","chr3",184273392,"-","17:C>T","AGG","CGAAGTCTCGCTCTGTCGCCAGG",1,18,NA,NA,"","",FALSE,"ADRA1A"

"3272","CGAAGTCTCGCTCTGTCACC","chr20",18503944,"-","17:C>T","AGG","CGAAGTCTCGCTCTGTCGCCAGG",1,18,NA,NA,"","",FALSE,"ADRA1A"

"3273","CGAAGTCTCGCTCTGTCACC","chr18",74383467,"-","17:G>T","AGG","CGAAGTCTCGCTCTGTCCCCAGG",1,18,NA,NA,"","",FALSE,"ADRA1A"

"3274","CGAAGTCTCGCTCTGTCACC","chr9",115365005,"-","16:A>G,17:C>T","AGG","CGAAGTCTCGCTCTGTTGCCAGG",2,17,18,NA,"GeneID:50514;GeneID:101928775","DEC1;LOC101928775",FALSE,"ADRA1A"

"3275","CGAAGTCTCGCTCTGTCACC","chr1",77965991,"-","16:A>G,17:C>T","AGG","CGAAGTCTCGCTCTGTTGCCAGG",2,17,18,NA,"GeneID:8880","FUBP1",FALSE,"ADRA1A"

"3276","CGAAGTCTCGCTCTGTCACC","chr5",156828901,"-","16:A>G,17:C>T","AGG","CGAAGTCTCGCTCTGTTGCCAGG",2,17,18,NA,"","",FALSE,"ADRA1A"

"3277","CGAAGTCTCGCTCTGTCACC","chr2",48354574,"-","16:A>G,17:C>T","AGG","CGAAGTCTCGCTCTGTTGCCAGG",2,17,18,NA,"GeneID:3344","FOXN2",FALSE,"ADRA1A"

"3278","CGAAGTCTCGCTCTGTCACC","chr13",109967690,"-","16:A>G,17:C>T","AGG","CGAAGTCTCGCTCTGTTGCCAGG",2,17,18,NA,"","",FALSE,"ADRA1A"

"3279","CGAAGTCTCGCTCTGTCACC","chr3",13110563,"-","16:A>G,17:C>T","AGG","CGAAGTCTCGCTCTGTTGCCAGG",2,17,18,NA,"","",FALSE,"ADRA1A"

"3280","CGAAGTCTCGCTCTGTCACC","chrX",136882041,"-","16:A>G,17:C>T","AGG","CGAAGTCTCGCTCTGTTGCCAGG",2,17,18,NA,"","",FALSE,"ADRA1A"

"3281","CGAAGTCTCGCTCTGTCACC","chr6",127378857,"-","16:A>G,17:C>T","AGG","CGAAGTCTCGCTCTGTTGCCAGG",2,17,18,NA,"","",FALSE,"ADRA1A"

"3282","CGAAGTCTCGCTCTGTCACC","chr3",20951520,"-","16:A>G,17:C>T","AGG","CGAAGTCTCGCTCTGTTGCCAGG",2,17,18,NA,"GeneID:100874028","SGOL1-AS1",FALSE,"ADRA1A"

"3283","CGAAGTCTCGCTCTGTCACC","chr4",2524312,"-","16:A>G,17:C>T","AGG","CGAAGTCTCGCTCTGTTGCCAGG",2,17,18,NA,"GeneID:6047","RNF4",FALSE,"ADRA1A"

"3284","CGAAGTCTCGCTCTGTCACC","chr12",31751917,"-","16:C>G,17:C>T","AGG","CGAAGTCTCGCTCTGTGGCCAGG",2,17,18,NA,"","",FALSE,"ADRA1A"

"3285","CGAAGTCTCGCTCTGTCACC","chr2",152592077,"-","15:G>A","AGG","CGAAGTCTCGCTCTGCCACCAGG",1,16,NA,NA,"GeneID:114793","FMNL2",FALSE,"ADRA1A"

"3286","CGAAGTCTCGCTCTGTCACC","chr4",40253237,"-","14:T>C,17:A>T","AGG","CGAAGTCTCGCTCTATCTCCAGG",2,15,18,NA,"","",FALSE,"ADRA1A"

"3287","CGAAGTCTCGCTCTGTCACC","chr19",37644508,"-","12:C>G,13:T>A","AGG","CGAAGTCTCGCTGAGTCACCAGG",2,13,14,NA,"GeneID:22835","ZFP30",TRUE,"ADRA1A"

"3288","CGAAGTCTCGCTCTGTCACC","chr2",192164702,"-","12:T>G","AGG","CGAAGTCTCGCTATGTCACCAGG",1,13,NA,NA,"GeneID:23671","TMEFF2",FALSE,"ADRA1A"

"3289","CGAAGTCTCGCTCTGTCACC","chr17",36363225,"-","12:A>G","AGG","CGAAGTCTCGCTTTGTCACCAGG",1,13,NA,NA,"","",FALSE,"ADRA1A"

"3290","CGAAGTCTCGCTCTGTCACC","chr16",60990817,"-","12:A>G","AGG","CGAAGTCTCGCTTTGTCACCAGG",1,13,NA,NA,"","",FALSE,"ADRA1A"

"3291","CGAAGTCTCGCTCTGTCACC","chr17",38167350,"-","12:A>G","AGG","CGAAGTCTCGCTTTGTCACCAGG",1,13,NA,NA,"","",FALSE,"ADRA1A"

"3292","CGAAGTCTCGCTCTGTCACC","chr17",38043379,"-","12:A>G","AGG","CGAAGTCTCGCTTTGTCACCAGG",1,13,NA,NA,"","",FALSE,"ADRA1A"

"3293","CGAAGTCTCGCTCTGTCACC","chr17",37963854,"-","12:A>G","AGG","CGAAGTCTCGCTTTGTCACCAGG",1,13,NA,NA,"","",FALSE,"ADRA1A"

"3294","CGAAGTCTCGCTCTGTCACC","chr17",36239342,"-","12:A>G","AGG","CGAAGTCTCGCTTTGTCACCAGG",1,13,NA,NA,"","",FALSE,"ADRA1A"

"3295","CGAAGTCTCGCTCTGTCACC","chr10",15123503,"-","12:A>G,17:C>T","AGG","CGAAGTCTCGCTTTGTCGCCAGG",2,13,18,NA,"GeneID:10557;GeneID:9397","RPP38;NMT2",FALSE,"ADRA1A"

"3296","CGAAGTCTCGCTCTGTCACC","chr3",11223159,"-","12:A>G,17:C>T","AGG","CGAAGTCTCGCTTTGTCGCCAGG",2,13,18,NA,"GeneID:3269","HRH1",FALSE,"ADRA1A"

"3297","CGAAGTCTCGCTCTGTCACC","chr14",65390297,"-","12:A>G,17:C>T","AGG","CGAAGTCTCGCTTTGTCGCCAGG",2,13,18,NA,"","",FALSE,"ADRA1A"

"3298","CGAAGTCTCGCTCTGTCACC","chr8",12845069,"-","11:G>A,17:C>T","AGG","CGAAGTCTCGCCCTGTCGCCAGG",2,12,18,NA,"","",FALSE,"ADRA1A"

"3299","CGAAGTCTCGCTCTGTCACC","chr9",129126124,"-","11:T>A,17:C>T","TGG","CGAAGTCTCGCACTGTCGCCTGG",2,12,18,NA,"GeneID:5524","PPP2R4",FALSE,"ADRA1A"

"3300","CGAAGTCTCGCTCTGTCACC","chr1",86909964,"-","11:T>A,12:A>G","CGG","CGAAGTCTCGCATTGTCACCCGG",2,12,13,NA,"GeneID:9403","SEP15",FALSE,"ADRA1A"

"3301","CGAAGTCTCGCTCTGTCACC","chr19",17627140,"-","10:A>G","AGG","CGAAGTCTCGTTCTGTCACCAGG",1,11,NA,NA,"GeneID:23025","UNC13A",FALSE,"ADRA1A"

"3302","CGAAGTCTCGCTCTGTCACC","chr11",86025374,"-","10:A>G","AGG","CGAAGTCTCGTTCTGTCACCAGG",1,11,NA,NA,"GeneID:8301","PICALM",FALSE,"ADRA1A"

"3303","CGAAGTCTCGCTCTGTCACC","chr13",28451398,"-","10:A>G","CGG","CGAAGTCTCGTTCTGTCACCCGG",1,11,NA,NA,"GeneID:2321","FLT1",FALSE,"ADRA1A"

"3304","CGAAGTCTCGCTCTGTCACC","chr20",34557903,"-","10:A>G,17:C>T","AGG","CGAAGTCTCGTTCTGTCGCCAGG",2,11,18,NA,"GeneID:84557","MAP1LC3A",FALSE,"ADRA1A"

"3305","CGAAGTCTCGCTCTGTCACC","chr6",40704286,"-","10:T>G,14:T>C","AGG","CGAAGTCTCGATCTATCACCAGG",2,11,15,NA,"","",FALSE,"ADRA1A"

"3306","CGAAGTCTCGCTCTGTCACC","chr19",1120691,"+","19:T>C","AGG","CGAAGTCTCGCTCTGTCACTAGG",1,20,NA,NA,"GeneID:22904","SBNO2",FALSE,"ADRA1A"

"3307","CGAAGTCTCGCTCTGTCACC","chr17",38456932,"+","19:T>C","AGG","CGAAGTCTCGCTCTGTCACTAGG",1,20,NA,NA,"GeneID:57636","ARHGAP23",FALSE,"ADRA1A"

"3308","CGAAGTCTCGCTCTGTCACC","chr11",78380230,"+","17:G>A","AGG","CGAAGTCTCGCTCTGTCGCCAGG",1,18,NA,NA,"GeneID:9846","GAB2",FALSE,"ADRA1A"

"3309","CGAAGTCTCGCTCTGTCACC","chr17",59102895,"+","17:G>A","AGG","CGAAGTCTCGCTCTGTCGCCAGG",1,18,NA,NA,"GeneID:4591","TRIM37",FALSE,"ADRA1A"

"3310","CGAAGTCTCGCTCTGTCACC","chr17",47477927,"+","17:G>A","AGG","CGAAGTCTCGCTCTGTCGCCAGG",1,18,NA,NA,"","",FALSE,"ADRA1A"

"3311","CGAAGTCTCGCTCTGTCACC","chr2",161859313,"+","17:G>A","AGG","CGAAGTCTCGCTCTGTCGCCAGG",1,18,NA,NA,"GeneID:57282","SLC4A10",FALSE,"ADRA1A"

"3312","CGAAGTCTCGCTCTGTCACC","chr12",55869701,"+","17:G>A","AGG","CGAAGTCTCGCTCTGTCGCCAGG",1,18,NA,NA,"","",FALSE,"ADRA1A"

"3313","CGAAGTCTCGCTCTGTCACC","chr14",28858800,"+","17:G>A","AGG","CGAAGTCTCGCTCTGTCGCCAGG",1,18,NA,NA,"","",FALSE,"ADRA1A"

"3314","CGAAGTCTCGCTCTGTCACC","chr7",23667256,"+","17:G>A","AGG","CGAAGTCTCGCTCTGTCGCCAGG",1,18,NA,NA,"","",FALSE,"ADRA1A"

"3315","CGAAGTCTCGCTCTGTCACC","chr17",44586578,"+","17:G>A","AGG","CGAAGTCTCGCTCTGTCGCCAGG",1,18,NA,NA,"","",FALSE,"ADRA1A"

"3316","CGAAGTCTCGCTCTGTCACC","chr16",85647609,"+","17:G>A","AGG","CGAAGTCTCGCTCTGTCGCCAGG",1,18,NA,NA,"GeneID:23199","GSE1",FALSE,"ADRA1A"

"3317","CGAAGTCTCGCTCTGTCACC","chr10",32784594,"+","17:G>A","AGG","CGAAGTCTCGCTCTGTCGCCAGG",1,18,NA,NA,"GeneID:79741","CCDC7",FALSE,"ADRA1A"

"3318","CGAAGTCTCGCTCTGTCACC","chr20",23446278,"+","17:G>A","AGG","CGAAGTCTCGCTCTGTCGCCAGG",1,18,NA,NA,"","",FALSE,"ADRA1A"

"3319","CGAAGTCTCGCTCTGTCACC","chr4",159430344,"+","17:G>A,19:G>C","AGG","CGAAGTCTCGCTCTGTCGCGAGG",2,18,20,NA,"","",FALSE,"ADRA1A"

"3320","CGAAGTCTCGCTCTGTCACC","chr4",186040553,"+","17:T>A","AGG","CGAAGTCTCGCTCTGTCTCCAGG",1,18,NA,NA,"","",FALSE,"ADRA1A"

"3321","CGAAGTCTCGCTCTGTCACC","chr16",76403114,"+","17:T>A","GGG","CGAAGTCTCGCTCTGTCTCCGGG",1,18,NA,NA,"GeneID:85445","CNTNAP4",FALSE,"ADRA1A"

"3322","CGAAGTCTCGCTCTGTCACC","chr17",54058073,"+","17:T>A","TGG","CGAAGTCTCGCTCTGTCTCCTGG",1,18,NA,NA,"","",FALSE,"ADRA1A"

"3323","CGAAGTCTCGCTCTGTCACC","chr13",32580154,"+","17:T>A","AGG","CGAAGTCTCGCTCTGTCTCCAGG",1,18,NA,NA,"","",FALSE,"ADRA1A"

"3324","CGAAGTCTCGCTCTGTCACC","chr2",160410766,"+","16:T>C","AGG","CGAAGTCTCGCTCTGTTACCAGG",1,17,NA,NA,"GeneID:5937","RBMS1",FALSE,"ADRA1A"

"3325","CGAAGTCTCGCTCTGTCACC","chr8",127956507,"+","16:T>C,17:G>A","AGG","CGAAGTCTCGCTCTGTTGCCAGG",2,17,18,NA,"GeneID:5820","PVT1",FALSE,"ADRA1A"

"3326","CGAAGTCTCGCTCTGTCACC","chr17",36568778,"+","16:T>C,17:G>A","AGG","CGAAGTCTCGCTCTGTTGCCAGG",2,17,18,NA,"GeneID:79893","GGNBP2",FALSE,"ADRA1A"

"3327","CGAAGTCTCGCTCTGTCACC","chr9",129119427,"+","16:T>C,17:G>A","TGG","CGAAGTCTCGCTCTGTTGCCTGG",2,17,18,NA,"GeneID:5524","PPP2R4",FALSE,"ADRA1A"

"3328","CGAAGTCTCGCTCTGTCACC","chr7",105418557,"+","16:T>C,17:G>A","AGG","CGAAGTCTCGCTCTGTTGCCAGG",2,17,18,NA,"","",FALSE,"ADRA1A"

"3329","CGAAGTCTCGCTCTGTCACC","chr19",19576248,"+","16:T>C,17:G>A","AGG","CGAAGTCTCGCTCTGTTGCCAGG",2,17,18,NA,"GeneID:80714","PBX4",FALSE,"ADRA1A"

"3330","CGAAGTCTCGCTCTGTCACC","chr1",168651106,"+","16:T>C,17:G>A","AGG","CGAAGTCTCGCTCTGTTGCCAGG",2,17,18,NA,"","",FALSE,"ADRA1A"

"3331","CGAAGTCTCGCTCTGTCACC","chr9",17281985,"+","16:T>C,17:G>A","AGG","CGAAGTCTCGCTCTGTTGCCAGG",2,17,18,NA,"GeneID:54875","CNTLN",FALSE,"ADRA1A"

"3332","CGAAGTCTCGCTCTGTCACC","chr7",67976969,"+","16:T>C,17:C>A","AGG","CGAAGTCTCGCTCTGTTCCCAGG",2,17,18,NA,"","",FALSE,"ADRA1A"

"3333","CGAAGTCTCGCTCTGTCACC","chr12",70933785,"+","16:G>C,17:C>A","AGG","CGAAGTCTCGCTCTGTGCCCAGG",2,17,18,NA,"","",FALSE,"ADRA1A"

"3334","CGAAGTCTCGCTCTGTCACC","chr3",118572101,"+","16:G>C,17:C>A","AGG","CGAAGTCTCGCTCTGTGCCCAGG",2,17,18,NA,"","",FALSE,"ADRA1A"

"3335","CGAAGTCTCGCTCTGTCACC","chr17",8036991,"+","14:A>G","AGG","CGAAGTCTCGCTCTATCACCAGG",1,15,NA,NA,"","",FALSE,"ADRA1A"

"3336","CGAAGTCTCGCTCTGTCACC","chr13",26341943,"+","14:T>G,17:C>A","AGG","CGAAGTCTCGCTCTTTCCCCAGG",2,15,18,NA,"GeneID:1024","CDK8",FALSE,"ADRA1A"

"3337","CGAAGTCTCGCTCTGTCACC","chr4",112853091,"+","14:C>G,17:G>A","AGG","CGAAGTCTCGCTCTCTCGCCAGG",2,15,18,NA,"GeneID:287","ANK2",FALSE,"ADRA1A"

"3338","CGAAGTCTCGCTCTGTCACC","chr18",49511304,"+","13:A>T","AGG","CGAAGTCTCGCTCAGTCACCAGG",1,14,NA,NA,"","",FALSE,"ADRA1A"

"3339","CGAAGTCTCGCTCTGTCACC","chr17",37424376,"+","12:G>C","AGG","CGAAGTCTCGCTGTGTCACCAGG",1,13,NA,NA,"GeneID:6871","TADA2A",FALSE,"ADRA1A"

"3340","CGAAGTCTCGCTCTGTCACC","chr22",19379831,"+","12:G>C,17:G>A","AGG","CGAAGTCTCGCTGTGTCGCCAGG",2,13,18,NA,"GeneID:7290;GeneID:128977","HIRA;C22orf39",FALSE,"ADRA1A"

"3341","CGAAGTCTCGCTCTGTCACC","chr8",79540154,"+","12:G>C,17:G>A","AGG","CGAAGTCTCGCTGTGTCGCCAGG",2,13,18,NA,"","",FALSE,"ADRA1A"

"3342","CGAAGTCTCGCTCTGTCACC","chr4",5040416,"+","12:G>C,17:G>A","AGG","CGAAGTCTCGCTGTGTCGCCAGG",2,13,18,NA,"","",FALSE,"ADRA1A"

"3343","CGAAGTCTCGCTCTGTCACC","chr1",23089614,"+","11:A>T,17:T>A","CGG","CGAAGTCTCGCACTGTCTCCCGG",2,12,18,NA,"GeneID:7798","LUZP1",FALSE,"ADRA1A"

"3344","CGAAGTCTCGCTCTGTCACC","chr6",110962800,"+","11:A>T,17:G>A","CGG","CGAAGTCTCGCACTGTCGCCCGG",2,12,18,NA,"GeneID:112495","GTF3C6",FALSE,"ADRA1A"

"3345","CGAAGTCTCGCTCTGTCACC","chr18",71277587,"+","11:C>T,17:G>A","AGG","CGAAGTCTCGCCCTGTCGCCAGG",2,12,18,NA,"","",FALSE,"ADRA1A"

"3346","CGAAGTCTCGCTCTGTCACC","chr1",36633336,"+","10:G>C,17:G>A","AGG","CGAAGTCTCGGTCTGTCGCCAGG",2,11,18,NA,"","",FALSE,"ADRA1A"

"3347","CGAAGTCTCGCTCTGTCACC","chr10",30534591,"-","0:A>G","CGG","TGAAGTCTCGCTCTGTCACCCGG",1,1,NA,NA,"","",FALSE,"ADRA1A"

"3348","CGAAGTCTCGCTCTGTCACC","chr10",121575830,"-","0:A>G","CGG","TGAAGTCTCGCTCTGTCACCCGG",1,1,NA,NA,"GeneID:2263","FGFR2",FALSE,"ADRA1A"

"3349","CGAAGTCTCGCTCTGTCACC","chr15",83875757,"-","0:A>G","CGG","TGAAGTCTCGCTCTGTCACCCGG",1,1,NA,NA,"GeneID:57188","ADAMTSL3",FALSE,"ADRA1A"

"3350","CGAAGTCTCGCTCTGTCACC","chr9",73718904,"-","0:A>G","AGG","TGAAGTCTCGCTCTGTCACCAGG",1,1,NA,NA,"","",FALSE,"ADRA1A"

"3351","CGAAGTCTCGCTCTGTCACC","chr19",18400445,"-","0:A>G","AGG","TGAAGTCTCGCTCTGTCACCAGG",1,1,NA,NA,"","",FALSE,"ADRA1A"

"3352","CGAAGTCTCGCTCTGTCACC","chr4",29443800,"-","0:A>G","AGG","TGAAGTCTCGCTCTGTCACCAGG",1,1,NA,NA,"","",FALSE,"ADRA1A"

"3353","CGAAGTCTCGCTCTGTCACC","chr17",39583450,"-","0:A>G","AGG","TGAAGTCTCGCTCTGTCACCAGG",1,1,NA,NA,"","",FALSE,"ADRA1A"

"3354","CGAAGTCTCGCTCTGTCACC","chr17",45494437,"-","0:A>G","AGG","TGAAGTCTCGCTCTGTCACCAGG",1,1,NA,NA,"","",FALSE,"ADRA1A"

"3355","CGAAGTCTCGCTCTGTCACC","chr17",64840842,"-","0:A>G","AGG","TGAAGTCTCGCTCTGTCACCAGG",1,1,NA,NA,"","",FALSE,"ADRA1A"

"3356","CGAAGTCTCGCTCTGTCACC","chr12",122787725,"-","0:A>G","AGG","TGAAGTCTCGCTCTGTCACCAGG",1,1,NA,NA,"GeneID:84660","CCDC62",FALSE,"ADRA1A"

"3357","CGAAGTCTCGCTCTGTCACC","chr14",68039402,"-","0:A>G","AGG","TGAAGTCTCGCTCTGTCACCAGG",1,1,NA,NA,"GeneID:5890","RAD51B",FALSE,"ADRA1A"

"3358","CGAAGTCTCGCTCTGTCACC","chr6",73290251,"-","0:A>G","AGG","TGAAGTCTCGCTCTGTCACCAGG",1,1,NA,NA,"GeneID:80759","KHDC1",FALSE,"ADRA1A"

"3359","CGAAGTCTCGCTCTGTCACC","chr4",39355976,"-","0:A>G","AGG","TGAAGTCTCGCTCTGTCACCAGG",1,1,NA,NA,"GeneID:5981","RFC1",TRUE,"ADRA1A"

"3360","CGAAGTCTCGCTCTGTCACC","chr10",117037979,"-","0:A>G","AGG","TGAAGTCTCGCTCTGTCACCAGG",1,1,NA,NA,"GeneID:57698","KIAA1598",FALSE,"ADRA1A"

"3361","CGAAGTCTCGCTCTGTCACC","chr3",196415756,"-","0:A>G","AGG","TGAAGTCTCGCTCTGTCACCAGG",1,1,NA,NA,"GeneID:26043","UBXN7",FALSE,"ADRA1A"

"3362","CGAAGTCTCGCTCTGTCACC","chr4",182696699,"-","0:A>G","AGG","TGAAGTCTCGCTCTGTCACCAGG",1,1,NA,NA,"GeneID:55714","TENM3",FALSE,"ADRA1A"

"3363","CGAAGTCTCGCTCTGTCACC","chr3",149077222,"-","0:A>G","AGG","TGAAGTCTCGCTCTGTCACCAGG",1,1,NA,NA,"GeneID:6596","HLTF",FALSE,"ADRA1A"

"3364","CGAAGTCTCGCTCTGTCACC","chr1",236507134,"-","0:A>G","AGG","TGAAGTCTCGCTCTGTCACCAGG",1,1,NA,NA,"","",FALSE,"ADRA1A"

"3365","CGAAGTCTCGCTCTGTCACC","chr4",44753083,"-","0:A>G","AGG","TGAAGTCTCGCTCTGTCACCAGG",1,1,NA,NA,"","",FALSE,"ADRA1A"

"3366","CGAAGTCTCGCTCTGTCACC","chr21",13295034,"-","0:A>G","AGG","TGAAGTCTCGCTCTGTCACCAGG",1,1,NA,NA,"","",FALSE,"ADRA1A"

"3367","CGAAGTCTCGCTCTGTCACC","chr20",5297163,"-","0:A>G","TGG","TGAAGTCTCGCTCTGTCACCTGG",1,1,NA,NA,"","",FALSE,"ADRA1A"

"3368","CGAAGTCTCGCTCTGTCACC","chr5",116892461,"-","1:T>C","AGG","CAAAGTCTCGCTCTGTCACCAGG",1,2,NA,NA,"","",FALSE,"ADRA1A"

"3369","CGAAGTCTCGCTCTGTCACC","chr5",155753311,"-","1:T>C","AGG","CAAAGTCTCGCTCTGTCACCAGG",1,2,NA,NA,"","",FALSE,"ADRA1A"

"3370","CGAAGTCTCGCTCTGTCACC","chr9",130966227,"-","1:T>C","AGG","CAAAGTCTCGCTCTGTCACCAGG",1,2,NA,NA,"","",FALSE,"ADRA1A"

"3371","CGAAGTCTCGCTCTGTCACC","chr15",29315026,"-","1:T>C","AGG","CAAAGTCTCGCTCTGTCACCAGG",1,2,NA,NA,"GeneID:23359","FAM189A1",FALSE,"ADRA1A"

"3372","CGAAGTCTCGCTCTGTCACC","chr8",42611743,"-","1:T>C","AGG","CAAAGTCTCGCTCTGTCACCAGG",1,2,NA,NA,"","",FALSE,"ADRA1A"

"3373","CGAAGTCTCGCTCTGTCACC","chr4",113470612,"-","1:T>C","AGG","CAAAGTCTCGCTCTGTCACCAGG",1,2,NA,NA,"GeneID:817","CAMK2D",FALSE,"ADRA1A"

"3374","CGAAGTCTCGCTCTGTCACC","chr22",40145816,"-","1:T>C","AGG","CAAAGTCTCGCTCTGTCACCAGG",1,2,NA,NA,"GeneID:23112","TNRC6B",FALSE,"ADRA1A"

"3375","CGAAGTCTCGCTCTGTCACC","chrX",48263108,"-","1:T>C","AGG","CAAAGTCTCGCTCTGTCACCAGG",1,2,NA,NA,"GeneID:6756","SSX1",FALSE,"ADRA1A"

"3376","CGAAGTCTCGCTCTGTCACC","chrX",52629598,"-","1:T>C","AGG","CAAAGTCTCGCTCTGTCACCAGG",1,2,NA,NA,"","",FALSE,"ADRA1A"

"3377","CGAAGTCTCGCTCTGTCACC","chr10",102336275,"-","1:T>C","AGG","CAAAGTCTCGCTCTGTCACCAGG",1,2,NA,NA,"GeneID:8729","GBF1",FALSE,"ADRA1A"

"3378","CGAAGTCTCGCTCTGTCACC","chr4",117474322,"-","1:T>C","TGG","CAAAGTCTCGCTCTGTCACCTGG",1,2,NA,NA,"","",FALSE,"ADRA1A"

"3379","CGAAGTCTCGCTCTGTCACC","chr11",17789960,"-","1:T>C","CGG","CAAAGTCTCGCTCTGTCACCCGG",1,2,NA,NA,"GeneID:3746;GeneID:26297","KCNC1;SERGEF",FALSE,"ADRA1A"

"3380","CGAAGTCTCGCTCTGTCACC","chr3",28053645,"-","1:T>C","CGG","CAAAGTCTCGCTCTGTCACCCGG",1,2,NA,NA,"","",FALSE,"ADRA1A"

"3381","CGAAGTCTCGCTCTGTCACC","chr8",104512291,"-","0:T>G,1:T>C","AGG","AAAAGTCTCGCTCTGTCACCAGG",2,1,2,NA,"GeneID:29967","LRP12",FALSE,"ADRA1A"

"3382","CGAAGTCTCGCTCTGTCACC","chr3",134336278,"-","0:T>G,1:G>C","AGG","ACAAGTCTCGCTCTGTCACCAGG",2,1,2,NA,"","",FALSE,"ADRA1A"

"3383","CGAAGTCTCGCTCTGTCACC","chr1",40243040,"-","0:T>G,1:G>C","TGG","ACAAGTCTCGCTCTGTCACCTGG",2,1,2,NA,"","",FALSE,"ADRA1A"

"3384","CGAAGTCTCGCTCTGTCACC","chr3",59783443,"-","0:A>G,1:G>C","AGG","TCAAGTCTCGCTCTGTCACCAGG",2,1,2,NA,"","",FALSE,"ADRA1A"

"3385","CGAAGTCTCGCTCTGTCACC","chr9",16659582,"-","2:C>T","GGG","CGGAGTCTCGCTCTGTCACCGGG",1,3,NA,NA,"GeneID:54796","BNC2",FALSE,"ADRA1A"

"3386","CGAAGTCTCGCTCTGTCACC","chr9",131368815,"-","2:C>T","GGG","CGGAGTCTCGCTCTGTCACCGGG",1,3,NA,NA,"","",FALSE,"ADRA1A"

"3387","CGAAGTCTCGCTCTGTCACC","chr18",26995413,"-","2:C>T","CGG","CGGAGTCTCGCTCTGTCACCCGG",1,3,NA,NA,"GeneID:83539","CHST9",FALSE,"ADRA1A"

"3388","CGAAGTCTCGCTCTGTCACC","chr10",13308903,"-","2:C>T","CGG","CGGAGTCTCGCTCTGTCACCCGG",1,3,NA,NA,"","",FALSE,"ADRA1A"

"3389","CGAAGTCTCGCTCTGTCACC","chr2",16918944,"-","2:C>T","CGG","CGGAGTCTCGCTCTGTCACCCGG",1,3,NA,NA,"","",FALSE,"ADRA1A"

"3390","CGAAGTCTCGCTCTGTCACC","chr17",7371866,"-","2:C>T","CGG","CGGAGTCTCGCTCTGTCACCCGG",1,3,NA,NA,"","",FALSE,"ADRA1A"

"3391","CGAAGTCTCGCTCTGTCACC","chr12",125589457,"-","2:C>T","CGG","CGGAGTCTCGCTCTGTCACCCGG",1,3,NA,NA,"GeneID:114795","TMEM132B",FALSE,"ADRA1A"

"3392","CGAAGTCTCGCTCTGTCACC","chr22",28170464,"-","2:C>T","CGG","CGGAGTCTCGCTCTGTCACCCGG",1,3,NA,NA,"GeneID:23331","TTC28",FALSE,"ADRA1A"

"3393","CGAAGTCTCGCTCTGTCACC","chr4",145141144,"-","2:C>T","CGG","CGGAGTCTCGCTCTGTCACCCGG",1,3,NA,NA,"GeneID:54726","OTUD4",FALSE,"ADRA1A"

"3394","CGAAGTCTCGCTCTGTCACC","chr22",50381883,"-","2:C>T","CGG","CGGAGTCTCGCTCTGTCACCCGG",1,3,NA,NA,"GeneID:9701","PPP6R2",FALSE,"ADRA1A"

"3395","CGAAGTCTCGCTCTGTCACC","chr12",123631782,"-","2:C>T","CGG","CGGAGTCTCGCTCTGTCACCCGG",1,3,NA,NA,"GeneID:1967","EIF2B1",FALSE,"ADRA1A"

"3396","CGAAGTCTCGCTCTGTCACC","chr17",73153654,"-","2:C>T","CGG","CGGAGTCTCGCTCTGTCACCCGG",1,3,NA,NA,"","",FALSE,"ADRA1A"

"3397","CGAAGTCTCGCTCTGTCACC","chr6",107113413,"-","2:C>T","CGG","CGGAGTCTCGCTCTGTCACCCGG",1,3,NA,NA,"GeneID:57673","BEND3",FALSE,"ADRA1A"

"3398","CGAAGTCTCGCTCTGTCACC","chr6",162072263,"-","2:C>T","CGG","CGGAGTCTCGCTCTGTCACCCGG",1,3,NA,NA,"GeneID:5071","PARK2",FALSE,"ADRA1A"

"3399","CGAAGTCTCGCTCTGTCACC","chr6",115815874,"-","2:C>T","CGG","CGGAGTCTCGCTCTGTCACCCGG",1,3,NA,NA,"","",FALSE,"ADRA1A"

"3400","CGAAGTCTCGCTCTGTCACC","chr2",40886026,"-","2:C>T","CGG","CGGAGTCTCGCTCTGTCACCCGG",1,3,NA,NA,"","",FALSE,"ADRA1A"

"3401","CGAAGTCTCGCTCTGTCACC","chr10",73505845,"-","2:C>T","CGG","CGGAGTCTCGCTCTGTCACCCGG",1,3,NA,NA,"GeneID:101929145;GeneID:159195","PPP3CB-AS1;USP54",TRUE,"ADRA1A"

"3402","CGAAGTCTCGCTCTGTCACC","chr14",57019120,"-","2:C>T","CGG","CGGAGTCTCGCTCTGTCACCCGG",1,3,NA,NA,"GeneID:100309464","OTX2-AS1",FALSE,"ADRA1A"

"3403","CGAAGTCTCGCTCTGTCACC","chr12",4724124,"-","2:C>T","CGG","CGGAGTCTCGCTCTGTCACCCGG",1,3,NA,NA,"GeneID:3742","KCNA6",FALSE,"ADRA1A"

"3404","CGAAGTCTCGCTCTGTCACC","chr14",81160853,"-","2:C>T","CGG","CGGAGTCTCGCTCTGTCACCCGG",1,3,NA,NA,"","",FALSE,"ADRA1A"

"3405","CGAAGTCTCGCTCTGTCACC","chr10",12545775,"-","2:C>T","CGG","CGGAGTCTCGCTCTGTCACCCGG",1,3,NA,NA,"GeneID:57118","CAMK1D",FALSE,"ADRA1A"

"3406","CGAAGTCTCGCTCTGTCACC","chr1",91691973,"-","2:C>T","CGG","CGGAGTCTCGCTCTGTCACCCGG",1,3,NA,NA,"GeneID:7049","TGFBR3",FALSE,"ADRA1A"

"3407","CGAAGTCTCGCTCTGTCACC","chr2",171971173,"-","2:C>T","CGG","CGGAGTCTCGCTCTGTCACCCGG",1,3,NA,NA,"GeneID:8604;GeneID:8520","SLC25A12;HAT1",FALSE,"ADRA1A"

"3408","CGAAGTCTCGCTCTGTCACC","chr22",19347914,"-","2:C>T","CGG","CGGAGTCTCGCTCTGTCACCCGG",1,3,NA,NA,"GeneID:7290","HIRA",FALSE,"ADRA1A"

"3409","CGAAGTCTCGCTCTGTCACC","chr18",2530550,"-","2:C>T","CGG","CGGAGTCTCGCTCTGTCACCCGG",1,3,NA,NA,"","",FALSE,"ADRA1A"

"3410","CGAAGTCTCGCTCTGTCACC","chr9",94326719,"-","2:C>T","CGG","CGGAGTCTCGCTCTGTCACCCGG",1,3,NA,NA,"GeneID:54754","NUTM2F",FALSE,"ADRA1A"

"3411","CGAAGTCTCGCTCTGTCACC","chr9",127339272,"-","2:C>T","CGG","CGGAGTCTCGCTCTGTCACCCGG",1,3,NA,NA,"GeneID:84253","GARNL3",FALSE,"ADRA1A"

"3412","CGAAGTCTCGCTCTGTCACC","chr12",65813179,"-","2:C>T","CGG","CGGAGTCTCGCTCTGTCACCCGG",1,3,NA,NA,"GeneID:204010","RPSAP52",FALSE,"ADRA1A"

"3413","CGAAGTCTCGCTCTGTCACC","chr6",108052408,"-","2:C>T","CGG","CGGAGTCTCGCTCTGTCACCCGG",1,3,NA,NA,"GeneID:28962","OSTM1",FALSE,"ADRA1A"

"3414","CGAAGTCTCGCTCTGTCACC","chr16",78914854,"-","2:C>T","CGG","CGGAGTCTCGCTCTGTCACCCGG",1,3,NA,NA,"GeneID:51741","WWOX",FALSE,"ADRA1A"

"3415","CGAAGTCTCGCTCTGTCACC","chr15",88824295,"-","2:C>T","CGG","CGGAGTCTCGCTCTGTCACCCGG",1,3,NA,NA,"GeneID:176","ACAN",FALSE,"ADRA1A"

"3416","CGAAGTCTCGCTCTGTCACC","chr6",42640002,"-","2:C>T","CGG","CGGAGTCTCGCTCTGTCACCCGG",1,3,NA,NA,"GeneID:23304","UBR2",FALSE,"ADRA1A"

"3417","CGAAGTCTCGCTCTGTCACC","chr3",72267721,"-","2:C>T","AGG","CGGAGTCTCGCTCTGTCACCAGG",1,3,NA,NA,"GeneID:285286","LINC00877",FALSE,"ADRA1A"

"3418","CGAAGTCTCGCTCTGTCACC","chr19",46400887,"-","2:C>T","AGG","CGGAGTCTCGCTCTGTCACCAGG",1,3,NA,NA,"","",FALSE,"ADRA1A"

"3419","CGAAGTCTCGCTCTGTCACC","chr17",5918962,"-","2:C>T","AGG","CGGAGTCTCGCTCTGTCACCAGG",1,3,NA,NA,"","",FALSE,"ADRA1A"

"3420","CGAAGTCTCGCTCTGTCACC","chr3",151313898,"-","2:C>T","AGG","CGGAGTCTCGCTCTGTCACCAGG",1,3,NA,NA,"GeneID:116931;GeneID:53836","MED12L;GPR87",FALSE,"ADRA1A"

"3421","CGAAGTCTCGCTCTGTCACC","chr22",20916521,"-","2:C>T","AGG","CGGAGTCTCGCTCTGTCACCAGG",1,3,NA,NA,"","",FALSE,"ADRA1A"

"3422","CGAAGTCTCGCTCTGTCACC","chr10",32183503,"-","2:C>T","AGG","CGGAGTCTCGCTCTGTCACCAGG",1,3,NA,NA,"","",FALSE,"ADRA1A"

"3423","CGAAGTCTCGCTCTGTCACC","chr3",129114007,"-","2:C>T","AGG","CGGAGTCTCGCTCTGTCACCAGG",1,3,NA,NA,"GeneID:339122;GeneID:100534599","RAB43;ISY1-RAB43",FALSE,"ADRA1A"

"3424","CGAAGTCTCGCTCTGTCACC","chr8",39974903,"-","2:C>T","AGG","CGGAGTCTCGCTCTGTCACCAGG",1,3,NA,NA,"GeneID:169355","IDO2",FALSE,"ADRA1A"

"3425","CGAAGTCTCGCTCTGTCACC","chr1",174190277,"-","2:C>T","AGG","CGGAGTCTCGCTCTGTCACCAGG",1,3,NA,NA,"GeneID:9910","RABGAP1L",FALSE,"ADRA1A"

"3426","CGAAGTCTCGCTCTGTCACC","chr16",20158881,"-","2:C>T","AGG","CGGAGTCTCGCTCTGTCACCAGG",1,3,NA,NA,"","",FALSE,"ADRA1A"

"3427","CGAAGTCTCGCTCTGTCACC","chr12",57678097,"-","2:C>T","AGG","CGGAGTCTCGCTCTGTCACCAGG",1,3,NA,NA,"","",FALSE,"ADRA1A"

"3428","CGAAGTCTCGCTCTGTCACC","chr5",55202261,"-","2:C>T","AGG","CGGAGTCTCGCTCTGTCACCAGG",1,3,NA,NA,"","",FALSE,"ADRA1A"

"3429","CGAAGTCTCGCTCTGTCACC","chr7",139348693,"-","2:C>T","AGG","CGGAGTCTCGCTCTGTCACCAGG",1,3,NA,NA,"GeneID:51631;GeneID:100996928","LUC7L2;C7orf55-LUC7L2",FALSE,"ADRA1A"

"3430","CGAAGTCTCGCTCTGTCACC","chr5",35993438,"-","2:C>T","AGG","CGGAGTCTCGCTCTGTCACCAGG",1,3,NA,NA,"GeneID:133688","UGT3A1",FALSE,"ADRA1A"

"3431","CGAAGTCTCGCTCTGTCACC","chr6",87759352,"-","2:C>T","AGG","CGGAGTCTCGCTCTGTCACCAGG",1,3,NA,NA,"","",FALSE,"ADRA1A"

"3432","CGAAGTCTCGCTCTGTCACC","chrX",71471295,"-","2:C>T","AGG","CGGAGTCTCGCTCTGTCACCAGG",1,3,NA,NA,"GeneID:6872","TAF1",FALSE,"ADRA1A"

"3433","CGAAGTCTCGCTCTGTCACC","chr2",48010143,"-","2:C>T","AGG","CGGAGTCTCGCTCTGTCACCAGG",1,3,NA,NA,"","",FALSE,"ADRA1A"

"3434","CGAAGTCTCGCTCTGTCACC","chr6",37796937,"-","2:C>T","AGG","CGGAGTCTCGCTCTGTCACCAGG",1,3,NA,NA,"","",FALSE,"ADRA1A"

"3435","CGAAGTCTCGCTCTGTCACC","chrX",146493244,"-","2:C>T","AGG","CGGAGTCTCGCTCTGTCACCAGG",1,3,NA,NA,"","",FALSE,"ADRA1A"

"3436","CGAAGTCTCGCTCTGTCACC","chr13",29597697,"-","2:C>T","AGG","CGGAGTCTCGCTCTGTCACCAGG",1,3,NA,NA,"","",FALSE,"ADRA1A"

"3437","CGAAGTCTCGCTCTGTCACC","chrX",114631278,"-","2:C>T","AGG","CGGAGTCTCGCTCTGTCACCAGG",1,3,NA,NA,"GeneID:3358","HTR2C",FALSE,"ADRA1A"

"3438","CGAAGTCTCGCTCTGTCACC","chr22",25866760,"-","2:C>T","AGG","CGGAGTCTCGCTCTGTCACCAGG",1,3,NA,NA,"GeneID:84700","MYO18B",FALSE,"ADRA1A"

"3439","CGAAGTCTCGCTCTGTCACC","chr5",54071947,"-","2:C>T","AGG","CGGAGTCTCGCTCTGTCACCAGG",1,3,NA,NA,"GeneID:54622","ARL15",FALSE,"ADRA1A"

"3440","CGAAGTCTCGCTCTGTCACC","chr3",30439946,"-","2:C>T","AGG","CGGAGTCTCGCTCTGTCACCAGG",1,3,NA,NA,"","",FALSE,"ADRA1A"

"3441","CGAAGTCTCGCTCTGTCACC","chr9",37155331,"-","2:C>T","AGG","CGGAGTCTCGCTCTGTCACCAGG",1,3,NA,NA,"GeneID:84186","ZCCHC7",FALSE,"ADRA1A"

"3442","CGAAGTCTCGCTCTGTCACC","chr2",31913224,"-","2:C>T","AGG","CGGAGTCTCGCTCTGTCACCAGG",1,3,NA,NA,"GeneID:51072;GeneID:84661","MEMO1;DPY30",FALSE,"ADRA1A"

"3443","CGAAGTCTCGCTCTGTCACC","chr5",155686834,"-","2:C>T","AGG","CGGAGTCTCGCTCTGTCACCAGG",1,3,NA,NA,"","",FALSE,"ADRA1A"

"3444","CGAAGTCTCGCTCTGTCACC","chr15",30835672,"-","2:C>T","AGG","CGGAGTCTCGCTCTGTCACCAGG",1,3,NA,NA,"","",FALSE,"ADRA1A"

"3445","CGAAGTCTCGCTCTGTCACC","chr1",29492534,"-","2:C>T","AGG","CGGAGTCTCGCTCTGTCACCAGG",1,3,NA,NA,"","",FALSE,"ADRA1A"

"3446","CGAAGTCTCGCTCTGTCACC","chr16",21492182,"-","2:C>T","AGG","CGGAGTCTCGCTCTGTCACCAGG",1,3,NA,NA,"GeneID:100271836","SMG1P3",FALSE,"ADRA1A"

"3447","CGAAGTCTCGCTCTGTCACC","chr16",21923672,"-","2:C>T","AGG","CGGAGTCTCGCTCTGTCACCAGG",1,3,NA,NA,"","",FALSE,"ADRA1A"

"3448","CGAAGTCTCGCTCTGTCACC","chr7",812383,"-","2:C>T","AGG","CGGAGTCTCGCTCTGTCACCAGG",1,3,NA,NA,"","",FALSE,"ADRA1A"

"3449","CGAAGTCTCGCTCTGTCACC","chr7",29771971,"-","2:C>T","AGG","CGGAGTCTCGCTCTGTCACCAGG",1,3,NA,NA,"","",FALSE,"ADRA1A"

"3450","CGAAGTCTCGCTCTGTCACC","chr5",108478100,"-","2:C>T","AGG","CGGAGTCTCGCTCTGTCACCAGG",1,3,NA,NA,"","",FALSE,"ADRA1A"

"3451","CGAAGTCTCGCTCTGTCACC","chr3",183226417,"-","2:C>T","AGG","CGGAGTCTCGCTCTGTCACCAGG",1,3,NA,NA,"GeneID:23101","MCF2L2",TRUE,"ADRA1A"

"3452","CGAAGTCTCGCTCTGTCACC","chr15",63577726,"-","2:C>T","AGG","CGGAGTCTCGCTCTGTCACCAGG",1,3,NA,NA,"GeneID:9960;GeneID:100130855","USP3;USP3-AS1",FALSE,"ADRA1A"

"3453","CGAAGTCTCGCTCTGTCACC","chr18",62444468,"-","2:C>T","AGG","CGGAGTCTCGCTCTGTCACCAGG",1,3,NA,NA,"","",FALSE,"ADRA1A"

"3454","CGAAGTCTCGCTCTGTCACC","chrX",5198925,"-","2:C>T","AGG","CGGAGTCTCGCTCTGTCACCAGG",1,3,NA,NA,"","",FALSE,"ADRA1A"

"3455","CGAAGTCTCGCTCTGTCACC","chr1",28913412,"-","2:C>T","AGG","CGGAGTCTCGCTCTGTCACCAGG",1,3,NA,NA,"GeneID:2035","EPB41",FALSE,"ADRA1A"

"3456","CGAAGTCTCGCTCTGTCACC","chr4",54640236,"-","2:C>T","AGG","CGGAGTCTCGCTCTGTCACCAGG",1,3,NA,NA,"","",FALSE,"ADRA1A"

"3457","CGAAGTCTCGCTCTGTCACC","chr11",116493211,"-","2:C>T","AGG","CGGAGTCTCGCTCTGTCACCAGG",1,3,NA,NA,"","",FALSE,"ADRA1A"

"3458","CGAAGTCTCGCTCTGTCACC","chr3",26642991,"-","2:C>T","AGG","CGGAGTCTCGCTCTGTCACCAGG",1,3,NA,NA,"GeneID:116135","LRRC3B",FALSE,"ADRA1A"

"3459","CGAAGTCTCGCTCTGTCACC","chr15",76113288,"-","2:C>T","AGG","CGGAGTCTCGCTCTGTCACCAGG",1,3,NA,NA,"GeneID:123591","C15orf27",TRUE,"ADRA1A"

"3460","CGAAGTCTCGCTCTGTCACC","chr6",118757268,"-","2:C>T","AGG","CGGAGTCTCGCTCTGTCACCAGG",1,3,NA,NA,"","",FALSE,"ADRA1A"

"3461","CGAAGTCTCGCTCTGTCACC","chr3",128000646,"-","2:C>T","AGG","CGGAGTCTCGCTCTGTCACCAGG",1,3,NA,NA,"","",FALSE,"ADRA1A"

"3462","CGAAGTCTCGCTCTGTCACC","chr22",42170602,"-","2:C>T","AGG","CGGAGTCTCGCTCTGTCACCAGG",1,3,NA,NA,"GeneID:6942","TCF20",FALSE,"ADRA1A"

"3463","CGAAGTCTCGCTCTGTCACC","chr1",201413402,"-","2:C>T","AGG","CGGAGTCTCGCTCTGTCACCAGG",1,3,NA,NA,"GeneID:7135","TNNI1",FALSE,"ADRA1A"

"3464","CGAAGTCTCGCTCTGTCACC","chr17",42012457,"-","2:C>T","AGG","CGGAGTCTCGCTCTGTCACCAGG",1,3,NA,NA,"GeneID:7266;GeneID:28511","DNAJC7;NKIRAS2",FALSE,"ADRA1A"

"3465","CGAAGTCTCGCTCTGTCACC","chr4",108734848,"-","2:C>T","AGG","CGGAGTCTCGCTCTGTCACCAGG",1,3,NA,NA,"","",FALSE,"ADRA1A"

"3466","CGAAGTCTCGCTCTGTCACC","chr4",8017967,"-","2:C>T","AGG","CGGAGTCTCGCTCTGTCACCAGG",1,3,NA,NA,"GeneID:84448","ABLIM2",FALSE,"ADRA1A"

"3467","CGAAGTCTCGCTCTGTCACC","chr2",32621793,"-","2:C>T","AGG","CGGAGTCTCGCTCTGTCACCAGG",1,3,NA,NA,"","",FALSE,"ADRA1A"

"3468","CGAAGTCTCGCTCTGTCACC","chr6",61852538,"-","2:C>T","AGG","CGGAGTCTCGCTCTGTCACCAGG",1,3,NA,NA,"GeneID:202559","KHDRBS2",FALSE,"ADRA1A"

"3469","CGAAGTCTCGCTCTGTCACC","chr18",72391101,"-","2:C>T","AGG","CGGAGTCTCGCTCTGTCACCAGG",1,3,NA,NA,"","",FALSE,"ADRA1A"

"3470","CGAAGTCTCGCTCTGTCACC","chr3",49127994,"-","2:C>T","AGG","CGGAGTCTCGCTCTGTCACCAGG",1,3,NA,NA,"GeneID:3913","LAMB2",FALSE,"ADRA1A"

"3471","CGAAGTCTCGCTCTGTCACC","chr12",1484279,"-","2:C>T","AGG","CGGAGTCTCGCTCTGTCACCAGG",1,3,NA,NA,"GeneID:23085","ERC1",FALSE,"ADRA1A"

"3472","CGAAGTCTCGCTCTGTCACC","chr2",107936029,"-","2:C>T","AGG","CGGAGTCTCGCTCTGTCACCAGG",1,3,NA,NA,"","",FALSE,"ADRA1A"

"3473","CGAAGTCTCGCTCTGTCACC","chr15",99557157,"-","2:C>T","AGG","CGGAGTCTCGCTCTGTCACCAGG",1,3,NA,NA,"","",FALSE,"ADRA1A"

"3474","CGAAGTCTCGCTCTGTCACC","chr3",102555366,"-","2:C>T","AGG","CGGAGTCTCGCTCTGTCACCAGG",1,3,NA,NA,"","",FALSE,"ADRA1A"

"3475","CGAAGTCTCGCTCTGTCACC","chr17",39505499,"-","2:C>T","AGG","CGGAGTCTCGCTCTGTCACCAGG",1,3,NA,NA,"GeneID:51755","CDK12",FALSE,"ADRA1A"

"3476","CGAAGTCTCGCTCTGTCACC","chr12",116271016,"-","2:C>T","AGG","CGGAGTCTCGCTCTGTCACCAGG",1,3,NA,NA,"GeneID:23389","MED13L",FALSE,"ADRA1A"

"3477","CGAAGTCTCGCTCTGTCACC","chr3",47144617,"-","2:C>T","AGG","CGGAGTCTCGCTCTGTCACCAGG",1,3,NA,NA,"GeneID:29072","SETD2",FALSE,"ADRA1A"

"3478","CGAAGTCTCGCTCTGTCACC","chr16",87380272,"-","2:C>T","AGG","CGGAGTCTCGCTCTGTCACCAGG",1,3,NA,NA,"GeneID:79791","FBXO31",FALSE,"ADRA1A"

"3479","CGAAGTCTCGCTCTGTCACC","chr2",118079454,"-","2:C>T","AGG","CGGAGTCTCGCTCTGTCACCAGG",1,3,NA,NA,"","",FALSE,"ADRA1A"

"3480","CGAAGTCTCGCTCTGTCACC","chr20",13855025,"-","2:C>T","AGG","CGGAGTCTCGCTCTGTCACCAGG",1,3,NA,NA,"GeneID:80343","SEL1L2",FALSE,"ADRA1A"

"3481","CGAAGTCTCGCTCTGTCACC","chr6",81850938,"-","2:C>T","AGG","CGGAGTCTCGCTCTGTCACCAGG",1,3,NA,NA,"","",FALSE,"ADRA1A"

"3482","CGAAGTCTCGCTCTGTCACC","chr7",139309041,"-","2:C>T","AGG","CGGAGTCTCGCTCTGTCACCAGG",1,3,NA,NA,"","",FALSE,"ADRA1A"

"3483","CGAAGTCTCGCTCTGTCACC","chr6",153622773,"-","2:C>T","AGG","CGGAGTCTCGCTCTGTCACCAGG",1,3,NA,NA,"","",FALSE,"ADRA1A"

"3484","CGAAGTCTCGCTCTGTCACC","chr14",80781431,"-","2:C>T","AGG","CGGAGTCTCGCTCTGTCACCAGG",1,3,NA,NA,"GeneID:145508","CEP128",FALSE,"ADRA1A"

"3485","CGAAGTCTCGCTCTGTCACC","chr3",9345011,"-","2:C>T","AGG","CGGAGTCTCGCTCTGTCACCAGG",1,3,NA,NA,"GeneID:9901","SRGAP3",FALSE,"ADRA1A"

"3486","CGAAGTCTCGCTCTGTCACC","chr19",12513130,"-","2:C>T","AGG","CGGAGTCTCGCTCTGTCACCAGG",1,3,NA,NA,"GeneID:163051","ZNF709",FALSE,"ADRA1A"

"3487","CGAAGTCTCGCTCTGTCACC","chr11",6551916,"-","2:C>T","AGG","CGGAGTCTCGCTCTGTCACCAGG",1,3,NA,NA,"GeneID:144132","DNHD1",FALSE,"ADRA1A"

"3488","CGAAGTCTCGCTCTGTCACC","chrX",118959892,"-","2:C>T","AGG","CGGAGTCTCGCTCTGTCACCAGG",1,3,NA,NA,"","",FALSE,"ADRA1A"

"3489","CGAAGTCTCGCTCTGTCACC","chr17",37338202,"-","2:C>T","AGG","CGGAGTCTCGCTCTGTCACCAGG",1,3,NA,NA,"GeneID:31","ACACA",FALSE,"ADRA1A"

"3490","CGAAGTCTCGCTCTGTCACC","chr3",122521897,"-","2:C>T","AGG","CGGAGTCTCGCTCTGTCACCAGG",1,3,NA,NA,"","",FALSE,"ADRA1A"

"3491","CGAAGTCTCGCTCTGTCACC","chr11",1807252,"-","2:C>T","AGG","CGGAGTCTCGCTCTGTCACCAGG",1,3,NA,NA,"","",FALSE,"ADRA1A"

"3492","CGAAGTCTCGCTCTGTCACC","chr2",161966711,"-","2:C>T","AGG","CGGAGTCTCGCTCTGTCACCAGG",1,3,NA,NA,"GeneID:57282","SLC4A10",FALSE,"ADRA1A"

"3493","CGAAGTCTCGCTCTGTCACC","chrX",68569753,"-","2:C>T","AGG","CGGAGTCTCGCTCTGTCACCAGG",1,3,NA,NA,"","",FALSE,"ADRA1A"

"3494","CGAAGTCTCGCTCTGTCACC","chr6",168899540,"-","2:C>T","AGG","CGGAGTCTCGCTCTGTCACCAGG",1,3,NA,NA,"","",FALSE,"ADRA1A"

"3495","CGAAGTCTCGCTCTGTCACC","chr3",134461887,"-","2:C>T","AGG","CGGAGTCTCGCTCTGTCACCAGG",1,3,NA,NA,"","",FALSE,"ADRA1A"

"3496","CGAAGTCTCGCTCTGTCACC","chr1",52444208,"-","2:C>T","AGG","CGGAGTCTCGCTCTGTCACCAGG",1,3,NA,NA,"GeneID:23318","ZCCHC11",FALSE,"ADRA1A"

"3497","CGAAGTCTCGCTCTGTCACC","chr7",100593602,"-","2:C>T","AGG","CGGAGTCTCGCTCTGTCACCAGG",1,3,NA,NA,"GeneID:26261;GeneID:100129845","FBXO24;PCOLCE-AS1",FALSE,"ADRA1A"

"3498","CGAAGTCTCGCTCTGTCACC","chr3",55303838,"-","2:C>T","AGG","CGGAGTCTCGCTCTGTCACCAGG",1,3,NA,NA,"","",FALSE,"ADRA1A"

"3499","CGAAGTCTCGCTCTGTCACC","chr21",21410981,"-","2:C>T","AGG","CGGAGTCTCGCTCTGTCACCAGG",1,3,NA,NA,"GeneID:4685","NCAM2",FALSE,"ADRA1A"

"3500","CGAAGTCTCGCTCTGTCACC","chr18",12733047,"-","2:C>T","AGG","CGGAGTCTCGCTCTGTCACCAGG",1,3,NA,NA,"","",FALSE,"ADRA1A"

"3501","CGAAGTCTCGCTCTGTCACC","chr9",98794124,"-","2:C>T","AGG","CGGAGTCTCGCTCTGTCACCAGG",1,3,NA,NA,"GeneID:203286","ANKS6",FALSE,"ADRA1A"

"3502","CGAAGTCTCGCTCTGTCACC","chr2",241251951,"-","2:C>T","AGG","CGGAGTCTCGCTCTGTCACCAGG",1,3,NA,NA,"GeneID:3069","HDLBP",FALSE,"ADRA1A"

"3503","CGAAGTCTCGCTCTGTCACC","chr2",102264806,"-","2:C>T","AGG","CGGAGTCTCGCTCTGTCACCAGG",1,3,NA,NA,"","",FALSE,"ADRA1A"

"3504","CGAAGTCTCGCTCTGTCACC","chr7",116916600,"-","2:C>T","AGG","CGGAGTCTCGCTCTGTCACCAGG",1,3,NA,NA,"GeneID:830","CAPZA2",FALSE,"ADRA1A"

"3505","CGAAGTCTCGCTCTGTCACC","chr4",82307298,"-","2:C>T","AGG","CGGAGTCTCGCTCTGTCACCAGG",1,3,NA,NA,"","",FALSE,"ADRA1A"

"3506","CGAAGTCTCGCTCTGTCACC","chr5",128350525,"-","2:C>T","AGG","CGGAGTCTCGCTCTGTCACCAGG",1,3,NA,NA,"GeneID:2201","FBN2",FALSE,"ADRA1A"

"3507","CGAAGTCTCGCTCTGTCACC","chr16",3353465,"-","2:C>T","AGG","CGGAGTCTCGCTCTGTCACCAGG",1,3,NA,NA,"","",FALSE,"ADRA1A"

"3508","CGAAGTCTCGCTCTGTCACC","chr6",30722417,"-","2:C>T","AGG","CGGAGTCTCGCTCTGTCACCAGG",1,3,NA,NA,"GeneID:203068","TUBB",FALSE,"ADRA1A"

"3509","CGAAGTCTCGCTCTGTCACC","chr22",20497257,"-","2:C>T","AGG","CGGAGTCTCGCTCTGTCACCAGG",1,3,NA,NA,"GeneID:51586","MED15",FALSE,"ADRA1A"

"3510","CGAAGTCTCGCTCTGTCACC","chr3",122792845,"-","2:C>T","AGG","CGGAGTCTCGCTCTGTCACCAGG",1,3,NA,NA,"GeneID:79663","HSPBAP1",FALSE,"ADRA1A"

"3511","CGAAGTCTCGCTCTGTCACC","chr4",21711456,"-","2:C>T","AGG","CGGAGTCTCGCTCTGTCACCAGG",1,3,NA,NA,"GeneID:80333","KCNIP4",FALSE,"ADRA1A"

"3512","CGAAGTCTCGCTCTGTCACC","chr13",71679917,"-","2:C>T","AGG","CGGAGTCTCGCTCTGTCACCAGG",1,3,NA,NA,"GeneID:1602","DACH1",FALSE,"ADRA1A"

"3513","CGAAGTCTCGCTCTGTCACC","chr9",91790484,"-","2:C>T","AGG","CGGAGTCTCGCTCTGTCACCAGG",1,3,NA,NA,"GeneID:4920","ROR2",FALSE,"ADRA1A"

"3514","CGAAGTCTCGCTCTGTCACC","chr9",4819781,"-","2:C>T","AGG","CGGAGTCTCGCTCTGTCACCAGG",1,3,NA,NA,"GeneID:10171","RCL1",FALSE,"ADRA1A"

"3515","CGAAGTCTCGCTCTGTCACC","chr6",41970313,"-","2:C>T","AGG","CGGAGTCTCGCTCTGTCACCAGG",1,3,NA,NA,"GeneID:896","CCND3",FALSE,"ADRA1A"

"3516","CGAAGTCTCGCTCTGTCACC","chr14",22790972,"-","2:C>T","AGG","CGGAGTCTCGCTCTGTCACCAGG",1,3,NA,NA,"GeneID:9056","SLC7A7",FALSE,"ADRA1A"

"3517","CGAAGTCTCGCTCTGTCACC","chr19",46480439,"-","2:C>T","AGG","CGGAGTCTCGCTCTGTCACCAGG",1,3,NA,NA,"","",FALSE,"ADRA1A"

"3518","CGAAGTCTCGCTCTGTCACC","chr9",71306847,"-","2:C>T","AGG","CGGAGTCTCGCTCTGTCACCAGG",1,3,NA,NA,"GeneID:80036","TRPM3",FALSE,"ADRA1A"

"3519","CGAAGTCTCGCTCTGTCACC","chr18",23854959,"-","2:C>T","AGG","CGGAGTCTCGCTCTGTCACCAGG",1,3,NA,NA,"GeneID:3909","LAMA3",FALSE,"ADRA1A"

"3520","CGAAGTCTCGCTCTGTCACC","chr1",177898922,"-","2:C>T","AGG","CGGAGTCTCGCTCTGTCACCAGG",1,3,NA,NA,"","",FALSE,"ADRA1A"

"3521","CGAAGTCTCGCTCTGTCACC","chr2",227373903,"-","2:C>T","AGG","CGGAGTCTCGCTCTGTCACCAGG",1,3,NA,NA,"GeneID:79853","TM4SF20",FALSE,"ADRA1A"

"3522","CGAAGTCTCGCTCTGTCACC","chr16",28689921,"-","2:C>T","AGG","CGGAGTCTCGCTCTGTCACCAGG",1,3,NA,NA,"GeneID:8663","EIF3C",FALSE,"ADRA1A"

"3523","CGAAGTCTCGCTCTGTCACC","chr16",28804107,"-","2:C>T","AGG","CGGAGTCTCGCTCTGTCACCAGG",1,3,NA,NA,"","",FALSE,"ADRA1A"

"3524","CGAAGTCTCGCTCTGTCACC","chr20",52144554,"-","2:C>T","AGG","CGGAGTCTCGCTCTGTCACCAGG",1,3,NA,NA,"GeneID:55734","ZFP64",FALSE,"ADRA1A"

"3525","CGAAGTCTCGCTCTGTCACC","chr11",118424817,"-","2:C>T","AGG","CGGAGTCTCGCTCTGTCACCAGG",1,3,NA,NA,"GeneID:10632","ATP5L",FALSE,"ADRA1A"

"3526","CGAAGTCTCGCTCTGTCACC","chr19",13791248,"-","2:C>T","AGG","CGGAGTCTCGCTCTGTCACCAGG",1,3,NA,NA,"","",FALSE,"ADRA1A"

"3527","CGAAGTCTCGCTCTGTCACC","chr3",4465453,"-","2:C>T","AGG","CGGAGTCTCGCTCTGTCACCAGG",1,3,NA,NA,"GeneID:285362","SUMF1",FALSE,"ADRA1A"

"3528","CGAAGTCTCGCTCTGTCACC","chr20",44117253,"-","2:C>T","AGG","CGGAGTCTCGCTCTGTCACCAGG",1,3,NA,NA,"GeneID:57158","JPH2",FALSE,"ADRA1A"

"3529","CGAAGTCTCGCTCTGTCACC","chr10",11777662,"-","2:C>T","AGG","CGGAGTCTCGCTCTGTCACCAGG",1,3,NA,NA,"","",FALSE,"ADRA1A"

"3530","CGAAGTCTCGCTCTGTCACC","chr8",25356174,"-","2:C>T","AGG","CGGAGTCTCGCTCTGTCACCAGG",1,3,NA,NA,"GeneID:80005","DOCK5",FALSE,"ADRA1A"

"3531","CGAAGTCTCGCTCTGTCACC","chr15",26966888,"-","2:C>T","AGG","CGGAGTCTCGCTCTGTCACCAGG",1,3,NA,NA,"","",FALSE,"ADRA1A"

"3532","CGAAGTCTCGCTCTGTCACC","chr13",31003560,"-","2:C>T","AGG","CGGAGTCTCGCTCTGTCACCAGG",1,3,NA,NA,"","",FALSE,"ADRA1A"

"3533","CGAAGTCTCGCTCTGTCACC","chrX",43905953,"-","2:C>T","AGG","CGGAGTCTCGCTCTGTCACCAGG",1,3,NA,NA,"","",FALSE,"ADRA1A"

"3534","CGAAGTCTCGCTCTGTCACC","chr9",130762885,"-","2:C>T","AGG","CGGAGTCTCGCTCTGTCACCAGG",1,3,NA,NA,"GeneID:25","ABL1",FALSE,"ADRA1A"

"3535","CGAAGTCTCGCTCTGTCACC","chr22",38367441,"-","2:C>T","AGG","CGGAGTCTCGCTCTGTCACCAGG",1,3,NA,NA,"GeneID:102800317","CSNK1E",FALSE,"ADRA1A"

"3536","CGAAGTCTCGCTCTGTCACC","chr10",107008971,"-","2:C>T","AGG","CGGAGTCTCGCTCTGTCACCAGG",1,3,NA,NA,"GeneID:114815","SORCS1",FALSE,"ADRA1A"

"3537","CGAAGTCTCGCTCTGTCACC","chr19",22711252,"-","2:C>T","AGG","CGGAGTCTCGCTCTGTCACCAGG",1,3,NA,NA,"","",FALSE,"ADRA1A"

"3538","CGAAGTCTCGCTCTGTCACC","chr6",152956602,"-","2:C>T","AGG","CGGAGTCTCGCTCTGTCACCAGG",1,3,NA,NA,"","",FALSE,"ADRA1A"

"3539","CGAAGTCTCGCTCTGTCACC","chrX",136202730,"-","2:C>T","AGG","CGGAGTCTCGCTCTGTCACCAGG",1,3,NA,NA,"GeneID:2273","FHL1",FALSE,"ADRA1A"

"3540","CGAAGTCTCGCTCTGTCACC","chr6",142157138,"-","2:C>T","AGG","CGGAGTCTCGCTCTGTCACCAGG",1,3,NA,NA,"GeneID:51534","VTA1",FALSE,"ADRA1A"

"3541","CGAAGTCTCGCTCTGTCACC","chr17",44491462,"-","2:C>T","AGG","CGGAGTCTCGCTCTGTCACCAGG",1,3,NA,NA,"GeneID:23131","GPATCH8",FALSE,"ADRA1A"

"3542","CGAAGTCTCGCTCTGTCACC","chr4",15991910,"-","2:C>T","AGG","CGGAGTCTCGCTCTGTCACCAGG",1,3,NA,NA,"GeneID:8842","PROM1",FALSE,"ADRA1A"

"3543","CGAAGTCTCGCTCTGTCACC","chr8",127957542,"-","2:C>T","AGG","CGGAGTCTCGCTCTGTCACCAGG",1,3,NA,NA,"GeneID:5820","PVT1",FALSE,"ADRA1A"

"3544","CGAAGTCTCGCTCTGTCACC","chr15",41190455,"-","2:C>T","AGG","CGGAGTCTCGCTCTGTCACCAGG",1,3,NA,NA,"GeneID:161829","EXD1",FALSE,"ADRA1A"

"3545","CGAAGTCTCGCTCTGTCACC","chr13",61054351,"-","2:C>T","AGG","CGGAGTCTCGCTCTGTCACCAGG",1,3,NA,NA,"","",FALSE,"ADRA1A"

"3546","CGAAGTCTCGCTCTGTCACC","chr2",106445756,"-","2:C>T","AGG","CGGAGTCTCGCTCTGTCACCAGG",1,3,NA,NA,"GeneID:653489","RGPD3",FALSE,"ADRA1A"

"3547","CGAAGTCTCGCTCTGTCACC","chr2",87805054,"-","2:C>T","AGG","CGGAGTCTCGCTCTGTCACCAGG",1,3,NA,NA,"GeneID:729857","RGPD2",FALSE,"ADRA1A"

"3548","CGAAGTCTCGCTCTGTCACC","chr14",22801749,"-","2:C>T","AGG","CGGAGTCTCGCTCTGTCACCAGG",1,3,NA,NA,"GeneID:9056","SLC7A7",FALSE,"ADRA1A"

"3549","CGAAGTCTCGCTCTGTCACC","chr7",77390923,"-","2:C>T","AGG","CGGAGTCTCGCTCTGTCACCAGG",1,3,NA,NA,"GeneID:54103","GSAP",FALSE,"ADRA1A"

"3550","CGAAGTCTCGCTCTGTCACC","chr1",206755856,"-","2:C>T","AGG","CGGAGTCTCGCTCTGTCACCAGG",1,3,NA,NA,"","",FALSE,"ADRA1A"

"3551","CGAAGTCTCGCTCTGTCACC","chr18",48940726,"-","2:C>T","AGG","CGGAGTCTCGCTCTGTCACCAGG",1,3,NA,NA,"GeneID:4092","SMAD7",FALSE,"ADRA1A"

"3552","CGAAGTCTCGCTCTGTCACC","chr4",82878468,"-","2:C>T","AGG","CGGAGTCTCGCTCTGTCACCAGG",1,3,NA,NA,"GeneID:22872","SEC31A",FALSE,"ADRA1A"

"3553","CGAAGTCTCGCTCTGTCACC","chr14",68886768,"-","2:C>T","AGG","CGGAGTCTCGCTCTGTCACCAGG",1,3,NA,NA,"GeneID:87","ACTN1",TRUE,"ADRA1A"

"3554","CGAAGTCTCGCTCTGTCACC","chr3",187920679,"-","2:C>T","AGG","CGGAGTCTCGCTCTGTCACCAGG",1,3,NA,NA,"","",FALSE,"ADRA1A"

"3555","CGAAGTCTCGCTCTGTCACC","chr14",35797327,"-","2:C>T","AGG","CGGAGTCTCGCTCTGTCACCAGG",1,3,NA,NA,"GeneID:253959","RALGAPA1",FALSE,"ADRA1A"

"3556","CGAAGTCTCGCTCTGTCACC","chr19",48779538,"-","2:C>T","AGG","CGGAGTCTCGCTCTGTCACCAGG",1,3,NA,NA,"","",FALSE,"ADRA1A"

"3557","CGAAGTCTCGCTCTGTCACC","chr3",156588436,"-","2:C>T","AGG","CGGAGTCTCGCTCTGTCACCAGG",1,3,NA,NA,"","",FALSE,"ADRA1A"

"3558","CGAAGTCTCGCTCTGTCACC","chr19",6783154,"-","2:C>T","AGG","CGGAGTCTCGCTCTGTCACCAGG",1,3,NA,NA,"GeneID:7409","VAV1",FALSE,"ADRA1A"

"3559","CGAAGTCTCGCTCTGTCACC","chr17",1887533,"-","2:C>T","AGG","CGGAGTCTCGCTCTGTCACCAGG",1,3,NA,NA,"GeneID:6117","RPA1",FALSE,"ADRA1A"

"3560","CGAAGTCTCGCTCTGTCACC","chr2",29231311,"-","2:C>T","AGG","CGGAGTCTCGCTCTGTCACCAGG",1,3,NA,NA,"GeneID:238","ALK",FALSE,"ADRA1A"

"3561","CGAAGTCTCGCTCTGTCACC","chr9",26992011,"-","2:C>T","AGG","CGGAGTCTCGCTCTGTCACCAGG",1,3,NA,NA,"GeneID:80173","IFT74",FALSE,"ADRA1A"

"3562","CGAAGTCTCGCTCTGTCACC","chr20",37097899,"-","2:C>T","AGG","CGGAGTCTCGCTCTGTCACCAGG",1,3,NA,NA,"","",FALSE,"ADRA1A"

"3563","CGAAGTCTCGCTCTGTCACC","chr17",76837109,"-","2:C>T","AGG","CGGAGTCTCGCTCTGTCACCAGG",1,3,NA,NA,"","",FALSE,"ADRA1A"

"3564","CGAAGTCTCGCTCTGTCACC","chr8",122634243,"-","2:C>T","AGG","CGGAGTCTCGCTCTGTCACCAGG",1,3,NA,NA,"","",FALSE,"ADRA1A"

"3565","CGAAGTCTCGCTCTGTCACC","chr9",34205966,"-","2:C>T","AGG","CGGAGTCTCGCTCTGTCACCAGG",1,3,NA,NA,"GeneID:51271","UBAP1",FALSE,"ADRA1A"

"3566","CGAAGTCTCGCTCTGTCACC","chr2",144307135,"-","2:C>T","AGG","CGGAGTCTCGCTCTGTCACCAGG",1,3,NA,NA,"GeneID:79712","GTDC1",FALSE,"ADRA1A"

"3567","CGAAGTCTCGCTCTGTCACC","chr5",17067635,"-","2:C>T","AGG","CGGAGTCTCGCTCTGTCACCAGG",1,3,NA,NA,"GeneID:10409","BASP1",FALSE,"ADRA1A"

"3568","CGAAGTCTCGCTCTGTCACC","chr12",118226508,"-","2:C>T","AGG","CGGAGTCTCGCTCTGTCACCAGG",1,3,NA,NA,"GeneID:51347","TAOK3",FALSE,"ADRA1A"

"3569","CGAAGTCTCGCTCTGTCACC","chr1",62147773,"-","2:C>T","AGG","CGGAGTCTCGCTCTGTCACCAGG",1,3,NA,NA,"GeneID:10207","INADL",FALSE,"ADRA1A"

"3570","CGAAGTCTCGCTCTGTCACC","chr1",2114748,"-","2:C>T","AGG","CGGAGTCTCGCTCTGTCACCAGG",1,3,NA,NA,"GeneID:5590","PRKCZ",FALSE,"ADRA1A"

"3571","CGAAGTCTCGCTCTGTCACC","chr8",97670598,"-","2:C>T","AGG","CGGAGTCTCGCTCTGTCACCAGG",1,3,NA,NA,"GeneID:92140","MTDH",FALSE,"ADRA1A"

"3572","CGAAGTCTCGCTCTGTCACC","chr12",56647501,"-","2:C>T","AGG","CGGAGTCTCGCTCTGTCACCAGG",1,3,NA,NA,"","",FALSE,"ADRA1A"

"3573","CGAAGTCTCGCTCTGTCACC","chr4",88555257,"-","2:C>T","AGG","CGGAGTCTCGCTCTGTCACCAGG",1,3,NA,NA,"","",FALSE,"ADRA1A"

"3574","CGAAGTCTCGCTCTGTCACC","chr3",147473487,"-","2:C>T","AGG","CGGAGTCTCGCTCTGTCACCAGG",1,3,NA,NA,"GeneID:7545","ZIC1",FALSE,"ADRA1A"

"3575","CGAAGTCTCGCTCTGTCACC","chr6",83187778,"-","2:C>T","AGG","CGGAGTCTCGCTCTGTCACCAGG",1,3,NA,NA,"GeneID:5238","PGM3",FALSE,"ADRA1A"

"3576","CGAAGTCTCGCTCTGTCACC","chr14",67152939,"-","2:C>T","AGG","CGGAGTCTCGCTCTGTCACCAGG",1,3,NA,NA,"GeneID:10243","GPHN",FALSE,"ADRA1A"

"3577","CGAAGTCTCGCTCTGTCACC","chrX",140082879,"-","2:C>T","AGG","CGGAGTCTCGCTCTGTCACCAGG",1,3,NA,NA,"","",FALSE,"ADRA1A"

"3578","CGAAGTCTCGCTCTGTCACC","chr7",72103047,"-","2:C>T","AGG","CGGAGTCTCGCTCTGTCACCAGG",1,3,NA,NA,"GeneID:83698","CALN1",FALSE,"ADRA1A"

"3579","CGAAGTCTCGCTCTGTCACC","chr6",43426995,"-","2:C>T","AGG","CGGAGTCTCGCTCTGTCACCAGG",1,3,NA,NA,"","",FALSE,"ADRA1A"

"3580","CGAAGTCTCGCTCTGTCACC","chr15",27831260,"-","2:C>T","AGG","CGGAGTCTCGCTCTGTCACCAGG",1,3,NA,NA,"GeneID:4948","OCA2",FALSE,"ADRA1A"

"3581","CGAAGTCTCGCTCTGTCACC","chr14",85185013,"-","2:C>T","AGG","CGGAGTCTCGCTCTGTCACCAGG",1,3,NA,NA,"","",FALSE,"ADRA1A"

"3582","CGAAGTCTCGCTCTGTCACC","chr5",109422663,"-","2:C>T","AGG","CGGAGTCTCGCTCTGTCACCAGG",1,3,NA,NA,"","",FALSE,"ADRA1A"

"3583","CGAAGTCTCGCTCTGTCACC","chr7",107162428,"-","2:C>T","AGG","CGGAGTCTCGCTCTGTCACCAGG",1,3,NA,NA,"","",FALSE,"ADRA1A"

"3584","CGAAGTCTCGCTCTGTCACC","chr6",16395989,"-","2:C>T","AGG","CGGAGTCTCGCTCTGTCACCAGG",1,3,NA,NA,"GeneID:6310","ATXN1",FALSE,"ADRA1A"

"3585","CGAAGTCTCGCTCTGTCACC","chr17",39988081,"-","2:C>T","AGG","CGGAGTCTCGCTCTGTCACCAGG",1,3,NA,NA,"GeneID:5709","PSMD3",FALSE,"ADRA1A"

"3586","CGAAGTCTCGCTCTGTCACC","chr10",101497994,"-","2:C>T","AGG","CGGAGTCTCGCTCTGTCACCAGG",1,3,NA,NA,"GeneID:8945","BTRC",FALSE,"ADRA1A"

"3587","CGAAGTCTCGCTCTGTCACC","chr3",193474298,"-","2:C>T","AGG","CGGAGTCTCGCTCTGTCACCAGG",1,3,NA,NA,"GeneID:84239","ATP13A4",FALSE,"ADRA1A"

"3588","CGAAGTCTCGCTCTGTCACC","chr7",9729497,"-","2:C>T","AGG","CGGAGTCTCGCTCTGTCACCAGG",1,3,NA,NA,"","",FALSE,"ADRA1A"

"3589","CGAAGTCTCGCTCTGTCACC","chr21",41607995,"-","2:C>T","AGG","CGGAGTCTCGCTCTGTCACCAGG",1,3,NA,NA,"","",FALSE,"ADRA1A"

"3590","CGAAGTCTCGCTCTGTCACC","chr8",74115848,"-","2:C>T","AGG","CGGAGTCTCGCTCTGTCACCAGG",1,3,NA,NA,"","",FALSE,"ADRA1A"

"3591","CGAAGTCTCGCTCTGTCACC","chr13",19479936,"-","2:C>T","AGG","CGGAGTCTCGCTCTGTCACCAGG",1,3,NA,NA,"GeneID:93492","TPTE2",FALSE,"ADRA1A"

"3592","CGAAGTCTCGCTCTGTCACC","chr18",26943082,"-","2:C>T","AGG","CGGAGTCTCGCTCTGTCACCAGG",1,3,NA,NA,"GeneID:83539","CHST9",FALSE,"ADRA1A"

"3593","CGAAGTCTCGCTCTGTCACC","chr11",8434222,"-","2:C>T","AGG","CGGAGTCTCGCTCTGTCACCAGG",1,3,NA,NA,"GeneID:65975","STK33",TRUE,"ADRA1A"

"3594","CGAAGTCTCGCTCTGTCACC","chr4",56751026,"-","2:C>T","AGG","CGGAGTCTCGCTCTGTCACCAGG",1,3,NA,NA,"","",FALSE,"ADRA1A"

"3595","CGAAGTCTCGCTCTGTCACC","chr16",10777985,"-","2:C>T","AGG","CGGAGTCTCGCTCTGTCACCAGG",1,3,NA,NA,"GeneID:780776","TVP23A",FALSE,"ADRA1A"

"3596","CGAAGTCTCGCTCTGTCACC","chr4",94989363,"-","2:C>T","AGG","CGGAGTCTCGCTCTGTCACCAGG",1,3,NA,NA,"GeneID:658","BMPR1B",FALSE,"ADRA1A"

"3597","CGAAGTCTCGCTCTGTCACC","chr20",63852215,"-","2:C>T","AGG","CGGAGTCTCGCTCTGTCACCAGG",1,3,NA,NA,"","",FALSE,"ADRA1A"

"3598","CGAAGTCTCGCTCTGTCACC","chrX",112321510,"-","2:C>T","AGG","CGGAGTCTCGCTCTGTCACCAGG",1,3,NA,NA,"","",FALSE,"ADRA1A"

"3599","CGAAGTCTCGCTCTGTCACC","chr1",2009093,"-","2:C>T","AGG","CGGAGTCTCGCTCTGTCACCAGG",1,3,NA,NA,"","",FALSE,"ADRA1A"

"3600","CGAAGTCTCGCTCTGTCACC","chr18",31939058,"-","2:C>T","AGG","CGGAGTCTCGCTCTGTCACCAGG",1,3,NA,NA,"GeneID:22878","TRAPPC8",FALSE,"ADRA1A"

"3601","CGAAGTCTCGCTCTGTCACC","chr16",57442320,"-","2:C>T","AGG","CGGAGTCTCGCTCTGTCACCAGG",1,3,NA,NA,"GeneID:57019","CIAPIN1",FALSE,"ADRA1A"

"3602","CGAAGTCTCGCTCTGTCACC","chr10",14964072,"-","2:C>T","AGG","CGGAGTCTCGCTCTGTCACCAGG",1,3,NA,NA,"GeneID:644890","MEIG1",FALSE,"ADRA1A"

"3603","CGAAGTCTCGCTCTGTCACC","chr19",41963840,"-","2:C>T","AGG","CGGAGTCTCGCTCTGTCACCAGG",1,3,NA,NA,"","",FALSE,"ADRA1A"

"3604","CGAAGTCTCGCTCTGTCACC","chr15",83085557,"-","2:C>T","AGG","CGGAGTCTCGCTCTGTCACCAGG",1,3,NA,NA,"","",FALSE,"ADRA1A"

"3605","CGAAGTCTCGCTCTGTCACC","chr1",64156681,"-","2:C>T","AGG","CGGAGTCTCGCTCTGTCACCAGG",1,3,NA,NA,"GeneID:4919;GeneID:101927034","ROR1;ROR1-AS1",FALSE,"ADRA1A"

"3606","CGAAGTCTCGCTCTGTCACC","chr1",23262985,"-","2:C>T","AGG","CGGAGTCTCGCTCTGTCACCAGG",1,3,NA,NA,"","",FALSE,"ADRA1A"

"3607","CGAAGTCTCGCTCTGTCACC","chr7",122608190,"-","2:C>T","AGG","CGGAGTCTCGCTCTGTCACCAGG",1,3,NA,NA,"GeneID:93664","CADPS2",FALSE,"ADRA1A"

"3608","CGAAGTCTCGCTCTGTCACC","chr6",15795788,"-","2:C>T","AGG","CGGAGTCTCGCTCTGTCACCAGG",1,3,NA,NA,"","",FALSE,"ADRA1A"

"3609","CGAAGTCTCGCTCTGTCACC","chr21",40670512,"-","2:C>T","AGG","CGGAGTCTCGCTCTGTCACCAGG",1,3,NA,NA,"GeneID:1826","DSCAM",FALSE,"ADRA1A"

"3610","CGAAGTCTCGCTCTGTCACC","chr1",117103927,"-","2:C>T","AGG","CGGAGTCTCGCTCTGTCACCAGG",1,3,NA,NA,"GeneID:8458","TTF2",TRUE,"ADRA1A"

"3611","CGAAGTCTCGCTCTGTCACC","chr4",75682958,"-","2:C>T","AGG","CGGAGTCTCGCTCTGTCACCAGG",1,3,NA,NA,"GeneID:9908","G3BP2",FALSE,"ADRA1A"

"3612","CGAAGTCTCGCTCTGTCACC","chr10",37586652,"-","2:C>T","AGG","CGGAGTCTCGCTCTGTCACCAGG",1,3,NA,NA,"","",FALSE,"ADRA1A"

"3613","CGAAGTCTCGCTCTGTCACC","chr2",37238425,"-","2:C>T","AGG","CGGAGTCTCGCTCTGTCACCAGG",1,3,NA,NA,"GeneID:55471","NDUFAF7",FALSE,"ADRA1A"

"3614","CGAAGTCTCGCTCTGTCACC","chr2",22845181,"-","2:C>T","AGG","CGGAGTCTCGCTCTGTCACCAGG",1,3,NA,NA,"","",FALSE,"ADRA1A"

"3615","CGAAGTCTCGCTCTGTCACC","chr19",51478173,"-","2:C>T","AGG","CGGAGTCTCGCTCTGTCACCAGG",1,3,NA,NA,"","",FALSE,"ADRA1A"

"3616","CGAAGTCTCGCTCTGTCACC","chr7",87910037,"-","2:C>T","AGG","CGGAGTCTCGCTCTGTCACCAGG",1,3,NA,NA,"","",FALSE,"ADRA1A"

"3617","CGAAGTCTCGCTCTGTCACC","chr20",43783486,"-","2:C>T","AGG","CGGAGTCTCGCTCTGTCACCAGG",1,3,NA,NA,"","",FALSE,"ADRA1A"

"3618","CGAAGTCTCGCTCTGTCACC","chr8",104799858,"-","2:C>T","AGG","CGGAGTCTCGCTCTGTCACCAGG",1,3,NA,NA,"GeneID:23414","ZFPM2",FALSE,"ADRA1A"

"3619","CGAAGTCTCGCTCTGTCACC","chr3",132752339,"-","2:C>T","AGG","CGGAGTCTCGCTCTGTCACCAGG",1,3,NA,NA,"GeneID:348808","NPHP3-AS1",FALSE,"ADRA1A"

"3620","CGAAGTCTCGCTCTGTCACC","chr8",54284786,"-","2:C>T","AGG","CGGAGTCTCGCTCTGTCACCAGG",1,3,NA,NA,"","",FALSE,"ADRA1A"

"3621","CGAAGTCTCGCTCTGTCACC","chr21",29089144,"-","2:C>T","AGG","CGGAGTCTCGCTCTGTCACCAGG",1,3,NA,NA,"GeneID:56911","MAP3K7CL",FALSE,"ADRA1A"

"3622","CGAAGTCTCGCTCTGTCACC","chr6",160901764,"-","2:C>T","AGG","CGGAGTCTCGCTCTGTCACCAGG",1,3,NA,NA,"","",FALSE,"ADRA1A"

"3623","CGAAGTCTCGCTCTGTCACC","chr15",44429841,"-","2:C>T","AGG","CGGAGTCTCGCTCTGTCACCAGG",1,3,NA,NA,"GeneID:51496","CTDSPL2",FALSE,"ADRA1A"

"3624","CGAAGTCTCGCTCTGTCACC","chr7",31262238,"-","2:C>T","AGG","CGGAGTCTCGCTCTGTCACCAGG",1,3,NA,NA,"","",FALSE,"ADRA1A"

"3625","CGAAGTCTCGCTCTGTCACC","chr9",37449018,"-","2:C>T","AGG","CGGAGTCTCGCTCTGTCACCAGG",1,3,NA,NA,"GeneID:9925","ZBTB5",FALSE,"ADRA1A"

"3626","CGAAGTCTCGCTCTGTCACC","chr22",40910982,"-","2:C>T","AGG","CGGAGTCTCGCTCTGTCACCAGG",1,3,NA,NA,"GeneID:63929","XPNPEP3",FALSE,"ADRA1A"

"3627","CGAAGTCTCGCTCTGTCACC","chr10",110127591,"-","2:C>T","AGG","CGGAGTCTCGCTCTGTCACCAGG",1,3,NA,NA,"GeneID:120","ADD3",FALSE,"ADRA1A"

"3628","CGAAGTCTCGCTCTGTCACC","chr6",17641509,"-","2:C>T","AGG","CGGAGTCTCGCTCTGTCACCAGG",1,3,NA,NA,"GeneID:9972","NUP153",FALSE,"ADRA1A"

"3629","CGAAGTCTCGCTCTGTCACC","chr1",220072337,"-","2:C>T","AGG","CGGAGTCTCGCTCTGTCACCAGG",1,3,NA,NA,"GeneID:10380","BPNT1",FALSE,"ADRA1A"

"3630","CGAAGTCTCGCTCTGTCACC","chr7",101291289,"-","2:C>T","AGG","CGGAGTCTCGCTCTGTCACCAGG",1,3,NA,NA,"","",FALSE,"ADRA1A"

"3631","CGAAGTCTCGCTCTGTCACC","chr10",37823308,"-","2:C>T","AGG","CGGAGTCTCGCTCTGTCACCAGG",1,3,NA,NA,"GeneID:57209","ZNF248",FALSE,"ADRA1A"

"3632","CGAAGTCTCGCTCTGTCACC","chr5",3337699,"-","2:C>T","AGG","CGGAGTCTCGCTCTGTCACCAGG",1,3,NA,NA,"","",FALSE,"ADRA1A"

"3633","CGAAGTCTCGCTCTGTCACC","chr10",94329752,"-","2:C>T","AGG","CGGAGTCTCGCTCTGTCACCAGG",1,3,NA,NA,"GeneID:51196","PLCE1",TRUE,"ADRA1A"

"3634","CGAAGTCTCGCTCTGTCACC","chr5",73778053,"-","2:C>T","AGG","CGGAGTCTCGCTCTGTCACCAGG",1,3,NA,NA,"GeneID:64283","ARHGEF28",TRUE,"ADRA1A"

"3635","CGAAGTCTCGCTCTGTCACC","chr4",352361,"-","2:C>T","AGG","CGGAGTCTCGCTCTGTCACCAGG",1,3,NA,NA,"GeneID:7700","ZNF141",FALSE,"ADRA1A"

"3636","CGAAGTCTCGCTCTGTCACC","chr5",173073941,"-","2:C>T","AGG","CGGAGTCTCGCTCTGTCACCAGG",1,3,NA,NA,"GeneID:153222","CREBRF",FALSE,"ADRA1A"

"3637","CGAAGTCTCGCTCTGTCACC","chr5",142189411,"-","2:C>T","AGG","CGGAGTCTCGCTCTGTCACCAGG",1,3,NA,NA,"","",FALSE,"ADRA1A"

"3638","CGAAGTCTCGCTCTGTCACC","chr2",134997793,"-","2:C>T","AGG","CGGAGTCTCGCTCTGTCACCAGG",1,3,NA,NA,"GeneID:80122","MAP3K19",FALSE,"ADRA1A"

"3639","CGAAGTCTCGCTCTGTCACC","chr20",58324841,"-","2:C>T","AGG","CGGAGTCTCGCTCTGTCACCAGG",1,3,NA,NA,"GeneID:57403","RAB22A",FALSE,"ADRA1A"

"3640","CGAAGTCTCGCTCTGTCACC","chr8",22438133,"-","2:C>T","AGG","CGGAGTCTCGCTCTGTCACCAGG",1,3,NA,NA,"","",FALSE,"ADRA1A"

"3641","CGAAGTCTCGCTCTGTCACC","chr2",34960651,"-","2:C>T","AGG","CGGAGTCTCGCTCTGTCACCAGG",1,3,NA,NA,"","",FALSE,"ADRA1A"

"3642","CGAAGTCTCGCTCTGTCACC","chr1",15788265,"-","2:C>T","AGG","CGGAGTCTCGCTCTGTCACCAGG",1,3,NA,NA,"","",FALSE,"ADRA1A"

"3643","CGAAGTCTCGCTCTGTCACC","chr19",1375197,"-","2:C>T","AGG","CGGAGTCTCGCTCTGTCACCAGG",1,3,NA,NA,"GeneID:84939","MUM1",FALSE,"ADRA1A"

"3644","CGAAGTCTCGCTCTGTCACC","chr3",186808097,"-","2:C>T","AGG","CGGAGTCTCGCTCTGTCACCAGG",1,3,NA,NA,"","",FALSE,"ADRA1A"

"3645","CGAAGTCTCGCTCTGTCACC","chr14",92025854,"-","2:C>T","AGG","CGGAGTCTCGCTCTGTCACCAGG",1,3,NA,NA,"GeneID:9321","TRIP11",FALSE,"ADRA1A"

"3646","CGAAGTCTCGCTCTGTCACC","chr20",49856796,"-","2:C>T","AGG","CGGAGTCTCGCTCTGTCACCAGG",1,3,NA,NA,"GeneID:23315","SLC9A8",FALSE,"ADRA1A"

"3647","CGAAGTCTCGCTCTGTCACC","chr19",37694238,"-","2:C>T","AGG","CGGAGTCTCGCTCTGTCACCAGG",1,3,NA,NA,"","",FALSE,"ADRA1A"

"3648","CGAAGTCTCGCTCTGTCACC","chr6",140706623,"-","2:C>T","AGG","CGGAGTCTCGCTCTGTCACCAGG",1,3,NA,NA,"","",FALSE,"ADRA1A"

"3649","CGAAGTCTCGCTCTGTCACC","chr2",152404042,"-","2:C>T","AGG","CGGAGTCTCGCTCTGTCACCAGG",1,3,NA,NA,"GeneID:114793","FMNL2",FALSE,"ADRA1A"

"3650","CGAAGTCTCGCTCTGTCACC","chr3",187349273,"-","2:C>T","AGG","CGGAGTCTCGCTCTGTCACCAGG",1,3,NA,NA,"","",FALSE,"ADRA1A"

"3651","CGAAGTCTCGCTCTGTCACC","chr1",43743064,"-","2:C>T","AGG","CGGAGTCTCGCTCTGTCACCAGG",1,3,NA,NA,"GeneID:6487","ST3GAL3",FALSE,"ADRA1A"

"3652","CGAAGTCTCGCTCTGTCACC","chr8",123384055,"-","2:C>T","AGG","CGGAGTCTCGCTCTGTCACCAGG",1,3,NA,NA,"GeneID:29028","ATAD2",FALSE,"ADRA1A"

"3653","CGAAGTCTCGCTCTGTCACC","chr5",42456285,"-","2:C>T","AGG","CGGAGTCTCGCTCTGTCACCAGG",1,3,NA,NA,"GeneID:2690","GHR",FALSE,"ADRA1A"

"3654","CGAAGTCTCGCTCTGTCACC","chr10",35088475,"-","2:C>T","AGG","CGGAGTCTCGCTCTGTCACCAGG",1,3,NA,NA,"GeneID:8453","CUL2",FALSE,"ADRA1A"

"3655","CGAAGTCTCGCTCTGTCACC","chr2",224858628,"-","2:C>T","AGG","CGGAGTCTCGCTCTGTCACCAGG",1,3,NA,NA,"GeneID:55619","DOCK10",FALSE,"ADRA1A"

"3656","CGAAGTCTCGCTCTGTCACC","chr3",186295476,"-","2:C>T","AGG","CGGAGTCTCGCTCTGTCACCAGG",1,3,NA,NA,"GeneID:1608","DGKG",FALSE,"ADRA1A"

"3657","CGAAGTCTCGCTCTGTCACC","chr5",15715465,"-","2:C>T","AGG","CGGAGTCTCGCTCTGTCACCAGG",1,3,NA,NA,"GeneID:23194","FBXL7",FALSE,"ADRA1A"

"3658","CGAAGTCTCGCTCTGTCACC","chr15",95896677,"-","2:C>T","AGG","CGGAGTCTCGCTCTGTCACCAGG",1,3,NA,NA,"","",FALSE,"ADRA1A"

"3659","CGAAGTCTCGCTCTGTCACC","chr19",35630288,"-","2:C>T","AGG","CGGAGTCTCGCTCTGTCACCAGG",1,3,NA,NA,"GeneID:79171","RBM42",FALSE,"ADRA1A"

"3660","CGAAGTCTCGCTCTGTCACC","chr16",22950742,"-","2:C>T","AGG","CGGAGTCTCGCTCTGTCACCAGG",1,3,NA,NA,"","",FALSE,"ADRA1A"

"3661","CGAAGTCTCGCTCTGTCACC","chr2",121168280,"-","2:C>T","AGG","CGGAGTCTCGCTCTGTCACCAGG",1,3,NA,NA,"","",FALSE,"ADRA1A"

"3662","CGAAGTCTCGCTCTGTCACC","chr16",59898680,"-","2:C>T","AGG","CGGAGTCTCGCTCTGTCACCAGG",1,3,NA,NA,"GeneID:101927580","LOC101927580",FALSE,"ADRA1A"

"3663","CGAAGTCTCGCTCTGTCACC","chr6",42359698,"-","2:C>T","AGG","CGGAGTCTCGCTCTGTCACCAGG",1,3,NA,NA,"GeneID:55809","TRERF1",FALSE,"ADRA1A"

"3664","CGAAGTCTCGCTCTGTCACC","chr20",56377761,"-","2:C>T","AGG","CGGAGTCTCGCTCTGTCACCAGG",1,3,NA,NA,"GeneID:6790","AURKA",FALSE,"ADRA1A"

"3665","CGAAGTCTCGCTCTGTCACC","chr5",138222693,"-","2:C>T","AGG","CGGAGTCTCGCTCTGTCACCAGG",1,3,NA,NA,"","",FALSE,"ADRA1A"

"3666","CGAAGTCTCGCTCTGTCACC","chr3",103501449,"-","2:C>T","AGG","CGGAGTCTCGCTCTGTCACCAGG",1,3,NA,NA,"","",FALSE,"ADRA1A"

"3667","CGAAGTCTCGCTCTGTCACC","chr3",28470468,"-","2:C>T","AGG","CGGAGTCTCGCTCTGTCACCAGG",1,3,NA,NA,"GeneID:152098","ZCWPW2",FALSE,"ADRA1A"

"3668","CGAAGTCTCGCTCTGTCACC","chr4",182892603,"-","2:C>T","AGG","CGGAGTCTCGCTCTGTCACCAGG",1,3,NA,NA,"GeneID:1635","DCTD",FALSE,"ADRA1A"

"3669","CGAAGTCTCGCTCTGTCACC","chr17",10322372,"-","2:C>T","AGG","CGGAGTCTCGCTCTGTCACCAGG",1,3,NA,NA,"GeneID:8735","MYH13",FALSE,"ADRA1A"

"3670","CGAAGTCTCGCTCTGTCACC","chr20",36979701,"-","2:C>T","AGG","CGGAGTCTCGCTCTGTCACCAGG",1,3,NA,NA,"","",FALSE,"ADRA1A"

"3671","CGAAGTCTCGCTCTGTCACC","chr10",102747334,"-","2:C>T","AGG","CGGAGTCTCGCTCTGTCACCAGG",1,3,NA,NA,"GeneID:54838","WBP1L",FALSE,"ADRA1A"

"3672","CGAAGTCTCGCTCTGTCACC","chr7",30060111,"-","2:C>T","AGG","CGGAGTCTCGCTCTGTCACCAGG",1,3,NA,NA,"GeneID:84725","PLEKHA8",FALSE,"ADRA1A"

"3673","CGAAGTCTCGCTCTGTCACC","chr20",47333700,"-","2:C>T","AGG","CGGAGTCTCGCTCTGTCACCAGG",1,3,NA,NA,"GeneID:23613","ZMYND8",FALSE,"ADRA1A"

"3674","CGAAGTCTCGCTCTGTCACC","chr2",136181928,"-","2:C>T","AGG","CGGAGTCTCGCTCTGTCACCAGG",1,3,NA,NA,"","",FALSE,"ADRA1A"

"3675","CGAAGTCTCGCTCTGTCACC","chr4",78751132,"-","2:C>T","AGG","CGGAGTCTCGCTCTGTCACCAGG",1,3,NA,NA,"","",FALSE,"ADRA1A"

"3676","CGAAGTCTCGCTCTGTCACC","chr1",69491206,"-","2:C>T","AGG","CGGAGTCTCGCTCTGTCACCAGG",1,3,NA,NA,"","",FALSE,"ADRA1A"

"3677","CGAAGTCTCGCTCTGTCACC","chr7",47764384,"-","2:C>T","AGG","CGGAGTCTCGCTCTGTCACCAGG",1,3,NA,NA,"GeneID:3364;GeneID:84847","HUS1;LINC00525",FALSE,"ADRA1A"

"3678","CGAAGTCTCGCTCTGTCACC","chr10",119874676,"-","2:C>T","AGG","CGGAGTCTCGCTCTGTCACCAGG",1,3,NA,NA,"GeneID:79892","MCMBP",FALSE,"ADRA1A"

"3679","CGAAGTCTCGCTCTGTCACC","chr9",84361319,"-","2:C>T","AGG","CGGAGTCTCGCTCTGTCACCAGG",1,3,NA,NA,"","",FALSE,"ADRA1A"

"3680","CGAAGTCTCGCTCTGTCACC","chr5",11454394,"-","2:C>T","AGG","CGGAGTCTCGCTCTGTCACCAGG",1,3,NA,NA,"GeneID:1501","CTNND2",FALSE,"ADRA1A"

"3681","CGAAGTCTCGCTCTGTCACC","chr7",48835477,"-","2:C>T","AGG","CGGAGTCTCGCTCTGTCACCAGG",1,3,NA,NA,"","",FALSE,"ADRA1A"

"3682","CGAAGTCTCGCTCTGTCACC","chr13",41227247,"-","2:C>T","AGG","CGGAGTCTCGCTCTGTCACCAGG",1,3,NA,NA,"GeneID:101929140;GeneID:9617","LOC101929140;MTRF1",FALSE,"ADRA1A"

"3683","CGAAGTCTCGCTCTGTCACC","chr1",231279704,"-","2:C>T","AGG","CGGAGTCTCGCTCTGTCACCAGG",1,3,NA,NA,"","",FALSE,"ADRA1A"

"3684","CGAAGTCTCGCTCTGTCACC","chrX",48205811,"-","2:C>T","AGG","CGGAGTCTCGCTCTGTCACCAGG",1,3,NA,NA,"","",FALSE,"ADRA1A"

"3685","CGAAGTCTCGCTCTGTCACC","chr14",94399542,"-","2:C>T","AGG","CGGAGTCTCGCTCTGTCACCAGG",1,3,NA,NA,"","",FALSE,"ADRA1A"

"3686","CGAAGTCTCGCTCTGTCACC","chr7",70062488,"-","2:C>T","TGG","CGGAGTCTCGCTCTGTCACCTGG",1,3,NA,NA,"GeneID:26053","AUTS2",FALSE,"ADRA1A"

"3687","CGAAGTCTCGCTCTGTCACC","chr12",115246926,"-","2:C>T","TGG","CGGAGTCTCGCTCTGTCACCTGG",1,3,NA,NA,"","",FALSE,"ADRA1A"

"3688","CGAAGTCTCGCTCTGTCACC","chr1",64822263,"-","2:C>T","TGG","CGGAGTCTCGCTCTGTCACCTGG",1,3,NA,NA,"GeneID:55225","RAVER2",FALSE,"ADRA1A"

"3689","CGAAGTCTCGCTCTGTCACC","chr17",5010722,"-","2:C>T","TGG","CGGAGTCTCGCTCTGTCACCTGG",1,3,NA,NA,"GeneID:10749","KIF1C",FALSE,"ADRA1A"

"3690","CGAAGTCTCGCTCTGTCACC","chr21",38286218,"-","2:C>T","TGG","CGGAGTCTCGCTCTGTCACCTGG",1,3,NA,NA,"GeneID:3772","KCNJ15",FALSE,"ADRA1A"

"3691","CGAAGTCTCGCTCTGTCACC","chr18",41909295,"-","2:C>T","TGG","CGGAGTCTCGCTCTGTCACCTGG",1,3,NA,NA,"","",FALSE,"ADRA1A"

"3692","CGAAGTCTCGCTCTGTCACC","chr10",8084568,"-","2:C>T","TGG","CGGAGTCTCGCTCTGTCACCTGG",1,3,NA,NA,"","",FALSE,"ADRA1A"

"3693","CGAAGTCTCGCTCTGTCACC","chr21",29081528,"-","2:C>T","TGG","CGGAGTCTCGCTCTGTCACCTGG",1,3,NA,NA,"GeneID:56911","MAP3K7CL",FALSE,"ADRA1A"

"3694","CGAAGTCTCGCTCTGTCACC","chr16",73936600,"-","2:C>T","TGG","CGGAGTCTCGCTCTGTCACCTGG",1,3,NA,NA,"","",FALSE,"ADRA1A"

"3695","CGAAGTCTCGCTCTGTCACC","chr13",84371581,"-","2:C>T","TGG","CGGAGTCTCGCTCTGTCACCTGG",1,3,NA,NA,"","",FALSE,"ADRA1A"

"3696","CGAAGTCTCGCTCTGTCACC","chr10",35051290,"-","2:C>T","TGG","CGGAGTCTCGCTCTGTCACCTGG",1,3,NA,NA,"GeneID:8453","CUL2",FALSE,"ADRA1A"

"3697","CGAAGTCTCGCTCTGTCACC","chr2",132787323,"-","2:C>T","TGG","CGGAGTCTCGCTCTGTCACCTGG",1,3,NA,NA,"GeneID:344148","NCKAP5",FALSE,"ADRA1A"

"3698","CGAAGTCTCGCTCTGTCACC","chr4",152852607,"-","2:C>T","TGG","CGGAGTCTCGCTCTGTCACCTGG",1,3,NA,NA,"GeneID:27236","ARFIP1",FALSE,"ADRA1A"

"3699","CGAAGTCTCGCTCTGTCACC","chr15",99048814,"-","2:C>T","TGG","CGGAGTCTCGCTCTGTCACCTGG",1,3,NA,NA,"","",FALSE,"ADRA1A"

"3700","CGAAGTCTCGCTCTGTCACC","chr10",100252810,"-","2:C>T","TGG","CGGAGTCTCGCTCTGTCACCTGG",1,3,NA,NA,"GeneID:55280","CWF19L1",FALSE,"ADRA1A"

"3701","CGAAGTCTCGCTCTGTCACC","chr3",33074284,"-","2:C>T","TGG","CGGAGTCTCGCTCTGTCACCTGG",1,3,NA,NA,"GeneID:2720","GLB1",FALSE,"ADRA1A"

"3702","CGAAGTCTCGCTCTGTCACC","chr22",33664833,"-","0:A>G,2:C>T","GGG","TGGAGTCTCGCTCTGTCACCGGG",2,1,3,NA,"GeneID:9215","LARGE",FALSE,"ADRA1A"

"3703","CGAAGTCTCGCTCTGTCACC","chr12",41430443,"-","0:A>G,2:C>T","GGG","TGGAGTCTCGCTCTGTCACCGGG",2,1,3,NA,"GeneID:29951","PDZRN4",FALSE,"ADRA1A"

"3704","CGAAGTCTCGCTCTGTCACC","chr3",183758332,"-","0:A>G,2:C>T","CGG","TGGAGTCTCGCTCTGTCACCCGG",2,1,3,NA,"GeneID:55689","YEATS2",FALSE,"ADRA1A"

"3705","CGAAGTCTCGCTCTGTCACC","chrX",134648661,"-","0:A>G,2:C>T","CGG","TGGAGTCTCGCTCTGTCACCCGG",2,1,3,NA,"GeneID:10761","PLAC1",FALSE,"ADRA1A"

"3706","CGAAGTCTCGCTCTGTCACC","chr16",68876796,"-","0:A>G,2:C>T","CGG","TGGAGTCTCGCTCTGTCACCCGG",2,1,3,NA,"GeneID:79613","TANGO6",FALSE,"ADRA1A"

"3707","CGAAGTCTCGCTCTGTCACC","chr6",12452361,"-","0:A>G,2:C>T","CGG","TGGAGTCTCGCTCTGTCACCCGG",2,1,3,NA,"","",FALSE,"ADRA1A"

"3708","CGAAGTCTCGCTCTGTCACC","chr18",9386429,"-","0:A>G,2:C>T","CGG","TGGAGTCTCGCTCTGTCACCCGG",2,1,3,NA,"GeneID:57045","TWSG1",FALSE,"ADRA1A"

"3709","CGAAGTCTCGCTCTGTCACC","chr2",60956950,"-","0:A>G,2:C>T","CGG","TGGAGTCTCGCTCTGTCACCCGG",2,1,3,NA,"GeneID:150962","PUS10",FALSE,"ADRA1A"

"3710","CGAAGTCTCGCTCTGTCACC","chr9",92626372,"-","0:A>G,2:C>T","CGG","TGGAGTCTCGCTCTGTCACCCGG",2,1,3,NA,"GeneID:64768","IPPK",FALSE,"ADRA1A"

"3711","CGAAGTCTCGCTCTGTCACC","chr7",2684153,"-","0:A>G,2:C>T","CGG","TGGAGTCTCGCTCTGTCACCCGG",2,1,3,NA,"GeneID:155185","AMZ1",FALSE,"ADRA1A"

"3712","CGAAGTCTCGCTCTGTCACC","chr16",69782258,"-","0:A>G,2:C>T","CGG","TGGAGTCTCGCTCTGTCACCCGG",2,1,3,NA,"GeneID:11060","WWP2",FALSE,"ADRA1A"

"3713","CGAAGTCTCGCTCTGTCACC","chr2",200640012,"-","0:A>G,2:C>T","CGG","TGGAGTCTCGCTCTGTCACCCGG",2,1,3,NA,"GeneID:316","AOX1",FALSE,"ADRA1A"

"3714","CGAAGTCTCGCTCTGTCACC","chr20",45007522,"-","0:A>G,2:C>T","CGG","TGGAGTCTCGCTCTGTCACCCGG",2,1,3,NA,"GeneID:6789","STK4",FALSE,"ADRA1A"

"3715","CGAAGTCTCGCTCTGTCACC","chr12",70928753,"-","0:A>G,2:C>T","CGG","TGGAGTCTCGCTCTGTCACCCGG",2,1,3,NA,"","",FALSE,"ADRA1A"

"3716","CGAAGTCTCGCTCTGTCACC","chr12",122404875,"-","0:A>G,2:C>T","CGG","TGGAGTCTCGCTCTGTCACCCGG",2,1,3,NA,"GeneID:6249","CLIP1",FALSE,"ADRA1A"

"3717","CGAAGTCTCGCTCTGTCACC","chr1",149207654,"-","0:A>G,2:C>T","CGG","TGGAGTCTCGCTCTGTCACCCGG",2,1,3,NA,"","",FALSE,"ADRA1A"

"3718","CGAAGTCTCGCTCTGTCACC","chr1",120587616,"-","0:A>G,2:C>T","CGG","TGGAGTCTCGCTCTGTCACCCGG",2,1,3,NA,"","",FALSE,"ADRA1A"

"3719","CGAAGTCTCGCTCTGTCACC","chr10",72118342,"-","0:A>G,2:C>T","CGG","TGGAGTCTCGCTCTGTCACCCGG",2,1,3,NA,"GeneID:51008","ASCC1",FALSE,"ADRA1A"

"3720","CGAAGTCTCGCTCTGTCACC","chr9",128642730,"-","0:A>G,2:C>T","CGG","TGGAGTCTCGCTCTGTCACCCGG",2,1,3,NA,"GeneID:89891","WDR34",FALSE,"ADRA1A"

"3721","CGAAGTCTCGCTCTGTCACC","chr8",118472262,"-","0:A>G,2:C>T","CGG","TGGAGTCTCGCTCTGTCACCCGG",2,1,3,NA,"GeneID:401474","SAMD12",FALSE,"ADRA1A"

"3722","CGAAGTCTCGCTCTGTCACC","chrX",130169183,"-","0:A>G,2:C>T","CGG","TGGAGTCTCGCTCTGTCACCCGG",2,1,3,NA,"","",FALSE,"ADRA1A"

"3723","CGAAGTCTCGCTCTGTCACC","chr4",1647310,"-","0:A>G,2:C>T","CGG","TGGAGTCTCGCTCTGTCACCCGG",2,1,3,NA,"GeneID:152877","FAM53A",FALSE,"ADRA1A"

"3724","CGAAGTCTCGCTCTGTCACC","chr17",3039610,"-","0:A>G,2:C>T","CGG","TGGAGTCTCGCTCTGTCACCCGG",2,1,3,NA,"","",FALSE,"ADRA1A"

"3725","CGAAGTCTCGCTCTGTCACC","chr8",46030570,"-","0:A>G,2:C>T","CGG","TGGAGTCTCGCTCTGTCACCCGG",2,1,3,NA,"","",FALSE,"ADRA1A"

"3726","CGAAGTCTCGCTCTGTCACC","chr18",114550,"-","0:A>G,2:C>T","CGG","TGGAGTCTCGCTCTGTCACCCGG",2,1,3,NA,"GeneID:727758","ROCK1P1",FALSE,"ADRA1A"

"3727","CGAAGTCTCGCTCTGTCACC","chr19",17820086,"-","0:A>G,2:C>T","CGG","TGGAGTCTCGCTCTGTCACCCGG",2,1,3,NA,"GeneID:3640","INSL3",FALSE,"ADRA1A"

"3728","CGAAGTCTCGCTCTGTCACC","chr14",90038813,"-","0:A>G,2:C>T","CGG","TGGAGTCTCGCTCTGTCACCCGG",2,1,3,NA,"GeneID:55775","TDP1",FALSE,"ADRA1A"

"3729","CGAAGTCTCGCTCTGTCACC","chr9",33208341,"-","0:A>G,2:C>T","CGG","TGGAGTCTCGCTCTGTCACCCGG",2,1,3,NA,"","",FALSE,"ADRA1A"

"3730","CGAAGTCTCGCTCTGTCACC","chr3",49592726,"-","0:A>G,2:C>T","CGG","TGGAGTCTCGCTCTGTCACCCGG",2,1,3,NA,"GeneID:8927","BSN",FALSE,"ADRA1A"

"3731","CGAAGTCTCGCTCTGTCACC","chr8",26189617,"-","0:A>G,2:C>T","CGG","TGGAGTCTCGCTCTGTCACCCGG",2,1,3,NA,"","",FALSE,"ADRA1A"

"3732","CGAAGTCTCGCTCTGTCACC","chr10",27057911,"-","0:A>G,2:C>T","CGG","TGGAGTCTCGCTCTGTCACCCGG",2,1,3,NA,"GeneID:22852","ANKRD26",FALSE,"ADRA1A"

"3733","CGAAGTCTCGCTCTGTCACC","chr4",14458358,"-","0:A>G,2:C>T","CGG","TGGAGTCTCGCTCTGTCACCCGG",2,1,3,NA,"","",FALSE,"ADRA1A"

"3734","CGAAGTCTCGCTCTGTCACC","chr9",626404,"-","0:A>G,2:C>T","CGG","TGGAGTCTCGCTCTGTCACCCGG",2,1,3,NA,"GeneID:23189","KANK1",FALSE,"ADRA1A"

"3735","CGAAGTCTCGCTCTGTCACC","chr7",137254271,"-","0:A>G,2:C>T","AGG","TGGAGTCTCGCTCTGTCACCAGG",2,1,3,NA,"GeneID:5764","PTN",FALSE,"ADRA1A"

"3736","CGAAGTCTCGCTCTGTCACC","chr1",58923867,"-","0:A>G,2:C>T","AGG","TGGAGTCTCGCTCTGTCACCAGG",2,1,3,NA,"","",FALSE,"ADRA1A"

"3737","CGAAGTCTCGCTCTGTCACC","chr6",99128771,"-","0:A>G,2:C>T","AGG","TGGAGTCTCGCTCTGTCACCAGG",2,1,3,NA,"","",FALSE,"ADRA1A"

"3738","CGAAGTCTCGCTCTGTCACC","chr19",55130220,"-","0:A>G,2:C>T","AGG","TGGAGTCTCGCTCTGTCACCAGG",2,1,3,NA,"","",FALSE,"ADRA1A"

"3739","CGAAGTCTCGCTCTGTCACC","chr6",66329262,"-","0:A>G,2:C>T","AGG","TGGAGTCTCGCTCTGTCACCAGG",2,1,3,NA,"","",FALSE,"ADRA1A"

"3740","CGAAGTCTCGCTCTGTCACC","chr21",17391308,"-","0:A>G,2:C>T","AGG","TGGAGTCTCGCTCTGTCACCAGG",2,1,3,NA,"","",FALSE,"ADRA1A"

"3741","CGAAGTCTCGCTCTGTCACC","chrUn_KI270750v1",39888,"-","0:A>G,2:C>T","AGG","TGGAGTCTCGCTCTGTCACCAGG",2,1,3,NA,"","",FALSE,"ADRA1A"

"3742","CGAAGTCTCGCTCTGTCACC","chr18",77758573,"-","0:A>G,2:C>T","AGG","TGGAGTCTCGCTCTGTCACCAGG",2,1,3,NA,"","",FALSE,"ADRA1A"

"3743","CGAAGTCTCGCTCTGTCACC","chr12",89290870,"-","0:A>G,2:C>T","AGG","TGGAGTCTCGCTCTGTCACCAGG",2,1,3,NA,"","",FALSE,"ADRA1A"

"3744","CGAAGTCTCGCTCTGTCACC","chr2",183898481,"-","0:A>G,2:C>T","AGG","TGGAGTCTCGCTCTGTCACCAGG",2,1,3,NA,"","",FALSE,"ADRA1A"

"3745","CGAAGTCTCGCTCTGTCACC","chrX",48075542,"-","0:A>G,2:C>T","AGG","TGGAGTCTCGCTCTGTCACCAGG",2,1,3,NA,"","",FALSE,"ADRA1A"

"3746","CGAAGTCTCGCTCTGTCACC","chr4",7749577,"-","0:A>G,2:C>T","AGG","TGGAGTCTCGCTCTGTCACCAGG",2,1,3,NA,"","",FALSE,"ADRA1A"

"3747","CGAAGTCTCGCTCTGTCACC","chr6",34904706,"-","0:A>G,2:C>T","AGG","TGGAGTCTCGCTCTGTCACCAGG",2,1,3,NA,"GeneID:23294","ANKS1A",FALSE,"ADRA1A"

"3748","CGAAGTCTCGCTCTGTCACC","chr17",43441125,"-","0:A>G,2:C>T","AGG","TGGAGTCTCGCTCTGTCACCAGG",2,1,3,NA,"","",FALSE,"ADRA1A"

"3749","CGAAGTCTCGCTCTGTCACC","chr11",63628810,"-","0:A>G,2:C>T","AGG","TGGAGTCTCGCTCTGTCACCAGG",2,1,3,NA,"GeneID:25923","ATL3",TRUE,"ADRA1A"

"3750","CGAAGTCTCGCTCTGTCACC","chr18",26258228,"-","0:A>G,2:C>T","AGG","TGGAGTCTCGCTCTGTCACCAGG",2,1,3,NA,"GeneID:6875","TAF4B",FALSE,"ADRA1A"

"3751","CGAAGTCTCGCTCTGTCACC","chr21",23468367,"-","0:A>G,2:C>T","AGG","TGGAGTCTCGCTCTGTCACCAGG",2,1,3,NA,"","",FALSE,"ADRA1A"

"3752","CGAAGTCTCGCTCTGTCACC","chr22",35164182,"-","0:A>G,2:C>T","AGG","TGGAGTCTCGCTCTGTCACCAGG",2,1,3,NA,"","",FALSE,"ADRA1A"

"3753","CGAAGTCTCGCTCTGTCACC","chr4",153203079,"-","0:A>G,2:C>T","AGG","TGGAGTCTCGCTCTGTCACCAGG",2,1,3,NA,"GeneID:23321","TRIM2",FALSE,"ADRA1A"

"3754","CGAAGTCTCGCTCTGTCACC","chr15",91586246,"-","0:A>G,2:C>T","AGG","TGGAGTCTCGCTCTGTCACCAGG",2,1,3,NA,"GeneID:101926928","LOC101926928",FALSE,"ADRA1A"

"3755","CGAAGTCTCGCTCTGTCACC","chr13",96386840,"-","0:A>G,2:C>T","AGG","TGGAGTCTCGCTCTGTCACCAGG",2,1,3,NA,"GeneID:266722","HS6ST3",FALSE,"ADRA1A"

"3756","CGAAGTCTCGCTCTGTCACC","chr14",102347555,"-","0:A>G,2:C>T","AGG","TGGAGTCTCGCTCTGTCACCAGG",2,1,3,NA,"GeneID:51550","CINP",FALSE,"ADRA1A"

"3757","CGAAGTCTCGCTCTGTCACC","chr17",5172505,"-","0:A>G,2:C>T","AGG","TGGAGTCTCGCTCTGTCACCAGG",2,1,3,NA,"GeneID:9098","USP6",FALSE,"ADRA1A"

"3758","CGAAGTCTCGCTCTGTCACC","chr19",10585661,"-","0:A>G,2:C>T","AGG","TGGAGTCTCGCTCTGTCACCAGG",2,1,3,NA,"GeneID:10053","AP1M2",FALSE,"ADRA1A"

"3759","CGAAGTCTCGCTCTGTCACC","chr3",196363897,"-","0:A>G,2:C>T","AGG","TGGAGTCTCGCTCTGTCACCAGG",2,1,3,NA,"GeneID:26043","UBXN7",FALSE,"ADRA1A"

"3760","CGAAGTCTCGCTCTGTCACC","chr12",46294995,"-","0:A>G,2:C>T","AGG","TGGAGTCTCGCTCTGTCACCAGG",2,1,3,NA,"","",FALSE,"ADRA1A"

"3761","CGAAGTCTCGCTCTGTCACC","chr11",126188066,"-","0:A>G,2:C>T","AGG","TGGAGTCTCGCTCTGTCACCAGG",2,1,3,NA,"","",FALSE,"ADRA1A"

"3762","CGAAGTCTCGCTCTGTCACC","chr22",41741918,"-","0:A>G,2:C>T","AGG","TGGAGTCTCGCTCTGTCACCAGG",2,1,3,NA,"GeneID:150365","MEI1",FALSE,"ADRA1A"

"3763","CGAAGTCTCGCTCTGTCACC","chr16",2120991,"-","0:A>G,2:C>T","AGG","TGGAGTCTCGCTCTGTCACCAGG",2,1,3,NA,"GeneID:5310","PKD1",FALSE,"ADRA1A"

"3764","CGAAGTCTCGCTCTGTCACC","chr8",104096842,"-","0:A>G,2:C>T","AGG","TGGAGTCTCGCTCTGTCACCAGG",2,1,3,NA,"GeneID:9699","RIMS2",FALSE,"ADRA1A"

"3765","CGAAGTCTCGCTCTGTCACC","chr8",83076234,"-","0:A>G,2:C>T","AGG","TGGAGTCTCGCTCTGTCACCAGG",2,1,3,NA,"","",FALSE,"ADRA1A"

"3766","CGAAGTCTCGCTCTGTCACC","chr7",85496035,"-","0:A>G,2:C>T","AGG","TGGAGTCTCGCTCTGTCACCAGG",2,1,3,NA,"","",FALSE,"ADRA1A"

"3767","CGAAGTCTCGCTCTGTCACC","chr19",48814814,"-","0:A>G,2:C>T","AGG","TGGAGTCTCGCTCTGTCACCAGG",2,1,3,NA,"GeneID:51171","HSD17B14",FALSE,"ADRA1A"

"3768","CGAAGTCTCGCTCTGTCACC","chr4",157967263,"-","0:A>G,2:C>T","AGG","TGGAGTCTCGCTCTGTCACCAGG",2,1,3,NA,"","",FALSE,"ADRA1A"

"3769","CGAAGTCTCGCTCTGTCACC","chr22",48656480,"-","0:A>G,2:C>T","AGG","TGGAGTCTCGCTCTGTCACCAGG",2,1,3,NA,"GeneID:25817","FAM19A5",FALSE,"ADRA1A"

"3770","CGAAGTCTCGCTCTGTCACC","chr21",30781945,"-","0:A>G,2:C>T","AGG","TGGAGTCTCGCTCTGTCACCAGG",2,1,3,NA,"","",FALSE,"ADRA1A"

"3771","CGAAGTCTCGCTCTGTCACC","chr3",104114664,"-","0:A>G,2:C>T","AGG","TGGAGTCTCGCTCTGTCACCAGG",2,1,3,NA,"","",FALSE,"ADRA1A"

"3772","CGAAGTCTCGCTCTGTCACC","chr8",55321963,"-","0:A>G,2:C>T","AGG","TGGAGTCTCGCTCTGTCACCAGG",2,1,3,NA,"GeneID:114786","XKR4",FALSE,"ADRA1A"

"3773","CGAAGTCTCGCTCTGTCACC","chr17",17254784,"-","0:A>G,2:C>T","AGG","TGGAGTCTCGCTCTGTCACCAGG",2,1,3,NA,"GeneID:8533","COPS3",FALSE,"ADRA1A"

"3774","CGAAGTCTCGCTCTGTCACC","chr3",50664870,"-","0:A>G,2:C>T","AGG","TGGAGTCTCGCTCTGTCACCAGG",2,1,3,NA,"","",FALSE,"ADRA1A"

"3775","CGAAGTCTCGCTCTGTCACC","chrX",51300874,"-","0:A>G,2:C>T","AGG","TGGAGTCTCGCTCTGTCACCAGG",2,1,3,NA,"","",FALSE,"ADRA1A"

"3776","CGAAGTCTCGCTCTGTCACC","chr8",124507136,"-","0:A>G,2:C>T","AGG","TGGAGTCTCGCTCTGTCACCAGG",2,1,3,NA,"GeneID:83940","TATDN1",FALSE,"ADRA1A"

"3777","CGAAGTCTCGCTCTGTCACC","chr2",111926706,"-","0:A>G,2:C>T","AGG","TGGAGTCTCGCTCTGTCACCAGG",2,1,3,NA,"GeneID:10461","MERTK",FALSE,"ADRA1A"

"3778","CGAAGTCTCGCTCTGTCACC","chr3",180113827,"-","0:A>G,2:C>T","AGG","TGGAGTCTCGCTCTGTCACCAGG",2,1,3,NA,"","",FALSE,"ADRA1A"

"3779","CGAAGTCTCGCTCTGTCACC","chr3",55126542,"-","0:A>G,2:C>T","AGG","TGGAGTCTCGCTCTGTCACCAGG",2,1,3,NA,"","",FALSE,"ADRA1A"

"3780","CGAAGTCTCGCTCTGTCACC","chr14",49506144,"-","0:A>G,2:C>T","AGG","TGGAGTCTCGCTCTGTCACCAGG",2,1,3,NA,"","",FALSE,"ADRA1A"

"3781","CGAAGTCTCGCTCTGTCACC","chr4",171464121,"-","0:A>G,2:C>T","AGG","TGGAGTCTCGCTCTGTCACCAGG",2,1,3,NA,"","",FALSE,"ADRA1A"

"3782","CGAAGTCTCGCTCTGTCACC","chr17",42950047,"-","0:A>G,2:C>T","AGG","TGGAGTCTCGCTCTGTCACCAGG",2,1,3,NA,"","",FALSE,"ADRA1A"

"3783","CGAAGTCTCGCTCTGTCACC","chr2",49978136,"-","0:A>G,2:C>T","AGG","TGGAGTCTCGCTCTGTCACCAGG",2,1,3,NA,"GeneID:9378","NRXN1",FALSE,"ADRA1A"

"3784","CGAAGTCTCGCTCTGTCACC","chr3",9022302,"-","0:A>G,2:C>T","AGG","TGGAGTCTCGCTCTGTCACCAGG",2,1,3,NA,"GeneID:9901","SRGAP3",FALSE,"ADRA1A"

"3785","CGAAGTCTCGCTCTGTCACC","chr2",34606336,"-","0:A>G,2:C>T","AGG","TGGAGTCTCGCTCTGTCACCAGG",2,1,3,NA,"","",FALSE,"ADRA1A"

"3786","CGAAGTCTCGCTCTGTCACC","chr9",101493228,"-","0:A>G,2:C>T","AGG","TGGAGTCTCGCTCTGTCACCAGG",2,1,3,NA,"GeneID:84302","TMEM246",FALSE,"ADRA1A"

"3787","CGAAGTCTCGCTCTGTCACC","chr17",669866,"-","0:A>G,2:C>T","AGG","TGGAGTCTCGCTCTGTCACCAGG",2,1,3,NA,"GeneID:55275","VPS53",FALSE,"ADRA1A"

"3788","CGAAGTCTCGCTCTGTCACC","chr20",2952094,"-","0:A>G,2:C>T","AGG","TGGAGTCTCGCTCTGTCACCAGG",2,1,3,NA,"GeneID:5786","PTPRA",FALSE,"ADRA1A"

"3789","CGAAGTCTCGCTCTGTCACC","chr9",96958137,"-","0:A>G,2:C>T","AGG","TGGAGTCTCGCTCTGTCACCAGG",2,1,3,NA,"GeneID:84278","HIATL2",FALSE,"ADRA1A"

"3790","CGAAGTCTCGCTCTGTCACC","chr11",112782821,"-","0:A>G,2:C>T","AGG","TGGAGTCTCGCTCTGTCACCAGG",2,1,3,NA,"","",FALSE,"ADRA1A"

"3791","CGAAGTCTCGCTCTGTCACC","chr1",237714967,"-","0:A>G,2:C>T","AGG","TGGAGTCTCGCTCTGTCACCAGG",2,1,3,NA,"GeneID:6262","RYR2",FALSE,"ADRA1A"

"3792","CGAAGTCTCGCTCTGTCACC","chr17",62858279,"-","0:A>G,2:C>T","AGG","TGGAGTCTCGCTCTGTCACCAGG",2,1,3,NA,"","",FALSE,"ADRA1A"

"3793","CGAAGTCTCGCTCTGTCACC","chr4",165090357,"-","0:A>G,2:C>T","AGG","TGGAGTCTCGCTCTGTCACCAGG",2,1,3,NA,"GeneID:201931","TMEM192",FALSE,"ADRA1A"

"3794","CGAAGTCTCGCTCTGTCACC","chr15",88475931,"-","0:A>G,2:C>T","AGG","TGGAGTCTCGCTCTGTCACCAGG",2,1,3,NA,"GeneID:64963","MRPS11",FALSE,"ADRA1A"

"3795","CGAAGTCTCGCTCTGTCACC","chr11",72323541,"-","0:A>G,2:C>T","AGG","TGGAGTCTCGCTCTGTCACCAGG",2,1,3,NA,"GeneID:81570","CLPB",FALSE,"ADRA1A"

"3796","CGAAGTCTCGCTCTGTCACC","chr16",89196920,"-","0:A>G,2:C>T","AGG","TGGAGTCTCGCTCTGTCACCAGG",2,1,3,NA,"GeneID:146429","SLC22A31",FALSE,"ADRA1A"

"3797","CGAAGTCTCGCTCTGTCACC","chr19",7313508,"-","0:A>G,2:C>T","AGG","TGGAGTCTCGCTCTGTCACCAGG",2,1,3,NA,"","",FALSE,"ADRA1A"

"3798","CGAAGTCTCGCTCTGTCACC","chr20",58325151,"-","0:A>G,2:C>T","AGG","TGGAGTCTCGCTCTGTCACCAGG",2,1,3,NA,"GeneID:57403","RAB22A",FALSE,"ADRA1A"

"3799","CGAAGTCTCGCTCTGTCACC","chr12",15544482,"-","0:A>G,2:C>T","AGG","TGGAGTCTCGCTCTGTCACCAGG",2,1,3,NA,"GeneID:5800","PTPRO",FALSE,"ADRA1A"

"3800","CGAAGTCTCGCTCTGTCACC","chr15",26394391,"-","0:A>G,2:C>T","AGG","TGGAGTCTCGCTCTGTCACCAGG",2,1,3,NA,"","",FALSE,"ADRA1A"

"3801","CGAAGTCTCGCTCTGTCACC","chr4",70219052,"-","0:A>G,2:C>T","AGG","TGGAGTCTCGCTCTGTCACCAGG",2,1,3,NA,"","",FALSE,"ADRA1A"

"3802","CGAAGTCTCGCTCTGTCACC","chr7",146574675,"-","0:A>G,2:C>T","AGG","TGGAGTCTCGCTCTGTCACCAGG",2,1,3,NA,"GeneID:26047","CNTNAP2",FALSE,"ADRA1A"

"3803","CGAAGTCTCGCTCTGTCACC","chr1",16288404,"-","0:A>G,2:C>T","AGG","TGGAGTCTCGCTCTGTCACCAGG",2,1,3,NA,"GeneID:54455","FBXO42",FALSE,"ADRA1A"

"3804","CGAAGTCTCGCTCTGTCACC","chr12",6032302,"-","0:A>G,2:C>T","AGG","TGGAGTCTCGCTCTGTCACCAGG",2,1,3,NA,"GeneID:7450","VWF",FALSE,"ADRA1A"

"3805","CGAAGTCTCGCTCTGTCACC","chr10",62612574,"-","0:A>G,2:C>T","AGG","TGGAGTCTCGCTCTGTCACCAGG",2,1,3,NA,"GeneID:22891","ZNF365",FALSE,"ADRA1A"

"3806","CGAAGTCTCGCTCTGTCACC","chr19",52362385,"-","0:A>G,2:C>T","AGG","TGGAGTCTCGCTCTGTCACCAGG",2,1,3,NA,"GeneID:162963","ZNF610",FALSE,"ADRA1A"

"3807","CGAAGTCTCGCTCTGTCACC","chr19",51563536,"-","0:A>G,2:C>T","AGG","TGGAGTCTCGCTCTGTCACCAGG",2,1,3,NA,"","",FALSE,"ADRA1A"

"3808","CGAAGTCTCGCTCTGTCACC","chr13",114287565,"-","0:A>G,2:C>T","AGG","TGGAGTCTCGCTCTGTCACCAGG",2,1,3,NA,"GeneID:65110","UPF3A",TRUE,"ADRA1A"

"3809","CGAAGTCTCGCTCTGTCACC","chr14",24246376,"-","0:A>G,2:C>T","AGG","TGGAGTCTCGCTCTGTCACCAGG",2,1,3,NA,"","",FALSE,"ADRA1A"

"3810","CGAAGTCTCGCTCTGTCACC","chr5",179546196,"-","0:A>G,2:C>T","AGG","TGGAGTCTCGCTCTGTCACCAGG",2,1,3,NA,"","",FALSE,"ADRA1A"

"3811","CGAAGTCTCGCTCTGTCACC","chrX",54001300,"-","0:A>G,2:C>T","AGG","TGGAGTCTCGCTCTGTCACCAGG",2,1,3,NA,"GeneID:23133","PHF8",FALSE,"ADRA1A"

"3812","CGAAGTCTCGCTCTGTCACC","chr14",22750347,"-","0:A>G,2:C>T","AGG","TGGAGTCTCGCTCTGTCACCAGG",2,1,3,NA,"","",FALSE,"ADRA1A"

"3813","CGAAGTCTCGCTCTGTCACC","chr9",6474680,"-","0:A>G,2:C>T","AGG","TGGAGTCTCGCTCTGTCACCAGG",2,1,3,NA,"GeneID:115426","UHRF2",FALSE,"ADRA1A"

"3814","CGAAGTCTCGCTCTGTCACC","chr1",31521581,"-","0:A>G,2:C>T","AGG","TGGAGTCTCGCTCTGTCACCAGG",2,1,3,NA,"","",FALSE,"ADRA1A"

"3815","CGAAGTCTCGCTCTGTCACC","chr1",17015970,"-","0:A>G,2:C>T","AGG","TGGAGTCTCGCTCTGTCACCAGG",2,1,3,NA,"","",FALSE,"ADRA1A"

"3816","CGAAGTCTCGCTCTGTCACC","chrUn_GL000216v2",100746,"-","0:A>G,2:C>T","AGG","TGGAGTCTCGCTCTGTCACCAGG",2,1,3,NA,"","",FALSE,"ADRA1A"

"3817","CGAAGTCTCGCTCTGTCACC","chr22",10743690,"-","0:A>G,2:C>T","AGG","TGGAGTCTCGCTCTGTCACCAGG",2,1,3,NA,"","",FALSE,"ADRA1A"

"3818","CGAAGTCTCGCTCTGTCACC","chrX",70534066,"-","0:A>G,2:C>T","AGG","TGGAGTCTCGCTCTGTCACCAGG",2,1,3,NA,"GeneID:56159","TEX11",FALSE,"ADRA1A"

"3819","CGAAGTCTCGCTCTGTCACC","chr14",63659487,"-","0:A>G,2:C>T","AGG","TGGAGTCTCGCTCTGTCACCAGG",2,1,3,NA,"","",FALSE,"ADRA1A"

"3820","CGAAGTCTCGCTCTGTCACC","chr20",6086116,"-","0:A>G,2:C>T","AGG","TGGAGTCTCGCTCTGTCACCAGG",2,1,3,NA,"GeneID:55612","FERMT1",FALSE,"ADRA1A"

"3821","CGAAGTCTCGCTCTGTCACC","chr8",63709309,"-","0:A>G,2:C>T","AGG","TGGAGTCTCGCTCTGTCACCAGG",2,1,3,NA,"GeneID:102724623","LOC102724623",FALSE,"ADRA1A"

"3822","CGAAGTCTCGCTCTGTCACC","chr12",88565599,"-","0:A>G,2:C>T","AGG","TGGAGTCTCGCTCTGTCACCAGG",2,1,3,NA,"GeneID:4254","KITLG",FALSE,"ADRA1A"

"3823","CGAAGTCTCGCTCTGTCACC","chr3",127313891,"-","0:A>G,2:C>T","AGG","TGGAGTCTCGCTCTGTCACCAGG",2,1,3,NA,"","",FALSE,"ADRA1A"

"3824","CGAAGTCTCGCTCTGTCACC","chr10",119530066,"-","0:A>G,2:C>T","AGG","TGGAGTCTCGCTCTGTCACCAGG",2,1,3,NA,"GeneID:6001","RGS10",FALSE,"ADRA1A"

"3825","CGAAGTCTCGCTCTGTCACC","chr3",193329215,"-","0:A>G,2:C>T","AGG","TGGAGTCTCGCTCTGTCACCAGG",2,1,3,NA,"GeneID:344905","ATP13A5",FALSE,"ADRA1A"

"3826","CGAAGTCTCGCTCTGTCACC","chr7",73459222,"-","0:A>G,2:C>T","AGG","TGGAGTCTCGCTCTGTCACCAGG",2,1,3,NA,"GeneID:9031","BAZ1B",FALSE,"ADRA1A"

"3827","CGAAGTCTCGCTCTGTCACC","chr7",81268443,"-","0:A>G,2:C>T","AGG","TGGAGTCTCGCTCTGTCACCAGG",2,1,3,NA,"","",FALSE,"ADRA1A"

"3828","CGAAGTCTCGCTCTGTCACC","chr5",145875523,"-","0:A>G,2:C>T","AGG","TGGAGTCTCGCTCTGTCACCAGG",2,1,3,NA,"GeneID:643226","GRXCR2",FALSE,"ADRA1A"

"3829","CGAAGTCTCGCTCTGTCACC","chr6",99396957,"-","0:A>G,2:C>T","AGG","TGGAGTCTCGCTCTGTCACCAGG",2,1,3,NA,"","",FALSE,"ADRA1A"

"3830","CGAAGTCTCGCTCTGTCACC","chr6",19555805,"-","0:A>G,2:C>T","AGG","TGGAGTCTCGCTCTGTCACCAGG",2,1,3,NA,"GeneID:100506885","LOC100506885",FALSE,"ADRA1A"

"3831","CGAAGTCTCGCTCTGTCACC","chr2",61391069,"-","0:A>G,2:C>T","AGG","TGGAGTCTCGCTCTGTCACCAGG",2,1,3,NA,"GeneID:9736","USP34",FALSE,"ADRA1A"

"3832","CGAAGTCTCGCTCTGTCACC","chr1",35587612,"-","0:A>G,2:C>T","AGG","TGGAGTCTCGCTCTGTCACCAGG",2,1,3,NA,"GeneID:339488","TFAP2E",FALSE,"ADRA1A"

"3833","CGAAGTCTCGCTCTGTCACC","chr12",12937647,"-","0:A>G,2:C>T","AGG","TGGAGTCTCGCTCTGTCACCAGG",2,1,3,NA,"GeneID:100506314","LOC100506314",FALSE,"ADRA1A"

"3834","CGAAGTCTCGCTCTGTCACC","chr21",40510099,"-","0:A>G,2:C>T","AGG","TGGAGTCTCGCTCTGTCACCAGG",2,1,3,NA,"GeneID:1826","DSCAM",FALSE,"ADRA1A"

"3835","CGAAGTCTCGCTCTGTCACC","chr1",176181817,"-","0:A>G,2:C>T","AGG","TGGAGTCTCGCTCTGTCACCAGG",2,1,3,NA,"GeneID:64326","RFWD2",FALSE,"ADRA1A"

"3836","CGAAGTCTCGCTCTGTCACC","chrX",1611859,"-","0:A>G,2:C>T","AGG","TGGAGTCTCGCTCTGTCACCAGG",2,1,3,NA,"","",FALSE,"ADRA1A"

"3837","CGAAGTCTCGCTCTGTCACC","chr15",33746728,"-","0:A>G,2:C>T","AGG","TGGAGTCTCGCTCTGTCACCAGG",2,1,3,NA,"GeneID:6263","RYR3",FALSE,"ADRA1A"

"3838","CGAAGTCTCGCTCTGTCACC","chrX",152955876,"-","0:A>G,2:C>T","AGG","TGGAGTCTCGCTCTGTCACCAGG",2,1,3,NA,"GeneID:7739","ZNF185",FALSE,"ADRA1A"

"3839","CGAAGTCTCGCTCTGTCACC","chr4",121533660,"-","0:A>G,2:C>T","AGG","TGGAGTCTCGCTCTGTCACCAGG",2,1,3,NA,"","",FALSE,"ADRA1A"

"3840","CGAAGTCTCGCTCTGTCACC","chr10",106269172,"-","0:A>G,2:C>T","AGG","TGGAGTCTCGCTCTGTCACCAGG",2,1,3,NA,"","",FALSE,"ADRA1A"

"3841","CGAAGTCTCGCTCTGTCACC","chr8",23184314,"-","0:A>G,2:C>T","AGG","TGGAGTCTCGCTCTGTCACCAGG",2,1,3,NA,"","",FALSE,"ADRA1A"

"3842","CGAAGTCTCGCTCTGTCACC","chr3",189070055,"-","0:A>G,2:C>T","AGG","TGGAGTCTCGCTCTGTCACCAGG",2,1,3,NA,"GeneID:285386","TPRG1",FALSE,"ADRA1A"

"3843","CGAAGTCTCGCTCTGTCACC","chr18",79562643,"-","0:A>G,2:C>T","AGG","TGGAGTCTCGCTCTGTCACCAGG",2,1,3,NA,"","",FALSE,"ADRA1A"

"3844","CGAAGTCTCGCTCTGTCACC","chr7",140466055,"-","0:A>G,2:C>T","AGG","TGGAGTCTCGCTCTGTCACCAGG",2,1,3,NA,"GeneID:23608","MKRN1",FALSE,"ADRA1A"

"3845","CGAAGTCTCGCTCTGTCACC","chr6",148600591,"-","0:A>G,2:C>T","AGG","TGGAGTCTCGCTCTGTCACCAGG",2,1,3,NA,"","",FALSE,"ADRA1A"

"3846","CGAAGTCTCGCTCTGTCACC","chr20",319615,"-","0:A>G,2:C>T","AGG","TGGAGTCTCGCTCTGTCACCAGG",2,1,3,NA,"","",FALSE,"ADRA1A"

"3847","CGAAGTCTCGCTCTGTCACC","chr19",38002693,"-","0:A>G,2:C>T","AGG","TGGAGTCTCGCTCTGTCACCAGG",2,1,3,NA,"GeneID:23094","SIPA1L3",FALSE,"ADRA1A"

"3848","CGAAGTCTCGCTCTGTCACC","chr19",34363794,"-","0:A>G,2:C>T","AGG","TGGAGTCTCGCTCTGTCACCAGG",2,1,3,NA,"GeneID:2821","GPI",FALSE,"ADRA1A"

"3849","CGAAGTCTCGCTCTGTCACC","chr3",120803662,"-","0:A>G,2:C>T","AGG","TGGAGTCTCGCTCTGTCACCAGG",2,1,3,NA,"","",FALSE,"ADRA1A"

"3850","CGAAGTCTCGCTCTGTCACC","chr14",54613091,"-","0:A>G,2:C>T","AGG","TGGAGTCTCGCTCTGTCACCAGG",2,1,3,NA,"GeneID:23034","SAMD4A",FALSE,"ADRA1A"

"3851","CGAAGTCTCGCTCTGTCACC","chr5",12698440,"-","0:A>G,2:C>T","AGG","TGGAGTCTCGCTCTGTCACCAGG",2,1,3,NA,"","",FALSE,"ADRA1A"

"3852","CGAAGTCTCGCTCTGTCACC","chr2",68444633,"-","0:A>G,2:C>T","AGG","TGGAGTCTCGCTCTGTCACCAGG",2,1,3,NA,"","",FALSE,"ADRA1A"

"3853","CGAAGTCTCGCTCTGTCACC","chr20",3120321,"-","0:A>G,2:C>T","AGG","TGGAGTCTCGCTCTGTCACCAGG",2,1,3,NA,"GeneID:100134015;GeneID:22888","UBOX5-AS1;UBOX5",FALSE,"ADRA1A"

"3854","CGAAGTCTCGCTCTGTCACC","chr11",130026701,"-","0:A>G,2:C>T","AGG","TGGAGTCTCGCTCTGTCACCAGG",2,1,3,NA,"","",FALSE,"ADRA1A"

"3855","CGAAGTCTCGCTCTGTCACC","chr8",22373237,"-","0:A>G,2:C>T","AGG","TGGAGTCTCGCTCTGTCACCAGG",2,1,3,NA,"GeneID:23516","SLC39A14",FALSE,"ADRA1A"

"3856","CGAAGTCTCGCTCTGTCACC","chr3",76934717,"-","0:A>G,2:C>T","AGG","TGGAGTCTCGCTCTGTCACCAGG",2,1,3,NA,"GeneID:6092","ROBO2",FALSE,"ADRA1A"

"3857","CGAAGTCTCGCTCTGTCACC","chr20",21306980,"-","0:A>G,2:C>T","AGG","TGGAGTCTCGCTCTGTCACCAGG",2,1,3,NA,"GeneID:22803","XRN2",FALSE,"ADRA1A"

"3858","CGAAGTCTCGCTCTGTCACC","chr2",5913612,"-","0:A>G,2:C>T","AGG","TGGAGTCTCGCTCTGTCACCAGG",2,1,3,NA,"","",FALSE,"ADRA1A"

"3859","CGAAGTCTCGCTCTGTCACC","chr5",34932395,"-","0:A>G,2:C>T","AGG","TGGAGTCTCGCTCTGTCACCAGG",2,1,3,NA,"GeneID:134218","DNAJC21",FALSE,"ADRA1A"

"3860","CGAAGTCTCGCTCTGTCACC","chr9",92168255,"-","0:A>G,2:C>T","AGG","TGGAGTCTCGCTCTGTCACCAGG",2,1,3,NA,"","",FALSE,"ADRA1A"

"3861","CGAAGTCTCGCTCTGTCACC","chr9",15200973,"-","0:A>G,2:C>T","AGG","TGGAGTCTCGCTCTGTCACCAGG",2,1,3,NA,"GeneID:158219","TTC39B",FALSE,"ADRA1A"

"3862","CGAAGTCTCGCTCTGTCACC","chr3",183741756,"-","0:A>G,2:C>T","AGG","TGGAGTCTCGCTCTGTCACCAGG",2,1,3,NA,"GeneID:55689","YEATS2",FALSE,"ADRA1A"

"3863","CGAAGTCTCGCTCTGTCACC","chr19",13751389,"-","0:A>G,2:C>T","AGG","TGGAGTCTCGCTCTGTCACCAGG",2,1,3,NA,"GeneID:81576","CCDC130",FALSE,"ADRA1A"

"3864","CGAAGTCTCGCTCTGTCACC","chr6",53271524,"-","0:A>G,2:C>T","AGG","TGGAGTCTCGCTCTGTCACCAGG",2,1,3,NA,"GeneID:60481","ELOVL5",FALSE,"ADRA1A"

"3865","CGAAGTCTCGCTCTGTCACC","chr5",160105529,"-","0:A>G,2:C>T","AGG","TGGAGTCTCGCTCTGTCACCAGG",2,1,3,NA,"GeneID:114825","PWWP2A",FALSE,"ADRA1A"

"3866","CGAAGTCTCGCTCTGTCACC","chr12",119965854,"-","0:A>G,2:C>T","AGG","TGGAGTCTCGCTCTGTCACCAGG",2,1,3,NA,"","",FALSE,"ADRA1A"

"3867","CGAAGTCTCGCTCTGTCACC","chr8",51198674,"-","0:A>G,2:C>T","AGG","TGGAGTCTCGCTCTGTCACCAGG",2,1,3,NA,"","",FALSE,"ADRA1A"

"3868","CGAAGTCTCGCTCTGTCACC","chr15",32052703,"-","0:A>G,2:C>T","AGG","TGGAGTCTCGCTCTGTCACCAGG",2,1,3,NA,"GeneID:1139","CHRNA7",FALSE,"ADRA1A"

"3869","CGAAGTCTCGCTCTGTCACC","chr2",70048083,"-","0:A>G,2:C>T","AGG","TGGAGTCTCGCTCTGTCACCAGG",2,1,3,NA,"GeneID:400960","PCBP1-AS1",FALSE,"ADRA1A"

"3870","CGAAGTCTCGCTCTGTCACC","chr2",138850123,"-","0:A>G,2:C>T","AGG","TGGAGTCTCGCTCTGTCACCAGG",2,1,3,NA,"","",FALSE,"ADRA1A"

"3871","CGAAGTCTCGCTCTGTCACC","chr17",48080895,"-","0:A>G,2:C>T","AGG","TGGAGTCTCGCTCTGTCACCAGG",2,1,3,NA,"GeneID:10951","CBX1",FALSE,"ADRA1A"

"3872","CGAAGTCTCGCTCTGTCACC","chr6",79921435,"-","0:A>G,2:C>T","AGG","TGGAGTCTCGCTCTGTCACCAGG",2,1,3,NA,"GeneID:6785","ELOVL4",FALSE,"ADRA1A"

"3873","CGAAGTCTCGCTCTGTCACC","chr12",48028462,"-","0:A>G,2:C>T","AGG","TGGAGTCTCGCTCTGTCACCAGG",2,1,3,NA,"","",FALSE,"ADRA1A"

"3874","CGAAGTCTCGCTCTGTCACC","chr11",46677918,"-","0:A>G,2:C>T","AGG","TGGAGTCTCGCTCTGTCACCAGG",2,1,3,NA,"GeneID:392","ARHGAP1",TRUE,"ADRA1A"

"3875","CGAAGTCTCGCTCTGTCACC","chr12",11887722,"-","0:A>G,2:C>T","AGG","TGGAGTCTCGCTCTGTCACCAGG",2,1,3,NA,"GeneID:2120","ETV6",FALSE,"ADRA1A"

"3876","CGAAGTCTCGCTCTGTCACC","chr9",120346752,"-","0:A>G,2:C>T","AGG","TGGAGTCTCGCTCTGTCACCAGG",2,1,3,NA,"","",FALSE,"ADRA1A"

"3877","CGAAGTCTCGCTCTGTCACC","chr11",32880657,"-","0:A>G,2:C>T","AGG","TGGAGTCTCGCTCTGTCACCAGG",2,1,3,NA,"","",FALSE,"ADRA1A"

"3878","CGAAGTCTCGCTCTGTCACC","chr20",401661,"-","0:A>G,2:C>T","AGG","TGGAGTCTCGCTCTGTCACCAGG",2,1,3,NA,"","",FALSE,"ADRA1A"

"3879","CGAAGTCTCGCTCTGTCACC","chr6",158651562,"-","0:A>G,2:C>T","AGG","TGGAGTCTCGCTCTGTCACCAGG",2,1,3,NA,"GeneID:94120","SYTL3",FALSE,"ADRA1A"

"3880","CGAAGTCTCGCTCTGTCACC","chr1",25184915,"-","0:A>G,2:C>T","AGG","TGGAGTCTCGCTCTGTCACCAGG",2,1,3,NA,"","",FALSE,"ADRA1A"

"3881","CGAAGTCTCGCTCTGTCACC","chr6",33530005,"-","0:A>G,2:C>T","AGG","TGGAGTCTCGCTCTGTCACCAGG",2,1,3,NA,"","",FALSE,"ADRA1A"

"3882","CGAAGTCTCGCTCTGTCACC","chr15",53293418,"-","0:A>G,2:C>T","AGG","TGGAGTCTCGCTCTGTCACCAGG",2,1,3,NA,"","",FALSE,"ADRA1A"

"3883","CGAAGTCTCGCTCTGTCACC","chr3",172877910,"-","0:A>G,2:C>T","AGG","TGGAGTCTCGCTCTGTCACCAGG",2,1,3,NA,"","",FALSE,"ADRA1A"

"3884","CGAAGTCTCGCTCTGTCACC","chr7",57586402,"-","0:A>G,2:C>T","AGG","TGGAGTCTCGCTCTGTCACCAGG",2,1,3,NA,"","",FALSE,"ADRA1A"

"3885","CGAAGTCTCGCTCTGTCACC","chr17",3786864,"-","0:A>G,2:C>T","AGG","TGGAGTCTCGCTCTGTCACCAGG",2,1,3,NA,"GeneID:3682","ITGAE",FALSE,"ADRA1A"

"3886","CGAAGTCTCGCTCTGTCACC","chr11",3644158,"-","0:A>G,2:C>T","AGG","TGGAGTCTCGCTCTGTCACCAGG",2,1,3,NA,"","",FALSE,"ADRA1A"

"3887","CGAAGTCTCGCTCTGTCACC","chr4",184723817,"-","0:A>G,2:C>T","AGG","TGGAGTCTCGCTCTGTCACCAGG",2,1,3,NA,"GeneID:79682","CENPU",FALSE,"ADRA1A"

"3888","CGAAGTCTCGCTCTGTCACC","chr19",44237654,"-","0:A>G,2:C>T","AGG","TGGAGTCTCGCTCTGTCACCAGG",2,1,3,NA,"GeneID:9310","ZNF235",FALSE,"ADRA1A"

"3889","CGAAGTCTCGCTCTGTCACC","chr18",66288288,"-","0:A>G,2:C>T","AGG","TGGAGTCTCGCTCTGTCACCAGG",2,1,3,NA,"","",FALSE,"ADRA1A"

"3890","CGAAGTCTCGCTCTGTCACC","chr5",78575523,"-","0:A>G,2:C>T","AGG","TGGAGTCTCGCTCTGTCACCAGG",2,1,3,NA,"GeneID:10184","LHFPL2",FALSE,"ADRA1A"

"3891","CGAAGTCTCGCTCTGTCACC","chr22",19384298,"-","0:A>G,2:C>T","AGG","TGGAGTCTCGCTCTGTCACCAGG",2,1,3,NA,"GeneID:7290;GeneID:128977","HIRA;C22orf39",FALSE,"ADRA1A"

"3892","CGAAGTCTCGCTCTGTCACC","chr20",54615875,"-","0:A>G,2:C>T","AGG","TGGAGTCTCGCTCTGTCACCAGG",2,1,3,NA,"GeneID:55816","DOK5",FALSE,"ADRA1A"

"3893","CGAAGTCTCGCTCTGTCACC","chr10",43008221,"-","0:A>G,2:C>T","AGG","TGGAGTCTCGCTCTGTCACCAGG",2,1,3,NA,"","",FALSE,"ADRA1A"

"3894","CGAAGTCTCGCTCTGTCACC","chr14",87031870,"-","0:A>G,2:C>T","AGG","TGGAGTCTCGCTCTGTCACCAGG",2,1,3,NA,"","",FALSE,"ADRA1A"

"3895","CGAAGTCTCGCTCTGTCACC","chr1",62665690,"-","0:A>G,2:C>T","AGG","TGGAGTCTCGCTCTGTCACCAGG",2,1,3,NA,"GeneID:85440","DOCK7",FALSE,"ADRA1A"

"3896","CGAAGTCTCGCTCTGTCACC","chr12",15766472,"-","0:A>G,2:C>T","AGG","TGGAGTCTCGCTCTGTCACCAGG",2,1,3,NA,"GeneID:2059","EPS8",FALSE,"ADRA1A"

"3897","CGAAGTCTCGCTCTGTCACC","chr16",73478240,"-","0:A>G,2:C>T","AGG","TGGAGTCTCGCTCTGTCACCAGG",2,1,3,NA,"GeneID:463;GeneID:100506172","ZFHX3;LOC100506172",FALSE,"ADRA1A"

"3898","CGAAGTCTCGCTCTGTCACC","chr18",13075540,"-","0:A>G,2:C>T","AGG","TGGAGTCTCGCTCTGTCACCAGG",2,1,3,NA,"GeneID:55125","CEP192",FALSE,"ADRA1A"

"3899","CGAAGTCTCGCTCTGTCACC","chr7",72226070,"-","0:A>G,2:C>T","AGG","TGGAGTCTCGCTCTGTCACCAGG",2,1,3,NA,"GeneID:83698","CALN1",FALSE,"ADRA1A"

"3900","CGAAGTCTCGCTCTGTCACC","chr11",102174558,"-","0:A>G,2:C>T","AGG","TGGAGTCTCGCTCTGTCACCAGG",2,1,3,NA,"GeneID:10413","YAP1",FALSE,"ADRA1A"

"3901","CGAAGTCTCGCTCTGTCACC","chr3",141649317,"-","0:A>G,2:C>T","AGG","TGGAGTCTCGCTCTGTCACCAGG",2,1,3,NA,"","",FALSE,"ADRA1A"

"3902","CGAAGTCTCGCTCTGTCACC","chr7",152763289,"-","0:A>G,2:C>T","AGG","TGGAGTCTCGCTCTGTCACCAGG",2,1,3,NA,"GeneID:57180","ACTR3B",FALSE,"ADRA1A"

"3903","CGAAGTCTCGCTCTGTCACC","chr9",77868769,"-","0:A>G,2:C>T","AGG","TGGAGTCTCGCTCTGTCACCAGG",2,1,3,NA,"GeneID:2776","GNAQ",FALSE,"ADRA1A"

"3904","CGAAGTCTCGCTCTGTCACC","chr12",79535258,"-","0:A>G,2:C>T","AGG","TGGAGTCTCGCTCTGTCACCAGG",2,1,3,NA,"","",FALSE,"ADRA1A"

"3905","CGAAGTCTCGCTCTGTCACC","chr7",71834193,"-","0:A>G,2:C>T","AGG","TGGAGTCTCGCTCTGTCACCAGG",2,1,3,NA,"GeneID:83698","CALN1",FALSE,"ADRA1A"

"3906","CGAAGTCTCGCTCTGTCACC","chr3",54365837,"-","0:A>G,2:C>T","AGG","TGGAGTCTCGCTCTGTCACCAGG",2,1,3,NA,"GeneID:55799","CACNA2D3",FALSE,"ADRA1A"

"3907","CGAAGTCTCGCTCTGTCACC","chr10",29161379,"-","0:A>G,2:C>T","AGG","TGGAGTCTCGCTCTGTCACCAGG",2,1,3,NA,"","",FALSE,"ADRA1A"

"3908","CGAAGTCTCGCTCTGTCACC","chr15",39861523,"-","0:A>G,2:C>T","AGG","TGGAGTCTCGCTCTGTCACCAGG",2,1,3,NA,"GeneID:11245","GPR176",FALSE,"ADRA1A"

"3909","CGAAGTCTCGCTCTGTCACC","chr9",110119317,"-","0:A>G,2:C>T","AGG","TGGAGTCTCGCTCTGTCACCAGG",2,1,3,NA,"GeneID:445815;GeneID:11217","PALM2-AKAP2;AKAP2",FALSE,"ADRA1A"

"3910","CGAAGTCTCGCTCTGTCACC","chr5",40875026,"-","0:A>G,2:C>T","AGG","TGGAGTCTCGCTCTGTCACCAGG",2,1,3,NA,"","",FALSE,"ADRA1A"

"3911","CGAAGTCTCGCTCTGTCACC","chr11",98006699,"-","0:A>G,2:C>T","AGG","TGGAGTCTCGCTCTGTCACCAGG",2,1,3,NA,"","",FALSE,"ADRA1A"

"3912","CGAAGTCTCGCTCTGTCACC","chr7",53176945,"-","0:A>G,2:C>T","AGG","TGGAGTCTCGCTCTGTCACCAGG",2,1,3,NA,"","",FALSE,"ADRA1A"

"3913","CGAAGTCTCGCTCTGTCACC","chr12",107121520,"-","0:A>G,2:C>T","AGG","TGGAGTCTCGCTCTGTCACCAGG",2,1,3,NA,"","",FALSE,"ADRA1A"

"3914","CGAAGTCTCGCTCTGTCACC","chr1",27197206,"-","0:A>G,2:C>T","AGG","TGGAGTCTCGCTCTGTCACCAGG",2,1,3,NA,"","",FALSE,"ADRA1A"

"3915","CGAAGTCTCGCTCTGTCACC","chr9",34218226,"-","0:A>G,2:C>T","AGG","TGGAGTCTCGCTCTGTCACCAGG",2,1,3,NA,"GeneID:51271","UBAP1",FALSE,"ADRA1A"

"3916","CGAAGTCTCGCTCTGTCACC","chr3",136242861,"-","0:A>G,2:C>T","AGG","TGGAGTCTCGCTCTGTCACCAGG",2,1,3,NA,"","",FALSE,"ADRA1A"

"3917","CGAAGTCTCGCTCTGTCACC","chr7",23677183,"-","0:A>G,2:C>T","AGG","TGGAGTCTCGCTCTGTCACCAGG",2,1,3,NA,"","",FALSE,"ADRA1A"

"3918","CGAAGTCTCGCTCTGTCACC","chr2",204880633,"-","0:A>G,2:C>T","AGG","TGGAGTCTCGCTCTGTCACCAGG",2,1,3,NA,"GeneID:117583","PARD3B",FALSE,"ADRA1A"

"3919","CGAAGTCTCGCTCTGTCACC","chr19",14828734,"-","0:A>G,2:C>T","AGG","TGGAGTCTCGCTCTGTCACCAGG",2,1,3,NA,"GeneID:26664;GeneID:26659","OR7C1;OR7A5",FALSE,"ADRA1A"

"3920","CGAAGTCTCGCTCTGTCACC","chr13",76648945,"-","0:A>G,2:C>T","AGG","TGGAGTCTCGCTCTGTCACCAGG",2,1,3,NA,"","",FALSE,"ADRA1A"

"3921","CGAAGTCTCGCTCTGTCACC","chr13",53582458,"-","0:A>G,2:C>T","AGG","TGGAGTCTCGCTCTGTCACCAGG",2,1,3,NA,"","",FALSE,"ADRA1A"

"3922","CGAAGTCTCGCTCTGTCACC","chr17",10095777,"-","0:A>G,2:C>T","AGG","TGGAGTCTCGCTCTGTCACCAGG",2,1,3,NA,"GeneID:8522","GAS7",FALSE,"ADRA1A"

"3923","CGAAGTCTCGCTCTGTCACC","chr22",23170707,"-","0:A>G,2:C>T","AGG","TGGAGTCTCGCTCTGTCACCAGG",2,1,3,NA,"","",FALSE,"ADRA1A"

"3924","CGAAGTCTCGCTCTGTCACC","chrX",2267519,"-","0:A>G,2:C>T","AGG","TGGAGTCTCGCTCTGTCACCAGG",2,1,3,NA,"GeneID:207063","DHRSX",FALSE,"ADRA1A"

"3925","CGAAGTCTCGCTCTGTCACC","chr1",29627563,"-","0:A>G,2:C>T","AGG","TGGAGTCTCGCTCTGTCACCAGG",2,1,3,NA,"","",FALSE,"ADRA1A"

"3926","CGAAGTCTCGCTCTGTCACC","chr16",85021386,"-","0:A>G,2:C>T","AGG","TGGAGTCTCGCTCTGTCACCAGG",2,1,3,NA,"","",FALSE,"ADRA1A"

"3927","CGAAGTCTCGCTCTGTCACC","chr11",4158069,"-","0:A>G,2:C>T","AGG","TGGAGTCTCGCTCTGTCACCAGG",2,1,3,NA,"","",FALSE,"ADRA1A"

"3928","CGAAGTCTCGCTCTGTCACC","chr17",39691518,"-","0:A>G,2:C>T","AGG","TGGAGTCTCGCTCTGTCACCAGG",2,1,3,NA,"GeneID:93210;GeneID:2064","PGAP3;ERBB2",FALSE,"ADRA1A"

"3929","CGAAGTCTCGCTCTGTCACC","chr4",43810552,"-","0:A>G,2:C>T","AGG","TGGAGTCTCGCTCTGTCACCAGG",2,1,3,NA,"","",FALSE,"ADRA1A"

"3930","CGAAGTCTCGCTCTGTCACC","chr19",43853449,"-","0:A>G,2:C>T","AGG","TGGAGTCTCGCTCTGTCACCAGG",2,1,3,NA,"","",FALSE,"ADRA1A"

"3931","CGAAGTCTCGCTCTGTCACC","chr11",107262189,"-","0:A>G,2:C>T","AGG","TGGAGTCTCGCTCTGTCACCAGG",2,1,3,NA,"","",FALSE,"ADRA1A"

"3932","CGAAGTCTCGCTCTGTCACC","chr2",133197924,"-","0:A>G,2:C>T","AGG","TGGAGTCTCGCTCTGTCACCAGG",2,1,3,NA,"GeneID:344148","NCKAP5",FALSE,"ADRA1A"

"3933","CGAAGTCTCGCTCTGTCACC","chr15",67765333,"-","0:A>G,2:C>T","AGG","TGGAGTCTCGCTCTGTCACCAGG",2,1,3,NA,"GeneID:5607","MAP2K5",FALSE,"ADRA1A"

"3934","CGAAGTCTCGCTCTGTCACC","chr15",35027251,"-","0:A>G,2:C>T","AGG","TGGAGTCTCGCTCTGTCACCAGG",2,1,3,NA,"","",FALSE,"ADRA1A"

"3935","CGAAGTCTCGCTCTGTCACC","chr5",108387667,"-","0:A>G,2:C>T","AGG","TGGAGTCTCGCTCTGTCACCAGG",2,1,3,NA,"","",FALSE,"ADRA1A"

"3936","CGAAGTCTCGCTCTGTCACC","chr3",27790646,"-","0:A>G,2:C>T","AGG","TGGAGTCTCGCTCTGTCACCAGG",2,1,3,NA,"","",FALSE,"ADRA1A"

"3937","CGAAGTCTCGCTCTGTCACC","chr8",23442657,"-","0:A>G,2:C>T","AGG","TGGAGTCTCGCTCTGTCACCAGG",2,1,3,NA,"GeneID:9583","ENTPD4",FALSE,"ADRA1A"

"3938","CGAAGTCTCGCTCTGTCACC","chr2",46677808,"-","0:A>G,2:C>T","AGG","TGGAGTCTCGCTCTGTCACCAGG",2,1,3,NA,"","",FALSE,"ADRA1A"

"3939","CGAAGTCTCGCTCTGTCACC","chr3",128686999,"-","0:A>G,2:C>T","AGG","TGGAGTCTCGCTCTGTCACCAGG",2,1,3,NA,"","",FALSE,"ADRA1A"

"3940","CGAAGTCTCGCTCTGTCACC","chr19",56878414,"-","0:A>G,2:C>T","AGG","TGGAGTCTCGCTCTGTCACCAGG",2,1,3,NA,"","",FALSE,"ADRA1A"

"3941","CGAAGTCTCGCTCTGTCACC","chr17",12353060,"-","0:A>G,2:C>T","AGG","TGGAGTCTCGCTCTGTCACCAGG",2,1,3,NA,"","",FALSE,"ADRA1A"

"3942","CGAAGTCTCGCTCTGTCACC","chrX",25990539,"-","0:A>G,2:C>T","AGG","TGGAGTCTCGCTCTGTCACCAGG",2,1,3,NA,"","",FALSE,"ADRA1A"

"3943","CGAAGTCTCGCTCTGTCACC","chr5",28620794,"-","0:A>G,2:C>T","AGG","TGGAGTCTCGCTCTGTCACCAGG",2,1,3,NA,"","",FALSE,"ADRA1A"

"3944","CGAAGTCTCGCTCTGTCACC","chr8",82567704,"-","0:A>G,2:C>T","AGG","TGGAGTCTCGCTCTGTCACCAGG",2,1,3,NA,"","",FALSE,"ADRA1A"

"3945","CGAAGTCTCGCTCTGTCACC","chr11",63384023,"-","0:A>G,2:C>T","AGG","TGGAGTCTCGCTCTGTCACCAGG",2,1,3,NA,"GeneID:114571","SLC22A9",FALSE,"ADRA1A"

"3946","CGAAGTCTCGCTCTGTCACC","chr1",99719383,"-","0:A>G,2:C>T","AGG","TGGAGTCTCGCTCTGTCACCAGG",2,1,3,NA,"GeneID:391059","FRRS1",FALSE,"ADRA1A"

"3947","CGAAGTCTCGCTCTGTCACC","chr17",60650525,"-","0:A>G,2:C>T","AGG","TGGAGTCTCGCTCTGTCACCAGG",2,1,3,NA,"GeneID:8493","PPM1D",FALSE,"ADRA1A"

"3948","CGAAGTCTCGCTCTGTCACC","chr12",111590659,"-","0:A>G,2:C>T","AGG","TGGAGTCTCGCTCTGTCACCAGG",2,1,3,NA,"GeneID:6311","ATXN2",FALSE,"ADRA1A"

"3949","CGAAGTCTCGCTCTGTCACC","chr9",106446482,"-","0:A>G,2:C>T","AGG","TGGAGTCTCGCTCTGTCACCAGG",2,1,3,NA,"GeneID:100996590","LINC01505",FALSE,"ADRA1A"

"3950","CGAAGTCTCGCTCTGTCACC","chr14",75217198,"-","0:A>G,2:C>T","AGG","TGGAGTCTCGCTCTGTCACCAGG",2,1,3,NA,"","",FALSE,"ADRA1A"

"3951","CGAAGTCTCGCTCTGTCACC","chr3",159863314,"-","0:A>G,2:C>T","AGG","TGGAGTCTCGCTCTGTCACCAGG",2,1,3,NA,"GeneID:100505385;GeneID:29970","IQCJ-SCHIP1;SCHIP1",FALSE,"ADRA1A"

"3952","CGAAGTCTCGCTCTGTCACC","chr8",41961727,"-","0:A>G,2:C>T","AGG","TGGAGTCTCGCTCTGTCACCAGG",2,1,3,NA,"GeneID:7994","KAT6A",FALSE,"ADRA1A"

"3953","CGAAGTCTCGCTCTGTCACC","chrX",43378734,"-","0:A>G,2:C>T","AGG","TGGAGTCTCGCTCTGTCACCAGG",2,1,3,NA,"","",FALSE,"ADRA1A"

"3954","CGAAGTCTCGCTCTGTCACC","chr1",27847705,"-","0:A>G,2:C>T","AGG","TGGAGTCTCGCTCTGTCACCAGG",2,1,3,NA,"GeneID:5511","PPP1R8",FALSE,"ADRA1A"

"3955","CGAAGTCTCGCTCTGTCACC","chr6",113375573,"-","0:A>G,2:C>T","AGG","TGGAGTCTCGCTCTGTCACCAGG",2,1,3,NA,"","",FALSE,"ADRA1A"

"3956","CGAAGTCTCGCTCTGTCACC","chr12",132858702,"-","0:A>G,2:C>T","AGG","TGGAGTCTCGCTCTGTCACCAGG",2,1,3,NA,"GeneID:55743","CHFR",FALSE,"ADRA1A"

"3957","CGAAGTCTCGCTCTGTCACC","chr11",48279376,"-","0:A>G,2:C>T","AGG","TGGAGTCTCGCTCTGTCACCAGG",2,1,3,NA,"","",FALSE,"ADRA1A"

"3958","CGAAGTCTCGCTCTGTCACC","chr9",24897792,"-","0:A>G,2:C>T","AGG","TGGAGTCTCGCTCTGTCACCAGG",2,1,3,NA,"","",FALSE,"ADRA1A"

"3959","CGAAGTCTCGCTCTGTCACC","chr19",21029939,"-","0:A>G,2:C>T","AGG","TGGAGTCTCGCTCTGTCACCAGG",2,1,3,NA,"GeneID:80264","ZNF430",FALSE,"ADRA1A"

"3960","CGAAGTCTCGCTCTGTCACC","chr12",94943497,"-","0:A>G,2:C>T","AGG","TGGAGTCTCGCTCTGTCACCAGG",2,1,3,NA,"GeneID:55967","NDUFA12",FALSE,"ADRA1A"

"3961","CGAAGTCTCGCTCTGTCACC","chr21",9536339,"-","0:A>G,2:C>T","AGG","TGGAGTCTCGCTCTGTCACCAGG",2,1,3,NA,"","",FALSE,"ADRA1A"

"3962","CGAAGTCTCGCTCTGTCACC","chr8",114346929,"-","0:A>G,2:C>T","AGG","TGGAGTCTCGCTCTGTCACCAGG",2,1,3,NA,"","",FALSE,"ADRA1A"

"3963","CGAAGTCTCGCTCTGTCACC","chr3",49934741,"-","0:A>G,2:C>T","AGG","TGGAGTCTCGCTCTGTCACCAGG",2,1,3,NA,"","",FALSE,"ADRA1A"

"3964","CGAAGTCTCGCTCTGTCACC","chr2",203429731,"-","0:A>G,2:C>T","AGG","TGGAGTCTCGCTCTGTCACCAGG",2,1,3,NA,"GeneID:10152;GeneID:65059","ABI2;RAPH1",TRUE,"ADRA1A"

"3965","CGAAGTCTCGCTCTGTCACC","chrX",123872075,"-","0:A>G,2:C>T","AGG","TGGAGTCTCGCTCTGTCACCAGG",2,1,3,NA,"GeneID:331","XIAP",FALSE,"ADRA1A"

"3966","CGAAGTCTCGCTCTGTCACC","chr5",166946341,"-","0:A>G,2:C>T","AGG","TGGAGTCTCGCTCTGTCACCAGG",2,1,3,NA,"","",FALSE,"ADRA1A"

"3967","CGAAGTCTCGCTCTGTCACC","chr1",40149016,"-","0:A>G,2:C>T","AGG","TGGAGTCTCGCTCTGTCACCAGG",2,1,3,NA,"","",FALSE,"ADRA1A"

"3968","CGAAGTCTCGCTCTGTCACC","chr15",75840392,"-","0:A>G,2:C>T","AGG","TGGAGTCTCGCTCTGTCACCAGG",2,1,3,NA,"","",FALSE,"ADRA1A"

"3969","CGAAGTCTCGCTCTGTCACC","chr7",101796643,"-","0:A>G,2:C>T","AGG","TGGAGTCTCGCTCTGTCACCAGG",2,1,3,NA,"","",FALSE,"ADRA1A"

"3970","CGAAGTCTCGCTCTGTCACC","chr4",172543071,"-","0:A>G,2:C>T","AGG","TGGAGTCTCGCTCTGTCACCAGG",2,1,3,NA,"GeneID:442117","GALNTL6",FALSE,"ADRA1A"

"3971","CGAAGTCTCGCTCTGTCACC","chr13",31974083,"-","0:A>G,2:C>T","AGG","TGGAGTCTCGCTCTGTCACCAGG",2,1,3,NA,"GeneID:10129","FRY",FALSE,"ADRA1A"

"3972","CGAAGTCTCGCTCTGTCACC","chr2",142939576,"-","0:A>G,2:C>T","AGG","TGGAGTCTCGCTCTGTCACCAGG",2,1,3,NA,"GeneID:8942","KYNU",FALSE,"ADRA1A"

"3973","CGAAGTCTCGCTCTGTCACC","chr16",75547753,"-","0:A>G,2:C>T","AGG","TGGAGTCTCGCTCTGTCACCAGG",2,1,3,NA,"GeneID:79583","TMEM231",FALSE,"ADRA1A"

"3974","CGAAGTCTCGCTCTGTCACC","chr11",9824537,"-","0:A>G,2:C>T","AGG","TGGAGTCTCGCTCTGTCACCAGG",2,1,3,NA,"GeneID:81846","SBF2",FALSE,"ADRA1A"

"3975","CGAAGTCTCGCTCTGTCACC","chr16",20261835,"-","0:A>G,2:C>T","AGG","TGGAGTCTCGCTCTGTCACCAGG",2,1,3,NA,"","",FALSE,"ADRA1A"

"3976","CGAAGTCTCGCTCTGTCACC","chr17",45032757,"-","0:A>G,2:C>T","AGG","TGGAGTCTCGCTCTGTCACCAGG",2,1,3,NA,"GeneID:79877","DCAKD",FALSE,"ADRA1A"

"3977","CGAAGTCTCGCTCTGTCACC","chr3",183113198,"-","0:A>G,2:C>T","AGG","TGGAGTCTCGCTCTGTCACCAGG",2,1,3,NA,"GeneID:56922","MCCC1",FALSE,"ADRA1A"

"3978","CGAAGTCTCGCTCTGTCACC","chr7",134174083,"-","0:A>G,2:C>T","AGG","TGGAGTCTCGCTCTGTCACCAGG",2,1,3,NA,"GeneID:136332","LRGUK",FALSE,"ADRA1A"

"3979","CGAAGTCTCGCTCTGTCACC","chr18",12696477,"-","0:A>G,2:C>T","AGG","TGGAGTCTCGCTCTGTCACCAGG",2,1,3,NA,"GeneID:56984;GeneID:79959","PSMG2;CEP76",FALSE,"ADRA1A"

"3980","CGAAGTCTCGCTCTGTCACC","chr13",32056165,"-","0:A>G,2:C>T","AGG","TGGAGTCTCGCTCTGTCACCAGG",2,1,3,NA,"GeneID:10129","FRY",FALSE,"ADRA1A"

"3981","CGAAGTCTCGCTCTGTCACC","chr2",14895030,"-","0:A>G,2:C>T","AGG","TGGAGTCTCGCTCTGTCACCAGG",2,1,3,NA,"","",FALSE,"ADRA1A"

"3982","CGAAGTCTCGCTCTGTCACC","chr9",107536107,"-","0:A>G,2:C>T","AGG","TGGAGTCTCGCTCTGTCACCAGG",2,1,3,NA,"","",FALSE,"ADRA1A"

"3983","CGAAGTCTCGCTCTGTCACC","chr11",133813359,"-","0:A>G,2:C>T","AGG","TGGAGTCTCGCTCTGTCACCAGG",2,1,3,NA,"","",FALSE,"ADRA1A"

"3984","CGAAGTCTCGCTCTGTCACC","chr9",32075456,"-","0:A>G,2:C>T","AGG","TGGAGTCTCGCTCTGTCACCAGG",2,1,3,NA,"","",FALSE,"ADRA1A"

"3985","CGAAGTCTCGCTCTGTCACC","chr1",239045994,"-","0:A>G,2:C>T","AGG","TGGAGTCTCGCTCTGTCACCAGG",2,1,3,NA,"","",FALSE,"ADRA1A"

"3986","CGAAGTCTCGCTCTGTCACC","chr12",101879965,"-","0:A>G,2:C>T","AGG","TGGAGTCTCGCTCTGTCACCAGG",2,1,3,NA,"GeneID:55332","DRAM1",FALSE,"ADRA1A"

"3987","CGAAGTCTCGCTCTGTCACC","chrX",33672847,"-","0:A>G,2:C>T","AGG","TGGAGTCTCGCTCTGTCACCAGG",2,1,3,NA,"","",FALSE,"ADRA1A"

"3988","CGAAGTCTCGCTCTGTCACC","chr2",235328233,"-","0:A>G,2:C>T","AGG","TGGAGTCTCGCTCTGTCACCAGG",2,1,3,NA,"","",FALSE,"ADRA1A"

"3989","CGAAGTCTCGCTCTGTCACC","chr2",21572743,"-","0:A>G,2:C>T","AGG","TGGAGTCTCGCTCTGTCACCAGG",2,1,3,NA,"","",FALSE,"ADRA1A"

"3990","CGAAGTCTCGCTCTGTCACC","chr15",89946395,"-","0:A>G,2:C>T","AGG","TGGAGTCTCGCTCTGTCACCAGG",2,1,3,NA,"","",FALSE,"ADRA1A"

"3991","CGAAGTCTCGCTCTGTCACC","chr17",56858047,"-","0:A>G,2:C>T","AGG","TGGAGTCTCGCTCTGTCACCAGG",2,1,3,NA,"GeneID:8526","DGKE",FALSE,"ADRA1A"

"3992","CGAAGTCTCGCTCTGTCACC","chr16",8480139,"-","0:A>G,2:C>T","AGG","TGGAGTCTCGCTCTGTCACCAGG",2,1,3,NA,"","",FALSE,"ADRA1A"

"3993","CGAAGTCTCGCTCTGTCACC","chr10",125898161,"-","0:A>G,2:C>T","AGG","TGGAGTCTCGCTCTGTCACCAGG",2,1,3,NA,"GeneID:92565","FANK1",FALSE,"ADRA1A"

"3994","CGAAGTCTCGCTCTGTCACC","chr21",44653132,"-","0:A>G,2:C>T","AGG","TGGAGTCTCGCTCTGTCACCAGG",2,1,3,NA,"GeneID:54084","TSPEAR",FALSE,"ADRA1A"

"3995","CGAAGTCTCGCTCTGTCACC","chr16",53785884,"-","0:A>G,2:C>T","AGG","TGGAGTCTCGCTCTGTCACCAGG",2,1,3,NA,"GeneID:79068","FTO",FALSE,"ADRA1A"

"3996","CGAAGTCTCGCTCTGTCACC","chr17",81671106,"-","0:A>G,2:C>T","AGG","TGGAGTCTCGCTCTGTCACCAGG",2,1,3,NA,"GeneID:339230","CCDC137",FALSE,"ADRA1A"

"3997","CGAAGTCTCGCTCTGTCACC","chr3",32834816,"-","0:A>G,2:C>T","AGG","TGGAGTCTCGCTCTGTCACCAGG",2,1,3,NA,"GeneID:131405","TRIM71",FALSE,"ADRA1A"

"3998","CGAAGTCTCGCTCTGTCACC","chr14",54455283,"-","0:A>G,2:C>T","AGG","TGGAGTCTCGCTCTGTCACCAGG",2,1,3,NA,"","",FALSE,"ADRA1A"

"3999","CGAAGTCTCGCTCTGTCACC","chr16",58627379,"-","0:A>G,2:C>T","AGG","TGGAGTCTCGCTCTGTCACCAGG",2,1,3,NA,"GeneID:23019","CNOT1",FALSE,"ADRA1A"

"4000","CGAAGTCTCGCTCTGTCACC","chr19",39862429,"-","0:A>G,2:C>T","AGG","TGGAGTCTCGCTCTGTCACCAGG",2,1,3,NA,"","",FALSE,"ADRA1A"

"4001","CGAAGTCTCGCTCTGTCACC","chr1",90123719,"-","0:A>G,2:C>T","AGG","TGGAGTCTCGCTCTGTCACCAGG",2,1,3,NA,"","",FALSE,"ADRA1A"

"4002","CGAAGTCTCGCTCTGTCACC","chr6",129217163,"-","0:A>G,2:C>T","AGG","TGGAGTCTCGCTCTGTCACCAGG",2,1,3,NA,"GeneID:3908","LAMA2",FALSE,"ADRA1A"

"4003","CGAAGTCTCGCTCTGTCACC","chr12",111876181,"-","0:A>G,2:C>T","AGG","TGGAGTCTCGCTCTGTCACCAGG",2,1,3,NA,"GeneID:8550","MAPKAPK5",FALSE,"ADRA1A"

"4004","CGAAGTCTCGCTCTGTCACC","chr8",51543686,"-","0:A>G,2:C>T","AGG","TGGAGTCTCGCTCTGTCACCAGG",2,1,3,NA,"GeneID:137902","PXDNL",FALSE,"ADRA1A"

"4005","CGAAGTCTCGCTCTGTCACC","chr18",44675878,"-","0:A>G,2:C>T","AGG","TGGAGTCTCGCTCTGTCACCAGG",2,1,3,NA,"","",FALSE,"ADRA1A"

"4006","CGAAGTCTCGCTCTGTCACC","chr10",18442799,"-","0:A>G,2:C>T","AGG","TGGAGTCTCGCTCTGTCACCAGG",2,1,3,NA,"GeneID:783","CACNB2",FALSE,"ADRA1A"

"4007","CGAAGTCTCGCTCTGTCACC","chr16",3487367,"-","0:A>G,2:C>T","AGG","TGGAGTCTCGCTCTGTCACCAGG",2,1,3,NA,"","",FALSE,"ADRA1A"

"4008","CGAAGTCTCGCTCTGTCACC","chr4",186729728,"-","0:A>G,2:C>T","AGG","TGGAGTCTCGCTCTGTCACCAGG",2,1,3,NA,"","",FALSE,"ADRA1A"

"4009","CGAAGTCTCGCTCTGTCACC","chr21",32877472,"-","0:A>G,2:C>T","AGG","TGGAGTCTCGCTCTGTCACCAGG",2,1,3,NA,"","",FALSE,"ADRA1A"

"4010","CGAAGTCTCGCTCTGTCACC","chr4",109423113,"-","0:A>G,2:C>T","AGG","TGGAGTCTCGCTCTGTCACCAGG",2,1,3,NA,"GeneID:100533182","SEC24B-AS1",FALSE,"ADRA1A"

"4011","CGAAGTCTCGCTCTGTCACC","chr16",56924558,"-","0:A>G,2:C>T","AGG","TGGAGTCTCGCTCTGTCACCAGG",2,1,3,NA,"","",FALSE,"ADRA1A"

"4012","CGAAGTCTCGCTCTGTCACC","chr7",80863206,"-","0:A>G,2:C>T","AGG","TGGAGTCTCGCTCTGTCACCAGG",2,1,3,NA,"GeneID:10512","SEMA3C",FALSE,"ADRA1A"

"4013","CGAAGTCTCGCTCTGTCACC","chr4",148647821,"-","0:A>G,2:C>T","AGG","TGGAGTCTCGCTCTGTCACCAGG",2,1,3,NA,"","",FALSE,"ADRA1A"

"4014","CGAAGTCTCGCTCTGTCACC","chr12",98851998,"-","0:A>G,2:C>T","AGG","TGGAGTCTCGCTCTGTCACCAGG",2,1,3,NA,"GeneID:56899","ANKS1B",FALSE,"ADRA1A"

"4015","CGAAGTCTCGCTCTGTCACC","chr2",185449497,"-","0:A>G,2:C>T","AGG","TGGAGTCTCGCTCTGTCACCAGG",2,1,3,NA,"","",FALSE,"ADRA1A"

"4016","CGAAGTCTCGCTCTGTCACC","chr22",44665001,"-","0:A>G,2:C>T","AGG","TGGAGTCTCGCTCTGTCACCAGG",2,1,3,NA,"","",FALSE,"ADRA1A"

"4017","CGAAGTCTCGCTCTGTCACC","chr15",72097884,"-","0:A>G,2:C>T","AGG","TGGAGTCTCGCTCTGTCACCAGG",2,1,3,NA,"GeneID:4649","MYO9A",FALSE,"ADRA1A"

"4018","CGAAGTCTCGCTCTGTCACC","chr7",145574364,"-","0:A>G,2:C>T","AGG","TGGAGTCTCGCTCTGTCACCAGG",2,1,3,NA,"","",FALSE,"ADRA1A"

"4019","CGAAGTCTCGCTCTGTCACC","chr12",111740758,"-","0:A>G,2:C>T","AGG","TGGAGTCTCGCTCTGTCACCAGG",2,1,3,NA,"GeneID:80724","ACAD10",FALSE,"ADRA1A"

"4020","CGAAGTCTCGCTCTGTCACC","chr7",153278588,"-","0:A>G,2:C>T","AGG","TGGAGTCTCGCTCTGTCACCAGG",2,1,3,NA,"","",FALSE,"ADRA1A"

"4021","CGAAGTCTCGCTCTGTCACC","chr6",137201647,"-","0:A>G,2:C>T","AGG","TGGAGTCTCGCTCTGTCACCAGG",2,1,3,NA,"GeneID:3459","IFNGR1",FALSE,"ADRA1A"

"4022","CGAAGTCTCGCTCTGTCACC","chr15",28206985,"-","0:A>G,2:C>T","AGG","TGGAGTCTCGCTCTGTCACCAGG",2,1,3,NA,"GeneID:8924","HERC2",FALSE,"ADRA1A"

"4023","CGAAGTCTCGCTCTGTCACC","chr10",100629516,"-","0:A>G,2:C>T","AGG","TGGAGTCTCGCTCTGTCACCAGG",2,1,3,NA,"","",FALSE,"ADRA1A"

"4024","CGAAGTCTCGCTCTGTCACC","chr17",53349403,"-","0:A>G,2:C>T","AGG","TGGAGTCTCGCTCTGTCACCAGG",2,1,3,NA,"","",FALSE,"ADRA1A"

"4025","CGAAGTCTCGCTCTGTCACC","chr8",102430387,"-","0:A>G,2:C>T","AGG","TGGAGTCTCGCTCTGTCACCAGG",2,1,3,NA,"","",FALSE,"ADRA1A"

"4026","CGAAGTCTCGCTCTGTCACC","chr1",205766535,"-","0:A>G,2:C>T","AGG","TGGAGTCTCGCTCTGTCACCAGG",2,1,3,NA,"","",FALSE,"ADRA1A"

"4027","CGAAGTCTCGCTCTGTCACC","chr3",21512121,"-","0:A>G,2:C>T","AGG","TGGAGTCTCGCTCTGTCACCAGG",2,1,3,NA,"GeneID:79750","ZNF385D",FALSE,"ADRA1A"

"4028","CGAAGTCTCGCTCTGTCACC","chr6",120494976,"-","0:A>G,2:C>T","AGG","TGGAGTCTCGCTCTGTCACCAGG",2,1,3,NA,"","",FALSE,"ADRA1A"

"4029","CGAAGTCTCGCTCTGTCACC","chr16",6807790,"-","0:A>G,2:C>T","AGG","TGGAGTCTCGCTCTGTCACCAGG",2,1,3,NA,"GeneID:54715","RBFOX1",FALSE,"ADRA1A"

"4030","CGAAGTCTCGCTCTGTCACC","chr8",80760630,"-","0:A>G,2:C>T","AGG","TGGAGTCTCGCTCTGTCACCAGG",2,1,3,NA,"GeneID:619279","ZNF704",FALSE,"ADRA1A"

"4031","CGAAGTCTCGCTCTGTCACC","chr1",17894924,"-","0:A>G,2:C>T","AGG","TGGAGTCTCGCTCTGTCACCAGG",2,1,3,NA,"","",FALSE,"ADRA1A"

"4032","CGAAGTCTCGCTCTGTCACC","chr20",344073,"-","0:A>G,2:C>T","AGG","TGGAGTCTCGCTCTGTCACCAGG",2,1,3,NA,"","",FALSE,"ADRA1A"

"4033","CGAAGTCTCGCTCTGTCACC","chr3",136139667,"-","0:A>G,2:C>T","AGG","TGGAGTCTCGCTCTGTCACCAGG",2,1,3,NA,"GeneID:5523","PPP2R3A",FALSE,"ADRA1A"

"4034","CGAAGTCTCGCTCTGTCACC","chr13",110885522,"-","0:A>G,2:C>T","AGG","TGGAGTCTCGCTCTGTCACCAGG",2,1,3,NA,"GeneID:55608","ANKRD10",FALSE,"ADRA1A"

"4035","CGAAGTCTCGCTCTGTCACC","chr2",100358417,"-","0:A>G,2:C>T","AGG","TGGAGTCTCGCTCTGTCACCAGG",2,1,3,NA,"","",FALSE,"ADRA1A"

"4036","CGAAGTCTCGCTCTGTCACC","chr10",115783987,"-","0:A>G,2:C>T","AGG","TGGAGTCTCGCTCTGTCACCAGG",2,1,3,NA,"GeneID:26033","ATRNL1",FALSE,"ADRA1A"

"4037","CGAAGTCTCGCTCTGTCACC","chr2",28876216,"-","0:A>G,2:C>T","AGG","TGGAGTCTCGCTCTGTCACCAGG",2,1,3,NA,"","",FALSE,"ADRA1A"

"4038","CGAAGTCTCGCTCTGTCACC","chr4",61392660,"-","0:A>G,2:C>T","AGG","TGGAGTCTCGCTCTGTCACCAGG",2,1,3,NA,"GeneID:23284","LPHN3",FALSE,"ADRA1A"

"4039","CGAAGTCTCGCTCTGTCACC","chr10",121934576,"-","0:A>G,2:C>T","AGG","TGGAGTCTCGCTCTGTCACCAGG",2,1,3,NA,"GeneID:100130887","ATE1-AS1",FALSE,"ADRA1A"

"4040","CGAAGTCTCGCTCTGTCACC","chr7",22811683,"-","0:A>G,2:C>T","AGG","TGGAGTCTCGCTCTGTCACCAGG",2,1,3,NA,"","",FALSE,"ADRA1A"

"4041","CGAAGTCTCGCTCTGTCACC","chr12",119922884,"-","0:A>G,2:C>T","AGG","TGGAGTCTCGCTCTGTCACCAGG",2,1,3,NA,"","",FALSE,"ADRA1A"

"4042","CGAAGTCTCGCTCTGTCACC","chr14",56489580,"-","0:A>G,2:C>T","AGG","TGGAGTCTCGCTCTGTCACCAGG",2,1,3,NA,"GeneID:54916","TMEM260",FALSE,"ADRA1A"

"4043","CGAAGTCTCGCTCTGTCACC","chrX",127645862,"-","0:A>G,2:C>T","AGG","TGGAGTCTCGCTCTGTCACCAGG",2,1,3,NA,"","",FALSE,"ADRA1A"

"4044","CGAAGTCTCGCTCTGTCACC","chr12",4196235,"-","0:A>G,2:C>T","AGG","TGGAGTCTCGCTCTGTCACCAGG",2,1,3,NA,"","",FALSE,"ADRA1A"

"4045","CGAAGTCTCGCTCTGTCACC","chr1",239413052,"-","0:A>G,2:C>T","AGG","TGGAGTCTCGCTCTGTCACCAGG",2,1,3,NA,"GeneID:1131","CHRM3",FALSE,"ADRA1A"

"4046","CGAAGTCTCGCTCTGTCACC","chr21",31852195,"-","0:A>G,2:C>T","AGG","TGGAGTCTCGCTCTGTCACCAGG",2,1,3,NA,"","",FALSE,"ADRA1A"

"4047","CGAAGTCTCGCTCTGTCACC","chr16",86853271,"-","0:A>G,2:C>T","AGG","TGGAGTCTCGCTCTGTCACCAGG",2,1,3,NA,"","",FALSE,"ADRA1A"

"4048","CGAAGTCTCGCTCTGTCACC","chr13",41353090,"-","0:A>G,2:C>T","AGG","TGGAGTCTCGCTCTGTCACCAGG",2,1,3,NA,"GeneID:79612","NAA16",FALSE,"ADRA1A"

"4049","CGAAGTCTCGCTCTGTCACC","chrX",89997291,"-","0:A>G,2:C>T","AGG","TGGAGTCTCGCTCTGTCACCAGG",2,1,3,NA,"","",FALSE,"ADRA1A"

"4050","CGAAGTCTCGCTCTGTCACC","chrY",3654906,"-","0:A>G,2:C>T","AGG","TGGAGTCTCGCTCTGTCACCAGG",2,1,3,NA,"","",FALSE,"ADRA1A"

"4051","CGAAGTCTCGCTCTGTCACC","chr4",2590439,"-","0:A>G,2:C>T","AGG","TGGAGTCTCGCTCTGTCACCAGG",2,1,3,NA,"GeneID:6047;GeneID:8603","RNF4;FAM193A",FALSE,"ADRA1A"

"4052","CGAAGTCTCGCTCTGTCACC","chr7",146269314,"-","0:A>G,2:C>T","AGG","TGGAGTCTCGCTCTGTCACCAGG",2,1,3,NA,"GeneID:26047","CNTNAP2",FALSE,"ADRA1A"

"4053","CGAAGTCTCGCTCTGTCACC","chr2",185483500,"-","0:A>G,2:C>T","AGG","TGGAGTCTCGCTCTGTCACCAGG",2,1,3,NA,"","",FALSE,"ADRA1A"

"4054","CGAAGTCTCGCTCTGTCACC","chr5",96942628,"-","0:A>G,2:C>T","AGG","TGGAGTCTCGCTCTGTCACCAGG",2,1,3,NA,"GeneID:4012","LNPEP",FALSE,"ADRA1A"

"4055","CGAAGTCTCGCTCTGTCACC","chr6",23336612,"-","0:A>G,2:C>T","AGG","TGGAGTCTCGCTCTGTCACCAGG",2,1,3,NA,"","",FALSE,"ADRA1A"

"4056","CGAAGTCTCGCTCTGTCACC","chr10",56951110,"-","0:A>G,2:C>T","AGG","TGGAGTCTCGCTCTGTCACCAGG",2,1,3,NA,"","",FALSE,"ADRA1A"

"4057","CGAAGTCTCGCTCTGTCACC","chr20",45183073,"-","0:A>G,2:C>T","AGG","TGGAGTCTCGCTCTGTCACCAGG",2,1,3,NA,"","",FALSE,"ADRA1A"

"4058","CGAAGTCTCGCTCTGTCACC","chr4",122237957,"-","0:A>G,2:C>T","AGG","TGGAGTCTCGCTCTGTCACCAGG",2,1,3,NA,"GeneID:84162","KIAA1109",FALSE,"ADRA1A"

"4059","CGAAGTCTCGCTCTGTCACC","chr10",89848921,"-","0:A>G,2:C>T","AGG","TGGAGTCTCGCTCTGTCACCAGG",2,1,3,NA,"","",FALSE,"ADRA1A"

"4060","CGAAGTCTCGCTCTGTCACC","chr1",13405600,"-","0:A>G,2:C>T","AGG","TGGAGTCTCGCTCTGTCACCAGG",2,1,3,NA,"","",FALSE,"ADRA1A"

"4061","CGAAGTCTCGCTCTGTCACC","chr17",42229840,"-","0:A>G,2:C>T","AGG","TGGAGTCTCGCTCTGTCACCAGG",2,1,3,NA,"GeneID:6777","STAT5B",FALSE,"ADRA1A"

"4062","CGAAGTCTCGCTCTGTCACC","chr6",129702973,"-","0:A>G,2:C>T","AGG","TGGAGTCTCGCTCTGTCACCAGG",2,1,3,NA,"GeneID:93663","ARHGAP18",FALSE,"ADRA1A"

"4063","CGAAGTCTCGCTCTGTCACC","chr4",182397375,"-","0:A>G,2:C>T","AGG","TGGAGTCTCGCTCTGTCACCAGG",2,1,3,NA,"GeneID:55714","TENM3",FALSE,"ADRA1A"

"4064","CGAAGTCTCGCTCTGTCACC","chr12",121929295,"-","0:A>G,2:C>T","AGG","TGGAGTCTCGCTCTGTCACCAGG",2,1,3,NA,"GeneID:144406","WDR66",FALSE,"ADRA1A"

"4065","CGAAGTCTCGCTCTGTCACC","chr3",107992783,"-","0:A>G,2:C>T","AGG","TGGAGTCTCGCTCTGTCACCAGG",2,1,3,NA,"","",FALSE,"ADRA1A"

"4066","CGAAGTCTCGCTCTGTCACC","chr10",71123767,"-","0:A>G,2:C>T","AGG","TGGAGTCTCGCTCTGTCACCAGG",2,1,3,NA,"","",FALSE,"ADRA1A"

"4067","CGAAGTCTCGCTCTGTCACC","chr19",56382597,"-","0:A>G,2:C>T","AGG","TGGAGTCTCGCTCTGTCACCAGG",2,1,3,NA,"GeneID:147948","ZNF582",FALSE,"ADRA1A"

"4068","CGAAGTCTCGCTCTGTCACC","chr12",51344665,"-","0:A>G,2:C>T","AGG","TGGAGTCTCGCTCTGTCACCAGG",2,1,3,NA,"GeneID:1990","CELA1",FALSE,"ADRA1A"

"4069","CGAAGTCTCGCTCTGTCACC","chr16",7003434,"-","0:A>G,2:C>T","AGG","TGGAGTCTCGCTCTGTCACCAGG",2,1,3,NA,"GeneID:54715","RBFOX1",FALSE,"ADRA1A"

"4070","CGAAGTCTCGCTCTGTCACC","chr9",80716527,"-","0:A>G,2:C>T","AGG","TGGAGTCTCGCTCTGTCACCAGG",2,1,3,NA,"","",FALSE,"ADRA1A"

"4071","CGAAGTCTCGCTCTGTCACC","chr19",8917354,"-","0:A>G,2:C>T","AGG","TGGAGTCTCGCTCTGTCACCAGG",2,1,3,NA,"GeneID:94025","MUC16",FALSE,"ADRA1A"

"4072","CGAAGTCTCGCTCTGTCACC","chr14",94961377,"-","0:A>G,2:C>T","AGG","TGGAGTCTCGCTCTGTCACCAGG",2,1,3,NA,"","",FALSE,"ADRA1A"

"4073","CGAAGTCTCGCTCTGTCACC","chr18",46428383,"-","0:A>G,2:C>T","AGG","TGGAGTCTCGCTCTGTCACCAGG",2,1,3,NA,"GeneID:494470","RNF165",FALSE,"ADRA1A"

"4074","CGAAGTCTCGCTCTGTCACC","chr1",203646737,"-","0:A>G,2:C>T","AGG","TGGAGTCTCGCTCTGTCACCAGG",2,1,3,NA,"GeneID:493","ATP2B4",FALSE,"ADRA1A"

"4075","CGAAGTCTCGCTCTGTCACC","chr1",209366120,"-","0:A>G,2:C>T","AGG","TGGAGTCTCGCTCTGTCACCAGG",2,1,3,NA,"","",FALSE,"ADRA1A"

"4076","CGAAGTCTCGCTCTGTCACC","chr2",161131056,"-","0:A>G,2:C>T","AGG","TGGAGTCTCGCTCTGTCACCAGG",2,1,3,NA,"","",FALSE,"ADRA1A"

"4077","CGAAGTCTCGCTCTGTCACC","chr22",33903799,"-","0:A>G,2:C>T","AGG","TGGAGTCTCGCTCTGTCACCAGG",2,1,3,NA,"GeneID:9215","LARGE",FALSE,"ADRA1A"

"4078","CGAAGTCTCGCTCTGTCACC","chr10",73399796,"-","0:A>G,2:C>T","AGG","TGGAGTCTCGCTCTGTCACCAGG",2,1,3,NA,"GeneID:310","ANXA7",FALSE,"ADRA1A"

"4079","CGAAGTCTCGCTCTGTCACC","chrX",131738393,"-","0:A>G,2:C>T","AGG","TGGAGTCTCGCTCTGTCACCAGG",2,1,3,NA,"GeneID:286467","FIRRE",FALSE,"ADRA1A"

"4080","CGAAGTCTCGCTCTGTCACC","chr19",8856506,"-","0:A>G,2:C>T","AGG","TGGAGTCTCGCTCTGTCACCAGG",2,1,3,NA,"GeneID:94025","MUC16",FALSE,"ADRA1A"

"4081","CGAAGTCTCGCTCTGTCACC","chr4",139339725,"-","0:A>G,2:C>T","AGG","TGGAGTCTCGCTCTGTCACCAGG",2,1,3,NA,"GeneID:80155","NAA15",FALSE,"ADRA1A"

"4082","CGAAGTCTCGCTCTGTCACC","chr16",61496996,"-","0:A>G,2:C>T","AGG","TGGAGTCTCGCTCTGTCACCAGG",2,1,3,NA,"","",FALSE,"ADRA1A"

"4083","CGAAGTCTCGCTCTGTCACC","chr19",47038491,"-","0:A>G,2:C>T","AGG","TGGAGTCTCGCTCTGTCACCAGG",2,1,3,NA,"GeneID:4861","NPAS1",FALSE,"ADRA1A"

"4084","CGAAGTCTCGCTCTGTCACC","chr17",43185063,"-","0:A>G,2:C>T","AGG","TGGAGTCTCGCTCTGTCACCAGG",2,1,3,NA,"GeneID:4077","NBR1",FALSE,"ADRA1A"

"4085","CGAAGTCTCGCTCTGTCACC","chr1",96563991,"-","0:A>G,2:C>T","AGG","TGGAGTCTCGCTCTGTCACCAGG",2,1,3,NA,"","",FALSE,"ADRA1A"

"4086","CGAAGTCTCGCTCTGTCACC","chr19",33100562,"-","0:A>G,2:C>T","AGG","TGGAGTCTCGCTCTGTCACCAGG",2,1,3,NA,"GeneID:55094","GPATCH1",FALSE,"ADRA1A"

"4087","CGAAGTCTCGCTCTGTCACC","chr2",61849759,"-","0:A>G,2:C>T","AGG","TGGAGTCTCGCTCTGTCACCAGG",2,1,3,NA,"GeneID:84140","FAM161A",FALSE,"ADRA1A"

"4088","CGAAGTCTCGCTCTGTCACC","chr5",76780494,"-","0:A>G,2:C>T","AGG","TGGAGTCTCGCTCTGTCACCAGG",2,1,3,NA,"","",FALSE,"ADRA1A"

"4089","CGAAGTCTCGCTCTGTCACC","chr15",55688808,"-","0:A>G,2:C>T","AGG","TGGAGTCTCGCTCTGTCACCAGG",2,1,3,NA,"GeneID:283659","PRTG",FALSE,"ADRA1A"

"4090","CGAAGTCTCGCTCTGTCACC","chr8",78481289,"-","0:A>G,2:C>T","AGG","TGGAGTCTCGCTCTGTCACCAGG",2,1,3,NA,"GeneID:101927003","LOC101927003",FALSE,"ADRA1A"

"4091","CGAAGTCTCGCTCTGTCACC","chr12",106320372,"-","0:A>G,2:C>T","AGG","TGGAGTCTCGCTCTGTCACCAGG",2,1,3,NA,"GeneID:255394","TCP11L2",FALSE,"ADRA1A"

"4092","CGAAGTCTCGCTCTGTCACC","chr1",20669840,"-","0:A>G,2:C>T","AGG","TGGAGTCTCGCTCTGTCACCAGG",2,1,3,NA,"GeneID:57576","KIF17",FALSE,"ADRA1A"

"4093","CGAAGTCTCGCTCTGTCACC","chr3",67422449,"-","0:A>G,2:C>T","AGG","TGGAGTCTCGCTCTGTCACCAGG",2,1,3,NA,"GeneID:8801","SUCLG2",FALSE,"ADRA1A"

"4094","CGAAGTCTCGCTCTGTCACC","chr17",6904710,"-","0:A>G,2:C>T","AGG","TGGAGTCTCGCTCTGTCACCAGG",2,1,3,NA,"GeneID:245;GeneID:100506713","ALOX12P2;LOC100506713",FALSE,"ADRA1A"

"4095","CGAAGTCTCGCTCTGTCACC","chr6",21726081,"-","0:A>G,2:C>T","AGG","TGGAGTCTCGCTCTGTCACCAGG",2,1,3,NA,"GeneID:401237","CASC15",FALSE,"ADRA1A"

"4096","CGAAGTCTCGCTCTGTCACC","chr17",42733915,"-","0:A>G,2:C>T","AGG","TGGAGTCTCGCTCTGTCACCAGG",2,1,3,NA,"GeneID:2145","EZH1",FALSE,"ADRA1A"

"4097","CGAAGTCTCGCTCTGTCACC","chr8",102780271,"-","0:A>G,2:C>T","AGG","TGGAGTCTCGCTCTGTCACCAGG",2,1,3,NA,"","",FALSE,"ADRA1A"

"4098","CGAAGTCTCGCTCTGTCACC","chr3",155564539,"-","0:A>G,2:C>T","AGG","TGGAGTCTCGCTCTGTCACCAGG",2,1,3,NA,"GeneID:23007","PLCH1",FALSE,"ADRA1A"

"4099","CGAAGTCTCGCTCTGTCACC","chr2",101453086,"-","0:A>G,2:C>T","AGG","TGGAGTCTCGCTCTGTCACCAGG",2,1,3,NA,"GeneID:731220","RFX8",FALSE,"ADRA1A"

"4100","CGAAGTCTCGCTCTGTCACC","chr13",41750407,"-","0:A>G,2:C>T","AGG","TGGAGTCTCGCTCTGTCACCAGG",2,1,3,NA,"GeneID:23078","VWA8",FALSE,"ADRA1A"

"4101","CGAAGTCTCGCTCTGTCACC","chr12",49079070,"-","0:A>G,2:C>T","AGG","TGGAGTCTCGCTCTGTCACCAGG",2,1,3,NA,"","",FALSE,"ADRA1A"

"4102","CGAAGTCTCGCTCTGTCACC","chr6",162556721,"-","0:A>G,2:C>T","TGG","TGGAGTCTCGCTCTGTCACCTGG",2,1,3,NA,"GeneID:5071","PARK2",FALSE,"ADRA1A"

"4103","CGAAGTCTCGCTCTGTCACC","chr12",56236017,"-","0:A>G,2:C>T","TGG","TGGAGTCTCGCTCTGTCACCTGG",2,1,3,NA,"GeneID:283375","SLC39A5",FALSE,"ADRA1A"

"4104","CGAAGTCTCGCTCTGTCACC","chr5",177628303,"-","0:A>G,2:C>T","TGG","TGGAGTCTCGCTCTGTCACCTGG",2,1,3,NA,"","",FALSE,"ADRA1A"

"4105","CGAAGTCTCGCTCTGTCACC","chr14",32562733,"-","0:A>G,2:C>T","TGG","TGGAGTCTCGCTCTGTCACCTGG",2,1,3,NA,"GeneID:9472","AKAP6",FALSE,"ADRA1A"

"4106","CGAAGTCTCGCTCTGTCACC","chr18",1772479,"-","0:A>G,2:C>T","TGG","TGGAGTCTCGCTCTGTCACCTGG",2,1,3,NA,"","",FALSE,"ADRA1A"

"4107","CGAAGTCTCGCTCTGTCACC","chr2",85118015,"-","0:A>G,2:C>T","TGG","TGGAGTCTCGCTCTGTCACCTGG",2,1,3,NA,"","",FALSE,"ADRA1A"

"4108","CGAAGTCTCGCTCTGTCACC","chr16",71635304,"-","0:A>G,2:C>T","TGG","TGGAGTCTCGCTCTGTCACCTGG",2,1,3,NA,"GeneID:91862","MARVELD3",TRUE,"ADRA1A"

"4109","CGAAGTCTCGCTCTGTCACC","chr19",3330788,"-","0:A>G,2:C>T","TGG","TGGAGTCTCGCTCTGTCACCTGG",2,1,3,NA,"GeneID:4782","NFIC",FALSE,"ADRA1A"

"4110","CGAAGTCTCGCTCTGTCACC","chr13",24648970,"-","0:A>G,2:C>T","TGG","TGGAGTCTCGCTCTGTCACCTGG",2,1,3,NA,"","",FALSE,"ADRA1A"

"4111","CGAAGTCTCGCTCTGTCACC","chr7",102034121,"-","0:A>G,2:C>T","TGG","TGGAGTCTCGCTCTGTCACCTGG",2,1,3,NA,"GeneID:1523","CUX1",FALSE,"ADRA1A"

"4112","CGAAGTCTCGCTCTGTCACC","chr5",19647505,"-","0:A>G,2:C>T","TGG","TGGAGTCTCGCTCTGTCACCTGG",2,1,3,NA,"GeneID:1016","CDH18",FALSE,"ADRA1A"

"4113","CGAAGTCTCGCTCTGTCACC","chr3",176945418,"-","0:A>G,2:C>T","TGG","TGGAGTCTCGCTCTGTCACCTGG",2,1,3,NA,"","",FALSE,"ADRA1A"

"4114","CGAAGTCTCGCTCTGTCACC","chrX",71274034,"-","0:A>G,2:C>T","TGG","TGGAGTCTCGCTCTGTCACCTGG",2,1,3,NA,"","",FALSE,"ADRA1A"

"4115","CGAAGTCTCGCTCTGTCACC","chr4",168896184,"-","0:A>G,2:C>T","TGG","TGGAGTCTCGCTCTGTCACCTGG",2,1,3,NA,"GeneID:23022;GeneID:84869","PALLD;CBR4",FALSE,"ADRA1A"

"4116","CGAAGTCTCGCTCTGTCACC","chr22",48126213,"-","0:T>G,2:C>T","AGG","AGGAGTCTCGCTCTGTCACCAGG",2,1,3,NA,"","",FALSE,"ADRA1A"

"4117","CGAAGTCTCGCTCTGTCACC","chr12",123368890,"-","0:T>G,2:C>T","AGG","AGGAGTCTCGCTCTGTCACCAGG",2,1,3,NA,"","",FALSE,"ADRA1A"

"4118","CGAAGTCTCGCTCTGTCACC","chr19",9131602,"-","0:T>G,2:C>T","AGG","AGGAGTCTCGCTCTGTCACCAGG",2,1,3,NA,"","",FALSE,"ADRA1A"

"4119","CGAAGTCTCGCTCTGTCACC","chr12",50486181,"-","0:T>G,2:C>T","AGG","AGGAGTCTCGCTCTGTCACCAGG",2,1,3,NA,"","",FALSE,"ADRA1A"

"4120","CGAAGTCTCGCTCTGTCACC","chr3",24556716,"-","0:T>G,2:C>T","AGG","AGGAGTCTCGCTCTGTCACCAGG",2,1,3,NA,"","",FALSE,"ADRA1A"

"4121","CGAAGTCTCGCTCTGTCACC","chr22",20098101,"-","0:T>G,2:C>T","AGG","AGGAGTCTCGCTCTGTCACCAGG",2,1,3,NA,"GeneID:54487","DGCR8",FALSE,"ADRA1A"

"4122","CGAAGTCTCGCTCTGTCACC","chr16",50504380,"-","0:T>G,2:C>T","AGG","AGGAGTCTCGCTCTGTCACCAGG",2,1,3,NA,"","",FALSE,"ADRA1A"

"4123","CGAAGTCTCGCTCTGTCACC","chr15",63649327,"-","0:T>G,2:C>T","AGG","AGGAGTCTCGCTCTGTCACCAGG",2,1,3,NA,"GeneID:8925","HERC1",FALSE,"ADRA1A"

"4124","CGAAGTCTCGCTCTGTCACC","chr11",63701064,"-","0:T>G,2:C>T","AGG","AGGAGTCTCGCTCTGTCACCAGG",2,1,3,NA,"GeneID:10313","RTN3",FALSE,"ADRA1A"

"4125","CGAAGTCTCGCTCTGTCACC","chr12",31912345,"-","0:T>G,2:C>T","CGG","AGGAGTCTCGCTCTGTCACCCGG",2,1,3,NA,"","",FALSE,"ADRA1A"

"4126","CGAAGTCTCGCTCTGTCACC","chr17",18772082,"-","0:T>G,2:C>T","CGG","AGGAGTCTCGCTCTGTCACCCGG",2,1,3,NA,"GeneID:10517","FBXW10",FALSE,"ADRA1A"

"4127","CGAAGTCTCGCTCTGTCACC","chr9",128413855,"-","0:T>G,2:C>T","CGG","AGGAGTCTCGCTCTGTCACCCGG",2,1,3,NA,"GeneID:51148","CERCAM",FALSE,"ADRA1A"

"4128","CGAAGTCTCGCTCTGTCACC","chr2",70159566,"-","0:C>G,2:C>T","AGG","GGGAGTCTCGCTCTGTCACCAGG",2,1,3,NA,"GeneID:54980","C2orf42",FALSE,"ADRA1A"

"4129","CGAAGTCTCGCTCTGTCACC","chr6",70710468,"-","0:C>G,2:C>T","AGG","GGGAGTCTCGCTCTGTCACCAGG",2,1,3,NA,"GeneID:60682","SMAP1",FALSE,"ADRA1A"

"4130","CGAAGTCTCGCTCTGTCACC","chr19",22828627,"-","0:C>G,2:C>T","AGG","GGGAGTCTCGCTCTGTCACCAGG",2,1,3,NA,"","",FALSE,"ADRA1A"

"4131","CGAAGTCTCGCTCTGTCACC","chr11",9325415,"-","0:C>G,2:C>T","AGG","GGGAGTCTCGCTCTGTCACCAGG",2,1,3,NA,"","",FALSE,"ADRA1A"

"4132","CGAAGTCTCGCTCTGTCACC","chr8",73686524,"-","0:C>G,2:C>T","AGG","GGGAGTCTCGCTCTGTCACCAGG",2,1,3,NA,"GeneID:27067","STAU2",FALSE,"ADRA1A"

"4133","CGAAGTCTCGCTCTGTCACC","chr9",131715839,"-","0:C>G,2:C>T","AGG","GGGAGTCTCGCTCTGTCACCAGG",2,1,3,NA,"GeneID:2889","RAPGEF1",FALSE,"ADRA1A"

"4134","CGAAGTCTCGCTCTGTCACC","chr9",96544193,"-","0:C>G,2:C>T","AGG","GGGAGTCTCGCTCTGTCACCAGG",2,1,3,NA,"GeneID:8555","CDC14B",FALSE,"ADRA1A"

"4135","CGAAGTCTCGCTCTGTCACC","chr15",50635292,"-","0:C>G,2:C>T","AGG","GGGAGTCTCGCTCTGTCACCAGG",2,1,3,NA,"GeneID:54822","TRPM7",FALSE,"ADRA1A"

"4136","CGAAGTCTCGCTCTGTCACC","chr5",43201933,"-","0:C>G,2:C>T","AGG","GGGAGTCTCGCTCTGTCACCAGG",2,1,3,NA,"GeneID:167359","NIM1K",FALSE,"ADRA1A"

"4137","CGAAGTCTCGCTCTGTCACC","chr7",106984644,"-","0:C>G,2:C>T","AGG","GGGAGTCTCGCTCTGTCACCAGG",2,1,3,NA,"","",FALSE,"ADRA1A"

"4138","CGAAGTCTCGCTCTGTCACC","chr1",14277637,"-","0:C>G,2:C>T","AGG","GGGAGTCTCGCTCTGTCACCAGG",2,1,3,NA,"GeneID:23254","KAZN",FALSE,"ADRA1A"

"4139","CGAAGTCTCGCTCTGTCACC","chr1",203775368,"-","0:C>G,2:C>T","AGG","GGGAGTCTCGCTCTGTCACCAGG",2,1,3,NA,"GeneID:54900","LAX1",TRUE,"ADRA1A"

"4140","CGAAGTCTCGCTCTGTCACC","chr5",28157659,"-","0:C>G,2:C>T","AGG","GGGAGTCTCGCTCTGTCACCAGG",2,1,3,NA,"","",FALSE,"ADRA1A"

"4141","CGAAGTCTCGCTCTGTCACC","chr20",34346419,"-","0:C>G,2:C>T","AGG","GGGAGTCTCGCTCTGTCACCAGG",2,1,3,NA,"","",FALSE,"ADRA1A"

"4142","CGAAGTCTCGCTCTGTCACC","chr17",78570832,"-","0:C>G,2:C>T","AGG","GGGAGTCTCGCTCTGTCACCAGG",2,1,3,NA,"GeneID:8632","DNAH17",FALSE,"ADRA1A"

"4143","CGAAGTCTCGCTCTGTCACC","chr7",56416135,"-","0:C>G,2:C>T","CGG","GGGAGTCTCGCTCTGTCACCCGG",2,1,3,NA,"","",FALSE,"ADRA1A"

"4144","CGAAGTCTCGCTCTGTCACC","chr7",55667621,"-","0:C>G,2:C>T","CGG","GGGAGTCTCGCTCTGTCACCCGG",2,1,3,NA,"","",FALSE,"ADRA1A"

"4145","CGAAGTCTCGCTCTGTCACC","chr9",126964344,"-","0:C>G,2:C>T","CGG","GGGAGTCTCGCTCTGTCACCCGG",2,1,3,NA,"GeneID:9649","RALGPS1",FALSE,"ADRA1A"

"4146","CGAAGTCTCGCTCTGTCACC","chr3",96085567,"-","0:C>G,2:C>T","CGG","GGGAGTCTCGCTCTGTCACCCGG",2,1,3,NA,"","",FALSE,"ADRA1A"

"4147","GATATGCTCTCTGCAATTCT","chr8",26766101,"+","none","TGG","GATATGCTCTCTGCAATTCTTGG",0,NA,NA,NA,"GeneID:148","ADRA1A",TRUE,"ADRA1A"

"4148","GATATGCTCTCTGCAATTCT","chr12",41210821,"-","6:C>G,8:C>G","GGG","GATATGGTGTCTGCAATTCTGGG",2,7,9,NA,"GeneID:29951","PDZRN4",FALSE,"ADRA1A"

"4149","GATCAAGAGAGACCTTCTAC","chr8",26770391,"-","none","AGG","GATCAAGAGAGACCTTCTACAGG",0,NA,NA,NA,"GeneID:148","ADRA1A",TRUE,"ADRA1A"

"4150","GGAAGCTGGCTTCATGTCAT","chr8",26756747,"+","none","GGG","GGAAGCTGGCTTCATGTCATGGG",0,NA,NA,NA,"GeneID:148","ADRA1A",TRUE,"ADRA1A"

"4151","TTCACAAACGCCATCCGTCT","chr8",26770361,"+","none","TGG","TTCACAAACGCCATCCGTCTTGG",0,NA,NA,NA,"GeneID:148","ADRA1A",TRUE,"ADRA1A"

"4152","AGCGAGCCGCCGCGCGTCCA","chr5",159972099,"-","none","CGG","AGCGAGCCGCCGCGCGTCCACGG",0,NA,NA,NA,"GeneID:147","ADRA1B",TRUE,"ADRA1B"

"4153","AGGCCCGATGGAGATGACGG","chr5",159917425,"-","none","TGG","AGGCCCGATGGAGATGACGGTGG",0,NA,NA,NA,"GeneID:101927766;GeneID:147","LOC101927766;ADRA1B",TRUE,"ADRA1B"

"4154","CCCGCACTCCTTGTCATCGT","chr5",159917473,"-","none","TGG","CCCGCACTCCTTGTCATCGTTGG",0,NA,NA,NA,"GeneID:101927766;GeneID:147","LOC101927766;ADRA1B",TRUE,"ADRA1B"

"4155","CGCGCTTTCGTGCGCATCCT","chr5",159971997,"+","none","CGG","CGCGCTTTCGTGCGCATCCTCGG",0,NA,NA,NA,"GeneID:147","ADRA1B",TRUE,"ADRA1B"

"4156","GTACTGCCGTGTCTATATAG","chr5",159917570,"+","none","TGG","GTACTGCCGTGTCTATATAGTGG",0,NA,NA,NA,"GeneID:101927766;GeneID:147","LOC101927766;ADRA1B",TRUE,"ADRA1B"

"4157","TTCTTCATCGCTCTACCGCT","chr5",159917832,"+","none","TGG","TTCTTCATCGCTCTACCGCTTGG",0,NA,NA,NA,"GeneID:101927766;GeneID:147","LOC101927766;ADRA1B",TRUE,"ADRA1B"

"4158","ACCTTGAAGACGCCCTCCGA","chr20",4222084,"+","none","TGG","ACCTTGAAGACGCCCTCCGATGG",0,NA,NA,NA,"GeneID:146","ADRA1D",TRUE,"ADRA1D"

"4159","ATACCGCAGAAGCGCTCGTC","chr20",4248214,"+","none","AGG","ATACCGCAGAAGCGCTCGTCAGG",0,NA,NA,NA,"GeneID:146","ADRA1D",TRUE,"ADRA1D"

"4160","CCCCCTGACGAGCGCTTCTG","chr20",4248220,"-","none","CGG","CCCCCTGACGAGCGCTTCTGCGG",0,NA,NA,NA,"GeneID:146","ADRA1D",TRUE,"ADRA1D"

"4161","CTGTCGCACAAGATCCGCGC","chr20",4221673,"-","none","CGG","CTGTCGCACAAGATCCGCGCCGG",0,NA,NA,NA,"GeneID:146","ADRA1D",TRUE,"ADRA1D"

"4162","GCCGCGCCTTCTGCGACGTA","chr20",4248444,"-","none","TGG","GCCGCGCCTTCTGCGACGTATGG",0,NA,NA,NA,"GeneID:146","ADRA1D",TRUE,"ADRA1D"

"4163","TGCGCGCGACCACGTACACG","chr20",4248110,"+","none","CGG","TGCGCGCGACCACGTACACGCGG",0,NA,NA,NA,"GeneID:146","ADRA1D",TRUE,"ADRA1D"

"4164","CCCGACGGCCGTGAGCGTGT","chr10",111079221,"-","none","AGG","CCCGACGGCCGTGAGCGTGTAGG",0,NA,NA,NA,"GeneID:150","ADRA2A",TRUE,"ADRA2A"

"4165","CGGACGCGGGCAACGCGAGC","chr10",111078057,"+","none","TGG","CGGACGCGGGCAACGCGAGCTGG",0,NA,NA,NA,"GeneID:150","ADRA2A",TRUE,"ADRA2A"

"4166","GCCGCGCGACACCGACGCGC","chr10",111078892,"+","none","TGG","GCCGCGCGACACCGACGCGCTGG",0,NA,NA,NA,"GeneID:150","ADRA2A",TRUE,"ADRA2A"

"4167","TACGTCATCTCGTCGTGCAT","chr10",111078626,"+","none","CGG","TACGTCATCTCGTCGTGCATCGG",0,NA,NA,NA,"GeneID:150","ADRA2A",TRUE,"ADRA2A"

"4168","TGAAGGCGCGGCGGAAATCG","chr10",111079333,"-","none","TGG","TGAAGGCGCGGCGGAAATCGTGG",0,NA,NA,NA,"GeneID:150","ADRA2A",TRUE,"ADRA2A"

"4169","TGGCACGCGGGTGCGACGCT","chr10",111078711,"-","none","TGG","TGGCACGCGGGTGCGACGCTTGG",0,NA,NA,NA,"GeneID:150","ADRA2A",TRUE,"ADRA2A"

"4170","AACGGACACTCGAAGTCCAC","chr2",96115391,"-","none","TGG","AACGGACACTCGAAGTCCACTGG",0,NA,NA,NA,"GeneID:151","ADRA2B",TRUE,"ADRA2B"

"4171","ACGGGCGGCACAGGATCCTC","chr2",96114816,"+","none","CGG","ACGGGCGGCACAGGATCCTCCGG",0,NA,NA,NA,"GeneID:151","ADRA2B",TRUE,"ADRA2B"

"4172","CGAGTGTCCGTTGACCTCTC","chr2",96115399,"+","none","TGG","CGAGTGTCCGTTGACCTCTCTGG",0,NA,NA,NA,"GeneID:151","ADRA2B",TRUE,"ADRA2B"

"4173","CTTCCGGCGCACGTGGTGCG","chr2",96115893,"-","none","AGG","CTTCCGGCGCACGTGGTGCGAGG",0,NA,NA,NA,"GeneID:151","ADRA2B",TRUE,"ADRA2B"

"4174","CTTCCGGCGCACGTGGTGCG","chr22",29268541,"+","10:C>A,16:C>T","CGG","CTTCCGGCGCCCGTGGCGCGCGG",2,11,17,NA,"GeneID:2130","EWSR1",FALSE,"ADRA2B"

"4175","TGGAGTTGTACTCCAGCGCG","chr2",96115782,"+","none","CGG","TGGAGTTGTACTCCAGCGCGCGG",0,NA,NA,NA,"GeneID:151","ADRA2B",TRUE,"ADRA2B"

"4176","TGGCGATCAGGTAGATGCGC","chr2",96115551,"+","none","AGG","TGGCGATCAGGTAGATGCGCAGG",0,NA,NA,NA,"GeneID:151","ADRA2B",TRUE,"ADRA2B"

"4177","CTACGCGCGCATCTACCGAG","chr4",3767292,"+","none","TGG","CTACGCGCGCATCTACCGAGTGG",0,NA,NA,NA,"GeneID:152","ADRA2C",TRUE,"ADRA2C"

"4178","GCGGCCTCAACGACGAGACC","chr4",3767210,"+","none","TGG","GCGGCCTCAACGACGAGACCTGG",0,NA,NA,NA,"GeneID:152","ADRA2C",TRUE,"ADRA2C"

"4179","GCGTCCCCGACTACCGAAAA","chr4",3767371,"+","none","CGG","GCGTCCCCGACTACCGAAAACGG",0,NA,NA,NA,"GeneID:152","ADRA2C",TRUE,"ADRA2C"

"4180","GGATGTGCTTAAAGGATCGC","chr4",3767940,"-","none","CGG","GGATGTGCTTAAAGGATCGCCGG",0,NA,NA,NA,"GeneID:152","ADRA2C",TRUE,"ADRA2C"

"4181","TAGTCGGGGACGCACCGTCG","chr4",3767364,"-","none","GGG","TAGTCGGGGACGCACCGTCGGGG",0,NA,NA,NA,"GeneID:152","ADRA2C",TRUE,"ADRA2C"

"4182","TCGTCGTTGAGGCCGCACTG","chr4",3767206,"-","none","CGG","TCGTCGTTGAGGCCGCACTGCGG",0,NA,NA,NA,"GeneID:152","ADRA2C",TRUE,"ADRA2C"

"4183","AGAGCCTGCTGACGCGCGCG","chr10",114044631,"+","none","CGG","AGAGCCTGCTGACGCGCGCGCGG",0,NA,NA,NA,"GeneID:153","ADRB1",TRUE,"ADRB1"

"4184","CCAACGGGCGTGCGGGTAAG","chr10",114045036,"+","none","CGG","CCAACGGGCGTGCGGGTAAGCGG",0,NA,NA,NA,"GeneID:153","ADRB1",TRUE,"ADRB1"

"4185","CCGCTTACCCGCACGCCCGT","chr10",114045039,"-","none","TGG","CCGCTTACCCGCACGCCCGTTGG",0,NA,NA,NA,"GeneID:153","ADRB1",TRUE,"ADRB1"

"4186","CGACGACAGGTTACCGGGCT","chr10",114044166,"-","none","CGG","CGACGACAGGTTACCGGGCTCGG",0,NA,NA,NA,"GeneID:153","ADRB1",TRUE,"ADRB1"

"4187","GCCGTTCGGGGCCACCATCG","chr10",114044467,"+","none","TGG","GCCGTTCGGGGCCACCATCGTGG",0,NA,NA,NA,"GeneID:153","ADRB1",TRUE,"ADRB1"

"4188","TACGGACGAGGCGATGGCGT","chr10",114044802,"-","none","AGG","TACGGACGAGGCGATGGCGTAGG",0,NA,NA,NA,"GeneID:153","ADRB1",TRUE,"ADRB1"

"4189","ACCACGACGTCACGCAGGAA","chr5",148826892,"+","none","AGG","ACCACGACGTCACGCAGGAAAGG",0,NA,NA,NA,"GeneID:154","ADRB2",TRUE,"ADRB2"

"4190","CCCTTTCCTGCGTGACGTCG","chr5",148826896,"-","none","TGG","CCCTTTCCTGCGTGACGTCGTGG",0,NA,NA,NA,"GeneID:154","ADRB2",TRUE,"ADRB2"

"4191","CGTCTGCAGACGCTCGAACT","chr5",148827009,"-","none","TGG","CGTCTGCAGACGCTCGAACTTGG",0,NA,NA,NA,"GeneID:154","ADRB2",TRUE,"ADRB2"

"4192","GCAACTTCTGGTGCGAGTTT","chr5",148827135,"+","none","TGG","GCAACTTCTGGTGCGAGTTTTGG",0,NA,NA,NA,"GeneID:154","ADRB2",TRUE,"ADRB2"

"4193","GCAACTTCTGGTGCGAGTTT","chr4",158459694,"-","14:A>C,18:G>A","AGG","GCAACTTCTGGTGCTAGTCTAGG",2,15,19,NA,"GeneID:59350","RXFP1",FALSE,"ADRB2"

"4194","TGTAAACTTCCTTACGGATG","chr5",148827736,"-","none","AGG","TGTAAACTTCCTTACGGATGAGG",0,NA,NA,NA,"GeneID:154","ADRB2",TRUE,"ADRB2"

"4195","TGTGAATCAATGTTATCGCT","chr5",148828016,"-","none","AGG","TGTGAATCAATGTTATCGCTAGG",0,NA,NA,NA,"GeneID:154","ADRB2",TRUE,"ADRB2"

"4196","CAACGTGTTCGTGACTTCGC","chr8",37966234,"-","none","TGG","CAACGTGTTCGTGACTTCGCTGG",0,NA,NA,NA,"GeneID:155","ADRB3",TRUE,"ADRB3"

"4197","CAGCGAAGTCACGAACACGT","chr8",37966232,"+","none","TGG","CAGCGAAGTCACGAACACGTTGG",0,NA,NA,NA,"GeneID:155","ADRB3",TRUE,"ADRB3"

"4198","CGGCCCCTACGCGCCACCAC","chr8",37965922,"+","none","TGG","CGGCCCCTACGCGCCACCACTGG",0,NA,NA,NA,"GeneID:155","ADRB3",TRUE,"ADRB3"

"4199","GTGACCAACCCGCTGCGTTA","chr8",37966035,"-","none","CGG","GTGACCAACCCGCTGCGTTACGG",0,NA,NA,NA,"GeneID:155","ADRB3",TRUE,"ADRB3"

"4200","TGATGCTCTTCGTCTACGCG","chr8",37965794,"-","none","CGG","TGATGCTCTTCGTCTACGCGCGG",0,NA,NA,NA,"GeneID:155","ADRB3",TRUE,"ADRB3"

"4201","TTACCTACCCGTCGAGCCGT","chr8",37965256,"+","none","TGG","TTACCTACCCGTCGAGCCGTTGG",0,NA,NA,NA,"GeneID:155","ADRB3",TRUE,"ADRB3"

"4202","CGCCGCCAAGATGGCGGACC","chr11",67266689,"+","none","TGG","CGCCGCCAAGATGGCGGACCTGG",0,NA,NA,NA,"GeneID:156","ADRBK1",TRUE,"ADRBK1"

"4203","CGCCGCCAAGATGGCGGACC","chr7",156063679,"-","1:A>C,3:T>G","AGG","CTCAGCCAAGATGGCGGACCAGG",2,2,4,NA,"","",FALSE,"ADRBK1"

"4204","CTCAGTGGCACTCTTCGAGA","chr11",67279629,"-","none","AGG","CTCAGTGGCACTCTTCGAGAAGG",0,NA,NA,NA,"GeneID:156","ADRBK1",TRUE,"ADRBK1"

"4205","CTTCGACTCATACATCATGA","chr11",67279476,"+","none","AGG","CTTCGACTCATACATCATGAAGG",0,NA,NA,NA,"GeneID:156","ADRBK1",TRUE,"ADRBK1"

"4206","GCACACACCTGGAAGAGATC","chr11",67279688,"-","none","CGG","GCACACACCTGGAAGAGATCCGG",0,NA,NA,NA,"GeneID:156","ADRBK1",TRUE,"ADRBK1"

"4207","GGCCGACGTGAGCTACCTGA","chr11",67266722,"+","none","TGG","GGCCGACGTGAGCTACCTGATGG",0,NA,NA,NA,"GeneID:156","ADRBK1",TRUE,"ADRBK1"

"4208","TGTATGAGTCGAAGATCTCC","chr11",67279470,"-","none","CGG","TGTATGAGTCGAAGATCTCCCGG",0,NA,NA,NA,"GeneID:156","ADRBK1",TRUE,"ADRBK1"

"4209","AAATTTGTCGACTTCTGCAA","chr22",25661616,"-","none","AGG","AAATTTGTCGACTTCTGCAAAGG",0,NA,NA,NA,"GeneID:157","ADRBK2",TRUE,"ADRBK2"

"4210","AAATTTGTCGACTTCTGCAA","chr7",89489226,"+","7:A>T,8:A>C","AGG","AAATTTGAAGACTTCTGCAAAGG",2,8,9,NA,"","",FALSE,"ADRBK2"

"4211","CAGCAAGAGGATCGTCCTGC","chr22",25565123,"+","none","CGG","CAGCAAGAGGATCGTCCTGCCGG",0,NA,NA,NA,"GeneID:157","ADRBK2",TRUE,"ADRBK2"

"4212","CAGCAAGAGGATCGTCCTGC","chr17",29043311,"+","3:G>C,13:C>G","TGG","CAGGAAGAGGATCCTCCTGCTGG",2,4,14,NA,"GeneID:51268","PIPOX",TRUE,"ADRBK2"

"4213","CTGATTGAAAATCTTGTCAA","chr22",25604426,"-","none","AGG","CTGATTGAAAATCTTGTCAAAGG",0,NA,NA,NA,"GeneID:157","ADRBK2",TRUE,"ADRBK2"

"4214","GCTCGCCAACATGGCGGACC","chr22",25565030,"+","none","TGG","GCTCGCCAACATGGCGGACCTGG",0,NA,NA,NA,"GeneID:157","ADRBK2",TRUE,"ADRBK2"

"4215","GCTCGCCAACATGGCGGACC","chr22",25421208,"+","14:T>C","TGG","GCTCGCCAACATGGTGGACCTGG",1,15,NA,NA,"","",FALSE,"ADRBK2"

"4216","GCTCGCCAACATGGCGGACC","chr22",25282932,"+","0:A>G,10:G>A","CGG","ACTCGCCAACGTGGCGGACCCGG",2,1,11,NA,"","",FALSE,"ADRBK2"

"4217","TTATGATGCCTACATCATGA","chr22",25661634,"+","none","AGG","TTATGATGCCTACATCATGAAGG",0,NA,NA,NA,"GeneID:157","ADRBK2",TRUE,"ADRBK2"

"4218","TTATTTCATTTCTCTCTGCA","chr22",25604403,"-","none","AGG","TTATTTCATTTCTCTCTGCAAGG",0,NA,NA,NA,"GeneID:157","ADRBK2",TRUE,"ADRBK2"

"4219","TTATTTCATTTCTCTCTGCA","chr7",133001093,"+","1:G>T,6:A>C","AGG","TGATTTAATTTCTCTCTGCAAGG",2,2,7,NA,"GeneID:54927","CHCHD3",FALSE,"ADRBK2"

"4220","ACATCCGCGTTGGCGAGGAT","chr20",62308061,"-","none","GGG","ACATCCGCGTTGGCGAGGATGGG",0,NA,NA,NA,"GeneID:11047;GeneID:3911","ADRM1;LAMA5",TRUE,"ADRM1"

"4221","ACCAGCCCTTTCCGCTTATC","chr20",62303688,"-","none","CGG","ACCAGCCCTTTCCGCTTATCCGG",0,NA,NA,NA,"GeneID:11047","ADRM1",TRUE,"ADRM1"

"4222","CACGAACTCTCTGCGCTAGG","chr20",62306298,"+","none","CGG","CACGAACTCTCTGCGCTAGGCGG",0,NA,NA,NA,"GeneID:11047","ADRM1",TRUE,"ADRM1"

"4223","CCACCGTGACTCCGGATAAG","chr20",62303674,"+","none","CGG","CCACCGTGACTCCGGATAAGCGG",0,NA,NA,NA,"GeneID:11047","ADRM1",TRUE,"ADRM1"

"4224","CGCCTAGCGCAGAGAGTTCG","chr20",62306300,"-","none","TGG","CGCCTAGCGCAGAGAGTTCGTGG",0,NA,NA,NA,"GeneID:11047","ADRM1",TRUE,"ADRM1"

"4225","CGCTTGAACTCACAGTCGTC","chr20",62304478,"-","none","AGG","CGCTTGAACTCACAGTCGTCAGG",0,NA,NA,NA,"GeneID:11047","ADRM1",TRUE,"ADRM1"

"4226","CGAGCCAGTCTACCCACATT","chr22",40354289,"+","none","AGG","CGAGCCAGTCTACCCACATTAGG",0,NA,NA,NA,"GeneID:158","ADSL",TRUE,"ADSL"

"4227","CTCGGACACGCTTCAAGTTC","chr22",40358932,"-","none","TGG","CTCGGACACGCTTCAAGTTCTGG",0,NA,NA,NA,"GeneID:158","ADSL",TRUE,"ADSL"

"4228","GGCTCGTTCCTTAGCAAAGT","chr22",40354275,"-","none","CGG","GGCTCGTTCCTTAGCAAAGTCGG",0,NA,NA,NA,"GeneID:158","ADSL",TRUE,"ADSL"

"4229","GGTGCTACTTCTTGCTATGT","chr22",40350002,"+","none","TGG","GGTGCTACTTCTTGCTATGTTGG",0,NA,NA,NA,"GeneID:158","ADSL",TRUE,"ADSL"

"4230","TCCGAGATGACCTGCGCTTC","chr22",40358946,"+","none","CGG","TCCGAGATGACCTGCGCTTCCGG",0,NA,NA,NA,"GeneID:158","ADSL",TRUE,"ADSL"

"4231","TCTTGAAGTCGATGTTCTCC","chr22",40349884,"-","none","AGG","TCTTGAAGTCGATGTTCTCCAGG",0,NA,NA,NA,"GeneID:158","ADSL",TRUE,"ADSL"

"4232","ATATCTGACAGAGCTCATAT","chr1",244432546,"-","none","TGG","ATATCTGACAGAGCTCATATTGG",0,NA,NA,NA,"GeneID:159","ADSS",TRUE,"ADSS"

"4233","CCAATGAATGCAGTGACATT","chr1",244437664,"+","none","TGG","CCAATGAATGCAGTGACATTTGG",0,NA,NA,NA,"GeneID:159","ADSS",TRUE,"ADSS"

"4234","CCAATGAATGCAGTGACATT","chr17",35460271,"+","3:T>A,14:A>G","TGG","CCATTGAATGCAGTAACATTTGG",2,4,15,NA,"","",TRUE,"ADSS"

"4235","CCTTTCTAGGACTAGAAGGC","chr1",244432584,"-","none","TGG","CCTTTCTAGGACTAGAAGGCTGG",0,NA,NA,NA,"GeneID:159","ADSS",TRUE,"ADSS"

"4236","GCAGAGAAAAATGTTCAAAA","chr1",244436832,"-","none","AGG","GCAGAGAAAAATGTTCAAAAAGG",0,NA,NA,NA,"GeneID:159","ADSS",TRUE,"ADSS"

"4237","GCAGAGAAAAATGTTCAAAA","chr17",35460211,"-","none","AGG","GCAGAGAAAAATGTTCAAAAAGG",0,NA,NA,NA,"","",TRUE,"ADSS"

"4238","GCAGAGAAAAATGTTCAAAA","chrY",17231963,"-","16:A>T,19:A>T","TGG","GCAGAGAAAAATGTTCTAATTGG",2,17,20,NA,"","",FALSE,"ADSS"

"4239","GCAGAGAAAAATGTTCAAAA","chr11",56809239,"-","2:A>T,3:T>C","AGG","GCTAAGAAAAATGTTCAAAAAGG",2,3,4,NA,"","",FALSE,"ADSS"

"4240","GCAGAGAAAAATGTTCAAAA","chr12",12944494,"+","1:A>C,5:A>G","AGG","GAAGAAAAAAATGTTCAAAAAGG",2,2,6,NA,"GeneID:100506314;GeneID:55507","LOC100506314;GPRC5D",TRUE,"ADSS"

"4241","GCAGAGAAAAATGTTCAAAA","chr4",95170693,"+","1:G>C,6:G>A","GGG","GGAGAGGAAAATGTTCAAAAGGG",2,2,7,NA,"GeneID:8633","UNC5C",FALSE,"ADSS"

"4242","GCAGAGAAAAATGTTCAAAA","chr3",43022296,"-","7:C>T,11:T>A","CGG","GCAGAGAGAAAAGTTCAAAACGG",2,8,12,NA,"GeneID:729085","FAM198A",FALSE,"ADSS"

"4243","GCAGAGAAAAATGTTCAAAA","chr15",75596347,"-","6:C>T,19:G>T","AGG","GCAGAGGAAAATGTTCAAACAGG",2,7,20,NA,"","",FALSE,"ADSS"

"4244","TACAGTTGTTGTGGATTCTG","chr1",244437728,"-","none","TGG","TACAGTTGTTGTGGATTCTGTGG",0,NA,NA,NA,"GeneID:159","ADSS",TRUE,"ADSS"

"4245","TACAGTTGTTGTGGATTCTG","chr17",35460335,"-","13:G>C","TGG","TACAGTTGTTGTGCATTCTGTGG",1,14,NA,NA,"","",TRUE,"ADSS"

"4246","TCTGCTTCTTCAAACAATCC","chr1",244436847,"+","none","AGG","TCTGCTTCTTCAAACAATCCAGG",0,NA,NA,NA,"GeneID:159","ADSS",TRUE,"ADSS"

"4247","TCTGCTTCTTCAAACAATCC","chr17",35460226,"+","none","AGG","TCTGCTTCTTCAAACAATCCAGG",0,NA,NA,NA,"","",TRUE,"ADSS"

"4248","TCTGCTTCTTCAAACAATCC","chr14",104738401,"-","8:G>A,17:C>A","TGG","TCTGCTTCCTCAAACAAGCCTGG",2,9,18,NA,"GeneID:122622","ADSSL1",TRUE,"ADSS"

"4249","ACATTGAAGGCCAACTCAAA","chr14",104740891,"+","none","AGG","ACATTGAAGGCCAACTCAAAAGG",0,NA,NA,NA,"GeneID:122622","ADSSL1",TRUE,"ADSSL1"

"4250","CAGTATAGGCACCACCAAGA","chr14",104740597,"+","none","AGG","CAGTATAGGCACCACCAAGAAGG",0,NA,NA,NA,"GeneID:122622","ADSSL1",TRUE,"ADSSL1"

"4251","CAGTATAGGCACCACCAAGA","chr18",52882436,"-","5:T>A,7:T>C","GGG","CAGTAAAAGCACCACCAAGAGGG",2,6,8,NA,"GeneID:1630","DCC",FALSE,"ADSSL1"

"4252","CCACTTGCCAGGCTTGTTTG","chr14",104738391,"+","none","AGG","CCACTTGCCAGGCTTGTTTGAGG",0,NA,NA,NA,"GeneID:122622","ADSSL1",TRUE,"ADSSL1"

"4253","CCAGTCGATGTTCCCCACCC","chr14",104740862,"+","none","TGG","CCAGTCGATGTTCCCCACCCTGG",0,NA,NA,NA,"GeneID:122622","ADSSL1",TRUE,"ADSSL1"

"4254","CGCAGATGCGGAGGCCTGTC","chr14",104740654,"-","none","CGG","CGCAGATGCGGAGGCCTGTCCGG",0,NA,NA,NA,"GeneID:122622","ADSSL1",TRUE,"ADSSL1"

"4255","CTCTCATGCAGGCAACGGGG","chr14",104738364,"+","none","TGG","CTCTCATGCAGGCAACGGGGTGG",0,NA,NA,NA,"GeneID:122622","ADSSL1",TRUE,"ADSSL1"

"4256","CCGGCTGATGTAAGCAATGC","chr6",11735566,"+","none","TGG","CCGGCTGATGTAAGCAATGCTGG",0,NA,NA,NA,"GeneID:84830","ADTRP",TRUE,"ADTRP"

"4257","CCGTCTCAAAGTAGAGCCAT","chr6",11723474,"+","none","AGG","CCGTCTCAAAGTAGAGCCATAGG",0,NA,NA,NA,"GeneID:84830","ADTRP",TRUE,"ADTRP"

"4258","GACGACTTCAGCCAATGTGA","chr6",11735644,"+","none","TGG","GACGACTTCAGCCAATGTGATGG",0,NA,NA,NA,"GeneID:84830","ADTRP",TRUE,"ADTRP"

"4259","GGCAAACACAGGATACACCC","chr6",11723448,"+","none","AGG","GGCAAACACAGGATACACCCAGG",0,NA,NA,NA,"GeneID:84830","ADTRP",TRUE,"ADTRP"

"4260","GGGTTTGACATCTACCAGCC","chr6",11770046,"-","none","AGG","GGGTTTGACATCTACCAGCCAGG",0,NA,NA,NA,"GeneID:84830","ADTRP",TRUE,"ADTRP"

"4261","TAGATACTGTCATCCCCGTG","chr6",11766291,"-","none","TGG","TAGATACTGTCATCCCCGTGTGG",0,NA,NA,NA,"GeneID:84830","ADTRP",TRUE,"ADTRP"

"4262","CCACCAAGAAGCCCCCGTCA","chr7",44106770,"+","none","GGG","CCACCAAGAAGCCCCCGTCAGGG",0,NA,NA,NA,"GeneID:165","AEBP1",TRUE,"AEBP1"

"4263","GAACTCCTCGATCTCGTCGT","chr7",44104756,"-","none","CGG","GAACTCCTCGATCTCGTCGTCGG",0,NA,NA,NA,"GeneID:165","AEBP1",TRUE,"AEBP1"

"4264","GAACTCCTCGATCTCGTCGT","chr14",104600026,"-","14:C>G,18:T>C","GGG","GAACTCCTCGATCTGGTCATGGG",2,15,19,NA,"GeneID:388021","TMEM179",FALSE,"AEBP1"

"4265","GCTGACCGACGACGAGATCG","chr7",44104748,"+","none","AGG","GCTGACCGACGACGAGATCGAGG",0,NA,NA,NA,"GeneID:165","AEBP1",TRUE,"AEBP1"

"4266","GGGCGCCCCTCTCAAATAAC","chr7",44107438,"+","none","TGG","GGGCGCCCCTCTCAAATAACTGG",0,NA,NA,NA,"GeneID:165","AEBP1",TRUE,"AEBP1"

"4267","GGTGCTCCCGTGCCTCCACA","chr7",44107484,"-","none","TGG","GGTGCTCCCGTGCCTCCACATGG",0,NA,NA,NA,"GeneID:165","AEBP1",TRUE,"AEBP1"

"4268","GTTTCTGAGGGAGCCAGAAT","chr7",44106805,"-","none","GGG","GTTTCTGAGGGAGCCAGAATGGG",0,NA,NA,NA,"GeneID:165","AEBP1",TRUE,"AEBP1"

"4269","AACTTTCTATATGAGCTGAG","chr12",19500166,"-","none","AGG","AACTTTCTATATGAGCTGAGAGG",0,NA,NA,NA,"GeneID:121536","AEBP2",TRUE,"AEBP2"

"4270","AACTTTCTATATGAGCTGAG","chr5",80672956,"+","6:T>C,9:G>T","AGG","AACTTTTTAGATGAGCTGAGAGG",2,7,10,NA,"GeneID:4437","MSH3",FALSE,"AEBP2"

"4271","AACTTTCTATATGAGCTGAG","chr11",25865560,"-","1:C>T,17:G>C","AGG","AGCTTTCTATATGAGCTCAGAGG",2,2,18,NA,"","",FALSE,"AEBP2"

"4272","GAAGAATCTCCTTCTAAAGC","chr12",19493916,"+","none","TGG","GAAGAATCTCCTTCTAAAGCTGG",0,NA,NA,NA,"GeneID:121536","AEBP2",TRUE,"AEBP2"

"4273","GCTAAGAGAAAAGAAGATTC","chr12",19512403,"+","none","TGG","GCTAAGAGAAAAGAAGATTCTGG",0,NA,NA,NA,"GeneID:121536","AEBP2",TRUE,"AEBP2"

"4274","GCTAAGAGAAAAGAAGATTC","chr2",163080862,"-","15:T>C,19:A>G","GGG","GCTAAGAGAAAAGAAAATTTGGG",2,16,20,NA,"","",FALSE,"AEBP2"

"4275","GCTAAGAGAAAAGAAGATTC","chr1",238064301,"+","10:T>A,13:C>A","TGG","GCTAAGAGAATAGCAGATTCTGG",2,11,14,NA,"","",FALSE,"AEBP2"

"4276","GCTAAGAGAAAAGAAGATTC","chrX",26035649,"-","2:T>A,3:C>T","AGG","GCAGAGAGAAAAGAAGATTCAGG",2,3,4,NA,"","",FALSE,"AEBP2"

"4277","GTTCTGCTGACTGAAGTGTG","chr12",19493863,"-","none","TGG","GTTCTGCTGACTGAAGTGTGTGG",0,NA,NA,NA,"GeneID:121536","AEBP2",TRUE,"AEBP2"

"4278","TGTGCATCGAAGAAATCATG","chr12",19500104,"-","none","TGG","TGTGCATCGAAGAAATCATGTGG",0,NA,NA,NA,"GeneID:121536","AEBP2",TRUE,"AEBP2"

"4279","TTTCTTCGATGCACAAACAC","chr12",19500109,"+","none","TGG","TTTCTTCGATGCACAAACACTGG",0,NA,NA,NA,"GeneID:121536","AEBP2",TRUE,"AEBP2"

"4280","AGTACATGGAGGACCAGTAC","chr15",88630208,"+","none","TGG","AGTACATGGAGGACCAGTACTGG",0,NA,NA,NA,"GeneID:64782","AEN",TRUE,"AEN"

"4281","CCGAGGGCGGGTAAGCGAGC","chr15",88626574,"+","none","TGG","CCGAGGGCGGGTAAGCGAGCTGG",0,NA,NA,NA,"GeneID:64782","AEN",TRUE,"AEN"

"4282","CTACTGATGAGTGCCCGTGC","chr15",88630065,"-","none","TGG","CTACTGATGAGTGCCCGTGCTGG",0,NA,NA,NA,"GeneID:64782","AEN",TRUE,"AEN"

"4283","GGTGGACATACTTGAGCGCC","chr15",88629284,"-","none","TGG","GGTGGACATACTTGAGCGCCTGG",0,NA,NA,NA,"GeneID:64782","AEN",TRUE,"AEN"

"4284","TCGCTGACTACCGTACCCGC","chr15",88626666,"+","none","TGG","TCGCTGACTACCGTACCCGCTGG",0,NA,NA,NA,"GeneID:64782","AEN",TRUE,"AEN"

"4285","TTGGGACATAGGTCGTATCC","chr15",88629320,"-","none","CGG","TTGGGACATAGGTCGTATCCCGG",0,NA,NA,NA,"GeneID:64782","AEN",TRUE,"AEN"

"4286","ACATGATGTTTCCACAAAGC","chr19",3062182,"-","none","AGG","ACATGATGTTTCCACAAAGCAGG",0,NA,NA,NA,"GeneID:166","AES",TRUE,"AES"

"4287","ACATGATGTTTCCACAAAGC","chr14",51481718,"-","10:G>A,12:T>G","TGG","ACATGATGTTCCAACAAAGCTGG",2,11,13,NA,"GeneID:100874185","FRMD6-AS2",FALSE,"AES"

"4288","CACGACCTGGACGCGTGCCA","chr19",3062785,"+","none","CGG","CACGACCTGGACGCGTGCCACGG",0,NA,NA,NA,"GeneID:166","AES",TRUE,"AES"

"4289","CACGTCCGTGGCACGCGTCC","chr19",3062793,"-","none","AGG","CACGTCCGTGGCACGCGTCCAGG",0,NA,NA,NA,"GeneID:166","AES",TRUE,"AES"

"4290","CTTACCGAATGCCTGCTTTG","chr19",3062168,"+","none","TGG","CTTACCGAATGCCTGCTTTGTGG",0,NA,NA,NA,"GeneID:166","AES",TRUE,"AES"

"4291","CTTACCGAATGCCTGCTTTG","chr10",105963856,"-","4:A>G,6:A>C","AGG","CTTATCTAATGCCTGCTTTGAGG",2,5,7,NA,"","",FALSE,"AES"

"4292","CTTACCGAATGCCTGCTTTG","chr5",37962538,"+","2:G>T,5:T>C","AGG","CTGACTGAATGCCTGCTTTGAGG",2,3,6,NA,"","",FALSE,"AES"

"4293","CTTACCGAATGCCTGCTTTG","chrX",14790059,"+","5:T>C,17:A>T","TGG","CTTACTGAATGCCTGCTATGTGG",2,6,18,NA,"","",FALSE,"AES"

"4294","GCTGAAATTCGTCTTTGATG","chr19",3061181,"+","none","CGG","GCTGAAATTCGTCTTTGATGCGG",0,NA,NA,NA,"GeneID:166","AES",TRUE,"AES"

"4295","TCCAGGCTGAGATCGTCAAA","chr19",3055711,"-","none","AGG","TCCAGGCTGAGATCGTCAAAAGG",0,NA,NA,NA,"GeneID:166","AES",TRUE,"AES"

"4296","TCCAGGCTGAGATCGTCAAA","chr3",98234051,"+","13:A>C,18:G>A","TGG","TCCAGGCTGAGATAGTCAGATGG",2,14,19,NA,"","",FALSE,"AES"

"4297","AGGGGCTGGCAGGCTGTTAG","chr4",7868660,"+","none","CGG","AGGGGCTGGCAGGCTGTTAGCGG",0,NA,NA,NA,"GeneID:60312","AFAP1",TRUE,"AFAP1"

"4298","AGGGGCTGGCAGGCTGTTAG","chr3",38355270,"-","10:A>T,15:G>C","GGG","AGGGGCTGGCTGGCTCTTAGGGG",2,11,16,NA,"GeneID:9942","XYLB",FALSE,"AFAP1"

"4299","ATAAGAGCTGCTCATCGCAT","chr4",7843318,"+","none","CGG","ATAAGAGCTGCTCATCGCATCGG",0,NA,NA,NA,"GeneID:60312","AFAP1",TRUE,"AFAP1"

"4300","ATCTCCGGCAGGGGCATCTG","chr4",7868637,"+","none","AGG","ATCTCCGGCAGGGGCATCTGAGG",0,NA,NA,NA,"GeneID:60312","AFAP1",TRUE,"AFAP1"

"4301","GAAGCTGTGCCGCTGAGCCC","chr4",7855494,"-","none","CGG","GAAGCTGTGCCGCTGAGCCCCGG",0,NA,NA,NA,"GeneID:60312","AFAP1",TRUE,"AFAP1"

"4302","GCTTCCTCATAATAACCTTC","chr4",7855509,"+","none","TGG","GCTTCCTCATAATAACCTTCTGG",0,NA,NA,NA,"GeneID:60312","AFAP1",TRUE,"AFAP1"

"4303","GCTTCCTCATAATAACCTTC","chr18",67157991,"+","15:G>C,16:A>C","AGG","GCTTCCTCATAATAAGATTCAGG",2,16,17,NA,"","",FALSE,"AFAP1"

"4304","GCTTCCTCATAATAACCTTC","chr6",30627225,"+","9:C>T,19:A>C","AGG","GCTTCCTCACAATAACCTTAAGG",2,10,20,NA,"GeneID:79969","ATAT1",TRUE,"AFAP1"

"4305","TTATGAGTCGTATGATGAAG","chr4",7843302,"-","none","AGG","TTATGAGTCGTATGATGAAGAGG",0,NA,NA,NA,"GeneID:60312","AFAP1",TRUE,"AFAP1"

"4306","CCACGAAGCTGGGCCCCGAG","chr5",149300313,"-","none","TGG","CCACGAAGCTGGGCCCCGAGTGG",0,NA,NA,NA,"GeneID:134265","AFAP1L1",TRUE,"AFAP1L1"

"4307","GCACGAGCTGCGTTTCACCC","chr5",149310050,"+","none","AGG","GCACGAGCTGCGTTTCACCCAGG",0,NA,NA,NA,"GeneID:134265","AFAP1L1",TRUE,"AFAP1L1"

"4308","GGGCACGTAGATGATGCTGC","chr5",149310010,"-","none","AGG","GGGCACGTAGATGATGCTGCAGG",0,NA,NA,NA,"GeneID:134265","AFAP1L1",TRUE,"AFAP1L1"

"4309","GGTCTGCTGTGTTCACATAC","chr5",149300289,"-","none","AGG","GGTCTGCTGTGTTCACATACAGG",0,NA,NA,NA,"GeneID:134265","AFAP1L1",TRUE,"AFAP1L1"

"4310","GTACCTCAGCGATACCACCC","chr5",149299563,"+","none","TGG","GTACCTCAGCGATACCACCCTGG",0,NA,NA,NA,"GeneID:134265","AFAP1L1",TRUE,"AFAP1L1"

"4311","TGGTATCGCTGAGGTACTCG","chr5",149299560,"-","none","TGG","TGGTATCGCTGAGGTACTCGTGG",0,NA,NA,NA,"GeneID:134265","AFAP1L1",TRUE,"AFAP1L1"

"4312","TGGTATCGCTGAGGTACTCG","chr14",23171146,"+","6:T>C,19:A>G","AGG","TGGTATTGCTGAGGTACTCAAGG",2,7,20,NA,"GeneID:23428","SLC7A8",FALSE,"AFAP1L1"

"4313","AAAGCCGGAGGAGCTCCGCC","chr10",114340617,"+","none","AGG","AAAGCCGGAGGAGCTCCGCCAGG",0,NA,NA,NA,"GeneID:84632","AFAP1L2",TRUE,"AFAP1L2"

"4314","CAACAGAATGCAGAGTCTCA","chr10",114333228,"-","none","AGG","CAACAGAATGCAGAGTCTCAAGG",0,NA,NA,NA,"GeneID:84632","AFAP1L2",TRUE,"AFAP1L2"

"4315","CAACAGAATGCAGAGTCTCA","chr4",147991274,"-","4:A>T,5:T>C","CGG","CAACTAAATGCAGAGTCTCACGG",2,5,6,NA,"GeneID:79658","ARHGAP10",FALSE,"AFAP1L2"

"4316","CTTCCTCAAGATTCTTGACC","chr10",114340680,"-","none","AGG","CTTCCTCAAGATTCTTGACCAGG",0,NA,NA,NA,"GeneID:84632","AFAP1L2",TRUE,"AFAP1L2"

"4317","CTTCCTCAAGATTCTTGACC","chr11",20918900,"+","0:A>C,6:T>C","TGG","ATTCCTTAAGATTCTTGACCTGG",2,1,7,NA,"GeneID:4745","NELL1",FALSE,"AFAP1L2"

"4318","GCCCCGATGGAGCGGTACAA","chr10",114404441,"-","none","AGG","GCCCCGATGGAGCGGTACAAAGG",0,NA,NA,NA,"GeneID:84632","AFAP1L2",TRUE,"AFAP1L2"

"4319","TCTCCTGGTCAAGAATCTTG","chr10",114340674,"+","none","AGG","TCTCCTGGTCAAGAATCTTGAGG",0,NA,NA,NA,"GeneID:84632","AFAP1L2",TRUE,"AFAP1L2"

"4320","TGCATTCTGTTGCTTGTTGA","chr10",114333236,"+","none","TGG","TGCATTCTGTTGCTTGTTGATGG",0,NA,NA,NA,"GeneID:84632","AFAP1L2",TRUE,"AFAP1L2"

"4321","TGCATTCTGTTGCTTGTTGA","chr10",35798006,"+","2:T>C,8:T>G","AGG","TGTATTCTTTTGCTTGTTGAAGG",2,3,9,NA,"GeneID:102578074","LINC01452",FALSE,"AFAP1L2"

"4322","TGCATTCTGTTGCTTGTTGA","chrX",80476140,"+","2:T>C,15:T>G","TGG","TGTATTCTGTTGCTTTTTGATGG",2,3,16,NA,"","",FALSE,"AFAP1L2"

"4323","ACAGAAAGAGTCAACAGCAG","chr4",86948542,"+","none","TGG","ACAGAAAGAGTCAACAGCAGTGG",0,NA,NA,NA,"GeneID:4299","AFF1",TRUE,"AFF1"

"4324","ACAGAAAGAGTCAACAGCAG","chr6",14371694,"+","0:T>A,5:C>A","GGG","TCAGACAGAGTCAACAGCAGGGG",2,1,6,NA,"","",FALSE,"AFF1"

"4325","ACAGAAAGAGTCAACAGCAG","chr1",33977895,"+","2:T>A,17:A>C","CGG","ACTGAAAGAGTCAACAGAAGCGG",2,3,18,NA,"GeneID:114784","CSMD2",FALSE,"AFF1"

"4326","CCAGTGCCTTCTTTGCCAAA","chr4",87127055,"+","none","TGG","CCAGTGCCTTCTTTGCCAAATGG",0,NA,NA,NA,"GeneID:4299","AFF1",TRUE,"AFF1"

"4327","CCAGTGCCTTCTTTGCCAAA","chr1",225769451,"+","14:C>G,15:T>C","CGG","CCAGTGCCTTCTTTCTCAAACGG",2,15,16,NA,"","",FALSE,"AFF1"

"4328","CCTCACTGTCACTGAGCTGA","chr4",87105645,"-","none","AGG","CCTCACTGTCACTGAGCTGAAGG",0,NA,NA,NA,"GeneID:4299","AFF1",TRUE,"AFF1"

"4329","CCTCACTGTCACTGAGCTGA","chr1",228917391,"-","10:G>T,14:A>T","AGG","CCTCACTGTCCCTGTGCTGAAGG",2,11,15,NA,"","",FALSE,"AFF1"

"4330","CCTGGTTTAGAGTTACCATT","chr4",87127073,"-","none","TGG","CCTGGTTTAGAGTTACCATTTGG",0,NA,NA,NA,"GeneID:4299","AFF1",TRUE,"AFF1"

"4331","CCTGGTTTAGAGTTACCATT","chr2",46451336,"-","7:G>A,17:A>T","CGG","CCTGGTTCAGAGTTACCTTTCGG",2,8,18,NA,"","",FALSE,"AFF1"

"4332","CCTGGTTTAGAGTTACCATT","chr17",81695390,"-","6:C>A,14:A>T","GGG","CCTGGTGTAGAGTTTCCATTGGG",2,7,15,NA,"GeneID:9146","HGS",FALSE,"AFF1"

"4333","CCTTCAGCTCAGTGACAGTG","chr4",87105642,"+","none","AGG","CCTTCAGCTCAGTGACAGTGAGG",0,NA,NA,NA,"GeneID:4299","AFF1",TRUE,"AFF1"

"4334","CCTTCAGCTCAGTGACAGTG","chr1",228917388,"+","8:A>T,12:G>T","AGG","CCTTCAGCACAGGGACAGTGAGG",2,9,13,NA,"","",FALSE,"AFF1"

"4335","TACTTGTCAAACTTCACTTG","chr4",87127100,"-","none","AGG","TACTTGTCAAACTTCACTTGAGG",0,NA,NA,NA,"GeneID:4299","AFF1",TRUE,"AFF1"

"4336","TACTTGTCAAACTTCACTTG","chr12",46178173,"+","5:T>G,9:T>A","TGG","TACTTTTCATACTTCACTTGTGG",2,6,10,NA,"","",FALSE,"AFF1"

"4337","AACCGAGTCCAGAACACGCT","chrX",148661931,"+","none","TGG","AACCGAGTCCAGAACACGCTTGG",0,NA,NA,NA,"GeneID:2334","AFF2",TRUE,"AFF2"

"4338","AAGATTTCTTCAACACAGCT","chrX",148809896,"-","none","TGG","AAGATTTCTTCAACACAGCTTGG",0,NA,NA,NA,"GeneID:2334","AFF2",TRUE,"AFF2"

"4339","ACTCTTCAACTTCTCCAATC","chrX",148662341,"-","none","TGG","ACTCTTCAACTTCTCCAATCTGG",0,NA,NA,NA,"GeneID:2334","AFF2",TRUE,"AFF2"

"4340","CATAGTTTCCAAGCGTGTTC","chrX",148661942,"-","none","TGG","CATAGTTTCCAAGCGTGTTCTGG",0,NA,NA,NA,"GeneID:2334","AFF2",TRUE,"AFF2"

"4341","GAATTCGCCGTGCAAGCGCC","chrX",148662453,"+","none","TGG","GAATTCGCCGTGCAAGCGCCTGG",0,NA,NA,NA,"GeneID:2334","AFF2",TRUE,"AFF2"

"4342","GCTGTGTTGAAGAAATCTTG","chrX",148809897,"+","none","CGG","GCTGTGTTGAAGAAATCTTGCGG",0,NA,NA,NA,"GeneID:2334","AFF2",TRUE,"AFF2"

"4343","GCTGTGTTGAAGAAATCTTG","chr8",61692034,"-","14:C>T","GGG","GCTGTGTTGAAGAAGTCTTGGGG",1,15,NA,NA,"GeneID:444","ASPH",FALSE,"AFF2"

"4344","GCTGTGTTGAAGAAATCTTG","chr17",9196224,"+","16:T>C,17:G>T","GGG","GCTGTGTTGAAGAAATTGTGGGG",2,17,18,NA,"GeneID:9423","NTN1",FALSE,"AFF2"

"4345","AACCGGATCCAGAACACTTT","chr2",100007416,"-","none","AGG","AACCGGATCCAGAACACTTTAGG",0,NA,NA,NA,"GeneID:3899","AFF3",TRUE,"AFF3"

"4346","AGAAACCGACCGCGTATGTG","chr2",100006799,"-","none","AGG","AGAAACCGACCGCGTATGTGAGG",0,NA,NA,NA,"GeneID:3899","AFF3",TRUE,"AFF3"

"4347","CTCCGTAATGCATTTCTATC","chr2",100008899,"+","none","TGG","CTCCGTAATGCATTTCTATCTGG",0,NA,NA,NA,"GeneID:3899","AFF3",TRUE,"AFF3"

"4348","CTCCGTAATGCATTTCTATC","chr8",104177891,"+","4:T>G,17:C>A","TGG","CTCCTTAATGCATTTCTCTCTGG",2,5,18,NA,"GeneID:9699","RIMS2",FALSE,"AFF3"

"4349","TCCGAAGACGACTTCAGCTT","chr2",100006737,"+","none","AGG","TCCGAAGACGACTTCAGCTTAGG",0,NA,NA,NA,"GeneID:3899","AFF3",TRUE,"AFF3"

"4350","TCTCTTCAGTGAGCCCTACA","chr2",100008813,"-","none","AGG","TCTCTTCAGTGAGCCCTACAAGG",0,NA,NA,NA,"GeneID:3899","AFF3",TRUE,"AFF3"

"4351","TGTAGTGCTACAGATAGACG","chr2",100007247,"+","none","AGG","TGTAGTGCTACAGATAGACGAGG",0,NA,NA,NA,"GeneID:3899","AFF3",TRUE,"AFF3"

"4352","CTTTCATACGCAGCACATTC","chr5",132937152,"+","none","CGG","CTTTCATACGCAGCACATTCCGG",0,NA,NA,NA,"GeneID:27125","AFF4",TRUE,"AFF4"

"4353","GAGTCTCAGCAGTCCAATTT","chr5",132904384,"-","none","TGG","GAGTCTCAGCAGTCCAATTTTGG",0,NA,NA,NA,"GeneID:27125","AFF4",TRUE,"AFF4"

"4354","GCAACATGAACCGTGAAGAC","chr5",132937174,"-","none","CGG","GCAACATGAACCGTGAAGACCGG",0,NA,NA,NA,"GeneID:27125","AFF4",TRUE,"AFF4"

"4355","GGGCAACTTACAACTTTGTA","chr5",132937055,"+","none","TGG","GGGCAACTTACAACTTTGTATGG",0,NA,NA,NA,"GeneID:27125","AFF4",TRUE,"AFF4"

"4356","TCTTTCAAGGCATCAGCTTC","chr5",132932211,"-","none","TGG","TCTTTCAAGGCATCAGCTTCTGG",0,NA,NA,NA,"GeneID:27125","AFF4",TRUE,"AFF4"

"4357","TCTTTCAAGGCATCAGCTTC","chr13",77317578,"+","3:A>T,9:T>G","TGG","TCTATCAAGTCATCAGCTTCTGG",2,4,10,NA,"GeneID:23077","MYCBP2",FALSE,"AFF4"

"4358","TCTTTCAAGGCATCAGCTTC","chr8",30056163,"+","4:A>T,14:T>A","AGG","TCTTACAAGGCATCTGCTTCAGG",2,5,15,NA,"","",FALSE,"AFF4"

"4359","TTGTTCTCCAGTGCCAAAAT","chr5",132904368,"+","none","TGG","TTGTTCTCCAGTGCCAAAATTGG",0,NA,NA,NA,"GeneID:27125","AFF4",TRUE,"AFF4"

"4360","TTGTTCTCCAGTGCCAAAAT","chr2",236089585,"-","18:C>T,19:T>A","AGG","TTGTTCTCCAGTGCCAAAGAAGG",2,19,20,NA,"GeneID:116987","AGAP1",FALSE,"AFF4"

"4361","TTGTTCTCCAGTGCCAAAAT","chr21",43287413,"+","16:C>A,18:C>A","TGG","TTGTTCTCCAGTGCCACACTTGG",2,17,19,NA,"","",FALSE,"AFF4"

"4362","AGTTTCCAGATTCCGTTCAA","chr18",12359994,"+","none","AGG","AGTTTCCAGATTCCGTTCAAAGG",0,NA,NA,NA,"GeneID:10939","AFG3L2",TRUE,"AFG3L2"

"4363","AGTTTCCAGATTCCGTTCAA","chr8",43271271,"+","14:A>G","AGG","AGTTTCCAGATTCCATTCAAAGG",1,15,NA,NA,"","",TRUE,"AFG3L2"

"4364","AGTTTCCAGATTCCGTTCAA","chr6",20111205,"+","13:A>C,15:G>T","AGG","AGTTTCCAGATTCAGGTCAAAGG",2,14,16,NA,"GeneID:154141","MBOAT1",FALSE,"AFG3L2"

"4365","ATGGGCGGACTCTTCAGTGT","chr18",12358823,"-","none","CGG","ATGGGCGGACTCTTCAGTGTCGG",0,NA,NA,NA,"GeneID:10939","AFG3L2",TRUE,"AFG3L2"

"4366","GTCTACATTGCTGAAAGTGA","chr18",12359929,"-","none","TGG","GTCTACATTGCTGAAAGTGATGG",0,NA,NA,NA,"GeneID:10939","AFG3L2",TRUE,"AFG3L2"

"4367","TAGTGAACCTAAAGAAGTTA","chr18",12370863,"-","none","TGG","TAGTGAACCTAAAGAAGTTATGG",0,NA,NA,NA,"GeneID:10939","AFG3L2",TRUE,"AFG3L2"

"4368","TAGTGAACCTAAAGAAGTTA","chr8",43271678,"-","2:T>C","TGG","TAATGAACCTAAAGAAGTTATGG",1,3,NA,NA,"","",TRUE,"AFG3L2"

"4369","TAGTGAACCTAAAGAAGTTA","chr15",86664634,"+","6:C>A,13:A>G","AGG","TAGTGACCCTAAAAAAGTTAAGG",2,7,14,NA,"GeneID:123624","AGBL1",FALSE,"AFG3L2"

"4370","TGAAGAGTCCGCCCATCCCT","chr18",12358827,"+","none","CGG","TGAAGAGTCCGCCCATCCCTCGG",0,NA,NA,NA,"GeneID:10939","AFG3L2",TRUE,"AFG3L2"

"4371","TTCTCTCCCATAACTTCTTT","chr18",12370853,"+","none","AGG","TTCTCTCCCATAACTTCTTTAGG",0,NA,NA,NA,"GeneID:10939","AFG3L2",TRUE,"AFG3L2"

"4372","TTCTCTCCCATAACTTCTTT","chr9",6030363,"+","12:G>A,15:C>T","AGG","TTCTCTCCCATAGCTCCTTTAGG",2,13,16,NA,"","",FALSE,"AFG3L2"

"4373","TTCTCTCCCATAACTTCTTT","chr8",43271668,"+","4:G>C","AGG","TTCTGTCCCATAACTTCTTTAGG",1,5,NA,NA,"","",TRUE,"AFG3L2"

"4374","AATTTCTTACCTATATCCCG","chr4",73481853,"-","none","AGG","AATTTCTTACCTATATCCCGAGG",0,NA,NA,NA,"GeneID:173","AFM",TRUE,"AFM"

"4375","AGAATACAAAGACAGATGTA","chr4",73484332,"+","none","TGG","AGAATACAAAGACAGATGTATGG",0,NA,NA,NA,"GeneID:173","AFM",TRUE,"AFM"

"4376","ATACTGAGCAAATGCAATGA","chr4",73484262,"-","none","TGG","ATACTGAGCAAATGCAATGATGG",0,NA,NA,NA,"GeneID:173","AFM",TRUE,"AFM"

"4377","ATACTGAGCAAATGCAATGA","chr5",156414865,"+","12:A>T,13:A>G","AGG","ATACTGAGCAAAAACAATGAAGG",2,13,14,NA,"GeneID:6444","SGCD",FALSE,"AFM"

"4378","ATGAAACTACTAAAACTTAC","chr4",73481775,"+","none","AGG","ATGAAACTACTAAAACTTACAGG",0,NA,NA,NA,"GeneID:173","AFM",TRUE,"AFM"

"4379","ATGAAACTACTAAAACTTAC","chr3",102313356,"+","15:T>C,17:C>T","TGG","ATGAAACTACTAAAATTCACTGG",2,16,18,NA,"GeneID:131368","ZPLD1",FALSE,"AFM"

"4380","TAACCCTGCCCACACAACCT","chr4",73481833,"+","none","CGG","TAACCCTGCCCACACAACCTCGG",0,NA,NA,NA,"GeneID:173","AFM",TRUE,"AFM"

"4381","TGCATTTGCTCAGTATGTTC","chr4",73484266,"+","none","AGG","TGCATTTGCTCAGTATGTTCAGG",0,NA,NA,NA,"GeneID:173","AFM",TRUE,"AFM"

"4382","TGCATTTGCTCAGTATGTTC","chr21",43506298,"-","14:A>T,19:A>G","TGG","TGCATTTGCTCAGTTTGTTTTGG",2,15,20,NA,"","",FALSE,"AFM"

"4383","TGCATTTGCTCAGTATGTTC","chr21",6032248,"+","14:T>A,19:T>C","TGG","TGCATTTGCTCAGTTTGTTTTGG",2,15,20,NA,"","",FALSE,"AFM"

"4384","ACAACCTTCAGACGACTCGT","chr17",78202588,"-","none","CGG","ACAACCTTCAGACGACTCGTCGG",0,NA,NA,NA,"GeneID:125061","AFMID",TRUE,"AFMID"

"4385","AGTATCCTCCGTGAAAGAAC","chr17",78202718,"-","none","AGG","AGTATCCTCCGTGAAAGAACAGG",0,NA,NA,NA,"GeneID:125061","AFMID",TRUE,"AFMID"

"4386","ATCAGTACTGTCCCAGCCGA","chr17",78190979,"+","none","TGG","ATCAGTACTGTCCCAGCCGATGG",0,NA,NA,NA,"GeneID:125061","AFMID",TRUE,"AFMID"

"4387","GCCGTAAGCCACTATTACCA","chr17",78204708,"-","none","CGG","GCCGTAAGCCACTATTACCACGG",0,NA,NA,NA,"GeneID:125061","AFMID",TRUE,"AFMID"

"4388","GCTTACGGCATCGCCCCCAA","chr17",78204719,"+","none","AGG","GCTTACGGCATCGCCCCCAAAGG",0,NA,NA,NA,"GeneID:125061","AFMID",TRUE,"AFMID"

"4389","TTCCCCGACGAGTCGTCTGA","chr17",78202581,"+","none","AGG","TTCCCCGACGAGTCGTCTGAAGG",0,NA,NA,NA,"GeneID:125061","AFMID",TRUE,"AFMID"

"4390","AACTTATCTCTGCAGTACAT","chr4",73437181,"-","none","TGG","AACTTATCTCTGCAGTACATTGG",0,NA,NA,NA,"GeneID:174","AFP",TRUE,"AFP"

"4391","AATTGATTCCACCCACTTCA","chr4",73436263,"-","none","TGG","AATTGATTCCACCCACTTCATGG",0,NA,NA,NA,"GeneID:174","AFP",TRUE,"AFP"

"4392","AATTGATTCCACCCACTTCA","chr11",27713564,"-","10:A>T,14:C>T","AGG","AATTGATTCCTCCCGCTTCAAGG",2,11,15,NA,"GeneID:627","BDNF",FALSE,"AFP"

"4393","ACTAGCAACCATGAAGTGGG","chr4",73436252,"+","none","TGG","ACTAGCAACCATGAAGTGGGTGG",0,NA,NA,NA,"GeneID:174","AFP",TRUE,"AFP"

"4394","TGAAGACTGTTCATCTCCAG","chr4",73438268,"-","none","TGG","TGAAGACTGTTCATCTCCAGTGG",0,NA,NA,NA,"GeneID:174","AFP",TRUE,"AFP"

"4395","TGAAGACTGTTCATCTCCAG","chr9",20320812,"+","13:G>T,19:C>G","AGG","TGAAGACTGTTCAGCTCCACAGG",2,14,20,NA,"","",FALSE,"AFP"

"4396","TTCAGGGTGTTTAGAAAACC","chr4",73438284,"+","none","AGG","TTCAGGGTGTTTAGAAAACCAGG",0,NA,NA,NA,"GeneID:174","AFP",TRUE,"AFP"

"4397","TTGCTTTCCAGCTTCCATAT","chr4",73437148,"+","none","TGG","TTGCTTTCCAGCTTCCATATTGG",0,NA,NA,NA,"GeneID:174","AFP",TRUE,"AFP"

"4398","TTGCTTTCCAGCTTCCATAT","chr3",140755568,"+","17:G>T,18:T>A","GGG","TTGCTTTCCAGCTTCCAGTTGGG",2,18,19,NA,"","",FALSE,"AFP"

"4399","TTGCTTTCCAGCTTCCATAT","chr10",58376590,"+","9:T>A,11:T>C","GGG","TTGCTTTCCTGTTTCCATATGGG",2,10,12,NA,"","",FALSE,"AFP"

"4400","ATAGTGCCCATGTATGCAGC","chr2",64569652,"+","none","AGG","ATAGTGCCCATGTATGCAGCAGG",0,NA,NA,NA,"GeneID:54812","AFTPH",TRUE,"AFTPH"

"4401","ATAGTGCCCATGTATGCAGC","chr17",10711952,"-","15:A>C,18:T>C","AGG","ATAGTGCCCATGTATTCAACAGG",2,16,19,NA,"GeneID:388335","TMEM220",FALSE,"AFTPH"

"4402","ATGCTTCGAAAATTCGCTCC","chr2",64567581,"-","none","AGG","ATGCTTCGAAAATTCGCTCCAGG",0,NA,NA,NA,"GeneID:54812","AFTPH",TRUE,"AFTPH"

"4403","ATGTACATCTGATCAGTTCC","chr2",64573046,"+","none","AGG","ATGTACATCTGATCAGTTCCAGG",0,NA,NA,NA,"GeneID:54812","AFTPH",TRUE,"AFTPH"

"4404","CATGGTGGCTGTCTGACCGA","chr2",64573009,"-","none","TGG","CATGGTGGCTGTCTGACCGATGG",0,NA,NA,NA,"GeneID:54812","AFTPH",TRUE,"AFTPH"

"4405","GACAGCTTTATTAAACCGCC","chr2",64567560,"+","none","TGG","GACAGCTTTATTAAACCGCCTGG",0,NA,NA,NA,"GeneID:54812","AFTPH",TRUE,"AFTPH"

"4406","GTTTCGGGTGTCTATTCCCA","chr2",64569195,"-","none","AGG","GTTTCGGGTGTCTATTCCCAAGG",0,NA,NA,NA,"GeneID:54812","AFTPH",TRUE,"AFTPH"

"4407","ACCACCTGGTATCTTAAAGC","chr4",177437458,"-","none","AGG","ACCACCTGGTATCTTAAAGCAGG",0,NA,NA,NA,"GeneID:175","AGA",TRUE,"AGA"

"4408","ATATCCTGCTTTAAGATACC","chr4",177437451,"+","none","AGG","ATATCCTGCTTTAAGATACCAGG",0,NA,NA,NA,"GeneID:175","AGA",TRUE,"AGA"

"4409","CACACTAGATGCCATGATCA","chr4",177440279,"-","none","TGG","CACACTAGATGCCATGATCATGG",0,NA,NA,NA,"GeneID:175","AGA",TRUE,"AGA"

"4410","CACACTAGATGCCATGATCA","chr1",10630011,"-","15:G>C,16:G>T","GGG","CACACTAGATGCCATCCTCAGGG",2,16,17,NA,"GeneID:5195","PEX14",TRUE,"AGA"

"4411","CACCACATTTGCTCAAAGTA","chr4",177438836,"-","none","TGG","CACCACATTTGCTCAAAGTATGG",0,NA,NA,NA,"GeneID:175","AGA",TRUE,"AGA"

"4412","GTGCATACCCTCCAATAATT","chr4",177438736,"+","none","TGG","GTGCATACCCTCCAATAATTTGG",0,NA,NA,NA,"GeneID:175","AGA",TRUE,"AGA"

"4413","TGCAGCACTACTATGGATGT","chr4",177439673,"-","none","AGG","TGCAGCACTACTATGGATGTAGG",0,NA,NA,NA,"GeneID:175","AGA",TRUE,"AGA"

"4414","AGATGCCTTCGTGAACAGCC","chr2",235709176,"+","none","AGG","AGATGCCTTCGTGAACAGCCAGG",0,NA,NA,NA,"GeneID:116987","AGAP1",TRUE,"AGAP1"

"4415","CATATGTGCCCGTCAGGTAC","chr2",235717603,"-","none","CGG","CATATGTGCCCGTCAGGTACCGG",0,NA,NA,NA,"GeneID:116987","AGAP1",TRUE,"AGAP1"

"4416","CCACTCACCACTTTGAGCTC","chr2",235709225,"-","none","CGG","CCACTCACCACTTTGAGCTCCGG",0,NA,NA,NA,"GeneID:116987","AGAP1",TRUE,"AGAP1"

"4417","CCACTCACCACTTTGAGCTC","chr7",68828872,"+","5:T>C,9:G>A","TGG","CCACTTACCGCTTTGAGCTCTGG",2,6,10,NA,"","",FALSE,"AGAP1"

"4418","CCACTCACCACTTTGAGCTC","chr5",110057735,"-","7:C>G,12:C>A","AGG","CCACTCAGCACTGTGAGCTCAGG",2,8,13,NA,"","",FALSE,"AGAP1"

"4419","CCACTCACCACTTTGAGCTC","chr7",151116839,"-","0:T>G,14:T>C","CGG","ACACTCACCACTTTAAGCTCCGG",2,1,15,NA,"GeneID:116988","AGAP3",TRUE,"AGAP1"

"4420","CCTGACGGGCACATATGTCC","chr2",235717606,"+","none","AGG","CCTGACGGGCACATATGTCCAGG",0,NA,NA,NA,"GeneID:116987","AGAP1",TRUE,"AGAP1"

"4421","CTACTCCATCTACGAGCTGC","chr2",235494766,"+","none","TGG","CTACTCCATCTACGAGCTGCTGG",0,NA,NA,NA,"GeneID:116987","AGAP1",TRUE,"AGAP1"

"4422","GGTGGACCGACTCGAAGCGC","chr2",235494739,"-","none","TGG","GGTGGACCGACTCGAAGCGCTGG",0,NA,NA,NA,"GeneID:116987","AGAP1",TRUE,"AGAP1"

"4423","GGTGGACCGACTCGAAGCGC","chr1",179586757,"-","8:T>C,17:A>G","TGG","GGTGGACCAACTCGAAGTGCTGG",2,9,18,NA,"","",TRUE,"AGAP1"

"4424","ACTTTGATATAATGCCTCAT","chr10",86993473,"+","none","TGG","ACTTTGATATAATGCCTCATTGG",0,NA,NA,NA,"","",TRUE,"AGAP11"

"4425","AGCATTGCTCAAATAACCTG","chr10",86983919,"+","none","TGG","AGCATTGCTCAAATAACCTGTGG",0,NA,NA,NA,"GeneID:10974","ADIRF",TRUE,"AGAP11"

"4426","AGCATTGCTCAAATAACCTG","chr2",201157938,"-","9:A>G,15:A>T","TGG","AGCATTGCTTAAATATCCTGTGG",2,10,16,NA,"GeneID:8837","CFLAR",FALSE,"AGAP11"

"4427","CATCTTCTTCATCCCCTAGC","chr10",86992382,"-","none","TGG","CATCTTCTTCATCCCCTAGCTGG",0,NA,NA,NA,"","",TRUE,"AGAP11"

"4428","CATCTTCTTCATCCCCTAGC","chr10",42784483,"-","10:C>T,13:A>G","TGG","CATCTTCTTCGTCTCCTAGCTGG",2,11,14,NA,"GeneID:9790","BMS1",TRUE,"AGAP11"

"4429","CATCTTCTTCATCCCCTAGC","chr10",79906693,"-","10:C>T,13:A>G","TGG","CATCTTCTTCGTCTCCTAGCTGG",2,11,14,NA,"","",TRUE,"AGAP11"

"4430","CATCTTCTTCATCCCCTAGC","chr10",73728445,"+","10:G>A,13:T>C","TGG","CATCTTCTTCGTCTCCTAGCTGG",2,11,14,NA,"GeneID:729096","BMS1P4",TRUE,"AGAP11"

"4431","GCCGCCACCAATAGTGATAG","chr10",86993421,"+","none","TGG","GCCGCCACCAATAGTGATAGTGG",0,NA,NA,NA,"","",TRUE,"AGAP11"

"4432","GCCGCCACCAATAGTGATAG","chr10",46789556,"+","14:C>T,16:G>A","TGG","GCCGCCACCAATAGCGGTAGTGG",2,15,17,NA,"GeneID:399761","BMS1P5",TRUE,"AGAP11"

"4433","GCCGCCACCAATAGTGATAG","chr10",42785541,"+","3:C>G,16:G>A","TGG","GCCCCCACCAATAGTGGTAGTGG",2,4,17,NA,"GeneID:9790","BMS1",TRUE,"AGAP11"

"4434","GCCGCCACCAATAGTGATAG","chr10",79907727,"+","3:C>G,16:G>A","TGG","GCCCCCACCAATAGTGGTAGTGG",2,4,17,NA,"","",TRUE,"AGAP11"

"4435","GCCGCCACCAATAGTGATAG","chr10",73727402,"-","3:G>C,16:C>T","TGG","GCCCCCACCAATAGTGGTAGTGG",2,4,17,NA,"GeneID:729096","BMS1P4",TRUE,"AGAP11"

"4436","GCTATACTTCTTGTAGTTCA","chr10",86983902,"-","none","TGG","GCTATACTTCTTGTAGTTCATGG",0,NA,NA,NA,"GeneID:10974","ADIRF",TRUE,"AGAP11"

"4437","TAGGGGATGAAGAAGATGTC","chr10",86992384,"+","none","TGG","TAGGGGATGAAGAAGATGTCTGG",0,NA,NA,NA,"","",TRUE,"AGAP11"

"4438","TAGGGGATGAAGAAGATGTC","chr2",157204229,"+","0:G>T,7:G>T","AGG","GAGGGGAGGAAGAAGATGTCAGG",2,1,8,NA,"","",FALSE,"AGAP11"

"4439","TAGGGGATGAAGAAGATGTC","chr14",99065733,"-","6:C>T,16:C>A","AGG","TAGGGGGTGAAGAAGAGGTCAGG",2,7,17,NA,"","",FALSE,"AGAP11"

"4440","TAGGGGATGAAGAAGATGTC","chr18",43979702,"-","5:T>C,16:T>A","AGG","TAGGGAATGAAGAAGAAGTCAGG",2,6,17,NA,"","",FALSE,"AGAP11"

"4441","ACATCTGGTGCTAATCCGAG","chr12",57734344,"-","none","AGG","ACATCTGGTGCTAATCCGAGAGG",0,NA,NA,NA,"GeneID:116986","AGAP2",TRUE,"AGAP2"

"4442","AGAGGCTGTGATCAATAGCC","chr12",57735409,"-","none","AGG","AGAGGCTGTGATCAATAGCCAGG",0,NA,NA,NA,"GeneID:116986","AGAP2",TRUE,"AGAP2"

"4443","AGCAGGCTCTAAACCGCCTC","chr12",57741984,"-","none","AGG","AGCAGGCTCTAAACCGCCTCAGG",0,NA,NA,NA,"GeneID:116986;GeneID:6302","AGAP2;TSPAN31",TRUE,"AGAP2"

"4444","GGTGGACGACCCCGAACTCC","chr12",57741947,"-","none","AGG","GGTGGACGACCCCGAACTCCAGG",0,NA,NA,NA,"GeneID:116986;GeneID:6302","AGAP2;TSPAN31",TRUE,"AGAP2"

"4445","TCCACCCTCACCTTGGCATC","chr12",57734307,"+","none","AGG","TCCACCCTCACCTTGGCATCAGG",0,NA,NA,NA,"GeneID:116986","AGAP2",TRUE,"AGAP2"

"4446","TCCACCCTCACCTTGGCATC","chr8",37763204,"-","13:G>A,17:G>T","CGG","TCCACCCTCACCTCGGCCTCCGG",2,14,18,NA,"GeneID:11212","PROSC",FALSE,"AGAP2"

"4447","TCCACCCTCACCTTGGCATC","chr3",171273247,"-","4:C>G,5:T>G","AGG","TCCAGACTCACCTTGGCATCAGG",2,5,6,NA,"GeneID:23043","TNIK",FALSE,"AGAP2"

"4448","TTACCTACCAGGCGCAGTTC","chr12",57735360,"+","none","AGG","TTACCTACCAGGCGCAGTTCAGG",0,NA,NA,NA,"GeneID:116986","AGAP2",TRUE,"AGAP2"

"4449","CAGCTTCCGCAACGCCAGCG","chr7",151117727,"+","none","AGG","CAGCTTCCGCAACGCCAGCGAGG",0,NA,NA,NA,"GeneID:116988","AGAP3",TRUE,"AGAP3"

"4450","CATAGGTCCCCGTCAGATAG","chr7",151117141,"-","none","CGG","CATAGGTCCCCGTCAGATAGCGG",0,NA,NA,NA,"GeneID:116988","AGAP3",TRUE,"AGAP3"

"4451","CATGGTGCTTGTGGGCACGC","chr7",151117754,"+","none","AGG","CATGGTGCTTGTGGGCACGCAGG",0,NA,NA,NA,"GeneID:116988","AGAP3",TRUE,"AGAP3"

"4452","CATGGTGCTTGTGGGCACGC","chrX",76996974,"-","16:G>T,17:T>G","TGG","CATGGTGCTTGTGGGCCAGCTGG",2,17,18,NA,"GeneID:101928469","MIR325HG",FALSE,"AGAP3"

"4453","CATGGTGCTTGTGGGCACGC","chr4",136929039,"+","16:T>A,17:A>C","GGG","CATGGTGCTTGTGGGCTAGCGGG",2,17,18,NA,"","",FALSE,"AGAP3"

"4454","CATGGTGCTTGTGGGCACGC","chr12",24866105,"+","16:C>A,17:A>C","TGG","CATGGTGCTTGTGGGCCAGCTGG",2,17,18,NA,"GeneID:586","BCAT1",FALSE,"AGAP3"

"4455","CCTAGGGGGGCGGTTTAAGA","chr7",151117365,"+","none","AGG","CCTAGGGGGGCGGTTTAAGAAGG",0,NA,NA,NA,"GeneID:116988","AGAP3",TRUE,"AGAP3"

"4456","CTTCATCTCGGATCAGCAGC","chr7",151117416,"-","none","AGG","CTTCATCTCGGATCAGCAGCAGG",0,NA,NA,NA,"GeneID:116988","AGAP3",TRUE,"AGAP3"

"4457","GGGCTGACTTCCCGCTAGAC","chr7",151117111,"-","none","AGG","GGGCTGACTTCCCGCTAGACAGG",0,NA,NA,NA,"GeneID:116988","AGAP3",TRUE,"AGAP3"

"4458","CACCTTTGTGTGATATGATG","chr10",45831390,"+","none","AGG","CACCTTTGTGTGATATGATGAGG",0,NA,NA,NA,"GeneID:119016","AGAP4",TRUE,"AGAP4"

"4459","CACCTTTGTGTGATATGATG","chr10",47507544,"+","none","AGG","CACCTTTGTGTGATATGATGAGG",0,NA,NA,NA,"GeneID:642517","AGAP9",TRUE,"AGAP4"

"4460","CACCTTTGTGTGATATGATG","chr10",73680067,"+","none","AGG","CACCTTTGTGTGATATGATGAGG",0,NA,NA,NA,"GeneID:729092","AGAP5",TRUE,"AGAP4"

"4461","CACCTTTGTGTGATATGATG","chr10",48015643,"+","none","AGG","CACCTTTGTGTGATATGATGAGG",0,NA,NA,NA,"GeneID:414224","CTGLF12P",TRUE,"AGAP4"

"4462","CACCTTTGTGTGATATGATG","chr10",46832459,"-","none","AGG","CACCTTTGTGTGATATGATGAGG",0,NA,NA,NA,"","",TRUE,"AGAP4"

"4463","CACCTTTGTGTGATATGATG","chr10",46125882,"-","none","AGG","CACCTTTGTGTGATATGATGAGG",0,NA,NA,NA,"","",TRUE,"AGAP4"

"4464","CACCTTTGTGTGATATGATG","chr10",46353224,"-","none","AGG","CACCTTTGTGTGATATGATGAGG",0,NA,NA,NA,"","",TRUE,"AGAP4"

"4465","CACCTTTGTGTGATATGATG","chr10",50004703,"-","none","AGG","CACCTTTGTGTGATATGATGAGG",0,NA,NA,NA,"GeneID:414189","AGAP6",TRUE,"AGAP4"

"4466","CACCTTTGTGTGATATGATG","chr10",45694766,"-","none","AGG","CACCTTTGTGTGATATGATGAGG",0,NA,NA,NA,"","",TRUE,"AGAP4"

"4467","CACCTTTGTGTGATATGATG","chr2",133138234,"-","0:C>G,10:C>A","AGG","GACCTTTGTGGGATATGATGAGG",2,1,11,NA,"GeneID:344148","NCKAP5",FALSE,"AGAP4"

"4468","CAGAGGAACTCTCAAACAGA","chr10",46110260,"+","none","TGG","CAGAGGAACTCTCAAACAGATGG",0,NA,NA,NA,"","",TRUE,"AGAP4"

"4469","CAGAGGAACTCTCAAACAGA","chr10",46817153,"+","none","TGG","CAGAGGAACTCTCAAACAGATGG",0,NA,NA,NA,"","",FALSE,"AGAP4"

"4470","CAGAGGAACTCTCAAACAGA","chr10",45679456,"+","none","TGG","CAGAGGAACTCTCAAACAGATGG",0,NA,NA,NA,"","",FALSE,"AGAP4"

"4471","CAGAGGAACTCTCAAACAGA","chr10",46340234,"+","none","TGG","CAGAGGAACTCTCAAACAGATGG",0,NA,NA,NA,"","",TRUE,"AGAP4"

"4472","CAGAGGAACTCTCAAACAGA","chr10",49991722,"+","none","TGG","CAGAGGAACTCTCAAACAGATGG",0,NA,NA,NA,"GeneID:414189","AGAP6",TRUE,"AGAP4"

"4473","CAGAGGAACTCTCAAACAGA","chr10",45681818,"+","none","TGG","CAGAGGAACTCTCAAACAGATGG",0,NA,NA,NA,"","",TRUE,"AGAP4"

"4474","CAGAGGAACTCTCAAACAGA","chr10",46112626,"+","none","TGG","CAGAGGAACTCTCAAACAGATGG",0,NA,NA,NA,"","",TRUE,"AGAP4"

"4475","CAGAGGAACTCTCAAACAGA","chr10",46819514,"+","none","TGG","CAGAGGAACTCTCAAACAGATGG",0,NA,NA,NA,"","",TRUE,"AGAP4"

"4476","CAGAGGAACTCTCAAACAGA","chr10",49989354,"+","none","AGG","CAGAGGAACTCTCAAACAGAAGG",0,NA,NA,NA,"GeneID:414189","AGAP6",TRUE,"AGAP4"

"4477","CAGAGGAACTCTCAAACAGA","chr10",46337862,"+","none","TGG","CAGAGGAACTCTCAAACAGATGG",0,NA,NA,NA,"","",TRUE,"AGAP4"

"4478","CAGAGGAACTCTCAAACAGA","chr10",45844327,"-","none","TGG","CAGAGGAACTCTCAAACAGATGG",0,NA,NA,NA,"GeneID:119016","AGAP4",TRUE,"AGAP4"

"4479","CAGAGGAACTCTCAAACAGA","chr10",48028573,"-","none","TGG","CAGAGGAACTCTCAAACAGATGG",0,NA,NA,NA,"GeneID:414224","CTGLF12P",TRUE,"AGAP4"

"4480","CAGAGGAACTCTCAAACAGA","chr10",73697096,"-","none","TGG","CAGAGGAACTCTCAAACAGATGG",0,NA,NA,NA,"GeneID:729092","AGAP5",TRUE,"AGAP4"

"4481","CAGAGGAACTCTCAAACAGA","chr10",45846688,"-","none","TGG","CAGAGGAACTCTCAAACAGATGG",0,NA,NA,NA,"GeneID:119016","AGAP4",TRUE,"AGAP4"

"4482","CAGAGGAACTCTCAAACAGA","chr10",48030939,"-","none","TGG","CAGAGGAACTCTCAAACAGATGG",0,NA,NA,NA,"GeneID:414224","CTGLF12P",TRUE,"AGAP4"

"4483","CAGAGGAACTCTCAAACAGA","chr10",47522867,"-","none","TGG","CAGAGGAACTCTCAAACAGATGG",0,NA,NA,NA,"GeneID:642517","AGAP9",TRUE,"AGAP4"

"4484","CAGAGGAACTCTCAAACAGA","chr10",47520500,"-","none","TGG","CAGAGGAACTCTCAAACAGATGG",0,NA,NA,NA,"GeneID:642517","AGAP9",TRUE,"AGAP4"

"4485","CAGAGGAACTCTCAAACAGA","chr10",73694737,"-","none","TGG","CAGAGGAACTCTCAAACAGATGG",0,NA,NA,NA,"GeneID:729092","AGAP5",TRUE,"AGAP4"

"4486","CAGAGGAACTCTCAAACAGA","chr5",72590696,"-","4:A>C,7:A>T","TGG","CAGATGATCTCTCAAACAGATGG",2,5,8,NA,"GeneID:102477328","LOC102477328",FALSE,"AGAP4"

"4487","CAGAGGAACTCTCAAACAGA","chr3",13256056,"+","1:T>A,5:A>G","TGG","CTGAGAAACTCTCAAACAGATGG",2,2,6,NA,"","",FALSE,"AGAP4"

"4488","CATCTGTTTGAGAGTTCCTC","chr10",73694735,"+","none","TGG","CATCTGTTTGAGAGTTCCTCTGG",0,NA,NA,NA,"GeneID:729092","AGAP5",TRUE,"AGAP4"

"4489","CATCTGTTTGAGAGTTCCTC","chr10",45844325,"+","none","TGG","CATCTGTTTGAGAGTTCCTCTGG",0,NA,NA,NA,"GeneID:119016","AGAP4",TRUE,"AGAP4"

"4490","CATCTGTTTGAGAGTTCCTC","chr10",48028571,"+","none","TGG","CATCTGTTTGAGAGTTCCTCTGG",0,NA,NA,NA,"GeneID:414224","CTGLF12P",TRUE,"AGAP4"

"4491","CATCTGTTTGAGAGTTCCTC","chr10",73697094,"+","none","TGG","CATCTGTTTGAGAGTTCCTCTGG",0,NA,NA,NA,"GeneID:729092","AGAP5",TRUE,"AGAP4"

"4492","CATCTGTTTGAGAGTTCCTC","chr10",45846686,"+","none","TGG","CATCTGTTTGAGAGTTCCTCTGG",0,NA,NA,NA,"GeneID:119016","AGAP4",TRUE,"AGAP4"

"4493","CATCTGTTTGAGAGTTCCTC","chr10",48030937,"+","none","TGG","CATCTGTTTGAGAGTTCCTCTGG",0,NA,NA,NA,"GeneID:414224","CTGLF12P",TRUE,"AGAP4"

"4494","CATCTGTTTGAGAGTTCCTC","chr10",47522865,"+","none","TGG","CATCTGTTTGAGAGTTCCTCTGG",0,NA,NA,NA,"GeneID:642517","AGAP9",TRUE,"AGAP4"

"4495","CATCTGTTTGAGAGTTCCTC","chr10",47520498,"+","none","TGG","CATCTGTTTGAGAGTTCCTCTGG",0,NA,NA,NA,"GeneID:642517","AGAP9",TRUE,"AGAP4"

"4496","CATCTGTTTGAGAGTTCCTC","chr10",46340236,"-","none","TGG","CATCTGTTTGAGAGTTCCTCTGG",0,NA,NA,NA,"","",TRUE,"AGAP4"

"4497","CATCTGTTTGAGAGTTCCTC","chr10",49991724,"-","none","TGG","CATCTGTTTGAGAGTTCCTCTGG",0,NA,NA,NA,"GeneID:414189","AGAP6",TRUE,"AGAP4"

"4498","CATCTGTTTGAGAGTTCCTC","chr10",45681820,"-","none","TGG","CATCTGTTTGAGAGTTCCTCTGG",0,NA,NA,NA,"","",TRUE,"AGAP4"

"4499","CATCTGTTTGAGAGTTCCTC","chr10",46112628,"-","none","TGG","CATCTGTTTGAGAGTTCCTCTGG",0,NA,NA,NA,"","",TRUE,"AGAP4"

"4500","CATCTGTTTGAGAGTTCCTC","chr10",46819516,"-","none","TGG","CATCTGTTTGAGAGTTCCTCTGG",0,NA,NA,NA,"","",TRUE,"AGAP4"

"4501","CATCTGTTTGAGAGTTCCTC","chr10",46817155,"-","none","TGG","CATCTGTTTGAGAGTTCCTCTGG",0,NA,NA,NA,"","",FALSE,"AGAP4"

"4502","CATCTGTTTGAGAGTTCCTC","chr10",45679458,"-","none","TGG","CATCTGTTTGAGAGTTCCTCTGG",0,NA,NA,NA,"","",FALSE,"AGAP4"

"4503","CATCTGTTTGAGAGTTCCTC","chr10",38982306,"-","14:G>A,17:A>G","AGG","CATCTGTTTGAGAGCTCTTCAGG",2,15,18,NA,"","",FALSE,"AGAP4"

"4504","CATCTGTTTGAGAGTTCCTC","chr10",42451608,"+","13:T>G,16:T>C","AGG","CATCTGTTTGAGATTTTCTCAGG",2,14,17,NA,"GeneID:414194","CCNYL2",TRUE,"AGAP4"

"4505","CATCTGTTTGAGAGTTCCTC","chr10",49989356,"-","1:A>T","TGG","CTTCTGTTTGAGAGTTCCTCTGG",1,2,NA,NA,"GeneID:414189","AGAP6",TRUE,"AGAP4"

"4506","CATCTGTTTGAGAGTTCCTC","chr4",116173792,"+","0:T>C,3:T>C","TGG","TATTTGTTTGAGAGTTCCTCTGG",2,1,4,NA,"","",FALSE,"AGAP4"

"4507","TAACCCTTCTGCCAATCCAG","chr10",45681781,"+","none","AGG","TAACCCTTCTGCCAATCCAGAGG",0,NA,NA,NA,"","",TRUE,"AGAP4"

"4508","TAACCCTTCTGCCAATCCAG","chr10",46112589,"+","none","AGG","TAACCCTTCTGCCAATCCAGAGG",0,NA,NA,NA,"","",TRUE,"AGAP4"

"4509","TAACCCTTCTGCCAATCCAG","chr10",46819477,"+","none","AGG","TAACCCTTCTGCCAATCCAGAGG",0,NA,NA,NA,"","",TRUE,"AGAP4"

"4510","TAACCCTTCTGCCAATCCAG","chr10",46340197,"+","none","AGG","TAACCCTTCTGCCAATCCAGAGG",0,NA,NA,NA,"","",TRUE,"AGAP4"

"4511","TAACCCTTCTGCCAATCCAG","chr10",49991685,"+","none","AGG","TAACCCTTCTGCCAATCCAGAGG",0,NA,NA,NA,"GeneID:414189","AGAP6",TRUE,"AGAP4"

"4512","TAACCCTTCTGCCAATCCAG","chr10",73694774,"-","none","AGG","TAACCCTTCTGCCAATCCAGAGG",0,NA,NA,NA,"GeneID:729092","AGAP5",TRUE,"AGAP4"

"4513","TAACCCTTCTGCCAATCCAG","chr10",45844364,"-","none","AGG","TAACCCTTCTGCCAATCCAGAGG",0,NA,NA,NA,"GeneID:119016","AGAP4",TRUE,"AGAP4"

"4514","TAACCCTTCTGCCAATCCAG","chr10",48028610,"-","none","AGG","TAACCCTTCTGCCAATCCAGAGG",0,NA,NA,NA,"GeneID:414224","CTGLF12P",TRUE,"AGAP4"

"4515","TAACCCTTCTGCCAATCCAG","chr10",47520537,"-","4:A>G","AGG","TAACTCTTCTGCCAATCCAGAGG",1,5,NA,NA,"GeneID:642517","AGAP9",TRUE,"AGAP4"

"4516","TAACCCTTCTGCCAATCCAG","chr10",47522904,"-","5:A>G","AGG","TAACCTTTCTGCCAATCCAGAGG",1,6,NA,NA,"GeneID:642517","AGAP9",TRUE,"AGAP4"

"4517","TAACCCTTCTGCCAATCCAG","chr10",45846725,"-","5:A>G","AGG","TAACCTTTCTGCCAATCCAGAGG",1,6,NA,NA,"GeneID:119016","AGAP4",TRUE,"AGAP4"

"4518","TAACCCTTCTGCCAATCCAG","chr10",48030976,"-","5:A>G","AGG","TAACCTTTCTGCCAATCCAGAGG",1,6,NA,NA,"GeneID:414224","CTGLF12P",TRUE,"AGAP4"

"4519","TAACCCTTCTGCCAATCCAG","chr10",73697133,"-","2:C>T,5:A>G","AGG","TAGCCTTTCTGCCAATCCAGAGG",2,3,6,NA,"GeneID:729092","AGAP5",TRUE,"AGAP4"

"4520","TAACCCTTCTGCCAATCCAG","chr10",46337825,"+","5:T>C","AGG","TAACCTTTCTGCCAATCCAGAGG",1,6,NA,NA,"","",TRUE,"AGAP4"

"4521","TAACCCTTCTGCCAATCCAG","chr10",46817116,"+","5:T>C","AGG","TAACCTTTCTGCCAATCCAGAGG",1,6,NA,NA,"","",FALSE,"AGAP4"

"4522","TAACCCTTCTGCCAATCCAG","chr10",45679419,"+","5:T>C","AGG","TAACCTTTCTGCCAATCCAGAGG",1,6,NA,NA,"","",FALSE,"AGAP4"

"4523","TAACCCTTCTGCCAATCCAG","chr10",46110222,"+","5:T>C","AGG","TAACCTTTCTGCCAATCCAGAGG",1,6,NA,NA,"","",TRUE,"AGAP4"

"4524","TAACCCTTCTGCCAATCCAG","chr11",10657769,"+","2:G>A,12:A>C","AGG","TAGCCCTTCTGCAAATCCAGAGG",2,3,13,NA,"GeneID:10335","MRVI1",FALSE,"AGAP4"

"4525","TACCTCATCATATCACACAA","chr10",46832454,"+","none","AGG","TACCTCATCATATCACACAAAGG",0,NA,NA,NA,"","",TRUE,"AGAP4"

"4526","TACCTCATCATATCACACAA","chr10",46125877,"+","none","AGG","TACCTCATCATATCACACAAAGG",0,NA,NA,NA,"","",TRUE,"AGAP4"

"4527","TACCTCATCATATCACACAA","chr10",46353219,"+","none","AGG","TACCTCATCATATCACACAAAGG",0,NA,NA,NA,"","",TRUE,"AGAP4"

"4528","TACCTCATCATATCACACAA","chr10",50004698,"+","none","AGG","TACCTCATCATATCACACAAAGG",0,NA,NA,NA,"GeneID:414189","AGAP6",TRUE,"AGAP4"

"4529","TACCTCATCATATCACACAA","chr10",45694761,"+","none","AGG","TACCTCATCATATCACACAAAGG",0,NA,NA,NA,"","",TRUE,"AGAP4"

"4530","TACCTCATCATATCACACAA","chr10",45831395,"-","none","AGG","TACCTCATCATATCACACAAAGG",0,NA,NA,NA,"GeneID:119016","AGAP4",TRUE,"AGAP4"

"4531","TACCTCATCATATCACACAA","chr10",47507549,"-","none","AGG","TACCTCATCATATCACACAAAGG",0,NA,NA,NA,"GeneID:642517","AGAP9",TRUE,"AGAP4"

"4532","TACCTCATCATATCACACAA","chr10",73680072,"-","none","AGG","TACCTCATCATATCACACAAAGG",0,NA,NA,NA,"GeneID:729092","AGAP5",TRUE,"AGAP4"

"4533","TACCTCATCATATCACACAA","chr10",48015648,"-","none","AGG","TACCTCATCATATCACACAAAGG",0,NA,NA,NA,"GeneID:414224","CTGLF12P",TRUE,"AGAP4"

"4534","TACCTCATCATATCACACAA","chr2",133138229,"+","0:A>T,14:C>A","AGG","AACCTCATCATATCCCACAAAGG",2,1,15,NA,"GeneID:344148","NCKAP5",FALSE,"AGAP4"

"4535","TACCTCATCATATCACACAA","chr1",24151623,"+","0:A>T,14:C>A","CGG","AACCTCATCATATCCCACAACGG",2,1,15,NA,"","",FALSE,"AGAP4"

"4536","TTGTCATTGTAAGATAATGC","chr10",45834022,"+","none","TGG","TTGTCATTGTAAGATAATGCTGG",0,NA,NA,NA,"GeneID:119016","AGAP4",TRUE,"AGAP4"

"4537","TTGTCATTGTAAGATAATGC","chr10",47510177,"+","none","TGG","TTGTCATTGTAAGATAATGCTGG",0,NA,NA,NA,"GeneID:642517","AGAP9",TRUE,"AGAP4"

"4538","TTGTCATTGTAAGATAATGC","chr10",48018274,"+","none","TGG","TTGTCATTGTAAGATAATGCTGG",0,NA,NA,NA,"GeneID:414224","CTGLF12P",TRUE,"AGAP4"

"4539","TTGTCATTGTAAGATAATGC","chr10",73682700,"+","none","TGG","TTGTCATTGTAAGATAATGCTGG",0,NA,NA,NA,"GeneID:729092","AGAP5",TRUE,"AGAP4"

"4540","TTGTCATTGTAAGATAATGC","chr10",50002069,"-","none","TGG","TTGTCATTGTAAGATAATGCTGG",0,NA,NA,NA,"GeneID:414189","AGAP6",TRUE,"AGAP4"

"4541","TTGTCATTGTAAGATAATGC","chr10",46829827,"-","none","TGG","TTGTCATTGTAAGATAATGCTGG",0,NA,NA,NA,"","",FALSE,"AGAP4"

"4542","TTGTCATTGTAAGATAATGC","chr10",46123243,"-","none","TGG","TTGTCATTGTAAGATAATGCTGG",0,NA,NA,NA,"","",TRUE,"AGAP4"

"4543","TTGTCATTGTAAGATAATGC","chr10",46350591,"-","none","TGG","TTGTCATTGTAAGATAATGCTGG",0,NA,NA,NA,"","",TRUE,"AGAP4"

"4544","TTGTCATTGTAAGATAATGC","chr10",87001709,"-","8:T>C","TGG","TTGTCATTATAAGATAATGCTGG",1,9,NA,NA,"","",TRUE,"AGAP4"

"4545","CAGAGGAACTCTCAAACAGA","chr10",46110260,"+","none","TGG","CAGAGGAACTCTCAAACAGATGG",0,NA,NA,NA,"","",TRUE,"AGAP5"

"4546","CAGAGGAACTCTCAAACAGA","chr10",46817153,"+","none","TGG","CAGAGGAACTCTCAAACAGATGG",0,NA,NA,NA,"","",FALSE,"AGAP5"

"4547","CAGAGGAACTCTCAAACAGA","chr10",45679456,"+","none","TGG","CAGAGGAACTCTCAAACAGATGG",0,NA,NA,NA,"","",FALSE,"AGAP5"

"4548","CAGAGGAACTCTCAAACAGA","chr10",46340234,"+","none","TGG","CAGAGGAACTCTCAAACAGATGG",0,NA,NA,NA,"","",TRUE,"AGAP5"

"4549","CAGAGGAACTCTCAAACAGA","chr10",49991722,"+","none","TGG","CAGAGGAACTCTCAAACAGATGG",0,NA,NA,NA,"GeneID:414189","AGAP6",TRUE,"AGAP5"

"4550","CAGAGGAACTCTCAAACAGA","chr10",45681818,"+","none","TGG","CAGAGGAACTCTCAAACAGATGG",0,NA,NA,NA,"","",TRUE,"AGAP5"

"4551","CAGAGGAACTCTCAAACAGA","chr10",46112626,"+","none","TGG","CAGAGGAACTCTCAAACAGATGG",0,NA,NA,NA,"","",TRUE,"AGAP5"

"4552","CAGAGGAACTCTCAAACAGA","chr10",46819514,"+","none","TGG","CAGAGGAACTCTCAAACAGATGG",0,NA,NA,NA,"","",TRUE,"AGAP5"

"4553","CAGAGGAACTCTCAAACAGA","chr10",49989354,"+","none","AGG","CAGAGGAACTCTCAAACAGAAGG",0,NA,NA,NA,"GeneID:414189","AGAP6",TRUE,"AGAP5"

"4554","CAGAGGAACTCTCAAACAGA","chr10",46337862,"+","none","TGG","CAGAGGAACTCTCAAACAGATGG",0,NA,NA,NA,"","",TRUE,"AGAP5"

"4555","CAGAGGAACTCTCAAACAGA","chr10",45844327,"-","none","TGG","CAGAGGAACTCTCAAACAGATGG",0,NA,NA,NA,"GeneID:119016","AGAP4",TRUE,"AGAP5"

"4556","CAGAGGAACTCTCAAACAGA","chr10",48028573,"-","none","TGG","CAGAGGAACTCTCAAACAGATGG",0,NA,NA,NA,"GeneID:414224","CTGLF12P",TRUE,"AGAP5"

"4557","CAGAGGAACTCTCAAACAGA","chr10",73697096,"-","none","TGG","CAGAGGAACTCTCAAACAGATGG",0,NA,NA,NA,"GeneID:729092","AGAP5",TRUE,"AGAP5"

"4558","CAGAGGAACTCTCAAACAGA","chr10",45846688,"-","none","TGG","CAGAGGAACTCTCAAACAGATGG",0,NA,NA,NA,"GeneID:119016","AGAP4",TRUE,"AGAP5"

"4559","CAGAGGAACTCTCAAACAGA","chr10",48030939,"-","none","TGG","CAGAGGAACTCTCAAACAGATGG",0,NA,NA,NA,"GeneID:414224","CTGLF12P",TRUE,"AGAP5"

"4560","CAGAGGAACTCTCAAACAGA","chr10",47522867,"-","none","TGG","CAGAGGAACTCTCAAACAGATGG",0,NA,NA,NA,"GeneID:642517","AGAP9",TRUE,"AGAP5"

"4561","CAGAGGAACTCTCAAACAGA","chr10",47520500,"-","none","TGG","CAGAGGAACTCTCAAACAGATGG",0,NA,NA,NA,"GeneID:642517","AGAP9",TRUE,"AGAP5"

"4562","CAGAGGAACTCTCAAACAGA","chr10",73694737,"-","none","TGG","CAGAGGAACTCTCAAACAGATGG",0,NA,NA,NA,"GeneID:729092","AGAP5",TRUE,"AGAP5"

"4563","CAGAGGAACTCTCAAACAGA","chr5",72590696,"-","4:A>C,7:A>T","TGG","CAGATGATCTCTCAAACAGATGG",2,5,8,NA,"GeneID:102477328","LOC102477328",FALSE,"AGAP5"

"4564","CAGAGGAACTCTCAAACAGA","chr3",13256056,"+","1:T>A,5:A>G","TGG","CTGAGAAACTCTCAAACAGATGG",2,2,6,NA,"","",FALSE,"AGAP5"

"4565","CATCTGTTTGAGAGTTCCTC","chr10",73694735,"+","none","TGG","CATCTGTTTGAGAGTTCCTCTGG",0,NA,NA,NA,"GeneID:729092","AGAP5",TRUE,"AGAP5"

"4566","CATCTGTTTGAGAGTTCCTC","chr10",45844325,"+","none","TGG","CATCTGTTTGAGAGTTCCTCTGG",0,NA,NA,NA,"GeneID:119016","AGAP4",TRUE,"AGAP5"

"4567","CATCTGTTTGAGAGTTCCTC","chr10",48028571,"+","none","TGG","CATCTGTTTGAGAGTTCCTCTGG",0,NA,NA,NA,"GeneID:414224","CTGLF12P",TRUE,"AGAP5"

"4568","CATCTGTTTGAGAGTTCCTC","chr10",73697094,"+","none","TGG","CATCTGTTTGAGAGTTCCTCTGG",0,NA,NA,NA,"GeneID:729092","AGAP5",TRUE,"AGAP5"

"4569","CATCTGTTTGAGAGTTCCTC","chr10",45846686,"+","none","TGG","CATCTGTTTGAGAGTTCCTCTGG",0,NA,NA,NA,"GeneID:119016","AGAP4",TRUE,"AGAP5"

"4570","CATCTGTTTGAGAGTTCCTC","chr10",48030937,"+","none","TGG","CATCTGTTTGAGAGTTCCTCTGG",0,NA,NA,NA,"GeneID:414224","CTGLF12P",TRUE,"AGAP5"

"4571","CATCTGTTTGAGAGTTCCTC","chr10",47522865,"+","none","TGG","CATCTGTTTGAGAGTTCCTCTGG",0,NA,NA,NA,"GeneID:642517","AGAP9",TRUE,"AGAP5"

"4572","CATCTGTTTGAGAGTTCCTC","chr10",47520498,"+","none","TGG","CATCTGTTTGAGAGTTCCTCTGG",0,NA,NA,NA,"GeneID:642517","AGAP9",TRUE,"AGAP5"

"4573","CATCTGTTTGAGAGTTCCTC","chr10",46340236,"-","none","TGG","CATCTGTTTGAGAGTTCCTCTGG",0,NA,NA,NA,"","",TRUE,"AGAP5"

"4574","CATCTGTTTGAGAGTTCCTC","chr10",49991724,"-","none","TGG","CATCTGTTTGAGAGTTCCTCTGG",0,NA,NA,NA,"GeneID:414189","AGAP6",TRUE,"AGAP5"

"4575","CATCTGTTTGAGAGTTCCTC","chr10",45681820,"-","none","TGG","CATCTGTTTGAGAGTTCCTCTGG",0,NA,NA,NA,"","",TRUE,"AGAP5"

"4576","CATCTGTTTGAGAGTTCCTC","chr10",46112628,"-","none","TGG","CATCTGTTTGAGAGTTCCTCTGG",0,NA,NA,NA,"","",TRUE,"AGAP5"

"4577","CATCTGTTTGAGAGTTCCTC","chr10",46819516,"-","none","TGG","CATCTGTTTGAGAGTTCCTCTGG",0,NA,NA,NA,"","",TRUE,"AGAP5"

"4578","CATCTGTTTGAGAGTTCCTC","chr10",46817155,"-","none","TGG","CATCTGTTTGAGAGTTCCTCTGG",0,NA,NA,NA,"","",FALSE,"AGAP5"

"4579","CATCTGTTTGAGAGTTCCTC","chr10",45679458,"-","none","TGG","CATCTGTTTGAGAGTTCCTCTGG",0,NA,NA,NA,"","",FALSE,"AGAP5"

"4580","CATCTGTTTGAGAGTTCCTC","chr10",38982306,"-","14:G>A,17:A>G","AGG","CATCTGTTTGAGAGCTCTTCAGG",2,15,18,NA,"","",FALSE,"AGAP5"

"4581","CATCTGTTTGAGAGTTCCTC","chr10",42451608,"+","13:T>G,16:T>C","AGG","CATCTGTTTGAGATTTTCTCAGG",2,14,17,NA,"GeneID:414194","CCNYL2",TRUE,"AGAP5"

"4582","CATCTGTTTGAGAGTTCCTC","chr10",49989356,"-","1:A>T","TGG","CTTCTGTTTGAGAGTTCCTCTGG",1,2,NA,NA,"GeneID:414189","AGAP6",TRUE,"AGAP5"

"4583","CATCTGTTTGAGAGTTCCTC","chr4",116173792,"+","0:T>C,3:T>C","TGG","TATTTGTTTGAGAGTTCCTCTGG",2,1,4,NA,"","",FALSE,"AGAP5"

"4584","TAACCCTTCTGCCAATCCAG","chr10",45681781,"+","none","AGG","TAACCCTTCTGCCAATCCAGAGG",0,NA,NA,NA,"","",TRUE,"AGAP5"

"4585","TAACCCTTCTGCCAATCCAG","chr10",46112589,"+","none","AGG","TAACCCTTCTGCCAATCCAGAGG",0,NA,NA,NA,"","",TRUE,"AGAP5"

"4586","TAACCCTTCTGCCAATCCAG","chr10",46819477,"+","none","AGG","TAACCCTTCTGCCAATCCAGAGG",0,NA,NA,NA,"","",TRUE,"AGAP5"

"4587","TAACCCTTCTGCCAATCCAG","chr10",46340197,"+","none","AGG","TAACCCTTCTGCCAATCCAGAGG",0,NA,NA,NA,"","",TRUE,"AGAP5"

"4588","TAACCCTTCTGCCAATCCAG","chr10",49991685,"+","none","AGG","TAACCCTTCTGCCAATCCAGAGG",0,NA,NA,NA,"GeneID:414189","AGAP6",TRUE,"AGAP5"

"4589","TAACCCTTCTGCCAATCCAG","chr10",73694774,"-","none","AGG","TAACCCTTCTGCCAATCCAGAGG",0,NA,NA,NA,"GeneID:729092","AGAP5",TRUE,"AGAP5"

"4590","TAACCCTTCTGCCAATCCAG","chr10",45844364,"-","none","AGG","TAACCCTTCTGCCAATCCAGAGG",0,NA,NA,NA,"GeneID:119016","AGAP4",TRUE,"AGAP5"

"4591","TAACCCTTCTGCCAATCCAG","chr10",48028610,"-","none","AGG","TAACCCTTCTGCCAATCCAGAGG",0,NA,NA,NA,"GeneID:414224","CTGLF12P",TRUE,"AGAP5"

"4592","TAACCCTTCTGCCAATCCAG","chr10",47520537,"-","4:A>G","AGG","TAACTCTTCTGCCAATCCAGAGG",1,5,NA,NA,"GeneID:642517","AGAP9",TRUE,"AGAP5"

"4593","TAACCCTTCTGCCAATCCAG","chr10",47522904,"-","5:A>G","AGG","TAACCTTTCTGCCAATCCAGAGG",1,6,NA,NA,"GeneID:642517","AGAP9",TRUE,"AGAP5"

"4594","TAACCCTTCTGCCAATCCAG","chr10",45846725,"-","5:A>G","AGG","TAACCTTTCTGCCAATCCAGAGG",1,6,NA,NA,"GeneID:119016","AGAP4",TRUE,"AGAP5"

"4595","TAACCCTTCTGCCAATCCAG","chr10",48030976,"-","5:A>G","AGG","TAACCTTTCTGCCAATCCAGAGG",1,6,NA,NA,"GeneID:414224","CTGLF12P",TRUE,"AGAP5"

"4596","TAACCCTTCTGCCAATCCAG","chr10",73697133,"-","2:C>T,5:A>G","AGG","TAGCCTTTCTGCCAATCCAGAGG",2,3,6,NA,"GeneID:729092","AGAP5",TRUE,"AGAP5"

"4597","TAACCCTTCTGCCAATCCAG","chr10",46337825,"+","5:T>C","AGG","TAACCTTTCTGCCAATCCAGAGG",1,6,NA,NA,"","",TRUE,"AGAP5"

"4598","TAACCCTTCTGCCAATCCAG","chr10",46817116,"+","5:T>C","AGG","TAACCTTTCTGCCAATCCAGAGG",1,6,NA,NA,"","",FALSE,"AGAP5"

"4599","TAACCCTTCTGCCAATCCAG","chr10",45679419,"+","5:T>C","AGG","TAACCTTTCTGCCAATCCAGAGG",1,6,NA,NA,"","",FALSE,"AGAP5"

"4600","TAACCCTTCTGCCAATCCAG","chr10",46110222,"+","5:T>C","AGG","TAACCTTTCTGCCAATCCAGAGG",1,6,NA,NA,"","",TRUE,"AGAP5"

"4601","TAACCCTTCTGCCAATCCAG","chr11",10657769,"+","2:G>A,12:A>C","AGG","TAGCCCTTCTGCAAATCCAGAGG",2,3,13,NA,"GeneID:10335","MRVI1",FALSE,"AGAP5"

"4602","TAGCCTTTCTGCCAATCCAG","chr10",73697133,"-","none","AGG","TAGCCTTTCTGCCAATCCAGAGG",0,NA,NA,NA,"GeneID:729092","AGAP5",TRUE,"AGAP5"

"4603","TAGCCTTTCTGCCAATCCAG","chr10",48030976,"-","2:T>C","AGG","TAACCTTTCTGCCAATCCAGAGG",1,3,NA,NA,"GeneID:414224","CTGLF12P",TRUE,"AGAP5"

"4604","TAGCCTTTCTGCCAATCCAG","chr10",47522904,"-","2:T>C","AGG","TAACCTTTCTGCCAATCCAGAGG",1,3,NA,NA,"GeneID:642517","AGAP9",TRUE,"AGAP5"

"4605","TAGCCTTTCTGCCAATCCAG","chr10",45846725,"-","2:T>C","AGG","TAACCTTTCTGCCAATCCAGAGG",1,3,NA,NA,"GeneID:119016","AGAP4",TRUE,"AGAP5"

"4606","TAGCCTTTCTGCCAATCCAG","chr10",73694774,"-","2:T>C,5:G>A","AGG","TAACCCTTCTGCCAATCCAGAGG",2,3,6,NA,"GeneID:729092","AGAP5",TRUE,"AGAP5"

"4607","TAGCCTTTCTGCCAATCCAG","chr10",45844364,"-","2:T>C,5:G>A","AGG","TAACCCTTCTGCCAATCCAGAGG",2,3,6,NA,"GeneID:119016","AGAP4",TRUE,"AGAP5"

"4608","TAGCCTTTCTGCCAATCCAG","chr10",48028610,"-","2:T>C,5:G>A","AGG","TAACCCTTCTGCCAATCCAGAGG",2,3,6,NA,"GeneID:414224","CTGLF12P",TRUE,"AGAP5"

"4609","TAGCCTTTCTGCCAATCCAG","chr10",46337825,"+","2:A>G","AGG","TAACCTTTCTGCCAATCCAGAGG",1,3,NA,NA,"","",TRUE,"AGAP5"

"4610","TAGCCTTTCTGCCAATCCAG","chr10",46817116,"+","2:A>G","AGG","TAACCTTTCTGCCAATCCAGAGG",1,3,NA,NA,"","",FALSE,"AGAP5"

"4611","TAGCCTTTCTGCCAATCCAG","chr10",45679419,"+","2:A>G","AGG","TAACCTTTCTGCCAATCCAGAGG",1,3,NA,NA,"","",FALSE,"AGAP5"

"4612","TAGCCTTTCTGCCAATCCAG","chr10",46110222,"+","2:A>G","AGG","TAACCTTTCTGCCAATCCAGAGG",1,3,NA,NA,"","",TRUE,"AGAP5"

"4613","TAGCCTTTCTGCCAATCCAG","chr10",46340197,"+","2:A>G,5:C>T","AGG","TAACCCTTCTGCCAATCCAGAGG",2,3,6,NA,"","",TRUE,"AGAP5"

"4614","TAGCCTTTCTGCCAATCCAG","chr10",49991685,"+","2:A>G,5:C>T","AGG","TAACCCTTCTGCCAATCCAGAGG",2,3,6,NA,"GeneID:414189","AGAP6",TRUE,"AGAP5"

"4615","TAGCCTTTCTGCCAATCCAG","chr10",45681781,"+","2:A>G,5:C>T","AGG","TAACCCTTCTGCCAATCCAGAGG",2,3,6,NA,"","",TRUE,"AGAP5"

"4616","TAGCCTTTCTGCCAATCCAG","chr10",46112589,"+","2:A>G,5:C>T","AGG","TAACCCTTCTGCCAATCCAGAGG",2,3,6,NA,"","",TRUE,"AGAP5"

"4617","TAGCCTTTCTGCCAATCCAG","chr10",46819477,"+","2:A>G,5:C>T","AGG","TAACCCTTCTGCCAATCCAGAGG",2,3,6,NA,"","",TRUE,"AGAP5"

"4618","TAGCCTTTCTGCCAATCCAG","chr11",10657769,"+","5:C>T,12:A>C","AGG","TAGCCCTTCTGCAAATCCAGAGG",2,6,13,NA,"GeneID:10335","MRVI1",FALSE,"AGAP5"

"4619","TATTGTGCTTGCCTCTGGAT","chr10",47522890,"+","none","TGG","TATTGTGCTTGCCTCTGGATTGG",0,NA,NA,NA,"GeneID:642517","AGAP9",TRUE,"AGAP5"

"4620","TATTGTGCTTGCCTCTGGAT","chr10",47520523,"+","none","TGG","TATTGTGCTTGCCTCTGGATTGG",0,NA,NA,NA,"GeneID:642517","AGAP9",TRUE,"AGAP5"

"4621","TATTGTGCTTGCCTCTGGAT","chr10",73694760,"+","none","TGG","TATTGTGCTTGCCTCTGGATTGG",0,NA,NA,NA,"GeneID:729092","AGAP5",TRUE,"AGAP5"

"4622","TATTGTGCTTGCCTCTGGAT","chr10",45844350,"+","none","TGG","TATTGTGCTTGCCTCTGGATTGG",0,NA,NA,NA,"GeneID:119016","AGAP4",TRUE,"AGAP5"

"4623","TATTGTGCTTGCCTCTGGAT","chr10",48028596,"+","none","TGG","TATTGTGCTTGCCTCTGGATTGG",0,NA,NA,NA,"GeneID:414224","CTGLF12P",TRUE,"AGAP5"

"4624","TATTGTGCTTGCCTCTGGAT","chr10",73697119,"+","none","TGG","TATTGTGCTTGCCTCTGGATTGG",0,NA,NA,NA,"GeneID:729092","AGAP5",TRUE,"AGAP5"

"4625","TATTGTGCTTGCCTCTGGAT","chr10",45846711,"+","none","TGG","TATTGTGCTTGCCTCTGGATTGG",0,NA,NA,NA,"GeneID:119016","AGAP4",TRUE,"AGAP5"

"4626","TATTGTGCTTGCCTCTGGAT","chr10",48030962,"+","none","TGG","TATTGTGCTTGCCTCTGGATTGG",0,NA,NA,NA,"GeneID:414224","CTGLF12P",TRUE,"AGAP5"

"4627","TATTGTGCTTGCCTCTGGAT","chr10",45679433,"-","none","TGG","TATTGTGCTTGCCTCTGGATTGG",0,NA,NA,NA,"","",FALSE,"AGAP5"

"4628","TATTGTGCTTGCCTCTGGAT","chr10",46340211,"-","none","TGG","TATTGTGCTTGCCTCTGGATTGG",0,NA,NA,NA,"","",TRUE,"AGAP5"

"4629","TATTGTGCTTGCCTCTGGAT","chr10",49991699,"-","none","TGG","TATTGTGCTTGCCTCTGGATTGG",0,NA,NA,NA,"GeneID:414189","AGAP6",TRUE,"AGAP5"

"4630","TATTGTGCTTGCCTCTGGAT","chr10",45681795,"-","none","TGG","TATTGTGCTTGCCTCTGGATTGG",0,NA,NA,NA,"","",TRUE,"AGAP5"

"4631","TATTGTGCTTGCCTCTGGAT","chr10",46112603,"-","none","TGG","TATTGTGCTTGCCTCTGGATTGG",0,NA,NA,NA,"","",TRUE,"AGAP5"

"4632","TATTGTGCTTGCCTCTGGAT","chr10",46819491,"-","none","TGG","TATTGTGCTTGCCTCTGGATTGG",0,NA,NA,NA,"","",TRUE,"AGAP5"

"4633","TATTGTGCTTGCCTCTGGAT","chr10",46337839,"-","none","TGG","TATTGTGCTTGCCTCTGGATTGG",0,NA,NA,NA,"","",TRUE,"AGAP5"

"4634","TATTGTGCTTGCCTCTGGAT","chr10",46817130,"-","none","TGG","TATTGTGCTTGCCTCTGGATTGG",0,NA,NA,NA,"","",FALSE,"AGAP5"

"4635","TATTGTGCTTGCCTCTGGAT","chr10",49989331,"-","11:T>G","TGG","TATTGTGCTTGACTCTGGATTGG",1,12,NA,NA,"GeneID:414189","AGAP6",TRUE,"AGAP5"

"4636","TATTGTGCTTGCCTCTGGAT","chr5",156631600,"+","0:A>T,2:A>T","TGG","AAATGTGCTTGCCTCTGGATTGG",2,1,3,NA,"GeneID:6444","SGCD",FALSE,"AGAP5"

"4637","TATTGTGCTTGCCTCTGGAT","chr10",38982281,"-","3:T>A,18:A>T","GGG","TATAGTGCTTGCCTCTGGTTGGG",2,4,19,NA,"","",FALSE,"AGAP5"

"4638","TTGTCATTGTAAGATAATGC","chr10",45834022,"+","none","TGG","TTGTCATTGTAAGATAATGCTGG",0,NA,NA,NA,"GeneID:119016","AGAP4",TRUE,"AGAP5"

"4639","TTGTCATTGTAAGATAATGC","chr10",47510177,"+","none","TGG","TTGTCATTGTAAGATAATGCTGG",0,NA,NA,NA,"GeneID:642517","AGAP9",TRUE,"AGAP5"

"4640","TTGTCATTGTAAGATAATGC","chr10",48018274,"+","none","TGG","TTGTCATTGTAAGATAATGCTGG",0,NA,NA,NA,"GeneID:414224","CTGLF12P",TRUE,"AGAP5"

"4641","TTGTCATTGTAAGATAATGC","chr10",73682700,"+","none","TGG","TTGTCATTGTAAGATAATGCTGG",0,NA,NA,NA,"GeneID:729092","AGAP5",TRUE,"AGAP5"

"4642","TTGTCATTGTAAGATAATGC","chr10",50002069,"-","none","TGG","TTGTCATTGTAAGATAATGCTGG",0,NA,NA,NA,"GeneID:414189","AGAP6",TRUE,"AGAP5"

"4643","TTGTCATTGTAAGATAATGC","chr10",46829827,"-","none","TGG","TTGTCATTGTAAGATAATGCTGG",0,NA,NA,NA,"","",FALSE,"AGAP5"

"4644","TTGTCATTGTAAGATAATGC","chr10",46123243,"-","none","TGG","TTGTCATTGTAAGATAATGCTGG",0,NA,NA,NA,"","",TRUE,"AGAP5"

"4645","TTGTCATTGTAAGATAATGC","chr10",46350591,"-","none","TGG","TTGTCATTGTAAGATAATGCTGG",0,NA,NA,NA,"","",TRUE,"AGAP5"

"4646","TTGTCATTGTAAGATAATGC","chr10",87001709,"-","8:T>C","TGG","TTGTCATTATAAGATAATGCTGG",1,9,NA,NA,"","",TRUE,"AGAP5"

"4647","AGTCAAGCACAATATTCCAG","chr10",49989337,"+","none","AGG","AGTCAAGCACAATATTCCAGAGG",0,NA,NA,NA,"GeneID:414189","AGAP6",TRUE,"AGAP6"

"4648","AGTCAAGCACAATATTCCAG","chr10",73694754,"-","2:C>A","AGG","AGGCAAGCACAATATTCCAGAGG",1,3,NA,NA,"GeneID:729092","AGAP5",TRUE,"AGAP6"

"4649","AGTCAAGCACAATATTCCAG","chr10",73697113,"-","2:C>A","AGG","AGGCAAGCACAATATTCCAGAGG",1,3,NA,NA,"GeneID:729092","AGAP5",TRUE,"AGAP6"

"4650","AGTCAAGCACAATATTCCAG","chr10",45846705,"-","2:C>A","AGG","AGGCAAGCACAATATTCCAGAGG",1,3,NA,NA,"GeneID:119016","AGAP4",TRUE,"AGAP6"

"4651","AGTCAAGCACAATATTCCAG","chr10",48030956,"-","2:C>A","AGG","AGGCAAGCACAATATTCCAGAGG",1,3,NA,NA,"GeneID:414224","CTGLF12P",TRUE,"AGAP6"

"4652","AGTCAAGCACAATATTCCAG","chr10",47522884,"-","2:C>A","AGG","AGGCAAGCACAATATTCCAGAGG",1,3,NA,NA,"GeneID:642517","AGAP9",TRUE,"AGAP6"

"4653","AGTCAAGCACAATATTCCAG","chr10",45844344,"-","2:C>A","AGG","AGGCAAGCACAATATTCCAGAGG",1,3,NA,NA,"GeneID:119016","AGAP4",TRUE,"AGAP6"

"4654","AGTCAAGCACAATATTCCAG","chr10",47520517,"-","2:C>A","AGG","AGGCAAGCACAATATTCCAGAGG",1,3,NA,NA,"GeneID:642517","AGAP9",TRUE,"AGAP6"

"4655","AGTCAAGCACAATATTCCAG","chr10",48028590,"-","2:C>A","AGG","AGGCAAGCACAATATTCCAGAGG",1,3,NA,NA,"GeneID:414224","CTGLF12P",TRUE,"AGAP6"

"4656","AGTCAAGCACAATATTCCAG","chr10",46819497,"+","2:G>T","AGG","AGGCAAGCACAATATTCCAGAGG",1,3,NA,NA,"","",TRUE,"AGAP6"

"4657","AGTCAAGCACAATATTCCAG","chr10",46817136,"+","2:G>T","AGG","AGGCAAGCACAATATTCCAGAGG",1,3,NA,NA,"","",FALSE,"AGAP6"

"4658","AGTCAAGCACAATATTCCAG","chr10",45679439,"+","2:G>T","AGG","AGGCAAGCACAATATTCCAGAGG",1,3,NA,NA,"","",FALSE,"AGAP6"

"4659","AGTCAAGCACAATATTCCAG","chr10",46340217,"+","2:G>T","AGG","AGGCAAGCACAATATTCCAGAGG",1,3,NA,NA,"","",TRUE,"AGAP6"

"4660","AGTCAAGCACAATATTCCAG","chr10",49991705,"+","2:G>T","AGG","AGGCAAGCACAATATTCCAGAGG",1,3,NA,NA,"GeneID:414189","AGAP6",TRUE,"AGAP6"

"4661","AGTCAAGCACAATATTCCAG","chr10",45681801,"+","2:G>T","AGG","AGGCAAGCACAATATTCCAGAGG",1,3,NA,NA,"","",TRUE,"AGAP6"

"4662","AGTCAAGCACAATATTCCAG","chr10",46112609,"+","2:G>T","AGG","AGGCAAGCACAATATTCCAGAGG",1,3,NA,NA,"","",TRUE,"AGAP6"

"4663","AGTCAAGCACAATATTCCAG","chr11",119339224,"+","5:T>A,6:T>G","GGG","AGTCATTCACAATATTCCAGGGG",2,6,7,NA,"GeneID:114902;GeneID:83552","C1QTNF5;MFRP",TRUE,"AGAP6"

"4664","AGTCAAGCACAATATTCCAG","chr10",46337845,"+","2:G>T,16:A>C","AGG","AGGCAAGCACAATATTACAGAGG",2,3,17,NA,"","",TRUE,"AGAP6"

"4665","CATCTGTTTGAGAGTTCCTC","chr10",73694735,"+","none","TGG","CATCTGTTTGAGAGTTCCTCTGG",0,NA,NA,NA,"GeneID:729092","AGAP5",TRUE,"AGAP6"

"4666","CATCTGTTTGAGAGTTCCTC","chr10",45844325,"+","none","TGG","CATCTGTTTGAGAGTTCCTCTGG",0,NA,NA,NA,"GeneID:119016","AGAP4",TRUE,"AGAP6"

"4667","CATCTGTTTGAGAGTTCCTC","chr10",48028571,"+","none","TGG","CATCTGTTTGAGAGTTCCTCTGG",0,NA,NA,NA,"GeneID:414224","CTGLF12P",TRUE,"AGAP6"

"4668","CATCTGTTTGAGAGTTCCTC","chr10",73697094,"+","none","TGG","CATCTGTTTGAGAGTTCCTCTGG",0,NA,NA,NA,"GeneID:729092","AGAP5",TRUE,"AGAP6"

"4669","CATCTGTTTGAGAGTTCCTC","chr10",45846686,"+","none","TGG","CATCTGTTTGAGAGTTCCTCTGG",0,NA,NA,NA,"GeneID:119016","AGAP4",TRUE,"AGAP6"

"4670","CATCTGTTTGAGAGTTCCTC","chr10",48030937,"+","none","TGG","CATCTGTTTGAGAGTTCCTCTGG",0,NA,NA,NA,"GeneID:414224","CTGLF12P",TRUE,"AGAP6"

"4671","CATCTGTTTGAGAGTTCCTC","chr10",47522865,"+","none","TGG","CATCTGTTTGAGAGTTCCTCTGG",0,NA,NA,NA,"GeneID:642517","AGAP9",TRUE,"AGAP6"

"4672","CATCTGTTTGAGAGTTCCTC","chr10",47520498,"+","none","TGG","CATCTGTTTGAGAGTTCCTCTGG",0,NA,NA,NA,"GeneID:642517","AGAP9",TRUE,"AGAP6"

"4673","CATCTGTTTGAGAGTTCCTC","chr10",46340236,"-","none","TGG","CATCTGTTTGAGAGTTCCTCTGG",0,NA,NA,NA,"","",TRUE,"AGAP6"

"4674","CATCTGTTTGAGAGTTCCTC","chr10",49991724,"-","none","TGG","CATCTGTTTGAGAGTTCCTCTGG",0,NA,NA,NA,"GeneID:414189","AGAP6",TRUE,"AGAP6"

"4675","CATCTGTTTGAGAGTTCCTC","chr10",45681820,"-","none","TGG","CATCTGTTTGAGAGTTCCTCTGG",0,NA,NA,NA,"","",TRUE,"AGAP6"

"4676","CATCTGTTTGAGAGTTCCTC","chr10",46112628,"-","none","TGG","CATCTGTTTGAGAGTTCCTCTGG",0,NA,NA,NA,"","",TRUE,"AGAP6"

"4677","CATCTGTTTGAGAGTTCCTC","chr10",46819516,"-","none","TGG","CATCTGTTTGAGAGTTCCTCTGG",0,NA,NA,NA,"","",TRUE,"AGAP6"

"4678","CATCTGTTTGAGAGTTCCTC","chr10",46817155,"-","none","TGG","CATCTGTTTGAGAGTTCCTCTGG",0,NA,NA,NA,"","",FALSE,"AGAP6"

"4679","CATCTGTTTGAGAGTTCCTC","chr10",45679458,"-","none","TGG","CATCTGTTTGAGAGTTCCTCTGG",0,NA,NA,NA,"","",FALSE,"AGAP6"

"4680","CATCTGTTTGAGAGTTCCTC","chr10",38982306,"-","14:G>A,17:A>G","AGG","CATCTGTTTGAGAGCTCTTCAGG",2,15,18,NA,"","",FALSE,"AGAP6"

"4681","CATCTGTTTGAGAGTTCCTC","chr10",42451608,"+","13:T>G,16:T>C","AGG","CATCTGTTTGAGATTTTCTCAGG",2,14,17,NA,"GeneID:414194","CCNYL2",TRUE,"AGAP6"

"4682","CATCTGTTTGAGAGTTCCTC","chr10",49989356,"-","1:A>T","TGG","CTTCTGTTTGAGAGTTCCTCTGG",1,2,NA,NA,"GeneID:414189","AGAP6",TRUE,"AGAP6"

"4683","CATCTGTTTGAGAGTTCCTC","chr4",116173792,"+","0:T>C,3:T>C","TGG","TATTTGTTTGAGAGTTCCTCTGG",2,1,4,NA,"","",FALSE,"AGAP6"

"4684","CTTCTGTTTGAGAGTTCCTC","chr10",49989356,"-","none","TGG","CTTCTGTTTGAGAGTTCCTCTGG",0,NA,NA,NA,"GeneID:414189","AGAP6",TRUE,"AGAP6"

"4685","CTTCTGTTTGAGAGTTCCTC","chr1",60275103,"+","18:A>T,19:G>C","TGG","CTTCTGTTTGAGAGTTCCAGTGG",2,19,20,NA,"","",FALSE,"AGAP6"

"4686","CTTCTGTTTGAGAGTTCCTC","chr10",46817155,"-","1:T>A","TGG","CATCTGTTTGAGAGTTCCTCTGG",1,2,NA,NA,"","",FALSE,"AGAP6"

"4687","CTTCTGTTTGAGAGTTCCTC","chr10",46340236,"-","1:T>A","TGG","CATCTGTTTGAGAGTTCCTCTGG",1,2,NA,NA,"","",TRUE,"AGAP6"

"4688","CTTCTGTTTGAGAGTTCCTC","chr10",45681820,"-","1:T>A","TGG","CATCTGTTTGAGAGTTCCTCTGG",1,2,NA,NA,"","",TRUE,"AGAP6"

"4689","CTTCTGTTTGAGAGTTCCTC","chr10",46112628,"-","1:T>A","TGG","CATCTGTTTGAGAGTTCCTCTGG",1,2,NA,NA,"","",TRUE,"AGAP6"

"4690","CTTCTGTTTGAGAGTTCCTC","chr10",46819516,"-","1:T>A","TGG","CATCTGTTTGAGAGTTCCTCTGG",1,2,NA,NA,"","",TRUE,"AGAP6"

"4691","CTTCTGTTTGAGAGTTCCTC","chr10",49991724,"-","1:T>A","TGG","CATCTGTTTGAGAGTTCCTCTGG",1,2,NA,NA,"GeneID:414189","AGAP6",TRUE,"AGAP6"

"4692","CTTCTGTTTGAGAGTTCCTC","chr10",45679458,"-","1:T>A","TGG","CATCTGTTTGAGAGTTCCTCTGG",1,2,NA,NA,"","",FALSE,"AGAP6"

"4693","CTTCTGTTTGAGAGTTCCTC","chr10",48028571,"+","1:A>T","TGG","CATCTGTTTGAGAGTTCCTCTGG",1,2,NA,NA,"GeneID:414224","CTGLF12P",TRUE,"AGAP6"

"4694","CTTCTGTTTGAGAGTTCCTC","chr10",73697094,"+","1:A>T","TGG","CATCTGTTTGAGAGTTCCTCTGG",1,2,NA,NA,"GeneID:729092","AGAP5",TRUE,"AGAP6"

"4695","CTTCTGTTTGAGAGTTCCTC","chr10",45846686,"+","1:A>T","TGG","CATCTGTTTGAGAGTTCCTCTGG",1,2,NA,NA,"GeneID:119016","AGAP4",TRUE,"AGAP6"

"4696","CTTCTGTTTGAGAGTTCCTC","chr10",48030937,"+","1:A>T","TGG","CATCTGTTTGAGAGTTCCTCTGG",1,2,NA,NA,"GeneID:414224","CTGLF12P",TRUE,"AGAP6"

"4697","CTTCTGTTTGAGAGTTCCTC","chr10",47522865,"+","1:A>T","TGG","CATCTGTTTGAGAGTTCCTCTGG",1,2,NA,NA,"GeneID:642517","AGAP9",TRUE,"AGAP6"

"4698","CTTCTGTTTGAGAGTTCCTC","chr10",47520498,"+","1:A>T","TGG","CATCTGTTTGAGAGTTCCTCTGG",1,2,NA,NA,"GeneID:642517","AGAP9",TRUE,"AGAP6"

"4699","CTTCTGTTTGAGAGTTCCTC","chr10",73694735,"+","1:A>T","TGG","CATCTGTTTGAGAGTTCCTCTGG",1,2,NA,NA,"GeneID:729092","AGAP5",TRUE,"AGAP6"

"4700","CTTCTGTTTGAGAGTTCCTC","chr10",45844325,"+","1:A>T","TGG","CATCTGTTTGAGAGTTCCTCTGG",1,2,NA,NA,"GeneID:119016","AGAP4",TRUE,"AGAP6"

"4701","TAACCCTTCTGCCAATCCAG","chr10",45681781,"+","none","AGG","TAACCCTTCTGCCAATCCAGAGG",0,NA,NA,NA,"","",TRUE,"AGAP6"

"4702","TAACCCTTCTGCCAATCCAG","chr10",46112589,"+","none","AGG","TAACCCTTCTGCCAATCCAGAGG",0,NA,NA,NA,"","",TRUE,"AGAP6"

"4703","TAACCCTTCTGCCAATCCAG","chr10",46819477,"+","none","AGG","TAACCCTTCTGCCAATCCAGAGG",0,NA,NA,NA,"","",TRUE,"AGAP6"

"4704","TAACCCTTCTGCCAATCCAG","chr10",46340197,"+","none","AGG","TAACCCTTCTGCCAATCCAGAGG",0,NA,NA,NA,"","",TRUE,"AGAP6"

"4705","TAACCCTTCTGCCAATCCAG","chr10",49991685,"+","none","AGG","TAACCCTTCTGCCAATCCAGAGG",0,NA,NA,NA,"GeneID:414189","AGAP6",TRUE,"AGAP6"

"4706","TAACCCTTCTGCCAATCCAG","chr10",73694774,"-","none","AGG","TAACCCTTCTGCCAATCCAGAGG",0,NA,NA,NA,"GeneID:729092","AGAP5",TRUE,"AGAP6"

"4707","TAACCCTTCTGCCAATCCAG","chr10",45844364,"-","none","AGG","TAACCCTTCTGCCAATCCAGAGG",0,NA,NA,NA,"GeneID:119016","AGAP4",TRUE,"AGAP6"

"4708","TAACCCTTCTGCCAATCCAG","chr10",48028610,"-","none","AGG","TAACCCTTCTGCCAATCCAGAGG",0,NA,NA,NA,"GeneID:414224","CTGLF12P",TRUE,"AGAP6"

"4709","TAACCCTTCTGCCAATCCAG","chr10",47520537,"-","4:A>G","AGG","TAACTCTTCTGCCAATCCAGAGG",1,5,NA,NA,"GeneID:642517","AGAP9",TRUE,"AGAP6"

"4710","TAACCCTTCTGCCAATCCAG","chr10",47522904,"-","5:A>G","AGG","TAACCTTTCTGCCAATCCAGAGG",1,6,NA,NA,"GeneID:642517","AGAP9",TRUE,"AGAP6"

"4711","TAACCCTTCTGCCAATCCAG","chr10",45846725,"-","5:A>G","AGG","TAACCTTTCTGCCAATCCAGAGG",1,6,NA,NA,"GeneID:119016","AGAP4",TRUE,"AGAP6"

"4712","TAACCCTTCTGCCAATCCAG","chr10",48030976,"-","5:A>G","AGG","TAACCTTTCTGCCAATCCAGAGG",1,6,NA,NA,"GeneID:414224","CTGLF12P",TRUE,"AGAP6"

"4713","TAACCCTTCTGCCAATCCAG","chr10",73697133,"-","2:C>T,5:A>G","AGG","TAGCCTTTCTGCCAATCCAGAGG",2,3,6,NA,"GeneID:729092","AGAP5",TRUE,"AGAP6"

"4714","TAACCCTTCTGCCAATCCAG","chr10",46337825,"+","5:T>C","AGG","TAACCTTTCTGCCAATCCAGAGG",1,6,NA,NA,"","",TRUE,"AGAP6"

"4715","TAACCCTTCTGCCAATCCAG","chr10",46817116,"+","5:T>C","AGG","TAACCTTTCTGCCAATCCAGAGG",1,6,NA,NA,"","",FALSE,"AGAP6"

"4716","TAACCCTTCTGCCAATCCAG","chr10",45679419,"+","5:T>C","AGG","TAACCTTTCTGCCAATCCAGAGG",1,6,NA,NA,"","",FALSE,"AGAP6"

"4717","TAACCCTTCTGCCAATCCAG","chr10",46110222,"+","5:T>C","AGG","TAACCTTTCTGCCAATCCAGAGG",1,6,NA,NA,"","",TRUE,"AGAP6"

"4718","TAACCCTTCTGCCAATCCAG","chr11",10657769,"+","2:G>A,12:A>C","AGG","TAGCCCTTCTGCAAATCCAGAGG",2,3,13,NA,"GeneID:10335","MRVI1",FALSE,"AGAP6"

"4719","TATTGTGCTTGACTCTGGAT","chr10",49989331,"-","none","TGG","TATTGTGCTTGACTCTGGATTGG",0,NA,NA,NA,"GeneID:414189","AGAP6",TRUE,"AGAP6"

"4720","TATTGTGCTTGACTCTGGAT","chr10",46112603,"-","11:G>T","TGG","TATTGTGCTTGCCTCTGGATTGG",1,12,NA,NA,"","",TRUE,"AGAP6"

"4721","TATTGTGCTTGACTCTGGAT","chr10",46819491,"-","11:G>T","TGG","TATTGTGCTTGCCTCTGGATTGG",1,12,NA,NA,"","",TRUE,"AGAP6"

"4722","TATTGTGCTTGACTCTGGAT","chr10",46337839,"-","11:G>T","TGG","TATTGTGCTTGCCTCTGGATTGG",1,12,NA,NA,"","",TRUE,"AGAP6"

"4723","TATTGTGCTTGACTCTGGAT","chr10",46817130,"-","11:G>T","TGG","TATTGTGCTTGCCTCTGGATTGG",1,12,NA,NA,"","",FALSE,"AGAP6"

"4724","TATTGTGCTTGACTCTGGAT","chr10",45679433,"-","11:G>T","TGG","TATTGTGCTTGCCTCTGGATTGG",1,12,NA,NA,"","",FALSE,"AGAP6"

"4725","TATTGTGCTTGACTCTGGAT","chr10",46340211,"-","11:G>T","TGG","TATTGTGCTTGCCTCTGGATTGG",1,12,NA,NA,"","",TRUE,"AGAP6"

"4726","TATTGTGCTTGACTCTGGAT","chr10",49991699,"-","11:G>T","TGG","TATTGTGCTTGCCTCTGGATTGG",1,12,NA,NA,"GeneID:414189","AGAP6",TRUE,"AGAP6"

"4727","TATTGTGCTTGACTCTGGAT","chr10",45681795,"-","11:G>T","TGG","TATTGTGCTTGCCTCTGGATTGG",1,12,NA,NA,"","",TRUE,"AGAP6"

"4728","TATTGTGCTTGACTCTGGAT","chr10",73694760,"+","11:C>A","TGG","TATTGTGCTTGCCTCTGGATTGG",1,12,NA,NA,"GeneID:729092","AGAP5",TRUE,"AGAP6"

"4729","TATTGTGCTTGACTCTGGAT","chr10",73697119,"+","11:C>A","TGG","TATTGTGCTTGCCTCTGGATTGG",1,12,NA,NA,"GeneID:729092","AGAP5",TRUE,"AGAP6"

"4730","TATTGTGCTTGACTCTGGAT","chr10",45846711,"+","11:C>A","TGG","TATTGTGCTTGCCTCTGGATTGG",1,12,NA,NA,"GeneID:119016","AGAP4",TRUE,"AGAP6"

"4731","TATTGTGCTTGACTCTGGAT","chr10",48030962,"+","11:C>A","TGG","TATTGTGCTTGCCTCTGGATTGG",1,12,NA,NA,"GeneID:414224","CTGLF12P",TRUE,"AGAP6"

"4732","TATTGTGCTTGACTCTGGAT","chr10",47522890,"+","11:C>A","TGG","TATTGTGCTTGCCTCTGGATTGG",1,12,NA,NA,"GeneID:642517","AGAP9",TRUE,"AGAP6"

"4733","TATTGTGCTTGACTCTGGAT","chr10",45844350,"+","11:C>A","TGG","TATTGTGCTTGCCTCTGGATTGG",1,12,NA,NA,"GeneID:119016","AGAP4",TRUE,"AGAP6"

"4734","TATTGTGCTTGACTCTGGAT","chr10",47520523,"+","11:C>A","TGG","TATTGTGCTTGCCTCTGGATTGG",1,12,NA,NA,"GeneID:642517","AGAP9",TRUE,"AGAP6"

"4735","TATTGTGCTTGACTCTGGAT","chr10",48028596,"+","11:C>A","TGG","TATTGTGCTTGCCTCTGGATTGG",1,12,NA,NA,"GeneID:414224","CTGLF12P",TRUE,"AGAP6"

"4736","TATTGTGCTTGCCTCTGGAT","chr10",47522890,"+","none","TGG","TATTGTGCTTGCCTCTGGATTGG",0,NA,NA,NA,"GeneID:642517","AGAP9",TRUE,"AGAP6"

"4737","TATTGTGCTTGCCTCTGGAT","chr10",47520523,"+","none","TGG","TATTGTGCTTGCCTCTGGATTGG",0,NA,NA,NA,"GeneID:642517","AGAP9",TRUE,"AGAP6"

"4738","TATTGTGCTTGCCTCTGGAT","chr10",73694760,"+","none","TGG","TATTGTGCTTGCCTCTGGATTGG",0,NA,NA,NA,"GeneID:729092","AGAP5",TRUE,"AGAP6"

"4739","TATTGTGCTTGCCTCTGGAT","chr10",45844350,"+","none","TGG","TATTGTGCTTGCCTCTGGATTGG",0,NA,NA,NA,"GeneID:119016","AGAP4",TRUE,"AGAP6"

"4740","TATTGTGCTTGCCTCTGGAT","chr10",48028596,"+","none","TGG","TATTGTGCTTGCCTCTGGATTGG",0,NA,NA,NA,"GeneID:414224","CTGLF12P",TRUE,"AGAP6"

"4741","TATTGTGCTTGCCTCTGGAT","chr10",73697119,"+","none","TGG","TATTGTGCTTGCCTCTGGATTGG",0,NA,NA,NA,"GeneID:729092","AGAP5",TRUE,"AGAP6"

"4742","TATTGTGCTTGCCTCTGGAT","chr10",45846711,"+","none","TGG","TATTGTGCTTGCCTCTGGATTGG",0,NA,NA,NA,"GeneID:119016","AGAP4",TRUE,"AGAP6"

"4743","TATTGTGCTTGCCTCTGGAT","chr10",48030962,"+","none","TGG","TATTGTGCTTGCCTCTGGATTGG",0,NA,NA,NA,"GeneID:414224","CTGLF12P",TRUE,"AGAP6"

"4744","TATTGTGCTTGCCTCTGGAT","chr10",45679433,"-","none","TGG","TATTGTGCTTGCCTCTGGATTGG",0,NA,NA,NA,"","",FALSE,"AGAP6"

"4745","TATTGTGCTTGCCTCTGGAT","chr10",46340211,"-","none","TGG","TATTGTGCTTGCCTCTGGATTGG",0,NA,NA,NA,"","",TRUE,"AGAP6"

"4746","TATTGTGCTTGCCTCTGGAT","chr10",49991699,"-","none","TGG","TATTGTGCTTGCCTCTGGATTGG",0,NA,NA,NA,"GeneID:414189","AGAP6",TRUE,"AGAP6"

"4747","TATTGTGCTTGCCTCTGGAT","chr10",45681795,"-","none","TGG","TATTGTGCTTGCCTCTGGATTGG",0,NA,NA,NA,"","",TRUE,"AGAP6"

"4748","TATTGTGCTTGCCTCTGGAT","chr10",46112603,"-","none","TGG","TATTGTGCTTGCCTCTGGATTGG",0,NA,NA,NA,"","",TRUE,"AGAP6"

"4749","TATTGTGCTTGCCTCTGGAT","chr10",46819491,"-","none","TGG","TATTGTGCTTGCCTCTGGATTGG",0,NA,NA,NA,"","",TRUE,"AGAP6"

"4750","TATTGTGCTTGCCTCTGGAT","chr10",46337839,"-","none","TGG","TATTGTGCTTGCCTCTGGATTGG",0,NA,NA,NA,"","",TRUE,"AGAP6"

"4751","TATTGTGCTTGCCTCTGGAT","chr10",46817130,"-","none","TGG","TATTGTGCTTGCCTCTGGATTGG",0,NA,NA,NA,"","",FALSE,"AGAP6"

"4752","TATTGTGCTTGCCTCTGGAT","chr10",49989331,"-","11:T>G","TGG","TATTGTGCTTGACTCTGGATTGG",1,12,NA,NA,"GeneID:414189","AGAP6",TRUE,"AGAP6"

"4753","TATTGTGCTTGCCTCTGGAT","chr5",156631600,"+","0:A>T,2:A>T","TGG","AAATGTGCTTGCCTCTGGATTGG",2,1,3,NA,"GeneID:6444","SGCD",FALSE,"AGAP6"

"4754","TATTGTGCTTGCCTCTGGAT","chr10",38982281,"-","3:T>A,18:A>T","GGG","TATAGTGCTTGCCTCTGGTTGGG",2,4,19,NA,"","",FALSE,"AGAP6"

"4755","CACCTTTGTGTGATATGATG","chr10",45831390,"+","none","AGG","CACCTTTGTGTGATATGATGAGG",0,NA,NA,NA,"GeneID:119016","AGAP4",TRUE,"AGAP7"

"4756","CACCTTTGTGTGATATGATG","chr10",47507544,"+","none","AGG","CACCTTTGTGTGATATGATGAGG",0,NA,NA,NA,"GeneID:642517","AGAP9",TRUE,"AGAP7"

"4757","CACCTTTGTGTGATATGATG","chr10",73680067,"+","none","AGG","CACCTTTGTGTGATATGATGAGG",0,NA,NA,NA,"GeneID:729092","AGAP5",TRUE,"AGAP7"

"4758","CACCTTTGTGTGATATGATG","chr10",48015643,"+","none","AGG","CACCTTTGTGTGATATGATGAGG",0,NA,NA,NA,"GeneID:414224","CTGLF12P",TRUE,"AGAP7"

"4759","CACCTTTGTGTGATATGATG","chr10",46832459,"-","none","AGG","CACCTTTGTGTGATATGATGAGG",0,NA,NA,NA,"","",TRUE,"AGAP7"

"4760","CACCTTTGTGTGATATGATG","chr10",46125882,"-","none","AGG","CACCTTTGTGTGATATGATGAGG",0,NA,NA,NA,"","",TRUE,"AGAP7"

"4761","CACCTTTGTGTGATATGATG","chr10",46353224,"-","none","AGG","CACCTTTGTGTGATATGATGAGG",0,NA,NA,NA,"","",TRUE,"AGAP7"

"4762","CACCTTTGTGTGATATGATG","chr10",50004703,"-","none","AGG","CACCTTTGTGTGATATGATGAGG",0,NA,NA,NA,"GeneID:414189","AGAP6",TRUE,"AGAP7"

"4763","CACCTTTGTGTGATATGATG","chr10",45694766,"-","none","AGG","CACCTTTGTGTGATATGATGAGG",0,NA,NA,NA,"","",TRUE,"AGAP7"

"4764","CACCTTTGTGTGATATGATG","chr2",133138234,"-","0:C>G,10:C>A","AGG","GACCTTTGTGGGATATGATGAGG",2,1,11,NA,"GeneID:344148","NCKAP5",FALSE,"AGAP7"

"4765","CAGAGGAACTCTCAAACAGA","chr10",46110260,"+","none","TGG","CAGAGGAACTCTCAAACAGATGG",0,NA,NA,NA,"","",TRUE,"AGAP7"

"4766","CAGAGGAACTCTCAAACAGA","chr10",46817153,"+","none","TGG","CAGAGGAACTCTCAAACAGATGG",0,NA,NA,NA,"","",FALSE,"AGAP7"

"4767","CAGAGGAACTCTCAAACAGA","chr10",45679456,"+","none","TGG","CAGAGGAACTCTCAAACAGATGG",0,NA,NA,NA,"","",FALSE,"AGAP7"

"4768","CAGAGGAACTCTCAAACAGA","chr10",46340234,"+","none","TGG","CAGAGGAACTCTCAAACAGATGG",0,NA,NA,NA,"","",TRUE,"AGAP7"

"4769","CAGAGGAACTCTCAAACAGA","chr10",49991722,"+","none","TGG","CAGAGGAACTCTCAAACAGATGG",0,NA,NA,NA,"GeneID:414189","AGAP6",TRUE,"AGAP7"

"4770","CAGAGGAACTCTCAAACAGA","chr10",45681818,"+","none","TGG","CAGAGGAACTCTCAAACAGATGG",0,NA,NA,NA,"","",TRUE,"AGAP7"

"4771","CAGAGGAACTCTCAAACAGA","chr10",46112626,"+","none","TGG","CAGAGGAACTCTCAAACAGATGG",0,NA,NA,NA,"","",TRUE,"AGAP7"

"4772","CAGAGGAACTCTCAAACAGA","chr10",46819514,"+","none","TGG","CAGAGGAACTCTCAAACAGATGG",0,NA,NA,NA,"","",TRUE,"AGAP7"

"4773","CAGAGGAACTCTCAAACAGA","chr10",49989354,"+","none","AGG","CAGAGGAACTCTCAAACAGAAGG",0,NA,NA,NA,"GeneID:414189","AGAP6",TRUE,"AGAP7"

"4774","CAGAGGAACTCTCAAACAGA","chr10",46337862,"+","none","TGG","CAGAGGAACTCTCAAACAGATGG",0,NA,NA,NA,"","",TRUE,"AGAP7"

"4775","CAGAGGAACTCTCAAACAGA","chr10",45844327,"-","none","TGG","CAGAGGAACTCTCAAACAGATGG",0,NA,NA,NA,"GeneID:119016","AGAP4",TRUE,"AGAP7"

"4776","CAGAGGAACTCTCAAACAGA","chr10",48028573,"-","none","TGG","CAGAGGAACTCTCAAACAGATGG",0,NA,NA,NA,"GeneID:414224","CTGLF12P",TRUE,"AGAP7"

"4777","CAGAGGAACTCTCAAACAGA","chr10",73697096,"-","none","TGG","CAGAGGAACTCTCAAACAGATGG",0,NA,NA,NA,"GeneID:729092","AGAP5",TRUE,"AGAP7"

"4778","CAGAGGAACTCTCAAACAGA","chr10",45846688,"-","none","TGG","CAGAGGAACTCTCAAACAGATGG",0,NA,NA,NA,"GeneID:119016","AGAP4",TRUE,"AGAP7"

"4779","CAGAGGAACTCTCAAACAGA","chr10",48030939,"-","none","TGG","CAGAGGAACTCTCAAACAGATGG",0,NA,NA,NA,"GeneID:414224","CTGLF12P",TRUE,"AGAP7"

"4780","CAGAGGAACTCTCAAACAGA","chr10",47522867,"-","none","TGG","CAGAGGAACTCTCAAACAGATGG",0,NA,NA,NA,"GeneID:642517","AGAP9",TRUE,"AGAP7"

"4781","CAGAGGAACTCTCAAACAGA","chr10",47520500,"-","none","TGG","CAGAGGAACTCTCAAACAGATGG",0,NA,NA,NA,"GeneID:642517","AGAP9",TRUE,"AGAP7"

"4782","CAGAGGAACTCTCAAACAGA","chr10",73694737,"-","none","TGG","CAGAGGAACTCTCAAACAGATGG",0,NA,NA,NA,"GeneID:729092","AGAP5",TRUE,"AGAP7"

"4783","CAGAGGAACTCTCAAACAGA","chr5",72590696,"-","4:A>C,7:A>T","TGG","CAGATGATCTCTCAAACAGATGG",2,5,8,NA,"GeneID:102477328","LOC102477328",FALSE,"AGAP7"

"4784","CAGAGGAACTCTCAAACAGA","chr3",13256056,"+","1:T>A,5:A>G","TGG","CTGAGAAACTCTCAAACAGATGG",2,2,6,NA,"","",FALSE,"AGAP7"

"4785","CATCTGTTTGAGAGTTCCTC","chr10",73694735,"+","none","TGG","CATCTGTTTGAGAGTTCCTCTGG",0,NA,NA,NA,"GeneID:729092","AGAP5",TRUE,"AGAP7"

"4786","CATCTGTTTGAGAGTTCCTC","chr10",45844325,"+","none","TGG","CATCTGTTTGAGAGTTCCTCTGG",0,NA,NA,NA,"GeneID:119016","AGAP4",TRUE,"AGAP7"

"4787","CATCTGTTTGAGAGTTCCTC","chr10",48028571,"+","none","TGG","CATCTGTTTGAGAGTTCCTCTGG",0,NA,NA,NA,"GeneID:414224","CTGLF12P",TRUE,"AGAP7"

"4788","CATCTGTTTGAGAGTTCCTC","chr10",73697094,"+","none","TGG","CATCTGTTTGAGAGTTCCTCTGG",0,NA,NA,NA,"GeneID:729092","AGAP5",TRUE,"AGAP7"

"4789","CATCTGTTTGAGAGTTCCTC","chr10",45846686,"+","none","TGG","CATCTGTTTGAGAGTTCCTCTGG",0,NA,NA,NA,"GeneID:119016","AGAP4",TRUE,"AGAP7"

"4790","CATCTGTTTGAGAGTTCCTC","chr10",48030937,"+","none","TGG","CATCTGTTTGAGAGTTCCTCTGG",0,NA,NA,NA,"GeneID:414224","CTGLF12P",TRUE,"AGAP7"

"4791","CATCTGTTTGAGAGTTCCTC","chr10",47522865,"+","none","TGG","CATCTGTTTGAGAGTTCCTCTGG",0,NA,NA,NA,"GeneID:642517","AGAP9",TRUE,"AGAP7"

"4792","CATCTGTTTGAGAGTTCCTC","chr10",47520498,"+","none","TGG","CATCTGTTTGAGAGTTCCTCTGG",0,NA,NA,NA,"GeneID:642517","AGAP9",TRUE,"AGAP7"

"4793","CATCTGTTTGAGAGTTCCTC","chr10",46340236,"-","none","TGG","CATCTGTTTGAGAGTTCCTCTGG",0,NA,NA,NA,"","",TRUE,"AGAP7"

"4794","CATCTGTTTGAGAGTTCCTC","chr10",49991724,"-","none","TGG","CATCTGTTTGAGAGTTCCTCTGG",0,NA,NA,NA,"GeneID:414189","AGAP6",TRUE,"AGAP7"

"4795","CATCTGTTTGAGAGTTCCTC","chr10",45681820,"-","none","TGG","CATCTGTTTGAGAGTTCCTCTGG",0,NA,NA,NA,"","",TRUE,"AGAP7"

"4796","CATCTGTTTGAGAGTTCCTC","chr10",46112628,"-","none","TGG","CATCTGTTTGAGAGTTCCTCTGG",0,NA,NA,NA,"","",TRUE,"AGAP7"

"4797","CATCTGTTTGAGAGTTCCTC","chr10",46819516,"-","none","TGG","CATCTGTTTGAGAGTTCCTCTGG",0,NA,NA,NA,"","",TRUE,"AGAP7"

"4798","CATCTGTTTGAGAGTTCCTC","chr10",46817155,"-","none","TGG","CATCTGTTTGAGAGTTCCTCTGG",0,NA,NA,NA,"","",FALSE,"AGAP7"

"4799","CATCTGTTTGAGAGTTCCTC","chr10",45679458,"-","none","TGG","CATCTGTTTGAGAGTTCCTCTGG",0,NA,NA,NA,"","",FALSE,"AGAP7"

"4800","CATCTGTTTGAGAGTTCCTC","chr10",38982306,"-","14:G>A,17:A>G","AGG","CATCTGTTTGAGAGCTCTTCAGG",2,15,18,NA,"","",FALSE,"AGAP7"

"4801","CATCTGTTTGAGAGTTCCTC","chr10",42451608,"+","13:T>G,16:T>C","AGG","CATCTGTTTGAGATTTTCTCAGG",2,14,17,NA,"GeneID:414194","CCNYL2",TRUE,"AGAP7"

"4802","CATCTGTTTGAGAGTTCCTC","chr10",49989356,"-","1:A>T","TGG","CTTCTGTTTGAGAGTTCCTCTGG",1,2,NA,NA,"GeneID:414189","AGAP6",TRUE,"AGAP7"

"4803","CATCTGTTTGAGAGTTCCTC","chr4",116173792,"+","0:T>C,3:T>C","TGG","TATTTGTTTGAGAGTTCCTCTGG",2,1,4,NA,"","",FALSE,"AGAP7"

"4804","TAACCCTTCTGCCAATCCAG","chr10",45681781,"+","none","AGG","TAACCCTTCTGCCAATCCAGAGG",0,NA,NA,NA,"","",TRUE,"AGAP7"

"4805","TAACCCTTCTGCCAATCCAG","chr10",46112589,"+","none","AGG","TAACCCTTCTGCCAATCCAGAGG",0,NA,NA,NA,"","",TRUE,"AGAP7"

"4806","TAACCCTTCTGCCAATCCAG","chr10",46819477,"+","none","AGG","TAACCCTTCTGCCAATCCAGAGG",0,NA,NA,NA,"","",TRUE,"AGAP7"

"4807","TAACCCTTCTGCCAATCCAG","chr10",46340197,"+","none","AGG","TAACCCTTCTGCCAATCCAGAGG",0,NA,NA,NA,"","",TRUE,"AGAP7"

"4808","TAACCCTTCTGCCAATCCAG","chr10",49991685,"+","none","AGG","TAACCCTTCTGCCAATCCAGAGG",0,NA,NA,NA,"GeneID:414189","AGAP6",TRUE,"AGAP7"

"4809","TAACCCTTCTGCCAATCCAG","chr10",73694774,"-","none","AGG","TAACCCTTCTGCCAATCCAGAGG",0,NA,NA,NA,"GeneID:729092","AGAP5",TRUE,"AGAP7"

"4810","TAACCCTTCTGCCAATCCAG","chr10",45844364,"-","none","AGG","TAACCCTTCTGCCAATCCAGAGG",0,NA,NA,NA,"GeneID:119016","AGAP4",TRUE,"AGAP7"

"4811","TAACCCTTCTGCCAATCCAG","chr10",48028610,"-","none","AGG","TAACCCTTCTGCCAATCCAGAGG",0,NA,NA,NA,"GeneID:414224","CTGLF12P",TRUE,"AGAP7"

"4812","TAACCCTTCTGCCAATCCAG","chr10",47520537,"-","4:A>G","AGG","TAACTCTTCTGCCAATCCAGAGG",1,5,NA,NA,"GeneID:642517","AGAP9",TRUE,"AGAP7"

"4813","TAACCCTTCTGCCAATCCAG","chr10",47522904,"-","5:A>G","AGG","TAACCTTTCTGCCAATCCAGAGG",1,6,NA,NA,"GeneID:642517","AGAP9",TRUE,"AGAP7"

"4814","TAACCCTTCTGCCAATCCAG","chr10",45846725,"-","5:A>G","AGG","TAACCTTTCTGCCAATCCAGAGG",1,6,NA,NA,"GeneID:119016","AGAP4",TRUE,"AGAP7"

"4815","TAACCCTTCTGCCAATCCAG","chr10",48030976,"-","5:A>G","AGG","TAACCTTTCTGCCAATCCAGAGG",1,6,NA,NA,"GeneID:414224","CTGLF12P",TRUE,"AGAP7"

"4816","TAACCCTTCTGCCAATCCAG","chr10",73697133,"-","2:C>T,5:A>G","AGG","TAGCCTTTCTGCCAATCCAGAGG",2,3,6,NA,"GeneID:729092","AGAP5",TRUE,"AGAP7"

"4817","TAACCCTTCTGCCAATCCAG","chr10",46337825,"+","5:T>C","AGG","TAACCTTTCTGCCAATCCAGAGG",1,6,NA,NA,"","",TRUE,"AGAP7"

"4818","TAACCCTTCTGCCAATCCAG","chr10",46817116,"+","5:T>C","AGG","TAACCTTTCTGCCAATCCAGAGG",1,6,NA,NA,"","",FALSE,"AGAP7"

"4819","TAACCCTTCTGCCAATCCAG","chr10",45679419,"+","5:T>C","AGG","TAACCTTTCTGCCAATCCAGAGG",1,6,NA,NA,"","",FALSE,"AGAP7"

"4820","TAACCCTTCTGCCAATCCAG","chr10",46110222,"+","5:T>C","AGG","TAACCTTTCTGCCAATCCAGAGG",1,6,NA,NA,"","",TRUE,"AGAP7"

"4821","TAACCCTTCTGCCAATCCAG","chr11",10657769,"+","2:G>A,12:A>C","AGG","TAGCCCTTCTGCAAATCCAGAGG",2,3,13,NA,"GeneID:10335","MRVI1",FALSE,"AGAP7"

"4822","TACCTCATCATATCACACAA","chr10",46832454,"+","none","AGG","TACCTCATCATATCACACAAAGG",0,NA,NA,NA,"","",TRUE,"AGAP7"

"4823","TACCTCATCATATCACACAA","chr10",46125877,"+","none","AGG","TACCTCATCATATCACACAAAGG",0,NA,NA,NA,"","",TRUE,"AGAP7"

"4824","TACCTCATCATATCACACAA","chr10",46353219,"+","none","AGG","TACCTCATCATATCACACAAAGG",0,NA,NA,NA,"","",TRUE,"AGAP7"

"4825","TACCTCATCATATCACACAA","chr10",50004698,"+","none","AGG","TACCTCATCATATCACACAAAGG",0,NA,NA,NA,"GeneID:414189","AGAP6",TRUE,"AGAP7"

"4826","TACCTCATCATATCACACAA","chr10",45694761,"+","none","AGG","TACCTCATCATATCACACAAAGG",0,NA,NA,NA,"","",TRUE,"AGAP7"

"4827","TACCTCATCATATCACACAA","chr10",45831395,"-","none","AGG","TACCTCATCATATCACACAAAGG",0,NA,NA,NA,"GeneID:119016","AGAP4",TRUE,"AGAP7"

"4828","TACCTCATCATATCACACAA","chr10",47507549,"-","none","AGG","TACCTCATCATATCACACAAAGG",0,NA,NA,NA,"GeneID:642517","AGAP9",TRUE,"AGAP7"

"4829","TACCTCATCATATCACACAA","chr10",73680072,"-","none","AGG","TACCTCATCATATCACACAAAGG",0,NA,NA,NA,"GeneID:729092","AGAP5",TRUE,"AGAP7"

"4830","TACCTCATCATATCACACAA","chr10",48015648,"-","none","AGG","TACCTCATCATATCACACAAAGG",0,NA,NA,NA,"GeneID:414224","CTGLF12P",TRUE,"AGAP7"

"4831","TACCTCATCATATCACACAA","chr2",133138229,"+","0:A>T,14:C>A","AGG","AACCTCATCATATCCCACAAAGG",2,1,15,NA,"GeneID:344148","NCKAP5",FALSE,"AGAP7"

"4832","TACCTCATCATATCACACAA","chr1",24151623,"+","0:A>T,14:C>A","CGG","AACCTCATCATATCCCACAACGG",2,1,15,NA,"","",FALSE,"AGAP7"

"4833","TTGTCATTGTAAGATAATGC","chr10",45834022,"+","none","TGG","TTGTCATTGTAAGATAATGCTGG",0,NA,NA,NA,"GeneID:119016","AGAP4",TRUE,"AGAP7"

"4834","TTGTCATTGTAAGATAATGC","chr10",47510177,"+","none","TGG","TTGTCATTGTAAGATAATGCTGG",0,NA,NA,NA,"GeneID:642517","AGAP9",TRUE,"AGAP7"

"4835","TTGTCATTGTAAGATAATGC","chr10",48018274,"+","none","TGG","TTGTCATTGTAAGATAATGCTGG",0,NA,NA,NA,"GeneID:414224","CTGLF12P",TRUE,"AGAP7"

"4836","TTGTCATTGTAAGATAATGC","chr10",73682700,"+","none","TGG","TTGTCATTGTAAGATAATGCTGG",0,NA,NA,NA,"GeneID:729092","AGAP5",TRUE,"AGAP7"

"4837","TTGTCATTGTAAGATAATGC","chr10",50002069,"-","none","TGG","TTGTCATTGTAAGATAATGCTGG",0,NA,NA,NA,"GeneID:414189","AGAP6",TRUE,"AGAP7"

"4838","TTGTCATTGTAAGATAATGC","chr10",46829827,"-","none","TGG","TTGTCATTGTAAGATAATGCTGG",0,NA,NA,NA,"","",FALSE,"AGAP7"

"4839","TTGTCATTGTAAGATAATGC","chr10",46123243,"-","none","TGG","TTGTCATTGTAAGATAATGCTGG",0,NA,NA,NA,"","",TRUE,"AGAP7"

"4840","TTGTCATTGTAAGATAATGC","chr10",46350591,"-","none","TGG","TTGTCATTGTAAGATAATGCTGG",0,NA,NA,NA,"","",TRUE,"AGAP7"

"4841","TTGTCATTGTAAGATAATGC","chr10",87001709,"-","8:T>C","TGG","TTGTCATTATAAGATAATGCTGG",1,9,NA,NA,"","",TRUE,"AGAP7"

"4842","CACCTTTGTGTGATATGATG","chr10",45831390,"+","none","AGG","CACCTTTGTGTGATATGATGAGG",0,NA,NA,NA,"GeneID:119016","AGAP4",TRUE,"AGAP8"

"4843","CACCTTTGTGTGATATGATG","chr10",47507544,"+","none","AGG","CACCTTTGTGTGATATGATGAGG",0,NA,NA,NA,"GeneID:642517","AGAP9",TRUE,"AGAP8"

"4844","CACCTTTGTGTGATATGATG","chr10",73680067,"+","none","AGG","CACCTTTGTGTGATATGATGAGG",0,NA,NA,NA,"GeneID:729092","AGAP5",TRUE,"AGAP8"

"4845","CACCTTTGTGTGATATGATG","chr10",48015643,"+","none","AGG","CACCTTTGTGTGATATGATGAGG",0,NA,NA,NA,"GeneID:414224","CTGLF12P",TRUE,"AGAP8"

"4846","CACCTTTGTGTGATATGATG","chr10",46832459,"-","none","AGG","CACCTTTGTGTGATATGATGAGG",0,NA,NA,NA,"","",TRUE,"AGAP8"

"4847","CACCTTTGTGTGATATGATG","chr10",46125882,"-","none","AGG","CACCTTTGTGTGATATGATGAGG",0,NA,NA,NA,"","",TRUE,"AGAP8"

"4848","CACCTTTGTGTGATATGATG","chr10",46353224,"-","none","AGG","CACCTTTGTGTGATATGATGAGG",0,NA,NA,NA,"","",TRUE,"AGAP8"

"4849","CACCTTTGTGTGATATGATG","chr10",50004703,"-","none","AGG","CACCTTTGTGTGATATGATGAGG",0,NA,NA,NA,"GeneID:414189","AGAP6",TRUE,"AGAP8"

"4850","CACCTTTGTGTGATATGATG","chr10",45694766,"-","none","AGG","CACCTTTGTGTGATATGATGAGG",0,NA,NA,NA,"","",TRUE,"AGAP8"

"4851","CACCTTTGTGTGATATGATG","chr2",133138234,"-","0:C>G,10:C>A","AGG","GACCTTTGTGGGATATGATGAGG",2,1,11,NA,"GeneID:344148","NCKAP5",FALSE,"AGAP8"

"4852","CAGAGGAACTCTCAAACAGA","chr10",46110260,"+","none","TGG","CAGAGGAACTCTCAAACAGATGG",0,NA,NA,NA,"","",TRUE,"AGAP8"

"4853","CAGAGGAACTCTCAAACAGA","chr10",46817153,"+","none","TGG","CAGAGGAACTCTCAAACAGATGG",0,NA,NA,NA,"","",FALSE,"AGAP8"

"4854","CAGAGGAACTCTCAAACAGA","chr10",45679456,"+","none","TGG","CAGAGGAACTCTCAAACAGATGG",0,NA,NA,NA,"","",FALSE,"AGAP8"

"4855","CAGAGGAACTCTCAAACAGA","chr10",46340234,"+","none","TGG","CAGAGGAACTCTCAAACAGATGG",0,NA,NA,NA,"","",TRUE,"AGAP8"

"4856","CAGAGGAACTCTCAAACAGA","chr10",49991722,"+","none","TGG","CAGAGGAACTCTCAAACAGATGG",0,NA,NA,NA,"GeneID:414189","AGAP6",TRUE,"AGAP8"

"4857","CAGAGGAACTCTCAAACAGA","chr10",45681818,"+","none","TGG","CAGAGGAACTCTCAAACAGATGG",0,NA,NA,NA,"","",TRUE,"AGAP8"

"4858","CAGAGGAACTCTCAAACAGA","chr10",46112626,"+","none","TGG","CAGAGGAACTCTCAAACAGATGG",0,NA,NA,NA,"","",TRUE,"AGAP8"

"4859","CAGAGGAACTCTCAAACAGA","chr10",46819514,"+","none","TGG","CAGAGGAACTCTCAAACAGATGG",0,NA,NA,NA,"","",TRUE,"AGAP8"

"4860","CAGAGGAACTCTCAAACAGA","chr10",49989354,"+","none","AGG","CAGAGGAACTCTCAAACAGAAGG",0,NA,NA,NA,"GeneID:414189","AGAP6",TRUE,"AGAP8"

"4861","CAGAGGAACTCTCAAACAGA","chr10",46337862,"+","none","TGG","CAGAGGAACTCTCAAACAGATGG",0,NA,NA,NA,"","",TRUE,"AGAP8"

"4862","CAGAGGAACTCTCAAACAGA","chr10",45844327,"-","none","TGG","CAGAGGAACTCTCAAACAGATGG",0,NA,NA,NA,"GeneID:119016","AGAP4",TRUE,"AGAP8"

"4863","CAGAGGAACTCTCAAACAGA","chr10",48028573,"-","none","TGG","CAGAGGAACTCTCAAACAGATGG",0,NA,NA,NA,"GeneID:414224","CTGLF12P",TRUE,"AGAP8"

"4864","CAGAGGAACTCTCAAACAGA","chr10",73697096,"-","none","TGG","CAGAGGAACTCTCAAACAGATGG",0,NA,NA,NA,"GeneID:729092","AGAP5",TRUE,"AGAP8"

"4865","CAGAGGAACTCTCAAACAGA","chr10",45846688,"-","none","TGG","CAGAGGAACTCTCAAACAGATGG",0,NA,NA,NA,"GeneID:119016","AGAP4",TRUE,"AGAP8"

"4866","CAGAGGAACTCTCAAACAGA","chr10",48030939,"-","none","TGG","CAGAGGAACTCTCAAACAGATGG",0,NA,NA,NA,"GeneID:414224","CTGLF12P",TRUE,"AGAP8"

"4867","CAGAGGAACTCTCAAACAGA","chr10",47522867,"-","none","TGG","CAGAGGAACTCTCAAACAGATGG",0,NA,NA,NA,"GeneID:642517","AGAP9",TRUE,"AGAP8"

"4868","CAGAGGAACTCTCAAACAGA","chr10",47520500,"-","none","TGG","CAGAGGAACTCTCAAACAGATGG",0,NA,NA,NA,"GeneID:642517","AGAP9",TRUE,"AGAP8"

"4869","CAGAGGAACTCTCAAACAGA","chr10",73694737,"-","none","TGG","CAGAGGAACTCTCAAACAGATGG",0,NA,NA,NA,"GeneID:729092","AGAP5",TRUE,"AGAP8"

"4870","CAGAGGAACTCTCAAACAGA","chr5",72590696,"-","4:A>C,7:A>T","TGG","CAGATGATCTCTCAAACAGATGG",2,5,8,NA,"GeneID:102477328","LOC102477328",FALSE,"AGAP8"

"4871","CAGAGGAACTCTCAAACAGA","chr3",13256056,"+","1:T>A,5:A>G","TGG","CTGAGAAACTCTCAAACAGATGG",2,2,6,NA,"","",FALSE,"AGAP8"

"4872","CATCTGTTTGAGAGTTCCTC","chr10",73694735,"+","none","TGG","CATCTGTTTGAGAGTTCCTCTGG",0,NA,NA,NA,"GeneID:729092","AGAP5",TRUE,"AGAP8"

"4873","CATCTGTTTGAGAGTTCCTC","chr10",45844325,"+","none","TGG","CATCTGTTTGAGAGTTCCTCTGG",0,NA,NA,NA,"GeneID:119016","AGAP4",TRUE,"AGAP8"

"4874","CATCTGTTTGAGAGTTCCTC","chr10",48028571,"+","none","TGG","CATCTGTTTGAGAGTTCCTCTGG",0,NA,NA,NA,"GeneID:414224","CTGLF12P",TRUE,"AGAP8"

"4875","CATCTGTTTGAGAGTTCCTC","chr10",73697094,"+","none","TGG","CATCTGTTTGAGAGTTCCTCTGG",0,NA,NA,NA,"GeneID:729092","AGAP5",TRUE,"AGAP8"

"4876","CATCTGTTTGAGAGTTCCTC","chr10",45846686,"+","none","TGG","CATCTGTTTGAGAGTTCCTCTGG",0,NA,NA,NA,"GeneID:119016","AGAP4",TRUE,"AGAP8"

"4877","CATCTGTTTGAGAGTTCCTC","chr10",48030937,"+","none","TGG","CATCTGTTTGAGAGTTCCTCTGG",0,NA,NA,NA,"GeneID:414224","CTGLF12P",TRUE,"AGAP8"

"4878","CATCTGTTTGAGAGTTCCTC","chr10",47522865,"+","none","TGG","CATCTGTTTGAGAGTTCCTCTGG",0,NA,NA,NA,"GeneID:642517","AGAP9",TRUE,"AGAP8"

"4879","CATCTGTTTGAGAGTTCCTC","chr10",47520498,"+","none","TGG","CATCTGTTTGAGAGTTCCTCTGG",0,NA,NA,NA,"GeneID:642517","AGAP9",TRUE,"AGAP8"

"4880","CATCTGTTTGAGAGTTCCTC","chr10",46340236,"-","none","TGG","CATCTGTTTGAGAGTTCCTCTGG",0,NA,NA,NA,"","",TRUE,"AGAP8"

"4881","CATCTGTTTGAGAGTTCCTC","chr10",49991724,"-","none","TGG","CATCTGTTTGAGAGTTCCTCTGG",0,NA,NA,NA,"GeneID:414189","AGAP6",TRUE,"AGAP8"

"4882","CATCTGTTTGAGAGTTCCTC","chr10",45681820,"-","none","TGG","CATCTGTTTGAGAGTTCCTCTGG",0,NA,NA,NA,"","",TRUE,"AGAP8"

"4883","CATCTGTTTGAGAGTTCCTC","chr10",46112628,"-","none","TGG","CATCTGTTTGAGAGTTCCTCTGG",0,NA,NA,NA,"","",TRUE,"AGAP8"

"4884","CATCTGTTTGAGAGTTCCTC","chr10",46819516,"-","none","TGG","CATCTGTTTGAGAGTTCCTCTGG",0,NA,NA,NA,"","",TRUE,"AGAP8"

"4885","CATCTGTTTGAGAGTTCCTC","chr10",46817155,"-","none","TGG","CATCTGTTTGAGAGTTCCTCTGG",0,NA,NA,NA,"","",FALSE,"AGAP8"

"4886","CATCTGTTTGAGAGTTCCTC","chr10",45679458,"-","none","TGG","CATCTGTTTGAGAGTTCCTCTGG",0,NA,NA,NA,"","",FALSE,"AGAP8"

"4887","CATCTGTTTGAGAGTTCCTC","chr10",38982306,"-","14:G>A,17:A>G","AGG","CATCTGTTTGAGAGCTCTTCAGG",2,15,18,NA,"","",FALSE,"AGAP8"

"4888","CATCTGTTTGAGAGTTCCTC","chr10",42451608,"+","13:T>G,16:T>C","AGG","CATCTGTTTGAGATTTTCTCAGG",2,14,17,NA,"GeneID:414194","CCNYL2",TRUE,"AGAP8"

"4889","CATCTGTTTGAGAGTTCCTC","chr10",49989356,"-","1:A>T","TGG","CTTCTGTTTGAGAGTTCCTCTGG",1,2,NA,NA,"GeneID:414189","AGAP6",TRUE,"AGAP8"

"4890","CATCTGTTTGAGAGTTCCTC","chr4",116173792,"+","0:T>C,3:T>C","TGG","TATTTGTTTGAGAGTTCCTCTGG",2,1,4,NA,"","",FALSE,"AGAP8"

"4891","TAACCCTTCTGCCAATCCAG","chr10",45681781,"+","none","AGG","TAACCCTTCTGCCAATCCAGAGG",0,NA,NA,NA,"","",TRUE,"AGAP8"

"4892","TAACCCTTCTGCCAATCCAG","chr10",46112589,"+","none","AGG","TAACCCTTCTGCCAATCCAGAGG",0,NA,NA,NA,"","",TRUE,"AGAP8"

"4893","TAACCCTTCTGCCAATCCAG","chr10",46819477,"+","none","AGG","TAACCCTTCTGCCAATCCAGAGG",0,NA,NA,NA,"","",TRUE,"AGAP8"

"4894","TAACCCTTCTGCCAATCCAG","chr10",46340197,"+","none","AGG","TAACCCTTCTGCCAATCCAGAGG",0,NA,NA,NA,"","",TRUE,"AGAP8"

"4895","TAACCCTTCTGCCAATCCAG","chr10",49991685,"+","none","AGG","TAACCCTTCTGCCAATCCAGAGG",0,NA,NA,NA,"GeneID:414189","AGAP6",TRUE,"AGAP8"

"4896","TAACCCTTCTGCCAATCCAG","chr10",73694774,"-","none","AGG","TAACCCTTCTGCCAATCCAGAGG",0,NA,NA,NA,"GeneID:729092","AGAP5",TRUE,"AGAP8"

"4897","TAACCCTTCTGCCAATCCAG","chr10",45844364,"-","none","AGG","TAACCCTTCTGCCAATCCAGAGG",0,NA,NA,NA,"GeneID:119016","AGAP4",TRUE,"AGAP8"

"4898","TAACCCTTCTGCCAATCCAG","chr10",48028610,"-","none","AGG","TAACCCTTCTGCCAATCCAGAGG",0,NA,NA,NA,"GeneID:414224","CTGLF12P",TRUE,"AGAP8"

"4899","TAACCCTTCTGCCAATCCAG","chr10",47520537,"-","4:A>G","AGG","TAACTCTTCTGCCAATCCAGAGG",1,5,NA,NA,"GeneID:642517","AGAP9",TRUE,"AGAP8"

"4900","TAACCCTTCTGCCAATCCAG","chr10",47522904,"-","5:A>G","AGG","TAACCTTTCTGCCAATCCAGAGG",1,6,NA,NA,"GeneID:642517","AGAP9",TRUE,"AGAP8"

"4901","TAACCCTTCTGCCAATCCAG","chr10",45846725,"-","5:A>G","AGG","TAACCTTTCTGCCAATCCAGAGG",1,6,NA,NA,"GeneID:119016","AGAP4",TRUE,"AGAP8"

"4902","TAACCCTTCTGCCAATCCAG","chr10",48030976,"-","5:A>G","AGG","TAACCTTTCTGCCAATCCAGAGG",1,6,NA,NA,"GeneID:414224","CTGLF12P",TRUE,"AGAP8"

"4903","TAACCCTTCTGCCAATCCAG","chr10",73697133,"-","2:C>T,5:A>G","AGG","TAGCCTTTCTGCCAATCCAGAGG",2,3,6,NA,"GeneID:729092","AGAP5",TRUE,"AGAP8"

"4904","TAACCCTTCTGCCAATCCAG","chr10",46337825,"+","5:T>C","AGG","TAACCTTTCTGCCAATCCAGAGG",1,6,NA,NA,"","",TRUE,"AGAP8"

"4905","TAACCCTTCTGCCAATCCAG","chr10",46817116,"+","5:T>C","AGG","TAACCTTTCTGCCAATCCAGAGG",1,6,NA,NA,"","",FALSE,"AGAP8"

"4906","TAACCCTTCTGCCAATCCAG","chr10",45679419,"+","5:T>C","AGG","TAACCTTTCTGCCAATCCAGAGG",1,6,NA,NA,"","",FALSE,"AGAP8"

"4907","TAACCCTTCTGCCAATCCAG","chr10",46110222,"+","5:T>C","AGG","TAACCTTTCTGCCAATCCAGAGG",1,6,NA,NA,"","",TRUE,"AGAP8"

"4908","TAACCCTTCTGCCAATCCAG","chr11",10657769,"+","2:G>A,12:A>C","AGG","TAGCCCTTCTGCAAATCCAGAGG",2,3,13,NA,"GeneID:10335","MRVI1",FALSE,"AGAP8"

"4909","TACCTCATCATATCACACAA","chr10",46832454,"+","none","AGG","TACCTCATCATATCACACAAAGG",0,NA,NA,NA,"","",TRUE,"AGAP8"

"4910","TACCTCATCATATCACACAA","chr10",46125877,"+","none","AGG","TACCTCATCATATCACACAAAGG",0,NA,NA,NA,"","",TRUE,"AGAP8"

"4911","TACCTCATCATATCACACAA","chr10",46353219,"+","none","AGG","TACCTCATCATATCACACAAAGG",0,NA,NA,NA,"","",TRUE,"AGAP8"

"4912","TACCTCATCATATCACACAA","chr10",50004698,"+","none","AGG","TACCTCATCATATCACACAAAGG",0,NA,NA,NA,"GeneID:414189","AGAP6",TRUE,"AGAP8"

"4913","TACCTCATCATATCACACAA","chr10",45694761,"+","none","AGG","TACCTCATCATATCACACAAAGG",0,NA,NA,NA,"","",TRUE,"AGAP8"

"4914","TACCTCATCATATCACACAA","chr10",45831395,"-","none","AGG","TACCTCATCATATCACACAAAGG",0,NA,NA,NA,"GeneID:119016","AGAP4",TRUE,"AGAP8"

"4915","TACCTCATCATATCACACAA","chr10",47507549,"-","none","AGG","TACCTCATCATATCACACAAAGG",0,NA,NA,NA,"GeneID:642517","AGAP9",TRUE,"AGAP8"

"4916","TACCTCATCATATCACACAA","chr10",73680072,"-","none","AGG","TACCTCATCATATCACACAAAGG",0,NA,NA,NA,"GeneID:729092","AGAP5",TRUE,"AGAP8"

"4917","TACCTCATCATATCACACAA","chr10",48015648,"-","none","AGG","TACCTCATCATATCACACAAAGG",0,NA,NA,NA,"GeneID:414224","CTGLF12P",TRUE,"AGAP8"

"4918","TACCTCATCATATCACACAA","chr2",133138229,"+","0:A>T,14:C>A","AGG","AACCTCATCATATCCCACAAAGG",2,1,15,NA,"GeneID:344148","NCKAP5",FALSE,"AGAP8"

"4919","TACCTCATCATATCACACAA","chr1",24151623,"+","0:A>T,14:C>A","CGG","AACCTCATCATATCCCACAACGG",2,1,15,NA,"","",FALSE,"AGAP8"

"4920","TTGTCATTGTAAGATAATGC","chr10",45834022,"+","none","TGG","TTGTCATTGTAAGATAATGCTGG",0,NA,NA,NA,"GeneID:119016","AGAP4",TRUE,"AGAP8"

"4921","TTGTCATTGTAAGATAATGC","chr10",47510177,"+","none","TGG","TTGTCATTGTAAGATAATGCTGG",0,NA,NA,NA,"GeneID:642517","AGAP9",TRUE,"AGAP8"

"4922","TTGTCATTGTAAGATAATGC","chr10",48018274,"+","none","TGG","TTGTCATTGTAAGATAATGCTGG",0,NA,NA,NA,"GeneID:414224","CTGLF12P",TRUE,"AGAP8"

"4923","TTGTCATTGTAAGATAATGC","chr10",73682700,"+","none","TGG","TTGTCATTGTAAGATAATGCTGG",0,NA,NA,NA,"GeneID:729092","AGAP5",TRUE,"AGAP8"

"4924","TTGTCATTGTAAGATAATGC","chr10",50002069,"-","none","TGG","TTGTCATTGTAAGATAATGCTGG",0,NA,NA,NA,"GeneID:414189","AGAP6",TRUE,"AGAP8"

"4925","TTGTCATTGTAAGATAATGC","chr10",46829827,"-","none","TGG","TTGTCATTGTAAGATAATGCTGG",0,NA,NA,NA,"","",FALSE,"AGAP8"

"4926","TTGTCATTGTAAGATAATGC","chr10",46123243,"-","none","TGG","TTGTCATTGTAAGATAATGCTGG",0,NA,NA,NA,"","",TRUE,"AGAP8"

"4927","TTGTCATTGTAAGATAATGC","chr10",46350591,"-","none","TGG","TTGTCATTGTAAGATAATGCTGG",0,NA,NA,NA,"","",TRUE,"AGAP8"

"4928","TTGTCATTGTAAGATAATGC","chr10",87001709,"-","8:T>C","TGG","TTGTCATTATAAGATAATGCTGG",1,9,NA,NA,"","",TRUE,"AGAP8"

"4929","CAGAGGAACTCTCAAACAGA","chr10",46110260,"+","none","TGG","CAGAGGAACTCTCAAACAGATGG",0,NA,NA,NA,"","",TRUE,"AGAP9"

"4930","CAGAGGAACTCTCAAACAGA","chr10",46817153,"+","none","TGG","CAGAGGAACTCTCAAACAGATGG",0,NA,NA,NA,"","",FALSE,"AGAP9"

"4931","CAGAGGAACTCTCAAACAGA","chr10",45679456,"+","none","TGG","CAGAGGAACTCTCAAACAGATGG",0,NA,NA,NA,"","",FALSE,"AGAP9"

"4932","CAGAGGAACTCTCAAACAGA","chr10",46340234,"+","none","TGG","CAGAGGAACTCTCAAACAGATGG",0,NA,NA,NA,"","",TRUE,"AGAP9"

"4933","CAGAGGAACTCTCAAACAGA","chr10",49991722,"+","none","TGG","CAGAGGAACTCTCAAACAGATGG",0,NA,NA,NA,"GeneID:414189","AGAP6",TRUE,"AGAP9"

"4934","CAGAGGAACTCTCAAACAGA","chr10",45681818,"+","none","TGG","CAGAGGAACTCTCAAACAGATGG",0,NA,NA,NA,"","",TRUE,"AGAP9"

"4935","CAGAGGAACTCTCAAACAGA","chr10",46112626,"+","none","TGG","CAGAGGAACTCTCAAACAGATGG",0,NA,NA,NA,"","",TRUE,"AGAP9"

"4936","CAGAGGAACTCTCAAACAGA","chr10",46819514,"+","none","TGG","CAGAGGAACTCTCAAACAGATGG",0,NA,NA,NA,"","",TRUE,"AGAP9"

"4937","CAGAGGAACTCTCAAACAGA","chr10",49989354,"+","none","AGG","CAGAGGAACTCTCAAACAGAAGG",0,NA,NA,NA,"GeneID:414189","AGAP6",TRUE,"AGAP9"

"4938","CAGAGGAACTCTCAAACAGA","chr10",46337862,"+","none","TGG","CAGAGGAACTCTCAAACAGATGG",0,NA,NA,NA,"","",TRUE,"AGAP9"

"4939","CAGAGGAACTCTCAAACAGA","chr10",45844327,"-","none","TGG","CAGAGGAACTCTCAAACAGATGG",0,NA,NA,NA,"GeneID:119016","AGAP4",TRUE,"AGAP9"

"4940","CAGAGGAACTCTCAAACAGA","chr10",48028573,"-","none","TGG","CAGAGGAACTCTCAAACAGATGG",0,NA,NA,NA,"GeneID:414224","CTGLF12P",TRUE,"AGAP9"

"4941","CAGAGGAACTCTCAAACAGA","chr10",73697096,"-","none","TGG","CAGAGGAACTCTCAAACAGATGG",0,NA,NA,NA,"GeneID:729092","AGAP5",TRUE,"AGAP9"

"4942","CAGAGGAACTCTCAAACAGA","chr10",45846688,"-","none","TGG","CAGAGGAACTCTCAAACAGATGG",0,NA,NA,NA,"GeneID:119016","AGAP4",TRUE,"AGAP9"

"4943","CAGAGGAACTCTCAAACAGA","chr10",48030939,"-","none","TGG","CAGAGGAACTCTCAAACAGATGG",0,NA,NA,NA,"GeneID:414224","CTGLF12P",TRUE,"AGAP9"

"4944","CAGAGGAACTCTCAAACAGA","chr10",47522867,"-","none","TGG","CAGAGGAACTCTCAAACAGATGG",0,NA,NA,NA,"GeneID:642517","AGAP9",TRUE,"AGAP9"

"4945","CAGAGGAACTCTCAAACAGA","chr10",47520500,"-","none","TGG","CAGAGGAACTCTCAAACAGATGG",0,NA,NA,NA,"GeneID:642517","AGAP9",TRUE,"AGAP9"

"4946","CAGAGGAACTCTCAAACAGA","chr10",73694737,"-","none","TGG","CAGAGGAACTCTCAAACAGATGG",0,NA,NA,NA,"GeneID:729092","AGAP5",TRUE,"AGAP9"

"4947","CAGAGGAACTCTCAAACAGA","chr5",72590696,"-","4:A>C,7:A>T","TGG","CAGATGATCTCTCAAACAGATGG",2,5,8,NA,"GeneID:102477328","LOC102477328",FALSE,"AGAP9"

"4948","CAGAGGAACTCTCAAACAGA","chr3",13256056,"+","1:T>A,5:A>G","TGG","CTGAGAAACTCTCAAACAGATGG",2,2,6,NA,"","",FALSE,"AGAP9"

"4949","CATCTGTTTGAGAGTTCCTC","chr10",73694735,"+","none","TGG","CATCTGTTTGAGAGTTCCTCTGG",0,NA,NA,NA,"GeneID:729092","AGAP5",TRUE,"AGAP9"

"4950","CATCTGTTTGAGAGTTCCTC","chr10",45844325,"+","none","TGG","CATCTGTTTGAGAGTTCCTCTGG",0,NA,NA,NA,"GeneID:119016","AGAP4",TRUE,"AGAP9"

"4951","CATCTGTTTGAGAGTTCCTC","chr10",48028571,"+","none","TGG","CATCTGTTTGAGAGTTCCTCTGG",0,NA,NA,NA,"GeneID:414224","CTGLF12P",TRUE,"AGAP9"

"4952","CATCTGTTTGAGAGTTCCTC","chr10",73697094,"+","none","TGG","CATCTGTTTGAGAGTTCCTCTGG",0,NA,NA,NA,"GeneID:729092","AGAP5",TRUE,"AGAP9"

"4953","CATCTGTTTGAGAGTTCCTC","chr10",45846686,"+","none","TGG","CATCTGTTTGAGAGTTCCTCTGG",0,NA,NA,NA,"GeneID:119016","AGAP4",TRUE,"AGAP9"

"4954","CATCTGTTTGAGAGTTCCTC","chr10",48030937,"+","none","TGG","CATCTGTTTGAGAGTTCCTCTGG",0,NA,NA,NA,"GeneID:414224","CTGLF12P",TRUE,"AGAP9"

"4955","CATCTGTTTGAGAGTTCCTC","chr10",47522865,"+","none","TGG","CATCTGTTTGAGAGTTCCTCTGG",0,NA,NA,NA,"GeneID:642517","AGAP9",TRUE,"AGAP9"

"4956","CATCTGTTTGAGAGTTCCTC","chr10",47520498,"+","none","TGG","CATCTGTTTGAGAGTTCCTCTGG",0,NA,NA,NA,"GeneID:642517","AGAP9",TRUE,"AGAP9"

"4957","CATCTGTTTGAGAGTTCCTC","chr10",46340236,"-","none","TGG","CATCTGTTTGAGAGTTCCTCTGG",0,NA,NA,NA,"","",TRUE,"AGAP9"

"4958","CATCTGTTTGAGAGTTCCTC","chr10",49991724,"-","none","TGG","CATCTGTTTGAGAGTTCCTCTGG",0,NA,NA,NA,"GeneID:414189","AGAP6",TRUE,"AGAP9"

"4959","CATCTGTTTGAGAGTTCCTC","chr10",45681820,"-","none","TGG","CATCTGTTTGAGAGTTCCTCTGG",0,NA,NA,NA,"","",TRUE,"AGAP9"

"4960","CATCTGTTTGAGAGTTCCTC","chr10",46112628,"-","none","TGG","CATCTGTTTGAGAGTTCCTCTGG",0,NA,NA,NA,"","",TRUE,"AGAP9"

"4961","CATCTGTTTGAGAGTTCCTC","chr10",46819516,"-","none","TGG","CATCTGTTTGAGAGTTCCTCTGG",0,NA,NA,NA,"","",TRUE,"AGAP9"

"4962","CATCTGTTTGAGAGTTCCTC","chr10",46817155,"-","none","TGG","CATCTGTTTGAGAGTTCCTCTGG",0,NA,NA,NA,"","",FALSE,"AGAP9"

"4963","CATCTGTTTGAGAGTTCCTC","chr10",45679458,"-","none","TGG","CATCTGTTTGAGAGTTCCTCTGG",0,NA,NA,NA,"","",FALSE,"AGAP9"

"4964","CATCTGTTTGAGAGTTCCTC","chr10",38982306,"-","14:G>A,17:A>G","AGG","CATCTGTTTGAGAGCTCTTCAGG",2,15,18,NA,"","",FALSE,"AGAP9"

"4965","CATCTGTTTGAGAGTTCCTC","chr10",42451608,"+","13:T>G,16:T>C","AGG","CATCTGTTTGAGATTTTCTCAGG",2,14,17,NA,"GeneID:414194","CCNYL2",TRUE,"AGAP9"

"4966","CATCTGTTTGAGAGTTCCTC","chr10",49989356,"-","1:A>T","TGG","CTTCTGTTTGAGAGTTCCTCTGG",1,2,NA,NA,"GeneID:414189","AGAP6",TRUE,"AGAP9"

"4967","CATCTGTTTGAGAGTTCCTC","chr4",116173792,"+","0:T>C,3:T>C","TGG","TATTTGTTTGAGAGTTCCTCTGG",2,1,4,NA,"","",FALSE,"AGAP9"

"4968","TAACCTTTCTGCCAATCCAG","chr10",46110222,"+","none","AGG","TAACCTTTCTGCCAATCCAGAGG",0,NA,NA,NA,"","",TRUE,"AGAP9"

"4969","TAACCTTTCTGCCAATCCAG","chr10",46337825,"+","none","AGG","TAACCTTTCTGCCAATCCAGAGG",0,NA,NA,NA,"","",TRUE,"AGAP9"

"4970","TAACCTTTCTGCCAATCCAG","chr10",46817116,"+","none","AGG","TAACCTTTCTGCCAATCCAGAGG",0,NA,NA,NA,"","",FALSE,"AGAP9"

"4971","TAACCTTTCTGCCAATCCAG","chr10",45679419,"+","none","AGG","TAACCTTTCTGCCAATCCAGAGG",0,NA,NA,NA,"","",FALSE,"AGAP9"

"4972","TAACCTTTCTGCCAATCCAG","chr10",48030976,"-","none","AGG","TAACCTTTCTGCCAATCCAGAGG",0,NA,NA,NA,"GeneID:414224","CTGLF12P",TRUE,"AGAP9"

"4973","TAACCTTTCTGCCAATCCAG","chr10",47522904,"-","none","AGG","TAACCTTTCTGCCAATCCAGAGG",0,NA,NA,NA,"GeneID:642517","AGAP9",TRUE,"AGAP9"

"4974","TAACCTTTCTGCCAATCCAG","chr10",45846725,"-","none","AGG","TAACCTTTCTGCCAATCCAGAGG",0,NA,NA,NA,"GeneID:119016","AGAP4",TRUE,"AGAP9"

"4975","TAACCTTTCTGCCAATCCAG","chr10",73697133,"-","2:C>T","AGG","TAGCCTTTCTGCCAATCCAGAGG",1,3,NA,NA,"GeneID:729092","AGAP5",TRUE,"AGAP9"

"4976","TAACCTTTCTGCCAATCCAG","chr10",45844364,"-","5:G>A","AGG","TAACCCTTCTGCCAATCCAGAGG",1,6,NA,NA,"GeneID:119016","AGAP4",TRUE,"AGAP9"

"4977","TAACCTTTCTGCCAATCCAG","chr10",48028610,"-","5:G>A","AGG","TAACCCTTCTGCCAATCCAGAGG",1,6,NA,NA,"GeneID:414224","CTGLF12P",TRUE,"AGAP9"

"4978","TAACCTTTCTGCCAATCCAG","chr10",73694774,"-","5:G>A","AGG","TAACCCTTCTGCCAATCCAGAGG",1,6,NA,NA,"GeneID:729092","AGAP5",TRUE,"AGAP9"

"4979","TAACCTTTCTGCCAATCCAG","chr10",47520537,"-","4:A>G,5:G>A","AGG","TAACTCTTCTGCCAATCCAGAGG",2,5,6,NA,"GeneID:642517","AGAP9",TRUE,"AGAP9"

"4980","TAACCTTTCTGCCAATCCAG","chr10",46340197,"+","5:C>T","AGG","TAACCCTTCTGCCAATCCAGAGG",1,6,NA,NA,"","",TRUE,"AGAP9"

"4981","TAACCTTTCTGCCAATCCAG","chr10",49991685,"+","5:C>T","AGG","TAACCCTTCTGCCAATCCAGAGG",1,6,NA,NA,"GeneID:414189","AGAP6",TRUE,"AGAP9"

"4982","TAACCTTTCTGCCAATCCAG","chr10",45681781,"+","5:C>T","AGG","TAACCCTTCTGCCAATCCAGAGG",1,6,NA,NA,"","",TRUE,"AGAP9"

"4983","TAACCTTTCTGCCAATCCAG","chr10",46112589,"+","5:C>T","AGG","TAACCCTTCTGCCAATCCAGAGG",1,6,NA,NA,"","",TRUE,"AGAP9"

"4984","TAACCTTTCTGCCAATCCAG","chr10",46819477,"+","5:C>T","AGG","TAACCCTTCTGCCAATCCAGAGG",1,6,NA,NA,"","",TRUE,"AGAP9"

"4985","TAACTCTTCTGCCAATCCAG","chr10",47520537,"-","none","AGG","TAACTCTTCTGCCAATCCAGAGG",0,NA,NA,NA,"GeneID:642517","AGAP9",TRUE,"AGAP9"

"4986","TAACTCTTCTGCCAATCCAG","chr10",73694774,"-","4:G>A","AGG","TAACCCTTCTGCCAATCCAGAGG",1,5,NA,NA,"GeneID:729092","AGAP5",TRUE,"AGAP9"

"4987","TAACTCTTCTGCCAATCCAG","chr10",45844364,"-","4:G>A","AGG","TAACCCTTCTGCCAATCCAGAGG",1,5,NA,NA,"GeneID:119016","AGAP4",TRUE,"AGAP9"

"4988","TAACTCTTCTGCCAATCCAG","chr10",48028610,"-","4:G>A","AGG","TAACCCTTCTGCCAATCCAGAGG",1,5,NA,NA,"GeneID:414224","CTGLF12P",TRUE,"AGAP9"

"4989","TAACTCTTCTGCCAATCCAG","chr10",47522904,"-","4:G>A,5:A>G","AGG","TAACCTTTCTGCCAATCCAGAGG",2,5,6,NA,"GeneID:642517","AGAP9",TRUE,"AGAP9"

"4990","TAACTCTTCTGCCAATCCAG","chr10",45846725,"-","4:G>A,5:A>G","AGG","TAACCTTTCTGCCAATCCAGAGG",2,5,6,NA,"GeneID:119016","AGAP4",TRUE,"AGAP9"

"4991","TAACTCTTCTGCCAATCCAG","chr10",48030976,"-","4:G>A,5:A>G","AGG","TAACCTTTCTGCCAATCCAGAGG",2,5,6,NA,"GeneID:414224","CTGLF12P",TRUE,"AGAP9"

"4992","TAACTCTTCTGCCAATCCAG","chr10",45681781,"+","4:C>T","AGG","TAACCCTTCTGCCAATCCAGAGG",1,5,NA,NA,"","",TRUE,"AGAP9"

"4993","TAACTCTTCTGCCAATCCAG","chr10",46112589,"+","4:C>T","AGG","TAACCCTTCTGCCAATCCAGAGG",1,5,NA,NA,"","",TRUE,"AGAP9"

"4994","TAACTCTTCTGCCAATCCAG","chr10",46819477,"+","4:C>T","AGG","TAACCCTTCTGCCAATCCAGAGG",1,5,NA,NA,"","",TRUE,"AGAP9"

"4995","TAACTCTTCTGCCAATCCAG","chr10",46340197,"+","4:C>T","AGG","TAACCCTTCTGCCAATCCAGAGG",1,5,NA,NA,"","",TRUE,"AGAP9"

"4996","TAACTCTTCTGCCAATCCAG","chr10",49991685,"+","4:C>T","AGG","TAACCCTTCTGCCAATCCAGAGG",1,5,NA,NA,"GeneID:414189","AGAP6",TRUE,"AGAP9"

"4997","TAACTCTTCTGCCAATCCAG","chr10",46337825,"+","4:C>T,5:T>C","AGG","TAACCTTTCTGCCAATCCAGAGG",2,5,6,NA,"","",TRUE,"AGAP9"

"4998","TAACTCTTCTGCCAATCCAG","chr10",46817116,"+","4:C>T,5:T>C","AGG","TAACCTTTCTGCCAATCCAGAGG",2,5,6,NA,"","",FALSE,"AGAP9"

"4999","TAACTCTTCTGCCAATCCAG","chr10",45679419,"+","4:C>T,5:T>C","AGG","TAACCTTTCTGCCAATCCAGAGG",2,5,6,NA,"","",FALSE,"AGAP9"

"5000","TAACTCTTCTGCCAATCCAG","chr10",46110222,"+","4:C>T,5:T>C","AGG","TAACCTTTCTGCCAATCCAGAGG",2,5,6,NA,"","",TRUE,"AGAP9"

"5001","TATTGTGCTTGCCTCTGGAT","chr10",47522890,"+","none","TGG","TATTGTGCTTGCCTCTGGATTGG",0,NA,NA,NA,"GeneID:642517","AGAP9",TRUE,"AGAP9"

"5002","TATTGTGCTTGCCTCTGGAT","chr10",47520523,"+","none","TGG","TATTGTGCTTGCCTCTGGATTGG",0,NA,NA,NA,"GeneID:642517","AGAP9",TRUE,"AGAP9"

"5003","TATTGTGCTTGCCTCTGGAT","chr10",73694760,"+","none","TGG","TATTGTGCTTGCCTCTGGATTGG",0,NA,NA,NA,"GeneID:729092","AGAP5",TRUE,"AGAP9"

"5004","TATTGTGCTTGCCTCTGGAT","chr10",45844350,"+","none","TGG","TATTGTGCTTGCCTCTGGATTGG",0,NA,NA,NA,"GeneID:119016","AGAP4",TRUE,"AGAP9"

"5005","TATTGTGCTTGCCTCTGGAT","chr10",48028596,"+","none","TGG","TATTGTGCTTGCCTCTGGATTGG",0,NA,NA,NA,"GeneID:414224","CTGLF12P",TRUE,"AGAP9"

"5006","TATTGTGCTTGCCTCTGGAT","chr10",73697119,"+","none","TGG","TATTGTGCTTGCCTCTGGATTGG",0,NA,NA,NA,"GeneID:729092","AGAP5",TRUE,"AGAP9"

"5007","TATTGTGCTTGCCTCTGGAT","chr10",45846711,"+","none","TGG","TATTGTGCTTGCCTCTGGATTGG",0,NA,NA,NA,"GeneID:119016","AGAP4",TRUE,"AGAP9"

"5008","TATTGTGCTTGCCTCTGGAT","chr10",48030962,"+","none","TGG","TATTGTGCTTGCCTCTGGATTGG",0,NA,NA,NA,"GeneID:414224","CTGLF12P",TRUE,"AGAP9"

"5009","TATTGTGCTTGCCTCTGGAT","chr10",45679433,"-","none","TGG","TATTGTGCTTGCCTCTGGATTGG",0,NA,NA,NA,"","",FALSE,"AGAP9"

"5010","TATTGTGCTTGCCTCTGGAT","chr10",46340211,"-","none","TGG","TATTGTGCTTGCCTCTGGATTGG",0,NA,NA,NA,"","",TRUE,"AGAP9"

"5011","TATTGTGCTTGCCTCTGGAT","chr10",49991699,"-","none","TGG","TATTGTGCTTGCCTCTGGATTGG",0,NA,NA,NA,"GeneID:414189","AGAP6",TRUE,"AGAP9"

"5012","TATTGTGCTTGCCTCTGGAT","chr10",45681795,"-","none","TGG","TATTGTGCTTGCCTCTGGATTGG",0,NA,NA,NA,"","",TRUE,"AGAP9"

"5013","TATTGTGCTTGCCTCTGGAT","chr10",46112603,"-","none","TGG","TATTGTGCTTGCCTCTGGATTGG",0,NA,NA,NA,"","",TRUE,"AGAP9"

"5014","TATTGTGCTTGCCTCTGGAT","chr10",46819491,"-","none","TGG","TATTGTGCTTGCCTCTGGATTGG",0,NA,NA,NA,"","",TRUE,"AGAP9"

"5015","TATTGTGCTTGCCTCTGGAT","chr10",46337839,"-","none","TGG","TATTGTGCTTGCCTCTGGATTGG",0,NA,NA,NA,"","",TRUE,"AGAP9"

"5016","TATTGTGCTTGCCTCTGGAT","chr10",46817130,"-","none","TGG","TATTGTGCTTGCCTCTGGATTGG",0,NA,NA,NA,"","",FALSE,"AGAP9"

"5017","TATTGTGCTTGCCTCTGGAT","chr10",49989331,"-","11:T>G","TGG","TATTGTGCTTGACTCTGGATTGG",1,12,NA,NA,"GeneID:414189","AGAP6",TRUE,"AGAP9"

"5018","TATTGTGCTTGCCTCTGGAT","chr5",156631600,"+","0:A>T,2:A>T","TGG","AAATGTGCTTGCCTCTGGATTGG",2,1,3,NA,"GeneID:6444","SGCD",FALSE,"AGAP9"

"5019","TATTGTGCTTGCCTCTGGAT","chr10",38982281,"-","3:T>A,18:A>T","GGG","TATAGTGCTTGCCTCTGGTTGGG",2,4,19,NA,"","",FALSE,"AGAP9"

"5020","TTGTCATTGTAAGATAATGC","chr10",45834022,"+","none","TGG","TTGTCATTGTAAGATAATGCTGG",0,NA,NA,NA,"GeneID:119016","AGAP4",TRUE,"AGAP9"

"5021","TTGTCATTGTAAGATAATGC","chr10",47510177,"+","none","TGG","TTGTCATTGTAAGATAATGCTGG",0,NA,NA,NA,"GeneID:642517","AGAP9",TRUE,"AGAP9"

"5022","TTGTCATTGTAAGATAATGC","chr10",48018274,"+","none","TGG","TTGTCATTGTAAGATAATGCTGG",0,NA,NA,NA,"GeneID:414224","CTGLF12P",TRUE,"AGAP9"

"5023","TTGTCATTGTAAGATAATGC","chr10",73682700,"+","none","TGG","TTGTCATTGTAAGATAATGCTGG",0,NA,NA,NA,"GeneID:729092","AGAP5",TRUE,"AGAP9"

"5024","TTGTCATTGTAAGATAATGC","chr10",50002069,"-","none","TGG","TTGTCATTGTAAGATAATGCTGG",0,NA,NA,NA,"GeneID:414189","AGAP6",TRUE,"AGAP9"

"5025","TTGTCATTGTAAGATAATGC","chr10",46829827,"-","none","TGG","TTGTCATTGTAAGATAATGCTGG",0,NA,NA,NA,"","",FALSE,"AGAP9"

"5026","TTGTCATTGTAAGATAATGC","chr10",46123243,"-","none","TGG","TTGTCATTGTAAGATAATGCTGG",0,NA,NA,NA,"","",TRUE,"AGAP9"

"5027","TTGTCATTGTAAGATAATGC","chr10",46350591,"-","none","TGG","TTGTCATTGTAAGATAATGCTGG",0,NA,NA,NA,"","",TRUE,"AGAP9"

"5028","TTGTCATTGTAAGATAATGC","chr10",87001709,"-","8:T>C","TGG","TTGTCATTATAAGATAATGCTGG",1,9,NA,NA,"","",TRUE,"AGAP9"

"5029","ACGCCTACGTGCAGATCCGA","chr15",86247757,"+","none","CGG","ACGCCTACGTGCAGATCCGACGG",0,NA,NA,NA,"GeneID:123624","AGBL1",TRUE,"AGBL1"

"5030","ACGCCTACGTGCAGATCCGA","chr14",81313404,"+","2:T>G,16:A>C","TGG","ACTCCTACGTGCAGATACGATGG",2,3,17,NA,"GeneID:85439","STON2",FALSE,"AGBL1"

"5031","ACTTGATGTGACATTGATTC","chr15",86154466,"+","none","TGG","ACTTGATGTGACATTGATTCTGG",0,NA,NA,NA,"GeneID:123624","AGBL1",TRUE,"AGBL1"

"5032","CGTAGGCGTTGGCTGTGTCA","chr15",86247746,"-","none","TGG","CGTAGGCGTTGGCTGTGTCATGG",0,NA,NA,NA,"GeneID:123624","AGBL1",TRUE,"AGBL1"

"5033","CTGCGTGTGTTTGCCTCCAG","chr15",86154539,"+","none","TGG","CTGCGTGTGTTTGCCTCCAGTGG",0,NA,NA,NA,"GeneID:123624","AGBL1",TRUE,"AGBL1"

"5034","CTGCGTGTGTTTGCCTCCAG","chr17",65345424,"-","14:A>G,18:A>T","TGG","CTGCGTGTGTTTGCTTCCTGTGG",2,15,19,NA,"","",FALSE,"AGBL1"

"5035","GAAAGCGCACCCAAGCAATC","chr15",86159004,"+","none","AGG","GAAAGCGCACCCAAGCAATCAGG",0,NA,NA,NA,"GeneID:123624","AGBL1",TRUE,"AGBL1"

"5036","GAAAGCGCACCCAAGCAATC","chr5",157957652,"+","6:C>G,10:T>C","TGG","GAAAGCCCACTCAAGCAATCTGG",2,7,11,NA,"","",FALSE,"AGBL1"

"5037","GCACTCTGTACCTGATTGCT","chr15",86159017,"-","none","TGG","GCACTCTGTACCTGATTGCTTGG",0,NA,NA,NA,"GeneID:123624","AGBL1",TRUE,"AGBL1"

"5038","AACCCTCAATGCCTGTTGAA","chr11",47710438,"-","none","TGG","AACCCTCAATGCCTGTTGAATGG",0,NA,NA,NA,"GeneID:79841","AGBL2",TRUE,"AGBL2"

"5039","AACCCTCAATGCCTGTTGAA","chr8",1116417,"-","14:G>C,18:A>T","AGG","AACCCTCAATGCCTCTTGTAAGG",2,15,19,NA,"GeneID:9228","DLGAP2",FALSE,"AGBL2"

"5040","AAGTAACATGTTCCCAGCTT","chr11",47714637,"-","none","TGG","AAGTAACATGTTCCCAGCTTTGG",0,NA,NA,NA,"GeneID:79841","AGBL2",TRUE,"AGBL2"

"5041","AAGTAACATGTTCCCAGCTT","chr7",49390870,"+","9:C>G,17:A>C","CGG","AAGTAACATCTTCCCAGATTCGG",2,10,18,NA,"","",FALSE,"AGBL2"

"5042","CCCCAAGAGAGCCATTCAAC","chr11",47710424,"+","none","AGG","CCCCAAGAGAGCCATTCAACAGG",0,NA,NA,NA,"GeneID:79841","AGBL2",TRUE,"AGBL2"

"5043","CGGTACATAAAGTCTTCATA","chr11",47714312,"+","none","AGG","CGGTACATAAAGTCTTCATAAGG",0,NA,NA,NA,"GeneID:79841","AGBL2",TRUE,"AGBL2"

"5044","CGGTACATAAAGTCTTCATA","chr10",5705921,"-","0:C>G,13:A>G","GGG","GGGTACATAAAGTTTTCATAGGG",2,1,14,NA,"GeneID:54906","FAM208B",FALSE,"AGBL2"

"5045","TACCGTCACCTCCAATATTA","chr11",47714297,"-","none","TGG","TACCGTCACCTCCAATATTATGG",0,NA,NA,NA,"GeneID:79841","AGBL2",TRUE,"AGBL2"

"5046","TTTAGGTGCGTTTCCAAAGC","chr11",47714621,"+","none","TGG","TTTAGGTGCGTTTCCAAAGCTGG",0,NA,NA,NA,"GeneID:79841","AGBL2",TRUE,"AGBL2"

"5047","CTAATAGTATCTGTGTAGTC","chr7",134993523,"-","none","CGG","CTAATAGTATCTGTGTAGTCCGG",0,NA,NA,NA,"GeneID:340351","AGBL3",TRUE,"AGBL3"

"5048","GGCCACCGTGTTGGGCTCAA","chr7",134993617,"-","none","TGG","GGCCACCGTGTTGGGCTCAATGG",0,NA,NA,NA,"GeneID:340351","AGBL3",TRUE,"AGBL3"

"5049","GGTTCCGTTTCTAAGCCCGT","chr7",135017089,"-","none","TGG","GGTTCCGTTTCTAAGCCCGTTGG",0,NA,NA,NA,"GeneID:340351","AGBL3",TRUE,"AGBL3"

"5050","GGTTCCGTTTCTAAGCCCGT","chr1",37665629,"-","1:A>C,5:T>G","TGG","GTTTCAGTTTCTAAGCCCGTTGG",2,2,6,NA,"","",FALSE,"AGBL3"

"5051","TACACTGGCTCAGGACAAGA","chr7",135017062,"-","none","AGG","TACACTGGCTCAGGACAAGAAGG",0,NA,NA,NA,"GeneID:340351","AGBL3",TRUE,"AGBL3"

"5052","TGAGCCAGTGTATATCCCAA","chr7",135017070,"+","none","CGG","TGAGCCAGTGTATATCCCAACGG",0,NA,NA,NA,"GeneID:340351","AGBL3",TRUE,"AGBL3"

"5053","TTGTAAGTGAAGATCTTCAT","chr7",134989271,"+","none","CGG","TTGTAAGTGAAGATCTTCATCGG",0,NA,NA,NA,"GeneID:340351","AGBL3",TRUE,"AGBL3"

"5054","ACCAAGAGTCTCTATAGAGA","chr1",49245811,"-","none","TGG","ACCAAGAGTCTCTATAGAGATGG",0,NA,NA,NA,"GeneID:84871","AGBL4",TRUE,"AGBL4"

"5055","ACCAAGAGTCTCTATAGAGA","chr5",1859992,"-","13:C>T,15:C>T","AGG","ACCAAGAGTCTCTGTGGAGAAGG",2,14,16,NA,"","",FALSE,"AGBL4"

"5056","CCCATCTCTATAGAGACTCT","chr1",49245807,"+","none","TGG","CCCATCTCTATAGAGACTCTTGG",0,NA,NA,NA,"GeneID:84871","AGBL4",TRUE,"AGBL4"

"5057","GTAATCCACGCTTCCGAGTC","chr1",49697351,"-","none","TGG","GTAATCCACGCTTCCGAGTCTGG",0,NA,NA,NA,"GeneID:84871","AGBL4",TRUE,"AGBL4"

"5058","TATTACCAGGTAACCTGGGC","chr1",49697426,"-","none","CGG","TATTACCAGGTAACCTGGGCCGG",0,NA,NA,NA,"GeneID:84871","AGBL4",TRUE,"AGBL4"

"5059","TGTTTACTACTACCGCTGCC","chr1",49045759,"-","none","CGG","TGTTTACTACTACCGCTGCCCGG",0,NA,NA,NA,"GeneID:84871","AGBL4",TRUE,"AGBL4"

"5060","TTACTTCTTTCGGGAGCAGC","chr1",49045597,"-","none","TGG","TTACTTCTTTCGGGAGCAGCTGG",0,NA,NA,NA,"GeneID:84871","AGBL4",TRUE,"AGBL4"

"5061","ATCCTTTGTTCATCGTTTCG","chr2",27053918,"+","none","TGG","ATCCTTTGTTCATCGTTTCGTGG",0,NA,NA,NA,"GeneID:60509","AGBL5",TRUE,"AGBL5"

"5062","CCTCGACCATTCCGTTTCGC","chr2",27054777,"+","none","AGG","CCTCGACCATTCCGTTTCGCAGG",0,NA,NA,NA,"GeneID:60509","AGBL5",TRUE,"AGBL5"

"5063","CTCCACGAAACGATGAACAA","chr2",27053923,"-","none","AGG","CTCCACGAAACGATGAACAAAGG",0,NA,NA,NA,"GeneID:60509","AGBL5",TRUE,"AGBL5"

"5064","GCTGGGAACGCATTCGAGAC","chr2",27053538,"+","none","CGG","GCTGGGAACGCATTCGAGACCGG",0,NA,NA,NA,"GeneID:60509","AGBL5",TRUE,"AGBL5"

"5065","TCACCCTCTTGCCTGCGAAA","chr2",27054791,"-","none","CGG","TCACCCTCTTGCCTGCGAAACGG",0,NA,NA,NA,"GeneID:60509","AGBL5",TRUE,"AGBL5"

"5066","TCTCGAATGCGTTCCCAGCG","chr2",27053537,"-","none","TGG","TCTCGAATGCGTTCCCAGCGTGG",0,NA,NA,NA,"GeneID:60509","AGBL5",TRUE,"AGBL5"

"5067","ATGGCTGCCGGAACAGCAGT","chr6",32184202,"-","none","TGG","ATGGCTGCCGGAACAGCAGTTGG",0,NA,NA,NA,"GeneID:177","AGER",TRUE,"AGER"

"5068","CAAAACATCACAGCCCGGAT","chr6",32183950,"-","none","TGG","CAAAACATCACAGCCCGGATTGG",0,NA,NA,NA,"GeneID:177","AGER",TRUE,"AGER"

"5069","GTGGCTCACCCCACAGACTG","chr6",32184161,"+","none","AGG","GTGGCTCACCCCACAGACTGAGG",0,NA,NA,NA,"GeneID:177","AGER",TRUE,"AGER"

"5070","TATCTCCCAGGGGCAGTAGT","chr6",32183977,"-","none","AGG","TATCTCCCAGGGGCAGTAGTAGG",0,NA,NA,NA,"GeneID:177","AGER",TRUE,"AGER"

"5071","TATCTCCCAGGGGCAGTAGT","chr13",97679261,"+","5:T>C,12:C>G","TGG","TATCTTCCAGGGCCAGTAGTTGG",2,6,13,NA,"","",FALSE,"AGER"

"5072","TCCCGACAGCCGGAAGGAAG","chr6",32183638,"+","none","AGG","TCCCGACAGCCGGAAGGAAGAGG",0,NA,NA,NA,"GeneID:177","AGER",TRUE,"AGER"

"5073","TCTTACGGTAGACACGGACT","chr6",32183548,"+","none","CGG","TCTTACGGTAGACACGGACTCGG",0,NA,NA,NA,"GeneID:177","AGER",TRUE,"AGER"

"5074","CAAAGGTCTGTAAACAGATT","chr2",227519942,"+","none","TGG","CAAAGGTCTGTAAACAGATTTGG",0,NA,NA,NA,"GeneID:3267","AGFG1",TRUE,"AGFG1"

"5075","CAAAGGTCTGTAAACAGATT","chrY",20592709,"-","4:T>C,6:T>A","AGG","CAAAAGACTGTAAACAGATTAGG",2,5,7,NA,"GeneID:9086","EIF1AY",TRUE,"AGFG1"

"5076","CAAAGGTCTGTAAACAGATT","chr11",115444814,"+","8:A>T,13:G>A","AGG","CAAAGGTCAGTAAGCAGATTAGG",2,9,14,NA,"GeneID:23705","CADM1",FALSE,"AGFG1"

"5077","CATGACAACATTCACACAAC","chr2",227491585,"+","none","AGG","CATGACAACATTCACACAACAGG",0,NA,NA,NA,"GeneID:3267","AGFG1",TRUE,"AGFG1"

"5078","CATGACAACATTCACACAAC","chr16",80753054,"+","12:A>C,19:A>C","AGG","CATGACAACATTAACACAAAAGG",2,13,20,NA,"GeneID:124359","CDYL2",FALSE,"AGFG1"

"5079","CCCCACCTACGTTAACATGA","chr2",227472528,"+","none","CGG","CCCCACCTACGTTAACATGACGG",0,NA,NA,NA,"GeneID:3267","AGFG1",TRUE,"AGFG1"

"5080","CTTCAGCAATTCCAGACTTC","chr2",227519987,"+","none","AGG","CTTCAGCAATTCCAGACTTCAGG",0,NA,NA,NA,"GeneID:3267","AGFG1",TRUE,"AGFG1"

"5081","CTTCAGCAATTCCAGACTTC","chr2",169964650,"-","1:G>A,14:T>C","AGG","CCTCAGCAATTCCAAACTTCAGG",2,2,15,NA,"GeneID:130507","UBR3",FALSE,"AGFG1"

"5082","GAGGATTAAATCCACCACAC","chr2",227491548,"+","none","AGG","GAGGATTAAATCCACCACACAGG",0,NA,NA,NA,"GeneID:3267","AGFG1",TRUE,"AGFG1"

"5083","TGCTTCGACTGCGACCAGCG","chr2",227472505,"+","none","CGG","TGCTTCGACTGCGACCAGCGCGG",0,NA,NA,NA,"GeneID:3267","AGFG1",TRUE,"AGFG1"

"5084","TGCTTCGACTGCGACCAGCG","chr7",100539484,"+","8:G>C,13:C>A","CGG","TGCTTCGAGTGCGCCCAGCGCGG",2,9,14,NA,"GeneID:3268","AGFG2",TRUE,"AGFG1"

"5085","ACAGACCCAACCAAATCTTC","chr7",100550403,"-","none","CGG","ACAGACCCAACCAAATCTTCCGG",0,NA,NA,NA,"GeneID:3268","AGFG2",TRUE,"AGFG2"

"5086","ACAGACCCAACCAAATCTTC","chr9",28900487,"+","10:T>C,15:A>T","TGG","ACAGACCCAATCAAAACTTCTGG",2,11,16,NA,"","",FALSE,"AGFG2"

"5087","ATCACCCAGAAGTGTCCGAA","chr7",100553438,"-","none","GGG","ATCACCCAGAAGTGTCCGAAGGG",0,NA,NA,NA,"GeneID:3268","AGFG2",TRUE,"AGFG2"

"5088","GAGAGTTACCTGGCTCGAGG","chr7",100553489,"-","none","TGG","GAGAGTTACCTGGCTCGAGGTGG",0,NA,NA,NA,"GeneID:3268","AGFG2",TRUE,"AGFG2"

"5089","GAGATTGACTTGACACGATG","chr7",100548840,"-","none","AGG","GAGATTGACTTGACACGATGAGG",0,NA,NA,NA,"GeneID:3268","AGFG2",TRUE,"AGFG2"

"5090","GTAGTATTCCTGCAATCCCG","chr7",100548885,"+","none","TGG","GTAGTATTCCTGCAATCCCGTGG",0,NA,NA,NA,"GeneID:3268","AGFG2",TRUE,"AGFG2"

"5091","TGGAACGAGGCTTGACCAGC","chr7",100555695,"-","none","TGG","TGGAACGAGGCTTGACCAGCTGG",0,NA,NA,NA,"GeneID:3268","AGFG2",TRUE,"AGFG2"

"5092","TGGAACGAGGCTTGACCAGC","chr5",74232520,"-","5:A>G,12:C>A","TGG","TGGAATGAGGCTGGACCAGCTGG",2,6,13,NA,"","",FALSE,"AGFG2"

"5093","ACAGAAGATACCTCCTTAGA","chr5",77036576,"+","none","AGG","ACAGAAGATACCTCCTTAGAAGG",0,NA,NA,NA,"GeneID:55109","AGGF1",TRUE,"AGGF1"

"5094","AGATTCTTCTGCAACAAATG","chr5",77039691,"+","none","AGG","AGATTCTTCTGCAACAAATGAGG",0,NA,NA,NA,"GeneID:55109","AGGF1",TRUE,"AGGF1"

"5095","AGATTCTTCTGCAACAAATG","chr20",53059162,"+","4:C>T,16:G>A","AGG","AGATCCTTCTGCAACAGATGAGG",2,5,17,NA,"GeneID:128553","TSHZ2",FALSE,"AGGF1"

"5096","AGCTAATGATGAGCCTTCTA","chr5",77036592,"-","none","AGG","AGCTAATGATGAGCCTTCTAAGG",0,NA,NA,NA,"GeneID:55109","AGGF1",TRUE,"AGGF1"

"5097","AGCTAATGATGAGCCTTCTA","chr20",29431328,"-","12:T>C","AGG","AGCTAATGATGAACCTTCTAAGG",1,13,NA,NA,"","",TRUE,"AGGF1"

"5098","AGCTAATGATGAGCCTTCTA","chr3",75655058,"+","9:C>T,12:A>G","AGG","AGCTAATGACGAACCTTCTAAGG",2,10,13,NA,"","",FALSE,"AGGF1"

"5099","AGCTAATGATGAGCCTTCTA","chr10",133641342,"-","9:G>A,12:T>C","AGG","AGCTAATGACGAACCTTCTAAGG",2,10,13,NA,"","",TRUE,"AGGF1"

"5100","AGCTAATGATGAGCCTTCTA","chr4",190041853,"-","9:G>A,12:T>C","AGG","AGCTAATGACGAACCTTCTAAGG",2,10,13,NA,"","",TRUE,"AGGF1"

"5101","CTCTATTATGATCCTTCCAC","chr5",77039539,"+","none","TGG","CTCTATTATGATCCTTCCACTGG",0,NA,NA,NA,"GeneID:55109","AGGF1",TRUE,"AGGF1"

"5102","CTCTATTATGATCCTTCCAC","chr5",104791913,"-","1:C>A,12:C>G","TGG","CGCTATTATGATGCTTCCACTGG",2,2,13,NA,"","",FALSE,"AGGF1"

"5103","GACGAAGCCATTACCAGTGA","chr5",77046622,"+","none","AGG","GACGAAGCCATTACCAGTGAAGG",0,NA,NA,NA,"GeneID:55109","AGGF1",TRUE,"AGGF1"

"5104","TCTATAGTATTTCCACTAGT","chr5",77046484,"-","none","TGG","TCTATAGTATTTCCACTAGTTGG",0,NA,NA,NA,"GeneID:55109","AGGF1",TRUE,"AGGF1"

"5105","AACTACCCTTTGCAAGCTGC","chr7",141596627,"-","none","AGG","AACTACCCTTTGCAAGCTGCAGG",0,NA,NA,NA,"GeneID:55750","AGK",TRUE,"AGK"

"5106","ACGGATGTGATCATTGTTGC","chr7",141611242,"+","none","AGG","ACGGATGTGATCATTGTTGCAGG",0,NA,NA,NA,"GeneID:55750","AGK",TRUE,"AGK"

"5107","CAAGAAACTCCTGGAACTGA","chr7",141611214,"+","none","TGG","CAAGAAACTCCTGGAACTGATGG",0,NA,NA,NA,"GeneID:55750","AGK",TRUE,"AGK"

"5108","CAAGAAACTCCTGGAACTGA","chr1",209639830,"+","15:C>A,19:C>A","AGG","CAAGAAACTCCTGGACCTGCAGG",2,16,20,NA,"GeneID:3914","LAMB3",FALSE,"AGK"

"5109","CAAGAAACTCCTGGAACTGA","chr4",180236804,"+","4:G>A,7:T>C","GGG","CAAGGAATTCCTGGAACTGAGGG",2,5,8,NA,"","",FALSE,"AGK"

"5110","CAAGAAACTCCTGGAACTGA","chr2",5915182,"-","7:C>G,16:T>G","AGG","CAAGAAAGTCCTGGAAATGAAGG",2,8,17,NA,"","",FALSE,"AGK"

"5111","GGCCTTCTTCACTTGTGCAT","chr7",141596589,"-","none","TGG","GGCCTTCTTCACTTGTGCATTGG",0,NA,NA,NA,"GeneID:55750","AGK",TRUE,"AGK"

"5112","GGCCTTCTTCACTTGTGCAT","chrX",132131163,"+","0:T>G,1:T>G","TGG","TTCCTTCTTCACTTGTGCATTGG",2,1,2,NA,"","",TRUE,"AGK"

"5113","GGTATTTCAGACAGATTATG","chr7",141611184,"+","none","AGG","GGTATTTCAGACAGATTATGAGG",0,NA,NA,NA,"GeneID:55750","AGK",TRUE,"AGK"

"5114","TGTTCTTCGACGAACAGATG","chr7",141614156,"+","none","AGG","TGTTCTTCGACGAACAGATGAGG",0,NA,NA,NA,"GeneID:55750","AGK",TRUE,"AGK"

"5115","TGTTCTTCGACGAACAGATG","chrX",132130930,"-","0:T>A,10:A>G","AGG","AGTTCTTCGATGAACAGATGAGG",2,1,11,NA,"","",TRUE,"AGK"

"5116","AAGTGCTCTGTCTAAGACCC","chr1",99870477,"-","none","AGG","AAGTGCTCTGTCTAAGACCCAGG",0,NA,NA,NA,"GeneID:178","AGL",TRUE,"AGL"

"5117","AAGTGCTCTGTCTAAGACCC","chr17",17892464,"+","1:C>A,15:A>G","AGG","ACGTGCTCTGTCTAAAACCCAGG",2,2,16,NA,"GeneID:146691","TOM1L2",FALSE,"AGL"

"5118","ATCAGCACCAACACGTAAAA","chr1",99862297,"-","none","TGG","ATCAGCACCAACACGTAAAATGG",0,NA,NA,NA,"GeneID:178","AGL",TRUE,"AGL"

"5119","CTTCAGCTGCTAATAGTAAA","chr1",99870393,"+","none","TGG","CTTCAGCTGCTAATAGTAAATGG",0,NA,NA,NA,"GeneID:178","AGL",TRUE,"AGL"

"5120","CTTCAGCTGCTAATAGTAAA","chr4",18895137,"+","12:T>A,15:A>G","TGG","CTTCAGCTGCTATTAATAAATGG",2,13,16,NA,"","",FALSE,"AGL"

"5121","TAGAGAAAAATTCCGTTCTC","chr1",99861593,"+","none","TGG","TAGAGAAAAATTCCGTTCTCTGG",0,NA,NA,NA,"GeneID:178","AGL",TRUE,"AGL"

"5122","TAGAGAAAAATTCCGTTCTC","chr9",133650888,"+","9:C>A,12:T>C","TGG","TAGAGAAAACTTTCGTTCTCTGG",2,10,13,NA,"GeneID:1621","DBH",FALSE,"AGL"

"5123","TATGAGCTACAGTTCCGATT","chr1",99861504,"+","none","AGG","TATGAGCTACAGTTCCGATTAGG",0,NA,NA,NA,"GeneID:178","AGL",TRUE,"AGL"

"5124","TGATAATCATGTGCTACCCT","chr1",99862313,"+","none","TGG","TGATAATCATGTGCTACCCTTGG",0,NA,NA,NA,"GeneID:178","AGL",TRUE,"AGL"

"5125","CACCGGCCGGGCCACGAACT","chr1",15584805,"+","none","CGG","CACCGGCCGGGCCACGAACTCGG",0,NA,NA,NA,"GeneID:23341;GeneID:79814","DNAJC16;AGMAT",TRUE,"AGMAT"

"5126","CAGCTGCCGGCGAATTCAAG","chr1",15583240,"-","none","AGG","CAGCTGCCGGCGAATTCAAGAGG",0,NA,NA,NA,"GeneID:23341;GeneID:79814","DNAJC16;AGMAT",TRUE,"AGMAT"

"5127","CTACAGATACAACCGGAGCC","chr1",15578860,"-","none","AGG","CTACAGATACAACCGGAGCCAGG",0,NA,NA,NA,"GeneID:23341;GeneID:79814","DNAJC16;AGMAT",TRUE,"AGMAT"

"5128","CTGCACCGGCAGGCGCATCA","chr1",15584772,"+","none","TGG","CTGCACCGGCAGGCGCATCATGG",0,NA,NA,NA,"GeneID:23341;GeneID:79814","DNAJC16;AGMAT",TRUE,"AGMAT"

"5129","CTGGCTCCGGTTGTATCTGT","chr1",15578858,"+","none","AGG","CTGGCTCCGGTTGTATCTGTAGG",0,NA,NA,NA,"GeneID:23341;GeneID:79814","DNAJC16;AGMAT",TRUE,"AGMAT"

"5130","GATTCGGACCTCGCCGCATC","chr1",15583376,"-","none","CGG","GATTCGGACCTCGCCGCATCCGG",0,NA,NA,NA,"GeneID:23341;GeneID:79814","DNAJC16;AGMAT",TRUE,"AGMAT"

"5131","AAAACAACATGCGAAATCCC","chr7",15561784,"+","none","TGG","AAAACAACATGCGAAATCCCTGG",0,NA,NA,NA,"GeneID:392636","AGMO",TRUE,"AGMO"

"5132","CTTCAGAACTATGATGTGTT","chr7",15431060,"+","none","TGG","CTTCAGAACTATGATGTGTTTGG",0,NA,NA,NA,"GeneID:392636","AGMO",TRUE,"AGMO"

"5133","GCTTTAACGTCAATCTCAGC","chr7",15560164,"-","none","TGG","GCTTTAACGTCAATCTCAGCTGG",0,NA,NA,NA,"GeneID:392636","AGMO",TRUE,"AGMO"

"5134","TGTTTGGAATGAAGTTTCAC","chr7",15561749,"+","none","TGG","TGTTTGGAATGAAGTTTCACTGG",0,NA,NA,NA,"GeneID:392636","AGMO",TRUE,"AGMO"

"5135","TGTTTGGAATGAAGTTTCAC","chr12",13826454,"-","1:A>C,6:T>C","TGG","TTTTTGAAATGAAGTTTCACTGG",2,2,7,NA,"GeneID:2904","GRIN2B",FALSE,"AGMO"

"5136","TGTTTGGAATGAAGTTTCAC","chr21",42096478,"-","9:T>A,15:G>A","AGG","TGTTTGGAAAGAAGTCTCACAGG",2,10,16,NA,"GeneID:89766","UMODL1",FALSE,"AGMO"

"5137","TTACCCAGGAAGTATATATC","chr7",15431000,"+","none","TGG","TTACCCAGGAAGTATATATCTGG",0,NA,NA,NA,"GeneID:392636","AGMO",TRUE,"AGMO"

"5138","TTGACGTTAAAGCATCATCC","chr7",15560171,"+","none","AGG","TTGACGTTAAAGCATCATCCAGG",0,NA,NA,NA,"GeneID:392636","AGMO",TRUE,"AGMO"

"5139","CAGCATTCGCCAGCTCACAA","chr1",35893169,"-","none","TGG","CAGCATTCGCCAGCTCACAATGG",0,NA,NA,NA,"GeneID:26523","AGO1",TRUE,"AGO1"

"5140","CCTCCTGAAGGTCGACTTTG","chr1",35893086,"+","none","AGG","CCTCCTGAAGGTCGACTTTGAGG",0,NA,NA,NA,"GeneID:26523","AGO1",TRUE,"AGO1"

"5141","GAACGCGCTGAGAGTCCGTG","chr1",35894130,"-","none","AGG","GAACGCGCTGAGAGTCCGTGAGG",0,NA,NA,NA,"GeneID:26523","AGO1",TRUE,"AGO1"

"5142","GTTGAGCATCATCTTCCACA","chr1",35893783,"-","none","TGG","GTTGAGCATCATCTTCCACATGG",0,NA,NA,NA,"GeneID:26523","AGO1",TRUE,"AGO1"

"5143","GTTGAGCATCATCTTCCACA","chr1",35825788,"-","12:T>G","TGG","GTTGAGCATCATATTCCACATGG",1,13,NA,NA,"GeneID:192670","AGO4",TRUE,"AGO1"

"5144","TACTACCACCCGCTGGGGGG","chr1",35893716,"+","none","TGG","TACTACCACCCGCTGGGGGGTGG",0,NA,NA,NA,"GeneID:26523","AGO1",TRUE,"AGO1"

"5145","TACTACCACCCGCTGGGGGG","chr1",35825721,"+","11:T>G,17:A>G","GGG","TACTACCACCCTCTGGGAGGGGG",2,12,18,NA,"GeneID:192670","AGO4",TRUE,"AGO1"

"5146","TCAGCGCGTTCGCTTCACCA","chr1",35894139,"+","none","AGG","TCAGCGCGTTCGCTTCACCAAGG",0,NA,NA,NA,"GeneID:26523","AGO1",TRUE,"AGO1"

"5147","ACCCTTTGGGAATCTGTCAG","chr8",140559417,"+","none","AGG","ACCCTTTGGGAATCTGTCAGAGG",0,NA,NA,NA,"GeneID:27161","AGO2",TRUE,"AGO2"

"5148","ACCCTTTGGGAATCTGTCAG","chr18",71752604,"-","3:A>G,13:T>G","GGG","ACCTTTTGGGAATATGTCAGGGG",2,4,14,NA,"GeneID:102724913","LOC102724913",FALSE,"AGO2"

"5149","GCCACCATGTACTCGGGAGC","chr8",140635492,"-","none","CGG","GCCACCATGTACTCGGGAGCCGG",0,NA,NA,NA,"GeneID:27161","AGO2",TRUE,"AGO2"

"5150","GCGTTACACGATGCACTTTC","chr8",140562527,"-","none","AGG","GCGTTACACGATGCACTTTCAGG",0,NA,NA,NA,"GeneID:27161","AGO2",TRUE,"AGO2"

"5151","GGGGCCGGCTCCCGAGTACA","chr8",140635485,"+","none","TGG","GGGGCCGGCTCCCGAGTACATGG",0,NA,NA,NA,"GeneID:27161","AGO2",TRUE,"AGO2"

"5152","TAACGCCTGCAAGCTCACGC","chr8",140562541,"+","none","AGG","TAACGCCTGCAAGCTCACGCAGG",0,NA,NA,NA,"GeneID:27161","AGO2",TRUE,"AGO2"

"5153","TCACAAACAAACTCGATTAC","chr8",140559477,"+","none","TGG","TCACAAACAAACTCGATTACTGG",0,NA,NA,NA,"GeneID:27161","AGO2",TRUE,"AGO2"

"5154","AAGGCATTACTTCTAGTGGC","chr1",36009034,"-","none","AGG","AAGGCATTACTTCTAGTGGCAGG",0,NA,NA,NA,"GeneID:192669","AGO3",TRUE,"AGO3"

"5155","CTACTGTTCTCTCCACAGTT","chr1",36008923,"-","none","TGG","CTACTGTTCTCTCCACAGTTTGG",0,NA,NA,NA,"GeneID:192669","AGO3",TRUE,"AGO3"

"5156","GATTGATGTCTACCTCTATG","chr1",35945800,"+","none","AGG","GATTGATGTCTACCTCTATGAGG",0,NA,NA,NA,"GeneID:192669","AGO3",TRUE,"AGO3"

"5157","GATTGATGTCTACCTCTATG","chr8",58814254,"-","0:T>C,17:G>T","TGG","AATTGATGTCTACCTCTCTGTGG",2,1,18,NA,"GeneID:9760","TOX",FALSE,"AGO3"

"5158","TCAGACTTCCACTATGATCA","chr1",36009524,"+","none","AGG","TCAGACTTCCACTATGATCAAGG",0,NA,NA,NA,"GeneID:192669","AGO3",TRUE,"AGO3"

"5159","TCTTCTGGGCACCATGAGTA","chr1",35945712,"-","none","GGG","TCTTCTGGGCACCATGAGTAGGG",0,NA,NA,NA,"GeneID:192669","AGO3",TRUE,"AGO3"

"5160","TGCTGTTGCCTTGATCATAG","chr1",36009535,"-","none","TGG","TGCTGTTGCCTTGATCATAGTGG",0,NA,NA,NA,"GeneID:192669","AGO3",TRUE,"AGO3"

"5161","ACCGGGTCCCAGCGCCTCCA","chr1",35808417,"-","none","TGG","ACCGGGTCCCAGCGCCTCCATGG",0,NA,NA,NA,"GeneID:192670","AGO4",TRUE,"AGO4"

"5162","CCAGCACCAATGCTGCAATA","chr1",35832155,"+","none","TGG","CCAGCACCAATGCTGCAATATGG",0,NA,NA,NA,"GeneID:192670","AGO4",TRUE,"AGO4"

"5163","CCAGCACCAATGCTGCAATA","chr2",37209551,"-","12:C>G,14:A>C","GGG","CCAGCACCAATGGTTCAATAGGG",2,13,15,NA,"GeneID:100505876;GeneID:10153","CEBPZOS;CEBPZ",TRUE,"AGO4"

"5164","CGCCGCCGCCATGGAGGCGC","chr1",35808406,"+","none","TGG","CGCCGCCGCCATGGAGGCGCTGG",0,NA,NA,NA,"GeneID:192670","AGO4",TRUE,"AGO4"

"5165","CGCCGCCGCCATGGAGGCGC","chr7",139360251,"+","13:T>G,14:C>A","AGG","CGCCGCCGCCATGTCGGCGCAGG",2,14,15,NA,"GeneID:51631;GeneID:100996928","LUC7L2;C7orf55-LUC7L2",TRUE,"AGO4"

"5166","CGCCGCCGCCATGGAGGCGC","chr4",15003489,"+","2:G>C,11:C>T","GGG","CGGCGCCGCCACGGAGGCGCGGG",2,3,12,NA,"GeneID:132864","CPEB2",TRUE,"AGO4"

"5167","GTCTAGTCACATTACAAACT","chr1",35826796,"-","none","CGG","GTCTAGTCACATTACAAACTCGG",0,NA,NA,NA,"GeneID:192670","AGO4",TRUE,"AGO4"

"5168","GTTAACATACGTTTGATGAC","chr1",35826825,"-","none","TGG","GTTAACATACGTTTGATGACTGG",0,NA,NA,NA,"GeneID:192670","AGO4",TRUE,"AGO4"

"5169","TTAGGTGAAGAGCAACAGTA","chr1",35832052,"+","none","TGG","TTAGGTGAAGAGCAACAGTATGG",0,NA,NA,NA,"GeneID:192670","AGO4",TRUE,"AGO4"

"5170","ATCCCTGTGTGTGCCGTGCG","chr6",32171320,"-","none","AGG","ATCCCTGTGTGTGCCGTGCGAGG",0,NA,NA,NA,"GeneID:100532746;GeneID:10554","PPT2-EGFL8;AGPAT1",TRUE,"AGPAT1"

"5171","CAGGAGTCATCTTCATCGAC","chr6",32170490,"-","none","CGG","CAGGAGTCATCTTCATCGACCGG",0,NA,NA,NA,"GeneID:100532746;GeneID:10554","PPT2-EGFL8;AGPAT1",TRUE,"AGPAT1"

"5172","CGCCTTCCATCTTGCAGTGC","chr6",32170172,"-","none","AGG","CGCCTTCCATCTTGCAGTGCAGG",0,NA,NA,NA,"GeneID:100532746;GeneID:10554","PPT2-EGFL8;AGPAT1",TRUE,"AGPAT1"

"5173","CTCGACGTTGCGTCCTCGCA","chr6",32171304,"+","none","CGG","CTCGACGTTGCGTCCTCGCACGG",0,NA,NA,NA,"GeneID:100532746;GeneID:10554","PPT2-EGFL8;AGPAT1",TRUE,"AGPAT1"

"5174","TCCCGTACAGGTATTTGATG","chr6",32171026,"+","none","TGG","TCCCGTACAGGTATTTGATGTGG",0,NA,NA,NA,"GeneID:100532746;GeneID:10554","PPT2-EGFL8;AGPAT1",TRUE,"AGPAT1"

"5175","TTGCCAAGCGCGAGCTACTG","chr6",32170544,"-","none","TGG","TTGCCAAGCGCGAGCTACTGTGG",0,NA,NA,NA,"GeneID:100532746;GeneID:10554","PPT2-EGFL8;AGPAT1",TRUE,"AGPAT1"

"5176","ATTGTCGTTGCGAGTACCCT","chr9",136676638,"+","none","CGG","ATTGTCGTTGCGAGTACCCTCGG",0,NA,NA,NA,"GeneID:10555","AGPAT2",TRUE,"AGPAT2"

"5177","GAGGGTACTCGCAACGACAA","chr9",136676639,"-","none","TGG","GAGGGTACTCGCAACGACAATGG",0,NA,NA,NA,"GeneID:10555","AGPAT2",TRUE,"AGPAT2"

"5178","GCGATCTGCACGCAGCGCTC","chr9",136677096,"+","none","CGG","GCGATCTGCACGCAGCGCTCCGG",0,NA,NA,NA,"GeneID:10555","AGPAT2",TRUE,"AGPAT2"

"5179","GCTGCGTGCAGATCGCCAAG","chr9",136677092,"-","none","CGG","GCTGCGTGCAGATCGCCAAGCGG",0,NA,NA,NA,"GeneID:10555","AGPAT2",TRUE,"AGPAT2"

"5180","GCTGCGTGCAGATCGCCAAG","chr4",1020224,"-","12:T>A,14:T>C","AGG","GCTGCGTGCAGAACACCAAGAGG",2,13,15,NA,"GeneID:53834","FGFRL1",FALSE,"AGPAT2"

"5181","TCCCCTGCAGCATCATCGGC","chr9",136677546,"-","none","TGG","TCCCCTGCAGCATCATCGGCTGG",0,NA,NA,NA,"GeneID:10555","AGPAT2",TRUE,"AGPAT2"

"5182","TGGCAGGTGTCCACGAGCGC","chr9",136673842,"+","none","AGG","TGGCAGGTGTCCACGAGCGCAGG",0,NA,NA,NA,"GeneID:10555","AGPAT2",TRUE,"AGPAT2"

"5183","AGCGCGTCCCCTCGCAGTAC","chr21",43970660,"-","none","AGG","AGCGCGTCCCCTCGCAGTACAGG",0,NA,NA,NA,"GeneID:56894","AGPAT3",TRUE,"AGPAT3"

"5184","AGTACCACGTCCAGCCGATG","chr21",43969161,"-","none","AGG","AGTACCACGTCCAGCCGATGAGG",0,NA,NA,NA,"GeneID:56894","AGPAT3",TRUE,"AGPAT3"

"5185","CAGGCCACGGTAGAGCGCTT","chr21",43968001,"+","none","TGG","CAGGCCACGGTAGAGCGCTTTGG",0,NA,NA,NA,"GeneID:56894","AGPAT3",TRUE,"AGPAT3"

"5186","CTCCAAGGTCCTCGCTAAGA","chr21",43969119,"+","none","AGG","CTCCAAGGTCCTCGCTAAGAAGG",0,NA,NA,NA,"GeneID:56894","AGPAT3",TRUE,"AGPAT3"

"5187","CTTCCCAAAGCGCTCTACCG","chr21",43968008,"-","none","TGG","CTTCCCAAAGCGCTCTACCGTGG",0,NA,NA,NA,"GeneID:56894","AGPAT3",TRUE,"AGPAT3"

"5188","GACCAAGCACCGCGTTAGCA","chr21",43970687,"+","none","TGG","GACCAAGCACCGCGTTAGCATGG",0,NA,NA,NA,"GeneID:56894","AGPAT3",TRUE,"AGPAT3"

"5189","ATGGCGAAGCCCTTGGTTCG","chr6",161153371,"+","none","TGG","ATGGCGAAGCCCTTGGTTCGTGG",0,NA,NA,NA,"GeneID:56895","AGPAT4",TRUE,"AGPAT4"

"5190","CCGCGCGCCTACCTCAAGTA","chr6",161166341,"-","none","TGG","CCGCGCGCCTACCTCAAGTATGG",0,NA,NA,NA,"GeneID:56895","AGPAT4",TRUE,"AGPAT4"

"5191","GAAAGGGTAGAGCACCAGCG","chr6",161139497,"+","none","AGG","GAAAGGGTAGAGCACCAGCGAGG",0,NA,NA,NA,"GeneID:56895","AGPAT4",TRUE,"AGPAT4"

"5192","GGCCCTGGACCCTCGTGAAC","chr6",161139533,"-","none","TGG","GGCCCTGGACCCTCGTGAACTGG",0,NA,NA,NA,"GeneID:56895","AGPAT4",TRUE,"AGPAT4"

"5193","TCACCTGTTGCCACGAACCA","chr6",161153384,"-","none","AGG","TCACCTGTTGCCACGAACCAAGG",0,NA,NA,NA,"GeneID:56895","AGPAT4",TRUE,"AGPAT4"

"5194","TGGAGCCTGTCCGAACGCTT","chr6",161166260,"-","none","TGG","TGGAGCCTGTCCGAACGCTTTGG",0,NA,NA,NA,"GeneID:56895","AGPAT4",TRUE,"AGPAT4"

"5195","TGGAGCCTGTCCGAACGCTT","chr18",32061691,"+","8:T>G,16:C>G","GGG","TGGAGCCTTTCCGAACCCTTGGG",2,9,17,NA,"GeneID:54941","RNF125",FALSE,"AGPAT4"

"5196","AAAAGTCCTTTCAGCTAGTC","chr8",6741710,"+","none","AGG","AAAAGTCCTTTCAGCTAGTCAGG",0,NA,NA,NA,"GeneID:55326","AGPAT5",TRUE,"AGPAT5"

"5197","AAACAAGTTGCAGAGCTACG","chr8",6732613,"+","none","TGG","AAACAAGTTGCAGAGCTACGTGG",0,NA,NA,NA,"GeneID:55326","AGPAT5",TRUE,"AGPAT5"

"5198","CAAATGCCTGACTAGCTGAA","chr8",6741719,"-","none","AGG","CAAATGCCTGACTAGCTGAAAGG",0,NA,NA,NA,"GeneID:55326","AGPAT5",TRUE,"AGPAT5"

"5199","CAAATGCCTGACTAGCTGAA","chr5",177360040,"-","3:G>T,12:T>A","TGG","CAACTGCCTGACAAGCTGAATGG",2,4,13,NA,"GeneID:10636","RGS14",FALSE,"AGPAT5"

"5200","CATCTCTTTCTCGTTAAATT","chr8",6732591,"-","none","TGG","CATCTCTTTCTCGTTAAATTTGG",0,NA,NA,NA,"GeneID:55326","AGPAT5",TRUE,"AGPAT5"

"5201","GCAAAGTAACACCCATACAA","chr8",6730802,"-","none","TGG","GCAAAGTAACACCCATACAATGG",0,NA,NA,NA,"GeneID:55326","AGPAT5",TRUE,"AGPAT5"

"5202","GTGCGCTACGTGCTGAAAGA","chr8",6730763,"+","none","AGG","GTGCGCTACGTGCTGAAAGAAGG",0,NA,NA,NA,"GeneID:55326","AGPAT5",TRUE,"AGPAT5"

"5203","ATGTCAGAGAGCTCGAACTC","chr8",41609743,"-","none","TGG","ATGTCAGAGAGCTCGAACTCTGG",0,NA,NA,NA,"GeneID:137964","AGPAT6",TRUE,"AGPAT6"

"5204","ATGTCAGAGAGCTCGAACTC","chr14",25432082,"+","13:G>C","TGG","ATGTCAGAGAGCTGGAACTCTGG",1,14,NA,NA,"","",TRUE,"AGPAT6"

"5205","CCCCATCTTACCGTTGGTGT","chr8",41609476,"-","none","AGG","CCCCATCTTACCGTTGGTGTAGG",0,NA,NA,NA,"GeneID:137964","AGPAT6",TRUE,"AGPAT6"

"5206","GCGCTCTCACCTGTCATGGT","chr8",41611982,"-","none","AGG","GCGCTCTCACCTGTCATGGTAGG",0,NA,NA,NA,"GeneID:137964","AGPAT6",TRUE,"AGPAT6"

"5207","TCAGCGCTCGCACGCAGATC","chr8",41611947,"-","none","CGG","TCAGCGCTCGCACGCAGATCCGG",0,NA,NA,NA,"GeneID:137964","AGPAT6",TRUE,"AGPAT6"

"5208","TGAGCCGAAGGCTGATGTAC","chr8",41609880,"-","none","TGG","TGAGCCGAAGGCTGATGTACTGG",0,NA,NA,NA,"GeneID:137964","AGPAT6",TRUE,"AGPAT6"

"5209","TGAGCCGAAGGCTGATGTAC","chr4",83581757,"-","7:G>T,10:T>C","TGG","TGAGCCGCAGACTGATGTACTGG",2,8,11,NA,"GeneID:84803","AGPAT9",TRUE,"AGPAT6"

"5210","TGGGATACTTGCCAAATGGG","chr8",41610788,"+","none","AGG","TGGGATACTTGCCAAATGGGAGG",0,NA,NA,NA,"GeneID:137964","AGPAT6",TRUE,"AGPAT6"

"5211","TGGGATACTTGCCAAATGGG","chr2",184738648,"-","16:C>A,18:A>C","GGG","TGGGATACTTGCCAAAGGTGGGG",2,17,19,NA,"GeneID:91752","ZNF804A",FALSE,"AGPAT6"

"5212","ACCTTCAGGAAAAATTAGTA","chr4",83596895,"-","none","TGG","ACCTTCAGGAAAAATTAGTATGG",0,NA,NA,NA,"GeneID:84803","AGPAT9",TRUE,"AGPAT9"

"5213","ACTGATCCCAATGAAAGCCA","chr4",83587262,"-","none","AGG","ACTGATCCCAATGAAAGCCAAGG",0,NA,NA,NA,"GeneID:84803","AGPAT9",TRUE,"AGPAT9"

"5214","AGGGTTACCTTGGCTTTCAT","chr4",83587252,"+","none","TGG","AGGGTTACCTTGGCTTTCATTGG",0,NA,NA,NA,"GeneID:84803","AGPAT9",TRUE,"AGPAT9"

"5215","AGGGTTACCTTGGCTTTCAT","chr5",11266544,"+","10:G>T","GGG","AGGGTTACCTGGGCTTTCATGGG",1,11,NA,NA,"GeneID:1501","CTNND2",FALSE,"AGPAT9"

"5216","CAACACAAATGCCTCCCTTC","chr4",83590213,"-","none","TGG","CAACACAAATGCCTCCCTTCTGG",0,NA,NA,NA,"GeneID:84803","AGPAT9",TRUE,"AGPAT9"

"5217","CAACACAAATGCCTCCCTTC","chr1",208000421,"-","7:C>T,11:T>G","TGG","CAACACAGATGACTCCCTTCTGG",2,8,12,NA,"","",FALSE,"AGPAT9"

"5218","CAGGCAGTACAGACCCCAGA","chr4",83590195,"+","none","AGG","CAGGCAGTACAGACCCCAGAAGG",0,NA,NA,NA,"GeneID:84803","AGPAT9",TRUE,"AGPAT9"

"5219","CAGGCAGTACAGACCCCAGA","chrX",55997331,"-","3:A>C","GGG","CAGTCAGTACAGACCCCAGAGGG",1,4,NA,NA,"","",FALSE,"AGPAT9"

"5220","GCTGGTTATAGGAACTACAC","chr4",83587284,"+","none","TGG","GCTGGTTATAGGAACTACACTGG",0,NA,NA,NA,"GeneID:84803","AGPAT9",TRUE,"AGPAT9"

"5221","AGCAAGAGTCCTATACAAGT","chr15",78533376,"+","none","AGG","AGCAAGAGTCCTATACAAGTAGG",0,NA,NA,NA,"GeneID:123688","HYKK",TRUE,"AGPHD1"

"5222","ATCATGTTTCTGAAAGCCGC","chr15",78513340,"+","none","TGG","ATCATGTTTCTGAAAGCCGCTGG",0,NA,NA,NA,"GeneID:123688","HYKK",TRUE,"AGPHD1"

"5223","CACTGACTCCACTAACGCAG","chr15",78513155,"-","none","AGG","CACTGACTCCACTAACGCAGAGG",0,NA,NA,NA,"GeneID:123688","HYKK",TRUE,"AGPHD1"

"5224","CCCCAGCTATTGTATGAAAT","chr15",78515050,"+","none","TGG","CCCCAGCTATTGTATGAAATTGG",0,NA,NA,NA,"GeneID:123688","HYKK",TRUE,"AGPHD1"

"5225","CCCCAGCTATTGTATGAAAT","chr16",87101688,"+","17:G>A,18:C>A","AGG","CCCCAGCTATTGTATGAGCTAGG",2,18,19,NA,"GeneID:100506581","C16orf95",FALSE,"AGPHD1"

"5226","CGATGAAGACTACTTAACTT","chr15",78527394,"-","none","TGG","CGATGAAGACTACTTAACTTTGG",0,NA,NA,NA,"GeneID:123688","HYKK",TRUE,"AGPHD1"

"5227","CTACTTTCAGGTATCAATCA","chr15",78533199,"+","none","CGG","CTACTTTCAGGTATCAATCACGG",0,NA,NA,NA,"GeneID:123688","HYKK",TRUE,"AGPHD1"

"5228","AAATACCCTTGGAGTAAATG","chr2",177434374,"+","none","TGG","AAATACCCTTGGAGTAAATGTGG",0,NA,NA,NA,"GeneID:8540","AGPS",TRUE,"AGPS"

"5229","AAATACCCTTGGAGTAAATG","chr3",137492142,"+","15:G>A,16:G>A","AGG","AAATACCCTTGGAGTGGATGAGG",2,16,17,NA,"","",FALSE,"AGPS"

"5230","AGGTGTATCACTAGGATTTA","chr2",177436770,"-","none","AGG","AGGTGTATCACTAGGATTTAAGG",0,NA,NA,NA,"GeneID:8540","AGPS",TRUE,"AGPS"

"5231","CTAGGCAAGAAGTTATGAAA","chr2",177420264,"+","none","TGG","CTAGGCAAGAAGTTATGAAATGG",0,NA,NA,NA,"GeneID:8540","AGPS",TRUE,"AGPS"

"5232","CTAGGCAAGAAGTTATGAAA","chr16",53854361,"-","11:A>C,19:A>T","AGG","CTAGGCAAGAATTTATGAATAGG",2,12,20,NA,"GeneID:79068","FTO",FALSE,"AGPS"

"5233","CTTGGCCATAAAACTATATC","chr2",177437035,"-","none","AGG","CTTGGCCATAAAACTATATCAGG",0,NA,NA,NA,"GeneID:8540","AGPS",TRUE,"AGPS"

"5234","CTTGGCCATAAAACTATATC","chr8",107213505,"+","10:T>A,17:T>A","TGG","CTTGGCCATATAACTATTTCTGG",2,11,18,NA,"","",FALSE,"AGPS"

"5235","GATCGAGTATTTAGAGCTCA","chr2",177436862,"+","none","TGG","GATCGAGTATTTAGAGCTCATGG",0,NA,NA,NA,"GeneID:8540","AGPS",TRUE,"AGPS"

"5236","TACCTTAGGTACCCTCTTAG","chr2",177434318,"+","none","TGG","TACCTTAGGTACCCTCTTAGTGG",0,NA,NA,NA,"GeneID:8540","AGPS",TRUE,"AGPS"

"5237","ATGTCTGAGTCCAGATGAGT","chr7",16801350,"+","none","TGG","ATGTCTGAGTCCAGATGAGTTGG",0,NA,NA,NA,"GeneID:10551","AGR2",TRUE,"AGR2"

"5238","CAAGTGATGAATAATCATCA","chr7",16801172,"+","none","AGG","CAAGTGATGAATAATCATCAAGG",0,NA,NA,NA,"GeneID:10551","AGR2",TRUE,"AGR2"

"5239","CAAGTGATGAATAATCATCA","chr1",69415260,"-","17:C>A,18:T>G","AGG","CAAGTGATGAATAATCAGAAAGG",2,18,19,NA,"","",FALSE,"AGR2"

"5240","CTCTATATAAATCCAAGACA","chr7",16801321,"-","none","AGG","CTCTATATAAATCCAAGACAAGG",0,NA,NA,NA,"GeneID:10551","AGR2",TRUE,"AGR2"

"5241","CTCTATATAAATCCAAGACA","chr4",128555185,"-","11:G>A,17:G>T","TGG","CTCTATATAAACCCAAGCCATGG",2,12,18,NA,"","",FALSE,"AGR2"

"5242","CTCTATATAAATCCAAGACA","chrX",14195504,"+","4:G>A,15:G>A","TGG","CTCTGTATAAATCCAGGACATGG",2,5,16,NA,"","",FALSE,"AGR2"

"5243","CTTGATGATTATTCATCACT","chr7",16801174,"-","none","TGG","CTTGATGATTATTCATCACTTGG",0,NA,NA,NA,"GeneID:10551","AGR2",TRUE,"AGR2"

"5244","GACAAACACCTTTCTCCTGA","chr7",16797662,"-","none","TGG","GACAAACACCTTTCTCCTGATGG",0,NA,NA,NA,"GeneID:10551","AGR2",TRUE,"AGR2"

"5245","GGAAACTTACAACCAGATTG","chr7",16799733,"+","none","AGG","GGAAACTTACAACCAGATTGAGG",0,NA,NA,NA,"GeneID:10551","AGR2",TRUE,"AGR2"

"5246","GAGAGTATTGACAATCCTCC","chr7",16862612,"+","none","AGG","GAGAGTATTGACAATCCTCCAGG",0,NA,NA,NA,"GeneID:155465","AGR3",TRUE,"AGR3"

"5247","GAGAGTATTGACAATCCTCC","chr1",27193073,"-","5:T>A,15:A>G","TGG","GAGAGAATTGACAATTCTCCTGG",2,6,16,NA,"","",FALSE,"AGR3"

"5248","GAGGATTGTCAATACTCTCA","chr7",16862611,"-","none","AGG","GAGGATTGTCAATACTCTCAAGG",0,NA,NA,NA,"GeneID:155465","AGR3",TRUE,"AGR3"

"5249","GATAAGAATTTATCACCTGA","chr7",16861415,"-","none","TGG","GATAAGAATTTATCACCTGATGG",0,NA,NA,NA,"GeneID:155465","AGR3",TRUE,"AGR3"

"5250","GATGGGGAGATGACATCACT","chr7",16873823,"-","none","TGG","GATGGGGAGATGACATCACTTGG",0,NA,NA,NA,"GeneID:155465","AGR3",TRUE,"AGR3"

"5251","GTGCCTAGAATCATGTTTGT","chr7",16861385,"-","none","AGG","GTGCCTAGAATCATGTTTGTAGG",0,NA,NA,NA,"GeneID:155465","AGR3",TRUE,"AGR3"

"5252","GTTCATCATGCTAAACCTTA","chr7",16861985,"-","none","TGG","GTTCATCATGCTAAACCTTATGG",0,NA,NA,NA,"GeneID:155465","AGR3",TRUE,"AGR3"

"5253","ATGTCCCGCCGGCTCCGGGC","chr1",1020243,"-","none","AGG","ATGTCCCGCCGGCTCCGGGCAGG",0,NA,NA,NA,"GeneID:375790","AGRN",TRUE,"AGRN"

"5254","CCTTGTTGCCGCCGTCCAGC","chr1",1022253,"-","none","AGG","CCTTGTTGCCGCCGTCCAGCAGG",0,NA,NA,NA,"GeneID:375790","AGRN",TRUE,"AGRN"

"5255","GAGGCGTCCGACCCACACAC","chr1",1040777,"-","none","AGG","GAGGCGTCCGACCCACACACAGG",0,NA,NA,NA,"GeneID:375790","AGRN",TRUE,"AGRN"

"5256","GCAACGAATGCGAGCTGCAG","chr1",1040805,"+","none","CGG","GCAACGAATGCGAGCTGCAGCGG",0,NA,NA,NA,"GeneID:375790","AGRN",TRUE,"AGRN"

"5257","TCCGCAGGGTGATCCGCATG","chr1",1022412,"-","none","AGG","TCCGCAGGGTGATCCGCATGAGG",0,NA,NA,NA,"GeneID:375790","AGRN",TRUE,"AGRN"

"5258","TCGCCTGCGCCATGGCCGGC","chr1",1020161,"+","none","CGG","TCGCCTGCGCCATGGCCGGCCGG",0,NA,NA,NA,"GeneID:375790","AGRN",TRUE,"AGRN"

"5259","AGAAGAGGATCTGTTGCAGG","chr16",67483044,"-","none","AGG","AGAAGAGGATCTGTTGCAGGAGG",0,NA,NA,NA,"GeneID:101927837;GeneID:181","LOC101927837;AGRP",TRUE,"AGRP"

"5260","AGAAGAGGATCTGTTGCAGG","chr22",48441591,"-","9:G>A,13:G>A","GGG","AGAAGAGGACCTGCTGCAGGGGG",2,10,14,NA,"","",FALSE,"AGRP"

"5261","CAGCCTTACGCAGCGACGTG","chr16",67482764,"+","none","AGG","CAGCCTTACGCAGCGACGTGAGG",0,NA,NA,NA,"GeneID:101927837;GeneID:181","LOC101927837;AGRP",TRUE,"AGRP"

"5262","GAAGAAGACAACTGCAGAAC","chr16",67483068,"-","none","AGG","GAAGAAGACAACTGCAGAACAGG",0,NA,NA,NA,"GeneID:101927837;GeneID:181","LOC101927837;AGRP",TRUE,"AGRP"

"5263","GCCCATCTGGGCTCCTCGCG","chr16",67483326,"+","none","TGG","GCCCATCTGGGCTCCTCGCGTGG",0,NA,NA,NA,"GeneID:101927837;GeneID:181","LOC101927837;AGRP",TRUE,"AGRP"

"5264","GCTCCTCACGTCGCTGCGTA","chr16",67482770,"-","none","AGG","GCTCCTCACGTCGCTGCGTAAGG",0,NA,NA,NA,"GeneID:101927837;GeneID:181","LOC101927837;AGRP",TRUE,"AGRP"

"5265","TGCCACGCGAGGAGCCCAGA","chr16",67483331,"-","none","TGG","TGCCACGCGAGGAGCCCAGATGG",0,NA,NA,NA,"GeneID:101927837;GeneID:181","LOC101927837;AGRP",TRUE,"AGRP"

"5266","AAGATCCTTGCAGCACCAGT","chr1",230704293,"+","none","TGG","AAGATCCTTGCAGCACCAGTTGG",0,NA,NA,NA,"GeneID:183","AGT",TRUE,"AGT"

"5267","AAGATCCTTGCAGCACCAGT","chr1",163963328,"-","9:G>C,18:G>C","GGG","AAGATCCTTCCAGCACCACTGGG",2,10,19,NA,"","",FALSE,"AGT"

"5268","AATTGAGCAATGACCGCATC","chr1",230704204,"-","none","AGG","AATTGAGCAATGACCGCATCAGG",0,NA,NA,NA,"GeneID:183","AGT",TRUE,"AGT"

"5269","GAGTCACCGAGAAGTTGTCC","chr1",230706059,"+","none","TGG","GAGTCACCGAGAAGTTGTCCTGG",0,NA,NA,NA,"GeneID:183","AGT",TRUE,"AGT"

"5270","GCCTCACTATGCCTCTGACC","chr1",230705999,"-","none","TGG","GCCTCACTATGCCTCTGACCTGG",0,NA,NA,NA,"GeneID:183","AGT",TRUE,"AGT"

"5271","GCCTCACTATGCCTCTGACC","chr7",136133749,"+","8:C>A,11:A>C","TGG","GCCTCACTCTGACTCTGACCTGG",2,9,12,NA,"","",FALSE,"AGT"

"5272","GGCCAGCATCCCGACCATTG","chr1",230710520,"+","none","CGG","GGCCAGCATCCCGACCATTGCGG",0,NA,NA,NA,"GeneID:183","AGT",TRUE,"AGT"

"5273","TTGCAGCGACTAGCACCAGC","chr1",230710570,"+","none","TGG","TTGCAGCGACTAGCACCAGCTGG",0,NA,NA,NA,"GeneID:183","AGT",TRUE,"AGT"

"5274","ATTTACTCACTTTATAAGAC","chr9",85669474,"+","none","TGG","ATTTACTCACTTTATAAGACTGG",0,NA,NA,NA,"GeneID:23287","AGTPBP1",TRUE,"AGTPBP1"

"5275","ATTTACTCACTTTATAAGAC","chr10",125258234,"-","10:G>A,13:C>T","AGG","ATTTACTCACCTTGTAAGACAGG",2,11,14,NA,"","",FALSE,"AGTPBP1"

"5276","ATTTACTCACTTTATAAGAC","chr16",76360021,"-","9:C>G,19:A>G","TGG","ATTTACTCAGTTTATAAGATTGG",2,10,20,NA,"GeneID:85445","CNTNAP4",FALSE,"AGTPBP1"

"5277","CAATATTTGTGAACCACCTT","chr9",85677529,"+","none","TGG","CAATATTTGTGAACCACCTTTGG",0,NA,NA,NA,"GeneID:23287","AGTPBP1",TRUE,"AGTPBP1"

"5278","CAATATTTGTGAACCACCTT","chr4",75048541,"+","4:G>A,13:A>C","TGG","CAATGTTTGTGAAACACCTTTGG",2,5,14,NA,"GeneID:25849","PARM1",TRUE,"AGTPBP1"

"5279","GCTGTGAATTCAGTATCCTT","chr9",85669559,"-","none","AGG","GCTGTGAATTCAGTATCCTTAGG",0,NA,NA,NA,"GeneID:23287","AGTPBP1",TRUE,"AGTPBP1"

"5280","TCCTACGATCCTAGAATTAT","chr9",85692785,"+","none","TGG","TCCTACGATCCTAGAATTATTGG",0,NA,NA,NA,"GeneID:23287","AGTPBP1",TRUE,"AGTPBP1"

"5281","TGATCTTCTCCAGTTGAGCC","chr9",85692760,"+","none","AGG","TGATCTTCTCCAGTTGAGCCAGG",0,NA,NA,NA,"GeneID:23287","AGTPBP1",TRUE,"AGTPBP1"

"5282","TGTACCATTAAGTCCTCATG","chr9",85677470,"+","none","TGG","TGTACCATTAAGTCCTCATGTGG",0,NA,NA,NA,"GeneID:23287","AGTPBP1",TRUE,"AGTPBP1"

"5283","ACACAGCTATGGAATACCGC","chr3",148741294,"+","none","TGG","ACACAGCTATGGAATACCGCTGG",0,NA,NA,NA,"GeneID:185","AGTR1",TRUE,"AGTR1"

"5284","CTACAAGCATTGTGCGTCGA","chr3",148741448,"-","none","AGG","CTACAAGCATTGTGCGTCGAAGG",0,NA,NA,NA,"GeneID:185","AGTR1",TRUE,"AGTR1"

"5285","GTAGAAACACACTAGCGTAC","chr3",148741370,"-","none","AGG","GTAGAAACACACTAGCGTACAGG",0,NA,NA,NA,"GeneID:185","AGTR1",TRUE,"AGTR1"

"5286","TCAGGCCCAGCCCTATCGGG","chr3",148741607,"-","none","AGG","TCAGGCCCAGCCCTATCGGGAGG",0,NA,NA,NA,"GeneID:185","AGTR1",TRUE,"AGTR1"

"5287","TCAGGCCCAGCCCTATCGGG","chr10",97498718,"+","15:G>T,16:G>C","CGG","TCAGGCCCAGCCCTAGGGGGCGG",2,16,17,NA,"GeneID:64210","MMS19",TRUE,"AGTR1"

"5288","TCATTGGGTGAACAATAGCC","chr3",148741418,"-","none","AGG","TCATTGGGTGAACAATAGCCAGG",0,NA,NA,NA,"GeneID:185","AGTR1",TRUE,"AGTR1"

"5289","TCTCAGCATTGATCGATACC","chr3",148741397,"+","none","TGG","TCTCAGCATTGATCGATACCTGG",0,NA,NA,NA,"GeneID:185","AGTR1",TRUE,"AGTR1"

"5290","AAGCCCGAAGTGAAGACCGC","chrX",116172326,"-","none","TGG","AAGCCCGAAGTGAAGACCGCTGG",0,NA,NA,NA,"GeneID:186","AGTR2",TRUE,"AGTR2"

"5291","AGCTGTTGGTGAATCCCAAG","chrX",116173194,"-","none","AGG","AGCTGTTGGTGAATCCCAAGAGG",0,NA,NA,NA,"GeneID:186","AGTR2",TRUE,"AGTR2"

"5292","ATTTCTGGTCAATATTGTCG","chrX",116172453,"+","none","TGG","ATTTCTGGTCAATATTGTCGTGG",0,NA,NA,NA,"GeneID:186","AGTR2",TRUE,"AGTR2"

"5293","GCTATTAATGACACCCATCC","chrX",116173127,"-","none","AGG","GCTATTAATGACACCCATCCAGG",0,NA,NA,NA,"GeneID:186","AGTR2",TRUE,"AGTR2"

"5294","TTACTGAAGACGAATAGCTA","chrX",116172991,"+","none","TGG","TTACTGAAGACGAATAGCTATGG",0,NA,NA,NA,"GeneID:186","AGTR2",TRUE,"AGTR2"

"5295","TTCGGGCTTGTGAACATCTC","chrX",116172337,"+","none","TGG","TTCGGGCTTGTGAACATCTCTGG",0,NA,NA,NA,"GeneID:186","AGTR2",TRUE,"AGTR2"

"5296","AACTTCGAAGTGCAGCGCAC","chr1",11746191,"+","none","AGG","AACTTCGAAGTGCAGCGCACAGG",0,NA,NA,NA,"GeneID:57085","AGTRAP",TRUE,"AGTRAP"

"5297","AGCTCAATGGTTTCCAGTCT","chr1",11746154,"-","none","TGG","AGCTCAATGGTTTCCAGTCTTGG",0,NA,NA,NA,"GeneID:57085","AGTRAP",TRUE,"AGTRAP"

"5298","AGCTCAATGGTTTCCAGTCT","chr2",186893006,"+","12:C>T,13:T>C","TGG","AGCTCAATGGTTCTCAGTCTTGG",2,13,14,NA,"","",FALSE,"AGTRAP"

"5299","AGCTCAATGGTTTCCAGTCT","chr5",138493994,"-","6:G>T,17:G>A","GGG","AGCTCACTGGTTTCCAGCCTGGG",2,7,18,NA,"","",FALSE,"AGTRAP"

"5300","CACCATGCTTATGGCGTCGA","chr1",11747528,"-","none","TGG","CACCATGCTTATGGCGTCGATGG",0,NA,NA,NA,"GeneID:57085","AGTRAP",TRUE,"AGTRAP"

"5301","CACCATGCTTATGGCGTCGA","chr21",44053159,"+","14:T>C,16:G>T","GGG","CACCATGCTTATGGTGGCGAGGG",2,15,17,NA,"GeneID:7109","TRAPPC10",FALSE,"AGTRAP"

"5302","CTCACGGACACGGGCCGCTT","chr1",11748486,"+","none","TGG","CTCACGGACACGGGCCGCTTTGG",0,NA,NA,NA,"GeneID:57085","AGTRAP",TRUE,"AGTRAP"

"5303","CTCCATCGACGCCATAAGCA","chr1",11747523,"+","none","TGG","CTCCATCGACGCCATAAGCATGG",0,NA,NA,NA,"GeneID:57085","AGTRAP",TRUE,"AGTRAP"

"5304","TGCTGATGTGCACGATGTCC","chr1",11748446,"-","none","AGG","TGCTGATGTGCACGATGTCCAGG",0,NA,NA,NA,"GeneID:57085","AGTRAP",TRUE,"AGTRAP"

"5305","CAACCTGCCTCCTCGCATCA","chr2",240868957,"+","none","TGG","CAACCTGCCTCCTCGCATCATGG",0,NA,NA,NA,"GeneID:189","AGXT",TRUE,"AGXT"

"5306","CATGAGCAAGGATATGTACC","chr2",240869008,"+","none","AGG","CATGAGCAAGGATATGTACCAGG",0,NA,NA,NA,"GeneID:189","AGXT",TRUE,"AGXT"

"5307","CCGAGTGCACCCGATGACCA","chr2",240870647,"+","none","AGG","CCGAGTGCACCCGATGACCAAGG",0,NA,NA,NA,"GeneID:189","AGXT",TRUE,"AGXT"

"5308","TATGCGCTCCCCGATGTCCA","chr2",240869341,"-","none","CGG","TATGCGCTCCCCGATGTCCACGG",0,NA,NA,NA,"GeneID:189","AGXT",TRUE,"AGXT"

"5309","TCCTGGTCTGGAACACGTAC","chr2",240869198,"-","none","TGG","TCCTGGTCTGGAACACGTACTGG",0,NA,NA,NA,"GeneID:189","AGXT",TRUE,"AGXT"

"5310","TGGAGGCCACTACACACTGC","chr2",240870674,"+","none","AGG","TGGAGGCCACTACACACTGCAGG",0,NA,NA,NA,"GeneID:189","AGXT",TRUE,"AGXT"

"5311","TGGAGGCCACTACACACTGC","chrX",41024990,"-","2:G>C,3:A>T","AGG","TGCTGGCCACTACACACTGCAGG",2,3,4,NA,"","",FALSE,"AGXT"

"5312","AAAAGCAGCTCGGCCGCCTG","chr5",35037025,"-","none","TGG","AAAAGCAGCTCGGCCGCCTGTGG",0,NA,NA,NA,"GeneID:64902","AGXT2",TRUE,"AGXT2"

"5313","AGACGGTGCTTGTATGCCAC","chr5",35037006,"+","none","AGG","AGACGGTGCTTGTATGCCACAGG",0,NA,NA,NA,"GeneID:64902","AGXT2",TRUE,"AGXT2"

"5314","AGGATACTCACGGGTGGCAA","chr5",35039312,"+","none","TGG","AGGATACTCACGGGTGGCAATGG",0,NA,NA,NA,"GeneID:64902","AGXT2",TRUE,"AGXT2"

"5315","CCTTGGCTACAACCGTGTCC","chr5",35039486,"-","none","TGG","CCTTGGCTACAACCGTGTCCTGG",0,NA,NA,NA,"GeneID:64902","AGXT2",TRUE,"AGXT2"

"5316","TATGTTGTTTGAGTGCGCCC","chr5",35035238,"+","none","TGG","TATGTTGTTTGAGTGCGCCCTGG",0,NA,NA,NA,"GeneID:64902","AGXT2",TRUE,"AGXT2"

"5317","TGGCTCAGAAGCCAATGAGC","chr5",35035276,"-","none","TGG","TGGCTCAGAAGCCAATGAGCTGG",0,NA,NA,NA,"GeneID:64902","AGXT2",TRUE,"AGXT2"

"5318","CAACGACTTAGCCTTACGCC","chr4",108756463,"-","none","TGG","CAACGACTTAGCCTTACGCCTGG",0,NA,NA,NA,"GeneID:64850","ETNPPL",TRUE,"AGXT2L1"

"5319","CAGAGTTGCTGAAAGGCGTT","chr4",108759786,"+","none","TGG","CAGAGTTGCTGAAAGGCGTTTGG",0,NA,NA,NA,"GeneID:64850","ETNPPL",TRUE,"AGXT2L1"

"5320","CAGAGTTGCTGAAAGGCGTT","chr7",70835562,"-","10:T>C,11:C>T","GGG","CAGAGTTGCTAGAAGGCGTTGGG",2,11,12,NA,"","",FALSE,"AGXT2L1"

"5321","CATACTCAACAATGTTGTCG","chr4",108759809,"+","none","TGG","CATACTCAACAATGTTGTCGTGG",0,NA,NA,NA,"GeneID:64850","ETNPPL",TRUE,"AGXT2L1"

"5322","CATCAAACATGTACTGCCTC","chr4",108760232,"+","none","TGG","CATCAAACATGTACTGCCTCTGG",0,NA,NA,NA,"GeneID:64850","ETNPPL",TRUE,"AGXT2L1"

"5323","GGTCAAGAGTGATCACATCC","chr4",108756418,"+","none","TGG","GGTCAAGAGTGATCACATCCTGG",0,NA,NA,NA,"GeneID:64850","ETNPPL",TRUE,"AGXT2L1"

"5324","TAATCTCAATTAAGGATGAT","chr4",108754672,"+","none","AGG","TAATCTCAATTAAGGATGATAGG",0,NA,NA,NA,"GeneID:64850","ETNPPL",TRUE,"AGXT2L1"

"5325","ATCCAGGTTGCGGAACTTGT","chr5",178224665,"+","none","AGG","ATCCAGGTTGCGGAACTTGTAGG",0,NA,NA,NA,"GeneID:85007","PHYKPL",TRUE,"AGXT2L2"

"5326","CATCGTACATGTACTGCCCT","chr5",178231449,"+","none","TGG","CATCGTACATGTACTGCCCTTGG",0,NA,NA,NA,"GeneID:85007","PHYKPL",TRUE,"AGXT2L2"

"5327","GAGGCTGGCTCGCCACTACA","chr5",178225385,"-","none","CGG","GAGGCTGGCTCGCCACTACACGG",0,NA,NA,NA,"GeneID:85007","PHYKPL",TRUE,"AGXT2L2"

"5328","GATCTAATACCACCACGTCC","chr5",178225355,"+","none","TGG","GATCTAATACCACCACGTCCTGG",0,NA,NA,NA,"GeneID:85007","PHYKPL",TRUE,"AGXT2L2"

"5329","GCAGTACATGTACGATGAAC","chr5",178231446,"-","none","AGG","GCAGTACATGTACGATGAACAGG",0,NA,NA,NA,"GeneID:85007","PHYKPL",TRUE,"AGXT2L2"

"5330","GTACCTGCATGACAACATCG","chr5",178230009,"-","none","TGG","GTACCTGCATGACAACATCGTGG",0,NA,NA,NA,"GeneID:85007","PHYKPL",TRUE,"AGXT2L2"

"5331","ATGTTATTTCGTCTTCTCCA","chr1",246918282,"+","none","AGG","ATGTTATTTCGTCTTCTCCAAGG",0,NA,NA,NA,"GeneID:25909","AHCTF1",TRUE,"AHCTF1"

"5332","ATGTTATTTCGTCTTCTCCA","chr2",161507590,"+","none","AGG","ATGTTATTTCGTCTTCTCCAAGG",0,NA,NA,NA,"GeneID:57282","SLC4A10",TRUE,"AHCTF1"

"5333","CAAAATGAAGTTGAAGCATC","chr1",246913233,"-","none","AGG","CAAAATGAAGTTGAAGCATCAGG",0,NA,NA,NA,"GeneID:25909","AHCTF1",TRUE,"AHCTF1"

"5334","CAAAATGAAGTTGAAGCATC","chr7",22588302,"+","15:A>G,16:A>C","AGG","CAAAATGAAGTTGAAAAATCAGG",2,16,17,NA,"GeneID:256227","STEAP1B",FALSE,"AHCTF1"

"5335","CAAAATGAAGTTGAAGCATC","chr18",24662553,"-","9:A>C,11:C>A","TGG","CAAAATGAATTGGAAGCATCTGG",2,10,12,NA,"","",FALSE,"AHCTF1"

"5336","CAAAATGAAGTTGAAGCATC","chrX",9183767,"-","4:A>T,15:A>C","AGG","CAAATTGAAGTTGAATCATCAGG",2,5,16,NA,"","",FALSE,"AHCTF1"

"5337","CCTTCTTGTTGACCTATGTT","chr1",246913273,"-","none","TGG","CCTTCTTGTTGACCTATGTTTGG",0,NA,NA,NA,"GeneID:25909","AHCTF1",TRUE,"AHCTF1"

"5338","CCTTCTTGTTGACCTATGTT","chr2",161507164,"-","7:T>C","TGG","CCTTCTTATTGACCTATGTTTGG",1,8,NA,NA,"GeneID:57282","SLC4A10",TRUE,"AHCTF1"

"5339","GAAAAATGGACTTGCTTGCT","chr1",246916374,"-","none","TGG","GAAAAATGGACTTGCTTGCTTGG",0,NA,NA,NA,"GeneID:25909","AHCTF1",TRUE,"AHCTF1"

"5340","GAAAAATGGACTTGCTTGCT","chr2",161507536,"-","none","TGG","GAAAAATGGACTTGCTTGCTTGG",0,NA,NA,NA,"GeneID:57282","SLC4A10",TRUE,"AHCTF1"

"5341","GAAAAATGGACTTGCTTGCT","chr7",33838943,"+","0:A>G,15:A>T","GGG","AAAAAATGGACTTGCATGCTGGG",2,1,16,NA,"","",FALSE,"AHCTF1"

"5342","GAAAAATGGACTTGCTTGCT","chr2",8230172,"-","0:T>C,16:C>A","AGG","AAAAAATGGACTTGCTGGCTAGG",2,1,17,NA,"GeneID:339788;GeneID:339789","LINC00298;LINC00299",FALSE,"AHCTF1"

"5343","GAGGTAGTAAACTCTATAAC","chr1",246916334,"-","none","AGG","GAGGTAGTAAACTCTATAACAGG",0,NA,NA,NA,"GeneID:25909","AHCTF1",TRUE,"AHCTF1"

"5344","GAGGTAGTAAACTCTATAAC","chr2",161507496,"-","none","AGG","GAGGTAGTAAACTCTATAACAGG",0,NA,NA,NA,"GeneID:57282","SLC4A10",TRUE,"AHCTF1"

"5345","TTAAGAGCTCAAGTGACTAG","chr2",161507649,"-","none","TGG","TTAAGAGCTCAAGTGACTAGTGG",0,NA,NA,NA,"GeneID:57282","SLC4A10",TRUE,"AHCTF1"

"5346","TTAAGAGCTCAAGTGACTAG","chr1",246918341,"-","none","TGG","TTAAGAGCTCAAGTGACTAGTGG",0,NA,NA,NA,"GeneID:25909","AHCTF1",TRUE,"AHCTF1"

"5347","ACCGCTCCCGCATACGCATC","chr20",34295498,"+","none","AGG","ACCGCTCCCGCATACGCATCAGG",0,NA,NA,NA,"GeneID:191","AHCY",TRUE,"AHCY"

"5348","ACGTGGAGATCGATGTCAAG","chr20",34290376,"-","none","TGG","ACGTGGAGATCGATGTCAAGTGG",0,NA,NA,NA,"GeneID:191","AHCY",TRUE,"AHCY"

"5349","ACGTGGAGATCGATGTCAAG","chr11",85857988,"+","2:A>G,10:T>C","TGG","ACATGGAGATTGATGTCAAGTGG",2,3,11,NA,"GeneID:220047","CCDC83",FALSE,"AHCY"

"5350","ACGTGGAGATCGATGTCAAG","chr10",46224032,"+","1:G>C,11:A>G","TGG","AGGTGGAGATCAATGTCAAGTGG",2,2,12,NA,"","",FALSE,"AHCY"

"5351","CAACGAGAACGCCGTGGAGA","chr20",34290351,"-","none","AGG","CAACGAGAACGCCGTGGAGAAGG",0,NA,NA,NA,"GeneID:191","AHCY",TRUE,"AHCY"

"5352","CCATCATCTTGTAGAGGTTG","chr20",34291471,"+","none","TGG","CCATCATCTTGTAGAGGTTGTGG",0,NA,NA,NA,"GeneID:191","AHCY",TRUE,"AHCY"

"5353","CCATCATCTTGTAGAGGTTG","chr15",50417867,"-","13:A>C","TGG","CCATCATCTTGTATAGGTTGTGG",1,14,NA,NA,"","",TRUE,"AHCY"

"5354","CCATCATCTTGTAGAGGTTG","chr9",120721160,"-","12:C>T,13:A>C","TGG","CCATCATCTTGTGTAGGTTGTGG",2,13,14,NA,"","",TRUE,"AHCY"

"5355","CCATCATCTTGTAGAGGTTG","chr2",138902225,"+","13:C>G","TGG","CCATCATCTTGTACAGGTTGTGG",1,14,NA,NA,"","",TRUE,"AHCY"

"5356","CCATCATCTTGTAGAGGTTG","chr20",19809837,"-","5:A>T,14:A>T","TGG","CCATCTTCTTGTAGTGGTTGTGG",2,6,15,NA,"","",FALSE,"AHCY"

"5357","TGCCGGGCCTGATGCGTATG","chr20",34295508,"-","none","CGG","TGCCGGGCCTGATGCGTATGCGG",0,NA,NA,NA,"GeneID:191","AHCY",TRUE,"AHCY"

"5358","TGTCAATGACTCCGTCACCA","chr20",34291420,"-","none","AGG","TGTCAATGACTCCGTCACCAAGG",0,NA,NA,NA,"GeneID:191","AHCY",TRUE,"AHCY"

"5359","ACCAAATTCCCCACCAAAAC","chr1",110009066,"+","none","TGG","ACCAAATTCCCCACCAAAACTGG",0,NA,NA,NA,"GeneID:10768","AHCYL1",TRUE,"AHCYL1"

"5360","ATCATCAGAGCTATCTGTGT","chr1",110011222,"-","none","AGG","ATCATCAGAGCTATCTGTGTAGG",0,NA,NA,NA,"GeneID:10768","AHCYL1",TRUE,"AHCYL1"

"5361","CACAGATAGCTCTGATGATG","chr1",110011223,"+","none","AGG","CACAGATAGCTCTGATGATGAGG",0,NA,NA,NA,"GeneID:10768","AHCYL1",TRUE,"AHCYL1"

"5362","CACAGATAGCTCTGATGATG","chr1",20899720,"-","9:A>G,14:C>T","GGG","CACAGATAGTTCTGGTGATGGGG",2,10,15,NA,"GeneID:8672","EIF4G3",TRUE,"AHCYL1"

"5363","CACAGATAGCTCTGATGATG","chr3",82067293,"-","6:C>A,14:C>T","GGG","CACAGAGAGCTCTGGTGATGGGG",2,7,15,NA,"","",FALSE,"AHCYL1"

"5364","CGCCGAGAAGTACTCCTTCA","chr1",109985123,"+","none","TGG","CGCCGAGAAGTACTCCTTCATGG",0,NA,NA,NA,"GeneID:10768","AHCYL1",TRUE,"AHCYL1"

"5365","CGTCACCAAGGCGCCCAAGA","chr1",109985150,"+","none","AGG","CGTCACCAAGGCGCCCAAGAAGG",0,NA,NA,NA,"GeneID:10768","AHCYL1",TRUE,"AHCYL1"

"5366","TCGAGCGAGACAAAGATCTT","chr1",110009092,"-","none","CGG","TCGAGCGAGACAAAGATCTTCGG",0,NA,NA,NA,"GeneID:10768","AHCYL1",TRUE,"AHCYL1"

"5367","AGATGTTGCAGGCAGCCCAT","chr7",129397262,"-","none","CGG","AGATGTTGCAGGCAGCCCATCGG",0,NA,NA,NA,"GeneID:23382","AHCYL2",TRUE,"AHCYL2"

"5368","AGCTCAAGGAGAAAAGCCTT","chr7",129389664,"+","none","TGG","AGCTCAAGGAGAAAAGCCTTTGG",0,NA,NA,NA,"GeneID:23382","AHCYL2",TRUE,"AHCYL2"

"5369","CGCCTCGGTGACGGTGCCGT","chr7",129225401,"-","none","CGG","CGCCTCGGTGACGGTGCCGTCGG",0,NA,NA,NA,"GeneID:23382","AHCYL2",TRUE,"AHCYL2"

"5370","CTCTGGGGGCCCAGTGCCGA","chr7",129397243,"+","none","TGG","CTCTGGGGGCCCAGTGCCGATGG",0,NA,NA,NA,"GeneID:23382","AHCYL2",TRUE,"AHCYL2"

"5371","CTCTGGGGGCCCAGTGCCGA","chr19",51026554,"+","16:G>C,19:G>A","CGG","CTCTGGGGGCCCAGTGGCGGCGG",2,17,20,NA,"GeneID:11012","KLK11",TRUE,"AHCYL2"

"5372","GTGTGTGCAACCCACGATTT","chr7",129389696,"-","none","TGG","GTGTGTGCAACCCACGATTTTGG",0,NA,NA,NA,"GeneID:23382","AHCYL2",TRUE,"AHCYL2"

"5373","GTGTGTGCAACCCACGATTT","chr5",145247585,"-","2:T>C,14:C>G","GGG","GTATGTGCAACCCAGGATTTGGG",2,3,15,NA,"","",FALSE,"AHCYL2"

"5374","TCCTAGTTGCGACTCCGCCT","chr7",129225152,"-","none","CGG","TCCTAGTTGCGACTCCGCCTCGG",0,NA,NA,NA,"GeneID:23382","AHCYL2",TRUE,"AHCYL2"

"5375","AGTGCGCCGCAAGGCGTACT","chr1",27550954,"+","none","TGG","AGTGCGCCGCAAGGCGTACTTGG",0,NA,NA,NA,"GeneID:27245","AHDC1",TRUE,"AHDC1"

"5376","CGAGCCACCATCGCCCGAGC","chr1",27551304,"-","none","CGG","CGAGCCACCATCGCCCGAGCCGG",0,NA,NA,NA,"GeneID:27245","AHDC1",TRUE,"AHDC1"

"5377","CTTTCGCTCGCTGCTCGATT","chr1",27549579,"-","none","CGG","CTTTCGCTCGCTGCTCGATTCGG",0,NA,NA,NA,"GeneID:27245","AHDC1",TRUE,"AHDC1"

"5378","GCGGGTGAACCTGCGACCAG","chr1",27551754,"-","none","TGG","GCGGGTGAACCTGCGACCAGTGG",0,NA,NA,NA,"GeneID:27245","AHDC1",TRUE,"AHDC1"

"5379","GGCCAAGATCCCCGTATCGC","chr1",27550662,"-","none","TGG","GGCCAAGATCCCCGTATCGCTGG",0,NA,NA,NA,"GeneID:27245","AHDC1",TRUE,"AHDC1"

"5380","GTTCCGCCGCCCCAGCGATA","chr1",27550648,"+","none","CGG","GTTCCGCCGCCCCAGCGATACGG",0,NA,NA,NA,"GeneID:27245","AHDC1",TRUE,"AHDC1"

"5381","AATAACAAGCCGAGATAGCC","chr6",135457650,"-","none","CGG","AATAACAAGCCGAGATAGCCCGG",0,NA,NA,NA,"GeneID:54806","AHI1",TRUE,"AHI1"

"5382","AATAACAAGCCGAGATAGCC","chr3",188232649,"-","10:T>G,14:A>T","AGG","AATAACAAGCAGAGTTAGCCAGG",2,11,15,NA,"GeneID:4026","LPP",FALSE,"AHI1"

"5383","CACTGCTTAGTTTGTCATCA","chr6",135465865,"+","none","TGG","CACTGCTTAGTTTGTCATCATGG",0,NA,NA,NA,"GeneID:54806","AHI1",TRUE,"AHI1"

"5384","CATGTGGTTGATGAGCATAC","chr6",135457520,"-","none","TGG","CATGTGGTTGATGAGCATACTGG",0,NA,NA,NA,"GeneID:54806","AHI1",TRUE,"AHI1"

"5385","GACTACACAAGACCTGAAAC","chr6",135466135,"-","none","CGG","GACTACACAAGACCTGAAACCGG",0,NA,NA,NA,"GeneID:54806","AHI1",TRUE,"AHI1"

"5386","TGTAGTACATTGACCATCTC","chr6",135463291,"-","none","TGG","TGTAGTACATTGACCATCTCTGG",0,NA,NA,NA,"GeneID:54806","AHI1",TRUE,"AHI1"

"5387","TGTAGTACATTGACCATCTC","chr20",46795541,"-","2:G>A,5:C>A","TGG","TGCAGGACATTGACCATCTCTGG",2,3,6,NA,"","",FALSE,"AHI1"

"5388","TTCAACTGTGTCACCAGAGA","chr6",135463275,"+","none","TGG","TTCAACTGTGTCACCAGAGATGG",0,NA,NA,NA,"GeneID:54806","AHI1",TRUE,"AHI1"

"5389","TTCAACTGTGTCACCAGAGA","chr16",82065720,"-","4:A>T,13:C>G","AGG","TTCATCTGTGTCAGCAGAGAAGG",2,5,14,NA,"GeneID:3294","HSD17B2",FALSE,"AHI1"

"5390","AGACCTTCGACGTGAATCGC","chr11",62519015,"+","none","TGG","AGACCTTCGACGTGAATCGCTGG",0,NA,NA,NA,"GeneID:79026","AHNAK",TRUE,"AHNAK"

"5391","AGACTGAAACTGCCCCGGCT","chr11",62491746,"+","none","TGG","AGACTGAAACTGCCCCGGCTTGG",0,NA,NA,NA,"GeneID:79026","AHNAK",TRUE,"AHNAK"

"5392","GCCCCATGGTGTTCAGCAGC","chr11",62535109,"+","none","TGG","GCCCCATGGTGTTCAGCAGCTGG",0,NA,NA,NA,"GeneID:79026","AHNAK",TRUE,"AHNAK"

"5393","GCTGAAGACTTCACGGGTCC","chr11",62535026,"+","none","AGG","GCTGAAGACTTCACGGGTCCAGG",0,NA,NA,NA,"GeneID:79026","AHNAK",TRUE,"AHNAK"

"5394","TCTTTGCATTCCAGTGCTGA","chr11",62491796,"+","none","TGG","TCTTTGCATTCCAGTGCTGATGG",0,NA,NA,NA,"GeneID:79026","AHNAK",TRUE,"AHNAK"

"5395","TCTTTGCATTCCAGTGCTGA","chr8",49740634,"+","0:A>T,15:A>G","AGG","ACTTTGCATTCCAGTACTGAAGG",2,1,16,NA,"","",TRUE,"AHNAK"

"5396","TCTTTGCATTCCAGTGCTGA","chr9",110818038,"-","6:C>G,16:C>G","TGG","TCTTTGGATTCCAGTGGTGATGG",2,7,17,NA,"","",FALSE,"AHNAK"

"5397","TTGCCAGCGATTCACGTCGA","chr11",62519021,"-","none","AGG","TTGCCAGCGATTCACGTCGAAGG",0,NA,NA,NA,"GeneID:79026","AHNAK",TRUE,"AHNAK"

"5398","CGGATGAGGGCATTCGACCA","chr14",104957472,"-","none","CGG","CGGATGAGGGCATTCGACCACGG",0,NA,NA,NA,"GeneID:113146","AHNAK2",TRUE,"AHNAK2"

"5399","GAGGGGCGCACCGCTGCCGG","chr14",104978171,"+","none","GGG","GAGGGGCGCACCGCTGCCGGGGG",0,NA,NA,NA,"GeneID:113146","AHNAK2",TRUE,"AHNAK2"

"5400","GAGGGGCGCACCGCTGCCGG","chr13",52738933,"-","9:C>T,11:C>G","CGG","GAGGGGCGCGCGGCTGCCGGCGG",2,10,12,NA,"GeneID:11061","LECT1",FALSE,"AHNAK2"

"5401","GATGTGCGACTGCTTCCACA","chr14",104978218,"-","none","TGG","GATGTGCGACTGCTTCCACATGG",0,NA,NA,NA,"GeneID:113146","AHNAK2",TRUE,"AHNAK2"

"5402","GGTCGAATGCCCTCATCCGC","chr14",104957473,"+","none","AGG","GGTCGAATGCCCTCATCCGCAGG",0,NA,NA,NA,"GeneID:113146","AHNAK2",TRUE,"AHNAK2"

"5403","TCCTGGTGGAAGCGAGATTC","chr14",104956627,"-","none","AGG","TCCTGGTGGAAGCGAGATTCAGG",0,NA,NA,NA,"GeneID:113146","AHNAK2",TRUE,"AHNAK2"

"5404","TCCTGGTGGAAGCGAGATTC","chr1",232272176,"+","0:A>T,13:A>G","TGG","ACCTGGTGGAAGCAAGATTCTGG",2,1,14,NA,"","",FALSE,"AHNAK2"

"5405","TTTCAGGATGAGTCGTCCAG","chr14",104956589,"-","none","AGG","TTTCAGGATGAGTCGTCCAGAGG",0,NA,NA,NA,"GeneID:113146","AHNAK2",TRUE,"AHNAK2"

"5406","AAGTCGGTCTCTATGCCGCT","chr7",17309979,"-","none","TGG","AAGTCGGTCTCTATGCCGCTTGG",0,NA,NA,NA,"GeneID:196","AHR",TRUE,"AHR"

"5407","AATTTCAGCGTCAGCTACAC","chr7",17330006,"+","none","TGG","AATTTCAGCGTCAGCTACACTGG",0,NA,NA,NA,"GeneID:196","AHR",TRUE,"AHR"

"5408","AGACCGACTTAATACAGAGT","chr7",17309989,"+","none","TGG","AGACCGACTTAATACAGAGTTGG",0,NA,NA,NA,"GeneID:196","AHR",TRUE,"AHR"

"5409","AGTTGTCACTACAGATGCTT","chr7",17327778,"+","none","TGG","AGTTGTCACTACAGATGCTTTGG",0,NA,NA,NA,"GeneID:196","AHR",TRUE,"AHR"

"5410","TCCGTTTCTTTCAGTAGGGG","chr7",17322515,"-","none","AGG","TCCGTTTCTTTCAGTAGGGGAGG",0,NA,NA,NA,"GeneID:196","AHR",TRUE,"AHR"

"5411","TTGCTGCTCTACAGTTATCC","chr7",17322540,"-","none","TGG","TTGCTGCTCTACAGTTATCCTGG",0,NA,NA,NA,"GeneID:196","AHR",TRUE,"AHR"

"5412","CCCGCGTACGTGCACTCCCC","chr5",343914,"-","none","CGG","CCCGCGTACGTGCACTCCCCCGG",0,NA,NA,NA,"GeneID:10016;GeneID:57491","PDCD6;AHRR",TRUE,"AHRR"

"5413","GCCGAGGACGATGATCCCGC","chr5",343892,"+","none","CGG","GCCGAGGACGATGATCCCGCCGG",0,NA,NA,NA,"GeneID:10016;GeneID:57491","PDCD6;AHRR",TRUE,"AHRR"

"5414","GGATACTTTACTGTTTCTGC","chr5",343955,"-","none","AGG","GGATACTTTACTGTTTCTGCAGG",0,NA,NA,NA,"GeneID:10016;GeneID:57491","PDCD6;AHRR",TRUE,"AHRR"

"5415","GGATACTTTACTGTTTCTGC","chr11",25159084,"+","6:A>T,8:A>T","AGG","GGATACATAACTGTTTCTGCAGG",2,7,9,NA,"","",FALSE,"AHRR"

"5416","GGATACTTTACTGTTTCTGC","chr5",168230874,"+","8:A>T,14:A>T","TGG","GGATACTTAACTGTATCTGCTGG",2,9,15,NA,"GeneID:57451;GeneID:101927862","TENM2;CTB-178M22.2",TRUE,"AHRR"

"5417","GGGAGTGCACGTACGCGGGC","chr5",343915,"+","none","CGG","GGGAGTGCACGTACGCGGGCCGG",0,NA,NA,NA,"GeneID:10016;GeneID:57491","PDCD6;AHRR",TRUE,"AHRR"

"5418","GTCTCTTAATGGCTTTGCTC","chr5",413342,"+","none","TGG","GTCTCTTAATGGCTTTGCTCTGG",0,NA,NA,NA,"GeneID:57491","AHRR",TRUE,"AHRR"

"5419","GTCTCTTAATGGCTTTGCTC","chr2",1851291,"+","7:G>A,16:T>G","AGG","GTCTCTTGATGGCTTTTCTCAGG",2,8,17,NA,"GeneID:23040","MYT1L",FALSE,"AHRR"

"5420","GTCTCTTAATGGCTTTGCTC","chr15",79543918,"-","8:A>T,16:G>C","AGG","GTCTCTTATTGGCTTTCCTCAGG",2,9,17,NA,"","",FALSE,"AHRR"

"5421","TTATGCATCAGCAACGATCG","chr5",413390,"+","none","TGG","TTATGCATCAGCAACGATCGTGG",0,NA,NA,NA,"GeneID:57491","AHRR",TRUE,"AHRR"

"5422","ACGGAAGTGAGTAAGCTTGA","chr14",77459709,"+","none","TGG","ACGGAAGTGAGTAAGCTTGATGG",0,NA,NA,NA,"GeneID:10598","AHSA1",TRUE,"AHSA1"

"5423","AGACGTTGCCATCTACCATG","chr14",77468144,"-","none","TGG","AGACGTTGCCATCTACCATGTGG",0,NA,NA,NA,"GeneID:10598","AHSA1",TRUE,"AHSA1"

"5424","GCATCCATTAACAATCGCAA","chr14",77459736,"+","none","AGG","GCATCCATTAACAATCGCAAAGG",0,NA,NA,NA,"GeneID:10598","AHSA1",TRUE,"AHSA1"

"5425","TACATCAGCACCCTCAAAAC","chr14",77462737,"+","none","AGG","TACATCAGCACCCTCAAAACAGG",0,NA,NA,NA,"GeneID:10598","AHSA1",TRUE,"AHSA1"

"5426","TGAGCCTGACACAAATCTCG","chr14",77462664,"+","none","TGG","TGAGCCTGACACAAATCTCGTGG",0,NA,NA,NA,"GeneID:10598","AHSA1",TRUE,"AHSA1"

"5427","TTGACAGGTACTTCTAAGTC","chr14",77462152,"+","none","AGG","TTGACAGGTACTTCTAAGTCAGG",0,NA,NA,NA,"GeneID:10598","AHSA1",TRUE,"AHSA1"

"5428","AATTCTGTTTGCCCTAGAGT","chr2",61186651,"-","none","AGG","AATTCTGTTTGCCCTAGAGTAGG",0,NA,NA,NA,"GeneID:130872","AHSA2",TRUE,"AHSA2"

"5429","AGATGTGGGAACTGGCCAGA","chr2",61186475,"+","none","AGG","AGATGTGGGAACTGGCCAGAAGG",0,NA,NA,NA,"GeneID:130872","AHSA2",TRUE,"AHSA2"

"5430","AGATGTGGGAACTGGCCAGA","chr1",46742997,"+","6:A>G","AGG","AGATGTAGGAACTGGCCAGAAGG",1,7,NA,NA,"","",TRUE,"AHSA2"

"5431","AGATGTGGGAACTGGCCAGA","chr3",44890546,"+","4:T>G,10:T>A","GGG","AGATTTGGGATCTGGCCAGAGGG",2,5,11,NA,"GeneID:7047","TGM4",TRUE,"AHSA2"

"5432","AGATGTGGGAACTGGCCAGA","chr1",184789998,"+","3:A>T,11:T>C","TGG","AGAAGTGGGAATTGGCCAGATGG",2,4,12,NA,"","",FALSE,"AHSA2"

"5433","ATAAGAAGATCATCATGAAA","chr2",61186452,"+","none","TGG","ATAAGAAGATCATCATGAAATGG",0,NA,NA,NA,"GeneID:130872","AHSA2",TRUE,"AHSA2"

"5434","ATAAGAAGATCATCATGAAA","chr18",45633437,"+","8:G>A,12:C>T","GGG","ATAAGAAGGTCACCATGAAAGGG",2,9,13,NA,"GeneID:8170","SLC14A2",FALSE,"AHSA2"

"5435","CTGCAGCTGACCCCCCTAAA","chr2",61186759,"+","none","TGG","CTGCAGCTGACCCCCCTAAATGG",0,NA,NA,NA,"GeneID:130872","AHSA2",TRUE,"AHSA2"

"5436","GGTTCAGGCCTCATCTCCAG","chr2",61185500,"+","none","TGG","GGTTCAGGCCTCATCTCCAGTGG",0,NA,NA,NA,"GeneID:130872","AHSA2",TRUE,"AHSA2"

"5437","GGTTCAGGCCTCATCTCCAG","chr1",46742747,"+","5:G>A","TGG","GGTTCGGGCCTCATCTCCAGTGG",1,6,NA,NA,"","",TRUE,"AHSA2"

"5438","GTATAGCATCTTCACTGTAA","chr2",61185593,"+","none","AGG","GTATAGCATCTTCACTGTAAAGG",0,NA,NA,NA,"GeneID:130872","AHSA2",TRUE,"AHSA2"

"5439","ACACCTTTACTTCATCAATC","chr3",186613326,"-","none","TGG","ACACCTTTACTTCATCAATCTGG",0,NA,NA,NA,"GeneID:197","AHSG",TRUE,"AHSG"

"5440","ACACCTTTACTTCATCAATC","chrX",37418059,"+","8:G>A,19:T>C","AGG","ACACCTTTGCTTCATCAATTAGG",2,9,20,NA,"GeneID:5638","PRRG1",FALSE,"AHSG"

"5441","CTCACGCTGCATCTTGCCAC","chr3",186615759,"-","none","AGG","CTCACGCTGCATCTTGCCACAGG",0,NA,NA,NA,"GeneID:197","AHSG",TRUE,"AHSG"

"5442","CTCACGCTGCATCTTGCCAC","chr5",178721697,"+","5:T>G,15:T>G","AGG","CTCACTCTGCATCTTTCCACAGG",2,6,16,NA,"GeneID:6940","ZNF354A",FALSE,"AHSG"

"5443","GCAAAATGTGATTCCAGTCC","chr3",186616505,"+","none","AGG","GCAAAATGTGATTCCAGTCCAGG",0,NA,NA,NA,"GeneID:197","AHSG",TRUE,"AHSG"

"5444","GGTTGTCTATAAATCAGCCC","chr3",186613210,"-","none","TGG","GGTTGTCTATAAATCAGCCCTGG",0,NA,NA,NA,"GeneID:197","AHSG",TRUE,"AHSG"

"5445","TGAGATTGAAATAGACACCC","chr3",186615704,"+","none","TGG","TGAGATTGAAATAGACACCCTGG",0,NA,NA,NA,"GeneID:197","AHSG",TRUE,"AHSG"

"5446","TGAGATTGAAATAGACACCC","chr5",160054193,"+","6:G>T,15:G>C","TGG","TGAGATGGAAATAGAGACCCTGG",2,7,16,NA,"GeneID:7265","TTC1",FALSE,"AHSG"

"5447","TTCCAGCTGTTGAAACTAGA","chr3",186616463,"+","none","TGG","TTCCAGCTGTTGAAACTAGATGG",0,NA,NA,NA,"GeneID:197","AHSG",TRUE,"AHSG"

"5448","TTCCAGCTGTTGAAACTAGA","chr7",72917606,"-","16:G>A,18:G>C","TGG","TTCCAGCTGTTGAAACCACATGG",2,17,19,NA,"GeneID:9883","POM121",FALSE,"AHSG"

"5449","TTCCAGCTGTTGAAACTAGA","chr10",108443363,"+","1:G>T,17:T>A","GGG","TGCCAGCTGTTGAAACTTGAGGG",2,2,18,NA,"","",FALSE,"AHSG"

"5450","AATAAGGATCTCATTTCCGC","chr16",31528157,"+","none","AGG","AATAAGGATCTCATTTCCGCAGG",0,NA,NA,NA,"GeneID:51327","AHSP",TRUE,"AHSP"

"5451","ACTTCTACATCAACTATTAC","chr16",31528518,"+","none","AGG","ACTTCTACATCAACTATTACAGG",0,NA,NA,NA,"GeneID:51327","AHSP",TRUE,"AHSP"

"5452","CAGGCAGATGGCTCTTCTTA","chr16",31528132,"+","none","AGG","CAGGCAGATGGCTCTTCTTAAGG",0,NA,NA,NA,"GeneID:51327","AHSP",TRUE,"AHSP"

"5453","GGAGCCCCAAGAGCGAGACA","chr16",31528555,"+","none","AGG","GGAGCCCCAAGAGCGAGACAAGG",0,NA,NA,NA,"GeneID:51327","AHSP",TRUE,"AHSP"

"5454","TCGGCAAGAGCTGAACACTC","chr16",31528591,"+","none","TGG","TCGGCAAGAGCTGAACACTCTGG",0,NA,NA,NA,"GeneID:51327","AHSP",TRUE,"AHSP"

"5455","TCGGGAGCTCATGAGACTTC","chr16",31528648,"-","none","AGG","TCGGGAGCTCATGAGACTTCAGG",0,NA,NA,NA,"GeneID:51327","AHSP",TRUE,"AHSP"

"5456","ACGAGACGCATTTCGTACTT","chr12",8604294,"-","none","TGG","ACGAGACGCATTTCGTACTTTGG",0,NA,NA,NA,"GeneID:57379","AICDA",TRUE,"AICDA"

"5457","CGCGCGCCTCTACTTCTGTG","chr12",8605292,"-","none","AGG","CGCGCGCCTCTACTTCTGTGAGG",0,NA,NA,NA,"GeneID:57379","AICDA",TRUE,"AICDA"

"5458","CTTTGGTTATCTTCGCAATA","chr12",8606866,"-","none","AGG","CTTTGGTTATCTTCGCAATAAGG",0,NA,NA,NA,"GeneID:57379","AICDA",TRUE,"AICDA"

"5459","GCCGCTGCTACCGCGTCACC","chr12",8605404,"-","none","TGG","GCCGCTGCTACCGCGTCACCTGG",0,NA,NA,NA,"GeneID:57379","AICDA",TRUE,"AICDA"

"5460","GCCGCTGCTACCGCGTCACC","chr16",84820067,"+","17:T>A,18:G>C","TGG","GCCGCTGCTACCGCGTCTGCTGG",2,18,19,NA,"GeneID:83716","CRISPLD2",TRUE,"AICDA"

"5461","GCCTCTTCACTACGTAGCAC","chr12",8606914,"+","none","AGG","GCCTCTTCACTACGTAGCACAGG",0,NA,NA,NA,"GeneID:57379","AICDA",TRUE,"AICDA"

"5462","GTAAGTCATCAACCTCATAC","chr12",8604312,"+","none","AGG","GTAAGTCATCAACCTCATACAGG",0,NA,NA,NA,"GeneID:57379","AICDA",TRUE,"AICDA"

"5463","ATGTTCAGCCTGTCCCATTA","chr1",222687596,"-","none","AGG","ATGTTCAGCCTGTCCCATTAAGG",0,NA,NA,NA,"GeneID:64853","AIDA",TRUE,"AIDA"

"5464","ATTAGCAAGACATCTACAAA","chr1",77811651,"-","none","AGG","ATTAGCAAGACATCTACAAAAGG",0,NA,NA,NA,"GeneID:374986","FAM73A",TRUE,"AIDA"

"5465","ATTAGCAAGACATCTACAAA","chr1",222703197,"-","none","AGG","ATTAGCAAGACATCTACAAAAGG",0,NA,NA,NA,"GeneID:64853","AIDA",TRUE,"AIDA"

"5466","GTCTCAAGAAGAATTTAAAC","chr4",158277999,"+","none","TGG","GTCTCAAGAAGAATTTAAACTGG",0,NA,NA,NA,"","",TRUE,"AIDA"

"5467","GTCTCAAGAAGAATTTAAAC","chr1",77811519,"-","none","TGG","GTCTCAAGAAGAATTTAAACTGG",0,NA,NA,NA,"GeneID:374986","FAM73A",TRUE,"AIDA"

"5468","GTCTCAAGAAGAATTTAAAC","chr1",222693815,"-","none","TGG","GTCTCAAGAAGAATTTAAACTGG",0,NA,NA,NA,"GeneID:64853","AIDA",TRUE,"AIDA"

"5469","GTCTCAAGAAGAATTTAAAC","chr2",11308262,"+","19:T>C","TGG","GTCTCAAGAAGAATTTAAATTGG",1,20,NA,NA,"GeneID:9475","ROCK2",TRUE,"AIDA"

"5470","TACTTGGTTCTAGCTTCTTC","chr1",222693785,"+","none","AGG","TACTTGGTTCTAGCTTCTTCAGG",0,NA,NA,NA,"GeneID:64853","AIDA",TRUE,"AIDA"

"5471","TACTTGGTTCTAGCTTCTTC","chr4",25882483,"-","0:T>A,6:A>C","AGG","AACTTGTTTCTAGCTTCTTCAGG",2,1,7,NA,"GeneID:389203","SMIM20",FALSE,"AIDA"

"5472","TATATGTAAGAATATTCTTT","chr1",222687634,"+","none","AGG","TATATGTAAGAATATTCTTTAGG",0,NA,NA,NA,"GeneID:64853","AIDA",TRUE,"AIDA"

"5473","TATATGTAAGAATATTCTTT","chr1",77811468,"+","none","AGG","TATATGTAAGAATATTCTTTAGG",0,NA,NA,NA,"GeneID:374986","FAM73A",TRUE,"AIDA"

"5474","TATATGTAAGAATATTCTTT","chr4",158278050,"-","none","AGG","TATATGTAAGAATATTCTTTAGG",0,NA,NA,NA,"","",TRUE,"AIDA"

"5475","TATATGTAAGAATATTCTTT","chr2",11308313,"-","none","AGG","TATATGTAAGAATATTCTTTAGG",0,NA,NA,NA,"GeneID:9475","ROCK2",TRUE,"AIDA"

"5476","TATATGTAAGAATATTCTTT","chr14",95073392,"-","15:T>A,16:A>G","AGG","TATATGTAAGAATATATTTTAGG",2,16,17,NA,"","",FALSE,"AIDA"

"5477","TATATGTAAGAATATTCTTT","chr17",47247594,"+","1:C>A","TGG","TCTATGTAAGAATATTCTTTTGG",1,2,NA,NA,"","",FALSE,"AIDA"

"5478","TATATGTAAGAATATTCTTT","chr3",163640573,"+","1:T>A","GGG","TTTATGTAAGAATATTCTTTGGG",1,2,NA,NA,"","",FALSE,"AIDA"

"5479","TATATGTAAGAATATTCTTT","chr18",1928790,"+","9:A>G","AGG","TATATGTAAAAATATTCTTTAGG",1,10,NA,NA,"","",TRUE,"AIDA"

"5480","TATATGTAAGAATATTCTTT","chr13",85492040,"+","1:T>A,11:T>A","TGG","TTTATGTAAGATTATTCTTTTGG",2,2,12,NA,"GeneID:100874137","LINC00351",FALSE,"AIDA"

"5481","TGAATTCACAGAAGAACAAA","chr1",222703149,"-","none","AGG","TGAATTCACAGAAGAACAAAAGG",0,NA,NA,NA,"GeneID:64853","AIDA",TRUE,"AIDA"

"5482","TGAATTCACAGAAGAACAAA","chr19",9115219,"+","0:C>T,3:C>A","GGG","CGACTTCACAGAAGAACAAAGGG",2,1,4,NA,"GeneID:125962","OR7G1",TRUE,"AIDA"

"5483","TGAATTCACAGAAGAACAAA","chr1",97915146,"+","1:C>G,16:G>C","TGG","TCAATTCACAGAAGAAGAAATGG",2,2,17,NA,"GeneID:1806","DPYD",FALSE,"AIDA"

"5484","TGAATTCACAGAAGAACAAA","chr18",65466409,"-","6:A>G,16:A>G","TGG","TGAATTTACAGAAGAATAAATGG",2,7,17,NA,"","",FALSE,"AIDA"

"5485","ATCCTCATCACTGCTATATT","chr6",31615688,"-","none","TGG","ATCCTCATCACTGCTATATTTGG",0,NA,NA,NA,"GeneID:199","AIF1",TRUE,"AIF1"

"5486","ATCCTCATCACTGCTATATT","chr3",119581985,"-","15:G>T,19:C>A","AGG","ATCCTCATCACTGCTCTATGAGG",2,16,20,NA,"GeneID:141","ADPRH",FALSE,"AIF1"

"5487","CAATATCGCCATTTCCATTA","chr6",31616125,"-","none","AGG","CAATATCGCCATTTCCATTAAGG",0,NA,NA,NA,"GeneID:199","AIF1",TRUE,"AIF1"

"5488","CAATATCGCCATTTCCATTA","chr11",100010002,"-","5:T>A,7:G>C","AGG","CAATAACCCCATTTCCATTAAGG",2,6,8,NA,"GeneID:53942","CNTN5",FALSE,"AIF1"

"5489","CATGTCCCTGAAACGAATGC","chr6",31616347,"+","none","TGG","CATGTCCCTGAAACGAATGCTGG",0,NA,NA,NA,"GeneID:199","AIF1",TRUE,"AIF1"

"5490","GAACGTCTCCCCGGAGCCAC","chr6",31616430,"-","none","TGG","GAACGTCTCCCCGGAGCCACTGG",0,NA,NA,NA,"GeneID:199","AIF1",TRUE,"AIF1"

"5491","TACATGGAGTTTGACCTTAA","chr6",31616108,"+","none","TGG","TACATGGAGTTTGACCTTAATGG",0,NA,NA,NA,"GeneID:199","AIF1",TRUE,"AIF1"

"5492","TACATGGAGTTTGACCTTAA","chr2",205694573,"+","9:A>T,13:G>A","GGG","TACATGGAGATTGGCCTTAAGGG",2,10,14,NA,"GeneID:8828","NRP2",FALSE,"AIF1"

"5493","TCCAAACTGGAAGGCTTCAA","chr6",31615714,"+","none","AGG","TCCAAACTGGAAGGCTTCAAAGG",0,NA,NA,NA,"GeneID:199","AIF1",TRUE,"AIF1"

"5494","CAATCTCGCCTTCATTGTTC","chr9",131114598,"-","none","AGG","CAATCTCGCCTTCATTGTTCAGG",0,NA,NA,NA,"GeneID:83543","AIF1L",TRUE,"AIF1L"

"5495","CATGAAATCCTCTGTAGCTG","chr9",131106208,"-","none","CGG","CATGAAATCCTCTGTAGCTGCGG",0,NA,NA,NA,"GeneID:83543","AIF1L",TRUE,"AIF1L"

"5496","CATGAAATCCTCTGTAGCTG","chr1",118825634,"-","1:A>T,7:T>A","TGG","CTTGAAAACCTCTGTAGCTGTGG",2,2,8,NA,"","",FALSE,"AIF1L"

"5497","CCTCCAGGAGGGAAGGCGTT","chr9",131096794,"+","none","CGG","CCTCCAGGAGGGAAGGCGTTCGG",0,NA,NA,NA,"GeneID:83543","AIF1L",TRUE,"AIF1L"

"5498","CTGAACAATGAAGGCGAGAT","chr9",131114596,"+","none","TGG","CTGAACAATGAAGGCGAGATTGG",0,NA,NA,NA,"GeneID:83543","AIF1L",TRUE,"AIF1L"

"5499","CTGAACAATGAAGGCGAGAT","chr6",33113091,"+","3:G>A,15:A>G","GGG","CTGGACAATGAAGGCAAGATGGG",2,4,16,NA,"GeneID:3116","HLA-DPB2",FALSE,"AIF1L"

"5500","GGGGCTTACCCGGTTGATCT","chr9",131096852,"-","none","CGG","GGGGCTTACCCGGTTGATCTCGG",0,NA,NA,NA,"GeneID:83543","AIF1L",TRUE,"AIF1L"

"5501","TTCCTCCTCAGTACTGTGCA","chr9",131106176,"+","none","GGG","TTCCTCCTCAGTACTGTGCAGGG",0,NA,NA,NA,"GeneID:83543","AIF1L",TRUE,"AIF1L"

"5502","AATAGCTTCATAGCCAACAT","chrX",130131762,"+","none","CGG","AATAGCTTCATAGCCAACATCGG",0,NA,NA,NA,"GeneID:9131","AIFM1",TRUE,"AIFM1"

"5503","AATGCTATTGTGCAATCCGT","chrX",130136691,"-","none","TGG","AATGCTATTGTGCAATCCGTTGG",0,NA,NA,NA,"GeneID:9131","AIFM1",TRUE,"AIFM1"

"5504","ACCATGGAAAAAGTCAGACG","chrX",130137079,"-","none","AGG","ACCATGGAAAAAGTCAGACGAGG",0,NA,NA,NA,"GeneID:9131","AIFM1",TRUE,"AIFM1"

"5505","ACCATGGAAAAAGTCAGACG","chr13",99080757,"-","10:C>T,16:T>C","TGG","ACCATGGAAAGAGTCAAACGTGG",2,11,17,NA,"GeneID:23348","DOCK9",FALSE,"AIFM1"

"5506","GACAGTAGTTTGCCCACAGT","chrX",130131733,"-","none","TGG","GACAGTAGTTTGCCCACAGTTGG",0,NA,NA,NA,"GeneID:9131","AIFM1",TRUE,"AIFM1"

"5507","TCTCTTCTTAGGAGTGATTT","chrX",130131790,"-","none","GGG","TCTCTTCTTAGGAGTGATTTGGG",0,NA,NA,NA,"GeneID:9131","AIFM1",TRUE,"AIFM1"

"5508","TCTCTTCTTAGGAGTGATTT","chrX",14750353,"+","8:A>T,11:C>G","GGG","TCTCTTCTAAGCAGTGATTTGGG",2,9,12,NA,"","",FALSE,"AIFM1"

"5509","TCTCTTCTTAGGAGTGATTT","chr12",108396663,"+","4:C>T,15:T>G","GGG","TCTCCTCTTAGGAGTTATTTGGG",2,5,16,NA,"","",FALSE,"AIFM1"

"5510","TCTCTTCTTAGGAGTGATTT","chr15",27470181,"+","6:A>C,17:A>T","TGG","TCTCTTATTAGGAGTGAATTTGG",2,7,18,NA,"GeneID:2567","GABRG3",FALSE,"AIFM1"

"5511","TGCCTTTACAGAGGGGGTTA","chrX",130136722,"-","none","AGG","TGCCTTTACAGAGGGGGTTAAGG",0,NA,NA,NA,"GeneID:9131","AIFM1",TRUE,"AIFM1"

"5512","AAACCTCATTAAACTTGCCC","chr10",70121138,"+","none","GGG","AAACCTCATTAAACTTGCCCGGG",0,NA,NA,NA,"GeneID:84883","AIFM2",TRUE,"AIFM2"

"5513","AAGTGAAGGAGATCCTCCTC","chr10",70117836,"-","none","CGG","AAGTGAAGGAGATCCTCCTCCGG",0,NA,NA,NA,"GeneID:84883","AIFM2",TRUE,"AIFM2"

"5514","AGGAGCTCCTGCCCTCCGTC","chr10",70117863,"-","none","CGG","AGGAGCTCCTGCCCTCCGTCCGG",0,NA,NA,NA,"GeneID:84883","AIFM2",TRUE,"AIFM2"

"5515","AGGAGCTCCTGCCCTCCGTC","chr7",151782615,"+","15:G>C,17:C>G","TGG","AGGAGCTCCTGCCCTGCCTCTGG",2,16,18,NA,"GeneID:51422","PRKAG2",FALSE,"AIFM2"

"5516","AGGAGCTCCTGCCCTCCGTC","chr9",86947885,"-","9:C>A,11:C>G","AGG","AGGAGCTCCGGGCCTCCGTCAGG",2,10,12,NA,"","",FALSE,"AIFM2"

"5517","CCACGATGAACCGTGAGCGC","chr10",70120574,"+","none","TGG","CCACGATGAACCGTGAGCGCTGG",0,NA,NA,NA,"GeneID:84883","AIFM2",TRUE,"AIFM2"

"5518","TCCCCACTACTAGCCCCTGC","chr10",70123445,"+","none","CGG","TCCCCACTACTAGCCCCTGCCGG",0,NA,NA,NA,"GeneID:84883","AIFM2",TRUE,"AIFM2"

"5519","TCCCCACTACTAGCCCCTGC","chr2",238019085,"+","13:T>C,19:T>C","GGG","TCCCCACTACTAGTCCCTGTGGG",2,14,20,NA,"GeneID:140739;GeneID:100533179","UBE2F;UBE2F-SCLY",FALSE,"AIFM2"

"5520","TCCCCACTACTAGCCCCTGC","chr16",78442451,"+","10:C>T,18:C>G","TGG","TCCCCACTACCAGCCCCTCCTGG",2,11,19,NA,"GeneID:51741","WWOX",FALSE,"AIFM2"

"5521","TTCTCCAGGTCCAGCGCTCA","chr10",70120587,"-","none","CGG","TTCTCCAGGTCCAGCGCTCACGG",0,NA,NA,NA,"GeneID:84883","AIFM2",TRUE,"AIFM2"

"5522","CGAGGTGCTCACCGAGGCTC","chr22",20975756,"+","none","AGG","CGAGGTGCTCACCGAGGCTCAGG",0,NA,NA,NA,"GeneID:150209","AIFM3",TRUE,"AIFM3"

"5523","CTAGCGTGCACAGGACGATC","chr22",20974758,"-","none","CGG","CTAGCGTGCACAGGACGATCCGG",0,NA,NA,NA,"GeneID:150209","AIFM3",TRUE,"AIFM3"

"5524","GAACCCACTGGCCATTCTCG","chr22",20973508,"-","none","AGG","GAACCCACTGGCCATTCTCGAGG",0,NA,NA,NA,"GeneID:150209","AIFM3",TRUE,"AIFM3"

"5525","GGCGCTCCTCCGTGTGGAAG","chr22",20973418,"-","none","TGG","GGCGCTCCTCCGTGTGGAAGTGG",0,NA,NA,NA,"GeneID:150209","AIFM3",TRUE,"AIFM3"

"5526","GGTGAGCACCTCGATGCCAT","chr22",20975749,"-","none","AGG","GGTGAGCACCTCGATGCCATAGG",0,NA,NA,NA,"GeneID:150209","AIFM3",TRUE,"AIFM3"

"5527","GGTGAGCACCTCGATGCCAT","chr8",58772534,"+","10:A>T,18:C>A","AGG","GGTGAGCACCACGATGCCCTAGG",2,11,19,NA,"","",FALSE,"AIFM3"

"5528","TCGTCCTGTGCACGCTAGAC","chr22",20974760,"+","none","CGG","TCGTCCTGTGCACGCTAGACCGG",0,NA,NA,NA,"GeneID:150209","AIFM3",TRUE,"AIFM3"

"5529","ATCATACTCACAACCCCAAC","chr6",143136981,"-","none","AGG","ATCATACTCACAACCCCAACAGG",0,NA,NA,NA,"GeneID:51390","AIG1",TRUE,"AIG1"

"5530","CATGTGGGTGTACCCTTTCC","chr6",143333308,"+","none","TGG","CATGTGGGTGTACCCTTTCCTGG",0,NA,NA,NA,"GeneID:51390","AIG1",TRUE,"AIG1"

"5531","CCTCATCTCGATTAATATAA","chr6",143284128,"-","none","AGG","CCTCATCTCGATTAATATAAAGG",0,NA,NA,NA,"GeneID:51390","AIG1",TRUE,"AIG1"

"5532","CGCACCATCAGTATCCCAGC","chr6",143284152,"+","none","AGG","CGCACCATCAGTATCCCAGCAGG",0,NA,NA,NA,"GeneID:51390","AIG1",TRUE,"AIG1"

"5533","CTTTCCAGTCTTCTGACTCG","chr6",143136876,"+","none","AGG","CTTTCCAGTCTTCTGACTCGAGG",0,NA,NA,NA,"GeneID:51390","AIG1",TRUE,"AIG1"

"5534","CTTTCCAGTCTTCTGACTCG","chr16",14211074,"+","16:A>C,19:A>G","TGG","CTTTCCAGTCTTCTGAATCATGG",2,17,20,NA,"GeneID:57496","MKL2",FALSE,"AIG1"

"5535","GATGATATACCCGAAGCTGC","chr6",143165125,"+","none","TGG","GATGATATACCCGAAGCTGCTGG",0,NA,NA,NA,"GeneID:51390","AIG1",TRUE,"AIG1"

"5536","AATAAACTCAATCCCCGACC","chr6",106525153,"+","none","TGG","AATAAACTCAATCCCCGACCTGG",0,NA,NA,NA,"GeneID:202","AIM1",TRUE,"AIM1"

"5537","ATAATCTTACCTTTCCAGGT","chr6",106525170,"-","none","CGG","ATAATCTTACCTTTCCAGGTCGG",0,NA,NA,NA,"GeneID:202","AIM1",TRUE,"AIM1"

"5538","CGACGCGGTGTTCGACGACG","chr6",106512448,"+","none","AGG","CGACGCGGTGTTCGACGACGAGG",0,NA,NA,NA,"GeneID:202","AIM1",TRUE,"AIM1"

"5539","CTGTTCAGCAGATGCACGTT","chr6",106520776,"-","none","TGG","CTGTTCAGCAGATGCACGTTTGG",0,NA,NA,NA,"GeneID:202","AIM1",TRUE,"AIM1"

"5540","GCCTCTAGTAAAACGTTTGT","chr6",106519323,"+","none","TGG","GCCTCTAGTAAAACGTTTGTTGG",0,NA,NA,NA,"GeneID:202","AIM1",TRUE,"AIM1"

"5541","TCGACCTGCCCACGCCGAAA","chr6",106512997,"-","none","TGG","TCGACCTGCCCACGCCGAAATGG",0,NA,NA,NA,"GeneID:202","AIM1",TRUE,"AIM1"

"5542","AAAAGTCTCTCCTCATGTTA","chr1",159068569,"-","none","AGG","AAAAGTCTCTCCTCATGTTAAGG",0,NA,NA,NA,"GeneID:9447","AIM2",TRUE,"AIM2"

"5543","AGTACTGGGGGTTAGAAACG","chr1",159063595,"-","none","AGG","AGTACTGGGGGTTAGAAACGAGG",0,NA,NA,NA,"GeneID:9447","AIM2",TRUE,"AIM2"

"5544","CCTTATCCTACCTTAACATG","chr1",159068556,"+","none","AGG","CCTTATCCTACCTTAACATGAGG",0,NA,NA,NA,"GeneID:9447","AIM2",TRUE,"AIM2"

"5545","TTCACGTTTGAGACCCAAGA","chr1",159066213,"-","none","AGG","TTCACGTTTGAGACCCAAGAAGG",0,NA,NA,NA,"GeneID:9447","AIM2",TRUE,"AIM2"

"5546","TTCAGCATCTAACACACGTG","chr1",159066032,"+","none","AGG","TTCAGCATCTAACACACGTGAGG",0,NA,NA,NA,"GeneID:9447","AIM2",TRUE,"AIM2"

"5547","TTTGACCTAAGTGACAACAC","chr1",159063627,"-","none","TGG","TTTGACCTAAGTGACAACACTGG",0,NA,NA,NA,"GeneID:9447","AIM2",TRUE,"AIM2"

"5548","TTTGACCTAAGTGACAACAC","chr9",118377671,"+","4:T>A,7:A>T","AGG","TTTGTCCAAAGTGACAACACAGG",2,5,8,NA,"","",FALSE,"AIM2"

"5549","TTTGACCTAAGTGACAACAC","chr2",134142417,"+","7:A>T,8:T>A","TGG","TTTGACCATAGTGACAACACTGG",2,8,9,NA,"GeneID:4249","MGAT5",FALSE,"AIM2"

"5550","ACCATAGAATTAGCGTGCAG","chr4",106328107,"-","none","TGG","ACCATAGAATTAGCGTGCAGTGG",0,NA,NA,NA,"GeneID:9255","AIMP1",TRUE,"AIMP1"

"5551","ACTGAAACAAGAGCTAATTC","chr4",106327523,"+","none","AGG","ACTGAAACAAGAGCTAATTCAGG",0,NA,NA,NA,"GeneID:9255","AIMP1",TRUE,"AIMP1"

"5552","ACTGAAACAAGAGCTAATTC","chr20",38055650,"-","10:T>C,18:C>A","CGG","ACTGAAACAAAAGCTAATGCCGG",2,11,19,NA,"GeneID:58490","RPRD1B",FALSE,"AIMP1"

"5553","ACTGAAACAAGAGCTAATTC","chr22",40242039,"-","0:A>T,4:C>T","TGG","TCTGGAACAAGAGCTAATTCTGG",2,1,5,NA,"GeneID:23112","TNRC6B",FALSE,"AIMP1"

"5554","GAATCATGTTCCTCTTGAAC","chr4",106331861,"+","none","AGG","GAATCATGTTCCTCTTGAACAGG",0,NA,NA,NA,"GeneID:9255","AIMP1",TRUE,"AIMP1"

"5555","GCAGCAAGTTTCTCTACTTA","chr4",106325086,"+","none","AGG","GCAGCAAGTTTCTCTACTTAAGG",0,NA,NA,NA,"GeneID:9255","AIMP1",TRUE,"AIMP1"

"5556","GCAGCAAGTTTCTCTACTTA","chr20",14127506,"+","10:G>T","AGG","GCAGCAAGTTGCTCTACTTAAGG",1,11,NA,NA,"GeneID:140733","MACROD2",TRUE,"AIMP1"

"5557","GCCAATAGATGTTTCCCGTC","chr4",106331723,"+","none","TGG","GCCAATAGATGTTTCCCGTCTGG",0,NA,NA,NA,"GeneID:9255","AIMP1",TRUE,"AIMP1"

"5558","TCCACTGCACGCTAATTCTA","chr4",106328103,"+","none","TGG","TCCACTGCACGCTAATTCTATGG",0,NA,NA,NA,"GeneID:9255","AIMP1",TRUE,"AIMP1"

"5559","AAGTCGTGGGCTCATCCGCT","chr7",6015291,"-","none","TGG","AAGTCGTGGGCTCATCCGCTTGG",0,NA,NA,NA,"GeneID:7965","AIMP2",TRUE,"AIMP2"

"5560","ACGCAACCCTTATAGATAGC","chr7",6023413,"+","none","TGG","ACGCAACCCTTATAGATAGCTGG",0,NA,NA,NA,"GeneID:7965;GeneID:27102","AIMP2;EIF2AK1",TRUE,"AIMP2"

"5561","GACTTTAACCACCAATGCGC","chr7",6015306,"+","none","TGG","GACTTTAACCACCAATGCGCTGG",0,NA,NA,NA,"GeneID:7965","AIMP2",TRUE,"AIMP2"

"5562","GCCCCGCCAAGACTATCAGC","chr7",6017998,"+","none","TGG","GCCCCGCCAAGACTATCAGCTGG",0,NA,NA,NA,"GeneID:7965","AIMP2",TRUE,"AIMP2"

"5563","TGAACTCACCGTAGCAGACG","chr7",6023543,"+","none","TGG","TGAACTCACCGTAGCAGACGTGG",0,NA,NA,NA,"GeneID:7965;GeneID:27102","AIMP2;EIF2AK1",TRUE,"AIMP2"

"5564","TTCAGGCACGCTCTTGACCG","chr7",6017940,"-","none","AGG","TTCAGGCACGCTCTTGACCGAGG",0,NA,NA,NA,"GeneID:7965","AIMP2",TRUE,"AIMP2"

"5565","ATCGTGTGCACCATGCGAGA","chr11",67487131,"+","none","AGG","ATCGTGTGCACCATGCGAGAAGG",0,NA,NA,NA,"GeneID:9049","AIP",TRUE,"AIP"

"5566","CCTTGCCCACCGCGATGTTG","chr11",67489304,"-","none","CGG","CCTTGCCCACCGCGATGTTGCGG",0,NA,NA,NA,"GeneID:9049","AIP",TRUE,"AIP"

"5567","CGTCGTCACTGTGCAGCGTC","chr11",67487022,"-","none","CGG","CGTCGTCACTGTGCAGCGTCCGG",0,NA,NA,NA,"GeneID:9049","AIP",TRUE,"AIP"

"5568","GCAACCGGTTGTACCGCGAG","chr11",67490125,"+","none","GGG","GCAACCGGTTGTACCGCGAGGGG",0,NA,NA,NA,"GeneID:9049","AIP",TRUE,"AIP"

"5569","GCTCACCGTCGTACTTGTTG","chr11",67490443,"-","none","AGG","GCTCACCGTCGTACTTGTTGAGG",0,NA,NA,NA,"GeneID:9049","AIP",TRUE,"AIP"

"5570","GGCAATGGCATCGTAGTACT","chr11",67490170,"-","none","TGG","GGCAATGGCATCGTAGTACTTGG",0,NA,NA,NA,"GeneID:9049","AIP",TRUE,"AIP"

"5571","AGAGCCGATTTCCCTCTCCG","chr17",6426957,"+","none","TGG","AGAGCCGATTTCCCTCTCCGTGG",0,NA,NA,NA,"GeneID:23746","AIPL1",TRUE,"AIPL1"

"5572","AGCTCGATCACAAAGACCAG","chr17",6428324,"+","none","AGG","AGCTCGATCACAAAGACCAGAGG",0,NA,NA,NA,"GeneID:23746","AIPL1",TRUE,"AIPL1"

"5573","ATGCCCCGAGTGATTACCAG","chr17",6427033,"-","none","AGG","ATGCCCCGAGTGATTACCAGAGG",0,NA,NA,NA,"GeneID:23746","AIPL1",TRUE,"AIPL1"

"5574","CATGTTCGCCTACCACACGC","chr17",6428388,"-","none","TGG","CATGTTCGCCTACCACACGCTGG",0,NA,NA,NA,"GeneID:23746","AIPL1",TRUE,"AIPL1"

"5575","GTTTCCGATGATGATGTGCA","chr17",6433999,"+","none","TGG","GTTTCCGATGATGATGTGCATGG",0,NA,NA,NA,"GeneID:23746","AIPL1",TRUE,"AIPL1"

"5576","TGCACGAGGTGGCCGAGTTC","chr17",6433933,"-","none","TGG","TGCACGAGGTGGCCGAGTTCTGG",0,NA,NA,NA,"GeneID:23746","AIPL1",TRUE,"AIPL1"

"5577","ACAACCTGGAGCGCTATGGC","chr21",44286677,"+","none","CGG","ACAACCTGGAGCGCTATGGCCGG",0,NA,NA,NA,"GeneID:326","AIRE",TRUE,"AIRE"

"5578","CCCCGCTCACCGTTCCCGAG","chr21",44287581,"-","none","TGG","CCCCGCTCACCGTTCCCGAGTGG",0,NA,NA,NA,"GeneID:326","AIRE",TRUE,"AIRE"

"5579","CGGGACGTCCCCGGAGGACA","chr21",44288386,"-","none","TGG","CGGGACGTCCCCGGAGGACATGG",0,NA,NA,NA,"GeneID:326","AIRE",TRUE,"AIRE"

"5580","GAAGCCCCCGGCCGTCCCCA","chr21",44287008,"+","none","AGG","GAAGCCCCCGGCCGTCCCCAAGG",0,NA,NA,NA,"GeneID:326","AIRE",TRUE,"AIRE"

"5581","GAAGCCCCCGGCCGTCCCCA","chr16",85627959,"-","9:T>C,13:G>C","GGG","GAAGCCCCCAGCCCTCCCCAGGG",2,10,14,NA,"GeneID:23199","GSE1",FALSE,"AIRE"

"5582","GCAGCCGGCCATAGCGCTCC","chr21",44286684,"-","none","AGG","GCAGCCGGCCATAGCGCTCCAGG",0,NA,NA,NA,"GeneID:326","AIRE",TRUE,"AIRE"

"5583","GTGGCGGTACCAAAGCCTTG","chr21",44287026,"-","none","GGG","GTGGCGGTACCAAAGCCTTGGGG",0,NA,NA,NA,"GeneID:326","AIRE",TRUE,"AIRE"

"5584","AGTGTTCTCACGAGTGCGTC","chr1",4772425,"+","none","AGG","AGTGTTCTCACGAGTGCGTCAGG",0,NA,NA,NA,"GeneID:55966","AJAP1",TRUE,"AJAP1"

"5585","CATCACCGTCTCCCTCATCA","chr1",4769877,"+","none","TGG","CATCACCGTCTCCCTCATCATGG",0,NA,NA,NA,"GeneID:55966","AJAP1",TRUE,"AJAP1"

"5586","CATCACCGTCTCCCTCATCA","chr11",70487442,"+","4:C>A,7:T>G","TGG","CATCCCCTTCTCCCTCATCATGG",2,5,8,NA,"GeneID:22941","SHANK2",TRUE,"AJAP1"

"5587","CGTCGAGTGCAGCGTCTCAT","chr1",4772470,"-","none","CGG","CGTCGAGTGCAGCGTCTCATCGG",0,NA,NA,NA,"GeneID:55966","AJAP1",TRUE,"AJAP1"

"5588","GAGCAGCTATGACCATGATG","chr1",4769892,"-","none","AGG","GAGCAGCTATGACCATGATGAGG",0,NA,NA,NA,"GeneID:55966","AJAP1",TRUE,"AJAP1"

"5589","TAAAAGCTGTTGAATCCACA","chr1",4655426,"-","none","TGG","TAAAAGCTGTTGAATCCACATGG",0,NA,NA,NA,"GeneID:55966","AJAP1",TRUE,"AJAP1"

"5590","TAAAAGCTGTTGAATCCACA","chr3",15315401,"-","15:C>G","GGG","TAAAAGCTGTTGAATGCACAGGG",1,16,NA,NA,"GeneID:9467","SH3BP5",FALSE,"AJAP1"

"5591","TAAAAGCTGTTGAATCCACA","chr9",20002087,"+","4:T>A,9:A>T","AGG","TAAATGCTGATGAATCCACAAGG",2,5,10,NA,"","",FALSE,"AJAP1"

"5592","TCCGACGAGTGTTCCCGCTT","chr1",4772288,"-","none","TGG","TCCGACGAGTGTTCCCGCTTTGG",0,NA,NA,NA,"GeneID:55966","AJAP1",TRUE,"AJAP1"

"5593","ACCCGACGGCTCAATCCCCG","chr14",22981342,"+","none","AGG","ACCCGACGGCTCAATCCCCGAGG",0,NA,NA,NA,"GeneID:84962","AJUBA",TRUE,"AJUBA"

"5594","ACCTGTATCAAGTGCAACAA","chr14",22978423,"-","none","AGG","ACCTGTATCAAGTGCAACAAAGG",0,NA,NA,NA,"GeneID:84962","AJUBA",TRUE,"AJUBA"

"5595","ACCTGTATCAAGTGCAACAA","chr5",99579125,"-","11:A>C,15:A>T","AGG","ACCTGTATCAATTGCTACAAAGG",2,12,16,NA,"","",FALSE,"AJUBA"

"5596","ACCTGTATCAAGTGCAACAA","chr5",99733112,"+","11:T>G,15:T>A","AGG","ACCTGTATCAATTGCTACAAAGG",2,12,16,NA,"","",FALSE,"AJUBA"

"5597","CGGGCCGCTGAGCGTACAAT","chr14",22981608,"+","none","CGG","CGGGCCGCTGAGCGTACAATCGG",0,NA,NA,NA,"GeneID:84962","AJUBA",TRUE,"AJUBA"

"5598","GCCTTTGTTGCACTTGATAC","chr14",22978419,"+","none","AGG","GCCTTTGTTGCACTTGATACAGG",0,NA,NA,NA,"GeneID:84962","AJUBA",TRUE,"AJUBA"

"5599","GCCTTTGTTGCACTTGATAC","chr4",119838613,"+","12:A>C,16:C>A","TGG","GCCTTTGTTGCAATTGCTACTGG",2,13,17,NA,"","",FALSE,"AJUBA"

"5600","GCTGAGAAATGCTGTGTCTG","chr14",22976477,"-","none","TGG","GCTGAGAAATGCTGTGTCTGTGG",0,NA,NA,NA,"GeneID:84962","AJUBA",TRUE,"AJUBA"

"5601","GCTGAGAAATGCTGTGTCTG","chr8",139140764,"-","0:A>C,2:T>A","TGG","TCAGAGAAATGCTGTGTCTGTGG",2,1,3,NA,"","",FALSE,"AJUBA"

"5602","GCTGAGAAATGCTGTGTCTG","chr11",117297999,"+","0:C>G,3:T>G","AGG","CCTTAGAAATGCTGTGTCTGAGG",2,1,4,NA,"GeneID:23621","BACE1",FALSE,"AJUBA"

"5603","TTTGAGGCGCCGCGCTACGA","chr14",22982009,"-","none","AGG","TTTGAGGCGCCGCGCTACGAAGG",0,NA,NA,NA,"GeneID:84962","AJUBA",TRUE,"AJUBA"

"5604","ACCCACCACAAAGATGATCT","chr9",127873023,"+","none","TGG","ACCCACCACAAAGATGATCTTGG",0,NA,NA,NA,"GeneID:203","AK1",TRUE,"AK1"

"5605","ACCCACCACAAAGATGATCT","chr7",132460750,"+","11:G>A,18:T>C","TGG","ACCCACCACAAGGATGATTTTGG",2,12,19,NA,"GeneID:91584","PLXNA4",FALSE,"AK1"

"5606","AGTATTGACTTTGGCCACCA","chr9",127871888,"+","none","TGG","AGTATTGACTTTGGCCACCATGG",0,NA,NA,NA,"GeneID:203","AK1",TRUE,"AK1"

"5607","ATTGTCGTCCACACGCCCGC","chr9",127868410,"+","none","TGG","ATTGTCGTCCACACGCCCGCTGG",0,NA,NA,NA,"GeneID:203","AK1",TRUE,"AK1"

"5608","CTCCACGTTTCAAGAGCCGC","chr9",127868436,"+","none","TGG","CTCCACGTTTCAAGAGCCGCTGG",0,NA,NA,NA,"GeneID:203","AK1",TRUE,"AK1"

"5609","GAAAACCAAGATCATCTTTG","chr9",127873031,"-","none","TGG","GAAAACCAAGATCATCTTTGTGG",0,NA,NA,NA,"GeneID:203","AK1",TRUE,"AK1"

"5610","TACCCGCGGGAGGTGCAGCA","chr9",127871844,"-","none","AGG","TACCCGCGGGAGGTGCAGCAAGG",0,NA,NA,NA,"GeneID:203","AK1",TRUE,"AK1"

"5611","TACCCGCGGGAGGTGCAGCA","chr3",159687118,"+","5:T>G,7:T>G","TGG","TACCCTCTGGAGGTGCAGCATGG",2,6,8,NA,"GeneID:100505385","IQCJ-SCHIP1",FALSE,"AK1"

"5612","TACCCGCGGGAGGTGCAGCA","chr20",62559334,"-","6:T>G,13:C>A","GGG","TACCCGAGGGAGGGGCAGCAGGG",2,7,14,NA,"","",FALSE,"AK1"

"5613","AATACCTTCCTGTGATTCTT","chr1",33021361,"+","none","CGG","AATACCTTCCTGTGATTCTTCGG",0,NA,NA,NA,"GeneID:204","AK2",TRUE,"AK2"

"5614","AATACCTTCCTGTGATTCTT","chr14",61384454,"+","3:C>A,8:T>C","TGG","AATCCCTTTCTGTGATTCTTTGG",2,4,9,NA,"GeneID:5583","PRKCH",FALSE,"AK2"

"5615","CCGGATGCCTTTAGGATACT","chr1",33036777,"+","none","CGG","CCGGATGCCTTTAGGATACTCGG",0,NA,NA,NA,"GeneID:204","AK2",TRUE,"AK2"

"5616","CCGGATGCCTTTAGGATACT","chr2",31823766,"+","1:T>C","CGG","CTGGATGCCTTTAGGATACTCGG",1,2,NA,NA,"","",TRUE,"AK2"

"5617","CCGGATGCCTTTAGGATACT","chr4",170034306,"+","2:A>G,16:A>T","TGG","CCAGATGCCTTTAGGAAACTTGG",2,3,17,NA,"","",FALSE,"AK2"

"5618","CTCTCTGTAGGCACCCAGAT","chr1",33024557,"-","none","TGG","CTCTCTGTAGGCACCCAGATTGG",0,NA,NA,NA,"GeneID:204","AK2",TRUE,"AK2"

"5619","CTTCCAGGTGAGTGATGAAA","chr1",33021690,"-","none","TGG","CTTCCAGGTGAGTGATGAAATGG",0,NA,NA,NA,"GeneID:204","AK2",TRUE,"AK2"

"5620","CTTCCAGGTGAGTGATGAAA","chr17",19414653,"+","14:G>A,17:C>A","GGG","CTTCCAGGTGAGTGGTGCAAGGG",2,15,18,NA,"GeneID:7732","RNF112",TRUE,"AK2"

"5621","CTTCCAGGTGAGTGATGAAA","chr4",46361224,"-","9:G>C,10:A>T","GGG","CTTCCAGGTCTGTGATGAAAGGG",2,10,11,NA,"GeneID:2555","GABRA2",FALSE,"AK2"

"5622","CTTCCAGGTGAGTGATGAAA","chr15",66416075,"-","1:T>A,18:C>T","TGG","CATCCAGGTGAGTGATGAGATGG",2,2,19,NA,"GeneID:5604","MAP2K1",FALSE,"AK2"

"5623","GCTCACCTGGGTCCCTTTAC","chr1",33036729,"+","none","CGG","GCTCACCTGGGTCCCTTTACCGG",0,NA,NA,NA,"GeneID:204","AK2",TRUE,"AK2"

"5624","GCTCACCTGGGTCCCTTTAC","chr7",808320,"+","4:T>A,18:C>A","TGG","GCTCTCCTGGGTCCCTTTCCTGG",2,5,19,NA,"","",FALSE,"AK2"

"5625","TTTACAGCTCGATGACCTCA","chr1",33021448,"-","none","TGG","TTTACAGCTCGATGACCTCATGG",0,NA,NA,NA,"GeneID:204","AK2",TRUE,"AK2"

"5626","AAACTCACTGGTAATATTCC","chr9",4718410,"+","none","AGG","AAACTCACTGGTAATATTCCAGG",0,NA,NA,NA,"GeneID:50808","AK3",TRUE,"AK3"

"5627","AACAACGCCTTACTGCTCGC","chr9",4719194,"-","none","TGG","AACAACGCCTTACTGCTCGCTGG",0,NA,NA,NA,"GeneID:50808","AK3",TRUE,"AK3"

"5628","AACAACGCCTTACTGCTCGC","chr12",128944567,"-","18:T>C","TGG","AACAACGCCTTACTGCTCACTGG",1,19,NA,NA,"GeneID:144423","GLT1D1",TRUE,"AK3"

"5629","AACAACGCCTTACTGCTCGC","chr8",143057687,"-","17:A>G","TGG","AACAACGCCTTACTGCTTGCTGG",1,18,NA,NA,"GeneID:286122","C8orf31",TRUE,"AK3"

"5630","AGATGATGTCATGACTCGGC","chr9",4722556,"-","none","TGG","AGATGATGTCATGACTCGGCTGG",0,NA,NA,NA,"GeneID:50808","AK3",TRUE,"AK3"

"5631","AGATGATGTCATGACTCGGC","chr10",32944583,"+","17:A>G","TGG","AGATGATGTCATGACTCAGCTGG",1,18,NA,NA,"GeneID:3688","ITGB1",TRUE,"AK3"

"5632","AGATGATGTCATGACTCGGC","chr16",2125077,"-","6:C>A,13:C>T","TGG","AGATGAGGTCATGGCTCGGCTGG",2,7,14,NA,"GeneID:5310","PKD1",FALSE,"AK3"

"5633","CATCCAACAGCCAGCTATAC","chr9",4722505,"+","none","TGG","CATCCAACAGCCAGCTATACTGG",0,NA,NA,NA,"GeneID:50808","AK3",TRUE,"AK3"

"5634","TAGGGCATTGATGACCTGAC","chr9",4718520,"-","none","TGG","TAGGGCATTGATGACCTGACTGG",0,NA,NA,NA,"GeneID:50808","AK3",TRUE,"AK3"

"5635","TAGGGCATTGATGACCTGAC","chr8",143057610,"-","0:C>A,1:A>T","TGG","GTGGGCATTGATGACCTGACTGG",2,1,2,NA,"GeneID:286122","C8orf31",TRUE,"AK3"

"5636","TGAATTCAATGTTATAGACT","chr9",4719151,"+","none","CGG","TGAATTCAATGTTATAGACTCGG",0,NA,NA,NA,"GeneID:50808","AK3",TRUE,"AK3"

"5637","TGAATTCAATGTTATAGACT","chr3",52734754,"-","13:C>T","TGG","TGAATTCAATGTTGTAGACTTGG",1,14,NA,NA,"GeneID:6787","NEK4",TRUE,"AK3"

"5638","TGAATTCAATGTTATAGACT","chr6",123512534,"+","14:A>T,17:T>A","AGG","TGAATTCAATGTTAAAGTCTAGG",2,15,18,NA,"GeneID:10345","TRDN",FALSE,"AK3"

"5639","TGAATTCAATGTTATAGACT","chr12",68362447,"+","7:C>A,10:T>G","TGG","TGAATTCCATTTTATAGACTTGG",2,8,11,NA,"GeneID:100507195","LOC100507195",FALSE,"AK3"

"5640","TGAATTCAATGTTATAGACT","chr12",128944524,"+","5:C>T,13:G>A","TGG","TGAATCCAATGTTGTAGACTTGG",2,6,14,NA,"GeneID:144423","GLT1D1",TRUE,"AK3"

"5641","TGAATTCAATGTTATAGACT","chr1",95267320,"+","7:T>A,19:A>T","TGG","TGAATTCTATGTTATAGACATGG",2,8,20,NA,"","",FALSE,"AK3"

"5642","AGGTTATATACCCTTCCGCT","chr6",80077975,"+","none","AGG","AGGTTATATACCCTTCCGCTAGG",0,NA,NA,NA,"","",TRUE,"AK4"

"5643","AGGTTATATACCCTTCCGCT","chr12",31616026,"+","none","AGG","AGGTTATATACCCTTCCGCTAGG",0,NA,NA,NA,"","",TRUE,"AK4"

"5644","AGGTTATATACCCTTCCGCT","chr1",65218881,"-","none","AGG","AGGTTATATACCCTTCCGCTAGG",0,NA,NA,NA,"GeneID:205","AK4",TRUE,"AK4"

"5645","AGGTTATATACCCTTCCGCT","chr4",151027345,"-","none","AGG","AGGTTATATACCCTTCCGCTAGG",0,NA,NA,NA,"","",TRUE,"AK4"

"5646","AGGTTATATACCCTTCCGCT","chr17",31345911,"-","none","AGG","AGGTTATATACCCTTCCGCTAGG",0,NA,NA,NA,"GeneID:4763","NF1",TRUE,"AK4"

"5647","AGGTTATATACCCTTCCGCT","chr5",55552140,"+","1:T>G","AGG","ATGTTATATACCCTTCCGCTAGG",1,2,NA,NA,"","",TRUE,"AK4"

"5648","CACCATCAAGGAGCCAGTGC","chr1",65190812,"-","none","TGG","CACCATCAAGGAGCCAGTGCTGG",0,NA,NA,NA,"GeneID:205","AK4",TRUE,"AK4"

"5649","CACCATCAAGGAGCCAGTGC","chr17",31345766,"-","0:T>G","TGG","AACCATCAAGGAGCCAGTGCTGG",1,1,NA,NA,"GeneID:4763","NF1",TRUE,"AK4"

"5650","CACCATCAAGGAGCCAGTGC","chr4",151027199,"-","0:T>G,7:C>T","TGG","AACCATCGAGGAGCCAGTGCTGG",2,1,8,NA,"","",TRUE,"AK4"

"5651","CACCATCAAGGAGCCAGTGC","chr12",31616171,"+","0:A>C","TGG","AACCATCAAGGAGCCAGTGCTGG",1,1,NA,NA,"","",TRUE,"AK4"

"5652","CACCATCAAGGAGCCAGTGC","chr5",55552286,"+","0:A>C,7:G>A","TGG","AACCATCGAGGAGCCAGTGCTGG",2,1,8,NA,"","",TRUE,"AK4"

"5653","CACTTGTATAATTCAATGAC","chr1",65224853,"-","none","TGG","CACTTGTATAATTCAATGACTGG",0,NA,NA,NA,"GeneID:205","AK4",TRUE,"AK4"

"5654","CACTTGTATAATTCAATGAC","chr17",31346058,"-","1:A>T","TGG","CTCTTGTATAATTCAATGACTGG",1,2,NA,NA,"GeneID:4763","NF1",TRUE,"AK4"

"5655","CACTTGTATAATTCAATGAC","chr4",151027488,"-","1:A>T,2:A>G","TGG","CTTTTGTATAATTCAATGACTGG",2,2,3,NA,"","",TRUE,"AK4"

"5656","CACTTGTATAATTCAATGAC","chr12",31615879,"+","1:T>A","TGG","CTCTTGTATAATTCAATGACTGG",1,2,NA,NA,"","",TRUE,"AK4"

"5657","TAGGGTATTGATGACGTCAC","chr1",65224748,"+","none","TGG","TAGGGTATTGATGACGTCACTGG",0,NA,NA,NA,"GeneID:205","AK4",TRUE,"AK4"

"5658","TAGGGTATTGATGACGTCAC","chr12",31615984,"-","0:G>A,2:A>C","TGG","CATGGTATTGATGACGTCACTGG",2,1,3,NA,"","",TRUE,"AK4"

"5659","TAGGGTATTGATGACGTCAC","chr17",31345953,"+","0:C>T,2:T>G","TGG","CATGGTATTGATGACGTCACTGG",2,1,3,NA,"GeneID:4763","NF1",TRUE,"AK4"

"5660","TATATTCTTACATGTACATG","chr1",65218917,"-","none","AGG","TATATTCTTACATGTACATGAGG",0,NA,NA,NA,"GeneID:205","AK4",TRUE,"AK4"

"5661","TATATTCTTACATGTACATG","chr12",47231220,"+","3:T>A,6:T>C","GGG","TATTTTTTTACATGTACATGGGG",2,4,7,NA,"GeneID:91523","PCED1B",FALSE,"AK4"

"5662","TATATTCTTACATGTACATG","chr10",44076903,"+","7:A>T,16:A>C","AGG","TATATTCATACATGTAAATGAGG",2,8,17,NA,"","",FALSE,"AK4"

"5663","TTTAATAGAAGTTGGTGAGA","chr1",65190701,"+","none","TGG","TTTAATAGAAGTTGGTGAGATGG",0,NA,NA,NA,"GeneID:205","AK4",TRUE,"AK4"

"5664","TTTAATAGAAGTTGGTGAGA","chrX",28125241,"+","10:C>G","AGG","TTTAATAGAACTTGGTGAGAAGG",1,11,NA,NA,"","",FALSE,"AK4"

"5665","TTTAATAGAAGTTGGTGAGA","chr7",12823382,"-","2:C>A","GGG","TTGAATAGAAGTTGGTGAGAGGG",1,3,NA,NA,"","",FALSE,"AK4"

"5666","TTTAATAGAAGTTGGTGAGA","chr11",90454662,"-","2:C>A,8:C>T","GGG","TTGAATAGGAGTTGGTGAGAGGG",2,3,9,NA,"GeneID:101929222","DISC1FP1",FALSE,"AK4"

"5667","ACAGATCTGTACCCCCGATT","chr1",77340372,"+","none","TGG","ACAGATCTGTACCCCCGATTTGG",0,NA,NA,NA,"GeneID:26289","AK5",TRUE,"AK5"

"5668","ATACTAGGTGGTCCAGGAAG","chr1",77297551,"+","none","TGG","ATACTAGGTGGTCCAGGAAGTGG",0,NA,NA,NA,"GeneID:26289","AK5",TRUE,"AK5"

"5669","GATGCAAATACCTGATGAAG","chr1",77297865,"+","none","AGG","GATGCAAATACCTGATGAAGAGG",0,NA,NA,NA,"GeneID:26289","AK5",TRUE,"AK5"

"5670","GGTCCTCAAAAGATAGAGCC","chr1",77297925,"-","none","TGG","GGTCCTCAAAAGATAGAGCCTGG",0,NA,NA,NA,"GeneID:26289","AK5",TRUE,"AK5"

"5671","GGTCCTCAAAAGATAGAGCC","chr5",107483489,"+","10:G>A,11:A>G","TGG","GGTCCTCAAAGAATAGAGCCTGG",2,11,12,NA,"GeneID:1946","EFNA5",FALSE,"AK5"

"5672","GTCTCTGATTAGCACAAGCC","chr1",77340405,"-","none","AGG","GTCTCTGATTAGCACAAGCCAGG",0,NA,NA,NA,"GeneID:26289","AK5",TRUE,"AK5"

"5673","TTGAAAATTGCAGAACGATA","chr1",77297590,"+","none","TGG","TTGAAAATTGCAGAACGATATGG",0,NA,NA,NA,"GeneID:26289","AK5",TRUE,"AK5"

"5674","TTGAAAATTGCAGAACGATA","chr18",69622812,"+","9:T>G,15:A>C","AGG","TTGAAAATTTCAGAAAGATAAGG",2,10,16,NA,"GeneID:220164","DOK6",FALSE,"AK5"

"5675","AAGTCTTTCGTACAATACAT","chr5",69355656,"+","none","TGG","AAGTCTTTCGTACAATACATTGG",0,NA,NA,NA,"GeneID:102157402","AK6",TRUE,"AK6"

"5676","AAGTCTTTCGTACAATACAT","chr12",38078687,"+","9:A>G","TGG","AAGTCTTTCATACAATACATTGG",1,10,NA,NA,"","",TRUE,"AK6"

"5677","AAGTCTTTCGTACAATACAT","chrY",18277180,"+","9:A>G,18:G>A","TGG","AAGTCTTTCATACAATACGTTGG",2,10,19,NA,"","",TRUE,"AK6"

"5678","AAGTCTTTCGTACAATACAT","chrY",17628807,"-","9:T>C,18:C>T","TGG","AAGTCTTTCATACAATACGTTGG",2,10,19,NA,"","",TRUE,"AK6"

"5679","AAGTCTTTCGTACAATACAT","chr8",66481268,"-","9:T>C,14:C>T","TGG","AAGTCTTTCATACAGTACATTGG",2,10,15,NA,"GeneID:254778","C8orf46",TRUE,"AK6"

"5680","AAGTCTTTCGTACAATACAT","chr12",34249779,"-","8:A>G,19:C>A","TGG","AAGTCTTTTGTACAATACAGTGG",2,9,20,NA,"","",TRUE,"AK6"

"5681","ACTATATGAAACCAGCGTTC","chr5",69355699,"+","none","AGG","ACTATATGAAACCAGCGTTCAGG",0,NA,NA,NA,"GeneID:102157402","AK6",TRUE,"AK6"

"5682","ACTATATGAAACCAGCGTTC","chr8",6474085,"+","5:G>A","AGG","ACTATGTGAAACCAGCGTTCAGG",1,6,NA,NA,"GeneID:79648","MCPH1",TRUE,"AK6"

"5683","ACTATATGAAACCAGCGTTC","chr12",38078730,"+","1:T>C,19:T>C","AGG","ATTATATGAAACCAGCGTTTAGG",2,2,20,NA,"","",TRUE,"AK6"

"5684","CTTCCGAACATCCTGCTCAC","chr12",34249488,"+","none","CGG","CTTCCGAACATCCTGCTCACCGG",0,NA,NA,NA,"","",TRUE,"AK6"

"5685","CTTCCGAACATCCTGCTCAC","chr5",69369464,"-","none","CGG","CTTCCGAACATCCTGCTCACCGG",0,NA,NA,NA,"GeneID:102157402;GeneID:6880;GeneID:5884","AK6;TAF9;RAD17",TRUE,"AK6"

"5686","CTTCCGAACATCCTGCTCAC","chrY",18277472,"-","3:C>G,4:A>G","CGG","CTTGTGAACATCCTGCTCACCGG",2,4,5,NA,"","",TRUE,"AK6"

"5687","CTTCCGAACATCCTGCTCAC","chrY",17628515,"+","3:G>C,4:T>C","CGG","CTTGTGAACATCCTGCTCACCGG",2,4,5,NA,"","",TRUE,"AK6"

"5688","CTTCCGAACATCCTGCTCAC","chr3",124862462,"+","5:C>G,19:A>C","AGG","CTTCCCAACATCCTGCTCAAAGG",2,6,20,NA,"GeneID:3693","ITGB5",TRUE,"AK6"

"5689","CTTCCGAACATCCTGCTCAC","chr12",38078976,"-","4:A>G,10:C>A","CGG","CTTCTGAACAGCCTGCTCACCGG",2,5,11,NA,"","",TRUE,"AK6"

"5690","GTGGGTGATTTAGCTCGAGA","chr5",69366504,"-","none","AGG","GTGGGTGATTTAGCTCGAGAAGG",0,NA,NA,NA,"GeneID:102157402;GeneID:6880","AK6;TAF9",TRUE,"AK6"

"5691","TGACCGGTGAGCAGGATGTT","chr5",69369458,"+","none","CGG","TGACCGGTGAGCAGGATGTTCGG",0,NA,NA,NA,"GeneID:102157402;GeneID:6880;GeneID:5884","AK6;TAF9;RAD17",TRUE,"AK6"

"5692","TGACCGGTGAGCAGGATGTT","chr11",76087305,"+","14:T>G,18:G>T","AGG","TGACCGGTGAGCAGTATGGTAGG",2,15,19,NA,"GeneID:7405","UVRAG",FALSE,"AK6"

"5693","TGACCGGTGAGCAGGATGTT","chr12",34249494,"-","0:C>A,1:A>C","CGG","GTACCGGTGAGCAGGATGTTCGG",2,1,2,NA,"","",TRUE,"AK6"

"5694","TGACCGGTGAGCAGGATGTT","chr8",66480661,"-","1:A>C,4:A>G","TGG","TTACTGGTGAGCAGGATGTTTGG",2,2,5,NA,"GeneID:254778","C8orf46",TRUE,"AK6"

"5695","TTGTCTTACAGGTACACCAG","chr5",69366586,"-","none","GGG","TTGTCTTACAGGTACACCAGGGG",0,NA,NA,NA,"GeneID:102157402;GeneID:6880","AK6;TAF9",TRUE,"AK6"

"5696","ACTTACGGGGTCCAGGGCTT","chr14",96408927,"-","none","TGG","ACTTACGGGGTCCAGGGCTTTGG",0,NA,NA,NA,"GeneID:122481","AK7",TRUE,"AK7"

"5697","ACTTACGGGGTCCAGGGCTT","chr4",7372753,"-","3:G>A,5:C>G","GGG","ACTCAGGGGGTCCAGGGCTTGGG",2,4,6,NA,"GeneID:57537","SORCS2",FALSE,"AK7"

"5698","CGATAATCTTCTTCAGTGAA","chr14",96420839,"-","none","TGG","CGATAATCTTCTTCAGTGAATGG",0,NA,NA,NA,"GeneID:122481","AK7",TRUE,"AK7"

"5699","CGATAATCTTCTTCAGTGAA","chr3",64589444,"-","9:T>A,19:A>T","AGG","CGATAATCTACTTCAGTGATAGG",2,10,20,NA,"GeneID:56999;GeneID:101929335","ADAMTS9;ADAMTS9-AS1",TRUE,"AK7"

"5700","CTCCAGTATGGAGCGGAAGG","chr14",96437870,"+","none","AGG","CTCCAGTATGGAGCGGAAGGAGG",0,NA,NA,NA,"GeneID:122481","AK7",TRUE,"AK7"

"5701","CTTATAATAGGAGGATTCTG","chr14",96420811,"+","none","AGG","CTTATAATAGGAGGATTCTGAGG",0,NA,NA,NA,"GeneID:122481","AK7",TRUE,"AK7"

"5702","CTTATAATAGGAGGATTCTG","chr4",58325831,"+","10:C>G","AGG","CTTATAATAGCAGGATTCTGAGG",1,11,NA,NA,"","",FALSE,"AK7"

"5703","CTTATAATAGGAGGATTCTG","chr20",41559596,"-","0:A>G,19:T>C","GGG","TTTATAATAGGAGGATTCTAGGG",2,1,20,NA,"GeneID:84181","CHD6",FALSE,"AK7"

"5704","GCAGCATACGTAGTTGCTGC","chr14",96437846,"+","none","TGG","GCAGCATACGTAGTTGCTGCTGG",0,NA,NA,NA,"GeneID:122481","AK7",TRUE,"AK7"

"5705","TTGGGCGCGCTCCAAAGCCC","chr14",96408913,"+","none","TGG","TTGGGCGCGCTCCAAAGCCCTGG",0,NA,NA,NA,"GeneID:122481","AK7",TRUE,"AK7"

"5706","CCACATCTTCGAGTTGATGC","chr9",132878173,"-","none","AGG","CCACATCTTCGAGTTGATGCAGG",0,NA,NA,NA,"GeneID:158067;GeneID:11092","AK8;C9orf9",TRUE,"AK8"

"5707","CCAGAAGGCTTTATCTGCAA","chr9",132863670,"-","none","AGG","CCAGAAGGCTTTATCTGCAAAGG",0,NA,NA,NA,"GeneID:158067","AK8",TRUE,"AK8"

"5708","CCAGAAGGCTTTATCTGCAA","chr3",37416030,"-","12:G>T,19:C>T","TGG","CCAGAAGGCTTTCTCTGCAGTGG",2,13,20,NA,"GeneID:339883","C3orf35",FALSE,"AK8"

"5709","GAACAGCAGTCTCCTCACCC","chr9",132863735,"-","none","TGG","GAACAGCAGTCTCCTCACCCTGG",0,NA,NA,NA,"GeneID:158067","AK8",TRUE,"AK8"

"5710","GAACAGCAGTCTCCTCACCC","chr4",5664645,"+","5:A>G,9:G>T","AGG","GAACAACAGGCTCCTCACCCAGG",2,6,10,NA,"GeneID:132884","EVC2",FALSE,"AK8"

"5711","GATACGGTGCGGGGCGATAG","chr9",132878225,"+","none","TGG","GATACGGTGCGGGGCGATAGTGG",0,NA,NA,NA,"GeneID:158067;GeneID:11092","AK8;C9orf9",TRUE,"AK8"

"5712","GTGCCCAGGATTGTAATATT","chr9",132866934,"-","none","AGG","GTGCCCAGGATTGTAATATTAGG",0,NA,NA,NA,"GeneID:158067","AK8",TRUE,"AK8"

"5713","GTGCCCAGGATTGTAATATT","chr17",31471383,"+","3:T>C,12:A>G","TGG","GTGTCCAGGATTATAATATTTGG",2,4,13,NA,"GeneID:84440","RAB11FIP4",FALSE,"AK8"

"5714","TGGACCTAATATTACAATCC","chr9",132866927,"+","none","TGG","TGGACCTAATATTACAATCCTGG",0,NA,NA,NA,"GeneID:158067","AK8",TRUE,"AK8"

"5715","TGGACCTAATATTACAATCC","chr2",163841424,"-","4:C>G,5:T>G","TGG","TGGAGATAATATTACAATCCTGG",2,5,6,NA,"","",FALSE,"AK8"

"5716","ACTTGTCATAAAGCTAATGT","chr6",109671954,"-","none","TGG","ACTTGTCATAAAGCTAATGTTGG",0,NA,NA,NA,"GeneID:221264","AK9",TRUE,"AK9"

"5717","ATTAGCCCGTTACATAACAC","chr6",109674224,"-","none","AGG","ATTAGCCCGTTACATAACACAGG",0,NA,NA,NA,"GeneID:221264","AK9",TRUE,"AK9"

"5718","ATTGCTGCTGAAACCGAATC","chr6",109672124,"-","none","AGG","ATTGCTGCTGAAACCGAATCAGG",0,NA,NA,NA,"GeneID:221264","AK9",TRUE,"AK9"

"5719","ATTGCTGCTGAAACCGAATC","chr1",215416371,"+","6:T>G,12:C>A","TGG","ATTGCTTCTGAACCCGAATCTGG",2,7,13,NA,"","",FALSE,"AK9"

"5720","GTTTACCATAACTCCTGATT","chr6",109672108,"+","none","CGG","GTTTACCATAACTCCTGATTCGG",0,NA,NA,NA,"GeneID:221264","AK9",TRUE,"AK9"

"5721","GTTTACCATAACTCCTGATT","chr13",91900948,"-","3:G>A,7:G>T","TGG","GTTCACCCTAACTCCTGATTTGG",2,4,8,NA,"GeneID:2262","GPC5",FALSE,"AK9"

"5722","TCCATGCCTGTGTTATGTAA","chr6",109674215,"+","none","CGG","TCCATGCCTGTGTTATGTAACGG",0,NA,NA,NA,"GeneID:221264","AK9",TRUE,"AK9"

"5723","TCCCCAGAAGTCTGTCACTT","chr6",109671920,"-","none","TGG","TCCCCAGAAGTCTGTCACTTTGG",0,NA,NA,NA,"GeneID:221264","AK9",TRUE,"AK9"

"5724","ACGGTCATCTGTGCCGCCCC","chr17",57116112,"+","none","TGG","ACGGTCATCTGTGCCGCCCCTGG",0,NA,NA,NA,"GeneID:8165","AKAP1",TRUE,"AKAP1"

"5725","AGAAGGTCGACCTCATCATC","chr17",57110117,"+","none","TGG","AGAAGGTCGACCTCATCATCTGG",0,NA,NA,NA,"GeneID:8165","AKAP1",TRUE,"AKAP1"

"5726","CATCACCGTGGAGGTCATTG","chr17",57114475,"+","none","TGG","CATCACCGTGGAGGTCATTGTGG",0,NA,NA,NA,"GeneID:8165","AKAP1",TRUE,"AKAP1"

"5727","CCTCGATCTCCCAGATGATG","chr17",57110130,"-","none","AGG","CCTCGATCTCCCAGATGATGAGG",0,NA,NA,NA,"GeneID:8165","AKAP1",TRUE,"AKAP1"

"5728","CTACGAGGAGACCAACGAAG","chr17",57116174,"+","none","TGG","CTACGAGGAGACCAACGAAGTGG",0,NA,NA,NA,"GeneID:8165","AKAP1",TRUE,"AKAP1"

"5729","GTGTGTGCTGCTGCACGAAC","chr17",57114523,"-","none","AGG","GTGTGTGCTGCTGCACGAACAGG",0,NA,NA,NA,"GeneID:8165","AKAP1",TRUE,"AKAP1"

"5730","ACCCAGGTCACCAAAATGAG","chr17",19962837,"+","none","CGG","ACCCAGGTCACCAAAATGAGCGG",0,NA,NA,NA,"GeneID:11216","AKAP10",TRUE,"AKAP10"

"5731","ACTTCGGCGAATGGAGCATT","chr17",19958439,"-","none","TGG","ACTTCGGCGAATGGAGCATTTGG",0,NA,NA,NA,"GeneID:11216","AKAP10",TRUE,"AKAP10"

"5732","CACTGGTCAGCACTTCAATC","chr17",19940945,"+","none","TGG","CACTGGTCAGCACTTCAATCTGG",0,NA,NA,NA,"GeneID:11216","AKAP10",TRUE,"AKAP10"

"5733","CACTGGTCAGCACTTCAATC","chr11",94967768,"+","10:A>C,15:A>C","AGG","CACTGGTCAGAACTTAAATCAGG",2,11,16,NA,"GeneID:51503","CWC15",FALSE,"AKAP10"

"5734","CAGATTGAAGTGCTGACCAG","chr17",19940947,"-","none","TGG","CAGATTGAAGTGCTGACCAGTGG",0,NA,NA,NA,"GeneID:11216","AKAP10",TRUE,"AKAP10"

"5735","CAGATTGAAGTGCTGACCAG","chr17",45946197,"+","6:A>G,12:G>C","GGG","CAGATTAAAGTGGTGACCAGGGG",2,7,13,NA,"GeneID:4137","MAPT",FALSE,"AKAP10"

"5736","GCATTGATTGCAACATGACT","chr17",19962934,"+","none","TGG","GCATTGATTGCAACATGACTTGG",0,NA,NA,NA,"GeneID:11216","AKAP10",TRUE,"AKAP10"

"5737","GCATTGATTGCAACATGACT","chr1",169566857,"-","1:A>G,5:T>C","GGG","GTATTAATTGCAACATGACTGGG",2,2,6,NA,"GeneID:2153","F5",FALSE,"AKAP10"

"5738","TGTCGTGCAAGACTTGTTCA","chr17",19958493,"+","none","AGG","TGTCGTGCAAGACTTGTTCAAGG",0,NA,NA,NA,"GeneID:11216","AKAP10",TRUE,"AKAP10"

"5739","AAGCAGTGATCCTCTAAATC","chr13",42297160,"+","none","AGG","AAGCAGTGATCCTCTAAATCAGG",0,NA,NA,NA,"GeneID:11215","AKAP11",TRUE,"AKAP11"

"5740","ACAAAAGTTACCTGATTTAG","chr13",42297173,"-","none","AGG","ACAAAAGTTACCTGATTTAGAGG",0,NA,NA,NA,"GeneID:11215","AKAP11",TRUE,"AKAP11"

"5741","ACAAAAGTTACCTGATTTAG","chr17",71979535,"+","14:G>A,16:G>T","TGG","ACAAAAGTTACCTGGTGTAGTGG",2,15,17,NA,"","",FALSE,"AKAP11"

"5742","AGTGACAAAGTCATCCTCTA","chr13",42298728,"-","none","TGG","AGTGACAAAGTCATCCTCTATGG",0,NA,NA,NA,"GeneID:11215","AKAP11",TRUE,"AKAP11"

"5743","AGTGACAAAGTCATCCTCTA","chr20",7503024,"+","3:T>G,5:A>C","TGG","AGTTAAAAAGTCATCCTCTATGG",2,4,6,NA,"","",FALSE,"AKAP11"

"5744","CACCTACATCACCAAGACTT","chr13",42298569,"+","none","AGG","CACCTACATCACCAAGACTTAGG",0,NA,NA,NA,"GeneID:11215","AKAP11",TRUE,"AKAP11"

"5745","CCAGTCTGTGTCATCCATAG","chr13",42298711,"+","none","AGG","CCAGTCTGTGTCATCCATAGAGG",0,NA,NA,NA,"GeneID:11215","AKAP11",TRUE,"AKAP11"

"5746","CTCCTAAGTAAATATGCTAC","chr13",42298610,"+","none","TGG","CTCCTAAGTAAATATGCTACTGG",0,NA,NA,NA,"GeneID:11215","AKAP11",TRUE,"AKAP11"

"5747","CTCCTAAGTAAATATGCTAC","chr4",11971827,"+","3:T>C,5:T>A","AGG","CTCTTTAGTAAATATGCTACAGG",2,4,6,NA,"","",FALSE,"AKAP11"

"5748","CACGCCCGTACCTTGGTGGC","chr6",151240715,"-","none","GGG","CACGCCCGTACCTTGGTGGCGGG",0,NA,NA,NA,"GeneID:9590","AKAP12",TRUE,"AKAP12"

"5749","CACGCCCGTACCTTGGTGGC","chr17",38705732,"-","3:G>C,19:T>G","CGG","CACCCCCGTACCTTGGTGGACGG",2,4,20,NA,"GeneID:4302","MLLT6",TRUE,"AKAP12"

"5750","GGGCGACACGGTCGTTAGTG","chr6",151351273,"+","none","AGG","GGGCGACACGGTCGTTAGTGAGG",0,NA,NA,NA,"GeneID:9590","AKAP12",TRUE,"AKAP12"

"5751","TGCCTCGGACCCCGCCACCA","chr6",151240702,"+","none","AGG","TGCCTCGGACCCCGCCACCAAGG",0,NA,NA,NA,"GeneID:9590","AKAP12",TRUE,"AKAP12"

"5752","TGCCTCGGACCCCGCCACCA","chr6",93419723,"-","16:G>T,19:C>T","TGG","TGCCTCGGACCCCGCCCCCGTGG",2,17,20,NA,"","",FALSE,"AKAP12"

"5753","TGTCAAGCCGAAACCTTAGC","chr6",151351805,"+","none","TGG","TGTCAAGCCGAAACCTTAGCTGG",0,NA,NA,NA,"GeneID:9590","AKAP12",TRUE,"AKAP12"

"5754","TTAGGGCTCCTTGACCGTTC","chr6",151305847,"-","none","AGG","TTAGGGCTCCTTGACCGTTCAGG",0,NA,NA,NA,"GeneID:9590","AKAP12",TRUE,"AKAP12"

"5755","TTGCTCAGCTACGCCATTGA","chr6",151305777,"-","none","TGG","TTGCTCAGCTACGCCATTGATGG",0,NA,NA,NA,"GeneID:9590","AKAP12",TRUE,"AKAP12"

"5756","ACATATAAGGGAGCTTGCTG","chr15",85485735,"-","none","TGG","ACATATAAGGGAGCTTGCTGTGG",0,NA,NA,NA,"GeneID:11214","AKAP13",TRUE,"AKAP13"

"5757","ACATATAAGGGAGCTTGCTG","chr15",82503521,"+","0:G>A,17:T>C","TGG","GCATATAAGGGAGCTTGTTGTGG",2,1,18,NA,"","",FALSE,"AKAP13"

"5758","ACATATAAGGGAGCTTGCTG","chr2",122519956,"+","3:G>T,18:A>T","AGG","ACAGATAAGGGAGCTTGCAGAGG",2,4,19,NA,"","",FALSE,"AKAP13"

"5759","ACATATAAGGGAGCTTGCTG","chr15",82365487,"-","0:C>T,17:A>G","TGG","GCATATAAGGGAGCTTGTTGTGG",2,1,18,NA,"GeneID:100134869","UBE2Q2P2",FALSE,"AKAP13"

"5760","CAATTCCTAGCAACCAGTGC","chr15",85533702,"+","none","TGG","CAATTCCTAGCAACCAGTGCTGG",0,NA,NA,NA,"GeneID:11214","AKAP13",TRUE,"AKAP13"

"5761","CAATTCCTAGCAACCAGTGC","chr1",22692721,"-","4:T>A,18:T>C","GGG","CAATACCTAGCAACCAGTACGGG",2,5,19,NA,"","",FALSE,"AKAP13"

"5762","CCCCAATACATTCCACTCCG","chr15",85533841,"-","none","TGG","CCCCAATACATTCCACTCCGTGG",0,NA,NA,NA,"GeneID:11214","AKAP13",TRUE,"AKAP13"

"5763","CCCCAATACATTCCACTCCG","chr2",197233044,"+","0:T>C,13:T>C","GGG","TCCCAATACATTCTACTCCGGGG",2,1,14,NA,"GeneID:91526","ANKRD44",FALSE,"AKAP13"

"5764","CTGCATCATACGCTTCATCC","chr15",85533680,"-","none","TGG","CTGCATCATACGCTTCATCCTGG",0,NA,NA,NA,"GeneID:11214","AKAP13",TRUE,"AKAP13"

"5765","TCCGAGTACTTGTACAGTGA","chr15",85521519,"-","none","CGG","TCCGAGTACTTGTACAGTGACGG",0,NA,NA,NA,"GeneID:11214","AKAP13",TRUE,"AKAP13"

"5766","TCCGTCACTGTACAAGTACT","chr15",85521515,"+","none","CGG","TCCGTCACTGTACAAGTACTCGG",0,NA,NA,NA,"GeneID:11214","AKAP13",TRUE,"AKAP13"

"5767","ACCACCAAGCATTAGTTCAC","chrX",119919941,"+","none","AGG","ACCACCAAGCATTAGTTCACAGG",0,NA,NA,NA,"GeneID:158798","AKAP14",TRUE,"AKAP14"

"5768","ACCAGCAGAGATTCGTGCTA","chrX",119914819,"-","none","CGG","ACCAGCAGAGATTCGTGCTACGG",0,NA,NA,NA,"GeneID:158798","AKAP14",TRUE,"AKAP14"

"5769","ACCAGCAGAGATTCGTGCTA","chr18",24598463,"-","16:A>C,19:A>T","TGG","ACCAGCAGAGATTCGTTCTTTGG",2,17,20,NA,"","",FALSE,"AKAP14"

"5770","ATTACCTGTGAACTAATGCT","chrX",119919948,"-","none","TGG","ATTACCTGTGAACTAATGCTTGG",0,NA,NA,NA,"GeneID:158798","AKAP14",TRUE,"AKAP14"

"5771","CCCGTAGCACGAATCTCTGC","chrX",119914815,"+","none","TGG","CCCGTAGCACGAATCTCTGCTGG",0,NA,NA,NA,"GeneID:158798","AKAP14",TRUE,"AKAP14"

"5772","GACTCACGGTGAATTCACTG","chrX",119903528,"+","none","TGG","GACTCACGGTGAATTCACTGTGG",0,NA,NA,NA,"GeneID:158798","AKAP14",TRUE,"AKAP14"

"5773","TACACAAGACAAGAACTACG","chrX",119903300,"+","none","AGG","TACACAAGACAAGAACTACGAGG",0,NA,NA,NA,"GeneID:158798","AKAP14",TRUE,"AKAP14"

"5774","ACACAGGAAACAAAAGGAGC","chrX",1599185,"+","none","TGG","ACACAGGAAACAAAAGGAGCTGG",0,NA,NA,NA,"GeneID:8227","AKAP17A",TRUE,"AKAP17A_X"

"5775","ACACAGGAAACAAAAGGAGC","chrY",7396317,"-","13:G>T,17:A>T","TGG","ACACAGGAAACAACAGGTGCTGG",2,14,18,NA,"","",FALSE,"AKAP17A_X"

"5776","ACACAGGAAACAAAAGGAGC","chrX",151675408,"-","7:C>T,9:C>T","AGG","ACACAGGGAGCAAAAGGAGCAGG",2,8,10,NA,"GeneID:139135","PASD1",FALSE,"AKAP17A_X"

"5777","ACACAGGAAACAAAAGGAGC","chr19",56161594,"-","9:G>T,18:G>C","AGG","ACACAGGAACCAAAAGGACCAGG",2,10,19,NA,"","",FALSE,"AKAP17A_X"

"5778","AGGACTCACCTTGATGTTGC","chrX",1594213,"-","none","AGG","AGGACTCACCTTGATGTTGCAGG",0,NA,NA,NA,"GeneID:8227","AKAP17A",TRUE,"AKAP17A_X"

"5779","AGGACTCACCTTGATGTTGC","chr1",77034509,"-","13:G>T,19:A>G","GGG","AGGACTCACCTTGCTGTTGTGGG",2,14,20,NA,"GeneID:81849","ST6GALNAC5",FALSE,"AKAP17A_X"

"5780","AGGACTCACCTTGATGTTGC","chr13",52036186,"-","8:C>G,10:G>A","TGG","AGGACTCAGCCTGATGTTGCTGG",2,9,11,NA,"GeneID:341676","NEK5",TRUE,"AKAP17A_X"

"5781","CAGCTGCCGCTTCTTAATTG","chrX",1595420,"-","none","AGG","CAGCTGCCGCTTCTTAATTGAGG",0,NA,NA,NA,"GeneID:8227","AKAP17A",TRUE,"AKAP17A_X"

"5782","CATCGTGCACGACACGTCTG","chrX",1593476,"+","none","AGG","CATCGTGCACGACACGTCTGAGG",0,NA,NA,NA,"GeneID:8227","AKAP17A",TRUE,"AKAP17A_X"

"5783","CTTGGCTCTGCTGAGCAGCT","chrX",1599412,"-","none","CGG","CTTGGCTCTGCTGAGCAGCTCGG",0,NA,NA,NA,"GeneID:8227","AKAP17A",TRUE,"AKAP17A_X"

"5784","CTTGGCTCTGCTGAGCAGCT","chr1",20500875,"+","8:G>T,12:C>G","GGG","CTTGGCTCGGCTCAGCAGCTGGG",2,9,13,NA,"GeneID:79594","MUL1",TRUE,"AKAP17A_X"

"5785","GTGATGCCTCAATTAAGAAG","chrX",1595411,"+","none","CGG","GTGATGCCTCAATTAAGAAGCGG",0,NA,NA,NA,"GeneID:8227","AKAP17A",TRUE,"AKAP17A_X"

"5786","GTGATGCCTCAATTAAGAAG","chr16",22062024,"-","15:A>T,16:T>C","TGG","GTGATGCCTCAATTATAAAGTGG",2,16,17,NA,"GeneID:730094","C16orf52",FALSE,"AKAP17A_X"

"5787","ACACAGGAAACAAAAGGAGC","chrX",1599185,"+","none","TGG","ACACAGGAAACAAAAGGAGCTGG",0,NA,NA,NA,"GeneID:8227","AKAP17A",TRUE,"AKAP17A_Y"

"5788","ACACAGGAAACAAAAGGAGC","chrY",7396317,"-","13:G>T,17:A>T","TGG","ACACAGGAAACAACAGGTGCTGG",2,14,18,NA,"","",FALSE,"AKAP17A_Y"

"5789","ACACAGGAAACAAAAGGAGC","chrX",151675408,"-","7:C>T,9:C>T","AGG","ACACAGGGAGCAAAAGGAGCAGG",2,8,10,NA,"GeneID:139135","PASD1",FALSE,"AKAP17A_Y"

"5790","ACACAGGAAACAAAAGGAGC","chr19",56161594,"-","9:G>T,18:G>C","AGG","ACACAGGAACCAAAAGGACCAGG",2,10,19,NA,"","",FALSE,"AKAP17A_Y"

"5791","AGGACTCACCTTGATGTTGC","chrX",1594213,"-","none","AGG","AGGACTCACCTTGATGTTGCAGG",0,NA,NA,NA,"GeneID:8227","AKAP17A",TRUE,"AKAP17A_Y"

"5792","AGGACTCACCTTGATGTTGC","chr1",77034509,"-","13:G>T,19:A>G","GGG","AGGACTCACCTTGCTGTTGTGGG",2,14,20,NA,"GeneID:81849","ST6GALNAC5",FALSE,"AKAP17A_Y"

"5793","AGGACTCACCTTGATGTTGC","chr13",52036186,"-","8:C>G,10:G>A","TGG","AGGACTCAGCCTGATGTTGCTGG",2,9,11,NA,"GeneID:341676","NEK5",TRUE,"AKAP17A_Y"

"5794","CAGCTGCCGCTTCTTAATTG","chrX",1595420,"-","none","AGG","CAGCTGCCGCTTCTTAATTGAGG",0,NA,NA,NA,"GeneID:8227","AKAP17A",TRUE,"AKAP17A_Y"

"5795","CATCGTGCACGACACGTCTG","chrX",1593476,"+","none","AGG","CATCGTGCACGACACGTCTGAGG",0,NA,NA,NA,"GeneID:8227","AKAP17A",TRUE,"AKAP17A_Y"

"5796","CTTGGCTCTGCTGAGCAGCT","chrX",1599412,"-","none","CGG","CTTGGCTCTGCTGAGCAGCTCGG",0,NA,NA,NA,"GeneID:8227","AKAP17A",TRUE,"AKAP17A_Y"

"5797","CTTGGCTCTGCTGAGCAGCT","chr1",20500875,"+","8:G>T,12:C>G","GGG","CTTGGCTCGGCTCAGCAGCTGGG",2,9,13,NA,"GeneID:79594","MUL1",TRUE,"AKAP17A_Y"

"5798","GTGATGCCTCAATTAAGAAG","chrX",1595411,"+","none","CGG","GTGATGCCTCAATTAAGAAGCGG",0,NA,NA,NA,"GeneID:8227","AKAP17A",TRUE,"AKAP17A_Y"

"5799","GTGATGCCTCAATTAAGAAG","chr16",22062024,"-","15:A>T,16:T>C","TGG","GTGATGCCTCAATTATAAAGTGG",2,16,17,NA,"GeneID:730094","C16orf52",FALSE,"AKAP17A_Y"

"5800","CTTTCTTCGATTAACCCGTG","chr9",110168411,"-","none","TGG","CTTTCTTCGATTAACCCGTGTGG",0,NA,NA,NA,"GeneID:445815;GeneID:11217","PALM2-AKAP2;AKAP2",TRUE,"AKAP2"

"5801","GCCTCTTCAGGGGCTAAGTC","chr9",110156380,"-","none","AGG","GCCTCTTCAGGGGCTAAGTCAGG",0,NA,NA,NA,"GeneID:445815;GeneID:11217","PALM2-AKAP2;AKAP2",TRUE,"AKAP2"

"5802","TAATCGAAGAAAGAGCGCAC","chr9",110168418,"+","none","TGG","TAATCGAAGAAAGAGCGCACTGG",0,NA,NA,NA,"GeneID:445815;GeneID:11217","PALM2-AKAP2;AKAP2",TRUE,"AKAP2"

"5803","TCAACAAGCCATAGCCGAGC","chr9",110137970,"+","none","AGG","TCAACAAGCCATAGCCGAGCAGG",0,NA,NA,NA,"GeneID:445815;GeneID:11217","PALM2-AKAP2;AKAP2",TRUE,"AKAP2"

"5804","TTCTAAGTTACTGTCTTGCA","chr9",110162098,"+","none","AGG","TTCTAAGTTACTGTCTTGCAAGG",0,NA,NA,NA,"GeneID:445815;GeneID:11217","PALM2-AKAP2;AKAP2",TRUE,"AKAP2"

"5805","TTCTATTCACCCCCGCATAA","chr9",110136321,"+","none","TGG","TTCTATTCACCCCCGCATAATGG",0,NA,NA,NA,"GeneID:445815;GeneID:11217","PALM2-AKAP2;AKAP2",TRUE,"AKAP2"

"5806","CAACTTTGCATACTCCATTT","chr12",4638144,"+","none","TGG","CAACTTTGCATACTCCATTTTGG",0,NA,NA,NA,"GeneID:10566","AKAP3",TRUE,"AKAP3"

"5807","CAACTTTGCATACTCCATTT","chr10",19526607,"-","3:T>G,15:T>G","TGG","CAAATTTGCATACTCAATTTTGG",2,4,16,NA,"GeneID:340895","MALRD1",FALSE,"AKAP3"

"5808","GAAGGCCGAAGTTAGCCTAC","chr12",4626771,"+","none","TGG","GAAGGCCGAAGTTAGCCTACTGG",0,NA,NA,NA,"GeneID:10566","AKAP3",TRUE,"AKAP3"

"5809","GACTGGTTACAAAGCCAAAA","chr12",4638161,"-","none","TGG","GACTGGTTACAAAGCCAAAATGG",0,NA,NA,NA,"GeneID:10566","AKAP3",TRUE,"AKAP3"

"5810","GTCGGTGCTGCGCTATGAGA","chr12",4615815,"-","none","AGG","GTCGGTGCTGCGCTATGAGAAGG",0,NA,NA,NA,"GeneID:10566","AKAP3",TRUE,"AKAP3"

"5811","GTGATTGCTGACAATCACCG","chr12",4626615,"+","none","TGG","GTGATTGCTGACAATCACCGTGG",0,NA,NA,NA,"GeneID:10566","AKAP3",TRUE,"AKAP3"

"5812","TCCCCACCGCCTCATTCAGC","chr12",4615785,"+","none","TGG","TCCCCACCGCCTCATTCAGCTGG",0,NA,NA,NA,"GeneID:10566","AKAP3",TRUE,"AKAP3"

"5813","TCCCCACCGCCTCATTCAGC","chr2",8629471,"+","14:C>T,15:C>T","AGG","TCCCCACCGCCTCACCCAGCAGG",2,15,16,NA,"","",FALSE,"AKAP3"

"5814","TCCCCACCGCCTCATTCAGC","chr19",40278789,"+","11:C>T,14:C>T","AGG","TCCCCACCGCCCCACTCAGCAGG",2,12,15,NA,"GeneID:208","AKT2",FALSE,"AKAP3"

"5815","AATCTTTATCTTCTACATTC","chrX",50197548,"+","none","AGG","AATCTTTATCTTCTACATTCAGG",0,NA,NA,NA,"GeneID:8852","AKAP4",TRUE,"AKAP4"

"5816","AGATGTCTGATGATATTGAC","chrX",50198734,"-","none","TGG","AGATGTCTGATGATATTGACTGG",0,NA,NA,NA,"GeneID:8852","AKAP4",TRUE,"AKAP4"

"5817","AGATGTCTGATGATATTGAC","chr3",150709999,"-","4:A>C,14:G>T","TGG","AGATTTCTGATGATCTTGACTGG",2,5,15,NA,"GeneID:101928085","ERICH6-AS1",FALSE,"AKAP4"

"5818","CCCAGAAGGACAGCAAGATC","chrX",50198670,"-","none","AGG","CCCAGAAGGACAGCAAGATCAGG",0,NA,NA,NA,"GeneID:8852","AKAP4",TRUE,"AKAP4"

"5819","CCCAGAAGGACAGCAAGATC","chr12",89421396,"-","8:G>C,17:A>T","TGG","CCCAGAAGCACAGCAAGTTCTGG",2,9,18,NA,"GeneID:282809","POC1B",FALSE,"AKAP4"

"5820","GGCGTACTCTGATACTACAA","chrX",50200864,"-","none","TGG","GGCGTACTCTGATACTACAATGG",0,NA,NA,NA,"GeneID:8852","AKAP4",TRUE,"AKAP4"

"5821","TCCTGATCTTGCTGTCCTTC","chrX",50198666,"+","none","TGG","TCCTGATCTTGCTGTCCTTCTGG",0,NA,NA,NA,"GeneID:8852","AKAP4",TRUE,"AKAP4"

"5822","TGTAGAAGATAAAGATTACA","chrX",50197545,"-","none","AGG","TGTAGAAGATAAAGATTACAAGG",0,NA,NA,NA,"GeneID:8852","AKAP4",TRUE,"AKAP4"

"5823","AACAAAGGCTAAGTCAACCC","chr14",64468912,"+","none","AGG","AACAAAGGCTAAGTCAACCCAGG",0,NA,NA,NA,"GeneID:7597;GeneID:9495","ZBTB25;AKAP5",TRUE,"AKAP5"

"5824","AACAAAGGCTAAGTCAACCC","chr4",55938383,"-","11:A>T,14:A>G","AGG","AACAAAGGCTATGTTAACCCAGG",2,12,15,NA,"","",FALSE,"AKAP5"

"5825","ACAGACCCCATTGAATGATC","chr14",64468888,"+","none","AGG","ACAGACCCCATTGAATGATCAGG",0,NA,NA,NA,"GeneID:7597;GeneID:9495","ZBTB25;AKAP5",TRUE,"AKAP5"

"5826","AGATGCCTTCACTTAGATCC","chr14",64468933,"-","none","TGG","AGATGCCTTCACTTAGATCCTGG",0,NA,NA,NA,"GeneID:7597;GeneID:9495","ZBTB25;AKAP5",TRUE,"AKAP5"

"5827","GCATCCTCAGCATTTATTGC","chr14",64468715,"-","none","AGG","GCATCCTCAGCATTTATTGCAGG",0,NA,NA,NA,"GeneID:7597;GeneID:9495","ZBTB25;AKAP5",TRUE,"AKAP5"

"5828","TCAAACGTCTTGTAACACGC","chr14",64468638,"+","none","AGG","TCAAACGTCTTGTAACACGCAGG",0,NA,NA,NA,"GeneID:7597;GeneID:9495","ZBTB25;AKAP5",TRUE,"AKAP5"

"5829","TCAAACGTCTTGTAACACGC","chr17",15119266,"-","5:A>G,12:G>A","AGG","TCAAATGTCTTGCAACACGCAGG",2,6,13,NA,"","",FALSE,"AKAP5"

"5830","TGTTACAAGACGTTTGAGTG","chr14",64468635,"-","none","AGG","TGTTACAAGACGTTTGAGTGAGG",0,NA,NA,NA,"GeneID:7597;GeneID:9495","ZBTB25;AKAP5",TRUE,"AKAP5"

"5831","ACTGTATCTGGAGACACACT","chr14",32577220,"+","none","TGG","ACTGTATCTGGAGACACACTTGG",0,NA,NA,NA,"GeneID:9472","AKAP6",TRUE,"AKAP6"

"5832","CCAGCACTGAGACTCGAAGC","chr14",32535687,"-","none","TGG","CCAGCACTGAGACTCGAAGCTGG",0,NA,NA,NA,"GeneID:9472","AKAP6",TRUE,"AKAP6"

"5833","CCATTCCTAGTGACGTGACT","chr14",32546486,"-","none","TGG","CCATTCCTAGTGACGTGACTTGG",0,NA,NA,NA,"GeneID:9472","AKAP6",TRUE,"AKAP6"

"5834","CCTGAGCTTATCCGAAGTGT","chr14",32545448,"+","none","TGG","CCTGAGCTTATCCGAAGTGTTGG",0,NA,NA,NA,"GeneID:9472","AKAP6",TRUE,"AKAP6"

"5835","GTGTCTCCAGATACAGTTTA","chr14",32577217,"-","none","AGG","GTGTCTCCAGATACAGTTTAAGG",0,NA,NA,NA,"GeneID:9472","AKAP6",TRUE,"AKAP6"

"5836","TTACTCTGTCAACGTGATAG","chr14",32535633,"+","none","TGG","TTACTCTGTCAACGTGATAGTGG",0,NA,NA,NA,"GeneID:9472","AKAP6",TRUE,"AKAP6"

"5837","AATACAACAAGATGAGCGAC","chr6",131165115,"+","none","TGG","AATACAACAAGATGAGCGACTGG",0,NA,NA,NA,"GeneID:9465","AKAP7",TRUE,"AKAP7"

"5838","AGCCAATACTATGGATTCTC","chr6",131145348,"+","none","TGG","AGCCAATACTATGGATTCTCTGG",0,NA,NA,NA,"GeneID:9465","AKAP7",TRUE,"AKAP7"

"5839","AGCCAATACTATGGATTCTC","chr8",16462064,"-","8:A>G,10:A>T","TGG","AGCCAATATTTTGGATTCTCTGG",2,9,11,NA,"GeneID:4481","MSR1",FALSE,"AKAP7"

"5840","ATTCATAGAAGAACTCCTCC","chr6",131169142,"+","none","AGG","ATTCATAGAAGAACTCCTCCAGG",0,NA,NA,NA,"GeneID:9465","AKAP7",TRUE,"AKAP7"

"5841","ATTCATAGAAGAACTCCTCC","chr2",127630881,"-","1:G>A,8:C>T","AGG","ACTCATAGGAGAACTCCTCCAGG",2,2,9,NA,"GeneID:4648","MYO7B",TRUE,"AKAP7"

"5842","CATTCCAATCACCAACAAAG","chr6",131160176,"+","none","AGG","CATTCCAATCACCAACAAAGAGG",0,NA,NA,NA,"GeneID:9465","AKAP7",TRUE,"AKAP7"

"5843","CATTCCAATCACCAACAAAG","chr1",65670466,"+","15:A>C,19:A>G","TGG","CATTCCAATCACCAAAAAAATGG",2,16,20,NA,"","",FALSE,"AKAP7"

"5844","TACCAGAGAATCCATAGTAT","chr6",131145353,"-","none","TGG","TACCAGAGAATCCATAGTATTGG",0,NA,NA,NA,"GeneID:9465","AKAP7",TRUE,"AKAP7"

"5845","TTAATAATTGCATCACCAGC","chr6",131165178,"-","none","AGG","TTAATAATTGCATCACCAGCAGG",0,NA,NA,NA,"GeneID:9465","AKAP7",TRUE,"AKAP7"

"5846","CCCTAAGGTGCATATGGAAC","chr19",15374622,"-","none","TGG","CCCTAAGGTGCATATGGAACTGG",0,NA,NA,NA,"GeneID:10270","AKAP8",TRUE,"AKAP8"

"5847","CCCTAAGGTGCATATGGAAC","chr1",48216992,"+","5:G>A,8:A>T","AGG","CCCTAGGGAGCATATGGAACAGG",2,6,9,NA,"","",FALSE,"AKAP8"

"5848","GAAGCAGTTCCAACTTTACG","chr19",15372274,"-","none","AGG","GAAGCAGTTCCAACTTTACGAGG",0,NA,NA,NA,"GeneID:10270","AKAP8",TRUE,"AKAP8"

"5849","GAAGGGCTTACCTTGCCAGC","chr19",15374591,"+","none","TGG","GAAGGGCTTACCTTGCCAGCTGG",0,NA,NA,NA,"GeneID:10270","AKAP8",TRUE,"AKAP8"

"5850","TGTCTGGCAGATGACGCAGC","chr19",15371988,"-","none","TGG","TGTCTGGCAGATGACGCAGCTGG",0,NA,NA,NA,"GeneID:10270","AKAP8",TRUE,"AKAP8"

"5851","TGTCTGGCAGATGACGCAGC","chrX",12233948,"-","1:G>C,19:T>G","AGG","TCTCTGGCAGATGACGCAGAAGG",2,2,20,NA,"GeneID:9758","FRMPD4",FALSE,"AKAP8"

"5852","TTCTTGCAGGCTACGGGGCG","chr19",15377003,"-","none","TGG","TTCTTGCAGGCTACGGGGCGTGG",0,NA,NA,NA,"GeneID:10270","AKAP8",TRUE,"AKAP8"

"5853","TTTGACCGCTTCGGACCAGA","chr19",15372312,"-","none","TGG","TTTGACCGCTTCGGACCAGATGG",0,NA,NA,NA,"GeneID:10270","AKAP8",TRUE,"AKAP8"

"5854","AATTAACCAGCGCTTAGATA","chr19",15403535,"-","none","TGG","AATTAACCAGCGCTTAGATATGG",0,NA,NA,NA,"GeneID:26993","AKAP8L",TRUE,"AKAP8L"

"5855","AGATAGCAAAGCCACCCGCA","chr19",15400979,"-","none","CGG","AGATAGCAAAGCCACCCGCACGG",0,NA,NA,NA,"GeneID:26993","AKAP8L",TRUE,"AKAP8L"

"5856","ATCTAAGCGCTGGTTAATTC","chr19",15403536,"+","none","TGG","ATCTAAGCGCTGGTTAATTCTGG",0,NA,NA,NA,"GeneID:26993","AKAP8L",TRUE,"AKAP8L"

"5857","CACTTTGCAGTCGACATACT","chr19",15410552,"-","none","CGG","CACTTTGCAGTCGACATACTCGG",0,NA,NA,NA,"GeneID:26993","AKAP8L",TRUE,"AKAP8L"

"5858","CGAGTATGTCGACTGCAAAG","chr19",15410550,"+","none","TGG","CGAGTATGTCGACTGCAAAGTGG",0,NA,NA,NA,"GeneID:26993","AKAP8L",TRUE,"AKAP8L"

"5859","TGTTGTCCGAGCAGTCCGTG","chr19",15400961,"+","none","CGG","TGTTGTCCGAGCAGTCCGTGCGG",0,NA,NA,NA,"GeneID:26993","AKAP8L",TRUE,"AKAP8L"

"5860","AGAAATAACCAGTCATGAGC","chr7",91973931,"+","none","AGG","AGAAATAACCAGTCATGAGCAGG",0,NA,NA,NA,"GeneID:10142","AKAP9",TRUE,"AKAP9"

"5861","AGAAATAACCAGTCATGAGC","chr3",149708080,"-","6:C>T,7:A>T","TGG","AGAAATGTCCAGTCATGAGCTGG",2,7,8,NA,"GeneID:25937","WWTR1",FALSE,"AKAP9"

"5862","AGCAGATGACTGCAGTTCAG","chr7",91980311,"+","none","AGG","AGCAGATGACTGCAGTTCAGAGG",0,NA,NA,NA,"GeneID:10142","AKAP9",TRUE,"AKAP9"

"5863","AGCAGATGACTGCAGTTCAG","chr10",34001427,"+","11:A>G,16:C>T","AGG","AGCAGATGACTACAGTCCAGAGG",2,12,17,NA,"","",FALSE,"AKAP9"

"5864","ATAATGAGAACTCTACATAG","chr7",91973908,"+","none","TGG","ATAATGAGAACTCTACATAGTGG",0,NA,NA,NA,"GeneID:10142","AKAP9",TRUE,"AKAP9"

"5865","ATAATGAGAACTCTACATAG","chr8",124423056,"-","5:G>C,11:T>A","AGG","ATAATCAGAACACTACATAGAGG",2,6,12,NA,"","",FALSE,"AKAP9"

"5866","TCCATCATCTCTAGATGAGT","chr7",91992941,"-","none","CGG","TCCATCATCTCTAGATGAGTCGG",0,NA,NA,NA,"GeneID:10142","AKAP9",TRUE,"AKAP9"

"5867","TCCATCATCTCTAGATGAGT","chr2",176113485,"-","10:A>G,16:G>C","TGG","TCCATCATCTTTAGATCAGTTGG",2,11,17,NA,"GeneID:3236","HOXD10",FALSE,"AKAP9"

"5868","TCCGACTCATCTAGAGATGA","chr7",91992937,"+","none","TGG","TCCGACTCATCTAGAGATGATGG",0,NA,NA,NA,"GeneID:10142","AKAP9",TRUE,"AKAP9"

"5869","TGAACTGCAGTCATCTGCTG","chr7",91980310,"-","none","TGG","TGAACTGCAGTCATCTGCTGTGG",0,NA,NA,NA,"GeneID:10142","AKAP9",TRUE,"AKAP9"

"5870","TGAACTGCAGTCATCTGCTG","chr7",102565798,"-","17:A>G,19:A>C","AGG","TGAACTGCAGTCATCTGTTTAGG",2,18,20,NA,"GeneID:548644","POLR2J3",TRUE,"AKAP9"

"5871","TGAACTGCAGTCATCTGCTG","chr7",75506585,"-","17:A>G,19:A>C","AGG","TGAACTGCAGTCATCTGTTTAGG",2,18,20,NA,"GeneID:5387","PMS2P3",FALSE,"AKAP9"

"5872","TGAACTGCAGTCATCTGCTG","chr7",102664882,"-","17:A>G,19:A>C","AGG","TGAACTGCAGTCATCTGTTTAGG",2,18,20,NA,"GeneID:246721","POLR2J2",FALSE,"AKAP9"

"5873","TGAACTGCAGTCATCTGCTG","chr12",9322253,"-","15:G>A,16:T>C","GGG","TGAACTGCAGTCATCCACTGGGG",2,16,17,NA,"","",FALSE,"AKAP9"

"5874","TGAACTGCAGTCATCTGCTG","chr7",102343448,"+","17:T>C,19:T>G","AGG","TGAACTGCAGTCATCTGTTTAGG",2,18,20,NA,"","",FALSE,"AKAP9"

"5875","TGAACTGCAGTCATCTGCTG","chr7",76529027,"+","17:T>C,19:T>G","AGG","TGAACTGCAGTCATCTGTTTAGG",2,18,20,NA,"","",FALSE,"AKAP9"

"5876","TGAACTGCAGTCATCTGCTG","chr12",9409505,"+","15:C>T,16:A>G","GGG","TGAACTGCAGTCATCCACTGGGG",2,16,17,NA,"","",FALSE,"AKAP9"

"5877","CATGCCCTGGAGCGTCCCAA","chr11",8911566,"+","none","AGG","CATGCCCTGGAGCGTCCCAAAGG",0,NA,NA,NA,"GeneID:56672","AKIP1",TRUE,"AKIP1"

"5878","CATGTCTATCGTTATCACAG","chr11",8914864,"+","none","AGG","CATGTCTATCGTTATCACAGAGG",0,NA,NA,NA,"GeneID:56672","AKIP1",TRUE,"AKIP1"

"5879","CATGTCTATCGTTATCACAG","chr6",90116955,"-","10:T>C,13:A>T","AGG","CATGTCTATCATTTTCACAGAGG",2,11,14,NA,"GeneID:60468","BACH2",TRUE,"AKIP1"

"5880","CTCTGTGATAACGATAGACA","chr11",8914866,"-","none","TGG","CTCTGTGATAACGATAGACATGG",0,NA,NA,NA,"GeneID:56672","AKIP1",TRUE,"AKIP1"

"5881","CTCTGTGATAACGATAGACA","chr6",90116953,"+","8:A>T,11:T>C","TGG","CTCTGTGAAAATGATAGACATGG",2,9,12,NA,"GeneID:60468","BACH2",TRUE,"AKIP1"

"5882","GACTATACTTCAAGTCAGTG","chr11",8912509,"+","none","TGG","GACTATACTTCAAGTCAGTGTGG",0,NA,NA,NA,"GeneID:56672","AKIP1",TRUE,"AKIP1"

"5883","GATATTTGATAGCTGCCTCC","chr11",8917319,"-","none","AGG","GATATTTGATAGCTGCCTCCAGG",0,NA,NA,NA,"GeneID:56672","AKIP1",TRUE,"AKIP1"

"5884","GATATTTGATAGCTGCCTCC","chr6",90116856,"+","12:A>C","AGG","GATATTTGATAGATGCCTCCAGG",1,13,NA,NA,"GeneID:60468","BACH2",TRUE,"AKIP1"

"5885","TCTCCCGGGAGAACGCGCTG","chr11",8911650,"-","none","CGG","TCTCCCGGGAGAACGCGCTGCGG",0,NA,NA,NA,"GeneID:56672","AKIP1",TRUE,"AKIP1"

"5886","AAGAATATAGTCGTTATCAG","chr1",38998194,"+","none","AGG","AAGAATATAGTCGTTATCAGAGG",0,NA,NA,NA,"GeneID:79647","AKIRIN1",TRUE,"AKIRIN1"

"5887","AAGAATATAGTCGTTATCAG","chr21",35389633,"+","12:T>G,13:G>T","TGG","AAGAATATAGTCTGTATCAGTGG",2,13,14,NA,"GeneID:861","RUNX1",FALSE,"AKIRIN1"

"5888","AAGAATATAGTCGTTATCAG","chrX",68133106,"+","0:T>A,7:C>T","AGG","TAGAATACAGTCGTTATCAGAGG",2,1,8,NA,"GeneID:4983","OPHN1",TRUE,"AKIRIN1"

"5889","AAGAATATAGTCGTTATCAG","chr9",100606835,"+","6:G>A,12:T>G","TGG","AAGAATGTAGTCTTTATCAGTGG",2,7,13,NA,"","",FALSE,"AKIRIN1"

"5890","ACATTTACCCTCCGACAAGT","chr1",39001000,"+","none","TGG","ACATTTACCCTCCGACAAGTTGG",0,NA,NA,NA,"GeneID:79647","AKIRIN1",TRUE,"AKIRIN1"

"5891","ACATTTACCCTCCGACAAGT","chrX",68133252,"+","12:T>C,18:A>G","CGG","ACATTTACCCTCTGACAAATCGG",2,13,19,NA,"GeneID:4983","OPHN1",TRUE,"AKIRIN1"

"5892","ATCCTCAATACCAAACTAGC","chr1",39001084,"+","none","AGG","ATCCTCAATACCAAACTAGCAGG",0,NA,NA,NA,"GeneID:79647","AKIRIN1",TRUE,"AKIRIN1"

"5893","ATCCTCAATACCAAACTAGC","chrX",147272222,"-","0:G>T,11:A>G","TGG","CTCCTCAATACTAAACTAGCTGG",2,1,12,NA,"","",FALSE,"AKIRIN1"

"5894","CACATGATCAGATTATGCGA","chr1",39003373,"+","none","CGG","CACATGATCAGATTATGCGACGG",0,NA,NA,NA,"GeneID:79647","AKIRIN1",TRUE,"AKIRIN1"

"5895","CACATGATCAGATTATGCGA","chrX",68133386,"+","18:A>G","CGG","CACATGATCAGATTATGCAACGG",1,19,NA,NA,"GeneID:4983","OPHN1",TRUE,"AKIRIN1"

"5896","CCTGGGCCTACCTGCTAGTT","chr1",39001097,"-","none","TGG","CCTGGGCCTACCTGCTAGTTTGG",0,NA,NA,NA,"GeneID:79647","AKIRIN1",TRUE,"AKIRIN1"

"5897","GTGGGCTTACCTGGAGAGCT","chr1",38998301,"-","none","AGG","GTGGGCTTACCTGGAGAGCTAGG",0,NA,NA,NA,"GeneID:79647","AKIRIN1",TRUE,"AKIRIN1"

"5898","AACCTGCCGTAGAGTAAATA","chr6",87677902,"+","none","AGG","AACCTGCCGTAGAGTAAATAAGG",0,NA,NA,NA,"GeneID:55122","AKIRIN2",TRUE,"AKIRIN2"

"5899","AACCTGCCGTAGAGTAAATA","chr4",149790115,"-","8:T>C","AGG","AACCTGCCATAGAGTAAATAAGG",1,9,NA,NA,"GeneID:285423","LOC285423",TRUE,"AKIRIN2"

"5900","ATATTGAACACAAAACTTGC","chr6",87677819,"-","none","AGG","ATATTGAACACAAAACTTGCAGG",0,NA,NA,NA,"GeneID:55122","AKIRIN2",TRUE,"AKIRIN2"

"5901","ATGTTATTACCTGGTGAAGC","chr6",87681609,"+","none","TGG","ATGTTATTACCTGGTGAAGCTGG",0,NA,NA,NA,"GeneID:55122","AKIRIN2",TRUE,"AKIRIN2"

"5902","ATGTTATTACCTGGTGAAGC","chr3",163227257,"-","0:A>T,6:T>A","TGG","TTGTTAATACCTGGTGAAGCTGG",2,1,7,NA,"GeneID:647107","LINC01192",FALSE,"AKIRIN2"

"5903","ATGTTATTACCTGGTGAAGC","chr2",100362233,"-","2:A>C,12:A>C","AGG","ATTTTATTACCTTGTGAAGCAGG",2,3,13,NA,"","",FALSE,"AKIRIN2"

"5904","ATGTTATTACCTGGTGAAGC","chr13",34998247,"-","2:G>C,12:A>C","AGG","ATCTTATTACCTTGTGAAGCAGG",2,3,13,NA,"GeneID:26960","NBEA",FALSE,"AKIRIN2"

"5905","CCACATGCATTTCTCCTCAG","chr6",87681636,"-","none","TGG","CCACATGCATTTCTCCTCAGTGG",0,NA,NA,NA,"GeneID:55122","AKIRIN2",TRUE,"AKIRIN2"

"5906","CCACATGCATTTCTCCTCAG","chr17",12713089,"-","8:A>T,9:G>A","AGG","CCACATGCTCTTCTCCTCAGAGG",2,9,10,NA,"GeneID:93649","MYOCD",FALSE,"AKIRIN2"

"5907","CCACATGCATTTCTCCTCAG","chr4",149790033,"+","0:G>C","TGG","GCACATGCATTTCTCCTCAGTGG",1,1,NA,NA,"GeneID:285423","LOC285423",TRUE,"AKIRIN2"

"5908","CCACATGCATTTCTCCTCAG","chr22",33417376,"-","4:G>T,16:G>A","AGG","CCACCTGCATTTCTCCCCAGAGG",2,5,17,NA,"GeneID:9215","LARGE",FALSE,"AKIRIN2"

"5909","CCACATGCATTTCTCCTCAG","chr1",248306034,"-","0:A>G,19:T>C","AGG","TCACATGCATTTCTCCTCAAAGG",2,1,20,NA,"","",FALSE,"AKIRIN2"

"5910","CCACTGAGGAGAAATGCATG","chr6",87681633,"+","none","TGG","CCACTGAGGAGAAATGCATGTGG",0,NA,NA,NA,"GeneID:55122","AKIRIN2",TRUE,"AKIRIN2"

"5911","CCACTGAGGAGAAATGCATG","chr11",100384147,"-","3:C>G,19:G>C","GGG","CCAGTGAGGAGAAATGCATCGGG",2,4,20,NA,"","",FALSE,"AKIRIN2"

"5912","CCACTGAGGAGAAATGCATG","chrX",128772543,"-","0:T>G,10:A>C","AGG","ACACTGAGGATAAATGCATGAGG",2,1,11,NA,"","",FALSE,"AKIRIN2"

"5913","GATCAAATAATGCGACGATA","chr6",87675879,"-","none","TGG","GATCAAATAATGCGACGATATGG",0,NA,NA,NA,"GeneID:55122","AKIRIN2",TRUE,"AKIRIN2"

"5914","GATCAAATAATGCGACGATA","chr4",149790252,"+","12:T>C,15:T>C","TGG","GATCAAATAATGTGATGATATGG",2,13,16,NA,"GeneID:285423","LOC285423",TRUE,"AKIRIN2"

"5915","ACCATTGACCAGCTACGCCT","chr9",114374102,"-","none","CGG","ACCATTGACCAGCTACGCCTCGG",0,NA,NA,NA,"GeneID:80709","AKNA",TRUE,"AKNA"

"5916","CCTGGGGCACCATTCCCGTG","chr9",114368531,"+","none","TGG","CCTGGGGCACCATTCCCGTGTGG",0,NA,NA,NA,"GeneID:80709","AKNA",TRUE,"AKNA"

"5917","CTCAAGTCTCCTCGAGCTGA","chr9",114367669,"+","none","TGG","CTCAAGTCTCCTCGAGCTGATGG",0,NA,NA,NA,"GeneID:80709","AKNA",TRUE,"AKNA"

"5918","GCCCAACCACAGCATCCACA","chr9",114368549,"-","none","CGG","GCCCAACCACAGCATCCACACGG",0,NA,NA,NA,"GeneID:80709","AKNA",TRUE,"AKNA"

"5919","GCTTACCAGCATGCCCACCC","chr9",114367640,"-","none","TGG","GCTTACCAGCATGCCCACCCTGG",0,NA,NA,NA,"GeneID:80709","AKNA",TRUE,"AKNA"

"5920","TAGCTGGTCAATGGTGTTCT","chr9",114374107,"+","none","CGG","TAGCTGGTCAATGGTGTTCTCGG",0,NA,NA,NA,"GeneID:80709","AKNA",TRUE,"AKNA"

"5921","ATATTTCAAAAGATATCCCA","chr1",108848966,"-","none","AGG","ATATTTCAAAAGATATCCCAAGG",0,NA,NA,NA,"GeneID:254268","AKNAD1",TRUE,"AKNAD1"

"5922","ATATTTCAAAAGATATCCCA","chr2",28783342,"-","13:G>A,14:A>T","AGG","ATATTTCAAAAGACTTCCCAAGG",2,14,15,NA,"GeneID:5500;GeneID:245711","PPP1CB;SPDYA",FALSE,"AKNAD1"

"5923","ATATTTCAAAAGATATCCCA","chr5",99232145,"-","5:T>A,10:G>T","GGG","ATATTACAAACGATATCCCAGGG",2,6,11,NA,"","",FALSE,"AKNAD1"

"5924","ATCCACCAAGAACTTCTCAC","chr1",108849538,"-","none","AGG","ATCCACCAAGAACTTCTCACAGG",0,NA,NA,NA,"GeneID:254268","AKNAD1",TRUE,"AKNAD1"

"5925","CTGACCAACTCAACCCGAAA","chr1",108852139,"-","none","AGG","CTGACCAACTCAACCCGAAAAGG",0,NA,NA,NA,"GeneID:254268","AKNAD1",TRUE,"AKNAD1"

"5926","TAGACTATCTTGCACAAGAG","chr1",108851755,"+","none","TGG","TAGACTATCTTGCACAAGAGTGG",0,NA,NA,NA,"GeneID:254268","AKNAD1",TRUE,"AKNAD1"

"5927","TAGTACCTGTGAGAAGTTCT","chr1",108849530,"+","none","TGG","TAGTACCTGTGAGAAGTTCTTGG",0,NA,NA,NA,"GeneID:254268","AKNAD1",TRUE,"AKNAD1"

"5928","TAGTACCTGTGAGAAGTTCT","chr4",29137908,"+","0:A>T,4:G>A","GGG","AAGTGCCTGTGAGAAGTTCTGGG",2,1,5,NA,"","",FALSE,"AKNAD1"

"5929","TTGTCACCAACCTCTCAGAA","chr1",108849011,"-","none","AGG","TTGTCACCAACCTCTCAGAAAGG",0,NA,NA,NA,"GeneID:254268","AKNAD1",TRUE,"AKNAD1"

"5930","TTGTCACCAACCTCTCAGAA","chrX",106036202,"+","8:C>A,16:T>A","AGG","TTGTCACCCACCTCTCTGAAAGG",2,9,17,NA,"GeneID:6906","SERPINA7",FALSE,"AKNAD1"

"5931","AATCTGCCGACTGTTGAAGT","chr1",45568112,"-","none","TGG","AATCTGCCGACTGTTGAAGTTGG",0,NA,NA,NA,"GeneID:10327","AKR1A1",TRUE,"AKR1A1"

"5932","ATCACGCCATGCACGATCAG","chr1",45568578,"-","none","AGG","ATCACGCCATGCACGATCAGAGG",0,NA,NA,NA,"GeneID:10327","AKR1A1",TRUE,"AKR1A1"

"5933","CAAGCACCACCCCGAGGATG","chr1",45566915,"+","none","TGG","CAAGCACCACCCCGAGGATGTGG",0,NA,NA,NA,"GeneID:10327","AKR1A1",TRUE,"AKR1A1"

"5934","GGTGGAATGCCACCCATACT","chr1",45568483,"+","none","TGG","GGTGGAATGCCACCCATACTTGG",0,NA,NA,NA,"GeneID:10327","AKR1A1",TRUE,"AKR1A1"

"5935","GTTAAGTATGCCCTTAGCGT","chr1",45566580,"+","none","AGG","GTTAAGTATGCCCTTAGCGTAGG",0,NA,NA,NA,"GeneID:10327","AKR1A1",TRUE,"AKR1A1"

"5936","TGACCTCCAGCTGGAGTATC","chr1",45566963,"+","none","TGG","TGACCTCCAGCTGGAGTATCTGG",0,NA,NA,NA,"GeneID:10327","AKR1A1",TRUE,"AKR1A1"

"5937","CGCCCACGTGTCCAGAATGT","chr7",134449719,"+","none","TGG","CGCCCACGTGTCCAGAATGTTGG",0,NA,NA,NA,"GeneID:231","AKR1B1",TRUE,"AKR1B1"

"5938","CGCCCACGTGTCCAGAATGT","chr11",16484242,"+","0:T>C,6:T>C","TGG","TGCCCATGTGTCCAGAATGTTGG",2,1,7,NA,"GeneID:55553","SOX6",TRUE,"AKR1B1"

"5939","CGCCCACGTGTCCAGAATGT","chr19",33361018,"+","0:T>C,6:T>C","TGG","TGCCCATGTGTCCAGAATGTTGG",2,1,7,NA,"","",TRUE,"AKR1B1"

"5940","GACGTCAATGGCCACCTTCA","chr7",134451705,"+","none","CGG","GACGTCAATGGCCACCTTCACGG",0,NA,NA,NA,"GeneID:231","AKR1B1",TRUE,"AKR1B1"

"5941","GGAGCTCTTCATCGTCAGCA","chr7",134451587,"-","none","AGG","GGAGCTCTTCATCGTCAGCAAGG",0,NA,NA,NA,"GeneID:231","AKR1B1",TRUE,"AKR1B1"

"5942","GGAGCTCTTCATCGTCAGCA","chr7",134568303,"+","3:C>G,6:G>C","AGG","GGACCTGTTCATCGTCAGCAAGG",2,4,7,NA,"GeneID:441282","AKR1B15",TRUE,"AKR1B1"

"5943","GGAGCTCTTCATCGTCAGCA","chr7",134530788,"+","3:C>G,6:G>C","AGG","GGACCTGTTCATCGTCAGCAAGG",2,4,7,NA,"GeneID:57016","AKR1B10",TRUE,"AKR1B1"

"5944","TCAGGTCGCTGAGTGTCTTC","chr7",134450835,"+","none","TGG","TCAGGTCGCTGAGTGTCTTCTGG",0,NA,NA,NA,"GeneID:231","AKR1B1",TRUE,"AKR1B1"

"5945","TCAGGTCGCTGAGTGTCTTC","chr3",74136232,"-","6:A>G,13:G>A","TGG","TCAGGTTGCTGAGCGTCTTCTGG",2,7,14,NA,"","",TRUE,"AKR1B1"

"5946","TCCCATACCTTAAAGCCAGT","chr7",134450777,"+","none","CGG","TCCCATACCTTAAAGCCAGTCGG",0,NA,NA,NA,"GeneID:231","AKR1B1",TRUE,"AKR1B1"

"5947","TGGGCTCACAGGTCCTGATC","chr7",134447372,"-","none","CGG","TGGGCTCACAGGTCCTGATCCGG",0,NA,NA,NA,"GeneID:231","AKR1B1",TRUE,"AKR1B1"

"5948","CATTACCTTTATCATCTTTG","chr18",68456319,"-","none","GGG","CATTACCTTTATCATCTTTGGGG",0,NA,NA,NA,"","",TRUE,"AKR1B10"

"5949","CATTACCTTTATCATCTTTG","chr7",134533023,"-","none","GGG","CATTACCTTTATCATCTTTGGGG",0,NA,NA,NA,"GeneID:57016","AKR1B10",TRUE,"AKR1B10"

"5950","CATTACCTTTATCATCTTTG","chr7",134571623,"-","0:A>G","GGG","TATTACCTTTATCATCTTTGGGG",1,1,NA,NA,"GeneID:441282","AKR1B15",TRUE,"AKR1B10"

"5951","CATTACCTTTATCATCTTTG","chr10",67750653,"-","0:A>G","CGG","TATTACCTTTATCATCTTTGCGG",1,1,NA,NA,"","",TRUE,"AKR1B10"

"5952","CATTACCTTTATCATCTTTG","chr5",29498075,"+","1:T>A,5:T>C","TGG","CTTTATCTTTATCATCTTTGTGG",2,2,6,NA,"","",FALSE,"AKR1B10"

"5953","CATTACCTTTATCATCTTTG","chrX",13643183,"+","2:A>T,18:A>T","TGG","CAATACCTTTATCATCTTAGTGG",2,3,19,NA,"","",FALSE,"AKR1B10"

"5954","CATTACCTTTATCATCTTTG","chr6",99848225,"-","7:T>A,16:G>A","GGG","CATTACCATTATCATCCTTGGGG",2,8,17,NA,"","",FALSE,"AKR1B10"

"5955","CATTACCTTTATCATCTTTG","chr4",180381511,"-","0:T>G,19:A>C","TGG","AATTACCTTTATCATCTTTTTGG",2,1,20,NA,"","",FALSE,"AKR1B10"

"5956","CGGTGGAAAAGCAACGTTCT","chr7",134533047,"+","none","TGG","CGGTGGAAAAGCAACGTTCTTGG",0,NA,NA,NA,"GeneID:57016","AKR1B10",TRUE,"AKR1B10"

"5957","CGGTGGAAAAGCAACGTTCT","chr10",67750677,"+","14:T>C","TGG","CGGTGGAAAAGCAATGTTCTTGG",1,15,NA,NA,"","",TRUE,"AKR1B10"

"5958","CGGTGGAAAAGCAACGTTCT","chr7",134571647,"+","1:A>G,11:G>C","TGG","CAGTGGAAAAGGAACGTTCTTGG",2,2,12,NA,"GeneID:441282","AKR1B15",TRUE,"AKR1B10"

"5959","GTGCCGATATCCTGCATCAA","chr7",134530682,"-","none","TGG","GTGCCGATATCCTGCATCAATGG",0,NA,NA,NA,"GeneID:57016","AKR1B10",TRUE,"AKR1B10"

"5960","GTGCCGATATCCTGCATCAA","chr10",67750389,"-","3:C>G","TGG","GTGGCGATATCCTGCATCAATGG",1,4,NA,NA,"","",TRUE,"AKR1B10"

"5961","GTGCCGATATCCTGCATCAA","chr7",134568197,"-","3:C>G,10:A>G","TGG","GTGGCGATATTCTGCATCAATGG",2,4,11,NA,"GeneID:441282","AKR1B15",TRUE,"AKR1B10"

"5962","TCTTAAACCTTGAATCCCTG","chr7",134532012,"-","none","TGG","TCTTAAACCTTGAATCCCTGTGG",0,NA,NA,NA,"GeneID:57016","AKR1B10",TRUE,"AKR1B10"

"5963","TCTTAAACCTTGAATCCCTG","chr8",142460582,"-","14:C>A","GGG","TCTTAAACCTTGAAGCCCTGGGG",1,15,NA,NA,"GeneID:575","BAI1",FALSE,"AKR1B10"

"5964","TCTTAAACCTTGAATCCCTG","chr7",134569517,"-","0:T>A,3:G>A","TGG","ACTCAAACCTTGAATCCCTGTGG",2,1,4,NA,"GeneID:441282","AKR1B15",TRUE,"AKR1B10"

"5965","TCTTAAACCTTGAATCCCTG","chr4",165493756,"-","1:C>G,4:A>T","CGG","TGTTTAACCTTGAATCCCTGCGG",2,2,5,NA,"GeneID:1363","CPE",FALSE,"AKR1B10"

"5966","TCTTTGAGAGACCCCTTGTG","chr7",134531920,"+","none","AGG","TCTTTGAGAGACCCCTTGTGAGG",0,NA,NA,NA,"GeneID:57016","AKR1B10",TRUE,"AKR1B10"

"5967","TCTTTGAGAGACCCCTTGTG","chr7",134569425,"+","none","AGG","TCTTTGAGAGACCCCTTGTGAGG",0,NA,NA,NA,"GeneID:441282","AKR1B15",TRUE,"AKR1B10"

"5968","TCTTTGAGAGACCCCTTGTG","chr10",67750530,"+","none","AGG","TCTTTGAGAGACCCCTTGTGAGG",0,NA,NA,NA,"","",TRUE,"AKR1B10"

"5969","TCTTTGAGAGACCCCTTGTG","chr3",134464711,"+","5:C>G,16:G>T","TGG","TCTTTCAGAGACCCCTGGTGTGG",2,6,17,NA,"","",FALSE,"AKR1B10"

"5970","TGGCCATTGATGCAGGATAT","chr7",134530676,"+","none","CGG","TGGCCATTGATGCAGGATATCGG",0,NA,NA,NA,"GeneID:57016","AKR1B10",TRUE,"AKR1B10"

"5971","AGTTGTTAGTTGAGTTCACT","chr7",134564642,"-","none","TGG","AGTTGTTAGTTGAGTTCACTTGG",0,NA,NA,NA,"GeneID:441282","AKR1B15",TRUE,"AKR1B15"

"5972","AGTTGTTAGTTGAGTTCACT","chr2",40677630,"-","18:A>G","TGG","AGTTGTTAGTTGAGTTCATTTGG",1,19,NA,NA,"","",FALSE,"AKR1B15"

"5973","AGTTGTTAGTTGAGTTCACT","chr10",54300423,"-","18:A>G","TGG","AGTTGTTAGTTGAGTTCATTTGG",1,19,NA,NA,"GeneID:65217","PCDH15",FALSE,"AKR1B15"

"5974","AGTTGTTAGTTGAGTTCACT","chr5",147007097,"-","14:G>A","TGG","AGTTGTTAGTTGAGCTCACTTGG",1,15,NA,NA,"GeneID:5521","PPP2R2B",FALSE,"AKR1B15"

"5975","AGTTGTTAGTTGAGTTCACT","chr1",199097264,"-","14:G>A,18:A>G","TGG","AGTTGTTAGTTGAGCTCATTTGG",2,15,19,NA,"","",FALSE,"AKR1B15"

"5976","AGTTGTTAGTTGAGTTCACT","chr8",128382225,"-","14:G>A,18:A>G","TGG","AGTTGTTAGTTGAGCTCATTTGG",2,15,19,NA,"","",FALSE,"AKR1B15"

"5977","AGTTGTTAGTTGAGTTCACT","chr14",91667981,"-","14:G>A,18:A>G","TGG","AGTTGTTAGTTGAGCTCATTTGG",2,15,19,NA,"GeneID:79820","CATSPERB",FALSE,"AKR1B15"

"5978","AGTTGTTAGTTGAGTTCACT","chr3",148577398,"-","14:G>A,18:A>G","TGG","AGTTGTTAGTTGAGCTCATTTGG",2,15,19,NA,"","",FALSE,"AKR1B15"

"5979","AGTTGTTAGTTGAGTTCACT","chr4",41021154,"-","14:G>A,18:A>G","TGG","AGTTGTTAGTTGAGCTCATTTGG",2,15,19,NA,"GeneID:323","APBB2",FALSE,"AKR1B15"

"5980","AGTTGTTAGTTGAGTTCACT","chr8",106793423,"-","14:G>A,18:A>G","TGG","AGTTGTTAGTTGAGCTCATTTGG",2,15,19,NA,"","",FALSE,"AKR1B15"

"5981","AGTTGTTAGTTGAGTTCACT","chr2",163072533,"-","14:G>A,18:A>G","TGG","AGTTGTTAGTTGAGCTCATTTGG",2,15,19,NA,"","",FALSE,"AKR1B15"

"5982","AGTTGTTAGTTGAGTTCACT","chr4",28309646,"-","14:G>A,18:A>G","TGG","AGTTGTTAGTTGAGCTCATTTGG",2,15,19,NA,"","",FALSE,"AKR1B15"

"5983","AGTTGTTAGTTGAGTTCACT","chr11",94125090,"-","14:G>A,18:A>G","TGG","AGTTGTTAGTTGAGCTCATTTGG",2,15,19,NA,"","",FALSE,"AKR1B15"

"5984","AGTTGTTAGTTGAGTTCACT","chr2",193456178,"-","14:G>A,18:A>G","TGG","AGTTGTTAGTTGAGCTCATTTGG",2,15,19,NA,"","",FALSE,"AKR1B15"

"5985","AGTTGTTAGTTGAGTTCACT","chr4",158664627,"-","14:G>A,18:A>G","TGG","AGTTGTTAGTTGAGCTCATTTGG",2,15,19,NA,"","",FALSE,"AKR1B15"

"5986","AGTTGTTAGTTGAGTTCACT","chr6",141183147,"-","14:G>A,18:A>G","TGG","AGTTGTTAGTTGAGCTCATTTGG",2,15,19,NA,"","",FALSE,"AKR1B15"

"5987","AGTTGTTAGTTGAGTTCACT","chr10",57841365,"-","14:G>A,18:A>G","TGG","AGTTGTTAGTTGAGCTCATTTGG",2,15,19,NA,"","",FALSE,"AKR1B15"

"5988","AGTTGTTAGTTGAGTTCACT","chr3",191192056,"-","14:G>A,18:A>G","TGG","AGTTGTTAGTTGAGCTCATTTGG",2,15,19,NA,"","",FALSE,"AKR1B15"

"5989","AGTTGTTAGTTGAGTTCACT","chr3",96302340,"-","14:G>A,18:A>G","TGG","AGTTGTTAGTTGAGCTCATTTGG",2,15,19,NA,"","",FALSE,"AKR1B15"

"5990","AGTTGTTAGTTGAGTTCACT","chr1",57693089,"-","14:G>A,18:A>G","TGG","AGTTGTTAGTTGAGCTCATTTGG",2,15,19,NA,"","",FALSE,"AKR1B15"

"5991","AGTTGTTAGTTGAGTTCACT","chr3",17781456,"-","14:G>A,18:A>G","TGG","AGTTGTTAGTTGAGCTCATTTGG",2,15,19,NA,"GeneID:9779","TBC1D5",FALSE,"AKR1B15"

"5992","AGTTGTTAGTTGAGTTCACT","chr7",109304110,"-","14:G>A,18:A>G","TGG","AGTTGTTAGTTGAGCTCATTTGG",2,15,19,NA,"","",FALSE,"AKR1B15"

"5993","AGTTGTTAGTTGAGTTCACT","chr15",27767196,"-","14:G>A,18:A>G","TGG","AGTTGTTAGTTGAGCTCATTTGG",2,15,19,NA,"GeneID:4948","OCA2",FALSE,"AKR1B15"

"5994","AGTTGTTAGTTGAGTTCACT","chr3",140878175,"-","14:G>A,18:A>G","TGG","AGTTGTTAGTTGAGCTCATTTGG",2,15,19,NA,"","",FALSE,"AKR1B15"

"5995","AGTTGTTAGTTGAGTTCACT","chr10",119890106,"-","14:G>A,18:A>G","GGG","AGTTGTTAGTTGAGCTCATTGGG",2,15,19,NA,"GeneID:79892","MCMBP",FALSE,"AKR1B15"

"5996","AGTTGTTAGTTGAGTTCACT","chrY",24428426,"-","14:G>A,18:A>G","TGG","AGTTGTTAGTTGAGCTCATTTGG",2,15,19,NA,"","",FALSE,"AKR1B15"

"5997","AGTTGTTAGTTGAGTTCACT","chrY",22794676,"-","14:G>A,18:A>G","TGG","AGTTGTTAGTTGAGCTCATTTGG",2,15,19,NA,"","",FALSE,"AKR1B15"

"5998","AGTTGTTAGTTGAGTTCACT","chr1",62761107,"-","14:G>A,18:A>G","TGG","AGTTGTTAGTTGAGCTCATTTGG",2,15,19,NA,"","",FALSE,"AKR1B15"

"5999","AGTTGTTAGTTGAGTTCACT","chr7",82645135,"-","14:G>A,18:A>G","TGG","AGTTGTTAGTTGAGCTCATTTGG",2,15,19,NA,"","",FALSE,"AKR1B15"

"6000","AGTTGTTAGTTGAGTTCACT","chr2",81040059,"-","14:G>A,18:A>G","TGG","AGTTGTTAGTTGAGCTCATTTGG",2,15,19,NA,"","",FALSE,"AKR1B15"

"6001","AGTTGTTAGTTGAGTTCACT","chrY",13636581,"-","14:G>A,18:A>G","TGG","AGTTGTTAGTTGAGCTCATTTGG",2,15,19,NA,"","",FALSE,"AKR1B15"

"6002","AGTTGTTAGTTGAGTTCACT","chr6",9727389,"-","14:G>A,18:A>G","TGG","AGTTGTTAGTTGAGCTCATTTGG",2,15,19,NA,"GeneID:266553","OFCC1",FALSE,"AKR1B15"

"6003","AGTTGTTAGTTGAGTTCACT","chr8",48242006,"-","14:G>A,18:A>G","TGG","AGTTGTTAGTTGAGCTCATTTGG",2,15,19,NA,"","",FALSE,"AKR1B15"

"6004","AGTTGTTAGTTGAGTTCACT","chrX",104896298,"-","14:G>A,18:A>G","TGG","AGTTGTTAGTTGAGCTCATTTGG",2,15,19,NA,"GeneID:26280","IL1RAPL2",FALSE,"AKR1B15"

"6005","AGTTGTTAGTTGAGTTCACT","chr10",115041940,"-","14:G>A,18:A>G","TGG","AGTTGTTAGTTGAGCTCATTTGG",2,15,19,NA,"","",FALSE,"AKR1B15"

"6006","AGTTGTTAGTTGAGTTCACT","chr11",110406288,"-","14:G>A,18:A>G","TGG","AGTTGTTAGTTGAGCTCATTTGG",2,15,19,NA,"","",TRUE,"AKR1B15"

"6007","AGTTGTTAGTTGAGTTCACT","chrY",6490175,"-","14:G>A,18:A>G","TGG","AGTTGTTAGTTGAGCTCATTTGG",2,15,19,NA,"","",FALSE,"AKR1B15"

"6008","AGTTGTTAGTTGAGTTCACT","chr1",171312209,"-","14:G>A,18:A>G","TGG","AGTTGTTAGTTGAGCTCATTTGG",2,15,19,NA,"","",FALSE,"AKR1B15"

"6009","AGTTGTTAGTTGAGTTCACT","chr1",173381559,"-","14:G>A,18:A>G","TGG","AGTTGTTAGTTGAGCTCATTTGG",2,15,19,NA,"","",FALSE,"AKR1B15"

"6010","AGTTGTTAGTTGAGTTCACT","chr3",51856048,"-","14:G>A,18:A>G","TGG","AGTTGTTAGTTGAGCTCATTTGG",2,15,19,NA,"","",FALSE,"AKR1B15"

"6011","AGTTGTTAGTTGAGTTCACT","chr20",34157338,"-","14:G>A,18:A>G","CGG","AGTTGTTAGTTGAGCTCATTCGG",2,15,19,NA,"","",FALSE,"AKR1B15"

"6012","AGTTGTTAGTTGAGTTCACT","chr8",35938645,"-","14:G>A,18:A>G","TGG","AGTTGTTAGTTGAGCTCATTTGG",2,15,19,NA,"","",FALSE,"AKR1B15"

"6013","AGTTGTTAGTTGAGTTCACT","chr20",23741909,"-","14:G>A,18:A>G","TGG","AGTTGTTAGTTGAGCTCATTTGG",2,15,19,NA,"","",FALSE,"AKR1B15"

"6014","AGTTGTTAGTTGAGTTCACT","chr20",23817639,"-","14:G>A,18:A>G","TGG","AGTTGTTAGTTGAGCTCATTTGG",2,15,19,NA,"","",FALSE,"AKR1B15"

"6015","AGTTGTTAGTTGAGTTCACT","chr1",172803469,"-","14:G>A,18:A>G","TGG","AGTTGTTAGTTGAGCTCATTTGG",2,15,19,NA,"","",FALSE,"AKR1B15"

"6016","AGTTGTTAGTTGAGTTCACT","chr8",53468251,"-","14:G>A,18:A>G","TGG","AGTTGTTAGTTGAGCTCATTTGG",2,15,19,NA,"","",FALSE,"AKR1B15"

"6017","AGTTGTTAGTTGAGTTCACT","chr3",20275536,"-","14:G>A,18:A>G","TGG","AGTTGTTAGTTGAGCTCATTTGG",2,15,19,NA,"GeneID:100874028","SGOL1-AS1",FALSE,"AKR1B15"

"6018","AGTTGTTAGTTGAGTTCACT","chrX",26504130,"-","14:G>A,18:A>G","TGG","AGTTGTTAGTTGAGCTCATTTGG",2,15,19,NA,"","",FALSE,"AKR1B15"

"6019","AGTTGTTAGTTGAGTTCACT","chr3",164818105,"-","14:C>A,18:A>G","TGG","AGTTGTTAGTTGAGGTCATTTGG",2,15,19,NA,"","",FALSE,"AKR1B15"

"6020","AGTTGTTAGTTGAGTTCACT","chrX",116250347,"-","14:C>A,18:A>G","TGG","AGTTGTTAGTTGAGGTCATTTGG",2,15,19,NA,"","",FALSE,"AKR1B15"

"6021","AGTTGTTAGTTGAGTTCACT","chr5",114998978,"-","14:C>A,18:A>G","TGG","AGTTGTTAGTTGAGGTCATTTGG",2,15,19,NA,"","",FALSE,"AKR1B15"

"6022","AGTTGTTAGTTGAGTTCACT","chr3",162579401,"-","14:C>A,18:A>G","TGG","AGTTGTTAGTTGAGGTCATTTGG",2,15,19,NA,"","",FALSE,"AKR1B15"

"6023","AGTTGTTAGTTGAGTTCACT","chr8",53437616,"+","18:T>C","TGG","AGTTGTTAGTTGAGTTCATTTGG",1,19,NA,NA,"","",FALSE,"AKR1B15"

"6024","AGTTGTTAGTTGAGTTCACT","chr2",172591094,"+","18:T>C","TGG","AGTTGTTAGTTGAGTTCATTTGG",1,19,NA,NA,"GeneID:5163","PDK1",FALSE,"AKR1B15"

"6025","AGTTGTTAGTTGAGTTCACT","chr8",92394279,"+","14:C>T,18:T>C","TGG","AGTTGTTAGTTGAGCTCATTTGG",2,15,19,NA,"","",FALSE,"AKR1B15"

"6026","AGTTGTTAGTTGAGTTCACT","chr8",42560411,"+","14:C>T,18:T>C","TGG","AGTTGTTAGTTGAGCTCATTTGG",2,15,19,NA,"","",FALSE,"AKR1B15"

"6027","AGTTGTTAGTTGAGTTCACT","chr2",183508079,"+","14:C>T,18:T>C","TGG","AGTTGTTAGTTGAGCTCATTTGG",2,15,19,NA,"","",FALSE,"AKR1B15"

"6028","AGTTGTTAGTTGAGTTCACT","chr2",166739213,"+","14:C>T,18:T>C","TGG","AGTTGTTAGTTGAGCTCATTTGG",2,15,19,NA,"","",FALSE,"AKR1B15"

"6029","AGTTGTTAGTTGAGTTCACT","chr3",34519594,"+","14:C>T,18:T>C","TGG","AGTTGTTAGTTGAGCTCATTTGG",2,15,19,NA,"","",FALSE,"AKR1B15"

"6030","AGTTGTTAGTTGAGTTCACT","chr1",188258188,"+","14:C>T,18:T>C","TGG","AGTTGTTAGTTGAGCTCATTTGG",2,15,19,NA,"","",FALSE,"AKR1B15"

"6031","AGTTGTTAGTTGAGTTCACT","chrY",9671806,"+","14:C>T,18:T>C","TGG","AGTTGTTAGTTGAGCTCATTTGG",2,15,19,NA,"","",FALSE,"AKR1B15"

"6032","AGTTGTTAGTTGAGTTCACT","chr10",94764699,"+","14:C>T,18:T>C","TGG","AGTTGTTAGTTGAGCTCATTTGG",2,15,19,NA,"GeneID:1557","CYP2C19",FALSE,"AKR1B15"

"6033","AGTTGTTAGTTGAGTTCACT","chr11",25000462,"+","14:C>T,18:T>C","TGG","AGTTGTTAGTTGAGCTCATTTGG",2,15,19,NA,"GeneID:338645","LUZP2",FALSE,"AKR1B15"

"6034","AGTTGTTAGTTGAGTTCACT","chr2",8895750,"+","14:C>T,18:T>C","TGG","AGTTGTTAGTTGAGCTCATTTGG",2,15,19,NA,"GeneID:129642","MBOAT2",FALSE,"AKR1B15"

"6035","AGTTGTTAGTTGAGTTCACT","chr10",56573513,"+","14:C>T,18:T>C","TGG","AGTTGTTAGTTGAGCTCATTTGG",2,15,19,NA,"","",FALSE,"AKR1B15"

"6036","AGTTGTTAGTTGAGTTCACT","chr8",53137081,"+","14:C>T,18:T>C","TGG","AGTTGTTAGTTGAGCTCATTTGG",2,15,19,NA,"","",FALSE,"AKR1B15"

"6037","AGTTGTTAGTTGAGTTCACT","chr5",144848209,"+","14:C>T,18:T>C","TGG","AGTTGTTAGTTGAGCTCATTTGG",2,15,19,NA,"","",FALSE,"AKR1B15"

"6038","AGTTGTTAGTTGAGTTCACT","chr3",73153599,"+","14:C>T,18:T>C","TGG","AGTTGTTAGTTGAGCTCATTTGG",2,15,19,NA,"","",FALSE,"AKR1B15"

"6039","AGTTGTTAGTTGAGTTCACT","chr1",38978480,"+","14:C>T,18:T>C","TGG","AGTTGTTAGTTGAGCTCATTTGG",2,15,19,NA,"","",FALSE,"AKR1B15"

"6040","AGTTGTTAGTTGAGTTCACT","chr10",17012106,"+","14:C>T,18:T>C","TGG","AGTTGTTAGTTGAGCTCATTTGG",2,15,19,NA,"GeneID:8029","CUBN",FALSE,"AKR1B15"

"6041","AGTTGTTAGTTGAGTTCACT","chr6",86672706,"+","14:C>T,18:T>C","TGG","AGTTGTTAGTTGAGCTCATTTGG",2,15,19,NA,"","",FALSE,"AKR1B15"

"6042","AGTTGTTAGTTGAGTTCACT","chr10",67684694,"+","14:C>T,18:T>C","TGG","AGTTGTTAGTTGAGCTCATTTGG",2,15,19,NA,"GeneID:29119","CTNNA3",FALSE,"AKR1B15"

"6043","AGTTGTTAGTTGAGTTCACT","chr8",82091860,"+","14:C>T,18:T>C","TGG","AGTTGTTAGTTGAGCTCATTTGG",2,15,19,NA,"","",FALSE,"AKR1B15"

"6044","AGTTGTTAGTTGAGTTCACT","chr9",76489917,"+","14:C>T,18:T>C","TGG","AGTTGTTAGTTGAGCTCATTTGG",2,15,19,NA,"GeneID:2650","GCNT1",FALSE,"AKR1B15"

"6045","AGTTGTTAGTTGAGTTCACT","chr7",91447679,"+","14:C>T,18:T>C","TGG","AGTTGTTAGTTGAGCTCATTTGG",2,15,19,NA,"","",FALSE,"AKR1B15"

"6046","AGTTGTTAGTTGAGTTCACT","chr19",5159039,"+","14:C>T,18:T>C","TGG","AGTTGTTAGTTGAGCTCATTTGG",2,15,19,NA,"GeneID:5802","PTPRS",TRUE,"AKR1B15"

"6047","AGTTGTTAGTTGAGTTCACT","chr4",184915662,"+","14:C>T,18:T>C","TGG","AGTTGTTAGTTGAGCTCATTTGG",2,15,19,NA,"","",FALSE,"AKR1B15"

"6048","AGTTGTTAGTTGAGTTCACT","chrY",25241667,"+","14:C>T,18:T>C","TGG","AGTTGTTAGTTGAGCTCATTTGG",2,15,19,NA,"","",FALSE,"AKR1B15"

"6049","AGTTGTTAGTTGAGTTCACT","chr7",64757832,"+","14:C>T,18:T>C","TGG","AGTTGTTAGTTGAGCTCATTTGG",2,15,19,NA,"","",FALSE,"AKR1B15"

"6050","AGTTGTTAGTTGAGTTCACT","chrY",12261645,"+","14:C>T,18:T>C","TGG","AGTTGTTAGTTGAGCTCATTTGG",2,15,19,NA,"","",FALSE,"AKR1B15"

"6051","AGTTGTTAGTTGAGTTCACT","chr2",218109434,"+","14:C>T,18:T>C","TGG","AGTTGTTAGTTGAGCTCATTTGG",2,15,19,NA,"","",FALSE,"AKR1B15"

"6052","AGTTGTTAGTTGAGTTCACT","chr16",57395637,"+","14:C>T,18:T>C","TGG","AGTTGTTAGTTGAGCTCATTTGG",2,15,19,NA,"","",FALSE,"AKR1B15"

"6053","AGTTGTTAGTTGAGTTCACT","chr7",19654501,"+","14:C>T,18:T>C","TGG","AGTTGTTAGTTGAGCTCATTTGG",2,15,19,NA,"","",FALSE,"AKR1B15"

"6054","AGTTGTTAGTTGAGTTCACT","chr14",31706327,"+","14:C>T,18:T>C","TGG","AGTTGTTAGTTGAGCTCATTTGG",2,15,19,NA,"GeneID:80224","NUBPL",FALSE,"AKR1B15"

"6055","AGTTGTTAGTTGAGTTCACT","chr19",32694029,"+","14:C>T,18:T>C","TGG","AGTTGTTAGTTGAGCTCATTTGG",2,15,19,NA,"GeneID:390916","NUDT19",FALSE,"AKR1B15"

"6056","AGTTGTTAGTTGAGTTCACT","chr4",101631719,"+","14:C>T,18:T>C","TGG","AGTTGTTAGTTGAGCTCATTTGG",2,15,19,NA,"GeneID:55024","BANK1",FALSE,"AKR1B15"

"6057","AGTTGTTAGTTGAGTTCACT","chr16",21630684,"+","14:C>T,18:T>C","TGG","AGTTGTTAGTTGAGCTCATTTGG",2,15,19,NA,"GeneID:51108","METTL9",FALSE,"AKR1B15"

"6058","AGTTGTTAGTTGAGTTCACT","chr13",47521539,"+","14:C>T,18:T>C","TGG","AGTTGTTAGTTGAGCTCATTTGG",2,15,19,NA,"","",FALSE,"AKR1B15"

"6059","AGTTGTTAGTTGAGTTCACT","chr5",32673822,"+","14:C>T,18:T>C","TGG","AGTTGTTAGTTGAGCTCATTTGG",2,15,19,NA,"","",FALSE,"AKR1B15"

"6060","AGTTGTTAGTTGAGTTCACT","chr13",111190116,"+","14:C>T,18:T>C","TGG","AGTTGTTAGTTGAGCTCATTTGG",2,15,19,NA,"GeneID:8874","ARHGEF7",FALSE,"AKR1B15"

"6061","AGTTGTTAGTTGAGTTCACT","chr8",53071867,"+","14:C>T,18:T>C","TGG","AGTTGTTAGTTGAGCTCATTTGG",2,15,19,NA,"","",FALSE,"AKR1B15"

"6062","AGTTGTTAGTTGAGTTCACT","chr12",29169340,"+","14:C>T,18:T>C","TGG","AGTTGTTAGTTGAGCTCATTTGG",2,15,19,NA,"GeneID:55711","FAR2",FALSE,"AKR1B15"

"6063","AGTTGTTAGTTGAGTTCACT","chr3",49221685,"+","14:C>T,18:T>C","TGG","AGTTGTTAGTTGAGCTCATTTGG",2,15,19,NA,"GeneID:339834","CCDC36",FALSE,"AKR1B15"

"6064","AGTTGTTAGTTGAGTTCACT","chr2",134804678,"+","14:C>T,18:T>C","TGG","AGTTGTTAGTTGAGCTCATTTGG",2,15,19,NA,"","",FALSE,"AKR1B15"

"6065","AGTTGTTAGTTGAGTTCACT","chrY",20007097,"+","14:G>T,18:T>C","TGG","AGTTGTTAGTTGAGGTCATTTGG",2,15,19,NA,"","",FALSE,"AKR1B15"

"6066","AGTTGTTAGTTGAGTTCACT","chr14",67463762,"-","0:A>T,3:T>A","GGG","TGTAGTTAGTTGAGTTCACTGGG",2,1,4,NA,"GeneID:161145","TMEM229B",FALSE,"AKR1B15"

"6067","AGTTGTTAGTTGAGTTCACT","chr1",212200750,"+","3:G>T,8:T>G","TGG","AGTGGTTATTTGAGTTCACTTGG",2,4,9,NA,"","",FALSE,"AKR1B15"

"6068","CAACCCGTTGGCCCTTTGAC","chr7",134564682,"+","none","TGG","CAACCCGTTGGCCCTTTGACTGG",0,NA,NA,NA,"GeneID:441282","AKR1B15",TRUE,"AKR1B15"

"6069","CAACCCGTTGGCCCTTTGAC","chr3",34519554,"-","5:A>G,7:G>A","TGG","CAACCTGCTGGCCCTTTGACTGG",2,6,8,NA,"","",FALSE,"AKR1B15"

"6070","CAACCCGTTGGCCCTTTGAC","chrY",12600438,"-","6:T>C,7:G>A","TGG","CAACCCACTGGCCCTTTGACTGG",2,7,8,NA,"","",FALSE,"AKR1B15"

"6071","CAACCCGTTGGCCCTTTGAC","chr18",26232359,"-","6:T>C,7:G>A","TGG","CAACCCACTGGCCCTTTGACTGG",2,7,8,NA,"GeneID:6875","TAF4B",FALSE,"AKR1B15"

"6072","CAACCCGTTGGCCCTTTGAC","chrY",20007057,"-","6:T>C,7:G>A","TGG","CAACCCACTGGCCCTTTGACTGG",2,7,8,NA,"","",FALSE,"AKR1B15"

"6073","CAACCCGTTGGCCCTTTGAC","chr10",119890146,"+","7:C>T","TGG","CAACCCGCTGGCCCTTTGACTGG",1,8,NA,NA,"GeneID:79892","MCMBP",FALSE,"AKR1B15"

"6074","CAACCCGTTGGCCCTTTGAC","chr8",106793463,"+","5:A>C,7:C>T","TGG","CAACCAGCTGGCCCTTTGACTGG",2,6,8,NA,"","",FALSE,"AKR1B15"

"6075","CAACCCGTTGGCCCTTTGAC","chr8",73913432,"+","6:A>G,7:C>T","TGG","CAACCCACTGGCCCTTTGACTGG",2,7,8,NA,"","",FALSE,"AKR1B15"

"6076","CAACCCGTTGGCCCTTTGAC","chr3",17781496,"+","6:A>G,7:C>T","TGG","CAACCCACTGGCCCTTTGACTGG",2,7,8,NA,"GeneID:9779","TBC1D5",FALSE,"AKR1B15"

"6077","CAACCCGTTGGCCCTTTGAC","chr12",127617542,"+","6:A>G,7:C>T","TGG","CAACCCACTGGCCCTTTGACTGG",2,7,8,NA,"","",FALSE,"AKR1B15"

"6078","CAACCCGTTGGCCCTTTGAC","chr8",72394765,"+","6:A>G,7:C>T","TGG","CAACCCACTGGCCCTTTGACTGG",2,7,8,NA,"","",FALSE,"AKR1B15"

"6079","CAACCCGTTGGCCCTTTGAC","chr4",184915622,"-","7:G>A,17:G>C","TGG","CAACCCGCTGGCCCTTTCACTGG",2,8,18,NA,"","",FALSE,"AKR1B15"

"6080","CAACCCGTTGGCCCTTTGAC","chr4",101631679,"-","5:A>G,17:G>C","TGG","CAACCTGTTGGCCCTTTCACTGG",2,6,18,NA,"GeneID:55024","BANK1",FALSE,"AKR1B15"

"6081","CAGTGGAAAAGGAACGTTCT","chr7",134571647,"+","none","TGG","CAGTGGAAAAGGAACGTTCTTGG",0,NA,NA,NA,"GeneID:441282","AKR1B15",TRUE,"AKR1B15"

"6082","CAGTGGAAAAGGAACGTTCT","chr7",134533047,"+","1:G>A,11:C>G","TGG","CGGTGGAAAAGCAACGTTCTTGG",2,2,12,NA,"GeneID:57016","AKR1B10",TRUE,"AKR1B15"

"6083","CATATTACCTTTATCATCTT","chr7",134571625,"-","none","TGG","CATATTACCTTTATCATCTTTGG",0,NA,NA,NA,"GeneID:441282","AKR1B15",TRUE,"AKR1B15"

"6084","CATATTACCTTTATCATCTT","chr18",68456321,"-","0:C>G,2:G>A","TGG","GACATTACCTTTATCATCTTTGG",2,1,3,NA,"","",TRUE,"AKR1B15"

"6085","CATATTACCTTTATCATCTT","chrX",126544219,"+","2:G>T,6:T>A","TGG","CAGATTTCCTTTATCATCTTTGG",2,3,7,NA,"","",FALSE,"AKR1B15"

"6086","CATATTACCTTTATCATCTT","chr4",80165615,"+","7:T>C,10:A>T","GGG","CATATTATCTATATCATCTTGGG",2,8,11,NA,"","",FALSE,"AKR1B15"

"6087","CATATTACCTTTATCATCTT","chr7",11633174,"+","6:T>A,13:A>T","TGG","CATATTTCCTTTAACATCTTTGG",2,7,14,NA,"GeneID:221981","THSD7A",FALSE,"AKR1B15"

"6088","CATATTACCTTTATCATCTT","chr8",36315395,"-","8:T>G,14:A>G","TGG","CATATTACATTTATTATCTTTGG",2,9,15,NA,"","",FALSE,"AKR1B15"

"6089","CATATTACCTTTATCATCTT","chrY",11805069,"-","5:C>A,17:C>G","AGG","CATATGACCTTTATCATGTTAGG",2,6,18,NA,"","",FALSE,"AKR1B15"

"6090","CATATTACCTTTATCATCTT","chr1",242906154,"-","5:C>A,17:C>G","AGG","CATATGACCTTTATCATGTTAGG",2,6,18,NA,"","",FALSE,"AKR1B15"

"6091","GTGGCGATATTCTGCATCAA","chr7",134568197,"-","none","TGG","GTGGCGATATTCTGCATCAATGG",0,NA,NA,NA,"GeneID:441282","AKR1B15",TRUE,"AKR1B15"

"6092","GTGGCGATATTCTGCATCAA","chr10",67750389,"-","10:G>A","TGG","GTGGCGATATCCTGCATCAATGG",1,11,NA,NA,"","",TRUE,"AKR1B15"

"6093","GTGGCGATATTCTGCATCAA","chr7",134530682,"-","3:G>C,10:G>A","TGG","GTGCCGATATCCTGCATCAATGG",2,4,11,NA,"GeneID:57016","AKR1B10",TRUE,"AKR1B15"

"6094","TTTCTATGAGAATCAACATG","chr7",134568231,"+","none","AGG","TTTCTATGAGAATCAACATGAGG",0,NA,NA,NA,"GeneID:441282","AKR1B15",TRUE,"AKR1B15"

"6095","ACCAAATTGGCAATTGAAGC","chr10",4965940,"+","none","TGG","ACCAAATTGGCAATTGAAGCTGG",0,NA,NA,NA,"GeneID:1645","AKR1C1",TRUE,"AKR1C1"

"6096","ACCAAATTGGCAATTGAAGC","chr20",22420651,"-","12:C>T,15:T>C","TGG","ACCAAATTGGCAGTTAAAGCTGG",2,13,16,NA,"GeneID:284788","LOC284788",TRUE,"AKR1C1"

"6097","ACCAAATTGGCAATTGAAGC","chr10",5200207,"+","8:A>G,14:A>T","TGG","ACCAAATTAGCAATAGAAGCTGG",2,9,15,NA,"GeneID:1109","AKR1C4",TRUE,"AKR1C1"

"6098","CAATTCCCATCGACCAGAGT","chr10",4966934,"+","none","TGG","CAATTCCCATCGACCAGAGTTGG",0,NA,NA,NA,"GeneID:1645","AKR1C1",TRUE,"AKR1C1"

"6099","CAATTCCCATCGACCAGAGT","chr10",5000638,"-","none","TGG","CAATTCCCATCGACCAGAGTTGG",0,NA,NA,NA,"GeneID:1646","AKR1C2",TRUE,"AKR1C1"

"6100","CAATTCCCATCGACCAGAGT","chr16",60979500,"+","3:G>T,10:G>C","GGG","CAAGTCCCATGGACCAGAGTGGG",2,4,11,NA,"","",FALSE,"AKR1C1"

"6101","CAATTCCCATCGACCAGAGT","chr6",21620479,"-","1:G>T,10:C>G","GGG","CCATTCCCATGGACCAGAGTGGG",2,2,11,NA,"","",FALSE,"AKR1C1"

"6102","CTGCCTACCTTTACAGACAC","chr10",5000541,"+","none","TGG","CTGCCTACCTTTACAGACACTGG",0,NA,NA,NA,"GeneID:1646","AKR1C2",TRUE,"AKR1C1"

"6103","CTGCCTACCTTTACAGACAC","chr10",4967031,"-","none","TGG","CTGCCTACCTTTACAGACACTGG",0,NA,NA,NA,"GeneID:1645","AKR1C1",TRUE,"AKR1C1"

"6104","CTTATTACCATGGTTCTTCT","chr10",4972298,"-","none","CGG","CTTATTACCATGGTTCTTCTCGG",0,NA,NA,NA,"GeneID:1645","AKR1C1",TRUE,"AKR1C1"

"6105","CTTATTACCATGGTTCTTCT","chr13",74740055,"+","10:C>T,11:T>G","AGG","CTTATTACCACTGTTCTTCTAGG",2,11,12,NA,"","",FALSE,"AKR1C1"

"6106","CTTATTACCATGGTTCTTCT","chr3",180108183,"+","4:C>T,7:G>C","GGG","CTTACTAGCATGGTTCTTCTGGG",2,5,8,NA,"","",FALSE,"AKR1C1"

"6107","GATCCCACCGAGAAGAACCA","chr10",4972288,"+","none","TGG","GATCCCACCGAGAAGAACCATGG",0,NA,NA,NA,"GeneID:1645","AKR1C1",TRUE,"AKR1C1"

"6108","GATCCCACCGAGAAGAACCA","chr10",4995757,"-","7:A>G","TGG","GATCCCATCGAGAAGAACCATGG",1,8,NA,NA,"GeneID:1646","AKR1C2",TRUE,"AKR1C1"

"6109","GCCAGCTTCAATTGCCAATT","chr10",4965944,"-","none","TGG","GCCAGCTTCAATTGCCAATTTGG",0,NA,NA,NA,"GeneID:1645","AKR1C1",TRUE,"AKR1C1"

"6110","GCCAGCTTCAATTGCCAATT","chr10",5200211,"-","9:A>T,15:A>G","TGG","GCCAGCTTCTATTGCTAATTTGG",2,10,16,NA,"GeneID:1109","AKR1C4",TRUE,"AKR1C1"

"6111","AGGAATTAGGAGTTATGTTT","chr10",5000404,"+","none","AGG","AGGAATTAGGAGTTATGTTTAGG",0,NA,NA,NA,"GeneID:1646","AKR1C2",TRUE,"AKR1C2"

"6112","AGGAATTAGGAGTTATGTTT","chr10",4967168,"-","15:C>A,16:T>C","AGG","AGGAATTAGGAGTTAGATTTAGG",2,16,17,NA,"GeneID:1645","AKR1C1",TRUE,"AKR1C2"

"6113","AGGAATTAGGAGTTATGTTT","chr17",54687788,"+","8:A>G","TGG","AGGAATTAAGAGTTATGTTTTGG",1,9,NA,NA,"","",FALSE,"AKR1C2"

"6114","ATTACCATGGTTCTTCTCGA","chr10",4995750,"+","none","TGG","ATTACCATGGTTCTTCTCGATGG",0,NA,NA,NA,"GeneID:1646","AKR1C2",TRUE,"AKR1C2"

"6115","ATTACCATGGTTCTTCTCGA","chr10",4972295,"-","19:C>T","TGG","ATTACCATGGTTCTTCTCGGTGG",1,20,NA,NA,"GeneID:1645","AKR1C1",TRUE,"AKR1C2"

"6116","CAATTCCCATCGACCAGAGT","chr10",4966934,"+","none","TGG","CAATTCCCATCGACCAGAGTTGG",0,NA,NA,NA,"GeneID:1645","AKR1C1",TRUE,"AKR1C2"

"6117","CAATTCCCATCGACCAGAGT","chr10",5000638,"-","none","TGG","CAATTCCCATCGACCAGAGTTGG",0,NA,NA,NA,"GeneID:1646","AKR1C2",TRUE,"AKR1C2"

"6118","CAATTCCCATCGACCAGAGT","chr16",60979500,"+","3:G>T,10:G>C","GGG","CAAGTCCCATGGACCAGAGTGGG",2,4,11,NA,"","",FALSE,"AKR1C2"

"6119","CAATTCCCATCGACCAGAGT","chr6",21620479,"-","1:G>T,10:C>G","GGG","CCATTCCCATGGACCAGAGTGGG",2,2,11,NA,"","",FALSE,"AKR1C2"

"6120","GATCCCATCGAGAAGAACCA","chr10",4995757,"-","none","TGG","GATCCCATCGAGAAGAACCATGG",0,NA,NA,NA,"GeneID:1646","AKR1C2",TRUE,"AKR1C2"

"6121","GATCCCATCGAGAAGAACCA","chr10",4972288,"+","7:C>T","TGG","GATCCCACCGAGAAGAACCATGG",1,8,NA,NA,"GeneID:1645","AKR1C1",TRUE,"AKR1C2"

"6122","GATCCCATCGAGAAGAACCA","chr1",11203989,"+","8:G>C,13:G>A","AGG","GATCCCATGGAGAGGAACCAAGG",2,9,14,NA,"GeneID:2475","MTOR",FALSE,"AKR1C2"

"6123","GATCCCATCGAGAAGAACCA","chr6",43221289,"+","1:C>A,18:G>C","GGG","GCTCCCATCGAGAAGAACGAGGG",2,2,19,NA,"GeneID:23113","CUL9",TRUE,"AKR1C2"

"6124","GGCTTCTATTGCCAATTTGA","chr10",5001633,"+","none","CGG","GGCTTCTATTGCCAATTTGACGG",0,NA,NA,NA,"GeneID:1646","AKR1C2",TRUE,"AKR1C2"

"6125","GTCAAATTGGCAATAGAAGC","chr10",5001634,"-","none","CGG","GTCAAATTGGCAATAGAAGCCGG",0,NA,NA,NA,"GeneID:1646","AKR1C2",TRUE,"AKR1C2"

"6126","ACCTCCAAAGCTTTACTTCT","chr10",5096415,"-","none","CGG","ACCTCCAAAGCTTTACTTCTCGG",0,NA,NA,NA,"GeneID:8644","AKR1C3",TRUE,"AKR1C3"

"6127","ACCTCCAAAGCTTTACTTCT","chr6",127450324,"+","0:T>A,19:C>T","CGG","TCCTCCAAAGCTTTACTTCCCGG",2,1,20,NA,"GeneID:387104;GeneID:9729","SOGA3;KIAA0408",TRUE,"AKR1C3"

"6128","CAACTCTGGTCGATGAAAAG","chr10",5097443,"-","none","TGG","CAACTCTGGTCGATGAAAAGTGG",0,NA,NA,NA,"GeneID:8644","AKR1C3",TRUE,"AKR1C3"

"6129","CAACTCTGGTCGATGAAAAG","chr6",43636365,"-","3:T>G,10:T>G","AGG","CAAATCTGGTAGATGAAAAGAGG",2,4,11,NA,"GeneID:9587","MAD2L1BP",TRUE,"AKR1C3"

"6130","CTGCATACCTTTAGAGACAT","chr10",5097538,"-","none","TGG","CTGCATACCTTTAGAGACATTGG",0,NA,NA,NA,"GeneID:8644","AKR1C3",TRUE,"AKR1C3"

"6131","CTTTCACCAACAGATGAAAA","chr10",5098813,"+","none","TGG","CTTTCACCAACAGATGAAAATGG",0,NA,NA,NA,"GeneID:8644","AKR1C3",TRUE,"AKR1C3"

"6132","CTTTCACCAACAGATGAAAA","chr17",8931317,"+","9:T>A,17:G>A","GGG","CTTTCACCATCAGATGAGAAGGG",2,10,18,NA,"GeneID:23533","PIK3R5",FALSE,"AKR1C3"

"6133","CTTTCACCAACAGATGAAAA","chrX",143734989,"-","5:G>T,11:A>T","TGG","CTTTCCCCAACTGATGAAAATGG",2,6,12,NA,"","",FALSE,"AKR1C3"

"6134","GGTTCCGAGAAGTAAAGCTT","chr10",5096408,"+","none","TGG","GGTTCCGAGAAGTAAAGCTTTGG",0,NA,NA,NA,"GeneID:8644","AKR1C3",TRUE,"AKR1C3"

"6135","GTTGAAGTTTGACACCCCAA","chr10",5099366,"-","none","TGG","GTTGAAGTTTGACACCCCAATGG",0,NA,NA,NA,"GeneID:8644","AKR1C3",TRUE,"AKR1C3"

"6136","GTTGAAGTTTGACACCCCAA","chr10",5206314,"-","18:C>T","TGG","GTTGAAGTTTGACACCCCGATGG",1,19,NA,NA,"GeneID:1109","AKR1C4",TRUE,"AKR1C3"

"6137","GTTGAAGTTTGACACCCCAA","chr1",14080829,"+","13:C>A,18:C>A","GGG","GTTGAAGTTTGACCCCCCCAGGG",2,14,19,NA,"GeneID:23254","KAZN",FALSE,"AKR1C3"

"6138","GTTGAAGTTTGACACCCCAA","chr10",4998687,"+","9:G>T,18:G>A","TGG","GTTGAAGTTGGACACCCCGATGG",2,10,19,NA,"GeneID:1646","AKR1C2",TRUE,"AKR1C3"

"6139","GTTGAAGTTTGACACCCCAA","chr10",4968861,"-","9:C>A,18:C>T","TGG","GTTGAAGTTGGACACCCCGATGG",2,10,19,NA,"GeneID:1645","AKR1C1",TRUE,"AKR1C3"

"6140","GTTGAAGTTTGACACCCCAA","chr7",134575459,"-","6:T>C,12:C>G","GGG","GTTGAAATTTGAGACCCCAAGGG",2,7,13,NA,"GeneID:441282","AKR1B15",TRUE,"AKR1C3"

"6141","AAAAGTAATATTCGACACAG","chr10",5205791,"+","none","TGG","AAAAGTAATATTCGACACAGTGG",0,NA,NA,NA,"GeneID:1109","AKR1C4",TRUE,"AKR1C4"

"6142","AAAAGTAATATTCGACACAG","chr8",55354853,"+","12:T>C,19:A>G","AGG","AAAAGTAATATTTGACACAAAGG",2,13,20,NA,"GeneID:114786","XKR4",FALSE,"AKR1C4"

"6143","AAAAGTAATATTCGACACAG","chr10",5098836,"+","12:T>C,17:T>C","TGG","AAAAGTAATATTTGACATAGTGG",2,13,18,NA,"GeneID:8644","AKR1C3",TRUE,"AKR1C4"

"6144","CACTTTCTTTCAACCACAGA","chr10",5204384,"+","none","TGG","CACTTTCTTTCAACCACAGATGG",0,NA,NA,NA,"GeneID:1109","AKR1C4",TRUE,"AKR1C4"

"6145","CCAGGTCATGGAGAAGTGTA","chr10",5206270,"+","none","AGG","CCAGGTCATGGAGAAGTGTAAGG",0,NA,NA,NA,"GeneID:1109","AKR1C4",TRUE,"AKR1C4"

"6146","CCAGGTCATGGAGAAGTGTA","chr15",26164710,"+","11:C>A,15:A>G","GGG","CCAGGTCATGGCGAAATGTAGGG",2,12,16,NA,"","",FALSE,"AKR1C4"

"6147","CCAGGTCATGGAGAAGTGTA","chr10",4885504,"-","5:G>A,7:G>T","AGG","CCAGGCCCTGGAGAAGTGTAAGG",2,6,8,NA,"","",TRUE,"AKR1C4"

"6148","CCAGGTCATGGAGAAGTGTA","chr10",5099322,"+","0:A>C,5:C>T","AGG","ACAGGCCATGGAGAAGTGTAAGG",2,1,6,NA,"GeneID:8644","AKR1C3",TRUE,"AKR1C4"

"6149","GCCAGCTTCTATTGCTAATT","chr10",5200211,"-","none","TGG","GCCAGCTTCTATTGCTAATTTGG",0,NA,NA,NA,"GeneID:1109","AKR1C4",TRUE,"AKR1C4"

"6150","GCCAGCTTCTATTGCTAATT","chr10",4965944,"-","9:T>A,15:G>A","TGG","GCCAGCTTCAATTGCCAATTTGG",2,10,16,NA,"GeneID:1645","AKR1C1",TRUE,"AKR1C4"

"6151","TCCGAGGAACAGAGCTGTAG","chr10",5200182,"+","none","AGG","TCCGAGGAACAGAGCTGTAGAGG",0,NA,NA,NA,"GeneID:1109","AKR1C4",TRUE,"AKR1C4"

"6152","TTCCCTACCTTGAGAGCCAT","chr10",5204481,"-","none","TGG","TTCCCTACCTTGAGAGCCATTGG",0,NA,NA,NA,"GeneID:1109","AKR1C4",TRUE,"AKR1C4"

"6153","AACCTTCACCGATGTTGCAC","chr7",138088616,"-","none","AGG","AACCTTCACCGATGTTGCACAGG",0,NA,NA,NA,"GeneID:6718","AKR1D1",TRUE,"AKR1D1"

"6154","ATATACCCTAGAGATGAGAA","chr7",138097877,"+","none","TGG","ATATACCCTAGAGATGAGAATGG",0,NA,NA,NA,"GeneID:6718","AKR1D1",TRUE,"AKR1D1"

"6155","ATATACCCTAGAGATGAGAA","chr16",71283437,"+","15:C>G","TGG","ATATACCCTAGAGATCAGAATGG",1,16,NA,NA,"GeneID:55783","CMTR2",TRUE,"AKR1D1"

"6156","CATTTGCCATTCTCATCTCT","chr7",138097886,"-","none","AGG","CATTTGCCATTCTCATCTCTAGG",0,NA,NA,NA,"GeneID:6718","AKR1D1",TRUE,"AKR1D1"

"6157","CATTTGCCATTCTCATCTCT","chr1",167520022,"-","15:G>A,18:T>G","AGG","CATTTGCCATTCTCACCTATAGG",2,16,19,NA,"","",TRUE,"AKR1D1"

"6158","CATTTGCCATTCTCATCTCT","chr20",44757786,"-","5:G>C,9:C>A","TGG","CATTTCCCAGTCTCATCTCTTGG",2,6,10,NA,"GeneID:140730","RIMS4",TRUE,"AKR1D1"

"6159","CATTTGCCATTCTCATCTCT","chr8",117110829,"-","8:A>T,9:C>A","CGG","CATTTGCCTGTCTCATCTCTCGG",2,9,10,NA,"GeneID:169026","SLC30A8",FALSE,"AKR1D1"

"6160","CATTTGCCATTCTCATCTCT","chr8",79766734,"-","1:A>T,18:A>G","AGG","CTTTTGCCATTCTCATCTTTAGG",2,2,19,NA,"GeneID:23462","HEY1",TRUE,"AKR1D1"

"6161","TGAACTCACCTTAAAGGCCA","chr7",138091873,"-","none","TGG","TGAACTCACCTTAAAGGCCATGG",0,NA,NA,NA,"GeneID:6718","AKR1D1",TRUE,"AKR1D1"

"6162","TGAACTCACCTTAAAGGCCA","chr1",221922782,"-","12:A>T,13:G>T","GGG","TGAACTCACCTTTCAGGCCAGGG",2,13,14,NA,"","",FALSE,"AKR1D1"

"6163","TGAACTCACCTTAAAGGCCA","chr11",34840581,"-","4:C>G,8:C>G","AGG","TGAAGTCAGCTTAAAGGCCAAGG",2,5,9,NA,"","",FALSE,"AKR1D1"

"6164","TGTAGGCCCCATCAATATGT","chr7",138088656,"-","none","CGG","TGTAGGCCCCATCAATATGTCGG",0,NA,NA,NA,"GeneID:6718","AKR1D1",TRUE,"AKR1D1"

"6165","TTACATCATTGAAGTACCCA","chr7",138091853,"+","none","TGG","TTACATCATTGAAGTACCCATGG",0,NA,NA,NA,"GeneID:6718","AKR1D1",TRUE,"AKR1D1"

"6166","TTACATCATTGAAGTACCCA","chr2",11921152,"-","0:C>A,2:A>T","CGG","GTTCATCATTGAAGTACCCACGG",2,1,3,NA,"GeneID:100506457","LOC100506457",FALSE,"AKR1D1"

"6167","TTACATCATTGAAGTACCCA","chr1",167519970,"+","8:C>T,13:A>G","TGG","TTACATCACTGAAATACCCATGG",2,9,14,NA,"","",TRUE,"AKR1D1"

"6168","ACCATGTTGCTCTCGTCCAG","chr10",4835752,"-","none","AGG","ACCATGTTGCTCTCGTCCAGAGG",0,NA,NA,NA,"GeneID:83592","AKR1E2",TRUE,"AKR1E2"

"6169","AGGGATCCGTTGCAAGATCA","chr10",4830778,"+","none","AGG","AGGGATCCGTTGCAAGATCAAGG",0,NA,NA,NA,"GeneID:83592","AKR1E2",TRUE,"AKR1E2"

"6170","AGGGATCCGTTGCAAGATCA","chr9",63860387,"+","8:A>G,10:C>T","TGG","AGGGATCCATCGCAAGATCATGG",2,9,11,NA,"","",FALSE,"AKR1E2"

"6171","AGTAAGCACAGTCGAAGTGC","chr10",4830730,"-","none","CGG","AGTAAGCACAGTCGAAGTGCCGG",0,NA,NA,NA,"GeneID:83592","AKR1E2",TRUE,"AKR1E2"

"6172","CCTCTCACATCCTCGAGTGC","chr10",4835721,"+","none","AGG","CCTCTCACATCCTCGAGTGCAGG",0,NA,NA,NA,"GeneID:83592","AKR1E2",TRUE,"AKR1E2"

"6173","GAACGGTACCTTGAAACCCA","chr10",4833455,"-","none","TGG","GAACGGTACCTTGAAACCCATGG",0,NA,NA,NA,"GeneID:83592","AKR1E2",TRUE,"AKR1E2"

"6174","TGGACCTCTACCTCATACAC","chr10",4833428,"+","none","TGG","TGGACCTCTACCTCATACACTGG",0,NA,NA,NA,"GeneID:83592","AKR1E2",TRUE,"AKR1E2"

"6175","TGGACCTCTACCTCATACAC","chr7",134450803,"-","13:A>G,16:A>T","TGG","TGGACCTCTACCTTATTCACTGG",2,14,17,NA,"GeneID:231","AKR1B1",TRUE,"AKR1E2"

"6176","ATAGCTGGGCTGAGACCTAC","chr1",19307009,"-","none","AGG","ATAGCTGGGCTGAGACCTACAGG",0,NA,NA,NA,"GeneID:8574","AKR7A2",TRUE,"AKR7A2"

"6177","ATAGCTGGGCTGAGACCTAC","chrX",118878183,"-","17:G>A","AGG","ATAGCTGGGCTGAGACCCACAGG",1,18,NA,NA,"GeneID:101928287","LINC01285",TRUE,"AKR7A2"

"6178","CACTTCCCAGCTAGCATAGT","chr1",19308214,"+","none","TGG","CACTTCCCAGCTAGCATAGTTGG",0,NA,NA,NA,"GeneID:8574","AKR7A2",TRUE,"AKR7A2"

"6179","CACTTCCCAGCTAGCATAGT","chr1",112923778,"-","10:C>G,12:C>T","TGG","CACTTCCCAGGTGGCATAGTTGG",2,11,13,NA,"GeneID:6566","SLC16A1",TRUE,"AKR7A2"

"6180","CCAAGTGAAAATTGCCACCA","chr1",19308627,"-","none","AGG","CCAAGTGAAAATTGCCACCAAGG",0,NA,NA,NA,"GeneID:8574","AKR7A2",TRUE,"AKR7A2"

"6181","CCCGGCTCACCGATTCCTGT","chr1",19306991,"+","none","AGG","CCCGGCTCACCGATTCCTGTAGG",0,NA,NA,NA,"GeneID:8574","AKR7A2",TRUE,"AKR7A2"

"6182","CCCGGCTCACCGATTCCTGT","chr13",23704807,"-","3:A>C,14:T>A","GGG","CCCTGCTCACCGATACCTGTGGG",2,4,15,NA,"","",FALSE,"AKR7A2"

"6183","GCCTCTCCAACTATGCTAGC","chr1",19308223,"-","none","TGG","GCCTCTCCAACTATGCTAGCTGG",0,NA,NA,NA,"GeneID:8574","AKR7A2",TRUE,"AKR7A2"

"6184","GCCTCTCCAACTATGCTAGC","chrX",118878778,"-","16:G>A,17:C>T","TGG","GCCTCTCCAACTATGCCGGCTGG",2,17,18,NA,"GeneID:101928287","LINC01285",TRUE,"AKR7A2"

"6185","GCCTCTCCAACTATGCTAGC","chr1",112923769,"+","16:C>T,18:C>G","TGG","GCCTCTCCAACTATGCCACCTGG",2,17,19,NA,"GeneID:6566","SLC16A1",TRUE,"AKR7A2"

"6186","TGCCGTGGTCAGGTGCGTGT","chr1",19308501,"+","none","AGG","TGCCGTGGTCAGGTGCGTGTAGG",0,NA,NA,NA,"GeneID:8574","AKR7A2",TRUE,"AKR7A2"

"6187","TGCCGTGGTCAGGTGCGTGT","chrX",118878864,"+","16:A>G","AGG","TGCCGTGGTCAGGTGCATGTAGG",1,17,NA,NA,"GeneID:101928287","LINC01285",TRUE,"AKR7A2"

"6188","ATACCTGGGCAGAGATGTAC","chr1",19284693,"-","none","AGG","ATACCTGGGCAGAGATGTACAGG",0,NA,NA,NA,"GeneID:22977","AKR7A3",TRUE,"AKR7A3"

"6189","ATACCTGGGCAGAGATGTAC","chr15",40203038,"-","16:G>C,18:C>T","AGG","ATACCTGGGCAGAGATCTGCAGG",2,17,19,NA,"GeneID:701","BUB1B",FALSE,"AKR7A3"

"6190","ATACCTGGGCAGAGATGTAC","chr10",67219392,"+","2:T>A,6:T>G","AGG","ATTCCTTGGCAGAGATGTACAGG",2,3,7,NA,"GeneID:29119","CTNNA3",FALSE,"AKR7A3"

"6191","ATTCCTGTACATCTCTGCCC","chr1",19284687,"+","none","AGG","ATTCCTGTACATCTCTGCCCAGG",0,NA,NA,NA,"GeneID:22977","AKR7A3",TRUE,"AKR7A3"

"6192","ATTCCTGTACATCTCTGCCC","chr20",15841969,"-","8:A>T,16:A>C","TGG","ATTCCTGTTCATCTCTTCCCTGG",2,9,17,NA,"GeneID:140733","MACROD2",FALSE,"AKR7A3"

"6193","ATTCCTGTACATCTCTGCCC","chr10",67219398,"-","1:G>A,19:T>G","AGG","ACTCCTGTACATCTCTGCCAAGG",2,2,20,NA,"GeneID:29119","CTNNA3",FALSE,"AKR7A3"

"6194","CCCAAACAGTGGAATGGCCT","chr1",19286337,"+","none","TGG","CCCAAACAGTGGAATGGCCTTGG",0,NA,NA,NA,"GeneID:22977","AKR7A3",TRUE,"AKR7A3"

"6195","CCCAAACAGTGGAATGGCCT","chr1",112923577,"-","8:T>C,13:A>T","TGG","CCCAAACAATGGATTGGCCTTGG",2,9,14,NA,"GeneID:6566","SLC16A1",TRUE,"AKR7A3"

"6196","GCATGTACAATGCCATCACC","chr1",19285093,"-","none","CGG","GCATGTACAATGCCATCACCCGG",0,NA,NA,NA,"GeneID:22977","AKR7A3",TRUE,"AKR7A3"

"6197","GCATGTACAATGCCATCACC","chrX",118878351,"-","15:G>A","CGG","GCATGTACAATGCCACCACCCGG",1,16,NA,NA,"GeneID:101928287","LINC01285",TRUE,"AKR7A3"

"6198","GCATGTACAATGCCATCACC","chr1",19307389,"-","10:G>A,15:G>A","CGG","GCATGTACAACGCCACCACCCGG",2,11,16,NA,"GeneID:8574","AKR7A2",TRUE,"AKR7A3"

"6199","GCATGTACAATGCCATCACC","chr11",44763710,"+","6:T>A,19:A>C","TGG","GCATGTTCAATGCCATCACATGG",2,7,20,NA,"GeneID:90139","TSPAN18",FALSE,"AKR7A3"

"6200","GCATGTACAATGCCATCACC","chr18",58041403,"-","9:A>T,13:T>G","TGG","GCATGTACATTGCAATCACCTGG",2,10,14,NA,"","",FALSE,"AKR7A3"

"6201","GCCTCTCCAACTATGCAGCC","chr1",19285953,"-","none","TGG","GCCTCTCCAACTATGCAGCCTGG",0,NA,NA,NA,"GeneID:22977","AKR7A3",TRUE,"AKR7A3"

"6202","GCCTCTCCAACTATGCAGCC","chr1",19270516,"-","16:G>T","TGG","GCCTCTCCAACTATGCCGCCTGG",1,17,NA,NA,"GeneID:246181","AKR7L",TRUE,"AKR7A3"

"6203","GCCTCTCCAACTATGCAGCC","chrX",118878778,"-","16:G>T,18:C>G","TGG","GCCTCTCCAACTATGCCGGCTGG",2,17,19,NA,"GeneID:101928287","LINC01285",TRUE,"AKR7A3"

"6204","GCCTCTCCAACTATGCAGCC","chr1",112923769,"+","16:C>A,17:A>G","TGG","GCCTCTCCAACTATGCCACCTGG",2,17,18,NA,"GeneID:6566","SLC16A1",TRUE,"AKR7A3"

"6205","TGCTGTGGTCTGGCATATGC","chr1",19286231,"+","none","AGG","TGCTGTGGTCTGGCATATGCAGG",0,NA,NA,NA,"GeneID:22977","AKR7A3",TRUE,"AKR7A3"

"6206","TGCTGTGGTCTGGCATATGC","chr6",143724930,"+","0:A>T,9:G>C","TGG","AGCTGTGGTGTGGCATATGCTGG",2,1,10,NA,"GeneID:9749","PHACTR2",FALSE,"AKR7A3"

"6207","ACCGCGTCCTGCAGAACTCC","chr14",104774955,"-","none","AGG","ACCGCGTCCTGCAGAACTCCAGG",0,NA,NA,NA,"GeneID:207","AKT1",TRUE,"AKT1"

"6208","GCAGGATGTGGACCAACGTG","chr14",104780117,"-","none","AGG","GCAGGATGTGGACCAACGTGAGG",0,NA,NA,NA,"GeneID:207","AKT1",TRUE,"AKT1"

"6209","GCCCGTCCTTGTCCAGCATG","chr14",104773348,"+","none","AGG","GCCCGTCCTTGTCCAGCATGAGG",0,NA,NA,NA,"GeneID:207","AKT1",TRUE,"AKT1"

"6210","GCCTGGAGTTCTGCAGGACG","chr14",104774951,"+","none","CGG","GCCTGGAGTTCTGCAGGACGCGG",0,NA,NA,NA,"GeneID:207","AKT1",TRUE,"AKT1"

"6211","GCCTGGAGTTCTGCAGGACG","chr7",45349591,"+","11:A>T,18:T>C","TGG","GCCTGGAGTTCAGCAGGATGTGG",2,12,19,NA,"","",FALSE,"AKT1"

"6212","GCCTGGAGTTCTGCAGGACG","chr20",63834525,"+","3:A>T,13:G>C","GGG","GCCAGGAGTTCTGGAGGACGGGG",2,4,14,NA,"","",FALSE,"AKT1"

"6213","TCACGTTGGTCCACATCCTG","chr14",104780116,"+","none","CGG","TCACGTTGGTCCACATCCTGCGG",0,NA,NA,NA,"GeneID:207","AKT1",TRUE,"AKT1"

"6214","TGTGCCGCAAAAGGTCTTCA","chr14",104773271,"+","none","TGG","TGTGCCGCAAAAGGTCTTCATGG",0,NA,NA,NA,"GeneID:207","AKT1",TRUE,"AKT1"

"6215","AACGGCGCGCTGCCTCCGCC","chr19",49873114,"+","none","AGG","AACGGCGCGCTGCCTCCGCCAGG",0,NA,NA,NA,"GeneID:11284;GeneID:84335","PNKP;AKT1S1",TRUE,"AKT1S1"

"6216","AAGTCGCTGGTGTTAAGCCG","chr19",49869941,"+","none","CGG","AAGTCGCTGGTGTTAAGCCGCGG",0,NA,NA,NA,"GeneID:11284;GeneID:84335","PNKP;AKT1S1",TRUE,"AKT1S1"

"6217","CGCGCATGCTCGCCGCGATG","chr19",49870017,"+","none","CGG","CGCGCATGCTCGCCGCGATGCGG",0,NA,NA,NA,"GeneID:11284;GeneID:84335","PNKP;AKT1S1",TRUE,"AKT1S1"

"6218","CGGCGGGGGTCTCCTCGCTC","chr19",49871686,"+","none","AGG","CGGCGGGGGTCTCCTCGCTCAGG",0,NA,NA,NA,"GeneID:11284;GeneID:84335","PNKP;AKT1S1",TRUE,"AKT1S1"

"6219","TCAGACCCCGAGAGTACAGA","chr19",49871813,"-","none","TGG","TCAGACCCCGAGAGTACAGATGG",0,NA,NA,NA,"GeneID:11284;GeneID:84335","PNKP;AKT1S1",TRUE,"AKT1S1"

"6220","TCAGACCCCGAGAGTACAGA","chr6",158761948,"-","14:T>A,15:C>T","TGG","TCAGACCCCGAGAGAGCAGATGG",2,15,16,NA,"GeneID:94120","SYTL3",FALSE,"AKT1S1"

"6221","TCAGACCCCGAGAGTACAGA","chr6",26131218,"-","6:A>G,8:T>G","TGG","TCAGACTCAGAGAGTACAGATGG",2,7,9,NA,"GeneID:8334","HIST1H2AC",FALSE,"AKT1S1"

"6222","TGTGGGCCAGTGCGATGTCG","chr19",49873087,"+","none","TGG","TGTGGGCCAGTGCGATGTCGTGG",0,NA,NA,NA,"GeneID:11284;GeneID:84335","PNKP;AKT1S1",TRUE,"AKT1S1"

"6223","CAATGATGACTTCCTTCCGC","chr19",40241942,"+","none","AGG","CAATGATGACTTCCTTCCGCAGG",0,NA,NA,NA,"GeneID:208","AKT2",TRUE,"AKT2"

"6224","CATCGAGAGGACCTTCCACG","chr19",40255176,"-","none","TGG","CATCGAGAGGACCTTCCACGTGG",0,NA,NA,NA,"GeneID:208","AKT2",TRUE,"AKT2"

"6225","GACCCCATGGACTACAAGTG","chr19",40242603,"-","none","TGG","GACCCCATGGACTACAAGTGTGG",0,NA,NA,NA,"GeneID:208","AKT2",TRUE,"AKT2"

"6226","TCGCCAACAGCCTCAAGCAG","chr19",40242638,"-","none","CGG","TCGCCAACAGCCTCAAGCAGCGG",0,NA,NA,NA,"GeneID:208","AKT2",TRUE,"AKT2"

"6227","TCTCGTCTGGAGAATCCACG","chr19",40255158,"+","none","TGG","TCTCGTCTGGAGAATCCACGTGG",0,NA,NA,NA,"GeneID:208","AKT2",TRUE,"AKT2"

"6228","TTAAACAACTTCTCCGTAGC","chr19",40256927,"-","none","AGG","TTAAACAACTTCTCCGTAGCAGG",0,NA,NA,NA,"GeneID:208","AKT2",TRUE,"AKT2"

"6229","AACCCAACCTTCTTTCACAA","chr1",243843134,"+","none","TGG","AACCCAACCTTCTTTCACAATGG",0,NA,NA,NA,"GeneID:10000","AKT3",TRUE,"AKT3"

"6230","AACCCAACCTTCTTTCACAA","chr2",19689601,"-","9:G>A,10:G>A","GGG","AACCCAACCCCCTTTCACAAGGG",2,10,11,NA,"","",FALSE,"AKT3"

"6231","AGAATGGACAGAAGCTATCC","chr1",243646014,"-","none","AGG","AGAATGGACAGAAGCTATCCAGG",0,NA,NA,NA,"GeneID:10000","AKT3",TRUE,"AKT3"

"6232","GATGTTACCATTGTGAAAGA","chr1",243843144,"-","none","AGG","GATGTTACCATTGTGAAAGAAGG",0,NA,NA,NA,"GeneID:10000","AKT3",TRUE,"AKT3"

"6233","GATGTTACCATTGTGAAAGA","chr18",50415221,"-","16:A>T,18:T>C","GGG","GATGTTACCATTGTGATAAAGGG",2,17,19,NA,"","",FALSE,"AKT3"

"6234","GATGTTACCATTGTGAAAGA","chr3",26344875,"-","15:C>T,18:T>C","TGG","GATGTTACCATTGTGGAAAATGG",2,16,19,NA,"","",FALSE,"AKT3"

"6235","GATGTTACCATTGTGAAAGA","chr11",42389424,"+","11:A>T,17:G>A","GGG","GATGTTACCATAGTGAAGGAGGG",2,12,18,NA,"","",FALSE,"AKT3"

"6236","GCAGTCTGTCTGCTACAGCC","chr1",243645993,"+","none","TGG","GCAGTCTGTCTGCTACAGCCTGG",0,NA,NA,NA,"GeneID:10000","AKT3",TRUE,"AKT3"

"6237","GCAGTCTGTCTGCTACAGCC","chr19",51249321,"+","6:A>T,19:T>C","AGG","GCAGTCAGTCTGCTACAGCTAGG",2,7,20,NA,"GeneID:284369","SIGLECL1",FALSE,"AKT3"

"6238","TAAGGTAAATCCACATCTTG","chr1",243695616,"+","none","AGG","TAAGGTAAATCCACATCTTGAGG",0,NA,NA,NA,"GeneID:10000","AKT3",TRUE,"AKT3"

"6239","TAAGGTAAATCCACATCTTG","chr4",106288677,"+","6:T>A,16:G>C","TGG","TAAGGTTAATCCACATGTTGTGG",2,7,17,NA,"GeneID:93627","TBCK",FALSE,"AKT3"

"6240","TTGCCACTGAAAAGTTGTTG","chr1",243695590,"+","none","AGG","TTGCCACTGAAAAGTTGTTGAGG",0,NA,NA,NA,"GeneID:10000","AKT3",TRUE,"AKT3"

"6241","CACCACTTACCTAATTGCAT","chr16",53494127,"+","none","AGG","CACCACTTACCTAATTGCATAGG",0,NA,NA,NA,"GeneID:64400","AKTIP",TRUE,"AKTIP"

"6242","CTGCAAGAAGAGAGTATTCC","chr16",53498394,"+","none","AGG","CTGCAAGAAGAGAGTATTCCAGG",0,NA,NA,NA,"GeneID:64400","AKTIP",TRUE,"AKTIP"

"6243","CTGCAAGAAGAGAGTATTCC","chr5",24170594,"-","none","AGG","CTGCAAGAAGAGAGTATTCCAGG",0,NA,NA,NA,"","",TRUE,"AKTIP"

"6244","CTGCAAGAAGAGAGTATTCC","chr4",183943216,"-","2:T>C,4:G>T","TGG","CTACCAGAAGAGAGTATTCCTGG",2,3,5,NA,"GeneID:56977","STOX2",FALSE,"AKTIP"

"6245","CTGCAAGAAGAGAGTATTCC","chr3",52753744,"-","1:T>A,18:A>G","AGG","CAGCAAGAAGAGAGTATTTCAGG",2,2,19,NA,"GeneID:6787","NEK4",TRUE,"AKTIP"

"6246","CTTAATGCAGAGCGATAAGA","chr16",53495260,"+","none","TGG","CTTAATGCAGAGCGATAAGATGG",0,NA,NA,NA,"GeneID:64400","AKTIP",TRUE,"AKTIP"

"6247","CTTAATGCAGAGCGATAAGA","chr5",24170664,"-","0:T>G","TGG","ATTAATGCAGAGCGATAAGATGG",1,1,NA,NA,"","",TRUE,"AKTIP"

"6248","CTTACTGGACAGTCACCATC","chr16",53495067,"+","none","TGG","CTTACTGGACAGTCACCATCTGG",0,NA,NA,NA,"GeneID:64400","AKTIP",TRUE,"AKTIP"

"6249","GGTGAAGAGAAGACATTAAC","chr5",24170421,"+","none","AGG","GGTGAAGAGAAGACATTAACAGG",0,NA,NA,NA,"","",TRUE,"AKTIP"

"6250","GGTGAAGAGAAGACATTAAC","chr16",53498567,"-","none","AGG","GGTGAAGAGAAGACATTAACAGG",0,NA,NA,NA,"GeneID:64400","AKTIP",TRUE,"AKTIP"

"6251","GGTGAAGAGAAGACATTAAC","chr7",123343688,"-","17:C>T,18:A>T","TGG","GGTGAAGAGAAGACATTGTCTGG",2,18,19,NA,"","",FALSE,"AKTIP"

"6252","GGTGAAGAGAAGACATTAAC","chr1",169996817,"+","16:G>T,19:A>C","TGG","GGTGAAGAGAAGACATGAAATGG",2,17,20,NA,"GeneID:22920","KIFAP3",FALSE,"AKTIP"

"6253","GGTGAAGAGAAGACATTAAC","chr18",75210934,"-","9:G>T,17:G>T","TGG","GGTGAAGAGCAGACATTCACTGG",2,10,18,NA,"GeneID:10194","TSHZ1",TRUE,"AKTIP"

"6254","TAGAAGACCCCTATGCAATT","chr16",53494139,"-","none","AGG","TAGAAGACCCCTATGCAATTAGG",0,NA,NA,NA,"GeneID:64400","AKTIP",TRUE,"AKTIP"

"6255","TAGAAGACCCCTATGCAATT","chr2",116170210,"+","16:C>A,17:T>A","AGG","TAGAAGACCCCTATGCCTTTAGG",2,17,18,NA,"","",FALSE,"AKTIP"

"6256","CGTCCATCCATCATGTCCGA","chr9",113390453,"+","none","CGG","CGTCCATCCATCATGTCCGACGG",0,NA,NA,NA,"GeneID:210","ALAD",TRUE,"ALAD"

"6257","CTCGCCGTGTACCACGTCTC","chr9",113389071,"-","none","TGG","CTCGCCGTGTACCACGTCTCTGG",0,NA,NA,NA,"GeneID:210","ALAD",TRUE,"ALAD"

"6258","CTCTCCAGAGACGTGGTACA","chr9",113389064,"+","none","CGG","CTCTCCAGAGACGTGGTACACGG",0,NA,NA,NA,"GeneID:210","ALAD",TRUE,"ALAD"

"6259","CTCTCCAGAGACGTGGTACA","chr2",27154650,"-","4:C>G,17:A>T","AGG","CTCTGCAGAGACGTGGTTCAAGG",2,5,18,NA,"GeneID:150921","TCF23",TRUE,"ALAD"

"6260","GCCCCGTCGGACATGATGGA","chr9",113390459,"-","none","TGG","GCCCCGTCGGACATGATGGATGG",0,NA,NA,NA,"GeneID:210","ALAD",TRUE,"ALAD"

"6261","GGTGGCATTGGCGTATGCCA","chr9",113390598,"-","none","AGG","GGTGGCATTGGCGTATGCCAAGG",0,NA,NA,NA,"GeneID:210","ALAD",TRUE,"ALAD"

"6262","GTGATAGGCTGTATGTCATC","chr9",113392139,"+","none","AGG","GTGATAGGCTGTATGTCATCAGG",0,NA,NA,NA,"GeneID:210","ALAD",TRUE,"ALAD"

"6263","AAGTTCTTCAGCAGTCCACT","chr3",52203924,"-","none","GGG","AAGTTCTTCAGCAGTCCACTGGG",0,NA,NA,NA,"GeneID:211","ALAS1",TRUE,"ALAS1"

"6264","ATAACTGCCCCACACACCCG","chr3",52204895,"-","none","TGG","ATAACTGCCCCACACACCCGTGG",0,NA,NA,NA,"GeneID:211","ALAS1",TRUE,"ALAS1"

"6265","GATGGCACACAGCTTCCGTC","chr3",52202565,"+","none","TGG","GATGGCACACAGCTTCCGTCTGG",0,NA,NA,NA,"GeneID:211","ALAS1",TRUE,"ALAS1"

"6266","GATGGCACACAGCTTCCGTC","chr5",126718664,"-","8:G>T,16:A>G","TGG","GATGGCACCCAGCTTCTGTCTGG",2,9,17,NA,"","",FALSE,"ALAS1"

"6267","GATGGCACACAGCTTCCGTC","chr11",128554281,"-","5:A>G,17:T>C","GGG","GATGGTACACAGCTTCCATCGGG",2,6,18,NA,"GeneID:2113","ETS1",FALSE,"ALAS1"

"6268","GTCATTGGCCACAAAGCACG","chr3",52205969,"-","none","AGG","GTCATTGGCCACAAAGCACGAGG",0,NA,NA,NA,"GeneID:211","ALAS1",TRUE,"ALAS1"

"6269","GTCATTGGCCACAAAGCACG","chrX",55020353,"+","9:A>C,18:G>C","AGG","GTCATTGGCAACAAAGCAGGAGG",2,10,19,NA,"GeneID:212","ALAS2",TRUE,"ALAS1"

"6270","GTGGTTAGTGTGAAAACCGA","chr3",52203891,"+","none","TGG","GTGGTTAGTGTGAAAACCGATGG",0,NA,NA,NA,"GeneID:211","ALAS1",TRUE,"ALAS1"

"6271","GTGGTTAGTGTGAAAACCGA","chr5",73890137,"+","9:A>G,14:T>A","TGG","GTGGTTAGTATGAATACCGATGG",2,10,15,NA,"GeneID:64283","ARHGEF28",FALSE,"ALAS1"

"6272","TACCTGGCATCATCTTAGCC","chr3",52206006,"-","none","AGG","TACCTGGCATCATCTTAGCCAGG",0,NA,NA,NA,"GeneID:211","ALAS1",TRUE,"ALAS1"

"6273","CCACTGTCCCTTCATGCTGT","chrX",55024798,"-","none","CGG","CCACTGTCCCTTCATGCTGTCGG",0,NA,NA,NA,"GeneID:212","ALAS2",TRUE,"ALAS2"

"6274","CCACTGTCCCTTCATGCTGT","chr10",96511073,"-","13:A>T,16:C>G","GGG","CCACTGTCCCTTCTTGGTGTGGG",2,14,17,NA,"GeneID:7093","TLL2",FALSE,"ALAS2"

"6275","CCGACAGCATGAAGGGACAG","chrX",55024795,"+","none","TGG","CCGACAGCATGAAGGGACAGTGG",0,NA,NA,NA,"GeneID:212","ALAS2",TRUE,"ALAS2"

"6276","CTCCACTGTTACGGATACCT","chrX",55017599,"+","none","TGG","CTCCACTGTTACGGATACCTTGG",0,NA,NA,NA,"GeneID:212","ALAS2",TRUE,"ALAS2"

"6277","GACAGCGTCCAATACCAAAC","chrX",55025885,"+","none","AGG","GACAGCGTCCAATACCAAACAGG",0,NA,NA,NA,"GeneID:212","ALAS2",TRUE,"ALAS2"

"6278","TATTGGACGCTGTCCCATCC","chrX",55025879,"-","none","TGG","TATTGGACGCTGTCCCATCCTGG",0,NA,NA,NA,"GeneID:212","ALAS2",TRUE,"ALAS2"

"6279","TGCGAGATTTACTCAGACGC","chrX",55017643,"-","none","AGG","TGCGAGATTTACTCAGACGCAGG",0,NA,NA,NA,"GeneID:212","ALAS2",TRUE,"ALAS2"

"6280","AATGTGATGTTATAAGCCTA","chr4",73420287,"-","none","AGG","AATGTGATGTTATAAGCCTAAGG",0,NA,NA,NA,"GeneID:213","ALB",TRUE,"ALB"

"6281","CCCTTGCCCAGACAAGAGTG","chr4",73405104,"+","none","AGG","CCCTTGCCCAGACAAGAGTGAGG",0,NA,NA,NA,"GeneID:213","ALB",TRUE,"ALB"

"6282","CCCTTGCCCAGACAAGAGTG","chr10",95663551,"-","2:A>G,14:G>T","GGG","CCTTTGCCCAGACACGAGTGGGG",2,3,15,NA,"GeneID:26123","TCTN3",TRUE,"ALB"

"6283","CGATGAATTTAAACCTCTTG","chr4",73416257,"+","none","TGG","CGATGAATTTAAACCTCTTGTGG",0,NA,NA,NA,"GeneID:213","ALB",TRUE,"ALB"

"6284","CTGCTGAAGATACTGAGCAA","chr4",73406642,"-","none","AGG","CTGCTGAAGATACTGAGCAAAGG",0,NA,NA,NA,"GeneID:213","ALB",TRUE,"ALB"

"6285","CTGCTGAAGATACTGAGCAA","chr4",1282623,"+","11:G>A,17:G>C","AGG","CTGCTGAAGATGCTGAGGAAAGG",2,12,18,NA,"","",FALSE,"ALB"

"6286","GCTGCAAGTCAAGCTGCCTT","chr4",73420268,"+","none","AGG","GCTGCAAGTCAAGCTGCCTTAGG",0,NA,NA,NA,"GeneID:213","ALB",TRUE,"ALB"

"6287","GCTGCAAGTCAAGCTGCCTT","chrX",45420810,"+","9:T>C,19:G>T","GGG","GCTGCAAGTTAAGCTGCCTGGGG",2,10,20,NA,"","",FALSE,"ALB"

"6288","TGCTCATCGGTTTAAAGATT","chr4",73405128,"+","none","TGG","TGCTCATCGGTTTAAAGATTTGG",0,NA,NA,NA,"GeneID:213","ALB",TRUE,"ALB"

"6289","AAAGTGCTACATCCCCTTGA","chr3",105533665,"+","none","AGG","AAAGTGCTACATCCCCTTGAAGG",0,NA,NA,NA,"GeneID:214","ALCAM",TRUE,"ALCAM"

"6290","AGTGCTTTGCTTACAATTTC","chr3",105532018,"-","none","AGG","AGTGCTTTGCTTACAATTTCAGG",0,NA,NA,NA,"GeneID:214","ALCAM",TRUE,"ALCAM"

"6291","CGAAACAGAGCAGCTAAAAA","chr3",105532044,"+","none","AGG","CGAAACAGAGCAGCTAAAAAAGG",0,NA,NA,NA,"GeneID:214","ALCAM",TRUE,"ALCAM"

"6292","TACCATGTGATATTGCCATC","chr3",105533635,"-","none","TGG","TACCATGTGATATTGCCATCTGG",0,NA,NA,NA,"GeneID:214","ALCAM",TRUE,"ALCAM"

"6293","TACCATGTGATATTGCCATC","chr19",40906852,"-","12:G>A,13:T>A","AGG","TACCATGTGATACAGCCATCAGG",2,13,14,NA,"","",FALSE,"ALCAM"

"6294","TCATTGACTCTCATTGTAGA","chr3",105547262,"+","none","AGG","TCATTGACTCTCATTGTAGAAGG",0,NA,NA,NA,"GeneID:214","ALCAM",TRUE,"ALCAM"

"6295","TGTCTGCGAAACTGCTCTGC","chr3",105547213,"+","none","AGG","TGTCTGCGAAACTGCTCTGCAGG",0,NA,NA,NA,"GeneID:214","ALCAM",TRUE,"ALCAM"

"6296","AAATCCTCCACATCATCTCA","chr19",49460861,"-","none","AGG","AAATCCTCCACATCATCTCAAGG",0,NA,NA,NA,"GeneID:126133","ALDH16A1",TRUE,"ALDH16A1"

"6297","AAATCCTCCACATCATCTCA","chr4",124607336,"+","8:T>C,18:A>C","TGG","AAATCCTCTACATCATCTAATGG",2,9,19,NA,"","",FALSE,"ALDH16A1"

"6298","AAGGGGGAGGCCACGACCAC","chr19",49464138,"-","none","AGG","AAGGGGGAGGCCACGACCACAGG",0,NA,NA,NA,"GeneID:126133","ALDH16A1",TRUE,"ALDH16A1"

"6299","CCATCATTTACCCACAGCCA","chr19",49460890,"-","none","GGG","CCATCATTTACCCACAGCCAGGG",0,NA,NA,NA,"GeneID:126133","ALDH16A1",TRUE,"ALDH16A1"

"6300","CCATCATTTACCCACAGCCA","chr8",118900867,"-","5:A>T,9:A>T","TGG","CCATCTTTTTCCCACAGCCATGG",2,6,10,NA,"","",FALSE,"ALDH16A1"

"6301","CCCATTCTATCCCCCAACCT","chr19",49463885,"+","none","TGG","CCCATTCTATCCCCCAACCTTGG",0,NA,NA,NA,"GeneID:126133","ALDH16A1",TRUE,"ALDH16A1"

"6302","CCCATTCTATCCCCCAACCT","chr9",19743490,"-","8:C>T,14:A>G","AGG","CCCATTCTGTCCCCTAACCTAGG",2,9,15,NA,"GeneID:25769","SLC24A2",FALSE,"ALDH16A1"

"6303","GCCCCCGCGGGGCGTCCCGT","chr19",49464190,"-","none","TGG","GCCCCCGCGGGGCGTCCCGTTGG",0,NA,NA,NA,"GeneID:126133","ALDH16A1",TRUE,"ALDH16A1"

"6304","GGATAGAATGGGCGTTCCGA","chr19",49463877,"-","none","AGG","GGATAGAATGGGCGTTCCGAAGG",0,NA,NA,NA,"GeneID:126133","ALDH16A1",TRUE,"ALDH16A1"

"6305","ACAGGCAATTCCAGTCTTAG","chr10",95637181,"-","none","AGG","ACAGGCAATTCCAGTCTTAGAGG",0,NA,NA,NA,"GeneID:5832","ALDH18A1",TRUE,"ALDH18A1"

"6306","ATTGTTCTTTCAGATGTAGA","chr10",95632960,"-","none","AGG","ATTGTTCTTTCAGATGTAGAAGG",0,NA,NA,NA,"GeneID:5832","ALDH18A1",TRUE,"ALDH18A1"

"6307","GCCGCACGCTCTGAGACAGA","chr10",95637324,"+","none","AGG","GCCGCACGCTCTGAGACAGAAGG",0,NA,NA,NA,"GeneID:5832","ALDH18A1",TRUE,"ALDH18A1"

"6308","GCGCAAGCGTTGTTTGCCAA","chr10",95637355,"+","none","AGG","GCGCAAGCGTTGTTTGCCAAAGG",0,NA,NA,NA,"GeneID:5832","ALDH18A1",TRUE,"ALDH18A1"

"6309","GGGCAGCACAGATGCTGTAC","chr10",95637094,"+","none","TGG","GGGCAGCACAGATGCTGTACTGG",0,NA,NA,NA,"GeneID:5832","ALDH18A1",TRUE,"ALDH18A1"

"6310","GGGCAGCACAGATGCTGTAC","chr18",23185558,"-","17:G>A,18:G>T","AGG","GGGCAGCACAGATGCTGCCCAGG",2,18,19,NA,"GeneID:91768","CABLES1",FALSE,"ALDH18A1"

"6311","TGTTAAAGATAATGATAGCC","chr10",95633021,"-","none","TGG","TGTTAAAGATAATGATAGCCTGG",0,NA,NA,NA,"GeneID:5832","ALDH18A1",TRUE,"ALDH18A1"

"6312","AAAACATTGCGCTACTGTGC","chr9",72928932,"-","none","AGG","AAAACATTGCGCTACTGTGCAGG",0,NA,NA,NA,"GeneID:216","ALDH1A1",TRUE,"ALDH1A1"

"6313","AAGCATCCATAGTACGCCAC","chr9",72930943,"+","none","GGG","AAGCATCCATAGTACGCCACGGG",0,NA,NA,NA,"GeneID:216","ALDH1A1",TRUE,"ALDH1A1"

"6314","ACTTACCAATTGGTATTGTA","chr9",72928885,"+","none","CGG","ACTTACCAATTGGTATTGTACGG",0,NA,NA,NA,"GeneID:216","ALDH1A1",TRUE,"ALDH1A1"

"6315","CAGAGCTCCTCCTCAGTTGC","chr9",72940169,"+","none","AGG","CAGAGCTCCTCCTCAGTTGCAGG",0,NA,NA,NA,"GeneID:216","ALDH1A1",TRUE,"ALDH1A1"

"6316","CAGAGCTCCTCCTCAGTTGC","chr6",37642880,"-","10:C>G,18:G>C","AGG","CAGAGCTCCTGCTCAGTTCCAGG",2,11,19,NA,"GeneID:266727","MDGA1",FALSE,"ALDH1A1"

"6317","GTATAATAGTCGCCCCCTCT","chr9",72930920,"+","none","CGG","GTATAATAGTCGCCCCCTCTCGG",0,NA,NA,NA,"GeneID:216","ALDH1A1",TRUE,"ALDH1A1"

"6318","TATACAAGACATGAACCTAT","chr9",72927140,"-","none","TGG","TATACAAGACATGAACCTATTGG",0,NA,NA,NA,"GeneID:216","ALDH1A1",TRUE,"ALDH1A1"

"6319","TATACAAGACATGAACCTAT","chr15",57995102,"-","1:A>T,17:G>A","TGG","TTTACAAGACATGAACCCATTGG",2,2,18,NA,"GeneID:8854","ALDH1A2",TRUE,"ALDH1A1"

"6320","AAAACCTTTCGATATTACGC","chr15",58010689,"-","none","AGG","AAAACCTTTCGATATTACGCAGG",0,NA,NA,NA,"GeneID:8854","ALDH1A2",TRUE,"ALDH1A2"

"6321","ACCACTCAGTGCACTCTACA","chr15",57992964,"-","none","TGG","ACCACTCAGTGCACTCTACATGG",0,NA,NA,NA,"GeneID:8854","ALDH1A2",TRUE,"ALDH1A2"

"6322","CCAGCCTGCGTAATATCGAA","chr15",58010682,"+","none","AGG","CCAGCCTGCGTAATATCGAAAGG",0,NA,NA,NA,"GeneID:8854","ALDH1A2",TRUE,"ALDH1A2"

"6323","CCCATGTAGAGTGCACTGAG","chr15",57992960,"+","none","TGG","CCCATGTAGAGTGCACTGAGTGG",0,NA,NA,NA,"GeneID:8854","ALDH1A2",TRUE,"ALDH1A2"

"6324","GATCTGTCCACACACTCCAA","chr15",57995083,"+","none","TGG","GATCTGTCCACACACTCCAATGG",0,NA,NA,NA,"GeneID:8854","ALDH1A2",TRUE,"ALDH1A2"

"6325","GATCTGTCCACACACTCCAA","chrX",12555294,"+","0:T>G,14:G>C","AGG","TATCTGTCCACACAGTCCAAAGG",2,1,15,NA,"GeneID:9758","FRMPD4",FALSE,"ALDH1A2"

"6326","TTTACAAGACATGAACCCAT","chr15",57995102,"-","none","TGG","TTTACAAGACATGAACCCATTGG",0,NA,NA,NA,"GeneID:8854","ALDH1A2",TRUE,"ALDH1A2"

"6327","TTTACAAGACATGAACCCAT","chr8",40216937,"+","9:A>C,15:G>C","TGG","TTTACAAGAAATGAAGCCATTGG",2,10,16,NA,"","",FALSE,"ALDH1A2"

"6328","TTTACAAGACATGAACCCAT","chr5",104792407,"+","7:C>G,16:T>C","AGG","TTTACAACACATGAACTCATAGG",2,8,17,NA,"","",FALSE,"ALDH1A2"

"6329","TTTACAAGACATGAACCCAT","chr9",72927140,"-","1:T>A,17:A>G","TGG","TATACAAGACATGAACCTATTGG",2,2,18,NA,"GeneID:216","ALDH1A1",TRUE,"ALDH1A2"

"6330","AGCGTCCGCACACACGATGC","chr15",100898161,"-","none","AGG","AGCGTCCGCACACACGATGCAGG",0,NA,NA,NA,"GeneID:220;GeneID:101927751","ALDH1A3;LOC101927751",TRUE,"ALDH1A3"

"6331","CATATTTGCTCCCGAGTTGA","chr15",100885329,"-","none","AGG","CATATTTGCTCCCGAGTTGAAGG",0,NA,NA,NA,"GeneID:220","ALDH1A3",TRUE,"ALDH1A3"

"6332","CGCTCAGCCCGACGTGGACA","chr15",100887564,"+","none","AGG","CGCTCAGCCCGACGTGGACAAGG",0,NA,NA,NA,"GeneID:220","ALDH1A3",TRUE,"ALDH1A3"

"6333","CGTCCCGGAGCAATCTGAAG","chr15",100898107,"+","none","CGG","CGTCCCGGAGCAATCTGAAGCGG",0,NA,NA,NA,"GeneID:220;GeneID:101927751","ALDH1A3;LOC101927751",TRUE,"ALDH1A3"

"6334","CTACATGTAACCCTTCAACT","chr15",100885315,"+","none","CGG","CTACATGTAACCCTTCAACTCGG",0,NA,NA,NA,"GeneID:220","ALDH1A3",TRUE,"ALDH1A3"

"6335","TACTCACGGCCAAGGTGGCG","chr15",100887699,"-","none","CGG","TACTCACGGCCAAGGTGGCGCGG",0,NA,NA,NA,"GeneID:220","ALDH1A3",TRUE,"ALDH1A3"

"6336","CGAAACGCTCTCCGCCACAG","chr9",38396902,"-","none","AGG","CGAAACGCTCTCCGCCACAGAGG",0,NA,NA,NA,"GeneID:219","ALDH1B1",TRUE,"ALDH1B1"

"6337","CGGCCCGATCCACATCAGCC","chr9",38395966,"-","none","CGG","CGGCCCGATCCACATCAGCCCGG",0,NA,NA,NA,"GeneID:219","ALDH1B1",TRUE,"ALDH1B1"

"6338","GAGCAGTTTGAACGAGTCCT","chr9",38396840,"+","none","AGG","GAGCAGTTTGAACGAGTCCTAGG",0,NA,NA,NA,"GeneID:219","ALDH1B1",TRUE,"ALDH1B1"

"6339","GGTAGGGTTGACCGTCGGGA","chr9",38395908,"-","none","AGG","GGTAGGGTTGACCGTCGGGAAGG",0,NA,NA,NA,"GeneID:219","ALDH1B1",TRUE,"ALDH1B1"

"6340","GTGAACATCATCACGGGGTA","chr9",38396450,"+","none","TGG","GTGAACATCATCACGGGGTATGG",0,NA,NA,NA,"GeneID:219","ALDH1B1",TRUE,"ALDH1B1"

"6341","GTGATGATGTTCACCACCCC","chr9",38396444,"-","none","AGG","GTGATGATGTTCACCACCCCAGG",0,NA,NA,NA,"GeneID:219","ALDH1B1",TRUE,"ALDH1B1"

"6342","AAGGAGGGCCACGAAGTGGT","chr3",126160899,"-","none","GGG","AAGGAGGGCCACGAAGTGGTGGG",0,NA,NA,NA,"GeneID:10840","ALDH1L1",TRUE,"ALDH1L1"

"6343","AAGGAGGGCCACGAAGTGGT","chrX",55171992,"+","13:G>A,19:A>T","GGG","AAGGAGGGCCACGGAGTGGAGGG",2,14,20,NA,"","",FALSE,"ALDH1L1"

"6344","AAGGAGGGCCACGAAGTGGT","chr12",94913375,"+","8:A>C,11:G>C","TGG","AAGGAGGGACAGGAAGTGGTTGG",2,9,12,NA,"GeneID:55967","NDUFA12",FALSE,"ALDH1L1"

"6345","CCAGGGGTGGTCACCAAAGC","chr3",126153477,"-","none","AGG","CCAGGGGTGGTCACCAAAGCAGG",0,NA,NA,NA,"GeneID:10840","ALDH1L1",TRUE,"ALDH1L1"

"6346","GCTGGTGAAGAATATTCAGC","chr3",126150509,"-","none","TGG","GCTGGTGAAGAATATTCAGCTGG",0,NA,NA,NA,"GeneID:10840","ALDH1L1",TRUE,"ALDH1L1"

"6347","GGAGACGCTTTGCCCATCCC","chr3",126153510,"-","none","AGG","GGAGACGCTTTGCCCATCCCAGG",0,NA,NA,NA,"GeneID:10840","ALDH1L1",TRUE,"ALDH1L1"

"6348","GGAGACGCTTTGCCCATCCC","chr7",24962489,"+","10:G>T,17:G>C","TGG","GGAGACGCTTGGCCCATGCCTGG",2,11,18,NA,"GeneID:26031","OSBPL3",FALSE,"ALDH1L1"

"6349","GGAGACGCTTTGCCCATCCC","chr2",137452467,"+","6:T>G,7:A>C","TGG","GGAGACTATTTGCCCATCCCTGG",2,7,8,NA,"GeneID:80731","THSD7B",FALSE,"ALDH1L1"

"6350","TGAACACACCCACCACTTCG","chr3",126160888,"+","none","TGG","TGAACACACCCACCACTTCGTGG",0,NA,NA,NA,"GeneID:10840","ALDH1L1",TRUE,"ALDH1L1"

"6351","TGCCCCCTTAAAGAAGTTCG","chr3",126150462,"+","none","AGG","TGCCCCCTTAAAGAAGTTCGAGG",0,NA,NA,NA,"GeneID:10840","ALDH1L1",TRUE,"ALDH1L1"

"6352","TGCCCCCTTAAAGAAGTTCG","chr17",59337151,"+","9:T>A,19:A>G","TGG","TGCCCCCTTTAAGAAGTTCATGG",2,10,20,NA,"GeneID:388403","YPEL2",FALSE,"ALDH1L1"

"6353","AAGTGCATCCACTGTATCAT","chr12",105068757,"+","none","TGG","AAGTGCATCCACTGTATCATTGG",0,NA,NA,NA,"GeneID:121053;GeneID:160428","C12orf45;ALDH1L2",TRUE,"ALDH1L2"

"6354","ACCATGGCCTTGATTCCTTC","chr12",105068716,"+","none","AGG","ACCATGGCCTTGATTCCTTCAGG",0,NA,NA,NA,"GeneID:121053;GeneID:160428","C12orf45;ALDH1L2",TRUE,"ALDH1L2"

"6355","ACCATGGCCTTGATTCCTTC","chr17",72109174,"-","12:A>T,16:A>G","TGG","ACCATGGCCTTGTTTCTTTCTGG",2,13,17,NA,"GeneID:400618","SOX9-AS1",FALSE,"ALDH1L2"

"6356","CTATAGCCACCTCCGCAAAG","chr12",105073923,"-","none","AGG","CTATAGCCACCTCCGCAAAGAGG",0,NA,NA,NA,"GeneID:121053;GeneID:160428","C12orf45;ALDH1L2",TRUE,"ALDH1L2"

"6357","CTGTGAACACCCCTACTACT","chr12",105073893,"+","none","CGG","CTGTGAACACCCCTACTACTCGG",0,NA,NA,NA,"GeneID:121053;GeneID:160428","C12orf45;ALDH1L2",TRUE,"ALDH1L2"

"6358","GCGCTCCGGCGCTTCTCCAC","chr12",105084395,"-","none","TGG","GCGCTCCGGCGCTTCTCCACTGG",0,NA,NA,NA,"GeneID:160428","ALDH1L2",TRUE,"ALDH1L2"

"6359","TCCTCTCCAGCATGCTGCGG","chr12",105084427,"-","none","CGG","TCCTCTCCAGCATGCTGCGGCGG",0,NA,NA,NA,"GeneID:160428","ALDH1L2",TRUE,"ALDH1L2"

"6360","TCCTCTCCAGCATGCTGCGG","chr12",94795666,"-","11:G>T,17:A>G","TGG","TCCTCTCCAGCCTGCTGTGGTGG",2,12,18,NA,"","",FALSE,"ALDH1L2"

"6361","CCCACCGTCAATCCGTCCAC","chr12",111781965,"+","none","TGG","CCCACCGTCAATCCGTCCACTGG",0,NA,NA,NA,"GeneID:217","ALDH2",TRUE,"ALDH2"

"6362","CCCGCTCGATCAGATCGGCC","chr12",111783261,"-","none","AGG","CCCGCTCGATCAGATCGGCCAGG",0,NA,NA,NA,"GeneID:217","ALDH2",TRUE,"ALDH2"

"6363","CTACACACGCCATGAACCTG","chr12",111789888,"+","none","TGG","CTACACACGCCATGAACCTGTGG",0,NA,NA,NA,"GeneID:217","ALDH2",TRUE,"ALDH2"

"6364","GCCCATACCGGAGACATTTG","chr12",111785334,"-","none","AGG","GCCCATACCGGAGACATTTGAGG",0,NA,NA,NA,"GeneID:217","ALDH2",TRUE,"ALDH2"

"6365","GCCTGGCCGATCTGATCGAG","chr12",111783257,"+","none","CGG","GCCTGGCCGATCTGATCGAGCGG",0,NA,NA,NA,"GeneID:217","ALDH2",TRUE,"ALDH2"

"6366","GCTGAAGAAGTCTCCGTCAA","chr12",111789869,"-","none","TGG","GCTGAAGAAGTCTCCGTCAATGG",0,NA,NA,NA,"GeneID:217","ALDH2",TRUE,"ALDH2"

"6367","AGTTCCAGGTGCCAATGACG","chr17",19743279,"+","none","AGG","AGTTCCAGGTGCCAATGACGAGG",0,NA,NA,NA,"GeneID:218","ALDH3A1",TRUE,"ALDH3A1"

"6368","CATGTTCTCACTCAGCTCCG","chr17",19742586,"+","none","AGG","CATGTTCTCACTCAGCTCCGAGG",0,NA,NA,NA,"GeneID:218","ALDH3A1",TRUE,"ALDH3A1"

"6369","CATGTTCTCACTCAGCTCCG","chr14",67572141,"+","5:C>T,12:G>C","AGG","CATGTCCTCACTGAGCTCCGAGG",2,6,13,NA,"GeneID:57475","PLEKHH1",TRUE,"ALDH3A1"

"6370","CTACATCCACTCGGAGCCAC","chr17",19743312,"-","none","TGG","CTACATCCACTCGGAGCCACTGG",0,NA,NA,NA,"GeneID:218","ALDH3A1",TRUE,"ALDH3A1"

"6371","GGGACACCCCCATTGATTAC","chr17",19742180,"+","none","TGG","GGGACACCCCCATTGATTACTGG",0,NA,NA,NA,"GeneID:218","ALDH3A1",TRUE,"ALDH3A1"

"6372","GTTCGACCATATCCTGTACA","chr17",19742133,"-","none","CGG","GTTCGACCATATCCTGTACACGG",0,NA,NA,NA,"GeneID:218","ALDH3A1",TRUE,"ALDH3A1"

"6373","TACCATCATCCCCCAGTACC","chr17",19742552,"-","none","TGG","TACCATCATCCCCCAGTACCTGG",0,NA,NA,NA,"GeneID:218","ALDH3A1",TRUE,"ALDH3A1"

"6374","CGATGTTGATCCTAAAACCA","chr17",19663348,"+","none","AGG","CGATGTTGATCCTAAAACCAAGG",0,NA,NA,NA,"GeneID:224","ALDH3A2",TRUE,"ALDH3A2"

"6375","CGTGCGTACCTTGCACAGGT","chr17",19649113,"-","none","CGG","CGTGCGTACCTTGCACAGGTCGG",0,NA,NA,NA,"GeneID:224","ALDH3A2",TRUE,"ALDH3A2"

"6376","GTCCGACAGGCGTTCCTGTC","chr17",19648992,"+","none","CGG","GTCCGACAGGCGTTCCTGTCCGG",0,NA,NA,NA,"GeneID:224","ALDH3A2",TRUE,"ALDH3A2"

"6377","TCAACATCGGTAAGTACTGT","chr17",19663337,"-","none","TGG","TCAACATCGGTAAGTACTGTTGG",0,NA,NA,NA,"GeneID:224","ALDH3A2",TRUE,"ALDH3A2"

"6378","TGGCTGAATGGTGAGAACGA","chr17",19651736,"-","none","AGG","TGGCTGAATGGTGAGAACGAAGG",0,NA,NA,NA,"GeneID:224","ALDH3A2",TRUE,"ALDH3A2"

"6379","TGGTGCTGATAATCGGAGCT","chr17",19651703,"+","none","TGG","TGGTGCTGATAATCGGAGCTTGG",0,NA,NA,NA,"GeneID:224","ALDH3A2",TRUE,"ALDH3A2"

"6380","AGTTCCAGGGCGCAATGATG","chr11",68018825,"-","none","AGG","AGTTCCAGGGCGCAATGATGAGG",0,NA,NA,NA,"GeneID:221","ALDH3B1",TRUE,"ALDH3B1"

"6381","AGTTCCAGGGCGCAATGATG","chr11",67666621,"+","10:T>C,13:G>A","AGG","AGTTCCAGGGTGCGATGATGAGG",2,11,14,NA,"GeneID:222","ALDH3B2",TRUE,"ALDH3B1"

"6382","CCACCCTAACTATCTAATCA","chr11",68008624,"+","none","CGG","CCACCCTAACTATCTAATCACGG",0,NA,NA,NA,"GeneID:221","ALDH3B1",TRUE,"ALDH3B1"

"6383","CCTTCCACGCGGGGCGCACG","chr11",68015337,"+","none","CGG","CCTTCCACGCGGGGCGCACGCGG",0,NA,NA,NA,"GeneID:221","ALDH3B1",TRUE,"ALDH3B1"

"6384","CTATCCGCTGAACCTGACGC","chr11",68018843,"+","none","TGG","CTATCCGCTGAACCTGACGCTGG",0,NA,NA,NA,"GeneID:221","ALDH3B1",TRUE,"ALDH3B1"

"6385","GCAGCTTCTGCACGACGCAC","chr11",68015419,"+","none","TGG","GCAGCTTCTGCACGACGCACTGG",0,NA,NA,NA,"GeneID:221","ALDH3B1",TRUE,"ALDH3B1"

"6386","TGGCCCGTGATTAGATAGTT","chr11",68008631,"-","none","AGG","TGGCCCGTGATTAGATAGTTAGG",0,NA,NA,NA,"GeneID:221","ALDH3B1",TRUE,"ALDH3B1"

"6387","AACGAGAGCGATCGCTACAT","chr11",67665286,"-","none","CGG","AACGAGAGCGATCGCTACATCGG",0,NA,NA,NA,"GeneID:222","ALDH3B2",TRUE,"ALDH3B2"

"6388","AACGAGAGCGATCGCTACAT","chr11",68021849,"+","0:G>A,2:T>C","CGG","GATGAGAGCGATCGCTACATCGG",2,1,3,NA,"GeneID:221","ALDH3B1",TRUE,"ALDH3B2"

"6389","AGCGATCGCTCTCGTTGCTC","chr11",67665290,"+","none","TGG","AGCGATCGCTCTCGTTGCTCTGG",0,NA,NA,NA,"GeneID:222","ALDH3B2",TRUE,"ALDH3B2"

"6390","AGCGATCGCTCTCGTTGCTC","chr11",68021845,"-","13:T>C,15:G>A","TGG","AGCGATCGCTCTCATCGCTCTGG",2,14,16,NA,"GeneID:221","ALDH3B1",TRUE,"ALDH3B2"

"6391","GAAGCCGTCAGAAATCAGCC","chr11",67666365,"-","none","AGG","GAAGCCGTCAGAAATCAGCCAGG",0,NA,NA,NA,"GeneID:222","ALDH3B2",TRUE,"ALDH3B2"

"6392","GAAGCCGTCAGAAATCAGCC","chr6",75610722,"+","6:A>G,9:T>A","TGG","GAAGCCATCTGAAATCAGCCTGG",2,7,10,NA,"GeneID:26054","SENP6",FALSE,"ALDH3B2"

"6393","GCCCTCCTCACCTGCGGCGA","chr11",67666562,"+","none","GGG","GCCCTCCTCACCTGCGGCGAGGG",0,NA,NA,NA,"GeneID:222","ALDH3B2",TRUE,"ALDH3B2"

"6394","TGCCCCTGCAGGGAGTTGCG","chr11",67666392,"-","none","TGG","TGCCCCTGCAGGGAGTTGCGTGG",0,NA,NA,NA,"GeneID:222","ALDH3B2",TRUE,"ALDH3B2"

"6395","TGCCCCTGCAGGGAGTTGCG","chr1",4325769,"+","15:C>T,17:C>G","GGG","TGCCCCTGCAGGGAGCTCCGGGG",2,16,18,NA,"","",FALSE,"ALDH3B2"

"6396","TGCCCCTGCAGGGAGTTGCG","chr13",51215422,"-","3:A>G,11:A>C","TGG","TGCTCCTGCAGTGAGTTGCGTGG",2,4,12,NA,"","",FALSE,"ALDH3B2"

"6397","TGGTCCTCATCATCGCACCC","chr11",67666628,"-","none","TGG","TGGTCCTCATCATCGCACCCTGG",0,NA,NA,NA,"GeneID:222","ALDH3B2",TRUE,"ALDH3B2"

"6398","TGGTCCTCATCATCGCACCC","chr11",68018818,"+","13:T>C,16:G>A","TGG","TGGTCCTCATCATTGCGCCCTGG",2,14,17,NA,"GeneID:221","ALDH3B1",TRUE,"ALDH3B2"

"6399","ATCTGGGCCCGGTCTGCAAT","chr1",18885545,"+","none","AGG","ATCTGGGCCCGGTCTGCAATAGG",0,NA,NA,NA,"GeneID:8659","ALDH4A1",TRUE,"ALDH4A1"

"6400","GATGGAAGCCATCCCATGCG","chr1",18889411,"-","none","TGG","GATGGAAGCCATCCCATGCGTGG",0,NA,NA,NA,"GeneID:8659","ALDH4A1",TRUE,"ALDH4A1"

"6401","GCCCGTCTTAGCCTTCACGC","chr1",18890043,"-","none","AGG","GCCCGTCTTAGCCTTCACGCAGG",0,NA,NA,NA,"GeneID:8659","ALDH4A1",TRUE,"ALDH4A1"

"6402","GGATGAGGAGGTGTGGACGT","chr1",18889384,"-","none","CGG","GGATGAGGAGGTGTGGACGTCGG",0,NA,NA,NA,"GeneID:8659","ALDH4A1",TRUE,"ALDH4A1"

"6403","GGATGAGGAGGTGTGGACGT","chr5",165549057,"-","13:C>A,18:G>C","TGG","GGATGAGGAGGTGGGGACCTTGG",2,14,19,NA,"","",FALSE,"ALDH4A1"

"6404","GGATGAGGAGGTGTGGACGT","chr2",239745685,"+","10:A>G,16:G>A","GGG","GGATGAGGAGATGTGGGCGTGGG",2,11,17,NA,"","",FALSE,"ALDH4A1"

"6405","GGATGAGGAGGTGTGGACGT","chr1",20969477,"+","3:G>T,7:T>G","GGG","GGAGGAGTAGGTGTGGACGTGGG",2,4,8,NA,"GeneID:8672","EIF4G3",TRUE,"ALDH4A1"

"6406","TCAGCATGTCTGCCGCCTTC","chr1",18885519,"+","none","AGG","TCAGCATGTCTGCCGCCTTCAGG",0,NA,NA,NA,"GeneID:8659","ALDH4A1",TRUE,"ALDH4A1"

"6407","TGAGCGAGATGCCCTGCAAA","chr1",18890013,"-","none","AGG","TGAGCGAGATGCCCTGCAAAAGG",0,NA,NA,NA,"GeneID:8659","ALDH4A1",TRUE,"ALDH4A1"

"6408","AGACACGCCCTTCTCCGCCC","chr6",24504951,"+","none","TGG","AGACACGCCCTTCTCCGCCCTGG",0,NA,NA,NA,"GeneID:7915","ALDH5A1",TRUE,"ALDH5A1"

"6409","CTCTTTATAGCTTGCAAGCC","chr6",24515156,"+","none","AGG","CTCTTTATAGCTTGCAAGCCAGG",0,NA,NA,NA,"GeneID:7915","ALDH5A1",TRUE,"ALDH5A1"

"6410","CTGTTCTCGAAAGAATGCCA","chr6",24515213,"+","none","AGG","CTGTTCTCGAAAGAATGCCAAGG",0,NA,NA,NA,"GeneID:7915","ALDH5A1",TRUE,"ALDH5A1"

"6411","GAGAAGGGCGTGTCTTCGGC","chr6",24504946,"-","none","AGG","GAGAAGGGCGTGTCTTCGGCAGG",0,NA,NA,NA,"GeneID:7915","ALDH5A1",TRUE,"ALDH5A1"

"6412","GAGGAAGCCCGCCGTGTTTA","chr6",24503328,"+","none","CGG","GAGGAAGCCCGCCGTGTTTACGG",0,NA,NA,NA,"GeneID:7915","ALDH5A1",TRUE,"ALDH5A1"

"6413","GATGACTGCAGCCACGCCTA","chr6",24503407,"-","none","TGG","GATGACTGCAGCCACGCCTATGG",0,NA,NA,NA,"GeneID:7915","ALDH5A1",TRUE,"ALDH5A1"

"6414","CCAACTGTAAAGCTCTTCAT","chr14",74072591,"-","none","TGG","CCAACTGTAAAGCTCTTCATTGG",0,NA,NA,NA,"GeneID:80127;GeneID:4329","CCDC176;ALDH6A1",TRUE,"ALDH6A1"

"6415","CCAATGAAGAGCTTTACAGT","chr14",74072588,"+","none","TGG","CCAATGAAGAGCTTTACAGTTGG",0,NA,NA,NA,"GeneID:80127;GeneID:4329","CCDC176;ALDH6A1",TRUE,"ALDH6A1"

"6416","CGCCGCCAATAGCGCCGCCA","chr14",74084373,"+","none","TGG","CGCCGCCAATAGCGCCGCCATGG",0,NA,NA,NA,"GeneID:4329","ALDH6A1",TRUE,"ALDH6A1"

"6417","GAGAGCCATGGCGGCGCTAT","chr14",74084381,"-","none","TGG","GAGAGCCATGGCGGCGCTATTGG",0,NA,NA,NA,"GeneID:4329","ALDH6A1",TRUE,"ALDH6A1"

"6418","TGCCTCACAGGTTTCTTCCA","chr14",74075007,"-","none","AGG","TGCCTCACAGGTTTCTTCCAAGG",0,NA,NA,NA,"GeneID:80127;GeneID:4329","CCDC176;ALDH6A1",TRUE,"ALDH6A1"

"6419","TGCTGAATACCAGGTGGGAC","chr14",74074975,"+","none","TGG","TGCTGAATACCAGGTGGGACTGG",0,NA,NA,NA,"GeneID:80127;GeneID:4329","CCDC176;ALDH6A1",TRUE,"ALDH6A1"

"6420","CATTAGGTTATGGATCGCCC","chr5",126552123,"-","none","TGG","CATTAGGTTATGGATCGCCCTGG",0,NA,NA,NA,"GeneID:501","ALDH7A1",TRUE,"ALDH7A1"

"6421","CCAAGACCTGTCACAATTGT","chr5",126552084,"+","none","CGG","CCAAGACCTGTCACAATTGTCGG",0,NA,NA,NA,"GeneID:501","ALDH7A1",TRUE,"ALDH7A1"

"6422","CCAAGACCTGTCACAATTGT","chr5",77291416,"-","0:C>G","CGG","GCAAGACCTGTCACAATTGTCGG",1,1,NA,NA,"GeneID:8622","PDE8B",TRUE,"ALDH7A1"

"6423","CCTTTCTAGGATAATAGCCA","chr5",126570848,"-","none","AGG","CCTTTCTAGGATAATAGCCAAGG",0,NA,NA,NA,"GeneID:501","ALDH7A1",TRUE,"ALDH7A1"

"6424","CTGGAGGACAACAAGCTGCC","chr5",126570823,"-","none","TGG","CTGGAGGACAACAAGCTGCCTGG",0,NA,NA,NA,"GeneID:501","ALDH7A1",TRUE,"ALDH7A1"

"6425","CTGGAGGACAACAAGCTGCC","chr5",77290894,"+","19:T>C","TGG","CTGGAGGACAACAAGCTGCTTGG",1,20,NA,NA,"GeneID:8622","PDE8B",TRUE,"ALDH7A1"

"6426","CTGGAGGACAACAAGCTGCC","chr18",59268742,"-","7:C>T,13:C>T","GGG","CTGGAGGGCAACAGGCTGCCGGG",2,8,14,NA,"GeneID:30062","RAX",TRUE,"ALDH7A1"

"6427","GCAGAATCTTTCGCTGGCTT","chr5",126550197,"-","none","GGG","GCAGAATCTTTCGCTGGCTTGGG",0,NA,NA,NA,"GeneID:501","ALDH7A1",TRUE,"ALDH7A1"

"6428","TAGCATCTTTACCAAAGATC","chr5",126550220,"-","none","TGG","TAGCATCTTTACCAAAGATCTGG",0,NA,NA,NA,"GeneID:501","ALDH7A1",TRUE,"ALDH7A1"

"6429","TAGCATCTTTACCAAAGATC","chr7",150541983,"-","19:A>G","TGG","TAGCATCTTTACCAAAGATTTGG",1,20,NA,NA,"","",TRUE,"ALDH7A1"

"6430","TAGCATCTTTACCAAAGATC","chr2",172893971,"-","19:A>G","TGG","TAGCATCTTTACCAAAGATTTGG",1,20,NA,NA,"GeneID:11069","RAPGEF4",TRUE,"ALDH7A1"

"6431","TAGCATCTTTACCAAAGATC","chr5",77291553,"+","5:A>T","TGG","TAGCAACTTTACCAAAGATCTGG",1,6,NA,NA,"GeneID:8622","PDE8B",TRUE,"ALDH7A1"

"6432","CAGCACTCACCGACTCCCAC","chr6",134942398,"+","none","CGG","CAGCACTCACCGACTCCCACCGG",0,NA,NA,NA,"GeneID:64577","ALDH8A1",TRUE,"ALDH8A1"

"6433","CTCCCGGTGAAGGAGATCAG","chr6",134932929,"+","none","GGG","CTCCCGGTGAAGGAGATCAGGGG",0,NA,NA,NA,"GeneID:64577","ALDH8A1",TRUE,"ALDH8A1"

"6434","GTGCCCCTGATCTCCTTCAC","chr6",134932935,"-","none","CGG","GTGCCCCTGATCTCCTTCACCGG",0,NA,NA,NA,"GeneID:64577","ALDH8A1",TRUE,"ALDH8A1"

"6435","TCAGGACCCGTGAGCGCTCC","chr6",134943884,"+","none","TGG","TCAGGACCCGTGAGCGCTCCTGG",0,NA,NA,NA,"GeneID:64577","ALDH8A1",TRUE,"ALDH8A1"

"6436","TCTGCGTGCACTCTGACGTG","chr6",134942459,"+","none","TGG","TCTGCGTGCACTCTGACGTGTGG",0,NA,NA,NA,"GeneID:64577","ALDH8A1",TRUE,"ALDH8A1"

"6437","TTCTCTGGCGGCCTTGACCG","chr6",134943936,"+","none","CGG","TTCTCTGGCGGCCTTGACCGCGG",0,NA,NA,NA,"GeneID:64577","ALDH8A1",TRUE,"ALDH8A1"

"6438","AAACCTGTTACCTTGGAACT","chr1",165679535,"-","none","TGG","AAACCTGTTACCTTGGAACTTGG",0,NA,NA,NA,"GeneID:223","ALDH9A1",TRUE,"ALDH9A1"

"6439","AAACCTGTTACCTTGGAACT","chr7",18240064,"+","6:T>G,10:A>C","TGG","AAACCTTTTAACTTGGAACTTGG",2,7,11,NA,"GeneID:9734","HDAC9",FALSE,"ALDH9A1"

"6440","AGTGAGGCTGGTGTACCTCC","chr1",165680595,"-","none","TGG","AGTGAGGCTGGTGTACCTCCTGG",0,NA,NA,NA,"GeneID:223","ALDH9A1",TRUE,"ALDH9A1"

"6441","AGTGAGGCTGGTGTACCTCC","chr12",120669053,"+","2:G>T,14:G>A","AGG","AGGGAGGCTGGTGTGCCTCCAGG",2,3,15,NA,"","",FALSE,"ALDH9A1"

"6442","GGTTTATGCAGGTAATGCCA","chr1",165680674,"-","none","TGG","GGTTTATGCAGGTAATGCCATGG",0,NA,NA,NA,"GeneID:223","ALDH9A1",TRUE,"ALDH9A1"

"6443","GTATAACCAAACGATCCACC","chr1",165682201,"+","none","TGG","GTATAACCAAACGATCCACCTGG",0,NA,NA,NA,"GeneID:223","ALDH9A1",TRUE,"ALDH9A1"

"6444","TACATACCTGGCCTTGTGTG","chr1",165679434,"+","none","AGG","TACATACCTGGCCTTGTGTGAGG",0,NA,NA,NA,"GeneID:223","ALDH9A1",TRUE,"ALDH9A1"

"6445","TCGGCTCCAGCATTAGCCTG","chr1",165682108,"-","none","TGG","TCGGCTCCAGCATTAGCCTGTGG",0,NA,NA,NA,"GeneID:223","ALDH9A1",TRUE,"ALDH9A1"

"6446","ACGGAGCTGACTTCGCCAAG","chr16",30068859,"+","none","TGG","ACGGAGCTGACTTCGCCAAGTGG",0,NA,NA,NA,"GeneID:226","ALDOA",TRUE,"ALDOA"

"6447","ACGGAGCTGACTTCGCCAAG","chr10",125667509,"-","1:A>G,14:T>C","TGG","ATGGAGCTGACTTCACCAAGTGG",2,2,15,NA,"GeneID:387718","TEX36",TRUE,"ALDOA"

"6448","CCAACAGGTAGACAAGGGCG","chr16",30068638,"+","none","TGG","CCAACAGGTAGACAAGGGCGTGG",0,NA,NA,NA,"GeneID:226","ALDOA",TRUE,"ALDOA"

"6449","CCAACAGGTAGACAAGGGCG","chr12",47846530,"-","8:C>A,12:C>G","AGG","CCAACAGGGAGAGAAGGGCGAGG",2,9,13,NA,"GeneID:7421","VDR",FALSE,"ALDOA"

"6450","CCTTGCCAGGTGCCACGATG","chr16",30067319,"-","none","CGG","CCTTGCCAGGTGCCACGATGCGG",0,NA,NA,NA,"GeneID:226","ALDOA",TRUE,"ALDOA"

"6451","CCTTGCCAGGTGCCACGATG","chr10",125667865,"+","7:C>A,8:A>G","CGG","CCTTGCCCAGTGCCACGATGCGG",2,8,9,NA,"GeneID:387718","TEX36",TRUE,"ALDOA"

"6452","CTTCAGCACACAACGCCACT","chr16",30068877,"-","none","TGG","CTTCAGCACACAACGCCACTTGG",0,NA,NA,NA,"GeneID:226","ALDOA",TRUE,"ALDOA"

"6453","CTTCAGCACACAACGCCACT","chr17",28574776,"+","0:T>C,12:G>A","TGG","TTTCAGCACACAGCGCCACTTGG",2,1,13,NA,"GeneID:230","ALDOC",TRUE,"ALDOA"

"6454","CTTCAGCACACAACGCCACT","chr10",125667491,"+","6:A>C,13:T>C","TGG","CTTCAGAACACAATGCCACTTGG",2,7,14,NA,"GeneID:387718","TEX36",TRUE,"ALDOA"

"6455","GGTCAGTGCTGGATATTGGT","chr16",30067261,"-","none","AGG","GGTCAGTGCTGGATATTGGTAGG",0,NA,NA,NA,"GeneID:226","ALDOA",TRUE,"ALDOA"

"6456","GGTCAGTGCTGGATATTGGT","chr16",54844852,"+","12:C>A,14:G>A","TGG","GGTCAGTGCTGGCTGTTGGTTGG",2,13,15,NA,"","",FALSE,"ALDOA"

"6457","GGTCAGTGCTGGATATTGGT","chr16",66289243,"+","12:C>A,13:C>T","GGG","GGTCAGTGCTGGCCATTGGTGGG",2,13,14,NA,"","",FALSE,"ALDOA"

"6458","GGTCAGTGCTGGATATTGGT","chr16",66288069,"+","12:C>A,13:C>T","GGG","GGTCAGTGCTGGCCATTGGTGGG",2,13,14,NA,"","",FALSE,"ALDOA"

"6459","GGTCAGTGCTGGATATTGGT","chr10",125667923,"+","5:A>G,16:C>T","AGG","GGTCAATGCTGGATATCGGTAGG",2,6,17,NA,"GeneID:387718","TEX36",TRUE,"ALDOA"

"6460","TGTGCGGCGCAGCGCTGTGA","chr16",30069634,"-","none","CGG","TGTGCGGCGCAGCGCTGTGACGG",0,NA,NA,NA,"GeneID:226","ALDOA",TRUE,"ALDOA"

"6461","AATATCCTTACCTTGAATGG","chr9",101428457,"+","none","TGG","AATATCCTTACCTTGAATGGTGG",0,NA,NA,NA,"GeneID:229","ALDOB",TRUE,"ALDOB"

"6462","AATATCCTTACCTTGAATGG","chr1",173660508,"+","3:T>A,19:T>G","GGG","AATTTCCTTACCTTGAATGTGGG",2,4,20,NA,"GeneID:339416","ANKRD45",FALSE,"ALDOB"

"6463","ATTACCTCTGGTTCAACAAT","chr9",101426603,"+","none","AGG","ATTACCTCTGGTTCAACAATAGG",0,NA,NA,NA,"GeneID:229","ALDOB",TRUE,"ALDOB"

"6464","GGTACCTATTGTTGAACCAG","chr9",101426610,"-","none","AGG","GGTACCTATTGTTGAACCAGAGG",0,NA,NA,NA,"GeneID:229","ALDOB",TRUE,"ALDOB"

"6465","TAGCGAGGCTGGATGGACAC","chr9",101427531,"+","none","TGG","TAGCGAGGCTGGATGGACACTGG",0,NA,NA,NA,"GeneID:229","ALDOB",TRUE,"ALDOB"

"6466","TATCCACAGTTAGACCAAGG","chr9",101428512,"-","none","AGG","TATCCACAGTTAGACCAAGGAGG",0,NA,NA,NA,"GeneID:229","ALDOB",TRUE,"ALDOB"

"6467","TCCATCCAGCCTCGCTATCC","chr9",101427528,"-","none","AGG","TCCATCCAGCCTCGCTATCCAGG",0,NA,NA,NA,"GeneID:229","ALDOB",TRUE,"ALDOB"

"6468","CACGTTGGCGTTCTCCAGAA","chr17",28574725,"+","none","TGG","CACGTTGGCGTTCTCCAGAATGG",0,NA,NA,NA,"GeneID:230","ALDOC",TRUE,"ALDOC"

"6469","CCACGACGATGCCCTTATCC","chr17",28575130,"+","none","TGG","CCACGACGATGCCCTTATCCTGG",0,NA,NA,NA,"GeneID:230","ALDOC",TRUE,"ALDOC"

"6470","CTCCTCTGGGGTATACTTGA","chr17",28574121,"+","none","TGG","CTCCTCTGGGGTATACTTGATGG",0,NA,NA,NA,"GeneID:230","ALDOC",TRUE,"ALDOC"

"6471","GAACCGCCGGCTGTACCGCC","chr17",28575265,"-","none","AGG","GAACCGCCGGCTGTACCGCCAGG",0,NA,NA,NA,"GeneID:230","ALDOC",TRUE,"ALDOC"

"6472","TCCCATCAAGTATACCCCAG","chr17",28574126,"-","none","AGG","TCCCATCAAGTATACCCCAGAGG",0,NA,NA,NA,"GeneID:230","ALDOC",TRUE,"ALDOC"

"6473","TTTCAGCACACAGCGCCACT","chr17",28574776,"+","none","TGG","TTTCAGCACACAGCGCCACTTGG",0,NA,NA,NA,"GeneID:230","ALDOC",TRUE,"ALDOC"

"6474","TTTCAGCACACAGCGCCACT","chr16",30068877,"-","0:G>A,12:T>C","TGG","CTTCAGCACACAACGCCACTTGG",2,1,13,NA,"GeneID:226","ALDOA",TRUE,"ALDOC"

"6475","ACAGAACTTCAGAGTCTTGC","chr16",5073006,"+","none","AGG","ACAGAACTTCAGAGTCTTGCAGG",0,NA,NA,NA,"GeneID:56052","ALG1",TRUE,"ALG1"

"6476","CAACCATCCCCTCGTTCTGC","chr16",5075506,"+","none","TGG","CAACCATCCCCTCGTTCTGCTGG",0,NA,NA,NA,"GeneID:56052","ALG1",TRUE,"ALG1"

"6477","GAAGCAAGCTCGTCATTGAC","chr16",5075442,"+","none","TGG","GAAGCAAGCTCGTCATTGACTGG",0,NA,NA,NA,"GeneID:56052","ALG1",TRUE,"ALG1"

"6478","GCACCACCGCTACTACATGC","chr16",5071947,"-","none","CGG","GCACCACCGCTACTACATGCCGG",0,NA,NA,NA,"GeneID:56052","ALG1",TRUE,"ALG1"

"6479","GCAGTACCACGCGCTGTCGT","chr16",5071995,"+","none","TGG","GCAGTACCACGCGCTGTCGTTGG",0,NA,NA,NA,"GeneID:56052","ALG1",TRUE,"ALG1"

"6480","TGTTCTGCAAGAGCTCATCA","chr16",5072963,"-","none","TGG","TGTTCTGCAAGAGCTCATCATGG",0,NA,NA,NA,"GeneID:56052","ALG1",TRUE,"ALG1"

"6481","AATTCTACGTTTCCAAACTA","chr12",34026445,"-","none","AGG","AATTCTACGTTTCCAAACTAAGG",0,NA,NA,NA,"GeneID:84920","ALG10",TRUE,"ALG10"

"6482","AATTCTACGTTTCCAAACTA","chr12",38320743,"-","5:G>A,7:A>G","AGG","AATTCCATGTTTCCAAACTAAGG",2,6,8,NA,"GeneID:144245","ALG10B",TRUE,"ALG10"

"6483","CTCTCGCAACGCCCGGCTGA","chr12",34022678,"-","none","AGG","CTCTCGCAACGCCCGGCTGAAGG",0,NA,NA,NA,"GeneID:84920","ALG10",TRUE,"ALG10"

"6484","CTCTCGCAACGCCCGGCTGA","chr12",38316972,"-","8:C>T","AGG","CTCTCGCAGCGCCCGGCTGAAGG",1,9,NA,NA,"GeneID:144245","ALG10B",TRUE,"ALG10"

"6485","GAGTGATCAAACCTGCCATT","chr12",34024010,"+","none","TGG","GAGTGATCAAACCTGCCATTTGG",0,NA,NA,NA,"GeneID:84920","ALG10",TRUE,"ALG10"

"6486","GAGTGATCAAACCTGCCATT","chr12",38318309,"+","5:G>A","TGG","GAGTGGTCAAACCTGCCATTTGG",1,6,NA,NA,"GeneID:144245","ALG10B",TRUE,"ALG10"

"6487","GCTCAGTTCACAAATGAGTC","chr12",34026799,"-","none","TGG","GCTCAGTTCACAAATGAGTCTGG",0,NA,NA,NA,"GeneID:84920","ALG10",TRUE,"ALG10"

"6488","GCTCAGTTCACAAATGAGTC","chr8",79724667,"+","8:T>C,19:T>C","CGG","GCTCAGTTTACAAATGAGTTCGG",2,9,20,NA,"","",FALSE,"ALG10"

"6489","GCTCAGTTCACAAATGAGTC","chr6",15507892,"-","6:G>A,10:C>G","AGG","GCTCAGCTCAGAAATGAGTCAGG",2,7,11,NA,"GeneID:3720","JARID2",FALSE,"ALG10"

"6490","GGGAGCTGGAATGGCGCAGC","chr12",34022589,"+","none","TGG","GGGAGCTGGAATGGCGCAGCTGG",0,NA,NA,NA,"GeneID:84920","ALG10",TRUE,"ALG10"

"6491","GGGAGCTGGAATGGCGCAGC","chr8",95232716,"+","17:T>A,19:G>C","AGG","GGGAGCTGGAATGGCGCTGGAGG",2,18,20,NA,"GeneID:100616530","C8orf37-AS1",FALSE,"ALG10"

"6492","GGGAGCTGGAATGGCGCAGC","chr6",36372529,"+","3:G>A,14:G>C","AGG","GGGGGCTGGAATGGGGCAGCAGG",2,4,15,NA,"GeneID:51513","ETV7",FALSE,"ALG10"

"6493","GGGAGCTGGAATGGCGCAGC","chr19",55695387,"-","6:G>A,14:C>G","CGG","GGGAGCCGGAATGGGGCAGCCGG",2,7,15,NA,"GeneID:29924","EPN1",TRUE,"ALG10"

"6494","TGATCACTCCAATTGACACC","chr12",34023999,"-","none","AGG","TGATCACTCCAATTGACACCAGG",0,NA,NA,NA,"GeneID:84920","ALG10",TRUE,"ALG10"

"6495","TGATCACTCCAATTGACACC","chr12",38318298,"-","3:G>A,12:G>A","AGG","TGACCACTCCAACTGACACCAGG",2,4,13,NA,"GeneID:144245","ALG10B",TRUE,"ALG10"

"6496","AATTCCATGTTTCCAAACTA","chr12",38320743,"-","none","AGG","AATTCCATGTTTCCAAACTAAGG",0,NA,NA,NA,"GeneID:144245","ALG10B",TRUE,"ALG10B"

"6497","AATTCCATGTTTCCAAACTA","chr12",34026445,"-","5:A>G,7:G>A","AGG","AATTCTACGTTTCCAAACTAAGG",2,6,8,NA,"GeneID:84920","ALG10",TRUE,"ALG10B"

"6498","ACTCAGTTCACAAACAAGTC","chr12",38321097,"-","none","TGG","ACTCAGTTCACAAACAAGTCTGG",0,NA,NA,NA,"GeneID:144245","ALG10B",TRUE,"ALG10B"

"6499","AGCAGGAATGGCGCAGCTAG","chr12",38316886,"+","none","AGG","AGCAGGAATGGCGCAGCTAGAGG",0,NA,NA,NA,"GeneID:144245","ALG10B",TRUE,"ALG10B"

"6500","CTCTCGCAGCGCCCGGCTGA","chr12",38316972,"-","none","AGG","CTCTCGCAGCGCCCGGCTGAAGG",0,NA,NA,NA,"GeneID:144245","ALG10B",TRUE,"ALG10B"

"6501","CTCTCGCAGCGCCCGGCTGA","chr7",105228780,"-","14:T>C,16:C>G","GGG","CTCTCGCAGCGCCCAGGTGAGGG",2,15,17,NA,"GeneID:6733","SRPK2",FALSE,"ALG10B"

"6502","CTCTCGCAGCGCCCGGCTGA","chr12",34022678,"-","8:T>C","AGG","CTCTCGCAACGCCCGGCTGAAGG",1,9,NA,NA,"GeneID:84920","ALG10",TRUE,"ALG10B"

"6503","GTACCTGGTGTCAGTTGGAG","chr12",38318292,"+","none","TGG","GTACCTGGTGTCAGTTGGAGTGG",0,NA,NA,NA,"GeneID:144245","ALG10B",TRUE,"ALG10B"

"6504","GTACCTGGTGTCAGTTGGAG","chr20",13516524,"-","0:G>C,7:G>C","AGG","CTACCTGCTGTCAGTTGGAGAGG",2,1,8,NA,"GeneID:55617","TASP1",FALSE,"ALG10B"

"6505","TGACCACTCCAACTGACACC","chr12",38318298,"-","none","AGG","TGACCACTCCAACTGACACCAGG",0,NA,NA,NA,"GeneID:144245","ALG10B",TRUE,"ALG10B"

"6506","TGACCACTCCAACTGACACC","chr12",1877571,"+","5:T>A,12:A>C","TGG","TGACCTCTCCAAATGACACCTGG",2,6,13,NA,"GeneID:93589","CACNA2D4",FALSE,"ALG10B"

"6507","TGACCACTCCAACTGACACC","chr12",34023999,"-","3:A>G,12:A>G","AGG","TGATCACTCCAATTGACACCAGG",2,4,13,NA,"GeneID:84920","ALG10",TRUE,"ALG10B"

"6508","CCCCCTATATACTTAAACAG","chr13",52024279,"-","none","AGG","CCCCCTATATACTTAAACAGAGG",0,NA,NA,NA,"GeneID:440138","ALG11",TRUE,"ALG11"

"6509","CCTCACGAAGGAGATATAAC","chr13",52028395,"+","none","TGG","CCTCACGAAGGAGATATAACTGG",0,NA,NA,NA,"GeneID:440138;GeneID:9724","ALG11;UTP14C",TRUE,"ALG11"

"6510","GGAAGATGGCGGCCGGCGAA","chr13",52012413,"+","none","AGG","GGAAGATGGCGGCCGGCGAAAGG",0,NA,NA,NA,"GeneID:440138","ALG11",TRUE,"ALG11"

"6511","GGCCCCCCGAATTGTGTGCA","chr13",52028355,"-","none","AGG","GGCCCCCCGAATTGTGTGCAAGG",0,NA,NA,NA,"GeneID:440138;GeneID:9724","ALG11;UTP14C",TRUE,"ALG11"

"6512","TGTTACGACAACCTCCAATG","chr13",52024752,"-","none","AGG","TGTTACGACAACCTCCAATGAGG",0,NA,NA,NA,"GeneID:440138;GeneID:9724","ALG11;UTP14C",TRUE,"ALG11"

"6513","TTAGTGTCAACTAGCAAAAA","chr13",52019021,"+","none","TGG","TTAGTGTCAACTAGCAAAAATGG",0,NA,NA,NA,"GeneID:440138","ALG11",TRUE,"ALG11"

"6514","TTAGTGTCAACTAGCAAAAA","chr18",71047991,"-","3:T>C,5:T>C","TGG","TTAATATCAACTAGCAAAAATGG",2,4,6,NA,"","",FALSE,"ALG11"

"6515","ACGAGTGGGCCCGCTTCATC","chr22",49910038,"-","none","TGG","ACGAGTGGGCCCGCTTCATCTGG",0,NA,NA,NA,"GeneID:79087","ALG12",TRUE,"ALG12"

"6516","CAGCGAAAGCACGTAAACCG","chr22",49913418,"+","none","CGG","CAGCGAAAGCACGTAAACCGCGG",0,NA,NA,NA,"GeneID:79087","ALG12",TRUE,"ALG12"

"6517","CAGCTACGTACCTACAGGCA","chr22",49910422,"+","none","GGG","CAGCTACGTACCTACAGGCAGGG",0,NA,NA,NA,"GeneID:79087","ALG12",TRUE,"ALG12"

"6518","CCTTCGCCATCATCGTGTTC","chr22",49910008,"-","none","AGG","CCTTCGCCATCATCGTGTTCAGG",0,NA,NA,NA,"GeneID:79087","ALG12",TRUE,"ALG12"

"6519","GAACGTCCTGGGGACGACTC","chr22",49913475,"+","none","CGG","GAACGTCCTGGGGACGACTCCGG",0,NA,NA,NA,"GeneID:79087","ALG12",TRUE,"ALG12"

"6520","TCCGCGTGCAGTAGAACATC","chr22",49910463,"+","none","AGG","TCCGCGTGCAGTAGAACATCAGG",0,NA,NA,NA,"GeneID:79087","ALG12",TRUE,"ALG12"

"6521","ACACCTGAAGTTTCATAGTC","chrX",111730529,"-","none","TGG","ACACCTGAAGTTTCATAGTCTGG",0,NA,NA,NA,"GeneID:79868","ALG13",TRUE,"ALG13"

"6522","CAGTACTGAGTCGTTTACTC","chrX",111682202,"+","none","TGG","CAGTACTGAGTCGTTTACTCTGG",0,NA,NA,NA,"GeneID:79868","ALG13",TRUE,"ALG13"

"6523","CTCATACAACTACCCCCAGA","chrX",111735100,"+","none","AGG","CTCATACAACTACCCCCAGAAGG",0,NA,NA,NA,"GeneID:79868","ALG13",TRUE,"ALG13"

"6524","CTTCTGGGGGTAGTTGTATG","chrX",111735102,"-","none","AGG","CTTCTGGGGGTAGTTGTATGAGG",0,NA,NA,NA,"GeneID:79868","ALG13",TRUE,"ALG13"

"6525","CTTCTGGGGGTAGTTGTATG","chr12",49764208,"-","13:G>A,17:G>T","TGG","CTTCTGGGGGTAGCTGTCTGTGG",2,14,18,NA,"GeneID:7009","TMBIM6",TRUE,"ALG13"

"6526","GAAGAATATCTTTATCGTGC","chrX",111730405,"+","none","AGG","GAAGAATATCTTTATCGTGCAGG",0,NA,NA,NA,"GeneID:79868","ALG13",TRUE,"ALG13"

"6527","GATCTTGTTATTAGTCACGC","chrX",111682272,"+","none","AGG","GATCTTGTTATTAGTCACGCAGG",0,NA,NA,NA,"GeneID:79868","ALG13",TRUE,"ALG13"

"6528","ACATGGATAACGTTTCTACA","chr1",94983176,"+","none","CGG","ACATGGATAACGTTTCTACACGG",0,NA,NA,NA,"GeneID:199857","ALG14",TRUE,"ALG14"

"6529","GTAGAAACGTTATCCATGTC","chr1",94983175,"-","none","CGG","GTAGAAACGTTATCCATGTCCGG",0,NA,NA,NA,"GeneID:199857","ALG14",TRUE,"ALG14"

"6530","GTGCGTTCTCGTTCTAGCTG","chr1",95072873,"-","none","CGG","GTGCGTTCTCGTTCTAGCTGCGG",0,NA,NA,NA,"GeneID:199857","ALG14",TRUE,"ALG14"

"6531","GTTCCATGGACGTTACGCCC","chr1",95072799,"-","none","CGG","GTTCCATGGACGTTACGCCCCGG",0,NA,NA,NA,"GeneID:199857","ALG14",TRUE,"ALG14"

"6532","TGATAGAGACCCTAGTAACA","chr1",95064867,"-","none","TGG","TGATAGAGACCCTAGTAACATGG",0,NA,NA,NA,"GeneID:199857;GeneID:101928098","ALG14;LOC101928098",TRUE,"ALG14"

"6533","TGCTGAACCTCCCGGCTTCT","chr1",95027210,"+","none","TGG","TGCTGAACCTCCCGGCTTCTTGG",0,NA,NA,NA,"GeneID:199857","ALG14",TRUE,"ALG14"

"6534","AGTTGTATGTCCATAACCAA","chr3",125929433,"+","none","AGG","AGTTGTATGTCCATAACCAAAGG",0,NA,NA,NA,"GeneID:100125556;GeneID:200810","FAM86JP;ALG1L",TRUE,"ALG1L"

"6535","AGTTGTATGTCCATAACCAA","chr16",5084863,"-","4:T>C,6:C>T","AGG","AGTTATGTGTCCATAACCAAAGG",2,5,7,NA,"GeneID:56052;GeneID:196483","ALG1;FAM86A",TRUE,"ALG1L"

"6536","AGTTGTATGTCCATAACCAA","chr4",3942503,"-","4:T>C,6:C>T","AGG","AGTTATGTGTCCATAACCAAAGG",2,5,7,NA,"GeneID:348926","FAM86EP",TRUE,"ALG1L"

"6537","AGTTGTATGTCCATAACCAA","chr7",6930820,"-","4:T>C,6:C>T","AGG","AGTTATGTGTCCATAACCAAAGG",2,5,7,NA,"","",TRUE,"ALG1L"

"6538","AGTTGTATGTCCATAACCAA","chr3",75422125,"-","4:T>C,6:C>T","AGG","AGTTATGTGTCCATAACCAAAGG",2,5,7,NA,"GeneID:692099","FAM86DP",TRUE,"ALG1L"

"6539","AGTTGTATGTCCATAACCAA","chr3",130098346,"-","4:T>C,6:C>T","AGG","AGTTATGTGTCCATAACCAAAGG",2,5,7,NA,"GeneID:644974","ALG1L2",TRUE,"ALG1L"

"6540","AGTTGTATGTCCATAACCAA","chr8",12424984,"-","4:T>C,6:C>T","AGG","AGTTATGTGTCCATAACCAAAGG",2,5,7,NA,"","",TRUE,"ALG1L"

"6541","AGTTGTATGTCCATAACCAA","chr8",12182701,"-","4:T>C,6:C>T","AGG","AGTTATGTGTCCATAACCAAAGG",2,5,7,NA,"GeneID:85002","FAM86B1",TRUE,"ALG1L"

"6542","AGTTGTATGTCCATAACCAA","chr11",3408799,"-","4:T>C,6:C>T","AGG","AGTTATGTGTCCATAACCAAAGG",2,5,7,NA,"GeneID:650368","TSSC2",TRUE,"ALG1L"

"6543","AGTTGTATGTCCATAACCAA","chr12",8231751,"-","4:T>C,6:C>T","AGG","AGTTATGTGTCCATAACCAAAGG",2,5,7,NA,"","",TRUE,"ALG1L"

"6544","AGTTGTATGTCCATAACCAA","chr11",71800653,"+","4:A>G,6:G>A","AGG","AGTTATGTGTCCATAACCAAAGG",2,5,7,NA,"GeneID:55199;GeneID:285407","FAM86C1;ALG1L9P",TRUE,"ALG1L"

"6545","AGTTGTATGTCCATAACCAA","chr4",9703744,"+","4:C>G,6:G>A","AGG","AGTTCTGTGTCCATAACCAAAGG",2,5,7,NA,"","",TRUE,"ALG1L"

"6546","CCCGCAGGTTCTTCCAAAAC","chr3",125929503,"+","none","TGG","CCCGCAGGTTCTTCCAAAACTGG",0,NA,NA,NA,"GeneID:100125556;GeneID:200810","FAM86JP;ALG1L",TRUE,"ALG1L"

"6547","CCCGCAGGTTCTTCCAAAAC","chr11",67792250,"-","16:C>T","TGG","CCCGCAGGTTCTTCCAGAACTGG",1,17,NA,NA,"GeneID:645332","FAM86C2P",TRUE,"ALG1L"

"6548","CCCGCAGGTTCTTCCAAAAC","chr16",5084793,"-","15:C>T,16:C>T","TGG","CCCGCAGGTTCTTCCGGAACTGG",2,16,17,NA,"GeneID:56052;GeneID:196483","ALG1;FAM86A",TRUE,"ALG1L"

"6549","CCCGCAGGTTCTTCCAAAAC","chr4",3942433,"-","15:C>T,16:C>T","AGG","CCCGCAGGTTCTTCCGGAACAGG",2,16,17,NA,"GeneID:348926","FAM86EP",TRUE,"ALG1L"

"6550","CCCGCAGGTTCTTCCAAAAC","chr11",3408729,"-","15:C>T,16:C>T","TGG","CCCGCAGGTTCTTCCGGAACTGG",2,16,17,NA,"GeneID:650368","TSSC2",TRUE,"ALG1L"

"6551","CCCGCAGGTTCTTCCAAAAC","chr3",130098276,"-","15:C>T,16:C>T","TGG","CCCGCAGGTTCTTCCGGAACTGG",2,16,17,NA,"GeneID:644974","ALG1L2",TRUE,"ALG1L"

"6552","CCCGCAGGTTCTTCCAAAAC","chr4",9166364,"+","16:G>A","TGG","CCCGCAGGTTCTTCCAGAACTGG",1,17,NA,NA,"","",TRUE,"ALG1L"

"6553","CCCGCAGGTTCTTCCAAAAC","chr11",71800723,"+","16:G>A","TGG","CCCGCAGGTTCTTCCAGAACTGG",1,17,NA,NA,"GeneID:55199;GeneID:285407","FAM86C1;ALG1L9P",TRUE,"ALG1L"

"6554","CCCGCAGGTTCTTCCAAAAC","chr3",156044222,"+","15:G>A,16:G>A","TGG","CCCGCAGGTTCTTCCGGAACTGG",2,16,17,NA,"GeneID:7881","KCNAB1",TRUE,"ALG1L"

"6555","CCCGCAGGTTCTTCCAAAAC","chr4",9703814,"+","15:G>A,16:G>A","TGG","CCCGCAGGTTCTTCCGGAACTGG",2,16,17,NA,"","",TRUE,"ALG1L"

"6556","CCTAGCAGGGTCGGTGGACC","chr3",130096040,"+","none","TGG","CCTAGCAGGGTCGGTGGACCTGG",0,NA,NA,NA,"GeneID:644974","ALG1L2",TRUE,"ALG1L"

"6557","CCTAGCAGGGTCGGTGGACC","chr4",3940186,"+","none","TGG","CCTAGCAGGGTCGGTGGACCTGG",0,NA,NA,NA,"","",TRUE,"ALG1L"

"6558","CCTAGCAGGGTCGGTGGACC","chr11",67790033,"+","none","TGG","CCTAGCAGGGTCGGTGGACCTGG",0,NA,NA,NA,"","",TRUE,"ALG1L"

"6559","CCTAGCAGGGTCGGTGGACC","chr7",6928511,"+","none","TGG","CCTAGCAGGGTCGGTGGACCTGG",0,NA,NA,NA,"","",TRUE,"ALG1L"

"6560","CCTAGCAGGGTCGGTGGACC","chr11",71802960,"-","none","TGG","CCTAGCAGGGTCGGTGGACCTGG",0,NA,NA,NA,"GeneID:285407","ALG1L9P",TRUE,"ALG1L"

"6561","CCTAGCAGGGTCGGTGGACC","chr3",125931740,"-","none","TGG","CCTAGCAGGGTCGGTGGACCTGG",0,NA,NA,NA,"GeneID:200810","ALG1L",TRUE,"ALG1L"

"6562","CCTAGCAGGGTCGGTGGACC","chr4",9168603,"-","none","TGG","CCTAGCAGGGTCGGTGGACCTGG",0,NA,NA,NA,"","",TRUE,"ALG1L"

"6563","CCTAGCAGGGTCGGTGGACC","chr16",5082550,"+","14:C>T","TGG","CCTAGCAGGGTCGGCGGACCTGG",1,15,NA,NA,"GeneID:56052","ALG1",TRUE,"ALG1L"

"6564","CCTAGCAGGGTCGGTGGACC","chr3",75419816,"+","14:C>T","TGG","CCTAGCAGGGTCGGCGGACCTGG",1,15,NA,NA,"","",TRUE,"ALG1L"

"6565","CCTAGCAGGGTCGGTGGACC","chr8",12422672,"+","11:T>C","TGG","CCTAGCAGGGTTGGTGGACCTGG",1,12,NA,NA,"","",TRUE,"ALG1L"

"6566","CCTAGCAGGGTCGGTGGACC","chr8",12180357,"+","11:T>C","TGG","CCTAGCAGGGTTGGTGGACCTGG",1,12,NA,NA,"","",TRUE,"ALG1L"

"6567","CCTAGCAGGGTCGGTGGACC","chr12",8229440,"+","11:T>C,12:A>G","TGG","CCTAGCAGGGTTAGTGGACCTGG",2,12,13,NA,"","",TRUE,"ALG1L"

"6568","CCTAGCAGGGTCGGTGGACC","chr4",9706076,"-","7:A>C","TGG","CCTAGCATGGTCGGTGGACCTGG",1,8,NA,NA,"","",TRUE,"ALG1L"

"6569","CCTAGCAGGGTCGGTGGACC","chr16",87186073,"+","5:G>C,10:A>T","TGG","CCTAGGAGGGACGGTGGACCTGG",2,6,11,NA,"GeneID:100506581","C16orf95",FALSE,"ALG1L"

"6570","CCTAGCAGGGTCGGTGGACC","chr11",3406490,"+","2:C>T,18:T>C","TGG","CCCAGCAGGGTCGGTGGATCTGG",2,3,19,NA,"GeneID:650368","TSSC2",TRUE,"ALG1L"

"6571","CTCCTACCACTTGAAGTTCA","chr3",125931630,"+","none","CGG","CTCCTACCACTTGAAGTTCACGG",0,NA,NA,NA,"GeneID:200810","ALG1L",TRUE,"ALG1L"

"6572","CTCCTACCACTTGAAGTTCA","chr4",9168493,"+","none","CGG","CTCCTACCACTTGAAGTTCACGG",0,NA,NA,NA,"","",TRUE,"ALG1L"

"6573","CTCCTACCACTTGAAGTTCA","chr4",9705966,"+","none","TGG","CTCCTACCACTTGAAGTTCATGG",0,NA,NA,NA,"","",TRUE,"ALG1L"

"6574","CTCCTACCACTTGAAGTTCA","chr12",8229550,"-","none","CGG","CTCCTACCACTTGAAGTTCACGG",0,NA,NA,NA,"","",TRUE,"ALG1L"

"6575","CTCCTACCACTTGAAGTTCA","chr7",6928621,"-","none","CGG","CTCCTACCACTTGAAGTTCACGG",0,NA,NA,NA,"","",TRUE,"ALG1L"

"6576","CTCCTACCACTTGAAGTTCA","chr8",12180467,"-","none","CGG","CTCCTACCACTTGAAGTTCACGG",0,NA,NA,NA,"","",TRUE,"ALG1L"

"6577","CTCCTACCACTTGAAGTTCA","chr8",12422782,"-","none","CGG","CTCCTACCACTTGAAGTTCACGG",0,NA,NA,NA,"","",TRUE,"ALG1L"

"6578","CTCCTACCACTTGAAGTTCA","chr3",75419926,"-","none","CGG","CTCCTACCACTTGAAGTTCACGG",0,NA,NA,NA,"","",TRUE,"ALG1L"

"6579","CTCCTACCACTTGAAGTTCA","chr3",130096150,"-","none","CGG","CTCCTACCACTTGAAGTTCACGG",0,NA,NA,NA,"GeneID:644974","ALG1L2",TRUE,"ALG1L"

"6580","CTCCTACCACTTGAAGTTCA","chrX",15179361,"+","11:A>T,16:C>T","GGG","CTCCTACCACTAGAAGCTCAGGG",2,12,17,NA,"","",FALSE,"ALG1L"

"6581","CTCCTACCACTTGAAGTTCA","chr11",3406600,"-","5:C>T","CGG","CTCCTGCCACTTGAAGTTCACGG",1,6,NA,NA,"GeneID:650368","TSSC2",TRUE,"ALG1L"

"6582","CTCCTACCACTTGAAGTTCA","chr2",240662697,"-","4:T>A,5:C>T","TGG","CTCCAGCCACTTGAAGTTCATGG",2,5,6,NA,"","",FALSE,"ALG1L"

"6583","CTCCTACCACTTGAAGTTCA","chr2",162345073,"+","8:C>A,13:G>A","TGG","CTCCTACCCCTTGGAGTTCATGG",2,9,14,NA,"GeneID:25801","GCA",FALSE,"ALG1L"

"6584","CTCCTACCACTTGAAGTTCA","chr1",14745022,"-","5:C>T,15:G>C","AGG","CTCCTGCCACTTGAACTTCAAGG",2,6,16,NA,"GeneID:23254","KAZN",FALSE,"ALG1L"

"6585","GTTATCACACAGACAAGAGA","chr8",8239496,"+","none","AGG","GTTATCACACAGACAAGAGAAGG",0,NA,NA,NA,"GeneID:286042","FAM86B3P",TRUE,"ALG1L"

"6586","GTTATCACACAGACAAGAGA","chr3",125932651,"+","none","AGG","GTTATCACACAGACAAGAGAAGG",0,NA,NA,NA,"GeneID:200810","ALG1L",TRUE,"ALG1L"

"6587","GTTATCACACAGACAAGAGA","chr16",5079785,"-","14:C>T","AGG","GTTATCACACAGACGAGAGAAGG",1,15,NA,NA,"GeneID:56052","ALG1",TRUE,"ALG1L"

"6588","GTTATCACACAGACAAGAGA","chr3",130093138,"-","14:C>T","AGG","GTTATCACACAGACGAGAGAAGG",1,15,NA,NA,"GeneID:644974","ALG1L2",TRUE,"ALG1L"

"6589","GTTATCACACAGACAAGAGA","chr11",3403630,"-","14:C>T","AGG","GTTATCACACAGACGAGAGAAGG",1,15,NA,NA,"GeneID:650368","TSSC2",TRUE,"ALG1L"

"6590","GTTATCACACAGACAAGAGA","chr11",67787146,"-","14:C>T","AGG","GTTATCACACAGACGAGAGAAGG",1,15,NA,NA,"","",TRUE,"ALG1L"

"6591","GTTATCACACAGACAAGAGA","chr3",75416949,"-","14:C>T","AGG","GTTATCACACAGACGAGAGAAGG",1,15,NA,NA,"","",TRUE,"ALG1L"

"6592","GTTATCACACAGACAAGAGA","chr4",3937324,"-","14:C>T","AGG","GTTATCACACAGACGAGAGAAGG",1,15,NA,NA,"","",TRUE,"ALG1L"

"6593","GTTATCACACAGACAAGAGA","chr4",9708905,"+","14:G>A","AGG","GTTATCACACAGACGAGAGAAGG",1,15,NA,NA,"","",TRUE,"ALG1L"

"6594","GTTATCACACAGACAAGAGA","chr3",156044590,"+","14:G>A","AGG","GTTATCACACAGACGAGAGAAGG",1,15,NA,NA,"GeneID:7881","KCNAB1",TRUE,"ALG1L"

"6595","GTTATCACACAGACAAGAGA","chr6",162100742,"-","7:T>G,9:T>G","TGG","GTTATCAAAAAGACAAGAGATGG",2,8,10,NA,"GeneID:5071","PARK2",FALSE,"ALG1L"

"6596","TTTGAACAACTTACTCTTGA","chr3",125932687,"-","none","TGG","TTTGAACAACTTACTCTTGATGG",0,NA,NA,NA,"GeneID:200810","ALG1L",TRUE,"ALG1L"

"6597","TTTGAACAACTTACTCTTGA","chr4",9708941,"-","11:C>A","CGG","TTTGAACAACTGACTCTTGACGG",1,12,NA,NA,"","",TRUE,"ALG1L"

"6598","TTTGAACAACTTACTCTTGA","chr8",8239532,"-","11:C>A,18:G>C","CGG","TTTGAACAACTGACTCTTCACGG",2,12,19,NA,"GeneID:286042","FAM86B3P",TRUE,"ALG1L"

"6599","TTTGAACAACTTACTCTTGA","chr2",225491261,"+","12:G>A,18:T>G","AGG","TTTGAACAACTTGCTCTTTAAGG",2,13,19,NA,"GeneID:57624","NYAP2",FALSE,"ALG1L"

"6600","TTTGAACAACTTACTCTTGA","chr3",130093102,"+","11:G>T","CGG","TTTGAACAACTGACTCTTGACGG",1,12,NA,NA,"GeneID:644974","ALG1L2",TRUE,"ALG1L"

"6601","TTTGAACAACTTACTCTTGA","chr11",3403594,"+","11:G>T","TGG","TTTGAACAACTGACTCTTGATGG",1,12,NA,NA,"GeneID:650368","TSSC2",TRUE,"ALG1L"

"6602","TTTGAACAACTTACTCTTGA","chr4",3937288,"+","11:G>T","CGG","TTTGAACAACTGACTCTTGACGG",1,12,NA,NA,"","",TRUE,"ALG1L"

"6603","TTTGAACAACTTACTCTTGA","chr16",5079749,"+","11:G>T","TGG","TTTGAACAACTGACTCTTGATGG",1,12,NA,NA,"GeneID:56052","ALG1",TRUE,"ALG1L"

"6604","TTTGAACAACTTACTCTTGA","chr3",75416913,"+","11:G>T,18:C>G","TGG","TTTGAACAACTGACTCTTCATGG",2,12,19,NA,"","",TRUE,"ALG1L"

"6605","TTTGAACAACTTACTCTTGA","chr7",6925655,"+","5:G>A,11:G>T","TGG","TTTGAGCAACTGACTCTTGATGG",2,6,12,NA,"","",TRUE,"ALG1L"

"6606","AGGGTTCATCTGATCGTGGC","chr9",99218384,"-","none","AGG","AGGGTTCATCTGATCGTGGCAGG",0,NA,NA,NA,"GeneID:85365","ALG2",TRUE,"ALG2"

"6607","CCGATATGTTACCAAACTGC","chr9",99217941,"-","none","TGG","CCGATATGTTACCAAACTGCTGG",0,NA,NA,NA,"GeneID:85365","ALG2",TRUE,"ALG2"

"6608","CGACGTGGTAGTGTGCGACC","chr9",99221548,"-","none","AGG","CGACGTGGTAGTGTGCGACCAGG",0,NA,NA,NA,"GeneID:85365","ALG2",TRUE,"ALG2"

"6609","CGTGCTGTTCCTCGCCGACG","chr9",99221575,"-","none","AGG","CGTGCTGTTCCTCGCCGACGAGG",0,NA,NA,NA,"GeneID:85365","ALG2",TRUE,"ALG2"

"6610","GGACTCGGTTCCCAAGCCGT","chr9",99221848,"-","none","CGG","GGACTCGGTTCCCAAGCCGTCGG",0,NA,NA,NA,"GeneID:85365","ALG2",TRUE,"ALG2"

"6611","TACCACGTCGAACTCCTCGT","chr9",99221558,"+","none","CGG","TACCACGTCGAACTCCTCGTCGG",0,NA,NA,NA,"GeneID:85365","ALG2",TRUE,"ALG2"

"6612","CTATGACTATACCCAACTGC","chr3",184245734,"-","none","AGG","CTATGACTATACCCAACTGCAGG",0,NA,NA,NA,"GeneID:10195","ALG3",TRUE,"ALG3"

"6613","CTGCGGAAACGCGGCCGGTC","chr3",184248908,"-","none","CGG","CTGCGGAAACGCGGCCGGTCCGG",0,NA,NA,NA,"GeneID:10195","ALG3",TRUE,"ALG3"

"6614","GATCTATCACCAGACCTGCA","chr3",184245469,"-","none","AGG","GATCTATCACCAGACCTGCAAGG",0,NA,NA,NA,"GeneID:10195","ALG3",TRUE,"ALG3"

"6615","GCCTACATGGCCGAGGTAGA","chr3",184245772,"-","none","AGG","GCCTACATGGCCGAGGTAGAAGG",0,NA,NA,NA,"GeneID:10195","ALG3",TRUE,"ALG3"

"6616","GCCTACATGGCCGAGGTAGA","chr5",5376797,"-","12:G>C,17:C>T","AGG","GCCTACATGGCCCAGGTGGAAGG",2,13,18,NA,"","",TRUE,"ALG3"

"6617","GGAGCCGCGCTACACGCTGC","chr3",184248810,"-","none","TGG","GGAGCCGCGCTACACGCTGCTGG",0,NA,NA,NA,"GeneID:10195","ALG3",TRUE,"ALG3"

"6618","TTGTACTATGCCACCAGCCG","chr3",184245561,"-","none","AGG","TTGTACTATGCCACCAGCCGAGG",0,NA,NA,NA,"GeneID:10195","ALG3",TRUE,"ALG3"

"6619","AATCAAATGGCTATAGCATG","chr13",36972016,"-","none","TGG","AATCAAATGGCTATAGCATGTGG",0,NA,NA,NA,"GeneID:29880","ALG5",TRUE,"ALG5"

"6620","AATCAAATGGCTATAGCATG","chr15",42373885,"-","0:C>T,7:C>A","GGG","GATCAAAGGGCTATAGCATGGGG",2,1,8,NA,"GeneID:825","CAPN3",FALSE,"ALG5"

"6621","AATCAAATGGCTATAGCATG","chr3",140136938,"+","3:G>C,15:A>G","TGG","AATGAAATGGCTATAACATGTGG",2,4,16,NA,"GeneID:64084","CLSTN2",FALSE,"ALG5"

"6622","AATCAAATGGCTATAGCATG","chr2",57367366,"+","9:T>G,17:C>A","AGG","AATCAAATGTCTATAGCCTGAGG",2,10,18,NA,"","",FALSE,"ALG5"

"6623","AATCAAATGGCTATAGCATG","chr4",129988558,"+","3:A>C,19:A>G","TGG","AATAAAATGGCTATAGCATATGG",2,4,20,NA,"","",FALSE,"ALG5"

"6624","AGAAAAAGAATCAATTGCTC","chr13",36971978,"-","none","AGG","AGAAAAAGAATCAATTGCTCAGG",0,NA,NA,NA,"GeneID:29880","ALG5",TRUE,"ALG5"

"6625","AGAAAAAGAATCAATTGCTC","chr1",66265710,"-","9:C>T,13:G>T","AGG","AGAAAAAGAGTCACTTGCTCAGG",2,10,14,NA,"GeneID:5142","PDE4B",FALSE,"ALG5"

"6626","AGCAGTTGTAAATGCAACGA","chr13",36995569,"+","none","TGG","AGCAGTTGTAAATGCAACGATGG",0,NA,NA,NA,"GeneID:29880","ALG5",TRUE,"ALG5"

"6627","CACAACGACAGAAAGTTGTT","chr13",36995449,"+","none","TGG","CACAACGACAGAAAGTTGTTTGG",0,NA,NA,NA,"GeneID:29880","ALG5",TRUE,"ALG5"

"6628","CAGGGTATATTCAGTTCTCG","chr13",36985723,"-","none","AGG","CAGGGTATATTCAGTTCTCGAGG",0,NA,NA,NA,"GeneID:29880","ALG5",TRUE,"ALG5"

"6629","GGCTAAATGATCTACAGCCT","chr13",36985632,"-","none","TGG","GGCTAAATGATCTACAGCCTTGG",0,NA,NA,NA,"GeneID:29880","ALG5",TRUE,"ALG5"

"6630","GGCTAAATGATCTACAGCCT","chr1",241347196,"+","0:T>G,1:T>G","TGG","TTCTAAATGATCTACAGCCTTGG",2,1,2,NA,"GeneID:6000","RGS7",FALSE,"ALG5"

"6631","AAACTTACACATATGCACAT","chr1",63402331,"-","none","AGG","AAACTTACACATATGCACATAGG",0,NA,NA,NA,"GeneID:29929","ALG6",TRUE,"ALG6"

"6632","AAACTTACACATATGCACAT","chr8",86025350,"+","5:A>T,7:A>C","TGG","AAACTAAAACATATGCACATTGG",2,6,8,NA,"GeneID:245972","ATP6V0D2",FALSE,"ALG6"

"6633","AAGCTCTTCATGCGTACAAC","chr1",63404519,"+","none","AGG","AAGCTCTTCATGCGTACAACAGG",0,NA,NA,NA,"GeneID:29929","ALG6",TRUE,"ALG6"

"6634","AGACTATGATAAGCTGTAAG","chr1",63402308,"-","none","AGG","AGACTATGATAAGCTGTAAGAGG",0,NA,NA,NA,"GeneID:29929","ALG6",TRUE,"ALG6"

"6635","GCTGGTAAACCGCCTATGTT","chr1",63396514,"+","none","TGG","GCTGGTAAACCGCCTATGTTTGG",0,NA,NA,NA,"GeneID:29929","ALG6",TRUE,"ALG6"

"6636","TCTCATATCCACGTGATGTA","chr1",63404488,"-","none","TGG","TCTCATATCCACGTGATGTATGG",0,NA,NA,NA,"GeneID:29929","ALG6",TRUE,"ALG6"

"6637","TCTCATATCCACGTGATGTA","chr13",77065349,"+","12:A>G,17:T>G","AGG","TCTCATATCCACATGATTTAAGG",2,13,18,NA,"GeneID:100874212;GeneID:23077","MYCBP2-AS1;MYCBP2",FALSE,"ALG6"

"6638","TTATCATACCATTGTTTGAC","chr1",63396586,"-","none","CGG","TTATCATACCATTGTTTGACCGG",0,NA,NA,NA,"GeneID:29929","ALG6",TRUE,"ALG6"

"6639","AAACCAATTGCCAGTACCCG","chr11",78139546,"+","none","TGG","AAACCAATTGCCAGTACCCGTGG",0,NA,NA,NA,"GeneID:79053","ALG8",TRUE,"ALG8"

"6640","AACCAAACCACTTGTCATTG","chr11",78109516,"+","none","AGG","AACCAAACCACTTGTCATTGAGG",0,NA,NA,NA,"GeneID:79053","ALG8",TRUE,"ALG8"

"6641","GAGGGAATAATGTCCTGTTG","chr11",78104380,"+","none","TGG","GAGGGAATAATGTCCTGTTGTGG",0,NA,NA,NA,"GeneID:79053","ALG8",TRUE,"ALG8"

"6642","GGCGGCGCTCACAATTGCCA","chr11",78139566,"-","none","CGG","GGCGGCGCTCACAATTGCCACGG",0,NA,NA,NA,"GeneID:79053","ALG8",TRUE,"ALG8"

"6643","GTTGCCAAGGGAGTCACTGA","chr11",78109469,"+","none","GGG","GTTGCCAAGGGAGTCACTGAGGG",0,NA,NA,NA,"GeneID:79053","ALG8",TRUE,"ALG8"

"6644","GTTGCCAAGGGAGTCACTGA","chr12",121713994,"+","11:T>A,15:T>A","TGG","GTTGCCAAGGGTGTCTCTGATGG",2,12,16,NA,"GeneID:144404","TMEM120B",FALSE,"ALG8"

"6645","TTTCTGATTCTGACCACAAC","chr11",78104396,"-","none","AGG","TTTCTGATTCTGACCACAACAGG",0,NA,NA,NA,"GeneID:79053","ALG8",TRUE,"ALG8"

"6646","TTTCTGATTCTGACCACAAC","chr14",87838851,"+","15:C>A,18:T>A","GGG","TTTCTGATTCTGACCCCATCGGG",2,16,19,NA,"GeneID:2581","GALC",FALSE,"ALG8"

"6647","TTTCTGATTCTGACCACAAC","chr1",66577482,"+","0:C>T,5:T>G","AGG","CTTCTTATTCTGACCACAACAGG",2,1,6,NA,"GeneID:84251","SGIP1",FALSE,"ALG8"

"6648","TTTCTGATTCTGACCACAAC","chr15",97967047,"-","3:T>G,19:C>G","AGG","TTTATGATTCTGACCACAAGAGG",2,4,20,NA,"GeneID:91947","ARRDC4",FALSE,"ALG8"

"6649","ACCCATTGCCTTTGATTTGC","chr11",111853713,"-","none","TGG","ACCCATTGCCTTTGATTTGCTGG",0,NA,NA,NA,"GeneID:79796","ALG9",TRUE,"ALG9"

"6650","ACTACGTTGATAGCCATGAC","chr11",111857688,"-","none","TGG","ACTACGTTGATAGCCATGACTGG",0,NA,NA,NA,"GeneID:79796","ALG9",TRUE,"ALG9"

"6651","GATGGCCCTCATACTATTTC","chr11",111853650,"-","none","TGG","GATGGCCCTCATACTATTTCTGG",0,NA,NA,NA,"GeneID:79796","ALG9",TRUE,"ALG9"

"6652","GATGGCCCTCATACTATTTC","chr11",123939379,"-","3:T>C,6:T>G","TGG","GATAGCACTCATACTATTTCTGG",2,4,7,NA,"","",FALSE,"ALG9"

"6653","GTCCATATACCATCCAGTCA","chr11",111857672,"+","none","TGG","GTCCATATACCATCCAGTCATGG",0,NA,NA,NA,"GeneID:79796","ALG9",TRUE,"ALG9"

"6654","TAGCTGTCAATGACCACCAC","chr11",111853459,"+","none","AGG","TAGCTGTCAATGACCACCACAGG",0,NA,NA,NA,"GeneID:79796","ALG9",TRUE,"ALG9"

"6655","TAGCTGTCAATGACCACCAC","chr15",19962414,"-","6:T>A,9:C>T","AGG","TAGCTGACAGTGACCACCACAGG",2,7,10,NA,"","",FALSE,"ALG9"

"6656","TCGAATGATGCTAGCCTTCT","chr11",111860582,"-","none","TGG","TCGAATGATGCTAGCCTTCTTGG",0,NA,NA,NA,"GeneID:79796","ALG9",TRUE,"ALG9"

"6657","AATGGGACAGTCCTCCAGCT","chr2",29328411,"-","none","TGG","AATGGGACAGTCCTCCAGCTTGG",0,NA,NA,NA,"GeneID:238","ALK",TRUE,"ALK"

"6658","AGGGTCCTGACCTGCCATTG","chr2",29320779,"+","none","AGG","AGGGTCCTGACCTGCCATTGAGG",0,NA,NA,NA,"GeneID:238","ALK",TRUE,"ALK"

"6659","AGGGTCCTGACCTGCCATTG","chr12",16034178,"+","5:T>C,7:A>T","GGG","AGGGTTCAGACCTGCCATTGGGG",2,6,8,NA,"GeneID:51071","DERA",FALSE,"ALK"

"6660","AGGGTCCTGACCTGCCATTG","chrX",130810602,"-","5:C>G,14:T>G","GGG","AGGGTGCTGACCTGACATTGGGG",2,6,15,NA,"GeneID:10495","ENOX2",FALSE,"ALK"

"6661","GATCAAGAGCTCTCCATGTG","chr2",29318305,"-","none","AGG","GATCAAGAGCTCTCCATGTGAGG",0,NA,NA,NA,"GeneID:238","ALK",TRUE,"ALK"

"6662","TCACATGGAGAGCTCTTGAT","chr2",29318304,"+","none","CGG","TCACATGGAGAGCTCTTGATCGG",0,NA,NA,NA,"GeneID:238","ALK",TRUE,"ALK"

"6663","TCACATGGAGAGCTCTTGAT","chr2",186891039,"+","11:T>G,12:A>C","TGG","TCACATGGAGATATCTTGATTGG",2,12,13,NA,"","",FALSE,"ALK"

"6664","TCAGGACCCTAAAGGATGCC","chr2",29320769,"-","none","CGG","TCAGGACCCTAAAGGATGCCCGG",0,NA,NA,NA,"GeneID:238","ALK",TRUE,"ALK"

"6665","TGTCCCATTCCAACAAGTGA","chr2",29328422,"+","none","AGG","TGTCCCATTCCAACAAGTGAAGG",0,NA,NA,NA,"GeneID:238","ALK",TRUE,"ALK"

"6666","ACCCTTGCCACGGGCTGCGT","chr14",77707836,"+","none","GGG","ACCCTTGCCACGGGCTGCGTGGG",0,NA,NA,NA,"GeneID:8846","ALKBH1",TRUE,"ALKBH1"

"6667","ACTGTTTCACCCAGTGCCAC","chr14",77694840,"+","none","TGG","ACTGTTTCACCCAGTGCCACTGG",0,NA,NA,NA,"GeneID:8846","ALKBH1",TRUE,"ALKBH1"

"6668","AGAAGTCGATGACCCCTTCC","chr14",77707865,"+","none","AGG","AGAAGTCGATGACCCCTTCCAGG",0,NA,NA,NA,"GeneID:8846","ALKBH1",TRUE,"ALKBH1"

"6669","AGGATAGCCTTTGAGTCCAT","chr14",77704369,"+","none","AGG","AGGATAGCCTTTGAGTCCATAGG",0,NA,NA,NA,"GeneID:8846","ALKBH1",TRUE,"ALKBH1"

"6670","GAAACCTAATGTATGTAACC","chr14",77694801,"-","none","TGG","GAAACCTAATGTATGTAACCTGG",0,NA,NA,NA,"GeneID:8846","ALKBH1",TRUE,"ALKBH1"

"6671","GTCAGCAAGTGGCAAGCCTA","chr14",77704388,"-","none","TGG","GTCAGCAAGTGGCAAGCCTATGG",0,NA,NA,NA,"GeneID:8846","ALKBH1",TRUE,"ALKBH1"

"6672","AAAGTCTCTGCAGGCACCGA","chr12",109088406,"+","none","AGG","AAAGTCTCTGCAGGCACCGAAGG",0,NA,NA,NA,"GeneID:121642","ALKBH2",TRUE,"ALKBH2"

"6673","CAGGCAACGTATGGCGACGC","chr12",109090134,"-","none","TGG","CAGGCAACGTATGGCGACGCTGG",0,NA,NA,NA,"GeneID:121642","ALKBH2",TRUE,"ALKBH2"

"6674","CTGCCTCAGCTTTGCCAAAC","chr12",109092551,"+","none","AGG","CTGCCTCAGCTTTGCCAAACAGG",0,NA,NA,NA,"GeneID:121642","ALKBH2",TRUE,"ALKBH2"

"6675","GCCCGAATGTGCCGCCAGCT","chr12",109092601,"+","none","AGG","GCCCGAATGTGCCGCCAGCTAGG",0,NA,NA,NA,"GeneID:121642","ALKBH2",TRUE,"ALKBH2"

"6676","TCCCACGGGAATCCTTATGC","chr12",109088378,"+","none","CGG","TCCCACGGGAATCCTTATGCCGG",0,NA,NA,NA,"GeneID:121642","ALKBH2",TRUE,"ALKBH2"

"6677","TGTGCCACTTCCCGAATACC","chr12",109090168,"+","none","TGG","TGTGCCACTTCCCGAATACCTGG",0,NA,NA,NA,"GeneID:121642","ALKBH2",TRUE,"ALKBH2"

"6678","AGCTTTGTCAAGATGTTCCC","chr11",43889780,"+","none","TGG","AGCTTTGTCAAGATGTTCCCTGG",0,NA,NA,NA,"GeneID:221120","ALKBH3",TRUE,"ALKBH3"

"6679","ATTACTTACTCAATCACTCG","chr11",43884006,"-","none","TGG","ATTACTTACTCAATCACTCGTGG",0,NA,NA,NA,"GeneID:221120","ALKBH3",TRUE,"ALKBH3"

"6680","ATTACTTACTCAATCACTCG","chr7",143826508,"+","6:C>T,19:T>G","AGG","ATTACTCACTCAATCACTCTAGG",2,7,20,NA,"GeneID:154761","LOC154761",FALSE,"ALKBH3"

"6681","ATTACTTACTCAATCACTCG","chr7",143710333,"-","6:G>A,19:A>C","AGG","ATTACTCACTCAATCACTCTAGG",2,7,20,NA,"GeneID:285966","FAM115C",FALSE,"ALKBH3"

"6682","ATTACTTACTCAATCACTCG","chr7",143631114,"-","6:G>A,19:A>C","AGG","ATTACTCACTCAATCACTCTAGG",2,7,20,NA,"GeneID:285966","FAM115C",FALSE,"ALKBH3"

"6683","GAAATCAGCCTGTCACCCAC","chr11",43886621,"+","none","AGG","GAAATCAGCCTGTCACCCACAGG",0,NA,NA,NA,"GeneID:221120","ALKBH3",TRUE,"ALKBH3"

"6684","GAAATCAGCCTGTCACCCAC","chr14",32825519,"-","17:A>G,18:A>T","AGG","GAAATCAGCCTGTCACCTTCAGG",2,18,19,NA,"GeneID:9472","AKAP6",FALSE,"ALKBH3"

"6685","TCTTTCACGTCAACAAAGCC","chr11",43889740,"-","none","AGG","TCTTTCACGTCAACAAAGCCAGG",0,NA,NA,NA,"GeneID:221120","ALKBH3",TRUE,"ALKBH3"

"6686","TGCTTACCTAGATACACCTG","chr11",43886640,"-","none","TGG","TGCTTACCTAGATACACCTGTGG",0,NA,NA,NA,"GeneID:221120","ALKBH3",TRUE,"ALKBH3"

"6687","TGCTTACCTAGATACACCTG","chr7",93727799,"+","5:C>A,7:T>C","TGG","TGCTTCCTTAGATACACCTGTGG",2,6,8,NA,"GeneID:2792","GNGT1",FALSE,"ALKBH3"

"6688","TGTGTTCAAAGAACCTCAGC","chr11",43883166,"+","none","AGG","TGTGTTCAAAGAACCTCAGCAGG",0,NA,NA,NA,"GeneID:221120","ALKBH3",TRUE,"ALKBH3"

"6689","TGTGTTCAAAGAACCTCAGC","chr7",1720595,"+","5:G>T,7:C>A","AGG","TGTGTGCCAAGAACCTCAGCAGG",2,6,8,NA,"GeneID:392617","ELFN1",FALSE,"ALKBH3"

"6690","CCGAGACCCCCGAAGTCCTT","chr7",102464800,"-","none","CGG","CCGAGACCCCCGAAGTCCTTCGG",0,NA,NA,NA,"GeneID:54784","ALKBH4",TRUE,"ALKBH4"

"6691","CGGTTCACACGGGTCTTCCC","chr7",102457389,"+","none","TGG","CGGTTCACACGGGTCTTCCCTGG",0,NA,NA,NA,"GeneID:54784","ALKBH4",TRUE,"ALKBH4"

"6692","CTGATCTGCGAGCGGCAGCG","chr7",102464747,"-","none","CGG","CTGATCTGCGAGCGGCAGCGCGG",0,NA,NA,NA,"GeneID:54784","ALKBH4",TRUE,"ALKBH4"

"6693","CTGATCTGCGAGCGGCAGCG","chr5",170716108,"+","5:G>C,8:A>C","TGG","CTGATGTGAGAGCGGCAGCGTGG",2,6,9,NA,"GeneID:30820","KCNIP1",FALSE,"ALKBH4"

"6694","GCCCGTCGAGCAGTGCAACC","chr7",102457836,"-","none","TGG","GCCCGTCGAGCAGTGCAACCTGG",0,NA,NA,NA,"GeneID:54784","ALKBH4",TRUE,"ALKBH4"

"6695","GTCCATGAGCCGCACCAACT","chr7",102459645,"+","none","CGG","GTCCATGAGCCGCACCAACTCGG",0,NA,NA,NA,"GeneID:54784","ALKBH4",TRUE,"ALKBH4"

"6696","TTCATTTACTGCTCCGACAC","chr7",102459769,"-","none","CGG","TTCATTTACTGCTCCGACACCGG",0,NA,NA,NA,"GeneID:54784","ALKBH4",TRUE,"ALKBH4"

"6697","ACTGGTTCCGACACCCGAAT","chr17",18184951,"-","none","AGG","ACTGGTTCCGACACCCGAATAGG",0,NA,NA,NA,"GeneID:54890","ALKBH5",TRUE,"ALKBH5"

"6698","AGCTGAAGCTACCCCTCCGC","chr17",18208281,"-","none","CGG","AGCTGAAGCTACCCCTCCGCCGG",0,NA,NA,NA,"GeneID:54890","ALKBH5",TRUE,"ALKBH5"

"6699","CCACCGTGTGCTCGTTGTAC","chr17",18184596,"-","none","AGG","CCACCGTGTGCTCGTTGTACAGG",0,NA,NA,NA,"GeneID:54890","ALKBH5",TRUE,"ALKBH5"

"6700","GCCGCCGCATCTTCACCTTT","chr17",18208371,"-","none","CGG","GCCGCCGCATCTTCACCTTTCGG",0,NA,NA,NA,"GeneID:54890","ALKBH5",TRUE,"ALKBH5"

"6701","GGACAAGATTAGATGCACCC","chr17",18206813,"+","none","CGG","GGACAAGATTAGATGCACCCCGG",0,NA,NA,NA,"GeneID:54890","ALKBH5",TRUE,"ALKBH5"

"6702","TATGCTTCAGATCGCCTGTC","chr17",18206878,"+","none","AGG","TATGCTTCAGATCGCCTGTCAGG",0,NA,NA,NA,"GeneID:54890","ALKBH5",TRUE,"ALKBH5"

"6703","TATGCTTCAGATCGCCTGTC","chr1",117707810,"-","13:A>C,18:G>A","AGG","TATGCTTCAGATCTCCTGCCAGG",2,14,19,NA,"","",FALSE,"ALKBH5"

"6704","ACAACCTGCTCCACTCTGAA","chr19",36013338,"+","none","CGG","ACAACCTGCTCCACTCTGAACGG",0,NA,NA,NA,"GeneID:84964","ALKBH6",TRUE,"ALKBH6"

"6705","ACAACCTGCTCCACTCTGAA","chr5",59898827,"-","1:T>G,16:T>A","GGG","AAAACCTGCTCCACTCAGAAGGG",2,2,17,NA,"GeneID:5144","PDE4D",FALSE,"ALKBH6"

"6706","ACACTTTGTCCACGTAGCGC","chr19",36010970,"+","none","TGG","ACACTTTGTCCACGTAGCGCTGG",0,NA,NA,NA,"GeneID:84964","ALKBH6",TRUE,"ALKBH6"

"6707","ACACTTTGTCCACGTAGCGC","chr11",64739637,"-","15:C>T,19:C>G","AGG","ACACTTTGTCCACGTGGCGGAGG",2,16,20,NA,"GeneID:10235","RASGRP2",TRUE,"ALKBH6"

"6708","AGCCCTGGAACCGTTCAGAG","chr19",36013351,"-","none","TGG","AGCCCTGGAACCGTTCAGAGTGG",0,NA,NA,NA,"GeneID:84964","ALKBH6",TRUE,"ALKBH6"

"6709","CTCGTGAACCAGTATCTGCC","chr19",36010909,"-","none","TGG","CTCGTGAACCAGTATCTGCCTGG",0,NA,NA,NA,"GeneID:84964","ALKBH6",TRUE,"ALKBH6"

"6710","TCAGGGACATAGTAGATTAC","chr19",36013060,"+","none","AGG","TCAGGGACATAGTAGATTACAGG",0,NA,NA,NA,"GeneID:84964","ALKBH6",TRUE,"ALKBH6"

"6711","TCAGGGACATAGTAGATTAC","chr2",187326825,"-","4:A>C,19:T>G","TGG","TCAGTGACATAGTAGATTAATGG",2,5,20,NA,"","",FALSE,"ALKBH6"

"6712","TTAATGCCCCAAAGCCAAAG","chr19",36011440,"-","none","TGG","TTAATGCCCCAAAGCCAAAGTGG",0,NA,NA,NA,"GeneID:84964","ALKBH6",TRUE,"ALKBH6"

"6713","CCCAGTGATCGTATTCGTAG","chr19",6372999,"-","none","CGG","CCCAGTGATCGTATTCGTAGCGG",0,NA,NA,NA,"GeneID:84266","ALKBH7",TRUE,"ALKBH7"

"6714","GCCGCTACGAATACGATCAC","chr19",6372995,"+","none","TGG","GCCGCTACGAATACGATCACTGG",0,NA,NA,NA,"GeneID:84266","ALKBH7",TRUE,"ALKBH7"

"6715","GCTCTCCTCCGTGCACGTGC","chr19",6374306,"+","none","TGG","GCTCTCCTCCGTGCACGTGCTGG",0,NA,NA,NA,"GeneID:84266","ALKBH7",TRUE,"ALKBH7"

"6716","GTGCACCAGCCGCATAACGC","chr19",6374507,"-","none","TGG","GTGCACCAGCCGCATAACGCTGG",0,NA,NA,NA,"GeneID:84266","ALKBH7",TRUE,"ALKBH7"

"6717","TCACGGAGATGCGCCGGCCC","chr19",6374888,"-","none","CGG","TCACGGAGATGCGCCGGCCCCGG",0,NA,NA,NA,"GeneID:84266","ALKBH7",TRUE,"ALKBH7"

"6718","TGATGTAGCCGCGGGCTTCC","chr19",6374333,"-","none","AGG","TGATGTAGCCGCGGGCTTCCAGG",0,NA,NA,NA,"GeneID:84266","ALKBH7",TRUE,"ALKBH7"

"6719","AATTACTTACCCCCAGATAA","chr11",107553097,"+","none","TGG","AATTACTTACCCCCAGATAATGG",0,NA,NA,NA,"GeneID:91801","ALKBH8",TRUE,"ALKBH8"

"6720","AATTACTTACCCCCAGATAA","chr4",146653558,"-","10:T>G,16:G>T","GGG","AATTACTTACACCCAGCTAAGGG",2,11,17,NA,"","",FALSE,"ALKBH8"

"6721","AGGCCTCAAGCCTTACCACC","chr11",107553941,"-","none","AGG","AGGCCTCAAGCCTTACCACCAGG",0,NA,NA,NA,"GeneID:91801","ALKBH8",TRUE,"ALKBH8"

"6722","AGGCCTCAAGCCTTACCACC","chr2",127093846,"+","12:C>T,18:T>C","TGG","AGGCCTCAAGCCCTACCATCTGG",2,13,19,NA,"GeneID:274","BIN1",FALSE,"ALKBH8"

"6723","AGGCCTCAAGCCTTACCACC","chrX",81477680,"-","5:T>A,14:G>T","TGG","AGGCCACAAGCCTTCCCACCTGG",2,6,15,NA,"","",FALSE,"ALKBH8"

"6724","ATGTCTCAGCAAAGTATGCT","chr11",107560797,"+","none","TGG","ATGTCTCAGCAAAGTATGCTTGG",0,NA,NA,NA,"GeneID:91801","ALKBH8",TRUE,"ALKBH8"

"6725","GACAGTATCCTATGCCACTC","chr11",107560766,"-","none","AGG","GACAGTATCCTATGCCACTCAGG",0,NA,NA,NA,"GeneID:91801","ALKBH8",TRUE,"ALKBH8"

"6726","GATAAAGATAAGCCATTATC","chr11",107553112,"-","none","TGG","GATAAAGATAAGCCATTATCTGG",0,NA,NA,NA,"GeneID:91801","ALKBH8",TRUE,"ALKBH8"

"6727","GATAAAGATAAGCCATTATC","chr19",36182427,"-","14:A>T,19:C>G","TGG","GATAAAGATAAGCCTTTATGTGG",2,15,20,NA,"GeneID:147929","ZNF565",TRUE,"ALKBH8"

"6728","TCTTCTACTACCATGAGTCC","chr11",107553920,"+","none","TGG","TCTTCTACTACCATGAGTCCTGG",0,NA,NA,NA,"GeneID:91801","ALKBH8",TRUE,"ALKBH8"

"6729","ACCAGGAGGAAAAGGATTCC","chr2",3678533,"+","none","AGG","ACCAGGAGGAAAAGGATTCCAGG",0,NA,NA,NA,"GeneID:55821","ALLC",TRUE,"ALLC"

"6730","ACCAGGAGGAAAAGGATTCC","chr1",62561171,"+","14:C>G","AGG","ACCAGGAGGAAAAGCATTCCAGG",1,15,NA,NA,"GeneID:85440","DOCK7",FALSE,"ALLC"

"6731","ACCAGGAGGAAAAGGATTCC","chr7",41075898,"+","6:G>A,14:C>G","AGG","ACCAGGGGGAAAAGCATTCCAGG",2,7,15,NA,"","",FALSE,"ALLC"

"6732","ACCAGGAGGAAAAGGATTCC","chr18",71140184,"-","3:A>T,14:G>C","AGG","ACCTGGAGGAAAAGCATTCCAGG",2,4,15,NA,"","",FALSE,"ALLC"

"6733","CTTCAAAGAGCATGAATATA","chr2",3678481,"+","none","CGG","CTTCAAAGAGCATGAATATACGG",0,NA,NA,NA,"GeneID:55821","ALLC",TRUE,"ALLC"

"6734","CTTCAAAGAGCATGAATATA","chr13",90678830,"+","14:G>A,19:T>A","TGG","CTTCAAAGAGCATGGATATTTGG",2,15,20,NA,"","",FALSE,"ALLC"

"6735","GACATGGCATCTGAATCCGT","chr2",3671160,"+","none","AGG","GACATGGCATCTGAATCCGTAGG",0,NA,NA,NA,"GeneID:55821","ALLC",TRUE,"ALLC"

"6736","TATTCATGCTCTTTGAAGCA","chr2",3678479,"-","none","CGG","TATTCATGCTCTTTGAAGCACGG",0,NA,NA,NA,"GeneID:55821","ALLC",TRUE,"ALLC"

"6737","TATTCATGCTCTTTGAAGCA","chr4",174506760,"+","15:G>A,16:G>A","AGG","TATTCATGCTCTTTGGGGCAAGG",2,16,17,NA,"GeneID:3248","HPGD",TRUE,"ALLC"

"6738","TCCTGCAGAAAACCTCATAA","chr2",3674103,"+","none","AGG","TCCTGCAGAAAACCTCATAAAGG",0,NA,NA,NA,"GeneID:55821","ALLC",TRUE,"ALLC"

"6739","TCCTGCAGAAAACCTCATAA","chr20",52636979,"+","15:G>C,18:G>A","AGG","TCCTGCAGAAAACCTGATGAAGG",2,16,19,NA,"GeneID:101927700","LINC01524",FALSE,"ALLC"

"6740","TCCTGCAGAAAACCTCATAA","chr14",103162136,"+","11:C>A,15:G>C","AGG","TCCTGCAGAAACCCTGATAAAGG",2,12,16,NA,"","",FALSE,"ALLC"

"6741","TTGGGAAATGGATGGATGGC","chr2",3678507,"+","none","TGG","TTGGGAAATGGATGGATGGCTGG",0,NA,NA,NA,"GeneID:55821","ALLC",TRUE,"ALLC"

"6742","TTGGGAAATGGATGGATGGC","chr22",30204255,"-","5:A>T,6:C>T","AGG","TTGGGTGATGGATGGATGGCAGG",2,6,7,NA,"","",FALSE,"ALLC"

"6743","TTGGGAAATGGATGGATGGC","chr16",88517452,"+","1:A>T,4:T>G","TGG","TAGGTAAATGGATGGATGGCTGG",2,2,5,NA,"GeneID:161882","ZFPM1",FALSE,"ALLC"

"6744","TTGGGAAATGGATGGATGGC","chr19",19627927,"+","5:G>A,8:G>T","CGG","TTGGGGAAGGGATGGATGGCCGG",2,6,9,NA,"GeneID:9170","LPAR2",TRUE,"ALLC"

"6745","TTGGGAAATGGATGGATGGC","chrX",1226266,"+","4:T>G,19:A>C","TGG","TTGGTAAATGGATGGATGGATGG",2,5,20,NA,"","",FALSE,"ALLC"

"6746","AATGGACTCTTCCCAAACCT","chr2",73419154,"+","none","TGG","AATGGACTCTTCCCAAACCTTGG",0,NA,NA,NA,"GeneID:7840","ALMS1",TRUE,"ALMS1"

"6747","CTTTGATGAAATAAAGAATC","chr2",73422930,"-","none","AGG","CTTTGATGAAATAAAGAATCAGG",0,NA,NA,NA,"GeneID:7840","ALMS1",TRUE,"ALMS1"

"6748","CTTTGATGAAATAAAGAATC","chr16",6540068,"+","13:G>A,18:G>T","AGG","CTTTGATGAAATAGAGAAGCAGG",2,14,19,NA,"GeneID:54715","RBFOX1",FALSE,"ALMS1"

"6749","CTTTGATGAAATAAAGAATC","chr15",95191506,"+","1:A>T,9:G>A","TGG","CATTGATGAGATAAAGAATCTGG",2,2,10,NA,"","",FALSE,"ALMS1"

"6750","CTTTGATGAAATAAAGAATC","chr13",47183390,"+","4:C>G,13:T>A","AGG","CTTTCATGAAATATAGAATCAGG",2,5,14,NA,"","",FALSE,"ALMS1"

"6751","CTTTGATGAAATAAAGAATC","chr3",54142333,"-","5:G>T,10:G>T","AGG","CTTTGCTGAACTAAAGAATCAGG",2,6,11,NA,"GeneID:55799","CACNA2D3",FALSE,"ALMS1"

"6752","CTTTGATGAAATAAAGAATC","chr5",65396097,"-","2:T>A,15:A>C","AGG","CTATGATGAAATAAATAATCAGG",2,3,16,NA,"GeneID:11174","ADAMTS6",FALSE,"ALMS1"

"6753","GCCCCCGCAGCACCGCTACT","chr2",73386143,"+","none","CGG","GCCCCCGCAGCACCGCTACTCGG",0,NA,NA,NA,"GeneID:7840","ALMS1",TRUE,"ALMS1"

"6754","GCCCCCGCAGCACCGCTACT","chr9",114882956,"+","5:G>C,13:T>C","TGG","GCCCCGGCAGCACTGCTACTTGG",2,6,14,NA,"","",FALSE,"ALMS1"

"6755","GCCCCCGCAGCACCGCTACT","chr1",39004954,"+","5:G>C,13:T>C","TGG","GCCCCGGCAGCACTGCTACTTGG",2,6,14,NA,"GeneID:79647","AKIRIN1",TRUE,"ALMS1"

"6756","TCCGCTCGCCCTCCGAGTAG","chr2",73386158,"-","none","CGG","TCCGCTCGCCCTCCGAGTAGCGG",0,NA,NA,NA,"GeneID:7840","ALMS1",TRUE,"ALMS1"

"6757","TTCTCACATTAAACCTAGTC","chr2",73419187,"-","none","TGG","TTCTCACATTAAACCTAGTCTGG",0,NA,NA,NA,"GeneID:7840","ALMS1",TRUE,"ALMS1"

"6758","TTCTCACATTAAACCTAGTC","chr20",13676888,"+","13:T>C,18:G>T","TGG","TTCTCACATTAAATCTAGGCTGG",2,14,19,NA,"","",FALSE,"ALMS1"

"6759","TTCTTGGTCTTGTGTCAAAC","chr2",73422901,"-","none","AGG","TTCTTGGTCTTGTGTCAAACAGG",0,NA,NA,NA,"GeneID:7840","ALMS1",TRUE,"ALMS1"

"6760","CGTACAACCGCGTGCAGCTT","chr17",6996169,"+","none","TGG","CGTACAACCGCGTGCAGCTTTGG",0,NA,NA,NA,"GeneID:100506713;GeneID:239","LOC100506713;ALOX12",TRUE,"ALOX12"

"6761","GACCATCGCTGCAGACCGTA","chr17",6998744,"+","none","AGG","GACCATCGCTGCAGACCGTAAGG",0,NA,NA,NA,"GeneID:100506713;GeneID:239","LOC100506713;ALOX12",TRUE,"ALOX12"

"6762","GGGCCGCTACCGCATCCGCG","chr17",6996119,"+","none","TGG","GGGCCGCTACCGCATCCGCGTGG",0,NA,NA,NA,"GeneID:100506713;GeneID:239","LOC100506713;ALOX12",TRUE,"ALOX12"

"6763","GTGGTTCTGCGACCGCATCA","chr17",6996914,"+","none","CGG","GTGGTTCTGCGACCGCATCACGG",0,NA,NA,NA,"GeneID:100506713;GeneID:239","LOC100506713;ALOX12",TRUE,"ALOX12"

"6764","GTTCCTTCTCTCGATGCTTC","chr17",6998545,"-","none","TGG","GTTCCTTCTCTCGATGCTTCTGG",0,NA,NA,NA,"GeneID:100506713;GeneID:239","LOC100506713;ALOX12",TRUE,"ALOX12"

"6765","TGGCCTTCCCGTGCTACCGC","chr17",6996964,"+","none","TGG","TGGCCTTCCCGTGCTACCGCTGG",0,NA,NA,NA,"GeneID:100506713;GeneID:239","LOC100506713;ALOX12",TRUE,"ALOX12"

"6766","CATGGAGGGCATCCCCACCG","chr17",8079487,"-","none","TGG","CATGGAGGGCATCCCCACCGTGG",0,NA,NA,NA,"GeneID:242","ALOX12B",TRUE,"ALOX12B"

"6767","CATGGAGGGCATCCCCACCG","chr10",127166533,"+","12:T>C,19:T>G","TGG","CATGGAGGGCATTCCCACCTTGG",2,13,20,NA,"GeneID:1793;GeneID:642938","DOCK1;FAM196A",FALSE,"ALOX12B"

"6768","GAGACGACAGATTTCTTGCC","chr17",8080236,"+","none","AGG","GAGACGACAGATTTCTTGCCAGG",0,NA,NA,NA,"GeneID:242","ALOX12B",TRUE,"ALOX12B"

"6769","GATGATGCCCATCGCCATCC","chr17",8079397,"-","none","AGG","GATGATGCCCATCGCCATCCAGG",0,NA,NA,NA,"GeneID:242","ALOX12B",TRUE,"ALOX12B"

"6770","GATGATGCCCATCGCCATCC","chr9",134433124,"-","2:T>A,13:A>C","CGG","GAAGATGCCCATCTCCATCCCGG",2,3,14,NA,"GeneID:6256","RXRA",FALSE,"ALOX12B"

"6771","GGCCGGGGTTGACGCCGTTG","chr17",8079869,"+","none","AGG","GGCCGGGGTTGACGCCGTTGAGG",0,NA,NA,NA,"GeneID:242","ALOX12B",TRUE,"ALOX12B"

"6772","GTTCCCCGTCACAGACGACA","chr17",8079818,"-","none","TGG","GTTCCCCGTCACAGACGACATGG",0,NA,NA,NA,"GeneID:242","ALOX12B",TRUE,"ALOX12B"

"6773","TGTTGGACTGCAAACATTCG","chr17",8080292,"-","none","TGG","TGTTGGACTGCAAACATTCGTGG",0,NA,NA,NA,"GeneID:242","ALOX12B",TRUE,"ALOX12B"

"6774","TGTTGGACTGCAAACATTCG","chr4",177373685,"-","8:T>A,19:G>C","TGG","TGTTGGACAGCAAACATTCCTGG",2,9,20,NA,"","",FALSE,"ALOX12B"

"6775","ACCTCCTTAAGGACGACGCC","chr17",4639541,"-","none","TGG","ACCTCCTTAAGGACGACGCCTGG",0,NA,NA,NA,"GeneID:246","ALOX15",TRUE,"ALOX15"

"6776","CTATGCCGGTTCCAACAACC","chr17",4641590,"-","none","AGG","CTATGCCGGTTCCAACAACCAGG",0,NA,NA,NA,"GeneID:246","ALOX15",TRUE,"ALOX15"

"6777","CTATGCCGGTTCCAACAACC","chr6",137478522,"+","6:T>C,13:C>A","TGG","CTATGCTGGTTCCCACAACCTGG",2,7,14,NA,"","",FALSE,"ALOX15"

"6778","GTTGGAACCGGCATAGAGCG","chr17",4641594,"+","none","AGG","GTTGGAACCGGCATAGAGCGAGG",0,NA,NA,NA,"GeneID:246","ALOX15",TRUE,"ALOX15"

"6779","TGGAAGTACCGGAGTATCTG","chr17",4639595,"-","none","GGG","TGGAAGTACCGGAGTATCTGGGG",0,NA,NA,NA,"GeneID:246","ALOX15",TRUE,"ALOX15"

"6780","TGGAAGTACCGGAGTATCTG","chr17",6657229,"+","16:C>T","GGG","TGGAAGTACCGGAGTACCTGGGG",1,17,NA,NA,"GeneID:388327","C17orf100",TRUE,"ALOX15"

"6781","TGTCCTTCTAGGCCGCACTG","chr17",4639123,"-","none","TGG","TGTCCTTCTAGGCCGCACTGTGG",0,NA,NA,NA,"GeneID:246","ALOX15",TRUE,"ALOX15"

"6782","TGTCCTTCTAGGCCGCACTG","chr17",6657690,"+","2:G>T,15:T>C","TGG","TGGCCTTCTAGGCCGTACTGTGG",2,3,16,NA,"GeneID:388327","C17orf100",TRUE,"ALOX15"

"6783","TGTTTCTGGAACAGGCCCTG","chr17",4639089,"+","none","AGG","TGTTTCTGGAACAGGCCCTGAGG",0,NA,NA,NA,"GeneID:246","ALOX15",TRUE,"ALOX15"

"6784","TGTTTCTGGAACAGGCCCTG","chr17",6657724,"-","17:A>G,18:G>A","AGG","TGTTTCTGGAACAGGCCTCGAGG",2,18,19,NA,"GeneID:388327","C17orf100",TRUE,"ALOX15"

"6785","TGTTTCTGGAACAGGCCCTG","chr13",79445369,"-","11:A>G,17:T>G","AGG","TGTTTCTGGAATAGGCCATGAGG",2,12,18,NA,"","",FALSE,"ALOX15"

"6786","AAGAGGAGGTGGCCGCCCCG","chr17",8039529,"-","none","CGG","AAGAGGAGGTGGCCGCCCCGCGG",0,NA,NA,NA,"GeneID:247","ALOX15B",TRUE,"ALOX15B"

"6787","AAGAGGAGGTGGCCGCCCCG","chr1",40824270,"-","13:T>G,19:T>C","CGG","AAGAGGAGGTGGCAGCCCCACGG",2,14,20,NA,"GeneID:9132","KCNQ4",TRUE,"ALOX15B"

"6788","AGGTGGAAGGCTTACAACCC","chr17",8042366,"+","none","AGG","AGGTGGAAGGCTTACAACCCAGG",0,NA,NA,NA,"GeneID:247","ALOX15B",TRUE,"ALOX15B"

"6789","AGGTGGAAGGCTTACAACCC","chr3",195843889,"+","7:T>A,14:G>C","AGG","AGGTGGATGGCTTAGAACCCAGG",2,8,15,NA,"","",FALSE,"ALOX15B"

"6790","GACTCCTCCAGAGCCCCTTG","chr17",8042816,"-","none","CGG","GACTCCTCCAGAGCCCCTTGCGG",0,NA,NA,NA,"GeneID:247","ALOX15B",TRUE,"ALOX15B"

"6791","GACTCCTCCAGAGCCCCTTG","chr7",100549414,"-","3:T>A,13:C>G","AGG","GACACCTCCAGAGGCCCTTGAGG",2,4,14,NA,"GeneID:3268","AGFG2",FALSE,"ALOX15B"

"6792","GCCAGCCTGTAGATAAAAGT","chr17",8042466,"-","none","TGG","GCCAGCCTGTAGATAAAAGTTGG",0,NA,NA,NA,"GeneID:247","ALOX15B",TRUE,"ALOX15B"

"6793","GCCAGCCTGTAGATAAAAGT","chr14",54054713,"-","9:T>A,11:G>C","AGG","GCCAGCCTGAACATAAAAGTAGG",2,10,12,NA,"","",FALSE,"ALOX15B"

"6794","GGTTCCAGCTGACACCGCCG","chr17",8039509,"+","none","CGG","GGTTCCAGCTGACACCGCCGCGG",0,NA,NA,NA,"GeneID:247","ALOX15B",TRUE,"ALOX15B"

"6795","TGTGCTCCAGCAACAGCGCC","chr17",8039929,"+","none","AGG","TGTGCTCCAGCAACAGCGCCAGG",0,NA,NA,NA,"GeneID:247","ALOX15B",TRUE,"ALOX15B"

"6796","TGTGCTCCAGCAACAGCGCC","chr11",114293165,"+","12:G>A,16:T>C","TGG","TGTGCTCCAGCAGCAGTGCCTGG",2,13,17,NA,"GeneID:4837","NNMT",FALSE,"ALOX15B"

"6797","CATTTGGCATCGATGCTCAA","chr10",45412218,"-","none","GGG","CATTTGGCATCGATGCTCAAGGG",0,NA,NA,NA,"GeneID:240","ALOX5",TRUE,"ALOX5"

"6798","GAATCGAGAAGCGCAAGTAC","chr10",45382537,"+","none","TGG","GAATCGAGAAGCGCAAGTACTGG",0,NA,NA,NA,"GeneID:240","ALOX5",TRUE,"ALOX5"

"6799","GCAACACCGACGTAAAGAAC","chr10",45395891,"+","none","TGG","GCAACACCGACGTAAAGAACTGG",0,NA,NA,NA,"GeneID:240","ALOX5",TRUE,"ALOX5"

"6800","GGATTCATACGACGTGACTG","chr10",45382484,"+","none","TGG","GGATTCATACGACGTGACTGTGG",0,NA,NA,NA,"GeneID:240","ALOX5",TRUE,"ALOX5"

"6801","GTGTTTCCAGTTCTTTACGT","chr10",45395900,"-","none","CGG","GTGTTTCCAGTTCTTTACGTCGG",0,NA,NA,NA,"GeneID:240","ALOX5",TRUE,"ALOX5"

"6802","GTGTTTCCAGTTCTTTACGT","chr12",61461073,"-","7:T>G,9:A>C","TGG","GTGTTTCAATTTCTTTACGTTGG",2,8,10,NA,"","",FALSE,"ALOX5"

"6803","TATCACGGGGTAAATCCTTG","chr10",45412241,"-","none","TGG","TATCACGGGGTAAATCCTTGTGG",0,NA,NA,NA,"GeneID:240","ALOX5",TRUE,"ALOX5"

"6804","ATCAGTCCAGCAAACGCAGC","chr13",30755948,"-","none","AGG","ATCAGTCCAGCAAACGCAGCAGG",0,NA,NA,NA,"GeneID:241","ALOX5AP",TRUE,"ALOX5AP"

"6805","ATCAGTCCAGCAAACGCAGC","chr2",84289003,"+","14:G>C,19:A>C","GGG","ATCAGTCCAGCAAAGGCAGAGGG",2,15,20,NA,"","",FALSE,"ALOX5AP"

"6806","CATCGTCACCCTCATCAGCG","chr13",30735643,"+","none","TGG","CATCGTCACCCTCATCAGCGTGG",0,NA,NA,NA,"GeneID:241","ALOX5AP",TRUE,"ALOX5AP"

"6807","CATTCTGGACCACGCTGATG","chr13",30735655,"-","none","AGG","CATTCTGGACCACGCTGATGAGG",0,NA,NA,NA,"GeneID:241","ALOX5AP",TRUE,"ALOX5AP"

"6808","GGCAGTGTAGACCCGCTCAA","chr13",30744137,"-","none","AGG","GGCAGTGTAGACCCGCTCAAAGG",0,NA,NA,NA,"GeneID:241","ALOX5AP",TRUE,"ALOX5AP"

"6809","GGTACGCATCTACACAGTTC","chr13",30752054,"-","none","TGG","GGTACGCATCTACACAGTTCTGG",0,NA,NA,NA,"GeneID:241","ALOX5AP",TRUE,"ALOX5AP"

"6810","TTAGTTCCTGCTGCGTTTGC","chr13",30755939,"+","none","TGG","TTAGTTCCTGCTGCGTTTGCTGG",0,NA,NA,NA,"GeneID:241","ALOX5AP",TRUE,"ALOX5AP"

"6811","AGAGTTGCGCACCCACGTCT","chr17",8110202,"+","none","TGG","AGAGTTGCGCACCCACGTCTTGG",0,NA,NA,NA,"GeneID:59344","ALOXE3",TRUE,"ALOXE3"

"6812","AGCAAAAATCTCGCCAGTCC","chr17",8108495,"+","none","AGG","AGCAAAAATCTCGCCAGTCCAGG",0,NA,NA,NA,"GeneID:59344","ALOXE3",TRUE,"ALOXE3"

"6813","CCACACTCGATACACGCTGC","chr17",8109971,"-","none","AGG","CCACACTCGATACACGCTGCAGG",0,NA,NA,NA,"GeneID:59344","ALOXE3",TRUE,"ALOXE3"

"6814","CCTGCAGCGTGTATCGAGTG","chr17",8109968,"+","none","TGG","CCTGCAGCGTGTATCGAGTGTGG",0,NA,NA,NA,"GeneID:59344","ALOXE3",TRUE,"ALOXE3"

"6815","CTGCTGCACAGATGCGTCAC","chr17",8108534,"+","none","TGG","CTGCTGCACAGATGCGTCACTGG",0,NA,NA,NA,"GeneID:59344","ALOXE3",TRUE,"ALOXE3"

"6816","GGGTCGCGGACCTTGTAGAT","chr17",8110080,"+","none","GGG","GGGTCGCGGACCTTGTAGATGGG",0,NA,NA,NA,"GeneID:59344","ALOXE3",TRUE,"ALOXE3"

"6817","AGCTGCCTGGCGGTTCCAGA","chr2",232456369,"-","none","AGG","AGCTGCCTGGCGGTTCCAGAAGG",0,NA,NA,NA,"GeneID:248","ALPI",TRUE,"ALPI"

"6818","AGCTGCCTGGCGGTTCCAGA","chr21",22561778,"-","10:A>G,12:G>C","GGG","AGCTGCCTGGTGCTTCCAGAGGG",2,11,13,NA,"","",FALSE,"ALPI"

"6819","ATCCTGGTGGCTGTCACCGT","chr2",232456593,"-","none","GGG","ATCCTGGTGGCTGTCACCGTGGG",0,NA,NA,NA,"GeneID:248","ALPI",TRUE,"ALPI"

"6820","ATCCTGGTGGCTGTCACCGT","chr19",7789100,"+","13:G>T,19:A>T","GGG","ATCCTGGTGGCTGGCACCGAGGG",2,14,20,NA,"","",FALSE,"ALPI"

"6821","CATGGACCGCTTCCCATACC","chr2",232456661,"+","none","TGG","CATGGACCGCTTCCCATACCTGG",0,NA,NA,NA,"GeneID:248","ALPI",TRUE,"ALPI"

"6822","CATGGACCGCTTCCCATACC","chr2",232379281,"+","18:T>C,19:G>C","TGG","CATGGACCGCTTCCCATATGTGG",2,19,20,NA,"GeneID:250","ALPP",TRUE,"ALPI"

"6823","CATGGACCGCTTCCCATACC","chr2",232407367,"+","15:G>A,19:G>C","TGG","CATGGACCGCTTCCCGTACGTGG",2,16,20,NA,"GeneID:251","ALPPL2",TRUE,"ALPI"

"6824","CATTACCTGGGATGACGCCC","chr2",232456252,"-","none","AGG","CATTACCTGGGATGACGCCCAGG",0,NA,NA,NA,"GeneID:248","ALPI",TRUE,"ALPI"

"6825","CATTACCTGGGATGACGCCC","chr2",232406947,"-","15:A>G","AGG","CATTACCTGGGATGATGCCCAGG",1,16,NA,NA,"GeneID:251","ALPPL2",TRUE,"ALPI"

"6826","CATTACCTGGGATGACGCCC","chr2",232378864,"-","15:A>G","AGG","CATTACCTGGGATGATGCCCAGG",1,16,NA,NA,"GeneID:250","ALPP",TRUE,"ALPI"

"6827","CTCTCCCTGGGCGTCATCCC","chr2",232456244,"+","none","AGG","CTCTCCCTGGGCGTCATCCCAGG",0,NA,NA,NA,"GeneID:248","ALPI",TRUE,"ALPI"

"6828","CTCTCCCTGGGCGTCATCCC","chr2",232378856,"+","12:A>G","AGG","CTCTCCCTGGGCATCATCCCAGG",1,13,NA,NA,"GeneID:250","ALPP",TRUE,"ALPI"

"6829","CTCTCCCTGGGCGTCATCCC","chr2",232406939,"+","12:A>G","AGG","CTCTCCCTGGGCATCATCCCAGG",1,13,NA,NA,"GeneID:251","ALPPL2",TRUE,"ALPI"

"6830","CTCTCCCTGGGCGTCATCCC","chr11",69035871,"+","7:A>T,12:T>G","AGG","CTCTCCCAGGGCTTCATCCCAGG",2,8,13,NA,"","",FALSE,"ALPI"

"6831","TGAGGTTCTTGGCGACCTTC","chr2",232456427,"-","none","TGG","TGAGGTTCTTGGCGACCTTCTGG",0,NA,NA,NA,"GeneID:248","ALPI",TRUE,"ALPI"

"6832","TGAGGTTCTTGGCGACCTTC","chr14",106514262,"-","12:T>G,16:T>G","AGG","TGAGGTTCTTGGAGACATTCAGG",2,13,17,NA,"GeneID:338005","LINC00221",FALSE,"ALPI"

"6833","AGCAGTCTAATAAGCAACAA","chr4",112423972,"+","none","TGG","AGCAGTCTAATAAGCAACAATGG",0,NA,NA,NA,"GeneID:80216","ALPK1",TRUE,"ALPK1"

"6834","AGCAGTCTAATAAGCAACAA","chr16",68289330,"-","10:C>A,13:A>C","GGG","AGCAGTCTAAGAATCAACAAGGG",2,11,14,NA,"GeneID:9057;GeneID:84138","SLC7A6;SLC7A6OS",FALSE,"ALPK1"

"6835","AGCCACGCCAATTGCCCCGC","chr4",112411966,"+","none","AGG","AGCCACGCCAATTGCCCCGCAGG",0,NA,NA,NA,"GeneID:80216","ALPK1",TRUE,"ALPK1"

"6836","CAGAGACGTCGAGCCCATAC","chr4",112411903,"-","none","AGG","CAGAGACGTCGAGCCCATACAGG",0,NA,NA,NA,"GeneID:80216","ALPK1",TRUE,"ALPK1"

"6837","GCTTGGCGAATAACCACCTG","chr4",112411985,"-","none","CGG","GCTTGGCGAATAACCACCTGCGG",0,NA,NA,NA,"GeneID:80216","ALPK1",TRUE,"ALPK1"

"6838","GTATACAGACCGACTGCACC","chr4",112425701,"-","none","AGG","GTATACAGACCGACTGCACCAGG",0,NA,NA,NA,"GeneID:80216","ALPK1",TRUE,"ALPK1"

"6839","TCGGTCTGTATACAGATCAG","chr4",112425708,"+","none","AGG","TCGGTCTGTATACAGATCAGAGG",0,NA,NA,NA,"GeneID:80216","ALPK1",TRUE,"ALPK1"

"6840","AATAATGCCACTCCCATCGA","chr18",58607374,"+","none","TGG","AATAATGCCACTCCCATCGATGG",0,NA,NA,NA,"GeneID:115701","ALPK2",TRUE,"ALPK2"

"6841","ATCCGTGAACTTTGATGCCA","chr18",58580043,"+","none","TGG","ATCCGTGAACTTTGATGCCATGG",0,NA,NA,NA,"GeneID:115701","ALPK2",TRUE,"ALPK2"

"6842","GAAAGGATGAAAGACTCCGA","chr18",58611783,"-","none","AGG","GAAAGGATGAAAGACTCCGAAGG",0,NA,NA,NA,"GeneID:115701","ALPK2",TRUE,"ALPK2"

"6843","GATAACCCTAACCTACACCG","chr18",58579817,"-","none","AGG","GATAACCCTAACCTACACCGAGG",0,NA,NA,NA,"GeneID:115701","ALPK2",TRUE,"ALPK2"

"6844","GTGCTTCGCTGCATAATATC","chr18",58611690,"-","none","TGG","GTGCTTCGCTGCATAATATCTGG",0,NA,NA,NA,"GeneID:115701","ALPK2",TRUE,"ALPK2"

"6845","TTCTTATACCAAGTTACCTC","chr18",58607405,"+","none","TGG","TTCTTATACCAAGTTACCTCTGG",0,NA,NA,NA,"GeneID:115701","ALPK2",TRUE,"ALPK2"

"6846","AAAATATGAGATCACTCATC","chr15",84839046,"+","none","AGG","AAAATATGAGATCACTCATCAGG",0,NA,NA,NA,"GeneID:57538","ALPK3",TRUE,"ALPK3"

"6847","CCCGGCCACCGGCTATAAAT","chr15",84817312,"+","none","AGG","CCCGGCCACCGGCTATAAATAGG",0,NA,NA,NA,"GeneID:57538","ALPK3",TRUE,"ALPK3"

"6848","CCGATACACACCGCAGTCTA","chr15",84859825,"-","none","CGG","CCGATACACACCGCAGTCTACGG",0,NA,NA,NA,"GeneID:57538","ALPK3",TRUE,"ALPK3"

"6849","CCGTAGACTGCGGTGTGTAT","chr15",84859822,"+","none","CGG","CCGTAGACTGCGGTGTGTATCGG",0,NA,NA,NA,"GeneID:57538","ALPK3",TRUE,"ALPK3"

"6850","CTCCGTATCATCCTTGTACC","chr15",84839003,"-","none","AGG","CTCCGTATCATCCTTGTACCAGG",0,NA,NA,NA,"GeneID:57538","ALPK3",TRUE,"ALPK3"

"6851","CTCCGTATCATCCTTGTACC","chr3",24753499,"+","4:T>G,18:G>C","TGG","CTCCTTATCATCCTTGTAGCTGG",2,5,19,NA,"","",FALSE,"ALPK3"

"6852","GTCTGCAGTAGCGCCGCGTC","chr15",84817157,"-","none","TGG","GTCTGCAGTAGCGCCGCGTCTGG",0,NA,NA,NA,"GeneID:57538","ALPK3",TRUE,"ALPK3"

"6853","CACCTTGGAGAGGGCCACGA","chr1",21561195,"-","none","AGG","CACCTTGGAGAGGGCCACGAAGG",0,NA,NA,NA,"GeneID:249","ALPL",TRUE,"ALPL"

"6854","CACCTTGGAGAGGGCCACGA","chr3",5797845,"-","14:T>G,17:T>G","TGG","CACCTTGGAGAGGGACAAGATGG",2,15,18,NA,"","",FALSE,"ALPL"

"6855","CAGCGCCGCCTACGCCCACT","chr1",21564089,"+","none","CGG","CAGCGCCGCCTACGCCCACTCGG",0,NA,NA,NA,"GeneID:249","ALPL",TRUE,"ALPL"

"6856","GATGCGGGCAGCCGTCACTG","chr1",21561111,"-","none","TGG","GATGCGGGCAGCCGTCACTGTGG",0,NA,NA,NA,"GeneID:249","ALPL",TRUE,"ALPL"

"6857","GATGCGGGCAGCCGTCACTG","chr11",33257394,"-","5:G>C,11:A>G","AGG","GATGCCGGCAGTCGTCACTGAGG",2,6,12,NA,"GeneID:10114","HIPK3",FALSE,"ALPL"

"6858","GGTGGCATGGTTCACTCTCG","chr1",21564067,"-","none","TGG","GGTGGCATGGTTCACTCTCGTGG",0,NA,NA,NA,"GeneID:249","ALPL",TRUE,"ALPL"

"6859","GTTGCACCGGGAACGCTCAG","chr1",21563212,"-","none","TGG","GTTGCACCGGGAACGCTCAGTGG",0,NA,NA,NA,"GeneID:249","ALPL",TRUE,"ALPL"

"6860","TGGCGGTGCCGGCACTGTCA","chr1",21563135,"-","none","GGG","TGGCGGTGCCGGCACTGTCAGGG",0,NA,NA,NA,"GeneID:249","ALPL",TRUE,"ALPL"

"6861","CACCGTGTGGGCGTAGGTGC","chr2",232379817,"-","none","CGG","CACCGTGTGGGCGTAGGTGCCGG",0,NA,NA,NA,"GeneID:250","ALPP",TRUE,"ALPP"

"6862","CACCGTGTGGGCGTAGGTGC","chr2",232407898,"-","17:G>A","CGG","CACCGTGTGGGCGTAGGCGCCGG",1,18,NA,NA,"GeneID:251","ALPPL2",TRUE,"ALPP"

"6863","CACCGTGTGGGCGTAGGTGC","chr2",232457203,"-","3:A>G,9:A>C","CGG","CACTGTGTGTGCGTAGGTGCCGG",2,4,10,NA,"GeneID:248","ALPI",TRUE,"ALPP"

"6864","CATCGCCCAGGAAGATGATG","chr2",232379067,"-","none","AGG","CATCGCCCAGGAAGATGATGAGG",0,NA,NA,NA,"GeneID:250","ALPP",TRUE,"ALPP"

"6865","CATCGCCCAGGAAGATGATG","chr2",232456445,"-","15:C>A","AGG","CATCGCCCAGGAAGAGGATGAGG",1,16,NA,NA,"GeneID:248","ALPI",TRUE,"ALPP"

"6866","CATCGCCCAGGAAGATGATG","chr19",313198,"-","12:C>T,17:C>T","TGG","CATCGCCCAGGAGGATGGTGTGG",2,13,18,NA,"GeneID:54531","MIER2",FALSE,"ALPP"

"6867","CATCGCCCAGGAAGATGATG","chr2",232407153,"-","1:C>T,4:T>C","AGG","CGTCACCCAGGAAGATGATGAGG",2,2,5,NA,"GeneID:251","ALPPL2",TRUE,"ALPP"

"6868","CATGGACCGCTTCCCATATG","chr2",232379281,"+","none","TGG","CATGGACCGCTTCCCATATGTGG",0,NA,NA,NA,"GeneID:250","ALPP",TRUE,"ALPP"

"6869","CATGGACCGCTTCCCATATG","chr2",232456661,"+","18:C>T,19:C>G","TGG","CATGGACCGCTTCCCATACCTGG",2,19,20,NA,"GeneID:248","ALPI",TRUE,"ALPP"

"6870","CATGGACCGCTTCCCATATG","chr2",232407367,"+","15:G>A,18:C>T","TGG","CATGGACCGCTTCCCGTACGTGG",2,16,19,NA,"GeneID:251","ALPPL2",TRUE,"ALPP"

"6871","CGAGGCGTGCTGCACTCGTG","chr2",232379790,"-","none","TGG","CGAGGCGTGCTGCACTCGTGTGG",0,NA,NA,NA,"GeneID:250","ALPP",TRUE,"ALPP"

"6872","CGAGGCGTGCTGCACTCGTG","chr2",232457176,"-","15:G>A","TGG","CGAGGCGTGCTGCACCCGTGTGG",1,16,NA,NA,"GeneID:248","ALPI",TRUE,"ALPP"

"6873","CGAGGCGTGCTGCACTCGTG","chr2",232407871,"-","6:T>C,15:G>A","TGG","CGAGGCATGCTGCACCCGTGTGG",2,7,16,NA,"GeneID:251","ALPPL2",TRUE,"ALPP"

"6874","CTGGAACCGCGAGGCAGCCG","chr2",232378992,"+","none","AGG","CTGGAACCGCGAGGCAGCCGAGG",0,NA,NA,NA,"GeneID:250","ALPP",TRUE,"ALPP"

"6875","CTGGAACCGCGAGGCAGCCG","chr2",232407078,"+","10:C>G","AGG","CTGGAACCGCCAGGCAGCCGAGG",1,11,NA,NA,"GeneID:251","ALPPL2",TRUE,"ALPP"

"6876","CTGGAACCGCGAGGCAGCCG","chr2",232456370,"+","10:C>G,18:T>C","AGG","CTGGAACCGCCAGGCAGCTGAGG",2,11,19,NA,"GeneID:248","ALPI",TRUE,"ALPP"

"6877","TCCATGGCCAGGGGTATCTC","chr2",232379267,"-","none","AGG","TCCATGGCCAGGGGTATCTCAGG",0,NA,NA,NA,"GeneID:250","ALPP",TRUE,"ALPP"

"6878","TCCATGGCCAGGGGTATCTC","chr2",232456647,"-","14:G>A,15:C>T","AGG","TCCATGGCCAGGGGCGTCTCAGG",2,15,16,NA,"GeneID:248","ALPI",TRUE,"ALPP"

"6879","TCCATGGCCAGGGGTATCTC","chr2",29033626,"+","15:G>A,16:G>T","TGG","TCCATGGCCAGGGGTGGCTCTGG",2,16,17,NA,"GeneID:165186","FAM179A",FALSE,"ALPP"

"6880","CACCGTGTGGGCGTAGGCGC","chr2",232407898,"-","none","CGG","CACCGTGTGGGCGTAGGCGCCGG",0,NA,NA,NA,"GeneID:251","ALPPL2",TRUE,"ALPPL2"

"6881","CACCGTGTGGGCGTAGGCGC","chr2",232379817,"-","17:A>G","CGG","CACCGTGTGGGCGTAGGTGCCGG",1,18,NA,NA,"GeneID:250","ALPP",TRUE,"ALPPL2"

"6882","CACCGTGTGGGCGTAGGCGC","chr7",1566888,"-","1:A>T,11:C>G","GGG","CTCCGTGTGGGGGTAGGCGCGGG",2,2,12,NA,"","",FALSE,"ALPPL2"

"6883","CATGGACCGCTTCCCGTACG","chr2",232407367,"+","none","TGG","CATGGACCGCTTCCCGTACGTGG",0,NA,NA,NA,"GeneID:251","ALPPL2",TRUE,"ALPPL2"

"6884","CATGGACCGCTTCCCGTACG","chr2",232456661,"+","15:A>G,19:C>G","TGG","CATGGACCGCTTCCCATACCTGG",2,16,20,NA,"GeneID:248","ALPI",TRUE,"ALPPL2"

"6885","CATGGACCGCTTCCCGTACG","chr2",232379281,"+","15:A>G,18:T>C","TGG","CATGGACCGCTTCCCATATGTGG",2,16,19,NA,"GeneID:250","ALPP",TRUE,"ALPPL2"

"6886","CGTCACCCAGGAAGATGATG","chr2",232407153,"-","none","AGG","CGTCACCCAGGAAGATGATGAGG",0,NA,NA,NA,"GeneID:251","ALPPL2",TRUE,"ALPPL2"

"6887","CGTCACCCAGGAAGATGATG","chr8",26624940,"-","2:T>A,3:C>G","TGG","CGAGACCCAGGAAGATGATGTGG",2,3,4,NA,"GeneID:1808","DPYSL2",FALSE,"ALPPL2"

"6888","CGTCACCCAGGAAGATGATG","chr2",232379067,"-","1:T>C,4:C>T","AGG","CATCGCCCAGGAAGATGATGAGG",2,2,5,NA,"GeneID:250","ALPP",TRUE,"ALPPL2"

"6889","GAAGCGGTCCATGGCCAGGA","chr2",232407360,"-","none","AGG","GAAGCGGTCCATGGCCAGGAAGG",0,NA,NA,NA,"GeneID:251","ALPPL2",TRUE,"ALPPL2"

"6890","GCCTGGCGGTTCCAGAAGTC","chr2",232407073,"-","none","CGG","GCCTGGCGGTTCCAGAAGTCCGG",0,NA,NA,NA,"GeneID:251","ALPPL2",TRUE,"ALPPL2"

"6891","GCCTGGCGGTTCCAGAAGTC","chr2",232456365,"-","18:C>A","CGG","GCCTGGCGGTTCCAGAAGGCCGG",1,19,NA,NA,"GeneID:248","ALPI",TRUE,"ALPPL2"

"6892","GCCTGGCGGTTCCAGAAGTC","chr9",136304952,"-","16:C>T,18:T>A","AGG","GCCTGGCGGTTCCAGAGGACAGG",2,17,19,NA,"GeneID:399693","MGC50722",FALSE,"ALPPL2"

"6893","GCCTGGCGGTTCCAGAAGTC","chr2",232378987,"-","4:G>C","CGG","GCCTCGCGGTTCCAGAAGTCCGG",1,5,NA,NA,"GeneID:250","ALPP",TRUE,"ALPPL2"

"6894","GGATGCCGACGTGCCTGCCT","chr2",232407935,"+","none","CGG","GGATGCCGACGTGCCTGCCTCGG",0,NA,NA,NA,"GeneID:251","ALPPL2",TRUE,"ALPPL2"

"6895","CAGATGCACAAGAGACCACC","chr2",201757668,"+","none","AGG","CAGATGCACAAGAGACCACCAGG",0,NA,NA,NA,"GeneID:57679","ALS2",TRUE,"ALS2"

"6896","CAGATGCACAAGAGACCACC","chr1",59138475,"-","15:T>G,19:C>G","GGG","CAGATGCACAAGAGAACACGGGG",2,16,20,NA,"GeneID:729467","HSD52",FALSE,"ALS2"

"6897","CTGCCTGCCAGATATGGACC","chr2",201767327,"+","none","AGG","CTGCCTGCCAGATATGGACCAGG",0,NA,NA,NA,"GeneID:57679","ALS2",TRUE,"ALS2"

"6898","CTGCCTGCCAGATATGGACC","chr1",154957569,"-","12:C>A,17:C>T","TGG","CTGCCTGCCAGAGATGGGCCTGG",2,13,18,NA,"GeneID:90780","PYGO2",TRUE,"ALS2"

"6899","GAACTCCATGTTTCACTCCG","chr2",201767240,"+","none","AGG","GAACTCCATGTTTCACTCCGAGG",0,NA,NA,NA,"GeneID:57679","ALS2",TRUE,"ALS2"

"6900","GGCACGGCGATGTTCTGCCT","chr2",201754504,"-","none","AGG","GGCACGGCGATGTTCTGCCTAGG",0,NA,NA,NA,"GeneID:57679","ALS2",TRUE,"ALS2"

"6901","GTGGCTGCTACTTATGAAGC","chr2",201757634,"-","none","TGG","GTGGCTGCTACTTATGAAGCTGG",0,NA,NA,NA,"GeneID:57679","ALS2",TRUE,"ALS2"

"6902","TTCCCCCAGGCTCTTAAGAA","chr2",201754650,"-","none","AGG","TTCCCCCAGGCTCTTAAGAAAGG",0,NA,NA,NA,"GeneID:57679","ALS2",TRUE,"ALS2"

"6903","CACCACCATGCAGCTTGTGT","chr3",46687650,"+","none","AGG","CACCACCATGCAGCTTGTGTAGG",0,NA,NA,NA,"GeneID:259173","ALS2CL",TRUE,"ALS2CL"

"6904","CCCTTCAGCATCACCCAACC","chr3",46686427,"-","none","CGG","CCCTTCAGCATCACCCAACCCGG",0,NA,NA,NA,"GeneID:259173","ALS2CL",TRUE,"ALS2CL"

"6905","CCCTTCAGCATCACCCAACC","chr2",66317812,"-","16:A>T,18:T>G","TGG","CCCTTCAGCATCACCCTAACTGG",2,17,19,NA,"","",FALSE,"ALS2CL"

"6906","CCCTTCAGCATCACCCAACC","chr1",44354873,"+","2:A>C,17:C>A","CGG","CCATTCAGCATCACCCACCCCGG",2,3,18,NA,"GeneID:79033","ERI3",TRUE,"ALS2CL"

"6907","CTACACGCAGCGAGTACTGG","chr3",46687138,"-","none","CGG","CTACACGCAGCGAGTACTGGCGG",0,NA,NA,NA,"GeneID:259173","ALS2CL",TRUE,"ALS2CL"

"6908","GCACGTACTGTTGCACGTGA","chr3",46687017,"+","none","TGG","GCACGTACTGTTGCACGTGATGG",0,NA,NA,NA,"GeneID:259173","ALS2CL",TRUE,"ALS2CL"

"6909","GTGAACGCAGTCACCCTCTT","chr3",46686395,"-","none","TGG","GTGAACGCAGTCACCCTCTTTGG",0,NA,NA,NA,"GeneID:259173","ALS2CL",TRUE,"ALS2CL"

"6910","GTGAACGCAGTCACCCTCTT","chr11",37200666,"-","9:T>C,10:C>A","GGG","GTGAACGCAAGCACCCTCTTGGG",2,10,11,NA,"","",FALSE,"ALS2CL"

"6911","GTGAACGCAGTCACCCTCTT","chr22",22686507,"-","4:A>T,15:A>G","GGG","GTGATCGCAGTCACCTTCTTGGG",2,5,16,NA,"","",FALSE,"ALS2CL"

"6912","TGAGTCCTACACAAGCTGCA","chr3",46687658,"-","none","TGG","TGAGTCCTACACAAGCTGCATGG",0,NA,NA,NA,"GeneID:259173","ALS2CL",TRUE,"ALS2CL"

"6913","TGAGTCCTACACAAGCTGCA","chr8",70991201,"-","14:G>C,15:T>G","GGG","TGAGTCCTACACAACATGCAGGG",2,15,16,NA,"","",FALSE,"ALS2CL"

"6914","TGAGTCCTACACAAGCTGCA","chr2",18066772,"-","8:A>T,10:A>T","CGG","TGAGTCCTTCTCAAGCTGCACGG",2,9,11,NA,"GeneID:3790","KCNS3",FALSE,"ALS2CL"

"6915","AACACAGGAGACGCCAAATT","chr2",201604630,"-","none","TGG","AACACAGGAGACGCCAAATTTGG",0,NA,NA,NA,"GeneID:151254","ALS2CR11",TRUE,"ALS2CR11"

"6916","AGCACACTTGATAACCGCAG","chr2",201619077,"-","none","CGG","AGCACACTTGATAACCGCAGCGG",0,NA,NA,NA,"GeneID:151254","ALS2CR11",TRUE,"ALS2CR11"

"6917","AGTTCTTTATATGAATGCCC","chr2",201603244,"+","none","AGG","AGTTCTTTATATGAATGCCCAGG",0,NA,NA,NA,"GeneID:151254","ALS2CR11",TRUE,"ALS2CR11"

"6918","ATACATACCACATCCCCAAA","chr2",201604602,"+","none","TGG","ATACATACCACATCCCCAAATGG",0,NA,NA,NA,"GeneID:151254","ALS2CR11",TRUE,"ALS2CR11"

"6919","ATACATACCACATCCCCAAA","chr6",21187864,"-","6:A>T,9:G>T","AGG","ATACATTCCCCATCCCCAAAAGG",2,7,10,NA,"GeneID:54901","CDKAL1",FALSE,"ALS2CR11"

"6920","ATTGTTGTAGGTTGGCTGCC","chr2",201603265,"-","none","TGG","ATTGTTGTAGGTTGGCTGCCTGG",0,NA,NA,NA,"GeneID:151254","ALS2CR11",TRUE,"ALS2CR11"

"6921","ATTGTTGTAGGTTGGCTGCC","chr9",134530579,"+","14:C>G,15:T>C","AGG","ATTGTTGTAGGTTGCTTGCCAGG",2,15,16,NA,"","",FALSE,"ALS2CR11"

"6922","CCGCCACCCCGCTATTGCAG","chr2",201619037,"-","none","AGG","CCGCCACCCCGCTATTGCAGAGG",0,NA,NA,NA,"GeneID:151254","ALS2CR11",TRUE,"ALS2CR11"

"6923","ACTTACTTTGGGGAAACAAC","chr2",201350704,"+","none","AGG","ACTTACTTTGGGGAAACAACAGG",0,NA,NA,NA,"GeneID:130540","ALS2CR12",TRUE,"ALS2CR12"

"6924","ACTTACTTTGGGGAAACAAC","chr16",53422275,"-","2:G>A,5:T>G","TGG","ACCTAATTTGGGGAAACAACTGG",2,3,6,NA,"","",FALSE,"ALS2CR12"

"6925","ACTTACTTTGGGGAAACAAC","chr3",133794081,"+","2:C>T,5:A>C","TGG","ACCTAATTTGGGGAAACAACTGG",2,3,6,NA,"GeneID:7018;GeneID:58477","TF;SRPRB",TRUE,"ALS2CR12"

"6926","AGTTCTTGCGTGGTAGTTGA","chr2",201351307,"+","none","GGG","AGTTCTTGCGTGGTAGTTGAGGG",0,NA,NA,NA,"GeneID:130540","ALS2CR12",TRUE,"ALS2CR12"

"6927","CTACCACGCAAGAACTCCAC","chr2",201351303,"-","none","AGG","CTACCACGCAAGAACTCCACAGG",0,NA,NA,NA,"GeneID:130540","ALS2CR12",TRUE,"ALS2CR12"

"6928","CTACCACGCAAGAACTCCAC","chr20",60994988,"+","10:T>A,13:G>A","GGG","CTACCACGCATGAGCTCCACGGG",2,11,14,NA,"","",FALSE,"ALS2CR12"

"6929","CTATCAACTTGTTCGGAATC","chr2",201346632,"-","none","GGG","CTATCAACTTGTTCGGAATCGGG",0,NA,NA,NA,"GeneID:130540","ALS2CR12",TRUE,"ALS2CR12"

"6930","GAATCGGAGATCCCGGACAA","chr2",201346556,"-","none","AGG","GAATCGGAGATCCCGGACAAAGG",0,NA,NA,NA,"GeneID:130540","ALS2CR12",TRUE,"ALS2CR12"

"6931","TTCCGAACAAGTTGATAGCC","chr2",201346634,"+","none","AGG","TTCCGAACAAGTTGATAGCCAGG",0,NA,NA,NA,"GeneID:130540","ALS2CR12",TRUE,"ALS2CR12"

"6932","CATGATTTAGTGCCCCCAAA","chr2",202917934,"+","none","AGG","CATGATTTAGTGCCCCCAAAAGG",0,NA,NA,NA,"GeneID:55759;GeneID:79800","WDR12;CARF",TRUE,"ALS2CR8"

"6933","GACCATTGAGAACAATACAC","chr2",202912931,"-","none","AGG","GACCATTGAGAACAATACACAGG",0,NA,NA,NA,"GeneID:55759;GeneID:79800","WDR12;CARF",TRUE,"ALS2CR8"

"6934","TGGGGGTAGTAGAATGCTTG","chr2",202917988,"+","none","AGG","TGGGGGTAGTAGAATGCTTGAGG",0,NA,NA,NA,"GeneID:55759;GeneID:79800","WDR12;CARF",TRUE,"ALS2CR8"

"6935","TGGGGGTAGTAGAATGCTTG","chr19",38317107,"-","7:A>T,19:A>C","TGG","TGGGGGTTGTAGAATGCTTTTGG",2,8,20,NA,"GeneID:90522","YIF1B",FALSE,"ALS2CR8"

"6936","TTAAGAGTCAACCATAATGA","chr2",202941923,"+","none","CGG","TTAAGAGTCAACCATAATGACGG",0,NA,NA,NA,"GeneID:55759;GeneID:79800","WDR12;CARF",TRUE,"ALS2CR8"

"6937","TTGACTCTTCACCGTCATTA","chr2",202941937,"-","none","TGG","TTGACTCTTCACCGTCATTATGG",0,NA,NA,NA,"GeneID:55759;GeneID:79800","WDR12;CARF",TRUE,"ALS2CR8"

"6938","TTTCAAGTTGATTTGCTATC","chr2",202924386,"+","none","TGG","TTTCAAGTTGATTTGCTATCTGG",0,NA,NA,NA,"GeneID:55759;GeneID:79800","WDR12;CARF",TRUE,"ALS2CR8"

"6939","AGGTCCTCTGGAGCACGTTA","chr12",85280335,"+","none","TGG","AGGTCCTCTGGAGCACGTTATGG",0,NA,NA,NA,"GeneID:8092","ALX1",TRUE,"ALX1"

"6940","CATTCGAAGAACTGCGATAC","chr12",85301413,"-","none","TGG","CATTCGAAGAACTGCGATACTGG",0,NA,NA,NA,"GeneID:8092","ALX1",TRUE,"ALX1"

"6941","CGCCGAGCATCACGTGCGCT","chr12",85280434,"+","none","TGG","CGCCGAGCATCACGTGCGCTTGG",0,NA,NA,NA,"GeneID:8092","ALX1",TRUE,"ALX1"

"6942","CTCCGAATGTCTCCCGTGAA","chr12",85283648,"+","none","AGG","CTCCGAATGTCTCCCGTGAAAGG",0,NA,NA,NA,"GeneID:8092","ALX1",TRUE,"ALX1"

"6943","GTCCATAGCTCTATTCAGTT","chr12",85283616,"-","none","CGG","GTCCATAGCTCTATTCAGTTCGG",0,NA,NA,NA,"GeneID:8092","ALX1",TRUE,"ALX1"

"6944","GTCCGAGGCGAGTGAGAATA","chr12",85301247,"-","none","AGG","GTCCGAGGCGAGTGAGAATAAGG",0,NA,NA,NA,"GeneID:8092","ALX1",TRUE,"ALX1"

"6945","ACTATCCTGATGTGTATGCC","chr1",110064634,"-","none","CGG","ACTATCCTGATGTGTATGCCCGG",0,NA,NA,NA,"GeneID:85369;GeneID:257","STRIP1;ALX3",TRUE,"ALX3"

"6946","ATCACGTGGTCCAGTTCAGA","chr1",110060731,"+","none","AGG","ATCACGTGGTCCAGTTCAGAAGG",0,NA,NA,NA,"GeneID:85369;GeneID:257","STRIP1;ALX3",TRUE,"ALX3"

"6947","CCGGAAAGCGGGTCAGCCGC","chr1",110070455,"+","none","GGG","CCGGAAAGCGGGTCAGCCGCGGG",0,NA,NA,NA,"GeneID:85369;GeneID:257","STRIP1;ALX3",TRUE,"ALX3"

"6948","CGAGCGTTATGGGAAGATCC","chr1",110061505,"-","none","AGG","CGAGCGTTATGGGAAGATCCAGG",0,NA,NA,NA,"GeneID:85369;GeneID:257","STRIP1;ALX3",TRUE,"ALX3"

"6949","GCACTGCGCGCCTTTCCGCG","chr1",110070581,"-","none","TGG","GCACTGCGCGCCTTTCCGCGTGG",0,NA,NA,NA,"GeneID:85369;GeneID:257","STRIP1;ALX3",TRUE,"ALX3"

"6950","GCTTTACCCTGAGCGAGACG","chr1",110060770,"+","none","AGG","GCTTTACCCTGAGCGAGACGAGG",0,NA,NA,NA,"GeneID:85369;GeneID:257","STRIP1;ALX3",TRUE,"ALX3"

"6951","ACTACCCAGACGTGTATGCG","chr11",44275395,"-","none","CGG","ACTACCCAGACGTGTATGCGCGG",0,NA,NA,NA,"GeneID:60529","ALX4",TRUE,"ALX4"

"6952","ACTACCCAGACGTGTATGCG","chr14",74245220,"+","13:C>G,19:C>G","CGG","ACTACCCAGACGTCTATGCCCGG",2,14,20,NA,"GeneID:338917","VSX2",TRUE,"ALX4"

"6953","CCGCAAGACCTCGAGCATCG","chr11",44264909,"-","none","CGG","CCGCAAGACCTCGAGCATCGCGG",0,NA,NA,NA,"GeneID:60529","ALX4",TRUE,"ALX4"

"6954","CCGTTACGGCGCTGGGCAGC","chr11",44309851,"-","none","AGG","CCGTTACGGCGCTGGGCAGCAGG",0,NA,NA,NA,"GeneID:60529","ALX4",TRUE,"ALX4"

"6955","CGTCTCTTACTGCGAGTCGC","chr11",44310025,"-","none","CGG","CGTCTCTTACTGCGAGTCGCCGG",0,NA,NA,NA,"GeneID:60529","ALX4",TRUE,"ALX4"

"6956","GCGGAGGGCCGCGATGCTCG","chr11",44264898,"+","none","AGG","GCGGAGGGCCGCGATGCTCGAGG",0,NA,NA,NA,"GeneID:60529","ALX4",TRUE,"ALX4"

"6957","TGTTCCCGCGCATACACGTC","chr11",44275387,"+","none","TGG","TGTTCCCGCGCATACACGTCTGG",0,NA,NA,NA,"GeneID:60529","ALX4",TRUE,"ALX4"

"6958","AGTCTCAGACGCCGATATTC","chr17",81890690,"-","none","AGG","AGTCTCAGACGCCGATATTCAGG",0,NA,NA,NA,"GeneID:10189","ALYREF",TRUE,"ALYREF"

"6959","CAAAACAACTTCCCGACAAG","chr17",81890799,"-","none","TGG","CAAAACAACTTCCCGACAAGTGG",0,NA,NA,NA,"GeneID:10189;GeneID:51529","ALYREF;ANAPC11",TRUE,"ALYREF"

"6960","CGTCACAGATTGACGCACAG","chr17",81888536,"-","none","CGG","CGTCACAGATTGACGCACAGCGG",0,NA,NA,NA,"GeneID:10189","ALYREF",TRUE,"ALYREF"

"6961","GACGCCGTTGTACTGCTTCA","chr17",81889191,"+","none","TGG","GACGCCGTTGTACTGCTTCATGG",0,NA,NA,NA,"GeneID:10189","ALYREF",TRUE,"ALYREF"

"6962","GGGACTCACTCTCGCATTAT","chr17",81888231,"+","none","AGG","GGGACTCACTCTCGCATTATAGG",0,NA,NA,NA,"GeneID:10189","ALYREF",TRUE,"ALYREF"

"6963","TTCCTTGCTAGCGTAAACAG","chr17",81888409,"-","none","AGG","TTCCTTGCTAGCGTAAACAGAGG",0,NA,NA,NA,"GeneID:10189","ALYREF",TRUE,"ALYREF"

"6964","AAGCCAAATAGTTGATATCG","chr5",34005761,"+","none","TGG","AAGCCAAATAGTTGATATCGTGG",0,NA,NA,NA,"GeneID:23600;GeneID:100534612","AMACR;C1QTNF3-AMACR",TRUE,"AMACR"

"6965","CACCTTTCTATACGACTTAC","chr5",33998710,"-","none","AGG","CACCTTTCTATACGACTTACAGG",0,NA,NA,NA,"GeneID:23600;GeneID:100534612","AMACR;C1QTNF3-AMACR",TRUE,"AMACR"

"6966","CACCTTTCTATACGACTTAC","chr12",93108466,"-","17:C>A,18:C>T","TGG","CACCTTTCTATACGACTGGCTGG",2,18,19,NA,"","",FALSE,"AMACR"

"6967","GACCCTTGCCAGTGCGTGTG","chr5",34004593,"+","none","CGG","GACCCTTGCCAGTGCGTGTGCGG",0,NA,NA,NA,"GeneID:23600;GeneID:100534612","AMACR;C1QTNF3-AMACR",TRUE,"AMACR"

"6968","GTATGCCCCGCTGAATCTCC","chr5",34004677,"-","none","TGG","GTATGCCCCGCTGAATCTCCTGG",0,NA,NA,NA,"GeneID:23600;GeneID:100534612","AMACR;C1QTNF3-AMACR",TRUE,"AMACR"

"6969","GTATGCCCCGCTGAATCTCC","chr17",43743783,"-","1:C>A,15:G>A","TGG","GGATGCCCCGCTGAACCTCCTGG",2,2,16,NA,"","",FALSE,"AMACR"

"6970","GTCCTGTAAGTCGTATAGAA","chr5",33998705,"+","none","AGG","GTCCTGTAAGTCGTATAGAAAGG",0,NA,NA,NA,"GeneID:23600;GeneID:100534612","AMACR;C1QTNF3-AMACR",TRUE,"AMACR"

"6971","TGGCCACGATATCAACTATT","chr5",34005767,"-","none","TGG","TGGCCACGATATCAACTATTTGG",0,NA,NA,NA,"GeneID:23600;GeneID:100534612","AMACR;C1QTNF3-AMACR",TRUE,"AMACR"

"6972","TGGCCACGATATCAACTATT","chr5",155756465,"-","6:T>G,16:T>A","TGG","TGGCCAAGATATCAACAATTTGG",2,7,17,NA,"","",FALSE,"AMACR"

"6973","AGGCTCAAACTAGCCATACC","chr4",70597025,"-","none","CGG","AGGCTCAAACTAGCCATACCCGG",0,NA,NA,NA,"GeneID:258","AMBN",TRUE,"AMBN"

"6974","GAGATTAAACACACTTTCTC","chr4",70598381,"+","none","AGG","GAGATTAAACACACTTTCTCAGG",0,NA,NA,NA,"GeneID:258","AMBN",TRUE,"AMBN"

"6975","GATACTGATCCTATGCCTCC","chr4",70593352,"+","none","TGG","GATACTGATCCTATGCCTCCTGG",0,NA,NA,NA,"GeneID:258","AMBN",TRUE,"AMBN"

"6976","GCACTGCAAAACTCATTTCC","chr4",70593373,"-","none","AGG","GCACTGCAAAACTCATTTCCAGG",0,NA,NA,NA,"GeneID:258","AMBN",TRUE,"AMBN"

"6977","GCACTGCAAAACTCATTTCC","chr8",39206658,"+","8:C>A,9:C>A","TGG","GCACTGCACCACTCATTTCCTGG",2,9,10,NA,"GeneID:203102","ADAM32",FALSE,"AMBN"

"6978","GCACTGCAAAACTCATTTCC","chr4",115602975,"+","6:A>C,13:T>C","AGG","GCACTGAAAAACTTATTTCCAGG",2,7,14,NA,"","",FALSE,"AMBN"

"6979","GCACTGCAAAACTCATTTCC","chr22",49366508,"-","0:G>C,11:A>G","GGG","CCACTGCAAAATTCATTTCCGGG",2,1,12,NA,"","",FALSE,"AMBN"

"6980","TATGGCTAGTTTGAGCCTTG","chr4",70597027,"+","none","AGG","TATGGCTAGTTTGAGCCTTGAGG",0,NA,NA,NA,"GeneID:258","AMBN",TRUE,"AMBN"

"6981","TTGATAGACAATGAGACAGT","chr4",70598348,"+","none","TGG","TTGATAGACAATGAGACAGTTGG",0,NA,NA,NA,"GeneID:258","AMBN",TRUE,"AMBN"

"6982","TTGATAGACAATGAGACAGT","chr12",126406240,"+","2:T>G,19:G>T","TGG","TTTATAGACAATGAGACAGGTGG",2,3,20,NA,"GeneID:101927531","LOC101927531",TRUE,"AMBN"

"6983","GCCCGACAACATCCAAGTGC","chr9",114078115,"-","none","AGG","GCCCGACAACATCCAAGTGCAGG",0,NA,NA,NA,"GeneID:259","AMBP",TRUE,"AMBP"

"6984","GCCCGACAACATCCAAGTGC","chr1",29971752,"+","2:A>C,4:C>G","AGG","GCACCACAACATCCAAGTGCAGG",2,3,5,NA,"","",FALSE,"AMBP"

"6985","GGCAGGTGGAACCGATGGCC","chr9",114076697,"+","none","AGG","GGCAGGTGGAACCGATGGCCAGG",0,NA,NA,NA,"GeneID:259","AMBP",TRUE,"AMBP"

"6986","TATGAGAAAACAGATACTGA","chr9",114074985,"-","none","TGG","TATGAGAAAACAGATACTGATGG",0,NA,NA,NA,"GeneID:259","AMBP",TRUE,"AMBP"

"6987","TATGAGAAAACAGATACTGA","chr9",17759552,"-","17:T>A","GGG","TATGAGAAAACAGATACAGAGGG",1,18,NA,NA,"GeneID:6456","SH3GL2",FALSE,"AMBP"

"6988","TATGAGAAAACAGATACTGA","chr6",23695262,"-","14:C>A,18:G>C","AGG","TATGAGAAAACAGAGACTCAAGG",2,15,19,NA,"","",FALSE,"AMBP"

"6989","TATGAGAAAACAGATACTGA","chr15",98679309,"-","1:G>T,17:T>A","GGG","TCTGAGAAAACAGATACAGAGGG",2,2,18,NA,"GeneID:3480","IGF1R",FALSE,"AMBP"

"6990","TATGAGAAAACAGATACTGA","chr11",85047584,"-","0:C>A,18:G>C","GGG","GATGAGAAAACAGATACTCAGGG",2,1,19,NA,"GeneID:1740","DLG2",FALSE,"AMBP"

"6991","TATGAGAAAACAGATACTGA","chr20",10325796,"-","0:C>A,14:T>A","AGG","GATGAGAAAACAGAAACTGAAGG",2,1,15,NA,"GeneID:100131208","SNAP25-AS1",FALSE,"AMBP"

"6992","TGCTCACCGCCAACGAGTGC","chr9",114076590,"+","none","TGG","TGCTCACCGCCAACGAGTGCTGG",0,NA,NA,NA,"GeneID:259","AMBP",TRUE,"AMBP"

"6993","TTCTAGGAAAGGTGTCTGTG","chr9",114075022,"-","none","AGG","TTCTAGGAAAGGTGTCTGTGAGG",0,NA,NA,NA,"GeneID:259","AMBP",TRUE,"AMBP"

"6994","TTGTCGGGCGGCGTTGGCAC","chr9",114078126,"+","none","AGG","TTGTCGGGCGGCGTTGGCACAGG",0,NA,NA,NA,"GeneID:259","AMBP",TRUE,"AMBP"

"6995","ACATTGCCAATACTACCTAC","chr11",46443539,"-","none","CGG","ACATTGCCAATACTACCTACCGG",0,NA,NA,NA,"GeneID:55626","AMBRA1",TRUE,"AMBRA1"

"6996","ACCGAGCTCCACGCAATGCC","chr11",46494161,"-","none","CGG","ACCGAGCTCCACGCAATGCCCGG",0,NA,NA,NA,"GeneID:55626","AMBRA1",TRUE,"AMBRA1"

"6997","ACCGAGCTCCACGCAATGCC","chr14",23514795,"-","2:T>G,12:G>C","TGG","ACAGAGCTCCACCCAATGCCTGG",2,3,13,NA,"","",FALSE,"AMBRA1"

"6998","CTACTCACTCTTCTGTTAAC","chr11",46493620,"-","none","AGG","CTACTCACTCTTCTGTTAACAGG",0,NA,NA,NA,"GeneID:55626","AMBRA1",TRUE,"AMBRA1"

"6999","CTACTCACTCTTCTGTTAAC","chrX",55917639,"+","15:C>T,18:G>A","AGG","CTACTCACTCTTCTGCTAGCAGG",2,16,19,NA,"","",FALSE,"AMBRA1"

"7000","GCAGACATCCGGGCATTGCG","chr11",46494150,"+","none","TGG","GCAGACATCCGGGCATTGCGTGG",0,NA,NA,NA,"GeneID:55626","AMBRA1",TRUE,"AMBRA1"

"7001","GGTAGTATTGGCAATGTTAC","chr11",46443542,"+","none","TGG","GGTAGTATTGGCAATGTTACTGG",0,NA,NA,NA,"GeneID:55626","AMBRA1",TRUE,"AMBRA1"

"7002","TTTCATGAACGTGGACAGCC","chr11",46493648,"-","none","TGG","TTTCATGAACGTGGACAGCCTGG",0,NA,NA,NA,"GeneID:55626","AMBRA1",TRUE,"AMBRA1"

"7003","TTTCATGAACGTGGACAGCC","chr7",83016034,"+","13:C>G","TGG","TTTCATGAACGTGCACAGCCTGG",1,14,NA,NA,"GeneID:27445","PCLO",FALSE,"AMBRA1"

"7004","ATTTAGGTACTTATATACTC","chr6",110892292,"+","none","TGG","ATTTAGGTACTTATATACTCTGG",0,NA,NA,NA,"GeneID:262","AMD1",TRUE,"AMD1"

"7005","ATTTAGGTACTTATATACTC","chr4",31649557,"-","1:T>A,5:T>C","AGG","AATTAAGTACTTATATACTCAGG",2,2,6,NA,"","",FALSE,"AMD1"

"7006","ATTTAGGTACTTATATACTC","chr10",17044382,"+","9:T>C,15:A>T","AGG","ATTTAGGTATTTATAAACTCAGG",2,10,16,NA,"GeneID:8029","CUBN",FALSE,"AMD1"

"7007","CCATTCATCGAATACCCACA","chr6",110892794,"-","none","AGG","CCATTCATCGAATACCCACAAGG",0,NA,NA,NA,"GeneID:262","AMD1",TRUE,"AMD1"

"7008","CCATTCATCGAATACCCACA","chr5",43587221,"+","18:T>C","AGG","CCATTCATCGAATACCCATAAGG",1,19,NA,NA,"","",TRUE,"AMD1"

"7009","CCATTCATCGAATACCCACA","chr6",11862233,"+","2:T>A,11:C>A","AGG","CCTTTCATCGACTACCCACAAGG",2,3,12,NA,"","",TRUE,"AMD1"

"7010","GACCACCTTGTTTGTTAATC","chr6",110893043,"+","none","AGG","GACCACCTTGTTTGTTAATCAGG",0,NA,NA,NA,"GeneID:262","AMD1",TRUE,"AMD1"

"7011","GACCACCTTGTTTGTTAATC","chr16",13009157,"-","3:A>G,15:T>A","AGG","GACTACCTTGTTTGTAAATCAGG",2,4,16,NA,"GeneID:729993","SHISA9",FALSE,"AMD1"

"7012","GGAATTCGTGACCTGATACC","chr6",110892740,"+","none","AGG","GGAATTCGTGACCTGATACCAGG",0,NA,NA,NA,"GeneID:262","AMD1",TRUE,"AMD1"

"7013","GGAATTCGTGACCTGATACC","chrX",155828934,"-","6:A>G,17:A>T","AGG","GGAATTTGTGACCTGATTCCAGG",2,7,18,NA,"","",TRUE,"AMD1"

"7014","GTTGTAGAAGTCTTCAAGCC","chr6",110893011,"+","none","AGG","GTTGTAGAAGTCTTCAAGCCAGG",0,NA,NA,NA,"GeneID:262","AMD1",TRUE,"AMD1"

"7015","TGACTGATTACCCGACTCTC","chr6",110892323,"-","none","TGG","TGACTGATTACCCGACTCTCTGG",0,NA,NA,NA,"GeneID:262","AMD1",TRUE,"AMD1"

"7016","TGACTGATTACCCGACTCTC","chr10",20350597,"-","13:G>C,18:G>A","TGG","TGACTGATTACCCCACTCCCTGG",2,14,19,NA,"","",TRUE,"AMD1"

"7017","TGACTGATTACCCGACTCTC","chrX",155829061,"+","6:G>A,13:A>G","TGG","TGACTGGTTACCCAACTCTCTGG",2,7,14,NA,"","",TRUE,"AMD1"

"7018","ACTCTTTCACCAGCCCATAC","chr12",95954936,"-","none","TGG","ACTCTTTCACCAGCCCATACTGG",0,NA,NA,NA,"GeneID:144193","AMDHD1",TRUE,"AMDHD1"

"7019","ACTCTTTCACCAGCCCATAC","chr14",30911330,"+","5:G>T,17:G>T","TGG","ACTCTGTCACCAGCCCAGACTGG",2,6,18,NA,"GeneID:29966","STRN3",FALSE,"AMDHD1"

"7020","AGTTCACGAATTTGCAATGA","chr12",95954953,"+","none","AGG","AGTTCACGAATTTGCAATGAAGG",0,NA,NA,NA,"GeneID:144193","AMDHD1",TRUE,"AMDHD1"

"7021","GAACGCGCAGCAAGTGGTGC","chr12",95943427,"+","none","TGG","GAACGCGCAGCAAGTGGTGCTGG",0,NA,NA,NA,"GeneID:144193","AMDHD1",TRUE,"AMDHD1"

"7022","GAAGAAATAATTGACTGCTC","chr12",95952783,"+","none","TGG","GAAGAAATAATTGACTGCTCTGG",0,NA,NA,NA,"GeneID:144193","AMDHD1",TRUE,"AMDHD1"

"7023","TGCGCAGCGCATCCCGCGCC","chr12",95943475,"-","none","AGG","TGCGCAGCGCATCCCGCGCCAGG",0,NA,NA,NA,"GeneID:144193","AMDHD1",TRUE,"AMDHD1"

"7024","TGCGCAGCGCATCCCGCGCC","chr15",23897815,"-","6:G>C,10:A>T","CGG","TGCGCACCGCTTCCCGCGCCCGG",2,7,11,NA,"","",FALSE,"AMDHD1"

"7025","TGTCTTTGAATAACATCAGC","chr12",95952744,"-","none","AGG","TGTCTTTGAATAACATCAGCAGG",0,NA,NA,NA,"GeneID:144193","AMDHD1",TRUE,"AMDHD1"

"7026","CAATGTCCGCATCGTGACGC","chr16",2527893,"+","none","TGG","CAATGTCCGCATCGTGACGCTGG",0,NA,NA,NA,"GeneID:51005","AMDHD2",TRUE,"AMDHD2"

"7027","CCCGCAGTCCCGCCGCTCGT","chr16",2520842,"-","none","CGG","CCCGCAGTCCCGCCGCTCGTCGG",0,NA,NA,NA,"GeneID:51005","AMDHD2",TRUE,"AMDHD2"

"7028","CGCCGTGCGACAGGATCCTC","chr16",2521050,"-","none","CGG","CGCCGTGCGACAGGATCCTCCGG",0,NA,NA,NA,"GeneID:51005","AMDHD2",TRUE,"AMDHD2"

"7029","CGCCGTGCGACAGGATCCTC","chr17",68601110,"-","5:T>A,13:G>C","AGG","CGCCGAGCGACAGCATCCTCAGG",2,6,14,NA,"GeneID:54757;GeneID:101928104","FAM20A;LINC01482",TRUE,"AMDHD2"

"7030","GATCTGCACGTCGATGAATC","chr16",2520881,"-","none","CGG","GATCTGCACGTCGATGAATCCGG",0,NA,NA,NA,"GeneID:51005","AMDHD2",TRUE,"AMDHD2"

"7031","GGGTTCGGGGGTTGCCCTCG","chr16",2521023,"+","none","TGG","GGGTTCGGGGGTTGCCCTCGTGG",0,NA,NA,NA,"GeneID:51005","AMDHD2",TRUE,"AMDHD2"

"7032","TCAGCGCCCGGATCACTTCG","chr16",2527938,"-","none","TGG","TCAGCGCCCGGATCACTTCGTGG",0,NA,NA,NA,"GeneID:51005","AMDHD2",TRUE,"AMDHD2"

"7033","AGGTGCTTACCCCTTTGAAG","chrX",11298233,"+","none","TGG","AGGTGCTTACCCCTTTGAAGTGG",0,NA,NA,NA,"GeneID:395;GeneID:265","ARHGAP6;AMELX",TRUE,"AMELX"

"7034","AGGTGCTTACCCCTTTGAAG","chrY",6868758,"-","7:G>A","TGG","AGGTGCTCACCCCTTTGAAGTGG",1,8,NA,NA,"GeneID:266","AMELY",TRUE,"AMELX"

"7035","AGGTGCTTACCCCTTTGAAG","chr3",61638359,"-","8:A>T,19:T>C","AGG","AGGTGCTTTCCCCTTTGAAAAGG",2,9,20,NA,"GeneID:5793","PTPRG",FALSE,"AMELX"

"7036","ATACCGGTGGCCTTATGCTC","chrX",11298261,"-","none","TGG","ATACCGGTGGCCTTATGCTCTGG",0,NA,NA,NA,"GeneID:395;GeneID:265","ARHGAP6;AMELX",TRUE,"AMELX"

"7037","CAACCAATGATGCCCGTTCC","chrX",11298694,"+","none","TGG","CAACCAATGATGCCCGTTCCTGG",0,NA,NA,NA,"GeneID:395;GeneID:265","ARHGAP6;AMELX",TRUE,"AMELX"

"7038","CAAGCTTCCAGAGTCAGATC","chrX",11298919,"-","none","AGG","CAAGCTTCCAGAGTCAGATCAGG",0,NA,NA,NA,"GeneID:395;GeneID:265","ARHGAP6;AMELX",TRUE,"AMELX"

"7039","CAAGCTTCCAGAGTCAGATC","chrY",6868070,"+","12:T>G,13:G>T","AGG","CAAGCTTCCAGATGCAGATCAGG",2,13,14,NA,"GeneID:266","AMELY",TRUE,"AMELX"

"7040","TAGCTGAAGTTGATATAACC","chrY",6870009,"+","none","AGG","TAGCTGAAGTTGATATAACCAGG",0,NA,NA,NA,"GeneID:266","AMELY",TRUE,"AMELX"

"7041","TAGCTGAAGTTGATATAACC","chrX",11296802,"-","none","AGG","TAGCTGAAGTTGATATAACCAGG",0,NA,NA,NA,"GeneID:395;GeneID:265","ARHGAP6;AMELX",TRUE,"AMELX"

"7042","TAGCTGAAGTTGATATAACC","chr1",237881677,"+","3:G>C,15:G>T","AGG","TAGGTGAAGTTGATAGAACCAGG",2,4,16,NA,"GeneID:100130331;GeneID:57829","LOC100130331;ZP4",FALSE,"AMELX"

"7043","TTTGTAGAACTCACATTCTC","chrX",11298095,"+","none","AGG","TTTGTAGAACTCACATTCTCAGG",0,NA,NA,NA,"GeneID:395;GeneID:265","ARHGAP6;AMELX",TRUE,"AMELX"

"7044","TTTGTAGAACTCACATTCTC","chr18",52840873,"+","13:T>C","AGG","TTTGTAGAACTCATATTCTCAGG",1,14,NA,NA,"GeneID:1630","DCC",FALSE,"AMELX"

"7045","TTTGTAGAACTCACATTCTC","chrY",6868896,"-","0:G>A","AGG","CTTGTAGAACTCACATTCTCAGG",1,1,NA,NA,"GeneID:266","AMELY",TRUE,"AMELX"

"7046","TTTGTAGAACTCACATTCTC","chr11",65227549,"+","3:C>G,8:G>A","AGG","TTTCTAGAGCTCACATTCTCAGG",2,4,9,NA,"GeneID:440044","SLC22A20",FALSE,"AMELX"

"7047","TTTGTAGAACTCACATTCTC","chr12",105400218,"-","7:G>T,19:T>G","TGG","TTTGTAGCACTCACATTCTATGG",2,8,20,NA,"","",FALSE,"AMELX"

"7048","TTTGTAGAACTCACATTCTC","chr13",85585524,"-","2:C>A,10:T>A","AGG","TTGGTAGAACACACATTCTCAGG",2,3,11,NA,"","",FALSE,"AMELX"

"7049","AGGTGCTCACCCCTTTGAAG","chrY",6868758,"-","none","TGG","AGGTGCTCACCCCTTTGAAGTGG",0,NA,NA,NA,"GeneID:266","AMELY",TRUE,"AMELY"

"7050","AGGTGCTCACCCCTTTGAAG","chrX",11298233,"+","7:T>C","TGG","AGGTGCTTACCCCTTTGAAGTGG",1,8,NA,NA,"GeneID:395;GeneID:265","ARHGAP6;AMELX",TRUE,"AMELY"

"7051","AGGTGCTCACCCCTTTGAAG","chr7",145535480,"-","2:G>C,10:T>G","TGG","AGCTGCTCACACCTTTGAAGTGG",2,3,11,NA,"","",FALSE,"AMELY"

"7052","CATACTTCCTGATCTGCATC","chrY",6868080,"-","none","TGG","CATACTTCCTGATCTGCATCTGG",0,NA,NA,NA,"GeneID:266","AMELY",TRUE,"AMELY"

"7053","CTGGTGGTCTTATCATGCTC","chrY",6868730,"+","none","TGG","CTGGTGGTCTTATCATGCTCTGG",0,NA,NA,NA,"GeneID:266","AMELY",TRUE,"AMELY"

"7054","CTGGTGGTCTTATCATGCTC","chr8",61015905,"-","10:T>A,16:T>C","AGG","CTGGTGGTCTAATCATACTCAGG",2,11,17,NA,"","",FALSE,"AMELY"

"7055","CTGGTGGTCTTATCATGCTC","chr2",20539910,"+","11:C>A,16:T>G","TGG","CTGGTGGTCTTCTCATTCTCTGG",2,12,17,NA,"","",FALSE,"AMELY"

"7056","CTGGTGGTCTTATCATGCTC","chr2",176975260,"-","8:C>G,10:T>A","TGG","CTGGTGGTGTAATCATGCTCTGG",2,9,11,NA,"","",FALSE,"AMELY"

"7057","GGGTTGGAGTCATGGATTGC","chrY",6868269,"+","none","TGG","GGGTTGGAGTCATGGATTGCTGG",0,NA,NA,NA,"GeneID:266","AMELY",TRUE,"AMELY"

"7058","TAAAAGCTACCACCTCATCC","chrX",11296772,"+","none","TGG","TAAAAGCTACCACCTCATCCTGG",0,NA,NA,NA,"GeneID:395;GeneID:265","ARHGAP6;AMELX",TRUE,"AMELY"

"7059","TAAAAGCTACCACCTCATCC","chrY",6870039,"-","none","TGG","TAAAAGCTACCACCTCATCCTGG",0,NA,NA,NA,"GeneID:266","AMELY",TRUE,"AMELY"

"7060","TAAAAGCTACCACCTCATCC","chr5",166245574,"+","7:A>T,10:A>C","TGG","TAAAAGCAACAACCTCATCCTGG",2,8,11,NA,"","",FALSE,"AMELY"

"7061","TAGCTGAAGTTGATATAACC","chrY",6870009,"+","none","AGG","TAGCTGAAGTTGATATAACCAGG",0,NA,NA,NA,"GeneID:266","AMELY",TRUE,"AMELY"

"7062","TAGCTGAAGTTGATATAACC","chrX",11296802,"-","none","AGG","TAGCTGAAGTTGATATAACCAGG",0,NA,NA,NA,"GeneID:395;GeneID:265","ARHGAP6;AMELX",TRUE,"AMELY"

"7063","TAGCTGAAGTTGATATAACC","chr1",237881677,"+","3:G>C,15:G>T","AGG","TAGGTGAAGTTGATAGAACCAGG",2,4,16,NA,"GeneID:100130331;GeneID:57829","LOC100130331;ZP4",FALSE,"AMELY"

"7064","CAGGGAGGCCTATACTCGAG","chrX",64191482,"-","none","AGG","CAGGGAGGCCTATACTCGAGAGG",0,NA,NA,NA,"GeneID:139285","AMER1",TRUE,"AMER1"

"7065","CCTCTGGAGAAGCGTTATGA","chrX",64191169,"-","none","AGG","CCTCTGGAGAAGCGTTATGAAGG",0,NA,NA,NA,"GeneID:139285","AMER1",TRUE,"AMER1"

"7066","GAGCAGCCTCCCTCGATACT","chrX",64190669,"-","none","TGG","GAGCAGCCTCCCTCGATACTTGG",0,NA,NA,NA,"GeneID:139285","AMER1",TRUE,"AMER1"

"7067","GGCCTTGGTAGAATCGACTA","chrX",64190702,"+","none","TGG","GGCCTTGGTAGAATCGACTATGG",0,NA,NA,NA,"GeneID:139285","AMER1",TRUE,"AMER1"

"7068","TACCATAGTCGATTCTACCA","chrX",64190707,"-","none","AGG","TACCATAGTCGATTCTACCAAGG",0,NA,NA,NA,"GeneID:139285","AMER1",TRUE,"AMER1"

"7069","TTAGCAGTATCCGCCGTCAC","chrX",64192749,"-","none","CGG","TTAGCAGTATCCGCCGTCACCGG",0,NA,NA,NA,"GeneID:139285","AMER1",TRUE,"AMER1"

"7070","AGCGGCCAAGGACGCGTCCT","chr13",25170160,"-","none","CGG","AGCGGCCAAGGACGCGTCCTCGG",0,NA,NA,NA,"GeneID:219287","AMER2",TRUE,"AMER2"

"7071","AGGCCGGCGGCAAACAAAAG","chr13",25171046,"-","none","CGG","AGGCCGGCGGCAAACAAAAGCGG",0,NA,NA,NA,"GeneID:219287","AMER2",TRUE,"AMER2"

"7072","CCAGTAGCCCTCGTCGCTGT","chr13",25170044,"+","none","TGG","CCAGTAGCCCTCGTCGCTGTTGG",0,NA,NA,NA,"GeneID:219287","AMER2",TRUE,"AMER2"

"7073","CGACGGACTTGCCGAGGTGC","chr13",25171282,"-","none","TGG","CGACGGACTTGCCGAGGTGCTGG",0,NA,NA,NA,"GeneID:219287","AMER2",TRUE,"AMER2"

"7074","CGGAGCTGGCGCGTCCGTGG","chr13",25171549,"-","none","GGG","CGGAGCTGGCGCGTCCGTGGGGG",0,NA,NA,NA,"GeneID:219287","AMER2",TRUE,"AMER2"

"7075","TGACTGTGCCGCCGAAACGC","chr13",25171459,"-","none","CGG","TGACTGTGCCGCCGAAACGCCGG",0,NA,NA,NA,"GeneID:219287","AMER2",TRUE,"AMER2"

"7076","ATAGTAGCCCTCGTCACTTG","chr2",130763216,"-","none","TGG","ATAGTAGCCCTCGTCACTTGTGG",0,NA,NA,NA,"GeneID:205147","AMER3",TRUE,"AMER3"

"7077","ATAGTAGCCCTCGTCACTTG","chr3",107055505,"+","12:A>G,15:T>A","GGG","ATAGTAGCCCTCATCTCTTGGGG",2,13,16,NA,"GeneID:100302640","LINC00882",FALSE,"AMER3"

"7078","ATCCGTGTCCACAAGTGACG","chr2",130763205,"+","none","AGG","ATCCGTGTCCACAAGTGACGAGG",0,NA,NA,NA,"GeneID:205147","AMER3",TRUE,"AMER3"

"7079","ATCGTCAGCTGGCTGCGCCG","chr2",130763593,"+","none","AGG","ATCGTCAGCTGGCTGCGCCGAGG",0,NA,NA,NA,"GeneID:205147","AMER3",TRUE,"AMER3"

"7080","CCAGAGCTTCGACTCGCTCA","chr2",130762794,"+","none","CGG","CCAGAGCTTCGACTCGCTCACGG",0,NA,NA,NA,"GeneID:205147","AMER3",TRUE,"AMER3"

"7081","CCGTGAGCGAGTCGAAGCTC","chr2",130762797,"-","none","TGG","CCGTGAGCGAGTCGAAGCTCTGG",0,NA,NA,NA,"GeneID:205147","AMER3",TRUE,"AMER3"

"7082","CCTGCTCGCCGGAGAGAGCA","chr2",130763799,"+","none","AGG","CCTGCTCGCCGGAGAGAGCAAGG",0,NA,NA,NA,"GeneID:205147","AMER3",TRUE,"AMER3"

"7083","AGCGAGGCCGTTAGCTGGTC","chr16",56425169,"+","none","CGG","AGCGAGGCCGTTAGCTGGTCCGG",0,NA,NA,NA,"GeneID:267","AMFR",TRUE,"AMFR"

"7084","AGTCTCTTCTTGTGACAGTG","chr16",56405226,"-","none","AGG","AGTCTCTTCTTGTGACAGTGAGG",0,NA,NA,NA,"GeneID:267","AMFR",TRUE,"AMFR"

"7085","AGTCTCTTCTTGTGACAGTG","chr1",242803980,"-","6:T>A,9:T>A","AGG","AGTCTCATCATGTGACAGTGAGG",2,7,10,NA,"","",FALSE,"AMFR"

"7086","CGCGCGGTAGGCGCTGATGA","chr16",56425222,"+","none","TGG","CGCGCGGTAGGCGCTGATGATGG",0,NA,NA,NA,"GeneID:267","AMFR",TRUE,"AMFR"

"7087","CGGCGAGCCGGACCAGCTAA","chr16",56425179,"-","none","CGG","CGGCGAGCCGGACCAGCTAACGG",0,NA,NA,NA,"GeneID:267","AMFR",TRUE,"AMFR"

"7088","TCTTCGAGTGAGTGAGAGAC","chr16",56414253,"-","none","AGG","TCTTCGAGTGAGTGAGAGACAGG",0,NA,NA,NA,"GeneID:267","AMFR",TRUE,"AMFR"

"7089","TCTTCGAGTGAGTGAGAGAC","chr13",44608922,"-","4:A>G,7:G>C","GGG","TCTTTGACTGAGTGAGAGACGGG",2,5,8,NA,"","",FALSE,"AMFR"

"7090","TGTCTCTCACTCACTCGAAG","chr16",56414252,"+","none","AGG","TGTCTCTCACTCACTCGAAGAGG",0,NA,NA,NA,"GeneID:267","AMFR",TRUE,"AMFR"

"7091","ACGACCACCGCTGCTTCACA","chr19",2250893,"+","none","CGG","ACGACCACCGCTGCTTCACACGG",0,NA,NA,NA,"GeneID:268","AMH",TRUE,"AMH"

"7092","ACTCCCGGCTGAGTACCGCC","chr19",2250848,"+","none","CGG","ACTCCCGGCTGAGTACCGCCCGG",0,NA,NA,NA,"GeneID:268","AMH",TRUE,"AMH"

"7093","AGACGCTCACGCGCCTGGTG","chr19",2251148,"+","none","CGG","AGACGCTCACGCGCCTGGTGCGG",0,NA,NA,NA,"GeneID:268","AMH",TRUE,"AMH"

"7094","CCACCGCTAACACCAGGTAG","chr19",2250677,"-","none","CGG","CCACCGCTAACACCAGGTAGCGG",0,NA,NA,NA,"GeneID:268;GeneID:100423031","AMH;MIR4321",TRUE,"AMH"

"7095","GTTAGCGGTGGACCGCCCTG","chr19",2250686,"+","none","CGG","GTTAGCGGTGGACCGCCCTGCGG",0,NA,NA,NA,"GeneID:268;GeneID:100423031","AMH;MIR4321",TRUE,"AMH"

"7096","TCCGACCGCAACCCGCGCTA","chr19",2251762,"+","none","CGG","TCCGACCGCAACCCGCGCTACGG",0,NA,NA,NA,"GeneID:268","AMH",TRUE,"AMH"

"7097","AACACACAGGTTCGCCTGTT","chr12",53424295,"-","none","TGG","AACACACAGGTTCGCCTGTTTGG",0,NA,NA,NA,"GeneID:269","AMHR2",TRUE,"AMHR2"

"7098","ATTGCAGAAGTCAGTGCCAC","chr12",53424807,"-","none","AGG","ATTGCAGAAGTCAGTGCCACAGG",0,NA,NA,NA,"GeneID:269","AMHR2",TRUE,"AMHR2"

"7099","CAGCAAGATGCTAGGGTCTT","chr12",53423927,"+","none","TGG","CAGCAAGATGCTAGGGTCTTTGG",0,NA,NA,NA,"GeneID:269","AMHR2",TRUE,"AMHR2"

"7100","GGCATTACTTCCCACAGCTG","chr12",53423957,"+","none","TGG","GGCATTACTTCCCACAGCTGTGG",0,NA,NA,NA,"GeneID:269","AMHR2",TRUE,"AMHR2"

"7101","TGCCGAGACAGTGATGAGCC","chr12",53424710,"+","none","AGG","TGCCGAGACAGTGATGAGCCAGG",0,NA,NA,NA,"GeneID:269","AMHR2",TRUE,"AMHR2"

"7102","TTTGTGCTTCCCCGCACTCC","chr12",53424328,"-","none","AGG","TTTGTGCTTCCCCGCACTCCAGG",0,NA,NA,NA,"GeneID:269","AMHR2",TRUE,"AMHR2"

"7103","CCAGAACCGCGTACACTTGA","chr11",118210639,"-","none","TGG","CCAGAACCGCGTACACTTGATGG",0,NA,NA,NA,"GeneID:120425","AMICA1",TRUE,"AMICA1"

"7104","GTCACCAGGAGAGCACGCCA","chr11",118212408,"-","none","AGG","GTCACCAGGAGAGCACGCCAAGG",0,NA,NA,NA,"GeneID:120425","AMICA1",TRUE,"AMICA1"

"7105","GTTACCTTGGCGTGCTCTCC","chr11",118212401,"+","none","TGG","GTTACCTTGGCGTGCTCTCCTGG",0,NA,NA,NA,"GeneID:120425","AMICA1",TRUE,"AMICA1"

"7106","TAGATACTTCGAGGCTCTTC","chr11",118203420,"+","none","CGG","TAGATACTTCGAGGCTCTTCCGG",0,NA,NA,NA,"GeneID:120425","AMICA1",TRUE,"AMICA1"

"7107","TGCGGAAAATGTCCCCCACC","chr11",118203556,"+","none","AGG","TGCGGAAAATGTCCCCCACCAGG",0,NA,NA,NA,"GeneID:120425","AMICA1",TRUE,"AMICA1"

"7108","TGTCCCCCATCAAGTGTACG","chr11",118210630,"+","none","CGG","TGTCCCCCATCAAGTGTACGCGG",0,NA,NA,NA,"GeneID:120425","AMICA1",TRUE,"AMICA1"

"7109","CACCCCCACCGTGACCCGAG","chr1",109508889,"-","none","AGG","CACCCCCACCGTGACCCGAGAGG",0,NA,NA,NA,"GeneID:57463","AMIGO1",TRUE,"AMIGO1"

"7110","CAGGACAGCTAACCACGGCT","chr1",109508806,"+","none","CGG","CAGGACAGCTAACCACGGCTCGG",0,NA,NA,NA,"GeneID:57463","AMIGO1",TRUE,"AMIGO1"

"7111","CCGGTGCGCCTTCGATGACA","chr1",109508443,"-","none","TGG","CCGGTGCGCCTTCGATGACATGG",0,NA,NA,NA,"GeneID:57463","AMIGO1",TRUE,"AMIGO1"

"7112","CTTCTCTGATACGCCCATTG","chr1",109507438,"-","none","TGG","CTTCTCTGATACGCCCATTGTGG",0,NA,NA,NA,"GeneID:57463","AMIGO1",TRUE,"AMIGO1"

"7113","GCCCAGTTACACAGCACTAC","chr1",109508716,"-","none","TGG","GCCCAGTTACACAGCACTACTGG",0,NA,NA,NA,"GeneID:57463","AMIGO1",TRUE,"AMIGO1"

"7114","GCTACCCAAACTAACGCTCC","chr1",109508344,"-","none","TGG","GCTACCCAAACTAACGCTCCTGG",0,NA,NA,NA,"GeneID:57463","AMIGO1",TRUE,"AMIGO1"

"7115","AAGTACCTGACGCGAGTGCC","chr12",47078183,"+","none","TGG","AAGTACCTGACGCGAGTGCCTGG",0,NA,NA,NA,"GeneID:347902","AMIGO2",TRUE,"AMIGO2"

"7116","AATAAAGCCAAGCGCACGAA","chr12",47078111,"+","none","AGG","AATAAAGCCAAGCGCACGAAAGG",0,NA,NA,NA,"GeneID:347902","AMIGO2",TRUE,"AMIGO2"

"7117","ACGATTACACCTGTCGCCTG","chr12",47078216,"-","none","TGG","ACGATTACACCTGTCGCCTGTGG",0,NA,NA,NA,"GeneID:347902","AMIGO2",TRUE,"AMIGO2"

"7118","AGGGCATCCTAAAGTCCACG","chr12",47077502,"-","none","AGG","AGGGCATCCTAAAGTCCACGAGG",0,NA,NA,NA,"GeneID:347902","AMIGO2",TRUE,"AMIGO2"

"7119","GAGAGCCCTCCAAACGCTGA","chr12",47078508,"+","none","AGG","GAGAGCCCTCCAAACGCTGAAGG",0,NA,NA,NA,"GeneID:347902","AMIGO2",TRUE,"AMIGO2"

"7120","TGACTCCAGGCACTCGCGTC","chr12",47078191,"-","none","AGG","TGACTCCAGGCACTCGCGTCAGG",0,NA,NA,NA,"GeneID:347902","AMIGO2",TRUE,"AMIGO2"

"7121","ACCGACCCACCAGCGCGTAC","chr3",49718582,"+","none","AGG","ACCGACCCACCAGCGCGTACAGG",0,NA,NA,NA,"GeneID:63891;GeneID:386724;GeneID:29925","RNF123;AMIGO3;GMPPB",TRUE,"AMIGO3"

"7122","CCAGCGCATCTAGTTCGTTG","chr3",49719164,"+","none","TGG","CCAGCGCATCTAGTTCGTTGTGG",0,NA,NA,NA,"GeneID:63891;GeneID:386724;GeneID:29925","RNF123;AMIGO3;GMPPB",TRUE,"AMIGO3"

"7123","CTCGCGCGCAAAGTCGCGCA","chr3",49718721,"+","none","CGG","CTCGCGCGCAAAGTCGCGCACGG",0,NA,NA,NA,"GeneID:63891;GeneID:386724;GeneID:29925","RNF123;AMIGO3;GMPPB",TRUE,"AMIGO3"

"7124","GGAAGAGCACCTGTACGCGC","chr3",49718594,"-","none","TGG","GGAAGAGCACCTGTACGCGCTGG",0,NA,NA,NA,"GeneID:63891;GeneID:386724;GeneID:29925","RNF123;AMIGO3;GMPPB",TRUE,"AMIGO3"

"7125","TCACGTTGTACTCGTGCGTC","chr3",49718357,"+","none","TGG","TCACGTTGTACTCGTGCGTCTGG",0,NA,NA,NA,"GeneID:63891;GeneID:386724;GeneID:29925","RNF123;AMIGO3;GMPPB",TRUE,"AMIGO3"

"7126","TGCGCGCGAGTACGTATGCT","chr3",49718711,"-","none","TGG","TGCGCGCGAGTACGTATGCTTGG",0,NA,NA,NA,"GeneID:63891;GeneID:386724;GeneID:29925","RNF123;AMIGO3;GMPPB",TRUE,"AMIGO3"

"7127","ACAGCCCACTGTTTGTAACA","chrX",110264583,"-","none","TGG","ACAGCCCACTGTTTGTAACATGG",0,NA,NA,NA,"GeneID:9949","AMMECR1",TRUE,"AMMECR1"

"7128","ACGCACCGCCACCTACCTAC","chrX",110202466,"-","none","CGG","ACGCACCGCCACCTACCTACCGG",0,NA,NA,NA,"GeneID:9949","AMMECR1",TRUE,"AMMECR1"

"7129","AGACCATAGACTCCTTATTG","chrX",110201015,"-","none","AGG","AGACCATAGACTCCTTATTGAGG",0,NA,NA,NA,"GeneID:9949","AMMECR1",TRUE,"AMMECR1"

"7130","CTCCGATTACTAATGAATTC","chrX",110200976,"-","none","AGG","CTCCGATTACTAATGAATTCAGG",0,NA,NA,NA,"GeneID:9949","AMMECR1",TRUE,"AMMECR1"

"7131","CTCCGATTACTAATGAATTC","chrX",150955855,"-","18:G>A,19:A>G","GGG","CTCCGATTACTAATGAATCTGGG",2,19,20,NA,"","",FALSE,"AMMECR1"

"7132","GGTCGAGACAAAAGATTACG","chrX",110264554,"-","none","TGG","GGTCGAGACAAAAGATTACGTGG",0,NA,NA,NA,"GeneID:9949","AMMECR1",TRUE,"AMMECR1"

"7133","TTCTTTCAGGTGGGTGTACA","chrX",110202525,"-","none","TGG","TTCTTTCAGGTGGGTGTACATGG",0,NA,NA,NA,"GeneID:9949","AMMECR1",TRUE,"AMMECR1"

"7134","TTCTTTCAGGTGGGTGTACA","chr15",91466199,"-","15:A>C,18:C>G","AGG","TTCTTTCAGGTGGGTTTAGAAGG",2,16,19,NA,"GeneID:101926928","LOC101926928",FALSE,"AMMECR1"

"7135","AGGGCTCAGCGCTCCCGATG","chr2",127873970,"+","none","CGG","AGGGCTCAGCGCTCCCGATGCGG",0,NA,NA,NA,"GeneID:83607","AMMECR1L",TRUE,"AMMECR1L"

"7136","ATTTCCCCCCCTGACCCGAG","chr2",127870890,"-","none","AGG","ATTTCCCCCCCTGACCCGAGAGG",0,NA,NA,NA,"GeneID:83607","AMMECR1L",TRUE,"AMMECR1L"

"7137","CACACGAATGAATCCCGCAT","chr2",127873987,"-","none","CGG","CACACGAATGAATCCCGCATCGG",0,NA,NA,NA,"GeneID:83607","AMMECR1L",TRUE,"AMMECR1L"

"7138","GGGCGGGACAAGCGGCTTCG","chr2",127871314,"-","none","TGG","GGGCGGGACAAGCGGCTTCGTGG",0,NA,NA,NA,"GeneID:83607","AMMECR1L",TRUE,"AMMECR1L"

"7139","TTAGTCCGCTCTTTGTGACG","chr2",127871343,"-","none","TGG","TTAGTCCGCTCTTTGTGACGTGG",0,NA,NA,NA,"GeneID:83607","AMMECR1L",TRUE,"AMMECR1L"

"7140","TTTCTATAGGTAGGGGTCCA","chr2",127869533,"-","none","TGG","TTTCTATAGGTAGGGGTCCATGG",0,NA,NA,NA,"GeneID:83607","AMMECR1L",TRUE,"AMMECR1L"

"7141","ACTTCGACGTCGCAGCCAAC","chr14",102923752,"+","none","TGG","ACTTCGACGTCGCAGCCAACTGG",0,NA,NA,NA,"GeneID:81693","AMN",TRUE,"AMN"

"7142","AGGTCACGCCGTCTCAGACA","chr14",102923957,"+","none","TGG","AGGTCACGCCGTCTCAGACATGG",0,NA,NA,NA,"GeneID:81693","AMN",TRUE,"AMN"

"7143","AGGTCACGCCGTCTCAGACA","chr8",125064658,"+","3:A>T,10:A>G","AGG","AGGACACGCCATCTCAGACAAGG",2,4,11,NA,"GeneID:9897","KIAA0196",FALSE,"AMN"

"7144","CCGAGGCGAGATGGGCGTCC","chr14",102922678,"+","none","TGG","CCGAGGCGAGATGGGCGTCCTGG",0,NA,NA,NA,"GeneID:81693","AMN",TRUE,"AMN"

"7145","CCGAGGCGAGATGGGCGTCC","chr1",44517304,"-","6:T>G,15:C>G","GGG","CCGAGGAGAGATGGGGGTCCGGG",2,7,16,NA,"GeneID:55182","RNF220",FALSE,"AMN"

"7146","CGCAGAGCTGCAGCCACAGC","chr14",102922711,"-","none","AGG","CGCAGAGCTGCAGCCACAGCAGG",0,NA,NA,NA,"GeneID:81693","AMN",TRUE,"AMN"

"7147","CGCAGAGCTGCAGCCACAGC","chr10",29394385,"-","15:G>T","CGG","CGCAGAGCTGCAGCCCCAGCCGG",1,16,NA,NA,"","",FALSE,"AMN"

"7148","CGCAGAGCTGCAGCCACAGC","chr1",179813025,"+","1:C>G,5:G>A","TGG","CCCAGGGCTGCAGCCACAGCTGG",2,2,6,NA,"GeneID:148753","FAM163A",FALSE,"AMN"

"7149","CGCAGAGCTGCAGCCACAGC","chr1",152709193,"-","1:G>C,10:C>G","AGG","CCCAGAGCTGGAGCCACAGCAGG",2,2,11,NA,"GeneID:199834","LCE4A",TRUE,"AMN"

"7150","CGCAGAGCTGCAGCCACAGC","chr16",89960009,"-","1:T>C,18:G>C","TGG","CACAGAGCTGCAGCCACACCTGG",2,2,19,NA,"GeneID:54849","DEF8",FALSE,"AMN"

"7151","CGGCCTTACCATGTCTGAGA","chr14",102923968,"-","none","CGG","CGGCCTTACCATGTCTGAGACGG",0,NA,NA,NA,"GeneID:81693","AMN",TRUE,"AMN"

"7152","TGCGACGTCGAAGTCCGTGT","chr14",102923746,"-","none","TGG","TGCGACGTCGAAGTCCGTGTTGG",0,NA,NA,NA,"GeneID:81693","AMN",TRUE,"AMN"

"7153","AAGTCCAAACTCTAGATCTA","chr12",31701974,"-","none","CGG","AAGTCCAAACTCTAGATCTACGG",0,NA,NA,NA,"GeneID:196394","AMN1",TRUE,"AMN1"

"7154","AAGTCCAAACTCTAGATCTA","chr16",26969381,"+","17:T>C,19:G>A","AGG","AAGTCCAAACTCTAGATTTGAGG",2,18,20,NA,"","",FALSE,"AMN1"

"7155","ACCTCTAATTTCTTCGCACA","chr12",31697358,"+","none","AGG","ACCTCTAATTTCTTCGCACAAGG",0,NA,NA,NA,"GeneID:196394","AMN1",TRUE,"AMN1"

"7156","ACCTTGTGCGAAGAAATTAG","chr12",31697362,"-","none","AGG","ACCTTGTGCGAAGAAATTAGAGG",0,NA,NA,NA,"GeneID:196394","AMN1",TRUE,"AMN1"

"7157","AGCTCCGTAGATCTAGAGTT","chr12",31701967,"+","none","TGG","AGCTCCGTAGATCTAGAGTTTGG",0,NA,NA,NA,"GeneID:196394","AMN1",TRUE,"AMN1"

"7158","CTGCTAAAGATCATCGATTT","chr12",31697830,"-","none","AGG","CTGCTAAAGATCATCGATTTAGG",0,NA,NA,NA,"GeneID:196394","AMN1",TRUE,"AMN1"

"7159","TGCTGCAATCTCACTGACGA","chr12",31697881,"-","none","AGG","TGCTGCAATCTCACTGACGAAGG",0,NA,NA,NA,"GeneID:196394","AMN1",TRUE,"AMN1"

"7160","AGAGGATCTCAACCATCTGC","chrX",112815425,"+","none","TGG","AGAGGATCTCAACCATCTGCTGG",0,NA,NA,NA,"GeneID:154796","AMOT",TRUE,"AMOT"

"7161","AGATCCTCTCAGACGAGAAC","chrX",112815414,"-","none","CGG","AGATCCTCTCAGACGAGAACCGG",0,NA,NA,NA,"GeneID:154796","AMOT",TRUE,"AMOT"

"7162","AGATCCTCTCAGACGAGAAC","chr21",37693563,"+","18:G>A,19:T>C","GGG","AGATCCTCTCAGACGAGAGTGGG",2,19,20,NA,"GeneID:3763","KCNJ6",FALSE,"AMOT"

"7163","CAACAAGCAGCTTGCAGAGA","chrX",112809950,"-","none","AGG","CAACAAGCAGCTTGCAGAGAAGG",0,NA,NA,NA,"GeneID:154796","AMOT",TRUE,"AMOT"

"7164","CAACAAGCAGCTTGCAGAGA","chr10",93346952,"-","16:T>C,19:G>T","AGG","CAACAAGCAGCTTGCAAAGCAGG",2,17,20,NA,"GeneID:26509","MYOF",FALSE,"AMOT"

"7165","CAACAAGCAGCTTGCAGAGA","chr1",7018594,"+","7:G>C,14:G>C","AGG","CAACAAGGAGCTTGGAGAGAAGG",2,8,15,NA,"GeneID:23261","CAMTA1",FALSE,"AMOT"

"7166","CAACAAGCAGCTTGCAGAGA","chr10",112599189,"+","3:G>C,19:T>A","AGG","CAAGAAGCAGCTTGCAGAGTAGG",2,4,20,NA,"GeneID:143187","VTI1A",FALSE,"AMOT"

"7167","CATATGCCTCCGAGACGCGC","chrX",112811356,"+","none","TGG","CATATGCCTCCGAGACGCGCTGG",0,NA,NA,NA,"GeneID:154796","AMOT",TRUE,"AMOT"

"7168","CTTCTCTGCAAGCTGCTTGT","chrX",112809948,"+","none","TGG","CTTCTCTGCAAGCTGCTTGTTGG",0,NA,NA,NA,"GeneID:154796","AMOT",TRUE,"AMOT"

"7169","CTTCTCTGCAAGCTGCTTGT","chr10",123477219,"-","8:T>G,9:C>T","AGG","CTTCTCTGAGAGCTGCTTGTAGG",2,9,10,NA,"","",FALSE,"AMOT"

"7170","GACAGAAATCCAGCGCGTCT","chrX",112811368,"-","none","CGG","GACAGAAATCCAGCGCGTCTCGG",0,NA,NA,NA,"GeneID:154796","AMOT",TRUE,"AMOT"

"7171","CCATCACCGTATTGTCCACC","chr11",94799687,"-","none","TGG","CCATCACCGTATTGTCCACCTGG",0,NA,NA,NA,"GeneID:154810","AMOTL1",TRUE,"AMOTL1"

"7172","CGTAGTAACCCTGAAGTTCC","chr11",94821778,"-","none","TGG","CGTAGTAACCCTGAAGTTCCTGG",0,NA,NA,NA,"GeneID:154810","AMOTL1",TRUE,"AMOTL1"

"7173","CTTGTCCAGCGATTCTCGCT","chr11",94830110,"-","none","TGG","CTTGTCCAGCGATTCTCGCTTGG",0,NA,NA,NA,"GeneID:154810","AMOTL1",TRUE,"AMOTL1"

"7174","GTCAGCACGCCAAGAACCGC","chr11",94799651,"+","none","AGG","GTCAGCACGCCAAGAACCGCAGG",0,NA,NA,NA,"GeneID:154810","AMOTL1",TRUE,"AMOTL1"

"7175","TACCACCAAGCGAGAATCGC","chr11",94830102,"+","none","TGG","TACCACCAAGCGAGAATCGCTGG",0,NA,NA,NA,"GeneID:154810","AMOTL1",TRUE,"AMOTL1"

"7176","TCCAGATGCCTTTGCGATTG","chr11",94821700,"+","none","TGG","TCCAGATGCCTTTGCGATTGTGG",0,NA,NA,NA,"GeneID:154810","AMOTL1",TRUE,"AMOTL1"

"7177","TCCAGATGCCTTTGCGATTG","chr12",55823333,"+","11:C>T,15:T>G","AGG","TCCAGATGCCTCTGCTATTGAGG",2,12,16,NA,"GeneID:85406","DNAJC14",FALSE,"AMOTL1"

"7178","ACTGTCCATCTTGTTCCGCA","chr3",134366322,"+","none","TGG","ACTGTCCATCTTGTTCCGCATGG",0,NA,NA,NA,"GeneID:51421","AMOTL2",TRUE,"AMOTL2"

"7179","CACCGACTTGGCAGGCCGCA","chr3",134359382,"+","none","GGG","CACCGACTTGGCAGGCCGCAGGG",0,NA,NA,NA,"GeneID:51421","AMOTL2",TRUE,"AMOTL2"

"7180","CAGAGCCTCCTCCAAGCGTG","chr3",134366360,"-","none","AGG","CAGAGCCTCCTCCAAGCGTGAGG",0,NA,NA,NA,"GeneID:51421","AMOTL2",TRUE,"AMOTL2"

"7181","CAGAGCCTCCTCCAAGCGTG","chr22",44712385,"+","12:G>C,17:A>G","TGG","CAGAGCCTCCTCGAAGCATGTGG",2,13,18,NA,"GeneID:55615;GeneID:553158","PRR5;PRR5-ARHGAP8",FALSE,"AMOTL2"

"7182","CCGTGCTGAGGGAGAATGCC","chr3",134367571,"-","none","AGG","CCGTGCTGAGGGAGAATGCCAGG",0,NA,NA,NA,"GeneID:51421","AMOTL2",TRUE,"AMOTL2"

"7183","GCCGCAGCCGCGAAAACAGA","chr3",134359359,"+","none","TGG","GCCGCAGCCGCGAAAACAGATGG",0,NA,NA,NA,"GeneID:51421","AMOTL2",TRUE,"AMOTL2"

"7184","GGAATCTGCAAATCGCCGCC","chr3",134365882,"-","none","TGG","GGAATCTGCAAATCGCCGCCTGG",0,NA,NA,NA,"GeneID:51421","AMOTL2",TRUE,"AMOTL2"

"7185","AATTAACAGTCTTCCGTCCT","chr1",114686875,"+","none","TGG","AATTAACAGTCTTCCGTCCTTGG",0,NA,NA,NA,"GeneID:270","AMPD1",TRUE,"AMPD1"

"7186","ACTGTGCTATCCTAGAATCA","chr1",114695486,"-","none","AGG","ACTGTGCTATCCTAGAATCAAGG",0,NA,NA,NA,"GeneID:270","AMPD1",TRUE,"AMPD1"

"7187","ATGCCTCTGTTCAAACTCCC","chr1",114695451,"-","none","AGG","ATGCCTCTGTTCAAACTCCCAGG",0,NA,NA,NA,"GeneID:270","AMPD1",TRUE,"AMPD1"

"7188","CCCAGAAAAAAGCGTTTCCA","chr1",114686895,"-","none","AGG","CCCAGAAAAAAGCGTTTCCAAGG",0,NA,NA,NA,"GeneID:270","AMPD1",TRUE,"AMPD1"

"7189","GCAAGCACACATATTCCATC","chr1",114688594,"-","none","TGG","GCAAGCACACATATTCCATCTGG",0,NA,NA,NA,"GeneID:270","AMPD1",TRUE,"AMPD1"

"7190","GCAAGCACACATATTCCATC","chr2",177878442,"-","0:G>C,15:A>G","AGG","CCAAGCACACATATTTCATCAGG",2,1,16,NA,"GeneID:50940","PDE11A",FALSE,"AMPD1"

"7191","GGAGATTTCCCCCTTTGATG","chr1",114688648,"-","none","TGG","GGAGATTTCCCCCTTTGATGTGG",0,NA,NA,NA,"GeneID:270","AMPD1",TRUE,"AMPD1"

"7192","ACATGGGGCCATTGATAATC","chr1",109627500,"-","none","AGG","ACATGGGGCCATTGATAATCAGG",0,NA,NA,NA,"GeneID:271","AMPD2",TRUE,"AMPD2"

"7193","CCTCCCGCAGCGTCTGTTCA","chr1",109628195,"-","none","CGG","CCTCCCGCAGCGTCTGTTCACGG",0,NA,NA,NA,"GeneID:271","AMPD2",TRUE,"AMPD2"

"7194","CCTCCCGCAGCGTCTGTTCA","chr21",39021648,"-","11:A>C,13:C>G","GGG","CCTCCCGCAGCTTGTGTTCAGGG",2,12,14,NA,"","",FALSE,"AMPD2"

"7195","CCTGGAGGAGATCGTGCACG","chr1",109628162,"+","none","TGG","CCTGGAGGAGATCGTGCACGTGG",0,NA,NA,NA,"GeneID:271","AMPD2",TRUE,"AMPD2"

"7196","GGCTGACGTCAATGTGCTGA","chr1",109627473,"+","none","TGG","GGCTGACGTCAATGTGCTGATGG",0,NA,NA,NA,"GeneID:271","AMPD2",TRUE,"AMPD2"

"7197","GGCTGACGTCAATGTGCTGA","chr17",17536113,"-","11:G>T,19:A>T","GGG","GGCTGACGTCACTGTGCTGTGGG",2,12,20,NA,"GeneID:10400","PEMT",FALSE,"AMPD2"

"7198","GGTGCCGTTCACAGACCTGC","chr1",109626724,"+","none","TGG","GGTGCCGTTCACAGACCTGCTGG",0,NA,NA,NA,"GeneID:271","AMPD2",TRUE,"AMPD2"

"7199","GGTGCCGTTCACAGACCTGC","chr2",106105957,"-","5:A>G,15:C>G","TGG","GGTGCTGTTCACAGAGCTGCTGG",2,6,16,NA,"GeneID:80146","UXS1",FALSE,"AMPD2"

"7200","GGTGTCGGGGCCCTGTTCAT","chr1",109626879,"-","none","AGG","GGTGTCGGGGCCCTGTTCATAGG",0,NA,NA,NA,"GeneID:271","AMPD2",TRUE,"AMPD2"

"7201","AGGAGAGTACTTTGCTCGGA","chr11",10495002,"+","none","TGG","AGGAGAGTACTTTGCTCGGATGG",0,NA,NA,NA,"GeneID:272","AMPD3",TRUE,"AMPD3"

"7202","AGTTGAAGAGTAACCCCCAC","chr11",10487320,"+","none","CGG","AGTTGAAGAGTAACCCCCACCGG",0,NA,NA,NA,"GeneID:272","AMPD3",TRUE,"AMPD3"

"7203","ATTCCAGAAAGTTCAGTCGC","chr11",10487252,"-","none","CGG","ATTCCAGAAAGTTCAGTCGCCGG",0,NA,NA,NA,"GeneID:272","AMPD3",TRUE,"AMPD3"

"7204","CAACTCCAAATACAACCCTG","chr11",10494930,"+","none","TGG","CAACTCCAAATACAACCCTGTGG",0,NA,NA,NA,"GeneID:272","AMPD3",TRUE,"AMPD3"

"7205","CAACTCCAAATACAACCCTG","chr3",59772813,"+","11:T>A,14:G>A","TGG","CAACTCCAAATTCAGCCCTGTGG",2,12,15,NA,"","",FALSE,"AMPD3"

"7206","CATCTACGGCCGCAGTCCTG","chr11",10495625,"+","none","AGG","CATCTACGGCCGCAGTCCTGAGG",0,NA,NA,NA,"GeneID:272","AMPD3",TRUE,"AMPD3"

"7207","CTTACTAAATCCGGGGCACC","chr11",10495718,"-","none","TGG","CTTACTAAATCCGGGGCACCTGG",0,NA,NA,NA,"GeneID:272","AMPD3",TRUE,"AMPD3"

"7208","AAATTAGGTCCTCCAAAAGC","chr7",38534998,"-","none","TGG","AAATTAGGTCCTCCAAAAGCTGG",0,NA,NA,NA,"GeneID:273","AMPH",TRUE,"AMPH"

"7209","AAATTAGGTCCTCCAAAAGC","chr7",29014075,"-","1:C>T,2:C>T","AGG","AGGTTAGGTCCTCCAAAAGCAGG",2,2,3,NA,"GeneID:54504","CPVL",FALSE,"AMPH"

"7210","AAATTAGGTCCTCCAAAAGC","chr16",22961985,"-","7:A>C,12:C>G","TGG","AAATTAGTTCCTGCAAAAGCTGG",2,8,13,NA,"","",FALSE,"AMPH"

"7211","AGACTTCAGCGAGAACTCCG","chr7",38503672,"-","none","AGG","AGACTTCAGCGAGAACTCCGAGG",0,NA,NA,NA,"GeneID:273","AMPH",TRUE,"AMPH"

"7212","AGACTTCAGCGAGAACTCCG","chr2",233446774,"+","13:G>A,15:T>C","AGG","AGACTTCAGCGAGGATTCCGAGG",2,14,16,NA,"GeneID:8527","DGKD",TRUE,"AMPH"

"7213","ATGAAGTCTATGAGCCTGAC","chr7",38494468,"-","none","TGG","ATGAAGTCTATGAGCCTGACTGG",0,NA,NA,NA,"GeneID:273","AMPH",TRUE,"AMPH"

"7214","CTTCTTGCCGTTTGAAGTTC","chr7",38534929,"+","none","TGG","CTTCTTGCCGTTTGAAGTTCTGG",0,NA,NA,NA,"GeneID:273","AMPH",TRUE,"AMPH"

"7215","CTTCTTGCCGTTTGAAGTTC","chr15",52008334,"+","6:T>G,8:G>C","TGG","CTTCTTTCGGTTTGAAGTTCTGG",2,7,9,NA,"GeneID:5597","MAPK6",FALSE,"AMPH"

"7216","TCTTCCCGCCCATACCAGTC","chr7",38494451,"+","none","AGG","TCTTCCCGCCCATACCAGTCAGG",0,NA,NA,NA,"GeneID:273","AMPH",TRUE,"AMPH"

"7217","TGATTGCTGCTAAATATCCT","chr7",38503652,"+","none","CGG","TGATTGCTGCTAAATATCCTCGG",0,NA,NA,NA,"GeneID:273","AMPH",TRUE,"AMPH"

"7218","TGATTGCTGCTAAATATCCT","chr17",64503383,"-","1:G>C,6:C>G","AGG","TCATTGGTGCTAAATATCCTAGG",2,2,7,NA,"GeneID:1655","DDX5",TRUE,"AMPH"

"7219","CCCTGCGAGTACAGTCGTCC","chr3",49417636,"-","none","AGG","CCCTGCGAGTACAGTCGTCCAGG",0,NA,NA,NA,"GeneID:275","AMT",TRUE,"AMT"

"7220","CGTCAAAGAGCGAGCAGTGC","chr3",49422120,"+","none","TGG","CGTCAAAGAGCGAGCAGTGCTGG",0,NA,NA,NA,"GeneID:275","AMT",TRUE,"AMT"

"7221","CTTCCCCAGACCAAGATACT","chr3",49421561,"-","none","TGG","CTTCCCCAGACCAAGATACTTGG",0,NA,NA,NA,"GeneID:275","AMT",TRUE,"AMT"

"7222","TAGTGACCGGGTGAAGCTGA","chr3",49421538,"-","none","TGG","TAGTGACCGGGTGAAGCTGATGG",0,NA,NA,NA,"GeneID:275","AMT",TRUE,"AMT"

"7223","TGACGTGTCTCATATGCTGC","chr3",49422105,"-","none","AGG","TGACGTGTCTCATATGCTGCAGG",0,NA,NA,NA,"GeneID:275","AMT",TRUE,"AMT"

"7224","TTGACAGGTACTGTGACTAG","chr3",49417705,"-","none","TGG","TTGACAGGTACTGTGACTAGTGG",0,NA,NA,NA,"GeneID:275","AMT",TRUE,"AMT"

"7225","AGTGTGAGCATCTGTGTTAA","chr4",70523894,"-","none","TGG","AGTGTGAGCATCTGTGTTAATGG",0,NA,NA,NA,"GeneID:401138","AMTN",TRUE,"AMTN"

"7226","CCAGACCCACCCATTGACCC","chr4",70524903,"+","none","TGG","CCAGACCCACCCATTGACCCTGG",0,NA,NA,NA,"GeneID:401138","AMTN",TRUE,"AMTN"

"7227","CCAGACCCACCCATTGACCC","chr1",22172628,"+","13:G>T,17:G>C","CGG","CCAGACCCACCCAGTGAGCCCGG",2,14,18,NA,"","",FALSE,"AMTN"

"7228","CTACAGTTAAATCCTGCTGC","chr4",70524865,"+","none","AGG","CTACAGTTAAATCCTGCTGCAGG",0,NA,NA,NA,"GeneID:401138","AMTN",TRUE,"AMTN"

"7229","TCACTTACCAGATGCAGATC","chr4",70523921,"-","none","TGG","TCACTTACCAGATGCAGATCTGG",0,NA,NA,NA,"GeneID:401138","AMTN",TRUE,"AMTN"

"7230","TCACTTACCAGATGCAGATC","chr19",48508579,"+","6:T>A,14:A>C","CGG","TCACTTTCCAGATGAAGATCCGG",2,7,15,NA,"GeneID:114783","LMTK3",FALSE,"AMTN"

"7231","TTACCTGATTTGACTGCTGT","chr4",70522822,"-","none","TGG","TTACCTGATTTGACTGCTGTTGG",0,NA,NA,NA,"GeneID:401138","AMTN",TRUE,"AMTN"

"7232","TTTGGTAGTGTTCCCTGATC","chr4",70522799,"-","none","CGG","TTTGGTAGTGTTCCCTGATCCGG",0,NA,NA,NA,"GeneID:401138","AMTN",TRUE,"AMTN"

"7233","CGTACCTCCTGGTAAATGAA","chr1",103693294,"+","none","AGG","CGTACCTCCTGGTAAATGAAAGG",0,NA,NA,NA,"GeneID:277","AMY1B",TRUE,"AMY1A"

"7234","CGTACCTCCTGGTAAATGAA","chr1",103658655,"-","none","AGG","CGTACCTCCTGGTAAATGAAAGG",0,NA,NA,NA,"GeneID:276","AMY1A",TRUE,"AMY1A"

"7235","CGTACCTCCTGGTAAATGAA","chr1",103752794,"-","none","AGG","CGTACCTCCTGGTAAATGAAAGG",0,NA,NA,NA,"","",TRUE,"AMY1A"

"7236","CGTACCTCCTGGTAAATGAA","chr1",103573923,"-","0:A>G","AGG","TGTACCTCCTGGTAAATGAAAGG",1,1,NA,NA,"GeneID:280","AMY2B",TRUE,"AMY1A"

"7237","CGTACCTCCTGGTAAATGAA","chr1",103713938,"-","0:A>G","AGG","TGTACCTCCTGGTAAATGAAAGG",1,1,NA,NA,"","",TRUE,"AMY1A"

"7238","CGTACCTCCTGGTAAATGAA","chr1",103619769,"-","0:A>G","AGG","TGTACCTCCTGGTAAATGAAAGG",1,1,NA,NA,"GeneID:279","AMY2A",TRUE,"AMY1A"

"7239","GTCAGAGATTGTCGTCTGTC","chr1",103752578,"+","none","TGG","GTCAGAGATTGTCGTCTGTCTGG",0,NA,NA,NA,"","",TRUE,"AMY1A"

"7240","GTCAGAGATTGTCGTCTGTC","chr1",103658439,"+","none","TGG","GTCAGAGATTGTCGTCTGTCTGG",0,NA,NA,NA,"GeneID:276","AMY1A",TRUE,"AMY1A"

"7241","GTCAGAGATTGTCGTCTGTC","chr1",103693510,"-","none","TGG","GTCAGAGATTGTCGTCTGTCTGG",0,NA,NA,NA,"GeneID:277","AMY1B",TRUE,"AMY1A"

"7242","GTCAGAGATTGTCGTCTGTC","chr1",103573707,"+","18:G>T,19:T>C","TGG","GTCAGAGATTGTCGTCTGGTTGG",2,19,20,NA,"GeneID:280","AMY2B",TRUE,"AMY1A"

"7243","GTCAGAGATTGTCGTCTGTC","chr1",103619553,"+","18:A>T","TGG","GTCAGAGATTGTCGTCTGACTGG",1,19,NA,NA,"GeneID:279","AMY2A",TRUE,"AMY1A"

"7244","GTCAGAGATTGTCGTCTGTC","chr1",103713722,"+","18:A>T","TGG","GTCAGAGATTGTCGTCTGACTGG",1,19,NA,NA,"","",TRUE,"AMY1A"

"7245","GTGCATAATTTATAGCTAAC","chr1",103695037,"+","none","TGG","GTGCATAATTTATAGCTAACTGG",0,NA,NA,NA,"GeneID:277","AMY1B",TRUE,"AMY1A"

"7246","GTGCATAATTTATAGCTAAC","chr1",103572178,"-","none","TGG","GTGCATAATTTATAGCTAACTGG",0,NA,NA,NA,"GeneID:280","AMY2B",TRUE,"AMY1A"

"7247","GTGCATAATTTATAGCTAAC","chr1",103618022,"-","none","TGG","GTGCATAATTTATAGCTAACTGG",0,NA,NA,NA,"GeneID:279","AMY2A",TRUE,"AMY1A"

"7248","GTGCATAATTTATAGCTAAC","chr1",103656912,"-","none","TGG","GTGCATAATTTATAGCTAACTGG",0,NA,NA,NA,"GeneID:276","AMY1A",TRUE,"AMY1A"

"7249","GTGCATAATTTATAGCTAAC","chr1",103751051,"-","none","TGG","GTGCATAATTTATAGCTAACTGG",0,NA,NA,NA,"","",TRUE,"AMY1A"

"7250","TTAAAATCCCATCCAGAATA","chr1",103694036,"+","none","TGG","TTAAAATCCCATCCAGAATATGG",0,NA,NA,NA,"GeneID:277","AMY1B",TRUE,"AMY1A"

"7251","TTAAAATCCCATCCAGAATA","chr1",103657913,"-","none","TGG","TTAAAATCCCATCCAGAATATGG",0,NA,NA,NA,"GeneID:276","AMY1A",TRUE,"AMY1A"

"7252","TTAAAATCCCATCCAGAATA","chr1",103752052,"-","none","TGG","TTAAAATCCCATCCAGAATATGG",0,NA,NA,NA,"","",TRUE,"AMY1A"

"7253","TTAAAATCCCATCCAGAATA","chr1",103573182,"-","none","TGG","TTAAAATCCCATCCAGAATATGG",0,NA,NA,NA,"GeneID:280","AMY2B",TRUE,"AMY1A"

"7254","TTAAAATCCCATCCAGAATA","chr2",160973451,"+","10:T>A,17:G>A","TGG","TTAAAATCCCTTCCAGAGTATGG",2,11,18,NA,"","",FALSE,"AMY1A"

"7255","TTAAAATCCCATCCAGAATA","chr1",103619030,"-","2:C>T","TGG","TTGAAATCCCATCCAGAATATGG",1,3,NA,NA,"GeneID:279","AMY2A",TRUE,"AMY1A"

"7256","TTAAAATCCCATCCAGAATA","chr1",103668128,"+","2:G>A","TGG","TTGAAATCCCATCCAGAATATGG",1,3,NA,NA,"","",TRUE,"AMY1A"

"7257","TTAAAATCCCATCCAGAATA","chr12",125581983,"+","7:T>C,8:T>C","GGG","TTAAAATTTCATCCAGAATAGGG",2,8,9,NA,"GeneID:114795","TMEM132B",FALSE,"AMY1A"

"7258","TTCACAACCCTTTCAGACCT","chr1",103572137,"+","none","TGG","TTCACAACCCTTTCAGACCTTGG",0,NA,NA,NA,"GeneID:280","AMY2B",TRUE,"AMY1A"

"7259","TTCACAACCCTTTCAGACCT","chr1",103656871,"+","none","TGG","TTCACAACCCTTTCAGACCTTGG",0,NA,NA,NA,"GeneID:276","AMY1A",TRUE,"AMY1A"

"7260","TTCACAACCCTTTCAGACCT","chr1",103751010,"+","none","TGG","TTCACAACCCTTTCAGACCTTGG",0,NA,NA,NA,"","",TRUE,"AMY1A"

"7261","TTCACAACCCTTTCAGACCT","chr1",103695078,"-","none","TGG","TTCACAACCCTTTCAGACCTTGG",0,NA,NA,NA,"GeneID:277","AMY1B",TRUE,"AMY1A"

"7262","TTCACAACCCTTTCAGACCT","chr1",103617981,"+","2:T>C","TGG","TTTACAACCCTTTCAGACCTTGG",1,3,NA,NA,"GeneID:279","AMY2A",TRUE,"AMY1A"

"7263","TTTCTAGGTTCGTATTTATG","chr1",103751925,"+","none","TGG","TTTCTAGGTTCGTATTTATGTGG",0,NA,NA,NA,"","",TRUE,"AMY1A"

"7264","TTTCTAGGTTCGTATTTATG","chr1",103573055,"+","none","TGG","TTTCTAGGTTCGTATTTATGTGG",0,NA,NA,NA,"GeneID:280","AMY2B",TRUE,"AMY1A"

"7265","TTTCTAGGTTCGTATTTATG","chr1",103618903,"+","none","TGG","TTTCTAGGTTCGTATTTATGTGG",0,NA,NA,NA,"GeneID:279","AMY2A",TRUE,"AMY1A"

"7266","TTTCTAGGTTCGTATTTATG","chr1",103657786,"+","none","TGG","TTTCTAGGTTCGTATTTATGTGG",0,NA,NA,NA,"GeneID:276","AMY1A",TRUE,"AMY1A"

"7267","TTTCTAGGTTCGTATTTATG","chr1",103668255,"-","none","TGG","TTTCTAGGTTCGTATTTATGTGG",0,NA,NA,NA,"","",TRUE,"AMY1A"

"7268","TTTCTAGGTTCGTATTTATG","chr1",103694163,"-","none","TGG","TTTCTAGGTTCGTATTTATGTGG",0,NA,NA,NA,"GeneID:277","AMY1B",TRUE,"AMY1A"

"7269","CGTACCTCCTGGTAAATGAA","chr1",103693294,"+","none","AGG","CGTACCTCCTGGTAAATGAAAGG",0,NA,NA,NA,"GeneID:277","AMY1B",TRUE,"AMY1B"

"7270","CGTACCTCCTGGTAAATGAA","chr1",103658655,"-","none","AGG","CGTACCTCCTGGTAAATGAAAGG",0,NA,NA,NA,"GeneID:276","AMY1A",TRUE,"AMY1B"

"7271","CGTACCTCCTGGTAAATGAA","chr1",103752794,"-","none","AGG","CGTACCTCCTGGTAAATGAAAGG",0,NA,NA,NA,"","",TRUE,"AMY1B"

"7272","CGTACCTCCTGGTAAATGAA","chr1",103573923,"-","0:A>G","AGG","TGTACCTCCTGGTAAATGAAAGG",1,1,NA,NA,"GeneID:280","AMY2B",TRUE,"AMY1B"

"7273","CGTACCTCCTGGTAAATGAA","chr1",103713938,"-","0:A>G","AGG","TGTACCTCCTGGTAAATGAAAGG",1,1,NA,NA,"","",TRUE,"AMY1B"

"7274","CGTACCTCCTGGTAAATGAA","chr1",103619769,"-","0:A>G","AGG","TGTACCTCCTGGTAAATGAAAGG",1,1,NA,NA,"GeneID:279","AMY2A",TRUE,"AMY1B"

"7275","GTCAGAGATTGTCGTCTGTC","chr1",103752578,"+","none","TGG","GTCAGAGATTGTCGTCTGTCTGG",0,NA,NA,NA,"","",TRUE,"AMY1B"

"7276","GTCAGAGATTGTCGTCTGTC","chr1",103658439,"+","none","TGG","GTCAGAGATTGTCGTCTGTCTGG",0,NA,NA,NA,"GeneID:276","AMY1A",TRUE,"AMY1B"

"7277","GTCAGAGATTGTCGTCTGTC","chr1",103693510,"-","none","TGG","GTCAGAGATTGTCGTCTGTCTGG",0,NA,NA,NA,"GeneID:277","AMY1B",TRUE,"AMY1B"

"7278","GTCAGAGATTGTCGTCTGTC","chr1",103573707,"+","18:G>T,19:T>C","TGG","GTCAGAGATTGTCGTCTGGTTGG",2,19,20,NA,"GeneID:280","AMY2B",TRUE,"AMY1B"

"7279","GTCAGAGATTGTCGTCTGTC","chr1",103619553,"+","18:A>T","TGG","GTCAGAGATTGTCGTCTGACTGG",1,19,NA,NA,"GeneID:279","AMY2A",TRUE,"AMY1B"

"7280","GTCAGAGATTGTCGTCTGTC","chr1",103713722,"+","18:A>T","TGG","GTCAGAGATTGTCGTCTGACTGG",1,19,NA,NA,"","",TRUE,"AMY1B"

"7281","GTGCATAATTTATAGCTAAC","chr1",103695037,"+","none","TGG","GTGCATAATTTATAGCTAACTGG",0,NA,NA,NA,"GeneID:277","AMY1B",TRUE,"AMY1B"

"7282","GTGCATAATTTATAGCTAAC","chr1",103572178,"-","none","TGG","GTGCATAATTTATAGCTAACTGG",0,NA,NA,NA,"GeneID:280","AMY2B",TRUE,"AMY1B"

"7283","GTGCATAATTTATAGCTAAC","chr1",103618022,"-","none","TGG","GTGCATAATTTATAGCTAACTGG",0,NA,NA,NA,"GeneID:279","AMY2A",TRUE,"AMY1B"

"7284","GTGCATAATTTATAGCTAAC","chr1",103656912,"-","none","TGG","GTGCATAATTTATAGCTAACTGG",0,NA,NA,NA,"GeneID:276","AMY1A",TRUE,"AMY1B"

"7285","GTGCATAATTTATAGCTAAC","chr1",103751051,"-","none","TGG","GTGCATAATTTATAGCTAACTGG",0,NA,NA,NA,"","",TRUE,"AMY1B"

"7286","TTAAAATCCCATCCAGAATA","chr1",103694036,"+","none","TGG","TTAAAATCCCATCCAGAATATGG",0,NA,NA,NA,"GeneID:277","AMY1B",TRUE,"AMY1B"

"7287","TTAAAATCCCATCCAGAATA","chr1",103657913,"-","none","TGG","TTAAAATCCCATCCAGAATATGG",0,NA,NA,NA,"GeneID:276","AMY1A",TRUE,"AMY1B"

"7288","TTAAAATCCCATCCAGAATA","chr1",103752052,"-","none","TGG","TTAAAATCCCATCCAGAATATGG",0,NA,NA,NA,"","",TRUE,"AMY1B"

"7289","TTAAAATCCCATCCAGAATA","chr1",103573182,"-","none","TGG","TTAAAATCCCATCCAGAATATGG",0,NA,NA,NA,"GeneID:280","AMY2B",TRUE,"AMY1B"

"7290","TTAAAATCCCATCCAGAATA","chr2",160973451,"+","10:T>A,17:G>A","TGG","TTAAAATCCCTTCCAGAGTATGG",2,11,18,NA,"","",FALSE,"AMY1B"

"7291","TTAAAATCCCATCCAGAATA","chr1",103619030,"-","2:C>T","TGG","TTGAAATCCCATCCAGAATATGG",1,3,NA,NA,"GeneID:279","AMY2A",TRUE,"AMY1B"

"7292","TTAAAATCCCATCCAGAATA","chr1",103668128,"+","2:G>A","TGG","TTGAAATCCCATCCAGAATATGG",1,3,NA,NA,"","",TRUE,"AMY1B"

"7293","TTAAAATCCCATCCAGAATA","chr12",125581983,"+","7:T>C,8:T>C","GGG","TTAAAATTTCATCCAGAATAGGG",2,8,9,NA,"GeneID:114795","TMEM132B",FALSE,"AMY1B"

"7294","TTCACAACCCTTTCAGACCT","chr1",103572137,"+","none","TGG","TTCACAACCCTTTCAGACCTTGG",0,NA,NA,NA,"GeneID:280","AMY2B",TRUE,"AMY1B"

"7295","TTCACAACCCTTTCAGACCT","chr1",103656871,"+","none","TGG","TTCACAACCCTTTCAGACCTTGG",0,NA,NA,NA,"GeneID:276","AMY1A",TRUE,"AMY1B"

"7296","TTCACAACCCTTTCAGACCT","chr1",103751010,"+","none","TGG","TTCACAACCCTTTCAGACCTTGG",0,NA,NA,NA,"","",TRUE,"AMY1B"

"7297","TTCACAACCCTTTCAGACCT","chr1",103695078,"-","none","TGG","TTCACAACCCTTTCAGACCTTGG",0,NA,NA,NA,"GeneID:277","AMY1B",TRUE,"AMY1B"

"7298","TTCACAACCCTTTCAGACCT","chr1",103617981,"+","2:T>C","TGG","TTTACAACCCTTTCAGACCTTGG",1,3,NA,NA,"GeneID:279","AMY2A",TRUE,"AMY1B"

"7299","TTTCTAGGTTCGTATTTATG","chr1",103751925,"+","none","TGG","TTTCTAGGTTCGTATTTATGTGG",0,NA,NA,NA,"","",TRUE,"AMY1B"

"7300","TTTCTAGGTTCGTATTTATG","chr1",103573055,"+","none","TGG","TTTCTAGGTTCGTATTTATGTGG",0,NA,NA,NA,"GeneID:280","AMY2B",TRUE,"AMY1B"

"7301","TTTCTAGGTTCGTATTTATG","chr1",103618903,"+","none","TGG","TTTCTAGGTTCGTATTTATGTGG",0,NA,NA,NA,"GeneID:279","AMY2A",TRUE,"AMY1B"

"7302","TTTCTAGGTTCGTATTTATG","chr1",103657786,"+","none","TGG","TTTCTAGGTTCGTATTTATGTGG",0,NA,NA,NA,"GeneID:276","AMY1A",TRUE,"AMY1B"

"7303","TTTCTAGGTTCGTATTTATG","chr1",103668255,"-","none","TGG","TTTCTAGGTTCGTATTTATGTGG",0,NA,NA,NA,"","",TRUE,"AMY1B"

"7304","TTTCTAGGTTCGTATTTATG","chr1",103694163,"-","none","TGG","TTTCTAGGTTCGTATTTATGTGG",0,NA,NA,NA,"GeneID:277","AMY1B",TRUE,"AMY1B"

"7305","AAAGCAAAATGAAGCTCTTT","chr1",103750461,"+","none","TGG","AAAGCAAAATGAAGCTCTTTTGG",0,NA,NA,NA,"","",TRUE,"AMY1C"

"7306","AAAGCAAAATGAAGCTCTTT","chr1",103656322,"+","none","TGG","AAAGCAAAATGAAGCTCTTTTGG",0,NA,NA,NA,"GeneID:276","AMY1A",TRUE,"AMY1C"

"7307","AAAGCAAAATGAAGCTCTTT","chr1",103695627,"-","none","TGG","AAAGCAAAATGAAGCTCTTTTGG",0,NA,NA,NA,"GeneID:277","AMY1B",TRUE,"AMY1C"

"7308","AAAGCAAAATGAAGCTCTTT","chr7",156241324,"-","0:A>T,1:G>T","GGG","TCAGCAAAATGAAGCTCTTTGGG",2,1,2,NA,"","",FALSE,"AMY1C"

"7309","AAAGCAAAATGAAGCTCTTT","chr13",31590167,"+","3:A>G,8:G>A","TGG","AAAACAAAGTGAAGCTCTTTTGG",2,4,9,NA,"","",FALSE,"AMY1C"

"7310","AAAGCAAAATGAAGCTCTTT","chr3",120584879,"-","4:A>G,14:A>G","GGG","AAAGTAAAATGAAGTTCTTTGGG",2,5,15,NA,"","",FALSE,"AMY1C"

"7311","AAAGCAAAATGAAGCTCTTT","chr3",83873625,"-","4:C>G,13:T>C","TGG","AAAGGAAAATGAAACTCTTTTGG",2,5,14,NA,"","",FALSE,"AMY1C"

"7312","CTGAACCCCTCCAAATCCCT","chr1",103695471,"+","none","TGG","CTGAACCCCTCCAAATCCCTTGG",0,NA,NA,NA,"GeneID:277","AMY1B",TRUE,"AMY1C"

"7313","CTGAACCCCTCCAAATCCCT","chr1",103571750,"-","none","TGG","CTGAACCCCTCCAAATCCCTTGG",0,NA,NA,NA,"GeneID:280","AMY2B",TRUE,"AMY1C"

"7314","CTGAACCCCTCCAAATCCCT","chr1",103656478,"-","none","TGG","CTGAACCCCTCCAAATCCCTTGG",0,NA,NA,NA,"GeneID:276","AMY1A",TRUE,"AMY1C"

"7315","CTGAACCCCTCCAAATCCCT","chr1",103750617,"-","none","TGG","CTGAACCCCTCCAAATCCCTTGG",0,NA,NA,NA,"","",TRUE,"AMY1C"

"7316","CTGAACCCCTCCAAATCCCT","chr10",104990571,"+","14:C>A,19:C>T","AGG","CTGAACCCCTCCAACTCCCCAGG",2,15,20,NA,"GeneID:22986","SORCS3",FALSE,"AMY1C"

"7317","GTGCATAATTTATAGCTAAC","chr1",103695037,"+","none","TGG","GTGCATAATTTATAGCTAACTGG",0,NA,NA,NA,"GeneID:277","AMY1B",TRUE,"AMY1C"

"7318","GTGCATAATTTATAGCTAAC","chr1",103572178,"-","none","TGG","GTGCATAATTTATAGCTAACTGG",0,NA,NA,NA,"GeneID:280","AMY2B",TRUE,"AMY1C"

"7319","GTGCATAATTTATAGCTAAC","chr1",103618022,"-","none","TGG","GTGCATAATTTATAGCTAACTGG",0,NA,NA,NA,"GeneID:279","AMY2A",TRUE,"AMY1C"

"7320","GTGCATAATTTATAGCTAAC","chr1",103656912,"-","none","TGG","GTGCATAATTTATAGCTAACTGG",0,NA,NA,NA,"GeneID:276","AMY1A",TRUE,"AMY1C"

"7321","GTGCATAATTTATAGCTAAC","chr1",103751051,"-","none","TGG","GTGCATAATTTATAGCTAACTGG",0,NA,NA,NA,"","",TRUE,"AMY1C"

"7322","TTAAAATCCCATCCAGAATA","chr1",103694036,"+","none","TGG","TTAAAATCCCATCCAGAATATGG",0,NA,NA,NA,"GeneID:277","AMY1B",TRUE,"AMY1C"

"7323","TTAAAATCCCATCCAGAATA","chr1",103657913,"-","none","TGG","TTAAAATCCCATCCAGAATATGG",0,NA,NA,NA,"GeneID:276","AMY1A",TRUE,"AMY1C"

"7324","TTAAAATCCCATCCAGAATA","chr1",103752052,"-","none","TGG","TTAAAATCCCATCCAGAATATGG",0,NA,NA,NA,"","",TRUE,"AMY1C"

"7325","TTAAAATCCCATCCAGAATA","chr1",103573182,"-","none","TGG","TTAAAATCCCATCCAGAATATGG",0,NA,NA,NA,"GeneID:280","AMY2B",TRUE,"AMY1C"

"7326","TTAAAATCCCATCCAGAATA","chr2",160973451,"+","10:T>A,17:G>A","TGG","TTAAAATCCCTTCCAGAGTATGG",2,11,18,NA,"","",FALSE,"AMY1C"

"7327","TTAAAATCCCATCCAGAATA","chr1",103619030,"-","2:C>T","TGG","TTGAAATCCCATCCAGAATATGG",1,3,NA,NA,"GeneID:279","AMY2A",TRUE,"AMY1C"

"7328","TTAAAATCCCATCCAGAATA","chr1",103668128,"+","2:G>A","TGG","TTGAAATCCCATCCAGAATATGG",1,3,NA,NA,"","",TRUE,"AMY1C"

"7329","TTAAAATCCCATCCAGAATA","chr12",125581983,"+","7:T>C,8:T>C","GGG","TTAAAATTTCATCCAGAATAGGG",2,8,9,NA,"GeneID:114795","TMEM132B",FALSE,"AMY1C"

"7330","TTCACAACCCTTTCAGACCT","chr1",103572137,"+","none","TGG","TTCACAACCCTTTCAGACCTTGG",0,NA,NA,NA,"GeneID:280","AMY2B",TRUE,"AMY1C"

"7331","TTCACAACCCTTTCAGACCT","chr1",103656871,"+","none","TGG","TTCACAACCCTTTCAGACCTTGG",0,NA,NA,NA,"GeneID:276","AMY1A",TRUE,"AMY1C"

"7332","TTCACAACCCTTTCAGACCT","chr1",103751010,"+","none","TGG","TTCACAACCCTTTCAGACCTTGG",0,NA,NA,NA,"","",TRUE,"AMY1C"

"7333","TTCACAACCCTTTCAGACCT","chr1",103695078,"-","none","TGG","TTCACAACCCTTTCAGACCTTGG",0,NA,NA,NA,"GeneID:277","AMY1B",TRUE,"AMY1C"

"7334","TTCACAACCCTTTCAGACCT","chr1",103617981,"+","2:T>C","TGG","TTTACAACCCTTTCAGACCTTGG",1,3,NA,NA,"GeneID:279","AMY2A",TRUE,"AMY1C"

"7335","TTTCTAGGTTCGTATTTATG","chr1",103751925,"+","none","TGG","TTTCTAGGTTCGTATTTATGTGG",0,NA,NA,NA,"","",TRUE,"AMY1C"

"7336","TTTCTAGGTTCGTATTTATG","chr1",103573055,"+","none","TGG","TTTCTAGGTTCGTATTTATGTGG",0,NA,NA,NA,"GeneID:280","AMY2B",TRUE,"AMY1C"

"7337","TTTCTAGGTTCGTATTTATG","chr1",103618903,"+","none","TGG","TTTCTAGGTTCGTATTTATGTGG",0,NA,NA,NA,"GeneID:279","AMY2A",TRUE,"AMY1C"

"7338","TTTCTAGGTTCGTATTTATG","chr1",103657786,"+","none","TGG","TTTCTAGGTTCGTATTTATGTGG",0,NA,NA,NA,"GeneID:276","AMY1A",TRUE,"AMY1C"

"7339","TTTCTAGGTTCGTATTTATG","chr1",103668255,"-","none","TGG","TTTCTAGGTTCGTATTTATGTGG",0,NA,NA,NA,"","",TRUE,"AMY1C"

"7340","TTTCTAGGTTCGTATTTATG","chr1",103694163,"-","none","TGG","TTTCTAGGTTCGTATTTATGTGG",0,NA,NA,NA,"GeneID:277","AMY1B",TRUE,"AMY1C"

"7341","GTCAGAGATTGTCGTCTGAC","chr1",103713722,"+","none","TGG","GTCAGAGATTGTCGTCTGACTGG",0,NA,NA,NA,"","",TRUE,"AMY2A"

"7342","GTCAGAGATTGTCGTCTGAC","chr1",103619553,"+","none","TGG","GTCAGAGATTGTCGTCTGACTGG",0,NA,NA,NA,"GeneID:279","AMY2A",TRUE,"AMY2A"

"7343","GTCAGAGATTGTCGTCTGAC","chr1",103693510,"-","18:A>T","TGG","GTCAGAGATTGTCGTCTGTCTGG",1,19,NA,NA,"GeneID:277","AMY1B",TRUE,"AMY2A"

"7344","GTCAGAGATTGTCGTCTGAC","chr1",103658439,"+","18:T>A","TGG","GTCAGAGATTGTCGTCTGTCTGG",1,19,NA,NA,"GeneID:276","AMY1A",TRUE,"AMY2A"

"7345","GTCAGAGATTGTCGTCTGAC","chr1",103752578,"+","18:T>A","TGG","GTCAGAGATTGTCGTCTGTCTGG",1,19,NA,NA,"","",TRUE,"AMY2A"

"7346","GTCAGAGATTGTCGTCTGAC","chr1",103573707,"+","18:G>A,19:T>C","TGG","GTCAGAGATTGTCGTCTGGTTGG",2,19,20,NA,"GeneID:280","AMY2B",TRUE,"AMY2A"

"7347","GTGCATAATTTATAGCTAAC","chr1",103695037,"+","none","TGG","GTGCATAATTTATAGCTAACTGG",0,NA,NA,NA,"GeneID:277","AMY1B",TRUE,"AMY2A"

"7348","GTGCATAATTTATAGCTAAC","chr1",103572178,"-","none","TGG","GTGCATAATTTATAGCTAACTGG",0,NA,NA,NA,"GeneID:280","AMY2B",TRUE,"AMY2A"

"7349","GTGCATAATTTATAGCTAAC","chr1",103618022,"-","none","TGG","GTGCATAATTTATAGCTAACTGG",0,NA,NA,NA,"GeneID:279","AMY2A",TRUE,"AMY2A"

"7350","GTGCATAATTTATAGCTAAC","chr1",103656912,"-","none","TGG","GTGCATAATTTATAGCTAACTGG",0,NA,NA,NA,"GeneID:276","AMY1A",TRUE,"AMY2A"

"7351","GTGCATAATTTATAGCTAAC","chr1",103751051,"-","none","TGG","GTGCATAATTTATAGCTAACTGG",0,NA,NA,NA,"","",TRUE,"AMY2A"

"7352","TGTACCTCCTGGTAAATGAA","chr1",103619769,"-","none","AGG","TGTACCTCCTGGTAAATGAAAGG",0,NA,NA,NA,"GeneID:279","AMY2A",TRUE,"AMY2A"

"7353","TGTACCTCCTGGTAAATGAA","chr1",103713938,"-","none","AGG","TGTACCTCCTGGTAAATGAAAGG",0,NA,NA,NA,"","",TRUE,"AMY2A"

"7354","TGTACCTCCTGGTAAATGAA","chr1",103573923,"-","none","AGG","TGTACCTCCTGGTAAATGAAAGG",0,NA,NA,NA,"GeneID:280","AMY2B",TRUE,"AMY2A"

"7355","TGTACCTCCTGGTAAATGAA","chr12",32680402,"-","12:T>A,19:A>T","GGG","TGTACCTCCTGGAAAATGATGGG",2,13,20,NA,"GeneID:10059","DNM1L",FALSE,"AMY2A"

"7356","TGTACCTCCTGGTAAATGAA","chr1",103752794,"-","0:G>A","AGG","CGTACCTCCTGGTAAATGAAAGG",1,1,NA,NA,"","",TRUE,"AMY2A"

"7357","TGTACCTCCTGGTAAATGAA","chr1",103658655,"-","0:G>A","AGG","CGTACCTCCTGGTAAATGAAAGG",1,1,NA,NA,"GeneID:276","AMY1A",TRUE,"AMY2A"

"7358","TGTACCTCCTGGTAAATGAA","chr1",103693294,"+","0:C>T","AGG","CGTACCTCCTGGTAAATGAAAGG",1,1,NA,NA,"GeneID:277","AMY1B",TRUE,"AMY2A"

"7359","TGTACCTCCTGGTAAATGAA","chr4",41641842,"+","5:T>C,14:C>A","AGG","TGTACTTCCTGGTACATGAAAGG",2,6,15,NA,"GeneID:22998","LIMCH1",FALSE,"AMY2A"

"7360","TTGAAATCCCATCCAGAATA","chr1",103668128,"+","none","TGG","TTGAAATCCCATCCAGAATATGG",0,NA,NA,NA,"","",TRUE,"AMY2A"

"7361","TTGAAATCCCATCCAGAATA","chr1",103619030,"-","none","TGG","TTGAAATCCCATCCAGAATATGG",0,NA,NA,NA,"GeneID:279","AMY2A",TRUE,"AMY2A"

"7362","TTGAAATCCCATCCAGAATA","chr1",103657913,"-","2:T>C","TGG","TTAAAATCCCATCCAGAATATGG",1,3,NA,NA,"GeneID:276","AMY1A",TRUE,"AMY2A"

"7363","TTGAAATCCCATCCAGAATA","chr1",103573182,"-","2:T>C","TGG","TTAAAATCCCATCCAGAATATGG",1,3,NA,NA,"GeneID:280","AMY2B",TRUE,"AMY2A"

"7364","TTGAAATCCCATCCAGAATA","chr1",103752052,"-","2:T>C","TGG","TTAAAATCCCATCCAGAATATGG",1,3,NA,NA,"","",TRUE,"AMY2A"

"7365","TTGAAATCCCATCCAGAATA","chr1",103694036,"+","2:A>G","TGG","TTAAAATCCCATCCAGAATATGG",1,3,NA,NA,"GeneID:277","AMY1B",TRUE,"AMY2A"

"7366","TTTACAACCCTTTCAGACCT","chr1",103617981,"+","none","TGG","TTTACAACCCTTTCAGACCTTGG",0,NA,NA,NA,"GeneID:279","AMY2A",TRUE,"AMY2A"

"7367","TTTACAACCCTTTCAGACCT","chr1",103695078,"-","2:G>A","TGG","TTCACAACCCTTTCAGACCTTGG",1,3,NA,NA,"GeneID:277","AMY1B",TRUE,"AMY2A"

"7368","TTTACAACCCTTTCAGACCT","chr1",103656871,"+","2:C>T","TGG","TTCACAACCCTTTCAGACCTTGG",1,3,NA,NA,"GeneID:276","AMY1A",TRUE,"AMY2A"

"7369","TTTACAACCCTTTCAGACCT","chr1",103751010,"+","2:C>T","TGG","TTCACAACCCTTTCAGACCTTGG",1,3,NA,NA,"","",TRUE,"AMY2A"

"7370","TTTACAACCCTTTCAGACCT","chr1",103572137,"+","2:C>T","TGG","TTCACAACCCTTTCAGACCTTGG",1,3,NA,NA,"GeneID:280","AMY2B",TRUE,"AMY2A"

"7371","TTTCTAGGTTCGTATTTATG","chr1",103751925,"+","none","TGG","TTTCTAGGTTCGTATTTATGTGG",0,NA,NA,NA,"","",TRUE,"AMY2A"

"7372","TTTCTAGGTTCGTATTTATG","chr1",103573055,"+","none","TGG","TTTCTAGGTTCGTATTTATGTGG",0,NA,NA,NA,"GeneID:280","AMY2B",TRUE,"AMY2A"

"7373","TTTCTAGGTTCGTATTTATG","chr1",103618903,"+","none","TGG","TTTCTAGGTTCGTATTTATGTGG",0,NA,NA,NA,"GeneID:279","AMY2A",TRUE,"AMY2A"

"7374","TTTCTAGGTTCGTATTTATG","chr1",103657786,"+","none","TGG","TTTCTAGGTTCGTATTTATGTGG",0,NA,NA,NA,"GeneID:276","AMY1A",TRUE,"AMY2A"

"7375","TTTCTAGGTTCGTATTTATG","chr1",103668255,"-","none","TGG","TTTCTAGGTTCGTATTTATGTGG",0,NA,NA,NA,"","",TRUE,"AMY2A"

"7376","TTTCTAGGTTCGTATTTATG","chr1",103694163,"-","none","TGG","TTTCTAGGTTCGTATTTATGTGG",0,NA,NA,NA,"GeneID:277","AMY1B",TRUE,"AMY2A"

"7377","ACAGGTCAGAGATTGTCGTC","chr1",103573703,"+","none","TGG","ACAGGTCAGAGATTGTCGTCTGG",0,NA,NA,NA,"GeneID:280","AMY2B",TRUE,"AMY2B"

"7378","ACAGGTCAGAGATTGTCGTC","chr6",116832481,"+","12:G>T,17:C>G","TGG","ACAGGTCAGAGAGTGTCCTCTGG",2,13,18,NA,"","",FALSE,"AMY2B"

"7379","ATGAATCATCTCATTGACAT","chr1",103573785,"+","none","TGG","ATGAATCATCTCATTGACATTGG",0,NA,NA,NA,"GeneID:280","AMY2B",TRUE,"AMY2B"

"7380","ATGAATCATCTCATTGACAT","chr1",103693432,"-","5:G>A","TGG","ATGAACCATCTCATTGACATTGG",1,6,NA,NA,"GeneID:277","AMY1B",TRUE,"AMY2B"

"7381","ATGAATCATCTCATTGACAT","chr1",103619631,"+","5:C>T","TGG","ATGAACCATCTCATTGACATTGG",1,6,NA,NA,"GeneID:279","AMY2A",TRUE,"AMY2B"

"7382","ATGAATCATCTCATTGACAT","chr1",103713800,"+","5:C>T","TGG","ATGAACCATCTCATTGACATTGG",1,6,NA,NA,"","",TRUE,"AMY2B"

"7383","ATGAATCATCTCATTGACAT","chr1",103658517,"+","5:C>T","TGG","ATGAACCATCTCATTGACATTGG",1,6,NA,NA,"GeneID:276","AMY1A",TRUE,"AMY2B"

"7384","ATGAATCATCTCATTGACAT","chr1",103752656,"+","5:C>T","TGG","ATGAACCATCTCATTGACATTGG",1,6,NA,NA,"","",TRUE,"AMY2B"

"7385","ATGAATCATCTCATTGACAT","chr10",49052928,"+","8:C>T,10:A>T","AGG","ATGAATCACCACATTGACATAGG",2,9,11,NA,"GeneID:196740","VSTM4",FALSE,"AMY2B"

"7386","GCTGTAATTAATCATATGTC","chr1",103573080,"+","none","TGG","GCTGTAATTAATCATATGTCTGG",0,NA,NA,NA,"GeneID:280","AMY2B",TRUE,"AMY2B"

"7387","GCTGTAATTAATCATATGTC","chr1",103694138,"-","19:C>G","TGG","GCTGTAATTAATCATATGTGTGG",1,20,NA,NA,"GeneID:277","AMY1B",TRUE,"AMY2B"

"7388","GCTGTAATTAATCATATGTC","chr1",103668230,"-","19:C>G","TGG","GCTGTAATTAATCATATGTGTGG",1,20,NA,NA,"","",TRUE,"AMY2B"

"7389","GCTGTAATTAATCATATGTC","chr1",103751950,"+","19:G>C","TGG","GCTGTAATTAATCATATGTGTGG",1,20,NA,NA,"","",TRUE,"AMY2B"

"7390","GCTGTAATTAATCATATGTC","chr1",103657811,"+","19:G>C","TGG","GCTGTAATTAATCATATGTGTGG",1,20,NA,NA,"GeneID:276","AMY1A",TRUE,"AMY2B"

"7391","GCTGTAATTAATCATATGTC","chr1",103618928,"+","19:G>C","TGG","GCTGTAATTAATCATATGTGTGG",1,20,NA,NA,"GeneID:279","AMY2A",TRUE,"AMY2B"

"7392","GTGCATAATTTATAGCTAAC","chr1",103695037,"+","none","TGG","GTGCATAATTTATAGCTAACTGG",0,NA,NA,NA,"GeneID:277","AMY1B",TRUE,"AMY2B"

"7393","GTGCATAATTTATAGCTAAC","chr1",103572178,"-","none","TGG","GTGCATAATTTATAGCTAACTGG",0,NA,NA,NA,"GeneID:280","AMY2B",TRUE,"AMY2B"

"7394","GTGCATAATTTATAGCTAAC","chr1",103618022,"-","none","TGG","GTGCATAATTTATAGCTAACTGG",0,NA,NA,NA,"GeneID:279","AMY2A",TRUE,"AMY2B"

"7395","GTGCATAATTTATAGCTAAC","chr1",103656912,"-","none","TGG","GTGCATAATTTATAGCTAACTGG",0,NA,NA,NA,"GeneID:276","AMY1A",TRUE,"AMY2B"

"7396","GTGCATAATTTATAGCTAAC","chr1",103751051,"-","none","TGG","GTGCATAATTTATAGCTAACTGG",0,NA,NA,NA,"","",TRUE,"AMY2B"

"7397","TTAAAATCCCATCCAGAATA","chr1",103694036,"+","none","TGG","TTAAAATCCCATCCAGAATATGG",0,NA,NA,NA,"GeneID:277","AMY1B",TRUE,"AMY2B"

"7398","TTAAAATCCCATCCAGAATA","chr1",103657913,"-","none","TGG","TTAAAATCCCATCCAGAATATGG",0,NA,NA,NA,"GeneID:276","AMY1A",TRUE,"AMY2B"

"7399","TTAAAATCCCATCCAGAATA","chr1",103752052,"-","none","TGG","TTAAAATCCCATCCAGAATATGG",0,NA,NA,NA,"","",TRUE,"AMY2B"

"7400","TTAAAATCCCATCCAGAATA","chr1",103573182,"-","none","TGG","TTAAAATCCCATCCAGAATATGG",0,NA,NA,NA,"GeneID:280","AMY2B",TRUE,"AMY2B"

"7401","TTAAAATCCCATCCAGAATA","chr2",160973451,"+","10:T>A,17:G>A","TGG","TTAAAATCCCTTCCAGAGTATGG",2,11,18,NA,"","",FALSE,"AMY2B"

"7402","TTAAAATCCCATCCAGAATA","chr1",103619030,"-","2:C>T","TGG","TTGAAATCCCATCCAGAATATGG",1,3,NA,NA,"GeneID:279","AMY2A",TRUE,"AMY2B"

"7403","TTAAAATCCCATCCAGAATA","chr1",103668128,"+","2:G>A","TGG","TTGAAATCCCATCCAGAATATGG",1,3,NA,NA,"","",TRUE,"AMY2B"

"7404","TTAAAATCCCATCCAGAATA","chr12",125581983,"+","7:T>C,8:T>C","GGG","TTAAAATTTCATCCAGAATAGGG",2,8,9,NA,"GeneID:114795","TMEM132B",FALSE,"AMY2B"

"7405","TTCACAACCCTTTCAGACCT","chr1",103572137,"+","none","TGG","TTCACAACCCTTTCAGACCTTGG",0,NA,NA,NA,"GeneID:280","AMY2B",TRUE,"AMY2B"

"7406","TTCACAACCCTTTCAGACCT","chr1",103656871,"+","none","TGG","TTCACAACCCTTTCAGACCTTGG",0,NA,NA,NA,"GeneID:276","AMY1A",TRUE,"AMY2B"

"7407","TTCACAACCCTTTCAGACCT","chr1",103751010,"+","none","TGG","TTCACAACCCTTTCAGACCTTGG",0,NA,NA,NA,"","",TRUE,"AMY2B"

"7408","TTCACAACCCTTTCAGACCT","chr1",103695078,"-","none","TGG","TTCACAACCCTTTCAGACCTTGG",0,NA,NA,NA,"GeneID:277","AMY1B",TRUE,"AMY2B"

"7409","TTCACAACCCTTTCAGACCT","chr1",103617981,"+","2:T>C","TGG","TTTACAACCCTTTCAGACCTTGG",1,3,NA,NA,"GeneID:279","AMY2A",TRUE,"AMY2B"

"7410","ACGGCAGGCACTTGACGCGC","chr7",2702797,"-","none","AGG","ACGGCAGGCACTTGACGCGCAGG",0,NA,NA,NA,"GeneID:155185","AMZ1",TRUE,"AMZ1"

"7411","GCGCGTCAAGTGCCTGCCGT","chr7",2702797,"+","none","CGG","GCGCGTCAAGTGCCTGCCGTCGG",0,NA,NA,NA,"GeneID:155185","AMZ1",TRUE,"AMZ1"

"7412","GGAGCCAGTCGAAGCCCGTG","chr7",2700633,"-","none","CGG","GGAGCCAGTCGAAGCCCGTGCGG",0,NA,NA,NA,"GeneID:155185","AMZ1",TRUE,"AMZ1"

"7413","GGTACAGGTCAGACAGTGTG","chr7",2708648,"-","none","AGG","GGTACAGGTCAGACAGTGTGAGG",0,NA,NA,NA,"GeneID:155185","AMZ1",TRUE,"AMZ1"

"7414","GTCCCGTACCTATCGGCTGT","chr7",2700744,"-","none","AGG","GTCCCGTACCTATCGGCTGTAGG",0,NA,NA,NA,"GeneID:155185","AMZ1",TRUE,"AMZ1"

"7415","GTCTGACCTGTACCCCCATG","chr7",2708654,"+","none","AGG","GTCTGACCTGTACCCCCATGAGG",0,NA,NA,NA,"GeneID:155185","AMZ1",TRUE,"AMZ1"

"7416","ATCTTTACCCAAGAGACTCG","chr17",68251121,"+","none","TGG","ATCTTTACCCAAGAGACTCGTGG",0,NA,NA,NA,"GeneID:51321","AMZ2",TRUE,"AMZ2"

"7417","ATCTTTACCCAAGAGACTCG","chr17",64972570,"-","18:A>G","TGG","ATCTTTACCCAAGAGACTTGTGG",1,19,NA,NA,"GeneID:201283","AMZ2P1",TRUE,"AMZ2"

"7418","CAAATATGTGTCCGATCTCA","chr17",68255710,"-","none","TGG","CAAATATGTGTCCGATCTCATGG",0,NA,NA,NA,"GeneID:51321","AMZ2",TRUE,"AMZ2"

"7419","CATGCAAGGCTCCAACCACT","chr17",68255761,"+","none","TGG","CATGCAAGGCTCCAACCACTTGG",0,NA,NA,NA,"GeneID:51321","AMZ2",TRUE,"AMZ2"

"7420","CTTGTTACAGAAACAGGAAC","chr17",68250897,"-","none","TGG","CTTGTTACAGAAACAGGAACTGG",0,NA,NA,NA,"GeneID:51321","AMZ2",TRUE,"AMZ2"

"7421","CTTGTTACAGAAACAGGAAC","chr17",64972803,"+","6:G>A,7:T>C","TGG","CTTGTTGTAGAAACAGGAACTGG",2,7,8,NA,"GeneID:201283","AMZ2P1",TRUE,"AMZ2"

"7422","TGGGGATATTCAGCTTTGCC","chr17",68254406,"+","none","AGG","TGGGGATATTCAGCTTTGCCAGG",0,NA,NA,NA,"GeneID:51321","AMZ2",TRUE,"AMZ2"

"7423","TTTACTACTTCGATCCTGTA","chr17",68254545,"+","none","AGG","TTTACTACTTCGATCCTGTAAGG",0,NA,NA,NA,"GeneID:51321","AMZ2",TRUE,"AMZ2"

"7424","AAACAGCAATCCATATTTAG","chr2",111872673,"+","none","TGG","AAACAGCAATCCATATTTAGTGG",0,NA,NA,NA,"GeneID:64682","ANAPC1",TRUE,"ANAPC1"

"7425","AAACAGCAATCCATATTTAG","chr2",86879487,"-","5:T>C","TGG","AAACAACAATCCATATTTAGTGG",1,6,NA,NA,"GeneID:100286979","ANAPC1P1",TRUE,"ANAPC1"

"7426","AATGAATACCTGGGTGAACC","chr2",111872620,"+","none","TGG","AATGAATACCTGGGTGAACCTGG",0,NA,NA,NA,"GeneID:64682","ANAPC1",TRUE,"ANAPC1"

"7427","AATGAATACCTGGGTGAACC","chr2",86879540,"-","none","TGG","AATGAATACCTGGGTGAACCTGG",0,NA,NA,NA,"GeneID:100286979","ANAPC1P1",TRUE,"ANAPC1"

"7428","CCTGAAAGGTTGCAAATGTT","chr2",86879460,"+","none","TGG","CCTGAAAGGTTGCAAATGTTTGG",0,NA,NA,NA,"GeneID:100286979","ANAPC1P1",TRUE,"ANAPC1"

"7429","CCTGAAAGGTTGCAAATGTT","chr2",111872700,"-","none","TGG","CCTGAAAGGTTGCAAATGTTTGG",0,NA,NA,NA,"GeneID:64682","ANAPC1",TRUE,"ANAPC1"

"7430","GTGTGACTTCATTATATCAC","chr2",86878524,"+","none","AGG","GTGTGACTTCATTATATCACAGG",0,NA,NA,NA,"GeneID:100286979","ANAPC1P1",TRUE,"ANAPC1"

"7431","GTGTGACTTCATTATATCAC","chr2",111873636,"-","none","AGG","GTGTGACTTCATTATATCACAGG",0,NA,NA,NA,"GeneID:64682","ANAPC1",TRUE,"ANAPC1"

"7432","GTGTGACTTCATTATATCAC","chr20",62055409,"-","6:T>G,9:C>G","AGG","GTGTGAATTGATTATATCACAGG",2,7,10,NA,"GeneID:6874","TAF4",FALSE,"ANAPC1"

"7433","TTACCTCTGATTTGACTCTC","chr2",111864801,"+","none","CGG","TTACCTCTGATTTGACTCTCCGG",0,NA,NA,NA,"GeneID:64682","ANAPC1",TRUE,"ANAPC1"

"7434","TTACCTCTGATTTGACTCTC","chr2",86886713,"-","none","CGG","TTACCTCTGATTTGACTCTCCGG",0,NA,NA,NA,"GeneID:100286979","ANAPC1P1",TRUE,"ANAPC1"

"7435","TTACCTCTGATTTGACTCTC","chr2",106369853,"-","none","CGG","TTACCTCTGATTTGACTCTCCGG",0,NA,NA,NA,"","",TRUE,"ANAPC1"

"7436","TTCAAAATGTGCATTCTGTG","chr2",86886682,"+","none","TGG","TTCAAAATGTGCATTCTGTGTGG",0,NA,NA,NA,"GeneID:100286979","ANAPC1P1",TRUE,"ANAPC1"

"7437","TTCAAAATGTGCATTCTGTG","chr2",106369822,"+","none","TGG","TTCAAAATGTGCATTCTGTGTGG",0,NA,NA,NA,"","",TRUE,"ANAPC1"

"7438","TTCAAAATGTGCATTCTGTG","chr2",111864832,"-","none","TGG","TTCAAAATGTGCATTCTGTGTGG",0,NA,NA,NA,"GeneID:64682","ANAPC1",TRUE,"ANAPC1"

"7439","TTCAAAATGTGCATTCTGTG","chr6",77234221,"-","5:G>T,15:A>G","GGG","TTCAACATGTGCATTTTGTGGGG",2,6,16,NA,"","",FALSE,"ANAPC1"

"7440","AGCAAGATCTCAGTCAGAGT","chr4",145064605,"-","none","AGG","AGCAAGATCTCAGTCAGAGTAGG",0,NA,NA,NA,"GeneID:10393","ANAPC10",TRUE,"ANAPC10"

"7441","AGCAAGATCTCAGTCAGAGT","chr5",151828439,"-","11:A>T,13:G>A","GGG","AGCAAGATCTCTGCCAGAGTGGG",2,12,14,NA,"GeneID:2741","GLRA1",FALSE,"ANAPC10"

"7442","ATTTAGTGAACATCCAATTC","chr4",145081661,"-","none","AGG","ATTTAGTGAACATCCAATTCAGG",0,NA,NA,NA,"GeneID:10393","ANAPC10",TRUE,"ANAPC10"

"7443","CCCTTGGCAGCAACTTGAGT","chr4",144995593,"-","none","TGG","CCCTTGGCAGCAACTTGAGTTGG",0,NA,NA,NA,"GeneID:10393","ANAPC10",TRUE,"ANAPC10"

"7444","CTGACCCCAAGCAGTTGGAA","chr4",145096048,"-","none","AGG","CTGACCCCAAGCAGTTGGAAAGG",0,NA,NA,NA,"GeneID:10393","ANAPC10",TRUE,"ANAPC10"

"7445","GACATACCCGAATTTCTTGA","chr4",145064564,"+","none","AGG","GACATACCCGAATTTCTTGAAGG",0,NA,NA,NA,"GeneID:10393","ANAPC10",TRUE,"ANAPC10"

"7446","TGTATCATGAATGTACGAGT","chr4",144995517,"+","none","TGG","TGTATCATGAATGTACGAGTTGG",0,NA,NA,NA,"GeneID:10393","ANAPC10",TRUE,"ANAPC10"

"7447","ACGGCGTGGCCACTTGGCTC","chr17",81894502,"+","none","TGG","ACGGCGTGGCCACTTGGCTCTGG",0,NA,NA,NA,"GeneID:51529","ANAPC11",TRUE,"ANAPC11"

"7448","CCCTCCGTAGGCAAGGTGCC","chr17",81899909,"+","none","CGG","CCCTCCGTAGGCAAGGTGCCCGG",0,NA,NA,NA,"GeneID:51529","ANAPC11",TRUE,"ANAPC11"

"7449","CGACGACTGCCCGCTGGTGT","chr17",81899932,"+","none","GGG","CGACGACTGCCCGCTGGTGTGGG",0,NA,NA,NA,"GeneID:51529","ANAPC11",TRUE,"ANAPC11"

"7450","CGTGCATCTGAGTAGTGAAC","chr17",81899442,"-","none","TGG","CGTGCATCTGAGTAGTGAACTGG",0,NA,NA,NA,"GeneID:51529","ANAPC11",TRUE,"ANAPC11"

"7451","GATGCCACAGTTCTCATCGT","chr17",81894532,"-","none","TGG","GATGCCACAGTTCTCATCGTTGG",0,NA,NA,NA,"GeneID:51529","ANAPC11",TRUE,"ANAPC11"

"7452","TACTCAGATGCACGTCTCCT","chr17",81899448,"+","none","TGG","TACTCAGATGCACGTCTCCTTGG",0,NA,NA,NA,"GeneID:51529","ANAPC11",TRUE,"ANAPC11"

"7453","TACTCAGATGCACGTCTCCT","chr12",2037548,"+","0:A>T,12:A>C","GGG","AACTCAGATGCAAGTCTCCTGGG",2,1,13,NA,"GeneID:775","CACNA1C",FALSE,"ANAPC11"

"7454","AGAAGACAAGCTGCCTTATG","chr3",134482825,"-","none","AGG","AGAAGACAAGCTGCCTTATGAGG",0,NA,NA,NA,"GeneID:25847","ANAPC13",TRUE,"ANAPC13"

"7455","AGAAGACAAGCTGCCTTATG","chr22",24420658,"+","13:G>C,17:G>A","GGG","AGAAGACAAGCTGGCTTGTGGGG",2,14,18,NA,"GeneID:101730217;GeneID:135","SPECC1L-ADORA2A;ADORA2A",FALSE,"ANAPC13"

"7456","AGAAGACAAGCTGCCTTATG","chr1",117700164,"-","0:C>T,1:A>C","AGG","GTAAGACAAGCTGCCTTATGAGG",2,1,2,NA,"","",TRUE,"ANAPC13"

"7457","AGCCAAGCGTCAGTTTCCAA","chr3",134478580,"+","none","TGG","AGCCAAGCGTCAGTTTCCAATGG",0,NA,NA,NA,"GeneID:25847","ANAPC13",TRUE,"ANAPC13"

"7458","ATTCTCATGGAGGTACTGTA","chr3",134478610,"+","none","AGG","ATTCTCATGGAGGTACTGTAAGG",0,NA,NA,NA,"GeneID:25847","ANAPC13",TRUE,"ANAPC13"

"7459","ATTCTCATGGAGGTACTGTA","chr20",11838537,"-","0:C>T,14:C>T","AGG","GTTCTCATGGAGGTGCTGTAAGG",2,1,15,NA,"GeneID:728450","LINC00687",TRUE,"ANAPC13"

"7460","CATGAGAATGTTCCCCCCAT","chr3",134478599,"-","none","TGG","CATGAGAATGTTCCCCCCATTGG",0,NA,NA,NA,"GeneID:25847","ANAPC13",TRUE,"ANAPC13"

"7461","CATGAGAATGTTCCCCCCAT","chr8",48952949,"-","11:C>A,12:A>G","AGG","CATGAGAATGTGTCCCCCATAGG",2,12,13,NA,"","",FALSE,"ANAPC13"

"7462","CCTGAACCTGAACAAGACAA","chr3",134478686,"-","none","TGG","CCTGAACCTGAACAAGACAATGG",0,NA,NA,NA,"GeneID:25847","ANAPC13",TRUE,"ANAPC13"

"7463","GGTATTGCGACATCCTCATA","chr3",134482809,"+","none","AGG","GGTATTGCGACATCCTCATAAGG",0,NA,NA,NA,"GeneID:25847","ANAPC13",TRUE,"ANAPC13"

"7464","GGTATTGCGACATCCTCATA","chr1",117700148,"+","3:G>A,8:A>G","AGG","GGTGTTGCAACATCCTCATAAGG",2,4,9,NA,"","",TRUE,"ANAPC13"

"7465","CACAGAGTCTCAGTCACACG","chr11",72111226,"+","none","AGG","CACAGAGTCTCAGTCACACGAGG",0,NA,NA,NA,"GeneID:25906","ANAPC15",TRUE,"ANAPC15"

"7466","CACAGAGTCTCAGTCACACG","chr10",130014075,"+","1:C>A,14:A>C","GGG","CCCAGAGTCTCAGTAACACGGGG",2,2,15,NA,"","",FALSE,"ANAPC15"

"7467","CCACCAGCACTATGATGACG","chr11",72110212,"-","none","AGG","CCACCAGCACTATGATGACGAGG",0,NA,NA,NA,"GeneID:220074;GeneID:25906","LRTOMT;ANAPC15",TRUE,"ANAPC15"

"7468","CCACCAGCACTATGATGACG","chr20",61958440,"+","10:G>T,16:T>G","TGG","CCACCAGCACGATGATTACGTGG",2,11,17,NA,"GeneID:6874","TAF4",FALSE,"ANAPC15"

"7469","CCACCAGCACTATGATGACG","chr20",61958496,"+","10:G>T,16:T>G","TGG","CCACCAGCACGATGATTACGTGG",2,11,17,NA,"GeneID:6874","TAF4",FALSE,"ANAPC15"

"7470","CCCACAGCTCCAAAGCATCG","chr11",72110590,"-","none","CGG","CCCACAGCTCCAAAGCATCGCGG",0,NA,NA,NA,"GeneID:220074;GeneID:25906","LRTOMT;ANAPC15",TRUE,"ANAPC15"

"7471","TAATCTGGATCGACCCTGTG","chr11",72111203,"-","none","TGG","TAATCTGGATCGACCCTGTGTGG",0,NA,NA,NA,"GeneID:25906","ANAPC15",TRUE,"ANAPC15"

"7472","TAATCTGGATCGACCCTGTG","chrX",8856953,"-","15:A>G,19:T>C","TGG","TAATCTGGATCGACCTTGTATGG",2,16,20,NA,"","",TRUE,"ANAPC15"

"7473","TGTCTACCTAGATCATCCAC","chr11",72109873,"+","none","TGG","TGTCTACCTAGATCATCCACTGG",0,NA,NA,NA,"GeneID:220074;GeneID:25906","LRTOMT;ANAPC15",TRUE,"ANAPC15"

"7474","TGTCTACCTAGATCATCCAC","chrX",8856654,"+","1:A>G,16:T>C","TGG","TATCTACCTAGATCATTCACTGG",2,2,17,NA,"","",TRUE,"ANAPC15"

"7475","TGTCTTTCTCCGCGATGCTT","chr11",72110578,"+","none","TGG","TGTCTTTCTCCGCGATGCTTTGG",0,NA,NA,NA,"GeneID:220074;GeneID:25906","LRTOMT;ANAPC15",TRUE,"ANAPC15"

"7476","AGCTAAAAACAGATTCGCAG","chr10",72230384,"-","none","AGG","AGCTAAAAACAGATTCGCAGAGG",0,NA,NA,NA,"GeneID:119504","ANAPC16",TRUE,"ANAPC16"

"7477","AGCTGGTTTGGTAGAAGAGC","chr10",72233028,"+","none","TGG","AGCTGGTTTGGTAGAAGAGCTGG",0,NA,NA,NA,"GeneID:119504","ANAPC16",TRUE,"ANAPC16"

"7478","ATGTTTCACCTGTTTAAGCG","chr10",72230419,"-","none","TGG","ATGTTTCACCTGTTTAAGCGTGG",0,NA,NA,NA,"GeneID:119504","ANAPC16",TRUE,"ANAPC16"

"7479","GTCAGTGGAAGTTCTGTCAC","chr10",72223950,"+","none","TGG","GTCAGTGGAAGTTCTGTCACTGG",0,NA,NA,NA,"GeneID:119504","ANAPC16",TRUE,"ANAPC16"

"7480","GTCAGTGGAAGTTCTGTCAC","chr13",96035040,"-","8:A>T,13:A>G","AGG","GTCAGTGGTAGTTTTGTCACAGG",2,9,14,NA,"GeneID:55757","UGGT2",FALSE,"ANAPC16"

"7481","TAAGCCCATCGAGCAGCTGC","chr10",72233070,"+","none","TGG","TAAGCCCATCGAGCAGCTGCTGG",0,NA,NA,NA,"GeneID:119504","ANAPC16",TRUE,"ANAPC16"

"7482","TCTCAGACCTTGCCCCACCA","chr10",72223987,"+","none","CGG","TCTCAGACCTTGCCCCACCACGG",0,NA,NA,NA,"GeneID:119504","ANAPC16",TRUE,"ANAPC16"

"7483","AGCCTATCCGCCGCTACCTG","chr9",137183126,"-","none","AGG","AGCCTATCCGCCGCTACCTGAGG",0,NA,NA,NA,"GeneID:29882","ANAPC2",TRUE,"ANAPC2"

"7484","AGGAGCGCTCGTACTCGCCC","chr9",137186243,"+","none","CGG","AGGAGCGCTCGTACTCGCCCCGG",0,NA,NA,NA,"GeneID:29882","ANAPC2",TRUE,"ANAPC2"

"7485","ATGCCCGTCGCCGGTACTAC","chr9",137187581,"-","none","CGG","ATGCCCGTCGCCGGTACTACCGG",0,NA,NA,NA,"GeneID:29882","ANAPC2",TRUE,"ANAPC2"

"7486","GAGATGATCCAGCGTCTGTA","chr9",137187690,"-","none","TGG","GAGATGATCCAGCGTCTGTATGG",0,NA,NA,NA,"GeneID:29882","ANAPC2",TRUE,"ANAPC2"

"7487","GCGCGTGCTGGACCCTTCCA","chr9",137183170,"-","none","TGG","GCGCGTGCTGGACCCTTCCATGG",0,NA,NA,NA,"GeneID:29882","ANAPC2",TRUE,"ANAPC2"

"7488","GGTAGAAGAACCTTTGCACG","chr9",137184960,"+","none","TGG","GGTAGAAGAACCTTTGCACGTGG",0,NA,NA,NA,"GeneID:29882","ANAPC2",TRUE,"ANAPC2"

"7489","ACAGGTCGGAAAACGCAACA","chr4",25377428,"-","none","TGG","ACAGGTCGGAAAACGCAACATGG",0,NA,NA,NA,"GeneID:29945","ANAPC4",TRUE,"ANAPC4"

"7490","CAAAGCAATGAGATCCCGCT","chr4",25377518,"-","none","TGG","CAAAGCAATGAGATCCCGCTTGG",0,NA,NA,NA,"GeneID:29945","ANAPC4",TRUE,"ANAPC4"

"7491","CACTTCCATCCAATGCATAC","chr4",25383359,"-","none","AGG","CACTTCCATCCAATGCATACAGG",0,NA,NA,NA,"GeneID:29945","ANAPC4",TRUE,"ANAPC4"

"7492","CACTTCCATCCAATGCATAC","chr13",61776826,"+","18:C>A","TGG","CACTTCCATCCAATGCATCCTGG",1,19,NA,NA,"","",FALSE,"ANAPC4"

"7493","CACTTCCATCCAATGCATAC","chr10",107658970,"-","2:A>G,15:C>G","GGG","CATTTCCATCCAATGGATACGGG",2,3,16,NA,"","",FALSE,"ANAPC4"

"7494","CGACCTGTTTCCCATCCTTC","chr4",25377440,"+","none","CGG","CGACCTGTTTCCCATCCTTCCGG",0,NA,NA,NA,"GeneID:29945","ANAPC4",TRUE,"ANAPC4"

"7495","CTCCAGTTTCCTGTATGCAT","chr4",25383347,"+","none","TGG","CTCCAGTTTCCTGTATGCATTGG",0,NA,NA,NA,"GeneID:29945","ANAPC4",TRUE,"ANAPC4"

"7496","TCCTGGTCTGGTCGCCCAAG","chr4",25377500,"+","none","CGG","TCCTGGTCTGGTCGCCCAAGCGG",0,NA,NA,NA,"GeneID:29945","ANAPC4",TRUE,"ANAPC4"

"7497","AAGAACTTGATGTATCTGTA","chr12",121345840,"-","none","AGG","AAGAACTTGATGTATCTGTAAGG",0,NA,NA,NA,"GeneID:51433","ANAPC5",TRUE,"ANAPC5"

"7498","CCACCTCGCAGAGCTACACG","chr12",121327103,"-","none","CGG","CCACCTCGCAGAGCTACACGCGG",0,NA,NA,NA,"GeneID:51433","ANAPC5",TRUE,"ANAPC5"

"7499","GCTCCGCGTGTAGCTCTGCG","chr12",121327097,"+","none","AGG","GCTCCGCGTGTAGCTCTGCGAGG",0,NA,NA,NA,"GeneID:51433","ANAPC5",TRUE,"ANAPC5"

"7500","GGTGACGCCGTACAAGATCG","chr12",121352234,"-","none","CGG","GGTGACGCCGTACAAGATCGCGG",0,NA,NA,NA,"GeneID:51433","ANAPC5",TRUE,"ANAPC5"

"7501","GTCCTTGATGCCGAACACAT","chr12",121352256,"+","none","TGG","GTCCTTGATGCCGAACACATTGG",0,NA,NA,NA,"GeneID:51433","ANAPC5",TRUE,"ANAPC5"

"7502","GTTTCTGCGTCACATGATCT","chr12",121346007,"-","none","TGG","GTTTCTGCGTCACATGATCTTGG",0,NA,NA,NA,"GeneID:51433","ANAPC5",TRUE,"ANAPC5"

"7503","CAAGAGCAATCAGTACCATC","chr12",110387740,"-","none","TGG","CAAGAGCAATCAGTACCATCTGG",0,NA,NA,NA,"GeneID:51434","ANAPC7",TRUE,"ANAPC7"

"7504","CCTGACCAGCCTTCTTGTAC","chr12",110388580,"+","none","AGG","CCTGACCAGCCTTCTTGTACAGG",0,NA,NA,NA,"GeneID:51434","ANAPC7",TRUE,"ANAPC7"

"7505","CCTGTACAAGAAGGCTGGTC","chr12",110388583,"-","none","AGG","CCTGTACAAGAAGGCTGGTCAGG",0,NA,NA,NA,"GeneID:51434","ANAPC7",TRUE,"ANAPC7"

"7506","GATCCAAACCGTGCCTAACT","chr12",110387817,"-","none","TGG","GATCCAAACCGTGCCTAACTTGG",0,NA,NA,NA,"GeneID:51434","ANAPC7",TRUE,"ANAPC7"

"7507","GATCCCATCAAGTATAGCAA","chr12",110395124,"+","none","TGG","GATCCCATCAAGTATAGCAATGG",0,NA,NA,NA,"GeneID:51434","ANAPC7",TRUE,"ANAPC7"

"7508","TGCTGTGAGTAAGTATACCA","chr12",110396351,"-","none","TGG","TGCTGTGAGTAAGTATACCATGG",0,NA,NA,NA,"GeneID:51434","ANAPC7",TRUE,"ANAPC7"

"7509","AACGTTTCTGAACCCCGCTG","chr14",20693925,"-","none","TGG","AACGTTTCTGAACCCCGCTGTGG",0,NA,NA,NA,"GeneID:6038;GeneID:283","RNASE4;ANG",TRUE,"ANG"

"7510","ACCTCCATGTAGCTTGCAAG","chr14",20693874,"-","none","TGG","ACCTCCATGTAGCTTGCAAGTGG",0,NA,NA,NA,"GeneID:6038;GeneID:283","RNASE4;ANG",TRUE,"ANG"

"7511","ATTGACTGATCCAAGTGGAC","chr14",20693972,"-","none","AGG","ATTGACTGATCCAAGTGGACAGG",0,NA,NA,NA,"GeneID:6038;GeneID:283","RNASE4;ANG",TRUE,"ANG"

"7512","ATTGACTGATCCAAGTGGAC","chr1",30678916,"-","13:C>T,16:A>C","TGG","ATTGACTGATCCAGGTTGACTGG",2,14,17,NA,"","",FALSE,"ANG"

"7513","GGCCCGCTGGTTACGGACGA","chr14",20693998,"-","none","CGG","GGCCCGCTGGTTACGGACGACGG",0,NA,NA,NA,"GeneID:6038;GeneID:283","RNASE4;ANG",TRUE,"ANG"

"7514","GGGTCTGACCCCACCGACCC","chr14",20693611,"+","none","TGG","GGGTCTGACCCCACCGACCCTGG",0,NA,NA,NA,"GeneID:6038;GeneID:283","RNASE4;ANG",TRUE,"ANG"

"7515","TTTGTTGTTGGTCTTCGTGC","chr14",20693584,"+","none","TGG","TTTGTTGTTGGTCTTCGTGCTGG",0,NA,NA,NA,"GeneID:6038;GeneID:283","RNASE4;ANG",TRUE,"ANG"

"7516","AGGACTGGGTGTAAAACCGA","chr14",76806806,"-","none","TGG","AGGACTGGGTGTAAAACCGATGG",0,NA,NA,NA,"GeneID:23357","ANGEL1",TRUE,"ANGEL1"

"7517","CGAAGCGATAGTTCCAATTG","chr14",76807959,"+","none","AGG","CGAAGCGATAGTTCCAATTGAGG",0,NA,NA,NA,"GeneID:23357","ANGEL1",TRUE,"ANGEL1"

"7518","GCTAGTCCTTTATCTATAAG","chr14",76809447,"+","none","GGG","GCTAGTCCTTTATCTATAAGGGG",0,NA,NA,NA,"GeneID:23357","ANGEL1",TRUE,"ANGEL1"

"7519","GGAACCCTCTCTGCGAATGA","chr14",76807435,"-","none","TGG","GGAACCCTCTCTGCGAATGATGG",0,NA,NA,NA,"GeneID:23357","ANGEL1",TRUE,"ANGEL1"

"7520","TAGGTCCCCGCACAAGATGA","chr14",76806493,"+","none","TGG","TAGGTCCCCGCACAAGATGATGG",0,NA,NA,NA,"GeneID:23357","ANGEL1",TRUE,"ANGEL1"

"7521","TCGCTTCGTGAACCTCATGC","chr14",76807947,"-","none","AGG","TCGCTTCGTGAACCTCATGCAGG",0,NA,NA,NA,"GeneID:23357","ANGEL1",TRUE,"ANGEL1"

"7522","AGCAGAGATCAGGCCAAGTT","chr1",213007140,"-","none","TGG","AGCAGAGATCAGGCCAAGTTTGG",0,NA,NA,NA,"GeneID:90806","ANGEL2",TRUE,"ANGEL2"

"7523","AGCAGAGATCAGGCCAAGTT","chr2",177792606,"+","2:G>C,15:T>A","TGG","AGGAGAGATCAGGCCTAGTTTGG",2,3,16,NA,"GeneID:50940","PDE11A",FALSE,"ANGEL2"

"7524","AGCAGAGATCAGGCCAAGTT","chr1",202601864,"-","9:C>G,16:C>T","GGG","AGCAGAGATGAGGCCAGGTTGGG",2,10,17,NA,"GeneID:127833","SYT2",FALSE,"ANGEL2"

"7525","AGCAGAGATCAGGCCAAGTT","chr20",496225,"-","6:G>C,17:T>C","TGG","AGCAGACATCAGGCCAAATTTGG",2,7,18,NA,"GeneID:1457","CSNK2A1",TRUE,"ANGEL2"

"7526","CAGGTCTCCAGTTACAGTAC","chr1",213013185,"+","none","TGG","CAGGTCTCCAGTTACAGTACTGG",0,NA,NA,NA,"GeneID:90806","ANGEL2",TRUE,"ANGEL2"

"7527","CTAAAACTCCAGTGTAATAC","chr1",213008252,"+","none","TGG","CTAAAACTCCAGTGTAATACTGG",0,NA,NA,NA,"GeneID:90806","ANGEL2",TRUE,"ANGEL2"

"7528","GTACCCAGTGATTCCAAACT","chr1",213007124,"+","none","TGG","GTACCCAGTGATTCCAAACTTGG",0,NA,NA,NA,"GeneID:90806","ANGEL2",TRUE,"ANGEL2"

"7529","TAGTTACGTCATGAACGCTG","chr1",213013131,"-","none","AGG","TAGTTACGTCATGAACGCTGAGG",0,NA,NA,NA,"GeneID:90806","ANGEL2",TRUE,"ANGEL2"

"7530","TATACTTTCACAAGATTTAC","chr1",213008313,"-","none","TGG","TATACTTTCACAAGATTTACTGG",0,NA,NA,NA,"GeneID:90806","ANGEL2",TRUE,"ANGEL2"

"7531","AAGTTGCTTCTCTAGCTTGT","chr8",107336193,"+","none","AGG","AAGTTGCTTCTCTAGCTTGTAGG",0,NA,NA,NA,"GeneID:284","ANGPT1",TRUE,"ANGPT1"

"7532","CGACAGTTGCCATCGTGTTC","chr8",107497394,"+","none","TGG","CGACAGTTGCCATCGTGTTCTGG",0,NA,NA,NA,"GeneID:284","ANGPT1",TRUE,"ANGPT1"

"7533","GAACTGCATTCTGCTGTATC","chr8",107347033,"+","none","TGG","GAACTGCATTCTGCTGTATCTGG",0,NA,NA,NA,"GeneID:284","ANGPT1",TRUE,"ANGPT1"

"7534","GAGAAATCCGGTTCCACGTG","chr8",107497322,"+","none","TGG","GAGAAATCCGGTTCCACGTGTGG",0,NA,NA,NA,"GeneID:284","ANGPT1",TRUE,"ANGPT1"

"7535","GCTGACAGATGTTGAGACCC","chr8",107346943,"-","none","AGG","GCTGACAGATGTTGAGACCCAGG",0,NA,NA,NA,"GeneID:284","ANGPT1",TRUE,"ANGPT1"

"7536","GCTGACAGATGTTGAGACCC","chrX",65731954,"-","3:G>C,5:A>G","AGG","GCTCATAGATGTTGAGACCCAGG",2,4,6,NA,"GeneID:4478","MSN",TRUE,"ANGPT1"

"7537","TCGACTTGAGATACAGCTGC","chr8",107336234,"-","none","TGG","TCGACTTGAGATACAGCTGCTGG",0,NA,NA,NA,"GeneID:284","ANGPT1",TRUE,"ANGPT1"

"7538","AACGCGGAAGTTAACTGATG","chr8",6532342,"-","none","TGG","AACGCGGAAGTTAACTGATGTGG",0,NA,NA,NA,"GeneID:79648;GeneID:285","MCPH1;ANGPT2",TRUE,"ANGPT2"

"7539","AAGAACTGAATTATTCACCG","chr8",6521244,"+","none","TGG","AAGAACTGAATTATTCACCGTGG",0,NA,NA,NA,"GeneID:79648;GeneID:285","MCPH1;ANGPT2",TRUE,"ANGPT2"

"7540","AAGAACTGAATTATTCACCG","chr10",28595300,"-","2:A>C,18:T>G","TGG","AATAACTGAATTATTCACAGTGG",2,3,19,NA,"GeneID:51322","WAC",FALSE,"ANGPT2"

"7541","GACAATTATTCAGCGACGTG","chr8",6514723,"-","none","AGG","GACAATTATTCAGCGACGTGAGG",0,NA,NA,NA,"GeneID:79648;GeneID:285","MCPH1;ANGPT2",TRUE,"ANGPT2"

"7542","GAGCTGAAGTTCAAGTCTCG","chr8",6527640,"+","none","TGG","GAGCTGAAGTTCAAGTCTCGTGG",0,NA,NA,NA,"GeneID:79648;GeneID:285","MCPH1;ANGPT2",TRUE,"ANGPT2"

"7543","GAGCTGAAGTTCAAGTCTCG","chr2",234270694,"-","6:A>T,19:G>C","TGG","GAGCTGTAGTTCAAGTCTCCTGG",2,7,20,NA,"","",FALSE,"ANGPT2"

"7544","TCCGCGTTTGCTCCGCTGTT","chr8",6532354,"+","none","TGG","TCCGCGTTTGCTCCGCTGTTTGG",0,NA,NA,NA,"GeneID:79648;GeneID:285","MCPH1;ANGPT2",TRUE,"ANGPT2"

"7545","TTTGCAGGCCTACTGTGACA","chr8",6514765,"-","none","TGG","TTTGCAGGCCTACTGTGACATGG",0,NA,NA,NA,"GeneID:79648;GeneID:285","MCPH1;ANGPT2",TRUE,"ANGPT2"

"7546","AGTTGTGCCTGACACCGCGC","chr20",885170,"+","none","AGG","AGTTGTGCCTGACACCGCGCAGG",0,NA,NA,NA,"GeneID:51378","ANGPT4",TRUE,"ANGPT4"

"7547","CTTCCTGGGCTTCGTTGCAT","chr20",881170,"+","none","TGG","CTTCCTGGGCTTCGTTGCATTGG",0,NA,NA,NA,"GeneID:51378","ANGPT4",TRUE,"ANGPT4"

"7548","GATCCGCAAGCTGACCGACA","chr20",890223,"-","none","TGG","GATCCGCAAGCTGACCGACATGG",0,NA,NA,NA,"GeneID:51378","ANGPT4",TRUE,"ANGPT4"

"7549","GCCCTCACCAACATCGAGCG","chr20",885196,"-","none","CGG","GCCCTCACCAACATCGAGCGCGG",0,NA,NA,NA,"GeneID:51378","ANGPT4",TRUE,"ANGPT4"

"7550","GGGCCATCAAGACGATCTTG","chr20",890341,"-","none","AGG","GGGCCATCAAGACGATCTTGAGG",0,NA,NA,NA,"GeneID:51378","ANGPT4",TRUE,"ANGPT4"

"7551","TGTCCAATGCAACGAAGCCC","chr20",881176,"-","none","AGG","TGTCCAATGCAACGAAGCCCAGG",0,NA,NA,NA,"GeneID:51378","ANGPT4",TRUE,"ANGPT4"

"7552","AACAGCCAACAGTATACTCC","chr1",178865084,"-","none","TGG","AACAGCCAACAGTATACTCCTGG",0,NA,NA,NA,"GeneID:55103;GeneID:9068","RALGPS2;ANGPTL1",TRUE,"ANGPTL1"

"7553","AACTGCGCCCACTTTCATAA","chr1",178851294,"-","none","AGG","AACTGCGCCCACTTTCATAAAGG",0,NA,NA,NA,"GeneID:55103;GeneID:9068","RALGPS2;ANGPTL1",TRUE,"ANGPTL1"

"7554","AGATACCCTCGTGCCACAGA","chr1",178865654,"-","none","TGG","AGATACCCTCGTGCCACAGATGG",0,NA,NA,NA,"GeneID:55103;GeneID:9068","RALGPS2;ANGPTL1",TRUE,"ANGPTL1"

"7555","AGATACCCTCGTGCCACAGA","chr3",136301294,"+","9:A>C,13:G>C","TGG","AGATACCCTAGTGGCACAGATGG",2,10,14,NA,"GeneID:5096","PCCB",FALSE,"ANGPTL1"

"7556","GTATGACCCGCCTCTGTATT","chr1",178851170,"+","none","CGG","GTATGACCCGCCTCTGTATTCGG",0,NA,NA,NA,"GeneID:55103;GeneID:9068","RALGPS2;ANGPTL1",TRUE,"ANGPTL1"

"7557","TGAATAACAGTCCAACCCCC","chr1",178853645,"+","none","AGG","TGAATAACAGTCCAACCCCCAGG",0,NA,NA,NA,"GeneID:55103;GeneID:9068","RALGPS2;ANGPTL1",TRUE,"ANGPTL1"

"7558","TGAATTCTATAGACTGCGCC","chr1",178852775,"-","none","TGG","TGAATTCTATAGACTGCGCCTGG",0,NA,NA,NA,"GeneID:55103;GeneID:9068","RALGPS2;ANGPTL1",TRUE,"ANGPTL1"

"7559","TGAATTCTATAGACTGCGCC","chr11",130895929,"-","3:G>T,16:C>G","AGG","TGACTTCTATAGACTGGGCCAGG",2,4,17,NA,"GeneID:399979","SNX19",FALSE,"ANGPTL1"

"7560","ATTTACTGGCTGACGAACCA","chr9",127091872,"-","none","AGG","ATTTACTGGCTGACGAACCAAGG",0,NA,NA,NA,"GeneID:9649;GeneID:23452","RALGPS1;ANGPTL2",TRUE,"ANGPTL2"

"7561","ATTTACTGGCTGACGAACCA","chr6",64220851,"+","13:G>C,14:T>G","TGG","ATTTACTGGCTGAGTAACCATGG",2,14,15,NA,"GeneID:346007","EYS",FALSE,"ANGPTL2"

"7562","CATTGACGGCGAATACTGGC","chr9",127091906,"-","none","TGG","CATTGACGGCGAATACTGGCTGG",0,NA,NA,NA,"GeneID:9649;GeneID:23452","RALGPS1;ANGPTL2",TRUE,"ANGPTL2"

"7563","CCAACGCGTTGTCCCGCTTG","chr9",127108271,"+","none","CGG","CCAACGCGTTGTCCCGCTTGCGG",0,NA,NA,NA,"GeneID:9649;GeneID:23452","RALGPS1;ANGPTL2",TRUE,"ANGPTL2"

"7564","GCACTTACCCGACGGCTTGT","chr9",127107906,"+","none","CGG","GCACTTACCCGACGGCTTGTCGG",0,NA,NA,NA,"GeneID:9649;GeneID:23452","RALGPS1;ANGPTL2",TRUE,"ANGPTL2"

"7565","GCTACCAGGACGGAGTCTAC","chr9",127089019,"-","none","TGG","GCTACCAGGACGGAGTCTACTGG",0,NA,NA,NA,"GeneID:9649;GeneID:23452","RALGPS1;ANGPTL2",TRUE,"ANGPTL2"

"7566","GCTACCAGGACGGAGTCTAC","chr19",10092660,"-","10:A>G,13:A>T","TGG","GCTACCAGGATGGTGTCTACTGG",2,11,14,NA,"GeneID:55337;GeneID:83854","C19orf66;ANGPTL6",TRUE,"ANGPTL2"

"7567","GGTACCGCGGGGGCCATTAC","chr9",127089046,"-","none","CGG","GGTACCGCGGGGGCCATTACCGG",0,NA,NA,NA,"GeneID:9649;GeneID:23452","RALGPS1;ANGPTL2",TRUE,"ANGPTL2"

"7568","AGACTTTGTCCATAAGACGA","chr1",62597739,"+","none","AGG","AGACTTTGTCCATAAGACGAAGG",0,NA,NA,NA,"GeneID:85440;GeneID:27329","DOCK7;ANGPTL3",TRUE,"ANGPTL3"

"7569","CAAAGACCTTCTCCAGACCG","chr1",62598724,"+","none","TGG","CAAAGACCTTCTCCAGACCGTGG",0,NA,NA,NA,"GeneID:85440;GeneID:27329","DOCK7;ANGPTL3",TRUE,"ANGPTL3"

"7570","CAAAGACCTTCTCCAGACCG","chr4",105616427,"+","15:C>G,18:T>C","TGG","CAAAGACCTTCTCCACACTGTGG",2,16,19,NA,"GeneID:54848","ARHGEF38",FALSE,"ANGPTL3"

"7571","GTCTACTGTGATGTTATATC","chr1",62601860,"+","none","AGG","GTCTACTGTGATGTTATATCAGG",0,NA,NA,NA,"GeneID:85440;GeneID:27329","DOCK7;ANGPTL3",TRUE,"ANGPTL3"

"7572","TAAGACCATGTCCCAACTGA","chr1",62597718,"-","none","AGG","TAAGACCATGTCCCAACTGAAGG",0,NA,NA,NA,"GeneID:85440;GeneID:27329","DOCK7;ANGPTL3",TRUE,"ANGPTL3"

"7573","TGTACCACCATTTATAACAG","chr1",62601782,"+","none","AGG","TGTACCACCATTTATAACAGAGG",0,NA,NA,NA,"GeneID:85440;GeneID:27329","DOCK7;ANGPTL3",TRUE,"ANGPTL3"

"7574","TTGTTTATATTGGTCTTCCA","chr1",62598744,"-","none","CGG","TTGTTTATATTGGTCTTCCACGG",0,NA,NA,NA,"GeneID:85440;GeneID:27329","DOCK7;ANGPTL3",TRUE,"ANGPTL3"

"7575","AACAGCTCCTGGCAATCCCT","chr19",8369226,"-","none","GGG","AACAGCTCCTGGCAATCCCTGGG",0,NA,NA,NA,"GeneID:51129","ANGPTL4",TRUE,"ANGPTL4"

"7576","AGGCGGCTGACATTGTGAGC","chr19",8366294,"-","none","CGG","AGGCGGCTGACATTGTGAGCCGG",0,NA,NA,NA,"GeneID:51129","ANGPTL4",TRUE,"ANGPTL4"

"7577","AGGCGGCTGACATTGTGAGC","chrY",7083670,"-","3:T>G,12:C>A","AGG","AGGAGGCTGACAGTGTGAGCAGG",2,4,13,NA,"GeneID:90665","TBL1Y",FALSE,"ANGPTL4"

"7578","ATCCCCAAACCCCGCCTTGT","chr19",8371124,"-","none","AGG","ATCCCCAAACCCCGCCTTGTAGG",0,NA,NA,NA,"GeneID:51129","ANGPTL4",TRUE,"ANGPTL4"

"7579","CCAAGCCTGCCCGAAGAAAG","chr19",8366241,"+","none","AGG","CCAAGCCTGCCCGAAGAAAGAGG",0,NA,NA,NA,"GeneID:51129","ANGPTL4",TRUE,"ANGPTL4"

"7580","CCAAGCCTGCCCGAAGAAAG","chr1",99263340,"+","9:G>C,11:A>C","TGG","CCAAGCCTGGCAGAAGAAAGTGG",2,10,12,NA,"","",FALSE,"ANGPTL4"

"7581","GTGAACTGCAAGATGACCTC","chr19",8369310,"+","none","AGG","GTGAACTGCAAGATGACCTCAGG",0,NA,NA,NA,"GeneID:51129","ANGPTL4",TRUE,"ANGPTL4"

"7582","TTTGCAGATGCTGAATTCGC","chr19",8366039,"-","none","AGG","TTTGCAGATGCTGAATTCGCAGG",0,NA,NA,NA,"GeneID:51129","ANGPTL4",TRUE,"ANGPTL4"

"7583","AAGTAATGATACTGTTTGTA","chr11",101907171,"-","none","AGG","AAGTAATGATACTGTTTGTAAGG",0,NA,NA,NA,"GeneID:253935","ANGPTL5",TRUE,"ANGPTL5"

"7584","AAGTAATGATACTGTTTGTA","chrX",64543153,"-","11:A>G,14:G>A","GGG","AAGTAATGATATTGCTTGTAGGG",2,12,15,NA,"","",FALSE,"ANGPTL5"

"7585","AAGTAATGATACTGTTTGTA","chrX",65154882,"+","11:T>C,14:C>T","GGG","AAGTAATGATATTGCTTGTAGGG",2,12,15,NA,"","",FALSE,"ANGPTL5"

"7586","GAAGTACCAAAAAACTACTA","chr11",101905794,"-","none","AGG","GAAGTACCAAAAAACTACTAAGG",0,NA,NA,NA,"GeneID:253935","ANGPTL5",TRUE,"ANGPTL5"

"7587","GGATGAGCAACAAGCTTCCT","chr11",101905763,"-","none","TGG","GGATGAGCAACAAGCTTCCTTGG",0,NA,NA,NA,"GeneID:253935","ANGPTL5",TRUE,"ANGPTL5"

"7588","GGATGAGCAACAAGCTTCCT","chr12",65368008,"-","4:T>C,10:C>G","TGG","GGATAAGCAAGAAGCTTCCTTGG",2,5,11,NA,"GeneID:253827","MSRB3",FALSE,"ANGPTL5"

"7589","GGATGAGCAACAAGCTTCCT","chr8",139984007,"-","3:C>A,12:A>T","GGG","GGAGGAGCAACATGCTTCCTGGG",2,4,13,NA,"GeneID:83696","TRAPPC9",FALSE,"ANGPTL5"

"7590","GTAGTTAACATTGTAGAAGA","chr11",101907218,"-","none","TGG","GTAGTTAACATTGTAGAAGATGG",0,NA,NA,NA,"GeneID:253935","ANGPTL5",TRUE,"ANGPTL5"

"7591","TAACTGTGTACATCATTCTA","chr11",101907815,"-","none","CGG","TAACTGTGTACATCATTCTACGG",0,NA,NA,NA,"GeneID:253935","ANGPTL5",TRUE,"ANGPTL5"

"7592","TACATTTAAGAATAAGAGTG","chr11",101907867,"+","none","AGG","TACATTTAAGAATAAGAGTGAGG",0,NA,NA,NA,"GeneID:253935","ANGPTL5",TRUE,"ANGPTL5"

"7593","TACATTTAAGAATAAGAGTG","chr12",77859055,"-","1:A>T,9:T>C","AGG","TTCATTTAAAAATAAGAGTGAGG",2,2,10,NA,"GeneID:89795","NAV3",FALSE,"ANGPTL5"

"7594","TACATTTAAGAATAAGAGTG","chr2",43668332,"-","9:T>C,17:T>C","TGG","TACATTTAAAAATAAGAATGTGG",2,10,18,NA,"GeneID:130271","PLEKHH2",FALSE,"ANGPTL5"

"7595","CTGAACCATCTTGCCTCCGC","chr19",10093724,"+","none","TGG","CTGAACCATCTTGCCTCCGCTGG",0,NA,NA,NA,"GeneID:83854","ANGPTL6",TRUE,"ANGPTL6"

"7596","GCCGTCACGTAGTGTCAGTA","chr19",10093788,"-","none","TGG","GCCGTCACGTAGTGTCAGTATGG",0,NA,NA,NA,"GeneID:83854","ANGPTL6",TRUE,"ANGPTL6"

"7597","GGCCAAGCCGCAGGCGGTAG","chr19",10093435,"+","none","TGG","GGCCAAGCCGCAGGCGGTAGTGG",0,NA,NA,NA,"GeneID:83854","ANGPTL6",TRUE,"ANGPTL6"

"7598","GGCTTCGGTAGTGGCCGCCG","chr19",10092680,"+","none","TGG","GGCTTCGGTAGTGGCCGCCGTGG",0,NA,NA,NA,"GeneID:55337;GeneID:83854","C19orf66;ANGPTL6",TRUE,"ANGPTL6"

"7599","TCAGCTGATACACGGGTTCA","chr19",10093558,"+","none","AGG","TCAGCTGATACACGGGTTCAAGG",0,NA,NA,NA,"GeneID:83854","ANGPTL6",TRUE,"ANGPTL6"

"7600","TGTGGTTCCGGTCCGTCTTG","chr19",10094892,"-","none","TGG","TGTGGTTCCGGTCCGTCTTGTGG",0,NA,NA,NA,"GeneID:83854","ANGPTL6",TRUE,"ANGPTL6"

"7601","ACGGTCACTCAGACCTCCGC","chr1",11189933,"+","none","AGG","ACGGTCACTCAGACCTCCGCAGG",0,NA,NA,NA,"GeneID:2475;GeneID:10218","MTOR;ANGPTL7",TRUE,"ANGPTL7"

"7602","ACGGTCACTCAGACCTCCGC","chr19",718318,"-","8:G>A,18:G>C","GGG","ACGGTCACCCAGACCTCCCCGGG",2,9,19,NA,"GeneID:5064","PALM",FALSE,"ANGPTL7"

"7603","AGGCTGTGTTGTTATGATAC","chr1",11194579,"-","none","TGG","AGGCTGTGTTGTTATGATACTGG",0,NA,NA,NA,"GeneID:2475;GeneID:10218","MTOR;ANGPTL7",TRUE,"ANGPTL7"

"7604","AGGCTGTGTTGTTATGATAC","chrX",42984447,"+","8:A>T,12:A>T","AGG","AGGCTGTGATGTAATGATACAGG",2,9,13,NA,"","",FALSE,"ANGPTL7"

"7605","CAGAAGAACTACCGCATCTC","chr1",11192298,"+","none","TGG","CAGAAGAACTACCGCATCTCTGG",0,NA,NA,NA,"GeneID:2475;GeneID:10218","MTOR;ANGPTL7",TRUE,"ANGPTL7"

"7606","CCTCCATCTCTACACGCAGC","chr1",11193755,"-","none","CGG","CCTCCATCTCTACACGCAGCCGG",0,NA,NA,NA,"GeneID:2475;GeneID:10218","MTOR;ANGPTL7",TRUE,"ANGPTL7"

"7607","CCTCCATCTCTACACGCAGC","chr1",159807574,"-","13:G>T,15:A>C","AGG","CCTCCATCTCTACCCTCAGCAGG",2,14,16,NA,"GeneID:343413","FCRL6",FALSE,"ANGPTL7"

"7608","GGAACTACACTGGCAATGTG","chr1",11194542,"+","none","GGG","GGAACTACACTGGCAATGTGGGG",0,NA,NA,NA,"GeneID:2475;GeneID:10218","MTOR;ANGPTL7",TRUE,"ANGPTL7"

"7609","TTGTCTCCTTCTACCGGGAC","chr1",11193643,"+","none","TGG","TTGTCTCCTTCTACCGGGACTGG",0,NA,NA,NA,"GeneID:2475;GeneID:10218","MTOR;ANGPTL7",TRUE,"ANGPTL7"

"7610","CAGCTTCTCACGAACCTCTC","chr12",133227871,"+","none","TGG","CAGCTTCTCACGAACCTCTCTGG",0,NA,NA,NA,"GeneID:647589","ANHX",TRUE,"ANHX"

"7611","CCTGGCGTGCGCCCGTGTCC","chr12",133234151,"-","none","TGG","CCTGGCGTGCGCCCGTGTCCTGG",0,NA,NA,NA,"GeneID:647589","ANHX",TRUE,"ANHX"

"7612","CTGCGTTGTCCAGGAGATGC","chr12",133234178,"+","none","AGG","CTGCGTTGTCCAGGAGATGCAGG",0,NA,NA,NA,"GeneID:647589","ANHX",TRUE,"ANHX"

"7613","GAAGCTGCACAATTTCGCTG","chr12",133227858,"-","none","TGG","GAAGCTGCACAATTTCGCTGTGG",0,NA,NA,NA,"GeneID:647589","ANHX",TRUE,"ANHX"

"7614","TCCACTACCGTCTGGTCATG","chr12",133231572,"-","none","AGG","TCCACTACCGTCTGGTCATGAGG",0,NA,NA,NA,"GeneID:647589","ANHX",TRUE,"ANHX"

"7615","TCCAGAGCTGCACTAACTCC","chr12",133231598,"+","none","TGG","TCCAGAGCTGCACTAACTCCTGG",0,NA,NA,NA,"GeneID:647589","ANHX",TRUE,"ANHX"

"7616","AGATATTAACACCTGTAACC","chr8",41758037,"-","none","AGG","AGATATTAACACCTGTAACCAGG",0,NA,NA,NA,"GeneID:286","ANK1",TRUE,"ANK1"

"7617","CTCCCGTGACCGAAAGAAGA","chr8",41896356,"-","none","AGG","CTCCCGTGACCGAAAGAAGAAGG",0,NA,NA,NA,"GeneID:286","ANK1",TRUE,"ANK1"

"7618","GCGGTTTCTCCGCTTCCTGT","chr8",41896375,"+","none","TGG","GCGGTTTCTCCGCTTCCTGTTGG",0,NA,NA,NA,"GeneID:286","ANK1",TRUE,"ANK1"

"7619","GCGGTTTCTCCGCTTCCTGT","chr6",114343133,"-","14:C>A,18:A>C","GGG","GCGGTTTCTCCGCTGCCTTTGGG",2,15,19,NA,"GeneID:101927768","LOC101927768",FALSE,"ANK1"

"7620","TAATATCTACCCCATTCCGC","chr8",41758049,"+","none","AGG","TAATATCTACCCCATTCCGCAGG",0,NA,NA,NA,"GeneID:286","ANK1",TRUE,"ANK1"

"7621","TCTTTACAGAATGGGTTGAA","chr8",41734058,"-","none","TGG","TCTTTACAGAATGGGTTGAATGG",0,NA,NA,NA,"GeneID:286","ANK1",TRUE,"ANK1"

"7622","TCTTTACAGAATGGGTTGAA","chr21",30778220,"+","14:C>G,19:T>A","AGG","TCTTTACAGAATGGCTTGATAGG",2,15,20,NA,"","",FALSE,"ANK1"

"7623","TCTTTACAGAATGGGTTGAA","chr3",73988397,"-","1:A>G,7:A>T","TGG","TTTTTACTGAATGGGTTGAATGG",2,2,8,NA,"","",FALSE,"ANK1"

"7624","TTGCATCTGGCTTCTAAGGA","chr8",41734034,"-","none","AGG","TTGCATCTGGCTTCTAAGGAAGG",0,NA,NA,NA,"GeneID:286","ANK1",TRUE,"ANK1"

"7625","AATGACCACCATGTTGCAAA","chr4",112904492,"+","none","AGG","AATGACCACCATGTTGCAAAAGG",0,NA,NA,NA,"GeneID:287","ANK2",TRUE,"ANK2"

"7626","CAATATTAATGCACAGTCTC","chr4",113199087,"+","none","AGG","CAATATTAATGCACAGTCTCAGG",0,NA,NA,NA,"GeneID:287","ANK2",TRUE,"ANK2"

"7627","CGCTCTTCACATTGCATCTT","chr4",113199021,"+","none","TGG","CGCTCTTCACATTGCATCTTTGG",0,NA,NA,NA,"GeneID:287","ANK2",TRUE,"ANK2"

"7628","CTGAGACTGTGCATTAATAT","chr4",113199089,"-","none","TGG","CTGAGACTGTGCATTAATATTGG",0,NA,NA,NA,"GeneID:287","ANK2",TRUE,"ANK2"

"7629","CTGAGACTGTGCATTAATAT","chr4",93757313,"-","9:T>A,14:C>A","GGG","CTGAGACTGAGCATGAATATGGG",2,10,15,NA,"GeneID:2895","GRID2",FALSE,"ANK2"

"7630","CTTAGTGGCAGAATCCACAG","chr4",113196446,"-","none","AGG","CTTAGTGGCAGAATCCACAGAGG",0,NA,NA,NA,"GeneID:287","ANK2",TRUE,"ANK2"

"7631","CTTAGTGGCAGAATCCACAG","chr14",93973955,"+","13:G>T,14:A>C","GGG","CTTAGTGGCAGAAGACACAGGGG",2,14,15,NA,"GeneID:51676","ASB2",TRUE,"ANK2"

"7632","TGGACTCAACGCTCTCCATC","chr4",113196369,"+","none","TGG","TGGACTCAACGCTCTCCATCTGG",0,NA,NA,NA,"GeneID:287","ANK2",TRUE,"ANK2"

"7633","AGCATTGCACATCGCATCTT","chr10",60278841,"-","none","TGG","AGCATTGCACATCGCATCTTTGG",0,NA,NA,NA,"GeneID:288","ANK3",TRUE,"ANK3"

"7634","CTGAGATTGTGCATTGACAT","chr10",60278773,"+","none","TGG","CTGAGATTGTGCATTGACATTGG",0,NA,NA,NA,"GeneID:288","ANK3",TRUE,"ANK3"

"7635","CTGAGATTGTGCATTGACAT","chr10",6025833,"+","1:A>T,5:C>A","TGG","CAGAGCTTGTGCATTGACATTGG",2,2,6,NA,"GeneID:3559","IL2RA",TRUE,"ANK3"

"7636","GAAGAAACTTGACAACTTCC","chr10",60270165,"+","none","AGG","GAAGAAACTTGACAACTTCCAGG",0,NA,NA,NA,"GeneID:288","ANK3",TRUE,"ANK3"

"7637","TCCTGGGCTGCCATATACAA","chr10",60270194,"+","none","TGG","TCCTGGGCTGCCATATACAATGG",0,NA,NA,NA,"GeneID:288","ANK3",TRUE,"ANK3"

"7638","TGGTTTCACGCCATTGTATA","chr10",60270207,"-","none","TGG","TGGTTTCACGCCATTGTATATGG",0,NA,NA,NA,"GeneID:288","ANK3",TRUE,"ANK3"

"7639","TTATTCACGAAGCTCTAGAA","chr10",60615187,"-","none","AGG","TTATTCACGAAGCTCTAGAAAGG",0,NA,NA,NA,"GeneID:288","ANK3",TRUE,"ANK3"

"7640","AACAAGGAGCTGTTGCACTT","chr2",189728288,"+","none","TGG","AACAAGGAGCTGTTGCACTTTGG",0,NA,NA,NA,"GeneID:150709","ANKAR",TRUE,"ANKAR"

"7641","AACAAGGAGCTGTTGCACTT","chr11",95499002,"+","12:G>T,15:A>C","GGG","AACAAGGAGCTGGTGAACTTGGG",2,13,16,NA,"","",FALSE,"ANKAR"

"7642","AACAAGGAGCTGTTGCACTT","chr15",42355468,"-","2:C>G,6:G>C","TGG","AAGAAGCAGCTGTTGCACTTTGG",2,3,7,NA,"GeneID:2595","GANC",TRUE,"ANKAR"

"7643","ACCAGTAGGTTGATTAGAGA","chr2",189719705,"-","none","TGG","ACCAGTAGGTTGATTAGAGATGG",0,NA,NA,NA,"GeneID:150709","ANKAR",TRUE,"ANKAR"

"7644","AGGTTTGACTGATATTACAA","chr2",189689524,"+","none","AGG","AGGTTTGACTGATATTACAAAGG",0,NA,NA,NA,"GeneID:150709","ANKAR",TRUE,"ANKAR"

"7645","AGGTTTGACTGATATTACAA","chrX",99021858,"-","0:C>T,10:A>C","AGG","GGGTTTGACTTATATTACAAAGG",2,1,11,NA,"","",FALSE,"ANKAR"

"7646","ATAATCTTTGCTGTAGTCTC","chr2",189689782,"-","none","TGG","ATAATCTTTGCTGTAGTCTCTGG",0,NA,NA,NA,"GeneID:150709","ANKAR",TRUE,"ANKAR"

"7647","ATAATCTTTGCTGTAGTCTC","chr19",36911724,"+","5:T>C,15:T>G","TGG","ATAATTTTTGCTGTATTCTCTGG",2,6,16,NA,"GeneID:25850;GeneID:374899","ZNF345;ZNF829",FALSE,"ANKAR"

"7648","ATAATCTTTGCTGTAGTCTC","chr1",99916663,"-","3:C>T,11:T>A","CGG","ATAGTCTTTGCAGTAGTCTCCGG",2,4,12,NA,"GeneID:178","AGL",TRUE,"ANKAR"

"7649","GCACAGCGAGAGTGTACTTC","chr2",189719738,"-","none","AGG","GCACAGCGAGAGTGTACTTCAGG",0,NA,NA,NA,"GeneID:150709","ANKAR",TRUE,"ANKAR"

"7650","GCACAGCGAGAGTGTACTTC","chr1",43206357,"-","6:T>G,14:C>A","TGG","GCACAGAGAGAGTGGACTTCTGG",2,7,15,NA,"GeneID:10969;GeneID:149465","EBNA1BP2;CFAP57",TRUE,"ANKAR"

"7651","TCAGCTAAAATGCAGTATGT","chr2",189728398,"+","none","TGG","TCAGCTAAAATGCAGTATGTTGG",0,NA,NA,NA,"GeneID:150709","ANKAR",TRUE,"ANKAR"

"7652","TCAGCTAAAATGCAGTATGT","chr14",16038711,"+","7:C>A,18:T>G","GGG","TCAGCTACAATGCAGTATTTGGG",2,8,19,NA,"","",FALSE,"ANKAR"

"7653","TCAGCTAAAATGCAGTATGT","chr22",11268811,"-","7:G>T,18:A>C","GGG","TCAGCTACAATGCAGTATTTGGG",2,8,19,NA,"","",FALSE,"ANKAR"

"7654","ACCTGTCCACTGTTGCTCGA","chr15",64947575,"-","none","TGG","ACCTGTCCACTGTTGCTCGATGG",0,NA,NA,NA,"GeneID:348094","ANKDD1A",TRUE,"ANKDD1A"

"7655","ACGCACGTGGTTTCTGGCCC","chr15",64915886,"-","none","TGG","ACGCACGTGGTTTCTGGCCCTGG",0,NA,NA,NA,"GeneID:348094","ANKDD1A",TRUE,"ANKDD1A"

"7656","GCCGCCCGCCAGAACAATGT","chr15",64915825,"+","none","CGG","GCCGCCCGCCAGAACAATGTCGG",0,NA,NA,NA,"GeneID:348094","ANKDD1A",TRUE,"ANKDD1A"

"7657","GGCTGTGCTGCAGCGACTTG","chr15",64930865,"+","none","TGG","GGCTGTGCTGCAGCGACTTGTGG",0,NA,NA,NA,"GeneID:348094","ANKDD1A",TRUE,"ANKDD1A"

"7658","GGGGAACACTGCCCTTCATC","chr15",64930823,"+","none","TGG","GGGGAACACTGCCCTTCATCTGG",0,NA,NA,NA,"GeneID:348094","ANKDD1A",TRUE,"ANKDD1A"

"7659","TCGACGCCATCGAGCAACAG","chr15",64947566,"+","none","TGG","TCGACGCCATCGAGCAACAGTGG",0,NA,NA,NA,"GeneID:348094","ANKDD1A",TRUE,"ANKDD1A"

"7660","CAGTAATTCACCTTGCAGCC","chr5",75625656,"+","none","TGG","CAGTAATTCACCTTGCAGCCTGG",0,NA,NA,NA,"GeneID:728780","ANKDD1B",TRUE,"ANKDD1B"

"7661","CCACATACCTACCTGATTCT","chr5",75625737,"-","none","TGG","CCACATACCTACCTGATTCTTGG",0,NA,NA,NA,"GeneID:728780","ANKDD1B",TRUE,"ANKDD1B"

"7662","CTTATCAGCAACATCCACCC","chr5",75620393,"-","none","TGG","CTTATCAGCAACATCCACCCTGG",0,NA,NA,NA,"GeneID:728780","ANKDD1B",TRUE,"ANKDD1B"

"7663","CTTATCAGCAACATCCACCC","chr5",76319901,"+","14:A>C,18:T>C","AGG","CTTATCAGCAACATACACTCAGG",2,15,19,NA,"GeneID:22987","SV2C",FALSE,"ANKDD1B"

"7664","GAGCAATCATGTGCGCATCG","chr5",75625882,"+","none","TGG","GAGCAATCATGTGCGCATCGTGG",0,NA,NA,NA,"GeneID:728780","ANKDD1B",TRUE,"ANKDD1B"

"7665","TATTCAAGATCTGCACCTCA","chr5",75625912,"+","none","AGG","TATTCAAGATCTGCACCTCAAGG",0,NA,NA,NA,"GeneID:728780","ANKDD1B",TRUE,"ANKDD1B"

"7666","TATTCAAGATCTGCACCTCA","chr7",57362829,"+","15:T>C,17:C>T","AGG","TATTCAAGATCTGCATCCCAAGG",2,16,18,NA,"","",FALSE,"ANKDD1B"

"7667","TCTTGCTTAAACACAAGGCC","chr5",75620372,"+","none","AGG","TCTTGCTTAAACACAAGGCCAGG",0,NA,NA,NA,"GeneID:728780","ANKDD1B",TRUE,"ANKDD1B"

"7668","ATATGCCTTTGCAACGTCCA","chr20",10053123,"-","none","TGG","ATATGCCTTTGCAACGTCCATGG",0,NA,NA,NA,"GeneID:63926;GeneID:100131208","ANKEF1;SNAP25-AS1",TRUE,"ANKEF1"

"7669","CAGCGATAATGCCGCTTAGT","chr20",10043147,"-","none","CGG","CAGCGATAATGCCGCTTAGTCGG",0,NA,NA,NA,"GeneID:63926;GeneID:100131208","ANKEF1;SNAP25-AS1",TRUE,"ANKEF1"

"7670","CGATTGGTCCGTAGAACGTG","chr20",10049558,"+","none","AGG","CGATTGGTCCGTAGAACGTGAGG",0,NA,NA,NA,"GeneID:63926;GeneID:100131208","ANKEF1;SNAP25-AS1",TRUE,"ANKEF1"

"7671","CTTGCACACGCCGTCATTAG","chr20",10050153,"-","none","CGG","CTTGCACACGCCGTCATTAGCGG",0,NA,NA,NA,"GeneID:63926;GeneID:100131208","ANKEF1;SNAP25-AS1",TRUE,"ANKEF1"

"7672","GCTCTGATCGCCCTTGAACA","chr20",10043168,"+","none","TGG","GCTCTGATCGCCCTTGAACATGG",0,NA,NA,NA,"GeneID:63926;GeneID:100131208","ANKEF1;SNAP25-AS1",TRUE,"ANKEF1"

"7673","TTCAACAGGGCATAGTGCCA","chr20",10053103,"+","none","TGG","TTCAACAGGGCATAGTGCCATGG",0,NA,NA,NA,"GeneID:63926;GeneID:100131208","ANKEF1;SNAP25-AS1",TRUE,"ANKEF1"

"7674","ACGCAGCCAAACGCCTGTAC","chr17",56350848,"+","none","AGG","ACGCAGCCAAACGCCTGTACAGG",0,NA,NA,NA,"GeneID:162282","ANKFN1",TRUE,"ANKFN1"

"7675","CCTCAACACACCTAACAGCG","chr17",56353933,"+","none","AGG","CCTCAACACACCTAACAGCGAGG",0,NA,NA,NA,"GeneID:162282","ANKFN1",TRUE,"ANKFN1"

"7676","GATTCGCTTGCTTTGCACAG","chr17",56326231,"+","none","AGG","GATTCGCTTGCTTTGCACAGAGG",0,NA,NA,NA,"GeneID:162282","ANKFN1",TRUE,"ANKFN1"

"7677","GGTGTCAAGCCCTCGCTGTT","chr17",56353946,"-","none","AGG","GGTGTCAAGCCCTCGCTGTTAGG",0,NA,NA,NA,"GeneID:162282","ANKFN1",TRUE,"ANKFN1"

"7678","GGTGTCAAGCCCTCGCTGTT","chr1",15103914,"+","2:G>T,13:T>C","GGG","GGGGTCAAGCCCTTGCTGTTGGG",2,3,14,NA,"GeneID:23254","KAZN",FALSE,"ANKFN1"

"7679","GTACAGGCGTTTGGCTGCGT","chr17",56350848,"-","none","TGG","GTACAGGCGTTTGGCTGCGTTGG",0,NA,NA,NA,"GeneID:162282","ANKFN1",TRUE,"ANKFN1"

"7680","TTACCAGTTTATGCTGTTCG","chr17",56326339,"-","none","AGG","TTACCAGTTTATGCTGTTCGAGG",0,NA,NA,NA,"GeneID:162282","ANKFN1",TRUE,"ANKFN1"

"7681","ACAGTAGTTCATCAGTGTGC","chr17",4209847,"+","none","TGG","ACAGTAGTTCATCAGTGTGCTGG",0,NA,NA,NA,"GeneID:51479","ANKFY1",TRUE,"ANKFY1"

"7682","AGCATTGTCATCGTCACCTC","chr17",4217087,"+","none","AGG","AGCATTGTCATCGTCACCTCAGG",0,NA,NA,NA,"GeneID:51479","ANKFY1",TRUE,"ANKFY1"

"7683","CATTCAGCTCCTCTGCCGTC","chr17",4209870,"+","none","TGG","CATTCAGCTCCTCTGCCGTCTGG",0,NA,NA,NA,"GeneID:51479","ANKFY1",TRUE,"ANKFY1"

"7684","CTAGTTTCATCAGTTCAGTC","chr17",4217016,"+","none","AGG","CTAGTTTCATCAGTTCAGTCAGG",0,NA,NA,NA,"GeneID:51479","ANKFY1",TRUE,"ANKFY1"

"7685","CTGTATTCGCTTCTACCAGA","chr17",4209888,"-","none","CGG","CTGTATTCGCTTCTACCAGACGG",0,NA,NA,NA,"GeneID:51479","ANKFY1",TRUE,"ANKFY1"

"7686","CTGTATTCGCTTCTACCAGA","chr12",104430280,"-","12:C>G,15:T>G","GGG","CTGTATTCGCTTGTAACAGAGGG",2,13,16,NA,"","",FALSE,"ANKFY1"

"7687","TGACGATGACAATGCTTCGC","chr17",4217083,"-","none","TGG","TGACGATGACAATGCTTCGCTGG",0,NA,NA,NA,"GeneID:51479","ANKFY1",TRUE,"ANKFY1"

"7688","CAAAGCCGTCCTGTGTATGG","chr5",14769016,"-","none","TGG","CAAAGCCGTCCTGTGTATGGTGG",0,NA,NA,NA,"GeneID:56172","ANKH",TRUE,"ANKH"

"7689","CGTGCTCACCAGTTTGTTGC","chr5",14741892,"+","none","TGG","CGTGCTCACCAGTTTGTTGCTGG",0,NA,NA,NA,"GeneID:56172","ANKH",TRUE,"ANKH"

"7690","CTCACCATTGCGTCCATGAA","chr5",14758474,"+","none","AGG","CTCACCATTGCGTCCATGAAAGG",0,NA,NA,NA,"GeneID:56172","ANKH",TRUE,"ANKH"

"7691","GAAGTTCACCTTCGTCTGCA","chr5",14741843,"-","none","TGG","GAAGTTCACCTTCGTCTGCATGG",0,NA,NA,NA,"GeneID:56172","ANKH",TRUE,"ANKH"

"7692","GAAGTTCACCTTCGTCTGCA","chr1",203975730,"+","13:C>G,18:G>C","AGG","GAAGTTCACCTTCCTCTGGAAGG",2,14,19,NA,"","",FALSE,"ANKH"

"7693","GCCGTCTTTCACACACTGAT","chr5",14768976,"-","none","AGG","GCCGTCTTTCACACACTGATAGG",0,NA,NA,NA,"GeneID:56172","ANKH",TRUE,"ANKH"

"7694","TCCCCACCGACTCGTCCACA","chr5",14758535,"+","none","TGG","TCCCCACCGACTCGTCCACATGG",0,NA,NA,NA,"GeneID:56172","ANKH",TRUE,"ANKH"

"7695","AAAGTACTCACCTATGAATC","chr5",140509803,"-","none","AGG","AAAGTACTCACCTATGAATCAGG",0,NA,NA,NA,"GeneID:54882;GeneID:404734","ANKHD1;ANKHD1-EIF4EBP3",TRUE,"ANKHD1"

"7696","CAGCCTCAGATTGCTATTAC","chr5",140497160,"+","none","TGG","CAGCCTCAGATTGCTATTACTGG",0,NA,NA,NA,"GeneID:54882;GeneID:404734","ANKHD1;ANKHD1-EIF4EBP3",TRUE,"ANKHD1"

"7697","CCCATTAGAACCTAAGTCAC","chr5",140497089,"-","none","TGG","CCCATTAGAACCTAAGTCACTGG",0,NA,NA,NA,"GeneID:54882;GeneID:404734","ANKHD1;ANKHD1-EIF4EBP3",TRUE,"ANKHD1"

"7698","CCCATTAGAACCTAAGTCAC","chr22",34978943,"-","2:A>G,15:G>C","TGG","CCTATTAGAACCTAACTCACTGG",2,3,16,NA,"","",FALSE,"ANKHD1"

"7699","GACGGGTCTTACCCCCTTGA","chr5",140509635,"+","none","TGG","GACGGGTCTTACCCCCTTGATGG",0,NA,NA,NA,"GeneID:54882;GeneID:404734","ANKHD1;ANKHD1-EIF4EBP3",TRUE,"ANKHD1"

"7700","GTGCAGTTGCTAGTGCAAGC","chr5",140510097,"+","none","AGG","GTGCAGTTGCTAGTGCAAGCAGG",0,NA,NA,NA,"GeneID:54882;GeneID:404734","ANKHD1;ANKHD1-EIF4EBP3",TRUE,"ANKHD1"

"7701","TCTTATGTCAGCATTTCGCA","chr5",140510159,"+","none","AGG","TCTTATGTCAGCATTTCGCAAGG",0,NA,NA,NA,"GeneID:54882;GeneID:404734","ANKHD1;ANKHD1-EIF4EBP3",TRUE,"ANKHD1"

"7702","ACCTGAGACTGATCTTGAGA","chr5",140485714,"-","none","AGG","ACCTGAGACTGATCTTGAGAAGG",0,NA,NA,NA,"GeneID:54882;GeneID:404734","ANKHD1;ANKHD1-EIF4EBP3",TRUE,"ANKHD1-EIF4EBP3"

"7703","ACCTTCTCAAGATCAGTCTC","chr5",140485710,"+","none","AGG","ACCTTCTCAAGATCAGTCTCAGG",0,NA,NA,NA,"GeneID:54882;GeneID:404734","ANKHD1;ANKHD1-EIF4EBP3",TRUE,"ANKHD1-EIF4EBP3"

"7704","ACCTTCTCAAGATCAGTCTC","chr11",116658955,"+","11:G>A,15:C>G","CGG","ACCTTCTCAAGGTCACTCTCCGG",2,12,16,NA,"","",FALSE,"ANKHD1-EIF4EBP3"

"7705","AGTGTATGCGTTGGCACACG","chr5",140486963,"-","none","TGG","AGTGTATGCGTTGGCACACGTGG",0,NA,NA,NA,"GeneID:54882;GeneID:404734","ANKHD1;ANKHD1-EIF4EBP3",TRUE,"ANKHD1-EIF4EBP3"

"7706","CAGCCTCAGATTGCTATTAC","chr5",140497160,"+","none","TGG","CAGCCTCAGATTGCTATTACTGG",0,NA,NA,NA,"GeneID:54882;GeneID:404734","ANKHD1;ANKHD1-EIF4EBP3",TRUE,"ANKHD1-EIF4EBP3"

"7707","CCCATTAGAACCTAAGTCAC","chr5",140497089,"-","none","TGG","CCCATTAGAACCTAAGTCACTGG",0,NA,NA,NA,"GeneID:54882;GeneID:404734","ANKHD1;ANKHD1-EIF4EBP3",TRUE,"ANKHD1-EIF4EBP3"

"7708","CCCATTAGAACCTAAGTCAC","chr22",34978943,"-","2:A>G,15:G>C","TGG","CCTATTAGAACCTAACTCACTGG",2,3,16,NA,"","",FALSE,"ANKHD1-EIF4EBP3"

"7709","GCCAACGCATACACTTGCCA","chr5",140486968,"+","none","TGG","GCCAACGCATACACTTGCCATGG",0,NA,NA,NA,"GeneID:54882;GeneID:404734","ANKHD1;ANKHD1-EIF4EBP3",TRUE,"ANKHD1-EIF4EBP3"

"7710","GAAGCTTTACTTCGAGCTCA","chr7",92327878,"+","none","TGG","GAAGCTTTACTTCGAGCTCATGG",0,NA,NA,NA,"GeneID:54467","ANKIB1",TRUE,"ANKIB1"

"7711","GGCGTGCCAAGCGAGGATGA","chr7",92307466,"-","none","AGG","GGCGTGCCAAGCGAGGATGAAGG",0,NA,NA,NA,"GeneID:54467","ANKIB1",TRUE,"ANKIB1"

"7712","GGCTCCCCATAAGATGTATT","chr7",92295086,"-","none","TGG","GGCTCCCCATAAGATGTATTTGG",0,NA,NA,NA,"GeneID:54467","ANKIB1",TRUE,"ANKIB1"

"7713","TTACATTATGCTGCTAGACA","chr7",92295125,"+","none","TGG","TTACATTATGCTGCTAGACATGG",0,NA,NA,NA,"GeneID:54467","ANKIB1",TRUE,"ANKIB1"

"7714","TTGTGCACATTCCGTTTATT","chr7",92307386,"-","none","TGG","TTGTGCACATTCCGTTTATTTGG",0,NA,NA,NA,"GeneID:54467","ANKIB1",TRUE,"ANKIB1"

"7715","TTTAGTCTACGAAGATCCTG","chr7",92327803,"-","none","AGG","TTTAGTCTACGAAGATCCTGAGG",0,NA,NA,NA,"GeneID:54467","ANKIB1",TRUE,"ANKIB1"

"7716","ACATCGAGAGGTCGGCTCTG","chr11",113394980,"+","none","CGG","ACATCGAGAGGTCGGCTCTGCGG",0,NA,NA,NA,"GeneID:255239","ANKK1",TRUE,"ANKK1"

"7717","ACCTCTCGATGTACTGCATC","chr11",113394972,"-","none","CGG","ACCTCTCGATGTACTGCATCCGG",0,NA,NA,NA,"GeneID:255239","ANKK1",TRUE,"ANKK1"

"7718","CAAGCCGGGCAACATACTCC","chr11",113393732,"+","none","TGG","CAAGCCGGGCAACATACTCCTGG",0,NA,NA,NA,"GeneID:255239","ANKK1",TRUE,"ANKK1"

"7719","GAGGCGGCTTAATGCTGTGC","chr11",113393696,"-","none","AGG","GAGGCGGCTTAATGCTGTGCAGG",0,NA,NA,NA,"GeneID:255239","ANKK1",TRUE,"ANKK1"

"7720","GCCGCATGCCTGCCGCCACT","chr11",113396094,"-","none","CGG","GCCGCATGCCTGCCGCCACTCGG",0,NA,NA,NA,"GeneID:255239","ANKK1",TRUE,"ANKK1"

"7721","GGTCCCAGCAGCGTTTCATC","chr11",113396172,"-","none","AGG","GGTCCCAGCAGCGTTTCATCAGG",0,NA,NA,NA,"GeneID:255239","ANKK1",TRUE,"ANKK1"

"7722","AACTTACAAGCAAAATGCAA","chr12",132743241,"+","none","CGG","AACTTACAAGCAAAATGCAACGG",0,NA,NA,NA,"GeneID:23141","ANKLE2",TRUE,"ANKLE2"

"7723","AACTTACAAGCAAAATGCAA","chr2",14127516,"+","12:G>A,15:G>T","AGG","AACTTACAAGCAGAAGGCAAAGG",2,13,16,NA,"GeneID:100499171","LINC00276",FALSE,"ANKLE2"

"7724","AACTTACAAGCAAAATGCAA","chr14",91752890,"+","10:T>C,18:C>A","CGG","AACTTACAAGTAAAATGCCACGG",2,11,19,NA,"GeneID:79820","CATSPERB",TRUE,"ANKLE2"

"7725","AACTTACAAGCAAAATGCAA","chr1",215937877,"-","3:T>A,4:T>A","TGG","AACAAACAAGCAAAATGCAATGG",2,4,5,NA,"GeneID:7399","USH2A",FALSE,"ANKLE2"

"7726","AACTTACAAGCAAAATGCAA","chr15",72923556,"-","2:A>G,5:G>T","GGG","AATTTCCAAGCAAAATGCAAGGG",2,3,6,NA,"","",FALSE,"ANKLE2"

"7727","AACTTACAAGCAAAATGCAA","chr7",24466122,"+","2:T>C,5:T>A","AGG","AATTTTCAAGCAAAATGCAAAGG",2,3,6,NA,"","",FALSE,"ANKLE2"

"7728","AACTTACAAGCAAAATGCAA","chr21",31767229,"-","9:A>C,17:C>G","GGG","AACTTACAATCAAAATGGAAGGG",2,10,18,NA,"","",FALSE,"ANKLE2"

"7729","AAGGAGCCGCCCCTGTACTA","chr12",132754715,"-","none","TGG","AAGGAGCCGCCCCTGTACTATGG",0,NA,NA,NA,"GeneID:23141","ANKLE2",TRUE,"ANKLE2"

"7730","ATTTGATTGTAAAAAACTCA","chr12",132743183,"-","none","AGG","ATTTGATTGTAAAAAACTCAAGG",0,NA,NA,NA,"GeneID:23141","ANKLE2",TRUE,"ANKLE2"

"7731","ATTTGATTGTAAAAAACTCA","chr4",97166836,"-","2:C>A,8:A>C","TGG","ATGTGATTTTAAAAAACTCATGG",2,3,9,NA,"","",FALSE,"ANKLE2"

"7732","ATTTGATTGTAAAAAACTCA","chr5",161082837,"+","8:T>G,9:A>T","TGG","ATTTGATTTAAAAAAACTCATGG",2,9,10,NA,"","",FALSE,"ANKLE2"

"7733","ATTTGATTGTAAAAAACTCA","chr2",203417262,"+","8:T>G,18:G>C","TGG","ATTTGATTTTAAAAAACTGATGG",2,9,19,NA,"GeneID:10152;GeneID:65059","ABI2;RAPH1",FALSE,"ANKLE2"

"7734","ATTTGATTGTAAAAAACTCA","chr7",12734767,"-","9:G>A,14:C>T","CGG","ATTTGATTGCAAAAGACTCACGG",2,10,15,NA,"","",FALSE,"ANKLE2"

"7735","CCCAGTTCCTCCGCCAAGCG","chr12",132761638,"-","none","CGG","CCCAGTTCCTCCGCCAAGCGCGG",0,NA,NA,NA,"GeneID:23141","ANKLE2",TRUE,"ANKLE2"

"7736","GACACCGACACCTACAGAGC","chr12",132754751,"-","none","TGG","GACACCGACACCTACAGAGCTGG",0,NA,NA,NA,"GeneID:23141","ANKLE2",TRUE,"ANKLE2"

"7737","TGCTGCTGATCGCTGTGCGG","chr12",132761711,"-","none","TGG","TGCTGCTGATCGCTGTGCGGTGG",0,NA,NA,NA,"GeneID:23141","ANKLE2",TRUE,"ANKLE2"

"7738","AGCAGAGCGCGAGACGTTCC","chr2",240500062,"-","none","TGG","AGCAGAGCGCGAGACGTTCCTGG",0,NA,NA,NA,"GeneID:51281","ANKMY1",TRUE,"ANKMY1"

"7739","AGGAACGTCTCGCGCTCTGC","chr2",240500061,"+","none","TGG","AGGAACGTCTCGCGCTCTGCTGG",0,NA,NA,NA,"GeneID:51281","ANKMY1",TRUE,"ANKMY1"

"7740","ATCTGAGCACTTGTTCACGT","chr2",240525780,"+","none","CGG","ATCTGAGCACTTGTTCACGTCGG",0,NA,NA,NA,"GeneID:51281","ANKMY1",TRUE,"ANKMY1"

"7741","GAAGCAGTTACCTCGCCTGT","chr2",240554854,"+","none","GGG","GAAGCAGTTACCTCGCCTGTGGG",0,NA,NA,NA,"GeneID:51281","ANKMY1",TRUE,"ANKMY1"

"7742","GAAGCAGTTACCTCGCCTGT","chr13",45564717,"+","13:A>C,18:A>G","TGG","GAAGCAGTTACCTAGCCTATTGG",2,14,19,NA,"GeneID:220081","ERICH6B",FALSE,"ANKMY1"

"7743","GAAGGTTGACAATGTCGTTG","chr2",240525818,"+","none","TGG","GAAGGTTGACAATGTCGTTGTGG",0,NA,NA,NA,"GeneID:51281","ANKMY1",TRUE,"ANKMY1"

"7744","TGTTCAACCCAAACTCCCCC","chr2",240554906,"+","none","TGG","TGTTCAACCCAAACTCCCCCTGG",0,NA,NA,NA,"GeneID:51281","ANKMY1",TRUE,"ANKMY1"

"7745","CTACTGGAAGTCATCGGGAA","chr7",16645508,"-","none","AGG","CTACTGGAAGTCATCGGGAAAGG",0,NA,NA,NA,"GeneID:57037","ANKMY2",TRUE,"ANKMY2"

"7746","CTACTGGAAGTCATCGGGAA","chr16",88885984,"-","13:T>A,16:T>C","CGG","CTACTGGAAGTCAACGAGAACGG",2,14,17,NA,"GeneID:863;GeneID:101927793","CBFA2T3;LOC101927793",TRUE,"ANKMY2"

"7747","CTACTGGAAGTCATCGGGAA","chr1",19033654,"-","0:C>G,13:G>A","AGG","GTACTGGAAGTCACCGGGAAAGG",2,1,14,NA,"","",FALSE,"ANKMY2"

"7748","GAATGTTCGTGTCAACTGTT","chr7",16636398,"-","none","TGG","GAATGTTCGTGTCAACTGTTTGG",0,NA,NA,NA,"GeneID:57037","ANKMY2",TRUE,"ANKMY2"

"7749","GCAATGGTTCACATAAAGAA","chr7",16645556,"-","none","AGG","GCAATGGTTCACATAAAGAAAGG",0,NA,NA,NA,"GeneID:57037","ANKMY2",TRUE,"ANKMY2"

"7750","GCAATGGTTCACATAAAGAA","chr8",75021195,"+","6:T>G,12:T>A","TGG","GCAATGTTTCACTTAAAGAATGG",2,7,13,NA,"GeneID:83690","CRISPLD1",FALSE,"ANKMY2"

"7751","GCAATGGTTCACATAAAGAA","chr4",86070088,"+","9:T>C,13:A>T","GGG","GCAATGGTTTACAAAAAGAAGGG",2,10,14,NA,"GeneID:5602","MAPK10",FALSE,"ANKMY2"

"7752","GTTGACACGAACATTCTTGC","chr7",16636402,"+","none","TGG","GTTGACACGAACATTCTTGCTGG",0,NA,NA,NA,"GeneID:57037","ANKMY2",TRUE,"ANKMY2"

"7753","TGCAAATTACTACTGCGACA","chr7",16627107,"-","none","TGG","TGCAAATTACTACTGCGACATGG",0,NA,NA,NA,"GeneID:57037","ANKMY2",TRUE,"ANKMY2"

"7754","TTATATGCTGCATGCATTAG","chr7",16627143,"+","none","AGG","TTATATGCTGCATGCATTAGAGG",0,NA,NA,NA,"GeneID:57037","ANKMY2",TRUE,"ANKMY2"

"7755","TTATATGCTGCATGCATTAG","chr3",85324094,"+","13:C>G,19:T>G","TGG","TTATATGCTGCATCCATTATTGG",2,14,20,NA,"GeneID:253559","CADM2",FALSE,"ANKMY2"

"7756","TTATATGCTGCATGCATTAG","chr1",81142430,"+","8:A>T,15:T>A","GGG","TTATATGCAGCATGCTTTAGGGG",2,9,16,NA,"","",FALSE,"ANKMY2"

"7757","AGCTTTGTCTGTTCACCAGT","chr5",73557622,"-","none","TGG","AGCTTTGTCTGTTCACCAGTTGG",0,NA,NA,NA,"GeneID:57763","ANKRA2",TRUE,"ANKRA2"

"7758","ATCTGTGTAGCCTTTACTAC","chr5",73554911,"+","none","AGG","ATCTGTGTAGCCTTTACTACAGG",0,NA,NA,NA,"GeneID:57763","ANKRA2",TRUE,"ANKRA2"

"7759","ATGGATCTAGCTGTAGCCCT","chr5",73553419,"-","none","AGG","ATGGATCTAGCTGTAGCCCTAGG",0,NA,NA,NA,"GeneID:57763","ANKRA2",TRUE,"ANKRA2"

"7760","CCAGAGTCAGTTTCAATTGT","chr5",73553449,"+","none","TGG","CCAGAGTCAGTTTCAATTGTTGG",0,NA,NA,NA,"GeneID:57763","ANKRA2",TRUE,"ANKRA2"

"7761","CCAGAGTCAGTTTCAATTGT","chrX",138524198,"-","14:A>T,15:C>T","TGG","CCAGAGTCAGTTTCTGTTGTTGG",2,15,16,NA,"","",FALSE,"ANKRA2"

"7762","CCAGAGTCAGTTTCAATTGT","chrX",20034692,"-","11:C>A,19:T>A","GGG","CCAGAGTCAGTGTCAATTGAGGG",2,12,20,NA,"GeneID:256714","MAP7D2",FALSE,"ANKRA2"

"7763","TCAGGGAGAGATGCTCTATC","chr5",73557595,"-","none","TGG","TCAGGGAGAGATGCTCTATCTGG",0,NA,NA,NA,"GeneID:57763","ANKRA2",TRUE,"ANKRA2"

"7764","TCGAGAAAGTGCACTGTCGT","chr5",73554937,"-","none","TGG","TCGAGAAAGTGCACTGTCGTTGG",0,NA,NA,NA,"GeneID:57763","ANKRA2",TRUE,"ANKRA2"

"7765","AACTAAAGTTCCAGTTGTAA","chr10",90919153,"-","none","AGG","AACTAAAGTTCCAGTTGTAAAGG",0,NA,NA,NA,"GeneID:27063","ANKRD1",TRUE,"ANKRD1"

"7766","AGAGCAGCCTTCAGAAACGT","chr10",90918931,"+","none","AGG","AGAGCAGCCTTCAGAAACGTAGG",0,NA,NA,NA,"GeneID:27063","ANKRD1",TRUE,"ANKRD1"

"7767","AGAGCAGCCTTCAGAAACGT","chr8",66551624,"+","13:A>G,15:G>A","GGG","AGAGCAGCCTTCAAAGACGTGGG",2,14,16,NA,"","",FALSE,"ANKRD1"

"7768","CCAGATCGAATTCCGTGATA","chr10",90917733,"-","none","TGG","CCAGATCGAATTCCGTGATATGG",0,NA,NA,NA,"GeneID:27063","ANKRD1",TRUE,"ANKRD1"

"7769","CCATATCACGGAATTCGATC","chr10",90917730,"+","none","TGG","CCATATCACGGAATTCGATCTGG",0,NA,NA,NA,"GeneID:27063","ANKRD1",TRUE,"ANKRD1"

"7770","GAAAAGCGAGAAACAACGAG","chr10",90920176,"-","none","AGG","GAAAAGCGAGAAACAACGAGAGG",0,NA,NA,NA,"GeneID:27063","ANKRD1",TRUE,"ANKRD1"

"7771","TTGGCTTTAGACGGAACCTG","chr10",90918962,"-","none","TGG","TTGGCTTTAGACGGAACCTGTGG",0,NA,NA,NA,"GeneID:27063","ANKRD1",TRUE,"ANKRD1"

"7772","AGAGCGAGCTGCCTTGTGAA","chr13",110906082,"+","none","TGG","AGAGCGAGCTGCCTTGTGAATGG",0,NA,NA,NA,"GeneID:55608","ANKRD10",TRUE,"ANKRD10"

"7773","AGAGCGAGCTGCCTTGTGAA","chr5",169146508,"-","5:G>C,11:C>G","GGG","AGAGCCAGCTGGCTTGTGAAGGG",2,6,12,NA,"GeneID:6586","SLIT3",FALSE,"ANKRD10"

"7774","CACTCAACGTCTCCACCACA","chr13",110910713,"-","none","CGG","CACTCAACGTCTCCACCACACGG",0,NA,NA,NA,"GeneID:55608","ANKRD10",TRUE,"ANKRD10"

"7775","CGTGAGCCCCATTCGCCACA","chr13",110906036,"+","none","AGG","CGTGAGCCCCATTCGCCACAAGG",0,NA,NA,NA,"GeneID:55608","ANKRD10",TRUE,"ANKRD10"

"7776","TCTGCCGTGCCACTCACGAA","chr13",110883759,"-","none","TGG","TCTGCCGTGCCACTCACGAATGG",0,NA,NA,NA,"GeneID:55608","ANKRD10",TRUE,"ANKRD10"

"7777","TCTGTGTCGCCATTCGTGAG","chr13",110883747,"+","none","TGG","TCTGTGTCGCCATTCGTGAGTGG",0,NA,NA,NA,"GeneID:55608","ANKRD10",TRUE,"ANKRD10"

"7778","TGGCGTCTGCGCGTACCGTG","chr13",110910695,"+","none","TGG","TGGCGTCTGCGCGTACCGTGTGG",0,NA,NA,NA,"GeneID:55608","ANKRD10",TRUE,"ANKRD10"

"7779","CCACCTGCTGGCGCTCGGAG","chr16",89291057,"+","none","AGG","CCACCTGCTGGCGCTCGGAGAGG",0,NA,NA,NA,"GeneID:29123","ANKRD11",TRUE,"ANKRD11"

"7780","CCACCTGCTGGCGCTCGGAG","chr11",45758414,"-","12:A>C,15:C>G","AGG","CCACCTGCTGGCTCTGGGAGAGG",2,13,16,NA,"","",FALSE,"ANKRD11"

"7781","CCCTTCACCGCGGGCGCCAA","chr16",89305231,"-","none","TGG","CCCTTCACCGCGGGCGCCAATGG",0,NA,NA,NA,"GeneID:29123","ANKRD11",TRUE,"ANKRD11"

"7782","CGGCCGTCATCTGCATGAGA","chr16",89291033,"+","none","AGG","CGGCCGTCATCTGCATGAGAAGG",0,NA,NA,NA,"GeneID:29123","ANKRD11",TRUE,"ANKRD11"

"7783","CGTCCCCGCGGATGGCGGCT","chr16",89290684,"+","none","CGG","CGTCCCCGCGGATGGCGGCTCGG",0,NA,NA,NA,"GeneID:29123","ANKRD11",TRUE,"ANKRD11"

"7784","GAGCAGAAGGACTCGGACAC","chr16",89305207,"-","none","AGG","GAGCAGAAGGACTCGGACACAGG",0,NA,NA,NA,"GeneID:29123","ANKRD11",TRUE,"ANKRD11"

"7785","GTCAACGTCAAGGACTTCGC","chr16",89290626,"-","none","AGG","GTCAACGTCAAGGACTTCGCAGG",0,NA,NA,NA,"GeneID:29123","ANKRD11",TRUE,"ANKRD11"

"7786","AAAAGGAAGTTTACGTTTCA","chr18",9195635,"-","none","TGG","AAAAGGAAGTTTACGTTTCATGG",0,NA,NA,NA,"GeneID:23253","ANKRD12",TRUE,"ANKRD12"

"7787","AAAAGGAAGTTTACGTTTCA","chr11",123283913,"-","12:C>T,13:T>G","AGG","AAAAGGAAGTTTGAGTTTCAAGG",2,13,14,NA,"","",FALSE,"ANKRD12"

"7788","ACAGCAAGAGACAACAGTCC","chr18",9208781,"+","none","AGG","ACAGCAAGAGACAACAGTCCAGG",0,NA,NA,NA,"GeneID:23253","ANKRD12",TRUE,"ANKRD12"

"7789","ACAGCAAGAGACAACAGTCC","chr3",130019072,"+","17:A>T,18:T>C","AGG","ACAGCAAGAGACAACAGATCAGG",2,18,19,NA,"","",FALSE,"ANKRD12"

"7790","ACAGCAAGAGACAACAGTCC","chr11",28636379,"+","4:G>C,7:C>G","TGG","ACAGGAACAGACAACAGTCCTGG",2,5,8,NA,"","",FALSE,"ANKRD12"

"7791","ACTCTCTGAGCGAAAACAGA","chr18",9208741,"+","none","TGG","ACTCTCTGAGCGAAAACAGATGG",0,NA,NA,NA,"GeneID:23253","ANKRD12",TRUE,"ANKRD12"

"7792","GAAGAACGAGATTCAGACAC","chr18",9195676,"+","none","AGG","GAAGAACGAGATTCAGACACAGG",0,NA,NA,NA,"GeneID:23253","ANKRD12",TRUE,"ANKRD12"

"7793","GAAGAACGAGATTCAGACAC","chr19",42021774,"+","6:T>C,11:C>T","AGG","GAAGAATGAGACTCAGACACAGG",2,7,12,NA,"GeneID:2901","GRIK5",FALSE,"ANKRD12"

"7794","TCATATACCTTTCTTCCATA","chr18",9182507,"-","none","TGG","TCATATACCTTTCTTCCATATGG",0,NA,NA,NA,"GeneID:23253","ANKRD12",TRUE,"ANKRD12"

"7795","TCATATACCTTTCTTCCATA","chr3",97394896,"+","0:C>T,15:A>C","AGG","CCATATACCTTTCTTACATAAGG",2,1,16,NA,"GeneID:285220","EPHA6",FALSE,"ANKRD12"

"7796","TCATATACCTTTCTTCCATA","chr11",79236159,"-","1:A>G,12:A>G","CGG","TTATATACCTTTTTTCCATACGG",2,2,13,NA,"GeneID:26011","TENM4",FALSE,"ANKRD12"

"7797","TTCTGACAGTGACAGCAATA","chr18",9182473,"+","none","TGG","TTCTGACAGTGACAGCAATATGG",0,NA,NA,NA,"GeneID:23253","ANKRD12",TRUE,"ANKRD12"

"7798","CATTCCCATCATTGACCTAA","chr12",110029534,"+","none","TGG","CATTCCCATCATTGACCTAATGG",0,NA,NA,NA,"GeneID:88455;GeneID:400073","ANKRD13A;C12orf76",TRUE,"ANKRD13A"

"7799","CATTCCCATCATTGACCTAA","chr14",45526584,"+","18:C>A,19:T>A","GGG","CATTCCCATCATTGACCTCTGGG",2,19,20,NA,"","",FALSE,"ANKRD13A"

"7800","GAGCACTCGTTCGAGCCATT","chr12",110029552,"-","none","AGG","GAGCACTCGTTCGAGCCATTAGG",0,NA,NA,NA,"GeneID:88455;GeneID:400073","ANKRD13A;C12orf76",TRUE,"ANKRD13A"

"7801","GCTGCTCCAAAAAATTCTCG","chr12",110013227,"+","none","AGG","GCTGCTCCAAAAAATTCTCGAGG",0,NA,NA,NA,"GeneID:88455","ANKRD13A",TRUE,"ANKRD13A"

"7802","TGCAATAATGTTCGACCTCG","chr12",110012025,"-","none","TGG","TGCAATAATGTTCGACCTCGTGG",0,NA,NA,NA,"GeneID:88455","ANKRD13A",TRUE,"ANKRD13A"

"7803","TGTGACAAAAGAAAATCGCC","chr12",110012105,"+","none","AGG","TGTGACAAAAGAAAATCGCCAGG",0,NA,NA,NA,"GeneID:88455","ANKRD13A",TRUE,"ANKRD13A"

"7804","TGTTGTGGTAGTCTCGATGT","chr12",110013179,"-","none","TGG","TGTTGTGGTAGTCTCGATGTTGG",0,NA,NA,NA,"GeneID:88455","ANKRD13A",TRUE,"ANKRD13A"

"7805","TGTTGTGGTAGTCTCGATGT","chr9",125958468,"+","7:A>G,14:A>C","TGG","TGTTGTGATAGTCTAGATGTTGG",2,8,15,NA,"GeneID:5090","PBX3",FALSE,"ANKRD13A"

"7806","ACTACCAGCGGGTGGTGAAG","chr17",29608048,"+","none","CGG","ACTACCAGCGGGTGGTGAAGCGG",0,NA,NA,NA,"GeneID:124930","ANKRD13B",TRUE,"ANKRD13B"

"7807","CCACGTCTGCGCCGTGCGCC","chr17",29607830,"-","none","AGG","CCACGTCTGCGCCGTGCGCCAGG",0,NA,NA,NA,"GeneID:124930","ANKRD13B",TRUE,"ANKRD13B"

"7808","CGCACGGCGCAGACGTGGGC","chr17",29607832,"+","none","AGG","CGCACGGCGCAGACGTGGGCAGG",0,NA,NA,NA,"GeneID:124930","ANKRD13B",TRUE,"ANKRD13B"

"7809","TCTGGCAGACACAAGCGCCG","chr17",29609077,"+","none","TGG","TCTGGCAGACACAAGCGCCGTGG",0,NA,NA,NA,"GeneID:124930","ANKRD13B",TRUE,"ANKRD13B"

"7810","TGCAGCTGGTGCTTCGGTAC","chr17",29608024,"+","none","CGG","TGCAGCTGGTGCTTCGGTACCGG",0,NA,NA,NA,"GeneID:124930","ANKRD13B",TRUE,"ANKRD13B"

"7811","TGCAGCTGGTGCTTCGGTAC","chr7",145884158,"-","3:A>T,15:A>C","TGG","TGCTGCTGGTGCTTCTGTACTGG",2,4,16,NA,"","",FALSE,"ANKRD13B"

"7812","TGGAGATTGACCACGACCGC","chr17",29609103,"+","none","CGG","TGGAGATTGACCACGACCGCCGG",0,NA,NA,NA,"GeneID:124930","ANKRD13B",TRUE,"ANKRD13B"

"7813","AGAATGTGCCCATTTACTTT","chr1",70324939,"-","none","TGG","AGAATGTGCCCATTTACTTTTGG",0,NA,NA,NA,"GeneID:81573","ANKRD13C",TRUE,"ANKRD13C"

"7814","CTAAAACAAGTCCATTCACC","chr1",70292494,"+","none","AGG","CTAAAACAAGTCCATTCACCAGG",0,NA,NA,NA,"GeneID:81573","ANKRD13C",TRUE,"ANKRD13C"

"7815","CTAAAACAAGTCCATTCACC","chr9",32648793,"+","2:G>A,16:T>C","TGG","CTGAAACAAGTCCATTTACCTGG",2,3,17,NA,"","",FALSE,"ANKRD13C"

"7816","CTGGCGGAAGCCATCAGCTA","chr1",70324869,"-","none","TGG","CTGGCGGAAGCCATCAGCTATGG",0,NA,NA,NA,"GeneID:81573","ANKRD13C",TRUE,"ANKRD13C"

"7817","GGCAGAATTCGGGAAAGTAA","chr1",70306265,"+","none","AGG","GGCAGAATTCGGGAAAGTAAAGG",0,NA,NA,NA,"GeneID:81573","ANKRD13C",TRUE,"ANKRD13C"

"7818","GGCAGAATTCGGGAAAGTAA","chr10",59763881,"-","9:C>G,17:C>A","AGG","GGCAGAATTGGGGAAAGGAAAGG",2,10,18,NA,"","",FALSE,"ANKRD13C"

"7819","TCTTCGAAATAAGGCCATCA","chr1",70292434,"-","none","TGG","TCTTCGAAATAAGGCCATCATGG",0,NA,NA,NA,"GeneID:81573","ANKRD13C",TRUE,"ANKRD13C"

"7820","TTACACCTTGCTGTGATGTT","chr1",70336068,"-","none","AGG","TTACACCTTGCTGTGATGTTAGG",0,NA,NA,NA,"GeneID:81573","ANKRD13C",TRUE,"ANKRD13C"

"7821","ACTGCTCAACAAACTTCGCC","chr11",67290424,"+","none","AGG","ACTGCTCAACAAACTTCGCCAGG",0,NA,NA,NA,"GeneID:338692","ANKRD13D",TRUE,"ANKRD13D"

"7822","CCACGTTGGCATTGTGTCGA","chr11",67290166,"-","none","AGG","CCACGTTGGCATTGTGTCGAAGG",0,NA,NA,NA,"GeneID:338692","ANKRD13D",TRUE,"ANKRD13D"

"7823","CCCCCAGCACGACATTGAAC","chr11",67290070,"+","none","AGG","CCCCCAGCACGACATTGAACAGG",0,NA,NA,NA,"GeneID:338692","ANKRD13D",TRUE,"ANKRD13D"

"7824","CCCTCTGGTAGTCCCGATAC","chr11",67290376,"-","none","TGG","CCCTCTGGTAGTCCCGATACTGG",0,NA,NA,NA,"GeneID:338692","ANKRD13D",TRUE,"ANKRD13D"

"7825","GCCGGTTCGCCCAGACGAGC","chr11",67289495,"-","none","CGG","GCCGGTTCGCCCAGACGAGCCGG",0,NA,NA,NA,"GeneID:338692","ANKRD13D",TRUE,"ANKRD13D"

"7826","GGCGAACCGGCATCGCGAAC","chr11",67289504,"+","none","TGG","GGCGAACCGGCATCGCGAACTGG",0,NA,NA,NA,"GeneID:338692","ANKRD13D",TRUE,"ANKRD13D"

"7827","AGCACTTCATTATGCAGCTA","chr10",5883007,"-","none","AGG","AGCACTTCATTATGCAGCTAAGG",0,NA,NA,NA,"GeneID:54522","ANKRD16",TRUE,"ANKRD16"

"7828","CAGAGGGTCGCCTTCTCGAC","chr10",5887928,"+","none","TGG","CAGAGGGTCGCCTTCTCGACTGG",0,NA,NA,NA,"GeneID:54522","ANKRD16",TRUE,"ANKRD16"

"7829","CGACGTCGATGTGCCCACAC","chr10",5883994,"+","none","TGG","CGACGTCGATGTGCCCACACTGG",0,NA,NA,NA,"GeneID:54522","ANKRD16",TRUE,"ANKRD16"

"7830","GCTGTACCTGCAGTATGCAG","chr10",5887839,"+","none","AGG","GCTGTACCTGCAGTATGCAGAGG",0,NA,NA,NA,"GeneID:54522","ANKRD16",TRUE,"ANKRD16"

"7831","GGACGAAGCCATCCGATTCT","chr10",5883082,"-","none","TGG","GGACGAAGCCATCCGATTCTTGG",0,NA,NA,NA,"GeneID:54522","ANKRD16",TRUE,"ANKRD16"

"7832","TTGATGGACGCAATCCAGTG","chr10",5884011,"-","none","TGG","TTGATGGACGCAATCCAGTGTGG",0,NA,NA,NA,"GeneID:54522","ANKRD16",TRUE,"ANKRD16"

"7833","AAAGGCTTTACCATCAGCCG","chr4",73161310,"+","none","TGG","AAAGGCTTTACCATCAGCCGTGG",0,NA,NA,NA,"GeneID:26057","ANKRD17",TRUE,"ANKRD17"

"7834","AGTACCTGATAAGAGCAACT","chr4",73177452,"+","none","TGG","AGTACCTGATAAGAGCAACTTGG",0,NA,NA,NA,"GeneID:26057","ANKRD17",TRUE,"ANKRD17"

"7835","ATCGTCTGTTAGTTGTGCGT","chr4",73161261,"-","none","TGG","ATCGTCTGTTAGTTGTGCGTTGG",0,NA,NA,NA,"GeneID:26057","ANKRD17",TRUE,"ANKRD17"

"7836","ATTTACATCTCCTTCTGAAC","chr4",73156126,"+","none","AGG","ATTTACATCTCCTTCTGAACAGG",0,NA,NA,NA,"GeneID:26057","ANKRD17",TRUE,"ANKRD17"

"7837","GCTTCCAAGTTGCTCTTATC","chr4",73177459,"-","none","AGG","GCTTCCAAGTTGCTCTTATCAGG",0,NA,NA,NA,"GeneID:26057","ANKRD17",TRUE,"ANKRD17"

"7838","GTGCGAAAGTTACTCATTGA","chr4",73156103,"-","none","AGG","GTGCGAAAGTTACTCATTGAAGG",0,NA,NA,NA,"GeneID:26057","ANKRD17",TRUE,"ANKRD17"

"7839","ATGCCGTTGACAATTTCAAA","chr9",33533523,"+","none","AGG","ATGCCGTTGACAATTTCAAAAGG",0,NA,NA,NA,"GeneID:441459","ANKRD18B",TRUE,"ANKRD18A"

"7840","ATGCCGTTGACAATTTCAAA","chr9",38611216,"-","none","AGG","ATGCCGTTGACAATTTCAAAAGG",0,NA,NA,NA,"GeneID:253650","ANKRD18A",TRUE,"ANKRD18A"

"7841","ATGCCGTTGACAATTTCAAA","chr9",97200460,"-","18:C>T","AGG","ATGCCGTTGACAATTTCAGAAGG",1,19,NA,NA,"","",TRUE,"ANKRD18A"

"7842","ATGCCGTTGACAATTTCAAA","chr4",163852563,"+","4:A>C,6:A>T","AGG","ATGCAGATGACAATTTCAAAAGG",2,5,7,NA,"GeneID:55016","MARCH1",FALSE,"ANKRD18A"

"7843","ATGCCGTTGACAATTTCAAA","chr9",92818402,"+","5:A>G,18:G>A","AGG","ATGCCATTGACAATTTCAGAAGG",2,6,19,NA,"GeneID:138649","ANKRD19P",TRUE,"ANKRD18A"

"7844","GCCACATTCCAGGAGAACGA","chr9",38615716,"+","none","TGG","GCCACATTCCAGGAGAACGATGG",0,NA,NA,NA,"GeneID:253650","ANKRD18A",TRUE,"ANKRD18A"

"7845","GCCACATTCCAGGAGAACGA","chr10",43393811,"-","17:T>G,18:T>C","AGG","GCCACATTCCAGGAGAAAAAAGG",2,18,19,NA,"GeneID:3185","HNRNPF",FALSE,"ANKRD18A"

"7846","GCCACATTCCAGGAGAACGA","chr3",9100911,"+","4:A>C,15:G>A","TGG","GCCAAATTCCAGGAGGACGATGG",2,5,16,NA,"GeneID:9901","SRGAP3",FALSE,"ANKRD18A"

"7847","GGCTTGTGCCATCGTTCTCC","chr9",38615727,"-","none","TGG","GGCTTGTGCCATCGTTCTCCTGG",0,NA,NA,NA,"GeneID:253650","ANKRD18A",TRUE,"ANKRD18A"

"7848","GGCTTGTGCCATCGTTCTCC","chr9",92813893,"+","12:T>C,13:A>G","TGG","GGCTTGTGCCATTATTCTCCTGG",2,13,14,NA,"GeneID:138649","ANKRD19P",TRUE,"ANKRD18A"

"7849","GTCTGTCACAGATGTCGATC","chr9",38615952,"+","none","TGG","GTCTGTCACAGATGTCGATCTGG",0,NA,NA,NA,"GeneID:253650","ANKRD18A",TRUE,"ANKRD18A"

"7850","GTCTGTCACAGATGTCGATC","chr9",33528798,"-","15:A>G","TGG","GTCTGTCACAGATGTTGATCTGG",1,16,NA,NA,"GeneID:441459","ANKRD18B",TRUE,"ANKRD18A"

"7851","GTCTGTCACAGATGTCGATC","chr9",97211395,"+","16:A>G","AGG","GTCTGTCACAGATGTCAATCAGG",1,17,NA,NA,"","",TRUE,"ANKRD18A"

"7852","TCCTAGGACTGTTCTACATT","chr9",33528720,"+","none","TGG","TCCTAGGACTGTTCTACATTTGG",0,NA,NA,NA,"GeneID:441459","ANKRD18B",TRUE,"ANKRD18A"

"7853","TCCTAGGACTGTTCTACATT","chr9",38616030,"-","none","TGG","TCCTAGGACTGTTCTACATTTGG",0,NA,NA,NA,"GeneID:253650","ANKRD18A",TRUE,"ANKRD18A"

"7854","TCCTAGGACTGTTCTACATT","chr3",198077274,"-","none","TGG","TCCTAGGACTGTTCTACATTTGG",0,NA,NA,NA,"GeneID:348840","ANKRD18DP",TRUE,"ANKRD18A"

"7855","TCCTAGGACTGTTCTACATT","chr9",97211473,"-","0:T>A,2:A>G","TGG","ACTTAGGACTGTTCTACATTTGG",2,1,3,NA,"","",TRUE,"ANKRD18A"

"7856","TCCTAGGACTGTTCTACATT","chr10",107106336,"-","1:C>G,6:G>C","TGG","TGCTAGCACTGTTCTACATTTGG",2,2,7,NA,"GeneID:114815","SORCS1",FALSE,"ANKRD18A"

"7857","TCCTAGGACTGTTCTACATT","chr8",57199105,"+","1:T>C,2:G>C","TGG","TTGTAGGACTGTTCTACATTTGG",2,2,3,NA,"GeneID:100507651","LOC100507651",FALSE,"ANKRD18A"

"7858","TCCTAGGACTGTTCTACATT","chr21",13615116,"+","2:A>C,11:C>T","TGG","TCATAGGACTGCTCTACATTTGG",2,3,12,NA,"GeneID:317754","POTED",TRUE,"ANKRD18A"

"7859","TCCTAGGACTGTTCTACATT","chr7",39816542,"+","2:A>C,11:C>T","TGG","TCATAGGACTGCTCTACATTTGG",2,3,12,NA,"","",TRUE,"ANKRD18A"

"7860","TCCTAGGACTGTTCTACATT","chr15",21435094,"-","2:T>G,11:G>A","TGG","TCATAGGACTGCTCTACATTTGG",2,3,12,NA,"GeneID:102723502","LOC102723502",TRUE,"ANKRD18A"

"7861","TCCTAGGACTGTTCTACATT","chrUn_GL000213v1",134376,"-","2:T>G,11:G>A","TGG","TCATAGGACTGCTCTACATTTGG",2,3,12,NA,"","",FALSE,"ANKRD18A"

"7862","TCCTAGGACTGTTCTACATT","chr18",14538235,"-","2:T>G,11:G>A","TGG","TCATAGGACTGCTCTACATTTGG",2,3,12,NA,"GeneID:388468","POTEC",TRUE,"ANKRD18A"

"7863","TCCTAGGACTGTTCTACATT","chr15",21872440,"-","2:T>G,11:G>A","TGG","TCATAGGACTGCTCTACATTTGG",2,3,12,NA,"GeneID:100996331","POTEB",TRUE,"ANKRD18A"

"7864","TCCTAGGACTGTTCTACATT","chr15",20861475,"-","2:T>G,11:G>A","TGG","TCATAGGACTGCTCTACATTTGG",2,3,12,NA,"GeneID:100287399","POTEB2",TRUE,"ANKRD18A"

"7865","TCCTAGGACTGTTCTACATT","chr15_KI270727v1_random",162297,"-","2:T>G,11:G>A","TGG","TCATAGGACTGCTCTACATTTGG",2,3,12,NA,"","",FALSE,"ANKRD18A"

"7866","TTCCACCATATGCTGTCTCC","chr9",38611264,"+","none","TGG","TTCCACCATATGCTGTCTCCTGG",0,NA,NA,NA,"GeneID:253650","ANKRD18A",TRUE,"ANKRD18A"

"7867","TTCCACCATATGCTGTCTCC","chr9",33533475,"-","none","TGG","TTCCACCATATGCTGTCTCCTGG",0,NA,NA,NA,"GeneID:441459","ANKRD18B",TRUE,"ANKRD18A"

"7868","TTCCACCATATGCTGTCTCC","chr7",1938339,"+","8:C>T,11:T>G","TGG","TTCCACCACATTCTGTCTCCTGG",2,9,12,NA,"GeneID:8379","MAD1L1",FALSE,"ANKRD18A"

"7869","ACCCCCGACCCGCATACTCT","chr9",33524551,"-","none","TGG","ACCCCCGACCCGCATACTCTTGG",0,NA,NA,NA,"GeneID:441459","ANKRD18B",TRUE,"ANKRD18B"

"7870","ACCCCCGACCCGCATACTCT","chr9",92809726,"-","10:T>G,13:C>T","TGG","ACCCCCGACCAGCGTACTCTTGG",2,11,14,NA,"GeneID:138649","ANKRD19P",TRUE,"ANKRD18B"

"7871","ACCCCCGACCCGCATACTCT","chr9",97221153,"+","13:G>A","TGG","ACCCCCGACCCGCGTACTCTTGG",1,14,NA,NA,"","",TRUE,"ANKRD18B"

"7872","ACCCCCGACCCGCATACTCT","chr9",38620203,"+","1:A>C,5:G>C","TGG","AACCCGGACCCGCATACTCTTGG",2,2,6,NA,"GeneID:253650","ANKRD18A",TRUE,"ANKRD18B"

"7873","CCAGGCGTCTCCCAAAACTG","chr9",33524503,"-","none","AGG","CCAGGCGTCTCCCAAAACTGAGG",0,NA,NA,NA,"GeneID:441459","ANKRD18B",TRUE,"ANKRD18B"

"7874","CCATTATGCCGTGTATAATG","chr9",33529091,"+","none","AGG","CCATTATGCCGTGTATAATGAGG",0,NA,NA,NA,"GeneID:441459","ANKRD18B",TRUE,"ANKRD18B"

"7875","CCATTATGCCGTGTATAATG","chr9",38615655,"-","19:T>C","AGG","CCATTATGCCGTGTATAATAAGG",1,20,NA,NA,"GeneID:253650","ANKRD18A",TRUE,"ANKRD18B"

"7876","CCATTATGCCGTGTATAATG","chr9",92813965,"+","9:T>C,19:A>G","AGG","CCATTATGCTGTGTATAATAAGG",2,10,20,NA,"GeneID:138649","ANKRD19P",TRUE,"ANKRD18B"

"7877","CTACTATATACCTTCATTAA","chr9",38615918,"+","none","AGG","CTACTATATACCTTCATTAAAGG",0,NA,NA,NA,"GeneID:253650","ANKRD18A",TRUE,"ANKRD18B"

"7878","CTACTATATACCTTCATTAA","chr9",33528832,"-","none","AGG","CTACTATATACCTTCATTAAAGG",0,NA,NA,NA,"GeneID:441459","ANKRD18B",TRUE,"ANKRD18B"

"7879","CTACTATATACCTTCATTAA","chr9",92813701,"-","none","AGG","CTACTATATACCTTCATTAAAGG",0,NA,NA,NA,"GeneID:138649","ANKRD19P",TRUE,"ANKRD18B"

"7880","CTACTATATACCTTCATTAA","chr8",57284266,"-","15:C>T,19:C>T","AGG","CTACTATATACCTTCGTTAGAGG",2,16,20,NA,"GeneID:26138","LINC00588",TRUE,"ANKRD18B"

"7881","CTACTATATACCTTCATTAA","chrY",26414153,"-","14:C>G,19:C>T","AGG","CTACTATATACCTTGATTAGAGG",2,15,20,NA,"","",TRUE,"ANKRD18B"

"7882","CTACTATATACCTTCATTAA","chr6",61153773,"-","0:A>G,6:T>A","AGG","TTACTAAATACCTTCATTAAAGG",2,1,7,NA,"","",FALSE,"ANKRD18B"

"7883","CTACTATATACCTTCATTAA","chr3",198077162,"+","2:G>A,5:G>A","AGG","CTGCTGTATACCTTCATTAAAGG",2,3,6,NA,"GeneID:348840","ANKRD18DP",TRUE,"ANKRD18B"

"7884","CTACTATATACCTTCATTAA","chr6",58296811,"+","0:T>C,6:A>T","AGG","TTACTAAATACCTTCATTAAAGG",2,1,7,NA,"","",FALSE,"ANKRD18B"

"7885","CTACTATATACCTTCATTAA","chr5",60783753,"+","8:A>T,17:A>T","AGG","CTACTATAAACCTTCATAAAAGG",2,9,18,NA,"GeneID:79993","ELOVL7",FALSE,"ANKRD18B"

"7886","GCCATTATTCTCCTGAAACG","chr9",33529026,"+","none","TGG","GCCATTATTCTCCTGAAACGTGG",0,NA,NA,NA,"GeneID:441459","ANKRD18B",TRUE,"ANKRD18B"

"7887","GCCATTATTCTCCTGAAACG","chr13",23941416,"-","11:C>G,15:C>T","TGG","GCCATTATTCTGCTGGAACGTGG",2,12,16,NA,"","",TRUE,"ANKRD18B"

"7888","GCCATTATTCTCCTGAAACG","chr9",92813900,"+","15:G>A,19:A>G","TGG","GCCATTATTCTCCTGGAACATGG",2,16,20,NA,"GeneID:138649","ANKRD19P",TRUE,"ANKRD18B"

"7889","GCCATTATTCTCCTGAAACG","chr5",61397419,"-","6:A>T,18:A>G","AGG","GCCATTTTTCTCCTGAAATGAGG",2,7,19,NA,"GeneID:57688","ZSWIM6",FALSE,"ANKRD18B"

"7890","GTCTGTCACAGATGTTGATC","chr9",33528798,"-","none","TGG","GTCTGTCACAGATGTTGATCTGG",0,NA,NA,NA,"GeneID:441459","ANKRD18B",TRUE,"ANKRD18B"

"7891","GTCTGTCACAGATGTTGATC","chr9",38615952,"+","15:C>T","TGG","GTCTGTCACAGATGTCGATCTGG",1,16,NA,NA,"GeneID:253650","ANKRD18A",TRUE,"ANKRD18B"

"7892","GTCTGTCACAGATGTTGATC","chr9",97211395,"+","15:C>T,16:A>G","AGG","GTCTGTCACAGATGTCAATCAGG",2,16,17,NA,"","",TRUE,"ANKRD18B"

"7893","ACGTCTTGCGCACGCGCTCT","chr10",97578244,"-","none","TGG","ACGTCTTGCGCACGCGCTCTTGG",0,NA,NA,NA,"GeneID:26287","ANKRD2",TRUE,"ANKRD2"

"7894","CATCACCTGGTCGCACGTGT","chr10",97578591,"-","none","CGG","CATCACCTGGTCGCACGTGTCGG",0,NA,NA,NA,"GeneID:26287","ANKRD2",TRUE,"ANKRD2"

"7895","CATCGATGATCTCCCGCCGC","chr10",97578274,"-","none","AGG","CATCGATGATCTCCCGCCGCAGG",0,NA,NA,NA,"GeneID:26287","ANKRD2",TRUE,"ANKRD2"

"7896","CGCACGCCAGAAGCTGCCCA","chr10",97577813,"+","none","TGG","CGCACGCCAGAAGCTGCCCATGG",0,NA,NA,NA,"GeneID:26287","ANKRD2",TRUE,"ANKRD2"

"7897","GCTTCTGGCGTGCGTCTCCT","chr10",97577807,"-","none","CGG","GCTTCTGGCGTGCGTCTCCTCGG",0,NA,NA,NA,"GeneID:26287","ANKRD2",TRUE,"ANKRD2"

"7898","GTCAGCCGACACGTGCGACC","chr10",97578583,"+","none","AGG","GTCAGCCGACACGTGCGACCAGG",0,NA,NA,NA,"GeneID:26287","ANKRD2",TRUE,"ANKRD2"

"7899","AATTTGCAAGAAAGAGAAAA","chr9",40231271,"+","none","TGG","AATTTGCAAGAAAGAGAAAATGG",0,NA,NA,NA,"GeneID:441430","ANKRD20A2",TRUE,"ANKRD20A1"

"7900","AATTTGCAAGAAAGAGAAAA","chr18",14188033,"+","none","TGG","AATTTGCAAGAAAGAGAAAATGG",0,NA,NA,NA,"","",TRUE,"ANKRD20A1"

"7901","AATTTGCAAGAAAGAGAAAA","chr9",42860564,"+","none","TGG","AATTTGCAAGAAAGAGAAAATGG",0,NA,NA,NA,"","",TRUE,"ANKRD20A1"

"7902","AATTTGCAAGAAAGAGAAAA","chr9",67867305,"+","none","TGG","AATTTGCAAGAAAGAGAAAATGG",0,NA,NA,NA,"GeneID:84210","ANKRD20A1",TRUE,"ANKRD20A1"

"7903","AATTTGCAAGAAAGAGAAAA","chr9",64377549,"+","none","TGG","AATTTGCAAGAAAGAGAAAATGG",0,NA,NA,NA,"GeneID:728747","ANKRD20A4",TRUE,"ANKRD20A1"

"7904","AATTTGCAAGAAAGAGAAAA","chr9",64079776,"+","none","TGG","AATTTGCAAGAAAGAGAAAATGG",0,NA,NA,NA,"","",FALSE,"ANKRD20A1"

"7905","AATTTGCAAGAAAGAGAAAA","chrUn_KI270744v1",53746,"-","none","TGG","AATTTGCAAGAAAGAGAAAATGG",0,NA,NA,NA,"","",FALSE,"ANKRD20A1"

"7906","AATTTGCAAGAAAGAGAAAA","chr9",66144956,"-","none","TGG","AATTTGCAAGAAAGAGAAAATGG",0,NA,NA,NA,"GeneID:441425","ANKRD20A3",TRUE,"ANKRD20A1"

"7907","AATTTGCAAGAAAGAGAAAA","chr21",13971224,"-","none","TGG","AATTTGCAAGAAAGAGAAAATGG",0,NA,NA,NA,"GeneID:391267","ANKRD20A11P",TRUE,"ANKRD20A1"

"7908","AATTTGCAAGAAAGAGAAAA","chr2",94849258,"-","none","TGG","AATTTGCAAGAAAGAGAAAATGG",0,NA,NA,NA,"","",TRUE,"ANKRD20A1"

"7909","AATTTGCAAGAAAGAGAAAA","chr13",18871918,"-","none","TGG","AATTTGCAAGAAAGAGAAAATGG",0,NA,NA,NA,"","",TRUE,"ANKRD20A1"

"7910","AATTTGCAAGAAAGAGAAAA","chr20",30716014,"-","none","TGG","AATTTGCAAGAAAGAGAAAATGG",0,NA,NA,NA,"","",TRUE,"ANKRD20A1"

"7911","AATTTGCAAGAAAGAGAAAA","chr2",100482342,"-","7:A>T,8:A>T","AGG","AATTTGCTTGAAAGAGAAAAAGG",2,8,9,NA,"GeneID:129521","NMS",FALSE,"ANKRD20A1"

"7912","AATTTGCAAGAAAGAGAAAA","chr10",32274114,"-","4:C>A,9:T>C","AGG","AATTGGCAAAAAAGAGAAAAAGG",2,5,10,NA,"GeneID:80314","EPC1",FALSE,"ANKRD20A1"

"7913","AATTTGCAAGAAAGAGAAAA","chr2",39540715,"+","1:G>A,6:A>C","GGG","AGTTTGAAAGAAAGAGAAAAGGG",2,2,7,NA,"GeneID:728730","LOC728730",FALSE,"ANKRD20A1"

"7914","AATTTGCAAGAAAGAGAAAA","chr21",22043188,"+","5:C>G,7:C>A","TGG","AATTTCCCAGAAAGAGAAAATGG",2,6,8,NA,"GeneID:101927843","LOC101927843",FALSE,"ANKRD20A1"

"7915","AATTTGCAAGAAAGAGAAAA","chr8",138921604,"+","6:T>C,13:A>G","TGG","AATTTGTAAGAAAAAGAAAATGG",2,7,14,NA,"","",FALSE,"ANKRD20A1"

"7916","AATTTGCAAGAAAGAGAAAA","chr1",173428325,"+","5:A>G,13:C>G","TGG","AATTTACAAGAAACAGAAAATGG",2,6,14,NA,"GeneID:101928673","LOC101928673",FALSE,"ANKRD20A1"

"7917","AATTTGCAAGAAAGAGAAAA","chr5",88435873,"+","6:A>C,17:G>A","AGG","AATTTGAAAGAAAGAGAGAAAGG",2,7,18,NA,"GeneID:100505894;GeneID:102546226","TMEM161B-AS1;LOC102546226",FALSE,"ANKRD20A1"

"7918","AATTTGCAAGAAAGAGAAAA","chr6",169415927,"+","6:A>C,17:G>A","AGG","AATTTGAAAGAAAGAGAGAAAGG",2,7,18,NA,"","",FALSE,"ANKRD20A1"

"7919","AATTTGCAAGAAAGAGAAAA","chr8",14652651,"-","9:G>C,15:G>C","TGG","AATTTGCAACAAAGACAAAATGG",2,10,16,NA,"GeneID:137868","SGCZ",FALSE,"ANKRD20A1"

"7920","AATTTGCAAGAAAGAGAAAA","chr11",91404361,"-","5:A>C,11:G>T","AGG","AATTTTCAAGACAGAGAAAAAGG",2,6,12,NA,"","",FALSE,"ANKRD20A1"

"7921","AATTTGCAAGAAAGAGAAAA","chr8",87856037,"-","4:C>A,18:A>T","TGG","AATTGGCAAGAAAGAGAATATGG",2,5,19,NA,"","",FALSE,"ANKRD20A1"

"7922","CGGACAGGCTGTCCATTGCC","chr18",14183921,"+","none","AGG","CGGACAGGCTGTCCATTGCCAGG",0,NA,NA,NA,"","",TRUE,"ANKRD20A1"

"7923","CGGACAGGCTGTCCATTGCC","chr9",42856467,"+","none","AGG","CGGACAGGCTGTCCATTGCCAGG",0,NA,NA,NA,"","",TRUE,"ANKRD20A1"

"7924","CGGACAGGCTGTCCATTGCC","chr21",14068906,"+","none","AGG","CGGACAGGCTGTCCATTGCCAGG",0,NA,NA,NA,"","",TRUE,"ANKRD20A1"

"7925","CGGACAGGCTGTCCATTGCC","chr9",67863210,"+","none","AGG","CGGACAGGCTGTCCATTGCCAGG",0,NA,NA,NA,"GeneID:84210","ANKRD20A1",TRUE,"ANKRD20A1"

"7926","CGGACAGGCTGTCCATTGCC","chr9",40227176,"+","none","AGG","CGGACAGGCTGTCCATTGCCAGG",0,NA,NA,NA,"GeneID:441430","ANKRD20A2",TRUE,"ANKRD20A1"

"7927","CGGACAGGCTGTCCATTGCC","chr9",64373456,"+","none","AGG","CGGACAGGCTGTCCATTGCCAGG",0,NA,NA,NA,"GeneID:728747","ANKRD20A4",TRUE,"ANKRD20A1"

"7928","CGGACAGGCTGTCCATTGCC","chrUn_KI270744v1",57836,"-","none","AGG","CGGACAGGCTGTCCATTGCCAGG",0,NA,NA,NA,"","",FALSE,"ANKRD20A1"

"7929","CGGACAGGCTGTCCATTGCC","chr2",94853362,"-","none","AGG","CGGACAGGCTGTCCATTGCCAGG",0,NA,NA,NA,"","",TRUE,"ANKRD20A1"

"7930","CGGACAGGCTGTCCATTGCC","chr9",66149049,"-","none","AGG","CGGACAGGCTGTCCATTGCCAGG",0,NA,NA,NA,"GeneID:441425","ANKRD20A3",TRUE,"ANKRD20A1"

"7931","CGGACAGGCTGTCCATTGCC","chr20",30720039,"-","none","AGG","CGGACAGGCTGTCCATTGCCAGG",0,NA,NA,NA,"","",TRUE,"ANKRD20A1"

"7932","CGGACAGGCTGTCCATTGCC","chr21",13975344,"-","4:T>G","AGG","CGGAAAGGCTGTCCATTGCCAGG",1,5,NA,NA,"GeneID:391267","ANKRD20A11P",TRUE,"ANKRD20A1"

"7933","CGGACAGGCTGTCCATTGCC","chr9",64075665,"+","1:A>G","AGG","CAGACAGGCTGTCCATTGCCAGG",1,2,NA,NA,"","",FALSE,"ANKRD20A1"

"7934","CTCTAATATACCTGTATCAA","chr9",66149213,"+","none","AGG","CTCTAATATACCTGTATCAAAGG",0,NA,NA,NA,"GeneID:441425","ANKRD20A3",TRUE,"ANKRD20A1"

"7935","CTCTAATATACCTGTATCAA","chr2",94853526,"+","none","AGG","CTCTAATATACCTGTATCAAAGG",0,NA,NA,NA,"","",TRUE,"ANKRD20A1"

"7936","CTCTAATATACCTGTATCAA","chr21",13975508,"+","none","AGG","CTCTAATATACCTGTATCAAAGG",0,NA,NA,NA,"GeneID:391267","ANKRD20A11P",TRUE,"ANKRD20A1"

"7937","CTCTAATATACCTGTATCAA","chrUn_KI270744v1",58001,"+","none","AGG","CTCTAATATACCTGTATCAAAGG",0,NA,NA,NA,"","",FALSE,"ANKRD20A1"

"7938","CTCTAATATACCTGTATCAA","chr18",14183757,"-","none","AGG","CTCTAATATACCTGTATCAAAGG",0,NA,NA,NA,"","",TRUE,"ANKRD20A1"

"7939","CTCTAATATACCTGTATCAA","chr9",64075500,"-","none","AGG","CTCTAATATACCTGTATCAAAGG",0,NA,NA,NA,"","",FALSE,"ANKRD20A1"

"7940","CTCTAATATACCTGTATCAA","chr9",42856303,"-","none","AGG","CTCTAATATACCTGTATCAAAGG",0,NA,NA,NA,"","",TRUE,"ANKRD20A1"

"7941","CTCTAATATACCTGTATCAA","chr9",67863046,"-","none","AGG","CTCTAATATACCTGTATCAAAGG",0,NA,NA,NA,"GeneID:84210","ANKRD20A1",TRUE,"ANKRD20A1"

"7942","CTCTAATATACCTGTATCAA","chr9",40227012,"-","none","AGG","CTCTAATATACCTGTATCAAAGG",0,NA,NA,NA,"GeneID:441430","ANKRD20A2",TRUE,"ANKRD20A1"

"7943","CTCTAATATACCTGTATCAA","chr9",64373292,"-","none","AGG","CTCTAATATACCTGTATCAAAGG",0,NA,NA,NA,"GeneID:728747","ANKRD20A4",TRUE,"ANKRD20A1"

"7944","CTCTAATATACCTGTATCAA","chr21",14068742,"-","none","AGG","CTCTAATATACCTGTATCAAAGG",0,NA,NA,NA,"","",TRUE,"ANKRD20A1"

"7945","CTCTAATATACCTGTATCAA","chr13",66317699,"-","2:A>G,3:C>A","AGG","CTTGAATATACCTGTATCAAAGG",2,3,4,NA,"GeneID:5101","PCDH9",FALSE,"ANKRD20A1"

"7946","GTACCGTCTCAGCCTATCAA","chr9",66144894,"+","none","CGG","GTACCGTCTCAGCCTATCAACGG",0,NA,NA,NA,"GeneID:441425","ANKRD20A3",TRUE,"ANKRD20A1"

"7947","GTACCGTCTCAGCCTATCAA","chr2",94849196,"+","none","CGG","GTACCGTCTCAGCCTATCAACGG",0,NA,NA,NA,"","",TRUE,"ANKRD20A1"

"7948","GTACCGTCTCAGCCTATCAA","chr9",40231333,"-","none","CGG","GTACCGTCTCAGCCTATCAACGG",0,NA,NA,NA,"GeneID:441430","ANKRD20A2",TRUE,"ANKRD20A1"

"7949","GTACCGTCTCAGCCTATCAA","chr9",42860627,"-","none","CGG","GTACCGTCTCAGCCTATCAACGG",0,NA,NA,NA,"","",TRUE,"ANKRD20A1"

"7950","GTACCGTCTCAGCCTATCAA","chr9",67867367,"-","none","CGG","GTACCGTCTCAGCCTATCAACGG",0,NA,NA,NA,"GeneID:84210","ANKRD20A1",TRUE,"ANKRD20A1"

"7951","GTACCGTCTCAGCCTATCAA","chr9",64377611,"-","none","CGG","GTACCGTCTCAGCCTATCAACGG",0,NA,NA,NA,"GeneID:728747","ANKRD20A4",TRUE,"ANKRD20A1"

"7952","GTACCGTCTCAGCCTATCAA","chr18",14188095,"-","5:T>C","CGG","GTACCATCTCAGCCTATCAACGG",1,6,NA,NA,"","",TRUE,"ANKRD20A1"

"7953","GTACCGTCTCAGCCTATCAA","chr21",13971162,"+","5:A>G,12:G>C","CGG","GTACCATCTCAGGCTATCAACGG",2,6,13,NA,"GeneID:391267","ANKRD20A11P",TRUE,"ANKRD20A1"

"7954","GTACCGTCTCAGCCTATCAA","chr13",18871856,"+","5:A>G,12:G>C","CGG","GTACCATCTCAGGCTATCAACGG",2,6,13,NA,"","",TRUE,"ANKRD20A1"

"7955","GTGCTTCAATATGTGCACCA","chr9",66148896,"+","none","TGG","GTGCTTCAATATGTGCACCATGG",0,NA,NA,NA,"GeneID:441425","ANKRD20A3",TRUE,"ANKRD20A1"

"7956","GTGCTTCAATATGTGCACCA","chr9",64373609,"-","none","TGG","GTGCTTCAATATGTGCACCATGG",0,NA,NA,NA,"GeneID:728747","ANKRD20A4",TRUE,"ANKRD20A1"

"7957","GTGCTTCAATATGTGCACCA","chr9",67863363,"-","none","TGG","GTGCTTCAATATGTGCACCATGG",0,NA,NA,NA,"GeneID:84210","ANKRD20A1",TRUE,"ANKRD20A1"

"7958","GTGCTTCAATATGTGCACCA","chr9",40227329,"-","none","TGG","GTGCTTCAATATGTGCACCATGG",0,NA,NA,NA,"GeneID:441430","ANKRD20A2",TRUE,"ANKRD20A1"

"7959","GTGCTTCAATATGTGCACCA","chr9",42856620,"-","none","TGG","GTGCTTCAATATGTGCACCATGG",0,NA,NA,NA,"","",TRUE,"ANKRD20A1"

"7960","GTGCTTCAATATGTGCACCA","chr9",64075818,"-","12:A>C","TGG","GTGCTTCAATATTTGCACCATGG",1,13,NA,NA,"","",FALSE,"ANKRD20A1"

"7961","GTGCTTCAATATGTGCACCA","chr18",14184074,"-","12:A>C,14:A>C","TGG","GTGCTTCAATATTTTCACCATGG",2,13,15,NA,"","",TRUE,"ANKRD20A1"

"7962","GTGCTTCAATATGTGCACCA","chrUn_KI270744v1",57683,"+","12:T>G","TGG","GTGCTTCAATATTTGCACCATGG",1,13,NA,NA,"","",FALSE,"ANKRD20A1"

"7963","GTGCTTCAATATGTGCACCA","chr21",13975191,"+","12:T>G","TGG","GTGCTTCAATATTTGCACCATGG",1,13,NA,NA,"GeneID:391267","ANKRD20A11P",TRUE,"ANKRD20A1"

"7964","GTGCTTCAATATGTGCACCA","chr20",30719886,"+","12:T>G","TGG","GTGCTTCAATATTTGCACCATGG",1,13,NA,NA,"","",TRUE,"ANKRD20A1"

"7965","GTGCTTCAATATGTGCACCA","chr2",94853209,"+","10:G>A","TGG","GTGCTTCAATGTGTGCACCATGG",1,11,NA,NA,"","",TRUE,"ANKRD20A1"

"7966","TACCAGTGGCCATGTGCAAG","chr9",67862961,"+","none","TGG","TACCAGTGGCCATGTGCAAGTGG",0,NA,NA,NA,"GeneID:84210","ANKRD20A1",TRUE,"ANKRD20A1"

"7967","TACCAGTGGCCATGTGCAAG","chr9",66149298,"-","1:C>T","TGG","TGCCAGTGGCCATGTGCAAGTGG",1,2,NA,NA,"GeneID:441425","ANKRD20A3",TRUE,"ANKRD20A1"

"7968","TACCAGTGGCCATGTGCAAG","chr2",94853611,"-","1:C>T","TGG","TGCCAGTGGCCATGTGCAAGTGG",1,2,NA,NA,"","",TRUE,"ANKRD20A1"

"7969","TACCAGTGGCCATGTGCAAG","chr9",64373207,"+","1:G>A","TGG","TGCCAGTGGCCATGTGCAAGTGG",1,2,NA,NA,"GeneID:728747","ANKRD20A4",TRUE,"ANKRD20A1"

"7970","TACCAGTGGCCATGTGCAAG","chr9",42856218,"+","1:G>A","TGG","TGCCAGTGGCCATGTGCAAGTGG",1,2,NA,NA,"","",TRUE,"ANKRD20A1"

"7971","TACCAGTGGCCATGTGCAAG","chr9",40226927,"+","1:G>A","TGG","TGCCAGTGGCCATGTGCAAGTGG",1,2,NA,NA,"GeneID:441430","ANKRD20A2",TRUE,"ANKRD20A1"

"7972","TACCAGTGGCCATGTGCAAG","chr21",14068657,"+","1:G>A,16:A>C","TGG","TGCCAGTGGCCATGTGAAAGTGG",2,2,17,NA,"","",TRUE,"ANKRD20A1"

"7973","TACCAGTGGCCATGTGCAAG","chr18",14183672,"+","1:G>A,16:A>C","TGG","TGCCAGTGGCCATGTGAAAGTGG",2,2,17,NA,"","",TRUE,"ANKRD20A1"

"7974","TACCAGTGGCCATGTGCAAG","chr21",13975593,"-","1:C>T,16:T>G","TGG","TGCCAGTGGCCATGTGAAAGTGG",2,2,17,NA,"GeneID:391267","ANKRD20A11P",TRUE,"ANKRD20A1"

"7975","TACCAGTGGCCATGTGCAAG","chr20",30720289,"-","1:C>T,16:T>G","TGG","TGCCAGTGGCCATGTGAAAGTGG",2,2,17,NA,"","",TRUE,"ANKRD20A1"

"7976","AATTTGCAAGAAAGAGAAAA","chr9",40231271,"+","none","TGG","AATTTGCAAGAAAGAGAAAATGG",0,NA,NA,NA,"GeneID:441430","ANKRD20A2",TRUE,"ANKRD20A2"

"7977","AATTTGCAAGAAAGAGAAAA","chr18",14188033,"+","none","TGG","AATTTGCAAGAAAGAGAAAATGG",0,NA,NA,NA,"","",TRUE,"ANKRD20A2"

"7978","AATTTGCAAGAAAGAGAAAA","chr9",42860564,"+","none","TGG","AATTTGCAAGAAAGAGAAAATGG",0,NA,NA,NA,"","",TRUE,"ANKRD20A2"

"7979","AATTTGCAAGAAAGAGAAAA","chr9",67867305,"+","none","TGG","AATTTGCAAGAAAGAGAAAATGG",0,NA,NA,NA,"GeneID:84210","ANKRD20A1",TRUE,"ANKRD20A2"

"7980","AATTTGCAAGAAAGAGAAAA","chr9",64377549,"+","none","TGG","AATTTGCAAGAAAGAGAAAATGG",0,NA,NA,NA,"GeneID:728747","ANKRD20A4",TRUE,"ANKRD20A2"

"7981","AATTTGCAAGAAAGAGAAAA","chr9",64079776,"+","none","TGG","AATTTGCAAGAAAGAGAAAATGG",0,NA,NA,NA,"","",FALSE,"ANKRD20A2"

"7982","AATTTGCAAGAAAGAGAAAA","chrUn_KI270744v1",53746,"-","none","TGG","AATTTGCAAGAAAGAGAAAATGG",0,NA,NA,NA,"","",FALSE,"ANKRD20A2"

"7983","AATTTGCAAGAAAGAGAAAA","chr9",66144956,"-","none","TGG","AATTTGCAAGAAAGAGAAAATGG",0,NA,NA,NA,"GeneID:441425","ANKRD20A3",TRUE,"ANKRD20A2"

"7984","AATTTGCAAGAAAGAGAAAA","chr21",13971224,"-","none","TGG","AATTTGCAAGAAAGAGAAAATGG",0,NA,NA,NA,"GeneID:391267","ANKRD20A11P",TRUE,"ANKRD20A2"

"7985","AATTTGCAAGAAAGAGAAAA","chr2",94849258,"-","none","TGG","AATTTGCAAGAAAGAGAAAATGG",0,NA,NA,NA,"","",TRUE,"ANKRD20A2"

"7986","AATTTGCAAGAAAGAGAAAA","chr13",18871918,"-","none","TGG","AATTTGCAAGAAAGAGAAAATGG",0,NA,NA,NA,"","",TRUE,"ANKRD20A2"

"7987","AATTTGCAAGAAAGAGAAAA","chr20",30716014,"-","none","TGG","AATTTGCAAGAAAGAGAAAATGG",0,NA,NA,NA,"","",TRUE,"ANKRD20A2"

"7988","AATTTGCAAGAAAGAGAAAA","chr2",100482342,"-","7:A>T,8:A>T","AGG","AATTTGCTTGAAAGAGAAAAAGG",2,8,9,NA,"GeneID:129521","NMS",FALSE,"ANKRD20A2"

"7989","AATTTGCAAGAAAGAGAAAA","chr10",32274114,"-","4:C>A,9:T>C","AGG","AATTGGCAAAAAAGAGAAAAAGG",2,5,10,NA,"GeneID:80314","EPC1",FALSE,"ANKRD20A2"

"7990","AATTTGCAAGAAAGAGAAAA","chr2",39540715,"+","1:G>A,6:A>C","GGG","AGTTTGAAAGAAAGAGAAAAGGG",2,2,7,NA,"GeneID:728730","LOC728730",FALSE,"ANKRD20A2"

"7991","AATTTGCAAGAAAGAGAAAA","chr21",22043188,"+","5:C>G,7:C>A","TGG","AATTTCCCAGAAAGAGAAAATGG",2,6,8,NA,"GeneID:101927843","LOC101927843",FALSE,"ANKRD20A2"

"7992","AATTTGCAAGAAAGAGAAAA","chr8",138921604,"+","6:T>C,13:A>G","TGG","AATTTGTAAGAAAAAGAAAATGG",2,7,14,NA,"","",FALSE,"ANKRD20A2"

"7993","AATTTGCAAGAAAGAGAAAA","chr1",173428325,"+","5:A>G,13:C>G","TGG","AATTTACAAGAAACAGAAAATGG",2,6,14,NA,"GeneID:101928673","LOC101928673",FALSE,"ANKRD20A2"

"7994","AATTTGCAAGAAAGAGAAAA","chr5",88435873,"+","6:A>C,17:G>A","AGG","AATTTGAAAGAAAGAGAGAAAGG",2,7,18,NA,"GeneID:100505894;GeneID:102546226","TMEM161B-AS1;LOC102546226",FALSE,"ANKRD20A2"

"7995","AATTTGCAAGAAAGAGAAAA","chr6",169415927,"+","6:A>C,17:G>A","AGG","AATTTGAAAGAAAGAGAGAAAGG",2,7,18,NA,"","",FALSE,"ANKRD20A2"

"7996","AATTTGCAAGAAAGAGAAAA","chr8",14652651,"-","9:G>C,15:G>C","TGG","AATTTGCAACAAAGACAAAATGG",2,10,16,NA,"GeneID:137868","SGCZ",FALSE,"ANKRD20A2"

"7997","AATTTGCAAGAAAGAGAAAA","chr11",91404361,"-","5:A>C,11:G>T","AGG","AATTTTCAAGACAGAGAAAAAGG",2,6,12,NA,"","",FALSE,"ANKRD20A2"

"7998","AATTTGCAAGAAAGAGAAAA","chr8",87856037,"-","4:C>A,18:A>T","TGG","AATTGGCAAGAAAGAGAATATGG",2,5,19,NA,"","",FALSE,"ANKRD20A2"

"7999","CGGACAGGCTGTCCATTGCC","chr18",14183921,"+","none","AGG","CGGACAGGCTGTCCATTGCCAGG",0,NA,NA,NA,"","",TRUE,"ANKRD20A2"

"8000","CGGACAGGCTGTCCATTGCC","chr9",42856467,"+","none","AGG","CGGACAGGCTGTCCATTGCCAGG",0,NA,NA,NA,"","",TRUE,"ANKRD20A2"

"8001","CGGACAGGCTGTCCATTGCC","chr21",14068906,"+","none","AGG","CGGACAGGCTGTCCATTGCCAGG",0,NA,NA,NA,"","",TRUE,"ANKRD20A2"

"8002","CGGACAGGCTGTCCATTGCC","chr9",67863210,"+","none","AGG","CGGACAGGCTGTCCATTGCCAGG",0,NA,NA,NA,"GeneID:84210","ANKRD20A1",TRUE,"ANKRD20A2"

"8003","CGGACAGGCTGTCCATTGCC","chr9",40227176,"+","none","AGG","CGGACAGGCTGTCCATTGCCAGG",0,NA,NA,NA,"GeneID:441430","ANKRD20A2",TRUE,"ANKRD20A2"

"8004","CGGACAGGCTGTCCATTGCC","chr9",64373456,"+","none","AGG","CGGACAGGCTGTCCATTGCCAGG",0,NA,NA,NA,"GeneID:728747","ANKRD20A4",TRUE,"ANKRD20A2"

"8005","CGGACAGGCTGTCCATTGCC","chrUn_KI270744v1",57836,"-","none","AGG","CGGACAGGCTGTCCATTGCCAGG",0,NA,NA,NA,"","",FALSE,"ANKRD20A2"

"8006","CGGACAGGCTGTCCATTGCC","chr2",94853362,"-","none","AGG","CGGACAGGCTGTCCATTGCCAGG",0,NA,NA,NA,"","",TRUE,"ANKRD20A2"

"8007","CGGACAGGCTGTCCATTGCC","chr9",66149049,"-","none","AGG","CGGACAGGCTGTCCATTGCCAGG",0,NA,NA,NA,"GeneID:441425","ANKRD20A3",TRUE,"ANKRD20A2"

"8008","CGGACAGGCTGTCCATTGCC","chr20",30720039,"-","none","AGG","CGGACAGGCTGTCCATTGCCAGG",0,NA,NA,NA,"","",TRUE,"ANKRD20A2"

"8009","CGGACAGGCTGTCCATTGCC","chr21",13975344,"-","4:T>G","AGG","CGGAAAGGCTGTCCATTGCCAGG",1,5,NA,NA,"GeneID:391267","ANKRD20A11P",TRUE,"ANKRD20A2"

"8010","CGGACAGGCTGTCCATTGCC","chr9",64075665,"+","1:A>G","AGG","CAGACAGGCTGTCCATTGCCAGG",1,2,NA,NA,"","",FALSE,"ANKRD20A2"

"8011","CTCTAATATACCTGTATCAA","chr9",66149213,"+","none","AGG","CTCTAATATACCTGTATCAAAGG",0,NA,NA,NA,"GeneID:441425","ANKRD20A3",TRUE,"ANKRD20A2"

"8012","CTCTAATATACCTGTATCAA","chr2",94853526,"+","none","AGG","CTCTAATATACCTGTATCAAAGG",0,NA,NA,NA,"","",TRUE,"ANKRD20A2"

"8013","CTCTAATATACCTGTATCAA","chr21",13975508,"+","none","AGG","CTCTAATATACCTGTATCAAAGG",0,NA,NA,NA,"GeneID:391267","ANKRD20A11P",TRUE,"ANKRD20A2"

"8014","CTCTAATATACCTGTATCAA","chrUn_KI270744v1",58001,"+","none","AGG","CTCTAATATACCTGTATCAAAGG",0,NA,NA,NA,"","",FALSE,"ANKRD20A2"

"8015","CTCTAATATACCTGTATCAA","chr18",14183757,"-","none","AGG","CTCTAATATACCTGTATCAAAGG",0,NA,NA,NA,"","",TRUE,"ANKRD20A2"

"8016","CTCTAATATACCTGTATCAA","chr9",64075500,"-","none","AGG","CTCTAATATACCTGTATCAAAGG",0,NA,NA,NA,"","",FALSE,"ANKRD20A2"

"8017","CTCTAATATACCTGTATCAA","chr9",42856303,"-","none","AGG","CTCTAATATACCTGTATCAAAGG",0,NA,NA,NA,"","",TRUE,"ANKRD20A2"

"8018","CTCTAATATACCTGTATCAA","chr9",67863046,"-","none","AGG","CTCTAATATACCTGTATCAAAGG",0,NA,NA,NA,"GeneID:84210","ANKRD20A1",TRUE,"ANKRD20A2"

"8019","CTCTAATATACCTGTATCAA","chr9",40227012,"-","none","AGG","CTCTAATATACCTGTATCAAAGG",0,NA,NA,NA,"GeneID:441430","ANKRD20A2",TRUE,"ANKRD20A2"

"8020","CTCTAATATACCTGTATCAA","chr9",64373292,"-","none","AGG","CTCTAATATACCTGTATCAAAGG",0,NA,NA,NA,"GeneID:728747","ANKRD20A4",TRUE,"ANKRD20A2"

"8021","CTCTAATATACCTGTATCAA","chr21",14068742,"-","none","AGG","CTCTAATATACCTGTATCAAAGG",0,NA,NA,NA,"","",TRUE,"ANKRD20A2"

"8022","CTCTAATATACCTGTATCAA","chr13",66317699,"-","2:A>G,3:C>A","AGG","CTTGAATATACCTGTATCAAAGG",2,3,4,NA,"GeneID:5101","PCDH9",FALSE,"ANKRD20A2"

"8023","GTACCGTCTCAGCCTATCAA","chr9",66144894,"+","none","CGG","GTACCGTCTCAGCCTATCAACGG",0,NA,NA,NA,"GeneID:441425","ANKRD20A3",TRUE,"ANKRD20A2"

"8024","GTACCGTCTCAGCCTATCAA","chr2",94849196,"+","none","CGG","GTACCGTCTCAGCCTATCAACGG",0,NA,NA,NA,"","",TRUE,"ANKRD20A2"

"8025","GTACCGTCTCAGCCTATCAA","chr9",40231333,"-","none","CGG","GTACCGTCTCAGCCTATCAACGG",0,NA,NA,NA,"GeneID:441430","ANKRD20A2",TRUE,"ANKRD20A2"

"8026","GTACCGTCTCAGCCTATCAA","chr9",42860627,"-","none","CGG","GTACCGTCTCAGCCTATCAACGG",0,NA,NA,NA,"","",TRUE,"ANKRD20A2"

"8027","GTACCGTCTCAGCCTATCAA","chr9",67867367,"-","none","CGG","GTACCGTCTCAGCCTATCAACGG",0,NA,NA,NA,"GeneID:84210","ANKRD20A1",TRUE,"ANKRD20A2"

"8028","GTACCGTCTCAGCCTATCAA","chr9",64377611,"-","none","CGG","GTACCGTCTCAGCCTATCAACGG",0,NA,NA,NA,"GeneID:728747","ANKRD20A4",TRUE,"ANKRD20A2"

"8029","GTACCGTCTCAGCCTATCAA","chr18",14188095,"-","5:T>C","CGG","GTACCATCTCAGCCTATCAACGG",1,6,NA,NA,"","",TRUE,"ANKRD20A2"

"8030","GTACCGTCTCAGCCTATCAA","chr21",13971162,"+","5:A>G,12:G>C","CGG","GTACCATCTCAGGCTATCAACGG",2,6,13,NA,"GeneID:391267","ANKRD20A11P",TRUE,"ANKRD20A2"

"8031","GTACCGTCTCAGCCTATCAA","chr13",18871856,"+","5:A>G,12:G>C","CGG","GTACCATCTCAGGCTATCAACGG",2,6,13,NA,"","",TRUE,"ANKRD20A2"

"8032","GTGCTTCAATATGTGCACCA","chr9",66148896,"+","none","TGG","GTGCTTCAATATGTGCACCATGG",0,NA,NA,NA,"GeneID:441425","ANKRD20A3",TRUE,"ANKRD20A2"

"8033","GTGCTTCAATATGTGCACCA","chr9",64373609,"-","none","TGG","GTGCTTCAATATGTGCACCATGG",0,NA,NA,NA,"GeneID:728747","ANKRD20A4",TRUE,"ANKRD20A2"

"8034","GTGCTTCAATATGTGCACCA","chr9",67863363,"-","none","TGG","GTGCTTCAATATGTGCACCATGG",0,NA,NA,NA,"GeneID:84210","ANKRD20A1",TRUE,"ANKRD20A2"

"8035","GTGCTTCAATATGTGCACCA","chr9",40227329,"-","none","TGG","GTGCTTCAATATGTGCACCATGG",0,NA,NA,NA,"GeneID:441430","ANKRD20A2",TRUE,"ANKRD20A2"

"8036","GTGCTTCAATATGTGCACCA","chr9",42856620,"-","none","TGG","GTGCTTCAATATGTGCACCATGG",0,NA,NA,NA,"","",TRUE,"ANKRD20A2"

"8037","GTGCTTCAATATGTGCACCA","chr9",64075818,"-","12:A>C","TGG","GTGCTTCAATATTTGCACCATGG",1,13,NA,NA,"","",FALSE,"ANKRD20A2"

"8038","GTGCTTCAATATGTGCACCA","chr18",14184074,"-","12:A>C,14:A>C","TGG","GTGCTTCAATATTTTCACCATGG",2,13,15,NA,"","",TRUE,"ANKRD20A2"

"8039","GTGCTTCAATATGTGCACCA","chrUn_KI270744v1",57683,"+","12:T>G","TGG","GTGCTTCAATATTTGCACCATGG",1,13,NA,NA,"","",FALSE,"ANKRD20A2"

"8040","GTGCTTCAATATGTGCACCA","chr21",13975191,"+","12:T>G","TGG","GTGCTTCAATATTTGCACCATGG",1,13,NA,NA,"GeneID:391267","ANKRD20A11P",TRUE,"ANKRD20A2"

"8041","GTGCTTCAATATGTGCACCA","chr20",30719886,"+","12:T>G","TGG","GTGCTTCAATATTTGCACCATGG",1,13,NA,NA,"","",TRUE,"ANKRD20A2"

"8042","GTGCTTCAATATGTGCACCA","chr2",94853209,"+","10:G>A","TGG","GTGCTTCAATGTGTGCACCATGG",1,11,NA,NA,"","",TRUE,"ANKRD20A2"

"8043","TCATAGAACTGCTCTACACT","chr9",67862934,"+","none","TGG","TCATAGAACTGCTCTACACTTGG",0,NA,NA,NA,"GeneID:84210","ANKRD20A1",TRUE,"ANKRD20A2"

"8044","TCATAGAACTGCTCTACACT","chr9",40226900,"+","none","TGG","TCATAGAACTGCTCTACACTTGG",0,NA,NA,NA,"GeneID:441430","ANKRD20A2",TRUE,"ANKRD20A2"

"8045","TCATAGAACTGCTCTACACT","chr9",64373180,"+","none","TGG","TCATAGAACTGCTCTACACTTGG",0,NA,NA,NA,"GeneID:728747","ANKRD20A4",TRUE,"ANKRD20A2"

"8046","TCATAGAACTGCTCTACACT","chr9",66149325,"-","none","TGG","TCATAGAACTGCTCTACACTTGG",0,NA,NA,NA,"GeneID:441425","ANKRD20A3",TRUE,"ANKRD20A2"

"8047","TCATAGAACTGCTCTACACT","chr21",13975620,"-","18:A>G","TGG","TCATAGAACTGCTCTACATTTGG",1,19,NA,NA,"GeneID:391267","ANKRD20A11P",TRUE,"ANKRD20A2"

"8048","TCATAGAACTGCTCTACACT","chr9",64075388,"+","18:T>C","TGG","TCATAGAACTGCTCTACATTTGG",1,19,NA,NA,"","",FALSE,"ANKRD20A2"

"8049","TCATAGAACTGCTCTACACT","chr21",14068630,"+","18:T>C","TGG","TCATAGAACTGCTCTACATTTGG",1,19,NA,NA,"","",TRUE,"ANKRD20A2"

"8050","TCATAGAACTGCTCTACACT","chr9",42856191,"+","16:T>C","TGG","TCATAGAACTGCTCTATACTTGG",1,17,NA,NA,"","",TRUE,"ANKRD20A2"

"8051","TCATAGAACTGCTCTACACT","chr2",94853638,"-","2:C>T","TGG","TCGTAGAACTGCTCTACACTTGG",1,3,NA,NA,"","",TRUE,"ANKRD20A2"

"8052","TCATAGAACTGCTCTACACT","chr9",64884504,"-","2:C>T,6:C>T","GGG","TCGTAGGACTGCTCTACACTGGG",2,3,7,NA,"","",TRUE,"ANKRD20A2"

"8053","TCATAGAACTGCTCTACACT","chr10",45194240,"-","2:C>T,6:C>T","GGG","TCGTAGGACTGCTCTACACTGGG",2,3,7,NA,"","",TRUE,"ANKRD20A2"

"8054","TCATAGAACTGCTCTACACT","chr18",15325993,"-","2:C>T,6:C>T","GGG","TCGTAGGACTGCTCTACACTGGG",2,3,7,NA,"GeneID:644669","LOC644669",TRUE,"ANKRD20A2"

"8055","TCATAGAACTGCTCTACACT","chr9",40763738,"-","2:C>T,6:C>T","GGG","TCGTAGGACTGCTCTACACTGGG",2,3,7,NA,"","",TRUE,"ANKRD20A2"

"8056","TCATAGAACTGCTCTACACT","chr8",43297013,"+","2:T>A,6:G>A","TGG","TCTTAGGACTGCTCTACACTTGG",2,3,7,NA,"GeneID:340441","POTEA",TRUE,"ANKRD20A2"

"8057","TCATAGAACTGCTCTACACT","chr21",13042495,"+","2:G>A,6:G>A","GGG","TCGTAGGACTGCTCTACACTGGG",2,3,7,NA,"","",TRUE,"ANKRD20A2"

"8058","TCATAGAACTGCTCTACACT","chr10",37129886,"+","2:G>A,6:G>A","GGG","TCGTAGGACTGCTCTACACTGGG",2,3,7,NA,"GeneID:91074","ANKRD30A",TRUE,"ANKRD20A2"

"8059","TCATAGAACTGCTCTACACT","chr18",14752559,"+","2:G>A,6:G>A","GGG","TCGTAGGACTGCTCTACACTGGG",2,3,7,NA,"GeneID:374860","ANKRD30B",TRUE,"ANKRD20A2"

"8060","TCATAGAACTGCTCTACACT","chr15",19878547,"+","2:G>A,6:G>A","GGG","TCGTAGGACTGCTCTACACTGGG",2,3,7,NA,"","",TRUE,"ANKRD20A2"

"8061","TCATAGAACTGCTCTACACT","chr22",16136722,"+","2:G>A,6:G>A","GGG","TCGTAGGACTGCTCTACACTGGG",2,3,7,NA,"","",FALSE,"ANKRD20A2"

"8062","TCATAGAACTGCTCTACACT","chr13",18413120,"+","2:G>A,6:G>A","GGG","TCGTAGGACTGCTCTACACTGGG",2,3,7,NA,"","",TRUE,"ANKRD20A2"

"8063","TCATAGAACTGCTCTACACT","chr18",14183645,"+","2:G>A,18:T>C","TGG","TCGTAGAACTGCTCTACATTTGG",2,3,19,NA,"","",TRUE,"ANKRD20A2"

"8064","TCATAGAACTGCTCTACACT","chr7",39816542,"+","6:G>A,18:T>C","TGG","TCATAGGACTGCTCTACATTTGG",2,7,19,NA,"","",TRUE,"ANKRD20A2"

"8065","TCATAGAACTGCTCTACACT","chr21",13615116,"+","6:G>A,18:T>C","TGG","TCATAGGACTGCTCTACATTTGG",2,7,19,NA,"GeneID:317754","POTED",TRUE,"ANKRD20A2"

"8066","TCATAGAACTGCTCTACACT","chr15",21435094,"-","6:C>T,18:A>G","TGG","TCATAGGACTGCTCTACATTTGG",2,7,19,NA,"GeneID:102723502","LOC102723502",TRUE,"ANKRD20A2"

"8067","TCATAGAACTGCTCTACACT","chrUn_GL000213v1",134376,"-","6:C>T,18:A>G","TGG","TCATAGGACTGCTCTACATTTGG",2,7,19,NA,"","",FALSE,"ANKRD20A2"

"8068","TCATAGAACTGCTCTACACT","chr18",14538235,"-","6:C>T,18:A>G","TGG","TCATAGGACTGCTCTACATTTGG",2,7,19,NA,"GeneID:388468","POTEC",TRUE,"ANKRD20A2"

"8069","TCATAGAACTGCTCTACACT","chr15",21872440,"-","6:C>T,18:A>G","TGG","TCATAGGACTGCTCTACATTTGG",2,7,19,NA,"GeneID:100996331","POTEB",TRUE,"ANKRD20A2"

"8070","TCATAGAACTGCTCTACACT","chr15",20861475,"-","6:C>T,18:A>G","TGG","TCATAGGACTGCTCTACATTTGG",2,7,19,NA,"GeneID:100287399","POTEB2",TRUE,"ANKRD20A2"

"8071","TCATAGAACTGCTCTACACT","chr15_KI270727v1_random",162297,"-","6:C>T,18:A>G","TGG","TCATAGGACTGCTCTACATTTGG",2,7,19,NA,"","",FALSE,"ANKRD20A2"

"8072","TCATAGAACTGCTCTACACT","chrUn_KI270744v1",58113,"-","2:C>T,18:A>G","TGG","TCGTAGAACTGCTCTACATTTGG",2,3,19,NA,"","",FALSE,"ANKRD20A2"

"8073","TCATAGAACTGCTCTACACT","chr20",30720316,"-","2:C>T,18:A>G","TGG","TCGTAGAACTGCTCTACATTTGG",2,3,19,NA,"","",TRUE,"ANKRD20A2"

"8074","AATTTGCAAGAAAGAGAAAA","chr9",40231271,"+","none","TGG","AATTTGCAAGAAAGAGAAAATGG",0,NA,NA,NA,"GeneID:441430","ANKRD20A2",TRUE,"ANKRD20A3"

"8075","AATTTGCAAGAAAGAGAAAA","chr18",14188033,"+","none","TGG","AATTTGCAAGAAAGAGAAAATGG",0,NA,NA,NA,"","",TRUE,"ANKRD20A3"

"8076","AATTTGCAAGAAAGAGAAAA","chr9",42860564,"+","none","TGG","AATTTGCAAGAAAGAGAAAATGG",0,NA,NA,NA,"","",TRUE,"ANKRD20A3"

"8077","AATTTGCAAGAAAGAGAAAA","chr9",67867305,"+","none","TGG","AATTTGCAAGAAAGAGAAAATGG",0,NA,NA,NA,"GeneID:84210","ANKRD20A1",TRUE,"ANKRD20A3"

"8078","AATTTGCAAGAAAGAGAAAA","chr9",64377549,"+","none","TGG","AATTTGCAAGAAAGAGAAAATGG",0,NA,NA,NA,"GeneID:728747","ANKRD20A4",TRUE,"ANKRD20A3"

"8079","AATTTGCAAGAAAGAGAAAA","chr9",64079776,"+","none","TGG","AATTTGCAAGAAAGAGAAAATGG",0,NA,NA,NA,"","",FALSE,"ANKRD20A3"

"8080","AATTTGCAAGAAAGAGAAAA","chrUn_KI270744v1",53746,"-","none","TGG","AATTTGCAAGAAAGAGAAAATGG",0,NA,NA,NA,"","",FALSE,"ANKRD20A3"

"8081","AATTTGCAAGAAAGAGAAAA","chr9",66144956,"-","none","TGG","AATTTGCAAGAAAGAGAAAATGG",0,NA,NA,NA,"GeneID:441425","ANKRD20A3",TRUE,"ANKRD20A3"

"8082","AATTTGCAAGAAAGAGAAAA","chr21",13971224,"-","none","TGG","AATTTGCAAGAAAGAGAAAATGG",0,NA,NA,NA,"GeneID:391267","ANKRD20A11P",TRUE,"ANKRD20A3"

"8083","AATTTGCAAGAAAGAGAAAA","chr2",94849258,"-","none","TGG","AATTTGCAAGAAAGAGAAAATGG",0,NA,NA,NA,"","",TRUE,"ANKRD20A3"

"8084","AATTTGCAAGAAAGAGAAAA","chr13",18871918,"-","none","TGG","AATTTGCAAGAAAGAGAAAATGG",0,NA,NA,NA,"","",TRUE,"ANKRD20A3"

"8085","AATTTGCAAGAAAGAGAAAA","chr20",30716014,"-","none","TGG","AATTTGCAAGAAAGAGAAAATGG",0,NA,NA,NA,"","",TRUE,"ANKRD20A3"

"8086","AATTTGCAAGAAAGAGAAAA","chr2",100482342,"-","7:A>T,8:A>T","AGG","AATTTGCTTGAAAGAGAAAAAGG",2,8,9,NA,"GeneID:129521","NMS",FALSE,"ANKRD20A3"

"8087","AATTTGCAAGAAAGAGAAAA","chr10",32274114,"-","4:C>A,9:T>C","AGG","AATTGGCAAAAAAGAGAAAAAGG",2,5,10,NA,"GeneID:80314","EPC1",FALSE,"ANKRD20A3"

"8088","AATTTGCAAGAAAGAGAAAA","chr2",39540715,"+","1:G>A,6:A>C","GGG","AGTTTGAAAGAAAGAGAAAAGGG",2,2,7,NA,"GeneID:728730","LOC728730",FALSE,"ANKRD20A3"

"8089","AATTTGCAAGAAAGAGAAAA","chr21",22043188,"+","5:C>G,7:C>A","TGG","AATTTCCCAGAAAGAGAAAATGG",2,6,8,NA,"GeneID:101927843","LOC101927843",FALSE,"ANKRD20A3"

"8090","AATTTGCAAGAAAGAGAAAA","chr8",138921604,"+","6:T>C,13:A>G","TGG","AATTTGTAAGAAAAAGAAAATGG",2,7,14,NA,"","",FALSE,"ANKRD20A3"

"8091","AATTTGCAAGAAAGAGAAAA","chr1",173428325,"+","5:A>G,13:C>G","TGG","AATTTACAAGAAACAGAAAATGG",2,6,14,NA,"GeneID:101928673","LOC101928673",FALSE,"ANKRD20A3"

"8092","AATTTGCAAGAAAGAGAAAA","chr5",88435873,"+","6:A>C,17:G>A","AGG","AATTTGAAAGAAAGAGAGAAAGG",2,7,18,NA,"GeneID:100505894;GeneID:102546226","TMEM161B-AS1;LOC102546226",FALSE,"ANKRD20A3"

"8093","AATTTGCAAGAAAGAGAAAA","chr6",169415927,"+","6:A>C,17:G>A","AGG","AATTTGAAAGAAAGAGAGAAAGG",2,7,18,NA,"","",FALSE,"ANKRD20A3"

"8094","AATTTGCAAGAAAGAGAAAA","chr8",14652651,"-","9:G>C,15:G>C","TGG","AATTTGCAACAAAGACAAAATGG",2,10,16,NA,"GeneID:137868","SGCZ",FALSE,"ANKRD20A3"

"8095","AATTTGCAAGAAAGAGAAAA","chr11",91404361,"-","5:A>C,11:G>T","AGG","AATTTTCAAGACAGAGAAAAAGG",2,6,12,NA,"","",FALSE,"ANKRD20A3"

"8096","AATTTGCAAGAAAGAGAAAA","chr8",87856037,"-","4:C>A,18:A>T","TGG","AATTGGCAAGAAAGAGAATATGG",2,5,19,NA,"","",FALSE,"ANKRD20A3"

"8097","CGGACAGGCTGTCCATTGCC","chr18",14183921,"+","none","AGG","CGGACAGGCTGTCCATTGCCAGG",0,NA,NA,NA,"","",TRUE,"ANKRD20A3"

"8098","CGGACAGGCTGTCCATTGCC","chr9",42856467,"+","none","AGG","CGGACAGGCTGTCCATTGCCAGG",0,NA,NA,NA,"","",TRUE,"ANKRD20A3"

"8099","CGGACAGGCTGTCCATTGCC","chr21",14068906,"+","none","AGG","CGGACAGGCTGTCCATTGCCAGG",0,NA,NA,NA,"","",TRUE,"ANKRD20A3"

"8100","CGGACAGGCTGTCCATTGCC","chr9",67863210,"+","none","AGG","CGGACAGGCTGTCCATTGCCAGG",0,NA,NA,NA,"GeneID:84210","ANKRD20A1",TRUE,"ANKRD20A3"

"8101","CGGACAGGCTGTCCATTGCC","chr9",40227176,"+","none","AGG","CGGACAGGCTGTCCATTGCCAGG",0,NA,NA,NA,"GeneID:441430","ANKRD20A2",TRUE,"ANKRD20A3"

"8102","CGGACAGGCTGTCCATTGCC","chr9",64373456,"+","none","AGG","CGGACAGGCTGTCCATTGCCAGG",0,NA,NA,NA,"GeneID:728747","ANKRD20A4",TRUE,"ANKRD20A3"

"8103","CGGACAGGCTGTCCATTGCC","chrUn_KI270744v1",57836,"-","none","AGG","CGGACAGGCTGTCCATTGCCAGG",0,NA,NA,NA,"","",FALSE,"ANKRD20A3"

"8104","CGGACAGGCTGTCCATTGCC","chr2",94853362,"-","none","AGG","CGGACAGGCTGTCCATTGCCAGG",0,NA,NA,NA,"","",TRUE,"ANKRD20A3"

"8105","CGGACAGGCTGTCCATTGCC","chr9",66149049,"-","none","AGG","CGGACAGGCTGTCCATTGCCAGG",0,NA,NA,NA,"GeneID:441425","ANKRD20A3",TRUE,"ANKRD20A3"

"8106","CGGACAGGCTGTCCATTGCC","chr20",30720039,"-","none","AGG","CGGACAGGCTGTCCATTGCCAGG",0,NA,NA,NA,"","",TRUE,"ANKRD20A3"

"8107","CGGACAGGCTGTCCATTGCC","chr21",13975344,"-","4:T>G","AGG","CGGAAAGGCTGTCCATTGCCAGG",1,5,NA,NA,"GeneID:391267","ANKRD20A11P",TRUE,"ANKRD20A3"

"8108","CGGACAGGCTGTCCATTGCC","chr9",64075665,"+","1:A>G","AGG","CAGACAGGCTGTCCATTGCCAGG",1,2,NA,NA,"","",FALSE,"ANKRD20A3"

"8109","CTCTAATATACCTGTATCAA","chr9",66149213,"+","none","AGG","CTCTAATATACCTGTATCAAAGG",0,NA,NA,NA,"GeneID:441425","ANKRD20A3",TRUE,"ANKRD20A3"

"8110","CTCTAATATACCTGTATCAA","chr2",94853526,"+","none","AGG","CTCTAATATACCTGTATCAAAGG",0,NA,NA,NA,"","",TRUE,"ANKRD20A3"

"8111","CTCTAATATACCTGTATCAA","chr21",13975508,"+","none","AGG","CTCTAATATACCTGTATCAAAGG",0,NA,NA,NA,"GeneID:391267","ANKRD20A11P",TRUE,"ANKRD20A3"

"8112","CTCTAATATACCTGTATCAA","chrUn_KI270744v1",58001,"+","none","AGG","CTCTAATATACCTGTATCAAAGG",0,NA,NA,NA,"","",FALSE,"ANKRD20A3"

"8113","CTCTAATATACCTGTATCAA","chr18",14183757,"-","none","AGG","CTCTAATATACCTGTATCAAAGG",0,NA,NA,NA,"","",TRUE,"ANKRD20A3"

"8114","CTCTAATATACCTGTATCAA","chr9",64075500,"-","none","AGG","CTCTAATATACCTGTATCAAAGG",0,NA,NA,NA,"","",FALSE,"ANKRD20A3"

"8115","CTCTAATATACCTGTATCAA","chr9",42856303,"-","none","AGG","CTCTAATATACCTGTATCAAAGG",0,NA,NA,NA,"","",TRUE,"ANKRD20A3"

"8116","CTCTAATATACCTGTATCAA","chr9",67863046,"-","none","AGG","CTCTAATATACCTGTATCAAAGG",0,NA,NA,NA,"GeneID:84210","ANKRD20A1",TRUE,"ANKRD20A3"

"8117","CTCTAATATACCTGTATCAA","chr9",40227012,"-","none","AGG","CTCTAATATACCTGTATCAAAGG",0,NA,NA,NA,"GeneID:441430","ANKRD20A2",TRUE,"ANKRD20A3"

"8118","CTCTAATATACCTGTATCAA","chr9",64373292,"-","none","AGG","CTCTAATATACCTGTATCAAAGG",0,NA,NA,NA,"GeneID:728747","ANKRD20A4",TRUE,"ANKRD20A3"

"8119","CTCTAATATACCTGTATCAA","chr21",14068742,"-","none","AGG","CTCTAATATACCTGTATCAAAGG",0,NA,NA,NA,"","",TRUE,"ANKRD20A3"

"8120","CTCTAATATACCTGTATCAA","chr13",66317699,"-","2:A>G,3:C>A","AGG","CTTGAATATACCTGTATCAAAGG",2,3,4,NA,"GeneID:5101","PCDH9",FALSE,"ANKRD20A3"

"8121","GTACCGTCTCAGCCTATCAA","chr9",66144894,"+","none","CGG","GTACCGTCTCAGCCTATCAACGG",0,NA,NA,NA,"GeneID:441425","ANKRD20A3",TRUE,"ANKRD20A3"

"8122","GTACCGTCTCAGCCTATCAA","chr2",94849196,"+","none","CGG","GTACCGTCTCAGCCTATCAACGG",0,NA,NA,NA,"","",TRUE,"ANKRD20A3"

"8123","GTACCGTCTCAGCCTATCAA","chr9",40231333,"-","none","CGG","GTACCGTCTCAGCCTATCAACGG",0,NA,NA,NA,"GeneID:441430","ANKRD20A2",TRUE,"ANKRD20A3"

"8124","GTACCGTCTCAGCCTATCAA","chr9",42860627,"-","none","CGG","GTACCGTCTCAGCCTATCAACGG",0,NA,NA,NA,"","",TRUE,"ANKRD20A3"

"8125","GTACCGTCTCAGCCTATCAA","chr9",67867367,"-","none","CGG","GTACCGTCTCAGCCTATCAACGG",0,NA,NA,NA,"GeneID:84210","ANKRD20A1",TRUE,"ANKRD20A3"

"8126","GTACCGTCTCAGCCTATCAA","chr9",64377611,"-","none","CGG","GTACCGTCTCAGCCTATCAACGG",0,NA,NA,NA,"GeneID:728747","ANKRD20A4",TRUE,"ANKRD20A3"

"8127","GTACCGTCTCAGCCTATCAA","chr18",14188095,"-","5:T>C","CGG","GTACCATCTCAGCCTATCAACGG",1,6,NA,NA,"","",TRUE,"ANKRD20A3"

"8128","GTACCGTCTCAGCCTATCAA","chr21",13971162,"+","5:A>G,12:G>C","CGG","GTACCATCTCAGGCTATCAACGG",2,6,13,NA,"GeneID:391267","ANKRD20A11P",TRUE,"ANKRD20A3"

"8129","GTACCGTCTCAGCCTATCAA","chr13",18871856,"+","5:A>G,12:G>C","CGG","GTACCATCTCAGGCTATCAACGG",2,6,13,NA,"","",TRUE,"ANKRD20A3"

"8130","GTGCTTCAATATGTGCACCA","chr9",66148896,"+","none","TGG","GTGCTTCAATATGTGCACCATGG",0,NA,NA,NA,"GeneID:441425","ANKRD20A3",TRUE,"ANKRD20A3"

"8131","GTGCTTCAATATGTGCACCA","chr9",64373609,"-","none","TGG","GTGCTTCAATATGTGCACCATGG",0,NA,NA,NA,"GeneID:728747","ANKRD20A4",TRUE,"ANKRD20A3"

"8132","GTGCTTCAATATGTGCACCA","chr9",67863363,"-","none","TGG","GTGCTTCAATATGTGCACCATGG",0,NA,NA,NA,"GeneID:84210","ANKRD20A1",TRUE,"ANKRD20A3"

"8133","GTGCTTCAATATGTGCACCA","chr9",40227329,"-","none","TGG","GTGCTTCAATATGTGCACCATGG",0,NA,NA,NA,"GeneID:441430","ANKRD20A2",TRUE,"ANKRD20A3"

"8134","GTGCTTCAATATGTGCACCA","chr9",42856620,"-","none","TGG","GTGCTTCAATATGTGCACCATGG",0,NA,NA,NA,"","",TRUE,"ANKRD20A3"

"8135","GTGCTTCAATATGTGCACCA","chr9",64075818,"-","12:A>C","TGG","GTGCTTCAATATTTGCACCATGG",1,13,NA,NA,"","",FALSE,"ANKRD20A3"

"8136","GTGCTTCAATATGTGCACCA","chr18",14184074,"-","12:A>C,14:A>C","TGG","GTGCTTCAATATTTTCACCATGG",2,13,15,NA,"","",TRUE,"ANKRD20A3"

"8137","GTGCTTCAATATGTGCACCA","chrUn_KI270744v1",57683,"+","12:T>G","TGG","GTGCTTCAATATTTGCACCATGG",1,13,NA,NA,"","",FALSE,"ANKRD20A3"

"8138","GTGCTTCAATATGTGCACCA","chr21",13975191,"+","12:T>G","TGG","GTGCTTCAATATTTGCACCATGG",1,13,NA,NA,"GeneID:391267","ANKRD20A11P",TRUE,"ANKRD20A3"

"8139","GTGCTTCAATATGTGCACCA","chr20",30719886,"+","12:T>G","TGG","GTGCTTCAATATTTGCACCATGG",1,13,NA,NA,"","",TRUE,"ANKRD20A3"

"8140","GTGCTTCAATATGTGCACCA","chr2",94853209,"+","10:G>A","TGG","GTGCTTCAATGTGTGCACCATGG",1,11,NA,NA,"","",TRUE,"ANKRD20A3"

"8141","TCATAGAACTGCTCTACACT","chr9",67862934,"+","none","TGG","TCATAGAACTGCTCTACACTTGG",0,NA,NA,NA,"GeneID:84210","ANKRD20A1",TRUE,"ANKRD20A3"

"8142","TCATAGAACTGCTCTACACT","chr9",40226900,"+","none","TGG","TCATAGAACTGCTCTACACTTGG",0,NA,NA,NA,"GeneID:441430","ANKRD20A2",TRUE,"ANKRD20A3"

"8143","TCATAGAACTGCTCTACACT","chr9",64373180,"+","none","TGG","TCATAGAACTGCTCTACACTTGG",0,NA,NA,NA,"GeneID:728747","ANKRD20A4",TRUE,"ANKRD20A3"

"8144","TCATAGAACTGCTCTACACT","chr9",66149325,"-","none","TGG","TCATAGAACTGCTCTACACTTGG",0,NA,NA,NA,"GeneID:441425","ANKRD20A3",TRUE,"ANKRD20A3"

"8145","TCATAGAACTGCTCTACACT","chr21",13975620,"-","18:A>G","TGG","TCATAGAACTGCTCTACATTTGG",1,19,NA,NA,"GeneID:391267","ANKRD20A11P",TRUE,"ANKRD20A3"

"8146","TCATAGAACTGCTCTACACT","chr9",64075388,"+","18:T>C","TGG","TCATAGAACTGCTCTACATTTGG",1,19,NA,NA,"","",FALSE,"ANKRD20A3"

"8147","TCATAGAACTGCTCTACACT","chr21",14068630,"+","18:T>C","TGG","TCATAGAACTGCTCTACATTTGG",1,19,NA,NA,"","",TRUE,"ANKRD20A3"

"8148","TCATAGAACTGCTCTACACT","chr9",42856191,"+","16:T>C","TGG","TCATAGAACTGCTCTATACTTGG",1,17,NA,NA,"","",TRUE,"ANKRD20A3"

"8149","TCATAGAACTGCTCTACACT","chr2",94853638,"-","2:C>T","TGG","TCGTAGAACTGCTCTACACTTGG",1,3,NA,NA,"","",TRUE,"ANKRD20A3"

"8150","TCATAGAACTGCTCTACACT","chr9",64884504,"-","2:C>T,6:C>T","GGG","TCGTAGGACTGCTCTACACTGGG",2,3,7,NA,"","",TRUE,"ANKRD20A3"

"8151","TCATAGAACTGCTCTACACT","chr10",45194240,"-","2:C>T,6:C>T","GGG","TCGTAGGACTGCTCTACACTGGG",2,3,7,NA,"","",TRUE,"ANKRD20A3"

"8152","TCATAGAACTGCTCTACACT","chr18",15325993,"-","2:C>T,6:C>T","GGG","TCGTAGGACTGCTCTACACTGGG",2,3,7,NA,"GeneID:644669","LOC644669",TRUE,"ANKRD20A3"

"8153","TCATAGAACTGCTCTACACT","chr9",40763738,"-","2:C>T,6:C>T","GGG","TCGTAGGACTGCTCTACACTGGG",2,3,7,NA,"","",TRUE,"ANKRD20A3"

"8154","TCATAGAACTGCTCTACACT","chr8",43297013,"+","2:T>A,6:G>A","TGG","TCTTAGGACTGCTCTACACTTGG",2,3,7,NA,"GeneID:340441","POTEA",TRUE,"ANKRD20A3"

"8155","TCATAGAACTGCTCTACACT","chr21",13042495,"+","2:G>A,6:G>A","GGG","TCGTAGGACTGCTCTACACTGGG",2,3,7,NA,"","",TRUE,"ANKRD20A3"

"8156","TCATAGAACTGCTCTACACT","chr10",37129886,"+","2:G>A,6:G>A","GGG","TCGTAGGACTGCTCTACACTGGG",2,3,7,NA,"GeneID:91074","ANKRD30A",TRUE,"ANKRD20A3"

"8157","TCATAGAACTGCTCTACACT","chr18",14752559,"+","2:G>A,6:G>A","GGG","TCGTAGGACTGCTCTACACTGGG",2,3,7,NA,"GeneID:374860","ANKRD30B",TRUE,"ANKRD20A3"

"8158","TCATAGAACTGCTCTACACT","chr15",19878547,"+","2:G>A,6:G>A","GGG","TCGTAGGACTGCTCTACACTGGG",2,3,7,NA,"","",TRUE,"ANKRD20A3"

"8159","TCATAGAACTGCTCTACACT","chr22",16136722,"+","2:G>A,6:G>A","GGG","TCGTAGGACTGCTCTACACTGGG",2,3,7,NA,"","",FALSE,"ANKRD20A3"

"8160","TCATAGAACTGCTCTACACT","chr13",18413120,"+","2:G>A,6:G>A","GGG","TCGTAGGACTGCTCTACACTGGG",2,3,7,NA,"","",TRUE,"ANKRD20A3"

"8161","TCATAGAACTGCTCTACACT","chr18",14183645,"+","2:G>A,18:T>C","TGG","TCGTAGAACTGCTCTACATTTGG",2,3,19,NA,"","",TRUE,"ANKRD20A3"

"8162","TCATAGAACTGCTCTACACT","chr7",39816542,"+","6:G>A,18:T>C","TGG","TCATAGGACTGCTCTACATTTGG",2,7,19,NA,"","",TRUE,"ANKRD20A3"

"8163","TCATAGAACTGCTCTACACT","chr21",13615116,"+","6:G>A,18:T>C","TGG","TCATAGGACTGCTCTACATTTGG",2,7,19,NA,"GeneID:317754","POTED",TRUE,"ANKRD20A3"

"8164","TCATAGAACTGCTCTACACT","chr15",21435094,"-","6:C>T,18:A>G","TGG","TCATAGGACTGCTCTACATTTGG",2,7,19,NA,"GeneID:102723502","LOC102723502",TRUE,"ANKRD20A3"

"8165","TCATAGAACTGCTCTACACT","chrUn_GL000213v1",134376,"-","6:C>T,18:A>G","TGG","TCATAGGACTGCTCTACATTTGG",2,7,19,NA,"","",FALSE,"ANKRD20A3"

"8166","TCATAGAACTGCTCTACACT","chr18",14538235,"-","6:C>T,18:A>G","TGG","TCATAGGACTGCTCTACATTTGG",2,7,19,NA,"GeneID:388468","POTEC",TRUE,"ANKRD20A3"

"8167","TCATAGAACTGCTCTACACT","chr15",21872440,"-","6:C>T,18:A>G","TGG","TCATAGGACTGCTCTACATTTGG",2,7,19,NA,"GeneID:100996331","POTEB",TRUE,"ANKRD20A3"

"8168","TCATAGAACTGCTCTACACT","chr15",20861475,"-","6:C>T,18:A>G","TGG","TCATAGGACTGCTCTACATTTGG",2,7,19,NA,"GeneID:100287399","POTEB2",TRUE,"ANKRD20A3"

"8169","TCATAGAACTGCTCTACACT","chr15_KI270727v1_random",162297,"-","6:C>T,18:A>G","TGG","TCATAGGACTGCTCTACATTTGG",2,7,19,NA,"","",FALSE,"ANKRD20A3"

"8170","TCATAGAACTGCTCTACACT","chrUn_KI270744v1",58113,"-","2:C>T,18:A>G","TGG","TCGTAGAACTGCTCTACATTTGG",2,3,19,NA,"","",FALSE,"ANKRD20A3"

"8171","TCATAGAACTGCTCTACACT","chr20",30720316,"-","2:C>T,18:A>G","TGG","TCGTAGAACTGCTCTACATTTGG",2,3,19,NA,"","",TRUE,"ANKRD20A3"

"8172","AATTTGCAAGAAAGAGAAAA","chr9",40231271,"+","none","TGG","AATTTGCAAGAAAGAGAAAATGG",0,NA,NA,NA,"GeneID:441430","ANKRD20A2",TRUE,"ANKRD20A4"

"8173","AATTTGCAAGAAAGAGAAAA","chr18",14188033,"+","none","TGG","AATTTGCAAGAAAGAGAAAATGG",0,NA,NA,NA,"","",TRUE,"ANKRD20A4"

"8174","AATTTGCAAGAAAGAGAAAA","chr9",42860564,"+","none","TGG","AATTTGCAAGAAAGAGAAAATGG",0,NA,NA,NA,"","",TRUE,"ANKRD20A4"

"8175","AATTTGCAAGAAAGAGAAAA","chr9",67867305,"+","none","TGG","AATTTGCAAGAAAGAGAAAATGG",0,NA,NA,NA,"GeneID:84210","ANKRD20A1",TRUE,"ANKRD20A4"

"8176","AATTTGCAAGAAAGAGAAAA","chr9",64377549,"+","none","TGG","AATTTGCAAGAAAGAGAAAATGG",0,NA,NA,NA,"GeneID:728747","ANKRD20A4",TRUE,"ANKRD20A4"

"8177","AATTTGCAAGAAAGAGAAAA","chr9",64079776,"+","none","TGG","AATTTGCAAGAAAGAGAAAATGG",0,NA,NA,NA,"","",FALSE,"ANKRD20A4"

"8178","AATTTGCAAGAAAGAGAAAA","chrUn_KI270744v1",53746,"-","none","TGG","AATTTGCAAGAAAGAGAAAATGG",0,NA,NA,NA,"","",FALSE,"ANKRD20A4"

"8179","AATTTGCAAGAAAGAGAAAA","chr9",66144956,"-","none","TGG","AATTTGCAAGAAAGAGAAAATGG",0,NA,NA,NA,"GeneID:441425","ANKRD20A3",TRUE,"ANKRD20A4"

"8180","AATTTGCAAGAAAGAGAAAA","chr21",13971224,"-","none","TGG","AATTTGCAAGAAAGAGAAAATGG",0,NA,NA,NA,"GeneID:391267","ANKRD20A11P",TRUE,"ANKRD20A4"

"8181","AATTTGCAAGAAAGAGAAAA","chr2",94849258,"-","none","TGG","AATTTGCAAGAAAGAGAAAATGG",0,NA,NA,NA,"","",TRUE,"ANKRD20A4"

"8182","AATTTGCAAGAAAGAGAAAA","chr13",18871918,"-","none","TGG","AATTTGCAAGAAAGAGAAAATGG",0,NA,NA,NA,"","",TRUE,"ANKRD20A4"

"8183","AATTTGCAAGAAAGAGAAAA","chr20",30716014,"-","none","TGG","AATTTGCAAGAAAGAGAAAATGG",0,NA,NA,NA,"","",TRUE,"ANKRD20A4"

"8184","AATTTGCAAGAAAGAGAAAA","chr2",100482342,"-","7:A>T,8:A>T","AGG","AATTTGCTTGAAAGAGAAAAAGG",2,8,9,NA,"GeneID:129521","NMS",FALSE,"ANKRD20A4"

"8185","AATTTGCAAGAAAGAGAAAA","chr10",32274114,"-","4:C>A,9:T>C","AGG","AATTGGCAAAAAAGAGAAAAAGG",2,5,10,NA,"GeneID:80314","EPC1",FALSE,"ANKRD20A4"

"8186","AATTTGCAAGAAAGAGAAAA","chr2",39540715,"+","1:G>A,6:A>C","GGG","AGTTTGAAAGAAAGAGAAAAGGG",2,2,7,NA,"GeneID:728730","LOC728730",FALSE,"ANKRD20A4"

"8187","AATTTGCAAGAAAGAGAAAA","chr21",22043188,"+","5:C>G,7:C>A","TGG","AATTTCCCAGAAAGAGAAAATGG",2,6,8,NA,"GeneID:101927843","LOC101927843",FALSE,"ANKRD20A4"

"8188","AATTTGCAAGAAAGAGAAAA","chr8",138921604,"+","6:T>C,13:A>G","TGG","AATTTGTAAGAAAAAGAAAATGG",2,7,14,NA,"","",FALSE,"ANKRD20A4"

"8189","AATTTGCAAGAAAGAGAAAA","chr1",173428325,"+","5:A>G,13:C>G","TGG","AATTTACAAGAAACAGAAAATGG",2,6,14,NA,"GeneID:101928673","LOC101928673",FALSE,"ANKRD20A4"

"8190","AATTTGCAAGAAAGAGAAAA","chr5",88435873,"+","6:A>C,17:G>A","AGG","AATTTGAAAGAAAGAGAGAAAGG",2,7,18,NA,"GeneID:100505894;GeneID:102546226","TMEM161B-AS1;LOC102546226",FALSE,"ANKRD20A4"

"8191","AATTTGCAAGAAAGAGAAAA","chr6",169415927,"+","6:A>C,17:G>A","AGG","AATTTGAAAGAAAGAGAGAAAGG",2,7,18,NA,"","",FALSE,"ANKRD20A4"

"8192","AATTTGCAAGAAAGAGAAAA","chr8",14652651,"-","9:G>C,15:G>C","TGG","AATTTGCAACAAAGACAAAATGG",2,10,16,NA,"GeneID:137868","SGCZ",FALSE,"ANKRD20A4"

"8193","AATTTGCAAGAAAGAGAAAA","chr11",91404361,"-","5:A>C,11:G>T","AGG","AATTTTCAAGACAGAGAAAAAGG",2,6,12,NA,"","",FALSE,"ANKRD20A4"

"8194","AATTTGCAAGAAAGAGAAAA","chr8",87856037,"-","4:C>A,18:A>T","TGG","AATTGGCAAGAAAGAGAATATGG",2,5,19,NA,"","",FALSE,"ANKRD20A4"

"8195","CGGACAGGCTGTCCATTGCC","chr18",14183921,"+","none","AGG","CGGACAGGCTGTCCATTGCCAGG",0,NA,NA,NA,"","",TRUE,"ANKRD20A4"

"8196","CGGACAGGCTGTCCATTGCC","chr9",42856467,"+","none","AGG","CGGACAGGCTGTCCATTGCCAGG",0,NA,NA,NA,"","",TRUE,"ANKRD20A4"

"8197","CGGACAGGCTGTCCATTGCC","chr21",14068906,"+","none","AGG","CGGACAGGCTGTCCATTGCCAGG",0,NA,NA,NA,"","",TRUE,"ANKRD20A4"

"8198","CGGACAGGCTGTCCATTGCC","chr9",67863210,"+","none","AGG","CGGACAGGCTGTCCATTGCCAGG",0,NA,NA,NA,"GeneID:84210","ANKRD20A1",TRUE,"ANKRD20A4"

"8199","CGGACAGGCTGTCCATTGCC","chr9",40227176,"+","none","AGG","CGGACAGGCTGTCCATTGCCAGG",0,NA,NA,NA,"GeneID:441430","ANKRD20A2",TRUE,"ANKRD20A4"

"8200","CGGACAGGCTGTCCATTGCC","chr9",64373456,"+","none","AGG","CGGACAGGCTGTCCATTGCCAGG",0,NA,NA,NA,"GeneID:728747","ANKRD20A4",TRUE,"ANKRD20A4"

"8201","CGGACAGGCTGTCCATTGCC","chrUn_KI270744v1",57836,"-","none","AGG","CGGACAGGCTGTCCATTGCCAGG",0,NA,NA,NA,"","",FALSE,"ANKRD20A4"

"8202","CGGACAGGCTGTCCATTGCC","chr2",94853362,"-","none","AGG","CGGACAGGCTGTCCATTGCCAGG",0,NA,NA,NA,"","",TRUE,"ANKRD20A4"

"8203","CGGACAGGCTGTCCATTGCC","chr9",66149049,"-","none","AGG","CGGACAGGCTGTCCATTGCCAGG",0,NA,NA,NA,"GeneID:441425","ANKRD20A3",TRUE,"ANKRD20A4"

"8204","CGGACAGGCTGTCCATTGCC","chr20",30720039,"-","none","AGG","CGGACAGGCTGTCCATTGCCAGG",0,NA,NA,NA,"","",TRUE,"ANKRD20A4"

"8205","CGGACAGGCTGTCCATTGCC","chr21",13975344,"-","4:T>G","AGG","CGGAAAGGCTGTCCATTGCCAGG",1,5,NA,NA,"GeneID:391267","ANKRD20A11P",TRUE,"ANKRD20A4"

"8206","CGGACAGGCTGTCCATTGCC","chr9",64075665,"+","1:A>G","AGG","CAGACAGGCTGTCCATTGCCAGG",1,2,NA,NA,"","",FALSE,"ANKRD20A4"

"8207","CTCTAATATACCTGTATCAA","chr9",66149213,"+","none","AGG","CTCTAATATACCTGTATCAAAGG",0,NA,NA,NA,"GeneID:441425","ANKRD20A3",TRUE,"ANKRD20A4"

"8208","CTCTAATATACCTGTATCAA","chr2",94853526,"+","none","AGG","CTCTAATATACCTGTATCAAAGG",0,NA,NA,NA,"","",TRUE,"ANKRD20A4"

"8209","CTCTAATATACCTGTATCAA","chr21",13975508,"+","none","AGG","CTCTAATATACCTGTATCAAAGG",0,NA,NA,NA,"GeneID:391267","ANKRD20A11P",TRUE,"ANKRD20A4"

"8210","CTCTAATATACCTGTATCAA","chrUn_KI270744v1",58001,"+","none","AGG","CTCTAATATACCTGTATCAAAGG",0,NA,NA,NA,"","",FALSE,"ANKRD20A4"

"8211","CTCTAATATACCTGTATCAA","chr18",14183757,"-","none","AGG","CTCTAATATACCTGTATCAAAGG",0,NA,NA,NA,"","",TRUE,"ANKRD20A4"

"8212","CTCTAATATACCTGTATCAA","chr9",64075500,"-","none","AGG","CTCTAATATACCTGTATCAAAGG",0,NA,NA,NA,"","",FALSE,"ANKRD20A4"

"8213","CTCTAATATACCTGTATCAA","chr9",42856303,"-","none","AGG","CTCTAATATACCTGTATCAAAGG",0,NA,NA,NA,"","",TRUE,"ANKRD20A4"

"8214","CTCTAATATACCTGTATCAA","chr9",67863046,"-","none","AGG","CTCTAATATACCTGTATCAAAGG",0,NA,NA,NA,"GeneID:84210","ANKRD20A1",TRUE,"ANKRD20A4"

"8215","CTCTAATATACCTGTATCAA","chr9",40227012,"-","none","AGG","CTCTAATATACCTGTATCAAAGG",0,NA,NA,NA,"GeneID:441430","ANKRD20A2",TRUE,"ANKRD20A4"

"8216","CTCTAATATACCTGTATCAA","chr9",64373292,"-","none","AGG","CTCTAATATACCTGTATCAAAGG",0,NA,NA,NA,"GeneID:728747","ANKRD20A4",TRUE,"ANKRD20A4"

"8217","CTCTAATATACCTGTATCAA","chr21",14068742,"-","none","AGG","CTCTAATATACCTGTATCAAAGG",0,NA,NA,NA,"","",TRUE,"ANKRD20A4"

"8218","CTCTAATATACCTGTATCAA","chr13",66317699,"-","2:A>G,3:C>A","AGG","CTTGAATATACCTGTATCAAAGG",2,3,4,NA,"GeneID:5101","PCDH9",FALSE,"ANKRD20A4"

"8219","GTACCGTCTCAGCCTATCAA","chr9",66144894,"+","none","CGG","GTACCGTCTCAGCCTATCAACGG",0,NA,NA,NA,"GeneID:441425","ANKRD20A3",TRUE,"ANKRD20A4"

"8220","GTACCGTCTCAGCCTATCAA","chr2",94849196,"+","none","CGG","GTACCGTCTCAGCCTATCAACGG",0,NA,NA,NA,"","",TRUE,"ANKRD20A4"

"8221","GTACCGTCTCAGCCTATCAA","chr9",40231333,"-","none","CGG","GTACCGTCTCAGCCTATCAACGG",0,NA,NA,NA,"GeneID:441430","ANKRD20A2",TRUE,"ANKRD20A4"

"8222","GTACCGTCTCAGCCTATCAA","chr9",42860627,"-","none","CGG","GTACCGTCTCAGCCTATCAACGG",0,NA,NA,NA,"","",TRUE,"ANKRD20A4"

"8223","GTACCGTCTCAGCCTATCAA","chr9",67867367,"-","none","CGG","GTACCGTCTCAGCCTATCAACGG",0,NA,NA,NA,"GeneID:84210","ANKRD20A1",TRUE,"ANKRD20A4"

"8224","GTACCGTCTCAGCCTATCAA","chr9",64377611,"-","none","CGG","GTACCGTCTCAGCCTATCAACGG",0,NA,NA,NA,"GeneID:728747","ANKRD20A4",TRUE,"ANKRD20A4"

"8225","GTACCGTCTCAGCCTATCAA","chr18",14188095,"-","5:T>C","CGG","GTACCATCTCAGCCTATCAACGG",1,6,NA,NA,"","",TRUE,"ANKRD20A4"

"8226","GTACCGTCTCAGCCTATCAA","chr21",13971162,"+","5:A>G,12:G>C","CGG","GTACCATCTCAGGCTATCAACGG",2,6,13,NA,"GeneID:391267","ANKRD20A11P",TRUE,"ANKRD20A4"

"8227","GTACCGTCTCAGCCTATCAA","chr13",18871856,"+","5:A>G,12:G>C","CGG","GTACCATCTCAGGCTATCAACGG",2,6,13,NA,"","",TRUE,"ANKRD20A4"

"8228","GTGCTTCAATATGTGCACCA","chr9",66148896,"+","none","TGG","GTGCTTCAATATGTGCACCATGG",0,NA,NA,NA,"GeneID:441425","ANKRD20A3",TRUE,"ANKRD20A4"

"8229","GTGCTTCAATATGTGCACCA","chr9",64373609,"-","none","TGG","GTGCTTCAATATGTGCACCATGG",0,NA,NA,NA,"GeneID:728747","ANKRD20A4",TRUE,"ANKRD20A4"

"8230","GTGCTTCAATATGTGCACCA","chr9",67863363,"-","none","TGG","GTGCTTCAATATGTGCACCATGG",0,NA,NA,NA,"GeneID:84210","ANKRD20A1",TRUE,"ANKRD20A4"

"8231","GTGCTTCAATATGTGCACCA","chr9",40227329,"-","none","TGG","GTGCTTCAATATGTGCACCATGG",0,NA,NA,NA,"GeneID:441430","ANKRD20A2",TRUE,"ANKRD20A4"

"8232","GTGCTTCAATATGTGCACCA","chr9",42856620,"-","none","TGG","GTGCTTCAATATGTGCACCATGG",0,NA,NA,NA,"","",TRUE,"ANKRD20A4"

"8233","GTGCTTCAATATGTGCACCA","chr9",64075818,"-","12:A>C","TGG","GTGCTTCAATATTTGCACCATGG",1,13,NA,NA,"","",FALSE,"ANKRD20A4"

"8234","GTGCTTCAATATGTGCACCA","chr18",14184074,"-","12:A>C,14:A>C","TGG","GTGCTTCAATATTTTCACCATGG",2,13,15,NA,"","",TRUE,"ANKRD20A4"

"8235","GTGCTTCAATATGTGCACCA","chrUn_KI270744v1",57683,"+","12:T>G","TGG","GTGCTTCAATATTTGCACCATGG",1,13,NA,NA,"","",FALSE,"ANKRD20A4"

"8236","GTGCTTCAATATGTGCACCA","chr21",13975191,"+","12:T>G","TGG","GTGCTTCAATATTTGCACCATGG",1,13,NA,NA,"GeneID:391267","ANKRD20A11P",TRUE,"ANKRD20A4"

"8237","GTGCTTCAATATGTGCACCA","chr20",30719886,"+","12:T>G","TGG","GTGCTTCAATATTTGCACCATGG",1,13,NA,NA,"","",TRUE,"ANKRD20A4"

"8238","GTGCTTCAATATGTGCACCA","chr2",94853209,"+","10:G>A","TGG","GTGCTTCAATGTGTGCACCATGG",1,11,NA,NA,"","",TRUE,"ANKRD20A4"

"8239","TCATAGAACTGCTCTACACT","chr9",67862934,"+","none","TGG","TCATAGAACTGCTCTACACTTGG",0,NA,NA,NA,"GeneID:84210","ANKRD20A1",TRUE,"ANKRD20A4"

"8240","TCATAGAACTGCTCTACACT","chr9",40226900,"+","none","TGG","TCATAGAACTGCTCTACACTTGG",0,NA,NA,NA,"GeneID:441430","ANKRD20A2",TRUE,"ANKRD20A4"

"8241","TCATAGAACTGCTCTACACT","chr9",64373180,"+","none","TGG","TCATAGAACTGCTCTACACTTGG",0,NA,NA,NA,"GeneID:728747","ANKRD20A4",TRUE,"ANKRD20A4"

"8242","TCATAGAACTGCTCTACACT","chr9",66149325,"-","none","TGG","TCATAGAACTGCTCTACACTTGG",0,NA,NA,NA,"GeneID:441425","ANKRD20A3",TRUE,"ANKRD20A4"

"8243","TCATAGAACTGCTCTACACT","chr21",13975620,"-","18:A>G","TGG","TCATAGAACTGCTCTACATTTGG",1,19,NA,NA,"GeneID:391267","ANKRD20A11P",TRUE,"ANKRD20A4"

"8244","TCATAGAACTGCTCTACACT","chr9",64075388,"+","18:T>C","TGG","TCATAGAACTGCTCTACATTTGG",1,19,NA,NA,"","",FALSE,"ANKRD20A4"

"8245","TCATAGAACTGCTCTACACT","chr21",14068630,"+","18:T>C","TGG","TCATAGAACTGCTCTACATTTGG",1,19,NA,NA,"","",TRUE,"ANKRD20A4"

"8246","TCATAGAACTGCTCTACACT","chr9",42856191,"+","16:T>C","TGG","TCATAGAACTGCTCTATACTTGG",1,17,NA,NA,"","",TRUE,"ANKRD20A4"

"8247","TCATAGAACTGCTCTACACT","chr2",94853638,"-","2:C>T","TGG","TCGTAGAACTGCTCTACACTTGG",1,3,NA,NA,"","",TRUE,"ANKRD20A4"

"8248","TCATAGAACTGCTCTACACT","chr9",64884504,"-","2:C>T,6:C>T","GGG","TCGTAGGACTGCTCTACACTGGG",2,3,7,NA,"","",TRUE,"ANKRD20A4"

"8249","TCATAGAACTGCTCTACACT","chr10",45194240,"-","2:C>T,6:C>T","GGG","TCGTAGGACTGCTCTACACTGGG",2,3,7,NA,"","",TRUE,"ANKRD20A4"

"8250","TCATAGAACTGCTCTACACT","chr18",15325993,"-","2:C>T,6:C>T","GGG","TCGTAGGACTGCTCTACACTGGG",2,3,7,NA,"GeneID:644669","LOC644669",TRUE,"ANKRD20A4"

"8251","TCATAGAACTGCTCTACACT","chr9",40763738,"-","2:C>T,6:C>T","GGG","TCGTAGGACTGCTCTACACTGGG",2,3,7,NA,"","",TRUE,"ANKRD20A4"

"8252","TCATAGAACTGCTCTACACT","chr8",43297013,"+","2:T>A,6:G>A","TGG","TCTTAGGACTGCTCTACACTTGG",2,3,7,NA,"GeneID:340441","POTEA",TRUE,"ANKRD20A4"

"8253","TCATAGAACTGCTCTACACT","chr21",13042495,"+","2:G>A,6:G>A","GGG","TCGTAGGACTGCTCTACACTGGG",2,3,7,NA,"","",TRUE,"ANKRD20A4"

"8254","TCATAGAACTGCTCTACACT","chr10",37129886,"+","2:G>A,6:G>A","GGG","TCGTAGGACTGCTCTACACTGGG",2,3,7,NA,"GeneID:91074","ANKRD30A",TRUE,"ANKRD20A4"

"8255","TCATAGAACTGCTCTACACT","chr18",14752559,"+","2:G>A,6:G>A","GGG","TCGTAGGACTGCTCTACACTGGG",2,3,7,NA,"GeneID:374860","ANKRD30B",TRUE,"ANKRD20A4"

"8256","TCATAGAACTGCTCTACACT","chr15",19878547,"+","2:G>A,6:G>A","GGG","TCGTAGGACTGCTCTACACTGGG",2,3,7,NA,"","",TRUE,"ANKRD20A4"

"8257","TCATAGAACTGCTCTACACT","chr22",16136722,"+","2:G>A,6:G>A","GGG","TCGTAGGACTGCTCTACACTGGG",2,3,7,NA,"","",FALSE,"ANKRD20A4"

"8258","TCATAGAACTGCTCTACACT","chr13",18413120,"+","2:G>A,6:G>A","GGG","TCGTAGGACTGCTCTACACTGGG",2,3,7,NA,"","",TRUE,"ANKRD20A4"

"8259","TCATAGAACTGCTCTACACT","chr18",14183645,"+","2:G>A,18:T>C","TGG","TCGTAGAACTGCTCTACATTTGG",2,3,19,NA,"","",TRUE,"ANKRD20A4"

"8260","TCATAGAACTGCTCTACACT","chr7",39816542,"+","6:G>A,18:T>C","TGG","TCATAGGACTGCTCTACATTTGG",2,7,19,NA,"","",TRUE,"ANKRD20A4"

"8261","TCATAGAACTGCTCTACACT","chr21",13615116,"+","6:G>A,18:T>C","TGG","TCATAGGACTGCTCTACATTTGG",2,7,19,NA,"GeneID:317754","POTED",TRUE,"ANKRD20A4"

"8262","TCATAGAACTGCTCTACACT","chr15",21435094,"-","6:C>T,18:A>G","TGG","TCATAGGACTGCTCTACATTTGG",2,7,19,NA,"GeneID:102723502","LOC102723502",TRUE,"ANKRD20A4"

"8263","TCATAGAACTGCTCTACACT","chrUn_GL000213v1",134376,"-","6:C>T,18:A>G","TGG","TCATAGGACTGCTCTACATTTGG",2,7,19,NA,"","",FALSE,"ANKRD20A4"

"8264","TCATAGAACTGCTCTACACT","chr18",14538235,"-","6:C>T,18:A>G","TGG","TCATAGGACTGCTCTACATTTGG",2,7,19,NA,"GeneID:388468","POTEC",TRUE,"ANKRD20A4"

"8265","TCATAGAACTGCTCTACACT","chr15",21872440,"-","6:C>T,18:A>G","TGG","TCATAGGACTGCTCTACATTTGG",2,7,19,NA,"GeneID:100996331","POTEB",TRUE,"ANKRD20A4"

"8266","TCATAGAACTGCTCTACACT","chr15",20861475,"-","6:C>T,18:A>G","TGG","TCATAGGACTGCTCTACATTTGG",2,7,19,NA,"GeneID:100287399","POTEB2",TRUE,"ANKRD20A4"

"8267","TCATAGAACTGCTCTACACT","chr15_KI270727v1_random",162297,"-","6:C>T,18:A>G","TGG","TCATAGGACTGCTCTACATTTGG",2,7,19,NA,"","",FALSE,"ANKRD20A4"

"8268","TCATAGAACTGCTCTACACT","chrUn_KI270744v1",58113,"-","2:C>T,18:A>G","TGG","TCGTAGAACTGCTCTACATTTGG",2,3,19,NA,"","",FALSE,"ANKRD20A4"

"8269","TCATAGAACTGCTCTACACT","chr20",30720316,"-","2:C>T,18:A>G","TGG","TCGTAGAACTGCTCTACATTTGG",2,3,19,NA,"","",TRUE,"ANKRD20A4"

"8270","AGCTATGCCAACGTTCAAGA","chr10",88831940,"-","none","TGG","AGCTATGCCAACGTTCAAGATGG",0,NA,NA,NA,"GeneID:118932","ANKRD22",TRUE,"ANKRD22"

"8271","ATTAAAGCCATCTTGAACGT","chr10",88831930,"+","none","TGG","ATTAAAGCCATCTTGAACGTTGG",0,NA,NA,NA,"GeneID:118932","ANKRD22",TRUE,"ANKRD22"

"8272","CTTCACAGCATAATGCAAGC","chr10",88828633,"+","none","AGG","CTTCACAGCATAATGCAAGCAGG",0,NA,NA,NA,"GeneID:118932","ANKRD22",TRUE,"ANKRD22"

"8273","GCACGGGCTTCCAAGAGCAG","chr10",88823301,"+","none","AGG","GCACGGGCTTCCAAGAGCAGAGG",0,NA,NA,NA,"GeneID:118932","ANKRD22",TRUE,"ANKRD22"

"8274","GCACGGGCTTCCAAGAGCAG","chr1",13829379,"-","12:A>T,18:A>T","GGG","GCACGGGCTTCCTAGAGCTGGGG",2,13,19,NA,"","",FALSE,"ANKRD22"

"8275","GTACGAATGCTACTTGATGC","chr10",88826065,"-","none","TGG","GTACGAATGCTACTTGATGCTGG",0,NA,NA,NA,"GeneID:118932","ANKRD22",TRUE,"ANKRD22"

"8276","TTAATGCCTGTTCTGCTTAT","chr10",88828574,"-","none","TGG","TTAATGCCTGTTCTGCTTATTGG",0,NA,NA,NA,"GeneID:118932","ANKRD22",TRUE,"ANKRD22"

"8277","TTAATGCCTGTTCTGCTTAT","chr6",47071033,"-","1:T>A,17:G>A","GGG","TAAATGCCTGTTCTGCTCATGGG",2,2,18,NA,"","",FALSE,"ANKRD22"

"8278","ACTGAACCAGCCGCAGTCCC","chr2",96840902,"-","none","AGG","ACTGAACCAGCCGCAGTCCCAGG",0,NA,NA,NA,"GeneID:200539;GeneID:51239","ANKRD23;ANKRD39",TRUE,"ANKRD23"

"8279","CAAGTCTCGCGCGTCCACTG","chr2",96840415,"+","none","TGG","CAAGTCTCGCGCGTCCACTGTGG",0,NA,NA,NA,"GeneID:200539;GeneID:51239","ANKRD23;ANKRD39",TRUE,"ANKRD23"

"8280","CTCCCACTCACCTTAACCAG","chr2",96842048,"+","none","GGG","CTCCCACTCACCTTAACCAGGGG",0,NA,NA,NA,"GeneID:200539;GeneID:51239","ANKRD23;ANKRD39",TRUE,"ANKRD23"

"8281","CTGTCAAGTACTTGTCAATC","chr2",96840815,"+","none","AGG","CTGTCAAGTACTTGTCAATCAGG",0,NA,NA,NA,"GeneID:200539;GeneID:51239","ANKRD23;ANKRD39",TRUE,"ANKRD23"

"8282","TCCACCGCACCGCCTTGCAC","chr2",96840493,"-","none","TGG","TCCACCGCACCGCCTTGCACTGG",0,NA,NA,NA,"GeneID:200539;GeneID:51239","ANKRD23;ANKRD39",TRUE,"ANKRD23"

"8283","TGAGACACAGAGTCCCCCCC","chr2",96842083,"-","none","AGG","TGAGACACAGAGTCCCCCCCAGG",0,NA,NA,NA,"GeneID:200539;GeneID:51239","ANKRD23;ANKRD39",TRUE,"ANKRD23"

"8284","CACATCTAAACCCCCAAGAT","chr19",4207289,"+","none","CGG","CACATCTAAACCCCCAAGATCGG",0,NA,NA,NA,"GeneID:170961","ANKRD24",TRUE,"ANKRD24"

"8285","CAGAAGCTTACCCGATCTTG","chr19",4207303,"-","none","GGG","CAGAAGCTTACCCGATCTTGGGG",0,NA,NA,NA,"GeneID:170961","ANKRD24",TRUE,"ANKRD24"

"8286","GCCCGTATTTGGCGGCCAGG","chr19",4202041,"-","none","TGG","GCCCGTATTTGGCGGCCAGGTGG",0,NA,NA,NA,"GeneID:170961","ANKRD24",TRUE,"ANKRD24"

"8287","GCGGGGTGCGGCCAGCTGTC","chr19",4200100,"+","none","TGG","GCGGGGTGCGGCCAGCTGTCTGG",0,NA,NA,NA,"GeneID:170961","ANKRD24",TRUE,"ANKRD24"

"8288","GTGCTTGAAGCAACTACTGC","chr19",4202068,"+","none","AGG","GTGCTTGAAGCAACTACTGCAGG",0,NA,NA,NA,"GeneID:170961","ANKRD24",TRUE,"ANKRD24"

"8289","TGGCAGCAATGTCATGAGCG","chr19",4200139,"+","none","CGG","TGGCAGCAATGTCATGAGCGCGG",0,NA,NA,NA,"GeneID:170961","ANKRD24",TRUE,"ANKRD24"

"8290","TGGCAGCAATGTCATGAGCG","chrX",39691314,"-","2:G>C,19:G>C","TGG","TGCCAGCAATGTCATGAGCCTGG",2,3,20,NA,"","",FALSE,"ANKRD24"

"8291","ATGTCTGTGACAACGAAAAC","chr10",27093702,"-","none","AGG","ATGTCTGTGACAACGAAAACAGG",0,NA,NA,NA,"GeneID:22852","ANKRD26",TRUE,"ANKRD26"

"8292","ATGTCTGTGACAACGAAAAC","chr9",42856274,"+","2:A>G,13:A>C","AGG","ATATCTGTGACAAAGAAAACAGG",2,3,14,NA,"","",TRUE,"ANKRD26"

"8293","ATGTCTGTGACAACGAAAAC","chr16",46564202,"-","6:A>C,14:T>C","AGG","ATGTCTTTGACAACAAAAACAGG",2,7,15,NA,"","",TRUE,"ANKRD26"

"8294","ATGTCTGTGACAACGAAAAC","chr2",129794518,"-","2:G>C,14:G>C","AGG","ATCTCTGTGACAACCAAAACAGG",2,3,15,NA,"","",FALSE,"ANKRD26"

"8295","CACGTCCGAGACCGAGATCT","chr10",27100186,"-","none","CGG","CACGTCCGAGACCGAGATCTCGG",0,NA,NA,NA,"GeneID:22852","ANKRD26",TRUE,"ANKRD26"

"8296","CCAAATCTTGCGGATGTCCA","chr10",27093445,"-","none","TGG","CCAAATCTTGCGGATGTCCATGG",0,NA,NA,NA,"GeneID:22852","ANKRD26",TRUE,"ANKRD26"

"8297","CGTTGTCACAGACATTGAGC","chr10",27093707,"+","none","TGG","CGTTGTCACAGACATTGAGCTGG",0,NA,NA,NA,"GeneID:22852","ANKRD26",TRUE,"ANKRD26"

"8298","GCAACTATTCTGCTAGAACA","chr10",27093475,"-","none","TGG","GCAACTATTCTGCTAGAACATGG",0,NA,NA,NA,"GeneID:22852","ANKRD26",TRUE,"ANKRD26"

"8299","GCAACTATTCTGCTAGAACA","chr16",46563977,"-","2:C>T,9:A>G","CGG","GCGACTATTTTGCTAGAACACGG",2,3,10,NA,"","",TRUE,"ANKRD26"

"8300","TGTGGATCTTGCCGAGATCT","chr10",27100172,"+","none","CGG","TGTGGATCTTGCCGAGATCTCGG",0,NA,NA,NA,"GeneID:22852","ANKRD26",TRUE,"ANKRD26"

"8301","AACAGGATTAAATTAGGAGC","chr19",32646577,"-","none","TGG","AACAGGATTAAATTAGGAGCTGG",0,NA,NA,NA,"GeneID:84079","ANKRD27",TRUE,"ANKRD27"

"8302","ACTCAAACTGACAAGTAGAC","chr19",32649731,"+","none","TGG","ACTCAAACTGACAAGTAGACTGG",0,NA,NA,NA,"GeneID:84079","ANKRD27",TRUE,"ANKRD27"

"8303","CAGGTCTTAGTACCCTGCAA","chr19",32649775,"-","none","AGG","CAGGTCTTAGTACCCTGCAAAGG",0,NA,NA,NA,"GeneID:84079","ANKRD27",TRUE,"ANKRD27"

"8304","CCTGTGTATAGCCCATCCTT","chr19",32646479,"-","none","TGG","CCTGTGTATAGCCCATCCTTTGG",0,NA,NA,NA,"GeneID:84079","ANKRD27",TRUE,"ANKRD27"

"8305","CCTGTGTATAGCCCATCCTT","chr16",77499324,"+","7:T>A,12:A>C","TGG","CCTGTGTTTAGCACATCCTTTGG",2,8,13,NA,"","",FALSE,"ANKRD27"

"8306","GACACTCCGAGCGATTTGAC","chr19",32644390,"-","none","AGG","GACACTCCGAGCGATTTGACAGG",0,NA,NA,NA,"GeneID:84079","ANKRD27",TRUE,"ANKRD27"

"8307","GGCTACTGACTATGTGGTGA","chr19",32644314,"+","none","CGG","GGCTACTGACTATGTGGTGACGG",0,NA,NA,NA,"GeneID:84079","ANKRD27",TRUE,"ANKRD27"

"8308","ATTAGTGCTCGAACTTCATC","chr3",15795250,"+","none","AGG","ATTAGTGCTCGAACTTCATCAGG",0,NA,NA,NA,"GeneID:23243","ANKRD28",TRUE,"ANKRD28"

"8309","CAATGATTTCTGCATCTCCA","chr3",15766251,"+","none","AGG","CAATGATTTCTGCATCTCCAAGG",0,NA,NA,NA,"GeneID:23243","ANKRD28",TRUE,"ANKRD28"

"8310","CTGGTGCAAGCTATATTTAA","chr3",15795280,"-","none","CGG","CTGGTGCAAGCTATATTTAACGG",0,NA,NA,NA,"GeneID:23243","ANKRD28",TRUE,"ANKRD28"

"8311","GTCAAACTACTCTTGTCTAG","chr3",15735474,"-","none","AGG","GTCAAACTACTCTTGTCTAGAGG",0,NA,NA,NA,"GeneID:23243","ANKRD28",TRUE,"ANKRD28"

"8312","TATCCATTGGGCAGCATATA","chr3",15735412,"-","none","TGG","TATCCATTGGGCAGCATATATGG",0,NA,NA,NA,"GeneID:23243","ANKRD28",TRUE,"ANKRD28"

"8313","TTGCACGCCGCAGCTTACCT","chr3",15766271,"-","none","TGG","TTGCACGCCGCAGCTTACCTTGG",0,NA,NA,NA,"GeneID:23243","ANKRD28",TRUE,"ANKRD28"

"8314","CAGCGTAGGCAGCAACCATC","chr18",23646250,"+","none","AGG","CAGCGTAGGCAGCAACCATCAGG",0,NA,NA,NA,"GeneID:147463","ANKRD29",TRUE,"ANKRD29"

"8315","CTATTCTTTGCCGCCCAGCA","chr18",23638912,"-","none","AGG","CTATTCTTTGCCGCCCAGCAAGG",0,NA,NA,NA,"GeneID:147463","ANKRD29",TRUE,"ANKRD29"

"8316","CTCTGCAGTCCACGTCCACC","chr18",23649087,"+","none","CGG","CTCTGCAGTCCACGTCCACCCGG",0,NA,NA,NA,"GeneID:147463","ANKRD29",TRUE,"ANKRD29"

"8317","GAAATCTCACGACATCATTA","chr18",23638886,"+","none","TGG","GAAATCTCACGACATCATTATGG",0,NA,NA,NA,"GeneID:147463","ANKRD29",TRUE,"ANKRD29"

"8318","GGACGGGGGCACCGCCCTGT","chr18",23634130,"-","none","TGG","GGACGGGGGCACCGCCCTGTTGG",0,NA,NA,NA,"GeneID:147463","ANKRD29",TRUE,"ANKRD29"

"8319","GTTTGCTCCGTGCTTCAGCA","chr18",23634068,"+","none","AGG","GTTTGCTCCGTGCTTCAGCAAGG",0,NA,NA,NA,"GeneID:147463","ANKRD29",TRUE,"ANKRD29"

"8320","ATAAATCTCGTAGATGTGTA","chr10",37130261,"+","none","TGG","ATAAATCTCGTAGATGTGTATGG",0,NA,NA,NA,"GeneID:91074","ANKRD30A",TRUE,"ANKRD30A"

"8321","ATGCAGTTAATAAGTATAAA","chr10",37132324,"+","none","TGG","ATGCAGTTAATAAGTATAAATGG",0,NA,NA,NA,"GeneID:91074","ANKRD30A",TRUE,"ANKRD30A"

"8322","ATGCAGTTAATAAGTATAAA","chr10",45191839,"-","15:A>T","TGG","ATGCAGTTAATAAGTTTAAATGG",1,16,NA,NA,"","",FALSE,"ANKRD30A"

"8323","ATGCAGTTAATAAGTATAAA","chr22",16139434,"+","8:G>A,15:T>A","TGG","ATGCAGTTGATAAGTTTAAATGG",2,9,16,NA,"","",FALSE,"ANKRD30A"

"8324","ATGCAGTTAATAAGTATAAA","chr15",19881285,"+","8:G>A,15:T>A","TGG","ATGCAGTTGATAAGTTTAAATGG",2,9,16,NA,"","",TRUE,"ANKRD30A"

"8325","ATGCAGTTAATAAGTATAAA","chr2",132154663,"-","8:C>T,15:A>T","TGG","ATGCAGTTGATAAGTTTAAATGG",2,9,16,NA,"GeneID:554226","ANKRD30BL",TRUE,"ANKRD30A"

"8326","ATGCAGTTAATAAGTATAAA","chr10",27338832,"-","8:C>T,15:A>T","TGG","ATGCAGTTGATAAGTTTAAATGG",2,9,16,NA,"","",TRUE,"ANKRD30A"

"8327","ATGCAGTTAATAAGTATAAA","chr3",132650857,"-","7:T>A,10:G>A","AGG","ATGCAGTAAACAAGTATAAAAGG",2,8,11,NA,"GeneID:84129;GeneID:100532724","ACAD11;NPHP3-ACAD11",FALSE,"ANKRD30A"

"8328","ATGCAGTTAATAAGTATAAA","chr15",27877621,"-","6:C>A,14:G>A","AGG","ATGCAGGTAATAAGCATAAAAGG",2,7,15,NA,"GeneID:4948","OCA2",FALSE,"ANKRD30A"

"8329","CACATCTACGAGATTTATAT","chr10",37130259,"-","none","CGG","CACATCTACGAGATTTATATCGG",0,NA,NA,NA,"GeneID:91074","ANKRD30A",TRUE,"ANKRD30A"

"8330","GAAATGTTACTACTTCCTCA","chr10",37129925,"-","none","TGG","GAAATGTTACTACTTCCTCATGG",0,NA,NA,NA,"GeneID:91074","ANKRD30A",TRUE,"ANKRD30A"

"8331","GAAATGTTACTACTTCCTCA","chr18",14752598,"-","16:A>G,17:C>A","TGG","GAAATGTTACTACTTCTGCATGG",2,17,18,NA,"GeneID:374860","ANKRD30B",TRUE,"ANKRD30A"

"8332","GAAATGTTACTACTTCCTCA","chr10",45194201,"+","17:G>T","TGG","GAAATGTTACTACTTCCGCATGG",1,18,NA,NA,"","",TRUE,"ANKRD30A"

"8333","GAACATTATGCTGTTACTTG","chr10",37134021,"+","none","TGG","GAACATTATGCTGTTACTTGTGG",0,NA,NA,NA,"GeneID:91074","ANKRD30A",TRUE,"ANKRD30A"

"8334","GAACATTATGCTGTTACTTG","chr14",18341799,"+","4:G>A,15:G>A","TGG","GAACGTTATGCTGTTGCTTGTGG",2,5,16,NA,"","",TRUE,"ANKRD30A"

"8335","GAACATTATGCTGTTACTTG","chr22",16140522,"+","4:G>A,15:G>A","TGG","GAACGTTATGCTGTTGCTTGTGG",2,5,16,NA,"","",FALSE,"ANKRD30A"

"8336","GAACATTATGCTGTTACTTG","chr15",19882382,"+","4:G>A,15:G>A","TGG","GAACGTTATGCTGTTGCTTGTGG",2,5,16,NA,"","",TRUE,"ANKRD30A"

"8337","GAACATTATGCTGTTACTTG","chr21",13046341,"+","4:G>A,15:G>A","TGG","GAACGTTATGCTGTTGCTTGTGG",2,5,16,NA,"","",TRUE,"ANKRD30A"

"8338","GAACATTATGCTGTTACTTG","chr13",18417284,"+","4:G>A,15:G>A","TGG","GAACGTTATGCTGTTGCTTGTGG",2,5,16,NA,"","",TRUE,"ANKRD30A"

"8339","GAACATTATGCTGTTACTTG","chr22",15272616,"+","4:G>A,15:G>A","TGG","GAACGTTATGCTGTTGCTTGTGG",2,5,16,NA,"","",FALSE,"ANKRD30A"

"8340","GAACATTATGCTGTTACTTG","chr18",15322165,"-","4:C>T,15:C>T","TGG","GAACGTTATGCTGTTGCTTGTGG",2,5,16,NA,"GeneID:644669","LOC644669",TRUE,"ANKRD30A"

"8341","GAACATTATGCTGTTACTTG","chr2",132153568,"-","4:C>T,15:C>T","TGG","GAACGTTATGCTGTTGCTTGTGG",2,5,16,NA,"GeneID:554226","ANKRD30BL",TRUE,"ANKRD30A"

"8342","GAACATTATGCTGTTACTTG","chr10",45190124,"-","4:C>T,15:C>T","TGG","GAACGTTATGCTGTTGCTTGTGG",2,5,16,NA,"","",TRUE,"ANKRD30A"

"8343","GTCTTTGCTGCAGATATATG","chr10",37133988,"+","none","TGG","GTCTTTGCTGCAGATATATGTGG",0,NA,NA,NA,"GeneID:91074","ANKRD30A",TRUE,"ANKRD30A"

"8344","GTCTTTGCTGCAGATATATG","chr11",32031144,"-","2:T>G,15:A>T","AGG","GTATTTGCTGCAGATTTATGAGG",2,3,16,NA,"","",FALSE,"ANKRD30A"

"8345","ACCATCTACTTCGGGGATCT","chr18",14748515,"+","none","AGG","ACCATCTACTTCGGGGATCTAGG",0,NA,NA,NA,"GeneID:374860","ANKRD30B",TRUE,"ANKRD30B"

"8346","AGTGTAGACCCGTTCGCTGA","chr18",14748480,"-","none","AGG","AGTGTAGACCCGTTCGCTGAAGG",0,NA,NA,NA,"GeneID:374860","ANKRD30B",TRUE,"ANKRD30B"

"8347","CGCCATCAAGGACATTAAGC","chr18",14752637,"-","none","TGG","CGCCATCAAGGACATTAAGCTGG",0,NA,NA,NA,"GeneID:374860","ANKRD30B",TRUE,"ANKRD30B"

"8348","CGCCATCAAGGACATTAAGC","chr13",18413198,"-","13:C>T,15:G>A","TGG","CGCCATCAAGGACGTCAAGCTGG",2,14,16,NA,"","",TRUE,"ANKRD30B"

"8349","CGCCATCAAGGACATTAAGC","chr21",13042573,"-","13:C>T,15:G>A","TGG","CGCCATCAAGGACGTCAAGCTGG",2,14,16,NA,"","",TRUE,"ANKRD30B"

"8350","CGCCATCAAGGACATTAAGC","chr10",37129964,"-","13:C>T,15:G>A","TGG","CGCCATCAAGGACGTCAAGCTGG",2,14,16,NA,"GeneID:91074","ANKRD30A",TRUE,"ANKRD30B"

"8351","GACAGGCTCTACAATGCGAG","chr18",14752833,"+","none","AGG","GACAGGCTCTACAATGCGAGAGG",0,NA,NA,NA,"GeneID:374860","ANKRD30B",TRUE,"ANKRD30B"

"8352","GACAGGCTCTACAATGCGAG","chr10",27340961,"-","17:A>C,18:C>T","AGG","GACAGGCTCTACAATGCTGGAGG",2,18,19,NA,"","",TRUE,"ANKRD30B"

"8353","GACAGGCTCTACAATGCGAG","chr10",45193963,"-","17:G>C","AGG","GACAGGCTCTACAATGCCAGAGG",1,18,NA,NA,"","",TRUE,"ANKRD30B"

"8354","GACAGGCTCTACAATGCGAG","chr2",132157131,"-","10:C>T,17:G>C","AGG","GACAGGCTCTGCAATGCCAGAGG",2,11,18,NA,"GeneID:554226","ANKRD30BL",TRUE,"ANKRD30B"

"8355","GACAGGCTCTACAATGCGAG","chr18",15325715,"-","10:C>T,17:G>C","AGG","GACAGGCTCTGCAATGCCAGAGG",2,11,18,NA,"GeneID:644669","LOC644669",TRUE,"ANKRD30B"

"8356","GACAGGCTCTACAATGCGAG","chr13",18413717,"+","10:G>A,17:C>G","AGG","GACAGGCTCTGCAATGCCAGAGG",2,11,18,NA,"","",TRUE,"ANKRD30B"

"8357","GACAGGCTCTACAATGCGAG","chr21",13042773,"+","10:G>A,17:C>G","AGG","GACAGGCTCTGCAATGCCAGAGG",2,11,18,NA,"","",TRUE,"ANKRD30B"

"8358","GTCCTATGGTGCAGTCATCG","chr18",14752978,"+","none","AGG","GTCCTATGGTGCAGTCATCGAGG",0,NA,NA,NA,"GeneID:374860","ANKRD30B",TRUE,"ANKRD30B"

"8359","TGCCAGCTTAATGTCCTTGA","chr18",14752632,"+","none","TGG","TGCCAGCTTAATGTCCTTGATGG",0,NA,NA,NA,"GeneID:374860","ANKRD30B",TRUE,"ANKRD30B"

"8360","TGCCAGCTTAATGTCCTTGA","chr13",18413193,"+","9:G>A,11:C>T","TGG","TGCCAGCTTGACGTCCTTGATGG",2,10,12,NA,"","",TRUE,"ANKRD30B"

"8361","TGCCAGCTTAATGTCCTTGA","chr21",13042568,"+","9:G>A,11:C>T","TGG","TGCCAGCTTGACGTCCTTGATGG",2,10,12,NA,"","",TRUE,"ANKRD30B"

"8362","TGCCAGCTTAATGTCCTTGA","chr10",37129959,"+","9:G>A,11:C>T","TGG","TGCCAGCTTGACGTCCTTGATGG",2,10,12,NA,"GeneID:91074","ANKRD30A",TRUE,"ANKRD30B"

"8363","TGCCAGCTTAATGTCCTTGA","chr14",18338032,"+","9:G>A,11:C>T","TGG","TGCCAGCTTGACGTCCTTGATGG",2,10,12,NA,"","",TRUE,"ANKRD30B"

"8364","TGCCAGCTTAATGTCCTTGA","chr22",15268849,"+","9:G>A,11:C>T","TGG","TGCCAGCTTGACGTCCTTGATGG",2,10,12,NA,"","",FALSE,"ANKRD30B"

"8365","AAGCATATCATCCAGATGAC","chr5",94628828,"+","none","AGG","AAGCATATCATCCAGATGACAGG",0,NA,NA,NA,"GeneID:84250","ANKRD32",TRUE,"ANKRD32"

"8366","ACTATGAATTATATAGTCCT","chr5",94630520,"-","none","TGG","ACTATGAATTATATAGTCCTTGG",0,NA,NA,NA,"GeneID:84250","ANKRD32",TRUE,"ANKRD32"

"8367","AGCGAAGTGATTCTCTTATA","chr5",94630721,"+","none","AGG","AGCGAAGTGATTCTCTTATAAGG",0,NA,NA,NA,"GeneID:84250","ANKRD32",TRUE,"ANKRD32"

"8368","AGGAAAGTGGATACTAACCA","chr5",94630500,"+","none","AGG","AGGAAAGTGGATACTAACCAAGG",0,NA,NA,NA,"GeneID:84250","ANKRD32",TRUE,"ANKRD32"

"8369","CCATCTTAAATCCTGTCATC","chr5",94628842,"-","none","TGG","CCATCTTAAATCCTGTCATCTGG",0,NA,NA,NA,"GeneID:84250","ANKRD32",TRUE,"ANKRD32"

"8370","CCATCTTAAATCCTGTCATC","chr4",90542278,"-","11:T>G,12:C>G","AGG","CCATCTTAAATAGTGTCATCAGG",2,12,13,NA,"GeneID:401145","CCSER1",FALSE,"ANKRD32"

"8371","CGTGAAGAACTGAAACGCAC","chr5",94630645,"+","none","TGG","CGTGAAGAACTGAAACGCACTGG",0,NA,NA,NA,"GeneID:84250","ANKRD32",TRUE,"ANKRD32"

"8372","CCGACAGCTTCGACACCGTG","chr12",51890646,"+","none","TGG","CCGACAGCTTCGACACCGTGTGG",0,NA,NA,NA,"GeneID:341405","ANKRD33",TRUE,"ANKRD33"

"8373","GCACTCACAGCGGTTCCGCA","chr12",51889470,"-","none","TGG","GCACTCACAGCGGTTCCGCATGG",0,NA,NA,NA,"GeneID:341405","ANKRD33",TRUE,"ANKRD33"

"8374","GCCACTCGGGCTTGTAGTGC","chr12",51890716,"-","none","TGG","GCCACTCGGGCTTGTAGTGCTGG",0,NA,NA,NA,"GeneID:341405","ANKRD33",TRUE,"ANKRD33"

"8375","GGAGACCCCACCATCCAGTA","chr12",51888762,"-","none","TGG","GGAGACCCCACCATCCAGTATGG",0,NA,NA,NA,"GeneID:341405","ANKRD33",TRUE,"ANKRD33"

"8376","GGCCCACATAGTAGTTGAGC","chr12",51889396,"-","none","AGG","GGCCCACATAGTAGTTGAGCAGG",0,NA,NA,NA,"GeneID:341405","ANKRD33",TRUE,"ANKRD33"

"8377","TGAGCAGGGCCACAACACTC","chr12",51889101,"-","none","TGG","TGAGCAGGGCCACAACACTCTGG",0,NA,NA,NA,"GeneID:341405","ANKRD33",TRUE,"ANKRD33"

"8378","ACGCTTTCCGGGACGCCCTC","chr5",10564695,"-","none","CGG","ACGCTTTCCGGGACGCCCTCCGG",0,NA,NA,NA,"GeneID:651746","ANKRD33B",TRUE,"ANKRD33B"

"8379","ATCCACGCGGCCCGTGTAAG","chr5",10649322,"-","none","TGG","ATCCACGCGGCCCGTGTAAGTGG",0,NA,NA,NA,"GeneID:651746","ANKRD33B",TRUE,"ANKRD33B"

"8380","CGAACGCGTTCCTCCTTTCA","chr5",10638079,"-","none","AGG","CGAACGCGTTCCTCCTTTCAAGG",0,NA,NA,NA,"GeneID:651746","ANKRD33B",TRUE,"ANKRD33B"

"8381","CTTGAAAGGAGGAACGCGTT","chr5",10638077,"+","none","CGG","CTTGAAAGGAGGAACGCGTTCGG",0,NA,NA,NA,"GeneID:651746","ANKRD33B",TRUE,"ANKRD33B"

"8382","GCCCGCTTACCCTGCCGTTG","chr5",10564823,"-","none","CGG","GCCCGCTTACCCTGCCGTTGCGG",0,NA,NA,NA,"GeneID:651746","ANKRD33B",TRUE,"ANKRD33B"

"8383","GGCCACTTACACGGGCCGCG","chr5",10649317,"+","none","TGG","GGCCACTTACACGGGCCGCGTGG",0,NA,NA,NA,"GeneID:651746","ANKRD33B",TRUE,"ANKRD33B"

"8384","CCCAATATCGCAGACCGATT","chr1",145961532,"-","none","AGG","CCCAATATCGCAGACCGATTAGG",0,NA,NA,NA,"GeneID:284615","ANKRD34A",TRUE,"ANKRD34A"

"8385","CCGGCCGCGTTTGCGAGATG","chr1",145960492,"+","none","TGG","CCGGCCGCGTTTGCGAGATGTGG",0,NA,NA,NA,"GeneID:284615","ANKRD34A",TRUE,"ANKRD34A"

"8386","CGCAGCTATGTTGCACACCG","chr1",145961746,"-","none","AGG","CGCAGCTATGTTGCACACCGAGG",0,NA,NA,NA,"GeneID:284615","ANKRD34A",TRUE,"ANKRD34A"

"8387","GCCCGCGTGATCTCGGACTG","chr1",145961426,"+","none","AGG","GCCCGCGTGATCTCGGACTGAGG",0,NA,NA,NA,"GeneID:284615","ANKRD34A",TRUE,"ANKRD34A"

"8388","GTCGTCGTAGCGGGCCCGAC","chr1",145961603,"+","none","AGG","GTCGTCGTAGCGGGCCCGACAGG",0,NA,NA,NA,"GeneID:284615","ANKRD34A",TRUE,"ANKRD34A"

"8389","TGAGCGCCGTGCGCCCTAAT","chr1",145961515,"+","none","CGG","TGAGCGCCGTGCGCCCTAATCGG",0,NA,NA,NA,"GeneID:284615","ANKRD34A",TRUE,"ANKRD34A"

"8390","ATTAATGAGAGCAACGACCG","chr5",80559894,"-","none","TGG","ATTAATGAGAGCAACGACCGTGG",0,NA,NA,NA,"GeneID:340120","ANKRD34B",TRUE,"ANKRD34B"

"8391","CAATGCCGATCCCAACATAC","chr5",80559781,"-","none","AGG","CAATGCCGATCCCAACATACAGG",0,NA,NA,NA,"GeneID:340120","ANKRD34B",TRUE,"ANKRD34B"

"8392","CTTCTAGCAAAAGTCTTGTG","chr5",80559925,"+","none","AGG","CTTCTAGCAAAAGTCTTGTGAGG",0,NA,NA,NA,"GeneID:340120","ANKRD34B",TRUE,"ANKRD34B"

"8393","GATCGGCATTGTTCTCTAAC","chr5",80559790,"+","none","AGG","GATCGGCATTGTTCTCTAACAGG",0,NA,NA,NA,"GeneID:340120","ANKRD34B",TRUE,"ANKRD34B"

"8394","TGACACCCATCTATATCCAC","chr5",80559486,"+","none","AGG","TGACACCCATCTATATCCACAGG",0,NA,NA,NA,"GeneID:340120","ANKRD34B",TRUE,"ANKRD34B"

"8395","TTTAGTAGTATGCTTCCCAC","chr5",80559527,"+","none","AGG","TTTAGTAGTATGCTTCCCACAGG",0,NA,NA,NA,"GeneID:340120","ANKRD34B",TRUE,"ANKRD34B"

"8396","ACAACAGATAAATCGTCTTC","chr15",79293740,"+","none","AGG","ACAACAGATAAATCGTCTTCAGG",0,NA,NA,NA,"GeneID:390616","ANKRD34C",TRUE,"ANKRD34C"

"8397","CCACATCTTCTAGAACGACG","chr15",79294598,"+","none","AGG","CCACATCTTCTAGAACGACGAGG",0,NA,NA,NA,"GeneID:390616","ANKRD34C",TRUE,"ANKRD34C"

"8398","CCAGCACCGAGGGCGCTATC","chr15",79294054,"-","none","AGG","CCAGCACCGAGGGCGCTATCAGG",0,NA,NA,NA,"GeneID:390616","ANKRD34C",TRUE,"ANKRD34C"

"8399","GCTGGCAGCCTCGACGCGTC","chr15",79294069,"+","none","AGG","GCTGGCAGCCTCGACGCGTCAGG",0,NA,NA,NA,"GeneID:390616","ANKRD34C",TRUE,"ANKRD34C"

"8400","GGTCTCATCCTGACGCGTCG","chr15",79294080,"-","none","AGG","GGTCTCATCCTGACGCGTCGAGG",0,NA,NA,NA,"GeneID:390616","ANKRD34C",TRUE,"ANKRD34C"

"8401","GTCTTTGCTATCGATACTGT","chr15",79294182,"-","none","TGG","GTCTTTGCTATCGATACTGTTGG",0,NA,NA,NA,"GeneID:390616","ANKRD34C",TRUE,"ANKRD34C"

"8402","ACCCCTGATGATCGCATCGC","chr1",145876215,"-","none","TGG","ACCCCTGATGATCGCATCGCTGG",0,NA,NA,NA,"GeneID:148741","ANKRD35",TRUE,"ANKRD35"

"8403","ACTGTGGAGGCACCTACAGC","chr1",145874848,"-","none","AGG","ACTGTGGAGGCACCTACAGCAGG",0,NA,NA,NA,"GeneID:148741","ANKRD35",TRUE,"ANKRD35"

"8404","CACCCAGCGATGCGATCATC","chr1",145876209,"+","none","AGG","CACCCAGCGATGCGATCATCAGG",0,NA,NA,NA,"GeneID:148741","ANKRD35",TRUE,"ANKRD35"

"8405","CCACAGATCGGCTTTGATCC","chr1",145874992,"-","none","TGG","CCACAGATCGGCTTTGATCCTGG",0,NA,NA,NA,"GeneID:148741","ANKRD35",TRUE,"ANKRD35"

"8406","GTGTGACCACGAAGCCTTCC","chr1",145876585,"-","none","TGG","GTGTGACCACGAAGCCTTCCTGG",0,NA,NA,NA,"GeneID:148741","ANKRD35",TRUE,"ANKRD35"

"8407","TCACATTATCCAACACGTCC","chr1",145876564,"+","none","AGG","TCACATTATCCAACACGTCCAGG",0,NA,NA,NA,"GeneID:148741","ANKRD35",TRUE,"ANKRD35"

"8408","CCAAAGAAATCCGTAATATT","chr2",97118403,"-","none","TGG","CCAAAGAAATCCGTAATATTTGG",0,NA,NA,NA,"GeneID:375248","ANKRD36",TRUE,"ANKRD36"

"8409","CCAAAGAAATCCGTAATATT","chrY",26414388,"-","12:T>C","TGG","CCAAAGAAATCCATAATATTTGG",1,13,NA,NA,"","",TRUE,"ANKRD36"

"8410","CCAAAGAAATCCGTAATATT","chr2",97585037,"+","7:C>A","TGG","CCAAAGACATCCGTAATATTTGG",1,8,NA,NA,"GeneID:57730","ANKRD36B",TRUE,"ANKRD36"

"8411","CCAAAGAAATCCGTAATATT","chr2",95986844,"+","7:C>A","TGG","CCAAAGACATCCGTAATATTTGG",1,8,NA,NA,"GeneID:400986","ANKRD36C",TRUE,"ANKRD36"

"8412","CCAAATATTACGGATTTCTT","chr2",97118400,"+","none","TGG","CCAAATATTACGGATTTCTTTGG",0,NA,NA,NA,"GeneID:375248","ANKRD36",TRUE,"ANKRD36"

"8413","CCAAATATTACGGATTTCTT","chr2",97585040,"-","15:C>A","TGG","CCAAATATTACGGATGTCTTTGG",1,16,NA,NA,"GeneID:57730","ANKRD36B",TRUE,"ANKRD36"

"8414","CCAAATATTACGGATTTCTT","chr2",95986847,"-","15:C>A","TGG","CCAAATATTACGGATGTCTTTGG",1,16,NA,NA,"GeneID:400986","ANKRD36C",TRUE,"ANKRD36"

"8415","CCAAATATTACGGATTTCTT","chrY",26414385,"+","10:T>C","TGG","CCAAATATTATGGATTTCTTTGG",1,11,NA,NA,"","",TRUE,"ANKRD36"

"8416","CGACTCACAGCAAATAACAG","chr2",97122901,"-","none","TGG","CGACTCACAGCAAATAACAGTGG",0,NA,NA,NA,"GeneID:375248","ANKRD36",TRUE,"ANKRD36"

"8417","CGACTCACAGCAAATAACAG","chr2",97580533,"+","13:G>A","TGG","CGACTCACAGCAAGTAACAGTGG",1,14,NA,NA,"GeneID:57730","ANKRD36B",TRUE,"ANKRD36"

"8418","CTACTATATACCTTGATCAG","chr2",97118169,"-","none","AGG","CTACTATATACCTTGATCAGAGG",0,NA,NA,NA,"GeneID:375248","ANKRD36",TRUE,"ANKRD36"

"8419","CTACTATATACCTTGATCAG","chrY",26414153,"-","17:A>G","AGG","CTACTATATACCTTGATTAGAGG",1,18,NA,NA,"","",TRUE,"ANKRD36"

"8420","CTACTATATACCTTGATCAG","chr8",57240200,"-","10:A>G,14:G>C","AGG","CTACTATATATCTTCATCAGAGG",2,11,15,NA,"GeneID:100507651","LOC100507651",TRUE,"ANKRD36"

"8421","CTACTATATACCTTGATCAG","chr2",97585272,"+","7:G>A","AGG","CTACTATGTACCTTGATCAGAGG",1,8,NA,NA,"GeneID:57730","ANKRD36B",TRUE,"ANKRD36"

"8422","CTACTATATACCTTGATCAG","chr2",95987080,"+","7:G>A","AGG","CTACTATGTACCTTGATCAGAGG",1,8,NA,NA,"GeneID:400986","ANKRD36C",TRUE,"ANKRD36"

"8423","CTACTATATACCTTGATCAG","chr10",27341127,"+","7:T>A,14:C>G","AGG","CTACTATTTACCTTCATCAGAGG",2,8,15,NA,"","",TRUE,"ANKRD36"

"8424","CTACTATATACCTTGATCAG","chr18",14752671,"-","7:A>T,14:G>C","AGG","CTACTATTTACCTTCATCAGAGG",2,8,15,NA,"GeneID:374860","ANKRD30B",TRUE,"ANKRD36"

"8425","CTACTATATACCTTGATCAG","chr10",37129998,"-","7:A>T,14:G>C","AGG","CTACTATTTACCTTCATCAGAGG",2,8,15,NA,"GeneID:91074","ANKRD30A",TRUE,"ANKRD36"

"8426","GTACCTGCCAAGATAATCAA","chr2",95982251,"+","none","CGG","GTACCTGCCAAGATAATCAACGG",0,NA,NA,NA,"GeneID:400986","ANKRD36C",TRUE,"ANKRD36"

"8427","GTACCTGCCAAGATAATCAA","chr2",97580457,"+","none","TGG","GTACCTGCCAAGATAATCAATGG",0,NA,NA,NA,"GeneID:57730","ANKRD36B",TRUE,"ANKRD36"

"8428","GTACCTGCCAAGATAATCAA","chr2",97122977,"-","none","TGG","GTACCTGCCAAGATAATCAATGG",0,NA,NA,NA,"GeneID:375248","ANKRD36",TRUE,"ANKRD36"

"8429","TCACAGGACCGCCCTACATT","chr2",97118057,"+","none","TGG","TCACAGGACCGCCCTACATTTGG",0,NA,NA,NA,"GeneID:375248","ANKRD36",TRUE,"ANKRD36"

"8430","TCACAGGACCGCCCTACATT","chr2",95987192,"-","none","TGG","TCACAGGACCGCCCTACATTTGG",0,NA,NA,NA,"GeneID:400986","ANKRD36C",TRUE,"ANKRD36"

"8431","TCACAGGACCGCCCTACATT","chr2",97585384,"-","9:A>G","TGG","TCACAGGACTGCCCTACATTTGG",1,10,NA,NA,"GeneID:57730","ANKRD36B",TRUE,"ANKRD36"

"8432","TCACAGGACCGCCCTACATT","chrY",26414041,"+","9:T>C","TGG","TCACAGGACTGCCCTACATTTGG",1,10,NA,NA,"","",TRUE,"ANKRD36"

"8433","AAACAGCTGAAGATCTCGCT","chr4",185399589,"+","none","TGG","AAACAGCTGAAGATCTCGCTTGG",0,NA,NA,NA,"GeneID:353322;GeneID:55325","ANKRD37;UFSP2",TRUE,"ANKRD37"

"8434","AAGCAAGTGAACACCCTGAC","chr4",185399673,"+","none","AGG","AAGCAAGTGAACACCCTGACAGG",0,NA,NA,NA,"GeneID:353322;GeneID:55325","ANKRD37;UFSP2",TRUE,"ANKRD37"

"8435","CAAGCAGGCTTAGGCACTCC","chr4",185398986,"-","none","AGG","CAAGCAGGCTTAGGCACTCCAGG",0,NA,NA,NA,"GeneID:353322","ANKRD37",TRUE,"ANKRD37"

"8436","GGCCTCGGTCAACGCACCCC","chr4",185397181,"+","none","CGG","GGCCTCGGTCAACGCACCCCCGG",0,NA,NA,NA,"GeneID:353322","ANKRD37",TRUE,"ANKRD37"

"8437","TCACTCAATTTGGGCATCAC","chr4",185399012,"-","none","TGG","TCACTCAATTTGGGCATCACTGG",0,NA,NA,NA,"GeneID:353322","ANKRD37",TRUE,"ANKRD37"

"8438","TCGCCTGTCCACTTAGCCGC","chr4",185397218,"+","none","AGG","TCGCCTGTCCACTTAGCCGCAGG",0,NA,NA,NA,"GeneID:353322","ANKRD37",TRUE,"ANKRD37"

"8439","CTGTACGCCGAGCACCGCGC","chr2",96857918,"+","none","TGG","CTGTACGCCGAGCACCGCGCTGG",0,NA,NA,NA,"GeneID:200539;GeneID:51239","ANKRD23;ANKRD39",TRUE,"ANKRD39"

"8440","GCGCGGCTCCTGCTATCACA","chr2",96853452,"-","none","TGG","GCGCGGCTCCTGCTATCACATGG",0,NA,NA,NA,"GeneID:200539;GeneID:51239","ANKRD23;ANKRD39",TRUE,"ANKRD39"

"8441","GCTCACCAGCGCAGTGTAGC","chr2",96854331,"+","none","CGG","GCTCACCAGCGCAGTGTAGCCGG",0,NA,NA,NA,"GeneID:200539;GeneID:51239","ANKRD23;ANKRD39",TRUE,"ANKRD39"

"8442","GCTCGGCGTACAGCAGACGC","chr2",96857911,"-","none","TGG","GCTCGGCGTACAGCAGACGCTGG",0,NA,NA,NA,"GeneID:200539;GeneID:51239","ANKRD23;ANKRD39",TRUE,"ANKRD39"

"8443","GGACCCAAGTCAGCCCGACT","chr2",96854357,"-","none","CGG","GGACCCAAGTCAGCCCGACTCGG",0,NA,NA,NA,"GeneID:200539;GeneID:51239","ANKRD23;ANKRD39",TRUE,"ANKRD39"

"8444","TCCCGCAGGTCACTGTTGCA","chr2",96848316,"+","none","AGG","TCCCGCAGGTCACTGTTGCAAGG",0,NA,NA,NA,"GeneID:200539;GeneID:51239","ANKRD23;ANKRD39",TRUE,"ANKRD39"

"8445","AGTTACCCAATACTCTGTTA","chr17",50696945,"-","none","AGG","AGTTACCCAATACTCTGTTAAGG",0,NA,NA,NA,"GeneID:91369","ANKRD40",TRUE,"ANKRD40"

"8446","ATGTAAACGAAACCATGGTC","chr17",50700678,"-","none","AGG","ATGTAAACGAAACCATGGTCAGG",0,NA,NA,NA,"GeneID:91369","ANKRD40",TRUE,"ANKRD40"

"8447","CCCGGGGAAAGGTCCCTAAC","chr17",50699644,"+","none","AGG","CCCGGGGAAAGGTCCCTAACAGG",0,NA,NA,NA,"GeneID:91369","ANKRD40",TRUE,"ANKRD40"

"8448","CCTCAAAATGGGACGTATGC","chr17",50699460,"-","none","AGG","CCTCAAAATGGGACGTATGCAGG",0,NA,NA,NA,"GeneID:91369","ANKRD40",TRUE,"ANKRD40"

"8449","TACCTTCCTTAACAGAGTAT","chr17",50696936,"+","none","TGG","TACCTTCCTTAACAGAGTATTGG",0,NA,NA,NA,"GeneID:91369","ANKRD40",TRUE,"ANKRD40"

"8450","TACCTTCCTTAACAGAGTAT","chr2",64452505,"-","12:T>G,14:G>C","TGG","TACCTTCCTTAAAACAGTATTGG",2,13,15,NA,"","",FALSE,"ANKRD40"

"8451","TGCCAGTCCAGTTAACATCA","chr17",50700595,"-","none","AGG","TGCCAGTCCAGTTAACATCAAGG",0,NA,NA,NA,"GeneID:91369","ANKRD40",TRUE,"ANKRD40"

"8452","AGTGCATCCCCGGTCATCCT","chr11",83210333,"-","none","GGG","AGTGCATCCCCGGTCATCCTGGG",0,NA,NA,NA,"GeneID:338699","ANKRD42",TRUE,"ANKRD42"

"8453","ATACATGATGCAGTACGAGC","chr11",83198507,"+","none","TGG","ATACATGATGCAGTACGAGCTGG",0,NA,NA,NA,"GeneID:338699","ANKRD42",TRUE,"ANKRD42"

"8454","ATCACACACGTAACAACGAG","chr11",83206090,"+","none","AGG","ATCACACACGTAACAACGAGAGG",0,NA,NA,NA,"GeneID:338699","ANKRD42",TRUE,"ANKRD42"

"8455","GCTTGCAACTTCTTGTTAAA","chr11",83211364,"+","none","TGG","GCTTGCAACTTCTTGTTAAATGG",0,NA,NA,NA,"GeneID:338699","ANKRD42",TRUE,"ANKRD42"

"8456","TGAAAAGCTGCATAATGCAC","chr11",83211330,"-","none","AGG","TGAAAAGCTGCATAATGCACAGG",0,NA,NA,NA,"GeneID:338699","ANKRD42",TRUE,"ANKRD42"

"8457","TTACAAATAATGCTCCGAAG","chr11",83210392,"+","none","TGG","TTACAAATAATGCTCCGAAGTGG",0,NA,NA,NA,"GeneID:338699","ANKRD42",TRUE,"ANKRD42"

"8458","TTACAAATAATGCTCCGAAG","chr5",173921926,"-","11:A>C,15:T>G","GGG","TTACAAATAATTCTCAGAAGGGG",2,12,16,NA,"GeneID:80315","CPEB4",FALSE,"ANKRD42"

"8459","AATCTTCAGCGGTGATCCAG","chr2",197187069,"-","none","AGG","AATCTTCAGCGGTGATCCAGAGG",0,NA,NA,NA,"GeneID:91526","ANKRD44",TRUE,"ANKRD44"

"8460","AGTGTCAATGTCTCCGACCG","chr2",197125885,"-","none","AGG","AGTGTCAATGTCTCCGACCGAGG",0,NA,NA,NA,"GeneID:91526","ANKRD44",TRUE,"ANKRD44"

"8461","ATGAGCATCCGGATCTCCTC","chr2",197187050,"+","none","TGG","ATGAGCATCCGGATCTCCTCTGG",0,NA,NA,NA,"GeneID:91526","ANKRD44",TRUE,"ANKRD44"

"8462","CGTGGCCGTTCAGAGCCGCA","chr2",197125841,"+","none","TGG","CGTGGCCGTTCAGAGCCGCATGG",0,NA,NA,NA,"GeneID:91526","ANKRD44",TRUE,"ANKRD44"

"8463","GAAACGAACCCCTCTTCATG","chr2",197147077,"-","none","TGG","GAAACGAACCCCTCTTCATGTGG",0,NA,NA,NA,"GeneID:91526","ANKRD44",TRUE,"ANKRD44"

"8464","GAAACGAACCCCTCTTCATG","chr1",2847946,"+","15:G>T,17:C>A","GGG","GAAACGAACCCCTCTGCCTGGGG",2,16,18,NA,"","",FALSE,"ANKRD44"

"8465","GTTATTTACCTGACAAAATC","chr2",197147017,"+","none","AGG","GTTATTTACCTGACAAAATCAGG",0,NA,NA,NA,"GeneID:91526","ANKRD44",TRUE,"ANKRD44"

"8466","GTTATTTACCTGACAAAATC","chr3",184927262,"-","0:A>C,4:G>A","TGG","TTTACTTACCTGACAAAATCTGG",2,1,5,NA,"GeneID:23355","VPS8",FALSE,"ANKRD44"

"8467","GTTATTTACCTGACAAAATC","chr8",93904701,"+","0:C>G,7:C>A","TGG","CTTATTTCCCTGACAAAATCTGG",2,1,8,NA,"GeneID:54704","PDP1",FALSE,"ANKRD44"

"8468","AAGTGACGTGATTAGAGCTT","chr1",173659132,"-","none","TGG","AAGTGACGTGATTAGAGCTTTGG",0,NA,NA,NA,"GeneID:339416","ANKRD45",TRUE,"ANKRD45"

"8469","AAGTGACGTGATTAGAGCTT","chr15",47113384,"-","10:G>T,17:T>G","AGG","AAGTGACGTGCTTAGAGATTAGG",2,11,18,NA,"","",FALSE,"ANKRD45"

"8470","ACAGTACCTTGTCTTCCTTA","chr1",173627057,"+","none","AGG","ACAGTACCTTGTCTTCCTTAAGG",0,NA,NA,NA,"GeneID:339416","ANKRD45",TRUE,"ANKRD45"

"8471","ACAGTACCTTGTCTTCCTTA","chr5",90488116,"-","15:A>G,19:G>T","AGG","ACAGTACCTTGTCTTTCTTCAGG",2,16,20,NA,"GeneID:10622","POLR3G",TRUE,"ANKRD45"

"8472","ACAGTACCTTGTCTTCCTTA","chrX",42118758,"-","3:G>C,7:T>G","AGG","ACACTACATTGTCTTCCTTAAGG",2,4,8,NA,"","",FALSE,"ANKRD45"

"8473","ACAGTACCTTGTCTTCCTTA","chr2",223920833,"-","1:C>G,17:C>A","CGG","AGAGTACCTTGTCTTCCGTACGG",2,2,18,NA,"GeneID:57590","WDFY1",FALSE,"ANKRD45"

"8474","ATATAGAAGCTTTGAACTTC","chr1",173646918,"-","none","CGG","ATATAGAAGCTTTGAACTTCCGG",0,NA,NA,NA,"GeneID:339416","ANKRD45",TRUE,"ANKRD45"

"8475","CAAAGTTTCCAAACGACCCC","chr1",173646963,"+","none","AGG","CAAAGTTTCCAAACGACCCCAGG",0,NA,NA,NA,"GeneID:339416","ANKRD45",TRUE,"ANKRD45"

"8476","GAAACTCCTTAAGGAAGACA","chr1",173627066,"-","none","AGG","GAAACTCCTTAAGGAAGACAAGG",0,NA,NA,NA,"GeneID:339416","ANKRD45",TRUE,"ANKRD45"

"8477","TTTGTTGTATGCAGCTTGCA","chr1",173659162,"-","none","TGG","TTTGTTGTATGCAGCTTGCATGG",0,NA,NA,NA,"GeneID:339416","ANKRD45",TRUE,"ANKRD45"

"8478","TTTGTTGTATGCAGCTTGCA","chr20",5659034,"-","7:T>A,17:G>C","TGG","TTTGTTGAATGCAGCTTCCATGG",2,8,18,NA,"","",FALSE,"ANKRD45"

"8479","CCCCTGCTGTCACGAATATT","chr8",100529699,"+","none","TGG","CCCCTGCTGTCACGAATATTTGG",0,NA,NA,NA,"GeneID:157567","ANKRD46",TRUE,"ANKRD46"

"8480","CTGCGCTTTGCCAGAACTAA","chr8",100527961,"+","none","AGG","CTGCGCTTTGCCAGAACTAAAGG",0,NA,NA,NA,"GeneID:157567","ANKRD46",TRUE,"ANKRD46"

"8481","GACCTAGAGATTAGATGGCC","chr8",100510566,"+","none","TGG","GACCTAGAGATTAGATGGCCTGG",0,NA,NA,NA,"GeneID:157567","ANKRD46",TRUE,"ANKRD46"

"8482","GCCACTTTCCAAAAGCCGCT","chr8",100529728,"+","none","TGG","GCCACTTTCCAAAAGCCGCTTGG",0,NA,NA,NA,"GeneID:157567","ANKRD46",TRUE,"ANKRD46"

"8483","TAAAGATGTCATCCGATTGC","chr8",100527932,"-","none","TGG","TAAAGATGTCATCCGATTGCTGG",0,NA,NA,NA,"GeneID:157567","ANKRD46",TRUE,"ANKRD46"

"8484","TCTCCAGCTTCCGAACCACG","chr8",100522695,"-","none","TGG","TCTCCAGCTTCCGAACCACGTGG",0,NA,NA,NA,"GeneID:157567","ANKRD46",TRUE,"ANKRD46"

"8485","AGAAAGGAGTCTCCGCACTG","chr11",94498077,"-","none","TGG","AGAAAGGAGTCTCCGCACTGTGG",0,NA,NA,NA,"GeneID:54851","ANKRD49",TRUE,"ANKRD49"

"8486","AGAAAGGAGTCTCCGCACTG","chr10",93849654,"+","9:A>T,10:A>C","GGG","AGAAAGGAGAATCCGCACTGGGG",2,10,11,NA,"","",FALSE,"ANKRD49"

"8487","AGGCCACTCACGTGAACACT","chr11",94498101,"+","none","AGG","AGGCCACTCACGTGAACACTAGG",0,NA,NA,NA,"GeneID:54851","ANKRD49",TRUE,"ANKRD49"

"8488","ATCCCTAGTGTTCACGTGAG","chr11",94498107,"-","none","TGG","ATCCCTAGTGTTCACGTGAGTGG",0,NA,NA,NA,"GeneID:54851","ANKRD49",TRUE,"ANKRD49"

"8489","ATCCCTAGTGTTCACGTGAG","chr2",224499792,"+","15:A>G,16:C>T","TGG","ATCCCTAGTGTTCACACGAGTGG",2,16,17,NA,"GeneID:8452","CUL3",TRUE,"ANKRD49"

"8490","ATGAACCGTTACGTCAAACC","chr11",94498397,"+","none","AGG","ATGAACCGTTACGTCAAACCAGG",0,NA,NA,NA,"GeneID:54851","ANKRD49",TRUE,"ANKRD49"

"8491","ATGAACCGTTACGTCAAACC","chr12",31534826,"-","7:T>C,12:T>C","AGG","ATGAACCATTACATCAAACCAGG",2,8,13,NA,"GeneID:160518","DENND5B",TRUE,"ANKRD49"

"8492","ATGAACCGTTACGTCAAACC","chr7",117440096,"-","7:G>C,12:T>C","AGG","ATGAACCCTTACATCAAACCAGG",2,8,13,NA,"","",TRUE,"ANKRD49"

"8493","GCCCAAAGAAGCAATCTGCT","chr11",94496915,"-","none","TGG","GCCCAAAGAAGCAATCTGCTTGG",0,NA,NA,NA,"GeneID:54851","ANKRD49",TRUE,"ANKRD49"

"8494","GCCCAAAGAAGCAATCTGCT","chr5",136857849,"-","15:A>G","TGG","GCCCAAAGAAGCAATTTGCTTGG",1,16,NA,NA,"","",TRUE,"ANKRD49"

"8495","GCCCAAAGAAGCAATCTGCT","chr14",21529375,"-","3:A>G,18:T>G","GGG","GCCTAAAGAAGCAATCTGATGGG",2,4,19,NA,"GeneID:6297","SALL2",FALSE,"ANKRD49"

"8496","GTGTTTCCAACAATTCAAGT","chr11",94496770,"-","none","TGG","GTGTTTCCAACAATTCAAGTTGG",0,NA,NA,NA,"GeneID:54851","ANKRD49",TRUE,"ANKRD49"

"8497","GTGTTTCCAACAATTCAAGT","chr5",136857704,"-","10:T>G","TGG","GTGTTTCCAAAAATTCAAGTTGG",1,11,NA,NA,"","",TRUE,"ANKRD49"

"8498","AACCCGTTATGACTAATCCT","chr4",124710499,"-","none","TGG","AACCCGTTATGACTAATCCTTGG",0,NA,NA,NA,"GeneID:57182","ANKRD50",TRUE,"ANKRD50"

"8499","CAAACGTCTACCAGCTTATC","chr4",124678785,"-","none","TGG","CAAACGTCTACCAGCTTATCTGG",0,NA,NA,NA,"GeneID:57182","ANKRD50",TRUE,"ANKRD50"

"8500","CATCAACAGAATCAACAAGC","chr4",124678831,"+","none","AGG","CATCAACAGAATCAACAAGCAGG",0,NA,NA,NA,"GeneID:57182","ANKRD50",TRUE,"ANKRD50"

"8501","GATATGCAGCCTTCGTTACG","chr4",124669824,"-","none","TGG","GATATGCAGCCTTCGTTACGTGG",0,NA,NA,NA,"GeneID:57182","ANKRD50",TRUE,"ANKRD50"

"8502","GTAGTCCACTGCGGCAGATC","chr4",124710093,"+","none","TGG","GTAGTCCACTGCGGCAGATCTGG",0,NA,NA,NA,"GeneID:57182","ANKRD50",TRUE,"ANKRD50"

"8503","TGCGTGATATAATTCCGTTA","chr4",124672160,"+","none","TGG","TGCGTGATATAATTCCGTTATGG",0,NA,NA,NA,"GeneID:57182","ANKRD50",TRUE,"ANKRD50"

"8504","CATGGGGATCCTCAGCATCA","chr12",56258250,"-","none","CGG","CATGGGGATCCTCAGCATCACGG",0,NA,NA,NA,"GeneID:283373","ANKRD52",TRUE,"ANKRD52"

"8505","CATGGGGATCCTCAGCATCA","chr7",16082573,"+","4:A>G,10:A>C","GGG","CATGAGGATCATCAGCATCAGGG",2,5,11,NA,"","",FALSE,"ANKRD52"

"8506","CATGGGGATCCTCAGCATCA","chr21",36072824,"-","8:G>A,19:A>T","GGG","CATGGGGACCCTCAGCATCTGGG",2,9,20,NA,"GeneID:54093;GeneID:100133286;GeneID:873","SETD4;LOC100133286;CBR1",TRUE,"ANKRD52"

"8507","CATGGGGATCCTCAGCATCA","chr11",18599355,"-","2:T>A,18:C>G","TGG","CAAGGGGATCCTCAGCATGATGG",2,3,19,NA,"GeneID:100506540","SPTY2D1-AS1",FALSE,"ANKRD52"

"8508","CCTGTTGAGCAGCCTCAACG","chr12",56255842,"-","none","TGG","CCTGTTGAGCAGCCTCAACGTGG",0,NA,NA,NA,"GeneID:283373","ANKRD52",TRUE,"ANKRD52"

"8509","GCGTTCCCTACTCTCGCAGA","chr12",56257847,"-","none","AGG","GCGTTCCCTACTCTCGCAGAAGG",0,NA,NA,NA,"GeneID:283373","ANKRD52",TRUE,"ANKRD52"

"8510","TGACCTGGTCCGTGATGCTG","chr12",56258238,"+","none","AGG","TGACCTGGTCCGTGATGCTGAGG",0,NA,NA,NA,"GeneID:283373","ANKRD52",TRUE,"ANKRD52"

"8511","TGACCTGGTCCGTGATGCTG","chr5",180279797,"+","3:G>C,8:C>T","CGG","TGAGCTGGCCCGTGATGCTGCGG",2,4,9,NA,"GeneID:5601","MAPK9",TRUE,"ANKRD52"

"8512","TGTCTGCCACAGCTTGTCCC","chr12",56255918,"+","none","GGG","TGTCTGCCACAGCTTGTCCCGGG",0,NA,NA,NA,"GeneID:283373","ANKRD52",TRUE,"ANKRD52"

"8513","TGTTCTCCTTCTGCGAGAGT","chr12",56257838,"+","none","AGG","TGTTCTCCTTCTGCGAGAGTAGG",0,NA,NA,NA,"GeneID:283373","ANKRD52",TRUE,"ANKRD52"

"8514","AGTAGCTCCCGATCGCCGTC","chr2",70979234,"-","none","TGG","AGTAGCTCCCGATCGCCGTCTGG",0,NA,NA,NA,"GeneID:79998","ANKRD53",TRUE,"ANKRD53"

"8515","CCAGACGGCAATCGACCAGA","chr2",70979216,"+","none","CGG","CCAGACGGCAATCGACCAGACGG",0,NA,NA,NA,"GeneID:79998","ANKRD53",TRUE,"ANKRD53"

"8516","CCATCCACTTCGCCGCCCAA","chr2",70979670,"+","none","TGG","CCATCCACTTCGCCGCCCAATGG",0,NA,NA,NA,"GeneID:79998","ANKRD53",TRUE,"ANKRD53"

"8517","CCCTTACTTGATACTCAATC","chr2",70982684,"-","none","AGG","CCCTTACTTGATACTCAATCAGG",0,NA,NA,NA,"GeneID:79998","ANKRD53",TRUE,"ANKRD53"

"8518","CGTTGGCGCCACTCTGCACC","chr2",70982007,"-","none","AGG","CGTTGGCGCCACTCTGCACCAGG",0,NA,NA,NA,"GeneID:79998","ANKRD53",TRUE,"ANKRD53"

"8519","TGTTGTCCCTGTGGATGACG","chr2",70979782,"-","none","AGG","TGTTGTCCCTGTGGATGACGAGG",0,NA,NA,NA,"GeneID:79998","ANKRD53",TRUE,"ANKRD53"

"8520","ACATGAGCAGCGAGAACGCC","chr22",37832683,"-","none","TGG","ACATGAGCAGCGAGAACGCCTGG",0,NA,NA,NA,"GeneID:129138","ANKRD54",TRUE,"ANKRD54"

"8521","ACATGAGCAGCGAGAACGCC","chr3",44170117,"-","2:G>T,10:T>G","AGG","ACCTGAGCAGAGAGAACGCCAGG",2,3,11,NA,"","",FALSE,"ANKRD54"

"8522","CCAGATACTCCCTCAGCATA","chr22",37832716,"+","none","TGG","CCAGATACTCCCTCAGCATATGG",0,NA,NA,NA,"GeneID:129138","ANKRD54",TRUE,"ANKRD54"

"8523","CTCGGCCAATGCCAATGATG","chr22",37840195,"-","none","TGG","CTCGGCCAATGCCAATGATGTGG",0,NA,NA,NA,"GeneID:129138","ANKRD54",TRUE,"ANKRD54"

"8524","CTGGTCATTGCCATTGCATG","chr22",37838503,"+","none","AGG","CTGGTCATTGCCATTGCATGAGG",0,NA,NA,NA,"GeneID:129138","ANKRD54",TRUE,"ANKRD54"

"8525","GCTGATCCTAACCAGCGAGA","chr22",37833712,"-","none","TGG","GCTGATCCTAACCAGCGAGATGG",0,NA,NA,NA,"GeneID:129138","ANKRD54",TRUE,"ANKRD54"

"8526","GCTGATCCTAACCAGCGAGA","chr1",158853273,"-","11:T>G,13:C>T","AGG","GCTGATCCTAAACGGCGAGAAGG",2,12,14,NA,"","",FALSE,"ANKRD54"

"8527","GCTGATCCTAACCAGCGAGA","chr4",123531921,"-","8:G>A,15:C>G","CGG","GCTGATCCCAACCAGGGAGACGG",2,9,16,NA,"","",FALSE,"ANKRD54"

"8528","TGTTCCCCAGCCCATCTCGC","chr22",37833698,"+","none","TGG","TGTTCCCCAGCCCATCTCGCTGG",0,NA,NA,NA,"GeneID:129138","ANKRD54",TRUE,"ANKRD54"

"8529","ATGCACGCCCTTGATGCATG","chr5",56176261,"-","none","CGG","ATGCACGCCCTTGATGCATGCGG",0,NA,NA,NA,"GeneID:79722","ANKRD55",TRUE,"ANKRD55"

"8530","ATGCACGCCCTTGATGCATG","chr17",34535215,"-","4:G>T,6:A>C","GGG","ATGCCCTCCCTTGATGCATGGGG",2,5,7,NA,"","",FALSE,"ANKRD55"

"8531","CCACTGCTGAGCCCGATATG","chr5",56170695,"-","none","AGG","CCACTGCTGAGCCCGATATGAGG",0,NA,NA,NA,"GeneID:79722","ANKRD55",TRUE,"ANKRD55"

"8532","CCACTGCTGAGCCCGATATG","chr9",84571038,"-","0:C>G,8:A>C","AGG","GCACTGCTTAGCCCGATATGAGG",2,1,9,NA,"","",FALSE,"ANKRD55"

"8533","CCTCATATCGGGCTCAGCAG","chr5",56170692,"+","none","TGG","CCTCATATCGGGCTCAGCAGTGG",0,NA,NA,NA,"GeneID:79722","ANKRD55",TRUE,"ANKRD55"

"8534","CTGCTTGATAAACCATGGTC","chr5",56183589,"+","none","AGG","CTGCTTGATAAACCATGGTCAGG",0,NA,NA,NA,"GeneID:79722","ANKRD55",TRUE,"ANKRD55"

"8535","GTTTATCAAGCAGCCTCTAA","chr5",56183582,"-","none","TGG","GTTTATCAAGCAGCCTCTAATGG",0,NA,NA,NA,"GeneID:79722","ANKRD55",TRUE,"ANKRD55"

"8536","GTTTATCAAGCAGCCTCTAA","chr15",57199137,"-","13:A>G,19:C>T","TGG","GTTTATCAAGCAGTCTCTAGTGG",2,14,20,NA,"GeneID:6938","TCF12",FALSE,"ANKRD55"

"8537","GTTTATCAAGCAGCCTCTAA","chr8",58986674,"+","0:A>G,17:C>T","GGG","ATTTATCAAGCAGCCTCCAAGGG",2,1,18,NA,"GeneID:9760","TOX",FALSE,"ANKRD55"

"8538","TTTATGCCTGGCCACCTACC","chr5",56176153,"-","none","TGG","TTTATGCCTGGCCACCTACCTGG",0,NA,NA,NA,"GeneID:79722","ANKRD55",TRUE,"ANKRD55"

"8539","CAGCCTTGCATGAAGCATCC","chr6",89606016,"+","none","TGG","CAGCCTTGCATGAAGCATCCTGG",0,NA,NA,NA,"GeneID:22881;GeneID:57226","ANKRD6;LYRM2",TRUE,"ANKRD6"

"8540","CAGCCTTGCATGAAGCATCC","chr12",131532911,"-","10:C>A,16:G>T","TGG","CAGCCTTGCAGGAAGCCTCCTGG",2,11,17,NA,"","",FALSE,"ANKRD6"

"8541","CAGCGCGGGACCCGGCCAGC","chr6",89612336,"-","none","AGG","CAGCGCGGGACCCGGCCAGCAGG",0,NA,NA,NA,"GeneID:22881;GeneID:57226","ANKRD6;LYRM2",TRUE,"ANKRD6"

"8542","CAGCGCGGGACCCGGCCAGC","chrX",41275850,"-","8:G>C,13:T>C","AGG","CAGCGCGGCACCCAGCCAGCAGG",2,9,14,NA,"","",TRUE,"ANKRD6"

"8543","CATCCACGAAGGGTGTGCCC","chr6",89603090,"+","none","TGG","CATCCACGAAGGGTGTGCCCTGG",0,NA,NA,NA,"GeneID:22881;GeneID:57226","ANKRD6;LYRM2",TRUE,"ANKRD6"

"8544","CTTGTTCTTGGCAAGCACGT","chr6",89606085,"-","none","TGG","CTTGTTCTTGGCAAGCACGTTGG",0,NA,NA,NA,"GeneID:22881;GeneID:57226","ANKRD6;LYRM2",TRUE,"ANKRD6"

"8545","GAGCACGCGCGTCCTCCTGC","chr6",89612318,"+","none","TGG","GAGCACGCGCGTCCTCCTGCTGG",0,NA,NA,NA,"GeneID:22881;GeneID:57226","ANKRD6;LYRM2",TRUE,"ANKRD6"

"8546","GGGACCAGACCGCCTTGCAC","chr6",89603029,"+","none","CGG","GGGACCAGACCGCCTTGCACCGG",0,NA,NA,NA,"GeneID:22881;GeneID:57226","ANKRD6;LYRM2",TRUE,"ANKRD6"

"8547","ACCCATTCTAGGGACACGAC","chr7",6035432,"+","none","AGG","ACCCATTCTAGGGACACGACAGG",0,NA,NA,NA,"GeneID:27102;GeneID:100310846","EIF2AK1;ANKRD61",TRUE,"ANKRD61"

"8548","AGAAGACTGCACTACGATCG","chr7",6031488,"+","none","AGG","AGAAGACTGCACTACGATCGAGG",0,NA,NA,NA,"GeneID:27102;GeneID:100310846","EIF2AK1;ANKRD61",TRUE,"ANKRD61"

"8549","ATGGGGATGATAGACTCTGT","chr7",6032841,"-","none","CGG","ATGGGGATGATAGACTCTGTCGG",0,NA,NA,NA,"GeneID:27102;GeneID:100310846","EIF2AK1;ANKRD61",TRUE,"ANKRD61"

"8550","ATGGGGATGATAGACTCTGT","chr9",136837197,"+","11:G>A,13:C>A","GGG","ATGGGGATGATGGCCTCTGTGGG",2,12,14,NA,"GeneID:55684","RABL6",TRUE,"ANKRD61"

"8551","GGTCAGAAGTAATCTGTTGC","chr7",6031568,"-","none","TGG","GGTCAGAAGTAATCTGTTGCTGG",0,NA,NA,NA,"GeneID:27102;GeneID:100310846","EIF2AK1;ANKRD61",TRUE,"ANKRD61"

"8552","GTCTATCATCCCCATCCATC","chr7",6032846,"+","none","TGG","GTCTATCATCCCCATCCATCTGG",0,NA,NA,NA,"GeneID:27102;GeneID:100310846","EIF2AK1;ANKRD61",TRUE,"ANKRD61"

"8553","TCCCTTAAGAGGCGGAATTC","chr7",6036200,"-","none","TGG","TCCCTTAAGAGGCGGAATTCTGG",0,NA,NA,NA,"GeneID:27102;GeneID:100310846","EIF2AK1;ANKRD61",TRUE,"ANKRD61"

"8554","CAAATCCCGGGTACCGAGTC","chr18",12094099,"+","none","CGG","CAAATCCCGGGTACCGAGTCCGG",0,NA,NA,NA,"GeneID:342850","ANKRD62",TRUE,"ANKRD62"

"8555","CCAAATGTTAGAGATATGTA","chr18",12095493,"+","none","TGG","CCAAATGTTAGAGATATGTATGG",0,NA,NA,NA,"GeneID:342850","ANKRD62",TRUE,"ANKRD62"

"8556","CCATACATATCTCTAACATT","chr18",12095496,"-","none","TGG","CCATACATATCTCTAACATTTGG",0,NA,NA,NA,"GeneID:342850","ANKRD62",TRUE,"ANKRD62"

"8557","CCATACATATCTCTAACATT","chr15",47124193,"-","0:A>G,19:C>A","AGG","TCATACATATCTCTAACATGAGG",2,1,20,NA,"","",FALSE,"ANKRD62"

"8558","CTACCACTCCTGGACGGCCA","chr18",12095197,"-","none","TGG","CTACCACTCCTGGACGGCCATGG",0,NA,NA,NA,"GeneID:342850","ANKRD62",TRUE,"ANKRD62"

"8559","TCCAGCCGCAACCTCCGTCA","chr18",12093893,"-","none","GGG","TCCAGCCGCAACCTCCGTCAGGG",0,NA,NA,NA,"GeneID:342850","ANKRD62",TRUE,"ANKRD62"

"8560","TGCCCATGGCCGTCCAGGAG","chr18",12095191,"+","none","TGG","TGCCCATGGCCGTCCAGGAGTGG",0,NA,NA,NA,"GeneID:342850","ANKRD62",TRUE,"ANKRD62"

"8561","AGAGGGGCCTAGTGTTGTCC","chr1",1420887,"-","none","AGG","AGAGGGGCCTAGTGTTGTCCAGG",0,NA,NA,NA,"GeneID:441869","ANKRD65",TRUE,"ANKRD65"

"8562","AGAGGGGCCTAGTGTTGTCC","chr5",9176310,"-","6:G>C,13:A>C","TGG","AGAGGGCCCTAGTTTTGTCCTGG",2,7,14,NA,"GeneID:9037","SEMA5A",FALSE,"ANKRD65"

"8563","CACCCGCGAGTGTCCGTGCC","chr1",1420435,"+","none","AGG","CACCCGCGAGTGTCCGTGCCAGG",0,NA,NA,NA,"GeneID:441869","ANKRD65",TRUE,"ANKRD65"

"8564","CCACGAGCAGGGCGCCGTCC","chr1",1420173,"+","none","AGG","CCACGAGCAGGGCGCCGTCCAGG",0,NA,NA,NA,"GeneID:441869","ANKRD65",TRUE,"ANKRD65"

"8565","CCCCGGTGGGCGCGGTGGAC","chr1",1420489,"-","none","CGG","CCCCGGTGGGCGCGGTGGACCGG",0,NA,NA,NA,"GeneID:441869","ANKRD65",TRUE,"ANKRD65"

"8566","CCGCCTGGCACGGACACTCG","chr1",1420441,"-","none","CGG","CCGCCTGGCACGGACACTCGCGG",0,NA,NA,NA,"GeneID:441869","ANKRD65",TRUE,"ANKRD65"

"8567","GGAACAGGAACTGCGGTGGA","chr1",1420944,"-","none","TGG","GGAACAGGAACTGCGGTGGATGG",0,NA,NA,NA,"GeneID:441869","ANKRD65",TRUE,"ANKRD65"

"8568","GGAACAGGAACTGCGGTGGA","chr3",14764967,"-","4:C>G,13:C>G","TGG","GGAAGAGGAACTGGGGTGGATGG",2,5,14,NA,"GeneID:84077","C3orf20",TRUE,"ANKRD65"

"8569","ATCGACGCCCCTGACTTCTT","chr6",46753864,"+","none","TGG","ATCGACGCCCCTGACTTCTTTGG",0,NA,NA,NA,"GeneID:100287718","ANKRD66",TRUE,"ANKRD66"

"8570","ATGACCGGACCCCACTTCAC","chr6",46752075,"+","none","TGG","ATGACCGGACCCCACTTCACTGG",0,NA,NA,NA,"GeneID:100287718","ANKRD66",TRUE,"ANKRD66"

"8571","CACATTTCAATGCACTCACC","chr6",46749904,"+","none","TGG","CACATTTCAATGCACTCACCTGG",0,NA,NA,NA,"GeneID:100287718","ANKRD66",TRUE,"ANKRD66"

"8572","CAGTCTACATCTTTGTAGTT","chr6",46752053,"-","none","TGG","CAGTCTACATCTTTGTAGTTTGG",0,NA,NA,NA,"GeneID:100287718","ANKRD66",TRUE,"ANKRD66"

"8573","CGATGGCAGCGTGCAATGCA","chr6",46753848,"-","none","TGG","CGATGGCAGCGTGCAATGCATGG",0,NA,NA,NA,"GeneID:100287718","ANKRD66",TRUE,"ANKRD66"

"8574","GGGTCCGGCCATCTTTGTTG","chr6",46749938,"-","none","TGG","GGGTCCGGCCATCTTTGTTGTGG",0,NA,NA,NA,"GeneID:100287718","ANKRD66",TRUE,"ANKRD66"

"8575","ATGCTTCAGATAATTATCAA","chr7",118236125,"+","none","AGG","ATGCTTCAGATAATTATCAAAGG",0,NA,NA,NA,"GeneID:56311","ANKRD7",TRUE,"ANKRD7"

"8576","ATGCTTCAGATAATTATCAA","chr18",2608107,"-","10:T>A,12:A>T","AGG","ATGCTTCAGAAATTTATCAAAGG",2,11,13,NA,"GeneID:10403","NDC80",FALSE,"ANKRD7"

"8577","ATGCTTCAGATAATTATCAA","chr4",22114395,"+","2:T>G,14:A>T","TGG","ATTCTTCAGATAATAATCAATGG",2,3,15,NA,"","",FALSE,"ANKRD7"

"8578","CTATGGCATACCTTAATCAA","chr7",118234536,"-","none","TGG","CTATGGCATACCTTAATCAATGG",0,NA,NA,NA,"GeneID:56311","ANKRD7",TRUE,"ANKRD7"

"8579","GCTACTATTCTTCTAAACTT","chr7",118234727,"+","none","TGG","GCTACTATTCTTCTAAACTTTGG",0,NA,NA,NA,"GeneID:56311","ANKRD7",TRUE,"ANKRD7"

"8580","TAACGAATATCCCTCAGATC","chr7",118234760,"-","none","TGG","TAACGAATATCCCTCAGATCTGG",0,NA,NA,NA,"GeneID:56311","ANKRD7",TRUE,"ANKRD7"

"8581","TTAATAACGGCAACTAATAG","chr7",118236055,"-","none","TGG","TTAATAACGGCAACTAATAGTGG",0,NA,NA,NA,"GeneID:56311","ANKRD7",TRUE,"ANKRD7"

"8582","TTGCACCTAGCCTGTGCTAA","chr7",118234437,"+","none","TGG","TTGCACCTAGCCTGTGCTAATGG",0,NA,NA,NA,"GeneID:56311","ANKRD7",TRUE,"ANKRD7"

"8583","AGAAGGCGAACGACGACTTG","chr14",102507777,"+","none","CGG","AGAAGGCGAACGACGACTTGCGG",0,NA,NA,NA,"GeneID:122416","ANKRD9",TRUE,"ANKRD9"

"8584","CATGGCGCGCGCGAAGAGCG","chr14",102507040,"+","none","AGG","CATGGCGCGCGCGAAGAGCGAGG",0,NA,NA,NA,"GeneID:122416","ANKRD9",TRUE,"ANKRD9"

"8585","GCAGGTAATGCGCGTACGCT","chr14",102507615,"+","none","TGG","GCAGGTAATGCGCGTACGCTTGG",0,NA,NA,NA,"GeneID:122416","ANKRD9",TRUE,"ANKRD9"

"8586","GCGCACCTTGCGCGACTTCC","chr14",102507471,"-","none","CGG","GCGCACCTTGCGCGACTTCCCGG",0,NA,NA,NA,"GeneID:122416","ANKRD9",TRUE,"ANKRD9"

"8587","GGAGGATATGCGCGCCAGCG","chr14",102507720,"-","none","AGG","GGAGGATATGCGCGCCAGCGAGG",0,NA,NA,NA,"GeneID:122416","ANKRD9",TRUE,"ANKRD9"

"8588","GGCAGTGCGCTACAACCGCG","chr14",102507513,"-","none","TGG","GGCAGTGCGCTACAACCGCGTGG",0,NA,NA,NA,"GeneID:122416","ANKRD9",TRUE,"ANKRD9"

"8589","CTCGAATTTGTTGTTGCGCA","chr6",34981783,"-","none","TGG","CTCGAATTTGTTGTTGCGCATGG",0,NA,NA,NA,"GeneID:23294","ANKS1A",TRUE,"ANKS1A"

"8590","CTGCACCATGCTGCTTTGAA","chr6",34967290,"+","none","TGG","CTGCACCATGCTGCTTTGAATGG",0,NA,NA,NA,"GeneID:23294","ANKS1A",TRUE,"ANKS1A"

"8591","CTGCACCATGCTGCTTTGAA","chr10",93302095,"+","16:G>T,19:G>A","TGG","CTGCACCATGCTGCTTGGAGTGG",2,17,20,NA,"","",FALSE,"ANKS1A"

"8592","CTGTCAACACAGTTCACATT","chr6",34967254,"-","none","TGG","CTGTCAACACAGTTCACATTTGG",0,NA,NA,NA,"GeneID:23294","ANKS1A",TRUE,"ANKS1A"

"8593","CTGTCAACACAGTTCACATT","chr12",78200071,"+","14:T>C,16:G>C","AGG","CTGTCAACACAGTTTAGATTAGG",2,15,17,NA,"GeneID:89795","NAV3",FALSE,"ANKS1A"

"8594","CTGTCAACACAGTTCACATT","chr1",118319550,"-","0:T>G,14:C>G","TGG","ATGTCAACACAGTTGACATTTGG",2,1,15,NA,"","",FALSE,"ANKS1A"

"8595","GATGGGCAGTGCTTTGCATG","chr6",34982756,"+","none","AGG","GATGGGCAGTGCTTTGCATGAGG",0,NA,NA,NA,"GeneID:23294","ANKS1A",TRUE,"ANKS1A"

"8596","GATGGGCAGTGCTTTGCATG","chr15",66760289,"-","8:G>C,13:G>A","GGG","GATGGGCACTGCTCTGCATGGGG",2,9,14,NA,"GeneID:4091","SMAD6",FALSE,"ANKS1A"

"8597","GTCGCCCGTACAGTGCTGCC","chr6",34981817,"-","none","AGG","GTCGCCCGTACAGTGCTGCCAGG",0,NA,NA,NA,"GeneID:23294","ANKS1A",TRUE,"ANKS1A"

"8598","TTTGTTTGGCAAGACCGATG","chr6",34982783,"+","none","TGG","TTTGTTTGGCAAGACCGATGTGG",0,NA,NA,NA,"GeneID:23294","ANKS1A",TRUE,"ANKS1A"

"8599","ACATTCCAGGGTCAATGAAC","chr12",99812156,"-","none","AGG","ACATTCCAGGGTCAATGAACAGG",0,NA,NA,NA,"GeneID:56899","ANKS1B",TRUE,"ANKS1B"

"8600","CACTTACTTATGTCCATTTA","chr12",99825301,"+","none","AGG","CACTTACTTATGTCCATTTAAGG",0,NA,NA,NA,"GeneID:56899","ANKS1B",TRUE,"ANKS1B"

"8601","CACTTACTTATGTCCATTTA","chr5",143267273,"+","8:A>T,13:A>C","AGG","CACTTACTAATGTACATTTAAGG",2,9,14,NA,"","",FALSE,"ANKS1B"

"8602","GTGCACCTGTTCATTGACCC","chr12",99812148,"+","none","TGG","GTGCACCTGTTCATTGACCCTGG",0,NA,NA,NA,"GeneID:56899","ANKS1B",TRUE,"ANKS1B"

"8603","TTACACCACGCAGCCTTAAA","chr12",99825317,"-","none","TGG","TTACACCACGCAGCCTTAAATGG",0,NA,NA,NA,"GeneID:56899","ANKS1B",TRUE,"ANKS1B"

"8604","TTGCGCGCAGCAAGGTGAAG","chr12",99806461,"+","none","TGG","TTGCGCGCAGCAAGGTGAAGTGG",0,NA,NA,NA,"GeneID:56899","ANKS1B",TRUE,"ANKS1B"

"8605","TTGGACTTGGCGGCACTCTA","chr12",99806560,"-","none","CGG","TTGGACTTGGCGGCACTCTACGG",0,NA,NA,NA,"GeneID:56899","ANKS1B",TRUE,"ANKS1B"

"8606","CAATCACCTCACGTTGGCAT","chr16",4726651,"+","none","TGG","CAATCACCTCACGTTGGCATTGG",0,NA,NA,NA,"GeneID:124401","ANKS3",TRUE,"ANKS3"

"8607","CAATCACCTCACGTTGGCAT","chr18",803381,"-","2:A>T,12:A>C","TGG","CATTCACCTCACTTTGGCATTGG",2,3,13,NA,"GeneID:7525","YES1",FALSE,"ANKS3"

"8608","CTGATGTATGCCTCCTACAT","chr16",4727111,"-","none","TGG","CTGATGTATGCCTCCTACATTGG",0,NA,NA,NA,"GeneID:124401","ANKS3",TRUE,"ANKS3"

"8609","GATTGTGTCGTGGCCAATGT","chr16",4727095,"+","none","AGG","GATTGTGTCGTGGCCAATGTAGG",0,NA,NA,NA,"GeneID:124401","ANKS3",TRUE,"ANKS3"

"8610","GGGTACCTGGGCTCCGATAG","chr16",4714044,"+","none","AGG","GGGTACCTGGGCTCCGATAGAGG",0,NA,NA,NA,"GeneID:124401","ANKS3",TRUE,"ANKS3"

"8611","GTACGGACACATGAAGATCG","chr16",4714107,"-","none","TGG","GTACGGACACATGAAGATCGTGG",0,NA,NA,NA,"GeneID:124401","ANKS3",TRUE,"ANKS3"

"8612","GTGGAGCCAATGCCAACGTG","chr16",4726660,"-","none","AGG","GTGGAGCCAATGCCAACGTGAGG",0,NA,NA,NA,"GeneID:124401","ANKS3",TRUE,"ANKS3"

"8613","CAAGCGAGATCTAAATCTTT","chr16",21233799,"+","none","CGG","CAAGCGAGATCTAAATCTTTCGG",0,NA,NA,NA,"GeneID:257629","ANKS4B",TRUE,"ANKS4B"

"8614","CACATTCGGGTCACTATCTA","chr16",21250141,"+","none","AGG","CACATTCGGGTCACTATCTAAGG",0,NA,NA,NA,"GeneID:257629;GeneID:1428","ANKS4B;CRYM",TRUE,"ANKS4B"

"8615","CGAAAGATTTAGATCTCGCT","chr16",21233801,"-","none","TGG","CGAAAGATTTAGATCTCGCTTGG",0,NA,NA,NA,"GeneID:257629","ANKS4B",TRUE,"ANKS4B"

"8616","GAATCCATTCTCAATCGTCC","chr16",21250352,"+","none","AGG","GAATCCATTCTCAATCGTCCAGG",0,NA,NA,NA,"GeneID:257629;GeneID:1428","ANKS4B;CRYM",TRUE,"ANKS4B"

"8617","GATAGTGACCCGAATGTGCC","chr16",21250139,"-","none","AGG","GATAGTGACCCGAATGTGCCAGG",0,NA,NA,NA,"GeneID:257629;GeneID:1428","ANKS4B;CRYM",TRUE,"ANKS4B"

"8618","TGTGGTCGATGCCACGCCCC","chr16",21250588,"+","none","TGG","TGTGGTCGATGCCACGCCCCTGG",0,NA,NA,NA,"GeneID:257629;GeneID:1428","ANKS4B;CRYM",TRUE,"ANKS4B"

"8619","CACGGAACTTGTTCGACTGC","chr9",98780316,"-","none","TGG","CACGGAACTTGTTCGACTGCTGG",0,NA,NA,NA,"GeneID:203286","ANKS6",TRUE,"ANKS6"

"8620","CCCAGTGCTGCCCGATGACA","chr9",98780202,"-","none","AGG","CCCAGTGCTGCCCGATGACAAGG",0,NA,NA,NA,"GeneID:203286","ANKS6",TRUE,"ANKS6"

"8621","CTGATGCAGACGCTGCCCCG","chr9",98778362,"-","none","TGG","CTGATGCAGACGCTGCCCCGTGG",0,NA,NA,NA,"GeneID:203286","ANKS6",TRUE,"ANKS6"

"8622","CTGATGCAGACGCTGCCCCG","chr2",239009168,"-","0:A>G,15:T>G","AGG","TTGATGCAGACGCTGACCCGAGG",2,1,16,NA,"","",FALSE,"ANKS6"

"8623","GGTGGAACCGAATGTCCAAT","chr9",98778400,"-","none","CGG","GGTGGAACCGAATGTCCAATCGG",0,NA,NA,NA,"GeneID:203286","ANKS6",TRUE,"ANKS6"

"8624","GGTGGAACCGAATGTCCAAT","chr4",40848570,"-","9:T>C,12:T>A","GGG","GGTGGAACCAAAAGTCCAATGGG",2,10,13,NA,"GeneID:323","APBB2",FALSE,"ANKS6"

"8625","GGTGGAACCGAATGTCCAAT","chr16",20051416,"-","8:T>G,18:A>T","AGG","GGTGGAACAGAATGTCCATTAGG",2,9,19,NA,"GeneID:124274","GPR139",FALSE,"ANKS6"

"8626","GTCACTCTTCGTGCAAAAAA","chr9",98782510,"-","none","TGG","GTCACTCTTCGTGCAAAAAATGG",0,NA,NA,NA,"GeneID:203286","ANKS6",TRUE,"ANKS6"

"8627","GTCACTCTTCGTGCAAAAAA","chr3",27272465,"+","0:C>G,10:C>G","GGG","CTCACTCTTCCTGCAAAAAAGGG",2,1,11,NA,"GeneID:152110","NEK10",FALSE,"ANKS6"

"8628","GTGAAATATCTGCTAAACCA","chr9",98782540,"-","none","AGG","GTGAAATATCTGCTAAACCAAGG",0,NA,NA,NA,"GeneID:203286","ANKS6",TRUE,"ANKS6"

"8629","GTGAAATATCTGCTAAACCA","chr10",20846215,"+","11:A>G,18:A>C","GGG","GTGAAATATCTACTAAACAAGGG",2,12,19,NA,"GeneID:10529","NEBL",FALSE,"ANKS6"

"8630","CATGAGCACCACGCAGTCTC","chr2",219233133,"-","none","TGG","CATGAGCACCACGCAGTCTCTGG",0,NA,NA,NA,"GeneID:55139","ANKZF1",TRUE,"ANKZF1"

"8631","CTACAGAACCTGCAAAGTAG","chr2",219233105,"+","none","AGG","CTACAGAACCTGCAAAGTAGAGG",0,NA,NA,NA,"GeneID:55139","ANKZF1",TRUE,"ANKZF1"

"8632","CTACAGAACCTGCAAAGTAG","chr2",165757744,"+","4:T>A,9:T>C","AGG","CTACTGAACTTGCAAAGTAGAGG",2,5,10,NA,"GeneID:2591","GALNT3",FALSE,"ANKZF1"

"8633","GAGAAGTTGAGCCGACCCCC","chr2",219232578,"+","none","AGG","GAGAAGTTGAGCCGACCCCCAGG",0,NA,NA,NA,"GeneID:55139","ANKZF1",TRUE,"ANKZF1"

"8634","GGGAACATTATAAGCTTGAC","chr2",219232260,"+","none","TGG","GGGAACATTATAAGCTTGACTGG",0,NA,NA,NA,"GeneID:55139","ANKZF1",TRUE,"ANKZF1"

"8635","GTCTTCTGATCCCGAGATGC","chr2",219232501,"-","none","TGG","GTCTTCTGATCCCGAGATGCTGG",0,NA,NA,NA,"GeneID:55139","ANKZF1",TRUE,"ANKZF1"

"8636","TAACCTAAAGCAACGTCTCA","chr2",219232291,"+","none","AGG","TAACCTAAAGCAACGTCTCAAGG",0,NA,NA,NA,"GeneID:55139","ANKZF1",TRUE,"ANKZF1"

"8637","TAACCTAAAGCAACGTCTCA","chr5",35341925,"+","11:C>A,13:T>C","AGG","TAACCTAAAGCCATGTCTCAAGG",2,12,14,NA,"","",FALSE,"ANKZF1"

"8638","TAACCTAAAGCAACGTCTCA","chr10",62353671,"-","0:T>A,13:A>G","GGG","AAACCTAAAGCAATGTCTCAGGG",2,1,14,NA,"","",FALSE,"ANKZF1"

"8639","AACTCACTCACCTCCGTAAA","chr7",36390035,"-","none","CGG","AACTCACTCACCTCCGTAAACGG",0,NA,NA,NA,"GeneID:23366;GeneID:54443","KIAA0895;ANLN",TRUE,"ANLN"

"8640","TCTCTGAAGATTCTCTCGCC","chr7",36396290,"-","none","TGG","TCTCTGAAGATTCTCTCGCCTGG",0,NA,NA,NA,"GeneID:54443","ANLN",TRUE,"ANLN"

"8641","TGCTGGAGCGAACCCGTGCC","chr7",36396269,"+","none","AGG","TGCTGGAGCGAACCCGTGCCAGG",0,NA,NA,NA,"GeneID:54443","ANLN",TRUE,"ANLN"

"8642","TGGGATAATGATGATATGAC","chr7",36399371,"+","none","AGG","TGGGATAATGATGATATGACAGG",0,NA,NA,NA,"GeneID:54443","ANLN",TRUE,"ANLN"

"8643","TGGGATAATGATGATATGAC","chr3",86560894,"+","14:A>T,16:A>T","AGG","TGGGATAATGATGAAAAGACAGG",2,15,17,NA,"","",FALSE,"ANLN"

"8644","TGGGGCGATGGATCCGTTTA","chr7",36390019,"+","none","CGG","TGGGGCGATGGATCCGTTTACGG",0,NA,NA,NA,"GeneID:23366;GeneID:54443","KIAA0895;ANLN",TRUE,"ANLN"

"8645","TTTGCAGATGTCGACTCAAC","chr7",36399167,"-","none","TGG","TTTGCAGATGTCGACTCAACTGG",0,NA,NA,NA,"GeneID:54443","ANLN",TRUE,"ANLN"

"8646","ACTGAAGATGCCGACGAAGA","chr11",70103142,"+","none","AGG","ACTGAAGATGCCGACGAAGAAGG",0,NA,NA,NA,"GeneID:55107","ANO1",TRUE,"ANO1"

"8647","CCTGATGCCGAGTGCAAGTA","chr11",70087775,"+","none","TGG","CCTGATGCCGAGTGCAAGTATGG",0,NA,NA,NA,"GeneID:55107","ANO1",TRUE,"ANO1"

"8648","GCCCCCGATGGACTACCACG","chr11",70087984,"+","none","AGG","GCCCCCGATGGACTACCACGAGG",0,NA,NA,NA,"GeneID:55107","ANO1",TRUE,"ANO1"

"8649","GGCCACGGGTCTCATTAATG","chr11",70104006,"-","none","TGG","GGCCACGGGTCTCATTAATGTGG",0,NA,NA,NA,"GeneID:55107","ANO1",TRUE,"ANO1"

"8650","TACCACATTAATGAGACCCG","chr11",70104001,"+","none","TGG","TACCACATTAATGAGACCCGTGG",0,NA,NA,NA,"GeneID:55107","ANO1",TRUE,"ANO1"

"8651","TCAACACCAACCTTCTTCGT","chr11",70103155,"-","none","CGG","TCAACACCAACCTTCTTCGTCGG",0,NA,NA,NA,"GeneID:55107","ANO1",TRUE,"ANO1"

"8652","AGAAGTATCCAAAGCTGATA","chr3",43605819,"+","none","AGG","AGAAGTATCCAAAGCTGATAAGG",0,NA,NA,NA,"GeneID:55129","ANO10",TRUE,"ANO10"

"8653","AGAAGTATCCAAAGCTGATA","chr11",122399485,"-","13:T>C,19:C>T","AGG","AGAAGTATCCAAAACTGATGAGG",2,14,20,NA,"GeneID:399959","MIR100HG",FALSE,"ANO10"

"8654","AGAAGTATCCAAAGCTGATA","chr8",134730945,"+","14:A>C,18:A>T","CGG","AGAAGTATCCAAAGATGAAACGG",2,15,19,NA,"","",FALSE,"ANO10"

"8655","AGAAGTATCCAAAGCTGATA","chr13",53920284,"-","6:A>T,15:T>A","AGG","AGAAGTTTCCAAAGCAGATAAGG",2,7,16,NA,"","",FALSE,"ANO10"

"8656","CAAAGCAAACCGAGTGTACC","chr3",43580368,"+","none","AGG","CAAAGCAAACCGAGTGTACCAGG",0,NA,NA,NA,"GeneID:55129","ANO10",TRUE,"ANO10"

"8657","CTAAAACTTTCAGAATTAGA","chr3",43574810,"+","none","TGG","CTAAAACTTTCAGAATTAGATGG",0,NA,NA,NA,"GeneID:55129","ANO10",TRUE,"ANO10"

"8658","CTAAAACTTTCAGAATTAGA","chr1",209278352,"+","6:A>C,15:C>T","AGG","CTAAAAATTTCAGAACTAGAAGG",2,7,16,NA,"","",FALSE,"ANO10"

"8659","GAAAGTGACCTTATCAGCTT","chr3",43605830,"-","none","TGG","GAAAGTGACCTTATCAGCTTTGG",0,NA,NA,NA,"GeneID:55129","ANO10",TRUE,"ANO10"

"8660","GAAAGTGACCTTATCAGCTT","chr7",111788856,"+","9:A>C,19:A>T","CGG","GAAAGTGACATTATCAGCTACGG",2,10,20,NA,"GeneID:9732","DOCK4",TRUE,"ANO10"

"8661","GCAAGTTCTATGACCACCAA","chr3",43605781,"+","none","AGG","GCAAGTTCTATGACCACCAAAGG",0,NA,NA,NA,"GeneID:55129","ANO10",TRUE,"ANO10"

"8662","GCTTTGAAGTATCAGCCCAT","chr3",43580354,"-","none","AGG","GCTTTGAAGTATCAGCCCATAGG",0,NA,NA,NA,"GeneID:55129","ANO10",TRUE,"ANO10"

"8663","AAACTTCGTTAAAACCGTCT","chr11",26441950,"+","none","CGG","AAACTTCGTTAAAACCGTCTCGG",0,NA,NA,NA,"GeneID:63982","ANO3",TRUE,"ANO3"

"8664","AGTCATTAATGAGACGAGAT","chr11",26463084,"-","none","CGG","AGTCATTAATGAGACGAGATCGG",0,NA,NA,NA,"GeneID:63982","ANO3",TRUE,"ANO3"

"8665","ATTCAAGACAAAATTATCTA","chr11",26463126,"+","none","AGG","ATTCAAGACAAAATTATCTAAGG",0,NA,NA,NA,"GeneID:63982","ANO3",TRUE,"ANO3"

"8666","GTCAACCGAGAGTGAATCTC","chr11",26442044,"+","none","AGG","GTCAACCGAGAGTGAATCTCAGG",0,NA,NA,NA,"GeneID:63982","ANO3",TRUE,"ANO3"

"8667","TACCCAAACAAAAATCGCTG","chr11",26443819,"-","none","AGG","TACCCAAACAAAAATCGCTGAGG",0,NA,NA,NA,"GeneID:63982","ANO3",TRUE,"ANO3"

"8668","TCTTTGAGTAAGCGTAGCTC","chr11",26441996,"-","none","TGG","TCTTTGAGTAAGCGTAGCTCTGG",0,NA,NA,NA,"GeneID:63982","ANO3",TRUE,"ANO3"

"8669","AAAATGGAGGCAAGCTCTTC","chr12",100901782,"+","none","TGG","AAAATGGAGGCAAGCTCTTCTGG",0,NA,NA,NA,"GeneID:121601","ANO4",TRUE,"ANO4"

"8670","AAAATGGAGGCAAGCTCTTC","chr2",233260783,"-","14:T>G,17:T>A","AGG","AAAATGGAGGCAAGATCATCAGG",2,15,18,NA,"GeneID:55054","ATG16L1",FALSE,"ANO4"

"8671","AGAGAATCGTCATCTTTGCA","chr12",100939367,"-","none","AGG","AGAGAATCGTCATCTTTGCAAGG",0,NA,NA,NA,"GeneID:121601","ANO4",TRUE,"ANO4"

"8672","ATCACCTGGGTGGAAGACTT","chr12",100901825,"-","none","TGG","ATCACCTGGGTGGAAGACTTTGG",0,NA,NA,NA,"GeneID:121601","ANO4",TRUE,"ANO4"

"8673","CATCATCTGAAGTACTAGTC","chr12",100939405,"-","none","AGG","CATCATCTGAAGTACTAGTCAGG",0,NA,NA,NA,"GeneID:121601","ANO4",TRUE,"ANO4"

"8674","CCATTTGATTTGTTTCTTTC","chr12",100942385,"-","none","TGG","CCATTTGATTTGTTTCTTTCTGG",0,NA,NA,NA,"GeneID:121601","ANO4",TRUE,"ANO4"

"8675","CCATTTGATTTGTTTCTTTC","chr3",65813444,"-","18:T>A,19:T>G","TGG","CCATTTGATTTGTTTCTTAATGG",2,19,20,NA,"GeneID:9223","MAGI1",FALSE,"ANO4"

"8676","CCATTTGATTTGTTTCTTTC","chr7",103748036,"-","0:A>G,4:T>A","AGG","TCATATGATTTGTTTCTTTCAGG",2,1,5,NA,"GeneID:5649","RELN",FALSE,"ANO4"

"8677","CCATTTGATTTGTTTCTTTC","chr12",113923335,"-","3:C>A,12:C>A","TGG","CCAGTTGATTTGGTTCTTTCTGG",2,4,13,NA,"GeneID:9904","RBM19",FALSE,"ANO4"

"8678","GAAAGAAACATTAGAGCAGA","chr12",100942493,"+","none","AGG","GAAAGAAACATTAGAGCAGAAGG",0,NA,NA,NA,"GeneID:121601","ANO4",TRUE,"ANO4"

"8679","GAAAGAAACATTAGAGCAGA","chr2",168362966,"+","10:G>T,11:G>T","GGG","GAAAGAAACAGGAGAGCAGAGGG",2,11,12,NA,"","",FALSE,"ANO4"

"8680","CTACTCTTTCCAAATGAGTG","chr11",22203828,"+","none","AGG","CTACTCTTTCCAAATGAGTGAGG",0,NA,NA,NA,"GeneID:203859","ANO5",TRUE,"ANO5"

"8681","GCTGGCGAAGATGGGCGACC","chr11",22193482,"+","none","CGG","GCTGGCGAAGATGGGCGACCCGG",0,NA,NA,NA,"GeneID:203859","ANO5",TRUE,"ANO5"

"8682","TCTCATCAATGAAGAAACAA","chr11",22211292,"+","none","TGG","TCTCATCAATGAAGAAACAATGG",0,NA,NA,NA,"GeneID:203859","ANO5",TRUE,"ANO5"

"8683","TCTCATCAATGAAGAAACAA","chr2",99821893,"+","5:C>T","AGG","TCTCACCAATGAAGAAACAAAGG",1,6,NA,NA,"GeneID:3899","AFF3",FALSE,"ANO5"

"8684","TCTCATCAATGAAGAAACAA","chrX",97184197,"+","2:C>T,8:T>A","GGG","TCCCATCATTGAAGAAACAAGGG",2,3,9,NA,"GeneID:1730","DIAPH2",FALSE,"ANO5"

"8685","TCTCATCAATGAAGAAACAA","chr8",32871549,"-","5:G>A,18:G>T","AGG","TCTCACCAATGAAGAAACCAAGG",2,6,19,NA,"","",FALSE,"ANO5"

"8686","TCTCCTGGAAGTGTTGGCGG","chr11",22193506,"+","none","AGG","TCTCCTGGAAGTGTTGGCGGAGG",0,NA,NA,NA,"GeneID:203859","ANO5",TRUE,"ANO5"

"8687","TTCTTCATTGATGAGAAAGC","chr11",22211288,"-","none","TGG","TTCTTCATTGATGAGAAAGCTGG",0,NA,NA,NA,"GeneID:203859","ANO5",TRUE,"ANO5"

"8688","TTCTTCATTGATGAGAAAGC","chr21",33131051,"+","18:C>G","TGG","TTCTTCATTGATGAGAAACCTGG",1,19,NA,NA,"","",FALSE,"ANO5"

"8689","TTTAACTTACCTCACTCATT","chr11",22203840,"-","none","TGG","TTTAACTTACCTCACTCATTTGG",0,NA,NA,NA,"GeneID:203859","ANO5",TRUE,"ANO5"

"8690","TTTAACTTACCTCACTCATT","chr19",46194436,"+","8:T>A,10:A>C","GGG","TTTAACTTTCATCACTCATTGGG",2,9,11,NA,"","",FALSE,"ANO5"

"8691","ATCTTGTTGCTTCTAACTGC","chr12",45347065,"-","none","AGG","ATCTTGTTGCTTCTAACTGCAGG",0,NA,NA,NA,"GeneID:196527","ANO6",TRUE,"ANO6"

"8692","ATCTTGTTGCTTCTAACTGC","chr10",81065802,"+","1:A>T,14:G>A","AGG","AACTTGTTGCTTCTGACTGCAGG",2,2,15,NA,"","",FALSE,"ANO6"

"8693","ATCTTGTTGCTTCTAACTGC","chr19",10329756,"+","7:G>T,15:G>A","AGG","ATCTTGTGGCTTCTAGCTGCAGG",2,8,16,NA,"GeneID:125950","RAVER1",FALSE,"ANO6"

"8694","CAGCCTTTGGTACACTCAAC","chr12",45348148,"+","none","TGG","CAGCCTTTGGTACACTCAACTGG",0,NA,NA,NA,"GeneID:196527","ANO6",TRUE,"ANO6"

"8695","GAATCTAACCTTATCTGTCA","chr12",45347039,"+","none","TGG","GAATCTAACCTTATCTGTCATGG",0,NA,NA,NA,"GeneID:196527","ANO6",TRUE,"ANO6"

"8696","GAATCTAACCTTATCTGTCA","chr11",79460364,"+","9:A>C,17:C>T","TGG","GAATCTAACATTATCTGCCATGG",2,10,18,NA,"","",FALSE,"ANO6"

"8697","GCAAGCCACTCCTACAACTG","chr12",45357363,"-","none","CGG","GCAAGCCACTCCTACAACTGCGG",0,NA,NA,NA,"GeneID:196527","ANO6",TRUE,"ANO6"

"8698","TAGACTTACAATGCGGCTTC","chr12",45348304,"-","none","TGG","TAGACTTACAATGCGGCTTCTGG",0,NA,NA,NA,"GeneID:196527","ANO6",TRUE,"ANO6"

"8699","TTAATCAAGATAACTGTACA","chr12",45357399,"+","none","TGG","TTAATCAAGATAACTGTACATGG",0,NA,NA,NA,"GeneID:196527","ANO6",TRUE,"ANO6"

"8700","CAGCAGACTTCGTCCTCGTT","chr2",241195699,"+","none","TGG","CAGCAGACTTCGTCCTCGTTTGG",0,NA,NA,NA,"GeneID:50636","ANO7",TRUE,"ANO7"

"8701","GAGGAGGGCGTAGTGCACTG","chr2",241199340,"-","none","TGG","GAGGAGGGCGTAGTGCACTGTGG",0,NA,NA,NA,"GeneID:50636","ANO7",TRUE,"ANO7"

"8702","GGGCTCACCGATCCGGGGCT","chr2",241191239,"-","none","TGG","GGGCTCACCGATCCGGGGCTTGG",0,NA,NA,NA,"GeneID:50636","ANO7",TRUE,"ANO7"

"8703","GGGGCTCACATCGATCAGGA","chr2",241190100,"-","none","CGG","GGGGCTCACATCGATCAGGACGG",0,NA,NA,NA,"GeneID:50636","ANO7",TRUE,"ANO7"

"8704","GTGCCTACAGCAGGACGTCC","chr2",241199305,"+","none","AGG","GTGCCTACAGCAGGACGTCCAGG",0,NA,NA,NA,"GeneID:50636","ANO7",TRUE,"ANO7"

"8705","GTGCTGTTACCTCCGAGGCG","chr2",241190161,"-","none","TGG","GTGCTGTTACCTCCGAGGCGTGG",0,NA,NA,NA,"GeneID:50636","ANO7",TRUE,"ANO7"

"8706","ACTCCACAACGTGCGCTTCC","chr19",17332947,"-","none","TGG","ACTCCACAACGTGCGCTTCCTGG",0,NA,NA,NA,"GeneID:57719","ANO8",TRUE,"ANO8"

"8707","ATAGATAAAGTCCTCCTCGC","chr19",17333139,"+","none","AGG","ATAGATAAAGTCCTCCTCGCAGG",0,NA,NA,NA,"GeneID:57719","ANO8",TRUE,"ANO8"

"8708","CCGATGACCACACGCTGCTA","chr19",17333528,"-","none","TGG","CCGATGACCACACGCTGCTATGG",0,NA,NA,NA,"GeneID:57719","ANO8",TRUE,"ANO8"

"8709","GCAGAATTTGCGTGCCAAGC","chr19",17332977,"-","none","AGG","GCAGAATTTGCGTGCCAAGCAGG",0,NA,NA,NA,"GeneID:57719","ANO8",TRUE,"ANO8"

"8710","GGGGACTCGCCTCTCATACG","chr19",17333411,"+","none","TGG","GGGGACTCGCCTCTCATACGTGG",0,NA,NA,NA,"GeneID:57719","ANO8",TRUE,"ANO8"

"8711","TTTGCGCAGACCCAGCTCGT","chr19",17333202,"+","none","CGG","TTTGCGCAGACCCAGCTCGTCGG",0,NA,NA,NA,"GeneID:57719","ANO8",TRUE,"ANO8"

"8712","AGACCGAGGCCTCCGAGCAG","chr11",433919,"-","none","TGG","AGACCGAGGCCTCCGAGCAGTGG",0,NA,NA,NA,"GeneID:338440","ANO9",TRUE,"ANO9"

"8713","AGGGGACAGCTTCCCGCTGA","chr11",434043,"-","none","TGG","AGGGGACAGCTTCCCGCTGATGG",0,NA,NA,NA,"GeneID:338440","ANO9",TRUE,"ANO9"

"8714","CAGGTGCTGATCTCCATCAG","chr11",434027,"+","none","CGG","CAGGTGCTGATCTCCATCAGCGG",0,NA,NA,NA,"GeneID:338440","ANO9",TRUE,"ANO9"

"8715","CGTGCTGACAACAGTGTCTT","chr11",433403,"-","none","TGG","CGTGCTGACAACAGTGTCTTTGG",0,NA,NA,NA,"GeneID:338440","ANO9",TRUE,"ANO9"

"8716","GGCGCGGCAGCAACAGTTCC","chr11",433849,"-","none","TGG","GGCGCGGCAGCAACAGTTCCTGG",0,NA,NA,NA,"GeneID:338440","ANO9",TRUE,"ANO9"

"8717","GGTGGTCGGCGCGGCCAGCT","chr11",433330,"+","none","CGG","GGTGGTCGGCGCGGCCAGCTCGG",0,NA,NA,NA,"GeneID:338440","ANO9",TRUE,"ANO9"

"8718","CCGTCGAGATATGTGAGTTG","chr15",68784482,"+","none","CGG","CCGTCGAGATATGTGAGTTGCGG",0,NA,NA,NA,"GeneID:8125","ANP32A",TRUE,"ANP32A"

"8719","CCTCAGCACAATAGAGCCAC","chr15",68787414,"-","none","TGG","CCTCAGCACAATAGAGCCACTGG",0,NA,NA,NA,"GeneID:8125","ANP32A",TRUE,"ANP32A"

"8720","CTCACGCATCTAAATTTAAG","chr15",68787452,"-","none","TGG","CTCACGCATCTAAATTTAAGTGG",0,NA,NA,NA,"GeneID:8125","ANP32A",TRUE,"ANP32A"

"8721","CTCACGCATCTAAATTTAAG","chr4",164197436,"-","12:A>T","TGG","CTCACGCATCTATATTTAAGTGG",1,13,NA,NA,"GeneID:55016","MARCH1",TRUE,"ANP32A"

"8722","CTCACGCATCTAAATTTAAG","chr15",35237592,"+","18:T>A","TGG","CTCACGCATCTAAATTTATGTGG",1,19,NA,NA,"GeneID:723972;GeneID:89978","ANP32AP1;DPH6",TRUE,"ANP32A"

"8723","CTCACGCATCTAAATTTAAG","chr12",48472931,"+","4:T>C,5:A>G","TGG","CTCATACATCTAAATTTAAGTGG",2,5,6,NA,"GeneID:23519","ANP32D",TRUE,"ANP32A"

"8724","CTCACGCATCTAAATTTAAG","chr9",97998618,"+","5:A>G,14:C>T","TGG","CTCACACATCTAAACTTAAGTGG",2,6,15,NA,"GeneID:10541","ANP32B",TRUE,"ANP32A"

"8725","TAGCCCTCAGCATCCGAGTC","chr15",68784434,"+","none","AGG","TAGCCCTCAGCATCCGAGTCAGG",0,NA,NA,NA,"GeneID:8125","ANP32A",TRUE,"ANP32A"

"8726","TAGCCCTCAGCATCCGAGTC","chr15",35237793,"-","15:T>C,19:A>G","AGG","TAGCCCTCAGCATCCAAGTTAGG",2,16,20,NA,"GeneID:723972;GeneID:89978","ANP32AP1;DPH6",TRUE,"ANP32A"

"8727","TCAGGTGAAAGAACTTGTCC","chr15",68787903,"-","none","TGG","TCAGGTGAAAGAACTTGTCCTGG",0,NA,NA,NA,"GeneID:8125","ANP32A",TRUE,"ANP32A"

"8728","TCAGGTGAAAGAACTTGTCC","chr15",35237375,"+","1:G>C,3:T>G","TGG","TGATGTGAAAGAACTTGTCCTGG",2,2,4,NA,"GeneID:723972;GeneID:89978","ANP32AP1;DPH6",TRUE,"ANP32A"

"8729","TCAGGTGAAAGAACTTGTCC","chr8",34649983,"-","8:C>T,18:C>G","AGG","TCAGGTGAGAGAACTTGTGCAGG",2,9,19,NA,"","",FALSE,"ANP32A"

"8730","TTCTTAAGTACAATCAACGT","chr15",68787818,"-","none","AGG","TTCTTAAGTACAATCAACGTAGG",0,NA,NA,NA,"GeneID:8125","ANP32A",TRUE,"ANP32A"

"8731","TTCTTAAGTACAATCAACGT","chr4",164197556,"-","10:T>G,19:C>A","AGG","TTCTTAAGTAAAATCAACGGAGG",2,11,20,NA,"GeneID:55016","MARCH1",TRUE,"ANP32A"

"8732","ACAGGTTCGAGAACTTGTCT","chr9",97994626,"+","none","TGG","ACAGGTTCGAGAACTTGTCTTGG",0,NA,NA,NA,"GeneID:10541","ANP32B",TRUE,"ANP32B"

"8733","ACAGGTTCGAGAACTTGTCT","chr4",176627109,"-","8:T>C,10:A>C","AGG","ACAGGTTCAATAACTTGTCTAGG",2,9,11,NA,"","",FALSE,"ANP32B"

"8734","CAAGCTGCCTAAATTGAAAA","chr9",97994758,"+","none","AGG","CAAGCTGCCTAAATTGAAAAAGG",0,NA,NA,NA,"GeneID:10541","ANP32B",TRUE,"ANP32B"

"8735","CAAGCTGCCTAAATTGAAAA","chrX",125850658,"-","13:T>A,19:C>T","AGG","CAAGCTGCCTAAAATGAAAGAGG",2,14,20,NA,"","",FALSE,"ANP32B"

"8736","CAAGCTGCCTAAATTGAAAA","chr1",184128023,"-","5:T>A,12:A>T","TGG","CAAGCAGCCTAATTTGAAAATGG",2,6,13,NA,"","",FALSE,"ANP32B"

"8737","CATCTGAGTCAGGTGCTTCC","chr9",98005094,"-","none","TGG","CATCTGAGTCAGGTGCTTCCTGG",0,NA,NA,NA,"GeneID:10541","ANP32B",TRUE,"ANP32B"

"8738","CATCTGAGTCAGGTGCTTCC","chr15",75322033,"+","14:T>G,16:C>T","TGG","CATCTGAGTCAGGTTCCTCCTGG",2,15,17,NA,"","",TRUE,"ANP32B"

"8739","CATCTGAGTCAGGTGCTTCC","chr15",81117916,"+","13:G>T","TGG","CATCTGAGTCAGGGGCTTCCTGG",1,14,NA,NA,"GeneID:161502","C15orf26",TRUE,"ANP32B"

"8740","CATCTGAGTCAGGTGCTTCC","chrX",102255473,"+","8:C>T,19:T>C","TGG","CATCTGAGCCAGGTGCTTCTTGG",2,9,20,NA,"GeneID:56001","NXF2",FALSE,"ANP32B"

"8741","CATCTGAGTCAGGTGCTTCC","chrX",102431628,"-","8:G>A,19:A>G","TGG","CATCTGAGCCAGGTGCTTCTTGG",2,9,20,NA,"GeneID:728343","NXF2B",FALSE,"ANP32B"

"8742","GGACGACGATGAAGTCAGTG","chr9",98011364,"+","none","AGG","GGACGACGATGAAGTCAGTGAGG",0,NA,NA,NA,"GeneID:10541","ANP32B",TRUE,"ANP32B"

"8743","GGACGACGATGAAGTCAGTG","chr15",75321895,"-","0:A>C,6:T>G","AGG","TGACGAAGATGAAGTCAGTGAGG",2,1,7,NA,"","",TRUE,"ANP32B"

"8744","GGACGACGATGAAGTCAGTG","chr11",115061004,"+","4:A>G,7:A>G","GGG","GGACAACAATGAAGTCAGTGGGG",2,5,8,NA,"","",FALSE,"ANP32B"

"8745","GTTACTTACCAAAGGTTCCA","chr9",97998667,"-","none","AGG","GTTACTTACCAAAGGTTCCAAGG",0,NA,NA,NA,"GeneID:10541","ANP32B",TRUE,"ANP32B"

"8746","GTTACTTACCAAAGGTTCCA","chr3",124200199,"-","13:A>C,14:T>C","CGG","GTTACTTACCAAATATTCCACGG",2,14,15,NA,"GeneID:8997","KALRN",FALSE,"ANP32B"

"8747","TGGCTATGACCGAGAGGACC","chr9",98005073,"+","none","AGG","TGGCTATGACCGAGAGGACCAGG",0,NA,NA,NA,"GeneID:10541","ANP32B",TRUE,"ANP32B"

"8748","TGGCTATGACCGAGAGGACC","chr15",75322054,"-","11:T>C","AGG","TGGCTATGACCAAGAGGACCAGG",1,12,NA,NA,"","",TRUE,"ANP32B"

"8749","AAAAGTTCTTTCACATCGGA","chr12",48472712,"-","none","GGG","AAAAGTTCTTTCACATCGGAGGG",0,NA,NA,NA,"GeneID:23519","ANP32D",TRUE,"ANP32D"

"8750","AAAAGTTCTTTCACATCGGA","chr15",35237373,"-","1:G>T,17:T>C","GGG","ACAAGTTCTTTCACATCAGAGGG",2,2,18,NA,"GeneID:723972;GeneID:89978","ANP32AP1;DPH6",TRUE,"ANP32D"

"8751","AAGCAGTAACAGAGCCTCAG","chr12",48472876,"+","none","TGG","AAGCAGTAACAGAGCCTCAGTGG",0,NA,NA,NA,"GeneID:23519","ANP32D",TRUE,"ANP32D"

"8752","AAGCAGTAACAGAGCCTCAG","chr1",9382492,"+","6:A>T,14:G>C","AGG","AAGCAGAAACAGAGGCTCAGAGG",2,7,15,NA,"","",FALSE,"ANP32D"

"8753","CACTTAAATTTAGATGTATG","chr12",48472933,"-","none","AGG","CACTTAAATTTAGATGTATGAGG",0,NA,NA,NA,"GeneID:23519","ANP32D",TRUE,"ANP32D"

"8754","CACTTAAATTTAGATGTATG","chr15",68787450,"+","16:C>T,17:G>A","AGG","CACTTAAATTTAGATGCGTGAGG",2,17,18,NA,"GeneID:8125","ANP32A",TRUE,"ANP32D"

"8755","CATTTGACTGACTGTTGTCC","chr12",48472735,"-","none","AGG","CATTTGACTGACTGTTGTCCAGG",0,NA,NA,NA,"GeneID:23519","ANP32D",TRUE,"ANP32D"

"8756","CATTTGACTGACTGTTGTCC","chr15",35237396,"-","4:G>A,8:G>A","AGG","CATTCGACCGACTGTTGTCCAGG",2,5,9,NA,"GeneID:723972;GeneID:89978","ANP32AP1;DPH6",TRUE,"ANP32D"

"8757","CATTTGACTGACTGTTGTCC","chr15",68787882,"+","4:C>T,8:C>T","AGG","CATTCGACCGACTGTTGTCCAGG",2,5,9,NA,"GeneID:8125","ANP32A",TRUE,"ANP32D"

"8758","CATTTGACTGACTGTTGTCC","chr4",164197620,"+","4:C>T,8:C>T","AGG","CATTCGACCGACTGTTGTCCAGG",2,5,9,NA,"GeneID:55016","MARCH1",TRUE,"ANP32D"

"8759","CATTTGACTGACTGTTGTCC","chr21",29269696,"+","6:T>A,10:T>A","AGG","CATTTGTCTGTCTGTTGTCCAGG",2,7,11,NA,"GeneID:193629;GeneID:571","LINC00189;BACH1",FALSE,"ANP32D"

"8760","CATTTGACTGACTGTTGTCC","chr3",11081268,"-","6:A>T,11:C>G","AGG","CATTTGTCTGAGTGTTGTCCAGG",2,7,12,NA,"","",FALSE,"ANP32D"

"8761","CATTTGACTGACTGTTGTCC","chr21",41952163,"-","2:C>A,13:A>C","TGG","CAGTTGACTGACTTTTGTCCTGG",2,3,14,NA,"GeneID:25966","C2CD2",FALSE,"ANP32D"

"8762","TCAGTCAAATGAAGGCAAAT","chr12",48472744,"+","none","TGG","TCAGTCAAATGAAGGCAAATTGG",0,NA,NA,NA,"GeneID:23519","ANP32D",TRUE,"ANP32D"

"8763","TGGTTACCTCGCAAGTGAAA","chr12",48473023,"-","none","AGG","TGGTTACCTCGCAAGTGAAAAGG",0,NA,NA,NA,"GeneID:23519","ANP32D",TRUE,"ANP32D"

"8764","TGGTTACCTCGCAAGTGAAA","chr15",35237684,"-","14:A>C","AGG","TGGTTACCTCGCAATTGAAAAGG",1,15,NA,NA,"GeneID:723972;GeneID:89978","ANP32AP1;DPH6",TRUE,"ANP32D"

"8765","TGGTTACCTCGCAAGTGAAA","chr15",68784543,"+","14:T>G","AGG","TGGTTACCTCGCAATTGAAAAGG",1,15,NA,NA,"GeneID:8125","ANP32A",TRUE,"ANP32D"

"8766","TGGTTACCTCGCAAGTGAAA","chr4",164197344,"+","14:T>G","AGG","TGGTTACCTCGCAATTGAAAAGG",1,15,NA,NA,"GeneID:55016","MARCH1",TRUE,"ANP32D"

"8767","ATTTCTGAGTATGGCTAATG","chr1",150231826,"-","none","TGG","ATTTCTGAGTATGGCTAATGTGG",0,NA,NA,NA,"GeneID:81611","ANP32E",TRUE,"ANP32E"

"8768","CTTACCTACCTCAATCTGAG","chr1",150230610,"-","none","TGG","CTTACCTACCTCAATCTGAGTGG",0,NA,NA,NA,"GeneID:81611","ANP32E",TRUE,"ANP32E"

"8769","GGAAAATGAAGCTGGTCCAC","chr1",150226747,"-","none","CGG","GGAAAATGAAGCTGGTCCACCGG",0,NA,NA,NA,"GeneID:81611","ANP32E",TRUE,"ANP32E"

"8770","TCAGGAGGATAATGAAGCGC","chr1",150229098,"-","none","CGG","TCAGGAGGATAATGAAGCGCCGG",0,NA,NA,NA,"GeneID:81611","ANP32E",TRUE,"ANP32E"

"8771","TCAGGAGGATAATGAAGCGC","chr9",88080655,"+","6:A>G,18:C>G","AGG","TCAGGAAGATAATGAAGCCCAGG",2,7,19,NA,"","",FALSE,"ANP32E"

"8772","TCTCAGTACAGTAGAAGCTC","chr1",150230572,"-","none","TGG","TCTCAGTACAGTAGAAGCTCTGG",0,NA,NA,NA,"GeneID:81611","ANP32E",TRUE,"ANP32E"

"8773","TTGTGATCTCACAGTTAAAC","chr1",150229185,"+","none","AGG","TTGTGATCTCACAGTTAAACAGG",0,NA,NA,NA,"GeneID:81611","ANP32E",TRUE,"ANP32E"

"8774","ATGGCCGGCTCATCGAAGCA","chr15",89805391,"+","none","TGG","ATGGCCGGCTCATCGAAGCATGG",0,NA,NA,NA,"GeneID:290","ANPEP",TRUE,"ANPEP"

"8775","CTACTGCAACGCTATCGCCC","chr15",89792280,"-","none","AGG","CTACTGCAACGCTATCGCCCAGG",0,NA,NA,NA,"GeneID:290","ANPEP",TRUE,"ANPEP"

"8776","CTTCATGGGGCCATAGACCT","chr15",89797574,"+","none","CGG","CTTCATGGGGCCATAGACCTCGG",0,NA,NA,NA,"GeneID:290","ANPEP",TRUE,"ANPEP"

"8777","CTTCCCATGCTTCGATGAGC","chr15",89805398,"-","none","CGG","CTTCCCATGCTTCGATGAGCCGG",0,NA,NA,NA,"GeneID:290","ANPEP",TRUE,"ANPEP"

"8778","GATAGCGTTGCAGTAGACGG","chr15",89792284,"+","none","TGG","GATAGCGTTGCAGTAGACGGTGG",0,NA,NA,NA,"GeneID:290","ANPEP",TRUE,"ANPEP"

"8779","GCTCATGTTTGACCGCTCCG","chr15",89797594,"-","none","AGG","GCTCATGTTTGACCGCTCCGAGG",0,NA,NA,NA,"GeneID:290","ANPEP",TRUE,"ANPEP"

"8780","CAGGAAGTGTGCTGCACCAC","chr2",69040045,"+","none","TGG","CAGGAAGTGTGCTGCACCACTGG",0,NA,NA,NA,"GeneID:84168","ANTXR1",TRUE,"ANTXR1"

"8781","CTATTACTTTGTGGAACAGT","chr2",69040076,"+","none","TGG","CTATTACTTTGTGGAACAGTTGG",0,NA,NA,NA,"GeneID:84168","ANTXR1",TRUE,"ANTXR1"

"8782","CTATTACTTTGTGGAACAGT","chr1",78447781,"-","5:A>T,8:T>A","GGG","CTATTTCTATGTGGAACAGTGGG",2,6,9,NA,"GeneID:5737","PTGFR",FALSE,"ANTXR1"

"8783","GGAGACACTTACATGCATGA","chr2",69070695,"+","none","AGG","GGAGACACTTACATGCATGAAGG",0,NA,NA,NA,"GeneID:84168","ANTXR1",TRUE,"ANTXR1"

"8784","GTCTTCTGTCAGTTTCATTA","chr2",69044791,"-","none","AGG","GTCTTCTGTCAGTTTCATTAAGG",0,NA,NA,NA,"GeneID:84168","ANTXR1",TRUE,"ANTXR1"

"8785","GTCTTCTGTCAGTTTCATTA","chr4",82388737,"-","17:C>A,19:A>T","TGG","GTCTTCTGTCAGTTTCAGTTTGG",2,18,20,NA,"","",FALSE,"ANTXR1"

"8786","GTCTTCTGTCAGTTTCATTA","chrX",105622447,"+","11:T>G,19:C>A","TGG","GTCTTCTGTCATTTTCATTCTGG",2,12,20,NA,"GeneID:26280","IL1RAPL2",FALSE,"ANTXR1"

"8787","TCGGGTGGAGAAAACAATAA","chr2",69044761,"-","none","AGG","TCGGGTGGAGAAAACAATAAAGG",0,NA,NA,NA,"GeneID:84168","ANTXR1",TRUE,"ANTXR1"

"8788","TCGGGTGGAGAAAACAATAA","chr1",9939729,"-","1:C>G,5:T>A","AGG","TGGGGAGGAGAAAACAATAAAGG",2,2,6,NA,"GeneID:84328","LZIC",FALSE,"ANTXR1"

"8789","TTCAGAGAACAAATCCGTCA","chr2",69070641,"+","none","AGG","TTCAGAGAACAAATCCGTCAAGG",0,NA,NA,NA,"GeneID:84168","ANTXR1",TRUE,"ANTXR1"

"8790","TTCAGAGAACAAATCCGTCA","chr14",73373492,"+","5:T>A,16:A>G","TGG","TTCAGTGAACAAATCCATCATGG",2,6,17,NA,"GeneID:8650","NUMB",FALSE,"ANTXR1"

"8791","AACACTAGCCCCAAGTGACC","chr4",80055185,"+","none","TGG","AACACTAGCCCCAAGTGACCTGG",0,NA,NA,NA,"GeneID:118429","ANTXR2",TRUE,"ANTXR2"

"8792","AACACTAGCCCCAAGTGACC","chr1",198913896,"+","4:T>C,5:C>T","AGG","AACATCAGCCCCAAGTGACCAGG",2,5,6,NA,"","",FALSE,"ANTXR2"

"8793","ATCATAATTGCTCTGACAGA","chr4",80055402,"-","none","TGG","ATCATAATTGCTCTGACAGATGG",0,NA,NA,NA,"GeneID:118429","ANTXR2",TRUE,"ANTXR2"

"8794","GGGGCTAGTGTTTATTGTGT","chr4",80055177,"-","none","TGG","GGGGCTAGTGTTTATTGTGTTGG",0,NA,NA,NA,"GeneID:118429","ANTXR2",TRUE,"ANTXR2"

"8795","GGGGCTAGTGTTTATTGTGT","chr6",105771965,"+","1:T>G,18:A>G","TGG","GTGGCTAGTGTTTATTGTATTGG",2,2,19,NA,"","",FALSE,"ANTXR2"

"8796","TGTCAGAGCAATTATGATAC","chr4",80055404,"+","none","TGG","TGTCAGAGCAATTATGATACTGG",0,NA,NA,NA,"GeneID:118429","ANTXR2",TRUE,"ANTXR2"

"8797","TTAAAACGTGTTAGTCCAGT","chr4",80055965,"-","none","AGG","TTAAAACGTGTTAGTCCAGTAGG",0,NA,NA,NA,"GeneID:118429","ANTXR2",TRUE,"ANTXR2"

"8798","TTAAAACGTGTTAGTCCAGT","chr7",27826862,"+","4:C>A,13:A>G","TGG","TTAACACGTGTTAATCCAGTTGG",2,5,14,NA,"GeneID:8887","TAX1BP1",FALSE,"ANTXR2"

"8799","TTAAAACGTGTTAGTCCAGT","chr3",156812334,"+","6:G>C,13:A>G","TGG","TTAAAAGGTGTTAATCCAGTTGG",2,7,14,NA,"GeneID:730091","LINC00886",FALSE,"ANTXR2"

"8800","TTACTAGCTTTAGTCCTTCA","chr4",80055927,"+","none","TGG","TTACTAGCTTTAGTCCTTCATGG",0,NA,NA,NA,"GeneID:118429","ANTXR2",TRUE,"ANTXR2"

"8801","ATGCAAGGCAGCGACATCCG","chr9",73158764,"-","none","AGG","ATGCAAGGCAGCGACATCCGAGG",0,NA,NA,NA,"GeneID:301","ANXA1",TRUE,"ANXA1"

"8802","CACGTTACCTTTAACCATTA","chr9",73158791,"-","none","TGG","CACGTTACCTTTAACCATTATGG",0,NA,NA,NA,"GeneID:301","ANXA1",TRUE,"ANXA1"

"8803","GATCAAAGCAGCATATCTCC","chr9",73159389,"+","none","AGG","GATCAAAGCAGCATATCTCCAGG",0,NA,NA,NA,"GeneID:301","ANXA1",TRUE,"ANXA1"

"8804","GGTATCAGAATTCCTCAAGC","chr9",73158543,"+","none","AGG","GGTATCAGAATTCCTCAAGCAGG",0,NA,NA,NA,"GeneID:301","ANXA1",TRUE,"ANXA1"

"8805","TCATCAGCATCAAATTGCGC","chr9",73160337,"-","none","TGG","TCATCAGCATCAAATTGCGCTGG",0,NA,NA,NA,"GeneID:301","ANXA1",TRUE,"ANXA1"

"8806","TGAACTTCGTGCTGCCATGA","chr9",73160354,"+","none","AGG","TGAACTTCGTGCTGCCATGAAGG",0,NA,NA,NA,"GeneID:301","ANXA1",TRUE,"ANXA1"

"8807","ATGCTAGGAGGAGCACTCCA","chr4",168128137,"+","none","AGG","ATGCTAGGAGGAGCACTCCAAGG",0,NA,NA,NA,"GeneID:11199","ANXA10",TRUE,"ANXA10"

"8808","CTCAGCGCTGCAATGCACAA","chr4",168139518,"+","none","AGG","CTCAGCGCTGCAATGCACAAAGG",0,NA,NA,NA,"GeneID:11199","ANXA10",TRUE,"ANXA10"

"8809","GAGGCATACCAGAGCATGTA","chr4",168139553,"+","none","TGG","GAGGCATACCAGAGCATGTATGG",0,NA,NA,NA,"GeneID:11199","ANXA10",TRUE,"ANXA10"

"8810","GAGGCATACCAGAGCATGTA","chr11",112762545,"-","6:T>A,19:G>T","TGG","GAGGCAAACCAGAGCATGTCTGG",2,7,20,NA,"","",FALSE,"ANXA10"

"8811","GGAGTGCTCCTCCTAGCATT","chr4",168128136,"-","none","TGG","GGAGTGCTCCTCCTAGCATTTGG",0,NA,NA,NA,"GeneID:11199","ANXA10",TRUE,"ANXA10"

"8812","TCCCAATTTCAATCCCATAA","chr4",168128106,"+","none","TGG","TCCCAATTTCAATCCCATAATGG",0,NA,NA,NA,"GeneID:11199","ANXA10",TRUE,"ANXA10"

"8813","TTACCCGGCCATACATGCTC","chr4",168139564,"-","none","TGG","TTACCCGGCCATACATGCTCTGG",0,NA,NA,NA,"GeneID:11199","ANXA10",TRUE,"ANXA10"

"8814","CCATTCCCGAGAGATAGTCC","chr10",80170798,"+","none","TGG","CCATTCCCGAGAGATAGTCCTGG",0,NA,NA,NA,"GeneID:311","ANXA11",TRUE,"ANXA11"

"8815","CTTGCCGTAAGCCGTCTTGA","chr10",80166889,"+","none","AGG","CTTGCCGTAAGCCGTCTTGAAGG",0,NA,NA,NA,"GeneID:311","ANXA11",TRUE,"ANXA11"

"8816","GCAGGACCTCGGCATCTCGC","chr10",80167246,"+","none","AGG","GCAGGACCTCGGCATCTCGCAGG",0,NA,NA,NA,"GeneID:311","ANXA11",TRUE,"ANXA11"

"8817","GCAGGACCTCGGCATCTCGC","chr2",229785788,"-","14:C>A,18:T>C","GGG","GCAGGACCTCGGCAGCTCACGGG",2,15,19,NA,"GeneID:9320","TRIP12",TRUE,"ANXA11"

"8818","GGAGTCGCTCCAACAAGCAG","chr10",80166931,"-","none","CGG","GGAGTCGCTCCAACAAGCAGCGG",0,NA,NA,NA,"GeneID:311","ANXA11",TRUE,"ANXA11"

"8819","GGCCACGTTATCCAGCCCGA","chr10",80170841,"+","none","TGG","GGCCACGTTATCCAGCCCGATGG",0,NA,NA,NA,"GeneID:311","ANXA11",TRUE,"ANXA11"

"8820","TGACCCCCTGCGAGATGCCG","chr10",80167255,"-","none","AGG","TGACCCCCTGCGAGATGCCGAGG",0,NA,NA,NA,"GeneID:311","ANXA11",TRUE,"ANXA11"

"8821","AGCCATCATTGAAATCTTAT","chr8",123702703,"-","none","CGG","AGCCATCATTGAAATCTTATCGG",0,NA,NA,NA,"GeneID:312","ANXA13",TRUE,"ANXA13"

"8822","CAGTCGTACACCCTCTCAGA","chr8",123735854,"-","none","AGG","CAGTCGTACACCCTCTCAGAAGG",0,NA,NA,NA,"GeneID:312","ANXA13",TRUE,"ANXA13"

"8823","CGATCCACATCAAAACCCTG","chr8",123712715,"+","none","AGG","CGATCCACATCAAAACCCTGAGG",0,NA,NA,NA,"GeneID:312","ANXA13",TRUE,"ANXA13"

"8824","CTGCACGACTGTCGAGGGTT","chr8",123735802,"+","none","GGG","CTGCACGACTGTCGAGGGTTGGG",0,NA,NA,NA,"GeneID:312","ANXA13",TRUE,"ANXA13"

"8825","GCCCGATAAGATTTCAATGA","chr8",123702698,"+","none","TGG","GCCCGATAAGATTTCAATGATGG",0,NA,NA,NA,"GeneID:312","ANXA13",TRUE,"ANXA13"

"8826","GGCTAAAGCGAGCAGTCCTC","chr8",123712734,"-","none","AGG","GGCTAAAGCGAGCAGTCCTCAGG",0,NA,NA,NA,"GeneID:312","ANXA13",TRUE,"ANXA13"

"8827","AATCCTGTGCAAGCTCAGCT","chr9",33624290,"+","none","TGG","AATCCTGTGCAAGCTCAGCTTGG",0,NA,NA,NA,"","",TRUE,"ANXA2"

"8828","AATCCTGTGCAAGCTCAGCT","chr10",64825588,"+","none","TGG","AATCCTGTGCAAGCTCAGCTTGG",0,NA,NA,NA,"","",TRUE,"ANXA2"

"8829","AATCCTGTGCAAGCTCAGCT","chr15",60386038,"-","none","TGG","AATCCTGTGCAAGCTCAGCTTGG",0,NA,NA,NA,"GeneID:302","ANXA2",TRUE,"ANXA2"

"8830","AATCCTGTGCAAGCTCAGCT","chr4",153308725,"-","none","TGG","AATCCTGTGCAAGCTCAGCTTGG",0,NA,NA,NA,"GeneID:23321","TRIM2",TRUE,"ANXA2"

"8831","CCTCCAAGCTGAGCTTGCAC","chr15",60386032,"+","none","AGG","CCTCCAAGCTGAGCTTGCACAGG",0,NA,NA,NA,"GeneID:302","ANXA2",TRUE,"ANXA2"

"8832","CCTCCAAGCTGAGCTTGCAC","chr4",153308719,"+","none","AGG","CCTCCAAGCTGAGCTTGCACAGG",0,NA,NA,NA,"GeneID:23321","TRIM2",TRUE,"ANXA2"

"8833","CCTCCAAGCTGAGCTTGCAC","chr9",33624296,"-","none","AGG","CCTCCAAGCTGAGCTTGCACAGG",0,NA,NA,NA,"","",TRUE,"ANXA2"

"8834","CCTCCAAGCTGAGCTTGCAC","chr10",64825594,"-","none","AGG","CCTCCAAGCTGAGCTTGCACAGG",0,NA,NA,NA,"","",TRUE,"ANXA2"

"8835","CCTCCAAGCTGAGCTTGCAC","chr19",5822688,"+","13:A>C,19:A>C","GGG","CCTCCAAGCTGAGATTGCAAGGG",2,14,20,NA,"","",FALSE,"ANXA2"

"8836","CCTCCAAGCTGAGCTTGCAC","chr11",13143814,"+","3:T>C,15:G>T","TGG","CCTTCAAGCTGAGCTGGCACTGG",2,4,16,NA,"","",FALSE,"ANXA2"

"8837","CTCAGCATCAAAGTTAGTAT","chr15",60382381,"+","none","AGG","CTCAGCATCAAAGTTAGTATAGG",0,NA,NA,NA,"GeneID:302","ANXA2",TRUE,"ANXA2"

"8838","CTCAGCATCAAAGTTAGTAT","chr9",33624361,"-","18:C>T","AGG","CTCAGCATCAAAGTTAGTGTAGG",1,19,NA,NA,"","",TRUE,"ANXA2"

"8839","CTCAGCATCAAAGTTAGTAT","chr10",64825659,"-","15:C>T,18:C>T","AGG","CTCAGCATCAAAGTTGGTGTAGG",2,16,19,NA,"","",TRUE,"ANXA2"

"8840","TATTTCCATAGGTGTGGATG","chr15",60364514,"-","none","AGG","TATTTCCATAGGTGTGGATGAGG",0,NA,NA,NA,"GeneID:302","ANXA2",TRUE,"ANXA2"

"8841","TATTTCCATAGGTGTGGATG","chr7",92748830,"-","2:T>A,6:A>G","GGG","TAATTCTATAGGTGTGGATGGGG",2,3,7,NA,"GeneID:1021","CDK6",FALSE,"ANXA2"

"8842","TATTTCCATAGGTGTGGATG","chr4",78577336,"+","3:A>T,11:T>G","AGG","TATATCCATAGTTGTGGATGAGG",2,4,12,NA,"GeneID:306","ANXA3",FALSE,"ANXA2"

"8843","TCACCTACCTTTGGTCTTGA","chr15",60382333,"+","none","TGG","TCACCTACCTTTGGTCTTGATGG",0,NA,NA,NA,"GeneID:302","ANXA2",TRUE,"ANXA2"

"8844","TCACCTACCTTTGGTCTTGA","chr3",98095546,"+","1:T>C,3:T>C","TGG","TTATCTACCTTTGGTCTTGATGG",2,2,4,NA,"","",FALSE,"ANXA2"

"8845","TTGCCTTCGCCTACCAGAGA","chr15",60364440,"-","none","AGG","TTGCCTTCGCCTACCAGAGAAGG",0,NA,NA,NA,"GeneID:302","ANXA2",TRUE,"ANXA2"

"8846","TTGCCTTCGCCTACCAGAGA","chr4",153308531,"-","8:T>C","AGG","TTGCCTTCACCTACCAGAGAAGG",1,9,NA,NA,"GeneID:23321","TRIM2",TRUE,"ANXA2"

"8847","TTGCCTTCGCCTACCAGAGA","chr9",33624484,"+","3:T>C,8:T>G","AGG","TTGTCTTCTCCTACCAGAGAAGG",2,4,9,NA,"","",TRUE,"ANXA2"

"8848","TTGCCTTCGCCTACCAGAGA","chr7",39206350,"+","7:T>C,8:C>G","AGG","TTGCCTTTCCCTACCAGAGAAGG",2,8,9,NA,"GeneID:11281","POU6F2",FALSE,"ANXA2"

"8849","TTGCCTTCGCCTACCAGAGA","chr10",64825782,"+","7:T>C,13:G>C","AGG","TTGCCTTTGCCTAGCAGAGAAGG",2,8,14,NA,"","",TRUE,"ANXA2"

"8850","AGTGAGTACTCTCCTAGTAC","chr5",43039900,"+","none","TGG","AGTGAGTACTCTCCTAGTACTGG",0,NA,NA,NA,"GeneID:648987;GeneID:389289","LOC648987;ANXA2R",TRUE,"ANXA2R"

"8851","AGTGAGTACTCTCCTAGTAC","chr5",43020695,"-","1:T>C,14:G>A","TGG","AATGAGTACTCTCCCAGTACTGG",2,2,15,NA,"GeneID:648987","LOC648987",FALSE,"ANXA2R"

"8852","CAACGCGAAGAATCCACTCC","chr5",43039529,"+","none","AGG","CAACGCGAAGAATCCACTCCAGG",0,NA,NA,NA,"GeneID:648987;GeneID:389289","LOC648987;ANXA2R",TRUE,"ANXA2R"

"8853","CCCGGAGTCTACTGGCAAAA","chr5",43039834,"-","none","CGG","CCCGGAGTCTACTGGCAAAACGG",0,NA,NA,NA,"GeneID:648987;GeneID:389289","LOC648987;ANXA2R",TRUE,"ANXA2R"

"8854","CGAAGCCCACTGAGTTCAGT","chr5",43039770,"-","none","TGG","CGAAGCCCACTGAGTTCAGTTGG",0,NA,NA,NA,"GeneID:648987;GeneID:389289","LOC648987;ANXA2R",TRUE,"ANXA2R"

"8855","GCGTGCTGTTCACGGATCTG","chr5",43039483,"-","none","TGG","GCGTGCTGTTCACGGATCTGTGG",0,NA,NA,NA,"GeneID:648987;GeneID:389289","LOC648987;ANXA2R",TRUE,"ANXA2R"

"8856","TTGCCGCGTTCTCTGTACTC","chr5",43039506,"-","none","TGG","TTGCCGCGTTCTCTGTACTCTGG",0,NA,NA,NA,"GeneID:648987;GeneID:389289","LOC648987;ANXA2R",TRUE,"ANXA2R"

"8857","ACAGCGGCAGCTGATTGTTA","chr4",78579075,"+","none","AGG","ACAGCGGCAGCTGATTGTTAAGG",0,NA,NA,NA,"GeneID:306","ANXA3",TRUE,"ANXA3"

"8858","CCACCATGAGATGCTCAAAG","chr4",78582214,"-","none","TGG","CCACCATGAGATGCTCAAAGTGG",0,NA,NA,NA,"GeneID:306","ANXA3",TRUE,"ANXA3"

"8859","GGGATATCATGGCATCTATC","chr4",78554465,"+","none","TGG","GGGATATCATGGCATCTATCTGG",0,NA,NA,NA,"GeneID:306","ANXA3",TRUE,"ANXA3"

"8860","TCATCAGCATTCTGACTGAG","chr4",78579044,"+","none","AGG","TCATCAGCATTCTGACTGAGAGG",0,NA,NA,NA,"GeneID:306","ANXA3",TRUE,"ANXA3"

"8861","TCATCAGCATTCTGACTGAG","chr5",27849536,"-","3:T>A,12:T>A","AGG","TCAACAGCATTCAGACTGAGAGG",2,4,13,NA,"","",FALSE,"ANXA3"

"8862","TGCTTTGCATCAAAGACTGC","chr4",78582251,"-","none","TGG","TGCTTTGCATCAAAGACTGCTGG",0,NA,NA,NA,"GeneID:306","ANXA3",TRUE,"ANXA3"

"8863","TGGCCACTTTGAGCATCTCA","chr4",78582208,"+","none","TGG","TGGCCACTTTGAGCATCTCATGG",0,NA,NA,NA,"GeneID:306","ANXA3",TRUE,"ANXA3"

"8864","TGGCCACTTTGAGCATCTCA","chr7",125805325,"+","14:T>A,15:C>T","TGG","TGGCCACTTTGAGCTCCTCATGG",2,15,16,NA,"","",FALSE,"ANXA3"

"8865","TGGCCACTTTGAGCATCTCA","chr9",125728783,"+","3:A>C,8:G>T","AGG","TGGACACTGTGAGCATCTCAAGG",2,4,9,NA,"","",FALSE,"ANXA3"

"8866","TGGCCACTTTGAGCATCTCA","chr17",8645031,"+","2:A>G,13:A>C","TGG","TGACCACTTTGAGAATCTCATGG",2,3,14,NA,"","",FALSE,"ANXA3"

"8867","AACCGAAATCACCTGTTGCA","chr2",69816172,"+","none","TGG","AACCGAAATCACCTGTTGCATGG",0,NA,NA,NA,"GeneID:307","ANXA4",TRUE,"ANXA4"

"8868","ATGTCACGTACGCTGCTGGT","chr2",69807987,"-","none","AGG","ATGTCACGTACGCTGCTGGTAGG",0,NA,NA,NA,"GeneID:307","ANXA4",TRUE,"ANXA4"

"8869","CCTGCCGATGGTGCTCTTGT","chr2",69804607,"-","none","AGG","CCTGCCGATGGTGCTCTTGTAGG",0,NA,NA,NA,"GeneID:307","ANXA4",TRUE,"ANXA4"

"8870","CTTATGCGCCGGATCTCCTC","chr2",69807959,"-","none","AGG","CTTATGCGCCGGATCTCCTCAGG",0,NA,NA,NA,"GeneID:307","ANXA4",TRUE,"ANXA4"

"8871","GTAGGCAAGGACGCTAATAA","chr2",69804550,"-","none","TGG","GTAGGCAAGGACGCTAATAATGG",0,NA,NA,NA,"GeneID:307","ANXA4",TRUE,"ANXA4"

"8872","TTGGCTTCAGGACCTGTATG","chr2",69816090,"+","none","AGG","TTGGCTTCAGGACCTGTATGAGG",0,NA,NA,NA,"GeneID:307","ANXA4",TRUE,"ANXA4"

"8873","ATAAGCATCATAAAGCCGAG","chr4",121683384,"+","none","AGG","ATAAGCATCATAAAGCCGAGAGG",0,NA,NA,NA,"GeneID:308","ANXA5",TRUE,"ANXA5"

"8874","CCGAAGTAATGCTCAGCGCC","chr4",121684714,"-","none","AGG","CCGAAGTAATGCTCAGCGCCAGG",0,NA,NA,NA,"GeneID:308","ANXA5",TRUE,"ANXA5"

"8875","GACCTGAAATCAGAACTAAC","chr4",121683445,"-","none","TGG","GACCTGAAATCAGAACTAACTGG",0,NA,NA,NA,"GeneID:308","ANXA5",TRUE,"ANXA5"

"8876","GCTAACAGAGACCCTGATGC","chr4",121677930,"-","none","TGG","GCTAACAGAGACCCTGATGCTGG",0,NA,NA,NA,"GeneID:308","ANXA5",TRUE,"ANXA5"

"8877","TCAGCCCGCTCATCAAATCC","chr4",121686322,"+","none","AGG","TCAGCCCGCTCATCAAATCCAGG",0,NA,NA,NA,"GeneID:308","ANXA5",TRUE,"ANXA5"

"8878","TTCGGGATGTCAACAGAGTC","chr4",121684729,"+","none","AGG","TTCGGGATGTCAACAGAGTCAGG",0,NA,NA,NA,"GeneID:308","ANXA5",TRUE,"ANXA5"

"8879","AATAGGCAGGTGGCCTCATC","chr5",151138709,"+","none","AGG","AATAGGCAGGTGGCCTCATCAGG",0,NA,NA,NA,"GeneID:309","ANXA6",TRUE,"ANXA6"

"8880","CAAGTTTGAACGGTTGATTG","chr5",151138736,"-","none","TGG","CAAGTTTGAACGGTTGATTGTGG",0,NA,NA,NA,"GeneID:309","ANXA6",TRUE,"ANXA6"

"8881","CCTCTGCCTGTTGCTCCGTG","chr5",151139391,"+","none","AGG","CCTCTGCCTGTTGCTCCGTGAGG",0,NA,NA,NA,"GeneID:309","ANXA6",TRUE,"ANXA6"

"8882","CCTCTGCCTGTTGCTCCGTG","chr1",2899475,"+","12:T>G,19:C>G","CGG","CCTCTGCCTGTTTCTCCGTCCGG",2,13,20,NA,"","",FALSE,"ANXA6"

"8883","CTGTGGTTACCTTGCCGTAG","chr5",151139342,"+","none","AGG","CTGTGGTTACCTTGCCGTAGAGG",0,NA,NA,NA,"GeneID:309","ANXA6",TRUE,"ANXA6"

"8884","GAAGTGCCTCATTGAGATCT","chr5",151137284,"-","none","TGG","GAAGTGCCTCATTGAGATCTTGG",0,NA,NA,NA,"GeneID:309","ANXA6",TRUE,"ANXA6"

"8885","TACACTGCCATGAAGGGCTT","chr5",151140154,"-","none","TGG","TACACTGCCATGAAGGGCTTTGG",0,NA,NA,NA,"GeneID:309","ANXA6",TRUE,"ANXA6"

"8886","AGATGCAGAAATTCTTCGTA","chr10",73388329,"-","none","AGG","AGATGCAGAAATTCTTCGTAAGG",0,NA,NA,NA,"GeneID:310","ANXA7",TRUE,"ANXA7"

"8887","ATCTCTTATAGCATCGAAGT","chr10",73388345,"+","none","TGG","ATCTCTTATAGCATCGAAGTTGG",0,NA,NA,NA,"GeneID:310","ANXA7",TRUE,"ANXA7"

"8888","CATTGCTTTCCGTAAGCTCC","chr10",73383579,"+","none","AGG","CATTGCTTTCCGTAAGCTCCAGG",0,NA,NA,NA,"GeneID:310","ANXA7",TRUE,"ANXA7"

"8889","CCAACCGTTCCAATGATCAG","chr10",73387730,"-","none","AGG","CCAACCGTTCCAATGATCAGAGG",0,NA,NA,NA,"GeneID:310","ANXA7",TRUE,"ANXA7"

"8890","CTCCTACGTATTACGATGCC","chr10",73383600,"-","none","TGG","CTCCTACGTATTACGATGCCTGG",0,NA,NA,NA,"GeneID:310","ANXA7",TRUE,"ANXA7"

"8891","GCAGCATTTAAGACCTCCTA","chr10",73387695,"-","none","TGG","GCAGCATTTAAGACCTCCTATGG",0,NA,NA,NA,"GeneID:310","ANXA7",TRUE,"ANXA7"

"8892","ACCTTGAAGTCTGAGCTCAG","chr10",46382590,"+","none","TGG","ACCTTGAAGTCTGAGCTCAGTGG",0,NA,NA,NA,"GeneID:643650","LINC00842",TRUE,"ANXA8"

"8893","ACCTTGAAGTCTGAGCTCAG","chr10",47477162,"-","none","TGG","ACCTTGAAGTCTGAGCTCAGTGG",0,NA,NA,NA,"GeneID:653145","ANXA8",TRUE,"ANXA8"

"8894","ACCTTGAAGTCTGAGCTCAG","chr9",124328045,"+","0:G>A,8:C>G","TGG","GCCTTGAACTCTGAGCTCAGTGG",2,1,9,NA,"GeneID:10783","NEK6",FALSE,"ANXA8"

"8895","ACCTTGAAGTCTGAGCTCAG","chr11",27947322,"-","5:G>C,12:G>C","GGG","ACCTTCAAGTCTCAGCTCAGGGG",2,6,13,NA,"","",FALSE,"ANXA8"

"8896","CATGGCGTCATGCAGCTCCT","chr10",47477083,"+","none","TGG","CATGGCGTCATGCAGCTCCTTGG",0,NA,NA,NA,"GeneID:653145","ANXA8",TRUE,"ANXA8"

"8897","CATGGCGTCATGCAGCTCCT","chr10",46382669,"-","none","TGG","CATGGCGTCATGCAGCTCCTTGG",0,NA,NA,NA,"GeneID:643650","LINC00842",TRUE,"ANXA8"

"8898","CCACTCACCGATCCCCTTCA","chr10",47479789,"+","none","TGG","CCACTCACCGATCCCCTTCATGG",0,NA,NA,NA,"GeneID:653145","ANXA8",TRUE,"ANXA8"

"8899","CCACTCACCGATCCCCTTCA","chr10",46379963,"-","none","TGG","CCACTCACCGATCCCCTTCATGG",0,NA,NA,NA,"GeneID:643650","LINC00842",TRUE,"ANXA8"

"8900","CCCTGCAGGGACCAACGAGC","chr10",46381137,"+","none","AGG","CCCTGCAGGGACCAACGAGCAGG",0,NA,NA,NA,"GeneID:643650","LINC00842",TRUE,"ANXA8"

"8901","CCCTGCAGGGACCAACGAGC","chr10",47478615,"-","none","AGG","CCCTGCAGGGACCAACGAGCAGG",0,NA,NA,NA,"GeneID:653145","ANXA8",TRUE,"ANXA8"

"8902","CCCTGCAGGGACCAACGAGC","chr21",6056722,"+","3:G>T,13:C>A","AGG","CCCGGCAGGGACCCACGAGCAGG",2,4,14,NA,"","",FALSE,"ANXA8"

"8903","CCCTGCAGGGACCAACGAGC","chr21",43481820,"-","3:C>A,13:G>T","AGG","CCCGGCAGGGACCCACGAGCAGG",2,4,14,NA,"","",FALSE,"ANXA8"

"8904","CTTGCCGAACTGAGCCTTGA","chr10",47478532,"+","none","AGG","CTTGCCGAACTGAGCCTTGAAGG",0,NA,NA,NA,"GeneID:653145","ANXA8",TRUE,"ANXA8"

"8905","CTTGCCGAACTGAGCCTTGA","chr10",46381220,"-","none","AGG","CTTGCCGAACTGAGCCTTGAAGG",0,NA,NA,NA,"GeneID:643650","LINC00842",TRUE,"ANXA8"

"8906","TCTCTTGCAGATTGAACAGG","chr10",46379874,"+","none","AGG","TCTCTTGCAGATTGAACAGGAGG",0,NA,NA,NA,"GeneID:643650","LINC00842",TRUE,"ANXA8"

"8907","TCTCTTGCAGATTGAACAGG","chr10",47479878,"-","none","AGG","TCTCTTGCAGATTGAACAGGAGG",0,NA,NA,NA,"GeneID:653145","ANXA8",TRUE,"ANXA8"

"8908","TCTCTTGCAGATTGAACAGG","chr10",6593100,"-","10:C>T,19:G>C","TGG","TCTCTTGCAGGTTGAACAGCTGG",2,11,20,NA,"","",FALSE,"ANXA8"

"8909","ACCTTGAAGTCTGAGCTCAG","chr10",46382590,"+","none","TGG","ACCTTGAAGTCTGAGCTCAGTGG",0,NA,NA,NA,"GeneID:643650","LINC00842",TRUE,"ANXA8L1"

"8910","ACCTTGAAGTCTGAGCTCAG","chr10",47477162,"-","none","TGG","ACCTTGAAGTCTGAGCTCAGTGG",0,NA,NA,NA,"GeneID:653145","ANXA8",TRUE,"ANXA8L1"

"8911","ACCTTGAAGTCTGAGCTCAG","chr9",124328045,"+","0:G>A,8:C>G","TGG","GCCTTGAACTCTGAGCTCAGTGG",2,1,9,NA,"GeneID:10783","NEK6",FALSE,"ANXA8L1"

"8912","ACCTTGAAGTCTGAGCTCAG","chr11",27947322,"-","5:G>C,12:G>C","GGG","ACCTTCAAGTCTCAGCTCAGGGG",2,6,13,NA,"","",FALSE,"ANXA8L1"

"8913","CATGGCGTCATGCAGCTCCT","chr10",47477083,"+","none","TGG","CATGGCGTCATGCAGCTCCTTGG",0,NA,NA,NA,"GeneID:653145","ANXA8",TRUE,"ANXA8L1"

"8914","CATGGCGTCATGCAGCTCCT","chr10",46382669,"-","none","TGG","CATGGCGTCATGCAGCTCCTTGG",0,NA,NA,NA,"GeneID:643650","LINC00842",TRUE,"ANXA8L1"

"8915","CCCTGCAGGGACCAACGAGC","chr10",46381137,"+","none","AGG","CCCTGCAGGGACCAACGAGCAGG",0,NA,NA,NA,"GeneID:643650","LINC00842",TRUE,"ANXA8L1"

"8916","CCCTGCAGGGACCAACGAGC","chr10",47478615,"-","none","AGG","CCCTGCAGGGACCAACGAGCAGG",0,NA,NA,NA,"GeneID:653145","ANXA8",TRUE,"ANXA8L1"

"8917","CCCTGCAGGGACCAACGAGC","chr21",6056722,"+","3:G>T,13:C>A","AGG","CCCGGCAGGGACCCACGAGCAGG",2,4,14,NA,"","",FALSE,"ANXA8L1"

"8918","CCCTGCAGGGACCAACGAGC","chr21",43481820,"-","3:C>A,13:G>T","AGG","CCCGGCAGGGACCCACGAGCAGG",2,4,14,NA,"","",FALSE,"ANXA8L1"

"8919","CTTGCCGAACTGAGCCTTGA","chr10",47478532,"+","none","AGG","CTTGCCGAACTGAGCCTTGAAGG",0,NA,NA,NA,"GeneID:653145","ANXA8",TRUE,"ANXA8L1"

"8920","CTTGCCGAACTGAGCCTTGA","chr10",46381220,"-","none","AGG","CTTGCCGAACTGAGCCTTGAAGG",0,NA,NA,NA,"GeneID:643650","LINC00842",TRUE,"ANXA8L1"

"8921","GACCCTCTACCAAGCCATGA","chr10",47479806,"-","10:T>G","AGG","GACCCTCTACAAAGCCATGAAGG",1,11,NA,NA,"GeneID:653145","ANXA8",TRUE,"ANXA8L1"

"8922","GACCCTCTACCAAGCCATGA","chr10",46379946,"+","10:A>C","AGG","GACCCTCTACAAAGCCATGAAGG",1,11,NA,NA,"GeneID:643650","LINC00842",TRUE,"ANXA8L1"

"8923","TGGTAGAGGGTCTCTGCATC","chr10",46379938,"-","1:A>C","AGG","TTGTAGAGGGTCTCTGCATCAGG",1,2,NA,NA,"GeneID:643650","LINC00842",TRUE,"ANXA8L1"

"8924","TGGTAGAGGGTCTCTGCATC","chr10",47479814,"+","1:T>G","AGG","TTGTAGAGGGTCTCTGCATCAGG",1,2,NA,NA,"GeneID:653145","ANXA8",TRUE,"ANXA8L1"

"8925","AAGTGAAAGAGATGGCCTGG","chr10",46375840,"+","none","TGG","AAGTGAAAGAGATGGCCTGGTGG",0,NA,NA,NA,"GeneID:643650","LINC00842",TRUE,"ANXA8L2"

"8926","AAGTGAAAGAGATGGCCTGG","chr10",47991691,"-","none","TGG","AAGTGAAAGAGATGGCCTGGTGG",0,NA,NA,NA,"","",TRUE,"ANXA8L2"

"8927","AAGTGAAAGAGATGGCCTGG","chr10",47483924,"-","1:C>T","TGG","AGGTGAAAGAGATGGCCTGGTGG",1,2,NA,NA,"GeneID:653145","ANXA8",TRUE,"ANXA8L2"

"8928","AAGTGAAAGAGATGGCCTGG","chr2",116924533,"-","0:C>T,2:T>C","AGG","GAATGAAAGAGATGGCCTGGAGG",2,1,3,NA,"","",FALSE,"ANXA8L2"

"8929","AAGTGAAAGAGATGGCCTGG","chr10",11859914,"-","2:T>C,3:T>A","GGG","AAAAGAAAGAGATGGCCTGGGGG",2,3,4,NA,"GeneID:254427;GeneID:219731","PROSER2;PROSER2-AS1",FALSE,"ANXA8L2"

"8930","AAGTGAAAGAGATGGCCTGG","chr7",70525788,"+","6:G>A,11:G>A","AGG","AAGTGAGAGAGGTGGCCTGGAGG",2,7,12,NA,"GeneID:26053","AUTS2",FALSE,"ANXA8L2"

"8931","AAGTGAAAGAGATGGCCTGG","chr1",208094722,"-","6:A>T,10:T>C","TGG","AAGTGATAGAAATGGCCTGGTGG",2,7,11,NA,"GeneID:5362","PLXNA2",FALSE,"ANXA8L2"

"8932","ACTTACCCAGGCTTTCCACC","chr10",47991673,"+","none","AGG","ACTTACCCAGGCTTTCCACCAGG",0,NA,NA,NA,"","",TRUE,"ANXA8L2"

"8933","ACTTACCCAGGCTTTCCACC","chr10",46375858,"-","none","AGG","ACTTACCCAGGCTTTCCACCAGG",0,NA,NA,NA,"GeneID:643650","LINC00842",TRUE,"ANXA8L2"

"8934","ACTTACCCAGGCTTTCCACC","chr10",47483906,"+","11:A>C","AGG","ACTTACCCAGGATTTCCACCAGG",1,12,NA,NA,"GeneID:653145","ANXA8",TRUE,"ANXA8L2"

"8935","CCACTCACCGATCCCCTTCA","chr10",47479789,"+","none","TGG","CCACTCACCGATCCCCTTCATGG",0,NA,NA,NA,"GeneID:653145","ANXA8",TRUE,"ANXA8L2"

"8936","CCACTCACCGATCCCCTTCA","chr10",46379963,"-","none","TGG","CCACTCACCGATCCCCTTCATGG",0,NA,NA,NA,"GeneID:643650","LINC00842",TRUE,"ANXA8L2"

"8937","CCCTGCAGGGACCAACGAGC","chr10",46381137,"+","none","AGG","CCCTGCAGGGACCAACGAGCAGG",0,NA,NA,NA,"GeneID:643650","LINC00842",TRUE,"ANXA8L2"

"8938","CCCTGCAGGGACCAACGAGC","chr10",47478615,"-","none","AGG","CCCTGCAGGGACCAACGAGCAGG",0,NA,NA,NA,"GeneID:653145","ANXA8",TRUE,"ANXA8L2"

"8939","CCCTGCAGGGACCAACGAGC","chr21",6056722,"+","3:G>T,13:C>A","AGG","CCCGGCAGGGACCCACGAGCAGG",2,4,14,NA,"","",FALSE,"ANXA8L2"

"8940","CCCTGCAGGGACCAACGAGC","chr21",43481820,"-","3:C>A,13:G>T","AGG","CCCGGCAGGGACCCACGAGCAGG",2,4,14,NA,"","",FALSE,"ANXA8L2"

"8941","CTTGCCGAACTGAGCCTTGA","chr10",47478532,"+","none","AGG","CTTGCCGAACTGAGCCTTGAAGG",0,NA,NA,NA,"GeneID:653145","ANXA8",TRUE,"ANXA8L2"

"8942","CTTGCCGAACTGAGCCTTGA","chr10",46381220,"-","none","AGG","CTTGCCGAACTGAGCCTTGAAGG",0,NA,NA,NA,"GeneID:643650","LINC00842",TRUE,"ANXA8L2"

"8943","TCTCTTGCAGATTGAACAGG","chr10",46379874,"+","none","AGG","TCTCTTGCAGATTGAACAGGAGG",0,NA,NA,NA,"GeneID:643650","LINC00842",TRUE,"ANXA8L2"

"8944","TCTCTTGCAGATTGAACAGG","chr10",47479878,"-","none","AGG","TCTCTTGCAGATTGAACAGGAGG",0,NA,NA,NA,"GeneID:653145","ANXA8",TRUE,"ANXA8L2"

"8945","TCTCTTGCAGATTGAACAGG","chr10",6593100,"-","10:C>T,19:G>C","TGG","TCTCTTGCAGGTTGAACAGCTGG",2,11,20,NA,"","",FALSE,"ANXA8L2"

"8946","CACGTCCACAGCAGAATCTG","chr1",150984589,"-","none","AGG","CACGTCCACAGCAGAATCTGAGG",0,NA,NA,NA,"GeneID:8416","ANXA9",TRUE,"ANXA9"

"8947","CACGTCCACAGCAGAATCTG","chr19",5832361,"+","14:G>A,18:A>T","GGG","CACGTCCACAGCAGGATCAGGGG",2,15,19,NA,"GeneID:2528","FUT6",TRUE,"ANXA9"

"8948","CACGTCCACAGCAGAATCTG","chr11",4917617,"-","4:C>A,16:C>A","AGG","CACGGCCACAGCAGAAGCTGAGG",2,5,17,NA,"GeneID:56547;GeneID:81282","MMP26;OR51G2",FALSE,"ANXA9"

"8949","GACCTTCTTGAACTTCAGCG","chr1",150983369,"+","none","TGG","GACCTTCTTGAACTTCAGCGTGG",0,NA,NA,NA,"GeneID:8416","ANXA9",TRUE,"ANXA9"

"8950","GACCTTCTTGAACTTCAGCG","chr12",123242641,"+","14:C>T,19:T>G","GGG","GACCTTCTTGAACTCCAGCTGGG",2,15,20,NA,"GeneID:10198;GeneID:91574","MPHOSPH9;C12orf65",FALSE,"ANXA9"

"8951","GACCTTCTTGAACTTCAGCG","chr4",101946822,"-","8:T>A,12:A>G","GGG","GACCTTCTAGAATTTCAGCGGGG",2,9,13,NA,"GeneID:55024","BANK1",FALSE,"ANXA9"

"8952","GCACGTCCACAATGGCACTG","chr1",150983984,"-","none","CGG","GCACGTCCACAATGGCACTGCGG",0,NA,NA,NA,"GeneID:8416","ANXA9",TRUE,"ANXA9"

"8953","GCTCATCTCACGAAACTTCC","chr1",150984032,"+","none","AGG","GCTCATCTCACGAAACTTCCAGG",0,NA,NA,NA,"GeneID:8416","ANXA9",TRUE,"ANXA9"

"8954","GTCCACGCTGAAGTTCAAGA","chr1",150983374,"-","none","AGG","GTCCACGCTGAAGTTCAAGAAGG",0,NA,NA,NA,"GeneID:8416","ANXA9",TRUE,"ANXA9"

"8955","TACTGTGTTTGTAGACTGCC","chr1",150984659,"-","none","AGG","TACTGTGTTTGTAGACTGCCAGG",0,NA,NA,NA,"GeneID:8416","ANXA9",TRUE,"ANXA9"

"8956","AGAATAGAGGCGCCACCGTA","chr7",36724105,"+","none","AGG","AGAATAGAGGCGCCACCGTAAGG",0,NA,NA,NA,"GeneID:313","AOAH",TRUE,"AOAH"

"8957","CACAGGTGTGCCCATTCGAG","chr7",36724024,"+","none","AGG","CACAGGTGTGCCCATTCGAGAGG",0,NA,NA,NA,"GeneID:313","AOAH",TRUE,"AOAH"

"8958","CGCAGATATGAATGCTGATG","chr7",36659239,"-","none","TGG","CGCAGATATGAATGCTGATGTGG",0,NA,NA,NA,"GeneID:313","AOAH",TRUE,"AOAH"

"8959","CTTGTCAATGACTAAATAGC","chr7",36673965,"+","none","AGG","CTTGTCAATGACTAAATAGCAGG",0,NA,NA,NA,"GeneID:313","AOAH",TRUE,"AOAH"

"8960","TACACACTCACCTTGGGAAG","chr7",36659154,"+","none","AGG","TACACACTCACCTTGGGAAGAGG",0,NA,NA,NA,"GeneID:313","AOAH",TRUE,"AOAH"

"8961","TACACACTCACCTTGGGAAG","chr2",232140084,"-","10:C>G,19:T>C","TGG","TACACACTCAGCTTGGGAAATGG",2,11,20,NA,"GeneID:129563","DIS3L2",FALSE,"AOAH"

"8962","TACACACTCACCTTGGGAAG","chr2",53754382,"+","10:T>C,19:A>G","AGG","TACACACTCATCTTGGGAAAAGG",2,11,20,NA,"GeneID:51130;GeneID:100302652","ASB3;GPR75-ASB3",FALSE,"AOAH"

"8963","TATTTAGTCATTGACAAGTT","chr7",36673963,"-","none","TGG","TATTTAGTCATTGACAAGTTTGG",0,NA,NA,NA,"GeneID:313","AOAH",TRUE,"AOAH"

"8964","TATTTAGTCATTGACAAGTT","chr11",24318477,"+","7:G>T,14:A>C","AGG","TATTTAGGCATTGAAAAGTTAGG",2,8,15,NA,"","",FALSE,"AOAH"

"8965","CGGAGAGCGCATTGCCTATG","chr7",150857495,"+","none","AGG","CGGAGAGCGCATTGCCTATGAGG",0,NA,NA,NA,"GeneID:26","AOC1",TRUE,"AOC1"

"8966","GATCCAGCGCTGGACGTAGT","chr7",150861123,"-","none","TGG","GATCCAGCGCTGGACGTAGTTGG",0,NA,NA,NA,"GeneID:26","AOC1",TRUE,"AOC1"

"8967","GCAGGCGGTACGTGCGCTTG","chr7",150858958,"-","none","TGG","GCAGGCGGTACGTGCGCTTGTGG",0,NA,NA,NA,"GeneID:26","AOC1",TRUE,"AOC1"

"8968","GCCCCAACTACGTCCAGCGC","chr7",150861117,"+","none","TGG","GCCCCAACTACGTCCAGCGCTGG",0,NA,NA,NA,"GeneID:26","AOC1",TRUE,"AOC1"

"8969","GGTGCACTACCGCGTAGACC","chr7",150858008,"+","none","TGG","GGTGCACTACCGCGTAGACCTGG",0,NA,NA,NA,"GeneID:26","AOC1",TRUE,"AOC1"

"8970","TCTACCACCAGAACGACCCC","chr7",150860553,"+","none","TGG","TCTACCACCAGAACGACCCCTGG",0,NA,NA,NA,"GeneID:26","AOC1",TRUE,"AOC1"

"8971","ACCCTCAGGACTAGAAGCCG","chr17",42850338,"-","none","TGG","ACCCTCAGGACTAGAAGCCGTGG",0,NA,NA,NA,"GeneID:314","AOC2",TRUE,"AOC2"

"8972","AGACGTGGTGTTTAAACCTG","chr17",42849110,"+","none","TGG","AGACGTGGTGTTTAAACCTGTGG",0,NA,NA,NA,"GeneID:314","AOC2",TRUE,"AOC2"

"8973","ATAGCGAGTCAGCATCGTCT","chr17",42845740,"-","none","TGG","ATAGCGAGTCAGCATCGTCTTGG",0,NA,NA,NA,"GeneID:314","AOC2",TRUE,"AOC2"

"8974","GGACTCGGCCGTAACAGCCG","chr17",42845775,"+","none","AGG","GGACTCGGCCGTAACAGCCGAGG",0,NA,NA,NA,"GeneID:314","AOC2",TRUE,"AOC2"

"8975","GGCAACCAACCTCTCCTAAG","chr17",42849720,"-","none","AGG","GGCAACCAACCTCTCCTAAGAGG",0,NA,NA,NA,"GeneID:314","AOC2",TRUE,"AOC2"

"8976","GGTGACCCCACGCATTAGTC","chr17",42849263,"-","none","TGG","GGTGACCCCACGCATTAGTCTGG",0,NA,NA,NA,"GeneID:314","AOC2",TRUE,"AOC2"

"8977","AGCAATGACGACCCGCTATG","chr17",42852476,"+","none","TGG","AGCAATGACGACCCGCTATGTGG",0,NA,NA,NA,"GeneID:8639","AOC3",TRUE,"AOC3"

"8978","ATTGAAAACGCTGCTGCTAC","chr17",42855484,"-","none","TGG","ATTGAAAACGCTGCTGCTACTGG",0,NA,NA,NA,"GeneID:8639","AOC3",TRUE,"AOC3"

"8979","ATTGAAAACGCTGCTGCTAC","chr14",81449718,"-","0:G>T,4:C>T","AGG","CTTGGAAACGCTGCTGCTACAGG",2,1,5,NA,"GeneID:100506700","LOC100506700",FALSE,"AOC3"

"8980","CCAGCTGACCTTTCCAGCAA","chr17",42855562,"-","none","TGG","CCAGCTGACCTTTCCAGCAATGG",0,NA,NA,NA,"GeneID:8639","AOC3",TRUE,"AOC3"

"8981","CTCCAGCAAAGCTGAGCATC","chr17",42854664,"-","none","TGG","CTCCAGCAAAGCTGAGCATCTGG",0,NA,NA,NA,"GeneID:8639","AOC3",TRUE,"AOC3"

"8982","GGTGGCGCTTCCCACGAGGA","chr17",42854576,"-","none","AGG","GGTGGCGCTTCCCACGAGGAAGG",0,NA,NA,NA,"GeneID:8639","AOC3",TRUE,"AOC3"

"8983","GGTGGCGCTTCCCACGAGGA","chr20",50609742,"+","9:G>T,16:C>A","GGG","GGTGGCGCTGCCCACGCGGAGGG",2,10,17,NA,"GeneID:140876","FAM65C",TRUE,"AOC3"

"8984","TACACCACGCCCCTGACCCG","chr17",42852522,"+","none","TGG","TACACCACGCCCCTGACCCGTGG",0,NA,NA,NA,"GeneID:8639","AOC3",TRUE,"AOC3"

"8985","ATACGCTTCTTCCTCAAATA","chr2",200593187,"-","none","AGG","ATACGCTTCTTCCTCAAATAAGG",0,NA,NA,NA,"GeneID:316","AOX1",TRUE,"AOX1"

"8986","ATACGCTTCTTCCTCAAATA","chr4",10225139,"+","1:C>T,4:C>G","AGG","ACACCCTTCTTCCTCAAATAAGG",2,2,5,NA,"","",FALSE,"AOX1"

"8987","CAATGCTGTTGCCTTATTTG","chr2",200593173,"+","none","AGG","CAATGCTGTTGCCTTATTTGAGG",0,NA,NA,NA,"GeneID:316","AOX1",TRUE,"AOX1"

"8988","CAATGCTGTTGCCTTATTTG","chr2",157996465,"+","5:T>C,14:A>T","GGG","CAATGTTGTTGCCTAATTTGGGG",2,6,15,NA,"GeneID:151531","UPP2",FALSE,"AOX1"

"8989","CAATGCTGTTGCCTTATTTG","chr17",72281205,"+","2:C>A,16:G>T","AGG","CACTGCTGTTGCCTTAGTTGAGG",2,3,17,NA,"","",FALSE,"AOX1"

"8990","CACCTGAACAGGATGAATTC","chr2",200597488,"-","none","TGG","CACCTGAACAGGATGAATTCTGG",0,NA,NA,NA,"GeneID:316","AOX1",TRUE,"AOX1"

"8991","CCTCTTCCAGTTCGACTCAC","chr2",200595261,"+","none","AGG","CCTCTTCCAGTTCGACTCACAGG",0,NA,NA,NA,"GeneID:316","AOX1",TRUE,"AOX1"

"8992","GCTGCCGTCACCACAGTAGA","chr2",200597445,"+","none","AGG","GCTGCCGTCACCACAGTAGAAGG",0,NA,NA,NA,"GeneID:316","AOX1",TRUE,"AOX1"

"8993","TGCACGGTACCTTATCCTCT","chr2",200595358,"-","none","TGG","TGCACGGTACCTTATCCTCTTGG",0,NA,NA,NA,"GeneID:316","AOX1",TRUE,"AOX1"

"8994","CAAGATTCGAAGTCATCTTC","chr4",112265743,"-","none","TGG","CAAGATTCGAAGTCATCTTCTGG",0,NA,NA,NA,"GeneID:55435","AP1AR",TRUE,"AP1AR"

"8995","CAAGATTCGAAGTCATCTTC","chr8",139504888,"-","7:T>G,16:A>G","TGG","CAAGATTAGAAGTCATTTTCTGG",2,8,17,NA,"","",FALSE,"AP1AR"

"8996","CCTTACCTATGACTGCTTCC","chr4",112257783,"-","none","TGG","CCTTACCTATGACTGCTTCCTGG",0,NA,NA,NA,"GeneID:55435","AP1AR",TRUE,"AP1AR"

"8997","TGAGCATGTTCTAAAATACT","chr4",112253212,"-","none","TGG","TGAGCATGTTCTAAAATACTTGG",0,NA,NA,NA,"GeneID:55435","AP1AR",TRUE,"AP1AR"

"8998","TGAGCATGTTCTAAAATACT","chrX",132018901,"-","14:A>T,19:G>A","AGG","TGAGCATGTTCTAATATACCAGG",2,15,20,NA,"","",FALSE,"AP1AR"

"8999","TGAGCATGTTCTAAAATACT","chr1",151569404,"+","9:C>T,18:A>C","TGG","TGAGCATGTCCTAAAATAATTGG",2,10,19,NA,"GeneID:7286","TUFT1",FALSE,"AP1AR"

"9000","TTTGTACAGGGGGAGAGCCC","chr4",112257762,"+","none","AGG","TTTGTACAGGGGGAGAGCCCAGG",0,NA,NA,NA,"GeneID:55435","AP1AR",TRUE,"AP1AR"

"9001","CCACCTGAGTGGCCAAACTG","chr22",29342280,"+","none","AGG","CCACCTGAGTGGCCAAACTGAGG",0,NA,NA,NA,"GeneID:162","AP1B1",TRUE,"AP1B1"

"9002","CCACCTGAGTGGCCAAACTG","chr2",236507531,"-","14:C>T,17:C>G","AGG","CCACCTGAGTGGCCGAAGTGAGG",2,15,18,NA,"GeneID:79781","IQCA1",TRUE,"AP1B1"

"9003","CCACCTGAGTGGCCAAACTG","chr4",182710622,"+","0:A>C,14:C>A","AGG","ACACCTGAGTGGCCCAACTGAGG",2,1,15,NA,"GeneID:55714","TENM3",FALSE,"AP1B1"

"9004","CGATGAGATTGATGTTGCGC","chr22",29354659,"+","none","AGG","CGATGAGATTGATGTTGCGCAGG",0,NA,NA,NA,"GeneID:162","AP1B1",TRUE,"AP1B1"

"9005","CGCACTTGTTGGGGTACTTG","chr22",29350029,"+","none","CGG","CGCACTTGTTGGGGTACTTGCGG",0,NA,NA,NA,"GeneID:162","AP1B1",TRUE,"AP1B1"

"9006","CTGGACCACATAGTTGACCT","chr22",29350081,"+","none","TGG","CTGGACCACATAGTTGACCTTGG",0,NA,NA,NA,"GeneID:162","AP1B1",TRUE,"AP1B1"

"9007","GGTGCAGCAGGTCCTCAGTT","chr22",29342295,"-","none","TGG","GGTGCAGCAGGTCCTCAGTTTGG",0,NA,NA,NA,"GeneID:162","AP1B1",TRUE,"AP1B1"

"9008","GGTGCAGCAGGTCCTCAGTT","chr19",38152396,"-","11:G>A,15:C>G","GGG","GGTGCAGCAGGCCCTGAGTTGGG",2,12,16,NA,"GeneID:23094","SIPA1L3",FALSE,"AP1B1"

"9009","TCTAAGGACTTGGACTACTA","chr22",29354757,"-","none","CGG","TCTAAGGACTTGGACTACTACGG",0,NA,NA,NA,"GeneID:162","AP1B1",TRUE,"AP1B1"

"9010","AAAAACATCTCCATAAGTTC","chr16",71771193,"+","none","AGG","AAAAACATCTCCATAAGTTCAGG",0,NA,NA,NA,"GeneID:164","AP1G1",TRUE,"AP1G1"

"9011","ATCAGCTCCCGCAATCTGAT","chr16",71789447,"+","none","GGG","ATCAGCTCCCGCAATCTGATGGG",0,NA,NA,NA,"GeneID:164","AP1G1",TRUE,"AP1G1"

"9012","TCTTTAAAATACGAACTAAT","chr16",71765553,"+","none","TGG","TCTTTAAAATACGAACTAATTGG",0,NA,NA,NA,"GeneID:164","AP1G1",TRUE,"AP1G1"

"9013","TGTGTGCTGTTCATGTCATC","chr16",71771225,"-","none","AGG","TGTGTGCTGTTCATGTCATCAGG",0,NA,NA,NA,"GeneID:164","AP1G1",TRUE,"AP1G1"

"9014","TGTGTGCTGTTCATGTCATC","chr5",83421417,"+","1:A>G,3:A>G","TGG","TATATGCTGTTCATGTCATCTGG",2,2,4,NA,"","",FALSE,"AP1G1"

"9015","TGTGTGCTGTTCATGTCATC","chr12",122326926,"-","7:G>A,10:C>A","TGG","TGTGTGCCGTGCATGTCATCTGG",2,8,11,NA,"GeneID:6249","CLIP1",FALSE,"AP1G1"

"9016","TTAAAGAACCTCATCATGTC","chr16",71765540,"-","none","CGG","TTAAAGAACCTCATCATGTCCGG",0,NA,NA,NA,"GeneID:164","AP1G1",TRUE,"AP1G1"

"9017","TTCGGGCTGTCCGGATGGTC","chr16",71789424,"+","none","CGG","TTCGGGCTGTCCGGATGGTCCGG",0,NA,NA,NA,"GeneID:164","AP1G1",TRUE,"AP1G1"

"9018","CACCCTCTTGTCTGTGAATC","chr14",23566635,"+","none","TGG","CACCCTCTTGTCTGTGAATCTGG",0,NA,NA,NA,"GeneID:8906;GeneID:102724814","AP1G2;LOC102724814",TRUE,"AP1G2"

"9019","CAGAATCCTCAATCCTAACA","chr14",23559939,"-","none","AGG","CAGAATCCTCAATCCTAACAAGG",0,NA,NA,NA,"GeneID:79178;GeneID:8906","THTPA;AP1G2",TRUE,"AP1G2"

"9020","CCTGGGGGCCATGCTTCTAT","chr14",23566610,"-","none","TGG","CCTGGGGGCCATGCTTCTATTGG",0,NA,NA,NA,"GeneID:8906;GeneID:102724814","AP1G2;LOC102724814",TRUE,"AP1G2"

"9021","CTGAGGCACAGTCAGCCCGT","chr14",23563737,"+","none","AGG","CTGAGGCACAGTCAGCCCGTAGG",0,NA,NA,NA,"GeneID:8906;GeneID:102724814","AP1G2;LOC102724814",TRUE,"AP1G2"

"9022","TCTCAAAGTGTTTGAGCGTG","chr14",23560385,"-","none","AGG","TCTCAAAGTGTTTGAGCGTGAGG",0,NA,NA,NA,"GeneID:8906","AP1G2",TRUE,"AP1G2"

"9023","TTGCATCATGGCTCGCACAT","chr14",23563795,"+","none","TGG","TTGCATCATGGCTCGCACATTGG",0,NA,NA,NA,"GeneID:8906;GeneID:102724814","AP1G2;LOC102724814",TRUE,"AP1G2"

"9024","ACGGGGGGGTCCGTTTCATG","chr19",16203570,"+","none","TGG","ACGGGGGGGTCCGTTTCATGTGG",0,NA,NA,NA,"GeneID:8907","AP1M1",TRUE,"AP1M1"

"9025","ATCTCGAACTTGACACTGAT","chr19",16233570,"-","none","CGG","ATCTCGAACTTGACACTGATCGG",0,NA,NA,NA,"GeneID:8907","AP1M1",TRUE,"AP1M1"

"9026","CGACACGCACGCGTTCTTCT","chr19",16206355,"-","none","TGG","CGACACGCACGCGTTCTTCTTGG",0,NA,NA,NA,"GeneID:8907","AP1M1",TRUE,"AP1M1"

"9027","CTGACATGTCCACGTCGCCA","chr19",16203481,"-","none","CGG","CTGACATGTCCACGTCGCCACGG",0,NA,NA,NA,"GeneID:8907","AP1M1",TRUE,"AP1M1"

"9028","CTTACGTACCTGGATGCCGG","chr19",16233607,"-","none","AGG","CTTACGTACCTGGATGCCGGAGG",0,NA,NA,NA,"GeneID:8907","AP1M1",TRUE,"AP1M1"

"9029","TGTTATCATCTACGAGCTGC","chr19",16208071,"+","none","TGG","TGTTATCATCTACGAGCTGCTGG",0,NA,NA,NA,"GeneID:8907","AP1M1",TRUE,"AP1M1"

"9030","ATTGATCAGCCGCAACTACA","chr19",10584048,"-","none","AGG","ATTGATCAGCCGCAACTACAAGG",0,NA,NA,NA,"GeneID:10053","AP1M2",TRUE,"AP1M2"

"9031","CCACATCGCCCTTGTAGTTG","chr19",10584036,"+","none","CGG","CCACATCGCCCTTGTAGTTGCGG",0,NA,NA,NA,"GeneID:10053","AP1M2",TRUE,"AP1M2"

"9032","CCACATCGCCCTTGTAGTTG","chr4",138744276,"-","7:A>C,14:G>A","GGG","CCACATCTCCCTTGCAGTTGGGG",2,8,15,NA,"","",FALSE,"AP1M2"

"9033","GGTATTCTGCGAATACTTCA","chr19",10581859,"-","none","AGG","GGTATTCTGCGAATACTTCAAGG",0,NA,NA,NA,"GeneID:10053","AP1M2",TRUE,"AP1M2"

"9034","GTCCTTCTGAGCGAAATCGT","chr19",10581354,"-","none","CGG","GTCCTTCTGAGCGAAATCGTCGG",0,NA,NA,NA,"GeneID:10053","AP1M2",TRUE,"AP1M2"

"9035","TACCGACGATTTCGCTCAGA","chr19",10581349,"+","none","AGG","TACCGACGATTTCGCTCAGAAGG",0,NA,NA,NA,"GeneID:10053","AP1M2",TRUE,"AP1M2"

"9036","TGTCATCGTCTACGAGTTGC","chr19",10581805,"-","none","TGG","TGTCATCGTCTACGAGTTGCTGG",0,NA,NA,NA,"GeneID:10053","AP1M2",TRUE,"AP1M2"

"9037","CGATGGCGCAGCAGAAGTAG","chr7",101157388,"-","none","AGG","CGATGGCGCAGCAGAAGTAGAGG",0,NA,NA,NA,"GeneID:1174","AP1S1",TRUE,"AP1S1"

"9038","CTTCCGTTCCTTGTCCGAAG","chr7",101156657,"-","none","TGG","CTTCCGTTCCTTGTCCGAAGTGG",0,NA,NA,NA,"GeneID:1174","AP1S1",TRUE,"AP1S1"

"9039","GATGGTGCGCGAGCTCATGC","chr7",101156679,"+","none","AGG","GATGGTGCGCGAGCTCATGCAGG",0,NA,NA,NA,"GeneID:1174","AP1S1",TRUE,"AP1S1"

"9040","GCCCTCCCAGGTGTGCGAGC","chr7",101159048,"+","none","TGG","GCCCTCCCAGGTGTGCGAGCTGG",0,NA,NA,NA,"GeneID:1174","AP1S1",TRUE,"AP1S1"

"9041","TGGCCCGTACCTCTTGCAGT","chr7",101159186,"-","none","AGG","TGGCCCGTACCTCTTGCAGTAGG",0,NA,NA,NA,"GeneID:1174","AP1S1",TRUE,"AP1S1"

"9042","TGTCTAAGAGCTCCACGTAT","chr7",101157451,"-","none","CGG","TGTCTAAGAGCTCCACGTATCGG",0,NA,NA,NA,"GeneID:1174","AP1S1",TRUE,"AP1S1"

"9043","TGTCTAAGAGCTCCACGTAT","chr2",190439256,"-","9:T>C,17:G>A","TGG","TGTCTAAGAACTCCACGCATTGG",2,10,18,NA,"GeneID:54842","MFSD6",FALSE,"AP1S1"

"9044","ATCTGAAGATTGTTTACAAA","chrX",15852347,"-","none","AGG","ATCTGAAGATTGTTTACAAAAGG",0,NA,NA,NA,"GeneID:8905","AP1S2",TRUE,"AP1S2"

"9045","ATCTGAAGATTGTTTACAAA","chr8",135683673,"+","2:G>C,6:T>A","AGG","ATGTGATGATTGTTTACAAAAGG",2,3,7,NA,"","",FALSE,"AP1S2"

"9046","ATCTGAAGATTGTTTACAAA","chr2",21344866,"+","6:G>A,11:T>G","TGG","ATCTGAGGATTTTTTACAAATGG",2,7,12,NA,"","",FALSE,"AP1S2"

"9047","ATCTGAAGATTGTTTACAAA","chr20",2338204,"+","7:C>G,15:C>A","GGG","ATCTGAACATTGTTTCCAAAGGG",2,8,16,NA,"GeneID:7053","TGM3",FALSE,"AP1S2"

"9048","ATTTCTTCAAGAACACTACG","chrX",15827337,"+","none","TGG","ATTTCTTCAAGAACACTACGTGG",0,NA,NA,NA,"GeneID:8905","AP1S2",TRUE,"AP1S2"

"9049","ATTTCTTCAAGAACACTACG","chr5",6208273,"-","15:C>G,18:A>G","AGG","ATTTCTTCAAGAACAGTATGAGG",2,16,19,NA,"","",FALSE,"AP1S2"

"9050","ATTTCTTCAAGAACACTACG","chr8",123949156,"-","2:T>A,19:A>C","TGG","ATATCTTCAAGAACACTACTTGG",2,3,20,NA,"GeneID:654463","FER1L6",FALSE,"AP1S2"

"9051","CAGCCTGCTCAATTGCTTTA","chrX",15845392,"+","none","AGG","CAGCCTGCTCAATTGCTTTAAGG",0,NA,NA,NA,"GeneID:8905","AP1S2",TRUE,"AP1S2"

"9052","CAGCCTGCTCAATTGCTTTA","chr17",60102250,"+","1:G>A","AGG","CGGCCTGCTCAATTGCTTTAAGG",1,2,NA,NA,"GeneID:653653","LOC653653",TRUE,"AP1S2"

"9053","CATAACGATGAATTATTTCC","chr17",60102403,"+","none","AGG","CATAACGATGAATTATTTCCAGG",0,NA,NA,NA,"GeneID:653653","LOC653653",TRUE,"AP1S2"

"9054","CATAACGATGAATTATTTCC","chrX",15845931,"+","none","AGG","CATAACGATGAATTATTTCCAGG",0,NA,NA,NA,"GeneID:8905","AP1S2",TRUE,"AP1S2"

"9055","CGTAGTGTTCTTGAAGAAAT","chrX",15827337,"-","none","TGG","CGTAGTGTTCTTGAAGAAATTGG",0,NA,NA,NA,"GeneID:8905","AP1S2",TRUE,"AP1S2"

"9056","TTCTTCTCTTTGTCTGATAG","chrX",15852441,"+","none","TGG","TTCTTCTCTTTGTCTGATAGTGG",0,NA,NA,NA,"GeneID:8905","AP1S2",TRUE,"AP1S2"

"9057","TTCTTCTCTTTGTCTGATAG","chr17",60102579,"+","14:C>T","TGG","TTCTTCTCTTTGTCCGATAGTGG",1,15,NA,NA,"GeneID:653653","LOC653653",TRUE,"AP1S2"

"9058","TTCTTCTCTTTGTCTGATAG","chr2",14706229,"+","0:G>T,10:A>T","TGG","GTCTTCTCTTAGTCTGATAGTGG",2,1,11,NA,"","",FALSE,"AP1S2"

"9059","TTCTTCTCTTTGTCTGATAG","chr20",54382911,"-","9:G>A,12:G>A","AGG","TTCTTCTCTCTGCCTGATAGAGG",2,10,13,NA,"","",FALSE,"AP1S2"

"9060","AGAGATTGTGCATCGTTACG","chr2",223775929,"-","none","TGG","AGAGATTGTGCATCGTTACGTGG",0,NA,NA,NA,"GeneID:130340","AP1S3",TRUE,"AP1S3"

"9061","ATATTGCTCTTCAGTCGACA","chr2",223777840,"-","none","AGG","ATATTGCTCTTCAGTCGACAAGG",0,NA,NA,NA,"GeneID:130340","AP1S3",TRUE,"AP1S3"

"9062","ATCCTGGACGAGTTTATAAT","chr2",223765285,"-","none","AGG","ATCCTGGACGAGTTTATAATAGG",0,NA,NA,NA,"GeneID:130340","AP1S3",TRUE,"AP1S3"

"9063","CACCTATTATAAACTCGTCC","chr2",223765280,"+","none","AGG","CACCTATTATAAACTCGTCCAGG",0,NA,NA,NA,"GeneID:130340","AP1S3",TRUE,"AP1S3"

"9064","TCACTCTCCCTGATAAAGAG","chr2",223777791,"-","none","AGG","TCACTCTCCCTGATAAAGAGAGG",0,NA,NA,NA,"GeneID:130340","AP1S3",TRUE,"AP1S3"

"9065","TCACTCTCCCTGATAAAGAG","chr1",151004403,"-","1:A>G,2:A>T","TGG","TTTCTCTCCCTGATAAAGAGTGG",2,2,3,NA,"GeneID:55793","FAM63A",FALSE,"AP1S3"

"9066","TCACTCTCCCTGATAAAGAG","chr20",48232428,"+","2:C>A,10:A>T","AGG","TCCCTCTCCCAGATAAAGAGAGG",2,3,11,NA,"","",FALSE,"AP1S3"

"9067","TTGCTGTGCAATAGAAAATC","chr2",223775971,"-","none","AGG","TTGCTGTGCAATAGAAAATCAGG",0,NA,NA,NA,"GeneID:130340","AP1S3",TRUE,"AP1S3"

"9068","ACGCGCCGTCCACTCGCCCA","chr19",49792014,"-","none","TGG","ACGCGCCGTCCACTCGCCCATGG",0,NA,NA,NA,"GeneID:160","AP2A1",TRUE,"AP2A1"

"9069","CGATGGGATGCGGGGGCTCG","chr19",49767153,"+","none","CGG","CGATGGGATGCGGGGGCTCGCGG",0,NA,NA,NA,"GeneID:160","AP2A1",TRUE,"AP2A1"

"9070","GCGAGGCCTTGTACAGTCGA","chr19",49791979,"-","none","AGG","GCGAGGCCTTGTACAGTCGAAGG",0,NA,NA,NA,"GeneID:160","AP2A1",TRUE,"AP2A1"

"9071","GCGCTCACAGTTCCGGATGT","chr19",49767188,"-","none","CGG","GCGCTCACAGTTCCGGATGTCGG",0,NA,NA,NA,"GeneID:160","AP2A1",TRUE,"AP2A1"

"9072","TGACATTGACTTTGGGCACA","chr19",49782019,"+","none","TGG","TGACATTGACTTTGGGCACATGG",0,NA,NA,NA,"GeneID:160","AP2A1",TRUE,"AP2A1"

"9073","TGACATTGACTTTGGGCACA","chr11",970241,"+","15:A>G","TGG","TGACATTGACTTTGGACACATGG",1,16,NA,NA,"GeneID:161","AP2A2",TRUE,"AP2A1"

"9074","TGACATTGACTTTGGGCACA","chr2",138060387,"-","8:A>T,11:C>A","TGG","TGACATTGTCTGTGGGCACATGG",2,9,12,NA,"","",FALSE,"AP2A1"

"9075","TGTGCCCAAAGTCAATGTCA","chr19",49782019,"-","none","TGG","TGTGCCCAAAGTCAATGTCATGG",0,NA,NA,NA,"GeneID:160","AP2A1",TRUE,"AP2A1"

"9076","ATCCCTAAGGTCCTCGTAGC","chr11",972232,"+","none","CGG","ATCCCTAAGGTCCTCGTAGCCGG",0,NA,NA,NA,"GeneID:161","AP2A2",TRUE,"AP2A2"

"9077","CTTAGGGATCTCCCCGGCGA","chr11",972221,"-","none","AGG","CTTAGGGATCTCCCCGGCGAAGG",0,NA,NA,NA,"GeneID:161","AP2A2",TRUE,"AP2A2"

"9078","GATCGGGGGACGTCCTGTAC","chr11",977145,"-","none","AGG","GATCGGGGGACGTCCTGTACAGG",0,NA,NA,NA,"GeneID:161","AP2A2",TRUE,"AP2A2"

"9079","GCTGAGTTCAAACAGATACA","chr11",970277,"+","none","CGG","GCTGAGTTCAAACAGATACACGG",0,NA,NA,NA,"GeneID:161","AP2A2",TRUE,"AP2A2"

"9080","GCTGAGTTCAAACAGATACA","chr12",13224752,"-","5:T>C,18:A>G","AGG","GCTGAATTCAAACAGATATAAGG",2,6,19,NA,"","",FALSE,"AP2A2"

"9081","GTAGGTGACAAGGCTCTTGA","chr11",970164,"+","none","TGG","GTAGGTGACAAGGCTCTTGATGG",0,NA,NA,NA,"GeneID:161","AP2A2",TRUE,"AP2A2"

"9082","TGTGCTTGCTGCGCCTGTAC","chr11",977129,"+","none","AGG","TGTGCTTGCTGCGCCTGTACAGG",0,NA,NA,NA,"GeneID:161","AP2A2",TRUE,"AP2A2"

"9083","AGTGATTGCTGCTATGACCG","chr17",35598299,"+","none","TGG","AGTGATTGCTGCTATGACCGTGG",0,NA,NA,NA,"GeneID:163","AP2B1",TRUE,"AP2B1"

"9084","AGTGATTGCTGCTATGACCG","chrX",93224888,"-","11:A>G,15:A>C","TGG","AGTGATTGCTGTTATTACCGTGG",2,12,16,NA,"","",TRUE,"AP2B1"

"9085","CATGTGCTGCAGAGCTTTAT","chr17",35674269,"-","none","TGG","CATGTGCTGCAGAGCTTTATTGG",0,NA,NA,NA,"GeneID:163","AP2B1",TRUE,"AP2B1"

"9086","CATGTGCTGCAGAGCTTTAT","chrX",93222767,"+","1:C>A,3:A>G","TGG","CCTATGCTGCAGAGCTTTATTGG",2,2,4,NA,"","",TRUE,"AP2B1"

"9087","CATGTGCTGCAGAGCTTTAT","chr1",103927261,"-","3:T>C,16:C>A","TGG","CATATGCTGCAGAGCTGTATTGG",2,4,17,NA,"","",TRUE,"AP2B1"

"9088","GGAACATTTACTCACCGCCA","chr17",35674217,"+","none","AGG","GGAACATTTACTCACCGCCAAGG",0,NA,NA,NA,"GeneID:163","AP2B1",TRUE,"AP2B1"

"9089","GGAACATTTACTCACCGCCA","chrX",93222819,"-","17:A>G","AGG","GGAACATTTACTCACCGTCAAGG",1,18,NA,NA,"","",TRUE,"AP2B1"

"9090","GGAACATTTACTCACCGCCA","chr3",158247825,"-","7:G>A,15:T>G","AGG","GGAACATCTACTCACAGCCAAGG",2,8,16,NA,"GeneID:51319","RSRC1",FALSE,"AP2B1"

"9091","GGTCTGGCTACCTGCAGTAA","chr17",35674174,"+","none","AGG","GGTCTGGCTACCTGCAGTAAAGG",0,NA,NA,NA,"GeneID:163","AP2B1",TRUE,"AP2B1"

"9092","GGTCTGGCTACCTGCAGTAA","chrX",93222862,"-","0:A>C","AGG","TGTCTGGCTACCTGCAGTAAAGG",1,1,NA,NA,"","",TRUE,"AP2B1"

"9093","TATTTCACAACCAATAAAAA","chr17",35594045,"+","none","AGG","TATTTCACAACCAATAAAAAAGG",0,NA,NA,NA,"GeneID:163","AP2B1",TRUE,"AP2B1"

"9094","TATTTCACAACCAATAAAAA","chr7",151474764,"+","10:A>C,15:G>A","TGG","TATTTCACAAACAATGAAAATGG",2,11,16,NA,"GeneID:6009","RHEB",FALSE,"AP2B1"

"9095","TATTTCACAACCAATAAAAA","chr4",133371266,"+","3:A>T","TGG","TATATCACAACCAATAAAAATGG",1,4,NA,NA,"","",FALSE,"AP2B1"

"9096","TATTTCACAACCAATAAAAA","chr8",94548066,"+","4:A>T,9:G>A","AGG","TATTACACAGCCAATAAAAAAGG",2,5,10,NA,"GeneID:25962","KIAA1429",FALSE,"AP2B1"

"9097","TATTTCACAACCAATAAAAA","chr17",56958773,"+","9:G>A,10:T>C","GGG","TATTTCACAGTCAATAAAAAGGG",2,10,11,NA,"GeneID:8161","COIL",FALSE,"AP2B1"

"9098","TTACCTAACATCCTTCCCCA","chr17",35598319,"-","none","CGG","TTACCTAACATCCTTCCCCACGG",0,NA,NA,NA,"GeneID:163","AP2B1",TRUE,"AP2B1"

"9099","AGCCCATTTCTTCTTGTCGT","chr3",184182821,"-","none","TGG","AGCCCATTTCTTCTTGTCGTTGG",0,NA,NA,NA,"GeneID:1173","AP2M1",TRUE,"AP2M1"

"9100","ATCAGCTTTATCCCGCCAGA","chr3",184181774,"+","none","TGG","ATCAGCTTTATCCCGCCAGATGG",0,NA,NA,NA,"GeneID:1173","AP2M1",TRUE,"AP2M1"

"9101","CCGCTTAACGTGGAAGAAGC","chr3",184178942,"-","none","TGG","CCGCTTAACGTGGAAGAAGCTGG",0,NA,NA,NA,"GeneID:1173","AP2M1",TRUE,"AP2M1"

"9102","CTATAAGATGTGTGACGTGA","chr3",184179027,"+","none","TGG","CTATAAGATGTGTGACGTGATGG",0,NA,NA,NA,"GeneID:1173","AP2M1",TRUE,"AP2M1"

"9103","GCGCCTGTCTCGGAATTCTG","chr3",184180191,"-","none","TGG","GCGCCTGTCTCGGAATTCTGTGG",0,NA,NA,NA,"GeneID:1173","AP2M1",TRUE,"AP2M1"

"9104","GCGCCTGTCTCGGAATTCTG","chr5",36066660,"+","10:G>C,19:C>G","CGG","GCGCCTGTCTGGGAATTCTCCGG",2,11,20,NA,"GeneID:167127","UGT3A2",FALSE,"AP2M1"

"9105","GCGCCTGTCTCGGAATTCTG","chr5",35991111,"+","10:G>C,19:C>G","CGG","GCGCCTGTCTGGGAATTCTCCGG",2,11,20,NA,"GeneID:133688","UGT3A1",TRUE,"AP2M1"

"9106","TACCCACAGAATTCCGAGAC","chr3",184180185,"+","none","AGG","TACCCACAGAATTCCGAGACAGG",0,NA,NA,NA,"GeneID:1173","AP2M1",TRUE,"AP2M1"

"9107","ATCAAACTGCATGTACCACT","chr19",46846073,"+","none","TGG","ATCAAACTGCATGTACCACTTGG",0,NA,NA,NA,"GeneID:1175","AP2S1",TRUE,"AP2S1"

"9108","ATCATTTACCGCCGCTATGC","chr19",46839543,"-","none","TGG","ATCATTTACCGCCGCTATGCTGG",0,NA,NA,NA,"GeneID:1175","AP2S1",TRUE,"AP2S1"

"9109","CACACAGGTTTACACGGTCG","chr19",46838535,"-","none","TGG","CACACAGGTTTACACGGTCGTGG",0,NA,NA,NA,"GeneID:1175","AP2S1",TRUE,"AP2S1"

"9110","CCGAGAGACCAGCCAGACGA","chr19",46838487,"-","none","AGG","CCGAGAGACCAGCCAGACGAAGG",0,NA,NA,NA,"GeneID:1175","AP2S1",TRUE,"AP2S1"

"9111","GGCCATTCACAACTTCGTGG","chr19",46839466,"-","none","AGG","GGCCATTCACAACTTCGTGGAGG",0,NA,NA,NA,"GeneID:1175","AP2S1",TRUE,"AP2S1"

"9112","GGCGTCTCGGACGGTGACCA","chr19",46846013,"+","none","CGG","GGCGTCTCGGACGGTGACCACGG",0,NA,NA,NA,"GeneID:1175","AP2S1",TRUE,"AP2S1"

"9113","AAAGAAGAAGCCGTATACTA","chr5",78181571,"-","none","TGG","AAAGAAGAAGCCGTATACTATGG",0,NA,NA,NA,"GeneID:8546","AP3B1",TRUE,"AP3B1"

"9114","AAAGAAGAAGCCGTATACTA","chr11",4863386,"-","10:T>G,12:T>C","TGG","AAAGAAGAAGACATATACTATGG",2,11,13,NA,"GeneID:56547","MMP26",FALSE,"AP3B1"

"9115","ACATGCTAACTCGATATGCT","chr5",78216084,"-","none","CGG","ACATGCTAACTCGATATGCTCGG",0,NA,NA,NA,"GeneID:8546","AP3B1",TRUE,"AP3B1"

"9116","ATTAACATTTCCTTCTGCTC","chr5",78225578,"+","none","TGG","ATTAACATTTCCTTCTGCTCTGG",0,NA,NA,NA,"GeneID:8546","AP3B1",TRUE,"AP3B1"

"9117","ATTAACATTTCCTTCTGCTC","chr6",122507257,"+","10:A>C,16:T>G","TGG","ATTAACATTTACTTCTTCTCTGG",2,11,17,NA,"GeneID:5570","PKIB",FALSE,"AP3B1"

"9118","ATTAACATTTCCTTCTGCTC","chr17",14049413,"+","7:C>T,18:C>T","TGG","ATTAACACTTCCTTCTGCCCTGG",2,8,19,NA,"GeneID:100874058","COX10-AS1",FALSE,"AP3B1"

"9119","CGAATCTGATGATGATCAGA","chr5",78181607,"-","none","AGG","CGAATCTGATGATGATCAGAAGG",0,NA,NA,NA,"GeneID:8546","AP3B1",TRUE,"AP3B1"

"9120","GAGCATATCGAGTTAGCATG","chr5",78216083,"+","none","TGG","GAGCATATCGAGTTAGCATGTGG",0,NA,NA,NA,"GeneID:8546","AP3B1",TRUE,"AP3B1"

"9121","TAGCCTTGATCCAGAGCAGA","chr5",78225591,"-","none","AGG","TAGCCTTGATCCAGAGCAGAAGG",0,NA,NA,NA,"GeneID:8546","AP3B1",TRUE,"AP3B1"

"9122","TAGCCTTGATCCAGAGCAGA","chr14",95486169,"-","8:G>T,9:G>A","TGG","TAGCCTTGCCCCAGAGCAGATGG",2,9,10,NA,"","",FALSE,"AP3B1"

"9123","CAACAAGGATTCTCTCAAGC","chr15",82689403,"-","none","TGG","CAACAAGGATTCTCTCAAGCTGG",0,NA,NA,NA,"GeneID:283692;GeneID:8120","LOC283692;AP3B2",TRUE,"AP3B2"

"9124","CAGCTTGAGAGAATCCTTGT","chr15",82689401,"+","none","TGG","CAGCTTGAGAGAATCCTTGTTGG",0,NA,NA,NA,"GeneID:283692;GeneID:8120","LOC283692;AP3B2",TRUE,"AP3B2"

"9125","CAGTTTGGACTCTGACCAGA","chr15",82681161,"-","none","AGG","CAGTTTGGACTCTGACCAGAAGG",0,NA,NA,NA,"GeneID:283692;GeneID:8120","LOC283692;AP3B2",TRUE,"AP3B2"

"9126","CGTGGCCTGTAAGAACATAG","chr15",82689159,"-","none","AGG","CGTGGCCTGTAAGAACATAGAGG",0,NA,NA,NA,"GeneID:283692;GeneID:8120","LOC283692;AP3B2",TRUE,"AP3B2"

"9127","CTTCTATCAGCTGATCCTTC","chr15",82681143,"+","none","TGG","CTTCTATCAGCTGATCCTTCTGG",0,NA,NA,NA,"GeneID:283692;GeneID:8120","LOC283692;AP3B2",TRUE,"AP3B2"

"9128","TCTTCACCACCGCGGGAAAC","chr15",82689180,"+","none","AGG","TCTTCACCACCGCGGGAAACAGG",0,NA,NA,NA,"GeneID:283692;GeneID:8120","LOC283692;AP3B2",TRUE,"AP3B2"

"9129","ACCCACCTTACGGATCTGAT","chr19",2137004,"+","none","TGG","ACCCACCTTACGGATCTGATTGG",0,NA,NA,NA,"GeneID:8943","AP3D1",TRUE,"AP3D1"

"9130","AGCTTTGTGTTCAGAAATTA","chr19",2123388,"-","none","AGG","AGCTTTGTGTTCAGAAATTAAGG",0,NA,NA,NA,"GeneID:8943","AP3D1",TRUE,"AP3D1"

"9131","AGCTTTGTGTTCAGAAATTA","chr2",128769619,"+","11:A>C,15:C>A","AGG","AGCTTTGTGTTAAGACATTAAGG",2,12,16,NA,"","",FALSE,"AP3D1"

"9132","AGCTTTGTGTTCAGAAATTA","chr18",13015713,"+","3:A>T,11:T>C","GGG","AGCATTGTGTTTAGAAATTAGGG",2,4,12,NA,"GeneID:55125","CEP192",FALSE,"AP3D1"

"9133","CCAGTTCTCACCTGCAATCA","chr19",2127140,"+","none","CGG","CCAGTTCTCACCTGCAATCACGG",0,NA,NA,NA,"GeneID:8943","AP3D1",TRUE,"AP3D1"

"9134","CCAGTTCTCACCTGCAATCA","chr15",29284600,"+","0:A>C,3:T>G","TGG","ACATTTCTCACCTGCAATCATGG",2,1,4,NA,"GeneID:23359","FAM189A1",FALSE,"AP3D1"

"9135","CGGTGCCTTCGTGAAAGCTC","chr19",2137045,"+","none","TGG","CGGTGCCTTCGTGAAAGCTCTGG",0,NA,NA,NA,"GeneID:8943","AP3D1",TRUE,"AP3D1"

"9136","GGATGACTCACAGTTCTGAT","chr19",2123346,"+","none","CGG","GGATGACTCACAGTTCTGATCGG",0,NA,NA,NA,"GeneID:8943","AP3D1",TRUE,"AP3D1"

"9137","TGTGTGAACACCGTGATTGC","chr19",2127153,"-","none","AGG","TGTGTGAACACCGTGATTGCAGG",0,NA,NA,NA,"GeneID:8943","AP3D1",TRUE,"AP3D1"

"9138","ATGAGTCGGAAATTTCCATC","chr10",74129131,"+","none","TGG","ATGAGTCGGAAATTTCCATCTGG",0,NA,NA,NA,"GeneID:26985","AP3M1",TRUE,"AP3M1"

"9139","ATGAGTCGGAAATTTCCATC","chr7",121557704,"-","7:T>C,18:G>A","AGG","ATGAGTCAGAAATTTCCACCAGG",2,8,19,NA,"","",FALSE,"AP3M1"

"9140","CAGATTCCCAACGCTTGAAC","chr10",74129177,"+","none","CGG","CAGATTCCCAACGCTTGAACCGG",0,NA,NA,NA,"GeneID:26985","AP3M1",TRUE,"AP3M1"

"9141","GATGCTTGCATTAAACTATC","chr10",74129937,"-","none","TGG","GATGCTTGCATTAAACTATCTGG",0,NA,NA,NA,"GeneID:26985","AP3M1",TRUE,"AP3M1"

"9142","TATGTTGGACAGCTGCCCGG","chr10",74134114,"+","none","TGG","TATGTTGGACAGCTGCCCGGTGG",0,NA,NA,NA,"GeneID:26985","AP3M1",TRUE,"AP3M1"

"9143","TCTGTTGTCAACTCTATTAC","chr10",74136633,"-","none","AGG","TCTGTTGTCAACTCTATTACAGG",0,NA,NA,NA,"GeneID:26985","AP3M1",TRUE,"AP3M1"

"9144","TCTGTTGTCAACTCTATTAC","chrX",143281813,"-","3:A>C,12:T>A","AGG","TCTTTTGTCAACACTATTACAGG",2,4,13,NA,"","",FALSE,"AP3M1"

"9145","TTTACCCCTGCCCGACGCCA","chr10",74134091,"+","none","TGG","TTTACCCCTGCCCGACGCCATGG",0,NA,NA,NA,"GeneID:26985","AP3M1",TRUE,"AP3M1"

"9146","CAGCGTTTGAAACGAACACA","chr8",42165462,"-","none","AGG","CAGCGTTTGAAACGAACACAAGG",0,NA,NA,NA,"GeneID:10947","AP3M2",TRUE,"AP3M2"

"9147","CTTGTGTTCGTTTCAAACGC","chr8",42165460,"+","none","TGG","CTTGTGTTCGTTTCAAACGCTGG",0,NA,NA,NA,"GeneID:10947","AP3M2",TRUE,"AP3M2"

"9148","GATGCCTGTGTCAAGCTGAC","chr8",42165105,"+","none","TGG","GATGCCTGTGTCAAGCTGACTGG",0,NA,NA,NA,"GeneID:10947","AP3M2",TRUE,"AP3M2"

"9149","GATGCCTGTGTCAAGCTGAC","chr5",56532716,"-","10:T>A,17:G>C","TGG","GATGCCTGTGACAAGCTCACTGG",2,11,18,NA,"GeneID:101928448","LOC101928448",FALSE,"AP3M2"

"9150","TGCCTCAGGAAGCACGAATG","chr8",42162272,"+","none","TGG","TGCCTCAGGAAGCACGAATGTGG",0,NA,NA,NA,"GeneID:10947","AP3M2",TRUE,"AP3M2"

"9151","TGGTGTTGACAACCGTTCGA","chr8",42158086,"-","none","AGG","TGGTGTTGACAACCGTTCGAAGG",0,NA,NA,NA,"GeneID:10947","AP3M2",TRUE,"AP3M2"

"9152","TGGTGTTGACAACCGTTCGA","chr9",68789222,"+","13:T>C","AGG","TGGTGTTGACAACTGTTCGAAGG",1,14,NA,NA,"GeneID:8395","PIP5K1B",TRUE,"AP3M2"

"9153","TTCACCCCAGTCCGTCGCCA","chr8",42162333,"-","none","AGG","TTCACCCCAGTCCGTCGCCAAGG",0,NA,NA,NA,"GeneID:10947","AP3M2",TRUE,"AP3M2"

"9154","TTCACCCCAGTCCGTCGCCA","chr9",68789143,"+","11:A>C,13:C>G","AGG","TTCACCCCAGTACCTCGCCAAGG",2,12,14,NA,"GeneID:8395","PIP5K1B",TRUE,"AP3M2"

"9155","AATTGATGCACAAAATAAGC","chr5",115902958,"+","none","TGG","AATTGATGCACAAAATAAGCTGG",0,NA,NA,NA,"GeneID:1176","AP3S1",TRUE,"AP3S1"

"9156","AATTGATGCACAAAATAAGC","chr1",214482949,"-","14:C>T","TGG","AATTGATGCACAAAGTAAGCTGG",1,15,NA,NA,"GeneID:5784","PTPN14",TRUE,"AP3S1"

"9157","AATTGATGCACAAAATAAGC","chr12",12452250,"-","5:C>T","TGG","AATTGGTGCACAAAATAAGCTGG",1,6,NA,NA,"GeneID:118426","LOH12CR1",TRUE,"AP3S1"

"9158","ATACACAACAGCAAATCATC","chr5",115866676,"+","none","AGG","ATACACAACAGCAAATCATCAGG",0,NA,NA,NA,"GeneID:1176","AP3S1",TRUE,"AP3S1"

"9159","ATACACAACAGCAAATCATC","chr1",214483291,"-","none","AGG","ATACACAACAGCAAATCATCAGG",0,NA,NA,NA,"GeneID:5784","PTPN14",TRUE,"AP3S1"

"9160","ATACACAACAGCAAATCATC","chr6",24751531,"-","1:G>A","AGG","ACACACAACAGCAAATCATCAGG",1,2,NA,NA,"","",TRUE,"AP3S1"

"9161","ATACACAACAGCAAATCATC","chr3",32786539,"-","3:A>G","AGG","ATATACAACAGCAAATCATCAGG",1,4,NA,NA,"","",FALSE,"AP3S1"

"9162","CAAATAGTACCTTGTCTACA","chr5",115895148,"-","none","TGG","CAAATAGTACCTTGTCTACATGG",0,NA,NA,NA,"GeneID:1176","AP3S1",TRUE,"AP3S1"

"9163","CAAATAGTACCTTGTCTACA","chr9",29011238,"+","14:A>T,15:G>C","CGG","CAAATAGTACCTTGAGTACACGG",2,15,16,NA,"","",FALSE,"AP3S1"

"9164","CAAATAGTACCTTGTCTACA","chrX",101621418,"-","3:C>T,9:C>G","GGG","CAAGTAGTAGCTTGTCTACAGGG",2,4,10,NA,"","",FALSE,"AP3S1"

"9165","TCACAATATTCTTGCAGAAA","chr5",115902886,"+","none","TGG","TCACAATATTCTTGCAGAAATGG",0,NA,NA,NA,"GeneID:1176","AP3S1",TRUE,"AP3S1"

"9166","TCACAATATTCTTGCAGAAA","chr2",238152888,"-","1:A>G,3:T>G","TGG","TTAAAATATTCTTGCAGAAATGG",2,2,4,NA,"GeneID:377007","KLHL30",TRUE,"AP3S1"

"9167","TTACTTACAATCCTCCTTCT","chr5",115866749,"-","none","AGG","TTACTTACAATCCTCCTTCTAGG",0,NA,NA,NA,"GeneID:1176","AP3S1",TRUE,"AP3S1"

"9168","TTACTTACAATCCTCCTTCT","chr2",149438136,"-","4:T>A,5:T>A","GGG","TTACAAACAATCCTCCTTCTGGG",2,5,6,NA,"GeneID:130574","LYPD6",FALSE,"AP3S1"

"9169","TTGTCATTTAGATTAATTGG","chr5",115870005,"+","none","AGG","TTGTCATTTAGATTAATTGGAGG",0,NA,NA,NA,"GeneID:1176","AP3S1",TRUE,"AP3S1"

"9170","TTGTCATTTAGATTAATTGG","chr2",218174670,"+","12:A>T,14:T>A","TGG","TTGTCATTTAGAATTATTGGTGG",2,13,15,NA,"","",FALSE,"AP3S1"

"9171","TTGTCATTTAGATTAATTGG","chr16",48670828,"+","7:G>T,10:A>G","AGG","TTGTCATGTAAATTAATTGGAGG",2,8,11,NA,"","",FALSE,"AP3S1"

"9172","TTGTCATTTAGATTAATTGG","chr5",166258989,"-","3:T>A,10:G>C","AGG","TTGACATTTACATTAATTGGAGG",2,4,11,NA,"","",FALSE,"AP3S1"

"9173","AGATGTTGTCATCCCGCTTG","chr15",89889070,"+","none","AGG","AGATGTTGTCATCCCGCTTGAGG",0,NA,NA,NA,"GeneID:10239;GeneID:100526783","AP3S2;C15orf38-AP3S2",TRUE,"AP3S2"

"9174","ATCTTCTGCAGTTTGATTGG","chr15",89888623,"-","none","TGG","ATCTTCTGCAGTTTGATTGGTGG",0,NA,NA,NA,"GeneID:10239;GeneID:100526783","AP3S2;C15orf38-AP3S2",TRUE,"AP3S2"

"9175","CTCAGATTGAGGCTCAAAAC","chr15",89837632,"-","none","AGG","CTCAGATTGAGGCTCAAAACAGG",0,NA,NA,NA,"GeneID:10239;GeneID:100526783","AP3S2;C15orf38-AP3S2",TRUE,"AP3S2"

"9176","CTTTCCATCTAGTCCTCAAG","chr15",89889086,"-","none","CGG","CTTTCCATCTAGTCCTCAAGCGG",0,NA,NA,NA,"GeneID:10239;GeneID:100526783","AP3S2;C15orf38-AP3S2",TRUE,"AP3S2"

"9177","GAGAATATACCTTATCCATA","chr15",89871464,"+","none","TGG","GAGAATATACCTTATCCATATGG",0,NA,NA,NA,"GeneID:10239;GeneID:100526783","AP3S2;C15orf38-AP3S2",TRUE,"AP3S2"

"9178","TTAGGTGCACTACATCCTCC","chr15",89837706,"-","none","AGG","TTAGGTGCACTACATCCTCCAGG",0,NA,NA,NA,"GeneID:10239;GeneID:100526783","AP3S2;C15orf38-AP3S2",TRUE,"AP3S2"

"9179","AACGCCAGCCCTCGCACCAT","chr1",113902658,"+","none","TGG","AACGCCAGCCCTCGCACCATTGG",0,NA,NA,NA,"GeneID:10717","AP4B1",TRUE,"AP4B1"

"9180","ACTTCATCCTTTGCCGCATC","chr1",113895758,"-","none","AGG","ACTTCATCCTTTGCCGCATCAGG",0,NA,NA,NA,"GeneID:100287722;GeneID:10717","AP4B1-AS1;AP4B1",TRUE,"AP4B1"

"9181","ATACAGTTTGCTGCGTGACC","chr1",113901341,"-","none","AGG","ATACAGTTTGCTGCGTGACCAGG",0,NA,NA,NA,"GeneID:10717","AP4B1",TRUE,"AP4B1"

"9182","CCTAAATCTGACCCTACTCT","chr1",113895923,"-","none","TGG","CCTAAATCTGACCCTACTCTTGG",0,NA,NA,NA,"GeneID:100287722;GeneID:10717","AP4B1-AS1;AP4B1",TRUE,"AP4B1"

"9183","GGGATAAGGCTTCATATGTC","chr1",113901815,"-","none","AGG","GGGATAAGGCTTCATATGTCAGG",0,NA,NA,NA,"GeneID:10717","AP4B1",TRUE,"AP4B1"

"9184","GTCTTTGCACAGCGTATTGA","chr1",113902696,"+","none","TGG","GTCTTTGCACAGCGTATTGATGG",0,NA,NA,NA,"GeneID:10717","AP4B1",TRUE,"AP4B1"

"9185","AAACCTACCAGTGTTGTAGT","chr15",50912137,"-","none","AGG","AAACCTACCAGTGTTGTAGTAGG",0,NA,NA,NA,"GeneID:23431","AP4E1",TRUE,"AP4E1"

"9186","CCTTGTGAATACAGTTGTAA","chr15",50923982,"+","none","AGG","CCTTGTGAATACAGTTGTAAAGG",0,NA,NA,NA,"GeneID:23431","AP4E1",TRUE,"AP4E1"

"9187","CCTTTACAACTGTATTCACA","chr15",50923985,"-","none","AGG","CCTTTACAACTGTATTCACAAGG",0,NA,NA,NA,"GeneID:23431","AP4E1",TRUE,"AP4E1"

"9188","GAAGGACGCCTTGGCGGCCG","chr15",50908854,"-","none","CGG","GAAGGACGCCTTGGCGGCCGCGG",0,NA,NA,NA,"GeneID:23431","AP4E1",TRUE,"AP4E1"

"9189","GAAGGACGCCTTGGCGGCCG","chr16_KI270728v1_random",1462083,"+","6:G>C,18:A>C","TGG","GAAGGAGGCCTTGGCGGCAGTGG",2,7,19,NA,"","",FALSE,"AP4E1"

"9190","GAAGGACGCCTTGGCGGCCG","chr16",33613622,"+","6:G>C,18:A>C","TGG","GAAGGAGGCCTTGGCGGCAGTGG",2,7,19,NA,"","",FALSE,"AP4E1"

"9191","GGCGGCGATGAGCGACATAG","chr15",50908771,"+","none","TGG","GGCGGCGATGAGCGACATAGTGG",0,NA,NA,NA,"GeneID:23431","AP4E1",TRUE,"AP4E1"

"9192","TCAGACTACTCAGTTCCTGC","chr15",50912097,"-","none","TGG","TCAGACTACTCAGTTCCTGCTGG",0,NA,NA,NA,"GeneID:23431","AP4E1",TRUE,"AP4E1"

"9193","CCAAAAAAACTTCATTCTTT","chr7",100104096,"-","none","TGG","CCAAAAAAACTTCATTCTTTTGG",0,NA,NA,NA,"GeneID:9179","AP4M1",TRUE,"AP4M1"

"9194","CCAAAAAAACTTCATTCTTT","chr8",71696006,"-","10:T>A,16:A>G","GGG","CCAAAAAAACATCATTTTTTGGG",2,11,17,NA,"","",TRUE,"AP4M1"

"9195","CCAAAAAAACTTCATTCTTT","chr14",65627144,"+","1:A>C,9:G>C","TGG","CAAAAAAAAGTTCATTCTTTTGG",2,2,10,NA,"GeneID:2530","FUT8",FALSE,"AP4M1"

"9196","CCTCACCTGGTCAGAGCGAC","chr7",100103678,"-","none","TGG","CCTCACCTGGTCAGAGCGACTGG",0,NA,NA,NA,"GeneID:9179","AP4M1",TRUE,"AP4M1"

"9197","CCTGTCCAGTCGCTCTGACC","chr7",100103670,"+","none","AGG","CCTGTCCAGTCGCTCTGACCAGG",0,NA,NA,NA,"GeneID:9179","AP4M1",TRUE,"AP4M1"

"9198","CTCTTTGACCTCAGCAGCGT","chr7",100103492,"+","none","TGG","CTCTTTGACCTCAGCAGCGTTGG",0,NA,NA,NA,"GeneID:9179","AP4M1",TRUE,"AP4M1"

"9199","GAAATTCCTCAGCATCTCCG","chr7",100103436,"-","none","TGG","GAAATTCCTCAGCATCTCCGTGG",0,NA,NA,NA,"GeneID:9179","AP4M1",TRUE,"AP4M1"

"9200","TGCTGACCACAGCTTCCGTC","chr7",100103461,"-","none","TGG","TGCTGACCACAGCTTCCGTCTGG",0,NA,NA,NA,"GeneID:9179","AP4M1",TRUE,"AP4M1"

"9201","ACCGTAGATAATGTTTAATT","chr14",31092899,"+","none","TGG","ACCGTAGATAATGTTTAATTTGG",0,NA,NA,NA,"GeneID:11154","AP4S1",TRUE,"AP4S1"

"9202","AGATGAGTATTTCAGCCGAG","chr14",31072951,"+","none","TGG","AGATGAGTATTTCAGCCGAGTGG",0,NA,NA,NA,"GeneID:11154","AP4S1",TRUE,"AP4S1"

"9203","AGATGAGTATTTCAGCCGAG","chr9",105331379,"-","16:A>G","AGG","AGATGAGTATTTCAGCTGAGAGG",1,17,NA,NA,"GeneID:23446","SLC44A1",FALSE,"AP4S1"

"9204","GCTGCATTGTGGAAACTAAC","chr14",31092961,"+","none","AGG","GCTGCATTGTGGAAACTAACAGG",0,NA,NA,NA,"GeneID:11154","AP4S1",TRUE,"AP4S1"

"9205","GCTGCATTGTGGAAACTAAC","chr6",89059990,"+","3:A>G","AGG","GCTACATTGTGGAAACTAACAGG",1,4,NA,NA,"","",TRUE,"AP4S1"

"9206","GTATGCAGCTCTCTTCATTG","chr14",31069886,"+","none","TGG","GTATGCAGCTCTCTTCATTGTGG",0,NA,NA,NA,"GeneID:11154","AP4S1",TRUE,"AP4S1"

"9207","GTATGCAGCTCTCTTCATTG","chr6",89059811,"+","7:T>G,16:G>A","TGG","GTATGCATCTCTCTTCGTTGTGG",2,8,17,NA,"","",TRUE,"AP4S1"

"9208","GTGCTCTTTCATTGAATATA","chr14",31069841,"+","none","AGG","GTGCTCTTTCATTGAATATAAGG",0,NA,NA,NA,"GeneID:11154","AP4S1",TRUE,"AP4S1"

"9209","TATTAATAAGCGTACACTTC","chr14",31066267,"+","none","TGG","TATTAATAAGCGTACACTTCTGG",0,NA,NA,NA,"GeneID:11154","AP4S1",TRUE,"AP4S1"

"9210","TATTAATAAGCGTACACTTC","chr6",89059698,"+","11:A>G","TGG","TATTAATAAGCATACACTTCTGG",1,12,NA,NA,"","",TRUE,"AP4S1"

"9211","AGGTCCCGAGGGGGAGGATT","chr11",65780497,"-","none","TGG","AGGTCCCGAGGGGGAGGATTTGG",0,NA,NA,NA,"GeneID:91056","AP5B1",TRUE,"AP5B1"

"9212","CCCGGAAGGCCCCCAAGCGC","chr11",65780539,"+","none","TGG","CCCGGAAGGCCCCCAAGCGCTGG",0,NA,NA,NA,"GeneID:91056","AP5B1",TRUE,"AP5B1"

"9213","CGGGCGTGGTCACGCCCATC","chr11",65778654,"+","none","AGG","CGGGCGTGGTCACGCCCATCAGG",0,NA,NA,NA,"GeneID:91056","AP5B1",TRUE,"AP5B1"

"9214","GCACGCCATGCTTGCGCTCA","chr11",65779501,"-","none","AGG","GCACGCCATGCTTGCGCTCAAGG",0,NA,NA,NA,"GeneID:91056","AP5B1",TRUE,"AP5B1"

"9215","GGTCGGCAAAGTTCACGAAC","chr11",65778196,"+","none","AGG","GGTCGGCAAAGTTCACGAACAGG",0,NA,NA,NA,"GeneID:91056","AP5B1",TRUE,"AP5B1"

"9216","TATCCGTGAGCAGTCCCCCC","chr11",65779852,"+","none","AGG","TATCCGTGAGCAGTCCCCCCAGG",0,NA,NA,NA,"GeneID:91056","AP5B1",TRUE,"AP5B1"

"9217","AGAGTTCTTCACCTCCTATC","chr14",57274429,"-","none","AGG","AGAGTTCTTCACCTCCTATCAGG",0,NA,NA,NA,"GeneID:55745","AP5M1",TRUE,"AP5M1"

"9218","AGAGTTCTTCACCTCCTATC","chr3",69378373,"+","4:C>T,14:T>C","AGG","AGAGCTCTTCACCTTCTATCAGG",2,5,15,NA,"GeneID:23150","FRMD4B",FALSE,"AP5M1"

"9219","AGCTTGAGTCTCCCCACCAA","chr14",57280230,"+","none","TGG","AGCTTGAGTCTCCCCACCAATGG",0,NA,NA,NA,"GeneID:55745","AP5M1",TRUE,"AP5M1"

"9220","AGCTTGAGTCTCCCCACCAA","chr2",121912379,"-","12:A>G,14:T>G","GGG","AGCTTGAGTCTCTCAACCAAGGG",2,13,15,NA,"","",FALSE,"AP5M1"

"9221","CCGCCACTAATTAGTGTCAG","chr14",57274526,"+","none","TGG","CCGCCACTAATTAGTGTCAGTGG",0,NA,NA,NA,"GeneID:55745","AP5M1",TRUE,"AP5M1"

"9222","GCTGTTTCTCCAGTACAAAT","chr14",57283200,"-","none","TGG","GCTGTTTCTCCAGTACAAATTGG",0,NA,NA,NA,"GeneID:55745","AP5M1",TRUE,"AP5M1"

"9223","TCTCTCGAAATACTTCAAGC","chr14",57282975,"-","none","TGG","TCTCTCGAAATACTTCAAGCTGG",0,NA,NA,NA,"GeneID:55745","AP5M1",TRUE,"AP5M1"

"9224","TTCTAGTAGTATTGATGCAA","chr14",57280316,"+","none","TGG","TTCTAGTAGTATTGATGCAATGG",0,NA,NA,NA,"GeneID:55745","AP5M1",TRUE,"AP5M1"

"9225","AGGCCCGTGTCCTCAGTATT","chr20",3822156,"-","none","CGG","AGGCCCGTGTCCTCAGTATTCGG",0,NA,NA,NA,"GeneID:55317","AP5S1",TRUE,"AP5S1"

"9226","CCCACGTGGGGCTTTCCGCC","chr20",3823972,"+","none","TGG","CCCACGTGGGGCTTTCCGCCTGG",0,NA,NA,NA,"GeneID:55317","AP5S1",TRUE,"AP5S1"

"9227","CCCACGTGGGGCTTTCCGCC","chr15",74388042,"+","10:C>G,19:T>C","GGG","CCCACGTGGGCCTTTCCGCTGGG",2,11,20,NA,"","",FALSE,"AP5S1"

"9228","CTCAACGACCAGTTTGTCCA","chr20",3824237,"+","none","AGG","CTCAACGACCAGTTTGTCCAAGG",0,NA,NA,NA,"GeneID:55317","AP5S1",TRUE,"AP5S1"

"9229","CTCAACGACCAGTTTGTCCA","chr5",61253092,"-","2:A>G,5:C>G","TGG","CTTAAGGACCAGTTTGTCCATGG",2,3,6,NA,"","",FALSE,"AP5S1"

"9230","CTTCTCAGCACCGAAGACGC","chr20",3822196,"-","none","AGG","CTTCTCAGCACCGAAGACGCAGG",0,NA,NA,NA,"GeneID:55317","AP5S1",TRUE,"AP5S1"

"9231","CTTCTCAGCACCGAAGACGC","chr9",124777493,"+","11:T>C,16:C>A","AGG","CTTCTCAGCACTGAAGCCGCAGG",2,12,17,NA,"GeneID:169611","OLFML2A",FALSE,"AP5S1"

"9232","TGAGCAAGTGCCGCTGCACG","chr20",3823948,"+","none","AGG","TGAGCAAGTGCCGCTGCACGAGG",0,NA,NA,NA,"GeneID:55317","AP5S1",TRUE,"AP5S1"

"9233","TGGCTGGGCGTGCTCTCGTT","chr20",3824033,"+","none","AGG","TGGCTGGGCGTGCTCTCGTTAGG",0,NA,NA,NA,"GeneID:55317","AP5S1",TRUE,"AP5S1"

"9234","CAGAGGGGGACATCTCTCGC","chr7",4781657,"-","none","AGG","CAGAGGGGGACATCTCTCGCAGG",0,NA,NA,NA,"GeneID:9907","AP5Z1",TRUE,"AP5Z1"

"9235","CAGAGGGGGACATCTCTCGC","chr5",176187441,"+","14:A>T,18:C>G","GGG","CAGAGGGGGACATCACTCCCGGG",2,15,19,NA,"","",TRUE,"AP5Z1"

"9236","CCAGCGCTCGTAGCACGCCC","chr7",4783350,"-","none","TGG","CCAGCGCTCGTAGCACGCCCTGG",0,NA,NA,NA,"GeneID:9907","AP5Z1",TRUE,"AP5Z1"

"9237","CCAGGGCGTGCTACGAGCGC","chr7",4783347,"+","none","TGG","CCAGGGCGTGCTACGAGCGCTGG",0,NA,NA,NA,"GeneID:9907","AP5Z1",TRUE,"AP5Z1"

"9238","GAGAAGAAGCCGCCGGAGTG","chr7",4783759,"-","none","TGG","GAGAAGAAGCCGCCGGAGTGTGG",0,NA,NA,NA,"GeneID:9907","AP5Z1",TRUE,"AP5Z1"

"9239","GTTTAGGGAGATCCAGGACG","chr7",4781168,"+","none","AGG","GTTTAGGGAGATCCAGGACGAGG",0,NA,NA,NA,"GeneID:9907","AP5Z1",TRUE,"AP5Z1"

"9240","TCACCTCCGGCTGTACTTCG","chr7",4781296,"-","none","TGG","TCACCTCCGGCTGTACTTCGTGG",0,NA,NA,NA,"GeneID:9907","AP5Z1",TRUE,"AP5Z1"

"9241","ACAGCCTGCCATTCCATGTA","chr12",98649612,"-","none","TGG","ACAGCCTGCCATTCCATGTATGG",0,NA,NA,NA,"GeneID:317","APAF1",TRUE,"APAF1"

"9242","AGCATTGTAGAATGATACGT","chr12",98648689,"-","none","AGG","AGCATTGTAGAATGATACGTAGG",0,NA,NA,NA,"GeneID:317","APAF1",TRUE,"APAF1"

"9243","CCAGGATGGGTCACCATACA","chr12",98649596,"+","none","TGG","CCAGGATGGGTCACCATACATGG",0,NA,NA,NA,"GeneID:317","APAF1",TRUE,"APAF1"

"9244","GAAACCCAATGCACTCCCCC","chr12",98659170,"-","none","TGG","GAAACCCAATGCACTCCCCCTGG",0,NA,NA,NA,"GeneID:317","APAF1",TRUE,"APAF1"

"9245","GTTTGCGAAGCATCAGAATG","chr12",98659316,"-","none","CGG","GTTTGCGAAGCATCAGAATGCGG",0,NA,NA,NA,"GeneID:317","APAF1",TRUE,"APAF1"

"9246","TACAATGCTCTACTACATGA","chr12",98648700,"+","none","AGG","TACAATGCTCTACTACATGAAGG",0,NA,NA,NA,"GeneID:317","APAF1",TRUE,"APAF1"

"9247","AGGCCGCCGTAGTCGGCATA","chr9",69516632,"+","none","GGG","AGGCCGCCGTAGTCGGCATAGGG",0,NA,NA,NA,"GeneID:320","APBA1",TRUE,"APBA1"

"9248","CGTCGGAGCGCTCGTCGTAA","chr9",69516480,"+","none","TGG","CGTCGGAGCGCTCGTCGTAATGG",0,NA,NA,NA,"GeneID:320","APBA1",TRUE,"APBA1"

"9249","GCACCCTACCTCTTTATTAG","chr9",69476038,"+","none","TGG","GCACCCTACCTCTTTATTAGTGG",0,NA,NA,NA,"GeneID:320","APBA1",TRUE,"APBA1"

"9250","GTGTTCTAGTCTCCGTCTCC","chr9",69476132,"-","none","TGG","GTGTTCTAGTCTCCGTCTCCTGG",0,NA,NA,NA,"GeneID:320","APBA1",TRUE,"APBA1"

"9251","GTGTTCTAGTCTCCGTCTCC","chr4",56085142,"-","13:A>G,17:T>A","TGG","GTGTTCTAGTCTCTGTCACCTGG",2,14,18,NA,"GeneID:57482","KIAA1211",FALSE,"APBA1"

"9252","TCAGCTCTTACCTTCAACGT","chr9",69471644,"+","none","AGG","TCAGCTCTTACCTTCAACGTAGG",0,NA,NA,NA,"GeneID:320","APBA1",TRUE,"APBA1"

"9253","TCAGCTCTTACCTTCAACGT","chr6",47427846,"+","5:C>T,18:A>G","TGG","TCAGCCCTTACCTTCAACATTGG",2,6,19,NA,"","",FALSE,"APBA1"

"9254","TCAGCTCTTACCTTCAACGT","chr13",64551532,"-","3:A>C,18:A>C","AGG","TCATCTCTTACCTTCAACTTAGG",2,4,19,NA,"","",FALSE,"APBA1"

"9255","TCATTCCCAACCTACGTTGA","chr9",69471657,"-","none","AGG","TCATTCCCAACCTACGTTGAAGG",0,NA,NA,NA,"GeneID:320","APBA1",TRUE,"APBA1"

"9256","CAAAGATGATCCCGTCGATG","chr15",29093102,"-","none","AGG","CAAAGATGATCCCGTCGATGAGG",0,NA,NA,NA,"GeneID:321","APBA2",TRUE,"APBA2"

"9257","CAGCAGCGCTCTGATCTCAA","chr15",29074950,"+","none","TGG","CAGCAGCGCTCTGATCTCAATGG",0,NA,NA,NA,"GeneID:321","APBA2",TRUE,"APBA2"

"9258","CAGCAGCGCTCTGATCTCAA","chr9",131276125,"+","3:G>C,18:C>A","GGG","CAGGAGCGCTCTGATCTCCAGGG",2,4,19,NA,"GeneID:286336","FAM78A",TRUE,"APBA2"

"9259","CATCAGAATGATGCAAGCGC","chr15",29093177,"+","none","AGG","CATCAGAATGATGCAAGCGCAGG",0,NA,NA,NA,"GeneID:321","APBA2",TRUE,"APBA2"

"9260","GAACGACTGCCATGGCCCAC","chr15",29053873,"+","none","CGG","GAACGACTGCCATGGCCCACCGG",0,NA,NA,NA,"GeneID:321","APBA2",TRUE,"APBA2"

"9261","GGAGGCGCCGGTGTTCCCGT","chr15",29054488,"-","none","TGG","GGAGGCGCCGGTGTTCCCGTTGG",0,NA,NA,NA,"GeneID:321","APBA2",TRUE,"APBA2"

"9262","TTTCCAGGTTTGCAATGGTC","chr15",29074913,"+","none","TGG","TTTCCAGGTTTGCAATGGTCTGG",0,NA,NA,NA,"GeneID:321","APBA2",TRUE,"APBA2"

"9263","TTTCCAGGTTTGCAATGGTC","chr3",44802929,"-","4:A>G,7:A>C","TGG","TTTCTAGTTTTGCAATGGTCTGG",2,5,8,NA,"GeneID:56992","KIF15",TRUE,"APBA2"

"9264","CATCAAGTTCCATCCGACTG","chr19",3760104,"+","none","AGG","CATCAAGTTCCATCCGACTGAGG",0,NA,NA,NA,"GeneID:9546","APBA3",TRUE,"APBA3"

"9265","CCTCACCTTGACGCGGTCCA","chr19",3754188,"+","none","TGG","CCTCACCTTGACGCGGTCCATGG",0,NA,NA,NA,"GeneID:9546","APBA3",TRUE,"APBA3"

"9266","CTTCCAGAGCCCCGAGACCC","chr19",3759587,"-","none","TGG","CTTCCAGAGCCCCGAGACCCTGG",0,NA,NA,NA,"GeneID:9546","APBA3",TRUE,"APBA3"

"9267","CTTCCAGAGCCCCGAGACCC","chr2",120867966,"-","13:G>C,16:A>T","TGG","CTTCCAGAGCCCCCAGTCCCTGG",2,14,17,NA,"GeneID:2736","GLI2",FALSE,"APBA3"

"9268","CTTCCAGAGCCCCGAGACCC","chr1",9425588,"+","11:T>C,12:G>C","TGG","CTTCCAGAGCCTGGAGACCCTGG",2,12,13,NA,"GeneID:100506022","LOC100506022",TRUE,"APBA3"

"9269","CTTCCAGAGCCCCGAGACCC","chr9",135029372,"+","2:G>T,18:A>C","TGG","CTGCCAGAGCCCCGAGACACTGG",2,3,19,NA,"","",FALSE,"APBA3"

"9270","GGCATTCCTCGCAGTGCAAG","chr19",3759900,"+","none","AGG","GGCATTCCTCGCAGTGCAAGAGG",0,NA,NA,NA,"GeneID:9546","APBA3",TRUE,"APBA3"

"9271","GGGACACTCACCCTCCTGGG","chr19",3759549,"+","none","GGG","GGGACACTCACCCTCCTGGGGGG",0,NA,NA,NA,"GeneID:9546","APBA3",TRUE,"APBA3"

"9272","GGGACACTCACCCTCCTGGG","chr1",220756804,"+","12:T>C,19:A>G","TGG","GGGACACTCACCTTCCTGGATGG",2,13,20,NA,"GeneID:54996","MARC2",FALSE,"APBA3"

"9273","GGGACACTCACCCTCCTGGG","chr10",70622665,"+","0:A>G,4:G>C","TGG","AGGAGACTCACCCTCCTGGGTGG",2,1,5,NA,"","",FALSE,"APBA3"

"9274","GGTTCCGTTCCGACACCAGC","chr19",3754247,"+","none","TGG","GGTTCCGTTCCGACACCAGCTGG",0,NA,NA,NA,"GeneID:9546","APBA3",TRUE,"APBA3"

"9275","GGTTCCGTTCCGACACCAGC","chr8",66429247,"-","7:C>A,14:C>T","AGG","GGTTCCGGTCCGACGCCAGCAGG",2,8,15,NA,"GeneID:23212","RRS1",TRUE,"APBA3"

"9276","CCCAGAGCCGTTGCCCCAAG","chr11",6403188,"-","none","AGG","CCCAGAGCCGTTGCCCCAAGAGG",0,NA,NA,NA,"GeneID:322","APBB1",TRUE,"APBB1"

"9277","CCCAGCGCCTAAGAATGAGT","chr11",6401045,"-","none","TGG","CCCAGCGCCTAAGAATGAGTTGG",0,NA,NA,NA,"GeneID:322","APBB1",TRUE,"APBB1"

"9278","CCCCCACGGAATACCAACCC","chr11",6403154,"-","none","AGG","CCCCCACGGAATACCAACCCAGG",0,NA,NA,NA,"GeneID:322","APBB1",TRUE,"APBB1"

"9279","GAAGTTCCAAGTCTATTACC","chr11",6401018,"-","none","TGG","GAAGTTCCAAGTCTATTACCTGG",0,NA,NA,NA,"GeneID:322","APBB1",TRUE,"APBB1"

"9280","GAAGTTCCAAGTCTATTACC","chr12",11016031,"-","13:G>A,17:A>T","TGG","GAAGTTCCAAGTCCATTTCCTGG",2,14,18,NA,"GeneID:100533464;GeneID:50840","PRH1-PRR4;TAS2R14",FALSE,"APBB1"

"9281","GCGTCGGGCGGGACAGTGGA","chr11",6402083,"-","none","AGG","GCGTCGGGCGGGACAGTGGAAGG",0,NA,NA,NA,"GeneID:322","APBB1",TRUE,"APBB1"

"9282","GCGTCGGGCGGGACAGTGGA","chr6",5084526,"+","16:G>T,19:G>A","AGG","GCGTCGGGCGGGACAGGGGGAGG",2,17,20,NA,"","",FALSE,"APBB1"

"9283","TTTCCTGGCCATTATCCTGC","chr11",6404759,"-","none","AGG","TTTCCTGGCCATTATCCTGCAGG",0,NA,NA,NA,"GeneID:322","APBB1",TRUE,"APBB1"

"9284","TTTCCTGGCCATTATCCTGC","chr3",50586299,"-","7:T>C,13:A>T","AGG","TTTCCTGACCATTTTCCTGCAGG",2,8,14,NA,"GeneID:51409","HEMK1",TRUE,"APBB1"

"9285","ATCTACCCGGAACTACAAAT","chr10",26511884,"+","none","TGG","ATCTACCCGGAACTACAAATTGG",0,NA,NA,NA,"GeneID:54518","APBB1IP",TRUE,"APBB1IP"

"9286","CAGAGGAAAAAAAGAAAGCA","chr10",26533455,"+","none","AGG","CAGAGGAAAAAAAGAAAGCAAGG",0,NA,NA,NA,"GeneID:54518","APBB1IP",TRUE,"APBB1IP"

"9287","CAGAGGAAAAAAAGAAAGCA","chr6",132428252,"-","16:C>T,18:C>G","AGG","CAGAGGAAAAAAAGAAGGGAAGG",2,17,19,NA,"","",FALSE,"APBB1IP"

"9288","CAGAGGAAAAAAAGAAAGCA","chr13",47842107,"+","19:C>A","AGG","CAGAGGAAAAAAAGAAAGCCAGG",1,20,NA,NA,"GeneID:8803","SUCLA2",FALSE,"APBB1IP"

"9289","CAGAGGAAAAAAAGAAAGCA","chr4",148264130,"+","16:T>A,17:C>G","GGG","CAGAGGAAAAAAAGAATCCAGGG",2,17,18,NA,"GeneID:4306","NR3C2",FALSE,"APBB1IP"

"9290","CAGAGGAAAAAAAGAAAGCA","chrX",31940147,"+","13:T>G,14:G>A","TGG","CAGAGGAAAAAAATGAAGCATGG",2,14,15,NA,"GeneID:1756","DMD",FALSE,"APBB1IP"

"9291","CAGAGGAAAAAAAGAAAGCA","chr6",29607996,"+","11:G>A,15:G>A","AGG","CAGAGGAAAAAGAGAGAGCAAGG",2,12,16,NA,"GeneID:2550","GABBR1",FALSE,"APBB1IP"

"9292","CAGAGGAAAAAAAGAAAGCA","chr8",87465249,"-","0:C>G,5:T>C","GGG","GAGAGAAAAAAAAGAAAGCAGGG",2,1,6,NA,"GeneID:168975","CNBD1",FALSE,"APBB1IP"

"9293","CAGAGGAAAAAAAGAAAGCA","chr2",154598195,"-","0:C>G,5:T>C","AGG","GAGAGAAAAAAAAGAAAGCAAGG",2,1,6,NA,"","",FALSE,"APBB1IP"

"9294","CAGAGGAAAAAAAGAAAGCA","chrX",138665876,"+","0:A>C,5:A>G","GGG","AAGAGAAAAAAAAGAAAGCAGGG",2,1,6,NA,"GeneID:2258","FGF13",FALSE,"APBB1IP"

"9295","CAGAGGAAAAAAAGAAAGCA","chr20",8260967,"+","6:T>A,8:T>A","CGG","CAGAGGTATAAAAGAAAGCACGG",2,7,9,NA,"GeneID:23236","PLCB1",FALSE,"APBB1IP"

"9296","CAGAGGAAAAAAAGAAAGCA","chr9",616807,"+","8:G>A,9:G>A","AGG","CAGAGGAAGGAAAGAAAGCAAGG",2,9,10,NA,"GeneID:23189","KANK1",FALSE,"APBB1IP"

"9297","CAGAGGAAAAAAAGAAAGCA","chr11",111366325,"+","7:G>A,15:C>A","GGG","CAGAGGAGAAAAAGACAGCAGGG",2,8,16,NA,"GeneID:5450","POU2AF1",FALSE,"APBB1IP"

"9298","CAGAGGAAAAAAAGAAAGCA","chr11",73692201,"+","5:C>G,17:T>G","AGG","CAGAGCAAAAAAAGAAATCAAGG",2,6,18,NA,"GeneID:5870","RAB6A",FALSE,"APBB1IP"

"9299","CAGAGGAAAAAAAGAAAGCA","chr8",23330574,"+","9:G>A,17:C>G","AGG","CAGAGGAAAGAAAGAAACCAAGG",2,10,18,NA,"GeneID:4017","LOXL2",FALSE,"APBB1IP"

"9300","CAGAGGAAAAAAAGAAAGCA","chr14",58851206,"+","0:A>C,18:G>C","AGG","AAGAGGAAAAAAAGAAAGGAAGG",2,1,19,NA,"GeneID:102723742","LINC01500",FALSE,"APBB1IP"

"9301","CAGAGGAAAAAAAGAAAGCA","chr20",6562698,"-","9:C>T,17:A>C","AGG","CAGAGGAAAGAAAGAAATCAAGG",2,10,18,NA,"","",FALSE,"APBB1IP"

"9302","CAGAGGAAAAAAAGAAAGCA","chr2",187249023,"-","6:G>T,17:T>C","TGG","CAGAGGCAAAAAAGAAAACATGG",2,7,18,NA,"","",FALSE,"APBB1IP"

"9303","CAGAGGAAAAAAAGAAAGCA","chr4",179358616,"-","4:T>C,13:T>C","AGG","CAGAAGAAAAAAAAAAAGCAAGG",2,5,14,NA,"","",FALSE,"APBB1IP"

"9304","CAGAGGAAAAAAAGAAAGCA","chr20",53670101,"-","4:A>C,12:A>T","GGG","CAGATGAAAAAATGAAAGCAGGG",2,5,13,NA,"","",FALSE,"APBB1IP"

"9305","CAGAGGAAAAAAAGAAAGCA","chrX",2732799,"-","0:C>G,18:C>G","AGG","GAGAGGAAAAAAAGAAAGGAAGG",2,1,19,NA,"GeneID:4267","CD99",FALSE,"APBB1IP"

"9306","CAGAGGAAAAAAAGAAAGCA","chr10",25690057,"-","0:C>G,10:C>T","AGG","GAGAGGAAAAGAAGAAAGCAAGG",2,1,11,NA,"GeneID:101929052","LINC00836",FALSE,"APBB1IP"

"9307","CAGAGGAAAAAAAGAAAGCA","chrX",29098168,"-","0:A>G,18:T>G","AGG","TAGAGGAAAAAAAGAAAGAAAGG",2,1,19,NA,"GeneID:11141","IL1RAPL1",FALSE,"APBB1IP"

"9308","CTATCCTCAGCTCGTCGTCA","chr10",26511736,"+","none","AGG","CTATCCTCAGCTCGTCGTCAAGG",0,NA,NA,NA,"GeneID:54518","APBB1IP",TRUE,"APBB1IP"

"9309","GAAAATGAATGCTAAGAACA","chr10",26533488,"+","none","AGG","GAAAATGAATGCTAAGAACAAGG",0,NA,NA,NA,"GeneID:54518","APBB1IP",TRUE,"APBB1IP"

"9310","GAAAATGAATGCTAAGAACA","chr6",103166479,"+","14:T>A,18:T>C","TGG","GAAAATGAATGCTATGAATATGG",2,15,19,NA,"","",FALSE,"APBB1IP"

"9311","GAAAATGAATGCTAAGAACA","chr6",142075706,"-","5:C>A,6:G>C","TGG","GAAAAGCAATGCTAAGAACATGG",2,6,7,NA,"GeneID:4829","NMBR",TRUE,"APBB1IP"

"9312","GAAAATGAATGCTAAGAACA","chr4",130464731,"+","8:T>A,12:A>T","AGG","GAAAATGATTGCAAAGAACAAGG",2,9,13,NA,"","",FALSE,"APBB1IP"

"9313","TGCTGTATTTAAAAACCCCC","chr10",26513638,"+","none","AGG","TGCTGTATTTAAAAACCCCCAGG",0,NA,NA,NA,"GeneID:54518","APBB1IP",TRUE,"APBB1IP"

"9314","TGCTGTATTTAAAAACCCCC","chr14",51360315,"+","1:T>G,13:T>A","AGG","TTCTGTATTTAAATACCCCCAGG",2,2,14,NA,"GeneID:283553","LINC00640",FALSE,"APBB1IP"

"9315","TTGTTGAAGTCTTATCAGAC","chr10",26513565,"+","none","TGG","TTGTTGAAGTCTTATCAGACTGG",0,NA,NA,NA,"GeneID:54518","APBB1IP",TRUE,"APBB1IP"

"9316","CCCAAATCCCGACTGTGTCT","chr4",40893269,"+","none","CGG","CCCAAATCCCGACTGTGTCTCGG",0,NA,NA,NA,"GeneID:323","APBB2",TRUE,"APBB2"

"9317","CCCCGGTAAAAGTAGTGTTG","chr4",40893338,"-","none","CGG","CCCCGGTAAAAGTAGTGTTGCGG",0,NA,NA,NA,"GeneID:323","APBB2",TRUE,"APBB2"

"9318","CTACAAGAATCATCATCATC","chr4",40934480,"+","none","AGG","CTACAAGAATCATCATCATCAGG",0,NA,NA,NA,"GeneID:323","APBB2",TRUE,"APBB2"

"9319","GCATGCAGCCACTGTTAACC","chr4",40934674,"-","none","CGG","GCATGCAGCCACTGTTAACCCGG",0,NA,NA,NA,"GeneID:323","APBB2",TRUE,"APBB2"

"9320","GCATGCAGCCACTGTTAACC","chr7",75721819,"+","1:T>C,2:G>A","AGG","GTGTGCAGCCACTGTTAACCAGG",2,2,3,NA,"GeneID:3092","HIP1",FALSE,"APBB2"

"9321","GTTTCAAAGATGCATAGCGT","chr4",40934617,"+","none","AGG","GTTTCAAAGATGCATAGCGTAGG",0,NA,NA,NA,"GeneID:323","APBB2",TRUE,"APBB2"

"9322","TCTTTACAAACCTTGGCTTC","chr4",40934444,"+","none","TGG","TCTTTACAAACCTTGGCTTCTGG",0,NA,NA,NA,"GeneID:323","APBB2",TRUE,"APBB2"

"9323","AGTAAGTACCTGCAGCATCG","chr5",140563828,"+","none","TGG","AGTAAGTACCTGCAGCATCGTGG",0,NA,NA,NA,"GeneID:10307","APBB3",TRUE,"APBB3"

"9324","CCACTCTACAGGGAACGGAG","chr5",140563661,"-","none","GGG","CCACTCTACAGGGAACGGAGGGG",0,NA,NA,NA,"GeneID:10307;GeneID:102465499","APBB3;MIR6831",TRUE,"APBB3"

"9325","CTGCCACTGGGTGCTACCGC","chr5",140563793,"+","none","TGG","CTGCCACTGGGTGCTACCGCTGG",0,NA,NA,NA,"GeneID:10307","APBB3",TRUE,"APBB3"

"9326","GATCTTGTCAGAGCGAGTAG","chr5",140561430,"-","none","AGG","GATCTTGTCAGAGCGAGTAGAGG",0,NA,NA,NA,"GeneID:10307","APBB3",TRUE,"APBB3"

"9327","TCCCCATACCTTGCCGTGGC","chr5",140561355,"+","none","AGG","TCCCCATACCTTGCCGTGGCAGG",0,NA,NA,NA,"GeneID:10307","APBB3",TRUE,"APBB3"

"9328","TCCGGTCCAGTGAACTCTCC","chr5",140563594,"+","none","AGG","TCCGGTCCAGTGAACTCTCCAGG",0,NA,NA,NA,"GeneID:10307","APBB3",TRUE,"APBB3"

"9329","AAAATGTCCCTCCGTTCTTA","chr5",112767235,"+","none","TGG","AAAATGTCCCTCCGTTCTTATGG",0,NA,NA,NA,"GeneID:324","APC",TRUE,"APC"

"9330","AGAGTGCAGTCCTGTTCCTA","chr5",112767291,"+","none","TGG","AGAGTGCAGTCCTGTTCCTATGG",0,NA,NA,NA,"GeneID:324","APC",TRUE,"APC"

"9331","GAAGTACTTAAACAACTACA","chr5",112766325,"+","none","AGG","GAAGTACTTAAACAACTACAAGG",0,NA,NA,NA,"GeneID:324","APC",TRUE,"APC"

"9332","GAAGTACTTAAACAACTACA","chr1",19266275,"-","6:C>G,14:A>T","AGG","GAAGTAGTTAAACATCTACAAGG",2,7,15,NA,"GeneID:246181","AKR7L",TRUE,"APC"

"9333","GATTTATTAGAGCGTCTTAA","chr5",112766388,"+","none","AGG","GATTTATTAGAGCGTCTTAAAGG",0,NA,NA,NA,"GeneID:324","APC",TRUE,"APC"

"9334","GGATCTGTATCAAGCCGTTC","chr5",112767268,"+","none","TGG","GGATCTGTATCAAGCCGTTCTGG",0,NA,NA,NA,"GeneID:324","APC",TRUE,"APC"

"9335","TGATCTTGACAAAGAAGAAA","chr5",112775643,"+","none","AGG","TGATCTTGACAAAGAAGAAAAGG",0,NA,NA,NA,"GeneID:324","APC",TRUE,"APC"

"9336","TGATCTTGACAAAGAAGAAA","chr4",70962406,"+","none","AGG","TGATCTTGACAAAGAAGAAAAGG",0,NA,NA,NA,"GeneID:92597","MOB1B",FALSE,"APC"

"9337","TGATCTTGACAAAGAAGAAA","chr2",163510231,"-","14:C>T,19:G>T","TGG","TGATCTTGACAAAGGAGAACTGG",2,15,20,NA,"","",FALSE,"APC"

"9338","TGATCTTGACAAAGAAGAAA","chr4",103962884,"-","14:C>T,17:G>T","AGG","TGATCTTGACAAAGGAGCAAAGG",2,15,18,NA,"","",FALSE,"APC"

"9339","TGATCTTGACAAAGAAGAAA","chr5",77676912,"+","17:T>A","AGG","TGATCTTGACAAAGAAGTAAAGG",1,18,NA,NA,"","",FALSE,"APC"

"9340","TGATCTTGACAAAGAAGAAA","chr18",9377155,"+","14:G>A,17:C>A","AGG","TGATCTTGACAAAGGAGCAAAGG",2,15,18,NA,"GeneID:57045","TWSG1",FALSE,"APC"

"9341","TGATCTTGACAAAGAAGAAA","chr8",108702404,"+","14:G>A,17:G>A","AGG","TGATCTTGACAAAGGAGGAAAGG",2,15,18,NA,"GeneID:157753","TMEM74",FALSE,"APC"

"9342","TGATCTTGACAAAGAAGAAA","chr2",128505816,"+","13:T>G,15:G>A","GGG","TGATCTTGACAAATAGGAAAGGG",2,14,16,NA,"","",FALSE,"APC"

"9343","TGATCTTGACAAAGAAGAAA","chr4",176017478,"-","2:A>T,9:C>G","AGG","TGTTCTTGAGAAAGAAGAAAAGG",2,3,10,NA,"","",FALSE,"APC"

"9344","TGATCTTGACAAAGAAGAAA","chr6",74791445,"+","1:T>G,6:A>T","AGG","TTATCTAGACAAAGAAGAAAAGG",2,2,7,NA,"","",FALSE,"APC"

"9345","TGATCTTGACAAAGAAGAAA","chrX",137090021,"+","5:C>T,9:T>C","GGG","TGATCCTGATAAAGAAGAAAGGG",2,6,10,NA,"","",FALSE,"APC"

"9346","TGATCTTGACAAAGAAGAAA","chr16",75898550,"+","5:A>T,9:A>C","AGG","TGATCATGAAAAAGAAGAAAAGG",2,6,10,NA,"","",FALSE,"APC"

"9347","TGATCTTGACAAAGAAGAAA","chr3",107150657,"+","6:G>T,9:A>C","AGG","TGATCTGGAAAAAGAAGAAAAGG",2,7,10,NA,"GeneID:100302640","LINC00882",FALSE,"APC"

"9348","TGATCTTGACAAAGAAGAAA","chr21",29687620,"+","3:A>T,15:T>A","AGG","TGAACTTGACAAAGATGAAAAGG",2,4,16,NA,"GeneID:2897","GRIK1",FALSE,"APC"

"9349","TGATCTTGACAAAGAAGAAA","chr11",23447469,"-","7:A>C,14:C>T","TGG","TGATCTTTACAAAGGAGAAATGG",2,8,15,NA,"","",FALSE,"APC"

"9350","TGATCTTGACAAAGAAGAAA","chr3",147801868,"-","1:T>C,19:G>T","AGG","TAATCTTGACAAAGAAGAACAGG",2,2,20,NA,"","",FALSE,"APC"

"9351","AGCGCTCCTCCATCAGCGAG","chr19",1455445,"-","none","CGG","AGCGCTCCTCCATCAGCGAGCGG",0,NA,NA,NA,"GeneID:10297","APC2",TRUE,"APC2"

"9352","AGGGCCCGGAGCCGTGTACT","chr19",1453524,"-","none","GGG","AGGGCCCGGAGCCGTGTACTGGG",0,NA,NA,NA,"GeneID:10297","APC2",TRUE,"APC2"

"9353","AGGGCCCGGAGCCGTGTACT","chr7",99504533,"-","11:A>G,17:G>T","CGG","AGGGCCCGGAGTCGTGTCCTCGG",2,12,18,NA,"","",FALSE,"APC2"

"9354","CGGCTCACCGTCTCCACGTG","chr19",1455245,"-","none","CGG","CGGCTCACCGTCTCCACGTGCGG",0,NA,NA,NA,"GeneID:10297","APC2",TRUE,"APC2"

"9355","CTTCGGCACCTCGGACGAGA","chr19",1455463,"+","none","TGG","CTTCGGCACCTCGGACGAGATGG",0,NA,NA,NA,"GeneID:10297","APC2",TRUE,"APC2"

"9356","GCAGCCCAGTACACGGCTCC","chr19",1453517,"+","none","GGG","GCAGCCCAGTACACGGCTCCGGG",0,NA,NA,NA,"GeneID:10297","APC2",TRUE,"APC2"

"9357","GCTCGTCCAGGCGCTTGGAC","chr19",1455220,"-","none","AGG","GCTCGTCCAGGCGCTTGGACAGG",0,NA,NA,NA,"GeneID:10297","APC2",TRUE,"APC2"

"9358","AGGGCCGGTAGAACATCCTC","chr18",10472009,"-","none","TGG","AGGGCCGGTAGAACATCCTCTGG",0,NA,NA,NA,"GeneID:147495","APCDD1",TRUE,"APCDD1"

"9359","CCTACCTTTGAACACGAACT","chr18",10485768,"-","none","CGG","CCTACCTTTGAACACGAACTCGG",0,NA,NA,NA,"GeneID:147495","APCDD1",TRUE,"APCDD1"

"9360","GCAGATGCCACCTACAATCG","chr18",10468610,"+","none","AGG","GCAGATGCCACCTACAATCGAGG",0,NA,NA,NA,"GeneID:147495","APCDD1",TRUE,"APCDD1"

"9361","GCTCCTCGACCAGGTGGTCG","chr18",10471955,"-","none","AGG","GCTCCTCGACCAGGTGGTCGAGG",0,NA,NA,NA,"GeneID:147495","APCDD1",TRUE,"APCDD1"

"9362","TGACCGATAGATGATCCGAC","chr18",10485486,"-","none","AGG","TGACCGATAGATGATCCGACAGG",0,NA,NA,NA,"GeneID:147495","APCDD1",TRUE,"APCDD1"

"9363","TGACTCCTTAAAAGCCCTCC","chr18",10468540,"-","none","AGG","TGACTCCTTAAAAGCCCTCCAGG",0,NA,NA,NA,"GeneID:147495","APCDD1",TRUE,"APCDD1"

"9364","ACCTTCACCGTGTATGCCGC","chr20",58461297,"-","none","CGG","ACCTTCACCGTGTATGCCGCCGG",0,NA,NA,NA,"GeneID:164284","APCDD1L",TRUE,"APCDD1L"

"9365","AGGGGTCCTCGTAGTAGAAC","chr20",58467560,"+","none","TGG","AGGGGTCCTCGTAGTAGAACTGG",0,NA,NA,NA,"GeneID:164284","APCDD1L",TRUE,"APCDD1L"

"9366","ATCCTGCCTCCACGCCTTAA","chr20",58470629,"-","none","TGG","ATCCTGCCTCCACGCCTTAATGG",0,NA,NA,NA,"GeneID:164284","APCDD1L",TRUE,"APCDD1L"

"9367","CGTGCCCCTGGTGTAGCGGC","chr20",58461275,"+","none","CGG","CGTGCCCCTGGTGTAGCGGCCGG",0,NA,NA,NA,"GeneID:164284","APCDD1L",TRUE,"APCDD1L"

"9368","GCGGGTCTGGTTGAGGCGCC","chr20",58467387,"+","none","CGG","GCGGGTCTGGTTGAGGCGCCCGG",0,NA,NA,NA,"GeneID:164284","APCDD1L",TRUE,"APCDD1L"

"9369","GCTCCCCTACGCTTGCGTCC","chr20",58514673,"-","none","TGG","GCTCCCCTACGCTTGCGTCCTGG",0,NA,NA,NA,"GeneID:164284","APCDD1L",TRUE,"APCDD1L"

"9370","ACTATAGGCTCGAAAACACA","chr1",159588196,"-","none","AGG","ACTATAGGCTCGAAAACACAAGG",0,NA,NA,NA,"GeneID:325","APCS",TRUE,"APCS"

"9371","AGCTCAGCCCAAGATTGTCC","chr1",159588470,"+","none","TGG","AGCTCAGCCCAAGATTGTCCTGG",0,NA,NA,NA,"GeneID:325","APCS",TRUE,"APCS"

"9372","ATATGAACAAGCCGCTGCTT","chr1",159587919,"+","none","TGG","ATATGAACAAGCCGCTGCTTTGG",0,NA,NA,NA,"GeneID:325","APCS",TRUE,"APCS"

"9373","ATATGAACAAGCCGCTGCTT","chr2",8368410,"+","2:C>A,12:A>C","TGG","ATCTGAACAAGCAGCTGCTTTGG",2,3,13,NA,"GeneID:339789","LINC00299",FALSE,"APCS"

"9374","CTCCTTACCTGTGTGAGCAA","chr1",159587973,"-","none","AGG","CTCCTTACCTGTGTGAGCAAAGG",0,NA,NA,NA,"GeneID:325","APCS",TRUE,"APCS"

"9375","GTAGCTCATTATCCCTGCCT","chr1",159588257,"-","none","TGG","GTAGCTCATTATCCCTGCCTTGG",0,NA,NA,NA,"GeneID:325","APCS",TRUE,"APCS"

"9376","TGTAAACTTGATCACACCGC","chr1",159588149,"+","none","TGG","TGTAAACTTGATCACACCGCTGG",0,NA,NA,NA,"GeneID:325","APCS",TRUE,"APCS"

"9377","TGTAAACTTGATCACACCGC","chr2",163424572,"-","3:C>T,17:C>G","TGG","TGTGAACTTGATCACACGGCTGG",2,4,18,NA,"","",FALSE,"APCS"

"9378","CATGAAAGCTGTGCTGCGCA","chr3",49675723,"+","none","AGG","CATGAAAGCTGTGCTGCGCAAGG",0,NA,NA,NA,"GeneID:327","APEH",TRUE,"APEH"

"9379","CATGAAAGCTGTGCTGCGCA","chr3",127134830,"+","5:G>A,17:C>G","TGG","CATGAGAGCTGTGCTGCCCATGG",2,6,18,NA,"","",FALSE,"APEH"

"9380","CATGTTTCTCCAGCGCTGAC","chr3",49675928,"-","none","AGG","CATGTTTCTCCAGCGCTGACAGG",0,NA,NA,NA,"GeneID:327","APEH",TRUE,"APEH"

"9381","CCTGGCTATCTCATCATCGC","chr3",49676172,"-","none","TGG","CCTGGCTATCTCATCATCGCTGG",0,NA,NA,NA,"GeneID:327","APEH",TRUE,"APEH"

"9382","CTTGCGCAGCACAGCTTTCA","chr3",49675725,"-","none","TGG","CTTGCGCAGCACAGCTTTCATGG",0,NA,NA,NA,"GeneID:327","APEH",TRUE,"APEH"

"9383","GAGCTTCAACCTGTCAGCGC","chr3",49675916,"+","none","TGG","GAGCTTCAACCTGTCAGCGCTGG",0,NA,NA,NA,"GeneID:327","APEH",TRUE,"APEH"

"9384","GTGTGTCTCCGAGTGCGACC","chr3",49676076,"-","none","AGG","GTGTGTCTCCGAGTGCGACCAGG",0,NA,NA,NA,"GeneID:327","APEH",TRUE,"APEH"

"9385","AATTGACCTTCGCAACCCCA","chr14",20457201,"+","none","AGG","AATTGACCTTCGCAACCCCAAGG",0,NA,NA,NA,"GeneID:328","APEX1",TRUE,"APEX1"

"9386","ATGCCGTAAGAAACTTTGAG","chr14",20456838,"-","none","TGG","ATGCCGTAAGAAACTTTGAGTGG",0,NA,NA,NA,"GeneID:328","APEX1",TRUE,"APEX1"

"9387","ATGCCGTAAGAAACTTTGAG","chr4",57007953,"+","4:T>C,13:T>C","GGG","ATGCTGTAAGAAATTTTGAGGGG",2,5,14,NA,"GeneID:5431","POLR2B",FALSE,"APEX1"

"9388","ATGTGGATGGGCTTCGAGCC","chr14",20456057,"+","none","TGG","ATGTGGATGGGCTTCGAGCCTGG",0,NA,NA,NA,"GeneID:328","APEX1",TRUE,"APEX1"

"9389","GTAACGGGAATGCCGAAGCG","chr14",20455636,"+","none","TGG","GTAACGGGAATGCCGAAGCGTGG",0,NA,NA,NA,"GeneID:328","APEX1",TRUE,"APEX1"

"9390","TCAGCTCCTTCGGACAAGGA","chr14",20456778,"+","none","AGG","TCAGCTCCTTCGGACAAGGAAGG",0,NA,NA,NA,"GeneID:328","APEX1",TRUE,"APEX1"

"9391","TCAGCTCCTTCGGACAAGGA","chr5",156446662,"-","13:G>T,16:C>T","AGG","TCAGCTCCTTCGGCCAGGGAAGG",2,14,17,NA,"GeneID:6444","SGCD",FALSE,"APEX1"

"9392","TTCACGCCACAAGAGCGCCA","chr14",20457244,"+","none","AGG","TTCACGCCACAAGAGCGCCAAGG",0,NA,NA,NA,"GeneID:328","APEX1",TRUE,"APEX1"

"9393","ACCGGATCTTATGCTGTGTG","chrX",55002413,"-","none","AGG","ACCGGATCTTATGCTGTGTGAGG",0,NA,NA,NA,"GeneID:27301","APEX2",TRUE,"APEX2"

"9394","AGGTGTAGCCACCTTCTGTA","chrX",55002248,"+","none","AGG","AGGTGTAGCCACCTTCTGTAAGG",0,NA,NA,NA,"GeneID:27301","APEX2",TRUE,"APEX2"

"9395","ATCTTAAAGACTAGCCGCTC","chrX",55003034,"-","none","AGG","ATCTTAAAGACTAGCCGCTCAGG",0,NA,NA,NA,"GeneID:27301","APEX2",TRUE,"APEX2"

"9396","ATTTACCAGAATAGCCGCTA","chrX",55001615,"-","none","CGG","ATTTACCAGAATAGCCGCTACGG",0,NA,NA,NA,"GeneID:27301","APEX2",TRUE,"APEX2"

"9397","GGGGGCAGTACACGTTGATT","chrX",55002994,"-","none","AGG","GGGGGCAGTACACGTTGATTAGG",0,NA,NA,NA,"GeneID:27301","APEX2",TRUE,"APEX2"

"9398","TATAACCCTCAACGATAGCC","chrX",55001567,"-","none","AGG","TATAACCCTCAACGATAGCCAGG",0,NA,NA,NA,"GeneID:27301","APEX2",TRUE,"APEX2"

"9399","ACCCATCTCCATCCGCCAGA","chr1",150267724,"-","none","TGG","ACCCATCTCCATCCGCCAGATGG",0,NA,NA,NA,"GeneID:51107","APH1A",TRUE,"APH1A"

"9400","ACCCATCTCCATCCGCCAGA","chr5",1018146,"+","10:C>A,13:T>C","AGG","ACCCATCTCCCTCTGCCAGAAGG",2,11,14,NA,"GeneID:85409","NKD2",FALSE,"APH1A"

"9401","ACCCATCTCCATCCGCCAGA","chr2",237179151,"-","1:T>G,14:A>C","GGG","AACCATCTCCATCCTCCAGAGGG",2,2,15,NA,"","",FALSE,"APH1A"

"9402","CCATGGATCCCAACCACACC","chr1",150267384,"+","none","TGG","CCATGGATCCCAACCACACCTGG",0,NA,NA,NA,"GeneID:51107","APH1A",TRUE,"APH1A"

"9403","CCATGGATCCCAACCACACC","chr11",65963378,"-","15:A>T,17:A>T","AGG","CCATGGATCCCAACCTCTCCAGG",2,16,18,NA,"GeneID:9092","SART1",FALSE,"APH1A"

"9404","TCACCAGTCCCGATGTCAGT","chr1",150267070,"+","none","AGG","TCACCAGTCCCGATGTCAGTAGG",0,NA,NA,NA,"GeneID:51107","APH1A",TRUE,"APH1A"

"9405","TGGGAGTCACCTACTGACAT","chr1",150267082,"-","none","CGG","TGGGAGTCACCTACTGACATCGG",0,NA,NA,NA,"GeneID:51107","APH1A",TRUE,"APH1A"

"9406","TGGGAGTCACCTACTGACAT","chr16",49555209,"-","6:C>A,7:C>G","AGG","TGGGAGGGACCTACTGACATAGG",2,7,8,NA,"GeneID:23090","ZNF423",FALSE,"APH1A"

"9407","TTACTTAAGCAGCTTGTAGT","chr1",150267952,"+","none","AGG","TTACTTAAGCAGCTTGTAGTAGG",0,NA,NA,NA,"GeneID:51107","APH1A",TRUE,"APH1A"

"9408","TTACTTAAGCAGCTTGTAGT","chr1",26001932,"+","16:G>T,18:C>G","TGG","TTACTTAAGCAGCTTGGACTTGG",2,17,19,NA,"","",FALSE,"APH1A"

"9409","TTAGCATCGCTGAGTGAGGA","chr1",150267753,"-","none","CGG","TTAGCATCGCTGAGTGAGGACGG",0,NA,NA,NA,"GeneID:51107","APH1A",TRUE,"APH1A"

"9410","AGCACCCTCTATGCGACTGC","chr15",63286599,"+","none","TGG","AGCACCCTCTATGCGACTGCTGG",0,NA,NA,NA,"GeneID:83464","APH1B",TRUE,"APH1B"

"9411","ATAGGCCAGCAGTCGCATAG","chr15",63286607,"-","none","AGG","ATAGGCCAGCAGTCGCATAGAGG",0,NA,NA,NA,"GeneID:83464","APH1B",TRUE,"APH1B"

"9412","GAATACCCTATCTGACTCCT","chr15",63287469,"+","none","TGG","GAATACCCTATCTGACTCCTTGG",0,NA,NA,NA,"GeneID:83464","APH1B",TRUE,"APH1B"

"9413","GTCAGATAGGGTATTCACAA","chr15",63287465,"-","none","AGG","GTCAGATAGGGTATTCACAAAGG",0,NA,NA,NA,"GeneID:83464","APH1B",TRUE,"APH1B"

"9414","TACTGATTTCGTCCCTTGTT","chr15",63279183,"+","none","TGG","TACTGATTTCGTCCCTTGTTTGG",0,NA,NA,NA,"GeneID:83464","APH1B",TRUE,"APH1B"

"9415","TCTTTCAGCTTTCATGACGC","chr15",63302336,"+","none","TGG","TCTTTCAGCTTTCATGACGCTGG",0,NA,NA,NA,"GeneID:83464","APH1B",TRUE,"APH1B"

"9416","ACCATCCAATATCACTTGAT","chr11",43318652,"-","none","AGG","ACCATCCAATATCACTTGATAGG",0,NA,NA,NA,"GeneID:8539","API5",TRUE,"API5"

"9417","ACCATCCAATATCACTTGAT","chr2",177998668,"+","8:G>A","AGG","ACCATCCAGTATCACTTGATAGG",1,9,NA,NA,"GeneID:50940","PDE11A",TRUE,"API5"

"9418","ATAGCAGAATCAGCCAATTC","chr11",43318741,"-","none","TGG","ATAGCAGAATCAGCCAATTCTGG",0,NA,NA,NA,"GeneID:8539","API5",TRUE,"API5"

"9419","ATAGCAGAATCAGCCAATTC","chr21",20020531,"-","10:C>G,19:A>G","AGG","ATAGCAGAATGAGCCAATTTAGG",2,11,20,NA,"","",FALSE,"API5"

"9420","ATAGCAGAATCAGCCAATTC","chrX",116108239,"+","8:C>A,12:A>G","TGG","ATAGCAGACTCAACCAATTCTGG",2,9,13,NA,"","",TRUE,"API5"

"9421","ATAGCAGAATCAGCCAATTC","chr22",22425285,"-","4:C>G,17:G>A","TGG","ATAGGAGAATCAGCCAACTCTGG",2,5,18,NA,"","",FALSE,"API5"

"9422","CCTATTAAGTATATTTAAAA","chr11",43321444,"+","none","TGG","CCTATTAAGTATATTTAAAATGG",0,NA,NA,NA,"GeneID:8539","API5",TRUE,"API5"

"9423","CCTATTAAGTATATTTAAAA","chrX",116108051,"-","3:C>T","TGG","CCTGTTAAGTATATTTAAAATGG",1,4,NA,NA,"","",TRUE,"API5"

"9424","CCTATTAAGTATATTTAAAA","chr2",229153826,"-","5:T>A,12:C>T","CGG","CCTATAAAGTATGTTTAAAACGG",2,6,13,NA,"GeneID:55022","PID1",FALSE,"API5"

"9425","GAACTGCCTCAATTTGCCAC","chr11",43320841,"+","none","TGG","GAACTGCCTCAATTTGCCACTGG",0,NA,NA,NA,"GeneID:8539","API5",TRUE,"API5"

"9426","GAACTGCCTCAATTTGCCAC","chrX",116108158,"-","none","TGG","GAACTGCCTCAATTTGCCACTGG",0,NA,NA,NA,"","",TRUE,"API5"

"9427","GAACTGCCTCAATTTGCCAC","chr2",177998498,"-","15:T>C","TGG","GAACTGCCTCAATTTACCACTGG",1,16,NA,NA,"GeneID:50940","PDE11A",TRUE,"API5"

"9428","GAACTGCCTCAATTTGCCAC","chr20",61487995,"-","10:G>T,18:A>T","TGG","GAACTGCCTCCATTTGCCTCTGG",2,11,19,NA,"GeneID:1002","CDH4",FALSE,"API5"

"9429","GTTAGTATATCTGCCACTCG","chr11",43320877,"-","none","AGG","GTTAGTATATCTGCCACTCGAGG",0,NA,NA,NA,"GeneID:8539","API5",TRUE,"API5"

"9430","GTTAGTATATCTGCCACTCG","chr2",177998462,"+","0:A>G","AGG","ATTAGTATATCTGCCACTCGAGG",1,1,NA,NA,"GeneID:50940","PDE11A",TRUE,"API5"

"9431","GTTAGTATATCTGCCACTCG","chrX",116108122,"+","0:A>G,4:A>G","AGG","ATTAATATATCTGCCACTCGAGG",2,1,5,NA,"","",TRUE,"API5"

"9432","TATACTAACTGAATCCAAAA","chr11",43322114,"+","none","AGG","TATACTAACTGAATCCAAAAAGG",0,NA,NA,NA,"GeneID:8539","API5",TRUE,"API5"

"9433","TATACTAACTGAATCCAAAA","chr2",7814994,"-","13:T>A,14:C>G","AGG","TATACTAACTGAAAGCAAAAAGG",2,14,15,NA,"","",FALSE,"API5"

"9434","AAAATACCTATAATACCCTC","chr11",34888285,"+","none","CGG","AAAATACCTATAATACCCTCCGG",0,NA,NA,NA,"GeneID:51074","APIP",TRUE,"APIP"

"9435","AAAATACCTATAATACCCTC","chr7",71451252,"+","2:T>A,3:T>A","TGG","AATTTACCTATAATACCCTCTGG",2,3,4,NA,"GeneID:64409","WBSCR17",FALSE,"APIP"

"9436","AAAATACCTATAATACCCTC","chr5",123162691,"-","5:A>T,19:A>G","GGG","AAAATTCCTATAATACCCTTGGG",2,6,20,NA,"GeneID:93166","PRDM6",FALSE,"APIP"

"9437","ATAAAGAAATGTACTTCCGG","chr11",34888304,"-","none","AGG","ATAAAGAAATGTACTTCCGGAGG",0,NA,NA,NA,"GeneID:51074","APIP",TRUE,"APIP"

"9438","ATAAAGAAATGTACTTCCGG","chr9",107117257,"+","17:T>C","AGG","ATAAAGAAATGTACTTCTGGAGG",1,18,NA,NA,"","",TRUE,"APIP"

"9439","ATAAAGAAATGTACTTCCGG","chr8",125579334,"+","11:G>T","AGG","ATAAAGAAATGGACTTCCGGAGG",1,12,NA,NA,"","",FALSE,"APIP"

"9440","ATAAAGAAATGTACTTCCGG","chr2",20113077,"-","9:C>A,17:A>G","CGG","ATAAAGAAAGGTACTTCTGGCGG",2,10,18,NA,"","",FALSE,"APIP"

"9441","CAGATATGATGATATGTTAG","chr11",34883487,"-","none","TGG","CAGATATGATGATATGTTAGTGG",0,NA,NA,NA,"GeneID:51074","APIP",TRUE,"APIP"

"9442","CAGATATGATGATATGTTAG","chr2",68805061,"+","15:T>G,17:G>T","AGG","CAGATATGATGATATTTGAGAGG",2,16,18,NA,"GeneID:9938","ARHGAP25",FALSE,"APIP"

"9443","CAGATATGATGATATGTTAG","chr8",141271693,"+","10:C>G,17:C>T","GGG","CAGATATGATCATATGTCAGGGG",2,11,18,NA,"GeneID:57210","SLC45A4",FALSE,"APIP"

"9444","CAGATATGATGATATGTTAG","chr9",107117286,"+","0:T>C","TGG","TAGATATGATGATATGTTAGTGG",1,1,NA,NA,"","",TRUE,"APIP"

"9445","GAAAGACCTCAAAGATAGAA","chr11",34883436,"-","none","TGG","GAAAGACCTCAAAGATAGAATGG",0,NA,NA,NA,"GeneID:51074","APIP",TRUE,"APIP"

"9446","GAAAGACCTCAAAGATAGAA","chr9",107117337,"+","7:T>C","TGG","GAAAGACTTCAAAGATAGAATGG",1,8,NA,NA,"","",TRUE,"APIP"

"9447","GAAAGACCTCAAAGATAGAA","chr8",93046633,"-","6:A>G,14:G>T","AGG","GAAAGATCTCAAAGCTAGAAAGG",2,7,15,NA,"","",FALSE,"APIP"

"9448","GAAAGACCTCAAAGATAGAA","chr3",125363571,"-","4:A>C,15:T>A","TGG","GAAATACCTCAAAGAAAGAATGG",2,5,16,NA,"GeneID:7707","ZNF148",FALSE,"APIP"

"9449","GTGTAAGCATTCATGAAAAG","chr11",34888759,"+","none","AGG","GTGTAAGCATTCATGAAAAGAGG",0,NA,NA,NA,"GeneID:51074","APIP",TRUE,"APIP"

"9450","GTGTAAGCATTCATGAAAAG","chr9",107117125,"-","16:C>T","AGG","GTGTAAGCATTCATGAGAAGAGG",1,17,NA,NA,"","",TRUE,"APIP"

"9451","GTGTAAGCATTCATGAAAAG","chrX",114609961,"+","6:A>G,14:C>G","TGG","GTGTAAACATTCATCAAAAGTGG",2,7,15,NA,"GeneID:3358","HTR2C",FALSE,"APIP"

"9452","TCCCAGTGACCCAGCCTAAA","chr11",34895037,"+","none","TGG","TCCCAGTGACCCAGCCTAAATGG",0,NA,NA,NA,"GeneID:51074","APIP",TRUE,"APIP"

"9453","TCCCAGTGACCCAGCCTAAA","chr9",107116938,"-","15:C>G,16:G>A","TGG","TCCCAGTGACCCAGCGCAAATGG",2,16,17,NA,"","",TRUE,"APIP"

"9454","TCCCAGTGACCCAGCCTAAA","chr11",68661194,"+","2:A>C,8:T>A","GGG","TCACAGTGTCCCAGCCTAAAGGG",2,3,9,NA,"","",FALSE,"APIP"

"9455","AAGACCTTGAAATGTTTGCA","chr1",10434668,"+","none","AGG","AAGACCTTGAAATGTTTGCAAGG",0,NA,NA,NA,"GeneID:100526739;GeneID:378708","APITD1-CORT;APITD1",TRUE,"APITD1"

"9456","AAGACCTTGAAATGTTTGCA","chr3",19367867,"-","10:A>T,11:C>T","AGG","AAGACCTTGATGTGTTTGCAAGG",2,11,12,NA,"GeneID:131096","KCNH8",FALSE,"APITD1"

"9457","AAGACCTTGAAATGTTTGCA","chr5",31614152,"+","16:G>T,17:C>G","GGG","AAGACCTTGAAATGTTGCCAGGG",2,17,18,NA,"","",FALSE,"APITD1"

"9458","AAGACCTTGAAATGTTTGCA","chr16",79411484,"+","3:C>A,7:G>T","GGG","AAGCCCTGGAAATGTTTGCAGGG",2,4,8,NA,"","",FALSE,"APITD1"

"9459","CACATCTTCAGTGTTAATTG","chr1",10440363,"-","none","TGG","CACATCTTCAGTGTTAATTGTGG",0,NA,NA,NA,"GeneID:100526739;GeneID:378708","APITD1-CORT;APITD1",TRUE,"APITD1"

"9460","CACATCTTCAGTGTTAATTG","chr7",12685845,"-","18:C>A","TGG","CACATCTTCAGTGTTAATGGTGG",1,19,NA,NA,"","",TRUE,"APITD1"

"9461","CACCAGTGAATTACTCCTCC","chr1",10440396,"-","none","TGG","CACCAGTGAATTACTCCTCCTGG",0,NA,NA,NA,"GeneID:100526739;GeneID:378708","APITD1-CORT;APITD1",TRUE,"APITD1"

"9462","CACCAGTGAATTACTCCTCC","chr2",165308822,"-","9:A>T,16:A>G","AGG","CACCAGTGATTTACTCTTCCAGG",2,10,17,NA,"GeneID:6326","SCN2A",FALSE,"APITD1"

"9463","CCCACCTTGCAAACATTTCA","chr1",10434675,"-","none","AGG","CCCACCTTGCAAACATTTCAAGG",0,NA,NA,NA,"GeneID:100526739;GeneID:378708","APITD1-CORT;APITD1",TRUE,"APITD1"

"9464","CCCACCTTGCAAACATTTCA","chr16",1977343,"+","11:G>A,18:G>C","GGG","CCCACCTTGCAGACATTTGAGGG",2,12,19,NA,"GeneID:10607","TBL3",TRUE,"APITD1"

"9465","GAGCTGACTTTCCGACAGTG","chr1",10433943,"+","none","TGG","GAGCTGACTTTCCGACAGTGTGG",0,NA,NA,NA,"GeneID:100526739;GeneID:378708","APITD1-CORT;APITD1",TRUE,"APITD1"

"9466","GGCAGCAGTTCACTATACTG","chr1",10433849,"+","none","TGG","GGCAGCAGTTCACTATACTGTGG",0,NA,NA,NA,"GeneID:100526739;GeneID:378708","APITD1-CORT;APITD1",TRUE,"APITD1"

"9467","AAGACCTTGAAATGTTTGCA","chr1",10434668,"+","none","AGG","AAGACCTTGAAATGTTTGCAAGG",0,NA,NA,NA,"GeneID:100526739;GeneID:378708","APITD1-CORT;APITD1",TRUE,"APITD1-CORT"

"9468","AAGACCTTGAAATGTTTGCA","chr3",19367867,"-","10:A>T,11:C>T","AGG","AAGACCTTGATGTGTTTGCAAGG",2,11,12,NA,"GeneID:131096","KCNH8",FALSE,"APITD1-CORT"

"9469","AAGACCTTGAAATGTTTGCA","chr5",31614152,"+","16:G>T,17:C>G","GGG","AAGACCTTGAAATGTTGCCAGGG",2,17,18,NA,"","",FALSE,"APITD1-CORT"

"9470","AAGACCTTGAAATGTTTGCA","chr16",79411484,"+","3:C>A,7:G>T","GGG","AAGCCCTGGAAATGTTTGCAGGG",2,4,8,NA,"","",FALSE,"APITD1-CORT"

"9471","AGAGAATCGCTGCTGCTCCT","chr1",10430539,"-","none","CGG","AGAGAATCGCTGCTGCTCCTCGG",0,NA,NA,NA,"GeneID:100526739;GeneID:378708","APITD1-CORT;APITD1",TRUE,"APITD1-CORT"

"9472","AGAGAATCGCTGCTGCTCCT","chr5",144483710,"+","5:T>A,8:C>G","TGG","AGAGATTCCCTGCTGCTCCTTGG",2,6,9,NA,"GeneID:57528","KCTD16",TRUE,"APITD1-CORT"

"9473","CCCACCTTGCAAACATTTCA","chr1",10434675,"-","none","AGG","CCCACCTTGCAAACATTTCAAGG",0,NA,NA,NA,"GeneID:100526739;GeneID:378708","APITD1-CORT;APITD1",TRUE,"APITD1-CORT"

"9474","CCCACCTTGCAAACATTTCA","chr16",1977343,"+","11:G>A,18:G>C","GGG","CCCACCTTGCAGACATTTGAGGG",2,12,19,NA,"GeneID:10607","TBL3",TRUE,"APITD1-CORT"

"9475","GAGCTGACTTTCCGACAGTG","chr1",10433943,"+","none","TGG","GAGCTGACTTTCCGACAGTGTGG",0,NA,NA,NA,"GeneID:100526739;GeneID:378708","APITD1-CORT;APITD1",TRUE,"APITD1-CORT"

"9476","GCAGCGATTCTCTTACCAAC","chr1",10430546,"+","none","AGG","GCAGCGATTCTCTTACCAACAGG",0,NA,NA,NA,"GeneID:100526739;GeneID:378708","APITD1-CORT;APITD1",TRUE,"APITD1-CORT"

"9477","GGCAGCAGTTCACTATACTG","chr1",10433849,"+","none","TGG","GGCAGCAGTTCACTATACTGTGG",0,NA,NA,NA,"GeneID:100526739;GeneID:378708","APITD1-CORT;APITD1",TRUE,"APITD1-CORT"

"9478","CAGAAGACATGCCATTCTTG","chr2",68490209,"+","none","AGG","CAGAAGACATGCCATTCTTGAGG",0,NA,NA,NA,"GeneID:10887","PROKR1",TRUE,"APLF"

"9479","CATCTAGAAAGCTGTCGTTC","chr2",68577877,"-","none","AGG","CATCTAGAAAGCTGTCGTTCAGG",0,NA,NA,NA,"GeneID:10887","PROKR1",TRUE,"APLF"

"9480","CATCTAGAAAGCTGTCGTTC","chr1",78367443,"-","10:A>C,15:A>G","CGG","CATCTAGAAATCTGTTGTTCCGG",2,11,16,NA,"GeneID:149047;GeneID:5737","MGC27382;PTGFR",FALSE,"APLF"

"9481","CATCTAGAAAGCTGTCGTTC","chr9",14736796,"-","6:G>C,15:T>G","AGG","CATCTACAAAGCTGTAGTTCAGG",2,7,16,NA,"GeneID:158326","FREM1",TRUE,"APLF"

"9482","GTCTATATTCTATCTTGTGC","chr2",68567352,"-","none","TGG","GTCTATATTCTATCTTGTGCTGG",0,NA,NA,NA,"GeneID:10887","PROKR1",TRUE,"APLF"

"9483","TACCATTGAAGCCAAATCTA","chr2",68502779,"+","none","TGG","TACCATTGAAGCCAAATCTATGG",0,NA,NA,NA,"GeneID:10887","PROKR1",TRUE,"APLF"

"9484","TATAGACATAATACGCTTCC","chr2",68567365,"+","none","AGG","TATAGACATAATACGCTTCCAGG",0,NA,NA,NA,"GeneID:10887","PROKR1",TRUE,"APLF"

"9485","TGGTCAGCTGCGAATCAAAC","chr2",68490239,"+","none","CGG","TGGTCAGCTGCGAATCAAACCGG",0,NA,NA,NA,"GeneID:10887","PROKR1",TRUE,"APLF"

"9486","AGACGGCAATGTCCGCCACC","chrX",129648735,"-","none","TGG","AGACGGCAATGTCCGCCACCTGG",0,NA,NA,NA,"GeneID:8862","APLN",TRUE,"APLN"

"9487","CTCTCCTTGACCGCGGTGTG","chrX",129654568,"-","none","TGG","CTCTCCTTGACCGCGGTGTGTGG",0,NA,NA,NA,"GeneID:8862","APLN",TRUE,"APLN"

"9488","GCACTCACCTCCACACACCG","chrX",129654555,"+","none","CGG","GCACTCACCTCCACACACCGCGG",0,NA,NA,NA,"GeneID:8862","APLN",TRUE,"APLN"

"9489","GCGGCCCCGCCTCTCCCATA","chrX",129648645,"-","none","AGG","GCGGCCCCGCCTCTCCCATAAGG",0,NA,NA,NA,"GeneID:8862","APLN",TRUE,"APLN"

"9490","GCGGCCCCGCCTCTCCCATA","chr19",35869823,"-","17:C>T,19:A>T","CGG","GCGGCCCCGCCTCTCCCGTTCGG",2,18,20,NA,"GeneID:333","APLP1",TRUE,"APLN"

"9491","TCCCTGATGCCGCTTCCCGA","chrX",129648770,"-","none","TGG","TCCCTGATGCCGCTTCCCGATGG",0,NA,NA,NA,"GeneID:8862","APLN",TRUE,"APLN"

"9492","TGGCCCATTCCTTGACCCTC","chrX",129648703,"+","none","TGG","TGGCCCATTCCTTGACCCTCTGG",0,NA,NA,NA,"GeneID:8862","APLN",TRUE,"APLN"

"9493","TGGCCCATTCCTTGACCCTC","chrX",153519956,"-","6:G>T,14:G>T","GGG","TGGCCCCTTCCTTGCCCCTCGGG",2,7,15,NA,"GeneID:492","ATP2B3",FALSE,"APLN"

"9494","CTTCCGCAAGGAACGCATCG","chr11",57236292,"-","none","AGG","CTTCCGCAAGGAACGCATCGAGG",0,NA,NA,NA,"GeneID:187","APLNR",TRUE,"APLNR"

"9495","GGCCCTCGATGCGTTCCTTG","chr11",57236286,"+","none","CGG","GGCCCTCGATGCGTTCCTTGCGG",0,NA,NA,NA,"GeneID:187","APLNR",TRUE,"APLNR"

"9496","GTTACGCACCACCGGGGACT","chr11",57236487,"-","none","TGG","GTTACGCACCACCGGGGACTTGG",0,NA,NA,NA,"GeneID:187","APLNR",TRUE,"APLNR"

"9497","TGTGGGCTACCTACACGTAC","chr11",57236734,"-","none","CGG","TGTGGGCTACCTACACGTACCGG",0,NA,NA,NA,"GeneID:187","APLNR",TRUE,"APLNR"

"9498","TTCATCGCCCAAACCATCGC","chr11",57236318,"-","none","TGG","TTCATCGCCCAAACCATCGCTGG",0,NA,NA,NA,"GeneID:187","APLNR",TRUE,"APLNR"

"9499","TTTCGACCCCCGCTTCCGCC","chr11",57236052,"-","none","AGG","TTTCGACCCCCGCTTCCGCCAGG",0,NA,NA,NA,"GeneID:187","APLNR",TRUE,"APLNR"

"9500","ACCCCCGCACCAGCGCTCCA","chr19",35870950,"-","none","TGG","ACCCCCGCACCAGCGCTCCATGG",0,NA,NA,NA,"GeneID:333","APLP1",TRUE,"APLP1"

"9501","GCCAGTAGATGATTACTTCG","chr19",35871938,"+","none","TGG","GCCAGTAGATGATTACTTCGTGG",0,NA,NA,NA,"GeneID:333","APLP1",TRUE,"APLP1"

"9502","GGCTACGCAGGCCATCCCCA","chr19",35870930,"+","none","TGG","GGCTACGCAGGCCATCCCCATGG",0,NA,NA,NA,"GeneID:333","APLP1",TRUE,"APLP1"

"9503","GGCTACGCAGGCCATCCCCA","chr3",33439676,"+","4:G>A,13:T>A","AGG","GGCTGCGCAGGCCTTCCCCAAGG",2,5,14,NA,"GeneID:7342","UBP1",FALSE,"APLP1"

"9504","GTAAATATCCACACCGTCTG","chr19",35872503,"-","none","TGG","GTAAATATCCACACCGTCTGTGG",0,NA,NA,NA,"GeneID:333","APLP1",TRUE,"APLP1"

"9505","TAGGATGCGCCAGATTAATG","chr19",35872591,"+","none","AGG","TAGGATGCGCCAGATTAATGAGG",0,NA,NA,NA,"GeneID:333","APLP1",TRUE,"APLP1"

"9506","TCCACGAAGTAATCATCTAC","chr19",35871942,"-","none","TGG","TCCACGAAGTAATCATCTACTGG",0,NA,NA,NA,"GeneID:333","APLP1",TRUE,"APLP1"

"9507","AAAAATGCGCGTCCGCGACC","chr11",130070678,"-","none","CGG","AAAAATGCGCGTCCGCGACCCGG",0,NA,NA,NA,"GeneID:334","APLP2",TRUE,"APLP2"

"9508","AGACTGAAGTCCTCCCGCAG","chr11",130141933,"-","none","TGG","AGACTGAAGTCCTCCCGCAGTGG",0,NA,NA,NA,"GeneID:334","APLP2",TRUE,"APLP2"

"9509","AGACTGAAGTCCTCCCGCAG","chr3",44404334,"-","11:A>G,16:A>C","TGG","AGACTGAAGTCTTCCCTCAGTGG",2,12,17,NA,"GeneID:285343","TCAIM",FALSE,"APLP2"

"9510","ATCTCCACGTGATTGAAGAA","chr11",130133638,"+","none","AGG","ATCTCCACGTGATTGAAGAAAGG",0,NA,NA,NA,"GeneID:334","APLP2",TRUE,"APLP2"

"9511","ATCTCCACGTGATTGAAGAA","chr1",7484374,"-","1:C>A,11:C>T","GGG","AGCTCCACGTGGTTGAAGAAGGG",2,2,12,NA,"GeneID:23261","CAMTA1",FALSE,"APLP2"

"9512","GATGACGATGACCGTGGCAA","chr11",130141997,"-","none","TGG","GATGACGATGACCGTGGCAATGG",0,NA,NA,NA,"GeneID:334","APLP2",TRUE,"APLP2"

"9513","TCCTCCTTTCTTCAATCACG","chr11",130133645,"-","none","TGG","TCCTCCTTTCTTCAATCACGTGG",0,NA,NA,NA,"GeneID:334","APLP2",TRUE,"APLP2"

"9514","TTGCCTGCGTCCGTAGACCG","chr11",130070711,"+","none","AGG","TTGCCTGCGTCCGTAGACCGAGG",0,NA,NA,NA,"GeneID:334","APLP2",TRUE,"APLP2"

"9515","ATAGAGACCATTGCCCGGTT","chr20",24973661,"-","none","TGG","ATAGAGACCATTGCCCGGTTTGG",0,NA,NA,NA,"GeneID:57136","APMAP",TRUE,"APMAP"

"9516","CGGGTCGTAAAACTTGAAAA","chr20",24973688,"-","none","TGG","CGGGTCGTAAAACTTGAAAATGG",0,NA,NA,NA,"GeneID:57136","APMAP",TRUE,"APMAP"

"9517","GCCTGTCGCAGCTTCGTATT","chr20",24978822,"+","none","TGG","GCCTGTCGCAGCTTCGTATTTGG",0,NA,NA,NA,"GeneID:57136","APMAP",TRUE,"APMAP"

"9518","GTCCTCCGAGACACCCATTG","chr20",24970332,"-","none","AGG","GTCCTCCGAGACACCCATTGAGG",0,NA,NA,NA,"GeneID:57136","APMAP",TRUE,"APMAP"

"9519","TCCAAATACGAAGCTGCGAC","chr20",24978826,"-","none","AGG","TCCAAATACGAAGCTGCGACAGG",0,NA,NA,NA,"GeneID:57136","APMAP",TRUE,"APMAP"

"9520","TCGGAAAACTCTGCCGCTAA","chr20",24983994,"+","none","AGG","TCGGAAAACTCTGCCGCTAAAGG",0,NA,NA,NA,"GeneID:57136","APMAP",TRUE,"APMAP"

"9521","CAGGTCCTTCACTCGATCCC","chr11",116837086,"+","none","AGG","CAGGTCCTTCACTCGATCCCAGG",0,NA,NA,NA,"GeneID:335","APOA1",TRUE,"APOA1"

"9522","CAGGTCCTTCACTCGATCCC","chr22",27259023,"-","7:G>A,14:G>C","TGG","CAGGTCCCTCACTCCATCCCTGG",2,8,15,NA,"","",FALSE,"APOA1"

"9523","CCTACCCGTCAGGAAGAGCA","chr11",116837339,"+","none","CGG","CCTACCCGTCAGGAAGAGCACGG",0,NA,NA,NA,"GeneID:335","APOA1",TRUE,"APOA1"

"9524","CCTACCCGTCAGGAAGAGCA","chr6",39369407,"+","3:T>A,7:C>G","GGG","CCTTCCCCTCAGGAAGAGCAGGG",2,4,8,NA,"GeneID:221458","KIF6",FALSE,"APOA1"

"9525","CTGGCGCAGCTCGTCGCTGT","chr11",116836023,"+","none","AGG","CTGGCGCAGCTCGTCGCTGTAGG",0,NA,NA,NA,"GeneID:335","APOA1",TRUE,"APOA1"

"9526","CTTGGCCGTGCTCTTCCTGA","chr11",116837347,"-","none","CGG","CTTGGCCGTGCTCTTCCTGACGG",0,NA,NA,NA,"GeneID:335","APOA1",TRUE,"APOA1"

"9527","CTTGGCCGTGCTCTTCCTGA","chr16",89370006,"-","7:A>C,8:C>A","AGG","CTTGGCCTGGCTCTTCCTGAAGG",2,8,9,NA,"GeneID:29123","ANKRD11",FALSE,"APOA1"

"9528","GCTGCCAGAAATGCCGAGCC","chr11",116837130,"+","none","TGG","GCTGCCAGAAATGCCGAGCCTGG",0,NA,NA,NA,"GeneID:335","APOA1",TRUE,"APOA1"

"9529","GGCGTGGTACTCGGCCAGTC","chr11",116835957,"+","none","TGG","GGCGTGGTACTCGGCCAGTCTGG",0,NA,NA,NA,"GeneID:335","APOA1",TRUE,"APOA1"

"9530","ACCTGAGGGAATGTCGATGC","chr1",156593537,"-","none","TGG","ACCTGAGGGAATGTCGATGCTGG",0,NA,NA,NA,"GeneID:128240","APOA1BP",TRUE,"APOA1BP"

"9531","ATACAGTTCATCAATCGTCA","chr1",156593411,"-","none","TGG","ATACAGTTCATCAATCGTCATGG",0,NA,NA,NA,"GeneID:128240","APOA1BP",TRUE,"APOA1BP"

"9532","CAAGCCCCTCTTCACTGCAT","chr1",156592594,"+","none","TGG","CAAGCCCCTCTTCACTGCATTGG",0,NA,NA,NA,"GeneID:128240","APOA1BP",TRUE,"APOA1BP"

"9533","CCTCTGCGGGCATTTCCCCA","chr1",156592651,"-","none","AGG","CCTCTGCGGGCATTTCCCCAAGG",0,NA,NA,NA,"GeneID:128240","APOA1BP",TRUE,"APOA1BP"

"9534","TAACGAATACCAGTTCAGCG","chr1",156592136,"+","none","TGG","TAACGAATACCAGTTCAGCGTGG",0,NA,NA,NA,"GeneID:128240","APOA1BP",TRUE,"APOA1BP"

"9535","TAAGTTGGTCCACGCTGAAC","chr1",156592148,"-","none","TGG","TAAGTTGGTCCACGCTGAACTGG",0,NA,NA,NA,"GeneID:128240","APOA1BP",TRUE,"APOA1BP"

"9536","CCCACCTTCAAGGCTGCAGA","chr1",161223344,"+","none","TGG","CCCACCTTCAAGGCTGCAGATGG",0,NA,NA,NA,"GeneID:336","APOA2",TRUE,"APOA2"

"9537","CCCACCTTCAAGGCTGCAGA","chr16",4234493,"+","0:A>C,10:G>A","GGG","ACCACCTTCAGGGCTGCAGAGGG",2,1,11,NA,"GeneID:6345","SRL",FALSE,"APOA2"

"9538","CCCACCTTCAAGGCTGCAGA","chrX",76875348,"-","4:T>G,14:T>A","AGG","CCCAACTTCAAGGCAGCAGAAGG",2,5,15,NA,"GeneID:101928469","MIR325HG",FALSE,"APOA2"

"9539","CCCACCTTCAAGGCTGCAGA","chr13",104303768,"-","0:C>G,16:C>G","AGG","GCCACCTTCAAGGCTGGAGAAGG",2,1,17,NA,"","",FALSE,"APOA2"

"9540","CCGTTCCAGCCTTCTTGATC","chr1",161222463,"+","none","AGG","CCGTTCCAGCCTTCTTGATCAGG",0,NA,NA,NA,"GeneID:336","APOA2",TRUE,"APOA2"

"9541","CTCACCATCTGCAGCCTTGA","chr1",161223351,"-","none","AGG","CTCACCATCTGCAGCCTTGAAGG",0,NA,NA,NA,"GeneID:336","APOA2",TRUE,"APOA2"

"9542","GTCCTTGCCATAGTCAGTCA","chr1",161222961,"+","none","CGG","GTCCTTGCCATAGTCAGTCACGG",0,NA,NA,NA,"GeneID:336","APOA2",TRUE,"APOA2"

"9543","TAACTTCTTGAGCTATTTCG","chr1",161222436,"-","none","TGG","TAACTTCTTGAGCTATTTCGTGG",0,NA,NA,NA,"GeneID:336","APOA2",TRUE,"APOA2"

"9544","TTCCAGACCGTGACTGACTA","chr1",161222971,"-","none","TGG","TTCCAGACCGTGACTGACTATGG",0,NA,NA,NA,"GeneID:336","APOA2",TRUE,"APOA2"

"9545","ACTCACCGGCGACAGCCACC","chr11",116823136,"+","none","AGG","ACTCACCGGCGACAGCCACCAGG",0,NA,NA,NA,"GeneID:337","APOA4",TRUE,"APOA4"

"9546","CAGGTTGCCACGCACGTCCT","chr11",116821223,"+","none","CGG","CAGGTTGCCACGCACGTCCTCGG",0,NA,NA,NA,"GeneID:337","APOA4",TRUE,"APOA4"

"9547","GAAGTAGTCCCACATCACCG","chr11",116822729,"+","none","TGG","GAAGTAGTCCCACATCACCGTGG",0,NA,NA,NA,"GeneID:337","APOA4",TRUE,"APOA4"

"9548","GACTTTGAATTCGTCAGCGT","chr11",116821427,"+","none","AGG","GACTTTGAATTCGTCAGCGTAGG",0,NA,NA,NA,"GeneID:337","APOA4",TRUE,"APOA4"

"9549","GGGTCAGGACCACGGCCTTC","chr11",116823163,"+","none","AGG","GGGTCAGGACCACGGCCTTCAGG",0,NA,NA,NA,"GeneID:337","APOA4",TRUE,"APOA4"

"9550","GTCCCTCTTACTTGAGTTGC","chr11",116822647,"+","none","TGG","GTCCCTCTTACTTGAGTTGCTGG",0,NA,NA,NA,"GeneID:337","APOA4",TRUE,"APOA4"

"9551","AGAGCAGGTAATGGCAAGCA","chr11",116791850,"-","none","TGG","AGAGCAGGTAATGGCAAGCATGG",0,NA,NA,NA,"GeneID:116519","APOA5",TRUE,"APOA5"

"9552","CCCAGAAGCCTTTCCGTGCC","chr11",116791661,"+","none","TGG","CCCAGAAGCCTTTCCGTGCCTGG",0,NA,NA,NA,"GeneID:116519","APOA5",TRUE,"APOA5"

"9553","CCCAGAAGCCTTTCCGTGCC","chr5",130892173,"-","5:C>T,13:T>G","CGG","CCCAGGAGCCTTTACGTGCCCGG",2,6,14,NA,"","",FALSE,"APOA5"

"9554","CGCGAGCCATCTTCTGCTGA","chr11",116791592,"+","none","TGG","CGCGAGCCATCTTCTGCTGATGG",0,NA,NA,NA,"GeneID:116519","APOA5",TRUE,"APOA5"

"9555","CTTCACTCGCGCCATCGACC","chr11",116790315,"-","none","AGG","CTTCACTCGCGCCATCGACCAGG",0,NA,NA,NA,"GeneID:116519","APOA5",TRUE,"APOA5"

"9556","GCGCCTCAGTCGCTGCGTGC","chr11",116790543,"-","none","AGG","GCGCCTCAGTCGCTGCGTGCAGG",0,NA,NA,NA,"GeneID:116519","APOA5",TRUE,"APOA5"

"9557","TGCCGTGCTCACCTGGGCTC","chr11",116791826,"-","none","TGG","TGCCGTGCTCACCTGGGCTCTGG",0,NA,NA,NA,"GeneID:116519","APOA5",TRUE,"APOA5"

"9558","AGCTGGCGATGGACCCGCCG","chr2",21043933,"-","none","AGG","AGCTGGCGATGGACCCGCCGAGG",0,NA,NA,NA,"GeneID:338","APOB",TRUE,"APOB"

"9559","AGCTGGCGATGGACCCGCCG","chr19",35248420,"-","0:C>T,19:A>C","AGG","GGCTGGCGATGGACCCGCCTAGG",2,1,20,NA,"GeneID:51599","LSR",TRUE,"APOB"

"9560","ATGCCTTACTTGGACAGACC","chr2",21043503,"+","none","AGG","ATGCCTTACTTGGACAGACCAGG",0,NA,NA,NA,"GeneID:338","APOB",TRUE,"APOB"

"9561","CGACCCGATTCAAGCACCTC","chr2",21042450,"-","none","CGG","CGACCCGATTCAAGCACCTCCGG",0,NA,NA,NA,"GeneID:338","APOB",TRUE,"APOB"

"9562","CGCCAGCAGCGCCAGCAGCG","chr2",21043906,"+","none","CGG","CGCCAGCAGCGCCAGCAGCGCGG",0,NA,NA,NA,"GeneID:338","APOB",TRUE,"APOB"

"9563","CGCCAGCAGCGCCAGCAGCG","chr4",987109,"-","0:C>G,6:C>G","CGG","GGCCAGGAGCGCCAGCAGCGCGG",2,1,7,NA,"GeneID:10861;GeneID:3425","SLC26A1;IDUA",TRUE,"APOB"

"9564","CGCCAGCAGCGCCAGCAGCG","chr9",137077629,"-","7:A>T,8:G>C","CGG","CGCCAGCTCCGCCAGCAGCGCGG",2,8,9,NA,"GeneID:91373","UAP1L1",TRUE,"APOB"

"9565","CGCCAGCAGCGCCAGCAGCG","chr17",29005818,"+","0:A>C,6:G>C","AGG","AGCCAGGAGCGCCAGCAGCGAGG",2,1,7,NA,"GeneID:51268;GeneID:124925","PIPOX;SEZ6",TRUE,"APOB"

"9566","CGCCAGCAGCGCCAGCAGCG","chr16",598892,"+","1:T>G,10:T>G","TGG","CTCCAGCAGCTCCAGCAGCGTGG",2,2,11,NA,"GeneID:57799","RAB40C",FALSE,"APOB"

"9567","CGCCAGCAGCGCCAGCAGCG","chr10",23199587,"+","3:T>C,11:A>C","GGG","CGCTAGCAGCGACAGCAGCGGGG",2,4,12,NA,"","",FALSE,"APOB"

"9568","CGCCAGCAGCGCCAGCAGCG","chr7",158829295,"+","7:T>A,19:C>G","CGG","CGCCAGCTGCGCCAGCAGCCCGG",2,8,20,NA,"GeneID:57488","ESYT2",TRUE,"APOB"

"9569","CGCCAGCAGCGCCAGCAGCG","chr11",64293031,"-","9:C>G,15:C>G","TGG","CGCCAGCAGGGCCAGGAGCGTGG",2,10,16,NA,"GeneID:50801","KCNK4",TRUE,"APOB"

"9570","CTGATTCAAGAAGTGCCACC","chr2",21042375,"-","none","AGG","CTGATTCAAGAAGTGCCACCAGG",0,NA,NA,NA,"GeneID:338","APOB",TRUE,"APOB"

"9571","TCTTACAGAAGAGGAAATGC","chr2",21043539,"-","none","TGG","TCTTACAGAAGAGGAAATGCTGG",0,NA,NA,NA,"GeneID:338","APOB",TRUE,"APOB"

"9572","TCTTACAGAAGAGGAAATGC","chr5",15181276,"-","19:C>G","AGG","TCTTACAGAAGAGGAAATGGAGG",1,20,NA,NA,"","",FALSE,"APOB"

"9573","TCTTACAGAAGAGGAAATGC","chr3",129114432,"-","19:T>G","AGG","TCTTACAGAAGAGGAAATGAAGG",1,20,NA,NA,"GeneID:339122;GeneID:100534599","RAB43;ISY1-RAB43",FALSE,"APOB"

"9574","TCTTACAGAAGAGGAAATGC","chr11",125729538,"-","13:G>C,17:T>A","TGG","TCTTACAGAAGAGCAAAAGCTGG",2,14,18,NA,"","",FALSE,"APOB"

"9575","TCTTACAGAAGAGGAAATGC","chr4",169547187,"-","10:T>C,13:T>C","TGG","TCTTACAGAAAAGAAAATGCTGG",2,11,14,NA,"GeneID:4750","NEK1",FALSE,"APOB"

"9576","TCTTACAGAAGAGGAAATGC","chr2",104029235,"+","18:A>G,19:T>C","AGG","TCTTACAGAAGAGGAAATATAGG",2,19,20,NA,"","",FALSE,"APOB"

"9577","TCTTACAGAAGAGGAAATGC","chr9",114093688,"+","17:C>T,18:C>G","AGG","TCTTACAGAAGAGGAAACCCAGG",2,18,19,NA,"GeneID:113220","KIF12",FALSE,"APOB"

"9578","TCTTACAGAAGAGGAAATGC","chr20",51034815,"+","15:C>A,19:G>C","AGG","TCTTACAGAAGAGGACATGGAGG",2,16,20,NA,"","",FALSE,"APOB"

"9579","TCTTACAGAAGAGGAAATGC","chr5",168014312,"+","10:T>G,19:G>C","AGG","TCTTACAGAATAGGAAATGGAGG",2,11,20,NA,"GeneID:57451","TENM2",FALSE,"APOB"

"9580","TCTTACAGAAGAGGAAATGC","chr15",91819284,"-","9:A>T","AGG","TCTTACAGATGAGGAAATGCAGG",1,10,NA,NA,"","",FALSE,"APOB"

"9581","TCTTACAGAAGAGGAAATGC","chr11",14342366,"-","2:G>A,9:A>T","AGG","TCCTACAGATGAGGAAATGCAGG",2,3,10,NA,"GeneID:22800","RRAS2",FALSE,"APOB"

"9582","TCTTACAGAAGAGGAAATGC","chr11",134422224,"+","1:T>C,7:A>G","AGG","TTTTACAAAAGAGGAAATGCAGG",2,2,8,NA,"","",FALSE,"APOB"

"9583","TCTTACAGAAGAGGAAATGC","chr15",51157436,"+","1:T>C,9:T>A","AGG","TTTTACAGATGAGGAAATGCAGG",2,2,10,NA,"","",FALSE,"APOB"

"9584","TCTTACAGAAGAGGAAATGC","chr5",141731564,"+","1:T>C,9:T>A","AGG","TTTTACAGATGAGGAAATGCAGG",2,2,10,NA,"","",FALSE,"APOB"

"9585","TCTTACAGAAGAGGAAATGC","chr1",32864302,"+","1:T>C,19:A>C","AGG","TTTTACAGAAGAGGAAATGAAGG",2,2,20,NA,"GeneID:252995","FNDC5",TRUE,"APOB"

"9586","TCTTACAGAAGAGGAAATGC","chr8",132276300,"+","1:T>C,19:A>C","AGG","TTTTACAGAAGAGGAAATGAAGG",2,2,20,NA,"GeneID:3786","KCNQ3",FALSE,"APOB"

"9587","TCTTACAGAAGAGGAAATGC","chr18",11993181,"+","1:A>C,19:A>C","AGG","TATTACAGAAGAGGAAATGAAGG",2,2,20,NA,"GeneID:3613","IMPA2",FALSE,"APOB"

"9588","TCTTACAGAAGAGGAAATGC","chr10",124156603,"+","8:C>A,19:A>C","GGG","TCTTACAGCAGAGGAAATGAGGG",2,9,20,NA,"","",FALSE,"APOB"

"9589","TCTTACAGAAGAGGAAATGC","chr20",43221854,"+","1:A>C,19:G>C","AGG","TATTACAGAAGAGGAAATGGAGG",2,2,20,NA,"","",FALSE,"APOB"

"9590","TCTTACAGAAGAGGAAATGC","chr3",187811104,"+","9:G>A,19:G>C","AGG","TCTTACAGAGGAGGAAATGGAGG",2,10,20,NA,"","",FALSE,"APOB"

"9591","TCTTACAGAAGAGGAAATGC","chr10",122485531,"+","9:T>A,19:G>C","AGG","TCTTACAGATGAGGAAATGGAGG",2,10,20,NA,"GeneID:5654","HTRA1",FALSE,"APOB"

"9592","TCTTACAGAAGAGGAAATGC","chr16",28599749,"+","9:T>A,19:G>C","AGG","TCTTACAGATGAGGAAATGGAGG",2,10,20,NA,"","",FALSE,"APOB"

"9593","TCTTACAGAAGAGGAAATGC","chr16",28612743,"+","9:T>A,19:G>C","AGG","TCTTACAGATGAGGAAATGGAGG",2,10,20,NA,"GeneID:6817","SULT1A1",TRUE,"APOB"

"9594","TCTTACAGAAGAGGAAATGC","chr10",71074682,"-","9:A>T,19:C>G","AGG","TCTTACAGATGAGGAAATGGAGG",2,10,20,NA,"","",FALSE,"APOB"

"9595","TCTTACAGAAGAGGAAATGC","chr14",73801841,"-","9:A>T,19:C>G","AGG","TCTTACAGATGAGGAAATGGAGG",2,10,20,NA,"","",FALSE,"APOB"

"9596","TCTTACAGAAGAGGAAATGC","chr6",138109271,"-","9:A>T,19:C>G","AGG","TCTTACAGATGAGGAAATGGAGG",2,10,20,NA,"","",FALSE,"APOB"

"9597","TCTTACAGAAGAGGAAATGC","chr2",10762085,"-","9:A>T,17:G>A","TGG","TCTTACAGATGAGGAAACGCTGG",2,10,18,NA,"GeneID:245973","ATP6V1C2",FALSE,"APOB"

"9598","TCTTACAGAAGAGGAAATGC","chr8",29781637,"-","1:A>G,19:C>G","AGG","TTTTACAGAAGAGGAAATGGAGG",2,2,20,NA,"GeneID:101929450","LOC101929450",FALSE,"APOB"

"9599","TCTTACAGAAGAGGAAATGC","chr13",99791592,"-","1:A>G,19:C>G","AGG","TTTTACAGAAGAGGAAATGGAGG",2,2,20,NA,"GeneID:171425","CLYBL",FALSE,"APOB"

"9600","TCTTACAGAAGAGGAAATGC","chr5",174655910,"-","1:A>G,19:C>G","AGG","TTTTACAGAAGAGGAAATGGAGG",2,2,20,NA,"","",FALSE,"APOB"

"9601","CCCCAGAGAACTTCGTAAAG","chr12",7652776,"-","none","AGG","CCCCAGAGAACTTCGTAAAGAGG",0,NA,NA,NA,"GeneID:339","APOBEC1",TRUE,"APOBEC1"

"9602","CGGAAGATCTGGCGAAGCTC","chr12",7652718,"-","none","AGG","CGGAAGATCTGGCGAAGCTCAGG",0,NA,NA,NA,"GeneID:339","APOBEC1",TRUE,"APOBEC1"

"9603","GCGTACAACATCATCCACAG","chr12",7651047,"+","none","AGG","GCGTACAACATCATCCACAGAGG",0,NA,NA,NA,"GeneID:339","APOBEC1",TRUE,"APOBEC1"

"9604","GTATCAGCCCTGTAGCTAAA","chr12",7649569,"+","none","AGG","GTATCAGCCCTGTAGCTAAAAGG",0,NA,NA,NA,"GeneID:339","APOBEC1",TRUE,"APOBEC1"

"9605","GTGGCGGAATCGTTTGGTAA","chr12",7649596,"+","none","TGG","GTGGCGGAATCGTTTGGTAATGG",0,NA,NA,NA,"GeneID:339","APOBEC1",TRUE,"APOBEC1"

"9606","TGGTCAACAGGTCCTTCAAC","chr12",7654622,"-","none","CGG","TGGTCAACAGGTCCTTCAACCGG",0,NA,NA,NA,"GeneID:339","APOBEC1",TRUE,"APOBEC1"

"9607","ACCAGGTGACATTGTACCGC","chr6",41061540,"-","none","AGG","ACCAGGTGACATTGTACCGCAGG",0,NA,NA,NA,"GeneID:221443;GeneID:10930","OARD1;APOBEC2",TRUE,"APOBEC2"

"9608","CCGCCCTTTGAGATTGTCAC","chr6",41053455,"+","none","AGG","CCGCCCTTTGAGATTGTCACAGG",0,NA,NA,NA,"GeneID:221443;GeneID:10930","OARD1;APOBEC2",TRUE,"APOBEC2"

"9609","CCGCCCTTTGAGATTGTCAC","chr6",15916052,"+","1:A>C,14:G>T","GGG","CAGCCCTTTGAGATGGTCACGGG",2,2,15,NA,"","",FALSE,"APOBEC2"

"9610","CCTATACTACGAGGAGAAGT","chr6",41061831,"+","none","TGG","CCTATACTACGAGGAGAAGTTGG",0,NA,NA,NA,"GeneID:221443;GeneID:10930","OARD1;APOBEC2",TRUE,"APOBEC2"

"9611","CGGAACTGTACTCCACATTC","chr6",41061366,"-","none","CGG","CGGAACTGTACTCCACATTCCGG",0,NA,NA,NA,"GeneID:221443;GeneID:10930","OARD1;APOBEC2",TRUE,"APOBEC2"

"9612","GCCAGACATATTCGAAGTCC","chr6",41061741,"-","none","TGG","GCCAGACATATTCGAAGTCCTGG",0,NA,NA,NA,"GeneID:221443;GeneID:10930","OARD1;APOBEC2",TRUE,"APOBEC2"

"9613","TTACCCTGTGACAATCTCAA","chr6",41053462,"-","none","AGG","TTACCCTGTGACAATCTCAAAGG",0,NA,NA,NA,"GeneID:221443;GeneID:10930","OARD1;APOBEC2",TRUE,"APOBEC2"

"9614","ATGGAAGCCAGCCCAGCATC","chr22",38957691,"+","none","CGG","ATGGAAGCCAGCCCAGCATCCGG",0,NA,NA,NA,"GeneID:200315","APOBEC3A",TRUE,"APOBEC3A"

"9615","ATGGAAGCCAGCCCAGCATC","chr16",85729971,"+","18:G>T,19:T>C","GGG","ATGGAAGCCAGCCCAGCAGTGGG",2,19,20,NA,"GeneID:404550","C16orf74",FALSE,"APOBEC3A"

"9616","CAGGGGCTTTCTACACAACC","chr22",38959664,"+","none","AGG","CAGGGGCTTTCTACACAACCAGG",0,NA,NA,NA,"GeneID:200315","APOBEC3A",TRUE,"APOBEC3A"

"9617","CAGGGGCTTTCTACACAACC","chr22",39083862,"+","13:T>C,14:G>A","AGG","CAGGGGCTTTCTATGCAACCAGG",2,14,15,NA,"GeneID:60489","APOBEC3G",TRUE,"APOBEC3A"

"9618","CCATACCTGGGCCCGGATGC","chr22",38957706,"-","none","TGG","CCATACCTGGGCCCGGATGCTGG",0,NA,NA,NA,"GeneID:200315","APOBEC3A",TRUE,"APOBEC3A"

"9619","CCATACCTGGGCCCGGATGC","chr2",135804760,"+","11:G>C,13:T>C","TGG","CCATACCTGGGGCTGGATGCTGG",2,12,14,NA,"GeneID:3938","LCT",TRUE,"APOBEC3A"

"9620","CCATACCTGGGCCCGGATGC","chr1",43532922,"+","0:G>C,15:C>G","CGG","GCATACCTGGGCCCGCATGCCGG",2,1,16,NA,"GeneID:5792","PTPRF",FALSE,"APOBEC3A"

"9621","CTTACCATCGTAGGTCATGA","chr22",38961666,"-","none","TGG","CTTACCATCGTAGGTCATGATGG",0,NA,NA,NA,"GeneID:200315","APOBEC3A",TRUE,"APOBEC3A"

"9622","CTTACCATCGTAGGTCATGA","chr22",38991611,"-","none","TGG","CTTACCATCGTAGGTCATGATGG",0,NA,NA,NA,"GeneID:200315;GeneID:9582;GeneID:100874530","APOBEC3A;APOBEC3B;APOBEC3B-AS1",TRUE,"APOBEC3A"

"9623","GACCTACCTGTGCTACGAAG","chr22",38959601,"+","none","TGG","GACCTACCTGTGCTACGAAGTGG",0,NA,NA,NA,"GeneID:200315","APOBEC3A",TRUE,"APOBEC3A"

"9624","TTTGCAGTGCCTCCTTATAT","chr22",38961616,"-","none","AGG","TTTGCAGTGCCTCCTTATATAGG",0,NA,NA,NA,"GeneID:200315","APOBEC3A",TRUE,"APOBEC3A"

"9625","TTTGCAGTGCCTCCTTATAT","chr22",38991561,"-","7:G>A","AGG","TTTGCAGCGCCTCCTTATATAGG",1,8,NA,NA,"GeneID:200315;GeneID:9582;GeneID:100874530","APOBEC3A;APOBEC3B;APOBEC3B-AS1",TRUE,"APOBEC3A"

"9626","CAGGGGCTTTCTACACAACC","chr22",38959664,"+","none","AGG","CAGGGGCTTTCTACACAACCAGG",0,NA,NA,NA,"GeneID:200315","APOBEC3A",TRUE,"APOBEC3A_B"

"9627","CAGGGGCTTTCTACACAACC","chr22",39083862,"+","13:T>C,14:G>A","AGG","CAGGGGCTTTCTATGCAACCAGG",2,14,15,NA,"GeneID:60489","APOBEC3G",TRUE,"APOBEC3A_B"

"9628","CTTACCATCGTAGGTCATGA","chr22",38961666,"-","none","TGG","CTTACCATCGTAGGTCATGATGG",0,NA,NA,NA,"GeneID:200315","APOBEC3A",TRUE,"APOBEC3A_B"

"9629","CTTACCATCGTAGGTCATGA","chr22",38991611,"-","none","TGG","CTTACCATCGTAGGTCATGATGG",0,NA,NA,NA,"GeneID:200315;GeneID:9582;GeneID:100874530","APOBEC3A;APOBEC3B;APOBEC3B-AS1",TRUE,"APOBEC3A_B"

"9630","GACCTACCTGTGCTACGAAG","chr22",38959601,"+","none","TGG","GACCTACCTGTGCTACGAAGTGG",0,NA,NA,NA,"GeneID:200315","APOBEC3A",TRUE,"APOBEC3A_B"

"9631","GTATTAATGAAGTGATTAAT","chr22",38992697,"+","none","TGG","GTATTAATGAAGTGATTAATTGG",0,NA,NA,NA,"GeneID:200315;GeneID:9582;GeneID:100874530","APOBEC3A;APOBEC3B;APOBEC3B-AS1",TRUE,"APOBEC3A_B"

"9632","GTATTAATGAAGTGATTAAT","chr5",54950318,"+","5:C>A,10:T>A","TGG","GTATTCATGATGTGATTAATTGG",2,6,11,NA,"","",FALSE,"APOBEC3A_B"

"9633","TAATCTTACTCTTGTACACT","chr22",38992648,"-","none","TGG","TAATCTTACTCTTGTACACTTGG",0,NA,NA,NA,"GeneID:200315;GeneID:9582;GeneID:100874530","APOBEC3A;APOBEC3B;APOBEC3B-AS1",TRUE,"APOBEC3A_B"

"9634","TTTGCAGTGCCTCCTTATAT","chr22",38961616,"-","none","AGG","TTTGCAGTGCCTCCTTATATAGG",0,NA,NA,NA,"GeneID:200315","APOBEC3A",TRUE,"APOBEC3A_B"

"9635","TTTGCAGTGCCTCCTTATAT","chr22",38991561,"-","7:G>A","AGG","TTTGCAGCGCCTCCTTATATAGG",1,8,NA,NA,"GeneID:200315;GeneID:9582;GeneID:100874530","APOBEC3A;APOBEC3B;APOBEC3B-AS1",TRUE,"APOBEC3A_B"

"9636","AGGAGCCCGCGTGACGATCA","chr22",38986059,"+","none","TGG","AGGAGCCCGCGTGACGATCATGG",0,NA,NA,NA,"GeneID:200315;GeneID:9582","APOBEC3A;APOBEC3B",TRUE,"APOBEC3B"

"9637","CATAGTCCATGATCGTCACG","chr22",38986068,"-","none","CGG","CATAGTCCATGATCGTCACGCGG",0,NA,NA,NA,"GeneID:200315;GeneID:9582","APOBEC3A;APOBEC3B",TRUE,"APOBEC3B"

"9638","CATAGTCCATGATCGTCACG","chr22",39025326,"-","14:A>C,19:T>C","CGG","CATAGTCCATGATCTTCACACGG",2,15,20,NA,"GeneID:140564","APOBEC3D",TRUE,"APOBEC3B"

"9639","CCAGACCTACTTGTGCTATG","chr22",38989522,"+","none","AGG","CCAGACCTACTTGTGCTATGAGG",0,NA,NA,NA,"GeneID:200315;GeneID:9582","APOBEC3A;APOBEC3B",TRUE,"APOBEC3B"

"9640","GAAAACTTTGTGTACAATGA","chr22",38986314,"+","none","AGG","GAAAACTTTGTGTACAATGAAGG",0,NA,NA,NA,"GeneID:200315;GeneID:9582","APOBEC3A;APOBEC3B",TRUE,"APOBEC3B"

"9641","GAAAACTTTGTGTACAATGA","chr22",39045444,"+","16:G>A","AGG","GAAAACTTTGTGTACAGTGAAGG",1,17,NA,NA,"GeneID:200316","APOBEC3F",TRUE,"APOBEC3B"

"9642","GAAAACTTTGTGTACAATGA","chr22",39025573,"+","13:G>A","AGG","GAAAACTTTGTGTGCAATGAAGG",1,14,NA,NA,"GeneID:140564","APOBEC3D",TRUE,"APOBEC3B"

"9643","GAAAGACCCCTGTGTCCCAA","chr22",38984203,"-","none","AGG","GAAAGACCCCTGTGTCCCAAAGG",0,NA,NA,NA,"GeneID:200315;GeneID:9582","APOBEC3A;APOBEC3B",TRUE,"APOBEC3B"

"9644","GAAAGACCCCTGTGTCCCAA","chr22",39022950,"-","none","AGG","GAAAGACCCCTGTGTCCCAAAGG",0,NA,NA,NA,"GeneID:140564","APOBEC3D",TRUE,"APOBEC3B"

"9645","GAAAGACCCCTGTGTCCCAA","chr3",42294889,"-","15:A>G,19:G>T","TGG","GAAAGACCCCTGTGTTCCACTGG",2,16,20,NA,"","",FALSE,"APOBEC3B"

"9646","GAAAGACCCCTGTGTCCCAA","chr10",79015191,"+","1:G>A","AGG","GGAAGACCCCTGTGTCCCAAAGG",1,2,NA,NA,"GeneID:283050","ZMIZ1-AS1",FALSE,"APOBEC3B"

"9647","GAAAGACCCCTGTGTCCCAA","chr8",2614576,"+","0:T>G,11:C>G","GGG","TAAAGACCCCTCTGTCCCAAGGG",2,1,12,NA,"","",FALSE,"APOBEC3B"

"9648","TGGCGCCGTCGAAGGACCAA","chr22",38989505,"-","none","AGG","TGGCGCCGTCGAAGGACCAAAGG",0,NA,NA,NA,"GeneID:200315;GeneID:9582","APOBEC3A;APOBEC3B",TRUE,"APOBEC3B"

"9649","AATCCCTTCCAAGGCTTGAA","chr22",39018315,"-","none","TGG","AATCCCTTCCAAGGCTTGAATGG",0,NA,NA,NA,"GeneID:27350","APOBEC3C",TRUE,"APOBEC3C"

"9650","AATCCCTTCCAAGGCTTGAA","chr22",39032244,"-","1:C>T","TGG","AGTCCCTTCCAAGGCTTGAATGG",1,2,NA,NA,"GeneID:140564","APOBEC3D",TRUE,"APOBEC3C"

"9651","AATCCCTTCCAAGGCTTGAA","chr22",39052623,"-","1:C>T,5:A>G","TGG","AGTCCTTTCCAAGGCTTGAATGG",2,2,6,NA,"GeneID:200316","APOBEC3F",TRUE,"APOBEC3C"

"9652","AATCCCTTCCAAGGCTTGAA","chrX",54346673,"-","4:A>G,14:T>G","AGG","AATCTCTTCCAAGGATTGAAAGG",2,5,15,NA,"GeneID:65267","WNK3",FALSE,"APOBEC3C"

"9653","CCAACGATCGGAACGAAACT","chr22",39015656,"+","none","TGG","CCAACGATCGGAACGAAACTTGG",0,NA,NA,NA,"GeneID:27350","APOBEC3C",TRUE,"APOBEC3C"

"9654","CCAAGTTTCGTTCCGATCGT","chr22",39015659,"-","none","TGG","CCAAGTTTCGTTCCGATCGTTGG",0,NA,NA,NA,"GeneID:27350","APOBEC3C",TRUE,"APOBEC3C"

"9655","CGGAGCCCCTCCTGGTAACA","chr22",39017978,"-","none","TGG","CGGAGCCCCTCCTGGTAACATGG",0,NA,NA,NA,"GeneID:27350","APOBEC3C",TRUE,"APOBEC3C"

"9656","CTACGGGAGAGTCTCCAGTG","chr22",39018366,"+","none","AGG","CTACGGGAGAGTCTCCAGTGAGG",0,NA,NA,NA,"GeneID:27350","APOBEC3C",TRUE,"APOBEC3C"

"9657","CTACGGGAGAGTCTCCAGTG","chr22",39032295,"+","10:T>G","AGG","CTACGGGAGATTCTCCAGTGAGG",1,11,NA,NA,"GeneID:140564","APOBEC3D",TRUE,"APOBEC3C"

"9658","CTACGGGAGAGTCTCCAGTG","chr15",73308608,"+","6:T>G,11:A>T","TGG","CTACGGTAGAGACTCCAGTGTGG",2,7,12,NA,"","",FALSE,"APOBEC3C"

"9659","GTCTCCTAACACAAAGTACC","chr22",39017830,"+","none","AGG","GTCTCCTAACACAAAGTACCAGG",0,NA,NA,NA,"GeneID:27350","APOBEC3C",TRUE,"APOBEC3C"

"9660","GTCTCCTAACACAAAGTACC","chr22",39052138,"+","15:C>G,19:G>C","AGG","GTCTCCTAACACAAACTACGAGG",2,16,20,NA,"GeneID:200316","APOBEC3F",TRUE,"APOBEC3C"

"9661","GTCTCCTAACACAAAGTACC","chr22",39031758,"+","15:C>G,19:G>C","AGG","GTCTCCTAACACAAACTACGAGG",2,16,20,NA,"GeneID:140564","APOBEC3D",TRUE,"APOBEC3C"

"9662","GTCTCCTAACACAAAGTACC","chr22",39070457,"+","11:A>C,15:C>G","AGG","GTCTCCTAACAAAAACTACCAGG",2,12,16,NA,"","",TRUE,"APOBEC3C"

"9663","GTCTCCTAACACAAAGTACC","chr7",132242958,"+","1:G>T,10:C>A","TGG","GGCTCCTAACCCAAAGTACCTGG",2,2,11,NA,"GeneID:91584","PLXNA4",FALSE,"APOBEC3C"

"9664","ATCTGGAAGCGCCTGTTAGC","chr22",39025138,"-","none","AGG","ATCTGGAAGCGCCTGTTAGCAGG",0,NA,NA,NA,"GeneID:140564","APOBEC3D",TRUE,"APOBEC3D"

"9665","ATTGGCGGTGGGTGCTCCTC","chr22",39025283,"+","none","AGG","ATTGGCGGTGGGTGCTCCTCAGG",0,NA,NA,NA,"GeneID:140564","APOBEC3D",TRUE,"APOBEC3D"

"9666","ATTGGCGGTGGGTGCTCCTC","chr1",157679315,"-","5:C>G,15:G>A","TGG","ATTGGGGGTGGGTGCCCCTCTGG",2,6,16,NA,"GeneID:115352","FCRL3",FALSE,"APOBEC3D"

"9667","CCAAACGTCAGTCGAATCAC","chr22",39022985,"+","none","AGG","CCAAACGTCAGTCGAATCACAGG",0,NA,NA,NA,"GeneID:140564","APOBEC3D",TRUE,"APOBEC3D"

"9668","CCAAACGTCAGTCGAATCAC","chr22",38984238,"+","6:T>G,10:A>G","AGG","CCAAACTTCAATCGAATCACAGG",2,7,11,NA,"GeneID:200315;GeneID:9582","APOBEC3A;APOBEC3B",TRUE,"APOBEC3D"

"9669","CCTGTGATTCGACTGACGTT","chr22",39022988,"-","none","TGG","CCTGTGATTCGACTGACGTTTGG",0,NA,NA,NA,"GeneID:140564","APOBEC3D",TRUE,"APOBEC3D"

"9670","CCTGTGATTCGACTGACGTT","chr22",38984241,"-","12:A>G,16:T>G","TGG","CCTGTGATTCGATTGAAGTTTGG",2,13,17,NA,"GeneID:200315;GeneID:9582","APOBEC3A;APOBEC3B",TRUE,"APOBEC3D"

"9671","GCAACCGACTGCCTGCTAAC","chr22",39025124,"+","none","AGG","GCAACCGACTGCCTGCTAACAGG",0,NA,NA,NA,"GeneID:140564","APOBEC3D",TRUE,"APOBEC3D"

"9672","TGAACATGAATCCACAGATC","chr22",39021514,"+","none","AGG","TGAACATGAATCCACAGATCAGG",0,NA,NA,NA,"GeneID:140564","APOBEC3D",TRUE,"APOBEC3D"

"9673","TGAACATGAATCCACAGATC","chr22",38982448,"+","none","AGG","TGAACATGAATCCACAGATCAGG",0,NA,NA,NA,"GeneID:200315;GeneID:9582","APOBEC3A;APOBEC3B",TRUE,"APOBEC3D"

"9674","TGAACATGAATCCACAGATC","chr22",39014357,"+","none","AGG","TGAACATGAATCCACAGATCAGG",0,NA,NA,NA,"GeneID:27350","APOBEC3C",TRUE,"APOBEC3D"

"9675","TGAACATGAATCCACAGATC","chr12",91499533,"+","11:G>C,14:G>C","AGG","TGAACATGAATGCAGAGATCAGG",2,12,15,NA,"","",FALSE,"APOBEC3D"

"9676","TGAACATGAATCCACAGATC","chr8",138918885,"+","8:G>A,17:C>A","TGG","TGAACATGGATCCACAGCTCTGG",2,9,18,NA,"","",FALSE,"APOBEC3D"

"9677","AAAAACCTACGCAAAGCCTA","chr22",39049470,"+","none","TGG","AAAAACCTACGCAAAGCCTATGG",0,NA,NA,NA,"GeneID:200316","APOBEC3F",TRUE,"APOBEC3F"

"9678","AAAAACCTACGCAAAGCCTA","chr20",52449532,"-","10:T>C,19:A>T","TGG","AAAAACCTACACAAAGCCTTTGG",2,11,20,NA,"GeneID:101927700","LINC01524",FALSE,"APOBEC3F"

"9679","AGGGGCCCGCGTGAAGATTA","chr22",39045188,"+","none","TGG","AGGGGCCCGCGTGAAGATTATGG",0,NA,NA,NA,"GeneID:200316","APOBEC3F",TRUE,"APOBEC3F"

"9680","AGGGGCCCGCGTGAAGATTA","chr22",39025317,"+","9:T>C,18:C>T","TGG","AGGGGCCCGTGTGAAGATCATGG",2,10,19,NA,"GeneID:140564","APOBEC3D",TRUE,"APOBEC3F"

"9681","CATCGTCCATAATCTTCACG","chr22",39045197,"-","none","CGG","CATCGTCCATAATCTTCACGCGG",0,NA,NA,NA,"GeneID:200316","APOBEC3F",TRUE,"APOBEC3F"

"9682","CATCGTCCATAATCTTCACG","chr15",98856305,"+","11:G>A,19:C>G","AGG","CATCGTCCATAGTCTTCACCAGG",2,12,20,NA,"GeneID:3480","IGF1R",FALSE,"APOBEC3F"

"9683","CCCACCACATGGGACAGCGC","chr22",39044144,"+","none","AGG","CCCACCACATGGGACAGCGCAGG",0,NA,NA,NA,"GeneID:200316","APOBEC3F",TRUE,"APOBEC3F"

"9684","CCCACCACATGGGACAGCGC","chr6",42336086,"+","0:G>C,2:T>C","AGG","GCTACCACATGGGACAGCGCAGG",2,1,3,NA,"GeneID:55809","TRERF1",FALSE,"APOBEC3F"

"9685","TCGGAAGACGCCCCTCTTCC","chr22",39049555,"-","none","AGG","TCGGAAGACGCCCCTCTTCCAGG",0,NA,NA,NA,"GeneID:200316","APOBEC3F",TRUE,"APOBEC3F"

"9686","TCGGAAGACGCCCCTCTTCC","chr22",39015725,"-","13:C>G","AGG","TCGGAAGACGCCCGTCTTCCAGG",1,14,NA,NA,"GeneID:27350","APOBEC3C",TRUE,"APOBEC3F"

"9687","TGGCCTCCCCAGCTGACCGC","chr22",39044172,"+","none","AGG","TGGCCTCCCCAGCTGACCGCAGG",0,NA,NA,NA,"GeneID:200316","APOBEC3F",TRUE,"APOBEC3F"

"9688","TGGCCTCCCCAGCTGACCGC","chr1",227733897,"+","7:A>C,15:C>A","AGG","TGGCCTCACCAGCTGCCCGCAGG",2,8,16,NA,"GeneID:116841;GeneID:65094","SNAP47;JMJD4",TRUE,"APOBEC3F"

"9689","TGGCCTCCCCAGCTGACCGC","chr10",49167423,"-","2:A>C,17:A>G","AGG","TGTCCTCCCCAGCTGACTGCAGG",2,3,18,NA,"GeneID:170371","C10orf128",FALSE,"APOBEC3F"

"9690","AAGGCTCAAATAGCTCTCTT","chr22",39081507,"-","none","TGG","AAGGCTCAAATAGCTCTCTTTGG",0,NA,NA,NA,"GeneID:60489","APOBEC3G",TRUE,"APOBEC3G"

"9691","CGAAAGATCTTTGCGTCCAA","chr22",39079058,"-","none","AGG","CGAAAGATCTTTGCGTCCAAAGG",0,NA,NA,NA,"GeneID:60489","APOBEC3G",TRUE,"APOBEC3G"

"9692","CGAAAGATCTTTGCGTCCAA","chr12",108282068,"+","1:C>G,9:C>T","AGG","CCAAAGATCCTTGCGTCCAAAGG",2,2,10,NA,"","",TRUE,"APOBEC3G"

"9693","CGAAGCGCCTCCTGGTAATC","chr22",39081148,"-","none","TGG","CGAAGCGCCTCCTGGTAATCTGG",0,NA,NA,NA,"GeneID:60489","APOBEC3G",TRUE,"APOBEC3G"

"9694","GAGCACAGCCAAGACCTGAG","chr22",39087081,"+","none","TGG","GAGCACAGCCAAGACCTGAGTGG",0,NA,NA,NA,"GeneID:60489","APOBEC3G",TRUE,"APOBEC3G"

"9695","GAGCACAGCCAAGACCTGAG","chr22",38962168,"+","13:C>A","TGG","GAGCACAGCCAAGCCCTGAGTGG",1,14,NA,NA,"GeneID:200315","APOBEC3A",TRUE,"APOBEC3G"

"9696","GAGCACAGCCAAGACCTGAG","chr22",38992104,"+","13:C>A","TGG","GAGCACAGCCAAGCCCTGAGTGG",1,14,NA,NA,"GeneID:200315;GeneID:9582;GeneID:100874530","APOBEC3A;APOBEC3B;APOBEC3B-AS1",TRUE,"APOBEC3G"

"9697","GAGCACAGCCAAGACCTGAG","chr16",87150288,"+","10:C>A,13:C>A","AGG","GAGCACAGCCCAGCCCTGAGAGG",2,11,14,NA,"GeneID:100506581","C16orf95",FALSE,"APOBEC3G"

"9698","GAGCACAGCCAAGACCTGAG","chr14",67527149,"+","5:G>C,8:G>C","TGG","GAGCAGAGGCAAGACCTGAGTGG",2,6,9,NA,"GeneID:161145","TMEM229B",FALSE,"APOBEC3G"

"9699","GAGCACAGCCAAGACCTGAG","chr11",72736587,"+","0:C>G,14:G>C","GGG","CAGCACAGCCAAGAGCTGAGGGG",2,1,15,NA,"GeneID:116985","ARAP1",FALSE,"APOBEC3G"

"9700","GAGCACAGCCAAGACCTGAG","chr4",88941735,"-","5:C>G,10:G>T","AGG","GAGCAGAGCCCAGACCTGAGAGG",2,6,11,NA,"GeneID:10144","FAM13A",FALSE,"APOBEC3G"

"9701","GAGGAAGCTGCATCGTGACC","chr22",39080991,"+","none","AGG","GAGGAAGCTGCATCGTGACCAGG",0,NA,NA,NA,"GeneID:60489","APOBEC3G",TRUE,"APOBEC3G"

"9702","TATATTACTGCACATCATGC","chr22",39081549,"+","none","TGG","TATATTACTGCACATCATGCTGG",0,NA,NA,NA,"GeneID:60489","APOBEC3G",TRUE,"APOBEC3G"

"9703","TATATTACTGCACATCATGC","chr6",128855784,"-","12:T>G,14:C>A","AGG","TATATTACTGCAAAGCATGCAGG",2,13,15,NA,"","",FALSE,"APOBEC3G"

"9704","AAGTCACCTGTTACCTCACG","chr22",39101309,"+","none","TGG","AAGTCACCTGTTACCTCACGTGG",0,NA,NA,NA,"GeneID:164668","APOBEC3H",TRUE,"APOBEC3H"

"9705","ACAGTCGAGCCATAAAGCGA","chr22",39102004,"+","none","CGG","ACAGTCGAGCCATAAAGCGACGG",0,NA,NA,NA,"GeneID:164668","APOBEC3H",TRUE,"APOBEC3H"

"9706","CCTCTCAAGCCGTCGCTTTA","chr22",39102016,"-","none","TGG","CCTCTCAAGCCGTCGCTTTATGG",0,NA,NA,NA,"GeneID:164668","APOBEC3H",TRUE,"APOBEC3H"

"9707","CCTCTCAAGCCGTCGCTTTA","chr2",239075296,"+","11:A>G,14:A>G","AGG","CCTCTCAAGCCATCACTTTAAGG",2,12,15,NA,"GeneID:9759","HDAC4",FALSE,"APOBEC3H"

"9708","CGACCCTGCGCACGTACCCC","chr22",39102517,"-","none","TGG","CGACCCTGCGCACGTACCCCTGG",0,NA,NA,NA,"GeneID:164668","APOBEC3H",TRUE,"APOBEC3H"

"9709","CGACCCTGCGCACGTACCCC","chr12",32878147,"-","2:C>T,9:A>C","AGG","CGGCCCTGCTCACGTACCCCAGG",2,3,10,NA,"GeneID:5318","PKP2",TRUE,"APOBEC3H"

"9710","CGACCCTGCGCACGTACCCC","chr12",6478418,"+","2:G>A,9:T>G","AGG","CGGCCCTGCTCACGTACCCCAGG",2,3,10,NA,"","",TRUE,"APOBEC3H"

"9711","GATTCCAGGGGTACGTGCGC","chr22",39102510,"+","none","AGG","GATTCCAGGGGTACGTGCGCAGG",0,NA,NA,NA,"GeneID:164668","APOBEC3H",TRUE,"APOBEC3H"

"9712","GATTCCAGGGGTACGTGCGC","chr10",32898703,"-","14:T>C,17:A>G","AGG","GATTCCAGGGGTACATGTGCAGG",2,15,18,NA,"","",FALSE,"APOBEC3H"

"9713","GATTCCAGGGGTACGTGCGC","chr9",85868535,"-","14:T>C,17:A>G","AGG","GATTCCAGGGGTACATGTGCAGG",2,15,18,NA,"","",FALSE,"APOBEC3H"

"9714","GATTCCAGGGGTACGTGCGC","chr8",118497685,"-","14:T>C,17:A>G","AGG","GATTCCAGGGGTACATGTGCAGG",2,15,18,NA,"GeneID:401474","SAMD12",FALSE,"APOBEC3H"

"9715","GATTCCAGGGGTACGTGCGC","chr14",44487780,"+","14:A>G,17:T>C","AGG","GATTCCAGGGGTACATGTGCAGG",2,15,18,NA,"","",FALSE,"APOBEC3H"

"9716","GATTCCAGGGGTACGTGCGC","chr5",172635708,"+","5:A>C,6:G>A","AGG","GATTCAGGGGGTACGTGCGCAGG",2,6,7,NA,"","",FALSE,"APOBEC3H"

"9717","GATTCCAGGGGTACGTGCGC","chr6",133281634,"+","4:T>C,17:T>C","AGG","GATTTCAGGGGTACGTGTGCAGG",2,5,18,NA,"GeneID:2070","EYA4",FALSE,"APOBEC3H"

"9718","GATTCCAGGGGTACGTGCGC","chr4",170074222,"+","5:A>C,17:T>C","AGG","GATTCAAGGGGTACGTGTGCAGG",2,6,18,NA,"GeneID:51166","AADAT",FALSE,"APOBEC3H"

"9719","GATTCCAGGGGTACGTGCGC","chrX",119195702,"-","5:T>G,14:T>C","AGG","GATTCAAGGGGTACATGCGCAGG",2,6,15,NA,"","",FALSE,"APOBEC3H"

"9720","GATTCCAGGGGTACGTGCGC","chr10",67368011,"-","5:T>G,14:T>C","AGG","GATTCAAGGGGTACATGCGCAGG",2,6,15,NA,"GeneID:29119","CTNNA3",FALSE,"APOBEC3H"

"9721","GCTTGCACCAGTGGTAGTAC","chr22",39101418,"-","none","AGG","GCTTGCACCAGTGGTAGTACAGG",0,NA,NA,NA,"GeneID:164668","APOBEC3H",TRUE,"APOBEC3H"

"9722","AACAACCCGCGGCCATAAGC","chr1",183648226,"+","none","TGG","AACAACCCGCGGCCATAAGCTGG",0,NA,NA,NA,"GeneID:23179;GeneID:403314","RGL1;APOBEC4",TRUE,"APOBEC4"

"9723","CTTCTTCACCTGTTCGAATA","chr1",183648660,"+","none","TGG","CTTCTTCACCTGTTCGAATATGG",0,NA,NA,NA,"GeneID:23179;GeneID:403314","RGL1;APOBEC4",TRUE,"APOBEC4"

"9724","GACTTACCACCAATGCATAT","chr1",183647864,"-","none","GGG","GACTTACCACCAATGCATATGGG",0,NA,NA,NA,"GeneID:23179;GeneID:403314","RGL1;APOBEC4",TRUE,"APOBEC4"

"9725","GACTTACCACCAATGCATAT","chr5",116525746,"-","15:T>G,16:G>T","AGG","GACTTACCACCAATGACTATAGG",2,16,17,NA,"GeneID:57556","SEMA6A",FALSE,"APOBEC4"

"9726","GCCTGGCCAGCTTATGGCCG","chr1",183648235,"-","none","CGG","GCCTGGCCAGCTTATGGCCGCGG",0,NA,NA,NA,"GeneID:23179;GeneID:403314","RGL1;APOBEC4",TRUE,"APOBEC4"

"9727","TAAGTGCCTTACGATATTCC","chr1",183647821,"+","none","TGG","TAAGTGCCTTACGATATTCCTGG",0,NA,NA,NA,"GeneID:23179;GeneID:403314","RGL1;APOBEC4",TRUE,"APOBEC4"

"9728","TGTCCTTACCATATTCGAAC","chr1",183648671,"-","none","AGG","TGTCCTTACCATATTCGAACAGG",0,NA,NA,NA,"GeneID:23179;GeneID:403314","RGL1;APOBEC4",TRUE,"APOBEC4"

"9729","AGGTTTGGCCTCGCGCACCC","chr16",28498433,"+","none","TGG","AGGTTTGGCCTCGCGCACCCTGG",0,NA,NA,NA,"GeneID:55911","APOBR",TRUE,"APOBR"

"9730","CTTTACAGACCCCGCGTGCC","chr16",28496860,"-","none","TGG","CTTTACAGACCCCGCGTGCCTGG",0,NA,NA,NA,"GeneID:55911","APOBR",TRUE,"APOBR"

"9731","GACCCCAACTAAGCAACCCG","chr16",28496753,"+","none","AGG","GACCCCAACTAAGCAACCCGAGG",0,NA,NA,NA,"GeneID:55911","APOBR",TRUE,"APOBR"

"9732","GAGTCCGACGAAAAGAGGGC","chr16",28498166,"-","none","CGG","GAGTCCGACGAAAAGAGGGCCGG",0,NA,NA,NA,"GeneID:55911","APOBR",TRUE,"APOBR"

"9733","GCAGGAGCTGCAAGCCCGTC","chr16",28498462,"+","none","TGG","GCAGGAGCTGCAAGCCCGTCTGG",0,NA,NA,NA,"GeneID:55911","APOBR",TRUE,"APOBR"

"9734","TCCTTGGGACAGAGACGTCT","chr16",28498103,"-","none","AGG","TCCTTGGGACAGAGACGTCTAGG",0,NA,NA,NA,"GeneID:55911","APOBR",TRUE,"APOBR"

"9735","AAACTCAAGATTGACTCATG","chr19",44919209,"+","none","AGG","AAACTCAAGATTGACTCATGAGG",0,NA,NA,NA,"GeneID:341","APOC1",TRUE,"APOC1"

"9736","AAACTCAAGATTGACTCATG","chr6",22588160,"-","11:T>A,17:G>T","TGG","AAACTCAAGATAGACTCCTGTGG",2,12,18,NA,"","",FALSE,"APOC1"

"9737","AAACTCAAGATTGACTCATG","chr19",44931036,"+","10:C>T","AGG","AAACTCAAGACTGACTCATGAGG",1,11,NA,NA,"GeneID:342","APOC1P1",TRUE,"APOC1"

"9738","AAACTCCTTCAGCTTATCCA","chr19",44916231,"-","none","AGG","AAACTCCTTCAGCTTATCCAAGG",0,NA,NA,NA,"GeneID:341","APOC1",TRUE,"APOC1"

"9739","AAACTCCTTCAGCTTATCCA","chrX",13408200,"+","14:G>T,17:A>C","AGG","AAACTCCTTCAGCTGATACAAGG",2,15,18,NA,"","",FALSE,"APOC1"

"9740","AGAGACATTTCAGAAAGTGA","chr19",44919184,"+","none","AGG","AGAGACATTTCAGAAAGTGAAGG",0,NA,NA,NA,"GeneID:341","APOC1",TRUE,"APOC1"

"9741","AGAGACATTTCAGAAAGTGA","chr1",65539202,"-","18:A>C,19:A>T","TGG","AGAGACATTTCAGAAAGTTTTGG",2,19,20,NA,"GeneID:3953","LEPR",FALSE,"APOC1"

"9742","AGAGACATTTCAGAAAGTGA","chr19",44931011,"+","11:G>A","AGG","AGAGACATTTCGGAAAGTGAAGG",1,12,NA,NA,"GeneID:342","APOC1P1",TRUE,"APOC1"

"9743","AGAGACATTTCAGAAAGTGA","chr17",49791948,"+","1:C>G,7:G>T","TGG","ACAGACAGTTCAGAAAGTGATGG",2,2,8,NA,"GeneID:11143","KAT7",TRUE,"APOC1"

"9744","AGAGACATTTCAGAAAGTGA","chr8",109201100,"+","8:G>T,9:G>T","GGG","AGAGACATGGCAGAAAGTGAGGG",2,9,10,NA,"","",FALSE,"APOC1"

"9745","AGAGACATTTCAGAAAGTGA","chrX",84369897,"-","3:A>C,18:G>C","AGG","AGATACATTTCAGAAAGTCAAGG",2,4,19,NA,"GeneID:139324","HDX",FALSE,"APOC1"

"9746","AGAGACATTTCAGAAAGTGA","chr17",64611936,"-","3:A>C,10:C>G","AGG","AGATACATTTGAGAAAGTGAAGG",2,4,11,NA,"GeneID:64750","SMURF2",FALSE,"APOC1"

"9747","CGATCGACAGAACCACCACC","chr19",44927004,"-","none","AGG","CGATCGACAGAACCACCACCAGG",0,NA,NA,NA,"GeneID:342","APOC1P1",TRUE,"APOC1"

"9748","CGATCGACAGAACCACCACC","chr19",44914920,"-","none","AGG","CGATCGACAGAACCACCACCAGG",0,NA,NA,NA,"GeneID:341","APOC1",TRUE,"APOC1"

"9749","GAGGCTCTTCCTGTCGCTCC","chr19",44926977,"+","none","CGG","GAGGCTCTTCCTGTCGCTCCCGG",0,NA,NA,NA,"GeneID:342","APOC1P1",TRUE,"APOC1"

"9750","GAGGCTCTTCCTGTCGCTCC","chr19",44914893,"+","none","CGG","GAGGCTCTTCCTGTCGCTCCCGG",0,NA,NA,NA,"GeneID:341","APOC1",TRUE,"APOC1"

"9751","GAGGCTCTTCCTGTCGCTCC","chr10",71738994,"+","5:G>T,9:G>C","AGG","GAGGCGCTTGCTGTCGCTCCAGG",2,6,10,NA,"GeneID:64072","CDH23",FALSE,"APOC1"

"9752","TGGAGACGTCTGGGGTCCCC","chr19",44916205,"-","none","TGG","TGGAGACGTCTGGGGTCCCCTGG",0,NA,NA,NA,"GeneID:341","APOC1",TRUE,"APOC1"

"9753","TGGAGACGTCTGGGGTCCCC","chr19",3457660,"+","6:T>C,9:A>C","AGG","TGGAGATGTATGGGGTCCCCAGG",2,7,10,NA,"GeneID:4782","NFIC",FALSE,"APOC1"

"9754","TGGAGACGTCTGGGGTCCCC","chr15",71706284,"-","7:G>C,14:A>C","TGG","TGGAGACCTCTGGGTTCCCCTGG",2,8,15,NA,"GeneID:79875","THSD4",FALSE,"APOC1"

"9755","AATGCCTGTGTAAGTGCTCA","chr19",44949187,"-","none","TGG","AATGCCTGTGTAAGTGCTCATGG",0,NA,NA,NA,"GeneID:344","APOC2",TRUE,"APOC2"

"9756","ACTCACCAAATCCCAATACC","chr19",44948519,"-","none","AGG","ACTCACCAAATCCCAATACCAGG",0,NA,NA,NA,"GeneID:344","APOC2",TRUE,"APOC2"

"9757","AGCTCTGTTTCTTGTCCTCC","chr19",44948498,"+","none","TGG","AGCTCTGTTTCTTGTCCTCCTGG",0,NA,NA,NA,"GeneID:344","APOC2",TRUE,"APOC2"

"9758","AGCTCTGTTTCTTGTCCTCC","chr1",40848376,"-","2:T>G,4:C>G","AGG","AGATGTGTTTCTTGTCCTCCAGG",2,3,5,NA,"","",FALSE,"APOC2"

"9759","GCAGCCATGAGCACTTACAC","chr19",44949180,"+","none","AGG","GCAGCCATGAGCACTTACACAGG",0,NA,NA,NA,"GeneID:344","APOC2",TRUE,"APOC2"

"9760","GCAGCCATGAGCACTTACAC","chr10",100878325,"+","6:C>A,15:C>T","TGG","GCAGCCCTGAGCACTCACACTGG",2,7,16,NA,"","",FALSE,"APOC2"

"9761","GCAGGTATGTCTTCTCGTAC","chr19",44948818,"-","none","AGG","GCAGGTATGTCTTCTCGTACAGG",0,NA,NA,NA,"GeneID:344","APOC2",TRUE,"APOC2"

"9762","TAGCCCGACCTTCCTCACCC","chr19",44948740,"+","none","AGG","TAGCCCGACCTTCCTCACCCAGG",0,NA,NA,NA,"GeneID:344","APOC2",TRUE,"APOC2"

"9763","ACTCCTTGTTGTTGCCCTCC","chr11",116830596,"+","none","TGG","ACTCCTTGTTGTTGCCCTCCTGG",0,NA,NA,NA,"GeneID:345","APOC3",TRUE,"APOC3"

"9764","ACTCCTTGTTGTTGCCCTCC","chr2",161066252,"+","14:A>C","AGG","ACTCCTTGTTGTTGACCTCCAGG",1,15,NA,NA,"","",FALSE,"APOC3"

"9765","ACTCCTTGTTGTTGCCCTCC","chr1",62886571,"+","11:A>T,14:T>C","AGG","ACTCCTTGTTGATGTCCTCCAGG",2,12,15,NA,"","",FALSE,"APOC3"

"9766","AGTGCTTACGGGCAGAGGCC","chr11",116830626,"-","none","AGG","AGTGCTTACGGGCAGAGGCCAGG",0,NA,NA,NA,"GeneID:345","APOC3",TRUE,"APOC3"

"9767","GAGCAGCGTGCAGGAGTCCC","chr11",116830857,"+","none","AGG","GAGCAGCGTGCAGGAGTCCCAGG",0,NA,NA,NA,"GeneID:345","APOC3",TRUE,"APOC3"

"9768","GAGCAGCGTGCAGGAGTCCC","chr1",180900101,"+","15:C>G,18:T>C","TGG","GAGCAGCGTGCAGGACTCTCTGG",2,16,19,NA,"","",FALSE,"APOC3"

"9769","GAGCAGCGTGCAGGAGTCCC","chr19",40630585,"+","6:G>C,8:A>T","TGG","GAGCAGGGAGCAGGAGTCCCTGG",2,7,9,NA,"","",FALSE,"APOC3"

"9770","GCACGCCACCAAGACCGCCA","chr11",116830827,"+","none","AGG","GCACGCCACCAAGACCGCCAAGG",0,NA,NA,NA,"GeneID:345","APOC3",TRUE,"APOC3"

"9771","GGTCAGACCAACTTCAGCCG","chr11",116832853,"+","none","TGG","GGTCAGACCAACTTCAGCCGTGG",0,NA,NA,NA,"GeneID:345","APOC3",TRUE,"APOC3"

"9772","TTTAGGGGCTGGGTGACCGA","chr11",116832758,"+","none","TGG","TTTAGGGGCTGGGTGACCGATGG",0,NA,NA,NA,"GeneID:345","APOC3",TRUE,"APOC3"

"9773","AAATGTCCCTCCTCAGAAAC","chr19",44942275,"+","none","AGG","AAATGTCCCTCCTCAGAAACAGG",0,NA,NA,NA,"GeneID:346","APOC4",TRUE,"APOC4"

"9774","CAGCGACTCATCTTTAGCTT","chr19",44944792,"-","none","TGG","CAGCGACTCATCTTTAGCTTTGG",0,NA,NA,NA,"GeneID:346","APOC4",TRUE,"APOC4"

"9775","CCTACTATGACGACCACCTG","chr19",44945171,"+","none","AGG","CCTACTATGACGACCACCTGAGG",0,NA,NA,NA,"GeneID:346","APOC4",TRUE,"APOC4"

"9776","GAACCATTGCCACCCGTCTC","chr19",44944868,"-","none","TGG","GAACCATTGCCACCCGTCTCTGG",0,NA,NA,NA,"GeneID:346","APOC4",TRUE,"APOC4"

"9777","GTCTTTGGATTCGAGGAACC","chr19",44945219,"-","none","AGG","GTCTTTGGATTCGAGGAACCAGG",0,NA,NA,NA,"GeneID:346","APOC4",TRUE,"APOC4"

"9778","TGCCCTGTGCCTCTGCGTGC","chr19",44942312,"+","none","TGG","TGCCCTGTGCCTCTGCGTGCTGG",0,NA,NA,NA,"GeneID:346","APOC4",TRUE,"APOC4"

"9779","TGCCCTGTGCCTCTGCGTGC","chr2",104736331,"-","14:T>C,18:A>C","TGG","TGCCCTGTGCCTCTACGTTCTGG",2,15,19,NA,"","",FALSE,"APOC4"

"9780","AGAACCTGGTTACGAGAGCT","chr3",195568890,"+","none","TGG","AGAACCTGGTTACGAGAGCTTGG",0,NA,NA,NA,"GeneID:347","APOD",TRUE,"APOD"

"9781","CTCTGCAGTATCTCGGAAGA","chr3",195573959,"-","none","TGG","CTCTGCAGTATCTCGGAAGATGG",0,NA,NA,NA,"GeneID:347","APOD",TRUE,"APOD"

"9782","GATGCAGCGTCCATTCTCAA","chr3",195573908,"+","none","AGG","GATGCAGCGTCCATTCTCAAAGG",0,NA,NA,NA,"GeneID:347","APOD",TRUE,"APOD"

"9783","GGAACTGTGAATCAAATCGA","chr3",195571338,"-","none","AGG","GGAACTGTGAATCAAATCGAAGG",0,NA,NA,NA,"GeneID:347","APOD",TRUE,"APOD"

"9784","GTCCCTCTGCCGCACCGAAG","chr3",195579400,"+","none","AGG","GTCCCTCTGCCGCACCGAAGAGG",0,NA,NA,NA,"GeneID:347","APOD",TRUE,"APOD"

"9785","TGCAGGTACAGGAATACACG","chr3",195569060,"+","none","AGG","TGCAGGTACAGGAATACACGAGG",0,NA,NA,NA,"GeneID:347","APOD",TRUE,"APOD"

"9786","ACAGTGTCTGCACCCAGCGC","chr19",44907880,"-","none","AGG","ACAGTGTCTGCACCCAGCGCAGG",0,NA,NA,NA,"GeneID:348","APOE",TRUE,"APOE"

"9787","ACAGTGTCTGCACCCAGCGC","chr17",80207000,"+","15:T>A,18:A>G","AGG","ACAGTGTCTGCACCCTGCACAGG",2,16,19,NA,"GeneID:79092;GeneID:6448","CARD14;SGSH",TRUE,"APOE"

"9788","CCTGGTGCAGTACCGCGGCG","chr19",44908691,"+","none","AGG","CCTGGTGCAGTACCGCGGCGAGG",0,NA,NA,NA,"GeneID:348","APOE",TRUE,"APOE"

"9789","CGCTCTGCCACTCGGTCTGC","chr19",44907817,"-","none","TGG","CGCTCTGCCACTCGGTCTGCTGG",0,NA,NA,NA,"GeneID:348","APOE",TRUE,"APOE"

"9790","CGCTCTGCCACTCGGTCTGC","chr6",35534051,"+","13:A>G,15:G>T","TGG","CGCTCTGCCACTCAGGCTGCTGG",2,14,16,NA,"","",FALSE,"APOE"

"9791","CGGCGTTCAGTGATTGTCGC","chr19",44909236,"-","none","TGG","CGGCGTTCAGTGATTGTCGCTGG",0,NA,NA,NA,"GeneID:348","APOE",TRUE,"APOE"

"9792","GCAGGAAGATGAAGGTTCTG","chr19",44906616,"+","none","TGG","GCAGGAAGATGAAGGTTCTGTGG",0,NA,NA,NA,"GeneID:348","APOE",TRUE,"APOE"

"9793","GCAGGAAGATGAAGGTTCTG","chr6",85005758,"-","12:C>T,16:C>A","AGG","GCAGGAAGATGAGGGTGCTGAGG",2,13,17,NA,"","",FALSE,"APOE"

"9794","GCAGGAAGATGAAGGTTCTG","chr22",46395187,"-","5:C>T,6:C>T","GGG","GCAGGGGGATGAAGGTTCTGGGG",2,6,7,NA,"GeneID:9620","CELSR1",FALSE,"APOE"

"9795","GCAGGAAGATGAAGGTTCTG","chr1",168084236,"-","5:G>T,10:G>C","TGG","GCAGGCAGATCAAGGTTCTGTGG",2,6,11,NA,"GeneID:23432","GPR161",TRUE,"APOE"

"9796","TGCGTTGCTGGTCACATTCC","chr19",44906641,"+","none","TGG","TGCGTTGCTGGTCACATTCCTGG",0,NA,NA,NA,"GeneID:348","APOE",TRUE,"APOE"

"9797","ACAGCTACAGCTCTACCGCC","chr12",56361865,"-","none","AGG","ACAGCTACAGCTCTACCGCCAGG",0,NA,NA,NA,"GeneID:319","APOF",TRUE,"APOF"

"9798","AGTGGGAACCTTCTACAACC","chr12",56361622,"-","none","TGG","AGTGGGAACCTTCTACAACCTGG",0,NA,NA,NA,"GeneID:319","APOF",TRUE,"APOF"

"9799","GCACTGATCGCTAGACCCAT","chr12",56361477,"+","none","AGG","GCACTGATCGCTAGACCCATAGG",0,NA,NA,NA,"GeneID:319","APOF",TRUE,"APOF"

"9800","GGCCCCTCTACCCAAGTTCT","chr12",56361949,"-","none","TGG","GGCCCCTCTACCCAAGTTCTTGG",0,NA,NA,NA,"GeneID:319","APOF",TRUE,"APOF"

"9801","TAGTAAGTTCAGCTCCCTAC","chr12",56361287,"-","none","TGG","TAGTAAGTTCAGCTCCCTACTGG",0,NA,NA,NA,"GeneID:319","APOF",TRUE,"APOF"

"9802","TTCTCAATGACTGGACTGTG","chr12",56362731,"-","none","TGG","TTCTCAATGACTGGACTGTGTGG",0,NA,NA,NA,"GeneID:319","APOF",TRUE,"APOF"

"9803","ACAAGAAAAACTGATCGTGT","chr17",66226035,"+","none","TGG","ACAAGAAAAACTGATCGTGTTGG",0,NA,NA,NA,"GeneID:350","APOH",TRUE,"APOH"

"9804","CTCGAGAACAAGATGAGCAC","chr17",66229347,"+","none","TGG","CTCGAGAACAAGATGAGCACTGG",0,NA,NA,NA,"GeneID:350","APOH",TRUE,"APOH"

"9805","GAATACGTAATCTCTTCTCC","chr17",66228111,"+","none","TGG","GAATACGTAATCTCTTCTCCTGG",0,NA,NA,NA,"GeneID:350","APOH",TRUE,"APOH"

"9806","GATTACGTATTCCTGCAAGC","chr17",66228103,"-","none","CGG","GATTACGTATTCCTGCAAGCCGG",0,NA,NA,NA,"GeneID:350","APOH",TRUE,"APOH"

"9807","TCCGTCCTGCAATAGCAACA","chr17",66229315,"+","none","TGG","TCCGTCCTGCAATAGCAACATGG",0,NA,NA,NA,"GeneID:350","APOH",TRUE,"APOH"

"9808","TTCAAAAGTCGTATAGCGTA","chr17",66226062,"+","none","CGG","TTCAAAAGTCGTATAGCGTACGG",0,NA,NA,NA,"GeneID:350","APOH",TRUE,"APOH"

"9809","ACTCCTGCTGACTGATAATG","chr22",36261662,"+","none","AGG","ACTCCTGCTGACTGATAATGAGG",0,NA,NA,NA,"GeneID:8542","APOL1",TRUE,"APOL1"

"9810","AGCAGCCACGAATCCGTTCC","chr22",36261688,"-","none","AGG","AGCAGCCACGAATCCGTTCCAGG",0,NA,NA,NA,"GeneID:8542","APOL1",TRUE,"APOL1"

"9811","AGCAGCCACGAATCCGTTCC","chr22",36231353,"+","15:A>G","AGG","AGCAGCCACGAATCCATTCCAGG",1,16,NA,NA,"GeneID:23780","APOL2",TRUE,"APOL1"

"9812","CTGCCCTCACTCCCACACCA","chr22",36257100,"-","none","AGG","CTGCCCTCACTCCCACACCAAGG",0,NA,NA,NA,"GeneID:8542","APOL1",TRUE,"APOL1"

"9813","CTGCCCTCACTCCCACACCA","chr15",28984227,"-","14:G>T,17:C>G","TGG","CTGCCCTCACTCCCCCAGCATGG",2,15,18,NA,"GeneID:321","APBA2",FALSE,"APOL1"

"9814","CTGCCCTCACTCCCACACCA","chr22",36149333,"+","12:T>C","AGG","CTGCCCTCACTCTCACACCAAGG",1,13,NA,NA,"GeneID:80833","APOL3",TRUE,"APOL1"

"9815","CTGCCCTCACTCCCACACCA","chr4",174446128,"+","3:G>C,4:G>C","TGG","CTGGGCTCACTCCCACACCATGG",2,4,5,NA,"","",FALSE,"APOL1"

"9816","CTGCCCTCACTCCCACACCA","chr22",36233417,"+","2:T>G,12:T>C","AGG","CTTCCCTCACTCTCACACCAAGG",2,3,13,NA,"GeneID:23780","APOL2",TRUE,"APOL1"

"9817","CTGCCCTCACTCCCACACCA","chr22",36199570,"+","2:T>G,12:T>C","AGG","CTTCCCTCACTCTCACACCAAGG",2,3,13,NA,"GeneID:80832","APOL4",TRUE,"APOL1"

"9818","CTGCCCTCACTCCCACACCA","chr1",1127994,"-","6:G>A,17:C>G","CGG","CTGCCCCCACTCCCACAGCACGG",2,7,18,NA,"","",FALSE,"APOL1"

"9819","CTGCCCTCACTCCCACACCA","chr1",41393404,"-","3:C>G,12:A>G","TGG","CTGGCCTCACTCTCACACCATGG",2,4,13,NA,"","",FALSE,"APOL1"

"9820","GATCCTCAAAGTAAGCCCCT","chr22",36257355,"+","none","CGG","GATCCTCAAAGTAAGCCCCTCGG",0,NA,NA,NA,"GeneID:8542","APOL1",TRUE,"APOL1"

"9821","GATCCTCAAAGTAAGCCCCT","chr10",30030760,"-","3:A>G,15:C>G","GGG","GATTCTCAAAGTAAGGCCCTGGG",2,4,16,NA,"GeneID:57608","KIAA1462",FALSE,"APOL1"

"9822","GCCAAGCTCACCAGATGCAG","chr22",36254990,"-","none","AGG","GCCAAGCTCACCAGATGCAGAGG",0,NA,NA,NA,"GeneID:8542","APOL1",TRUE,"APOL1"

"9823","GCCAAGCTCACCAGATGCAG","chr22",36155963,"+","9:T>A","AGG","GCCAAGCTCTCCAGATGCAGAGG",1,10,NA,NA,"GeneID:80833","APOL3",TRUE,"APOL1"

"9824","GCCAAGCTCACCAGATGCAG","chr22",36237289,"+","9:T>A","AGG","GCCAAGCTCTCCAGATGCAGAGG",1,10,NA,NA,"GeneID:23780","APOL2",TRUE,"APOL1"

"9825","GCCAAGCTCACCAGATGCAG","chr10",6649822,"-","4:A>T,17:C>G","TGG","GCCATGCTCACCAGATGGAGTGG",2,5,18,NA,"","",FALSE,"APOL1"

"9826","GCCAAGCTCACCAGATGCAG","chr12",106067442,"-","0:G>C,15:C>A","AGG","CCCAAGCTCACCAGAGGCAGAGG",2,1,16,NA,"GeneID:9891","NUAK1",TRUE,"APOL1"

"9827","GTGCAACAAAACGTTCCAAG","chr22",36257319,"+","none","TGG","GTGCAACAAAACGTTCCAAGTGG",0,NA,NA,NA,"GeneID:8542","APOL1",TRUE,"APOL1"

"9828","AAGCGGCACCAATGTAGCAA","chr22",36227840,"-","none","AGG","AAGCGGCACCAATGTAGCAAAGG",0,NA,NA,NA,"GeneID:23780","APOL2",TRUE,"APOL2"

"9829","ACGAGCCCAAGCCCGCAACT","chr22",36227867,"-","none","TGG","ACGAGCCCAAGCCCGCAACTTGG",0,NA,NA,NA,"GeneID:23780","APOL2",TRUE,"APOL2"

"9830","AGCAGCCACGAATCCATTCC","chr22",36231353,"+","none","AGG","AGCAGCCACGAATCCATTCCAGG",0,NA,NA,NA,"GeneID:23780","APOL2",TRUE,"APOL2"

"9831","AGCAGCCACGAATCCATTCC","chr22",36261688,"-","15:C>T","AGG","AGCAGCCACGAATCCGTTCCAGG",1,16,NA,NA,"GeneID:8542","APOL1",TRUE,"APOL2"

"9832","AGCGAGCCTACCTGGGTTCA","chr22",36233141,"+","none","TGG","AGCGAGCCTACCTGGGTTCATGG",0,NA,NA,NA,"GeneID:23780","APOL2",TRUE,"APOL2"

"9833","GCAGATTCTCTCTGCTCACT","chr22",36231400,"+","none","TGG","GCAGATTCTCTCTGCTCACTTGG",0,NA,NA,NA,"GeneID:23780","APOL2",TRUE,"APOL2"

"9834","GGGGCATACGCTCCTAACTG","chr22",36227695,"+","none","AGG","GGGGCATACGCTCCTAACTGAGG",0,NA,NA,NA,"GeneID:23780","APOL2",TRUE,"APOL2"

"9835","AACCAGCATTGACCGATTGA","chr22",36141618,"-","none","AGG","AACCAGCATTGACCGATTGAAGG",0,NA,NA,NA,"GeneID:80833","APOL3",TRUE,"APOL3"

"9836","AATAACCAGACACGTTCTCC","chr22",36160752,"+","none","AGG","AATAACCAGACACGTTCTCCAGG",0,NA,NA,NA,"GeneID:80833","APOL3",TRUE,"APOL3"

"9837","AGTGCTTTGACTCGTATACA","chr22",36141321,"+","none","AGG","AGTGCTTTGACTCGTATACAAGG",0,NA,NA,NA,"GeneID:80833","APOL3",TRUE,"APOL3"

"9838","AGTGCTTTGACTCGTATACA","chr22",36227525,"+","13:A>G,17:G>A","AGG","AGTGCTTTGACTCATATGCAAGG",2,14,18,NA,"GeneID:23780","APOL2",TRUE,"APOL3"

"9839","CGCAGTCACGAATCTCTTCC","chr22",36145486,"+","none","AGG","CGCAGTCACGAATCTCTTCCAGG",0,NA,NA,NA,"GeneID:80833","APOL3",TRUE,"APOL3"

"9840","GACTCTCTCCCGGAAGTATT","chr22",36145549,"+","none","TGG","GACTCTCTCCCGGAAGTATTTGG",0,NA,NA,NA,"GeneID:80833","APOL3",TRUE,"APOL3"

"9841","GACTCTCTCCCGGAAGTATT","chr16",66163646,"+","10:T>C,11:A>G","TGG","GACTCTCTCCTAGAAGTATTTGG",2,11,12,NA,"","",FALSE,"APOL3"

"9842","GATATACCCTGGAACCCAAA","chr22",36160781,"+","none","AGG","GATATACCCTGGAACCCAAAAGG",0,NA,NA,NA,"GeneID:80833","APOL3",TRUE,"APOL3"

"9843","AAATCTTTGTCGTCTGCATC","chr22",36201994,"-","none","TGG","AAATCTTTGTCGTCTGCATCTGG",0,NA,NA,NA,"GeneID:80832","APOL4",TRUE,"APOL4"

"9844","AGGCTGCGTCATCGCCAATG","chr22",36191697,"-","none","TGG","AGGCTGCGTCATCGCCAATGTGG",0,NA,NA,NA,"GeneID:80832","APOL4",TRUE,"APOL4"

"9845","AGGGTGCAGCAAAACCATCC","chr22",36199358,"-","none","AGG","AGGGTGCAGCAAAACCATCCAGG",0,NA,NA,NA,"GeneID:80832","APOL4",TRUE,"APOL4"

"9846","TGCGTGTGGCTGAATTGCCC","chr22",36195312,"-","none","AGG","TGCGTGTGGCTGAATTGCCCAGG",0,NA,NA,NA,"GeneID:80832","APOL4",TRUE,"APOL4"

"9847","TGCTGACTAGCGATGAAGCC","chr22",36195345,"-","none","TGG","TGCTGACTAGCGATGAAGCCTGG",0,NA,NA,NA,"GeneID:80832","APOL4",TRUE,"APOL4"

"9848","TGCTGACTAGCGATGAAGCC","chr22",36145507,"-","9:T>C,11:T>C","TGG","TGCTGACTAACAATGAAGCCTGG",2,10,12,NA,"GeneID:80833","APOL3",TRUE,"APOL4"

"9849","TTGGACGCCCTTTGATTGCT","chr22",36191341,"-","none","TGG","TTGGACGCCCTTTGATTGCTTGG",0,NA,NA,NA,"GeneID:80832","APOL4",TRUE,"APOL4"

"9850","ACCCCTCTAGCCGTCCAGAA","chr22",35726875,"-","none","AGG","ACCCCTCTAGCCGTCCAGAAAGG",0,NA,NA,NA,"","",TRUE,"APOL5"

"9851","CTAGGCTGGTGCTCCACAAC","chr22",35728748,"-","none","AGG","CTAGGCTGGTGCTCCACAACAGG",0,NA,NA,NA,"","",TRUE,"APOL5"

"9852","GAACTTACCAGGTAACACCT","chr22",35717914,"-","none","TGG","GAACTTACCAGGTAACACCTTGG",0,NA,NA,NA,"","",TRUE,"APOL5"

"9853","GGCTGTTCGAGGATCCCGTG","chr22",35727159,"+","none","TGG","GGCTGTTCGAGGATCCCGTGTGG",0,NA,NA,NA,"","",TRUE,"APOL5"

"9854","TAATCTACGGAGGTGAGGTC","chr22",35720606,"+","none","TGG","TAATCTACGGAGGTGAGGTCTGG",0,NA,NA,NA,"","",TRUE,"APOL5"

"9855","TTTGCAAGTTCCCGGTTCCA","chr22",35717894,"+","none","AGG","TTTGCAAGTTCCCGGTTCCAAGG",0,NA,NA,NA,"","",TRUE,"APOL5"

"9856","ACAGAGGCTGATGGACAACC","chr22",35656415,"+","none","AGG","ACAGAGGCTGATGGACAACCAGG",0,NA,NA,NA,"GeneID:80830","APOL6",TRUE,"APOL6"

"9857","ACAGAGGCTGATGGACAACC","chrX",114842663,"-","0:A>T,17:C>T","CGG","TCAGAGGCTGATGGACAGCCCGG",2,1,18,NA,"GeneID:3358","HTR2C",FALSE,"APOL6"

"9858","CAACGTACCACTCACGATGC","chr22",35658934,"-","none","TGG","CAACGTACCACTCACGATGCTGG",0,NA,NA,NA,"GeneID:80830","APOL6",TRUE,"APOL6"

"9859","CCTCACTTTCTCTCTCCGCC","chr22",35656436,"-","none","TGG","CCTCACTTTCTCTCTCCGCCTGG",0,NA,NA,NA,"GeneID:80830","APOL6",TRUE,"APOL6"

"9860","CCTCACTTTCTCTCTCCGCC","chr14",81786083,"+","16:T>C","TGG","CCTCACTTTCTCTCTCTGCCTGG",1,17,NA,NA,"","",FALSE,"APOL6"

"9861","CCTCACTTTCTCTCTCCGCC","chr15",40853101,"+","0:T>C,14:C>T","AGG","TCTCACTTTCTCTCCCCGCCAGG",2,1,15,NA,"GeneID:6692","SPINT1",FALSE,"APOL6"

"9862","CCTCACTTTCTCTCTCCGCC","chr17",3759261,"+","6:G>T,16:G>C","AGG","CCTCACGTTCTCTCTCGGCCAGG",2,7,17,NA,"GeneID:3682","ITGAE",FALSE,"APOL6"

"9863","CCTCACTTTCTCTCTCCGCC","chr2",100660322,"-","0:A>G,13:A>G","TGG","TCTCACTTTCTCTTTCCGCCTGG",2,1,14,NA,"","",TRUE,"APOL6"

"9864","CGCCAGTGTTGTTCCCGCAA","chr22",35659228,"-","none","AGG","CGCCAGTGTTGTTCCCGCAAAGG",0,NA,NA,NA,"GeneID:80830","APOL6",TRUE,"APOL6"

"9865","GACCAAAAATGCTCGCGTGC","chr22",35659250,"+","none","TGG","GACCAAAAATGCTCGCGTGCTGG",0,NA,NA,NA,"GeneID:80830","APOL6",TRUE,"APOL6"

"9866","TTTGCACCTGCACGCGGCTC","chr22",35659202,"-","none","CGG","TTTGCACCTGCACGCGGCTCCGG",0,NA,NA,NA,"GeneID:80830","APOL6",TRUE,"APOL6"

"9867","CCGCGCCTTCCGAGTACCCC","chr12",12726034,"-","none","TGG","CCGCGCCTTCCGAGTACCCCTGG",0,NA,NA,NA,"GeneID:81575","APOLD1",TRUE,"APOLD1"

"9868","CGTTGCGCTCAGCGAGCTGC","chr12",12787080,"-","none","CGG","CGTTGCGCTCAGCGAGCTGCCGG",0,NA,NA,NA,"GeneID:81575","APOLD1",TRUE,"APOLD1"

"9869","CTACGAGGGAGCGCCTGCGC","chr12",12787048,"-","none","AGG","CTACGAGGGAGCGCCTGCGCAGG",0,NA,NA,NA,"GeneID:81575","APOLD1",TRUE,"APOLD1"

"9870","GCGGGCCAGGGGTACTCGGA","chr12",12726026,"+","none","AGG","GCGGGCCAGGGGTACTCGGAAGG",0,NA,NA,NA,"GeneID:81575","APOLD1",TRUE,"APOLD1"

"9871","TCCCTCGTAGCCAACGTGGC","chr12",12787058,"+","none","CGG","TCCCTCGTAGCCAACGTGGCCGG",0,NA,NA,NA,"GeneID:81575","APOLD1",TRUE,"APOLD1"

"9872","TGTTCCGCGCGCCGTGTCAC","chr12",12726001,"+","none","CGG","TGTTCCGCGCGCCGTGTCACCGG",0,NA,NA,NA,"GeneID:81575","APOLD1",TRUE,"APOLD1"

"9873","AACCCCTTACCTTGATTCCT","chr6",31657713,"-","none","AGG","AACCCCTTACCTTGATTCCTAGG",0,NA,NA,NA,"GeneID:55937","APOM",TRUE,"APOM"

"9874","AAGCCTTCTTATTGACTCCT","chr6",31657693,"+","none","AGG","AAGCCTTCTTATTGACTCCTAGG",0,NA,NA,NA,"GeneID:55937","APOM",TRUE,"APOM"

"9875","ACATGCGGATGGTAGCACGA","chr6",31656608,"-","none","AGG","ACATGCGGATGGTAGCACGAAGG",0,NA,NA,NA,"GeneID:55937","APOM",TRUE,"APOM"

"9876","CACTGTAGAGGAGAAAGCGC","chr6",31657461,"-","none","TGG","CACTGTAGAGGAGAAAGCGCTGG",0,NA,NA,NA,"GeneID:55937","APOM",TRUE,"APOM"

"9877","TCATTCAGCATGATTCCACC","chr6",31657423,"-","none","TGG","TCATTCAGCATGATTCCACCTGG",0,NA,NA,NA,"GeneID:55937","APOM",TRUE,"APOM"

"9878","TGGATCTACCACCTGACTGA","chr6",31657252,"+","none","AGG","TGGATCTACCACCTGACTGAAGG",0,NA,NA,NA,"GeneID:55937","APOM",TRUE,"APOM"

"9879","ATTGCGAGCCATACACAACC","chrX",23878923,"-","none","TGG","ATTGCGAGCCATACACAACCTGG",0,NA,NA,NA,"GeneID:79135","APOO",TRUE,"APOO"

"9880","ATTGCGAGCCATACACAACC","chr16",59754809,"-","18:C>G","TGG","ATTGCGAGCCATACACAAGCTGG",1,19,NA,NA,"","",TRUE,"APOO"

"9881","CTGCATTTCAGGTAATTCAG","chrX",23880943,"-","none","AGG","CTGCATTTCAGGTAATTCAGAGG",0,NA,NA,NA,"GeneID:79135","APOO",TRUE,"APOO"

"9882","CTGCATTTCAGGTAATTCAG","chr2",10354316,"+","12:C>T,14:C>A","AGG","CTGCATTTCAGGCACTTCAGAGG",2,13,15,NA,"GeneID:3241","HPCAL1",TRUE,"APOO"

"9883","CTGCATTTCAGGTAATTCAG","chr17",36818427,"+","11:T>G,17:A>C","GGG","CTGCATTTCAGTTAATTAAGGGG",2,12,18,NA,"","",FALSE,"APOO"

"9884","CTGCATTTCAGGTAATTCAG","chr5",126634478,"+","10:T>G,17:T>C","TGG","CTGCATTTCATGTAATTTAGTGG",2,11,18,NA,"GeneID:389320","TEX43",FALSE,"APOO"

"9885","CTGCATTTCAGGTAATTCAG","chr11",129879868,"-","8:C>G,14:A>T","TGG","CTGCATTTGAGGTATTTCAGTGG",2,9,15,NA,"GeneID:4798","NFRKB",FALSE,"APOO"

"9886","TCAATCGAAGTATGTGGAGG","chrX",23878988,"-","none","AGG","TCAATCGAAGTATGTGGAGGAGG",0,NA,NA,NA,"GeneID:79135","APOO",TRUE,"APOO"

"9887","TGCACCTACCTCTAGCCAAA","chrX",23868583,"+","none","AGG","TGCACCTACCTCTAGCCAAAAGG",0,NA,NA,NA,"GeneID:79135","APOO",TRUE,"APOO"

"9888","TGCACCTACCTCTAGCCAAA","chr3",38844373,"+","1:C>G,3:C>A","AGG","TCCCCCTACCTCTAGCCAAAAGG",2,2,4,NA,"","",FALSE,"APOO"

"9889","TGGCCTTATTGGACTCCTTT","chrX",23868601,"-","none","TGG","TGGCCTTATTGGACTCCTTTTGG",0,NA,NA,NA,"GeneID:79135","APOO",TRUE,"APOO"

"9890","TGGCCTTATTGGACTCCTTT","chr3",87595192,"+","7:T>A","TGG","TGGCCTTTTTGGACTCCTTTTGG",1,8,NA,NA,"","",TRUE,"APOO"

"9891","TGGCCTTATTGGACTCCTTT","chr12",65135047,"+","7:G>A","TGG","TGGCCTTGTTGGACTCCTTTTGG",1,8,NA,NA,"","",TRUE,"APOO"

"9892","TGGCCTTATTGGACTCCTTT","chr4",68304560,"-","8:T>A,16:A>G","TGG","TGGCCTTAATGGACTCTTTTTGG",2,9,17,NA,"","",TRUE,"APOO"

"9893","TGTTACCTCATCAACCTTCA","chrX",23880838,"+","none","CGG","TGTTACCTCATCAACCTTCACGG",0,NA,NA,NA,"GeneID:79135","APOO",TRUE,"APOO"

"9894","TGTTACCTCATCAACCTTCA","chr1",191614609,"-","14:C>G,15:A>G","TGG","TGTTACCTCATCAAGTTTCATGG",2,15,16,NA,"","",FALSE,"APOO"

"9895","AGCGGTGGTGCAGTATATAT","chrX",85051394,"-","none","GGG","AGCGGTGGTGCAGTATATATGGG",0,NA,NA,NA,"GeneID:139322","APOOL",TRUE,"APOOL"

"9896","AGCGGTGGTGCAGTATATAT","chr5",145997328,"-","19:G>A","GGG","AGCGGTGGTGCAGTATATACGGG",1,20,NA,NA,"GeneID:153769","SH3RF2",TRUE,"APOOL"

"9897","AGCGGTGGTGCAGTATATAT","chr20",5823013,"-","3:T>C,10:C>G","GGG","AGCAGTGGTGGAGTATATATGGG",2,4,11,NA,"GeneID:149840","C20orf196",FALSE,"APOOL"

"9898","GATGCATATATCAGACCTGC","chrX",85046469,"-","none","AGG","GATGCATATATCAGACCTGCAGG",0,NA,NA,NA,"GeneID:139322","APOOL",TRUE,"APOOL"

"9899","GATGCATATATCAGACCTGC","chr5",145997241,"-","7:C>T,16:A>G","AGG","GATGCATGTATCAGACTTGCAGG",2,8,17,NA,"GeneID:153769","SH3RF2",TRUE,"APOOL"

"9900","GCAGCTAGTGAAACCAGAGC","chrX",85046528,"+","none","AGG","GCAGCTAGTGAAACCAGAGCAGG",0,NA,NA,NA,"GeneID:139322","APOOL",TRUE,"APOOL"

"9901","GCTTCCATCCGCACTGCAAC","chrX",85051463,"+","none","TGG","GCTTCCATCCGCACTGCAACTGG",0,NA,NA,NA,"GeneID:139322","APOOL",TRUE,"APOOL"

"9902","GCTTCCATCCGCACTGCAAC","chr5",145997397,"+","9:T>C,10:A>G","TGG","GCTTCCATCTACACTGCAACTGG",2,10,11,NA,"GeneID:153769","SH3RF2",TRUE,"APOOL"

"9903","TTGTAGACATGGCGGCCATC","chrX",85003904,"+","none","AGG","TTGTAGACATGGCGGCCATCAGG",0,NA,NA,NA,"GeneID:139322","APOOL",TRUE,"APOOL"

"9904","TTGTTACATTGGCTGGTGCA","chrX",85051486,"+","none","AGG","TTGTTACATTGGCTGGTGCAAGG",0,NA,NA,NA,"GeneID:139322","APOOL",TRUE,"APOOL"

"9905","AAGTGGCCCTGGAAAGGATT","chr14",103590224,"+","none","TGG","AAGTGGCCCTGGAAAGGATTTGG",0,NA,NA,NA,"GeneID:3831;GeneID:84334","KLC1;APOPT1",TRUE,"APOPT1"

"9906","AAGTGGCCCTGGAAAGGATT","chr11",94528080,"+","0:C>A,14:G>A","TGG","CAGTGGCCCTGGAAGGGATTTGG",2,1,15,NA,"GeneID:643037","C11orf97",TRUE,"APOPT1"

"9907","ACTAACCTGATTCAGTTCTC","chr14",103574156,"-","none","AGG","ACTAACCTGATTCAGTTCTCAGG",0,NA,NA,NA,"GeneID:3831;GeneID:84334","KLC1;APOPT1",TRUE,"APOPT1"

"9908","ACTAACCTGATTCAGTTCTC","chr1",105982958,"+","0:C>A,3:T>A","AGG","CCTTACCTGATTCAGTTCTCAGG",2,1,4,NA,"","",FALSE,"APOPT1"

"9909","AGAAGCACATGTATTATAAC","chr14",103587342,"+","none","AGG","AGAAGCACATGTATTATAACAGG",0,NA,NA,NA,"GeneID:3831;GeneID:84334","KLC1;APOPT1",TRUE,"APOPT1"

"9910","CAAGAAAGTCTTGCCATGAT","chr14",103571641,"+","none","TGG","CAAGAAAGTCTTGCCATGATTGG",0,NA,NA,NA,"GeneID:3831;GeneID:84334","KLC1;APOPT1",TRUE,"APOPT1"

"9911","CAAGAAAGTCTTGCCATGAT","chr6",98724358,"+","5:G>A,14:T>C","TGG","CAAGAGAGTCTTGCTATGATTGG",2,6,15,NA,"","",FALSE,"APOPT1"

"9912","CGAAGGTTTGAATATTTATC","chr14",103571676,"-","none","TGG","CGAAGGTTTGAATATTTATCTGG",0,NA,NA,NA,"GeneID:3831;GeneID:84334","KLC1;APOPT1",TRUE,"APOPT1"

"9913","CTGGGCCTGAGAACTGAATC","chr14",103574148,"+","none","AGG","CTGGGCCTGAGAACTGAATCAGG",0,NA,NA,NA,"GeneID:3831;GeneID:84334","KLC1;APOPT1",TRUE,"APOPT1"

"9914","CTGGGCCTGAGAACTGAATC","chr8",26139029,"-","17:G>T,19:T>G","TGG","CTGGGCCTGAGAACTGACTATGG",2,18,20,NA,"","",FALSE,"APOPT1"

"9915","ACTGCCTTCTTATCAGCTTT","chr21",25975960,"+","none","AGG","ACTGCCTTCTTATCAGCTTTAGG",0,NA,NA,NA,"GeneID:351","APP",TRUE,"APP"

"9916","ACTGCCTTCTTATCAGCTTT","chr6",47436553,"-","17:G>A,18:G>A","TGG","ACTGCCTTCTTATCAGCCCTTGG",2,18,19,NA,"","",FALSE,"APP"

"9917","ACTGCCTTCTTATCAGCTTT","chr4",28187476,"+","11:T>A,18:C>T","AGG","ACTGCCTTCTTTTCAGCTCTAGG",2,12,19,NA,"","",FALSE,"APP"

"9918","ACTGCCTTCTTATCAGCTTT","chr4",189323969,"-","0:G>T,17:T>A","TGG","CCTGCCTTCTTATCAGCATTTGG",2,1,18,NA,"","",FALSE,"APP"

"9919","CTTGCCTAAAGCTGATAAGA","chr21",25975967,"-","none","AGG","CTTGCCTAAAGCTGATAAGAAGG",0,NA,NA,NA,"GeneID:351","APP",TRUE,"APP"

"9920","CTTGCCTAAAGCTGATAAGA","chr10",91211097,"-","5:T>G,6:T>A","TGG","CTTGCAAAAAGCTGATAAGATGG",2,6,7,NA,"GeneID:84333","PCGF5",FALSE,"APP"

"9921","GAACTACATCACCGCTCTGC","chr21",25975086,"-","none","AGG","GAACTACATCACCGCTCTGCAGG",0,NA,NA,NA,"GeneID:351","APP",TRUE,"APP"

"9922","GACAAGTATCTCGAGACACC","chr21",25982422,"-","none","TGG","GACAAGTATCTCGAGACACCTGG",0,NA,NA,NA,"GeneID:351","APP",TRUE,"APP"

"9923","GCTCAATGACCGCCGCCGCC","chr21",25975116,"-","none","TGG","GCTCAATGACCGCCGCCGCCTGG",0,NA,NA,NA,"GeneID:351","APP",TRUE,"APP"

"9924","TGTCTCGAGATACTTGTCAA","chr21",25982424,"+","none","CGG","TGTCTCGAGATACTTGTCAACGG",0,NA,NA,NA,"GeneID:351","APP",TRUE,"APP"

"9925","AGGTTGCTTCATGTGCGAAA","chr17",60466441,"-","none","TGG","AGGTTGCTTCATGTGCGAAATGG",0,NA,NA,NA,"GeneID:10513","APPBP2",TRUE,"APPBP2"

"9926","AGGTTGCTTCATGTGCGAAA","chr17",63926837,"-","12:G>C","TGG","AGGTTGCTTCATCTGCGAAATGG",1,13,NA,NA,"","",FALSE,"APPBP2"

"9927","CTTACCAGTGAAAGTTGTGG","chr17",60464046,"-","none","TGG","CTTACCAGTGAAAGTTGTGGTGG",0,NA,NA,NA,"GeneID:10513","APPBP2",TRUE,"APPBP2"

"9928","CTTACCAGTGAAAGTTGTGG","chr2",189153869,"-","15:C>A,16:T>C","AGG","CTTACCAGTGAAAGTGATGGAGG",2,16,17,NA,"GeneID:1290","COL5A2",FALSE,"APPBP2"

"9929","GCAAATAAAGCTGCACTCTA","chr17",60466336,"-","none","TGG","GCAAATAAAGCTGCACTCTATGG",0,NA,NA,NA,"GeneID:10513","APPBP2",TRUE,"APPBP2"

"9930","GCAAATAAAGCTGCACTCTA","chr14",65649581,"+","1:G>C,13:A>C","AGG","GGAAATAAAGCTGAACTCTAAGG",2,2,14,NA,"GeneID:2530","FUT8",FALSE,"APPBP2"

"9931","GCAAATAAAGCTGCACTCTA","chrX",118373207,"+","9:T>G,17:T>C","GGG","GCAAATAAATCTGCACTTTAGGG",2,10,18,NA,"GeneID:54521","WDR44",FALSE,"APPBP2"

"9932","TATTTGCGATCTATAGCAAT","chr17",60451881,"-","none","TGG","TATTTGCGATCTATAGCAATTGG",0,NA,NA,NA,"GeneID:10513","APPBP2",TRUE,"APPBP2"

"9933","TATTTGCGATCTATAGCAAT","chr5",170680658,"+","3:C>T,13:A>T","GGG","TATCTGCGATCTAAAGCAATGGG",2,4,14,NA,"GeneID:30820","KCNIP1",TRUE,"APPBP2"

"9934","TGTCTTAAGACAAGCTTCTA","chr17",60464022,"-","none","AGG","TGTCTTAAGACAAGCTTCTAAGG",0,NA,NA,NA,"GeneID:10513","APPBP2",TRUE,"APPBP2"

"9935","TGTCTTAAGACAAGCTTCTA","chr3",121667712,"-","3:C>G,6:A>T","TGG","TGTGTTTAGACAAGCTTCTATGG",2,4,7,NA,"GeneID:2804","GOLGB1",FALSE,"APPBP2"

"9936","TGTCTTAAGACAAGCTTCTA","chr8",138050500,"+","7:G>A,12:T>A","AGG","TGTCTTAGGACATGCTTCTAAGG",2,8,13,NA,"","",FALSE,"APPBP2"

"9937","TTATGAAGTAGCCCTTTCAG","chr17",60451963,"-","none","TGG","TTATGAAGTAGCCCTTTCAGTGG",0,NA,NA,NA,"GeneID:10513","APPBP2",TRUE,"APPBP2"

"9938","TTATGAAGTAGCCCTTTCAG","chr2",44972651,"+","2:C>A,17:G>C","AGG","TTCTGAAGTAGCCCTTTGAGAGG",2,3,18,NA,"","",FALSE,"APPBP2"

"9939","AGTTGTATCAAGCTATGCAT","chr3",57235626,"+","none","CGG","AGTTGTATCAAGCTATGCATCGG",0,NA,NA,NA,"GeneID:26060","APPL1",TRUE,"APPL1"

"9940","CATATAGTTGGAAATAGCTG","chr3",57235602,"-","none","TGG","CATATAGTTGGAAATAGCTGTGG",0,NA,NA,NA,"GeneID:26060","APPL1",TRUE,"APPL1"

"9941","CATATAGTTGGAAATAGCTG","chr12",102803206,"-","5:C>T,16:G>C","GGG","CATATGGTTGGAAATACCTGGGG",2,6,17,NA,"","",FALSE,"APPL1"

"9942","GATGCATAGCTTGATACAAC","chr3",57235627,"-","none","TGG","GATGCATAGCTTGATACAACTGG",0,NA,NA,NA,"GeneID:26060","APPL1",TRUE,"APPL1"

"9943","TCAGATCTCTTTCTTTAAAC","chr3",57240529,"-","none","TGG","TCAGATCTCTTTCTTTAAACTGG",0,NA,NA,NA,"GeneID:26060","APPL1",TRUE,"APPL1"

"9944","TCAGATCTCTTTCTTTAAAC","chr14",45729498,"+","16:C>A,19:G>C","AGG","TCAGATCTCTTTCTTTCAAGAGG",2,17,20,NA,"","",FALSE,"APPL1"

"9945","TCAGATCTCTTTCTTTAAAC","chr11",122790635,"+","15:A>T,19:G>C","TGG","TCAGATCTCTTTCTTAAAAGTGG",2,16,20,NA,"GeneID:84959","UBASH3B",FALSE,"APPL1"

"9946","TCAGATCTCTTTCTTTAAAC","chr20",17270289,"+","3:C>G,14:A>T","AGG","TCACATCTCTTTCTATAAACAGG",2,4,15,NA,"GeneID:5126","PCSK2",FALSE,"APPL1"

"9947","TCAGATCTCTTTCTTTAAAC","chr9",26629225,"-","8:C>G,19:A>G","TGG","TCAGATCTGTTTCTTTAAATTGG",2,9,20,NA,"","",FALSE,"APPL1"

"9948","TTTAAAAGAATATGAAAAAC","chr3",57237529,"+","none","AGG","TTTAAAAGAATATGAAAAACAGG",0,NA,NA,NA,"GeneID:26060","APPL1",TRUE,"APPL1"

"9949","TTTAAAAGAATATGAAAAAC","chr12",81383263,"+","14:T>A,19:A>C","TGG","TTTAAAAGAATATGTAAAAATGG",2,15,20,NA,"GeneID:8499","PPFIA2",FALSE,"APPL1"

"9950","TTTAAAAGAATATGAAAAAC","chr7",13654410,"+","12:A>T,19:A>C","AGG","TTTAAAAGAATAAGAAAAAAAGG",2,13,20,NA,"","",FALSE,"APPL1"

"9951","TTTAAAAGAATATGAAAAAC","chr4",40136840,"-","0:G>A,4:G>T","TGG","CTTACAAGAATATGAAAAACTGG",2,1,5,NA,"GeneID:55728","N4BP2",FALSE,"APPL1"

"9952","TTTAAAAGAATATGAAAAAC","chrX",108873940,"-","1:T>A,5:G>T","TGG","TATAACAGAATATGAAAAACTGG",2,2,6,NA,"","",FALSE,"APPL1"

"9953","TTTAAAAGAATATGAAAAAC","chr18",32242106,"+","7:A>G,8:T>A","TGG","TTTAAAAATATATGAAAAACTGG",2,8,9,NA,"GeneID:64762","GAREM",FALSE,"APPL1"

"9954","TTTAAAAGAATATGAAAAAC","chr6",136144010,"+","3:T>A,19:A>C","AGG","TTTTAAAGAATATGAAAAAAAGG",2,4,20,NA,"GeneID:27115","PDE7B",FALSE,"APPL1"

"9955","TTTAAAAGAATATGAAAAAC","chr3",105398542,"-","9:G>T,14:C>T","TGG","TTTAAAAGACTATGGAAAACTGG",2,10,15,NA,"GeneID:214","ALCAM",FALSE,"APPL1"

"9956","TTTAAAAGAATATGAAAAAC","chr5",166208536,"-","5:G>T,15:G>T","AGG","TTTAACAGAATATGACAAACAGG",2,6,16,NA,"","",FALSE,"APPL1"

"9957","TTTAAAGAAAGAGATCTGAA","chr3",57240530,"+","none","AGG","TTTAAAGAAAGAGATCTGAAAGG",0,NA,NA,NA,"GeneID:26060","APPL1",TRUE,"APPL1"

"9958","TTTAAAGAAAGAGATCTGAA","chr15",44179582,"-","15:C>G,17:G>C","AGG","TTTAAAGAAAGAGATGTCAAAGG",2,16,18,NA,"GeneID:84978","FRMD5",FALSE,"APPL1"

"9959","TTTAAAGAAAGAGATCTGAA","chr7",69676355,"-","11:A>T,17:G>C","TGG","TTTAAAGAAAGTGATCTCAATGG",2,12,18,NA,"GeneID:26053","AUTS2",FALSE,"APPL1"

"9960","TTTAAAGAAAGAGATCTGAA","chr11",112569830,"-","4:G>T,9:G>T","AGG","TTTACAGAACGAGATCTGAAAGG",2,5,10,NA,"GeneID:101928823","LOC101928823",FALSE,"APPL1"

"9961","TTTAAAGAAAGAGATCTGAA","chrX",113462357,"-","1:G>A,17:G>C","CGG","TCTAAAGAAAGAGATCTCAACGG",2,2,18,NA,"","",FALSE,"APPL1"

"9962","ACAGCTTTCTAAGCAACTGC","chr12",105217682,"-","none","TGG","ACAGCTTTCTAAGCAACTGCTGG",0,NA,NA,NA,"GeneID:55198","APPL2",TRUE,"APPL2"

"9963","ACAGCTTTCTAAGCAACTGC","chr6",153488962,"+","6:C>T,12:A>G","AGG","ACAGCTCTCTAAACAACTGCAGG",2,7,13,NA,"","",FALSE,"APPL2"

"9964","CCTGCAACGCCTCCTCTAGC","chr12",105235969,"+","none","AGG","CCTGCAACGCCTCCTCTAGCAGG",0,NA,NA,NA,"GeneID:55198;GeneID:387882","APPL2;C12orf75",TRUE,"APPL2"

"9965","CCTGCTAGAGGAGGCGTTGC","chr12",105235972,"-","none","AGG","CCTGCTAGAGGAGGCGTTGCAGG",0,NA,NA,NA,"GeneID:55198;GeneID:387882","APPL2;C12orf75",TRUE,"APPL2"

"9966","TCCACAGAATGAGATGTGCC","chr12",105217712,"-","none","TGG","TCCACAGAATGAGATGTGCCTGG",0,NA,NA,NA,"GeneID:55198","APPL2",TRUE,"APPL2"

"9967","TCTTACCTCATCCACCACTT","chr12",105217062,"+","none","TGG","TCTTACCTCATCCACCACTTTGG",0,NA,NA,NA,"GeneID:55198","APPL2",TRUE,"APPL2"

"9968","TGGTCTTAGAACTTTGCTCT","chr12",105217129,"-","none","TGG","TGGTCTTAGAACTTTGCTCTTGG",0,NA,NA,NA,"GeneID:55198","APPL2",TRUE,"APPL2"

"9969","ACGACGACCACCCTCTGTCC","chr16",88810092,"+","none","TGG","ACGACGACCACCCTCTGTCCTGG",0,NA,NA,NA,"GeneID:353","APRT",TRUE,"APRT"

"9970","AGGACAGAGGGTGGTCGTCG","chr16",88810093,"-","none","TGG","AGGACAGAGGGTGGTCGTCGTGG",0,NA,NA,NA,"GeneID:353","APRT",TRUE,"APRT"

"9971","CGCCTGCGATGTAGTCGATG","chr16",88811546,"+","none","CGG","CGCCTGCGATGTAGTCGATGCGG",0,NA,NA,NA,"GeneID:353","APRT",TRUE,"APRT"

"9972","CTGACAGGCCTAGACTCCCG","chr16",88810543,"-","none","AGG","CTGACAGGCCTAGACTCCCGAGG",0,NA,NA,NA,"GeneID:353","APRT",TRUE,"APRT"

"9973","GCTGCGTGCTCATCCGAAAG","chr16",88810479,"-","none","CGG","GCTGCGTGCTCATCCGAAAGCGG",0,NA,NA,NA,"GeneID:353","APRT",TRUE,"APRT"

"9974","GGCCGCATCGACTACATCGC","chr16",88811551,"-","none","AGG","GGCCGCATCGACTACATCGCAGG",0,NA,NA,NA,"GeneID:353","APRT",TRUE,"APRT"

"9975","AAGAAATGTTCTCGACAGCA","chr9",32989760,"-","none","AGG","AAGAAATGTTCTCGACAGCAAGG",0,NA,NA,NA,"GeneID:54840","APTX",TRUE,"APTX"

"9976","AAGAAATGTTCTCGACAGCA","chrX",24622186,"-","3:G>T,12:C>G","AGG","AAGCAATGTTCTGGACAGCAAGG",2,4,13,NA,"GeneID:9468","PCYT1B",FALSE,"APTX"

"9977","ACATCTTCATGTGATCAGCC","chr9",32974535,"-","none","AGG","ACATCTTCATGTGATCAGCCAGG",0,NA,NA,NA,"GeneID:54840","APTX",TRUE,"APTX"

"9978","AGGCTTGAAGATTTCTATGC","chr9",32985987,"-","none","AGG","AGGCTTGAAGATTTCTATGCAGG",0,NA,NA,NA,"GeneID:54840","APTX",TRUE,"APTX"

"9979","AGGCTTGAAGATTTCTATGC","chr5",5937529,"-","5:C>A,16:G>T","AGG","AGGCTGGAAGATTTCTCTGCAGG",2,6,17,NA,"","",FALSE,"APTX"

"9980","GAGTGATGATGCGGGTGTGC","chr9",32989876,"-","none","TGG","GAGTGATGATGCGGGTGTGCTGG",0,NA,NA,NA,"GeneID:54840","APTX",TRUE,"APTX"

"9981","GAGTGATGATGCGGGTGTGC","chr11",64708334,"-","12:T>C,18:A>C","AGG","GAGTGATGATGCAGGTGTTCAGG",2,13,19,NA,"GeneID:9379","NRXN2",FALSE,"APTX"

"9982","GTTGAAAGCAGAGTGTAACA","chr9",32988105,"-","none","AGG","GTTGAAAGCAGAGTGTAACAAGG",0,NA,NA,NA,"GeneID:54840","APTX",TRUE,"APTX"

"9983","TGATCACATGAAGATGTACA","chr9",32974538,"+","none","TGG","TGATCACATGAAGATGTACATGG",0,NA,NA,NA,"GeneID:54840","APTX",TRUE,"APTX"

"9984","ATTAACCCTGCTCGGTCCTT","chr7",30922584,"+","none","TGG","ATTAACCCTGCTCGGTCCTTTGG",0,NA,NA,NA,"GeneID:358","AQP1",TRUE,"AQP1"

"9985","CCCTCACAGATTGACTACAC","chr7",30922554,"+","none","TGG","CCCTCACAGATTGACTACACTGG",0,NA,NA,NA,"GeneID:358","AQP1",TRUE,"AQP1"

"9986","CCTAATACCTGCCTAGACCC","chr7",30921826,"-","none","AGG","CCTAATACCTGCCTAGACCCAGG",0,NA,NA,NA,"GeneID:358","AQP1",TRUE,"AQP1"

"9987","GGCCTGGGCATCGAGATCAT","chr7",30922092,"+","none","CGG","GGCCTGGGCATCGAGATCATCGG",0,NA,NA,NA,"GeneID:358","AQP1",TRUE,"AQP1"

"9988","TCCCGATGATCTCGATGCCC","chr7",30922097,"-","none","AGG","TCCCGATGATCTCGATGCCCAGG",0,NA,NA,NA,"GeneID:358","AQP1",TRUE,"AQP1"

"9989","TTCTGGGGTACCAAGACCAG","chr7",30921727,"-","none","AGG","TTCTGGGGTACCAAGACCAGAGG",0,NA,NA,NA,"GeneID:358","AQP1",TRUE,"AQP1"

"9990","CCCACGTCTCTTCACCTACG","chr1",154323758,"+","none","TGG","CCCACGTCTCTTCACCTACGTGG",0,NA,NA,NA,"GeneID:89872","AQP10",TRUE,"AQP10"

"9991","GAGGCGTCCAACGATGCACA","chr1",154323014,"-","none","TGG","GAGGCGTCCAACGATGCACATGG",0,NA,NA,NA,"GeneID:89872","AQP10",TRUE,"AQP10"

"9992","GATGCCCTACAGAACTATAC","chr1",154323239,"+","none","AGG","GATGCCCTACAGAACTATACAGG",0,NA,NA,NA,"GeneID:89872","AQP10",TRUE,"AQP10"

"9993","GCATCGTTGGACGCCTCCCC","chr1",154323017,"+","none","TGG","GCATCGTTGGACGCCTCCCCTGG",0,NA,NA,NA,"GeneID:89872","AQP10",TRUE,"AQP10"

"9994","GCCACGTAGGTGAAGAGACG","chr1",154323762,"-","none","TGG","GCCACGTAGGTGAAGAGACGTGG",0,NA,NA,NA,"GeneID:89872","AQP10",TRUE,"AQP10"

"9995","TCCCACCTGTATAGTTCTGT","chr1",154323247,"-","none","AGG","TCCCACCTGTATAGTTCTGTAGG",0,NA,NA,NA,"GeneID:89872","AQP10",TRUE,"AQP10"

"9996","TCCCACCTGTATAGTTCTGT","chr4",101906919,"-","2:T>G,13:A>C","GGG","TCACACCTGTATATTTCTGTGGG",2,3,14,NA,"GeneID:55024","BANK1",FALSE,"AQP10"

"9997","CAACCATACAATTAATAAAA","chr11",77609349,"+","none","AGG","CAACCATACAATTAATAAAAAGG",0,NA,NA,NA,"GeneID:1207;GeneID:282679","CLNS1A;AQP11",TRUE,"AQP11"

"9998","CAACCATACAATTAATAAAA","chr4",19247185,"-","6:C>A,8:T>G","TGG","CAACCAGAAAATTAATAAAATGG",2,7,9,NA,"","",FALSE,"AQP11"

"9999","CATGTCCCCCGAGACGGGTG","chr11",77590324,"+","none","CGG","CATGTCCCCCGAGACGGGTGCGG",0,NA,NA,NA,"GeneID:1207;GeneID:282679","CLNS1A;AQP11",TRUE,"AQP11"

"10000","CATGTCCCCCGAGACGGGTG","chr8",143491555,"-","5:C>G,13:G>T","TGG","CATGTGCCCCGAGCCGGGTGTGG",2,6,14,NA,"GeneID:23144","ZC3H3",FALSE,"AQP11"

"10001","CCGCTTTGAGCAAGTCGACT","chr11",77590471,"-","none","CGG","CCGCTTTGAGCAAGTCGACTCGG",0,NA,NA,NA,"GeneID:1207;GeneID:282679","CLNS1A;AQP11",TRUE,"AQP11"

"10002","GGCTGGCCCGCGTAGTCGCC","chr11",77590077,"+","none","CGG","GGCTGGCCCGCGTAGTCGCCCGG",0,NA,NA,NA,"GeneID:1207;GeneID:282679","CLNS1A;AQP11",TRUE,"AQP11"

"10003","GTTACAGGAGGAAGTCTAAC","chr11",77603548,"+","none","AGG","GTTACAGGAGGAAGTCTAACAGG",0,NA,NA,NA,"GeneID:1207;GeneID:282679","CLNS1A;AQP11",TRUE,"AQP11"

"10004","GTTACAGGAGGAAGTCTAAC","chr8",118688156,"-","6:T>C,18:G>T","AGG","GTTACAAGAGGAAGTCTACCAGG",2,7,19,NA,"GeneID:552860","SAMD12-AS1",FALSE,"AQP11"

"10005","TGTAGCGAAAGTGCCAAAGC","chr11",77603587,"-","none","TGG","TGTAGCGAAAGTGCCAAAGCTGG",0,NA,NA,NA,"GeneID:1207;GeneID:282679","CLNS1A;AQP11",TRUE,"AQP11"

"10006","AAGGCCACTCAGCTGGAATG","chr2",240676536,"+","none","TGG","AAGGCCACTCAGCTGGAATGTGG",0,NA,NA,NA,"GeneID:653437","AQP12B",TRUE,"AQP12B"

"10007","AAGGCCACTCAGCTGGAATG","chr2",240698344,"-","none","TGG","AAGGCCACTCAGCTGGAATGTGG",0,NA,NA,NA,"GeneID:375318","AQP12A",TRUE,"AQP12B"

"10008","AAGGCCACTCAGCTGGAATG","chr9",98809071,"+","6:C>A","TGG","AAGGCCCCTCAGCTGGAATGTGG",1,7,NA,NA,"GeneID:79695","GALNT12",FALSE,"AQP12B"

"10009","AAGGCCACTCAGCTGGAATG","chr13",20168989,"+","8:A>T,14:A>G","GGG","AAGGCCACACAGCTAGAATGGGG",2,9,15,NA,"","",FALSE,"AQP12B"

"10010","AAGGCCACTCAGCTGGAATG","chr17",74904872,"+","8:A>T,18:A>T","GGG","AAGGCCACACAGCTGGAAAGGGG",2,9,19,NA,"","",FALSE,"AQP12B"

"10011","AAGGCCACTCAGCTGGAATG","chr5",174715040,"+","8:C>T,18:G>T","AGG","AAGGCCACCCAGCTGGAAGGAGG",2,9,19,NA,"","",FALSE,"AQP12B"

"10012","AAGGCCACTCAGCTGGAATG","chr18",41758347,"-","9:A>G,18:C>A","TGG","AAGGCCACTTAGCTGGAAGGTGG",2,10,19,NA,"","",FALSE,"AQP12B"

"10013","AAGGCCACTCAGCTGGAATG","chr8",138269688,"-","8:T>A,18:T>A","TGG","AAGGCCACACAGCTGGAAAGTGG",2,9,19,NA,"GeneID:51059","FAM135B",FALSE,"AQP12B"

"10014","CACCTGCACGTACTCCAGTA","chr2",240680373,"+","none","AGG","CACCTGCACGTACTCCAGTAAGG",0,NA,NA,NA,"GeneID:653437","AQP12B",TRUE,"AQP12B"

"10015","CACCTGCACGTACTCCAGTA","chr2",240694507,"-","none","AGG","CACCTGCACGTACTCCAGTAAGG",0,NA,NA,NA,"GeneID:375318","AQP12A",TRUE,"AQP12B"

"10016","GCCAGCTTCAGCAGCGTGCC","chr2",240682490,"+","none","AGG","GCCAGCTTCAGCAGCGTGCCAGG",0,NA,NA,NA,"GeneID:653437","AQP12B",TRUE,"AQP12B"

"10017","GCCAGCTTCAGCAGCGTGCC","chr2",240692241,"-","10:T>C","AGG","GCCAGCTTCAACAGCGTGCCAGG",1,11,NA,NA,"GeneID:375318","AQP12A",TRUE,"AQP12B"

"10018","GCGGTGGGCGCGGTGCAGCT","chr2",240682697,"-","none","CGG","GCGGTGGGCGCGGTGCAGCTCGG",0,NA,NA,NA,"GeneID:653437","AQP12B",TRUE,"AQP12B"

"10019","TGCCTTCTTCAACCCTGCCC","chr2",240694448,"+","none","TGG","TGCCTTCTTCAACCCTGCCCTGG",0,NA,NA,NA,"GeneID:375318","AQP12A",TRUE,"AQP12B"

"10020","TGCCTTCTTCAACCCTGCCC","chr2",240680432,"-","none","TGG","TGCCTTCTTCAACCCTGCCCTGG",0,NA,NA,NA,"GeneID:653437","AQP12B",TRUE,"AQP12B"

"10021","TTCTCTTGCAGGGATGGTCC","chr2",240698145,"+","none","TGG","TTCTCTTGCAGGGATGGTCCTGG",0,NA,NA,NA,"GeneID:375318","AQP12A",TRUE,"AQP12B"

"10022","TTCTCTTGCAGGGATGGTCC","chr2",240676735,"-","none","TGG","TTCTCTTGCAGGGATGGTCCTGG",0,NA,NA,NA,"GeneID:653437","AQP12B",TRUE,"AQP12B"

"10023","TTCTCTTGCAGGGATGGTCC","chr1",147509354,"-","6:C>A,10:T>C","AGG","TTCTCTGGCAAGGATGGTCCAGG",2,7,11,NA,"GeneID:100289211","LINC00624",FALSE,"AQP12B"

"10024","AGAGCTCCACAGTCACCGCC","chr12",49954180,"-","none","TGG","AGAGCTCCACAGTCACCGCCTGG",0,NA,NA,NA,"GeneID:359;GeneID:101927318","AQP2;LOC101927318",TRUE,"AQP2"

"10025","AGAGCTCCACAGTCACCGCC","chr10",129774377,"-","12:G>A,17:T>C","AGG","AGAGCTCCACAGCCACCACCAGG",2,13,18,NA,"","",FALSE,"AQP2"

"10026","ATCACGCCAGCAGACATCCG","chr12",49951148,"+","none","CGG","ATCACGCCAGCAGACATCCGCGG",0,NA,NA,NA,"GeneID:359","AQP2",TRUE,"AQP2"

"10027","CACCGATTGGGAGGAGCGCG","chr12",49955520,"+","none","AGG","CACCGATTGGGAGGAGCGCGAGG",0,NA,NA,NA,"GeneID:359;GeneID:101927318","AQP2;LOC101927318",TRUE,"AQP2"

"10028","CCTCAGGCCTTGGTACCCCG","chr12",49955590,"-","none","TGG","CCTCAGGCCTTGGTACCCCGTGG",0,NA,NA,NA,"GeneID:359;GeneID:101927318","AQP2;LOC101927318",TRUE,"AQP2"

"10029","GCCTCCACCGATGAGCGCCG","chr12",49954232,"+","none","CGG","GCCTCCACCGATGAGCGCCGCGG",0,NA,NA,NA,"GeneID:359;GeneID:101927318","AQP2;LOC101927318",TRUE,"AQP2"

"10030","TCTCCGAGCCGCCTTCTACG","chr12",49951078,"+","none","TGG","TCTCCGAGCCGCCTTCTACGTGG",0,NA,NA,NA,"GeneID:359","AQP2",TRUE,"AQP2"

"10031","AGATGCTCCACATCCGCTAC","chr9",33447473,"-","none","CGG","AGATGCTCCACATCCGCTACCGG",0,NA,NA,NA,"GeneID:360","AQP3",TRUE,"AQP3"

"10032","ATCTTTGCTACCTACCCCTC","chr9",33442888,"-","none","TGG","ATCTTTGCTACCTACCCCTCTGG",0,NA,NA,NA,"GeneID:360","AQP3",TRUE,"AQP3"

"10033","CAAGCTGCCCATCTACACCC","chr9",33443377,"-","none","TGG","CAAGCTGCCCATCTACACCCTGG",0,NA,NA,NA,"GeneID:360","AQP3",TRUE,"AQP3"

"10034","CCCCGCCATGGGTCGACAGA","chr9",33447517,"-","none","AGG","CCCCGCCATGGGTCGACAGAAGG",0,NA,NA,NA,"GeneID:360","AQP3",TRUE,"AQP3"

"10035","TGATCCAGGGCTCACGAGCC","chr9",33443395,"+","none","AGG","TGATCCAGGGCTCACGAGCCAGG",0,NA,NA,NA,"GeneID:360","AQP3",TRUE,"AQP3"

"10036","TGCCCCGGCTGAGCACAACC","chr9",33443846,"+","none","TGG","TGCCCCGGCTGAGCACAACCTGG",0,NA,NA,NA,"GeneID:360","AQP3",TRUE,"AQP3"

"10037","ATGAATCCCGCCCGATCCTT","chr18",26860811,"-","none","TGG","ATGAATCCCGCCCGATCCTTTGG",0,NA,NA,NA,"GeneID:361","AQP4",TRUE,"AQP4"

"10038","ATGAATCCCGCCCGATCCTT","chr12",49974766,"+","8:A>C,14:C>A","CGG","ATGAATCCAGCCCGCTCCTTCGG",2,9,15,NA,"GeneID:363","AQP6",TRUE,"AQP4"

"10039","ATGTGATTATCAACTCAACC","chr18",26861240,"+","none","AGG","ATGTGATTATCAACTCAACCAGG",0,NA,NA,NA,"GeneID:361","AQP4",TRUE,"AQP4"

"10040","GAGATGAGAACCATGTCGAC","chr18",26862407,"+","none","CGG","GAGATGAGAACCATGTCGACCGG",0,NA,NA,NA,"GeneID:361","AQP4",TRUE,"AQP4"

"10041","GAGATGAGAACCATGTCGAC","chr8",139357024,"+","11:T>C,17:A>G","TGG","GAGATGAGAACTATGTCAACTGG",2,12,18,NA,"","",FALSE,"AQP4"

"10042","GGATCGGGCGGGATTCATGC","chr18",26860813,"+","none","TGG","GGATCGGGCGGGATTCATGCTGG",0,NA,NA,NA,"GeneID:361","AQP4",TRUE,"AQP4"

"10043","GTTCATGGAAATCTTACCGC","chr18",26861275,"-","none","TGG","GTTCATGGAAATCTTACCGCTGG",0,NA,NA,NA,"GeneID:361","AQP4",TRUE,"AQP4"

"10044","TGCATGTGATTGACGTTGAC","chr18",26856267,"-","none","CGG","TGCATGTGATTGACGTTGACCGG",0,NA,NA,NA,"GeneID:361","AQP4",TRUE,"AQP4"

"10045","AACCGATTCATGACCACCGC","chr12",49964139,"-","none","AGG","AACCGATTCATGACCACCGCAGG",0,NA,NA,NA,"GeneID:362","AQP5",TRUE,"AQP5"

"10046","AGGCACGTATGAGCCTGACG","chr12",49965098,"+","none","AGG","AGGCACGTATGAGCCTGACGAGG",0,NA,NA,NA,"GeneID:362","AQP5",TRUE,"AQP5"

"10047","GCAGCGCCGACGGCCACTTG","chr12",49962115,"-","none","AGG","GCAGCGCCGACGGCCACTTGAGG",0,NA,NA,NA,"GeneID:101927318;GeneID:362","LOC101927318;AQP5",TRUE,"AQP5"

"10048","GCCCATCGTGGGGGCGGTCC","chr12",49965005,"+","none","TGG","GCCCATCGTGGGGGCGGTCCTGG",0,NA,NA,NA,"GeneID:362","AQP5",TRUE,"AQP5"

"10049","TACCATCCTGCAGATCGCGC","chr12",49962136,"+","none","TGG","TACCATCCTGCAGATCGCGCTGG",0,NA,NA,NA,"GeneID:101927318;GeneID:362","LOC101927318;AQP5",TRUE,"AQP5"

"10050","TACTGCTCACTCCGACAAGG","chr12",49963646,"-","none","TGG","TACTGCTCACTCCGACAAGGTGG",0,NA,NA,NA,"GeneID:362","AQP5",TRUE,"AQP5"

"10051","AAGCCTGCACGCCAACATGC","chr12",49973210,"-","none","TGG","AAGCCTGCACGCCAACATGCTGG",0,NA,NA,NA,"GeneID:363","AQP6",TRUE,"AQP6"

"10052","ACCGACAGCCGTCAGACATC","chr12",49974407,"+","none","AGG","ACCGACAGCCGTCAGACATCAGG",0,NA,NA,NA,"GeneID:363","AQP6",TRUE,"AQP6"

"10053","ATGAATCCAGCCCGCTCCTT","chr12",49974766,"+","none","CGG","ATGAATCCAGCCCGCTCCTTCGG",0,NA,NA,NA,"GeneID:363","AQP6",TRUE,"AQP6"

"10054","ATGAATCCAGCCCGCTCCTT","chr12",49964112,"+","5:C>T,17:T>C","TGG","ATGAACCCAGCCCGCTCTTTTGG",2,6,18,NA,"GeneID:362","AQP5",TRUE,"AQP6"

"10055","ATGAATCCAGCCCGCTCCTT","chr18",26860811,"-","8:G>T,14:T>G","TGG","ATGAATCCCGCCCGATCCTTTGG",2,9,15,NA,"GeneID:361","AQP4",TRUE,"AQP6"

"10056","CACCTACCACGTTGATCCCA","chr12",49973562,"-","none","AGG","CACCTACCACGTTGATCCCAAGG",0,NA,NA,NA,"GeneID:363","AQP6",TRUE,"AQP6"

"10057","GGATAGCCAGCCGCTGCGCC","chr12",49975547,"-","none","AGG","GGATAGCCAGCCGCTGCGCCAGG",0,NA,NA,NA,"GeneID:363","AQP6",TRUE,"AQP6"

"10058","GTCCGGAACAGTGTCTCAAC","chr12",49974323,"+","none","TGG","GTCCGGAACAGTGTCTCAACTGG",0,NA,NA,NA,"GeneID:363","AQP6",TRUE,"AQP6"

"10059","CACGTGGCAGGCCGCATCTC","chr2",94602593,"+","none","TGG","CACGTGGCAGGCCGCATCTCTGG",0,NA,NA,NA,"GeneID:100509620","LOC100509620",TRUE,"AQP7"

"10060","CACGTGGCAGGCCGCATCTC","chr9",33386970,"-","none","TGG","CACGTGGCAGGCCGCATCTCTGG",0,NA,NA,NA,"GeneID:364","AQP7",TRUE,"AQP7"

"10061","CACGTGGCAGGCCGCATCTC","chr9",43111833,"-","none","TGG","CACGTGGCAGGCCGCATCTCTGG",0,NA,NA,NA,"","",TRUE,"AQP7"

"10062","CACGTGGCAGGCCGCATCTC","chr9",65892804,"+","3:A>G,16:C>T","TGG","CACATGGCAGGCCGCACCTCTGG",2,4,17,NA,"","",TRUE,"AQP7"

"10063","CACGTGGCAGGCCGCATCTC","chr9",63141415,"+","3:A>G,16:C>T","TGG","CACATGGCAGGCCGCACCTCTGG",2,4,17,NA,"","",TRUE,"AQP7"

"10064","CACGTGGCAGGCCGCATCTC","chr9",64623525,"-","3:T>C,16:G>A","TGG","CACATGGCAGGCCGCACCTCTGG",2,4,17,NA,"","",TRUE,"AQP7"

"10065","CACGTGGCAGGCCGCATCTC","chr9",63319036,"-","3:T>C,16:G>A","TGG","CACATGGCAGGCCGCACCTCTGG",2,4,17,NA,"","",TRUE,"AQP7"

"10066","CACGTGGCAGGCCGCATCTC","chr9",40483521,"-","3:T>C,16:G>A","TGG","CACATGGCAGGCCGCACCTCTGG",2,4,17,NA,"","",TRUE,"AQP7"

"10067","CACTGTCTTAGGTCCACCCG","chr9",33395186,"-","none","TGG","CACTGTCTTAGGTCCACCCGTGG",0,NA,NA,NA,"GeneID:364","AQP7",TRUE,"AQP7"

"10068","CACTGTCTTAGGTCCACCCG","chr9",65136560,"-","6:T>G,8:G>A","TGG","CACTGTATCAGGTCCACCCGTGG",2,7,9,NA,"","",FALSE,"AQP7"

"10069","CACTGTCTTAGGTCCACCCG","chr9",63326850,"-","6:T>G,8:G>A","TGG","CACTGTATCAGGTCCACCCGTGG",2,7,9,NA,"","",TRUE,"AQP7"

"10070","CACTGTCTTAGGTCCACCCG","chr9",43119677,"-","6:T>G,8:G>A","TGG","CACTGTATCAGGTCCACCCGTGG",2,7,9,NA,"","",TRUE,"AQP7"

"10071","CACTGTCTTAGGTCCACCCG","chr9",40491347,"-","6:T>G,8:G>A","TGG","CACTGTATCAGGTCCACCCGTGG",2,7,9,NA,"","",TRUE,"AQP7"

"10072","CACTGTCTTAGGTCCACCCG","chr9",64631350,"-","6:T>G,8:G>A","TGG","CACTGTATCAGGTCCACCCGTGG",2,7,9,NA,"","",TRUE,"AQP7"

"10073","CACTGTCTTAGGTCCACCCG","chr2",94594732,"+","8:C>T","TGG","CACTGTCTCAGGTCCACCCGTGG",1,9,NA,NA,"GeneID:100509620","LOC100509620",TRUE,"AQP7"

"10074","CACTGTCTTAGGTCCACCCG","chr9",63133586,"+","6:A>C,8:C>T","TGG","CACTGTATCAGGTCCACCCGTGG",2,7,9,NA,"","",TRUE,"AQP7"

"10075","CACTGTCTTAGGTCCACCCG","chr9",65884979,"+","6:A>C,8:C>T","TGG","CACTGTATCAGGTCCACCCGTGG",2,7,9,NA,"","",TRUE,"AQP7"

"10076","GACCTTTGCTAACTGTGCGC","chr9",33386499,"-","none","TGG","GACCTTTGCTAACTGTGCGCTGG",0,NA,NA,NA,"GeneID:364","AQP7",TRUE,"AQP7"

"10077","GACCTTTGCTAACTGTGCGC","chr2",94603058,"+","6:C>T,7:A>G","TGG","GACCTTCACTAACTGTGCGCTGG",2,7,8,NA,"GeneID:100509620","LOC100509620",TRUE,"AQP7"

"10078","GACCTTTGCTAACTGTGCGC","chr7",102170732,"+","2:G>C,19:T>C","GGG","GAGCTTTGCTAACTGTGCGTGGG",2,3,20,NA,"GeneID:1523","CUX1",FALSE,"AQP7"

"10079","GCCCAGCGCACAGTTAGCAA","chr9",33386494,"+","none","AGG","GCCCAGCGCACAGTTAGCAAAGG",0,NA,NA,NA,"GeneID:364","AQP7",TRUE,"AQP7"

"10080","GCCCAGCGCACAGTTAGCAA","chr2",94603063,"-","17:A>G,18:C>T","AGG","GCCCAGCGCACAGTTAGTGAAGG",2,18,19,NA,"GeneID:100509620","LOC100509620",TRUE,"AQP7"

"10081","GCCTGCCACGTGCACTCCCA","chr9",43111841,"+","none","TGG","GCCTGCCACGTGCACTCCCATGG",0,NA,NA,NA,"","",TRUE,"AQP7"

"10082","GCCTGCCACGTGCACTCCCA","chr9",33386978,"+","none","TGG","GCCTGCCACGTGCACTCCCATGG",0,NA,NA,NA,"GeneID:364","AQP7",TRUE,"AQP7"

"10083","GCCTGCCACGTGCACTCCCA","chr2",94602585,"-","12:C>G","TGG","GCCTGCCACGTGGACTCCCATGG",1,13,NA,NA,"GeneID:100509620","LOC100509620",TRUE,"AQP7"

"10084","GCCTGCCACGTGCACTCCCA","chr9",65892796,"-","8:A>G","TGG","GCCTGCCATGTGCACTCCCATGG",1,9,NA,NA,"","",TRUE,"AQP7"

"10085","GCCTGCCACGTGCACTCCCA","chr9",63141407,"-","8:A>G","TGG","GCCTGCCATGTGCACTCCCATGG",1,9,NA,NA,"","",TRUE,"AQP7"

"10086","GCCTGCCACGTGCACTCCCA","chr9",63319044,"+","8:T>C","TGG","GCCTGCCATGTGCACTCCCATGG",1,9,NA,NA,"","",TRUE,"AQP7"

"10087","GCCTGCCACGTGCACTCCCA","chr9",40483529,"+","8:T>C","TGG","GCCTGCCATGTGCACTCCCATGG",1,9,NA,NA,"","",TRUE,"AQP7"

"10088","GCCTGCCACGTGCACTCCCA","chr9",64623533,"+","8:T>C","TGG","GCCTGCCATGTGCACTCCCATGG",1,9,NA,NA,"","",TRUE,"AQP7"

"10089","TCTTCCTCTGCAGTATTTCC","chr9",33395124,"+","none","TGG","TCTTCCTCTGCAGTATTTCCTGG",0,NA,NA,NA,"GeneID:364","AQP7",TRUE,"AQP7"

"10090","TCTTCCTCTGCAGTATTTCC","chr4",146923140,"-","14:G>T,19:A>G","GGG","TCTTCCTCTGCAGTCTTTCTGGG",2,15,20,NA,"GeneID:83894","TTC29",FALSE,"AQP7"

"10091","TCTTCCTCTGCAGTATTTCC","chr20",45970230,"+","1:T>C,11:T>A","AGG","TTTTCCTCTGCTGTATTTCCAGG",2,2,12,NA,"GeneID:63925","ZNF335",TRUE,"AQP7"

"10092","TCTTCCTCTGCAGTATTTCC","chr17",13573545,"+","9:C>G,14:C>A","TGG","TCTTCCTCTCCAGTCTTTCCTGG",2,10,15,NA,"GeneID:9955","HS3ST3A1",FALSE,"AQP7"

"10093","ATGAATCCCGCCCGTGCTTT","chr16",25227089,"+","none","TGG","ATGAATCCCGCCCGTGCTTTTGG",0,NA,NA,NA,"GeneID:343","AQP8",TRUE,"AQP8"

"10094","CTGCACAAACCGTTCGTACC","chr16",25217273,"-","none","AGG","CTGCACAAACCGTTCGTACCAGG",0,NA,NA,NA,"GeneID:343","AQP8",TRUE,"AQP8"

"10095","GATCATCCTGACGACGCTGC","chr16",25224456,"+","none","TGG","GATCATCCTGACGACGCTGCTGG",0,NA,NA,NA,"GeneID:343","AQP8",TRUE,"AQP8"

"10096","GATCATCCTGACGACGCTGC","chr11",65111352,"+","5:G>T,11:A>C","TGG","GATCAGCCTGAAGACGCTGCTGG",2,6,12,NA,"GeneID:738","VPS51",TRUE,"AQP8"

"10097","TCCCATTCTCAATGACCGAC","chr16",25217346,"-","none","AGG","TCCCATTCTCAATGACCGACAGG",0,NA,NA,NA,"GeneID:343","AQP8",TRUE,"AQP8"

"10098","TGCCGTCACCGTGGATATCC","chr16",25224549,"+","none","TGG","TGCCGTCACCGTGGATATCCTGG",0,NA,NA,NA,"GeneID:343","AQP8",TRUE,"AQP8"

"10099","TGGTGATGCTCCTCCCGTAC","chr16",25221515,"+","none","TGG","TGGTGATGCTCCTCCCGTACTGG",0,NA,NA,NA,"GeneID:343","AQP8",TRUE,"AQP8"

"10100","AAGCCCCAAGATGCAGCCTG","chr15",58138555,"+","none","AGG","AAGCCCCAAGATGCAGCCTGAGG",0,NA,NA,NA,"GeneID:8854;GeneID:366","ALDH1A2;AQP9",TRUE,"AQP9"

"10101","AAGCCCCAAGATGCAGCCTG","chr2",111920004,"+","11:A>T","TGG","AAGCCCCAAGAAGCAGCCTGTGG",1,12,NA,NA,"GeneID:10461","MERTK",FALSE,"AQP9"

"10102","ACCGTCTTTGGCATTTACTA","chr15",58173183,"+","none","TGG","ACCGTCTTTGGCATTTACTATGG",0,NA,NA,NA,"GeneID:8854;GeneID:366","ALDH1A2;AQP9",TRUE,"AQP9"

"10103","ACCGTCTTTGGCATTTACTA","chr5",145860529,"-","2:T>G,8:C>A","TGG","ACAGTCTTGGGCATTTACTATGG",2,3,9,NA,"GeneID:643226","GRXCR2",FALSE,"AQP9"

"10104","ACGTGCCCAAGAACTCAGAG","chr15",58138645,"-","none","AGG","ACGTGCCCAAGAACTCAGAGAGG",0,NA,NA,NA,"GeneID:8854;GeneID:366","ALDH1A2;AQP9",TRUE,"AQP9"

"10105","CCACAAAGGCTCCCAAGAAC","chr15",58173155,"-","none","TGG","CCACAAAGGCTCCCAAGAACTGG",0,NA,NA,NA,"GeneID:8854;GeneID:366","ALDH1A2;AQP9",TRUE,"AQP9"

"10106","GACACCGCCAGCCACATAAA","chr15",58166775,"-","none","TGG","GACACCGCCAGCCACATAAATGG",0,NA,NA,NA,"GeneID:8854;GeneID:366","ALDH1A2;AQP9",TRUE,"AQP9"

"10107","GTCCTCGACTGAGAATAGCT","chr15",58166698,"-","none","TGG","GTCCTCGACTGAGAATAGCTTGG",0,NA,NA,NA,"GeneID:8854;GeneID:366","ALDH1A2;AQP9",TRUE,"AQP9"

"10108","ACAAATGCGACTAAGTAAGT","chr5",115984643,"-","none","TGG","ACAAATGCGACTAAGTAAGTTGG",0,NA,NA,NA,"GeneID:206338","AQPEP",TRUE,"AQPEP"

"10109","ATGCCTGGTGTCCAATCTCG","chr5",115992261,"-","none","TGG","ATGCCTGGTGTCCAATCTCGTGG",0,NA,NA,NA,"GeneID:206338","AQPEP",TRUE,"AQPEP"

"10110","CCCTTTCTGTTCTGTTGACG","chr5",115984681,"-","none","TGG","CCCTTTCTGTTCTGTTGACGTGG",0,NA,NA,NA,"GeneID:206338","AQPEP",TRUE,"AQPEP"

"10111","GGCCCTGTTAGCGTCCCAGC","chr5",115983286,"+","none","TGG","GGCCCTGTTAGCGTCCCAGCTGG",0,NA,NA,NA,"GeneID:206338","AQPEP",TRUE,"AQPEP"

"10112","GTTCCAGCTGGGACGCTAAC","chr5",115983292,"-","none","AGG","GTTCCAGCTGGGACGCTAACAGG",0,NA,NA,NA,"GeneID:206338","AQPEP",TRUE,"AQPEP"

"10113","TATGTTGTCTCCCACGAGAT","chr5",115992247,"+","none","TGG","TATGTTGTCTCCCACGAGATTGG",0,NA,NA,NA,"GeneID:206338","AQPEP",TRUE,"AQPEP"

"10114","AAAAAGAGATTGTCAAATCA","chr15",34960775,"-","none","AGG","AAAAAGAGATTGTCAAATCAAGG",0,NA,NA,NA,"GeneID:9716","AQR",TRUE,"AQR"

"10115","AAAAAGAGATTGTCAAATCA","chr4",31375868,"-","11:T>C,12:T>A","CGG","AAAAAGAGATTAACAAATCACGG",2,12,13,NA,"","",FALSE,"AQR"

"10116","AAAAAGAGATTGTCAAATCA","chrX",138410559,"+","15:G>A,18:A>C","AGG","AAAAAGAGATTGTCAGATAAAGG",2,16,19,NA,"","",FALSE,"AQR"

"10117","AAAAAGAGATTGTCAAATCA","chr4",139068019,"-","5:T>C,12:C>A","AGG","AAAAAAAGATTGGCAAATCAAGG",2,6,13,NA,"GeneID:1998","ELF2",FALSE,"AQR"

"10118","ATACTTACCTTTATATCAAA","chr15",34964225,"+","none","AGG","ATACTTACCTTTATATCAAAAGG",0,NA,NA,NA,"GeneID:9716","AQR",TRUE,"AQR"

"10119","TATTAGAAAGATAATGCTCT","chr15",34952894,"-","none","TGG","TATTAGAAAGATAATGCTCTTGG",0,NA,NA,NA,"GeneID:9716","AQR",TRUE,"AQR"

"10120","TGCAGTTAGCATGTAAATAC","chr15",34964275,"-","none","TGG","TGCAGTTAGCATGTAAATACTGG",0,NA,NA,NA,"GeneID:9716","AQR",TRUE,"AQR"

"10121","TGCAGTTAGCATGTAAATAC","chr4",57273995,"-","8:T>C,17:T>A","TGG","TGCAGTTAACATGTAAAAACTGG",2,9,18,NA,"","",FALSE,"AQR"

"10122","AAGCTTCATCTCCACAGATC","chrX",67643321,"-","none","AGG","AAGCTTCATCTCCACAGATCAGG",0,NA,NA,NA,"GeneID:367","AR",TRUE,"AR"

"10123","AGAGAGACAGCTTGTACACG","chrX",67711642,"+","none","TGG","AGAGAGACAGCTTGTACACGTGG",0,NA,NA,NA,"GeneID:367","AR",TRUE,"AR"

"10124","CATTTCCGAAGACGACAAGA","chrX",67686080,"-","none","TGG","CATTTCCGAAGACGACAAGATGG",0,NA,NA,NA,"GeneID:367","AR",TRUE,"AR"

"10125","GCACTATTGATAAATTCCGA","chrX",67686045,"+","none","AGG","GCACTATTGATAAATTCCGAAGG",0,NA,NA,NA,"GeneID:367","AR",TRUE,"AR"

"10126","TGTCCAGCACACACTACACC","chrX",67711565,"-","none","TGG","TGTCCAGCACACACTACACCTGG",0,NA,NA,NA,"GeneID:367","AR",TRUE,"AR"

"10127","TTCTTCAAAAGAGCCGCTGA","chrX",67643385,"+","none","AGG","TTCTTCAAAAGAGCCGCTGAAGG",0,NA,NA,NA,"GeneID:367","AR",TRUE,"AR"

"10128","ACGGAAGACCTTCTTCAGCC","chrX",47564986,"+","none","TGG","ACGGAAGACCTTCTTCAGCCTGG",0,NA,NA,NA,"GeneID:369","ARAF",TRUE,"ARAF"

"10129","CACCATCAGTGCTGAAACTC","chrX",47566894,"-","none","TGG","CACCATCAGTGCTGAAACTCTGG",0,NA,NA,NA,"GeneID:369","ARAF",TRUE,"ARAF"

"10130","CGTAGACACTCATGCCATCC","chrX",47563236,"-","none","CGG","CGTAGACACTCATGCCATCCCGG",0,NA,NA,NA,"GeneID:369","ARAF",TRUE,"ARAF"

"10131","CTGGGCTCACTGTTGGCGGT","chrX",47565129,"-","none","TGG","CTGGGCTCACTGTTGGCGGTTGG",0,NA,NA,NA,"GeneID:369","ARAF",TRUE,"ARAF"

"10132","CTGGGCTCACTGTTGGCGGT","chr7",138030725,"-","9:T>G,16:T>G","GGG","CTGGGCTCAATGTTGGAGGTGGG",2,10,17,NA,"GeneID:6718","AKR1D1",FALSE,"ARAF"

"10133","CTGGGCTCACTGTTGGCGGT","chr11",12764223,"-","9:T>G,11:A>C","GGG","CTGGGCTCAATTTTGGCGGTGGG",2,10,12,NA,"GeneID:7003","TEAD1",TRUE,"ARAF"

"10134","GGCCAGAGTTTCAGCACTGA","chrX",47566889,"+","none","TGG","GGCCAGAGTTTCAGCACTGATGG",0,NA,NA,NA,"GeneID:369","ARAF",TRUE,"ARAF"

"10135","GGCCAGAGTTTCAGCACTGA","chr4",156972321,"+","14:A>C,17:A>T","AGG","GGCCAGAGTTTCAGAACAGAAGG",2,15,18,NA,"","",FALSE,"ARAF"

"10136","TGTGGTCTACCGACTCATCA","chrX",47563305,"+","none","AGG","TGTGGTCTACCGACTCATCAAGG",0,NA,NA,NA,"GeneID:369","ARAF",TRUE,"ARAF"

"10137","AATAGCTGCGCCACACCCCA","chr11",72712274,"-","none","TGG","AATAGCTGCGCCACACCCCATGG",0,NA,NA,NA,"GeneID:116985","ARAP1",TRUE,"ARAP1"

"10138","AGCCAGAGTGATGACCAAGA","chr11",72713177,"-","none","AGG","AGCCAGAGTGATGACCAAGAAGG",0,NA,NA,NA,"GeneID:116985","ARAP1",TRUE,"ARAP1"

"10139","AGCCAGAGTGATGACCAAGA","chr12",94939938,"+","11:A>T,13:C>A","GGG","AGCCAGAGTGAAGCCCAAGAGGG",2,12,14,NA,"GeneID:55967","NDUFA12",FALSE,"ARAP1"

"10140","CCACCCGTGCCGCCCCGCAC","chr11",72726643,"-","none","CGG","CCACCCGTGCCGCCCCGCACCGG",0,NA,NA,NA,"GeneID:116985","ARAP1",TRUE,"ARAP1"

"10141","GTAGCATGCCCATGTCCATC","chr11",72726971,"+","none","AGG","GTAGCATGCCCATGTCCATCAGG",0,NA,NA,NA,"GeneID:116985","ARAP1",TRUE,"ARAP1"

"10142","GTAGCATGCCCATGTCCATC","chr1",85314029,"-","14:T>A,17:A>T","TGG","GTAGCATGCCCATGACCTTCTGG",2,15,18,NA,"","",FALSE,"ARAP1"

"10143","GTGTGGCGCAGCTATTGTGC","chr11",72712278,"+","none","TGG","GTGTGGCGCAGCTATTGTGCTGG",0,NA,NA,NA,"GeneID:116985","ARAP1",TRUE,"ARAP1"

"10144","TCTGCCAAATGCCGCTTAGC","chr11",72726673,"+","none","AGG","TCTGCCAAATGCCGCTTAGCAGG",0,NA,NA,NA,"GeneID:116985","ARAP1",TRUE,"ARAP1"

"10145","AAGAGAAAGGTCTCTCCATA","chr4",36213264,"+","none","AGG","AAGAGAAAGGTCTCTCCATAAGG",0,NA,NA,NA,"GeneID:116984","ARAP2",TRUE,"ARAP2"

"10146","GACATCTAGCCCACCGCAGT","chr4",36229128,"-","none","TGG","GACATCTAGCCCACCGCAGTTGG",0,NA,NA,NA,"GeneID:116984","ARAP2",TRUE,"ARAP2"

"10147","GCAGTGGGGAAGTCGCGTTT","chr4",36229019,"+","none","AGG","GCAGTGGGGAAGTCGCGTTTAGG",0,NA,NA,NA,"GeneID:116984","ARAP2",TRUE,"ARAP2"

"10148","TACCTCAACTGAAAAATCTG","chr4",36214433,"-","none","TGG","TACCTCAACTGAAAAATCTGTGG",0,NA,NA,NA,"GeneID:116984","ARAP2",TRUE,"ARAP2"

"10149","TACCTCAACTGAAAAATCTG","chr6",31562375,"-","1:G>T,11:A>T","TGG","TCCCTCAACTGTAAAATCTGTGG",2,2,12,NA,"","",FALSE,"ARAP2"

"10150","TAGCAACATTTCTTCTTTCA","chr4",36214451,"+","none","CGG","TAGCAACATTTCTTCTTTCACGG",0,NA,NA,NA,"GeneID:116984","ARAP2",TRUE,"ARAP2"

"10151","TAGCAACATTTCTTCTTTCA","chr3",14609175,"-","5:A>T,7:G>T","TGG","TAGCATCCTTTCTTCTTTCATGG",2,6,8,NA,"","",FALSE,"ARAP2"

"10152","TAGCAACATTTCTTCTTTCA","chr1",236990024,"+","3:A>C,9:C>T","TGG","TAGAAACATCTCTTCTTTCATGG",2,4,10,NA,"","",FALSE,"ARAP2"

"10153","TAGCAACATTTCTTCTTTCA","chr4",14682543,"+","2:T>G,13:A>T","AGG","TATCAACATTTCTACTTTCAAGG",2,3,14,NA,"","",FALSE,"ARAP2"

"10154","TCCCCTAAAAGATTCAAATG","chr4",36213310,"-","none","AGG","TCCCCTAAAAGATTCAAATGAGG",0,NA,NA,NA,"GeneID:116984","ARAP2",TRUE,"ARAP2"

"10155","CAGGGGTGGGGGCAGAGATT","chr5",141679784,"+","none","TGG","CAGGGGTGGGGGCAGAGATTTGG",0,NA,NA,NA,"GeneID:64411","ARAP3",TRUE,"ARAP3"

"10156","CAGGGGTGGGGGCAGAGATT","chr4",107939777,"-","15:A>T,19:T>A","AGG","CAGGGGTGGGGGCAGTGATAAGG",2,16,20,NA,"GeneID:101929595;GeneID:113612","LOC101929595;CYP2U1",FALSE,"ARAP3"

"10157","CAGGGGTGGGGGCAGAGATT","chr12",124141249,"-","12:C>G,19:G>A","TGG","CAGGGGTGGGGGGAGAGATCTGG",2,13,20,NA,"GeneID:144347","FAM101A",FALSE,"ARAP3"

"10158","CAGGGGTGGGGGCAGAGATT","chr1",223196913,"-","0:C>G,9:T>C","GGG","GAGGGGTGGAGGCAGAGATTGGG",2,1,10,NA,"","",FALSE,"ARAP3"

"10159","CAGGGGTGGGGGCAGAGATT","chr16",51055118,"+","0:T>C,7:A>G","AGG","TAGGGGTAGGGGCAGAGATTAGG",2,1,8,NA,"","",TRUE,"ARAP3"

"10160","CAGGGGTGGGGGCAGAGATT","chr10",8118568,"+","4:A>G,9:A>G","GGG","CAGGAGTGGAGGCAGAGATTGGG",2,5,10,NA,"","",FALSE,"ARAP3"

"10161","CAGGGGTGGGGGCAGAGATT","chr13",28190424,"+","1:G>A,12:T>C","GGG","CGGGGGTGGGGGTAGAGATTGGG",2,2,13,NA,"GeneID:255967","PAN3",FALSE,"ARAP3"

"10162","CAGGGGTGGGGGCAGAGATT","chr8",26681595,"+","1:G>A,15:T>A","AGG","CGGGGGTGGGGGCAGTGATTAGG",2,2,16,NA,"","",FALSE,"ARAP3"

"10163","CAGGGGTGGGGGCAGAGATT","chr16",80873999,"+","7:T>G,19:A>T","GGG","CAGGGGTTGGGGCAGAGATAGGG",2,8,20,NA,"GeneID:100996425","LOC100996425",FALSE,"ARAP3"

"10164","CAGGGGTGGGGGCAGAGATT","chr4",74546064,"-","5:T>C,18:T>A","GGG","CAGGGATGGGGGCAGAGAATGGG",2,6,19,NA,"","",FALSE,"ARAP3"

"10165","CCAGTTACCTGTGCCTGTTG","chr5",141679752,"+","none","TGG","CCAGTTACCTGTGCCTGTTGTGG",0,NA,NA,NA,"GeneID:64411","ARAP3",TRUE,"ARAP3"

"10166","CCAGTTACCTGTGCCTGTTG","chr5",29955425,"+","8:T>C,17:C>T","GGG","CCAGTTACTTGTGCCTGCTGGGG",2,9,18,NA,"","",FALSE,"ARAP3"

"10167","CCTCCTGCCAGTGCACATCA","chr5",141679647,"-","none","TGG","CCTCCTGCCAGTGCACATCATGG",0,NA,NA,NA,"GeneID:64411","ARAP3",TRUE,"ARAP3"

"10168","GATGTACTTTGGCAGTGACA","chr5",141673402,"-","none","AGG","GATGTACTTTGGCAGTGACAAGG",0,NA,NA,NA,"GeneID:64411","ARAP3",TRUE,"ARAP3"

"10169","GTACTATGGTGTCCAACCTG","chr5",141679611,"-","none","TGG","GTACTATGGTGTCCAACCTGTGG",0,NA,NA,NA,"GeneID:64411","ARAP3",TRUE,"ARAP3"

"10170","TGAACTGCACAAAGCGTCTC","chr5",141673435,"+","none","TGG","TGAACTGCACAAAGCGTCTCTGG",0,NA,NA,NA,"GeneID:64411","ARAP3",TRUE,"ARAP3"

"10171","ACAGGTCCCGCTTGCGCCAC","chr8",142613346,"+","none","AGG","ACAGGTCCCGCTTGCGCCACAGG",0,NA,NA,NA,"GeneID:23237","ARC",TRUE,"ARC"

"10172","AGCACGTGCGGCGGACGCAC","chr8",142614133,"-","none","CGG","AGCACGTGCGGCGGACGCACCGG",0,NA,NA,NA,"GeneID:23237","ARC",TRUE,"ARC"

"10173","CCACGAGCGACTCGCAGCGC","chr8",142614013,"-","none","TGG","CCACGAGCGACTCGCAGCGCTGG",0,NA,NA,NA,"GeneID:23237","ARC",TRUE,"ARC"

"10174","CCAGCGCTGCGAGTCGCTCG","chr8",142614010,"+","none","TGG","CCAGCGCTGCGAGTCGCTCGTGG",0,NA,NA,NA,"GeneID:23237","ARC",TRUE,"ARC"

"10175","CCGCGTCCACGTAGAGCGTC","chr8",142613322,"+","none","TGG","CCGCGTCCACGTAGAGCGTCTGG",0,NA,NA,NA,"GeneID:23237","ARC",TRUE,"ARC"

"10176","CCTGTACCAGACGCTCTACG","chr8",142613331,"-","none","TGG","CCTGTACCAGACGCTCTACGTGG",0,NA,NA,NA,"GeneID:23237","ARC",TRUE,"ARC"

"10177","AATGACCCGAACTCGGATTG","chr11",118581310,"+","none","AGG","AATGACCCGAACTCGGATTGAGG",0,NA,NA,NA,"GeneID:372","ARCN1",TRUE,"ARCN1"

"10178","AGTACGCTTGCCCATACTAG","chr11",118584580,"-","none","AGG","AGTACGCTTGCCCATACTAGAGG",0,NA,NA,NA,"GeneID:372","ARCN1",TRUE,"ARCN1"

"10179","ATAAGCCCTCAATCCGAGTT","chr11",118581319,"-","none","CGG","ATAAGCCCTCAATCCGAGTTCGG",0,NA,NA,NA,"GeneID:372","ARCN1",TRUE,"ARCN1"

"10180","ATACCGGGAGAATGTTAACT","chr11",118583270,"+","none","TGG","ATACCGGGAGAATGTTAACTTGG",0,NA,NA,NA,"GeneID:372","ARCN1",TRUE,"ARCN1"

"10181","GACTCAAGAACGTGAAGCTA","chr11",118583807,"+","none","AGG","GACTCAAGAACGTGAAGCTAAGG",0,NA,NA,NA,"GeneID:372","ARCN1",TRUE,"ARCN1"

"10182","GCATGCTCCACCCATTAATA","chr11",118584617,"+","none","TGG","GCATGCTCCACCCATTAATATGG",0,NA,NA,NA,"GeneID:372","ARCN1",TRUE,"ARCN1"

"10183","ACTTGCATGTTACTGCTTCC","chr4",74449230,"-","none","AGG","ACTTGCATGTTACTGCTTCCAGG",0,NA,NA,NA,"GeneID:374","AREG",TRUE,"AREG"

"10184","ACTTGCATGTTACTGCTTCC","chr9",124711130,"-","11:G>T,18:A>G","CGG","ACTTGCATGTTCCTGCTTTCCGG",2,12,19,NA,"GeneID:2649","NR6A1",FALSE,"AREG"

"10185","ACTTGCATGTTACTGCTTCC","chr4",64487613,"-","1:C>G,8:G>C","AGG","AGTTGCATCTTACTGCTTCCAGG",2,2,9,NA,"","",FALSE,"AREG"

"10186","ATCAAGAGCGACAGCACCAC","chr4",74445375,"-","none","CGG","ATCAAGAGCGACAGCACCACCGG",0,NA,NA,NA,"GeneID:374","AREG",TRUE,"AREG"

"10187","CCTGCAGGCCATTATGCTGC","chr4",74446526,"+","none","TGG","CCTGCAGGCCATTATGCTGCTGG",0,NA,NA,NA,"GeneID:374","AREG",TRUE,"AREG"

"10188","CCTGCAGGCCATTATGCTGC","chr5",142049241,"-","7:G>C,14:C>A","TGG","CCTGCAGCCCATTAGGCTGCTGG",2,8,15,NA,"","",FALSE,"AREG"

"10189","GAATCATCGACAATATAGCC","chr4",74446754,"-","none","AGG","GAATCATCGACAATATAGCCAGG",0,NA,NA,NA,"GeneID:374","AREG",TRUE,"AREG"

"10190","GAGAGCCCCGCTGCTACCGC","chr4",74445347,"+","none","CGG","GAGAGCCCCGCTGCTACCGCCGG",0,NA,NA,NA,"GeneID:374","AREG",TRUE,"AREG"

"10191","TAAGCCCCCCCAAAACAAGA","chr4",74449059,"+","none","CGG","TAAGCCCCCCCAAAACAAGACGG",0,NA,NA,NA,"GeneID:374","AREG",TRUE,"AREG"

"10192","ACATAGCAGTACACCTTCTT","chr14",74675708,"+","none","CGG","ACATAGCAGTACACCTTCTTCGG",0,NA,NA,NA,"GeneID:9870","AREL1",TRUE,"AREL1"

"10193","ATGTGAACTCTTAGTCCCAC","chr14",74683471,"+","none","AGG","ATGTGAACTCTTAGTCCCACAGG",0,NA,NA,NA,"GeneID:9870","AREL1",TRUE,"AREL1"

"10194","CAATTCCAACGTAGTAAAAG","chr14",74683394,"-","none","TGG","CAATTCCAACGTAGTAAAAGTGG",0,NA,NA,NA,"GeneID:9870","AREL1",TRUE,"AREL1"

"10195","CTCTCATGTCGAGCTAGCAG","chr14",74683451,"-","none","TGG","CTCTCATGTCGAGCTAGCAGTGG",0,NA,NA,NA,"GeneID:9870","AREL1",TRUE,"AREL1"

"10196","GTAAATGCTCACGCCTGAAG","chr14",74675890,"+","none","TGG","GTAAATGCTCACGCCTGAAGTGG",0,NA,NA,NA,"GeneID:9870","AREL1",TRUE,"AREL1"

"10197","GTACTGCTATGTGTCACCAA","chr14",74675700,"-","none","AGG","GTACTGCTATGTGTCACCAAAGG",0,NA,NA,NA,"GeneID:9870","AREL1",TRUE,"AREL1"

"10198","CCCGCACCCAGGCTTCAACG","chr1",228097330,"+","none","TGG","CCCGCACCCAGGCTTCAACGTGG",0,NA,NA,NA,"GeneID:375;GeneID:100500810","ARF1;MIR3620",TRUE,"ARF1"

"10199","CCCGGCAGGCCTGATCTTCG","chr1",228097582,"+","none","TGG","CCCGGCAGGCCTGATCTTCGTGG",0,NA,NA,NA,"GeneID:375","ARF1",TRUE,"ARF1"

"10200","CCGCATTCATGGCGTTGGGG","chr1",228097856,"-","none","AGG","CCGCATTCATGGCGTTGGGGAGG",0,NA,NA,NA,"GeneID:375","ARF1",TRUE,"ARF1"

"10201","GATCCTCTACAAGCTTAAGC","chr1",228097209,"+","none","TGG","GATCCTCTACAAGCTTAAGCTGG",0,NA,NA,NA,"GeneID:375","ARF1",TRUE,"ARF1"

"10202","TGCTGTCCACCACGAAGATC","chr1",228097594,"-","none","AGG","TGCTGTCCACCACGAAGATCAGG",0,NA,NA,NA,"GeneID:375","ARF1",TRUE,"ARF1"

"10203","TGCTGTCCACCACGAAGATC","chr8",124152170,"-","3:T>A,15:C>T","AGG","TGCAGTCCACCACGAGGATCAGG",2,4,16,NA,"GeneID:157376","FER1L6-AS2",TRUE,"ARF1"

"10204","TTCATAGAGCCCGTCGCCGC","chr1",228097951,"-","none","TGG","TTCATAGAGCCCGTCGCCGCTGG",0,NA,NA,NA,"GeneID:375","ARF1",TRUE,"ARF1"

"10205","TTCATAGAGCCCGTCGCCGC","chr12",120845150,"+","2:G>C,18:A>G","TGG","TTGATAGAGCCCGTCGCCACTGG",2,3,19,NA,"GeneID:121665","SPPL3",TRUE,"ARF1"

"10206","AGGACAAGATTCGACCCCTC","chr12",48940024,"-","none","TGG","AGGACAAGATTCGACCCCTCTGG",0,NA,NA,NA,"GeneID:377","ARF3",TRUE,"ARF3"

"10207","ATTCCCTTCGTCACCGTAAC","chr12",48939036,"-","none","TGG","ATTCCCTTCGTCACCGTAACTGG",0,NA,NA,NA,"GeneID:377","ARF3",TRUE,"ARF3"

"10208","CCCCGAGTTTCAGCTTGTAT","chr12",48940974,"+","none","AGG","CCCCGAGTTTCAGCTTGTATAGG",0,NA,NA,NA,"GeneID:377","ARF3",TRUE,"ARF3"

"10209","GAAGGAGATGCGCATCCTGA","chr12",48941031,"-","none","TGG","GAAGGAGATGCGCATCCTGATGG",0,NA,NA,NA,"GeneID:377","ARF3",TRUE,"ARF3"

"10210","TGATCGGGAGCGAGTAAATG","chr12",48939734,"-","none","AGG","TGATCGGGAGCGAGTAAATGAGG",0,NA,NA,NA,"GeneID:377","ARF3",TRUE,"ARF3"

"10211","TTCGTACAGCCCGTCCCCGC","chr12",48938988,"+","none","TGG","TTCGTACAGCCCGTCCCCGCTGG",0,NA,NA,NA,"GeneID:377","ARF3",TRUE,"ARF3"

"10212","CTAGTTGGATTGGATGCTGC","chr3",57584448,"-","none","TGG","CTAGTTGGATTGGATGCTGCTGG",0,NA,NA,NA,"GeneID:378","ARF4",TRUE,"ARF4"

"10213","CTAGTTGGATTGGATGCTGC","chr9",134078966,"+","3:T>G","TGG","CTATTTGGATTGGATGCTGCTGG",1,4,NA,NA,"","",TRUE,"ARF4"

"10214","CTAGTTGGATTGGATGCTGC","chr18",39304865,"-","3:A>C,19:A>G","TGG","CTATTTGGATTGGATGCTGTTGG",2,4,20,NA,"GeneID:647946","LINC00669",FALSE,"ARF4"

"10215","TATCCCTTACCTGGGTATTC","chr3",57583887,"+","none","TGG","TATCCCTTACCTGGGTATTCTGG",0,NA,NA,NA,"GeneID:378","ARF4",TRUE,"ARF4"

"10216","TCCCTCTTCTCCCGACTATT","chr3",57597102,"-","none","TGG","TCCCTCTTCTCCCGACTATTTGG",0,NA,NA,NA,"GeneID:378","ARF4",TRUE,"ARF4"

"10217","TCCCTCTTCTCCCGACTATT","chr10",130405553,"-","10:A>G,12:A>G","TGG","TCCCTCTTCTTCTGACTATTTGG",2,11,13,NA,"","",FALSE,"ARF4"

"10218","TCTGCTTCTTGCCAAATAGT","chr3",57597088,"+","none","CGG","TCTGCTTCTTGCCAAATAGTCGG",0,NA,NA,NA,"GeneID:378","ARF4",TRUE,"ARF4"

"10219","TTGGTGGTCAAGATAGAATT","chr3",57583933,"-","none","AGG","TTGGTGGTCAAGATAGAATTAGG",0,NA,NA,NA,"GeneID:378","ARF4",TRUE,"ARF4"

"10220","TTGGTGGTCAAGATAGAATT","chr9",134079105,"+","13:C>T","AGG","TTGGTGGTCAAGACAGAATTAGG",1,14,NA,NA,"","",TRUE,"ARF4"

"10221","TTGGTGGTCAAGATAGAATT","chr13",100756837,"-","0:G>A","AGG","CTGGTGGTCAAGATAGAATTAGG",1,1,NA,NA,"GeneID:100885778","NALCN-AS1",TRUE,"ARF4"

"10222","TTGGTGGTCAAGATAGAATT","chr2",193436557,"+","6:T>G,15:T>G","AGG","TTGGTGTTCAAGATATAATTAGG",2,7,16,NA,"","",FALSE,"ARF4"

"10223","TTGGTGGTCAAGATAGAATT","chr5",180775637,"-","0:G>A,15:T>C","AGG","CTGGTGGTCAAGATAAAATTAGG",2,1,16,NA,"","",FALSE,"ARF4"

"10224","TTTCTTACCAATGGTAGGAA","chr3",57584375,"+","none","TGG","TTTCTTACCAATGGTAGGAATGG",0,NA,NA,NA,"GeneID:378","ARF4",TRUE,"ARF4"

"10225","TTTCTTACCAATGGTAGGAA","chr2",214930841,"-","5:C>A,7:C>G","AGG","TTTCTGAGCAATGGTAGGAAAGG",2,6,8,NA,"GeneID:101928103","LOC101928103",FALSE,"ARF4"

"10226","AACACTCCACCTGAGTGTTC","chr7",127589584,"-","none","TGG","AACACTCCACCTGAGTGTTCTGG",0,NA,NA,NA,"GeneID:79571;GeneID:381","GCC1;ARF5",TRUE,"ARF5"

"10227","CCCCCAACTTCAGTTTGTAC","chr7",127589116,"-","none","AGG","CCCCCAACTTCAGTTTGTACAGG",0,NA,NA,NA,"GeneID:79571;GeneID:381","GCC1;ARF5",TRUE,"ARF5"

"10228","CCCCCAACTTCAGTTTGTAC","chr8",124152024,"-","1:C>G,13:G>A","AGG","CGCCCAACTTCAGCTTGTACAGG",2,2,14,NA,"GeneID:157376","FER1L6-AS2",TRUE,"ARF5"

"10229","GAAGATCCGCGAAAAGAGCG","chr7",127588517,"-","none","CGG","GAAGATCCGCGAAAAGAGCGCGG",0,NA,NA,NA,"GeneID:79571;GeneID:381","GCC1;ARF5",TRUE,"ARF5"

"10230","GGGCTCACCTATGGTTGGGA","chr7",127589151,"-","none","TGG","GGGCTCACCTATGGTTGGGATGG",0,NA,NA,NA,"GeneID:79571;GeneID:381","GCC1;ARF5",TRUE,"ARF5"

"10231","TACTGTCCACCACAAAGATG","chr7",127590070,"-","none","AGG","TACTGTCCACCACAAAGATGAGG",0,NA,NA,NA,"GeneID:79571;GeneID:381","GCC1;ARF5",TRUE,"ARF5"

"10232","TGGACAGTAATGACCGGGAG","chr7",127590081,"+","none","CGG","TGGACAGTAATGACCGGGAGCGG",0,NA,NA,NA,"GeneID:79571;GeneID:381","GCC1;ARF5",TRUE,"ARF5"

"10233","AACTGGGCCTGACCCGGATT","chr14",49894148,"+","none","CGG","AACTGGGCCTGACCCGGATTCGG",0,NA,NA,NA,"GeneID:382","ARF6",TRUE,"ARF6"

"10234","ACCAGTTCCTGTCCCGAATC","chr14",49894164,"-","none","CGG","ACCAGTTCCTGTCCCGAATCCGG",0,NA,NA,NA,"GeneID:382","ARF6",TRUE,"ARF6"

"10235","CGACCGCGACCGCATCGATG","chr14",49894008,"+","none","AGG","CGACCGCGACCGCATCGATGAGG",0,NA,NA,NA,"GeneID:382","ARF6",TRUE,"ARF6"

"10236","CTCTGGCGGCATTACTACAC","chr14",49893952,"+","none","TGG","CTCTGGCGGCATTACTACACTGG",0,NA,NA,NA,"GeneID:382","ARF6",TRUE,"ARF6"

"10237","CTCTGGCGGCATTACTACAC","chr7",99638531,"-","4:G>C,12:T>A","TGG","CTCTCGCGGCATAACTACACTGG",2,5,13,NA,"","",TRUE,"ARF6"

"10238","GAGCCTCATCGATGCGGTCG","chr14",49894014,"-","none","CGG","GAGCCTCATCGATGCGGTCGCGG",0,NA,NA,NA,"GeneID:382","ARF6",TRUE,"ARF6"

"10239","GTGTAGTAATGCCGCCAGAG","chr14",49893952,"-","none","CGG","GTGTAGTAATGCCGCCAGAGCGG",0,NA,NA,NA,"GeneID:382","ARF6",TRUE,"ARF6"

"10240","AGGAGGATTACGATCCTTGC","chr20",63276577,"+","none","TGG","AGGAGGATTACGATCCTTGCTGG",0,NA,NA,NA,"GeneID:55738","ARFGAP1",TRUE,"ARFGAP1"

"10241","AGGCGGTCACACTCTGCGGC","chr20",63278128,"-","none","TGG","AGGCGGTCACACTCTGCGGCTGG",0,NA,NA,NA,"GeneID:55738","ARFGAP1",TRUE,"ARFGAP1"

"10242","CAGGCAGATCCAGATGCCGT","chr20",63276139,"-","none","AGG","CAGGCAGATCCAGATGCCGTAGG",0,NA,NA,NA,"GeneID:55738","ARFGAP1",TRUE,"ARFGAP1"

"10243","CAGGCAGATCCAGATGCCGT","chr15",65927799,"+","8:G>T,15:C>G","GGG","CAGGCAGAGCCAGATCCCGTGGG",2,9,16,NA,"GeneID:84465","MEGF11",FALSE,"ARFGAP1"

"10244","CTTACCCTTGATAGGAGCCG","chr20",63278188,"-","none","AGG","CTTACCCTTGATAGGAGCCGAGG",0,NA,NA,NA,"GeneID:55738","ARFGAP1",TRUE,"ARFGAP1"

"10245","TCCTTGTCAGGTGGTCGCTC","chr20",63277194,"+","none","TGG","TCCTTGTCAGGTGGTCGCTCTGG",0,NA,NA,NA,"GeneID:55738","ARFGAP1",TRUE,"ARFGAP1"

"10246","TGCTAAGTTCCGAGAGTTCC","chr20",63276548,"+","none","TGG","TGCTAAGTTCCGAGAGTTCCTGG",0,NA,NA,NA,"GeneID:55738","ARFGAP1",TRUE,"ARFGAP1"

"10247","ACTTGACACCCTGCTTAAAC","chr11",47166320,"+","none","TGG","ACTTGACACCCTGCTTAAACTGG",0,NA,NA,NA,"GeneID:84364","ARFGAP2",TRUE,"ARFGAP2"

"10248","CGACCTGCATACACCTCAGC","chr11",47175867,"+","none","TGG","CGACCTGCATACACCTCAGCTGG",0,NA,NA,NA,"GeneID:84364","ARFGAP2",TRUE,"ARFGAP2"

"10249","CTTGGCGCCGCAGTCGAAAC","chr11",47176610,"+","none","AGG","CTTGGCGCCGCAGTCGAAACAGG",0,NA,NA,NA,"GeneID:84364","ARFGAP2",TRUE,"ARFGAP2"

"10250","GAAAACACCGTACGTGATGC","chr11",47176574,"+","none","TGG","GAAAACACCGTACGTGATGCTGG",0,NA,NA,NA,"GeneID:84364","ARFGAP2",TRUE,"ARFGAP2"

"10251","GGTCGGCGGGAATGCCAATG","chr11",47175852,"-","none","CGG","GGTCGGCGGGAATGCCAATGCGG",0,NA,NA,NA,"GeneID:84364","ARFGAP2",TRUE,"ARFGAP2"

"10252","TGGTGTGATGAATTCCTTGC","chr11",47166269,"-","none","AGG","TGGTGTGATGAATTCCTTGCAGG",0,NA,NA,NA,"GeneID:84364","ARFGAP2",TRUE,"ARFGAP2"

"10253","TGGTGTGATGAATTCCTTGC","chr10",69233376,"+","8:G>T,19:G>C","AGG","TGGTGTGAGGAATTCCTTGGAGG",2,9,20,NA,"GeneID:80201","HKDC1",FALSE,"ARFGAP2"

"10254","AAACCAAATCAAGCTAAAAA","chr22",42823659,"-","none","AGG","AAACCAAATCAAGCTAAAAAAGG",0,NA,NA,NA,"GeneID:26286","ARFGAP3",TRUE,"ARFGAP3"

"10255","AAACCAAATCAAGCTAAAAA","chr22",46806639,"+","3:G>C,4:A>C","AGG","AAAGAAAATCAAGCTAAAAAAGG",2,4,5,NA,"GeneID:25771","TBC1D22A",FALSE,"ARFGAP3"

"10256","AAACCAAATCAAGCTAAAAA","chr11",39858208,"-","9:T>G,18:C>T","AGG","AAACCAAATAAAGCTAAAGAAGG",2,10,19,NA,"","",FALSE,"ARFGAP3"

"10257","AACCATCTTCTTTAACATCA","chr22",42831582,"-","none","AGG","AACCATCTTCTTTAACATCAAGG",0,NA,NA,NA,"GeneID:26286","ARFGAP3",TRUE,"ARFGAP3"

"10258","AACCATCTTCTTTAACATCA","chr3",169424539,"-","13:G>T,18:C>G","TGG","AACCATCTTCTTTCACATGATGG",2,14,19,NA,"GeneID:2122","MECOM",FALSE,"ARFGAP3"

"10259","AACCATCTTCTTTAACATCA","chr2",71841195,"+","5:A>T,13:T>A","TGG","AACCAACTTCTTTTACATCATGG",2,6,14,NA,"","",FALSE,"ARFGAP3"

"10260","ATCATTACGATTAGCCTATA","chr22",42817831,"-","none","AGG","ATCATTACGATTAGCCTATAAGG",0,NA,NA,NA,"GeneID:26286","ARFGAP3",TRUE,"ARFGAP3"

"10261","GACGAAAAGATGAACATTAG","chr22",42817785,"-","none","TGG","GACGAAAAGATGAACATTAGTGG",0,NA,NA,NA,"GeneID:26286","ARFGAP3",TRUE,"ARFGAP3"

"10262","GACGAAAAGATGAACATTAG","chr5",82541586,"+","2:A>C,14:T>C","TGG","GAAGAAAAGATGAATATTAGTGG",2,3,15,NA,"","",FALSE,"ARFGAP3"

"10263","GGCCTTGATGTTAAAGAAGA","chr22",42831577,"+","none","TGG","GGCCTTGATGTTAAAGAAGATGG",0,NA,NA,NA,"GeneID:26286","ARFGAP3",TRUE,"ARFGAP3"

"10264","TTCAGGTGAGTGACACAGCG","chr22",42831621,"-","none","TGG","TTCAGGTGAGTGACACAGCGTGG",0,NA,NA,NA,"GeneID:26286","ARFGAP3",TRUE,"ARFGAP3"

"10265","AGAGATGTACTAACTATGCG","chr8",67301236,"+","none","AGG","AGAGATGTACTAACTATGCGAGG",0,NA,NA,NA,"GeneID:10565","ARFGEF1",TRUE,"ARFGEF1"

"10266","CCCCTCAGAGGAAATAAAAG","chr8",67302454,"-","none","CGG","CCCCTCAGAGGAAATAAAAGCGG",0,NA,NA,NA,"GeneID:10565","ARFGEF1",TRUE,"ARFGEF1"

"10267","CCTCGCAAGCTTTGCGCAGC","chr8",67343172,"+","none","TGG","CCTCGCAAGCTTTGCGCAGCTGG",0,NA,NA,NA,"GeneID:10565","ARFGEF1",TRUE,"ARFGEF1"

"10268","CGTCTTCTTCCCCTCATACA","chr8",67343266,"+","none","TGG","CGTCTTCTTCCCCTCATACATGG",0,NA,NA,NA,"GeneID:10565","ARFGEF1",TRUE,"ARFGEF1"

"10269","TACATCTCTAGATTGCTTAC","chr8",67301225,"-","none","AGG","TACATCTCTAGATTGCTTACAGG",0,NA,NA,NA,"GeneID:10565","ARFGEF1",TRUE,"ARFGEF1"

"10270","TCTCTGCGTCCATGTATGAG","chr8",67343278,"-","none","GGG","TCTCTGCGTCCATGTATGAGGGG",0,NA,NA,NA,"GeneID:10565","ARFGEF1",TRUE,"ARFGEF1"

"10271","ACTGGCAACGCCCCTGACAG","chr20",48951346,"+","none","TGG","ACTGGCAACGCCCCTGACAGTGG",0,NA,NA,NA,"GeneID:10564","ARFGEF2",TRUE,"ARFGEF2"

"10272","ACTGGCAACGCCCCTGACAG","chr19",13223703,"-","8:T>G,10:C>G","AGG","ACTGGCAAAGGCCCTGACAGAGG",2,9,11,NA,"GeneID:773","CACNA1A",FALSE,"ARFGEF2"

"10273","CACATCCCTTGACTGCTTGC","chr20",48941965,"+","none","AGG","CACATCCCTTGACTGCTTGCAGG",0,NA,NA,NA,"GeneID:10564","ARFGEF2",TRUE,"ARFGEF2"

"10274","CATCAATCAAACCACTGCCA","chr20",48952808,"+","none","AGG","CATCAATCAAACCACTGCCAAGG",0,NA,NA,NA,"GeneID:10564","ARFGEF2",TRUE,"ARFGEF2"

"10275","CATCAATCAAACCACTGCCA","chr5",42488565,"-","11:T>G,13:G>T","AGG","CATCAATCAAAACCCTGCCAAGG",2,12,14,NA,"GeneID:2690","GHR",FALSE,"ARFGEF2"

"10276","CTTTAGAAACTCATCGCATA","chr20",48951316,"+","none","CGG","CTTTAGAAACTCATCGCATACGG",0,NA,NA,NA,"GeneID:10564","ARFGEF2",TRUE,"ARFGEF2"

"10277","TGACGTTCAGCATCTGAGTA","chr20",48952838,"-","none","AGG","TGACGTTCAGCATCTGAGTAAGG",0,NA,NA,NA,"GeneID:10564","ARFGEF2",TRUE,"ARFGEF2"

"10278","TGGCACTGCTGCACCACCAA","chr20",48941866,"+","none","AGG","TGGCACTGCTGCACCACCAAAGG",0,NA,NA,NA,"GeneID:10564","ARFGEF2",TRUE,"ARFGEF2"

"10279","TGGCACTGCTGCACCACCAA","chr4",146567704,"-","6:T>A,15:G>T","GGG","TGGCACAGCTGCACCCCCAAGGG",2,7,16,NA,"","",FALSE,"ARFGEF2"

"10280","CATTGCTAGTGTGAACACTT","chr4",152882802,"+","none","TGG","CATTGCTAGTGTGAACACTTTGG",0,NA,NA,NA,"GeneID:27236","ARFIP1",TRUE,"ARFIP1"

"10281","CGACTAGCTGCAACCCTGCT","chr4",152870790,"-","none","AGG","CGACTAGCTGCAACCCTGCTAGG",0,NA,NA,NA,"GeneID:27236","ARFIP1",TRUE,"ARFIP1"

"10282","CTCGTGAACATAGCTTTAAT","chr4",152829703,"+","none","AGG","CTCGTGAACATAGCTTTAATAGG",0,NA,NA,NA,"GeneID:27236","ARFIP1",TRUE,"ARFIP1"

"10283","CTCGTGAACATAGCTTTAAT","chr7",101277547,"+","3:A>G,11:G>A","AGG","CTCATGAACATGGCTTTAATAGG",2,4,12,NA,"","",FALSE,"ARFIP1"

"10284","GCTAGTCGACTGGCTCAGCA","chr4",152870802,"+","none","AGG","GCTAGTCGACTGGCTCAGCAAGG",0,NA,NA,NA,"GeneID:27236","ARFIP1",TRUE,"ARFIP1"

"10285","TGCCGATACCCAGAAACTGC","chr4",152882739,"+","none","TGG","TGCCGATACCCAGAAACTGCTGG",0,NA,NA,NA,"GeneID:27236","ARFIP1",TRUE,"ARFIP1"

"10286","TGCTTCAATAACACCCTCTT","chr4",152863681,"-","none","TGG","TGCTTCAATAACACCCTCTTTGG",0,NA,NA,NA,"GeneID:27236","ARFIP1",TRUE,"ARFIP1"

"10287","AGTTTGACATCGTCAAGAAA","chr11",6479160,"-","none","TGG","AGTTTGACATCGTCAAGAAATGG",0,NA,NA,NA,"GeneID:23647","ARFIP2",TRUE,"ARFIP2"

"10288","ATAGCCACCAGACACAATGC","chr11",6480002,"+","none","TGG","ATAGCCACCAGACACAATGCTGG",0,NA,NA,NA,"GeneID:23647","ARFIP2",TRUE,"ARFIP2"

"10289","CAACTGTTATCAGAACGATT","chr11",6478930,"-","none","TGG","CAACTGTTATCAGAACGATTTGG",0,NA,NA,NA,"GeneID:23647","ARFIP2",TRUE,"ARFIP2"

"10290","GTATGAGAGTGTCCTGCAGC","chr11",6478838,"-","none","TGG","GTATGAGAGTGTCCTGCAGCTGG",0,NA,NA,NA,"GeneID:23647","ARFIP2",TRUE,"ARFIP2"

"10291","GTATGAGAGTGTCCTGCAGC","chrX",117862435,"+","8:A>G,12:T>C","AGG","GTATGAGAATGTTCTGCAGCAGG",2,9,13,NA,"","",FALSE,"ARFIP2"

"10292","TGCCTACCGAACAGACTTAG","chr11",6477860,"-","none","AGG","TGCCTACCGAACAGACTTAGAGG",0,NA,NA,NA,"GeneID:23647","ARFIP2",TRUE,"ARFIP2"

"10293","TGGTCGACTTGAGAGTGCCC","chr11",6477803,"-","none","AGG","TGGTCGACTTGAGAGTGCCCAGG",0,NA,NA,NA,"GeneID:23647","ARFIP2",TRUE,"ARFIP2"

"10294","CAGTATTATGCGGAGTGTCA","chr20",63702200,"-","none","CGG","CAGTATTATGCGGAGTGTCACGG",0,NA,NA,NA,"GeneID:10139","ARFRP1",TRUE,"ARFRP1"

"10295","CCTCTCCCCAGTCGGCACTG","chr20",63706430,"-","none","TGG","CCTCTCCCCAGTCGGCACTGTGG",0,NA,NA,NA,"GeneID:10139","ARFRP1",TRUE,"ARFRP1"

"10296","CGTCATTGACTCCACCGACG","chr20",63702168,"-","none","AGG","CGTCATTGACTCCACCGACGAGG",0,NA,NA,NA,"GeneID:10139","ARFRP1",TRUE,"ARFRP1"

"10297","GAAAGGCTCGGCTCATGTTC","chr20",63706401,"-","none","TGG","GAAAGGCTCGGCTCATGTTCTGG",0,NA,NA,NA,"GeneID:10139","ARFRP1",TRUE,"ARFRP1"

"10298","GACACCGCACAGCGCCTCGC","chr20",63701865,"+","none","TGG","GACACCGCACAGCGCCTCGCTGG",0,NA,NA,NA,"GeneID:10139","ARFRP1",TRUE,"ARFRP1"

"10299","GCTGTGCGGTGTCCCCGTCT","chr20",63701858,"-","none","TGG","GCTGTGCGGTGTCCCCGTCTTGG",0,NA,NA,NA,"GeneID:10139","ARFRP1",TRUE,"ARFRP1"

"10300","ACCCAGATGACTCCAAGATC","chr6",131581261,"-","none","AGG","ACCCAGATGACTCCAAGATCAGG",0,NA,NA,NA,"GeneID:383;GeneID:9439","ARG1;MED23",TRUE,"ARG1"

"10301","ACCCTGATCTTGGAGTCATC","chr6",131581256,"+","none","TGG","ACCCTGATCTTGGAGTCATCTGG",0,NA,NA,NA,"GeneID:383;GeneID:9439","ARG1;MED23",TRUE,"ARG1"

"10302","AGATATACAGGGAGTCACCC","chr6",131582645,"-","none","AGG","AGATATACAGGGAGTCACCCAGG",0,NA,NA,NA,"GeneID:383;GeneID:9439","ARG1;MED23",TRUE,"ARG1"

"10303","CCAGCCTTTCTCAATACTGT","chr6",131576689,"-","none","AGG","CCAGCCTTTCTCAATACTGTAGG",0,NA,NA,NA,"GeneID:383;GeneID:9439","ARG1;MED23",TRUE,"ARG1"

"10304","GTATATTGGCTTGAGAGACG","chr6",131582679,"+","none","TGG","GTATATTGGCTTGAGAGACGTGG",0,NA,NA,NA,"GeneID:383;GeneID:9439","ARG1;MED23",TRUE,"ARG1"

"10305","GTATATTGGCTTGAGAGACG","chr14",67646702,"+","9:T>C,10:C>T","TGG","GTATATTGGTCTGAGAGACGTGG",2,10,11,NA,"GeneID:384","ARG2",TRUE,"ARG1"

"10306","TAATTGTTCAGCCACGAGGA","chr6",131576651,"+","none","GGG","TAATTGTTCAGCCACGAGGAGGG",0,NA,NA,NA,"GeneID:383;GeneID:9439","ARG1;MED23",TRUE,"ARG1"

"10307","TAATTGTTCAGCCACGAGGA","chr9",108045194,"-","14:A>G,18:T>C","AGG","TAATTGTTCAGCCATGAGAAAGG",2,15,19,NA,"","",FALSE,"ARG1"

"10308","ATGAGAGATATTGATCGACT","chr14",67646957,"+","none","TGG","ATGAGAGATATTGATCGACTTGG",0,NA,NA,NA,"GeneID:384","ARG2",TRUE,"ARG2"

"10309","ATGTTCGTTCCATGACCTTC","chr14",67646986,"-","none","TGG","ATGTTCGTTCCATGACCTTCTGG",0,NA,NA,NA,"GeneID:384","ARG2",TRUE,"ARG2"

"10310","GAAGAAATCCGTCCACTCCG","chr14",67620033,"+","none","TGG","GAAGAAATCCGTCCACTCCGTGG",0,NA,NA,NA,"GeneID:384","ARG2",TRUE,"ARG2"

"10311","GATAGTGAATCCACGCTCAG","chr14",67642255,"+","none","TGG","GATAGTGAATCCACGCTCAGTGG",0,NA,NA,NA,"GeneID:384","ARG2",TRUE,"ARG2"

"10312","GGCTGCCACCTAAAAGACTT","chr14",67642184,"+","none","TGG","GGCTGCCACCTAAAAGACTTTGG",0,NA,NA,NA,"GeneID:384","ARG2",TRUE,"ARG2"

"10313","GGCTGCCACCTAAAAGACTT","chr2",60534332,"-","5:C>G,14:C>T","GGG","GGCTGGCACCTAAAGGACTTGGG",2,6,15,NA,"GeneID:53335","BCL11A",TRUE,"ARG2"

"10314","TGCTGCGGATCATGTCCCTA","chr14",67619966,"+","none","AGG","TGCTGCGGATCATGTCCCTAAGG",0,NA,NA,NA,"GeneID:384","ARG2",TRUE,"ARG2"

"10315","AAAGGCTTGGCTTGTCATAT","chr3",121586508,"-","none","TGG","AAAGGCTTGGCTTGTCATATTGG",0,NA,NA,NA,"GeneID:503582","ARGFX",TRUE,"ARGFX"

"10316","ACTGGTAGAACTCTCAGTGA","chr3",121586235,"-","none","AGG","ACTGGTAGAACTCTCAGTGAAGG",0,NA,NA,NA,"GeneID:503582","ARGFX",TRUE,"ARGFX"

"10317","AGCCAGACCCTTTCATCAAT","chr3",121570744,"+","none","AGG","AGCCAGACCCTTTCATCAATAGG",0,NA,NA,NA,"GeneID:503582","ARGFX",TRUE,"ARGFX"

"10318","AGCCAGACCCTTTCATCAAT","chr5",122675825,"+","19:C>T","AGG","AGCCAGACCCTTTCATCAACAGG",1,20,NA,NA,"GeneID:101927379","LOC101927379",TRUE,"ARGFX"

"10319","AGCTTTGAGACTCGACCTAC","chr3",121585028,"+","none","CGG","AGCTTTGAGACTCGACCTACCGG",0,NA,NA,NA,"GeneID:503582","ARGFX",TRUE,"ARGFX"

"10320","AGCTTTGAGACTCGACCTAC","chr20",17292751,"-","3:G>A,12:T>G","AGG","AGCCTTGAGACTAGACCTACAGG",2,4,13,NA,"GeneID:5126","PCSK2",FALSE,"ARGFX"

"10321","GCCCTGAAGCAATACGGAGA","chr3",121584907,"+","none","AGG","GCCCTGAAGCAATACGGAGAAGG",0,NA,NA,NA,"GeneID:503582","ARGFX",TRUE,"ARGFX"

"10322","GCCCTGAAGCAATACGGAGA","chr12",48377820,"-","15:A>C,17:A>T","AGG","GCCCTGAAGCAATACTGTGAAGG",2,16,18,NA,"","",FALSE,"ARGFX"

"10323","TGATACTCACTGGGACTAGC","chr3",121570806,"-","none","TGG","TGATACTCACTGGGACTAGCTGG",0,NA,NA,NA,"GeneID:503582","ARGFX",TRUE,"ARGFX"

"10324","TGATACTCACTGGGACTAGC","chr13",74160036,"-","11:A>C,19:A>G","TGG","TGATACTCACTTGGACTAGTTGG",2,12,20,NA,"","",FALSE,"ARGFX"

"10325","CCCGACCGCATCGACATCTT","chr13",106567665,"-","none","CGG","CCCGACCGCATCGACATCTTCGG",0,NA,NA,NA,"GeneID:55082","ARGLU1",TRUE,"ARGLU1"

"10326","CCGTTCGCGCTCCACCAACA","chr13",106567732,"-","none","CGG","CCGTTCGCGCTCCACCAACACGG",0,NA,NA,NA,"GeneID:55082","ARGLU1",TRUE,"ARGLU1"

"10327","GGAAGAACGTGCAAAACGTG","chr13",106557109,"-","none","AGG","GGAAGAACGTGCAAAACGTGAGG",0,NA,NA,NA,"GeneID:55082","ARGLU1",TRUE,"ARGLU1"

"10328","GTCCTTCTCATTAAAAACCC","chr13",106544003,"-","none","AGG","GTCCTTCTCATTAAAAACCCAGG",0,NA,NA,NA,"GeneID:55082","ARGLU1",TRUE,"ARGLU1"

"10329","TGCCGCACAAAAAGCTAGAG","chr13",106559433,"-","none","AGG","TGCCGCACAAAAAGCTAGAGAGG",0,NA,NA,NA,"GeneID:55082","ARGLU1",TRUE,"ARGLU1"

"10330","TGCCGCACAAAAAGCTAGAG","chr1",88367862,"+","15:A>T,16:T>A","GGG","TGCCGCACAAAAAGCATGAGGGG",2,16,17,NA,"GeneID:101927891","PKN2-AS1",FALSE,"ARGLU1"

"10331","TTGAACGAGAAGTTCTCCGA","chr13",106559527,"-","none","AGG","TTGAACGAGAAGTTCTCCGAAGG",0,NA,NA,NA,"GeneID:55082","ARGLU1",TRUE,"ARGLU1"

"10332","AACAGTCTCCCTGAGTACAA","chr11",46680505,"+","none","TGG","AACAGTCTCCCTGAGTACAATGG",0,NA,NA,NA,"GeneID:392","ARHGAP1",TRUE,"ARHGAP1"

"10333","ACCCATACTATGACATCGCC","chr11",46695684,"-","none","CGG","ACCCATACTATGACATCGCCCGG",0,NA,NA,NA,"GeneID:392","ARHGAP1",TRUE,"ARHGAP1"

"10334","CGTGCTCGCTCAGCTCGCTC","chr11",46681050,"+","none","AGG","CGTGCTCGCTCAGCTCGCTCAGG",0,NA,NA,NA,"GeneID:392","ARHGAP1",TRUE,"ARHGAP1"

"10335","CTCACCGTGGGCCTGTAAGT","chr11",46680481,"+","none","AGG","CTCACCGTGGGCCTGTAAGTAGG",0,NA,NA,NA,"GeneID:392","ARHGAP1",TRUE,"ARHGAP1"

"10336","TCCACCAACAGCTTCAAGTT","chr11",46681100,"-","none","CGG","TCCACCAACAGCTTCAAGTTCGG",0,NA,NA,NA,"GeneID:392","ARHGAP1",TRUE,"ARHGAP1"

"10337","TGCCGGGCGATGTCATAGTA","chr11",46695679,"+","none","TGG","TGCCGGGCGATGTCATAGTATGG",0,NA,NA,NA,"GeneID:392","ARHGAP1",TRUE,"ARHGAP1"

"10338","AAAATTTGAAAATTCACGTA","chr4",147822904,"-","none","AGG","AAAATTTGAAAATTCACGTAAGG",0,NA,NA,NA,"GeneID:79658","ARHGAP10",TRUE,"ARHGAP10"

"10339","AAAATTTGAAAATTCACGTA","chr7",112362694,"+","8:G>A,17:C>G","TGG","AAAATTTGGAAATTCACCTATGG",2,9,18,NA,"","",FALSE,"ARHGAP10"

"10340","AAATTCAGAAAAGAGCAACT","chr4",147847189,"+","none","TGG","AAATTCAGAAAAGAGCAACTTGG",0,NA,NA,NA,"GeneID:79658","ARHGAP10",TRUE,"ARHGAP10"

"10341","AAATTCAGAAAAGAGCAACT","chr4",110229740,"-","0:C>T,5:C>G","GGG","GAATTGAGAAAAGAGCAACTGGG",2,1,6,NA,"","",FALSE,"ARHGAP10"

"10342","AAATTCAGAAAAGAGCAACT","chr11",35174132,"+","4:A>T,10:C>A","GGG","AAATACAGAACAGAGCAACTGGG",2,5,11,NA,"GeneID:960","CD44",FALSE,"ARHGAP10"

"10343","GAAAGACTCACATTTACAAG","chr4",147857632,"+","none","AGG","GAAAGACTCACATTTACAAGAGG",0,NA,NA,NA,"GeneID:79658","ARHGAP10",TRUE,"ARHGAP10"

"10344","TGAAACCCTGATTAAACCCT","chr4",147847164,"+","none","TGG","TGAAACCCTGATTAAACCCTTGG",0,NA,NA,NA,"GeneID:79658","ARHGAP10",TRUE,"ARHGAP10"

"10345","ATCTTAATACATGAACTGTG","chr15",30634369,"-","none","TGG","ATCTTAATACATGAACTGTGTGG",0,NA,NA,NA,"","",TRUE,"ARHGAP11A"

"10346","ATCTTAATACATGAACTGTG","chr15",32624372,"-","none","TGG","ATCTTAATACATGAACTGTGTGG",0,NA,NA,NA,"GeneID:9824","ARHGAP11A",TRUE,"ARHGAP11A"

"10347","ATCTTAATACATGAACTGTG","chr1",5122298,"-","15:C>G,18:C>A","AGG","ATCTTAATACATGAAGTGGGAGG",2,16,19,NA,"","",FALSE,"ARHGAP11A"

"10348","ATCTTAATACATGAACTGTG","chrX",119369691,"-","14:A>T,16:T>A","TGG","ATCTTAATACATGATCAGTGTGG",2,15,17,NA,"","",FALSE,"ARHGAP11A"

"10349","CTTGGAATGTGTCCATATTC","chr15",30630753,"-","none","TGG","CTTGGAATGTGTCCATATTCTGG",0,NA,NA,NA,"","",TRUE,"ARHGAP11A"

"10350","CTTGGAATGTGTCCATATTC","chr15",32620158,"-","none","TGG","CTTGGAATGTGTCCATATTCTGG",0,NA,NA,NA,"GeneID:9824","ARHGAP11A",TRUE,"ARHGAP11A"

"10351","CTTGGAATGTGTCCATATTC","chr11",42593769,"-","11:T>A,13:T>G","AGG","CTTGGAATGTGACAATATTCAGG",2,12,14,NA,"","",FALSE,"ARHGAP11A"

"10352","CTTGGAATGTGTCCATATTC","chr3",144851340,"+","10:T>G,16:C>A","TGG","CTTGGAATGTTTCCATCTTCTGG",2,11,17,NA,"","",FALSE,"ARHGAP11A"

"10353","CTTGGAATGTGTCCATATTC","chr22",47033399,"+","2:G>T,16:C>A","CGG","CTGGGAATGTGTCCATCTTCCGG",2,3,17,NA,"GeneID:25771","TBC1D22A",FALSE,"ARHGAP11A"

"10354","CTTGGAATGTGTCCATATTC","chr3",31576911,"-","0:A>G,10:G>C","AGG","TTTGGAATGTCTCCATATTCAGG",2,1,11,NA,"GeneID:201595","STT3B",FALSE,"ARHGAP11A"

"10355","GAAGACCATATTCATACCGA","chr15",32623519,"+","none","AGG","GAAGACCATATTCATACCGAAGG",0,NA,NA,NA,"GeneID:9824","ARHGAP11A",TRUE,"ARHGAP11A"

"10356","GAAGACCATATTCATACCGA","chr15",30633517,"+","5:A>C","AGG","GAAGAACATATTCATACCGAAGG",1,6,NA,NA,"","",TRUE,"ARHGAP11A"

"10357","GAATGGGGCAGTGCATTAAA","chr15",30630723,"-","none","AGG","GAATGGGGCAGTGCATTAAAAGG",0,NA,NA,NA,"","",TRUE,"ARHGAP11A"

"10358","GAATGGGGCAGTGCATTAAA","chr15",32620128,"-","none","AGG","GAATGGGGCAGTGCATTAAAAGG",0,NA,NA,NA,"GeneID:9824","ARHGAP11A",TRUE,"ARHGAP11A"

"10359","TGATTTCCGAAAAAGCCCTT","chr15",32623538,"-","none","CGG","TGATTTCCGAAAAAGCCCTTCGG",0,NA,NA,NA,"GeneID:9824","ARHGAP11A",TRUE,"ARHGAP11A"

"10360","TGATTTCCGAAAAAGCCCTT","chr15",30633536,"-","none","CGG","TGATTTCCGAAAAAGCCCTTCGG",0,NA,NA,NA,"","",TRUE,"ARHGAP11A"

"10361","TTTCAGAATAAAGTGGATCA","chr15",30634163,"+","none","TGG","TTTCAGAATAAAGTGGATCATGG",0,NA,NA,NA,"","",TRUE,"ARHGAP11A"

"10362","TTTCAGAATAAAGTGGATCA","chr15",32624166,"+","none","TGG","TTTCAGAATAAAGTGGATCATGG",0,NA,NA,NA,"GeneID:9824","ARHGAP11A",TRUE,"ARHGAP11A"

"10363","AGAACTTACCCCTATTTCCG","chr15",30626938,"-","none","TGG","AGAACTTACCCCTATTTCCGTGG",0,NA,NA,NA,"","",TRUE,"ARHGAP11B"

"10364","AGAACTTACCCCTATTTCCG","chr15",32616329,"-","none","TGG","AGAACTTACCCCTATTTCCGTGG",0,NA,NA,NA,"GeneID:9824","ARHGAP11A",TRUE,"ARHGAP11B"

"10365","CTTGGAATGTGTCCATATTC","chr15",30630753,"-","none","TGG","CTTGGAATGTGTCCATATTCTGG",0,NA,NA,NA,"","",TRUE,"ARHGAP11B"

"10366","CTTGGAATGTGTCCATATTC","chr15",32620158,"-","none","TGG","CTTGGAATGTGTCCATATTCTGG",0,NA,NA,NA,"GeneID:9824","ARHGAP11A",TRUE,"ARHGAP11B"

"10367","CTTGGAATGTGTCCATATTC","chr11",42593769,"-","11:T>A,13:T>G","AGG","CTTGGAATGTGACAATATTCAGG",2,12,14,NA,"","",FALSE,"ARHGAP11B"

"10368","CTTGGAATGTGTCCATATTC","chr3",144851340,"+","10:T>G,16:C>A","TGG","CTTGGAATGTTTCCATCTTCTGG",2,11,17,NA,"","",FALSE,"ARHGAP11B"

"10369","CTTGGAATGTGTCCATATTC","chr22",47033399,"+","2:G>T,16:C>A","CGG","CTGGGAATGTGTCCATCTTCCGG",2,3,17,NA,"GeneID:25771","TBC1D22A",FALSE,"ARHGAP11B"

"10370","CTTGGAATGTGTCCATATTC","chr3",31576911,"-","0:A>G,10:G>C","AGG","TTTGGAATGTCTCCATATTCAGG",2,1,11,NA,"GeneID:201595","STT3B",FALSE,"ARHGAP11B"

"10371","GAAGAACATATTCATACCGA","chr15",30633517,"+","none","AGG","GAAGAACATATTCATACCGAAGG",0,NA,NA,NA,"","",TRUE,"ARHGAP11B"

"10372","GAAGAACATATTCATACCGA","chr15",32623519,"+","5:C>A","AGG","GAAGACCATATTCATACCGAAGG",1,6,NA,NA,"GeneID:9824","ARHGAP11A",TRUE,"ARHGAP11B"

"10373","GAATGGGGCAGTGCATTAAA","chr15",30630723,"-","none","AGG","GAATGGGGCAGTGCATTAAAAGG",0,NA,NA,NA,"","",TRUE,"ARHGAP11B"

"10374","GAATGGGGCAGTGCATTAAA","chr15",32620128,"-","none","AGG","GAATGGGGCAGTGCATTAAAAGG",0,NA,NA,NA,"GeneID:9824","ARHGAP11A",TRUE,"ARHGAP11B"

"10375","TATCCGGAATGTGGGATCAG","chr15",32616203,"+","none","AGG","TATCCGGAATGTGGGATCAGAGG",0,NA,NA,NA,"GeneID:9824","ARHGAP11A",TRUE,"ARHGAP11B"

"10376","TATCCGGAATGTGGGATCAG","chr15",30626812,"+","none","AGG","TATCCGGAATGTGGGATCAGAGG",0,NA,NA,NA,"","",TRUE,"ARHGAP11B"

"10377","TGATTTCCGAAAAAGCCCTT","chr15",32623538,"-","none","CGG","TGATTTCCGAAAAAGCCCTTCGG",0,NA,NA,NA,"GeneID:9824","ARHGAP11A",TRUE,"ARHGAP11B"

"10378","TGATTTCCGAAAAAGCCCTT","chr15",30633536,"-","none","CGG","TGATTTCCGAAAAAGCCCTTCGG",0,NA,NA,NA,"","",TRUE,"ARHGAP11B"

"10379","CAAGGGGGCAACAATTGAGA","chr10",31820427,"-","none","TGG","CAAGGGGGCAACAATTGAGATGG",0,NA,NA,NA,"GeneID:94134","ARHGAP12",TRUE,"ARHGAP12"

"10380","CAAGGGGGCAACAATTGAGA","chr6",55453647,"+","6:A>G,11:T>C","TGG","CAAGGGAGCAATAATTGAGATGG",2,7,12,NA,"GeneID:54511","HMGCLL1",FALSE,"ARHGAP12"

"10381","CTCAAGCATGTTGATGATCA","chr10",31852574,"-","none","AGG","CTCAAGCATGTTGATGATCAAGG",0,NA,NA,NA,"GeneID:94134","ARHGAP12",TRUE,"ARHGAP12"

"10382","CTCAAGCATGTTGATGATCA","chr20",40488111,"-","1:G>A,17:G>A","TGG","CCCAAGCATGTTGATGACCATGG",2,2,18,NA,"","",FALSE,"ARHGAP12"

"10383","GAAAGAACTGGTTGTCTTCT","chr10",31826366,"-","none","TGG","GAAAGAACTGGTTGTCTTCTTGG",0,NA,NA,NA,"GeneID:94134","ARHGAP12",TRUE,"ARHGAP12"

"10384","GTCAGAATGGGAATTGCCAA","chr10",31852518,"-","none","AGG","GTCAGAATGGGAATTGCCAAAGG",0,NA,NA,NA,"GeneID:94134","ARHGAP12",TRUE,"ARHGAP12"

"10385","GTCAGAATGGGAATTGCCAA","chrX",121839341,"-","13:T>A,15:A>C","GGG","GTCAGAATGGGAAATTCCAAGGG",2,14,16,NA,"","",FALSE,"ARHGAP12"

"10386","GTCCAAACCAGAGTTCACAG","chr10",31820454,"-","none","TGG","GTCCAAACCAGAGTTCACAGTGG",0,NA,NA,NA,"GeneID:94134","ARHGAP12",TRUE,"ARHGAP12"

"10387","GTCCAAACCAGAGTTCACAG","chr16",4402684,"+","11:G>A,17:G>C","TGG","GTCCAAACCAGGGTTCAGAGTGG",2,12,18,NA,"GeneID:100529144;GeneID:79585","CORO7-PAM16;CORO7",FALSE,"ARHGAP12"

"10388","GTCCAAACCAGAGTTCACAG","chr14",36311008,"+","6:G>A,13:A>T","TGG","GTCCAAGCCAGAGATCACAGTGG",2,7,14,NA,"GeneID:51562","MBIP",FALSE,"ARHGAP12"

"10389","GTCCAAACCAGAGTTCACAG","chr10",48554215,"-","5:A>T,16:C>T","GGG","GTCCATACCAGAGTTCGCAGGGG",2,6,17,NA,"GeneID:58504","ARHGAP22",FALSE,"ARHGAP12"

"10390","TGTGCTACTTCCTTGAGTTT","chr10",31826309,"+","none","TGG","TGTGCTACTTCCTTGAGTTTTGG",0,NA,NA,NA,"GeneID:94134","ARHGAP12",TRUE,"ARHGAP12"

"10391","ACACTCACCAGTTGTTCCAA","chr2",143202190,"-","none","TGG","ACACTCACCAGTTGTTCCAATGG",0,NA,NA,NA,"GeneID:55843","ARHGAP15",TRUE,"ARHGAP15"

"10392","CAAAAAGCTAAAATTGCAGA","chr2",143216407,"+","none","TGG","CAAAAAGCTAAAATTGCAGATGG",0,NA,NA,NA,"GeneID:55843","ARHGAP15",TRUE,"ARHGAP15"

"10393","CAAAAAGCTAAAATTGCAGA","chr1",88385065,"+","6:C>G,16:G>C","AGG","CAAAAACCTAAAATTGGAGAAGG",2,7,17,NA,"GeneID:101927891","PKN2-AS1",FALSE,"ARHGAP15"

"10394","GAAAGATGTCATTCCTCCAT","chr2",143202171,"+","none","TGG","GAAAGATGTCATTCCTCCATTGG",0,NA,NA,NA,"GeneID:55843","ARHGAP15",TRUE,"ARHGAP15"

"10395","GAAAGATGTCATTCCTCCAT","chr7",51313617,"+","4:C>G,10:T>A","AGG","GAAACATGTCTTTCCTCCATAGG",2,5,11,NA,"GeneID:23242","COBL",FALSE,"ARHGAP15"

"10396","TCCATGATCCTCACCGATGT","chr2",143155616,"+","none","CGG","TCCATGATCCTCACCGATGTCGG",0,NA,NA,NA,"GeneID:55843","ARHGAP15",TRUE,"ARHGAP15"

"10397","TTCAGTGACCTTCCCGACAT","chr2",143155632,"-","none","CGG","TTCAGTGACCTTCCCGACATCGG",0,NA,NA,NA,"GeneID:55843","ARHGAP15",TRUE,"ARHGAP15"

"10398","TTGCAGATGGTTGAAAAAGA","chr2",143216377,"+","none","AGG","TTGCAGATGGTTGAAAAAGAAGG",0,NA,NA,NA,"GeneID:55843","ARHGAP15",TRUE,"ARHGAP15"

"10399","TTGCAGATGGTTGAAAAAGA","chr1",122461277,"-","11:T>A,16:C>T","GGG","TTGCAGATGGTAGAAAGAGAGGG",2,12,17,NA,"","",FALSE,"ARHGAP15"

"10400","TTGCAGATGGTTGAAAAAGA","chr7",61504345,"-","11:T>A,12:G>C","GGG","TTGCAGATGGTACAAAAAGAGGG",2,12,13,NA,"","",FALSE,"ARHGAP15"

"10401","TTGCAGATGGTTGAAAAAGA","chr7",61509813,"-","11:T>A,12:G>C","GGG","TTGCAGATGGTACAAAAAGAGGG",2,12,13,NA,"","",FALSE,"ARHGAP15"

"10402","TTGCAGATGGTTGAAAAAGA","chr11",53445750,"-","8:A>C,9:G>C","GGG","TTGCAGATTCTTGAAAAAGAGGG",2,9,10,NA,"","",FALSE,"ARHGAP15"

"10403","TTGCAGATGGTTGAAAAAGA","chr20",15160153,"+","3:A>C,6:G>A","TGG","TTGAAGGTGGTTGAAAAAGATGG",2,4,7,NA,"GeneID:140733","MACROD2",FALSE,"ARHGAP15"

"10404","TTGCAGATGGTTGAAAAAGA","chr20",29238624,"+","8:T>G,11:A>T","TGG","TTGCAGATTGTAGAAAAAGATGG",2,9,12,NA,"","",FALSE,"ARHGAP15"

"10405","TTGCAGATGGTTGAAAAAGA","chr11",52864911,"-","8:A>C,11:T>A","GGG","TTGCAGATTGTAGAAAAAGAGGG",2,9,12,NA,"","",FALSE,"ARHGAP15"

"10406","TTGCAGATGGTTGAAAAAGA","chr11",52472229,"-","8:A>C,11:T>A","GGG","TTGCAGATTGTAGAAAAAGAGGG",2,9,12,NA,"","",FALSE,"ARHGAP15"

"10407","TTGCAGATGGTTGAAAAAGA","chr11",54033685,"-","8:A>C,11:T>A","GGG","TTGCAGATTGTAGAAAAAGAGGG",2,9,12,NA,"","",FALSE,"ARHGAP15"

"10408","TTGCAGATGGTTGAAAAAGA","chr11",51784241,"-","8:A>C,11:T>A","GGG","TTGCAGATTGTAGAAAAAGAGGG",2,9,12,NA,"","",FALSE,"ARHGAP15"

"10409","TTGCAGATGGTTGAAAAAGA","chr11",52853009,"-","8:A>C,11:T>A","GGG","TTGCAGATTGTAGAAAAAGAGGG",2,9,12,NA,"","",FALSE,"ARHGAP15"

"10410","TTGCAGATGGTTGAAAAAGA","chr11",51561508,"-","8:A>C,11:T>A","GGG","TTGCAGATTGTAGAAAAAGAGGG",2,9,12,NA,"","",FALSE,"ARHGAP15"

"10411","TTGCAGATGGTTGAAAAAGA","chr3",71589039,"-","5:T>C,18:A>C","AGG","TTGCAAATGGTTGAAAAATAAGG",2,6,19,NA,"","",FALSE,"ARHGAP15"

"10412","ACCTCAGCTATGCCGTACAG","chr16",24968658,"+","none","AGG","ACCTCAGCTATGCCGTACAGAGG",0,NA,NA,NA,"GeneID:55114","ARHGAP17",TRUE,"ARHGAP17"

"10413","ATATTGACCGCACCGTGTCC","chr16",24977285,"+","none","AGG","ATATTGACCGCACCGTGTCCAGG",0,NA,NA,NA,"GeneID:55114","ARHGAP17",TRUE,"ARHGAP17"

"10414","CCTTAGTGAAGATCTATTAC","chr16",24978967,"-","none","AGG","CCTTAGTGAAGATCTATTACAGG",0,NA,NA,NA,"GeneID:55114","ARHGAP17",TRUE,"ARHGAP17"

"10415","GCAAGAAGCATCGACTCAGC","chr16",24970525,"-","none","TGG","GCAAGAAGCATCGACTCAGCTGG",0,NA,NA,NA,"GeneID:55114","ARHGAP17",TRUE,"ARHGAP17"

"10416","TCTCAACAAAGACTTCGTGC","chr16",24968695,"+","none","TGG","TCTCAACAAAGACTTCGTGCTGG",0,NA,NA,NA,"GeneID:55114","ARHGAP17",TRUE,"ARHGAP17"

"10417","TCTCAACAAAGACTTCGTGC","chr10",2093524,"-","10:G>C,15:A>G","AGG","TCTCAACAAACACTTTGTGCAGG",2,11,16,NA,"","",FALSE,"ARHGAP17"

"10418","TTGTCAGATTGAGAGACGCC","chr16",24977306,"-","none","TGG","TTGTCAGATTGAGAGACGCCTGG",0,NA,NA,NA,"GeneID:55114","ARHGAP17",TRUE,"ARHGAP17"

"10419","AATCTCTTCGGAGAGTCTGC","chr6",129638565,"-","none","TGG","AATCTCTTCGGAGAGTCTGCTGG",0,NA,NA,NA,"GeneID:93663","ARHGAP18",TRUE,"ARHGAP18"

"10420","AGCAGACTCTCCGAAGAGAT","chr6",129638564,"+","none","TGG","AGCAGACTCTCCGAAGAGATTGG",0,NA,NA,NA,"GeneID:93663","ARHGAP18",TRUE,"ARHGAP18"

"10421","AGCAGACTCTCCGAAGAGAT","chr9",99459891,"+","11:G>C,15:C>G","GGG","AGCAGACTCTCGGAACAGATGGG",2,12,16,NA,"","",FALSE,"ARHGAP18"

"10422","AGGCTGTTAGTACCACTCCC","chr6",129710093,"+","none","TGG","AGGCTGTTAGTACCACTCCCTGG",0,NA,NA,NA,"GeneID:93663","ARHGAP18",TRUE,"ARHGAP18"

"10423","GACGGTCTGGTCCTTGCCGC","chr6",129710064,"+","none","TGG","GACGGTCTGGTCCTTGCCGCTGG",0,NA,NA,NA,"GeneID:93663","ARHGAP18",TRUE,"ARHGAP18"

"10424","GACTGCGATTCAGTGCCACC","chr6",129634079,"+","none","TGG","GACTGCGATTCAGTGCCACCTGG",0,NA,NA,NA,"GeneID:93663","ARHGAP18",TRUE,"ARHGAP18"

"10425","TCAACATACCATCTCTTCCT","chr6",129634032,"+","none","TGG","TCAACATACCATCTCTTCCTTGG",0,NA,NA,NA,"GeneID:93663","ARHGAP18",TRUE,"ARHGAP18"

"10426","TCAACATACCATCTCTTCCT","chr9",108340352,"+","11:C>T,16:A>T","TGG","TCAACATACCACCTCTACCTTGG",2,12,17,NA,"","",FALSE,"ARHGAP18"

"10427","TCAACATACCATCTCTTCCT","chr2",20019008,"+","2:C>A,6:G>T","TGG","TCCACAGACCATCTCTTCCTTGG",2,3,7,NA,"","",FALSE,"ARHGAP18"

"10428","AATGCACACCTCAAAATCGC","chr10",97263421,"-","none","TGG","AATGCACACCTCAAAATCGCTGG",0,NA,NA,NA,"GeneID:100533184;GeneID:84986","ARHGAP19-SLIT1;ARHGAP19",TRUE,"ARHGAP19"

"10429","CAAGACCAATATACCAGACA","chr10",97259580,"-","none","AGG","CAAGACCAATATACCAGACAAGG",0,NA,NA,NA,"GeneID:100533184;GeneID:84986","ARHGAP19-SLIT1;ARHGAP19",TRUE,"ARHGAP19"

"10430","GCAATGCCTTCCTCCGTCAG","chr10",97264860,"+","none","TGG","GCAATGCCTTCCTCCGTCAGTGG",0,NA,NA,NA,"GeneID:100533184;GeneID:84986","ARHGAP19-SLIT1;ARHGAP19",TRUE,"ARHGAP19"

"10431","GGGGTGCAAACATAAGGGCA","chr10",97259421,"+","none","AGG","GGGGTGCAAACATAAGGGCAAGG",0,NA,NA,NA,"GeneID:100533184;GeneID:84986","ARHGAP19-SLIT1;ARHGAP19",TRUE,"ARHGAP19"

"10432","GGGGTGCAAACATAAGGGCA","chrX",77437381,"+","9:T>A,14:G>A","TGG","GGGGTGCAATCATAGGGGCATGG",2,10,15,NA,"","",FALSE,"ARHGAP19"

"10433","TAATGGAACTGACATTGACT","chr10",97263536,"-","none","TGG","TAATGGAACTGACATTGACTTGG",0,NA,NA,NA,"GeneID:100533184;GeneID:84986","ARHGAP19-SLIT1;ARHGAP19",TRUE,"ARHGAP19"

"10434","TGTGTAGATACTCAATCAGT","chr10",97264828,"+","none","TGG","TGTGTAGATACTCAATCAGTTGG",0,NA,NA,NA,"GeneID:100533184;GeneID:84986","ARHGAP19-SLIT1;ARHGAP19",TRUE,"ARHGAP19"

"10435","AGTGGCCACAAAGTTCACTG","chr11",110624166,"+","none","TGG","AGTGGCCACAAAGTTCACTGTGG",0,NA,NA,NA,"GeneID:57569","ARHGAP20",TRUE,"ARHGAP20"

"10436","CATGTGTCAACACTAGCAGA","chr11",110630762,"+","none","AGG","CATGTGTCAACACTAGCAGAAGG",0,NA,NA,NA,"GeneID:57569","ARHGAP20",TRUE,"ARHGAP20"

"10437","GAGCGCTCCATCTCTTATCC","chr11",110690580,"-","none","TGG","GAGCGCTCCATCTCTTATCCTGG",0,NA,NA,NA,"GeneID:57569","ARHGAP20",TRUE,"ARHGAP20"

"10438","GCCCAAAACAAAGGATTTCA","chr11",110624193,"+","none","TGG","GCCCAAAACAAAGGATTTCATGG",0,NA,NA,NA,"GeneID:57569","ARHGAP20",TRUE,"ARHGAP20"

"10439","GCCCAAAACAAAGGATTTCA","chr22",30286572,"+","3:A>C,17:C>T","CGG","GCCAAAAACAAAGGATTCCACGG",2,4,18,NA,"GeneID:652968","GATSL3",TRUE,"ARHGAP20"

"10440","GCCCAAAACAAAGGATTTCA","chr14",72333654,"-","5:C>T,13:G>C","GGG","GCCCAGAACAAAGCATTTCAGGG",2,6,14,NA,"GeneID:9628","RGS6",FALSE,"ARHGAP20"

"10441","GCCCAAAACAAAGGATTTCA","chr7",130861655,"-","1:C>G,17:T>A","GGG","GGCCAAAACAAAGGATTACAGGG",2,2,18,NA,"","",FALSE,"ARHGAP20"

"10442","GGCCGGGCAGAACTCAAAAG","chr11",110630693,"-","none","AGG","GGCCGGGCAGAACTCAAAAGAGG",0,NA,NA,NA,"GeneID:57569","ARHGAP20",TRUE,"ARHGAP20"

"10443","TACAAAAACGGCCTACTACC","chr11",110690548,"-","none","AGG","TACAAAAACGGCCTACTACCAGG",0,NA,NA,NA,"GeneID:57569","ARHGAP20",TRUE,"ARHGAP20"

"10444","ACGTTGAAAAGAACATCTCA","chr10",24670287,"-","none","AGG","ACGTTGAAAAGAACATCTCAAGG",0,NA,NA,NA,"GeneID:57584","ARHGAP21",TRUE,"ARHGAP21"

"10445","ACGTTGAAAAGAACATCTCA","chr18",40318390,"+","7:G>A,12:C>A","TGG","ACGTTGAGAAGACCATCTCATGG",2,8,13,NA,"","",FALSE,"ARHGAP21"

"10446","AGGAAAACAAAGAAACCGCT","chr6",80069839,"-","none","TGG","AGGAAAACAAAGAAACCGCTTGG",0,NA,NA,NA,"","",TRUE,"ARHGAP21"

"10447","AGGAAAACAAAGAAACCGCT","chr10",24635085,"-","none","TGG","AGGAAAACAAAGAAACCGCTTGG",0,NA,NA,NA,"GeneID:57584","ARHGAP21",TRUE,"ARHGAP21"

"10448","CATTTGTAGGATGAAGAAAA","chr10",24666998,"-","none","TGG","CATTTGTAGGATGAAGAAAATGG",0,NA,NA,NA,"GeneID:57584","ARHGAP21",TRUE,"ARHGAP21"

"10449","CATTTGTAGGATGAAGAAAA","chr1",86833600,"+","13:G>A,17:T>A","TGG","CATTTGTAGGATGGAGATAATGG",2,14,18,NA,"","",FALSE,"ARHGAP21"

"10450","CATTTGTAGGATGAAGAAAA","chr1",239416999,"+","13:G>A,15:A>G","AGG","CATTTGTAGGATGGAAAAAAAGG",2,14,16,NA,"GeneID:1131","CHRM3",FALSE,"ARHGAP21"

"10451","CTGAAGATGAAACATTCTCC","chr10",24670328,"-","none","TGG","CTGAAGATGAAACATTCTCCTGG",0,NA,NA,NA,"GeneID:57584","ARHGAP21",TRUE,"ARHGAP21"

"10452","CTGAAGATGAAACATTCTCC","chr12",77389083,"+","11:T>A,14:G>T","TGG","CTGAAGATGAATCAGTCTCCTGG",2,12,15,NA,"GeneID:89795","NAV3",FALSE,"ARHGAP21"

"10453","CTGAAGATGAAACATTCTCC","chr2",38431318,"+","11:C>A,15:G>T","TGG","CTGAAGATGAACCATGCTCCTGG",2,12,16,NA,"GeneID:100996902","LOC100996902",TRUE,"ARHGAP21"

"10454","CTGAAGATGAAACATTCTCC","chr6",80070070,"-","1:G>A,2:T>C","TGG","CCAAAGATGAAACATTCTCCTGG",2,2,3,NA,"","",TRUE,"ARHGAP21"

"10455","CTGAAGATGAAACATTCTCC","chr16",53149188,"-","0:C>G,3:G>T","AGG","GTGCAGATGAAACATTCTCCAGG",2,1,4,NA,"GeneID:80205","CHD9",FALSE,"ARHGAP21"

"10456","CTGAAGATGAAACATTCTCC","chr15",49227058,"-","1:T>A,3:G>T","AGG","CAGCAGATGAAACATTCTCCAGG",2,2,4,NA,"GeneID:2585","GALK2",FALSE,"ARHGAP21"

"10457","CTGAAGATGAAACATTCTCC","chr20",45233852,"-","3:G>T,5:G>C","AGG","CTGCACATGAAACATTCTCCAGG",2,4,6,NA,"","",FALSE,"ARHGAP21"

"10458","CTGAAGATGAAACATTCTCC","chr5",29791410,"-","3:G>T,5:G>C","AGG","CTGCACATGAAACATTCTCCAGG",2,4,6,NA,"","",FALSE,"ARHGAP21"

"10459","CTGAAGATGAAACATTCTCC","chr4",123801055,"-","3:A>T,5:G>C","AGG","CTGTACATGAAACATTCTCCAGG",2,4,6,NA,"GeneID:285419","LINC01091",FALSE,"ARHGAP21"

"10460","CTGAAGATGAAACATTCTCC","chr11",25171360,"+","1:A>T,5:C>G","AGG","CAGAACATGAAACATTCTCCAGG",2,2,6,NA,"","",FALSE,"ARHGAP21"

"10461","CTGAAGATGAAACATTCTCC","chr5",11354810,"+","3:C>A,5:C>G","AGG","CTGCACATGAAACATTCTCCAGG",2,4,6,NA,"GeneID:1501","CTNND2",FALSE,"ARHGAP21"

"10462","CTGAAGATGAAACATTCTCC","chr5",136609673,"+","5:C>G,9:G>A","AGG","CTGAACATGGAACATTCTCCAGG",2,6,10,NA,"","",FALSE,"ARHGAP21"

"10463","CTGAAGATGAAACATTCTCC","chr18",74559598,"+","0:A>C,17:C>T","TGG","ATGAAGATGAAACATTCCCCTGG",2,1,18,NA,"GeneID:84735","CNDP1",FALSE,"ARHGAP21"

"10464","TAGGTCTCAAAAAATAAAGA","chr10",24670380,"-","none","TGG","TAGGTCTCAAAAAATAAAGATGG",0,NA,NA,NA,"GeneID:57584","ARHGAP21",TRUE,"ARHGAP21"

"10465","TAGGTCTCAAAAAATAAAGA","chr7",40377248,"-","14:C>A","AGG","TAGGTCTCAAAAAAGAAAGAAGG",1,15,NA,NA,"GeneID:79783","SUGCT",FALSE,"ARHGAP21"

"10466","TAGGTCTCAAAAAATAAAGA","chr14",71647728,"-","10:G>T,18:T>C","GGG","TAGGTCTCAACAAATAAAAAGGG",2,11,19,NA,"GeneID:26037","SIPA1L1",FALSE,"ARHGAP21"

"10467","TAGGTCTCAAAAAATAAAGA","chr4",161945743,"+","1:C>A,2:T>G","AGG","TCTGTCTCAAAAAATAAAGAAGG",2,2,3,NA,"GeneID:56884","FSTL5",FALSE,"ARHGAP21"

"10468","TAGGTCTCAAAAAATAAAGA","chr16",11567975,"+","1:C>A,2:T>G","CGG","TCTGTCTCAAAAAATAAAGACGG",2,2,3,NA,"GeneID:9516","LITAF",FALSE,"ARHGAP21"

"10469","TAGGTCTCAAAAAATAAAGA","chr17",76218979,"+","0:C>T,3:C>G","AGG","CAGCTCTCAAAAAATAAAGAAGG",2,1,4,NA,"GeneID:114804","RNF157",FALSE,"ARHGAP21"

"10470","TAGGTCTCAAAAAATAAAGA","chrX",84897313,"+","5:T>C,7:T>C","TGG","TAGGTTTTAAAAAATAAAGATGG",2,6,8,NA,"","",FALSE,"ARHGAP21"

"10471","TAGGTCTCAAAAAATAAAGA","chr2",96554285,"+","1:C>A,14:A>T","AGG","TCGGTCTCAAAAAAAAAAGAAGG",2,2,15,NA,"","",FALSE,"ARHGAP21"

"10472","TTTAACTTGCTTAACAAATA","chr10",24635047,"+","none","TGG","TTTAACTTGCTTAACAAATATGG",0,NA,NA,NA,"GeneID:57584","ARHGAP21",TRUE,"ARHGAP21"

"10473","TTTAACTTGCTTAACAAATA","chr6",146463373,"-","0:T>A,12:A>T","AGG","ATTAACTTGCTTTACAAATAAGG",2,1,13,NA,"","",FALSE,"ARHGAP21"

"10474","ACATGCTGAGCCCAAAGATC","chr10",48604778,"-","none","AGG","ACATGCTGAGCCCAAAGATCAGG",0,NA,NA,NA,"GeneID:58504","ARHGAP22",TRUE,"ARHGAP22"

"10475","ACATGCTGAGCCCAAAGATC","chr14",105523755,"-","1:T>G,13:A>T","AGG","AAATGCTGAGCCCTAAGATCAGG",2,2,14,NA,"","",FALSE,"ARHGAP22"

"10476","CCGCTCCAAAAGCCTAGTGA","chr10",48583131,"-","none","TGG","CCGCTCCAAAAGCCTAGTGATGG",0,NA,NA,NA,"GeneID:58504","ARHGAP22",TRUE,"ARHGAP22"

"10477","TACCTGGGCTGATCTCAAAG","chr10",48555459,"+","none","AGG","TACCTGGGCTGATCTCAAAGAGG",0,NA,NA,NA,"GeneID:58504","ARHGAP22",TRUE,"ARHGAP22"

"10478","TAGGGATTTATTTCTCTACA","chr10",48555533,"-","none","AGG","TAGGGATTTATTTCTCTACAAGG",0,NA,NA,NA,"GeneID:58504","ARHGAP22",TRUE,"ARHGAP22"

"10479","TGAGGGCACGGCATCCGCCC","chr10",48583088,"+","none","AGG","TGAGGGCACGGCATCCGCCCAGG",0,NA,NA,NA,"GeneID:58504","ARHGAP22",TRUE,"ARHGAP22"

"10480","TGAGGGCACGGCATCCGCCC","chr17",76947606,"-","0:T>A,5:A>C","AGG","AGAGGTCACGGCATCCGCCCAGG",2,1,6,NA,"GeneID:146664","MGAT5B",FALSE,"ARHGAP22"

"10481","TGCCGACAGCTTCCAGCAAA","chr10",48652261,"-","none","AGG","TGCCGACAGCTTCCAGCAAAAGG",0,NA,NA,NA,"GeneID:58504","ARHGAP22",TRUE,"ARHGAP22"

"10482","CATCGTGTACCCACCCGAGT","chr17",38458220,"+","none","CGG","CATCGTGTACCCACCCGAGTCGG",0,NA,NA,NA,"GeneID:57636","ARHGAP23",TRUE,"ARHGAP23"

"10483","CGGGTGGGTACACGATGAAG","chr17",38458217,"-","none","TGG","CGGGTGGGTACACGATGAAGTGG",0,NA,NA,NA,"GeneID:57636","ARHGAP23",TRUE,"ARHGAP23"

"10484","CTGGAGGATGTCCTCGTCCT","chr17",38463359,"-","none","TGG","CTGGAGGATGTCCTCGTCCTTGG",0,NA,NA,NA,"GeneID:57636","ARHGAP23",TRUE,"ARHGAP23"

"10485","GAGTGAAGCCAAAGCCGTCC","chr17",38458190,"-","none","TGG","GAGTGAAGCCAAAGCCGTCCTGG",0,NA,NA,NA,"GeneID:57636","ARHGAP23",TRUE,"ARHGAP23"

"10486","GTCTATCATGCCCAAGGACG","chr17",38463345,"+","none","AGG","GTCTATCATGCCCAAGGACGAGG",0,NA,NA,NA,"GeneID:57636","ARHGAP23",TRUE,"ARHGAP23"

"10487","TCCCTGCAGGAGGAAGAGAA","chr17",38460895,"+","none","TGG","TCCCTGCAGGAGGAAGAGAATGG",0,NA,NA,NA,"GeneID:57636","ARHGAP23",TRUE,"ARHGAP23"

"10488","TCCCTGCAGGAGGAAGAGAA","chr20",62580967,"+","13:G>A,19:T>A","GGG","TCCCTGCAGGAGGGAGAGATGGG",2,14,20,NA,"","",FALSE,"ARHGAP23"

"10489","TCCCTGCAGGAGGAAGAGAA","chr17",4327946,"-","1:A>G,9:T>C","GGG","TTCCTGCAGAAGGAAGAGAAGGG",2,2,10,NA,"GeneID:7326","UBE2G1",FALSE,"ARHGAP23"

"10490","TCCCTGCAGGAGGAAGAGAA","chr4",4366311,"+","2:A>C,7:C>A","AGG","TCACTGCCGGAGGAAGAGAAAGG",2,3,8,NA,"GeneID:27065","NSG1",FALSE,"ARHGAP23"

"10491","TCCCTGCAGGAGGAAGAGAA","chr17",15466017,"+","6:A>C,13:G>A","AGG","TCCCTGAAGGAGGGAGAGAAAGG",2,7,14,NA,"GeneID:284040;GeneID:100533496","CDRT4;TVP23C-CDRT4",FALSE,"ARHGAP23"

"10492","TCCCTGCAGGAGGAAGAGAA","chr1",248222310,"-","3:A>G,19:G>T","TGG","TCCTTGCAGGAGGAAGAGACTGG",2,4,20,NA,"","",FALSE,"ARHGAP23"

"10493","AAATCTTCATACTTCGCATA","chr4",85972108,"-","none","AGG","AAATCTTCATACTTCGCATAAGG",0,NA,NA,NA,"GeneID:83478","ARHGAP24",TRUE,"ARHGAP24"

"10494","ACCTCCTCAAGTATATTTGC","chr4",85974939,"+","none","AGG","ACCTCCTCAAGTATATTTGCAGG",0,NA,NA,NA,"GeneID:83478","ARHGAP24",TRUE,"ARHGAP24"

"10495","AGTCAATCCGCCGAGTCATA","chr4",85923731,"+","none","TGG","AGTCAATCCGCCGAGTCATATGG",0,NA,NA,NA,"GeneID:83478","ARHGAP24",TRUE,"ARHGAP24"

"10496","ATGTGCCAAACTGCTCAGCA","chr4",85972134,"+","none","AGG","ATGTGCCAAACTGCTCAGCAAGG",0,NA,NA,NA,"GeneID:83478","ARHGAP24",TRUE,"ARHGAP24"

"10497","ATGTGCCAAACTGCTCAGCA","chr14",76305008,"+","15:G>C,16:G>A","TGG","ATGTGCCAAACTGCTGGGCATGG",2,16,17,NA,"","",FALSE,"ARHGAP24"

"10498","CCCTGCAATGAAGAGAACCC","chr4",85721923,"+","none","AGG","CCCTGCAATGAAGAGAACCCAGG",0,NA,NA,NA,"GeneID:83478","ARHGAP24",TRUE,"ARHGAP24"

"10499","GTACACTCACCTCCTCCGAA","chr4",85923760,"-","none","AGG","GTACACTCACCTCCTCCGAAAGG",0,NA,NA,NA,"GeneID:83478","ARHGAP24",TRUE,"ARHGAP24"

"10500","AAATGTCCCTAAAATTGCCA","chr2",68735197,"+","none","AGG","AAATGTCCCTAAAATTGCCAAGG",0,NA,NA,NA,"GeneID:9938","ARHGAP25",TRUE,"ARHGAP25"

"10501","AGTCCTGTCCCATGCGATTC","chr2",68787852,"-","none","TGG","AGTCCTGTCCCATGCGATTCTGG",0,NA,NA,NA,"GeneID:9938","ARHGAP25",TRUE,"ARHGAP25"

"10502","ATCTCCTTGATTGTACATCC","chr2",68782250,"-","none","TGG","ATCTCCTTGATTGTACATCCTGG",0,NA,NA,NA,"GeneID:9938","ARHGAP25",TRUE,"ARHGAP25"

"10503","ATCTCCTTGATTGTACATCC","chr21",40568488,"-","10:T>A,17:C>A","TGG","ATCTCCTTGAATGTACAGCCTGG",2,11,18,NA,"GeneID:1826","DSCAM",FALSE,"ARHGAP25"

"10504","CAGGGCTGCATGTATCTACC","chr2",68782229,"+","none","AGG","CAGGGCTGCATGTATCTACCAGG",0,NA,NA,NA,"GeneID:9938","ARHGAP25",TRUE,"ARHGAP25"

"10505","CTCATGGGACCAGAATCGCA","chr2",68787840,"+","none","TGG","CTCATGGGACCAGAATCGCATGG",0,NA,NA,NA,"GeneID:9938","ARHGAP25",TRUE,"ARHGAP25"

"10506","CTGGGATTTCAACCTGAAAG","chr2",68735222,"+","none","TGG","CTGGGATTTCAACCTGAAAGTGG",0,NA,NA,NA,"GeneID:9938","ARHGAP25",TRUE,"ARHGAP25"

"10507","AAAGAAAGAATCTCAGCTTC","chr5",142885374,"+","none","AGG","AAAGAAAGAATCTCAGCTTCAGG",0,NA,NA,NA,"GeneID:23092","ARHGAP26",TRUE,"ARHGAP26"

"10508","AAAGAAAGAATCTCAGCTTC","chr6",71175203,"-","7:A>C,9:C>T","TGG","AAAGAAATAGTCTCAGCTTCTGG",2,8,10,NA,"","",FALSE,"ARHGAP26"

"10509","AAAGAAAGAATCTCAGCTTC","chr15",93098068,"-","3:G>C,9:G>T","TGG","AAACAAAGACTCTCAGCTTCTGG",2,4,10,NA,"","",FALSE,"ARHGAP26"

"10510","AAAGAAAGAATCTCAGCTTC","chr13",87895273,"+","0:C>A,18:G>T","AGG","CAAGAAAGAATCTCAGCTGCAGG",2,1,19,NA,"","",FALSE,"ARHGAP26"

"10511","AAAGAAAGAATCTCAGCTTC","chr15",96401620,"+","2:G>A,19:T>C","GGG","AAGGAAAGAATCTCAGCTTTGGG",2,3,20,NA,"","",FALSE,"ARHGAP26"

"10512","AAAGAAAGAATCTCAGCTTC","chrX",5170758,"-","3:T>C,19:A>G","GGG","AAAAAAAGAATCTCAGCTTTGGG",2,4,20,NA,"","",FALSE,"ARHGAP26"

"10513","AAAGAAAGAATCTCAGCTTC","chr18",439485,"-","3:T>C,14:A>T","TGG","AAAAAAAGAATCTCTGCTTCTGG",2,4,15,NA,"GeneID:81035","COLEC12",FALSE,"ARHGAP26"

"10514","AAAGAAAGAATCTCAGCTTC","chr11",110727710,"-","2:C>T,19:C>G","AGG","AAGGAAAGAATCTCAGCTTGAGG",2,3,20,NA,"","",FALSE,"ARHGAP26"

"10515","AAAGAGACAGAAAAGTATTG","chr5",142885318,"+","none","TGG","AAAGAGACAGAAAAGTATTGTGG",0,NA,NA,NA,"GeneID:23092","ARHGAP26",TRUE,"ARHGAP26"

"10516","AAAGAGACAGAAAAGTATTG","chr3",42739358,"-","13:A>T","GGG","AAAGAGACAGAAATGTATTGGGG",1,14,NA,NA,"GeneID:152206;GeneID:100874114","CCDC13;CCDC13-AS1",FALSE,"ARHGAP26"

"10517","AAAGAGACAGAAAAGTATTG","chr2",49341669,"+","1:C>A,15:A>T","TGG","ACAGAGACAGAAAAGAATTGTGG",2,2,16,NA,"","",FALSE,"ARHGAP26"

"10518","AAAGAGACAGAAAAGTATTG","chr11",124490377,"-","2:C>T,19:T>C","TGG","AAGGAGACAGAAAAGTATTATGG",2,3,20,NA,"","",FALSE,"ARHGAP26"

"10519","ACAGATGATGAGATGTGTAT","chr5",142873473,"+","none","AGG","ACAGATGATGAGATGTGTATAGG",0,NA,NA,NA,"GeneID:23092","ARHGAP26",TRUE,"ARHGAP26"

"10520","ACAGATGATGAGATGTGTAT","chr5",39972303,"-","3:G>C,6:G>C","AGG","ACACATCATGAGATGTGTATAGG",2,4,7,NA,"","",FALSE,"ARHGAP26"

"10521","ACAGATGATGAGATGTGTAT","chr3",42340711,"+","2:T>A,3:A>G","AGG","ACTAATGATGAGATGTGTATAGG",2,3,4,NA,"","",TRUE,"ARHGAP26"

"10522","CCAGATTGAGAATGCCAGCG","chr5",142879369,"+","none","AGG","CCAGATTGAGAATGCCAGCGAGG",0,NA,NA,NA,"GeneID:23092","ARHGAP26",TRUE,"ARHGAP26"

"10523","CCAGATTGAGAATGCCAGCG","chr3",113270831,"+","8:T>A,19:C>G","AGG","CCAGATTGTGAATGCCAGCCAGG",2,9,20,NA,"GeneID:91653","BOC",TRUE,"ARHGAP26"

"10524","TAGATTTGTCTTCAGCGAAG","chr5",142873396,"+","none","CGG","TAGATTTGTCTTCAGCGAAGCGG",0,NA,NA,NA,"GeneID:23092","ARHGAP26",TRUE,"ARHGAP26"

"10525","TCCCTTGGAGAAGTTTCGAA","chr5",142879402,"+","none","AGG","TCCCTTGGAGAAGTTTCGAAAGG",0,NA,NA,NA,"GeneID:23092","ARHGAP26",TRUE,"ARHGAP26"

"10526","AAGCATCCATAAATCCAGCC","chr17",45404488,"-","none","AGG","AAGCATCCATAAATCCAGCCAGG",0,NA,NA,NA,"GeneID:201176","ARHGAP27",TRUE,"ARHGAP27"

"10527","AAGCATCCATAAATCCAGCC","chr16",87493260,"-","5:T>A,9:C>A","CGG","AAGCAACCAGAAATCCAGCCCGG",2,6,10,NA,"GeneID:101928737","LOC101928737",TRUE,"ARHGAP27"

"10528","AAGCATCCATAAATCCAGCC","chr15",59778479,"+","2:C>G,6:A>C","AGG","AACCATACATAAATCCAGCCAGG",2,3,7,NA,"","",FALSE,"ARHGAP27"

"10529","ATGCAGCCGGGCCTGAGCCC","chr17",45405697,"-","none","TGG","ATGCAGCCGGGCCTGAGCCCTGG",0,NA,NA,NA,"GeneID:201176","ARHGAP27",TRUE,"ARHGAP27"

"10530","ATGCAGCCGGGCCTGAGCCC","chr12",129336785,"+","15:G>A,19:G>C","AGG","ATGCAGCCGGGCCTGGGCCGAGG",2,16,20,NA,"GeneID:121256","TMEM132D",FALSE,"ARHGAP27"

"10531","ATGCAGCCGGGCCTGAGCCC","chr12",124359101,"-","3:T>G,8:T>C","CGG","ATGAAGCCAGGCCTGAGCCCCGG",2,4,9,NA,"GeneID:9612","NCOR2",FALSE,"ARHGAP27"

"10532","CCCCTCACCCGCGGGTCCCC","chr17",45405667,"+","none","TGG","CCCCTCACCCGCGGGTCCCCTGG",0,NA,NA,NA,"GeneID:201176","ARHGAP27",TRUE,"ARHGAP27"

"10533","CCCCTCACCCGCGGGTCCCC","chr9",113688121,"+","15:A>T,16:A>C","TGG","CCCCTCACCCGCGGGAACCCTGG",2,16,17,NA,"","",FALSE,"ARHGAP27"

"10534","CTGGATTTATGGATGCTTCG","chr17",45404490,"+","none","AGG","CTGGATTTATGGATGCTTCGAGG",0,NA,NA,NA,"GeneID:201176","ARHGAP27",TRUE,"ARHGAP27"

"10535","TCAAAGACCTCGGCTGCAGG","chr17",45403625,"-","none","CGG","TCAAAGACCTCGGCTGCAGGCGG",0,NA,NA,NA,"GeneID:201176","ARHGAP27",TRUE,"ARHGAP27"

"10536","TCAAAGACCTCGGCTGCAGG","chr1",206023286,"-","11:T>C,15:G>C","AGG","TCAAAGACCTCAGCTCCAGGAGG",2,12,16,NA,"GeneID:1510","CTSE",FALSE,"ARHGAP27"

"10537","TTGAGTCCTTGAAGAATGTC","chr17",45403641,"+","none","AGG","TTGAGTCCTTGAAGAATGTCAGG",0,NA,NA,NA,"GeneID:201176","ARHGAP27",TRUE,"ARHGAP27"

"10538","AATGACAGCTCTTCAGCCTC","chr18",6870621,"-","none","TGG","AATGACAGCTCTTCAGCCTCTGG",0,NA,NA,NA,"GeneID:79822","ARHGAP28",TRUE,"ARHGAP28"

"10539","AATGTTCAGAAAACCAGATT","chr18",6873414,"+","none","TGG","AATGTTCAGAAAACCAGATTTGG",0,NA,NA,NA,"GeneID:79822","ARHGAP28",TRUE,"ARHGAP28"

"10540","AATGTTCAGAAAACCAGATT","chr5",164409400,"-","2:T>A,11:C>T","TGG","AAAGTTCAGAAGACCAGATTTGG",2,3,12,NA,"GeneID:102546299","LOC102546299",FALSE,"ARHGAP28"

"10541","CCACTTATCGCATTCTGAAC","chr18",6868215,"-","none","TGG","CCACTTATCGCATTCTGAACTGG",0,NA,NA,NA,"GeneID:79822","ARHGAP28",TRUE,"ARHGAP28"

"10542","CCAGTTCAGAATGCGATAAG","chr18",6868212,"+","none","TGG","CCAGTTCAGAATGCGATAAGTGG",0,NA,NA,NA,"GeneID:79822","ARHGAP28",TRUE,"ARHGAP28"

"10543","TGAAGTGTCTTATTCAGAAA","chr18",6870641,"+","none","TGG","TGAAGTGTCTTATTCAGAAATGG",0,NA,NA,NA,"GeneID:79822","ARHGAP28",TRUE,"ARHGAP28"

"10544","TGAAGTGTCTTATTCAGAAA","chr16",65126227,"-","10:C>A","TGG","TGAAGTGTCTGATTCAGAAATGG",1,11,NA,NA,"","",FALSE,"ARHGAP28"

"10545","TGAAGTGTCTTATTCAGAAA","chr1",108587034,"+","1:C>G,8:T>C","GGG","TCAAGTGTTTTATTCAGAAAGGG",2,2,9,NA,"GeneID:284611","FAM102B",FALSE,"ARHGAP28"

"10546","TGCTTCAGTTAAGCCAAATC","chr18",6873430,"-","none","TGG","TGCTTCAGTTAAGCCAAATCTGG",0,NA,NA,NA,"GeneID:79822","ARHGAP28",TRUE,"ARHGAP28"

"10547","TGCTTCAGTTAAGCCAAATC","chr2",88413702,"+","8:C>T,11:T>A","AGG","TGCTTCAGCTATGCCAAATCAGG",2,9,12,NA,"","",FALSE,"ARHGAP28"

"10548","AAACAAAAACGATCTCTTCC","chr1",94209302,"-","none","AGG","AAACAAAAACGATCTCTTCCAGG",0,NA,NA,NA,"GeneID:9411","ARHGAP29",TRUE,"ARHGAP29"

"10549","ACCAACTCCTTGATGTAATC","chr1",94231465,"+","none","CGG","ACCAACTCCTTGATGTAATCCGG",0,NA,NA,NA,"GeneID:9411","ARHGAP29",TRUE,"ARHGAP29"

"10550","AGGAGTTGGTGAATGATATC","chr1",94231455,"-","none","AGG","AGGAGTTGGTGAATGATATCAGG",0,NA,NA,NA,"GeneID:9411","ARHGAP29",TRUE,"ARHGAP29"

"10551","AGGAGTTGGTGAATGATATC","chr4",124905352,"-","5:C>A,9:T>A","AGG","AGGAGGTGGAGAATGATATCAGG",2,6,10,NA,"","",FALSE,"ARHGAP29"

"10552","CTCTACTTACATATTTCCAA","chr1",94209243,"+","none","AGG","CTCTACTTACATATTTCCAAAGG",0,NA,NA,NA,"GeneID:9411","ARHGAP29",TRUE,"ARHGAP29"

"10553","CTCTACTTACATATTTCCAA","chr13",39390079,"+","4:T>A,17:T>C","GGG","CTCTTCTTACATATTTCTAAGGG",2,5,18,NA,"GeneID:10186","LHFP",FALSE,"ARHGAP29"

"10554","GCCTGTTTCTCGAGAAACTA","chr1",94208833,"-","none","AGG","GCCTGTTTCTCGAGAAACTAAGG",0,NA,NA,NA,"GeneID:9411","ARHGAP29",TRUE,"ARHGAP29"

"10555","TAGCCTTACAAACTTCCTTA","chr1",94208887,"-","none","TGG","TAGCCTTACAAACTTCCTTATGG",0,NA,NA,NA,"GeneID:9411","ARHGAP29",TRUE,"ARHGAP29"

"10556","ACACACGTGGACCAGCTCTT","chr1",161053277,"-","none","TGG","ACACACGTGGACCAGCTCTTTGG",0,NA,NA,NA,"GeneID:257106","ARHGAP30",TRUE,"ARHGAP30"

"10557","AGACTAAGCGTAAACTTCCA","chr1",161052458,"-","none","CGG","AGACTAAGCGTAAACTTCCACGG",0,NA,NA,NA,"GeneID:257106","ARHGAP30",TRUE,"ARHGAP30"

"10558","AGTGGGTGGCGATCGCTTCC","chr1",161052763,"-","none","AGG","AGTGGGTGGCGATCGCTTCCAGG",0,NA,NA,NA,"GeneID:257106","ARHGAP30",TRUE,"ARHGAP30"

"10559","CTGCAATCTCGATGATAGTA","chr1",161052632,"+","none","TGG","CTGCAATCTCGATGATAGTATGG",0,NA,NA,NA,"GeneID:257106","ARHGAP30",TRUE,"ARHGAP30"

"10560","GCGGGTACAATCCATCGTCG","chr1",161053311,"-","none","TGG","GCGGGTACAATCCATCGTCGTGG",0,NA,NA,NA,"GeneID:257106","ARHGAP30",TRUE,"ARHGAP30"

"10561","TGGAGGTCTATCTTCAATTT","chr1",161052495,"-","none","AGG","TGGAGGTCTATCTTCAATTTAGG",0,NA,NA,NA,"GeneID:257106","ARHGAP30",TRUE,"ARHGAP30"

"10562","TGGAGGTCTATCTTCAATTT","chr6",112924178,"-","6:G>A,9:A>T","AGG","TGGAGGCCTTTCTTCAATTTAGG",2,7,10,NA,"","",FALSE,"ARHGAP30"

"10563","TGGAGGTCTATCTTCAATTT","chr11",8383129,"+","9:G>A,10:A>T","TGG","TGGAGGTCTGACTTCAATTTTGG",2,10,11,NA,"","",FALSE,"ARHGAP30"

"10564","CACTAACCATCCTGCTCGCA","chr3",119390878,"+","none","AGG","CACTAACCATCCTGCTCGCAAGG",0,NA,NA,NA,"GeneID:57514","ARHGAP31",TRUE,"ARHGAP31"

"10565","CCTCAAACATACAACGGCTA","chr3",119365396,"+","none","AGG","CCTCAAACATACAACGGCTAAGG",0,NA,NA,NA,"GeneID:57514","ARHGAP31",TRUE,"ARHGAP31"

"10566","GCACAGCTCTTCAAAACGTA","chr3",119365320,"-","none","TGG","GCACAGCTCTTCAAAACGTATGG",0,NA,NA,NA,"GeneID:57514","ARHGAP31",TRUE,"ARHGAP31"

"10567","TCCCTCCTTTCCTTGCGAGC","chr3",119390891,"-","none","AGG","TCCCTCCTTTCCTTGCGAGCAGG",0,NA,NA,NA,"GeneID:57514","ARHGAP31",TRUE,"ARHGAP31"

"10568","TCCCTCCTTTCCTTGCGAGC","chr1",18090763,"+","16:C>G,18:C>G","TGG","TCCCTCCTTTCCTTGCCACCTGG",2,17,19,NA,"","",FALSE,"ARHGAP31"

"10569","TGAACTCAATCACCACCTGC","chr3",119383146,"-","none","TGG","TGAACTCAATCACCACCTGCTGG",0,NA,NA,NA,"GeneID:57514","ARHGAP31",TRUE,"ARHGAP31"

"10570","TGCATCTCCATTGCAACCAG","chr3",119383103,"-","none","TGG","TGCATCTCCATTGCAACCAGTGG",0,NA,NA,NA,"GeneID:57514","ARHGAP31",TRUE,"ARHGAP31"

"10571","AACGGGGAAGTTCTGAGAGC","chr11",129066756,"+","none","TGG","AACGGGGAAGTTCTGAGAGCTGG",0,NA,NA,NA,"GeneID:9743","ARHGAP32",TRUE,"ARHGAP32"

"10572","CCTCGTGCAGATTGCTTGTC","chr11",129093622,"-","none","AGG","CCTCGTGCAGATTGCTTGTCAGG",0,NA,NA,NA,"GeneID:9743","ARHGAP32",TRUE,"ARHGAP32"

"10573","CCTGACAAGCAATCTGCACG","chr11",129093619,"+","none","AGG","CCTGACAAGCAATCTGCACGAGG",0,NA,NA,NA,"GeneID:9743","ARHGAP32",TRUE,"ARHGAP32"

"10574","CGAACTCAACATTCTCATAA","chr11",129123456,"+","none","TGG","CGAACTCAACATTCTCATAATGG",0,NA,NA,NA,"GeneID:9743","ARHGAP32",TRUE,"ARHGAP32"

"10575","TCCCCGTTCTGACACCCTGA","chr11",129066744,"-","none","AGG","TCCCCGTTCTGACACCCTGAAGG",0,NA,NA,NA,"GeneID:9743","ARHGAP32",TRUE,"ARHGAP32"

"10576","TCCCCGTTCTGACACCCTGA","chr2",113337455,"+","3:T>C,4:T>C","GGG","TCCTTGTTCTGACACCCTGAGGG",2,4,5,NA,"","",FALSE,"ARHGAP32"

"10577","TTCAGGTTACCCTTCACTAA","chr11",129123515,"-","none","AGG","TTCAGGTTACCCTTCACTAAAGG",0,NA,NA,NA,"GeneID:9743","ARHGAP32",TRUE,"ARHGAP32"

"10578","AACGAAAGTCATCGTAACTC","chr19",35778486,"-","none","CGG","AACGAAAGTCATCGTAACTCCGG",0,NA,NA,NA,"GeneID:115703","ARHGAP33",TRUE,"ARHGAP33"

"10579","CACTACGAGAACGTTGACTT","chr19",35777875,"+","none","TGG","CACTACGAGAACGTTGACTTTGG",0,NA,NA,NA,"GeneID:115703","ARHGAP33",TRUE,"ARHGAP33"

"10580","CACTACGAGAACGTTGACTT","chr9",108653683,"+","1:T>A,5:T>C","TGG","CTCTATGAGAACGTTGACTTTGG",2,2,6,NA,"","",FALSE,"ARHGAP33"

"10581","CGGGGTGCAGGTGACCTGTC","chr19",35778338,"+","none","AGG","CGGGGTGCAGGTGACCTGTCAGG",0,NA,NA,NA,"GeneID:115703","ARHGAP33",TRUE,"ARHGAP33"

"10582","CGGGGTGCAGGTGACCTGTC","chr19",47341245,"+","13:G>A,19:G>C","GGG","CGGGGTGCAGGTGGCCTGTGGGG",2,14,20,NA,"GeneID:27202","C5AR2",TRUE,"ARHGAP33"

"10583","CGGGGTGCAGGTGACCTGTC","chr16",1182848,"+","0:T>C,17:C>G","TGG","TGGGGTGCAGGTGACCTCTCTGG",2,1,18,NA,"GeneID:8912","CACNA1H",FALSE,"ARHGAP33"

"10584","CTGCTGTCTCCAGACCGTGA","chr19",35778282,"+","none","AGG","CTGCTGTCTCCAGACCGTGAAGG",0,NA,NA,NA,"GeneID:115703","ARHGAP33",TRUE,"ARHGAP33"

"10585","CTGCTGTCTCCAGACCGTGA","chr5",179409856,"+","13:C>A,16:C>G","TGG","CTGCTGTCTCCAGCCCCTGATGG",2,14,17,NA,"","",FALSE,"ARHGAP33"

"10586","TCCACCGGTGCATATTTGAC","chr19",35778521,"+","none","CGG","TCCACCGGTGCATATTTGACCGG",0,NA,NA,NA,"GeneID:115703","ARHGAP33",TRUE,"ARHGAP33"

"10587","TGGGCGCAGTCAGCCAGCCG","chr19",35777851,"-","none","CGG","TGGGCGCAGTCAGCCAGCCGCGG",0,NA,NA,NA,"GeneID:115703","ARHGAP33",TRUE,"ARHGAP33"

"10588","TGGGCGCAGTCAGCCAGCCG","chr13",99983218,"+","9:A>T,11:C>A","GGG","TGGGCGCAGACCGCCAGCCGGGG",2,10,12,NA,"GeneID:7546","ZIC2",FALSE,"ARHGAP33"

"10589","TGGGCGCAGTCAGCCAGCCG","chr1",119975354,"+","5:A>G,17:G>C","TGG","TGGGCACAGTCAGCCAGGCGTGG",2,6,18,NA,"GeneID:4853","NOTCH2",FALSE,"ARHGAP33"

"10590","ATTGAGTACATTGAAGCCAC","chr19",46937386,"+","none","AGG","ATTGAGTACATTGAAGCCACAGG",0,NA,NA,NA,"GeneID:2909","ARHGAP35",TRUE,"ARHGAP35"

"10591","CAGACTTGTTCCCGCTGACC","chr19",46988013,"-","none","CGG","CAGACTTGTTCCCGCTGACCCGG",0,NA,NA,NA,"GeneID:2909","ARHGAP35",TRUE,"ARHGAP35"

"10592","GTATAACATGCAGATCGACT","chr19",46989640,"+","none","TGG","GTATAACATGCAGATCGACTTGG",0,NA,NA,NA,"GeneID:2909","ARHGAP35",TRUE,"ARHGAP35"

"10593","TCTCCTCAGAAATCAACGAC","chr19",46999294,"+","none","CGG","TCTCCTCAGAAATCAACGACCGG",0,NA,NA,NA,"GeneID:2909","ARHGAP35",TRUE,"ARHGAP35"

"10594","TGACATACTTGAAGACTTCG","chr19",46999373,"-","none","TGG","TGACATACTTGAAGACTTCGTGG",0,NA,NA,NA,"GeneID:2909","ARHGAP35",TRUE,"ARHGAP35"

"10595","TGGAGTCACGACAGTTGTTA","chr19",46937333,"-","none","AGG","TGGAGTCACGACAGTTGTTAAGG",0,NA,NA,NA,"GeneID:2909","ARHGAP35",TRUE,"ARHGAP35"

"10596","CCCAGAAGTCGTTGGCCAAT","chrX",131084301,"-","none","TGG","CCCAGAAGTCGTTGGCCAATTGG",0,NA,NA,NA,"GeneID:158763","ARHGAP36",TRUE,"ARHGAP36"

"10597","CTCCGTGCAGCGAGTGCGTC","chrX",131084659,"+","none","AGG","CTCCGTGCAGCGAGTGCGTCAGG",0,NA,NA,NA,"GeneID:158763","ARHGAP36",TRUE,"ARHGAP36"

"10598","GATTGCGAAACAAATCCCCC","chrX",131084348,"+","none","AGG","GATTGCGAAACAAATCCCCCAGG",0,NA,NA,NA,"GeneID:158763","ARHGAP36",TRUE,"ARHGAP36"

"10599","GCCTGCGACTGAAAAAGCGC","chrX",131083902,"-","none","CGG","GCCTGCGACTGAAAAAGCGCCGG",0,NA,NA,NA,"GeneID:158763","ARHGAP36",TRUE,"ARHGAP36"

"10600","TACCTGACGCACTCGCTGCA","chrX",131084664,"-","none","CGG","TACCTGACGCACTCGCTGCACGG",0,NA,NA,NA,"GeneID:158763","ARHGAP36",TRUE,"ARHGAP36"

"10601","TTTAACTCACTCGACGCCCA","chrX",131083959,"-","none","CGG","TTTAACTCACTCGACGCCCACGG",0,NA,NA,NA,"GeneID:158763","ARHGAP36",TRUE,"ARHGAP36"

"10602","AAATCTCATAGTCCTTCTCA","chr8",144555599,"+","none","AGG","AAATCTCATAGTCCTTCTCAAGG",0,NA,NA,NA,"GeneID:80728","ARHGAP39",TRUE,"ARHGAP39"

"10603","ACGGCCAGCTTCTTCACTAC","chr8",144555561,"-","none","AGG","ACGGCCAGCTTCTTCACTACAGG",0,NA,NA,NA,"GeneID:80728","ARHGAP39",TRUE,"ARHGAP39"

"10604","ACGGCCAGCTTCTTCACTAC","chr3",168071470,"-","2:T>C,18:G>T","AGG","ACAGCCAGCTTCTTCACTCCAGG",2,3,19,NA,"GeneID:27333","GOLIM4",FALSE,"ARHGAP39"

"10605","CAAGGAGTACGAACCACCGC","chr8",144547798,"+","none","CGG","CAAGGAGTACGAACCACCGCCGG",0,NA,NA,NA,"GeneID:80728","ARHGAP39",TRUE,"ARHGAP39"

"10606","GGGTTCATAGCCATAGCGCG","chr8",144548155,"+","none","GGG","GGGTTCATAGCCATAGCGCGGGG",0,NA,NA,NA,"GeneID:80728","ARHGAP39",TRUE,"ARHGAP39"

"10607","GGTTGGCGTACATGCGCTCG","chr8",144581221,"+","none","CGG","GGTTGGCGTACATGCGCTCGCGG",0,NA,NA,NA,"GeneID:80728","ARHGAP39",TRUE,"ARHGAP39"

"10608","TCTCGCTGGTGCGCTTGATG","chr8",144581158,"+","none","CGG","TCTCGCTGGTGCGCTTGATGCGG",0,NA,NA,NA,"GeneID:80728","ARHGAP39",TRUE,"ARHGAP39"

"10609","AAGCCAGCAGCTCGCCGAAC","chrX",153910705,"+","none","AGG","AAGCCAGCAGCTCGCCGAACAGG",0,NA,NA,NA,"GeneID:393","ARHGAP4",TRUE,"ARHGAP4"

"10610","ACATCACCTACCGGAAGCTT","chrX",153921593,"+","none","TGG","ACATCACCTACCGGAAGCTTTGG",0,NA,NA,NA,"GeneID:393","ARHGAP4",TRUE,"ARHGAP4"

"10611","AGCACGTGAGCCGCCTGCTG","chrX",153910564,"-","none","TGG","AGCACGTGAGCCGCCTGCTGTGG",0,NA,NA,NA,"GeneID:393","ARHGAP4",TRUE,"ARHGAP4"

"10612","GCCGAGCGCTTCTCCAGCCG","chrX",153921646,"-","none","TGG","GCCGAGCGCTTCTCCAGCCGTGG",0,NA,NA,NA,"GeneID:393","ARHGAP4",TRUE,"ARHGAP4"

"10613","GCCGAGCGCTTCTCCAGCCG","chr9",136200617,"-","10:C>A,13:A>G","AGG","GCCGAGCGCTGCTTCAGCCGAGG",2,11,14,NA,"GeneID:8022","LHX3",TRUE,"ARHGAP4"

"10614","GGGTGCTGAAGCTCTACTTC","chrX",153910760,"-","none","CGG","GGGTGCTGAAGCTCTACTTCCGG",0,NA,NA,NA,"GeneID:393","ARHGAP4",TRUE,"ARHGAP4"

"10615","GTGCTCCACCCTCTCCGCTG","chrX",153910579,"+","none","TGG","GTGCTCCACCCTCTCCGCTGTGG",0,NA,NA,NA,"GeneID:393","ARHGAP4",TRUE,"ARHGAP4"

"10616","GTGCTCCACCCTCTCCGCTG","chr2",5666388,"+","11:A>T,16:T>G","AGG","GTGCTCCACCCACTCCTCTGAGG",2,12,17,NA,"","",FALSE,"ARHGAP4"

"10617","AAGCTGATCCATTCGGCCTG","chr20",38623372,"-","none","AGG","AAGCTGATCCATTCGGCCTGAGG",0,NA,NA,NA,"GeneID:343578","ARHGAP40",TRUE,"ARHGAP40"

"10618","ATGCGGGCCCGCGGGATCCG","chr20",38602017,"-","none","AGG","ATGCGGGCCCGCGGGATCCGAGG",0,NA,NA,NA,"GeneID:343578","ARHGAP40",TRUE,"ARHGAP40"

"10619","GGCCGCTGTGTGCCGCCGGC","chr20",38627109,"+","none","TGG","GGCCGCTGTGTGCCGCCGGCTGG",0,NA,NA,NA,"GeneID:343578","ARHGAP40",TRUE,"ARHGAP40"

"10620","GGTGATCACCATCCAAGCCC","chr20",38627055,"-","none","AGG","GGTGATCACCATCCAAGCCCAGG",0,NA,NA,NA,"GeneID:343578","ARHGAP40",TRUE,"ARHGAP40"

"10621","GTAGTCGCAGGAGATTCTTC","chr20",38623397,"-","none","TGG","GTAGTCGCAGGAGATTCTTCTGG",0,NA,NA,NA,"GeneID:343578","ARHGAP40",TRUE,"ARHGAP40"

"10622","GTAGTCGCAGGAGATTCTTC","chr1",175642385,"+","1:G>T,6:A>G","TGG","GGAGTCACAGGAGATTCTTCTGG",2,2,7,NA,"GeneID:7143","TNR",FALSE,"ARHGAP40"

"10623","TCCCGCGGGCCCGCATTGCC","chr20",38602021,"+","none","AGG","TCCCGCGGGCCCGCATTGCCAGG",0,NA,NA,NA,"GeneID:343578","ARHGAP40",TRUE,"ARHGAP40"

"10624","GAAGCTCACCGCATGTCTGC","chr17",12896456,"+","none","AGG","GAAGCTCACCGCATGTCTGCAGG",0,NA,NA,NA,"GeneID:9912","ARHGAP44",TRUE,"ARHGAP44"

"10625","GTTCAATCGCATGCGCCAGC","chr17",12789849,"+","none","TGG","GTTCAATCGCATGCGCCAGCTGG",0,NA,NA,NA,"GeneID:100128006;GeneID:9912","LOC100128006;ARHGAP44",TRUE,"ARHGAP44"

"10626","TCTGCAGGTGGAGAAGCGTC","chr17",12896399,"+","none","TGG","TCTGCAGGTGGAGAAGCGTCTGG",0,NA,NA,NA,"GeneID:9912","ARHGAP44",TRUE,"ARHGAP44"

"10627","TCTGCAGGTGGAGAAGCGTC","chr15",65356040,"-","16:C>G,17:G>C","GGG","TCTGCAGGTGGAGAAGGCTCGGG",2,17,18,NA,"GeneID:9543","IGDCC3",FALSE,"ARHGAP44"

"10628","TGACCTACCTGCCCACCGTC","chr17",12789879,"-","none","TGG","TGACCTACCTGCCCACCGTCTGG",0,NA,NA,NA,"GeneID:100128006;GeneID:9912","LOC100128006;ARHGAP44",TRUE,"ARHGAP44"

"10629","TGAGCTTCTTGTGCGTGCTG","chr17",12896444,"-","none","TGG","TGAGCTTCTTGTGCGTGCTGTGG",0,NA,NA,NA,"GeneID:9912","ARHGAP44",TRUE,"ARHGAP44"

"10630","TGAGCTTCTTGTGCGTGCTG","chr12",12902652,"+","1:C>G,13:G>C","AGG","TCAGCTTCTTGTGGGTGCTGAGG",2,2,14,NA,"GeneID:9052","GPRC5A",FALSE,"ARHGAP44"

"10631","TTTGAGTGAAGACCTTCTTC","chr17",12894957,"+","none","AGG","TTTGAGTGAAGACCTTCTTCAGG",0,NA,NA,NA,"GeneID:9912","ARHGAP44",TRUE,"ARHGAP44"

"10632","ACGTGATAACACATCTAAAC","chr14",32152506,"+","none","AGG","ACGTGATAACACATCTAAACAGG",0,NA,NA,NA,"GeneID:394","ARHGAP5",TRUE,"ARHGAP5"

"10633","ACGTGATAACACATCTAAAC","chr7",54937833,"+","2:A>G,16:G>A","AGG","ACATGATAACACATCTGAACAGG",2,3,17,NA,"","",TRUE,"ARHGAP5"

"10634","ATCCACCAACACGTAGAAAT","chr14",32117164,"+","none","TGG","ATCCACCAACACGTAGAAATTGG",0,NA,NA,NA,"GeneID:394","ARHGAP5",TRUE,"ARHGAP5"

"10635","ATCCACCAACACGTAGAAAT","chr7",54937416,"+","11:T>C","TGG","ATCCACCAACATGTAGAAATTGG",1,12,NA,NA,"","",TRUE,"ARHGAP5"

"10636","ATCCACCAACACGTAGAAAT","chr8",4098632,"-","1:T>A,9:A>G","GGG","AACCACCAATACGTAGAAATGGG",2,2,10,NA,"GeneID:64478","CSMD1",FALSE,"ARHGAP5"

"10637","ATCCACCAACACGTAGAAAT","chr6",168039687,"+","6:T>C,13:G>T","GGG","ATCCACTAACACGGAGAAATGGG",2,7,14,NA,"GeneID:3834","KIF25",FALSE,"ARHGAP5"

"10638","ATTCAAAAGCAGTTTGATCA","chr14",32146318,"+","none","AGG","ATTCAAAAGCAGTTTGATCAAGG",0,NA,NA,NA,"GeneID:394","ARHGAP5",TRUE,"ARHGAP5"

"10639","ATTCAAAAGCAGTTTGATCA","chr7",90653575,"-","7:A>T,9:C>G","AGG","ATTCAAATGGAGTTTGATCAAGG",2,8,10,NA,"GeneID:5218","CDK14",FALSE,"ARHGAP5"

"10640","CTCTTAGGGTTATGTACCGA","chr14",32146255,"+","none","AGG","CTCTTAGGGTTATGTACCGAAGG",0,NA,NA,NA,"GeneID:394","ARHGAP5",TRUE,"ARHGAP5"

"10641","CTTGCTGCTTCCAATAGTTC","chr14",32150014,"-","none","TGG","CTTGCTGCTTCCAATAGTTCTGG",0,NA,NA,NA,"GeneID:394","ARHGAP5",TRUE,"ARHGAP5"

"10642","CTTTCCCAATTTCTACGTGT","chr14",32117172,"-","none","TGG","CTTTCCCAATTTCTACGTGTTGG",0,NA,NA,NA,"GeneID:394","ARHGAP5",TRUE,"ARHGAP5"

"10643","CTTTCCCAATTTCTACGTGT","chr7",54937424,"-","16:T>C","TGG","CTTTCCCAATTTCTACATGTTGG",1,17,NA,NA,"","",TRUE,"ARHGAP5"

"10644","AAGGAGAAAAACAAAGACAA","chrX",11196926,"-","none","AGG","AAGGAGAAAAACAAAGACAAAGG",0,NA,NA,NA,"GeneID:395","ARHGAP6",TRUE,"ARHGAP6"

"10645","AAGGAGAAAAACAAAGACAA","chr13",27313760,"-","16:G>T,19:C>T","AGG","AAGGAGAAAAACAAAGCCAGAGG",2,17,20,NA,"","",FALSE,"ARHGAP6"

"10646","AAGGAGAAAAACAAAGACAA","chr3",33177031,"-","16:A>T","AGG","AAGGAGAAAAACAAAGTCAAAGG",1,17,NA,NA,"GeneID:26032","SUSD5",FALSE,"ARHGAP6"

"10647","AAGGAGAAAAACAAAGACAA","chr18",42590190,"-","16:A>T,17:C>G","TGG","AAGGAGAAAAACAAAGTGAATGG",2,17,18,NA,"GeneID:284260","LINC00907",FALSE,"ARHGAP6"

"10648","AAGGAGAAAAACAAAGACAA","chr4",153471286,"-","12:C>T,19:A>T","GGG","AAGGAGAAAAACGAAGACATGGG",2,13,20,NA,"GeneID:23240","KIAA0922",FALSE,"ARHGAP6"

"10649","AAGGAGAAAAACAAAGACAA","chr6",136552703,"-","12:G>T,16:G>T","TGG","AAGGAGAAAAACCAAGCCAATGG",2,13,17,NA,"","",FALSE,"ARHGAP6"

"10650","AAGGAGAAAAACAAAGACAA","chr11",80812043,"-","11:T>G,17:C>G","AGG","AAGGAGAAAAAAAAAGAGAAAGG",2,12,18,NA,"","",FALSE,"ARHGAP6"

"10651","AAGGAGAAAAACAAAGACAA","chrX",54542931,"-","11:T>G,17:C>G","TGG","AAGGAGAAAAAAAAAGAGAATGG",2,12,18,NA,"GeneID:54552","GNL3L",FALSE,"ARHGAP6"

"10652","AAGGAGAAAAACAAAGACAA","chr4",177530,"-","11:C>G,12:C>T","AGG","AAGGAGAAAAAGGAAGACAAAGG",2,12,13,NA,"GeneID:255403","ZNF718",FALSE,"ARHGAP6"

"10653","AAGGAGAAAAACAAAGACAA","chr3",178069585,"-","11:A>G,16:G>T","GGG","AAGGAGAAAAATAAAGCCAAGGG",2,12,17,NA,"","",FALSE,"ARHGAP6"

"10654","AAGGAGAAAAACAAAGACAA","chr7",8269973,"+","17:T>C,18:G>A","GGG","AAGGAGAAAAACAAAGATGAGGG",2,18,19,NA,"","",FALSE,"ARHGAP6"

"10655","AAGGAGAAAAACAAAGACAA","chr15",47148020,"+","16:T>A,19:G>A","AGG","AAGGAGAAAAACAAAGTCAGAGG",2,17,20,NA,"","",FALSE,"ARHGAP6"

"10656","AAGGAGAAAAACAAAGACAA","chr15",26810340,"+","16:T>A,19:G>A","AGG","AAGGAGAAAAACAAAGTCAGAGG",2,17,20,NA,"GeneID:2562","GABRB3",FALSE,"ARHGAP6"

"10657","AAGGAGAAAAACAAAGACAA","chr9",31152361,"+","16:T>A,19:G>A","AGG","AAGGAGAAAAACAAAGTCAGAGG",2,17,20,NA,"","",FALSE,"ARHGAP6"

"10658","AAGGAGAAAAACAAAGACAA","chr4",57818622,"+","13:G>A,16:T>A","AGG","AAGGAGAAAAACAGAGTCAAAGG",2,14,17,NA,"","",FALSE,"ARHGAP6"

"10659","AAGGAGAAAAACAAAGACAA","chr6",113466196,"+","11:A>C,16:T>A","AGG","AAGGAGAAAAAAAAAGTCAAAGG",2,12,17,NA,"","",FALSE,"ARHGAP6"

"10660","AAGGAGAAAAACAAAGACAA","chr1",198531968,"+","11:A>C,16:G>A","GGG","AAGGAGAAAAAAAAAGGCAAGGG",2,12,17,NA,"GeneID:127124","ATP6V1G3",FALSE,"ARHGAP6"

"10661","AAGGAGAAAAACAAAGACAA","chr3",124259794,"+","11:T>C,17:G>C","GGG","AAGGAGAAAAATAAAGAGAAGGG",2,12,18,NA,"GeneID:8997","KALRN",FALSE,"ARHGAP6"

"10662","AAGGAGAAAAACAAAGACAA","chr16",8828969,"-","2:T>C,3:T>C","AGG","AAAAAGAAAAACAAAGACAAAGG",2,3,4,NA,"GeneID:5373","PMM2",FALSE,"ARHGAP6"

"10663","AAGGAGAAAAACAAAGACAA","chr5",56337955,"-","7:C>T","AGG","AAGGAGAGAAACAAAGACAAAGG",1,8,NA,NA,"","",FALSE,"ARHGAP6"

"10664","AAGGAGAAAAACAAAGACAA","chr2",137338827,"+","1:T>A,9:G>A","AGG","ATGGAGAAAGACAAAGACAAAGG",2,2,10,NA,"GeneID:80731","THSD7B",FALSE,"ARHGAP6"

"10665","AAGGAGAAAAACAAAGACAA","chr4",153148668,"+","3:A>G,9:G>A","AGG","AAGAAGAAAGACAAAGACAAAGG",2,4,10,NA,"","",FALSE,"ARHGAP6"

"10666","AAGGAGAAAAACAAAGACAA","chr12",30506385,"+","3:A>G,11:A>C","AGG","AAGAAGAAAAAAAAAGACAAAGG",2,4,12,NA,"","",FALSE,"ARHGAP6"

"10667","AAGGAGAAAAACAAAGACAA","chr16",7775397,"+","2:A>G,11:G>C","AGG","AAAGAGAAAAAGAAAGACAAAGG",2,3,12,NA,"","",FALSE,"ARHGAP6"

"10668","AAGGAGAAAAACAAAGACAA","chr10",22550780,"+","2:T>G,14:T>A","AGG","AATGAGAAAAACAATGACAAAGG",2,3,15,NA,"GeneID:5305","PIP4K2A",TRUE,"ARHGAP6"

"10669","AAGGAGAAAAACAAAGACAA","chr3",157640107,"+","3:A>G,15:C>G","TGG","AAGAAGAAAAACAAACACAATGG",2,4,16,NA,"GeneID:152078","C3orf55",FALSE,"ARHGAP6"

"10670","AAGGAGAAAAACAAAGACAA","chr5",158347172,"+","0:T>A,15:A>G","TGG","TAGGAGAAAAACAAAAACAATGG",2,1,16,NA,"GeneID:101927697","LOC101927697",FALSE,"ARHGAP6"

"10671","AAGGAGAAAAACAAAGACAA","chr6",111990542,"+","1:T>A,15:A>G","GGG","ATGGAGAAAAACAAAAACAAGGG",2,2,16,NA,"","",FALSE,"ARHGAP6"

"10672","AAGGAGAAAAACAAAGACAA","chr2",188770005,"+","6:G>A,17:T>C","AGG","AAGGAGGAAAACAAAGATAAAGG",2,7,18,NA,"GeneID:116093","DIRC1",FALSE,"ARHGAP6"

"10673","AAGGAGAAAAACAAAGACAA","chr22",46669532,"-","9:C>T,17:A>G","AGG","AAGGAGAAAGACAAAGATAAAGG",2,10,18,NA,"GeneID:23151","GRAMD4",FALSE,"ARHGAP6"

"10674","AAGGAGAAAAACAAAGACAA","chr11",10389855,"-","8:C>T,17:T>G","TGG","AAGGAGAAGAACAAAGAAAATGG",2,9,18,NA,"GeneID:272","AMPD3",FALSE,"ARHGAP6"

"10675","AAGGAGAAAAACAAAGACAA","chr19",32553956,"-","5:T>C,15:A>C","AGG","AAGGAAAAAAACAAATACAAAGG",2,6,16,NA,"","",FALSE,"ARHGAP6"

"10676","AAGGAGAAAAACAAAGACAA","chr22",33102158,"-","5:T>C,11:T>G","AGG","AAGGAAAAAAAAAAAGACAAAGG",2,6,12,NA,"","",FALSE,"ARHGAP6"

"10677","AAGGAGAAAAACAAAGACAA","chr4",128478828,"-","0:G>T,19:C>T","AGG","CAGGAGAAAAACAAAGACAGAGG",2,1,20,NA,"GeneID:100507487","LOC100507487",FALSE,"ARHGAP6"

"10678","CTCGCCCGCTTCAAGTAGCG","chrX",11664767,"-","none","CGG","CTCGCCCGCTTCAAGTAGCGCGG",0,NA,NA,NA,"GeneID:395","ARHGAP6",TRUE,"ARHGAP6"

"10679","CTCGGATTTCCACACGACGT","chrX",11664240,"+","none","AGG","CTCGGATTTCCACACGACGTAGG",0,NA,NA,NA,"GeneID:395","ARHGAP6",TRUE,"ARHGAP6"

"10680","CTGACCTCAGCCGTACACTG","chrX",11254655,"+","none","CGG","CTGACCTCAGCCGTACACTGCGG",0,NA,NA,NA,"GeneID:395","ARHGAP6",TRUE,"ARHGAP6"

"10681","GAAATCTTTAAGAAAGAAAC","chrX",11196960,"-","none","TGG","GAAATCTTTAAGAAAGAAACTGG",0,NA,NA,NA,"GeneID:395","ARHGAP6",TRUE,"ARHGAP6"

"10682","GAAATCTTTAAGAAAGAAAC","chr10",119212236,"-","16:G>T,19:T>G","AGG","GAAATCTTTAAGAAAGCAAAAGG",2,17,20,NA,"GeneID:2869","GRK5",FALSE,"ARHGAP6"

"10683","GAAATCTTTAAGAAAGAAAC","chrX",146997269,"+","11:T>G,15:T>G","TGG","GAAATCTTTAATAAATAAACTGG",2,12,16,NA,"","",FALSE,"ARHGAP6"

"10684","GAAATCTTTAAGAAAGAAAC","chr1",56552716,"+","2:C>A,4:A>T","AGG","GACAACTTTAAGAAAGAAACAGG",2,3,5,NA,"GeneID:8613","PPAP2B",FALSE,"ARHGAP6"

"10685","GAAATCTTTAAGAAAGAAAC","chr12",32708429,"-","4:C>A,19:T>G","GGG","GAAAGCTTTAAGAAAGAAAAGGG",2,5,20,NA,"GeneID:10059","DNM1L",FALSE,"ARHGAP6"

"10686","GCATGTCAGGCCGCAGTGTA","chrX",11254668,"-","none","CGG","GCATGTCAGGCCGCAGTGTACGG",0,NA,NA,NA,"GeneID:395","ARHGAP6",TRUE,"ARHGAP6"

"10687","AGACCGTCCGCGAGATCCAG","chr22",44848014,"+","none","AGG","AGACCGTCCGCGAGATCCAGAGG",0,NA,NA,NA,"GeneID:553158;GeneID:23779","PRR5-ARHGAP8;ARHGAP8",TRUE,"ARHGAP8"

"10688","AGGACGACGTAGTTGTGCTC","chr22",44859786,"-","none","TGG","AGGACGACGTAGTTGTGCTCTGG",0,NA,NA,NA,"GeneID:553158;GeneID:23779","PRR5-ARHGAP8;ARHGAP8",TRUE,"ARHGAP8"

"10689","CCCGTAGTCGTCAAAGTTCA","chr22",44848940,"-","none","CGG","CCCGTAGTCGTCAAAGTTCACGG",0,NA,NA,NA,"GeneID:553158;GeneID:23779","PRR5-ARHGAP8;ARHGAP8",TRUE,"ARHGAP8"

"10690","CCCGTGAACTTTGACGACTA","chr22",44848936,"+","none","CGG","CCCGTGAACTTTGACGACTACGG",0,NA,NA,NA,"GeneID:553158;GeneID:23779","PRR5-ARHGAP8;ARHGAP8",TRUE,"ARHGAP8"

"10691","CTCGCGGACGGTCTGCACGC","chr22",44848008,"-","none","TGG","CTCGCGGACGGTCTGCACGCTGG",0,NA,NA,NA,"GeneID:553158;GeneID:23779","PRR5-ARHGAP8;ARHGAP8",TRUE,"ARHGAP8"

"10692","GGCGGCAGCCAGTGACACGC","chr22",44859746,"-","none","AGG","GGCGGCAGCCAGTGACACGCAGG",0,NA,NA,NA,"GeneID:553158;GeneID:23779","PRR5-ARHGAP8;ARHGAP8",TRUE,"ARHGAP8"

"10693","GGCGGCAGCCAGTGACACGC","chr22",44865859,"+","9:G>C,16:G>A","AGG","GGCGGCAGCGAGTGACGCGCAGG",2,10,17,NA,"","",FALSE,"ARHGAP8"

"10694","AAGCGGAAGATCCCTCTCCC","chr12",57478574,"-","none","AGG","AAGCGGAAGATCCCTCTCCCAGG",0,NA,NA,NA,"GeneID:64333;GeneID:4141","ARHGAP9;MARS",TRUE,"ARHGAP9"

"10695","CTTCTGACGGCAAGTCTTCC","chr12",57478553,"+","none","TGG","CTTCTGACGGCAAGTCTTCCTGG",0,NA,NA,NA,"GeneID:64333;GeneID:4141","ARHGAP9;MARS",TRUE,"ARHGAP9"

"10696","GACAGCGGCGAAGGTCCACC","chr12",57477613,"+","none","AGG","GACAGCGGCGAAGGTCCACCAGG",0,NA,NA,NA,"GeneID:64333;GeneID:4141","ARHGAP9;MARS",TRUE,"ARHGAP9"

"10697","TCACCGTCTCGCTGCGACTG","chr12",57477454,"+","none","CGG","TCACCGTCTCGCTGCGACTGCGG",0,NA,NA,NA,"GeneID:64333;GeneID:4141","ARHGAP9;MARS",TRUE,"ARHGAP9"

"10698","TGGCTACAATGCTATCCAGC","chr12",57479394,"-","none","CGG","TGGCTACAATGCTATCCAGCCGG",0,NA,NA,NA,"GeneID:64333;GeneID:4141","ARHGAP9;MARS",TRUE,"ARHGAP9"

"10699","TTCGAAAGACCAACTCCGAC","chr12",57479226,"-","none","TGG","TTCGAAAGACCAACTCCGACTGG",0,NA,NA,NA,"GeneID:64333;GeneID:4141","ARHGAP9;MARS",TRUE,"ARHGAP9"

"10700","ACAAGACCGACCACCTGTCC","chr17",81868917,"-","none","TGG","ACAAGACCGACCACCTGTCCTGG",0,NA,NA,NA,"GeneID:396","ARHGDIA",TRUE,"ARHGDIA"

"10701","AGTACATCCAGCATACGTAC","chr17",81869188,"-","none","AGG","AGTACATCCAGCATACGTACAGG",0,NA,NA,NA,"GeneID:396","ARHGDIA",TRUE,"ARHGDIA"

"10702","CAGGCCAGTCACCACGACGT","chr17",81869590,"+","none","TGG","CAGGCCAGTCACCACGACGTTGG",0,NA,NA,NA,"GeneID:396","ARHGDIA",TRUE,"ARHGDIA"

"10703","CCCCAACGTCCCCAACGTCG","chr17",81869604,"-","none","TGG","CCCCAACGTCCCCAACGTCGTGG",0,NA,NA,NA,"GeneID:396","ARHGDIA",TRUE,"ARHGDIA"

"10704","GAAGCAGTCGTTTGTGCTGA","chr17",81869367,"-","none","AGG","GAAGCAGTCGTTTGTGCTGAAGG",0,NA,NA,NA,"GeneID:396","ARHGDIA",TRUE,"ARHGDIA"

"10705","GAAGCAGTCGTTTGTGCTGA","chr15",65055723,"+","14:G>T,15:A>G","AGG","GAAGCAGTCGTTTGGACTGAAGG",2,15,16,NA,"GeneID:51285","RASL12",FALSE,"ARHGDIA"

"10706","GTTAACCGAGAGATAGTGTC","chr17",81869216,"-","none","CGG","GTTAACCGAGAGATAGTGTCCGG",0,NA,NA,NA,"GeneID:396","ARHGDIA",TRUE,"ARHGDIA"

"10707","AATACGTTCAGCACACCTAC","chr12",14944791,"-","none","AGG","AATACGTTCAGCACACCTACAGG",0,NA,NA,NA,"GeneID:397","ARHGDIB",TRUE,"ARHGDIB"

"10708","CAGCTATGGACCTCGGCCTG","chr12",14942676,"-","none","AGG","CAGCTATGGACCTCGGCCTGAGG",0,NA,NA,NA,"GeneID:397","ARHGDIB",TRUE,"ARHGDIB"

"10709","CCGGGGCACTCTCACAAACC","chr12",14949825,"+","none","AGG","CCGGGGCACTCTCACAAACCAGG",0,NA,NA,NA,"GeneID:397","ARHGDIB",TRUE,"ARHGDIB"

"10710","GAGCCGGGTGACAACGACAT","chr12",14949850,"+","none","TGG","GAGCCGGGTGACAACGACATTGG",0,NA,NA,NA,"GeneID:397","ARHGDIB",TRUE,"ARHGDIB"

"10711","GTGAACAGGGATATTGTGTC","chr12",14944819,"-","none","AGG","GTGAACAGGGATATTGTGTCAGG",0,NA,NA,NA,"GeneID:397","ARHGDIB",TRUE,"ARHGDIB"

"10712","TCCACTCCTTCTTAATCGAC","chr12",14942529,"+","none","AGG","TCCACTCCTTCTTAATCGACAGG",0,NA,NA,NA,"GeneID:397","ARHGDIB",TRUE,"ARHGDIB"

"10713","ATGAGGCTGTGCCCGAGTAC","chr16",281796,"+","none","CGG","ATGAGGCTGTGCCCGAGTACCGG",0,NA,NA,NA,"GeneID:398","ARHGDIG",TRUE,"ARHGDIG"

"10714","CAAGTACAAGCGGGTGCTGC","chr16",281881,"+","none","TGG","CAAGTACAAGCGGGTGCTGCTGG",0,NA,NA,NA,"GeneID:398","ARHGDIG",TRUE,"ARHGDIG"

"10715","GCCCCGGAGCCTGTTCCGAC","chr16",282067,"-","none","AGG","GCCCCGGAGCCTGTTCCGACAGG",0,NA,NA,NA,"GeneID:398","ARHGDIG",TRUE,"ARHGDIG"

"10716","GGCTCCGGGGCCCGTCGTCA","chr16",282076,"+","none","TGG","GGCTCCGGGGCCCGTCGTCATGG",0,NA,NA,NA,"GeneID:398","ARHGDIG",TRUE,"ARHGDIG"

"10717","GTCCACAGGGAGATTGTCAG","chr16",282466,"+","none","CGG","GTCCACAGGGAGATTGTCAGCGG",0,NA,NA,NA,"GeneID:398","ARHGDIG",TRUE,"ARHGDIG"

"10718","GTCTGCACCACACCTACCGC","chr16",282497,"+","none","CGG","GTCTGCACCACACCTACCGCCGG",0,NA,NA,NA,"GeneID:398","ARHGDIG",TRUE,"ARHGDIG"

"10719","CTCAGCCCCGATGATGCTGA","chr19",41888228,"-","none","CGG","CTCAGCCCCGATGATGCTGACGG",0,NA,NA,NA,"GeneID:9138","ARHGEF1",TRUE,"ARHGEF1"

"10720","CTGGTTCCCGTCAGCATCAT","chr19",41888218,"+","none","CGG","CTGGTTCCCGTCAGCATCATCGG",0,NA,NA,NA,"GeneID:9138","ARHGEF1",TRUE,"ARHGEF1"

"10721","CTGTCTGCATGCCGACATGC","chr19",41892029,"+","none","TGG","CTGTCTGCATGCCGACATGCTGG",0,NA,NA,NA,"GeneID:9138","ARHGEF1",TRUE,"ARHGEF1"

"10722","GAGATGGAAGACTTCGCCCG","chr19",41888079,"+","none","AGG","GAGATGGAAGACTTCGCCCGAGG",0,NA,NA,NA,"GeneID:9138","ARHGEF1",TRUE,"ARHGEF1"

"10723","GCGGCGCCCAGCCCACCTCA","chr19",41888798,"+","none","TGG","GCGGCGCCCAGCCCACCTCATGG",0,NA,NA,NA,"GeneID:9138","ARHGEF1",TRUE,"ARHGEF1"

"10724","GCGGCGCCCAGCCCACCTCA","chr12",57177482,"-","14:G>T,15:T>G","TGG","GCGGCGCCCAGCCCCACTCATGG",2,15,16,NA,"GeneID:4035","LRP1",TRUE,"ARHGEF1"

"10725","GCGGCGCCCAGCCCACCTCA","chr17",36457852,"-","5:G>C,10:A>C","GGG","GCGGCCCCCATCCCACCTCAGGG",2,6,11,NA,"","",FALSE,"ARHGEF1"

"10726","GTGGCCCTGCAGTTTGAGCC","chr19",41888835,"+","none","AGG","GTGGCCCTGCAGTTTGAGCCAGG",0,NA,NA,NA,"GeneID:9138","ARHGEF1",TRUE,"ARHGEF1"

"10727","GTGGCCCTGCAGTTTGAGCC","chr16",29176074,"-","15:G>C,17:G>C","TGG","GTGGCCCTGCAGTTTCACCCTGG",2,16,18,NA,"","",FALSE,"ARHGEF1"

"10728","GTGGCCCTGCAGTTTGAGCC","chr16",88142142,"-","15:G>C,17:G>C","TGG","GTGGCCCTGCAGTTTCACCCTGG",2,16,18,NA,"","",FALSE,"ARHGEF1"

"10729","GTGGCCCTGCAGTTTGAGCC","chr2",5494020,"+","2:T>G,16:G>A","TGG","GTTGCCCTGCAGTTTGGGCCTGG",2,3,17,NA,"","",FALSE,"ARHGEF1"

"10730","AATCCCAGAAGCGGACAGAC","chr8",1858035,"+","none","AGG","AATCCCAGAAGCGGACAGACAGG",0,NA,NA,NA,"GeneID:9639","ARHGEF10",TRUE,"ARHGEF10"

"10731","AGGACAGCGCACTTGCCCGC","chr8",1866545,"+","none","TGG","AGGACAGCGCACTTGCCCGCTGG",0,NA,NA,NA,"GeneID:9639","ARHGEF10",TRUE,"ARHGEF10"

"10732","CAATTTACGATGACGTTCCA","chr8",1869205,"+","none","AGG","CAATTTACGATGACGTTCCAAGG",0,NA,NA,NA,"GeneID:9639","ARHGEF10",TRUE,"ARHGEF10"

"10733","CAGACCCGGCCAACACAGCC","chr8",1866572,"+","none","TGG","CAGACCCGGCCAACACAGCCTGG",0,NA,NA,NA,"GeneID:9639","ARHGEF10",TRUE,"ARHGEF10"

"10734","GGGGCCTGTCTGTCCGCTTC","chr8",1858042,"-","none","TGG","GGGGCCTGTCTGTCCGCTTCTGG",0,NA,NA,NA,"GeneID:9639","ARHGEF10",TRUE,"ARHGEF10"

"10735","TCATCGTAAATTGCTTCCTC","chr8",1869198,"-","none","TGG","TCATCGTAAATTGCTTCCTCTGG",0,NA,NA,NA,"GeneID:9639","ARHGEF10",TRUE,"ARHGEF10"

"10736","TCATCGTAAATTGCTTCCTC","chr9",110276774,"-","5:T>C,16:A>G","TGG","TCATCATAAATTGCTTTCTCTGG",2,6,17,NA,"","",FALSE,"ARHGEF10"

"10737","AGACCCCCCACTGATCCACT","chr1",17587604,"+","none","TGG","AGACCCCCCACTGATCCACTTGG",0,NA,NA,NA,"GeneID:55160","ARHGEF10L",TRUE,"ARHGEF10L"

"10738","AGCCCAGATGCGCATCAGCC","chr1",17603569,"+","none","CGG","AGCCCAGATGCGCATCAGCCCGG",0,NA,NA,NA,"GeneID:55160","ARHGEF10L",TRUE,"ARHGEF10L"

"10739","AGCCCAGATGCGCATCAGCC","chr16",22421701,"+","11:A>G,13:T>A","TGG","AGCCCAGATGCACTTCAGCCTGG",2,12,14,NA,"GeneID:1039","CDR2",FALSE,"ARHGEF10L"

"10740","AGTCCAAGTGGATCAGTGGG","chr1",17587610,"-","none","GGG","AGTCCAAGTGGATCAGTGGGGGG",0,NA,NA,NA,"GeneID:55160","ARHGEF10L",TRUE,"ARHGEF10L"

"10741","ATACCGGGCTGATGCGCATC","chr1",17603575,"-","none","TGG","ATACCGGGCTGATGCGCATCTGG",0,NA,NA,NA,"GeneID:55160","ARHGEF10L",TRUE,"ARHGEF10L"

"10742","GGAACTCTTCCGCTTCCAGC","chr1",17602191,"-","none","TGG","GGAACTCTTCCGCTTCCAGCTGG",0,NA,NA,NA,"GeneID:55160","ARHGEF10L",TRUE,"ARHGEF10L"

"10743","TGCATCCCCATTGCTCACCC","chr1",17602137,"-","none","AGG","TGCATCCCCATTGCTCACCCAGG",0,NA,NA,NA,"GeneID:55160","ARHGEF10L",TRUE,"ARHGEF10L"

"10744","TGCATCCCCATTGCTCACCC","chr4",8204421,"+","16:C>A,19:G>C","AGG","TGCATCCCCATTGCTCCCCGAGG",2,17,20,NA,"GeneID:54436","SH3TC1",FALSE,"ARHGEF10L"

"10745","CATCCGAAGGCTGGCGATGG","chr1",156986096,"+","none","TGG","CATCCGAAGGCTGGCGATGGTGG",0,NA,NA,NA,"GeneID:9826","ARHGEF11",TRUE,"ARHGEF11"

"10746","CATCCGAAGGCTGGCGATGG","chr6",68928235,"+","5:C>G,15:C>G","TGG","CATCCCAAGGCTGGCCATGGTGG",2,6,16,NA,"GeneID:577","BAI3",FALSE,"ARHGEF11"

"10747","CCAACAGGTCAACGGCACCA","chr1",156979273,"-","none","TGG","CCAACAGGTCAACGGCACCATGG",0,NA,NA,NA,"GeneID:9826","ARHGEF11",TRUE,"ARHGEF11"

"10748","CCGGTGTGAAAGAGGGCGAC","chr1",156980448,"-","none","CGG","CCGGTGTGAAAGAGGGCGACCGG",0,NA,NA,NA,"GeneID:9826","ARHGEF11",TRUE,"ARHGEF11"

"10749","CTCTTTCACACCGGCCTTCA","chr1",156980454,"+","none","TGG","CTCTTTCACACCGGCCTTCATGG",0,NA,NA,NA,"GeneID:9826","ARHGEF11",TRUE,"ARHGEF11"

"10750","GAGGTTACCTGTTGTCTCAG","chr1",156986073,"+","none","AGG","GAGGTTACCTGTTGTCTCAGAGG",0,NA,NA,NA,"GeneID:9826","ARHGEF11",TRUE,"ARHGEF11"

"10751","TGATCAGCTTTACCACTTCC","chr1",156979231,"+","none","AGG","TGATCAGCTTTACCACTTCCAGG",0,NA,NA,NA,"GeneID:9826","ARHGEF11",TRUE,"ARHGEF11"

"10752","TGATCAGCTTTACCACTTCC","chr13",30581641,"-","16:G>A,18:C>G","TGG","TGATCAGCTTTACCACCTGCTGG",2,17,19,NA,"GeneID:3146","HMGB1",FALSE,"ARHGEF11"

"10753","ACTTTCTGCTTCTTATCTGT","chr11",120407768,"-","none","TGG","ACTTTCTGCTTCTTATCTGTTGG",0,NA,NA,NA,"GeneID:23365","ARHGEF12",TRUE,"ARHGEF12"

"10754","ACTTTCTGCTTCTTATCTGT","chr6",2395240,"+","12:C>T,16:A>C","GGG","ACTTTCTGCTTCCTATATGTGGG",2,13,17,NA,"","",TRUE,"ARHGEF12"

"10755","ACTTTCTGCTTCTTATCTGT","chr3",57776334,"+","5:G>C,8:G>C","TGG","ACTTTGTGGTTCTTATCTGTTGG",2,6,9,NA,"GeneID:7871","SLMAP",FALSE,"ARHGEF12"

"10756","ATCATCCAGAAAGATGACAA","chr11",120420772,"+","none","TGG","ATCATCCAGAAAGATGACAATGG",0,NA,NA,NA,"GeneID:23365","ARHGEF12",TRUE,"ARHGEF12"

"10757","ATCATCCAGAAAGATGACAA","chr14",74048180,"-","10:G>T,17:T>G","TGG","ATCATCCAGACAGATGAAAATGG",2,11,18,NA,"GeneID:80127","CCDC176",TRUE,"ARHGEF12"

"10758","CAAATCCATTGTCATCTTTC","chr11",120420780,"-","none","TGG","CAAATCCATTGTCATCTTTCTGG",0,NA,NA,NA,"GeneID:23365","ARHGEF12",TRUE,"ARHGEF12"

"10759","CAAATCCATTGTCATCTTTC","chr6",97897618,"-","2:G>T,5:A>G","CGG","CACATTCATTGTCATCTTTCCGG",2,3,6,NA,"GeneID:101927314","LOC101927314",FALSE,"ARHGEF12"

"10760","CAAATCCATTGTCATCTTTC","chr12",46553355,"-","7:A>T,18:T>A","CGG","CAAATCCTTTGTCATCTTACCGG",2,8,19,NA,"","",FALSE,"ARHGEF12"

"10761","CAAATCCATTGTCATCTTTC","chrX",138117330,"-","0:C>G,12:T>G","TGG","GAAATCCATTGTAATCTTTCTGG",2,1,13,NA,"","",FALSE,"ARHGEF12"

"10762","TTCCCCTCAAAAAACCTATA","chr11",120406119,"+","none","AGG","TTCCCCTCAAAAAACCTATAAGG",0,NA,NA,NA,"GeneID:23365","ARHGEF12",TRUE,"ARHGEF12"

"10763","TTCCCCTCAAAAAACCTATA","chr11",30316143,"+","6:A>T,13:C>A","AGG","TTCCCCACAAAAACCCTATAAGG",2,7,14,NA,"","",FALSE,"ARHGEF12"

"10764","TTCGTACAGTCTGTCAAAGA","chr11",120420829,"+","none","AGG","TTCGTACAGTCTGTCAAAGAAGG",0,NA,NA,NA,"GeneID:23365","ARHGEF12",TRUE,"ARHGEF12"

"10765","TTCGTACAGTCTGTCAAAGA","chrX",88706992,"-","3:T>C,16:A>T","TGG","TTCATACAGTCTGTCATAGATGG",2,4,17,NA,"","",FALSE,"ARHGEF12"

"10766","TTGACAGACTGTACGAAGAC","chr11",120420826,"-","none","TGG","TTGACAGACTGTACGAAGACTGG",0,NA,NA,NA,"GeneID:23365","ARHGEF12",TRUE,"ARHGEF12"

"10767","CATCACACAAGTCGCTGATG","chr17",8315796,"-","none","TGG","CATCACACAAGTCGCTGATGTGG",0,NA,NA,NA,"GeneID:22899","ARHGEF15",TRUE,"ARHGEF15"

"10768","CTCCCAAACCAACTCGTGTC","chr17",8313158,"+","none","AGG","CTCCCAAACCAACTCGTGTCAGG",0,NA,NA,NA,"GeneID:22899","ARHGEF15",TRUE,"ARHGEF15"

"10769","CTGTGGGGGGTCCCCGAAAA","chr17",8313194,"-","none","TGG","CTGTGGGGGGTCCCCGAAAATGG",0,NA,NA,NA,"GeneID:22899","ARHGEF15",TRUE,"ARHGEF15"

"10770","GCTTCTCCGCCGAGCTGCGC","chr17",8316032,"+","none","CGG","GCTTCTCCGCCGAGCTGCGCCGG",0,NA,NA,NA,"GeneID:22899","ARHGEF15",TRUE,"ARHGEF15"

"10771","GGGAAGAGCGCACACGGGAC","chr17",8315772,"-","none","AGG","GGGAAGAGCGCACACGGGACAGG",0,NA,NA,NA,"GeneID:22899","ARHGEF15",TRUE,"ARHGEF15"

"10772","GGGAAGAGCGCACACGGGAC","chr16",31271106,"+","9:C>G,18:G>A","TGG","GGGAAGAGCCCACACGGGGCTGG",2,10,19,NA,"GeneID:3684","ITGAM",FALSE,"ARHGEF15"

"10773","TGCCCTTCCAGCGCATCACC","chr17",8316107,"+","none","CGG","TGCCCTTCCAGCGCATCACCCGG",0,NA,NA,NA,"GeneID:22899","ARHGEF15",TRUE,"ARHGEF15"

"10774","CATGTTCGAGATCCTCACGT","chr1",3469434,"+","none","CGG","CATGTTCGAGATCCTCACGTCGG",0,NA,NA,NA,"GeneID:27237","ARHGEF16",TRUE,"ARHGEF16"

"10775","CCCGGTAGGATTGGTTCCGC","chr1",3463530,"-","none","AGG","CCCGGTAGGATTGGTTCCGCAGG",0,NA,NA,NA,"GeneID:27237","ARHGEF16",TRUE,"ARHGEF16"

"10776","CCTGGACCAGCTCTCCACTG","chr1",3468896,"+","none","AGG","CCTGGACCAGCTCTCCACTGAGG",0,NA,NA,NA,"GeneID:27237","ARHGEF16",TRUE,"ARHGEF16"

"10777","GCGGCATCGTCTCTAACACG","chr1",3463210,"-","none","CGG","GCGGCATCGTCTCTAACACGCGG",0,NA,NA,NA,"GeneID:27237","ARHGEF16",TRUE,"ARHGEF16"

"10778","GCGGGCGACCGTGACCCAGA","chr1",3469518,"+","none","TGG","GCGGGCGACCGTGACCCAGATGG",0,NA,NA,NA,"GeneID:27237","ARHGEF16",TRUE,"ARHGEF16"

"10779","GTCCCCCCAGGTGGTGGAAT","chr1",3468869,"+","none","TGG","GTCCCCCCAGGTGGTGGAATTGG",0,NA,NA,NA,"GeneID:27237","ARHGEF16",TRUE,"ARHGEF16"

"10780","AAGATCTCGTCCACCAGTGA","chr11",73352880,"-","none","AGG","AAGATCTCGTCCACCAGTGAAGG",0,NA,NA,NA,"GeneID:9828","ARHGEF17",TRUE,"ARHGEF17"

"10781","ACAGGACATGCGGAAGCACG","chr11",73346878,"+","none","TGG","ACAGGACATGCGGAAGCACGTGG",0,NA,NA,NA,"GeneID:9828","ARHGEF17",TRUE,"ARHGEF17"

"10782","ACGCTACGAGCTTCTGGTGA","chr11",73355931,"+","none","AGG","ACGCTACGAGCTTCTGGTGAAGG",0,NA,NA,NA,"GeneID:9828","ARHGEF17",TRUE,"ARHGEF17"

"10783","CACTCACCGACTGGACGAGC","chr11",73352999,"-","none","AGG","CACTCACCGACTGGACGAGCAGG",0,NA,NA,NA,"GeneID:9828","ARHGEF17",TRUE,"ARHGEF17"

"10784","GGACACAGAGCAGTCGTATG","chr11",73346914,"+","none","TGG","GGACACAGAGCAGTCGTATGTGG",0,NA,NA,NA,"GeneID:9828","ARHGEF17",TRUE,"ARHGEF17"

"10785","TTCACCAGAAGCTCGTAGCG","chr11",73355932,"-","none","TGG","TTCACCAGAAGCTCGTAGCGTGG",0,NA,NA,NA,"GeneID:9828","ARHGEF17",TRUE,"ARHGEF17"

"10786","ACTCGGTGTTTGGAGATGTA","chr19",7441730,"-","none","AGG","ACTCGGTGTTTGGAGATGTAAGG",0,NA,NA,NA,"GeneID:23370","ARHGEF18",TRUE,"ARHGEF18"

"10787","ATGATGCGCTCCACCAGCAC","chr19",7451232,"-","none","TGG","ATGATGCGCTCCACCAGCACTGG",0,NA,NA,NA,"GeneID:23370","ARHGEF18",TRUE,"ARHGEF18"

"10788","CACGCCAAGCCGCCGCACGA","chr19",7451167,"-","none","TGG","CACGCCAAGCCGCCGCACGATGG",0,NA,NA,NA,"GeneID:23370","ARHGEF18",TRUE,"ARHGEF18"

"10789","CCCATGCGCTGACGACCTGC","chr19",7444318,"+","none","TGG","CCCATGCGCTGACGACCTGCTGG",0,NA,NA,NA,"GeneID:23370","ARHGEF18",TRUE,"ARHGEF18"

"10790","TGATCTTGAGCGTCCGCACG","chr19",7444228,"-","none","TGG","TGATCTTGAGCGTCCGCACGTGG",0,NA,NA,NA,"GeneID:23370","ARHGEF18",TRUE,"ARHGEF18"

"10791","TTTGCACTCAGACCAATCAC","chr19",7441641,"+","none","AGG","TTTGCACTCAGACCAATCACAGG",0,NA,NA,NA,"GeneID:23370","ARHGEF18",TRUE,"ARHGEF18"

"10792","TTTGCACTCAGACCAATCAC","chr6",158061110,"+","3:A>G,14:T>A","AGG","TTTACACTCAGACCTATCACAGG",2,4,15,NA,"GeneID:8871","SYNJ2",FALSE,"ARHGEF18"

"10793","AGGTGCGCTCCTGGTAGGCC","chr1",16205943,"+","none","TGG","AGGTGCGCTCCTGGTAGGCCTGG",0,NA,NA,NA,"GeneID:128272","ARHGEF19",TRUE,"ARHGEF19"

"10794","AGGTGCGCTCCTGGTAGGCC","chr11",47597815,"-","0:C>T,6:G>C","TGG","GGGTGCCCTCCTGGTAGGCCTGG",2,1,7,NA,"","",FALSE,"ARHGEF19"

"10795","CACCCGCCTCAAGATGTTGG","chr1",16205542,"-","none","TGG","CACCCGCCTCAAGATGTTGGTGG",0,NA,NA,NA,"GeneID:128272","ARHGEF19",TRUE,"ARHGEF19"

"10796","CTAGTGTACAGTCCATGAAG","chr1",16205137,"-","none","AGG","CTAGTGTACAGTCCATGAAGAGG",0,NA,NA,NA,"GeneID:128272","ARHGEF19",TRUE,"ARHGEF19"

"10797","CTAGTGTACAGTCCATGAAG","chrX",66682825,"+","1:A>T,8:A>C","TGG","CAAGTGTAAAGTCCATGAAGTGG",2,2,9,NA,"","",FALSE,"ARHGEF19"

"10798","GAGCAAGAAGATCCACTTTG","chr1",16205094,"-","none","AGG","GAGCAAGAAGATCCACTTTGAGG",0,NA,NA,NA,"GeneID:128272","ARHGEF19",TRUE,"ARHGEF19"

"10799","GCGCTTCAGCGTGTGCGACG","chr1",16206021,"-","none","TGG","GCGCTTCAGCGTGTGCGACGTGG",0,NA,NA,NA,"GeneID:128272","ARHGEF19",TRUE,"ARHGEF19"

"10800","GTACCTCCACCAACATCTTG","chr1",16205533,"+","none","AGG","GTACCTCCACCAACATCTTGAGG",0,NA,NA,NA,"GeneID:128272","ARHGEF19",TRUE,"ARHGEF19"

"10801","GTACCTCCACCAACATCTTG","chr6",137735493,"-","12:G>T,13:A>G","TGG","GTACCTCCACCACTATCTTGTGG",2,13,14,NA,"GeneID:100507406","LOC100507406",FALSE,"ARHGEF19"

"10802","AGAGGTGCCCATTGGTATAG","chr1",155969239,"+","none","CGG","AGAGGTGCCCATTGGTATAGCGG",0,NA,NA,NA,"GeneID:9181","ARHGEF2",TRUE,"ARHGEF2"

"10803","AGGCGGTGTTGTTCTTCAGC","chr1",155966442,"+","none","AGG","AGGCGGTGTTGTTCTTCAGCAGG",0,NA,NA,NA,"GeneID:9181","ARHGEF2",TRUE,"ARHGEF2"

"10804","CACCCTCGCCAACTGTACCA","chr1",155966833,"-","none","AGG","CACCCTCGCCAACTGTACCAAGG",0,NA,NA,NA,"GeneID:9181","ARHGEF2",TRUE,"ARHGEF2"

"10805","GCTATTAGAACGCCGACGCC","chr1",155963000,"-","none","AGG","GCTATTAGAACGCCGACGCCAGG",0,NA,NA,NA,"GeneID:9181","ARHGEF2",TRUE,"ARHGEF2"

"10806","GGCGTCGGCGTTCTAATAGC","chr1",155963000,"+","none","TGG","GGCGTCGGCGTTCTAATAGCTGG",0,NA,NA,NA,"GeneID:9181","ARHGEF2",TRUE,"ARHGEF2"

"10807","GGCTGTGATGCTCTTGTTAC","chr1",155969177,"+","none","AGG","GGCTGTGATGCTCTTGTTACAGG",0,NA,NA,NA,"GeneID:9181","ARHGEF2",TRUE,"ARHGEF2"

"10808","CAGAAGGGATCGCGAACATG","chr12",57610637,"+","none","AGG","CAGAAGGGATCGCGAACATGAGG",0,NA,NA,NA,"GeneID:115557","ARHGEF25",TRUE,"ARHGEF25"

"10809","CAGAAGGGATCGCGAACATG","chr9",18328563,"+","10:A>C,12:A>C","TGG","CAGAAGGGATAGAGAACATGTGG",2,11,13,NA,"GeneID:92949","ADAMTSL1",FALSE,"ARHGEF25"

"10810","CCCTCCGTACGTTCAGACTC","chr12",57610611,"-","none","TGG","CCCTCCGTACGTTCAGACTCTGG",0,NA,NA,NA,"GeneID:115557","ARHGEF25",TRUE,"ARHGEF25"

"10811","CGACTTGGGGCAGATTGTGG","chr12",57613738,"+","none","AGG","CGACTTGGGGCAGATTGTGGAGG",0,NA,NA,NA,"GeneID:115557","ARHGEF25",TRUE,"ARHGEF25"

"10812","TAGGTATGTCCTGAGTGAAC","chr12",57613690,"+","none","TGG","TAGGTATGTCCTGAGTGAACTGG",0,NA,NA,NA,"GeneID:115557","ARHGEF25",TRUE,"ARHGEF25"

"10813","TGGGACAAAGTCTCCTCTTC","chr12",57613445,"-","none","AGG","TGGGACAAAGTCTCCTCTTCAGG",0,NA,NA,NA,"GeneID:115557","ARHGEF25",TRUE,"ARHGEF25"

"10814","TGGGACAAAGTCTCCTCTTC","chr7",136026462,"+","4:C>A,17:C>T","AGG","TGGGCCAAAGTCTCCTCCTCAGG",2,5,18,NA,"","",FALSE,"ARHGEF25"

"10815","TTCCTTCCAGCCACCTGAAG","chr12",57613429,"+","none","AGG","TTCCTTCCAGCCACCTGAAGAGG",0,NA,NA,NA,"GeneID:115557","ARHGEF25",TRUE,"ARHGEF25"

"10816","AAACACTCACCGCAGAGAGT","chr3",154129709,"-","none","TGG","AAACACTCACCGCAGAGAGTTGG",0,NA,NA,NA,"GeneID:26084","ARHGEF26",TRUE,"ARHGEF26"

"10817","ATACAATGACTAAAACCGAG","chr3",154152862,"+","none","AGG","ATACAATGACTAAAACCGAGAGG",0,NA,NA,NA,"GeneID:26084","ARHGEF26",TRUE,"ARHGEF26"

"10818","ATACAATGACTAAAACCGAG","chr16",26232374,"+","11:G>A,16:T>C","AGG","ATACAATGACTGAAACTGAGAGG",2,12,17,NA,"","",FALSE,"ARHGEF26"

"10819","ATACAATGACTAAAACCGAG","chr16",64985872,"-","7:T>C,9:A>G","AGG","ATACAATAATTAAAACCGAGAGG",2,8,10,NA,"GeneID:1009","CDH11",TRUE,"ARHGEF26"

"10820","ATGTGGATCTCAATGGCTTA","chr3",154129682,"-","none","TGG","ATGTGGATCTCAATGGCTTATGG",0,NA,NA,NA,"GeneID:26084","ARHGEF26",TRUE,"ARHGEF26"

"10821","ATTATCTCAGACAGTAAGCC","chr3",154149402,"+","none","AGG","ATTATCTCAGACAGTAAGCCAGG",0,NA,NA,NA,"GeneID:26084","ARHGEF26",TRUE,"ARHGEF26"

"10822","CAAGGAACATTTGATGGGGA","chr3",154124427,"+","none","AGG","CAAGGAACATTTGATGGGGAAGG",0,NA,NA,NA,"GeneID:26084","ARHGEF26",TRUE,"ARHGEF26"

"10823","CAAGGAACATTTGATGGGGA","chrX",136887326,"+","0:G>C,4:A>G","GGG","GAAGAAACATTTGATGGGGAGGG",2,1,5,NA,"","",FALSE,"ARHGEF26"

"10824","CAATATTACAGATGTCTGTG","chr3",154152899,"+","none","AGG","CAATATTACAGATGTCTGTGAGG",0,NA,NA,NA,"GeneID:26084","ARHGEF26",TRUE,"ARHGEF26"

"10825","CATGATTGCAGAGCGCATCG","chr5",73749934,"+","none","AGG","CATGATTGCAGAGCGCATCGAGG",0,NA,NA,NA,"GeneID:64283","ARHGEF28",TRUE,"ARHGEF28"

"10826","CCACACAGCCGAGCATTTGT","chr5",73776617,"+","none","TGG","CCACACAGCCGAGCATTTGTTGG",0,NA,NA,NA,"GeneID:64283","ARHGEF28",TRUE,"ARHGEF28"

"10827","CCACACAGCCGAGCATTTGT","chr3",99326428,"-","10:T>C,13:A>G","TGG","CCACACAGCCAAGTATTTGTTGG",2,11,14,NA,"","",TRUE,"ARHGEF28"

"10828","CCACACAGCCGAGCATTTGT","chr22",25852692,"+","10:A>G,11:G>A","GGG","CCACACAGCCAGGCATTTGTGGG",2,11,12,NA,"GeneID:84700","MYO18B",FALSE,"ARHGEF28"

"10829","CGATGAGTGAATGTAATGCA","chr5",73776574,"-","none","AGG","CGATGAGTGAATGTAATGCAAGG",0,NA,NA,NA,"GeneID:64283","ARHGEF28",TRUE,"ARHGEF28"

"10830","CTTGGCTTTACCCAACGAAG","chr5",73773951,"+","none","AGG","CTTGGCTTTACCCAACGAAGAGG",0,NA,NA,NA,"GeneID:64283","ARHGEF28",TRUE,"ARHGEF28"

"10831","GTTCTCCAGTCCAGCGTCCC","chr5",73749962,"+","none","AGG","GTTCTCCAGTCCAGCGTCCCAGG",0,NA,NA,NA,"GeneID:64283","ARHGEF28",TRUE,"ARHGEF28"

"10832","TTAGACTTAGCTTTACGTGA","chr5",73773985,"+","none","AGG","TTAGACTTAGCTTTACGTGAAGG",0,NA,NA,NA,"GeneID:64283","ARHGEF28",TRUE,"ARHGEF28"

"10833","ACTTACCCCTCTGAAATTCC","chr3",56958816,"+","none","AGG","ACTTACCCCTCTGAAATTCCAGG",0,NA,NA,NA,"GeneID:50650;GeneID:100874200","ARHGEF3;ARHGEF3-AS1",TRUE,"ARHGEF3"

"10834","ACTTACCCCTCTGAAATTCC","chr2",209101000,"-","0:G>T,16:T>A","TGG","CCTTACCCCTCTGAAAATCCTGG",2,1,17,NA,"","",FALSE,"ARHGEF3"

"10835","ATGAATCAATGCAGCTGTAG","chr3",57035111,"-","none","AGG","ATGAATCAATGCAGCTGTAGAGG",0,NA,NA,NA,"GeneID:50650","ARHGEF3",TRUE,"ARHGEF3"

"10836","ATGAATCAATGCAGCTGTAG","chr2",88087813,"+","1:G>T,6:G>C","TGG","AGGAATGAATGCAGCTGTAGTGG",2,2,7,NA,"GeneID:150572","SMYD1",FALSE,"ARHGEF3"

"10837","CACTTATGTCGAGACTGCAA","chr3",56882290,"+","none","AGG","CACTTATGTCGAGACTGCAAAGG",0,NA,NA,NA,"GeneID:50650","ARHGEF3",TRUE,"ARHGEF3"

"10838","CTTCTAGGCCTAAACGGCAG","chr3",56958876,"-","none","CGG","CTTCTAGGCCTAAACGGCAGCGG",0,NA,NA,NA,"GeneID:50650;GeneID:100874200","ARHGEF3;ARHGEF3-AS1",TRUE,"ARHGEF3"

"10839","GCTGCATTGATTCATTGCTG","chr3",57035116,"+","none","TGG","GCTGCATTGATTCATTGCTGTGG",0,NA,NA,NA,"GeneID:50650","ARHGEF3",TRUE,"ARHGEF3"

"10840","GCTGCATTGATTCATTGCTG","chrY",14162533,"-","4:A>G,8:G>C","AGG","GCTGTATTCATTCATTGCTGAGG",2,5,9,NA,"","",FALSE,"ARHGEF3"

"10841","GCTGCATTGATTCATTGCTG","chrY",14817437,"+","8:T>G,13:T>A","AGG","GCTGCATTTATTCTTTGCTGAGG",2,9,14,NA,"GeneID:22829","NLGN4Y",FALSE,"ARHGEF3"

"10842","GCTGCATTGATTCATTGCTG","chrY",22258623,"+","8:C>G,13:C>A","AGG","GCTGCATTCATTCCTTGCTGAGG",2,9,14,NA,"","",FALSE,"ARHGEF3"

"10843","TGTTTCCCTCTCCTAAAGCC","chr3",56958837,"-","none","TGG","TGTTTCCCTCTCCTAAAGCCTGG",0,NA,NA,NA,"GeneID:50650;GeneID:100874200","ARHGEF3;ARHGEF3-AS1",TRUE,"ARHGEF3"

"10844","TGTTTCCCTCTCCTAAAGCC","chr10",79003809,"-","2:T>A,14:C>T","TGG","TGATTCCCTCTCCTGAAGCCTGG",2,3,15,NA,"GeneID:283050","ZMIZ1-AS1",TRUE,"ARHGEF3"

"10845","AATCTGACCTTGCTTTGTTT","chr2",38919460,"-","none","TGG","AATCTGACCTTGCTTTGTTTTGG",0,NA,NA,NA,"GeneID:729967;GeneID:100271715","MORN2;ARHGEF33",TRUE,"ARHGEF33"

"10846","AATCTGACCTTGCTTTGTTT","chr3",174349690,"-","10:C>A,12:C>G","CGG","AATCTGACCTGGGTTTGTTTCGG",2,11,13,NA,"","",FALSE,"ARHGEF33"

"10847","CTAGCCTCCGAACTCAAAAC","chr2",38928915,"+","none","TGG","CTAGCCTCCGAACTCAAAACTGG",0,NA,NA,NA,"GeneID:729967;GeneID:100271715","MORN2;ARHGEF33",TRUE,"ARHGEF33"

"10848","CTTTACCTGGTAAATCTGCG","chr2",38921409,"-","none","TGG","CTTTACCTGGTAAATCTGCGTGG",0,NA,NA,NA,"GeneID:729967;GeneID:100271715","MORN2;ARHGEF33",TRUE,"ARHGEF33"

"10849","GAAAAAACCAAAACAAAGCA","chr2",38919450,"+","none","AGG","GAAAAAACCAAAACAAAGCAAGG",0,NA,NA,NA,"GeneID:729967;GeneID:100271715","MORN2;ARHGEF33",TRUE,"ARHGEF33"

"10850","GAAAAAACCAAAACAAAGCA","chr2",131511547,"-","0:T>C,8:T>G","CGG","AAAAAAACAAAAACAAAGCACGG",2,1,9,NA,"GeneID:150776","LOC150776",FALSE,"ARHGEF33"

"10851","GAAAAAACCAAAACAAAGCA","chr9",90806133,"-","5:G>T,8:T>G","AGG","GAAAACACAAAAACAAAGCAAGG",2,6,9,NA,"GeneID:6850","SYK",FALSE,"ARHGEF33"

"10852","GAAAAAACCAAAACAAAGCA","chr7",41018626,"+","0:A>G,7:A>C","TGG","AAAAAAAACAAAACAAAGCATGG",2,1,8,NA,"","",FALSE,"ARHGEF33"

"10853","GAAAAAACCAAAACAAAGCA","chrX",66840321,"+","0:C>G,7:G>C","GGG","CAAAAAAGCAAAACAAAGCAGGG",2,1,8,NA,"","",FALSE,"ARHGEF33"

"10854","GAAAAAACCAAAACAAAGCA","chr15",48139537,"+","0:A>G,8:A>C","AGG","AAAAAAACAAAAACAAAGCAAGG",2,1,9,NA,"GeneID:283652;GeneID:50804","SLC24A5;MYEF2",TRUE,"ARHGEF33"

"10855","GAAAAAACCAAAACAAAGCA","chr2",130161419,"+","0:A>G,8:A>C","CGG","AAAAAAACAAAAACAAAGCACGG",2,1,9,NA,"GeneID:55627","SMPD4",FALSE,"ARHGEF33"

"10856","GAAAAAACCAAAACAAAGCA","chr18",13167616,"+","0:T>G,17:A>G","AGG","TAAAAAACCAAAACAAAACAAGG",2,1,18,NA,"","",FALSE,"ARHGEF33"

"10857","GAAAAAACCAAAACAAAGCA","chr10",34872847,"+","7:A>C,17:A>G","CGG","GAAAAAAACAAAACAAAACACGG",2,8,18,NA,"","",FALSE,"ARHGEF33"

"10858","GAAAAAACCAAAACAAAGCA","chr22",18033909,"+","7:A>C,17:A>G","GGG","GAAAAAAACAAAACAAAACAGGG",2,8,18,NA,"GeneID:100192420","FLJ41941",FALSE,"ARHGEF33"

"10859","GAAAAAACCAAAACAAAGCA","chr13",33614619,"+","7:A>C,17:A>G","GGG","GAAAAAAACAAAACAAAACAGGG",2,8,18,NA,"","",FALSE,"ARHGEF33"

"10860","GAAAAAACCAAAACAAAGCA","chr8",23928974,"+","7:A>C,17:A>G","GGG","GAAAAAAACAAAACAAAACAGGG",2,8,18,NA,"","",FALSE,"ARHGEF33"

"10861","GAAAAAACCAAAACAAAGCA","chr20",36882385,"+","8:A>C,17:A>G","CGG","GAAAAAACAAAAACAAAACACGG",2,9,18,NA,"GeneID:140711","TLDC2",FALSE,"ARHGEF33"

"10862","GAAAAAACCAAAACAAAGCA","chr18",58597293,"+","8:A>C,18:A>C","TGG","GAAAAAACAAAAACAAAGAATGG",2,9,19,NA,"GeneID:115701","ALPK2",FALSE,"ARHGEF33"

"10863","GAAAAAACCAAAACAAAGCA","chr3",157238231,"-","8:T>G,13:T>G","GGG","GAAAAAACAAAAAAAAAGCAGGG",2,9,14,NA,"","",FALSE,"ARHGEF33"

"10864","GAAAAAACCAAAACAAAGCA","chr14",70538795,"-","7:T>G,17:T>C","GGG","GAAAAAAACAAAACAAAACAGGG",2,8,18,NA,"","",FALSE,"ARHGEF33"

"10865","GAAAAAACCAAAACAAAGCA","chr1",35721385,"-","7:T>G,17:T>C","TGG","GAAAAAAACAAAACAAAACATGG",2,8,18,NA,"GeneID:63967","CLSPN",FALSE,"ARHGEF33"

"10866","GAAAAAACCAAAACAAAGCA","chr1",54411143,"-","7:T>G,17:T>C","AGG","GAAAAAAACAAAACAAAACAAGG",2,8,18,NA,"GeneID:23648","SSBP3",FALSE,"ARHGEF33"

"10867","GAAAAAACCAAAACAAAGCA","chr5",101418821,"-","7:T>G,17:T>C","GGG","GAAAAAAACAAAACAAAACAGGG",2,8,18,NA,"","",FALSE,"ARHGEF33"

"10868","GAAAAAACCAAAACAAAGCA","chr1",88773133,"-","2:G>T,18:A>G","GGG","GACAAAACCAAAACAAAGTAGGG",2,3,19,NA,"GeneID:5586","PKN2",FALSE,"ARHGEF33"

"10869","GAACTGTCAAGAATTCAACA","chr2",38928957,"+","none","TGG","GAACTGTCAAGAATTCAACATGG",0,NA,NA,NA,"GeneID:729967;GeneID:100271715","MORN2;ARHGEF33",TRUE,"ARHGEF33"

"10870","TCCTTCCACGCAGATTTACC","chr2",38921401,"+","none","AGG","TCCTTCCACGCAGATTTACCAGG",0,NA,NA,NA,"GeneID:729967;GeneID:100271715","MORN2;ARHGEF33",TRUE,"ARHGEF33"

"10871","TCCTTCCACGCAGATTTACC","chr15",23481775,"-","2:A>G,18:A>G","AGG","TCTTTCCACGCAGATTTATCAGG",2,3,19,NA,"","",FALSE,"ARHGEF33"

"10872","GAGAAAGGGGTCCCAGTCAG","chr7",144187268,"-","none","CGG","GAGAAAGGGGTCCCAGTCAGCGG",0,NA,NA,NA,"GeneID:445328","ARHGEF35",TRUE,"ARHGEF35"

"10873","GAGAAAGGGGTCCCAGTCAG","chr1",15482240,"-","16:C>A,17:C>G","AGG","GAGAAAGGGGTCCCAGGGAGAGG",2,17,18,NA,"GeneID:51032","CELA2B",FALSE,"ARHGEF35"

"10874","GAGAAAGGGGTCCCAGTCAG","chr7",144285851,"-","11:C>G","CGG","GAGAAAGGGGTGCCAGTCAGCGG",1,12,NA,NA,"","",TRUE,"ARHGEF35"

"10875","GAGAAAGGGGTCCCAGTCAG","chr7",144363764,"+","11:G>C","CGG","GAGAAAGGGGTGCCAGTCAGCGG",1,12,NA,NA,"GeneID:7984","ARHGEF5",TRUE,"ARHGEF35"

"10876","GAGAAAGGGGTCCCAGTCAG","chrX",111898883,"+","9:A>G,16:G>T","AGG","GAGAAAGGGATCCCAGGCAGAGG",2,10,17,NA,"GeneID:7224;GeneID:100329135","TRPC5;TRPC5OS",FALSE,"ARHGEF35"

"10877","GCAGTACCCCAGAGCCTAGC","chr7",144362984,"+","none","AGG","GCAGTACCCCAGAGCCTAGCAGG",0,NA,NA,NA,"GeneID:7984","ARHGEF5",TRUE,"ARHGEF35"

"10878","GCAGTACCCCAGAGCCTAGC","chr7",144286631,"-","none","AGG","GCAGTACCCCAGAGCCTAGCAGG",0,NA,NA,NA,"","",TRUE,"ARHGEF35"

"10879","GCAGTACCCCAGAGCCTAGC","chr7",144188048,"-","none","AGG","GCAGTACCCCAGAGCCTAGCAGG",0,NA,NA,NA,"GeneID:445328","ARHGEF35",TRUE,"ARHGEF35"

"10880","GCATCTGATGGGCTCTAGCA","chr7",144187009,"+","none","AGG","GCATCTGATGGGCTCTAGCAAGG",0,NA,NA,NA,"GeneID:445328","ARHGEF35",TRUE,"ARHGEF35"

"10881","GCATCTGATGGGCTCTAGCA","chr7",144285592,"+","3:C>T,19:G>A","AGG","GCACCTGATGGGCTCTAGCGAGG",2,4,20,NA,"","",TRUE,"ARHGEF35"

"10882","GCATCTGATGGGCTCTAGCA","chr7",144364023,"-","3:G>A,19:C>T","AGG","GCACCTGATGGGCTCTAGCGAGG",2,4,20,NA,"GeneID:7984","ARHGEF5",TRUE,"ARHGEF35"

"10883","GGCTGAGGCCTGTGACACTC","chr7",144362950,"+","none","CGG","GGCTGAGGCCTGTGACACTCCGG",0,NA,NA,NA,"GeneID:7984","ARHGEF5",TRUE,"ARHGEF35"

"10884","GGCTGAGGCCTGTGACACTC","chr7",144286665,"-","none","CGG","GGCTGAGGCCTGTGACACTCCGG",0,NA,NA,NA,"","",TRUE,"ARHGEF35"

"10885","GGCTGAGGCCTGTGACACTC","chr7",144188082,"-","none","CGG","GGCTGAGGCCTGTGACACTCCGG",0,NA,NA,NA,"GeneID:445328","ARHGEF35",TRUE,"ARHGEF35"

"10886","GGCTGAGGCCTGTGACACTC","chr3",196215210,"+","10:A>T,16:C>A","TGG","GGCTGAGGCCAGTGACCCTCTGG",2,11,17,NA,"GeneID:200931;GeneID:5130","SLC51A;PCYT1A",FALSE,"ARHGEF35"

"10887","GGCTGAGGCCTGTGACACTC","chr8",16759023,"-","2:T>G,8:T>G","AGG","GGATGAGGACTGTGACACTCAGG",2,3,9,NA,"","",FALSE,"ARHGEF35"

"10888","TGGGAGGAGAGGCTCCATGC","chr7",144188343,"+","none","TGG","TGGGAGGAGAGGCTCCATGCTGG",0,NA,NA,NA,"GeneID:445328","ARHGEF35",TRUE,"ARHGEF35"

"10889","TGGGAGGAGAGGCTCCATGC","chr9",135246721,"-","17:C>A,18:G>C","AGG","TGGGAGGAGAGGCTCCAGCCAGG",2,18,19,NA,"","",FALSE,"ARHGEF35"

"10890","TGGGAGGAGAGGCTCCATGC","chr7",144362689,"-","17:G>A","TGG","TGGGAGGAGAGGCTCCACGCTGG",1,18,NA,NA,"GeneID:7984","ARHGEF5",TRUE,"ARHGEF35"

"10891","TGGGAGGAGAGGCTCCATGC","chr2",4206725,"-","14:T>G,17:C>A","AGG","TGGGAGGAGAGGCTACAGGCAGG",2,15,18,NA,"","",FALSE,"ARHGEF35"

"10892","TGGGAGGAGAGGCTCCATGC","chr7",144286926,"+","17:C>T","TGG","TGGGAGGAGAGGCTCCACGCTGG",1,18,NA,NA,"","",TRUE,"ARHGEF35"

"10893","TGGGAGGAGAGGCTCCATGC","chr5",172582271,"+","10:A>G,18:C>G","CGG","TGGGAGGAGAAGCTCCATCCCGG",2,11,19,NA,"","",FALSE,"ARHGEF35"

"10894","TGGGAGGAGAGGCTCCATGC","chr7",2513549,"-","9:C>T,17:C>A","CGG","TGGGAGGAGGGGCTCCAGGCCGG",2,10,18,NA,"GeneID:3955","LFNG",FALSE,"ARHGEF35"

"10895","TTCAGCCCTGATGGAGGCTG","chr7",144362659,"+","none","AGG","TTCAGCCCTGATGGAGGCTGAGG",0,NA,NA,NA,"GeneID:7984","ARHGEF5",TRUE,"ARHGEF35"

"10896","TTCAGCCCTGATGGAGGCTG","chr7",144286956,"-","none","AGG","TTCAGCCCTGATGGAGGCTGAGG",0,NA,NA,NA,"","",TRUE,"ARHGEF35"

"10897","TTCAGCCCTGATGGAGGCTG","chr7",144188373,"-","none","AGG","TTCAGCCCTGATGGAGGCTGAGG",0,NA,NA,NA,"GeneID:445328","ARHGEF35",TRUE,"ARHGEF35"

"10898","TTCAGCCCTGATGGAGGCTG","chr1",6450063,"-","10:G>T,13:G>C","TGG","TTCAGCCCTGCTGCAGGCTGTGG",2,11,14,NA,"GeneID:83715","ESPN",FALSE,"ARHGEF35"

"10899","TTCAGCCCTGATGGAGGCTG","chr1",29594492,"+","11:C>T,19:A>G","AGG","TTCAGCCCTGACGGAGGCTAAGG",2,12,20,NA,"","",FALSE,"ARHGEF35"

"10900","TTCAGCCCTGATGGAGGCTG","chr22",29212386,"-","9:A>C,10:A>T","AGG","TTCAGCCCTTTTGGAGGCTGAGG",2,10,11,NA,"GeneID:129080","EMID1",FALSE,"ARHGEF35"

"10901","CAGATTCCTCCATGATCTGC","chr5",149601169,"+","none","AGG","CAGATTCCTCCATGATCTGCAGG",0,NA,NA,NA,"GeneID:389337","ARHGEF37",TRUE,"ARHGEF37"

"10902","CAGATTCCTCCATGATCTGC","chr11",36250193,"+","4:G>T,11:T>A","AGG","CAGAGTCCTCCTTGATCTGCAGG",2,5,12,NA,"","",FALSE,"ARHGEF37"

"10903","CAGATTCCTCCATGATCTGC","chrX",4787667,"-","9:C>G,13:A>C","AGG","CAGATTCCTGCATTATCTGCAGG",2,10,14,NA,"","",FALSE,"ARHGEF37"

"10904","CCGGGAGCTCATCGACACTG","chr5",149597873,"+","none","AGG","CCGGGAGCTCATCGACACTGAGG",0,NA,NA,NA,"GeneID:389337","ARHGEF37",TRUE,"ARHGEF37"

"10905","CTACTGTGCCAGCTACGACC","chr5",149609596,"+","none","AGG","CTACTGTGCCAGCTACGACCAGG",0,NA,NA,NA,"GeneID:389337","ARHGEF37",TRUE,"ARHGEF37"

"10906","GACGAGCCATCCTCCAGGTC","chr5",149597787,"+","none","AGG","GACGAGCCATCCTCCAGGTCAGG",0,NA,NA,NA,"GeneID:389337","ARHGEF37",TRUE,"ARHGEF37"

"10907","GAGGAACAAGTGCAGCTAGT","chr5",149601209,"+","none","TGG","GAGGAACAAGTGCAGCTAGTTGG",0,NA,NA,NA,"GeneID:389337","ARHGEF37",TRUE,"ARHGEF37"

"10908","GAGGAACAAGTGCAGCTAGT","chr4",123906402,"-","0:T>C,11:T>C","TGG","AAGGAACAAGTACAGCTAGTTGG",2,1,12,NA,"GeneID:285419","LINC01091",FALSE,"ARHGEF37"

"10909","TGCTACTGGTGGACACGTAC","chr5",149609622,"+","none","CGG","TGCTACTGGTGGACACGTACCGG",0,NA,NA,NA,"GeneID:389337","ARHGEF37",TRUE,"ARHGEF37"

"10910","AGCTGAGACCTTAACCCCAG","chr4",105589278,"+","none","AGG","AGCTGAGACCTTAACCCCAGAGG",0,NA,NA,NA,"GeneID:54848","ARHGEF38",TRUE,"ARHGEF38"

"10911","CCCTATTACTGTGCGAACTT","chr4",105645255,"+","none","CGG","CCCTATTACTGTGCGAACTTCGG",0,NA,NA,NA,"GeneID:54848","ARHGEF38",TRUE,"ARHGEF38"

"10912","GCTACAGAACACTCACCCTG","chr4",105589261,"-","none","TGG","GCTACAGAACACTCACCCTGTGG",0,NA,NA,NA,"GeneID:54848","ARHGEF38",TRUE,"ARHGEF38"

"10913","GGCCACAACAGACGTGGAAC","chr4",105613469,"+","none","CGG","GGCCACAACAGACGTGGAACCGG",0,NA,NA,NA,"GeneID:54848","ARHGEF38",TRUE,"ARHGEF38"

"10914","GGCCACAACAGACGTGGAAC","chr5",179306993,"-","12:A>G,17:C>T","TGG","GGCCACAACAGATGTGGGACTGG",2,13,18,NA,"GeneID:9509","ADAMTS2",FALSE,"ARHGEF38"

"10915","GTTTCTTCAGACTGATAGGC","chr4",105613373,"+","none","TGG","GTTTCTTCAGACTGATAGGCTGG",0,NA,NA,NA,"GeneID:54848","ARHGEF38",TRUE,"ARHGEF38"

"10916","TCCGAAGTTCGCACAGTAAT","chr4",105645259,"-","none","AGG","TCCGAAGTTCGCACAGTAATAGG",0,NA,NA,NA,"GeneID:54848","ARHGEF38",TRUE,"ARHGEF38"

"10917","ACTGCTTATGTCCACAGACC","chr9",35662224,"-","none","AGG","ACTGCTTATGTCCACAGACCAGG",0,NA,NA,NA,"GeneID:84904","ARHGEF39",TRUE,"ARHGEF39"

"10918","CCGGAGGTTTGTACGGCTTC","chr9",35664080,"-","none","AGG","CCGGAGGTTTGTACGGCTTCAGG",0,NA,NA,NA,"GeneID:84904","ARHGEF39",TRUE,"ARHGEF39"

"10919","CCTGAAGCCGTACAAACCTC","chr9",35664077,"+","none","CGG","CCTGAAGCCGTACAAACCTCCGG",0,NA,NA,NA,"GeneID:84904","ARHGEF39",TRUE,"ARHGEF39"

"10920","GCTGGTACCACAGTCTGACT","chr9",35662189,"-","none","TGG","GCTGGTACCACAGTCTGACTTGG",0,NA,NA,NA,"GeneID:84904","ARHGEF39",TRUE,"ARHGEF39"

"10921","GGAGGGCTTCTGCCGCCACT","chr9",35664427,"-","none","TGG","GGAGGGCTTCTGCCGCCACTTGG",0,NA,NA,NA,"GeneID:84904","ARHGEF39",TRUE,"ARHGEF39"

"10922","TCTCTGAGTTGGCAGCAAAT","chr9",35664391,"+","none","TGG","TCTCTGAGTTGGCAGCAAATTGG",0,NA,NA,NA,"GeneID:84904","ARHGEF39",TRUE,"ARHGEF39"

"10923","AGCTGTCTGCGCTGACGAAG","chr2",131027969,"+","none","TGG","AGCTGTCTGCGCTGACGAAGTGG",0,NA,NA,NA,"GeneID:50649","ARHGEF4",TRUE,"ARHGEF4"

"10924","CCATGTCACCATGGACGACC","chr2",131038896,"+","none","AGG","CCATGTCACCATGGACGACCAGG",0,NA,NA,NA,"GeneID:50649","ARHGEF4",TRUE,"ARHGEF4"

"10925","CCATGTCACCATGGACGACC","chr13",24286257,"+","6:G>C,15:T>C","AGG","CCATGTGACCATGGATGACCAGG",2,7,16,NA,"GeneID:221178","SPATA13",TRUE,"ARHGEF4"

"10926","CCCATGTGGTTCAGTCCCGT","chr2",130946589,"-","none","TGG","CCCATGTGGTTCAGTCCCGTTGG",0,NA,NA,NA,"GeneID:50649","ARHGEF4",TRUE,"ARHGEF4"

"10927","GCACCTGCGCGACATCTGCG","chr2",131040170,"+","none","AGG","GCACCTGCGCGACATCTGCGAGG",0,NA,NA,NA,"GeneID:50649","ARHGEF4",TRUE,"ARHGEF4"

"10928","TCACCTCGCAGATGTCGCGC","chr2",131040176,"-","none","AGG","TCACCTCGCAGATGTCGCGCAGG",0,NA,NA,NA,"GeneID:50649","ARHGEF4",TRUE,"ARHGEF4"

"10929","TGGGGACGTCATCGAAGTGA","chr2",131038935,"+","none","TGG","TGGGGACGTCATCGAAGTGATGG",0,NA,NA,NA,"GeneID:50649","ARHGEF4",TRUE,"ARHGEF4"

"10930","ACTCACGTGGCGAAGGAAGC","chr14",21082464,"-","none","AGG","ACTCACGTGGCGAAGGAAGCAGG",0,NA,NA,NA,"GeneID:55701","ARHGEF40",TRUE,"ARHGEF40"

"10931","GAGGCGCACAAGCTGGTGAA","chr14",21078933,"-","none","TGG","GAGGCGCACAAGCTGGTGAATGG",0,NA,NA,NA,"GeneID:55701","ARHGEF40",TRUE,"ARHGEF40"

"10932","GCCCAGGTGCCCCGAAGTTC","chr14",21082343,"-","none","AGG","GCCCAGGTGCCCCGAAGTTCAGG",0,NA,NA,NA,"GeneID:55701","ARHGEF40",TRUE,"ARHGEF40"

"10933","GGACGCACTGAGGTACACGC","chr14",21073166,"+","none","TGG","GGACGCACTGAGGTACACGCTGG",0,NA,NA,NA,"GeneID:55701","ARHGEF40",TRUE,"ARHGEF40"

"10934","TCACCTGCTGGAGTCGCCGC","chr14",21078994,"-","none","AGG","TCACCTGCTGGAGTCGCCGCAGG",0,NA,NA,NA,"GeneID:55701","ARHGEF40",TRUE,"ARHGEF40"

"10935","TTGCCTCAAAGGGTGGATAC","chr14",21073091,"-","none","AGG","TTGCCTCAAAGGGTGGATACAGG",0,NA,NA,NA,"GeneID:55701","ARHGEF40",TRUE,"ARHGEF40"

"10936","AGATTCAAGAGGTCCAGCCG","chr7",144366352,"+","none","TGG","AGATTCAAGAGGTCCAGCCGTGG",0,NA,NA,NA,"GeneID:7984","ARHGEF5",TRUE,"ARHGEF5"

"10937","AGATTCAAGAGGTCCAGCCG","chr7",144283263,"-","none","TGG","AGATTCAAGAGGTCCAGCCGTGG",0,NA,NA,NA,"","",TRUE,"ARHGEF5"

"10938","ATTTGAGCTGATTGTGTCAG","chr7",144371165,"+","none","AGG","ATTTGAGCTGATTGTGTCAGAGG",0,NA,NA,NA,"GeneID:7984","ARHGEF5",TRUE,"ARHGEF5"

"10939","ATTTGAGCTGATTGTGTCAG","chr7",144278450,"-","none","AGG","ATTTGAGCTGATTGTGTCAGAGG",0,NA,NA,NA,"","",TRUE,"ARHGEF5"

"10940","ATTTGAGCTGATTGTGTCAG","chr18",4124551,"-","1:T>A,12:T>A","TGG","AATTGAGCTGATAGTGTCAGTGG",2,2,13,NA,"GeneID:9229","DLGAP1",FALSE,"ARHGEF5"

"10941","CCACAGGGAGAAAAAACCAA","chr7",144366839,"+","none","AGG","CCACAGGGAGAAAAAACCAAAGG",0,NA,NA,NA,"GeneID:7984","ARHGEF5",TRUE,"ARHGEF5"

"10942","CCACAGGGAGAAAAAACCAA","chr7",144282776,"-","none","AGG","CCACAGGGAGAAAAAACCAAAGG",0,NA,NA,NA,"","",TRUE,"ARHGEF5"

"10943","CCACAGGGAGAAAAAACCAA","chr5",81929189,"-","17:T>G,19:G>T","AGG","CCACAGGGAGAAAAAACAACAGG",2,18,20,NA,"","",FALSE,"ARHGEF5"

"10944","CCACAGGGAGAAAAAACCAA","chr20",62151419,"-","11:G>T,15:G>T","GGG","CCACAGGGAGACAAACCCAAGGG",2,12,16,NA,"GeneID:26039","SS18L1",FALSE,"ARHGEF5"

"10945","CCACAGGGAGAAAAAACCAA","chr12",120678387,"-","2:G>T,3:T>G","AGG","CCCAAGGGAGAAAAAACCAAAGG",2,3,4,NA,"","",FALSE,"ARHGEF5"

"10946","CCACAGGGAGAAAAAACCAA","chrX",65642869,"+","2:T>A,9:A>G","TGG","CCTCAGGGAAAAAAAACCAATGG",2,3,10,NA,"GeneID:4478","MSN",FALSE,"ARHGEF5"

"10947","CCACAGGGAGAAAAAACCAA","chr19",35292409,"+","6:A>G,17:A>C","GGG","CCACAGAGAGAAAAAACAAAGGG",2,7,18,NA,"GeneID:4099","MAG",FALSE,"ARHGEF5"

"10948","CCACAGGGAGAAAAAACCAA","chr2",118314032,"-","3:C>G,19:A>T","TGG","CCAGAGGGAGAAAAAACCATTGG",2,4,20,NA,"","",FALSE,"ARHGEF5"

"10949","CCACAGGGAGAAAAAACCAA","chr21",39861915,"-","0:T>G,15:C>T","GGG","ACACAGGGAGAAAAAGCCAAGGG",2,1,16,NA,"","",FALSE,"ARHGEF5"

"10950","CCGAAAAACAACAGTGTCTG","chr7",144283222,"+","none","AGG","CCGAAAAACAACAGTGTCTGAGG",0,NA,NA,NA,"","",TRUE,"ARHGEF5"

"10951","CCGAAAAACAACAGTGTCTG","chr7",144366393,"-","none","AGG","CCGAAAAACAACAGTGTCTGAGG",0,NA,NA,NA,"GeneID:7984","ARHGEF5",TRUE,"ARHGEF5"

"10952","CTCACAGGAGTTGATGAGTT","chr7",144282720,"+","none","TGG","CTCACAGGAGTTGATGAGTTTGG",0,NA,NA,NA,"","",TRUE,"ARHGEF5"

"10953","CTCACAGGAGTTGATGAGTT","chr7",144366895,"-","none","TGG","CTCACAGGAGTTGATGAGTTTGG",0,NA,NA,NA,"GeneID:7984","ARHGEF5",TRUE,"ARHGEF5"

"10954","CTCACAGGAGTTGATGAGTT","chr8",69528295,"-","9:T>C,13:G>T","GGG","CTCACAGGAATTGCTGAGTTGGG",2,10,14,NA,"GeneID:23213","SULF1",FALSE,"ARHGEF5"

"10955","GTAAACGAGAGAAGAGCCAT","chr7",144278339,"+","none","TGG","GTAAACGAGAGAAGAGCCATTGG",0,NA,NA,NA,"","",TRUE,"ARHGEF5"

"10956","GTAAACGAGAGAAGAGCCAT","chr7",144371276,"-","none","TGG","GTAAACGAGAGAAGAGCCATTGG",0,NA,NA,NA,"GeneID:7984","ARHGEF5",TRUE,"ARHGEF5"

"10957","GATCCTGATGACCTTTATTC","chrX",136747566,"-","none","AGG","GATCCTGATGACCTTTATTCAGG",0,NA,NA,NA,"GeneID:9459","ARHGEF6",TRUE,"ARHGEF6"

"10958","GTCCGTGAAATTAAATCCAG","chrX",136743586,"-","none","TGG","GTCCGTGAAATTAAATCCAGTGG",0,NA,NA,NA,"GeneID:9459","ARHGEF6",TRUE,"ARHGEF6"

"10959","GTGCTAGAAACTGCTCCCTG","chrX",136745256,"+","none","TGG","GTGCTAGAAACTGCTCCCTGTGG",0,NA,NA,NA,"GeneID:9459","ARHGEF6",TRUE,"ARHGEF6"

"10960","GTGCTAGAAACTGCTCCCTG","chrX",114889665,"-","6:T>C,12:A>C","AGG","GTGCTAAAAACTTCTCCCTGAGG",2,7,13,NA,"GeneID:3358","HTR2C",FALSE,"ARHGEF6"

"10961","TAAAAGAGTACTCAGTACCT","chrX",136747529,"+","none","TGG","TAAAAGAGTACTCAGTACCTTGG",0,NA,NA,NA,"GeneID:9459","ARHGEF6",TRUE,"ARHGEF6"

"10962","TAAAAGAGTACTCAGTACCT","chr5",115645216,"+","17:A>C,18:G>C","GGG","TAAAAGAGTACTCAGTAAGTGGG",2,18,19,NA,"","",FALSE,"ARHGEF6"

"10963","TACGTCACACGAGTTGAAGA","chrX",136743664,"-","none","AGG","TACGTCACACGAGTTGAAGAAGG",0,NA,NA,NA,"GeneID:9459","ARHGEF6",TRUE,"ARHGEF6"

"10964","TACTTCTCAGACAAACCCAC","chrX",136745275,"-","none","AGG","TACTTCTCAGACAAACCCACAGG",0,NA,NA,NA,"GeneID:9459","ARHGEF6",TRUE,"ARHGEF6"

"10965","AACATCCGCGAGTTCCTGCG","chr13",111153943,"+","none","CGG","AACATCCGCGAGTTCCTGCGCGG",0,NA,NA,NA,"GeneID:8874","ARHGEF7",TRUE,"ARHGEF7"

"10966","ACTCTAAATAAAGTAACAGC","chr13",111205351,"+","none","AGG","ACTCTAAATAAAGTAACAGCAGG",0,NA,NA,NA,"GeneID:8874","ARHGEF7",TRUE,"ARHGEF7"

"10967","ACTCTAAATAAAGTAACAGC","chr18",72079041,"-","9:A>T,19:T>G","AGG","ACTCTAAATTAAGTAACAGAAGG",2,10,20,NA,"","",FALSE,"ARHGEF7"

"10968","ACTGCACCAGCGCCAAAACA","chr13",111288404,"+","none","AGG","ACTGCACCAGCGCCAAAACAAGG",0,NA,NA,NA,"GeneID:8874","ARHGEF7",TRUE,"ARHGEF7"

"10969","CGCAGCCGCGCAGGAACTCG","chr13",111153951,"-","none","CGG","CGCAGCCGCGCAGGAACTCGCGG",0,NA,NA,NA,"GeneID:8874","ARHGEF7",TRUE,"ARHGEF7"

"10970","CTTGACAGGCACAGCTGCTT","chr13",111288345,"+","none","TGG","CTTGACAGGCACAGCTGCTTTGG",0,NA,NA,NA,"GeneID:8874","ARHGEF7",TRUE,"ARHGEF7"

"10971","CTTGACAGGCACAGCTGCTT","chr2",4784503,"-","8:T>C,14:T>G","AGG","CTTGACAGACACAGATGCTTAGG",2,9,15,NA,"","",FALSE,"ARHGEF7"

"10972","CTTGACAGGCACAGCTGCTT","chr1",85313937,"-","4:C>T,17:A>G","TGG","CTTGGCAGGCACAGCTGTTTTGG",2,5,18,NA,"","",FALSE,"ARHGEF7"

"10973","TACTTTATTTAGAGTCACTA","chr13",111205346,"-","none","AGG","TACTTTATTTAGAGTCACTAAGG",0,NA,NA,NA,"GeneID:8874","ARHGEF7",TRUE,"ARHGEF7"

"10974","ACTTACGTGCTCTAGGAAGC","chrX",63697118,"+","none","AGG","ACTTACGTGCTCTAGGAAGCAGG",0,NA,NA,NA,"GeneID:23229","ARHGEF9",TRUE,"ARHGEF9"

"10975","ACTTACGTGCTCTAGGAAGC","chr2",139123985,"+","0:T>A,6:T>G","CGG","TCTTACTTGCTCTAGGAAGCCGG",2,1,7,NA,"","",FALSE,"ARHGEF9"

"10976","CAAGCACCATGCAGTGGATA","chrX",63785133,"-","none","AGG","CAAGCACCATGCAGTGGATAAGG",0,NA,NA,NA,"GeneID:23229","ARHGEF9",TRUE,"ARHGEF9"

"10977","CAAGCACCATGCAGTGGATA","chr12",111049928,"-","14:T>A,17:A>T","GGG","CAAGCACCATGCAGAGGTTAGGG",2,15,18,NA,"GeneID:23316","CUX2",FALSE,"ARHGEF9"

"10978","GACCCCCACCTCAGCGAGAT","chrX",63697146,"-","none","AGG","GACCCCCACCTCAGCGAGATAGG",0,NA,NA,NA,"GeneID:23229","ARHGEF9",TRUE,"ARHGEF9"

"10979","TCACTACATCAAGCACCTCA","chrX",63706271,"-","none","AGG","TCACTACATCAAGCACCTCAAGG",0,NA,NA,NA,"GeneID:23229","ARHGEF9",TRUE,"ARHGEF9"

"10980","TCCGCCCCTTATCCACTGCA","chrX",63785124,"+","none","TGG","TCCGCCCCTTATCCACTGCATGG",0,NA,NA,NA,"GeneID:23229","ARHGEF9",TRUE,"ARHGEF9"

"10981","TTACCTCACAAATATCCTTG","chrX",63706253,"+","none","AGG","TTACCTCACAAATATCCTTGAGG",0,NA,NA,NA,"GeneID:23229","ARHGEF9",TRUE,"ARHGEF9"

"10982","TTACCTCACAAATATCCTTG","chr12",45445896,"+","10:G>A,12:G>T","GGG","TTACCTCACAGAGATCCTTGGGG",2,11,13,NA,"","",FALSE,"ARHGEF9"

"10983","AATACTCACAGGCAAGCTGG","chr1",26732781,"-","none","AGG","AATACTCACAGGCAAGCTGGAGG",0,NA,NA,NA,"GeneID:8289","ARID1A",TRUE,"ARID1A"

"10984","AATACTCACAGGCAAGCTGG","chr6",75049811,"-","6:A>G,12:C>G","AGG","AATACTTACAGGGAAGCTGGAGG",2,7,13,NA,"","",FALSE,"ARID1A"

"10985","ATGGTCATCGGGTACCGCTG","chr1",26729794,"-","none","CGG","ATGGTCATCGGGTACCGCTGCGG",0,NA,NA,NA,"GeneID:8289","ARID1A",TRUE,"ARID1A"

"10986","CCCCTCAATGACCTCCAGTA","chr1",26732716,"+","none","AGG","CCCCTCAATGACCTCCAGTAAGG",0,NA,NA,NA,"GeneID:8289","ARID1A",TRUE,"ARID1A"

"10987","GCGGTACCCGATGACCATGC","chr1",26729796,"+","none","AGG","GCGGTACCCGATGACCATGCAGG",0,NA,NA,NA,"GeneID:8289","ARID1A",TRUE,"ARID1A"

"10988","TGAGCGAGACTGAGCAACAC","chr1",26761048,"-","none","TGG","TGAGCGAGACTGAGCAACACTGG",0,NA,NA,NA,"GeneID:8289","ARID1A",TRUE,"ARID1A"

"10989","TTCAATAGATGACCTCCCCA","chr1",26760866,"+","none","TGG","TTCAATAGATGACCTCCCCATGG",0,NA,NA,NA,"GeneID:8289","ARID1A",TRUE,"ARID1A"

"10990","CAAAGTTGCTTCCGTTCCCG","chr6",157084692,"-","none","TGG","CAAAGTTGCTTCCGTTCCCGTGG",0,NA,NA,NA,"GeneID:57492","ARID1B",TRUE,"ARID1B"

"10991","CAGCAGAGCAGTCCGTACCC","chr6",156829301,"+","none","AGG","CAGCAGAGCAGTCCGTACCCAGG",0,NA,NA,NA,"GeneID:57492","ARID1B",TRUE,"ARID1B"

"10992","CTCTAGCCTGATGAACACGC","chr6",157167117,"+","none","AGG","CTCTAGCCTGATGAACACGCAGG",0,NA,NA,NA,"GeneID:57492","ARID1B",TRUE,"ARID1B"

"10993","GCGGCGCCTGCGTGTTCATC","chr6",157167126,"-","none","AGG","GCGGCGCCTGCGTGTTCATCAGG",0,NA,NA,NA,"GeneID:57492","ARID1B",TRUE,"ARID1B"

"10994","GGAAGCAACCAGTCTCGATC","chr6",157084856,"+","none","TGG","GGAAGCAACCAGTCTCGATCTGG",0,NA,NA,NA,"GeneID:57492","ARID1B",TRUE,"ARID1B"

"10995","GTCCGACCCTGGATGCCAAT","chr6",156829361,"-","none","TGG","GTCCGACCCTGGATGCCAATTGG",0,NA,NA,NA,"GeneID:57492","ARID1B",TRUE,"ARID1B"

"10996","AGACACATGAAGTTCTGTCC","chr12",45860911,"-","none","AGG","AGACACATGAAGTTCTGTCCAGG",0,NA,NA,NA,"GeneID:196528","ARID2",TRUE,"ARID2"

"10997","AGACACATGAAGTTCTGTCC","chr15",40277280,"-","6:G>T,16:A>C","TGG","AGACACCTGAAGTTCTTTCCTGG",2,7,17,NA,"GeneID:56924","PAK6",TRUE,"ARID2"

"10998","AGCTGTGGCTTTGGATTGCC","chr12",45811478,"-","none","TGG","AGCTGTGGCTTTGGATTGCCTGG",0,NA,NA,NA,"GeneID:196528","ARID2",TRUE,"ARID2"

"10999","AGGTTTCTGAGAAGAATCAG","chr12",45731214,"+","none","TGG","AGGTTTCTGAGAAGAATCAGTGG",0,NA,NA,NA,"GeneID:196528","ARID2",TRUE,"ARID2"

"11000","CTTTAAAACAGTATTACTTG","chr12",45731292,"+","none","CGG","CTTTAAAACAGTATTACTTGCGG",0,NA,NA,NA,"GeneID:196528","ARID2",TRUE,"ARID2"

"11001","TACCCGACACACTGTGTTGC","chr12",45811534,"-","none","TGG","TACCCGACACACTGTGTTGCTGG",0,NA,NA,NA,"GeneID:196528","ARID2",TRUE,"ARID2"

"11002","TGGCCCTGCACTTGTACAGC","chr12",45860824,"-","none","TGG","TGGCCCTGCACTTGTACAGCTGG",0,NA,NA,NA,"GeneID:196528","ARID2",TRUE,"ARID2"

"11003","CCCAACTCACATGTCCTCGT","chr19",929886,"-","none","CGG","CCCAACTCACATGTCCTCGTCGG",0,NA,NA,NA,"GeneID:1820","ARID3A",TRUE,"ARID3A"

"11004","CCTGTTCATGCTGTACGTGC","chr19",964293,"+","none","TGG","CCTGTTCATGCTGTACGTGCTGG",0,NA,NA,NA,"GeneID:1820","ARID3A",TRUE,"ARID3A"

"11005","CTTTCGGGGGAACAGTGCCG","chr19",932557,"-","none","TGG","CTTTCGGGGGAACAGTGCCGTGG",0,NA,NA,NA,"GeneID:1820","ARID3A",TRUE,"ARID3A"

"11006","TCACAGCTCTACGAACTCGA","chr19",960085,"+","none","CGG","TCACAGCTCTACGAACTCGACGG",0,NA,NA,NA,"GeneID:1820","ARID3A",TRUE,"ARID3A"

"11007","TCCGCGGCGATGGCGTTCCC","chr19",932596,"+","none","AGG","TCCGCGGCGATGGCGTTCCCAGG",0,NA,NA,NA,"GeneID:1820","ARID3A",TRUE,"ARID3A"

"11008","TGAGGACATGGCCTCCGACG","chr19",929869,"+","none","AGG","TGAGGACATGGCCTCCGACGAGG",0,NA,NA,NA,"GeneID:1820","ARID3A",TRUE,"ARID3A"

"11009","CCAGCGCCGCCTTCACCCTC","chr15",74589975,"+","none","AGG","CCAGCGCCGCCTTCACCCTCAGG",0,NA,NA,NA,"GeneID:10620","ARID3B",TRUE,"ARID3B"

"11010","CCAGCGCCGCCTTCACCCTC","chr21",25562157,"+","12:G>T,15:G>C","GGG","CCAGCGCCGCCTGCAGCCTCGGG",2,13,16,NA,"GeneID:114614","MIR155HG",TRUE,"ARID3B"

"11011","CTCGAGAGATCTCTCTTCCC","chr1",81502374,"-","none","CGG","CTCGAGAGATCTCTCTTCCCCGG",0,NA,NA,NA,"GeneID:23266","LPHN2",TRUE,"ARID3B"

"11012","CTCGAGAGATCTCTCTTCCC","chr15",74572902,"-","none","CGG","CTCGAGAGATCTCTCTTCCCCGG",0,NA,NA,NA,"GeneID:10620","ARID3B",TRUE,"ARID3B"

"11013","GGGATTGCAGCTGTATGAAC","chr15",74573121,"+","none","TGG","GGGATTGCAGCTGTATGAACTGG",0,NA,NA,NA,"GeneID:10620","ARID3B",TRUE,"ARID3B"

"11014","GGGATTGCAGCTGTATGAAC","chr5",128441015,"-","10:T>G,18:G>T","TGG","GGGATTGCAGATGTATGACCTGG",2,11,19,NA,"GeneID:2201","FBN2",FALSE,"ARID3B"

"11015","GTACCAATACCAGTAGCCCT","chr15",74591383,"+","none","CGG","GTACCAATACCAGTAGCCCTCGG",0,NA,NA,NA,"GeneID:10620","ARID3B",TRUE,"ARID3B"

"11016","TGGCCACTCACTGCGTCCTG","chr15",74589994,"-","none","AGG","TGGCCACTCACTGCGTCCTGAGG",0,NA,NA,NA,"GeneID:10620","ARID3B",TRUE,"ARID3B"

"11017","TGGCCACTCACTGCGTCCTG","chr1",148351425,"-","13:T>G","GGG","TGGCCACTCACTGAGTCCTGGGG",1,14,NA,NA,"GeneID:388685","LINC01138",FALSE,"ARID3B"

"11018","TGGCCACTCACTGCGTCCTG","chr19",34393678,"-","7:T>A,9:G>T","TGG","TGGCCACACCCTGCGTCCTGTGG",2,8,10,NA,"GeneID:2821","GPI",TRUE,"ARID3B"

"11019","TGGGGATATCCGAGGGCTAC","chr15",74591395,"-","none","TGG","TGGGGATATCCGAGGGCTACTGG",0,NA,NA,NA,"GeneID:10620","ARID3B",TRUE,"ARID3B"

"11020","TGGGGATATCCGAGGGCTAC","chr1",81502912,"-","9:A>G","TGG","TGGGGATATTCGAGGGCTACTGG",1,10,NA,NA,"GeneID:23266","LPHN2",TRUE,"ARID3B"

"11021","AGCTCGATGCAGACCCCAAG","chr9",34625787,"-","none","AGG","AGCTCGATGCAGACCCCAAGAGG",0,NA,NA,NA,"GeneID:138715","ARID3C",TRUE,"ARID3C"

"11022","AGTGAACCGCGTGCCCATCA","chr9",34624020,"-","none","TGG","AGTGAACCGCGTGCCCATCATGG",0,NA,NA,NA,"GeneID:138715","ARID3C",TRUE,"ARID3C"

"11023","CCTGTACGCTCTGTTTCGCC","chr9",34623981,"-","none","TGG","CCTGTACGCTCTGTTTCGCCTGG",0,NA,NA,NA,"GeneID:138715","ARID3C",TRUE,"ARID3C"

"11024","CTGCTTGAATTGTTCCTCGT","chr9",34627696,"+","none","AGG","CTGCTTGAATTGTTCCTCGTAGG",0,NA,NA,NA,"GeneID:138715","ARID3C",TRUE,"ARID3C"

"11025","CTGCTTGAATTGTTCCTCGT","chr12",26685795,"+","9:A>T,18:C>G","AGG","CTGCTTGAAATGTTCCTCCTAGG",2,10,19,NA,"GeneID:3709","ITPR2",FALSE,"ARID3C"

"11026","TCCCCACGAGTGGACCTACG","chr9",34627713,"-","none","AGG","TCCCCACGAGTGGACCTACGAGG",0,NA,NA,NA,"GeneID:138715","ARID3C",TRUE,"ARID3C"

"11027","TGTTTAGCTTCATGCAAAAG","chr9",34625745,"-","none","AGG","TGTTTAGCTTCATGCAAAAGAGG",0,NA,NA,NA,"GeneID:138715","ARID3C",TRUE,"ARID3C"

"11028","CTCTTAAAGGACCCTTTACT","chr14",58304990,"-","none","TGG","CTCTTAAAGGACCCTTTACTTGG",0,NA,NA,NA,"GeneID:5926","ARID4A",TRUE,"ARID4A"

"11029","GATGTCAGTGCCAAGTACCG","chr14",58301615,"+","none","AGG","GATGTCAGTGCCAAGTACCGAGG",0,NA,NA,NA,"GeneID:5926","ARID4A",TRUE,"ARID4A"

"11030","GATGTCAGTGCCAAGTACCG","chr9",113491971,"+","3:C>G,18:A>C","TGG","GATCTCAGTGCCAAGTACAGTGG",2,4,19,NA,"GeneID:5998","RGS3",FALSE,"ARID4A"

"11031","GGTACAAGATGACCAAGTAA","chr14",58304975,"+","none","AGG","GGTACAAGATGACCAAGTAAAGG",0,NA,NA,NA,"GeneID:5926","ARID4A",TRUE,"ARID4A"

"11032","GTGTGGTATTATCCTGTTTC","chr14",58304951,"-","none","AGG","GTGTGGTATTATCCTGTTTCAGG",0,NA,NA,NA,"GeneID:5926","ARID4A",TRUE,"ARID4A"

"11033","GTGTGGTATTATCCTGTTTC","chr8",5677111,"-","10:A>T,12:A>G","TGG","GTGTGGTATTTTTCTGTTTCTGG",2,11,13,NA,"","",FALSE,"ARID4A"

"11034","TCGGTACTTGGCACTGACAT","chr14",58301616,"-","none","CGG","TCGGTACTTGGCACTGACATCGG",0,NA,NA,NA,"GeneID:5926","ARID4A",TRUE,"ARID4A"

"11035","TGAGCCTGCCTACCTGACAG","chr14",58301587,"+","none","TGG","TGAGCCTGCCTACCTGACAGTGG",0,NA,NA,NA,"GeneID:5926","ARID4A",TRUE,"ARID4A"

"11036","TGAGCCTGCCTACCTGACAG","chr18",22350442,"+","1:C>G,2:T>A","TGG","TCTGCCTGCCTACCTGACAGTGG",2,2,3,NA,"","",FALSE,"ARID4A"

"11037","AGTTCAGGATGACCACATAA","chr1",235257173,"-","none","AGG","AGTTCAGGATGACCACATAAAGG",0,NA,NA,NA,"GeneID:51742","ARID4B",TRUE,"ARID4B"

"11038","ATCAGTGCCCACTGTCAAAT","chr1",235260713,"+","none","AGG","ATCAGTGCCCACTGTCAAATAGG",0,NA,NA,NA,"GeneID:51742","ARID4B",TRUE,"ARID4B"

"11039","CCTTTAGTGGGCCCTTTATG","chr1",235257158,"+","none","TGG","CCTTTAGTGGGCCCTTTATGTGG",0,NA,NA,NA,"GeneID:51742","ARID4B",TRUE,"ARID4B"

"11040","CCTTTAGTGGGCCCTTTATG","chr6",121260128,"-","0:T>G,17:G>T","TGG","ACTTTAGTGGGCCCTTTCTGTGG",2,1,18,NA,"GeneID:221322","TBC1D32",FALSE,"ARID4B"

"11041","GATGTGAGTGCTAAATACAG","chr1",235260696,"-","none","AGG","GATGTGAGTGCTAAATACAGAGG",0,NA,NA,NA,"GeneID:51742","ARID4B",TRUE,"ARID4B"

"11042","GATGTGAGTGCTAAATACAG","chr22",19966011,"+","6:G>A,14:T>A","GGG","GATGTGGGTGCTAATTACAGGGG",2,7,15,NA,"GeneID:1312","COMT",FALSE,"ARID4B"

"11043","TAGACATGATTCTTCAACAG","chr1",235257197,"-","none","TGG","TAGACATGATTCTTCAACAGTGG",0,NA,NA,NA,"GeneID:51742","ARID4B",TRUE,"ARID4B"

"11044","TAGACATGATTCTTCAACAG","chr9",95499207,"+","0:A>T,3:T>A","GGG","AAGTCATGATTCTTCAACAGGGG",2,1,4,NA,"GeneID:5727","PTCH1",FALSE,"ARID4B"

"11045","TGAGCCTCCCTATTTGACAG","chr1",235260724,"-","none","TGG","TGAGCCTCCCTATTTGACAGTGG",0,NA,NA,NA,"GeneID:51742","ARID4B",TRUE,"ARID4B"

"11046","AAGAACGTGTACGACGAGCT","chr2",96550208,"+","none","GGG","AAGAACGTGTACGACGAGCTGGG",0,NA,NA,NA,"GeneID:10865","ARID5A",TRUE,"ARID5A"

"11047","AATCCAGAACCCCATCTCGC","chr2",96547492,"+","none","TGG","AATCCAGAACCCCATCTCGCTGG",0,NA,NA,NA,"GeneID:10865","ARID5A",TRUE,"ARID5A"

"11048","AGCGACACACGCCCATCGAG","chr2",96549411,"+","none","AGG","AGCGACACACGCCCATCGAGAGG",0,NA,NA,NA,"GeneID:10865","ARID5A",TRUE,"ARID5A"

"11049","CCCCGCCGTACCTCTCGTAG","chr2",96550276,"-","none","TGG","CCCCGCCGTACCTCTCGTAGTGG",0,NA,NA,NA,"GeneID:10865","ARID5A",TRUE,"ARID5A"

"11050","GCTCCTTCATGAACTTGTAG","chr2",96549394,"-","none","AGG","GCTCCTTCATGAACTTGTAGAGG",0,NA,NA,NA,"GeneID:10865","ARID5A",TRUE,"ARID5A"

"11051","TCAACTCACCTCCAGCGAGA","chr2",96547506,"-","none","TGG","TCAACTCACCTCCAGCGAGATGG",0,NA,NA,NA,"GeneID:10865","ARID5A",TRUE,"ARID5A"

"11052","AGGCAGTCAACCCTAAACAG","chr10",61940344,"+","none","TGG","AGGCAGTCAACCCTAAACAGTGG",0,NA,NA,NA,"GeneID:84159","ARID5B",TRUE,"ARID5B"

"11053","CAAAGGTGTCCCGACAGTAC","chr10",62000256,"-","none","AGG","CAAAGGTGTCCCGACAGTACAGG",0,NA,NA,NA,"GeneID:84159","ARID5B",TRUE,"ARID5B"

"11054","CAGAAATAGCGACCATGGCG","chr10",61902391,"+","none","AGG","CAGAAATAGCGACCATGGCGAGG",0,NA,NA,NA,"GeneID:84159","ARID5B",TRUE,"ARID5B"

"11055","CAGAATGTACCCACTTGACC","chr10",61940232,"-","none","AGG","CAGAATGTACCCACTTGACCAGG",0,NA,NA,NA,"GeneID:84159","ARID5B",TRUE,"ARID5B"

"11056","GCTATGCAAATCGGATCCTT","chr10",61902281,"-","none","TGG","GCTATGCAAATCGGATCCTTTGG",0,NA,NA,NA,"GeneID:84159","ARID5B",TRUE,"ARID5B"

"11057","GGATGCGTTTCAGCATCGAG","chr10",62000151,"-","none","CGG","GGATGCGTTTCAGCATCGAGCGG",0,NA,NA,NA,"GeneID:84159","ARID5B",TRUE,"ARID5B"

"11058","AAAACTTATGTCCACATTCA","chr15",72555284,"-","none","AGG","AAAACTTATGTCCACATTCAAGG",0,NA,NA,NA,"GeneID:25820","ARIH1",TRUE,"ARIH1"

"11059","AAGATATCACAACCATGAGC","chr15",72555866,"-","none","AGG","AAGATATCACAACCATGAGCAGG",0,NA,NA,NA,"GeneID:25820","ARIH1",TRUE,"ARIH1"

"11060","AATAACAAATAGCTTTGTAG","chr15",72561527,"+","none","AGG","AATAACAAATAGCTTTGTAGAGG",0,NA,NA,NA,"GeneID:25820","ARIH1",TRUE,"ARIH1"

"11061","AATAACAAATAGCTTTGTAG","chr17",69647408,"+","16:A>G,18:T>A","AGG","AATAACAAATAGCTTTATTGAGG",2,17,19,NA,"GeneID:101928122","LINC01483",FALSE,"ARIH1"

"11062","AATAACAAATAGCTTTGTAG","chr11",101279079,"+","11:A>G,13:A>T","AGG","AATAACAAATAACATTGTAGAGG",2,12,14,NA,"","",FALSE,"ARIH1"

"11063","AATAACAAATAGCTTTGTAG","chr20",13212809,"-","3:C>T,5:C>G","AGG","AATGAGAAATAGCTTTGTAGAGG",2,4,6,NA,"","",FALSE,"ARIH1"

"11064","ACTATTTCGTGTCCTGCTCA","chr15",72555851,"+","none","TGG","ACTATTTCGTGTCCTGCTCATGG",0,NA,NA,NA,"GeneID:25820","ARIH1",TRUE,"ARIH1"

"11065","CATGCCTTCTTCCATTATTT","chr15",72555337,"-","none","TGG","CATGCCTTCTTCCATTATTTTGG",0,NA,NA,NA,"GeneID:25820","ARIH1",TRUE,"ARIH1"

"11066","CATGCCTTCTTCCATTATTT","chr11",84285408,"+","2:G>T,6:C>T","AGG","CAGGCCCTCTTCCATTATTTAGG",2,3,7,NA,"GeneID:1740","DLG2",FALSE,"ARIH1"

"11067","CATGCCTTCTTCCATTATTT","chrX",101000521,"-","3:A>C,10:C>A","TGG","CATTCCTTCTGCCATTATTTTGG",2,4,11,NA,"","",FALSE,"ARIH1"

"11068","TATTTCACTGGCCTTGAATG","chr15",72555270,"+","none","TGG","TATTTCACTGGCCTTGAATGTGG",0,NA,NA,NA,"GeneID:25820","ARIH1",TRUE,"ARIH1"

"11069","AACAAGCAGTTGAGCAGAAT","chr3",48964929,"-","none","TGG","AACAAGCAGTTGAGCAGAATTGG",0,NA,NA,NA,"GeneID:10425","ARIH2",TRUE,"ARIH2"

"11070","AACAAGCAGTTGAGCAGAAT","chr18",28652064,"-","3:C>T","TGG","AACGAGCAGTTGAGCAGAATTGG",1,4,NA,NA,"","",TRUE,"ARIH2"

"11071","AACAAGCAGTTGAGCAGAAT","chr18",28669957,"+","6:A>C,19:A>T","AGG","AACAAGAAGTTGAGCAGAAAAGG",2,7,20,NA,"","",FALSE,"ARIH2"

"11072","AGTGTGAGCACTAATGTAGT","chr3",48973790,"-","none","TGG","AGTGTGAGCACTAATGTAGTTGG",0,NA,NA,NA,"GeneID:10425","ARIH2",TRUE,"ARIH2"

"11073","AGTGTGAGCACTAATGTAGT","chr18",28652593,"-","13:G>T","CGG","AGTGTGAGCACTACTGTAGTCGG",1,14,NA,NA,"","",TRUE,"ARIH2"

"11074","CACACTTCGTGAGCCATTTC","chr3",48973749,"-","none","CGG","CACACTTCGTGAGCCATTTCCGG",0,NA,NA,NA,"GeneID:10425","ARIH2",TRUE,"ARIH2"

"11075","CACACTTCGTGAGCCATTTC","chr18",28652552,"-","8:A>C,19:A>G","CGG","CACACTTCTTGAGCCATTTTCGG",2,9,20,NA,"","",TRUE,"ARIH2"

"11076","CAGACTGCCCCATGGTTATT","chr3",48970625,"+","none","CGG","CAGACTGCCCCATGGTTATTCGG",0,NA,NA,NA,"GeneID:10425","ARIH2",TRUE,"ARIH2"

"11077","CAGACTGCCCCATGGTTATT","chr18",28652422,"+","9:T>C,17:G>A","CGG","CAGACTGCCTCATGGTTGTTCGG",2,10,18,NA,"","",TRUE,"ARIH2"

"11078","TGCACCGATTGCACTGTACT","chr3",48970671,"-","none","CGG","TGCACCGATTGCACTGTACTCGG",0,NA,NA,NA,"GeneID:10425","ARIH2",TRUE,"ARIH2"

"11079","TGCACCGATTGCACTGTACT","chr18",28652468,"-","15:T>C","CGG","TGCACCGATTGCACTATACTCGG",1,16,NA,NA,"","",TRUE,"ARIH2"

"11080","TTCTGCTCAACTGCTTGTTG","chr3",48964930,"+","none","AGG","TTCTGCTCAACTGCTTGTTGAGG",0,NA,NA,NA,"GeneID:10425","ARIH2",TRUE,"ARIH2"

"11081","TTCTGCTCAACTGCTTGTTG","chr18",28652065,"+","15:C>T","AGG","TTCTGCTCAACTGCTCGTTGAGG",1,16,NA,NA,"","",TRUE,"ARIH2"

"11082","TTCTGCTCAACTGCTTGTTG","chr12",118995185,"+","0:A>T,16:T>G","AGG","ATCTGCTCAACTGCTTTTTGAGG",2,1,17,NA,"GeneID:84530","SRRM4",FALSE,"ARIH2"

"11083","CCGATCCGGAAGTGACGCAA","chr3",48918804,"+","none","CGG","CCGATCCGGAAGTGACGCAACGG",0,NA,NA,NA,"GeneID:646450;GeneID:10425","ARIH2OS;ARIH2",TRUE,"ARIH2OS"

"11084","CCGCGCAGTTCTCCGACTTA","chr3",48918386,"-","none","CGG","CCGCGCAGTTCTCCGACTTACGG",0,NA,NA,NA,"GeneID:646450","ARIH2OS",TRUE,"ARIH2OS"

"11085","CGCTAAACAGGCCCTCTAGC","chr3",48918746,"+","none","CGG","CGCTAAACAGGCCCTCTAGCCGG",0,NA,NA,NA,"GeneID:646450","ARIH2OS",TRUE,"ARIH2OS"

"11086","GTAATGCTCGGACAACGTGC","chr3",48919132,"-","none","CGG","GTAATGCTCGGACAACGTGCCGG",0,NA,NA,NA,"GeneID:646450;GeneID:10425","ARIH2OS;ARIH2",TRUE,"ARIH2OS"

"11087","TGGCGCCGTTGCGTCACTTC","chr3",48918812,"-","none","CGG","TGGCGCCGTTGCGTCACTTCCGG",0,NA,NA,NA,"GeneID:646450;GeneID:10425","ARIH2OS;ARIH2",TRUE,"ARIH2OS"

"11088","TGTCGCGACCCGAGCGACCG","chr3",48918774,"+","none","TGG","TGTCGCGACCCGAGCGACCGTGG",0,NA,NA,NA,"GeneID:646450","ARIH2OS",TRUE,"ARIH2OS"

"11089","ATCAGAGTTAGTTGCCATGT","chr12",101401066,"-","none","TGG","ATCAGAGTTAGTTGCCATGTTGG",0,NA,NA,NA,"GeneID:400","ARL1",TRUE,"ARL1"

"11090","CATGGCAACTAACTCTGATT","chr12",101401067,"+","none","TGG","CATGGCAACTAACTCTGATTTGG",0,NA,NA,NA,"GeneID:400","ARL1",TRUE,"ARL1"

"11091","CATGGCAACTAACTCTGATT","chr2",8757720,"+","13:A>T,18:C>T","TGG","CATGGCAACTAACACTGACTTGG",2,14,19,NA,"GeneID:57498","KIDINS220",TRUE,"ARL1"

"11092","CGTCTCTACATTAAATCCAA","chr12",101402923,"+","none","TGG","CGTCTCTACATTAAATCCAATGG",0,NA,NA,NA,"GeneID:400","ARL1",TRUE,"ARL1"

"11093","TAGGAGGACAGACAAGTATC","chr12",101402866,"-","none","AGG","TAGGAGGACAGACAAGTATCAGG",0,NA,NA,NA,"GeneID:400","ARL1",TRUE,"ARL1"

"11094","TAGGAGGACAGACAAGTATC","chr14",88577112,"-","0:G>A,2:T>C","AGG","CAAGAGGACAGACAAGTATCAGG",2,1,3,NA,"GeneID:79882","ZC3H14",FALSE,"ARL1"

"11095","TTCCCGAGTTCCAAACAGAC","chr12",101405934,"+","none","TGG","TTCCCGAGTTCCAAACAGACTGG",0,NA,NA,NA,"GeneID:400","ARL1",TRUE,"ARL1"

"11096","TTGTAATCTGTACAAAATTG","chr12",101405871,"+","none","TGG","TTGTAATCTGTACAAAATTGTGG",0,NA,NA,NA,"GeneID:400","ARL1",TRUE,"ARL1"

"11097","TTGTAATCTGTACAAAATTG","chr11",15953118,"+","0:A>T,15:G>A","GGG","ATGTAATCTGTACAAGATTGGGG",2,1,16,NA,"","",FALSE,"ARL1"

"11098","ACGCACGGAGTTGAAGCCCC","chr5",176366524,"-","none","AGG","ACGCACGGAGTTGAAGCCCCAGG",0,NA,NA,NA,"GeneID:285598","ARL10",TRUE,"ARL10"

"11099","GCTGGAACAGCGCGAGGTGC","chr5",176366417,"+","none","TGG","GCTGGAACAGCGCGAGGTGCTGG",0,NA,NA,NA,"GeneID:285598","ARL10",TRUE,"ARL10"

"11100","GGCCGAAGTAGGTCTTCCAG","chr5",176365643,"-","none","AGG","GGCCGAAGTAGGTCTTCCAGAGG",0,NA,NA,NA,"GeneID:285598","ARL10",TRUE,"ARL10"

"11101","TACAGGCTATCGATAACCAG","chr5",176371770,"+","none","CGG","TACAGGCTATCGATAACCAGCGG",0,NA,NA,NA,"GeneID:285598","ARL10",TRUE,"ARL10"

"11102","TACAGGCTATCGATAACCAG","chr10",121838813,"+","10:G>C,16:G>C","AGG","TACAGGCTATGGATAAGCAGAGG",2,11,17,NA,"GeneID:11101","ATE1",FALSE,"ARL10"

"11103","TGGACTCGGCTGACCGACTG","chr5",176368878,"+","none","CGG","TGGACTCGGCTGACCGACTGCGG",0,NA,NA,NA,"GeneID:285598","ARL10",TRUE,"ARL10"

"11104","TTGTTGGCCACCACGACGAC","chr5",176368958,"-","none","AGG","TTGTTGGCCACCACGACGACAGG",0,NA,NA,NA,"GeneID:285598","ARL10",TRUE,"ARL10"

"11105","ACATGCAGCTGCGAGATTTC","chr13",49630965,"-","none","AGG","ACATGCAGCTGCGAGATTTCAGG",0,NA,NA,NA,"GeneID:115761","ARL11",TRUE,"ARL11"

"11106","AGATATCCTCGTGTACGTGC","chr13",49630689,"+","none","TGG","AGATATCCTCGTGTACGTGCTGG",0,NA,NA,NA,"GeneID:115761","ARL11",TRUE,"ARL11"

"11107","AGTCCTGAACGACCCCAACA","chr13",49630761,"+","none","TGG","AGTCCTGAACGACCCCAACATGG",0,NA,NA,NA,"GeneID:115761","ARL11",TRUE,"ARL11"

"11108","TGAAGCCCGCTTACCCGAGT","chr13",49630722,"+","none","CGG","TGAAGCCCGCTTACCCGAGTCGG",0,NA,NA,NA,"GeneID:115761","ARL11",TRUE,"ARL11"

"11109","TGCTGTCCAGCACGTACACG","chr13",49630698,"-","none","AGG","TGCTGTCCAGCACGTACACGAGG",0,NA,NA,NA,"GeneID:115761","ARL11",TRUE,"ARL11"

"11110","TGTGAGCTCAGCCGCCGACT","chr13",49630739,"-","none","CGG","TGTGAGCTCAGCCGCCGACTCGG",0,NA,NA,NA,"GeneID:115761","ARL11",TRUE,"ARL11"

"11111","AGCAGCAGGAGGACAAAAGC","chrX",100973697,"-","none","CGG","AGCAGCAGGAGGACAAAAGCCGG",0,NA,NA,NA,"GeneID:392509","ARL13A",TRUE,"ARL13A"

"11112","AGCAGCAGGAGGACAAAAGC","chr13",99752613,"-","15:C>T,16:G>T","AGG","AGCAGCAGGAGGACAGCAGCAGG",2,16,17,NA,"GeneID:171425","CLYBL",FALSE,"ARL13A"

"11113","AGCAGCAGGAGGACAAAAGC","chr10",4499808,"+","15:C>A,16:C>A","AGG","AGCAGCAGGAGGACACCAGCAGG",2,16,17,NA,"","",FALSE,"ARL13A"

"11114","AGCAGCAGGAGGACAAAAGC","chr3",55692352,"+","0:T>A,5:A>C","AGG","TGCAGAAGGAGGACAAAAGCAGG",2,1,6,NA,"GeneID:26059","ERC2",FALSE,"ARL13A"

"11115","AGCAGCAGGAGGACAAAAGC","chr19",33552939,"-","2:T>G,17:C>T","AGG","AGAAGCAGGAGGACAAAGGCAGG",2,3,18,NA,"","",FALSE,"ARL13A"

"11116","CAGTGACATAAGACGCATGC","chrX",100985829,"+","none","AGG","CAGTGACATAAGACGCATGCAGG",0,NA,NA,NA,"GeneID:392509","ARL13A",TRUE,"ARL13A"

"11117","CAGTGACATAAGACGCATGC","chrX",137982362,"+","8:C>T,12:T>A","AGG","CAGTGACACAAGTCGCATGCAGG",2,9,13,NA,"","",FALSE,"ARL13A"

"11118","CCTCCTGCTGCTCTTGCCTA","chrX",100973705,"+","none","AGG","CCTCCTGCTGCTCTTGCCTAAGG",0,NA,NA,NA,"GeneID:392509","ARL13A",TRUE,"ARL13A"

"11119","CCTCCTGCTGCTCTTGCCTA","chr8",96522937,"+","2:A>T,12:T>C","GGG","CCACCTGCTGCTTTTGCCTAGGG",2,3,13,NA,"GeneID:6383","SDC2",FALSE,"ARL13A"

"11120","CTCTGGCAAAACTGTTCTTG","chrX",100974159,"+","none","TGG","CTCTGGCAAAACTGTTCTTGTGG",0,NA,NA,NA,"GeneID:392509","ARL13A",TRUE,"ARL13A"

"11121","CTCTGGCAAAACTGTTCTTG","chr3",82912703,"+","11:T>C,15:C>T","AGG","CTCTGGCAAAATTGTCCTTGAGG",2,12,16,NA,"","",FALSE,"ARL13A"

"11122","CTCTGGCAAAACTGTTCTTG","chrX",33048827,"-","9:A>T,13:A>C","CGG","CTCTGGCAATACTTTTCTTGCGG",2,10,14,NA,"GeneID:1756","DMD",FALSE,"ARL13A"

"11123","GAGTTGTTCAAGCCAATGAT","chrX",100974142,"-","none","AGG","GAGTTGTTCAAGCCAATGATAGG",0,NA,NA,NA,"GeneID:392509","ARL13A",TRUE,"ARL13A"

"11124","GAGTTGTTCAAGCCAATGAT","chr11",115849057,"-","12:C>G,17:G>C","GGG","GAGTTGTTCAAGGCAATCATGGG",2,13,18,NA,"","",FALSE,"ARL13A"

"11125","GAGTTGTTCAAGCCAATGAT","chr2",87178182,"+","8:A>C,17:T>G","TGG","GAGTTGTTAAAGCCAATTATTGG",2,9,18,NA,"","",FALSE,"ARL13A"

"11126","GAGTTGTTCAAGCCAATGAT","chr2",85595575,"+","2:C>G,19:G>T","AGG","GACTTGTTCAAGCCAATGAGAGG",2,3,20,NA,"","",FALSE,"ARL13A"

"11127","GAGTTGTTCAAGCCAATGAT","chr2",111787265,"-","8:T>G,17:A>C","TGG","GAGTTGTTAAAGCCAATTATTGG",2,9,18,NA,"GeneID:64682","ANAPC1",FALSE,"ARL13A"

"11128","TCTGCTGTCCGATAAAAGAG","chrX",100985874,"+","none","TGG","TCTGCTGTCCGATAAAAGAGTGG",0,NA,NA,NA,"GeneID:392509","ARL13A",TRUE,"ARL13A"

"11129","ATAGGCTTTCCCGATATCCT","chr3",94003882,"-","none","AGG","ATAGGCTTTCCCGATATCCTAGG",0,NA,NA,NA,"GeneID:200894","ARL13B",TRUE,"ARL13B"

"11130","CTGCCACCTGAAACATAAAA","chr3",94043073,"+","none","TGG","CTGCCACCTGAAACATAAAATGG",0,NA,NA,NA,"GeneID:200894","ARL13B",TRUE,"ARL13B"

"11131","CTGCCACCTGAAACATAAAA","chr12",112324545,"-","6:A>G,13:T>G","AGG","CTGCCATCTGAAAAATAAAAAGG",2,7,14,NA,"GeneID:283450","HECTD4",FALSE,"ARL13B"

"11132","CTTCCAAAACTTGAGCCTCT","chr3",94050852,"+","none","TGG","CTTCCAAAACTTGAGCCTCTTGG",0,NA,NA,NA,"GeneID:200894;GeneID:200895","ARL13B;DHFRL1",TRUE,"ARL13B"

"11133","CTTCCAAAACTTGAGCCTCT","chr9",96373385,"+","3:T>C,4:A>C","CGG","CTTTAAAAACTTGAGCCTCTCGG",2,4,5,NA,"GeneID:11046","SLC35D2",FALSE,"ARL13B"

"11134","CTTCCAAAACTTGAGCCTCT","chr5",10161888,"-","9:C>G,14:T>C","GGG","CTTCCAAAAGTTGAACCTCTGGG",2,10,15,NA,"","",FALSE,"ARL13B"

"11135","TGGTAAAACCGCAACAGCAA","chr3",93995909,"+","none","AGG","TGGTAAAACCGCAACAGCAAAGG",0,NA,NA,NA,"GeneID:200894","ARL13B",TRUE,"ARL13B"

"11136","TTATGATGTGTTTCACCAAG","chr3",94050870,"-","none","AGG","TTATGATGTGTTTCACCAAGAGG",0,NA,NA,NA,"GeneID:200894;GeneID:200895","ARL13B;DHFRL1",TRUE,"ARL13B"

"11137","TTATTACCTGATTCCAATGA","chr3",94043227,"-","none","TGG","TTATTACCTGATTCCAATGATGG",0,NA,NA,NA,"GeneID:200894","ARL13B",TRUE,"ARL13B"

"11138","CAATGTGGAAATGATCGAGT","chr3",160677486,"+","none","TGG","CAATGTGGAAATGATCGAGTTGG",0,NA,NA,NA,"GeneID:80117","ARL14",TRUE,"ARL14"

"11139","CAGTACAGACAAACAGCGAC","chr3",160677615,"+","none","TGG","CAGTACAGACAAACAGCGACTGG",0,NA,NA,NA,"GeneID:80117","ARL14",TRUE,"ARL14"

"11140","CAGTACAGACAAACAGCGAC","chr6",143973281,"+","3:C>T,4:C>A","AGG","CAGCCCAGACAAACAGCGACAGG",2,4,5,NA,"GeneID:5325","PLAGL1",FALSE,"ARL14"

"11141","GCGTTCTTCAAGCAGAACTG","chr3",160677904,"+","none","AGG","GCGTTCTTCAAGCAGAACTGAGG",0,NA,NA,NA,"GeneID:80117","ARL14",TRUE,"ARL14"

"11142","GGAATCTTTCACTCACAGTC","chr3",160677512,"+","none","TGG","GGAATCTTTCACTCACAGTCTGG",0,NA,NA,NA,"GeneID:80117","ARL14",TRUE,"ARL14"

"11143","TGTTACTGTGAGAACACCGA","chr3",160677574,"+","none","TGG","TGTTACTGTGAGAACACCGATGG",0,NA,NA,NA,"GeneID:80117","ARL14",TRUE,"ARL14"

"11144","TGTTACTGTGAGAACACCGA","chr11",8158374,"+","10:G>A,18:A>G","AGG","TGTTACTGTGGGAACACCAAAGG",2,11,19,NA,"GeneID:79608","RIC3",FALSE,"ARL14"

"11145","TTGTTGGCTAATAGAACAAC","chr3",160677697,"-","none","AGG","TTGTTGGCTAATAGAACAACAGG",0,NA,NA,NA,"GeneID:80117","ARL14",TRUE,"ARL14"

"11146","AATGCCGCTGTGACCGCAAG","chr11",30336712,"+","none","TGG","AATGCCGCTGTGACCGCAAGTGG",0,NA,NA,NA,"GeneID:120534","ARL14EP",TRUE,"ARL14EP"

"11147","AGCCTACCTGTCTGAACCAG","chr11",30332980,"-","none","CGG","AGCCTACCTGTCTGAACCAGCGG",0,NA,NA,NA,"GeneID:120534","ARL14EP",TRUE,"ARL14EP"

"11148","ATCCTAAGCAGTCTTCATCC","chr11",30336647,"-","none","AGG","ATCCTAAGCAGTCTTCATCCAGG",0,NA,NA,NA,"GeneID:120534","ARL14EP",TRUE,"ARL14EP"

"11149","ATCCTAAGCAGTCTTCATCC","chr17",74182861,"-","8:T>G,17:C>A","AGG","ATCCTAAGAAGTCTTCAGCCAGG",2,9,18,NA,"","",FALSE,"ARL14EP"

"11150","ATGATGGATCCATGTTCAGT","chr11",30330948,"+","none","TGG","ATGATGGATCCATGTTCAGTTGG",0,NA,NA,NA,"GeneID:120534","ARL14EP",TRUE,"ARL14EP"

"11151","ATTACACATACATCTGACTC","chr11",30331365,"-","none","AGG","ATTACACATACATCTGACTCAGG",0,NA,NA,NA,"GeneID:120534","ARL14EP",TRUE,"ARL14EP"

"11152","CTTACAAAAAATGCAACCGC","chr11",30332961,"+","none","TGG","CTTACAAAAAATGCAACCGCTGG",0,NA,NA,NA,"GeneID:120534","ARL14EP",TRUE,"ARL14EP"

"11153","AAACCCACCGTCGGTTGCAG","chr5",116058862,"-","none","CGG","AAACCCACCGTCGGTTGCAGCGG",0,NA,NA,NA,"GeneID:644100","ARL14EPL",TRUE,"ARL14EPL"

"11154","AAACCCACCGTCGGTTGCAG","chr9",133950480,"-","14:G>A,15:T>A","AGG","AAACCCACCGTCGGCAGCAGAGG",2,15,16,NA,"GeneID:7410","VAV2",FALSE,"ARL14EPL"

"11155","AAGTCTGCTACCTGTGGTCC","chr5",116054058,"-","none","AGG","AAGTCTGCTACCTGTGGTCCAGG",0,NA,NA,NA,"GeneID:644100","ARL14EPL",TRUE,"ARL14EPL"

"11156","CCAATTTGACAGTTCTTCTC","chr5",116051528,"-","none","AGG","CCAATTTGACAGTTCTTCTCAGG",0,NA,NA,NA,"GeneID:644100","ARL14EPL",TRUE,"ARL14EPL"

"11157","CCTGAGAAGAACTGTCAAAT","chr5",116051525,"+","none","TGG","CCTGAGAAGAACTGTCAAATTGG",0,NA,NA,NA,"GeneID:644100","ARL14EPL",TRUE,"ARL14EPL"

"11158","GCCGTTTAATGTTCCTGACT","chr5",116058925,"+","none","AGG","GCCGTTTAATGTTCCTGACTAGG",0,NA,NA,NA,"GeneID:644100","ARL14EPL",TRUE,"ARL14EPL"

"11159","TGCTTGGCATTTCGAAACCC","chr5",116054037,"+","none","TGG","TGCTTGGCATTTCGAAACCCTGG",0,NA,NA,NA,"GeneID:644100","ARL14EPL",TRUE,"ARL14EPL"

"11160","AGTAGCGGCTCCAGTATTTC","chr5",54113374,"+","none","CGG","AGTAGCGGCTCCAGTATTTCCGG",0,NA,NA,NA,"GeneID:54622","ARL15",TRUE,"ARL15"

"11161","ATCCATGTCATCCAGTGAGC","chr5",53886632,"+","none","AGG","ATCCATGTCATCCAGTGAGCAGG",0,NA,NA,NA,"GeneID:54622","ARL15",TRUE,"ARL15"

"11162","GAACTTGAACCACTTGCACG","chr5",53886678,"-","none","TGG","GAACTTGAACCACTTGCACGTGG",0,NA,NA,NA,"GeneID:54622","ARL15",TRUE,"ARL15"

"11163","GATAACGTCGTGTCGACCAC","chr5",54171785,"-","none","AGG","GATAACGTCGTGTCGACCACAGG",0,NA,NA,NA,"GeneID:54622","ARL15",TRUE,"ARL15"

"11164","TCTTGTACTGAGCGAGCTGC","chr5",54113202,"+","none","TGG","TCTTGTACTGAGCGAGCTGCTGG",0,NA,NA,NA,"GeneID:54622","ARL15",TRUE,"ARL15"

"11165","TGTGGTCGACACGACGTTAT","chr5",54171784,"+","none","CGG","TGTGGTCGACACGACGTTATCGG",0,NA,NA,NA,"GeneID:54622","ARL15",TRUE,"ARL15"

"11166","ACACCTACCGTGGGCCGTGT","chr17",81683527,"+","none","CGG","ACACCTACCGTGGGCCGTGTCGG",0,NA,NA,NA,"GeneID:339231;GeneID:9146","ARL16;HGS",TRUE,"ARL16"

"11167","ACACCTACCGTGGGCCGTGT","chr13",76991731,"+","7:G>C,16:C>G","GGG","ACACCTAGCGTGGGCCCTGTGGG",2,8,17,NA,"GeneID:1203","CLN5",TRUE,"ARL16"

"11168","CACCAATCTTACTGACATCG","chr17",81683101,"-","none","TGG","CACCAATCTTACTGACATCGTGG",0,NA,NA,NA,"GeneID:339231","ARL16",TRUE,"ARL16"

"11169","GCAGTCAATCGTTGGCTCTG","chr17",81681703,"+","none","TGG","GCAGTCAATCGTTGGCTCTGTGG",0,NA,NA,NA,"GeneID:339231","ARL16",TRUE,"ARL16"

"11170","GCAGTCAATCGTTGGCTCTG","chr1",26219075,"+","8:C>T,17:T>C","TGG","GCAGTCAACCGTTGGCTTTGTGG",2,9,18,NA,"GeneID:101928303","LOC101928303",TRUE,"ARL16"

"11171","GCAGTCAATCGTTGGCTCTG","chr2",3842304,"-","0:T>C,14:A>C","TGG","ACAGTCAATCGTTGTCTCTGTGG",2,1,15,NA,"GeneID:728597","DCDC2C",FALSE,"ARL16"

"11172","TGCCACGATGTCAGTAAGAT","chr17",81683096,"+","none","TGG","TGCCACGATGTCAGTAAGATTGG",0,NA,NA,NA,"GeneID:339231","ARL16",TRUE,"ARL16"

"11173","TGCCGTGGTGATGTTCTGCT","chr17",81681785,"+","none","TGG","TGCCGTGGTGATGTTCTGCTTGG",0,NA,NA,NA,"GeneID:339231","ARL16",TRUE,"ARL16"

"11174","TGCCGTGGTGATGTTCTGCT","chr1",26219157,"+","7:A>G,12:A>G","TGG","TGCCGTGATGATATTCTGCTTGG",2,8,13,NA,"GeneID:101928303","LOC101928303",TRUE,"ARL16"

"11175","TGGGGGAGCCGCCCCCGACA","chr17",81683544,"-","none","CGG","TGGGGGAGCCGCCCCCGACACGG",0,NA,NA,NA,"GeneID:339231;GeneID:9146","ARL16;HGS",TRUE,"ARL16"

"11176","TGGGGGAGCCGCCCCCGACA","chr19",5042020,"+","2:A>G,8:A>C","TGG","TGAGGGAGACGCCCCCGACATGG",2,3,9,NA,"GeneID:23030","KDM4B",FALSE,"ARL16"

"11177","AAAATAACACCTTCGCTGTC","chr17",46352883,"-","none","TGG","AAAATAACACCTTCGCTGTCTGG",0,NA,NA,NA,"GeneID:100506084","ARL17B",TRUE,"ARL17A"

"11178","AAAATAACACCTTCGCTGTC","chr17",46570822,"-","none","TGG","AAAATAACACCTTCGCTGTCTGG",0,NA,NA,NA,"GeneID:51326","ARL17A",TRUE,"ARL17A"

"11179","AAGCTCTTTAAAAGTCTACT","chr17",46576284,"-","none","TGG","AAGCTCTTTAAAAGTCTACTTGG",0,NA,NA,NA,"GeneID:51326","ARL17A",TRUE,"ARL17A"

"11180","AAGCTCTTTAAAAGTCTACT","chr17",46358359,"-","none","TGG","AAGCTCTTTAAAAGTCTACTTGG",0,NA,NA,NA,"GeneID:100506084","ARL17B",TRUE,"ARL17A"

"11181","AAGCTCTTTAAAAGTCTACT","chr3",123035320,"-","18:T>G,19:C>A","TGG","AAGCTCTTTAAAAGTCTAAGTGG",2,19,20,NA,"","",FALSE,"ARL17A"

"11182","GGTCACCGGCACAAATGATG","chr17",46517167,"-","none","AGG","GGTCACCGGCACAAATGATGAGG",0,NA,NA,NA,"GeneID:474170;GeneID:51326","LRRC37A2;ARL17A",TRUE,"ARL17A"

"11183","GGTCACCGGCACAAATGATG","chr17",46299599,"-","6:A>G","AGG","GGTCACTGGCACAAATGATGAGG",1,7,NA,NA,"GeneID:100506084;GeneID:9884","ARL17B;LRRC37A",TRUE,"ARL17A"

"11184","GGTCACCGGCACAAATGATG","chr17",47021655,"-","6:A>G","AGG","GGTCACTGGCACAAATGATGAGG",1,7,NA,NA,"","",TRUE,"ARL17A"

"11185","GGTCACCGGCACAAATGATG","chr17",32024516,"-","2:G>A,6:A>G","AGG","GGCCACTGGCACAAATGATGAGG",2,3,7,NA,"GeneID:114659","LRRC37B",TRUE,"ARL17A"

"11186","GGTCACCGGCACAAATGATG","chr1",32422809,"+","6:T>C","AGG","GGTCACTGGCACAAATGATGAGG",1,7,NA,NA,"","",FALSE,"ARL17A"

"11187","GGTCACCGGCACAAATGATG","chr1",32421816,"+","6:T>C","AGG","GGTCACTGGCACAAATGATGAGG",1,7,NA,NA,"","",FALSE,"ARL17A"

"11188","GGTCACCGGCACAAATGATG","chr17",64892774,"+","6:T>C","AGG","GGTCACTGGCACAAATGATGAGG",1,7,NA,NA,"GeneID:374819","LRRC37A3",TRUE,"ARL17A"

"11189","GGTCACCGGCACAAATGATG","chr17",45545850,"+","6:T>C","AGG","GGTCACTGGCACAAATGATGAGG",1,7,NA,NA,"","",TRUE,"ARL17A"

"11190","GGTCACCGGCACAAATGATG","chr17",32095107,"+","2:C>T,6:T>C","AGG","GGCCACTGGCACAAATGATGAGG",2,3,7,NA,"","",TRUE,"ARL17A"

"11191","GGTCACCGGCACAAATGATG","chr17",68147697,"+","2:C>T,6:T>C","AGG","GGCCACTGGCACAAATGATGAGG",2,3,7,NA,"","",FALSE,"ARL17A"

"11192","GTTTCATTTAGGTTTCTGTG","chr17",46570860,"-","none","TGG","GTTTCATTTAGGTTTCTGTGTGG",0,NA,NA,NA,"GeneID:51326","ARL17A",TRUE,"ARL17A"

"11193","GTTTCATTTAGGTTTCTGTG","chr17",46352921,"-","none","TGG","GTTTCATTTAGGTTTCTGTGTGG",0,NA,NA,NA,"GeneID:100506084","ARL17B",TRUE,"ARL17A"

"11194","GTTTCATTTAGGTTTCTGTG","chr17",82788999,"+","12:G>T,16:A>T","TGG","GTTTCATTTAGGGTTCAGTGTGG",2,13,17,NA,"GeneID:6904","TBCD",FALSE,"ARL17A"

"11195","TAACTTACCTACTGTAGGGA","chr17",46358241,"+","none","CGG","TAACTTACCTACTGTAGGGACGG",0,NA,NA,NA,"GeneID:100506084","ARL17B",TRUE,"ARL17A"

"11196","TAACTTACCTACTGTAGGGA","chr17",46576166,"+","none","CGG","TAACTTACCTACTGTAGGGACGG",0,NA,NA,NA,"GeneID:51326","ARL17A",TRUE,"ARL17A"

"11197","TGTGCAGGTGCCAGAAGCCC","chr17",45503253,"+","none","AGG","TGTGCAGGTGCCAGAAGCCCAGG",0,NA,NA,NA,"","",FALSE,"ARL17A"

"11198","TGTGCAGGTGCCAGAAGCCC","chr17",64849714,"+","none","AGG","TGTGCAGGTGCCAGAAGCCCAGG",0,NA,NA,NA,"","",FALSE,"ARL17A"

"11199","TGTGCAGGTGCCAGAAGCCC","chr17",47060134,"-","none","AGG","TGTGCAGGTGCCAGAAGCCCAGG",0,NA,NA,NA,"","",FALSE,"ARL17A"

"11200","TGTGCAGGTGCCAGAAGCCC","chr17",46557617,"-","none","AGG","TGTGCAGGTGCCAGAAGCCCAGG",0,NA,NA,NA,"GeneID:51326","ARL17A",TRUE,"ARL17A"

"11201","TGTGCAGGTGCCAGAAGCCC","chr17",46339761,"-","none","AGG","TGTGCAGGTGCCAGAAGCCCAGG",0,NA,NA,NA,"GeneID:100506084","ARL17B",TRUE,"ARL17A"

"11202","TGTGCAGGTGCCAGAAGCCC","chr17",39045784,"-","10:T>G","AGG","TGTGCAGGTGACAGAAGCCCAGG",1,11,NA,NA,"GeneID:342666","LRRC37A11P",FALSE,"ARL17A"

"11203","TGTGCAGGTGCCAGAAGCCC","chr17",43138649,"+","11:T>C,12:G>A","AGG","TGTGCAGGTGCTGGAAGCCCAGG",2,12,13,NA,"GeneID:672;GeneID:10230","BRCA1;NBR2",TRUE,"ARL17A"

"11204","AAAATAACACCTTCGCTGTC","chr17",46352883,"-","none","TGG","AAAATAACACCTTCGCTGTCTGG",0,NA,NA,NA,"GeneID:100506084","ARL17B",TRUE,"ARL17B"

"11205","AAAATAACACCTTCGCTGTC","chr17",46570822,"-","none","TGG","AAAATAACACCTTCGCTGTCTGG",0,NA,NA,NA,"GeneID:51326","ARL17A",TRUE,"ARL17B"

"11206","AAGCTCTTTAAAAGTCTACT","chr17",46576284,"-","none","TGG","AAGCTCTTTAAAAGTCTACTTGG",0,NA,NA,NA,"GeneID:51326","ARL17A",TRUE,"ARL17B"

"11207","AAGCTCTTTAAAAGTCTACT","chr17",46358359,"-","none","TGG","AAGCTCTTTAAAAGTCTACTTGG",0,NA,NA,NA,"GeneID:100506084","ARL17B",TRUE,"ARL17B"

"11208","AAGCTCTTTAAAAGTCTACT","chr3",123035320,"-","18:T>G,19:C>A","TGG","AAGCTCTTTAAAAGTCTAAGTGG",2,19,20,NA,"","",FALSE,"ARL17B"

"11209","GTTTCATTTAGGTTTCTGTG","chr17",46570860,"-","none","TGG","GTTTCATTTAGGTTTCTGTGTGG",0,NA,NA,NA,"GeneID:51326","ARL17A",TRUE,"ARL17B"

"11210","GTTTCATTTAGGTTTCTGTG","chr17",46352921,"-","none","TGG","GTTTCATTTAGGTTTCTGTGTGG",0,NA,NA,NA,"GeneID:100506084","ARL17B",TRUE,"ARL17B"

"11211","GTTTCATTTAGGTTTCTGTG","chr17",82788999,"+","12:G>T,16:A>T","TGG","GTTTCATTTAGGGTTCAGTGTGG",2,13,17,NA,"GeneID:6904","TBCD",FALSE,"ARL17B"

"11212","TAACTTACCTACTGTAGGGA","chr17",46358241,"+","none","CGG","TAACTTACCTACTGTAGGGACGG",0,NA,NA,NA,"GeneID:100506084","ARL17B",TRUE,"ARL17B"

"11213","TAACTTACCTACTGTAGGGA","chr17",46576166,"+","none","CGG","TAACTTACCTACTGTAGGGACGG",0,NA,NA,NA,"GeneID:51326","ARL17A",TRUE,"ARL17B"

"11214","TGTGCAGGTGCCAGAAGCCC","chr17",45503253,"+","none","AGG","TGTGCAGGTGCCAGAAGCCCAGG",0,NA,NA,NA,"","",FALSE,"ARL17B"

"11215","TGTGCAGGTGCCAGAAGCCC","chr17",64849714,"+","none","AGG","TGTGCAGGTGCCAGAAGCCCAGG",0,NA,NA,NA,"","",FALSE,"ARL17B"

"11216","TGTGCAGGTGCCAGAAGCCC","chr17",47060134,"-","none","AGG","TGTGCAGGTGCCAGAAGCCCAGG",0,NA,NA,NA,"","",FALSE,"ARL17B"

"11217","TGTGCAGGTGCCAGAAGCCC","chr17",46557617,"-","none","AGG","TGTGCAGGTGCCAGAAGCCCAGG",0,NA,NA,NA,"GeneID:51326","ARL17A",TRUE,"ARL17B"

"11218","TGTGCAGGTGCCAGAAGCCC","chr17",46339761,"-","none","AGG","TGTGCAGGTGCCAGAAGCCCAGG",0,NA,NA,NA,"GeneID:100506084","ARL17B",TRUE,"ARL17B"

"11219","TGTGCAGGTGCCAGAAGCCC","chr17",39045784,"-","10:T>G","AGG","TGTGCAGGTGACAGAAGCCCAGG",1,11,NA,NA,"GeneID:342666","LRRC37A11P",FALSE,"ARL17B"

"11220","TGTGCAGGTGCCAGAAGCCC","chr17",43138649,"+","11:T>C,12:G>A","AGG","TGTGCAGGTGCTGGAAGCCCAGG",2,12,13,NA,"GeneID:672;GeneID:10230","BRCA1;NBR2",TRUE,"ARL17B"

"11221","TTACCACAGCTGATTTCCAG","chr17",46339504,"+","none","AGG","TTACCACAGCTGATTTCCAGAGG",0,NA,NA,NA,"GeneID:100506084","ARL17B",TRUE,"ARL17B"

"11222","TTACCACAGCTGATTTCCAG","chr17",46557360,"+","none","AGG","TTACCACAGCTGATTTCCAGAGG",0,NA,NA,NA,"GeneID:51326","ARL17A",TRUE,"ARL17B"

"11223","TTACCACAGCTGATTTCCAG","chr17",45503510,"-","none","AGG","TTACCACAGCTGATTTCCAGAGG",0,NA,NA,NA,"","",FALSE,"ARL17B"

"11224","TTACCACAGCTGATTTCCAG","chr17",43138906,"-","none","AGG","TTACCACAGCTGATTTCCAGAGG",0,NA,NA,NA,"GeneID:672;GeneID:10230","BRCA1;NBR2",TRUE,"ARL17B"

"11225","TTACCACAGCTGATTTCCAG","chr17",64849971,"-","none","AGG","TTACCACAGCTGATTTCCAGAGG",0,NA,NA,NA,"","",FALSE,"ARL17B"

"11226","TTACCACAGCTGATTTCCAG","chr17",43919483,"-","none","AGG","TTACCACAGCTGATTTCCAGAGG",0,NA,NA,NA,"","",FALSE,"ARL17B"

"11227","AACTACTTTGAGAGCACCGA","chr11",65018628,"+","none","TGG","AACTACTTTGAGAGCACCGATGG",0,NA,NA,NA,"GeneID:402;GeneID:100528018","ARL2;ARL2-SNX15",TRUE,"ARL2"

"11228","ACCTGCTGCCGGGCATCGAC","chr11",65021790,"+","none","TGG","ACCTGCTGCCGGGCATCGACTGG",0,NA,NA,NA,"GeneID:402;GeneID:100528018","ARL2;ARL2-SNX15",TRUE,"ARL2"

"11229","ATCAAGACCCTGGAGCACCG","chr11",65018451,"+","none","AGG","ATCAAGACCCTGGAGCACCGAGG",0,NA,NA,NA,"GeneID:402;GeneID:100528018","ARL2;ARL2-SNX15",TRUE,"ARL2"

"11230","CACCTCGCGGATGGCGTTAG","chr11",65020482,"-","none","AGG","CACCTCGCGGATGGCGTTAGAGG",0,NA,NA,NA,"GeneID:402;GeneID:100528018","ARL2;ARL2-SNX15",TRUE,"ARL2"

"11231","CAGCAGGTTCTCCCCGGTGA","chr11",65021778,"-","none","CGG","CAGCAGGTTCTCCCCGGTGACGG",0,NA,NA,NA,"GeneID:402;GeneID:100528018","ARL2;ARL2-SNX15",TRUE,"ARL2"

"11232","GTCCTCTAACGCCATCCGCG","chr11",65020477,"+","none","AGG","GTCCTCTAACGCCATCCGCGAGG",0,NA,NA,NA,"GeneID:402;GeneID:100528018","ARL2;ARL2-SNX15",TRUE,"ARL2"

"11233","AAAAGCCAGAAAATCTGTGA","chr16",57250460,"-","none","AGG","AAAAGCCAGAAAATCTGTGAAGG",0,NA,NA,NA,"GeneID:23568;GeneID:51090","ARL2BP;PLLP",TRUE,"ARL2BP"

"11234","AAGGCTGCCATGTTGAACTC","chr16",57249817,"-","none","AGG","AAGGCTGCCATGTTGAACTCAGG",0,NA,NA,NA,"GeneID:23568;GeneID:51090","ARL2BP;PLLP",TRUE,"ARL2BP"

"11235","AAGGCTGCCATGTTGAACTC","chr8",19605003,"+","15:G>A,16:C>A","AGG","AAGGCTGCCATGTTGGCCTCAGG",2,16,17,NA,"GeneID:55790","CSGALNACT1",FALSE,"ARL2BP"

"11236","AAGGCTGCCATGTTGAACTC","chr1",233604013,"+","1:T>A,17:T>C","AGG","ATGGCTGCCATGTTGAATTCAGG",2,2,18,NA,"","",FALSE,"ARL2BP"

"11237","ACTGCGATGGACGCCTTAGA","chr16",57245361,"+","none","AGG","ACTGCGATGGACGCCTTAGAAGG",0,NA,NA,NA,"GeneID:23568","ARL2BP",TRUE,"ARL2BP"

"11238","CAGGCACCATAAGGATGAAG","chr16",57250407,"+","none","TGG","CAGGCACCATAAGGATGAAGTGG",0,NA,NA,NA,"GeneID:23568;GeneID:51090","ARL2BP;PLLP",TRUE,"ARL2BP"

"11239","CAGGCACCATAAGGATGAAG","chr5",15821856,"-","16:T>C,18:C>T","GGG","CAGGCACCATAAGGATAAGGGGG",2,17,19,NA,"GeneID:23194","FBXL7",FALSE,"ARL2BP"

"11240","CAGGCACCATAAGGATGAAG","chr11",121462397,"-","1:A>T,3:T>C","AGG","CTGACACCATAAGGATGAAGAGG",2,2,4,NA,"GeneID:6653","SORL1",FALSE,"ARL2BP"

"11241","CAGGCACCATAAGGATGAAG","chr11",70133565,"-","0:C>G,5:C>T","AGG","GAGGCGCCATAAGGATGAAGAGG",2,1,6,NA,"GeneID:55107","ANO1",FALSE,"ARL2BP"

"11242","CAGGCACCATAAGGATGAAG","chr6",86365574,"+","3:T>G,18:T>A","TGG","CAGTCACCATAAGGATGATGTGG",2,4,19,NA,"","",FALSE,"ARL2BP"

"11243","CAGGCACCATAAGGATGAAG","chr7",95938029,"-","9:T>A,15:T>A","TGG","CAGGCACCAAAAGGAAGAAGTGG",2,10,16,NA,"GeneID:1780","DYNC1I1",FALSE,"ARL2BP"

"11244","CGCAAAGCTCTCTCCTTCTA","chr16",57245377,"-","none","AGG","CGCAAAGCTCTCTCCTTCTAAGG",0,NA,NA,NA,"GeneID:23568","ARL2BP",TRUE,"ARL2BP"

"11245","CGCAAAGCTCTCTCCTTCTA","chr17",82577362,"+","1:A>G,13:T>C","AGG","CACAAAGCTCTCTTCTTCTAAGG",2,2,14,NA,"GeneID:3607","FOXK2",TRUE,"ARL2BP"

"11246","CTCAACTCACTGTAATGTTG","chr16",57249842,"-","none","TGG","CTCAACTCACTGTAATGTTGTGG",0,NA,NA,NA,"GeneID:23568;GeneID:51090","ARL2BP;PLLP",TRUE,"ARL2BP"

"11247","AAACTGAATGTATGGGACAT","chr10",102699433,"-","none","TGG","AAACTGAATGTATGGGACATTGG",0,NA,NA,NA,"GeneID:403","ARL3",TRUE,"ARL3"

"11248","AAACTGAATGTATGGGACAT","chr2",65209623,"-","10:T>A,12:C>A","GGG","AAACTGAATGAAGGGGACATGGG",2,11,13,NA,"","",FALSE,"ARL3"

"11249","AGAGGAAAATCAGACCATAC","chr10",102699405,"-","none","TGG","AGAGGAAAATCAGACCATACTGG",0,NA,NA,NA,"GeneID:403","ARL3",TRUE,"ARL3"

"11250","AGTATTCTCACCTCCTGGTC","chr10",102705430,"+","none","TGG","AGTATTCTCACCTCCTGGTCTGG",0,NA,NA,NA,"GeneID:403","ARL3",TRUE,"ARL3"

"11251","ATACCATCCGCGACCGAGTC","chr10",102685860,"-","none","TGG","ATACCATCCGCGACCGAGTCTGG",0,NA,NA,NA,"GeneID:403","ARL3",TRUE,"ARL3"

"11252","CCAGGAACTAGCGGAATTAC","chr10",102685985,"-","none","TGG","CCAGGAACTAGCGGAATTACTGG",0,NA,NA,NA,"GeneID:403","ARL3",TRUE,"ARL3"

"11253","TGCCAGCATTATCCAAGCCC","chr10",102705404,"+","none","AGG","TGCCAGCATTATCCAAGCCCAGG",0,NA,NA,NA,"GeneID:403","ARL3",TRUE,"ARL3"

"11254","AGACTGAAATGAAGGCAGGT","chr7",12688294,"-","none","TGG","AGACTGAAATGAAGGCAGGTTGG",0,NA,NA,NA,"GeneID:10124","ARL4A",TRUE,"ARL4A"

"11255","AGACTGAAATGAAGGCAGGT","chr17",38782316,"-","12:C>T,19:C>A","AGG","AGACTGAAATGAGGGCAGGGAGG",2,13,20,NA,"GeneID:8396","PIP4K2B",FALSE,"ARL4A"

"11256","AGACTGAAATGAAGGCAGGT","chr4",40786151,"-","0:C>T","TGG","GGACTGAAATGAAGGCAGGTTGG",1,1,NA,NA,"GeneID:79730","NSUN7",TRUE,"ARL4A"

"11257","AGACTGAAATGAAGGCAGGT","chr20",33310073,"-","2:C>T,7:G>T","GGG","AGGCTGACATGAAGGCAGGTGGG",2,3,8,NA,"","",FALSE,"ARL4A"

"11258","AGACTGAAATGAAGGCAGGT","chr16",13230120,"+","4:A>T,9:A>T","TGG","AGACAGAAAAGAAGGCAGGTTGG",2,5,10,NA,"GeneID:729993","SHISA9",FALSE,"ARL4A"

"11259","CTGAACTTCACAAAATAACT","chr10",60684841,"+","none","AGG","CTGAACTTCACAAAATAACTAGG",0,NA,NA,NA,"GeneID:288","ANK3",TRUE,"ARL4A"

"11260","CTGAACTTCACAAAATAACT","chr6",150935303,"+","none","AGG","CTGAACTTCACAAAATAACTAGG",0,NA,NA,NA,"GeneID:25902","MTHFD1L",TRUE,"ARL4A"

"11261","CTGAACTTCACAAAATAACT","chr7",12688591,"+","none","AGG","CTGAACTTCACAAAATAACTAGG",0,NA,NA,NA,"GeneID:10124","ARL4A",TRUE,"ARL4A"

"11262","CTGAACTTCACAAAATAACT","chr4",40786448,"+","18:A>C","AGG","CTGAACTTCACAAAATAAATAGG",1,19,NA,NA,"GeneID:79730","NSUN7",TRUE,"ARL4A"

"11263","CTGAACTTCACAAAATAACT","chr12",77201545,"+","14:T>A,19:A>T","TGG","CTGAACTTCACAAATTAACATGG",2,15,20,NA,"","",FALSE,"ARL4A"

"11264","CTGAACTTCACAAAATAACT","chr3",23879145,"-","2:A>C,10:A>G","AGG","CTTAACTTCATAAAATAACTAGG",2,3,11,NA,"GeneID:7324","UBE2E1",TRUE,"ARL4A"

"11265","GTCTTTCCACATTGTTATTC","chr7",12688310,"+","none","TGG","GTCTTTCCACATTGTTATTCTGG",0,NA,NA,NA,"GeneID:10124","ARL4A",TRUE,"ARL4A"

"11266","GTCTTTCCACATTGTTATTC","chr4",40786167,"+","3:C>T","TGG","GTCCTTCCACATTGTTATTCTGG",1,4,NA,NA,"GeneID:79730","NSUN7",TRUE,"ARL4A"

"11267","GTCTTTCCACATTGTTATTC","chr6",150935022,"+","3:C>T","TGG","GTCCTTCCACATTGTTATTCTGG",1,4,NA,NA,"GeneID:25902","MTHFD1L",TRUE,"ARL4A"

"11268","GTTTCTCAAGTCCTTCCTTT","chr4",40786635,"-","none","AGG","GTTTCTCAAGTCCTTCCTTTAGG",0,NA,NA,NA,"GeneID:79730","NSUN7",TRUE,"ARL4A"

"11269","GTTTCTCAAGTCCTTCCTTT","chr10",60685028,"-","none","AGG","GTTTCTCAAGTCCTTCCTTTAGG",0,NA,NA,NA,"GeneID:288","ANK3",TRUE,"ARL4A"

"11270","GTTTCTCAAGTCCTTCCTTT","chr6",150935490,"-","none","AGG","GTTTCTCAAGTCCTTCCTTTAGG",0,NA,NA,NA,"GeneID:25902","MTHFD1L",TRUE,"ARL4A"

"11271","GTTTCTCAAGTCCTTCCTTT","chr7",12688778,"-","none","AGG","GTTTCTCAAGTCCTTCCTTTAGG",0,NA,NA,NA,"GeneID:10124","ARL4A",TRUE,"ARL4A"

"11272","GTTTCTCAAGTCCTTCCTTT","chr3",130367254,"-","11:A>G,16:A>G","TGG","GTTTCTCAAGTTCTTCTTTTTGG",2,12,17,NA,"GeneID:256076","COL6A5",FALSE,"ARL4A"

"11273","GTTTCTCAAGTCCTTCCTTT","chr11",95650419,"-","3:G>A,9:A>C","TGG","GTTCCTCAATTCCTTCCTTTTGG",2,4,10,NA,"","",FALSE,"ARL4A"

"11274","TGCACCTGGAAATGGGGAAT","chr6",150934956,"+","none","GGG","TGCACCTGGAAATGGGGAATGGG",0,NA,NA,NA,"GeneID:25902","MTHFD1L",TRUE,"ARL4A"

"11275","TGCACCTGGAAATGGGGAAT","chr4",40786100,"+","none","GGG","TGCACCTGGAAATGGGGAATGGG",0,NA,NA,NA,"GeneID:79730","NSUN7",TRUE,"ARL4A"

"11276","TGCACCTGGAAATGGGGAAT","chr7",12688243,"+","none","GGG","TGCACCTGGAAATGGGGAATGGG",0,NA,NA,NA,"GeneID:10124","ARL4A",TRUE,"ARL4A"

"11277","TTGCTAACAAACAAGATTTG","chr7",12688648,"+","none","AGG","TTGCTAACAAACAAGATTTGAGG",0,NA,NA,NA,"GeneID:10124","ARL4A",TRUE,"ARL4A"

"11278","TTGCTAACAAACAAGATTTG","chr6",154628675,"-","12:C>T,19:A>C","AGG","TTGCTAACAAACGAGATTTTAGG",2,13,20,NA,"","",FALSE,"ARL4A"

"11279","TTGCTAACAAACAAGATTTG","chr6",150935360,"+","16:C>T","AGG","TTGCTAACAAACAAGACTTGAGG",1,17,NA,NA,"GeneID:25902","MTHFD1L",TRUE,"ARL4A"

"11280","TTGCTAACAAACAAGATTTG","chr2",136798223,"-","9:A>T,10:C>T","AGG","TTGCTAACATGCAAGATTTGAGG",2,10,11,NA,"GeneID:80731","THSD7B",FALSE,"ARL4A"

"11281","AGATGATGCCGTCCGTGCAG","chr2",234496315,"+","none","CGG","AGATGATGCCGTCCGTGCAGCGG",0,NA,NA,NA,"GeneID:10123","ARL4C",TRUE,"ARL4C"

"11282","AGCCCAACATGACGATATGC","chr2",234496525,"+","none","AGG","AGCCCAACATGACGATATGCAGG",0,NA,NA,NA,"GeneID:10123","ARL4C",TRUE,"ARL4C"

"11283","CACCACCTATCACGTCCAGC","chr2",234496111,"-","none","CGG","CACCACCTATCACGTCCAGCCGG",0,NA,NA,NA,"GeneID:10123","ARL4C",TRUE,"ARL4C"

"11284","GACCAAGTTCGCCGAGAACC","chr2",234496234,"-","none","AGG","GACCAAGTTCGCCGAGAACCAGG",0,NA,NA,NA,"GeneID:10123","ARL4C",TRUE,"ARL4C"

"11285","GGCGCTGCACGAGCTTATCC","chr2",234496135,"-","none","CGG","GGCGCTGCACGAGCTTATCCCGG",0,NA,NA,NA,"GeneID:10123","ARL4C",TRUE,"ARL4C"

"11286","TCGCCGATGATGGCGCACGC","chr2",234496089,"+","none","CGG","TCGCCGATGATGGCGCACGCCGG",0,NA,NA,NA,"GeneID:10123","ARL4C",TRUE,"ARL4C"

"11287","CGGGTATAAGAGCGCCACAG","chr17",43399987,"-","none","TGG","CGGGTATAAGAGCGCCACAGTGG",0,NA,NA,NA,"GeneID:379","ARL4D",TRUE,"ARL4D"

"11288","GATTCGGTGCAACTCCACCT","chr17",43400069,"-","none","TGG","GATTCGGTGCAACTCCACCTTGG",0,NA,NA,NA,"GeneID:379","ARL4D",TRUE,"ARL4D"

"11289","GCCCAATGACCACGACATGC","chr17",43399797,"-","none","AGG","GCCCAATGACCACGACATGCAGG",0,NA,NA,NA,"GeneID:379","ARL4D",TRUE,"ARL4D"

"11290","TCTTATACCCGCCGGACAGA","chr17",43399996,"+","none","CGG","TCTTATACCCGCCGGACAGACGG",0,NA,NA,NA,"GeneID:379","ARL4D",TRUE,"ARL4D"

"11291","TGATGCCACGCGATCCCCCG","chr17",43399923,"-","none","AGG","TGATGCCACGCGATCCCCCGAGG",0,NA,NA,NA,"GeneID:379","ARL4D",TRUE,"ARL4D"

"11292","TGGGCGCCATCTCAGTCAAG","chr17",43399743,"-","none","TGG","TGGGCGCCATCTCAGTCAAGTGG",0,NA,NA,NA,"GeneID:379","ARL4D",TRUE,"ARL4D"

"11293","ATAATACACGTTTCCTAATG","chr2",151814231,"-","none","TGG","ATAATACACGTTTCCTAATGTGG",0,NA,NA,NA,"GeneID:26225","ARL5A",TRUE,"ARL5A"

"11294","ATAATACACGTTTCCTAATG","chrX",119186656,"-","14:A>G,17:G>T","TGG","ATAATACACGTTTCTTACTGTGG",2,15,18,NA,"","",TRUE,"ARL5A"

"11295","ATAATACACGTTTCCTAATG","chrX",76029970,"+","19:A>G","TGG","ATAATACACGTTTCCTAATATGG",1,20,NA,NA,"","",TRUE,"ARL5A"

"11296","ATAATACACGTTTCCTAATG","chr22",24686321,"+","7:G>A","TGG","ATAATACGCGTTTCCTAATGTGG",1,8,NA,NA,"","",TRUE,"ARL5A"

"11297","CACTTACTATACTAACACAG","chr2",151814170,"-","none","AGG","CACTTACTATACTAACACAGAGG",0,NA,NA,NA,"GeneID:26225","ARL5A",TRUE,"ARL5A"

"11298","CTATAAAATGTTAGCGCATG","chr2",151812358,"-","none","AGG","CTATAAAATGTTAGCGCATGAGG",0,NA,NA,NA,"GeneID:26225","ARL5A",TRUE,"ARL5A"

"11299","CTATAAAATGTTAGCGCATG","chr10",99013302,"-","15:T>C","AGG","CTATAAAATGTTAGCACATGAGG",1,16,NA,NA,"GeneID:60495","HPSE2",TRUE,"ARL5A"

"11300","CTATAAAATGTTAGCGCATG","chrX",76030107,"+","13:C>G,15:A>G","AGG","CTATAAAATGTTACCACATGAGG",2,14,16,NA,"","",TRUE,"ARL5A"

"11301","CTATAAAATGTTAGCGCATG","chr22",24686459,"+","8:C>T","AGG","CTATAAAACGTTAGCGCATGAGG",1,9,NA,NA,"","",TRUE,"ARL5A"

"11302","CTATAAAATGTTAGCGCATG","chrX",119186518,"-","2:C>T,15:T>C","AGG","CTGTAAAATGTTAGCACATGAGG",2,3,16,NA,"","",TRUE,"ARL5A"

"11303","GAGCACAAAGTTATCATTGT","chr22",24686194,"+","none","TGG","GAGCACAAAGTTATCATTGTTGG",0,NA,NA,NA,"","",TRUE,"ARL5A"

"11304","GAGCACAAAGTTATCATTGT","chr2",151815180,"-","none","TGG","GAGCACAAAGTTATCATTGTTGG",0,NA,NA,NA,"GeneID:26225","ARL5A",TRUE,"ARL5A"

"11305","GAGCACAAAGTTATCATTGT","chrX",119186783,"-","14:A>G,17:G>A","GGG","GAGCACAAAGTTATTATCGTGGG",2,15,18,NA,"","",TRUE,"ARL5A"

"11306","TAATAGGACCTAAGAAAAGC","chr2",151806958,"-","none","TGG","TAATAGGACCTAAGAAAAGCTGG",0,NA,NA,NA,"GeneID:26225","ARL5A",TRUE,"ARL5A"

"11307","TTAGTTTGTAATAGTTGTTG","chr2",151812424,"-","none","TGG","TTAGTTTGTAATAGTTGTTGTGG",0,NA,NA,NA,"GeneID:26225","ARL5A",TRUE,"ARL5A"

"11308","AGAACACAAAGTAATTATAG","chr10",18666572,"+","none","TGG","AGAACACAAAGTAATTATAGTGG",0,NA,NA,NA,"GeneID:221079","ARL5B",TRUE,"ARL5B"

"11309","AGAACACAAAGTAATTATAG","chr10",123955792,"-","17:G>A,19:A>C","GGG","AGAACACAAAGTAATTACATGGG",2,18,20,NA,"","",FALSE,"ARL5B"

"11310","AGAACACAAAGTAATTATAG","chr10",128815314,"+","13:G>A,16:C>A","AGG","AGAACACAAAGTAGTTCTAGAGG",2,14,17,NA,"","",FALSE,"ARL5B"

"11311","AGAACACAAAGTAATTATAG","chr15",58254821,"-","5:A>T,16:C>T","TGG","AGAACTCAAAGTAATTGTAGTGG",2,6,17,NA,"GeneID:8854","ALDH1A2",FALSE,"ARL5B"

"11312","AGGAGTCTCTGCGATCATCC","chr10",18668630,"+","none","TGG","AGGAGTCTCTGCGATCATCCTGG",0,NA,NA,NA,"GeneID:221079","ARL5B",TRUE,"ARL5B"

"11313","AGGTCATCCACTCTAGACCT","chr10",18675176,"-","none","TGG","AGGTCATCCACTCTAGACCTTGG",0,NA,NA,NA,"GeneID:221079","ARL5B",TRUE,"ARL5B"

"11314","ATTTGAGTAATATGTGTTCC","chr10",18668651,"-","none","AGG","ATTTGAGTAATATGTGTTCCAGG",0,NA,NA,NA,"GeneID:221079","ARL5B",TRUE,"ARL5B"

"11315","GAGTGGATGACCTCCCGGAT","chr10",18675183,"+","none","TGG","GAGTGGATGACCTCCCGGATTGG",0,NA,NA,NA,"GeneID:221079","ARL5B",TRUE,"ARL5B"

"11316","TTGTTGTTGATAGCATTGAC","chr10",18672631,"+","none","AGG","TTGTTGTTGATAGCATTGACAGG",0,NA,NA,NA,"GeneID:221079","ARL5B",TRUE,"ARL5B"

"11317","AGAGCACACGGTCATCATCG","chr17",39165121,"-","none","TGG","AGAGCACACGGTCATCATCGTGG",0,NA,NA,NA,"GeneID:390790","ARL5C",TRUE,"ARL5C"

"11318","AGAGCACACGGTCATCATCG","chr10",32881888,"+","9:A>G,10:T>G","AGG","AGAGCACACATTCATCATCGAGG",2,10,11,NA,"GeneID:79741","CCDC7",FALSE,"ARL5C"

"11319","AGCCTTGTATATGCCACGAG","chr17",39160612,"+","none","TGG","AGCCTTGTATATGCCACGAGTGG",0,NA,NA,NA,"GeneID:390790","ARL5C",TRUE,"ARL5C"

"11320","CACTTACAACCGGTAGAGAA","chr17",39165071,"+","none","TGG","CACTTACAACCGGTAGAGAATGG",0,NA,NA,NA,"GeneID:390790","ARL5C",TRUE,"ARL5C"

"11321","GCACCATCAAAGACCACTCG","chr17",39160628,"-","none","TGG","GCACCATCAAAGACCACTCGTGG",0,NA,NA,NA,"GeneID:390790","ARL5C",TRUE,"ARL5C"

"11322","GGTCCGTGCTGTCAATCACA","chr17",39161320,"+","none","AGG","GGTCCGTGCTGTCAATCACAAGG",0,NA,NA,NA,"GeneID:390790","ARL5C",TRUE,"ARL5C"

"11323","TTTATATAGCTCCTCCCGAG","chr17",39161282,"+","none","TGG","TTTATATAGCTCCTCCCGAGTGG",0,NA,NA,NA,"GeneID:390790","ARL5C",TRUE,"ARL5C"

"11324","AAGAGTATCGAGTTCTTCTT","chr3",97785016,"-","none","TGG","AAGAGTATCGAGTTCTTCTTTGG",0,NA,NA,NA,"GeneID:84100","ARL6",TRUE,"ARL6"

"11325","AAGGAAGATACAGAAATCTC","chr3",97780646,"+","none","TGG","AAGGAAGATACAGAAATCTCTGG",0,NA,NA,NA,"GeneID:84100","ARL6",TRUE,"ARL6"

"11326","AAGGAAGATACAGAAATCTC","chr6",155250870,"-","11:C>T,18:G>A","AGG","AAGGAAGATACGGAAATCCCAGG",2,12,19,NA,"GeneID:26230","TIAM2",FALSE,"ARL6"

"11327","AAGGAAGATACAGAAATCTC","chr4",145561930,"+","2:A>G,8:G>T","AGG","AAAGAAGAGACAGAAATCTCAGG",2,3,9,NA,"","",FALSE,"ARL6"

"11328","AAGGAAGATACAGAAATCTC","chr2",67555616,"+","4:C>A,10:T>C","AGG","AAGGCAGATATAGAAATCTCAGG",2,5,11,NA,"","",FALSE,"ARL6"

"11329","AAGGAAGATACAGAAATCTC","chr10",90125400,"+","6:A>G,19:T>C","AGG","AAGGAAAATACAGAAATCTTAGG",2,7,20,NA,"","",FALSE,"ARL6"

"11330","AAGGAAGATACAGAAATCTC","chr14",82245437,"-","9:C>T,19:C>G","AGG","AAGGAAGATGCAGAAATCTGAGG",2,10,20,NA,"","",FALSE,"ARL6"

"11331","GATACTCTTCTGAATCATCC","chr3",97785027,"+","none","AGG","GATACTCTTCTGAATCATCCAGG",0,NA,NA,NA,"GeneID:84100","ARL6",TRUE,"ARL6"

"11332","GATACTCTTCTGAATCATCC","chr6",96315719,"+","14:A>T,18:T>C","AGG","GATACTCTTCTGAAACATTCAGG",2,15,19,NA,"","",FALSE,"ARL6"

"11333","TAGAGAAATTCAAATCATCC","chr3",97780198,"+","none","AGG","TAGAGAAATTCAAATCATCCAGG",0,NA,NA,NA,"GeneID:84100","ARL6",TRUE,"ARL6"

"11334","TCTATGCTGAATCCTATTGT","chr3",97780182,"-","none","TGG","TCTATGCTGAATCCTATTGTTGG",0,NA,NA,NA,"GeneID:84100","ARL6",TRUE,"ARL6"

"11335","TCTATGCTGAATCCTATTGT","chr4",120709106,"-","12:C>G,15:A>T","TGG","TCTATGCTGAATGCTTTTGTTGG",2,13,16,NA,"GeneID:11107","PRDM5",FALSE,"ARL6"

"11336","TTTACAGTGTTTGACATGTC","chr3",97780621,"+","none","AGG","TTTACAGTGTTTGACATGTCAGG",0,NA,NA,NA,"GeneID:84100","ARL6",TRUE,"ARL6"

"11337","TTTACAGTGTTTGACATGTC","chr6",139431423,"+","5:T>A,18:A>T","TGG","TTTACTGTGTTTGACATGACTGG",2,6,19,NA,"","",FALSE,"ARL6"

"11338","CCACCCAAGCAACCGCAGCA","chr16",18794637,"+","none","AGG","CCACCCAAGCAACCGCAGCAAGG",0,NA,NA,NA,"GeneID:23204","ARL6IP1",TRUE,"ARL6IP1"

"11339","CCACCCAAGCAACCGCAGCA","chr10",36995412,"-","5:A>G,14:A>C","AGG","CCACCTAAGCAACCTCAGCAAGG",2,6,15,NA,"","",TRUE,"ARL6IP1"

"11340","CCACCCAAGCAACCGCAGCA","chr3",138649967,"-","0:T>G,13:A>G","TGG","ACACCCAAGCAACTGCAGCATGG",2,1,14,NA,"","",FALSE,"ARL6IP1"

"11341","CTAGATCCATCTGTTCTGTC","chr16",18798011,"-","none","CGG","CTAGATCCATCTGTTCTGTCCGG",0,NA,NA,NA,"GeneID:23204","ARL6IP1",TRUE,"ARL6IP1"

"11342","CTGCTTCTCACCTACCTGAT","chr16",18794600,"-","none","AGG","CTGCTTCTCACCTACCTGATAGG",0,NA,NA,NA,"GeneID:23204","ARL6IP1",TRUE,"ARL6IP1"

"11343","GAAACCAGGCTCTTTCCCAT","chr16",18798737,"+","none","CGG","GAAACCAGGCTCTTTCCCATCGG",0,NA,NA,NA,"GeneID:23204","ARL6IP1",TRUE,"ARL6IP1"

"11344","GAAACCAGGCTCTTTCCCAT","chr10",124314512,"-","4:C>G,19:C>A","TGG","GAAAGCAGGCTCTTTCCCAGTGG",2,5,20,NA,"","",FALSE,"ARL6IP1"

"11345","GCACAAAAACATAACAAAAC","chr16",18797980,"+","none","AGG","GCACAAAAACATAACAAAACAGG",0,NA,NA,NA,"GeneID:23204","ARL6IP1",TRUE,"ARL6IP1"

"11346","GCACAAAAACATAACAAAAC","chr9",3550151,"-","1:C>G,8:C>T","AGG","GGACAAAAGCATAACAAAACAGG",2,2,9,NA,"GeneID:101929302","RFX3-AS1",FALSE,"ARL6IP1"

"11347","GCACAAAAACATAACAAAAC","chr1",50010518,"+","3:A>C,11:A>T","TGG","GCAAAAAAACAAAACAAAACTGG",2,4,12,NA,"GeneID:84871","AGBL4",FALSE,"ARL6IP1"

"11348","GCACAAAAACATAACAAAAC","chr17",65573295,"-","9:T>G,13:A>T","GGG","GCACAAAAAAATATCAAAACGGG",2,10,14,NA,"","",FALSE,"ARL6IP1"

"11349","GCACAAAAACATAACAAAAC","chr1",247216667,"-","5:C>T,14:T>G","AGG","GCACAGAAACATAAAAAAACAGG",2,6,15,NA,"","",FALSE,"ARL6IP1"

"11350","GCACAAAAACATAACAAAAC","chr4",116271671,"-","3:T>G,11:T>A","TGG","GCAAAAAAACAAAACAAAACTGG",2,4,12,NA,"","",FALSE,"ARL6IP1"

"11351","GCACAAAAACATAACAAAAC","chr19",36942728,"-","3:T>G,11:T>A","TGG","GCAAAAAAACAAAACAAAACTGG",2,4,12,NA,"GeneID:374900","ZNF568",FALSE,"ARL6IP1"

"11352","GCACAAAAACATAACAAAAC","chr15",72735734,"-","0:T>C,11:T>A","TGG","ACACAAAAACAAAACAAAACTGG",2,1,12,NA,"GeneID:585","BBS4",TRUE,"ARL6IP1"

"11353","TCCTCCGATGGGAAAGAGCC","chr16",18798744,"-","none","TGG","TCCTCCGATGGGAAAGAGCCTGG",0,NA,NA,NA,"GeneID:23204","ARL6IP1",TRUE,"ARL6IP1"

"11354","AGAGCAGGTTGCTCACTACG","chr3",69085151,"-","none","CGG","AGAGCAGGTTGCTCACTACGCGG",0,NA,NA,NA,"GeneID:10550","ARL6IP5",TRUE,"ARL6IP5"

"11355","CGGGCAAAGCGATCGGAACC","chr3",69085095,"-","none","CGG","CGGGCAAAGCGATCGGAACCCGG",0,NA,NA,NA,"GeneID:10550","ARL6IP5",TRUE,"ARL6IP5"

"11356","CTACCCCACGACGTTCGTTA","chr3",69101949,"+","none","TGG","CTACCCCACGACGTTCGTTATGG",0,NA,NA,NA,"GeneID:10550","ARL6IP5",TRUE,"ARL6IP5"

"11357","GACCACCATAACGAACGTCG","chr3",69101957,"-","none","TGG","GACCACCATAACGAACGTCGTGG",0,NA,NA,NA,"GeneID:10550","ARL6IP5",TRUE,"ARL6IP5"

"11358","TCCATGCATCGTTGAGACTT","chr3",69104472,"+","none","CGG","TCCATGCATCGTTGAGACTTCGG",0,NA,NA,NA,"GeneID:10550","ARL6IP5",TRUE,"ARL6IP5"

"11359","TCCGAAGTCTCAACGATGCA","chr3",69104476,"-","none","TGG","TCCGAAGTCTCAACGATGCATGG",0,NA,NA,NA,"GeneID:10550","ARL6IP5",TRUE,"ARL6IP5"

"11360","ACGGGCGACCCGTTCCCGTC","chr2",152718849,"-","none","CGG","ACGGGCGACCCGTTCCCGTCCGG",0,NA,NA,NA,"GeneID:151188","ARL6IP6",TRUE,"ARL6IP6"

"11361","ATCGCCTACTTGATCGTTAA","chr2",152719002,"+","none","AGG","ATCGCCTACTTGATCGTTAAAGG",0,NA,NA,NA,"GeneID:151188","ARL6IP6",TRUE,"ARL6IP6"

"11362","CTTATAATATCCCTAACTGC","chr2",152735007,"+","none","TGG","CTTATAATATCCCTAACTGCTGG",0,NA,NA,NA,"GeneID:151188","ARL6IP6",TRUE,"ARL6IP6"

"11363","CTTATAATATCCCTAACTGC","chr8",80351205,"+","17:C>T","TGG","CTTATAATATCCCTAACCGCTGG",1,18,NA,NA,"","",TRUE,"ARL6IP6"

"11364","CTTATAATATCCCTAACTGC","chr2",128594119,"+","6:C>A,8:T>A","GGG","CTTATACTTTCCCTAACTGCGGG",2,7,9,NA,"","",FALSE,"ARL6IP6"

"11365","TAGCTGCTCTTACTGTAGCA","chr2",152759805,"+","none","TGG","TAGCTGCTCTTACTGTAGCATGG",0,NA,NA,NA,"GeneID:151188","ARL6IP6",TRUE,"ARL6IP6"

"11366","TAGCTGCTCTTACTGTAGCA","chr8",80351383,"+","18:T>C,19:G>A","TGG","TAGCTGCTCTTACTGTAGTGTGG",2,19,20,NA,"","",TRUE,"ARL6IP6"

"11367","TAGCTGCTCTTACTGTAGCA","chr17",41888309,"-","8:T>G,10:C>A","GGG","TAGCTGCTATGACTGTAGCAGGG",2,9,11,NA,"GeneID:47","ACLY",FALSE,"ARL6IP6"

"11368","TCGCCATGCTATAGCCCATG","chr2",152759770,"-","none","TGG","TCGCCATGCTATAGCCCATGTGG",0,NA,NA,NA,"GeneID:151188","ARL6IP6",TRUE,"ARL6IP6"

"11369","TCGCCATGCTATAGCCCATG","chr8",80351348,"-","19:T>C","TGG","TCGCCATGCTATAGCCCATATGG",1,20,NA,NA,"","",TRUE,"ARL6IP6"

"11370","TGTCCAAGAAAAGCTGCAAC","chr2",152735038,"-","none","AGG","TGTCCAAGAAAAGCTGCAACAGG",0,NA,NA,NA,"GeneID:151188","ARL6IP6",TRUE,"ARL6IP6"

"11371","TGTCCAAGAAAAGCTGCAAC","chr8",80351236,"-","17:G>T","AGG","TGTCCAAGAAAAGCTGCCACAGG",1,18,NA,NA,"","",TRUE,"ARL6IP6"

"11372","ACACGATGGCGCTCACTCCT","chr1",202137962,"+","none","CGG","ACACGATGGCGCTCACTCCTCGG",0,NA,NA,NA,"GeneID:127829","ARL8A",TRUE,"ARL8A"

"11373","AGTAGCGCTCCCACATGCTG","chr1",202137986,"+","none","CGG","AGTAGCGCTCCCACATGCTGCGG",0,NA,NA,NA,"GeneID:127829","ARL8A",TRUE,"ARL8A"

"11374","CCAGTCAGGACAGTTCAACG","chr1",202138432,"-","none","AGG","CCAGTCAGGACAGTTCAACGAGG",0,NA,NA,NA,"GeneID:127829","ARL8A",TRUE,"ARL8A"

"11375","GAACGAGCTCCACAACCTAC","chr1",202135735,"-","none","TGG","GAACGAGCTCCACAACCTACTGG",0,NA,NA,NA,"GeneID:127829","ARL8A",TRUE,"ARL8A"

"11376","GTTGTGGAGCTCGTTCTTAG","chr1",202135739,"+","none","AGG","GTTGTGGAGCTCGTTCTTAGAGG",0,NA,NA,NA,"GeneID:127829","ARL8A",TRUE,"ARL8A"

"11377","TTTGCGCATGTTGAAACCCA","chr1",202138394,"+","none","CGG","TTTGCGCATGTTGAAACCCACGG",0,NA,NA,NA,"GeneID:127829","ARL8A",TRUE,"ARL8A"

"11378","AACATGAGGAAGGTAACTAA","chr3",5170544,"+","none","AGG","AACATGAGGAAGGTAACTAAAGG",0,NA,NA,NA,"GeneID:55207","ARL8B",TRUE,"ARL8B"

"11379","AACATGAGGAAGGTAACTAA","chr3",196044857,"+","4:C>T,11:A>G","AGG","AACACGAGGAAAGTAACTAAAGG",2,5,12,NA,"GeneID:7037","TFRC",TRUE,"ARL8B"

"11380","AACATGAGGAAGGTAACTAA","chr2",71880427,"-","1:C>T,13:T>A","AGG","AGCATGAGGAAGGAAACTAAAGG",2,2,14,NA,"","",FALSE,"ARL8B"

"11381","AACCCCGATTTCGAAGCATG","chr3",5172168,"+","none","TGG","AACCCCGATTTCGAAGCATGTGG",0,NA,NA,NA,"GeneID:55207","ARL8B",TRUE,"ARL8B"

"11382","AACCCCGATTTCGAAGCATG","chr1",28121080,"-","2:C>G","TGG","AAGCCCGATTTCGAAGCATGTGG",1,3,NA,NA,"","",TRUE,"ARL8B"

"11383","CACCGCGATGACATTGACGA","chr3",5122571,"-","none","AGG","CACCGCGATGACATTGACGAAGG",0,NA,NA,NA,"GeneID:55207","ARL8B",TRUE,"ARL8B"

"11384","CACCTTCGTCAATGTCATCG","chr3",5122566,"+","none","CGG","CACCTTCGTCAATGTCATCGCGG",0,NA,NA,NA,"GeneID:55207","ARL8B",TRUE,"ARL8B"

"11385","CACCTTCGTCAATGTCATCG","chr1",202144451,"-","12:G>A,15:C>G","CGG","CACCTTCGTCAACGTGATCGCGG",2,13,16,NA,"GeneID:127829","ARL8A",TRUE,"ARL8B"

"11386","CCTCATGTTGAAGCCCACTG","chr1",28121147,"+","none","TGG","CCTCATGTTGAAGCCCACTGTGG",0,NA,NA,NA,"","",TRUE,"ARL8B"

"11387","CCTCATGTTGAAGCCCACTG","chr3",5170533,"-","none","TGG","CCTCATGTTGAAGCCCACTGTGG",0,NA,NA,NA,"GeneID:55207","ARL8B",TRUE,"ARL8B"

"11388","CCTCATGTTGAAGCCCACTG","chr8",118025267,"-","7:C>A,18:T>A","CGG","CCTCATGGTGAAGCCCACAGCGG",2,8,19,NA,"GeneID:2131","EXT1",FALSE,"ARL8B"

"11389","CCTCATGTTGAAGCCCACTG","chr3",196044846,"-","4:C>T,12:T>C","TGG","CCTCGTGTTGAAACCCACTGTGG",2,5,13,NA,"GeneID:7037","TFRC",TRUE,"ARL8B"

"11390","GATTTAAAGATCTGGGACAT","chr3",5172140,"+","none","AGG","GATTTAAAGATCTGGGACATAGG",0,NA,NA,NA,"GeneID:55207","ARL8B",TRUE,"ARL8B"

"11391","GATTTAAAGATCTGGGACAT","chr8",111558900,"-","14:A>C,15:G>C","AGG","GATTTAAAGATCTGTCACATAGG",2,15,16,NA,"","",FALSE,"ARL8B"

"11392","AAAGATCAGCAGCAATCCCT","chr4",56518725,"-","none","TGG","AAAGATCAGCAGCAATCCCTTGG",0,NA,NA,NA,"GeneID:132946","ARL9",TRUE,"ARL9"

"11393","AGACAGCCAGATGGAGTTCC","chr4",56511321,"+","none","TGG","AGACAGCCAGATGGAGTTCCTGG",0,NA,NA,NA,"GeneID:132946","ARL9",TRUE,"ARL9"

"11394","AGACAGCCAGATGGAGTTCC","chr11",17421910,"-","2:C>T,11:T>A","AGG","AGGCAGCCAGAAGGAGTTCCAGG",2,3,12,NA,"GeneID:6833","ABCC8",FALSE,"ARL9"

"11395","ATTTCCCAGTAGGACCGAAA","chr4",56518694,"-","none","AGG","ATTTCCCAGTAGGACCGAAAAGG",0,NA,NA,NA,"GeneID:132946","ARL9",TRUE,"ARL9"

"11396","ATTTCCCAGTAGGACCGAAA","chr8",105808059,"+","5:A>C,16:C>G","GGG","ATTTCACAGTAGGACCCAAAGGG",2,6,17,NA,"","",FALSE,"ARL9"

"11397","CAAAGACTTGATTGCACAGC","chr4",56523836,"+","none","TGG","CAAAGACTTGATTGCACAGCTGG",0,NA,NA,NA,"GeneID:132946","ARL9",TRUE,"ARL9"

"11398","CAGCTGTGCAATCAAGTCTT","chr4",56523838,"-","none","TGG","CAGCTGTGCAATCAAGTCTTTGG",0,NA,NA,NA,"GeneID:132946","ARL9",TRUE,"ARL9"

"11399","CAGCTGTGCAATCAAGTCTT","chr3",37958915,"+","5:C>G,8:G>C","GGG","CAGCTCTGGAATCAAGTCTTGGG",2,6,9,NA,"GeneID:10217","CTDSPL",FALSE,"ARL9"

"11400","GGAACCTACCTGACTAAGAA","chr4",56523783,"+","none","TGG","GGAACCTACCTGACTAAGAATGG",0,NA,NA,NA,"GeneID:132946","ARL9",TRUE,"ARL9"

"11401","ATCAGCAATAGCATCAACCA","chr8",65605309,"-","none","AGG","ATCAGCAATAGCATCAACCAAGG",0,NA,NA,NA,"GeneID:55156","ARMC1",TRUE,"ARMC1"

"11402","ATCAGCAATAGCATCAACCA","chr4",138481491,"-","6:C>T,8:C>A","AGG","ATCAGCGAGAGCATCAACCAAGG",2,7,9,NA,"","",TRUE,"ARMC1"

"11403","ATCAGCAATAGCATCAACCA","chr8",101837254,"-","5:C>G,9:C>T","AGG","ATCAGGAATGGCATCAACCAAGG",2,6,10,NA,"GeneID:83988","NCALD",FALSE,"ARMC1"

"11404","ATCAGCAATAGCATCAACCA","chr6",163490535,"+","5:A>C,11:A>C","GGG","ATCAGAAATAGAATCAACCAGGG",2,6,12,NA,"GeneID:9444","QKI",FALSE,"ARMC1"

"11405","ATCAGCAATAGCATCAACCA","chr6",103632819,"+","0:C>A,17:A>C","AGG","CTCAGCAATAGCATCAAACAAGG",2,1,18,NA,"","",FALSE,"ARMC1"

"11406","ATGAGATGAATTCACGTCGA","chr8",65613324,"-","none","AGG","ATGAGATGAATTCACGTCGAAGG",0,NA,NA,NA,"GeneID:55156","ARMC1",TRUE,"ARMC1"

"11407","ATGAGATGAATTCACGTCGA","chr1",17831686,"+","15:A>G,17:T>C","GGG","ATGAGATGAATTCACATTGAGGG",2,16,18,NA,"","",FALSE,"ARMC1"

"11408","CAGACCTTACCGTATCATCA","chr8",65613233,"+","none","AGG","CAGACCTTACCGTATCATCAAGG",0,NA,NA,NA,"GeneID:55156","ARMC1",TRUE,"ARMC1"

"11409","CAGACCTTACCGTATCATCA","chr13",33657429,"+","10:A>C,12:A>T","GGG","CAGACCTTACAGAATCATCAGGG",2,11,13,NA,"","",TRUE,"ARMC1"

"11410","GCTGTAGGCTCTTCGATACT","chr8",65622341,"-","none","TGG","GCTGTAGGCTCTTCGATACTTGG",0,NA,NA,NA,"GeneID:55156","ARMC1",TRUE,"ARMC1"

"11411","TCGGAGAAATCTATGTGAAG","chr8",65605516,"-","none","AGG","TCGGAGAAATCTATGTGAAGAGG",0,NA,NA,NA,"GeneID:55156","ARMC1",TRUE,"ARMC1"

"11412","TTGCTGAGCTTTCATAACCT","chr8",65605289,"+","none","TGG","TTGCTGAGCTTTCATAACCTTGG",0,NA,NA,NA,"GeneID:55156","ARMC1",TRUE,"ARMC1"

"11413","TTGCTGAGCTTTCATAACCT","chr4",138481471,"+","2:A>G,4:G>T","TGG","TTACGGAGCTTTCATAACCTTGG",2,3,5,NA,"","",TRUE,"ARMC1"

"11414","AAGCTTTCTCTTTAATACTC","chr7",103086691,"-","none","TGG","AAGCTTTCTCTTTAATACTCTGG",0,NA,NA,NA,"GeneID:83787","ARMC10",TRUE,"ARMC10"

"11415","AAGCTTTCTCTTTAATACTC","chr4",18582150,"+","15:A>T,17:A>C","TGG","AAGCTTTCTCTTTAAAAATCTGG",2,16,18,NA,"","",FALSE,"ARMC10"

"11416","AAGCTTTCTCTTTAATACTC","chr3",94507250,"+","7:G>C","TGG","AAGCTTTGTCTTTAATACTCTGG",1,8,NA,NA,"","",TRUE,"ARMC10"

"11417","ACGTCAGAGGGTCAGTTGTG","chr7",103075793,"+","none","CGG","ACGTCAGAGGGTCAGTTGTGCGG",0,NA,NA,NA,"GeneID:83787","ARMC10",TRUE,"ARMC10"

"11418","CCGTTGACTCCAGCAGGTAA","chr7",103083742,"-","none","AGG","CCGTTGACTCCAGCAGGTAAAGG",0,NA,NA,NA,"GeneID:83787","ARMC10",TRUE,"ARMC10"

"11419","CCGTTGACTCCAGCAGGTAA","chr3",94507400,"+","1:A>C","AGG","CAGTTGACTCCAGCAGGTAAAGG",1,2,NA,NA,"","",TRUE,"ARMC10"

"11420","GCGCTCGGCCCGGCCTCAGA","chr7",103075816,"+","none","CGG","GCGCTCGGCCCGGCCTCAGACGG",0,NA,NA,NA,"GeneID:83787","ARMC10",TRUE,"ARMC10"

"11421","GTAGGCTATTATTCGTGAAT","chr7",103086625,"+","none","TGG","GTAGGCTATTATTCGTGAATTGG",0,NA,NA,NA,"GeneID:83787","ARMC10",TRUE,"ARMC10"

"11422","TATGCAGAAGACTTAACTGA","chr7",103083674,"+","none","TGG","TATGCAGAAGACTTAACTGATGG",0,NA,NA,NA,"GeneID:83787","ARMC10",TRUE,"ARMC10"

"11423","TATGCAGAAGACTTAACTGA","chr3",94507468,"-","1:G>T,2:G>A","TGG","TCCGCAGAAGACTTAACTGATGG",2,2,3,NA,"","",TRUE,"ARMC10"

"11424","CACTCGCTGTGTGTACTTGC","chr6",35738141,"+","none","TGG","CACTCGCTGTGTGTACTTGCTGG",0,NA,NA,NA,"GeneID:221481","ARMC12",TRUE,"ARMC12"

"11425","CAGGTAGCTCAGCAGTCGTA","chr6",35747585,"-","none","CGG","CAGGTAGCTCAGCAGTCGTACGG",0,NA,NA,NA,"GeneID:221481","ARMC12",TRUE,"ARMC12"

"11426","CCAGGCCTCTGCTTGTACTA","chr6",35738379,"+","none","CGG","CCAGGCCTCTGCTTGTACTACGG",0,NA,NA,NA,"GeneID:221481","ARMC12",TRUE,"ARMC12"

"11427","GCCGCTCTCGCTCGACTGCC","chr6",35738030,"-","none","AGG","GCCGCTCTCGCTCGACTGCCAGG",0,NA,NA,NA,"GeneID:221481","ARMC12",TRUE,"ARMC12"

"11428","GGCATCACCCGTCGCAGCTG","chr6",35747371,"-","none","TGG","GGCATCACCCGTCGCAGCTGTGG",0,NA,NA,NA,"GeneID:221481","ARMC12",TRUE,"ARMC12"

"11429","GTCATCCGTAGTACAAGCAG","chr6",35738387,"-","none","AGG","GTCATCCGTAGTACAAGCAGAGG",0,NA,NA,NA,"GeneID:221481","ARMC12",TRUE,"ARMC12"

"11430","ACCAGGGGAAGAGACATGCG","chr6",108894468,"+","none","AGG","ACCAGGGGAAGAGACATGCGAGG",0,NA,NA,NA,"GeneID:84071","ARMC2",TRUE,"ARMC2"

"11431","ACCAGGGGAAGAGACATGCG","chr9",117508257,"+","1:A>C,8:C>A","TGG","AACAGGGGCAGAGACATGCGTGG",2,2,9,NA,"","",FALSE,"ARMC2"

"11432","AGCCCTGAATAAGCGAGCCC","chr6",108868926,"-","none","TGG","AGCCCTGAATAAGCGAGCCCTGG",0,NA,NA,NA,"GeneID:84071","ARMC2",TRUE,"ARMC2"

"11433","ATGGCCTTACAGTCGGAGGG","chr6",108868985,"-","none","AGG","ATGGCCTTACAGTCGGAGGGAGG",0,NA,NA,NA,"GeneID:84071","ARMC2",TRUE,"ARMC2"

"11434","CAACATCAACTAGTTTACAC","chr6",108904341,"-","none","AGG","CAACATCAACTAGTTTACACAGG",0,NA,NA,NA,"GeneID:84071","ARMC2",TRUE,"ARMC2"

"11435","CAACATCAACTAGTTTACAC","chr19",57606458,"+","10:G>T,15:C>T","TGG","CAACATCAACGAGTTCACACTGG",2,11,16,NA,"GeneID:348327","ZNF530",TRUE,"ARMC2"

"11436","CCAGTAGCTCAGACCTGAGC","chr6",108894504,"+","none","AGG","CCAGTAGCTCAGACCTGAGCAGG",0,NA,NA,NA,"GeneID:84071","ARMC2",TRUE,"ARMC2"

"11437","CTGTGTAAACTAGTTGATGT","chr6",108904339,"+","none","TGG","CTGTGTAAACTAGTTGATGTTGG",0,NA,NA,NA,"GeneID:84071","ARMC2",TRUE,"ARMC2"

"11438","AAAACAACCCTCCTTGAACT","chr10",22955817,"+","none","TGG","AAAACAACCCTCCTTGAACTTGG",0,NA,NA,NA,"GeneID:219681","ARMC3",TRUE,"ARMC3"

"11439","AGAGCCTCCTCCTAAGGATG","chr10",22932022,"+","none","TGG","AGAGCCTCCTCCTAAGGATGTGG",0,NA,NA,NA,"GeneID:219681","ARMC3",TRUE,"ARMC3"

"11440","AGAGCCTCCTCCTAAGGATG","chr7",157835525,"+","4:A>C,8:G>C","GGG","AGAGACTCGTCCTAAGGATGGGG",2,5,9,NA,"GeneID:5799","PTPRN2",FALSE,"ARMC3"

"11441","AGTTACCTTCTGGAGCGAGC","chr10",22959124,"-","none","TGG","AGTTACCTTCTGGAGCGAGCTGG",0,NA,NA,NA,"GeneID:219681","ARMC3",TRUE,"ARMC3"

"11442","AGTTACCTTCTGGAGCGAGC","chr1",241535799,"-","14:T>C,16:A>C","AGG","AGTTACCTTCTGGAACTAGCAGG",2,15,17,NA,"GeneID:8564","KMO",FALSE,"ARMC3"

"11443","AGTTACCTTCTGGAGCGAGC","chr6",166521092,"+","4:G>A,15:G>C","AGG","AGTTGCCTTCTGGAGGGAGCAGG",2,5,16,NA,"GeneID:6196","RPS6KA2",FALSE,"ARMC3"

"11444","ATTGCCCAGCTCGCTCCAGA","chr10",22959116,"+","none","AGG","ATTGCCCAGCTCGCTCCAGAAGG",0,NA,NA,NA,"GeneID:219681","ARMC3",TRUE,"ARMC3"

"11445","CAGAAACTTACCACATCCTT","chr10",22932035,"-","none","AGG","CAGAAACTTACCACATCCTTAGG",0,NA,NA,NA,"GeneID:219681","ARMC3",TRUE,"ARMC3"

"11446","CAGAAACTTACCACATCCTT","chr3",85363,"-","6:A>G,13:C>G","GGG","CAGAAATTTACCAGATCCTTGGG",2,7,14,NA,"","",FALSE,"ARMC3"

"11447","GATACTTACTATTAGAAGCC","chr10",22955921,"-","none","AGG","GATACTTACTATTAGAAGCCAGG",0,NA,NA,NA,"GeneID:219681","ARMC3",TRUE,"ARMC3"

"11448","GATACTTACTATTAGAAGCC","chr8",125006043,"+","7:T>A,18:T>C","AGG","GATACTTTCTATTAGAAGTCAGG",2,8,19,NA,"GeneID:6713","SQLE",FALSE,"ARMC3"

"11449","AAACTTGTGTTCCATTCAAG","chr10",27994954,"+","none","TGG","AAACTTGTGTTCCATTCAAGTGG",0,NA,NA,NA,"GeneID:55130","ARMC4",TRUE,"ARMC4"

"11450","AAACTTGTGTTCCATTCAAG","chr10",27263161,"-","14:G>A","TGG","AAACTTGTGTTCCACTCAAGTGG",1,15,NA,NA,"","",TRUE,"ARMC4"

"11451","AAACTTGTGTTCCATTCAAG","chr19",27544427,"+","8:T>G,12:A>C","GGG","AAACTTGTTTTCAATTCAAGGGG",2,9,13,NA,"","",FALSE,"ARMC4"

"11452","AGACCCCATAGTAAAAATCC","chr10",27985184,"-","none","TGG","AGACCCCATAGTAAAAATCCTGG",0,NA,NA,NA,"GeneID:55130","ARMC4",TRUE,"ARMC4"

"11453","GAAGCCCAAGCATGTGTTGA","chr10",27987387,"-","none","AGG","GAAGCCCAAGCATGTGTTGAAGG",0,NA,NA,NA,"GeneID:55130","ARMC4",TRUE,"ARMC4"

"11454","GAAGCCCAAGCATGTGTTGA","chr4",4286296,"-","12:T>A,18:A>C","TGG","GAAGCCCAAGCAAGTGTTTATGG",2,13,19,NA,"GeneID:55646","LYAR",FALSE,"ARMC4"

"11455","GATCACCTTCAACACATGCT","chr10",27987379,"+","none","TGG","GATCACCTTCAACACATGCTTGG",0,NA,NA,NA,"GeneID:55130","ARMC4",TRUE,"ARMC4"

"11456","GCGCCCTCAGCATTTGAATC","chr10",27994933,"-","none","AGG","GCGCCCTCAGCATTTGAATCAGG",0,NA,NA,NA,"GeneID:55130","ARMC4",TRUE,"ARMC4"

"11457","GCGCCCTCAGCATTTGAATC","chr10",27263182,"+","8:C>A","AGG","GCGCCCTCCGCATTTGAATCAGG",1,9,NA,NA,"","",TRUE,"ARMC4"

"11458","TGCTATGCTGCTTAAGCAAT","chr10",27985061,"-","none","TGG","TGCTATGCTGCTTAAGCAATTGG",0,NA,NA,NA,"GeneID:55130","ARMC4",TRUE,"ARMC4"

"11459","ACCACGCACCCAACGCACTC","chr16",31464522,"+","none","CGG","ACCACGCACCCAACGCACTCCGG",0,NA,NA,NA,"GeneID:79798","ARMC5",TRUE,"ARMC5"

"11460","CCCCAAGCGGGCAGTACGCG","chr16",31462422,"+","none","AGG","CCCCAAGCGGGCAGTACGCGAGG",0,NA,NA,NA,"GeneID:79798","ARMC5",TRUE,"ARMC5"

"11461","CGCCCCTTCCGTACAGCAAT","chr16",31459936,"-","none","CGG","CGCCCCTTCCGTACAGCAATCGG",0,NA,NA,NA,"GeneID:79798","ARMC5",TRUE,"ARMC5"

"11462","CGCGCCATAGCTGCGCACGA","chr16",31464770,"-","none","AGG","CGCGCCATAGCTGCGCACGAAGG",0,NA,NA,NA,"GeneID:79798","ARMC5",TRUE,"ARMC5"

"11463","CTCGCCGTAGCAGCGCGAGT","chr16",31459733,"-","none","AGG","CTCGCCGTAGCAGCGCGAGTAGG",0,NA,NA,NA,"GeneID:79798","ARMC5",TRUE,"ARMC5"

"11464","TCCCTCGCGTACTGCCCGCT","chr16",31462427,"-","none","TGG","TCCCTCGCGTACTGCCCGCTTGG",0,NA,NA,NA,"GeneID:79798","ARMC5",TRUE,"ARMC5"

"11465","AGAACTCGTTGCGAATGGCC","chr19",19054206,"-","none","AGG","AGAACTCGTTGCGAATGGCCAGG",0,NA,NA,NA,"GeneID:93436","ARMC6",TRUE,"ARMC6"

"11466","AGCTGTGCGCGAGAACATCG","chr19",19042794,"+","none","AGG","AGCTGTGCGCGAGAACATCGAGG",0,NA,NA,NA,"GeneID:93436","ARMC6",TRUE,"ARMC6"

"11467","CACACGGATGTCGTCATCGA","chr19",19052093,"-","none","AGG","CACACGGATGTCGTCATCGAAGG",0,NA,NA,NA,"GeneID:93436","ARMC6",TRUE,"ARMC6"

"11468","CCCCGAGGTCGACGACCTCC","chr19",19054230,"-","none","TGG","CCCCGAGGTCGACGACCTCCTGG",0,NA,NA,NA,"GeneID:93436","ARMC6",TRUE,"ARMC6"

"11469","GACGACATCCGTGTGCCCTT","chr19",19052098,"+","none","TGG","GACGACATCCGTGTGCCCTTTGG",0,NA,NA,NA,"GeneID:93436","ARMC6",TRUE,"ARMC6"

"11470","TTGCGATTCAAACTGCTCCA","chr19",19042856,"-","none","CGG","TTGCGATTCAAACTGCTCCACGG",0,NA,NA,NA,"GeneID:93436","ARMC6",TRUE,"ARMC6"

"11471","AGAGCTGACCGCCACGCCCG","chr17",75128842,"+","none","TGG","AGAGCTGACCGCCACGCCCGTGG",0,NA,NA,NA,"GeneID:79637","ARMC7",TRUE,"ARMC7"

"11472","ATTCCGTGACCAGCGCCTGC","chr17",75110338,"-","none","AGG","ATTCCGTGACCAGCGCCTGCAGG",0,NA,NA,NA,"GeneID:79637","ARMC7",TRUE,"ARMC7"

"11473","CCAGCAACTACGAGTATCTG","chr17",75110510,"+","none","CGG","CCAGCAACTACGAGTATCTGCGG",0,NA,NA,NA,"GeneID:79637","ARMC7",TRUE,"ARMC7"

"11474","CCGCAGATACTCGTAGTTGC","chr17",75110513,"-","none","TGG","CCGCAGATACTCGTAGTTGCTGG",0,NA,NA,NA,"GeneID:79637","ARMC7",TRUE,"ARMC7"

"11475","TATCCCAGCCGCCCGACGTG","chr17",75110315,"-","none","GGG","TATCCCAGCCGCCCGACGTGGGG",0,NA,NA,NA,"GeneID:79637","ARMC7",TRUE,"ARMC7"

"11476","TCCATGGATCAGCGCTGCCG","chr17",75129026,"-","none","TGG","TCCATGGATCAGCGCTGCCGTGG",0,NA,NA,NA,"GeneID:79637","ARMC7",TRUE,"ARMC7"

"11477","TCCATGGATCAGCGCTGCCG","chr1",117239921,"-","7:C>T,18:A>G","TGG","TCCATGGGTCAGCGCTGCTGTGG",2,8,19,NA,"","",FALSE,"ARMC7"

"11478","AGCTCCTAAAACAATGAGAT","chr3",138221969,"-","none","TGG","AGCTCCTAAAACAATGAGATTGG",0,NA,NA,NA,"GeneID:25852","ARMC8",TRUE,"ARMC8"

"11479","AGCTCCTAAAACAATGAGAT","chr1",210526909,"+","13:C>A,15:T>G","GGG","AGCTCCTAAAACACTTAGATGGG",2,14,16,NA,"GeneID:55733","HHAT",FALSE,"ARMC8"

"11480","AGCTCCTAAAACAATGAGAT","chr10",101955180,"+","11:A>C,14:A>T","GGG","AGCTCCTAAAAAAAAGAGATGGG",2,12,15,NA,"GeneID:79591","C10orf76",FALSE,"ARMC8"

"11481","AGCTCCTAAAACAATGAGAT","chr2",211928864,"+","6:A>T,18:C>A","AGG","AGCTCCAAAAACAATGAGCTAGG",2,7,19,NA,"GeneID:2066","ERBB4",FALSE,"ARMC8"

"11482","CAATATTCTGAACTGCACCG","chr3",138235065,"-","none","TGG","CAATATTCTGAACTGCACCGTGG",0,NA,NA,NA,"GeneID:25852","ARMC8",TRUE,"ARMC8"

"11483","CAATATTCTGAACTGCACCG","chr9",38454185,"+","9:T>G,19:A>G","CGG","CAATATTCTTAACTGCACCACGG",2,10,20,NA,"","",FALSE,"ARMC8"

"11484","GACATGAAAAATGCTGTAAT","chr3",138221926,"+","none","TGG","GACATGAAAAATGCTGTAATTGG",0,NA,NA,NA,"GeneID:25852","ARMC8",TRUE,"ARMC8"

"11485","GCTCAAGATGGCGTGCTTGT","chr3",138187547,"+","none","TGG","GCTCAAGATGGCGTGCTTGTTGG",0,NA,NA,NA,"GeneID:25852","ARMC8",TRUE,"ARMC8"

"11486","GCTCAAGATGGCGTGCTTGT","chr9",38454426,"-","11:A>G,16:G>A","TGG","GCTCAAGATGGTGTGCCTGTTGG",2,12,17,NA,"","",FALSE,"ARMC8"

"11487","TCACCGAAAGGACGCTCATG","chr3",138187583,"-","none","CGG","TCACCGAAAGGACGCTCATGCGG",0,NA,NA,NA,"GeneID:25852","ARMC8",TRUE,"ARMC8"

"11488","TCTTACTTTGTAGGACAGTG","chr3",138235100,"-","none","AGG","TCTTACTTTGTAGGACAGTGAGG",0,NA,NA,NA,"GeneID:25852","ARMC8",TRUE,"ARMC8"

"11489","TCTTACTTTGTAGGACAGTG","chr12",28988868,"-","5:A>G,15:C>G","AGG","TCTTATTTTGTAGGAGAGTGAGG",2,6,16,NA,"","",FALSE,"ARMC8"

"11490","ACCATAGGGTTGGGAACAAA","chr2",231216745,"-","none","AGG","ACCATAGGGTTGGGAACAAAAGG",0,NA,NA,NA,"GeneID:80210","ARMC9",TRUE,"ARMC9"

"11491","ATTACTTGGACTAGTGAAAG","chr2",231206267,"+","none","AGG","ATTACTTGGACTAGTGAAAGAGG",0,NA,NA,NA,"GeneID:80210","ARMC9",TRUE,"ARMC9"

"11492","ATTACTTGGACTAGTGAAAG","chr5",6963775,"-","1:T>A,5:G>A","AGG","AATACCTGGACTAGTGAAAGAGG",2,2,6,NA,"","",FALSE,"ARMC9"

"11493","ATTACTTGGACTAGTGAAAG","chr10",45041986,"+","5:A>T,7:A>G","GGG","ATTACATAGACTAGTGAAAGGGG",2,6,8,NA,"","",FALSE,"ARMC9"

"11494","CTCCAAATCATTGACAATTC","chr2",231208230,"+","none","AGG","CTCCAAATCATTGACAATTCAGG",0,NA,NA,NA,"GeneID:80210","ARMC9",TRUE,"ARMC9"

"11495","CTCCAAATCATTGACAATTC","chr5",110389953,"+","1:C>T,7:A>T","TGG","CCCCAAAACATTGACAATTCTGG",2,2,8,NA,"GeneID:642987","TMEM232",FALSE,"ARMC9"

"11496","CTCCAAATCATTGACAATTC","chr13",100235577,"+","1:A>T,13:G>A","TGG","CACCAAATCATTGGCAATTCTGG",2,2,14,NA,"GeneID:5095","PCCA",FALSE,"ARMC9"

"11497","GTTCAATATGGGGGACATTC","chr2",231206231,"+","none","TGG","GTTCAATATGGGGGACATTCTGG",0,NA,NA,NA,"GeneID:80210","ARMC9",TRUE,"ARMC9"

"11498","GTTCAATATGGGGGACATTC","chr1",240662085,"-","0:T>C,12:T>C","TGG","ATTCAATATGGGAGACATTCTGG",2,1,13,NA,"","",FALSE,"ARMC9"

"11499","TACCTGAATTGTCAATGATT","chr2",231208235,"-","none","TGG","TACCTGAATTGTCAATGATTTGG",0,NA,NA,NA,"GeneID:80210","ARMC9",TRUE,"ARMC9"

"11500","TACCTGAATTGTCAATGATT","chr5",110389958,"-","4:T>A,17:A>T","TGG","TACCAGAATTGTCAATGTTTTGG",2,5,18,NA,"GeneID:642987","TMEM232",FALSE,"ARMC9"

"11501","TACCTGAATTGTCAATGATT","chr13",100235582,"-","4:T>A,11:G>A","TGG","TACCAGAATTGCCAATGATTTGG",2,5,12,NA,"GeneID:5095","PCCA",FALSE,"ARMC9"

"11502","TTCCTACTTCAAAACCTACC","chr2",231216663,"+","none","TGG","TTCCTACTTCAAAACCTACCTGG",0,NA,NA,NA,"GeneID:80210","ARMC9",TRUE,"ARMC9"

"11503","TTCCTACTTCAAAACCTACC","chr16",82047268,"+","2:A>C,11:G>A","TGG","TTACTACTTCAGAACCTACCTGG",2,3,12,NA,"GeneID:3294","HSD17B2",FALSE,"ARMC9"

"11504","AAAGGAGCTAAAACTAACGC","chrX",101553095,"+","none","TGG","AAAGGAGCTAAAACTAACGCTGG",0,NA,NA,NA,"GeneID:51309","ARMCX1",TRUE,"ARMCX1"

"11505","AAGGATCATTTGTTCGCTCC","chrX",101553571,"-","none","AGG","AAGGATCATTTGTTCGCTCCAGG",0,NA,NA,NA,"GeneID:51309","ARMCX1",TRUE,"ARMCX1"

"11506","GCACCGAGTTTACCCTGCCC","chrX",101553344,"+","none","AGG","GCACCGAGTTTACCCTGCCCAGG",0,NA,NA,NA,"GeneID:51309","ARMCX1",TRUE,"ARMCX1"

"11507","GCAGGGTAAACTCGGTGCAA","chrX",101553342,"-","none","GGG","GCAGGGTAAACTCGGTGCAAGGG",0,NA,NA,NA,"GeneID:51309","ARMCX1",TRUE,"ARMCX1"

"11508","GCTATACCATGGGCCGCACT","chrX",101552922,"+","none","CGG","GCTATACCATGGGCCGCACTCGG",0,NA,NA,NA,"GeneID:51309","ARMCX1",TRUE,"ARMCX1"

"11509","TTTGGGACTCGAGGATTGTC","chrX",101553172,"+","none","CGG","TTTGGGACTCGAGGATTGTCCGG",0,NA,NA,NA,"GeneID:51309","ARMCX1",TRUE,"ARMCX1"

"11510","CACAGCCAGCATCCCGAACG","chrX",101657560,"+","none","CGG","CACAGCCAGCATCCCGAACGCGG",0,NA,NA,NA,"GeneID:9823","ARMCX2",TRUE,"ARMCX2"

"11511","CCTAAGGCCGAATCAGTAGT","chrX",101657196,"-","none","TGG","CCTAAGGCCGAATCAGTAGTTGG",0,NA,NA,NA,"GeneID:9823","ARMCX2",TRUE,"ARMCX2"

"11512","CGGGGTGGCATCGCCTACCG","chrX",101656942,"-","none","AGG","CGGGGTGGCATCGCCTACCGAGG",0,NA,NA,NA,"GeneID:9823","ARMCX2",TRUE,"ARMCX2"

"11513","GCCGGATTCACAATCGACCT","chrX",101657394,"-","none","TGG","GCCGGATTCACAATCGACCTTGG",0,NA,NA,NA,"GeneID:9823","ARMCX2",TRUE,"ARMCX2"

"11514","GCTGCACCATGAGCCGCGTT","chrX",101657576,"-","none","CGG","GCTGCACCATGAGCCGCGTTCGG",0,NA,NA,NA,"GeneID:9823","ARMCX2",TRUE,"ARMCX2"

"11515","TACCGCTCCAGTCGCTGATG","chrX",101656754,"+","none","TGG","TACCGCTCCAGTCGCTGATGTGG",0,NA,NA,NA,"GeneID:9823","ARMCX2",TRUE,"ARMCX2"

"11516","ACGATTGCATGGGCTACGCC","chrX",101624971,"+","none","AGG","ACGATTGCATGGGCTACGCCAGG",0,NA,NA,NA,"GeneID:51566","ARMCX3",TRUE,"ARMCX3"

"11517","ATCAGACCAGTCATTATACC","chrX",101625145,"-","none","TGG","ATCAGACCAGTCATTATACCTGG",0,NA,NA,NA,"GeneID:51566","ARMCX3",TRUE,"ARMCX3"

"11518","CAGAGATATTATTCGTGATC","chrX",101625443,"+","none","TGG","CAGAGATATTATTCGTGATCTGG",0,NA,NA,NA,"GeneID:51566","ARMCX3",TRUE,"ARMCX3"

"11519","TCACCGAATTGATTCTGAGT","chrX",101625948,"-","none","AGG","TCACCGAATTGATTCTGAGTAGG",0,NA,NA,NA,"GeneID:51566","ARMCX3",TRUE,"ARMCX3"

"11520","TCGCAAAGATTCTCAATACT","chrX",101625481,"+","none","CGG","TCGCAAAGATTCTCAATACTCGG",0,NA,NA,NA,"GeneID:51566","ARMCX3",TRUE,"ARMCX3"

"11521","TTGAGAATCTTTGCGACAAT","chrX",101625477,"-","none","TGG","TTGAGAATCTTTGCGACAATTGG",0,NA,NA,NA,"GeneID:51566","ARMCX3",TRUE,"ARMCX3"

"11522","ACTAAACCCCAAGTCGAGAT","chrX",101488787,"+","none","TGG","ACTAAACCCCAAGTCGAGATTGG",0,NA,NA,NA,"GeneID:100131755","ARMCX4",TRUE,"ARMCX4"

"11523","CACCACTGGACTGATCTACG","chrX",101492692,"-","none","TGG","CACCACTGGACTGATCTACGTGG",0,NA,NA,NA,"GeneID:100131755","ARMCX4",TRUE,"ARMCX4"

"11524","CATGAAGTTGTTCGTAATGT","chrX",101494814,"+","none","AGG","CATGAAGTTGTTCGTAATGTAGG",0,NA,NA,NA,"GeneID:100131755","ARMCX4",TRUE,"ARMCX4"

"11525","CGGGCAGAGTCTGTTGTATA","chrX",101490815,"-","none","TGG","CGGGCAGAGTCTGTTGTATATGG",0,NA,NA,NA,"GeneID:100131755","ARMCX4",TRUE,"ARMCX4"

"11526","CTTAGTTTCCGACCAGATCA","chrX",101494569,"-","none","TGG","CTTAGTTTCCGACCAGATCATGG",0,NA,NA,NA,"GeneID:100131755","ARMCX4",TRUE,"ARMCX4"

"11527","GCTGGGACTAGTGATCAATC","chrX",101492501,"+","none","TGG","GCTGGGACTAGTGATCAATCTGG",0,NA,NA,NA,"GeneID:100131755","ARMCX4",TRUE,"ARMCX4"

"11528","AAAGTATGGGCCTAATCCGA","chrX",102603007,"+","none","AGG","AAAGTATGGGCCTAATCCGAAGG",0,NA,NA,NA,"GeneID:64860","ARMCX5",TRUE,"ARMCX5"

"11529","ACAGTACTGACCCTAGACAT","chrX",102602477,"-","none","AGG","ACAGTACTGACCCTAGACATAGG",0,NA,NA,NA,"GeneID:64860","ARMCX5",TRUE,"ARMCX5"

"11530","CCCAATGTTAAAGAACACCC","chrX",102603230,"+","none","TGG","CCCAATGTTAAAGAACACCCTGG",0,NA,NA,NA,"GeneID:64860","ARMCX5",TRUE,"ARMCX5"

"11531","GTCAAAGGTCGAATCCCCAG","chrX",102602915,"-","none","AGG","GTCAAAGGTCGAATCCCCAGAGG",0,NA,NA,NA,"GeneID:64860","ARMCX5",TRUE,"ARMCX5"

"11532","TAAGCCCCTACCTAAGATCC","chrX",102602731,"+","none","AGG","TAAGCCCCTACCTAAGATCCAGG",0,NA,NA,NA,"GeneID:64860","ARMCX5",TRUE,"ARMCX5"

"11533","TGGTAATAAGGCCCATGATA","chrX",102602948,"-","none","AGG","TGGTAATAAGGCCCATGATAAGG",0,NA,NA,NA,"GeneID:64860","ARMCX5",TRUE,"ARMCX5"

"11534","AAGCCCTACAGCAGATCTAC","chrX",102601471,"-","none","AGG","AAGCCCTACAGCAGATCTACAGG",0,NA,NA,NA,"GeneID:64860","ARMCX5",TRUE,"ARMCX5-GPRASP2"

"11535","CTGTATGACACAGCACCTCG","chrX",102599784,"+","none","AGG","CTGTATGACACAGCACCTCGAGG",0,NA,NA,NA,"GeneID:64860","ARMCX5",TRUE,"ARMCX5-GPRASP2"

"11536","CTGTATGACACAGCACCTCG","chrX",102595894,"-","3:T>A,19:T>C","GGG","CTGAATGACACAGCACCTCAGGG",2,4,20,NA,"","",FALSE,"ARMCX5-GPRASP2"

"11537","GCAATGGCGGCGGCTGTGTA","chrX",102599729,"-","none","TGG","GCAATGGCGGCGGCTGTGTATGG",0,NA,NA,NA,"GeneID:64860","ARMCX5",TRUE,"ARMCX5-GPRASP2"

"11538","GCAATGGCGGCGGCTGTGTA","chr17",64779044,"-","13:T>G,18:C>A","GGG","GCAATGGCGGCGGATGTGGAGGG",2,14,19,NA,"GeneID:146880","LOC146880",FALSE,"ARMCX5-GPRASP2"

"11539","GCTTGTCACCATTGGAAGCA","chrX",102601487,"+","none","AGG","GCTTGTCACCATTGGAAGCAAGG",0,NA,NA,NA,"GeneID:64860","ARMCX5",TRUE,"ARMCX5-GPRASP2"

"11540","TGATTAAGTGTGTGCTCCAC","chrX",102605591,"-","none","AGG","TGATTAAGTGTGTGCTCCACAGG",0,NA,NA,NA,"","",TRUE,"ARMCX5-GPRASP2"

"11541","TTGTCCATAGGCCTACTTTA","chrX",102713771,"+","none","AGG","TTGTCCATAGGCCTACTTTAAGG",0,NA,NA,NA,"GeneID:114928","GPRASP2",TRUE,"ARMCX5-GPRASP2"

"11542","TTGTCCATAGGCCTACTTTA","chrX",102652820,"+","14:G>A","AGG","TTGTCCATAGGCCTGCTTTAAGG",1,15,NA,NA,"GeneID:9737","GPRASP1",TRUE,"ARMCX5-GPRASP2"

"11543","CAGTTTGTAAACGCAGTAGC","chrX",101616539,"+","none","AGG","CAGTTTGTAAACGCAGTAGCAGG",0,NA,NA,NA,"GeneID:54470","ARMCX6",TRUE,"ARMCX6"

"11544","CTCGGCCCTGGACTGAGGAT","chrX",101616404,"-","none","GGG","CTCGGCCCTGGACTGAGGATGGG",0,NA,NA,NA,"GeneID:54470","ARMCX6",TRUE,"ARMCX6"

"11545","GAAGTGGGTTGGATGGCGGC","chrX",101616585,"-","none","AGG","GAAGTGGGTTGGATGGCGGCAGG",0,NA,NA,NA,"GeneID:54470","ARMCX6",TRUE,"ARMCX6"

"11546","GAAGTGGGTTGGATGGCGGC","chr7",71921833,"-","4:C>A,9:T>A","GGG","GAAGGGGGTAGGATGGCGGCGGG",2,5,10,NA,"GeneID:83698","CALN1",FALSE,"ARMCX6"

"11547","GAGCACACCCAATAAAACAG","chrX",101616314,"-","none","CGG","GAGCACACCCAATAAAACAGCGG",0,NA,NA,NA,"GeneID:54470","ARMCX6",TRUE,"ARMCX6"

"11548","TCCAACCCACTTCCCGAGCC","chrX",101616592,"+","none","CGG","TCCAACCCACTTCCCGAGCCCGG",0,NA,NA,NA,"GeneID:54470","ARMCX6",TRUE,"ARMCX6"

"11549","TGCGTTTACAAACTGACCAT","chrX",101616534,"-","none","AGG","TGCGTTTACAAACTGACCATAGG",0,NA,NA,NA,"GeneID:54470","ARMCX6",TRUE,"ARMCX6"

"11550","TGCGTTTACAAACTGACCAT","chr9",35750901,"+","1:A>G,5:C>T","TGG","TACGTCTACAAACTGACCATTGG",2,2,6,NA,"GeneID:9827","RGP1",TRUE,"ARMCX6"

"11551","ACCGATGGTAACTGAGGCGG","chr10",122454747,"+","none","AGG","ACCGATGGTAACTGAGGCGGAGG",0,NA,NA,NA,"GeneID:387715","ARMS2",TRUE,"ARMS2"

"11552","AGAATCACCTTGCTGCAGTG","chr10",122456917,"-","none","TGG","AGAATCACCTTGCTGCAGTGTGG",0,NA,NA,NA,"GeneID:387715","ARMS2",TRUE,"ARMS2"

"11553","AGAATCACCTTGCTGCAGTG","chr2",20669585,"-","0:C>T,5:C>G","AGG","GGAATGACCTTGCTGCAGTGAGG",2,1,6,NA,"GeneID:151449","GDF7",FALSE,"ARMS2"

"11554","AGAATCACCTTGCTGCAGTG","chr6",48307870,"+","9:A>T,14:A>G","TGG","AGAATCACCATGCTACAGTGTGG",2,10,15,NA,"","",FALSE,"ARMS2"

"11555","CAGCACAGACTCTCGCAGAG","chr10",122454833,"-","none","TGG","CAGCACAGACTCTCGCAGAGTGG",0,NA,NA,NA,"GeneID:387715","ARMS2",TRUE,"ARMS2"

"11556","CCCTCCGCCTCAGTTACCAT","chr10",122454751,"-","none","CGG","CCCTCCGCCTCAGTTACCATCGG",0,NA,NA,NA,"GeneID:387715","ARMS2",TRUE,"ARMS2"

"11557","CTATCATCCACACTGCAGCA","chr10",122456907,"+","none","AGG","CTATCATCCACACTGCAGCAAGG",0,NA,NA,NA,"GeneID:387715","ARMS2",TRUE,"ARMS2"

"11558","CTATCATCCACACTGCAGCA","chr17",34573122,"+","3:G>T,17:T>G","GGG","CTAGCATCCACACTGCATCAGGG",2,4,18,NA,"","",FALSE,"ARMS2"

"11559","TACATGCTGCGCCTATACCC","chr10",122454724,"+","none","AGG","TACATGCTGCGCCTATACCCAGG",0,NA,NA,NA,"GeneID:387715","ARMS2",TRUE,"ARMS2"

"11560","TACATGCTGCGCCTATACCC","chr14",59073183,"+","5:C>G,10:A>G","TGG","TACATCCTGCACCTATACCCTGG",2,6,11,NA,"","",FALSE,"ARMS2"

"11561","CTCAGCCTATTCACAGAAAC","chr1",150832354,"+","none","TGG","CTCAGCCTATTCACAGAAACTGG",0,NA,NA,NA,"GeneID:405","ARNT",TRUE,"ARNT"

"11562","GACATCAGATGTACCATCAC","chr1",150858436,"-","none","TGG","GACATCAGATGTACCATCACTGG",0,NA,NA,NA,"GeneID:405","ARNT",TRUE,"ARNT"

"11563","GTCGCCGCTTAATAGCCCTC","chr1",150858352,"+","none","TGG","GTCGCCGCTTAATAGCCCTCTGG",0,NA,NA,NA,"GeneID:405","ARNT",TRUE,"ARNT"

"11564","GTGGAGGAGCCATTGTCCAG","chr1",150858371,"-","none","AGG","GTGGAGGAGCCATTGTCCAGAGG",0,NA,NA,NA,"GeneID:405","ARNT",TRUE,"ARNT"

"11565","TGAATAGGCTGAGCTTTGTG","chr1",150832347,"-","none","AGG","TGAATAGGCTGAGCTTTGTGAGG",0,NA,NA,NA,"GeneID:405","ARNT",TRUE,"ARNT"

"11566","TGAATAGGCTGAGCTTTGTG","chr8",1888747,"+","8:G>C,13:G>C","AGG","TGAATAGGGTGAGGTTTGTGAGG",2,9,14,NA,"GeneID:9639","ARHGEF10",FALSE,"ARNT"

"11567","TGAATAGGCTGAGCTTTGTG","chr8",1888689,"+","8:G>C,13:G>C","AGG","TGAATAGGGTGAGGTTTGTGAGG",2,9,14,NA,"GeneID:9639","ARHGEF10",FALSE,"ARNT"

"11568","TGAATAGGCTGAGCTTTGTG","chr8",1888718,"+","8:G>C,13:G>C","AGG","TGAATAGGGTGAGGTTTGTGAGG",2,9,14,NA,"GeneID:9639","ARHGEF10",FALSE,"ARNT"

"11569","TGAATAGGCTGAGCTTTGTG","chr8",1888834,"+","8:G>C,13:G>C","AGG","TGAATAGGGTGAGGTTTGTGAGG",2,9,14,NA,"GeneID:9639","ARHGEF10",FALSE,"ARNT"

"11570","TGAATAGGCTGAGCTTTGTG","chr2",237206504,"+","5:T>A,17:C>G","AGG","TGAATTGGCTGAGCTTTCTGAGG",2,6,18,NA,"","",FALSE,"ARNT"

"11571","TGATCAGATGTCTAACGATA","chr1",150846278,"-","none","AGG","TGATCAGATGTCTAACGATAAGG",0,NA,NA,NA,"GeneID:405","ARNT",TRUE,"ARNT"

"11572","CCCGGAGGGTTGACCGCCGC","chr15",80404527,"-","none","CGG","CCCGGAGGGTTGACCGCCGCCGG",0,NA,NA,NA,"GeneID:9915","ARNT2",TRUE,"ARNT2"

"11573","CGCGGCGGTCTTTCATCTGC","chr15",80508230,"+","none","AGG","CGCGGCGGTCTTTCATCTGCAGG",0,NA,NA,NA,"GeneID:9915","ARNT2",TRUE,"ARNT2"

"11574","CTCTCTCTTAGGCCGGATCT","chr15",80508144,"+","none","TGG","CTCTCTCTTAGGCCGGATCTTGG",0,NA,NA,NA,"GeneID:9915","ARNT2",TRUE,"ARNT2"

"11575","TAGGTGTGGAAATGCTCCTT","chr15",80513907,"+","none","TGG","TAGGTGTGGAAATGCTCCTTTGG",0,NA,NA,NA,"GeneID:9915","ARNT2",TRUE,"ARNT2"

"11576","TAGGTGTGGAAATGCTCCTT","chr16",64548362,"+","8:T>G,17:A>C","AGG","TAGGTGTGTAAATGCTCATTAGG",2,9,18,NA,"","",FALSE,"ARNT2"

"11577","TAGGTGTGGAAATGCTCCTT","chr20",53699360,"-","2:A>C,18:G>A","GGG","TATGTGTGGAAATGCTCCCTGGG",2,3,19,NA,"","",FALSE,"ARNT2"

"11578","TCCGAGCAAGATGGCAACCC","chr15",80404505,"+","none","CGG","TCCGAGCAAGATGGCAACCCCGG",0,NA,NA,NA,"GeneID:9915","ARNT2",TRUE,"ARNT2"

"11579","TGGTTATTCTGTTTAGAGGA","chr15",80513937,"-","none","AGG","TGGTTATTCTGTTTAGAGGAAGG",0,NA,NA,NA,"GeneID:9915","ARNT2",TRUE,"ARNT2"

"11580","ACAGACAAAGATGACCCTCA","chr11",13356751,"+","none","TGG","ACAGACAAAGATGACCCTCATGG",0,NA,NA,NA,"GeneID:406","ARNTL",TRUE,"ARNTL"

"11581","ACAGACAAAGATGACCCTCA","chr3",148605497,"+","13:G>A,14:T>C","AGG","ACAGACAAAGATGGTCCTCAAGG",2,14,15,NA,"","",FALSE,"ARNTL"

"11582","GGCGTACTCGTGATGTTCAA","chr11",13381200,"-","none","TGG","GGCGTACTCGTGATGTTCAATGG",0,NA,NA,NA,"GeneID:406","ARNTL",TRUE,"ARNTL"

"11583","GGGTCATCGCCTTCTAGCTG","chr11",13381167,"+","none","TGG","GGGTCATCGCCTTCTAGCTGTGG",0,NA,NA,NA,"GeneID:406","ARNTL",TRUE,"ARNTL"

"11584","GGGTCATCGCCTTCTAGCTG","chrX",42778612,"-","0:T>C,8:T>C","AGG","AGGTCATCACCTTCTAGCTGAGG",2,1,9,NA,"","",FALSE,"ARNTL"

"11585","GTTCATGGTACCTTCCATGA","chr11",13356768,"-","none","GGG","GTTCATGGTACCTTCCATGAGGG",0,NA,NA,NA,"GeneID:406","ARNTL",TRUE,"ARNTL"

"11586","TTATCACACTACGGAGTCGA","chr11",13375674,"+","none","TGG","TTATCACACTACGGAGTCGATGG",0,NA,NA,NA,"GeneID:406","ARNTL",TRUE,"ARNTL"

"11587","TTTCATGAACCCTTGGACCA","chr11",13375702,"+","none","AGG","TTTCATGAACCCTTGGACCAAGG",0,NA,NA,NA,"GeneID:406","ARNTL",TRUE,"ARNTL"

"11588","TTTCATGAACCCTTGGACCA","chr3",133217199,"+","11:T>C,12:C>T","TGG","TTTCATGAACCTCTGGACCATGG",2,12,13,NA,"GeneID:66000","TMEM108",FALSE,"ARNTL"

"11589","TTTCATGAACCCTTGGACCA","chr20",45913123,"+","3:A>C,17:G>C","AGG","TTTAATGAACCCTTGGAGCAAGG",2,4,18,NA,"","",FALSE,"ARNTL"

"11590","AGAAAGTTGTTCCTTTACTT","chr12",27389233,"-","none","TGG","AGAAAGTTGTTCCTTTACTTTGG",0,NA,NA,NA,"GeneID:56938","ARNTL2",TRUE,"ARNTL2"

"11591","AGGCTTGACAAATTCTTATG","chr12",27385478,"+","none","TGG","AGGCTTGACAAATTCTTATGTGG",0,NA,NA,NA,"GeneID:56938","ARNTL2",TRUE,"ARNTL2"

"11592","AGGCTTGACAAATTCTTATG","chr3",190910939,"+","6:T>G,8:A>C","AGG","AGGCTTTAAAAATTCTTATGAGG",2,7,9,NA,"","",FALSE,"ARNTL2"

"11593","CAAAATACTTAATTATGATC","chr12",27387298,"+","none","AGG","CAAAATACTTAATTATGATCAGG",0,NA,NA,NA,"GeneID:56938","ARNTL2",TRUE,"ARNTL2"

"11594","GCTCAGACATTTAATCCTTA","chr12",27385538,"+","none","AGG","GCTCAGACATTTAATCCTTAAGG",0,NA,NA,NA,"GeneID:56938","ARNTL2",TRUE,"ARNTL2"

"11595","TATGAATAGGCTAGTTTGAC","chr12",27389169,"+","none","TGG","TATGAATAGGCTAGTTTGACTGG",0,NA,NA,NA,"GeneID:56938","ARNTL2",TRUE,"ARNTL2"

"11596","TATGAATAGGCTAGTTTGAC","chr7",117917867,"+","1:T>A,9:A>G","AGG","TTTGAATAGACTAGTTTGACAGG",2,2,10,NA,"","",FALSE,"ARNTL2"

"11597","TGCAGAAGGCTTCTTATTTG","chr12",27387229,"+","none","TGG","TGCAGAAGGCTTCTTATTTGTGG",0,NA,NA,NA,"GeneID:56938","ARNTL2",TRUE,"ARNTL2"

"11598","CTCACTTGCAGGTATTGACT","chr7",99344281,"+","none","GGG","CTCACTTGCAGGTATTGACTGGG",0,NA,NA,NA,"GeneID:10552","ARPC1A",TRUE,"ARPC1A"

"11599","GATCATGTGACTTCAAATGC","chr7",99348937,"+","none","AGG","GATCATGTGACTTCAAATGCAGG",0,NA,NA,NA,"GeneID:10552","ARPC1A",TRUE,"ARPC1A"

"11600","GATCATGTGACTTCAAATGC","chr2",221501288,"+","9:G>A,13:G>C","AGG","GATCATGTGGCTTGAAATGCAGG",2,10,14,NA,"GeneID:2043","EPHA4",TRUE,"ARPC1A"

"11601","GATGTGCACTTCGTGATTAT","chr7",99338198,"-","none","TGG","GATGTGCACTTCGTGATTATTGG",0,NA,NA,NA,"GeneID:10552","ARPC1A",TRUE,"ARPC1A"

"11602","GATGTGCACTTCGTGATTAT","chr4",86914714,"+","11:G>C","TGG","GATGTGCACTTGGTGATTATTGG",1,12,NA,NA,"","",TRUE,"ARPC1A"

"11603","GTTCATGAGCTTTCACCCAC","chr7",99338238,"-","none","TGG","GTTCATGAGCTTTCACCCACTGG",0,NA,NA,NA,"GeneID:10552","ARPC1A",TRUE,"ARPC1A"

"11604","GTTCATGAGCTTTCACCCAC","chr4",86914349,"+","18:C>A","CGG","GTTCATGAGCTTTCACCCCCCGG",1,19,NA,NA,"","",TRUE,"ARPC1A"

"11605","GTTCATGAGCTTTCACCCAC","chr7",107872300,"+","4:T>A,15:T>C","CGG","GTTCTTGAGCTTTCATCCACCGG",2,5,16,NA,"","",FALSE,"ARPC1A"

"11606","GTTCATGAGCTTTCACCCAC","chr5",74799115,"-","4:C>T,16:T>G","TGG","GTTCGTGAGCTTTCACACACTGG",2,5,17,NA,"GeneID:26049","FAM169A",TRUE,"ARPC1A"

"11607","TAGCTGCGCGATTAATTCTC","chr7",99344401,"-","none","AGG","TAGCTGCGCGATTAATTCTCAGG",0,NA,NA,NA,"GeneID:10552","ARPC1A",TRUE,"ARPC1A"

"11608","TAGCTGCGCGATTAATTCTC","chr5",74799271,"-","6:A>G","AGG","TAGCTGTGCGATTAATTCTCAGG",1,7,NA,NA,"GeneID:26049","FAM169A",TRUE,"ARPC1A"

"11609","TAGCTGCGCGATTAATTCTC","chr19",53004317,"-","7:T>C,19:T>G","AGG","TAGCTGCACGATTAATTCTAAGG",2,8,20,NA,"GeneID:79986","ZNF702P",TRUE,"ARPC1A"

"11610","TGCTGCCAGCAAAACGTTGT","chr7",99348916,"-","none","TGG","TGCTGCCAGCAAAACGTTGTTGG",0,NA,NA,NA,"GeneID:10552","ARPC1A",TRUE,"ARPC1A"

"11611","TGCTGCCAGCAAAACGTTGT","chr19",53004494,"-","15:T>C","TGG","TGCTGCCAGCAAAACATTGTTGG",1,16,NA,NA,"GeneID:79986","ZNF702P",TRUE,"ARPC1A"

"11612","TGCTGCCAGCAAAACGTTGT","chr5",74799450,"-","15:T>C","TGG","TGCTGCCAGCAAAACATTGTTGG",1,16,NA,NA,"GeneID:26049","FAM169A",TRUE,"ARPC1A"

"11613","TGCTGCCAGCAAAACGTTGT","chr4",86914014,"+","17:C>T","TGG","TGCTGCCAGCAAAACGTCGTTGG",1,18,NA,NA,"","",TRUE,"ARPC1A"

"11614","TGCTGCCAGCAAAACGTTGT","chr4",170451904,"-","2:A>G,14:T>G","AGG","TGTTGCCAGCAAAAAGTTGTAGG",2,3,15,NA,"","",FALSE,"ARPC1A"

"11615","CAGACCGCAACGCCTACGTG","chr7",99388083,"+","none","TGG","CAGACCGCAACGCCTACGTGTGG",0,NA,NA,NA,"GeneID:10095","ARPC1B",TRUE,"ARPC1B"

"11616","CATCTGCCCCAACAACCATG","chr7",99386691,"+","none","AGG","CATCTGCCCCAACAACCATGAGG",0,NA,NA,NA,"GeneID:10095","ARPC1B",TRUE,"ARPC1B"

"11617","CATGCCGCCATCCATGCCAG","chr7",99394081,"-","none","TGG","CATGCCGCCATCCATGCCAGTGG",0,NA,NA,NA,"GeneID:10095;GeneID:11333","ARPC1B;PDAP1",TRUE,"ARPC1B"

"11618","CCGCACATGGAAGCCCACGC","chr7",99388117,"+","none","TGG","CCGCACATGGAAGCCCACGCTGG",0,NA,NA,NA,"GeneID:10095","ARPC1B",TRUE,"ARPC1B"

"11619","CGCCGCTGAGCACCGAGATC","chr7",99394031,"-","none","TGG","CGCCGCTGAGCACCGAGATCTGG",0,NA,NA,NA,"GeneID:10095;GeneID:11333","ARPC1B;PDAP1",TRUE,"ARPC1B"

"11620","GCACCCCAACAATGTGCTGC","chr7",99389961,"+","none","TGG","GCACCCCAACAATGTGCTGCTGG",0,NA,NA,NA,"GeneID:10095","ARPC1B",TRUE,"ARPC1B"

"11621","AATGTTACTTCTACTGCTTC","chr2",218225929,"-","none","CGG","AATGTTACTTCTACTGCTTCCGG",0,NA,NA,NA,"GeneID:10109","ARPC2",TRUE,"ARPC2"

"11622","AATGTTACTTCTACTGCTTC","chr14",88977531,"+","1:C>A,10:T>C","TGG","ACTGTTACTTTTACTGCTTCTGG",2,2,11,NA,"","",FALSE,"ARPC2"

"11623","GATTCCATTGTGCATCAAGC","chr2",218238713,"+","none","TGG","GATTCCATTGTGCATCAAGCTGG",0,NA,NA,NA,"GeneID:10109","ARPC2",TRUE,"ARPC2"

"11624","GCAGTAGAAGTAACATTTGC","chr2",218225932,"+","none","AGG","GCAGTAGAAGTAACATTTGCAGG",0,NA,NA,NA,"GeneID:10109","ARPC2",TRUE,"ARPC2"

"11625","GCAGTAGAAGTAACATTTGC","chr4",168389872,"+","9:C>G,18:T>G","TGG","GCAGTAGAACTAACATTTTCTGG",2,10,19,NA,"GeneID:91351","DDX60L",FALSE,"ARPC2"

"11626","TCAGGCACATGGTGCTGATG","chr2",218228828,"+","none","AGG","TCAGGCACATGGTGCTGATGAGG",0,NA,NA,NA,"GeneID:10109","ARPC2",TRUE,"ARPC2"

"11627","TCAGGCACATGGTGCTGATG","chr18",36309686,"-","0:G>A,17:G>T","AGG","CCAGGCACATGGTGCTGCTGAGG",2,1,18,NA,"GeneID:80206","FHOD3",FALSE,"ARPC2"

"11628","TGGTCTCATCATCCCTATAA","chr2",218238829,"-","none","TGG","TGGTCTCATCATCCCTATAATGG",0,NA,NA,NA,"GeneID:10109","ARPC2",TRUE,"ARPC2"

"11629","TTTCCTCACAGATTTCGATG","chr2",218228726,"+","none","GGG","TTTCCTCACAGATTTCGATGGGG",0,NA,NA,NA,"GeneID:10109","ARPC2",TRUE,"ARPC2"

"11630","AGCAAAAGATACAGATATTG","chr12",110440370,"-","none","TGG","AGCAAAAGATACAGATATTGTGG",0,NA,NA,NA,"GeneID:10094","ARPC3",TRUE,"ARPC3"

"11631","AGCAAAAGATACAGATATTG","chr11",94188873,"-","0:C>T,1:T>C","TGG","GACAAAAGATACAGATATTGTGG",2,1,2,NA,"","",TRUE,"ARPC3"

"11632","AGCAAAAGATACAGATATTG","chr20",49134583,"+","0:G>A,1:A>G","TGG","GACAAAAGATACAGATATTGTGG",2,1,2,NA,"GeneID:6780","STAU1",TRUE,"ARPC3"

"11633","AGCAAAAGATACAGATATTG","chr6",15934885,"+","0:G>A,1:A>G","TGG","GACAAAAGATACAGATATTGTGG",2,1,2,NA,"","",TRUE,"ARPC3"

"11634","AGCAAAAGATACAGATATTG","chr12",127249883,"+","2:G>C,18:A>T","AGG","AGGAAAAGATACAGATATAGAGG",2,3,19,NA,"","",FALSE,"ARPC3"

"11635","AGCAAAAGATACAGATATTG","chr1",61292535,"+","6:G>A,19:T>G","AGG","AGCAAAGGATACAGATATTTAGG",2,7,20,NA,"GeneID:4774","NFIA",FALSE,"ARPC3"

"11636","CCCTGTAGAATGAAGCTGAT","chr12",110437140,"-","none","AGG","CCCTGTAGAATGAAGCTGATAGG",0,NA,NA,NA,"GeneID:10094","ARPC3",TRUE,"ARPC3"

"11637","CCCTGTAGAATGAAGCTGAT","chr13",91760523,"-","0:A>G,4:G>C","GGG","TCCTCTAGAATGAAGCTGATGGG",2,1,5,NA,"GeneID:2262","GPC5",FALSE,"ARPC3"

"11638","CTTCAAAAACTATGAAATTA","chr12",110440313,"-","none","AGG","CTTCAAAAACTATGAAATTAAGG",0,NA,NA,NA,"GeneID:10094","ARPC3",TRUE,"ARPC3"

"11639","CTTCAAAAACTATGAAATTA","chr11",40460647,"-","9:A>G,19:C>T","TGG","CTTCAAAAATTATGAAATTGTGG",2,10,20,NA,"GeneID:57689","LRRC4C",FALSE,"ARPC3"

"11640","TACCAAACTCATCGGAAACA","chr12",110445502,"-","none","TGG","TACCAAACTCATCGGAAACATGG",0,NA,NA,NA,"GeneID:10094","ARPC3",TRUE,"ARPC3"

"11641","TACCAAACTCATCGGAAACA","chr11",94188942,"-","0:G>A,2:A>G","AGG","CATCAAACTCATCGGAAACAAGG",2,1,3,NA,"","",TRUE,"ARPC3"

"11642","TGAGAAAGAAATGTATACGC","chr12",110436643,"-","none","TGG","TGAGAAAGAAATGTATACGCTGG",0,NA,NA,NA,"GeneID:10094","ARPC3",TRUE,"ARPC3"

"11643","TGAGAAAGAAATGTATACGC","chr11",94188693,"-","18:T>C","TGG","TGAGAAAGAAATGTATACACTGG",1,19,NA,NA,"","",TRUE,"ARPC3"

"11644","TGAGAAAGAAATGTATACGC","chr11",73921898,"-","18:T>C","TGG","TGAGAAAGAAATGTATACACTGG",1,19,NA,NA,"GeneID:80227","PAAF1",TRUE,"ARPC3"

"11645","TGAGAAAGAAATGTATACGC","chr20",49134751,"+","18:A>G","TGG","TGAGAAAGAAATGTATACACTGG",1,19,NA,NA,"GeneID:6780","STAU1",TRUE,"ARPC3"

"11646","TGAGAAAGAAATGTATACGC","chr1",211442545,"+","18:A>G","AGG","TGAGAAAGAAATGTATACACAGG",1,19,NA,NA,"","",TRUE,"ARPC3"

"11647","TGAGAAAGAAATGTATACGC","chr6",15935053,"+","18:A>G,19:T>C","TGG","TGAGAAAGAAATGTATACATTGG",2,19,20,NA,"","",TRUE,"ARPC3"

"11648","TTTAGACTTACTCTCTCTGG","chr12",110445440,"+","none","GGG","TTTAGACTTACTCTCTCTGGGGG",0,NA,NA,NA,"GeneID:10094","ARPC3",TRUE,"ARPC3"

"11649","TTTAGACTTACTCTCTCTGG","chr8",124842440,"-","0:T>A,3:A>T","AGG","ATTTGACTTACTCTCTCTGGAGG",2,1,4,NA,"","",FALSE,"ARPC3"

"11650","TTTAGACTTACTCTCTCTGG","chr5",170351341,"+","5:T>A,11:C>T","TGG","TTTAGTCTTACCCTCTCTGGTGG",2,6,12,NA,"","",FALSE,"ARPC3"

"11651","ACGACACAACAAGCCGGAAG","chr3",9797747,"+","none","TGG","ACGACACAACAAGCCGGAAGTGG",0,NA,NA,NA,"GeneID:10093;GeneID:100526693","ARPC4;ARPC4-TTLL3",TRUE,"ARPC4"

"11652","AGAACTTCTTTATCCTTCGA","chr3",9801721,"+","none","AGG","AGAACTTCTTTATCCTTCGAAGG",0,NA,NA,NA,"GeneID:10093;GeneID:100526693","ARPC4;ARPC4-TTLL3",TRUE,"ARPC4"

"11653","AGAACTTCTTTATCCTTCGA","chr3",164553703,"-","14:A>G,17:A>G","AGG","AGAACTTCTTTATCTTTTGAAGG",2,15,18,NA,"","",FALSE,"ARPC4"

"11654","AGAACTTCTTTATCCTTCGA","chr2",114242285,"+","14:T>C,17:A>C","TGG","AGAACTTCTTTATCTTTAGATGG",2,15,18,NA,"","",FALSE,"ARPC4"

"11655","CAATGCCCGTGCCCGCATTG","chr3",9803970,"+","none","TGG","CAATGCCCGTGCCCGCATTGTGG",0,NA,NA,NA,"GeneID:10093;GeneID:100526693","ARPC4;ARPC4-TTLL3",TRUE,"ARPC4"

"11656","GTTTCACAGCAATGCTGACC","chr3",9800274,"-","none","CGG","GTTTCACAGCAATGCTGACCCGG",0,NA,NA,NA,"GeneID:10093;GeneID:100526693","ARPC4;ARPC4-TTLL3",TRUE,"ARPC4"

"11657","TACAACCTGTGACCATCAGC","chr3",9800204,"+","none","AGG","TACAACCTGTGACCATCAGCAGG",0,NA,NA,NA,"GeneID:10093;GeneID:100526693","ARPC4;ARPC4-TTLL3",TRUE,"ARPC4"

"11658","TGTTGTGTCGTTCCACAACC","chr3",9797738,"-","none","TGG","TGTTGTGTCGTTCCACAACCTGG",0,NA,NA,NA,"GeneID:10093;GeneID:100526693","ARPC4;ARPC4-TTLL3",TRUE,"ARPC4"

"11659","ACGACACAACAAGCCGGAAG","chr3",9797747,"+","none","TGG","ACGACACAACAAGCCGGAAGTGG",0,NA,NA,NA,"GeneID:10093;GeneID:100526693","ARPC4;ARPC4-TTLL3",TRUE,"ARPC4-TTLL3"

"11660","CGCAAGAGACACCGGATCAC","chr3",9812978,"-","none","CGG","CGCAAGAGACACCGGATCACCGG",0,NA,NA,NA,"GeneID:100526693;GeneID:26140","ARPC4-TTLL3;TTLL3",TRUE,"ARPC4-TTLL3"

"11661","GTTTCACAGCAATGCTGACC","chr3",9800274,"-","none","CGG","GTTTCACAGCAATGCTGACCCGG",0,NA,NA,NA,"GeneID:10093;GeneID:100526693","ARPC4;ARPC4-TTLL3",TRUE,"ARPC4-TTLL3"

"11662","TCATGAAGCGCATGAACTTG","chr3",9801689,"-","none","TGG","TCATGAAGCGCATGAACTTGTGG",0,NA,NA,NA,"GeneID:10093;GeneID:100526693","ARPC4;ARPC4-TTLL3",TRUE,"ARPC4-TTLL3"

"11663","TGATCCGGTGTCTCTTGCGC","chr3",9812979,"+","none","CGG","TGATCCGGTGTCTCTTGCGCCGG",0,NA,NA,NA,"GeneID:100526693;GeneID:26140","ARPC4-TTLL3;TTLL3",TRUE,"ARPC4-TTLL3"

"11664","TGTTGTGTCGTTCCACAACC","chr3",9797738,"-","none","TGG","TGTTGTGTCGTTCCACAACCTGG",0,NA,NA,NA,"GeneID:10093;GeneID:100526693","ARPC4;ARPC4-TTLL3",TRUE,"ARPC4-TTLL3"

"11665","ATAGCACTGCTATTGTCAGA","chr1",183630482,"+","none","CGG","ATAGCACTGCTATTGTCAGACGG",0,NA,NA,NA,"GeneID:10092","ARPC5",TRUE,"ARPC5"

"11666","ATAGCACTGCTATTGTCAGA","chr3",100256584,"+","10:C>T,17:T>A","AGG","ATAGCACTGCCATTGTCTGAAGG",2,11,18,NA,"","",FALSE,"ARPC5"

"11667","CCCTATCAACACCAAGAGTC","chr1",183633092,"-","none","AGG","CCCTATCAACACCAAGAGTCAGG",0,NA,NA,NA,"GeneID:10092","ARPC5",TRUE,"ARPC5"

"11668","CCCTATCAACACCAAGAGTC","chr5",137658099,"-","3:T>A,8:G>T","AGG","CCCAATCACCACCAAGAGTCAGG",2,4,9,NA,"GeneID:26249","KLHL3",FALSE,"ARPC5"

"11669","CCCTATCAACACCAAGAGTC","chr2",134880803,"-","5:T>A,10:A>T","AGG","CCCTAACAACTCCAAGAGTCAGG",2,6,11,NA,"GeneID:130013","ACMSD",FALSE,"ARPC5"

"11670","GCAAGGATATTGCCGCAGGC","chr1",183635506,"+","none","AGG","GCAAGGATATTGCCGCAGGCAGG",0,NA,NA,NA,"GeneID:10092","ARPC5",TRUE,"ARPC5"

"11671","GTCGAAGAACACAGTGTCGT","chr1",183635637,"-","none","CGG","GTCGAAGAACACAGTGTCGTCGG",0,NA,NA,NA,"GeneID:10092","ARPC5",TRUE,"ARPC5"

"11672","TCAAGACAATGCTGCCTGCC","chr1",183630612,"+","none","CGG","TCAAGACAATGCTGCCTGCCCGG",0,NA,NA,NA,"GeneID:10092","ARPC5",TRUE,"ARPC5"

"11673","TCAAGACAATGCTGCCTGCC","chr18",73041183,"-","9:T>A,10:T>C","AGG","TCAAGACAAAACTGCCTGCCAGG",2,10,11,NA,"","",FALSE,"ARPC5"

"11674","TGCAGTCAAGACACGAACAA","chr1",183627546,"+","none","TGG","TGCAGTCAAGACACGAACAATGG",0,NA,NA,NA,"GeneID:10092","ARPC5",TRUE,"ARPC5"

"11675","AGCGCGAGGACAGCGTGTTC","chr9",124869298,"-","none","CGG","AGCGCGAGGACAGCGTGTTCCGG",0,NA,NA,NA,"GeneID:81873","ARPC5L",TRUE,"ARPC5L"

"11676","CAAATTCGTCGATGTCCACC","chr9",124869325,"-","none","CGG","CAAATTCGTCGATGTCCACCCGG",0,NA,NA,NA,"GeneID:81873","ARPC5L",TRUE,"ARPC5L"

"11677","CCCCCACAGGCCTTAGCAGT","chr9",124876868,"+","none","AGG","CCCCCACAGGCCTTAGCAGTAGG",0,NA,NA,NA,"GeneID:81873","ARPC5L",TRUE,"ARPC5L"

"11678","GCAGCGCAGTGTTACTCCAG","chr9",124875119,"+","none","TGG","GCAGCGCAGTGTTACTCCAGTGG",0,NA,NA,NA,"GeneID:81873","ARPC5L",TRUE,"ARPC5L"

"11679","GCCTAGTCCTCCTACTGCTA","chr9",124876881,"-","none","AGG","GCCTAGTCCTCCTACTGCTAAGG",0,NA,NA,NA,"GeneID:81873","ARPC5L",TRUE,"ARPC5L"

"11680","GCCTAGTCCTCCTACTGCTA","chr10",55718716,"-","15:T>A,16:G>C","AGG","GCCTAGTCCTCCTACACCTAAGG",2,16,17,NA,"","",FALSE,"ARPC5L"

"11681","GCCTAGTCCTCCTACTGCTA","chr7",50706409,"-","10:A>G,16:A>C","GGG","GCCTAGTCCTTCTACTTCTAGGG",2,11,17,NA,"GeneID:2887","GRB10",FALSE,"ARPC5L"

"11682","GTTGACGGGAGAGTTCCGCA","chr9",124873723,"-","none","AGG","GTTGACGGGAGAGTTCCGCAAGG",0,NA,NA,NA,"GeneID:81873","ARPC5L",TRUE,"ARPC5L"

"11683","AAAAGCAAGATATCCTCATC","chr2",100509371,"+","none","TGG","AAAAGCAAGATATCCTCATCTGG",0,NA,NA,NA,"","",TRUE,"ARPP19"

"11684","AAAAGCAAGATATCCTCATC","chr15",52557152,"-","none","TGG","AAAAGCAAGATATCCTCATCTGG",0,NA,NA,NA,"GeneID:10776","ARPP19",TRUE,"ARPP19"

"11685","AAAAGCAAGATATCCTCATC","chr12",2914591,"-","13:T>G,18:T>A","AGG","AAAAGCAAGATATACTCAACAGG",2,14,19,NA,"GeneID:7289","TULP3",FALSE,"ARPP19"

"11686","AAAAGCAAGATATCCTCATC","chr5",180982367,"+","9:G>A","TGG","AAAAGCAAGGTATCCTCATCTGG",1,10,NA,NA,"","",TRUE,"ARPP19"

"11687","GCTCACCTTCTGCTCCTCCG","chr15",52568841,"+","none","CGG","GCTCACCTTCTGCTCCTCCGCGG",0,NA,NA,NA,"GeneID:10776","ARPP19",TRUE,"ARPP19"

"11688","GCTCACCTTCTGCTCCTCCG","chr16",31428246,"+","0:A>G,8:C>T","TGG","ACTCACCTCCTGCTCCTCCGTGG",2,1,9,NA,"GeneID:1339","COX6A2",TRUE,"ARPP19"

"11689","GCTCACCTTCTGCTCCTCCG","chr14",92915909,"+","7:C>T,18:A>C","TGG","GCTCACCCTCTGCTCCTCAGTGG",2,8,19,NA,"","",FALSE,"ARPP19"

"11690","GTCCCTTGTTGCTAGCAAGC","chr15",52551944,"-","none","TGG","GTCCCTTGTTGCTAGCAAGCTGG",0,NA,NA,NA,"GeneID:10776","ARPP19",TRUE,"ARPP19"

"11691","GTCCCTTGTTGCTAGCAAGC","chr2",100509584,"+","0:A>G,4:A>C","TGG","ATCCATTGTTGCTAGCAAGCTGG",2,1,5,NA,"","",TRUE,"ARPP19"

"11692","GTCCCTTGTTGCTAGCAAGC","chr5",180982580,"+","0:A>G,14:A>G","TGG","ATCCCTTGTTGCTAACAAGCTGG",2,1,15,NA,"","",TRUE,"ARPP19"

"11693","TATGTCTGCGGAAGTCCCCG","chr15",52568873,"-","none","AGG","TATGTCTGCGGAAGTCCCCGAGG",0,NA,NA,NA,"GeneID:10776","ARPP19",TRUE,"ARPP19"

"11694","TATGTCTGCGGAAGTCCCCG","chr2",100509275,"+","8:T>C,16:T>C","AGG","TATGTCTGTGGAAGTCTCCGAGG",2,9,17,NA,"","",TRUE,"ARPP19"

"11695","TTCCGTTGAGGAAGGTCTTG","chr15",52551967,"+","none","CGG","TTCCGTTGAGGAAGGTCTTGCGG",0,NA,NA,NA,"GeneID:10776","ARPP19",TRUE,"ARPP19"

"11696","TTCCGTTGAGGAAGGTCTTG","chr5",180982557,"-","17:G>A","TGG","TTCCGTTGAGGAAGGTCCTGTGG",1,18,NA,NA,"","",TRUE,"ARPP19"

"11697","TTTGCTTCTTCTGCTTTCTC","chr15",52557175,"+","none","TGG","TTTGCTTCTTCTGCTTTCTCTGG",0,NA,NA,NA,"GeneID:10776","ARPP19",TRUE,"ARPP19"

"11698","TTTGCTTCTTCTGCTTTCTC","chrX",125629860,"-","12:A>C","AGG","TTTGCTTCTTCTTCTTTCTCAGG",1,13,NA,NA,"","",FALSE,"ARPP19"

"11699","TTTGCTTCTTCTGCTTTCTC","chr5",180982344,"-","11:T>A","TGG","TTTGCTTCTTCAGCTTTCTCTGG",1,12,NA,NA,"","",TRUE,"ARPP19"

"11700","TTTGCTTCTTCTGCTTTCTC","chr4",177257073,"+","10:T>C,18:A>T","GGG","TTTGCTTCTTTTGCTTTCACGGG",2,11,19,NA,"","",FALSE,"ARPP19"

"11701","TTTGCTTCTTCTGCTTTCTC","chrX",130000947,"+","10:G>C,13:A>C","AGG","TTTGCTTCTTGTGATTTCTCAGG",2,11,14,NA,"GeneID:63035","BCORL1",FALSE,"ARPP19"

"11702","TTTGCTTCTTCTGCTTTCTC","chr9",132859742,"-","3:G>C,8:T>A","TGG","TTTCCTTCATCTGCTTTCTCTGG",2,4,9,NA,"GeneID:158067","AK8",FALSE,"ARPP19"

"11703","TTTGCTTCTTCTGCTTTCTC","chr6",111755983,"-","3:A>C,19:A>G","TGG","TTTTCTTCTTCTGCTTTCTTTGG",2,4,20,NA,"GeneID:2534","FYN",FALSE,"ARPP19"

"11704","TTTGCTTCTTCTGCTTTCTC","chr7",32425659,"-","3:A>C,18:T>A","CGG","TTTTCTTCTTCTGCTTTCACCGG",2,4,19,NA,"","",FALSE,"ARPP19"

"11705","AGATTGCAGCCAAGAATACA","chr3",35689304,"+","none","CGG","AGATTGCAGCCAAGAATACACGG",0,NA,NA,NA,"GeneID:10777","ARPP21",TRUE,"ARPP21"

"11706","AGATTGCAGCCAAGAATACA","chr6",165695421,"+","3:C>T,16:C>T","GGG","AGACTGCAGCCAAGAACACAGGG",2,4,17,NA,"GeneID:10846","PDE10A",FALSE,"ARPP21"

"11707","AGATTGCAGCCAAGAATACA","chr6",38036029,"+","3:G>T,17:T>A","GGG","AGAGTGCAGCCAAGAATTCAGGG",2,4,18,NA,"GeneID:60685","ZFAND3",FALSE,"ARPP21"

"11708","AGATTGCAGCCAAGAATACA","chr6",120833607,"-","2:C>T,16:G>A","GGG","AGGTTGCAGCCAAGAACACAGGG",2,3,17,NA,"","",FALSE,"ARPP21"

"11709","CAAAATAAGCTGCCACTCGA","chr3",35690918,"-","none","TGG","CAAAATAAGCTGCCACTCGATGG",0,NA,NA,NA,"GeneID:10777","ARPP21",TRUE,"ARPP21"

"11710","CAACATACAGAGGCGGCTGG","chr3",35682837,"+","none","AGG","CAACATACAGAGGCGGCTGGAGG",0,NA,NA,NA,"GeneID:10777","ARPP21",TRUE,"ARPP21"

"11711","CATCGAGTGGCAGCTTATTT","chr3",35690916,"+","none","TGG","CATCGAGTGGCAGCTTATTTTGG",0,NA,NA,NA,"GeneID:10777","ARPP21",TRUE,"ARPP21"

"11712","CTGTAGAATCCGTGTATTCT","chr3",35689316,"-","none","TGG","CTGTAGAATCCGTGTATTCTTGG",0,NA,NA,NA,"GeneID:10777","ARPP21",TRUE,"ARPP21"

"11713","CTGTAGAATCCGTGTATTCT","chr14",93590308,"-","11:G>C,15:A>T","TGG","CTGTAGAATCCCTGTTTTCTTGG",2,12,16,NA,"GeneID:57578","UNC79",FALSE,"ARPP21"

"11714","TCAAGAAAGAAGAAAATCCA","chr3",35682867,"+","none","AGG","TCAAGAAAGAAGAAAATCCAAGG",0,NA,NA,NA,"GeneID:10777","ARPP21",TRUE,"ARPP21"

"11715","TCAAGAAAGAAGAAAATCCA","chrX",141273302,"+","12:G>A,17:A>C","TGG","TCAAGAAAGAAGGAAATACATGG",2,13,18,NA,"","",FALSE,"ARPP21"

"11716","TCAAGAAAGAAGAAAATCCA","chr12",132184672,"+","1:G>C,5:C>A","AGG","TGAAGCAAGAAGAAAATCCAAGG",2,2,6,NA,"","",FALSE,"ARPP21"

"11717","TCAAGAAAGAAGAAAATCCA","chr16",59808390,"+","0:G>T,8:C>G","AGG","GCAAGAAACAAGAAAATCCAAGG",2,1,9,NA,"","",FALSE,"ARPP21"

"11718","TCAAGAAAGAAGAAAATCCA","chr5",131524178,"+","0:A>T,16:A>T","AGG","ACAAGAAAGAAGAAAAACCAAGG",2,1,17,NA,"GeneID:51735","RAPGEF6",FALSE,"ARPP21"

"11719","TCAAGAAAGAAGAAAATCCA","chr3",169306696,"-","7:C>T,10:A>T","GGG","TCAAGAAGGATGAAAATCCAGGG",2,8,11,NA,"GeneID:2122","MECOM",FALSE,"ARPP21"

"11720","TCAAGAAAGAAGAAAATCCA","chr18",54161641,"-","6:C>T,18:T>G","TGG","TCAAGAGAGAAGAAAATCAATGG",2,7,19,NA,"GeneID:8932","MBD2",FALSE,"ARPP21"

"11721","ACGGGACTTCGTGGACCATG","chrX",70269862,"+","none","TGG","ACGGGACTTCGTGGACCATGTGG",0,NA,NA,NA,"GeneID:407","ARR3",TRUE,"ARR3"

"11722","CCACGAAGTCCCGTTTCCCC","chrX",70269856,"-","none","AGG","CCACGAAGTCCCGTTTCCCCAGG",0,NA,NA,NA,"GeneID:407","ARR3",TRUE,"ARR3"

"11723","CTCCACAGACGGTGTTGTCC","chrX",70270091,"+","none","TGG","CTCCACAGACGGTGTTGTCCTGG",0,NA,NA,NA,"GeneID:407","ARR3",TRUE,"ARR3"

"11724","CTCCACAGACGGTGTTGTCC","chr5",174291917,"+","2:G>C,6:T>A","AGG","CTGCACTGACGGTGTTGTCCAGG",2,3,7,NA,"","",FALSE,"ARR3"

"11725","GAGCGACTACTGCACAAGCT","chrX",70276230,"+","none","AGG","GAGCGACTACTGCACAAGCTAGG",0,NA,NA,NA,"GeneID:407","ARR3",TRUE,"ARR3"

"11726","TCTCACCTTCCCATTGGAGC","chrX",70269678,"-","none","TGG","TCTCACCTTCCCATTGGAGCTGG",0,NA,NA,NA,"GeneID:407","ARR3",TRUE,"ARR3"

"11727","TGTGCAGTAGTCGCTCCTGT","chrX",70276226,"-","none","AGG","TGTGCAGTAGTCGCTCCTGTAGG",0,NA,NA,NA,"GeneID:407","ARR3",TRUE,"ARR3"

"11728","ACTCACCCACAGGGTCCACG","chr11",75287308,"+","none","AGG","ACTCACCCACAGGGTCCACGAGG",0,NA,NA,NA,"GeneID:408","ARRB1",TRUE,"ARRB1"

"11729","CAACGTACAGTCGTTCCCAC","chr11",75283375,"-","none","CGG","CAACGTACAGTCGTTCCCACCGG",0,NA,NA,NA,"GeneID:408","ARRB1",TRUE,"ARRB1"

"11730","CAACGTACAGTCGTTCCCAC","chr7",30020446,"+","11:A>C,14:C>T","TGG","CAACGTACAGTAGTCCCCACTGG",2,12,15,NA,"GeneID:55033","FKBP14",FALSE,"ARRB1"

"11731","CCGGGTCCCTTTGTCGCCCA","chr11",75351586,"+","none","TGG","CCGGGTCCCTTTGTCGCCCATGG",0,NA,NA,NA,"GeneID:408","ARRB1",TRUE,"ARRB1"

"11732","TCGCGGACCATGGGCGACAA","chr11",75351596,"-","none","AGG","TCGCGGACCATGGGCGACAAAGG",0,NA,NA,NA,"GeneID:408","ARRB1",TRUE,"ARRB1"

"11733","TGTGGACCACATCGACCTCG","chr11",75287326,"-","none","TGG","TGTGGACCACATCGACCTCGTGG",0,NA,NA,NA,"GeneID:408","ARRB1",TRUE,"ARRB1"

"11734","TTCTTGTCCTCGGGGGCCGG","chr11",75283356,"+","none","TGG","TTCTTGTCCTCGGGGGCCGGTGG",0,NA,NA,NA,"GeneID:408","ARRB1",TRUE,"ARRB1"

"11735","CAGTACCTTTGCGGTCCTTC","chr17",4716177,"-","none","AGG","CAGTACCTTTGCGGTCCTTCAGG",0,NA,NA,NA,"GeneID:409","ARRB2",TRUE,"ARRB2"

"11736","CCTGGTAGGTGGCGATGAAC","chr17",4716490,"-","none","AGG","CCTGGTAGGTGGCGATGAACAGG",0,NA,NA,NA,"GeneID:409","ARRB2",TRUE,"ARRB2"

"11737","CCTGTTCATCGCCACCTACC","chr17",4716487,"+","none","AGG","CCTGTTCATCGCCACCTACCAGG",0,NA,NA,NA,"GeneID:409","ARRB2",TRUE,"ARRB2"

"11738","CCTGTTCATCGCCACCTACC","chr13",76219378,"+","13:T>A,16:G>T","TGG","CCTGTTCATCGCCTCCGACCTGG",2,14,17,NA,"","",FALSE,"ARRB2"

"11739","GAAGTCGAGCCCTAACTGCA","chr17",4715021,"+","none","AGG","GAAGTCGAGCCCTAACTGCAAGG",0,NA,NA,NA,"GeneID:409","ARRB2",TRUE,"ARRB2"

"11740","GACTACCTGAAGGACCGCAA","chr17",4716169,"+","none","AGG","GACTACCTGAAGGACCGCAAAGG",0,NA,NA,NA,"GeneID:409","ARRB2",TRUE,"ARRB2"

"11741","GCGGGACTTCGTAGATCACC","chr17",4715992,"+","none","TGG","GCGGGACTTCGTAGATCACCTGG",0,NA,NA,NA,"GeneID:409","ARRB2",TRUE,"ARRB2"

"11742","GACCCCGCAGGAACCTATGC","chr9",137612910,"-","none","AGG","GACCCCGCAGGAACCTATGCAGG",0,NA,NA,NA,"GeneID:92714","ARRDC1",TRUE,"ARRDC1"

"11743","GCCATCCGGGTGACCTGCAT","chr9",137612894,"+","none","AGG","GCCATCCGGGTGACCTGCATAGG",0,NA,NA,NA,"GeneID:92714","ARRDC1",TRUE,"ARRDC1"

"11744","GGCTCAAGATATAGAACACG","chr9",137613717,"-","none","AGG","GGCTCAAGATATAGAACACGAGG",0,NA,NA,NA,"GeneID:92714","ARRDC1",TRUE,"ARRDC1"

"11745","GTGGTGCGGAAGGCACCACC","chr9",137614680,"-","none","AGG","GTGGTGCGGAAGGCACCACCAGG",0,NA,NA,NA,"GeneID:92714","ARRDC1",TRUE,"ARRDC1"

"11746","TAGCCCCAAGAACTGTACTC","chr9",137614972,"-","none","TGG","TAGCCCCAAGAACTGTACTCTGG",0,NA,NA,NA,"GeneID:92714","ARRDC1",TRUE,"ARRDC1"

"11747","TTGTGATCCTTGGAAAACCG","chr9",137613688,"-","none","TGG","TTGTGATCCTTGGAAAACCGTGG",0,NA,NA,NA,"GeneID:92714","ARRDC1",TRUE,"ARRDC1"

"11748","AACAGGAACTCATGGCGCCC","chr19",18008739,"-","none","AGG","AACAGGAACTCATGGCGCCCAGG",0,NA,NA,NA,"GeneID:27106","ARRDC2",TRUE,"ARRDC2"

"11749","ACTACCGTGTTTGCCCTCGA","chr19",18008987,"-","none","AGG","ACTACCGTGTTTGCCCTCGAAGG",0,NA,NA,NA,"GeneID:27106","ARRDC2",TRUE,"ARRDC2"

"11750","AGAGGACTTACGGGGGCAGC","chr19",18008768,"-","none","TGG","AGAGGACTTACGGGGGCAGCTGG",0,NA,NA,NA,"GeneID:27106","ARRDC2",TRUE,"ARRDC2"

"11751","CGCCGTATCCAGCGACTACG","chr19",18001508,"+","none","CGG","CGCCGTATCCAGCGACTACGCGG",0,NA,NA,NA,"GeneID:27106","ARRDC2",TRUE,"ARRDC2"

"11752","CTCCGCGGCCGCGTAGTCGC","chr19",18001519,"-","none","TGG","CTCCGCGGCCGCGTAGTCGCTGG",0,NA,NA,NA,"GeneID:27106","ARRDC2",TRUE,"ARRDC2"

"11753","TAGTGTCCGCTACTGTATCA","chr19",18009003,"+","none","AGG","TAGTGTCCGCTACTGTATCAAGG",0,NA,NA,NA,"GeneID:27106","ARRDC2",TRUE,"ARRDC2"

"11754","ACACTGACTGCTTACACTTG","chr5",91374162,"+","none","AGG","ACACTGACTGCTTACACTTGAGG",0,NA,NA,NA,"GeneID:57561","ARRDC3",TRUE,"ARRDC3"

"11755","ACCATTCCTCTACATCCATT","chr5",91374196,"-","none","TGG","ACCATTCCTCTACATCCATTTGG",0,NA,NA,NA,"GeneID:57561","ARRDC3",TRUE,"ARRDC3"

"11756","CCACTCGCTACCTCATTCGA","chr5",91376747,"-","none","AGG","CCACTCGCTACCTCATTCGAAGG",0,NA,NA,NA,"GeneID:57561","ARRDC3",TRUE,"ARRDC3"

"11757","CCTTAAGTGCCAAAATTGAA","chr5",91375529,"-","none","AGG","CCTTAAGTGCCAAAATTGAAAGG",0,NA,NA,NA,"GeneID:57561","ARRDC3",TRUE,"ARRDC3"

"11758","CCTTCGAATGAGGTAGCGAG","chr5",91376744,"+","none","TGG","CCTTCGAATGAGGTAGCGAGTGG",0,NA,NA,NA,"GeneID:57561","ARRDC3",TRUE,"ARRDC3"

"11759","GCCTTCCTGAATGAATAGTG","chr5",91378727,"+","none","TGG","GCCTTCCTGAATGAATAGTGTGG",0,NA,NA,NA,"GeneID:57561","ARRDC3",TRUE,"ARRDC3"

"11760","ATTGGCGACCATGTGTCGAA","chr15",97969236,"-","none","TGG","ATTGGCGACCATGTGTCGAATGG",0,NA,NA,NA,"GeneID:91947","ARRDC4",TRUE,"ARRDC4"

"11761","CATCGACATGACTAACAACC","chr15",97966002,"-","none","TGG","CATCGACATGACTAACAACCTGG",0,NA,NA,NA,"GeneID:91947","ARRDC4",TRUE,"ARRDC4"

"11762","CCTTTGGTCACCTCGTTTAC","chr15",97965895,"+","none","TGG","CCTTTGGTCACCTCGTTTACTGG",0,NA,NA,NA,"GeneID:91947","ARRDC4",TRUE,"ARRDC4"

"11763","CGAGGAAACCACATCGCTTC","chr15",97969259,"+","none","TGG","CGAGGAAACCACATCGCTTCTGG",0,NA,NA,NA,"GeneID:91947","ARRDC4",TRUE,"ARRDC4"

"11764","CGAGGAAACCACATCGCTTC","chr8",126332205,"+","15:C>G,18:C>T","TGG","CGAGGAAACCACATCCCTCCTGG",2,16,19,NA,"","",FALSE,"ARRDC4"

"11765","GAGTGCCAAAATTGAAAGAA","chr15",97968081,"+","none","AGG","GAGTGCCAAAATTGAAAGAAAGG",0,NA,NA,NA,"GeneID:91947","ARRDC4",TRUE,"ARRDC4"

"11766","GAGTGCCAAAATTGAAAGAA","chr5",91375525,"-","0:T>C,18:C>T","AGG","AAGTGCCAAAATTGAAAGGAAGG",2,1,19,NA,"GeneID:57561","ARRDC3",TRUE,"ARRDC4"

"11767","GAGTGCCAAAATTGAAAGAA","chr20",52884747,"-","0:G>C,19:G>T","AGG","CAGTGCCAAAATTGAAAGACAGG",2,1,20,NA,"","",FALSE,"ARRDC4"

"11768","GATGGAAGTTGAAAGCGAAA","chr15",97965643,"-","none","TGG","GATGGAAGTTGAAAGCGAAATGG",0,NA,NA,NA,"GeneID:91947","ARRDC4",TRUE,"ARRDC4"

"11769","GATGGAAGTTGAAAGCGAAA","chr8",12079271,"+","8:A>T,16:C>G","GGG","GATGGAAGATGAAAGCCAAAGGG",2,9,17,NA,"","",FALSE,"ARRDC4"

"11770","GATGGAAGTTGAAAGCGAAA","chr8",12325517,"+","8:A>T,16:C>G","GGG","GATGGAAGATGAAAGCCAAAGGG",2,9,17,NA,"","",FALSE,"ARRDC4"

"11771","GATGGAAGTTGAAAGCGAAA","chr11",71849147,"-","8:T>A,16:G>C","GGG","GATGGAAGATGAAAGCCAAAGGG",2,9,17,NA,"","",FALSE,"ARRDC4"

"11772","GATGGAAGTTGAAAGCGAAA","chr4",9415173,"-","8:T>A,16:G>C","GGG","GATGGAAGATGAAAGCCAAAGGG",2,9,17,NA,"","",FALSE,"ARRDC4"

"11773","CACGAGCTCCACCTTCACTA","chr19",4902690,"+","none","TGG","CACGAGCTCCACCTTCACTATGG",0,NA,NA,NA,"GeneID:645432","ARRDC5",TRUE,"ARRDC5"

"11774","CATGCACACTCGCTACGAGC","chr19",4891191,"-","none","TGG","CATGCACACTCGCTACGAGCTGG",0,NA,NA,NA,"GeneID:645432","ARRDC5",TRUE,"ARRDC5"

"11775","GATCGAATTAGTGCTGCCCG","chr19",4902788,"-","none","AGG","GATCGAATTAGTGCTGCCCGAGG",0,NA,NA,NA,"GeneID:645432","ARRDC5",TRUE,"ARRDC5"

"11776","GTATGCCCACATACAGTACG","chr19",4891377,"-","none","AGG","GTATGCCCACATACAGTACGAGG",0,NA,NA,NA,"GeneID:645432","ARRDC5",TRUE,"ARRDC5"

"11777","TCCATTTCAACTTACCTCCC","chr19",4896820,"-","none","AGG","TCCATTTCAACTTACCTCCCAGG",0,NA,NA,NA,"GeneID:645432","ARRDC5",TRUE,"ARRDC5"

"11778","TCCATTTCAACTTACCTCCC","chr19",48857498,"-","11:C>A,13:A>T","AGG","TCCATTTCAACGTTCCTCCCAGG",2,12,14,NA,"GeneID:57664","PLEKHA4",TRUE,"ARRDC5"

"11779","TTTCGTACAAGCTTCCTGCA","chr19",4896756,"-","none","TGG","TTTCGTACAAGCTTCCTGCATGG",0,NA,NA,NA,"GeneID:645432","ARRDC5",TRUE,"ARRDC5"

"11780","CATCCCGTACTCCCACGACC","chr22",50627167,"-","none","AGG","CATCCCGTACTCCCACGACCAGG",0,NA,NA,NA,"GeneID:410","ARSA",TRUE,"ARSA"

"11781","CATCCCGTACTCCCACGACC","chrX",119513877,"-","5:A>G,9:T>G","TGG","CATCCTGTAATCCCACGACCTGG",2,6,10,NA,"","",FALSE,"ARSA"

"11782","CGGACTAGAGGCCCGCTACA","chr22",50626907,"-","none","TGG","CGGACTAGAGGCCCGCTACATGG",0,NA,NA,NA,"GeneID:410","ARSA",TRUE,"ARSA"

"11783","GAGACCATGCGTATGTCCCG","chr22",50626254,"-","none","AGG","GAGACCATGCGTATGTCCCGAGG",0,NA,NA,NA,"GeneID:410","ARSA",TRUE,"ARSA"

"11784","GTTCGGATGGGCATGTACCC","chr22",50627358,"-","none","TGG","GTTCGGATGGGCATGTACCCTGG",0,NA,NA,NA,"GeneID:410","ARSA",TRUE,"ARSA"

"11785","TGCTCCGGTCTCTTGCGGTG","chr22",50626227,"-","none","TGG","TGCTCCGGTCTCTTGCGGTGTGG",0,NA,NA,NA,"GeneID:410","ARSA",TRUE,"ARSA"

"11786","TTACGTGAGAGGCATAGTAC","chr22",50626829,"+","none","AGG","TTACGTGAGAGGCATAGTACAGG",0,NA,NA,NA,"GeneID:410","ARSA",TRUE,"ARSA"

"11787","CAATTAATGTACAGCGTTCA","chr5",78964552,"+","none","TGG","CAATTAATGTACAGCGTTCATGG",0,NA,NA,NA,"GeneID:411","ARSB",TRUE,"ARSB"

"11788","CTACCCATATGGTCGGAAAA","chr5",78969069,"-","none","TGG","CTACCCATATGGTCGGAAAATGG",0,NA,NA,NA,"GeneID:411","ARSB",TRUE,"ARSB"

"11789","CTACCCATATGGTCGGAAAA","chr4",113903545,"-","1:T>A,4:C>G","TGG","CAACGCATATGGTCGGAAAATGG",2,2,5,NA,"GeneID:79642","ARSJ",TRUE,"ARSB"

"11790","GAATGCCTTCCAACCCGCCG","chr5",78969025,"-","none","AGG","GAATGCCTTCCAACCCGCCGAGG",0,NA,NA,NA,"GeneID:411","ARSB",TRUE,"ARSB"

"11791","GTCTGTGCATGAGCCCCTTC","chr5",78955456,"-","none","AGG","GTCTGTGCATGAGCCCCTTCAGG",0,NA,NA,NA,"GeneID:411","ARSB",TRUE,"ARSB"

"11792","TATGAGGGCTATAGCCCTTT","chr5",78964436,"+","none","TGG","TATGAGGGCTATAGCCCTTTTGG",0,NA,NA,NA,"GeneID:411","ARSB",TRUE,"ARSB"

"11793","TGCAGGAATGGTGTCCCTTA","chr5",78955375,"-","none","TGG","TGCAGGAATGGTGTCCCTTATGG",0,NA,NA,NA,"GeneID:411","ARSB",TRUE,"ARSB"

"11794","CATATCCACACCTATGAGGC","chrX",2920589,"+","none","CGG","CATATCCACACCTATGAGGCCGG",0,NA,NA,NA,"GeneID:414","ARSD",TRUE,"ARSD"

"11795","CCTCCGCGCGGCGGATCGCA","chrX",2929254,"+","none","TGG","CCTCCGCGCGGCGGATCGCATGG",0,NA,NA,NA,"GeneID:414","ARSD",TRUE,"ARSD"

"11796","CGGCCATGCGATCCGCCGCG","chrX",2929260,"-","none","CGG","CGGCCATGCGATCCGCCGCGCGG",0,NA,NA,NA,"GeneID:414","ARSD",TRUE,"ARSD"

"11797","CGGGCCCTTCAGTGGAACGC","chrX",2920680,"-","none","AGG","CGGGCCCTTCAGTGGAACGCAGG",0,NA,NA,NA,"GeneID:414","ARSD",TRUE,"ARSD"

"11798","CGGGCCCTTCAGTGGAACGC","chr6",14935232,"-","0:C>G,3:T>C","TGG","GGGACCCTTCAGTGGAACGCTGG",2,1,4,NA,"","",FALSE,"ARSD"

"11799","CGGGCCCTTCAGTGGAACGC","chrY",12367219,"+","0:T>C,17:T>C","AGG","TGGGCCCTTCAGTGGAATGCAGG",2,1,18,NA,"GeneID:352887","GYG2P1",TRUE,"ARSD"

"11800","GTGACACCAACCTGATCTGA","chrX",2921891,"+","none","AGG","GTGACACCAACCTGATCTGAAGG",0,NA,NA,NA,"GeneID:414","ARSD",TRUE,"ARSD"

"11801","TCCCTGTGAGGAATGCAGCT","chrX",2921920,"+","none","CGG","TCCCTGTGAGGAATGCAGCTCGG",0,NA,NA,NA,"GeneID:414","ARSD",TRUE,"ARSD"

"11802","TCCCTGTGAGGAATGCAGCT","chr16",25868775,"-","13:G>A,19:T>A","GGG","TCCCTGTGAGGAACGCAGCAGGG",2,14,20,NA,"GeneID:9951","HS3ST4",FALSE,"ARSD"

"11803","TCCCTGTGAGGAATGCAGCT","chr2",240972407,"-","11:G>T,13:T>A","GGG","TCCCTGTGAGGCAAGCAGCTGGG",2,12,14,NA,"GeneID:728763","LOC728763",FALSE,"ARSD"

"11804","TCCCTGTGAGGAATGCAGCT","chrY",12366023,"-","4:G>A,5:T>C","CGG","TCCCCATGAGGAATGCAGCTCGG",2,5,6,NA,"GeneID:352887","GYG2P1",TRUE,"ARSD"

"11805","AACGACACCCCTTATTCTGC","chrX",2949328,"-","none","AGG","AACGACACCCCTTATTCTGCAGG",0,NA,NA,NA,"GeneID:415","ARSE",TRUE,"ARSE"

"11806","AGAGACTGACCTGATCGCAC","chrX",2955405,"+","none","AGG","AGAGACTGACCTGATCGCACAGG",0,NA,NA,NA,"GeneID:415","ARSE",TRUE,"ARSE"

"11807","CACACCTGATACCCGTCTCG","chrX",2949488,"-","none","TGG","CACACCTGATACCCGTCTCGTGG",0,NA,NA,NA,"GeneID:415","ARSE",TRUE,"ARSE"

"11808","GAATATTGACCGCCTTGCAG","chrX",2955511,"-","none","AGG","GAATATTGACCGCCTTGCAGAGG",0,NA,NA,NA,"GeneID:415","ARSE",TRUE,"ARSE"

"11809","GAATATTGACCGCCTTGCAG","chrX",2921998,"-","11:T>C,12:C>G","AGG","GAATATTGACCAGCTTGCAGAGG",2,12,13,NA,"GeneID:414","ARSD",TRUE,"ARSE"

"11810","GTACATACCAATGAGTCCAG","chrX",2953134,"+","none","TGG","GTACATACCAATGAGTCCAGTGG",0,NA,NA,NA,"GeneID:415","ARSE",TRUE,"ARSE"

"11811","TACCGTGTTCTTCAGTGGAC","chrX",2953225,"-","none","CGG","TACCGTGTTCTTCAGTGGACCGG",0,NA,NA,NA,"GeneID:415","ARSE",TRUE,"ARSE"

"11812","ACCATGATTAGGACAATATT","chrX",3072104,"-","none","AGG","ACCATGATTAGGACAATATTAGG",0,NA,NA,NA,"GeneID:416","ARSF",TRUE,"ARSF"

"11813","ACCATGATTAGGACAATATT","chrY",12257932,"+","8:C>T,13:T>C","AGG","ACCATGATCAGGATAATATTAGG",2,9,14,NA,"","",TRUE,"ARSF"

"11814","AGTTTGCGCACCTGATCGGA","chrX",3076660,"-","none","TGG","AGTTTGCGCACCTGATCGGATGG",0,NA,NA,NA,"GeneID:416","ARSF",TRUE,"ARSF"

"11815","CAAAATCTTGCAGTCCCCGC","chrX",3080922,"+","none","AGG","CAAAATCTTGCAGTCCCCGCAGG",0,NA,NA,NA,"GeneID:416","ARSF",TRUE,"ARSF"

"11816","CATCTAGGTATGGTTTCTAG","chrX",3080883,"+","none","TGG","CATCTAGGTATGGTTTCTAGTGG",0,NA,NA,NA,"GeneID:416","ARSF",TRUE,"ARSF"

"11817","GCCTAATATTGTCCTAATCA","chrX",3072100,"+","none","TGG","GCCTAATATTGTCCTAATCATGG",0,NA,NA,NA,"GeneID:416","ARSF",TRUE,"ARSF"

"11818","GCCTAATATTGTCCTAATCA","chrY",12257936,"-","10:T>C,15:C>T","TGG","GCCTAATATTATCCTGATCATGG",2,11,16,NA,"","",TRUE,"ARSF"

"11819","GCCTAATATTGTCCTAATCA","chr5",56224798,"-","5:A>T,16:A>T","AGG","GCCTATTATTGTCCTATTCAAGG",2,6,17,NA,"GeneID:79722","ANKRD55",FALSE,"ARSF"

"11820","GGAAGATACCCCATCCGATC","chrX",3076647,"+","none","AGG","GGAAGATACCCCATCCGATCAGG",0,NA,NA,NA,"GeneID:416","ARSF",TRUE,"ARSF"

"11821","GGAAGATACCCCATCCGATC","chrY",12253987,"-","11:C>G,15:A>G","AGG","GGAAGATACCCGATCTGATCAGG",2,12,16,NA,"","",TRUE,"ARSF"

"11822","AGAATTCTTACCACGGAAGT","chr17",68347163,"-","none","TGG","AGAATTCTTACCACGGAAGTTGG",0,NA,NA,NA,"GeneID:22901","ARSG",TRUE,"ARSG"

"11823","CAGCAGGCGGGTTACGTCAC","chr17",68343760,"+","none","TGG","CAGCAGGCGGGTTACGTCACTGG",0,NA,NA,NA,"GeneID:22901","ARSG",TRUE,"ARSG"

"11824","CCTCCGAAGCCATCTTATCA","chr17",68307684,"-","none","AGG","CCTCCGAAGCCATCTTATCAAGG",0,NA,NA,NA,"GeneID:22901","ARSG",TRUE,"ARSG"

"11825","TCTTATCACCCCAACTTCCG","chr17",68347150,"+","none","TGG","TCTTATCACCCCAACTTCCGTGG",0,NA,NA,NA,"GeneID:22901","ARSG",TRUE,"ARSG"

"11826","TGCGTGTGACTCCATTGCGA","chr17",68343678,"-","none","AGG","TGCGTGTGACTCCATTGCGAAGG",0,NA,NA,NA,"GeneID:22901","ARSG",TRUE,"ARSG"

"11827","TTGGCGGGAGTGAGTTTCTC","chr17",68307520,"+","none","AGG","TTGGCGGGAGTGAGTTTCTCAGG",0,NA,NA,NA,"GeneID:22901","ARSG",TRUE,"ARSG"

"11828","TTGGCGGGAGTGAGTTTCTC","chr1",167151737,"-","2:A>C,4:T>G","TGG","TTTGAGGGAGTGAGTTTCTCTGG",2,3,5,NA,"GeneID:10223","GPA33",FALSE,"ARSG"

"11829","ACCTGAACCGTGCCTTCACG","chrX",3013064,"+","none","TGG","ACCTGAACCGTGCCTTCACGTGG",0,NA,NA,NA,"GeneID:347527","ARSH",TRUE,"ARSH"

"11830","CACACCTAATATTGACCGCC","chrX",3010029,"+","none","TGG","CACACCTAATATTGACCGCCTGG",0,NA,NA,NA,"GeneID:347527","ARSH",TRUE,"ARSH"

"11831","CCATACCTATGAGTCCCGTG","chrX",3013158,"-","none","CGG","CCATACCTATGAGTCCCGTGCGG",0,NA,NA,NA,"GeneID:347527","ARSH",TRUE,"ARSH"

"11832","GTTATGGATTTACTCGACGT","chrX",3015269,"+","none","TGG","GTTATGGATTTACTCGACGTTGG",0,NA,NA,NA,"GeneID:347527","ARSH",TRUE,"ARSH"

"11833","TCATTCCCAAGTTCGCCCGC","chrX",3015173,"+","none","TGG","TCATTCCCAAGTTCGCCCGCTGG",0,NA,NA,NA,"GeneID:347527","ARSH",TRUE,"ARSH"

"11834","TGAGTGGAGTATGTACGTGC","chrX",3018572,"-","none","AGG","TGAGTGGAGTATGTACGTGCAGG",0,NA,NA,NA,"GeneID:347527","ARSH",TRUE,"ARSH"

"11835","AACCATGGTAGCCCACGTCG","chr5",150302174,"+","none","TGG","AACCATGGTAGCCCACGTCGTGG",0,NA,NA,NA,"GeneID:340075","ARSI",TRUE,"ARSI"

"11836","AGATATCGAGACCCCTACGC","chr5",150302153,"-","none","TGG","AGATATCGAGACCCCTACGCTGG",0,NA,NA,NA,"GeneID:340075","ARSI",TRUE,"ARSI"

"11837","ATTCGGCCAGGCGAGCCAGC","chr5",150297440,"+","none","AGG","ATTCGGCCAGGCGAGCCAGCAGG",0,NA,NA,NA,"GeneID:340075","ARSI",TRUE,"ARSI"

"11838","TCCGTTGCTTTCGCTTGAGC","chr5",150297905,"+","none","AGG","TCCGTTGCTTTCGCTTGAGCAGG",0,NA,NA,NA,"GeneID:340075","ARSI",TRUE,"ARSI"

"11839","TGTGGCCCGGCGGAAGTACG","chr5",150298133,"-","none","CGG","TGTGGCCCGGCGGAAGTACGCGG",0,NA,NA,NA,"GeneID:340075","ARSI",TRUE,"ARSI"

"11840","TTGTGCAGGATCTCCGTGCG","chr5",150297751,"+","none","TGG","TTGTGCAGGATCTCCGTGCGTGG",0,NA,NA,NA,"GeneID:340075","ARSI",TRUE,"ARSI"

"11841","CGAACCGGTGGCACAATGAA","chr4",113902624,"-","none","CGG","CGAACCGGTGGCACAATGAACGG",0,NA,NA,NA,"GeneID:79642","ARSJ",TRUE,"ARSJ"

"11842","CTTGACAAGCTCGCTGCCGA","chr4",113978508,"-","none","AGG","CTTGACAAGCTCGCTGCCGAAGG",0,NA,NA,NA,"GeneID:79642","ARSJ",TRUE,"ARSJ"

"11843","CTTGACAAGCTCGCTGCCGA","chr9",21079372,"-","12:G>C,17:A>G","TGG","CTTGACAAGCTCCCTGCTGATGG",2,13,18,NA,"","",FALSE,"ARSJ"

"11844","GGGACCCAGTCGCTGTAGCC","chr4",113902670,"+","none","AGG","GGGACCCAGTCGCTGTAGCCAGG",0,NA,NA,NA,"GeneID:79642","ARSJ",TRUE,"ARSJ"

"11845","GGTTGGATATTCAACGCATA","chr4",113903556,"-","none","TGG","GGTTGGATATTCAACGCATATGG",0,NA,NA,NA,"GeneID:79642","ARSJ",TRUE,"ARSJ"

"11846","TAAATCCCTGATCATCCGCT","chr4",113978569,"+","none","AGG","TAAATCCCTGATCATCCGCTAGG",0,NA,NA,NA,"GeneID:79642","ARSJ",TRUE,"ARSJ"

"11847","TCGTGAAGAAGCTCCTACGG","chr4",113902510,"-","none","AGG","TCGTGAAGAAGCTCCTACGGAGG",0,NA,NA,NA,"GeneID:79642","ARSJ",TRUE,"ARSJ"

"11848","AGCAGCCAAAGCGCCCAATG","chr5",95555358,"+","none","TGG","AGCAGCCAAAGCGCCCAATGTGG",0,NA,NA,NA,"GeneID:153642","ARSK",TRUE,"ARSK"

"11849","ATGTTCATACCTGCGCGTGA","chr5",95566117,"-","none","TGG","ATGTTCATACCTGCGCGTGATGG",0,NA,NA,NA,"GeneID:153642","ARSK",TRUE,"ARSK"

"11850","ATTTGTTGCCCATCACGCGC","chr5",95566105,"+","none","AGG","ATTTGTTGCCCATCACGCGCAGG",0,NA,NA,NA,"GeneID:153642","ARSK",TRUE,"ARSK"

"11851","CTTCAGGACATCACTCCATT","chr5",95568027,"+","none","AGG","CTTCAGGACATCACTCCATTAGG",0,NA,NA,NA,"GeneID:153642","ARSK",TRUE,"ARSK"

"11852","GGTGGTCGCAGCCTTGGCGC","chr5",95555298,"+","none","TGG","GGTGGTCGCAGCCTTGGCGCTGG",0,NA,NA,NA,"GeneID:153642","ARSK",TRUE,"ARSK"

"11853","TCACTCACTTAACAGAATCT","chr5",95567907,"+","none","TGG","TCACTCACTTAACAGAATCTTGG",0,NA,NA,NA,"GeneID:153642","ARSK",TRUE,"ARSK"

"11854","TCACTCACTTAACAGAATCT","chr6",22830023,"-","12:T>G,19:T>A","AGG","TCACTCACTTAAAAGAATCAAGG",2,13,20,NA,"","",FALSE,"ARSK"

"11855","TCACTCACTTAACAGAATCT","chr3",55631070,"-","5:A>G,17:T>A","AGG","TCACTTACTTAACAGAAACTAGG",2,6,18,NA,"GeneID:26059","ERC2",FALSE,"ARSK"

"11856","ATAGACAAGAAGTGCAAGTC","chr11",3661367,"+","none","TGG","ATAGACAAGAAGTGCAAGTCTGG",0,NA,NA,NA,"GeneID:417","ART1",TRUE,"ART1"

"11857","CCTTACCTGAATTATCCAGA","chr11",3661399,"-","none","TGG","CCTTACCTGAATTATCCAGATGG",0,NA,NA,NA,"GeneID:417","ART1",TRUE,"ART1"

"11858","CTGGAAAGGCCCTCACCACG","chr11",3664149,"-","none","AGG","CTGGAAAGGCCCTCACCACGAGG",0,NA,NA,NA,"GeneID:417","ART1",TRUE,"ART1"

"11859","CTGGAAAGGCCCTCACCACG","chr7",157841605,"+","6:G>A,12:C>T","GGG","CTGGAAGGGCCCCCACCACGGGG",2,7,13,NA,"GeneID:5799","PTPRN2",FALSE,"ART1"

"11860","GCTGCTGCTCTGGTTCCTCG","chr11",3664131,"+","none","TGG","GCTGCTGCTCTGGTTCCTCGTGG",0,NA,NA,NA,"GeneID:417","ART1",TRUE,"ART1"

"11861","GCTGCTGCTCTGGTTCCTCG","chr19",18606568,"-","12:G>C,13:C>A","GGG","GCTGCTGCTCTGCGTCCTCGGGG",2,13,14,NA,"GeneID:9244","CRLF1",TRUE,"ART1"

"11862","GCTGCTGCTCTGGTTCCTCG","chr12",6824044,"+","12:C>G,18:A>C","CGG","GCTGCTGCTCTGCTTCCTAGCGG",2,13,19,NA,"GeneID:27239","GPR162",TRUE,"ART1"

"11863","GCTGCTGCTCTGGTTCCTCG","chr6",163782899,"-","8:G>A,18:A>G","TGG","GCTGCTGCCCTGGTTCCTTGTGG",2,9,19,NA,"","",FALSE,"ART1"

"11864","GGGCCACCCCATGCTCATCG","chr11",3659851,"-","none","CGG","GGGCCACCCCATGCTCATCGCGG",0,NA,NA,NA,"GeneID:417","ART1",TRUE,"ART1"

"11865","TCCTTTGATGACCAGTACGC","chr11",3659651,"+","none","TGG","TCCTTTGATGACCAGTACGCTGG",0,NA,NA,NA,"GeneID:417","ART1",TRUE,"ART1"

"11866","AGCTGTCTTACCAGGTATTT","chr4",76103960,"-","none","GGG","AGCTGTCTTACCAGGTATTTGGG",0,NA,NA,NA,"GeneID:419","ART3",TRUE,"ART3"

"11867","AGCTGTCTTACCAGGTATTT","chr5",158333057,"+","6:A>C,11:T>C","TGG","AGCTGTATTACTAGGTATTTTGG",2,7,12,NA,"GeneID:101927697","LOC101927697",FALSE,"ART3"

"11868","ATGCCATCAGGGCTATTCCA","chr4",76082035,"-","none","TGG","ATGCCATCAGGGCTATTCCATGG",0,NA,NA,NA,"GeneID:419","ART3",TRUE,"ART3"

"11869","CTTGAACCCACCCAAATACC","chr4",76103947,"+","none","TGG","CTTGAACCCACCCAAATACCTGG",0,NA,NA,NA,"GeneID:419","ART3",TRUE,"ART3"

"11870","GAAAACTGTATTGAGAACCT","chr4",76097654,"+","none","AGG","GAAAACTGTATTGAGAACCTAGG",0,NA,NA,NA,"GeneID:419","ART3",TRUE,"ART3"

"11871","GAAAACTGTATTGAGAACCT","chr2",27496076,"+","4:T>A,8:A>T","GGG","GAAATCTGAATTGAGAACCTGGG",2,5,9,NA,"","",FALSE,"ART3"

"11872","TAACCATGGAATAGCCCTGA","chr4",76082029,"+","none","TGG","TAACCATGGAATAGCCCTGATGG",0,NA,NA,NA,"GeneID:419","ART3",TRUE,"ART3"

"11873","TACCATGGTCTTCAAGCTTC","chr4",76101002,"-","none","TGG","TACCATGGTCTTCAAGCTTCTGG",0,NA,NA,NA,"GeneID:419","ART3",TRUE,"ART3"

"11874","TACCATGGTCTTCAAGCTTC","chr8",101094723,"-","10:T>A,13:G>T","TGG","TACCATGGTCATCCAGCTTCTGG",2,11,14,NA,"","",FALSE,"ART3"

"11875","TACCATGGTCTTCAAGCTTC","chr4",76100303,"-","3:A>G","TGG","TACTATGGTCTTCAAGCTTCTGG",1,4,NA,NA,"GeneID:419","ART3",TRUE,"ART3"

"11876","TACCATGGTCTTCAAGCTTC","chr20",62765569,"-","0:C>A,19:T>G","GGG","GACCATGGTCTTCAAGCTTAGGG",2,1,20,NA,"","",FALSE,"ART3"

"11877","AGCCAGATTCTCATCGTTGC","chr12",14843036,"+","none","AGG","AGCCAGATTCTCATCGTTGCAGG",0,NA,NA,NA,"GeneID:144608;GeneID:420","C12orf60;ART4",TRUE,"ART4"

"11878","ATCGACTTCGACTTCGCACC","chr12",14841121,"-","none","AGG","ATCGACTTCGACTTCGCACCAGG",0,NA,NA,NA,"GeneID:144608;GeneID:420","C12orf60;ART4",TRUE,"ART4"

"11879","CTCCTGCAACGATGAGAATC","chr12",14843041,"-","none","TGG","CTCCTGCAACGATGAGAATCTGG",0,NA,NA,NA,"GeneID:144608;GeneID:420","C12orf60;ART4",TRUE,"ART4"

"11880","GATGATGACACTGGTCAAAA","chr12",14829391,"+","none","AGG","GATGATGACACTGGTCAAAAAGG",0,NA,NA,NA,"GeneID:144608;GeneID:420","C12orf60;ART4",TRUE,"ART4"

"11881","GATGATGACACTGGTCAAAA","chr22",47246207,"-","2:T>A,15:T>G","TGG","GAAGATGACACTGGTAAAAATGG",2,3,16,NA,"","",FALSE,"ART4"

"11882","GATGCAATAGCTATAGGATC","chr12",14829419,"+","none","AGG","GATGCAATAGCTATAGGATCAGG",0,NA,NA,NA,"GeneID:144608;GeneID:420","C12orf60;ART4",TRUE,"ART4"

"11883","GATGCAATAGCTATAGGATC","chr6",136341269,"-","4:C>G,14:A>T","AGG","GATGGAATAGCTATTGGATCAGG",2,5,15,NA,"","",FALSE,"ART4"

"11884","GTCCACTTTAATGCCTACAC","chr12",14840704,"-","none","AGG","GTCCACTTTAATGCCTACACAGG",0,NA,NA,NA,"GeneID:144608;GeneID:420","C12orf60;ART4",TRUE,"ART4"

"11885","ATGATCGCCCTCGGCAGCCT","chr11",3641824,"-","none","CGG","ATGATCGCCCTCGGCAGCCTCGG",0,NA,NA,NA,"GeneID:116969","ART5",TRUE,"ART5"

"11886","ATGATCGCCCTCGGCAGCCT","chr19",10888004,"+","6:C>G,12:A>G","GGG","ATGATCCCCCTCAGCAGCCTGGG",2,7,13,NA,"GeneID:10498","CARM1",FALSE,"ART5"

"11887","CACCTTTGACGATACCTATG","chr11",3640310,"-","none","TGG","CACCTTTGACGATACCTATGTGG",0,NA,NA,NA,"GeneID:116969","ART5",TRUE,"ART5"

"11888","CTGGAGTTACCTGCCAGGTG","chr11",3641795,"+","none","TGG","CTGGAGTTACCTGCCAGGTGTGG",0,NA,NA,NA,"GeneID:116969","ART5",TRUE,"ART5"

"11889","GGTGCCTCTTCGTCATATGA","chr11",3638751,"+","none","AGG","GGTGCCTCTTCGTCATATGAAGG",0,NA,NA,NA,"GeneID:116969","ART5",TRUE,"ART5"

"11890","GTGACCTTCATATGACGAAG","chr11",3638758,"-","none","AGG","GTGACCTTCATATGACGAAGAGG",0,NA,NA,NA,"GeneID:116969","ART5",TRUE,"ART5"

"11891","GTTGAATCAGGCCGTGCGGA","chr11",3640082,"-","none","CGG","GTTGAATCAGGCCGTGCGGACGG",0,NA,NA,NA,"GeneID:116969","ART5",TRUE,"ART5"

"11892","ATAGAGATGGAACTTGGACT","chr1",43935650,"+","none","TGG","ATAGAGATGGAACTTGGACTTGG",0,NA,NA,NA,"GeneID:9048","ARTN",TRUE,"ARTN"

"11893","ATAGAGATGGAACTTGGACT","chr2",3726177,"+","13:G>T,19:A>T","GGG","ATAGAGATGGAACGTGGACAGGG",2,14,20,NA,"GeneID:728597","DCDC2C",FALSE,"ARTN"

"11894","CCGACCCACGCGCTACGAAG","chr1",43936665,"+","none","CGG","CCGACCCACGCGCTACGAAGCGG",0,NA,NA,NA,"GeneID:9048","ARTN",TRUE,"ARTN"

"11895","CCGCTTCGTAGCGCGTGGGT","chr1",43936668,"-","none","CGG","CCGCTTCGTAGCGCGTGGGTCGG",0,NA,NA,NA,"GeneID:9048","ARTN",TRUE,"ARTN"

"11896","GAACCACTCACCTGCTGCCT","chr1",43935707,"-","none","AGG","GAACCACTCACCTGCTGCCTAGG",0,NA,NA,NA,"GeneID:9048","ARTN",TRUE,"ARTN"

"11897","GAACCACTCACCTGCTGCCT","chr14",75236954,"+","4:A>C,6:A>C","CGG","GAACAAATCACCTGCTGCCTCGG",2,5,7,NA,"","",FALSE,"ARTN"

"11898","TCACCTACCCGGCAGGTGGC","chr1",43936219,"-","none","CGG","TCACCTACCCGGCAGGTGGCCGG",0,NA,NA,NA,"GeneID:9048","ARTN",TRUE,"ARTN"

"11899","TCTGCTGAGCAGCGTCGCAG","chr1",43936124,"+","none","AGG","TCTGCTGAGCAGCGTCGCAGAGG",0,NA,NA,NA,"GeneID:9048","ARTN",TRUE,"ARTN"

"11900","AGAGCTTACGGTTGATGTTT","chr1",230997127,"-","none","AGG","AGAGCTTACGGTTGATGTTTAGG",0,NA,NA,NA,"GeneID:64801","ARV1",TRUE,"ARV1"

"11901","AGAGCTTACGGTTGATGTTT","chr1",21138128,"-","10:A>C,19:C>A","TGG","AGAGCTTACGTTTGATGTTGTGG",2,11,20,NA,"GeneID:8672","EIF4G3",FALSE,"ARV1"

"11902","CGTGTTGAGTGGCTTACTGC","chr1",230997157,"+","none","TGG","CGTGTTGAGTGGCTTACTGCTGG",0,NA,NA,NA,"GeneID:64801","ARV1",TRUE,"ARV1"

"11903","GCATATCTGATCAAGTCATC","chr1",230990199,"-","none","AGG","GCATATCTGATCAAGTCATCAGG",0,NA,NA,NA,"GeneID:64801","ARV1",TRUE,"ARV1"

"11904","GCATATCTGATCAAGTCATC","chrX",118909419,"+","6:A>C,13:T>A","AGG","GCATATATGATCATGTCATCAGG",2,7,14,NA,"GeneID:101928287","LINC01285",FALSE,"ARV1"

"11905","TCATGGGCCGTTCTACCCAC","chr1",230995796,"-","none","AGG","TCATGGGCCGTTCTACCCACAGG",0,NA,NA,NA,"GeneID:64801","ARV1",TRUE,"ARV1"

"11906","TGCTATATTGTGCAAAGCTC","chr1",230988381,"+","none","AGG","TGCTATATTGTGCAAAGCTCAGG",0,NA,NA,NA,"GeneID:64801","ARV1",TRUE,"ARV1"

"11907","TGCTGATTCCAGCTGTCATT","chr1",230995885,"+","none","TGG","TGCTGATTCCAGCTGTCATTTGG",0,NA,NA,NA,"GeneID:64801","ARV1",TRUE,"ARV1"

"11908","AGCAGGAGGCCCGCTTCGAG","chr22",19990722,"-","none","AGG","AGCAGGAGGCCCGCTTCGAGAGG",0,NA,NA,NA,"GeneID:421","ARVCF",TRUE,"ARVCF"

"11909","CACCTGGCGTACTGTCCGAG","chr22",19981689,"+","none","TGG","CACCTGGCGTACTGTCCGAGTGG",0,NA,NA,NA,"GeneID:421","ARVCF",TRUE,"ARVCF"

"11910","CCGGAAGGACACTGACAACA","chr22",19977959,"-","none","AGG","CCGGAAGGACACTGACAACAAGG",0,NA,NA,NA,"GeneID:421","ARVCF",TRUE,"ARVCF"

"11911","CTGAGGCCCGGCGGCGACTC","chr22",19978035,"-","none","CGG","CTGAGGCCCGGCGGCGACTCCGG",0,NA,NA,NA,"GeneID:421","ARVCF",TRUE,"ARVCF"

"11912","GACCACTCGGACAGTACGCC","chr22",19981694,"-","none","AGG","GACCACTCGGACAGTACGCCAGG",0,NA,NA,NA,"GeneID:421","ARVCF",TRUE,"ARVCF"

"11913","GGAGGACTGCAATGTGCACT","chr22",19990772,"-","none","CGG","GGAGGACTGCAATGTGCACTCGG",0,NA,NA,NA,"GeneID:421","ARVCF",TRUE,"ARVCF"

"11914","AGCAAGTCGTACCGCGAGAA","chrX",25013461,"-","none","CGG","AGCAAGTCGTACCGCGAGAACGG",0,NA,NA,NA,"GeneID:170302","ARX",TRUE,"ARX"

"11915","CTTGACCGAGGCCCGAGTCC","chrX",25010261,"-","none","AGG","CTTGACCGAGGCCCGAGTCCAGG",0,NA,NA,NA,"GeneID:170302","ARX",TRUE,"ARX"

"11916","GCGCGGCTGGTCAGCGGAGC","chrX",25015564,"+","none","AGG","GCGCGGCTGGTCAGCGGAGCAGG",0,NA,NA,NA,"GeneID:170302","ARX",TRUE,"ARX"

"11917","GCTCACCTGGACTCGGGCCT","chrX",25010253,"+","none","CGG","GCTCACCTGGACTCGGGCCTCGG",0,NA,NA,NA,"GeneID:170302","ARX",TRUE,"ARX"

"11918","GCTGACCAGCCGCGCCGACC","chrX",25015559,"-","none","CGG","GCTGACCAGCCGCGCCGACCCGG",0,NA,NA,NA,"GeneID:170302","ARX",TRUE,"ARX"

"11919","TGCGCCGTCCGGCCGTTCCC","chrX",25013568,"+","none","CGG","TGCGCCGTCCGGCCGTTCCCCGG",0,NA,NA,NA,"GeneID:170302","ARX",TRUE,"ARX"

"11920","CGCTGGGTCAGACCTACTAC","chr10",102870072,"+","none","GGG","CGCTGGGTCAGACCTACTACGGG",0,NA,NA,NA,"GeneID:57412","AS3MT",TRUE,"AS3MT"

"11921","CTTCAGCACCTGCCCGTAGT","chr10",102870087,"-","none","AGG","CTTCAGCACCTGCCCGTAGTAGG",0,NA,NA,NA,"GeneID:57412","AS3MT",TRUE,"AS3MT"

"11922","TCGTGACGCTGAGATACAGA","chr10",102869814,"+","none","AGG","TCGTGACGCTGAGATACAGAAGG",0,NA,NA,NA,"GeneID:57412","AS3MT",TRUE,"AS3MT"

"11923","TGAAGCACTTGTTGTTTATC","chr10",102874619,"-","none","AGG","TGAAGCACTTGTTGTTTATCAGG",0,NA,NA,NA,"GeneID:57412","AS3MT",TRUE,"AS3MT"

"11924","TGAAGCACTTGTTGTTTATC","chr9",81630554,"-","5:T>G,10:A>C","TGG","TGAAGAACTTTTTGTTTATCTGG",2,6,11,NA,"GeneID:7088","TLE1",FALSE,"AS3MT"

"11925","TGATAAACAACAAGTGCTTC","chr10",102874618,"+","none","AGG","TGATAAACAACAAGTGCTTCAGG",0,NA,NA,NA,"GeneID:57412","AS3MT",TRUE,"AS3MT"

"11926","TTGAACTGCCAGAAGAAATC","chr10",102876993,"+","none","AGG","TTGAACTGCCAGAAGAAATCAGG",0,NA,NA,NA,"GeneID:57412","AS3MT",TRUE,"AS3MT"

"11927","AGAAAGCTCGCGGGTCCCAC","chr8",18084756,"-","none","CGG","AGAAAGCTCGCGGGTCCCACCGG",0,NA,NA,NA,"GeneID:427","ASAH1",TRUE,"ASAH1"

"11928","GGACTAAGGCGACGCAACTC","chr8",18084027,"+","none","CGG","GGACTAAGGCGACGCAACTCCGG",0,NA,NA,NA,"GeneID:427","ASAH1",TRUE,"ASAH1"

"11929","GTGAAAAGCGCGCTGAGACT","chr8",18084721,"+","none","TGG","GTGAAAAGCGCGCTGAGACTTGG",0,NA,NA,NA,"GeneID:427","ASAH1",TRUE,"ASAH1"

"11930","GTTCCTCACTAGAACAGTTC","chr8",18061392,"-","none","TGG","GTTCCTCACTAGAACAGTTCTGG",0,NA,NA,NA,"GeneID:427","ASAH1",TRUE,"ASAH1"

"11931","TTGCGTCGCCTTAGTCCTCC","chr8",18084024,"-","none","TGG","TTGCGTCGCCTTAGTCCTCCTGG",0,NA,NA,NA,"GeneID:427","ASAH1",TRUE,"ASAH1"

"11932","TTTACTCACGTTGGTCCTGA","chr8",18075531,"+","none","AGG","TTTACTCACGTTGGTCCTGAAGG",0,NA,NA,NA,"GeneID:427","ASAH1",TRUE,"ASAH1"

"11933","AACAGACTGCAGAGTAAATA","chr10",50236038,"-","none","TGG","AACAGACTGCAGAGTAAATATGG",0,NA,NA,NA,"GeneID:56624","ASAH2",TRUE,"ASAH2"

"11934","CACGACTGTATAGCCTGGTG","chr10",50243290,"+","none","AGG","CACGACTGTATAGCCTGGTGAGG",0,NA,NA,NA,"GeneID:56624","ASAH2",TRUE,"ASAH2"

"11935","CAGCTCGTCCAACACCAATA","chr10",50245253,"+","none","TGG","CAGCTCGTCCAACACCAATATGG",0,NA,NA,NA,"GeneID:56624","ASAH2",TRUE,"ASAH2"

"11936","CAGCTCGTCCAACACCAATA","chr6",157155757,"-","6:G>C,14:C>G","CGG","CAGCTCCTCCAACAGCAATACGG",2,7,15,NA,"GeneID:57492","ARID1B",FALSE,"ASAH2"

"11937","CAGCTCGTCCAACACCAATA","chr20",36269270,"-","5:C>G,19:C>T","TGG","CAGCTGGTCCAACACCAATGTGG",2,6,20,NA,"GeneID:25980","AAR2",FALSE,"ASAH2"

"11938","GTGTTTGTCAGCATCGACAT","chr10",50243232,"-","none","AGG","GTGTTTGTCAGCATCGACATAGG",0,NA,NA,NA,"GeneID:56624","ASAH2",TRUE,"ASAH2"

"11939","GTTGGACGAGCTGACTGCAC","chr10",50245246,"-","none","AGG","GTTGGACGAGCTGACTGCACAGG",0,NA,NA,NA,"GeneID:56624","ASAH2",TRUE,"ASAH2"

"11940","TTGATTGCTAAATCCTTCAC","chr10",50235920,"+","none","TGG","TTGATTGCTAAATCCTTCACTGG",0,NA,NA,NA,"GeneID:56624","ASAH2",TRUE,"ASAH2"

"11941","AGTCTTACCACTCTGTATTC","chr10",50196764,"+","none","AGG","AGTCTTACCACTCTGTATTCAGG",0,NA,NA,NA,"GeneID:56624","ASAH2",TRUE,"ASAH2B"

"11942","AGTCTTACCACTCTGTATTC","chr10",50745262,"-","none","AGG","AGTCTTACCACTCTGTATTCAGG",0,NA,NA,NA,"GeneID:653308","ASAH2B",TRUE,"ASAH2B"

"11943","ATGTAAGCAGATAATGCGTG","chr10",50742953,"-","none","CGG","ATGTAAGCAGATAATGCGTGCGG",0,NA,NA,NA,"GeneID:653308","ASAH2B",TRUE,"ASAH2B"

"11944","ATGTAAGCAGATAATGCGTG","chr10",50199087,"+","16:T>C","CGG","ATGTAAGCAGATAATGTGTGCGG",1,17,NA,NA,"GeneID:56624","ASAH2",TRUE,"ASAH2B"

"11945","ATTGTGGATAGAGCACCAAA","chr10",50745202,"+","none","AGG","ATTGTGGATAGAGCACCAAAAGG",0,NA,NA,NA,"GeneID:653308","ASAH2B",TRUE,"ASAH2B"

"11946","ATTGTGGATAGAGCACCAAA","chr10",50196824,"-","none","AGG","ATTGTGGATAGAGCACCAAAAGG",0,NA,NA,NA,"GeneID:56624","ASAH2",TRUE,"ASAH2B"

"11947","ATTGTGGATAGAGCACCAAA","chr3",99278958,"+","2:G>T,7:G>A","TGG","ATGGTGGGTAGAGCACCAAATGG",2,3,8,NA,"","",FALSE,"ASAH2B"

"11948","ATTGTGGATAGAGCACCAAA","chr7",21919743,"-","5:T>C,18:G>T","TGG","ATTGTAGATAGAGCACCACATGG",2,6,19,NA,"GeneID:55536","CDCA7L",FALSE,"ASAH2B"

"11949","CCGTAGCAATAGCCTTAGCA","chr10",50199049,"+","none","AGG","CCGTAGCAATAGCCTTAGCAAGG",0,NA,NA,NA,"GeneID:56624","ASAH2",TRUE,"ASAH2B"

"11950","CCGTAGCAATAGCCTTAGCA","chr10",50742991,"-","none","AGG","CCGTAGCAATAGCCTTAGCAAGG",0,NA,NA,NA,"GeneID:653308","ASAH2B",TRUE,"ASAH2B"

"11951","GAAGAATTCAGTACAAAACC","chr10",50749380,"+","none","AGG","GAAGAATTCAGTACAAAACCAGG",0,NA,NA,NA,"GeneID:653308","ASAH2B",TRUE,"ASAH2B"

"11952","GAAGAATTCAGTACAAAACC","chr10",50192654,"-","none","AGG","GAAGAATTCAGTACAAAACCAGG",0,NA,NA,NA,"GeneID:56624","ASAH2",TRUE,"ASAH2B"

"11953","GAAGAATTCAGTACAAAACC","chr13",103956682,"-","10:T>C,12:A>T","AGG","GAAGAATTCAATTCAAAACCAGG",2,11,13,NA,"","",FALSE,"ASAH2B"

"11954","GAAGAATTCAGTACAAAACC","chr12",88854483,"-","4:G>T,13:C>G","CGG","GAAGCATTCAGTAGAAAACCCGG",2,5,14,NA,"","",FALSE,"ASAH2B"

"11955","GTTGCTGAAGTTATATTTGT","chr10",50749348,"+","none","AGG","GTTGCTGAAGTTATATTTGTAGG",0,NA,NA,NA,"GeneID:653308","ASAH2B",TRUE,"ASAH2B"

"11956","GTTGCTGAAGTTATATTTGT","chr10",50192686,"-","none","AGG","GTTGCTGAAGTTATATTTGTAGG",0,NA,NA,NA,"GeneID:56624","ASAH2",TRUE,"ASAH2B"

"11957","GTTGCTGAAGTTATATTTGT","chr15",81098954,"-","9:T>C,13:G>A","GGG","GTTGCTGAAATTACATTTGTGGG",2,10,14,NA,"GeneID:161502","C15orf26",FALSE,"ASAH2B"

"11958","AGAAGCCATTTGACAAAGCC","chr8",130187258,"-","none","TGG","AGAAGCCATTTGACAAAGCCTGG",0,NA,NA,NA,"GeneID:50807","ASAP1",TRUE,"ASAP1"

"11959","AGAAGCCATTTGACAAAGCC","chr9",84584764,"-","5:T>G,18:A>G","TGG","AGAAGACATTTGACAAAGTCTGG",2,6,19,NA,"","",FALSE,"ASAP1"

"11960","AGTCGAGACAACCCCGACCT","chr8",130214619,"-","none","TGG","AGTCGAGACAACCCCGACCTTGG",0,NA,NA,NA,"GeneID:50807","ASAP1",TRUE,"ASAP1"

"11961","CTTTGTCTCATAATCTTTCC","chr8",130187237,"+","none","AGG","CTTTGTCTCATAATCTTTCCAGG",0,NA,NA,NA,"GeneID:50807","ASAP1",TRUE,"ASAP1"

"11962","CTTTGTCTCATAATCTTTCC","chr12",76989278,"-","12:A>T,19:T>G","GGG","CTTTGTCTCATATTCTTTCAGGG",2,13,20,NA,"","",FALSE,"ASAP1"

"11963","CTTTGTCTCATAATCTTTCC","chr9",104076131,"-","10:C>A,18:A>G","TGG","CTTTGTCTCAGAATCTTTTCTGG",2,11,19,NA,"","",FALSE,"ASAP1"

"11964","CTTTGTCTCATAATCTTTCC","chr9",6101977,"+","6:G>C,17:A>T","TGG","CTTTGTGTCATAATCTTACCTGG",2,7,18,NA,"","",FALSE,"ASAP1"

"11965","CTTTGTCTCATAATCTTTCC","chr22",34961248,"-","6:T>G,10:T>A","TGG","CTTTGTATCAAAATCTTTCCTGG",2,7,11,NA,"","",FALSE,"ASAP1"

"11966","GCACAAGTTCTTGATAAGTT","chr8",130214655,"-","none","TGG","GCACAAGTTCTTGATAAGTTTGG",0,NA,NA,NA,"GeneID:50807","ASAP1",TRUE,"ASAP1"

"11967","TCACATTGTGGCTCAAACCC","chr8",130188158,"+","none","TGG","TCACATTGTGGCTCAAACCCTGG",0,NA,NA,NA,"GeneID:50807","ASAP1",TRUE,"ASAP1"

"11968","TTACTCCTTTGACTCCCTTT","chr8",130188104,"+","none","AGG","TTACTCCTTTGACTCCCTTTAGG",0,NA,NA,NA,"GeneID:50807","ASAP1",TRUE,"ASAP1"

"11969","TTACTCCTTTGACTCCCTTT","chr6",89695521,"-","10:A>C,12:A>G","TGG","TTACTCCTTTTATTCCCTTTTGG",2,11,13,NA,"GeneID:23195","MDN1",FALSE,"ASAP1"

"11970","AAATGAAGAGCAGTACACCC","chr2",9297309,"+","none","AGG","AAATGAAGAGCAGTACACCCAGG",0,NA,NA,NA,"GeneID:8853","ASAP2",TRUE,"ASAP2"

"11971","AAATGAAGAGCAGTACACCC","chr10",11285464,"+","10:G>C,13:G>T","AGG","AAATGAAGAGGAGGACACCCAGG",2,11,14,NA,"GeneID:10659","CELF2",FALSE,"ASAP2"

"11972","AGAGCTGTTGATTGCTTTCA","chr2",9279362,"-","none","CGG","AGAGCTGTTGATTGCTTTCACGG",0,NA,NA,NA,"GeneID:8853","ASAP2",TRUE,"ASAP2"

"11973","ATTCGTGGCCGAGACCCATG","chr2",9207130,"+","none","AGG","ATTCGTGGCCGAGACCCATGAGG",0,NA,NA,NA,"GeneID:8853","ASAP2",TRUE,"ASAP2"

"11974","CGCCAAACTTCTCCAGAGCC","chr2",9297330,"-","none","TGG","CGCCAAACTTCTCCAGAGCCTGG",0,NA,NA,NA,"GeneID:8853","ASAP2",TRUE,"ASAP2"

"11975","CTCGGCCACGAATTCCGACA","chr2",9207123,"-","none","CGG","CTCGGCCACGAATTCCGACACGG",0,NA,NA,NA,"GeneID:8853","ASAP2",TRUE,"ASAP2"

"11976","GTGAAAGCAATCAACAGCTC","chr2",9279361,"+","none","TGG","GTGAAAGCAATCAACAGCTCTGG",0,NA,NA,NA,"GeneID:8853","ASAP2",TRUE,"ASAP2"

"11977","ACTTACCAAGGCCGGAGCTA","chr1",23456115,"+","none","TGG","ACTTACCAAGGCCGGAGCTATGG",0,NA,NA,NA,"GeneID:55616","ASAP3",TRUE,"ASAP3"

"11978","CAACATTGTCTCTTTCCCCC","chr1",23452737,"-","none","TGG","CAACATTGTCTCTTTCCCCCTGG",0,NA,NA,NA,"GeneID:55616","ASAP3",TRUE,"ASAP3"

"11979","CAACATTGTCTCTTTCCCCC","chr6",85790252,"-","3:C>G,15:A>G","AGG","CAAGATTGTCTCTTTTCCCCAGG",2,4,16,NA,"","",FALSE,"ASAP3"

"11980","CTTGGCCGTGTTCACCCGCG","chr1",23455906,"-","none","AGG","CTTGGCCGTGTTCACCCGCGAGG",0,NA,NA,NA,"GeneID:55616","ASAP3",TRUE,"ASAP3"

"11981","GCCCCTTCATCAGACTGTCC","chr1",23452716,"+","none","AGG","GCCCCTTCATCAGACTGTCCAGG",0,NA,NA,NA,"GeneID:55616","ASAP3",TRUE,"ASAP3"

"11982","GTGCGGGCAATCCATAGCTC","chr1",23456129,"-","none","CGG","GTGCGGGCAATCCATAGCTCCGG",0,NA,NA,NA,"GeneID:55616","ASAP3",TRUE,"ASAP3"

"11983","GTGCGGGCAATCCATAGCTC","chr1",119329185,"-","1:T>A,7:C>G","TGG","GAGCGGGGAATCCATAGCTCTGG",2,2,8,NA,"","",FALSE,"ASAP3"

"11984","TGAAGAGCGCAGCAACCTCG","chr1",23455888,"+","none","CGG","TGAAGAGCGCAGCAACCTCGCGG",0,NA,NA,NA,"GeneID:55616","ASAP3",TRUE,"ASAP3"

"11985","AGTCAGGGTTCGCGCCAGCC","chr2",238444485,"-","none","AGG","AGTCAGGGTTCGCGCCAGCCAGG",0,NA,NA,NA,"GeneID:51665","ASB1",TRUE,"ASB1"

"11986","CAGTGCAGGTCGTAATCTGA","chr2",238433545,"+","none","AGG","CAGTGCAGGTCGTAATCTGAAGG",0,NA,NA,NA,"GeneID:51665","ASB1",TRUE,"ASB1"

"11987","CTCCATGATGCAGCTTACGT","chr2",238433624,"+","none","CGG","CTCCATGATGCAGCTTACGTCGG",0,NA,NA,NA,"GeneID:51665","ASB1",TRUE,"ASB1"

"11988","CTGCACACCGTTGCGAATCG","chr2",238435752,"+","none","CGG","CTGCACACCGTTGCGAATCGCGG",0,NA,NA,NA,"GeneID:51665","ASB1",TRUE,"ASB1"

"11989","GTAGGCTGCGCTGATGTACA","chr2",238444433,"-","none","AGG","GTAGGCTGCGCTGATGTACAAGG",0,NA,NA,NA,"GeneID:51665","ASB1",TRUE,"ASB1"

"11990","TGTCTACCACGCCTCTCGCG","chr2",238435959,"+","none","TGG","TGTCTACCACGCCTCTCGCGTGG",0,NA,NA,NA,"GeneID:51665","ASB1",TRUE,"ASB1"

"11991","ATACCAGCGACCCAGAGCGA","chr7",151186848,"-","none","TGG","ATACCAGCGACCCAGAGCGATGG",0,NA,NA,NA,"GeneID:136371","ASB10",TRUE,"ASB10"

"11992","ATACCAGCGACCCAGAGCGA","chr22",23539685,"+","8:A>G,14:C>G","TGG","ATACCAGCAACCCACAGCGATGG",2,9,15,NA,"","",FALSE,"ASB10"

"11993","CACGTGCCCTCGGACCATCG","chr7",151176639,"-","none","AGG","CACGTGCCCTCGGACCATCGAGG",0,NA,NA,NA,"GeneID:136371","ASB10",TRUE,"ASB10"

"11994","CCTCCATCGCTCTGGGTCGC","chr7",151186842,"+","none","TGG","CCTCCATCGCTCTGGGTCGCTGG",0,NA,NA,NA,"GeneID:136371","ASB10",TRUE,"ASB10"

"11995","CGACCCCAACATCGCTGACC","chr7",151186440,"-","none","AGG","CGACCCCAACATCGCTGACCAGG",0,NA,NA,NA,"GeneID:136371","ASB10",TRUE,"ASB10"

"11996","CTGGTCTCTGACATACGAAG","chr7",151186635,"-","none","AGG","CTGGTCTCTGACATACGAAGAGG",0,NA,NA,NA,"GeneID:136371","ASB10",TRUE,"ASB10"

"11997","CTGGTCTCTGACATACGAAG","chr10",101333797,"-","2:A>C,4:T>A","GGG","CTTGACTCTGACATACGAAGGGG",2,3,5,NA,"","",FALSE,"ASB10"

"11998","GGTGACTCCTGAAACTCTGC","chr7",151176564,"-","none","AGG","GGTGACTCCTGAAACTCTGCAGG",0,NA,NA,NA,"GeneID:136371","ASB10",TRUE,"ASB10"

"11999","GGTGACTCCTGAAACTCTGC","chr1",5990011,"+","6:A>T,12:G>A","AGG","GGTGACACCTGAGACTCTGCAGG",2,7,13,NA,"GeneID:261734","NPHP4",FALSE,"ASB10"

"12000","ACTTCATGAAGCTGCAGCTC","chrX",15302765,"-","none","AGG","ACTTCATGAAGCTGCAGCTCAGG",0,NA,NA,NA,"GeneID:140456","ASB11",TRUE,"ASB11"

"12001","AGGCCAGGTGCACCTCCAAC","chrX",15293196,"+","none","TGG","AGGCCAGGTGCACCTCCAACTGG",0,NA,NA,NA,"GeneID:140456","ASB11",TRUE,"ASB11"

"12002","GCTCGGAACTCCCCTATATG","chrX",15289554,"-","none","TGG","GCTCGGAACTCCCCTATATGTGG",0,NA,NA,NA,"GeneID:140456","ASB11",TRUE,"ASB11"

"12003","GTTCGGAGCCAAGGCCCAGT","chrX",15293214,"-","none","TGG","GTTCGGAGCCAAGGCCCAGTTGG",0,NA,NA,NA,"GeneID:140456","ASB11",TRUE,"ASB11"

"12004","TGAGCTGCAGCTTCATGAAG","chrX",15302764,"+","none","TGG","TGAGCTGCAGCTTCATGAAGTGG",0,NA,NA,NA,"GeneID:140456","ASB11",TRUE,"ASB11"

"12005","TTCCGAGCTGAGGCACCTCA","chrX",15289566,"+","none","TGG","TTCCGAGCTGAGGCACCTCATGG",0,NA,NA,NA,"GeneID:140456","ASB11",TRUE,"ASB11"

"12006","AGGCTCTGCGGACGACTAAA","chrX",64224417,"+","none","CGG","AGGCTCTGCGGACGACTAAACGG",0,NA,NA,NA,"GeneID:142689","ASB12",TRUE,"ASB12"

"12007","CACCAAAATCGATTAACAGC","chrX",64224908,"+","none","TGG","CACCAAAATCGATTAACAGCTGG",0,NA,NA,NA,"GeneID:142689","ASB12",TRUE,"ASB12"

"12008","GTCTTGGGACACGAGCCAAT","chrX",64224983,"+","none","AGG","GTCTTGGGACACGAGCCAATAGG",0,NA,NA,NA,"GeneID:142689","ASB12",TRUE,"ASB12"

"12009","TCCACGGTCACTTCTATCAC","chrX",64224444,"-","none","AGG","TCCACGGTCACTTCTATCACAGG",0,NA,NA,NA,"GeneID:142689","ASB12",TRUE,"ASB12"

"12010","TGAAACGTTTGTAACGCTCC","chrX",64225460,"+","none","TGG","TGAAACGTTTGTAACGCTCCTGG",0,NA,NA,NA,"GeneID:142689","ASB12",TRUE,"ASB12"

"12011","TGACAACGACTCCTATACTT","chrX",64225499,"-","none","TGG","TGACAACGACTCCTATACTTTGG",0,NA,NA,NA,"GeneID:142689","ASB12",TRUE,"ASB12"

"12012","CACACACTCGATGCTGCCCG","chr10",5651291,"+","none","AGG","CACACACTCGATGCTGCCCGAGG",0,NA,NA,NA,"GeneID:79754","ASB13",TRUE,"ASB13"

"12013","CCAAGTTAAACATCCCGCCC","chr10",5640732,"-","none","CGG","CCAAGTTAAACATCCCGCCCCGG",0,NA,NA,NA,"GeneID:79754","ASB13",TRUE,"ASB13"

"12014","CCGGGGCGGGATGTTTAACT","chr10",5640729,"+","none","TGG","CCGGGGCGGGATGTTTAACTTGG",0,NA,NA,NA,"GeneID:79754","ASB13",TRUE,"ASB13"

"12015","CTGCGTGAACCAGGTCACCG","chr10",5652945,"-","none","TGG","CTGCGTGAACCAGGTCACCGTGG",0,NA,NA,NA,"GeneID:79754","ASB13",TRUE,"ASB13"

"12016","GTGGATGCTCGCAACATCGA","chr10",5651343,"-","none","CGG","GTGGATGCTCGCAACATCGACGG",0,NA,NA,NA,"GeneID:79754","ASB13",TRUE,"ASB13"

"12017","TATTGACGTCGGGGCCAATC","chr10",5649059,"-","none","TGG","TATTGACGTCGGGGCCAATCTGG",0,NA,NA,NA,"GeneID:79754","ASB13",TRUE,"ASB13"

"12018","AACAGCACAACATGCACCTA","chr3",57291921,"-","none","AGG","AACAGCACAACATGCACCTAAGG",0,NA,NA,NA,"GeneID:142686","ASB14",TRUE,"ASB14"

"12019","AACTCTGTTGAATGATGAGC","chr3",57291963,"+","none","TGG","AACTCTGTTGAATGATGAGCTGG",0,NA,NA,NA,"GeneID:142686","ASB14",TRUE,"ASB14"

"12020","ACCAAGTACCATTCCGCATT","chr3",57288240,"-","none","TGG","ACCAAGTACCATTCCGCATTTGG",0,NA,NA,NA,"GeneID:142686","ASB14",TRUE,"ASB14"

"12021","CTTATAGTCAGCGCTCAAAA","chr3",57289092,"+","none","AGG","CTTATAGTCAGCGCTCAAAAAGG",0,NA,NA,NA,"GeneID:142686","ASB14",TRUE,"ASB14"

"12022","TGCACTGCAGCCTTATGCAG","chr3",57288189,"+","none","AGG","TGCACTGCAGCCTTATGCAGAGG",0,NA,NA,NA,"GeneID:142686","ASB14",TRUE,"ASB14"

"12023","TGCACTGCAGCCTTATGCAG","chr12",59585050,"-","16:G>C,19:G>C","GGG","TGCACTGCAGCCTTATCCACGGG",2,17,20,NA,"","",FALSE,"ASB14"

"12024","TTCATCTATGTCTTCATCGC","chr3",57291997,"+","none","TGG","TTCATCTATGTCTTCATCGCTGG",0,NA,NA,NA,"GeneID:142686","ASB14",TRUE,"ASB14"

"12025","ACAAATACTTGAGATTGTTC","chr7",123616469,"+","none","TGG","ACAAATACTTGAGATTGTTCTGG",0,NA,NA,NA,"GeneID:142685;GeneID:102724555","ASB15;LOC102724555",TRUE,"ASB15"

"12026","ACAAATACTTGAGATTGTTC","chr17",77282837,"+","0:C>A,19:G>C","TGG","CCAAATACTTGAGATTGTTGTGG",2,1,20,NA,"GeneID:10801","SEPT9",FALSE,"ASB15"

"12027","CAGCATCCTATAAGACACTC","chr7",123617575,"+","none","TGG","CAGCATCCTATAAGACACTCTGG",0,NA,NA,NA,"GeneID:142685;GeneID:102724555","ASB15;LOC102724555",TRUE,"ASB15"

"12028","CTAGGCGTCGCTGCCGAGTA","chr7",123624756,"+","none","TGG","CTAGGCGTCGCTGCCGAGTATGG",0,NA,NA,NA,"GeneID:142685;GeneID:102724555","ASB15;LOC102724555",TRUE,"ASB15"

"12029","GAATTCCCAGAGTGTCTTAT","chr7",123617584,"-","none","AGG","GAATTCCCAGAGTGTCTTATAGG",0,NA,NA,NA,"GeneID:142685;GeneID:102724555","ASB15;LOC102724555",TRUE,"ASB15"

"12030","GAATTCCCAGAGTGTCTTAT","chr1",176952174,"+","15:G>C,18:C>A","GGG","GAATTCCCAGAGTGTGTTCTGGG",2,16,19,NA,"GeneID:460","ASTN1",FALSE,"ASB15"

"12031","TGAACAACAGCTTCATGCAA","chr7",123616440,"-","none","TGG","TGAACAACAGCTTCATGCAATGG",0,NA,NA,NA,"GeneID:142685;GeneID:102724555","ASB15;LOC102724555",TRUE,"ASB15"

"12032","TGAACAACAGCTTCATGCAA","chr13",77101359,"+","2:C>A,12:G>T","AGG","TGCACAACAGCTGCATGCAAAGG",2,3,13,NA,"GeneID:100874212;GeneID:23077","MYCBP2-AS1;MYCBP2",FALSE,"ASB15"

"12033","TGCTGACCATCGCTTGACAC","chr7",123624637,"-","none","AGG","TGCTGACCATCGCTTGACACAGG",0,NA,NA,NA,"GeneID:142685;GeneID:102724555","ASB15;LOC102724555",TRUE,"ASB15"

"12034","AGCAGGAGCGACGGTGAACC","chr17",44176758,"+","none","TGG","AGCAGGAGCGACGGTGAACCTGG",0,NA,NA,NA,"GeneID:92591;GeneID:339201","ASB16;ASB16-AS1",TRUE,"ASB16"

"12035","CACAAGGATAGGCATTAAGC","chr17",44177652,"-","none","AGG","CACAAGGATAGGCATTAAGCAGG",0,NA,NA,NA,"GeneID:92591;GeneID:339201","ASB16;ASB16-AS1",TRUE,"ASB16"

"12036","CCCGACTAGCTGTGCGCGCT","chr17",44178271,"+","none","CGG","CCCGACTAGCTGTGCGCGCTCGG",0,NA,NA,NA,"GeneID:92591;GeneID:339201","ASB16;ASB16-AS1",TRUE,"ASB16"

"12037","CCTGGAGCATGGCGCCGACG","chr17",44176854,"+","none","TGG","CCTGGAGCATGGCGCCGACGTGG",0,NA,NA,NA,"GeneID:92591;GeneID:339201","ASB16;ASB16-AS1",TRUE,"ASB16"

"12038","CTCGCCATCGCTACAGCCCG","chr17",44172086,"+","none","AGG","CTCGCCATCGCTACAGCCCGAGG",0,NA,NA,NA,"GeneID:92591","ASB16",TRUE,"ASB16"

"12039","GGGTCGCGCTGCCTTGCATG","chr17",44172169,"+","none","AGG","GGGTCGCGCTGCCTTGCATGAGG",0,NA,NA,NA,"GeneID:92591","ASB16",TRUE,"ASB16"

"12040","ACCAACAATATGCTCCCAGA","chr1",75919012,"-","none","TGG","ACCAACAATATGCTCCCAGATGG",0,NA,NA,NA,"GeneID:127247","ASB17",TRUE,"ASB17"

"12041","ACCATTACTCTTACTCTCGA","chr1",75922173,"+","none","AGG","ACCATTACTCTTACTCTCGAAGG",0,NA,NA,NA,"GeneID:127247","ASB17",TRUE,"ASB17"

"12042","CCCTTCGAGAGTAAGAGTAA","chr1",75922177,"-","none","TGG","CCCTTCGAGAGTAAGAGTAATGG",0,NA,NA,NA,"GeneID:127247","ASB17",TRUE,"ASB17"

"12043","GATATCACTGTTACGAACCA","chr1",75932156,"-","none","AGG","GATATCACTGTTACGAACCAAGG",0,NA,NA,NA,"GeneID:127247","ASB17",TRUE,"ASB17"

"12044","GCCAGTGATCTGTAAATCCT","chr1",75932136,"+","none","TGG","GCCAGTGATCTGTAAATCCTTGG",0,NA,NA,NA,"GeneID:127247","ASB17",TRUE,"ASB17"

"12045","GGTTAATAGTTGATTCCTGA","chr1",75919029,"+","none","TGG","GGTTAATAGTTGATTCCTGATGG",0,NA,NA,NA,"GeneID:127247","ASB17",TRUE,"ASB17"

"12046","GGTTAATAGTTGATTCCTGA","chr10",130639540,"-","4:A>T,12:A>T","AGG","GGTTTATAGTTGTTTCCTGAAGG",2,5,13,NA,"","",FALSE,"ASB17"

"12047","ATAGTCGAGCGCGCCCGCGT","chr2",236214490,"+","none","CGG","ATAGTCGAGCGCGCCCGCGTCGG",0,NA,NA,NA,"GeneID:401036","ASB18",TRUE,"ASB18"

"12048","ATCTGTCCCCGCAGTCATCG","chr2",236196348,"-","none","AGG","ATCTGTCCCCGCAGTCATCGAGG",0,NA,NA,NA,"GeneID:401036","ASB18",TRUE,"ASB18"

"12049","ATCTGTCCCCGCAGTCATCG","chr16",31265796,"+","16:C>A,17:C>T","TGG","ATCTGTCCCCGCAGTCCCCGTGG",2,17,18,NA,"GeneID:3684","ITGAM",TRUE,"ASB18"

"12050","CACGCCCCTGTGCATCGCCG","chr2",236237902,"-","none","CGG","CACGCCCCTGTGCATCGCCGCGG",0,NA,NA,NA,"GeneID:401036","ASB18",TRUE,"ASB18"

"12051","GACGCGGGCGCGCTCGACTA","chr2",236214491,"-","none","TGG","GACGCGGGCGCGCTCGACTATGG",0,NA,NA,NA,"GeneID:401036","ASB18",TRUE,"ASB18"

"12052","GTGAAATCTCCAGCCACGTT","chr2",236241290,"-","none","TGG","GTGAAATCTCCAGCCACGTTTGG",0,NA,NA,NA,"GeneID:401036","ASB18",TRUE,"ASB18"

"12053","GTTCTTCCAGGATGCCAACG","chr2",236241348,"-","none","TGG","GTTCTTCCAGGATGCCAACGTGG",0,NA,NA,NA,"GeneID:401036","ASB18",TRUE,"ASB18"

"12054","AAGCCCATTGGGGCCCGGGC","chr14",93956822,"+","none","CGG","AAGCCCATTGGGGCCCGGGCCGG",0,NA,NA,NA,"GeneID:51676","ASB2",TRUE,"ASB2"

"12055","AAGCCCATTGGGGCCCGGGC","chr16",85474277,"-","11:T>C,14:T>G","CGG","AAGCCCATTGGAGCACGGGCCGG",2,12,15,NA,"GeneID:23199","GSE1",FALSE,"ASB2"

"12056","AGCCTGCGAGCGCAAGAACG","chr14",93951226,"-","none","CGG","AGCCTGCGAGCGCAAGAACGCGG",0,NA,NA,NA,"GeneID:51676","ASB2",TRUE,"ASB2"

"12057","CCGTTTACTTGGCAACGTGC","chr14",93953442,"-","none","AGG","CCGTTTACTTGGCAACGTGCAGG",0,NA,NA,NA,"GeneID:51676","ASB2",TRUE,"ASB2"

"12058","CGAGAGACACCGCTCTACAA","chr14",93953353,"-","none","AGG","CGAGAGACACCGCTCTACAAAGG",0,NA,NA,NA,"GeneID:51676","ASB2",TRUE,"ASB2"

"12059","CTCCGCGTTCTTGCGCTCGC","chr14",93951221,"+","none","AGG","CTCCGCGTTCTTGCGCTCGCAGG",0,NA,NA,NA,"GeneID:51676","ASB2",TRUE,"ASB2"

"12060","GCCTGCGGACCCCTTGATAA","chr14",93954463,"-","none","AGG","GCCTGCGGACCCCTTGATAAAGG",0,NA,NA,NA,"GeneID:51676","ASB2",TRUE,"ASB2"

"12061","AATGGATCCCATTCTATGTG","chr2",53729491,"-","none","TGG","AATGGATCCCATTCTATGTGTGG",0,NA,NA,NA,"GeneID:51130;GeneID:100302652","ASB3;GPR75-ASB3",TRUE,"ASB3"

"12062","CTTGTTCATTGCTGCTCAAG","chr2",53716683,"-","none","AGG","CTTGTTCATTGCTGCTCAAGAGG",0,NA,NA,NA,"GeneID:51130;GeneID:100302652","ASB3;GPR75-ASB3",TRUE,"ASB3"

"12063","CTTGTTCATTGCTGCTCAAG","chr20",47997272,"+","16:A>C,19:T>G","AGG","CTTGTTCATTGCTGCTAAATAGG",2,17,20,NA,"","",FALSE,"ASB3"

"12064","TCCTCATTACAGTAAAGATC","chr2",53716613,"+","none","AGG","TCCTCATTACAGTAAAGATCAGG",0,NA,NA,NA,"GeneID:51130;GeneID:100302652","ASB3;GPR75-ASB3",TRUE,"ASB3"

"12065","TCCTCATTACAGTAAAGATC","chr16",85372718,"-","14:G>T,19:C>G","AGG","TCCTCATTACAGTACAGATGAGG",2,15,20,NA,"GeneID:23199","GSE1",FALSE,"ASB3"

"12066","TTAAGGCTGTTGCTTCAACA","chr2",53729524,"-","none","CGG","TTAAGGCTGTTGCTTCAACACGG",0,NA,NA,NA,"GeneID:51130;GeneID:100302652","ASB3;GPR75-ASB3",TRUE,"ASB3"

"12067","TTATTTGTGGCTGCTCAGTA","chr2",53728749,"-","none","TGG","TTATTTGTGGCTGCTCAGTATGG",0,NA,NA,NA,"GeneID:51130;GeneID:100302652","ASB3;GPR75-ASB3",TRUE,"ASB3"

"12068","TTATTTGTGGCTGCTCAGTA","chr21",15008488,"-","10:T>G,12:T>C","AGG","TTATTTGTGGATACTCAGTAAGG",2,11,13,NA,"GeneID:8204","NRIP1",FALSE,"ASB3"

"12069","TTGCATCTCGCTGCAAGTCA","chr2",53750871,"-","none","AGG","TTGCATCTCGCTGCAAGTCAAGG",0,NA,NA,NA,"GeneID:51130;GeneID:100302652","ASB3;GPR75-ASB3",TRUE,"ASB3"

"12070","AGCTATCCCCGATGATGACT","chr7",95536525,"+","none","TGG","AGCTATCCCCGATGATGACTTGG",0,NA,NA,NA,"GeneID:51666","ASB4",TRUE,"ASB4"

"12071","ATTTACCTCCAAGTCATCAT","chr7",95536536,"-","none","CGG","ATTTACCTCCAAGTCATCATCGG",0,NA,NA,NA,"GeneID:51666","ASB4",TRUE,"ASB4"

"12072","ATTTACCTCCAAGTCATCAT","chr16",16884457,"+","0:T>A,8:T>C","GGG","TTTTACCTTCAAGTCATCATGGG",2,1,9,NA,"","",FALSE,"ASB4"

"12073","GCATCATGTGCATGAGCACG","chr7",95528119,"-","none","TGG","GCATCATGTGCATGAGCACGTGG",0,NA,NA,NA,"GeneID:51666","ASB4",TRUE,"ASB4"

"12074","GCTTTGCCCCACGATCACAG","chr7",95495947,"-","none","AGG","GCTTTGCCCCACGATCACAGAGG",0,NA,NA,NA,"GeneID:51666","ASB4",TRUE,"ASB4"

"12075","GGCATTCACGCTGTCCACTA","chr7",95527923,"-","none","TGG","GGCATTCACGCTGTCCACTATGG",0,NA,NA,NA,"GeneID:51666","ASB4",TRUE,"ASB4"

"12076","GTTAAGATCCTCTGTGATCG","chr7",95495936,"+","none","TGG","GTTAAGATCCTCTGTGATCGTGG",0,NA,NA,NA,"GeneID:51666","ASB4",TRUE,"ASB4"

"12077","CATCTATGCCCCAGGATATC","chr4",176221244,"+","none","AGG","CATCTATGCCCCAGGATATCAGG",0,NA,NA,NA,"GeneID:140458","ASB5",TRUE,"ASB5"

"12078","CATCTATGCCCCAGGATATC","chr14",36880010,"-","5:A>T,17:A>T","TGG","CATCTTTGCCCCAGGATTTCTGG",2,6,18,NA,"GeneID:89874","SLC25A21",FALSE,"ASB5"

"12079","CCATTGCACGAAGCCTGCCT","chr4",176222361,"-","none","TGG","CCATTGCACGAAGCCTGCCTTGG",0,NA,NA,NA,"GeneID:140458","ASB5",TRUE,"ASB5"

"12080","CTACATGAAGCAGCAAGTCA","chr4",176225298,"-","none","AGG","CTACATGAAGCAGCAAGTCAAGG",0,NA,NA,NA,"GeneID:140458","ASB5",TRUE,"ASB5"

"12081","CTACATGAAGCAGCAAGTCA","chr2",228647957,"+","10:G>C,15:C>A","TGG","CTACATGAAGGAGCACGTCATGG",2,11,16,NA,"","",FALSE,"ASB5"

"12082","CTACATGAAGCAGCAAGTCA","chr1",160091840,"+","2:T>A,18:G>C","TGG","CTTCATGAAGCAGCAAGTGATGG",2,3,19,NA,"GeneID:93185","IGSF8",TRUE,"ASB5"

"12083","CTTCGTGCAATGGGGTGACA","chr4",176222368,"+","none","TGG","CTTCGTGCAATGGGGTGACATGG",0,NA,NA,NA,"GeneID:140458","ASB5",TRUE,"ASB5"

"12084","GACATACAAGCTACATAGAG","chr4",176221192,"+","none","AGG","GACATACAAGCTACATAGAGAGG",0,NA,NA,NA,"GeneID:140458","ASB5",TRUE,"ASB5"

"12085","TGACTTGCTGCTTCATGTAG","chr4",176225298,"+","none","TGG","TGACTTGCTGCTTCATGTAGTGG",0,NA,NA,NA,"GeneID:140458","ASB5",TRUE,"ASB5"

"12086","TGACTTGCTGCTTCATGTAG","chr3",40293962,"+","1:T>G,10:G>C","GGG","TTACTTGCTGGTTCATGTAGGGG",2,2,11,NA,"GeneID:440952","EIF1B-AS1",FALSE,"ASB5"

"12087","TGACTTGCTGCTTCATGTAG","chr1",160091840,"-","1:G>C,17:T>A","AGG","TCACTTGCTGCTTCATGAAGAGG",2,2,18,NA,"GeneID:93185","IGSF8",TRUE,"ASB5"

"12088","ATCACGGGGCCGACGTTAAT","chr9",129639413,"-","none","CGG","ATCACGGGGCCGACGTTAATCGG",0,NA,NA,NA,"GeneID:140459","ASB6",TRUE,"ASB6"

"12089","CCGACGTTCTCTTGCGGCAT","chr9",129640562,"-","none","GGG","CCGACGTTCTCTTGCGGCATGGG",0,NA,NA,NA,"GeneID:140459","ASB6",TRUE,"ASB6"

"12090","CCGGTCCCTCCGATTAACGT","chr9",129639401,"+","none","CGG","CCGGTCCCTCCGATTAACGTCGG",0,NA,NA,NA,"GeneID:140459","ASB6",TRUE,"ASB6"

"12091","GATCCACGAGAGTAGCCCCT","chr9",129639291,"-","none","TGG","GATCCACGAGAGTAGCCCCTTGG",0,NA,NA,NA,"GeneID:140459","ASB6",TRUE,"ASB6"

"12092","GCCCAGTTATGTCGCCAGCG","chr9",129640702,"-","none","AGG","GCCCAGTTATGTCGCCAGCGAGG",0,NA,NA,NA,"GeneID:140459","ASB6",TRUE,"ASB6"

"12093","GTCAATGCCGCTGACAAGCA","chr9",129639203,"-","none","TGG","GTCAATGCCGCTGACAAGCATGG",0,NA,NA,NA,"GeneID:140459","ASB6",TRUE,"ASB6"

"12094","AGCTCGCCATTATCCGAGAG","chr15",100629688,"+","none","AGG","AGCTCGCCATTATCCGAGAGAGG",0,NA,NA,NA,"GeneID:140460","ASB7",TRUE,"ASB7"

"12095","CCGAATGGCCGAGATGCGAA","chr15",100612333,"+","none","TGG","CCGAATGGCCGAGATGCGAATGG",0,NA,NA,NA,"GeneID:140460","ASB7",TRUE,"ASB7"

"12096","CTAACATCAAGCGTGCAATG","chr15",100629509,"-","none","CGG","CTAACATCAAGCGTGCAATGCGG",0,NA,NA,NA,"GeneID:140460","ASB7",TRUE,"ASB7"

"12097","GAGTCCAGCCATTCGCATCT","chr15",100612344,"-","none","CGG","GAGTCCAGCCATTCGCATCTCGG",0,NA,NA,NA,"GeneID:140460","ASB7",TRUE,"ASB7"

"12098","GTAGTTCATCAAGTAGCTTT","chr15",100648389,"-","none","AGG","GTAGTTCATCAAGTAGCTTTAGG",0,NA,NA,NA,"GeneID:140460","ASB7",TRUE,"ASB7"

"12099","GTAGTTCATCAAGTAGCTTT","chr16",65373474,"-","10:A>T,11:G>T","GGG","GTAGTTCATCTCGTAGCTTTGGG",2,11,12,NA,"GeneID:283867","LINC00922",FALSE,"ASB7"

"12100","TGATGAACTACCAATTGCCA","chr15",100648398,"+","none","AGG","TGATGAACTACCAATTGCCAAGG",0,NA,NA,NA,"GeneID:140460","ASB7",TRUE,"ASB7"

"12101","CCGGCTATTCTAAAAGTAAC","chr12",48149361,"+","none","AGG","CCGGCTATTCTAAAAGTAACAGG",0,NA,NA,NA,"GeneID:140461","ASB8",TRUE,"ASB8"

"12102","CCTTGATGAGTTCCAGTATG","chr12",48153481,"-","none","TGG","CCTTGATGAGTTCCAGTATGTGG",0,NA,NA,NA,"GeneID:140461","ASB8",TRUE,"ASB8"

"12103","CTATGCCGTGCGCCGTAGCC","chr12",48149448,"-","none","TGG","CTATGCCGTGCGCCGTAGCCTGG",0,NA,NA,NA,"GeneID:140461","ASB8",TRUE,"ASB8"

"12104","CTTCCCACATGATAATGTAG","chr12",48153384,"-","none","AGG","CTTCCCACATGATAATGTAGAGG",0,NA,NA,NA,"GeneID:140461","ASB8",TRUE,"ASB8"

"12105","GCAGATGTGAACTGCACTCA","chr12",48151282,"-","none","TGG","GCAGATGTGAACTGCACTCATGG",0,NA,NA,NA,"GeneID:140461","ASB8",TRUE,"ASB8"

"12106","GTCAGCATCTGACACCATAC","chr12",48151233,"+","none","AGG","GTCAGCATCTGACACCATACAGG",0,NA,NA,NA,"GeneID:140461","ASB8",TRUE,"ASB8"

"12107","GTCAGCATCTGACACCATAC","chr18",44559421,"+","5:A>C,15:A>C","AGG","GTCAGAATCTGACACAATACAGG",2,6,16,NA,"","",FALSE,"ASB8"

"12108","AAAAAGCTACCTGGCTGATG","chrX",15258855,"+","none","AGG","AAAAAGCTACCTGGCTGATGAGG",0,NA,NA,NA,"GeneID:140462","ASB9",TRUE,"ASB9"

"12109","AGAGAGATGACCTCCAAGAC","chrX",15254769,"+","none","AGG","AGAGAGATGACCTCCAAGACAGG",0,NA,NA,NA,"GeneID:140462","ASB9",TRUE,"ASB9"

"12110","AGAGAGATGACCTCCAAGAC","chr4",145405292,"+","5:C>G,10:A>C","TGG","AGAGACATGAACTCCAAGACTGG",2,6,11,NA,"","",FALSE,"ASB9"

"12111","CAAGGGACTTTCCTGGCATC","chrX",15269808,"-","none","AGG","CAAGGGACTTTCCTGGCATCAGG",0,NA,NA,NA,"GeneID:140462","ASB9",TRUE,"ASB9"

"12112","CAAGGGACTTTCCTGGCATC","chr6",16491034,"-","11:T>G,15:A>C","AGG","CAAGGGACTTTACTGTCATCAGG",2,12,16,NA,"GeneID:6310","ATXN1",FALSE,"ASB9"

"12113","CTGACACAAGCATTAAACAG","chrX",15252351,"+","none","TGG","CTGACACAAGCATTAAACAGTGG",0,NA,NA,NA,"GeneID:140462","ASB9",TRUE,"ASB9"

"12114","CTGACACAAGCATTAAACAG","chr15",92795961,"-","4:A>G,9:T>C","TGG","CTGATACAAACATTAAACAGTGG",2,5,10,NA,"GeneID:400451","FAM174B",TRUE,"ASB9"

"12115","TATGACTCACCGCCCATCAA","chrX",15269770,"+","none","TGG","TATGACTCACCGCCCATCAATGG",0,NA,NA,NA,"GeneID:140462","ASB9",TRUE,"ASB9"

"12116","TGAATGGTGTGACAGCAGAC","chrX",15252383,"-","none","TGG","TGAATGGTGTGACAGCAGACTGG",0,NA,NA,NA,"GeneID:140462","ASB9",TRUE,"ASB9"

"12117","TGAATGGTGTGACAGCAGAC","chr15",92795929,"+","7:C>T,14:A>G","TGG","TGAATGGCGTGACAACAGACTGG",2,8,15,NA,"GeneID:400451","FAM174B",TRUE,"ASB9"

"12118","AAAGTAGACCGGAATCCTTG","chr10",72210755,"+","none","TGG","AAAGTAGACCGGAATCCTTGTGG",0,NA,NA,NA,"GeneID:51008","ASCC1",TRUE,"ASCC1"

"12119","AGAATTAGTTGATCGAGTGC","chr10",72133155,"-","none","TGG","AGAATTAGTTGATCGAGTGCTGG",0,NA,NA,NA,"GeneID:51008","ASCC1",TRUE,"ASCC1"

"12120","CTCCACCTCGTAGGCATCAC","chr10",72210784,"+","none","AGG","CTCCACCTCGTAGGCATCACAGG",0,NA,NA,NA,"GeneID:51008","ASCC1",TRUE,"ASCC1"

"12121","CTGGACTAATAGTGAAAGAG","chr10",72133117,"-","none","TGG","CTGGACTAATAGTGAAAGAGTGG",0,NA,NA,NA,"GeneID:51008","ASCC1",TRUE,"ASCC1"

"12122","GTAGCATCTCACATGTCTGC","chr10",72161562,"+","none","TGG","GTAGCATCTCACATGTCTGCTGG",0,NA,NA,NA,"GeneID:51008","ASCC1",TRUE,"ASCC1"

"12123","TTGAACACATCAAACGACTG","chr10",72204475,"-","none","TGG","TTGAACACATCAAACGACTGTGG",0,NA,NA,NA,"GeneID:51008","ASCC1",TRUE,"ASCC1"

"12124","CCACCCCCTCGTCGAATTTG","chr22",29825178,"+","none","CGG","CCACCCCCTCGTCGAATTTGCGG",0,NA,NA,NA,"GeneID:84164","ASCC2",TRUE,"ASCC2"

"12125","CTACGAAGGTGGCGCGTTCC","chr22",29825674,"+","none","AGG","CTACGAAGGTGGCGCGTTCCAGG",0,NA,NA,NA,"GeneID:84164","ASCC2",TRUE,"ASCC2"

"12126","GCCAAGTTACTACAGTGACC","chr22",29816034,"-","none","TGG","GCCAAGTTACTACAGTGACCTGG",0,NA,NA,NA,"GeneID:84164","ASCC2",TRUE,"ASCC2"

"12127","TCCAGGTCACTGTAGTAACT","chr22",29816030,"+","none","TGG","TCCAGGTCACTGTAGTAACTTGG",0,NA,NA,NA,"GeneID:84164","ASCC2",TRUE,"ASCC2"

"12128","TCGTAGCCAATGACCTCGAC","chr22",29825660,"-","none","TGG","TCGTAGCCAATGACCTCGACTGG",0,NA,NA,NA,"GeneID:84164","ASCC2",TRUE,"ASCC2"

"12129","TGTCCCCCGCAAATTCGACG","chr22",29825187,"-","none","AGG","TGTCCCCCGCAAATTCGACGAGG",0,NA,NA,NA,"GeneID:84164","ASCC2",TRUE,"ASCC2"

"12130","AGTGACTTGACAACCATAAT","chr6",100800445,"+","none","TGG","AGTGACTTGACAACCATAATTGG",0,NA,NA,NA,"GeneID:10973","ASCC3",TRUE,"ASCC3"

"12131","CAGCAAAGATCTTCTACTCT","chr6",100848202,"+","none","TGG","CAGCAAAGATCTTCTACTCTTGG",0,NA,NA,NA,"GeneID:10973","ASCC3",TRUE,"ASCC3"

"12132","CCAGAACAGAATTACAATTG","chr6",100805810,"-","none","TGG","CCAGAACAGAATTACAATTGTGG",0,NA,NA,NA,"GeneID:10973","ASCC3",TRUE,"ASCC3"

"12133","CTTTGTATCCGCAATTCCTT","chr6",100800299,"+","none","AGG","CTTTGTATCCGCAATTCCTTAGG",0,NA,NA,NA,"GeneID:10973","ASCC3",TRUE,"ASCC3"

"12134","CTTTGTATCCGCAATTCCTT","chr12",90414745,"+","10:C>G,12:G>A","TGG","CTTTGTATCCCCGATTCCTTTGG",2,11,13,NA,"","",FALSE,"ASCC3"

"12135","TCAATAAGTTCAAGTCCTTC","chr6",100805839,"+","none","AGG","TCAATAAGTTCAAGTCCTTCAGG",0,NA,NA,NA,"GeneID:10973","ASCC3",TRUE,"ASCC3"

"12136","TGAATAGTTGGAACTGATAA","chr6",100848694,"-","none","TGG","TGAATAGTTGGAACTGATAATGG",0,NA,NA,NA,"GeneID:10973","ASCC3",TRUE,"ASCC3"

"12137","AGAGCCCTCGTCCGACGAGT","chr12",102958878,"-","none","AGG","AGAGCCCTCGTCCGACGAGTAGG",0,NA,NA,NA,"GeneID:429","ASCL1",TRUE,"ASCL1"

"12138","CGAGCGCAACCGCGTCAAGT","chr12",102958621,"+","none","TGG","CGAGCGCAACCGCGTCAAGTTGG",0,NA,NA,NA,"GeneID:429","ASCL1",TRUE,"ASCL1"

"12139","CGAGCGCAACCGCGTCAAGT","chr11",2270139,"-","15:C>G,19:G>A","TGG","CGAGCGCAACCGCGTGAAGCTGG",2,16,20,NA,"GeneID:430","ASCL2",TRUE,"ASCL1"

"12140","CGTTGGGGACGTGCTCCCGA","chr12",102958666,"-","none","AGG","CGTTGGGGACGTGCTCCCGAAGG",0,NA,NA,NA,"GeneID:429","ASCL1",TRUE,"ASCL1"

"12141","GCCGGTCTCATCCTACTCGT","chr12",102958864,"+","none","CGG","GCCGGTCTCATCCTACTCGTCGG",0,NA,NA,NA,"GeneID:429","ASCL1",TRUE,"ASCL1"

"12142","GCGCTCGTTGCGGCGCGCCA","chr12",102958602,"-","none","CGG","GCGCTCGTTGCGGCGCGCCACGG",0,NA,NA,NA,"GeneID:429","ASCL1",TRUE,"ASCL1"

"12143","GCGCTGTCGCTTGACTTGCT","chr12",102958494,"-","none","TGG","GCGCTGTCGCTTGACTTGCTTGG",0,NA,NA,NA,"GeneID:429","ASCL1",TRUE,"ASCL1"

"12144","AACTGTTGCGCTGCAGCCGG","chr11",2270224,"-","none","CGG","AACTGTTGCGCTGCAGCCGGCGG",0,NA,NA,NA,"GeneID:430","ASCL2",TRUE,"ASCL2"

"12145","CCCGCGTTCCGCCTACTCGT","chr11",2269833,"-","none","CGG","CCCGCGTTCCGCCTACTCGTCGG",0,NA,NA,NA,"GeneID:430","ASCL2",TRUE,"ASCL2"

"12146","GCCGCTGTCGTCCGACGAGT","chr11",2269819,"+","none","AGG","GCCGCTGTCGTCCGACGAGTAGG",0,NA,NA,NA,"GeneID:430","ASCL2",TRUE,"ASCL2"

"12147","GCGCTCATTGCGCCGCGCTA","chr11",2270158,"+","none","CGG","GCGCTCATTGCGCCGCGCTACGG",0,NA,NA,NA,"GeneID:430","ASCL2",TRUE,"ASCL2"

"12148","TCCCGCCAGCGCGTTGCGCA","chr11",2269978,"+","none","CGG","TCCCGCCAGCGCGTTGCGCACGG",0,NA,NA,NA,"GeneID:430","ASCL2",TRUE,"ASCL2"

"12149","TCGAGTAGCTCGCGCTCCGC","chr11",2269778,"+","none","AGG","TCGAGTAGCTCGCGCTCCGCAGG",0,NA,NA,NA,"GeneID:430","ASCL2",TRUE,"ASCL2"

"12150","AGTGGCAAGCGGGCAGAATC","chr11",8938087,"+","none","AGG","AGTGGCAAGCGGGCAGAATCAGG",0,NA,NA,NA,"GeneID:56676","ASCL3",TRUE,"ASCL3"

"12151","CCTACGGGCCAGCCTTCACC","chr11",8937876,"-","none","CGG","CCTACGGGCCAGCCTTCACCCGG",0,NA,NA,NA,"GeneID:56676","ASCL3",TRUE,"ASCL3"

"12152","CGCTTCTCCAAATACTCCTC","chr11",8937778,"+","none","TGG","CGCTTCTCCAAATACTCCTCTGG",0,NA,NA,NA,"GeneID:56676","ASCL3",TRUE,"ASCL3"

"12153","CGCTTCTCCAAATACTCCTC","chr18",78096056,"-","13:C>T,19:T>G","CGG","CGCTTCTCCAAATGCTCCTACGG",2,14,20,NA,"","",FALSE,"ASCL3"

"12154","GGTGTCATCCCCTTACTCTG","chr11",8938010,"-","none","AGG","GGTGTCATCCCCTTACTCTGAGG",0,NA,NA,NA,"GeneID:56676","ASCL3",TRUE,"ASCL3"

"12155","GGTGTCATCCCCTTACTCTG","chr5",153364215,"-","4:G>A,10:C>G","TGG","GGTGCCATCCGCTTACTCTGTGG",2,5,11,NA,"","",FALSE,"ASCL3"

"12156","TACTCGCACCCTCTGTAATT","chr11",8937898,"+","none","TGG","TACTCGCACCCTCTGTAATTTGG",0,NA,NA,NA,"GeneID:56676","ASCL3",TRUE,"ASCL3"

"12157","TCCCAGCGACTCTCTTATCC","chr11",8937968,"-","none","TGG","TCCCAGCGACTCTCTTATCCTGG",0,NA,NA,NA,"GeneID:56676","ASCL3",TRUE,"ASCL3"

"12158","AATGATGGAGACGCGTAAAC","chr12",107775214,"+","none","CGG","AATGATGGAGACGCGTAAACCGG",0,NA,NA,NA,"GeneID:121549","ASCL4",TRUE,"ASCL4"

"12159","GCAGTACTTGCCCGTGCCGC","chr12",107775391,"+","none","TGG","GCAGTACTTGCCCGTGCCGCTGG",0,NA,NA,NA,"GeneID:121549","ASCL4",TRUE,"ASCL4"

"12160","GCGGGTGCGCTGCGTGAACG","chr12",107775466,"+","none","AGG","GCGGGTGCGCTGCGTGAACGAGG",0,NA,NA,NA,"GeneID:121549","ASCL4",TRUE,"ASCL4"

"12161","TGATGTAGTCGATGGCAGCG","chr12",107775562,"-","none","CGG","TGATGTAGTCGATGGCAGCGCGG",0,NA,NA,NA,"GeneID:121549","ASCL4",TRUE,"ASCL4"

"12162","TGTTGCATTCCGCCCTGCGC","chr12",107775649,"-","none","TGG","TGTTGCATTCCGCCCTGCGCTGG",0,NA,NA,NA,"GeneID:121549","ASCL4",TRUE,"ASCL4"

"12163","TTCGCCCAGCAGCGAGCCCG","chr12",107775700,"+","none","AGG","TTCGCCCAGCAGCGAGCCCGAGG",0,NA,NA,NA,"GeneID:121549","ASCL4",TRUE,"ASCL4"

"12164","AGCGGGGGGCGTCGAGCCGT","chr1",201114922,"+","none","CGG","AGCGGGGGGCGTCGAGCCGTCGG",0,NA,NA,NA,"GeneID:647219","ASCL5",TRUE,"ASCL5"

"12165","CCCCGCATAGGCGTCGTAGT","chr1",201115186,"+","none","AGG","CCCCGCATAGGCGTCGTAGTAGG",0,NA,NA,NA,"GeneID:647219","ASCL5",TRUE,"ASCL5"

"12166","GCCCTACTACGACGCCTATG","chr1",201115191,"-","none","CGG","GCCCTACTACGACGCCTATGCGG",0,NA,NA,NA,"GeneID:647219","ASCL5",TRUE,"ASCL5"

"12167","GCGAGTCAAGTGCGTCAACG","chr1",201115071,"-","none","AGG","GCGAGTCAAGTGCGTCAACGAGG",0,NA,NA,NA,"GeneID:647219","ASCL5",TRUE,"ASCL5"

"12168","GGGGTATTCGTAGACCCCGA","chr1",201115135,"+","none","AGG","GGGGTATTCGTAGACCCCGAAGG",0,NA,NA,NA,"GeneID:647219","ASCL5",TRUE,"ASCL5"

"12169","TCGACGCCCCCCGCTTCCCG","chr1",201114917,"-","none","CGG","TCGACGCCCCCCGCTTCCCGCGG",0,NA,NA,NA,"GeneID:647219","ASCL5",TRUE,"ASCL5"

"12170","AATAAATTCTTGTCCTCGAT","chr6",118905727,"-","none","AGG","AATAAATTCTTGTCCTCGATAGG",0,NA,NA,NA,"GeneID:254394;GeneID:25842","MCM9;ASF1A",TRUE,"ASF1A"

"12171","CAGGTCCTCGATGCACTCGA","chr6",118894495,"-","none","AGG","CAGGTCCTCGATGCACTCGAAGG",0,NA,NA,NA,"GeneID:254394;GeneID:25842","MCM9;ASF1A",TRUE,"ASF1A"

"12172","CTAATTACTTGTACCTATCG","chr6",118905711,"+","none","AGG","CTAATTACTTGTACCTATCGAGG",0,NA,NA,NA,"GeneID:254394;GeneID:25842","MCM9;ASF1A",TRUE,"ASF1A"

"12173","GATCACCTTCGAGTGCATCG","chr6",118894487,"+","none","AGG","GATCACCTTCGAGTGCATCGAGG",0,NA,NA,NA,"GeneID:254394;GeneID:25842","MCM9;ASF1A",TRUE,"ASF1A"

"12174","GTGGTCACATGCAGTCCATG","chr6",118907598,"-","none","TGG","GTGGTCACATGCAGTCCATGTGG",0,NA,NA,NA,"GeneID:254394;GeneID:25842","MCM9;ASF1A",TRUE,"ASF1A"

"12175","TCACAAGATTCCACATTAAT","chr6",118907435,"+","none","TGG","TCACAAGATTCCACATTAATTGG",0,NA,NA,NA,"GeneID:254394;GeneID:25842","MCM9;ASF1A",TRUE,"ASF1A"

"12176","CACGTTCAGCACCGACACCT","chr19",14136429,"+","none","TGG","CACGTTCAGCACCGACACCTTGG",0,NA,NA,NA,"GeneID:55723","ASF1B",TRUE,"ASF1B"

"12177","CCAGCTCCAGCGGAACATCT","chr19",14120649,"-","none","TGG","CCAGCTCCAGCGGAACATCTTGG",0,NA,NA,NA,"GeneID:55723","ASF1B",TRUE,"ASF1B"

"12178","CCAGCTCCAGCGGAACATCT","chr6",88268925,"+","15:T>C,17:A>T","TGG","CCAGCTCCAGCGGAATAACTTGG",2,16,18,NA,"","",FALSE,"ASF1B"

"12179","CGAGGCGATGGCCAAGGTGT","chr19",14136443,"-","none","CGG","CGAGGCGATGGCCAAGGTGTCGG",0,NA,NA,NA,"GeneID:55723","ASF1B",TRUE,"ASF1B"

"12180","CTCCTGTCCATGGTAGGTGC","chr19",14121618,"+","none","AGG","CTCCTGTCCATGGTAGGTGCAGG",0,NA,NA,NA,"GeneID:55723","ASF1B",TRUE,"ASF1B"

"12181","GCGGGTCACCCGGGGGTTCG","chr19",14120623,"+","none","AGG","GCGGGTCACCCGGGGGTTCGAGG",0,NA,NA,NA,"GeneID:55723","ASF1B",TRUE,"ASF1B"

"12182","TGTTGACGTAGTAGCCCACT","chr19",14121590,"+","none","CGG","TGTTGACGTAGTAGCCCACTCGG",0,NA,NA,NA,"GeneID:55723","ASF1B",TRUE,"ASF1B"

"12183","CAGACACGAACTGCTTCACG","chr17",7174418,"+","none","TGG","CAGACACGAACTGCTTCACGTGG",0,NA,NA,NA,"GeneID:432","ASGR1",TRUE,"ASGR1"

"12184","CAGCAACTTCACAGCGAGCA","chr17",7177013,"-","none","CGG","CAGCAACTTCACAGCGAGCACGG",0,NA,NA,NA,"GeneID:432","ASGR1",TRUE,"ASGR1"

"12185","CTGTCAGATGGCGGCGCTCC","chr17",7174382,"-","none","AGG","CTGTCAGATGGCGGCGCTCCAGG",0,NA,NA,NA,"GeneID:432","ASGR1",TRUE,"ASGR1"

"12186","GAAGTCGCTAGAGTCCCAGC","chr17",7176859,"-","none","TGG","GAAGTCGCTAGAGTCCCAGCTGG",0,NA,NA,NA,"GeneID:432","ASGR1",TRUE,"ASGR1"

"12187","GGGTGCTCAAGCCCTTGACC","chr17",7176983,"+","none","TGG","GGGTGCTCAAGCCCTTGACCTGG",0,NA,NA,NA,"GeneID:432","ASGR1",TRUE,"ASGR1"

"12188","GTCCGGAGCAGAGACGCTGC","chr17",7177281,"+","none","AGG","GTCCGGAGCAGAGACGCTGCAGG",0,NA,NA,NA,"GeneID:432","ASGR1",TRUE,"ASGR1"

"12189","GTCCGGAGCAGAGACGCTGC","chr17",7078068,"+","1:C>T,4:A>G","AGG","GCCCAGAGCAGAGACGCTGCAGG",2,2,5,NA,"GeneID:10462","CLEC10A",TRUE,"ASGR1"

"12190","CACCCCTACCGTTGCTGTGG","chr17",7107221,"+","none","AGG","CACCCCTACCGTTGCTGTGGAGG",0,NA,NA,NA,"GeneID:433","ASGR2",TRUE,"ASGR2"

"12191","CACCCCTACCGTTGCTGTGG","chr16",872116,"+","7:G>A,15:C>T","AGG","CACCCCTGCCGTTGCCGTGGAGG",2,8,16,NA,"GeneID:64788","LMF1",TRUE,"ASGR2"

"12192","GAGCTTCAACATCCTGCTGC","chr17",7108801,"-","none","TGG","GAGCTTCAACATCCTGCTGCTGG",0,NA,NA,NA,"GeneID:433","ASGR2",TRUE,"ASGR2"

"12193","GAGCTTCAACATCCTGCTGC","chr11",36161095,"-","9:C>G,18:A>C","TGG","GAGCTTCAAGATCCTGCTTCTGG",2,10,19,NA,"GeneID:143458","LDLRAD3",FALSE,"ASGR2"

"12194","GGACCTGCTGCCCCGTCAAC","chr17",7107122,"-","none","TGG","GGACCTGCTGCCCCGTCAACTGG",0,NA,NA,NA,"GeneID:433","ASGR2",TRUE,"ASGR2"

"12195","GGACCTGCTGCCCCGTCAAC","chr17",7174260,"-","13:C>G","TGG","GGACCTGCTGCCCGGTCAACTGG",1,14,NA,NA,"GeneID:432","ASGR1",TRUE,"ASGR2"

"12196","GGACCTGCTGCCCCGTCAAC","chr17",7075853,"-","13:A>G","TGG","GGACCTGCTGCCCTGTCAACTGG",1,14,NA,NA,"GeneID:10462","CLEC10A",TRUE,"ASGR2"

"12197","GGCGGAGAAGTACTGCCAGC","chr17",7107043,"-","none","TGG","GGCGGAGAAGTACTGCCAGCTGG",0,NA,NA,NA,"GeneID:433","ASGR2",TRUE,"ASGR2"

"12198","TCCGCTTCCAGGAGGCAGCG","chr17",7107898,"-","none","TGG","TCCGCTTCCAGGAGGCAGCGTGG",0,NA,NA,NA,"GeneID:433","ASGR2",TRUE,"ASGR2"

"12199","TCTGGCAGGCCACGAAGCGC","chr17",7107251,"+","none","AGG","TCTGGCAGGCCACGAAGCGCAGG",0,NA,NA,NA,"GeneID:433","ASGR2",TRUE,"ASGR2"

"12200","TCTGGCAGGCCACGAAGCGC","chr16",2088102,"-","6:C>T,17:C>G","AGG","TCTGGCGGGCCACGAAGGGCAGG",2,7,18,NA,"GeneID:7249","TSC2",TRUE,"ASGR2"

"12201","AGGAAGAGGACCTTCGCAAA","chr1",155521366,"-","none","CGG","AGGAAGAGGACCTTCGCAAACGG",0,NA,NA,NA,"GeneID:55870","ASH1L",TRUE,"ASH1L"

"12202","CCGCTGGGAGGGTTTATCAG","chr1",155459863,"+","none","AGG","CCGCTGGGAGGGTTTATCAGAGG",0,NA,NA,NA,"GeneID:55870","ASH1L",TRUE,"ASH1L"

"12203","GAAGGACTGGGAATCTCAAC","chr1",155415887,"+","none","TGG","GAAGGACTGGGAATCTCAACTGG",0,NA,NA,NA,"GeneID:55870","ASH1L",TRUE,"ASH1L"

"12204","GCACAAATTGTAGCCCTACC","chr1",155459833,"-","none","CGG","GCACAAATTGTAGCCCTACCCGG",0,NA,NA,NA,"GeneID:55870","ASH1L",TRUE,"ASH1L"

"12205","TCAGGTTCAGAAGGTTTAGC","chr1",155415857,"+","none","TGG","TCAGGTTCAGAAGGTTTAGCTGG",0,NA,NA,NA,"GeneID:55870","ASH1L",TRUE,"ASH1L"

"12206","TCAGGTTCAGAAGGTTTAGC","chr6",34284668,"+","2:T>A,7:A>C","AGG","TCTGGTTAAGAAGGTTTAGCAGG",2,3,8,NA,"GeneID:11165","NUDT3",TRUE,"ASH1L"

"12207","TTTCTCGATTCCGTTTGCGA","chr1",155521353,"+","none","AGG","TTTCTCGATTCCGTTTGCGAAGG",0,NA,NA,NA,"GeneID:55870","ASH1L",TRUE,"ASH1L"

"12208","ACCTTTCCGTACTCCATGAG","chr8",38128362,"-","none","AGG","ACCTTTCCGTACTCCATGAGAGG",0,NA,NA,NA,"GeneID:9070","ASH2L",TRUE,"ASH2L"

"12209","CCATGACTCACCTCACTATG","chr8",38133537,"-","none","GGG","CCATGACTCACCTCACTATGGGG",0,NA,NA,NA,"GeneID:9070","ASH2L",TRUE,"ASH2L"

"12210","CCCAGTCTGGCAGCGGTATC","chr8",38128421,"-","none","TGG","CCCAGTCTGGCAGCGGTATCTGG",0,NA,NA,NA,"GeneID:9070","ASH2L",TRUE,"ASH2L"

"12211","CCCAGTCTGGCAGCGGTATC","chr22",22340631,"-","14:T>C,17:C>T","TGG","CCCAGTCTGGCAGCAGTGTCTGG",2,15,18,NA,"","",TRUE,"ASH2L"

"12212","CCCAGTCTGGCAGCGGTATC","chr22",22447768,"-","13:A>G,17:C>T","TGG","CCCAGTCTGGCAGTGGTGTCTGG",2,14,18,NA,"","",TRUE,"ASH2L"

"12213","GAAGCAGACTCCCCATAGTG","chr8",38133524,"+","none","AGG","GAAGCAGACTCCCCATAGTGAGG",0,NA,NA,NA,"GeneID:9070","ASH2L",TRUE,"ASH2L"

"12214","GCTCTACCTCACCCAACTGT","chr8",38107088,"-","none","CGG","GCTCTACCTCACCCAACTGTCGG",0,NA,NA,NA,"GeneID:9070","ASH2L",TRUE,"ASH2L"

"12215","GCTCTACCTCACCCAACTGT","chr1",55580427,"-","3:A>G,12:T>G","GGG","GCTTTACCTCACACAACTGTGGG",2,4,13,NA,"","",FALSE,"ASH2L"

"12216","GGTGAGGTAGAGCTGCAATG","chr8",38107095,"+","none","TGG","GGTGAGGTAGAGCTGCAATGTGG",0,NA,NA,NA,"GeneID:9070","ASH2L",TRUE,"ASH2L"

"12217","ACCTCCTTTCATCGACCAGC","chr12",50078051,"+","none","TGG","ACCTCCTTTCATCGACCAGCTGG",0,NA,NA,NA,"GeneID:41","ASIC1",TRUE,"ASIC1"

"12218","CGTGAGTTCTACGACCGAGC","chr12",50059858,"+","none","TGG","CGTGAGTTCTACGACCGAGCTGG",0,NA,NA,NA,"GeneID:41","ASIC1",TRUE,"ASIC1"

"12219","CTTCAGAGACAGCCGCTCGT","chr12",50058872,"-","none","AGG","CTTCAGAGACAGCCGCTCGTAGG",0,NA,NA,NA,"GeneID:41","ASIC1",TRUE,"ASIC1"

"12220","GACGAGACGTCCTTCGAAGC","chr12",50077998,"+","none","AGG","GACGAGACGTCCTTCGAAGCAGG",0,NA,NA,NA,"GeneID:41","ASIC1",TRUE,"ASIC1"

"12221","GGCAGCCACCTCGTCGAGCT","chr12",50058992,"-","none","TGG","GGCAGCCACCTCGTCGAGCTTGG",0,NA,NA,NA,"GeneID:41","ASIC1",TRUE,"ASIC1"

"12222","GGCAGCCACCTCGTCGAGCT","chr8",144417755,"+","11:G>C,13:C>T","TGG","GGCAGCCACCTGGCCGAGCTTGG",2,12,14,NA,"","",FALSE,"ASIC1"

"12223","GTAGAACTCACGCATGTTGA","chr12",50059850,"-","none","AGG","GTAGAACTCACGCATGTTGAAGG",0,NA,NA,NA,"GeneID:41","ASIC1",TRUE,"ASIC1"

"12224","AAGGTGGCTCAGACTGACTG","chr17",33088930,"+","none","TGG","AAGGTGGCTCAGACTGACTGTGG",0,NA,NA,NA,"GeneID:40","ASIC2",TRUE,"ASIC2"

"12225","ATCCGCCACATCTTCGTGTA","chr17",34156428,"-","none","TGG","ATCCGCCACATCTTCGTGTATGG",0,NA,NA,NA,"GeneID:40","ASIC2",TRUE,"ASIC2"

"12226","CGGGGTCCCGATGGACCTCA","chr17",34156522,"-","none","AGG","CGGGGTCCCGATGGACCTCAAGG",0,NA,NA,NA,"GeneID:40","ASIC2",TRUE,"ASIC2"

"12227","CGGGGTCCCGATGGACCTCA","chr22",44278715,"+","0:A>C,13:C>G","GGG","AGGGGTCCCGATGCACCTCAGGG",2,1,14,NA,"GeneID:85352","KIAA1644",FALSE,"ASIC2"

"12228","GATCATGCTGGACATTCAGC","chr17",33111949,"-","none","AGG","GATCATGCTGGACATTCAGCAGG",0,NA,NA,NA,"GeneID:40","ASIC2",TRUE,"ASIC2"

"12229","GATCATGCTGGACATTCAGC","chr2",219531849,"+","15:C>T","AGG","GATCATGCTGGACATCCAGCAGG",1,16,NA,NA,"GeneID:55515","ASIC4",TRUE,"ASIC2"

"12230","GATCATGCTGGACATTCAGC","chr12",50077310,"+","0:A>G,15:C>T","AGG","AATCATGCTGGACATCCAGCAGG",2,1,16,NA,"GeneID:41","ASIC1",TRUE,"ASIC2"

"12231","GCCACCTTTCATCCAAGAGC","chr17",33088918,"-","none","TGG","GCCACCTTTCATCCAAGAGCTGG",0,NA,NA,NA,"GeneID:40","ASIC2",TRUE,"ASIC2"

"12232","GCCACCTTTCATCCAAGAGC","chr16",71430254,"+","9:A>C,12:A>C","TGG","GCCACCTTTAATACAAGAGCTGG",2,10,13,NA,"","",TRUE,"ASIC2"

"12233","GCCTGTCCCCCCCTTGACCG","chr17",33111980,"+","none","TGG","GCCTGTCCCCCCCTTGACCGTGG",0,NA,NA,NA,"GeneID:40","ASIC2",TRUE,"ASIC2"

"12234","AGGCCAGGCGACACCCCATA","chr7",151050879,"-","none","AGG","AGGCCAGGCGACACCCCATAAGG",0,NA,NA,NA,"GeneID:9311","ASIC3",TRUE,"ASIC3"

"12235","CCACCTCACAGAGGTAGTCT","chr7",151052046,"-","none","AGG","CCACCTCACAGAGGTAGTCTAGG",0,NA,NA,NA,"GeneID:9311","ASIC3",TRUE,"ASIC3"

"12236","CCACCTCACAGAGGTAGTCT","chr21",43329751,"-","11:C>T,15:G>T","GGG","CCACCTCACAGGGGTCGTCTGGG",2,12,16,NA,"","",FALSE,"ASIC3"

"12237","CGCCCTTACCTATGGCCGGG","chr7",151051084,"-","none","TGG","CGCCCTTACCTATGGCCGGGTGG",0,NA,NA,NA,"GeneID:9311","ASIC3",TRUE,"ASIC3"

"12238","GATCCTAGACTACCTCTGTG","chr7",151052040,"+","none","AGG","GATCCTAGACTACCTCTGTGAGG",0,NA,NA,NA,"GeneID:9311","ASIC3",TRUE,"ASIC3"

"12239","GCACAGTTCTTGTACTGCTG","chr7",151051061,"-","none","GGG","GCACAGTTCTTGTACTGCTGGGG",0,NA,NA,NA,"GeneID:9311","ASIC3",TRUE,"ASIC3"

"12240","GGCCTGCGAAACCCGCTACG","chr7",151050894,"+","none","TGG","GGCCTGCGAAACCCGCTACGTGG",0,NA,NA,NA,"GeneID:9311","ASIC3",TRUE,"ASIC3"

"12241","AGATGCTCACCCGCTGTTCC","chr2",219532118,"-","none","TGG","AGATGCTCACCCGCTGTTCCTGG",0,NA,NA,NA,"GeneID:55515","ASIC4",TRUE,"ASIC4"

"12242","AGATGCTCACCCGCTGTTCC","chr7",75055009,"-","12:A>C,17:T>A","AGG","AGATGCTCACCCTCTGTACCAGG",2,13,18,NA,"GeneID:81554","WBSCR16",FALSE,"ASIC4"

"12243","CATCGCTGAGTGCCGAATGC","chr2",219515095,"-","none","CGG","CATCGCTGAGTGCCGAATGCCGG",0,NA,NA,NA,"GeneID:55515","ASIC4",TRUE,"ASIC4"

"12244","GCTCCGCGGGTCCGCGTTGA","chr2",219531788,"-","none","AGG","GCTCCGCGGGTCCGCGTTGAAGG",0,NA,NA,NA,"GeneID:55515","ASIC4",TRUE,"ASIC4"

"12245","TCCCAGGTCTATACTCGCTA","chr2",219531751,"+","none","TGG","TCCCAGGTCTATACTCGCTATGG",0,NA,NA,NA,"GeneID:55515","ASIC4",TRUE,"ASIC4"

"12246","TCTGCAATATCAACCGCTTC","chr2",219515073,"+","none","CGG","TCTGCAATATCAACCGCTTCCGG",0,NA,NA,NA,"GeneID:55515","ASIC4",TRUE,"ASIC4"

"12247","TGCAGATGAGACGTCGTTTG","chr2",219531995,"+","none","AGG","TGCAGATGAGACGTCGTTTGAGG",0,NA,NA,NA,"GeneID:55515","ASIC4",TRUE,"ASIC4"

"12248","ATCGTTACCTTTGGGCTACA","chr4",155854068,"+","none","TGG","ATCGTTACCTTTGGGCTACATGG",0,NA,NA,NA,"GeneID:51802","ASIC5",TRUE,"ASIC5"

"12249","GGTAACCATCCGCCAAGTGA","chr4",155843682,"-","none","AGG","GGTAACCATCCGCCAAGTGAAGG",0,NA,NA,NA,"GeneID:51802","ASIC5",TRUE,"ASIC5"

"12250","GTCCATTGAGGTTCAATATG","chr4",155863493,"-","none","TGG","GTCCATTGAGGTTCAATATGTGG",0,NA,NA,NA,"GeneID:51802;GeneID:6999","ASIC5;TDO2",TRUE,"ASIC5"

"12251","TCAAACTTCTTTCGCTCAGT","chr4",155863681,"+","none","GGG","TCAAACTTCTTTCGCTCAGTGGG",0,NA,NA,NA,"GeneID:51802;GeneID:6999","ASIC5;TDO2",TRUE,"ASIC5"

"12252","TCACCAAAACTTCAGCATTG","chr4",155854162,"-","none","TGG","TCACCAAAACTTCAGCATTGTGG",0,NA,NA,NA,"GeneID:51802","ASIC5",TRUE,"ASIC5"

"12253","TCCCAGCATCAACGAAACCA","chr4",155843784,"+","none","AGG","TCCCAGCATCAACGAAACCAAGG",0,NA,NA,NA,"GeneID:51802","ASIC5",TRUE,"ASIC5"

"12254","AACAAGAAATCCAAACAGAT","chr20",34262836,"+","none","CGG","AACAAGAAATCCAAACAGATCGG",0,NA,NA,NA,"GeneID:434","ASIP",TRUE,"ASIP"

"12255","AACAAGAAATCCAAACAGAT","chr21",38790432,"-","2:T>G,4:A>T","GGG","AAAATGAAATCCAAACAGATGGG",2,3,5,NA,"","",FALSE,"ASIP"

"12256","AACAAGAAATCCAAACAGAT","chr7",98614002,"+","7:G>A,9:A>T","GGG","AACAAGAGAACCAAACAGATGGG",2,8,10,NA,"","",FALSE,"ASIP"

"12257","AACAAGAAATCCAAACAGAT","chr11",61191770,"+","1:T>A,10:A>C","AGG","ATCAAGAAATACAAACAGATAGG",2,2,11,NA,"","",FALSE,"ASIP"

"12258","AGAAAAGAAAAGATCTTCTA","chr20",34262871,"+","none","AGG","AGAAAAGAAAAGATCTTCTAAGG",0,NA,NA,NA,"GeneID:434","ASIP",TRUE,"ASIP"

"12259","AGAAAAGAAAAGATCTTCTA","chr15",58707047,"+","13:A>T,14:A>C","GGG","AGAAAAGAAAAGAAATTCTAGGG",2,14,15,NA,"GeneID:102","ADAM10",FALSE,"ASIP"

"12260","AGAAAAGAAAAGATCTTCTA","chr16",66761479,"+","13:G>T,15:A>T","GGG","AGAAAAGAAAAGAGCATCTAGGG",2,14,16,NA,"GeneID:283847","CCDC79",FALSE,"ASIP"

"12261","AGAAAAGAAAAGATCTTCTA","chr14",103447556,"-","5:C>T,6:T>C","AGG","AGAAAGAAAAAGATCTTCTAAGG",2,6,7,NA,"GeneID:4140","MARK3",FALSE,"ASIP"

"12262","AGAAAAGAAAAGATCTTCTA","chr13",71578676,"+","9:G>A,11:A>G","GGG","AGAAAAGAAGAAATCTTCTAGGG",2,10,12,NA,"GeneID:1602","DACH1",FALSE,"ASIP"

"12263","AGAAAAGAAAAGATCTTCTA","chr12",114472280,"-","5:A>T,12:G>T","TGG","AGAAATGAAAAGCTCTTCTATGG",2,6,13,NA,"","",FALSE,"ASIP"

"12264","AGAAAAGAAAAGATCTTCTA","chr18",41163691,"-","2:G>T,17:A>G","AGG","AGCAAAGAAAAGATCTTTTAAGG",2,3,18,NA,"","",FALSE,"ASIP"

"12265","CAACTCCTCTGTGAACCTAC","chr20",34260487,"+","none","TGG","CAACTCCTCTGTGAACCTACTGG",0,NA,NA,NA,"GeneID:434","ASIP",TRUE,"ASIP"

"12266","CCCCCTATCTGCGCCCTGCG","chr20",34269028,"+","none","TGG","CCCCCTATCTGCGCCCTGCGTGG",0,NA,NA,NA,"GeneID:434","ASIP",TRUE,"ASIP"

"12267","CCCCCTATCTGCGCCCTGCG","chr15",42070718,"-","1:A>G,17:A>C","AGG","CTCCCTATCTGCGCCCTTCGAGG",2,2,18,NA,"GeneID:283748","PLA2G4D",TRUE,"ASIP"

"12268","CCGCAACAGCTGCAAGCCGC","chr20",34269055,"+","none","CGG","CCGCAACAGCTGCAAGCCGCCGG",0,NA,NA,NA,"GeneID:434","ASIP",TRUE,"ASIP"

"12269","GGATGTCACCCGCTTACTCC","chr20",34260376,"+","none","TGG","GGATGTCACCCGCTTACTCCTGG",0,NA,NA,NA,"GeneID:434","ASIP",TRUE,"ASIP"

"12270","ACCGCCCAACATGGCCTCGG","chr7",66076071,"+","none","AGG","ACCGCCCAACATGGCCTCGGAGG",0,NA,NA,NA,"GeneID:435","ASL",TRUE,"ASL"

"12271","ACCGCCCAACATGGCCTCGG","chr16",69089018,"+","3:C>G,10:T>A","TGG","ACCCCCCAACTTGGCCTCGGTGG",2,4,11,NA,"","",FALSE,"ASL"

"12272","ACCGCCCAACATGGCCTCGG","chr8",1944842,"-","3:G>C,18:T>C","GGG","ACCCCCCAACATGGCCTCAGGGG",2,4,19,NA,"GeneID:9639","ARHGEF10",FALSE,"ASL"

"12273","ACGCGTCCATTGCCTACGAC","chr7",66081863,"+","none","CGG","ACGCGTCCATTGCCTACGACCGG",0,NA,NA,NA,"GeneID:435","ASL",TRUE,"ASL"

"12274","CAAGTACCTTGTCTAGGCCA","chr7",66081984,"-","none","TGG","CAAGTACCTTGTCTAGGCCATGG",0,NA,NA,NA,"GeneID:435","ASL",TRUE,"ASL"

"12275","GGAACGTGATGTTCTCTTCC","chr7",66086584,"+","none","CGG","GGAACGTGATGTTCTCTTCCCGG",0,NA,NA,NA,"GeneID:435","ASL",TRUE,"ASL"

"12276","TATCTGGTCCATCTCGGCCT","chr7",66081959,"-","none","TGG","TATCTGGTCCATCTCGGCCTTGG",0,NA,NA,NA,"GeneID:435","ASL",TRUE,"ASL"

"12277","TGGCTCACCTCAGAATCCAG","chr7",66086650,"-","none","TGG","TGGCTCACCTCAGAATCCAGTGG",0,NA,NA,NA,"GeneID:435","ASL",TRUE,"ASL"

"12278","TGGCTCACCTCAGAATCCAG","chr8",48763254,"-","5:C>G,11:A>T","GGG","TGGCTGACCTCTGAATCCAGGGG",2,6,12,NA,"","",FALSE,"ASL"

"12279","AGAAGGAAGGAACCAGTACC","chrX",1627702,"+","none","TGG","AGAAGGAAGGAACCAGTACCTGG",0,NA,NA,NA,"GeneID:438","ASMT",TRUE,"ASMT_X"

"12280","AGAAGGAAGGAACCAGTACC","chr3",64631380,"+","12:G>C,18:T>C","TGG","AGAAGGAAGGAAGCAGTATCTGG",2,13,19,NA,"GeneID:56999","ADAMTS9",FALSE,"ASMT_X"

"12281","CCCACCTCACGGCGTCTGCC","chrX",1624383,"-","none","AGG","CCCACCTCACGGCGTCTGCCAGG",0,NA,NA,NA,"GeneID:438","ASMT",TRUE,"ASMT_X"

"12282","CCCACCTCACGGCGTCTGCC","chr6",79631096,"+","10:A>G,13:T>G","CGG","CCCACCTCACAGCTTCTGCCCGG",2,11,14,NA,"","",FALSE,"ASMT_X"

"12283","GGCCGTAAAAAGCTCTTCAG","chrX",1627743,"-","none","CGG","GGCCGTAAAAAGCTCTTCAGCGG",0,NA,NA,NA,"GeneID:438","ASMT",TRUE,"ASMT_X"

"12284","GTCCAGCGACTACCTGACCA","chrX",1624290,"+","none","CGG","GTCCAGCGACTACCTGACCACGG",0,NA,NA,NA,"GeneID:438","ASMT",TRUE,"ASMT_X"

"12285","TCTCCACTTTCAGCAGCTTC","chrX",1623278,"-","none","AGG","TCTCCACTTTCAGCAGCTTCAGG",0,NA,NA,NA,"GeneID:438","ASMT",TRUE,"ASMT_X"

"12286","TCTCTTCGCCGCCTGCGAGC","chrX",1623140,"+","none","TGG","TCTCTTCGCCGCCTGCGAGCTGG",0,NA,NA,NA,"GeneID:438","ASMT",TRUE,"ASMT_X"

"12287","AGAAGGAAGGAACCAGTACC","chrX",1627702,"+","none","TGG","AGAAGGAAGGAACCAGTACCTGG",0,NA,NA,NA,"GeneID:438","ASMT",TRUE,"ASMT_Y"

"12288","AGAAGGAAGGAACCAGTACC","chr3",64631380,"+","12:G>C,18:T>C","TGG","AGAAGGAAGGAAGCAGTATCTGG",2,13,19,NA,"GeneID:56999","ADAMTS9",FALSE,"ASMT_Y"

"12289","CCCACCTCACGGCGTCTGCC","chrX",1624383,"-","none","AGG","CCCACCTCACGGCGTCTGCCAGG",0,NA,NA,NA,"GeneID:438","ASMT",TRUE,"ASMT_Y"

"12290","CCCACCTCACGGCGTCTGCC","chr6",79631096,"+","10:A>G,13:T>G","CGG","CCCACCTCACAGCTTCTGCCCGG",2,11,14,NA,"","",FALSE,"ASMT_Y"

"12291","GGCCGTAAAAAGCTCTTCAG","chrX",1627743,"-","none","CGG","GGCCGTAAAAAGCTCTTCAGCGG",0,NA,NA,NA,"GeneID:438","ASMT",TRUE,"ASMT_Y"

"12292","GTCCAGCGACTACCTGACCA","chrX",1624290,"+","none","CGG","GTCCAGCGACTACCTGACCACGG",0,NA,NA,NA,"GeneID:438","ASMT",TRUE,"ASMT_Y"

"12293","TCTCCACTTTCAGCAGCTTC","chrX",1623278,"-","none","AGG","TCTCCACTTTCAGCAGCTTCAGG",0,NA,NA,NA,"GeneID:438","ASMT",TRUE,"ASMT_Y"

"12294","TCTCTTCGCCGCCTGCGAGC","chrX",1623140,"+","none","TGG","TCTCTTCGCCGCCTGCGAGCTGG",0,NA,NA,NA,"GeneID:438","ASMT",TRUE,"ASMT_Y"

"12295","AGAGAACACAGCGTGTTCAC","chrX",1435053,"-","none","AGG","AGAGAACACAGCGTGTTCACAGG",0,NA,NA,NA,"GeneID:8623","ASMTL",TRUE,"ASMTL_X"

"12296","AGAGAACACAGCGTGTTCAC","chrX",941840,"+","5:G>A,11:G>C","TGG","AGAGAGCACAGGGTGTTCACTGG",2,6,12,NA,"","",FALSE,"ASMTL_X"

"12297","GGCCCCTTGCCTGGTACAGC","chrX",1442175,"+","none","CGG","GGCCCCTTGCCTGGTACAGCCGG",0,NA,NA,NA,"GeneID:8623","ASMTL",TRUE,"ASMTL_X"

"12298","GGCCCCTTGCCTGGTACAGC","chr6",101106630,"-","6:T>A,10:A>G","GGG","GGCCCCATGCTTGGTACAGCGGG",2,7,11,NA,"","",FALSE,"ASMTL_X"

"12299","GTCCGCTCCAATGACCACGT","chrX",1439105,"+","none","CGG","GTCCGCTCCAATGACCACGTCGG",0,NA,NA,NA,"GeneID:8623","ASMTL",TRUE,"ASMTL_X"

"12300","GTTGCAGGGTCTCAGGTTTG","chrX",1442304,"-","none","AGG","GTTGCAGGGTCTCAGGTTTGAGG",0,NA,NA,NA,"GeneID:8623","ASMTL",TRUE,"ASMTL_X"

"12301","GTTGCAGGGTCTCAGGTTTG","chr6",161635999,"-","10:C>G,16:C>A","AGG","GTTGCAGGGTGTCAGGGTTGAGG",2,11,17,NA,"GeneID:5071","PARK2",FALSE,"ASMTL_X"

"12302","GTTGCAGGGTCTCAGGTTTG","chr3",50349570,"+","0:T>G,11:C>T","GGG","TTTGCAGGGTCCCAGGTTTGGGG",2,1,12,NA,"GeneID:10641","NPRL2",TRUE,"ASMTL_X"

"12303","GTTGCAGGGTCTCAGGTTTG","chr5",2866013,"-","0:A>C,15:A>C","AGG","TTTGCAGGGTCTCAGTTTTGAGG",2,1,16,NA,"","",FALSE,"ASMTL_X"

"12304","TCCTCTCACAGACAGTCGGG","chrX",1435749,"-","none","GGG","TCCTCTCACAGACAGTCGGGGGG",0,NA,NA,NA,"GeneID:8623","ASMTL",TRUE,"ASMTL_X"

"12305","TTACCGGGACAGCATCCTGT","chrX",1435689,"+","none","AGG","TTACCGGGACAGCATCCTGTAGG",0,NA,NA,NA,"GeneID:8623","ASMTL",TRUE,"ASMTL_X"

"12306","AGAGAACACAGCGTGTTCAC","chrX",1435053,"-","none","AGG","AGAGAACACAGCGTGTTCACAGG",0,NA,NA,NA,"GeneID:8623","ASMTL",TRUE,"ASMTL_Y"

"12307","AGAGAACACAGCGTGTTCAC","chrX",941840,"+","5:G>A,11:G>C","TGG","AGAGAGCACAGGGTGTTCACTGG",2,6,12,NA,"","",FALSE,"ASMTL_Y"

"12308","GGCCCCTTGCCTGGTACAGC","chrX",1442175,"+","none","CGG","GGCCCCTTGCCTGGTACAGCCGG",0,NA,NA,NA,"GeneID:8623","ASMTL",TRUE,"ASMTL_Y"

"12309","GGCCCCTTGCCTGGTACAGC","chr6",101106630,"-","6:T>A,10:A>G","GGG","GGCCCCATGCTTGGTACAGCGGG",2,7,11,NA,"","",FALSE,"ASMTL_Y"

"12310","GTCCGCTCCAATGACCACGT","chrX",1439105,"+","none","CGG","GTCCGCTCCAATGACCACGTCGG",0,NA,NA,NA,"GeneID:8623","ASMTL",TRUE,"ASMTL_Y"

"12311","GTTGCAGGGTCTCAGGTTTG","chrX",1442304,"-","none","AGG","GTTGCAGGGTCTCAGGTTTGAGG",0,NA,NA,NA,"GeneID:8623","ASMTL",TRUE,"ASMTL_Y"

"12312","GTTGCAGGGTCTCAGGTTTG","chr6",161635999,"-","10:C>G,16:C>A","AGG","GTTGCAGGGTGTCAGGGTTGAGG",2,11,17,NA,"GeneID:5071","PARK2",FALSE,"ASMTL_Y"

"12313","GTTGCAGGGTCTCAGGTTTG","chr3",50349570,"+","0:T>G,11:C>T","GGG","TTTGCAGGGTCCCAGGTTTGGGG",2,1,12,NA,"GeneID:10641","NPRL2",TRUE,"ASMTL_Y"

"12314","GTTGCAGGGTCTCAGGTTTG","chr5",2866013,"-","0:A>C,15:A>C","AGG","TTTGCAGGGTCTCAGTTTTGAGG",2,1,16,NA,"","",FALSE,"ASMTL_Y"

"12315","TCCTCTCACAGACAGTCGGG","chrX",1435749,"-","none","GGG","TCCTCTCACAGACAGTCGGGGGG",0,NA,NA,NA,"GeneID:8623","ASMTL",TRUE,"ASMTL_Y"

"12316","TTACCGGGACAGCATCCTGT","chrX",1435689,"+","none","AGG","TTACCGGGACAGCATCCTGTAGG",0,NA,NA,NA,"GeneID:8623","ASMTL",TRUE,"ASMTL_Y"

"12317","CAGAACACTCTCACGCCCCT","chr19",12738536,"-","none","TGG","CAGAACACTCTCACGCCCCTTGG",0,NA,NA,NA,"GeneID:439","ASNA1",TRUE,"ASNA1"

"12318","CAGGCCCCGCTCCACGATGG","chr19",12745691,"-","none","TGG","CAGGCCCCGCTCCACGATGGTGG",0,NA,NA,NA,"GeneID:439","ASNA1",TRUE,"ASNA1"

"12319","GCTGCCTGACGAGTTCTTCG","chr19",12745405,"+","none","AGG","GCTGCCTGACGAGTTCTTCGAGG",0,NA,NA,NA,"GeneID:439","ASNA1",TRUE,"ASNA1"

"12320","GGTCTGCGTTCATGTCCCCC","chr19",12747216,"-","none","AGG","GGTCTGCGTTCATGTCCCCCAGG",0,NA,NA,NA,"GeneID:439","ASNA1",TRUE,"ASNA1"

"12321","TCCTCCTCGAAGAACTCGTC","chr19",12745412,"-","none","AGG","TCCTCCTCGAAGAACTCGTCAGG",0,NA,NA,NA,"GeneID:439","ASNA1",TRUE,"ASNA1"

"12322","TGAACTGTTCGCTGACTGAG","chr19",12747276,"-","none","CGG","TGAACTGTTCGCTGACTGAGCGG",0,NA,NA,NA,"GeneID:439","ASNA1",TRUE,"ASNA1"

"12323","AAACTGTGAAGAACAACCTC","chr7",97858923,"-","none","AGG","AAACTGTGAAGAACAACCTCAGG",0,NA,NA,NA,"GeneID:440","ASNS",TRUE,"ASNS"

"12324","AAACTGTGAAGAACAACCTC","chr8",46595748,"-","16:T>G","CGG","AAACTGTGAAGAACAAACTCCGG",1,17,NA,NA,"","",TRUE,"ASNS"

"12325","AAACTGTGAAGAACAACCTC","chr13",100063091,"-","12:C>T","TGG","AAACTGTGAAGAGCAACCTCTGG",1,13,NA,NA,"","",TRUE,"ASNS"

"12326","AACGTTTGATGACAGACAGA","chr7",97858875,"-","none","AGG","AACGTTTGATGACAGACAGAAGG",0,NA,NA,NA,"GeneID:440","ASNS",TRUE,"ASNS"

"12327","ATTTGAATACCAGACCAAAG","chr14_GL000194v1_random",44061,"+","none","TGG","ATTTGAATACCAGACCAAAGTGG",0,NA,NA,NA,"","",FALSE,"ASNS"

"12328","ATTTGAATACCAGACCAAAG","chr21",9058839,"+","none","TGG","ATTTGAATACCAGACCAAAGTGG",0,NA,NA,NA,"","",TRUE,"ASNS"

"12329","ATTTGAATACCAGACCAAAG","chrUn_GL000218v1",29287,"+","none","TGG","ATTTGAATACCAGACCAAAGTGG",0,NA,NA,NA,"","",FALSE,"ASNS"

"12330","ATTTGAATACCAGACCAAAG","chrY",11144269,"+","none","TGG","ATTTGAATACCAGACCAAAGTGG",0,NA,NA,NA,"","",TRUE,"ASNS"

"12331","ATTTGAATACCAGACCAAAG","chr9",64060160,"+","none","TGG","ATTTGAATACCAGACCAAAGTGG",0,NA,NA,NA,"","",FALSE,"ASNS"

"12332","ATTTGAATACCAGACCAAAG","chr2",94887059,"-","none","TGG","ATTTGAATACCAGACCAAAGTGG",0,NA,NA,NA,"GeneID:442028","LOC442028",TRUE,"ASNS"

"12333","ATTTGAATACCAGACCAAAG","chr7",97864459,"-","none","TGG","ATTTGAATACCAGACCAAAGTGG",0,NA,NA,NA,"GeneID:440","ASNS",TRUE,"ASNS"

"12334","ATTTGAATACCAGACCAAAG","chrUn_KI270744v1",90252,"-","none","TGG","ATTTGAATACCAGACCAAAGTGG",0,NA,NA,NA,"","",FALSE,"ASNS"

"12335","ATTTGAATACCAGACCAAAG","chr8",46600637,"-","9:A>G,19:T>C","TGG","ATTTGAATATCAGACCAAAATGG",2,10,20,NA,"","",TRUE,"ASNS"

"12336","CCATGCCAATTGCAAATGTC","chr7",97858309,"+","none","TGG","CCATGCCAATTGCAAATGTCTGG",0,NA,NA,NA,"GeneID:440","ASNS",TRUE,"ASNS"

"12337","CCATGCCAATTGCAAATGTC","chr8",46595146,"+","11:A>G","TGG","CCATGCCAATTACAAATGTCTGG",1,12,NA,NA,"","",TRUE,"ASNS"

"12338","CCATGCCAATTGCAAATGTC","chr21",25016235,"-","4:T>C,14:C>T","AGG","CCATACCAATTGCAGATGTCAGG",2,5,15,NA,"GeneID:339622","LOC339622",FALSE,"ASNS"

"12339","CTCCATATGTATCTCTACCC","chr7",97864321,"+","none","AGG","CTCCATATGTATCTCTACCCAGG",0,NA,NA,NA,"GeneID:440","ASNS",TRUE,"ASNS"

"12340","CTCCATATGTATCTCTACCC","chr4",12872036,"-","14:A>G,18:A>G","TGG","CTCCATATGTATCTTTACTCTGG",2,15,19,NA,"","",FALSE,"ASNS"

"12341","CTCCATATGTATCTCTACCC","chr2",133353350,"+","0:T>C,8:C>G","TGG","TTCCATATCTATCTCTACCCTGG",2,1,9,NA,"GeneID:344148","NCKAP5",FALSE,"ASNS"

"12342","CTCCATATGTATCTCTACCC","chr9",64060298,"-","4:A>T,16:C>T","AGG","CTCCTTATGTATCTCTGCCCAGG",2,5,17,NA,"","",FALSE,"ASNS"

"12343","GGAAGACAGCCCCGATTTAC","chr7",97858291,"-","none","TGG","GGAAGACAGCCCCGATTTACTGG",0,NA,NA,NA,"GeneID:440","ASNS",TRUE,"ASNS"

"12344","GGAAGACAGCCCCGATTTAC","chr8",46595128,"-","12:A>G","TGG","GGAAGACAGCCCTGATTTACTGG",1,13,NA,NA,"","",TRUE,"ASNS"

"12345","ACCTCATAGGTAGTTCTCAC","chr2",189667754,"+","none","TGG","ACCTCATAGGTAGTTCTCACTGG",0,NA,NA,NA,"GeneID:54529","ASNSD1",TRUE,"ASNSD1"

"12346","AGTGTACCAGATCGAATCAC","chr2",189667335,"+","none","AGG","AGTGTACCAGATCGAATCACAGG",0,NA,NA,NA,"GeneID:54529","ASNSD1",TRUE,"ASNSD1"

"12347","CGTGTCCGCTTTCAGTCGCA","chr2",189667817,"+","none","TGG","CGTGTCCGCTTTCAGTCGCATGG",0,NA,NA,NA,"GeneID:54529","ASNSD1",TRUE,"ASNSD1"

"12348","GCAAACTTGACTTTACCCCG","chr2",189670501,"+","none","AGG","GCAAACTTGACTTTACCCCGAGG",0,NA,NA,NA,"GeneID:54529","ASNSD1",TRUE,"ASNSD1"

"12349","GCCCTTCCTGTGATTCGATC","chr2",189667344,"-","none","TGG","GCCCTTCCTGTGATTCGATCTGG",0,NA,NA,NA,"GeneID:54529","ASNSD1",TRUE,"ASNSD1"

"12350","TTCTAAATTCTCTGCCGATT","chr2",189670472,"+","none","TGG","TTCTAAATTCTCTGCCGATTTGG",0,NA,NA,NA,"GeneID:54529","ASNSD1",TRUE,"ASNSD1"

"12351","AACGAGTGGTCGCATATTTG","chr17",3483551,"-","none","AGG","AACGAGTGGTCGCATATTTGAGG",0,NA,NA,NA,"GeneID:84690;GeneID:443","SPATA22;ASPA",TRUE,"ASPA"

"12352","AAGAGTGCACCCCATGTTAG","chr17",3481724,"-","none","AGG","AAGAGTGCACCCCATGTTAGAGG",0,NA,NA,NA,"GeneID:84690;GeneID:443","SPATA22;ASPA",TRUE,"ASPA"

"12353","ATTTCCTCCCTGCGCCATTG","chr17",3494356,"+","none","AGG","ATTTCCTCCCTGCGCCATTGAGG",0,NA,NA,NA,"GeneID:84690;GeneID:443","SPATA22;ASPA",TRUE,"ASPA"

"12354","ATTTCCTCCCTGCGCCATTG","chr4",30007938,"+","12:A>C,14:A>C","GGG","ATTTCCTCCCTGAGACATTGGGG",2,13,15,NA,"","",TRUE,"ASPA"

"12355","CTCAATCAGATAAACGTAGC","chr17",3483520,"-","none","AGG","CTCAATCAGATAAACGTAGCAGG",0,NA,NA,NA,"GeneID:84690;GeneID:443","SPATA22;ASPA",TRUE,"ASPA"

"12356","TAGAGAAAGTTGATTACCCC","chr17",3494391,"+","none","CGG","TAGAGAAAGTTGATTACCCCCGG",0,NA,NA,NA,"GeneID:84690;GeneID:443","SPATA22;ASPA",TRUE,"ASPA"

"12357","TCACAACACCACCTCTAACA","chr17",3481710,"+","none","TGG","TCACAACACCACCTCTAACATGG",0,NA,NA,NA,"GeneID:84690;GeneID:443","SPATA22;ASPA",TRUE,"ASPA"

"12358","ACGATGGGGGACAGAGTGAA","chr19",50514492,"-","none","AGG","ACGATGGGGGACAGAGTGAAAGG",0,NA,NA,NA,"GeneID:554235","ASPDH",TRUE,"ASPDH"

"12359","AGGAGGCTTCTCAGCAGAGA","chr19",50511719,"+","none","TGG","AGGAGGCTTCTCAGCAGAGATGG",0,NA,NA,NA,"GeneID:554235","ASPDH",TRUE,"ASPDH"

"12360","AGGAGGCTTCTCAGCAGAGA","chr9",119229586,"+","14:A>C,19:G>A","TGG","AGGAGGCTTCTCAGAAGAGGTGG",2,15,20,NA,"GeneID:1620","BRINP1",FALSE,"ASPDH"

"12361","AGGAGGCTTCTCAGCAGAGA","chr6",43888934,"-","0:C>T,7:T>A","TGG","GGGAGGCATCTCAGCAGAGATGG",2,1,8,NA,"","",FALSE,"ASPDH"

"12362","AGGAGGCTTCTCAGCAGAGA","chr4",141004404,"+","5:A>G,6:T>C","TGG","AGGAGATTTCTCAGCAGAGATGG",2,6,7,NA,"GeneID:57484","RNF150",FALSE,"ASPDH"

"12363","AGGAGGCTTCTCAGCAGAGA","chr1",86549428,"+","6:A>C,11:G>C","AGG","AGGAGGATTCTGAGCAGAGAAGG",2,7,12,NA,"GeneID:22802","CLCA4",FALSE,"ASPDH"

"12364","AGGAGGCTTCTCAGCAGAGA","chr2",98907506,"-","6:C>G,11:C>G","AGG","AGGAGGGTTCTGAGCAGAGAAGG",2,7,12,NA,"GeneID:343990","KIAA1211L",FALSE,"ASPDH"

"12365","CAATCACCCCATCGAAGCCC","chr19",50512381,"+","none","AGG","CAATCACCCCATCGAAGCCCAGG",0,NA,NA,NA,"GeneID:554235","ASPDH",TRUE,"ASPDH"

"12366","CACTCAGGGCCATGGCCGAC","chr19",50513814,"-","none","AGG","CACTCAGGGCCATGGCCGACAGG",0,NA,NA,NA,"GeneID:554235","ASPDH",TRUE,"ASPDH"

"12367","CACTCAGGGCCATGGCCGAC","chr20",7885520,"+","7:A>G,17:A>G","CGG","CACTCAGAGCCATGGCCAACCGG",2,8,18,NA,"GeneID:54363","HAO1",TRUE,"ASPDH"

"12368","GTTGGAATTTCGCGGGGCAA","chr19",50512436,"+","none","AGG","GTTGGAATTTCGCGGGGCAAAGG",0,NA,NA,NA,"GeneID:554235","ASPDH",TRUE,"ASPDH"

"12369","TCCCCGGCCTGGAGGGGAGC","chr19",50511743,"+","none","TGG","TCCCCGGCCTGGAGGGGAGCTGG",0,NA,NA,NA,"GeneID:554235","ASPDH",TRUE,"ASPDH"

"12370","CCCACACTCACGGTAGCACC","chr14",104092732,"-","none","AGG","CCCACACTCACGGTAGCACCAGG",0,NA,NA,NA,"GeneID:374569","ASPG",TRUE,"ASPG"

"12371","GAACCTCCGAGCGTCTACCT","chr14",104098901,"-","none","TGG","GAACCTCCGAGCGTCTACCTTGG",0,NA,NA,NA,"GeneID:374569","ASPG",TRUE,"ASPG"

"12372","GCGGTCCCTACCAGGGCGGC","chr14",104103666,"-","none","AGG","GCGGTCCCTACCAGGGCGGCAGG",0,NA,NA,NA,"GeneID:374569","ASPG",TRUE,"ASPG"

"12373","GGCCTGCTGCGCCTCTACCC","chr14",104103636,"+","none","TGG","GGCCTGCTGCGCCTCTACCCTGG",0,NA,NA,NA,"GeneID:374569","ASPG",TRUE,"ASPG"

"12374","GGGCAACCAAGGTAGACGCT","chr14",104098892,"+","none","CGG","GGGCAACCAAGGTAGACGCTCGG",0,NA,NA,NA,"GeneID:374569","ASPG",TRUE,"ASPG"

"12375","GTGCTCCTCGTCATGGAACA","chr14",104092680,"-","none","TGG","GTGCTCCTCGTCATGGAACATGG",0,NA,NA,NA,"GeneID:374569","ASPG",TRUE,"ASPG"

"12376","CAGGAACTTCATTCTTCACG","chr8",61684123,"-","none","TGG","CAGGAACTTCATTCTTCACGTGG",0,NA,NA,NA,"GeneID:444","ASPH",TRUE,"ASPH"

"12377","CTAGGAATCTATGATGCTGA","chr8",61681011,"-","none","TGG","CTAGGAATCTATGATGCTGATGG",0,NA,NA,NA,"GeneID:444","ASPH",TRUE,"ASPH"

"12378","CTGCAGAAAAACTCCGTAAA","chr8",61583946,"-","none","AGG","CTGCAGAAAAACTCCGTAAAAGG",0,NA,NA,NA,"GeneID:444","ASPH",TRUE,"ASPH"

"12379","GGACATCTGTAGCTGTCGTT","chr8",61684072,"-","none","TGG","GGACATCTGTAGCTGTCGTTTGG",0,NA,NA,NA,"GeneID:444","ASPH",TRUE,"ASPH"

"12380","GTACTTGCCTAATAAAACTT","chr8",61680959,"+","none","TGG","GTACTTGCCTAATAAAACTTTGG",0,NA,NA,NA,"GeneID:444","ASPH",TRUE,"ASPH"

"12381","GTACTTGCCTAATAAAACTT","chr6",84581950,"+","2:T>A,13:G>A","TGG","GTTCTTGCCTAATGAAACTTTGG",2,3,14,NA,"","",FALSE,"ASPH"

"12382","TCAAATTTATTTAAAAGTTT","chr8",61583992,"+","none","AGG","TCAAATTTATTTAAAAGTTTAGG",0,NA,NA,NA,"GeneID:444","ASPH",TRUE,"ASPH"

"12383","TCAAATTTATTTAAAAGTTT","chrX",1122803,"-","15:G>T,16:G>C","GGG","TCAAATTTATTTAAACCTTTGGG",2,16,17,NA,"","",FALSE,"ASPH"

"12384","TCAAATTTATTTAAAAGTTT","chr10",67115705,"-","0:T>A,8:G>T","TGG","ACAAATTTCTTTAAAAGTTTTGG",2,1,9,NA,"GeneID:29119","CTNNA3",FALSE,"ASPH"

"12385","TCAAATTTATTTAAAAGTTT","chr2",148768259,"-","1:T>G,8:A>T","TGG","TAAAATTTTTTTAAAAGTTTTGG",2,2,9,NA,"GeneID:26122","EPC2",FALSE,"ASPH"

"12386","TCAAATTTATTTAAAAGTTT","chrX",105028486,"+","1:T>C,8:T>A","TGG","TTAAATTTTTTTAAAAGTTTTGG",2,2,9,NA,"GeneID:26280","IL1RAPL2",FALSE,"ASPH"

"12387","TCAAATTTATTTAAAAGTTT","chr14",61690715,"+","3:T>A,16:T>G","TGG","TCATATTTATTTAAAATTTTTGG",2,4,17,NA,"GeneID:100750246","HIF1A-AS1",FALSE,"ASPH"

"12388","ACAGGCCTAAAGATCCCTCC","chr16",29904847,"+","none","TGG","ACAGGCCTAAAGATCCCTCCTGG",0,NA,NA,NA,"GeneID:253982","ASPHD1",TRUE,"ASPHD1"

"12389","GCCTCGAGTGGTCTTCATCG","chr16",29905803,"+","none","TGG","GCCTCGAGTGGTCTTCATCGTGG",0,NA,NA,NA,"GeneID:253982;GeneID:253980","ASPHD1;KCTD13",TRUE,"ASPHD1"

"12390","GGAGCTCCACGTCGTGCCGC","chr16",29901588,"-","none","TGG","GGAGCTCCACGTCGTGCCGCTGG",0,NA,NA,NA,"GeneID:253982","ASPHD1",TRUE,"ASPHD1"

"12391","TCCACGATGAAGACCACTCG","chr16",29905807,"-","none","AGG","TCCACGATGAAGACCACTCGAGG",0,NA,NA,NA,"GeneID:253982;GeneID:253980","ASPHD1;KCTD13",TRUE,"ASPHD1"

"12392","TCGGCGGTGAGCCCCAGTGC","chr16",29904884,"+","none","TGG","TCGGCGGTGAGCCCCAGTGCTGG",0,NA,NA,NA,"GeneID:253982","ASPHD1",TRUE,"ASPHD1"

"12393","TTGCGTAGGCCCGAAGCCGC","chr16",29901414,"-","none","CGG","TTGCGTAGGCCCGAAGCCGCCGG",0,NA,NA,NA,"GeneID:253982","ASPHD1",TRUE,"ASPHD1"

"12394","CCACGGACGTACCGCTTGCT","chr22",26434356,"+","none","CGG","CCACGGACGTACCGCTTGCTCGG",0,NA,NA,NA,"GeneID:57168","ASPHD2",TRUE,"ASPHD2"

"12395","CCCCCAAGATGTCGCTCGAG","chr22",26433685,"+","none","TGG","CCCCCAAGATGTCGCTCGAGTGG",0,NA,NA,NA,"GeneID:57168","ASPHD2",TRUE,"ASPHD2"

"12396","CTGCATGCTGCGTTCCATGA","chr22",26442550,"+","none","AGG","CTGCATGCTGCGTTCCATGAAGG",0,NA,NA,NA,"GeneID:57168","ASPHD2",TRUE,"ASPHD2"

"12397","CTTCATGGAACGCAGCATGC","chr22",26442552,"-","none","AGG","CTTCATGGAACGCAGCATGCAGG",0,NA,NA,NA,"GeneID:57168","ASPHD2",TRUE,"ASPHD2"

"12398","CTTCATGGAACGCAGCATGC","chr17",17553029,"+","10:A>C,11:C>G","AGG","CTTCATGGAAACCAGCATGCAGG",2,11,12,NA,"GeneID:10400","PEMT",FALSE,"ASPHD2"

"12399","CTTCATGGAACGCAGCATGC","chr3",188403143,"+","9:G>A,10:G>C","GGG","CTTCATGGAGGGCAGCATGCGGG",2,10,11,NA,"GeneID:4026","LPP",FALSE,"ASPHD2"

"12400","GGGAAATACTCTCATCGTCC","chr22",26443197,"-","none","CGG","GGGAAATACTCTCATCGTCCCGG",0,NA,NA,NA,"GeneID:57168","ASPHD2",TRUE,"ASPHD2"

"12401","GTGGCATCCAAACGTCGCAG","chr22",26443139,"+","none","CGG","GTGGCATCCAAACGTCGCAGCGG",0,NA,NA,NA,"GeneID:57168","ASPHD2",TRUE,"ASPHD2"

"12402","AAACACCAAGCTATATTACT","chr1",197143987,"-","none","AGG","AAACACCAAGCTATATTACTAGG",0,NA,NA,NA,"GeneID:259266","ASPM",TRUE,"ASPM"

"12403","AAACACCAAGCTATATTACT","chr6",5031675,"+","6:A>C,12:C>A","TGG","AAACACAAAGCTCTATTACTTGG",2,7,13,NA,"GeneID:100129461","LOC100129461",FALSE,"ASPM"

"12404","ATGTCATAATCTCTCTTACT","chr1",197144027,"+","none","CGG","ATGTCATAATCTCTCTTACTCGG",0,NA,NA,NA,"GeneID:259266","ASPM",TRUE,"ASPM"

"12405","GGGAAGTGAGCCCGACCGAG","chr1",197146386,"-","none","CGG","GGGAAGTGAGCCCGACCGAGCGG",0,NA,NA,NA,"GeneID:259266","ASPM",TRUE,"ASPM"

"12406","TCGAATCTGCCATGGCGAAC","chr1",197146428,"-","none","CGG","TCGAATCTGCCATGGCGAACCGG",0,NA,NA,NA,"GeneID:259266","ASPM",TRUE,"ASPM"

"12407","TGCTCTCCAACATCTATACC","chr1",197096074,"+","none","AGG","TGCTCTCCAACATCTATACCAGG",0,NA,NA,NA,"GeneID:259266","ASPM",TRUE,"ASPM"

"12408","TTACCGTGTTCTCTCTAGTT","chr1",197095993,"+","none","TGG","TTACCGTGTTCTCTCTAGTTTGG",0,NA,NA,NA,"GeneID:259266","ASPM",TRUE,"ASPM"

"12409","ACGACATTGGCCCCATCAAA","chr2",69961353,"+","none","AGG","ACGACATTGGCCCCATCAAAAGG",0,NA,NA,NA,"GeneID:151516","ASPRV1",TRUE,"ASPRV1"

"12410","ATGTGCGGTGCTCAAAGTCC","chr2",69960781,"+","none","AGG","ATGTGCGGTGCTCAAAGTCCAGG",0,NA,NA,NA,"GeneID:151516","ASPRV1",TRUE,"ASPRV1"

"12411","CAAAGACGATCTCTTTGGGC","chr2",69961123,"+","none","AGG","CAAAGACGATCTCTTTGGGCAGG",0,NA,NA,NA,"GeneID:151516","ASPRV1",TRUE,"ASPRV1"

"12412","CGGCACTCGCATTGGCCACT","chr2",69960850,"+","none","AGG","CGGCACTCGCATTGGCCACTAGG",0,NA,NA,NA,"GeneID:151516","ASPRV1",TRUE,"ASPRV1"

"12413","GTAAGGGCTACTATCTCAAG","chr2",69961091,"-","none","GGG","GTAAGGGCTACTATCTCAAGGGG",0,NA,NA,NA,"GeneID:151516","ASPRV1",TRUE,"ASPRV1"

"12414","TCATCAATGACCTCAACCAT","chr2",69961304,"-","none","TGG","TCATCAATGACCTCAACCATTGG",0,NA,NA,NA,"GeneID:151516","ASPRV1",TRUE,"ASPRV1"

"12415","TCATCAATGACCTCAACCAT","chr5",108414444,"-","12:T>A,16:T>G","CGG","TCATCAATGACCACAAACATCGG",2,13,17,NA,"","",FALSE,"ASPRV1"

"12416","ACTGCAGCGTCGTGCCCCGC","chr17",81996011,"-","none","AGG","ACTGCAGCGTCGTGCCCCGCAGG",0,NA,NA,NA,"GeneID:79058","ASPSCR1",TRUE,"ASPSCR1"

"12417","AGGTTCTGGAGGACACGTGC","chr17",81979181,"+","none","CGG","AGGTTCTGGAGGACACGTGCCGG",0,NA,NA,NA,"GeneID:79058","ASPSCR1",TRUE,"ASPSCR1"

"12418","AGGTTCTGGAGGACACGTGC","chr18",66239458,"-","11:A>C,15:A>G","AGG","AGGTTCTGGAGTACATGTGCAGG",2,12,16,NA,"","",FALSE,"ASPSCR1"

"12419","AGGTTCTGGAGGACACGTGC","chr6",74380570,"+","11:T>G,15:T>C","AGG","AGGTTCTGGAGTACATGTGCAGG",2,12,16,NA,"GeneID:101928516","LOC101928516",FALSE,"ASPSCR1"

"12420","AGGTTCTGGAGGACACGTGC","chr2",229660139,"+","1:A>G,6:A>T","AGG","AAGTTCAGGAGGACACGTGCAGG",2,2,7,NA,"GeneID:92737","DNER",FALSE,"ASPSCR1"

"12421","AGGTTCTGGAGGACACGTGC","chr2",181759555,"+","1:A>G,11:T>G","AGG","AAGTTCTGGAGTACACGTGCAGG",2,2,12,NA,"","",FALSE,"ASPSCR1"

"12422","AGGTTCTGGAGGACACGTGC","chr5",21873645,"+","1:A>G,15:T>C","AGG","AAGTTCTGGAGGACATGTGCAGG",2,2,16,NA,"GeneID:1010","CDH12",FALSE,"ASPSCR1"

"12423","AGGTTCTGGAGGACACGTGC","chr10",91253199,"+","6:A>T,15:T>C","AGG","AGGTTCAGGAGGACATGTGCAGG",2,7,16,NA,"GeneID:84333","PCGF5",FALSE,"ASPSCR1"

"12424","AGGTTCTGGAGGACACGTGC","chr2",240757804,"+","6:C>T,16:C>G","AGG","AGGTTCCGGAGGACACCTGCAGG",2,7,17,NA,"GeneID:547","KIF1A",FALSE,"ASPSCR1"

"12425","AGGTTCTGGAGGACACGTGC","chr2",181604283,"-","9:C>T,11:A>C","AGG","AGGTTCTGGGGTACACGTGCAGG",2,10,12,NA,"GeneID:375298","CERKL",FALSE,"ASPSCR1"

"12426","AGGTTCTGGAGGACACGTGC","chr18",76560828,"-","6:T>A,11:A>C","AGG","AGGTTCAGGAGTACACGTGCAGG",2,7,12,NA,"GeneID:284276","LINC00908",FALSE,"ASPSCR1"

"12427","AGGTTCTGGAGGACACGTGC","chr5",114619306,"-","6:T>A,11:A>C","AGG","AGGTTCAGGAGTACACGTGCAGG",2,7,12,NA,"","",FALSE,"ASPSCR1"

"12428","AGGTTCTGGAGGACACGTGC","chr3",98976046,"-","1:T>C,11:A>C","AGG","AAGTTCTGGAGTACACGTGCAGG",2,2,12,NA,"","",FALSE,"ASPSCR1"

"12429","GCACGACCCACCATGTTCTC","chr17",81983659,"-","none","AGG","GCACGACCCACCATGTTCTCAGG",0,NA,NA,NA,"GeneID:79058","ASPSCR1",TRUE,"ASPSCR1"

"12430","GCACGACCCACCATGTTCTC","chr6",149918365,"-","4:G>C,16:G>A","AGG","GCACCACCCACCATGTCCTCAGG",2,5,17,NA,"GeneID:100652739;GeneID:353091","RAET1E-AS1;RAET1G",TRUE,"ASPSCR1"

"12431","GCCCGCTTCCCGGAGCCGTG","chr17",81983631,"+","none","AGG","GCCCGCTTCCCGGAGCCGTGAGG",0,NA,NA,NA,"GeneID:79058","ASPSCR1",TRUE,"ASPSCR1"

"12432","GGCACGACGCTGCAGTCGCT","chr17",81996015,"+","none","GGG","GGCACGACGCTGCAGTCGCTGGG",0,NA,NA,NA,"GeneID:79058","ASPSCR1",TRUE,"ASPSCR1"

"12433","TCACTTCAGATCATATTCAC","chr17",81979223,"-","none","AGG","TCACTTCAGATCATATTCACAGG",0,NA,NA,NA,"GeneID:79058","ASPSCR1",TRUE,"ASPSCR1"

"12434","AGCGCAGTTTGCAGCAGCTA","chr11",62357018,"+","none","TGG","AGCGCAGTTTGCAGCAGCTATGG",0,NA,NA,NA,"GeneID:80150","ASRGL1",TRUE,"ASRGL1"

"12435","CTGCAAACTGCGCTGCGCCT","chr11",62357012,"-","none","TGG","CTGCAAACTGCGCTGCGCCTTGG",0,NA,NA,NA,"GeneID:80150","ASRGL1",TRUE,"ASRGL1"

"12436","GACAAGCCGAGCAAGTTTAA","chr11",62356438,"-","none","TGG","GACAAGCCGAGCAAGTTTAATGG",0,NA,NA,NA,"GeneID:80150","ASRGL1",TRUE,"ASRGL1"

"12437","GACACGGTGAGTCCCCAACG","chr11",62389228,"-","none","CGG","GACACGGTGAGTCCCCAACGCGG",0,NA,NA,NA,"GeneID:80150","ASRGL1",TRUE,"ASRGL1"

"12438","GGTTATGCCGACAATGACAT","chr11",62391529,"+","none","CGG","GGTTATGCCGACAATGACATCGG",0,NA,NA,NA,"GeneID:80150","ASRGL1",TRUE,"ASRGL1"

"12439","TTTATTAACGATACCGCCTG","chr11",62389197,"-","none","TGG","TTTATTAACGATACCGCCTGTGG",0,NA,NA,NA,"GeneID:80150","ASRGL1",TRUE,"ASRGL1"

"12440","AAGCTTCAGTGCCTTCTTCC","chr9",130454341,"-","none","TGG","AAGCTTCAGTGCCTTCTTCCTGG",0,NA,NA,NA,"GeneID:445","ASS1",TRUE,"ASS1"

"12441","AAGCTTCAGTGCCTTCTTCC","chr9",78178403,"-","none","TGG","AAGCTTCAGTGCCTTCTTCCTGG",0,NA,NA,NA,"","",TRUE,"ASS1"

"12442","AAGCTTCAGTGCCTTCTTCC","chrX",115665036,"-","none","TGG","AAGCTTCAGTGCCTTCTTCCTGG",0,NA,NA,NA,"","",TRUE,"ASS1"

"12443","AAGCTTCAGTGCCTTCTTCC","chr5",145228954,"-","none","TGG","AAGCTTCAGTGCCTTCTTCCTGG",0,NA,NA,NA,"","",TRUE,"ASS1"

"12444","AAGCTTCAGTGCCTTCTTCC","chr9",32946137,"-","none","AGG","AAGCTTCAGTGCCTTCTTCCAGG",0,NA,NA,NA,"","",TRUE,"ASS1"

"12445","AAGCTTCAGTGCCTTCTTCC","chr4",22388543,"-","12:C>G,19:C>G","TGG","AAGCTTCAGTGCGTTCTTCGTGG",2,13,20,NA,"GeneID:166647","GPR125",TRUE,"ASS1"

"12446","AAGCTTCAGTGCCTTCTTCC","chr11",107176427,"-","1:C>T","TGG","AGGCTTCAGTGCCTTCTTCCTGG",1,2,NA,NA,"","",TRUE,"ASS1"

"12447","AAGCTTCAGTGCCTTCTTCC","chr6",25024319,"+","3:T>C","TGG","AAGTTTCAGTGCCTTCTTCCTGG",1,4,NA,NA,"GeneID:9750","FAM65B",TRUE,"ASS1"

"12448","AAGCTTCAGTGCCTTCTTCC","chr2",38811492,"+","3:G>C","TGG","AAGGTTCAGTGCCTTCTTCCTGG",1,4,NA,NA,"GeneID:90957","DHX57",TRUE,"ASS1"

"12449","AAGCTTCAGTGCCTTCTTCC","chr1",2321438,"+","1:G>A,9:G>T","AGG","AGGCTTCAGGGCCTTCTTCCAGG",2,2,10,NA,"GeneID:79906","MORN1",TRUE,"ASS1"

"12450","AAGCTTCAGTGCCTTCTTCC","chr5",53859307,"-","2:T>C,12:T>G","TGG","AAACTTCAGTGCATTCTTCCTGG",2,3,13,NA,"","",TRUE,"ASS1"

"12451","CCATGCTCATTTAGACATCG","chr9",130489366,"+","none","AGG","CCATGCTCATTTAGACATCGAGG",0,NA,NA,NA,"GeneID:445","ASS1",TRUE,"ASS1"

"12452","CCATGCTCATTTAGACATCG","chr5",145229687,"+","19:A>G","AGG","CCATGCTCATTTAGACATCAAGG",1,20,NA,NA,"","",TRUE,"ASS1"

"12453","CCATGCTCATTTAGACATCG","chrX",115665767,"+","19:A>G","GGG","CCATGCTCATTTAGACATCAGGG",1,20,NA,NA,"","",TRUE,"ASS1"

"12454","CCATGCTCATTTAGACATCG","chr11",107177158,"+","1:T>C,3:C>T","AGG","CTACGCTCATTTAGACATCGAGG",2,2,4,NA,"","",TRUE,"ASS1"

"12455","CCATGCTCATTTAGACATCG","chr12",32986703,"+","3:C>T,19:A>G","AGG","CCACGCTCATTTAGACATCAAGG",2,4,20,NA,"","",TRUE,"ASS1"

"12456","CCATGCTCATTTAGACATCG","chr9",78179145,"+","3:C>T,19:A>G","AGG","CCACGCTCATTTAGACATCAAGG",2,4,20,NA,"","",TRUE,"ASS1"

"12457","CCATGCTCATTTAGACATCG","chr11",133429818,"+","0:G>C,19:T>G","AGG","GCATGCTCATTTAGACATCTAGG",2,1,20,NA,"GeneID:4978","OPCML",FALSE,"ASS1"

"12458","CCATGCTCATTTAGACATCG","chr3",177297508,"-","3:G>A,18:A>G","AGG","CCACGCTCATTTAGACATTGAGG",2,4,19,NA,"GeneID:100820709","LINC00501",TRUE,"ASS1"

"12459","CCATGCTCATTTAGACATCG","chrY",11922879,"-","1:A>G,13:G>C","AGG","CTATGCTCATTTACACATCGAGG",2,2,14,NA,"","",TRUE,"ASS1"

"12460","CCCGGCCGAGGATGTACACC","chr9",130494966,"-","none","TGG","CCCGGCCGAGGATGTACACCTGG",0,NA,NA,NA,"GeneID:445","ASS1",TRUE,"ASS1"

"12461","CCCGGCCGAGGATGTACACC","chr12",32986901,"-","0:T>G","TGG","ACCGGCCGAGGATGTACACCTGG",1,1,NA,NA,"","",TRUE,"ASS1"

"12462","CCCGGCCGAGGATGTACACC","chr9",78179343,"-","3:T>C,6:A>G","TGG","CCCAGCTGAGGATGTACACCTGG",2,4,7,NA,"","",TRUE,"ASS1"

"12463","CCCGGCCGAGGATGTACACC","chr5",53860232,"-","2:A>G,7:T>C","TGG","CCTGGCCAAGGATGTACACCTGG",2,3,8,NA,"","",TRUE,"ASS1"

"12464","CCCGGCCGAGGATGTACACC","chr5",145229885,"-","3:T>C,7:T>C","TGG","CCCAGCCAAGGATGTACACCTGG",2,4,8,NA,"","",TRUE,"ASS1"

"12465","CCCGGCCGAGGATGTACACC","chr11",107177364,"-","3:T>C,7:T>C","TGG","CCCAGCCAAGGATGTACACCTGG",2,4,8,NA,"","",TRUE,"ASS1"

"12466","CCCGGCCGAGGATGTACACC","chrX",115665965,"-","2:A>G,7:A>C","AGG","CCTGGCCTAGGATGTACACCAGG",2,3,8,NA,"","",TRUE,"ASS1"

"12467","CCCGGCCGAGGATGTACACC","chr7",21220357,"+","3:A>G","TGG","CCCAGCCGAGGATGTACACCTGG",1,4,NA,NA,"","",TRUE,"ASS1"

"12468","CCCGGCCGAGGATGTACACC","chr2",38810586,"+","2:T>C,6:A>C","TGG","CCTGGCAGAGGATGTACACCTGG",2,3,7,NA,"GeneID:90957","DHX57",TRUE,"ASS1"

"12469","CCCGGCCGAGGATGTACACC","chr6",25023402,"+","3:A>G,7:A>G","CGG","CCCAGCCAAGGATGTACACCCGG",2,4,8,NA,"GeneID:9750","FAM65B",TRUE,"ASS1"

"12470","CCCGGCCGAGGATGTACACC","chr4",148526638,"+","6:T>C,7:A>G","TGG","CCCGGCTAAGGATGTACACCTGG",2,7,8,NA,"","",TRUE,"ASS1"

"12471","CCCGGCCGAGGATGTACACC","chr9",32947063,"-","4:T>C,13:T>C","TGG","CCCGACCGAGGATATACACCTGG",2,5,14,NA,"","",TRUE,"ASS1"

"12472","CCTCGATGTCTAAATGAGCA","chr9",130489369,"-","none","TGG","CCTCGATGTCTAAATGAGCATGG",0,NA,NA,NA,"GeneID:445","ASS1",TRUE,"ASS1"

"12473","CCTCGATGTCTAAATGAGCA","chr5",145229690,"-","3:A>G","TGG","CCTTGATGTCTAAATGAGCATGG",1,4,NA,NA,"","",TRUE,"ASS1"

"12474","CCTCGATGTCTAAATGAGCA","chrX",115665770,"-","2:G>A,3:A>G","TGG","CCCTGATGTCTAAATGAGCATGG",2,3,4,NA,"","",TRUE,"ASS1"

"12475","CCTCGATGTCTAAATGAGCA","chr7",21220552,"+","0:T>C,19:G>A","TGG","TCTCGATGTCTAAATGAGCGTGG",2,1,20,NA,"","",TRUE,"ASS1"

"12476","CCTCGATGTCTAAATGAGCA","chr3",177297505,"+","4:A>G,19:G>A","TGG","CCTCAATGTCTAAATGAGCGTGG",2,5,20,NA,"GeneID:100820709","LINC00501",TRUE,"ASS1"

"12477","CCTCGATGTCTAAATGAGCA","chr9",78179148,"-","3:A>G,19:C>T","TGG","CCTTGATGTCTAAATGAGCGTGG",2,4,20,NA,"","",TRUE,"ASS1"

"12478","CCTCGATGTCTAAATGAGCA","chr12",32986706,"-","3:A>G,19:C>T","TGG","CCTTGATGTCTAAATGAGCGTGG",2,4,20,NA,"","",TRUE,"ASS1"

"12479","GCGAGTGGAAGGGAAAGTGC","chr9",130494924,"+","none","AGG","GCGAGTGGAAGGGAAAGTGCAGG",0,NA,NA,NA,"GeneID:445","ASS1",TRUE,"ASS1"

"12480","GCGAGTGGAAGGGAAAGTGC","chr4",148526680,"-","18:T>C","AGG","GCGAGTGGAAGGGAAAGTACAGG",1,19,NA,NA,"","",TRUE,"ASS1"

"12481","GCGAGTGGAAGGGAAAGTGC","chr7",21220399,"-","10:T>C,18:T>C","AGG","GCGAGTGGAAAGGAAAGTACAGG",2,11,19,NA,"","",TRUE,"ASS1"

"12482","GCGAGTGGAAGGGAAAGTGC","chr2",38810628,"-","0:G>C","AGG","CCGAGTGGAAGGGAAAGTGCAGG",1,1,NA,NA,"GeneID:90957","DHX57",TRUE,"ASS1"

"12483","GCGAGTGGAAGGGAAAGTGC","chrY",11922723,"-","1:A>G","AGG","GTGAGTGGAAGGGAAAGTGCAGG",1,2,NA,NA,"","",TRUE,"ASS1"

"12484","GCGAGTGGAAGGGAAAGTGC","chr3",177297352,"-","0:T>C,1:A>G","AGG","ATGAGTGGAAGGGAAAGTGCAGG",2,1,2,NA,"GeneID:100820709","LINC00501",TRUE,"ASS1"

"12485","GCGAGTGGAAGGGAAAGTGC","chrX",115665923,"+","1:T>C","AGG","GTGAGTGGAAGGGAAAGTGCAGG",1,2,NA,NA,"","",TRUE,"ASS1"

"12486","GCGAGTGGAAGGGAAAGTGC","chr11",107177322,"+","1:T>C","AGG","GTGAGTGGAAGGGAAAGTGCAGG",1,2,NA,NA,"","",TRUE,"ASS1"

"12487","GCGAGTGGAAGGGAAAGTGC","chr9",32947021,"+","0:A>G,1:T>C","AGG","ATGAGTGGAAGGGAAAGTGCAGG",2,1,2,NA,"","",TRUE,"ASS1"

"12488","GCGAGTGGAAGGGAAAGTGC","chr5",53860190,"+","2:A>G","AGG","GCAAGTGGAAGGGAAAGTGCAGG",1,3,NA,NA,"","",TRUE,"ASS1"

"12489","GCGAGTGGAAGGGAAAGTGC","chrX",3368174,"+","2:T>G","AGG","GCTAGTGGAAGGGAAAGTGCAGG",1,3,NA,NA,"","",TRUE,"ASS1"

"12490","GCGAGTGGAAGGGAAAGTGC","chr12",32986859,"+","2:C>G","AGG","GCCAGTGGAAGGGAAAGTGCAGG",1,3,NA,NA,"","",TRUE,"ASS1"

"12491","GCGAGTGGAAGGGAAAGTGC","chr5",128395792,"+","2:A>G,3:T>A","AGG","GCATGTGGAAGGGAAAGTGCAGG",2,3,4,NA,"GeneID:2201","FBN2",FALSE,"ASS1"

"12492","GCGAGTGGAAGGGAAAGTGC","chr1",32728953,"+","2:T>G,4:A>G","TGG","GCTAATGGAAGGGAAAGTGCTGG",2,3,5,NA,"","",FALSE,"ASS1"

"12493","GCGAGTGGAAGGGAAAGTGC","chr9",78179301,"+","6:A>G","AGG","GCGAGTAGAAGGGAAAGTGCAGG",1,7,NA,NA,"","",TRUE,"ASS1"

"12494","TCAGGCCAACATTGGCCAGA","chr9",130454300,"+","none","AGG","TCAGGCCAACATTGGCCAGAAGG",0,NA,NA,NA,"GeneID:445","ASS1",TRUE,"ASS1"

"12495","TCAGGCCAACATTGGCCAGA","chr4",136811648,"+","17:T>A","AGG","TCAGGCCAACATTGGCCTGAAGG",1,18,NA,NA,"","",FALSE,"ASS1"

"12496","TCAGGCCAACATTGGCCAGA","chr11",45098287,"-","0:G>A,1:T>G","GGG","CAAGGCCAACATTGGCCAGAGGG",2,1,2,NA,"GeneID:56981","PRDM11",FALSE,"ASS1"

"12497","TCAGGCCAACATTGGCCAGA","chrY",11923648,"-","0:G>A,2:A>T","AGG","CCTGGCCAACATTGGCCAGAAGG",2,1,3,NA,"","",TRUE,"ASS1"

"12498","TCAGGCCAACATTGGCCAGA","chr7",21221307,"-","0:G>A,2:A>T","AGG","CCTGGCCAACATTGGCCAGAAGG",2,1,3,NA,"","",TRUE,"ASS1"

"12499","TCAGGCCAACATTGGCCAGA","chr5",53859266,"+","0:C>T,2:T>A","AGG","CCTGGCCAACATTGGCCAGAAGG",2,1,3,NA,"","",TRUE,"ASS1"

"12500","TCAGGCCAACATTGGCCAGA","chr14",98069666,"-","8:G>T,11:G>A","AGG","TCAGGCCACCACTGGCCAGAAGG",2,9,12,NA,"","",FALSE,"ASS1"

"12501","ATTGCCATAATCAACAGCCC","chr3",131018507,"-","none","TGG","ATTGCCATAATCAACAGCCCTGG",0,NA,NA,NA,"GeneID:28990","ASTE1",TRUE,"ASTE1"

"12502","CCACATAGCACCGCAAAGAG","chr3",131024638,"+","none","AGG","CCACATAGCACCGCAAAGAGAGG",0,NA,NA,NA,"GeneID:28990","ASTE1",TRUE,"ASTE1"

"12503","GCTGGCATAGTCCGTGCACC","chr3",131014349,"+","none","AGG","GCTGGCATAGTCCGTGCACCAGG",0,NA,NA,NA,"GeneID:27032;GeneID:28990","ATP2C1;ASTE1",TRUE,"ASTE1"

"12504","GGTGCTTACCGAGTTAGGTC","chr3",131016134,"+","none","TGG","GGTGCTTACCGAGTTAGGTCTGG",0,NA,NA,NA,"GeneID:27032;GeneID:28990","ATP2C1;ASTE1",TRUE,"ASTE1"

"12505","GGTGCTTACCGAGTTAGGTC","chr2",160578783,"+","10:T>G,12:A>G","AGG","GGTGCTTACCTAATTAGGTCAGG",2,11,13,NA,"","",FALSE,"ASTE1"

"12506","TATGAGGGAAACAACCGGTT","chr3",131014114,"-","none","TGG","TATGAGGGAAACAACCGGTTTGG",0,NA,NA,NA,"GeneID:27032;GeneID:28990","ATP2C1;ASTE1",TRUE,"ASTE1"

"12507","TTTACCAGAATGGGTATTAG","chr3",131024315,"-","none","TGG","TTTACCAGAATGGGTATTAGTGG",0,NA,NA,NA,"GeneID:28990","ASTE1",TRUE,"ASTE1"

"12508","AGAGAGCAGCTTCCTCATCG","chr2",96135370,"-","none","AGG","AGAGAGCAGCTTCCTCATCGAGG",0,NA,NA,NA,"GeneID:431705","ASTL",TRUE,"ASTL"

"12509","AGAGAGCAGCTTCCTCATCG","chr10",90598850,"-","17:T>A,18:A>G","TGG","AGAGAGCAGCTTCCTCAATGTGG",2,18,19,NA,"","",FALSE,"ASTL"

"12510","CAAACTCCGCAAGAGCCTCC","chr2",96133497,"+","none","AGG","CAAACTCCGCAAGAGCCTCCAGG",0,NA,NA,NA,"GeneID:431705","ASTL",TRUE,"ASTL"

"12511","CATGGGTGGTAGTGGTGTCG","chr2",96133997,"-","none","TGG","CATGGGTGGTAGTGGTGTCGTGG",0,NA,NA,NA,"GeneID:431705","ASTL",TRUE,"ASTL"

"12512","CCCTCACCTTGGTTAATTGC","chr2",96137567,"+","none","AGG","CCCTCACCTTGGTTAATTGCAGG",0,NA,NA,NA,"GeneID:431705","ASTL",TRUE,"ASTL"

"12513","TGCTCACTCACCGTACTTGC","chr2",96133953,"+","none","TGG","TGCTCACTCACCGTACTTGCTGG",0,NA,NA,NA,"GeneID:431705","ASTL",TRUE,"ASTL"

"12514","TTGAACGTTCCACGTGCATC","chr2",96133480,"-","none","AGG","TTGAACGTTCCACGTGCATCAGG",0,NA,NA,NA,"GeneID:431705","ASTL",TRUE,"ASTL"

"12515","AGTCGAGGGTGCGCGTGACC","chr1",177032500,"+","none","TGG","AGTCGAGGGTGCGCGTGACCTGG",0,NA,NA,NA,"GeneID:460","ASTN1",TRUE,"ASTN1"

"12516","ATATCCCTTTGGTGCGCTGG","chr1",177061224,"-","none","AGG","ATATCCCTTTGGTGCGCTGGAGG",0,NA,NA,NA,"GeneID:460","ASTN1",TRUE,"ASTN1"

"12517","TATTATAGCCACTAGCCCTG","chr1",177030886,"-","none","TGG","TATTATAGCCACTAGCCCTGTGG",0,NA,NA,NA,"GeneID:460","ASTN1",TRUE,"ASTN1"

"12518","TGATGATCCTGTATACTCGC","chr1",177032789,"-","none","CGG","TGATGATCCTGTATACTCGCCGG",0,NA,NA,NA,"GeneID:460","ASTN1",TRUE,"ASTN1"

"12519","TGCTGCCTCCAGCGCACCAA","chr1",177061216,"+","none","AGG","TGCTGCCTCCAGCGCACCAAAGG",0,NA,NA,NA,"GeneID:460","ASTN1",TRUE,"ASTN1"

"12520","TGTTGTTGATCCGCTTTCTC","chr1",177030814,"+","none","TGG","TGTTGTTGATCCGCTTTCTCTGG",0,NA,NA,NA,"GeneID:460","ASTN1",TRUE,"ASTN1"

"12521","CACGTCTCCATGAGCAGCTC","chr9",117291416,"-","none","CGG","CACGTCTCCATGAGCAGCTCCGG",0,NA,NA,NA,"GeneID:23245","ASTN2",TRUE,"ASTN2"

"12522","CCGGATGGGCACGCCAATGA","chr9",117214517,"+","none","CGG","CCGGATGGGCACGCCAATGACGG",0,NA,NA,NA,"GeneID:23245","ASTN2",TRUE,"ASTN2"

"12523","CTGCTCCTCAACAATCTCCG","chr9",117291352,"+","none","AGG","CTGCTCCTCAACAATCTCCGAGG",0,NA,NA,NA,"GeneID:23245","ASTN2",TRUE,"ASTN2"

"12524","GACGCTTCTGCCAACGTCGC","chr9",117214663,"+","none","TGG","GACGCTTCTGCCAACGTCGCTGG",0,NA,NA,NA,"GeneID:23245","ASTN2",TRUE,"ASTN2"

"12525","GCTCGTTACGCACAAAGAGC","chr9",117414559,"+","none","AGG","GCTCGTTACGCACAAAGAGCAGG",0,NA,NA,NA,"GeneID:23245","ASTN2",TRUE,"ASTN2"

"12526","GTGCCGGCTGAAGACCGTCA","chr9",117414733,"-","none","CGG","GTGCCGGCTGAAGACCGTCACGG",0,NA,NA,NA,"GeneID:23245","ASTN2",TRUE,"ASTN2"

"12527","AATGCTTGAAGACTGTGTCC","chr12",26928250,"-","none","AGG","AATGCTTGAAGACTGTGTCCAGG",0,NA,NA,NA,"GeneID:55726","ASUN",TRUE,"ASUN"

"12528","AATGCTTGAAGACTGTGTCC","chr11",127651553,"+","10:C>G,16:T>G","TGG","AATGCTTGAACACTGTTTCCTGG",2,11,17,NA,"","",FALSE,"ASUN"

"12529","AATGCTTGAAGACTGTGTCC","chr9",27450466,"-","5:G>A,9:C>T","AGG","AATGCCTGAGGACTGTGTCCAGG",2,6,10,NA,"GeneID:79817","MOB3B",FALSE,"ASUN"

"12530","AATGCTTGAAGACTGTGTCC","chr17",73358584,"+","2:G>T,11:G>A","TGG","AAGGCTTGAAGGCTGTGTCCTGG",2,3,12,NA,"GeneID:54549","SDK2",FALSE,"ASUN"

"12531","ATGGCAGCATTAGCCGCTGT","chr12",26928882,"-","none","TGG","ATGGCAGCATTAGCCGCTGTTGG",0,NA,NA,NA,"GeneID:55726","ASUN",TRUE,"ASUN"

"12532","CTTCACCAACTGGGTAGGTG","chr12",26925792,"+","none","TGG","CTTCACCAACTGGGTAGGTGTGG",0,NA,NA,NA,"GeneID:55726","ASUN",TRUE,"ASUN"

"12533","GTAGAGTACGAGCCTCATGT","chr12",26928766,"+","none","TGG","GTAGAGTACGAGCCTCATGTTGG",0,NA,NA,NA,"GeneID:55726","ASUN",TRUE,"ASUN"

"12534","TATGTTCATGAATCGTTTCC","chr12",26928229,"+","none","TGG","TATGTTCATGAATCGTTTCCTGG",0,NA,NA,NA,"GeneID:55726","ASUN",TRUE,"ASUN"

"12535","TTGATCCACACCTACCCAGT","chr12",26925800,"-","none","TGG","TTGATCCACACCTACCCAGTTGG",0,NA,NA,NA,"GeneID:55726","ASUN",TRUE,"ASUN"

"12536","CACTGCTGCTGAGACGCAAC","chr20",32431599,"-","none","AGG","CACTGCTGCTGAGACGCAACAGG",0,NA,NA,NA,"GeneID:171023","ASXL1",TRUE,"ASXL1"

"12537","CCTCACCAGGCGCGCGGCCT","chr20",32358818,"-","none","CGG","CCTCACCAGGCGCGCGGCCTCGG",0,NA,NA,NA,"GeneID:171023","ASXL1",TRUE,"ASXL1"

"12538","GTGGGCCGAGGCCGCGCGCC","chr20",32358810,"+","none","TGG","GTGGGCCGAGGCCGCGCGCCTGG",0,NA,NA,NA,"GeneID:171023","ASXL1",TRUE,"ASXL1"

"12539","TCAGAGCTGGCGGGAGCGCC","chr20",32431650,"+","none","TGG","TCAGAGCTGGCGGGAGCGCCTGG",0,NA,NA,NA,"GeneID:171023","ASXL1",TRUE,"ASXL1"

"12540","TCAGAGCTGGCGGGAGCGCC","chr2",11130378,"+","16:G>C,18:G>C","AGG","TCAGAGCTGGCGGGAGGGGCAGG",2,17,19,NA,"GeneID:285150","FLJ33534",FALSE,"ASXL1"

"12541","TCCACATCAGCCGTGTCCTC","chr20",32428181,"-","none","TGG","TCCACATCAGCCGTGTCCTCTGG",0,NA,NA,NA,"GeneID:171023","ASXL1",TRUE,"ASXL1"

"12542","TCCACATCAGCCGTGTCCTC","chr14",75539621,"-","0:C>A,15:G>A","AGG","GCCACATCAGCCGTGCCCTCAGG",2,1,16,NA,"GeneID:10538","BATF",FALSE,"ASXL1"

"12543","TCGCCATCCAGCTACAGTGG","chr20",32428147,"+","none","AGG","TCGCCATCCAGCTACAGTGGAGG",0,NA,NA,NA,"GeneID:171023","ASXL1",TRUE,"ASXL1"

"12544","AAGAATTTCTTTATGACTCA","chr2",25845515,"+","none","TGG","AAGAATTTCTTTATGACTCATGG",0,NA,NA,NA,"GeneID:55252","ASXL2",TRUE,"ASXL2"

"12545","AAGAATTTCTTTATGACTCA","chr8",131617228,"+","2:C>G,8:A>C","TGG","AACAATTTATTTATGACTCATGG",2,3,9,NA,"","",FALSE,"ASXL2"

"12546","AAGAATTTCTTTATGACTCA","chr10",91441716,"+","7:G>T,19:T>A","GGG","AAGAATTGCTTTATGACTCTGGG",2,8,20,NA,"GeneID:143279","HECTD2",FALSE,"ASXL2"

"12547","AAGAATTTCTTTATGACTCA","chr10",91447880,"-","7:C>A,19:A>T","GGG","AAGAATTGCTTTATGACTCTGGG",2,8,20,NA,"GeneID:143279","HECTD2",FALSE,"ASXL2"

"12548","CACCGCGGGAAAAGCAGCGC","chr2",25878292,"-","none","AGG","CACCGCGGGAAAAGCAGCGCAGG",0,NA,NA,NA,"GeneID:55252","ASXL2",TRUE,"ASXL2"

"12549","CTTCAAGTTATCCAGAGAGA","chr2",25845498,"-","none","AGG","CTTCAAGTTATCCAGAGAGAAGG",0,NA,NA,NA,"GeneID:55252","ASXL2",TRUE,"ASXL2"

"12550","GACAGCTCTTTCACCCCATC","chr2",25799503,"+","none","CGG","GACAGCTCTTTCACCCCATCCGG",0,NA,NA,NA,"GeneID:55252","ASXL2",TRUE,"ASXL2"

"12551","GACAGCTCTTTCACCCCATC","chr3",45763191,"-","13:T>G,19:C>G","TGG","GACAGCTCTTTCAACCCATGTGG",2,14,20,NA,"GeneID:54716","SLC6A20",FALSE,"ASXL2"

"12552","GACAGCTCTTTCACCCCATC","chr3",128283050,"+","0:C>G,12:T>A","AGG","CACAGCTCTTTCTCCCCATCAGG",2,1,13,NA,"GeneID:60678","EEFSEC",FALSE,"ASXL2"

"12553","GGGGTGAAAGAGCTGTCAGA","chr2",25799500,"-","none","AGG","GGGGTGAAAGAGCTGTCAGAAGG",0,NA,NA,NA,"GeneID:55252","ASXL2",TRUE,"ASXL2"

"12554","GGGGTGAAAGAGCTGTCAGA","chr3",135531961,"+","7:G>A,18:T>G","GGG","GGGGTGAGAGAGCTGTCATAGGG",2,8,19,NA,"","",FALSE,"ASXL2"

"12555","TCAGTTCAAGACCCGACATG","chr2",25878219,"-","none","AGG","TCAGTTCAAGACCCGACATGAGG",0,NA,NA,NA,"GeneID:55252","ASXL2",TRUE,"ASXL2"

"12556","AAATCCAACGTGCCATCTGC","chr18",33646265,"-","none","TGG","AAATCCAACGTGCCATCTGCTGG",0,NA,NA,NA,"GeneID:80816","ASXL3",TRUE,"ASXL3"

"12557","AATATCTGCTTTGCTGTCAT","chr18",33607620,"-","none","TGG","AATATCTGCTTTGCTGTCATTGG",0,NA,NA,NA,"GeneID:80816","ASXL3",TRUE,"ASXL3"

"12558","AATATCTGCTTTGCTGTCAT","chr10",129122926,"-","16:T>A,18:A>T","TGG","AATATCTGCTTTGCTGACTTTGG",2,17,19,NA,"","",FALSE,"ASXL3"

"12559","AATGACAGCAAAGCAGATAT","chr18",33607619,"+","none","TGG","AATGACAGCAAAGCAGATATTGG",0,NA,NA,NA,"GeneID:80816","ASXL3",TRUE,"ASXL3"

"12560","ATGGCCGAGGCAAATGCCCA","chr18",33646316,"+","none","TGG","ATGGCCGAGGCAAATGCCCATGG",0,NA,NA,NA,"GeneID:80816","ASXL3",TRUE,"ASXL3"

"12561","CTTCACACTAACACTCGAAT","chr18",33644927,"+","none","AGG","CTTCACACTAACACTCGAATAGG",0,NA,NA,NA,"GeneID:80816","ASXL3",TRUE,"ASXL3"

"12562","GTGAAGCATTGCATTCAGAC","chr18",33644913,"-","none","AGG","GTGAAGCATTGCATTCAGACAGG",0,NA,NA,NA,"GeneID:80816","ASXL3",TRUE,"ASXL3"

"12563","CCTCCGCTACCTGAGACGTC","chr7",117427345,"+","none","CGG","CCTCCGCTACCTGAGACGTCCGG",0,NA,NA,NA,"GeneID:136991","ASZ1",TRUE,"ASZ1"

"12564","CTGATCCAAATGTTGCTTGT","chr7",117420164,"-","none","AGG","CTGATCCAAATGTTGCTTGTAGG",0,NA,NA,NA,"GeneID:136991","ASZ1",TRUE,"ASZ1"

"12565","GGGAGATTGGGTATCTCGAC","chr7",117427367,"-","none","CGG","GGGAGATTGGGTATCTCGACCGG",0,NA,NA,NA,"GeneID:136991","ASZ1",TRUE,"ASZ1"

"12566","GTCCAGGAGCTCCTAGATTC","chr7",117426837,"-","none","TGG","GTCCAGGAGCTCCTAGATTCTGG",0,NA,NA,NA,"GeneID:136991","ASZ1",TRUE,"ASZ1"

"12567","GTCCAGGAGCTCCTAGATTC","chr14",95787619,"+","12:T>C,16:G>A","AGG","GTCCAGGAGCTCTTAGGTTCAGG",2,13,17,NA,"","",FALSE,"ASZ1"

"12568","GTCCAGGAGCTCCTAGATTC","chr14",95787527,"+","12:T>C,16:G>A","AGG","GTCCAGGAGCTCTTAGGTTCAGG",2,13,17,NA,"","",FALSE,"ASZ1"

"12569","TCTTACCTACAAGCAACATT","chr7",117420156,"+","none","TGG","TCTTACCTACAAGCAACATTTGG",0,NA,NA,NA,"GeneID:136991","ASZ1",TRUE,"ASZ1"

"12570","TCTTACCTACAAGCAACATT","chr10",14205108,"+","9:A>C,16:G>C","TGG","TCTTACCTAAAAGCAAGATTTGG",2,10,17,NA,"GeneID:55691","FRMD4A",FALSE,"ASZ1"

"12571","TCTTACCTACAAGCAACATT","chr2",24883621,"+","9:T>C,18:C>T","GGG","TCTTACCTATAAGCAACACTGGG",2,10,19,NA,"GeneID:109","ADCY3",FALSE,"ASZ1"

"12572","TCTTACCTACAAGCAACATT","chr6",133353394,"-","9:C>G,16:T>G","TGG","TCTTACCTAGAAGCAAAATTTGG",2,10,17,NA,"GeneID:2070","EYA4",FALSE,"ASZ1"

"12573","TCTTACCTACAAGCAACATT","chr8",90665586,"-","9:A>G,19:G>A","TGG","TCTTACCTATAAGCAACATCTGG",2,10,20,NA,"GeneID:169200","TMEM64",FALSE,"ASZ1"

"12574","TTTAAGAAAGCAATGACCAT","chr7",117426873,"-","none","CGG","TTTAAGAAAGCAATGACCATCGG",0,NA,NA,NA,"GeneID:136991","ASZ1",TRUE,"ASZ1"

"12575","TTTAAGAAAGCAATGACCAT","chr11",40665291,"-","18:G>T,19:G>A","AGG","TTTAAGAAAGCAATGACCCCAGG",2,19,20,NA,"GeneID:57689","LRRC4C",FALSE,"ASZ1"

"12576","ATGGTTGTAGCTTTATGGCA","chr10",87784493,"+","none","AGG","ATGGTTGTAGCTTTATGGCAAGG",0,NA,NA,NA,"GeneID:84896","ATAD1",TRUE,"ATAD1"

"12577","ATGGTTGTAGCTTTATGGCA","chr2",220910098,"-","4:T>A,5:C>A","AGG","ATGGAGGTAGCTTTATGGCAAGG",2,5,6,NA,"","",FALSE,"ATAD1"

"12578","ATGGTTGTAGCTTTATGGCA","chr18",1362997,"+","1:C>T,2:A>G","AGG","ACAGTTGTAGCTTTATGGCAAGG",2,2,3,NA,"","",TRUE,"ATAD1"

"12579","CATAAACTGAGCTTTCATCA","chr10",87776359,"+","none","TGG","CATAAACTGAGCTTTCATCATGG",0,NA,NA,NA,"GeneID:84896","ATAD1",TRUE,"ATAD1"

"12580","CATAAACTGAGCTTTCATCA","chr18",1362904,"+","18:T>C","TGG","CATAAACTGAGCTTTCATTATGG",1,19,NA,NA,"","",TRUE,"ATAD1"

"12581","CTTCATGGTCAGAACTTGAA","chr10",87776388,"+","none","CGG","CTTCATGGTCAGAACTTGAACGG",0,NA,NA,NA,"GeneID:84896","ATAD1",TRUE,"ATAD1"

"12582","CTTCATGGTCAGAACTTGAA","chr7",39156944,"+","11:A>G,17:T>G","GGG","CTTCATGGTCAAAACTTTAAGGG",2,12,18,NA,"GeneID:11281","POU6F2",FALSE,"ATAD1"

"12583","CTTCATGGTCAGAACTTGAA","chr21",15359786,"+","0:G>C,12:C>A","GGG","GTTCATGGTCAGCACTTGAAGGG",2,1,13,NA,"","",FALSE,"ATAD1"

"12584","CTTCATGGTCAGAACTTGAA","chr1",224090722,"-","0:T>G,12:C>T","AGG","ATTCATGGTCAGGACTTGAAAGG",2,1,13,NA,"","",FALSE,"ATAD1"

"12585","CTTCGACACTGACCGATAAG","chr10",87784557,"-","none","TGG","CTTCGACACTGACCGATAAGTGG",0,NA,NA,NA,"GeneID:84896","ATAD1",TRUE,"ATAD1"

"12586","CTTCGACACTGACCGATAAG","chr18",1363061,"-","14:T>C","TGG","CTTCGACACTGACCAATAAGTGG",1,15,NA,NA,"","",TRUE,"ATAD1"

"12587","GAAAAACTAATGAAGCAAAT","chr10",87792732,"-","none","TGG","GAAAAACTAATGAAGCAAATTGG",0,NA,NA,NA,"GeneID:84896","ATAD1",TRUE,"ATAD1"

"12588","GAAAAACTAATGAAGCAAAT","chr18",1363688,"-","16:A>T","TGG","GAAAAACTAATGAAGCTAATTGG",1,17,NA,NA,"","",TRUE,"ATAD1"

"12589","GAAAAACTAATGAAGCAAAT","chr6",81968129,"-","16:G>T,18:G>T","GGG","GAAAAACTAATGAAGCCACTGGG",2,17,19,NA,"","",FALSE,"ATAD1"

"12590","GAAAAACTAATGAAGCAAAT","chr17",58936092,"+","15:A>C,16:G>A","CGG","GAAAAACTAATGAAGAGAATCGG",2,16,17,NA,"GeneID:22843","PPM1E",FALSE,"ATAD1"

"12591","GAAAAACTAATGAAGCAAAT","chr4",147123460,"+","7:A>T,12:G>A","GGG","GAAAAACAAATGGAGCAAATGGG",2,8,13,NA,"","",FALSE,"ATAD1"

"12592","GTTACTTGGAGTGATATAGC","chr10",87790410,"-","none","AGG","GTTACTTGGAGTGATATAGCAGG",0,NA,NA,NA,"GeneID:84896","ATAD1",TRUE,"ATAD1"

"12593","GTTACTTGGAGTGATATAGC","chrX",137738822,"-","8:G>C,19:T>G","TGG","GTTACTTGCAGTGATATAGATGG",2,9,20,NA,"","",FALSE,"ATAD1"

"12594","AAAAAAGAAGAGCACAGAGA","chr8",123372638,"-","none","AGG","AAAAAAGAAGAGCACAGAGAAGG",0,NA,NA,NA,"GeneID:29028","ATAD2",TRUE,"ATAD2"

"12595","AAAAAAGAAGAGCACAGAGA","chr1",68616405,"-","11:T>C,12:C>G","TGG","AAAAAAGAAGAAGACAGAGATGG",2,12,13,NA,"","",FALSE,"ATAD2"

"12596","AAAAAAGAAGAGCACAGAGA","chr17",75041364,"+","14:G>C,19:G>A","TGG","AAAAAAGAAGAGCAGAGAGGTGG",2,15,20,NA,"GeneID:23510;GeneID:10476","KCTD2;ATP5H",FALSE,"ATAD2"

"12597","AAAAAAGAAGAGCACAGAGA","chr16",81246862,"+","12:T>C,19:C>A","TGG","AAAAAAGAAGAGTACAGAGCTGG",2,13,20,NA,"GeneID:53630","BCO1",FALSE,"ATAD2"

"12598","AAAAAAGAAGAGCACAGAGA","chr3",28083519,"-","5:A>T,9:A>C","AGG","AAAAATGAATAGCACAGAGAAGG",2,6,10,NA,"","",FALSE,"ATAD2"

"12599","AAAAAAGAAGAGCACAGAGA","chr4",112687132,"+","6:A>G","AGG","AAAAAAAAAGAGCACAGAGAAGG",1,7,NA,NA,"","",FALSE,"ATAD2"

"12600","AAAAAAGAAGAGCACAGAGA","chr12",2201218,"+","2:T>A,10:G>A","AGG","AATAAAGAAGGGCACAGAGAAGG",2,3,11,NA,"GeneID:775","CACNA1C",FALSE,"ATAD2"

"12601","AAAAAAGAAGAGCACAGAGA","chr3",5014824,"+","5:G>A,14:G>C","TGG","AAAAAGGAAGAGCAGAGAGATGG",2,6,15,NA,"","",FALSE,"ATAD2"

"12602","AAAAAAGAAGAGCACAGAGA","chr8",75217020,"+","9:A>G,18:A>G","AGG","AAAAAAGAAAAGCACAGAAAAGG",2,10,19,NA,"","",FALSE,"ATAD2"

"12603","AAAAAAGAAGAGCACAGAGA","chr11",36746273,"-","9:G>C,18:A>C","TGG","AAAAAAGAACAGCACAGATATGG",2,10,19,NA,"","",FALSE,"ATAD2"

"12604","AAAAAAGAAGAGCACAGAGA","chr18",10390569,"-","8:C>T,19:A>T","GGG","AAAAAAGAGGAGCACAGAGTGGG",2,9,20,NA,"","",FALSE,"ATAD2"

"12605","AAAAAAGAAGAGCACAGAGA","chr1",227514325,"-","6:T>C,19:C>T","AGG","AAAAAAAAAGAGCACAGAGGAGG",2,7,20,NA,"","",FALSE,"ATAD2"

"12606","AAAAAAGAAGAGCACAGAGA","chr1",242070814,"-","6:T>C,11:T>C","AGG","AAAAAAAAAGAACACAGAGAAGG",2,7,12,NA,"","",FALSE,"ATAD2"

"12607","AAAAAAGAAGAGCACAGAGA","chr1",247499138,"-","6:A>C,11:T>C","AGG","AAAAAATAAGAACACAGAGAAGG",2,7,12,NA,"","",FALSE,"ATAD2"

"12608","AAAAAAGAAGAGCACAGAGA","chr5",109809342,"-","5:A>T,16:T>C","GGG","AAAAATGAAGAGCACAAAGAGGG",2,6,17,NA,"GeneID:4124","MAN2A1",FALSE,"ATAD2"

"12609","AAAAAAGAAGAGCACAGAGA","chr5",14675262,"-","4:G>T,18:G>C","AGG","AAAACAGAAGAGCACAGACAAGG",2,5,19,NA,"GeneID:90268","OTULIN",FALSE,"ATAD2"

"12610","AAAAAAGAAGAGCACAGAGA","chr4",118411620,"-","0:A>T,14:T>G","GGG","TAAAAAGAAGAGCAAAGAGAGGG",2,1,15,NA,"","",FALSE,"ATAD2"

"12611","AAAAAAGAAGAGCACAGAGA","chr12",93142333,"-","0:C>T,14:T>G","AGG","GAAAAAGAAGAGCAAAGAGAAGG",2,1,15,NA,"","",FALSE,"ATAD2"

"12612","AATCTTAATATGTACACAAG","chr8",123370967,"-","none","AGG","AATCTTAATATGTACACAAGAGG",0,NA,NA,NA,"GeneID:29028","ATAD2",TRUE,"ATAD2"

"12613","AATGAGAGAACTTGAAGACT","chr8",123371261,"-","none","TGG","AATGAGAGAACTTGAAGACTTGG",0,NA,NA,NA,"GeneID:29028","ATAD2",TRUE,"ATAD2"

"12614","AATGAGAGAACTTGAAGACT","chr7",113013558,"-","3:G>C,6:A>T","TGG","AATCAGTGAACTTGAAGACTTGG",2,4,7,NA,"","",FALSE,"ATAD2"

"12615","GAAACAACTGATAATCAAGA","chr8",123370913,"-","none","AGG","GAAACAACTGATAATCAAGAAGG",0,NA,NA,NA,"GeneID:29028","ATAD2",TRUE,"ATAD2"

"12616","GAAACAACTGATAATCAAGA","chr12",90274144,"+","11:A>T,16:T>A","GGG","GAAACAACTGAAAATCTAGAGGG",2,12,17,NA,"","",FALSE,"ATAD2"

"12617","GAAACAACTGATAATCAAGA","chr8",47067274,"+","10:G>A,13:T>A","GGG","GAAACAACTGGTATTCAAGAGGG",2,11,14,NA,"","",FALSE,"ATAD2"

"12618","GAAACAACTGATAATCAAGA","chr11",125559989,"+","10:G>A,13:T>A","AGG","GAAACAACTGGTATTCAAGAAGG",2,11,14,NA,"","",FALSE,"ATAD2"

"12619","GAAACAACTGATAATCAAGA","chr4",141981823,"+","10:G>A,13:T>A","AGG","GAAACAACTGGTATTCAAGAAGG",2,11,14,NA,"","",FALSE,"ATAD2"

"12620","TAGGCAGTTGGCCAGACAGC","chr8",123372669,"-","none","AGG","TAGGCAGTTGGCCAGACAGCAGG",0,NA,NA,NA,"GeneID:29028","ATAD2",TRUE,"ATAD2"

"12621","TAGGCAGTTGGCCAGACAGC","chr11",79047741,"+","7:G>T,13:T>A","TGG","TAGGCAGGTGGCCTGACAGCTGG",2,8,14,NA,"GeneID:26011","TENM4",FALSE,"ATAD2"

"12622","TGAAGCTGTACTTCAAAAAA","chr8",123371312,"-","none","TGG","TGAAGCTGTACTTCAAAAAATGG",0,NA,NA,NA,"GeneID:29028","ATAD2",TRUE,"ATAD2"

"12623","TGAAGCTGTACTTCAAAAAA","chr7",125094710,"-","8:C>A,16:G>T","GGG","TGAAGCTGGACTTCAACAAAGGG",2,9,17,NA,"GeneID:401398","POT1-AS1",FALSE,"ATAD2"

"12624","ACATTACCAAATGGACATAG","chr2",23888357,"-","none","TGG","ACATTACCAAATGGACATAGTGG",0,NA,NA,NA,"GeneID:54454","ATAD2B",TRUE,"ATAD2B"

"12625","GAGGAATGGAACTTATCAAC","chr2",23895830,"-","none","TGG","GAGGAATGGAACTTATCAACTGG",0,NA,NA,NA,"GeneID:54454","ATAD2B",TRUE,"ATAD2B"

"12626","GAGGAATGGAACTTATCAAC","chr4",25536315,"-","14:A>T,19:C>G","AGG","GAGGAATGGAACTTTTCAAGAGG",2,15,20,NA,"","",FALSE,"ATAD2B"

"12627","GGGGACCTTTCTTGTATAAA","chr2",23887924,"-","none","TGG","GGGGACCTTTCTTGTATAAATGG",0,NA,NA,NA,"GeneID:54454","ATAD2B",TRUE,"ATAD2B"

"12628","TCAGAAAAAGTTGTCGTTCC","chr2",23887890,"-","none","AGG","TCAGAAAAAGTTGTCGTTCCAGG",0,NA,NA,NA,"GeneID:54454","ATAD2B",TRUE,"ATAD2B"

"12629","TCTAGGTTAACTTCTCAGCC","chr2",23888384,"-","none","TGG","TCTAGGTTAACTTCTCAGCCTGG",0,NA,NA,NA,"GeneID:54454","ATAD2B",TRUE,"ATAD2B"

"12630","TCTAGGTTAACTTCTCAGCC","chr3",166297865,"-","4:A>C,9:A>T","TGG","TCTATGTTATCTTCTCAGCCTGG",2,5,10,NA,"","",FALSE,"ATAD2B"

"12631","TGTTTCAAAGTGCGTTTGGC","chr2",23895902,"+","none","TGG","TGTTTCAAAGTGCGTTTGGCTGG",0,NA,NA,NA,"GeneID:54454","ATAD2B",TRUE,"ATAD2B"

"12632","AGACAGCCTACCTCGCCGCA","chr1",1518981,"-","none","TGG","AGACAGCCTACCTCGCCGCATGG",0,NA,NA,NA,"GeneID:55210","ATAD3A",TRUE,"ATAD3A"

"12633","AGACAGCCTACCTCGCCGCA","chr1",1480927,"-","none","TGG","AGACAGCCTACCTCGCCGCATGG",0,NA,NA,NA,"GeneID:83858","ATAD3B",TRUE,"ATAD3A"

"12634","AGGACGGCTGGCACCTTGTT","chr1",1482542,"+","none","TGG","AGGACGGCTGGCACCTTGTTTGG",0,NA,NA,NA,"GeneID:83858","ATAD3B",TRUE,"ATAD3A"

"12635","AGGACGGCTGGCACCTTGTT","chr1",1520545,"+","none","TGG","AGGACGGCTGGCACCTTGTTTGG",0,NA,NA,NA,"GeneID:55210","ATAD3A",TRUE,"ATAD3A"

"12636","AGGACGGCTGGCACCTTGTT","chr1",1452362,"+","3:G>A","TGG","AGGGCGGCTGGCACCTTGTTTGG",1,4,NA,NA,"GeneID:219293","ATAD3C",TRUE,"ATAD3A"

"12637","GACGGTGCTCGGCCGCCTTC","chr1",1520264,"-","none","AGG","GACGGTGCTCGGCCGCCTTCAGG",0,NA,NA,NA,"GeneID:55210","ATAD3A",TRUE,"ATAD3A"

"12638","GACGGTGCTCGGCCGCCTTC","chr1",1482261,"-","12:T>G","AGG","GACGGTGCTCGGACGCCTTCAGG",1,13,NA,NA,"GeneID:83858","ATAD3B",TRUE,"ATAD3A"

"12639","TCACCTGATGGACTCCAAGA","chr1",1520290,"-","none","CGG","TCACCTGATGGACTCCAAGACGG",0,NA,NA,NA,"GeneID:55210","ATAD3A",TRUE,"ATAD3A"

"12640","TCACCTGATGGACTCCAAGA","chr1",1452106,"-","none","AGG","TCACCTGATGGACTCCAAGAAGG",0,NA,NA,NA,"GeneID:219293","ATAD3C",TRUE,"ATAD3A"

"12641","TCACCTGATGGACTCCAAGA","chr1",1482287,"-","none","CGG","TCACCTGATGGACTCCAAGACGG",0,NA,NA,NA,"GeneID:83858","ATAD3B",TRUE,"ATAD3A"

"12642","TTCTCAGCAACTTCTCAATG","chr1",1518913,"+","none","AGG","TTCTCAGCAACTTCTCAATGAGG",0,NA,NA,NA,"GeneID:55210","ATAD3A",TRUE,"ATAD3A"

"12643","TTCTCAGCAACTTCTCAATG","chr1",1480859,"+","none","AGG","TTCTCAGCAACTTCTCAATGAGG",0,NA,NA,NA,"GeneID:83858","ATAD3B",TRUE,"ATAD3A"

"12644","TTCTCAGCAACTTCTCAATG","chr1",1452038,"+","13:G>C","AGG","TTCTCAGCAACTTGTCAATGAGG",1,14,NA,NA,"GeneID:219293","ATAD3C",TRUE,"ATAD3A"

"12645","TTCTCAGCAACTTCTCAATG","chr19",19256415,"-","7:C>G,17:A>T","TGG","TTCTCAGGAACTTCTCATTGTGG",2,8,18,NA,"GeneID:404037","HAPLN4",TRUE,"ATAD3A"

"12646","TTCTCAGCAACTTCTCAATG","chr8",25180946,"-","1:G>A,16:G>T","TGG","TCCTCAGCAACTTCTCCATGTGG",2,2,17,NA,"","",FALSE,"ATAD3A"

"12647","TTTGTCCCAGTCTGTCACAA","chr1",1520585,"-","none","AGG","TTTGTCCCAGTCTGTCACAAAGG",0,NA,NA,NA,"GeneID:55210","ATAD3A",TRUE,"ATAD3A"

"12648","TTTGTCCCAGTCTGTCACAA","chr1",1482582,"-","8:C>T","AGG","TTTGTCCCGGTCTGTCACAAAGG",1,9,NA,NA,"GeneID:83858","ATAD3B",TRUE,"ATAD3A"

"12649","TTTGTCCCAGTCTGTCACAA","chr1",1452402,"-","8:C>T","AGG","TTTGTCCCGGTCTGTCACAAAGG",1,9,NA,NA,"GeneID:219293","ATAD3C",TRUE,"ATAD3A"

"12650","TTTGTCCCAGTCTGTCACAA","chr4",135839392,"+","3:T>G,13:T>G","AGG","TTTTTCCCAGTCTTTCACAAAGG",2,4,14,NA,"","",FALSE,"ATAD3A"

"12651","CAGAAACTCGCCCTGCACTC","chr1",1486540,"+","none","AGG","CAGAAACTCGCCCTGCACTCAGG",0,NA,NA,NA,"GeneID:83858","ATAD3B",TRUE,"ATAD3B"

"12652","CAGAAACTCGCCCTGCACTC","chr1",1524269,"+","none","AGG","CAGAAACTCGCCCTGCACTCAGG",0,NA,NA,NA,"GeneID:55210","ATAD3A",TRUE,"ATAD3B"

"12653","CAGAAACTCGCCCTGCACTC","chr1",1456221,"+","none","AGG","CAGAAACTCGCCCTGCACTCAGG",0,NA,NA,NA,"GeneID:219293","ATAD3C",TRUE,"ATAD3B"

"12654","CCTGCATCTGCGCCAGATTC","chr1",1450700,"-","none","AGG","CCTGCATCTGCGCCAGATTCAGG",0,NA,NA,NA,"GeneID:219293","ATAD3C",TRUE,"ATAD3B"

"12655","CCTGCATCTGCGCCAGATTC","chr1",1477292,"-","none","AGG","CCTGCATCTGCGCCAGATTCAGG",0,NA,NA,NA,"GeneID:83858","ATAD3B",TRUE,"ATAD3B"

"12656","CCTGCATCTGCGCCAGATTC","chr1",1516030,"-","10:A>G","AGG","CCTGCATCTGTGCCAGATTCAGG",1,11,NA,NA,"GeneID:55210","ATAD3A",TRUE,"ATAD3B"

"12657","CCTGCATCTGCGCCAGATTC","chr1",1503603,"-","10:A>G,15:G>C","AGG","CCTGCATCTGTGCCACATTCAGG",2,11,16,NA,"","",TRUE,"ATAD3B"

"12658","CCTGCATCTGCGCCAGATTC","chr12",131834051,"+","12:T>C,17:G>T","AGG","CCTGCATCTGCGTCAGAGTCAGG",2,13,18,NA,"GeneID:4326","MMP17",FALSE,"ATAD3B"

"12659","GACGTCTCACCCGCGCCGGC","chr1",1486658,"-","none","TGG","GACGTCTCACCCGCGCCGGCTGG",0,NA,NA,NA,"GeneID:83858","ATAD3B",TRUE,"ATAD3B"

"12660","GACGTCTCACCCGCGCCGGC","chr1",1524387,"-","none","TGG","GACGTCTCACCCGCGCCGGCTGG",0,NA,NA,NA,"GeneID:55210","ATAD3A",TRUE,"ATAD3B"

"12661","GACGTCTCACCCGCGCCGGC","chr1",1456339,"-","none","TGG","GACGTCTCACCCGCGCCGGCTGG",0,NA,NA,NA,"GeneID:219293","ATAD3C",TRUE,"ATAD3B"

"12662","GCGACATCGCCATAGCAACC","chr1",1486131,"+","none","AGG","GCGACATCGCCATAGCAACCAGG",0,NA,NA,NA,"GeneID:83858","ATAD3B",TRUE,"ATAD3B"

"12663","GCGACATCGCCATAGCAACC","chr1",1523860,"+","19:A>C","AGG","GCGACATCGCCATAGCAACAAGG",1,20,NA,NA,"GeneID:55210","ATAD3A",TRUE,"ATAD3B"

"12664","GTACAGGCCCCGGTTCTTCT","chr1",1486161,"-","none","TGG","GTACAGGCCCCGGTTCTTCTTGG",0,NA,NA,NA,"GeneID:83858","ATAD3B",TRUE,"ATAD3B"

"12665","GTACAGGCCCCGGTTCTTCT","chr1",1523890,"-","8:A>G,9:C>G","TGG","GTACAGGCTGCGGTTCTTCTTGG",2,9,10,NA,"GeneID:55210","ATAD3A",TRUE,"ATAD3B"

"12666","TGCGCCCGCAGGTTACGCCA","chr1",1477262,"+","none","AGG","TGCGCCCGCAGGTTACGCCAAGG",0,NA,NA,NA,"GeneID:83858","ATAD3B",TRUE,"ATAD3B"

"12667","CAACACCCTCCAGCACGTCC","chr1",1485810,"-","none","TGG","CAACACCCTCCAGCACGTCCTGG",0,NA,NA,NA,"GeneID:83858","ATAD3B",TRUE,"ATAD3C"

"12668","CAACACCCTCCAGCACGTCC","chr1",1455491,"-","none","TGG","CAACACCCTCCAGCACGTCCTGG",0,NA,NA,NA,"GeneID:219293","ATAD3C",TRUE,"ATAD3C"

"12669","CAACACCCTCCAGCACGTCC","chr1",1523539,"-","14:C>T","TGG","CAACACCCTCCAGCGCGTCCTGG",1,15,NA,NA,"GeneID:55210","ATAD3A",TRUE,"ATAD3C"

"12670","CAACACCCTCCAGCACGTCC","chr1",228287730,"-","2:G>T,16:G>C","AGG","CACCACCCTCCAGCACCTCCAGG",2,3,17,NA,"GeneID:84033","OBSCN",TRUE,"ATAD3C"

"12671","CCAGCCGAGCCTCGATGTAG","chr1",1454412,"-","none","CGG","CCAGCCGAGCCTCGATGTAGCGG",0,NA,NA,NA,"GeneID:219293","ATAD3C",TRUE,"ATAD3C"

"12672","CCAGCCGAGCCTCGATGTAG","chr1",1485083,"-","17:T>A","CGG","CCAGCCGAGCCTCGATGAAGCGG",1,18,NA,NA,"GeneID:83858","ATAD3B",TRUE,"ATAD3C"

"12673","CCAGCCGAGCCTCGATGTAG","chr1",1522811,"-","17:T>A","CGG","CCAGCCGAGCCTCGATGAAGCGG",1,18,NA,NA,"GeneID:55210","ATAD3A",TRUE,"ATAD3C"

"12674","CCGCTACATCGAGGCTCGGC","chr1",1454409,"+","none","TGG","CCGCTACATCGAGGCTCGGCTGG",0,NA,NA,NA,"GeneID:219293","ATAD3C",TRUE,"ATAD3C"

"12675","CCGCTACATCGAGGCTCGGC","chr1",1522808,"+","5:T>A","TGG","CCGCTTCATCGAGGCTCGGCTGG",1,6,NA,NA,"GeneID:55210","ATAD3A",TRUE,"ATAD3C"

"12676","CCGCTACATCGAGGCTCGGC","chr1",1485080,"+","5:T>A","TGG","CCGCTTCATCGAGGCTCGGCTGG",1,6,NA,NA,"GeneID:83858","ATAD3B",TRUE,"ATAD3C"

"12677","GCGACATCGCCATAATGACA","chr1",1455812,"+","none","AGG","GCGACATCGCCATAATGACAAGG",0,NA,NA,NA,"GeneID:219293","ATAD3C",TRUE,"ATAD3C"

"12678","TGCCTGGTGGCCCGTACAGC","chr1",1455873,"-","none","AGG","TGCCTGGTGGCCCGTACAGCAGG",0,NA,NA,NA,"GeneID:219293","ATAD3C",TRUE,"ATAD3C"

"12679","TGCCTGGTGGCCCGTACAGC","chr1",1523921,"-","18:A>C","AGG","TGCCTGGTGGCCCGTACATCAGG",1,19,NA,NA,"GeneID:55210","ATAD3A",TRUE,"ATAD3C"

"12680","TGCCTGGTGGCCCGTACAGC","chr1",1486192,"-","13:T>C","AGG","TGCCTGGTGGCCCATACAGCAGG",1,14,NA,NA,"GeneID:83858","ATAD3B",TRUE,"ATAD3C"

"12681","TTCCCCTCCAGCAGGTCAGC","chr1",1455448,"+","none","CGG","TTCCCCTCCAGCAGGTCAGCCGG",0,NA,NA,NA,"GeneID:219293","ATAD3C",TRUE,"ATAD3C"

"12682","TTCCCCTCCAGCAGGTCAGC","chr16",68402929,"-","13:T>C","AGG","TTCCCCTCCAGCAAGTCAGCAGG",1,14,NA,NA,"GeneID:55512","SMPD3",FALSE,"ATAD3C"

"12683","TTCCCCTCCAGCAGGTCAGC","chr1",1523496,"+","9:G>A","CGG","TTCCCCTCCGGCAGGTCAGCCGG",1,10,NA,NA,"GeneID:55210","ATAD3A",TRUE,"ATAD3C"

"12684","TTCCCCTCCAGCAGGTCAGC","chr1",1485767,"+","9:G>A","CGG","TTCCCCTCCGGCAGGTCAGCCGG",1,10,NA,NA,"GeneID:83858","ATAD3B",TRUE,"ATAD3C"

"12685","AGCTGCTCCGCCTCCCGTGA","chr17",30832376,"+","none","AGG","AGCTGCTCCGCCTCCCGTGAAGG",0,NA,NA,NA,"GeneID:79915","ATAD5",TRUE,"ATAD5"

"12686","CAAAGACTGTACGACACCTT","chr17",30834365,"+","none","TGG","CAAAGACTGTACGACACCTTTGG",0,NA,NA,NA,"GeneID:79915","ATAD5",TRUE,"ATAD5"

"12687","CTCAATCTCGCAGTCCTTCA","chr17",30832393,"-","none","CGG","CTCAATCTCGCAGTCCTTCACGG",0,NA,NA,NA,"GeneID:79915","ATAD5",TRUE,"ATAD5"

"12688","GAGAAATCTGAAGCAACTGA","chr17",30837266,"+","none","TGG","GAGAAATCTGAAGCAACTGATGG",0,NA,NA,NA,"GeneID:79915","ATAD5",TRUE,"ATAD5"

"12689","GAGAAATCTGAAGCAACTGA","chr20",61006469,"+","6:C>T,16:G>C","AGG","GAGAAACCTGAAGCAAGTGAAGG",2,7,17,NA,"","",FALSE,"ATAD5"

"12690","GAGAAATCTGAAGCAACTGA","chr7",34737071,"+","6:G>T,17:A>T","AGG","GAGAAAGCTGAAGCAACAGAAGG",2,7,18,NA,"GeneID:404744;GeneID:387129","NPSR1-AS1;NPSR1",FALSE,"ATAD5"

"12691","GTCGACGAAATAGTCAAAAG","chr17",30834966,"+","none","TGG","GTCGACGAAATAGTCAAAAGTGG",0,NA,NA,NA,"GeneID:79915","ATAD5",TRUE,"ATAD5"

"12692","TCGTCGCTACTAATTTCAAT","chr17",30834480,"-","none","TGG","TCGTCGCTACTAATTTCAATTGG",0,NA,NA,NA,"GeneID:79915","ATAD5",TRUE,"ATAD5"

"12693","ATCCTTGATGCACTAGTGAT","chr6",30627656,"-","none","AGG","ATCCTTGATGCACTAGTGATAGG",0,NA,NA,NA,"GeneID:79969","ATAT1",TRUE,"ATAT1"

"12694","CATGAGTCTGTGCAACGCCA","chr6",30628088,"+","none","TGG","CATGAGTCTGTGCAACGCCATGG",0,NA,NA,NA,"GeneID:79969","ATAT1",TRUE,"ATAT1"

"12695","CTCCTATCACTAGTGCATCA","chr6",30627651,"+","none","AGG","CTCCTATCACTAGTGCATCAAGG",0,NA,NA,NA,"GeneID:79969","ATAT1",TRUE,"ATAT1"

"12696","CTTGCCCAGTTCATCTATAA","chr6",30627488,"-","none","TGG","CTTGCCCAGTTCATCTATAATGG",0,NA,NA,NA,"GeneID:79969","ATAT1",TRUE,"ATAT1"

"12697","CTTGCCCAGTTCATCTATAA","chr6",119306503,"+","9:A>T,11:T>C","AGG","CTTGCCCAGATTATCTATAAAGG",2,10,12,NA,"GeneID:4121","MAN1A1",FALSE,"ATAT1"

"12698","CTTGCCCAGTTCATCTATAA","chr17",39774076,"+","3:C>G,16:C>A","TGG","CTTCCCCAGTTCATCTCTAATGG",2,4,17,NA,"GeneID:22806","IKZF3",FALSE,"ATAT1"

"12699","TATGACCATTATAGATGAAC","chr6",30627480,"+","none","TGG","TATGACCATTATAGATGAACTGG",0,NA,NA,NA,"GeneID:79969","ATAT1",TRUE,"ATAT1"

"12700","TCTCTCTTAGGATGATCGTG","chr6",30628021,"+","none","AGG","TCTCTCTTAGGATGATCGTGAGG",0,NA,NA,NA,"GeneID:79969","ATAT1",TRUE,"ATAT1"

"12701","TCTCTCTTAGGATGATCGTG","chr20",51157420,"+","6:A>T,16:T>C","AGG","TCTCTCATAGGATGATTGTGAGG",2,7,17,NA,"","",FALSE,"ATAT1"

"12702","ACGCCCCGGCGGAGGATGCC","chr19",3909552,"+","none","TGG","ACGCCCCGGCGGAGGATGCCTGG",0,NA,NA,NA,"GeneID:85300","ATCAY",TRUE,"ATCAY"

"12703","CCCCGATGAGACCGACTCGC","chr19",3905596,"+","none","TGG","CCCCGATGAGACCGACTCGCTGG",0,NA,NA,NA,"GeneID:85300","ATCAY",TRUE,"ATCAY"

"12704","CTCACCTCCGGTCGATCATC","chr19",3909602,"-","none","TGG","CTCACCTCCGGTCGATCATCTGG",0,NA,NA,NA,"GeneID:85300","ATCAY",TRUE,"ATCAY"

"12705","CTCCAGCGAGTCGGTCTCAT","chr19",3905601,"-","none","CGG","CTCCAGCGAGTCGGTCTCATCGG",0,NA,NA,NA,"GeneID:85300","ATCAY",TRUE,"ATCAY"

"12706","GCAGGCTGCGAAGACGATGA","chr19",3908294,"-","none","TGG","GCAGGCTGCGAAGACGATGATGG",0,NA,NA,NA,"GeneID:85300","ATCAY",TRUE,"ATCAY"

"12707","TCCTCAGGGTACTACGGCGA","chr19",3908260,"+","none","AGG","TCCTCAGGGTACTACGGCGAAGG",0,NA,NA,NA,"GeneID:85300","ATCAY",TRUE,"ATCAY"

"12708","CGCAACAGCATCATCCATTG","chr10",121911113,"+","none","TGG","CGCAACAGCATCATCCATTGTGG",0,NA,NA,NA,"GeneID:11101","ATE1",TRUE,"ATE1"

"12709","CTTCAGTCACAAGATTTCGT","chr10",121910970,"-","none","AGG","CTTCAGTCACAAGATTTCGTAGG",0,NA,NA,NA,"GeneID:11101","ATE1",TRUE,"ATE1"

"12710","CTTCAGTCACAAGATTTCGT","chr21",30737005,"-","10:A>T,12:A>C","AGG","CTTCAGTCACTATATTTCGTAGG",2,11,13,NA,"","",FALSE,"ATE1"

"12711","GGCACATTCCATGACAGTAC","chr10",121924302,"-","none","AGG","GGCACATTCCATGACAGTACAGG",0,NA,NA,NA,"GeneID:11101","ATE1",TRUE,"ATE1"

"12712","TATCAGGATCTCATAGACCG","chr10",121924277,"-","none","AGG","TATCAGGATCTCATAGACCGAGG",0,NA,NA,NA,"GeneID:11101","ATE1",TRUE,"ATE1"

"12713","TGACGGAAAGATCATTGCTG","chr10",121841181,"-","none","TGG","TGACGGAAAGATCATTGCTGTGG",0,NA,NA,NA,"GeneID:11101","ATE1",TRUE,"ATE1"

"12714","TTCCGTCAAGCCAGTACTGC","chr10",121841193,"+","none","TGG","TTCCGTCAAGCCAGTACTGCTGG",0,NA,NA,NA,"GeneID:11101","ATE1",TRUE,"ATE1"

"12715","ATCTGTCTTAGTTGTCTGAG","chr12",50814378,"-","none","AGG","ATCTGTCTTAGTTGTCTGAGAGG",0,NA,NA,NA,"GeneID:466","ATF1",TRUE,"ATF1"

"12716","CAGGACTCATCCGACAGCAT","chr12",50795938,"+","none","AGG","CAGGACTCATCCGACAGCATAGG",0,NA,NA,NA,"GeneID:466","ATF1",TRUE,"ATF1"

"12717","CCCATCTATCAGACTAGCAG","chr12",50809558,"+","none","CGG","CCCATCTATCAGACTAGCAGCGG",0,NA,NA,NA,"GeneID:466","ATF1",TRUE,"ATF1"

"12718","CTATGCTGTCGGATGAGTCC","chr12",50795940,"-","none","TGG","CTATGCTGTCGGATGAGTCCTGG",0,NA,NA,NA,"GeneID:466","ATF1",TRUE,"ATF1"

"12719","GATACTTGTGCCCAGCAATC","chr12",50814157,"+","none","AGG","GATACTTGTGCCCAGCAATCAGG",0,NA,NA,NA,"GeneID:466","ATF1",TRUE,"ATF1"

"12720","TTGTACGACCACCTGATTGC","chr12",50814171,"-","none","TGG","TTGTACGACCACCTGATTGCTGG",0,NA,NA,NA,"GeneID:466","ATF1",TRUE,"ATF1"

"12721","ACGTAATGACAGTGTCATTG","chr2",175121449,"-","none","TGG","ACGTAATGACAGTGTCATTGTGG",0,NA,NA,NA,"GeneID:1386","ATF2",TRUE,"ATF2"

"12722","AGTTACATGTGAATTCTGCC","chr2",175136413,"-","none","AGG","AGTTACATGTGAATTCTGCCAGG",0,NA,NA,NA,"GeneID:1386","ATF2",TRUE,"ATF2"

"12723","AGTTACATGTGAATTCTGCC","chr3",135920201,"+","15:T>C,16:C>T","AGG","AGTTACATGTGAATTTCGCCAGG",2,16,17,NA,"","",FALSE,"ATF2"

"12724","AGTTACATGTGAATTCTGCC","chr5",24166325,"-","1:T>C,4:C>T","AGG","AATTGCATGTGAATTCTGCCAGG",2,2,5,NA,"","",FALSE,"ATF2"

"12725","AGTTACATGTGAATTCTGCC","chr1",103153062,"-","1:T>C,18:A>G","TGG","AATTACATGTGAATTCTGTCTGG",2,2,19,NA,"","",FALSE,"ATF2"

"12726","ATGACACTGTCATTACGTGC","chr2",175121451,"+","none","TGG","ATGACACTGTCATTACGTGCTGG",0,NA,NA,NA,"GeneID:1386","ATF2",TRUE,"ATF2"

"12727","CCCTTTCTATGTACTGCGCC","chr2",175130150,"-","none","TGG","CCCTTTCTATGTACTGCGCCTGG",0,NA,NA,NA,"GeneID:1386","ATF2",TRUE,"ATF2"

"12728","TATATTACCTGGCCACATCC","chr2",175130129,"+","none","AGG","TATATTACCTGGCCACATCCAGG",0,NA,NA,NA,"GeneID:1386","ATF2",TRUE,"ATF2"

"12729","TTCTTGAATTCATTCTCAAA","chr2",175118275,"+","none","TGG","TTCTTGAATTCATTCTCAAATGG",0,NA,NA,NA,"GeneID:1386","ATF2",TRUE,"ATF2"

"12730","TTCTTGAATTCATTCTCAAA","chr19",36968761,"+","10:T>C,14:T>C","TGG","TTCTTGAATTTATTTTCAAATGG",2,11,15,NA,"GeneID:374900","ZNF568",FALSE,"ATF2"

"12731","CCACCGGATGTCCTCTGCGC","chr1",212615185,"+","none","TGG","CCACCGGATGTCCTCTGCGCTGG",0,NA,NA,NA,"GeneID:467","ATF3",TRUE,"ATF3"

"12732","CTGAGCCCGGACAATACACG","chr1",212619463,"-","none","TGG","CTGAGCCCGGACAATACACGTGG",0,NA,NA,NA,"GeneID:467","ATF3",TRUE,"ATF3"

"12733","GGTGTCCATCACAAAAGCCG","chr1",212615239,"+","none","AGG","GGTGTCCATCACAAAAGCCGAGG",0,NA,NA,NA,"GeneID:467","ATF3",TRUE,"ATF3"

"12734","TATACATGCTCAACCTTCAT","chr1",212619436,"+","none","CGG","TATACATGCTCAACCTTCATCGG",0,NA,NA,NA,"GeneID:467","ATF3",TRUE,"ATF3"

"12735","TGAGTCAACAGCCCATATGC","chr1",212619076,"-","none","AGG","TGAGTCAACAGCCCATATGCAGG",0,NA,NA,NA,"GeneID:467","ATF3",TRUE,"ATF3"

"12736","TGAGTCAACAGCCCATATGC","chr3",112511280,"+","3:C>G,8:G>C","TGG","TGACTCAAGAGCCCATATGCTGG",2,4,9,NA,"","",FALSE,"ATF3"

"12737","TTCTGGACCCAAAAGGGCCT","chr1",212619051,"-","none","TGG","TTCTGGACCCAAAAGGGCCTTGG",0,NA,NA,NA,"GeneID:467","ATF3",TRUE,"ATF3"

"12738","TTCTGGACCCAAAAGGGCCT","chr5",158586454,"-","11:G>T,12:G>T","AGG","TTCTGGACCCACCAGGGCCTAGG",2,12,13,NA,"","",FALSE,"ATF3"

"12739","TTCTGGACCCAAAAGGGCCT","chr8",35996826,"-","11:G>T,12:G>T","GGG","TTCTGGACCCACCAGGGCCTGGG",2,12,13,NA,"","",FALSE,"ATF3"

"12740","TTCTGGACCCAAAAGGGCCT","chr12",100608482,"-","0:G>A,12:G>T","GGG","CTCTGGACCCAACAGGGCCTGGG",2,1,13,NA,"GeneID:283431","GAS2L3",FALSE,"ATF3"

"12741","AGGATCGTAAGGTTTGGGAC","chr22",39522315,"-","none","GGG","AGGATCGTAAGGTTTGGGACGGG",0,NA,NA,NA,"GeneID:468","ATF4",TRUE,"ATF4"

"12742","AGGATCGTAAGGTTTGGGAC","chr17",76226017,"+","18:G>A,19:T>C","GGG","AGGATCGTAAGGTTTGGGGTGGG",2,19,20,NA,"GeneID:114804","RNF157",TRUE,"ATF4"

"12743","AGGATCGTAAGGTTTGGGAC","chr11",113791084,"-","5:A>G,18:C>T","GGG","AGGATTGTAAGGTTTGGGGCGGG",2,6,19,NA,"","",TRUE,"ATF4"

"12744","CCACTCACCCTTGCTGTTGT","chr22",39521659,"-","none","TGG","CCACTCACCCTTGCTGTTGTTGG",0,NA,NA,NA,"GeneID:468","ATF4",TRUE,"ATF4"

"12745","GCAACGTAAGCAGTGTAGTC","chr22",39522131,"-","none","TGG","GCAACGTAAGCAGTGTAGTCTGG",0,NA,NA,NA,"GeneID:468","ATF4",TRUE,"ATF4"

"12746","GCAACGTAAGCAGTGTAGTC","chr11",113790900,"-","15:C>A","TGG","GCAACGTAAGCAGTGGAGTCTGG",1,16,NA,NA,"","",TRUE,"ATF4"

"12747","GCAACGTAAGCAGTGTAGTC","chr17",76226201,"+","15:G>T","CGG","GCAACGTAAGCAGTGGAGTCCGG",1,16,NA,NA,"GeneID:114804","RNF157",TRUE,"ATF4"

"12748","TAATAAGCAGCCCCCCCAGA","chr22",39521935,"+","none","CGG","TAATAAGCAGCCCCCCCAGACGG",0,NA,NA,NA,"GeneID:468","ATF4",TRUE,"ATF4"

"12749","TAATAAGCAGCCCCCCCAGA","chr17",76226397,"-","7:C>G,8:A>T","TGG","TAATAAGGTGCCCCCCCAGATGG",2,8,9,NA,"GeneID:114804","RNF157",TRUE,"ATF4"

"12750","TCTCTTAGATGATTACCTGG","chr22",39521540,"+","none","AGG","TCTCTTAGATGATTACCTGGAGG",0,NA,NA,NA,"GeneID:468","ATF4",TRUE,"ATF4"

"12751","TCTCTTAGATGATTACCTGG","chrX",154623386,"-","12:G>A","AGG","TCTCTTAGATGACTACCTGGAGG",1,13,NA,NA,"","",TRUE,"ATF4"

"12752","TCTCTTAGATGATTACCTGG","chr17",76226691,"-","12:G>A,16:C>G","AGG","TCTCTTAGATGACTACGTGGAGG",2,13,17,NA,"GeneID:114804","RNF157",TRUE,"ATF4"

"12753","TCTCTTAGATGATTACCTGG","chrX",154581019,"+","12:C>T","AGG","TCTCTTAGATGACTACCTGGAGG",1,13,NA,NA,"","",TRUE,"ATF4"

"12754","TCTCTTAGATGATTACCTGG","chr11",113790412,"+","10:A>G,12:C>T","AGG","TCTCTTAGATAACTACCTGGAGG",2,11,13,NA,"","",TRUE,"ATF4"

"12755","TTTGCAGAGGATGCCTTCTC","chr22",39521765,"+","none","CGG","TTTGCAGAGGATGCCTTCTCCGG",0,NA,NA,NA,"GeneID:468","ATF4",TRUE,"ATF4"

"12756","TTTGCAGAGGATGCCTTCTC","chr7",3189465,"+","15:C>T,18:C>T","TGG","TTTGCAGAGGATGCCCTCCCTGG",2,16,19,NA,"","",FALSE,"ATF4"

"12757","AGACTATGGGAAACTCCCCC","chr19",49930924,"+","none","CGG","AGACTATGGGAAACTCCCCCCGG",0,NA,NA,NA,"GeneID:22809","ATF5",TRUE,"ATF5"

"12758","AGCCATCCCAGCCCACTAGC","chr19",49930901,"-","none","TGG","AGCCATCCCAGCCCACTAGCTGG",0,NA,NA,NA,"GeneID:22809","ATF5",TRUE,"ATF5"

"12759","CGGCTCTGAGGTACCGCCAG","chr19",49932904,"+","none","CGG","CGGCTCTGAGGTACCGCCAGCGG",0,NA,NA,NA,"GeneID:22809","ATF5",TRUE,"ATF5"

"12760","GCGCGAGATCCAGTACGTCA","chr19",49933016,"+","none","AGG","GCGCGAGATCCAGTACGTCAAGG",0,NA,NA,NA,"GeneID:22809","ATF5",TRUE,"ATF5"

"12761","GGCCATCTACTGCCGCAACG","chr19",49932728,"+","none","AGG","GGCCATCTACTGCCGCAACGAGG",0,NA,NA,NA,"GeneID:22809","ATF5",TRUE,"ATF5"

"12762","GTACTGGATCTCGCGCTCCA","chr19",49933012,"-","none","CGG","GTACTGGATCTCGCGCTCCACGG",0,NA,NA,NA,"GeneID:22809","ATF5",TRUE,"ATF5"

"12763","GTACTGGATCTCGCGCTCCA","chr11",75667901,"+","7:G>A,18:G>C","AGG","GTACTGGGTCTCGCGCTCGAAGG",2,8,19,NA,"GeneID:4135","MAP6",TRUE,"ATF5"

"12764","ACAGGCTTATCTTCCTTCAG","chr1",161791494,"-","none","TGG","ACAGGCTTATCTTCCTTCAGTGG",0,NA,NA,NA,"GeneID:22926","ATF6",TRUE,"ATF6"

"12765","AGACGAGACTGAATAACTTG","chr1",161784034,"-","none","AGG","AGACGAGACTGAATAACTTGAGG",0,NA,NA,NA,"GeneID:22926","ATF6",TRUE,"ATF6"

"12766","ATCAACAACCAAATCTGTAC","chr1",161781977,"+","none","AGG","ATCAACAACCAAATCTGTACAGG",0,NA,NA,NA,"GeneID:22926","ATF6",TRUE,"ATF6"

"12767","ATCAACAACCAAATCTGTAC","chr2",102029260,"-","8:C>G,16:T>C","TGG","ATCAACAAGCAAATCTATACTGG",2,9,17,NA,"","",FALSE,"ATF6"

"12768","CATAATTACCTGTACAGATT","chr1",161781988,"-","none","TGG","CATAATTACCTGTACAGATTTGG",0,NA,NA,NA,"GeneID:22926","ATF6",TRUE,"ATF6"

"12769","GTAATACCAGTCTTGTTCCT","chr1",161791524,"-","none","AGG","GTAATACCAGTCTTGTTCCTAGG",0,NA,NA,NA,"GeneID:22926","ATF6",TRUE,"ATF6"

"12770","GTTATTCAGTCTCGTCTCCT","chr1",161784037,"+","none","CGG","GTTATTCAGTCTCGTCTCCTCGG",0,NA,NA,NA,"GeneID:22926","ATF6",TRUE,"ATF6"

"12771","AATACCGGGACATCCTGCTC","chr6",32127665,"+","none","CGG","AATACCGGGACATCCTGCTCCGG",0,NA,NA,NA,"GeneID:1388","ATF6B",TRUE,"ATF6B"

"12772","CCCTCTGAGGGATCTTCCTC","chr6",32117336,"+","none","CGG","CCCTCTGAGGGATCTTCCTCCGG",0,NA,NA,NA,"GeneID:1388","ATF6B",TRUE,"ATF6B"

"12773","GACGCAGCTCTTCCGTTGCC","chr6",32127687,"-","none","CGG","GACGCAGCTCTTCCGTTGCCCGG",0,NA,NA,NA,"GeneID:1388","ATF6B",TRUE,"ATF6B"

"12774","GTCTCTTAGTAGTAGCTCCT","chr6",32117645,"+","none","TGG","GTCTCTTAGTAGTAGCTCCTTGG",0,NA,NA,NA,"GeneID:1388","ATF6B",TRUE,"ATF6B"

"12775","TCAACCGCACTGAGTCCCTG","chr6",32117588,"-","none","AGG","TCAACCGCACTGAGTCCCTGAGG",0,NA,NA,NA,"GeneID:1388","ATF6B",TRUE,"ATF6B"

"12776","TGGGTCCAGCGCCACCAGAG","chr6",32117362,"-","none","AGG","TGGGTCCAGCGCCACCAGAGAGG",0,NA,NA,NA,"GeneID:1388","ATF6B",TRUE,"ATF6B"

"12777","ACACCCACCATTGTACGTCC","chr12",53534603,"-","none","TGG","ACACCCACCATTGTACGTCCTGG",0,NA,NA,NA,"GeneID:11016","ATF7",TRUE,"ATF7"

"12778","GAATTTACCTTCTCCTTCAG","chr12",53537406,"+","none","TGG","GAATTTACCTTCTCCTTCAGTGG",0,NA,NA,NA,"GeneID:11016","ATF7",TRUE,"ATF7"

"12779","GAATTTACCTTCTCCTTCAG","chr10",93264382,"-","7:C>G,14:A>G","TGG","GAATTTAGCTTCTCTTTCAGTGG",2,8,15,NA,"","",FALSE,"ATF7"

"12780","GAATTTACCTTCTCCTTCAG","chr4",122096525,"-","5:G>A,19:G>C","AGG","GAATTCACCTTCTCCTTCACAGG",2,6,20,NA,"","",FALSE,"ATF7"

"12781","GCTTGTGTTTATGAACTGCC","chr12",53552594,"+","none","AGG","GCTTGTGTTTATGAACTGCCAGG",0,NA,NA,NA,"GeneID:11016","ATF7",TRUE,"ATF7"

"12782","GGGGATGAGTCTACCTCCAC","chr12",53537463,"+","none","TGG","GGGGATGAGTCTACCTCCACTGG",0,NA,NA,NA,"GeneID:11016","ATF7",TRUE,"ATF7"

"12783","GTTTCAGAGATTTACAAACG","chr12",53552624,"-","none","AGG","GTTTCAGAGATTTACAAACGAGG",0,NA,NA,NA,"GeneID:11016","ATF7",TRUE,"ATF7"

"12784","TACTTACCCCATTTGCCTGT","chr12",53534494,"+","none","TGG","TACTTACCCCATTTGCCTGTTGG",0,NA,NA,NA,"GeneID:11016","ATF7",TRUE,"ATF7"

"12785","AACAGTTCTCACTGAACTAC","chr12",14438245,"+","none","AGG","AACAGTTCTCACTGAACTACAGG",0,NA,NA,NA,"GeneID:55729","ATF7IP",TRUE,"ATF7IP"

"12786","AACAGTTCTCACTGAACTAC","chr10",60782867,"-","1:A>T,4:G>C","TGG","ATCACTTCTCACTGAACTACTGG",2,2,5,NA,"GeneID:983","CDK1",FALSE,"ATF7IP"

"12787","AACAGTTCTCACTGAACTAC","chr11",123385012,"-","0:C>T,7:A>G","TGG","GACAGTTTTCACTGAACTACTGG",2,1,8,NA,"GeneID:57476","GRAMD1B",FALSE,"ATF7IP"

"12788","CCCAACCCACCAGTATCACC","chr12",14456566,"+","none","AGG","CCCAACCCACCAGTATCACCAGG",0,NA,NA,NA,"GeneID:55729","ATF7IP",TRUE,"ATF7IP"

"12789","CCCAACCCACCAGTATCACC","chr1",209134538,"-","4:C>T","AGG","CCCAGCCCACCAGTATCACCAGG",1,5,NA,NA,"","",FALSE,"ATF7IP"

"12790","CCCAACCCACCAGTATCACC","chr15",80467391,"-","3:G>T,9:A>G","AGG","CCCCACCCATCAGTATCACCAGG",2,4,10,NA,"GeneID:9915","ARNT2",FALSE,"ATF7IP"

"12791","CCCAACCCACCAGTATCACC","chr1",31723277,"+","3:T>A,17:C>A","AGG","CCCTACCCACCAGTATCCCCAGG",2,4,18,NA,"","",FALSE,"ATF7IP"

"12792","GCAATAATAACATGTCTTAC","chr12",14456612,"+","none","AGG","GCAATAATAACATGTCTTACAGG",0,NA,NA,NA,"GeneID:55729","ATF7IP",TRUE,"ATF7IP"

"12793","GCAATAATAACATGTCTTAC","chr12",17630724,"-","12:C>A,13:G>C","TGG","GCAATAATAACAGCTCTTACTGG",2,13,14,NA,"","",FALSE,"ATF7IP"

"12794","GCAATAATAACATGTCTTAC","chr1",68587591,"-","11:A>T,16:T>A","AGG","GCAATAATAACTTGTCATACAGG",2,12,17,NA,"","",FALSE,"ATF7IP"

"12795","GCAATAATAACATGTCTTAC","chr13",75119578,"+","10:A>C,13:C>G","AGG","GCAATAATAAAATCTCTTACAGG",2,11,14,NA,"","",FALSE,"ATF7IP"

"12796","TGCTGTATTTGATAAGACTT","chr12",14438173,"+","none","TGG","TGCTGTATTTGATAAGACTTTGG",0,NA,NA,NA,"GeneID:55729","ATF7IP",TRUE,"ATF7IP"

"12797","TGCTGTATTTGATAAGACTT","chr8",98576069,"+","15:A>G,18:A>T","AGG","TGCTGTATTTGATAAAACATAGG",2,16,19,NA,"GeneID:6788","STK3",FALSE,"ATF7IP"

"12798","TGCTGTATTTGATAAGACTT","chr9",27991000,"-","0:T>A,11:C>T","AGG","AGCTGTATTTGGTAAGACTTAGG",2,1,12,NA,"GeneID:158038","LINGO2",FALSE,"ATF7IP"

"12799","TGTTTCTTTAGGTTATACAG","chr12",14438118,"+","none","TGG","TGTTTCTTTAGGTTATACAGTGG",0,NA,NA,NA,"GeneID:55729","ATF7IP",TRUE,"ATF7IP"

"12800","TGTTTCTTTAGGTTATACAG","chr6",78052057,"-","16:C>T,19:T>C","AGG","TGTTTCTTTAGGTTATGCAAAGG",2,17,20,NA,"","",FALSE,"ATF7IP"

"12801","TGTTTCTTTAGGTTATACAG","chr1",222694914,"-","13:G>A,17:A>G","TGG","TGTTTCTTTAGGTCATATAGTGG",2,14,18,NA,"GeneID:64853","AIDA",FALSE,"ATF7IP"

"12802","TTCAAAGCGTTTGGTTAACC","chr12",14447000,"-","none","TGG","TTCAAAGCGTTTGGTTAACCTGG",0,NA,NA,NA,"GeneID:55729","ATF7IP",TRUE,"ATF7IP"

"12803","AACCTTAGAGGCTCCATTAC","chr16",10440445,"-","none","TGG","AACCTTAGAGGCTCCATTACTGG",0,NA,NA,NA,"GeneID:80063","ATF7IP2",TRUE,"ATF7IP2"

"12804","AACCTTAGAGGCTCCATTAC","chr11",11136677,"-","7:G>C","TGG","AACCTTACAGGCTCCATTACTGG",1,8,NA,NA,"","",FALSE,"ATF7IP2"

"12805","AAGGCCATTATCAAAAGAAG","chr16",10433521,"+","none","AGG","AAGGCCATTATCAAAAGAAGAGG",0,NA,NA,NA,"GeneID:80063","ATF7IP2",TRUE,"ATF7IP2"

"12806","AAGGCCATTATCAAAAGAAG","chr14",68425423,"+","2:T>G,14:G>A","TGG","AATGCCATTATCAAGAGAAGTGG",2,3,15,NA,"GeneID:5890","RAD51B",FALSE,"ATF7IP2"

"12807","ACTCACCATAACTTCAGCAT","chr16",10473520,"-","none","TGG","ACTCACCATAACTTCAGCATTGG",0,NA,NA,NA,"GeneID:80063","ATF7IP2",TRUE,"ATF7IP2"

"12808","ACTCACCATAACTTCAGCAT","chr5",145967624,"-","11:T>G,18:A>T","CGG","ACTCACCATAAATTCAGCTTCGG",2,12,19,NA,"GeneID:153769","SH3RF2",FALSE,"ATF7IP2"

"12809","CAGCTTTCGAATGTGACTCC","chr16",10480897,"-","none","AGG","CAGCTTTCGAATGTGACTCCAGG",0,NA,NA,NA,"GeneID:80063","ATF7IP2",TRUE,"ATF7IP2"

"12810","GAAAGCTGCTTCAAACTCAA","chr16",10480909,"+","none","AGG","GAAAGCTGCTTCAAACTCAAAGG",0,NA,NA,NA,"GeneID:80063","ATF7IP2",TRUE,"ATF7IP2"

"12811","GATACCAGAAAAATTACATC","chr16",10473477,"+","none","AGG","GATACCAGAAAAATTACATCAGG",0,NA,NA,NA,"GeneID:80063","ATF7IP2",TRUE,"ATF7IP2"

"12812","GATACCAGAAAAATTACATC","chr7",32076336,"+","0:T>G,14:G>T","AGG","TATACCAGAAAAATGACATCAGG",2,1,15,NA,"GeneID:5137","PDE1C",FALSE,"ATF7IP2"

"12813","ATGGGACACTATTACGCAAC","chr5",82178565,"+","none","AGG","ATGGGACACTATTACGCAACAGG",0,NA,NA,NA,"GeneID:83734","ATG10",TRUE,"ATG10"

"12814","CATCTAGGAGCATCTACCCA","chr5",82058557,"+","none","TGG","CATCTAGGAGCATCTACCCATGG",0,NA,NA,NA,"GeneID:83734","ATG10",TRUE,"ATG10"

"12815","CATGAATTCATTCGTCTTGC","chr5",82252604,"-","none","AGG","CATGAATTCATTCGTCTTGCAGG",0,NA,NA,NA,"GeneID:83734","ATG10",TRUE,"ATG10"

"12816","CTATAAGATGCGACTGCTAC","chr5",82178538,"+","none","AGG","CTATAAGATGCGACTGCTACAGG",0,NA,NA,NA,"GeneID:83734","ATG10",TRUE,"ATG10"

"12817","CTTGCCCTAAAGTAAAGTAC","chr5",82164509,"-","none","AGG","CTTGCCCTAAAGTAAAGTACAGG",0,NA,NA,NA,"GeneID:83734","ATG10",TRUE,"ATG10"

"12818","CTTGCCCTAAAGTAAAGTAC","chr15",24066562,"-","4:A>G,16:T>C","TGG","CTTGTCCTAAAGTAAAATACTGG",2,5,17,NA,"","",FALSE,"ATG10"

"12819","CTTGCCCTAAAGTAAAGTAC","chr11",11941305,"-","3:T>C,15:A>T","TGG","CTTACCCTAAAGTAATGTACTGG",2,4,16,NA,"GeneID:55031","USP47",FALSE,"ATG10"

"12820","GGAGGCTTTCGAGCTACCCT","chr5",82164397,"+","none","TGG","GGAGGCTTTCGAGCTACCCTTGG",0,NA,NA,NA,"GeneID:83734","ATG10",TRUE,"ATG10"

"12821","CATAGAGAGTTCCAACTTCT","chr5",115832603,"+","none","TGG","CATAGAGAGTTCCAACTTCTTGG",0,NA,NA,NA,"GeneID:9140","ATG12",TRUE,"ATG12"

"12822","CATAGAGAGTTCCAACTTCT","chr17",73067928,"+","2:G>T","TGG","CAGAGAGAGTTCCAACTTCTTGG",1,3,NA,NA,"GeneID:201266","SLC39A11",TRUE,"ATG12"

"12823","CATAGAGAGTTCCAACTTCT","chr5",74137843,"-","4:T>C,16:T>A","TGG","CATAAAGAGTTCCAACATCTTGG",2,5,17,NA,"","",FALSE,"ATG12"

"12824","CCTCCAGCAGCAATTGAAGT","chr5",115841496,"+","none","AGG","CCTCCAGCAGCAATTGAAGTAGG",0,NA,NA,NA,"GeneID:9140;GeneID:1176","ATG12;AP3S1",TRUE,"ATG12"

"12825","CCTTCCCCAGACCAAGAAGT","chr17",73067942,"-","none","TGG","CCTTCCCCAGACCAAGAAGTTGG",0,NA,NA,NA,"GeneID:201266","SLC39A11",TRUE,"ATG12"

"12826","CCTTCCCCAGACCAAGAAGT","chr5",115832617,"-","none","TGG","CCTTCCCCAGACCAAGAAGTTGG",0,NA,NA,NA,"GeneID:9140","ATG12",TRUE,"ATG12"

"12827","CCTTCCCCAGACCAAGAAGT","chr6",43915096,"-","3:C>A,5:A>G","GGG","CCTGCTCCAGACCAAGAAGTGGG",2,4,6,NA,"","",FALSE,"ATG12"

"12828","CCTTCCCCAGACCAAGAAGT","chr19",10765676,"-","5:T>G,6:C>G","GGG","CCTTCAGCAGACCAAGAAGTGGG",2,6,7,NA,"GeneID:1785","DNM2",FALSE,"ATG12"

"12829","CCTTCCCCAGACCAAGAAGT","chr11",109662992,"-","3:G>A,12:T>G","GGG","CCTCCCCCAGACAAAGAAGTGGG",2,4,13,NA,"","",FALSE,"ATG12"

"12830","CCTTCCCCAGACCAAGAAGT","chr11",35288301,"-","3:T>A,19:C>A","AGG","CCTACCCCAGACCAAGAAGGAGG",2,4,20,NA,"GeneID:6506","SLC1A2",FALSE,"ATG12"

"12831","CTTCCTACTTCAATTGCTGC","chr5",115841502,"-","none","TGG","CTTCCTACTTCAATTGCTGCTGG",0,NA,NA,NA,"GeneID:9140;GeneID:1176","ATG12;AP3S1",TRUE,"ATG12"

"12832","GAGCGAACACGAACCATCCA","chr5",115837682,"-","none","AGG","GAGCGAACACGAACCATCCAAGG",0,NA,NA,NA,"GeneID:9140","ATG12",TRUE,"ATG12"

"12833","GAGCGAACACGAACCATCCA","chr17",73068044,"-","9:A>G","AGG","GAGCGAACATGAACCATCCAAGG",1,10,NA,NA,"GeneID:201266","SLC39A11",TRUE,"ATG12"

"12834","GTTTCATACCAACTGTTCTG","chr5",115837618,"+","none","AGG","GTTTCATACCAACTGTTCTGAGG",0,NA,NA,NA,"GeneID:9140","ATG12",TRUE,"ATG12"

"12835","ACACCATATTTCCAGCTCCA","chr11",46649146,"-","none","TGG","ACACCATATTTCCAGCTCCATGG",0,NA,NA,NA,"GeneID:9776","ATG13",TRUE,"ATG13"

"12836","ACACCATATTTCCAGCTCCA","chr18",2362426,"+","14:A>G,15:A>C","AGG","ACACCATATTTCCAAATCCAAGG",2,15,16,NA,"","",FALSE,"ATG13"

"12837","ACACCATATTTCCAGCTCCA","chr8",19929970,"+","0:C>A,15:G>C","AGG","CCACCATATTTCCAGGTCCAAGG",2,1,16,NA,"GeneID:4023","LPL",FALSE,"ATG13"

"12838","CAGTCTGTTGTACACCGTGT","chr11",46650202,"-","none","AGG","CAGTCTGTTGTACACCGTGTAGG",0,NA,NA,NA,"GeneID:9776","ATG13",TRUE,"ATG13"

"12839","CTTTCTACAGGGAGATTCCA","chr11",46649126,"+","none","TGG","CTTTCTACAGGGAGATTCCATGG",0,NA,NA,NA,"GeneID:9776","ATG13",TRUE,"ATG13"

"12840","CTTTCTACAGGGAGATTCCA","chr10",68222512,"+","6:G>A,7:T>C","GGG","CTTTCTGTAGGGAGATTCCAGGG",2,7,8,NA,"","",FALSE,"ATG13"

"12841","GACTGTCCAAGTGATTGTCC","chr11",46645337,"+","none","AGG","GACTGTCCAAGTGATTGTCCAGG",0,NA,NA,NA,"GeneID:9776","ATG13",TRUE,"ATG13"

"12842","TCACCCTAGTTATAGCAAGA","chr11",46650242,"-","none","AGG","TCACCCTAGTTATAGCAAGAAGG",0,NA,NA,NA,"GeneID:9776","ATG13",TRUE,"ATG13"

"12843","TTTACCCAATCTGAACCCGT","chr11",46645404,"-","none","TGG","TTTACCCAATCTGAACCCGTTGG",0,NA,NA,NA,"GeneID:9776","ATG13",TRUE,"ATG13"

"12844","AAAATGGATAACAGATCAGT","chr14",55395941,"-","none","TGG","AAAATGGATAACAGATCAGTTGG",0,NA,NA,NA,"GeneID:22863","ATG14",TRUE,"ATG14"

"12845","AAAATGGATAACAGATCAGT","chr1",96879949,"+","5:T>G,15:A>T","GGG","AAAATTGATAACAGAACAGTGGG",2,6,16,NA,"","",FALSE,"ATG14"

"12846","ATCGCCGCTCTGAACGCATT","chr14",55411633,"+","none","TGG","ATCGCCGCTCTGAACGCATTTGG",0,NA,NA,NA,"GeneID:22863","ATG14",TRUE,"ATG14"

"12847","CGGCGGGTAGTGTTGCACAG","chr14",55411670,"+","none","CGG","CGGCGGGTAGTGTTGCACAGCGG",0,NA,NA,NA,"GeneID:22863","ATG14",TRUE,"ATG14"

"12848","TCACAGAGTGTTAAAAGCTA","chr14",55395968,"-","none","TGG","TCACAGAGTGTTAAAAGCTATGG",0,NA,NA,NA,"GeneID:22863","ATG14",TRUE,"ATG14"

"12849","TCTACTTCGACGGCCGCGAC","chr14",55411609,"-","none","CGG","TCTACTTCGACGGCCGCGACCGG",0,NA,NA,NA,"GeneID:22863","ATG14",TRUE,"ATG14"

"12850","TTCATACCTGAGAAAAACAA","chr14",55377976,"+","none","AGG","TTCATACCTGAGAAAAACAAAGG",0,NA,NA,NA,"GeneID:22863","ATG14",TRUE,"ATG14"

"12851","TTCATACCTGAGAAAAACAA","chr3",77935327,"-","5:A>T,6:A>G","TGG","TTCATTTCTGAGAAAAACAATGG",2,6,7,NA,"","",FALSE,"ATG14"

"12852","TTCATACCTGAGAAAAACAA","chr16",71753880,"+","1:G>T,11:A>G","TGG","TGCATACCTGAAAAAAACAATGG",2,2,12,NA,"GeneID:164","AP1G1",TRUE,"ATG14"

"12853","TTCATACCTGAGAAAAACAA","chr2",38843203,"+","1:A>T,15:G>A","AGG","TACATACCTGAGAAAGACAAAGG",2,2,16,NA,"GeneID:90957","DHX57",TRUE,"ATG14"

"12854","TTCATACCTGAGAAAAACAA","chr10",122681267,"-","7:T>G,18:G>T","TGG","TTCATACATGAGAAAAACCATGG",2,8,19,NA,"","",FALSE,"ATG14"

"12855","AACGCTGTGCAGTTCAGTCC","chr2",233277588,"+","none","AGG","AACGCTGTGCAGTTCAGTCCAGG",0,NA,NA,NA,"GeneID:55054","ATG16L1",TRUE,"ATG16L1"

"12856","ACTGAATTACACAAGAAACG","chr2",233263208,"+","none","TGG","ACTGAATTACACAAGAAACGTGG",0,NA,NA,NA,"GeneID:55054","ATG16L1",TRUE,"ATG16L1"

"12857","CAATTTAGTCCCGGACATGA","chr2",233263121,"+","none","TGG","CAATTTAGTCCCGGACATGATGG",0,NA,NA,NA,"GeneID:55054","ATG16L1",TRUE,"ATG16L1"

"12858","TGGACCGCAGGGTTAAGCTT","chr2",233277637,"+","none","TGG","TGGACCGCAGGGTTAAGCTTTGG",0,NA,NA,NA,"GeneID:55054","ATG16L1",TRUE,"ATG16L1"

"12859","TTACGTGGCTGCTCTGCTGA","chr2",233273036,"-","none","TGG","TTACGTGGCTGCTCTGCTGATGG",0,NA,NA,NA,"GeneID:55054","ATG16L1",TRUE,"ATG16L1"

"12860","TTCCAGGGATGATGACATTG","chr2",233272959,"+","none","AGG","TTCCAGGGATGATGACATTGAGG",0,NA,NA,NA,"GeneID:55054","ATG16L1",TRUE,"ATG16L1"

"12861","ACCCTCAGTGCGACCAGTGA","chr11",72817789,"-","none","TGG","ACCCTCAGTGCGACCAGTGATGG",0,NA,NA,NA,"GeneID:89849","ATG16L2",TRUE,"ATG16L2"

"12862","AGGGCCGATGGCTTCAAGTG","chr11",72828726,"+","none","TGG","AGGGCCGATGGCTTCAAGTGTGG",0,NA,NA,NA,"GeneID:89849","ATG16L2",TRUE,"ATG16L2"

"12863","AGGGCCGATGGCTTCAAGTG","chr19",48203078,"-","5:C>G,19:G>C","AGG","AGGGCGGATGGCTTCAAGTCAGG",2,6,20,NA,"GeneID:22900","CARD8",FALSE,"ATG16L2"

"12864","CCGCCCCCAGATGGCCTACC","chr11",72821657,"+","none","AGG","CCGCCCCCAGATGGCCTACCAGG",0,NA,NA,NA,"GeneID:89849","ATG16L2",TRUE,"ATG16L2"

"12865","CCGCCCCCAGATGGCCTACC","chr14",24507449,"-","7:A>G,9:G>C","TGG","CCGCCCCTACATGGCCTACCTGG",2,8,10,NA,"GeneID:1215","CMA1",TRUE,"ATG16L2"

"12866","CGCACTGAGGGTGAAGTGGC","chr11",72817797,"+","none","AGG","CGCACTGAGGGTGAAGTGGCAGG",0,NA,NA,NA,"GeneID:89849","ATG16L2",TRUE,"ATG16L2"

"12867","GACATACCTGAACACAGCTT","chr11",72828763,"-","none","TGG","GACATACCTGAACACAGCTTTGG",0,NA,NA,NA,"GeneID:89849","ATG16L2",TRUE,"ATG16L2"

"12868","GCTCCGACTCCAGCGTGCCC","chr11",72821702,"-","none","AGG","GCTCCGACTCCAGCGTGCCCAGG",0,NA,NA,NA,"GeneID:89849","ATG16L2",TRUE,"ATG16L2"

"12869","GCTCCGACTCCAGCGTGCCC","chr10",69837072,"+","6:G>A,13:G>C","AGG","GCTCCGGCTCCAGGGTGCCCAGG",2,7,14,NA,"GeneID:1305","COL13A1",FALSE,"ATG16L2"

"12870","GCTCCGACTCCAGCGTGCCC","chr10",133258007,"+","5:C>G,17:G>C","TGG","GCTCCCACTCCAGCGTGGCCTGG",2,6,18,NA,"","",FALSE,"ATG16L2"

"12871","AGGGGTCCGCCTGCACTACG","chr11",64913282,"-","none","AGG","AGGGGTCCGCCTGCACTACGAGG",0,NA,NA,NA,"GeneID:23130","ATG2A",TRUE,"ATG2A"

"12872","CACTGCACAGTGCGCGTGTC","chr11",64914375,"-","none","CGG","CACTGCACAGTGCGCGTGTCCGG",0,NA,NA,NA,"GeneID:23130","ATG2A",TRUE,"ATG2A"

"12873","GGAACGTGGTGTGGCCGTCG","chr11",64913836,"-","none","AGG","GGAACGTGGTGTGGCCGTCGAGG",0,NA,NA,NA,"GeneID:23130","ATG2A",TRUE,"ATG2A"

"12874","GGAGTCAATGGAGTCACCGC","chr11",64914463,"-","none","TGG","GGAGTCAATGGAGTCACCGCTGG",0,NA,NA,NA,"GeneID:23130","ATG2A",TRUE,"ATG2A"

"12875","GGCGGCTGATGCACGTCCAC","chr11",64913332,"+","none","CGG","GGCGGCTGATGCACGTCCACCGG",0,NA,NA,NA,"GeneID:23130","ATG2A",TRUE,"ATG2A"

"12876","TTTGCCCAGACCATTGAGAC","chr11",64914082,"-","none","TGG","TTTGCCCAGACCATTGAGACTGG",0,NA,NA,NA,"GeneID:23130","ATG2A",TRUE,"ATG2A"

"12877","CACCAACCTGGGCGAGGTCT","chr14",96347171,"+","none","AGG","CACCAACCTGGGCGAGGTCTAGG",0,NA,NA,NA,"GeneID:55102","ATG2B",TRUE,"ATG2B"

"12878","CACCAACCTGGGCGAGGTCT","chr10",1216057,"+","7:G>C,12:G>C","CGG","CACCAACGTGGGGGAGGTCTCGG",2,8,13,NA,"GeneID:105","ADARB2",TRUE,"ATG2B"

"12879","CACCAACCTGGGCGAGGTCT","chr9",33262749,"+","2:A>C,13:C>G","TGG","CAACAACCTGGGCCAGGTCTTGG",2,3,14,NA,"GeneID:573","BAG1",TRUE,"ATG2B"

"12880","CACCAACCTGGGCGAGGTCT","chr8",20245193,"+","5:G>A,13:C>G","AGG","CACCAGCCTGGGCCAGGTCTAGG",2,6,14,NA,"","",FALSE,"ATG2B"

"12881","CACTTGAAATTCGAATAGAA","chr14",96344655,"-","none","AGG","CACTTGAAATTCGAATAGAAAGG",0,NA,NA,NA,"GeneID:55102","ATG2B",TRUE,"ATG2B"

"12882","CTGGTTCTGAGCCTATGTAT","chr14",96345362,"-","none","TGG","CTGGTTCTGAGCCTATGTATTGG",0,NA,NA,NA,"GeneID:55102","ATG2B",TRUE,"ATG2B"

"12883","CTGGTTCTGAGCCTATGTAT","chr4",142331963,"-","6:T>G,16:A>C","GGG","CTGGTTATGAGCCTATTTATGGG",2,7,17,NA,"GeneID:8821","INPP4B",FALSE,"ATG2B"

"12884","TAACACCAAACCTGTTTCAA","chr14",96345221,"+","none","TGG","TAACACCAAACCTGTTTCAATGG",0,NA,NA,NA,"GeneID:55102","ATG2B",TRUE,"ATG2B"

"12885","TTAGTGTCTCAATGAGATCT","chr14",96347325,"-","none","TGG","TTAGTGTCTCAATGAGATCTTGG",0,NA,NA,NA,"GeneID:55102","ATG2B",TRUE,"ATG2B"

"12886","TTCAAGTGCAGTTCCAGTTT","chr14",96344667,"+","none","TGG","TTCAAGTGCAGTTCCAGTTTTGG",0,NA,NA,NA,"GeneID:55102","ATG2B",TRUE,"ATG2B"

"12887","AGGTGTAATTACCCCAGAAG","chr3",112558377,"-","none","AGG","AGGTGTAATTACCCCAGAAGAGG",0,NA,NA,NA,"GeneID:64422","ATG3",TRUE,"ATG3"

"12888","ATTACTTACCATTGCCATGT","chr3",112553270,"+","none","TGG","ATTACTTACCATTGCCATGTTGG",0,NA,NA,NA,"GeneID:64422","ATG3",TRUE,"ATG3"

"12889","ATTACTTACCATTGCCATGT","chr21",30557827,"+","0:G>A,15:T>C","AGG","GTTACTTACCATTGCTATGTAGG",2,1,16,NA,"","",FALSE,"ATG3"

"12890","CCTGAATAGTTTGTGGCAGC","chr3",112553318,"-","none","TGG","CCTGAATAGTTTGTGGCAGCTGG",0,NA,NA,NA,"GeneID:64422","ATG3",TRUE,"ATG3"

"12891","TAATTTCAAAGGACAATATA","chr3",112541875,"-","none","AGG","TAATTTCAAAGGACAATATAAGG",0,NA,NA,NA,"GeneID:64422","ATG3",TRUE,"ATG3"

"12892","TAATTTCAAAGGACAATATA","chr3",166276375,"-","13:T>G,18:T>A","AGG","TAATTTCAAAGGAAAATAAAAGG",2,14,19,NA,"","",FALSE,"ATG3"

"12893","TAGTCCACCACTGTCCAACA","chr3",112553287,"-","none","TGG","TAGTCCACCACTGTCCAACATGG",0,NA,NA,NA,"GeneID:64422","ATG3",TRUE,"ATG3"

"12894","TAGTCCACCACTGTCCAACA","chr12",116211211,"+","3:A>T,7:G>C","AGG","TAGACCAGCACTGTCCAACAAGG",2,4,8,NA,"GeneID:23389","MED13L",FALSE,"ATG3"

"12895","TTGCTCAGCACTATGTGAAG","chr3",112541844,"-","none","AGG","TTGCTCAGCACTATGTGAAGAGG",0,NA,NA,NA,"GeneID:64422","ATG3",TRUE,"ATG3"

"12896","AAGCAGCATCTCCTTAAAAC","chrX",108126165,"+","none","AGG","AAGCAGCATCTCCTTAAAACAGG",0,NA,NA,NA,"GeneID:115201","ATG4A",TRUE,"ATG4A"

"12897","AAGCAGCATCTCCTTAAAAC","chr3",132349108,"+","5:A>G,9:G>C","AGG","AAGCAACATGTCCTTAAAACAGG",2,6,10,NA,"GeneID:55","ACPP",FALSE,"ATG4A"

"12898","AGCCCTTATCTGTAGACACT","chrX",108131329,"+","none","TGG","AGCCCTTATCTGTAGACACTTGG",0,NA,NA,NA,"GeneID:115201","ATG4A",TRUE,"ATG4A"

"12899","AGCCCTTATCTGTAGACACT","chr6",110639307,"+","0:C>A,1:A>G","TGG","CACCCTTATCTGTAGACACTTGG",2,1,2,NA,"GeneID:23097","CDK19",FALSE,"ATG4A"

"12900","AGCCCTTATCTGTAGACACT","chrX",82998961,"+","9:A>C","TGG","AGCCCTTATATGTAGACACTTGG",1,10,NA,NA,"","",TRUE,"ATG4A"

"12901","CATACCAGCTCATCTGTATC","chrX",108126135,"-","none","TGG","CATACCAGCTCATCTGTATCTGG",0,NA,NA,NA,"GeneID:115201","ATG4A",TRUE,"ATG4A"

"12902","CTGATATAAGTGCTCGTCTA","chrX",108128798,"+","none","TGG","CTGATATAAGTGCTCGTCTATGG",0,NA,NA,NA,"GeneID:115201","ATG4A",TRUE,"ATG4A"

"12903","TGGGGATGTATGCTACGCTG","chrX",108131288,"+","none","TGG","TGGGGATGTATGCTACGCTGTGG",0,NA,NA,NA,"GeneID:115201","ATG4A",TRUE,"ATG4A"

"12904","TGGGGATGTATGCTACGCTG","chrX",82998920,"+","15:T>C","TGG","TGGGGATGTATGCTATGCTGTGG",1,16,NA,NA,"","",TRUE,"ATG4A"

"12905","TTCTTAAAAAGACTGGAGCT","chrX",108134045,"+","none","GGG","TTCTTAAAAAGACTGGAGCTGGG",0,NA,NA,NA,"GeneID:115201","ATG4A",TRUE,"ATG4A"

"12906","TTCTTAAAAAGACTGGAGCT","chr1",39990149,"+","2:T>C,17:T>G","CGG","TTTTTAAAAAGACTGGATCTCGG",2,3,18,NA,"","",FALSE,"ATG4A"

"12907","TTCTTAAAAAGACTGGAGCT","chr1",177192570,"-","7:A>T,14:T>C","AGG","TTCTTAATAAGACTAGAGCTAGG",2,8,15,NA,"GeneID:57795","BRINP2",FALSE,"ATG4A"

"12908","ACTCACCTCGGCCTAGGTGC","chr2",241653596,"-","none","CGG","ACTCACCTCGGCCTAGGTGCCGG",0,NA,NA,NA,"GeneID:23192","ATG4B",TRUE,"ATG4B"

"12909","ACTCACCTCGGCCTAGGTGC","chr1",185353362,"-","13:T>A,17:T>A","AGG","ACTCACCTCGGCCAAGGAGCAGG",2,14,18,NA,"","",FALSE,"ATG4B"

"12910","ACTCACCTCGGCCTAGGTGC","chr2",46851724,"+","2:C>T,14:G>A","TGG","ACCCACCTCGGCCTGGGTGCTGG",2,3,15,NA,"GeneID:100134259","LINC01119",FALSE,"ATG4B"

"12911","ACTCACCTCGGCCTAGGTGC","chr6",75655969,"-","2:G>A,15:T>C","TGG","ACCCACCTCGGCCTAAGTGCTGG",2,3,16,NA,"GeneID:26054","SENP6",FALSE,"ATG4B"

"12912","ACTCACCTCGGCCTAGGTGC","chr10",132662673,"-","0:G>T,13:T>A","GGG","CCTCACCTCGGCCAAGGTGCGGG",2,1,14,NA,"GeneID:3632","INPP5A",FALSE,"ATG4B"

"12913","CTCTGACCTACGACACTCTC","chr2",241651012,"+","none","CGG","CTCTGACCTACGACACTCTCCGG",0,NA,NA,NA,"GeneID:23192","ATG4B",TRUE,"ATG4B"

"12914","CTGAGACCTCAGAGCCCGTT","chr2",241651057,"+","none","TGG","CTGAGACCTCAGAGCCCGTTTGG",0,NA,NA,NA,"GeneID:23192","ATG4B",TRUE,"ATG4B"

"12915","CTGAGACCTCAGAGCCCGTT","chr20",6296460,"+","8:C>T,17:T>G","GGG","CTGAGACCCCAGAGCCCTTTGGG",2,9,18,NA,"","",FALSE,"ATG4B"

"12916","GGACGAGATCTTGTCTGATG","chr2",241651267,"+","none","TGG","GGACGAGATCTTGTCTGATGTGG",0,NA,NA,NA,"GeneID:23192","ATG4B",TRUE,"ATG4B"

"12917","TCCTCAACGCATTCATCGAC","chr2",241654593,"+","none","AGG","TCCTCAACGCATTCATCGACAGG",0,NA,NA,NA,"GeneID:23192","ATG4B",TRUE,"ATG4B"

"12918","TCCTGTCGATGAATGCGTTG","chr2",241654597,"-","none","AGG","TCCTGTCGATGAATGCGTTGAGG",0,NA,NA,NA,"GeneID:23192","ATG4B",TRUE,"ATG4B"

"12919","AATTCTCCTGTATTATTGCT","chr1",62805206,"+","none","TGG","AATTCTCCTGTATTATTGCTTGG",0,NA,NA,NA,"GeneID:84938","ATG4C",TRUE,"ATG4C"

"12920","ACTTACCTTCATATTTAAAA","chr1",62805241,"-","none","TGG","ACTTACCTTCATATTTAAAATGG",0,NA,NA,NA,"GeneID:84938","ATG4C",TRUE,"ATG4C"

"12921","ACTTACCTTCATATTTAAAA","chr11",132259201,"-","6:T>G","TGG","ACTTACATTCATATTTAAAATGG",1,7,NA,NA,"GeneID:50863","NTM",FALSE,"ATG4C"

"12922","ACTTACCTTCATATTTAAAA","chr9",74975346,"-","2:G>A,9:T>G","TGG","ACCTACCTTAATATTTAAAATGG",2,3,10,NA,"","",FALSE,"ATG4C"

"12923","ACTTACCTTCATATTTAAAA","chr2",45544910,"-","4:A>T,12:A>T","TGG","ACTTTCCTTCATTTTTAAAATGG",2,5,13,NA,"GeneID:55133","SRBD1",FALSE,"ATG4C"

"12924","ACTTACCTTCATATTTAAAA","chr3",26626272,"-","1:A>G,10:G>T","AGG","ATTTACCTTCCTATTTAAAAAGG",2,2,11,NA,"GeneID:116135","LRRC3B",FALSE,"ATG4C"

"12925","AGATTGGTATGGACCAGCTG","chr1",62819296,"+","none","TGG","AGATTGGTATGGACCAGCTGTGG",0,NA,NA,NA,"GeneID:84938","ATG4C",TRUE,"ATG4C"

"12926","ATAGAGGATCACGTAATTGC","chr1",62816612,"+","none","AGG","ATAGAGGATCACGTAATTGCAGG",0,NA,NA,NA,"GeneID:84938","ATG4C",TRUE,"ATG4C"

"12927","ATTGTACATCCCGACTCTGC","chr1",62816594,"-","none","AGG","ATTGTACATCCCGACTCTGCAGG",0,NA,NA,NA,"GeneID:84938","ATG4C",TRUE,"ATG4C"

"12928","TTGGTTTGGTGATTCCCCCT","chr1",62819215,"+","none","TGG","TTGGTTTGGTGATTCCCCCTTGG",0,NA,NA,NA,"GeneID:84938","ATG4C",TRUE,"ATG4C"

"12929","TTGGTTTGGTGATTCCCCCT","chr22",50461916,"-","4:C>A,5:G>A","TGG","TTGGGCTGGTGATTCCCCCTTGG",2,5,6,NA,"GeneID:6305","SBF1",FALSE,"ATG4C"

"12930","ACCGTACTTGACGTTGTTCC","chr19",10544307,"-","none","AGG","ACCGTACTTGACGTTGTTCCAGG",0,NA,NA,NA,"GeneID:84971","ATG4D",TRUE,"ATG4D"

"12931","CCCGAGTGAGCCGGACGAAG","chr19",10544254,"+","none","TGG","CCCGAGTGAGCCGGACGAAGTGG",0,NA,NA,NA,"GeneID:84971","ATG4D",TRUE,"ATG4D"

"12932","CCGCTACCGTTTCGAGGGCG","chr19",10544843,"+","none","AGG","CCGCTACCGTTTCGAGGGCGAGG",0,NA,NA,NA,"GeneID:84971","ATG4D",TRUE,"ATG4D"

"12933","CTGCACCTCGCCCCGCAAGA","chr19",10552212,"+","none","TGG","CTGCACCTCGCCCCGCAAGATGG",0,NA,NA,NA,"GeneID:84971;GeneID:100302226","ATG4D;MIR1238",TRUE,"ATG4D"

"12934","GCTCACCCTCGCCCTCGAAA","chr19",10544852,"-","none","CGG","GCTCACCCTCGCCCTCGAAACGG",0,NA,NA,NA,"GeneID:84971","ATG4D",TRUE,"ATG4D"

"12935","GCTTGGGTCCATCTTGGCAA","chr19",10552238,"-","none","AGG","GCTTGGGTCCATCTTGGCAAAGG",0,NA,NA,NA,"GeneID:84971","ATG4D",TRUE,"ATG4D"

"12936","AAATGTACTGTGATGTTCCA","chr6",106293031,"+","none","AGG","AAATGTACTGTGATGTTCCAAGG",0,NA,NA,NA,"GeneID:9474","ATG5",TRUE,"ATG5"

"12937","AACTTGTTTCACGCTATATC","chr6",106316138,"-","none","AGG","AACTTGTTTCACGCTATATCAGG",0,NA,NA,NA,"GeneID:9474","ATG5",TRUE,"ATG5"

"12938","AAGAGTAAGTTATTTGACGT","chr6",106308460,"-","none","TGG","AAGAGTAAGTTATTTGACGTTGG",0,NA,NA,NA,"GeneID:9474","ATG5",TRUE,"ATG5"

"12939","AAGAGTAAGTTATTTGACGT","chr4",148451796,"-","0:A>T,4:T>C","GGG","TAGAATAAGTTATTTGACGTGGG",2,1,5,NA,"","",FALSE,"ATG5"

"12940","AGATCAAATAGCAAACCAAT","chr6",106293076,"+","none","TGG","AGATCAAATAGCAAACCAATTGG",0,NA,NA,NA,"GeneID:9474","ATG5",TRUE,"ATG5"

"12941","TGATATAGCGTGAAACAAGT","chr6",106316137,"+","none","TGG","TGATATAGCGTGAAACAAGTTGG",0,NA,NA,NA,"GeneID:9474","ATG5",TRUE,"ATG5"

"12942","TTCCATGAGTTTCCGATTGA","chr6",106248200,"+","none","TGG","TTCCATGAGTTTCCGATTGATGG",0,NA,NA,NA,"GeneID:9474","ATG5",TRUE,"ATG5"

"12943","AGAAGAAGCTGAACGAGTAT","chr3",11298789,"+","none","CGG","AGAAGAAGCTGAACGAGTATCGG",0,NA,NA,NA,"GeneID:10533","ATG7",TRUE,"ATG7"

"12944","CTTGAAAGACTCGAGTGTGT","chr3",11306998,"-","none","TGG","CTTGAAAGACTCGAGTGTGTTGG",0,NA,NA,NA,"GeneID:10533","ATG7",TRUE,"ATG7"

"12945","GATACTCGTTCAGCTTCTTC","chr3",11298790,"-","none","TGG","GATACTCGTTCAGCTTCTTCTGG",0,NA,NA,NA,"GeneID:10533","ATG7",TRUE,"ATG7"

"12946","GCCCGTTGCTGCCCAGCTAT","chr3",11306958,"+","none","TGG","GCCCGTTGCTGCCCAGCTATTGG",0,NA,NA,NA,"GeneID:10533","ATG7",TRUE,"ATG7"

"12947","GGTGAACCTCAGTGAATGTA","chr3",11340702,"+","none","TGG","GGTGAACCTCAGTGAATGTATGG",0,NA,NA,NA,"GeneID:10533","ATG7",TRUE,"ATG7"

"12948","GGTGAACCTCAGTGAATGTA","chr7",8865776,"-","14:A>T,15:G>T","AGG","GGTGAACCTCAGTGTCTGTAAGG",2,15,16,NA,"","",FALSE,"ATG7"

"12949","TAGGGTCCATACATTCACTG","chr3",11340711,"-","none","AGG","TAGGGTCCATACATTCACTGAGG",0,NA,NA,NA,"GeneID:10533","ATG7",TRUE,"ATG7"

"12950","AGGATATTCGAGAGAAGAAG","chr2",219227762,"+","none","AGG","AGGATATTCGAGAGAAGAAGAGG",0,NA,NA,NA,"GeneID:79065","ATG9A",TRUE,"ATG9A"

"12951","AGTTAGCGATGCCAATCCAC","chr2",219224478,"+","none","AGG","AGTTAGCGATGCCAATCCACAGG",0,NA,NA,NA,"GeneID:79065","ATG9A",TRUE,"ATG9A"

"12952","ATCACCATCCTGGTCATTGC","chr2",219225170,"-","none","TGG","ATCACCATCCTGGTCATTGCTGG",0,NA,NA,NA,"GeneID:79065","ATG9A",TRUE,"ATG9A"

"12953","ATCACCATCCTGGTCATTGC","chr3",129940248,"-","15:G>T,17:T>A","TGG","ATCACCATCCTGGTCCTAGCTGG",2,16,18,NA,"","",FALSE,"ATG9A"

"12954","ATCACCATCCTGGTCATTGC","chr16",85439180,"-","10:T>A,16:C>A","CGG","ATCACCATCCAGGTCAGTGCCGG",2,11,17,NA,"GeneID:23199","GSE1",FALSE,"ATG9A"

"12955","CTCAAGGCCGAGTACAAACG","chr2",219224546,"-","none","TGG","CTCAAGGCCGAGTACAAACGTGG",0,NA,NA,NA,"GeneID:79065","ATG9A",TRUE,"ATG9A"

"12956","CTGCCCTTCCGTATTGCACG","chr2",219224833,"-","none","TGG","CTGCCCTTCCGTATTGCACGTGG",0,NA,NA,NA,"GeneID:79065","ATG9A",TRUE,"ATG9A"

"12957","GCACGCTCTGCGCATCCCTA","chr2",219225072,"-","none","TGG","GCACGCTCTGCGCATCCCTATGG",0,NA,NA,NA,"GeneID:79065","ATG9A",TRUE,"ATG9A"

"12958","GCACGCTCTGCGCATCCCTA","chr1",2438148,"-","10:T>G,18:G>A","GGG","GCACGCTCTGAGCATCCCCAGGG",2,11,19,NA,"GeneID:9651","PLCH2",FALSE,"ATG9A"

"12959","CCGAGTGTCCATCGCCCGCC","chr11",290477,"+","none","TGG","CCGAGTGTCCATCGCCCGCCTGG",0,NA,NA,NA,"GeneID:80162","ATHL1",TRUE,"ATHL1"

"12960","GGAGTACCGCATCCGCACGC","chr11",292034,"+","none","TGG","GGAGTACCGCATCCGCACGCTGG",0,NA,NA,NA,"GeneID:80162","ATHL1",TRUE,"ATHL1"

"12961","GGTCCACGTCAACGGGGCCG","chr11",292619,"+","none","TGG","GGTCCACGTCAACGGGGCCGTGG",0,NA,NA,NA,"GeneID:80162","ATHL1",TRUE,"ATHL1"

"12962","TGAGGACATTTACGGAGTCC","chr11",292595,"+","none","AGG","TGAGGACATTTACGGAGTCCAGG",0,NA,NA,NA,"GeneID:80162","ATHL1",TRUE,"ATHL1"

"12963","TGCGATGCGCATAGATGCAC","chr11",290435,"-","none","TGG","TGCGATGCGCATAGATGCACTGG",0,NA,NA,NA,"GeneID:80162","ATHL1",TRUE,"ATHL1"

"12964","TGTTCCACCCAGAAGCCGCC","chr11",292003,"+","none","AGG","TGTTCCACCCAGAAGCCGCCAGG",0,NA,NA,NA,"GeneID:80162","ATHL1",TRUE,"ATHL1"

"12965","TGTTCCACCCAGAAGCCGCC","chr6",149965054,"-","2:C>A,17:A>C","TGG","TGGTCCACCCAGAAGCCTCCTGG",2,3,18,NA,"GeneID:80329","ULBP1",FALSE,"ATHL1"

"12966","AAGGCTTCTTCTTCATATCC","chr2",215336058,"-","none","TGG","AAGGCTTCTTCTTCATATCCTGG",0,NA,NA,NA,"GeneID:471","ATIC",TRUE,"ATIC"

"12967","AAGGCTTCTTCTTCATATCC","chr20",923021,"-","0:G>T,7:T>G","TGG","CAGGCTTATTCTTCATATCCTGG",2,1,8,NA,"","",FALSE,"ATIC"

"12968","AAGGCTTCTTCTTCATATCC","chrX",8632744,"-","7:T>G,13:A>G","AGG","AAGGCTTATTCTTTATATCCAGG",2,8,14,NA,"GeneID:3730","KAL1",FALSE,"ATIC"

"12969","AAGGCTTCTTCTTCATATCC","chr13",44133478,"-","4:T>G,15:C>A","TGG","AAGGATTCTTCTTCAGATCCTGG",2,5,16,NA,"GeneID:101929212","SMIM2-AS1",FALSE,"ATIC"

"12970","TCGCACAAGTTTATAAATCC","chr2",215332398,"-","none","AGG","TCGCACAAGTTTATAAATCCAGG",0,NA,NA,NA,"GeneID:471","ATIC",TRUE,"ATIC"

"12971","TCTGATGGTATAATTGCCCC","chr2",215336037,"+","none","AGG","TCTGATGGTATAATTGCCCCAGG",0,NA,NA,NA,"GeneID:471","ATIC",TRUE,"ATIC"

"12972","TCTGATGGTATAATTGCCCC","chr3",120693993,"-","4:A>T,19:T>G","AGG","TCTGTTGGTATAATTGCCCAAGG",2,5,20,NA,"GeneID:285282","RABL3",FALSE,"ATIC"

"12973","TCTTGCATATGCCGCTGAGA","chr2",215333430,"-","none","TGG","TCTTGCATATGCCGCTGAGATGG",0,NA,NA,NA,"GeneID:471","ATIC",TRUE,"ATIC"

"12974","TGTGCGATGCTTTGAACGCC","chr2",215332411,"+","none","TGG","TGTGCGATGCTTTGAACGCCTGG",0,NA,NA,NA,"GeneID:471","ATIC",TRUE,"ATIC"

"12975","TTCTAACCAGGTGCTGCTGT","chr2",215333339,"+","none","TGG","TTCTAACCAGGTGCTGCTGTTGG",0,NA,NA,NA,"GeneID:471","ATIC",TRUE,"ATIC"

"12976","ATCTCAACATGAAGTCCATC","chr14",50588044,"-","none","AGG","ATCTCAACATGAAGTCCATCAGG",0,NA,NA,NA,"GeneID:51062","ATL1",TRUE,"ATL1"

"12977","CACAATGATCAGCTCAATAC","chr14",50591617,"+","none","AGG","CACAATGATCAGCTCAATACAGG",0,NA,NA,NA,"GeneID:51062","ATL1",TRUE,"ATL1"

"12978","GACTACAATGAACCATTGAC","chr14",50590961,"+","none","TGG","GACTACAATGAACCATTGACTGG",0,NA,NA,NA,"GeneID:51062","ATL1",TRUE,"ATL1"

"12979","GGATCTGAGCGAGAGACCAC","chr14",50591000,"+","none","AGG","GGATCTGAGCGAGAGACCACAGG",0,NA,NA,NA,"GeneID:51062","ATL1",TRUE,"ATL1"

"12980","GTAGGTTGCAGTGTTATTGA","chr14",50591530,"+","none","TGG","GTAGGTTGCAGTGTTATTGATGG",0,NA,NA,NA,"GeneID:51062","ATL1",TRUE,"ATL1"

"12981","GTTGAGATACATGTACAACC","chr14",50588056,"+","none","AGG","GTTGAGATACATGTACAACCAGG",0,NA,NA,NA,"GeneID:51062","ATL1",TRUE,"ATL1"

"12982","ACGAGATCTTAACATAGTAG","chr2",38343344,"-","none","TGG","ACGAGATCTTAACATAGTAGTGG",0,NA,NA,NA,"GeneID:64225","ATL2",TRUE,"ATL2"

"12983","CACAGTCTTTGATAGTTGAC","chr2",38318575,"+","none","TGG","CACAGTCTTTGATAGTTGACTGG",0,NA,NA,NA,"GeneID:64225","ATL2",TRUE,"ATL2"

"12984","CACAGTCTTTGATAGTTGAC","chr11",63652511,"+","10:C>G,12:C>T","TGG","CACAGTCTTTCACAGTTGACTGG",2,11,13,NA,"GeneID:25923","ATL3",TRUE,"ATL2"

"12985","CGCCATGTAAAGCCTGTCAA","chr2",38318963,"+","none","TGG","CGCCATGTAAAGCCTGTCAATGG",0,NA,NA,NA,"GeneID:64225","ATL2",TRUE,"ATL2"

"12986","CTAGGTTGCTGTGCTGCTTA","chr2",38318623,"-","none","TGG","CTAGGTTGCTGTGCTGCTTATGG",0,NA,NA,NA,"GeneID:64225","ATL2",TRUE,"ATL2"

"12987","GGAAACAATGAACCATTGAC","chr2",38318978,"-","none","AGG","GGAAACAATGAACCATTGACAGG",0,NA,NA,NA,"GeneID:64225","ATL2",TRUE,"ATL2"

"12988","TGAGCAAGAACAATCTGTAC","chr2",38343430,"+","none","TGG","TGAGCAAGAACAATCTGTACTGG",0,NA,NA,NA,"GeneID:64225","ATL2",TRUE,"ATL2"

"12989","AATCCTGTTAACGGTTCTTC","chr11",63658848,"+","none","TGG","AATCCTGTTAACGGTTCTTCTGG",0,NA,NA,NA,"GeneID:25923","ATL3",TRUE,"ATL3"

"12990","AGATGATCTTCAACAGCTGC","chr11",63651937,"-","none","AGG","AGATGATCTTCAACAGCTGCAGG",0,NA,NA,NA,"GeneID:25923","ATL3",TRUE,"ATL3"

"12991","CACAGTCTTTCACAGTTGAC","chr11",63652511,"+","none","TGG","CACAGTCTTTCACAGTTGACTGG",0,NA,NA,NA,"GeneID:25923","ATL3",TRUE,"ATL3"

"12992","CACAGTCTTTCACAGTTGAC","chr2",38318575,"+","10:G>C,12:T>C","TGG","CACAGTCTTTGATAGTTGACTGG",2,11,13,NA,"GeneID:64225","ATL2",TRUE,"ATL3"

"12993","CACTATGACTAGTTCTGTTC","chr11",63652472,"-","none","AGG","CACTATGACTAGTTCTGTTCAGG",0,NA,NA,NA,"GeneID:25923","ATL3",TRUE,"ATL3"

"12994","CACTATGACTAGTTCTGTTC","chr2",38318536,"-","12:G>A,18:G>A","AGG","CACTATGACTAGCTCTGTCCAGG",2,13,19,NA,"GeneID:64225","ATL2",TRUE,"ATL3"

"12995","GACCCAGAAGAACCGTTAAC","chr11",63658854,"-","none","AGG","GACCCAGAAGAACCGTTAACAGG",0,NA,NA,NA,"GeneID:25923","ATL3",TRUE,"ATL3"

"12996","TGGATCAGATCCCCCTCTCC","chr11",63658823,"+","none","AGG","TGGATCAGATCCCCCTCTCCAGG",0,NA,NA,NA,"GeneID:25923","ATL3",TRUE,"ATL3"

"12997","TGGATCAGATCCCCCTCTCC","chr17",40084725,"-","13:T>G,18:A>G","AGG","TGGATCAGATCCCACTCTTCAGG",2,14,19,NA,"GeneID:7067","THRA",TRUE,"ATL3"

"12998","AAACAATTAAACATCTAGAT","chr11",108227815,"+","none","CGG","AAACAATTAAACATCTAGATCGG",0,NA,NA,NA,"GeneID:472","ATM",TRUE,"ATM"

"12999","CCAAGGCTATTCAGTGTGCG","chr11",108244096,"+","none","AGG","CCAAGGCTATTCAGTGTGCGAGG",0,NA,NA,NA,"GeneID:472","ATM",TRUE,"ATM"

"13000","CCTCGCACACTGAATAGCCT","chr11",108244099,"-","none","TGG","CCTCGCACACTGAATAGCCTTGG",0,NA,NA,NA,"GeneID:472","ATM",TRUE,"ATM"

"13001","CTCTATCATGTTCTAGTTGA","chr11",108227662,"-","none","CGG","CTCTATCATGTTCTAGTTGACGG",0,NA,NA,NA,"GeneID:472","ATM",TRUE,"ATM"

"13002","TCTAGATGTTTAATTGTTTC","chr11",108227814,"-","none","AGG","TCTAGATGTTTAATTGTTTCAGG",0,NA,NA,NA,"GeneID:472","ATM",TRUE,"ATM"

"13003","TCTAGATGTTTAATTGTTTC","chr5",105802355,"-","1:T>G,4:G>C","TGG","TATACATGTTTAATTGTTTCTGG",2,2,5,NA,"","",FALSE,"ATM"

"13004","TCTAGATGTTTAATTGTTTC","chr2",51256570,"-","5:A>T","AGG","TCTAGTTGTTTAATTGTTTCAGG",1,6,NA,NA,"GeneID:730100","LOC730100",FALSE,"ATM"

"13005","TCTAGATGTTTAATTGTTTC","chr1",116281333,"+","6:A>T,11:T>A","AGG","TCTAGAAGTTTTATTGTTTCAGG",2,7,12,NA,"","",FALSE,"ATM"

"13006","TCTAGATGTTTAATTGTTTC","chr14",27894350,"+","7:T>G,11:T>A","AGG","TCTAGATTTTTTATTGTTTCAGG",2,8,12,NA,"","",FALSE,"ATM"

"13007","TCTAGATGTTTAATTGTTTC","chr12",86752934,"-","6:T>A,11:A>T","AGG","TCTAGAAGTTTTATTGTTTCAGG",2,7,12,NA,"GeneID:25834","MGAT4C",FALSE,"ATM"

"13008","TGATAGAGCTACAGAACGAA","chr11",108227674,"+","none","AGG","TGATAGAGCTACAGAACGAAAGG",0,NA,NA,NA,"GeneID:472","ATM",TRUE,"ATM"

"13009","AGACTGCAGTGCTGTTCTAC","chr16",81042419,"-","none","TGG","AGACTGCAGTGCTGTTCTACTGG",0,NA,NA,NA,"GeneID:23300","ATMIN",TRUE,"ATMIN"

"13010","AGACTGCAGTGCTGTTCTAC","chr9",95292935,"-","8:T>C,13:A>C","TGG","AGACTGCAATGCTTTTCTACTGG",2,9,14,NA,"GeneID:2176","FANCC",TRUE,"ATMIN"

"13011","CGGGACAGCTCGCTCACCGA","chr16",81036083,"-","none","CGG","CGGGACAGCTCGCTCACCGACGG",0,NA,NA,NA,"GeneID:23300","ATMIN",TRUE,"ATMIN"

"13012","GCACAGGATGTTGGTCCGCA","chr16",81036108,"-","none","CGG","GCACAGGATGTTGGTCCGCACGG",0,NA,NA,NA,"GeneID:23300","ATMIN",TRUE,"ATMIN"

"13013","TGCTGTCTGAGTTTCGTTGC","chr16",81044842,"-","none","TGG","TGCTGTCTGAGTTTCGTTGCTGG",0,NA,NA,NA,"GeneID:23300","ATMIN",TRUE,"ATMIN"

"13014","TTCGATATCAAAATCGATTC","chr16",81044395,"-","none","CGG","TTCGATATCAAAATCGATTCCGG",0,NA,NA,NA,"GeneID:23300","ATMIN",TRUE,"ATMIN"

"13015","TTCTACTGCTGTCCAATTGA","chr16",81041406,"+","none","AGG","TTCTACTGCTGTCCAATTGAAGG",0,NA,NA,NA,"GeneID:23300","ATMIN",TRUE,"ATMIN"

"13016","TTCTACTGCTGTCCAATTGA","chr7",95753559,"+","12:T>C,17:G>T","AGG","TTCTACTGCTGTTCAATGGAAGG",2,13,18,NA,"","",FALSE,"ATMIN"

"13017","TTCTACTGCTGTCCAATTGA","chr9",95292721,"+","8:T>C,17:C>T","AGG","TTCTACTGTTGTCCAATCGAAGG",2,9,18,NA,"GeneID:2176","FANCC",TRUE,"ATMIN"

"13018","TTCTACTGCTGTCCAATTGA","chr4",166502012,"+","9:A>T,17:A>T","TGG","TTCTACTGCAGTCCAATAGATGG",2,10,18,NA,"","",FALSE,"ATMIN"

"13019","ACGAGTTGAAGCCGCGATCC","chr12",6937859,"-","none","AGG","ACGAGTTGAAGCCGCGATCCAGG",0,NA,NA,NA,"GeneID:1822","ATN1",TRUE,"ATN1"

"13020","CCCGGGAAGAACTGAGATCG","chr12",6934215,"+","none","AGG","CCCGGGAAGAACTGAGATCGAGG",0,NA,NA,NA,"GeneID:1822","ATN1",TRUE,"ATN1"

"13021","CGACCCTGCTTGTTGACCTT","chr12",6934494,"-","none","TGG","CGACCCTGCTTGTTGACCTTTGG",0,NA,NA,NA,"GeneID:1822","ATN1",TRUE,"ATN1"

"13022","CTAACAGAAGGCCCGAGTAG","chr12",6934457,"+","none","AGG","CTAACAGAAGGCCCGAGTAGAGG",0,NA,NA,NA,"GeneID:1822","ATN1",TRUE,"ATN1"

"13023","TCCTCCTGTAGATGTCAATG","chr12",6934164,"+","none","AGG","TCCTCCTGTAGATGTCAATGAGG",0,NA,NA,NA,"GeneID:1822","ATN1",TRUE,"ATN1"

"13024","TTCTTCGCGCGCGCGCTGCT","chr12",6937972,"-","none","CGG","TTCTTCGCGCGCGCGCTGCTCGG",0,NA,NA,NA,"GeneID:1822","ATN1",TRUE,"ATN1"

"13025","AACATTGCGCAGCTGGTCGA","chr4",93829461,"-","none","AGG","AACATTGCGCAGCTGGTCGAAGG",0,NA,NA,NA,"GeneID:474","ATOH1",TRUE,"ATOH1"

"13026","CGTTTGTAGCAGCTCGGACA","chr4",93829557,"-","none","AGG","CGTTTGTAGCAGCTCGGACAAGG",0,NA,NA,NA,"GeneID:474","ATOH1",TRUE,"ATOH1"

"13027","GAAGCGAGTCCGGCAACTCC","chr4",93829734,"-","none","CGG","GAAGCGAGTCCGGCAACTCCCGG",0,NA,NA,NA,"GeneID:474","ATOH1",TRUE,"ATOH1"

"13028","GAGTACCCTCCCGCAGAAGC","chr4",93829763,"-","none","TGG","GAGTACCCTCCCGCAGAAGCTGG",0,NA,NA,NA,"GeneID:474","ATOH1",TRUE,"ATOH1"

"13029","GCGGGCAACGCGACCGCAGC","chr4",93829667,"+","none","TGG","GCGGGCAACGCGACCGCAGCTGG",0,NA,NA,NA,"GeneID:474","ATOH1",TRUE,"ATOH1"

"13030","GTTGCCCGCGCCCCCTTCAT","chr4",93829656,"-","none","AGG","GTTGCCCGCGCCCCCTTCATAGG",0,NA,NA,NA,"GeneID:474","ATOH1",TRUE,"ATOH1"

"13031","CACCCTGCGTAAGCGGTCGA","chr10",68231479,"+","none","AGG","CACCCTGCGTAAGCGGTCGAAGG",0,NA,NA,NA,"GeneID:220202","ATOH7",TRUE,"ATOH7"

"13032","CCGCTCCGAGCCGAATCGCT","chr10",68231359,"+","none","CGG","CCGCTCCGAGCCGAATCGCTCGG",0,NA,NA,NA,"GeneID:220202","ATOH7",TRUE,"ATOH7"

"13033","CTGCCTTCGACCGCTTACGC","chr10",68231485,"-","none","AGG","CTGCCTTCGACCGCTTACGCAGG",0,NA,NA,NA,"GeneID:220202","ATOH7",TRUE,"ATOH7"

"13034","CTGGCCGAGGCCGAGCGATT","chr10",68231372,"-","none","CGG","CTGGCCGAGGCCGAGCGATTCGG",0,NA,NA,NA,"GeneID:220202","ATOH7",TRUE,"ATOH7"

"13035","GCGCGCCCTAGGTGGCCATC","chr10",68231211,"+","none","TGG","GCGCGCCCTAGGTGGCCATCTGG",0,NA,NA,NA,"GeneID:220202","ATOH7",TRUE,"ATOH7"

"13036","GCGCGTTGCACCCCCGTGCG","chr10",68231619,"-","none","CGG","GCGCGTTGCACCCCCGTGCGCGG",0,NA,NA,NA,"GeneID:220202","ATOH7",TRUE,"ATOH7"

"13037","CGCCCGAGGTCTCCGACGCG","chr2",85754490,"+","none","CGG","CGCCCGAGGTCTCCGACGCGCGG",0,NA,NA,NA,"GeneID:84913","ATOH8",TRUE,"ATOH8"

"13038","GAAGCAGCGTTTCCGCGCGT","chr2",85754505,"-","none","CGG","GAAGCAGCGTTTCCGCGCGTCGG",0,NA,NA,NA,"GeneID:84913","ATOH8",TRUE,"ATOH8"

"13039","GGGACGTGCCAAGAAGCGCA","chr2",85764160,"+","none","AGG","GGGACGTGCCAAGAAGCGCAAGG",0,NA,NA,NA,"GeneID:84913","ATOH8",TRUE,"ATOH8"

"13040","GGTGCATACCTTGCGCTTCT","chr2",85764171,"-","none","TGG","GGTGCATACCTTGCGCTTCTTGG",0,NA,NA,NA,"GeneID:84913","ATOH8",TRUE,"ATOH8"

"13041","TAGCCGTTCGCGCGCCGCGC","chr2",85754288,"-","none","CGG","TAGCCGTTCGCGCGCCGCGCCGG",0,NA,NA,NA,"GeneID:84913","ATOH8",TRUE,"ATOH8"

"13042","TCTTCCACGGCCCGTCCTCG","chr2",85754209,"-","none","AGG","TCTTCCACGGCCCGTCCTCGAGG",0,NA,NA,NA,"GeneID:84913","ATOH8",TRUE,"ATOH8"

"13043","ACTCACCTCCAAGCTTATTG","chr5",151751697,"+","none","AGG","ACTCACCTCCAAGCTTATTGAGG",0,NA,NA,NA,"GeneID:475","ATOX1",TRUE,"ATOX1"

"13044","CATTGACCTGCCCAACAAGA","chr5",151746416,"-","none","AGG","CATTGACCTGCCCAACAAGAAGG",0,NA,NA,NA,"GeneID:475","ATOX1",TRUE,"ATOX1"

"13045","CTTGCAACCCTGAAGAAAAC","chr5",151746358,"-","none","AGG","CTTGCAACCCTGAAGAAAACAGG",0,NA,NA,NA,"GeneID:475","ATOX1",TRUE,"ATOX1"

"13046","GCTGTGCTGAAGCTGTCTCT","chr5",151751725,"-","none","CGG","GCTGTGCTGAAGCTGTCTCTCGG",0,NA,NA,NA,"GeneID:475","ATOX1",TRUE,"ATOX1"

"13047","GCTGTGCTGAAGCTGTCTCT","chr12",3291763,"-","16:C>G,17:G>A","TGG","GCTGTGCTGAAGCTGTGCCTTGG",2,17,18,NA,"","",FALSE,"ATOX1"

"13048","GCTGTGCTGAAGCTGTCTCT","chr17",81230801,"-","6:C>G,9:C>T","GGG","GCTGTGGTGGAGCTGTCTCTGGG",2,7,10,NA,"GeneID:146705","ENTHD2",TRUE,"ATOX1"

"13049","TCAGAAGCACGAGTTCTCTG","chr5",151751763,"-","none","TGG","TCAGAAGCACGAGTTCTCTGTGG",0,NA,NA,NA,"GeneID:475","ATOX1",TRUE,"ATOX1"

"13050","TCAGAAGCACGAGTTCTCTG","chr4",110749778,"-","9:A>G,12:G>C","AGG","TCAGAAGCATGACTTCTCTGAGG",2,10,13,NA,"","",FALSE,"ATOX1"

"13051","TTCAATGCAGACCTTCTTGT","chr5",151746402,"+","none","TGG","TTCAATGCAGACCTTCTTGTTGG",0,NA,NA,NA,"GeneID:475","ATOX1",TRUE,"ATOX1"

"13052","CCACATCGAGACCGCCAACC","chr15",25781074,"-","none","TGG","CCACATCGAGACCGCCAACCTGG",0,NA,NA,NA,"GeneID:57194","ATP10A",TRUE,"ATP10A"

"13053","CCAGGTTGGCGGTCTCGATG","chr15",25781071,"+","none","TGG","CCAGGTTGGCGGTCTCGATGTGG",0,NA,NA,NA,"GeneID:57194","ATP10A",TRUE,"ATP10A"

"13054","GTGTCCCAGCGCGGCAGCAT","chr15",25718311,"-","none","CGG","GTGTCCCAGCGCGGCAGCATCGG",0,NA,NA,NA,"GeneID:57194","ATP10A",TRUE,"ATP10A"

"13055","TAAGGAGCTTCCATCAGACT","chr15",25723944,"+","none","TGG","TAAGGAGCTTCCATCAGACTTGG",0,NA,NA,NA,"GeneID:57194","ATP10A",TRUE,"ATP10A"

"13056","TAAGGAGCTTCCATCAGACT","chr7",158965211,"+","12:C>A,14:G>C","GGG","TAAGGAGCTTCCCTGAGACTGGG",2,13,15,NA,"","",FALSE,"ATP10A"

"13057","TAAGGAGCTTCCATCAGACT","chr2",176960993,"+","1:G>A,13:C>T","TGG","TGAGGAGCTTCCACCAGACTTGG",2,2,14,NA,"","",FALSE,"ATP10A"

"13058","TCTCCCCGCAGCGCAGCGTC","chr15",25718390,"-","none","TGG","TCTCCCCGCAGCGCAGCGTCTGG",0,NA,NA,NA,"GeneID:57194","ATP10A",TRUE,"ATP10A"

"13059","TCTCCCCGCAGCGCAGCGTC","chr7",100451006,"+","11:T>C,17:C>G","CGG","TCTCCCCGCAGTGCAGCCTCCGG",2,12,18,NA,"","",FALSE,"ATP10A"

"13060","TTACTACAGGACATGGACTG","chr15",25724010,"-","none","TGG","TTACTACAGGACATGGACTGTGG",0,NA,NA,NA,"GeneID:57194","ATP10A",TRUE,"ATP10A"

"13061","TTACTACAGGACATGGACTG","chr10",98169455,"-","8:A>C,13:C>A","TGG","TTACTACATGACAGGGACTGTGG",2,9,14,NA,"GeneID:27291","R3HCC1L",FALSE,"ATP10A"

"13062","AGCAGCGGTGTGGTTTCCGA","chr5",160687982,"+","none","TGG","AGCAGCGGTGTGGTTTCCGATGG",0,NA,NA,NA,"GeneID:23120","ATP10B",TRUE,"ATP10B"

"13063","CAACCACCTCAACAAATTTA","chr5",160649180,"-","none","AGG","CAACCACCTCAACAAATTTAAGG",0,NA,NA,NA,"GeneID:23120","ATP10B",TRUE,"ATP10B"

"13064","CAAGCAAAGATGTGTCGTGA","chr5",160670479,"-","none","AGG","CAAGCAAAGATGTGTCGTGAAGG",0,NA,NA,NA,"GeneID:23120","ATP10B",TRUE,"ATP10B"

"13065","GCCACGATGAGTCCACTGAG","chr5",160688046,"+","none","AGG","GCCACGATGAGTCCACTGAGAGG",0,NA,NA,NA,"GeneID:23120","ATP10B",TRUE,"ATP10B"

"13066","GTTGGGTTTCTCACACACGA","chr5",160649199,"+","none","TGG","GTTGGGTTTCTCACACACGATGG",0,NA,NA,NA,"GeneID:23120","ATP10B",TRUE,"ATP10B"

"13067","TCACGACACATCTTTGCTTG","chr5",160670479,"+","none","AGG","TCACGACACATCTTTGCTTGAGG",0,NA,NA,NA,"GeneID:23120","ATP10B",TRUE,"ATP10B"

"13068","AAAAATACATTGACCGATGC","chr4",47523019,"+","none","TGG","AAAAATACATTGACCGATGCTGG",0,NA,NA,NA,"GeneID:57205","ATP10D",TRUE,"ATP10D"

"13069","CACTGGCGACGGCTGATCAG","chr4",47512570,"+","none","AGG","CACTGGCGACGGCTGATCAGAGG",0,NA,NA,NA,"GeneID:57205","ATP10D",TRUE,"ATP10D"

"13070","CTACCAAAGGTACCCAGTTC","chr4",47515511,"-","none","AGG","CTACCAAAGGTACCCAGTTCAGG",0,NA,NA,NA,"GeneID:57205","ATP10D",TRUE,"ATP10D"

"13071","TATTCCTCGTTGCTCGCCTG","chr4",47512627,"+","none","TGG","TATTCCTCGTTGCTCGCCTGTGG",0,NA,NA,NA,"GeneID:57205","ATP10D",TRUE,"ATP10D"

"13072","TATTCGCCTCTCCTGCAACG","chr4",47523065,"+","none","AGG","TATTCGCCTCTCCTGCAACGAGG",0,NA,NA,NA,"GeneID:57205","ATP10D",TRUE,"ATP10D"

"13073","TGTATTTCCGATAATCTTCC","chr4",47515604,"-","none","AGG","TGTATTTCCGATAATCTTCCAGG",0,NA,NA,NA,"GeneID:57205","ATP10D",TRUE,"ATP10D"

"13074","CCCACAAGTCCAGTGACAAG","chr13",112806227,"+","none","CGG","CCCACAAGTCCAGTGACAAGCGG",0,NA,NA,NA,"GeneID:23250","ATP11A",TRUE,"ATP11A"

"13075","CCTGCACCAGAAATATGATA","chr13",112805027,"-","none","AGG","CCTGCACCAGAAATATGATAAGG",0,NA,NA,NA,"GeneID:23250","ATP11A",TRUE,"ATP11A"

"13076","CCTGCACCAGAAATATGATA","chr4",118718514,"+","7:A>C,13:C>T","GGG","CCTGCACAAGAAACATGATAGGG",2,8,14,NA,"","",FALSE,"ATP11A"

"13077","GAATTGTTCAAATAAATTCT","chr13",112804981,"-","none","TGG","GAATTGTTCAAATAAATTCTTGG",0,NA,NA,NA,"GeneID:23250","ATP11A",TRUE,"ATP11A"

"13078","GACGAGACGATCCTGTTGTC","chr13",112785233,"-","none","TGG","GACGAGACGATCCTGTTGTCTGG",0,NA,NA,NA,"GeneID:23250","ATP11A",TRUE,"ATP11A"

"13079","GGACAGCAGGACCATCTACG","chr13",112785157,"+","none","TGG","GGACAGCAGGACCATCTACGTGG",0,NA,NA,NA,"GeneID:23250","ATP11A",TRUE,"ATP11A"

"13080","TCCGCTTGTCACTGGACTTG","chr13",112806231,"-","none","TGG","TCCGCTTGTCACTGGACTTGTGG",0,NA,NA,NA,"GeneID:23250","ATP11A",TRUE,"ATP11A"

"13081","ATCTTTCCCTATTAGTAAAT","chr3",182889504,"-","none","AGG","ATCTTTCCCTATTAGTAAATAGG",0,NA,NA,NA,"GeneID:23200","ATP11B",TRUE,"ATP11B"

"13082","ATCTTTCCCTATTAGTAAAT","chr13",95266692,"+","5:C>T,9:C>T","GGG","ATCTTCCCCCATTAGTAAATGGG",2,6,10,NA,"GeneID:10257","ABCC4",FALSE,"ATP11B"

"13083","ATCTTTCCCTATTAGTAAAT","chr1",239960696,"+","1:A>T,11:G>T","CGG","AACTTTCCCTAGTAGTAAATCGG",2,2,12,NA,"","",FALSE,"ATP11B"

"13084","ATCTTTCCCTATTAGTAAAT","chr11",114034228,"+","9:C>T,13:T>A","GGG","ATCTTTCCCCATTTGTAAATGGG",2,10,14,NA,"","",FALSE,"ATP11B"

"13085","CACTTTGGTCTTCACAGTCA","chr3",182896722,"+","none","TGG","CACTTTGGTCTTCACAGTCATGG",0,NA,NA,NA,"GeneID:23200","ATP11B",TRUE,"ATP11B"

"13086","CTGTTTAGATGTTTGGAAAC","chr3",182896691,"+","none","TGG","CTGTTTAGATGTTTGGAAACTGG",0,NA,NA,NA,"GeneID:23200","ATP11B",TRUE,"ATP11B"

"13087","GAACCATCTACGTAGCCAAC","chr3",182820290,"+","none","AGG","GAACCATCTACGTAGCCAACAGG",0,NA,NA,NA,"GeneID:23200","ATP11B",TRUE,"ATP11B"

"13088","GCCATTCTGAGGAAACCTGT","chr3",182820308,"-","none","TGG","GCCATTCTGAGGAAACCTGTTGG",0,NA,NA,NA,"GeneID:23200","ATP11B",TRUE,"ATP11B"

"13089","GGGAAAGATACATCTCTGCT","chr3",182889515,"+","none","TGG","GGGAAAGATACATCTCTGCTTGG",0,NA,NA,NA,"GeneID:23200","ATP11B",TRUE,"ATP11B"

"13090","CCAACTAGCCCAGTTACCAG","chrX",139816908,"-","none","TGG","CCAACTAGCCCAGTTACCAGTGG",0,NA,NA,NA,"GeneID:286410","ATP11C",TRUE,"ATP11C"

"13091","CCACTGGTAACTGGGCTAGT","chrX",139816905,"+","none","TGG","CCACTGGTAACTGGGCTAGTTGG",0,NA,NA,NA,"GeneID:286410","ATP11C",TRUE,"ATP11C"

"13092","CCACTGGTAACTGGGCTAGT","chr3",182829689,"-","14:T>C,17:C>T","AGG","CCACTGGTAACTGGACTGGTAGG",2,15,18,NA,"GeneID:23200","ATP11B",TRUE,"ATP11C"

"13093","CTCCCTTACAGTATACACTT","chrX",139819418,"-","none","TGG","CTCCCTTACAGTATACACTTTGG",0,NA,NA,NA,"GeneID:286410","ATP11C",TRUE,"ATP11C"

"13094","TAAGCTTCTGTTTCCGAAAC","chrX",139826746,"+","none","TGG","TAAGCTTCTGTTTCCGAAACTGG",0,NA,NA,NA,"GeneID:286410","ATP11C",TRUE,"ATP11C"

"13095","TAATAGAATAGTCTCATCTA","chrX",139826705,"-","none","AGG","TAATAGAATAGTCTCATCTAAGG",0,NA,NA,NA,"GeneID:286410","ATP11C",TRUE,"ATP11C"

"13096","TAATAGAATAGTCTCATCTA","chr5",36660721,"-","3:T>A,16:G>A","GGG","TAAAAGAATAGTCTCACCTAGGG",2,4,17,NA,"GeneID:6507","SLC1A3",FALSE,"ATP11C"

"13097","TCTCATAATCTTCCTTGTAC","chrX",139819339,"-","none","AGG","TCTCATAATCTTCCTTGTACAGG",0,NA,NA,NA,"GeneID:286410","ATP11C",TRUE,"ATP11C"

"13098","TCTCATAATCTTCCTTGTAC","chr21",16851007,"-","6:A>T,13:T>G","AGG","TCTCATTATCTTCATTGTACAGG",2,7,14,NA,"","",FALSE,"ATP11C"

"13099","AACCCCAGAAATTTACTCCG","chr13",24681563,"+","none","TGG","AACCCCAGAAATTTACTCCGTGG",0,NA,NA,NA,"GeneID:479","ATP12A",TRUE,"ATP12A"

"13100","ATGACCACAAACTCAGCAAT","chr13",24685314,"+","none","AGG","ATGACCACAAACTCAGCAATAGG",0,NA,NA,NA,"GeneID:479","ATP12A",TRUE,"ATP12A"

"13101","ATGACCACAAACTCAGCAAT","chr4",161700313,"-","4:C>G,16:C>G","TGG","ATGAGCACAAACTCAGGAATTGG",2,5,17,NA,"GeneID:56884","FSTL5",FALSE,"ATP12A"

"13102","ATTCCCTATTGCTGAGTTTG","chr13",24685321,"-","none","TGG","ATTCCCTATTGCTGAGTTTGTGG",0,NA,NA,NA,"GeneID:479","ATP12A",TRUE,"ATP12A"

"13103","ATTCCCTATTGCTGAGTTTG","chr2",217694072,"-","5:A>G,15:T>C","TGG","ATTCCTTATTGCTGAATTTGTGG",2,6,16,NA,"","",TRUE,"ATP12A"

"13104","CTTAGTTCCGCTGAGCTCCA","chr13",24681583,"-","none","CGG","CTTAGTTCCGCTGAGCTCCACGG",0,NA,NA,NA,"GeneID:479","ATP12A",TRUE,"ATP12A"

"13105","GGACATCGTGAAAACAGACA","chr13",24681602,"+","none","AGG","GGACATCGTGAAAACAGACAAGG",0,NA,NA,NA,"GeneID:479","ATP12A",TRUE,"ATP12A"

"13106","GGACATCGTGAAAACAGACA","chr2",120210414,"+","7:C>G,18:G>C","AGG","GGACATCCTGAAAACAGAGAAGG",2,8,19,NA,"","",FALSE,"ATP12A"

"13107","GTTTCAGAAAGAACTCCATC","chr13",24681698,"+","none","TGG","GTTTCAGAAAGAACTCCATCTGG",0,NA,NA,NA,"GeneID:479","ATP12A",TRUE,"ATP12A"

"13108","CAAGTATTCCTACGATGCCC","chr19",19659727,"-","none","TGG","CAAGTATTCCTACGATGCCCTGG",0,NA,NA,NA,"GeneID:57130","ATP13A1",TRUE,"ATP13A1"

"13109","CATAGCGAACGGTGACGAGC","chr19",19663521,"-","none","TGG","CATAGCGAACGGTGACGAGCTGG",0,NA,NA,NA,"GeneID:57130","ATP13A1",TRUE,"ATP13A1"

"13110","CGTGGCCCTGCACCGCAATG","chr19",19659899,"-","none","AGG","CGTGGCCCTGCACCGCAATGAGG",0,NA,NA,NA,"GeneID:57130","ATP13A1",TRUE,"ATP13A1"

"13111","TCACCGTTCGCTATGAGCGC","chr19",19663526,"+","none","CGG","TCACCGTTCGCTATGAGCGCCGG",0,NA,NA,NA,"GeneID:57130","ATP13A1",TRUE,"ATP13A1"

"13112","TCCCTACCTCATTGCGGTGC","chr19",19659890,"+","none","AGG","TCCCTACCTCATTGCGGTGCAGG",0,NA,NA,NA,"GeneID:57130","ATP13A1",TRUE,"ATP13A1"

"13113","TCCCTACCTCATTGCGGTGC","chr14",53152617,"+","2:A>C,12:A>T","CGG","TCACTACCTCATAGCGGTGCCGG",2,3,13,NA,"GeneID:80821","DDHD1",TRUE,"ATP13A1"

"13114","TTCTACCGCAGGGCGAAGAC","chr19",19659782,"-","none","GGG","TTCTACCGCAGGGCGAAGACGGG",0,NA,NA,NA,"GeneID:57130","ATP13A1",TRUE,"ATP13A1"

"13115","TTCTACCGCAGGGCGAAGAC","chr7",1523792,"-","6:A>G,13:T>G","AGG","TTCTACTGCAGGGAGAAGACAGG",2,7,14,NA,"","",FALSE,"ATP13A1"

"13116","CCCCAACGTGATCAGCATAC","chr1",17002068,"-","none","CGG","CCCCAACGTGATCAGCATACCGG",0,NA,NA,NA,"GeneID:23400","ATP13A2",TRUE,"ATP13A2"

"13117","CCCCAACGTGATCAGCATAC","chr7",96121303,"+","15:G>C,18:T>A","GGG","CCCCAACGTGATCAGGATTCGGG",2,16,19,NA,"GeneID:10165","SLC25A13",TRUE,"ATP13A2"

"13118","CTGTGTTCCAGGATAGTTCC","chr1",17005063,"-","none","TGG","CTGTGTTCCAGGATAGTTCCTGG",0,NA,NA,NA,"GeneID:23400","ATP13A2",TRUE,"ATP13A2"

"13119","GACCGGTATGCTGATCACGT","chr1",17002063,"+","none","TGG","GACCGGTATGCTGATCACGTTGG",0,NA,NA,NA,"GeneID:23400","ATP13A2",TRUE,"ATP13A2"

"13120","GCCTCACCTGCCCTCGCCGA","chr1",17005006,"+","none","TGG","GCCTCACCTGCCCTCGCCGATGG",0,NA,NA,NA,"GeneID:23400","ATP13A2",TRUE,"ATP13A2"

"13121","GCCTCACCTGCCCTCGCCGA","chr19",57578631,"-","15:A>C,19:G>T","CGG","GCCTCACCTGCCCTCTCCGCCGG",2,16,20,NA,"GeneID:55659;GeneID:284307","ZNF416;ZIK1",TRUE,"ATP13A2"

"13122","GCCTCACCTGCCCTCGCCGA","chrX",150984088,"-","5:G>T,19:C>T","AGG","GCCTCCCCTGCCCTCGCCGGAGG",2,6,20,NA,"GeneID:3149","HMGB3",FALSE,"ATP13A2"

"13123","GGTCATCGGCTATCACGTCG","chr1",17005504,"-","none","TGG","GGTCATCGGCTATCACGTCGTGG",0,NA,NA,NA,"GeneID:23400","ATP13A2",TRUE,"ATP13A2"

"13124","TGATAGCCGATGACCCTCCA","chr1",17005509,"+","none","TGG","TGATAGCCGATGACCCTCCATGG",0,NA,NA,NA,"GeneID:23400","ATP13A2",TRUE,"ATP13A2"

"13125","TGATAGCCGATGACCCTCCA","chr17",73899942,"+","10:A>T,15:T>C","TGG","TGATAGCCGAAGACCTTCCATGG",2,11,16,NA,"","",FALSE,"ATP13A2"

"13126","AGCTTATTAGACATAGATTT","chr3",194459879,"+","none","TGG","AGCTTATTAGACATAGATTTTGG",0,NA,NA,NA,"GeneID:79572","ATP13A3",TRUE,"ATP13A3"

"13127","ATCTTCTTGACCCTGATTGA","chr3",194462145,"+","none","TGG","ATCTTCTTGACCCTGATTGATGG",0,NA,NA,NA,"GeneID:79572","ATP13A3",TRUE,"ATP13A3"

"13128","ATCTTCTTGACCCTGATTGA","chr1",78139206,"+","11:A>C,13:A>T","AGG","ATCTTCTTGACACAGATTGAAGG",2,12,14,NA,"","",FALSE,"ATP13A3"

"13129","ATGGTTACAATTTGAGTCGC","chr3",194460804,"-","none","TGG","ATGGTTACAATTTGAGTCGCTGG",0,NA,NA,NA,"GeneID:79572","ATP13A3",TRUE,"ATP13A3"

"13130","GCCACTCAGGCATCCAATAG","chr3",194460722,"+","none","AGG","GCCACTCAGGCATCCAATAGAGG",0,NA,NA,NA,"GeneID:79572","ATP13A3",TRUE,"ATP13A3"

"13131","TAGGTATGGACAGGGAAGAA","chr3",194462175,"-","none","AGG","TAGGTATGGACAGGGAAGAAAGG",0,NA,NA,NA,"GeneID:79572","ATP13A3",TRUE,"ATP13A3"

"13132","TAGGTATGGACAGGGAAGAA","chr6",45446691,"-","14:A>C,16:A>T","TGG","TAGGTATGGACAGGTATGAATGG",2,15,17,NA,"GeneID:860","RUNX2",FALSE,"ATP13A3"

"13133","TAGGTATGGACAGGGAAGAA","chr13",36849649,"-","0:G>A,17:T>C","GGG","CAGGTATGGACAGGGAAAAAGGG",2,1,18,NA,"GeneID:4093","SMAD9",TRUE,"ATP13A3"

"13134","TTCACAGACTGAATCACAAC","chr3",194459790,"-","none","AGG","TTCACAGACTGAATCACAACAGG",0,NA,NA,NA,"GeneID:79572","ATP13A3",TRUE,"ATP13A3"

"13135","TTCACAGACTGAATCACAAC","chr4",148103538,"+","8:G>C,14:A>C","TGG","TTCACAGAGTGAATAACAACTGG",2,9,15,NA,"GeneID:4306","NR3C2",FALSE,"ATP13A3"

"13136","AGTTTACTTACCTTTAGGTC","chr3",193502481,"+","none","TGG","AGTTTACTTACCTTTAGGTCTGG",0,NA,NA,NA,"GeneID:84239","ATP13A4",TRUE,"ATP13A4"

"13137","CTCATTTGGAATCCTCCCCT","chr3",193514789,"-","none","TGG","CTCATTTGGAATCCTCCCCTTGG",0,NA,NA,NA,"GeneID:84239","ATP13A4",TRUE,"ATP13A4"

"13138","GAAGGACAGTTCCAGAAAAT","chr3",193493092,"-","none","TGG","GAAGGACAGTTCCAGAAAATTGG",0,NA,NA,NA,"GeneID:84239","ATP13A4",TRUE,"ATP13A4"

"13139","GAAGGACAGTTCCAGAAAAT","chr6",126901254,"-","1:A>T,6:T>G","AGG","GTAGGAAAGTTCCAGAAAATAGG",2,2,7,NA,"","",FALSE,"ATP13A4"

"13140","TCAGCATTAAACAGCGCATT","chr3",193502571,"-","none","TGG","TCAGCATTAAACAGCGCATTTGG",0,NA,NA,NA,"GeneID:84239","ATP13A4",TRUE,"ATP13A4"

"13141","TGCAGAAAATAAGATATGTT","chr3",193493124,"-","none","TGG","TGCAGAAAATAAGATATGTTTGG",0,NA,NA,NA,"GeneID:84239","ATP13A4",TRUE,"ATP13A4"

"13142","TGCAGAAAATAAGATATGTT","chr3",193362601,"-","10:G>T,13:C>T","TGG","TGCAGAAAATCAGGTATGTTTGG",2,11,14,NA,"GeneID:344905","ATP13A5",TRUE,"ATP13A4"

"13143","TGCAGAAAATAAGATATGTT","chr5",167000550,"+","12:C>G,14:G>T","TGG","TGCAGAAAATAACAGATGTTTGG",2,13,15,NA,"","",FALSE,"ATP13A4"

"13144","TGCAGAAAATAAGATATGTT","chr4",135329708,"-","1:T>C,5:G>T","GGG","TACAGCAAATAAGATATGTTGGG",2,2,6,NA,"","",FALSE,"ATP13A4"

"13145","TGTGCCCATACGTGCCATGC","chr3",193514749,"+","none","TGG","TGTGCCCATACGTGCCATGCTGG",0,NA,NA,NA,"GeneID:84239","ATP13A4",TRUE,"ATP13A4"

"13146","ACTTCAGTGTGGATAAGTAG","chr3",193363321,"+","none","AGG","ACTTCAGTGTGGATAAGTAGAGG",0,NA,NA,NA,"GeneID:344905","ATP13A5",TRUE,"ATP13A5"

"13147","ACTTCAGTGTGGATAAGTAG","chr12",9186294,"-","12:A>T,15:A>T","TGG","ACTTCAGTGTGGTTATGTAGTGG",2,13,16,NA,"GeneID:5858","PZP",FALSE,"ATP13A5"

"13148","AGGTCAGCACGGATGCGACA","chr3",193364213,"+","none","AGG","AGGTCAGCACGGATGCGACAAGG",0,NA,NA,NA,"GeneID:344905","ATP13A5",TRUE,"ATP13A5"

"13149","CTTTCTGAAACCGCTTCTCC","chr3",193362570,"+","none","AGG","CTTTCTGAAACCGCTTCTCCAGG",0,NA,NA,NA,"GeneID:344905","ATP13A5",TRUE,"ATP13A5"

"13150","CTTTCTGAAACCGCTTCTCC","chr9",135456468,"-","11:T>G,12:G>C","TGG","CTTTCTGAAACACCTTCTCCTGG",2,12,13,NA,"","",FALSE,"ATP13A5"

"13151","GACCCAATCCAAATGTCTGA","chr3",193362405,"+","none","TGG","GACCCAATCCAAATGTCTGATGG",0,NA,NA,NA,"GeneID:344905","ATP13A5",TRUE,"ATP13A5"

"13152","GCAATCGTAGCTGCGGTGCA","chr3",193362627,"-","none","TGG","GCAATCGTAGCTGCGGTGCATGG",0,NA,NA,NA,"GeneID:344905","ATP13A5",TRUE,"ATP13A5"

"13153","GCAATCGTAGCTGCGGTGCA","chr19",6725038,"+","6:C>G,13:G>C","AGG","GCAATCCTAGCTGGGGTGCAAGG",2,7,14,NA,"GeneID:718","C3",FALSE,"ATP13A5"

"13154","GTCGCATCCGTGCTGACCTG","chr3",193364212,"-","none","TGG","GTCGCATCCGTGCTGACCTGTGG",0,NA,NA,NA,"GeneID:344905","ATP13A5",TRUE,"ATP13A5"

"13155","ATTTACGATGAAGTTCATCA","chr1",116384802,"-","none","AGG","ATTTACGATGAAGTTCATCAAGG",0,NA,NA,NA,"GeneID:476","ATP1A1",TRUE,"ATP1A1"

"13156","CGACAAAACTTGATCCATTC","chr1",116387365,"-","none","AGG","CGACAAAACTTGATCCATTCAGG",0,NA,NA,NA,"GeneID:476","ATP1A1",TRUE,"ATP1A1"

"13157","CGTTGGGACCATCTCGCGCC","chr1",116387319,"-","none","AGG","CGTTGGGACCATCTCGCGCCAGG",0,NA,NA,NA,"GeneID:476","ATP1A1",TRUE,"ATP1A1"

"13158","GATGAACTTCATCGTAAATA","chr1",116384803,"+","none","TGG","GATGAACTTCATCGTAAATATGG",0,NA,NA,NA,"GeneID:476","ATP1A1",TRUE,"ATP1A1"

"13159","GCAACCAGTTATGATTACAA","chr1",116388158,"-","none","CGG","GCAACCAGTTATGATTACAACGG",0,NA,NA,NA,"GeneID:476","ATP1A1",TRUE,"ATP1A1"

"13160","TCAGCCGTTGTAATCATAAC","chr1",116388151,"+","none","TGG","TCAGCCGTTGTAATCATAACTGG",0,NA,NA,NA,"GeneID:476","ATP1A1",TRUE,"ATP1A1"

"13161","AAGTGCATTGAGCTCTCCTG","chr1",160129304,"+","none","TGG","AAGTGCATTGAGCTCTCCTGTGG",0,NA,NA,NA,"GeneID:477","ATP1A2",TRUE,"ATP1A2"

"13162","CAAGCAGACCTGGTACTTGT","chr1",160129389,"-","none","TGG","CAAGCAGACCTGGTACTTGTTGG",0,NA,NA,NA,"GeneID:477","ATP1A2",TRUE,"ATP1A2"

"13163","CAAGCAGACCTGGTACTTGT","chr20",25037147,"-","11:A>C,19:T>A","TGG","CAAGCAGACCTTGTACTTGATGG",2,12,20,NA,"GeneID:84532","ACSS1",FALSE,"ATP1A2"

"13164","CAAGCAGACCTGGTACTTGT","chr8",3898650,"+","2:T>A,15:T>C","TGG","CATGCAGACCTGGTATTTGTTGG",2,3,16,NA,"GeneID:64478","CSMD1",FALSE,"ATP1A2"

"13165","CACAGCAAGCCCTTGTGATC","chr1",160124290,"+","none","CGG","CACAGCAAGCCCTTGTGATCCGG",0,NA,NA,NA,"GeneID:477","ATP1A2",TRUE,"ATP1A2"

"13166","CACAGCAAGCCCTTGTGATC","chr19",41985983,"-","1:A>T,13:C>A","CGG","CTCAGCAAGCCCTGGTGATCCGG",2,2,14,NA,"GeneID:478","ATP1A3",TRUE,"ATP1A2"

"13167","CTGCAACCGCGCCGTCTTCA","chr1",160129037,"+","none","AGG","CTGCAACCGCGCCGTCTTCAAGG",0,NA,NA,NA,"GeneID:477","ATP1A2",TRUE,"ATP1A2"

"13168","CTGCAACCGCGCCGTCTTCA","chr19",41981753,"-","6:A>G,12:A>G","AGG","CTGCAATCGCGCTGTCTTCAAGG",2,7,13,NA,"GeneID:478","ATP1A3",TRUE,"ATP1A2"

"13169","GAAGATGCAGATCAACGCAG","chr1",160124321,"+","none","AGG","GAAGATGCAGATCAACGCAGAGG",0,NA,NA,NA,"GeneID:477","ATP1A2",TRUE,"ATP1A2"

"13170","TTGACAAACGATCCCCTACG","chr1",160128988,"+","none","TGG","TTGACAAACGATCCCCTACGTGG",0,NA,NA,NA,"GeneID:477","ATP1A2",TRUE,"ATP1A2"

"13171","ATACAACACAGACTGTGTGC","chr19",41988319,"-","none","AGG","ATACAACACAGACTGTGTGCAGG",0,NA,NA,NA,"GeneID:478","ATP1A3",TRUE,"ATP1A3"

"13172","ATACAACACAGACTGTGTGC","chr19",2489963,"+","6:A>C,9:C>A","AGG","ATACAAAACCGACTGTGTGCAGG",2,7,10,NA,"","",FALSE,"ATP1A3"

"13173","CACAGTCTGTGTTGTATTTC","chr19",41988322,"+","none","CGG","CACAGTCTGTGTTGTATTTCCGG",0,NA,NA,NA,"GeneID:478","ATP1A3",TRUE,"ATP1A3"

"13174","CACAGTCTGTGTTGTATTTC","chrX",4442025,"+","8:T>G,9:A>T","AGG","CACAGTCTTAGTTGTATTTCAGG",2,9,10,NA,"","",FALSE,"ATP1A3"

"13175","CCACGAGGCTGACACCACTG","chr19",41981919,"-","none","AGG","CCACGAGGCTGACACCACTGAGG",0,NA,NA,NA,"GeneID:478","ATP1A3",TRUE,"ATP1A3"

"13176","CGTATCGCCACCCTGGCATC","chr19",41985116,"-","none","AGG","CGTATCGCCACCCTGGCATCAGG",0,NA,NA,NA,"GeneID:478","ATP1A3",TRUE,"ATP1A3"

"13177","GACGCTGACCGCCAAGCGCA","chr19",41982078,"-","none","TGG","GACGCTGACCGCCAAGCGCATGG",0,NA,NA,NA,"GeneID:478","ATP1A3",TRUE,"ATP1A3"

"13178","GACGCTGACCGCCAAGCGCA","chr1",160128659,"+","3:C>G,9:A>C","TGG","GACCCTGACAGCCAAGCGCATGG",2,4,10,NA,"GeneID:477","ATP1A2",TRUE,"ATP1A3"

"13179","GTGCTCAATCTCGATGGCGA","chr19",41985070,"+","none","TGG","GTGCTCAATCTCGATGGCGATGG",0,NA,NA,NA,"GeneID:478","ATP1A3",TRUE,"ATP1A3"

"13180","AAAACAGAAGCGCAATATGG","chr1",160152141,"+","none","AGG","AAAACAGAAGCGCAATATGGAGG",0,NA,NA,NA,"GeneID:480","ATP1A4",TRUE,"ATP1A4"

"13181","AAAACAGAAGCGCAATATGG","chr14",22038250,"+","10:G>C,17:G>T","TGG","AAAACAGAAGGGCAATAGGGTGG",2,11,18,NA,"","",FALSE,"ATP1A4"

"13182","GCAAAGGAAATCCTGACTCG","chr1",160155062,"+","none","AGG","GCAAAGGAAATCCTGACTCGAGG",0,NA,NA,NA,"GeneID:480","ATP1A4",TRUE,"ATP1A4"

"13183","GCTGAGCACCAAGTACTCCG","chr1",160153190,"+","none","TGG","GCTGAGCACCAAGTACTCCGTGG",0,NA,NA,NA,"GeneID:480","ATP1A4",TRUE,"ATP1A4"

"13184","GGATGATCACAAATTAACCT","chr1",160153163,"+","none","TGG","GGATGATCACAAATTAACCTTGG",0,NA,NA,NA,"GeneID:480","ATP1A4",TRUE,"ATP1A4"

"13185","GGATGATCACAAATTAACCT","chr4",124886086,"-","10:A>T,13:C>A","TGG","GGATGATCACTAAGTAACCTTGG",2,11,14,NA,"","",FALSE,"ATP1A4"

"13186","GGGGGGTGGGGTAACAGTAT","chr1",160155093,"-","none","TGG","GGGGGGTGGGGTAACAGTATTGG",0,NA,NA,NA,"GeneID:480","ATP1A4",TRUE,"ATP1A4"

"13187","TTCGTCTTGGACTCTGGTCA","chr1",160152078,"-","none","TGG","TTCGTCTTGGACTCTGGTCATGG",0,NA,NA,NA,"GeneID:480","ATP1A4",TRUE,"ATP1A4"

"13188","ATAAAGCTCAACCGAGTTCT","chr1",169127372,"+","none","AGG","ATAAAGCTCAACCGAGTTCTAGG",0,NA,NA,NA,"GeneID:481","ATP1B1",TRUE,"ATP1B1"

"13189","ATAAAGCTCAACCGAGTTCT","chr4",42029744,"+","11:G>C,12:T>C","AGG","ATAAAGCTCAAGTGAGTTCTAGG",2,12,13,NA,"GeneID:10463","SLC30A9",TRUE,"ATP1B1"

"13190","ATAAAGCTCAACCGAGTTCT","chr7",130664543,"+","9:T>A,13:A>G","AGG","ATAAAGCTCTACCAAGTTCTAGG",2,10,14,NA,"GeneID:26958","COPG2",FALSE,"ATP1B1"

"13191","CATATGTACTGAACATAGTT","chr1",169124955,"+","none","AGG","CATATGTACTGAACATAGTTAGG",0,NA,NA,NA,"GeneID:481","ATP1B1",TRUE,"ATP1B1"

"13192","CATATGTACTGAACATAGTT","chr4",42029512,"+","7:G>A,14:G>A","AGG","CATATGTGCTGAACGTAGTTAGG",2,8,15,NA,"GeneID:10463","SLC30A9",TRUE,"ATP1B1"

"13193","GTCCTTCCCGTTCAGTGCAC","chr1",169130063,"+","none","TGG","GTCCTTCCCGTTCAGTGCACTGG",0,NA,NA,NA,"GeneID:481","ATP1B1",TRUE,"ATP1B1"

"13194","TAATGATCCCAAGAGCTATG","chr1",169124932,"+","none","AGG","TAATGATCCCAAGAGCTATGAGG",0,NA,NA,NA,"GeneID:481","ATP1B1",TRUE,"ATP1B1"

"13195","TCATGAACGAGGAGAGCGAA","chr1",169127260,"+","none","AGG","TCATGAACGAGGAGAGCGAAAGG",0,NA,NA,NA,"GeneID:481","ATP1B1",TRUE,"ATP1B1"

"13196","TGAATTTAAGCCCACATATC","chr1",169111457,"+","none","AGG","TGAATTTAAGCCCACATATCAGG",0,NA,NA,NA,"GeneID:481","ATP1B1",TRUE,"ATP1B1"

"13197","AAGAATGATGTCTGCCGCCC","chr17",7654077,"+","none","TGG","AAGAATGATGTCTGCCGCCCTGG",0,NA,NA,NA,"GeneID:482","ATP1B2",TRUE,"ATP1B2"

"13198","ACTCACCCGGTGTGGCCAGT","chr17",7653488,"-","none","CGG","ACTCACCCGGTGTGGCCAGTCGG",0,NA,NA,NA,"GeneID:482","ATP1B2",TRUE,"ATP1B2"

"13199","ACTCACCCGGTGTGGCCAGT","chr22",44659215,"+","5:G>C,9:C>G","GGG","ACTCAGCCGCTGTGGCCAGTGGG",2,6,10,NA,"","",FALSE,"ATP1B2"

"13200","AGCATGAATGTTACCTGTGC","chr17",7654657,"+","none","TGG","AGCATGAATGTTACCTGTGCTGG",0,NA,NA,NA,"GeneID:482","ATP1B2",TRUE,"ATP1B2"

"13201","CAGGTCATCAACTTCTATGC","chr17",7654624,"+","none","AGG","CAGGTCATCAACTTCTATGCAGG",0,NA,NA,NA,"GeneID:482","ATP1B2",TRUE,"ATP1B2"

"13202","CGACCATACCCCCAAGTACC","chr17",7653461,"+","none","AGG","CGACCATACCCCCAAGTACCAGG",0,NA,NA,NA,"GeneID:482","ATP1B2",TRUE,"ATP1B2"

"13203","GTTCGTAATAGCGTCCAGGG","chr17",7654094,"-","none","CGG","GTTCGTAATAGCGTCCAGGGCGG",0,NA,NA,NA,"GeneID:482","ATP1B2",TRUE,"ATP1B2"

"13204","AACTGACATGCAACATAAAC","chr3",141913722,"-","none","TGG","AACTGACATGCAACATAAACTGG",0,NA,NA,NA,"GeneID:483","ATP1B3",TRUE,"ATP1B3"

"13205","ATAATTGGATTAAAGCCTGA","chr3",141915969,"+","none","AGG","ATAATTGGATTAAAGCCTGAAGG",0,NA,NA,NA,"GeneID:483","ATP1B3",TRUE,"ATP1B3"

"13206","ATAATTGGATTAAAGCCTGA","chr2",60735178,"-","15:A>G","AGG","ATAATTGGATTAAAGTCTGAAGG",1,16,NA,NA,"","",TRUE,"ATP1B3"

"13207","ATGTACCCTGCATACGAAGT","chr3",141907228,"-","none","TGG","ATGTACCCTGCATACGAAGTTGG",0,NA,NA,NA,"GeneID:483","ATP1B3",TRUE,"ATP1B3"

"13208","CGTGACCAGATTCCTAGCCC","chr3",141903726,"+","none","AGG","CGTGACCAGATTCCTAGCCCAGG",0,NA,NA,NA,"GeneID:483","ATP1B3",TRUE,"ATP1B3"

"13209","CGTGACCAGATTCCTAGCCC","chr2",60735493,"-","13:A>G,16:T>C","AGG","CGTGACCAGATTCTTAACCCAGG",2,14,17,NA,"","",TRUE,"ATP1B3"

"13210","GCTTCAGACTCTCAACGATG","chr3",141903692,"+","none","AGG","GCTTCAGACTCTCAACGATGAGG",0,NA,NA,NA,"GeneID:483","ATP1B3",TRUE,"ATP1B3"

"13211","TCCAAAACCAGTGACCGCAT","chr3",141907179,"+","none","TGG","TCCAAAACCAGTGACCGCATTGG",0,NA,NA,NA,"GeneID:483","ATP1B3",TRUE,"ATP1B3"

"13212","TCCAAAACCAGTGACCGCAT","chr2",60735458,"-","12:G>C,15:A>G","TGG","TCCAAAACCAGTCACTGCATTGG",2,13,16,NA,"","",TRUE,"ATP1B3"

"13213","ACGTTTCTGAACCCGACACT","chrX",120371150,"+","none","TGG","ACGTTTCTGAACCCGACACTTGG",0,NA,NA,NA,"GeneID:23439","ATP1B4",TRUE,"ATP1B4"

"13214","AGATCATGAGTGAATACCTG","chrX",120366720,"+","none","TGG","AGATCATGAGTGAATACCTGTGG",0,NA,NA,NA,"GeneID:23439","ATP1B4",TRUE,"ATP1B4"

"13215","AGATCATGAGTGAATACCTG","chr16",25169136,"+","9:T>G,14:A>T","CGG","AGATCATGATTGAAAACCTGCGG",2,10,15,NA,"GeneID:51451","LCMT1",TRUE,"ATP1B4"

"13216","AGGCTTTACCCGCTCCGTGA","chrX",120370819,"-","none","AGG","AGGCTTTACCCGCTCCGTGAAGG",0,NA,NA,NA,"GeneID:23439","ATP1B4",TRUE,"ATP1B4"

"13217","CCAGACCAGAGCAGTTCTTT","chrX",120375489,"-","none","AGG","CCAGACCAGAGCAGTTCTTTAGG",0,NA,NA,NA,"GeneID:23439","ATP1B4",TRUE,"ATP1B4"

"13218","CCAGACCAGAGCAGTTCTTT","chr5",6553033,"-","14:G>A,16:T>G","GGG","CCAGACCAGAGCAGCTATTTGGG",2,15,17,NA,"","",FALSE,"ATP1B4"

"13219","CCAGACCAGAGCAGTTCTTT","chr3",54142038,"-","14:C>A,19:G>A","AGG","CCAGACCAGAGCAGGTCTTCAGG",2,15,20,NA,"GeneID:55799","CACNA2D3",FALSE,"ATP1B4"

"13220","GACGGTGGTGCCCAAATCGG","chrX",120366595,"+","none","AGG","GACGGTGGTGCCCAAATCGGAGG",0,NA,NA,NA,"GeneID:23439","ATP1B4",TRUE,"ATP1B4"

"13221","TCCCTATATACCAACCTTCA","chrX",120370802,"+","none","CGG","TCCCTATATACCAACCTTCACGG",0,NA,NA,NA,"GeneID:23439","ATP1B4",TRUE,"ATP1B4"

"13222","AACCTGCCAAACCCCCACGA","chr16",28881002,"-","none","TGG","AACCTGCCAAACCCCCACGATGG",0,NA,NA,NA,"GeneID:487","ATP2A1",TRUE,"ATP2A1"

"13223","CCTCATTGCCAATGCCATCG","chr16",28880985,"+","none","TGG","CCTCATTGCCAATGCCATCGTGG",0,NA,NA,NA,"GeneID:487","ATP2A1",TRUE,"ATP2A1"

"13224","CTGACCGCAAGTCAGTGCAA","chr16",28882520,"+","none","AGG","CTGACCGCAAGTCAGTGCAAAGG",0,NA,NA,NA,"GeneID:487","ATP2A1",TRUE,"ATP2A1"

"13225","CTGGTCAACCCGCAGCGTGG","chr16",28884622,"-","none","TGG","CTGGTCAACCCGCAGCGTGGTGG",0,NA,NA,NA,"GeneID:487","ATP2A1",TRUE,"ATP2A1"

"13226","CTGGTCAACCCGCAGCGTGG","chr17",3950705,"+","6:C>A,9:T>C","TGG","CTGGTCCACTCGCAGCGTGGTGG",2,7,10,NA,"GeneID:489","ATP2A3",TRUE,"ATP2A1"

"13227","GAACGCAGAGAACGCCATCG","chr16",28882455,"+","none","AGG","GAACGCAGAGAACGCCATCGAGG",0,NA,NA,NA,"GeneID:487","ATP2A1",TRUE,"ATP2A1"

"13228","GCGAGGATTCGGATGTCTGC","chr16",28884591,"-","none","AGG","GCGAGGATTCGGATGTCTGCAGG",0,NA,NA,NA,"GeneID:487","ATP2A1",TRUE,"ATP2A1"

"13229","ACAGAGTTACCGGCTGAAGA","chr12",110282599,"+","none","AGG","ACAGAGTTACCGGCTGAAGAAGG",0,NA,NA,NA,"GeneID:488","ATP2A2",TRUE,"ATP2A2"

"13230","AGTTTGAAGACTTGCTAGTT","chr12",110282742,"+","none","AGG","AGTTTGAAGACTTGCTAGTTAGG",0,NA,NA,NA,"GeneID:488","ATP2A2",TRUE,"ATP2A2"

"13231","AGTTTGAAGACTTGCTAGTT","chr8",49372378,"-","2:T>A,15:T>A","AGG","AGATTGAAGACTTGCAAGTTAGG",2,3,16,NA,"","",FALSE,"ATP2A2"

"13232","ATTAGTAGCCAATGCAATTG","chr12",110292090,"+","none","TGG","ATTAGTAGCCAATGCAATTGTGG",0,NA,NA,NA,"GeneID:488","ATP2A2",TRUE,"ATP2A2"

"13233","CCATACACCCACAATTGCAT","chr12",110292101,"-","none","TGG","CCATACACCCACAATTGCATTGG",0,NA,NA,NA,"GeneID:488","ATP2A2",TRUE,"ATP2A2"

"13234","CCATACACCCACAATTGCAT","chrX",105319794,"+","0:T>C,14:G>T","GGG","TCATACACCCACAAGTGCATGGG",2,1,15,NA,"GeneID:26280","IL1RAPL2",FALSE,"ATP2A2"

"13235","CTCAATCACAAGTTCCAGCA","chr12",110282721,"-","none","AGG","CTCAATCACAAGTTCCAGCAAGG",0,NA,NA,NA,"GeneID:488","ATP2A2",TRUE,"ATP2A2"

"13236","TTAAGATTACCTTCTTCAGC","chr12",110282611,"-","none","CGG","TTAAGATTACCTTCTTCAGCCGG",0,NA,NA,NA,"GeneID:488","ATP2A2",TRUE,"ATP2A2"

"13237","TTAAGATTACCTTCTTCAGC","chr17",29383668,"+","4:T>G,19:A>C","GGG","TTAATATTACCTTCTTCAGAGGG",2,5,20,NA,"","",FALSE,"ATP2A2"

"13238","AGACCCCAGAGCTGTGAACC","chr17",3950536,"-","none","AGG","AGACCCCAGAGCTGTGAACCAGG",0,NA,NA,NA,"GeneID:489","ATP2A3",TRUE,"ATP2A3"

"13239","CAAGTCCACCACGCTGCGAG","chr17",3950713,"-","none","TGG","CAAGTCCACCACGCTGCGAGTGG",0,NA,NA,NA,"GeneID:489","ATP2A3",TRUE,"ATP2A3"

"13240","CATCGGCCACTTCGCCGACC","chr17",3947641,"-","none","CGG","CATCGGCCACTTCGCCGACCCGG",0,NA,NA,NA,"GeneID:489","ATP2A3",TRUE,"ATP2A3"

"13241","GGACGGCAGGCTTCGCACGA","chr17",3947471,"+","none","TGG","GGACGGCAGGCTTCGCACGATGG",0,NA,NA,NA,"GeneID:489","ATP2A3",TRUE,"ATP2A3"

"13242","GGGGGCCTCACCCGTCAGGA","chr17",3950681,"+","none","TGG","GGGGGCCTCACCCGTCAGGATGG",0,NA,NA,NA,"GeneID:489","ATP2A3",TRUE,"ATP2A3"

"13243","GGGGGCCTCACCCGTCAGGA","chr11",65577225,"+","14:C>T,19:C>A","GGG","GGGGGCCTCACCCGCCAGGCGGG",2,15,20,NA,"GeneID:254102","EHBP1L1",FALSE,"ATP2A3"

"13244","GTCCGTGACCAAGCACACAG","chr17",3950566,"-","none","AGG","GTCCGTGACCAAGCACACAGAGG",0,NA,NA,NA,"GeneID:489","ATP2A3",TRUE,"ATP2A3"

"13245","GTCCGTGACCAAGCACACAG","chr8",141119805,"+","0:C>G,4:C>G","GGG","CTCCCTGACCAAGCACACAGGGG",2,1,5,NA,"GeneID:22898","DENND3",FALSE,"ATP2A3"

"13246","AGAAAGGCCCAATGATACTA","chr12",89642188,"+","none","TGG","AGAAAGGCCCAATGATACTATGG",0,NA,NA,NA,"GeneID:490","ATP2B1",TRUE,"ATP2B1"

"13247","AGAAAGGCCCAATGATACTA","chr12",21178656,"-","6:G>C,14:C>T","TGG","AGAAAGCCCCAATGGTACTATGG",2,7,15,NA,"GeneID:10599","SLCO1B1",TRUE,"ATP2B1"

"13248","ATTGATGAAAGCTCATTGAC","chr12",89634833,"-","none","TGG","ATTGATGAAAGCTCATTGACTGG",0,NA,NA,NA,"GeneID:490","ATP2B1",TRUE,"ATP2B1"

"13249","CCACACACACTACAGACAAG","chr12",89635158,"+","none","AGG","CCACACACACTACAGACAAGAGG",0,NA,NA,NA,"GeneID:490","ATP2B1",TRUE,"ATP2B1"

"13250","GATATTGCTCAAGTGAAATA","chr12",89634998,"-","none","TGG","GATATTGCTCAAGTGAAATATGG",0,NA,NA,NA,"GeneID:490","ATP2B1",TRUE,"ATP2B1"

"13251","GATATTGCTCAAGTGAAATA","chr1",203699695,"+","8:C>T,14:C>G","CGG","GATATTGCCCAAGTCAAATACGG",2,9,15,NA,"GeneID:493","ATP2B4",TRUE,"ATP2B1"

"13252","TGAATAAGTATGCCGTCAGC","chr12",89634869,"+","none","TGG","TGAATAAGTATGCCGTCAGCTGG",0,NA,NA,NA,"GeneID:490","ATP2B1",TRUE,"ATP2B1"

"13253","TTACGTGCATTATCCCCTTC","chr12",89642153,"+","none","TGG","TTACGTGCATTATCCCCTTCTGG",0,NA,NA,NA,"GeneID:490","ATP2B1",TRUE,"ATP2B1"

"13254","AACAGAAATTTACCGTGGTC","chr3",10402160,"-","none","CGG","AACAGAAATTTACCGTGGTCCGG",0,NA,NA,NA,"GeneID:491","ATP2B2",TRUE,"ATP2B2"

"13255","CCTGCAGAGCCGCATCGAGC","chr3",10402183,"-","none","AGG","CCTGCAGAGCCGCATCGAGCAGG",0,NA,NA,NA,"GeneID:491","ATP2B2",TRUE,"ATP2B2"

"13256","CCTGCAGAGCCGCATCGAGC","chrX",153541813,"+","12:A>C,15:T>C","AGG","CCTGCAGAGCCGAATTGAGCAGG",2,13,16,NA,"GeneID:492","ATP2B3",TRUE,"ATP2B2"

"13257","CTGTCCTTCTACCACCCGCC","chr3",10410634,"-","none","CGG","CTGTCCTTCTACCACCCGCCCGG",0,NA,NA,NA,"GeneID:491","ATP2B2",TRUE,"ATP2B2"

"13258","CTGTCCTTCTACCACCCGCC","chr2",237218481,"+","13:C>A,17:A>G","TGG","CTGTCCTTCTACCCCCCACCTGG",2,14,18,NA,"","",FALSE,"ATP2B2"

"13259","CTTACCTTCGTTGCCCTCGC","chr3",10410612,"+","none","CGG","CTTACCTTCGTTGCCCTCGCCGG",0,NA,NA,NA,"GeneID:491","ATP2B2",TRUE,"ATP2B2"

"13260","GGGCGAGTTCGGGTGCACAA","chr3",10449461,"-","none","TGG","GGGCGAGTTCGGGTGCACAATGG",0,NA,NA,NA,"GeneID:491","ATP2B2",TRUE,"ATP2B2"

"13261","TGCACCCGAACTCGCCCCCA","chr3",10449464,"+","none","TGG","TGCACCCGAACTCGCCCCCATGG",0,NA,NA,NA,"GeneID:491","ATP2B2",TRUE,"ATP2B2"

"13262","AGAAGCGCAGGCAGATCTAC","chrX",153541385,"+","none","GGG","AGAAGCGCAGGCAGATCTACGGG",0,NA,NA,NA,"GeneID:492","ATP2B3",TRUE,"ATP2B3"

"13263","CCTGCAGAGCCGAATTGAGC","chrX",153541813,"+","none","AGG","CCTGCAGAGCCGAATTGAGCAGG",0,NA,NA,NA,"GeneID:492","ATP2B3",TRUE,"ATP2B3"

"13264","CCTGCAGAGCCGAATTGAGC","chr3",10402183,"-","12:G>T,15:G>A","AGG","CCTGCAGAGCCGCATCGAGCAGG",2,13,16,NA,"GeneID:491","ATP2B2",TRUE,"ATP2B3"

"13265","CGAGGCGCTGCAGAAGATCG","chrX",153536375,"+","none","AGG","CGAGGCGCTGCAGAAGATCGAGG",0,NA,NA,NA,"GeneID:492","ATP2B3",TRUE,"ATP2B3"

"13266","CTCTCGTTCTATGCGCCGCC","chrX",153541519,"+","none","AGG","CTCTCGTTCTATGCGCCGCCAGG",0,NA,NA,NA,"GeneID:492","ATP2B3",TRUE,"ATP2B3"

"13267","GAGCCCGCTGACATCCCCGT","chrX",153536404,"-","none","AGG","GAGCCCGCTGACATCCCCGTAGG",0,NA,NA,NA,"GeneID:492","ATP2B3",TRUE,"ATP2B3"

"13268","GGCACTCACCGTACTTGACC","chrX",153541915,"-","none","TGG","GGCACTCACCGTACTTGACCTGG",0,NA,NA,NA,"GeneID:492","ATP2B3",TRUE,"ATP2B3"

"13269","AAAGACCCCATGTTGCTCTC","chr1",203700309,"+","none","AGG","AAAGACCCCATGTTGCTCTCAGG",0,NA,NA,NA,"GeneID:493","ATP2B4",TRUE,"ATP2B4"

"13270","AAAGACCCCATGTTGCTCTC","chrX",153542426,"+","5:T>C,12:C>T","AGG","AAAGATCCCATGCTGCTCTCAGG",2,6,13,NA,"GeneID:492","ATP2B3",TRUE,"ATP2B4"

"13271","CTCTCGGCCATCGAGTTGGC","chr1",203683235,"-","none","AGG","CTCTCGGCCATCGAGTTGGCAGG",0,NA,NA,NA,"GeneID:493","ATP2B4",TRUE,"ATP2B4"

"13272","GCTGCAGTGCCGCATTGAAC","chr1",203699604,"+","none","AGG","GCTGCAGTGCCGCATTGAACAGG",0,NA,NA,NA,"GeneID:493","ATP2B4",TRUE,"ATP2B4"

"13273","GCTGGATGAGTTGACCGTTT","chr1",203699649,"-","none","CGG","GCTGGATGAGTTGACCGTTTCGG",0,NA,NA,NA,"GeneID:493","ATP2B4",TRUE,"ATP2B4"

"13274","GCTGGATGAGTTGACCGTTT","chr6",119484084,"+","13:T>A,16:T>G","GGG","GCTGGATGAGTTGTCCTTTTGGG",2,14,17,NA,"","",FALSE,"ATP2B4"

"13275","TGTCCAGGGACTTCTTGACA","chr1",203700290,"-","none","TGG","TGTCCAGGGACTTCTTGACATGG",0,NA,NA,NA,"GeneID:493","ATP2B4",TRUE,"ATP2B4"

"13276","TGTCTTGCCTGCCAACTCGA","chr1",203683225,"+","none","TGG","TGTCTTGCCTGCCAACTCGATGG",0,NA,NA,NA,"GeneID:493","ATP2B4",TRUE,"ATP2B4"

"13277","AAACCAAAGAGCCGAAGAAC","chr3",130850906,"+","none","AGG","AAACCAAAGAGCCGAAGAACAGG",0,NA,NA,NA,"GeneID:27032","ATP2C1",TRUE,"ATP2C1"

"13278","AATATCCTCTCCATGCAATT","chr3",130850863,"+","none","AGG","AATATCCTCTCCATGCAATTAGG",0,NA,NA,NA,"GeneID:27032","ATP2C1",TRUE,"ATP2C1"

"13279","ATACCTGTTCTTCGGCTCTT","chr3",130850912,"-","none","TGG","ATACCTGTTCTTCGGCTCTTTGG",0,NA,NA,NA,"GeneID:27032","ATP2C1",TRUE,"ATP2C1"

"13280","ATGCTTGCAACTTCACTGAC","chr3",130930499,"-","none","TGG","ATGCTTGCAACTTCACTGACTGG",0,NA,NA,NA,"GeneID:27032","ATP2C1",TRUE,"ATP2C1"

"13281","TCCCCTTATTATGCTGCTTC","chr3",130934629,"+","none","TGG","TCCCCTTATTATGCTGCTTCTGG",0,NA,NA,NA,"GeneID:27032","ATP2C1",TRUE,"ATP2C1"

"13282","TGATGCCGTCAGTATCACTG","chr3",130934689,"+","none","TGG","TGATGCCGTCAGTATCACTGTGG",0,NA,NA,NA,"GeneID:27032","ATP2C1",TRUE,"ATP2C1"

"13283","AATTCTTACACAAAACGCTC","chr16",84398598,"-","none","TGG","AATTCTTACACAAAACGCTCTGG",0,NA,NA,NA,"GeneID:9914","ATP2C2",TRUE,"ATP2C2"

"13284","ACTCACCGTGGCGATGCTGA","chr16",84408480,"-","none","CGG","ACTCACCGTGGCGATGCTGACGG",0,NA,NA,NA,"GeneID:9914","ATP2C2",TRUE,"ATP2C2"

"13285","CCGAGAAGCCGAGTTTCTTC","chr16",84368644,"-","none","AGG","CCGAGAAGCCGAGTTTCTTCAGG",0,NA,NA,NA,"GeneID:9914","ATP2C2",TRUE,"ATP2C2"

"13286","CTGGCATTTGCACGCTTCCT","chr16",84398562,"-","none","TGG","CTGGCATTTGCACGCTTCCTTGG",0,NA,NA,NA,"GeneID:9914","ATP2C2",TRUE,"ATP2C2"

"13287","CTGGCATTTGCACGCTTCCT","chr12",5245961,"+","11:C>A,13:A>G","AGG","CTGGCATTTGCCCACTTCCTAGG",2,12,14,NA,"","",FALSE,"ATP2C2"

"13288","CTGGCATTTGCACGCTTCCT","chr5",179870183,"+","10:G>C,12:G>C","GGG","CTGGCATTTGGAGGCTTCCTGGG",2,11,13,NA,"GeneID:23061","TBC1D9B",FALSE,"ATP2C2"

"13289","GGACGCCGTCAGCATCGCCA","chr16",84408472,"+","none","CGG","GGACGCCGTCAGCATCGCCACGG",0,NA,NA,NA,"GeneID:9914","ATP2C2",TRUE,"ATP2C2"

"13290","GGAGACGCGTCCCTCGACCA","chr16",84368616,"-","none","TGG","GGAGACGCGTCCCTCGACCATGG",0,NA,NA,NA,"GeneID:9914","ATP2C2",TRUE,"ATP2C2"

"13291","AGCTGTCCTGCAGATCTTGT","chr19",35553050,"+","none","AGG","AGCTGTCCTGCAGATCTTGTAGG",0,NA,NA,NA,"GeneID:495","ATP4A",TRUE,"ATP4A"

"13292","AGCTGTCCTGCAGATCTTGT","chrY",19407056,"-","14:C>A,15:C>G","GGG","AGCTGTCCTGCAGAGGTTGTGGG",2,15,16,NA,"","",FALSE,"ATP4A"

"13293","CAACCCAAAGCGTGACAGAT","chr19",35553747,"-","none","TGG","CAACCCAAAGCGTGACAGATTGG",0,NA,NA,NA,"GeneID:495","ATP4A",TRUE,"ATP4A"

"13294","CACTGACTACTTCACGGCAA","chr19",35553137,"-","none","TGG","CACTGACTACTTCACGGCAATGG",0,NA,NA,NA,"GeneID:495","ATP4A",TRUE,"ATP4A"

"13295","CAGTGCAGAGTTCGATGAAG","chr19",35554926,"+","none","AGG","CAGTGCAGAGTTCGATGAAGAGG",0,NA,NA,NA,"GeneID:495","ATP4A",TRUE,"ATP4A"

"13296","CGCTGACGGTGATGTAGATG","chr19",35554977,"+","none","AGG","CGCTGACGGTGATGTAGATGAGG",0,NA,NA,NA,"GeneID:495","ATP4A",TRUE,"ATP4A"

"13297","TTGACCAATCTGTCACGCTT","chr19",35553740,"+","none","TGG","TTGACCAATCTGTCACGCTTTGG",0,NA,NA,NA,"GeneID:495","ATP4A",TRUE,"ATP4A"

"13298","CAGAGTGGACTGCGCCTTCC","chr13",113651672,"-","none","TGG","CAGAGTGGACTGCGCCTTCCTGG",0,NA,NA,NA,"GeneID:496","ATP4B",TRUE,"ATP4B"

"13299","CAGGAAGGCGCAGTCCACTC","chr13",113651670,"+","none","TGG","CAGGAAGGCGCAGTCCACTCTGG",0,NA,NA,NA,"GeneID:496","ATP4B",TRUE,"ATP4B"

"13300","CCGGATGTTTACGGGGAGAA","chr13",113653400,"-","none","AGG","CCGGATGTTTACGGGGAGAAAGG",0,NA,NA,NA,"GeneID:496","ATP4B",TRUE,"ATP4B"

"13301","GTGGATCAGCCTGTACTACG","chr13",113654921,"-","none","TGG","GTGGATCAGCCTGTACTACGTGG",0,NA,NA,NA,"GeneID:496","ATP4B",TRUE,"ATP4B"

"13302","TCTCTCCAGGGGTAACCTTA","chr13",113653423,"-","none","AGG","TCTCTCCAGGGGTAACCTTAAGG",0,NA,NA,NA,"GeneID:496","ATP4B",TRUE,"ATP4B"

"13303","TCTTGGTAGTCCGGTGTGTA","chr13",113654830,"+","none","CGG","TCTTGGTAGTCCGGTGTGTACGG",0,NA,NA,NA,"GeneID:496","ATP4B",TRUE,"ATP4B"

"13304","ACCAACTCGCCTACGCGTCT","chr18",46089696,"+","none","TGG","ACCAACTCGCCTACGCGTCTTGG",0,NA,NA,NA,"GeneID:498","ATP5A1",TRUE,"ATP5A1"

"13305","ATCCGCCCTGCAATTAACGT","chr18",46086441,"-","none","TGG","ATCCGCCCTGCAATTAACGTTGG",0,NA,NA,NA,"GeneID:498","ATP5A1",TRUE,"ATP5A1"

"13306","CAATTGGTTCCAAGACGCGT","chr18",46089708,"-","none","AGG","CAATTGGTTCCAAGACGCGTAGG",0,NA,NA,NA,"GeneID:498","ATP5A1",TRUE,"ATP5A1"

"13307","CACCACAATGGTGTACTTCA","chr18",46087466,"+","none","TGG","CACCACAATGGTGTACTTCATGG",0,NA,NA,NA,"GeneID:498","ATP5A1",TRUE,"ATP5A1"

"13308","CACCACAATGGTGTACTTCA","chr16",72005871,"+","7:G>A","CGG","CACCACAGTGGTGTACTTCACGG",1,8,NA,NA,"","",TRUE,"ATP5A1"

"13309","CACCACAATGGTGTACTTCA","chr9",42411661,"+","6:G>A,15:A>C","TGG","CACCACGATGGTGTAATTCATGG",2,7,16,NA,"","",TRUE,"ATP5A1"

"13310","CACCACAATGGTGTACTTCA","chr9",39655031,"+","6:G>A,15:A>C","TGG","CACCACGATGGTGTAATTCATGG",2,7,16,NA,"","",TRUE,"ATP5A1"

"13311","CACCACAATGGTGTACTTCA","chr9",62687385,"-","6:C>T,15:T>G","TGG","CACCACGATGGTGTAATTCATGG",2,7,16,NA,"","",TRUE,"ATP5A1"

"13312","CACCACAATGGTGTACTTCA","chr9",66721910,"-","6:C>T,15:T>G","TGG","CACCACGATGGTGTAATTCATGG",2,7,16,NA,"","",TRUE,"ATP5A1"

"13313","GACAGACCAACGTTAATTGC","chr18",46086432,"+","none","AGG","GACAGACCAACGTTAATTGCAGG",0,NA,NA,NA,"GeneID:498","ATP5A1",TRUE,"ATP5A1"

"13314","TGCCATGAAGTACACCATTG","chr18",46087471,"-","none","TGG","TGCCATGAAGTACACCATTGTGG",0,NA,NA,NA,"GeneID:498","ATP5A1",TRUE,"ATP5A1"

"13315","TGCCATGAAGTACACCATTG","chr9",62687380,"+","9:T>G,18:C>T","TGG","TGCCATGAATTACACCATCGTGG",2,10,19,NA,"","",TRUE,"ATP5A1"

"13316","TGCCATGAAGTACACCATTG","chr9",66721905,"+","9:T>G,18:C>T","TGG","TGCCATGAATTACACCATCGTGG",2,10,19,NA,"","",TRUE,"ATP5A1"

"13317","TGCCATGAAGTACACCATTG","chr9",42411666,"-","9:A>C,18:G>A","TGG","TGCCATGAATTACACCATCGTGG",2,10,19,NA,"","",TRUE,"ATP5A1"

"13318","TGCCATGAAGTACACCATTG","chr9",39655036,"-","9:A>C,18:G>A","TGG","TGCCATGAATTACACCATCGTGG",2,10,19,NA,"","",TRUE,"ATP5A1"

"13319","TGCCATGAAGTACACCATTG","chr16",72005876,"-","4:C>T,17:G>A","TGG","TGCCGTGAAGTACACCACTGTGG",2,5,18,NA,"","",TRUE,"ATP5A1"

"13320","AATGACCGCCACGATGCGCC","chr12",56645279,"+","none","CGG","AATGACCGCCACGATGCGCCCGG",0,NA,NA,NA,"GeneID:506","ATP5B",TRUE,"ATP5B"

"13321","AATTCTGGTGACTGGTATCA","chr12",56643881,"-","none","AGG","AATTCTGGTGACTGGTATCAAGG",0,NA,NA,NA,"GeneID:506","ATP5B",TRUE,"ATP5B"

"13322","CACCAGAATCCAGTACTTTC","chr12",56644877,"+","none","TGG","CACCAGAATCCAGTACTTTCTGG",0,NA,NA,NA,"GeneID:506","ATP5B",TRUE,"ATP5B"

"13323","CACCAGAATCCAGTACTTTC","chr2",98206514,"-","13:C>A","TGG","CACCAGAATCCAGGACTTTCTGG",1,14,NA,NA,"GeneID:200403","VWA3B",TRUE,"ATP5B"

"13324","CGCCGCCACCGGGCGCATCG","chr12",56645290,"-","none","TGG","CGCCGCCACCGGGCGCATCGTGG",0,NA,NA,NA,"GeneID:506","ATP5B",TRUE,"ATP5B"

"13325","TCTGCTAGCTCCCTATGCCA","chr12",56643851,"-","none","AGG","TCTGCTAGCTCCCTATGCCAAGG",0,NA,NA,NA,"GeneID:506","ATP5B",TRUE,"ATP5B"

"13326","TGGCAAGACTGTACTGATCA","chr12",56643545,"-","none","TGG","TGGCAAGACTGTACTGATCATGG",0,NA,NA,NA,"GeneID:506","ATP5B",TRUE,"ATP5B"

"13327","AAATCAGAGGCATACTTTAT","chr10",7799172,"+","none","AGG","AAATCAGAGGCATACTTTATAGG",0,NA,NA,NA,"GeneID:509","ATP5C1",TRUE,"ATP5C1"

"13328","CAGGATTCAAGTTCGAAATA","chr10",7796117,"+","none","TGG","CAGGATTCAAGTTCGAAATATGG",0,NA,NA,NA,"GeneID:509","ATP5C1",TRUE,"ATP5C1"

"13329","CCCAATCCATATATTCGAGC","chr10",7797150,"-","none","TGG","CCCAATCCATATATTCGAGCTGG",0,NA,NA,NA,"GeneID:509","ATP5C1",TRUE,"ATP5C1"

"13330","CCCAATCCATATATTCGAGC","chr14",53991780,"+","16:A>G","TGG","CCCAATCCATATATTCAAGCTGG",1,17,NA,NA,"","",TRUE,"ATP5C1"

"13331","CTGAAACCAGCTCGAATATA","chr10",7797141,"+","none","TGG","CTGAAACCAGCTCGAATATATGG",0,NA,NA,NA,"GeneID:509","ATP5C1",TRUE,"ATP5C1"

"13332","CTGAAACCAGCTCGAATATA","chr14",53991789,"-","12:A>G","TGG","CTGAAACCAGCTTGAATATATGG",1,13,NA,NA,"","",TRUE,"ATP5C1"

"13333","GACTGACGCATCTCCAAAAG","chr10",7799827,"-","none","TGG","GACTGACGCATCTCCAAAAGTGG",0,NA,NA,NA,"GeneID:509","ATP5C1",TRUE,"ATP5C1"

"13334","GACTGACGCATCTCCAAAAG","chr7",1913117,"-","5:G>T,7:T>C","GGG","GACTGCCACATCTCCAAAAGGGG",2,6,8,NA,"GeneID:8379","MAD1L1",FALSE,"ATP5C1"

"13335","GGAAGAAAGCCCCCCACTTT","chr10",7799811,"+","none","TGG","GGAAGAAAGCCCCCCACTTTTGG",0,NA,NA,NA,"GeneID:509","ATP5C1",TRUE,"ATP5C1"

"13336","CCACGCCCGTGCCTATGCCG","chr19",1241900,"+","none","AGG","CCACGCCCGTGCCTATGCCGAGG",0,NA,NA,NA,"GeneID:513","ATP5D",TRUE,"ATP5D"

"13337","CGCAGTGAACGCCGACTCTT","chr19",1244112,"+","none","CGG","CGCAGTGAACGCCGACTCTTCGG",0,NA,NA,NA,"GeneID:513","ATP5D",TRUE,"ATP5D"

"13338","CGCAGTGAACGCCGACTCTT","chr17",5002514,"-","5:T>A,10:G>C","GGG","CGCAGAGAACCCCGACTCTTGGG",2,6,11,NA,"GeneID:10749","KIF1C",TRUE,"ATP5D"

"13339","CGGCGGCCTCGGCATAGGCA","chr19",1241909,"-","none","CGG","CGGCGGCCTCGGCATAGGCACGG",0,NA,NA,NA,"GeneID:513","ATP5D",TRUE,"ATP5D"

"13340","CGGCGGCCTCGGCATAGGCA","chr12",122130427,"-","4:G>C,13:A>T","CGG","CGGCCGCCTCGGCTTAGGCACGG",2,5,14,NA,"GeneID:22877","MLXIP",FALSE,"ATP5D"

"13341","CGTTGGCCTCGATTCGGATC","chr19",1244391,"-","none","TGG","CGTTGGCCTCGATTCGGATCTGG",0,NA,NA,NA,"GeneID:513","ATP5D",TRUE,"ATP5D"

"13342","GGCCGAAGAGGCCGTGACGC","chr19",1244145,"+","none","TGG","GGCCGAAGAGGCCGTGACGCTGG",0,NA,NA,NA,"GeneID:513","ATP5D",TRUE,"ATP5D"

"13343","GTGGACGTGCCCACGCTGAC","chr19",1242485,"+","none","CGG","GTGGACGTGCCCACGCTGACCGG",0,NA,NA,NA,"GeneID:513","ATP5D",TRUE,"ATP5D"

"13344","AAAAATTGTGAAAGTAAAGA","chr13",27945387,"+","none","AGG","AAAAATTGTGAAAGTAAAGAAGG",0,NA,NA,NA,"GeneID:432369","ATP5EP2",TRUE,"ATP5E"

"13345","AAAAATTGTGAAAGTAAAGA","chr20",59030313,"-","none","AGG","AAAAATTGTGAAAGTAAAGAAGG",0,NA,NA,NA,"GeneID:514","ATP5E",TRUE,"ATP5E"

"13346","AAAAATTGTGAAAGTAAAGA","chr11",14540618,"+","12:C>A,18:A>G","AGG","AAAAATTGTGAACGTAAAAAAGG",2,13,19,NA,"GeneID:5682","PSMA1",FALSE,"ATP5E"

"13347","AAAAATTGTGAAAGTAAAGA","chr8",87899860,"-","5:G>A,6:C>A","AGG","AAAAACGGTGAAAGTAAAGAAGG",2,6,7,NA,"","",FALSE,"ATP5E"

"13348","AAAAATTGTGAAAGTAAAGA","chr8",81854836,"+","8:A>T,10:T>A","AGG","AAAAATTGAGTAAGTAAAGAAGG",2,9,11,NA,"","",FALSE,"ATP5E"

"13349","AAAAATTGTGAAAGTAAAGA","chrX",86963022,"+","6:A>T,15:T>A","TGG","AAAAATAGTGAAAGTTAAGATGG",2,7,16,NA,"","",FALSE,"ATP5E"

"13350","AAAAATTGTGAAAGTAAAGA","chr16",66632482,"-","7:T>C,18:A>C","TGG","AAAAATTATGAAAGTAAATATGG",2,8,19,NA,"GeneID:146223","CMTM4",FALSE,"ATP5E"

"13351","GAGTCCAGCCTGTCTCCAGT","chr20",59032221,"+","none","AGG","GAGTCCAGCCTGTCTCCAGTAGG",0,NA,NA,NA,"GeneID:514","ATP5E",TRUE,"ATP5E"

"13352","GAGTCCAGCCTGTCTCCAGT","chr13",27945269,"-","none","AGG","GAGTCCAGCCTGTCTCCAGTAGG",0,NA,NA,NA,"GeneID:432369","ATP5EP2",TRUE,"ATP5E"

"13353","GAGTCCAGCCTGTCTCCAGT","chr9",5147294,"-","8:T>G,17:A>T","GGG","GAGTCCAGACTGTCTCCTGTGGG",2,9,18,NA,"GeneID:11172","INSL6",FALSE,"ATP5E"

"13354","GCAAATGCTGAGAAGACTTC","chr20",59030345,"-","none","TGG","GCAAATGCTGAGAAGACTTCTGG",0,NA,NA,NA,"GeneID:514","ATP5E",TRUE,"ATP5E"

"13355","GCAAATGCTGAGAAGACTTC","chr3",195451316,"-","12:C>T,17:C>A","AGG","GCAAATGCTGAGGAGACGTCAGG",2,13,18,NA,"","",FALSE,"ATP5E"

"13356","GCTACAGCATGGTGGCCTAC","chr13",27945251,"+","none","TGG","GCTACAGCATGGTGGCCTACTGG",0,NA,NA,NA,"GeneID:432369","ATP5EP2",TRUE,"ATP5E"

"13357","GCTACAGCATGGTGGCCTAC","chr20",59032239,"-","none","TGG","GCTACAGCATGGTGGCCTACTGG",0,NA,NA,NA,"GeneID:514","ATP5E",TRUE,"ATP5E"

"13358","AAGCCCAGTTCCGAGTACAT","chr1",111456091,"-","none","AGG","AAGCCCAGTTCCGAGTACATAGG",0,NA,NA,NA,"GeneID:515","ATP5F1",TRUE,"ATP5F1"

"13359","AAGCCCAGTTCCGAGTACAT","chr7",152749196,"-","15:G>A,19:G>A","AGG","AAGCCCAGTTCCGAGCACACAGG",2,16,20,NA,"","",TRUE,"ATP5F1"

"13360","AAGCCCAGTTCCGAGTACAT","chr6",139614955,"+","11:T>C,15:C>T","AGG","AAGCCCAGTTCTGAGCACATAGG",2,12,16,NA,"","",TRUE,"ATP5F1"

"13361","AAGCCCAGTTCCGAGTACAT","chr9",125730333,"+","0:T>A,15:C>T","AGG","TAGCCCAGTTCCGAGCACATAGG",2,1,16,NA,"","",FALSE,"ATP5F1"

"13362","CTCACACTTACCCTGGACCT","chr1",111449864,"-","none","AGG","CTCACACTTACCCTGGACCTAGG",0,NA,NA,NA,"GeneID:515","ATP5F1",TRUE,"ATP5F1"

"13363","GCAGAACATGATGCGTCGAA","chr1",111459551,"+","none","AGG","GCAGAACATGATGCGTCGAAAGG",0,NA,NA,NA,"GeneID:515","ATP5F1",TRUE,"ATP5F1"

"13364","GCAGAACATGATGCGTCGAA","chr16",75709199,"-","14:T>C,17:T>C","AGG","GCAGAACATGATGCATCAAAAGG",2,15,18,NA,"GeneID:54386","TERF2IP",TRUE,"ATP5F1"

"13365","GCAGAACATGATGCGTCGAA","chr18",68277882,"-","13:A>G,16:A>G","AGG","GCAGAACATGATGTGTTGAAAGG",2,14,17,NA,"","",TRUE,"ATP5F1"

"13366","GCAGAACATGATGCGTCGAA","chr11",122960940,"+","16:T>C","AGG","GCAGAACATGATGCGTTGAAAGG",1,17,NA,NA,"","",TRUE,"ATP5F1"

"13367","GCAGAACATGATGCGTCGAA","chr7",152749575,"+","14:A>G,16:T>C","AGG","GCAGAACATGATGCATTGAAAGG",2,15,17,NA,"","",TRUE,"ATP5F1"

"13368","GCCCCAGAATAACATTGCTA","chr1",111459449,"+","none","TGG","GCCCCAGAATAACATTGCTATGG",0,NA,NA,NA,"GeneID:515","ATP5F1",TRUE,"ATP5F1"

"13369","GTAGGGTATTGCAGGCAACA","chr1",111454206,"+","none","AGG","GTAGGGTATTGCAGGCAACAAGG",0,NA,NA,NA,"GeneID:515","ATP5F1",TRUE,"ATP5F1"

"13370","TCGTTATGGACTGATCCCTG","chr1",111454294,"+","none","AGG","TCGTTATGGACTGATCCCTGAGG",0,NA,NA,NA,"GeneID:515","ATP5F1",TRUE,"ATP5F1"

"13371","TCGTTATGGACTGATCCCTG","chr11",122960493,"+","5:T>A,9:G>A","AGG","TCGTTTTGGGCTGATCCCTGAGG",2,6,10,NA,"","",TRUE,"ATP5F1"

"13372","AATGATCAAGCTGCCAAACA","chr17",48895303,"-","none","CGG","AATGATCAAGCTGCCAAACACGG",0,NA,NA,NA,"GeneID:516","ATP5G1",TRUE,"ATP5G1"

"13373","AATGATCAAGCTGCCAAACA","chr11",23974910,"-","7:A>T,9:T>C","AGG","AATGATCTAACTGCCAAACAAGG",2,8,10,NA,"","",FALSE,"ATP5G1"

"13374","ACTGAAAAAATGCAGACCGC","chr17",48893408,"+","none","CGG","ACTGAAAAAATGCAGACCGCCGG",0,NA,NA,NA,"GeneID:516","ATP5G1",TRUE,"ATP5G1"

"13375","ACTGAAAAAATGCAGACCGC","chr3",172829161,"-","18:T>C","TGG","ACTGAAAAAATGCAGACCACTGG",1,19,NA,NA,"","",TRUE,"ATP5G1"

"13376","ACTGAAAAAATGCAGACCGC","chr14",31320623,"-","18:T>C","CGG","ACTGAAAAAATGCAGACCACCGG",1,19,NA,NA,"GeneID:25938","HEATR5A",TRUE,"ATP5G1"

"13377","ACTGAAAAAATGCAGACCGC","chr3",138889946,"-","18:G>C","TGG","ACTGAAAAAATGCAGACCCCTGG",1,19,NA,NA,"","",TRUE,"ATP5G1"

"13378","ACTGAAAAAATGCAGACCGC","chr13",106663427,"+","7:C>A,18:A>G","AGG","ACTGAAACAATGCAGACCACAGG",2,8,19,NA,"GeneID:100874173","LINC00443",TRUE,"ATP5G1"

"13379","ACTGAAAAAATGCAGACCGC","chr12",104514019,"+","3:T>G,18:C>G","CGG","ACTTAAAAAATGCAGACCCCCGG",2,4,19,NA,"GeneID:50515","CHST11",TRUE,"ATP5G1"

"13380","ACTGAAAAAATGCAGACCGC","chr1",44088504,"-","8:C>T,18:T>C","TGG","ACTGAAAAGATGCAGACCACTGG",2,9,19,NA,"","",FALSE,"ATP5G1"

"13381","ATTATTCATTTCTCCAGCTC","chr17",48893434,"+","none","TGG","ATTATTCATTTCTCCAGCTCTGG",0,NA,NA,NA,"GeneID:516","ATP5G1",TRUE,"ATP5G1"

"13382","ATTATTCATTTCTCCAGCTC","chr2",118783164,"-","0:A>T,7:A>T","TGG","TTTATTCTTTTCTCCAGCTCTGG",2,1,8,NA,"","",FALSE,"ATP5G1"

"13383","CCCAGTGAATTCATCTAAAC","chr17",48894427,"+","none","AGG","CCCAGTGAATTCATCTAAACAGG",0,NA,NA,NA,"GeneID:516","ATP5G1",TRUE,"ATP5G1"

"13384","GCTGGCATTGGAACCGTGTT","chr17",48895287,"+","none","TGG","GCTGGCATTGGAACCGTGTTTGG",0,NA,NA,NA,"GeneID:516","ATP5G1",TRUE,"ATP5G1"

"13385","GCTGGCATTGGAACCGTGTT","chr14",31320365,"-","12:C>T,14:C>G","TGG","GCTGGCATTGGAGCGGTGTTTGG",2,13,15,NA,"GeneID:25938","HEATR5A",TRUE,"ATP5G1"

"13386","GCTGGCATTGGAACCGTGTT","chr13",106663685,"+","13:A>C,14:A>C","TGG","GCTGGCATTGGAAAAGTGTTTGG",2,14,15,NA,"GeneID:100874173","LINC00443",TRUE,"ATP5G1"

"13387","GCTGGCATTGGAACCGTGTT","chr12",104514277,"+","12:G>A,14:G>C","TGG","GCTGGCATTGGAGCGGTGTTTGG",2,13,15,NA,"GeneID:50515","CHST11",TRUE,"ATP5G1"

"13388","GCTGGCATTGGAACCGTGTT","chr10",76836709,"+","1:T>C,14:A>C","TGG","GTTGGCATTGGAACAGTGTTTGG",2,2,15,NA,"","",TRUE,"ATP5G1"

"13389","GCTGGCATTGGAACCGTGTT","chr12",53669174,"-","5:C>G,14:A>G","TGG","GCTGGGATTGGAACTGTGTTTGG",2,6,15,NA,"GeneID:517","ATP5G2",TRUE,"ATP5G1"

"13390","GCTGGCATTGGAACCGTGTT","chr10",69433110,"-","4:T>C,14:A>G","TGG","GCTGACATTGGAACTGTGTTTGG",2,5,15,NA,"","",TRUE,"ATP5G1"

"13391","TACAGATCCGCTGTTGTACC","chr17",48894366,"+","none","AGG","TACAGATCCGCTGTTGTACCAGG",0,NA,NA,NA,"GeneID:516","ATP5G1",TRUE,"ATP5G1"

"13392","ACCGGAGATACTGACAGATG","chr12",53669872,"-","none","AGG","ACCGGAGATACTGACAGATGAGG",0,NA,NA,NA,"GeneID:517","ATP5G2",TRUE,"ATP5G2"

"13393","ACCTCATCTGTCAGTATCTC","chr12",53669868,"+","none","CGG","ACCTCATCTGTCAGTATCTCCGG",0,NA,NA,NA,"GeneID:517","ATP5G2",TRUE,"ATP5G2"

"13394","ACCTCATCTGTCAGTATCTC","chrX",42099057,"+","0:C>A,1:T>C","CGG","CTCTCATCTGTCAGTATCTCCGG",2,1,2,NA,"","",TRUE,"ATP5G2"

"13395","AGCCCCAGCTCCAATGAACT","chr12",53669221,"+","none","TGG","AGCCCCAGCTCCAATGAACTTGG",0,NA,NA,NA,"GeneID:517","ATP5G2",TRUE,"ATP5G2"

"13396","AGCCCCAGCTCCAATGAACT","chrX",42098939,"+","13:G>A","TGG","AGCCCCAGCTCCAGTGAACTTGG",1,14,NA,NA,"","",TRUE,"ATP5G2"

"13397","AGCCCCAGCTCCAATGAACT","chr2",197263327,"-","0:G>T,9:G>A","TGG","CGCCCCAGCCCCAATGAACTTGG",2,1,10,NA,"GeneID:91526;GeneID:101927547","ANKRD44;ANKRD44-IT1",TRUE,"ATP5G2"

"13398","AGCCCCAGCTCCAATGAACT","chr1",209267986,"+","9:C>T,18:T>C","TGG","AGCCCCAGCCCCAATGAATTTGG",2,10,19,NA,"","",TRUE,"ATP5G2"

"13399","AGCCCCAGCTCCAATGAACT","chr8",137699036,"-","8:T>G,18:A>G","AGG","AGCCCCAGATCCAATGAATTAGG",2,9,19,NA,"","",FALSE,"ATP5G2"

"13400","AGCCCCAGCTCCAATGAACT","chr3",130293552,"-","7:T>C,15:T>C","TGG","AGCCCCAACTCCAATAAACTTGG",2,8,16,NA,"","",TRUE,"ATP5G2"

"13401","GCAACATACAGGATCAGCTC","chr12",53676166,"+","none","AGG","GCAACATACAGGATCAGCTCAGG",0,NA,NA,NA,"GeneID:517","ATP5G2",TRUE,"ATP5G2"

"13402","GGTACTCACCAAGGAGGGAG","chr12",53672566,"+","none","TGG","GGTACTCACCAAGGAGGGAGTGG",0,NA,NA,NA,"GeneID:517","ATP5G2",TRUE,"ATP5G2"

"13403","GGTACTCACCAAGGAGGGAG","chr7",131947584,"+","4:A>C,10:C>A","AGG","GGTAATCACCCAGGAGGGAGAGG",2,5,11,NA,"GeneID:101928782","LOC101928782",FALSE,"ATP5G2"

"13404","TTCACAGAGCCTCAGCAGCT","chr12",53669328,"-","none","TGG","TTCACAGAGCCTCAGCAGCTTGG",0,NA,NA,NA,"GeneID:517","ATP5G2",TRUE,"ATP5G2"

"13405","TTCACAGAGCCTCAGCAGCT","chr8",138762315,"-","1:G>A,17:G>C","GGG","TCCACAGAGCCTCAGCACCTGGG",2,2,18,NA,"GeneID:169044","COL22A1",FALSE,"ATP5G2"

"13406","ATCAGTGTTATCTCGACCAG","chr2",175180117,"-","none","AGG","ATCAGTGTTATCTCGACCAGAGG",0,NA,NA,NA,"GeneID:518","ATP5G3",TRUE,"ATP5G3"

"13407","ATGCAACTCTGGATCCAGCT","chr2",175180153,"+","none","CGG","ATGCAACTCTGGATCCAGCTCGG",0,NA,NA,NA,"GeneID:518","ATP5G3",TRUE,"ATP5G3"

"13408","ATGCAACTCTGGATCCAGCT","chr22",26904340,"+","5:T>A,11:C>G","GGG","ATGCATCTCTGCATCCAGCTGGG",2,6,12,NA,"GeneID:101929539","LINC01422",FALSE,"ATP5G3"

"13409","CAGGGCTCTACGGTATTTAA","chr2",175179233,"-","none","TGG","CAGGGCTCTACGGTATTTAATGG",0,NA,NA,NA,"GeneID:518","ATP5G3",TRUE,"ATP5G3"

"13410","GAGACCCATAGCTTCAGACA","chr2",175178329,"+","none","AGG","GAGACCCATAGCTTCAGACAAGG",0,NA,NA,NA,"GeneID:518","ATP5G3",TRUE,"ATP5G3"

"13411","GCTCGCCTGCACCCCCTCTC","chr2",175181356,"-","none","TGG","GCTCGCCTGCACCCCCTCTCTGG",0,NA,NA,NA,"GeneID:518","ATP5G3",TRUE,"ATP5G3"

"13412","GTGTGTCTCAGCTAATCCAA","chr2",175179199,"-","none","AGG","GTGTGTCTCAGCTAATCCAAAGG",0,NA,NA,NA,"GeneID:518","ATP5G3",TRUE,"ATP5G3"

"13413","GTGTGTCTCAGCTAATCCAA","chr7",46553507,"-","1:T>A,13:C>T","TGG","GAGTGTCTCAGCTGATCCAATGG",2,2,14,NA,"","",FALSE,"ATP5G3"

"13414","ACAAGAAAAAGTATCCCTAT","chr17",75038961,"-","none","TGG","ACAAGAAAAAGTATCCCTATTGG",0,NA,NA,NA,"GeneID:23510;GeneID:10476","KCTD2;ATP5H",TRUE,"ATP5H"

"13415","ACAAGAAAAAGTATCCCTAT","chr15",43267933,"-","15:C>G","TGG","ACAAGAAAAAGTATCGCTATTGG",1,16,NA,NA,"","",TRUE,"ATP5H"

"13416","ACAAGAAAAAGTATCCCTAT","chr9",14069367,"+","6:G>A","TGG","ACAAGAGAAAGTATCCCTATTGG",1,7,NA,NA,"","",TRUE,"ATP5H"

"13417","ACAAGAAAAAGTATCCCTAT","chr12",68642543,"-","2:C>T,16:A>G","TGG","ACGAGAAAAAGTATCCTTATTGG",2,3,17,NA,"GeneID:5908","RAP1B",TRUE,"ATP5H"

"13418","AGAATCCACCAGCTATCGAC","chr17",75042240,"-","none","TGG","AGAATCCACCAGCTATCGACTGG",0,NA,NA,NA,"GeneID:23510;GeneID:10476","KCTD2;ATP5H",TRUE,"ATP5H"

"13419","AGAATCCACCAGCTATCGAC","chr15",43268229,"-","16:A>G","TGG","AGAATCCACCAGCTATTGACTGG",1,17,NA,NA,"","",TRUE,"ATP5H"

"13420","AGAATCCACCAGCTATCGAC","chr12",68642834,"-","10:C>T,12:A>G","TGG","AGAATCCACCGGTTATCGACTGG",2,11,13,NA,"GeneID:5908","RAP1B",TRUE,"ATP5H"

"13421","AGCTTCATTCAAGTCCTCAA","chr17",75039000,"+","none","TGG","AGCTTCATTCAAGTCCTCAATGG",0,NA,NA,NA,"GeneID:23510;GeneID:10476","KCTD2;ATP5H",TRUE,"ATP5H"

"13422","AGCTTCATTCAAGTCCTCAA","chr9",14069328,"-","11:C>T,19:C>T","TGG","AGCTTCATTCAGGTCCTCAGTGG",2,12,20,NA,"","",TRUE,"ATP5H"

"13423","AGCTTCATTCAAGTCCTCAA","chr15",43267972,"+","6:G>A,15:G>C","TGG","AGCTTCGTTCAAGTCGTCAATGG",2,7,16,NA,"","",TRUE,"ATP5H"

"13424","AGCTTCATTCAAGTCCTCAA","chr12",68642582,"+","6:G>A,19:G>A","TGG","AGCTTCGTTCAAGTCCTCAGTGG",2,7,20,NA,"GeneID:5908","RAP1B",TRUE,"ATP5H"

"13425","AGCTTCATTCAAGTCCTCAA","chr11",76057938,"-","3:G>A,18:G>T","GGG","AGCCTCATTCAAGTCCTCCAGGG",2,4,19,NA,"GeneID:7405","UVRAG",FALSE,"ATP5H"

"13426","AGGTGAAATCTTGTGCTGAG","chr17",75039253,"-","none","TGG","AGGTGAAATCTTGTGCTGAGTGG",0,NA,NA,NA,"GeneID:23510;GeneID:10476","KCTD2;ATP5H",TRUE,"ATP5H"

"13427","AGGTGAAATCTTGTGCTGAG","chr9",2900496,"-","1:A>C","TGG","ATGTGAAATCTTGTGCTGAGTGG",1,2,NA,NA,"","",TRUE,"ATP5H"

"13428","AGGTGAAATCTTGTGCTGAG","chr12",68642690,"-","1:A>C","TGG","ATGTGAAATCTTGTGCTGAGTGG",1,2,NA,NA,"GeneID:5908","RAP1B",TRUE,"ATP5H"

"13429","AGGTGAAATCTTGTGCTGAG","chr9",14069220,"+","1:T>G,8:A>T","TGG","ATGTGAAAACTTGTGCTGAGTGG",2,2,9,NA,"","",TRUE,"ATP5H"

"13430","GATTGTAGAATATGAGAAAG","chr17",75039210,"-","none","AGG","GATTGTAGAATATGAGAAAGAGG",0,NA,NA,NA,"GeneID:23510;GeneID:10476","KCTD2;ATP5H",TRUE,"ATP5H"

"13431","TAGTAAGCCCAGTCGATAGC","chr17",75042229,"+","none","TGG","TAGTAAGCCCAGTCGATAGCTGG",0,NA,NA,NA,"GeneID:23510;GeneID:10476","KCTD2;ATP5H",TRUE,"ATP5H"

"13432","TAGTAAGCCCAGTCGATAGC","chr12",68642823,"+","18:A>G","CGG","TAGTAAGCCCAGTCGATAACCGG",1,19,NA,NA,"GeneID:5908","RAP1B",TRUE,"ATP5H"

"13433","TAGTAAGCCCAGTCGATAGC","chr15",43268218,"+","7:T>C,14:A>G","TGG","TAGTAAGTCCAGTCAATAGCTGG",2,8,15,NA,"","",TRUE,"ATP5H"

"13434","TAGTAAGCCCAGTCGATAGC","chr9",2900634,"+","9:T>C,14:A>G","TGG","TAGTAAGCCTAGTCAATAGCTGG",2,10,15,NA,"","",TRUE,"ATP5H"

"13435","CGGTCACTCACTGTAGCGCG","chr4",673900,"+","none","TGG","CGGTCACTCACTGTAGCGCGTGG",0,NA,NA,NA,"GeneID:521;GeneID:4636","ATP5I;MYL5",TRUE,"ATP5I"

"13436","CGTACCTTCTGCCAATTCTC","chr4",673297,"+","none","TGG","CGTACCTTCTGCCAATTCTCTGG",0,NA,NA,NA,"GeneID:521","ATP5I",TRUE,"ATP5I"

"13437","CGTAGGCCACACCGAGGAAC","chr4",673926,"+","none","AGG","CGTAGGCCACACCGAGGAACAGG",0,NA,NA,NA,"GeneID:521;GeneID:4636","ATP5I;MYL5",TRUE,"ATP5I"

"13438","GAAACGGATTGCCAGAGAAT","chr4",673311,"-","none","TGG","GAAACGGATTGCCAGAGAATTGG",0,NA,NA,NA,"GeneID:521","ATP5I",TRUE,"ATP5I"

"13439","GAAACGGATTGCCAGAGAAT","chr9",11586388,"+","5:A>G,8:A>T","GGG","GAAACAGAATGCCAGAGAATGGG",2,6,9,NA,"","",FALSE,"ATP5I"

"13440","GCAGGTCTCTCCGCTCATCA","chr4",674213,"-","none","AGG","GCAGGTCTCTCCGCTCATCAAGG",0,NA,NA,NA,"GeneID:521;GeneID:4636","ATP5I;MYL5",TRUE,"ATP5I"

"13441","GCCTGGATCACCTTGATGAG","chr4",674200,"+","none","CGG","GCCTGGATCACCTTGATGAGCGG",0,NA,NA,NA,"GeneID:521;GeneID:4636","ATP5I;MYL5",TRUE,"ATP5I"

"13442","AGACGTACCTTCAAATTTGA","chr21",25725217,"+","none","AGG","AGACGTACCTTCAAATTTGAAGG",0,NA,NA,NA,"GeneID:522","ATP5J",TRUE,"ATP5J"

"13443","CGGCCCATAATGCATTGCGA","chr21",25734944,"-","none","TGG","CGGCCCATAATGCATTGCGATGG",0,NA,NA,NA,"GeneID:522;GeneID:2551","ATP5J;GABPA",TRUE,"ATP5J"

"13444","CGGCCCATAATGCATTGCGA","chrY",6899314,"+","0:T>C,7:G>T","TGG","TGGCCCAGAATGCATTGCGATGG",2,1,8,NA,"","",FALSE,"ATP5J"

"13445","CGGCCCATAATGCATTGCGA","chr9",105158189,"+","0:T>C,8:C>A","TGG","TGGCCCATCATGCATTGCGATGG",2,1,9,NA,"","",FALSE,"ATP5J"

"13446","CGGCCCATAATGCATTGCGA","chr14",94382490,"+","0:T>C,18:C>G","AGG","TGGCCCATAATGCATTGCCAAGG",2,1,19,NA,"GeneID:5265","SERPINA1",FALSE,"ATP5J"

"13447","GACGGCTGACCGAATGACAG","chr21",25729743,"+","none","AGG","GACGGCTGACCGAATGACAGAGG",0,NA,NA,NA,"GeneID:522","ATP5J",TRUE,"ATP5J"

"13448","GACGGCTGACCGAATGACAG","chr5",73803548,"+","3:T>G,4:A>G","AGG","GACTACTGACCGAATGACAGAGG",2,4,5,NA,"GeneID:64283","ARHGEF28",TRUE,"ATP5J"

"13449","GACGGCTGACCGAATGACAG","chr22",22988294,"-","3:A>C,10:T>G","AGG","GACTGCTGACAGAATGACAGAGG",2,4,11,NA,"","",TRUE,"ATP5J"

"13450","TACTCTGAACTAGCATCAAC","chr21",25725314,"+","none","AGG","TACTCTGAACTAGCATCAACAGG",0,NA,NA,NA,"GeneID:522","ATP5J",TRUE,"ATP5J"

"13451","TCCACAAAGAGTTTCTGTAT","chr21",25729660,"+","none","AGG","TCCACAAAGAGTTTCTGTATAGG",0,NA,NA,NA,"GeneID:522","ATP5J",TRUE,"ATP5J"

"13452","TCCACAAAGAGTTTCTGTAT","chr5",73803466,"+","5:G>A","GGG","TCCACGAAGAGTTTCTGTATGGG",1,6,NA,NA,"GeneID:64283","ARHGEF28",TRUE,"ATP5J"

"13453","TCCACAAAGAGTTTCTGTAT","chrY",6901161,"-","4:A>G,19:C>A","AGG","TCCATAAAGAGTTTCTGTAGAGG",2,5,20,NA,"","",TRUE,"ATP5J"

"13454","TGAAGTCATCGAAAAACCCC","chr22",22988561,"+","none","AGG","TGAAGTCATCGAAAAACCCCAGG",0,NA,NA,NA,"","",TRUE,"ATP5J"

"13455","TGAAGTCATCGAAAAACCCC","chr21",25724647,"-","none","AGG","TGAAGTCATCGAAAAACCCCAGG",0,NA,NA,NA,"GeneID:522","ATP5J",TRUE,"ATP5J"

"13456","TGAAGTCATCGAAAAACCCC","chr8",98633844,"+","10:A>G","AGG","TGAAGTCATCAAAAAACCCCAGG",1,11,NA,NA,"GeneID:6788","STK3",TRUE,"ATP5J"

"13457","TGAAGTCATCGAAAAACCCC","chrY",6901344,"+","9:T>C","AGG","TGAAGTCATTGAAAAACCCCAGG",1,10,NA,NA,"","",TRUE,"ATP5J"

"13458","GACTCCAAAATGGCGTCAGT","chr7",99466130,"-","none","TGG","GACTCCAAAATGGCGTCAGTTGG",0,NA,NA,NA,"GeneID:26024;GeneID:100526740;GeneID:9551","PTCD1;ATP5J2-PTCD1;ATP5J2",TRUE,"ATP5J2"

"13459","GACTCCAAAATGGCGTCAGT","chr12",88971257,"-","10:C>A,13:C>G","GGG","GACTCCAAAAGGGGGTCAGTGGG",2,11,14,NA,"","",FALSE,"ATP5J2"

"13460","GAGCATCTCGGGGATTACCA","chr7",99459200,"-","none","TGG","GAGCATCTCGGGGATTACCATGG",0,NA,NA,NA,"GeneID:26024;GeneID:100526740;GeneID:9551","PTCD1;ATP5J2-PTCD1;ATP5J2",TRUE,"ATP5J2"

"13461","GAGCATCTCGGGGATTACCA","chr12",6270241,"-","13:C>T","TGG","GAGCATCTCGGGGGTTACCATGG",1,14,NA,NA,"","",TRUE,"ATP5J2"

"13462","GAGCATCTCGGGGATTACCA","chr6",108907691,"-","13:C>T","TGG","GAGCATCTCGGGGGTTACCATGG",1,14,NA,NA,"GeneID:84071","ARMC2",TRUE,"ATP5J2"

"13463","GAGCATCTCGGGGATTACCA","chr17",64491686,"+","13:T>A","TGG","GAGCATCTCGGGGTTTACCATGG",1,14,NA,NA,"GeneID:11232","POLG2",TRUE,"ATP5J2"

"13464","GAGCATCTCGGGGATTACCA","chr9",77040570,"+","13:C>A,15:G>T","TGG","GAGCATCTCGGGGCTGACCATGG",2,14,16,NA,"","",TRUE,"ATP5J2"

"13465","GAGCATCTCGGGGATTACCA","chr8",94594503,"+","9:T>G,13:T>A","TGG","GAGCATCTCTGGGTTTACCATGG",2,10,14,NA,"","",TRUE,"ATP5J2"

"13466","GAGCATCTCGGGGATTACCA","chr15",66415015,"-","9:T>C,13:C>T","TGG","GAGCATCTCAGGGGTTACCATGG",2,10,14,NA,"GeneID:5604","MAP2K1",TRUE,"ATP5J2"

"13467","GCACTCACTGAGATGCTTGT","chr7",99459138,"+","none","AGG","GCACTCACTGAGATGCTTGTAGG",0,NA,NA,NA,"GeneID:26024;GeneID:100526740;GeneID:9551","PTCD1;ATP5J2-PTCD1;ATP5J2",TRUE,"ATP5J2"

"13468","GCACTCACTGAGATGCTTGT","chr12",122842795,"+","17:C>T,19:C>T","TGG","GCACTCACTGAGATGCTCGCTGG",2,18,20,NA,"GeneID:9026","HIP1R",FALSE,"ATP5J2"

"13469","GCACTCACTGAGATGCTTGT","chr9",4868306,"-","1:T>G,16:G>A","AGG","GAACTCACTGAGATGCCTGTAGG",2,2,17,NA,"GeneID:10171","RCL1",FALSE,"ATP5J2"

"13470","GCACTCACTGAGATGCTTGT","chr9",4866518,"-","1:T>G,16:G>A","AGG","GAACTCACTGAGATGCCTGTAGG",2,2,17,NA,"GeneID:10171","RCL1",FALSE,"ATP5J2"

"13471","GTCCTCTTCAGTGGTATTTG","chr6",108907601,"+","none","CGG","GTCCTCTTCAGTGGTATTTGCGG",0,NA,NA,NA,"GeneID:84071","ARMC2",TRUE,"ATP5J2"

"13472","GTCCTCTTCAGTGGTATTTG","chr7",99458319,"+","none","CGG","GTCCTCTTCAGTGGTATTTGCGG",0,NA,NA,NA,"GeneID:26024;GeneID:100526740;GeneID:9551","PTCD1;ATP5J2-PTCD1;ATP5J2",TRUE,"ATP5J2"

"13473","GTCCTCTTCAGTGGTATTTG","chr12",6270151,"+","12:A>G,14:C>T","CGG","GTCCTCTTCAGTAGCATTTGCGG",2,13,15,NA,"","",TRUE,"ATP5J2"

"13474","TCCCGCATCAAGATCCAGCT","chr7",99460123,"+","none","TGG","TCCCGCATCAAGATCCAGCTTGG",0,NA,NA,NA,"GeneID:26024;GeneID:100526740;GeneID:9551","PTCD1;ATP5J2-PTCD1;ATP5J2",TRUE,"ATP5J2"

"13475","TCCCGCATCAAGATCCAGCT","chr9",77040478,"-","3:A>G","TGG","TCCTGCATCAAGATCCAGCTTGG",1,4,NA,NA,"","",TRUE,"ATP5J2"

"13476","TCCCGCATCAAGATCCAGCT","chr17",64491585,"-","4:T>C","TGG","TCCCACATCAAGATCCAGCTTGG",1,5,NA,NA,"GeneID:11232","POLG2",TRUE,"ATP5J2"

"13477","TCCCGCATCAAGATCCAGCT","chr6",108907789,"+","4:C>G","TGG","TCCCCCATCAAGATCCAGCTTGG",1,5,NA,NA,"GeneID:84071","ARMC2",TRUE,"ATP5J2"

"13478","TCCCGCATCAAGATCCAGCT","chr12",6270342,"+","6:G>A","TGG","TCCCGCGTCAAGATCCAGCTTGG",1,7,NA,NA,"","",TRUE,"ATP5J2"

"13479","TCCCGCATCAAGATCCAGCT","chr15",66415116,"+","3:T>C,12:C>A","TGG","TCCTGCATCAAGCTCCAGCTTGG",2,4,13,NA,"GeneID:5604","MAP2K1",TRUE,"ATP5J2"

"13480","TTCCGTTTCAGTACCAGTGA","chr7",99460184,"-","none","AGG","TTCCGTTTCAGTACCAGTGAAGG",0,NA,NA,NA,"GeneID:26024;GeneID:100526740;GeneID:9551","PTCD1;ATP5J2-PTCD1;ATP5J2",TRUE,"ATP5J2"

"13481","ATACCACGCGCTGCTGAAGA","chr7",99429634,"-","none","TGG","ATACCACGCGCTGCTGAAGATGG",0,NA,NA,NA,"GeneID:26024;GeneID:100526740","PTCD1;ATP5J2-PTCD1",TRUE,"ATP5J2-PTCD1"

"13482","GACTCGCTCGACTGTTCGCC","chr7",99435209,"-","none","AGG","GACTCGCTCGACTGTTCGCCAGG",0,NA,NA,NA,"GeneID:26024;GeneID:100526740","PTCD1;ATP5J2-PTCD1",TRUE,"ATP5J2-PTCD1"

"13483","GCCCATGGAGAGCAACTACA","chr7",99433342,"-","none","CGG","GCCCATGGAGAGCAACTACACGG",0,NA,NA,NA,"GeneID:26024;GeneID:100526740","PTCD1;ATP5J2-PTCD1",TRUE,"ATP5J2-PTCD1"

"13484","GCCGTGTAGGTGGCGTCCGA","chr7",99429762,"+","none","GGG","GCCGTGTAGGTGGCGTCCGAGGG",0,NA,NA,NA,"GeneID:26024;GeneID:100526740","PTCD1;ATP5J2-PTCD1",TRUE,"ATP5J2-PTCD1"

"13485","TCTCCGGCCTCGCCATAATT","chr7",99434843,"+","none","TGG","TCTCCGGCCTCGCCATAATTTGG",0,NA,NA,NA,"GeneID:26024;GeneID:100526740","PTCD1;ATP5J2-PTCD1",TRUE,"ATP5J2-PTCD1"

"13486","TTCCGTTTCAGTACCAGTGA","chr7",99460184,"-","none","AGG","TTCCGTTTCAGTACCAGTGAAGG",0,NA,NA,NA,"GeneID:26024;GeneID:100526740;GeneID:9551","PTCD1;ATP5J2-PTCD1;ATP5J2",TRUE,"ATP5J2-PTCD1"

"13487","AAGACCCCGGCGCTGGTGAA","chr11",118401695,"+","none","CGG","AAGACCCCGGCGCTGGTGAACGG",0,NA,NA,NA,"GeneID:10632","ATP5L",TRUE,"ATP5L"

"13488","AAGACCCCGGCGCTGGTGAA","chr16",4228781,"+","10:G>C,16:G>T","AGG","AAGACCCCGGGGCTGGGGAAAGG",2,11,17,NA,"GeneID:6345","SRL",FALSE,"ATP5L"

"13489","CGCTCACCGTTCACCAGCGC","chr11",118401704,"-","none","CGG","CGCTCACCGTTCACCAGCGCCGG",0,NA,NA,NA,"GeneID:10632","ATP5L",TRUE,"ATP5L"

"13490","GACTTACTCGAAGCCTCGAT","chr11",118406943,"+","none","TGG","GACTTACTCGAAGCCTCGATTGG",0,NA,NA,NA,"GeneID:10632","ATP5L",TRUE,"ATP5L"

"13491","GACTTACTCGAAGCCTCGAT","chr22",42640195,"-","8:A>G","TGG","GACTTACTTGAAGCCTCGATTGG",1,9,NA,NA,"GeneID:1727;GeneID:267020","CYB5R3;ATP5L2",TRUE,"ATP5L"

"13492","GACTTACTCGAAGCCTCGAT","chr6",122859909,"-","9:T>C,16:A>G","TGG","GACTTACTCAAAGCCTTGATTGG",2,10,17,NA,"","",TRUE,"ATP5L"

"13493","TACCAAAATGTGGCCAATCG","chr11",118406959,"-","none","AGG","TACCAAAATGTGGCCAATCGAGG",0,NA,NA,NA,"GeneID:10632","ATP5L",TRUE,"ATP5L"

"13494","TACCAAAATGTGGCCAATCG","chr17",42647906,"-","16:C>T,18:A>G","AGG","TACCAAAATGTGGCCAGTTGAGG",2,17,19,NA,"","",TRUE,"ATP5L"

"13495","TACCAAAATGTGGCCAATCG","chr10",1365162,"-","16:C>T,17:C>A","CGG","TACCAAAATGTGGCCAGGCGCGG",2,17,18,NA,"GeneID:105","ADARB2",FALSE,"ATP5L"

"13496","TACCAAAATGTGGCCAATCG","chr4",24658279,"+","19:C>G","AGG","TACCAAAATGTGGCCAATCCAGG",1,20,NA,NA,"","",TRUE,"ATP5L"

"13497","TACCAAAATGTGGCCAATCG","chr6",122859893,"+","19:A>G","AGG","TACCAAAATGTGGCCAATCAAGG",1,20,NA,NA,"","",TRUE,"ATP5L"

"13498","TACCAAAATGTGGCCAATCG","chr22",42640179,"+","10:C>T","AGG","TACCAAAATGCGGCCAATCGAGG",1,11,NA,NA,"GeneID:1727;GeneID:267020","CYB5R3;ATP5L2",TRUE,"ATP5L"

"13499","TACCAAAATGTGGCCAATCG","chr17",76202713,"+","8:A>T","AGG","TACCAAAAAGTGGCCAATCGAGG",1,9,NA,NA,"GeneID:114804","RNF157",TRUE,"ATP5L"

"13500","TGGCCACTGAGGTGTTGATG","chr11",118409021,"+","none","TGG","TGGCCACTGAGGTGTTGATGTGG",0,NA,NA,NA,"GeneID:10632","ATP5L",TRUE,"ATP5L"

"13501","TGGCCACTGAGGTGTTGATG","chr22",42640019,"-","15:G>A,18:G>A","TGG","TGGCCACTGAGGTGTCGACGTGG",2,16,19,NA,"GeneID:1727;GeneID:267020","CYB5R3;ATP5L2",TRUE,"ATP5L"

"13502","TGGCCACTGAGGTGTTGATG","chr11",28144950,"-","15:T>A,16:T>C","TGG","TGGCCACTGAGGTGTAAATGTGG",2,16,17,NA,"GeneID:196074","METTL15",TRUE,"ATP5L"

"13503","TGGCCACTGAGGTGTTGATG","chr17",42648070,"+","15:G>T,16:A>G","TGG","TGGCCACTGAGGTGTGAATGTGG",2,16,17,NA,"","",TRUE,"ATP5L"

"13504","TTTCAGGAAGCTGTGCTGAA","chr11",118408993,"+","none","TGG","TTTCAGGAAGCTGTGCTGAATGG",0,NA,NA,NA,"GeneID:10632","ATP5L",TRUE,"ATP5L"

"13505","TTTCAGGAAGCTGTGCTGAA","chr2",36398215,"+","14:C>G,17:A>G","TGG","TTTCAGGAAGCTGTCCTAAATGG",2,15,18,NA,"GeneID:51232","CRIM1",FALSE,"ATP5L"

"13506","TTTCAGGAAGCTGTGCTGAA","chr19",29599939,"+","12:T>G,19:G>A","GGG","TTTCAGGAAGCTTTGCTGAGGGG",2,13,20,NA,"","",FALSE,"ATP5L"

"13507","TTTCAGGAAGCTGTGCTGAA","chr4",24658146,"-","0:C>A,3:T>G","TGG","GTTAAGGAAGCTGTGCTGAATGG",2,1,4,NA,"","",TRUE,"ATP5L"

"13508","TTTCAGGAAGCTGTGCTGAA","chr11",28144978,"-","0:C>A,3:T>G","TGG","GTTAAGGAAGCTGTGCTGAATGG",2,1,4,NA,"GeneID:196074","METTL15",TRUE,"ATP5L"

"13509","TTTCAGGAAGCTGTGCTGAA","chr6",122859761,"-","0:C>A,3:T>G","TGG","GTTAAGGAAGCTGTGCTGAATGG",2,1,4,NA,"","",TRUE,"ATP5L"

"13510","TTTCAGGAAGCTGTGCTGAA","chr4",148703610,"-","0:C>A,3:T>G","TGG","GTTAAGGAAGCTGTGCTGAATGG",2,1,4,NA,"","",TRUE,"ATP5L"

"13511","AAACCACGTCGACACCTCAG","chr22",42640013,"+","none","TGG","AAACCACGTCGACACCTCAGTGG",0,NA,NA,NA,"GeneID:1727;GeneID:267020","CYB5R3;ATP5L2",TRUE,"ATP5L2"

"13512","AAACCACGTCGACACCTCAG","chr11",118409027,"-","7:T>C,10:T>C","TGG","AAACCACATCAACACCTCAGTGG",2,8,11,NA,"GeneID:10632","ATP5L",TRUE,"ATP5L2"

"13513","ACAGCAGCATTCACCAGTGC","chr22",42640215,"+","none","TGG","ACAGCAGCATTCACCAGTGCTGG",0,NA,NA,NA,"GeneID:1727;GeneID:267020","CYB5R3;ATP5L2",TRUE,"ATP5L2"

"13514","ACAGCAGCATTCACCAGTGC","chr9",131577606,"+","13:A>C,17:A>T","TGG","ACAGCAGCATTCAACAGAGCTGG",2,14,18,NA,"GeneID:2889","RAPGEF1",TRUE,"ATP5L2"

"13515","GACTTACTTGAAGCCTCGAT","chr22",42640195,"-","none","TGG","GACTTACTTGAAGCCTCGATTGG",0,NA,NA,NA,"GeneID:1727;GeneID:267020","CYB5R3;ATP5L2",TRUE,"ATP5L2"

"13516","GACTTACTTGAAGCCTCGAT","chr17",76202729,"-","5:C>T,6:A>G","TGG","GACTTGTTTGAAGCCTCGATTGG",2,6,7,NA,"GeneID:114804","RNF157",TRUE,"ATP5L2"

"13517","GACTTACTTGAAGCCTCGAT","chr11",118406943,"+","8:C>T","TGG","GACTTACTCGAAGCCTCGATTGG",1,9,NA,NA,"GeneID:10632","ATP5L",TRUE,"ATP5L2"

"13518","TACCAAAATGCGGCCAATCG","chr22",42640179,"+","none","AGG","TACCAAAATGCGGCCAATCGAGG",0,NA,NA,NA,"GeneID:1727;GeneID:267020","CYB5R3;ATP5L2",TRUE,"ATP5L2"

"13519","TACCAAAATGCGGCCAATCG","chr11",118406959,"-","10:A>G","AGG","TACCAAAATGTGGCCAATCGAGG",1,11,NA,NA,"GeneID:10632","ATP5L",TRUE,"ATP5L2"

"13520","TACCAAAATGCGGCCAATCG","chr4",24658279,"+","10:T>C,19:C>G","AGG","TACCAAAATGTGGCCAATCCAGG",2,11,20,NA,"","",TRUE,"ATP5L2"

"13521","TACCAAAATGCGGCCAATCG","chr6",122859893,"+","10:T>C,19:A>G","AGG","TACCAAAATGTGGCCAATCAAGG",2,11,20,NA,"","",TRUE,"ATP5L2"

"13522","TACCAAAATGCGGCCAATCG","chr17",76202713,"+","8:A>T,10:T>C","AGG","TACCAAAAAGTGGCCAATCGAGG",2,9,11,NA,"GeneID:114804","RNF157",TRUE,"ATP5L2"

"13523","TCCACAAGGTTACGGACAAA","chr22",42640245,"+","none","TGG","TCCACAAGGTTACGGACAAATGG",0,NA,NA,NA,"GeneID:1727;GeneID:267020","CYB5R3;ATP5L2",TRUE,"ATP5L2"

"13524","TCCATTTGTCCGTAACCTTG","chr22",42640249,"-","none","TGG","TCCATTTGTCCGTAACCTTGTGG",0,NA,NA,NA,"GeneID:1727;GeneID:267020","CYB5R3;ATP5L2",TRUE,"ATP5L2"

"13525","TCCATTTGTCCGTAACCTTG","chr11",118401670,"+","0:C>T,2:A>C","TGG","CCAATTTGTCCGTAACCTTGTGG",2,1,3,NA,"GeneID:10632","ATP5L",TRUE,"ATP5L2"

"13526","ACCATGATGAGTGTCCATCG","chr21",33907374,"-","none","CGG","ACCATGATGAGTGTCCATCGCGG",0,NA,NA,NA,"GeneID:539","ATP5O",TRUE,"ATP5O"

"13527","ACCATGATGAGTGTCCATCG","chr1",33952047,"-","6:G>T,18:C>G","AGG","ACCATGCTGAGTGTCCATGGAGG",2,7,19,NA,"GeneID:114784","CSMD2",FALSE,"ATP5O"

"13528","ACCATGATGAGTGTCCATCG","chr2",206236470,"-","1:A>G,17:G>A","TGG","ATCATGATGAGTGTCCACCGTGG",2,2,18,NA,"GeneID:101669764","GPR1-AS",TRUE,"ATP5O"

"13529","CTCAAGAGCTTCCTAAGTCA","chr21",33903962,"-","none","AGG","CTCAAGAGCTTCCTAAGTCAAGG",0,NA,NA,NA,"GeneID:539","ATP5O",TRUE,"ATP5O"

"13530","GCGATGCTTCAGTACCTCTG","chr21",33914478,"-","none","TGG","GCGATGCTTCAGTACCTCTGTGG",0,NA,NA,NA,"GeneID:539","ATP5O",TRUE,"ATP5O"

"13531","GCGATGCTTCAGTACCTCTG","chr2",206236811,"-","1:A>G,15:T>G","TGG","GTGATGCTTCAGTACATCTGTGG",2,2,16,NA,"GeneID:101669764","GPR1-AS",TRUE,"ATP5O"

"13532","TCAATACCGTATACCTGAAC","chr21",33912373,"+","none","AGG","TCAATACCGTATACCTGAACAGG",0,NA,NA,NA,"GeneID:539","ATP5O",TRUE,"ATP5O"

"13533","TCCGCGATGGACACTCATCA","chr21",33907370,"+","none","TGG","TCCGCGATGGACACTCATCATGG",0,NA,NA,NA,"GeneID:539","ATP5O",TRUE,"ATP5O"

"13534","TTAACATACCCTCACAAGCT","chr21",33914440,"+","none","TGG","TTAACATACCCTCACAAGCTTGG",0,NA,NA,NA,"GeneID:539","ATP5O",TRUE,"ATP5O"

"13535","CTAGATCAAATGATGCCGTT","chr14",50321478,"+","none","TGG","CTAGATCAAATGATGCCGTTTGG",0,NA,NA,NA,"GeneID:27109","ATP5S",TRUE,"ATP5S"

"13536","GGGGCCATGGTGCGCTACCA","chr14",50322577,"+","none","TGG","GGGGCCATGGTGCGCTACCATGG",0,NA,NA,NA,"GeneID:27109","ATP5S",TRUE,"ATP5S"

"13537","GGGTGGATTATGATCGCATC","chr14",50322509,"+","none","AGG","GGGTGGATTATGATCGCATCAGG",0,NA,NA,NA,"GeneID:27109","ATP5S",TRUE,"ATP5S"

"13538","GTGCAAGTGTCATTATATCG","chr14",50323975,"+","none","AGG","GTGCAAGTGTCATTATATCGAGG",0,NA,NA,NA,"GeneID:27109","ATP5S",TRUE,"ATP5S"

"13539","TCATTGCTTTGCGTCATTTA","chr14",50324094,"+","none","AGG","TCATTGCTTTGCGTCATTTAAGG",0,NA,NA,NA,"GeneID:27109","ATP5S",TRUE,"ATP5S"

"13540","TCTTTACGCCACACAACTGC","chr14",50321513,"-","none","TGG","TCTTTACGCCACACAACTGCTGG",0,NA,NA,NA,"GeneID:27109","ATP5S",TRUE,"ATP5S"

"13541","AAAAAGGCACCTGCGCCGTA","chr19",41436418,"+","none","TGG","AAAAAGGCACCTGCGCCGTATGG",0,NA,NA,NA,"GeneID:55101","ATP5SL",TRUE,"ATP5SL"

"13542","CCATGTGGAATGGGCGTATC","chr19",41438375,"-","none","AGG","CCATGTGGAATGGGCGTATCAGG",0,NA,NA,NA,"GeneID:55101","ATP5SL",TRUE,"ATP5SL"

"13543","CGCACTCACGGAGGTTATCC","chr19",41433527,"+","none","AGG","CGCACTCACGGAGGTTATCCAGG",0,NA,NA,NA,"GeneID:55101","ATP5SL",TRUE,"ATP5SL"

"13544","CGTTTCCAGGTTTCGAGACA","chr19",41433662,"-","none","AGG","CGTTTCCAGGTTTCGAGACAAGG",0,NA,NA,NA,"GeneID:55101","ATP5SL",TRUE,"ATP5SL"

"13545","CTCCACATCGTAGAAATAGT","chr19",41438273,"+","none","TGG","CTCCACATCGTAGAAATAGTTGG",0,NA,NA,NA,"GeneID:55101","ATP5SL",TRUE,"ATP5SL"

"13546","TTCTCTGCAGATCTTACACC","chr19",41436462,"-","none","TGG","TTCTCTGCAGATCTTACACCTGG",0,NA,NA,NA,"GeneID:55101","ATP5SL",TRUE,"ATP5SL"

"13547","CGCCTCGAAGTCCACAGCAA","chrX",154435331,"+","none","TGG","CGCCTCGAAGTCCACAGCAATGG",0,NA,NA,NA,"GeneID:537","ATP6AP1",TRUE,"ATP6AP1"

"13548","GCTGACTGCATACCAGTCGA","chrX",154432308,"-","none","CGG","GCTGACTGCATACCAGTCGACGG",0,NA,NA,NA,"GeneID:537","ATP6AP1",TRUE,"ATP6AP1"

"13549","GGCTGCTGACATACTCGCAG","chrX",154435411,"-","none","TGG","GGCTGCTGACATACTCGCAGTGG",0,NA,NA,NA,"GeneID:537","ATP6AP1",TRUE,"ATP6AP1"

"13550","GGTAGAGAGCTGCAAGTCGC","chrX",154429094,"-","none","TGG","GGTAGAGAGCTGCAAGTCGCTGG",0,NA,NA,NA,"GeneID:537","ATP6AP1",TRUE,"ATP6AP1"

"13551","TACCTACTTAGATCCCGCCC","chrX",154429110,"+","none","TGG","TACCTACTTAGATCCCGCCCTGG",0,NA,NA,NA,"GeneID:537","ATP6AP1",TRUE,"ATP6AP1"

"13552","TGGTGCTTCCTGCCGTCGAC","chrX",154432293,"+","none","TGG","TGGTGCTTCCTGCCGTCGACTGG",0,NA,NA,NA,"GeneID:537","ATP6AP1",TRUE,"ATP6AP1"

"13553","ACCCCAGAAGTCTTGCTATA","chr5",82310185,"+","none","AGG","ACCCCAGAAGTCTTGCTATAAGG",0,NA,NA,NA,"GeneID:92270","ATP6AP1L",TRUE,"ATP6AP1L"

"13554","AGGCGATGAGATCATATTGT","chr5",82318158,"-","none","TGG","AGGCGATGAGATCATATTGTTGG",0,NA,NA,NA,"GeneID:92270","ATP6AP1L",TRUE,"ATP6AP1L"

"13555","CGACCCATCATCCGTGTCGC","chr5",82312798,"-","none","TGG","CGACCCATCATCCGTGTCGCTGG",0,NA,NA,NA,"GeneID:92270","ATP6AP1L",TRUE,"ATP6AP1L"

"13556","GCCCACGCGTTGGCAGCGGT","chr5",82312744,"-","none","AGG","GCCCACGCGTTGGCAGCGGTAGG",0,NA,NA,NA,"GeneID:92270","ATP6AP1L",TRUE,"ATP6AP1L"

"13557","GGCTTGGTCTCAACTCCAAA","chr5",82305459,"+","none","TGG","GGCTTGGTCTCAACTCCAAATGG",0,NA,NA,NA,"GeneID:92270","ATP6AP1L",TRUE,"ATP6AP1L"

"13558","GGTAAATCGTCCCCCCTTGA","chr5",82318013,"-","none","TGG","GGTAAATCGTCCCCCCTTGATGG",0,NA,NA,NA,"GeneID:92270","ATP6AP1L",TRUE,"ATP6AP1L"

"13559","AACAAACTGGCTCTACCCCC","chrX",40591314,"+","none","AGG","AACAAACTGGCTCTACCCCCAGG",0,NA,NA,NA,"GeneID:10159","ATP6AP2",TRUE,"ATP6AP2"

"13560","AACAAACTGGCTCTACCCCC","chr1",197222458,"+","0:G>A,17:A>C","AGG","GACAAACTGGCTCTACCACCAGG",2,1,18,NA,"","",TRUE,"ATP6AP2"

"13561","ACGTCTGGGATCCGCTCTCC","chrX",40589062,"-","none","TGG","ACGTCTGGGATCCGCTCTCCTGG",0,NA,NA,NA,"GeneID:10159","ATP6AP2",TRUE,"ATP6AP2"

"13562","ACGTCTGGGATCCGCTCTCC","chr1",197222323,"-","11:A>G,13:T>C","CGG","ACGTCTGGGATTCACTCTCCCGG",2,12,14,NA,"","",TRUE,"ATP6AP2"

"13563","CTTCTTACAGAGAGTGTATA","chrX",40597516,"+","none","TGG","CTTCTTACAGAGAGTGTATATGG",0,NA,NA,NA,"GeneID:10159","ATP6AP2",TRUE,"ATP6AP2"

"13564","GCTGGCGCAAGGTGACTGAA","chrX",40597570,"-","none","AGG","GCTGGCGCAAGGTGACTGAAAGG",0,NA,NA,NA,"GeneID:10159","ATP6AP2",TRUE,"ATP6AP2"

"13565","GCTGGCGCAAGGTGACTGAA","chr2",48959638,"-","1:A>G,5:T>G","TGG","GTTGGAGCAAGGTGACTGAATGG",2,2,6,NA,"","",FALSE,"ATP6AP2"

"13566","GTAACCTGTTTCATCGTCCT","chrX",40591261,"+","none","CGG","GTAACCTGTTTCATCGTCCTCGG",0,NA,NA,NA,"GeneID:10159","ATP6AP2",TRUE,"ATP6AP2"

"13567","GTAACCTGTTTCATCGTCCT","chr1",197222405,"+","2:G>A,7:A>G","CGG","GTGACCTATTTCATCGTCCTCGG",2,3,8,NA,"","",TRUE,"ATP6AP2"

"13568","TTTCGGAAAACAACAGACCC","chrX",40589023,"-","none","TGG","TTTCGGAAAACAACAGACCCTGG",0,NA,NA,NA,"GeneID:10159","ATP6AP2",TRUE,"ATP6AP2"

"13569","TTTCGGAAAACAACAGACCC","chr10",112662763,"-","10:T>G,15:T>C","AGG","TTTCGGAAAAAAACAAACCCAGG",2,11,16,NA,"GeneID:143187","VTI1A",FALSE,"ATP6AP2"

"13570","AAAACCAACAAGTTTACCTA","chr17",42490558,"+","none","TGG","AAAACCAACAAGTTTACCTATGG",0,NA,NA,NA,"GeneID:535","ATP6V0A1",TRUE,"ATP6V0A1"

"13571","AAGCCCCTGAATATCTACTT","chr17",42500716,"+","none","TGG","AAGCCCCTGAATATCTACTTTGG",0,NA,NA,NA,"GeneID:535","ATP6V0A1",TRUE,"ATP6V0A1"

"13572","ACTTACCGAGAGATAAATCC","chr17",42490615,"+","none","AGG","ACTTACCGAGAGATAAATCCAGG",0,NA,NA,NA,"GeneID:535","ATP6V0A1",TRUE,"ATP6V0A1"

"13573","ACTTACCGAGAGATAAATCC","chr18",66242636,"+","5:T>C,6:G>C","AGG","ACTTATGGAGAGATAAATCCAGG",2,6,7,NA,"","",FALSE,"ATP6V0A1"

"13574","CCGTCCACTTGTAAAAAATA","chr17",42500793,"-","none","AGG","CCGTCCACTTGTAAAAAATAAGG",0,NA,NA,NA,"GeneID:535","ATP6V0A1",TRUE,"ATP6V0A1"

"13575","GTACCATCCACACAGCAAAA","chr17",42494412,"-","none","AGG","GTACCATCCACACAGCAAAAAGG",0,NA,NA,NA,"GeneID:535","ATP6V0A1",TRUE,"ATP6V0A1"

"13576","GTACCATCCACACAGCAAAA","chr22",35434083,"-","0:G>C,1:G>A","TGG","CCACCATCCACACAGCAAAATGG",2,1,2,NA,"","",FALSE,"ATP6V0A1"

"13577","TTCCCAGAAGAATGAGAATG","chr17",42494451,"+","none","AGG","TTCCCAGAAGAATGAGAATGAGG",0,NA,NA,NA,"GeneID:535","ATP6V0A1",TRUE,"ATP6V0A1"

"13578","TTCCCAGAAGAATGAGAATG","chr10",113977850,"-","14:C>T,18:T>A","AGG","TTCCCAGAAGAATGGGAAAGAGG",2,15,19,NA,"","",FALSE,"ATP6V0A1"

"13579","TTCCCAGAAGAATGAGAATG","chr13",30420506,"+","14:G>A,19:T>G","TGG","TTCCCAGAAGAATGGGAATTTGG",2,15,20,NA,"GeneID:103724387","LINC01058",FALSE,"ATP6V0A1"

"13580","TTCCCAGAAGAATGAGAATG","chr7",122263461,"+","5:C>A,11:T>A","AGG","TTCCCCGAAGATTGAGAATGAGG",2,6,12,NA,"","",FALSE,"ATP6V0A1"

"13581","TTCCCAGAAGAATGAGAATG","chr1",31686763,"+","2:A>C,19:A>G","AGG","TTACCAGAAGAATGAGAATAAGG",2,3,20,NA,"GeneID:1307","COL16A1",FALSE,"ATP6V0A1"

"13582","TTCCCAGAAGAATGAGAATG","chr17",33387489,"-","2:C>G,18:T>A","AGG","TTGCCAGAAGAATGAGAAAGAGG",2,3,19,NA,"GeneID:40","ASIC2",FALSE,"ATP6V0A1"

"13583","CGACACTCACGTCTCGGAAC","chr12",123712672,"-","none","TGG","CGACACTCACGTCTCGGAACTGG",0,NA,NA,NA,"GeneID:23545","ATP6V0A2",TRUE,"ATP6V0A2"

"13584","CGTAGGCCGTGCCCGACTGC","chr12",123712615,"-","none","AGG","CGTAGGCCGTGCCCGACTGCAGG",0,NA,NA,NA,"GeneID:23545","ATP6V0A2",TRUE,"ATP6V0A2"

"13585","TCCTGCGCCACCCCTGAAAC","chr12",123722411,"+","none","AGG","TCCTGCGCCACCCCTGAAACAGG",0,NA,NA,NA,"GeneID:23545","ATP6V0A2",TRUE,"ATP6V0A2"

"13586","TCTTTCCAAAGAAAATTTGT","chr12",123718640,"+","none","TGG","TCTTTCCAAAGAAAATTTGTTGG",0,NA,NA,NA,"GeneID:23545","ATP6V0A2",TRUE,"ATP6V0A2"

"13587","TCTTTCCAAAGAAAATTTGT","chr2",174821019,"-","15:T>A,17:C>A","GGG","TCTTTCCAAAGAAAAATGGTGGG",2,16,18,NA,"GeneID:1123","CHN1",FALSE,"ATP6V0A2"

"13588","TGAAGAGCTAGAGCGAATAT","chr12",123718678,"+","none","TGG","TGAAGAGCTAGAGCGAATATTGG",0,NA,NA,NA,"GeneID:23545","ATP6V0A2",TRUE,"ATP6V0A2"

"13589","TGAAGAGCTAGAGCGAATAT","chr13",106557091,"-","3:C>T,19:G>A","TGG","TGAGGAGCTAGAGCGAATACTGG",2,4,20,NA,"GeneID:55082","ARGLU1",TRUE,"ATP6V0A2"

"13590","TTTCACAGTGTATTTGGTAC","chr12",123722342,"+","none","AGG","TTTCACAGTGTATTTGGTACAGG",0,NA,NA,NA,"GeneID:23545","ATP6V0A2",TRUE,"ATP6V0A2"

"13591","TTTCACAGTGTATTTGGTAC","chr7",104427094,"+","15:A>G","AGG","TTTCACAGTGTATTTAGTACAGG",1,16,NA,NA,"GeneID:375612","LHFPL3",FALSE,"ATP6V0A2"

"13592","TTTCACAGTGTATTTGGTAC","chr12",84684794,"-","5:A>G,17:C>A","TGG","TTTCATAGTGTATTTGGGACTGG",2,6,18,NA,"","",FALSE,"ATP6V0A2"

"13593","AATTTGTGAATGAAGTCAGA","chr7",138769200,"-","none","AGG","AATTTGTGAATGAAGTCAGAAGG",0,NA,NA,NA,"GeneID:50617","ATP6V0A4",TRUE,"ATP6V0A4"

"13594","AATTTGTGAATGAAGTCAGA","chrX",23870170,"-","1:A>T,3:T>A","AGG","ATTATGTGAATGAAGTCAGAAGG",2,2,4,NA,"GeneID:79135","APOO",FALSE,"ATP6V0A4"

"13595","AATTTGTGAATGAAGTCAGA","chr1",118131612,"+","5:T>G,14:A>G","TGG","AATTTTTGAATGAAATCAGATGG",2,6,15,NA,"GeneID:200162","SPAG17",FALSE,"ATP6V0A4"

"13596","GCAGTGCCTGCATATATGAC","chr7",138762351,"-","none","CGG","GCAGTGCCTGCATATATGACCGG",0,NA,NA,NA,"GeneID:50617","ATP6V0A4",TRUE,"ATP6V0A4"

"13597","GCAGTGCCTGCATATATGAC","chr22",35959156,"-","15:C>T,17:G>C","TGG","GCAGTGCCTGCATATGTCACTGG",2,16,18,NA,"GeneID:23543","RBFOX2",FALSE,"ATP6V0A4"

"13598","GCCCACTGACCCCGCTCCCA","chr7",138768800,"-","none","CGG","GCCCACTGACCCCGCTCCCACGG",0,NA,NA,NA,"GeneID:50617","ATP6V0A4",TRUE,"ATP6V0A4"

"13599","GCCCACTGACCCCGCTCCCA","chr5",139690919,"+","0:C>G,8:G>A","TGG","CCCCACTGGCCCCGCTCCCATGG",2,1,9,NA,"","",FALSE,"ATP6V0A4"

"13600","TCCAGGGTAATCATTTCCCG","chr7",138768780,"+","none","TGG","TCCAGGGTAATCATTTCCCGTGG",0,NA,NA,NA,"GeneID:50617","ATP6V0A4",TRUE,"ATP6V0A4"

"13601","TCCAGGGTAATCATTTCCCG","chr9",78487354,"+","1:T>C,16:T>C","TGG","TTCAGGGTAATCATTTTCCGTGG",2,2,17,NA,"","",FALSE,"ATP6V0A4"

"13602","TGAATGTGAACAGCTTTCAA","chr7",138769224,"-","none","AGG","TGAATGTGAACAGCTTTCAAAGG",0,NA,NA,NA,"GeneID:50617","ATP6V0A4",TRUE,"ATP6V0A4"

"13603","TGAATGTGAACAGCTTTCAA","chr19",45600907,"-","12:A>C,17:C>G","TGG","TGAATGTGAACATCTTTGAATGG",2,13,18,NA,"GeneID:80207;GeneID:2828","OPA3;GPR4",FALSE,"ATP6V0A4"

"13604","TGAATGTGAACAGCTTTCAA","chr9",106858250,"-","3:A>T,5:G>C","AGG","TGATTCTGAACAGCTTTCAAAGG",2,4,6,NA,"","",FALSE,"ATP6V0A4"

"13605","TGAATGTGAACAGCTTTCAA","chrY",11055924,"-","2:G>T,15:T>A","AGG","TGCATGTGAACAGCTATCAAAGG",2,3,16,NA,"","",FALSE,"ATP6V0A4"

"13606","TTACACTAACCCCAACTTTC","chr7",138762329,"+","none","CGG","TTACACTAACCCCAACTTTCCGG",0,NA,NA,NA,"GeneID:50617","ATP6V0A4",TRUE,"ATP6V0A4"

"13607","ACCAGGGGCATCTATATTAC","chr1",43976296,"+","none","CGG","ACCAGGGGCATCTATATTACCGG",0,NA,NA,NA,"GeneID:533","ATP6V0B",TRUE,"ATP6V0B"

"13608","CATCATTGGTGGAGGAGTGA","chr1",43976325,"+","none","AGG","CATCATTGGTGGAGGAGTGAAGG",0,NA,NA,NA,"GeneID:533","ATP6V0B",TRUE,"ATP6V0B"

"13609","GCCCTTCATGTGGTCCAACC","chr1",43976107,"+","none","TGG","GCCCTTCATGTGGTCCAACCTGG",0,NA,NA,NA,"GeneID:533","ATP6V0B",TRUE,"ATP6V0B"

"13610","GCCCTTCATGTGGTCCAACC","chr19",48592736,"-","6:T>G,15:C>G","AGG","GCCCTTAATGTGGTCGAACCAGG",2,7,16,NA,"GeneID:6820","SULT2B1",TRUE,"ATP6V0B"

"13611","GCTTCCGCTTTGATGTGGCA","chr1",43975826,"+","none","TGG","GCTTCCGCTTTGATGTGGCATGG",0,NA,NA,NA,"GeneID:533","ATP6V0B",TRUE,"ATP6V0B"

"13612","GGCCATCTACGGCATCATCA","chr1",43976610,"+","none","TGG","GGCCATCTACGGCATCATCATGG",0,NA,NA,NA,"GeneID:533","ATP6V0B",TRUE,"ATP6V0B"

"13613","GGCCATCTACGGCATCATCA","chr19",51382501,"-","0:G>C,8:G>T","TGG","CGCCATCTCCGGCATCATCATGG",2,1,9,NA,"GeneID:3982","LIM2",TRUE,"ATP6V0B"

"13614","TGCCATGATGATGCCGTAGA","chr1",43976615,"-","none","TGG","TGCCATGATGATGCCGTAGATGG",0,NA,NA,NA,"GeneID:533","ATP6V0B",TRUE,"ATP6V0B"

"13615","ACCGCTCTTGGCTGTGCCAT","chr16",2519232,"-","none","AGG","ACCGCTCTTGGCTGTGCCATAGG",0,NA,NA,NA,"GeneID:527","ATP6V0C",TRUE,"ATP6V0C"

"13616","CAAGAGCGGTACCGGCATTG","chr16",2519242,"+","none","CGG","CAAGAGCGGTACCGGCATTGCGG",0,NA,NA,NA,"GeneID:527","ATP6V0C",TRUE,"ATP6V0C"

"13617","CCTGATTCTCATCTTCGCCG","chr16",2519672,"+","none","AGG","CCTGATTCTCATCTTCGCCGAGG",0,NA,NA,NA,"GeneID:527","ATP6V0C",TRUE,"ATP6V0C"

"13618","CCTGATTCTCATCTTCGCCG","chr6",42727627,"+","6:C>T,16:C>G","AGG","CCTGATCCTCATCTTCCCCGAGG",2,7,17,NA,"","",TRUE,"ATP6V0C"

"13619","GACATGTCCGAGTCCAAGAG","chr16",2514100,"+","none","CGG","GACATGTCCGAGTCCAAGAGCGG",0,NA,NA,NA,"GeneID:527","ATP6V0C",TRUE,"ATP6V0C"

"13620","GCGCTCACCGCTGAAGACCA","chr16",2514170,"-","none","TGG","GCGCTCACCGCTGAAGACCATGG",0,NA,NA,NA,"GeneID:527","ATP6V0C",TRUE,"ATP6V0C"

"13621","GGGCGACGATGAGACCGTAG","chr16",2519705,"-","none","AGG","GGGCGACGATGAGACCGTAGAGG",0,NA,NA,NA,"GeneID:527","ATP6V0C",TRUE,"ATP6V0C"

"13622","AACGTGATCCTGCTCATCAC","chr16",67444667,"-","none","AGG","AACGTGATCCTGCTCATCACAGG",0,NA,NA,NA,"GeneID:9114","ATP6V0D1",TRUE,"ATP6V0D1"

"13623","AGTCATCGATGACCGGCTCA","chr16",67453622,"-","none","AGG","AGTCATCGATGACCGGCTCAAGG",0,NA,NA,NA,"GeneID:9114","ATP6V0D1",TRUE,"ATP6V0D1"

"13624","ATCTGCTCGAAGCTGCCTAG","chr16",67444595,"+","none","TGG","ATCTGCTCGAAGCTGCCTAGTGG",0,NA,NA,NA,"GeneID:9114","ATP6V0D1",TRUE,"ATP6V0D1"

"13625","GGTGCAGTGCGAGACGCTAG","chr16",67480959,"-","none","AGG","GGTGCAGTGCGAGACGCTAGAGG",0,NA,NA,NA,"GeneID:9114","ATP6V0D1",TRUE,"ATP6V0D1"

"13626","TCGATGACTGACACCGTCAG","chr16",67453633,"+","none","AGG","TCGATGACTGACACCGTCAGAGG",0,NA,NA,NA,"GeneID:9114","ATP6V0D1",TRUE,"ATP6V0D1"

"13627","TCTCGATGTTCATCTCGTCA","chr16",67443121,"+","none","AGG","TCTCGATGTTCATCTCGTCAAGG",0,NA,NA,NA,"GeneID:9114","ATP6V0D1",TRUE,"ATP6V0D1"

"13628","AGTTACCATAATCAGTAGTC","chr8",86113727,"-","none","TGG","AGTTACCATAATCAGTAGTCTGG",0,NA,NA,NA,"GeneID:245972","ATP6V0D2",TRUE,"ATP6V0D2"

"13629","ATTCTGATCGAAACGCCATT","chr8",86139613,"+","none","AGG","ATTCTGATCGAAACGCCATTAGG",0,NA,NA,NA,"GeneID:245972","ATP6V0D2",TRUE,"ATP6V0D2"

"13630","CAGCTCCGCACCTTCGAGCA","chr8",86098979,"-","none","TGG","CAGCTCCGCACCTTCGAGCATGG",0,NA,NA,NA,"GeneID:245972","ATP6V0D2",TRUE,"ATP6V0D2"

"13631","CATCTCCAGACTACTGATTA","chr8",86113719,"+","none","TGG","CATCTCCAGACTACTGATTATGG",0,NA,NA,NA,"GeneID:245972","ATP6V0D2",TRUE,"ATP6V0D2"

"13632","TAATGGCGTTTCGATCAGAA","chr8",86139614,"-","none","TGG","TAATGGCGTTTCGATCAGAATGG",0,NA,NA,NA,"GeneID:245972","ATP6V0D2",TRUE,"ATP6V0D2"

"13633","TTCCCCCATGCTCGAAGGTG","chr8",86098971,"+","none","CGG","TTCCCCCATGCTCGAAGGTGCGG",0,NA,NA,NA,"GeneID:245972","ATP6V0D2",TRUE,"ATP6V0D2"

"13634","AGTGAGGCCGTGATACGCCA","chr5",172983861,"-","none","TGG","AGTGAGGCCGTGATACGCCATGG",0,NA,NA,NA,"GeneID:8992","ATP6V0E1",TRUE,"ATP6V0E1"

"13635","GAGATAGCAGCAAACTGAAC","chr5",172994800,"-","none","AGG","GAGATAGCAGCAAACTGAACAGG",0,NA,NA,NA,"GeneID:8992","ATP6V0E1",TRUE,"ATP6V0E1"

"13636","GAGATAGCAGCAAACTGAAC","chr15",74015280,"+","10:G>C,18:T>A","TGG","GAGATAGCAGGAAACTGATCTGG",2,11,19,NA,"GeneID:5371","PML",FALSE,"ATP6V0E1"

"13637","GAGATAGCAGCAAACTGAAC","chr4",87622057,"-","0:T>C,4:G>A","AGG","AAGACAGCAGCAAACTGAACAGG",2,1,5,NA,"","",FALSE,"ATP6V0E1"

"13638","GAGATAGCAGCAAACTGAAC","chr2",33602171,"-","4:G>A,9:T>C","AGG","GAGACAGCAACAAACTGAACAGG",2,5,10,NA,"","",TRUE,"ATP6V0E1"

"13639","GAGATAGCAGCAAACTGAAC","chr8",139399481,"+","2:C>G,16:A>G","AGG","GACATAGCAGCAAACTAAACAGG",2,3,17,NA,"","",FALSE,"ATP6V0E1"

"13640","GAGATAGCAGCAAACTGAAC","chr10",17063525,"-","7:T>G,17:C>T","AGG","GAGATAGAAGCAAACTGGACAGG",2,8,18,NA,"GeneID:8029","CUBN",FALSE,"ATP6V0E1"

"13641","TACCCCCGGTTAGGACCCTT","chr5",172983947,"-","none","AGG","TACCCCCGGTTAGGACCCTTAGG",0,NA,NA,NA,"GeneID:8992","ATP6V0E1",TRUE,"ATP6V0E1"

"13642","TAGAGTTATCATTACCATGT","chr5",172994771,"+","none","TGG","TAGAGTTATCATTACCATGTTGG",0,NA,NA,NA,"GeneID:8992","ATP6V0E1",TRUE,"ATP6V0E1"

"13643","TAGAGTTATCATTACCATGT","chr2",33602142,"+","0:G>T,1:G>A","AGG","GGGAGTTATCATTACCATGTAGG",2,1,2,NA,"","",TRUE,"ATP6V0E1"

"13644","TAGTTGGCTGATTGCAATTC","chr5",173020234,"+","none","TGG","TAGTTGGCTGATTGCAATTCTGG",0,NA,NA,NA,"GeneID:8992","ATP6V0E1",TRUE,"ATP6V0E1"

"13645","TTCAATTGCGGTCCAAAGAG","chr5",173020271,"-","none","AGG","TTCAATTGCGGTCCAAAGAGAGG",0,NA,NA,NA,"GeneID:8992","ATP6V0E1",TRUE,"ATP6V0E1"

"13646","TTCAATTGCGGTCCAAAGAG","chr1",47550693,"-","5:G>A,8:A>G","AGG","TTCAACTGTGGTCCAAAGAGAGG",2,6,9,NA,"","",TRUE,"ATP6V0E1"

"13647","CAGCTGGCTCATCGCCATCC","chr7",149878674,"+","none","TGG","CAGCTGGCTCATCGCCATCCTGG",0,NA,NA,NA,"GeneID:401431;GeneID:155066","ATP6V0E2-AS1;ATP6V0E2",TRUE,"ATP6V0E2"

"13648","CGCAGCTGAACCCCCTGTTC","chr7",149878697,"+","none","GGG","CGCAGCTGAACCCCCTGTTCGGG",0,NA,NA,NA,"GeneID:401431;GeneID:155066","ATP6V0E2-AS1;ATP6V0E2",TRUE,"ATP6V0E2"

"13649","CTCCAATACCCGCACTGCTC","chr7",149879554,"+","none","TGG","CTCCAATACCCGCACTGCTCTGG",0,NA,NA,NA,"GeneID:401431;GeneID:155066","ATP6V0E2-AS1;ATP6V0E2",TRUE,"ATP6V0E2"

"13650","GGCGCACTCATTCGCCCTCC","chr7",149874070,"+","none","CGG","GGCGCACTCATTCGCCCTCCCGG",0,NA,NA,NA,"GeneID:401431;GeneID:155066","ATP6V0E2-AS1;ATP6V0E2",TRUE,"ATP6V0E2"

"13651","GTGCCGAAGGGACCCAACCG","chr7",149874146,"+","none","CGG","GTGCCGAAGGGACCCAACCGCGG",0,NA,NA,NA,"GeneID:401431;GeneID:155066","ATP6V0E2-AS1;ATP6V0E2",TRUE,"ATP6V0E2"

"13652","TCCCTGACAACCCCTTCGTC","chr7",149879368,"+","none","CGG","TCCCTGACAACCCCTTCGTCCGG",0,NA,NA,NA,"GeneID:401431;GeneID:155066","ATP6V0E2-AS1;ATP6V0E2",TRUE,"ATP6V0E2"

"13653","ATGTTACCCCCACGAAACAG","chr3",113784767,"+","none","AGG","ATGTTACCCCCACGAAACAGAGG",0,NA,NA,NA,"GeneID:523","ATP6V1A",TRUE,"ATP6V1A"

"13654","GAGAGTGGGCCACAGCGAAT","chr3",113781095,"+","none","TGG","GAGAGTGGGCCACAGCGAATTGG",0,NA,NA,NA,"GeneID:523","ATP6V1A",TRUE,"ATP6V1A"

"13655","GTAACTTACATTGCTCCACC","chr3",113784794,"+","none","TGG","GTAACTTACATTGCTCCACCTGG",0,NA,NA,NA,"GeneID:523","ATP6V1A",TRUE,"ATP6V1A"

"13656","GTGCATGGGGTCTCAGGACC","chr3",113778813,"+","none","TGG","GTGCATGGGGTCTCAGGACCTGG",0,NA,NA,NA,"GeneID:523","ATP6V1A",TRUE,"ATP6V1A"

"13657","GTGCATGGGGTCTCAGGACC","chr3",123290729,"-","12:G>A,17:C>T","TGG","GTGCATGGGGTCCCAGGGCCTGG",2,13,18,NA,"GeneID:111","ADCY5",FALSE,"ATP6V1A"

"13658","GTGCATGGGGTCTCAGGACC","chr10",81498190,"+","1:G>T,5:C>T","AGG","GGGCACGGGGTCTCAGGACCAGG",2,2,6,NA,"","",FALSE,"ATP6V1A"

"13659","GTGCATGGGGTCTCAGGACC","chr7",100577950,"+","2:A>G,10:G>T","TGG","GTACATGGGGGCTCAGGACCTGG",2,3,11,NA,"GeneID:4034","LRCH4",TRUE,"ATP6V1A"

"13660","TGGAGAGATTATTCGATTGG","chr3",113781119,"+","none","AGG","TGGAGAGATTATTCGATTGGAGG",0,NA,NA,NA,"GeneID:523","ATP6V1A",TRUE,"ATP6V1A"

"13661","TTTATCTTCATCGAGTATTT","chr3",113778775,"-","none","TGG","TTTATCTTCATCGAGTATTTTGG",0,NA,NA,NA,"GeneID:523","ATP6V1A",TRUE,"ATP6V1A"

"13662","AGGACTGTGTGCAGCGTGAA","chr2",70943662,"+","none","CGG","AGGACTGTGTGCAGCGTGAACGG",0,NA,NA,NA,"GeneID:25806;GeneID:525;GeneID:101927750","VAX2;ATP6V1B1;ATP6V1B1-AS1",TRUE,"ATP6V1B1"

"13663","CCTACGAACTCCGGTGTCAG","chr2",70958394,"+","none","AGG","CCTACGAACTCCGGTGTCAGAGG",0,NA,NA,NA,"GeneID:25806;GeneID:525","VAX2;ATP6V1B1",TRUE,"ATP6V1B1"

"13664","CCTACGAACTCCGGTGTCAG","chr18",65861434,"-","5:G>C,13:T>C","AGG","CCTACCAACTCCGATGTCAGAGG",2,6,14,NA,"GeneID:1005","CDH7",FALSE,"ATP6V1B1"

"13665","CTCCGGCAAGCCCATTGACA","chr2",70959033,"+","none","AGG","CTCCGGCAAGCCCATTGACAAGG",0,NA,NA,NA,"GeneID:25806;GeneID:525","VAX2;ATP6V1B1",TRUE,"ATP6V1B1"

"13666","GGTCATGGCGGAGGACTTTC","chr2",70959063,"+","none","TGG","GGTCATGGCGGAGGACTTTCTGG",0,NA,NA,NA,"GeneID:25806;GeneID:525","VAX2;ATP6V1B1",TRUE,"ATP6V1B1"

"13667","GTCCCCTGTAAATTCGCAAG","chr2",70958372,"-","none","TGG","GTCCCCTGTAAATTCGCAAGTGG",0,NA,NA,NA,"GeneID:25806;GeneID:525","VAX2;ATP6V1B1",TRUE,"ATP6V1B1"

"13668","TGACCCGGTCCAGCACCACC","chr2",70943691,"-","none","AGG","TGACCCGGTCCAGCACCACCAGG",0,NA,NA,NA,"GeneID:25806;GeneID:525;GeneID:101927750","VAX2;ATP6V1B1;ATP6V1B1-AS1",TRUE,"ATP6V1B1"

"13669","ATGGACAATTTCAGCATACC","chr8",20209439,"-","none","TGG","ATGGACAATTTCAGCATACCTGG",0,NA,NA,NA,"GeneID:526","ATP6V1B2",TRUE,"ATP6V1B2"

"13670","ATGGACAATTTCAGCATACC","chr6",51648060,"-","15:A>T,17:G>T","TGG","ATGGACAATTTCAGCTTCCCTGG",2,16,18,NA,"GeneID:5314","PKHD1",TRUE,"ATP6V1B2"

"13671","CTTCATTAACCGTGATAGTG","chr8",20217247,"-","none","AGG","CTTCATTAACCGTGATAGTGAGG",0,NA,NA,NA,"GeneID:526","ATP6V1B2",TRUE,"ATP6V1B2"

"13672","CTTCATTAACCGTGATAGTG","chr7",43541964,"-","10:T>G,15:T>A","AGG","CTTCATTAACAGTGAAAGTGAGG",2,11,16,NA,"GeneID:23072","HECW1",TRUE,"ATP6V1B2"

"13673","GTAATGTAGCCAGTCAAGTC","chr8",20216432,"-","none","TGG","GTAATGTAGCCAGTCAAGTCTGG",0,NA,NA,NA,"GeneID:526","ATP6V1B2",TRUE,"ATP6V1B2"

"13674","GTAATGTAGCCAGTCAAGTC","chr5",73724655,"+","2:T>A,12:T>G","TGG","GTTATGTAGCCATTCAAGTCTGG",2,3,13,NA,"GeneID:64283","ARHGEF28",FALSE,"ATP6V1B2"

"13675","TACAGAGGGGCAGATCTATG","chr8",20216449,"+","none","TGG","TACAGAGGGGCAGATCTATGTGG",0,NA,NA,NA,"GeneID:526","ATP6V1B2",TRUE,"ATP6V1B2"

"13676","TACTAGCTGGTTAGATACAT","chr8",20217307,"-","none","CGG","TACTAGCTGGTTAGATACATCGG",0,NA,NA,NA,"GeneID:526","ATP6V1B2",TRUE,"ATP6V1B2"

"13677","TACTAGCTGGTTAGATACAT","chr12",88072825,"+","6:T>C,17:G>C","TGG","TACTAGTTGGTTAGATAGATTGG",2,7,18,NA,"GeneID:80184","CEP290",FALSE,"ATP6V1B2"

"13678","TGGTTCCAAGGCAGTAGTTC","chr8",20209509,"+","none","AGG","TGGTTCCAAGGCAGTAGTTCAGG",0,NA,NA,NA,"GeneID:526","ATP6V1B2",TRUE,"ATP6V1B2"

"13679","TGGTTCCAAGGCAGTAGTTC","chr9",121239800,"-","3:G>A,15:C>T","AGG","TGGCTCCAAGGCAGTGGTTCAGG",2,4,16,NA,"GeneID:2934","GSN",TRUE,"ATP6V1B2"

"13680","AGAGTATCTCGTCACATTAC","chr8",103053946,"+","none","TGG","AGAGTATCTCGTCACATTACTGG",0,NA,NA,NA,"GeneID:528","ATP6V1C1",TRUE,"ATP6V1C1"

"13681","ATGCAGATGCTCGAGATTTC","chr8",103052756,"-","none","AGG","ATGCAGATGCTCGAGATTTCAGG",0,NA,NA,NA,"GeneID:528","ATP6V1C1",TRUE,"ATP6V1C1"

"13682","CAAAGTTCAAGAGAATCTGT","chr8",103048923,"+","none","TGG","CAAAGTTCAAGAGAATCTGTTGG",0,NA,NA,NA,"GeneID:528","ATP6V1C1",TRUE,"ATP6V1C1"

"13683","TAAGAAAGTAGCTCAATACA","chr8",103048875,"+","none","TGG","TAAGAAAGTAGCTCAATACATGG",0,NA,NA,NA,"GeneID:528","ATP6V1C1",TRUE,"ATP6V1C1"

"13684","TAAGAAAGTAGCTCAATACA","chr7",8476753,"+","7:A>G,9:G>A","TGG","TAAGAAAATGGCTCAATACATGG",2,8,10,NA,"GeneID:30010","NXPH1",FALSE,"ATP6V1C1"

"13685","TCTAGCAGAAATTGTGAAGA","chr8",103053904,"+","none","AGG","TCTAGCAGAAATTGTGAAGAAGG",0,NA,NA,NA,"GeneID:528","ATP6V1C1",TRUE,"ATP6V1C1"

"13686","TCTAGCAGAAATTGTGAAGA","chr8",110692470,"-","2:T>A","TGG","TCAAGCAGAAATTGTGAAGATGG",1,3,NA,NA,"","",FALSE,"ATP6V1C1"

"13687","TCTGCATACAATAACCTGAA","chr8",103052769,"+","none","AGG","TCTGCATACAATAACCTGAAAGG",0,NA,NA,NA,"GeneID:528","ATP6V1C1",TRUE,"ATP6V1C1"

"13688","AACTCGACACCTTTGCTGAA","chr2",10726547,"+","none","AGG","AACTCGACACCTTTGCTGAAAGG",0,NA,NA,NA,"GeneID:245973","ATP6V1C2",TRUE,"ATP6V1C2"

"13689","AATTTCTGCCCCTGGCGATA","chr2",10722866,"+","none","AGG","AATTTCTGCCCCTGGCGATAAGG",0,NA,NA,NA,"GeneID:245973","ATP6V1C2",TRUE,"ATP6V1C2"

"13690","ATCTGTCTAGGTGGGGACCT","chr2",10726491,"+","none","TGG","ATCTGTCTAGGTGGGGACCTTGG",0,NA,NA,NA,"GeneID:245973","ATP6V1C2",TRUE,"ATP6V1C2"

"13691","CCTACCTACCTCCGTTTGCC","chr2",10754055,"-","none","AGG","CCTACCTACCTCCGTTTGCCAGG",0,NA,NA,NA,"GeneID:245973","ATP6V1C2",TRUE,"ATP6V1C2"

"13692","CTGGAAGCATGTCGGAGTTT","chr2",10722841,"+","none","TGG","CTGGAAGCATGTCGGAGTTTTGG",0,NA,NA,NA,"GeneID:245973","ATP6V1C2",TRUE,"ATP6V1C2"

"13693","TCTGAGCCATTCTCCTTATG","chr2",10753983,"-","none","AGG","TCTGAGCCATTCTCCTTATGAGG",0,NA,NA,NA,"GeneID:245973","ATP6V1C2",TRUE,"ATP6V1C2"

"13694","CTGTCGAAATCGAAGAGTTA","chr14",67352943,"+","none","AGG","CTGTCGAAATCGAAGAGTTAAGG",0,NA,NA,NA,"GeneID:51382","ATP6V1D",TRUE,"ATP6V1D"

"13695","CTTGCGCTTTATTGACATTT","chr14",67349069,"+","none","TGG","CTTGCGCTTTATTGACATTTTGG",0,NA,NA,NA,"GeneID:51382","ATP6V1D",TRUE,"ATP6V1D"

"13696","CTTGCGCTTTATTGACATTT","chr3",30389328,"+","4:G>C,9:C>T","TGG","CTTGGGCTTCATTGACATTTTGG",2,5,10,NA,"","",FALSE,"ATP6V1D"

"13697","CTTTAAACGAGCCTTCATGA","chr14",67353009,"+","none","TGG","CTTTAAACGAGCCTTCATGATGG",0,NA,NA,NA,"GeneID:51382","ATP6V1D",TRUE,"ATP6V1D"

"13698","GAAGTCACCTGCTGTGAACT","chr14",67350612,"+","none","TGG","GAAGTCACCTGCTGTGAACTTGG",0,NA,NA,NA,"GeneID:51382","ATP6V1D",TRUE,"ATP6V1D"

"13699","GCGAAGAAAGATAATGTAGC","chr14",67349038,"-","none","AGG","GCGAAGAAAGATAATGTAGCAGG",0,NA,NA,NA,"GeneID:51382","ATP6V1D",TRUE,"ATP6V1D"

"13700","GCGAAGAAAGATAATGTAGC","chr2",143790645,"+","1:A>C,16:G>T","AGG","GAGAAGAAAGATAATGGAGCAGG",2,2,17,NA,"","",FALSE,"ATP6V1D"

"13701","GCTGAAGCCAAGTTCACAGC","chr14",67350622,"-","none","AGG","GCTGAAGCCAAGTTCACAGCAGG",0,NA,NA,NA,"GeneID:51382","ATP6V1D",TRUE,"ATP6V1D"

"13702","GCTGAAGCCAAGTTCACAGC","chr7",91941667,"-","0:G>C,3:T>C","TGG","CCTAAAGCCAAGTTCACAGCTGG",2,1,4,NA,"GeneID:10142","AKAP9",FALSE,"ATP6V1D"

"13703","ATTCCATAATCTTTAGTCTT","chr22",17613253,"+","none","TGG","ATTCCATAATCTTTAGTCTTTGG",0,NA,NA,NA,"GeneID:529","ATP6V1E1",TRUE,"ATP6V1E1"

"13704","ATTCCATAATCTTTAGTCTT","chr12",64338627,"+","none","TGG","ATTCCATAATCTTTAGTCTTTGG",0,NA,NA,NA,"GeneID:115749","C12orf56",TRUE,"ATP6V1E1"

"13705","ATTCCATAATCTTTAGTCTT","chr1",42903377,"-","none","TGG","ATTCCATAATCTTTAGTCTTTGG",0,NA,NA,NA,"","",TRUE,"ATP6V1E1"

"13706","ATTCCATAATCTTTAGTCTT","chr3",167719059,"-","4:A>G,13:T>A","AGG","ATTCTATAATCTTAAGTCTTAGG",2,5,14,NA,"GeneID:11235","PDCD10",FALSE,"ATP6V1E1"

"13707","ATTCCATAATCTTTAGTCTT","chrX",27139171,"-","2:C>A,16:G>A","TGG","ATGCCATAATCTTTAGCCTTTGG",2,3,17,NA,"","",FALSE,"ATP6V1E1"

"13708","ATTCCATAATCTTTAGTCTT","chr19",7096269,"-","0:G>T,12:G>A","AGG","CTTCCATAATCTCTAGTCTTAGG",2,1,13,NA,"","",FALSE,"ATP6V1E1"

"13709","CTTATAGATAAAGCATATGA","chr22",17619513,"-","none","TGG","CTTATAGATAAAGCATATGATGG",0,NA,NA,NA,"GeneID:529","ATP6V1E1",TRUE,"ATP6V1E1"

"13710","CTTATAGATAAAGCATATGA","chr13",107076111,"-","12:A>C,16:C>T","TGG","CTTATAGATAAATCATGTGATGG",2,13,17,NA,"","",FALSE,"ATP6V1E1"

"13711","GAAGAGTTCAACATAGAGAA","chr1",42903336,"+","none","AGG","GAAGAGTTCAACATAGAGAAAGG",0,NA,NA,NA,"","",TRUE,"ATP6V1E1"

"13712","GAAGAGTTCAACATAGAGAA","chr22",17613294,"-","none","AGG","GAAGAGTTCAACATAGAGAAAGG",0,NA,NA,NA,"GeneID:529","ATP6V1E1",TRUE,"ATP6V1E1"

"13713","GAAGAGTTCAACATAGAGAA","chr12",64338668,"-","none","AGG","GAAGAGTTCAACATAGAGAAAGG",0,NA,NA,NA,"GeneID:115749","C12orf56",TRUE,"ATP6V1E1"

"13714","GAAGAGTTCAACATAGAGAA","chr11",84665722,"-","14:G>T,15:A>C","AGG","GAAGAGTTCAACATCTAGAAAGG",2,15,16,NA,"GeneID:1740","DLG2",FALSE,"ATP6V1E1"

"13715","GAAGAGTTCAACATAGAGAA","chr2",237202409,"+","5:C>G","TGG","GAAGACTTCAACATAGAGAATGG",1,6,NA,NA,"","",FALSE,"ATP6V1E1"

"13716","GAAGAGTTCAACATAGAGAA","chr2",22628588,"+","4:G>A,7:C>T","TGG","GAAGGGTCCAACATAGAGAATGG",2,5,8,NA,"","",FALSE,"ATP6V1E1"

"13717","GAAGAGTTCAACATAGAGAA","chr2",46512586,"-","8:A>G,14:A>T","AGG","GAAGAGTTTAACATTGAGAAAGG",2,9,15,NA,"GeneID:90423","ATP6V1E2",TRUE,"ATP6V1E1"

"13718","TAAGGTCATCTCTTGCTCTG","chr22",17612816,"+","none","AGG","TAAGGTCATCTCTTGCTCTGAGG",0,NA,NA,NA,"GeneID:529","ATP6V1E1",TRUE,"ATP6V1E1"

"13719","TAAGGTCATCTCTTGCTCTG","chr1",42903482,"-","13:G>A","AGG","TAAGGTCATCTCTCGCTCTGAGG",1,14,NA,NA,"","",TRUE,"ATP6V1E1"

"13720","TAAGGTCATCTCTTGCTCTG","chr4",150609940,"+","14:T>G,17:T>C","TGG","TAAGGTCATCTCTTTCTTTGTGG",2,15,18,NA,"GeneID:987","LRBA",FALSE,"ATP6V1E1"

"13721","TAAGGTCATCTCTTGCTCTG","chr6",105541033,"-","5:G>A","AGG","TAAGGCCATCTCTTGCTCTGAGG",1,6,NA,NA,"","",FALSE,"ATP6V1E1"

"13722","TATTTCTTCTGCTTTCTCAT","chr22",17619469,"+","none","TGG","TATTTCTTCTGCTTTCTCATTGG",0,NA,NA,NA,"GeneID:529","ATP6V1E1",TRUE,"ATP6V1E1"

"13723","TATTTCTTCTGCTTTCTCAT","chr1",42903301,"-","none","TGG","TATTTCTTCTGCTTTCTCATTGG",0,NA,NA,NA,"","",TRUE,"ATP6V1E1"

"13724","TATTTCTTCTGCTTTCTCAT","chr18",78306093,"-","12:T>A","GGG","TATTTCTTCTGCATTCTCATGGG",1,13,NA,NA,"","",FALSE,"ATP6V1E1"

"13725","TATTTCTTCTGCTTTCTCAT","chr3",14491512,"-","11:C>G,12:G>A","GGG","TATTTCTTCTGGCTTCTCATGGG",2,12,13,NA,"GeneID:80852","GRIP2",TRUE,"ATP6V1E1"

"13726","TATTTCTTCTGCTTTCTCAT","chr11",62065085,"+","17:T>C,18:G>A","GGG","TATTTCTTCTGCTTTCTTGTGGG",2,18,19,NA,"","",FALSE,"ATP6V1E1"

"13727","TATTTCTTCTGCTTTCTCAT","chr16",55250436,"+","11:T>C,14:G>T","GGG","TATTTCTTCTGTTTGCTCATGGG",2,12,15,NA,"","",FALSE,"ATP6V1E1"

"13728","TATTTCTTCTGCTTTCTCAT","chr5",118435497,"+","10:T>G,11:A>C","AGG","TATTTCTTCTTATTTCTCATAGG",2,11,12,NA,"","",FALSE,"ATP6V1E1"

"13729","TATTTCTTCTGCTTTCTCAT","chr12",64338703,"+","4:C>T","TGG","TATTCCTTCTGCTTTCTCATTGG",1,5,NA,NA,"GeneID:115749","C12orf56",TRUE,"ATP6V1E1"

"13730","TATTTCTTCTGCTTTCTCAT","chr2",46512621,"+","0:G>T,6:C>T","TGG","GATTTCCTCTGCTTTCTCATTGG",2,1,7,NA,"GeneID:90423","ATP6V1E2",TRUE,"ATP6V1E1"

"13731","TATTTCTTCTGCTTTCTCAT","chr2",228294677,"-","5:A>G,17:A>G","GGG","TATTTTTTCTGCTTTCTTATGGG",2,6,18,NA,"","",FALSE,"ATP6V1E1"

"13732","TCTCGCTTGATTCATCAAAT","chr22",17612847,"+","none","TGG","TCTCGCTTGATTCATCAAATTGG",0,NA,NA,NA,"GeneID:529","ATP6V1E1",TRUE,"ATP6V1E1"

"13733","TCTCGCTTGATTCATCAAAT","chr1",42903451,"-","0:G>A,3:A>G","TGG","CCTTGCTTGATTCATCAAATTGG",2,1,4,NA,"","",TRUE,"ATP6V1E1"

"13734","TCTCGCTTGATTCATCAAAT","chr12",64338553,"+","3:T>C","TGG","TCTTGCTTGATTCATCAAATTGG",1,4,NA,NA,"GeneID:115749","C12orf56",TRUE,"ATP6V1E1"

"13735","AAATACCTTGGAAAGCCGAC","chr2",46512113,"-","none","TGG","AAATACCTTGGAAAGCCGACTGG",0,NA,NA,NA,"GeneID:90423","ATP6V1E2",TRUE,"ATP6V1E2"

"13736","AACAAGGCCATTCGTATTTC","chr2",46512064,"+","none","TGG","AACAAGGCCATTCGTATTTCTGG",0,NA,NA,NA,"GeneID:90423","ATP6V1E2",TRUE,"ATP6V1E2"

"13737","AATTGTCATGTACTCGGGGA","chr2",46512237,"+","none","TGG","AATTGTCATGTACTCGGGGATGG",0,NA,NA,NA,"GeneID:90423","ATP6V1E2",TRUE,"ATP6V1E2"

"13738","AGGACCCAGAGGTCTACCAG","chr2",46512372,"-","none","GGG","AGGACCCAGAGGTCTACCAGGGG",0,NA,NA,NA,"GeneID:90423","ATP6V1E2",TRUE,"ATP6V1E2"

"13739","AGGACCCAGAGGTCTACCAG","chr2",112764383,"+","10:C>G,18:T>A","GGG","AGGACCCAGACGTCTACCTGGGG",2,11,19,NA,"GeneID:150468","CKAP2L",FALSE,"ATP6V1E2"

"13740","CGGCAGCGTACAATCATCAC","chr2",46512301,"+","none","AGG","CGGCAGCGTACAATCATCACAGG",0,NA,NA,NA,"GeneID:90423","ATP6V1E2",TRUE,"ATP6V1E2"

"13741","CTGTGATGATTGTACGCTGC","chr2",46512303,"-","none","CGG","CTGTGATGATTGTACGCTGCCGG",0,NA,NA,NA,"GeneID:90423","ATP6V1E2",TRUE,"ATP6V1E2"

"13742","AGGGTCACGCTGATTAGGGT","chr7",128865157,"-","none","TGG","AGGGTCACGCTGATTAGGGTTGG",0,NA,NA,NA,"GeneID:375616;GeneID:9296","KCP;ATP6V1F",TRUE,"ATP6V1F"

"13743","ATCAGCGTGACCCTCCGCTT","chr7",128865163,"+","none","TGG","ATCAGCGTGACCCTCCGCTTTGG",0,NA,NA,NA,"GeneID:375616;GeneID:9296","KCP;ATP6V1F",TRUE,"ATP6V1F"

"13744","GAACCGCCATCCCAATTTCC","chr7",128862993,"+","none","TGG","GAACCGCCATCCCAATTTCCTGG",0,NA,NA,NA,"GeneID:375616;GeneID:9296","KCP;ATP6V1F",TRUE,"ATP6V1F"

"13745","GAACCGCCATCCCAATTTCC","chr12",48085150,"+","4:T>C,9:C>T","TGG","GAACTGCCACCCCAATTTCCTGG",2,5,10,NA,"GeneID:29843","SENP1",TRUE,"ATP6V1F"

"13746","GAGTCCTTGGCGGCGTCATA","chr7",128865509,"-","none","TGG","GAGTCCTTGGCGGCGTCATATGG",0,NA,NA,NA,"GeneID:375616;GeneID:9296","KCP;ATP6V1F",TRUE,"ATP6V1F"

"13747","GAGTCCTTGGCGGCGTCATA","chr12",48085352,"-","12:A>C,14:G>C","TGG","GAGTCCTTGGCGTCCTCATATGG",2,13,15,NA,"GeneID:29843","SENP1",TRUE,"ATP6V1F"

"13748","GCACCCATATGACGCCGCCA","chr7",128865502,"+","none","AGG","GCACCCATATGACGCCGCCAAGG",0,NA,NA,NA,"GeneID:375616;GeneID:9296","KCP;ATP6V1F",TRUE,"ATP6V1F"

"13749","GCACCCATATGACGCCGCCA","chr12",48085345,"+","12:G>C,14:A>C","AGG","GCACCCATATGAGGACGCCAAGG",2,13,15,NA,"GeneID:29843","SENP1",TRUE,"ATP6V1F"

"13750","GGTAAGCTCATCGCAGTGAT","chr7",128862916,"+","none","CGG","GGTAAGCTCATCGCAGTGATCGG",0,NA,NA,NA,"GeneID:375616;GeneID:9296","KCP;ATP6V1F",TRUE,"ATP6V1F"

"13751","CCATCCTCCAGACATACTTC","chr9",114597630,"+","none","CGG","CCATCCTCCAGACATACTTCCGG",0,NA,NA,NA,"GeneID:9550","ATP6V1G1",TRUE,"ATP6V1G1"

"13752","CCATCCTCCAGACATACTTC","chr6",65631258,"-","14:C>A,16:T>G","AGG","CCATCCTCCAGACAGAATTCAGG",2,15,17,NA,"GeneID:346007","EYS",FALSE,"ATP6V1G1"

"13753","CCATCCTCCAGACATACTTC","chr5",154868755,"-","3:C>A,13:G>T","TGG","CCAGCCTCCAGACCTACTTCTGG",2,4,14,NA,"GeneID:9337","CNOT8",FALSE,"ATP6V1G1"

"13754","CCCCTGAGACTGACTAGCCA","chr9",114587839,"-","none","TGG","CCCCTGAGACTGACTAGCCATGG",0,NA,NA,NA,"GeneID:9550","ATP6V1G1",TRUE,"ATP6V1G1"

"13755","CCCCTGAGACTGACTAGCCA","chr5",154868993,"+","11:C>G,15:G>A","TGG","CCCCTGAGACTCACTGGCCATGG",2,12,16,NA,"GeneID:9337","CNOT8",FALSE,"ATP6V1G1"

"13756","CGCCATGGCTAGTCAGTCTC","chr9",114587834,"+","none","AGG","CGCCATGGCTAGTCAGTCTCAGG",0,NA,NA,NA,"GeneID:9550","ATP6V1G1",TRUE,"ATP6V1G1"

"13757","CGCCATGGCTAGTCAGTCTC","chrX",19782499,"-","1:T>C,12:G>A","TGG","CACCATGGCTAGCCAGTCTCTGG",2,2,13,NA,"GeneID:30011","SH3KBP1",FALSE,"ATP6V1G1"

"13758","CTCCAGGCATTGGGATCCCG","chr9",114597563,"+","none","TGG","CTCCAGGCATTGGGATCCCGTGG",0,NA,NA,NA,"GeneID:9550","ATP6V1G1",TRUE,"ATP6V1G1"

"13759","GCAGGCCAAAGAAGAAGCTC","chr9",114592570,"+","none","AGG","GCAGGCCAAAGAAGAAGCTCAGG",0,NA,NA,NA,"GeneID:9550","ATP6V1G1",TRUE,"ATP6V1G1"

"13760","GCAGGCCAAAGAAGAAGCTC","chr10",35158406,"+","14:T>A,19:T>C","AGG","GCAGGCCAAAGAAGTAGCTTAGG",2,15,20,NA,"GeneID:1390","CREM",TRUE,"ATP6V1G1"

"13761","GCAGGCCAAAGAAGAAGCTC","chr9",136779963,"+","0:A>G,14:C>A","AGG","ACAGGCCAAAGAAGCAGCTCAGG",2,1,15,NA,"","",TRUE,"ATP6V1G1"

"13762","TGAAAACAGGAAAGAACCGG","chr9",114592542,"+","none","AGG","TGAAAACAGGAAAGAACCGGAGG",0,NA,NA,NA,"GeneID:9550","ATP6V1G1",TRUE,"ATP6V1G1"

"13763","TGAAAACAGGAAAGAACCGG","chr6",42490437,"-","0:T>A,17:A>G","GGG","AGAAAACAGGAAAGAACTGGGGG",2,1,18,NA,"","",FALSE,"ATP6V1G1"

"13764","ATTCGTGCTCTCGCTCTCTG","chr6",31546128,"+","none","CGG","ATTCGTGCTCTCGCTCTCTGCGG",0,NA,NA,NA,"GeneID:100532737;GeneID:534","ATP6V1G2-DDX39B;ATP6V1G2",TRUE,"ATP6V1G2"

"13765","CCTTTGCCTGCTTCAGTCGC","chr6",31546179,"+","none","CGG","CCTTTGCCTGCTTCAGTCGCCGG",0,NA,NA,NA,"GeneID:100532737;GeneID:534","ATP6V1G2-DDX39B;ATP6V1G2",TRUE,"ATP6V1G2"

"13766","GAAATGGCCAGTCAGTCCCA","chr6",31546542,"-","none","AGG","GAAATGGCCAGTCAGTCCCAAGG",0,NA,NA,NA,"GeneID:100532737;GeneID:534","ATP6V1G2-DDX39B;ATP6V1G2",TRUE,"ATP6V1G2"

"13767","GAAGGCCCGGCGACTGAAGC","chr6",31546188,"-","none","AGG","GAAGGCCCGGCGACTGAAGCAGG",0,NA,NA,NA,"GeneID:100532737;GeneID:534","ATP6V1G2-DDX39B;ATP6V1G2",TRUE,"ATP6V1G2"

"13768","GAGAAACCGAGAGCGTGTCC","chr6",31545478,"-","none","TGG","GAGAAACCGAGAGCGTGTCCTGG",0,NA,NA,NA,"GeneID:100532737;GeneID:534","ATP6V1G2-DDX39B;ATP6V1G2",TRUE,"ATP6V1G2"

"13769","GAGAAACCGAGAGCGTGTCC","chr7",27097556,"+","6:T>C,14:T>G","AGG","GAGAAATCGAGAGCTTGTCCAGG",2,7,15,NA,"GeneID:100506311","HOTAIRM1",FALSE,"ATP6V1G2"

"13770","TTGGCATGGTCTGCGACGTC","chr6",31545446,"-","none","AGG","TTGGCATGGTCTGCGACGTCAGG",0,NA,NA,NA,"GeneID:100532737;GeneID:534","ATP6V1G2-DDX39B;ATP6V1G2",TRUE,"ATP6V1G2"

"13771","AAAGCGATTGAAGCAAGCCA","chr1",198529154,"-","none","AGG","AAAGCGATTGAAGCAAGCCAAGG",0,NA,NA,NA,"GeneID:127124","ATP6V1G3",TRUE,"ATP6V1G3"

"13772","CACAGACCATGCTCAAGAGC","chr1",198523434,"+","none","TGG","CACAGACCATGCTCAAGAGCTGG",0,NA,NA,NA,"GeneID:127124","ATP6V1G3",TRUE,"ATP6V1G3"

"13773","CCCCTGAGACTGGCTTGTCA","chr1",198540629,"+","none","TGG","CCCCTGAGACTGGCTTGTCATGG",0,NA,NA,NA,"GeneID:127124","ATP6V1G3",TRUE,"ATP6V1G3"

"13774","CCCCTGAGACTGGCTTGTCA","chr19",49574226,"-","8:C>T,13:A>G","AGG","CCCCTGAGGCTGGTTTGTCAAGG",2,9,14,NA,"GeneID:51070","NOSIP",FALSE,"ATP6V1G3"

"13775","CCCCTGAGACTGGCTTGTCA","chr9",32889373,"-","1:A>G,11:A>C","TGG","CTCCTGAGACTTGCTTGTCATGG",2,2,12,NA,"","",FALSE,"ATP6V1G3"

"13776","GATGAACCAGCTCTTGAGCA","chr1",198523443,"-","none","TGG","GATGAACCAGCTCTTGAGCATGG",0,NA,NA,NA,"GeneID:127124","ATP6V1G3",TRUE,"ATP6V1G3"

"13777","TACCATGACAAGCCAGTCTC","chr1",198540634,"-","none","AGG","TACCATGACAAGCCAGTCTCAGG",0,NA,NA,NA,"GeneID:127124","ATP6V1G3",TRUE,"ATP6V1G3"

"13778","TTCTACCATTGCTTCCTCCT","chr1",198529134,"+","none","TGG","TTCTACCATTGCTTCCTCCTTGG",0,NA,NA,NA,"GeneID:127124","ATP6V1G3",TRUE,"ATP6V1G3"

"13779","TTCTACCATTGCTTCCTCCT","chr1",115752555,"-","2:T>G,10:A>C","GGG","TTATACCATTTCTTCCTCCTGGG",2,3,11,NA,"GeneID:845","CASQ2",FALSE,"ATP6V1G3"

"13780","CAAGAGATGCTTCAAACTGA","chr8",53832993,"-","none","AGG","CAAGAGATGCTTCAAACTGAAGG",0,NA,NA,NA,"GeneID:51606","ATP6V1H",TRUE,"ATP6V1H"

"13781","CATAGTTAGTATATACTGAA","chr8",53829461,"+","none","CGG","CATAGTTAGTATATACTGAACGG",0,NA,NA,NA,"GeneID:51606","ATP6V1H",TRUE,"ATP6V1H"

"13782","CATAGTTAGTATATACTGAA","chr14",70493452,"+","2:C>T,5:C>T","TGG","CACAGCTAGTATATACTGAATGG",2,3,6,NA,"","",FALSE,"ATP6V1H"

"13783","CGTTCAGTATATACTAACTA","chr8",53829463,"-","none","TGG","CGTTCAGTATATACTAACTATGG",0,NA,NA,NA,"GeneID:51606","ATP6V1H",TRUE,"ATP6V1H"

"13784","GATGTAGCAAGAACACTGCG","chr8",53817470,"-","none","TGG","GATGTAGCAAGAACACTGCGTGG",0,NA,NA,NA,"GeneID:51606","ATP6V1H",TRUE,"ATP6V1H"

"13785","TCTGCCAATGTTGAATCGCC","chr8",53817439,"-","none","AGG","TCTGCCAATGTTGAATCGCCAGG",0,NA,NA,NA,"GeneID:51606","ATP6V1H",TRUE,"ATP6V1H"

"13786","TTATTGCCAAGTTAGCAGCT","chr8",53814736,"-","none","TGG","TTATTGCCAAGTTAGCAGCTTGG",0,NA,NA,NA,"GeneID:51606","ATP6V1H",TRUE,"ATP6V1H"

"13787","TTATTGCCAAGTTAGCAGCT","chr4",91299975,"+","0:A>T,1:C>T","CGG","ACATTGCCAAGTTAGCAGCTCGG",2,1,2,NA,"GeneID:401145","CCSER1",FALSE,"ATP6V1H"

"13788","ACCAGACACGAATGAGCCGT","chrX",77998472,"+","none","TGG","ACCAGACACGAATGAGCCGTTGG",0,NA,NA,NA,"GeneID:538;GeneID:5230","ATP7A;PGK1",TRUE,"ATP7A"

"13789","ATACTAACTCTATATAGCCC","chrX",77989642,"-","none","CGG","ATACTAACTCTATATAGCCCCGG",0,NA,NA,NA,"GeneID:538;GeneID:5230","ATP7A;PGK1",TRUE,"ATP7A"

"13790","ATACTAACTCTATATAGCCC","chr17",58663879,"+","13:T>A,17:G>C","AGG","ATACTAACTCTATTTAGGCCAGG",2,14,18,NA,"GeneID:56155","TEX14",FALSE,"ATP7A"

"13791","ATTGCTCCTCTCAACGTTTC","chrX",77989906,"-","none","TGG","ATTGCTCCTCTCAACGTTTCTGG",0,NA,NA,NA,"GeneID:538;GeneID:5230","ATP7A;PGK1",TRUE,"ATP7A"

"13792","CCGTTCAATGTTTGCTACAC","chrX",77998645,"-","none","AGG","CCGTTCAATGTTTGCTACACAGG",0,NA,NA,NA,"GeneID:538;GeneID:5230","ATP7A;PGK1",TRUE,"ATP7A"

"13793","CGCCGTAACAGTCAGAAACA","chrX",77988383,"-","none","AGG","CGCCGTAACAGTCAGAAACAAGG",0,NA,NA,NA,"GeneID:538;GeneID:5230","ATP7A;PGK1",TRUE,"ATP7A"

"13794","TCAGAAATCAGCGTTCATGT","chrX",78046360,"+","none","TGG","TCAGAAATCAGCGTTCATGTTGG",0,NA,NA,NA,"GeneID:538;GeneID:5230","ATP7A;PGK1",TRUE,"ATP7A"

"13795","GATAAGTGATGACGGCCTCT","chr13",51974657,"+","none","TGG","GATAAGTGATGACGGCCTCTTGG",0,NA,NA,NA,"GeneID:540","ATP7B",TRUE,"ATP7B"

"13796","GCAGCAAATATCGGTGTCTT","chr13",51974039,"-","none","TGG","GCAGCAAATATCGGTGTCTTTGG",0,NA,NA,NA,"GeneID:540","ATP7B",TRUE,"ATP7B"

"13797","GCGGGGTCTATAAATGCACA","chr13",51950097,"-","none","TGG","GCGGGGTCTATAAATGCACATGG",0,NA,NA,NA,"GeneID:540","ATP7B",TRUE,"ATP7B"

"13798","GCTCATTAAAGCTACCCACG","chr13",51950068,"-","none","TGG","GCTCATTAAAGCTACCCACGTGG",0,NA,NA,NA,"GeneID:540","ATP7B",TRUE,"ATP7B"

"13799","TGTTGCAGGCACACAACCGA","chr13",51974887,"+","none","TGG","TGTTGCAGGCACACAACCGATGG",0,NA,NA,NA,"GeneID:540","ATP7B",TRUE,"ATP7B"

"13800","TTTGAGGCTTCAGTCGTTTC","chr13",51973936,"-","none","TGG","TTTGAGGCTTCAGTCGTTTCTGG",0,NA,NA,NA,"GeneID:540","ATP7B",TRUE,"ATP7B"

"13801","AACTGAGAGTAGAGAAATCT","chr4",42625659,"+","none","TGG","AACTGAGAGTAGAGAAATCTTGG",0,NA,NA,NA,"GeneID:10396","ATP8A1",TRUE,"ATP8A1"

"13802","CCTTACCTTCGGCGCGCGAG","chr4",42656818,"+","none","CGG","CCTTACCTTCGGCGCGCGAGCGG",0,NA,NA,NA,"GeneID:10396","ATP8A1",TRUE,"ATP8A1"

"13803","CTCCGACACGGTCCTCCGCA","chr4",42656843,"+","none","TGG","CTCCGACACGGTCCTCCGCATGG",0,NA,NA,NA,"GeneID:10396","ATP8A1",TRUE,"ATP8A1"

"13804","GAGATCCGCTCGCGCGCCGA","chr4",42656826,"-","none","AGG","GAGATCCGCTCGCGCGCCGAAGG",0,NA,NA,NA,"GeneID:10396","ATP8A1",TRUE,"ATP8A1"

"13805","GAGATCCGCTCGCGCGCCGA","chr15",91916226,"+","10:G>C,12:G>C","GGG","GAGATCCGCTGGGGCGCCGAGGG",2,11,13,NA,"GeneID:28232","SLCO3A1",TRUE,"ATP8A1"

"13806","GTGAACAAGAAACAAACGCA","chr4",42616034,"-","none","AGG","GTGAACAAGAAACAAACGCAAGG",0,NA,NA,NA,"GeneID:10396","ATP8A1",TRUE,"ATP8A1"

"13807","GTGAACAAGAAACAAACGCA","chr12",6134552,"+","8:A>G,16:A>C","AGG","GTGAACAAAAAACAAAAGCAAGG",2,9,17,NA,"","",FALSE,"ATP8A1"

"13808","TCTCTTTATTGCACTGCTGC","chr4",42625615,"-","none","AGG","TCTCTTTATTGCACTGCTGCAGG",0,NA,NA,NA,"GeneID:10396","ATP8A1",TRUE,"ATP8A1"

"13809","ACACGACACCAAACTCATGC","chr13",25543380,"+","none","AGG","ACACGACACCAAACTCATGCAGG",0,NA,NA,NA,"GeneID:51761","ATP8A2",TRUE,"ATP8A2"

"13810","CATGAGACCTGTTCCAGTAC","chr13",25551453,"-","none","AGG","CATGAGACCTGTTCCAGTACAGG",0,NA,NA,NA,"GeneID:51761","ATP8A2",TRUE,"ATP8A2"

"13811","CATGAGACCTGTTCCAGTAC","chr12",65662211,"-","0:T>G,2:T>A","AGG","AAAGAGACCTGTTCCAGTACAGG",2,1,3,NA,"","",FALSE,"ATP8A2"

"13812","GCTCGGCGGGGGCCCTGTAC","chr13",25551437,"+","none","TGG","GCTCGGCGGGGGCCCTGTACTGG",0,NA,NA,NA,"GeneID:51761","ATP8A2",TRUE,"ATP8A2"

"13813","GCTCGGCGGGGGCCCTGTAC","chr19",10166720,"+","6:G>C,17:C>T","CGG","GCTCGGGGGGGGCCCTGCACCGG",2,7,18,NA,"GeneID:1786","DNMT1",FALSE,"ATP8A2"

"13814","GGCGAGATGCTGAACGGCGC","chr13",25372206,"+","none","AGG","GGCGAGATGCTGAACGGCGCAGG",0,NA,NA,NA,"GeneID:51761","ATP8A2",TRUE,"ATP8A2"

"13815","GTCGAGGATCCGCTCGTCCG","chr13",25372265,"+","none","TGG","GTCGAGGATCCGCTCGTCCGTGG",0,NA,NA,NA,"GeneID:51761","ATP8A2",TRUE,"ATP8A2"

"13816","GTGTACCTCTTAATAAGATC","chr13",25543314,"-","none","TGG","GTGTACCTCTTAATAAGATCTGG",0,NA,NA,NA,"GeneID:51761","ATP8A2",TRUE,"ATP8A2"

"13817","CACAAAATTCTTGTGTATTA","chr18",57706506,"-","none","AGG","CACAAAATTCTTGTGTATTAAGG",0,NA,NA,NA,"GeneID:5205","ATP8B1",TRUE,"ATP8B1"

"13818","CACAAAATTCTTGTGTATTA","chr9",100428717,"+","16:T>A,18:C>T","GGG","CACAAAATTCTTGTGTTTCAGGG",2,17,19,NA,"GeneID:91283","MSANTD3",FALSE,"ATP8B1"

"13819","CACAAAATTCTTGTGTATTA","chr9",16136544,"+","2:G>C,7:C>T","AGG","CAGAAAACTCTTGTGTATTAAGG",2,3,8,NA,"","",FALSE,"ATP8B1"

"13820","TAGGACGTGTGAAGTCATTA","chr18",57701048,"-","none","AGG","TAGGACGTGTGAAGTCATTAAGG",0,NA,NA,NA,"GeneID:5205","ATP8B1",TRUE,"ATP8B1"

"13821","TCTCAAGGCTCGCCATAAAA","chr18",57701087,"-","none","TGG","TCTCAAGGCTCGCCATAAAATGG",0,NA,NA,NA,"GeneID:5205","ATP8B1",TRUE,"ATP8B1"

"13822","TTAGGTTCAAAGTTGCTAAG","chr18",57697851,"-","none","TGG","TTAGGTTCAAAGTTGCTAAGTGG",0,NA,NA,NA,"GeneID:5205","ATP8B1",TRUE,"ATP8B1"

"13823","AACTGCTCAAAGAGGTTGAC","chr1",154330862,"-","none","AGG","AACTGCTCAAAGAGGTTGACAGG",0,NA,NA,NA,"GeneID:57198","ATP8B2",TRUE,"ATP8B2"

"13824","ACCACTCACATAGTCATCAG","chr1",154331135,"-","none","TGG","ACCACTCACATAGTCATCAGTGG",0,NA,NA,NA,"GeneID:57198","ATP8B2",TRUE,"ATP8B2"

"13825","AGTAAGTGTTGGCAACTTCC","chr1",154330885,"-","none","TGG","AGTAAGTGTTGGCAACTTCCTGG",0,NA,NA,NA,"GeneID:57198","ATP8B2",TRUE,"ATP8B2"

"13826","CTTTAACAGCTGTGATGGTG","chr1",154331109,"-","none","AGG","CTTTAACAGCTGTGATGGTGAGG",0,NA,NA,NA,"GeneID:57198","ATP8B2",TRUE,"ATP8B2"

"13827","CTTTAACAGCTGTGATGGTG","chr18",59461216,"-","17:G>C,18:T>A","TGG","CTTTAACAGCTGTGATGCAGTGG",2,18,19,NA,"GeneID:147372","CCBE1",FALSE,"ATP8B2"

"13828","CTTTAACAGCTGTGATGGTG","chr21",41579935,"+","5:G>A,16:T>G","CGG","CTTTAGCAGCTGTGATTGTGCGG",2,6,17,NA,"","",FALSE,"ATP8B2"

"13829","GAAGGTGAGAATATTGTACT","chr1",154330836,"-","none","TGG","GAAGGTGAGAATATTGTACTTGG",0,NA,NA,NA,"GeneID:57198","ATP8B2",TRUE,"ATP8B2"

"13830","TGTGCAAAAAAGCGCCCCCC","chr1",154328150,"+","none","AGG","TGTGCAAAAAAGCGCCCCCCAGG",0,NA,NA,NA,"GeneID:57198","ATP8B2",TRUE,"ATP8B2"

"13831","AGGGGTACTGAGCGAGAACC","chr19",1807218,"+","none","AGG","AGGGGTACTGAGCGAGAACCAGG",0,NA,NA,NA,"GeneID:148229","ATP8B3",TRUE,"ATP8B3"

"13832","CTCCTTGAACTGCCCGTTGT","chr19",1809678,"+","none","AGG","CTCCTTGAACTGCCCGTTGTAGG",0,NA,NA,NA,"GeneID:148229","ATP8B3",TRUE,"ATP8B3"

"13833","CTGGCCGAGGCTGCCCATGC","chr19",1810655,"+","none","TGG","CTGGCCGAGGCTGCCCATGCTGG",0,NA,NA,NA,"GeneID:148229","ATP8B3",TRUE,"ATP8B3"

"13834","GAGAGAAGATCTCCAAGATG","chr19",1810636,"-","none","AGG","GAGAGAAGATCTCCAAGATGAGG",0,NA,NA,NA,"GeneID:148229","ATP8B3",TRUE,"ATP8B3"

"13835","GAGAGAAGATCTCCAAGATG","chr4",64207376,"+","6:G>A,9:G>T","AGG","GAGAGAGGAGCTCCAAGATGAGG",2,7,10,NA,"","",FALSE,"ATP8B3"

"13836","GCCAACAACCGTGCCTACAA","chr19",1809694,"-","none","CGG","GCCAACAACCGTGCCTACAACGG",0,NA,NA,NA,"GeneID:148229","ATP8B3",TRUE,"ATP8B3"

"13837","TCCTCTTCATCCGTGCCACC","chr19",1807188,"-","none","CGG","TCCTCTTCATCCGTGCCACCCGG",0,NA,NA,NA,"GeneID:148229","ATP8B3",TRUE,"ATP8B3"

"13838","TCCTCTTCATCCGTGCCACC","chr12",124954140,"+","11:T>C,13:G>T","CGG","TCCTCTTCATCTGGGCCACCCGG",2,12,14,NA,"GeneID:57647","DHX37",TRUE,"ATP8B3"

"13839","AATAGGCATTTGCCACTCTT","chr15",50047373,"+","none","TGG","AATAGGCATTTGCCACTCTTTGG",0,NA,NA,NA,"GeneID:79895","ATP8B4",TRUE,"ATP8B4"

"13840","AATAGGCATTTGCCACTCTT","chr7",146342986,"+","11:T>G,16:A>T","GGG","AATAGGCATTTTCCACACTTGGG",2,12,17,NA,"GeneID:26047","CNTNAP2",FALSE,"ATP8B4"

"13841","ATATTACCTGTAAAATCAGA","chr15",50047343,"+","none","AGG","ATATTACCTGTAAAATCAGAAGG",0,NA,NA,NA,"GeneID:79895","ATP8B4",TRUE,"ATP8B4"

"13842","ATATTACCTGTAAAATCAGA","chr6",58263869,"-","13:A>T,18:G>C","CGG","ATATTACCTGTAATATCACACGG",2,14,19,NA,"","",FALSE,"ATP8B4"

"13843","ATATTACCTGTAAAATCAGA","chr21",21461334,"-","8:G>A,9:A>C","TGG","ATATTACCCTTAAAATCAGATGG",2,9,10,NA,"GeneID:4685","NCAM2",FALSE,"ATP8B4"

"13844","ATTTGAACAGTTCCAAAGAG","chr15",50047388,"-","none","TGG","ATTTGAACAGTTCCAAAGAGTGG",0,NA,NA,NA,"GeneID:79895","ATP8B4",TRUE,"ATP8B4"

"13845","ATTTGAACAGTTCCAAAGAG","chr3",182828151,"+","13:A>C,14:G>A","TGG","ATTTGAACAGTTCAGAAGAGTGG",2,14,15,NA,"GeneID:23200","ATP11B",TRUE,"ATP8B4"

"13846","ATTTGAACAGTTCCAAAGAG","chr14",70807942,"+","6:C>A,19:T>G","AGG","ATTTGACCAGTTCCAAAGATAGG",2,7,20,NA,"GeneID:4293","MAP3K9",FALSE,"ATP8B4"

"13847","GAATATTATATTTCGATGTG","chr15",50047430,"+","none","TGG","GAATATTATATTTCGATGTGTGG",0,NA,NA,NA,"GeneID:79895","ATP8B4",TRUE,"ATP8B4"

"13848","TAATGAAAAGTTCCAGTATG","chr15",50074128,"-","none","CGG","TAATGAAAAGTTCCAGTATGCGG",0,NA,NA,NA,"GeneID:79895","ATP8B4",TRUE,"ATP8B4"

"13849","TAATGAAAAGTTCCAGTATG","chrX",116821433,"-","1:A>T,17:A>T","GGG","TTATGAAAAGTTCCAGTTTGGGG",2,2,18,NA,"","",FALSE,"ATP8B4"

"13850","TGCAGTGAAAAGAAATTGCG","chr15",50106940,"-","none","TGG","TGCAGTGAAAAGAAATTGCGTGG",0,NA,NA,NA,"GeneID:79895","ATP8B4",TRUE,"ATP8B4"

"13851","TGCAGTGAAAAGAAATTGCG","chr6",114077401,"-","17:A>C,18:C>G","GGG","TGCAGTGAAAAGAAATTTGGGGG",2,18,19,NA,"GeneID:101927768;GeneID:222537","LOC101927768;HS3ST5",FALSE,"ATP8B4"

"13852","TGCAGTGAAAAGAAATTGCG","chr20",61159587,"-","5:T>A,7:A>T","TGG","TGCAGAGTAAAGAAATTGCGTGG",2,6,8,NA,"","",FALSE,"ATP8B4"

"13853","TGCAGTGAAAAGAAATTGCG","chr6",124825767,"+","0:A>T,19:C>G","GGG","AGCAGTGAAAAGAAATTGCCGGG",2,1,20,NA,"","",FALSE,"ATP8B4"

"13854","CTATACCTACTGGGTTCCCC","chr20",51725820,"-","none","TGG","CTATACCTACTGGGTTCCCCTGG",0,NA,NA,NA,"GeneID:10079","ATP9A",TRUE,"ATP9A"

"13855","GGACAACATCCCGCTGCAGC","chr20",51768344,"-","none","CGG","GGACAACATCCCGCTGCAGCCGG",0,NA,NA,NA,"GeneID:10079","ATP9A",TRUE,"ATP9A"

"13856","GGACAACATCCCGCTGCAGC","chr14",100316495,"-","8:C>A,11:T>G","AGG","GGACAACAGCCAGCTGCAGCAGG",2,9,12,NA,"","",FALSE,"ATP9A"

"13857","TGATTGTTGATGACATTCCG","chr20",51729864,"+","none","AGG","TGATTGTTGATGACATTCCGAGG",0,NA,NA,NA,"GeneID:10079","ATP9A",TRUE,"ATP9A"

"13858","TGCTGTCCATCCGCTTCTTC","chr20",51768314,"+","none","TGG","TGCTGTCCATCCGCTTCTTCTGG",0,NA,NA,NA,"GeneID:10079","ATP9A",TRUE,"ATP9A"

"13859","TTTCCCACAGGTGCTGCGAG","chr20",51729968,"-","none","TGG","TTTCCCACAGGTGCTGCGAGTGG",0,NA,NA,NA,"GeneID:10079","ATP9A",TRUE,"ATP9A"

"13860","TTTCCCACAGGTGCTGCGAG","chr7",103879696,"+","4:T>C,16:G>C","TGG","TTTCTCACAGGTGCTGGGAGTGG",2,5,17,NA,"GeneID:5649","RELN",FALSE,"ATP9A"

"13861","TTTGTTCCCGAAATGAGACT","chr20",51725849,"-","none","TGG","TTTGTTCCCGAAATGAGACTTGG",0,NA,NA,NA,"GeneID:10079","ATP9A",TRUE,"ATP9A"

"13862","AAGAGTTCAGACATACAAGT","chr18",79143815,"+","none","TGG","AAGAGTTCAGACATACAAGTTGG",0,NA,NA,NA,"GeneID:374868","ATP9B",TRUE,"ATP9B"

"13863","AAGAGTTCAGACATACAAGT","chr2",54416077,"+","2:A>G,18:A>G","GGG","AAAAGTTCAGACATACAAATGGG",2,3,19,NA,"","",FALSE,"ATP9B"

"13864","AGACCTCATCATAGTGGAAA","chr18",79143838,"+","none","AGG","AGACCTCATCATAGTGGAAAAGG",0,NA,NA,NA,"GeneID:374868","ATP9B",TRUE,"ATP9B"

"13865","AGACCTCATCATAGTGGAAA","chr2",18480377,"+","3:G>C,13:C>G","GGG","AGAGCTCATCATACTGGAAAGGG",2,4,14,NA,"","",FALSE,"ATP9B"

"13866","CTACACCTACTGGGCTCCTC","chr18",79113332,"+","none","TGG","CTACACCTACTGGGCTCCTCTGG",0,NA,NA,NA,"GeneID:374868","ATP9B",TRUE,"ATP9B"

"13867","GGGAAGCAATTGATGAATTT","chr18",79126294,"+","none","CGG","GGGAAGCAATTGATGAATTTCGG",0,NA,NA,NA,"GeneID:374868","ATP9B",TRUE,"ATP9B"

"13868","GGGAAGCAATTGATGAATTT","chr14",59597996,"+","5:A>G,7:C>A","AGG","GGGAAACCATTGATGAATTTAGG",2,6,8,NA,"GeneID:6252","RTN1",FALSE,"ATP9B"

"13869","GGGAAGCAATTGATGAATTT","chr13",105484386,"+","7:T>A,10:A>T","GGG","GGGAAGCTATAGATGAATTTGGG",2,8,11,NA,"GeneID:282706;GeneID:267012","DAOA-AS1;DAOA",FALSE,"ATP9B"

"13870","TATAGCAAGCTTACAGTAAG","chr18",79126353,"+","none","AGG","TATAGCAAGCTTACAGTAAGAGG",0,NA,NA,NA,"GeneID:374868","ATP9B",TRUE,"ATP9B"

"13871","TATAGCAAGCTTACAGTAAG","chr4",170000477,"-","11:G>A,12:A>T","TGG","TATAGCAAGCTCTCAGTAAGTGG",2,12,13,NA,"GeneID:9848","MFAP3L",FALSE,"ATP9B"

"13872","TATAGCAAGCTTACAGTAAG","chr9",82600041,"-","11:T>A,17:C>T","AGG","TATAGCAAGCTAACAGTGAGAGG",2,12,18,NA,"","",FALSE,"ATP9B"

"13873","TATAGCAAGCTTACAGTAAG","chr6",68162686,"-","10:T>A,12:A>T","TGG","TATAGCAAGCATTCAGTAAGTGG",2,11,13,NA,"","",FALSE,"ATP9B"

"13874","TCTTACCAGAGGAGCCCAGT","chr18",79113340,"-","none","AGG","TCTTACCAGAGGAGCCCAGTAGG",0,NA,NA,NA,"GeneID:374868","ATP9B",TRUE,"ATP9B"

"13875","TCTTACCAGAGGAGCCCAGT","chr19",18266082,"-","2:T>A,4:G>T","TGG","TCATCCCAGAGGAGCCCAGTTGG",2,3,5,NA,"GeneID:80726","KIAA1683",TRUE,"ATP9B"

"13876","AACTGCAGAAGAAATAAAAC","chr1",46658128,"-","none","AGG","AACTGCAGAAGAAATAAAACAGG",0,NA,NA,NA,"GeneID:64756","ATPAF1",TRUE,"ATPAF1"

"13877","AACTGCAGAAGAAATAAAAC","chr3",180615791,"+","14:A>T,19:A>C","GGG","AACTGCAGAAGAAAAAAAAAGGG",2,15,20,NA,"GeneID:151613;GeneID:339829","TTC14;CCDC39",FALSE,"ATPAF1"

"13878","AACTGCAGAAGAAATAAAAC","chrY",6892261,"+","0:C>A,3:A>T","TGG","CACAGCAGAAGAAATAAAACTGG",2,1,4,NA,"","",FALSE,"ATPAF1"

"13879","AACTGCAGAAGAAATAAAAC","chr9",108213683,"+","2:A>C,19:G>C","GGG","AAATGCAGAAGAAATAAAAGGGG",2,3,20,NA,"","",FALSE,"ATPAF1"

"13880","GAAATTCACTGCGTTTCTCC","chr1",46665314,"+","none","AGG","GAAATTCACTGCGTTTCTCCAGG",0,NA,NA,NA,"GeneID:64756","ATPAF1",TRUE,"ATPAF1"

"13881","GTTCACAGACTGTTTCCCCA","chr1",46658707,"+","none","AGG","GTTCACAGACTGTTTCCCCAAGG",0,NA,NA,NA,"GeneID:64756","ATPAF1",TRUE,"ATPAF1"

"13882","GTTCACAGACTGTTTCCCCA","chr18",433119,"+","5:T>C,6:G>A","TGG","GTTCATGGACTGTTTCCCCATGG",2,6,7,NA,"GeneID:81035","COLEC12",FALSE,"ATPAF1"

"13883","TATATTTCAGACAGATGCCT","chr1",46658727,"-","none","TGG","TATATTTCAGACAGATGCCTTGG",0,NA,NA,NA,"GeneID:64756","ATPAF1",TRUE,"ATPAF1"

"13884","TGAATTTCGGAAGCAGCCAG","chr1",46665302,"-","none","TGG","TGAATTTCGGAAGCAGCCAGTGG",0,NA,NA,NA,"GeneID:64756","ATPAF1",TRUE,"ATPAF1"

"13885","TGAATTTCGGAAGCAGCCAG","chr5",127183821,"+","3:T>A,8:A>G","TGG","TGATTTTCAGAAGCAGCCAGTGG",2,4,9,NA,"","",FALSE,"ATPAF1"

"13886","TGTGAACAGAGGATTCACTA","chr1",46658694,"-","none","AGG","TGTGAACAGAGGATTCACTAAGG",0,NA,NA,NA,"GeneID:64756","ATPAF1",TRUE,"ATPAF1"

"13887","TGTGAACAGAGGATTCACTA","chr10",107342775,"+","11:A>G,18:C>T","GGG","TGTGAACAGAGAATTCACCAGGG",2,12,19,NA,"","",FALSE,"ATPAF1"

"13888","CAAGTACTACACCATGCACC","chr17",18028233,"-","none","TGG","CAAGTACTACACCATGCACCTGG",0,NA,NA,NA,"GeneID:91647","ATPAF2",TRUE,"ATPAF2"

"13889","CAAGTACTACACCATGCACC","chr1",218293852,"-","16:T>G,18:A>G","AGG","CAAGTACTACACCATGAATCAGG",2,17,19,NA,"GeneID:51018","RRP15",FALSE,"ATPAF2"

"13890","CATGGTCTTACCTGTCGGCG","chr17",18038869,"+","none","GGG","CATGGTCTTACCTGTCGGCGGGG",0,NA,NA,NA,"GeneID:91647","ATPAF2",TRUE,"ATPAF2"

"13891","GCTCTTTACCGTCCCCAGCG","chr17",18028305,"-","none","AGG","GCTCTTTACCGTCCCCAGCGAGG",0,NA,NA,NA,"GeneID:91647","ATPAF2",TRUE,"ATPAF2"

"13892","GGCCACCCGCCGGCCGATTC","chr17",18038946,"+","none","AGG","GGCCACCCGCCGGCCGATTCAGG",0,NA,NA,NA,"GeneID:91647","ATPAF2",TRUE,"ATPAF2"

"13893","GGGGACGCCGTCTCCTGAAT","chr17",18038962,"-","none","CGG","GGGGACGCCGTCTCCTGAATCGG",0,NA,NA,NA,"GeneID:91647","ATPAF2",TRUE,"ATPAF2"

"13894","TCAGAATGTCAGCATCACAC","chr17",18028623,"-","none","AGG","TCAGAATGTCAGCATCACACAGG",0,NA,NA,NA,"GeneID:91647","ATPAF2",TRUE,"ATPAF2"

"13895","TCAGAATGTCAGCATCACAC","chr12",111197390,"+","1:T>C,5:C>A","AGG","TTAGACTGTCAGCATCACACAGG",2,2,6,NA,"GeneID:23316","CUX2",FALSE,"ATPAF2"

"13896","TCAGAATGTCAGCATCACAC","chr18",73660331,"+","0:A>T,17:G>C","AGG","ACAGAATGTCAGCATCAGACAGG",2,1,18,NA,"","",FALSE,"ATPAF2"

"13897","ATGATGCAGTGCATTGCTGC","chr15",35542462,"-","none","TGG","ATGATGCAGTGCATTGCTGCTGG",0,NA,NA,NA,"GeneID:89978","DPH6",TRUE,"ATPBD4"

"13898","CTCTATCGCCGAACCATAAG","chr15",35538361,"-","none","AGG","CTCTATCGCCGAACCATAAGAGG",0,NA,NA,NA,"GeneID:89978","DPH6",TRUE,"ATPBD4"

"13899","GCAGCCAGGCGATTGTTTAC","chr15",35522182,"-","none","AGG","GCAGCCAGGCGATTGTTTACAGG",0,NA,NA,NA,"GeneID:89978","DPH6",TRUE,"ATPBD4"

"13900","TAGCTACATGTATCAGACAG","chr15",35538428,"-","none","TGG","TAGCTACATGTATCAGACAGTGG",0,NA,NA,NA,"GeneID:89978","DPH6",TRUE,"ATPBD4"

"13901","TGACTATCAGCGTATTCGAG","chr15",35454756,"-","none","TGG","TGACTATCAGCGTATTCGAGTGG",0,NA,NA,NA,"GeneID:89978","DPH6",TRUE,"ATPBD4"

"13902","TTATCCAATATGTGATATGG","chr15",35520799,"-","none","TGG","TTATCCAATATGTGATATGGTGG",0,NA,NA,NA,"GeneID:89978","DPH6",TRUE,"ATPBD4"

"13903","AGAAATCGTTCATCATAAGA","chr1",28237884,"+","none","AGG","AGAAATCGTTCATCATAAGAAGG",0,NA,NA,NA,"GeneID:93974","ATPIF1",TRUE,"ATPIF1"

"13904","AGAAATCGTTCATCATAAGA","chr16",5699768,"+","7:C>G,13:G>C","TGG","AGAAATCCTTCATGATAAGATGG",2,8,14,NA,"GeneID:54715","RBFOX1",FALSE,"ATPIF1"

"13905","AGAAATCGTTCATCATAAGA","chr17",67431012,"-","6:T>G,18:T>C","TGG","AGAAATAGTTCATCATAAAATGG",2,7,19,NA,"GeneID:26207","PITPNC1",FALSE,"ATPIF1"

"13906","CCTGATCCGAGCCGAAGCCT","chr1",28236251,"-","none","CGG","CCTGATCCGAGCCGAAGCCTCGG",0,NA,NA,NA,"GeneID:93974","ATPIF1",TRUE,"ATPIF1"

"13907","CTGAAGAGGAACGATATTTC","chr1",28236430,"+","none","CGG","CTGAAGAGGAACGATATTTCCGG",0,NA,NA,NA,"GeneID:93974","ATPIF1",TRUE,"ATPIF1"

"13908","GATGAACGATTTCTTCTTCA","chr1",28237878,"-","none","TGG","GATGAACGATTTCTTCTTCATGG",0,NA,NA,NA,"GeneID:93974","ATPIF1",TRUE,"ATPIF1"

"13909","TGCAGTCCGAGAATGTCGAC","chr1",28236355,"+","none","CGG","TGCAGTCCGAGAATGTCGACCGG",0,NA,NA,NA,"GeneID:93974","ATPIF1",TRUE,"ATPIF1"

"13910","TTGGCGGCGCGGACGTGGCT","chr1",28236198,"+","none","TGG","TTGGCGGCGCGGACGTGGCTTGG",0,NA,NA,NA,"GeneID:93974","ATPIF1",TRUE,"ATPIF1"

"13911","ACAACTGTATTATATTCCTC","chr3",142568124,"+","none","TGG","ACAACTGTATTATATTCCTCTGG",0,NA,NA,NA,"GeneID:545","ATR",TRUE,"ATR"

"13912","CTTACCAATACAACTGCCTT","chr3",142566115,"+","none","TGG","CTTACCAATACAACTGCCTTTGG",0,NA,NA,NA,"GeneID:545","ATR",TRUE,"ATR"

"13913","CTTCCATGATCCCCGCCCTG","chr3",142578659,"-","none","CGG","CTTCCATGATCCCCGCCCTGCGG",0,NA,NA,NA,"GeneID:545","ATR",TRUE,"ATR"

"13914","CTTCCATGATCCCCGCCCTG","chr10",66883294,"-","11:A>G,13:A>G","AGG","CTTCCATGATCTCTGCCCTGAGG",2,12,14,NA,"GeneID:29119","CTNNA3",FALSE,"ATR"

"13915","CTTCCATGATCCCCGCCCTG","chr1",203364808,"+","1:A>T,13:A>C","GGG","CATCCATGATCCCAGCCCTGGGG",2,2,14,NA,"","",FALSE,"ATR"

"13916","CTTCCATGATCCCCGCCCTG","chr3",138459292,"-","5:G>T,14:T>C","GGG","CTTCCCTGATCCCCACCCTGGGG",2,6,15,NA,"GeneID:83850","ESYT3",FALSE,"ATR"

"13917","GAGGCCAAAGGCAGTTGTAT","chr3",142566122,"-","none","TGG","GAGGCCAAAGGCAGTTGTATTGG",0,NA,NA,NA,"GeneID:545","ATR",TRUE,"ATR"

"13918","GAGGCCAAAGGCAGTTGTAT","chrX",153474516,"+","7:G>A,19:G>T","GGG","GAGGCCAGAGGCAGTTGTAGGGG",2,8,20,NA,"GeneID:55559","HAUS7",FALSE,"ATR"

"13919","GGATCATGGAAGCCAGCTCC","chr3",142578667,"+","none","AGG","GGATCATGGAAGCCAGCTCCAGG",0,NA,NA,NA,"GeneID:545","ATR",TRUE,"ATR"

"13920","GGATCATGGAAGCCAGCTCC","chr14",50951969,"-","0:A>C,6:T>A","AGG","TGATCAAGGAAGCCAGCTCCAGG",2,1,7,NA,"","",FALSE,"ATR"

"13921","GGATCATGGAAGCCAGCTCC","chr2",233171834,"+","6:G>T,17:C>T","TGG","GGATCAGGGAAGCCAGCCCCTGG",2,7,18,NA,"GeneID:3635","INPP5D",FALSE,"ATR"

"13922","TTCTGTGTCAATTCATTGAC","chr3",142568087,"-","none","CGG","TTCTGTGTCAATTCATTGACCGG",0,NA,NA,NA,"GeneID:545","ATR",TRUE,"ATR"

"13923","TTCTGTGTCAATTCATTGAC","chr7",99140743,"+","0:C>T,9:T>A","TGG","CTCTGTGTCTATTCATTGACTGG",2,1,10,NA,"GeneID:57154","SMURF1",FALSE,"ATR"

"13924","TTCTGTGTCAATTCATTGAC","chr14",86902136,"+","8:G>C,13:G>C","TGG","TTCTGTGTGAATTGATTGACTGG",2,9,14,NA,"","",FALSE,"ATR"

"13925","TTCTGTGTCAATTCATTGAC","chr10",33238261,"-","7:G>A,10:A>T","AGG","TTCTGTGCCATTTCATTGACAGG",2,8,11,NA,"GeneID:8829","NRP1",FALSE,"ATR"

"13926","TTCTGTGTCAATTCATTGAC","chr8",72833820,"-","5:T>A,13:C>G","AGG","TTCTGAGTCAATTGATTGACAGG",2,6,14,NA,"GeneID:9312","KCNB2",FALSE,"ATR"

"13927","ATAAGAGGTGATAGTATTCC","chr2",27215675,"-","none","AGG","ATAAGAGGTGATAGTATTCCAGG",0,NA,NA,NA,"GeneID:51374","ATRAID",TRUE,"ATRAID"

"13928","CCCTGGTCCAAACTTTCATC","chr2",27215350,"+","none","AGG","CCCTGGTCCAAACTTTCATCAGG",0,NA,NA,NA,"GeneID:51374","ATRAID",TRUE,"ATRAID"

"13929","CTGCAAGCAAACCCCCTCAA","chr2",27215477,"+","none","AGG","CTGCAAGCAAACCCCCTCAAAGG",0,NA,NA,NA,"GeneID:51374","ATRAID",TRUE,"ATRAID"

"13930","GACTTGGCCAACACCTTCCG","chr2",27215501,"+","none","TGG","GACTTGGCCAACACCTTCCGTGG",0,NA,NA,NA,"GeneID:51374","ATRAID",TRUE,"ATRAID"

"13931","GCCTGATGAAAGTTTGGACC","chr2",27215354,"-","none","AGG","GCCTGATGAAAGTTTGGACCAGG",0,NA,NA,NA,"GeneID:51374","ATRAID",TRUE,"ATRAID"

"13932","GCCTGATGAAAGTTTGGACC","chr7",109100176,"-","17:A>T,19:T>G","TGG","GCCTGATGAAAGTTTGGTCATGG",2,18,20,NA,"","",FALSE,"ATRAID"

"13933","GGTCCCCAGTGTTATTGCAA","chr2",27215730,"-","none","AGG","GGTCCCCAGTGTTATTGCAAAGG",0,NA,NA,NA,"GeneID:51374","ATRAID",TRUE,"ATRAID"

"13934","CAGTGAACGAGCAAATAAAC","chr3",48454370,"+","none","TGG","CAGTGAACGAGCAAATAAACTGG",0,NA,NA,NA,"GeneID:84126","ATRIP",TRUE,"ATRIP"

"13935","CAGTGAACGAGCAAATAAAC","chr7",18906639,"-","1:A>T,7:C>G","TGG","CTGTGAAGGAGCAAATAAACTGG",2,2,8,NA,"GeneID:9734","HDAC9",FALSE,"ATRIP"

"13936","CAGTTTATTTGCTCGTTCAC","chr3",48454372,"-","none","TGG","CAGTTTATTTGCTCGTTCACTGG",0,NA,NA,NA,"GeneID:84126","ATRIP",TRUE,"ATRIP"

"13937","GCGAGACTCACTACATCAGA","chr3",48451781,"+","none","CGG","GCGAGACTCACTACATCAGACGG",0,NA,NA,NA,"GeneID:84126","ATRIP",TRUE,"ATRIP"

"13938","GGCATGTCAAAAAATCCTTC","chr3",48450065,"+","none","AGG","GGCATGTCAAAAAATCCTTCAGG",0,NA,NA,NA,"GeneID:84126","ATRIP",TRUE,"ATRIP"

"13939","GGCATGTCAAAAAATCCTTC","chrX",135398200,"+","11:C>A,14:A>T","AGG","GGCATGTCAAACAAACCTTCAGG",2,12,15,NA,"","",FALSE,"ATRIP"

"13940","GGCATGTCAAAAAATCCTTC","chr18",51987101,"+","5:A>G,8:C>A","AGG","GGCATATCCAAAAATCCTTCAGG",2,6,9,NA,"","",FALSE,"ATRIP"

"13941","TAAAGATAATTTCGAATTAG","chr3",48450112,"+","none","AGG","TAAAGATAATTTCGAATTAGAGG",0,NA,NA,NA,"GeneID:84126","ATRIP",TRUE,"ATRIP"

"13942","TTTCCTTGTCACTGAGTGCT","chr3",48451862,"-","none","TGG","TTTCCTTGTCACTGAGTGCTTGG",0,NA,NA,NA,"GeneID:84126","ATRIP",TRUE,"ATRIP"

"13943","TTTCCTTGTCACTGAGTGCT","chr14",39442463,"+","0:A>T,2:C>T","TGG","ATCCCTTGTCACTGAGTGCTTGG",2,1,3,NA,"","",FALSE,"ATRIP"

"13944","TTTCCTTGTCACTGAGTGCT","chr1",168203558,"-","9:A>G,13:A>C","AGG","TTTCCTTGTTACTTAGTGCTAGG",2,10,14,NA,"","",FALSE,"ATRIP"

"13945","TTTCCTTGTCACTGAGTGCT","chr22",33234429,"-","3:A>G,13:T>C","AGG","TTTTCTTGTCACTAAGTGCTAGG",2,4,14,NA,"GeneID:9215","LARGE",FALSE,"ATRIP"

"13946","AATACAAAACGAAGTGCACG","chr20",3535299,"+","none","TGG","AATACAAAACGAAGTGCACGTGG",0,NA,NA,NA,"GeneID:8455","ATRN",TRUE,"ATRN"

"13947","CACCGCTAGTTGCTGCATTT","chr20",3540313,"+","none","AGG","CACCGCTAGTTGCTGCATTTAGG",0,NA,NA,NA,"GeneID:8455","ATRN",TRUE,"ATRN"

"13948","CCTGATGTGGCAACAACCTC","chr20",3545804,"-","none","AGG","CCTGATGTGGCAACAACCTCAGG",0,NA,NA,NA,"GeneID:8455","ATRN",TRUE,"ATRN"

"13949","GATGCTGCTTATAATTTGAC","chr20",3545849,"+","none","TGG","GATGCTGCTTATAATTTGACTGG",0,NA,NA,NA,"GeneID:8455","ATRN",TRUE,"ATRN"

"13950","TACAGACTAACTGGATCTTC","chr20",3535247,"+","none","TGG","TACAGACTAACTGGATCTTCTGG",0,NA,NA,NA,"GeneID:8455","ATRN",TRUE,"ATRN"

"13951","TACCTAAATGCAGCAACTAG","chr20",3540318,"-","none","CGG","TACCTAAATGCAGCAACTAGCGG",0,NA,NA,NA,"GeneID:8455","ATRN",TRUE,"ATRN"

"13952","CACCTAAGTACAGCTATTAA","chr10",115121795,"-","none","AGG","CACCTAAGTACAGCTATTAAAGG",0,NA,NA,NA,"GeneID:26033","ATRNL1",TRUE,"ATRNL1"

"13953","CACCTAAGTACAGCTATTAA","chr19",32091912,"+","13:T>C","AGG","CACCTAAGTACAGTTATTAAAGG",1,14,NA,NA,"","",FALSE,"ATRNL1"

"13954","CCAGATGTAGTAACAACTTC","chr10",115127635,"-","none","AGG","CCAGATGTAGTAACAACTTCAGG",0,NA,NA,NA,"GeneID:26033","ATRNL1",TRUE,"ATRNL1"

"13955","CCATCTGTTAAATATCCAGA","chr10",115120197,"-","none","AGG","CCATCTGTTAAATATCCAGAAGG",0,NA,NA,NA,"GeneID:26033","ATRNL1",TRUE,"ATRNL1"

"13956","CTTAATCTTAACACTGCATT","chr10",115121702,"-","none","TGG","CTTAATCTTAACACTGCATTTGG",0,NA,NA,NA,"GeneID:26033","ATRNL1",TRUE,"ATRNL1"

"13957","GATGCTGCGTATAATCTAAC","chr10",115127680,"+","none","TGG","GATGCTGCGTATAATCTAACTGG",0,NA,NA,NA,"GeneID:26033","ATRNL1",TRUE,"ATRNL1"

"13958","TCCAGGTTAACAGAACCTTC","chr10",115120179,"+","none","TGG","TCCAGGTTAACAGAACCTTCTGG",0,NA,NA,NA,"GeneID:26033","ATRNL1",TRUE,"ATRNL1"

"13959","TCCAGGTTAACAGAACCTTC","chr19",55555114,"-","3:A>T,8:G>T","TGG","TCCTGGTTCACAGAACCTTCTGG",2,4,9,NA,"","",FALSE,"ATRNL1"

"13960","TCCAGGTTAACAGAACCTTC","chr10",1195330,"+","5:C>G,8:T>A","AGG","TCCAGCTTTACAGAACCTTCAGG",2,6,9,NA,"GeneID:105","ADARB2",FALSE,"ATRNL1"

"13961","ACATACTTTGTTACAATTGA","chrX",77696677,"+","none","AGG","ACATACTTTGTTACAATTGAAGG",0,NA,NA,NA,"GeneID:546","ATRX",TRUE,"ATRX"

"13962","ACATACTTTGTTACAATTGA","chr11",34843879,"-","3:C>A,5:A>G","GGG","ACAGATTTTGTTACAATTGAGGG",2,4,6,NA,"","",FALSE,"ATRX"

"13963","ACTATGCAGAGCTTGCCAAA","chrX",77696578,"-","none","AGG","ACTATGCAGAGCTTGCCAAAAGG",0,NA,NA,NA,"GeneID:546","ATRX",TRUE,"ATRX"

"13964","ATTCTTCTGATGAGTGTGCA","chrX",77717178,"+","none","AGG","ATTCTTCTGATGAGTGTGCAAGG",0,NA,NA,NA,"GeneID:546","ATRX",TRUE,"ATRX"

"13965","ATTCTTCTGATGAGTGTGCA","chrX",63973100,"-","12:G>T,13:T>C","TGG","ATTCTTCTGATGCATGTGCATGG",2,13,14,NA,"","",FALSE,"ATRX"

"13966","ATTCTTCTGATGAGTGTGCA","chr12",89505142,"-","2:G>A,3:T>G","CGG","ATCATTCTGATGAGTGTGCACGG",2,3,4,NA,"GeneID:282809","POC1B",FALSE,"ATRX"

"13967","ATTCTTCTGATGAGTGTGCA","chr7",35945803,"+","3:T>C,13:A>G","GGG","ATTTTTCTGATGAATGTGCAGGG",2,4,14,NA,"","",FALSE,"ATRX"

"13968","ATTCTTCTGATGAGTGTGCA","chr7",45749181,"-","3:A>G,13:T>C","GGG","ATTTTTCTGATGAATGTGCAGGG",2,4,14,NA,"GeneID:641977","SEPT7P2",FALSE,"ATRX"

"13969","CTTGCAATGAATCAAAACAC","chrX",77717132,"-","none","AGG","CTTGCAATGAATCAAAACACAGG",0,NA,NA,NA,"GeneID:546","ATRX",TRUE,"ATRX"

"13970","CTTGCAATGAATCAAAACAC","chr16",83848051,"+","11:C>T,12:A>C","AGG","CTTGCAATGAACAAAAACACAGG",2,12,13,NA,"","",FALSE,"ATRX"

"13971","CTTGCAATGAATCAAAACAC","chr4",10539975,"+","1:C>T,7:A>T","AGG","CCTGCAAAGAATCAAAACACAGG",2,2,8,NA,"GeneID:116449","CLNK",FALSE,"ATRX"

"13972","CTTGCAATGAATCAAAACAC","chr1",234988558,"+","1:C>T,16:C>A","TGG","CCTGCAATGAATCAAACCACTGG",2,2,17,NA,"","",FALSE,"ATRX"

"13973","TCAGAAAAATCCAAGTCTTC","chrX",77697603,"-","none","AGG","TCAGAAAAATCCAAGTCTTCAGG",0,NA,NA,NA,"GeneID:546","ATRX",TRUE,"ATRX"

"13974","TCAGAAAAATCCAAGTCTTC","chr10",92265209,"+","15:A>T,19:G>C","TGG","TCAGAAAAATCCAAGACTTGTGG",2,16,20,NA,"GeneID:22849","CPEB3",FALSE,"ATRX"

"13975","TCAGAAAAATCCAAGTCTTC","chr2",241289156,"+","4:G>A,9:C>T","AGG","TCAGGAAAACCCAAGTCTTCAGG",2,5,10,NA,"GeneID:3069","HDLBP",FALSE,"ATRX"

"13976","TCAGAAAAATCCAAGTCTTC","chr5",35673488,"-","1:A>G,14:T>C","AGG","TTAGAAAAATCCAAATCTTCAGG",2,2,15,NA,"GeneID:79925","SPEF2",FALSE,"ATRX"

"13977","TCGTGACGATCCTGAAGACT","chrX",77697590,"+","none","TGG","TCGTGACGATCCTGAAGACTTGG",0,NA,NA,NA,"GeneID:546","ATRX",TRUE,"ATRX"

"13978","AATAGAACCCAGCAAGCCCG","chr6",16306477,"-","none","CGG","AATAGAACCCAGCAAGCCCGCGG",0,NA,NA,NA,"GeneID:6310","ATXN1",TRUE,"ATXN1"

"13979","CCGCTCAGGTAGCCGATGAC","chr6",16326910,"+","none","AGG","CCGCTCAGGTAGCCGATGACAGG",0,NA,NA,NA,"GeneID:6310","ATXN1",TRUE,"ATXN1"

"13980","GCCGGTGTTCTGCGGAGAAC","chr6",16327536,"+","none","TGG","GCCGGTGTTCTGCGGAGAACTGG",0,NA,NA,NA,"GeneID:6310","ATXN1",TRUE,"ATXN1"

"13981","GCCGTGATACAGTTCGCCGT","chr6",16326412,"-","none","CGG","GCCGTGATACAGTTCGCCGTCGG",0,NA,NA,NA,"GeneID:6310","ATXN1",TRUE,"ATXN1"

"13982","GTCATGCAATACGCCGACTC","chr6",16327423,"-","none","CGG","GTCATGCAATACGCCGACTCCGG",0,NA,NA,NA,"GeneID:6310","ATXN1",TRUE,"ATXN1"

"13983","TCTACTTGCCTACATTAGAC","chr6",16306327,"+","none","CGG","TCTACTTGCCTACATTAGACCGG",0,NA,NA,NA,"GeneID:6310","ATXN1",TRUE,"ATXN1"

"13984","AAGATTCCCAGTCTATTGTT","chr22",45700329,"+","none","TGG","AAGATTCCCAGTCTATTGTTTGG",0,NA,NA,NA,"GeneID:25814","ATXN10",TRUE,"ATXN10"

"13985","ACTTACGGCCATTCTGATTC","chr22",45702833,"-","none","AGG","ACTTACGGCCATTCTGATTCAGG",0,NA,NA,NA,"GeneID:25814","ATXN10",TRUE,"ATXN10"

"13986","CCCAGGAACTTGGATACGAT","chr22",45692990,"+","none","TGG","CCCAGGAACTTGGATACGATTGG",0,NA,NA,NA,"GeneID:25814","ATXN10",TRUE,"ATXN10"

"13987","GTAACTACCTGATTGAATTC","chr22",45689891,"-","none","TGG","GTAACTACCTGATTGAATTCTGG",0,NA,NA,NA,"GeneID:25814","ATXN10",TRUE,"ATXN10"

"13988","GTAACTACCTGATTGAATTC","chr10",115063331,"-","9:T>A,13:T>A","TGG","GTAACTACCAGATAGAATTCTGG",2,10,14,NA,"","",FALSE,"ATXN10"

"13989","TCTGTTTCGTGAACTGCGAG","chr22",45693034,"+","none","TGG","TCTGTTTCGTGAACTGCGAGTGG",0,NA,NA,NA,"GeneID:25814","ATXN10",TRUE,"ATXN10"

"13990","TTCTGTTATTAACTGCAGAC","chr22",45689824,"-","none","TGG","TTCTGTTATTAACTGCAGACTGG",0,NA,NA,NA,"GeneID:25814","ATXN10",TRUE,"ATXN10"

"13991","AAGAGTTGTGTCGTCCGCCC","chr16",71851411,"-","none","AGG","AAGAGTTGTGTCGTCCGCCCAGG",0,NA,NA,NA,"GeneID:342371;GeneID:9798","ATXN1L;IST1",TRUE,"ATXN1L"

"13992","CATCTCCCACCTGTAGCCGA","chr16",71851446,"-","none","TGG","CATCTCCCACCTGTAGCCGATGG",0,NA,NA,NA,"GeneID:342371;GeneID:9798","ATXN1L;IST1",TRUE,"ATXN1L"

"13993","CATCTCCCACCTGTAGCCGA","chr1",118345407,"+","14:G>A,17:T>C","AGG","CATCTCCCACCTGTGGCTGAAGG",2,15,18,NA,"","",FALSE,"ATXN1L"

"13994","CTAAAGCGCCAACCACCCGC","chr16",71850753,"-","none","TGG","CTAAAGCGCCAACCACCCGCTGG",0,NA,NA,NA,"GeneID:342371;GeneID:9798","ATXN1L;IST1",TRUE,"ATXN1L"

"13995","CTTACTAAATTTCGCTCTCG","chr16",71850562,"-","none","TGG","CTTACTAAATTTCGCTCTCGTGG",0,NA,NA,NA,"GeneID:342371;GeneID:9798","ATXN1L;IST1",TRUE,"ATXN1L"

"13996","GAAGACGCTACATTAAACGT","chr16",71850064,"-","none","TGG","GAAGACGCTACATTAAACGTTGG",0,NA,NA,NA,"GeneID:342371;GeneID:9798","ATXN1L;IST1",TRUE,"ATXN1L"

"13997","GTGCGCAGTGCCGAAGTGAG","chr16",71851219,"+","none","CGG","GTGCGCAGTGCCGAAGTGAGCGG",0,NA,NA,NA,"GeneID:342371;GeneID:9798","ATXN1L;IST1",TRUE,"ATXN1L"

"13998","AATCTATGCAAATATGAGGA","chr12",111554183,"-","none","TGG","AATCTATGCAAATATGAGGATGG",0,NA,NA,NA,"GeneID:6311","ATXN2",TRUE,"ATXN2"

"13999","AATCTATGCAAATATGAGGA","chr6",105464461,"+","12:G>T,17:T>G","TGG","AATCTATGCAAAGATGATGATGG",2,13,18,NA,"","",FALSE,"ATXN2"

"14000","AATCTATGCAAATATGAGGA","chrX",135202480,"+","5:T>A,18:A>G","GGG","AATCTTTGCAAATATGAGAAGGG",2,6,19,NA,"","",FALSE,"ATXN2"

"14001","AATCTATGCAAATATGAGGA","chrX",135192273,"-","5:A>T,18:T>C","GGG","AATCTTTGCAAATATGAGAAGGG",2,6,19,NA,"","",FALSE,"ATXN2"

"14002","AATCTATGCAAATATGAGGA","chr18",42626270,"-","3:A>G,16:A>T","AGG","AATTTATGCAAATATGTGGAAGG",2,4,17,NA,"GeneID:284260","LINC00907",FALSE,"ATXN2"

"14003","AGGTGAACTCACAGCCAATG","chr12",111525220,"-","none","AGG","AGGTGAACTCACAGCCAATGAGG",0,NA,NA,NA,"GeneID:6311","ATXN2",TRUE,"ATXN2"

"14004","AGGTGAACTCACAGCCAATG","chr1",14918334,"+","3:A>T,6:T>A","AGG","AGGAGATCTCACAGCCAATGAGG",2,4,7,NA,"GeneID:23254","KAZN",FALSE,"ATXN2"

"14005","ATTATATCGAAACATATCAT","chr12",111520934,"+","none","TGG","ATTATATCGAAACATATCATTGG",0,NA,NA,NA,"GeneID:6311","ATXN2",TRUE,"ATXN2"

"14006","CGAACACAAAGAGAAGGACC","chr12",111525256,"-","none","TGG","CGAACACAAAGAGAAGGACCTGG",0,NA,NA,NA,"GeneID:6311","ATXN2",TRUE,"ATXN2"

"14007","CGAACACAAAGAGAAGGACC","chr1",8656254,"-","1:T>C,4:C>G","GGG","CAAAGACAAAGAGAAGGACCGGG",2,2,5,NA,"GeneID:473","RERE",TRUE,"ATXN2"

"14008","CGAACACAAAGAGAAGGACC","chr15",22278972,"+","1:A>G,4:G>C","GGG","CAAAGACAAAGAGAAGGACCGGG",2,2,5,NA,"","",TRUE,"ATXN2"

"14009","CGAACACAAAGAGAAGGACC","chr14",106695627,"-","3:G>T,15:T>C","TGG","CGACCACAAAGAGAAAGACCTGG",2,4,16,NA,"","",FALSE,"ATXN2"

"14010","CGATATAATGAAGAAAATTA","chr12",111520923,"-","none","TGG","CGATATAATGAAGAAAATTATGG",0,NA,NA,NA,"GeneID:6311","ATXN2",TRUE,"ATXN2"

"14011","CGATATAATGAAGAAAATTA","chr5",118128633,"+","1:C>G,5:C>T","AGG","CCATACAATGAAGAAAATTAAGG",2,2,6,NA,"","",FALSE,"ATXN2"

"14012","TGTGAGTTCACCTGCATCCC","chr12",111525227,"+","none","AGG","TGTGAGTTCACCTGCATCCCAGG",0,NA,NA,NA,"GeneID:6311","ATXN2",TRUE,"ATXN2"

"14013","TGTGAGTTCACCTGCATCCC","chr9",32702352,"+","11:T>C,18:T>C","AGG","TGTGAGTTCACTTGCATCTCAGG",2,12,19,NA,"","",FALSE,"ATXN2"

"14014","AACTTACCACAACAGCTGTA","chr16",28825667,"-","none","AGG","AACTTACCACAACAGCTGTAAGG",0,NA,NA,NA,"GeneID:11273","ATXN2L",TRUE,"ATXN2L"

"14015","AACTTACCACAACAGCTGTA","chr6",107911338,"+","14:A>G,18:G>T","AGG","AACTTACCACAACAACTGGAAGG",2,15,19,NA,"GeneID:11231","SEC63",TRUE,"ATXN2L"

"14016","AACTTACCACAACAGCTGTA","chr20",7850750,"+","6:A>C,12:T>C","AGG","AACTTAACACAATAGCTGTAAGG",2,7,13,NA,"","",FALSE,"ATXN2L"

"14017","AAGACACTCACAGGTGACTG","chr16",28825393,"-","none","TGG","AAGACACTCACAGGTGACTGTGG",0,NA,NA,NA,"GeneID:11273","ATXN2L",TRUE,"ATXN2L"

"14018","AAGACACTCACAGGTGACTG","chr4",116511514,"+","4:G>C,13:A>G","AGG","AAGAGACTCACAGATGACTGAGG",2,5,14,NA,"","",FALSE,"ATXN2L"

"14019","CCCTCGTCGGGAGGACATTG","chr16",28826292,"+","none","TGG","CCCTCGTCGGGAGGACATTGTGG",0,NA,NA,NA,"GeneID:11273","ATXN2L",TRUE,"ATXN2L"

"14020","CTTCAAGACGCTAAGCTCAA","chr16",28825819,"+","none","AGG","CTTCAAGACGCTAAGCTCAAAGG",0,NA,NA,NA,"GeneID:11273","ATXN2L",TRUE,"ATXN2L"

"14021","CTTGAAGATACCCTCATAAG","chr16",28825806,"-","none","TGG","CTTGAAGATACCCTCATAAGTGG",0,NA,NA,NA,"GeneID:11273","ATXN2L",TRUE,"ATXN2L"

"14022","CTTGAAGATACCCTCATAAG","chr7",16586368,"+","12:T>C,18:G>A","TGG","CTTGAAGATACCTTCATAGGTGG",2,13,19,NA,"","",TRUE,"ATXN2L"

"14023","TCCACAATGTCCTCCCGACG","chr16",28826296,"-","none","AGG","TCCACAATGTCCTCCCGACGAGG",0,NA,NA,NA,"GeneID:11273","ATXN2L",TRUE,"ATXN2L"

"14024","TCCACAATGTCCTCCCGACG","chr7",16586276,"+","2:T>C,6:G>A","AGG","TCTACAGTGTCCTCCCGACGAGG",2,3,7,NA,"","",TRUE,"ATXN2L"

"14025","ACAGCAGCCTTCTGGAAATA","chr14",92096121,"-","none","TGG","ACAGCAGCCTTCTGGAAATATGG",0,NA,NA,NA,"GeneID:4287","ATXN3",TRUE,"ATXN3"

"14026","ACAGCAGCCTTCTGGAAATA","chr18",75890909,"-","8:T>G,9:G>A","AGG","ACAGCAGCACTCTGGAAATAAGG",2,9,10,NA,"","",FALSE,"ATXN3"

"14027","ACAGCAGCCTTCTGGAAATA","chr5",90113285,"+","2:T>A,9:A>T","AGG","ACTGCAGCCATCTGGAAATAAGG",2,3,10,NA,"","",FALSE,"ATXN3"

"14028","ATCCTCAATTGCACATCAGC","chr14",92096744,"-","none","TGG","ATCCTCAATTGCACATCAGCTGG",0,NA,NA,NA,"GeneID:4287","ATXN3",TRUE,"ATXN3"

"14029","ATCCTGAGCCTCTGATACTC","chr14",92093754,"+","none","TGG","ATCCTGAGCCTCTGATACTCTGG",0,NA,NA,NA,"GeneID:4287","ATXN3",TRUE,"ATXN3"

"14030","ATCCTGAGCCTCTGATACTC","chr8",110555708,"-","11:A>G","TGG","ATCCTGAGCCTTTGATACTCTGG",1,12,NA,NA,"","",TRUE,"ATXN3"

"14031","CTGTCATCCATATTTCCAGA","chr14",92096111,"+","none","AGG","CTGTCATCCATATTTCCAGAAGG",0,NA,NA,NA,"GeneID:4287","ATXN3",TRUE,"ATXN3"

"14032","CTGTCATCCATATTTCCAGA","chr5",40260374,"-","19:G>T","AGG","CTGTCATCCATATTTCCAGCAGG",1,20,NA,NA,"","",FALSE,"ATXN3"

"14033","CTGTCATCCATATTTCCAGA","chr13",103231964,"+","0:A>C,8:A>C","TGG","ATGTCATCAATATTTCCAGATGG",2,1,9,NA,"","",FALSE,"ATXN3"

"14034","CTGTCATCCATATTTCCAGA","chr2",131486560,"-","9:A>T,11:C>T","AGG","CTGTCATCCTTGTTTCCAGAAGG",2,10,12,NA,"GeneID:653784","MZT2A",TRUE,"ATXN3"

"14035","TCAACAGTCCAGAGTATCAG","chr14",92093765,"-","none","AGG","TCAACAGTCCAGAGTATCAGAGG",0,NA,NA,NA,"GeneID:4287","ATXN3",TRUE,"ATXN3"

"14036","TCAACAGTCCAGAGTATCAG","chr8",110555697,"+","4:T>C,19:A>G","AGG","TCAATAGTCCAGAGTATCAAAGG",2,5,20,NA,"","",TRUE,"ATXN3"

"14037","TGCCTGAATAACTTATTGCA","chr14",92096791,"-","none","AGG","TGCCTGAATAACTTATTGCAAGG",0,NA,NA,NA,"GeneID:4287","ATXN3",TRUE,"ATXN3"

"14038","TGCCTGAATAACTTATTGCA","chr15",45530580,"+","0:A>T,14:C>A","TGG","AGCCTGAATAACTTCTTGCATGG",2,1,15,NA,"","",FALSE,"ATXN3"

"14039","ACAGTTCATACCTACACGAA","chrX",13318998,"-","none","AGG","ACAGTTCATACCTACACGAAAGG",0,NA,NA,NA,"GeneID:100093698;GeneID:92552","GS1-600G8.3;ATXN3L",TRUE,"ATXN3L"

"14040","ATCGAGCCAAGAAATTTGCA","chrX",13319471,"+","none","AGG","ATCGAGCCAAGAAATTTGCAAGG",0,NA,NA,NA,"GeneID:100093698;GeneID:92552","GS1-600G8.3;ATXN3L",TRUE,"ATXN3L"

"14041","ATGCCGAGCTTCTGATATTC","chrX",13319623,"+","none","AGG","ATGCCGAGCTTCTGATATTCAGG",0,NA,NA,NA,"GeneID:100093698;GeneID:92552","GS1-600G8.3;ATXN3L",TRUE,"ATXN3L"

"14042","ATTGCTCTCGAACTTGTTGT","chrX",13318972,"+","none","TGG","ATTGCTCTCGAACTTGTTGTTGG",0,NA,NA,NA,"GeneID:100093698;GeneID:92552","GS1-600G8.3;ATXN3L",TRUE,"ATXN3L"

"14043","CTTCGACACTGATGATCTGC","chrX",13319381,"+","none","AGG","CTTCGACACTGATGATCTGCAGG",0,NA,NA,NA,"GeneID:100093698;GeneID:92552","GS1-600G8.3;ATXN3L",TRUE,"ATXN3L"

"14044","TTTCGTGTAGGTATGAACTG","chrX",13318997,"+","none","TGG","TTTCGTGTAGGTATGAACTGTGG",0,NA,NA,NA,"GeneID:100093698;GeneID:92552","GS1-600G8.3;ATXN3L",TRUE,"ATXN3L"

"14045","TTTCGTGTAGGTATGAACTG","chr5",168668033,"+","14:A>G,17:A>C","TGG","TTTCGTGTAGGTATAAAATGTGG",2,15,18,NA,"GeneID:6586","SLIT3",FALSE,"ATXN3L"

"14046","AACGGCCAAAGGCGGCTTGC","chr3",63979935,"-","none","TGG","AACGGCCAAAGGCGGCTTGCTGG",0,NA,NA,NA,"GeneID:6314","ATXN7",TRUE,"ATXN7"

"14047","ATATCTCCTAGGGACAGAAT","chr3",63913145,"+","none","TGG","ATATCTCCTAGGGACAGAATTGG",0,NA,NA,NA,"GeneID:6314","ATXN7",TRUE,"ATXN7"

"14048","CAGGCATTTCAATCACATTA","chr3",63952461,"+","none","TGG","CAGGCATTTCAATCACATTATGG",0,NA,NA,NA,"GeneID:6314","ATXN7",TRUE,"ATXN7"

"14049","CAGGCATTTCAATCACATTA","chr18",26016345,"+","2:A>G,17:A>T","AGG","CAAGCATTTCAATCACAATAAGG",2,3,18,NA,"GeneID:6760","SS18",TRUE,"ATXN7"

"14050","GACAGAGCCCCATGACTTCG","chr3",63913199,"-","none","CGG","GACAGAGCCCCATGACTTCGCGG",0,NA,NA,NA,"GeneID:6314","ATXN7",TRUE,"ATXN7"

"14051","GACAGAGCCCCATGACTTCG","chr18",77468670,"-","11:G>T,14:G>T","TGG","GACAGAGCCCCCTGCCTTCGTGG",2,12,15,NA,"","",FALSE,"ATXN7"

"14052","GGTGTGTAACGACTGTAATC","chr3",63952427,"+","none","AGG","GGTGTGTAACGACTGTAATCAGG",0,NA,NA,NA,"GeneID:6314","ATXN7",TRUE,"ATXN7"

"14053","TGCGCTAAGAACACCTCCAC","chr3",63980037,"-","none","TGG","TGCGCTAAGAACACCTCCACTGG",0,NA,NA,NA,"GeneID:6314","ATXN7",TRUE,"ATXN7"

"14054","AAACCTGTGGCTTGACGACC","chr7",105788618,"+","none","TGG","AAACCTGTGGCTTGACGACCTGG",0,NA,NA,NA,"GeneID:222255","ATXN7L1",TRUE,"ATXN7L1"

"14055","ACTGTCGGAGCAGTGTAATT","chr7",105876375,"+","none","TGG","ACTGTCGGAGCAGTGTAATTTGG",0,NA,NA,NA,"GeneID:222255","ATXN7L1",TRUE,"ATXN7L1"

"14056","CACACAGTCTTTCCTGCGAA","chr7",105665086,"-","none","AGG","CACACAGTCTTTCCTGCGAAAGG",0,NA,NA,NA,"GeneID:222255","ATXN7L1",TRUE,"ATXN7L1"

"14057","GGCGTCGATCCAGGAGGACC","chr7",105876399,"+","none","AGG","GGCGTCGATCCAGGAGGACCAGG",0,NA,NA,NA,"GeneID:222255","ATXN7L1",TRUE,"ATXN7L1"

"14058","TCCACTTACCGCAGTGCGAC","chr7",105788594,"+","none","TGG","TCCACTTACCGCAGTGCGACTGG",0,NA,NA,NA,"GeneID:222255","ATXN7L1",TRUE,"ATXN7L1"

"14059","TTATCCCTTGATCCTTTCGC","chr7",105665071,"+","none","AGG","TTATCCCTTGATCCTTTCGCAGG",0,NA,NA,NA,"GeneID:222255","ATXN7L1",TRUE,"ATXN7L1"

"14060","CTTACCAGAGGTTGTCCTTC","chr1",109487202,"-","none","TGG","CTTACCAGAGGTTGTCCTTCTGG",0,NA,NA,NA,"GeneID:127002","ATXN7L2",TRUE,"ATXN7L2"

"14061","GGAAGGAGTGCGACCTCAAC","chr1",109488382,"+","none","AGG","GGAAGGAGTGCGACCTCAACAGG",0,NA,NA,NA,"GeneID:127002","ATXN7L2",TRUE,"ATXN7L2"

"14062","GGCCCTGCCCATTCACTACA","chr1",109487088,"-","none","TGG","GGCCCTGCCCATTCACTACATGG",0,NA,NA,NA,"GeneID:127002","ATXN7L2",TRUE,"ATXN7L2"

"14063","GGGTTACCTTGCAGGTCAAC","chr1",109488452,"-","none","AGG","GGGTTACCTTGCAGGTCAACAGG",0,NA,NA,NA,"GeneID:127002","ATXN7L2",TRUE,"ATXN7L2"

"14064","GGGTTACCTTGCAGGTCAAC","chr18",45513115,"+","11:A>C,18:T>A","AGG","GGGTTACCTTGAAGGTCATCAGG",2,12,19,NA,"GeneID:8170","SLC14A2",FALSE,"ATXN7L2"

"14065","GTGTAACCACTGCAGCCAAG","chr1",109486557,"+","none","TGG","GTGTAACCACTGCAGCCAAGTGG",0,NA,NA,NA,"GeneID:127002","ATXN7L2",TRUE,"ATXN7L2"

"14066","GTGTAACCACTGCAGCCAAG","chr2",339847,"+","3:G>T,14:T>G","AGG","GTGGAACCACTGCATCCAAGAGG",2,4,15,NA,"","",TRUE,"ATXN7L2"

"14067","TGCCCATGATGACTTCTACT","chr1",109486530,"+","none","TGG","TGCCCATGATGACTTCTACTTGG",0,NA,NA,NA,"GeneID:127002","ATXN7L2",TRUE,"ATXN7L2"

"14068","TGCCCATGATGACTTCTACT","chr3",63952403,"+","0:A>T,12:T>C","TGG","AGCCCATGATGATTTCTACTTGG",2,1,13,NA,"GeneID:6314","ATXN7",TRUE,"ATXN7L2"

"14069","TGCCCATGATGACTTCTACT","chrX",82197868,"+","2:A>C,12:G>C","AGG","TGACCATGATGAGTTCTACTAGG",2,3,13,NA,"","",FALSE,"ATXN7L2"

"14070","CCAAACAAGAATCCTCGACC","chr17",44197681,"+","none","AGG","CCAAACAAGAATCCTCGACCAGG",0,NA,NA,NA,"GeneID:56970","ATXN7L3",TRUE,"ATXN7L3"

"14071","CCAAACAAGAATCCTCGACC","chr12",74538186,"-","16:A>C,18:A>G","AGG","CCAAACAAGAATCCTCTATCAGG",2,17,19,NA,"GeneID:552889","ATXN7L3B",TRUE,"ATXN7L3"

"14072","CCCGAGAAAATAAATCCGTA","chr17",44194775,"+","none","CGG","CCCGAGAAAATAAATCCGTACGG",0,NA,NA,NA,"GeneID:56970","ATXN7L3",TRUE,"ATXN7L3"

"14073","CTCACCGGCGGTTGGCGATT","chr17",44197222,"+","none","CGG","CTCACCGGCGGTTGGCGATTCGG",0,NA,NA,NA,"GeneID:56970","ATXN7L3",TRUE,"ATXN7L3"

"14074","CTCCAAGACGAGTGAAAATC","chr17",44194531,"-","none","AGG","CTCCAAGACGAGTGAAAATCAGG",0,NA,NA,NA,"GeneID:56970","ATXN7L3",TRUE,"ATXN7L3"

"14075","CTCCAAGACGAGTGAAAATC","chrX",66055747,"-","1:G>A,12:G>A","AGG","CCCCAAGACGAGCGAAAATCAGG",2,2,13,NA,"","",TRUE,"ATXN7L3"

"14076","GATATACGCGGACCTGGTCG","chr17",44197696,"-","none","AGG","GATATACGCGGACCTGGTCGAGG",0,NA,NA,NA,"GeneID:56970","ATXN7L3",TRUE,"ATXN7L3"

"14077","GCAGCCGAATCGCCAACCGC","chr17",44197229,"-","none","CGG","GCAGCCGAATCGCCAACCGCCGG",0,NA,NA,NA,"GeneID:56970","ATXN7L3",TRUE,"ATXN7L3"

"14078","CCTCTAGCTTGTTAGTATCC","chr12",74538138,"-","none","AGG","CCTCTAGCTTGTTAGTATCCAGG",0,NA,NA,NA,"GeneID:552889","ATXN7L3B",TRUE,"ATXN7L3B"

"14079","CCTCTAGCTTGTTAGTATCC","chr21",37646391,"+","3:G>C,14:T>G","CGG","CCTGTAGCTTGTTATTATCCCGG",2,4,15,NA,"GeneID:3763","KCNJ6",FALSE,"ATXN7L3B"

"14080","CGCAGAGACTGGTAGCGTGA","chr12",74538261,"+","none","AGG","CGCAGAGACTGGTAGCGTGAAGG",0,NA,NA,NA,"GeneID:552889","ATXN7L3B",TRUE,"ATXN7L3B"

"14081","GATTTACGTAGACCTGATAG","chr12",74538171,"+","none","AGG","GATTTACGTAGACCTGATAGAGG",0,NA,NA,NA,"GeneID:552889","ATXN7L3B",TRUE,"ATXN7L3B"

"14082","TAAAATGGAGGAAATTTCGT","chr12",74538108,"+","none","TGG","TAAAATGGAGGAAATTTCGTTGG",0,NA,NA,NA,"GeneID:552889","ATXN7L3B",TRUE,"ATXN7L3B"

"14083","TACCAGTCTCTGCGAACTCC","chr12",74538255,"-","none","AGG","TACCAGTCTCTGCGAACTCCAGG",0,NA,NA,NA,"GeneID:552889","ATXN7L3B",TRUE,"ATXN7L3B"

"14084","TACCAGTCTCTGCGAACTCC","chr2",108983607,"-","4:A>T,12:C>G","AGG","TACCTGTCTCTGGGAACTCCAGG",2,5,13,NA,"GeneID:10913","EDAR",FALSE,"ATXN7L3B"

"14085","TACGTAAATCTCCTGAGCGA","chr12",74538161,"-","none","TGG","TACGTAAATCTCCTGAGCGATGG",0,NA,NA,NA,"GeneID:552889","ATXN7L3B",TRUE,"ATXN7L3B"

"14086","ATACCAGCACAGAATATCCC","chr9",91355878,"+","none","TGG","ATACCAGCACAGAATATCCCTGG",0,NA,NA,NA,"GeneID:549","AUH",TRUE,"AUH"

"14087","CAGTAAAAATCTTATAAAAA","chr9",91356089,"-","none","TGG","CAGTAAAAATCTTATAAAAATGG",0,NA,NA,NA,"GeneID:549","AUH",TRUE,"AUH"

"14088","CAGTAAAAATCTTATAAAAA","chr14",58832339,"+","5:G>A,9:G>T","AGG","CAGTAGAAAGCTTATAAAAAAGG",2,6,10,NA,"GeneID:102723742","LINC01500",FALSE,"AUH"

"14089","CCATCTATTGCTGCAATTGT","chr9",91298039,"+","none","TGG","CCATCTATTGCTGCAATTGTTGG",0,NA,NA,NA,"GeneID:549","AUH",TRUE,"AUH"

"14090","GACTTCACTCCTGATTATTA","chr9",91355901,"+","none","TGG","GACTTCACTCCTGATTATTATGG",0,NA,NA,NA,"GeneID:549","AUH",TRUE,"AUH"

"14091","GCCTGTGATATACGAGTAGC","chr9",91297985,"-","none","AGG","GCCTGTGATATACGAGTAGCAGG",0,NA,NA,NA,"GeneID:549","AUH",TRUE,"AUH"

"14092","TAACTAGGAATTGTGGTGCT","chr9",91356142,"-","none","TGG","TAACTAGGAATTGTGGTGCTTGG",0,NA,NA,NA,"GeneID:549","AUH",TRUE,"AUH"

"14093","TAACTAGGAATTGTGGTGCT","chrY",21218443,"-","11:C>A,19:T>A","TGG","TAACTAGGAATGGTGGTGCATGG",2,12,20,NA,"","",FALSE,"AUH"

"14094","AAAATGCTAACACTCCTTCC","chr1",25837510,"-","none","TGG","AAAATGCTAACACTCCTTCCTGG",0,NA,NA,NA,"GeneID:79000","AUNIP",TRUE,"AUNIP"

"14095","AAAATGCTAACACTCCTTCC","chr4",12853143,"+","4:A>T,9:T>A","TGG","AAAAAGCTATCACTCCTTCCTGG",2,5,10,NA,"","",FALSE,"AUNIP"

"14096","CAAAGAAGAGCTCCATCTAC","chr1",25837459,"-","none","AGG","CAAAGAAGAGCTCCATCTACAGG",0,NA,NA,NA,"GeneID:79000","AUNIP",TRUE,"AUNIP"

"14097","CGCGTCCAGCCACACGCCGC","chr1",25859306,"+","none","AGG","CGCGTCCAGCCACACGCCGCAGG",0,NA,NA,NA,"GeneID:79000","AUNIP",TRUE,"AUNIP"

"14098","GCCTGATTTGCTCTTTACCC","chr1",25835033,"-","none","AGG","GCCTGATTTGCTCTTTACCCAGG",0,NA,NA,NA,"GeneID:79000","AUNIP",TRUE,"AUNIP"

"14099","TCAGCGGCCATGAGGCGGAC","chr1",25859346,"-","none","AGG","TCAGCGGCCATGAGGCGGACAGG",0,NA,NA,NA,"GeneID:79000","AUNIP",TRUE,"AUNIP"

"14100","TTCTTGTTTGCTAGACCGAA","chr1",25835504,"-","none","AGG","TTCTTGTTTGCTAGACCGAAAGG",0,NA,NA,NA,"GeneID:79000","AUNIP",TRUE,"AUNIP"

"14101","TTCTTGTTTGCTAGACCGAA","chr10",103205170,"+","14:C>A,17:A>G","GGG","TTCTTGTTTGCTAGCCCAAAGGG",2,15,18,NA,"","",FALSE,"AUNIP"

"14102","AACCTGCGAAGGACGCTGTC","chr2",74529358,"+","none","TGG","AACCTGCGAAGGACGCTGTCTGG",0,NA,NA,NA,"GeneID:550;GeneID:27429","AUP1;HTRA2",TRUE,"AUP1"

"14103","AGAGATTCTGTGCTTCCACG","chr2",74528827,"-","none","AGG","AGAGATTCTGTGCTTCCACGAGG",0,NA,NA,NA,"GeneID:550","AUP1",TRUE,"AUP1"

"14104","AGAGATTCTGTGCTTCCACG","chr12",112794983,"+","12:A>C,19:A>G","GGG","AGAGATTCTGTGATTCCACAGGG",2,13,20,NA,"GeneID:22895","RPH3A",FALSE,"AUP1"

"14105","CTGCTGCTCTACGCGCCAGT","chr2",74529448,"-","none","CGG","CTGCTGCTCTACGCGCCAGTCGG",0,NA,NA,NA,"GeneID:550;GeneID:27429","AUP1;HTRA2",TRUE,"AUP1"

"14106","GAGCCCTAGCACCGCACACA","chr2",74529245,"+","none","TGG","GAGCCCTAGCACCGCACACATGG",0,NA,NA,NA,"GeneID:550","AUP1",TRUE,"AUP1"

"14107","GCTAAACCCACCTGAAGCGC","chr2",74528739,"+","none","AGG","GCTAAACCCACCTGAAGCGCAGG",0,NA,NA,NA,"GeneID:550","AUP1",TRUE,"AUP1"

"14108","GTGTCCCCAGATTCGTAGTG","chr2",74529272,"-","none","CGG","GTGTCCCCAGATTCGTAGTGCGG",0,NA,NA,NA,"GeneID:550","AUP1",TRUE,"AUP1"

"14109","GTGTCCCCAGATTCGTAGTG","chr19",6362404,"-","15:C>A,16:C>T","GGG","GTGTCCCCAGATTCGGGGTGGGG",2,16,17,NA,"GeneID:8192","CLPP",TRUE,"AUP1"

"14110","CCATATAGAAAATAATCCTG","chr20",56384312,"-","none","AGG","CCATATAGAAAATAATCCTGAGG",0,NA,NA,NA,"GeneID:6790","AURKA",TRUE,"AURKA"

"14111","CTTTGAAATTGGTCGCCCTC","chr20",56383135,"-","none","TGG","CTTTGAAATTGGTCGCCCTCTGG",0,NA,NA,NA,"GeneID:6790","AURKA",TRUE,"AURKA"

"14112","CTTTGAAATTGGTCGCCCTC","chr1",220267502,"-","1:G>A","CGG","CCTTGAAATTGGTCGCCCTCCGG",1,2,NA,NA,"GeneID:25782","RAB3GAP2",TRUE,"AURKA"

"14113","GCTAGTTTACCAGGTGCCGA","chr20",56386246,"+","none","TGG","GCTAGTTTACCAGGTGCCGATGG",0,NA,NA,NA,"GeneID:6790","AURKA",TRUE,"AURKA"

"14114","TCTGAGCTGATGCTCCACTC","chr20",56383013,"+","none","CGG","TCTGAGCTGATGCTCCACTCCGG",0,NA,NA,NA,"GeneID:6790","AURKA",TRUE,"AURKA"

"14115","TCTGAGCTGATGCTCCACTC","chr1",74630490,"+","9:T>A,11:C>G","TGG","TCTGAGCTGTTCCTCCACTCTGG",2,10,12,NA,"GeneID:127254","ERICH3",FALSE,"AURKA"

"14116","TGAGTCACGAGAACACGTTT","chr20",56386489,"+","none","TGG","TGAGTCACGAGAACACGTTTTGG",0,NA,NA,NA,"GeneID:6790","AURKA",TRUE,"AURKA"

"14117","TGAGTCACGAGAACACGTTT","chr1",220267830,"+","15:T>C,19:C>T","TGG","TGAGTCACGAGAACATGTTCTGG",2,16,20,NA,"GeneID:25782","RAB3GAP2",TRUE,"AURKA"

"14118","TTTGATGCCAGTTCCTCCTC","chr20",56384293,"+","none","AGG","TTTGATGCCAGTTCCTCCTCAGG",0,NA,NA,NA,"GeneID:6790","AURKA",TRUE,"AURKA"

"14119","TTTGATGCCAGTTCCTCCTC","chr1",220267566,"+","none","AGG","TTTGATGCCAGTTCCTCCTCAGG",0,NA,NA,NA,"GeneID:25782","RAB3GAP2",TRUE,"AURKA"

"14120","TTTGATGCCAGTTCCTCCTC","chr6",47359981,"+","3:T>G,16:G>C","TGG","TTTTATGCCAGTTCCTGCTCTGG",2,4,17,NA,"","",FALSE,"AURKA"

"14121","CCTTCAGCCAGATGCGCCTC","chr1",1373862,"+","none","AGG","CCTTCAGCCAGATGCGCCTCAGG",0,NA,NA,NA,"GeneID:54998","AURKAIP1",TRUE,"AURKAIP1"

"14122","CCTTCAGCCAGATGCGCCTC","chr12",76670101,"+","4:G>C,15:A>G","AGG","CCTTGAGCCAGATGCACCTCAGG",2,5,16,NA,"","",FALSE,"AURKAIP1"

"14123","CGGCCGGCGATGTGCTGTAA","chr1",1374367,"+","none","AGG","CGGCCGGCGATGTGCTGTAAAGG",0,NA,NA,NA,"GeneID:54998","AURKAIP1",TRUE,"AURKAIP1"

"14124","CTTCCTACCTGCCCAAGGAA","chr1",1374696,"+","none","CGG","CTTCCTACCTGCCCAAGGAACGG",0,NA,NA,NA,"GeneID:54998","AURKAIP1",TRUE,"AURKAIP1"

"14125","CTTCCTACCTGCCCAAGGAA","chr13",55013093,"-","11:T>G,12:A>G","TGG","CTTCCTACCTGATCAAGGAATGG",2,12,13,NA,"","",FALSE,"AURKAIP1"

"14126","CTTCCTACCTGCCCAAGGAA","chr15",100655032,"-","0:A>G,5:G>A","GGG","TTTCCCACCTGCCCAAGGAAGGG",2,1,6,NA,"","",FALSE,"AURKAIP1"

"14127","CTTCCTACCTGCCCAAGGAA","chr12",92766119,"+","4:T>C,18:G>A","TGG","CTTCTTACCTGCCCAAGGGATGG",2,5,19,NA,"GeneID:440107","PLEKHG7",FALSE,"AURKAIP1"

"14128","GCCAGACTCATTTGCCCCGC","chr1",1373793,"+","none","AGG","GCCAGACTCATTTGCCCCGCAGG",0,NA,NA,NA,"GeneID:54998","AURKAIP1",TRUE,"AURKAIP1"

"14129","GCCCACAGACCATGCTCCTG","chr1",1374747,"-","none","GGG","GCCCACAGACCATGCTCCTGGGG",0,NA,NA,NA,"GeneID:54998","AURKAIP1",TRUE,"AURKAIP1"

"14130","GCCCACAGACCATGCTCCTG","chr12",107508893,"+","1:G>C,8:T>A","GGG","GGCCACAGTCCATGCTCCTGGGG",2,2,9,NA,"GeneID:121551","BTBD11",FALSE,"AURKAIP1"

"14131","GCCCACAGACCATGCTCCTG","chr9",135137472,"-","1:A>G,11:A>T","TGG","GTCCACAGACCTTGCTCCTGTGG",2,2,12,NA,"","",FALSE,"AURKAIP1"

"14132","GCCCCTTTACAGCACATCGC","chr1",1374373,"-","none","CGG","GCCCCTTTACAGCACATCGCCGG",0,NA,NA,NA,"GeneID:54998","AURKAIP1",TRUE,"AURKAIP1"

"14133","ACCCATACTGCAGGTGGGCC","chr17",8207167,"+","none","TGG","ACCCATACTGCAGGTGGGCCTGG",0,NA,NA,NA,"GeneID:9212","AURKB",TRUE,"AURKB"

"14134","ACCCATACTGCAGGTGGGCC","chr12",49654223,"+","2:T>C,17:A>G","CGG","ACTCATACTGCAGGTGGACCCGG",2,3,18,NA,"GeneID:91010","FMNL3",TRUE,"AURKB"

"14135","ATTCTAGAGTATGCCCCCCG","chr17",8206810,"-","none","CGG","ATTCTAGAGTATGCCCCCCGCGG",0,NA,NA,NA,"GeneID:9212","AURKB",TRUE,"AURKB"

"14136","CGCTCCAATGTCCAGCCCAC","chr17",8207739,"-","none","AGG","CGCTCCAATGTCCAGCCCACAGG",0,NA,NA,NA,"GeneID:9212","AURKB",TRUE,"AURKB"

"14137","GCGCAGAGAGATCGAAATCC","chr17",8207188,"-","none","AGG","GCGCAGAGAGATCGAAATCCAGG",0,NA,NA,NA,"GeneID:9212","AURKB",TRUE,"AURKB"

"14138","GGGCACTTACGTTAAGATGT","chr17",8207560,"+","none","CGG","GGGCACTTACGTTAAGATGTCGG",0,NA,NA,NA,"GeneID:9212","AURKB",TRUE,"AURKB"

"14139","TGACGAGCAGCGAACAGCCA","chr17",8206751,"-","none","CGG","TGACGAGCAGCGAACAGCCACGG",0,NA,NA,NA,"GeneID:9212","AURKB",TRUE,"AURKB"

"14140","ACTATTTCCATGATGCACGC","chr19",57232567,"+","none","CGG","ACTATTTCCATGATGCACGCCGG",0,NA,NA,NA,"GeneID:6795","AURKC",TRUE,"AURKC"

"14141","AGATGAACAGCGCACAGCCA","chr19",57232658,"+","none","CGG","AGATGAACAGCGCACAGCCACGG",0,NA,NA,NA,"GeneID:6795","AURKC",TRUE,"AURKC"

"14142","CTCTCACACTCTTCGCCTGC","chr19",57231293,"-","none","AGG","CTCTCACACTCTTCGCCTGCAGG",0,NA,NA,NA,"GeneID:6795","AURKC",TRUE,"AURKC"

"14143","GACTTACAGACGCATCCTCA","chr19",57235036,"+","none","AGG","GACTTACAGACGCATCCTCAAGG",0,NA,NA,NA,"GeneID:6795","AURKC",TRUE,"AURKC"

"14144","TCTCAATTTCCCGGCGCAGC","chr19",57232188,"-","none","TGG","TCTCAATTTCCCGGCGCAGCTGG",0,NA,NA,NA,"GeneID:6795","AURKC",TRUE,"AURKC"

"14145","TCTCAATTTCCCGGCGCAGC","chrX",40832845,"+","11:T>C,18:T>G","TGG","TCTCAATTTCCTGGCGCATCTGG",2,12,19,NA,"","",FALSE,"AURKC"

"14146","TTTCAAAGTCATCGACTGTG","chr19",57232041,"-","none","AGG","TTTCAAAGTCATCGACTGTGAGG",0,NA,NA,NA,"GeneID:6795","AURKC",TRUE,"AURKC"

"14147","CGTTTCTCCACACGTTCCTG","chr7",69899306,"-","none","AGG","CGTTTCTCCACACGTTCCTGAGG",0,NA,NA,NA,"GeneID:26053","AUTS2",TRUE,"AUTS2"

"14148","GAAGCCCTTGGATTCTCCAC","chr7",70118180,"-","none","TGG","GAAGCCCTTGGATTCTCCACTGG",0,NA,NA,NA,"GeneID:26053","AUTS2",TRUE,"AUTS2"

"14149","GAAGTAGCCTGTTCCCAAGC","chr7",70134551,"-","none","TGG","GAAGTAGCCTGTTCCCAAGCTGG",0,NA,NA,NA,"GeneID:26053","AUTS2",TRUE,"AUTS2"

"14150","GAGTTCAGCTCCTTCCAGCT","chr7",70134534,"+","none","TGG","GAGTTCAGCTCCTTCCAGCTTGG",0,NA,NA,NA,"GeneID:26053","AUTS2",TRUE,"AUTS2"

"14151","GAGTTCAGCTCCTTCCAGCT","chr12",112803698,"+","1:T>A,2:C>G","TGG","GTCTTCAGCTCCTTCCAGCTTGG",2,2,3,NA,"GeneID:22895","RPH3A",FALSE,"AUTS2"

"14152","GAGTTCAGCTCCTTCCAGCT","chr3",169820771,"+","5:A>C,19:C>T","AGG","GAGTTAAGCTCCTTCCAGCCAGG",2,6,20,NA,"","",FALSE,"AUTS2"

"14153","GCTTCCACCGGAGCAGCTCT","chr7",70118195,"+","none","CGG","GCTTCCACCGGAGCAGCTCTCGG",0,NA,NA,NA,"GeneID:26053","AUTS2",TRUE,"AUTS2"

"14154","TTTCTCGATCTGAGCTGTAG","chr7",69899416,"-","none","TGG","TTTCTCGATCTGAGCTGTAGTGG",0,NA,NA,NA,"GeneID:26053","AUTS2",TRUE,"AUTS2"

"14155","AAAGAAAGATTGTCTCTAAC","chr15",34003125,"-","none","TGG","AAAGAAAGATTGTCTCTAACTGG",0,NA,NA,NA,"GeneID:57099;GeneID:1133","AVEN;CHRM5",TRUE,"AVEN"

"14156","AGTGTCCTCCTTAGCTCTGC","chr15",34003033,"-","none","AGG","AGTGTCCTCCTTAGCTCTGCAGG",0,NA,NA,NA,"GeneID:57099;GeneID:1133","AVEN;CHRM5",TRUE,"AVEN"

"14157","GATCGCCACAGCGAGCGGCC","chr15",34038960,"-","none","CGG","GATCGCCACAGCGAGCGGCCCGG",0,NA,NA,NA,"GeneID:57099;GeneID:1133","AVEN;CHRM5",TRUE,"AVEN"

"14158","GATCTCCGCCAGGCCGGCCG","chr15",34038976,"+","none","CGG","GATCTCCGCCAGGCCGGCCGCGG",0,NA,NA,NA,"GeneID:57099;GeneID:1133","AVEN;CHRM5",TRUE,"AVEN"

"14159","GGGACTCATTCTCACAGTTC","chr15",33875975,"-","none","CGG","GGGACTCATTCTCACAGTTCCGG",0,NA,NA,NA,"GeneID:57099","AVEN",TRUE,"AVEN"

"14160","TGAAGCTTCTTGTCCAAAAC","chr15",33875926,"-","none","AGG","TGAAGCTTCTTGTCCAAAACAGG",0,NA,NA,NA,"GeneID:57099","AVEN",TRUE,"AVEN"

"14161","TGAAGCTTCTTGTCCAAAAC","chr18",76504966,"-","2:G>T,5:A>G","TGG","TGCAGTTTCTTGTCCAAAACTGG",2,3,6,NA,"","",FALSE,"AVEN"

"14162","TGAAGCTTCTTGTCCAAAAC","chr21",40202628,"+","5:A>C,19:T>C","GGG","TGAAGATTCTTGTCCAAAATGGG",2,6,20,NA,"GeneID:1826","DSCAM",FALSE,"AVEN"

"14163","AGTCCTTCCCGATCCAGAAG","chr12",57813365,"+","none","TGG","AGTCCTTCCCGATCCAGAAGTGG",0,NA,NA,NA,"GeneID:10677","AVIL",TRUE,"AVIL"

"14164","CATCATGGTTCAGTAAGTCC","chr12",57809810,"+","none","TGG","CATCATGGTTCAGTAAGTCCTGG",0,NA,NA,NA,"GeneID:10677","AVIL",TRUE,"AVIL"

"14165","CATCATGGTTCAGTAAGTCC","chr10",93338623,"+","14:T>A,17:A>T","AGG","CATCATGGTTCAGTTAGACCAGG",2,15,18,NA,"GeneID:26509","MYOF",FALSE,"AVIL"

"14166","CCAATACCTACGACGTGAAG","chr12",57811063,"-","none","CGG","CCAATACCTACGACGTGAAGCGG",0,NA,NA,NA,"GeneID:10677","AVIL",TRUE,"AVIL"

"14167","GTCCAGCTGTGTGGTATATA","chr12",57813315,"+","none","TGG","GTCCAGCTGTGTGGTATATATGG",0,NA,NA,NA,"GeneID:10677","AVIL",TRUE,"AVIL"

"14168","TAGCAGCCGCTTCACGTCGT","chr12",57811054,"+","none","AGG","TAGCAGCCGCTTCACGTCGTAGG",0,NA,NA,NA,"GeneID:10677","AVIL",TRUE,"AVIL"

"14169","TCCAGTATCTCAGATTCAGC","chr12",57809875,"-","none","TGG","TCCAGTATCTCAGATTCAGCTGG",0,NA,NA,NA,"GeneID:10677","AVIL",TRUE,"AVIL"

"14170","GAAGGATCCCAAGTATATCT","chr7",32552273,"+","none","TGG","GAAGGATCCCAAGTATATCTTGG",0,NA,NA,NA,"GeneID:23080","AVL9",TRUE,"AVL9"

"14171","GAAGGATCCCAAGTATATCT","chr16",51276313,"-","4:A>C,16:C>T","TGG","GAAGTATCCCAAGTATGTCTTGG",2,5,17,NA,"","",FALSE,"AVL9"

"14172","GACCACCACGATGTGCAGTA","chr7",32495755,"-","none","CGG","GACCACCACGATGTGCAGTACGG",0,NA,NA,NA,"GeneID:23080","AVL9",TRUE,"AVL9"

"14173","GCCCGTACTGCACATCGTGG","chr7",32495750,"+","none","TGG","GCCCGTACTGCACATCGTGGTGG",0,NA,NA,NA,"GeneID:23080","AVL9",TRUE,"AVL9"

"14174","GCTTGAAGTAAACCATACAG","chr7",32551336,"-","none","AGG","GCTTGAAGTAAACCATACAGAGG",0,NA,NA,NA,"GeneID:23080","AVL9",TRUE,"AVL9"

"14175","GTTACTTACCAAGATATACT","chr7",32552284,"-","none","TGG","GTTACTTACCAAGATATACTTGG",0,NA,NA,NA,"GeneID:23080","AVL9",TRUE,"AVL9"

"14176","TTCCCAAATTTCTATTCTAA","chr7",32551401,"+","none","AGG","TTCCCAAATTTCTATTCTAAAGG",0,NA,NA,NA,"GeneID:23080","AVL9",TRUE,"AVL9"

"14177","TTCCCAAATTTCTATTCTAA","chrY",6201174,"+","13:T>A,18:T>A","GGG","TTCCCAAATTTCTTTTCTTAGGG",2,14,19,NA,"","",FALSE,"AVL9"

"14178","TTCCCAAATTTCTATTCTAA","chrX",93087132,"+","13:T>A,18:T>A","GGG","TTCCCAAATTTCTTTTCTTAGGG",2,14,19,NA,"","",FALSE,"AVL9"

"14179","TTCCCAAATTTCTATTCTAA","chr11",10151039,"+","7:C>A,15:G>T","AGG","TTCCCAACTTTCTATGCTAAAGG",2,8,16,NA,"GeneID:81846","SBF2",FALSE,"AVL9"

"14180","TTCCCAAATTTCTATTCTAA","chr4",35890337,"-","9:T>A,19:A>T","GGG","TTCCCAAATATCTATTCTATGGG",2,10,20,NA,"","",FALSE,"AVL9"

"14181","TTCCCAAATTTCTATTCTAA","chr3",142541715,"-","5:G>T,10:C>A","GGG","TTCCCCAATTGCTATTCTAAGGG",2,6,11,NA,"GeneID:545","ATR",FALSE,"AVL9"

"14182","CAAGAGGGCCATGTCCGACC","chr20",3084568,"-","none","TGG","CAAGAGGGCCATGTCCGACCTGG",0,NA,NA,NA,"GeneID:551","AVP",TRUE,"AVP"

"14183","CGTTGCTCCGGTCGCTGGCG","chr20",3082730,"+","none","CGG","CGTTGCTCCGGTCGCTGGCGCGG",0,NA,NA,NA,"GeneID:551","AVP",TRUE,"AVP"

"14184","GCAGTTCTGGAAGTAGCACG","chr20",3084599,"+","none","CGG","GCAGTTCTGGAAGTAGCACGCGG",0,NA,NA,NA,"GeneID:551","AVP",TRUE,"AVP"

"14185","GCAGTTCTGGAAGTAGCACG","chr20",3071710,"-","11:A>T,18:C>G","CGG","GCAGTTCTGGATGTAGCAGGCGG",2,12,19,NA,"GeneID:5020","OXT",TRUE,"AVP"

"14186","GCGCGGGGCTCAGTAGGCGT","chr20",3082620,"+","none","CGG","GCGCGGGGCTCAGTAGGCGTCGG",0,NA,NA,NA,"GeneID:551","AVP",TRUE,"AVP"

"14187","GCGCGGGGCTCAGTAGGCGT","chr20",38727632,"-","9:C>A,13:G>A","TGG","GCGCGGGGCGCAGCAGGCGTTGG",2,10,14,NA,"GeneID:140679","SLC32A1",TRUE,"AVP"

"14188","GTCGTTGCAGCAAACGCCGA","chr20",3082977,"+","none","AGG","GTCGTTGCAGCAAACGCCGAAGG",0,NA,NA,NA,"GeneID:551","AVP",TRUE,"AVP"

"14189","TTCGGCGTTTGCTGCAACGA","chr20",3082978,"-","none","CGG","TTCGGCGTTTGCTGCAACGACGG",0,NA,NA,NA,"GeneID:551","AVP",TRUE,"AVP"

"14190","AGAAAAGTGCCAGGATCCGC","chr10",97677922,"-","none","CGG","AGAAAAGTGCCAGGATCCGCCGG",0,NA,NA,NA,"GeneID:60370","AVPI1",TRUE,"AVPI1"

"14191","CAAGCCCTGTTTCAACGCAG","chr10",97679768,"-","none","CGG","CAAGCCCTGTTTCAACGCAGCGG",0,NA,NA,NA,"GeneID:60370","AVPI1",TRUE,"AVPI1"

"14192","CAAGCCCTGTTTCAACGCAG","chr7",49395420,"-","11:C>A,15:T>G","AGG","CAAGCCCTGTTGCAAAGCAGAGG",2,12,16,NA,"","",FALSE,"AVPI1"

"14193","CAGGCCCCGATTGAGGCCCG","chr10",97679843,"-","none","GGG","CAGGCCCCGATTGAGGCCCGGGG",0,NA,NA,NA,"GeneID:60370","AVPI1",TRUE,"AVPI1"

"14194","CCCTGGATCAGTGTCTGATC","chr10",97677861,"+","none","TGG","CCCTGGATCAGTGTCTGATCTGG",0,NA,NA,NA,"GeneID:60370","AVPI1",TRUE,"AVPI1"

"14195","GATGATCTGTGCCCGTTCCT","chr10",97679731,"+","none","CGG","GATGATCTGTGCCCGTTCCTCGG",0,NA,NA,NA,"GeneID:60370","AVPI1",TRUE,"AVPI1"

"14196","GGTCCCCGCTGCGTTGAAAC","chr10",97679760,"+","none","AGG","GGTCCCCGCTGCGTTGAAACAGG",0,NA,NA,NA,"GeneID:60370","AVPI1",TRUE,"AVPI1"

"14197","CACATACCCGTACTGTTTGT","chr12",63147413,"+","none","TGG","CACATACCCGTACTGTTTGTTGG",0,NA,NA,NA,"GeneID:552","AVPR1A",TRUE,"AVPR1A"

"14198","CCGCTCGCGCCTCATGATCG","chr12",63150322,"-","none","CGG","CCGCTCGCGCCTCATGATCGCGG",0,NA,NA,NA,"GeneID:552","AVPR1A",TRUE,"AVPR1A"

"14199","CGGCATCTTTGTGGCGCCCG","chr12",63150151,"-","none","TGG","CGGCATCTTTGTGGCGCCCGTGG",0,NA,NA,NA,"GeneID:552","AVPR1A",TRUE,"AVPR1A"

"14200","GCACACCGCGATGTAGCGGT","chr12",63150374,"+","none","CGG","GCACACCGCGATGTAGCGGTCGG",0,NA,NA,NA,"GeneID:552","AVPR1A",TRUE,"AVPR1A"

"14201","GGTGTTCGGCATGTTTGCGT","chr12",63150424,"-","none","CGG","GGTGTTCGGCATGTTTGCGTCGG",0,NA,NA,NA,"GeneID:552","AVPR1A",TRUE,"AVPR1A"

"14202","TCGAAGCCCAACAAACAGTA","chr12",63147423,"-","none","CGG","TCGAAGCCCAACAAACAGTACGG",0,NA,NA,NA,"GeneID:552","AVPR1A",TRUE,"AVPR1A"

"14203","GCTGGCGACCGGGGGCAACC","chr1",206116733,"-","none","TGG","GCTGGCGACCGGGGGCAACCTGG",0,NA,NA,NA,"GeneID:553","AVPR1B",TRUE,"AVPR1B"

"14204","GGCCACGGGCCTACCTCACC","chr1",206116287,"-","none","TGG","GGCCACGGGCCTACCTCACCTGG",0,NA,NA,NA,"GeneID:553","AVPR1B",TRUE,"AVPR1B"

"14205","GGGCTAAGTGCAGCACGAAC","chr1",206116658,"+","none","AGG","GGGCTAAGTGCAGCACGAACAGG",0,NA,NA,NA,"GeneID:553","AVPR1B",TRUE,"AVPR1B"

"14206","GTCCAGGTGAGGTAGGCCCG","chr1",206116282,"+","none","TGG","GTCCAGGTGAGGTAGGCCCGTGG",0,NA,NA,NA,"GeneID:553","AVPR1B",TRUE,"AVPR1B"

"14207","GTTGAAGCCCATGTAGATCC","chr1",206110437,"+","none","AGG","GTTGAAGCCCATGTAGATCCAGG",0,NA,NA,NA,"GeneID:553","AVPR1B",TRUE,"AVPR1B"

"14208","GTTGAAGCCCATGTAGATCC","chr6",90316243,"+","0:A>G,15:A>G","AGG","ATTGAAGCCCATGTAAATCCAGG",2,1,16,NA,"","",FALSE,"AVPR1B"

"14209","GTTGAAGCCCATGTAGATCC","chr10",98510421,"-","4:G>T,13:G>A","TGG","GTTGCAGCCCATGCAGATCCTGG",2,5,14,NA,"GeneID:60495","HPSE2",FALSE,"AVPR1B"

"14210","TCAACAGCCACCTGTTACCG","chr1",206110422,"-","none","CGG","TCAACAGCCACCTGTTACCGCGG",0,NA,NA,NA,"GeneID:553","AVPR1B",TRUE,"AVPR1B"

"14211","AAGGCCACCGACCGCTTCCG","chrX",153905803,"+","none","TGG","AAGGCCACCGACCGCTTCCGTGG",0,NA,NA,NA,"GeneID:554","AVPR2",TRUE,"AVPR2"

"14212","CGCCGCAGGGGACGCCGGAC","chrX",153906244,"+","none","AGG","CGCCGCAGGGGACGCCGGACAGG",0,NA,NA,NA,"GeneID:554","AVPR2",TRUE,"AVPR2"

"14213","CTCATGGCGTCCACCACTTC","chrX",153905148,"+","none","CGG","CTCATGGCGTCCACCACTTCCGG",0,NA,NA,NA,"GeneID:554","AVPR2",TRUE,"AVPR2"

"14214","GCAAGCCTTACCGGAAGTGG","chrX",153905161,"-","none","TGG","GCAAGCCTTACCGGAAGTGGTGG",0,NA,NA,NA,"GeneID:554","AVPR2",TRUE,"AVPR2"

"14215","GCTGAAAGATGCATAGATCC","chrX",153906579,"-","none","AGG","GCTGAAAGATGCATAGATCCAGG",0,NA,NA,NA,"GeneID:554","AVPR2",TRUE,"AVPR2"

"14216","TCCTCACGATGAAGTGTCCT","chrX",153906711,"-","none","TGG","TCCTCACGATGAAGTGTCCTTGG",0,NA,NA,NA,"GeneID:554","AVPR2",TRUE,"AVPR2"

"14217","TCCTCACGATGAAGTGTCCT","chr1",11390682,"+","7:C>G,16:C>T","GGG","TCCTCACCATGAAGTGCCCTGGG",2,8,17,NA,"","",FALSE,"AVPR2"

"14218","AACAGCAGGTAGACGAACAA","chrX",70235732,"-","none","TGG","AACAGCAGGTAGACGAACAATGG",0,NA,NA,NA,"GeneID:158833","AWAT1",TRUE,"AWAT1"

"14219","ACCTGAGCACAACTACCTCA","chrX",70237066,"+","none","TGG","ACCTGAGCACAACTACCTCATGG",0,NA,NA,NA,"GeneID:158833","AWAT1",TRUE,"AWAT1"

"14220","CAACCTCGTGGGCATTGTAG","chrX",70238266,"+","none","TGG","CAACCTCGTGGGCATTGTAGTGG",0,NA,NA,NA,"GeneID:158833","AWAT1",TRUE,"AWAT1"

"14221","CAACCTCGTGGGCATTGTAG","chr14",101152551,"+","10:A>G,14:A>T","GGG","CAACCTCGTGAGCAATGTAGGGG",2,11,15,NA,"","",FALSE,"AWAT1"

"14222","CCCATGAGGTAGTTGTGCTC","chrX",70237070,"-","none","AGG","CCCATGAGGTAGTTGTGCTCAGG",0,NA,NA,NA,"GeneID:158833","AWAT1",TRUE,"AWAT1"

"14223","GACACACCAGTTCCTTACCC","chrX",70236086,"-","none","AGG","GACACACCAGTTCCTTACCCAGG",0,NA,NA,NA,"GeneID:158833","AWAT1",TRUE,"AWAT1"

"14224","GTGAGTCTTACCTCGCTCTG","chrX",70235814,"-","none","GGG","GTGAGTCTTACCTCGCTCTGGGG",0,NA,NA,NA,"GeneID:158833","AWAT1",TRUE,"AWAT1"

"14225","AGGATGCTTACCTCGCTGAG","chrX",70044340,"+","none","GGG","AGGATGCTTACCTCGCTGAGGGG",0,NA,NA,NA,"GeneID:158835","AWAT2",TRUE,"AWAT2"

"14226","AGGATGCTTACCTCGCTGAG","chr1",59402896,"+","1:A>G,14:C>G","AGG","AAGATGCTTACCTCCCTGAGAGG",2,2,15,NA,"GeneID:55277","FGGY",FALSE,"AWAT2"

"14227","CACAGGCAACATGGTCATTG","chrX",70043171,"-","none","TGG","CACAGGCAACATGGTCATTGTGG",0,NA,NA,NA,"GeneID:158835","AWAT2",TRUE,"AWAT2"

"14228","GACTGGAAGACCCCTCAGCG","chrX",70044353,"-","none","AGG","GACTGGAAGACCCCTCAGCGAGG",0,NA,NA,NA,"GeneID:158835","AWAT2",TRUE,"AWAT2"

"14229","GACTGGAAGACCCCTCAGCG","chr13",52509127,"+","14:A>T,19:A>G","TGG","GACTGGAAGACCCCACAGCATGG",2,15,20,NA,"","",FALSE,"AWAT2"

"14230","GACTGGAAGACCCCTCAGCG","chrX",70235801,"+","14:A>T,15:G>C","AGG","GACTGGAAGACCCCAGAGCGAGG",2,15,16,NA,"GeneID:158833","AWAT1",TRUE,"AWAT2"

"14231","GCCGCCGGTTTACCTGTGTG","chrX",70043973,"-","none","AGG","GCCGCCGGTTTACCTGTGTGAGG",0,NA,NA,NA,"GeneID:158835","AWAT2",TRUE,"AWAT2"

"14232","GCTCCCAGTGTGAGTATGTA","chrX",70043521,"+","none","AGG","GCTCCCAGTGTGAGTATGTAAGG",0,NA,NA,NA,"GeneID:158835","AWAT2",TRUE,"AWAT2"

"14233","GGCAGACGAGGATGTAGTTG","chrX",70043630,"+","none","CGG","GGCAGACGAGGATGTAGTTGCGG",0,NA,NA,NA,"GeneID:158835","AWAT2",TRUE,"AWAT2"

"14234","CGTTCATTACAGTCACATGA","chr1",179378715,"+","none","TGG","CGTTCATTACAGTCACATGATGG",0,NA,NA,NA,"GeneID:126859","AXDND1",TRUE,"AXDND1"

"14235","GACACAATATGAAATTCTTC","chr1",179379426,"-","none","AGG","GACACAATATGAAATTCTTCAGG",0,NA,NA,NA,"GeneID:126859","AXDND1",TRUE,"AXDND1"

"14236","GACACAATATGAAATTCTTC","chr2",12407164,"-","6:G>T,8:A>T","AGG","GACACACTTTGAAATTCTTCAGG",2,7,9,NA,"GeneID:100506457","LOC100506457",FALSE,"AXDND1"

"14237","GACACAATATGAAATTCTTC","chr10",131125435,"+","8:G>A,18:G>T","AGG","GACACAATGTGAAATTCTGCAGG",2,9,19,NA,"GeneID:256536","TCERG1L",FALSE,"AXDND1"

"14238","GACACAATATGAAATTCTTC","chr6",99984102,"-","4:T>G,15:T>A","TGG","GACAAAATATGAAATACTTCTGG",2,5,16,NA,"GeneID:84539","MCHR2",FALSE,"AXDND1"

"14239","GGCAGTTTGTCCCCCACATT","chr1",179378690,"+","none","TGG","GGCAGTTTGTCCCCCACATTTGG",0,NA,NA,NA,"GeneID:126859","AXDND1",TRUE,"AXDND1"

"14240","GGCAGTTTGTCCCCCACATT","chr1",229399524,"+","9:G>T,11:T>C","TGG","GGCAGTTTGGCTCCCACATTTGG",2,10,12,NA,"","",FALSE,"AXDND1"

"14241","GGCAGTTTGTCCCCCACATT","chr18",6369167,"+","6:G>T,19:A>T","CGG","GGCAGTGTGTCCCCCACATACGG",2,7,20,NA,"GeneID:91133","L3MBTL4",FALSE,"AXDND1"

"14242","TACGATGTAACATATGCCAA","chr1",179378652,"+","none","AGG","TACGATGTAACATATGCCAAAGG",0,NA,NA,NA,"GeneID:126859","AXDND1",TRUE,"AXDND1"

"14243","TACGATGTAACATATGCCAA","chr12",70248508,"-","2:A>G,13:G>T","TGG","TATGATGTAACATCTGCCAATGG",2,3,14,NA,"GeneID:4848","CNOT2",FALSE,"AXDND1"

"14244","TCCTCACAGATAGTGAAAAC","chr1",179382713,"+","none","AGG","TCCTCACAGATAGTGAAAACAGG",0,NA,NA,NA,"GeneID:126859","AXDND1",TRUE,"AXDND1"

"14245","TCCTCACAGATAGTGAAAAC","chr1",108554863,"-","12:T>C,15:C>T","AGG","TCCTCACAGATAATGGAAACAGG",2,13,16,NA,"","",FALSE,"AXDND1"

"14246","TTTCATATTGTGTCAAGTAC","chr1",179379432,"+","none","AGG","TTTCATATTGTGTCAAGTACAGG",0,NA,NA,NA,"GeneID:126859","AXDND1",TRUE,"AXDND1"

"14247","CCCCAAGGTCTTCGGGGACG","chr16",293705,"-","none","AGG","CCCCAAGGTCTTCGGGGACGAGG",0,NA,NA,NA,"GeneID:8312","AXIN1",TRUE,"AXIN1"

"14248","CCGGTACTTACGGGAATGTG","chr16",309961,"+","none","AGG","CCGGTACTTACGGGAATGTGAGG",0,NA,NA,NA,"GeneID:8312","AXIN1",TRUE,"AXIN1"

"14249","CTGCTCGCTGTCGTTGGCAC","chr16",314592,"+","none","TGG","CTGCTCGCTGTCGTTGGCACTGG",0,NA,NA,NA,"GeneID:8312","AXIN1",TRUE,"AXIN1"

"14250","GGAGGCGCGCCGACGTCTGG","chr16",293530,"-","none","AGG","GGAGGCGCGCCGACGTCTGGAGG",0,NA,NA,NA,"GeneID:8312","AXIN1",TRUE,"AXIN1"

"14251","GGGATGGGATCCCCCCATAC","chr16",310050,"-","none","AGG","GGGATGGGATCCCCCCATACAGG",0,NA,NA,NA,"GeneID:8312","AXIN1",TRUE,"AXIN1"

"14252","GGGTGTCTGCATCGCTGGAC","chr16",314564,"+","none","AGG","GGGTGTCTGCATCGCTGGACAGG",0,NA,NA,NA,"GeneID:8312","AXIN1",TRUE,"AXIN1"

"14253","CAACCCATCTTCGTTCCGCC","chr17",65558434,"+","none","TGG","CAACCCATCTTCGTTCCGCCTGG",0,NA,NA,NA,"GeneID:8313","AXIN2",TRUE,"AXIN2"

"14254","CACCAACATAGCGCTACTCA","chr17",65558599,"+","none","TGG","CACCAACATAGCGCTACTCATGG",0,NA,NA,NA,"GeneID:8313","AXIN2",TRUE,"AXIN2"

"14255","GATATCCAGTGATGCGCTGA","chr17",65549553,"-","none","CGG","GATATCCAGTGATGCGCTGACGG",0,NA,NA,NA,"GeneID:8313","AXIN2",TRUE,"AXIN2"

"14256","GGGCTATAGCGGCCTACGCC","chr17",65537650,"+","none","TGG","GGGCTATAGCGGCCTACGCCTGG",0,NA,NA,NA,"GeneID:8313","AXIN2",TRUE,"AXIN2"

"14257","TATCTCACTGTCGTTGGCGC","chr17",65549569,"+","none","TGG","TATCTCACTGTCGTTGGCGCTGG",0,NA,NA,NA,"GeneID:8313","AXIN2",TRUE,"AXIN2"

"14258","TGATCGTCCAGTATCGTCTG","chr17",65537710,"+","none","CGG","TGATCGTCCAGTATCGTCTGCGG",0,NA,NA,NA,"GeneID:8313","AXIN2",TRUE,"AXIN2"

"14259","CAGAGCCCGTGGACCTACTC","chr19",41221966,"+","none","TGG","CAGAGCCCGTGGACCTACTCTGG",0,NA,NA,NA,"GeneID:558","AXL",TRUE,"AXL"

"14260","CAGAGCCCGTGGACCTACTC","chr5",36814344,"+","7:T>C,14:A>C","TGG","CAGAGCCTGTGGACATACTCTGG",2,8,15,NA,"","",FALSE,"AXL"

"14261","CGGGCACCTGTGATATTCCC","chr19",41220673,"-","none","TGG","CGGGCACCTGTGATATTCCCTGG",0,NA,NA,NA,"GeneID:558","AXL",TRUE,"AXL"

"14262","CTGCGAAGCCCATAACGCCA","chr19",41230991,"+","none","AGG","CTGCGAAGCCCATAACGCCAAGG",0,NA,NA,NA,"GeneID:558","AXL",TRUE,"AXL"

"14263","GAGAGCCCCCCGAGGTACAT","chr19",41220734,"+","none","TGG","GAGAGCCCCCCGAGGTACATTGG",0,NA,NA,NA,"GeneID:558","AXL",TRUE,"AXL"

"14264","GAGAGCCCCCCGAGGTACAT","chr4",150254100,"-","1:G>T,12:C>T","GGG","GCGAGCCCCCCGGGGTACATGGG",2,2,13,NA,"GeneID:166614","DCLK2",FALSE,"AXL"

"14265","GCGGGATGTGGTGACCCCCT","chr19",41231011,"-","none","TGG","GCGGGATGTGGTGACCCCCTTGG",0,NA,NA,NA,"GeneID:558","AXL",TRUE,"AXL"

"14266","GCTAGGCTTGCCTTACTTCC","chr19",41221874,"+","none","TGG","GCTAGGCTTGCCTTACTTCCTGG",0,NA,NA,NA,"GeneID:558","AXL",TRUE,"AXL"

"14267","AATACTCCACGATGTCTTTC","chr7",99971760,"+","none","AGG","AATACTCCACGATGTCTTTCAGG",0,NA,NA,NA,"GeneID:563","AZGP1",TRUE,"AZGP1"

"14268","ACTCACCATCTTGGTTCTCC","chr7",99975938,"+","none","TGG","ACTCACCATCTTGGTTCTCCTGG",0,NA,NA,NA,"GeneID:563","AZGP1",TRUE,"AZGP1"

"14269","ACTCACCATCTTGGTTCTCC","chr7",99980834,"-","11:G>A,14:C>A","TGG","ACTCACCATCTCGGGTCTCCTGG",2,12,15,NA,"GeneID:646282","AZGP1P1",TRUE,"AZGP1"

"14270","ACTCACCATCTTGGTTCTCC","chr3",14406417,"-","10:T>A,11:C>A","TGG","ACTCACCATCAGGGTTCTCCTGG",2,11,12,NA,"GeneID:6533","SLC6A6",FALSE,"AZGP1"

"14271","ACTCACCATCTTGGTTCTCC","chr7",101289682,"+","14:G>T","TGG","ACTCACCATCTTGGGTCTCCTGG",1,15,NA,NA,"","",TRUE,"AZGP1"

"14272","ACTCACCATCTTGGTTCTCC","chr4",77401339,"+","0:T>A,11:A>T","TGG","TCTCACCATCTAGGTTCTCCTGG",2,1,12,NA,"GeneID:901","CCNG2",FALSE,"AZGP1"

"14273","AGGTATTTCCGCAGAGTCGC","chr7",99968186,"+","none","AGG","AGGTATTTCCGCAGAGTCGCAGG",0,NA,NA,NA,"GeneID:563","AZGP1",TRUE,"AZGP1"

"14274","AGGTATTTCCGCAGAGTCGC","chr7",99985996,"-","17:A>G","AGG","AGGTATTTCCGCAGAGTTGCAGG",1,18,NA,NA,"GeneID:646282","AZGP1P1",TRUE,"AZGP1"

"14275","GTCCCCCAGGAGAACCAAGA","chr7",99975946,"-","none","TGG","GTCCCCCAGGAGAACCAAGATGG",0,NA,NA,NA,"GeneID:563","AZGP1",TRUE,"AZGP1"

"14276","GTCCCCCAGGAGAACCAAGA","chr7",101289690,"-","0:T>C,13:G>T","TGG","ATCCCCCAGGAGACCCAAGATGG",2,1,14,NA,"","",TRUE,"AZGP1"

"14277","TGAAGACGTCCCCGCGTTTC","chr7",99971940,"-","none","AGG","TGAAGACGTCCCCGCGTTTCAGG",0,NA,NA,NA,"GeneID:563","AZGP1",TRUE,"AZGP1"

"14278","TTCAGGGTCTCACGTATTGC","chr7",99968415,"-","none","AGG","TTCAGGGTCTCACGTATTGCAGG",0,NA,NA,NA,"GeneID:563","AZGP1",TRUE,"AZGP1"

"14279","AAGATCCAACAGCACCACGC","chr17",81208958,"-","none","AGG","AAGATCCAACAGCACCACGCAGG",0,NA,NA,NA,"GeneID:22994","CEP131",TRUE,"AZI1"

"14280","CCACCTTACCAGGACGTTCC","chr17",81207115,"+","none","AGG","CCACCTTACCAGGACGTTCCAGG",0,NA,NA,NA,"GeneID:22994","CEP131",TRUE,"AZI1"

"14281","GCGGAGGAAAGAATGCACCG","chr17",81206777,"-","none","TGG","GCGGAGGAAAGAATGCACCGTGG",0,NA,NA,NA,"GeneID:22994","CEP131",TRUE,"AZI1"

"14282","GGCTGCCCTCGAAGAGCATC","chr17",81207195,"+","none","AGG","GGCTGCCCTCGAAGAGCATCAGG",0,NA,NA,NA,"GeneID:22994","CEP131",TRUE,"AZI1"

"14283","TACCTGGGGGAGCCGCTCCG","chr17",81208924,"+","none","AGG","TACCTGGGGGAGCCGCTCCGAGG",0,NA,NA,NA,"GeneID:22994","CEP131",TRUE,"AZI1"

"14284","TCACCTTGCCATCCAATGCC","chr17",81206832,"-","none","CGG","TCACCTTGCCATCCAATGCCCGG",0,NA,NA,NA,"GeneID:22994","CEP131",TRUE,"AZI1"

"14285","AACCTCTCGATATGCATGAT","chr3",28338534,"+","none","AGG","AACCTCTCGATATGCATGATAGG",0,NA,NA,NA,"GeneID:64343","AZI2",TRUE,"AZI2"

"14286","AAGTAGAACTCCTCCAGCTG","chr3",28337961,"-","none","AGG","AAGTAGAACTCCTCCAGCTGAGG",0,NA,NA,NA,"GeneID:64343","AZI2",TRUE,"AZI2"

"14287","AAGTAGAACTCCTCCAGCTG","chr13",20424923,"+","1:T>A,9:C>T","AGG","ATGTAGAACCCCTCCAGCTGAGG",2,2,10,NA,"GeneID:51084","CRYL1",FALSE,"AZI2"

"14288","CAAATGACAGGATCCATATC","chr3",28332417,"-","none","AGG","CAAATGACAGGATCCATATCAGG",0,NA,NA,NA,"GeneID:64343","AZI2",TRUE,"AZI2"

"14289","CCAGCTCTTGTTCCAAACCA","chr3",28336795,"+","none","TGG","CCAGCTCTTGTTCCAAACCATGG",0,NA,NA,NA,"GeneID:64343","AZI2",TRUE,"AZI2"

"14290","CCAGCTCTTGTTCCAAACCA","chr9",112773039,"+","3:C>G,17:G>C","AGG","CCACCTCTTGTTCCAAAGCAAGG",2,4,18,NA,"GeneID:401548","SNX30",FALSE,"AZI2"

"14291","CCAGCTCTTGTTCCAAACCA","chr15",44920173,"-","0:A>G,19:A>T","TGG","TCAGCTCTTGTTCCAAACCTTGG",2,1,20,NA,"","",TRUE,"AZI2"

"14292","GGCCTATCATGCATATCGAG","chr3",28338539,"-","none","AGG","GGCCTATCATGCATATCGAGAGG",0,NA,NA,NA,"GeneID:64343","AZI2",TRUE,"AZI2"

"14293","TTCAGATTGTCTTCCTGATA","chr3",28332401,"+","none","TGG","TTCAGATTGTCTTCCTGATATGG",0,NA,NA,NA,"GeneID:64343","AZI2",TRUE,"AZI2"

"14294","TTCAGATTGTCTTCCTGATA","chr3",167991894,"+","3:T>A,15:A>T","GGG","TTCTGATTGTCTTCCAGATAGGG",2,4,16,NA,"","",FALSE,"AZI2"

"14295","ATCTGAGACACTTGCTTGCA","chr8",102838833,"+","none","AGG","ATCTGAGACACTTGCTTGCAAGG",0,NA,NA,NA,"GeneID:51582","AZIN1",TRUE,"AZIN1"

"14296","CAATTTCATTGTCACATGTC","chr8",102838780,"+","none","AGG","CAATTTCATTGTCACATGTCAGG",0,NA,NA,NA,"GeneID:51582","AZIN1",TRUE,"AZIN1"

"14297","CTCACTTAACCCCAATTATT","chr8",102836250,"+","none","TGG","CTCACTTAACCCCAATTATTTGG",0,NA,NA,NA,"GeneID:51582","AZIN1",TRUE,"AZIN1"

"14298","GAAGAACTGTAGGCATCTCT","chr8",102836298,"-","none","TGG","GAAGAACTGTAGGCATCTCTTGG",0,NA,NA,NA,"GeneID:51582","AZIN1",TRUE,"AZIN1"

"14299","GCCAAAATCTCAAGTACAGC","chr8",102839683,"+","none","TGG","GCCAAAATCTCAAGTACAGCTGG",0,NA,NA,NA,"GeneID:51582","AZIN1",TRUE,"AZIN1"

"14300","TTTACTGGAACAAGCAAATC","chr8",102839649,"+","none","CGG","TTTACTGGAACAAGCAAATCCGG",0,NA,NA,NA,"GeneID:51582","AZIN1",TRUE,"AZIN1"

"14301","TTTACTGGAACAAGCAAATC","chr7",119324690,"-","0:T>A,10:T>G","AGG","ATTACTGGAAAAAGCAAATCAGG",2,1,11,NA,"","",FALSE,"AZIN1"

"14302","CGCCCCAACAACGTGTGCAC","chr19",830887,"+","none","CGG","CGCCCCAACAACGTGTGCACCGG",0,NA,NA,NA,"GeneID:566","AZU1",TRUE,"AZU1"

"14303","CGGTCATCACGAAGCGGGCA","chr19",828344,"-","none","TGG","CGGTCATCACGAAGCGGGCATGG",0,NA,NA,NA,"GeneID:566","AZU1",TRUE,"AZU1"

"14304","GGGCACCCCCCTCGTCTGCG","chr19",831723,"+","none","AGG","GGGCACCCCCCTCGTCTGCGAGG",0,NA,NA,NA,"GeneID:566","AZU1",TRUE,"AZU1"

"14305","TGCCCGCTTCGTGATGACCG","chr19",828344,"+","none","CGG","TGCCCGCTTCGTGATGACCGCGG",0,NA,NA,NA,"GeneID:566","AZU1",TRUE,"AZU1"

"14306","TGGCAGTATCGTCACGCTGC","chr19",830732,"-","none","TGG","TGGCAGTATCGTCACGCTGCTGG",0,NA,NA,NA,"GeneID:566","AZU1",TRUE,"AZU1"

"14307","TGTTCTCAACAACCCGGGAC","chr19",831843,"+","none","CGG","TGTTCTCAACAACCCGGGACCGG",0,NA,NA,NA,"GeneID:566","AZU1",TRUE,"AZU1"

"14308","ACCCAGACACATAGCAATTC","chr15",44715483,"-","none","AGG","ACCCAGACACATAGCAATTCAGG",0,NA,NA,NA,"GeneID:567","B2M",TRUE,"B2M"

"14309","ACTCACGCTGGATAGCCTCC","chr15",44711599,"-","none","AGG","ACTCACGCTGGATAGCCTCCAGG",0,NA,NA,NA,"GeneID:567","B2M",TRUE,"B2M"

"14310","CAGTAAGTCAACTTCAATGT","chr15",44715515,"-","none","CGG","CAGTAAGTCAACTTCAATGTCGG",0,NA,NA,NA,"GeneID:567","B2M",TRUE,"B2M"

"14311","CGTGAGTAAACCTGAATCTT","chr15",44715430,"-","none","TGG","CGTGAGTAAACCTGAATCTTTGG",0,NA,NA,NA,"GeneID:567","B2M",TRUE,"B2M"

"14312","CGTGAGTAAACCTGAATCTT","chr9",72461015,"-","1:G>C,19:C>A","AGG","CCTGAGTAAACCTGAATCTGAGG",2,2,20,NA,"","",FALSE,"B2M"

"14313","GAGTAGCGCGAGCACAGCTA","chr15",44711565,"-","none","AGG","GAGTAGCGCGAGCACAGCTAAGG",0,NA,NA,NA,"GeneID:567","B2M",TRUE,"B2M"

"14314","GGCCGAGATGTCTCGCTCCG","chr15",44711539,"+","none","TGG","GGCCGAGATGTCTCGCTCCGTGG",0,NA,NA,NA,"GeneID:567","B2M",TRUE,"B2M"

"14315","AAGAAGGTGTTCATCCTCTA","chr3",161086337,"+","none","AGG","AAGAAGGTGTTCATCCTCTAAGG",0,NA,NA,NA,"GeneID:8706","B3GALNT1",TRUE,"B3GALNT1"

"14316","ATGTGATAGAACGCGTGAAC","chr3",161086610,"-","none","TGG","ATGTGATAGAACGCGTGAACTGG",0,NA,NA,NA,"GeneID:8706","B3GALNT1",TRUE,"B3GALNT1"

"14317","CCAAAAAGTGATGATCTCCT","chr3",161085794,"+","none","TGG","CCAAAAAGTGATGATCTCCTTGG",0,NA,NA,NA,"GeneID:8706","B3GALNT1",TRUE,"B3GALNT1"

"14318","CCAAAAAGTGATGATCTCCT","chr1",179222072,"-","1:A>G,12:G>C","TGG","CTAAAAAGTGATCATCTCCTTGG",2,2,13,NA,"GeneID:27","ABL2",TRUE,"B3GALNT1"

"14319","CGCGTTCTATCACATTGTAG","chr3",161086615,"+","none","TGG","CGCGTTCTATCACATTGTAGTGG",0,NA,NA,NA,"GeneID:8706","B3GALNT1",TRUE,"B3GALNT1"

"14320","GTCTGTCTTCATTACGTACT","chr3",161086214,"+","none","TGG","GTCTGTCTTCATTACGTACTTGG",0,NA,NA,NA,"GeneID:8706","B3GALNT1",TRUE,"B3GALNT1"

"14321","GTCTGTCTTCATTACGTACT","chr1",202711745,"+","3:A>T,11:A>T","AGG","GTCAGTCTTCAATACGTACTAGG",2,4,12,NA,"","",FALSE,"B3GALNT1"

"14322","TGTGGATGGCCTCGGCTCTC","chr3",161086739,"-","none","TGG","TGTGGATGGCCTCGGCTCTCTGG",0,NA,NA,NA,"GeneID:8706","B3GALNT1",TRUE,"B3GALNT1"

"14323","TGTGGATGGCCTCGGCTCTC","chr1",2075735,"-","9:C>G,12:C>G","AGG","TGTGGATGGGCTGGGCTCTCAGG",2,10,13,NA,"GeneID:5590","PRKCZ",FALSE,"B3GALNT1"

"14324","AACCTACCGTTGACTTAATG","chr1",235494673,"+","none","TGG","AACCTACCGTTGACTTAATGTGG",0,NA,NA,NA,"GeneID:148789","B3GALNT2",TRUE,"B3GALNT2"

"14325","ACGCTGACAACTCGATCCTC","chr1",235484436,"+","none","AGG","ACGCTGACAACTCGATCCTCAGG",0,NA,NA,NA,"GeneID:148789","B3GALNT2",TRUE,"B3GALNT2"

"14326","CAGCCCCGATGAAGTGTCTT","chr1",235484459,"+","none","CGG","CAGCCCCGATGAAGTGTCTTCGG",0,NA,NA,NA,"GeneID:148789","B3GALNT2",TRUE,"B3GALNT2"

"14327","CCGCAGCCAGAGGTGCAGCG","chr1",235504186,"+","none","CGG","CCGCAGCCAGAGGTGCAGCGCGG",0,NA,NA,NA,"GeneID:148789","B3GALNT2",TRUE,"B3GALNT2"

"14328","CCGCAGCCAGAGGTGCAGCG","chr9",124817549,"+","2:C>G,14:C>G","TGG","CCCCAGCCAGAGGTCCAGCGTGG",2,3,15,NA,"","",FALSE,"B3GALNT2"

"14329","GGGGGCGGCCAATGCGAAAC","chr1",235504243,"-","none","TGG","GGGGGCGGCCAATGCGAAACTGG",0,NA,NA,NA,"GeneID:148789","B3GALNT2",TRUE,"B3GALNT2"

"14330","TTATCACGTTTCGAAGTTCA","chr1",235494729,"+","none","TGG","TTATCACGTTTCGAAGTTCATGG",0,NA,NA,NA,"GeneID:148789","B3GALNT2",TRUE,"B3GALNT2"

"14331","AATCTTCCATGATATCATCG","chr2",167869443,"+","none","TGG","AATCTTCCATGATATCATCGTGG",0,NA,NA,NA,"GeneID:8708","B3GALT1",TRUE,"B3GALT1"

"14332","GTCCCACATATACGTCTTCA","chr2",167869827,"-","none","AGG","GTCCCACATATACGTCTTCAAGG",0,NA,NA,NA,"GeneID:8708","B3GALT1",TRUE,"B3GALT1"

"14333","TAGCCAGTCCCCGAACAGAA","chr2",167869741,"-","none","AGG","TAGCCAGTCCCCGAACAGAAAGG",0,NA,NA,NA,"GeneID:8708","B3GALT1",TRUE,"B3GALT1"

"14334","TCACCTTGAAGACGTATATG","chr2",167869821,"+","none","TGG","TCACCTTGAAGACGTATATGTGG",0,NA,NA,NA,"GeneID:8708","B3GALT1",TRUE,"B3GALT1"

"14335","TTACTGCGGACATCCCGAAT","chr2",167869675,"-","none","CGG","TTACTGCGGACATCCCGAATCGG",0,NA,NA,NA,"GeneID:8708","B3GALT1",TRUE,"B3GALT1"

"14336","TTCGGGATGTCCGCAGTAAG","chr2",167869676,"+","none","TGG","TTCGGGATGTCCGCAGTAAGTGG",0,NA,NA,NA,"GeneID:8708","B3GALT1",TRUE,"B3GALT1"

"14337","ATGAGTTTGTGTTCAATCAC","chr1",193180464,"-","none","TGG","ATGAGTTTGTGTTCAATCACTGG",0,NA,NA,NA,"GeneID:79577;GeneID:8707","CDC73;B3GALT2",TRUE,"B3GALT2"

"14338","ATTCTTGTGATTTGAATACC","chr1",193180999,"+","none","AGG","ATTCTTGTGATTTGAATACCAGG",0,NA,NA,NA,"GeneID:79577;GeneID:8707","CDC73;B3GALT2",TRUE,"B3GALT2"

"14339","CCTGTGACATACACTTTCCG","chr1",193181368,"-","none","AGG","CCTGTGACATACACTTTCCGAGG",0,NA,NA,NA,"GeneID:79577;GeneID:8707","CDC73;B3GALT2",TRUE,"B3GALT2"

"14340","CTACCTTCAACGTGCAATAC","chr1",193180946,"-","none","TGG","CTACCTTCAACGTGCAATACTGG",0,NA,NA,NA,"GeneID:79577;GeneID:8707","CDC73;B3GALT2",TRUE,"B3GALT2"

"14341","CTGTTCCGCACTCATCTTAT","chr1",193181479,"-","none","TGG","CTGTTCCGCACTCATCTTATTGG",0,NA,NA,NA,"GeneID:79577;GeneID:8707","CDC73;B3GALT2",TRUE,"B3GALT2"

"14342","CTTCCAGTATTGCACGTTGA","chr1",193180940,"+","none","AGG","CTTCCAGTATTGCACGTTGAAGG",0,NA,NA,NA,"GeneID:79577;GeneID:8707","CDC73;B3GALT2",TRUE,"B3GALT2"

"14343","AATTCCTGCTGACGTCCCAC","chr6",33278395,"+","none","AGG","AATTCCTGCTGACGTCCCACAGG",0,NA,NA,NA,"GeneID:8705","B3GALT4",TRUE,"B3GALT4"

"14344","CACACGGGGTGCTGTGCGTT","chr6",33277761,"-","none","CGG","CACACGGGGTGCTGTGCGTTCGG",0,NA,NA,NA,"GeneID:8705","B3GALT4",TRUE,"B3GALT4"

"14345","CTCGACACCGCAGGATGCCC","chr6",33278513,"-","none","AGG","CTCGACACCGCAGGATGCCCAGG",0,NA,NA,NA,"GeneID:8705","B3GALT4",TRUE,"B3GALT4"

"14346","GGGCAGGCACCGCGTATCAG","chr6",33278141,"+","none","AGG","GGGCAGGCACCGCGTATCAGAGG",0,NA,NA,NA,"GeneID:8705","B3GALT4",TRUE,"B3GALT4"

"14347","GGTTCACGCGCCAGTGCACC","chr6",33278105,"-","none","CGG","GGTTCACGCGCCAGTGCACCCGG",0,NA,NA,NA,"GeneID:8705","B3GALT4",TRUE,"B3GALT4"

"14348","GTGGGGGTAAGTGCCCGACG","chr6",33278298,"+","none","AGG","GTGGGGGTAAGTGCCCGACGAGG",0,NA,NA,NA,"GeneID:8705","B3GALT4",TRUE,"B3GALT4"

"14349","ACGGAGAAGCGTAAGCCCCC","chr21",39661363,"-","none","TGG","ACGGAGAAGCGTAAGCCCCCTGG",0,NA,NA,NA,"GeneID:10317","B3GALT5",TRUE,"B3GALT5"

"14350","CAGTAGTCCAAGAGAGTCCG","chr21",39661426,"-","none","AGG","CAGTAGTCCAAGAGAGTCCGAGG",0,NA,NA,NA,"GeneID:10317","B3GALT5",TRUE,"B3GALT5"

"14351","GGATAATGTCCCCGTGTCGC","chr21",39660900,"-","none","TGG","GGATAATGTCCCCGTGTCGCTGG",0,NA,NA,NA,"GeneID:10317","B3GALT5",TRUE,"B3GALT5"

"14352","GTCGCCAGAAAACACGTAGC","chr21",39661211,"-","none","CGG","GTCGCCAGAAAACACGTAGCCGG",0,NA,NA,NA,"GeneID:10317","B3GALT5",TRUE,"B3GALT5"

"14353","TTCATCACAAACGCCGCCTG","chr21",39661000,"-","none","AGG","TTCATCACAAACGCCGCCTGAGG",0,NA,NA,NA,"GeneID:10317","B3GALT5",TRUE,"B3GALT5"

"14354","TTGTGGGGCTCTGCCTCGAA","chr21",39661292,"+","none","AGG","TTGTGGGGCTCTGCCTCGAAAGG",0,NA,NA,NA,"GeneID:10317","B3GALT5",TRUE,"B3GALT5"

"14355","CGACGCCTACGAAAACCTCA","chr1",1232655,"+","none","CGG","CGACGCCTACGAAAACCTCACGG",0,NA,NA,NA,"GeneID:126792","B3GALT6",TRUE,"B3GALT6"

"14356","CGCCTTGAGCACGAACTCGA","chr1",1232723,"-","none","AGG","CGCCTTGAGCACGAACTCGAAGG",0,NA,NA,NA,"GeneID:126792","B3GALT6",TRUE,"B3GALT6"

"14357","CGCGCTTCGACACCGAATAC","chr1",1233056,"+","none","CGG","CGCGCTTCGACACCGAATACCGG",0,NA,NA,NA,"GeneID:126792","B3GALT6",TRUE,"B3GALT6"

"14358","CGCGGACCAGTCGTACACGT","chr1",1233206,"-","none","AGG","CGCGGACCAGTCGTACACGTAGG",0,NA,NA,NA,"GeneID:126792","B3GALT6",TRUE,"B3GALT6"

"14359","CGGACGACGACTCCTTCGCG","chr1",1232741,"+","none","CGG","CGGACGACGACTCCTTCGCGCGG",0,NA,NA,NA,"GeneID:126792","B3GALT6",TRUE,"B3GALT6"

"14360","CGTGTGGGCGCGCTTTGCCG","chr1",1232544,"+","none","TGG","CGTGTGGGCGCGCTTTGCCGTGG",0,NA,NA,NA,"GeneID:126792","B3GALT6",TRUE,"B3GALT6"

"14361","AGGAGCGCGAGCAGCGCCGG","chr13",31200120,"-","none","CGG","AGGAGCGCGAGCAGCGCCGGCGG",0,NA,NA,NA,"GeneID:145173","B3GALTL",TRUE,"B3GALTL"

"14362","AGGAGCGCGAGCAGCGCCGG","chr14",32934741,"-","2:G>C,16:C>G","CGG","AGCAGCGCGAGCAGCGGCGGCGG",2,3,17,NA,"","",FALSE,"B3GALTL"

"14363","ATACAGGATTTGGAGAAAAG","chr13",31222945,"+","none","TGG","ATACAGGATTTGGAGAAAAGTGG",0,NA,NA,NA,"GeneID:145173","B3GALTL",TRUE,"B3GALTL"

"14364","ATACAGGATTTGGAGAAAAG","chr7",43554546,"-","1:C>A,9:C>A","AGG","AGACAGGATGTGGAGAAAAGAGG",2,2,10,NA,"GeneID:23072","HECW1",FALSE,"B3GALTL"

"14365","ATATCAAGGAAAAATGACAT","chr13",31222969,"+","none","AGG","ATATCAAGGAAAAATGACATAGG",0,NA,NA,NA,"GeneID:145173","B3GALTL",TRUE,"B3GALTL"

"14366","ATATCAAGGAAAAATGACAT","chr6",78149457,"-","2:A>T,7:A>C","TGG","ATTTCAATGAAAAATGACATTGG",2,3,8,NA,"","",FALSE,"B3GALTL"

"14367","ATATCAAGGAAAAATGACAT","chr10",38243117,"-","5:A>T,7:A>C","TGG","ATATCTATGAAAAATGACATTGG",2,6,8,NA,"","",FALSE,"B3GALTL"

"14368","ATATCAAGGAAAAATGACAT","chr1",77611450,"-","5:A>T,7:A>C","TGG","ATATCTATGAAAAATGACATTGG",2,6,8,NA,"GeneID:26009","ZZZ3",FALSE,"B3GALTL"

"14369","ATATCAAGGAAAAATGACAT","chr1",177215276,"+","5:T>A,7:T>G","TGG","ATATCTATGAAAAATGACATTGG",2,6,8,NA,"GeneID:57795","BRINP2",FALSE,"B3GALTL"

"14370","ATATCAAGGAAAAATGACAT","chrX",98203709,"+","4:G>C,16:T>A","TGG","ATATGAAGGAAAAATGTCATTGG",2,5,17,NA,"","",FALSE,"B3GALTL"
[truncated: 32,119,287 more chars]
